# Distinct stage-specific transcriptional states of B cells derived from human tonsillar tissue

**DOI:** 10.1172/jci.insight.155199

**Published:** 2023-04-10

**Authors:** Diego A. Espinoza, Carole Le Coz, Emylette Cruz Cabrera, Neil Romberg, Amit Bar-Or, Rui Li

**Affiliations:** 1 Center for Neuroinflammation and Experimental Therapeutics, and; 2 Department of Neurology, Perelman School of Medicine, University of Pennsylvania, Philadelphia, Pennsylvania, USA.; 3 Division of Immunology and Allergy, Children’s Hospital of Philadelphia, Philadelphia, Pennsylvania, USA.; 4 Department of Pediatrics, Perelman School of Medicine, Philadelphia, Pennsylvania, USA.

**Keywords:** Cell Biology, Immunology, Bioinformatics

## Abstract

B cells within secondary lymphoid tissues encompass a diversity of activation states and multiple maturation processes that reflect antigen recognition and transition through the germinal center (GC) reaction, in which mature B cells differentiate into memory and antibody-secreting cells (ASCs). Here, utilizing single-cell RNA-seq, we identify a range of distinct activation and maturation states of tonsil-derived B cells. In particular, we identify what we believe is a previously uncharacterized *CCL4*/*CCL3* chemokine–expressing B cell population with an expression pattern consistent with B cell receptor/CD40 activation. Furthermore, we present a computational method that leverages regulatory network inference and pseudotemporal modeling to identify upstream transcription factor modulation along a GC-to-ASC axis of transcriptional maturation. Our data set provides valuable insight into diverse B cell functional profiles and will be a useful resource for further studies into the B cell immune compartment.

## Introduction

Within secondary lymphoid tissues (SLTs), mature B cells encounter antigen and, across multiple maturation processes incorporating the germinal center (GC) reaction, undergo a number of activation and fate-determining states as they ultimately differentiate into either antigen-specific memory B cells or antibody-secreting cells (ASCs). These intramaturation states likely reflect a diversity of B cell functions inclusive of, but likely also beyond, the well-characterized B cell functions of humoral memory development and antibody production. Currently, the functional capabilities of B cells beyond antibody secretion remain incompletely understood, but are of interest given the growing recognition of antibody-independent roles of B cells in both normal immune responses and in autoimmune disease (1–4). Previous insights into the SLT B cell compartment and its dynamics have largely relied on a priori–defined phenotypic markers to identify and further characterize cells of interest. To this end, high-throughput phenotypic characterization of B cells through single-cell RNA-seq (scRNA-seq) provides an alternative venue by which the diverse phenotypic and functional states of lymphoid tissue–derived B cells can be explored in a more unbiased manner. Such an approach would not only permit unbiased characterization of B cell functional states but also help better define existing B cell maturation processes at a higher, transcriptomic resolution. Advances in single-cell technology have already contributed to better understanding of B cell maturation, as demonstrated in a recent study that leveraged scRNA-seq along with repertoire analysis to better understand the interplay between antibody class switching and gene expression in human tonsillar B cells (5).

Here, we utilize scRNA-seq to better characterize the diversity of distinct B cell activation and maturation states of SLT-derived B cells from the human tonsil. We identify a *CCL4*/*CCL3*-expressing B cell population with an activation state consistent with B cell receptor (BCR) and CD40 costimulation and transcriptional processes likely associated with activity of the transcription factor (TF) MYC. Furthermore, we leverage regulatory network analyses to provide TF-level insight into the differentiation of ASCs from GC B cells. This data set provides valuable insight into the diverse functional profiles of B cells and their maturation processes and will be a useful resource on which to base further studies into the B cell immune compartment in healthy and diseased states.

## Results

### scRNA-seq of B cells derived from human tonsils identifies GC and non-GC cellular states and ASCs.

**Figure 1:**
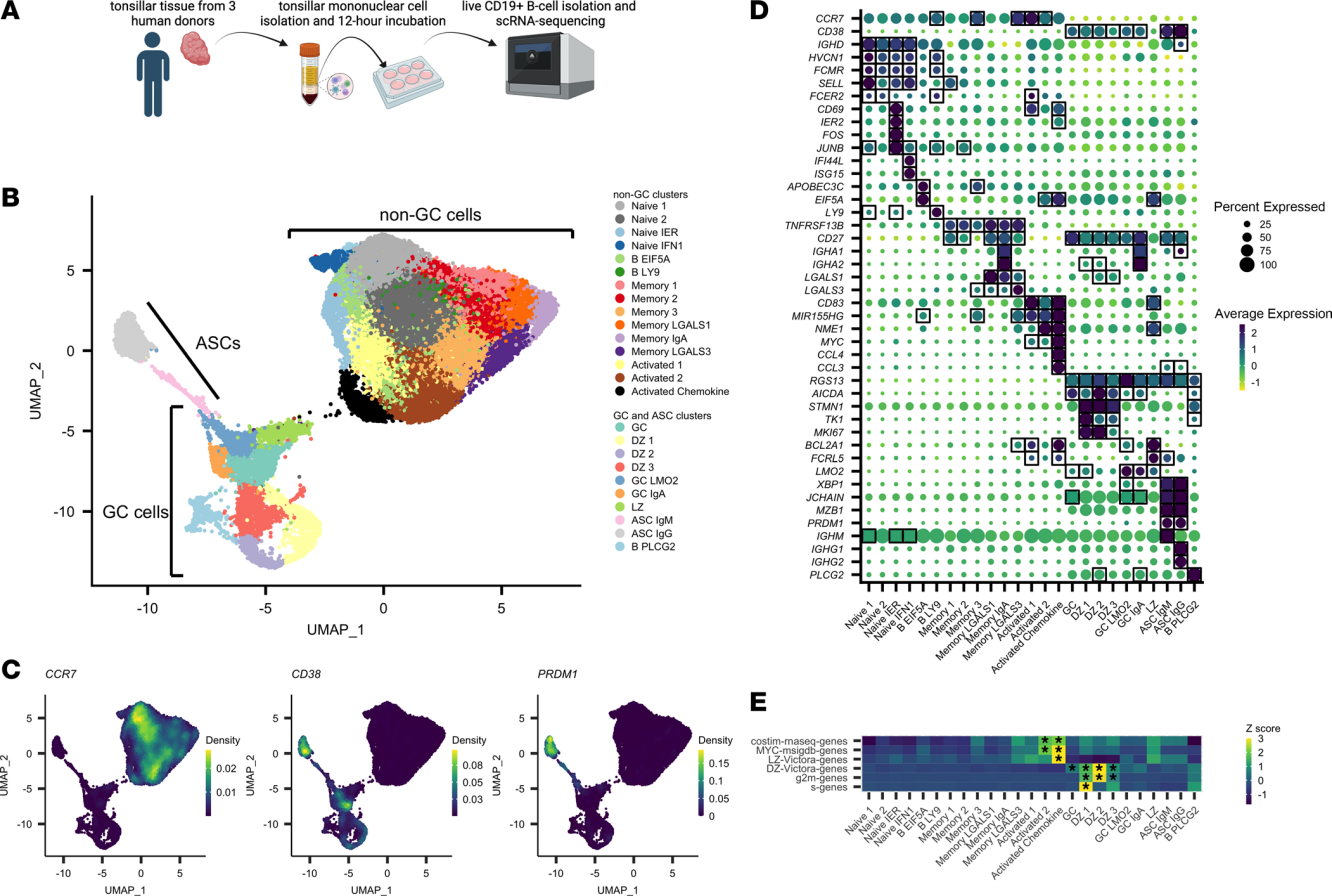
scRNA-seq of human tonsillar B cells identifies distinct B cell maturation and activation states. (**A**) Experimental design for tonsillar B cell isolation, culture, and scRNA-seq. (**B**) UMAP plot of 45,376 single B cell transcriptomes from 3 human donors. (**C**) Visualization of *CCR7*, *CD38*, and *PRDM1* expression patterns in UMAP space using the kernel density estimation method in the R package Nebulosa. (**D**) Selected marker gene average expression (scaled log-normalized counts) for each cluster and proportion of cells with transcript detected. Genes were determined to be marker genes if their average log_2_(fold change) was greater than 0.3 for the cluster of interest and adjusted *P* value was less than 0.01. Marker-gene expression is denoted by square boxes on gene-cluster pairs. (**E**) Selected scaled average AUCell scores for 5 gene sets across each cluster. Heatmap cells were labeled with an asterisk (*) if the gene set was enriched within that cluster (Wilcoxon’s rank-sum test, AUC > 0.85).

We sorted live CD3^–^CD14^–^CD19^+^ B cells from short-term (12-hour) cultured tonsillar mononuclear cells (3 human donors, information in Supplemental Table 1; supplemental material available online with this article; https://doi.org/10.1172/jci.insight.155199DS1) and recovered single-cell transcriptomes using the 10× Genomics Chromium 3′ scRNA-seq platform (Figure 1A). Following data preprocessing (see Methods and Supplemental Figure 1, A–C) to enrich for live B cells and to integrate data across 3 donors, we analyzed 45,376 B cell transcriptomes. We performed marker gene identification using a one-versus-all approach for each cluster to identify marker genes for each cluster (Supplemental Figure 2A, cluster and grouping counts by donor in Supplemental Tables 2 and 3, full marker list in Supplemental Table 4). Based on prior work, we initially used the overall RNA expression patterns of *CCR7*, *CD38*, and *PRDM1* (Figure 1, B and C) to split our clusters into 3 partitions, with each partition named according to transcriptional similarity to known B cell states in SLTs: (i) non-GC B cell clusters (*CCR7*^+^*CD38*^–^), defined by high levels of the tissue emigrant marker gene *CCR7* (6) and low expression of the *CD38* (7) human GC and plasma cell marker gene; (ii) GC B cell clusters (*CCR7*^–^*CD38*^+^*PRDM1*^–^), defined by high levels of CD38 and low levels of *PRDM1*; and (iii) ASC clusters (*CCR7*^–^*CD38*^+^*PRDM1*^+^), defined by high levels of *CD38* and *PRDM1*, a plasma cell marker gene (8)*,* and low levels of *CCR7*.

### Clusters of non-GC B cells capture multiple distinct states of B cell activation.

We first focused on characterizing the transcriptional phenotypes of clusters within the non-GC B cell clusters. Using *IGHD*, *FCMR*, and *HVCN1* (9) expression to define the naive pool, we identified 4 B cell subclusters (Naive 1, Naive 2, Naive IER [immediate early response activated], and Naive IFN1 [type 1 interferon activated]*;* Figure 1B). The Naive IER cluster’s marker genes included activation markers *IER2*, *FOS*, *JUNB*, and *CD69* (Figure 1D). CD69 is an early marker of B cell activation, suggesting that cells within this cluster shared an expression pattern consistent with an early form of B cell activation. In contrast, the Naive IFN1 cluster’s marker genes included IFN-stimulated genes such as *IFI44L* and *ISG15*, suggesting that cells within this cluster shared an expression pattern indicative of cellular type 1 IFN activation. A fifth cluster, B EIF5A, colocalized partially with the naive clusters in the data set (Figure 1B); however, the B EIF5A cluster expressed no naive-like or memory-like marker genes, and instead showed enrichment for genes involved in transcriptional regulation, such as *APOBEC3C* and *EIF5A* (Figure 1D), suggesting a B cell state with increased transcriptional activity. Proximal to the naive clusters in the uniform manifold approximation and projection (UMAP) space, we identified a cluster (B LY9) that shared marker genes with Naive B cells (*HVCN1*, *FCMR*, and *FCER2*), but expressed uniquely high levels of *LY9* (Figure 1D).

We next defined 6 memory B cell clusters based on enrichment for the memory B cell marker gene *TNFRSF13B* (10) (Figure 1D). Five of these 6 clusters (the exception being Memory 3) expressed CD27, another memory B cell marker, as a marker gene as well. Limiting the differential expression analysis to these 6 memory B cell clusters (Supplemental Figure 3), we found that the Memory 2 and Memory 3 showed relative enrichment for *IGHD* and *IGHM*, indicating that these may represent non–class-switched memory B cells. In contrast, Memory 1 showed enrichment for *IGHG1* and *IGHG3* (Supplemental Figure 2B), indicating that their heavy-chain expression was more representative of a class-switched memory identity. The Memory IgA cluster expressed marker genes *IGHA1* and *IGHA2*, identifying what is likely a mucosal B cell memory cell subset (Figure 1D). Two other memory B cell clusters, Memory LGALS1 and Memory LGALS3, exhibited high expression of galectin transcripts *LGALS1* (encoding galectin-1) and *LGALS3* (encoding galectin-3), respectively. The B cell–specific roles of galectin-1 and galectin-3, particularly in memory B cells, have not been fully determined (11).

Three clusters (Activated 1, Activated 2, and Activated Chemokine) expressed the same set of 3 cellular activation–associated genes as marker genes: *CD83*, *MIR155HG*, and *NFKB1* (Figure 1, B and D). CD83 surface expression is known to be upregulated on B cells in a number of activation contexts, including LPS, CD40, and IgM stimulation (12). *MIR155HG* encodes the microRNA miR-155, which has also been shown to be upregulated on B cells upon TLR, CD40, and BCR stimulation (13) in mice and upon BCR cross-linking in Ramos cells (14) and shown to enhance BCR signaling in CD40-stimulated human B cells (15). Additionally, miR-155 is downregulated in GC B cells by BCL6, a GC-specific TF (16), suggesting that the Activated 1, Activated 2, and Activated Chemokine clusters potentially represent cellular states primed for the GC reaction. Overall, the coexpression pattern of *CD83* and *MIR155HG* in these clusters suggests that cells within these clusters may represent B cells receiving BCR and/or CD40 signaling prior to entry into the GC reaction. In contrast to the Activated 1 cluster, however, Activated 2 and Activated Chemokine expressed higher levels of *NME1* (Figure 1B).

Next, in order to better determine the similarity of the 3 Activated clusters to B cells stimulated through the BCR alone (as a proxy for B cells encountering antigen but not receiving T cell help), CD40 alone (as a proxy for B cells encountering activated T cells expressing CD40L, but not encountering their antigen), or both the BCR and CD40 (as a proxy for B cells encountering both their antigen and receiving costimulation through interaction with activated T cells), we stimulated peripheral blood B cells in vitro (*n* = 3; donor information in Supplemental Table 5) to derive gene signatures for each stimulus (BCR-only signature, CD40-only signature, costimulation signature; Supplemental Table 6). We found that the Activated 2 and Activated Chemokine clusters showed enrichment for only one of our in vitro stimulation signatures, the costimulation signature (Figure 1E), corroborating our marker-gene observations. Of note, we also observed marker-gene expression of *MYC* in our Activated clusters (Figure 1E); MYC is a TF known to be necessary for GC formation (17, 18) and only expressed in a small subset of light zone (LZ) cells in the GC (17, 18). Thus, given the expression of the activation markers above, the enrichment for a BCR/CD40 costimulation signature and elevated MYC transcript expression, it is plausible that cells within these Activated clusters are representative of cells primed for entry into the GC reaction. The Activated Chemokine cluster, in addition to sharing marker gene expression of *CD83*, *MIR155HG*, *NFKB1*, and *MYC* and showing enrichment for our BCR/CD40 costimulation signature, also showed cluster-specific marker-gene expression of the chemokine transcripts *CCL4* and *CCL3* (Figure 1D). These chemokines are known to be secreted by B cells and are chemoattractant for T cells (in particular, regulatory T cells; see refs. 3, 4). Enrichment analysis of literature-defined gene sets, including MYC signature– and LZ-specific gene sets, found that the Activated Chemokine cluster had the highest enrichment for these gene sets, in line with its upregulation of activation-associated transcripts (Figure 1E). The functional roles and necessity of this chemokine-expressing B cell population has not yet been elucidated with regard to the GC reaction.

### Clusters of GC B cells capture multiple distinct states of B cell maturation.

We next characterized clusters with transcriptional states corresponding to stages of the GC reaction. Clusters were denoted as GC clusters if they were significantly enriched for the marker genes *CD38* (save for one cluster) and *RGS13*, another marker shown to be enriched in GC B cells (19) (Figure 1D). Next, using prior gene signatures for LZ and dark zone (DZ) genes (20), we found 3 clusters, DZ 1, DZ 2, and DZ 3, with the highest enrichment for the DZ transcriptional program (Figure 1E), which included genes such as *AICDA*, *STMN1*, *TK1*, and *MKI67* (Figure 1D). These cells also represented actively cycling cells, a process that appeared to be represented by a cyclical structure in the UMAP plot (Figure 1B), with DZ 1 representing cells with gene expression most consistent with S-phase, DZ 2 representing cells with gene expression most consistent with G_2_/M-phase, and DZ 3 showing no particular skewing toward S- or G_2_/M-phase gene expression (Figure 1E). Clusters GC and GC IgA represented cells without clear enrichment for DZ or LZ programs and likely represent a mixture of cells in both programs (Figure 1, D and E) or, alternatively, may represent a temporally intermediate LZ-DZ state previously described as the “intermediate zone” or “gray zone” (21–23), with transcriptional features shared across DZ and LZ programs. GC IgA uniquely showed enrichment for the *IGHA1* and *IGHA2* heavy chain genes. Cluster LZ showed relatively high enrichment for the LZ program (Figure 1E) compared with other GC clusters. The LZ markers *BCL2A1* and *FCRL5* were also enriched within this cluster (Figure 1D). Notably, the Activated 2 and Activated Chemokine clusters also showed high enrichment for the LZ program and MYC gene set (Figure 1E), while likely representing cells not in the GC reaction given their expression of *CCR7* and lack of *CD38* expression. The underlying transcriptional similarity between these 2 cellular phenotypes is likely because the LZ transcriptional program is expected to share intracellular signaling mechanisms with the overall B cell activation transcriptional program, as some LZ B cells are expected to undergo BCR/CD40 costimulation. Finally, the GC LMO2 cluster showed enrichment for the GC DZ and LZ marker genes *AICDA* and *BCL2A1*, along with the gene *LMO2*, while expressing marker genes *XBP1* and *JCHAIN* (albeit at a subtle, but statistically significant level), which are instead associated with ASCs, suggesting an intermediate cellular state between GC and antibody-secreting phenotypes (Figure 1D). Finally, another cluster, B PLCG2, showed particularly high marker-gene expression of *PLCG2* and a number of histone genes (Supplemental Figure 2), while sharing expression programs with the DZ program and largely colocalizing with DZ cells in the UMAP plot (Figure 1B). The high expression of histone transcripts, expected to be restricted to the nucleus, suggests that these cells likely represent dead or dying, recently cell-cycling DZ cells whose cytoplasmic RNA has leaked to the extracellular environment, resulting in enrichment of nucleus-associated transcripts.

### Clusters of antibody-secreting B cells are segregated by IgM or IgG heavy chain expression.

We identified 2 clusters likely representing ASCs: ASC IgM and ASC IgG. These clusters both showed marker-gene expression of canonical ASC genes *XBP1*, *JCHAIN*, *MZB1*, and *PRDM1*, but differed in terms of their antibody isotype (Figure 1D).

In summary, our scRNA-seq approach resolved multiple transcriptionally defined populations of SLT-derived B cells, recapitulating heterogeneous naive B cell, memory B cell, GC cell, and ASC subsets, some with distinct signatures of activation and maturation. Of particular interest, we identified a cluster (Activated Chemokine) with high expression of *CCL4* and *CCL3* transcripts and a transcriptional profile consistent with BCR/CD40 activation, potentially representing a B cell population secreting T cell–attractant chemokines and interacting with T cells prior to entry into the GC reaction within human tonsils.

### TF regulatory network analysis identifies MYC, REL, and FOSL1 as TFs driving the production of chemokine transcripts in the Activated Chemokine cluster.

We next sought to use gene regulatory network (GRN) analysis to identify TF signatures associated with each cluster in order to hypothesize the upstream transcriptional drivers of the observed cellular identities. Using the SCENIC (24) pipeline, and a given list of curated TFs and corresponding motif information, we derived TF GRNs (regulons) from our scRNA-seq data. Hereafter, a regulon refers to a TF and its set of SCENIC-predicted downstream targets. We next inferred the single-cell activity of each regulon using the AUCell algorithm, which quantifies a measure of enrichment for a regulon’s predicted targets within the expressed genes for each cell. Differential activity of regulons was then identified between clusters by using Wilcoxon’s rank-sum test (see Methods) in order to investigate differential regulon activity (analogous to TF activity) within our data set.

**Figure 2:**
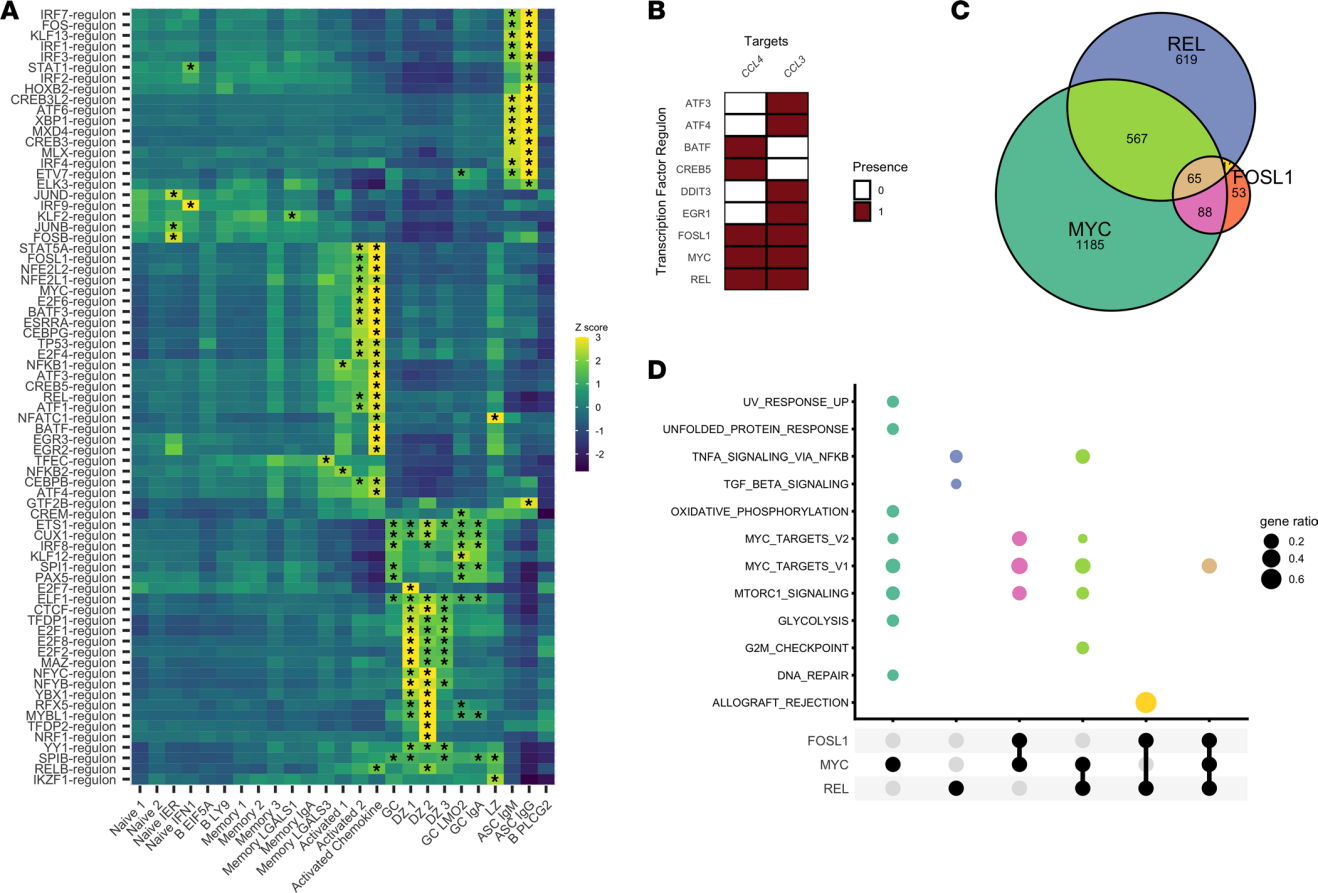
SCENIC analysis identifies MYC and REL as transcription factors predicted to regulate chemokine expression in activated B cells. (**A**) Average AUCell scores for selected regulons across all clusters. Regulons were selected for plotting if they were enriched (Wilcoxon’s rank-sum test, AUC > 0.85) in at least 1 cluster. Heatmap cells were labeled with an asterisk (*) if the gene set was enriched within that cluster. (**B**) Predicted targets for all regulons identified that target at least one of the *CCL4* or *CCL3* genes. (**C**) Venn diagram of the REL, MYC, and FOSL1 regulon targets. (**D**) Gene enrichment analysis of the various gene set intersections shown in **C** (hypergeometric test, clusterProfiler). Gene enrichment results only shown for enrichment tests with adjusted *P* values of less than 0.05. Colors match the corresponding sets with the identical color in **B**.

We first found that the IRF9 (IFN regulatory factor 9) regulon exhibited increased activity only in the Naive IFN1 cluster (Figure 2A), suggesting a role for the IRF9 TF downstream of type 1 IFN signaling and upstream of IFN-response gene transcription in B cells. In line with known literature, we also found that the XBP1 (25) and IRF4 (25) regulon activities were enriched in both ASC clusters (Figure 2A). In murine models, XBP1 is a TF necessary for terminal differentiation of plasma cells (26) and, similarly, IRF4 is a TF necessary for the generation of plasma cells (27). Among other regulons, we also found enrichment of the ATF6 regulon in ASC clusters (Figure 2A). ATF6 is involved in the unfolded-protein response and is activated by PRDM1 (28), another TF that controls plasma cell development. Meanwhile, within the GC, GC IgA, and GC LMO2 clusters, we found upregulation of the regulon for IRF8, the canonical GC TF (8) (Figure 2A).

We next inspected the regulons enriched within the Activated Chemokine cluster, aiming to gain additional insight into the transcriptional processes driving chemokine expression. We found that this cluster showed overall enrichment for a relatively large number of regulons when compared with other clusters (Figure 2A). We chose to identify the TFs whose regulons were predicted to target the chemokine genes *CCL4* and *CCL3*. Among chemokine-targeting TFs, we found that 3 TFs were predicted to target both of the chemokine transcripts: FOSL1, MYC, and REL (Figure 2B); all 3 furthermore showed enrichment (AUC > 0.85) in the Activated Chemokine cluster (Figure 2A, Supplemental Figure 4, and Supplemental Table 7). Among these regulons, both REL and FOSL1 shared considerable overlap of predicted downstream targets with MYC (Figure 2C), suggesting overlapping targets for these 3 TFs.

We next partitioned the network structure of the REL, FOSL1, and MYC regulons to classify genes into 1 of 7 gene sets (MYC-only, REL-only, FOSL1-only, MYC-REL, MYC-FOSL1, FOSL1-REL, and FOSL1-MYC-REL; Figure 2C) and utilized a hypergeometric test to determine whether individual gene sets from the Molecular Signatures Database’s (29) Hallmark gene sets (v7.2) collection were enriched within each of our gene sets. MYC-only targets were enriched for genes involved in metabolic processes (glycolysis and oxidative phosphorylation) along with cellular processes such as DNA repair and mTORc1 signaling (in addition to being enriched for genes from previously derived MYC target gene sets) (Figure 2D). On the other hand, REL-only genes had a target gene set that was most enriched for genes involved in NF-κB signaling and TGF-β signaling. (Figure 2D). Enrichment analyses of remaining gene sets (MYC-FOSL1, MYC-REL, and FOSL1-MYC-REL) showed overlapping functions with the MYC-only and REL-only gene sets, and 1 gene set (FOSL1-REL) enriched for an allograft rejection pathway, potentially indicating a role for these TF networks in the cellular reaction against non-self (allo)antigens.

Overall, these enrichment analyses suggest that while the TFs MYC and REL both are predicted to target similar genes (including chemokine genes) and may have overlapping functions, there are particular processes, such as cellular metabolism, in which MYC may have a more overt regulatory role, and other processes, such as the downstream enactment of the NF-κB signaling pathway, in which REL may play a more overt regulatory role. In fact, the predicted regulon networks identify MYC as a target of REL and REL as a target of MYC (Supplemental Table 8), underscoring a potential relationship between these 2 TF networks in BCR/CD40-activated human B cells.

### Gene expression and TF network pseudotemporal modeling identifies genes and TF network expression changes associated with the GC-to-ASC transition.

**Figure 3:**
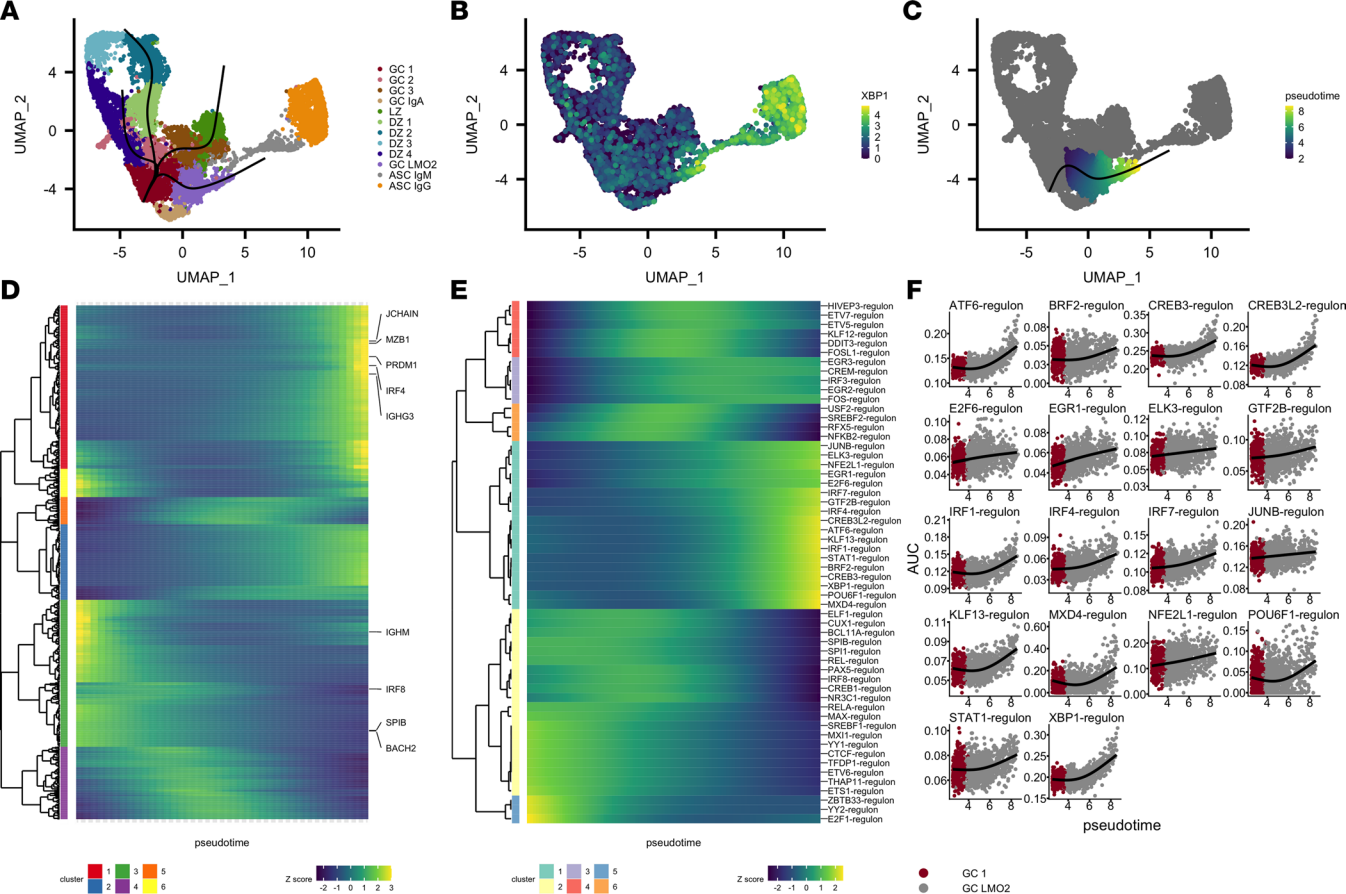
Trajectory inference of tonsillar B cell scRNA-seq models transcriptional dynamics of a GC-to-ASC transition. (**A**) UMAP plot and cluster labels for the reanalysis (normalization, integration, dimensionality reduction) of the GC and ASC clusters. Slingshot trajectories (black) are overlaid on the UMAP plot. (**B**) Gene expression levels of *XBP1* (log-normalized counts) overlaid on cells in the UMAP plot. (**C**) Pseudotemporal ordering (pseudotime) results for trajectory of interest. (**D**) Modeled expression values across increasing pseudotime (left to right) for the cellular trajectory shown in **C**. Heatmap shows genes deemed associated with pseudotime based on an adjusted *P* value of less than 0.01 from tradeSeq’s associationTest. Selected genes are labeled. Expression patterns were clustered using Manhattan distance of the modeled gene expression values across pseudotime and hierarchical clustering was performed with a 6-cluster cutoff. (**E**) Modeled regulon activity values across increasing pseudotime (left to right) for the cellular trajectory shown in **C**. Regulons with adjusted *P* values of less than 1 × 10^–6^ determined by fitting generalized additive models are shown. Expression patterns were clustered using Manhattan distance of the modeled gene expression values across pseudotime and hierarchical clustering was performed with a 6-cluster cutoff. (**F**) Generalized additive model results alongside regulon activity values are displayed for cluster 1 from **E**.

While the analyses above revealed identities and states of discrete B cell populations, we considered whether we could explore the continuum of B cell maturation using our scRNA-seq transcription-level data. We chose to focus on GC-to-ASC maturation, and so we reanalyzed and reclustered the GC and ASC clusters (Figure 3A) independently of non-GC cells. We identified 1 GC cluster, GC LMO2 (which in fact was most similar to the GC LMO2 cluster identified in the full analysis; Figure 3A and Supplemental Figure 5), that showed a gradient of ASC marker expression (*XBP1*, *PRDM1*, *MZB1*, and *IGHG4*; Figure 3B and Supplemental Figure 6) across its 2-dimensional UMAP representation, consistent with a cellular trajectory of differentiation from GC-like cells to ASC-like cells. We inferred that these cells captured a continuum of states across GC-to-ASC differentiation rather than a terminal cell type, and modeled transcriptional dynamics across this continuum based on these assumptions.

We leveraged the slingshot (30) trajectory inference method and the tradeSeq gene expression modeling software to build slingshot differentiation trajectories on our UMAP plot (Figure 3A) and order cells on the GC 1–to–GC LMO2 trajectory (Figure 3C) before modeling gene expression across this pseudotemporal axis. We then plotted the smoothed model expression values from the genes that with most significant gene expression variability over pseudotime (967 genes with *P* value <0.01, Figure 3D; full list of 10,487 genes in Supplemental Table 9) and identified 6 clusters of gene expression dynamics present in the data. One cluster (red, Figure 3D) displayed an increasing gene expression pattern along the pseudotemporal axis. This cluster included canonical ASC genes such as *IRF4*, *MZB1*, *PRDM1*, *JCHAIN*, and *IGHG3* (Figure 3D). One other cluster of genes (green, Figure 3D) displayed a decreasing gene expression pattern along the pseudotemporal axis, and included genes associated with the GC reaction such as *IGHM*, *IRF8*, *SPIB*, and *BACH2* (31).

In total, these gene expression patterns suggested that the pseudotemporal axis was consistent with a GC-to-ASC differentiation axis. We therefore chose to leverage our prior TF regulon approach to develop a method by which we could model TF dynamics in the GC-to-ASC transition. We considered that by modeling TF regulon activity, rather than TF gene expression, we may more optimally infer TF dynamics, assuming that predicted TF regulon activity (rather than TF gene expression) might be more consistent with true TF activity. Because TFs are known regulators of cell fate, this analysis may provide more salient targets for further study and/or inference. Using our TF pipeline in conjunction with our pseudotemporal modeling, we found a number of well-characterized GC-associated TFs, such as PAX5, IRF8, and SPIB (31) that decreased in regulon activity along the GC-to-ASC transition (yellow and blue, Figure 3E). The canonical ASC TFs, XBP1 and IRF4 (31), instead increased in regulon activity along the transition (Figure 3, E and F). The ATF6, CREB3, and CREB3L2 TFs, which are associated with protein folding and cellular secretory function (32, 33), also increased in regulon activity along the GC-to-ASC transition (Figure 3, E and F). In addition to IRF4, two other members of the IFN-regulated factor family of TFs, IRF1 and IRF7, increased in regulon activity along the GC-to-ASC transition, and regulon activity of the TF STAT1, a molecule activated upon IFN stimulation, also increased along this transition (Figure 3, E and F). Moreover, we identified a number of other TFs whose regulon activities increased along the pseudotemporal axis but whose roles have not been well described in GCs, ASCs, or B cells in general: KLF13, MXD4, and POU6F1 (Figure 3F). Gene-level analyses of the TFs associated with increasing activity along the GC-to-ASC transition showed considerable variability and poor detection, in line with our postulate that TF regulatory network analyses would more optimally identify important TFs in cellular transitions (Supplemental Figure 7).

## Discussion

The antibody-independent functional capabilities of B cells in human health and disease remain incompletely defined. Here, we present a single-cell transcriptomic snapshot of human B cell maturation and functional states utilizing tonsil-derived B cells to infer potential B cell functions beyond antibody secretion. Among our findings, we identify a dual *CCL4* and *CCL3* chemokine-expressing B cell population with enrichment for a BCR/CD40 costimulatory transcriptomic signature, and implicate the transcription factors MYC, REL, and FOSL1 in regulation of this subset’s CCL4 and CCL3 chemokine expression. Furthermore, using a joint trajectory inference plus regulatory network computational approach, we model the transcriptional dynamics of the GC-to-ASC transition to identify associated candidate TFs involved in the GC-to-ASC transition. Overall, our data represent a resource for the probing of B cell functional heterogeneity and maturation pathways, and implicates associated TFs in driving the captured phenotypes.

The identification of a distinct B cell population expressing *CCL4* and *CCL3* with a transcriptomic profile of BCR and CD40 costimulation is consistent with prior reports demonstrating the potential for CCL4 and CCL3 production by in vitro–stimulated healthy naive, memory, and GC B cells (34) as well as malignant B cells (35, 36). While the ontogeny of these cells cannot be solely determined from our transcriptomic data, the lack of gene expression of typical GC markers (*RGS13* and *CD38*; Figure 1D) in these cells and the distinct colocalization of the cells in the UMAP space with what are deemed non-GC cells (Figure 1B) suggest that these cells share more transcriptomic similarities with cells outside of the GC reaction than with cells within the GC reaction. Our regulon analysis in particular highlights MYC as a potential TF driving expression of these chemokines, further suggesting a temporal association of these B cells with a state immediately prior to entry of the GC reaction. Further studies that recover B cell repertoires in these cells and others with sufficient resolution within the SLTs may provide insight into the ontogeny of these *CCL4*- and *CCL3*-coexpressing B cells.

Our delineation of transcriptionally defined B cell states complements previously published single-cell investigations of human tonsillar B cell populations. Certain observations remain consistent among published investigations (5, 21–23) and ours; for example, existing studies (5, 21–23) into the human and/or murine SLT B cell compartment identify a transcriptional continuum between LZ-like B cells and DZ-like B cells, similar to the continuum observed in our data, with DZ-like B cells adopting transcriptomic patterns associated with cell cycling. Though we do not define distinct “intermediate” or “gray-zone” cell populations as prior studies have (21–23), we identify a population of cells with transcriptional features suggestive of GC identity, but without definitive LZ-like or DZ-like features (cluster GC), which potentially includes cells with “intermediate” or “gray-zone” cell states. A previous study (5) also identified a distinct tonsillar naive B cell population with an early-activation B cell gene expression pattern (exhibiting characteristic expression of *JUN* and *CD69*). However, analysis of this existing tonsillar cell atlas (5) of more than 32,000 immune cells profiled with scRNA-seq/scBCR-seq did not identify a *CCL4*/*CCL3*-coexpressing B cell population (Supplemental Figure 8). We further identify distinct expression patterns within memory B cell subsets (*LGALS1* and *LGALS3*) not detailed elsewhere. Explanations for such differences in observed cell states among these studies are likely multifactorial in nature. Prior studies performed single-cell assays on SLT B cells with the goal of minimizing time between the extraction of cells from their in vivo niche and execution of the single-cell assay. In contrast, we opted to perform a 12-hour tonsillar mononuclear cell culture, which may allow for more frequent cell-cell interactions, revealing a wider spectrum of potential states that otherwise may not be appreciated when interrogating cell immediately after extraction (37, 38). Differences in sample processing parameters as well as donor variability may also affect transcriptomic profiles and may contribute to the differences observed across studies, as might lack of synchrony of immune responses in different GCs. While our study examined only 3 human tonsils and more than 50% of cells analyzed came from a single donor, one notes that each sample represents an aggregate of B cell populations that likely reflect nonsynchronized GCs within each tonsil, and that the GCs are likely further nonsynchronized across tonsils, both contributing to further diversity.

We also present what we believe is a novel approach for interrogating transcriptional dynamics along cellular differentiation pathway, which leverages an existing trajectory inference algorithm, slingshot (30) but, rather than modeling gene expression levels along the derived pseudotemporal axis, we instead leverage an existing gene regulatory network approach (SCENIC; ref. 24) to infer TF-target networks, and then model the activities of these networks along the pseudotemporal axis. While the SCENIC approach itself (in the absence of pseudotemporal modeling) identifies candidate TFs implicated in the cellular identity of various clusters in our data, we envision the addition of pseudotemporal modeling approaches as further implicating TFs in cellular differentiation pathways. Moreover, as the approach leverages TF-target information, rather than TF gene expression, we find potential TF candidates that otherwise may not have been identified utilizing gene-centric approaches. One notes a potential limitation of this approach is that certain TFs may share similar binding motifs, which could implicate multiple candidate TFs when in fact, in vivo, only a subset of the identified TFs actually modulate a given expression program. While our pseudotemporal approach relies on a priori knowledge and assumptions for choosing the direction of the pseudotemporal axis, we envision this approach as being a worthwhile addition to the growing tool set used in single-cell transcriptomics and a useful tool for identifying candidate targets for further study.

Ultimately, we believe that the B cell transcriptional landscape analysis provided here will contribute to building a better understanding of B cell function and maturation. In addition to transcriptomically characterizing a chemokine-expressing B cell population that potentially represents a pre-GC population, our pseudotemporal modeling findings, which coincide with well-studied transcriptome dynamics involved in the GC-to-ASC transition, further suggest a potential role for other, understudied TFs. Furthermore, our regulon pseudotemporal modeling approach represents a viable alternative to gene expression–based pseudotemporal modeling approaches, as incorporation of a regulatory network provides stronger evidence for candidate TFs involved in cellular state transitions. We hope these analyses will provide a resource motivating further studies into the diverse functional roles of specific B cell subsets, as well as the potential roles and utility of specific molecules implicated in B cell identity and maturations.

## Methods

### 10× Chromium scRNA-seq.

We isolated tonsillar mononuclear cells using Ficoll-gradient centrifugation from 3 healthy human tonsil donors (4 year-old male, 17 year-old female, 5 year-old male). Whole tonsillar mononuclear cells were incubated overnight (12 hours at 37°C in 6-well plates at 6 million cells per well, 4 mL per well) following isolation. Following overnight incubation, FACS purification of CD3^–^CD14^–^CD19^+^ live B cells was performed, utilizing an aqua dye for viability staining. scRNA-seq libraries were then generated utilizing 10× Chromium v2 chemistry (3′ end) with the goal of recovering 10,000 cells per reaction. Resulting libraries were sequenced utilizing an Illumina HiSeq 2500 (read 1, 26 base pairs; read 2, 98 base pairs; index read, 8 base pairs).

### scRNA-seq preprocessing and clustering.

For bash, R, and python code implementation, see *Data availability and reproducibility*. Briefly, for our pipeline, we mapped scRNA-seq data to the human genome using CellRanger version 3.1.0. We next kept cells with at least 200 unique genes expressed and kept genes expressed in at least 3 cells. We then discarded cells with greater than 5% read fraction deriving from mitochondrial counts, greater than 4% read fraction deriving from a dissociation signature (39), or greater than 40,000 unique RNA features (Supplemental Figure 1A). We furthermore excluded contaminating non-B cells (predicted to be T cells based on detection of 2 or more of the markers CD3D, IL-32, and CD2. We next used SCTransform (40) to normalize and scale our data by regularized negative binomial regression (using 3000 variable genes per donor). We used the Seurat integration pipeline to integrate our data across the 3 donors using 40 CCA dimensions, followed by PCA on the scaled/integrated data, excluding immunoglobulin kappa/lambda chain genes (gene names beginning with IGKC or IGLC). We chose the first 35 PCs to build a kNN (30 neighbors, euclidean distance) and a UMAP 2-dimensional projection. We identified clusters using the Louvain algorithm (resolution = 1.3) on a shared nearest-neighbors graph derived from the kNN (minimum Jaccard distance prior to trimming = 1/15).

### scRNA-seq marker gene identification.

We performed marker-gene identification using a one-versus-all approach for each cluster and a likelihood ratio test in which “donor” was used as a latent variable in Seurat; those genes that showed an adjusted *P* value of less than 0.01 and an average log_2_(fold change) of greater than 0.3 were considered “marker” genes for each cluster.

### scRNA-seq gene set sources and module enrichment.

We obtained LZ and DZ signature genes from previous microarray profiling work on human tonsillar GC sorted B cells (20). The G_2_/M and S gene sets were obtained directly from Seurat (cc.genes.updated.2019). The MYC signature and the remaining Hallmark gene sets were obtained from the Molecular Signature Database. Gene sets were scored at a single-cell level using AUCell, part of the SCENIC tool set (24).

### In vitro peripheral B cell stimulation and bulk RNA-seq.

Ficoll- and FACS-isolated (CD3^–^CD14^–^CD19^+^) human peripheral B cells were stimulated with anti-BCR cross-linking antibodies (Jackson Immunoresearch, catalog 109-006-129, 10 μg/mL) or anti-CD40L (Enzo Life Sciences, catalog ALX-522-110-C010, 1 μg/mL) or the combination of anti-BCR and anti-CD40L. After 18 hours, RNA was isolated from the differentially stimulated B cells using the RNeasy Plus Micro Kit (Qiagen) according to the manufacturer’s protocol. RNA quantity and quality were assessed by an Agilent TapeStation and Thermo Fisher Scientific Nanodrop. Samples were run on a Bioanalyzer (Agilent) with the RNA 6000 Pico kit (Agilent) in order to calculate RNA integrity. Bulk RNA-seq was performed using an Illumina NovaSeq 6000 instrument (60 million reads/sample, 2 × 100 bp). Reads were pseudoaligned to GENCODE reference genome v33 using kallisto (41). DESeq2 (42) was used for differential gene expression analysis and a false discovery rate of 0.05 was utilized. See the *Data availability and reproducibility* section for further kallisto and DESeq2 parameters utilized.

### TF gene regulatory network analysis.

We used the python implementation of SCENIC (pySCENIC) (24) on our full scRNA-seq count matrix. In brief, TF gene target networks are determined by construction of GRNs (using GRNBoost2, a random forest regression method) for each TF and pruning of their targets based on TF motif enrichment. Activity of the resulting TF regulatory target networks, or regulons for short, was inferred on the whole single-cell data set by using the AUCell algorithm, which scores the TF regulons independently for each cell, based on the enrichment for each regulon’s targets at the top of the gene expression rankings for each cell. Regulon activities were tested for differential enrichment in clusters using Wilcoxon’s rank-sum test using the Presto (43) package. Motifs, genomic sequences, and table of TFs interrogated are included in a previous report (44), and the list of TFs is also included as Supplemental Table 10.

### Gene enrichment analysis.

The hypergeometric test clusterprofiler (45) (function enricher) was utilized to determine whether individual gene sets from the Molecular Signatures Database’s (29) Hallmark gene sets (v7.2) collection were enriched within each of our specified gene sets (Figure 2C). An adjusted *P* value of 0.05 was utilized as a cutoff to determine statistical significance for enrichment. See the *Data availability and reproducibility* section for access to GitHub repository, which includes the code and files utilized for the enrichment analysis.

### Trajectory inference analysis.

Transcriptomes belonging to the GC or ASC cluster partitions (Figure 1) were reanalyzed from the sctransform step through marker-gene identification. For specific analysis parameters, please refer to the *Data availability and reproducibility* section. The resulting UMAP 2-dimensional reduction was used to construct differentiation trajectories using a modified form of slingshot as follows: (a) a predefined tree was defined on GC clusters such that a branch existed from the GC to GC-LMO2 cluster, (b) principal curves were built on this branch using slingshot, (c) pseudotemporal ordering was performed, setting GC 1 as the root cluster. Gene expression–centric pseudotemporal analysis was performed using the tradeSeq package (knots = 3, see *Data availability and reproducibility* for full code), while regulon activity-centric pseudotemporal analysis was performed using the mgcv package (knots = 3, see *Data availability and reproducibility* for full code).

### Statistics.

For marker gene identification (Figure 1D), a likelihood ratio test with “donor” as a covariate was utilized and marker genes were deemed significant if they had an adjusted *P* value of less than 0.01 and log_2_(fold change) of greater than 0.3, using the FindAllMarkers function in Seurat (46). For regulon enrichment (Figure 2A), Wilcoxon’s rank-sum test was performed, and an AUC cutoff of greater than 0.85 was used to determine whether regulons were enriched per cluster. For gene set enrichment (Figure 2D), an adjusted *P*-value cutoff of 0.05 was utilized to determine gene sets as enriched after using a hypergeometric test. For pseudotime-associated genes (Figure 3D), a *P* value of 0.01 was utilized to identify significantly associated with pseudotime resulting after using the tradeSeq function associationTest. For pseudotime-associated regulons (Figure 3E), an adjusted *P* value of 1 × 10^–5^ was utilized to identify the top regulons significantly associated with the pseudotemporal general additive model after employing the mgcv function gam and adjusting for multiple comparisons with the Benjamini-Hochberg procedure (47).

### Study approval.

Human tonsillar tissue samples obtained from the Children’s Hospital of Philadelphia were deidentified and thus exempt from human subject requirements.

### Data availability and reproducibility.

Complete R, python, and bash code (including R markdown notebooks with clear documentation) used to analyze and visualize our results have been deposited at https://github.com/diegoalexespi/espinozada-tonsil-paper-2021 Raw FASTQ files and processed CellRanger files for scRNA-seq have been deposited in the NCBI Gene Expression Omnibus database (GEO GSE182221). Raw FASTQ files and processed kallisto files for bulk RNA-seq have been deposited in the NCBI GEO database with accession number GSE182266.

## Author contributions

DAE performed analysis and wrote the manuscript. CLC, ECC, and RL performed experiments. NR, RL, and ABO supervised the project and edited the manuscript.

## Supplementary Material

Supplemental data

Supplemental table 1

Supplemental table 2

Supplemental table 3

Supplemental table 4

Supplemental table 5

Supplemental table 6

Supplemental table 7

Supplemental table 8

Supplemental table 9

## Version 1 02/21/2023

In-Press Preview

## Version 2 04/10/2023

Electronic publication
