# Supplementary material for: Distinct stage-specific transcriptional states of B cells derived from human tonsillar tissue
Source: JCI Insight. 2023 Apr 10;8(7):e155199. doi: 10.1172/jci.insight.155199 (PMC10132144; doi:10.1172/jci.insight.155199)
Supplement: Supplemental data [file jciinsight-8-155199-s225.pdf]

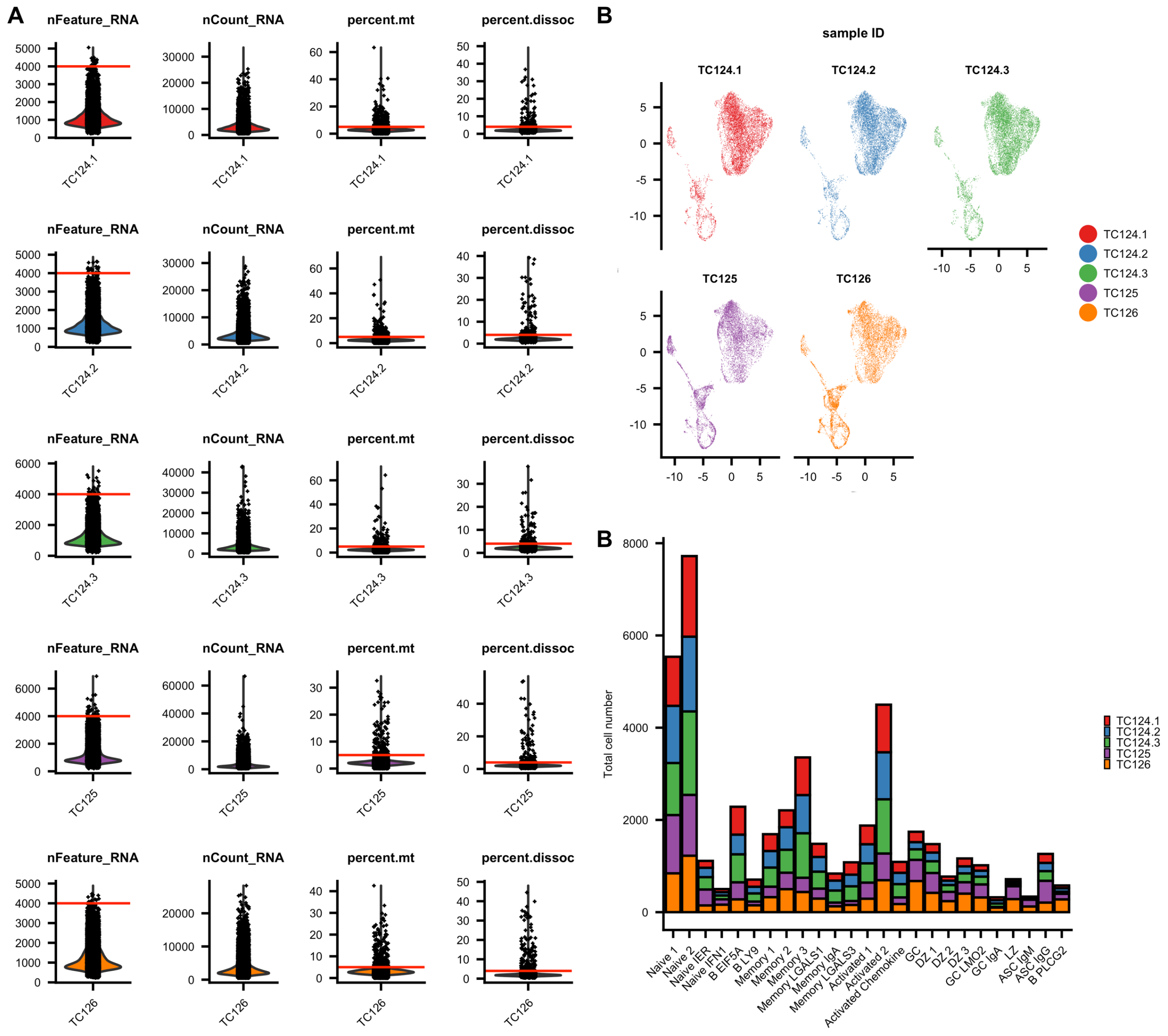

Figure S1. **Preprocessing of scRNA-seq data**. (**A**) Quality control metrics from five samples (3 biological replicates of TC124) with red horizontal lines denoting cutoffs used for downstream processing of scRNA-seq data. (**B**) UMAP projection of 45,376 single B-cell transcriptomes, colored and split by sample and/or donor. (**C**) Distribution of sample origin for cells within each cluster identified from **Fig. 1C**.

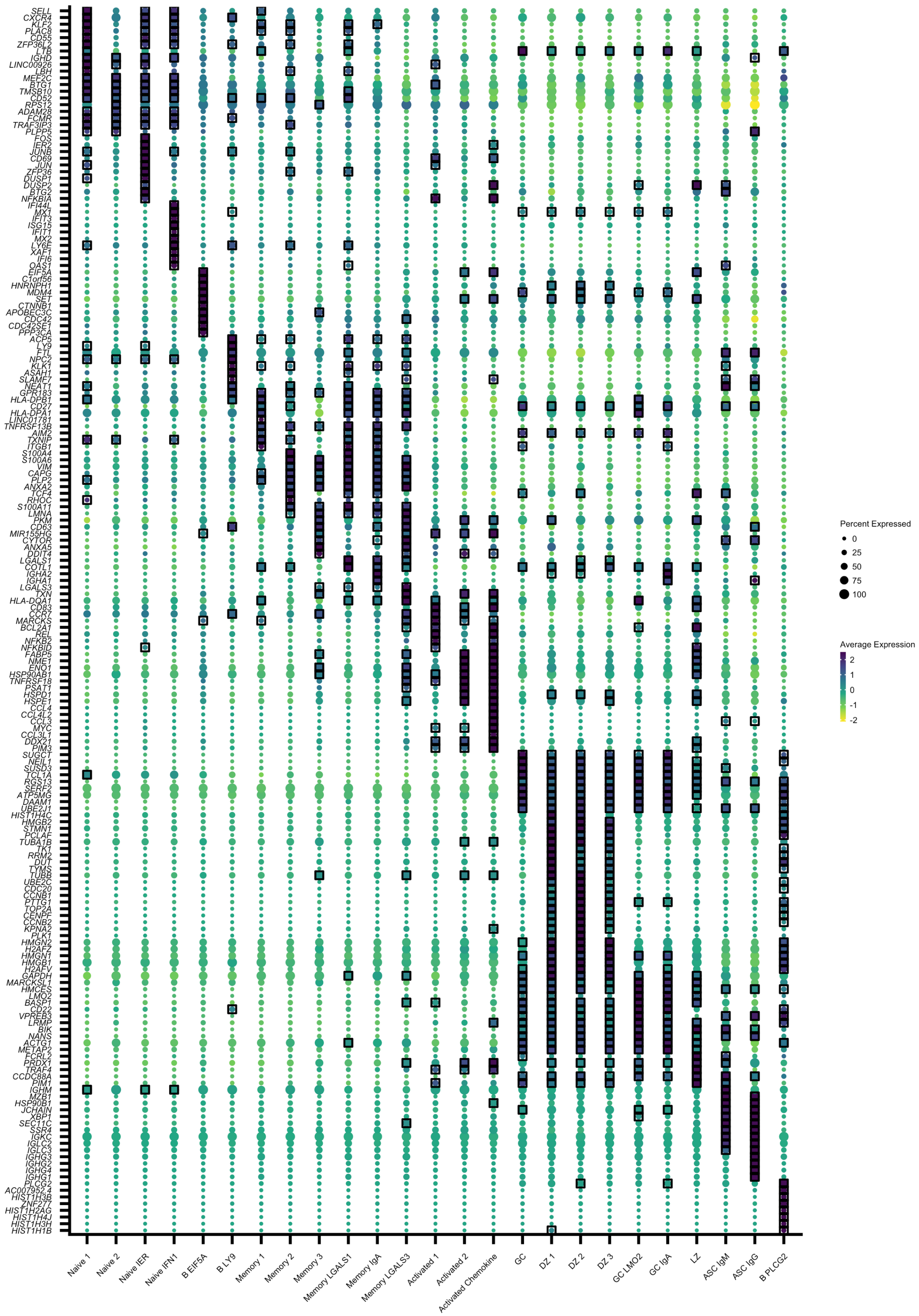
SELL
CXCR4
KLF2
PLAC8
CD55
ZFP36L2
LTB
IGHD
LINC00926
LBH
MEF2C
BTG1
TMSB10
CD52
RPS12
ADAM28
FCMR
TRAF3IP3
PLPP5
FOS
IER2
JUNB
CD69
JUN
ZFP36
DUSP1
DUSP2
BTG2
NFKBIA
IFI44L
MX1
IFIT3
ISG15
IFIT1
MX2
LY6E
XAF1
IFI6
OAS1
EIF5A
C1orf56
HNRNPH1
MDM4
SET
CTNNB1
APOBEC3C
CDC42
CDC42SE1
PPP3CA
ACP5
LY9
FTL
NPC2
KLK1
ASAH1
SLAMF7
NEAT1
GPR183
HLA-DPB1
CD27
HLA-DPA1
LINC01781
TNFRSF13B
AIM2
TXNIP
ITGB1
S100A4
S100A6
VIM
CAPG
PLP2
ANXA2
TCF4
RHOC
S100A11
LMNA
PKM
CD63
MIR155HG
CYTOR
ANXA5
DDIT4
LGALS1
COTL1
IGHA2
IGHA1
LGALS3
TXN
HLA-DQA1
CD83
CCR7
MARCKS
BCL2A1
REL
NFKB2
NFKBID
FABP5
NME1
ENO1
HSP90AB1
TNFRSF18
PSAT1
HSPD1
HSPE1
CCL4
CCL4L2
CCL3
MYC
CCL3L1
DDX21
PIM3
SUGCT
NEIL1
SUSD3
TCL1A
RGS13
SERF2
ATP5MG
DAAM1
UBE2J1
HIST1H4C
HMGB2
STMN1
PCLAF
TUBA1B
TK1
RRM2
DUT
TYMS
TUBB
UBE2C
CDC20
CCNB1
PTTG1
TOP2A
CENPF
CCNB2
KPNA2
PLK1
HMGN2
H2AFZ
HMGN1
HMGB1
H2AFV
GAPDH
MARCKSL1
HMCES
LMO2
BASP1
CD22
VPREB3
LRMP
BIK
NANS
ACTG1
METAP2
FCRL2
PRDX1
TRAF4
CCDC88A
PIM1
IGHM
MZB1
HSP90B1
JCHAIN
XBP1
SEC11C
SSR4
IGKC
IGLC2
IGLC3
IGHG3
IGHG2
IGHG4
IGHG1
PLCG2
AC007952.4
HIST1H3B
ZNF277
HIST1H2AG
HIST1H4J
HIST1H3H
HIST1H1B
Naive 1
Naive 2
Naive IER
Naive IFN1
B EIF5A
B LY9
Memory 1
Memory 2
Memory 3
Memory LGALS1
Memory IgA
Memory LGALS3
Activated 1
Activated 2
Activated Chemokine
GC
DZ 1
DZ 2
DZ 3
GC LMO2
GC IgA
LZ
ASC IgM
ASC IgG
B PLCG2
Percent Expressed
0
25
50
75
100
Average Expression
2
1
0
-1
-2

Figure S2. **Average expression of top 10 marker-genes for each cluster**. Top 10 marker-gene average expression (scaled log normalized counts) for each cluster. At least 10 marker-genes will be shown for each cluster. Genes were denoted as marker-genes if their average log2 fold-change was > 0.3 for the cluster of interest and adjusted p-value was < 0.01 (likelihood-ratio test). Marker-gene expression is denoted by square boxes on gene-cluster pairs.

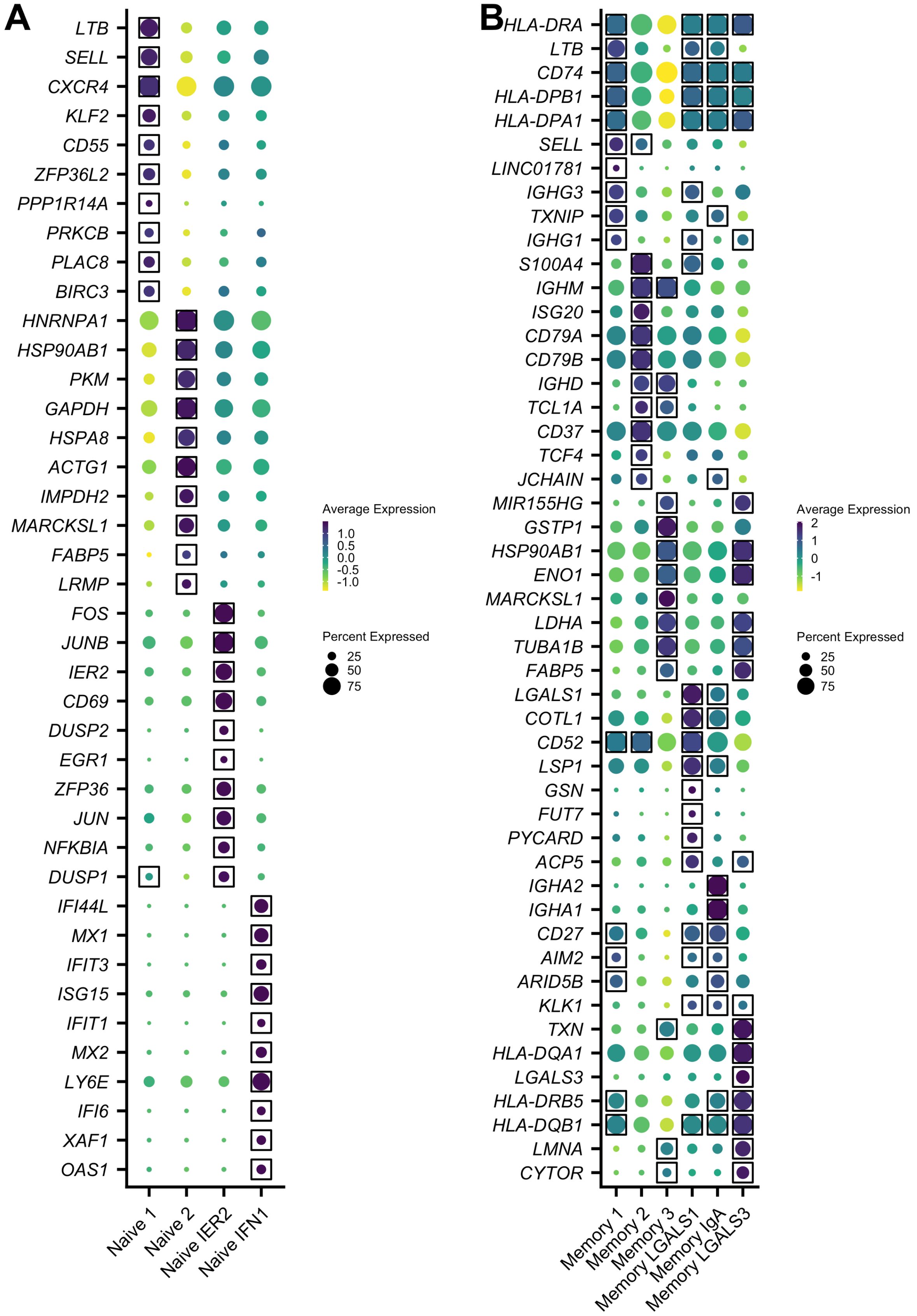
A
LTB
SELL
CXCR4
KLF2
CD55
ZFP36L2
PPP1R14A
PRKCB
PLAC8
BIRC3
HNRNPA1
HSP90AB1
PKM
GAPDH
HSPA8
ACTG1
IMPDH2
MARCKSL1
FABP5
LRMP
FOS
JUNB
IER2
CD69
DUSP2
EGR1
ZFP36
JUN
NFKBIA
DUSP1
IFI44L
MX1
IFIT3
ISG15
IFIT1
MX2
LY6E
IFI6
XAF1
OAS1
Naive 1
Naive 2
Naive IER2
Naive IFN1
Average Expression
1.0
0.5
0.0
-0.5
-1.0
Percent Expressed
25
50
75
B
HLA-DRA
LTB
CD74
HLA-DPB1
HLA-DPA1
SELL
LINC01781
IGHG3
TXNIP
IGHG1
S100A4
IGHM
ISG20
CD79A
CD79B
IGHD
TCL1A
CD37
TCF4
JCHAIN
MIR155HG
GSTP1
HSP90AB1
ENO1
MARCKSL1
LDHA
TUBA1B
FABP5
LGALS1
COTL1
CD52
LSP1
GSN
FUT7
PYCARD
ACP5
IGHA2
IGHA1
CD27
AIM2
ARID5B
KLK1
TXN
HLA-DQA1
LGALS3
HLA-DRB5
HLA-DQB1
LMNA
CYTOR
Memory 1
Memory 2
Memory 3
Memory LGALS1
Memory IgA
Memory LGALS3
Average Expression
2
1
0
-1
Percent Expressed
25
50
75

Figure S3. Memory-cell differential expression analysis. Marker-genes were determined for solely the memory-cell clusters (restricting comparisons to within the 6 clusters). Top 10 marker-gene average expression (scaled log normalized counts) for each cluster. At least 10 marker-genes will be shown for each cluster. Genes were denoted as marker-genes if their average log2 fold-change was > 0.3 for the cluster of interest and adjusted p-value was < 0.01 (likelihood-ratio test). Marker-gene expression is denoted by square boxes on gene-cluster pairs.

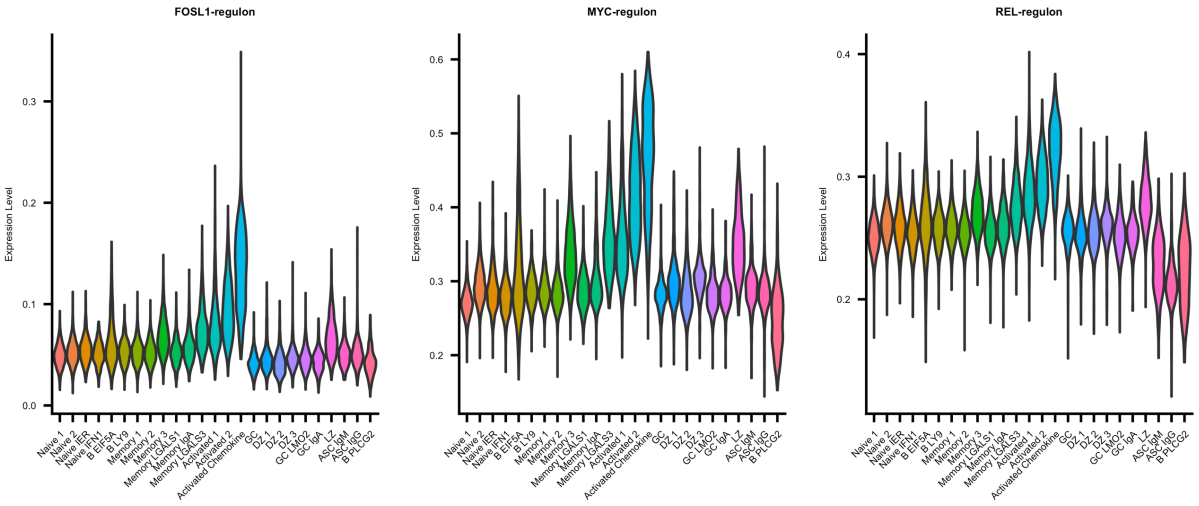

Figure S4. MYC, REL, and FOSL1 regulon activities across clusters. Violin plots of AUCell scores for the MYC, REL, and FOSL1 regulons across all clusters identified in **Fig. 1B**.

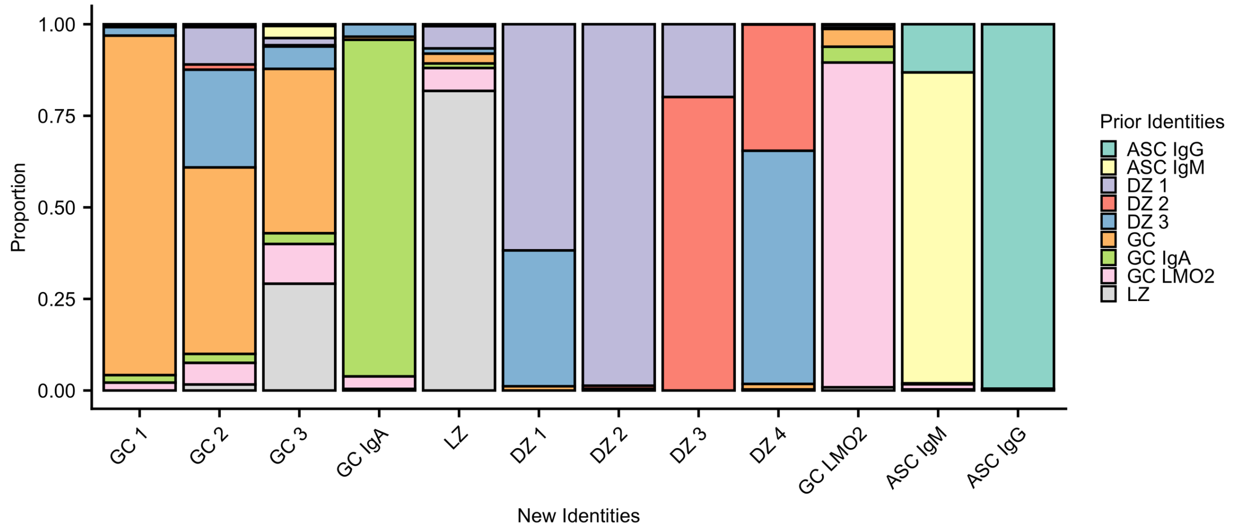

Figure S5. **Proportions of original clusters in new clusters.** Proportion of original cluster identities (from **Fig. 1B**) across new cluster identities (from **Fig. 3A**).

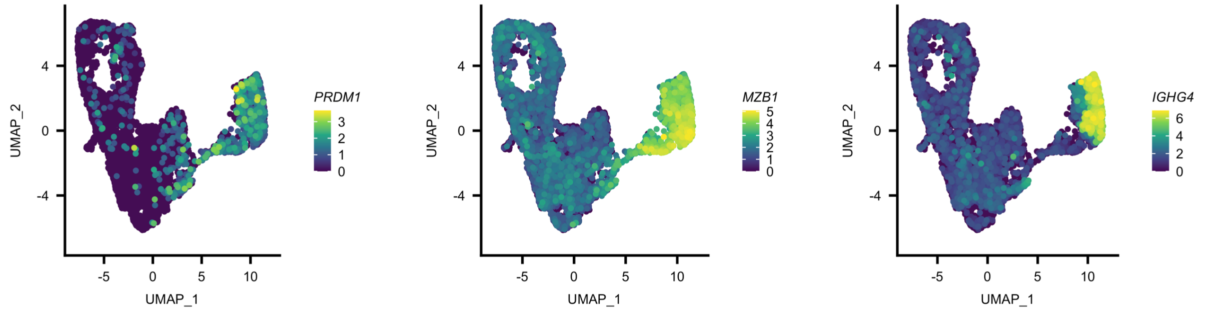

Figure S6. Gene expression levels of *PRDM1*, *MZB1*, and *IGHG4* in GC and ASC clusters. Gene expression levels of *PRDM1*, *MZB1*, and *IGHG4* (log normalized counts) gene expression overlaid on cells in the UMAP projection in **Fig. 3A-B**.

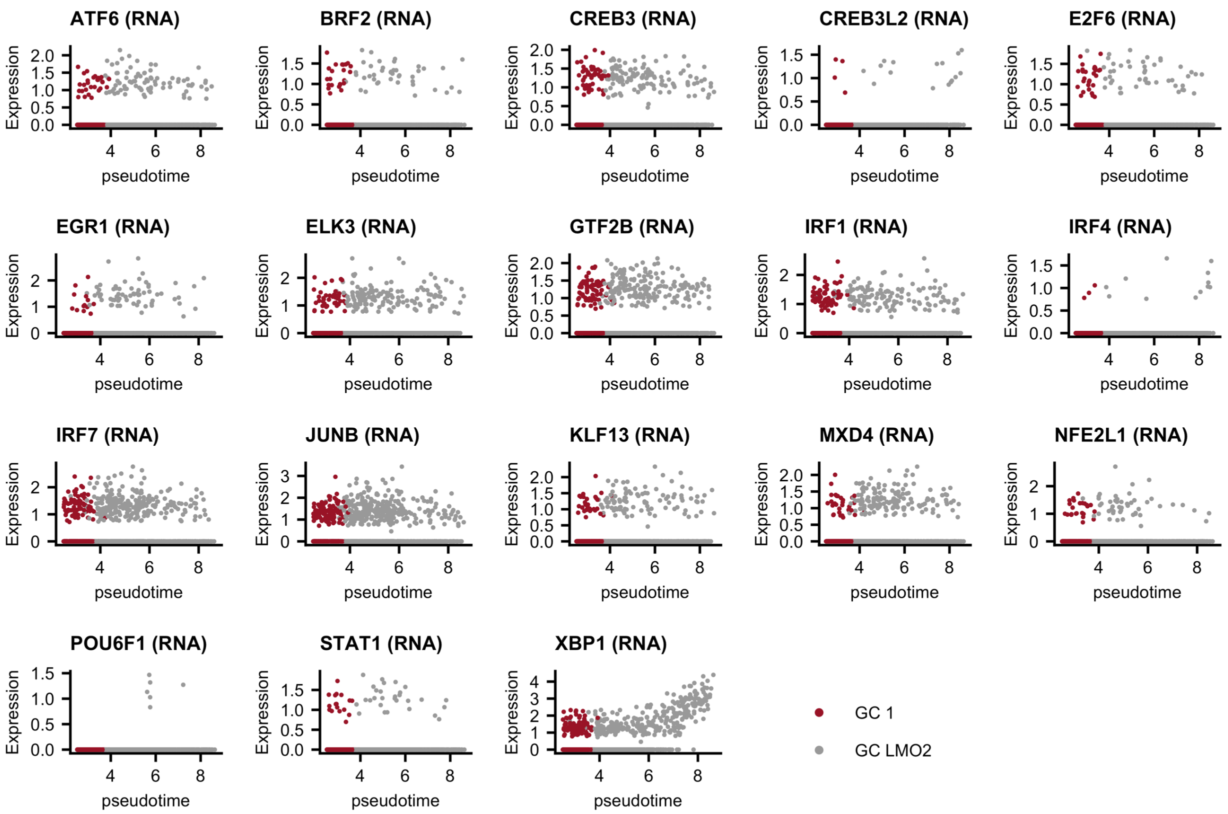

Figure S7. Gene-level expression of select TFs along the GC-to-ASC pseudotemporal axis. Gene expression levels along the GC-to-ASC pseudotemporal axis for the TF regulon activities plotted in **Fig. 3f**.

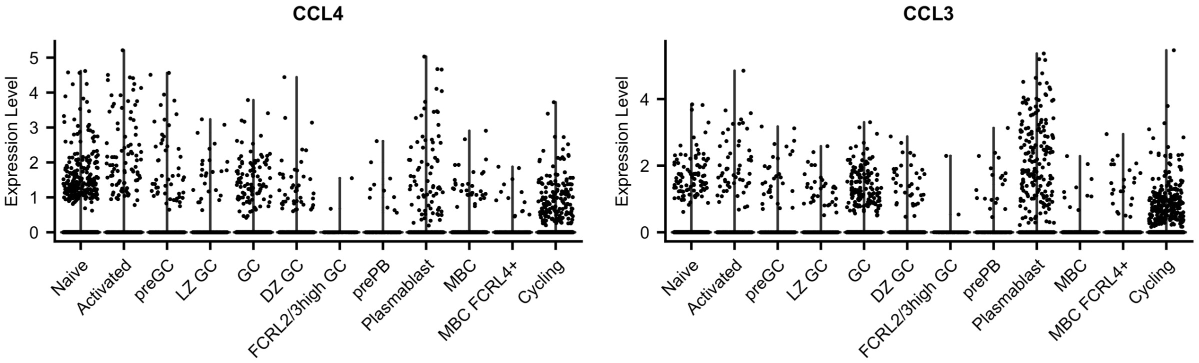

Figure S8. *CCL4* and *CCL3* gene expression within the King HW et al. dataset.

Gene expression levels across defined clusters in the dataset provided in King HW et al (5). A distinct *CCL4*/*CCL3* co-expressing population is not identified.

TABLE LEGENDS

Table S1. Donor information (n = 3) and cells recovered per 10X sample.

Table S2. Cluster distribution across donors.

Table S3. Coarse group distribution across donors.

Table S4. Likelihood-ratio test gene differential expression results.

Table S5. Donor information (n=3) for each bulk RNA-seq sample.

Table S6. Identified gene signatures (BCR-only, CD40-only, BCR/CD40) from bulk RNA-seq.

Table S7. Wilcoxon rank sum test regulon differential expression results.

Table S8. TF-Target pairs from SCENIC pipeline.

Table S9. *tradeSeq* association test results for GC to ASC trajectory gene differential expression.

Table S10. List of transcription factors tested in SCENIC pipeline.
