# Supplementary material for: Distinct stage-specific transcriptional states of B cells derived from human tonsillar tissue
Source: JCI Insight. 2023 Apr 10;8(7):e155199. doi: 10.1172/jci.insight.155199 (PMC10132144; doi:10.1172/jci.insight.155199)
Supplement: Supplemental table 1 [file jciinsight-8-155199-s226.pdf]

Sample Name	Sex	Age	10X Lanes	Final Cells Recovered post-processing
TC124	Male	4	3	"8912, 8962, 10183"
TC125	Female	17	1	8491
TC126	Male	5	1	8828
