# Supplementary material for: Distinct stage-specific transcriptional states of B cells derived from human tonsillar tissue
Source: JCI Insight. 2023 Apr 10;8(7):e155199. doi: 10.1172/jci.insight.155199 (PMC10132144; doi:10.1172/jci.insight.155199)
Supplement: Supplemental table 2 [file jciinsight-8-155199-s227.pdf]

donor	cluster_name	count
TC124	Naive 1	3433
TC124	Naive 2	5179
TC124	Naive IER2	622
TC124	Naive IFN1	223
TC124	B EIF5A	1640
TC124	B LY9	477
TC124	Memory 1	1140
TC124	Memory 2	1353
TC124	Memory 3	2609
TC124	Memory LGALS1	973
TC124	Memory IgA	630
TC124	Memory LGALS3	839
TC124	Activated	1238
TC124	Activated NME1	3228
TC124	Activated Chemokine	771
TC124	GC	611
TC124	DZ 1	627
TC124	DZ 2	332
TC124	DZ 3	517
TC124	GC LMO2	417
TC124	GC IgA	211
TC124	LZ	154
TC124	ASC IgM	68
TC124	ASC IgG	584
TC124	B PLCG2	181
TC125	Naive 1	1263
TC125	Naive 2	1319
TC125	Naive IER2	344
TC125	Naive IFN1	117
TC125	B EIF5A	369
TC125	B LY9	81
TC125	Memory 1	226
TC125	Memory 2	357
TC125	Memory 3	310
TC125	Memory LGALS1	216
TC125	Memory IgA	80
TC125	Memory LGALS3	86
TC125	Activated	349
TC125	Activated NME1	578
TC125	Activated Chemokine	143
TC125	GC	458
TC125	DZ 1	431
TC125	DZ 2	202
TC125	DZ 3	247
TC125	GC LMO2	282
TC125	GC IgA	14
TC125	LZ	278
TC125	ASC IgM	147
TC125	ASC IgG	472

TC125	B PLCG2	122
TC126	Naive 1	842
TC126	Naive 2	1223
TC126	Naive IER2	146
TC126	Naive IFN1	161
TC126	B EIF5A	277
TC126	B LY9	148
TC126	Memory 1	325
TC126	Memory 2	498
TC126	Memory 3	436
TC126	Memory LGALS1	293
TC126	Memory IgA	127
TC126	Memory LGALS3	156
TC126	Activated	291
TC126	Activated NME1	693
TC126	Activated Chemokine	176
TC126	GC	675
TC126	DZ 1	418
TC126	DZ 2	238
TC126	DZ 3	401
TC126	GC LMO2	318
TC126	GC IgA	98
TC126	LZ	283
TC126	ASC IgM	122
TC126	ASC IgG	207
TC126	B PLCG2	276
