# Supplementary material for: Distinct stage-specific transcriptional states of B cells derived from human tonsillar tissue
Source: JCI Insight. 2023 Apr 10;8(7):e155199. doi: 10.1172/jci.insight.155199 (PMC10132144; doi:10.1172/jci.insight.155199)
Supplement: Supplemental table 3 [file jciinsight-8-155199-s228.pdf]

donor	grouping	count
TC124	ASC	652
TC124	GC	3050
TC124	non−GC	24355
TC125	ASC	619
TC125	GC	2034
TC125	non−GC	5838
TC126	ASC	329
TC126	GC	2707
TC126	non−GC	5792
