# Supplementary material for: Distinct stage-specific transcriptional states of B cells derived from human tonsillar tissue
Source: JCI Insight. 2023 Apr 10;8(7):e155199. doi: 10.1172/jci.insight.155199 (PMC10132144; doi:10.1172/jci.insight.155199)
Supplement: Supplemental table 4 [file jciinsight-8-155199-s229.pdf]

p_val	avg_log2FC	pct.1	pct.2	p_val_adj	cluster	gene	cluster_names
0	0.678390738596959	0.749	0.589	0	0	MEF2C	Naive 2
0	0.530388809598243	0.98	0.926	0	0	BTG1	Naive 2
0	0.512668912712584	0.998	0.983	0	0	TMSB10	Naive 2
0	0.463631133151577	0.793	0.568	0	0	IGHD	Naive 2
0	0.457319967123718	0.966	0.848	0	0	CD52	Naive 2
0	0.456441976276855	1	0.996	0	0	RPS12	Naive 2
0	0.38643701689355	1	0.995	0	0	RPL12	Naive 2
0	0.358927633561675	0.997	0.978	0	0	RPSA	Naive 2
0	0.350786678171197	1	0.999	0	0	RPL13A	Naive 2
0	0.348634828999385	1	0.999	0	0	RPL13	Naive 2
0	0.348292274506282	1	0.999	0	0	RPL34	Naive 2
0	0.345244639173151	1	0.999	0	0	RPS19	Naive 2
0	0.34128560603671	1	0.998	0	0	RPL18A	Naive 2
0	0.336591582070157	0.991	0.964	0	0	MT-ND3	Naive 2
0	0.334537702040415	1	0.999	0	0	EEF1A1	Naive 2
0	0.323497476443285	0.999	0.995	0	0	RPL31	Naive 2
0	0.322687618811019	0.999	0.992	0	0	RPS5	Naive 2
0	0.318340795978724	1	0.997	0	0	RPL3	Naive 2
0	0.317095820018712	1	0.998	0	0	RPL39	Naive 2
0	0.316438328131834	1	0.999	0	0	RPLP1	Naive 2
0	0.314628817101718	1	0.997	0	0	RPS3A	Naive 2
0	0.313827803406306	1	0.998	0	0	RPL32	Naive 2
0	0.306401766555309	1	0.997	0	0	RPL11	Naive 2
0	0.304122219997052	1	0.998	0	0	RPS29	Naive 2

0 -0.702271708611225 0.2 0.433 0 0
YWHAE Naive 2
0 -0.77166769747628 0.019 0.175 0 0 BIK
Naive 2
0 -0.878454810393786 0.234 0.444 0 0 TXN
Naive 2
0 -0.920744890330294 0.12 0.341 0 0
CD27 Naive 2
0 -0.924659823757891 0.11 0.338 0 0
NME1 Naive 2
0 -1.06626849671682 0.401 0.64 0 0
PRDX1 Naive 2
0 -1.46019588978664 0.031 0.242 0 0
RGS13 Naive 2
3.93351528004083e-312 -0.793232556682932 0.079 0.266
7.01306439278479e-308 0 UBE2J1 Naive 2
1.09912637786038e-304 -1.32749146340088 0.15 0.315
1.95963241908728e-300 0 MIR155HG Naive 2
4.04408723782934e-298 -0.646361748595568 0.017 0.158
7.21020313632592e-294 0 NEIL1 Naive 2
4.97069832583844e-298 -0.679626354135813 0.111 0.32
8.86225804513735e-294 0 CD82 Naive 2
4.05652560706256e-294 -0.552807411845522 0.012 0.14
7.23237950483185e-290 0 CD38 Naive 2
2.26012629285412e-281 -0.879478039486636 0.61 0.737
4.02957916752961e-277 0 ENO1 Naive 2
5.67387474236778e-279 -0.763373990501685 0.645 0.769
1.01159512781675e-274 0 RAN Naive 2
5.3321705139146e-277 -0.853925099305617 0.217 0.427
9.50672680925833e-273 0 EIF5A Naive 2
1.60523178799568e-269 -0.522096656346134 0.026 0.164
2.8619677548175e-265 0 AIM2 Naive 2
9.49465344010225e-266 -0.9117474571946 0.059 0.217
1.69280176183583e-261 0 PTTG1 Naive 2
3.6677740137285e-258 -0.679375322244635 0.389 0.586
6.53927428907655e-254 0 TCEA1 Naive 2
2.939813159295e-257 -1.05145458884914 0.059 0.207
5.24139288170705e-253 0 XBP1 Naive 2
3.20380674104574e-254 -0.46665246703159 0.011 0.126
5.71206703861045e-250 0 HRK Naive 2
6.16325685357657e-247 -0.656295115545579 0.131 0.31
1.09884706442417e-242 0 SRM Naive 2
1.65515805988057e-243 -0.419476666645065 0.009 0.12
2.95098130496106e-239 0 MYBL2 Naive 2
2.76204064140238e-243 -0.682563577073565 0.455 0.626
4.92444225955631e-239 0 LDHA Naive 2
1.80569044480991e-242 -0.684359620938062 0.063 0.217
3.21936549405159e-238 0 DAAM1 Naive 2
3.66763091927274e-239 -0.602747656000651 0.3 0.503
6.53901916597138e-235 0 CYCS Naive 2

4.67325918985822e-239	-0.931449232971528			0.256	0.434
8.33195380959823e-235	0	FABP5	Naive 2		
2.35810443200139e-238	-0.54268675761147			0.451	0.64
4.20426439181527e-234	0	POLR2L	Naive 2		
2.116506565979e-236	-0.495448543939493			0.029	0.153
3.77351955648396e-232	0	CYTOR	Naive 2		
2.34251553352791e-236	-0.584508774087843			0.117	0.306
4.1764709447269e-232	0	POU2AF1	Naive 2		
5.86349208225612e-235	-0.561613087852725			0.094	0.262
1.04540200334544e-230	0	MTHFD2	Naive 2		
2.70759452272396e-233	-0.567385924815124			0.078	0.247
4.82737027456454e-229	0	NANS	Naive 2		
1.22024983157875e-230	-0.678557512344495			0.408	0.59
2.17558342472176e-226	0	NCL	Naive 2		
3.4770020894129e-230	-0.846889868299928			0.58	0.72
6.19914702521426e-226	0	H2AFZ	Naive 2		
2.3151416709366e-229	-1.11872530010183			0.089	0.23
4.12766608511286e-225	0	LGALS1	Naive 2		
1.1693114834579e-225	0.362808000492376			0.954	0.908
2.0847654438571e-221	0	EEF1B2	Naive 2		
1.76166170976002e-223	-0.634091158196456			0.06	0.201
3.14086666233114e-219	0	BCL2A1	Naive 2		
2.21559736377127e-223	-0.572100237305106			0.45	0.628
3.95018853986779e-219	0	SEC61B	Naive 2		
1.3231945954618e-222	-1.4562583414322		0.06	0.199	
2.35912364424884e-218	0	MZB1	Naive 2		
1.39820524390124e-221	-0.659063337860054			0.169	0.348
2.49286012935153e-217	0	DDX21	Naive 2		
1.97388203804017e-221	-0.588165200412115			0.154	0.333
3.51923428562182e-217	0	ODC1	Naive 2		
4.28122905075246e-220	-0.587658862894689			0.863	0.907
7.63300327458656e-216	0	PFN1	Naive 2		
4.7714421634011e-215	-0.393359870368968			0.027	0.146
8.50700423312782e-211	0	SLC1A5	Naive 2		
1.08257390292862e-214	-0.663948799283596			0.169	0.35
1.93012101153144e-210	0	SEC11C	Naive 2		
1.16043284096804e-214	-0.586947238918603			0.317	0.506
2.06893571216192e-210	0	SNRPD1	Naive 2		
1.56349881390555e-213	-1.68616918908409			0.181	0.327
2.78756203531221e-209	0	HMGB2	Naive 2		
8.61281257924391e-212	-0.425547424356215			0.007	0.1
1.5355783547534e-207	0	AICDA	Naive 2		
2.11190557279149e-211	-0.648758575355528			0.013	0.111
3.76531644572995e-207	0	PCLAF	Naive 2		
5.5024743115616e-209	-0.598784432983381			0.501	0.665
9.81036145008318e-205	0	SET	Naive 2		
4.20210588221509e-205	-0.685493956194869			0.045	0.173
7.49193457740128e-201	0	DUSP2	Naive 2		
4.65437887103251e-205	-0.465073246561079			0.031	0.152
8.29829208916386e-201	0	PHGDH	Naive 2		

7.40742099852209e-204	-0.503087348720931	0.166	0.356
1.3206690898265e-199	0	IMP4	Naive 2
6.48132664782935e-203	-0.519391680387714	0.267	0.464
1.15555572804149e-198	0	SLIRP	Naive 2
2.9483540685463e-202	-0.52157349719306	0.51	0.673
5.2566204688112e-198	0	SNRPG	Naive 2
4.71552071442391e-198	-0.401094235551833	0.019	0.131
8.40730188174639e-194	0	CPNE5	Naive 2
4.64030565866213e-197	-0.608260253880144	0.575	0.718
8.27320095882871e-193	0	EZR	Naive 2
1.40373068372039e-196	-0.587989453751977	0.099	0.257
2.50271143600509e-192	0	CKS2	Naive 2
8.8819942463181e-192	-1.30346989800321	0.057	0.166
1.58357075417605e-187	0	IGHA1	Naive 2
1.48991265435158e-191	-0.612796856498941	0.063	0.178
2.65636527144343e-187	0	LMNA	Naive 2
1.71965395793544e-190	-0.521397769091099	0.049	0.184
3.06597104160309e-186	0	SUSD3	Naive 2
2.72613320726792e-190	-0.499616838810366	0.235	0.421
4.86042289523797e-186	0	CCT5	Naive 2
7.20792580497445e-190	-0.459982955112255	0.057	0.184
1.28510109176889e-185	0	CD58	Naive 2
3.05710004666044e-189	-0.522441839830012	0.474	0.635
5.45050367319089e-185	0	POMP	Naive 2
4.95434661924525e-188	-0.524764731871451	0.388	0.565
8.83310458745235e-184	0	SRSF9	Naive 2
2.35674612975937e-187	-0.40828375647492	0.022	0.13
4.20184267474799e-183	0	AC023590.1	Naive 2
1.22597073201979e-184	0.525485639251723	0.399	0.253
2.18578321811808e-180	0	ADAM28	Naive 2
2.81812544058627e-184	-0.475540223258994	0.32	0.507
5.02443584802125e-180	0	PRELID1	Naive 2
3.46972887508507e-182	-0.629000440867381	0.07	0.21
6.18617961138917e-178	0	RGS2	Naive 2
3.55902197357262e-181	-0.598808004949098	0.36	0.526
6.34538027668263e-177	0	HSPE1	Naive 2
3.53927534380741e-180	-0.386279903932012	0.03	0.132
6.31017401047424e-176	0	PYCR1	Naive 2
5.85333613775258e-180	-0.324687291127661	0.017	0.116
1.04359129999991e-175	0	FAM241A	Naive 2
1.88948702876126e-178	-0.41378610835748	0.718	0.821
3.36876642357845e-174	0	RPS26	Naive 2
2.7284253549078e-178	-0.493304360548897	0.135	0.299
4.86450956526512e-174	0	SMS	Naive 2
7.21605739855868e-176	-0.380724175380114	0.058	0.183
1.28655087358903e-171	0	PPP1R14B	Naive 2
1.22289301946977e-175	0.462765779868323	0.378	0.226
2.18029596441265e-171	0	FCMR	Naive 2
1.15201859612965e-174	-0.545166549886194	0.025	0.126
2.05393395503955e-170	0	VPREB3	Naive 2

1.0486482030778e-173 -0.476380777005271 0.049 0.175
1.86963488126741e-169 0 CD81 Naive 2
1.51886852728129e-171 -0.592467929595527 0.514 0.66
2.70799069728982e-167 0 HERPUD1 Naive 2
8.60902876192954e-171 -0.671549366820418 0.151 0.303
1.53490373796442e-166 0 HSPA5 Naive 2
3.7540747348648e-170 -0.439029306543821 0.11 0.264
6.69313984479045e-166 0 HNRNPAB Naive 2
1.43901366283247e-169 -0.48193944784361 0.673 0.777
2.56561745946401e-165 0 PSMA7 Naive 2
2.92596174282589e-169 -0.562571666083926 0.303 0.473
5.21669719128427e-165 0 ANXA2 Naive 2
4.03086312638055e-169 -0.329125210352789 0.019 0.12
7.18662586802389e-165 0 SEMA4A Naive 2
1.17739747317129e-167 -0.595464402897456 0.22 0.38
2.0991819549171e-163 0 TUBB4B Naive 2
2.91886325891583e-167 -0.39850903739057 0.048 0.166
5.20404130432104e-163 0 PRDX4 Naive 2
4.1005306886476e-167 -0.766127345001153 0.472 0.598
7.31083616478981e-163 0 TUBB Naive 2
1.54591053352095e-165 -0.479001953805833 0.522 0.664
2.75620389021451e-161 0 ERH Naive 2
1.29569277890801e-164 -0.564570212197987 0.357 0.511
2.3100906555151e-160 0 RANBP1 Naive 2
2.90129858579178e-164 -1.11819292671419 0.392 0.509
5.17272524860817e-160 0 LTB Naive 2
1.55053953534428e-162 0.39790350886969 0.633 0.487
2.76445693756532e-158 0 BANK1 Naive 2
2.63038286676082e-162 0.45251458540115 0.463 0.324
4.68970961314787e-158 0 TRAF3IP3 Naive 2
1.00325250509794e-161 -0.44825748407256 0.504 0.653
1.78869889133911e-157 0 ATP5F1B Naive 2
2.55039577713873e-161 -0.474012025442794 0.306 0.487
4.54710063106065e-157 0 COX17 Naive 2
2.65312040492307e-160 -0.476850049777428 0.422 0.581
4.73024836993735e-156 0 SEC61G Naive 2
3.26746285389893e-160 -0.409438569300533 0.124 0.285
5.82555952221641e-156 0 CDV3 Naive 2
5.67266460007175e-160 -0.497245524055165 0.251 0.421
1.01137937154679e-155 0 FNBP1 Naive 2
5.79559564193242e-160 -0.475655526246549 0.184 0.359
1.03329674700013e-155 0 RFTN1 Naive 2
2.4058485760089e-159 0.510811908237922 0.276 0.164
4.28938742616627e-155 0 PLPP5 Naive 2
2.63601603742897e-159 -0.496669839153754 0.12 0.268
4.69975299313211e-155 0 SLBP Naive 2
2.70174461542167e-159 -0.524372486002343 0.117 0.269
4.8169404748353e-155 0 KPNA2 Naive 2
2.49848443025557e-158 -0.396094637687183 0.075 0.205
4.45454789070266e-154 0 TUBA1C Naive 2

5.41798301953358e-158	-0.424040441727871	0.19	0.362
9.65972192552642e-154	0	EIF3A	Naive 2
1.11703858053107e-157	-0.462347666639165	0.187	0.35
1.99156808522885e-153	0	NOP56	Naive 2
5.26583134479157e-157	-0.429894453008766	0.098	0.25
9.3884507046289e-153	0	SIAH2	Naive 2
6.54392777316069e-157	-0.481890901739424	0.39	0.558
1.16671688267682e-152	0	SRSF7	Naive 2
3.53280066355835e-156	-0.637962574397977	0.129	0.264
6.29863030305818e-152	0	RGS1	Naive 2
6.59909689547775e-156	-0.590848191310736	0.489	0.628
1.17655298549473e-151	0	DYNLL1	Naive 2
1.96157179561073e-154	-1.48129550698844	0.115	0.237
3.49728635439437e-150	0	IGHG1	Naive 2
3.06634982354212e-154	0.445839672235066	0.454	0.314
5.46699510039325e-150	0	HVCN1	Naive 2
7.466628887909e-154	-0.38668300477234	0.07	0.206
1.3312252644253e-149	0	TRABD	Naive 2
8.65025061352107e-153	-0.452854600862763	0.228	0.405
1.54225318188467e-148	0	PTP4A2	Naive 2
1.17389421402182e-152	-0.466038628490541	0.723	0.822
2.0929359941795e-148	0	CALM2	Naive 2
2.40104038572913e-152	-0.385340934624689	0.019	0.111
4.28081490371646e-148	0	WDR66	Naive 2
2.62785743121245e-151	-0.457496657275999	0.103	0.244
4.68520701410868e-147	0	TRAF4	Naive 2
1.85183139809327e-150	-0.594996967172013	0.23	0.386
3.30163019966048e-146	0	H2AFV	Naive 2
5.54068559712016e-150	-0.432674565589953	0.034	0.136
9.87848835110554e-146	0	UBE2S	Naive 2
2.10326275873063e-149	-0.528520557700052	0.261	0.426
3.74990717254084e-145	0	BASP1	Naive 2
4.52876728886819e-149	-0.48804797730888	0.308	0.467
8.07433919932309e-145	0	HMGA1	Naive 2
1.47097409114852e-148	-0.432649973688061	0.29	0.464
2.6225997071087e-144	0	EIF5B	Naive 2
2.53473747034839e-148	-0.388490237040152	0.167	0.331
4.51918343588414e-144	0	GLRX3	Naive 2
4.05662677687499e-148	-0.510524009591372	0.14	0.28
7.23255988049042e-144	0	SRGN	Naive 2
1.16348509045678e-146	-0.431017878394177	0.171	0.329
2.07437756777539e-142	0	ATP1B3	Naive 2
2.0945360421811e-146	-0.433890201069759	0.197	0.363
3.73434830960469e-142	0	VOPP1	Naive 2
4.118078087686e-146	-0.438349222799407	0.29	0.457
7.34212142253537e-142	0	CCT2	Naive 2
5.05698596245172e-146	-0.467610438380094	0.154	0.307
9.01610027245518e-142	0	JPT1	Naive 2
1.88710030735271e-145	-0.387941178871805	0.089	0.221
3.36451113797915e-141	0	EBNA1BP2	Naive 2

1.74855220747684e-144 -0.535586814567194 0.336 0.487
3.11749373071047e-140 0 HSPD1 Naive 2
4.90551377085045e-144 -0.432573852157897 0.431 0.585
8.74604050204926e-140 0 ATP5PF Naive 2
2.75303171647322e-143 -0.421671640221049 0.171 0.328
4.9083802473001e-139 0 NOP58 Naive 2
1.246449782839e-141 -0.402033334750744 0.09 0.227
2.22229531782366e-137 0 UBALD2 Naive 2
3.51456888720045e-141 -0.447025362345157 0.064 0.19
6.26612486898969e-137 0 CCDC88A Naive 2
4.2840640691205e-141 -0.42600126953569 0.212 0.377
7.63805782883494e-137 0 TIMM13 Naive 2
8.57003938196342e-141 0.321480323020306 0.708 0.609
1.52795232141026e-136 0 HLA-DMB Naive 2
8.8639957267873e-141 -0.457718948531694 0.255 0.412
1.58036179812891e-136 0 NHP2 Naive 2
2.23009799492638e-140 -0.394241000634178 0.245 0.417
3.97604171515424e-136 0 NDUFS6 Naive 2
2.25871440104741e-140 -0.394523906171784 0.131 0.282
4.02706190562742e-136 0 GSPT1 Naive 2
9.07012599174177e-140 -0.413648686989372 0.098 0.232
1.61711276306764e-135 0 CFLAR Naive 2
9.76725228287817e-140 -1.70775821075511 0.313 0.422
1.74140340951435e-135 0 JCHAIN Naive 2
1.04077824465086e-139 -0.424939700627126 0.664 0.76
1.85560353238803e-135 0 CHCHD2 Naive 2
1.51022759103946e-139 -0.398877598097664 0.17 0.334
2.69258477206425e-135 0 TIMM8B Naive 2
1.57351417565327e-139 -0.416403221035721 0.28 0.445
2.80541842377222e-135 0 DDX39A Naive 2
1.8375744876449e-138 -0.661017009851247 0.054 0.146
3.2762115540221e-134 0 MYC Naive 2
1.20325344463389e-137 -0.454999945631402 0.103 0.23
2.14528056643776e-133 0 PIM3 Naive 2
1.67230282842517e-137 0.36663828435372 0.665 0.558
2.98154871279923e-133 0 SARAF Naive 2
7.45202400216458e-136 -0.41694841114345 0.211 0.374
1.32862135934592e-131 0 H2AFY Naive 2
1.88997437186607e-135 -0.327324520907134 0.059 0.174
3.36963530760002e-131 0 PAICS Naive 2
3.61453014352998e-135 -0.401024997402051 0.135 0.274
6.44434579289961e-131 0 GARS Naive 2
6.3882597912016e-135 -0.485802109787311 0.39 0.537
1.13896283817333e-130 0 PA2G4 Naive 2
1.79102004886048e-134 -0.312617138819406 0.074 0.206
3.19320964511334e-130 0 LRRC59 Naive 2
4.99717485662905e-134 -0.334870903572615 0.064 0.189
8.90946305188393e-130 0 CCDC167 Naive 2
7.04057381597536e-134 -0.366909307207319 0.063 0.176
1.25526390565025e-129 0 NOLC1 Naive 2

7.49367911357416e-134	-0.42257433153722	0.312	0.476
1.33604804915914e-129	0	SEM1	Naive 2
1.59542654315807e-133	-0.485677390606171	0.144	0.295
2.84448598379653e-129	0	PIM1	Naive 2
4.59268799992135e-133	-0.37542911858552	0.837	0.892
8.18830343505978e-129	0	H3F3B	Naive 2
5.10794833568991e-133	-0.421426679642668	0.332	0.49
9.10696108770153e-129	0	PRMT1	Naive 2
9.55111559030671e-133	-0.386003759932179	0.156	0.31
1.70286839859578e-128	0	PRDX3	Naive 2
7.96935967215129e-132	-0.421020706599444	0.522	0.657
1.42085713594785e-127	0	PSMA4	Naive 2
8.28548524654628e-132	-0.361148256822458	0.106	0.241
1.47721916460674e-127	0	MRPL12	Naive 2
3.48219906211405e-131	-0.458806541931283	0.466	0.603
6.20841270784314e-127	0	LDHB	Naive 2
7.60103234585551e-131	-0.408184285998127	0.476	0.619
1.35518805694258e-126	0	SRSF2	Naive 2
5.51766664117847e-130	-0.355262480635228	0.056	0.16
9.83744785455709e-126	0	NOP16	Naive 2
7.24902582517408e-130	-0.349254210630712	0.051	0.166
1.29242881437029e-125	0	SEC14L1	Naive 2
5.33077332068557e-129	-0.380534522416138	0.364	0.525
9.50423575345031e-125	0	COX5A	Naive 2
4.49780161096324e-128	-0.38212550781982	0.344	0.513
8.01913049218637e-124	0	GTF3A	Naive 2
5.53623686919305e-128	-0.446762114755541	0.409	0.554
9.87055671408429e-124	0	PSME2	Naive 2
2.29132037044301e-127	-0.359082076428611	0.096	0.223
4.08519508846285e-123	0	TOMM40	Naive 2
4.2325557792075e-127	-0.480154851783219	0.212	0.35
7.54622369874905e-123	0	SLC3A2	Naive 2
2.36517192587652e-126	0.330135057403763	0.854	0.808
4.21686502664525e-122	0	NOP53	Naive 2
4.08858261668092e-125	-0.359142932456588	0.089	0.208
7.28953394728042e-121	0	GPATCH4	Naive 2
7.72232155283055e-125	-0.452946441285371	0.421	0.565
1.37681270965416e-120	0	PARP1	Naive 2
3.52279968610287e-123	-0.38821414558485	0.313	0.469
6.28079956035281e-119	0	CCT6A	Naive 2
5.2731101518837e-123	-0.391831526406361	0.335	0.497
9.40142808979346e-119	0	MTDH	Naive 2
1.1548449908e-122	-0.382307695900252	0.53	0.662
2.05897313409732e-118	0	ATP5MD	Naive 2
2.79564637961009e-122	-0.390904647228734	0.325	0.486
4.98435793020682e-118	0	RAP1B	Naive 2
4.34374022677178e-122	-0.424499180059735	0.458	0.595
7.74445445031141e-118	0	SNRPE	Naive 2
5.45548820811468e-122	0.406689796082829	0.144	0.061
9.72658992624766e-118	0	KCNG1	Naive 2

2.03374925914916e-121	-0.452353466166912	0.475	0.606	3.62597155413703e-117	0	ANP32B	Naive 2
4.91823634950487e-121	-0.380927987097052	0.069	0.189	8.76872358753224e-117	0	SEL1L3	Naive 2
5.50738369194845e-121	-0.415043372007947	0.471	0.608	9.81911438437489e-117	0	ATP5MC3	Naive 2
1.11485236529612e-120	-0.551926844703396	0.235	0.372	1.98767028208644e-116	0	S100A10	Naive 2
1.42542215201869e-120	-0.435160374683046	0.095	0.213	2.54138515483412e-116	0	DNMT1	Naive 2
2.16277318645165e-120	-0.344302008864408	0.232	0.394	3.85600831412465e-116	0	PSMA5	Naive 2
2.56096824560375e-120	-0.352451941975408	0.252	0.417	4.56595028508693e-116	0	NOL7	Naive 2
3.21794441091531e-120	-3.1837812204481	0.361	0.469	5.73727309022091e-116	0	IGHG3	Naive 2
3.56721537579464e-119	-0.443401760454591	0.081	0.195	6.35998829350426e-115	0	PDIA4	Naive 2
3.94761050211796e-119	-0.341193474364253	0.268	0.432	7.03819476422612e-115	0	PSMB2	Naive 2
6.36438410084054e-119	-0.32393525405862	0.061	0.168	1.13470604133886e-114	0	RPF2	Naive 2
7.92752982340643e-119	-0.502400767058873	0.679	0.762	1.41339929221513e-114	0	HSP90AA1	Naive 2
1.68739058452707e-118	-0.324236106214387	0.124	0.263	3.00844867315331e-114	0	MRPL15	Naive 2
1.84694406711785e-118	-0.376714827788683	0.135	0.269	3.29291657726442e-114	0	DKC1	Naive 2
2.53836691032141e-118	-0.389070466649756	0.252	0.411	4.52565436441205e-114	0	SMARCB1	Naive 2
2.745894452673e-118	-0.362203961200564	0.237	0.395	4.89565521967069e-114	0	PFDN2	Naive 2
3.04737213340424e-118	-0.345917812282622	0.144	0.289	5.43315977664642e-114	0	SYNCRIP	Naive 2
4.86841367377268e-118	-0.353200666026838	0.101	0.23	8.67989473896932e-114	0	GNL3	Naive 2
6.13293937742433e-118	-0.328622693875256	0.115	0.248	1.09344176160098e-113	0	DCAF13	Naive 2
4.75412519450087e-117	-0.477524077693555	0.094	0.188	8.47612980927561e-113	0	TNFRSF18	Naive 2
6.55278686195541e-117	-0.404335574075498	0.252	0.4	1.16829636961803e-112	0	LCP1	Naive 2
6.36207341994328e-116	-0.33936099823529	0.211	0.366	1.13429407004169e-111	0	STRAP	Naive 2
1.4127650495364e-115	-0.494482827928184	0.103	0.222	2.51881880681844e-111	0	HMCES	Naive 2
1.68396211481206e-115	-0.463912175763532	0.209	0.356	3.00233605449842e-111	0	GRHPR	Naive 2
1.7798843255434e-115	-0.432331831529519	0.594	0.697	3.17335576401133e-111	0	SRSF3	Naive 2

4.12033303780114e-115	-0.343446144202871	0.144	0.284
7.34614177309565e-111	0	AK6	Naive 2
6.30354565002308e-115	-0.387211615314906	0.323	0.478
1.12385915394261e-110	0	MRPL51	Naive 2
8.13245609622676e-115	-0.356013125545102	0.188	0.338
1.44993559739627e-110	0	LPXN	Naive 2
2.39496470103368e-114	-0.39874218556988	0.062	0.164
4.26998256547295e-110	0	SDF2L1	Naive 2
2.96576850478878e-114	-0.82229146062589	0.602	0.679
5.28766866718792e-110	0	TUBA1B	Naive 2
3.55540339689692e-114	-0.569646736423823	0.382	0.521
6.33892871632751e-110	0	SAT1	Naive 2
9.93178425170065e-114	-0.332657932407196	0.154	0.301
1.77073781423571e-109	0	ATOX1	Naive 2
1.69335520618887e-113	-0.343759583768557	0.254	0.415
3.01908299711413e-109	0	MRPL20	Naive 2
3.32129551838509e-113	-0.354497063723348	0.174	0.314
5.92153777972878e-109	0	DCTPP1	Naive 2
5.33407030616227e-113	-0.349146158948758	0.246	0.401
9.51011394885671e-109	0	PSMC1	Naive 2
5.3504521207984e-113	-0.396378407335051	0.063	0.151
9.53932108617147e-109	0	PSAT1	Naive 2
8.90005075547611e-113	-0.358726610352072	0.266	0.419
1.58679004919384e-108	0	PHB	Naive 2
2.81765172828822e-112	-0.347265403385524	0.296	0.454
5.02359126636508e-108	0	MINOS1	Naive 2
2.85496009374824e-111	-0.350716449827561	0.113	0.25
5.09010835114373e-107	0	UBE2G1	Naive 2
3.90826249896845e-111	-0.654022087699391	0.212	0.333
6.96804120941084e-107	0	HSP90B1	Naive 2
1.57071760778966e-110	0.34160568461154	0.469	0.329
2.80043242292818e-106	0	TXNIP	Naive 2
1.66252184436311e-110	-0.316507840441275	0.096	0.217
2.96411019631499e-106	0	GTPBP4	Naive 2
3.3763920718602e-110	0.37633792088519	0.285	0.177
6.01976942491954e-106	0	CLEC2B	Naive 2
7.52612566932572e-110	-0.320132440962183	0.504	0.641
1.34183294558408e-105	0	NDUFA13	Naive 2
1.60748497643463e-109	-0.335071789486501	0.263	0.422
2.86598496448531e-105	0	NDUFAB1	Naive 2
2.00489455497992e-109	-0.335809883515672	0.039	0.132
3.5745265020737e-105	0	GMDS	Naive 2
2.0424066913354e-109	-0.349787790867952	0.18	0.32
3.64140688998189e-105	0	PDCD5	Naive 2
2.21958791207272e-109	-0.388879280116845	0.23	0.374
3.95730328843444e-105	0	PDIA6	Naive 2
3.67959532141588e-109	-0.339965045626646	0.499	0.634
6.56035049855238e-105	0	PTGES3	Naive 2
3.93266351409703e-109	-0.342589178519347	0.093	0.205
7.01154577928359e-105	0	WDR43	Naive 2

1.52976896614555e-108 -0.366296589469993 0.472 0.609
2.72742508974091e-104 0 EIF2S2 Naive 2
2.33415483871917e-108 -0.315344584734887 0.244 0.399
4.16156466195241e-104 0 ARHGDIA Naive 2
4.52887365916405e-108 -0.317098542930114 0.076 0.181
8.07452884692359e-104 0 MRTO4 Naive 2
4.8831206145035e-108 -0.333970301503022 0.432 0.581
8.70611574359829e-104 0 SNU13 Naive 2
7.04154906597501e-108 -0.447952484119699 0.133 0.259
1.25543778297268e-103 0 GCHFR Naive 2
8.38064248241725e-108 -0.328750110801724 0.159 0.298
1.49418474819017e-103 0 NDUFAF8 Naive 2
1.66237065124201e-107 -0.321497872291878 0.245 0.401
2.96384063409938e-103 0 PSMC3 Naive 2
1.87939048233292e-107 -0.357637216773126 0.412 0.559
3.35076529095135e-103 0 SOD1 Naive 2
2.1240663783288e-107 -0.345870064760073 0.556 0.677
3.78699794592242e-103 0 PSMB1 Naive 2
2.94854411647134e-107 -0.4531491358053 0.327 0.46
5.25695930525676e-103 0 COTL1 Naive 2
5.09100338959581e-107 -0.363120379055392 0.327 0.474
9.07674994331036e-103 0 ILF2 Naive 2
8.82345122497584e-107 -0.306425581652496 0.085 0.203
1.57313311890094e-102 0 ZNF593 Naive 2
7.56809123056518e-106 -0.374885994735187 0.512 0.637
1.34931498549747e-101 0 SERBP1 Naive 2
1.23639722695683e-105 -0.309353092148817 0.308 0.467
2.20437261594134e-101 0 AURKAIP1 Naive 2
1.51671708637235e-105 -0.317877835209088 0.242 0.397
2.70415489329327e-101 0 PSMD7 Naive 2
2.83060036852681e-105 -0.369424096337912 0.545 0.665
5.04667739704646e-101 0 HNRNPC Naive 2
2.40190337051928e-104 -0.327905197058833 0.191 0.341
4.28235351929883e-100 0 NDUFAF3 Naive 2
3.15880586944307e-104 -0.307434891837635 0.129 0.258
5.63183498463005e-100 0 EIF2S1 Naive 2
5.07021527914768e-104 -0.341837702301474 0.547 0.674
9.0396868211924e-100 0 YWHAB Naive 2
1.01056825199941e-103 -0.367973575919229 0.234 0.377
1.80174213648975e-99 0 ANXA5 Naive 2
1.07675297141881e-103 -0.350016567678531 0.454 0.588
1.91974287274259e-99 0 SNRPB Naive 2
1.17150830160135e-103 -0.335539551105933 0.44 0.58
2.08868215092505e-99 0 PGK1 Naive 2
1.29896422732615e-103 -0.424734103319881 0.937 0.943
2.3159233208998e-99 0 YBX1 Naive 2
1.53996627125549e-103 -0.324692511379353 0.38 0.529
2.74560586502142e-99 0 EIF3I Naive 2
1.71501052327532e-103 -0.361990223338144 0.081 0.186
3.05769226194756e-99 0 SAMSN1 Naive 2

1.88170179067159e-103	-0.33492043939428	0.608	0.719	3.35488612258838e-99	0	ELOB	Naive 2
2.71364271035449e-103	-0.33745708068786	0.24	0.389	4.83815358829102e-99	0	EIF3J	Naive 2
5.24523358335266e-103	-0.770260833654761	0.405	0.477	9.35172695575946e-99	0	S100A6	Naive 2
2.08664424397875e-102	-0.460271512512489	0.308	0.44	3.72027802258971e-98	0	METAP2	Naive 2
4.88402655301667e-102	-0.332937594429934	0.498	0.627	8.70773094137342e-98	0	RBM3	Naive 2
5.69485280842455e-102	-0.329234309721532	0.056	0.157	1.01533530721401e-97	0	SOCS1	Naive 2
7.03025120410244e-102	-0.303156711800191	0.29	0.447	1.25342348717942e-97	0	ADRM1	Naive 2
9.36532631338819e-102	-0.326224437807857	0.36	0.511	1.66974402841398e-97	0	UBE2L3	Naive 2
1.02898845404212e-101	-0.318444294263863	0.213	0.365	1.8345835147117e-97	0	EIF1AX	Naive 2
2.28306422614454e-101	-0.344619437438365	0.456	0.59	4.07047520879311e-97	0	UQCRQ	Naive 2
3.7478499277912e-101	0.346252436858324	0.125	0.064	6.68204163625893e-97	0	CHI3L2	Naive 2
8.95985954458987e-101	0.350949475300749	0.604	0.506	1.59745335820493e-96	0	ID3	Naive 2
1.84020258558366e-100	-0.313945011518514	0.4	0.548	3.28089718983711e-96	0	SELENOT	Naive 2
2.05671225371131e-100	-0.367527378776257	0.249	0.385	3.6669122771419e-96	0	HSPA9	Naive 2
3.39590158204233e-100	-0.333229874475736	0.536	0.662	6.05455293062328e-96	0	ATP5MF	Naive 2
4.86548779805881e-100	-0.310891157297466	0.953	0.963	8.67467819515905e-96	0	OAZ1	Naive 2
5.41708075437589e-100	-0.327859280109456	0.321	0.47	9.65811327697678e-96	0	CCT7	Naive 2
6.29446290732689e-100	-0.332670067578315	0.458	0.596	1.12223979174731e-95	0	PARK7	Naive 2
6.71791129977669e-100	-0.355212284604376	0.307	0.453	1.19773640563719e-95	0	PPP1R15A	Naive 2
7.7912569730663e-100	-0.383902992841292	0.196	0.331	1.38910320572799e-95	0	CALR	Naive 2
1.48993849664949e-99	-0.300923813795117	0.222	0.375	2.65641134567637e-95	0	POLR2K	Naive 2
2.04921517979046e-99	-0.44808007199424	0.057	0.14	3.65354574404841e-95	0	LGALS3	Naive 2
2.09362205278775e-99	-0.337904321916411	0.224	0.372	3.73271875791528e-95	0	RBM17	Naive 2
2.25132157350475e-99	-0.318930063700865	0.273	0.426	4.01388123340162e-95	0	ENY2	Naive 2
2.65300566629594e-99	-0.325684549901697	0.32	0.472	4.73004380243903e-95	0	ATP5F1C	Naive 2

3.05024546235465e-99	-0.356841080718384	0.624	0.72	5.4382826348321e-95	0	TPI1	Naive 2
5.92995904314639e-99	-0.331204734583946	0.201	0.348	1.05725239780257e-94	0	HSBP1	Naive 2
1.01576441376093e-98	-0.323543599279801	0.297	0.443	1.81100637329437e-94	0	NUDC	Naive 2
1.28174209099979e-98	-0.303270323940814	0.2	0.34	2.28521797404353e-94	0	TCP1	Naive 2
1.69994326425716e-98	-0.384896472107333	0.217	0.349	3.03082884584409e-94	0	PMAIP1	Naive 2
2.4597473077777e-98	-0.304424241853673	0.18	0.316	4.38548347503686e-94	0	SSRP1	Naive 2
2.95687536513637e-98	-0.426531462080715	0.32	0.445	5.27181308850164e-94	0	DEK	Naive 2
3.32011751174032e-98	-0.338681531107084	0.076	0.183	5.91943751168182e-94	0	DEF8	Naive 2
7.57688866072504e-98	-0.301907303125131	0.044	0.136	1.35088347932067e-93	0	VNN2	Naive 2
7.64080607684238e-98	0.388785820150198	0.406	0.314	1.36227931544023e-93	0	SMC6	Naive 2
9.35190145424879e-98	-0.345597091086671	0.143	0.271	1.66735051027802e-93	0	SH3KBP1	Naive 2
1.12735963281863e-97	-0.320510815309792	0.497	0.627	2.00996948935233e-93	0	PSMB3	Naive 2
1.86220504833143e-97	-0.306773884116873	0.538	0.67	3.32012538067011e-93	0	UQCR11	Naive 2
1.94356190823102e-97	-0.303195568590873	0.214	0.363	3.46517652618509e-93	0	BUD31	Naive 2
5.28794935834733e-97	-0.340164999335369	0.311	0.461	9.42788491099746e-93	0	ROMO1	Naive 2
7.59651788740201e-97	-0.329674514930067	0.123	0.247	1.3543831741449e-92	0	KDELR2	Naive 2
2.21248205074565e-96	-0.329775482172134	0.231	0.376	3.94463424827441e-92	0	MRPS6	Naive 2
3.08910384694713e-96	-0.306910575581688	0.175	0.319	5.50756324872204e-92	0	LYPLA1	Naive 2
2.254802878332e-95	-0.351846195020612	0.033	0.103	4.02008805177811e-91	0	FKBP11	Naive 2
2.94165762349739e-95	-0.302984424892813	0.258	0.409	5.24468137693349e-91	0	ATP6V0B	Naive 2
3.14288166033946e-95	-0.409370293636856	0.226	0.361	5.60344371221922e-91	0	SPIB	Naive 2
7.09251074358654e-95	-0.306509101686393	0.39	0.534	1.26452374047404e-90	0	PSMD8	Naive 2
1.18393874926084e-94	-0.369763802517733	0.075	0.178	2.11084439605715e-90	0	C12orf75	Naive 2
1.3837637001816e-94	-0.409046964361775	0.033	0.106	2.46711230105378e-90	0	CCNB1	Naive 2
3.04848890116442e-94	-0.314065141007738	0.134	0.257	5.43515086188605e-90	0	NIFK	Naive 2

3.33572398992171e-94	0.365549289157347	0.388	0.306
5.94726230163141e-90	0	LTA4H	Naive 2
5.63046224766228e-94	-0.488535991799791	0.82	0.858
1.00385511413571e-89	0	ATP5MG	Naive 2
1.32768357300049e-93	-0.305718798589789	0.234	0.376
2.36712704230258e-89	0	CHMP4B	Naive 2
1.85358240357268e-93	-0.405905428317171	0.296	0.431
3.30475206732972e-89	0	PPIB	Naive 2
3.09173213726367e-93	-0.375101904246877	0.077	0.17
5.5122492275274e-89	0	MCM7	Naive 2
1.26855495013194e-92	-0.314880924833786	0.057	0.155
2.26170662059024e-88	0	SIT1	Naive 2
3.19876852907444e-92	-0.303022033388347	0.399	0.544
5.70308441048681e-88	0	PSMB6	Naive 2
4.50167663122015e-92	-0.382057330982585	0.132	0.236
8.0260392658024e-88	0	EIF4EBP1	Naive 2
7.25020192225382e-92	-0.408967197565962	0.221	0.351
1.29263850071863e-87	0	HNRNPH1	Naive 2
9.01409488513671e-92	-0.565624547841384	0.723	0.777
1.60712297707102e-87	0	HMGN1	Naive 2
1.52271477163695e-91	-0.304971020992355	0.133	0.261
2.71484816635152e-87	0	ZBTB8OS	Naive 2
1.89089424519966e-91	-0.34015357463778	0.193	0.327
3.37127534976647e-87	0	CD99	Naive 2
2.00361402245567e-91	-0.306398261959759	0.156	0.293
3.57224344063621e-87	0	TOP1	Naive 2
3.18193303597814e-91	-0.348628246427279	0.336	0.467
5.67306840984542e-87	0	C1QBP	Naive 2
3.32421096674651e-91	-0.328141732498587	0.04	0.117
5.92673573261235e-87	0	RMI2	Naive 2
4.33357295791126e-91	0.334602472763803	0.388	0.278
7.72632722665998e-87	0	LINC00926	Naive 2
8.15263761234858e-91	-0.309313408696392	0.092	0.208
1.45353375990563e-86	0	PRPSAP2	Naive 2
1.49590602205506e-90	-0.301794054376029	0.371	0.514
2.66705084672197e-86	0	CCT8	Naive 2
1.98952673997619e-90	0.329176319029662	0.153	0.075
3.54712722470355e-86	0	CNN3	Naive 2
3.18023890288155e-90	-0.354038534920783	0.142	0.261
5.67004793994751e-86	0	MYDGF	Naive 2
7.23180560192307e-90	-0.311837132048675	0.128	0.249
1.28935862076686e-85	0	SINHCAF	Naive 2
1.57363909757472e-89	-0.309988801440635	0.329	0.469
2.80564114706598e-85	0	PGAM1	Naive 2
4.70812591002838e-89	-0.322551967029141	0.627	0.724
8.39411768498959e-85	0	NDUFA4	Naive 2
1.28908293847347e-88	-0.381703950480696	0.086	0.186
2.29830597100436e-84	0	SMC4	Naive 2
1.39876479432941e-88	-0.382647958095558	0.374	0.507
2.4938577518099e-84	0	RPS27L	Naive 2

3.53111793162589e-88 -0.321573991066908 0.302 0.444
6.2956301602958e-84 0 HNRNPD Naive 2
3.21382406591108e-87 0.308780113904367 0.709 0.636
5.72992692711287e-83 0 ZFP36L1 Naive 2
3.43034246675621e-86 -0.410829826028031 0.269 0.397
6.11595758397965e-82 0 ACADM Naive 2
4.40699538028221e-86 0.343402101503514 0.171 0.104
7.85723206350515e-82 0 ENTPD1 Naive 2
1.35909959679921e-85 -0.33746899258238 0.396 0.53
2.4231386711333e-81 0 MZT2B Naive 2
2.28945631270336e-85 0.332846008006851 0.162 0.098
4.08187165991883e-81 0 ETHE1 Naive 2
3.46399271010578e-85 -0.303121158657959 0.232 0.369
6.1759526028476e-81 0 LSM6 Naive 2
5.27440850468674e-85 -0.31263822509545 0.336 0.481
9.403742923006e-81 0 CAPZA1 Naive 2
8.72791919698108e-85 -0.622248921487094 0.89 0.887
1.55610071362976e-80 0 GAPDH Naive 2
1.8728443149249e-84 -0.304698256838658 0.599 0.702
3.3390941290796e-80 0 COX6A1 Naive 2
2.96383713394259e-84 -0.330847454966468 0.164 0.283
5.2842252261062 5e-80 0 ANP32E Naive 2
1.87706924350726e-83 -0.35611480984669 0.055 0.134
3.34662675424909e-79 0 LPP Naive 2
2.52145369538396e-83 -0.331637082612759 0.311 0.453
4.49549979350006e-79 0 DNAJA1 Naive 2
7.1241285943354e-83 0.304760279573011 0.523 0.432
1.27016088708406e-78 0 PNRC1 Naive 2
5.96074094568294e-81 0.321768409404631 0.261 0.181
1.06274050320581e-76 0 FAM129C Naive 2
2.53753856080354e-80 0.348996721823768 0.4 0.312
4.52417750005662e-76 0 PTPN6 Naive 2
4.01631545146071e-79 -0.319177589229938 0.334 0.464
7.16068881840929e-75 0 PPA1 Naive 2
2.37465101885361e-78 0.353251985609684 0.405 0.328
4.23376530151411e-74 0 PKIG Naive 2
3.21028596989318e-78 -0.315050885433126 0.193 0.311
5.72361885572255e-74 0 YWHAH Naive 2
6.44122875124009e-78 -0.321748811095151 0.182 0.311
1.1484066740586e-73 0 TUBA4A Naive 2
1.04517092584677e-77 0.334099246190617 0.23 0.143
1.8634352436922e-73 0 SLC2A3 Naive 2
1.26254752570235e-77 -0.371791309069589 0.581 0.673
2.25099598357471e-73 0 SLC25A5 Naive 2
2.00920271863716e-76 -0.530090601498319 0.455 0.55
3.5822075270582e-72 0 ISG20 Naive 2
2.05888321039362e-75 -0.368753830186924 0.846 0.874
3.67078287581078e-71 0 ARPC2 Naive 2
6.48341009896065e-75 -0.340477236654589 0.293 0.424
1.15592718654369e-70 0 RPL22L1 Naive 2

1.3640003866057e-73 -0.318164985396187 0.389 0.51
2.4318762892793e-69 0 CBX3 Naive 2
3.45220219899905e-73 0.357499372627659 0.202 0.123
6.1549313005954e-69 0 FCER2 Naive 2
1.85680229089929e-71 -0.348962675720916 0.167 0.278
3.31049280444435e-67 0 SYNE2 Naive 2
2.2638707607658e-71 -0.424829265507558 0.726 0.768
4.03625517936934e-67 0 PKM Naive 2
1.13722168273506e-70 0.313386737245121 0.346 0.265
2.02755253814834e-66 0 IFITM2 Naive 2
5.98780605504133e-70 -0.359703076616626 0.395 0.512
1.06756594155332e-65 0 C4orf3 Naive 2
6.58165848359373e-70 -0.337832702307134 0.097 0.177
1.17344389103993e-65 0 MARCKS Naive 2
1.39073935953147e-69 -0.318136471358799 0.263 0.388
2.47954920410867e-65 0 SELENOK Naive 2
2.4503595157766e-69 -0.542967356582975 0.517 0.581
4.3687459806781e-65 0 VIM Naive 2
5.67818683369627e-69 -0.430216241862279 0.689 0.745
1.01236393057971e-64 0 HSPA8 Naive 2
7.94777409495159e-69 -0.371488643642828 0.086 0.165
1.41700864338892e-64 0 NFKBID Naive 2
1.1219969410354e-68 -0.318026064984386 0.59 0.695
2.00040834617201e-64 0 TAGLN2 Naive 2
3.14902040784627e-68 -0.305317518109875 0.047 0.123
5.61438848514911e-64 0 CD9 Naive 2
2.6270630781557e-66 0.30469574424962 0.475 0.401
4.68379076204379e-62 0 NPC2 Naive 2
1.12418832601127e-65 -0.40918333342462 0.248 0.353
2.00431536644549e-61 0 DUT Naive 2
1.62527423896454e-65 0.309988313628007 0.286 0.217
2.89770144064989e-61 0 NT5C3A Naive 2
4.4337843895781e-65 -0.328873415863836 0.3 0.417
7.9049941881788e-61 0 NUCKS1 Naive 2
7.20993892156435e-62 -0.806437925784797 0.373 0.439
1.28546001032571e-57 0 STMN1 Naive 2
3.42985599422985e-61 -0.831482551502514 0.215 0.273
6.11509025211241e-57 0 S100A4 Naive 2
9.03834906463721e-59 -0.450361004630198 0.931 0.934
1.61144725473417e-54 0 SERF2 Naive 2
1.20067767333745e-57 -0.305200759040001 0.127 0.223
2.14068822379333e-53 0 EAF2 Naive 2
2.16722428762994e-57 -1.32856706123278 0.351 0.437
3.86394418241543e-53 0 HIST1H4C Naive 2
5.87497142800676e-56 -0.323108505011021 0.119 0.202
1.04744865589932e-51 0 PCNA Naive 2
5.05762487869535e-55 -0.308230164648268 0.291 0.418
9.01723939622595e-51 0 CSTB Naive 2
2.87761845012175e-54 -0.317094626671055 0.206 0.317
5.13050593472207e-50 0 TCF4 Naive 2

3.04689122828152e-54	-0.345170850469339	0.213	0.321	5.43230237090313e-50	0	BCL7A	Naive 2
3.56666362233008e-53	-0.325404957310689	0.052	0.104	6.3590045722523e-49	0	KLK1	Naive 2
2.98750940374627e-52	-0.34303837566308	0.218	0.311	5.32643051593923e-48	0	NFKBIA	Naive 2
4.49440797833476e-52	-0.333769340728705	0.178	0.273	8.01307998457305e-48	0	MDM4	Naive 2
6.54884345481784e-51	-2.30965926361715	0.073	0.137	1.16759329955947e-46	0	IGHG2	Naive 2
4.95499151704199e-50	-0.322682304400916	0.066	0.121	8.83425437573416e-46	0	C1orf56	Naive 2
1.10957381503347e-49	-1.95456999966591	0.18	0.256	1.97825915482317e-45	0	IGHG4	Naive 2
5.2322292300358e-49	-0.354030582344416	0.72	0.771	9.32854149423083e-45	0	CYBA	Naive 2
8.39108954920451e-47	-0.726688039765604	0.685	0.722	1.49604735572767e-42	0	HMGN2	Naive 2
6.82914483147198e-44	-0.450248485276305	0.929	0.932	1.21756823200314e-39	0	HMGB1	Naive 2
1.03884998281284e-38	-0.441942210889819	0.432	0.513	1.85216563435701e-34	0	SSR4	Naive 2
7.02666432151862e-27	-0.309953049509448	0.939	0.928	1.25278398188355e-22	0	NPM1	Naive 2
2.06565561794793e-20	-0.727219417164255	0.156	0.223	3.68285740123936e-16	0	PLCG2	Naive 2
9.89846946890283e-17	-0.394894831554688	0.86	0.846	1.76479812161069e-12	0	HSP90AB1	Naive 2
2.26493136415457e-08	-0.310944810330708	0.793	0.784	0.000403814612915119	0	ACTG1	Naive 2
2.9275809859743e-08	-1.15966971785123	0.403	0.434	0.000521958413989359	0	IGLC3	Naive 2
0.000490229942859223	-1.03825869235442	0.912	0.929	1	0	IGKC	Naive 2
0.00582875899481827	-1.61244335182059	0.608	0.652	1	0	IGLC2	Naive 2
0	1.62640448940425	0.691	0.327	0	1	SELL	Naive 1
0	1.4569475157633	0.95	0.723	0	1	CXCR4	Naive 1
0	1.37014617049403	0.515	0.24	0	1	KLF2	Naive 1
0	1.12052718981081	0.401	0.185	0	1	PLAC8	Naive 1
0	1.07245100439787	0.386	0.217	0	1	CD55	Naive 1
0	1.04807980200102	0.449	0.282	0	1	ZFP36L2	Naive 1
0	0.920022166919104	0.75	0.452	0	1	LTB	Naive 1

0 0.896449716084155 0.77 0.583 0 1
IGHD Naive 1
0 0.769311376153668 0.968 0.931 0 1
BTG1 Naive 1
0 0.755657277672165 0.904 0.84 0 1
CD37 Naive 1
0 0.730873185117909 0.998 0.984 0 1
TMSB10 Naive 1
0 0.670396024214154 1 1 0 1
MALAT1 Naive 1
0 0.660507967673452 0.946 0.857 0 1
CD52 Naive 1
0 0.59892571787207 0.998 0.894 0 1 IGHM
Naive 1
0 0.342707348770136 1 0.998 0 1 B2M
Naive 1
0 0.319782592951137 1 1 0 1
RPS27 Naive 1
0 -0.513550939309875 0.566 0.836 0 1
RPS26 Naive 1
0 -0.567286162008272 0.697 0.894 0 1
ARPC2 Naive 1
0 -0.567990110131293 0.318 0.645 0 1
PSMB3 Naive 1
0 -0.573125057462614 0.516 0.793 0 1
PSMA7 Naive 1
0 -0.5756266690887 0.281 0.606 0 1 UQCRQ
Naive 1
0 -0.582548280444209 0.368 0.696 0 1
PSMB1 Naive 1
0 -0.585836981928602 0.456 0.76 0 1
COX6C Naive 1
0 -0.588788279772904 0.923 0.984 0 1
RPS24 Naive 1
0 -0.603624201798462 0.34 0.673 0 1
COX7B Naive 1
0 -0.604539490290965 0.331 0.646 0 1
POMP Naive 1
0 -0.626157610317502 0.316 0.649 0 1
POLR2L Naive 1
0 -0.638654654358515 0.807 0.944 0 1
HNRNPA1 Naive 1
0 -0.655321862135901 0.434 0.741 0 1
TPI1 Naive 1
0 -0.657586286237126 0.425 0.746 0 1
NDUFA4 Naive 1
0 -0.669003181483911 0.347 0.674 0 1
PSMA4 Naive 1
0 -0.672390040848006 0.263 0.595 0 1
SEC61G Naive 1

0	-0.681655026267257	0.374	0.726	0	1	
SLC25A3	Naive 1					
0	-0.692871884665234	0.292	0.649	0	1	
RBM3	Naive 1					
0	-0.699262095483764	0.164	0.481	0	1	
PPA1	Naive 1					
0	-0.699264986439557	0.856	0.954	0	1	
YBX1	Naive 1					
0	-0.710311962647316	0.275	0.613	0	1	
SNRPE	Naive 1					
0	-0.719069265614345	0.034	0.262	0	1	
MTHFD2	Naive 1					
0	-0.725950554264534	0.348	0.677	0	1	SET
	Naive 1					
0	-0.734684478048692	0.745	0.921	0	1	
PFN1	Naive 1					
0	-0.738632760245737	0.299	0.639	0	1	
SEC61B	Naive 1					
0	-0.751253438288004	0.243	0.569	0	1	
PSME2	Naive 1					
0	-0.755454925827904	0.072	0.335	0	1	
ODC1	Naive 1					
0	-0.759516031728475	0.274	0.628	0	1	
ATP5MC3	Naive 1					
0	-0.774145198035019	0.537	0.8	0	1	
HMGN1	Naive 1					
0	-0.777663593018135	0.218	0.536	0	1	
HSPE1	Naive 1					
0	-0.781688536896334	0.383	0.721	0	1	
SRSF3	Naive 1					
0	-0.784346024161547	0.322	0.635	0	1	
LDHA	Naive 1					
0	-0.784430743230117	0.183	0.514	0	1	
SNRPD1	Naive 1					
0	-0.799535525898772	0.272	0.622	0	1	
LDHB	Naive 1					
0	-0.816593859975745	0.133	0.435	0	1	
BASP1	Naive 1					
0	-0.833968069223933	0.107	0.433	0	1	
YWHAE	Naive 1					
0	-0.835905411143201	0.8	0.948	0	1	
NPM1	Naive 1					
0	-0.864634788962167	0.501	0.782	0	1	RAN
	Naive 1					
0	-0.866146860276722	0.144	0.481	0	1	
HMGA1	Naive 1					
0	-0.921560897319187	0.161	0.503	0	1	
HSPD1	Naive 1					
0	-1.06064792761773	0.05	0.334	0	1	
NME1	Naive 1					

0	-1.06680456548166	0.556	0.818	0	1
ACTG1	Naive 1
0	-1.0722828797469 0.287	0.617	0	1	TUBB
Naive 1
0	-1.11996806331772	0.053	0.338	0	1
CD27	Naive 1
0	-1.14251028249994	0.111	0.45	0	1	TXN
Naive 1
0	-1.15775951142291	0.272	0.644	0	1
PRDX1	Naive 1
0	-1.23767549092402	0.405	0.782	0	1
HSPA8	Naive 1
0	-1.30442695651224	0.397	0.759	0	1
ENO1	Naive 1
0	-1.33831984411749	0.596	0.883	0	1
HSP90AB1	Naive 1
0	-1.39139373768553	0.672	0.918	0	1
GAPDH	Naive 1
0	-1.40969168148133	0.023	0.232	0	1
RGS13	Naive 1
0	-1.41401053744144	0.391	0.812	0	1	PKM
Naive 1
0	-1.63640415521685	0.055	0.319	0	1
MIR155HG	Naive 1
0	-1.67313077943232	0.066	0.451	0	1
FABP5	Naive 1
8.39911597930119e-322	-0.554888129003236	0.5	0.778
1.49747838794961e-317	1	CHCHD2	Naive 1
5.06911352633119e-321	-0.588049387247989	0.217	0.537
9.03772250609588e-317	1	COX5A	Naive 1
4.74401833136765e-320	-0.583276896683403	0.352	0.666
8.45811028299538e-316	1	ATP5F1B	Naive 1
5.00043840155926e-320	-0.640486374055294	0.047	0.281
8.91528162614e-316	1	RGS10	Naive 1
3.08074633464309e-319	0.946543474692797	0.45	0.275
5.49266264003517e-315	1	LINC00926	Naive 1
3.35709701298794e-318	-0.530942375237538	0.415	0.722
5.98536826445619e-314	1	COX6A1	Naive 1
3.44651873485246e-315	-0.743835456371955	0.221	0.521
6.14479825236846e-311	1	RANBP1	Naive 1
4.22474285254963e-315	-0.881806273081223	0.449	0.73
7.53229403181073e-311	1	H2AFZ	Naive 1
1.6370964590839e-313	-0.577592852796168	0.375	0.683
2.91877927690068e-309	1	SNRPG	Naive 1
3.82232337080437e-312	-0.514070150462151	0.436	0.737
6.81482033780712e-308	1	ELOB	Naive 1
2.45705531124706e-311	-0.880943051030122	0.111	0.372
4.38068391442238e-307	1	LRMP	Naive 1
2.67583056535981e-311	-0.613676875871861	0.178	0.487
4.77073831498e-307	1	ILF2	Naive 1

9.11286855029533e-311 0.842956498529853 0.667 0.564
1.62473333383215e-306 1 SARAF Naive 1
4.69449083772804e-310 0.951343122430512 0.351 0.192
8.36980771458533e-306 1 LBH Naive 1
9.92239386633841e-308 -0.591601231928349 0.286 0.597
1.76906360242947e-303 1 ATP5PF Naive 1
2.1334338131615e-307 -0.690280612431656 0.518 0.779
3.80369914548563e-303 1 HSP90AA1 Naive 1
1.18759722982466e-301 -0.532926500785889 0.369 0.678
2.11736710105439e-297 1 ATP5MF Naive 1
1.93305298564758e-300 -0.602513913283698 0.182 0.485
3.44644016811108e-296 1 SEM1 Naive 1
6.65337327606713e-299 -0.506219658803067 0.363 0.677
1.18622992139001e-294 1 ATP5MD Naive 1
6.12949437687091e-296 -0.558463821181197 0.144 0.441
1.09282755245231e-291 1 PSMB2 Naive 1
1.76890950159658e-293 0.641187853588227 0.867 0.791
3.15378875039654e-289 1 LAPTM5 Naive 1
7.74715532644385e-293 -0.537471253966111 0.228 0.542
1.38124032315167e-288 1 EIF3I Naive 1
4.08839387032352e-292 -0.422747103527583 0.965 0.991
7.28919743139981e-288 1 RPLP0 Naive 1
6.06158507330107e-290 -0.51979865836736 0.8 0.935
1.08072000271885e-285 1 CFL1 Naive 1
1.18642787729777e-289 -0.609087780576865 0.14 0.428
2.11528226243419e-285 1 PHB Naive 1
1.068972850369e-287 -0.582574129060375 0.261 0.57
1.90587169492288e-283 1 IMPDH2 Naive 1
7.67999163712551e-286 -0.650480531014504 0.1 0.361
1.36926570898311e-281 1 RFTN1 Naive 1
1.20647066244058e-283 -0.658697193960214 0.14 0.419
2.15101654406531e-279 1 NHP2 Naive 1
2.42498547916923e-283 -0.951288688725845 0.027 0.213
4.32350661081083e-279 1 PTTG1 Naive 1
6.90591228119831e-283 -0.570520600908627 0.27 0.571
1.23125510061485e-278 1 SRSF9 Naive 1
8.62358898711935e-282 -0.61686813410379 0.322 0.62
1.53749968051351e-277 1 ANP32B Naive 1
1.23989729513497e-281 -0.666555620585054 0.353 0.64
2.21061288749614e-277 1 DYNLL1 Naive 1
5.62915268961274e-281 -0.594957421581009 0.186 0.481
1.00362163303106e-276 1 C1QBP Naive 1
1.10753747007704e-279 -0.513667593051258 0.379 0.678
1.97462855540035e-275 1 HNRNPM Naive 1
3.31474598442218e-273 -0.512317925040429 0.304 0.61
5.9098606156263e-269 1 PARK7 Naive 1
4.84787017153483e-272 -0.718410581630341 0.175 0.421
8.64326772882944e-268 1 EIF5A Naive 1
1.79810068706812e-268 -0.51204942061466 0.388 0.675
3.20583371497375e-264 1 ERH Naive 1

4.79761716062092e-268 -0.602486784735089 0.137 0.408
8.55367163567103e-264 1 LCP1 Naive 1
6.86145847672436e-267 -0.468916449556134 0.332 0.638
1.22332943181519e-262 1 PSMA2 Naive 1
2.61964276127256e-265 -0.425049471542781 0.464 0.752
4.67056107907284e-261 1 COX6B1 Naive 1
8.24546676850959e-265 -0.5648699916594 0.098 0.355
1.47008427015758e-260 1 IMP4 Naive 1
9.94004461256748e-265 0.88102404970121 0.48 0.335
1.77221055397466e-260 1 TXNIP Naive 1
2.01225007479265e-264 -0.550563708117174 0.205 0.499
3.58764065834781e-260 1 PRMT1 Naive 1
3.46795152809091e-263 -0.549847528880775 0.411 0.691
6.18301077943329e-259 1 SLC25A5 Naive 1
4.86314391017109e-263 -0.450194268811659 0.352 0.654
8.67049927744404e-259 1 NDUFA13 Naive 1
6.43564340364358e-263 -0.569987035791326 0.147 0.423
1.14741086243561e-258 1 CCT5 Naive 1
3.60851350484479e-262 0.401275033164866 0.976 0.968
6.43361872778778e-258 1 MT-ND3 Naive 1
2.00924943048928e-261 -0.724416284127308 0.014 0.167
3.58229080961933e-257 1 BIK Naive 1
8.88232758055433e-261 -0.483408763078249 0.235 0.54
1.58363018433703e-256 1 NDUFA11 Naive 1
4.39037890954585e-257 0.856825679182085 0.597 0.49
7.8276065578293e-253 1 TSC22D3 Naive 1
6.97984299021688e-257 -0.523216021515843 0.133 0.408
1.24443620672577e-252 1 PSMC1 Naive 1
5.74128845728032e-256 0.502966479044291 0.914 0.859
1.02361431904851e-251 1 CD79A Naive 1
2.04342172600622e-254 -0.473458869589132 0.293 0.593
3.6432165952965e-250 1 PGK1 Naive 1
3.0482553438975e-253 -0.786306427487123 0.355 0.621
5.43473445263486e-249 1 MARCKSL1 Naive 1
1.60146290271356e-251 0.894256736297183 0.454 0.321
2.85524820924801e-247 1 HVCN1 Naive 1
1.00626910641419e-250 -0.535066111942806 0.2 0.487
1.79407718982586e-246 1 MRPL51 Naive 1
1.73347397535501e-250 -0.626323460554118 0.098 0.338
3.09061075066045e-246 1 CALR Naive 1
2.5942011965994e-250 -0.533937650019308 0.192 0.477
4.62520131341707e-246 1 CCT6A Naive 1
5.86300362530585e-250 -0.509084133884455 0.19 0.479
1.04531491635578e-245 1 ATP5MC1 Naive 1
1.03188973539424e-249 -0.487369746882761 0.149 0.429
1.8397562092344e-245 1 NDUFAB1 Naive 1
3.3433938886041e-249 0.844602012379745 0.158 0.045
5.96093696399225e-245 1 PPP1R14A Naive 1
1.05356777273733e-247 -0.383249998244085 0.804 0.94
1.87840598201339e-243 1 BTF3 Naive 1

1.06947822473125e-247	-0.756420719382697	0.017	0.17
1.90677272687335e-243	1	DUSP2	Naive 1
3.67703026547673e-247	-0.453612630683746	0.246	0.546
6.55577726031847e-243	1	PSMD8	Naive 1
8.71398952154086e-246	-0.645588839962328	0.314	0.593
1.55361719179552e-241	1	NCL	Naive 1
3.80500210863509e-245	-0.444152616851685	0.253	0.556
6.7839382594855e-241	1	PSMB6	Naive 1
8.98131754019814e-245	-0.400907386973082	0.444	0.731
1.60127910424193e-240	1	ATP5MPL	Naive 1
1.12479584061216e-244	-0.966259798167656	0.455	0.695
2.00539850422742e-240	1	TUBA1B	Naive 1
3.43647996623998e-244	-0.666005012860469	0.085	0.307
6.12690013180927e-240	1	SRM	Naive 1
6.10404591583814e-244	-0.502135020298955	0.076	0.31
1.08829034633478e-239	1	PSMD14	Naive 1
3.96598471261863e-243	-0.551745099445847	0.123	0.38
7.07095414412776e-239	1	TIMM13	Naive 1
6.66916488578761e-242	-0.53653163653908	0.686	0.874
1.18904540748707e-237	1	ATP5MG	Naive 1
2.41126731536208e-241	-0.482912052690098	0.233	0.525
4.29904849655905e-237	1	CCT3	Naive 1
6.50312809682778e-241	-0.525145887751666	0.01	0.148
1.15944270838343e-236	1	PHGDH	Naive 1
7.66219658253455e-241	0.783567686294046	0.182	0.065
1.36609302870008e-236	1	CALHM6	Naive 1
5.64821036114591e-240	-0.49194898235579	0.256	0.55
1.00701942528871e-235	1	SSBP1	Naive 1
1.62024717033227e-239	-0.532075772223012	0.185	0.462
2.8887386799854e-235	1	CCT2	Naive 1
4.52935551513923e-239	0.793925078018066	0.19	0.073
8.07538794794173e-235	1	RNASE6	Naive 1
5.3085210878371e-237	-0.460219755219692	0.29	0.586
9.46456224750476e-233	1	PSMA1	Naive 1
1.00301956189869e-236	-0.485410870627579	0.202	0.487
1.78828357690918e-232	1	VDAC1	Naive 1
4.01452626138684e-236	-0.533444978244973	0.01	0.149
7.1574988714266e-232	1	CYTOR	Naive 1
6.68160262008308e-236	-0.472477409301528	0.006	0.13
1.19126293113461e-231	1	PYCR1	Naive 1
3.96352758556341e-235	-0.51661031650591	0.209	0.491
7.06657333230101e-231	1	COX17	Naive 1
8.009222804382e-235	-0.387866323733597	0.401	0.688
1.42796433379327e-230	1	ATP5F1A	Naive 1
1.30721402496336e-234	-0.339448392699118	0.764	0.923
2.33063188510718e-230	1	SRP14	Naive 1
2.39628706486293e-234	-0.591773278044618	0.014	0.153
4.27234020794411e-230	1	PSAT1	Naive 1
2.18555342607371e-232	-0.554783793381902	0.181	0.451
3.89662320334681e-228	1	CTSH	Naive 1

5.64287618330758e-232 -0.552084403558824 0.065 0.276
1.00606839472191e-227 1 GARS Naive 1
3.01507468881447e-231 -0.45744281007142 0.311 0.6
5.37557666268732e-227 1 SNRPB Naive 1
1.55146436951723e-230 -0.688182046653927 0.064 0.258
2.76610582441227e-226 1 UBE2J1 Naive 1
4.27869982928319e-230 -0.482690263825186 0.192 0.469
7.62849392562899e-226 1 ROMO1 Naive 1
4.44692385711256e-230 -0.470152716304852 0.14 0.407
7.92842054484599e-226 1 PSMC3 Naive 1
1.78887199374239e-229 -0.500148424145577 0.395 0.665
3.18937987764331e-225 1 GSTP1 Naive 1
2.73644981013118e-228 -0.508332793012413 0.076 0.304
4.87881636648289e-224 1 ATOX1 Naive 1
2.78450758243095e-228 -0.553086601259479 0.309 0.586
4.96449856871613e-224 1 TCEA1 Naive 1
1.17434300444088e-227 0.439640102462439 0.915 0.848
2.09373614261765e-223 1 CD79B Naive 1
8.10637718678663e-227 -0.480933811514854 0.186 0.461
1.44528598863219e-222 1 CCT4 Naive 1
2.59326702947207e-226 -0.507299639473106 0.031 0.204
4.62353578684575e-222 1 TUBA1C Naive 1
6.5275527798863e-226 -0.379385485626208 0.405 0.692
1.16379738512593e-221 1 RBX1 Naive 1
4.48892944590691e-225 -0.491669306518367 0.189 0.464
8.00331230910742e-221 1 SLIRP Naive 1
2.96230501567415e-224 -0.477590127876761 0.257 0.545
5.28149361244545e-220 1 TALDO1 Naive 1
6.66746464558385e-224 -0.457185317483892 0.162 0.432
1.18874227166115e-219 1 PSMA3 Naive 1
1.07677967551637e-223 -0.430274937243585 0.33 0.621
1.91979048347814e-219 1 EIF2S2 Naive 1
3.71664630964888e-223 -0.683105609795668 0.036 0.212
6.62640870547298e-219 1 DAAM1 Naive 1
5.48702006202948e-223 0.822255791183614 0.543 0.456
9.78280806859237e-219 1 ITM2B Naive 1
6.54871770515755e-223 -0.931526669130196 0.51 0.744
1.16757087965254e-218 1 HMGN2 Naive 1
9.77207218865914e-223 -0.4317805785576 0.198 0.48
1.74226275051604e-218 1 PSMB7 Naive 1
1.18584341174097e-221 -0.569017528955873 0.053 0.243
2.11424021879298e-217 1 TRAF4 Naive 1
4.02904841462758e-221 -0.707643379591699 0.026 0.177
7.18339041843951e-217 1 LMNA Naive 1
4.1890041023563e-221 -0.457171154976487 0.153 0.42
7.46857541409106e-217 1 NDUFS6 Naive 1
1.50659145210619e-220 -0.466790103382543 0.229 0.51
2.68610189996013e-216 1 PRELID1 Naive 1
2.8693420376223e-220 -0.428211816754384 0.372 0.655
5.1157499188768e-216 1 ACTR3 Naive 1

1.21606832093727e-219	-0.561202523330437		0.174	0.433
2.16812820939907e-215	1	RPL22L1	Naive 1	
1.62054518649326e-219	-0.705049404339786		0.033	0.191
2.88927001299884e-215	1	TNFRSF18	Naive 1	
5.10458653611931e-219	-0.636662065345184		0.074	0.281
9.10096733524712e-215	1	SRGN	Naive 1	
6.68641384115676e-217	-0.452378590487615		0.162	0.432
1.19212072373984e-212	1	NDUFS5	Naive 1	
2.15746940623377e-216	-0.503930064164707		0.022	0.169
3.84655220437419e-212	1	CLECL1	Naive 1	
5.60623389801432e-216	-0.507891662990762		0.009	0.134
9.99535441676973e-212	1	CD38	Naive 1	
8.61921780276023e-216	-0.410056300462404		0.195	0.474
1.53672034205412e-211	1	AURKAIP1	Naive 1	
3.5772485074995e-215	-0.555439288110872		0.843	0.946
6.37787636402087e-211	1	SERF2	Naive 1	
3.25497177283718e-213	-0.536036996563282		0.083	0.297
5.8032891737914e-209	1	SMS	Naive 1	
5.08772212871774e-213	-0.610716583358992		0.059	0.254
9.07089978329087e-209	1	CKS2	Naive 1	
1.17436806511329e-212	0.811669326894626		0.365	0.237
2.09378082329048e-208	1	FCMR	Naive 1	
2.57702753363837e-211	0.827634898609569		0.421	0.319
4.59458238972385e-207	1	RIPOR2	Naive 1	
4.20947339187945e-211	-0.365041942732791		0.434	0.708
7.50507011038187e-207	1	ATP5ME	Naive 1	
4.41105302169422e-211	-0.409081802674598		0.276	0.558
7.86446643237862e-207	1	FKBP1A	Naive 1	
1.11117132432755e-210	-0.43450863568135		0.204	0.479
1.9811073541436e-206	1	PGAM1	Naive 1	
3.29713207226125e-210	-0.540117243991999		0.275	0.545
5.87845677163458e-206	1	PA2G4	Naive 1	
3.21018571474024e-209	-0.48095609094855		0.187	0.449
5.72344011081037e-205	1	DDX39A	Naive 1	
6.19069671059036e-209	-0.409630860671442		0.248	0.532
1.10373931653116e-204	1	TUFM	Naive 1	
3.5227362018433e-208	-0.994331138917261		0.041	0.201
6.28068637426641e-204	1	XBP1	Naive 1	
6.47136678333365e-208	-0.499111843677601		0.017	0.157
1.15377998380056e-203	1	AIM2	Naive 1	
2.02179687837409e-206	-0.418311889251417		0.207	0.479
3.60466165445317e-202	1	ATP5F1C	Naive 1	
3.013403578376e-206	-0.509017218635785		0.17	0.423
5.37259723988657e-202	1	FNBP1	Naive 1	
7.41276109035857e-206	-0.450531920625036		0.229	0.502
1.32162117480003e-201	1	SNRPF	Naive 1	
3.4578212429469e-205	-0.382889891254022		0.271	0.558
6.16494949405002e-201	1	NDUFB8	Naive 1	
1.07593548274293e-204	-0.451673305091768		0.101	0.334
1.91828537218237e-200	1	TIMM8B	Naive 1	

1.45614896524027e-203	-0.413928550879083	0.249	0.523
2.59616799012687e-199	1	CCT8	Naive 1
1.61036182904055e-203	-0.505723981631233	0.031	0.188
2.87111410499639e-199	1	CCDC88A	Naive 1
3.48629630860503e-203	-0.344517630712828	0.408	0.687
6.2157176886119e-199	1	UBE2D3	Naive 1
3.14149019821713e-202	-0.43312220003266	0.192	0.46
5.60096287440133e-198	1	MINOS1	Naive 1
6.20830095219544e-201	-0.456442396741566	0.306	0.573
1.10687797676693e-196	1	PARP1	Naive 1
1.27354816841203e-200	-0.416476750637878	0.013	0.141
2.27060902946181e-196	1	SLC1A5	Naive 1
1.42666700430107e-198	-0.448110085205993	0.164	0.422
2.54360460196837e-194	1	ARPC5L	Naive 1
4.58443408723358e-198	-0.425166092302583	0.131	0.379
8.17358753412875e-194	1	CYC1	Naive 1
5.07593672565317e-198	-0.408890828736916	0.378	0.649
9.04988758816704e-194	1	SERBP1	Naive 1
8.64168268129452e-198	-0.448996210408154	0.201	0.467
1.540725605248e-193	1	EIF5B	Naive 1
2.29312163106561e-197	-0.427950714012992	0.221	0.49
4.08840655602688e-193	1	SFPQ	Naive 1
3.17753044637152e-197	-0.47880204120558	0.14	0.378
5.66521903283578e-193	1	PDIA6	Naive 1
1.2875379540447e-196	-0.44373418855483	0.128	0.367
2.29555141826629e-192	1	MIF	Naive 1
1.36696315077098e-196	-0.42666234496103	0.132	0.376
2.43715860150958e-192	1	LSM6	Naive 1
2.37441460433092e-196	-0.477012100094022	0.111	0.341
4.2333437980616e-192	1	LPXN	Naive 1
3.48882419714399e-196	-0.36526312703648	0.335	0.619
6.22022466108802e-192	1	NDUFB2	Naive 1
6.76716639681715e-196	-0.340516345380455	0.617	0.834
1.20651809688853e-191	1	TMA7	Naive 1
2.47558295653977e-195	-0.434875855101089	0.221	0.489
4.41371685321475e-191	1	LSM5	Naive 1
3.32568451950751e-195	-0.409695413439605	0.173	0.434
5.92936292982995e-191	1	MDH2	Naive 1
3.60095923474356e-195	-0.360089671330569	0.337	0.612
6.42015021962429e-191	1	COPE	Naive 1
2.41375107854083e-194	-0.413919855417835	0.147	0.397
4.30347679793045e-190	1	PSMA5	Naive 1
3.66619360567029e-194	-0.395291242769031	0.244	0.518
6.53645657954956e-190	1	UBE2L3	Naive 1
5.75909813321987e-194	-0.385203219492486	0.411	0.677
1.02678960617177e-189	1	HNRNPC	Naive 1
8.20043218074698e-194	-0.422724420153617	0.179	0.439
1.46205505350538e-189	1	BANF1	Naive 1
1.66584357546426e-193	-0.382759516892935	0.287	0.565
2.97003251069524e-189	1	GHITM	Naive 1

2.44172438029512e-193 -0.478809495834194 0.048 0.223
4.35335039762816e-189 1 NPM3 Naive 1
1.31241690497193e-191 -0.574479462326155 0.123 0.346
2.33990809987445e-187 1 SEC11C Naive 1
7.53572546027383e-191 -0.422088027427189 0.104 0.331
1.34354449231222e-186 1 GLRX3 Naive 1
1.0342167426315e-190 -0.358193700856646 0.395 0.669
1.8439050304377e-186 1 UQCRH Naive 1
1.73058807366521e-190 -0.435949827819593 0.054 0.24
3.0854654765377e-186 1 MRPL12 Naive 1
4.66656781790296e-190 -0.44217797073224 0.061 0.252
8.32002376253919e-186 1 MRPL3 Naive 1
1.33610190365943e-189 -0.367724147963482 0.371 0.644
2.3821360840344e-185 1 PTGES3 Naive 1
2.30955053360434e-189 -0.426977687110957 0.115 0.344
4.11769764636318e-185 1 TCP1 Naive 1
2.34475156117696e-189 -0.52609520834484 0.134 0.358
4.1804575584224e-185 1 GRHPR Naive 1
2.83640916610069e-189 -0.376207272974803 0.773 0.912
5.05703390224092e-185 1 PPIA Naive 1
1.03270367298146e-188 0.32093099515773 0.988 0.979
1.84120737855864e-184 1 MT-CO1 Naive 1
1.7689049283736e-188 -0.406060756595672 0.149 0.397
3.1537805967973e-184 1 EWSR1 Naive 1
7.21230956449821e-188 -0.434794245086222 0.376 0.643
1.28588267225439e-183 1 SYNGR2 Naive 1
8.82682181716735e-188 -0.351647711818939 0.357 0.629
1.57373406178277e-183 1 XRCC6 Naive 1
5.98561032069055e-187 -0.468581700171385 0.042 0.208
1.06717446407592e-182 1 GPATCH4 Naive 1
9.66411656175485e-187 -0.401264457362442 0.152 0.401
1.72301534179527e-182 1 PSMD7 Naive 1
1.04675665878405e-186 -0.398212936002641 0.335 0.608
1.86626244694609e-182 1 APRT Naive 1
5.07284625096772e-184 -0.31989890810648 0.783 0.922
9.04437758085034e-180 1 ATP5F1E Naive 1
6.45138868201689e-184 -0.352473448769626 0.456 0.708
1.15021808811679e-179 1 HNRNPA3 Naive 1
1.19547498828376e-183 0.73405423029174 0.552 0.486
2.13141235661111e-179 1 FXYD5 Naive 1
2.09122424417473e-183 -0.361418676195812 0.218 0.484
3.72844370493913e-179 1 VCP Naive 1
9.28395543269239e-183 -0.310623244261365 0.485 0.738
1.65523641409473e-178 1 COX7A2 Naive 1
9.31973191190584e-183 -0.504613713849471 0.171 0.403
1.66161500257369e-178 1 ACADM Naive 1
5.96387442581589e-182 -0.490096284087711 0.061 0.24
1.06329917137871e-177 1 NANS Naive 1
5.01907218226114e-181 0.78891801810665 0.289 0.194
8.94850379375339e-177 1 PRKCB Naive 1

1.85775347398456e-180 0.744556368040643 0.299 0.194
3.31218866876706e-176 1 C16orf74 Naive 1
1.76165919802694e-179 -0.39381117714846 0.197 0.45
3.14086218416223e-175 1 HNRNPD Naive 1
1.9252373708045e-179 -0.428429351359168 0.036 0.191
3.43250570840734e-175 1 ASNS Naive 1
3.47164825877879e-179 -0.39733973912281 0.125 0.36
6.18960168057671e-175 1 PDAP1 Naive 1
1.338697707799e-178 0.737175305994956 0.516 0.438
2.38676414323484e-174 1 PNRC1 Naive 1
2.81764539846441e-178 -0.396401490114951 0.095 0.313
5.02357998092219e-174 1 MRPS15 Naive 1
1.09365065814138e-177 -0.39347710440525 0.247 0.512
1.94986975840028e-173 1 RSL1D1 Naive 1
6.55681480634578e-177 -0.407924395771653 0.092 0.304
1.16901451182339e-172 1 PHPT1 Naive 1
1.84559956188937e-176 -0.395562627453841 0.114 0.341
3.29051945889255e-172 1 STOML2 Naive 1
2.55816184925212e-176 -0.363224967211083 0.215 0.476
4.56094676103161e-172 1 CCT7 Naive 1
7.74600251533785e-176 -0.414375859545349 0.097 0.31
1.38103478845958e-171 1 PRDX3 Naive 1
9.13759098203598e-176 0.462876357566367 0.767 0.64
1.6291410961872e-171 1 TCL1A Naive 1
1.02148063132193e-175 -0.494937287960488 0.204 0.447
1.82119781758387e-171 1 METAP2 Naive 1
2.98971962855134e-175 -0.416019838921604 0.086 0.289
5.33037112574418e-171 1 SYNCRIP Naive 1
5.90986958493202e-175 -0.315386892729363 0.495 0.75
1.05367064829753e-170 1 ATP5MC2 Naive 1
4.8012879870096e-174 -0.367179412510571 0.283 0.55
8.56021635203942e-170 1 COX8A Naive 1
6.34436858814115e-174 -0.395690159581323 0.122 0.351
1.13113747557968e-169 1 HSBP1 Naive 1
7.90145362323332e-174 -0.306562931905062 0.381 0.653
1.40875016648627e-169 1 NDUFA1 Naive 1
1.06549553413208e-173 -0.441592235446494 0.111 0.328
1.89967198780408e-169 1 NOP58 Naive 1
1.74519555508908e-173 -0.330443914198077 0.281 0.55
3.11150915516832e-169 1 ATP5PB Naive 1
2.30321911363823e-173 -0.337859744733713 0.202 0.466
4.1064093577056e-169 1 NDUFB7 Naive 1
8.85121359641656e-173 -0.411335767500783 0.093 0.3
1.57808287210511e-168 1 NDUFAF8 Naive 1
6.13327734859679e-172 -0.403170824196042 0.153 0.389
1.09350201848132e-167 1 TKT Naive 1
1.55878917719561e-171 -0.35800660931937 0.22 0.482
2.77916522402206e-167 1 NDUFB9 Naive 1
4.19172990059116e-171 -0.428091640912635 0.135 0.363
7.47343523976399e-167 1 VOPP1 Naive 1

4.59479159977743e-171 -0.437906314723423 0.247 0.499
8.19205394324317e-167 1 CYCS Naive 1
5.72310072597822e-171 -0.384332851530898 0.034 0.186
1.02037162843466e-166 1 CCDC167 Naive 1
7.62947370441526e-170 -0.4975951850157 0.227 0.467
1.3602588667602e-165 1 COTL1 Naive 1
9.3160594675839e-170 -0.442708628319899 0.114 0.328
1.66096024247553e-165 1 ATP1B3 Naive 1
2.00268829496664e-169 -0.365514078787815 0.129 0.359
3.57059296109601e-165 1 PSMD2 Naive 1
4.4816474782219e-169 -0.620875855468043 0.107 0.3
7.99032928892183e-165 1 HSPA5 Naive 1
7.62670609464462e-169 -0.367719719794794 0.133 0.366
1.35976542961419e-164 1 HSD17B10 Naive 1
1.06405181082273e-168 -0.382171971477752 0.029 0.172
1.89709797351584e-164 1 PAICS Naive 1
1.92895476202827e-168 -0.550139344413344 0.159 0.38
3.4391334452202e-164 1 TUBB4B Naive 1
3.72439830002149e-168 -0.325479952006554 0.36 0.628
6.64022972910832e-164 1 TPM3 Naive 1
4.09712585394454e-168 -0.36249808370175 0.303 0.566
7.30476568499773e-164 1 SOD1 Naive 1
5.24563150840843e-168 -0.39201155802703 0.033 0.18
9.35243641634139e-164 1 PPP1R14B Naive 1
1.72374477869665e-167 -0.397642228367876 0.28 0.539
3.07326456593826e-163 1 MZT2B Naive 1
2.06057456039436e-167 -0.360638022304218 0.168 0.416
3.6737983837271e-163 1 SNRPC Naive 1
4.95433523140896e-166 -0.38254770781944 0.135 0.366
8.83308428407903e-162 1 BUD31 Naive 1
5.5040154695168e-166 -0.418302390267335 0.051 0.219
9.8131091806015e-162 1 EBNA1BP2 Naive 1
1.78447021510511e-165 -0.376541997639125 0.146 0.382
3.1815319465109e-161 1 PHB2 Naive 1
2.01284598916402e-165 -0.382169442989964 0.21 0.459
3.58870311408052e-161 1 DNAJA1 Naive 1
3.80301834752711e-165 -0.323118698552872 0.268 0.534
6.78040141180608e-161 1 RTRAF Naive 1
2.06884526256145e-164 -0.672773637650149 0.275 0.499
3.6885442186208e-160 1 CD83 Naive 1
3.94614401396576e-164 -0.423875450777966 0.16 0.39
7.03558016249956e-160 1 HSPA9 Naive 1
7.27524381236582e-164 -0.386040866499861 0.06 0.239
1.2971032193067e-159 1 PSMD1 Naive 1
8.32962688585696e-163 -0.379681835857176 0.336 0.587
1.48508917747944e-158 1 ATP5IF1 Naive 1
8.89325147544768e-163 -0.360640404900212 0.01 0.114
1.58557780555757e-158 1 MYBL2 Naive 1
3.10946027367912e-162 -0.370985461141846 0.114 0.331
5.5438567219425e-158 1 PSMD11 Naive 1

6.05583751164026e-162	-0.461623825375693	0.04	0.186
1.07969526995034e-157	1	SAMSN1	Naive 1
7.2874412581893e-162	-0.52768273071319	0.141	0.352
1.29927790192257e-157	1	SLC3A2	Naive 1
1.3860644865892e-161	-0.383373666493741	0.108	0.319
2.47121437313989e-157	1	SSRP1	Naive 1
3.19306554147319e-161	-0.399277137694426	0.118	0.333
5.69291655389255e-157	1	FERMT3	Naive 1
6.55838645326218e-161	-0.320751020155116	0.341	0.603
1.16929472075211e-156	1	RBM8A	Naive 1
2.06178520055355e-160	-0.3855327434828	0.084	0.282
3.67595683406693e-156	1	CDV3	Naive 1
3.32833203771496e-160	-0.33949043138731	0.173	0.418
5.934083190042e-156	1	MRPL20	Naive 1
4.87539107975431e-160	-0.403080004774764	0.073	0.256
8.69233475609396e-156	1	CDK4	Naive 1
4.98928693418408e-160	-0.430562791946683	0.06	0.231
8.8953996749568e-156	1	C19orf48	Naive 1
5.17514467735133e-160	-0.331986080311696	0.233	0.492
9.22676544524968e-156	1	GADD45GIP1	Naive 1
1.26635239381363e-159	-0.432234541468091	0.062	0.23
2.25777968293032e-155	1	CFLAR	Naive 1
1.71700300615739e-159	0.660635351635633	0.189	0.094
3.06124465967802e-155	1	C1orf162	Naive 1
3.28396932687091e-159	-0.349765709720519	0.161	0.396
5.85498891287815e-155	1	CACYBP	Naive 1
8.18337823060948e-159	-0.384451363655745	0.159	0.392
1.45901450473536e-154	1	EIF3J	Naive 1
8.55511286889998e-159	-0.334898221570421	0.382	0.642
1.52529107339618e-154	1	RSL24D1	Naive 1
9.57621973340456e-159	-0.354554788173271	0.064	0.245
1.7073442162687e-154	1	PSMB5	Naive 1
1.42348357718414e-158	-0.396037441595452	0.067	0.245
2.5379288697616e-154	1	HSPH1	Naive 1
2.27638882888847e-158	-0.320605588841421	0.25	0.509
4.05857364302525e-154	1	UBE2N	Naive 1
3.42852980270489e-158	-0.580684911194604	0.094	0.27
6.11272578524254e-154	1	TNFRSF13B	Naive 1
7.49871779498377e-158	-0.346200314176249	0.078	0.273
1.33694639566766e-153	1	MRPL13	Naive 1
9.05918065608561e-158	-0.472926935125882	0.101	0.292
1.6151613191735e-153	1	PIM1	Naive 1
1.70323316993633e-157	-0.376198769969805	0.044	0.202
3.03669441867948e-153	1	ZNF593	Naive 1
2.74431772552274e-157	-0.341077375409172	0.201	0.45
4.89284407283449e-153	1	TOMM22	Naive 1
4.0248907843689e-157	-0.344645737907337	0.186	0.43
7.17597777945132e-153	1	ENY2	Naive 1
9.36686379910417e-157	-0.324643340282024	0.01	0.116
1.67001814674228e-152	1	SEMA4A	Naive 1

1.31403945303576e-156 -0.359550322798191 0.064 0.244
2.34280094081745e-152 1 MRPL4 Naive 1
1.69921832003979e-156 -0.391267259860206 0.108 0.315
3.02953634279894e-152 1 DCTPP1 Naive 1
2.32227409355198e-156 0.72901108962067 0.324 0.233
4.14038248139382e-152 1 RASGRP2 Naive 1
3.48733242835344e-155 -0.48960371839802 0.082 0.265
6.21756498651135e-151 1 KPNA2 Naive 1
1.27797814042111e-154 -0.330927553669105 0.158 0.395
2.2785072265568e-150 1 POLR2E Naive 1
8.48870334439951e-154 -0.322044213679989 0.952 0.985
1.51345091927299e-149 1 RPL5 Naive 1
9.26603376081598e-154 -0.311529590895688 0.261 0.52
1.65204115921588e-149 1 EIF3M Naive 1
2.22566567345036e-153 -0.344276917740458 0.374 0.625
3.96813932919465e-149 1 SRSF2 Naive 1
2.72074242541537e-152 -0.311777669825005 0.186 0.432
4.85081167027306e-148 1 UQCRFS1 Naive 1
8.14144885256998e-152 -0.392497254628041 0.056 0.221
1.4515389159247e-147 1 TOMM40 Naive 1
9.36107758810699e-152 0.503766545393058 0.823 0.768
1.66898652318359e-147 1 HLA-DPB1 Naive 1
1.61014271235898e-151 -0.381463899491057 0.05 0.207
2.87072344186482e-147 1 YARS Naive 1
4.03050710183958e-151 -0.326607992817014 0.215 0.461
7.18599111186979e-147 1 ZNF706 Naive 1
4.15791149462986e-151 -0.334862659036405 0.17 0.408
7.41314040377558e-147 1 ANAPC11 Naive 1
4.68354870268031e-151 -0.333785736240474 0.7 0.866
8.35029898200873e-147 1 HNRNPA2B1 Naive 1
2.53494899441198e-150 -0.517652868499731 0.138 0.342
4.51956056213712e-146 1 DDX21 Naive 1
4.51137972825204e-150 -0.350518889523895 0.028 0.16
8.04333891750056e-146 1 MDFIC Naive 1
1.48449790391582e-149 -0.346968342604158 0.097 0.297
2.64671131289152e-145 1 OLA1 Naive 1
1.7944404731859e-149 -0.378526069575174 0.016 0.13
3.19930791964314e-145 1 GMDS Naive 1
2.69734121512989e-149 -0.348844460726486 0.117 0.328
4.80908965245508e-145 1 AHSA1 Naive 1
3.36072856521403e-149 -0.342562206345495 0.089 0.287
5.99184295892009e-145 1 THOC7 Naive 1
1.73043237007109e-148 -0.999598181036638 0.073 0.224
3.08518787259974e-144 1 LGALS1 Naive 1
1.7414816731621e-148 -0.320836738243374 0.161 0.395
3.10488767508072e-144 1 RPS19BP1 Naive 1
5.07819375919518e-148 -0.346239306762244 0.144 0.367
9.0539116532691e-144 1 STRAP Naive 1
5.62804886274552e-148 -0.423336736827362 0.041 0.18
1.0034248317389e-143 1 CD58 Naive 1

1.37064456793639e-147	-0.356411584165072	0.04	0.186
2.44372220017379e-143	1	TFDP1	Naive 1
3.6632489196697e-146	-0.337673709276303	0.207	0.449
6.53120649887912e-142	1	EIF6	Naive 1
3.74842677264396e-146	-0.342002183860945	0.154	0.379
6.68307009294691e-142	1	CHMP4B	Naive 1
1.2130061130556e-145	-0.329514612446169	0.089	0.286
2.16266859896683e-141	1	PSMC2	Naive 1
1.79888620059144e-145	-0.351532970386586	0.077	0.259
3.20723420703447e-141	1	STIP1	Naive 1
2.00780453572545e-145	-0.358214664743386	0.084	0.27
3.5797147067449e-141	1	HNRNPA1P48	Naive 1
2.07224477798187e-145	-0.360650382673148	0.071	0.247
3.69460521466387e-141	1	DCAF13	Naive 1
3.41999981292823e-145	-0.323619009398896	0.179	0.415
6.09751766646974e-141	1	NDUFB3	Naive 1
1.30579236661e-144	-0.374477186930859	0.157	0.372
2.32809721042898e-140	1	H2AFY	Naive 1
1.49845595600147e-144	-0.331609021528791	0.073	0.256
2.67159712395501e-140	1	MRPL27	Naive 1
1.86916558795986e-144	-0.33968099444292	0.077	0.262
3.33253532677364e-140	1	MRPL15	Naive 1
4.73016112935071e-144	0.751932173945682	0.377	0.316
8.43340427751939e-140	1	AES	Naive 1
4.88008814450063e-144	-0.378923040270553	0.039	0.181
8.70070915283017e-140	1	MRTO4	Naive 1
9.79362794745677e-144	-0.333642023850143	0.071	0.246
1.74610592675207e-139	1	DAZAP1	Naive 1
3.39402487251407e-143	-0.333341800834587	0.07	0.248
6.05120694520534e-139	1	TIMM17A	Naive 1
4.6024822101332e-143	0.589028249319485	0.117	0.046
8.20576553244648e-139	1	KLF3	Naive 1
6.07002247945175e-143	-0.328782703336721	0.185	0.42
1.08222430786145e-138	1	SSB	Naive 1
1.05051038229146e-142	-0.322735249031411	0.083	0.275
1.87295496058744e-138	1	MCTS1	Naive 1
1.74492379851817e-141	-0.305535549422326	0.221	0.464
3.11102464037805e-137	1	LSM3	Naive 1
2.13739981738932e-141	-0.307724256496724	0.203	0.44
3.81077013442342e-137	1	MDH1	Naive 1
4.25608541487304e-141	-0.370532885309052	0.016	0.125
7.58817468617714e-137	1	AC023590.1	Naive 1
9.47041787991788e-141	-0.33012235975844	0.096	0.292
1.68848080381056e-136	1	TXNDC17	Naive 1
1.03077646349826e-140	-0.318686327876139	0.167	0.393
1.83777135677105e-136	1	VPS29	Naive 1
4.82122801731387e-140	-0.325517294627516	0.101	0.299
8.5957674320689e-136	1	MRPL47	Naive 1
6.8797564237549e-140	-0.315848785938579	0.148	0.371
1.22659177279126e-135	1	PSMC4	Naive 1

8.59232623026491e-140 -0.361442059971962 0.189 0.416
1.53192584359393e-135 1 SHMT2 Naive 1
1.05370449401387e-139 -0.306022330504827 0.181 0.418
1.87864974237732e-135 1 NOL7 Naive 1
1.09847637066519e-139 0.7288855456925 0.353 0.256
1.95847352125897e-135 1 JUN Naive 1
1.27245105274745e-139 -0.335324537488511 0.212 0.443
2.26865298194342e-135 1 HDAC1 Naive 1
2.4960949650916e-139 -0.580632894668229 0.013 0.106
4.45028771326182e-135 1 PCLAF Naive 1
6.45058375853871e-139 -0.303127428620474 0.012 0.112
1.15007457830987e-134 1 FAM241A Naive 1
7.97631355422749e-139 -0.587505163666506 0.985 0.99
1.42209694358322e-134 1 ACTB Naive 1
9.45009806738605e-139 -0.319401564375225 0.131 0.344
1.68485798443426e-134 1 GTF3C6 Naive 1
1.39497183473054e-138 -0.535358730084684 0.026 0.139
2.48709528414108e-134 1 LGALS3 Naive 1
2.44627929551081e-138 -0.316939283841549 0.105 0.308
4.36147135596623e-134 1 MRPS7 Naive 1
6.89081278252753e-138 -0.319445659475876 0.059 0.223
1.22856301099683e-133 1 MRPL23 Naive 1
1.10251501871329e-137 -0.335691776703589 0.117 0.319
1.96567402686393e-133 1 ILF3 Naive 1
2.10545344980233e-137 -0.33129335527327 0.144 0.359
3.75381295565257e-133 1 EIF3A Naive 1
6.69979148460443e-137 -0.317068559076362 0.097 0.291
1.19450582379012e-132 1 PSMD3 Naive 1
8.08211142611608e-137 0.668788613085526 0.258 0.181
1.44095964616224e-132 1 S1PR4 Naive 1
5.90812754377101e-135 -0.379989942188123 0.044 0.181
1.05336005977893e-130 1 NFKB1 Naive 1
2.72938040096263e-134 -0.380228665746119 0.102 0.285
4.86621231687628e-130 1 ANP32E Naive 1
6.85845337062181e-134 -0.303655399431946 0.136 0.351
1.22279365144816e-129 1 NDUFB6 Naive 1
7.53125439211165e-134 0.678760139957042 0.415 0.338
1.34274734556959e-129 1 TRAF3IP3 Naive 1
1.0518305445399e-133 -0.437788484236361 0.061 0.211
1.87530867786018e-129 1 DNMT1 Naive 1
1.61859666360076e-133 -0.375899563964323 0.082 0.252
2.88579599153379e-129 1 TARS Naive 1
3.08840000687959e-133 -0.331717236540871 0.075 0.249
5.50630837226562e-129 1 FUBP1 Naive 1
3.40682947724331e-133 -0.31447345145889 0.098 0.289
6.0740362749771e-129 1 MTCH2 Naive 1
3.41436109097378e-133 -0.321342756589481 0.163 0.383
6.08746438909715e-129 1 SARS Naive 1
4.04494736547476e-133 -0.470393371788116 0.029 0.149
7.21173665790496e-129 1 NEIL1 Naive 1

9.59329047648431e-133	-0.321229659992462		0.042	0.183
1.71038775905239e-128	1	ETF1	Naive 1	
3.8982959940362e-132	-0.480729497223344		0.159	0.36
6.95027192776714e-128	1	DUT	Naive 1	
2.32516455181025e-131	-0.304266299283476		0.132	0.341
4.14553587942249e-127	1	NDUFAF3	Naive 1	
2.75350295705588e-131	-0.341758871886781		0.12	0.321
4.90922042213493e-127	1	PDCD5	Naive 1	
8.5345196307397e-131	-0.35297461454106		0.139	0.344
1.52161950496458e-126	1	PRDX2	Naive 1	
1.09192144544251e-130	-0.317497954839706		0.045	0.189
1.94678674507945e-126	1	RARS	Naive 1	
1.16319441588986e-130	-0.315862445297323		0.174	0.392
2.07385932409003e-126	1	RBBP7	Naive 1	
1.27491093870114e-130	-0.333065583874871		0.139	0.347
2.27303871261026e-126	1	NAA38	Naive 1	
1.3899213426657e-130	-0.4879836730266	0.021	0.121	
2.47809076183868e-126	1	VPREB3	Naive 1	
1.62979363622497e-130	-0.484300029964902		0.07	0.22
2.9057590740255e-126	1	HMCES	Naive 1	
3.05117026929257e-130	-0.337281131127872		0.051	0.202
5.43993147312171e-126	1	BID	Naive 1	
4.50617474759938e-130	-0.335241999861046		0.038	0.173
8.03405895749493e-126	1	GAR1	Naive 1	
2.51506091850412e-129	-0.382255014494846		0.109	0.297
4.484102111601e-125	1	POU2AF1	Naive 1	
3.61865175961579e-129	-0.36150808510729		0.116	0.301
6.451694222219e-125	1	WARS	Naive 1	
5.06880097910505e-129	-0.336350619114244		0.062	0.215
9.0371652656464e-125	1	ATIC	Naive 1	
2.71293833255855e-128	0.611314751006094		0.57	0.504
4.83689775311864e-124	1	BANK1	Naive 1	
5.10738397331037e-128	-0.302118919229471		0.185	0.411
9.10595488601506e-124	1	C19orf70	Naive 1	
8.60350619060239e-128	-0.348173165049212		0.036	0.161
1.5339191187225e-123	1	PRDX4	Naive 1	
9.11125356177922e-128	-0.357061139105706		0.038	0.167
1.62444539752962e-123	1	PPAN	Naive 1	
2.61041808250843e-127	-0.540388707742722		0.098	0.261
4.65411439930428e-123	1	RGS1	Naive 1	
2.85483099433867e-127	-0.391069118083798		0.115	0.304
5.08987817980641e-123	1	JPT1	Naive 1	
4.26089557841687e-127	-0.327594189890042		0.038	0.168
7.59675072675943e-123	1	PEA15	Naive 1	
8.07761696652225e-127	-0.317098686667353		0.082	0.257
1.44015832896125e-122	1	EIF2S1	Naive 1	
8.46553081987218e-127	-0.355652968671758		0.136	0.334
1.50931948987501e-122	1	CALM3	Naive 1	
2.63843685811982e-125	-0.342935939864736		0.087	0.259
4.70406907434183e-121	1	HNRNPAB	Naive 1	

3.2671604332742e-125	-1.24277044581502	0.064	0.191	5.82502033648458e-121	1	MZB1	Naive 1
4.60442313237661e-125	-0.459504701628229	0.182	0.384	8.20922600271426e-121	1	H2AFV	Naive 1
8.86201346221391e-125	-2.98804828335161	0.289	0.473	1.58000838017812e-120	1	IGHG3	Naive 1
3.99321760868095e-124	-1.40543155010548	0.088	0.234	7.11950767451727e-120	1	IGHG1	Naive 1
5.56876923875506e-124	-0.347708085473588	0.076	0.245	9.9285586757764e-120	1	SIAH2	Naive 1
6.94836994952109e-123	-0.404110937215415	0.045	0.177	1.23882487830011e-118	1	SUSD3	Naive 1
8.27014078813195e-122	-0.586587315724537	0.153	0.334	1.47448340111604e-117	1	HSP90B1	Naive 1
1.20169082150106e-121	-0.330557175361678	0.069	0.227	2.14249456565424e-117	1	GNL3	Naive 1
1.8170881661309e-121	-0.373870963963745	0.079	0.236	3.23968649139478e-117	1	NFKBIE	Naive 1
6.15144520331458e-121	-0.31522823651313	0.165	0.377	1.09674116529896e-116	1	MRPS6	Naive 1
7.89590812860781e-121	-0.341651146201268	0.067	0.223	1.40776146024949e-116	1	UBALD2	Naive 1
1.24433293830805e-120	-0.349176415664184	0.035	0.157	2.21852119570942e-116	1	NOP16	Naive 1
1.37168092744356e-120	-0.351776750402108	0.058	0.203	2.44556992553913e-116	1	WDR43	Naive 1
2.02578617642775e-120	-0.363516081643228	0.22	0.434	3.61177417395303e-116	1	PPIB	Naive 1
1.03873944293742e-119	-0.302005823670753	0.072	0.233	1.85196855281312e-115	1	EPRS	Naive 1
1.34835573241938e-119	-0.314372324281288	0.098	0.278	2.40398343533051e-115	1	GSPT1	Naive 1
2.24165043049589e-118	-0.404296684589033	0.155	0.353	3.99663855253112e-114	1	HNRNPH1	Naive 1
3.2507161514935e-118	-0.357387932814039	0.116	0.3	5.79570182649776e-114	1	GADD45B	Naive 1
1.59925943846704e-117	-0.307817803333071	0.076	0.238	2.85131965284289e-113	1	WDR46	Naive 1
1.64451853599266e-117	-0.321046703612397	0.168	0.378	2.93201209782131e-113	1	ANXA5	Naive 1
7.13627254221766e-117	-0.316097449374654	0.086	0.257	1.27232603155199e-112	1	NIFK	Naive 1
4.96074688585232e-116	-1.30406142031916	0.247	0.447	8.8445156227861e-112	1	HIST1H4C	Naive 1
8.0343538677064e-115	0.410759325420039	0.763	0.689	1.43244495107337e-110	1	EMP3	Naive 1
1.19220351020745e-114	-0.3128072580775	0.21	0.423	2.12557963834886e-110	1	NUCKS1	Naive 1
1.61619969074463e-114	-1.18537524485324	0.047	0.162	2.8815224286286e-110	1	IGHA1	Naive 1

6.33298828372277e-114	0.546483346106926		0.151	0.079
1.12910848110493e-109	1	PIK3IP1	Naive	1
1.61871883063873e-113	-0.470825051871364		0.061	0.193
2.8860138031458e-109	1	BCL2A1	Naive	1
2.27421369237548e-112	-0.326380128421268		0.094	0.262
4.05469559213624e-108	1	SSR3	Naive	1
1.95699845392969e-111	-0.310232527038082		0.065	0.214
3.48913254351124e-107	1	GTPBP4	Naive	1
2.87480218238925e-110	0.622204943380427		0.254	0.188
5.1254848109818e-106	1	CHPT1	Naive	1
6.12302417457924e-108	-0.306023466365361		0.086	0.253
1.09167398008573e-103	1	CHCHD10	Naive	1
1.16229118150707e-107	-0.493777187717281		0.019	0.106
2.07224894750896e-103	1	KLK1	Naive	1
1.52796252700482e-107	0.671771393515918		0.388	0.341
2.72420438939689e-103	1	BIRC3	Naive	1
3.5528685852065e-107	-0.371172671248975		0.027	0.131
6.33440940056466e-103	1	UBE2S	Naive	1
3.93052834935838e-107	0.635455629293122		0.399	0.359
7.00773899407105e-103	1	RCSD1	Naive	1
8.8829180962781e-107	0.596984013965075		0.203	0.137
1.58373546738542e-102	1	SUN2	Naive	1
6.17936045362385e-104	-0.385761240906433		0.099	0.256
1.1017181752766e-99	1	GCHFR	Naive	1
2.83064940168261e-103	-0.305396700702903		0.094	0.255
5.04676481825993e-99	1	EIF4A1	Naive	1
5.19642441593859e-103	-0.308366651184569		0.101	0.267
9.26470509117691e-99	1	DKC1	Naive	1
1.76635536941347e-100	-0.322011557389177		0.101	0.26
3.14923498812728e-96	1	MYDGF	Naive	1
3.55315874208453e-100	-0.310175415284821		0.06	0.197
6.33492672126251e-96	1	LCK	Naive	1
9.14283271547444e-100	-0.353825351617176		0.044	0.16
1.63007564484194e-95	1	SDF2L1	Naive	1
3.62060452626765e-99	0.419069171567057		0.6	0.525
6.4551758098826e-95	1	ISG20	Naive	1
1.6543117743062e-97	0.624292399975939		0.198	0.136
2.94947246241053e-93	1	DUSP1	Naive	1
3.37293144966823e-96	-0.367580007299911		0.09	0.236
6.01359948161348e-92	1	EIF4EBP1	Naive	1
2.26716488599611e-94	0.5863274356685	0.574	0.57	
4.04212827524247e-90	1	SNX2	Naive	1
1.77057004115724e-93	-0.384297273065555		0.058	0.174
3.15674932637924e-89	1	DDIT4	Naive	1
5.71882461432052e-93	-0.318853647357098		0.464	0.659
1.01960924048721e-88	1	HERPUD1	Naive	1
1.05988855293249e-91	0.603252189316503		0.504	0.492
1.88967530102333e-87	1	SP100	Naive	1
1.60552663716369e-91	-0.345362548886131		0.061	0.184
2.86249344139914e-87	1	SMC4	Naive	1

5.42226831501177e-91 0.547957612031868 0.206 0.151
9.66736217883448e-87 1 GLIPR1 Naive 1
1.65988354587028e-90 0.555303684950134 0.233 0.166
2.95940637393212e-86 1 MARCH1 Naive 1
2.51725188578286e-90 0.520255246441165 0.158 0.106
4.48800838716227e-86 1 PBXIP1 Naive 1
8.15592571021321e-89 0.584385759115916 0.315 0.26
1.45411999487391e-84 1 HHEX Naive 1
5.29802796408522e-88 0.633133968207989 0.484 0.452
9.44585405716754e-84 1 JUNB Naive 1
3.62600142271328e-87 -1.31336175390098 0.188 0.318
6.46479793655551e-83 1 HMGB2 Naive 1
1.50359034006891e-84 -0.572758059083832 0.437 0.589
2.68075121730887e-80 1 VIM Naive 1
2.91713607967177e-83 0.525779057959478 0.196 0.141
5.2009619164468e-79 1 ARRDC2 Naive 1
3.55412169070119e-83 0.578100738339032 0.241 0.203
6.33664356235114e-79 1 CCDC32 Naive 1
5.53018748411751e-83 0.643960352305003 0.264 0.228
9.8597712654331e-79 1 IL4R Naive 1
7.95041658271004e-83 -0.385665650034842 0.022 0.103
1.41747977253137e-78 1 CCNB1 Naive 1
1.22470353023914e-82 0.613768141764445 0.389 0.356
2.18352392406335e-78 1 ARID5B Naive 1
1.87271038438389e-82 -0.323905391867029 0.064 0.18
3.33885534431803e-78 1 CBX6 Naive 1
2.56836678685309e-82 0.595375858530205 0.318 0.281
4.57914114428037e-78 1 PPM1K Naive 1
4.97057094466838e-81 -0.412653963004497 0.325 0.523
8.86203093724925e-77 1 SSR4 Naive 1
1.90841795961744e-80 0.514499216242307 0.159 0.102
3.40251838020194e-76 1 LINC02397 Naive 1
3.92480466308483e-79 0.573955060049476 0.237 0.198
6.99753423381394e-75 1 IRF1 Naive 1
5.23841314719228e-79 0.515034132970507 0.515 0.485
9.33956680012912e-75 1 UCP2 Naive 1
7.33130528690201e-78 0.477503704828391 0.149 0.094
1.30709841960176e-73 1 RHOC Naive 1
2.97791669843937e-74 -0.300515851066726 0.072 0.19
5.30932768164756e-70 1 PDIA4 Naive 1
1.21753867379385e-73 0.555023659170967 0.321 0.272
2.17074970150705e-69 1 ADAM28 Naive 1
2.58737166135722e-73 -0.319711390928767 0.068 0.177
4.61302493503378e-69 1 MARCKS Naive 1
1.1836435837257e-72 -0.364686287890151 0.082 0.2
2.11031814542454e-68 1 RGS2 Naive 1
4.87287554118638e-71 0.555671549419576 0.248 0.206
8.68784980238119e-67 1 FAM111B Naive 1
3.71790174895993e-69 0.513829779532563 0.385 0.346
6.62864702822065e-65 1 LY6E Naive 1

2.4957384078449e-68	0.52213902552277 0.177	0.15
4.44965200734668e-64	1	TSPAN13	Naive 1
3.1599813036111e-67	0.418358221090099	0.872	0.903
5.63393066620824e-63	1	DDX5	Naive 1
3.23877100597645e-66	-2.14873407380297	0.063	0.135
5.77440482655541e-62	1	IGHG2	Naive 1
9.06023118309394e-65	0.36623587377172 0.882	0.922
1.61534861763382e-60	1	PFDN5	Naive 1
1.03419441257998e-64	0.548959318168675	0.402	0.397
1.84386521818884e-60	1	ORAI2	Naive 1
2.14455730923058e-63	0.516888749772786	0.611	0.617
3.82353122662719e-59	1	MEF2C	Naive 1
3.0005560310863e-62	0.438012327612094	0.132	0.097
5.34969134782377e-58	1	CDKN2D	Naive 1
4.90922420728197e-62	0.433267278639628	0.13	0.091
8.75265583916302e-58	1	TMBIM1	Naive 1
6.92934716650753e-61	0.495324031728106	0.228	0.19
1.23543330631663e-56	1	FAM129C	Naive 1
1.72266670746784e-58	-0.487904072757828	0.059	0.141
3.0713424727444e-54	1	MYC	Naive 1
1.73295763853983e-57	0.363763001430767	0.775	0.776
3.08969017375266e-53	1	ARHGDIB	Naive 1
1.8144826411379e-57	0.494623840158114	0.233	0.212
3.23504110088477e-53	1	YPEL3	Naive 1
1.02427667563836e-55	0.484433841695771	0.418	0.413
1.82618288499562e-51	1	NPC2	Naive 1
3.20216459339239e-55	0.423493306988753	0.77	0.822
5.70913925355928e-51	1	NOP53	Naive 1
3.33716218520585e-55	0.394207807386142	0.104	0.073
5.94982646000351e-51	1	ACSS1	Naive 1
9.20632643392997e-54	0.468572921957417	0.209	0.182
1.64139593990537e-49	1	ANKRD44	Naive 1
3.12446456487657e-52	0.455973423353112	0.228	0.191
5.57060787271844e-48	1	CLEC2B	Naive 1
8.75478104109616e-52	-1.31867268953714	0.316	0.415
1.56088991181703e-47	1	JCHAIN	Naive 1
1.51016564765212e-50	0.504217890473385	0.278	0.273
2.69247433319896e-46	1	RSRP1	Naive 1
1.27956691292742e-49	0.503011369784101	0.38	0.384
2.28133984905829e-45	1	EVL	Naive 1
4.90121124365193e-49	0.50789509067089 0.224	0.194
8.73836952630702e-45	1	YBX3	Naive 1
1.4601249539601e-48	0.403242199315609	0.118	0.095
2.60325678041546e-44	1	GUCD1	Naive 1
2.25551284605624e-48	0.444189134576133	0.646	0.681
4.02135385323368e-44	1	TAGLN2	Naive 1
1.32699463576133e-47	0.492760649981137	0.292	0.277
2.36589873609888e-43	1	RNASET2	Naive 1
1.39639215632872e-47	0.467720187080258	0.246	0.23
2.48962757551848e-43	1	HSD17B11 Naive 1

5.06455972506146e-47 0.491882447041372 0.151 0.119
9.02960353381207e-43 1 AL139020.1 Naive 1
1.53593540291213e-46 0.367933056451496 0.124 0.085
2.73841922985204e-42 1 CCR6 Naive 1
2.58631768120578e-41 -1.70480777821326 0.156 0.255
4.61114579382178e-37 1 IGHG4 Naive 1
3.57473591979388e-41 0.462157052493459 0.208 0.197
6.37339667140051e-37 1 CLK1 Naive 1
6.85094842109634e-41 0.464126313373216 0.386 0.389
1.22145559399727e-36 1 EVI2B Naive 1
7.22540114561774e-41 0.491917684332777 0.274 0.281
1.28821677025219e-36 1 SMCHD1 Naive 1
9.17669629438024e-41 0.360664044030593 0.116 0.088
1.63611318232505e-36 1 ANXA4 Naive 1
8.95252809545225e-40 0.377521394479161 0.165 0.133
1.59614623413818e-35 1 FCER2 Naive 1
1.24983267566729e-39 0.441173535439501 0.195 0.185
2.22832667744722e-35 1 CDCA7L Naive 1
1.50748531482247e-38 0.374871080513737 0.138 0.109
2.68769556779699e-34 1 P2RY10 Naive 1
2.2597906540108e-38 0.420146006496083 0.178 0.166
4.02898075703586e-34 1 BNIP3L Naive 1
5.75658389551013e-38 0.360796515337452 0.134 0.112
1.0263413427305e-33 1 CLN8 Naive 1
1.23745348832966e-37 0.348168106491253 0.112 0.093
2.20625582434296e-33 1 ABTB1 Naive 1
2.60432280608512e-37 -0.678247752951414 0.114 0.225
4.64324713096917e-33 1 PLCG2 Naive 1
9.72828128222944e-37 0.336699374066957 0.101 0.082
1.73445526980869e-32 1 KLHL24 Naive 1
2.10414664299867e-36 0.381431828138291 0.136 0.114
3.75148304980233e-32 1 SAMD9 Naive 1
4.72149212334189e-36 0.424129701016041 0.165 0.158
8.41794830670625e-32 1 APLP2 Naive 1
7.9775046367111e-35 0.436822339460063 0.283 0.286
1.42230930167922e-30 1 SP110 Naive 1
5.09124001505653e-34 0.458184322416846 0.442 0.48
9.07717182284429e-30 1 N4BP2L2 Naive 1
5.26050799681519e-34 0.36570232463441 0.127 0.102
9.3789597075218e-30 1 MTSS1 Naive 1
4.98964841872689e-33 0.467767375747936 0.383 0.406
8.89604416574817e-29 1 STX7 Naive 1
6.86123609718285e-33 0.434222920845326 0.214 0.212
1.22328978376673e-28 1 WASHC4 Naive 1
3.41748860855328e-32 0.474628682290618 0.453 0.489
6.09304044018964e-28 1 NEAT1 Naive 1
5.35246697904884e-32 0.373930805004213 0.162 0.151
9.54291337694618e-28 1 SRPK2 Naive 1
5.6109576413669e-32 0.376026716940895 0.192 0.182
1.00037763787931e-27 1 MBP Naive 1

4.36905766941395e-31 0.330091515541145 0.106 0.09
7.78959291879812e-27 1 RALB Naive 1
1.59908718252648e-30 0.366897968183151 0.164 0.147
2.85101253772647e-26 1 CELF2 Naive 1
4.59317669274273e-30 0.408285266744913 0.289 0.298
8.18917472549102e-26 1 PDLIM1 Naive 1
1.23761207941653e-29 0.388100823749914 0.285 0.283
2.20653857639173e-25 1 GNG7 Naive 1
4.75560908853439e-29 0.424716799106395 0.458 0.497
8.47877544394797e-25 1 ANKRD12 Naive 1
6.28235339102014e-29 0.358889819537004 0.602 0.629
1.12008078608498e-24 1 HLA-DMB Naive 1
8.04002110924303e-29 0.39549328275825 0.149 0.145
1.43345536356694e-24 1 TAGAP Naive 1
1.95988800403162e-28 0.329436659453187 0.126 0.116
3.49428432238798e-24 1 HPCAL1 Naive 1
2.35452823159166e-28 0.336327016360852 0.133 0.128
4.19788838410477e-24 1 ICAM2 Naive 1
5.02103398012711e-28 0.343106457798126 0.132 0.121
8.95200148316862e-24 1 RNF213 Naive 1
5.33305092553502e-27 0.367941767080956 0.166 0.165
9.50829649513638e-23 1 RNF13 Naive 1
9.62200156223618e-27 0.316671131436709 0.115 0.095
1.71550665853109e-22 1 BTLA Naive 1
3.08454681629228e-26 0.414876407927626 0.268 0.287
5.49943851876751e-22 1 YPEL5 Naive 1
4.30766974591977e-26 0.411176092868865 0.188 0.183
7.68014439000036e-22 1 PLPP5 Naive 1
6.38748611254232e-26 0.314809564936152 0.117 0.103
1.13882489900517e-21 1 PDE4B Naive 1
7.07912964373066e-26 0.418761216856234 0.457 0.496
1.26213802418074e-21 1 CYTIP Naive 1
2.87386714424915e-25 0.430058283918197 0.301 0.321
5.12381773148181e-21 1 SNHG7 Naive 1
5.73090497097586e-25 0.36523641946386 0.168 0.174
1.02176304727529e-20 1 BLCAP Naive 1
1.23551878204468e-24 0.332094432441813 0.14 0.132
2.20280643650747e-20 1 ODF2L Naive 1
4.28452360657715e-24 0.360532180064319 0.172 0.168
7.6388771381664e-20 1 TMEM154 Naive 1
4.58045656872265e-24 0.421334111079179 0.363 0.403
8.16649601637561e-20 1 BLOC1S2 Naive 1
7.95303393978238e-24 0.374470756391555 0.198 0.203
1.4179464211238e-19 1 TRIM38 Naive 1
1.7213581415355e-23 0.333706268937725 0.124 0.116
3.06900943054364e-19 1 CARD16 Naive 1
3.07434867389317e-23 0.365792383412025 0.404 0.43
5.48125625068413e-19 1 PLP2 Naive 1
6.67907636549855e-23 0.411279370881261 0.296 0.323
1.19081252520474e-18 1 LTA4H Naive 1

7.15843147659597e-23 0.411812886767737 0.555 0.624
1.2762767479623e-18 1 PNISR Naive 1
1.58628985923173e-22 0.339307097873711 0.166 0.156
2.82819619002425e-18 1 SLC2A3 Naive 1
2.56491829529714e-22 0.336285580219772 0.144 0.142
4.57299282868527e-18 1 LY9 Naive 1
5.59163267738889e-22 0.374770490402403 0.281 0.295
9.96932190051666e-18 1 BCL11A Naive 1
1.5316360378674e-21 -0.566833685143389 0.203 0.272
2.73075389191379e-17 1 S100A4 Naive 1
6.19528136258675e-21 0.334561938503144 0.138 0.14
1.10455671413559e-16 1 ADD3 Naive 1
1.88537007643745e-20 0.400559935493674 0.352 0.391
3.36142630928034e-16 1 PSAP Naive 1
2.1381977793956e-20 0.355773571088391 0.219 0.23
3.81219282088442e-16 1 NT5C3A Naive 1
3.50154244403182e-20 0.372298501239616 0.288 0.305
6.24290002346432e-16 1 BCL7A Naive 1
1.42318544427569e-19 0.3488744092344 0.194 0.207
2.53739732859912e-15 1 RALGPS2 Naive 1
1.56025707574144e-19 0.338385784050345 0.154 0.16
2.78178234033942e-15 1 AIDA Naive 1
1.65910554087585e-19 0.36728024867792 0.233 0.257
2.95801926882755e-15 1 TBC1D10C Naive 1
4.10231104283668e-19 0.315198008155307 0.161 0.164
7.31401035827351e-15 1 TNFRSF14 Naive 1
4.85479131124705e-19 0.325072682847798 0.156 0.163
8.65560742882236e-15 1 FAM215B Naive 1
6.2490756931196e-19 0.347163836373956 0.577 0.624
1.11414770532629e-14 1 CLEC2D Naive 1
7.59370549864249e-19 0.301151532522673 0.126 0.13
1.35388175335297e-14 1 SIPA1 Naive 1
8.3372803148814e-19 0.377929795736983 0.296 0.324
1.4864537073402e-14 1 TNFRSF13C Naive 1
1.19993668108479e-18 0.315436031158055 0.13 0.129
2.13936710870607e-14 1 CLK4 Naive 1
2.19635027030152e-18 0.38237995961181 0.566 0.627
3.91587289692058e-14 1 IFI16 Naive 1
1.26829521549409e-17 0.301925750870236 0.125 0.125
2.26124353970441e-13 1 AC025164.1 Naive 1
1.47957361652956e-17 0.393139202371467 0.455 0.524
2.63793180091056e-13 1 VAMP2 Naive 1
7.57872672388586e-17 0.401365800830859 0.403 0.462
1.35121118760161e-12 1 CTSS Naive 1
2.20533518851435e-16 0.31896434650793 0.19 0.204
3.93189210760224e-12 1 BIN1 Naive 1
5.23001522884323e-16 0.310173971727903 0.141 0.153
9.32459415150459e-12 1 CHMP1B Naive 1
6.25575844868523e-16 0.351652280871792 0.266 0.292
1.11533917381609e-11 1 RUBCNL Naive 1

1.10619748779586e-15	0.30571011756988 0.134	0.142
1.97223950099123e-11	1	ADD1	Naive 1
1.82406072068086e-15	0.334710143428501	0.163	0.181
3.2521178589019e-11	1	HEXA	Naive 1
2.23035160701524e-13	0.33812805449818 0.349	0.395
3.97649388014746e-09	1	ARL6IP5	Naive 1
5.65209001998892e-13	0.348866746818169	0.647	0.733
1.00771112966382e-08	1	SRSF5	Naive 1
2.8435084234513e-12	0.314014355059635	0.168	0.19
5.06969116817132e-08	1	HLA-DOB	Naive 1
7.38963988026414e-12	0.337424102888242	0.232	0.268
1.31749889425229e-07	1	TTC3	Naive 1
6.36449055652854e-11	0.333160655555796	0.227	0.265
1.13472502132347e-06	1	MAT2B	Naive 1
7.31255810416117e-11	0.344530649003238	0.283	0.335
1.3037559843909e-06	1	CCNDBP1	Naive 1
1.61609412916884e-10	-0.67657554536946	0.903	0.93
2.88133422289513e-06	1	IGKC	Naive 1
5.12127369107352e-10	0.30279809655579 0.229	0.26
9.13071886381498e-06	1	LYSMD2	Naive 1
9.4585491060971e-10	0.326960746936434	0.247	0.291
1.68636472012605e-05	1	PLEKHF2	Naive 1
1.05041575636553e-09	0.300387307688269	0.25	0.29
1.87278625202411e-05	1	ACAP1	Naive 1
1.44173898528347e-09	0.325093354950701	0.32	0.368
2.5704764368619e-05	1	ANXA6	Naive 1
3.37473738534413e-09	0.307014661389604	0.219	0.258
6.01681928433005e-05	1	PRDM2	Naive 1
1.17548316723843e-08	0.321706132555183	0.21	0.249
0.000209576893886939	1	GCC2	Naive 1
3.01854413704197e-08	0.320649805442686	0.249	0.305
0.000538176234193213	1	MPHOSPH8	Naive 1
4.10481508239294e-08	0.320727171583904	0.327	0.391
0.000731847481039837	1	LAPTM4A	Naive 1
7.17735868656787e-08	0.324990031802727	0.283	0.341
0.00127965128022819	1	BTK	Naive 1
1.18867611445953e-07	0.305908407093395	0.409	0.48
0.00211929064446989	1	GPSM3	Naive 1
1.98363523300766e-07	0.317597839591154	0.442	0.521
0.00353662325692935	1	C6orf48	Naive 1
2.07956584959837e-07	-1.29203942140165	0.579	0.654
0.00370765795324893	1	IGLC2	Naive 1
6.4322275577815e-06	-0.364110476843682	0.381	0.435
0.114680185127686	1	STMN1	Naive 1
5.25736742276033e-05	0.307527540232204	0.349	0.43
0.937336037803939	1	KMT2E	Naive 1
0	1.75929818018284 0.813	0.359	0	2	FABP5
Activated NME1
0	1.65254654934553 0.775	0.247	0	2	NME1
Activated NME1

0	1.59306350013214	0.747	0.236	0	2	MIR155HG	Activated NME1
0	1.58868802055569	0.982	0.686	0	2	ENO1	Activated NME1
0	1.51102989742955	0.998	0.832	0	2	HSP90AB1	Activated NME1
0	1.36452783443915	0.811	0.364	0	2	TXN	Activated NME1
0	1.33455895208333	0.552	0.13	0	2	TNFRSF18	Activated NME1
0	1.32749862557487	0.481	0.098	0	2	PSAT1	Activated NME1
0	1.26949978561741	0.823	0.421	0	2	HSPD1	Activated NME1
0	1.2650070341703	0.861	0.458	0	2	HSPE1	Activated NME1
0	1.2531057286248	0.673	0.237	0	2	SRM	Activated NME1
0	1.24162293005019	0.897	0.564	0	2	LDHA	Activated NME1
0	1.23604394435331	0.912	0.487	0	2	PSME2	Activated NME1
0	1.23507671092199	0.79	0.34	0	2	NHP2	Activated NME1
0	1.22381688934689	0.868	0.443	0	2	RANBP1	Activated NME1
0	1.19611496911836	0.993	0.922	0	2	NPM1	Activated NME1
0	1.18606931282761	0.852	0.475	0	2	PA2G4	Activated NME1
0	1.18442222971449	0.549	0.181	0	2	EIF4EBP1	Activated NME1
0	1.17869919163989	0.998	0.936	0	2	YBX1	Activated NME1
0	1.17566721691676	0.857	0.526	0	2	NCL	Activated NME1
0	1.17240846611717	0.901	0.566	0	2	PRDX1	Activated NME1
0	1.12792459214079	0.285	0.041	0	2	NPW	Activated NME1
0	1.12556009726922	0.466	0.076	0	2	PYCR1	Activated NME1
0	1.11956543833073	0.957	0.711	0	2	HSPA8	Activated NME1
0	1.10159320194221	0.961	0.725	0	2	RAN	Activated NME1
0	1.03668511077577	0.752	0.353	0	2	PHB	Activated NME1
0	1.02368721872715	0.658	0.28	0	2	DDX21	Activated NME1

0 0.995019439842536 0.605 0.27 0 2
ODC1 Activated NME1
0 0.985813368346043 0.524 0.162 0 2
EBNA1BP2 Activated NME1
0 0.982967285305793 0.538 0.164 0 2
NPM3 Activated NME1
0 0.976649819219753 0.683 0.312 0 2
TIMM13 Activated NME1
0 0.975345441560952 0.708 0.324 0 2
HSPA9 Activated NME1
0 0.965503713751972 0.531 0.201 0 2
MTHFD2 Activated NME1
0 0.950963626782393 0.65 0.291 0 2
SLC3A2 Activated NME1
0 0.949030154110251 0.711 0.367 0 2
RPL22L1 Activated NME1
0 0.948836003114843 0.875 0.538 0 2
SNRPE Activated NME1
0 0.942186344683227 0.745 0.409 0 2
PPA1 Activated NME1
0 0.931964596009407 0.768 0.407 0 2
CCT6A Activated NME1
0 0.929764699116137 0.768 0.409 0 2
C1QBP Activated NME1
0 0.916100394154654 0.789 0.428 0 2
PRMT1 Activated NME1
0 0.91118629770386 0.554 0.217 0 2 GARS
Activated NME1
0 0.908347729040697 0.938 0.651 0 2
SRSF3 Activated NME1
0 0.901209983687697 0.82 0.481 0 2
SSBP1 Activated NME1
0 0.898788901580502 0.608 0.269 0 2
ATP1B3 Activated NME1
0 0.893798528959079 0.967 0.738 0 2 PKM
Activated NME1
0 0.889940330010455 0.767 0.414 0 2
ILF2 Activated NME1
0 0.883040505004033 0.766 0.435 0 2
CYCS Activated NME1
0 0.872914528299025 0.513 0.185 0 2
MRPL12 Activated NME1
0 0.87171891879613 0.733 0.395 0 2 CCT2
Activated NME1
0 0.870641738039086 0.493 0.15 0 2
FKBP4 Activated NME1
0 0.865403504393098 0.703 0.355 0 2
CCT5 Activated NME1
0 0.857350045498905 0.473 0.15 0 2
CMSS1 Activated NME1

0 0.852591696397914 0.483 0.155 0 2
GPATCH4 Activated NME1
0 0.845092365693125 0.526 0.196 0 2
MRPL3 Activated NME1
0 0.841500621426634 0.953 0.725 0 2
HSP90AA1 Activated NME1
0 0.837969703910173 0.851 0.545 0 2
APRT Activated NME1
0 0.836926518305684 0.443 0.133 0 2
MRTO4 Activated NME1
0 0.836072104363487 0.521 0.216 0 2
DKC1 Activated NME1
0 0.833455508084056 0.96 0.738 0 2
PSMA7 Activated NME1
0 0.831163999474123 0.622 0.289 0 2
NOP56 Activated NME1
0 0.825836232813778 0.479 0.171 0 2
TOMM40 Activated NME1
0 0.821569890589266 0.932 0.678 0 2
TPI1 Activated NME1
0 0.806992787718712 0.717 0.399 0 2
SLIRP Activated NME1
0 0.80277254559768 0.418 0.128 0 2 NOLC1
Activated NME1
0 0.802072015108018 0.41 0.113 0 2
NOP16 Activated NME1
0 0.800496592094825 0.588 0.264 0 2
PDCD5 Activated NME1
0 0.794086253065539 0.638 0.305 0 2 MIF
Activated NME1
0 0.787313965898391 0.509 0.203 0 2
CDK4 Activated NME1
0 0.786155062710006 0.724 0.395 0 2
CCT4 Activated NME1
0 0.785705471054837 0.438 0.143 0 2
ASNS Activated NME1
0 0.781956888729269 0.396 0.108 0 2
IFRD2 Activated NME1
0 0.776511038014416 0.687 0.355 0 2
SHMT2 Activated NME1
0 0.759762318192504 0.825 0.52 0 2
PSMA1 Activated NME1
0 0.759662538655413 0.748 0.411 0 2
ATP5MC1 Activated NME1
0 0.757744564399663 0.707 0.404 0 2
EIF5B Activated NME1
0 0.755392433993726 0.573 0.271 0 2
NOP58 Activated NME1
0 0.755212434911577 0.607 0.273 0 2
TMEM147 Activated NME1

0	0.75187598607029	0.643	0.333	0	2	EIF3J	Activated	NME1
0	0.750657681531205	0.856	0.59	0	2	SERBP1	Activated	NME1
0	0.743953744923215	0.413	0.128	0	2	GAR1	Activated	NME1
0	0.740183838370068	0.885	0.604	0	2	COX7B	Activated	NME1
0	0.740171118288095	0.406	0.121	0	2	RPF2	Activated	NME1
0	0.739267022498804	0.923	0.672	0	2	HNRNPDL	Activated	NME1
0	0.738715410578102	0.763	0.442	0	2	SNRPD1	Activated	NME1
0	0.736573217742486	0.485	0.166	0	2	YBX3	Activated	NME1
0	0.72900151208405	0.719	0.423	0	2	VDAC1	Activated	NME1
0	0.728226783724328	0.783	0.438	0	2	CD83	Activated	NME1
0	0.727377250459855	0.709	0.388	0	2	EIF6	Activated	NME1
0	0.7248565827602	0.654	0.344	0	2	PSMC1	Activated	NME1
0	0.719502926789199	0.39	0.129	0	2	PAICS	Activated	NME1
0	0.715573472001298	0.465	0.179	0	2	GNL3	Activated	NME1
0	0.713662713613876	0.586	0.274	0	2	PSMD11	Activated	NME1
0	0.711770132104899	0.494	0.196	0	2	DCAF13	Activated	NME1
0	0.703244141658572	0.757	0.482	0	2	TALDO1	Activated	NME1
0	0.702193190833997	0.77	0.459	0	2	CCT3	Activated	NME1
0	0.702123958417456	0.369	0.109	0	2	NDUFAF2	Activated	NME1
0	0.701613287448405	0.507	0.213	0	2	POLD2	Activated	NME1
0	0.701446483099054	0.456	0.168	0	2	ATIC	Activated	NME1
0	0.699309158612151	0.85	0.566	0	2	SRSF2	Activated	NME1
0	0.698581663739805	0.896	0.63	0	2	PSMB1	Activated	NME1
0	0.697059600059505	0.833	0.544	0	2	PARK7	Activated	NME1
0	0.693044361382298	0.76	0.469	0	2	TUFM	Activated	NME1

0 0.690472432548453 0.846 0.557 0 2
EIF2S2 Activated NME1
0 0.689549147848193 0.651 0.344 0 2
PSMC3 Activated NME1
0 0.685509124589675 0.695 0.388 0 2
NUDC Activated NME1
0 0.675554713996501 0.816 0.501 0 2
IMPDH2 Activated NME1
0 0.674779177222157 0.748 0.45 0 2
RSL1D1 Activated NME1
0 0.671082748966124 0.295 0.052 0 2
MTHFD1L Activated NME1
0 0.668089300437995 0.772 0.473 0 2
XRCC5 Activated NME1
0 0.663972698871452 0.701 0.391 0 2
APEX1 Activated NME1
0 0.662564974940533 0.715 0.414 0 2
CCT7 Activated NME1
0 0.658220162950305 0.893 0.605 0 2
PSMA4 Activated NME1
0 0.652742762174688 0.658 0.358 0 2
MRPL20 Activated NME1
0 0.645005350795531 0.723 0.431 0 2
GADD45GIP1 Activated NME1
0 0.644609636204774 0.847 0.568 0 2
XRCC6 Activated NME1
0 0.639648693693526 0.938 0.722 0 2
CHCHD2 Activated NME1
0 0.63027765747603 0.887 0.619 0 2 SNRPG
Activated NME1
0 0.621861364895447 0.837 0.586 0 2
PTGES3 Activated NME1
0 0.618989707398919 0.864 0.545 0 2
TUBB Activated NME1
0 0.616016641988287 0.852 0.578 0 2
PSMB3 Activated NME1
0 0.615798150611332 0.827 0.538 0 2
UQCRQ Activated NME1
0 0.615777128382085 0.863 0.614 0 2
ATP5MD Activated NME1
0 0.615714187351679 0.857 0.58 0 2
POMP Activated NME1
0 0.602309957524541 0.878 0.614 0 2 ERH
Activated NME1
0 0.565149998259701 0.999 0.974 0 2
RPS24 Activated NME1
0 0.561735263541512 0.881 0.615 0 2
HNRNPM Activated NME1
0 0.543633893687697 0.992 0.92 0 2
HNRNPA1 Activated NME1

0 0.538798682991414 0.985 0.885 0 2
PPIA Activated NME1
0 0.510653605645595 0.926 0.637 0 2
TUBA1B Activated NME1
0 0.417628941296528 1 0.987 0 2
RPLP0 Activated NME1
0 0.383350560923144 0.967 0.785 0 2
RPS26 Activated NME1
0 0.377475224593746 1 0.992 0 2
RPL35 Activated NME1
0 0.372381563519448 0.999 0.979 0 2
RPL5 Activated NME1
0 0.36332726495773 0.999 0.985 0 2 RPL14
Activated NME1
0 -0.824529324954662 0.876 0.954 0 2
HLA-DRA Activated NME1
0 -0.891889239019266 0.982 0.99 0 2
CD74 Activated NME1
0 -0.973130092922331 0.998 0.998 0 2
TMSB4X Activated NME1
0 -1.01876906224984 0.619 0.794 0 2
ARHGDIB Activated NME1
0 -1.04979602631778 0.281 0.541 0 2
LSP1 Activated NME1
0 -1.05111301682946 0.742 0.882 0 2
CD52 Activated NME1
0 -1.20486265962847 0.072 0.329 0 2
CD27 Activated NME1
0 -1.27677952486064 0.559 0.772 0 2
CXCR4 Activated NME1
0 -1.39800507435322 0.565 0.826 0 2
LAPTM5 Activated NME1
0 -1.49232031505862 0.502 0.805 0 2
HLA-DPB1 Activated NME1
0 -1.49783865656607 0.244 0.566 0 2
ISG20 Activated NME1
0 -2.34804241329546 0.146 0.527 0 2 LTB
Activated NME1
0 -2.45931013049873 0.191 0.427 0 2
JCHAIN Activated NME1
4.94065645841247e-324 0.675366568376515 0.568 0.285
8.80869639970358e-320 2 STOML2 Activated NME1
1.48219693752374e-323 0.671803806945551 0.54 0.253
2.64260891991108e-319 2 PSMD14 Activated NME1
6.32404026676796e-322 0.644324764726917 0.662 0.37
1.12751313916206e-317 2 PSMA3 Activated NME1
1.80729213248728e-320 0.430282725105352 0.997 0.961
3.22222114301157e-316 2 EIF1 Activated NME1
6.75032010599978e-319 0.677900726978687 0.379 0.123
1.2035145716987e-314 2 TSEN15 Activated NME1

7.58331478488812e-319 0.621451921727891 0.653 0.362
1.3520291929977e-314 2 EIF5A Activated NME1
2.02594772883978e-314 0.717096432313836 0.561 0.248
3.61206220574845e-310 2 WARS Activated NME1
7.9640260711553e-314 0.520418030185214 0.907 0.678
1.41990620822628e-309 2 ELOB Activated NME1
6.22829453397428e-312 0.661393945060384 0.56 0.264
1.11044263246227e-307 2 SSRP1 Activated NME1
6.90554200977621e-311 0.595629656443575 0.759 0.476
1.231189084923e-306 2 EIF3I Activated NME1
8.51638627463805e-309 0.661278093418063 0.425 0.152
1.51838650890522e-304 2 ABCE1 Activated NME1
2.22643675954777e-307 0.610944203169549 0.78 0.504
3.96951409859772e-303 2 GHITM Activated NME1
4.05094059265257e-305 0.578731263328223 0.294 0.08
7.22242198264027e-301 2 BOP1 Activated NME1
1.29251682909028e-302 0.65921866976795 0.378 0.127
2.30442825458506e-298 2 PPAN Activated NME1
2.13809790572675e-300 -1.07355453103392 0.272 0.513
3.81201475612023e-296 2 UCP2 Activated NME1
2.5193502992437e-298 0.562013986834364 0.812 0.538
4.49174964852159e-294 2 SNRPB Activated NME1
9.49981701725848e-297 0.602383912529793 0.801 0.555
1.69372237600701e-292 2 LDHB Activated NME1
2.53344097307704e-296 0.658590742162609 0.523 0.247
4.51687191089906e-292 2 NDUFAF8 Activated NME1
1.8888487917155e-294 0.615289362054063 0.665 0.373
3.36762851074957e-290 2 PSMC5 Activated NME1
2.04633785584505e-294 0.608682489179639 0.673 0.392
3.64841576318614e-290 2 TOMM22 Activated NME1
5.95391831362088e-293 0.592432045118456 0.681 0.401
1.06152409613547e-288 2 DNAJA1 Activated NME1
6.9933652494451e-293 -0.432914988015512 1 1
1.24684709032357e-288 2 RPS27 Activated NME1
1.53952127443916e-292 0.669379641705498 0.485 0.207
2.74481248019757e-288 2 EIF4A1 Activated NME1
3.31710343591112e-292 0.540673338562522 0.834 0.575
5.91406371588594e-288 2 PSMA2 Activated NME1
5.49103089098344e-292 0.666719682928853 0.647 0.363
9.78995897553437e-288 2 ARPC5L Activated NME1
1.20052598726048e-290 0.621797426150929 0.653 0.354
2.14041778268671e-286 2 C19orf70 Activated NME1
5.47916979623595e-290 0.621345888869063 0.632 0.361
9.76881182970907e-286 2 NDUFS6 Activated NME1
3.42440221217148e-289 0.657960202702288 0.542 0.262
6.10536670408053e-285 2 DCTPP1 Activated NME1
1.63445935807908e-288 0.624238822064993 0.708 0.428
2.91407758951918e-284 2 DDX18 Activated NME1
4.58373020469496e-287 0.590719188532685 0.702 0.423
8.17233258195064e-283 2 NDUFB9 Activated NME1

1.43575319235698e-286 0.621387822448013 0.603 0.32
2.55980436665326e-282 2 CYC1 Activated NME1
1.65539008074234e-286 0.616670482702222 0.621 0.343
2.95139497495551e-282 2 PSMD7 Activated NME1
2.31841402910831e-285 0.629816068655396 0.568 0.289
4.13350037249721e-281 2 TCP1 Activated NME1
1.68189433790147e-284 0.516661567876963 0.856 0.594
2.99864941504453e-280 2 SUB1 Activated NME1
6.91718741483989e-284 0.639505979456489 0.37 0.126
1.2332653441918e-279 2 PUM3 Activated NME1
1.7991980176655e-283 0.386060043221747 0.989 0.916
3.20779014569582e-279 2 BTF3 Activated NME1
4.13995392995005e-283 0.375431149676978 0.995 0.965
7.38112386170795e-279 2 RPS17 Activated NME1
1.23267987781575e-282 0.666304200991016 0.423 0.16
2.19774495415771e-278 2 WDR43 Activated NME1
2.87199602601135e-282 0.675693273659541 0.486 0.204
5.12048171477564e-278 2 TARS Activated NME1
1.70314981827605e-281 0.640327613620534 0.43 0.17
3.03654581100437e-277 2 GTPBP4 Activated NME1
8.43420014524248e-278 0.567900003104092 0.727 0.458
1.50373354389528e-273 2 GTF3A Activated NME1
2.42207169726773e-277 0.63390818494279 0.478 0.21
4.31831162905863e-273 2 NIFK Activated NME1
6.15296395900539e-275 0.580640283002239 0.713 0.442
1.09701194425107e-270 2 SNRPF Activated NME1
5.11118485698164e-274 -1.43056820570014 0.035 0.225
9.11273148151257e-270 2 RGS13 Activated NME1
5.5122745285188e-274 0.566908704086416 0.692 0.419
9.82783425689617e-270 2 PSMB7 Activated NME1
6.28756679736675e-273 0.572117540886256 0.642 0.368
1.12101028430252e-268 2 NDUFAB1 Activated NME1
1.25223235633137e-272 0.581753487038394 0.707 0.429
2.23260506810319e-268 2 SFPQ Activated NME1
1.87038908259937e-272 0.602362796364589 0.586 0.313
3.33471669536642e-268 2 STRAP Activated NME1
1.10646800195806e-269 -0.77008816170024 0.704 0.827
1.97272180069102e-265 2 HLA-DPA1 Activated NME1
4.05123180065636e-269 0.601600156103492 0.581 0.304
7.22294117739022e-265 2 PDAP1 Activated NME1
2.06607280585514e-268 0.488790516993324 0.832 0.556
3.68360120555912e-264 2 ANP32B Activated NME1
6.17717202682639e-267 0.506074292487382 0.222 0.047
1.10132800066288e-262 2 METTL1 Activated NME1
1.98798319954205e-266 0.526253897637691 0.218 0.037
3.54437524646351e-262 2 CCND2 Activated NME1
3.56963340345537e-266 0.585229364006833 0.658 0.38
6.36429939502058e-262 2 BANF1 Activated NME1
1.60003821725224e-265 0.578864932523976 0.665 0.383
2.85270813753901e-261 2 HNRNPR Activated NME1

2.36541487256783e-265	0.613838307699162	0.45	0.192	
4.21729817630118e-261	2	WDR46	Activated	NME1
9.79706787369737e-265	0.585471411626857	0.319	0.099	
1.7467192312015e-260	2	PAK1IP1	Activated	NME1
2.37106750393478e-264	0.516718222677419	0.792	0.534	
4.22737625276531e-260	2	ATP5PF	Activated	NME1
7.22331414800339e-263	0.578714210030648	0.383	0.139	
1.28784467944753e-258	2	MRPL24	Activated	NME1
9.97944272946049e-263	0.544043331574106	0.717	0.428	
1.77923484423551e-258	2	LSM5	Activated	NME1
2.42029182255239e-262	0.560074163520036	0.726	0.449	
4.31513829042866e-258	2	FUS	Activated	NME1
5.13455308388035e-262	0.596343442881314	0.821	0.587	
9.15439469325027e-258	2	RSL24D1	Activated	NME1
5.36512987381659e-262	-0.833791374460375	0.75	0.858	
9.56549005202759e-258	2	CD37	Activated	NME1
2.18114550011556e-261	0.590045489314274	0.675	0.402	
3.88876431215603e-257	2	NSA2	Activated	NME1
4.36088743036017e-258	0.550635839981822	0.309	0.096	
7.77502619958914e-254	2	LYAR	Activated	NME1
6.76932318602688e-258	-1.03626834400097	0.151	0.37	
1.20690263083673e-253	2	S100A10	Activated	NME1
7.09866361292745e-258	0.545513684548542	0.791	0.547	
1.26562073554883e-253	2	RBM8A	Activated	NME1
1.63032711171142e-257	0.514089992211341	0.762	0.492	
2.90671020747029e-253	2	PSMB6	Activated	NME1
5.3900567924133e-256	0.505270445043175	0.822	0.573	
9.60993225519367e-252	2	SEC61B	Activated	NME1
3.04809138518356e-254	0.568951399223238	0.694	0.418	
5.43444213064376e-250	2	FBL	Activated	NME1
4.20198987232743e-254	0.48103526800669	0.846	0.592	
7.49172774337258e-250	2	NDUFA13	Activated	NME1
5.41222111163023e-254	0.627740912878431	0.499	0.229	
9.64944901992554e-250	2	NARS	Activated	NME1
2.18089150383759e-253	0.584834288661424	0.401	0.155	
3.88831146219204e-249	2	DNPEP	Activated	NME1
2.32848864598206e-253	0.502815316149379	0.996	0.974	
4.15146240692141e-249	2	FTH1	Activated	NME1
2.66628429291758e-252	0.589745920647046	0.435	0.194	
4.75371826584275e-248	2	PSMD1	Activated	NME1
3.55503590663833e-252	-0.634889914454038	0.977	0.986	
6.33827351794548e-248	2	TMSB10	Activated	NME1
7.65251481906492e-252	0.450839705292803	0.862	0.616	
1.36436686709108e-247	2	ATP5MF	Activated	NME1
5.61227518755049e-250	0.600648798441241	0.391	0.148	
1.00061254318838e-245	2	TIMM44	Activated	NME1
9.92350304720974e-250	0.37621504432312	0.979	0.893	
1.76926135828702e-245	2	COX7C	Activated	NME1
1.05846814940918e-248	0.55181587166358	0.627	0.359	
1.88714286358163e-244	2	SNRPC	Activated	NME1

1.77344929543379e-248	0.58422791973225		0.461	0.212	
3.1618827488289e-244	2	STIP1	Activated	NME1	
5.81334158596469e-248	0.522681310050269			0.746	0.484
1.03646067136164e-243	2	PSMD8	Activated	NME1	
7.80388880309672e-247	0.55997899560386		0.625	0.356	
1.39135533470411e-242	2	TXNL1	Activated	NME1	
8.72514244900826e-247	0.62125664526134		0.599	0.329	
1.55560564723368e-242	2	SARS	Activated	NME1	
1.97456676113094e-246	0.568208916134882			0.367	0.144
3.52045507842035e-242	2	ETF1	Activated	NME1	
3.48238152835227e-246	0.551986925346462			0.644	0.378
6.20873802689925e-242	2	PSMB2	Activated	NME1	
3.81369133427512e-246	0.588925044559497			0.461	0.211
6.79943027987911e-242	2	EIF2S1	Activated	NME1	
8.14625993261637e-246	0.511636787092796			0.738	0.471
1.45239668338617e-241	2	COX5A	Activated	NME1	
1.5046707737494e-245	-0.861782643564267			0.105	0.303
2.6826775225178e-241	2	GNG7	Activated	NME1	
5.48975676373381e-244	0.572434995321911			0.367	0.135
9.787687334061e-240	2	TRMT1	Activated	NME1	
1.1355179672113e-242	-0.608233963764627			1	1
2.02451498374102e-238	2	MALAT1	Activated	NME1	
5.2730823186684e-241	0.584302045909985			0.495	0.252
9.4013784659539e-237	2	ATOX1	Activated	NME1	
1.87362273937931e-239	0.556007556051223			0.581	0.318
3.34048198203938e-235	2	PSMC4	Activated	NME1	
3.27364723842144e-239	0.558322398183632			0.508	0.258
5.83658566138159e-235	2	MRPS7	Activated	NME1	
4.57844830240235e-239	0.572346391531289			0.497	0.249
8.16291547835314e-235	2	NAA10	Activated	NME1	
6.73603859034725e-239	0.595528203699288			0.305	0.106
1.20096832027301e-234	2	SLC1A5	Activated	NME1	
8.15930685494297e-239	0.480302978876023			0.857	0.63
1.45472281916778e-234	2	UBE2D3	Activated	NME1	
1.66216855204966e-238	0.522618871809093			0.673	0.407
2.96348031144933e-234	2	NDUFB7	Activated	NME1	
4.73910827152842e-238	0.565240567854008			0.423	0.201
8.44935613730801e-234	2	PSMB5	Activated	NME1	
1.07638675821058e-237	0.442272614939369			0.819	0.579
1.91908995121364e-233	2	LSM7	Activated	NME1	
1.17457051083487e-237	0.583170910642691			0.483	0.236
2.09414176376749e-233	2	AK6	Activated	NME1	
2.35212801540709e-237	0.556060039919494			0.439	0.19
4.1936090386693e-233	2	EMG1	Activated	NME1	
1.52145269295741e-236	-0.771944433294592			0.055	0.237
2.71259800627377e-232	2	LY86	Activated	NME1	
6.58145685693138e-236	0.451463605078593			0.88	0.655
1.1734079430223e-231	2	HNRNPA3	Activated	NME1	
1.87028708501233e-235	0.439171667357786			0.811	0.559
3.33453484386848e-231	2	ATP5MC3	Activated	NME1	

6.16660032485176e-235 0.414129414940859 0.898 0.686
1.09944317191782e-230 2 NDUFA4 Activated NME1
1.76802879914622e-234 0.550873532227108 0.605 0.341
3.15221854599779e-230 2 CACYBP Activated NME1
9.72527496419103e-234 0.452536718875392 0.852 0.613
1.73391927336562e-229 2 SET Activated NME1
1.94647029759596e-233 0.342425811886018 0.989 0.939
3.47036189358384e-229 2 NACA Activated NME1
5.26016080566552e-233 0.566760031884987 0.415 0.168
9.37834070042106e-229 2 WDR74 Activated NME1
1.41314926929007e-232 0.626811810994002 0.483 0.247
2.51950383221727e-228 2 SMS Activated NME1
1.04696143456143e-231 0.546238638570623 0.487 0.243
1.86662754167958e-227 2 PSMD3 Activated NME1
5.86718223252287e-231 0.535547557417129 0.702 0.451
1.0460599202365e-226 2 PRELID1 Activated NME1
2.56100323171377e-230 0.543468614846566 0.37 0.15
4.56601266182248e-226 2 RARS Activated NME1
2.56553726425276e-230 -0.820180274907362 0.084 0.278
4.57409638843624e-226 2 SYNE2 Activated NME1
5.9737891724863e-229 0.508974620766818 0.665 0.393
1.06506687156258e-224 2 MAGOH Activated NME1
1.27249105439327e-228 0.461391267376158 0.849 0.622
2.26872430087776e-224 2 HNRNPC Activated NME1
1.44194755454278e-228 0.521542148415125 0.309 0.107
2.57084829499432e-224 2 GART Activated NME1
2.66268826713952e-228 0.532166160134619 0.307 0.098
4.74730691148306e-224 2 PNO1 Activated NME1
1.01817642030629e-227 0.550438677897952 0.533 0.278
1.81530673976408e-223 2 KARS Activated NME1
3.72928973678227e-227 -0.830705544745522 0.804 0.873
6.64895067170911e-223 2 CD79A Activated NME1
4.11972887915483e-227 0.499903176632615 0.717 0.465
7.34506461864514e-223 2 CCT8 Activated NME1
5.69441581447181e-226 0.546512703591976 0.497 0.248
1.01525739556218e-221 2 OLA1 Activated NME1
6.82834737326211e-225 0.536862762586695 0.493 0.238
1.2174260531789e-220 2 MRPL11 Activated NME1
1.09312825220731e-224 0.379195641458403 0.91 0.702
1.9489383608604e-220 2 COX6C Activated NME1
7.62381672526462e-224 0.534774745166876 0.558 0.306
1.35925028394743e-219 2 PSMD2 Activated NME1
7.64859447393617e-224 0.495736090729328 0.818 0.582
1.36366790875808e-219 2 RBM3 Activated NME1
3.73493411226763e-221 0.566246125336173 0.392 0.166
6.65901402876196e-217 2 YARS Activated NME1
3.7835057995444e-220 0.541850237482256 0.42 0.189
6.74561249000772e-216 2 PRPF19 Activated NME1
6.97825598939592e-220 -1.05770619605377 0.11 0.292
1.2441532603494e-215 2 KLF2 Activated NME1

3.57347574197848e-219	0.498600595403934	0.719	0.465	6.37114990037344e-215	2	MRPL52	Activated	NME1
1.05921225731044e-218	-0.573999675854641	0.013	0.154	1.88846953355879e-214	2	AIM2	Activated	NME1
3.47235265384345e-218	-1.38499944028732	0.115	0.28	6.19085754653748e-214	2	S100A4	Activated	NME1
1.12916965108749e-217	0.545278323771966	0.371	0.162	2.01319657092388e-213	2	ZNF593	Activated	NME1
2.56319598265993e-217	-0.829121562970766	0.096	0.294	4.56992211748439e-213	2	NCF1	Activated	NME1
4.63664111132366e-217	0.546168685735791	0.391	0.16	8.26666743737895e-213	2	TUBA1C	Activated	NME1
6.9975637779758e-216	0.527040541004794	0.578	0.329	1.24759564597531e-211	2	PHB2	Activated	NME1
3.76342404740175e-215	0.40516018823313	0.181	0.035	6.70980873411258e-211	2	SLC38A5	Activated	NME1
1.60600874054175e-213	0.419958126142859	0.875	0.662	2.86335298351188e-209	2	SLC25A3	Activated	NME1
6.34503009607411e-213	0.503123979766079	0.274	0.087	1.13125541582905e-208	2	CD320	Activated	NME1
7.08389750187233e-213	0.53612293095288	0.352	0.141	1.26298808560882e-208	2	PPP1R14B	Activated	NME1
8.72331051775681e-213	0.450206825719363	0.829	0.606	1.55527903221086e-208	2	ATP5F1B	Activated	NME1
1.18357398309833e-212	0.542690010692291	0.445	0.212	2.11019405446602e-208	2	BUD23	Activated	NME1
1.26873333048426e-212	0.440384285621146	0.747	0.499	2.26202465492038e-208	2	FKBP1A	Activated	NME1
1.58751965284107e-212	0.509847465411363	0.648	0.395	2.83038878905034e-208	2	ADRM1	Activated	NME1
2.09622580441094e-212	0.43297582722444	0.828	0.584	3.73736098668426e-208	2	POLR2L	Activated	NME1
1.0782988307227e-211	0.523046950823296	0.537	0.294	1.92249898529551e-207	2	GTF3C6	Activated	NME1
3.03574726091838e-209	0.508293194195073	0.559	0.314	5.41243379149138e-205	2	EIF1AX	Activated	NME1
3.14755246874474e-209	0.507419570454098	0.624	0.378	5.61177129652500e-205	2	MDH2	Activated	NME1
8.53039618194746e-209	0.553491102015108	0.458	0.234	1.52088433527941e-204	2	GSPT1	Activated	NME1
9.72785311124845e-209	0.461539621051127	0.772	0.533	1.73437893120449e-204	2	PGK1	Activated	NME1
1.36270822902875e-208	0.468592397387027	0.263	0.083	2.42957250153535e-204	2	FARSB	Activated	NME1
5.05191945810241e-208	0.512014472600794	0.416	0.188	9.00706720185078e-204	2	EEF1E1	Activated	NME1
5.16151249761387e-208	0.460615261141469	0.747	0.509	9.20246063199577e-204	2	SNRPB2	Activated	NME1
7.1694799870299e-208	-0.832590138686893	0.667	0.773	1.27824658688756e-203	2	CYBA	Activated	NME1

1.99096411937401e-207 0.484714648617145 0.305 0.111
3.54968992843192e-203 2 TIMM50 Activated NME1
6.90336597492599e-207 0.524821664925445 0.256 0.078
1.23080111966955e-202 2 STAT5A Activated NME1
2.03211208973045e-206 -0.871016027398988 0.278 0.473
3.62305264478041e-202 2 PTPRC Activated NME1
6.97861706221788e-206 0.520214660281853 0.738 0.507
1.24421763602283e-201 2 SRSF7 Activated NME1
9.57551450339164e-206 0.48236171838016 0.661 0.416
1.70721848080969e-201 2 AURKAIP1 Activated NME1
3.17251262107833e-205 0.513986412686728 0.503 0.264
5.65627275212056e-201 2 PFDN6 Activated NME1
5.38217642889437e-205 0.484483792820251 0.264 0.083
9.59588235507577e-201 2 RRS1 Activated NME1
1.19116581293318e-203 0.378939651852524 0.879 0.663
2.12372952787857e-199 2 COX6A1 Activated NME1
2.02559700052383e-203 0.44521389944702 0.806 0.57
3.61143689223394e-199 2 HNRNPU Activated NME1
1.33391785616354e-202 0.526184376269157 0.363 0.144
2.37824214575397e-198 2 C8orf33 Activated NME1
3.40120705649764e-202 0.567456636463407 0.351 0.144
6.06401206102965e-198 2 NFKB1 Activated NME1
3.10432576412355e-201 0.400973667860977 0.778 0.53
5.53470240485587e-197 2 SEC61G Activated NME1
8.05739348550988e-201 0.541807749515053 0.464 0.228
1.43655268453156e-196 2 DNPH1 Activated NME1
1.15667048882331e-200 0.487962947417656 0.316 0.116
2.06222781452308e-196 2 PES1 Activated NME1
1.41287053533256e-200 0.402064187516664 0.169 0.034
2.51900687744442e-196 2 RCN1 Activated NME1
1.69813691862075e-200 0.550875120784277 0.429 0.193
3.02760831220893e-196 2 NFKBIE Activated NME1
2.92342566339567e-200 0.554485051031376 0.333 0.126
5.21217561526813e-196 2 ARL8B Activated NME1
9.94151174061462e-200 0.484374370345589 0.822 0.587
1.77247212823418e-195 2 SYNGR2 Activated NME1
3.87359967103465e-199 0.519247628484514 0.416 0.191
6.90624085348768e-195 2 EPRS Activated NME1
5.92576961199311e-199 0.52493728184824 0.515 0.278
1.05650546412225e-194 2 POLR2I Activated NME1
6.07826622715273e-199 0.531004820804292 0.396 0.174
1.08369408563906e-194 2 MRPL1 Activated NME1
6.57025798233832e-199 0.472007668536379 0.597 0.326
1.1714112956711e-194 2 TUBB4B Activated NME1
1.18040102003866e-197 0.533715258068318 0.317 0.108
2.10453697862693e-193 2 CCDC58 Activated NME1
3.17761951113701e-197 -0.652543179539252 0.024 0.176
5.66537782640618e-193 2 SUSD3 Activated NME1
1.56719490613749e-196 0.522091638213815 0.362 0.146
2.79415179815253e-192 2 SNHG15 Activated NME1

2.22348777215502e-196 -0.65723927624013 0.012 0.148
3.96425634897519e-192 2 NEIL1 Activated NME1
2.94272274711329e-196 0.480356011527562 0.572 0.329
5.24658038582828e-192 2 PSMD13 Activated NME1
1.49286062630245e-195 0.519041976705428 0.358 0.148
2.66162121063464e-191 2 MARS Activated NME1
2.12998473515813e-195 0.502591687401002 0.415 0.201
3.79754978431343e-191 2 MRPL4 Activated NME1
3.17417154103217e-195 0.48077692243371 0.305 0.123
5.65923044050626e-191 2 RUVBL1 Activated NME1
3.94289854706988e-195 -0.771734143205224 0.06 0.223
7.02979381957088e-191 2 C16orf74 Activated NME1
1.57662360531923e-194 -0.86749151367152 0.347 0.52
2.81096222592366e-190 2 TSC22D3 Activated NME1
2.99463869818313e-194 0.496001153889937 0.368 0.169
5.33914133499071e-190 2 RUVBL2 Activated NME1
3.28579264041722e-194 0.395542750737921 0.897 0.73
5.85823969859987e-190 2 SNRPD2 Activated NME1
1.60632787227948e-193 0.442257164129259 0.746 0.506
2.86392196348709e-189 2 JTB Activated NME1
1.61578002250523e-193 0.491536376953289 0.289 0.105
2.88077420212457e-189 2 ABCF2.1 Activated NME1
3.99202239264701e-193 0.581685766268553 0.408 0.186
7.11737672385036e-189 2 PIM3 Activated NME1
4.52812052028477e-193 0.449670360043023 0.237 0.072
8.07318607561572e-189 2 TSR1 Activated NME1
4.74203590720881e-193 0.361591196164646 0.929 0.76
8.45457581896258e-189 2 NAP1L1 Activated NME1
2.19225267981562e-191 0.477382475460418 0.281 0.103
3.90856730284328e-187 2 TRAP1 Activated NME1
1.3918370147585e-190 -1.16805776107446 0.37 0.475
2.48150621361292e-186 2 S100A6 Activated NME1
1.58458948751566e-190 0.49447962247016 0.574 0.33
2.82516459729167e-186 2 KPNB1 Activated NME1
2.88243822009265e-190 0.513686805802168 0.562 0.33
5.13909910260318e-186 2 RWDD1 Activated NME1
3.0698351590717e-190 0.492082048069229 0.371 0.162
5.47320910510893e-186 2 AHCY Activated NME1
4.44459195085016e-190 0.494427723353958 0.512 0.279
7.92426298917076e-186 2 AHSA1 Activated NME1
1.12001311993057e-189 0.489981810227019 0.491 0.264
1.99687139152422e-185 2 MRPS15 Activated NME1
1.89754889132602e-189 0.509779803197342 0.415 0.186
3.38313991834517e-185 2 LARS Activated NME1
1.97082594551879e-189 0.539010905304872 0.387 0.175
3.51378557826545e-185 2 AARS Activated NME1
1.38310032589951e-188 -0.880818003832514 0.145 0.32
2.46592957104624e-184 2 ZFP36L2 Activated NME1
5.81128628849752e-188 0.522040249387959 0.466 0.229
1.03609423237622e-183 2 RRP7A Activated NME1

1.04400215845049e-187 0.456468126065516 0.257 0.089
1.86135144830138e-183 2 AIMP2 Activated NME1
1.4048555846899e-187 0.403542681225304 0.182 0.044
2.50471702194363e-183 2 BCAT1 Activated NME1
1.82618140516443e-183 0.507845525443912 0.438 0.201
3.25589882726766e-179 2 TMEM109 Activated NME1
3.01788221424978e-183 -1.09427146387367 0.212 0.389
5.38058219978594e-179 2 SELL Activated NME1
3.38091744058251e-183 0.48797440394656 0.373 0.172
6.02783770481455e-179 2 CCDC124 Activated NME1
9.93784714884575e-183 -0.953081135995441 0.356 0.513
1.77181876816771e-178 2 SAT1 Activated NME1
1.27761837013837e-182 0.459102081436798 0.298 0.118
2.27786579211969e-178 2 RRP1 Activated NME1
2.54361846591513e-182 -0.844067993210201 0.168 0.349
4.53501736288008e-178 2 RIPOR2 Activated NME1
4.58015392340696e-182 0.436229016421751 0.744 0.51
8.16595643004227e-178 2 SOD1 Activated NME1
4.98617262103314e-182 0.420639707653227 0.226 0.07
8.88984716603999e-178 2 POLR3H Activated NME1
8.16857124414168e-182 0.478014556084909 0.559 0.325
1.45637456711802e-177 2 ZC3H15 Activated NME1
8.62851605915577e-182 0.471062771246115 0.537 0.301
1.53837812818688e-177 2 PUF60 Activated NME1
4.6450077649187e-181 0.464470086817706 0.287 0.116
8.28158434407355e-177 2 EXOSC4 Activated NME1
3.40609095015834e-180 0.435523450575833 0.754 0.534
6.07271955503731e-176 2 SNU13 Activated NME1
1.58969709100118e-179 0.501959675259477 0.431 0.217
2.834270943546e-175 2 HNRNPAB Activated NME1
4.3657465523743e-179 0.460970895501972 0.479 0.253
7.78368952822814e-175 2 MRPL47 Activated NME1
1.49192483436141e-178 0.439485931519137 0.28 0.105
2.65995278718297e-174 2 HSPBP1 Activated NME1
3.03402695113108e-178 0.502985858915227 0.476 0.247
5.4093666511716e-174 2 TIMM10 Activated NME1
3.32996085361214e-178 0.476947513114582 0.597 0.367
5.93698720590509e-174 2 NOL7 Activated NME1
9.35039028017453e-178 0.479248157280206 0.502 0.272
1.66708108305232e-173 2 ILF3 Activated NME1
1.23716931219428e-177 0.489692783926198 0.497 0.265
2.20574916671119e-173 2 AIMP1 Activated NME1
3.74650147602246e-177 0.471524425827328 0.399 0.192
6.67963748160045e-173 2 SLC25A39 Activated NME1
1.05069417321218e-176 -1.1956681620059 0.084 0.219
1.87328264142e-172 2 LGALS1 Activated NME1
1.411856501208e-176 0.519933481932681 0.513 0.286
2.51719895600374e-172 2 CALR Activated NME1
5.23593374740829e-176 -0.877760104668125 0.626 0.701
9.33514627825425e-172 2 EZR Activated NME1

1.45536187062565e-175 0.444961810145168 0.641 0.392
2.59476467913846e-171 2 DDX39A Activated NME1
2.03458219980299e-175 0.463610856068333 0.548 0.324
3.62745660402876e-171 2 ABRACL Activated NME1
3.20810393185161e-175 0.480557495420426 0.426 0.216
5.71972850009823e-171 2 MRPL21 Activated NME1
1.14394836386909e-172 0.467890519670256 0.338 0.133
2.03954553794221e-168 2 RRP15 Activated NME1
1.57398428526867e-172 0.356726703225274 0.857 0.654
2.80625658220551e-168 2 ATP5ME Activated NME1
1.79318750237914e-172 0.433708000929897 0.541 0.315
3.19707399799177e-168 2 HSD17B10 Activated NME1
7.13895780958955e-172 0.434344082402266 0.72 0.494
1.27280478787172e-167 2 EIF3G Activated NME1
1.06572438724602e-171 0.466741731004999 0.324 0.144
1.90008001002092e-167 2 DCUN1D5 Activated NME1
1.40121202607739e-171 0.447029494647342 0.633 0.405
2.49822092129338e-167 2 MINOS1 Activated NME1
1.71678152570437e-171 0.4889717071743 0.453 0.243
3.06084978217832e-167 2 SYNCRIP Activated NME1
1.18576487226239e-170 0.4832787807212 0.499 0.282
2.11410019075661e-166 2 GLRX3 Activated NME1
1.43178579663982e-170 0.472445392092869 0.429 0.219
2.55273089682913e-166 2 DDX1 Activated NME1
3.15454697362758e-170 0.378137909868588 0.193 0.052
5.62424179928061e-166 2 WDR12 Activated NME1
5.42946247040839e-170 0.450324241625527 0.648 0.423
9.68018863849112e-166 2 PGAM1 Activated NME1
7.35450818371758e-170 0.42056188607868 0.26 0.095
1.31123526407501e-165 2 PRMT5 Activated NME1
1.55272870539338e-168 0.450325263997756 0.463 0.245
2.76836000884586e-164 2 MRPS12 Activated NME1
3.20963982325326e-168 0.475031180310024 0.445 0.226
5.72246684087824e-164 2 EXOSC8 Activated NME1
3.47888618424617e-168 0.473179718915792 0.408 0.192
6.20250617789249e-164 2 COX20 Activated NME1
4.17241398192541e-168 0.439131478125019 0.538 0.31
7.43899688837482e-164 2 LSM2 Activated NME1
1.12896629044616e-167 0.431340582857247 0.662 0.433
2.01283399923645e-163 2 PSMD4 Activated NME1
1.77078723756475e-167 0.46659203212384 0.539 0.315
3.15713656585419e-163 2 BUD31 Activated NME1
4.20510714470406e-167 -0.732871259021332 0.026 0.162
7.49728552829288e-163 2 BIK Activated NME1
4.56963576380658e-167 0.457531689821473 0.419 0.206
8.14720360329075e-163 2 FUBP1 Activated NME1
1.75175244305199e-166 0.469293918363466 0.569 0.346
3.12319943071739e-162 2 PFDN2 Activated NME1
2.03916970992765e-166 -0.691350162170547 0.78 0.865
3.63563567583002e-162 2 CD79B Activated NME1

2.34625739696064e-166	0.457425528355832		0.303	0.125
4.18314231304113e-162	2	NIP7	Activated	NME1
2.60936314604653e-166	-0.627090470285462		0.051	0.202
4.65223355308635e-162	2	HLA-DOB	Activated	NME1
7.49122700302015e-166	0.442630738708144		0.277	0.108
1.33561086236846e-161	2	BZW2	Activated	NME1
1.59962967436614e-165	0.360221390014962		0.828	0.628
2.85197974642739e-161	2	UQCR11	Activated	NME1
3.82818412713102e-165	0.3870735390946	0.789	0.572	
6.82526948026189e-161	2	ATP5F1D	Activated	NME1
2.43111824825641e-163	0.466515231042623		0.423	0.226
4.33444072481635e-159	2	AK2	Activated	NME1
3.41876199576542e-163	0.371828121348437		0.785	0.562
6.09531076225016e-159	2	NDUFB2	Activated	NME1
4.516539229678e-163	0.456013974426705		0.371	0.173
8.0525377925929e-159	2	TFAM	Activated	NME1
1.25714356480247e-162	0.450688330745741		0.395	0.186
2.24136126168632e-158	2	BRIX1	Activated	NME1
2.91795115048118e-162	0.431012857833246		0.257	0.097
5.2024151061929e-158	2	MCRIP2	Activated	NME1
9.89715996230779e-162	0.417400178337802		0.264	0.094
1.76456464967986e-157	2	C20orf27	Activated	NME1
1.51732859620511e-161	0.432648301971892		0.61	0.381
2.70524515417409e-157	2	BZW1	Activated	NME1
2.79754715237004e-161	-0.638818451366305		0.099	0.256
4.98774681796055e-157	2	MCUB	Activated	NME1
5.11247435638418e-161	0.444779428159448		0.411	0.202
9.11503052999735e-157	2	RPIA	Activated	NME1
5.32448643172297e-161	0.461762959292796		0.398	0.196
9.49302685911889e-157	2	AATF	Activated	NME1
6.72592690739967e-161	0.443108569208736		0.567	0.344
1.19916550832029e-156	2	PSMA5	Activated	NME1
1.00070442099631e-160	0.456117517489911		0.369	0.171
1.78415591219432e-156	2	FAM136A	Activated	NME1
1.54769027133073e-160	-0.747788018338524		0.15	0.313
2.75937698475556e-156	2	GGA2	Activated	NME1
1.61220177023072e-160	0.640407191394582		0.33	0.142
2.87439453614436e-156	2	DDIT4	Activated	NME1
1.71323065073081e-160	0.429909234981029		0.324	0.135
3.05451892718797e-156	2	NDUFAF4	Activated	NME1
1.76983181920021e-160	-0.659869697169545		0.109	0.27
3.15543315045205e-156	2	TBC1D10C	Activated	NME1
4.45801893989695e-160	0.441676956231689		0.406	0.203
7.94820196794227e-156	2	MRPS23	Activated	NME1
1.03460961118394e-159	0.355662372026216		0.19	0.059
1.84460547577985e-155	2	BYSL	Activated	NME1
1.69030304931535e-159	0.30652163422984	0.936	0.791	
3.01364130662433e-155	2	HNRNPK	Activated	NME1
2.57488489520993e-159	0.437925272112678		0.318	0.133
4.59076227966979e-155	2	GNL2	Activated	NME1

1.24501949286659e-158	0.442638176966221	0.733	0.524	2.21974525383184e-154	2	CNBP	Activated	NME1
2.12381807711529e-158	0.329257138442769	0.865	0.678	3.78655524968885e-154	2	ATP5MPL	Activated	NME1
2.95073714407951e-158	0.386240074105554	0.729	0.5	5.26086925417935e-154	2	NDUFB8	Activated	NME1
3.80514466378338e-158	0.435208157787011	0.361	0.17	6.78419242105939e-154	2	TSTA3	Activated	NME1
6.05575556810546e-158	-0.704259195194461	0.07	0.227	1.07968066023752e-153	2	FAM111B	Activated	NME1
7.28302268037146e-158	-0.754095438676379	0.235	0.405	1.29849011368343e-153	2	EVI2B	Activated	NME1
5.917149730187e-157	0.441593086949923	0.404	0.203	1.05496862539504e-152	2	HSPH1	Activated	NME1
3.73320304474745e-156	0.425075744283126	0.257	0.08	6.65592770848022e-152	2	PARVB	Activated	NME1
1.22857685438642e-155	0.430642282899313	0.651	0.429	2.19042967368556e-151	2	VCP	Activated	NME1
6.18558541470609e-155	0.407830505977605	0.262	0.111	1.10282802358795e-150	2	ISOC2	Activated	NME1
6.69737581371411e-155	0.439786903919057	0.401	0.2	1.19407513382709e-150	2	NUTF2	Activated	NME1
7.6985923523035e-155	0.422806235441703	0.216	0.071	1.37258203049219e-150	2	SLC43A3	Activated	NME1
1.16534155558442e-154	-0.440061167945126	0.01	0.123	2.07768745945146e-150	2	CPNE5	Activated	NME1
1.45335198076079e-154	-0.691791079981211	0.332	0.487	2.59118124649841e-150	2	GPSM3	Activated	NME1
2.16809897579019e-154	0.425032424724156	0.534	0.309	3.86550366393634e-150	2	PSMC6	Activated	NME1
3.51565806945512e-154	0.453810548354055	0.435	0.226	6.26806677203153e-150	2	PABPC4	Activated	NME1
1.93451160866188e-153	0.38925048717489	0.235	0.087	3.44904074708327e-149	2	BOLA3	Activated	NME1
9.98209654525667e-153	0.354429703446991	0.874	0.701	1.77970799305381e-148	2	ATP5MC2	Activated	NME1
1.49219574392232e-152	0.417915874250531	0.36	0.162	2.6604357918391e-148	2	TMEM126A	Activated	NME1
1.89821564158808e-152	0.446011133956452	0.4	0.2	3.38432866738739e-148	2	TRAF4	Activated	NME1
2.72529773920008e-152	0.394858674533185	0.23	0.081	4.85893333921982e-148	2	TBL3	Activated	NME1
3.26899092881262e-152	0.430830086216674	0.367	0.173	5.82828392698002e-148	2	GGCT	Activated	NME1
3.53241070970344e-152	-0.524594433164942	0.016	0.13	6.29793505433026e-148	2	CD38	Activated	NME1
4.25479977058054e-152	-0.714310394059203	0.068	0.204	7.58588251096804e-148	2	DAAM1	Activated	NME1
8.99561172172702e-152	0.420192048685699	0.283	0.112	1.60382761386671e-147	2	PTGES2	Activated	NME1

2.87855981760347e-151	0.426124590899462	0.447	0.236
5.13218429880523e-147	2	MRPS26	Activated	NME1
6.16398461245317e-150	0.410598768490844	0.645	0.421
1.09897681655428e-145	2	RAD23A	Activated	NME1
9.80949037492123e-150	0.553285993399219	0.312	0.111
1.74893403894471e-145	2	MYC	Activated	NME1
1.38598862945024e-149	0.429659071657437	0.317	0.144
2.47107912744683e-145	2	POLR2H	Activated	NME1
1.83629251687688e-149	0.387715862572421	0.705	0.484
3.27392592833979e-145	2	SRRM1	Activated	NME1
2.01488105745848e-149	0.389292666971636	0.704	0.48
3.59233143734273e-145	2	NDUFA11	Activated	NME1
2.44573518816492e-148	0.379594461318612	0.603	0.371
4.36050126697923e-144	2	YWHAE	Activated	NME1
3.05993121023916e-148	0.403261929629391	0.283	0.112
5.4555513547354e-144	2	GRWD1	Activated	NME1
3.24107538171652e-148	0.422308247776051	0.291	0.12
5.77851329806238e-144	2	QTRT1	Activated	NME1
3.26568716366169e-148	-0.678165459772295	0.126	0.25
5.82239364409242e-144	2	CAPG	Activated	NME1
3.59880580136559e-148	-0.574160124915883	0.037	0.166
6.41631086325471e-144	2	CD81	Activated	NME1
4.65555399859794e-148	-0.653792793740608	0.146	0.3
8.30038722410028e-144	2	ACAP1	Activated	NME1
4.81081659500637e-148	0.441453020285379	0.431	0.232
8.57720490723685e-144	2	NAA20	Activated	NME1
7.75487184359935e-148	0.425879928937608	0.405	0.205
1.38261610099533e-143	2	C19orf24	Activated	NME1
8.34505936683359e-148	0.3847208654604	0.773	0.567
1.48784063451276e-143	2	HNRNPF	Activated	NME1
1.00242878517513e-147	0.36696342250401	0.78	0.574
1.78723028108874e-143	2	ST13	Activated	NME1
1.09164858497394e-147	-0.724676814729489	0.258	0.409
1.94630026215003e-143	2	SYPL1	Activated	NME1
1.18001224806036e-147	0.422938691217223	0.246	0.09
2.10384383706681e-143	2	PCK2	Activated	NME1
2.22505795467145e-147	0.389088801081843	0.583	0.361
3.96705582738372e-143	2	SNRPD3	Activated	NME1
2.88016511794207e-147	0.343054848149499	0.823	0.624
5.13504638877892e-143	2	TRMT112	Activated	NME1
3.31937317624406e-147	0.427347321508607	0.398	0.197
5.91811043592554e-143	2	HPRT1	Activated	NME1
3.48583459179745e-147	-0.74925002331343	0.205	0.353
6.21489449371567e-143	2	STK17A	Activated	NME1
5.88917620197244e-147	0.368118545211695	0.195	0.064
1.04998122504967e-142	2	ATAD3A	Activated	NME1
6.08109547152849e-147	0.393276911239719	0.682	0.468
1.08419851161882e-142	2	EIF3M	Activated	NME1
7.79777311840267e-147	0.361895880708689	0.202	0.063
1.39026496928001e-142	2	ALKBH2	Activated	NME1

1.13414093690922e-146	0.580207681698476	0.435	0.236	2.02205987641544e-142	2	SRGN	Activated	NME1
2.03756656420043e-146	0.421642170694627	0.587	0.37	3.63277742731295e-142	2	SSB	Activated	NME1
3.4613200942553e-146	0.438497425795609	0.343	0.165	6.17118759604777e-142	2	TMA16	Activated	NME1
1.02219341045243e-145	0.439516988182307	0.459	0.258	1.82246863149564e-141	2	PHPT1	Activated	NME1
2.90497739991265e-145	-0.79684825772833	0.116	0.254	5.17928420630426e-141	2	RGS1	Activated	NME1
6.04343863127188e-145	0.411620456913256	0.304	0.132	1.07748467356946e-140	2	MAGOHB	Activated	NME1
9.3065977540715e-145	0.431496867019531	0.463	0.255	1.65927331357341e-140	2	NCBP2	Activated	NME1
1.15027443215808e-144	0.441990488811076	0.369	0.185	2.05082428509465e-140	2	MRPL23	Activated	NME1
1.91443330178017e-144	0.429269122590167	0.61	0.355	3.41324313374386e-140	2	PDCD4	Activated	NME1
2.95198956660623e-144	0.371582677752679	0.199	0.063	5.26310219830225e-140	2	RRP9	Activated	NME1
1.14487791565144e-143	-0.624446756622106	0.084	0.229	2.04120283581495e-139	2	YPEL3	Activated	NME1
7.55989331219137e-142	0.415702045747189	0.552	0.338	1.3478533786306e-137	2	BSG	Activated	NME1
8.76332894761277e-142	0.385228850823958	0.624	0.405	1.56241391806988e-137	2	ANP32A	Activated	NME1
9.34036177471692e-142	-0.503458455489208	0.03	0.152	1.66529310081428e-137	2	SOCS1	Activated	NME1
3.62055267091262e-141	-0.677413075839105	0.719	0.769	6.4550833569701e-137	2	MYL12A	Activated	NME1
7.22351335550024e-141	0.434177777354745	0.497	0.287	1.28788019615214e-136	2	HAX1	Activated	NME1
1.02783953283367e-140	0.39185303747507	0.267	0.109	1.83253510308915e-136	2	NUP93	Activated	NME1
1.89166988685868e-140	0.416512160352106	0.342	0.163	3.37265824128034e-136	2	NOC2L	Activated	NME1
4.523724872579e-140	0.420280011203178	0.352	0.167	8.0653490753211e-136	2	YIF1A	Activated	NME1
6.30933987747794e-140	0.381159160268284	0.227	0.078	1.12489220675554e-135	2	PDCD11	Activated	NME1
1.54189212430862e-139	0.377601197380901	0.517	0.302	2.74903946842984e-135	2	IMP4	Activated	NME1
3.75122282069695e-139	-0.655930119737543	0.061	0.187	6.68805516702059e-135	2	MARCH1	Activated	NME1
1.05945302906481e-138	-0.462472760249643	0.02	0.13	1.88889880551965e-134	2	PYCARD	Activated	NME1
1.3874575306645e-138	0.39920563358176	0.304	0.137	2.47369803142174e-134	2	PPIH	Activated	NME1
1.99255937196017e-138	0.411946812734448	0.271	0.106	3.55253410426779e-134	2	IARS	Activated	NME1

3.54148130497662e-138 0.41483114788368 0.441 0.242
6.31410701864282e-134 2 HDAC2 Activated NME1
3.93455169165137e-138 0.407002949431804 0.335 0.163
7.01491221104523e-134 2 FARSA Activated NME1
5.70022929722624e-138 -0.590208872731163 0.015 0.12
1.01629388140247e-133 2 VPREB3 Activated NME1
1.06548897362739e-137 -0.578410054997708 0.048 0.173
1.89966029108027e-133 2 C12orf75 Activated NME1
1.93258444371199e-137 -1.20929103297188 0.319 0.44
3.4456048046941e-133 2 STMN1 Activated NME1
5.58385522557468e-137 0.373122603239366 0.217 0.074
9.95545548167709e-133 2 POLR1C Activated NME1
1.16132806244342e-136 0.392280999107356 0.322 0.153
2.07053180253037e-132 2 RPL26L1 Activated NME1
1.26366770995129e-136 0.385832027484674 0.559 0.348
2.25299316007215e-132 2 CDC37 Activated NME1
3.53668577918036e-136 -0.73872856404408 0.064 0.199
6.30555707570066e-132 2 RGS2 Activated NME1
3.67627489903396e-136 0.32656679073534 0.896 0.742
6.55443051748766e-132 2 MT-ND5 Activated NME1
3.89846671419656e-136 0.379077873786036 0.42 0.23
6.95057630474104e-132 2 MRPL13 Activated NME1
8.17609108492962e-136 0.399545939036088 0.432 0.243
1.4577152795321e-131 2 PSMC2 Activated NME1
8.33371342257155e-136 -0.614615962793242 0.146 0.3
1.48581776611028e-131 2 YPEL5 Activated NME1
1.38130755437301e-135 -0.753504801285921 0.269 0.421
2.46273323869164e-131 2 SMIM14 Activated NME1
7.53025187099789e-135 0.41709196875634 0.424 0.23
1.34256860608021e-130 2 SUPT16H Activated NME1
8.82114387493864e-135 0.34102816791447 0.184 0.063
1.57272174146281e-130 2 PPIL1 Activated NME1
1.68936267822684e-134 0.319108627837476 0.153 0.046
3.01196471901063e-130 2 CTPS1 Activated NME1
2.09268699654805e-134 0.40229960569665 0.481 0.281
3.73105164614553e-130 2 SRP72 Activated NME1
5.25982082789728e-134 0.476854527637255 0.309 0.135
9.37773455405805e-130 2 CEBPB Activated NME1
1.18487218467991e-133 -0.539173154188504 0.05 0.181
2.1125086180658e-129 2 SEL1L3 Activated NME1
1.22236754287925e-133 0.403748584202052 0.416 0.223
2.17935909219942e-129 2 ABCF1 Activated NME1
1.95754597251814e-133 0.373842559560355 0.558 0.346
3.4901087144026e-129 2 EWSR1 Activated NME1
5.45318492583279e-133 0.379324444316923 0.308 0.135
9.72248340426728e-129 2 DDX56 Activated NME1
6.7946642783708e-133 -0.714704866944524 0.326 0.461
1.21142069419073e-128 2 PNRC1 Activated NME1
7.57808383612332e-133 0.384556174773904 0.278 0.122
1.35109656714243e-128 2 PSME3 Activated NME1

9.20859157992645e-133	0.394508135742774	0.316	0.137	1.64179979278509e-128	2	FAM162A	Activated	NME1
9.89654584589005e-133	0.378087984707232	0.47	0.27	1.76445515886374e-128	2	CDC123	Activated	NME1
1.91166744867868e-132	0.334768712703344	0.645	0.427	3.40831189424922e-128	2	SEM1	Activated	NME1
6.0872124914562e-132	0.403316143761912	0.405	0.22	1.08528911510173e-127	2	MRPS16	Activated	NME1
7.860312618275e-132	0.406524839066691	0.375	0.196	1.40141513671225e-127	2	PSMD12	Activated	NME1
5.86049066613777e-131	0.41033223130124	0.265	0.116	1.0448668808657e-126	2	GOT1	Activated	NME1
1.98100434974611e-130	0.362924440074933	0.637	0.429	3.53193265516233e-126	2	SF3B5	Activated	NME1
2.07506236397543e-130	-0.456422713820763	0.016	0.114	3.69962868873179e-126	2	RMI2	Activated	NME1
2.30825131747811e-130	0.409764647121225	0.369	0.192	4.11538127393173e-126	2	CHORDC1	Activated	NME1
8.39860495567652e-130	0.392220318501877	0.298	0.138	1.49738727754757e-125	2	UTP11	Activated	NME1
7.23953157802159e-129	0.37762408247346	0.262	0.113	1.29073608504547e-124	2	TOMM5	Activated	NME1
1.28072003405957e-128	0.345607361775934	0.582	0.379	2.28339574872482e-124	2	NDUFS5	Activated	NME1
5.2649802842708e-128	0.38590755438936	0.343	0.167	9.38693334882641e-124	2	USP14	Activated	NME1
1.96235934272974e-127	0.382613953884606	0.325	0.159	3.49869047215285e-123	2	SSSCA1	Activated	NME1
3.70421323138218e-127	-0.423551142567677	0.009	0.104	6.60424177023129e-123	2	WDR66	Activated	NME1
9.70455203647171e-127	0.371220963464041	0.284	0.128	1.73022458258254e-122	2	MPHOSPH6	Activated	NME1
1.09905711922544e-126	-0.715483012400848	0.191	0.338	1.95950893786704e-122	2	AES	Activated	NME1
1.49456347136125e-126	-0.588555636385212	0.112	0.253	2.66465721308997e-122	2	NCF4	Activated	NME1
1.89101915283903e-126	0.374098823577974	0.521	0.317	3.37149804759671e-122	2	TTC1	Activated	NME1
2.07770864095304e-126	0.38253138023479	0.386	0.207	3.70434673595517e-122	2	NUDT5	Activated	NME1
2.86117419562747e-126	0.376926636691861	0.284	0.13	5.10118747338422e-122	2	ADSL	Activated	NME1
8.38164970350142e-126	0.37214535170549	0.274	0.127	1.49436432563727e-121	2	FAM207A	Activated	NME1
1.85201713939377e-125	0.334902232109334	0.777	0.586	3.30196135782516e-121	2	NOP10	Activated	NME1
3.28259902002977e-125	0.391054817737065	0.368	0.175	5.85254579281107e-121	2	ESF1	Activated	NME1
8.81850689170945e-125	0.373333785479985	0.311	0.143	1.57225159372288e-120	2	DNAJC2	Activated	NME1

9.02565796765357e-125	-0.515602519531976		0.045	0.159
1.60918455905295e-120	2	ALOX5	Activated	NME1
2.05387583148271e-124	-0.611726197905061		0.083	0.22
3.66185521995052e-120	2	EAF2	Activated	NME1
2.54089479757381e-124	0.361968180428822		0.551	0.337
4.53016133459435e-120	2	C8orf59	Activated	NME1
4.54997781002161e-124	0.397020991185488		0.459	0.272
8.11215543748753e-120	2	G3BP1	Activated	NME1
5.93649302618688e-123	-0.428187576683807		0.02	0.121
1.05841734163886e-118	2	AC023590.1	Activated	NME1
8.2449455901669e-123	-0.432141993216145		0.016	0.116
1.46999134927086e-118	2	HRK	Activated	NME1
1.10931541621306e-122	0.385951894731778		0.397	0.21
1.97779845556627e-118	2	NOB1	Activated	NME1
3.16431025646152e-122	0.367146588042435		0.299	0.129
5.64164875624524e-118	2	PRDX4	Activated	NME1
5.6599846515773e-122	0.395014885419512		0.402	0.221
1.00911866352972e-117	2	MRPL15	Activated	NME1
5.83873856431047e-122	-0.63575499407548		0.371	0.505
1.04098869863091e-117	2	CYTIP	Activated	NME1
3.6397810831279e-121	0.326954576148843		0.196	0.066
6.48936569310873e-117	2	NAT10	Activated	NME1
4.07956999160225e-121	-0.39283758753236		0.013	0.111
7.27346533802765e-117	2	MYBL2	Activated	NME1
5.06768407737222e-121	0.385163659703118		0.378	0.21
9.03517394154693e-117	2	TIMM17A	Activated	NME1
5.32175306323877e-121	-0.686055719388216		0.15	0.28
9.48815353644841e-117	2	HHEX	Activated	NME1
7.78995824462536e-121	0.361221658639278		0.543	0.346
1.38887165543426e-116	2	POLR2E	Activated	NME1
1.44772920258095e-120	0.391872177559596		0.713	0.502
2.58115639528158e-116	2	ID3	Activated	NME1
1.71800055586839e-120	-0.739133931492141		0.172	0.318
3.06302319105776e-116	2	BCL7A	Activated	NME1
3.41157411555975e-120	-0.738374409491133		0.123	0.247
6.08249549063148e-116	2	UBE2J1	Activated	NME1
3.75212922916241e-120	-0.538786359142618		0.925	0.936
6.68967120267367e-116	2	BTG1	Activated	NME1
1.49147478372128e-119	-0.654448830405644		0.118	0.25
2.65915039189667e-115	2	GCHFR	Activated	NME1
5.8871328642156e-119	0.378918610251521		0.332	0.163
1.049616918361e-114	2	SDAD1	Activated	NME1
6.58674666594697e-119	0.360554061214269		0.317	0.146
1.17435106307168e-114	2	NOL11	Activated	NME1
1.93769491062149e-118	0.368478642497632		0.478	0.29
3.45471625614706e-114	2	PSMD6	Activated	NME1
2.29177524925537e-118	0.347901818061729		0.235	0.095
4.08600609189741e-114	2	LTV1	Activated	NME1
3.91992013148918e-118	0.378712149285198		0.319	0.151
6.98882560243207e-114	2	MMAB	Activated	NME1

4.31049048123927e-118 0.324218431888362 0.199 0.068
7.6851734790015e-114 2 TIMM8A Activated NME1
7.87490104865581e-118 0.361303599798757 0.372 0.191
1.40401610796484e-113 2 MRPS35 Activated NME1
1.03859693162916e-117 -0.503221305760404 0.945 0.937
1.85171446940162e-113 2 EEF2 Activated NME1
2.02281524861907e-117 0.36588792122408 0.297 0.138
3.60647730676293e-113 2 GRPEL1 Activated NME1
2.07024881762412e-117 0.330868942401594 0.2 0.073
3.69104661694204e-113 2 UTP4 Activated NME1
5.55215719120386e-117 0.318063471457774 0.704 0.493
9.89894105619737e-113 2 DNAJC8 Activated NME1
1.3105807261891e-116 0.333477056179957 0.267 0.129
2.33663437672254e-112 2 WDR18 Activated NME1
2.14138550530834e-116 0.358290521991472 0.305 0.146
3.81787621741425e-112 2 WDR77 Activated NME1
4.69113765961035e-116 0.34780938588255 0.263 0.12
8.36382933331929e-112 2 EXOSC7 Activated NME1
7.6345509167071e-116 0.365921687575809 0.457 0.269
1.36116408293971e-111 2 MRPL14 Activated NME1
1.313720040048e-114 0.316700808691997 0.151 0.048
2.34223145940157e-110 2 MT1X Activated NME1
1.34039264657892e-114 0.339591673235246 0.523 0.324
2.38978604958555e-110 2 MRPS18C Activated NME1
5.92779943728044e-114 -0.441070667405865 0.021 0.11
1.05686736167273e-109 2 CDKN2D Activated NME1
1.029882686614e-113 0.354571991155451 0.53 0.324
1.83617784196411e-109 2 RNH1 Activated NME1
1.78220834124645e-113 0.355676445371975 0.456 0.257
3.17749925160829e-109 2 HSPA5 Activated NME1
2.39325221990596e-113 0.344742814232089 0.406 0.229
4.26692938287033e-109 2 PRPF31 Activated NME1
4.32432662352827e-113 0.36106631502493 0.316 0.152
7.70984193708855e-109 2 SMIM37 Activated NME1
5.43347904347052e-113 0.322980429205978 0.183 0.065
9.68734978660358e-109 2 DPH2 Activated NME1
8.30538241286095e-113 -0.668355508471776 0.237 0.377
1.48076663038898e-108 2 RCSD1 Activated NME1
1.58146341792875e-112 -0.415342795242507 0.042 0.151
2.81959112782518e-108 2 CORO1B Activated NME1
3.88202049768624e-112 0.348843512097977 0.505 0.313
6.92125434532479e-108 2 ACP1 Activated NME1
4.7439144269543e-112 -0.648334102596258 0.127 0.257
8.45792503181683e-108 2 RASGRP2 Activated NME1
7.68054479539606e-112 0.354980088911809 0.407 0.231
1.36936433157116e-107 2 NDUFA9 Activated NME1
1.93714725232752e-111 0.357044649270139 0.318 0.169
3.45373983617474e-107 2 LRRC59 Activated NME1
1.98858432832296e-111 -0.705472625677786 0.346 0.447
3.545446998967e-107 2 COTL1 Activated NME1

3.53228426732897e-111 -0.673135495609439 0.506 0.628
6.29770962022083e-107 2 MEF2C Activated NME1
3.55444434672855e-110 0.33408754868834 0.61 0.407
6.33721882578233e-106 2 RNPS1 Activated NME1
4.38104648598264e-110 0.337759526683379 0.509 0.304
7.81096777985846e-106 2 P4HB Activated NME1
4.10057124607833e-109 0.349297628432205 0.414 0.238
7.31090847463305e-105 2 EIF4A3 Activated NME1
9.40615162778701e-109 -0.645681635159699 0.176 0.301
1.67702277371815e-104 2 BCAS4 Activated NME1
1.03163373587623e-108 -0.411893498655033 0.028 0.131
1.83929978769374e-104 2 VNN2 Activated NME1
2.53210037839205e-108 0.358942338027668 0.46 0.272
4.51448176463518e-104 2 PYURF Activated NME1
3.61691583362935e-108 0.354534575647907 0.261 0.116
6.44859923977777e-104 2 SRPRB Activated NME1
4.43326396376168e-108 0.343817145975533 0.493 0.306
7.90406632099071e-104 2 NDUFB6 Activated NME1
4.4868618529205e-108 -0.45173994234011 0.04 0.149
7.99962599757196e-104 2 SIT1 Activated NME1
4.94516181646001e-108 0.302115246281916 0.618 0.414
8.81672900256655e-104 2 LSM3 Activated NME1
5.27348735214993e-108 0.335767214860591 0.531 0.342
9.40210060014811e-104 2 TKT Activated NME1
1.17030798378753e-107 -0.648466727745731 0.093 0.214
2.08654210429478e-103 2 HMCES Activated NME1
1.75620786354884e-107 -1.41433430475421 0.076 0.186
3.13114299992122e-103 2 MZB1 Activated NME1
4.02841557261838e-107 0.340500769430748 0.24 0.095
7.1822621244213e-103 2 SRFBP1 Activated NME1
6.23949513944792e-107 0.337580652197373 0.545 0.353
1.11243958841217e-102 2 NDUFA6 Activated NME1
8.08661770339502e-106 0.300426838276657 0.618 0.427
1.4417630703383e-101 2 ATP5F1C Activated NME1
9.45566165456387e-106 0.317712954786397 0.656 0.465
1.68584991639219e-101 2 WDR83OS Activated NME1
1.05571291646751e-105 -0.313360378523118 1 0.996
1.88223055876992e-101 2 RPS15A Activated NME1
1.52137129240342e-105 -0.515351264780517 0.092 0.221
2.71245287722606e-101 2 GLRX Activated NME1
1.94872329143936e-105 0.342662362628158 0.756 0.52
3.47437875630723e-101 2 CCR7 Activated NME1
2.74521597159844e-105 -0.583011735539886 0.266 0.389
4.89444555576285e-101 2 ZFAND6 Activated NME1
3.40569972288788e-105 -0.547454101334328 0.792 0.818
6.07202203593679e-101 2 NOP53 Activated NME1
3.50821378035006e-105 0.341838657175005 0.312 0.16
6.25479434898611e-101 2 MRPL19 Activated NME1
3.92766134439359e-105 0.343928695249033 0.332 0.173
7.00262741091933e-101 2 METTL5 Activated NME1

4.88864558574276e-105 0.360345473307697 0.308 0.15
8.71596621482077e-101 2 NXT1 Activated NME1
7.66870040920014e-105 -0.696142091039389 0.662 0.763
1.36725259595629e-100 2 HLA-DRB1 Activated NME1
8.5091537653404e-105 0.341426347094047 0.367 0.19
1.51709702482254e-100 2 PITHD1 Activated NME1
1.09124661600274e-104 0.325097576425894 0.537 0.348
1.94558359167128e-100 2 RPS19BP1 Activated NME1
1.27703210358941e-104 -0.30760325187788 1 0.999
2.27682053748957e-100 2 RPL34 Activated NME1
7.79157192450699e-104 -0.585666459947868 0.169 0.297
1.38915935842035e-99 2 CARHSP1 Activated NME1
1.08651788903152e-103 0.410659294974894 0.307 0.147
1.93715274435429e-99 2 CD58 Activated NME1
2.11766364239381e-103 0.335573564622687 0.416 0.24
3.77558250802393e-99 2 NSFL1C Activated NME1
7.27021508591759e-103 0.325911512860823 0.235 0.107
1.29620664766825e-98 2 GCSH Activated NME1
9.84779039673827e-103 -0.605090725228082 0.02 0.103
1.75576254983447e-98 2 PCLAF Activated NME1
1.9166444250127e-102 0.341166257593874 0.222 0.101
3.41718534535513e-98 2 DESI1 Activated NME1
2.10818319429989e-102 -0.643639419844401 0.117 0.222
3.75867981711727e-98 2 LBH Activated NME1
2.84356533768994e-102 0.314293206768091 0.191 0.073
5.0697926405674e-98 2 EXOSC5 Activated NME1
1.36409543277342e-101 -0.681361534430756 0.397 0.494
2.43204574709173e-97 2 NEAT1 Activated NME1
2.25769275724572e-101 0.329369988655003 0.419 0.249
4.02524041689339e-97 2 MTCH2 Activated NME1
2.98775454330194e-101 -0.444738869658509 0.032 0.122
5.32686757525303e-97 2 PBXIP1 Activated NME1
5.12332926712798e-101 0.330231545021803 0.298 0.156
9.13438375036247e-97 2 POLDIP2 Activated NME1
9.34931735705908e-101 0.324686494611906 0.315 0.157
1.66688979159006e-96 2 GTPBP6 Activated NME1
2.00791482870287e-100 0.314444418446639 0.206 0.082
3.57991134809434e-96 2 NOP14 Activated NME1
4.55092803823723e-100 0.306248543722691 0.622 0.43
8.11384959937317e-96 2 CSNK2B Activated NME1
1.19248157772176e-99 0.312359212312412 0.461 0.289
2.12607540492013e-95 2 TIMM8B Activated NME1
1.59146086626844e-99 0.326671479534588 0.276 0.133
2.83741557847e-95 2 TIMM9 Activated NME1
3.90260626556324e-99 -0.557296224044651 0.122 0.241
6.9579567108727e-95 2 ATM Activated NME1
3.91786228150329e-99 -0.493888592588341 0.059 0.154
6.98515666169221e-95 2 SUN2 Activated NME1
4.33914485416025e-99 0.305635120149108 0.603 0.412
7.73626136048231e-95 2 ZNF706 Activated NME1

7.56108463037035e-99	-0.503958475710388			0.065	0.168
1.34806577874873e-94	2	GLIPR1	Activated	NME1	
8.02718298947608e-99	-0.647575918029423			0.292	0.416
1.43116645519369e-94	2	STX7	Activated	NME1	
1.01565130476335e-98	0.327155633468089			0.283	0.138
1.81080471126257e-94	2	BCCIP	Activated	NME1	
3.01200165381706e-98	0.318676856344364			0.521	0.331
5.37009774859043e-94	2	PDIA6	Activated	NME1	
3.25376871293042e-98	-0.531702853517238			0.148	0.271
5.80114423828365e-94	2	CNN2	Activated	NME1	
4.93927044736778e-98	0.345142920225964			0.427	0.25
8.80622528061202e-94	2	CEBPZ	Activated	NME1	
1.27475460910755e-97	-0.486272150949157			0.04	0.13
2.27275999257784e-93	2	LPP	Activated	NME1	
1.70257785349974e-97	0.304316279871957			0.196	0.077
3.03552605500468e-93	2	RRP1B	Activated	NME1	
1.96440408880794e-97	0.335180755614419			0.441	0.257
3.50233604993567e-93	2	SRI	Activated	NME1	
4.00239220620341e-97	-0.597713908392158			0.321	0.424
7.13586506444005e-93	2	NPC2	Activated	NME1	
4.75298646801865e-97	0.314564737415264			0.502	0.318
8.47409957383045e-93	2	PPM1G	Activated	NME1	
4.96518049651161e-97	-0.602786683099836			0.269	0.396
8.85242030723055e-93	2	EVL	Activated	NME1	
5.24487818648254e-97	0.310844160491129			0.562	0.368
9.35109331867973e-93	2	EIF4H	Activated	NME1	
5.25436582925556e-97	0.323291437265421			0.392	0.217
9.36800883697975e-93	2	DNAJC19	Activated	NME1	
1.11713196729163e-96	0.307128969698353			0.553	0.363
1.99173458448426e-92	2	SRSF10	Activated	NME1	
1.17180365166353e-96	0.323640223094944			0.294	0.142
2.08920873055091e-92	2	NAA15	Activated	NME1	
1.23316006562885e-96	0.343987928227577			0.469	0.296
2.19860108100967e-92	2	AKR1A1	Activated	NME1	
1.4756972378614e-96	0.321425324646467			0.553	0.362
2.6310206053831e-92	2	ANXA7	Activated	NME1	
1.59324453731745e-96	-0.480418496849175			0.095	0.209
2.84059568558329e-92	2	POLD4	Activated	NME1	
1.97497507228577e-96	0.335233035208897			0.355	0.188
3.5211830563783e-92	2	NIPA2	Activated	NME1	
3.5143958581135e-96	-0.337094095029313			1	0.998
6.26581637543056e-92	2	B2M	Activated	NME1	
8.26064968446899e-96	-0.605998871926128			0.186	0.3
1.47279123224398e-91	2	DCK	Activated	NME1	
1.20270964242582e-95	0.315434436027847			0.248	0.12
2.144311021481e-91	2	EIF4G1	Activated	NME1	
1.69736815878365e-95	0.308536593671197			0.25	0.119
3.02623769029536e-91	2	FH	Activated	NME1	
1.85843967493083e-95	0.317396994305073			0.611	0.421
3.31341209643418e-91	2	FDPS	Activated	NME1	

2.10755761351709e-95 -0.487994675177488 0.995 0.979
3.75756446913963e-91 2 MT-CO1 Activated NME1
3.48925536991314e-95 0.322230786186182 0.409 0.236
6.22099339901815e-91 2 DDX27 Activated NME1
8.46527933152007e-95 0.342331021839352 0.326 0.166
1.50927465201671e-90 2 ARL2 Activated NME1
1.17922044397125e-94 0.390237225614207 0.431 0.26
2.10243212955635e-90 2 GADD45B Activated NME1
1.76825574892066e-94 0.330857410859782 0.449 0.265
3.15262317475065e-90 2 DPP7 Activated NME1
2.61190541569699e-94 -0.504842875653213 0.06 0.18
4.65676616564616e-90 2 CCDC88A Activated NME1
4.39221359018484e-94 0.326698614731054 0.361 0.205
7.83087760994054e-90 2 SF3A3 Activated NME1
7.24476145094726e-94 0.316992389798292 0.208 0.085
1.29166851908939e-89 2 PLGRKT Activated NME1
7.63197534463156e-94 0.302325504847056 0.396 0.233
1.36070488419436e-89 2 MRPL16 Activated NME1
8.06268379924787e-94 0.317908766159308 0.294 0.145
1.4374958945679e-89 2 MPHOSPH10 Activated NME1
1.35554208523671e-93 -1.22265967223072 0.08 0.155
2.41679598376853e-89 2 IGHA1 Activated NME1
2.41741199916362e-93 0.326703004276053 0.346 0.192
4.31000385330882e-89 2 ITPA Activated NME1
2.51604277782418e-93 -0.51936484324734 0.199 0.316
4.48585266858274e-89 2 CD99 Activated NME1
3.61457017272193e-93 0.305123587491497 0.458 0.286
6.44441716094593e-89 2 SNF8 Activated NME1
4.0335529815908e-93 0.324114581425273 0.458 0.288
7.19142161087824e-89 2 ELAVL1 Activated NME1
4.68360265623676e-93 0.317823744729982 0.346 0.186
8.35039517580451e-89 2 SNRNP40 Activated NME1
6.35722081212708e-93 0.311097819093605 0.45 0.271
1.13342889859414e-88 2 ZMAT2 Activated NME1
2.5237546013855e-92 0.303194837425036 0.422 0.256
4.4996020788102e-88 2 POLR2F Activated NME1
3.27795973039544e-92 -0.642162543193293 0.611 0.686
5.84427440332202e-88 2 HLA-DMA Activated NME1
3.28901441932184e-92 0.310722513919647 0.154 0.052
5.86398380820891e-88 2 TRIB3 Activated NME1
4.63166078299665e-92 0.346214908904382 0.286 0.142
8.25778801000472e-88 2 NAMPT Activated NME1
9.3910281290526e-92 0.305789808881122 0.325 0.175
1.67432640512879e-87 2 CHCHD3 Activated NME1
1.67629608406555e-91 0.322867281962401 0.362 0.209
2.98866828828047e-87 2 DAZAP1 Activated NME1
3.0985072316281e-91 0.308987118671241 0.497 0.328
5.52432854326974e-87 2 MRPS34 Activated NME1
3.21345532712758e-91 -0.594547588919716 0.183 0.289
5.72926950273576e-87 2 ADAM28 Activated NME1

3.7113701246217e-91 0.342801637181622 0.371 0.214
6.61700179518803e-87 2 CCDC85B Activated NME1
7.3984528468435e-91 0.313010774639198 0.285 0.146
1.31907015806373e-86 2 COA4 Activated NME1
1.80840045818883e-90 0.325725436481971 0.329 0.183
3.22419717690487e-86 2 UQCC2 Activated NME1
2.50307129520533e-90 0.303277735127896 0.612 0.432
4.46272581222159e-86 2 EIF5 Activated NME1
7.46425057468166e-90 0.34484087251307 0.378 0.217
1.33080123495999e-85 2 NAXE Activated NME1
1.15909308596133e-89 0.302210510314041 0.362 0.214
2.06654706296045e-85 2 HDDC2 Activated NME1
1.61319715434533e-89 0.30745909553185 0.241 0.114
2.87616920648229e-85 2 BMS1 Activated NME1
1.7759576969488e-89 0.31868554872495 0.325 0.169
3.16635497789002e-85 2 PEPD Activated NME1
2.37168931140128e-89 -0.684837881801627 0.691 0.729
4.22848487329735e-85 2 LIMD2 Activated NME1
2.83333920690875e-89 0.34453511945004 0.306 0.162
5.05156047199761e-85 2 CARS Activated NME1
3.6555484925161e-89 -0.722066145409201 0.128 0.22
6.51747740730696e-85 2 PLAC8 Activated NME1
6.92653415384986e-89 0.307673227760059 0.391 0.231
1.23493177428989e-84 2 SNRPA1 Activated NME1
6.95029320132052e-89 0.307312499564864 0.201 0.083
1.23916777486344e-84 2 SURF6 Activated NME1
2.11505807839354e-88 0.30618230040059 0.388 0.237
3.77093704796785e-84 2 MCTS1 Activated NME1
2.44449484084794e-88 0.30638227128076 0.328 0.179
4.35828985174779e-84 2 TRMT10C Activated NME1
3.01043993416248e-88 0.313924677077081 0.345 0.192
5.36731335861828e-84 2 TMEM208 Activated NME1
5.14971314323447e-88 -0.562742024570944 0.186 0.278
9.18142356307273e-84 2 GPR183 Activated NME1
5.59473408113681e-88 0.324072630122331 0.653 0.458
9.97485139325882e-84 2 SQSTM1 Activated NME1
6.71121343449876e-88 0.307172950852575 0.395 0.231
1.19654224323678e-83 2 RPL7L1 Activated NME1
7.14931388905622e-88 -0.413193127341519 0.047 0.135
1.27465117327983e-83 2 ZBTB20 Activated NME1
2.40782188759865e-87 0.31191288932222 0.309 0.155
4.29290564339963e-83 2 RABGGTB Activated NME1
3.52750628997764e-87 0.311739508966016 0.374 0.221
6.28919096440114e-83 2 DCXR Activated NME1
3.72504966435747e-87 0.30340953646448 0.366 0.213
6.64139104658293e-83 2 DDX54 Activated NME1
4.78078188996488e-87 0.310500264968734 0.346 0.198
8.52365603161839e-83 2 COPS3 Activated NME1
7.05941451254977e-87 0.314186610225916 0.454 0.283
1.2586230134425e-82 2 MRPS18B Activated NME1

8.77661806686115e-87 0.311389842826274 0.378 0.225
1.56478323514068e-82 2 MORF4L2 Activated NME1
9.6752536196353e-87 0.329031486284082 0.498 0.308
1.72500096784478e-82 2 PMAIP1 Activated NME1
1.35661218020253e-86 0.304530742652361 0.319 0.18
2.41870385608309e-82 2 HARS Activated NME1
2.95655534450166e-86 0.306000237080375 0.277 0.137
5.27124252371201e-82 2 AFG3L2 Activated NME1
3.3477191302157e-86 -0.581215195469449 0.362 0.434
5.96864843726158e-82 2 PLP2 Activated NME1
4.67208590482346e-86 0.306846632504601 0.258 0.126
8.32986195970975e-82 2 ZPR1 Activated NME1
1.20496959755099e-85 -0.598959741570568 0.298 0.409
2.14834029547366e-81 2 ORAI2 Activated NME1
1.05370329230384e-84 0.321899776990327 0.394 0.228
1.87864759984852e-80 2 DENR Activated NME1
2.19380485144613e-84 0.304807794752447 0.353 0.195
3.9113346696433e-80 2 DNTTIP2 Activated NME1
2.31807857109048e-84 0.326072928819176 0.322 0.174
4.13290228439721e-80 2 ATP1A1 Activated NME1
6.51308281099462e-83 -0.399968935028656 0.057 0.157
1.16121753437223e-78 2 SH2B2 Activated NME1
1.26495946488961e-82 0.302135725049219 0.285 0.14
2.25529622995168e-78 2 CFAP97 Activated NME1
8.29539804785567e-82 0.310263113821458 0.38 0.227
1.47898651795219e-77 2 PTBP1 Activated NME1
1.67903497373661e-81 -0.573492972935058 0.188 0.296
2.993551454675e-77 2 PPM1K Activated NME1
3.02196235764848e-81 -0.400967058418814 0.053 0.13
5.38785668745147e-77 2 DDAH2 Activated NME1
3.09686887312332e-81 -0.474966057136603 0.077 0.183
5.52140751389156e-77 2 TMEM156 Activated NME1
3.8716072971762e-81 0.316717381621747 0.507 0.335
6.90268865013544e-77 2 CHMP4B Activated NME1
9.04616086546675e-81 0.3024417074241 0.393 0.231
1.61284002070407e-76 2 HNRNPA1P48 Activated NME1
6.71550143010451e-80 -0.344910613493614 0.04 0.119
1.19730674997333e-75 2 CRIP1 Activated NME1
3.26520780114613e-79 -0.540250915932803 0.146 0.246
5.82153898866343e-75 2 CCND3 Activated NME1
5.70087987619477e-78 0.304523958232879 0.442 0.277
1.01640987312677e-73 2 TNIP1 Activated NME1
6.69589529797897e-77 -0.68193041104757 0.119 0.218
1.19381117267667e-72 2 FOS Activated NME1
1.83183674899398e-76 0.362729304767404 0.474 0.321
3.26598173978136e-72 2 ACAT2 Activated NME1
6.08081720877685e-76 -0.569426174449419 0.223 0.332
1.08414890015282e-71 2 TNFRSF13C Activated NME1
1.69727732042871e-73 -0.390367941340936 0.052 0.136
3.02607573459234e-69 2 CCNG2 Activated NME1

5.63288439714118e-73	-0.344891045554967	0.065	0.153	1.0042869591663e-68	2	FLOT1	Activated	NME1
6.50360342988686e-73	-0.504023513027617	0.236	0.339	1.15952745551453e-68	2	CCNDBP1	Activated	NME1
4.08330916403259e-72	-0.587681392912288	0.452	0.519	7.28013190855371e-68	2	BANK1	Activated	NME1
2.82430140225322e-71	-0.507345837152341	0.184	0.284	5.03544697007727e-67	2	RSRP1	Activated	NME1
4.30589662297349e-71	-0.469434064980145	0.161	0.259	7.67698308909943e-67	2	SH3KBP1	Activated	NME1
1.53398895335878e-69	-0.555935443979036	0.148	0.242	2.73494890494337e-65	2	IL4R	Activated	NME1
1.99936939259046e-69	-0.56149373074984	0.394	0.475	3.56467569004953e-65	2	ITM2B	Activated	NME1
1.15336617178451e-68	-0.517105074236409	0.194	0.309	2.0563365476746e-64	2	TCF4	Activated	NME1
1.91390943824535e-68	-0.383009043147755	0.064	0.153	3.41230913744763e-64	2	HSH2D	Activated	NME1
2.67605967663059e-68	-0.494363842257928	0.191	0.288	4.77114679746468e-64	2	STK17B	Activated	NME1
5.28754622244559e-68	0.313409599548179	0.286	0.15	9.42716615999825e-64	2	MARCKS	Activated	NME1
5.02220485165694e-67	-0.410179906942766	0.093	0.176	8.95408903001916e-63	2	BNIP3L	Activated	NME1
1.25854994394102e-66	0.317190569028391	0.345	0.203	2.24386869505245e-62	2	HMGCS1	Activated	NME1
7.0562470025491e-66	-0.42305886958187	0.088	0.175	1.25805827808448e-61	2	SNX10	Activated	NME1
3.01175450705413e-65	0.342675594760767	0.21	0.123	5.3696571106268e-61	2	PHGDH	Activated	NME1
5.84733721492309e-65	-0.303645957591996	0.046	0.125	1.04252175204864e-60	2	ABI3	Activated	NME1
5.04148403891268e-64	0.311311929807175	0.334	0.197	8.98846189297742e-60	2	C19orf48	Activated	NME1
8.16093209727679e-64	-0.506761514618367	0.214	0.302	1.45501258362348e-59	2	BCL11A	Activated	NME1
9.28227454866376e-63	-1.46060819608233	0.16	0.222	1.65493672928126e-58	2	IGHG1	Activated	NME1
1.30420034347849e-62	-0.322370030826796	0.035	0.101	2.3252587923878e-58	2	ABTB1	Activated	NME1
3.49553890025574e-62	-0.485296796577855	0.076	0.15	6.23219630526596e-58	2	DUSP1	Activated	NME1
4.66029638862682e-62	-0.44291977505239	0.16	0.251	8.30884243128276e-58	2	NCOA3	Activated	NME1
9.45770321819032e-62	-0.376411554511344	0.048	0.106	1.68621390677115e-57	2	RHOC	Activated	NME1
2.7691609926832e-61	-0.30056296263375	0.49	0.505	4.93713713385488e-57	2	HLA-DRB5	Activated	NME1
7.23039808382609e-61	-0.41816154232751	0.652	0.702	1.28910767436535e-56	2	HLA-DQB1	Activated	NME1

7.49790436965463e-61	-0.530498302240063	0.137	0.213	
1.33680137006572e-56	2	PRKCB	Activated	NME1
2.05880945480255e-60	-0.413110516741047	0.174	0.269	
3.67065137696747e-56	2	ILK	Activated	NME1
5.9909308895868e-59	-0.54551605224324	0.189	0.264	
1.06812306830443e-54	2	MDM4	Activated	NME1
7.54817852135911e-59	-0.968777845396706	0.126	0.221	
1.34576474857312e-54	2	PLCG2	Activated	NME1
8.48352267398016e-59	-0.459506996689824	0.162	0.239	
1.51252725754392e-54	2	HSD17B11	Activated	NME1
1.00424810996935e-58	-0.424566355447409	0.186	0.275	
1.79047395526436e-54	2	GRN	Activated	NME1
2.66809730242344e-58	-0.433712238728308	0.17	0.259	
4.75695068049075e-54	2	WIPF1	Activated	NME1
4.70960155470062e-58	-0.432666238828936	0.167	0.249	
8.39674861187573e-54	2	ARHGAP45	Activated	NME1
1.51866109414541e-57	-0.433818577236352	0.131	0.21	
2.70762086475186e-53	2	TRIM38	Activated	NME1
3.41703291985037e-57	-0.475480426328181	0.229	0.305	
6.09222799280123e-53	2	PDLIM1	Activated	NME1
9.78400065971045e-57	-0.498792975275297	0.683	0.701	
1.74438947761978e-52	2	CCNI	Activated	NME1
1.94599004536246e-55	-0.505058385651795	0.302	0.366	
3.46950565187673e-51	2	ARID5B	Activated	NME1
6.72296856650396e-55	-0.443663878748888	0.188	0.276	
1.19863806572199e-50	2	ALOX5AP	Activated	NME1
1.26749506555486e-54	-0.372761620091331	0.098	0.185	
2.25981695237776e-50	2	SYK	Activated	NME1
5.95326010637743e-54	-0.529059608467219	0.175	0.245	
1.06140674436603e-49	2	CD55	Activated	NME1
1.05194706134185e-53	-1.52080869088947	0.279	0.305	
1.87551641566638e-49	2	HMGB2	Activated	NME1
2.08458429752646e-53	-0.314441471157427	0.04	0.105	
3.71660534405992e-49	2	MX1	Activated	NME1
2.82514853462855e-53	-0.459893462612256	0.319	0.399	
5.03695732238924e-49	2	SH3BGRL	Activated	NME1
5.9340470082253e-53	-0.387755876715906	0.93	0.899	
1.05798124109649e-48	2	MYL6	Activated	NME1
8.56589955192965e-53	-0.333098077767513	0.078	0.157	
1.52721423111354e-48	2	PTPN18	Activated	NME1
8.71084185273901e-53	-0.506621643206216	0.397	0.451	
1.55305599392484e-48	2	KLF6	Activated	NME1
2.97382727673226e-52	-0.353834126028578	0.082	0.151	
5.30203665168595e-48	2	GNB5	Activated	NME1
1.07248328972786e-51	-0.497646611935533	0.341	0.428	
1.91213045725581e-47	2	AC114760.2	Activated	NME1
5.26697322105052e-51	-0.400907116217335	0.206	0.285	
9.39048655581098e-47	2	CCNG1	Activated	NME1
1.52507767544152e-50	-0.303753430195746	0.077	0.153	
2.71906098754469e-46	2	P2RY8	Activated	NME1

5.20699427512772e-50 -0.317331895255215 0.044 0.106
9.28355009312521e-46 2 CENPM Activated NME1
1.38075349110524e-49 -0.3849679879503 0.138 0.216
2.46174539929152e-45 2 MIS18BP1 Activated NME1
1.60453762432336e-49 -0.401019446873082 0.125 0.22
2.86073013040612e-45 2 ADA Activated NME1
2.13201540885037e-49 -0.536497611249029 0.246 0.302
3.80117027243933e-45 2 LINC00926 Activated NME1
2.57367027141218e-49 -0.439077425474015 0.225 0.308
4.58859672690078e-45 2 ITSN2 Activated NME1
9.99828794024086e-49 -0.300000827498627 0.049 0.111
1.78259475686554e-44 2 SCIMP Activated NME1
3.34145433633086e-48 -0.465346506002241 0.218 0.286
5.95747893624429e-44 2 RNASET2 Activated NME1
4.07757659199629e-48 -0.459317686679048 0.445 0.497
7.26991130587019e-44 2 ANKRD12 Activated NME1
4.38102430690772e-48 -0.505946864318186 0.829 0.812
7.81092823678577e-44 2 CORO1A Activated NME1
4.38192060118831e-48 -0.311995270557337 0.084 0.153
7.81252623985864e-44 2 ANKRD13A Activated NME1
4.92386102383622e-48 -0.477805781014665 0.601 0.624
8.77875181939759e-44 2 ARPC1B Activated NME1
7.38938649177735e-48 -0.488570322578035 0.341 0.409
1.31745371761898e-43 2 MBD4 Activated NME1
1.05336643706519e-47 -0.361361165544466 0.106 0.18
1.87804702064353e-43 2 IRF7 Activated NME1
1.36454808483637e-47 -0.315729785602007 0.074 0.154
2.43285278045476e-43 2 SEC14L1 Activated NME1
1.59541005467127e-47 -0.348163181745099 0.996 0.977
2.84445658647342e-43 2 MT-CO2 Activated NME1
2.40790476175743e-47 -0.41563008743674 0.189 0.264
4.29305339973732e-43 2 LYSMD2 Activated NME1
5.21641995699913e-47 -0.312345616473222 0.049 0.121
9.30035514133374e-43 2 TNFRSF17 Activated NME1
8.12328006227532e-47 -0.594525674084326 0.421 0.459
1.44829960230307e-42 2 JUNB Activated NME1
9.98076782968524e-47 -0.467429854399914 0.659 0.671
1.77947109635458e-42 2 SNX3 Activated NME1
1.08538001651318e-46 -0.382349690248875 0.196 0.282
1.93512403144136e-42 2 POU2AF1 Activated NME1
1.7300734296859e-46 -0.44910689057068 0.364 0.43
3.08454791778699e-42 2 GABARAPL2 Activated NME1
1.90974987388626e-46 -0.373757396706587 0.167 0.215
3.40489305015182e-42 2 MGST3 Activated NME1
3.13723030913858e-46 -0.352234846926084 0.101 0.172
5.59336791816317e-42 2 DEF8 Activated NME1
6.17292780624347e-46 -0.404921719014291 0.254 0.328
1.10057129857515e-41 2 IGBP1 Activated NME1
1.06448140881949e-45 -0.31362594627171 0.092 0.167
1.89786390378427e-41 2 IL10RA Activated NME1

1.4653658335514e-45 -0.480206359170144 0.255 0.319
2.61260074463878e-41 2 PSIP1 Activated NME1
9.38699915774046e-45 -0.330788339893162 0.111 0.19
1.67360807983355e-40 2 RASGRP3 Activated NME1
9.90052577979769e-45 -0.338237859365308 0.081 0.146
1.76516474128013e-40 2 ADD3 Activated NME1
1.73842491599651e-44 -0.410631250277086 0.228 0.292
3.09943778273018e-40 2 PLEKHF2 Activated NME1
2.07078161352817e-44 -0.32446394604143 0.087 0.147
3.69199653875937e-40 2 MYO1G Activated NME1
4.46941390976989e-44 -0.447647048000217 0.451 0.498
7.96851805972873e-40 2 SP100 Activated NME1
8.79229752111571e-44 -0.31535500027963 0.075 0.136
1.56757872503972e-39 2 SIPA1 Activated NME1
1.14460758212195e-43 -0.353584884042457 0.049 0.117
2.04072085816523e-39 2 CD9 Activated NME1
1.30970718246421e-43 -0.312844052348281 0.106 0.176
2.33507693561545e-39 2 CYB5R3 Activated NME1
2.50858983579832e-43 -0.434810247853904 0.197 0.271
4.47256481824482e-39 2 CD22 Activated NME1
5.34534972597438e-43 -0.36829653717428 0.133 0.189
9.53022402643972e-39 2 MBP Activated NME1
5.53995380036069e-43 -0.34993763393441 0.062 0.11
9.87718363066307e-39 2 C1orf162 Activated NME1
1.51108290095256e-42 -0.45116483494666 0.693 0.704
2.69410970410833e-38 2 COMMD6 Activated NME1
2.16263447275979e-41 -0.350631554155338 0.126 0.189
3.85576100148343e-37 2 INPP5D Activated NME1
4.05058945677043e-41 -0.418476427347159 0.266 0.337
7.22179594247601e-37 2 TSPO Activated NME1
1.14615334356398e-40 -0.311133735828624 0.09 0.154
2.04347679624021e-36 2 SPSB3 Activated NME1
1.70721442586062e-40 -0.43434606838819 0.439 0.497
3.0437925998669e-36 2 RAC2 Activated NME1
2.42076823958651e-40 -0.390507392732972 0.072 0.116
4.31598769435879e-36 2 C1orf56 Activated NME1
3.30175609464785e-40 -0.65878181536583 0.142 0.195
5.88670094114765e-36 2 PTTG1 Activated NME1
3.91155314694661e-40 -0.44609757482205 0.231 0.291
6.97390810569112e-36 2 SP110 Activated NME1
4.03322194387121e-40 -0.372860482119963 0.115 0.194
7.19083140372798e-36 2 P2RX5 Activated NME1
5.59110055759143e-40 -0.302560944500337 0.074 0.133
9.96837318412976e-36 2 AMFR Activated NME1
8.88855448419422e-40 -0.507865203054649 0.336 0.354
1.58474037898699e-35 2 TXNIP Activated NME1
1.43986693368574e-39 -0.342439589550795 0.151 0.224
2.56713875606831e-35 2 PNKD Activated NME1
3.24192807475611e-39 -0.313460692871297 0.076 0.141
5.78003356448266e-35 2 PLEKHA2 Activated NME1

8.77052592052494e-39 -0.365347088922044 0.192 0.268
1.56369706637039e-34 2 CCDC69 Activated NME1
1.05965255279857e-38 -0.483122569144644 0.294 0.352
1.88925453638458e-34 2 BIRC3 Activated NME1
2.57737185331737e-38 -0.411604442447002 0.138 0.196
4.59519627727953e-34 2 S1PR4 Activated NME1
5.97527710638692e-38 -0.315621586747153 0.074 0.128
1.06533215529772e-33 2 RNF213 Activated NME1
8.25510520135624e-38 -0.332983670587617 0.189 0.252
1.4718027063498e-33 2 TPM4 Activated NME1
1.7113752152419e-37 -3.12081445194393 0.447 0.451
3.05121087125479e-33 2 IGHG3 Activated NME1
2.56873284742179e-37 -0.34607713579383 0.167 0.241
4.5797937936683e-33 2 CPNE3 Activated NME1
3.38988777202763e-37 -0.402170768887498 0.279 0.34
6.04383090874806e-33 2 FAM96A Activated NME1
9.6601002140413e-37 -0.354859731114949 0.151 0.208
1.72229926716142e-32 2 BIN1 Activated NME1
1.34215832876977e-36 -0.36921948164465 0.143 0.21
2.39293408436362e-32 2 IRF1 Activated NME1
3.48226626743979e-36 -0.469600438587105 0.561 0.571
6.2085325282184e-32 2 VIM Activated NME1
4.15151699513343e-36 -0.3427301088288 0.146 0.213
7.40173965062339e-32 2 CAT Activated NME1
5.44798510540312e-36 -0.3291768742892 0.066 0.114
9.71321264442321e-32 2 LINC02397 Activated NME1
7.85592226666223e-36 -0.385888669792185 0.207 0.271
1.40063238092321e-31 2 RBM6 Activated NME1
1.11877659957558e-35 -0.360011365371689 0.15 0.214
1.9946667993833e-31 2 SNHG25 Activated NME1
1.5262688392932e-35 -0.469001943234088 0.301 0.342
2.72118471357584e-31 2 HVCN1 Activated NME1
5.11727362321912e-35 -0.347896337139685 0.237 0.308
9.12358714283736e-31 2 CDK2AP2 Activated NME1
6.44559189444716e-35 -0.344748081781178 0.109 0.152
1.14918457886098e-30 2 ARRDC2 Activated NME1
6.50137236861857e-35 -0.397565769451709 0.368 0.424
1.159129679601e-30 2 CTSH Activated NME1
6.78236596942393e-34 -0.351826292029161 0.163 0.218
1.20922802868859e-29 2 WASHC4 Activated NME1
2.02716418867201e-33 -0.479381293906503 0.29 0.343
3.61423103198332e-29 2 IER2 Activated NME1
4.53207018378423e-33 -0.303267356430779 0.101 0.146
8.08022793066891e-29 2 LY9 Activated NME1
7.44317980688123e-33 -0.38717363901758 0.28 0.338
1.32704452776886e-28 2 BPTF Activated NME1
7.81981692236436e-33 -0.397326945437189 0.233 0.279
1.39419515908834e-28 2 TNRC6B Activated NME1
2.65141663503733e-32 -0.313164505505768 0.144 0.199
4.72721071860805e-28 2 CYTH1 Activated NME1

3.69528813421855e-32	-0.383748439411206	0.35	0.393	6.58832921449825e-28	2	ARL6IP5	Activated	NME1
6.20824862731377e-32	-0.405149646358088	0.599	0.617	1.10686864776377e-27	2	PNISR	Activated	NME1
8.52405285598723e-32	-0.397172944972668	0.49	0.494	1.51975338369396e-27	2	FXYD5	Activated	NME1
1.08182876700232e-31	-0.319191173908665	0.092	0.152	1.92879250868844e-27	2	TAGAP	Activated	NME1
1.45887530012822e-31	-0.300104893383818	0.125	0.182	2.6010287725986e-27	2	RGS19	Activated	NME1
6.52053763143201e-31	-0.342325038844442	0.227	0.283	1.16254665430801e-26	2	TANK	Activated	NME1
1.09675362460162e-30	-0.37453233039427	0.461	0.507	1.95540203730222e-26	2	PSMB9	Activated	NME1
1.79447845979712e-30	-0.319007038450696	0.178	0.229	3.19937564597229e-26	2	MAGED2	Activated	NME1
1.21594651446495e-29	-0.314255110532397	0.172	0.227	2.16791104063956e-25	2	SKAP2	Activated	NME1
1.68211809809251e-29	-0.345311836482737	0.251	0.295	2.99904835708914e-25	2	YWHAH	Activated	NME1
2.36772527553935e-29	-0.318534692336489	0.941	0.915	4.2214173937591e-25	2	PFDN5	Activated	NME1
4.3093382804343e-29	-0.323936297988108	0.142	0.19	7.68311922018631e-25	2	ANKRD44	Activated	NME1
9.67052489927332e-29	-0.330219019406863	0.186	0.245	1.72415788429144e-24	2	SP140	Activated	NME1
3.51696560929258e-28	-0.323457196708764	0.17	0.233	6.27039798480773e-24	2	UBE2G1	Activated	NME1
5.84117861557303e-28	-0.391659021271962	0.261	0.32	1.04142373537052e-23	2	HMGN3	Activated	NME1
6.3527026452084e-28	-0.365460243315971	0.124	0.174	1.13262335461421e-23	2	SMC4	Activated	NME1
1.26022983218895e-27	-0.334146766920466	0.205	0.259	2.24686376780968e-23	2	PRMT2	Activated	NME1
1.61329530205691e-27	-0.472588132115282	0.232	0.272	2.87634419403726e-23	2	JUN	Activated	NME1
1.39711207496666e-26	-0.343366368131593	0.278	0.329	2.49091111845806e-22	2	BLNK	Activated	NME1
1.64531586177576e-26	-0.406655046357424	0.317	0.351	2.93343364996001e-22	2	TRAF3IP3	Activated	NME1
3.70184041433674e-26	-0.347473108901451	0.237	0.288	6.60001127472098e-22	2	LAT2	Activated	NME1
4.52890246169904e-26	-0.331161782910938	0.147	0.2	8.07458019896322e-22	2	FAM129C	Activated	NME1
6.71629474163954e-26	-0.34312182057263	0.343	0.388	1.19744818948691e-21	2	JAK1	Activated	NME1
1.7583584323904e-25	-0.36519885409691	0.436	0.462	3.13497724910884e-21	2	SWAP70	Activated	NME1
1.81171142417104e-25	-0.325899955594766	0.19	0.241	3.23010029815455e-21	2	DSTN	Activated	NME1

2.26520984230689e-25 -0.315387431317637 0.207 0.249
4.03864262784895e-21 2 GCC2 Activated NME1
5.08037201445766e-25 -0.354844631814005 0.495 0.518
9.05779526457657e-21 2 VAMP2 Activated NME1
5.66051711458664e-25 -0.331779028107855 0.216 0.258
1.00921359635965e-20 2 PRDM2 Activated NME1
6.38792398160417e-25 -0.386909873660564 0.429 0.446
1.13890296668021e-20 2 ANXA2 Activated NME1
6.43265252908309e-25 -0.386798069918618 0.867 0.699
1.14687761941022e-20 2 HMGN2 Activated NME1
1.01765411285426e-24 -0.304315504241698 0.167 0.209
1.81437551780787e-20 2 RALGPS2 Activated NME1
1.16148696112437e-24 -0.318969978847153 0.222 0.267
2.07081510298864e-20 2 DAPP1 Activated NME1
1.75563190567646e-24 -0.363193474351609 0.451 0.489
3.13011612463055e-20 2 GMFG Activated NME1
2.81050096332946e-24 -0.344429381622036 0.511 0.533
5.01084216752009e-20 2 PAIP2 Activated NME1
3.11542688825835e-24 -0.31416080615279 0.196 0.246
5.5544945990758e-20 2 DRAM2 Activated NME1
5.17536275225738e-24 -0.300564034893864 0.202 0.254
9.22715425099969e-20 2 PPP2R5C Activated NME1
7.13808919741921e-24 -0.301887672018433 0.102 0.165
1.27264992300787e-19 2 KIAA0040 Activated NME1
9.4906115986882e-24 -0.310602465186689 0.164 0.203
1.69208114193012e-19 2 CLK1 Activated NME1
1.10557686114991e-23 -0.312859778393487 0.228 0.275
1.97113298574417e-19 2 SEPT9 Activated NME1
2.96019002948027e-23 -0.302084843668533 0.146 0.195
5.27772280356038e-19 2 LBR Activated NME1
7.39916908469814e-23 -0.308433585670709 0.385 0.427
1.31919785611083e-18 2 ECH1 Activated NME1
3.89241069418539e-22 -0.319157488619477 0.287 0.335
6.93977902666312e-18 2 CCM2 Activated NME1
5.8808236115339e-22 -0.329729029314771 0.16 0.196
1.04849204170038e-17 2 DNMT1 Activated NME1
1.43158894441085e-21 -0.310914660329315 0.282 0.317
2.55237992899011e-17 2 SPI1 Activated NME1
2.44392295284508e-21 -0.312271153421648 0.212 0.252
4.35727023262749e-17 2 OFD1 Activated NME1
5.0535294508006e-21 -0.315235504732349 0.273 0.316
9.00993765783239e-17 2 IL16 Activated NME1
1.0277017478395e-20 -1.29325869747352 0.936 0.925
1.83228944622305e-16 2 IGKC Activated NME1
1.15667308031669e-20 -0.334967192713129 0.369 0.389
2.06223243489662e-16 2 PSAP Activated NME1
1.77122777689937e-20 -0.343784232171417 0.598 0.629
3.15792200343388e-16 2 HLA-DMB Activated NME1
3.82263605486427e-20 -0.33096088268511 0.233 0.279
6.81537782221752e-16 2 ITGAE Activated NME1

7.17379048169615e-20 -2.30043507428884 0.08 0.131
1.27901510498161e-15 2 IGHG2 Activated NME1
2.80081486524063e-19 -0.427220455801364 0.82 0.782
4.99357282323752e-15 2 ACTG1 Activated NME1
1.70836579734804e-18 -0.400011235776471 0.492 0.492
3.04584538009182e-14 2 C4orf3 Activated NME1
2.70752572410596e-18 -0.301476833658515 0.74 0.721
4.82724761350852e-14 2 SRSF5 Activated NME1
3.4571974383303e-18 -0.301444165320141 0.184 0.201
6.1638373127991e-14 2 PPP3CA Activated NME1
5.1299397247337e-18 -0.307099832105195 0.964 0.937
9.14616953522771e-14 2 H3F3A Activated NME1
1.77654639655355e-17 -0.334504713607671 0.417 0.431
3.16740457041533e-13 2 IRF8 Activated NME1
2.78174119482017e-17 -1.96407649488558 0.229 0.245
4.95956637624488e-13 2 IGHG4 Activated NME1
4.0201612759378e-17 -0.334518647539581 0.345 0.368
7.1675455388695e-13 2 SNAP23 Activated NME1
5.44744066460871e-17 -0.324844722456488 0.24 0.249
9.71224196093087e-13 2 TNFRSF13B Activated NME1
7.26695739383096e-17 -0.447110707044216 0.358 0.36
1.29562583374612e-12 2 H2AFV Activated NME1
1.4085073889815e-16 -0.483836173966986 0.964 0.928
2.51122782381512e-12 2 HMGB1 Activated NME1
1.5183080279374e-16 -0.309143929549269 0.333 0.361
2.70699138300959e-12 2 TMEM243 Activated NME1
1.77735776485387e-16 -0.333886152241812 0.451 0.456
3.16885115895796e-12 2 CTSS Activated NME1
4.6473605689216e-16 -0.470714725002935 0.976 0.928
8.28577915833032e-12 2 SERF2 Activated NME1
9.4727484823466e-16 -1.33719254152411 0.439 0.42
1.68889632691757e-11 2 HIST1H4C Activated NME1
6.8954490798643e-15 -0.306942887788435 0.195 0.221
1.22938961644901e-10 2 NANS Activated NME1
1.13824885200955e-14 -0.31844586177574 0.346 0.402
2.02938387824783e-10 2 CSTB Activated NME1
1.59359143388835e-14 -0.304752145757468 0.481 0.477
2.84121416747953e-10 2 SSR2 Activated NME1
4.1441877756776e-12 -0.353760845374357 0.71 0.673
7.38867238525558e-08 2 TAGLN2 Activated NME1
7.22625753609454e-12 -0.347329246316132 0.611 0.573
1.2883694561103e-07 2 SARAF Activated NME1
2.71777626681081e-11 -0.564655190452624 0.716 0.649
4.845523306097e-07 2 TCL1A Activated NME1
1.04704997956577e-10 -0.30745214828717 0.64 0.618
1.86678540856782e-06 2 IFI16 Activated NME1
9.73303490475578e-10 -0.302639442606208 0.923 0.863
1.73530279316891e-05 2 ARPC3 Activated NME1
1.26790925790234e-09 -0.321939262255053 0.258 0.252
2.26055541591408e-05 2 FCMR Activated NME1

5.87894962249768e-09	-0.350458372886502	0.382	0.374
0.000104815792819511	2	ACADM	Activated NME1
1.17586311047708e-08	-1.790334962483	0.634	0.646
0.000209644633966958	2	IGLC2	Activated NME1
1.50493839350038e-08	-0.439006078583126	0.635	0.583
0.000268315466177183	2	MARCKSL1	Activated NME1
1.38573348859247e-07	-0.324177052704794	0.463	0.419
0.00247062423681152	2	DEK	Activated NME1
0.00125607773535111	-1.0929631922858	0.475	0.424	1	2
IGLC3	Activated NME1
0	1.51383922834952	0.866	0.433	0	3	S100A6
Memory 3
0	1.42581374466845	0.92	0.542	0	3	VIM
Memory 3
0	1.18886742022012	0.494	0.132	0	3	LMNA
Memory 3
0	0.92033568397809	0.739	0.421	0	3	ANXA2
Memory 3
0	0.785753280141066	0.948	0.746	0	3	PKM
Memory 3
0	0.43339459230157	1	0.999	0	3	EEF1A1
Memory 3
0	0.342451045812923	1	0.999	0	3
RPLP1	Memory 3
0	-1.22526433615044	0.973	0.99	0	3
CD74	Memory 3
0	-1.33391071858181	0.846	0.954	0	3
HLA-DRA	Memory 3
3.53084738611371e-300	-1.93311380213739	0.213	0.511
6.29514780470213e-296	3	LTB	Memory 3
3.19543255361979e-262	-1.05679839194767	0.429	0.699
5.69713669984872e-258	3	HLA-DMA	Memory 3
8.07688701071621e-247	0.567437867211527	0.931	0.679
1.44002818514059e-242	3	EMP3	Memory 3
1.81648450049589e-239	0.340367564139393	1	0.996
3.23861021593412e-235	3	RPS12	Memory 3
1.99310385481476e-223	0.802270818079906	0.709	0.443
3.55350486274923e-219	3	CD63	Memory 3
1.54145120813904e-221	0.630099588315721	0.897	0.701
2.7482533589911e-217	3	ENO1	Memory 3
7.12920445840673e-217	-0.797483077971027	0.395	0.513
1.27106586288934e-212	3	HLA-DRB5	Memory 3
1.53750714703782e-211	0.333089647022187	0.999	0.987
2.74122149245373e-207	3	RPLP0	Memory 3
1.86920791139769e-207	0.680257383280585	0.796	0.523
3.33261078523094e-203	3	CCR7	Memory 3
1.50989463782102e-204	-0.987538461512705	0.626	0.787
2.6919911497711e-200	3	HLA-DPB1	Memory 3
6.81893118057244e-200	0.750589207951138	0.573	0.264
1.21574724018426e-195	3	MIR155HG	Memory 3

1.02821356534029e-195 0.67574031305919 0.294 0.103
1.8332019656452e-191 3 VRK2 Memory 3
4.81500717715173e-193 -1.25421704186932 0.565 0.768
8.58467629614383e-189 3 HLA-DRB1 Memory 3
2.4775290676853e-192 0.344894097224215 0.997 0.979
4.41718657477612e-188 3 RPL5 Memory 3
1.01343584592688e-185 0.316624250048718 0.999 0.991
1.80685476970303e-181 3 RPL9 Memory 3
1.40357986147939e-180 0.548072779008598 0.821 0.617
2.50244253503161e-176 3 GSTP1 Memory 3
8.22845796671843e-180 -1.3881375174078 0.032 0.22
1.46705177088623e-175 3 RGS13 Memory 3
2.24407702938289e-177 0.653578836144039 0.306 0.108
4.00096493568676e-173 3 RAB13 Memory 3
2.42916954774031e-177 -0.808881821516201 0.721 0.822
4.33096638666619e-173 3 HLA-DPA1 Memory 3
6.61446884016196e-177 0.310524907389575 0.995 0.975
1.17929364951248e-172 3 RPS24 Memory 3
5.12238068461464e-173 0.765094836639347 0.288 0.12
9.13269252259944e-169 3 CYTOR Memory 3
2.4485829268864e-169 0.684300249798119 0.379 0.174
4.36557850034577e-165 3 IFNGR1 Memory 3
1.14474101976702e-164 0.716578611308181 0.543 0.337
2.04095876414263e-160 3 ANXA5 Memory 3
1.26167289483098e-163 0.48759125348899 0.94 0.848
2.24943660419415e-159 3 SH3BGRL3 Memory 3
3.49973932828947e-159 -0.821525996557097 0.559 0.708
6.2396852484073e-155 3 HLA-DQB1 Memory 3
1.54820928822403e-157 0.678927951292775 0.428 0.193
2.76030233997463e-153 3 MGST3 Memory 3
4.39777289802469e-155 0.631058808001267 0.525 0.304
7.84078929988823e-151 3 TUBA1A Memory 3
1.98645852943349e-153 0.51634085667428 0.856 0.702
3.54165691212697e-149 3 CLIC1 Memory 3
2.41230738530276e-153 -0.803770793855444 0.706 0.808
4.3009028372563e-149 3 LAPTM5 Memory 3
3.38157771589959e-148 0.657047128068333 0.628 0.391
6.02901490967739e-144 3 TXN Memory 3
1.64377331619374e-138 0.366649691763183 0.959 0.839
2.93068344544182e-134 3 HSP90AB1 Memory 3
5.20255292443447e-137 0.52597609433995 0.78 0.582
9.27563160897422e-133 3 LDHA Memory 3
1.56751662832789e-136 0.600363602484293 0.453 0.232
2.79472539664579e-132 3 TNFRSF13B Memory 3
1.70531339450122e-134 -2.05616345622137 0.24 0.416
3.04040325105622e-130 3 JCHAIN Memory 3
1.72094356699063e-132 0.625521665384375 0.323 0.15
3.0682702855876e-128 3 CD58 Memory 3
2.11106569304041e-132 0.3722705367834 0.988 0.959
3.76381902412174e-128 3 MT-ND2 Memory 3

4.60737533731745e-132	-0.395070849288585	0.999	0.999
8.21448948890328e-128	3	PTMA	Memory 3
9.70453195522449e-131	-0.630428889632448	0.023	0.172
1.73022100229697e-126	3	SUSD3	Memory 3
7.33384812621492e-130	0.572123131194066	0.184	0.055
1.30755178242286e-125	3	MACROD2	Memory 3
2.07846833887132e-127	0.445014455264614	0.824	0.665
3.70570120137368e-123	3	TAGLN2	Memory 3
3.18786780195774e-122	0.543242924753175	0.477	0.3
5.68364950411045e-118	3	LPXN	Memory 3
1.29764139133488e-121	0.347396752276707	0.776	0.561
2.31356483661095e-117	3	TUBB	Memory 3
6.14109876209548e-120	0.506123721808393	0.606	0.414
1.094896498294e-115	3	PPP1R15A	Memory 3
1.2695756510479e-115	0.412309870151048	0.814	0.635
2.2635264282533e-111	3	ZFP36L1	Memory 3
3.13602734058596e-115	-0.615565013412569	0.016	0.144
5.59122314553071e-111	3	NEIL1	Memory 3
6.59252887130017e-115	0.510824290142604	0.591	0.366
1.17538197246411e-110	3	CD44	Memory 3
2.48006026985844e-114	0.692594475433683	0.317	0.148
4.42169945513061e-110	3	DDIT4	Memory 3
6.75563454111671e-113	-0.706946376881553	0.021	0.159
1.2044620823357e-108	3	BIK	Memory 3
3.79670498069013e-110	-0.721975883684748	0.665	0.77
6.76914531007244e-106	3	CYBA	Memory 3
4.28756246283646e-110	0.568566883577167	0.411	0.205
7.64429511499113e-106	3	GBP2	Memory 3
1.15367473876853e-109	0.496806873813087	0.513	0.338
2.05688669175042e-105	3	APOBEC3G	Memory 3
8.15220142068958e-108	-0.509544271352419	0.012	0.127
1.45345599129475e-103	3	CD38	Memory 3
1.58044004432214e-106	0.342016053455722	0.974	0.912
2.81776655502194e-102	3	EEF1B2	Memory 3
3.60984945781606e-106	0.513309519752913	0.492	0.324
6.43600059834025e-102	3	S100A11	Memory 3
8.65941131543296e-99	-0.744066496738257	0.096	0.246
1.54388644342854e-94	3	UBE2J1	Memory 3
1.02147784106375e-98	-0.359569757460797	0.007	0.11
1.82119284283256e-94	3	SEMA4A	Memory 3
4.34354501562743e-98	-0.400707934181601	0.007	0.109
7.74410640836215e-94	3	MYBL2	Memory 3
2.72257469212503e-96	-0.665032407463223	0.462	0.639
4.85407841858971e-92	3	HLA-DMB	Memory 3
5.65918593836394e-96	0.442519156225897	0.615	0.432
1.00897626095091e-91	3	KLF6	Memory 3
1.80271093518056e-94	0.4588003178874	0.199	0.066
3.21405332633342e-90	3	CAPN2	Memory 3
2.06794710447654e-92	0.535018777725629	0.458	0.253
3.68694289257122e-88	3	GPR183	Memory 3

5.83763268671199e-92	-0.690377126778649	0.057	0.196
1.04079153171388e-87	3	RGS2	Memory 3
7.79841849255034e-92	0.551555381727091	0.315	0.154
1.3903800330368e-87	3	CBX6	Memory 3
9.49265484591823e-92	-0.428462107492903	0.01	0.114
1.69244543247876e-87	3	HRK	Memory 3
9.53599574213707e-91	0.400064535472592	0.995	0.974
1.70017268086562e-86	3	FTH1	Memory 3
4.02517421092665e-88	-0.933977095462572	0.557	0.663
7.17648310066112e-84	3	TCL1A	Memory 3
7.26376474633068e-88	-0.413134290923454	0.008	0.102
1.2950566166233e-83	3	WDR66	Memory 3
3.72051274436879e-87	-0.670757839549468	0.784	0.862
6.63330217193511e-83	3	CD79B	Memory 3
1.1275970022453e-85	0.455349213624032	0.571	0.391
2.01039269530314e-81	3	FABP5	Memory 3
4.20921029126281e-85	0.453382972897871	0.611	0.412
7.50460102829246e-81	3	PLP2	Memory 3
4.89387680275141e-85	-0.503891475561849	0.049	0.178
8.72529295162548e-81	3	SEL1L3	Memory 3
6.71976069581964e-85	-0.62043056761657	0.134	0.274
1.19806613445768e-80	3	CD22	Memory 3
8.60496114010295e-85	-0.406871174713346	0.017	0.119
1.53417852166895e-80	3	AC023590.1	Memory 3
5.48249830571138e-84	-0.552800558898903	0.016	0.117
9.77474622925281e-80	3	VPREB3	Memory 3
1.63673918988176e-83	-0.583444709544194	0.075	0.217
2.91814230164019e-79	3	EAF2	Memory 3
2.87698266917947e-83	0.355768279565572	0.934	0.86
5.12937240088007e-79	3	UBC	Memory 3
3.50309459344361e-83	-0.616437343090692	0.362	0.527
6.2456673506506e-79	3	LSP1	Memory 3
3.85697577470868e-83	-0.722267525180112	0.204	0.351
6.87660210872811e-79	3	LRMP	Memory 3
2.11110007728827e-82	0.452722857881259	0.518	0.331
3.76388032779726e-78	3	CAST	Memory 3
6.65458907461659e-82	-0.648854706167169	0.213	0.348
1.18644668611339e-77	3	HVCN1	Memory 3
8.24522214388174e-82	-0.635763250824788	0.076	0.212
1.47004065603268e-77	3	HMCES	Memory 3
8.69640776043827e-82	-1.4102138692694	0.06	0.185
1.55048253960854e-77	3	MZB1	Memory 3
9.4495532539881e-80	0.483217985884463	0.629	0.466
1.68476084965354e-75	3	SQSTM1	Memory 3
1.45715270162887e-79	-0.602507063566949	0.013	0.101
2.59795755173412e-75	3	PCLAF	Memory 3
2.37133005329298e-79	0.44571288824255	0.489	0.324
4.22784435201606e-75	3	APOBEC3C	Memory 3
3.39478586551894e-79	-0.646501905192284	0.305	0.439
6.05256371963371e-75	3	IRF8	Memory 3

1.7418608554208e-78 -0.537324734014957 0.829 0.886
3.10556371912974e-74 3 MS4A1 Memory 3
2.89385386995272e-78 0.361104800904016 0.613 0.468
5.15945206473871e-74 3 CD47 Memory 3
1.23626785397636e-77 -0.806854047609177 0.076 0.199
2.20414195685444e-73 3 PTTG1 Memory 3
3.53140386436153e-77 -0.543630537906975 0.486 0.629
6.29613994977018e-73 3 CLEC2D Memory 3
3.28473594931096e-76 -0.679898453692851 0.157 0.315
5.85635572402651e-72 3 BCL7A Memory 3
4.95145770144539e-75 -0.379272874177777 0.018 0.119
8.82795393590699e-71 3 CPNE5 Memory 3
2.82742142566148e-74 -0.386684886878552 0.024 0.111
5.04100965981186e-70 3 SCIMP Memory 3
1.1087166472024e-72 -0.791191838143878 0.414 0.543
1.97673091029716e-68 3 ISG20 Memory 3
5.96185870675846e-72 -0.401004072960997 0.021 0.11
1.06293978882797e-67 3 RMI2 Memory 3
9.0740922512045e-72 -0.381376850994156 0.018 0.107
1.61781990746725e-67 3 CENPM Memory 3
9.82344997788716e-72 -0.593900857315565 0.165 0.299
1.7514228965575e-67 3 BCAS4 Memory 3
3.15686658307945e-71 0.397936319879627 0.521 0.382
5.62837743097234e-67 3 FNBP1 Memory 3
5.34532676059071e-71 -0.482577002340272 0.042 0.162
9.53018308145717e-67 3 CD81 Memory 3
3.11288486615509e-70 -0.492558955018856 0.068 0.197
5.54996242786791e-66 3 HLA-DOB Memory 3
5.65372531900061e-70 0.383545743940093 0.679 0.521
1.00800268712462e-65 3 IMPDH2 Memory 3
5.99635966215839e-70 -0.418838788721977 0.04 0.148
1.06909096416622e-65 3 SOCS1 Memory 3
3.46907527815905e-69 0.317684793162623 0.79 0.669
6.18501431342978e-65 3 MORF4L1 Memory 3
5.19817336873524e-69 0.44530160588362 0.309 0.181
9.26782329911805e-65 3 RNF145 Memory 3
1.37401313495616e-68 -0.501070308337818 0.105 0.228
2.44972801831334e-64 3 LY86 Memory 3
7.8982989685697e-68 -0.637194586701732 0.557 0.66
1.40818772310629e-63 3 HLA-DQA1 Memory 3
4.29167826632735e-67 0.323008316466395 0.508 0.341
7.65163318103504e-63 3 TUBB4B Memory 3
6.52747570892379e-67 0.35834027397587 0.203 0.101
1.16378364414402e-62 3 GALNT2 Memory 3
7.80246973581649e-67 0.312583151205814 0.813 0.687
1.39110232919872e-62 3 HNRNPDL Memory 3
8.04359698897661e-67 0.406797611054522 0.296 0.174
1.43409290716464e-62 3 TUBA1C Memory 3
1.18606800557712e-66 -0.445107829047783 0.069 0.189
2.11464064714345e-62 3 LCK Memory 3

3.06305731174773e-66 -0.561052319911687 0.189 0.332
5.46112488111502e-62 3 TNFRSF13C Memory 3
3.75383422113864e-66 -0.578106362282837 0.813 0.873
6.69271103286809e-62 3 CD52 Memory 3
1.12729692544712e-64 -0.470587272885798 0.933 0.94
2.00985768837966e-60 3 H3F3A Memory 3
1.25841059831974e-63 -0.612769896862557 0.185 0.313
2.24362025574426e-59 3 CD27 Memory 3
1.62041316091436e-63 -3.21269913535666 0.351 0.458
2.8890346245942e-59 3 IGHG3 Memory 3
1.00390309795208e-62 -0.596495369504915 0.558 0.641
1.78985883333876e-58 3 HERPUD1 Memory 3
1.20054368817282e-62 -1.57174163574835 0.212 0.31
2.14044934164332e-58 3 HMGB2 Memory 3
1.53705686810998e-61 -0.55878707803964 0.122 0.242
2.74041869015328e-57 3 IL4R Memory 3
1.74432585599794e-61 0.346033102365794 0.16 0.079
3.10995856865872e-57 3 LRRFIP2 Memory 3
2.67586070094998e-61 0.410582768804274 0.469 0.343
4.77079204372372e-57 3 CHMP4B Memory 3
9.60628674064796e-61 0.404800107018094 0.39 0.225
1.71270486299012e-56 3 CAPG Memory 3
1.23719604796169e-60 -0.378240312464084 0.029 0.12
2.20579683391089e-56 3 TNFRSF17 Memory 3
1.45457961719097e-60 0.359692809657893 0.453 0.326
2.59336999948979e-56 3 VOPP1 Memory 3
7.53025377017219e-60 -0.523234519763658 0.395 0.512
1.342568944684e-55 3 TSC22D3 Memory 3
8.2982456447854e-60 0.378599440463978 0.622 0.5
1.47949421600879e-55 3 TALDO1 Memory 3
1.28183387054908e-57 0.348090436505315 0.256 0.148
2.28538160780196e-53 3 DDB2 Memory 3
4.11159896772726e-56 -0.445538636504064 0.145 0.27
7.33056979956094e-52 3 CCDC69 Memory 3
6.34053955049693e-56 -0.39809276534189 0.07 0.173
1.1304547964581e-51 3 DEF8 Memory 3
6.58304294582514e-56 -0.357664678783529 0.034 0.127
1.17369072681116e-51 3 VNN2 Memory 3
1.90694445526252e-54 -0.54060138796314 0.24 0.373
3.39989126928755e-50 3 RCSD1 Memory 3
3.52777856331438e-54 0.32772427337345 0.161 0.067
6.28967640053322e-50 3 TRAF1 Memory 3
3.64021249648834e-54 0.356538661524389 0.496 0.371
6.49013485998906e-50 3 ANXA7 Memory 3
4.10491312222021e-54 -0.381672481048059 0.044 0.145
7.31864960560641e-50 3 SIT1 Memory 3
4.80631385385876e-54 -0.478669146291354 0.3 0.408
8.56917697004479e-50 3 REL Memory 3
4.80718850239413e-54 0.340274614056282 0.179 0.074
8.5707363809185e-50 3 FGR Memory 3

3.25204454215872e-53 0.310527931096447 0.147 0.066
5.79807021421478e-49 3 RAB34 Memory 3
1.94340634395906e-52 -0.461245634377946 0.058 0.177
3.46489917064461e-48 3 CCDC88A Memory 3
5.11226682210366e-52 -0.509671380942242 0.231 0.347
9.11466051712861e-48 3 STK17A Memory 3
4.97598544906385e-51 0.397954651726525 0.309 0.187
8.87168445713593e-47 3 TYMP Memory 3
6.34415725415594e-51 0.309205801317079 0.692 0.566
1.13109979684346e-46 3 APRT Memory 3
1.30535136920011e-50 -0.406539807133125 0.067 0.159
2.32731095614687e-46 3 CD72 Memory 3
1.40965446170604e-50 0.361094021154081 0.182 0.093
2.5132729397757e-46 3 SGK1 Memory 3
2.42988123442891e-50 -0.926330223096569 0.101 0.22
4.3322352528633e-46 3 PLCG2 Memory 3
4.49934880620937e-50 0.328414672696807 0.198 0.104
8.02188898659068e-46 3 ZNF267 Memory 3
1.7849848084011e-49 -1.09266055477728 0.094 0.152
3.18244941489832e-45 3 IGHA1 Memory 3
2.13571006353962e-49 -0.500182839018463 0.168 0.275
3.80775747228479e-45 3 HHEX Memory 3
3.43190009210693e-49 -0.30980218112013 0.028 0.102
6.11873467421745e-45 3 STAG3 Memory 3
4.22299632764951e-49 -0.530803666018867 0.219 0.34
7.5291801525663e-45 3 RIPOR2 Memory 3
6.15848238575334e-49 -0.5469821233103 0.154 0.248
1.09799582455596e-44 3 RGS1 Memory 3
1.57072403781316e-48 -0.411377135202448 0.114 0.236
2.80044388701708e-44 3 UBE2G1 Memory 3
5.358486297999e-48 -0.429702230660813 0.171 0.292
9.55364522070241e-44 3 LAT2 Memory 3
3.79327513226762e-47 -0.656285568947306 0.179 0.282
6.76303023331995e-43 3 KLF2 Memory 3
4.28492667545061e-47 -0.53912765426598 0.267 0.384
7.6395957696609e-43 3 ACADM Memory 3
5.29527265701098e-47 -0.511572966479114 0.158 0.284
9.44094162018488e-43 3 NCF1 Memory 3
6.6568200715238e-47 -0.481622222375326 0.142 0.252
1.18684445055198e-42 3 RASGRP2 Memory 3
9.91069592257261e-47 -0.438940154660006 0.106 0.2
1.76697797603547e-42 3 DNMT1 Memory 3
1.004260918091e-46 0.345081617119895 0.269 0.16
1.79049679086444e-42 3 TNIP2 Memory 3
2.62951884239138e-46 -0.425364053195052 0.084 0.194
4.68816914409959e-42 3 P2RX5 Memory 3
1.01403111672229e-45 0.300111411182849 0.152 0.07
1.80791607800417e-41 3 RDX Memory 3
1.278842448719e-45 -0.445977992677632 0.242 0.334
2.2800482018211e-41 3 PTPN6 Memory 3

4.13126966203853e-45	0.314716627642293		0.253	0.155
7.36564068044849e-41	3	PPP1R14B Memory 3		
4.48389045143089e-45	0.326798488685428		0.52	0.369
7.99432828585614e-41	3	PDCD4 Memory 3		
7.3507278258122e-45	0.345377079902318		0.278	0.181
1.31056126406406e-40	3	YARS Memory 3		
1.12660311204029e-44	0.435789217872431		0.356	0.248
2.00862068845663e-40	3	SRGN Memory 3		
1.67570612245423e-44	-0.531572732962196		0.207	0.31
2.98761644572365e-40	3	ZFP36L2 Memory 3		
3.39973319103161e-44	-0.511634455751322		0.216	0.332
6.06138430629025e-40	3	AES Memory 3		
4.19105817437407e-44	0.307256493879936		0.28	0.162
7.47223761909153e-40	3	PARP14 Memory 3		
5.53586441973724e-44	0.455940959024364		0.203	0.119
9.86989267394952e-40	3	LGALS3 Memory 3		
6.97144171660609e-44	-0.424545833413015		0.12	0.226
1.2429383436537e-39	3	NANS Memory 3		
4.28940326894297e-43	-0.328623563224653		0.058	0.154
7.64757708819842e-39	3	SH2B2 Memory 3		
6.77532834074052e-43	-0.415131067543537		0.081	0.18
1.20797328987063e-38	3	TMEM156 Memory 3		
6.90114932261055e-42	-0.532336922298285		0.848	0.867
1.23040591272823e-37	3	CD79A Memory 3		
7.05023397933385e-42	-0.304287318895682		0.028	0.1
1.25698621617543e-37	3	FCRL5 Memory 3		
9.39915554018592e-42	-0.350969637048937		0.081	0.172
1.67577544125975e-37	3	SCPEP1 Memory 3		
1.17366009391796e-41	0.352149532492582		0.336	0.223
2.09251858144633e-37	3	TARS Memory 3		
1.18845416067845e-41	-0.34379384159211		0.079	0.147
2.11889492307361e-37	3	LY9 Memory 3		
8.4450453949954e-41	-0.413667195886945		0.108	0.201
1.50566714347373e-36	3	FAM129C Memory 3		
2.00187261386777e-40	-0.577247450531345		0.125	0.215
3.56913868326484e-36	3	FOS Memory 3		
4.6687854423672e-40	-0.441118249623867		0.149	0.243
8.32397756519648e-36	3	CCND3 Memory 3		
2.36264022298257e-39	-0.541975658789326		0.672	0.729
4.21235125355563e-35	3	LIMD2 Memory 3		
3.33248538601319e-39	-0.325605433898461		0.062	0.15
5.94148819472291e-35	3	HSH2D Memory 3		
3.42878723757758e-39	-0.382636362853155		0.079	0.167
6.11318476587707e-35	3	C12orf75 Memory 3		
9.49914068153626e-39	-0.447157777509359		0.199	0.305
1.6936017921111e-34	3	GGA2 Memory 3		
5.23094090309422e-38	-0.31972435451195		0.057	0.133
9.32624453612669e-34	3	CCNG2 Memory 3		
5.52127823447709e-38	-1.35834769363035		0.152	0.221
9.84388696424921e-34	3	IGHG1 Memory 3		

5.58041562350064e-38	-0.421155790506895	0.217	0.337
9.94932301513929e-34	3	GYPC	Memory 3
6.67365354702239e-38	0.353990661028891	0.443	0.317
1.18984569089862e-33	3	SLC3A2	Memory 3
9.19966926534895e-38	-0.307433035469274	0.056	0.129
1.64020903331906e-33	3	PLEK	Memory 3
2.05818083791836e-37	-0.317181073010933	0.093	0.189
3.66953061592465e-33	3	RASGRP3	Memory 3
2.44876439242169e-37	0.395040410965711	0.313	0.21
4.36590203524863e-33	3	EIF4EBP1	Memory 3
3.08879136144486e-37	-0.414466076008437	0.333	0.441
5.50700611832004e-33	3	CYB561A3	Memory 3
1.17956226519231e-36	-0.321299990515755	0.064	0.153
2.10304156261137e-32	3	RBM38	Memory 3
2.44238627075625e-35	-0.361516882408837	0.118	0.216
4.35453048213133e-31	3	GLRX	Memory 3
6.49824558634433e-35	-0.312257922473455	0.094	0.182
1.15857220558933e-30	3	EHMT1	Memory 3
7.08357912547847e-35	-0.456987567263214	0.184	0.258
1.26293132228156e-30	3	FCMR	Memory 3
1.97067815267949e-34	-0.447627019587184	0.13	0.212
3.51352207841226e-30	3	PRKCB	Memory 3
4.03637712972547e-34	-0.359330218376206	0.202	0.29
7.19645678458753e-30	3	GNG7	Memory 3
1.71570693317851e-33	-0.359959851468183	0.254	0.363
3.05893389116396e-29	3	CD19	Memory 3
5.04350457875737e-33	-0.40017782735313	0.104	0.18
8.99206431346651e-29	3	MARCH1	Memory 3
2.79612775993582e-32	-0.397795946661621	0.291	0.391
4.98521618318957e-28	3	EVL	Memory 3
5.51634148022253e-32	-0.338385055258624	0.072	0.152
9.83508522508874e-28	3	TAGAP	Memory 3
6.67179149380201e-32	-0.352022011451175	0.928	0.936
1.18951370542996e-27	3	BTG1	Memory 3
1.53115669745255e-31	-0.46595941916881	0.084	0.157
2.72989927588815e-27	3	DUSP2	Memory 3
1.73118388229281e-31	-0.345743531938629	0.062	0.125
3.08652774373985e-27	3	LPP	Memory 3
2.2553522609017e-30	-0.338232091176996	0.15	0.246
4.02106754596163e-26	3	NCF4	Memory 3
2.55449877326847e-30	-0.386797656133975	0.402	0.498
4.55441586286035e-26	3	RAC2	Memory 3
2.73650316631584e-30	-0.344997258630694	0.629	0.679
4.87891149522452e-26	3	ATP6V1G1	Memory 3
4.52931489897756e-30	-0.37263510939803	0.174	0.266
8.07531553338708e-26	3	SYNE2	Memory 3
5.20215265639145e-30	-0.887558342423007	0.376	0.432
9.27491797108032e-26	3	STMN1	Memory 3
6.83112347490319e-29	-0.46344283800573	0.415	0.495
1.21792100434049e-24	3	UCP2	Memory 3

3.31006753934819e-28 -0.381302226736902 0.107 0.181
5.90151941590389e-24 3 PDIA4 Memory 3
7.06154120450555e-28 -0.385004726303908 0.213 0.295
1.2590021813513e-23 3 DCK Memory 3
1.28831097574027e-27 -0.340083125870152 0.183 0.271
2.29692963864732e-23 3 RBM6 Memory 3
1.43176071099106e-27 -0.438174697369023 0.74 0.779
2.55268617162596e-23 3 ARHGDIB Memory 3
1.46296674305987e-27 -0.373333577813087 0.187 0.281
2.60832340620144e-23 3 ITGAE Memory 3
4.43076559597849e-27 -0.393637553025711 0.15 0.217
7.89961198107006e-23 3 LBH Memory 3
4.46894954395479e-27 -0.389794473657554 0.38 0.442
7.967690141917e-23 3 COTL1 Memory 3
2.07756343380234e-26 -0.302442375927562 0.159 0.239
3.70408784612619e-22 3 ZNF581 Memory 3
2.42942069752223e-26 -0.388527762158729 0.355 0.446
4.33141416161239e-22 3 RHOH Memory 3
4.10550688718892e-26 -0.367809959347404 0.28 0.367
7.31970822916912e-22 3 TPD52 Memory 3
4.91214477694591e-26 -0.391765156059637 0.253 0.345
8.75786292281687e-22 3 SPIB Memory 3
1.09285135064856e-25 -0.338426608113913 0.064 0.127
1.94844467307131e-21 3 AL139020.1 Memory 3
2.36614029920186e-24 -0.336572831065652 0.336 0.429
4.21859153944699e-20 3 TMEM123 Memory 3
4.29688634920392e-24 -0.322843525466936 0.204 0.284
7.66091867199567e-20 3 STK17B Memory 3
1.17969792221831e-23 -0.304058104489411 0.231 0.318
2.10328342552303e-19 3 IL16 Memory 3
4.58227659598603e-23 -0.622264005284856 0.308 0.377
8.16974094298349e-19 3 SELL Memory 3
8.75636471477906e-23 -0.310897193206369 0.309 0.364
1.56117226499796e-18 3 ARID5B Memory 3
2.721374447443e-22 -1.37383241478915 0.372 0.426
4.85193850234613e-18 3 HIST1H4C Memory 3
6.22587601972342e-22 -0.345086477943977 0.322 0.404
1.11001143555649e-17 3 ORAI2 Memory 3
8.12330310545749e-22 -0.478265718156086 0.158 0.215
1.44830371067202e-17 3 PLAC8 Memory 3
1.15893157521165e-21 -0.389802118454189 0.366 0.421
2.06625910544485e-17 3 METAP2 Memory 3
3.47372097153425e-21 -0.348408018826288 0.137 0.217
6.19329712014842e-17 3 FAM111B Memory 3
3.62660855604974e-21 -0.30883698081663 0.183 0.246
6.46588039458108e-17 3 PHACTR1 Memory 3
1.38750069121092e-20 -0.392150337466699 0.549 0.621
2.47377498235995e-16 3 MEF2C Memory 3
4.35594754383751e-20 -0.301718284328377 0.161 0.235
7.76621887590789e-16 3 ATM Memory 3

1.06971016442492e-19	-0.311358073743486	0.668	0.702
1.9071862521532e-15	3	CCNI	Memory 3
1.36299894212158e-19	-0.349050351610509	0.233	0.32
2.43009081390856e-15	3	HMGN3	Memory 3
1.66046482273613e-19	-0.361668268271769	0.337	0.409
2.96044273245624e-15	3	STX7	Memory 3
2.56632762715896e-19	-0.462192504483066	0.868	0.85
4.57550552646171e-15	3	ATP5MG	Memory 3
1.67919796911585e-18	-0.306209968688175	0.211	0.295
2.99384205913665e-14	3	TUBA4A	Memory 3
2.11468459126248e-18	-2.32819654552424	0.056	0.131
3.77027115776188e-14	3	IGHG2	Memory 3
3.42465818720687e-18	-0.426402975872694	0.742	0.751
6.10582308197112e-14	3	CXCR4	Memory 3
8.8996626814277e-18	-0.329659147753743	0.111	0.174
1.58672085947175e-13	3	SMC4	Memory 3
1.14478838164093e-17	-0.309719711333549	0.221	0.289
2.04104320562761e-13	3	CARHSP1	Memory 3
1.94784242019869e-17	-1.95946049093645	0.187	0.247
3.47280825097224e-13	3	IGHG4	Memory 3
3.60858564989323e-17	-0.304187633147891	0.189	0.247
6.43374735519463e-13	3	SLBP	Memory 3
4.94119788354078e-17	-0.339622597853625	0.182	0.234
8.80966170656486e-13	3	CKS2	Memory 3
1.28240255927099e-16	-0.308280475425867	0.095	0.147
2.28639552292426e-12	3	DUSP1	Memory 3
8.34486921087041e-16	-0.380814651934017	0.226	0.281
1.48780673160609e-11	3	HSPA5	Memory 3
2.80009922910891e-15	-0.363009327911616	0.339	0.403
4.99229691557827e-11	3	BASP1	Memory 3
2.25482649733181e-14	-0.311460038485207	0.345	0.353
4.02013016209289e-10	3	TXNIP	Memory 3
3.31765744216604e-14	-0.307125142895323	0.502	0.532
5.91505145363783e-10	3	SRSF7	Memory 3
6.48212979951018e-14	-0.386680523227064	0.83	0.813
1.15569892195467e-09	3	CORO1A	Memory 3
8.93101830328661e-14	-0.406762394780497	0.322	0.362
1.59231125329297e-09	3	H2AFV	Memory 3
1.2586059607133e-13	-0.365741013265315	0.7	0.693
2.24396856735574e-09	3	EZR	Memory 3
1.91711440991513e-13	-0.444351719774823	0.955	0.931
3.41802328143769e-09	3	SERF2	Memory 3
6.38110329778988e-13	-0.318350432479683	0.217	0.26
1.13768690696296e-08	3	MDM4	Memory 3
1.7163759442208e-12	-0.300936399788277	0.265	0.317
3.06012667095126e-08	3	PSIP1	Memory 3
4.33884435921773e-11	-0.327191527218357	0.272	0.299
7.73572560804929e-07	3	LINC00926	Memory 3
1.37331104832082e-10	-0.446706896918366	0.293	0.314
2.4484762680512e-06	3	HSP90B1	Memory 3

2.165344225275e-10	-0.324236841012226	0.525	0.542
3.86059221924279e-06	3	PARP1	Memory 3
3.42338209381611e-10	-0.705426740053976	0.749	0.713
6.10354793506474e-06	3	HMGN2	Memory 3
3.5101717557729e-10	-0.316408991406751	0.663	0.657
6.2582852233675e-06	3	SLC25A5	Memory 3
4.24352330078976e-10	-0.577485507638184	0.208	0.206
7.56577769297807e-06	3	LGALS1	Memory 3
6.4449889926834e-10	-0.314912756936269	0.118	0.132
1.14907708750552e-05	3	MYC	Memory 3
9.90726634717619e-10	-0.315182735206735	0.463	0.495
1.76636651703804e-05	3	C4orf3	Memory 3
3.60915400478753e-06	-1.05253090613305	0.919	0.927
0.0643476067513569	3	IGKC	Memory 3
4.21659268495993e-06	-0.320380683625002	0.313	0.351
0.0751776309801506	3	S100A10	Memory 3
0.000117171346942177	-1.01789115492362	0.458	0.427	1
3	IGLC3	Memory 3
0.000163216780868077	-1.7384000321183	0.562	0.651	1	3
IGLC2	Memory 3
0.000787134708372115	-0.341755019884837	0.957	0.93	1
3	HMGB1	Memory 3
0.00107677132317933	-0.372251307962412	0.812	0.764	1
3	HMGN1	Memory 3
0	1.80671979806381	0.66	0.377	0	4	EIF5A	B
EIF5A
0	1.71353810762302	0.478	0.092	0	4	C1orf56	B
EIF5A
0	1.43983714033053	0.612	0.313	0	4	HNRNPH1	B
EIF5A
0	1.35438140753933	0.524	0.243	0	4	MDM4	B
EIF5A
0	1.24043768347899	0.797	0.628	0	4	SET	B
EIF5A
3.50048120186269e-306	1.2388644151459	0.395	0.138
6.24100793480099e-302	4	CTNNB1	B EIF5A
2.05207041906464e-250	1.190523145277	0.547	0.325
3.65863635015034e-246	4	APOBEC3C	B EIF5A
1.10823117834095e-237	1.05477695864127	0.747	0.596
1.97586536786407e-233	4	CDC42	B EIF5A
1.30803273387281e-231	1.00749910836956	0.238	0.055
2.33209156122183e-227	4	B4GALT1	B EIF5A
1.56402467596949e-226	1.14978553438941	0.382	0.17
2.788499594786e-222	4	CDC42SE1	B EIF5A
2.9707087034906e-209	1.10389994045448	0.404	0.188
5.29647654745339e-205	4	PPP3CA	B EIF5A
6.30188889724146e-204	1.036401553979	0.342	0.135
1.12356377148918e-199	4	TSPYL1	B EIF5A
2.41311215126247e-202	0.827115977714131	0.172	0.029
4.30233765448587e-198	4	PHKG1	B EIF5A

5.73107829307632e-163 1.01279150007939 0.337 0.157
1.02179394887258e-158 4 CBX6 B EIF5A
1.62213566648727e-155 0.934553590565273 0.633 0.494
2.89210567978015e-151 4 FOXP1 B EIF5A
3.30771008330858e-130 0.877267087831733 0.245 0.099
5.89731630753088e-126 4 AC058791.1 B EIF5A
6.74540131512141e-130 0.936797087718015 0.411 0.267
1.202637600473e-125 4 TNRC6B B EIF5A
7.04448736499111e-111 -1.23518998507384 0.037 0.215
1.25596165230426e-106 4 RGS13 B EIF5A
2.54202188578618e-110 0.602862664702743 0.128 0.031
4.53217082016818e-106 4 GIGYF1 B EIF5A
5.59038881007158e-102 -0.788555998239054 0.098 0.314
9.96710420947662e-98 4 CD27 B EIF5A
3.83713805806636e-91 0.497055180356402 0.106 0.026
6.84123344372651e-87 4 LRRC75A B EIF5A
1.50481047743989e-85 0.596612218430918 0.119 0.038
2.68292660022757e-81 4 C16orf54 B EIF5A
3.82071856124688e-83 0.654975538113456 0.175 0.074
6.81195912284705e-79 4 FGD2 B EIF5A
4.9526662468978e-81 0.725903700202533 0.296 0.196
8.83010865159408e-77 4 SRSF6 B EIF5A
4.73706703508991e-75 -1.6980002457049 0.222 0.413
8.44571681686181e-71 4 JCHAIN B EIF5A
5.89747126332335e-74 -0.625498606057505 0.075 0.243
1.05146015153792e-69 4 UBE2J1 B EIF5A
5.43102404153417e-72 -0.466535320087055 0.011 0.124
9.68297276365127e-68 4 CD38 B EIF5A
1.45519323208018e-69 -0.426456689532047 0.65 0.823
2.59446401347575e-65 4 CORO1A B EIF5A
1.80891262061477e-68 0.498615125164807 0.112 0.044
3.22511031129408e-64 4 MTA2 B EIF5A
1.10204438660227e-64 -0.395592445223552 0.582 0.772
1.96483493687319e-60 4 CYBA B EIF5A
1.95006289107353e-64 -0.456794614784704 0.842 0.938
3.476767128495e-60 4 SERF2 B EIF5A
5.78242260873186e-64 -0.773865959500774 0.056 0.197
1.0309481269108e-59 4 PTTG1 B EIF5A
6.60373916582208e-64 0.642475729338665 0.325 0.238
1.17738065587442e-59 4 PPP1CB B EIF5A
2.10904189826628e-63 -0.584083297817793 0.027 0.155
3.76021080041895e-59 4 BIK B EIF5A
1.99470370138349e-61 -0.327285107408282 0.711 0.874
3.55635722919663e-57 4 CD79A B EIF5A
5.85807475043953e-61 -0.483016767622031 0.035 0.168
1.04443614725586e-56 4 SUSD3 B EIF5A
3.5204749572738e-58 0.642895519236853 0.224 0.141
6.27665480132346e-54 4 SERPINB9 B EIF5A
9.29421421591653e-58 -0.395662146180587 0.03 0.146
1.65706545255576e-53 4 AIM2 B EIF5A

8.28210471026572e-57 -0.461384483393377 0.421 0.614
1.47661644879328e-52 4 DYNLL1 B EIF5A
1.01245062411655e-56 -0.428467536274516 0.97 0.991
1.8050982177374e-52 4 ACTB B EIF5A
1.60113888791475e-56 0.523965034402004 0.14 0.072
2.8546705232632e-52 4 RASSF3 B EIF5A
3.08032540031453e-56 -0.508675599063047 0.017 0.114
5.49191215622078e-52 4 VPREB3 B EIF5A
8.38515291007033e-55 -0.362474169514934 0.306 0.526
1.49498891233644e-50 4 LSP1 B EIF5A
1.14560438116031e-54 -0.513271736381142 0.064 0.209
2.04249805117071e-50 4 HMCES B EIF5A
8.99349814098819e-54 -0.363517161601301 0.874 0.95
1.60345078355678e-49 4 HLA-DRA B EIF5A
1.15219042634097e-53 -0.535410636234826 0.662 0.792
2.05424031112331e-49 4 ACTG1 B EIF5A
2.42062602639024e-53 -0.534248587430632 0.102 0.248
4.31573414245116e-49 4 RGS1 B EIF5A
8.32785334604476e-53 -0.473819307064278 0.026 0.14
1.48477297306632e-48 4 NEIL1 B EIF5A
1.27594549016688e-52 -0.48772449257456 0.178 0.357
2.27488321441854e-48 4 S100A10 B EIF5A
1.84951089475787e-51 -0.381003097849162 0.113 0.282
3.29749297426381e-47 4 POU2AF1 B EIF5A
1.13368629755873e-50 -0.434551641557453 0.038 0.159
2.02124929991746e-46 4 CD81 B EIF5A
1.56452273091613e-50 -0.493344359112102 0.8 0.893
2.78938757695037e-46 4 GAPDH B EIF5A
7.20730070565768e-49 -1.22776063955102 0.06 0.182
1.28498964281171e-44 4 MZB1 B EIF5A
7.68240450485972e-49 -0.409321987134386 0.07 0.214
1.36969589917144e-44 4 EAF2 B EIF5A
8.73496094535714e-49 -0.3767448431581 0.728 0.858
1.55735618694772e-44 4 ATP5MG B EIF5A
1.50315316889297e-48 0.457632605344742 0.11 0.05
2.67997178481928e-44 4 FAM76A B EIF5A
2.25741461326293e-48 -1.37469231711699 0.164 0.31
4.02474451398648e-44 4 HMGB2 B EIF5A
2.04215901921911e-47 -0.313162541417346 0.015 0.106
3.64096531536574e-43 4 MYBL2 B EIF5A
2.32428039186335e-47 -0.433791932632498 0.345 0.544
4.14395951065317e-43 4 ISG20 B EIF5A
2.4771307760376e-47 -0.309932171193096 0.526 0.703
4.41647646059743e-43 4 EZR B EIF5A
3.19939835424691e-47 0.49042825135337 0.128 0.072
5.70420732578682e-43 4 ADAM17 B EIF5A
8.93155365775317e-47 0.485669678837905 0.143 0.086
1.59240670164081e-42 4 CNOT9 B EIF5A
1.11691766961676e-46 0.49418812434855 0.156 0.093
1.99135251315972e-42 4 CHP1 B EIF5A

3.11097413776558e-45 -0.33451966982747 0.162 0.327
5.54655579022226e-41 4 PRDX2 B EIF5A
1.30970373286768e-44 0.58094229283307 0.341 0.285
2.33507078532978e-40 4 WTAP B EIF5A
6.16500044865121e-44 -0.347105679368515 0.577 0.763
1.09915792999002e-39 4 HLA-DRB1 B EIF5A
7.8064066888288e-43 0.46254166424962 0.12 0.067
1.39180424855129e-38 4 THAP5 B EIF5A
1.62715451266924e-42 -0.39123041022664 0.278 0.446
2.90105378063799e-38 4 COTL1 B EIF5A
2.77270432531771e-42 -0.30888351413481 0.479 0.662
4.94345454160895e-38 4 HLA-DQA1 B EIF5A
4.36289880870322e-42 -0.820200541143887 0.1 0.212
7.77861228603697e-38 4 LGALS1 B EIF5A
5.39568154491881e-42 -0.333657473549316 0.018 0.111
9.61996062643575e-38 4 HRK B EIF5A
1.79731083917529e-41 -0.311160407775938 0.022 0.116
3.20442549516562e-37 4 AC023590.1 B EIF5A
3.13187985058323e-41 -0.376882963952558 0.141 0.297
5.58382858560485e-37 4 BCAS4 B EIF5A
4.65604295613105e-41 -0.396194816872705 0.215 0.384
8.30125898648604e-37 4 ACADM B EIF5A
1.67456661554621e-40 -0.437575068861273 0.07 0.197
2.98558481885734e-36 4 DAAM1 B EIF5A
1.20693881798394e-39 0.440745169627334 0.12 0.064
2.15185121858356e-35 4 RASGEF1B B EIF5A
3.71779524200503e-38 0.514841587619366 0.494 0.454
6.62845713697077e-34 4 CAPZA1 B EIF5A
2.76880640127485e-37 0.458739257288523 0.154 0.104
4.93650493283294e-33 4 TP53 B EIF5A
2.98092601747828e-37 -0.339523858956129 0.093 0.225
5.31469299656202e-33 4 NANS B EIF5A
7.45221546015399e-37 0.551130050753909 0.38 0.338
1.32865549439086e-32 4 EIF2S3 B EIF5A
1.4971537490353e-36 -0.401039555940648 0.207 0.367
2.66927541915504e-32 4 H2AFV B EIF5A
3.42019456212921e-36 -0.306064430241936 0.17 0.33
6.09786488482017e-32 4 NAA38 B EIF5A
3.9135076323261e-36 -0.313952766016861 0.04 0.143
6.97739275767421e-32 4 SIT1 B EIF5A
5.00628995168167e-36 -0.725776158731242 0.285 0.436
8.92571435485325e-32 4 STMN1 B EIF5A
5.82901748110457e-36 -0.302329347657802 0.057 0.171
1.03925552670613e-31 4 DEF8 B EIF5A
9.53474152364978e-36 -0.428289278008537 0.071 0.192
1.69994906625152e-31 4 RGS2 B EIF5A
2.4232824686047e-35 0.404428886586888 0.126 0.078
4.32047031327531e-31 4 TOR1AIP2 B EIF5A
1.05487845313765e-34 -0.330166110272096 0.127 0.282
1.88074279409912e-30 4 NCF1 B EIF5A

1.20970138081622e-34	-0.319702830964283		0.111	0.243
2.15677659185724e-30	4	DSTN	B EIF5A	
6.23051392781068e-34	0.504781288221171		0.216	0.163
1.11083832818937e-29	4	SUMF2	B EIF5A	
7.54970660023365e-34	-0.31198979932325		0.08	0.199
1.34603718975566e-29	4	DNMT1	B EIF5A	
1.61410302080865e-33	-0.416387122856765		0.153	0.283
2.87778427579975e-29	4	HSPA5	B EIF5A	
2.90049027265815e-33	-0.312939457050839		0.133	0.271
5.17128410712222e-29	4	CD22	B EIF5A	
3.63916085070491e-33	-0.345260550902871		0.056	0.174
6.48825988072179e-29	4	CCDC88A	B EIF5A	
4.07390290381614e-33	-0.303250525357072		0.972	0.99
7.2633614872138e-29	4	CD74	B EIF5A	
7.52137971674093e-33	-0.998285831672415		0.067	0.152
1.34098678969774e-28	4	IGHA1	B EIF5A	
1.04180366115979e-32	-0.666306236038445		0.344	0.496
1.85743174748179e-28	4	LTB	B EIF5A	
8.18738791078744e-32	0.500459534660335		0.249	0.21
1.45972939061429e-27	4	TRA2A	B EIF5A	
9.01280312966189e-32	0.539233000390051		0.348	0.32
1.60689266998742e-27	4	TNFRSF13C		B EIF5A
1.2105045117992e-31	0.487620007653653		0.192	0.13
2.1582084940868e-27	4	AC016831.5		B EIF5A
2.817783423177e-31	-1.24287184787796		0.121	0.221
5.02382606518228e-27	4	IGHG1	B EIF5A	
4.49363049847458e-31	-0.316227590546101		0.061	0.166
8.01169381573034e-27	4	C12orf75	B EIF5A	
4.95133084969263e-31	-0.347789684739399		0.114	0.244
8.82772777191699e-27	4	GCHFR	B EIF5A	
9.82278229692625e-31	0.441917745177113		0.165	0.125
1.75130385571898e-26	4	AMFR	B EIF5A	
4.46478392307472e-30	0.359177233053429		0.115	0.071
7.96026325644992e-26	4	C2orf68	B EIF5A	
7.28045158744293e-30	-0.322754933364355		0.127	0.249
1.2980317135252e-25	4	SLBP	B EIF5A	
5.15270976895005e-29	-1.20614089322251		0.276	0.43
9.18676624706104e-25	4	HIST1H4C	B EIF5A	
8.1950490994921e-29	-0.406299752137274		0.459	0.595
1.46109530394845e-24	4	MARCKSL1	B EIF5A	
8.9066257837615e-29	0.499070658179602		0.332	0.291
1.58796231098684e-24	4	BCL11A	B EIF5A	
2.42744013644728e-27	-0.331806333616892		0.651	0.774
4.32788301927185e-23	4	HMGN1	B EIF5A	
2.87418184695893e-27	-2.79715213045848		0.332	0.457
5.12437881494308e-23	4	IGHG3	B EIF5A	
4.27234445892637e-27	0.415148735072688		0.12	0.073
7.61716293581982e-23	4	JARID2	B EIF5A	
1.26210710005273e-26	0.4459131288615	0.172	0.138	
2.25021074868401e-22	4	SEC22B	B EIF5A	

1.66236264207476e-25	-0.305456764884948		0.194	0.326
2.96382635455508e-21	4	SEC11C	B EIF5A	
1.65221687888461e-24	0.411701624720151		0.14	0.11
2.94573747336337e-20	4	SETD5	B EIF5A	
8.56524817972037e-23	-0.617755247956567		0.102	0.186
1.52709809796234e-18	4	XBP1	B EIF5A	
1.11514603919242e-22	-0.562925482576115		0.604	0.722
1.98819387327616e-18	4	HMGN2	B EIF5A	
2.38170965335274e-22	0.427233983240834		0.207	0.174
4.2463501409626e-18	4	C6orf62	B EIF5A	
2.53661427290056e-22	0.36199603454267	0.123	0.086	
4.52252958715441e-18	4	PAN3	B EIF5A	
2.27133631996754e-21	0.38913937178342	0.151	0.121	
4.04956552487012e-17	4	ZC3HAV1	B EIF5A	
3.17546238270935e-20	0.402355915535607		0.179	0.144
5.6615318821325e-16	4	PAFAH1B2	B EIF5A	
4.76524525908407e-20	-0.346469372427227		0.583	0.702
8.49595577242099e-16	4	H2AFZ	B EIF5A	
7.02124083308083e-20	-0.709661885778061		0.104	0.217
1.25181702812998e-15	4	PLCG2	B EIF5A	
2.00829270734496e-19	-0.301098949058995		0.366	0.506
3.58058506792532e-15	4	SSR4	B EIF5A	
3.03886020978778e-19	0.475981197387815		0.21	0.184
5.41798386803064e-15	4	WDR43	B EIF5A	
1.00016780952257e-18	0.454317738399383		0.292	0.276
1.78319918759779e-14	4	PIM2	B EIF5A	
1.26084551886916e-18	0.431436555346624		0.166	0.151
2.24796147559182e-14	4	HNRNPL	B EIF5A	
6.50313710964236e-18	0.370039637999997		0.168	0.141
1.15944431527814e-13	4	TLK1	B EIF5A	
1.56716219369302e-17	0.422794830148011		0.261	0.241
2.79409347513528e-13	4	CDC42SE2	B EIF5A	
1.39409155568884e-15	-1.72591653586744		0.159	0.247
2.48552583463763e-11	4	IGHG4	B EIF5A	
1.63099406732984e-15	0.386608440137504		0.242	0.224
2.90789932264237e-11	4	PSMA3-AS1		B EIF5A
4.58229272734354e-15	0.333721694842354		0.117	0.1
8.1697697035808e-11	4	SYNC	B EIF5A	
7.46961504925784e-15	0.416473023648528		0.266	0.265
1.33175766713218e-10	4	RBM6	B EIF5A	
1.31953993161074e-13	-2.05833474493746		0.064	0.129
2.35260774406879e-09	4	IGHG2	B EIF5A	
1.48458492302909e-13	0.323467732758637		0.4	0.397
2.64686645926856e-09	4	NUCKS1	B EIF5A	
1.77899215477028e-13	0.382984141970685		0.171	0.156
3.17176511273994e-09	4	TMED4	B EIF5A	
3.48495154194477e-13	-0.527065112347284		0.198	0.267
6.21332010413334e-09	4	S100A4	B EIF5A	
3.98481703040051e-13	0.357403187284215		0.206	0.194
7.10453028350107e-09	4	DDX6	B EIF5A	

4.94653479880072e-13	0.329246937035435	0.111	0.097
8.81917689278181e-09	4	CYBB	B EIF5A
7.18858932986979e-13	0.308051581817021	0.133	0.112
1.28165359162248e-08	4	PIP4K2A	B EIF5A
1.30534801244289e-12	0.365612938428537	0.19	0.162
2.32730497138443e-08	4	MARCKS	B EIF5A
2.01508327755489e-11	0.348407944992568	0.211	0.202
3.59269197555261e-07	4	TRIM38	B EIF5A
3.0906349188411e-10	-0.360897617314005	0.586	0.67
5.5102929968018e-06	4	TUBA1B	B EIF5A
8.56263452723716e-10	0.383393752202145	0.322	0.285
1.52663210986111e-05	4	MIR155HG B EIF5A
1.59474787613845e-08	0.331602324472684	0.913	0.944
0.000284327598836725	4	YBX1	B EIF5A
2.04474842867913e-07	0.321195251846932	0.263	0.279
0.00364558197349203	4	STK17B	B EIF5A
3.43675726941398e-07	0.325851722787903	0.216	0.197
0.00612739453563818	4	YBX3	B EIF5A
1.00251275694197e-06	0.33295319414354 0.177	0.17
0.0178737999435184	4	PARP14	B EIF5A
3.95439748252296e-06	0.318656342579901	0.189	0.171
0.0705029527159018	4	TNFRSF18 B EIF5A
1.16496642984153e-05	0.346871401457456	0.554	0.605
0.207701864776447	4	ARF6	B EIF5A
3.30085964984116e-05	0.332489942596813	0.362	0.405
0.58851026697018 4	STX7	B EIF5A
0.0003533428728211	-1.60057005385102	0.566	0.649	1
4	IGLC2	B EIF5A
0.000689760508946231	0.305717748539551	0.199	0.212	1
4	GABPB1-AS1	B EIF5A
0	2.97295509157723 0.98	0.227	0	5	S100A4
Memory 2
0	1.65266038900283 0.894	0.443	0	5	S100A6
Memory 2
1.62096631507948e-252	0.373204575925755	1	0.998
2.89002084315521e-248	5	B2M	Memory 2
2.5150366829886e-237	0.906477731654774	0.82	0.557
4.48405890210038e-233	5	VIM	Memory 2
3.82327805411133e-225	0.962785281877715	0.538	0.233
6.81652244267508e-221	5	TNFRSF13B	Memory 2
5.89632500791359e-193	0.302052752846715	1	0.999
1.05125578566091e-188	5	RPL34	Memory 2
6.72203453492864e-193	0.31602851788192 1	0.998
1.19847153723243e-188	5	RPS14	Memory 2
3.54884776920105e-180	0.824140821885645	0.489	0.225
6.32724068770855e-176	5	CAPG	Memory 2
9.83727198192241e-171	0.530344502489679	0.971	0.863
1.75388722165695e-166	5	CD52	Memory 2
3.47675349630411e-161	0.732976823649837	0.656	0.415
6.1987038085606e-157	5	PLP2	Memory 2

3.87317984055094e-142 -1.30476841956879 0.037 0.215
6.90549233771827e-138 5 RGS13 Memory 2
7.51361270659208e-137 0.492800938378158 0.893 0.688
1.3396020094583e-132 5 EMP3 Memory 2
6.21263156650239e-136 -0.96086143897731 0.089 0.31
1.10765008199171e-131 5 NME1 Memory 2
9.23927534278122e-133 0.338834031564248 0.996 0.98
1.64727040086446e-128 5 MT-CO1 Memory 2
2.34535273158503e-125 0.687439576073771 0.639 0.434
4.18152938514296e-121 5 ANXA2 Memory 2
6.64538173903796e-121 0.399410107161745 0.962 0.902
1.18480511025308e-116 5 HLA-A Memory 2
9.53019693935021e-120 0.475943907495906 0.933 0.851
1.69913881231675e-115 5 SH3BGRL3 Memory 2
5.57948646070887e-107 0.760146563716532 0.446 0.29
9.94766641079784e-103 5 TCF4 Memory 2
2.96744166973394e-99 0.608171105661231 0.253 0.093
5.29065175296864e-95 5 RHOC Memory 2
7.33089681543031e-95 0.337604669730327 0.992 0.967
1.30702559322307e-90 5 MT-ND3 Memory 2
4.8917950487257e-85 -0.923262961323748 0.297 0.481
8.72158139237305e-81 5 CD83 Memory 2
5.40081992860128e-85 -0.754828131452953 0.312 0.494
9.62912185070323e-81 5 RANBP1 Memory 2
1.02909364596694e-82 -0.611876840332953 0.15 0.338
1.83477106139445e-78 5 RFTN1 Memory 2
8.22477916141488e-82 -0.48805171112987 0.013 0.124
1.46639587668866e-77 5 CD38 Memory 2
4.27823930746193e-80 -0.717928025773878 0.323 0.506
7.62767286127388e-76 5 HSPE1 Memory 2
4.88592072889865e-80 -0.85681154130743 0.777 0.852
8.7111080675534e-76 5 HSP90AB1 Memory 2
5.06086956657831e-79 -0.67302206731617 0.355 0.538
9.02302435025247e-75 5 PSME2 Memory 2
1.32844841917306e-78 0.5629467137059 0.482 0.329
2.36849068654365e-74 5 S100A11 Memory 2
1.61694795299189e-78 0.519498311517153 0.532 0.342
2.88285650538923e-74 5 LY6E Memory 2
3.02960266375964e-75 0.48524150231733 0.212 0.085
5.40147858921705e-71 5 AHNAK Memory 2
4.80803571808464e-74 -0.706980400121991 0.296 0.47
8.57224688177311e-70 5 HSPD1 Memory 2
1.39646888108026e-71 -0.670250713809168 0.643 0.753
2.489764368078e-67 5 HSP90AA1 Memory 2
2.97580916762153e-71 0.482099182965441 0.451 0.265
5.30557016495243e-67 5 KLF2 Memory 2
3.97042173784027e-71 -0.416839655879549 0.015 0.12
7.07886491639541e-67 5 PYCR1 Memory 2
5.41566739232613e-70 -0.559105290141858 0.274 0.458
9.65559339377825e-66 5 ILF2 Memory 2

6.47818074768228e-70	0.488563219913201	0.547	0.374
1.15499484550427e-65	5 CD44 Memory 2		
1.35644349914537e-68	-0.589887234860569	0.144	0.311
2.41840311462628e-64	5 ODC1 Memory 2		
1.69185114055115e-68	-0.651791236850622	0.099	0.241
3.01640139848865e-64	5 UBE2J1 Memory 2		
8.21782509083255e-68	-1.01751677200645	0.622	0.721
1.46515603544454e-63	5 HMGN2 Memory 2		
9.09225326351878e-68	-0.522801263624347	0.03	0.14
1.62105783435276e-63	5 NEIL1 Memory 2		
9.41240503104079e-66	-0.55400052033059	0.227	0.402
1.67813769298426e-61	5 YWHAE Memory 2		
1.11072503122493e-65	-0.791132927370297	0.072	0.196
1.98031165817093e-61	5 PTTG1 Memory 2		
1.20692622233693e-65	-1.12020293466709	0.156	0.293
2.1518287618045e-61	5 MIR155HG Memory 2		
3.82927666502837e-64	-0.589643891830248	0.442	0.591
6.82721736607908e-60	5 ANP32B Memory 2		
6.03868425816732e-64	0.556359090379622	0.345	0.189
1.07663701638865e-59	5 ACP5 Memory 2		
9.12391351054102e-64	-0.776690797565247	0.487	0.605
1.62670253979436e-59	5 PRDX1 Memory 2		
1.74964596624806e-63	-0.440711950919563	0.028	0.137
3.11944379322366e-59	5 PHGDH Memory 2		
2.62532406895377e-63	-0.706865789737379	0.684	0.772
4.68069028253767e-59	5 HMGN1 Memory 2		
3.13517933461051e-63	-0.607755639274274	0.324	0.482
5.58971123567707e-59	5 SNRPD1 Memory 2		
3.87790426503718e-63	0.446077851863476	0.507	0.339
6.91391551413479e-59	5 TRAF3IP3 Memory 2		
1.4365088786241e-62	-0.594872714351876	0.93	0.943
2.56115167969892e-58	5 YBX1 Memory 2		
9.66505531118378e-61	-0.561623133781156	0.563	0.686
1.72318271143096e-56	5 SRSF3 Memory 2		
1.34953968838499e-60	-0.54765599260791	0.5	0.663
2.4060943104216e-56	5 TCL1A Memory 2		
1.97858288118044e-60	-0.506770396951322	0.035	0.141
3.52761541885661e-56	5 PSAT1 Memory 2		
7.71485619204841e-60	0.461315147280273	0.579	0.43
1.37548171048031e-55	5 COTL1 Memory 2		
3.19030210832828e-59	-0.667181129676607	0.67	0.752
5.6879896289385e-55	5 RAN Memory 2		
4.56567663344887e-59	0.411638800747532	0.632	0.487
8.140144869776e-55	5 FXYD5 Memory 2		
6.98026240622059e-59	-0.57512177120858	0.101	0.237
1.24451098440507e-54	5 CKS2 Memory 2		
1.96344634703006e-58	-0.76717758162824	0.239	0.399
3.50062849211989e-54	5 EIF5A Memory 2		
2.20473848126521e-58	-0.582791425684454	0.14	0.287
3.93082823824774e-54	5 SRM Memory 2		

4.8281065914724e-58	-0.514882030684444	0.271	0.439
8.60803124193615e-54	5	SLIRP	Memory 2
7.59374878640138e-58	-0.55680789840121	0.232	0.393
1.3538894711275e-53	5	NHP2	Memory 2
6.87349115302057e-57	-0.573972276513233	0.248	0.406
1.22547473767204e-52	5	BASP1	Memory 2
1.11661351534581e-56	-0.313195950654939	0.015	0.107
1.99081023651004e-52	5	SEMA4A	Memory 2
1.54484466585674e-56	-0.369130375986697	0.022	0.116
2.75430355475599e-52	5	AC023590.1	Memory 2
5.64491391175019e-56	0.45643894039279 0.336	0.204
1.00643170132594e-51	5	MGST3	Memory 2
1.50060617203814e-55	-0.556547479231314	0.365	0.52
2.67543074412679e-51	5	PA2G4	Memory 2
3.36627879647185e-54	-0.891562168969994	0.072	0.187
6.00173846622966e-50	5	XBP1	Memory 2
6.04531666734757e-54	-0.473083687872081	0.554	0.684
1.0778195086214e-49	5	HNRNPA3	Memory 2
6.75645760046334e-54	-0.493890611913652	0.197	0.357
1.20460882558661e-49	5	TIMM13	Memory 2
1.0928604413293e-53	0.433926153551957	0.227	0.112
1.94846088084602e-49	5	FCGR2B	Memory 2
1.5025047628498e-53	0.386285350837698	0.173	0.072
2.6788157416849e-49	5	TIMP1	Memory 2
3.36856539011074e-53	-0.782619484902944	0.61	0.7
6.00581523402844e-49	5	H2AFZ	Memory 2
5.88419346070493e-53	-0.670427109109718	0.462	0.595
1.04909285210908e-48	5	MARCKSL1	Memory 2
6.01185756025928e-53	-0.515144980348987	0.53	0.652
1.07185408441863e-48	5	SNRPG	Memory 2
8.29974393279296e-53	0.462436773759392	0.404	0.261
1.47976134577766e-48	5	GPR183	Memory 2
3.45599822792852e-52	-0.335926152172609	0.019	0.105
6.16169924057375e-48	5	MYBL2	Memory 2
6.12838250960382e-52	-0.509674874978758	0.486	0.614
1.09262931763726e-47	5	POMP	Memory 2
8.65216823413937e-52	0.341682796181737	0.84	0.76
1.54259507446471e-47	5	MYL12A	Memory 2
8.8453747043463e-52	0.350594004138153	0.655	0.505
1.5770418560379e-47	5	BANK1	Memory 2
2.82348269351387e-51	0.365089349005034	0.779	0.672
5.03398729426588e-47	5	TAGLN2	Memory 2
3.20121102969273e-51	-0.652125644890462	0.208	0.347
5.70743914483917e-47	5	LRMP	Memory 2
4.2036940090575e-51	0.316285518004331	0.882	0.812
7.49476604874862e-47	5	NOP53	Memory 2
5.05111887257489e-51	-0.478579199568619	0.457	0.601
9.00563983791378e-47	5	SRSF2	Memory 2
7.16527129695366e-51	-0.466925396742951	0.302	0.465
1.27749621953387e-46	5	SFPQ	Memory 2

2.94106540751859e-50	0.422006996570006		0.255	0.142
5.2436255150649e-46	5	ALOX5	Memory 2	
3.87304884896714e-50	-0.479219280799502		0.107	0.24
6.90525879282352e-46	5	MTHFD2	Memory 2	
7.13474555334101e-50	-0.481436674843267		0.297	0.449
1.27205378470517e-45	5	PPA1	Memory 2	
1.28442901364976e-49	-0.713058932640955		0.647	0.719
2.29000848843617e-45	5	ENO1	Memory 2	
1.44937491498895e-49	-0.579163043852964		0.182	0.324
2.5840905359338e-45	5	DDX21	Memory 2	
2.29026222668764e-49	-0.457506550119733		0.672	0.764
4.08330852396139e-45	5	PSMA7	Memory 2	
2.40587333234216e-49	-0.455048488202995		0.427	0.566
4.28943156423284e-45	5	ATP5PF	Memory 2	
5.28481244791272e-49	-0.447815621816107		0.476	0.611
9.42229211338359e-45	5	PSMB3	Memory 2	
1.94093243653142e-48	-0.489149849624738		0.318	0.471
3.46048844109187e-44	5	PRMT1	Memory 2	
3.27256932829531e-48	-0.462134706842071		0.272	0.424
5.8346638554177e-44	5	DDX39A	Memory 2	
3.67203323583893e-48	-0.453328037512418		0.206	0.353
6.54686805617723e-44	5	H2AFY	Memory 2	
8.60646990516479e-48	-0.361415103292956		0.03	0.12
1.53444751939183e-43	5	GMDS	Memory 2	
9.67785539798738e-48	0.490478083833513		0.43	0.297
1.72546483890717e-43	5	CD27	Memory 2	
1.65995594853937e-47	-0.418695766092325		0.11	0.245
2.95953546065085e-43	5	HNRNPAB	Memory 2	
2.78062299289597e-47	-0.466206736875658		0.525	0.646
4.95757273403423e-43	5	ERH	Memory 2	
3.68893511870357e-47	0.332346222572638		0.119	0.042
6.5770024231366e-43	5	HCK	Memory 2	
5.3218905985945e-47	-0.427178141415044		0.183	0.331
9.48839874823413e-43	5	IMP4	Memory 2	
1.71895201572352e-46	0.410292985811288		0.418	0.291
3.06471954883346e-42	5	PDLIM1	Memory 2	
3.16681255264261e-46	-0.519720007557315		0.282	0.424
5.64611010010651e-42	5	METAP2	Memory 2	
5.09299179952259e-46	-0.338139937693925		0.05	0.16
9.08029507936882e-42	5	PAICS	Memory 2	
6.20857883285905e-46	0.399809890390212		0.431	0.296
1.10692752011044e-41	5	ZFP36L2	Memory 2	
9.52440378738377e-46	-0.488634540858491		0.453	0.577
1.69810595125265e-41	5	SNRPE	Memory 2	
2.14287770188535e-45	0.395604670404785		0.142	0.054
3.82053665469139e-41	5	PPP1R14A	Memory 2	
2.67490138357139e-45	-0.510481278096235		0.203	0.338
4.76908167676943e-41	5	GRHPR	Memory 2	
3.470693771333e-45	-0.480776910044343		0.329	0.475
6.1878999249096e-41	5	CYCS	Memory 2	

5.47902519899456e-45	-0.396904963929332		0.086	0.204
9.7685540272874e-41	5	EBNA1BP2 Memory 2		
5.72050412369333e-45	-0.469950444292587		0.236	0.382
1.01990868021328e-40	5	LCP1 Memory 2		
6.47380331143775e-45	-0.561999159247878		0.054	0.157
1.15421439239624e-40	5	DUSP2 Memory 2		
7.25779884416139e-45	0.511933175976604		0.181	0.091
1.29399295592553e-40	5	KLK1 Memory 2		
1.03650603970317e-44	0.396933177532464		0.178	0.087
1.84798661818678e-40	5	ANXA4 Memory 2		
1.4299487549978e-44	-0.430301198706316		0.126	0.257
2.54945563528559e-40	5	GARS Memory 2		
1.7640084465629e-44	-0.398279327919627		0.089	0.214
3.14505065937699e-40	5	GNL3 Memory 2		
3.11314015556712e-44	0.352305105083049		0.828	0.748
5.55041758336061e-40	5	HLA-C Memory 2		
4.07893354071504e-44	-0.460124215129122		0.525	0.639
7.27233060974084e-40	5	PSMA4 Memory 2		
1.23841610165454e-43	0.450307746520437		0.222	0.123
2.20797206763987e-39	5	CD24 Memory 2		
1.95803319080411e-43	-0.49742838498497		0.266	0.408
3.49097737588464e-39	5	RPL22L1 Memory 2		
4.65021458889836e-43	-0.418040713480664		0.166	0.308
8.29086759054688e-39	5	NOP58 Memory 2		
5.35266866056424e-43	-0.631561466947114		0.163	0.283
9.54327295491998e-39	5	HSPA5 Memory 2		
6.57917327713829e-43	-0.431951893198667		0.447	0.573
1.17300080358099e-38	5	UQCRQ Memory 2		
9.84645032427836e-43	-0.350630397889371		0.058	0.161
1.75552362831559e-38	5	GAR1 Memory 2		
9.94171647845765e-43	-0.505630167254177		0.057	0.153
1.77250863094421e-38	5	BIK Memory 2		
1.01786629372541e-42	-0.372898971538535		0.101	0.224
1.81475381508303e-38	5	MRPL12 Memory 2		
1.23219512566106e-42	-1.43884432732575		0.218	0.307
2.1968806895411e-38	5	HMGB2 Memory 2		
1.81661363468854e-42	-0.412485572478995		0.544	0.662
3.23884044928619e-38	5	PSMB1 Memory 2		
2.16512961489659e-42	-0.441667594828576		0.293	0.435
3.86020959039913e-38	5	CCT2 Memory 2		
2.24103212879357e-42	-0.439224992789326		0.254	0.396
3.99553618242606e-38	5	CCT5 Memory 2		
4.30195813609403e-42	-0.494716918490453		0.522	0.643
7.66996116084205e-38	5	SET Memory 2		
6.73217073719235e-42	-0.565768063662162		0.447	0.565
1.20027872073402e-37	5	NCL Memory 2		
1.09671516708213e-41	-0.441874695555317		0.309	0.452
1.95533347139074e-37	5	C1QBP Memory 2		
2.30796611664193e-41	0.367036606169121		0.339	0.205
4.11487278936089e-37	5	PLAC8 Memory 2		

3.21861912811297e-41	-0.44019788947201			0.31	0.449
5.73847604351262e-37	5	CCT6A	Memory 2		
3.27773108956913e-41	-0.410452970966419			0.527	0.646
5.84386675959281e-37	5	ATP5MF	Memory 2		
3.30428128580483e-41	-0.410965164594659			0.17	0.309
5.89120310446143e-37	5	ATP1B3	Memory 2		
3.83815655414194e-41	-0.410864040097239			0.106	0.224
6.84304932037966e-37	5	NANS	Memory 2		
6.50477644726879e-41	-0.430030760386385			0.089	0.198
1.15973659278355e-36	5	DNMT1	Memory 2		
6.65158088386384e-41	-0.351435979153989			0.051	0.147
1.18591035578408e-36	5	NOP16	Memory 2		
1.32737969917317e-40	-0.396925587506071			0.521	0.647
2.36658526565585e-36	5	HNRNPM	Memory 2		
1.69494064237403e-40	-0.41204265661205			0.666	0.748
3.02190967128866e-36	5	CHCHD2	Memory 2		
1.90236819646688e-40	-0.371381283653355			0.15	0.288
3.39173225748081e-36	5	PSMD14	Memory 2		
2.90468642768687e-40	-0.637231798510368			0.05	0.135
5.17876543192292e-36	5	MYC	Memory 2		
4.96652473468426e-40	-0.359670087125996			0.071	0.177
8.85481694946858e-36	5	ASNS	Memory 2		
5.97568079098193e-40	-0.435258101791882			0.212	0.353
1.06540412822417e-35	5	ABRACL	Memory 2		
6.64990906494614e-40	-0.41632284805029			0.291	0.436
1.18561228718925e-35	5	DNAJA1	Memory 2		
2.97219370068928e-39	-0.409761455049344			0.157	0.285
5.29912414895891e-35	5	WARS	Memory 2		
3.81369185073381e-39	-0.388373587947895			0.439	0.577
6.79943120067331e-35	5	RBM8A	Memory 2		
3.98154717897712e-39	-0.434482435236691			0.469	0.585
7.0987004653983e-35	5	LDHB	Memory 2		
4.17039875498313e-39	-0.374912186854773			0.085	0.193
7.43540394025942e-35	5	GPATCH4	Memory 2		
5.46175591462336e-39	0.338384914615055			0.153	0.072
9.737764620182e-35	5	CAPN2	Memory 2		
5.49647719703375e-39	-0.301707639232146			0.035	0.12
9.79966919459148e-35	5	LMNB1	Memory 2		
7.28852698686759e-39	-0.369860279680842			0.138	0.271
1.29947147648862e-34	5	SYNCRIP	Memory 2		
1.04878086296175e-38	-0.367021050453376			0.079	0.185
1.86987140057451e-34	5	LCK	Memory 2		
2.04099208588167e-38	-0.360280804503101			0.385	0.53
3.63888478991843e-34	5	SELENOT	Memory 2		
2.53730902442726e-38	-0.372939778902147			0.119	0.242
4.52376825965136e-34	5	NIFK	Memory 2		
3.09435444513647e-38	-0.383419974320538			0.371	0.504
5.51692454023382e-34	5	COX5A	Memory 2		
3.53658747298253e-38	-0.355556167005846			0.067	0.17
6.30538180558056e-34	5	NFKB1	Memory 2		

3.87895452207238e-38	-0.403274751344352	0.32	0.455	6.91578801740284e-34	5	SEM1	Memory 2
4.33712588679409e-38	-0.435702614462337	0.425	0.54	7.73266174356518e-34	5	SRSF9	Memory 2
1.24116947381666e-37	0.374850732412382	0.44	0.34	2.21288105486772e-33	5	CAST	Memory 2
1.24539299680943e-37	-0.372493907471586	0.263	0.406	2.22041117401154e-33	5	PSMA3	Memory 2
1.81269366719981e-37	0.341827516976371	0.601	0.498	3.23185153925054e-33	5	TSC22D3	Memory 2
2.15861918532663e-37	0.363649576517869	0.323	0.206	3.84860214551885e-33	5	LBH	Memory 2
2.25516521743745e-37	-0.398121249920624	0.312	0.451	4.02073406616924e-33	5	ATP5MC1	Memory 2
2.69702130071822e-37	-0.370635526451127	0.111	0.23	4.80851927705051e-33	5	SIAH2	Memory 2
2.78319212740098e-37	-0.444967112355908	0.122	0.238	4.9621532439432e-33	5	IL4R	Memory 2
2.83375460199174e-37	-0.481728150910896	0.915	0.93	5.05230107989108e-33	5	NPM1	Memory 2
3.2522649177636e-37	-0.457928541689052	0.102	0.207	5.79846312188073e-33	5	HMCES	Memory 2
6.89394564693085e-37	-0.424875646183989	0.305	0.441	1.2291215693913e-32	5	EIF5B	Memory 2
7.2246582873585e-37	-0.347116026086085	0.268	0.411	1.28808432605315e-32	5	PSMB2	Memory 2
1.45996942677609e-36	0.365478290589481	0.384	0.28	2.60297949099909e-32	5	ACAP1	Memory 2
1.5477422579555e-36	-0.3094105111528	0.029	0.104	2.75946967170885e-32	5	CENPM	Memory 2
1.58121321743146e-36	-0.381838511421305	0.278	0.418	2.81914504535855e-32	5	HNRNPR	Memory 2
1.95989690322892e-36	-0.362405652227281	0.233	0.374	3.49430018876685e-32	5	EWSR1	Memory 2
2.0555593415099e-36	0.316108433340922	0.704	0.616	3.664856749978e-32	5	IFI16	Memory 2
2.35449046534054e-36	0.367821036366821	0.414	0.3	4.19782105065565e-32	5	ARHGAP24	Memory 2
2.54921251405222e-36	-0.438679860267858	0.192	0.314	4.54499099130371e-32	5	CALR	Memory 2
2.79971539623285e-36	-0.634155449700214	0.293	0.41	4.99161257994355e-32	5	FABP5	Memory 2
2.82842757533892e-36	-0.386374369316314	0.27	0.41	5.04280352407176e-32	5	BZW1	Memory 2
4.48604441314274e-36	-0.330783320226497	0.069	0.168	7.99816858419219e-32	5	MRTO4	Memory 2
4.63813709999956e-36	-0.413274069585494	0.407	0.536	8.26933463558922e-32	5	SRSF7	Memory 2
6.44653280493572e-36	-0.39962535549104	0.287	0.429	1.14935233379199e-31	5	TMEM123	Memory 2

8.60758303018893e-36 -0.393112311498799 0.106 0.212
1.53464597845238e-31 5 EAF2 Memory 2
1.01993607825686e-35 0.363695767385378 0.393 0.273
1.81844403392416e-31 5 IFITM2 Memory 2
1.03732412785422e-35 -0.499956937465957 0.21 0.333
1.84944518755128e-31 5 SLC3A2 Memory 2
1.04864229387142e-35 -0.337875168444651 0.07 0.173
1.86962434574336e-31 5 SEL1L3 Memory 2
1.35178290468957e-35 -0.355299791021591 0.406 0.538
2.41009374077104e-31 5 GHITM Memory 2
1.79446288945777e-35 -0.406992856298554 0.48 0.59
3.19934788561427e-31 5 ATP5MC3 Memory 2
1.96975270441455e-35 -0.34100311155069 0.072 0.17
3.5118720967007e-31 5 DEF8 Memory 2
2.09727862555909e-35 -0.365250560372445 0.611 0.705
3.7392380615093e-31 5 ELOB Memory 2
2.55389296110481e-35 -0.423694551948124 0.035 0.113
4.55333576035377e-31 5 VPREB3 Memory 2
2.68012847547204e-35 -0.344190188378903 0.187 0.322
4.7784010589191e-31 5 NDUFAF3 Memory 2
4.14868076217458e-35 0.312203298170533 0.592 0.487
7.39668293088105e-31 5 ANKRD12 Memory 2
4.85269467492404e-35 0.335794732468751 0.618 0.51
8.65186933592207e-31 5 LSP1 Memory 2
6.09867111692658e-35 -0.423675376997977 0.451 0.559
1.08733207343684e-30 5 SEC61G Memory 2
6.10775497623304e-35 -0.332265156425519 0.137 0.266
1.08895163471259e-30 5 AK6 Memory 2
1.98406446626335e-34 -0.324846104996389 0.063 0.156
3.53738853690093e-30 5 PPAN Memory 2
2.59212906434792e-34 -0.350687963836539 0.125 0.239
4.6215069088259e-30 5 CDK4 Memory 2
3.36084631998184e-34 -0.395272592200216 0.396 0.52
5.99205290389562e-30 5 SSBP1 Memory 2
3.50966085405673e-34 0.32849730883336 0.16 0.087
6.25737433669774e-30 5 ITGB7 Memory 2
5.91585713404628e-34 -0.37354863796519 0.461 0.579
1.05473816842911e-29 5 PARK7 Memory 2
7.63052218552279e-34 -0.371480520163955 0.642 0.727
1.36044580045686e-29 5 COX6C Memory 2
1.21320026159537e-33 -0.332453531387402 0.122 0.241
2.16301474639839e-29 5 BUD23 Memory 2
1.40535639108866e-33 0.310009570345188 0.149 0.069
2.50560990967198e-29 5 MYO1F Memory 2
1.58282962283102e-33 -0.517974295638517 0.678 0.739
2.82202693454543e-29 5 HSPA8 Memory 2
1.76307509502762e-33 -0.496304592987904 0.542 0.64
3.14338658692474e-29 5 HERPUD1 Memory 2
2.96952317815948e-33 -0.327309280758119 0.097 0.206
5.29436287434054e-29 5 TOMM40 Memory 2

3.01391217737798e-33 -0.362424807147372 0.072 0.165
5.3735040210472e-29 5 SUSD3 Memory 2
3.26564747396628e-33 -0.359777698212287 0.596 0.689
5.82232288133449e-29 5 COX6A1 Memory 2
3.46407717706369e-33 -0.369132502830673 0.335 0.463
6.17610319898685e-29 5 LSM5 Memory 2
4.2931956987642e-33 -0.385196805175991 0.229 0.356
7.65433861132669e-29 5 PDIA6 Memory 2
5.62680982766581e-33 -0.329811349955457 0.142 0.259
1.00320392417454e-28 5 RRP7A Memory 2
5.63650428111949e-33 -0.41748767980137 0.154 0.269
1.00493234828079e-28 5 CD22 Memory 2
5.68491187623401e-33 -0.382292496234475 0.305 0.434
1.01356293841376e-28 5 CCT4 Memory 2
9.47435658306793e-33 0.369168447953051 0.407 0.314
1.68918303519518e-28 5 FCRLA Memory 2
1.46507685443529e-32 -0.3609365995503 0.263 0.397
2.61208552377268e-28 5 ARPC5L Memory 2
1.8817070741644e-32 -0.32906296662456 0.067 0.161
3.35489554252772e-28 5 NOLC1 Memory 2
2.5626998539545e-32 -0.339186034191741 0.183 0.311
4.56903756961547e-28 5 PSMD11 Memory 2
2.81710783888771e-32 -0.328510831833159 0.175 0.299
5.02262156595291e-28 5 SSRP1 Memory 2
2.94521309878572e-32 -0.404451499447596 0.283 0.403
5.25102043382505e-28 5 NUCKS1 Memory 2
3.29172916431117e-32 -0.369790203073025 0.148 0.271
5.86882392705038e-28 5 FAM3C Memory 2
3.63153438750412e-32 -0.381281338490469 0.515 0.621
6.47466265948109e-28 5 SERBP1 Memory 2
3.9823587645733e-32 -0.327954036981009 0.395 0.525
7.10014744135773e-28 5 PSMB6 Memory 2
4.07145501980001e-32 -0.4351813444562 0.139 0.248
7.25899715480144e-28 5 KPNA2 Memory 2
4.08560546608693e-32 -0.341444563469133 0.269 0.402
7.28422598548639e-28 5 NDUFAB1 Memory 2
4.14404633091845e-32 0.331658720370674 0.188 0.116
7.3884202033945e-28 5 PYCARD Memory 2
4.99352370327366e-32 -0.316886026754314 0.069 0.167
8.9029534105666e-28 5 LYPLAL1 Memory 2
6.96664195012158e-32 -0.372138122328272 0.337 0.457
1.24208259328718e-27 5 MRPL51 Memory 2
8.56691505237063e-32 -0.334626806574547 0.043 0.122
1.52739528468716e-27 5 UBE2S Memory 2
1.28179360674869e-31 -0.344707018084675 0.501 0.614
2.28530982147223e-27 5 POLR2L Memory 2
2.23927181408409e-31 -0.323881055363584 0.157 0.281
3.99239771733052e-27 5 NDUFAF8 Memory 2
2.95549478724499e-31 -0.618485074022443 0.5 0.581
5.26935165617909e-27 5 TUBB Memory 2

4.1142800011274e-31 -0.330691542833799 0.087 0.187
7.33534981401004e-27 5 CMSS1 Memory 2
5.54600145308822e-31 -0.386374575477947 0.244 0.368
9.88796599071099e-27 5 HSPA9 Memory 2
6.01606188179547e-31 0.354331972994023 0.295 0.201
1.07260367290532e-26 5 PRKCB Memory 2
8.28284491706954e-31 -0.321299872430282 0.226 0.355
1.47674842026433e-26 5 CYC1 Memory 2
9.51983611556419e-31 0.337189163543532 0.424 0.327
1.69729158104394e-26 5 RIPOR2 Memory 2
1.0367072842395e-30 -0.347241344541868 0.361 0.481
1.8483454170706e-26 5 PRELID1 Memory 2
1.95525784531081e-30 -0.316334647699131 0.104 0.209
3.48602921240465e-26 5 UBALD2 Memory 2
1.99667293100078e-30 -0.361749515878157 0.474 0.591
3.55986816868129e-26 5 EIF2S2 Memory 2
2.03718060880292e-30 -0.329084690822622 0.37 0.496
3.63208930743473e-26 5 CCT8 Memory 2
2.58964932357447e-30 0.31525692111603 0.461 0.347
4.61708577900092e-26 5 TXNIP Memory 2
3.00242755555831e-30 -0.4786818722935 0.832 0.852
5.35302808880491e-26 5 ATP5MG Memory 2
4.73056801679557e-30 -0.459900911781864 0.096 0.176
8.43412971714482e-26 5 TNFRSF18 Memory 2
5.51118416425505e-30 -0.412583178510387 0.515 0.615
9.82589024645032e-26 5 SYNGR2 Memory 2
6.50974148096931e-30 0.340441947157316 0.531 0.441
1.16062180864202e-25 5 KLF6 Memory 2
7.99902607445131e-30 -0.538700006291698 0.311 0.414
1.42614635881392e-25 5 TXN Memory 2
9.58938437527215e-30 -0.314966112937147 0.265 0.394
1.70969134026727e-25 5 NDUFS6 Memory 2
9.59989047103818e-30 0.343847198270103 0.569 0.48
1.7115644720814e-25 5 NEAT1 Memory 2
1.13535453448487e-29 -0.365779935336151 0.641 0.711
2.02422359953308e-25 5 NDUFA4 Memory 2
1.1593137386947e-29 0.315311005126159 0.54 0.451
2.06694046471877e-25 5 JUNB Memory 2
1.1907493058731e-29 -0.321221688210511 0.493 0.606
2.12298693744115e-25 5 PSMA2 Memory 2
1.46309635046311e-29 -0.34939406314737 0.564 0.657
2.60855448324067e-25 5 UBE2D3 Memory 2
1.67634252680436e-29 -0.326906597285429 0.396 0.516
2.9887510910395e-25 5 PSMD8 Memory 2
2.11531149035741e-29 -0.38075785417689 0.506 0.602
3.77138885615822e-25 5 SEC61B Memory 2
3.29441321906123e-29 -0.31533810213243 0.104 0.206
5.87360932826426e-25 5 RELB Memory 2
3.3393487171623e-29 -0.344993539416654 0.241 0.37
5.95372482782867e-25 5 EIF3J Memory 2

3.70852413298124e-29 -0.304423647625348 0.12 0.229
6.61192767669225e-25 5 HSPH1 Memory 2
3.71396963197124e-29 -0.331314578766346 0.374 0.496
6.62163645684152e-25 5 CCT3 Memory 2
3.88370237935669e-29 -0.328509787193517 0.329 0.451
6.92425297215505e-25 5 PGAM1 Memory 2
4.03667351353548e-29 -0.341790299620308 0.303 0.426
7.19698520728241e-25 5 HNRNPD Memory 2
4.67025103706759e-29 -0.334899165581425 0.567 0.668
8.3265905739878e-25 5 DBI Memory 2
5.38666820969199e-29 -0.311799039236142 0.095 0.193
9.60389075105986e-25 5 PRPSAP2 Memory 2
5.40526620783454e-29 -0.381512319565001 0.636 0.707
9.6370491219482e-25 5 TPI1 Memory 2
6.15078233870808e-29 -0.3895238915228 0.29 0.406
1.09662298316826e-24 5 REL Memory 2
6.17389180212825e-29 -0.310914096003256 0.147 0.262
1.10074316940145e-24 5 GSPT1 Memory 2
8.97375908202608e-29 -0.309063972315803 0.126 0.233
1.59993150673443e-24 5 MRPL3 Memory 2
1.0532015613706e-28 -0.341586573711975 0.292 0.415
1.87775306376764e-24 5 TMED9 Memory 2
1.24872696943851e-28 -0.350889396400486 0.5 0.625
2.22635531381192e-24 5 CLEC2D Memory 2
1.27182617988627e-28 -0.357483535457708 0.167 0.276
2.26753889611924e-24 5 SMS Memory 2
1.65676200786046e-28 -0.304318471370116 0.101 0.201
2.95384098381442e-24 5 GTPBP4 Memory 2
1.93463425748605e-28 0.328436152325323 0.473 0.384
3.44925941767188e-24 5 EVI2B Memory 2
3.61189912383051e-28 -0.315002501985062 0.288 0.413
6.43965494787741e-24 5 BANF1 Memory 2
6.04857501195416e-28 0.31890543824186 0.303 0.215
1.07840043888131e-23 5 LY86 Memory 2
7.42675528393765e-28 0.326276701924439 0.446 0.363
1.32411619957324e-23 5 CXXC5 Memory 2
7.43284752450669e-28 -0.322581089015459 0.096 0.19
1.3252023851443e-23 5 WDR43 Memory 2
7.4337673692419e-28 -0.323620975927676 0.301 0.426
1.32536638426214e-23 5 MAGOH Memory 2
7.65218619950841e-28 -0.325285450170987 0.545 0.644
1.36430827751036e-23 5 ATP5MD Memory 2
8.4150763642217e-28 -0.334987121003469 0.36 0.474
1.50032396497709e-23 5 SNRPF Memory 2
9.01282358449293e-28 0.31840900246043 0.415 0.323
1.60689631687924e-23 5 ZFP36 Memory 2
9.75950217734968e-28 -0.349025502448767 0.076 0.158
1.74002164319967e-23 5 MCM7 Memory 2
1.12555621184698e-27 0.364967835636218 0.334 0.263
2.00675417010198e-23 5 GRN Memory 2

1.40146379753087e-27	-0.336349616474073	0.346	0.462
2.49866980461779e-23	5	COX17	Memory 2
2.18041365565461e-27	-0.321634916751052	0.539	0.637
3.88745950666661e-23	5	COX7B	Memory 2
2.80750437650493e-27	-0.37601248147967	0.088	0.18
5.00549955287063e-23	5	PDIA4	Memory 2
3.33956209026231e-27	-0.308944120824404	0.319	0.441
5.95410525072867e-23	5	LSM3	Memory 2
7.17465803713279e-27	-0.340160144932848	0.283	0.399
1.27916978144041e-22	5	PHB	Memory 2
7.90114163413066e-27	-0.30797687542898	0.085	0.173
1.40869454194915e-22	5	CCDC88A	Memory 2
1.08825391149842e-26	-0.315832275972428	0.337	0.458
1.94024789881054e-22	5	VDAC1	Memory 2
2.14468809841857e-26	-0.313899120752921	0.165	0.276
3.82376441067048e-22	5	ARF4	Memory 2
2.83683670560902e-26	-0.328669334584056	0.47	0.57
5.05779616243032e-22	5	SNRPB	Memory 2
3.03117784925916e-26	-0.307650927873224	0.274	0.398
5.40428698744416e-22	5	SSB	Memory 2
3.05840727685647e-26	-0.324086076671939	0.245	0.367
5.45283433390741e-22	5	TPD52	Memory 2
5.39928018278911e-26	-0.316883714098846	0.317	0.433
9.62637663789471e-22	5	MINOS1	Memory 2
5.50576951317258e-26	-1.1259252672299 0.109	0.179
9.8162364650354e-22	5	MZB1	Memory 2
8.16757140161642e-26	-0.391544951539278	0.59	0.66
1.45619630519419e-21	5	SLC25A5	Memory 2
9.58261836673341e-26	0.32920815268009 0.219	0.156
1.7084850286049e-21	5	IL10RA	Memory 2
1.41589780270965e-25	-0.731910550490356	0.624	0.668
2.52440419245104e-21	5	TUBA1B	Memory 2
1.41995853406571e-25	-0.300328021367033	0.303	0.424
2.53164407038575e-21	5	NUDC	Memory 2
2.61855674058956e-25	-0.307667225865872	0.432	0.539
4.66862481279713e-21	5	SOD1	Memory 2
3.53751306992493e-25	-0.345034772974045	0.15	0.247
6.30703205236916e-21	5	SLBP	Memory 2
4.72093180475568e-25	-0.323029850790808	0.563	0.649
8.41694931469891e-21	5	HNRNPC	Memory 2
7.43295021885657e-25	-0.303878581414195	0.354	0.471
1.32522069451994e-20	5	PRDX6	Memory 2
8.15169701388592e-25	-0.339361778472766	0.174	0.283
1.45336606060572e-20	5	GADD45B	Memory 2
1.6638856962892e-24	-0.376016656439293	0.303	0.413
2.96654180791401e-20	5	PPIB	Memory 2
1.84476502797732e-24	0.316880624098292	0.285	0.211
3.28903156838077e-20	5	YPEL3	Memory 2
1.96544483520775e-24	-0.349162626126222	0.345	0.444
3.50419159669191e-20	5	HMGA1	Memory 2

1.15768293910881e-23	0.316065589598322		0.271	0.199
2.06403291213709e-19	5	BIN1	Memory 2	
1.53473381067009e-23	0.322588634937134		0.155	0.092
2.7362769110437e-19	5	MT2A	Memory 2	
1.67291592941941e-23	-0.385532515816746		0.103	0.181
2.98264181056186e-19	5	BCL2A1	Memory 2	
2.53673795420052e-23	-0.334912278657198		0.814	0.847
4.52275009854411e-19	5	HNRNPA2B1	Memory 2	
4.06382271083504e-23	-0.386292750884964		0.337	0.434
7.24538951114779e-19	5	IRF8	Memory 2	
5.5920892454843e-23	-0.301087862304224		0.332	0.445
9.97013591577395e-19	5	FDPS	Memory 2	
8.33061401178618e-23	-0.306148353227328		0.335	0.44
1.48526517216136e-18	5	ROMO1	Memory 2	
1.4596294883789e-22	-0.315966146249705		0.109	0.196
2.60237341483074e-18	5	MANF	Memory 2	
1.94545338590323e-22	-0.302108207622117		0.429	0.534
3.46854884172687e-18	5	SPCS2	Memory 2	
2.17640300644564e-22	-0.310690703393572		0.879	0.896
3.88030892019194e-18	5	PPIA	Memory 2	
3.33023855408454e-22	-0.316750488457681		0.172	0.267
5.93748231807732e-18	5	ANP32E	Memory 2	
4.10563388612068e-22	-0.492935973272227		0.926	0.932
7.31993465556456e-18	5	HMGB1	Memory 2	
4.50932141252592e-22	-0.340527037930102		0.325	0.431
8.03966914639247e-18	5	CD40	Memory 2	
4.97524733193053e-22	-0.326232918912849		0.124	0.212
8.87036846809895e-18	5	PIM3	Memory 2	
5.42903198038469e-22	-0.300744467655216		0.155	0.251
9.67942111782787e-18	5	DKC1	Memory 2	
2.17957041234392e-21	-0.343423051209689		0.462	0.544
3.88595608816797e-17	5	PARP1	Memory 2	
2.64652531769424e-21	-0.311818520686173		0.191	0.286
4.71848998891707e-17	5	JPT1	Memory 2	
5.02114266694408e-21	-0.304700601335716		0.062	0.124
8.95219526089459e-17	5	LPP	Memory 2	
6.52821273772523e-21	-0.555667869342711		0.235	0.316
1.16391504900903e-16	5	HSP90B1	Memory 2	
8.98720185272002e-21	-0.312387610697209		0.383	0.484
1.60232821832145e-16	5	RSL1D1	Memory 2	
1.43537668815899e-20	-0.301095090223586		0.221	0.314
2.55913309731867e-16	5	CALM3	Memory 2	
2.47091644525537e-20	-0.327800410647429		0.115	0.192
4.4053969302458e-16	5	PCNA	Memory 2	
3.66378793744732e-20	-0.333579292027423		0.138	0.222
6.53216751367484e-16	5	EIF4EBP1	Memory 2	
4.0736680216085e-20	-0.303899252722106		0.08	0.15
7.26294271572579e-16	5	SDF2L1	Memory 2	
5.99669378833286e-20	-0.376494839576641		0.27	0.357
1.06915053552187e-15	5	TUBB4B	Memory 2	

2.81331111826925e-19 -0.311576178777263 0.16 0.245
5.01585239276225e-15 5 MYDGF Memory 2
6.56275491494715e-19 -0.326640649034753 0.233 0.333
1.17007357378593e-14 5 HNRNPH1 Memory 2
2.37158815366383e-18 -0.307125693867583 0.218 0.307
4.22830451916724e-14 5 BCL7A Memory 2
3.92326891388963e-18 0.313230142202388 0.216 0.156
6.99479614657383e-14 5 LMNA Memory 2
5.64178391418282e-18 -0.372118721900643 0.286 0.363
1.00587365405966e-13 5 H2AFV Memory 2
2.43810777258184e-16 -0.333527506932851 0.18 0.26
4.34690234773617e-12 5 SRGN Memory 2
1.45552645558174e-15 -1.18074332440225 0.372 0.425
2.59505811765668e-11 5 HIST1H4C Memory 2
1.48823005151973e-15 -0.336170224101926 0.496 0.555
2.65336535885453e-11 5 TCEA1 Memory 2
1.70654309754392e-14 -0.707542398566339 0.143 0.215
3.04259568861105e-10 5 PLCG2 Memory 2
1.95130144740403e-13 -0.306367362342757 0.223 0.299
3.47897535057664e-09 5 NFKBIA Memory 2
1.09795853629894e-12 -0.375192522641948 0.946 0.932
1.95755027436738e-08 5 SERF2 Memory 2
3.78051368956583e-12 -0.306207725437154 0.549 0.599
6.74027785712693e-08 5 LDHA Memory 2
5.74863588601693e-12 -0.307320333168369 0.273 0.338
1.02492429211796e-07 5 DUT Memory 2
6.9918980213068e-11 -2.80988827290323 0.415 0.452
1.24658549821879e-06 5 IGHG3 Memory 2
1.20408344568254e-08 -0.404712146944625 0.45 0.502
0.00021467603753074 5 SSR4 Memory 2
8.24632703104549e-08 -1.79201914520005 0.202 0.245
0.0014702376463651 5 IGHG4 Memory 2
8.65272949800815e-07 -0.392702218188867 0.917 0.886
0.0154269514219987 5 GAPDH Memory 2
2.46948560312665e-06 -1.10009158254028 0.194 0.217
0.0440284588181451 5 IGHG1 Memory 2
5.44735213563976e-06 -1.44366581449754 0.58 0.648
0.0971208412263213 5 IGLC2 Memory 2
0.000450654996379107 -1.16186517260333 0.389 0.431 1
5 IGLC3 Memory 2
0.00651123411709557 -0.936178757082279 0.409 0.403 1
5 JCHAIN Memory 2
0 1.76330988680316 0.837 0.456 0 6 CD83
Activated
9.54509764543913e-295 1.66689410718752 0.611 0.273
1.70179545920534e-290 6 MIR155HG Activated
2.56257449255987e-250 0.691505835243175 0.984 0.963
4.56881406278499e-246 6 EIF1 Activated
2.5545739835501e-240 1.25223423551443 0.559 0.284
4.55454995527147e-236 6 NFKBIA Activated

4.65201977829976e-214 1.11971619913042 0.775 0.533
8.29408606273063e-210 6 CCR7 Activated
5.09260347972239e-194 1.12635227880653 0.388 0.154
9.07960274399705e-190 6 MARCKS Activated
1.73675855352929e-184 1.17133894595117 0.559 0.305
3.09646682508738e-180 6 CD69 Activated
3.08071395959373e-181 1.1599921478556 0.38 0.168
5.49260491855967e-177 6 BCL2A1 Activated
6.72758239198122e-169 1.00820653309209 0.601 0.391
1.19946066466633e-164 6 REL Activated
4.35540181100626e-159 0.920143601521351 0.331 0.125
7.76524588884307e-155 6 NFKB2 Activated
1.18911799339941e-145 -1.00012116714407 0.757 0.894
2.1200784704318e-141 6 GAPDH Activated
2.39156861208135e-144 -1.0543668668376 0.063 0.314
4.26392767847984e-140 6 CD27 Activated
2.53186490231316e-128 0.853123500307269 0.331 0.157
4.51406193433413e-124 6 NFKB1 Activated
1.47406104627242e-120 0.800571754152299 0.208 0.068
2.6281034393991e-116 6 TRAF1 Activated
3.12942117011764e-120 0.555070530923867 0.956 0.903
5.57944500420274e-116 6 HLA-A Activated
3.05980781450157e-117 0.710672786122217 0.842 0.692
5.45533135247485e-113 6 EMP3 Activated
4.53991493009897e-114 0.829090211635042 0.574 0.419
8.09421432887346e-110 6 CD40 Activated
2.62409989490249e-110 -0.650235876738798 0.68 0.876
4.67850770262166e-106 6 CD52 Activated
4.6511075049957e-103 -0.802744052400285 0.611 0.793
8.29245957065684e-99 6 ACTG1 Activated
6.2937604342416e-99 0.820363681246196 0.473 0.311
1.12211454782093e-94 6 DDX21 Activated
8.79406292878326e-99 -1.22684736796917 0.042 0.213
1.56789347957277e-94 6 RGS13 Activated
9.78153598946906e-98 -0.59976569186289 0.576 0.785
1.74395005156244e-93 6 ARHGDIB Activated
1.28963775313861e-96 0.81214842103345 0.331 0.184
2.29929515007083e-92 6 RNF145 Activated
4.1401092736364e-95 0.883907067118808 0.294 0.124
7.38140082396634e-91 6 MYC Activated
2.04038791943317e-93 -0.73458420323735 0.211 0.447
3.6378076215574e-89 6 COTL1 Activated
1.05555184069628e-92 0.678613074486254 0.733 0.605
1.8819433767774e-88 6 SYNGR2 Activated
2.13048187840004e-91 0.892739511398516 0.282 0.146
3.79843614099944e-87 6 NFKBID Activated
2.35691110894162e-91 -0.621518931436461 0.578 0.783
4.20213681613202e-87 6 HLA-DPB1 Activated
1.1701123624479e-89 0.422739356097769 0.931 0.844
2.08619333100835e-85 6 HSP90AB1 Activated

3.13857526453603e-89 0.737785968184714 0.633 0.496
5.59576583914129e-85 6 FOXP1 Activated
1.34203914338274e-83 0.713667029629386 0.487 0.342
2.39272158873708e-79 6 LITAF Activated
1.58907872027982e-81 -0.499054883812594 0.694 0.863
2.83316845038689e-77 6 CD79B Activated
1.87642043202712e-81 0.719780495628823 0.311 0.165
3.34546998826116e-77 6 BCL2 Activated
2.66571082195996e-81 0.653672936560244 0.751 0.644
4.75269582447242e-77 6 ZFP36L1 Activated
3.56141889606716e-80 0.44863940471655 0.106 0.022
6.34965374979813e-76 6 IL2RA Activated
3.52750200221624e-79 -0.578450792342877 0.022 0.167
6.28918331975134e-75 6 SUSD3 Activated
6.22795496669767e-77 0.760894464454589 0.311 0.166
1.11038209101253e-72 6 TNFRSF18 Activated
2.34569716906941e-75 -0.657656618657107 0.07 0.244
4.18214348273385e-71 6 GCHFR Activated
5.0360498719947e-75 -0.369586750440784 0.954 0.991
8.97877331677935e-71 6 CD74 Activated
2.49520791012267e-74 0.743982070667126 0.456 0.321
4.4487061829577e-70 6 PMAIP1 Activated
1.39343922965446e-73 0.643490541482651 0.351 0.214
2.48436280255093e-69 6 TRAF4 Activated
3.76146336192362e-73 -0.741215591856361 0.334 0.542
6.70631302797363e-69 6 ISG20 Activated
4.57388426608629e-72 -0.628034216260117 0.109 0.296
8.15477825800524e-68 6 BCAS4 Activated
2.52099360720705e-71 -0.66064359502905 0.021 0.154
4.49467950228945e-67 6 BIK Activated
5.21755014158019e-70 0.731655180644138 0.447 0.321
9.30237014742332e-66 6 SLC3A2 Activated
5.80300751338683e-70 -0.576014628946939 0.301 0.525
1.03461820956174e-65 6 LSP1 Activated
1.20540263374002e-69 -0.495525603642033 0.011 0.123
2.14911235569509e-65 6 CD38 Activated
4.90729413025706e-69 0.736201532156346 0.373 0.264
8.74921470483532e-65 6 PIM1 Activated
5.70044000827809e-69 -0.506606538650843 0.024 0.159
1.0163314490759e-64 6 CD81 Activated
4.43415055194658e-65 -0.537864895309503 0.358 0.565
7.90564701906555e-61 6 ATP5IF1 Activated
1.29080834659847e-62 0.592518331052948 0.217 0.104
2.30138220115041e-58 6 SNX9 Activated
4.77247068605929e-61 -0.537158078014528 0.011 0.113
8.5088379861751e-57 6 VPREB3 Activated
2.0856565684012e-58 0.619465581510375 0.267 0.158
3.7185170958025e-54 6 CD58 Activated
2.06637150560794e-57 0.597389691569123 0.347 0.227
3.68413375734839e-53 6 TARS Activated

3.38907767865746e-57 0.59499125338998 0.575 0.471
6.04238659327839e-53 6 TNFAIP8 Activated
3.9994306168758e-56 -0.328202745861522 0.661 0.821
7.13058484682787e-52 6 HLA-DPA1 Activated
1.09419036692958e-55 0.596004475772695 0.324 0.212
1.95083200519874e-51 6 NFKBIE Activated
1.46758980508761e-55 -0.45906672223125 0.062 0.215
2.61656586349071e-51 6 GLRX Activated
1.01088576438032e-54 -1.02023016736904 0.124 0.269
1.80230822931367e-50 6 S100A4 Activated
1.10453000055778e-54 0.550742992117331 0.216 0.113
1.96926653799447e-50 6 SLAMF1 Activated
1.25016007550052e-54 -0.498890684340658 0.404 0.587
2.22891039860987e-50 6 LDHB Activated
1.80084844610819e-54 -0.515565422579718 0.024 0.139
3.21073269456629e-50 6 NEIL1 Activated
3.20115778703457e-54 -1.30596902338242 0.053 0.181
5.70734421850393e-50 6 MZB1 Activated
7.03897302610154e-54 0.552195621741369 0.168 0.083
1.25497850082364e-49 6 EBI3 Activated
2.2868503291623e-53 -1.54680932862325 0.166 0.308
4.07722545186346e-49 6 HMGB2 Activated
1.83576904641468e-52 -0.52800644936148 0.304 0.497
3.27299263285273e-48 6 UCP2 Activated
2.87626175487539e-52 0.536849504593752 0.203 0.115
5.12808708276733e-48 6 PNO1 Activated
3.54755288639395e-52 0.608650312226024 0.256 0.164
6.32493204115178e-48 6 SNHG15 Activated
4.12044849726963e-52 -0.414431398256204 0.029 0.145
7.34634762578203e-48 6 AIM2 Activated
8.85621816525667e-52 0.770619050854771 0.336 0.252
1.57897513668361e-47 6 SRGN Activated
1.04068654387143e-51 -0.32567990709656 0.013 0.107
1.85544003906837e-47 6 SEMA4A Activated
1.47538176281013e-51 -0.662551491620371 0.129 0.28
2.63045814491418e-47 6 KLF2 Activated
2.29023845547552e-51 -0.545685347303282 0.873 0.934
4.0832661422673e-47 6 HMGB1 Activated
2.3924104284256e-51 -0.538807530975041 0.438 0.612
4.26542855284001e-47 6 DYNLL1 Activated
4.57814489185346e-51 0.441397752211991 0.107 0.032
8.16237452768554e-47 6 IL13RA1 Activated
1.69460104183304e-50 -0.493521008309863 0.082 0.225
3.02130419748412e-46 6 LY86 Activated
7.1043308567257e-50 0.59474919728404 0.296 0.206
1.26663114844563e-45 6 CFLAR Activated
1.59977055183502e-49 -0.338783462679419 0.018 0.121
2.85223091686665e-45 6 ABI3 Activated
2.86184686938909e-49 0.66298015579841 0.552 0.475
5.10238678343381e-45 6 SQSTM1 Activated

3.25667570484216e-49	-0.496687551521118	0.118	0.281
5.8063271141631e-45	6	NCF1	Activated
3.88892688107755e-48	0.433906656140508	0.138	0.06
6.93356773627316e-44	6	ZC3H12A	Activated
9.84058593961547e-48	0.37068313270537	0.1	0.035
1.75447806717404e-43	6	TNFAIP3	Activated
2.89148040905017e-47	-1.64709279058012	0.274	0.409
5.15522042129555e-43	6	JCHAIN	Activated
1.10477491522741e-46	0.539586986748585	0.308	0.204
1.96970319635895e-42	6	PIM3	Activated
1.31183811569754e-46	-0.415800949580075	0.047	0.174
2.33887617647714e-42	6	SEL1L3	Activated
2.37166654962178e-46	-0.352196331003539	0.021	0.125
4.22844429132067e-42	6	VNN2	Activated
6.84590646381849e-46	0.602993309237911	0.369	0.281
1.2205566634342e-41	6	CD82	Activated
7.30875561393145e-46	0.603058785605809	0.234	0.133
1.30307803840784e-41	6	FCER2	Activated
2.86797268981337e-45	-0.339626613265466	0.974	0.99
5.11330850866826e-41	6	ACTB	Activated
3.64434506252916e-45	-0.554252976864404	0.102	0.24
6.49750281198323e-41	6	UBE2J1	Activated
3.99300076398675e-45	0.52324015665268	0.198	0.119
7.11912106211198e-41	6	MAP3K8	Activated
4.8264813058622e-45	-0.510857970330092	0.072	0.208
8.60513352022171e-41	6	HMCES	Activated
6.30587312673591e-45	-0.35531408229218	0.015	0.11
1.12427411976575e-40	6	HRK	Activated
8.99433936250957e-45	-0.384566728714864	0.032	0.143
1.60360076494183e-40	6	SIT1	Activated
1.8792411273165e-44	-0.334671232357808	0.015	0.105
3.35049900589259e-40	6	MYBL2	Activated
2.9469107765716e-44	0.401640161950095	0.119	0.051
5.25404722354951e-40	6	NFKBIZ	Activated
6.12023388139715e-44	-0.406982750829599	0.193	0.374
1.0911764987143e-39	6	CXXC5	Activated
9.95316999401905e-44	-0.352259868199507	0.498	0.677
1.77455067823366e-39	6	SNX3	Activated
1.35239807158522e-43	-0.314638624276895	0.88	0.94
2.41119052182929e-39	6	EEF2	Activated
1.92704427125861e-43	0.372599917602869	0.942	0.935
3.43572723122697e-39	6	BTG1	Activated
5.83392394968837e-43	-0.36994881519648	0.026	0.123
1.04013030098994e-38	6	PYCARD	Activated
5.84426177869035e-43	-0.433611833916316	0.327	0.497
1.0419734325227e-38	6	CBX3	Activated
1.11904144844516e-42	-0.374004690234648	0.075	0.214
1.99513899843287e-38	6	TCEAL8	Activated
1.45652404189674e-42	-0.374995187501687	0.036	0.144
2.5968367142977e-38	6	SOCS1	Activated

5.27963370356288e-42 -0.444976245840095 0.704 0.819
9.41305893008226e-38 6 CORO1A Activated
6.09459241880887e-42 -0.462444406301645 0.083 0.217
1.08660488234943e-37 6 FAM111B Activated
8.9400648993298e-42 0.484820778484194 0.162 0.092
1.59392417090151e-37 6 FCRL5 Activated
9.54694453703074e-42 0.410000360686793 0.147 0.073
1.70212474150721e-37 6 GPR137 Activated
1.53789310384494e-41 0.526276162474787 0.52 0.434
2.74190961484514e-37 6 IL2RG Activated
1.56945294793244e-41 -0.371136242654841 0.019 0.107
2.79817766086874e-37 6 RMI2 Activated
1.59893333819109e-41 -0.342685976292006 0.022 0.115
2.85073824866089e-37 6 AC023590.1 Activated
2.23397073862975e-41 0.580101623494554 0.331 0.247
3.98294642990298e-37 6 SDCBP Activated
4.16210137411253e-41 -0.505402284152386 0.071 0.196
7.42061053990523e-37 6 DAAM1 Activated
5.56856300669953e-41 -0.472409434863173 0.219 0.382
9.92819098464459e-37 6 ACADM Activated
9.97236910396607e-41 -0.315168612603578 0.623 0.77
1.77797368754611e-36 6 MYL12A Activated
1.05518928495499e-40 -0.380241919718778 0.05 0.17
1.88129697614625e-36 6 DEF8 Activated
3.55043789705422e-40 -0.457445119181167 0.107 0.242
6.33007572665797e-36 6 CCND3 Activated
4.70528635098836e-40 -0.434334798157473 0.388 0.547
8.38905503517715e-36 6 PARP1 Activated
5.32780764849205e-40 0.417101334179959 0.158 0.083
9.49894825649648e-36 6 IER5 Activated
6.75970004087662e-40 -0.413598590466707 0.236 0.411
1.20518692028789e-35 6 STX7 Activated
9.04839275990658e-40 0.47477004546375 0.614 0.526
1.61323794516374e-35 6 PSME2 Activated
1.37006266687239e-39 0.38461097250105 0.978 0.976
2.44268472876679e-35 6 FTH1 Activated
1.51488345804456e-39 0.549470123252749 0.473 0.379
2.70088571734765e-35 6 CD44 Activated
3.19452371050676e-39 -0.428548131178469 0.2 0.371
5.69551632346249e-35 6 RCSD1 Activated
3.38612529534979e-39 0.503597077363592 0.279 0.198
6.03712278907914e-35 6 RELB Activated
5.25276719126525e-39 -0.522609648714473 0.207 0.355
9.36515862530682e-35 6 S100A10 Activated
9.32041110303954e-39 0.411660216368061 0.127 0.06
1.66173609556092e-34 6 PTGER4 Activated
9.59102759042488e-39 -0.52295527098738 0.09 0.202
1.70998430909685e-34 6 ACP5 Activated
1.09540042069095e-38 0.467937303425898 0.222 0.139
1.9529894100499e-34 6 NOP16 Activated

1.48868189005096e-38	-0.374595933283387	0.684	0.805	2.65417094177186e-34	6	LAPTM5	Activated
1.81638262491592e-38	0.500420743032411	0.266	0.182	3.2384285819626e-34	6	WDR43	Activated
6.00508731871987e-38	-0.330692608437629	0.044	0.154	1.07064701805457e-33	6	PTPN18	Activated
8.95599313279248e-38	-2.99028390082579	0.325	0.456	1.59676401564557e-33	6	IGHG3	Activated
1.19239738883102e-37	-0.393444313592141	0.146	0.295	2.12592530454683e-33	6	C7orf50	Activated
1.79344627451807e-37	0.468548632904548	0.182	0.108	3.19753536283827e-33	6	ZNF267	Activated
5.71524828694973e-37	0.698905649718433	0.403	0.342	1.01897161708027e-32	6	INSIG1	Activated
5.76260504684112e-37	-0.378405339856883	0.326	0.499	1.0274148538013e-32	6	RAC2	Activated
6.64273349709781e-37	0.475018686814876	0.272	0.201	1.18433295519757e-32	6	UBALD2	Activated
7.26753932685356e-37	0.483785169810473	0.645	0.583	1.29572958658472e-32	6	EIF2S2	Activated
7.36696542724549e-37	-0.399349522283157	0.054	0.165	1.3134562660236e-32	6	C12orf75	Activated
1.39742455910346e-36	-0.388257526053088	0.168	0.325	2.49146824642555e-32	6	FCRLA	Activated
2.24541862485811e-36	0.436445861831315	0.216	0.138	4.00335686625953e-32	6	TM2D3	Activated
2.4005311303689e-36	0.535887890124844	0.394	0.319	4.27990695233471e-32	6	NOP56	Activated
5.015184330241e-36	-0.382682185932403	0.143	0.29	8.94157214238668e-32	6	CARHSP1	Activated
8.24562787041861e-36	-0.393652378757721	0.087	0.212	1.47011299301693e-31	6	EAF2	Activated
9.9688347331256e-36	0.422112263204257	0.192	0.118	1.77734354456896e-31	6	PAK1IP1	Activated
4.4391059885012e-35	-0.322288747378622	0.028	0.117	7.9144820668988e-31	6	TNFRSF17	Activated
7.38726376227729e-35	0.406235625479666	0.144	0.083	1.31707525617642e-30	6	SLC43A3	Activated
7.97146562462276e-35	-0.654389330993687	0.084	0.195	1.42123260621399e-30	6	PTTG1	Activated
9.17500974522252e-35	-0.346346905984628	0.21	0.367	1.63581248747572e-30	6	TKT	Activated
9.39010812328505e-35	-0.365560259291517	0.173	0.328	1.67416237730049e-30	6	NAA38	Activated
1.40654903988562e-34	0.494188825742857	0.539	0.477	2.50773628321207e-30	6	RSL1D1	Activated
1.7353935875847e-34	0.4407999051456	0.818	0.792	3.09403322730476e-30	6	HLA-E	Activated
2.84004888566984e-34	-0.357234536406682	0.118	0.26	5.06352315826075e-30	6	TBC1D10C	Activated

3.22579700463292e-34	-0.348866059533829	0.175	0.335	5.75127347956003e-30	6	GYPC	Activated
3.73118506088424e-34	-0.432416985019054	0.14	0.272	6.65232984505052e-30	6	HHEX	Activated
3.78482943010891e-34	-0.325578319764328	0.044	0.148	6.74797239094117e-30	6	HSH2D	Activated
4.59291020584464e-34	-0.337987932078725	0.093	0.222	8.18869960600041e-30	6	PNKD	Activated
4.90680797353089e-34	-0.336095588791394	0.184	0.343	8.74834793600823e-30	6	SEPHS2	Activated
7.59496211838131e-34	-0.472259731930115	0.169	0.308	1.3541057960862e-29	6	ZFP36L2	Activated
8.36495747259032e-34	-0.338965313014217	0.179	0.337	1.49138826778813e-29	6	CCM2	Activated
8.86917490665312e-34	-0.367877571548401	0.169	0.32	1.58128519410718e-29	6	HMGN3	Activated
9.04980026264023e-34	-0.300433118023695	0.397	0.571	1.61348888882613e-29	6	ERP29	Activated
1.188571895501e-33	0.46188170370319	0.526	0.454	2.11910483248873e-29	6	SFPQ	Activated
7.30556020436964e-33	-0.368920287485568	0.072	0.191	1.30250832883706e-28	6	P2RX5	Activated
1.41213513906915e-32	-0.338499774785496	0.113	0.246	2.51769573944638e-28	6	MCUB	Activated
2.16539205643714e-32	-0.377261057359486	0.037	0.124	3.86067749742178e-28	6	LPP	Activated
3.17291529740903e-32	0.46193829120766	0.469	0.401	5.65699068375056e-28	6	BZW1	Activated
5.4815341709806e-32	-0.459537390170818	0.106	0.216	9.77302727344131e-28	6	LBH	Activated
7.13862798934643e-32	0.506852693597243	0.132	0.079	1.27274598422058e-27	6	SNHG12	Activated
9.29823167604983e-32	-0.353614992588757	0.061	0.173	1.65778172552292e-27	6	CCDC88A	Activated
1.08692386776718e-31	-0.329431997227524	0.178	0.325	1.9378765638421e-27	6	PRDX2	Activated
1.29895412076463e-31	-0.404516487686459	0.166	0.309	2.31590530191126e-27	6	BCL7A	Activated
1.4264137885334e-31	-0.348342465640202	0.251	0.409	2.54315314357621e-27	6	MBD4	Activated
2.0025604610898e-31	0.470393982091084	0.495	0.432	3.57036504607701e-27	6	EIF5B	Activated
2.2143909939994e-31	-0.352977510170255	0.359	0.513	3.94803770320153e-27	6	MZT2B	Activated
4.4044770748026e-31	-1.33991700341741	0.119	0.22	7.85274217666556e-27	6	IGHG1	Activated
1.01738885994723e-30	0.455873625648127	0.252	0.185	1.81390259839992e-26	6	GPATCH4	Activated
1.08838904269344e-30	-0.314968473776672	0.111	0.238	1.94048882421813e-26	6	CHCHD10	Activated

1.38962254574289e-30 -0.433081910068876 0.898 0.935
2.47755803680499e-26 6 SERF2 Activated
2.40431236846439e-30 0.478223111428586 0.377 0.317
4.28664852173516e-26 6 RHOG Activated
2.56792631654416e-30 -0.316672176164162 0.035 0.119
4.57835582976659e-26 6 GMDS Activated
5.36213707891688e-30 -0.354688769609061 0.162 0.299
9.5601541980009e-26 6 BCL11A Activated
7.00472117796108e-30 0.43340756862046 0.271 0.205
1.24887173881868e-25 6 GNL3 Activated
8.27748018740551e-30 -0.353225107351113 0.303 0.46
1.47579194261253e-25 6 PTPRC Activated
8.43554903789935e-30 -0.537147074700788 0.549 0.66
1.50397403796707e-25 6 TCL1A Activated
1.22430471716116e-29 0.349772104975606 0.122 0.065
2.18281288022664e-25 6 PIKFYVE Activated
1.58081781878138e-29 0.3697259687926 0.159 0.093
2.81844008910532e-25 6 STAT5A Activated
1.76874989252801e-29 0.466474143909574 0.242 0.167
3.15350418338819e-25 6 PARP14 Activated
2.16995898606344e-29 0.455825933793145 0.438 0.378
3.86881987625251e-25 6 ANXA7 Activated
2.36360622509436e-29 -0.312650153185008 0.152 0.292
4.21407353872073e-25 6 GSTK1 Activated
3.25368035007823e-29 0.333024244413288 0.109 0.057
5.80098669615447e-25 6 DUSP10 Activated
3.31971555237333e-29 0.397904063696388 0.151 0.094
5.91872085832641e-25 6 ATF7IP Activated
3.69169628339827e-29 0.44582650025537 0.247 0.186
6.58192530367078e-25 6 YARS Activated
3.70294969073886e-29 -0.336003429939006 0.191 0.336
6.60198900361831e-25 6 TSPO Activated
4.58952368293923e-29 -0.404929835461379 0.138 0.253
8.18266177431236e-25 6 TNFRSF13B Activated
5.57852226177351e-29 -0.78862281536234 0.381 0.493
9.94594734051599e-25 6 LTB Activated
6.08642344728577e-29 0.467827781495834 0.403 0.34
1.08514843641658e-24 6 CMTM6 Activated
6.80129035776617e-29 0.465827595038484 0.658 0.617
1.21260205788613e-24 6 CLEC2D Activated
1.0135743869534e-28 0.427125535563461 0.679 0.633
1.80710177449921e-24 6 GPX4 Activated
2.06225593235037e-28 -0.303145658090845 0.044 0.135
3.67679610178747e-24 6 PHGDH Activated
2.34247741209796e-28 -0.323486393829023 0.133 0.266
4.17640297802946e-24 6 CCDC69 Activated
2.54850910361193e-28 -0.334872249534709 0.119 0.244
4.54373688082971e-24 6 NCF4 Activated
8.01134872277577e-28 0.314086133076783 0.105 0.062
1.42834336378369e-23 6 IL21R Activated

1.53242681935557e-27	0.493975998589817	0.3	0.216	2.73216377622904e-23	6	GBP2	Activated
2.42690008629784e-27	0.418652249856825	0.222	0.163	4.32692016386041e-23	6	NXT1	Activated
3.35743429928542e-27	-0.358876401497235	0.16	0.284	5.98596961219598e-23	6	RNASET2	Activated
4.66442604673877e-27	-0.341267844723228	0.168	0.303	8.31620519873056e-23	6	GGA2	Activated
4.68869865025086e-27	0.51616202222134	0.471	0.426	8.35948082353225e-23	6	PPP1R15A	Activated
7.41423710981866e-27	-0.845650517083559	0.1	0.216	1.32188433430957e-22	6	PLCG2	Activated
1.21816443439158e-26	-0.321181882488889	0.083	0.192	2.17186537007675e-22	6	HLA-DOB	Activated
1.71656912747513e-26	0.330455813346389	0.14	0.085	3.06047109737541e-22	6	POLR1C	Activated
2.54525654295749e-26	0.448398916665417	0.287	0.234	4.53793789043892e-22	6	NIFK	Activated
3.25507749987134e-26	0.43657653144228	0.312	0.249	5.8034776745206e-22	6	NFE2L2	Activated
3.87169307294092e-26	0.37086448220644	0.166	0.104	6.90284157974636e-22	6	ARID5A	Activated
9.17644159325527e-26	0.411059711341901	0.215	0.15	1.63606777166148e-21	6	CEBPB	Activated
1.00682841553343e-25	0.402352710834254	0.225	0.166	1.79507438205456e-21	6	TNIP2	Activated
1.80237710890546e-25	-0.349690744269883	0.178	0.302	3.21345814746754e-21	6	PDLIM1	Activated
2.22311229966213e-25	-0.33183230587554	0.192	0.329	3.96358691906762e-21	6	AES	Activated
2.60334871569553e-25	0.37737921908681	0.209	0.147	4.64151042521356e-21	6	RPF2	Activated
3.37550697393666e-25	0.449227729149068	0.35	0.299	6.01819138383167e-21	6	NOP58	Activated
3.70430141560264e-25	-0.332620460719325	0.142	0.264	6.60439899387795e-21	6	SYNE2	Activated
4.46549262617907e-25	0.386406167235494	0.201	0.137	7.96152680321467e-21	6	RAB29	Activated
6.89698968977986e-25	-0.32048170477513	0.111	0.223	1.22966429179085e-20	6	NANS	Activated
9.09068119541996e-25	0.311294269160839	0.132	0.08	1.62077755033142e-20	6	SNX11	Activated
1.20487493555481e-24	0.383437783699223	0.211	0.151	2.14817152260068e-20	6	RRP15	Activated
1.24013230280653e-24	-0.313520150446857	0.19	0.318	2.21103188267376e-20	6	PSIP1	Activated
2.16759976624903e-24	0.454999722929657	0.271	0.217	3.8646136232454e-20	6	CCDC50	Activated
3.65123607931276e-24	-0.380364138060014	0.235	0.365	6.50978880580672e-20	6	H2AFV	Activated

4.21648343639458e-24	0.374423275553546	0.206	0.149
7.5175683187479e-20	6	PPAN	Activated
4.57054730748386e-24	0.37069136516353	0.176	0.121
8.14882879451298e-20	6	PLEK	Activated
5.38746534730832e-24	-0.399722979457103	0.089	0.19
9.60531196771601e-20	6	RGS2	Activated
7.1067955962612e-24	-0.319824505002611	0.192	0.327
1.26707058685741e-19	6	TNFRSF13C	Activated
9.61014430230472e-24	0.466615213891906	0.433	0.39
1.71339262765791e-19	6	FNBP1	Activated
1.18572994240197e-23	0.423398745514195	0.391	0.345
2.11403791430847e-19	6	RBM17	Activated
1.55326058320706e-23	-0.328460522473275	0.17	0.289
2.76930829379987e-19	6	GNG7	Activated
1.63354063308639e-23	-0.35280514078745	0.108	0.211
2.91243959472972e-19	6	C16orf74	Activated
1.9766845975593e-23	0.42121525576295	0.34	0.276
3.52423096898848e-19	6	WARS	Activated
2.53724766796608e-23	-0.317915739439244	0.083	0.178
4.52365886721673e-19	6	MARCH1	Activated
4.79243825719033e-23	0.395701323135229	0.417	0.359
8.54443816874463e-19	6	HSPA9	Activated
5.86706025794451e-23	0.330449962639942	0.576	0.509
1.04603817338893e-18	6	PA2G4	Activated
8.0134427176118e-23	0.371796754876638	0.318	0.253
1.42871670212301e-18	6	NARS	Activated
8.7467539916632e-23	0.393192476709911	0.634	0.592
1.55945876917363e-18	6	HNRNPU	Activated
8.90388368243991e-23	0.388518237178024	0.23	0.176
1.58747342174221e-18	6	ABCE1	Activated
1.41990970474427e-22	0.446789381943547	0.299	0.239
2.53155701258857e-18	6	PHACTR1	Activated
1.52497024560896e-22	0.408646653131032	0.291	0.238
2.71886945089621e-18	6	SLC38A1	Activated
2.10438259338909e-22	0.369510766082662	0.209	0.154
3.75190372575342e-18	6	NOLC1	Activated
2.19344163194265e-22	0.534715672649338	0.201	0.167
3.91068708559056e-18	6	CREM	Activated
2.65627007696956e-22	0.35886948957721	0.724	0.701
4.73586392022903e-18	6	SON	Activated
4.33750877374992e-22	0.342349281366772	0.176	0.127
7.73334439271873e-18	6	GRWD1	Activated
4.84904447213869e-22	0.407271414315433	0.277	0.226
8.64536138937606e-18	6	SINHCAF	Activated
8.35758868103891e-22	0.402844313828152	0.247	0.192
1.49007448594243e-17	6	ESF1	Activated
1.0639472586953e-21	0.376394932589148	0.321	0.265
1.89691156752786e-17	6	CEBPZ	Activated
1.16151255836466e-21	0.534019826393496	0.241	0.191
2.07086074030835e-17	6	HSPB1	Activated

1.1622733368159e-21	0.330962288553732	0.153	0.107	2.07221713220907e-17	6	GALNT2	Activated
2.45479327299733e-21	0.346283973406075	0.204	0.154	4.37665092642694e-17	6	GAR1	Activated
2.53555561827357e-21	0.339653851027447	0.162	0.111	4.52064211181994e-17	6	NOC3L	Activated
2.91353889473371e-21	0.337768947829517	0.146	0.105	5.19454849542073e-17	6	MAT2A	Activated
3.42370934268407e-21	0.369812795976696	0.612	0.569	6.10413138707143e-17	6	RBM8A	Activated
3.48171952066351e-21	0.341989053371314	0.175	0.127	6.20755773339098e-17	6	CEBPG	Activated
3.51853249567203e-21	-0.316783262736646	0.439	0.557	6.27319158653366e-17	6	TCEA1	Activated
4.44696450438564e-21	-1.22675945016618	0.316	0.427	7.92849301486916e-17	6	HIST1H4C	Activated
6.27620333983462e-21	0.333383381096831	0.137	0.095	1.11898429345911e-16	6	FCRL3	Activated
7.59039304009826e-21	-0.311868804027526	0.102	0.197	1.35329117511912e-16	6	DNMT1	Activated
8.95358118124607e-21	0.326821130582001	0.158	0.108	1.59633398880436e-16	6	SRFBP1	Activated
1.10566774397805e-20	0.372691553772717	0.366	0.303	1.97129502073847e-16	6	ARHGAP24	Activated
1.23646349197872e-20	0.339856924085489	0.215	0.161	2.20449075984886e-16	6	MRTO4	Activated
1.25085842455912e-20	0.372947702077189	0.462	0.418	2.23015548514645e-16	6	EIF6	Activated
1.36270117992874e-20	0.368209087284048	0.507	0.461	2.42955993369496e-16	6	PRMT1	Activated
1.84256827411071e-20	0.37873741675473	0.284	0.233	3.28511497591198e-16	6	EIF4A1	Activated
2.14763181221959e-20	0.31973050355795	0.109	0.064	3.82901275800631e-16	6	CD86	Activated
2.33647788462017e-20	0.375518913711324	0.248	0.187	4.16570642048931e-16	6	IFNGR1	Activated
6.70323136200626e-20	0.359144971003648	0.503	0.453	1.1951191195321e-15	6	DDX18	Activated
8.19789491557677e-20	-0.975772069364461	0.083	0.15	1.46160268449818e-15	6	IGHA1	Activated
8.53554898511984e-20	0.376669991144392	0.284	0.24	1.52180302855702e-15	6	ABCF1	Activated
1.30138517271192e-19	0.349965808251215	0.2	0.155	2.32023962442808e-15	6	NAMPT	Activated
1.93247539091401e-19	0.336897924784441	0.169	0.129	3.4454103744606e-15	6	SRPRB	Activated
2.39581365384402e-19	-0.381723105316619	0.808	0.853	4.2714961634385e-15	6	ATP5MG	Activated
3.04020097589685e-19	0.360943981700163	0.278	0.234	5.42037431992649e-15	6	EIF2S1	Activated

4.38652464602445e-19	0.376695383037929		0.19	0.143
7.82073479139699e-15	6	SERPINB9	Activated	
4.65786050577683e-19	-0.50459677159436		0.384	0.468
8.30449949574951e-15	6	S100A6	Activated	
4.8234997463141e-19	0.369647518276327		0.3	0.248
8.59981769770341e-15	6	GARS	Activated	
4.89825098370878e-19	0.416512008179695		0.411	0.374
8.73309167885438e-15	6	TAF1D	Activated	
5.15769788156334e-19	0.337280901850916		0.683	0.652
9.19565955303927e-15	6	TMBIM6	Activated	
6.09494510427783e-19	0.303318533163379		0.142	0.102
1.08666776264169e-14	6	RIOK1	Activated	
7.5410364929192e-19	-2.13698827542228		0.061	0.129
1.34449139632256e-14	6	IGHG2	Activated	
8.58408713854764e-19	0.337516237127957		0.251	0.205
1.53045689593166e-14	6	BRIX1	Activated	
2.18948251337876e-18	0.355697920795094		0.366	0.294
3.903628373103e-14	6	LINC00926	Activated	
2.41424652451607e-18	0.34490899443101	0.636	0.609	
4.30436012855969e-14	6	RSL24D1	Activated	
2.5950035350401e-18	-0.306887571463121		0.118	0.209
4.62663180262299e-14	6	PRKCB	Activated	
2.73236789539562e-18	0.362977023313343		0.198	0.152
4.87153872070086e-14	6	RILPL2	Activated	
3.88523327020103e-18	0.370320766339839		0.506	0.466
6.92698239744142e-14	6	CYCS	Activated	
4.87603742873987e-18	0.303218984914999		0.199	0.151
8.69348713170031e-14	6	NDUFAF4	Activated	
6.08673662456055e-18	-0.373925893442242		0.132	0.215
1.0852042727929e-13	6	PLAC8	Activated	
8.26344107552507e-18	0.378273585003891		0.325	0.287
1.47328890935537e-13	6	RRAS2	Activated	
8.69465398104929e-18	0.362909385387318		0.292	0.251
1.55016985828128e-13	6	SLC50A1	Activated	
1.82988573321799e-17	-0.300765191953002		0.224	0.336
3.26250327375436e-13	6	RIPOR2	Activated	
2.80786182965929e-17	0.376132003404917		0.201	0.172
5.00613685609955e-13	6	SNX8	Activated	
3.24579695349274e-17	0.343166232702957		0.236	0.194
5.78693138838221e-13	6	GTPBP4	Activated	
4.7451162569449e-17	0.332860938023819		0.323	0.287
8.46006777450706e-13	6	MRPL14	Activated	
6.49887310477365e-17	0.416861601736908		0.422	0.396
1.15868408585009e-12	6	ATF4	Activated	
9.35990736400033e-17	0.464835790067097		0.658	0.653
1.66877788392762e-12	6	HLA-DQA1	Activated	
1.14900022162816e-16	0.347124557287168		0.433	0.378
2.04855249514084e-12	6	PDCD4	Activated	
1.21000303217447e-16	0.310622718656927		0.177	0.135
2.15731440606387e-12	6	IFRD2	Activated	

1.22200854350006e-16 0.355653925145261 0.231 0.191
2.17871903220627e-12 6 WDR74 Activated
1.45137217106669e-16 -0.311139045970643 0.152 0.245
2.5876514437948e-12 6 RGS1 Activated
3.03319819365445e-16 0.328613868182478 0.561 0.528
5.40788905946652e-12 6 SRSF7 Activated
3.25349049333235e-16 0.375100559012244 0.343 0.312
5.80064820056224e-12 6 ATP2B1 Activated
4.47402129968393e-16 0.36017336696919 0.461 0.437
7.97673257520648e-12 6 DAD1 Activated
6.96072538930793e-16 0.346441905864629 0.241 0.209
1.24102772965971e-11 6 ARPP19 Activated
7.57709931717682e-16 0.339982327956064 0.454 0.421
1.35092103725946e-11 6 GPBP1 Activated
7.9027646691886e-16 0.34570979217999 0.234 0.194
1.40898391286964e-11 6 AARS Activated
8.53659062007316e-16 0.358767818799501 0.283 0.24
1.52198874165284e-11 6 HLA-F Activated
9.6249479890245e-16 0.435953869080906 0.47 0.444
1.71603197696318e-11 6 KLF6 Activated
1.33527382471482e-15 0.317894965975206 0.438 0.396
2.38065970208405e-11 6 BASP1 Activated
1.57108602526788e-15 0.354658583887532 0.438 0.415
2.8010892744501e-11 6 PRRC2C Activated
1.96517263815325e-15 0.337221898346224 0.326 0.266
3.50370629656342e-11 6 JUN Activated
2.00808475635903e-15 0.337424035240653 0.451 0.421
3.58021431211251e-11 6 TMEM123 Activated
2.29408681001226e-15 -0.646716638738252 0.35 0.431
4.09012737357087e-11 6 STMN1 Activated
2.47881939708122e-15 0.320780569868596 0.485 0.448
4.41948710305611e-11 6 ILF2 Activated
2.5594574300093e-15 0.314733380531381 0.624 0.593
4.56325665196358e-11 6 SRSF2 Activated
2.62677135205441e-15 0.32209440912689 0.208 0.171
4.68327064357781e-11 6 TIMM44 Activated
3.03322514880898e-15 0.317941002936463 0.66 0.634
5.40793711781153e-11 6 HERPUD1 Activated
2.4215980222412e-14 0.371376957062317 0.37 0.351
4.31746711385384e-10 6 CHMP4B Activated
4.05136772377928e-14 -0.367695640408295 0.507 0.592
7.22318351472607e-10 6 MARCKSL1 Activated
5.63448282864587e-14 0.312898922208239 0.373 0.327
1.00457194351927e-09 6 TRBC2 Activated
6.01900292002249e-14 0.340510901801426 0.405 0.373
1.07312803061081e-09 6 LCP1 Activated
6.55542472179746e-14 0.311945744373178 0.182 0.146
1.16876667364927e-09 6 SVIP Activated
6.84695566823772e-14 0.305912326741708 0.49 0.459
1.2207437260901e-09 6 SWAP70 Activated

6.90329821696101e-14	0.300822487299883	0.184	0.157	1.23078903910198e-09	6	TRIAP1	Activated
8.05540091019695e-14	0.367185807838544	0.492	0.471	1.43619742827902e-09	6	SNHG8	Activated
1.58180686854907e-13	0.32379298567783	0.469	0.442	2.82020346593614e-09	6	CD164	Activated
1.71658426062627e-13	0.302512516061959	0.588	0.558	3.06049807827058e-09	6	NCL	Activated
1.79336042511546e-13	-0.379592509733538	0.717	0.77	3.19738230193835e-09	6	HMGN1	Activated
2.21139894019024e-13	0.307914388312543	0.326	0.299	3.94270317046518e-09	6	POLE4	Activated
2.63428288289127e-13	-0.577987622010854	0.145	0.208	4.69666295190685e-09	6	LGALS1	Activated
2.90323246596635e-13	0.348272067560063	0.361	0.346	5.17617316357141e-09	6	UBE2A	Activated
4.07356889709889e-13	0.307821884375853	0.231	0.203	7.2627659866376e-09	6	NIPA2	Activated
5.28788685047244e-13	0.309479927326624	0.276	0.244	9.42777346570732e-09	6	LAMP1	Activated
7.67501290514985e-13	0.360914401470245	0.298	0.276	1.36837805085917e-08	6	TANK	Activated
8.0782860907613e-13	-0.313053249867196	0.25	0.339	1.44027762712183e-08	6	DUT	Activated
8.70709494900189e-13	0.33869442580435	0.443	0.426	1.55238795845755e-08	6	RNPS1	Activated
9.33089350680245e-13	0.307331617757018	0.479	0.459	1.66360500332781e-08	6	GADD45GIP1	Activated
1.10936378554628e-12	0.30245795329324	0.203	0.178	1.97788469325046e-08	6	SDF4	Activated
1.57295872244535e-12	-0.621495659192622	0.669	0.718	2.80442810624782e-08	6	HMGN2	Activated
1.99062459800206e-12	0.321240873942172	0.423	0.4	3.54908459577788e-08	6	RPL22L1	Activated
3.43619792883442e-12	0.337732438520417	0.307	0.29	6.12639728731889e-08	6	G3BP1	Activated
7.97554508446785e-12	0.342368177964232	0.347	0.33	1.42195993310977e-07	6	GRHPR	Activated
1.08185455886732e-11	0.343815796659444	0.249	0.233	1.92883849300455e-07	6	RALA	Activated
1.65976138185506e-11	0.303082019344867	0.23	0.209	2.95918856770938e-07	6	STX5	Activated
1.86162506960605e-11	0.308708813719363	0.248	0.228	3.31909133660062e-07	6	NOB1	Activated
2.69526630626238e-11	0.358902883099559	0.297	0.276	4.80539029743519e-07	6	GADD45B	Activated
4.28312448055213e-11	0.337167027486024	0.375	0.368	7.63638263637639e-07	6	PFDN2	Activated
6.02038350427957e-11	-0.326092485418535	0.323	0.407	1.073374174978e-06	6	FABP5	Activated

9.80105735550887e-11 0.30035667192414 0.51 0.491
1.74743051591368e-06 6 ANKRD12 Activated
1.02346386938299e-10 0.300014241095577 0.483 0.475
1.82473373272294e-06 6 VAPA Activated
1.29451075238751e-10 0.30118454633149 0.351 0.334
2.3079832204317e-06 6 VOPP1 Activated
1.3068608204629e-10 0.301366965185489 0.379 0.363
2.3300021568033e-06 6 EIF3J Activated
5.439855759893e-10 0.302408158601825 0.341 0.333
9.69871883431323e-06 6 DARS Activated
1.55336435053288e-09 0.326903296126167 0.364 0.346
2.76949330056508e-05 6 BIRC3 Activated
1.62339557528491e-09 0.301093823587456 0.256 0.239
2.89435197117546e-05 6 ANKRD11 Activated
2.13670316112591e-09 0.351522490988719 0.179 0.168
3.80952806597139e-05 6 SAMSN1 Activated
2.28933652625988e-09 0.329180786905498 0.849 0.866
4.08165809266874e-05 6 UBC Activated
2.59371796033208e-09 0.521100853464525 0.614 0.649
4.62433975147607e-05 6 ZFAS1 Activated
2.79977320213303e-09 0.304197520022841 0.43 0.424
4.99171564208298e-05 6 STK4 Activated
5.00621935230883e-09 0.301935923940106 0.452 0.45
8.92558848323141e-05 6 EIF5 Activated
3.74810238818829e-07 -1.67852998314754 0.198 0.245
0.00668249174790091 6 IGHG4 Activated
0.00302584672651886 -0.55255428677803 0.914 0.927 1
6 IGKC Activated
0 2.26635411586193 0.964 0.47 0 7 LTB GC
0 1.86778556539439 0.596 0.057 0 7 SUGCT GC
0 1.86254580185668 0.74 0.11 0 7 NEIL1 GC
0 1.58801281568944 0.693 0.14 0 7 SUSD3 GC
0 1.55948049178023 0.984 0.642 0 7 TCL1A GC
0 1.52933239525062 0.811 0.182 0 7 RGS13 GC
0 1.50535371612632 0.998 0.931 0 7 SERF2 GC
0 1.49287315204014 0.995 0.846 0 7 ATP5MG GC
0 1.47873167937677 0.639 0.173 0 7 DAAM1 GC
0 1.46367297739174 0.759 0.214 0 7 UBE2J1 GC
0 1.44750754333442 0.934 0.518 0 7 ISG20 GC
0 1.44621423184704 0.537 0.066 0 7 AICDA GC
0 1.44462988064429 0.58 0.087 0 7 HRK GC
0 1.43175711510476 0.814 0.283 0 7 CD27 GC
0 1.40296123735752 0.978 0.715 0 7 LIMD2 GC
0 1.38260111104397 0.433 0.07 0 7 SH3TC1 GC
0 1.35514995958237 0.969 0.683 0 7 EZR GC
0 1.30162438075945 0.49 0.106 0 7 LPP GC
0 1.29933104731061 0.987 0.851 0 7 CD79B GC
0 1.28149289935136 0.495 0.079 0 7 WDR66 GC
0 1.26347526909206 0.915 0.592 0 7 DYNLL1 GC
0 1.25669254776155 0.98 0.778 0 7 ACTG1 GC

0 1.2378722954761 0.766 0.27 0 7 BCAS4 GC
0 1.21798192923729 0.85 0.478 0 7 C4orf3 GC
0 1.19008127293181 0.782 0.311 0 7 RFTN1 GC
0 1.18375560252529 0.851 0.412 0 7 IRF8 GC
0 1.18197886665823 0.708 0.256 0 7 POU2AF1 GC
0 1.11131323358414 0.873 0.521 0 7 SRSF9 GC
0 1.08831999823743 0.905 0.538 0 7 TCEA1 GC
0 1.04200469338051 0.993 0.884 0 7 GAPDH GC
0 1.01074609959194 0.972 0.808 0 7 CORO1A GC
0 1.00185594362717 0.922 0.382 0 7 JCHAIN GC
0 0.906336365409336 0.976 0.793 0 7
LAPTM5 GC
0 0.800890252258652 0.999 0.989 0 7
ACTB GC
0 -1.0076578075775 0.998 0.998 0 7 B2M GC
0 -2.07999214495098 0.097 0.561 0 7
CCR7 GC
0 -2.28904825275246 0.193 0.718 0 7
EMP3 GC
1.88387230759267e-320 1.21660901845263 0.726 0.256
3.35875593720698e-316 7 NCF1 GC
1.30575299771356e-315 1.09785696967751 0.917 0.575
2.3280270196235e-311 7 MARCKSL1 GC
3.96655130918406e-308 1.17517946955828 0.769 0.314
7.07196432914425e-304 7 GRHPR GC
2.21150890977601e-306 1.12444253525267 0.474 0.097
3.94289923523965e-302 7 CPNE5 GC
5.48125031832227e-305 1.10811814734128 0.488 0.096
9.77252119253678e-301 7 AC023590.1 GC
1.0849877764424e-303 1.18424830631733 0.561 0.153
1.93442470661915e-299 7 SEL1L3 GC
3.01092652454291e-300 1.08684896116833 0.812 0.381
5.36818090060756e-296 7 BASP1 GC
1.28746179417638e-297 0.969534803359605 0.961 0.76
2.29541563283706e-293 7 HMGN1 GC
1.32276311311123e-293 1.16041600198305 0.763 0.285
2.35835435436601e-289 7 BCL7A GC
1.25086211495189e-290 -1.210520136064 0.729 0.912
2.23016206474773e-286 7 HLA-A GC
3.63144834268859e-286 1.11283561661313 0.787 0.359
6.47450925017949e-282 7 ACADM GC
3.23887763351305e-285 0.752134600985913 0.986 0.865
5.77459493279041e-281 7 ARPC2 GC
1.11224134660338e-281 1.09963121560025 0.502 0.103
1.98301509685917e-277 7 CD38 GC
1.25793161243984e-280 1.13190227644734 0.626 0.19
2.242766271819e-276 7 EAF2 GC
1.38993805413712e-275 0.955900454567789 0.366 0.057
2.47812055672106e-271 7 SNTA1 GC
2.60823150398112e-271 0.638051864877064 0.993 0.96

4.65021594844794e-267 7 OAZ1 GC
8.99293634791177e-270 1.11069506684722 0.727 0.346
1.60335062146919e-265 7 TPD52 GC
1.32927888525225e-269 1.03763451570589 0.735 0.323
2.36997132451624e-265 7 STK17A GC
5.19719156082254e-269 0.736394788726956 0.976 0.865
9.26607283379051e-265 7 ARPC3 GC
2.24253716373597e-267 1.01199633708841 0.342 0.062
3.99821950922486e-263 7 S1PR2 GC
1.04754664427038e-265 1.12786943328139 0.553 0.137
1.86767091206965e-261 7 CD81 GC
9.53539752998382e-265 1.08712825111177 0.647 0.243
1.70006602562082e-260 7 SYNE2 GC
9.03784685155983e-256 1.07915280006557 0.506 0.151
1.6113577151646e-251 7 DEF8 GC
3.42114936943042e-255 0.868323737368597 0.322 0.046
6.0995672107575e-251 7 MME GC
9.55045448257203e-255 0.975989471354102 0.817 0.424
1.70275052969777e-250 7 RHOH GC
1.58781698579361e-244 0.977905635266832 0.438 0.112
2.83091890397143e-240 7 DLAT GC
8.01129524115397e-243 1.0274023098837 0.839 0.475
1.42833382854534e-238 7 UCP2 GC
4.31927987731837e-241 1.02124375549881 0.481 0.126
7.70084409327092e-237 7 SOCS1 GC
1.17797276567249e-234 0.968107172625397 0.636 0.246
2.10020764391748e-230 7 CCDC69 GC
5.54414015656888e-233 0.979980099355435 0.458 0.128
9.88464748514666e-229 7 AIM2 GC
3.80080253632704e-232 0.961040441241142 0.492 0.133
6.77645084201748e-228 7 SEC14L1 GC
1.26572342180107e-229 0.879890940835452 0.86 0.528
2.25665828872913e-225 7 PARP1 GC
2.01965239243243e-229 1.08309007438049 0.585 0.187
3.60083825046778e-225 7 HMCES GC
2.55341480835876e-227 0.715247452654997 0.244 0.032
4.55248326182284e-223 7 SYBU GC
3.10099630636962e-224 1.02756355879137 0.745 0.404
5.52876631462639e-220 7 METAP2 GC
1.37383001051671e-222 0.479303422119716 0.999 0.998
2.44940152575025e-218 7 TMSB4X GC
4.2212839913711e-221 0.919282892665346 0.745 0.393
7.52612722821554e-217 7 LSM10 GC
2.27191395345346e-215 0.944162715052269 0.683 0.282
4.05059538761217e-211 7 GGA2 GC
3.0591971447004e-215 0.974670591528248 0.478 0.124
5.45424258928634e-211 7 SIT1 GC
1.77703664580189e-214 -0.969145283249775 0.999 1
3.16827863580018e-210 7 MALAT1 GC
3.22678045848319e-213 0.908552050704889 0.385 0.091

5.75302687942969e-209 7 SEMA4A GC
4.31884986033168e-211 0.774444556968282 0.916 0.647
7.70007741598535e-207 7 SLC25A5 GC
8.29421002046727e-208 0.789892242774041 0.323 0.06
1.47877470454911e-203 7 ST14 GC
1.54626426098935e-205 0.909832868947679 0.445 0.108
2.75683455091791e-201 7 VNN2 GC
1.31722323212022e-204 0.586064748607164 0.99 0.916
2.34847730054713e-200 7 CFL1 GC
5.05740752636572e-199 0.878156653383388 0.717 0.37
9.01685187875744e-195 7 SMARCB1 GC
2.27960047545622e-198 0.981380668386899 0.61 0.222
4.06429968769089e-194 7 GCHFR GC
2.28650730151376e-195 0.780804342136453 0.874 0.614
4.07661386786888e-191 7 LAMTOR5 GC
3.67369664213769e-195 -1.70689078903251 0.263 0.582
6.5498337432673e-191 7 VIM GC
9.53640703198199e-193 0.928271124126214 0.573 0.213
1.70024600973207e-188 7 UBE2G1 GC
1.92169311881867e-192 -1.33048797103375 0.066 0.362
3.4261866615418e-188 7 LY6E GC
4.27590911057823e-192 0.769649260020523 0.89 0.643
7.62351835324993e-188 7 YWHAB GC
3.5341087218947e-191 0.903076015391606 0.707 0.352
6.30096244026607e-187 7 SNAP23 GC
6.44002983912425e-191 0.600235787925996 0.982 0.902
1.14819292001746e-186 7 ATP5F1E GC
1.30906877232406e-190 0.856627726248032 0.453 0.145
2.33393871417656e-186 7 SMIM20 GC
3.31191853359364e-188 0.715937156695771 0.24 0.03
5.9048195535441e-184 7 SLC2A5 GC
5.82565850301689e-186 0.885008250018826 0.353 0.087
1.03865665450288e-181 7 STAG3 GC
1.2647184069031e-185 0.536234025560046 0.982 0.936
2.25486644766754e-181 7 EEF2 GC
7.63465337252034e-184 0.753045862175605 0.886 0.612
1.36118234978665e-179 7 ARPC1B GC
7.71111042065669e-184 0.870850152164129 0.595 0.218
1.37481387689888e-179 7 IL4R GC
1.38924418650878e-183 0.818336186095547 0.71 0.379
2.4768834601265e-179 7 FNBP1 GC
1.95901658172419e-183 -1.88155596902848 0.158 0.477
3.49273066355606e-179 7 S100A6 GC
1.9115762555776e-182 0.610775997321094 0.999 0.989
3.4081493060693e-178 7 CD74 GC
4.38377086905527e-182 0.868808733872051 0.547 0.22
7.81582508243864e-178 7 ZNF581 GC
2.26467341104919e-179 0.888855491769604 0.518 0.159
4.0376862245596e-175 7 TMEM156 GC
2.58191384475947e-179 1.06377620975826 0.519 0.173

4.60329419382166e-175	7	RGS2	GC		
8.85521004037797e-178	0.850834388281323			0.461	0.16
1.57879539809899e-173	7	BLOC1S6	GC		
1.48884946969029e-176	0.641125297339014			0.244	0.037
2.65446971951082e-172	7	ASB2	GC		
9.02599750410722e-176	0.796329207349761			0.483	0.17
1.60924509500728e-171	7	RASGRP3	GC		
5.8923785440678e-175	1.12195302558514		0.783	0.486	
1.05055217062185e-170	7	SAT1	GC		
3.39670551593129e-174	0.743395100822417			0.279	0.053
6.05598626435389e-170	7	DCAF12	GC		
5.63284815641186e-174	-0.677156616961232			0.998	0.999
1.00428049780667e-169	7	RPS29	GC		
1.77187286456509e-172	0.520164873381007			0.987	0.896
3.15907213023309e-168	7	PFN1	GC		
3.00142527759986e-172	-0.731851976050875			0.965	0.987
5.35124112743279e-168	7	HLA-B	GC		
3.07440957343387e-172	0.656769861700971			0.244	0.049
5.48136482847524e-168	7	BFSP2	GC		
8.09911642720956e-172	0.796646257285213			0.718	0.381
1.44399146780719e-167	7	YWHAE	GC		
1.00045609808327e-171	0.713178465759676			0.881	0.625
1.78371317727266e-167	7	BRK1	GC		
5.69331901960372e-171	0.82050172743433		0.607	0.253	
1.01506184800515e-166	7	ALOX5AP	GC		
1.69891730232439e-170	0.686338335792603			0.951	0.755
3.02899965831416e-166	7	CYBA	GC		
3.9748315310634e-170	-1.22064192708284			0.309	0.587
7.08672713673293e-166	7	SARAF	GC		
6.76601520271629e-169	0.707385513077698			0.309	0.07
1.20631285049229e-164	7	MYO1E	GC		
4.20431564860304e-166	0.918126370287577			0.688	0.335
7.49587436989436e-162	7	S100A10	GC		
1.21219574229071e-161	0.958836446819723			0.497	0.155
2.16122378893011e-157	7	CCDC88A	GC		
1.4127462414253e-160	0.848328529334552			0.693	0.324
2.51878527383717e-156	7	SPIB	GC		
8.33592736925373e-159	0.775318985012848			0.83	0.503
1.48621249066425e-154	7	LSP1	GC		
2.89067425074489e-158	0.827290060529378			0.475	0.176
5.15378312165307e-154	7	PRPSAP2	GC		
1.66735616777884e-157	0.795102406504327			0.444	0.151
2.97272931153289e-153	7	LYPLAL1	GC		
9.52427191725415e-157	-1.15103720694769			0.379	0.63
1.69808244012724e-152	7	SUB1	GC		
1.27964979245481e-155	-0.750007285628487			0.992	0.997
2.28148761496767e-151	7	RPS12	GC		
1.61763631087482e-155	0.795405525085196			0.548	0.238
2.88408377865872e-151	7	SH3KBP1	GC		
2.1006930426406e-154	0.617032782666916			0.231	0.044

3.74532562572392e-150 7 ASB13 GC
1.35223235190747e-152 0.666396178062789 0.252 0.041
2.41089506021583e-148 7 LINC01991 GC
5.13740800475118e-152 0.776543774920862 0.542 0.23
9.15948473167088e-148 7 NCOA3 GC
2.88899742097286e-150 0.735727776634422 0.728 0.413
5.15079350185251e-146 7 CD40 GC
2.74882096659048e-149 0.850616542184384 0.493 0.173
4.90087290133416e-145 7 P2RX5 GC
8.23915372917832e-149 0.626939611213931 0.242 0.046
1.4689587183752e-144 7 A4GALT GC
1.32136103832418e-148 0.731759242003389 0.259 0.044
2.35585459522817e-144 7 SERPINA9 GC
3.03387384112113e-148 0.782674104390462 0.684 0.362
5.40909367133486e-144 7 PTP4A2 GC
5.38938066014548e-148 -1.53573989629633 0.483 0.673
9.60872677897337e-144 7 TUBA1B GC
3.67130535409096e-147 0.79417527658627 0.537 0.238
6.54557031580878e-143 7 PPP2R5C GC
8.85824052833383e-147 0.69655512175072 0.802 0.512
1.57933570379664e-142 7 FKBP1A GC
1.35013286453238e-146 0.660117669108025 0.845 0.569
2.40715188417477e-142 7 LDHB GC
3.25615012447974e-146 0.775692859223671 0.542 0.229
5.80539005693492e-142 7 RGS1 GC
3.37586357078767e-146 0.743768898282443 0.733 0.389
6.01882716035733e-142 7 MBD4 GC
8.17106491981819e-146 -0.834845900622772 0.977 0.986
1.45681916455439e-141 7 TMSB10 GC
1.33121205033554e-145 0.749939299981354 0.526 0.207
2.37341796454323e-141 7 LY86 GC
2.51116466323552e-145 0.890161945320587 0.506 0.199
4.47715547808261e-141 7 ADA GC
1.14099861883425e-144 0.639263881233109 0.866 0.623
2.03428643751958e-140 7 EIF3H GC
2.31764055959456e-143 -1.15849442508024 0.12 0.393
4.13212135370114e-139 7 CD44 GC
6.80800725960007e-142 -1.26244879780251 0.099 0.363
1.2137996143141e-137 7 TXNIP GC
5.550902691562e-141 0.621407870679714 0.278 0.062
9.89670440878589e-137 7 MTA3 GC
1.16214082897819e-140 0.806493615604547 0.554 0.252
2.07198088398522e-136 7 CD22 GC
2.38905039831249e-139 0.748772663052149 0.359 0.091
4.25943795515134e-135 7 MYBL2 GC
7.0853167672442e-139 0.749603005497663 0.471 0.184
1.26324112643197e-134 7 USP34 GC
4.36336208403031e-138 0.769968683279825 0.466 0.169
7.77943825961763e-134 7 LCK GC
4.81609365155011e-137 -0.618284218917672 0.779 0.911

8.5866133713487e-133	7	IGHM	GC
4.11936951016682e-135	0.644536838940321	0.743	0.425
7.34442389967643e-131	7	COTL1	GC
2.17470777915762e-134	0.412660331492937	0.983	0.921
3.87728649946013e-130	7	BTF3	GC
9.19324874946818e-134	0.633913086995242	0.856	0.574
1.63906431954268e-129	7	ATP5MC3	GC
1.27187903900646e-132	0.6796879256541	0.706	0.404
2.26763313864461e-128	7	PPP1CC	GC
1.24232582828703e-131	0.807897251243008	0.475	0.135
2.21494271925295e-127	7	BIK	GC
2.35350989781072e-131	0.599464633103134	0.195	0.04
4.19607279680673e-127	7	CCDC144A	GC
5.67488358412624e-131	0.680801103654924	0.76	0.458
1.01177499421387e-126	7	MTDH	GC
1.0077968265037e-130	0.67191016821987	0.742	0.448
1.79680096197344e-126	7	RAP1B	GC
2.03754918469062e-130	0.637088378943618	0.795	0.505
3.63274644138491e-126	7	SMDT1	GC
2.66990783295358e-130	0.633193882049322	0.247	0.056
4.76017867537294e-126	7	PITPNC1	GC
7.57222331165831e-130	0.718658422445634	0.323	0.082
1.35005169423556e-125	7	GCSAM	GC
4.28400120551917e-128	0.683062011953562	0.31	0.091
7.63794574932013e-124	7	FAM241A	GC
6.93134322447901e-128	0.655478502354659	0.818	0.546
1.23578918349236e-123	7	ATP5IF1	GC
1.91748487007792e-127	-0.995952954926405	0.04	0.218
3.41868377486193e-123	7	PLAC8	GC
1.40364319340579e-126	0.456462875273802	0.976	0.899
2.50255544952318e-122	7	MYL6	GC
1.64594430460565e-125	0.723099009869801	0.554	0.273
2.93455410068141e-121	7	GNG7	GC
1.33119794218586e-124	0.685689643286308	0.46	0.187
2.37339281112317e-120	7	POLD4	GC
1.76709638170761e-124	0.728881743141129	0.585	0.262
3.15055613894651e-120	7	ITGAE	GC
3.72802751015565e-124	-0.849074543239589	0.924	0.965
6.64670024785651e-120	7	FTL	GC
2.08272082433766e-123	0.689114071424954	0.714	0.424
3.71328295771162e-119	7	ROMO1	GC
5.5797487986931e-123	0.663302841599192	0.685	0.382
9.94813413318992e-119	7	SYPL1	GC
1.4219075991708e-122	0.674561147423987	0.635	0.349
2.53511905856162e-118	7	TKT	GC
4.11797400845735e-122	0.732119802456685	0.411	0.136
7.34193585967862e-118	7	RBM38	GC
2.47953120177927e-121	0.684241073723627	0.898	0.67
4.42075617965227e-117	7	HLA-DMA	GC
2.86700947507958e-121	0.609022502895182	0.796	0.512

5.11159119311939e-117 7 TRAPPC1 GC
2.94533889668808e-121 0.675029953510667 0.565 0.278
5.25124471890517e-117 7 RRAS2 GC
1.61296390603325e-120 0.734559132957132 0.281 0.068
2.87575334806668e-116 7 RGCC GC
2.0274613822992e-120 0.456370655751209 0.145 0.016
3.61476089850125e-116 7 ANK1 GC
4.68644203750732e-120 0.450420066991301 0.169 0.026
8.35545750867181e-116 7 MIR3681HG GC
1.74613488370731e-119 0.528690258278373 0.835 0.612
3.11318388416177e-115 7 ACTR3 GC
2.7380165671011e-119 0.697001457654945 0.583 0.295
4.88160973748455e-115 7 TIMM8B GC
2.77386650297365e-119 -1.16039919763852 0.252 0.504
4.94552658815173e-115 7 FXYD5 GC
1.43595559785269e-118 0.738619579772443 0.462 0.182
2.56016523541156e-114 7 DNMT1 GC
3.68521601295439e-118 0.752470487347986 0.484 0.207
6.57037162949638e-114 7 NANS GC
1.3782566569769e-117 0.643633982193884 0.641 0.342
2.45729379372412e-113 7 DBNL GC
1.39276724414111e-117 0.690741372362048 0.54 0.254
2.48316471957919e-113 7 RBM6 GC
2.61242159698052e-117 0.637096989329398 0.294 0.087
4.65768646525657e-113 7 FAM76B GC
4.28880185190065e-117 -0.968919638855867 0.029 0.227
7.64650482175366e-113 7 GBP2 GC
1.21827954726093e-116 0.55186153660944 0.848 0.598
2.17207060481151e-112 7 POLR2L GC
3.11422906086388e-116 0.582675875871545 0.243 0.055
5.55235899261421e-112 7 FANCA GC
4.56127487529253e-116 0.650990858525873 0.712 0.446
8.13229697515905e-112 7 CAPZA1 GC
1.32066784258407e-115 0.859318465438415 0.552 0.257
2.35461869654313e-111 7 PIM1 GC
2.02744706944622e-115 0.713239479813727 0.338 0.118
3.61473538011567e-111 7 AMFR GC
6.34123281482394e-115 0.6041758093764 0.318 0.1
1.13057839855496e-110 7 OGG1 GC
1.27301142170548e-114 -1.1036514952447 0.053 0.277
2.26965206375871e-110 7 GPR183 GC
1.39284931739076e-114 0.55615711394738 0.946 0.77
2.48331104797599e-110 7 ARHGDIB GC
1.39475797515623e-114 0.512403915177368 0.211 0.048
2.48671399390604e-110 7 BORCS8-MEF2B GC
2.56006516229229e-113 0.396080252880994 0.128 0.014
4.56434017785092e-109 7 AL512631.1 GC
4.69739653141435e-113 0.527824224465602 0.819 0.585
8.37498827585864e-109 7 TBCA GC
1.6328023731026e-112 -1.11159915594988 0.314 0.531

2.91112335100462e-108 7 ID3 GC
1.85023704954565e-112 -0.96578224847728 0.061 0.256
3.29878763563494e-108 7 TNFRSF13B GC
2.98375621573437e-112 -1.64813422803269 0.075 0.295
5.3197389570328e-108 7 MIR155HG GC
1.69911851616931e-111 0.649992511406481 0.396 0.139
3.02935840247827e-107 7 PTPN18 GC
3.41173869407778e-111 0.691402304438024 0.67 0.387
6.08278891767127e-107 7 ORAI2 GC
4.8185497333451e-111 0.664788029070134 0.352 0.118
8.59099231958098e-107 7 MSI2 GC
6.56516790089298e-111 0.654263389289684 0.595 0.326
1.17050378505021e-106 7 SEPHS2 GC
1.3353308834552e-109 -1.04661731870377 0.104 0.281
2.38076143211228e-105 7 KLF2 GC
1.81187536413167e-109 0.443202157277648 0.989 0.937
3.23039258671036e-105 7 H3F3A GC
1.07210266324238e-108 0.68830547639486 0.434 0.171
1.91145183829484e-104 7 ZNF106 GC
1.53047972676671e-108 0.65687996391321 0.601 0.301
2.72869230485236e-104 7 PSIP1 GC
1.83218880221692e-108 0.675820175258148 0.583 0.313
3.26660941547254e-104 7 IMP4 GC
5.2680955919262e-108 0.663996489569323 0.386 0.134
9.39248763084522e-104 7 HSH2D GC
5.65520490776462e-108 0.664931540441972 0.592 0.312
1.00826648300535e-103 7 HSBP1 GC
1.32476199473187e-107 0.665263439770796 0.6 0.326
2.36191816040745e-103 7 COX14 GC
2.79676805848663e-107 0.638759896926644 0.516 0.252
4.98635777147582e-103 7 ATXN10 GC
2.2597836764217e-105 0.600351773577313 0.382 0.124
4.02896831669224e-101 7 PLEKHA2 GC
8.26396654960042e-105 0.658183091225688 0.315 0.099
1.47338259612826e-100 7 E2F5 GC
1.3937950427998e-104 0.438149973197313 0.163 0.028
2.48499718180777e-100 7 RAPGEF5 GC
8.28685101214541e-104 0.625251441525856 0.304 0.087
1.47746266695541e-99 7 PXMP2 GC
1.8118262547215e-103 0.633777437766802 0.638 0.328
3.23030502954295e-99 7 LRMP GC
7.22292526582574e-102 -0.82495352712043 0.396 0.614
1.28777534564407e-97 7 IGHD GC
7.48685435026727e-102 0.589920908385939 0.455 0.213
1.33483126210915e-97 7 RPIA GC
8.4780926834961e-102 0.471657550551478 0.179 0.035
1.51155914454052e-97 7 SLC30A4 GC
8.94338637645303e-102 -1.10849580351331 0.219 0.378
1.59451635705781e-97 7 SELL GC
1.42679005448935e-101 0.679465891727262 0.473 0.217

2.54382398814907e-97	7	NSMCE1	GC		
4.40933256684787e-101	0.676625059432608			0.571	0.278
7.86139903343306e-97	7	TUBA4A	GC		
5.47342517251265e-101	0.565051988589954			0.234	0.064
9.75856974007281e-97	7	RPRD1B	GC		
1.18963417691975e-100	0.650361850436361			0.424	0.167
2.12099877403022e-96	7	SYK	GC		
3.45868888835763e-100	0.521227056783603			0.225	0.056
6.16649641905282e-96	7	DTX1	GC		
9.25304609173167e-100	0.614797376739405			0.732	0.442
1.64972558769484e-95	7	PTPRC	GC		
1.7844322355374e-99	0.498705339796796			0.903	0.716
3.18146423273963e-95	7	COX6C	GC		
2.18197693482917e-99	0.688081631989148			0.554	0.278
3.89024667710693e-95	7	RUBCNL	GC		
2.3717448626397e-99	-1.0824623580157		0.224	0.472	
4.22858391560033e-95	7	CD63	GC		
3.82210208459715e-99	0.535977097698356			0.231	0.064
6.81442580662825e-95	7	TERF2	GC		
4.76193281583435e-99	0.592678986635418			0.673	0.404
8.49005001735106e-95	7	HDAC1	GC		
6.75930448930784e-99	0.651096851318771			0.459	0.197
1.2051163973987e-94	7	SNHG25	GC		
1.08334148805802e-98	-0.92346702045157			0.666	0.798
1.93148953905865e-94	7	HLA-E	GC		
2.56370829499548e-98	-0.797481005381877			0.947	0.961
4.57083551914743e-94	7	MT-ND2	GC		
3.13096122392525e-98	0.552830254272553			0.697	0.429
5.58219076613632e-94	7	HMGA1	GC		
5.19436312845933e-98	0.593780579664351			0.7	0.436
9.26103002173014e-94	7	ATP5F1C	GC		
7.45986998229958e-98	0.563909961323332			0.725	0.437
1.33002021914419e-93	7	SEM1	GC		
1.20933542436239e-97	-0.996749571681222			0.324	0.52
2.15612412809571e-93	7	BANK1	GC		
6.49547958769587e-97	0.558584521134887			0.359	0.137
1.1580790556903e-92	7	P2RY8	GC		
9.35974308023394e-97	0.454506088228904			0.971	0.862
1.66874859377491e-92	7	CD79A	GC		
5.52257056627164e-96	0.453800480380902			0.963	0.843
9.8461910626057e-92	7	CD37	GC		
8.94136636872526e-96	0.640832426878179			0.503	0.226
1.59415620988003e-91	7	CCND3	GC		
1.02490179321803e-95	-0.605217198170235			0.988	0.991
1.82729740712843e-91	7	RPL9	GC		
3.48980181773329e-95	-0.832303858858906			0.967	0.976
6.22196766083668e-91	7	FTH1	GC		
5.38739349230531e-95	0.581845571928934			0.568	0.287
9.60518385743113e-91	7	BLK	GC		
7.10384178586192e-95	0.369072286111293			0.131	0.02

1.26654395200132e-90 7 SERP2 GC
8.42828823937165e-95 0.499381044662444 0.853 0.636
1.50267951019757e-90 7 HNRNPC GC
2.1130373945339e-94 0.585297476234159 0.338 0.123
3.76733437071448e-90 7 KMT2A GC
5.72943409534541e-94 -1.02216181186972 0.209 0.435
1.02150080485913e-89 7 PLP2 GC
1.1183868598468e-93 -0.681592455167625 0.961 0.969
1.99397193242086e-89 7 MT-ND3 GC
1.33295402999325e-93 0.503087887493074 0.865 0.662
2.37652374007497e-89 7 SNX3 GC
1.6379025361186e-93 0.461288879415068 0.901 0.699
2.92021643164584e-89 7 NDUFA4 GC
1.64094292220203e-92 -0.873503868813878 0.093 0.259
2.92563713599399e-88 7 FCMR GC
4.96823076781232e-92 0.55098667114343 0.287 0.098
8.85785863593258e-88 7 ACSF3 GC
5.72385048274288e-92 0.593322609834979 0.333 0.12
1.02050530256823e-87 7 RABGAP1L GC
6.77274720565324e-92 0.568646521247016 0.489 0.219
1.20751309929592e-87 7 ATM GC
1.64448028251607e-91 0.738978214786215 0.488 0.248
2.9319438956979e-87 7 MDM4 GC
1.66418629657496e-91 0.477933684475051 0.854 0.632
2.96707774816349e-87 7 ATP5MF GC
4.92969881302469e-91 0.54737039509383 0.299 0.102
8.78916001374171e-87 7 MAP4K2 GC
1.7005302668229e-90 0.635329054333685 0.448 0.2
3.03187541271855e-86 7 SYAP1 GC
2.68565342408404e-90 0.621652445244026 0.272 0.088
4.78825148979943e-86 7 RNGTT GC
3.43892721382009e-89 0.647587494843646 0.599 0.317
6.13126332951985e-85 7 GYPC GC
3.53447306568134e-89 -1.36560921622209 0.107 0.27
6.30161202880326e-85 7 S100A4 GC
3.78446792287733e-89 0.585968378754008 0.373 0.153
6.74732785969798e-85 7 UBE2R2 GC
8.65546616945993e-89 0.582877410130643 0.493 0.233
1.54318306335301e-84 7 SLBP GC
1.17894919984358e-88 0.395779438673067 0.135 0.021
2.10194852840111e-84 7 IQCD GC
1.4276542609858e-88 0.526432963345312 0.831 0.606
2.54536478191157e-84 7 UQCR10 GC
1.93215256734327e-88 0.550331283959854 0.562 0.304
3.44483481231631e-84 7 SPI1 GC
2.57251285625005e-88 0.465640096907734 0.884 0.679
4.58653317140821e-84 7 PPP1CA GC
3.19681097679451e-88 0.373421125078516 0.973 0.901
5.69959429052692e-84 7 SRP14 GC
3.58547176558587e-88 0.428687154093221 0.935 0.802

6.39253761086304e-84 7 TMA7 GC
4.01278047670214e-88 0.57594412180094 0.53 0.285
7.15438631191224e-84 7 LYPLA1 GC
4.17319529756205e-88 0.443178089885004 0.804 0.597
7.44038989602338e-84 7 RBM3 GC
7.14456546975005e-88 0.461913162377118 0.881 0.677
1.27380457760174e-83 7 COX6A1 GC
1.04072002220906e-87 0.378111093087198 0.963 0.892
1.85549972759654e-83 7 SLC25A6 GC
5.1156439688243e-87 0.556663808328298 0.584 0.329
9.12068163201684e-83 7 RBBP4 GC
6.00062042731008e-87 0.583460162292306 0.393 0.164
1.06985061598511e-82 7 CDK13 GC
6.50804454386118e-87 0.57904932109108 0.313 0.116
1.16031926172501e-82 7 SPATS2 GC
1.10893174496996e-86 -1.14406374886033 0.269 0.463
1.97711440810694e-82 7 JUNB GC
1.98027383927144e-86 0.502138825622337 0.261 0.088
3.53063022803705e-82 7 STK40 GC
3.07281406891603e-86 0.555697729495679 0.331 0.127
5.4785202034704e-82 7 MAP4K1 GC
3.15242967223362e-86 0.508598650379392 0.626 0.367
5.62046686262531e-82 7 ZFAND6 GC
3.20488738246398e-86 0.771131885629258 0.618 0.397
5.71399371419504e-82 7 SMIM14 GC
3.82883158753427e-86 0.513696905235819 0.662 0.41
6.82642383741484e-82 7 FAM49B GC
4.23280031022397e-86 0.470357215128192 0.853 0.639
7.54665967309832e-82 7 UQCR11 GC
4.86131962850283e-86 0.509712518314987 0.691 0.442
8.6672467656577e-82 7 MAP1LC3B GC
8.79794686687975e-86 0.422185591010094 0.146 0.026
1.56858594689599e-81 7 RNF144B GC
1.37255152253919e-85 0.56713880709675 0.537 0.273
2.44712210953512e-81 7 LAT2 GC
1.52777078678215e-85 0.595182778788472 0.493 0.229
2.72386253575389e-81 7 SP140 GC
1.7240847843398e-85 0.456935123018947 0.872 0.69
3.07387076199944e-81 7 RHOA GC
3.86292737383469e-85 0.597051533502488 0.579 0.311
6.88721321480987e-81 7 NAA38 GC
7.14025434724534e-85 0.604430280602944 0.173 0.029
1.27303594757037e-80 7 PEG10 GC
7.26834949498826e-85 0.551941222007347 0.299 0.109
1.29587403146146e-80 7 C12orf49 GC
8.01913564289708e-85 0.578552144012248 0.505 0.275
1.42973169377212e-80 7 PRDX3 GC
1.16911383042893e-84 0.574259049257916 0.549 0.29
2.08441304827175e-80 7 ITSN2 GC
2.0312292060681e-84 0.436221392994685 0.873 0.667

3.62147855149881e-80 7 ATP5ME GC
2.2095352494098e-84 0.486751227424248 0.894 0.695
3.93938039617274e-80 7 COMMD6 GC
2.54220944787943e-84 0.413252675059061 0.153 0.035
4.53250522462423e-80 7 EEPD1 GC
6.16364726542875e-84 0.55477025846727 0.342 0.133
1.09891667095329e-79 7 CUL3 GC
7.59837062975063e-84 0.381574730173715 0.935 0.8
1.35471349957824e-79 7 HNRNPK GC
9.4566283555749e-84 0.536850566781477 0.597 0.354
1.68602226951545e-79 7 PPP2CA GC
1.54253647095276e-83 0.500627284805937 0.748 0.492
2.75018827406168e-79 7 RTRAF GC
1.6231116226028e-83 0.436874294749516 0.853 0.645
2.89384571193853e-79 7 ATP5F1A GC
4.34418172882329e-83 0.70147504491664 0.696 0.384
7.74524160431905e-79 7 CSTB GC
7.19724807415659e-83 0.440439561478343 0.935 0.826
1.28319735914138e-78 7 UQCRB GC
8.82118932202528e-83 0.424250735955064 0.901 0.723
1.57272984422389e-78 7 OST4 GC
2.17715202573739e-82 0.532179308041798 0.37 0.161
3.8816443466872e-78 7 CYB5R3 GC
3.66664436861585e-82 -0.870016271919811 0.188 0.354
6.5372602448052e-78 7 TRAF3IP3 GC
3.89992433740127e-82 0.565062381335877 0.604 0.345
6.95317510115272e-78 7 CD19 GC
4.17068750147901e-82 0.465028591179836 0.236 0.077
7.43591874638693e-78 7 PARN GC
4.53077748919812e-82 0.528386139589184 0.493 0.254
8.07792318549132e-78 7 THOC7 GC
1.54054443113612e-81 -0.836841595067278 0.023 0.178
2.74663666627258e-77 7 TNFRSF18 GC
1.93583595152014e-81 0.380589205884032 0.135 0.03
3.45140191796526e-77 7 KANK1 GC
2.25649454550075e-81 0.340900292492394 0.11 0.017
4.02310412517328e-77 7 LOXL2 GC
4.42121804705649e-81 0.585611474914472 0.643 0.352
7.88258965609702e-77 7 RCSD1 GC
1.33704362083365e-80 0.565244076247571 0.459 0.25
2.38381507158432e-76 7 CDV3 GC
3.14560977939582e-80 0.540162555682111 0.296 0.11
5.60830767568481e-76 7 ABI3 GC
5.29419819144427e-80 0.430918670667356 0.884 0.693
9.439025955526e-76 7 ELOB GC
6.00464147992308e-80 0.537574683625978 0.183 0.042
1.07056752945549e-75 7 COL9A3 GC
7.9929661566405e-80 0.599230197976799 0.501 0.261
1.42506593606743e-75 7 TOP1 GC
1.25940405460098e-79 0.596999111835066 0.356 0.138

2.24539148894808e-75	7	SH2B2	GC
1.26372718796028e-79	0.512558559970628	0.573	0.341
2.25309920341439e-75	7	LSM14A	GC
3.67973235952631e-79	0.43469898009515	0.208	0.062
6.56059482379946e-75	7	MFHAS1	GC
4.37290274884713e-79	0.405770614313639	0.9	0.709
7.79644831091954e-75	7	COX6B1	GC
6.62966395609334e-79	0.437617679244763	0.851	0.625
1.18200278673188e-74	7	PSMA4	GC
1.17994350183584e-78	0.544078065201038	0.591	0.32
2.10372126942311e-74	7	CCM2	GC
2.33345394006506e-78	0.512533167329116	0.366	0.163
4.160315029742e-74	7	COMMD7	GC
2.50447278239028e-78	0.550466979617532	0.509	0.26
4.46522452372363e-74	7	SEPT9	GC
2.77477693423198e-78	0.562462567034163	0.474	0.23
4.9471497960422e-74	7	PTRHD1	GC
3.25779622760801e-78	-0.763706353665177	0.121	0.285
5.80832489420232e-74	7	IFITM2	GC
4.62805652062634e-78	0.563988541423138	0.459	0.225
8.2513619706247e-74	7	ZCCHC7	GC
4.86232929292957e-78	0.508023538754832	0.296	0.103
8.66904689636412e-74	7	MOB3A	GC
8.12246616635756e-78	0.62350899726732	0.452	0.228
1.44815449279989e-73	7	DSTN	GC
1.52071587031417e-77	0.459402719774032	0.715	0.476
2.71128432518313e-73	7	UBE2L3	GC
2.33262524649988e-77	0.53878600751793	0.431	0.201
4.15883755198463e-73	7	EHD1	GC
3.08017326864843e-77	0.504553179715012	0.244	0.066
5.49164092067329e-73	7	CDCA7	GC
7.57701131143916e-77	0.38403053599053	0.153	0.038
1.35090534671649e-72	7	ACY3	GC
9.8330665494693e-77	-0.498841504711969	0.978	0.986
1.75313743510488e-72	7	RPL27	GC
1.74446578133495e-76	0.417453590188227	0.173	0.04
3.11020804154208e-72	7	MYBL1	GC
3.72756932935448e-76	0.494744240958742	0.194	0.056
6.6458833573061e-72	7	TESC	GC
7.40753467805298e-76	0.437551416414448	0.792	0.545
1.32068935775007e-71	7	SEC61G	GC
1.06390057451989e-75	0.510218421560761	0.286	0.094
1.89682833431152e-71	7	BACH2	GC
1.26451957873779e-75	0.414997360298456	0.175	0.045
2.25451195693161e-71	7	PEX5	GC
3.15124842821301e-75	0.466148439837933	0.247	0.085
5.61836082266098e-71	7	XPNPEP1	GC
1.01442912233913e-74	0.405897296245313	0.881	0.689
1.80862568221843e-70	7	ATP5MPL	GC
1.28849502293778e-74	0.402885757605419	0.833	0.599

2.29725777639577e-70 7 POMP GC
2.46368583291135e-74 0.568403383378546 0.415 0.178
4.39250547149765e-70 7 HLA-DOB GC
1.03380426650861e-73 0.422130892532136 0.851 0.661
1.84316962675821e-69 7 GDI2 GC
1.10108427456099e-73 0.493815362387815 0.726 0.475
1.96312315311478e-69 7 NDUFB1 GC
1.43122393849443e-73 0.603136670719166 0.236 0.064
2.55172915994172e-69 7 RASSF6 GC
1.70468163794804e-73 0.533820450963418 0.357 0.137
3.03927689229755e-69 7 TAGAP GC
2.54508406956415e-73 0.373157231390015 0.132 0.034
4.53763038762592e-69 7 IGLC5 GC
7.47371696910946e-73 0.417058308926624 0.881 0.692
1.33248899842253e-68 7 CCNI GC
8.38566217462727e-73 0.54198200284145 0.581 0.347
1.4950797091143e-68 7 RAB11A GC
1.05369985495057e-72 -0.785849831124971 0.048 0.204
1.87864147139137e-68 7 YBX3 GC
1.14411544346399e-72 0.471729133619293 0.669 0.421
2.03984342415195e-68 7 CLTA GC
2.15970137275355e-72 0.497501823645568 0.578 0.337
3.85053157748231e-68 7 LSM6 GC
8.1241291412952e-72 0.488010960562108 0.694 0.469
1.44845098460152e-67 7 UBE2N GC
1.0493102730825e-71 0.51961779650325 0.283 0.097
1.87081528587878e-67 7 SORL1 GC
1.12149041288181e-71 0.515226010881803 0.299 0.107
1.99950525712698e-67 7 HDAC7 GC
1.6691146451711e-71 -0.697256769775202 0.05 0.189
2.97586450087556e-67 7 PLPP5 GC
1.81182145593631e-71 0.434492028406875 0.167 0.046
3.23029647378885e-67 7 UCHL1 GC
2.63831861114537e-71 0.373496061397838 0.153 0.034
4.70385825181108e-67 7 RTN4IP1 GC
3.43993378811564e-71 0.549815131355145 0.529 0.301
6.13305795083138e-67 7 PGLS GC
6.54523209782636e-71 0.55297319035153 0.465 0.242
1.16694943072146e-66 7 TIFA GC
9.01108010860192e-71 0.550348544165559 0.62 0.372
1.60658547256264e-66 7 LSM8 GC
9.19633893221842e-71 0.522658133943676 0.497 0.269
1.63961526822522e-66 7 STK17B GC
1.31324673072117e-70 0.53671705862521 0.298 0.096
2.34138759620278e-66 7 RMI2 GC
1.53762153394039e-70 0.487537989805867 0.351 0.158
2.74142543286231e-66 7 COX11 GC
1.96322098825703e-70 0.536510267963626 0.267 0.092
3.50022669996347e-66 7 MX1 GC
4.16906015449842e-70 -0.650060830924274 0.032 0.169

7.43301734945523e-66	7	MARCKS	GC		
9.90482387548657e-70	0.545892145461981			0.47	0.23
1.7659310487605e-65	7	NCF4	GC		
1.08882920104426e-69	0.444696383620426			0.691	0.467
1.9412735825418e-65	7	PRELID1	GC		
2.87096324463111e-69	0.497173855800481			0.43	0.22
5.11864036885281e-65	7	IAH1	GC		
7.67501951979417e-69	0.482273028118561			0.598	0.356
1.3683792301841e-64	7	TERF2IP	GC		
7.97231128263993e-69	0.449546574598459			0.244	0.082
1.42138337858187e-64	7	MAP4K4	GC		
1.87555718500467e-68	0.478144295690341			0.315	0.13
3.34393090514483e-64	7	CLIC4	GC		
2.08891835880405e-68	0.336928009744619			0.134	0.033
3.72433254191173e-64	7	PRDM15	GC		
2.32190584002359e-68	0.581734833634098			0.304	0.115
4.13972592217807e-64	7	AL139020.1		GC	
2.87351712162929e-68	0.474011828741185			0.321	0.139
5.12319367615286e-64	7	ANKRD13A	GC		
4.02706766935327e-68	0.431098042234734			0.82	0.628
7.17985894768995e-64	7	UQCRH	GC		
4.29955259597794e-68	0.522416908670629			0.343	0.14
7.66567232336907e-64	7	ALOX5	GC		
1.66231501653786e-67	0.496432803598676			0.458	0.235
2.96374144298535e-63	7	WAS	GC		
2.29790035791818e-67	0.480308110621619			0.471	0.241
4.09692654813233e-63	7	WIPF1	GC		
2.52051319236286e-67	0.478379067497909			0.389	0.185
4.49382297066374e-63	7	REPIN1	GC		
5.86603974276946e-67	-0.88974883572028			0.057	0.203
1.04585622573837e-62	7	ACP5	GC		
1.99294632478477e-66	0.490842728914092			0.299	0.114
3.55322400245876e-62	7	EPS15	GC		
2.6215368644774e-66	0.525238968508281			0.325	0.122
4.67393807567675e-62	7	STIM2	GC		
2.78122318009659e-66	0.401630109467431			0.184	0.049
4.95864280779421e-62	7	BCL6	GC		
3.06174984304587e-66	0.471387840825376			0.467	0.231
5.45879379516648e-62	7	MCUB	GC		
3.12664976636025e-66	-0.995013146197009			0.16	0.322
5.57450386844369e-62	7	CD69	GC		
4.7564851602427e-66	0.486635772900402			0.587	0.356
8.48033739219671e-62	7	RBBP7	GC		
5.07287169353018e-66	0.487146189774357			0.467	0.238
9.04442294239496e-62	7	CSK	GC		
6.89379170376723e-66	0.335741252316486			0.138	0.029
1.22909412286466e-61	7	CTPS2	GC		
8.12414255101946e-66	0.496314483576993			0.439	0.224
1.44845337542126e-61	7	MTF2	GC		
9.16680380770946e-66	0.413839305316205			0.788	0.55

1.63434945087652e-61 7 ATP5PF GC
1.01469812605087e-65 0.494464606485201 0.525 0.307
1.8091052889361e-61 7 NDUFAF3 GC
2.05930425482078e-65 0.530614302504492 0.558 0.323
3.67153355591998e-61 7 BPTF GC
3.76182629615217e-65 0.462744861832957 0.3 0.115
6.7069601034097e-61 7 SSBP2 GC
4.45754221506968e-65 0.476259335935048 0.543 0.312
7.94735201524773e-61 7 IGBP1 GC
7.5739389549675e-65 0.468382484755226 0.463 0.25
1.35035757628116e-60 7 ABI1 GC
8.32405599889442e-65 0.461356405537872 0.534 0.279
1.48409594404289e-60 7 DCK GC
8.44347000215613e-65 0.425991468909387 0.692 0.451
1.50538626668442e-60 7 SWAP70 GC
1.37549748729899e-64 -0.678716799361171 0.063 0.2
2.45237447010536e-60 7 CLEC2B GC
1.40601914238475e-64 0.48171014266951 0.398 0.207
2.50679152895777e-60 7 PHAX GC
1.66742151709826e-64 0.616090147036832 0.3 0.101
2.97284582283448e-60 7 VPREB3 GC
1.87112381202789e-64 0.501039550857384 0.36 0.157
3.33602664446452e-60 7 PPP1R18 GC
3.05377061208363e-64 0.487249933124144 0.587 0.349
5.4445676242839e-60 7 TMEM243 GC
4.11323179546875e-64 0.438103326760852 0.271 0.111
7.33348096814123e-60 7 ACYP2 GC
6.22620748951042e-64 0.40997456771174 0.706 0.481
1.11007053330481e-59 7 CCT8 GC
6.33364393420016e-64 0.401196612177215 0.725 0.515
1.12922537702855e-59 7 SELENOT GC
8.56172671128252e-64 0.377793120876596 0.869 0.714
1.52647025535456e-59 7 EIF3F GC
1.18266399853699e-63 0.438879205680086 0.311 0.133
2.10857164299159e-59 7 ITGB1 GC
1.83963964590744e-63 0.411770840910713 0.648 0.432
3.27989352468838e-59 7 ACTR2 GC
3.45069680831197e-63 0.399634578305082 0.592 0.366
6.1522473395394e-59 7 LCP1 GC
3.96379474293345e-63 0.425917103374797 0.623 0.409
7.06704964717605e-59 7 COX7A2L GC
4.35736270549464e-63 -0.748751442924898 0.059 0.216
7.76874196762639e-59 7 MGST3 GC
5.45508011342157e-63 -0.414904538212165 0.998 0.999
9.72586233421931e-59 7 RPL13 GC
5.65494850255302e-63 0.362112305003669 0.798 0.593
1.00822076852018e-58 7 PSMA2 GC
8.24271366853896e-63 0.500551381825695 0.644 0.409
1.46959341996381e-58 7 CTSH GC
1.27404766396212e-62 0.361173323633064 0.825 0.62

2.27149958007806e-58	7	ATP5F1B	GC
1.43700768831357e-62	-0.599968722240722	0.03	0.154
2.56204100749427e-58	7	CELF2	GC
2.09701614849045e-62	0.357551722999309	0.137	0.035
3.73877009114362e-58	7	TOX	GC
3.33291973738277e-62	-0.494025795374542	0.972	0.983
5.94226259977974e-58	7	RPS21	GC
3.64135905647379e-62	0.502934300021643	0.567	0.329
6.49217906178712e-58	7	NDUFC1	GC
3.92663200985985e-62	0.364504676337487	0.825	0.64
7.00079221037913e-58	7	ZFAS1	GC
5.26060845866828e-62	0.430457412939564	0.495	0.281
9.37913882095968e-58	7	PFDN4	GC
5.30985121350141e-62	0.453468795483568	0.226	0.087
9.46693372855167e-58	7	FAM208B	GC
5.907982201839e-62	0.497118364752859	0.365	0.168
1.05333414676588e-57	7	EHMT1	GC
7.11789103047356e-62	0.517241164823745	0.482	0.285
1.26904879182313e-57	7	HSPA4	GC
1.72474392860767e-61	0.444086938472688	0.626	0.349
3.07504595031462e-57	7	H2AFV	GC
2.25289724190287e-61	0.427315164951496	0.193	0.058
4.01669049258862e-57	7	ZNF608	GC
2.36598989428274e-61	0.487458159221823	0.424	0.225
4.21832338251671e-57	7	CHCHD10	GC
5.36357922759696e-61	0.43742334943825	0.212	0.07
9.56272540488262e-57	7	DNASE1	GC
8.05421257005376e-61	-0.408530208400026	0.999	0.998
1.43598555911488e-56	7	RPLP2	GC
9.58372103491157e-61	0.337582327096375	0.127	0.026
1.70868162331438e-56	7	FGD6	GC
1.06642897151199e-60	0.436093168977175	0.424	0.213
1.90133621330873e-56	7	MPLKIP	GC
1.40833495742298e-60	0.493535813012961	0.436	0.226
2.51092039558943e-56	7	CPNE3	GC
1.65542806985461e-60	-0.800114086748515	0.089	0.243
2.95146270574378e-56	7	CAPG	GC
2.1728427777202e-60	0.475489265732443	0.31	0.135
3.87396138839735e-56	7	INO80C	GC
2.86368147569356e-60	0.400621626904817	0.725	0.509
5.10565770301405e-56	7	ATP5PB	GC
3.02860041244633e-60	0.314445912030679	0.117	0.024
5.39969167535056e-56	7	LHFPL2	GC
3.29136851825285e-60	0.33937108687466	0.843	0.691
5.868180931193e-56	7	CIRBP	GC
3.57890097622202e-60	0.502138544440131	0.373	0.17
6.38082255050625e-56	7	PLIN3	GC
3.85900326381095e-60	0.440506217561767	0.176	0.053
6.88021691904854e-56	7	RGS16	GC
7.7437718305607e-60	0.408142787823666	0.185	0.061

1.38063707967067e-55	7	QPCT	GC	
8.77466433469201e-60	0.32773959020603	0.119	0.024	
1.56443490423224e-55	7	HTR3A	GC	
1.04129582855865e-59	0.447898278685051		0.404	0.201
1.85652633273721e-55	7	TCEAL8	GC	
1.13556031252328e-59	0.332154258346286		0.136	0.037
2.02459048119775e-55	7	DENND6B	GC	
2.50169726909724e-59	0.482842742887663		0.313	0.142
4.46027606107346e-55	7	SMARCA4	GC	
3.93569327165415e-59	0.324137107059488		0.119	0.03
7.01694753403219e-55	7	PDGFD	GC	
5.20769685690586e-59	0.339924165360974		0.805	0.61
9.28480272617746e-55	7	NDUFA13	GC	
6.71461865462043e-59	-0.613303372487972		0.049	0.176
1.19714935993228e-54	7	BCL2	GC	
1.0116895422572e-58	0.362864048681685		0.157	0.049
1.80374128489036e-54	7	CDKN2C	GC	
1.14398618721877e-58	0.379735614588919		0.153	0.038
2.03961297319235e-54	7	IGF2BP3	GC	
1.29577776565824e-58	0.446766305822293		0.479	0.266
2.31024217839208e-54	7	RNF7	GC	
1.40019950311629e-58	0.496971819486059		0.366	0.171
2.49641569410604e-54	7	MTERF4	GC	
1.46511123229695e-58	0.448769042696726		0.294	0.12
2.61214681606222e-54	7	COMMD4	GC	
2.30780088178199e-58	-0.787544387387854		0.215	0.387
4.11457819212911e-54	7	PDCD4	GC	
2.59251239767864e-58	0.426916007490356		0.58	0.337
4.62219035382125e-54	7	H2AFY	GC	
4.16964863847846e-58	0.46130125485969	0.606	0.377	
7.43406655754324e-54	7	NDUFB3	GC	
5.03539446586481e-58	0.483249632772131		0.374	0.183
8.97760479319037e-54	7	LBR	GC	
5.07911770983081e-58	0.436954794988023		0.647	0.41
9.05555896485735e-54	7	HNRNPD	GC	
5.25383218132476e-58	0.436457262826028		0.558	0.349
9.36705739608391e-54	7	POLR1D	GC	
5.33504509647664e-58	0.370505941946617		0.71	0.496
9.51185190250819e-54	7	EIF3I	GC	
8.87942688778978e-58	-0.325713371530302		1	1
1.58311301982404e-53	7	RPL41	GC	
1.07720757721287e-57	0.434490333786803		0.506	0.295
1.92055338941283e-53	7	UGP2	GC	
1.10529444449468e-57	-0.755421492142277		0.143	0.273
1.97062946508957e-53	7	JUN	GC	
2.21519814448452e-57	0.447570784495831		0.327	0.149
3.94947677180145e-53	7	ACAP2	GC	
2.23942039219396e-57	0.446924196579344		0.303	0.134
3.99266261724261e-53	7	CORO1B	GC	
2.30435815066765e-57	0.455831396262703		0.385	0.189

4.10844014682535e-53 7 UBE2E1 GC
2.45611588171571e-57 -0.771907355253998 0.263 0.42
4.37900900551093e-53 7 NPC2 GC
2.90181666095514e-57 0.395802618512733 0.196 0.077
5.17364892481693e-53 7 GPR160 GC
3.52592256749985e-57 -0.72890704261342 0.186 0.325
6.28636734559548e-53 7 TUBA1A GC
1.03419028203941e-56 0.464265515306655 0.518 0.308
1.84385785384807e-52 7 SCAND1 GC
1.44993756638711e-56 -0.782603511006 0.174 0.302
2.58509368711158e-52 7 LINC00926 GC
2.22108668304198e-56 0.43407123486744 0.474 0.27
3.95997544719554e-52 7 CNIH1 GC
2.23360106388923e-56 0.379299359357072 0.201 0.07
3.9822873368081e-52 7 CUX1 GC
2.45802837764846e-56 0.419625339479107 0.465 0.267
4.38241879450945e-52 7 SEPT2 GC
2.9241391149724e-56 -0.753002487471302 0.032 0.164
5.21344762808429e-52 7 LMNA GC
4.01364188786499e-56 0.432120546179281 0.268 0.111
7.1559221218745e-52 7 CHMP7 GC
4.45805141189436e-56 -0.367719514429124 0.999 0.999
7.94825986226645e-52 7 PTMA GC
4.5635831204212e-56 0.416731065943361 0.209 0.072
8.13641234539896e-52 7 KLHL6 GC
4.85824602687219e-56 0.371620361315008 0.174 0.059
8.66176684131043e-52 7 FAM129A GC
6.37896657432706e-56 -0.77188475549635 0.224 0.358
1.13730595053677e-51 7 TUBB4B GC
6.83128293948121e-56 0.41151873530409 0.472 0.269
1.21794943528011e-51 7 TANK GC
2.47686749642205e-55 0.449317890401365 0.259 0.107
4.41600705937087e-51 7 BRI3BP GC
2.62356144119013e-55 0.438306977695226 0.281 0.123
4.67754769349788e-51 7 DNAJB2 GC
2.82187182824087e-55 -0.329954381166222 1 0.999
5.03111528257065e-51 7 RPLP1 GC
3.87688943311645e-55 0.467913577101009 0.315 0.146
6.91210617030332e-51 7 MAD1L1 GC
5.60355185479511e-55 0.415617966303199 0.635 0.394
9.9905726019142e-51 7 STX7 GC
7.96922878237334e-55 0.344352935354012 0.183 0.067
1.42083379960934e-50 7 NEK6 GC
8.69717760605429e-55 0.402672779441544 0.224 0.082
1.55061979538342e-50 7 PTK2B GC
9.0909015212992e-55 0.436978938884933 0.265 0.111
1.62081683223244e-50 7 KLHL5 GC
1.07557234075457e-54 0.370381654704637 0.714 0.508
1.91763792633132e-50 7 CAPZB GC
1.61589544155717e-54 0.42411658401681 0.212 0.072

2.88097998275227e-50 7 LMO2 GC
1.8003129965317e-54 0.393483952197401 0.224 0.082
3.20977804151637e-50 7 DENND2D GC
1.9031971957732e-54 0.436093703824523 0.714 0.499
3.39321028034404e-50 7 MZT2B GC
2.95653308099741e-54 0.395784815473514 0.573 0.359
5.27120283011028e-50 7 EWSR1 GC
3.22679354004877e-54 0.372631713602908 0.185 0.062
5.75305020255295e-50 7 TEX9 GC
3.49154557824722e-54 0.319897237007241 0.885 0.709
6.22507661145697e-50 7 HMGN2 GC
3.71642572695964e-54 0.405644087627326 0.341 0.171
6.62601542859634e-50 7 TRAPPC6A GC
4.00098408918366e-54 0.44588243126116 0.244 0.098
7.13335453260554e-50 7 OTULIN GC
4.05408867617433e-54 0.346759389207153 0.769 0.571
7.22803470075121e-50 7 COPE GC
5.94317170628049e-54 -0.395677298656341 0.999 0.999
1.05960808351275e-49 7 RPL34 GC
2.17513067212304e-53 0.388056566947213 0.231 0.091
3.87804047532817e-49 7 RAD17 GC
5.12608513024582e-53 0.444442436241253 0.263 0.111
9.13929717871528e-49 7 BRWD1 GC
6.18994949343755e-53 0.359498034172797 0.696 0.481
1.10360609518498e-48 7 CBX3 GC
7.39764867698807e-53 0.38714973443772 0.699 0.486
1.3189267826202e-48 7 ARL6IP4 GC
9.53938133696717e-53 0.361210713337153 0.78 0.576
1.70077629856788e-48 7 NDUFB2 GC
1.3541212357969e-52 0.386808560368214 0.37 0.199
2.41426275130229e-48 7 ARL2BP GC
1.51727663502818e-52 0.344613193681192 0.167 0.054
2.70515251259174e-48 7 SLC15A4 GC
1.84816688162409e-52 0.314711394632794 0.824 0.633
3.29509673324759e-48 7 ERH GC
2.21592016114004e-52 0.382856004495026 0.796 0.62
3.95076405529659e-48 7 ATP5PO GC
2.2278564132955e-52 0.441560405145127 0.295 0.121
3.97204519926454e-48 7 STAP1 GC
2.24490157564814e-52 0.373451278267795 0.748 0.547
4.00243501922307e-48 7 NEDD8 GC
3.65449614962851e-52 0.392773478002838 0.618 0.402
6.51560118517268e-48 7 EID1 GC
4.5543070172362e-52 0.401333036594744 0.512 0.325
8.11987398103042e-48 7 EIF3A GC
4.80694943600662e-52 0.392915383271853 0.612 0.404
8.57031014945621e-48 7 MOB1A GC
5.45266910985774e-52 0.430199889967298 0.416 0.223
9.72156375596537e-48 7 SEPT1 GC
5.62692226206427e-52 -0.386297409938119 0.994 0.996

1.00322397010344e-47 7 RPL35A GC
9.10998527070621e-52 0.374353982855206 0.755 0.559
1.62421927391421e-47 7 RAC1 GC
2.47609046552813e-51 0.349397940359639 0.751 0.548
4.4146216909901e-47 7 SNU13 GC
2.96497765700931e-51 -0.378106696256324 0.999 0.999
5.28625866468189e-47 7 RPL32 GC
3.17189124730476e-51 0.447968949216703 0.499 0.289
5.65516490481966e-47 7 PDHB GC
3.70116938741611e-51 0.406461485129252 0.252 0.107
6.59881490082419e-47 7 ZNF296 GC
4.58907136802993e-51 -0.430837911574342 0.997 0.998
8.18185534206055e-47 7 RPL11 GC
1.53919459945087e-50 0.417351985918247 0.345 0.18
2.74423005136096e-46 7 CLINT1 GC
2.60905390487087e-50 0.397897508101713 0.157 0.041
4.65168220699428e-46 7 EML6 GC
2.73178149249902e-50 0.429062199949153 0.549 0.312
4.8704932229765e-46 7 TNFRSF13C GC
3.43475195105059e-50 0.369766862708042 0.731 0.523
6.1238192535281e-46 7 PAIP2 GC
4.11611308411899e-50 -0.373546075811086 0.994 0.995
7.33861801767575e-46 7 RPS25 GC
6.07015699158822e-50 0.430867562967486 0.323 0.146
1.08224829003026e-45 7 MYL6B GC
6.99703847451105e-50 0.326545140908076 0.15 0.043
1.24750198962058e-45 7 GRAP GC
7.41981554443943e-50 0.346389594646661 0.14 0.039
1.32287891341811e-45 7 IKBKE GC
7.74484130582835e-50 0.407757898079559 0.283 0.125
1.38082775641614e-45 7 C16orf87 GC
1.10962138344473e-49 0.417322201053986 0.394 0.22
1.9783439645436e-45 7 PLEKHJ1 GC
1.16605171069365e-49 0.420096794069375 0.448 0.259
2.0789535949957e-45 7 PPP1R7 GC
1.17847187976538e-49 0.455954418652734 0.342 0.176
2.1010975144337e-45 7 TRABD GC
1.18511620495867e-49 0.435031393416526 0.471 0.278
2.11294368182081e-45 7 GSTK1 GC
1.19227595759456e-49 0.388036206803072 0.816 0.653
2.12570880479535e-45 7 UBL5 GC
1.82423907494702e-49 0.353696743106035 0.707 0.507
3.25243584672304e-45 7 SRP9 GC
1.993996156868e-49 -0.397891901611471 0.998 0.999
3.55509574807996e-45 7 RPL39 GC
2.0018732187086e-49 0.403871094339756 0.289 0.128
3.56913976163556e-45 7 CERS4 GC
2.01961175666981e-49 0.353672666433482 0.685 0.481
3.6007658009666e-45 7 EIF3M GC
2.35276571320635e-49 0.468262803690627 0.346 0.153

4.19474599007561e-45 7 C12orf75 GC
3.49083229703721e-49 0.443004700809082 0.645 0.41
6.22380490238764e-45 7 NDUFA12 GC
4.39487722542066e-49 0.360810552659187 0.19 0.074
7.8356266052025e-45 7 WEE1 GC
6.84830540490068e-49 0.373593867485929 0.2 0.07
1.22098437063974e-44 7 TMED8 GC
6.87918564062672e-49 0.382282401655157 0.388 0.199
1.22649000786734e-44 7 ARHGEF1 GC
7.68221615526666e-49 0.337145664827964 0.659 0.448
1.36966231832249e-44 7 PPP4C GC
1.12094375485598e-48 0.395611068304014 0.216 0.086
1.99853062053272e-44 7 RITA1 GC
1.26169561368231e-48 0.430808236590041 0.478 0.259
2.24947710963418e-44 7 HHEX GC
1.27814674763533e-48 0.405072601239397 0.268 0.114
2.27880783635904e-44 7 CD180 GC
1.29292433203054e-48 -0.962753752838512 0.275 0.414
2.30515479157726e-44 7 TXN GC
2.00786708645618e-48 0.360556572215827 0.802 0.613
3.57982622844272e-44 7 NDUFA1 GC
2.13624726716336e-48 0.413480760108787 0.509 0.315
3.80871525262556e-44 7 GNB2 GC
2.25532739055428e-48 0.397534089506093 0.45 0.246
4.02102320461923e-44 7 ZCCHC10 GC
2.94089186942782e-48 0.392471179229936 0.443 0.259
5.24331611400286e-44 7 GRN GC
3.82579675260376e-48 0.368238021594814 0.162 0.065
6.82101303021725e-44 7 EYA3 GC
3.91894406730032e-48 0.332308008950027 0.71 0.489
6.98708537758974e-44 7 COX5A GC
7.58396619961107e-48 0.375246087264372 0.638 0.413
1.35214533372866e-43 7 TMEM123 GC
8.36002556769198e-48 0.416704076766168 0.154 0.044
1.4905089584638e-43 7 HES6 GC
9.16387515656915e-48 0.359948175026623 0.182 0.071
1.63382730166471e-43 7 EZH2 GC
1.31968297615663e-47 0.385408580878073 0.635 0.415
2.35286277818965e-43 7 GABARAPL2 GC
1.6892780210015e-47 0.335774245892775 0.679 0.46
3.01181378364357e-43 7 PRDX5 GC
1.7085531509786e-47 0.39713717980287 0.423 0.245
3.04617941287975e-43 7 MCTS1 GC
2.13913329959955e-47 0.354167833502282 0.631 0.427
3.81386075985604e-43 7 KHDRBS1 GC
2.59223622272376e-47 0.396746915724936 0.25 0.106
4.62169796149419e-43 7 TRAC GC
3.13428170836538e-47 -0.617162311502536 0.894 0.917
5.58811085784464e-43 7 EEF1B2 GC
7.34899065007187e-47 0.3432425490963 0.706 0.516

1.31025154300131e-42 7 NDUFB8 GC
8.06570149562517e-47 0.33213321857942 0.196 0.068
1.43803391965501e-42 7 DHTKD1 GC
8.36022944307726e-47 0.325368325213004 0.149 0.045
1.49054530740624e-42 7 ATP8A1 GC
8.47914749084261e-47 0.312150789855348 0.143 0.042
1.51174720614233e-42 7 LINC00467 GC
9.44232426181869e-47 0.383389069760848 0.575 0.383
1.68347199263965e-42 7 ARPC4 GC
1.18264853298426e-46 0.385004522455649 0.561 0.349
2.10854406945763e-42 7 GNAS GC
1.76750279606545e-46 0.323129020760878 0.147 0.045
3.15128073510509e-42 7 MYL5 GC
1.82083993057629e-46 0.375220267199693 0.509 0.305
3.24637551222446e-42 7 ATP2B1 GC
1.98392919529374e-46 0.398613744458179 0.229 0.094
3.5371473622892e-42 7 SYVN1 GC
1.99514032241448e-46 -0.629922524553803 0.12 0.246
3.55713568083278e-42 7 PHACTR1 GC
3.66185393036372e-46 -0.467271666872682 0.027 0.116
6.52871937244547e-42 7 P2RY10 GC
3.70469546018554e-46 -0.330580530273974 0.999 0.999
6.6051015359648e-42 7 RPS19 GC
4.34587065359381e-46 0.321746801553525 0.626 0.422
7.74825278829241e-42 7 KTN1 GC
4.53505989026911e-46 0.384021308751269 0.21 0.077
8.08555827836079e-42 7 GPR18 GC
7.30702342513082e-46 0.38768229087823 0.36 0.191
1.30276920646657e-41 7 ETFB GC
7.48399180096137e-46 0.336751427238002 0.732 0.538
1.3343208981934e-41 7 UBE2D2 GC
9.9826582058963e-46 0.378152071143803 0.47 0.301
1.77980813152925e-41 7 FERMT3 GC
1.31193797332152e-45 0.397300279736104 0.403 0.222
2.33905421263495e-41 7 NGLY1 GC
1.33036717893331e-45 0.389075166782141 0.512 0.311
2.3719116433202e-41 7 PRDX2 GC
1.39550078668207e-45 0.359390417705696 0.526 0.337
2.48803835257546e-41 7 SNX17 GC
1.51756286982178e-45 -0.599342473568851 0.329 0.455
2.70566284060526e-41 7 DRAP1 GC
1.61192344772958e-45 -0.522795006189631 0.085 0.198
2.87389831495706e-41 7 HSPB1 GC
1.71866385888283e-45 0.321501218279599 0.162 0.054
3.0642057940022e-41 7 KIF3A GC
1.85702704224676e-45 -0.473119535122275 0.981 0.974
3.31089351362175e-41 7 MT-ND4 GC
2.68653010710502e-45 -0.926162519888831 0.524 0.579
4.78981452795755e-41 7 TUBB GC
2.89273847591901e-45 0.392343985908462 0.348 0.166

5.157463428716e-41	7	BLCAP	GC		
3.72143808695644e-45	0.365953884823488			0.189	0.07
6.63495196523463e-41	7	PAG1	GC		
3.99120811149656e-45	0.328816852280351			0.176	0.063
7.11592494198722e-41	7	ST20	GC		
4.57325113720494e-45	0.329253414355542			0.687	0.488
8.15364945252268e-41	7	ARGLU1	GC		
5.31485436180842e-45	0.370528777704408			0.666	0.448
9.47585384166823e-41	7	COX17	GC		
8.01163424809568e-45	0.403900416674742			0.372	0.179
1.42839427009298e-40	7	CDCA7L	GC		
9.26777381305734e-45	0.381657715983346			0.407	0.223
1.65235139312999e-40	7	OAZ2	GC		
1.04152369598166e-44	0.407389365200924			0.47	0.27
1.85693259756569e-40	7	PIM2	GC		
1.13771530643423e-44	-0.451138169389978			0.019	0.112
2.02843261984159e-40	7	SNX9	GC		
1.81819116361187e-44	-0.640486054208785			0.21	0.334
3.24165302560361e-40	7	SMC6	GC		
2.52626530175056e-44	0.423325939093518			0.399	0.199
4.50407840649107e-40	7	C16orf74	GC		
2.73470495845161e-44	0.421884233829735			0.349	0.183
4.87570547042337e-40	7	RFC1	GC		
4.89055476934328e-44	0.317960550999402			0.688	0.495
8.71937009826214e-40	7	NDUFA11	GC		
4.96435446963303e-44	0.388132842749008			0.475	0.29
8.85094758390872e-40	7	WASF2	GC		
7.6701331041018e-44	0.422085898587304			0.397	0.217
1.36750803113031e-39	7	SIAH2	GC		
9.44909408342744e-44	-0.457413881279178			0.021	0.104
1.68467898413428e-39	7	RHOC	GC		
1.22306978546365e-43	0.354743925884358			0.406	0.233
2.18061112050315e-39	7	CPSF6	GC		
1.40264561586924e-43	0.371587316144561			0.558	0.37
2.50077686853327e-39	7	SUPT4H1	GC		
1.96264887588268e-43	0.414380633893536			0.376	0.222
3.49920668081123e-39	7	DDX54	GC		
2.20614108323118e-43	0.338011809839141			0.21	0.09
3.93332893729287e-39	7	TUT7	GC		
3.99077276117206e-43	0.318148632627326			0.148	0.045
7.11514875589366e-39	7	SYNE1	GC		
4.26829158594048e-43	0.397336738246555			0.404	0.226
7.60993706857329e-39	7	AKAP13	GC		
4.67618391440495e-43	0.350384954129561			0.575	0.381
8.33716830099259e-39	7	CUTA	GC		
4.94423879407922e-43	0.315573931853997			0.869	0.729
8.81508334596384e-39	7	SKP1	GC		
5.41397879778072e-43	0.367570133012683			0.243	0.099
9.65258279856325e-39	7	RHBDF2	GC		
6.36377898466053e-43	0.397943905288703			0.488	0.301

1.13459815517513e-38	7	FKBP3	GC
6.41839966004198e-43	-0.62485330448701	0.24	0.367
1.14433647538889e-38	7	ANXA6	GC
9.73388898849614e-43	0.356352068189457	0.221	0.083
1.73545506775898e-38	7	VEZT	GC
9.94116849133471e-43	0.362745236663843	0.496	0.304
1.77241093032006e-38	7	MZT2A	GC
1.13219294623928e-42	0.353255615612667	0.291	0.14
2.01858680385001e-38	7	PTEN	GC
1.27417738947512e-42	0.327626345323308	0.115	0.031
2.27173086769519e-38	7	AC012368.1	GC
1.6998379418353e-42	-0.56334655235427	0.121	0.246
3.03064106649815e-38	7	HLA-F	GC
1.75136699742161e-42	0.369824407215794	0.383	0.209
3.12251221970299e-38	7	DMAC1	GC
1.8645014310989e-42	0.384895884579701	0.396	0.221
3.32421960150623e-38	7	GTF2I	GC
1.96613299637715e-42	-0.370563394243051	0.014	0.101
3.50541851924083e-38	7	PARVB	GC
2.26132277345006e-42	0.379128386472874	0.498	0.276
4.03171237278412e-38	7	CARHSP1	GC
2.8332234419975e-42	0.395930499719568	0.61	0.402
5.05135407473734e-38	7	SEPT6	GC
3.90097542647532e-42	0.370059787127144	0.474	0.302
6.95504908786284e-38	7	MMADHC	GC
5.14217084347683e-42	0.374179769880772	0.294	0.142
9.16797639683485e-38	7	ZNF580	GC
1.03935673171495e-41	0.401292149821101	0.432	0.247
1.85306911697458e-37	7	MPC2	GC
1.09517459448295e-41	0.42738148083588	0.249	0.113
1.95258678450365e-37	7	TBC1D1	GC
1.2269751908807e-41	0.412788946279988	0.392	0.235
2.1875740678212e-37	7	NSRP1	GC
1.94697091160627e-41	0.360657086972295	0.528	0.338
3.47125443830281e-37	7	POLR2J	GC
2.21036889477632e-41	0.323398657373643	0.193	0.078
3.94086670249671e-37	7	BPNT1	GC
2.2336368059483e-41	0.339647735200676	0.21	0.084
3.98235106132523e-37	7	TPST2	GC
2.25528426739041e-41	0.34392485074736	0.144	0.034
4.02094632033035e-37	7	MILR1	GC
2.73803531186891e-41	0.304989919913942	0.174	0.063
4.88164315753107e-37	7	SYT17	GC
4.97900443642434e-41	0.346472786353626	0.239	0.107
8.87706700970095e-37	7	HPS1	GC
8.5704319306609e-41	0.316751447463402	0.186	0.074
1.52802230891753e-36	7	FAM45A	GC
1.02191772298873e-40	0.315484714822196	0.163	0.064
1.82197710831661e-36	7	FUCA1	GC
1.40044024042422e-40	0.406156213037364	0.417	0.246

2.49684490465235e-36	7	RGS10	GC
1.43183224653053e-40	0.3553500754813	0.331	0.187
2.55281371233928e-36	7	FYTTD1	GC
1.46533832971133e-40	0.346110976555274	0.191	0.077
2.61255170804232e-36	7	VGLL4	GC
1.48654308251334e-40	0.369435383471556	0.651	0.441
2.65035766181303e-36	7	HIGD2A	GC
1.53863669207276e-40	0.355380797541579	0.435	0.262
2.74323535829653e-36	7	STMP1	GC
1.82521433179619e-40	0.393965807598172	0.172	0.071
3.25417463215942e-36	7	ADAM17	GC
1.87629447732898e-40	0.373179258330615	0.315	0.162
3.34524542362984e-36	7	TFDP1	GC
2.37099865383314e-40	-0.680380085534953	0.346	0.452
4.2272534999191e-36	7	PNRC1	GC
2.48639456144185e-40	-0.416287211113437	0.989	0.991
4.43299286359467e-36	7	RPL30	GC
4.42611002034981e-40	0.333691533689002	0.251	0.117
7.89131155528168e-36	7	NOA1	GC
4.76084267314335e-40	0.599221206612919	0.95	0.797
8.48810640194727e-36	7	RPS26	GC
4.9446635506599e-40	0.364437902235839	0.442	0.252
8.81584064447153e-36	7	ILK	GC
5.80722083274287e-40	0.332839659650247	0.468	0.278
1.03536940226973e-35	7	YPEL5	GC
6.82665083282253e-40	0.364650367383836	0.267	0.122
1.21712357698393e-35	7	SASH3	GC
7.36956111031885e-40	0.351300719359146	0.479	0.309
1.31391905035875e-35	7	WDR1	GC
8.62208135178409e-40	-0.363236087111884	0.99	0.993
1.53723088420959e-35	7	RPL35	GC
9.27672332154379e-40	0.330401773331064	0.192	0.078
1.65394700099804e-35	7	N4BP3	GC
1.58264314654171e-39	0.342600043303234	0.194	0.076
2.82169446596922e-35	7	SLC35E3	GC
2.0452558517112e-39	0.302234127850666	0.682	0.483
3.6464866580159e-35	7	SF3B6	GC
3.00542572244514e-39	0.340591144205655	0.401	0.237
5.35837352054744e-35	7	ETFA	GC
3.02910533466792e-39	0.328890109121184	0.592	0.375
5.40059190117944e-35	7	EVL	GC
3.20246798936234e-39	0.306481113180886	0.485	0.306
5.70968017823412e-35	7	LPXN	GC
4.42730090000596e-39	0.372924219357301	0.228	0.092
7.89343477462063e-35	7	CCDC28B	GC
6.91461843790404e-39	0.345321865666775	0.454	0.269
1.23280732129391e-34	7	QARS	GC
7.77346586349423e-39	0.356083614023782	0.486	0.289
1.38593122880239e-34	7	IFT57	GC
9.88065323314445e-39	0.357802643428358	0.508	0.313

1.76162166493732e-34	7	AP3S1	GC		
9.90560219198105e-39	-0.717019775219829			0.357	0.483
1.7660698148083e-34	7	SQSTM1	GC		
1.06297860828595e-38	0.333279293451615			0.449	0.246
1.89518456071303e-34	7	TBC1D10C	GC		
1.42353619830564e-38	0.311493436514208			0.585	0.397
2.53802268795912e-34	7	PSMB2	GC		
1.59382925360815e-38	0.36209317891826		0.376	0.211	
2.84163817625796e-34	7	IFI27L2	GC		
1.75941505634004e-38	-0.64633730041006			0.389	0.496
3.13686110394867e-34	7	ANKRD12	GC		
1.85257981922554e-38	0.445045954249209			0.32	0.152
3.30296455969721e-34	7	KIAA0040	GC		
1.89993175903474e-38	0.333652747148928			0.36	0.201
3.38738833318304e-34	7	MRPS14	GC		
1.95071879623785e-38	0.345811241953867			0.26	0.127
3.47793654181245e-34	7	PAPSS1	GC		
1.99176401523206e-38	-0.79986306207714			0.213	0.316
3.55111606275724e-34	7	HSP90B1	GC		
2.85831587254995e-38	0.375651893600272			0.696	0.483
5.09609136916931e-34	7	RAC2	GC		
2.95572828760821e-38	0.317060359998938			0.381	0.222
5.26976796397667e-34	7	SINHCAF	GC		
3.00423934645759e-38	0.341467179855967			0.305	0.164
5.35625833079923e-34	7	MTX1	GC		
3.01942178948017e-38	0.314507599543841			0.415	0.257
5.3833271084642e-34	7	CD2BP2	GC		
3.16406297407548e-38	0.352931771521912			0.335	0.185
5.64120787647917e-34	7	NDUFA8	GC		
3.55532504057621e-38	0.342742983706898			0.475	0.286
6.33878901484333e-34	7	BCL11A	GC		
6.15952006537094e-38	0.303750632126944			0.709	0.51
1.09818083245498e-33	7	COX8A	GC		
8.10960355355956e-38	0.317271711970015			0.622	0.418
1.44586121756413e-33	7	TAF7	GC		
8.77049910097911e-38	0.328011504772512			0.421	0.251
1.56369228471357e-33	7	UPF3A	GC		
9.21766220315238e-38	0.302542927446586			0.65	0.471
1.64341699420004e-33	7	GRB2	GC		
5.39786003693965e-37	-0.558873069004457			0.682	0.755
9.62384465985971e-33	7	HLA-C	GC		
5.52897720348361e-37	0.315884093650685			0.662	0.486
9.85761345609093e-33	7	THRAP3	GC		
5.85403429541711e-37	-0.490732544197104			0.122	0.223
1.04371577452992e-32	7	CCDC50	GC		
6.10968189769629e-37	-0.352865893656992			0.999	0.999
1.08929518554027e-32	7	EEF1A1	GC		
6.53843470656633e-37	0.334001097442421			0.483	0.303
1.16573752383371e-32	7	SCP2	GC		
7.76487510303047e-37	0.314035100787294			0.136	0.047

1.3843995821193e-32 7 CPM GC
7.81622964399267e-37 0.319056309721453 0.57 0.408
1.39355558322745e-32 7 HCLS1 GC
7.82515955180483e-37 0.32977857961258 0.294 0.16
1.39514769649128e-32 7 TOR3A GC
7.8544880162862e-37 0.309244710651627 0.33 0.173
1.40037666842367e-32 7 TMC6 GC
1.0272858085702e-36 0.381281932682719 0.822 0.618
1.83154786809981e-32 7 HLA-DMB GC
1.10145462591854e-36 0.349000891461788 0.765 0.596
1.96378345255017e-32 7 LSM7 GC
1.21117351112563e-36 0.345154671788998 0.3 0.153
2.15940125298589e-32 7 APLP2 GC
1.9344304885814e-36 -0.597390875994838 0.22 0.332
3.44889611809178e-32 7 PTPN6 GC
2.10044417724777e-36 0.324512712364858 0.343 0.197
3.74488192361505e-32 7 URM1 GC
3.1765120007277e-36 0.33734785231527 0.448 0.278
5.66340324609741e-32 7 RTF2 GC
4.29012927412275e-36 0.345770118207163 0.284 0.15
7.64887148283345e-32 7 SVBP GC
4.99326758846633e-36 -0.599630540060748 0.155 0.241
8.90249678347662e-32 7 CD55 GC
5.17020339053834e-36 -0.429481860837408 0.933 0.934
9.2179556249908e-32 7 MT-ATP6 GC
6.07772312586334e-36 -0.437597725067735 0.024 0.108
1.08359725611018e-31 7 MTSS1 GC
6.12507427399646e-36 0.344176751133109 0.249 0.128
1.09203949231083e-31 7 SULF2 GC
6.72580608217849e-36 0.336225520115137 0.439 0.251
1.1991439663916e-31 7 CNN2 GC
6.82335196514102e-36 0.317600402875486 0.448 0.281
1.21653542186499e-31 7 NDUFB5 GC
1.08024286773769e-35 0.336266089446786 0.41 0.238
1.92596500888953e-31 7 TMEM134 GC
1.77154868756057e-35 0.344324867547256 0.368 0.2
3.15849415505174e-31 7 IKZF3 GC
2.46121764005975e-35 0.306014549338911 0.207 0.094
4.38810493046253e-31 7 RAB30-AS1 GC
3.5773352188575e-35 0.359826364456692 0.313 0.176
6.37803096170104e-31 7 MRPS33 GC
4.62923560060865e-35 0.307493243858784 0.357 0.216
8.25346415232517e-31 7 PSMD9 GC
5.1796137236531e-35 0.349831459620772 0.408 0.238
9.2347333079011e-31 7 DCTN3 GC
5.40185589986424e-35 0.390742957607936 0.29 0.134
9.63096888386796e-31 7 MKNK2 GC
5.67425154467292e-35 -0.557381668124545 0.571 0.638
1.01166230789973e-30 7 GPX4 GC
7.10468096739358e-35 0.334424534829286 0.251 0.135

1.2666935696766e-30 7 MRPS27 GC
9.85428970955403e-35 0.326170858538395 0.53 0.36
1.75692131231639e-30 7 RPS19BP1 GC
1.01784175741222e-34 0.304154850043155 0.222 0.109
1.81471006929025e-30 7 COA1 GC
1.11487225045128e-34 0.314566515761691 0.509 0.331
1.98770573532959e-30 7 HSD17B10 GC
1.22925880514087e-34 -0.386962896084144 0.026 0.118
2.19164552368565e-30 7 PYCR1 GC
1.8729008003935e-34 0.30311562376178 0.47 0.302
3.33919483702158e-30 7 PSMD6 GC
1.91022591170905e-34 0.320187054406065 0.223 0.105
3.40574177798606e-30 7 RAB11FIP1 GC
2.01890550384889e-34 0.31648727485916 0.33 0.195
3.59950662281219e-30 7 NDUFA7 GC
2.07497602896614e-34 0.310973737164934 0.485 0.309
3.69947476204373e-30 7 SFT2D1 GC
2.65425835483157e-34 0.309581043800369 0.406 0.241
4.7322772208292e-30 7 OTUB1 GC
2.66793318189891e-34 0.324696454672375 0.417 0.259
4.75665807000756e-30 7 MTCH2 GC
2.73835093350179e-34 0.320996314959866 0.29 0.156
4.88220587934034e-30 7 DCPS GC
2.99916023786363e-34 -0.560719827138957 0.052 0.14
5.34720278808706e-30 7 FCER2 GC
3.01262580053873e-34 0.328249158631793 0.612 0.439
5.3712105397805e-30 7 NDUFB10 GC
3.38759960425715e-34 0.357679605118506 0.186 0.068
6.03975133443008e-30 7 LINC01857 GC
3.6433062965212e-34 0.309982182995607 0.573 0.396
6.49565079606765e-30 7 MDH2 GC
4.10806572065805e-34 0.313774443419705 0.538 0.363
7.32427037336123e-30 7 FAM96B GC
5.547968401259e-34 -0.397558481002133 0.037 0.12
9.89147286260467e-30 7 SLAMF1 GC
5.91396750502564e-34 0.356896028453248 0.263 0.124
1.05440126647102e-29 7 ETS1 GC
6.92300427995414e-34 0.430815067690566 0.224 0.087
1.23430243307302e-29 7 FCRL2 GC
7.76900179822828e-34 0.323279914095458 0.522 0.34
1.38513533060612e-29 7 RBM17 GC
9.50533449838987e-34 0.330177113785509 0.524 0.347
1.69470608771793e-29 7 DGUOK GC
9.93977607520734e-34 -0.320658921074218 0.993 0.993
1.77216267644872e-29 7 RPS5 GC
1.00278237920962e-33 0.315441471809001 0.385 0.228
1.78786070389283e-29 7 MRPL27 GC
1.14912755577191e-33 0.314035441375286 0.232 0.115
2.04877951918574e-29 7 SS18 GC
1.1989187736641e-33 -0.432215362842792 0.039 0.121

2.13755228156572e-29 7 FCGR2B GC
1.66011247931191e-33 0.32380495337912 0.338 0.187
2.95981453936521e-29 7 RFXANK GC
2.32370537448398e-33 0.314850500257155 0.47 0.307
4.1429343121675e-29 7 ZNF207 GC
2.81125685456674e-33 0.319514035535509 0.247 0.12
5.01218984600703e-29 7 ARL3 GC
2.82299508089087e-33 0.303685830631694 0.274 0.144
5.03311792972032e-29 7 MRPL10 GC
2.82308383546983e-33 0.319046811278863 0.374 0.22
5.03327617025916e-29 7 SREK1 GC
2.90198682690931e-33 0.316085213042103 0.606 0.415
5.1739523136966e-29 7 ECH1 GC
3.68462514132227e-33 0.329742953653442 0.498 0.317
6.56931816446348e-29 7 BLNK GC
5.87453643194213e-33 0.342931531324693 0.419 0.25
1.04737110045096e-28 7 LYSMD2 GC
6.96027484679921e-33 0.328335280355311 0.512 0.34
1.24094740243583e-28 7 LAMTOR2 GC
8.03180645765612e-33 0.32461193654308 0.208 0.095
1.43199077333551e-28 7 TACC1 GC
1.22403187935213e-32 -0.376748299024178 0.99 0.993
2.18232643769691e-28 7 RPL36 GC
1.24858687616938e-32 -0.525704591923633 0.224 0.309
2.22610554152238e-28 7 ARHGAP24 GC
1.29047338367048e-32 -0.643546242747264 0.184 0.282
2.3007849957461e-28 7 ADAM28 GC
2.27187578589567e-32 0.301831000645085 0.505 0.338
4.05052733867339e-28 7 MRPS34 GC
2.27425578911961e-32 -0.532332440568302 0.061 0.161
4.05477064642135e-28 7 SLC2A3 GC
2.2988730283796e-32 0.342945430618658 0.352 0.201
4.09866072229799e-28 7 EBLN3P GC
3.83193123637693e-32 -0.368840665428242 0.048 0.123
6.83195020133643e-28 7 IL27RA GC
4.2876977877197e-32 0.397447535773994 0.245 0.111
7.64453638572544e-28 7 GMDS GC
5.53632185567284e-32 0.30585926530968 0.479 0.328
9.87070823647911e-28 7 NDUFS7 GC
9.90213616021805e-32 0.313238861058775 0.452 0.274
1.76545185600528e-27 7 JPT1 GC
9.95544947469503e-32 0.300169951163373 0.204 0.095
1.77495708684338e-27 7 KLC1 GC
1.89662703705066e-31 0.303821236332588 0.485 0.311
3.38149634435762e-27 7 SMIM26 GC
1.93155356167194e-31 0.315812393786768 0.401 0.24
3.4437668451049e-27 7 NBDY GC
2.037783608292e-31 0.305532086959855 0.322 0.19
3.6331643952238e-27 7 INTS11 GC
2.11566365292949e-31 -0.525663150860962 0.224 0.333

3.77201672680798e-27 7 TRBC2 GC
2.33136575245276e-31 0.312557370088625 0.48 0.327
4.15659200004802e-27 7 BAX GC
3.09951574372763e-31 0.337504972847176 0.28 0.138
5.52612661949198e-27 7 RAB30 GC
3.40308642334776e-31 0.348059329181783 0.495 0.302
6.06736278418671e-27 7 CALM3 GC
3.51574715174972e-31 0.333841136722844 0.211 0.097
6.26822559685458e-27 7 AC084033.3 GC
4.39568878266233e-31 0.414597917370102 0.104 0.024
7.83707353060867e-27 7 TCL1B GC
5.51626388144829e-31 -0.374526046134555 0.034 0.118
9.83494687423415e-27 7 ENTPD1 GC
5.9300966901272e-31 0.340587460363434 0.221 0.1
1.05727693888278e-26 7 SCIMP GC
7.1524869803925e-31 -0.420888853759188 0.068 0.151
1.27521690373418e-26 7 SVIP GC
8.04707182395676e-31 0.326357086662391 0.308 0.178
1.43471243549325e-26 7 MAP2K2 GC
9.57802953242513e-31 0.329541256330243 0.37 0.223
1.70766688533608e-26 7 HDDC2 GC
1.55286767346552e-30 0.366226848025338 0.51 0.306
2.76860777502168e-26 7 HMGN3 GC
2.07337178873108e-30 0.311933350123505 0.431 0.275
3.69661456212864e-26 7 BABAM1 GC
2.47148441108012e-30 0.316040717018042 0.205 0.096
4.40640955651474e-26 7 PTPN7 GC
2.59058224348566e-30 0.301102011239319 0.298 0.172
4.61874908191059e-26 7 LAP3 GC
2.72581946803593e-30 -0.553713921127222 0.252 0.34
4.85986352956126e-26 7 CD48 GC
2.92063499986024e-30 -0.487556446900825 0.165 0.249
5.20720014125082e-26 7 CKS1B GC
4.15839966842475e-30 0.30844491923084 0.464 0.3
7.41401076883448e-26 7 OCIAD2 GC
5.52484842337807e-30 0.319590989033991 0.296 0.171
9.85025225404076e-26 7 EIF4E2 GC
6.9029086992041e-30 0.334007015384761 0.16 0.059
1.2307195919811e-25 7 HOPX GC
9.37945381614425e-30 0.305857648369682 0.491 0.327
1.67226282088036e-25 7 DARS GC
1.43829384156781e-29 0.30151663857827 0.501 0.343
2.56433409013125e-25 7 DDX17 GC
2.6532028463981e-29 0.307443510816175 0.35 0.189
4.73039535484317e-25 7 AFF3 GC
3.34490702809913e-29 -0.528382876521587 0.71 0.759
5.96363474039794e-25 7 MT-ND5 GC
4.56673132908189e-29 0.308399541172639 0.37 0.213
8.14202528662011e-25 7 GNAI2 GC
4.97835333759094e-29 0.322107469640073 0.264 0.137

8.87590616559088e-25	7	WDR18	GC		
5.34614009809551e-29	-0.583643423809776			0.237	0.305
9.53163318089449e-25	7	ZFP36L2	GC		
5.54961067947439e-29	-0.453767843569982			0.165	0.255
9.89440088043489e-25	7	DNPH1	GC		
9.77984447031008e-29	-0.603537289033592			0.591	0.65
1.74364847061158e-24	7	ZFP36L1	GC		
1.05541143950535e-28	0.307016554735075			0.291	0.169
1.88169305549409e-24	7	AKIRIN2	GC		
1.20330332911584e-28	-0.407537018878164			0.967	0.965
2.14536950548063e-24	7	RPL38	GC		
1.6969069254006e-28	-0.667994224011284			0.213	0.331
3.02541535729673e-24	7	SLC3A2	GC		
1.74225899359584e-28	0.378774009258864			0.33	0.169
3.10627355968202e-24	7	QRSL1	GC		
1.75356981619129e-28	-0.452176019028573			0.106	0.205
3.12643962528745e-24	7	NPM3	GC		
3.82965193057562e-28	0.304194904599806			0.669	0.478
6.82788642702327e-24	7	GMFG	GC		
5.49667164811476e-28	-0.598427529749435			0.467	0.532
9.80001588142381e-24	7	PSME2	GC		
9.47942693591699e-28	0.353076898565535			0.375	0.234
1.69008702840464e-23	7	ZBTB8OS	GC		
1.20803939706854e-27	-0.386298471804154			0.05	0.126
2.15381344103351e-23	7	RAB13	GC		
1.39102490019397e-27	0.300004429987485			0.228	0.109
2.48005829455584e-23	7	PAX5	GC		
1.70172510196497e-27	0.316048878561009			0.314	0.171
3.03400568429334e-23	7	RGS19	GC		
2.43589469522669e-27	-0.468162094871442			0.125	0.229
4.34295665211967e-23	7	TMEM109	GC		
3.46763931991439e-27	0.304283732712499			0.457	0.278
6.18245414347536e-23	7	SP110	GC		
3.62062073570398e-27	-0.608944110206936			0.208	0.299
6.45520470968662e-23	7	NFKBIA	GC		
1.61272985664683e-26	0.321270048357149			0.386	0.241
2.87533606141564e-22	7	TRAPPC2L	GC		
1.75225553225792e-26	-0.520234618880155			0.354	0.441
3.12409638846265e-22	7	IL2RG	GC		
5.61164996378645e-26	-0.336683042062128			0.98	0.981
1.00050107204349e-21	7	RPSA	GC		
6.89668439991152e-26	-0.46464771134557			0.205	0.297
1.22960986166022e-21	7	ACADVL	GC		
7.80546371876464e-26	-0.361972139231036			0.964	0.959
1.39163612641855e-21	7	MT-CYB	GC		
1.10978561642349e-25	-0.475899063861331			0.231	0.3
1.97863677552144e-21	7	PDLIM1	GC		
1.5372164023832e-25	-0.442798238522128			0.975	0.968
2.740703123809e-21	7	RPS17	GC		
1.94715891558687e-25	-0.413831117079951			0.135	0.193

3.47158963059983e-21 7 S1PR4 GC
5.54562465158695e-25 -0.421440799936045 0.102 0.188
9.88729419131438e-21 7 FKBP4 GC
5.99700298275063e-25 -0.498174990114949 0.116 0.199
1.06920566179461e-20 7 CHPT1 GC
7.10474819406874e-25 -0.30630578822288 0.999 0.998
1.26670555552052e-20 7 RPL18A GC
9.51374755845087e-25 -0.554391418861927 0.243 0.331
1.69620605219621e-20 7 ZFP36 GC
4.64586941063413e-24 -0.357474994896785 0.041 0.1
8.28312057221959e-20 7 BTLA GC
1.83591257152475e-23 -0.531865959564548 0.39 0.455
3.27324852377149e-19 7 ARL6IP1 GC
3.10670979712216e-23 -0.402054466553493 0.119 0.197
5.53895289728909e-19 7 ADK GC
3.23284701269287e-23 0.311609250214681 0.416 0.265
5.76384293893012e-19 7 TXNL4A GC
4.50265822876363e-23 0.303584835209508 0.522 0.36
8.02778935606268e-19 7 CXXC5 GC
6.99649068149796e-23 0.311798858035522 0.475 0.291
1.24740432360427e-18 7 TCF4 GC
7.48526091155749e-23 -0.418302780638662 0.24 0.328
1.33454716792158e-18 7 P4HB GC
1.63878901034514e-22 -0.572712289865882 0.276 0.34
2.92179692654435e-18 7 HVCN1 GC
2.11625021143607e-22 -0.475152654130298 0.112 0.2
3.77306250196936e-18 7 TYMP GC
4.08600216382606e-22 -0.406775229190524 0.118 0.189
7.28493325788548e-18 7 KIF20B GC
4.08666426617132e-22 -0.463464903075343 0.303 0.387
7.28611372015684e-18 7 LAPTM4A GC
4.91519040585298e-22 -0.441032873722778 0.055 0.139
8.76329297459529e-18 7 PSAT1 GC
6.8499763629347e-21 -0.445392510657586 0.154 0.231
1.22128228574763e-16 7 NT5C3A GC
1.12251076332481e-20 -0.422601483247309 0.122 0.192
2.00132443993181e-16 7 IFNGR1 GC
1.32528282803844e-20 -0.449718811678018 0.14 0.235
2.36284675410973e-16 7 TARS GC
1.36834269134157e-20 -0.373193910165664 0.059 0.107
2.43961818439289e-16 7 C1orf162 GC
1.53353658705007e-20 -0.327437537123696 0.073 0.151
2.73414238105157e-16 7 TSEN15 GC
1.54031476242266e-20 -0.421452131865882 0.174 0.234
2.74622718992337e-16 7 HSD17B11 GC
1.78744689340755e-20 -0.413389906969218 0.135 0.19
3.18683906625632e-16 7 PCNA GC
2.17765295133565e-20 -0.650371691397793 0.573 0.598
3.88253744693634e-16 7 LDHA GC
2.58231209634336e-20 -0.478301605940831 0.196 0.285

4.60400423657058e-16	7	MT-ND4L	GC
3.16974902305098e-20	-0.385318544702153	0.118	0.207
5.6513455331976e-16	7	NDFIP1	GC
1.09207381068147e-19	-0.438774888367664	0.274	0.351
1.947058397064e-15	7	LITAF	GC
1.13689909957833e-19	0.400372806563834	0.217	0.106
2.02697740463821e-15	7	CD9	GC
1.92146522482994e-19	-0.492264260184876	0.421	0.469
3.42578034934931e-15	7	ITM2B	GC
1.97994288428478e-19	-0.399117830923348	0.108	0.177
3.53004016839134e-15	7	ASAH1	GC
5.19360170338922e-19	-0.68438666054584	0.48	0.5
9.25967247697264e-15	7	SSR4	GC
3.92909524304734e-18	-0.355364443789783	0.062	0.125
7.00518390882911e-14	7	DDAH2	GC
5.26167904933858e-18	-0.416218078712299	0.225	0.309
9.38104757706576e-14	7	TMEM147	GC
6.35439860774002e-18	-0.317777090891407	0.085	0.148
1.13292572777397e-13	7	PRDX4	GC
6.41318667456297e-18	-0.521400236526457	0.252	0.349
1.14340705220783e-13	7	CAST	GC
8.19462277044776e-18	-0.511945759222573	0.181	0.283
1.46101929374313e-13	7	WARS	GC
1.75549348262211e-17	-0.476150243532026	0.469	0.535
3.12986933016696e-13	7	IMPDH2	GC
2.67955688759317e-17	-0.626999578023736	0.076	0.133
4.77738197488986e-13	7	MYC	GC
2.94175197387962e-17	-0.4601823624469	0.423	0.483
5.24484959422998e-13	7	ISCU	GC
3.9642003236823e-17	-0.453524818483835	0.174	0.213
7.06777275709317e-13	7	LBH	GC
6.11534347672347e-17	0.30951903203743	0.595	0.413
1.09030458846503e-12	7	AC114760.2	GC
1.0611896383149e-16	-0.368298443446549	0.145	0.207
1.89199500615164e-12	7	AP1S2	GC
1.16825315120419e-16	-0.431802735832216	0.849	0.855
2.08287854328195e-12	7	SH3BGRL3	GC
1.74781324520143e-16	-0.319309002411005	0.073	0.119
3.11617623486962e-12	7	SAMD9	GC
3.68977502454784e-16	-0.506822187955961	0.192	0.258
6.57849989126635e-12	7	SRGN	GC
3.74857896884413e-16	-0.443753058423522	0.967	0.978
6.6833414435522e-12	7	RPS10	GC
4.193188740398e-16	-0.399920053545072	0.107	0.173
7.47603620525559e-12	7	PARP14	GC
7.20359805864675e-16	-0.524598196466286	0.341	0.211
1.28432949787613e-11	7	IGHG1	GC
8.09662485763711e-16	-0.404226699025364	0.237	0.281
1.44354724586812e-11	7	RNASET2	GC
8.37693934890406e-16	-0.468599250080092	0.088	0.163

1.49352451651611e-11	7	DDIT4	GC	
9.49393092739213e-16	-0.379776914622971		0.099	0.149
1.69267294504474e-11	7	ARRDC2	GC	
1.49799276246791e-15	-0.677694979886916		0.435	0.473
2.67077129620404e-11	7	CD83	GC	
1.56998495392623e-15	-0.431047820110872		0.068	0.128
2.79912617435508e-11	7	LGALS3	GC	
1.7605883886225e-15	-0.378460663506361		0.184	0.254
3.13895303807505e-11	7	NFE2L2	GC	
1.82819921787692e-15	-0.445186517702014		0.677	0.697
3.25949638555277e-11	7	HNRNPDL	GC	
2.63570001430058e-15	-0.426446804211037		0.29	0.338
4.69918955549651e-11	7	S100A11	GC	
8.12568771275857e-15	-0.34021086930718		0.095	0.159
1.44872886230772e-10	7	NOLC1	GC	
8.83171823136217e-15	-0.328869677712607		0.11	0.16
1.57460704346956e-10	7	TGIF1	GC	
1.57730792585078e-14	-0.364618888633674		0.982	0.976
2.81218230099935e-10	7	RPL36A	GC	
7.11696635069817e-14	-0.389598591992864		0.439	0.477
1.26888393066598e-09	7	N4BP2L2	GC	
8.08409003369858e-14	-0.466211654184337		0.154	0.22
1.44131241210812e-09	7	EIF4EBP1	GC	
1.24225752791075e-13	-0.362794214548509		0.743	0.753
2.21482094651208e-09	7	TOMM7	GC	
2.39615334548589e-13	-0.334650712656381		0.128	0.199
4.27210179966679e-09	7	AARS	GC	
3.22441505508942e-13	-0.425770595033022		0.31	0.364
5.74880960171893e-09	7	HSPA9	GC	
3.56378122857147e-13	-0.445489935609504		0.237	0.282
6.35386555242008e-09	7	SRM	GC	
4.09611946696549e-13	-0.3971530373065	0.284	0.344	
7.30297139765278e-09	7	PKIG	GC	
5.34209812674848e-13	-0.303369244137959		0.144	0.201
9.52442675017986e-09	7	NENF	GC	
6.55565747586998e-13	-2.15193420112187		0.63	0.443
1.16880817137286e-08	7	IGHG3	GC	
7.96475057827908e-13	-0.388164845891362		0.303	0.339
1.42003538060138e-08	7	IER2	GC	
1.09070963450646e-12	-0.318820441552175		0.143	0.191
1.94462620736156e-08	7	CWF19L2	GC	
1.21852031212327e-12	-0.403327232740779		0.106	0.168
2.17249986448457e-08	7	CBX6	GC	
1.38533497018356e-12	-0.327343211313887		0.128	0.184
2.46991371834026e-08	7	CMSS1	GC	
2.7933802441585e-12	-0.348411985106111		0.11	0.146
4.98031763731019e-08	7	SUN2	GC	
3.76733422313246e-12	-0.312412775966991		0.261	0.299
6.71678018642287e-08	7	MPHOSPH8	GC	
6.38020776117533e-12	-0.327095685363387		0.167	0.219

1.13752724173995e-07 7 NFKBIE GC
1.67489503858215e-11 -0.340353379278459 0.103 0.165
2.98617036428811e-07 7 CD58 GC
1.92712199731549e-11 -0.330471628272964 0.147 0.205
3.43586580901378e-07 7 PTPN1 GC
3.21893515758512e-11 -0.49556555722635 0.308 0.362
5.73903949245851e-07 7 ARID5B GC
6.04124969312851e-11 -0.347163212535956 0.22 0.265
1.07709440778788e-06 7 TTC3 GC
9.04455704014901e-11 -0.655925746306461 0.447 0.428
1.61255407468817e-06 7 IGLC3 GC
1.24559707954741e-10 -0.357656237775172 0.36 0.39
2.22077503312507e-06 7 ARL6IP5 GC
5.58020732796098e-10 -0.307214339364833 0.267 0.299
9.94895164502162e-06 7 RNASEH2B GC
9.65034135745872e-10 -0.394002309521911 0.417 0.445
1.72055936062132e-05 7 ANXA2 GC
1.1731505350235e-09 -0.494981565954984 0.495 0.484
2.09161008889339e-05 7 RANBP1 GC
2.49178615798716e-09 -0.333910768265198 0.145 0.192
4.44260554107531e-05 7 RNF145 GC
4.63345503888431e-09 -0.357085713599044 0.169 0.21
8.26098698882684e-05 7 PIM3 GC
6.78409747734027e-09 -0.344200449389926 0.351 0.395
0.0001209536739235 7 TMED2 GC
8.76708047044595e-09 -0.319255954956998 0.157 0.184
0.000156308277707581 7 MBP GC
8.87884463465121e-09 -0.369041658035198 0.815 0.815
0.000158300920991196 7 NOP53 GC
2.76244385384116e-08 -0.34814166931123 0.832 0.817
0.000492516114701341 7 SEPT7 GC
3.04744886845864e-08 -0.348981413947068 0.37 0.403
0.000543329658757492 7 CMPK1 GC
1.05383588299749e-07 -0.402342429265526 0.42 0.446
0.00187888399579622 7 KLF6 GC
4.4272848373439e-07 -0.340708452924818 0.297 0.323
0.00789340613650043 7 NOP56 GC
1.40680265629529e-06 -0.406627380296407 0.524 0.512
0.0250818845590888 7 PA2G4 GC
2.21410810378326e-06 -0.303899114015397 0.376 0.387
0.0394753333823518 7 PSAP GC
3.68195806561729e-06 -0.64458677785558 0.892 0.846
0.0656456303518907 7 HSP90AB1 GC
4.88645783159442e-06 -0.333822906059229 0.499 0.494
0.087120656679497 7 SP100 GC
6.4454095094098e-06 -0.607221171768344 0.603 0.415
0.114915206143267 7 HIST1H4C GC
1.57552537702154e-05 -0.344554850235906 0.761 0.769
0.28090041946917 7 RPL17 GC
3.98704778187867e-05 -0.330269166408148 0.476 0.468

0.710850749031148	7	CYCS	GC
8.44338381238344e-05	-0.40395573941876	0.304	0.318	1
	7	DDX21	GC
0.00017014318241972	-0.30659497799711	0.315	0.337	1
	7	APOBEC3C	GC
0.00017398684579542	-0.304764775606539	0.666	0.652	1
	7	TMBIM6	GC
0.000824343145575944	-1.54060985281035	0.196	0.123	1
	7	IGHG2	GC
0.00127285704132409	-0.610165922056035	0.475	0.401	1
	7	FABP5	GC
0.00480681446902977	0.337439698789602	0.857	0.749	1
	7	HLA-DRB1	GC
0.00719744888396772	-0.862178098002958	0.133	0.148	1
	7	IGHA1	GC
0	0.476358932388696	1	0.999	0	8
RPS29	Memory 1
0	-2.38558010299426	0.147	0.675	0	8
TCL1A	Memory 1
1.5607533752125e-320	0.487241293980978	1	0.998
2.78266719266636e-316	8	B2M	Memory 1
2.17969024214942e-312	0.406263418039281	1	1
3.8861697327282e-308	8	RPS27	Memory 1
1.09767364658713e-306	-1.70528316846002	0.668	0.916
1.95704234450019e-302	8	IGHM	Memory 1
1.03960700984482e-294	0.431109948314588	1	0.999
1.85351533785233e-290	8	RPL34	Memory 1
3.31570587071306e-293	0.44852744185538 1	0.998
5.91157199689431e-289	8	RPS14	Memory 1
5.58704912415278e-282	0.892421550906211	0.952	0.768
9.961149883452e-278	8	HLA-DPB1	Memory 1
5.08145844283475e-279	0.37838122158222 1	1
9.05973225773008e-275	8	RPL41	Memory 1
1.67111818299745e-253	0.367101745532138	1	1
2.97943660846616e-249	8	RPL21	Memory 1
4.77711520842862e-252	0.66620095633633 0.997	0.944
8.51711870510738e-248	8	HLA-DRA	Memory 1
1.48039685974775e-239	0.43879876851449 1	0.996
2.63939956124426e-235	8	RPS15A	Memory 1
8.80317633276127e-238	-1.4553073414292 0.23	0.621
1.56951830836801e-233	8	IGHD	Memory 1
1.73429070786402e-210	0.377102992393938	1	0.998
3.09206690305077e-206	8	RPS28	Memory 1
4.6569327828468e-198	0.353555658666749	1	0.998
8.30284545853756e-194	8	RPL39	Memory 1
3.46359828884082e-195	0.334633954779413	1	0.999
6.17524938917429e-191	8	RPL13	Memory 1
1.00400120120273e-182	0.359758924035121	1	0.998
1.79003374162435e-178	8	RPL11	Memory 1
9.9497038502606e-174	0.924182040863996	0.597	0.292

1.77393269946296e-169 8 CD27 Memory 1
3.14629144664208e-173 0.531584532565359 0.979 0.902
5.60952302021817e-169 8 HLA-A Memory 1
7.01848866056638e-170 0.692070732463304 0.946 0.809
1.25132634329238e-165 8 HLA-DPA1 Memory 1
5.13947521368742e-165 0.866775090493976 0.155 0.014
9.1631703584833e-161 8 LINC01781 Memory 1
2.1188458947982e-161 0.31655699520884 1 0.999
3.77769034583571e-157 8 RPS18 Memory 1
1.93457623072831e-160 0.398582010794327 0.998 0.991
3.4491559617655e-156 8 RPL30 Memory 1
5.23530979964956e-146 0.311798070289128 1 0.998
9.3340338417952e-142 8 RPL23A Memory 1
7.23055168658697e-145 0.305873776010345 1 0.998
1.28913506020159e-140 8 RPLP2 Memory 1
3.62966212457959e-143 0.365072583628983 0.998 0.986
6.47132460191296e-139 8 HLA-B Memory 1
1.43998273397279e-141 0.864489326148504 0.516 0.238
2.56734521640009e-137 8 TNFRSF13B Memory 1
7.16026785007357e-131 0.710324680855484 0.749 0.479
1.27660415498962e-126 8 LTB Memory 1
2.29236865612704e-121 0.324531145926917 0.999 0.995
4.0870640770089e-117 8 RPS25 Memory 1
3.62127734540287e-117 0.85182049578299 0.504 0.259
6.45637537911877e-113 8 GPR183 Memory 1
1.20608708047575e-112 0.767523060532184 0.315 0.133
2.15033265578022e-108 8 AIM2 Memory 1
2.84048530090503e-108 0.32499538053701 0.999 0.992
5.06430124298358e-104 8 RPL36 Memory 1
1.68224629505685e-105 0.642085280421069 0.665 0.429
2.99927691945686e-101 8 COTL1 Memory 1
1.72419686399794e-105 -0.928920317872067 0.086 0.307
3.07407058882192e-101 8 NME1 Memory 1
1.09687130329605e-101 0.699636954959236 0.59 0.343
1.95561184664653e-97 8 TXNIP Memory 1
5.55476041508549e-96 0.783511058862296 0.281 0.134
9.90358234405592e-92 8 ITGB1 Memory 1
5.27093336476026e-94 0.3845062495616 0.963 0.865
9.39754709603107e-90 8 CD52 Memory 1
1.44715356491144e-93 -1.28644684611088 0.04 0.213
2.5801300908806e-89 8 RGS13 Memory 1
2.0855859821487e-91 -0.765450304211893 0.039 0.204
3.71839124757292e-87 8 YBX3 Memory 1
2.48831813867077e-90 -0.753349848410696 0.403 0.616
4.43642240943612e-86 8 POMP Memory 1
2.49085225720875e-90 0.499066896583314 0.885 0.691
4.44094048937749e-86 8 EMP3 Memory 1
9.13574073458124e-88 0.566663952865195 0.497 0.265
1.62881121556849e-83 8 KLF2 Memory 1
2.09876951544624e-85 -1.07586251465615 0.203 0.412

3.7418961690891e-81	8	FABP5	Memory 1	
9.2310196498163e-85	0.483889513587032		0.898	0.789
1.64579849336575e-80	8	HLA-E	Memory 1	
1.83931952633716e-83	-0.936026833001236		0.427	0.606
3.27932278350652e-79	8	PRDX1	Memory 1	
8.35177162351197e-81	0.530929397144078		0.867	0.747
1.48903736275595e-76	8	HLA-C	Memory 1	
1.48154803159962e-80	-0.728413081663965		0.137	0.355
2.64145198553896e-76	8	ABRACL	Memory 1	
2.85111768567721e-79	0.532828699973475		0.698	0.505
5.08325772179389e-75	8	BANK1	Memory 1	
3.58824036257116e-78	0.34963325996132	0.986	0.964	
6.39747374242812e-74	8	RPL38	Memory 1	
1.64062571629282e-77	-1.33096987306361		0.132	0.293
2.92507158957847e-73	8	MIR155HG	Memory 1	
4.30393447335897e-76	-0.526392341963462		0.005	0.123
7.67348477255171e-72	8	CD38	Memory 1	
7.06103937897807e-73	0.539694404179118		0.157	0.064
1.258912710878e-68	8	TEX9	Memory 1	
8.2634862624819e-71	0.585387177606831		0.283	0.146
1.4732969657379e-66	8	CLECL1	Memory 1	
2.6738594668211e-70	0.480679725387467		0.847	0.749
4.76722404339533e-66	8	TOMM7	Memory 1	
7.70739229736894e-69	0.377700309664275		0.959	0.916
1.37415097269791e-64	8	PFDN5	Memory 1	
1.39951148409097e-68	0.452098244182089		0.831	0.692
2.49518902498579e-64	8	HLA-DQB1	Memory 1	
1.09746288839504e-67	-0.618892149414825		0.192	0.401
1.95666658371951e-63	8	YWHAE	Memory 1	
2.77664419023532e-67	0.470244301875363		0.558	0.364
4.95047892677056e-63	8	SELL	Memory 1	
1.20237105134142e-66	0.519004547053941		0.663	0.497
2.14370734743661e-62	8	TSC22D3	Memory 1	
2.33954147647078e-65	-0.717367682575268		0.304	0.492
4.17116849839975e-61	8	RANBP1	Memory 1	
4.73919776241571e-65	-0.674016712793707		0.052	0.208
8.44951569061098e-61	8	HMCES	Memory 1	
7.30147348530923e-65	-0.364433839674759		0.004	0.107
1.30177970769578e-60	8	SEMA4A	Memory 1	
3.72888859049428e-64	-0.872832375807065		0.609	0.719
6.64823546799225e-60	8	ENO1	Memory 1	
8.74768995437129e-64	-1.04525810070724		0.541	0.671
1.55962564196486e-59	8	TUBA1B	Memory 1	
1.01715476575851e-63	-0.629185173218136		0.468	0.64
1.81348523187085e-59	8	PSMA4	Memory 1	
1.29881279014052e-63	-0.867244250542623		0.429	0.594
2.31565332354154e-59	8	MARCKSL1	Memory 1	
1.68638208999451e-63	-0.576027401642503		0.452	0.639
3.00665062825122e-59	8	GSTP1	Memory 1	
3.17367636355531e-63	-0.650272671933088		0.289	0.481

5.65834758858275e-59 8 SNRPD1 Memory 1
7.40092901316716e-63 -0.658853590887202 0.074 0.239
1.31951163375757e-58 8 IL4R Memory 1
2.37392299542443e-61 -0.746613533571824 0.649 0.752
4.23246730854222e-57 8 RAN Memory 1
4.56289339827844e-61 0.352036325170562 0.94 0.879
8.13518263979063e-57 8 MS4A1 Memory 1
5.19731261370408e-60 0.563330397521543 0.297 0.163
9.266288658973e-56 8 SAMSN1 Memory 1
3.01762019092977e-59 0.324364785526928 0.982 0.968
5.38011503840868e-55 8 MT-ND3 Memory 1
8.87442201693761e-59 -0.705010196430665 0.451 0.602
1.58222070139981e-54 8 LDHA Memory 1
1.01628066691884e-58 -0.390570742917546 0.005 0.105
1.8119268010496e-54 8 MYBL2 Memory 1
1.19433536974158e-58 -0.98407854494989 0.051 0.187
2.12938053071227e-54 8 XBP1 Memory 1
3.06370673170752e-57 -0.796232213600496 0.785 0.85
5.46228273196134e-53 8 HSP90AB1 Memory 1
8.94607617590186e-57 0.39831223160314 0.782 0.648
1.59499592140154e-52 8 HLA-DQA1 Memory 1
9.03982469542951e-57 -0.57794590722011 0.214 0.396
1.61171034494813e-52 8 CCT5 Memory 1
9.91920003582827e-57 -0.608709569196405 0.497 0.651
1.76849417438782e-52 8 SNRPG Memory 1
4.37434212642011e-56 -0.597933945591883 0.407 0.578
7.79901457719441e-52 8 SNRPE Memory 1
1.199421972507e-55 -0.569737925048252 0.019 0.127
2.13844943478273e-51 8 AL139020.1 Memory 1
1.40424029395091e-55 -0.674772222168536 0.643 0.752
2.50362002008508e-51 8 HSP90AA1 Memory 1
1.32240574288326e-54 -1.00643242652992 0.605 0.72
2.35771719898656e-50 8 HMGN2 Memory 1
2.81555308563522e-54 -0.586364685146066 0.509 0.669
5.01984959637903e-50 8 DBI Memory 1
6.70879566776146e-54 -0.755144065052873 0.179 0.346
1.19611117960519e-49 8 LRMP Memory 1
1.82967639647672e-53 -0.540438194434288 0.272 0.456
3.26213004727834e-49 8 ILF2 Memory 1
6.31655172466307e-53 0.445342059723423 0.154 0.065
1.12617800699018e-48 8 THEMIS2 Memory 1
6.91563459902694e-53 -0.649963425422306 0.166 0.338
1.23298849266051e-48 8 GRHPR Memory 1
2.05655508117831e-52 -0.505923194435 0.076 0.23
3.66663205423281e-48 8 SIAH2 Memory 1
2.89178047363702e-52 -0.597086530145546 0.364 0.536
5.15575540644744e-48 8 PSME2 Memory 1
3.43397848061646e-52 -0.550922035069317 0.255 0.435
6.12244023309109e-48 8 CCT2 Memory 1
1.21319005385119e-51 -0.499832083406861 0.031 0.14

2.16299654701129e-47	8	PSAT1	Memory 1
1.62542453829933e-51	-0.629119674892763	0.033	0.153
2.89796940933387e-47	8	BIK	Memory 1
2.38271251517497e-51	-0.828404916130254	0.586	0.7
4.24813814330545e-47	8	H2AFZ	Memory 1
3.0916996649337e-51	-0.563225519001366	0.371	0.541
5.51219133261029e-47	8	SRSF9	Memory 1
4.15006957221302e-51	0.416613238556874	0.659	0.51
7.39915904029859e-47	8	LSP1	Memory 1
3.4078012039015e-50	-0.538562461411948	0.389	0.561
6.07576876643598e-46	8	SEC61G	Memory 1
7.18980025906489e-50	-0.514212822779576	0.092	0.239
1.28186948818868e-45	8	MTHFD2	Memory 1
9.30030727321719e-50	-0.531286721333044	0.027	0.139
1.65815178374189e-45	8	NEIL1	Memory 1
1.76449651953312e-49	0.445927373831597	0.182	0.068
3.1459208446756e-45	8	MYO1F	Memory 1
1.96861531990766e-49	-0.505233155277629	0.344	0.521
3.50984425386338e-45	8	SSBP1	Memory 1
2.44455690467195e-49	0.50716507404458 0.507	0.354
4.35840050533962e-45	8	ARID5B	Memory 1
4.96471911014549e-49	-0.497731683402619	0.325	0.504
8.85159770147839e-45	8	COX5A	Memory 1
8.81497689725946e-49	-0.530308588262906	0.4	0.565
1.57162223101239e-44	8	ATP5PF	Memory 1
1.05236396218851e-48	-0.384452355382327	0.021	0.118
1.87625970818589e-44	8	PYCR1	Memory 1
1.5561808632617e-48	-0.503105973233016	0.256	0.442
2.77451486110928e-44	8	ROMO1	Memory 1
2.27077601886363e-48	-0.614708567687478	0.909	0.934
4.04856656403197e-44	8	SERF2	Memory 1
8.805802131966e-48	0.458816950222597	0.219	0.115
1.56998646210822e-43	8	PYCARD	Memory 1
1.73003321964152e-47	0.438753255245464	0.19	0.087
3.08447622729887e-43	8	ITGB7	Memory 1
2.72651338402983e-47	-0.436244675461956	0.027	0.135
4.86110071238679e-43	8	PHGDH	Memory 1
7.06000788156007e-47	-0.554366893717111	0.232	0.391
1.25872880520334e-42	8	NHP2	Memory 1
3.12386493412009e-46	0.455764337062591	0.242	0.124
5.56953879104271e-42	8	CD24	Memory 1
7.48320996629048e-46	-0.515880980517738	0.187	0.355
1.33418150488993e-41	8	TIMM13	Memory 1
1.19502108206684e-45	-0.48094065528338	0.451	0.611
2.13060308721697e-41	8	PSMB3	Memory 1
1.24124401776266e-45	-0.554305705814373	0.526	0.662
2.21301395926905e-41	8	SLC25A5	Memory 1
1.50329032853239e-45	0.463647354916073	0.409	0.274
2.6802163267404e-41	8	RNASET2	Memory 1
2.95524599272876e-45	-0.492493065170676	0.361	0.53

5.26890808043611e-41	8	FKBP1A	Memory 1		
3.69797048794782e-45	0.467902264459173			0.572	0.421
6.59311158296216e-41	8	PLP2	Memory 1		
6.716546831192e-45	-0.711825635747179			0.327	0.477
1.19749313453322e-40	8	CD83	Memory 1		
8.47277082998748e-45	-0.529112547755373			0.173	0.335
1.51061031127847e-40	8	RFTN1	Memory 1		
9.88398225851777e-45	-0.501278820052391			0.152	0.309
1.76221519687113e-40	8	ODC1	Memory 1		
1.08512549093259e-44	-0.517187048405589			0.452	0.603
1.93467023778372e-40	8	SEC61B	Memory 1		
1.17554462655339e-44	-0.479479002646262			0.429	0.592
2.09587851468203e-40	8	EIF2S2	Memory 1		
1.75496365387928e-44	-0.537537210898121			0.562	0.684
3.12892469850136e-40	8	SRSF3	Memory 1		
1.88555791576673e-44	-0.605056061071773			0.312	0.467
3.36176120802051e-40	8	HSPD1	Memory 1		
1.98576434041646e-44	-0.609945631145948			0.419	0.564
3.54041924252851e-40	8	NCL	Memory 1		
2.93550355900318e-44	0.576388062987039			0.199	0.091
5.23370929534677e-40	8	KLK1	Memory 1		
3.44244715953102e-44	0.403177867655314			0.147	0.059
6.13753904072785e-40	8	KYNU	Memory 1		
3.66547925009275e-44	-0.498506978571522			0.287	0.458
6.53518295499036e-40	8	MRPL51	Memory 1		
4.54804814040525e-44	-0.465114271273466			0.496	0.646
8.10871502952852e-40	8	ATP5MF	Memory 1		
5.65463327830041e-44	-0.488149627308639			0.292	0.463
1.00816456718818e-39	8	SFPQ	Memory 1		
9.87478598006495e-44	-0.452999262549312			0.493	0.647
1.76057559238578e-39	8	HNRNPM	Memory 1		
1.78616137684676e-43	-0.45798336638161			0.238	0.411
3.1845471187801e-39	8	PSMB2	Memory 1		
2.41857356017167e-43	-0.770515205857839			0.224	0.398
4.31207480043007e-39	8	EIF5A	Memory 1		
2.91251671723826e-43	-0.493999809377469			0.271	0.437
5.19272605516409e-39	8	SLIRP	Memory 1		
9.97200858164354e-43	0.445670455119896			0.387	0.232
1.77790941002123e-38	8	CAPG	Memory 1		
6.94283856992253e-42	-0.504197381162734			0.514	0.645
1.23783868863149e-37	8	ERH	Memory 1		
9.37632775112456e-42	-0.429703855463891			0.146	0.312
1.671705474748e-37	8	TIMM8B	Memory 1		
1.23523233339537e-41	0.445811037585661			0.657	0.498
2.2022957272106e-37	8	HLA-DRB5	Memory 1		
1.41968788375786e-41	-0.502879189960928			0.675	0.763
2.53116152795189e-37	8	PSMA7	Memory 1		
1.45238388003778e-41	-0.486026476014739			0.263	0.423
2.58945521971936e-37	8	DDX39A	Memory 1		
6.88218696659595e-41	-0.596193976278424			0.694	0.764

1.22702511427439e-36 8 PKM Memory 1
9.56968172722962e-41 -0.442556224870283 0.559 0.69
1.70617855514777e-36 8 COX6A1 Memory 1
2.24101231821887e-40 -0.570626706441605 0.18 0.323
3.99550086215242e-36 8 DDX21 Memory 1
3.55587362035889e-40 -0.493218790527977 0.081 0.212
6.33976707773787e-36 8 EAF2 Memory 1
3.65766328280098e-40 -0.515918008956022 0.072 0.188
6.52124786690586e-36 8 PLPP5 Memory 1
6.6029279958911e-40 -0.475120369110695 0.088 0.223
1.17723603238742e-35 8 NANS Memory 1
6.83645516544907e-40 -0.499947111975908 0.448 0.59
1.21887159144791e-35 8 ATP5MC3 Memory 1
9.82599251138513e-40 -0.556919237194785 0.193 0.332
1.75187620485486e-35 8 SLC3A2 Memory 1
1.26181104373976e-39 -0.456808806047118 0.288 0.455
2.24968290988362e-35 8 SEM1 Memory 1
1.38110175629859e-39 0.428683935557984 0.504 0.385
2.46236632130476e-35 8 ARL6IP5 Memory 1
4.42022881240301e-39 -0.981008821548529 0.308 0.433
7.88082594963333e-35 8 STMN1 Memory 1
4.57986951022397e-39 -0.725497440421681 0.078 0.194
8.16544934977832e-35 8 PTTG1 Memory 1
5.34901459184245e-39 -0.395771262833415 0.096 0.23
9.53675811579591e-35 8 DCAF13 Memory 1
6.75382526693508e-39 -0.405170087995907 0.235 0.401
1.20413950684186e-34 8 NDUFAB1 Memory 1
7.18469054745107e-39 -0.524397910557841 0.103 0.235
1.28095847770505e-34 8 CKS2 Memory 1
2.09342986072763e-38 -0.468999040058843 0.2 0.352
3.7323760986913e-34 8 H2AFY Memory 1
2.10074908383393e-38 -0.425615779059548 0.368 0.529
3.74542554156752e-34 8 NDUFB8 Memory 1
2.92729593290721e-38 -0.529065890225473 0.153 0.285
5.21907591878026e-34 8 SRM Memory 1
6.95458616765165e-38 -0.424596308740748 0.529 0.661
1.23993316783061e-33 8 PSMB1 Memory 1
6.98301138817246e-38 -0.730107508596902 0.474 0.581
1.24500110039727e-33 8 TUBB Memory 1
1.02112545240205e-37 -0.506544335218663 0.934 0.942
1.82056456908761e-33 8 YBX1 Memory 1
1.42673773558929e-37 -0.413169067906638 0.585 0.705
2.54373070878214e-33 8 ELOB Memory 1
1.54863058268437e-37 -0.415381101898352 0.1 0.232
2.76105346586797e-33 8 NSMCE1 Memory 1
1.69795442540506e-37 0.425063683200183 0.195 0.108
3.02728294505467e-33 8 CRIP1 Memory 1
1.71908482512395e-37 -0.409029453651224 0.497 0.637
3.06495633471349e-33 8 COX7B Memory 1
1.85622549929857e-37 -0.444753208651574 0.658 0.747

3.30946444269943e-33 8 CHCHD2 Memory 1
2.03853113449268e-37 -0.44733654111576 0.051 0.156
3.634497159687e-33 8 CD72 Memory 1
2.61444971768855e-37 -0.332321079346501 0.047 0.159
4.66130240166692e-33 8 PAICS Memory 1
2.76400922158243e-37 -0.500936308634028 0.112 0.224
4.92795204115931e-33 8 GBP2 Memory 1
5.70199503748017e-37 -0.430517787044627 0.144 0.294
1.01660869523234e-32 8 RRAS2 Memory 1
5.77225439815335e-37 -0.500498058622534 0.457 0.588
1.02913523664676e-32 8 ANP32B Memory 1
9.47610001219041e-37 0.434081041812939 0.189 0.101
1.68949387117343e-32 8 SCIMP Memory 1
1.56376318361109e-36 -0.419335200968896 0.51 0.644
2.78803338006021e-32 8 ATP5MD Memory 1
1.85440976803406e-36 -0.436883646646364 0.253 0.407
3.30622717542792e-32 8 RPA3 Memory 1
2.64199353850581e-36 -0.469143650678448 0.319 0.469
4.71041027980202e-32 8 PRMT1 Memory 1
2.82828975949034e-36 -0.391631414998516 0.364 0.525
5.04255781219533e-32 8 PSMB6 Memory 1
2.84213745831234e-36 -0.49407288244499 0.509 0.642
5.06724687442507e-32 8 SET Memory 1
3.84834752973245e-36 0.445346415837613 0.383 0.28
6.86121881075998e-32 8 CD82 Memory 1
6.53962558851184e-36 -0.484617409329076 0.053 0.14
1.16594984617578e-31 8 FCER2 Memory 1
7.25959546646221e-36 -0.495437979580086 0.207 0.35
1.29431327571555e-31 8 INSIG1 Memory 1
8.35247267688529e-36 0.456296948806715 0.51 0.415
1.48916235356188e-31 8 CTSH Memory 1
9.36038279157061e-36 -0.477093164425763 0.286 0.445
1.66886264790912e-31 8 RHOH Memory 1
1.13133812075692e-35 -0.462657899997534 0.145 0.282
2.01706273549752e-31 8 GADD45B Memory 1
1.84056135063901e-35 -0.422443213114365 0.281 0.433
3.28153683205429e-31 8 CCT4 Memory 1
2.14603017851655e-35 -0.427103662217007 0.41 0.556
3.82615720527715e-31 8 PSMA1 Memory 1
2.48827662391693e-35 -0.396178580474611 0.132 0.275
4.4363483927815e-31 8 TOP1 Memory 1
3.62327823485137e-35 0.430276236510728 0.401 0.279
6.45994276491651e-31 8 GNG7 Memory 1
4.21033289317325e-35 -0.510794255189364 0.276 0.423
7.50660251523859e-31 8 METAP2 Memory 1
5.74942998660114e-35 0.383441367488792 0.187 0.092
1.02506587231112e-30 8 CYSLTR1 Memory 1
6.37127312184507e-35 -0.412198141030762 0.471 0.606
1.13593428489376e-30 8 PSMA2 Memory 1
6.42238547223848e-35 -0.424864930877138 0.436 0.578

1.1450471058454e-30	8	PARK7	Memory 1
9.56938194703019e-35	-0.416125675353767	0.265	0.416
1.70612510733601e-30	8	HNRNPR	Memory 1
9.84174066243075e-35	-0.494625785142895	0.116	0.242
1.75468394270478e-30	8	GCHFR	Memory 1
1.09811581749007e-34	-0.404304752452556	0.179	0.329
1.95783069100304e-30	8	IMP4	Memory 1
1.44494017934176e-34	-0.415979568486432	0.249	0.399
2.57618384574842e-30	8	PHB	Memory 1
1.46102992232041e-34	-0.419515900232783	0.24	0.394
2.60487024850506e-30	8	SHMT2	Memory 1
2.02494848804845e-34	-0.488351206019177	0.381	0.517
3.61028065934159e-30	8	PA2G4	Memory 1
2.16764986242079e-34	-0.542262419366843	0.374	0.502
3.86470293971002e-30	8	HSPE1	Memory 1
2.44364529250948e-34	-0.387799332392013	0.338	0.496
4.35677519201515e-30	8	CCT8	Memory 1
2.54243540350633e-34	-0.39263967278236	0.226	0.38
4.53290808091143e-30	8	PSMC3	Memory 1
4.78751453094212e-34	-0.346574139064831	0.378	0.21
8.53565965721671e-30	8	IGHG1	Memory 1
5.34121148226547e-34	0.394256498094962	0.496	0.384
9.5228459517311e-30	8	EVI2B	Memory 1
6.27768560640339e-34	-0.410095992657615	0.313	0.462
1.11924856676566e-29	8	LSM5	Memory 1
7.33913438623043e-34	-0.350021600086262	0.127	0.267
1.30849426972102e-29	8	PSMC2	Memory 1
7.63036708177514e-34	-0.440790709545092	0.306	0.448
1.36041814700969e-29	8	CCT6A	Memory 1
7.87068439392434e-34	-0.415026608119432	0.51	0.633
1.40326432059277e-29	8	ATP5F1B	Memory 1
1.04070592633511e-33	-0.438357128005668	0.234	0.38
1.85547459606286e-29	8	LCP1	Memory 1
1.07438755093759e-33	-0.368196135878703	0.235	0.391
1.91552556456663e-29	8	SNRPC	Memory 1
1.24417541585112e-33	-0.346330076485371	0.08	0.202
2.21824034892096e-29	8	UQCC2	Memory 1
2.11882637125398e-33	-0.38315906083115	0.378	0.539
3.77765553730871e-29	8	SNRPB2	Memory 1
3.23894668779055e-33	-0.605541368215277	0.704	0.77
5.77471804966177e-29	8	HMGN1	Memory 1
6.92093937655309e-33	-0.379703424335754	0.3	0.451
1.23393428144565e-28	8	PGAM1	Memory 1
7.06039576493073e-33	-0.311069676424008	0.069	0.188
1.2587979609295e-28	8	LRRC59	Memory 1
7.47572390891144e-33	-0.527464750756742	0.116	0.239
1.33284681571982e-28	8	UBE2J1	Memory 1
1.0355485110006e-32	-0.388963836746684	0.17	0.31
1.84627944026298e-28	8	PSMD11	Memory 1
1.77713382412664e-32	-0.480746620560147	0.139	0.269

3.16845189503538e-28 8 CD22 Memory 1
2.05855322231046e-32 -0.411919175545099 0.629 0.726
3.67019454005732e-28 8 COX6C Memory 1
2.09638558336681e-32 -0.388165943421739 0.309 0.458
3.73764585658468e-28 8 VDAC1 Memory 1
2.85665625824239e-32 -0.383254924438575 0.229 0.38
5.09313244282036e-28 8 PSMC1 Memory 1
4.40770834965789e-32 -0.350225308251017 0.085 0.203
7.85850321660506e-28 8 EBNA1BP2 Memory 1
5.82351117035398e-32 0.384777292815027 0.321 0.215
1.03827380656241e-27 8 LY86 Memory 1
6.86383227133739e-32 0.421066676139391 0.471 0.373
1.22375265565674e-27 8 ZFAND6 Memory 1
8.35854310026039e-32 -0.383709509129581 0.058 0.163
1.49024464934542e-27 8 APLP2 Memory 1
9.98147914304777e-32 -0.326358340111075 0.057 0.16
1.77959791641399e-27 8 GAR1 Memory 1
1.10112805783491e-31 0.301349117744279 0.869 0.813
1.96320121431386e-27 8 NOP53 Memory 1
1.20315582226049e-31 -0.470652517901959 0.261 0.407
2.14510651550822e-27 8 RPL22L1 Memory 1
1.34418888109143e-31 -0.402418274116834 0.171 0.307
2.39655435609791e-27 8 ATP1B3 Memory 1
1.39540418560995e-31 0.336425656069567 0.329 0.207
2.48786612252397e-27 8 PLAC8 Memory 1
1.48819928530269e-31 -0.420069663567074 0.303 0.445
2.65331050576617e-27 8 FDPS Memory 1
1.719590443434e-31 0.379869006935756 0.201 0.108
3.06585780159847e-27 8 SESN3 Memory 1
2.0411080373893e-31 -0.389184155680834 0.131 0.255
3.63909151986139e-27 8 GARS Memory 1
2.45376390370661e-31 -0.343363068387353 0.147 0.287
4.37481566391851e-27 8 PSMD14 Memory 1
2.95939057145574e-31 -0.383095957976797 0.47 0.601
5.27629744984844e-27 8 XRCC6 Memory 1
3.68702534112455e-31 -0.353156113044823 0.342 0.496
6.57359748069096e-27 8 MRPL52 Memory 1
4.48968026158792e-31 -0.344032369437199 0.067 0.167
8.00465093838511e-27 8 MRTO4 Memory 1
4.55321535074593e-31 -0.438524100283213 0.312 0.445
8.11792764884492e-27 8 HMGA1 Memory 1
5.26823252849759e-31 -0.334741022319051 0.664 0.775
9.39273177505835e-27 8 SEC62 Memory 1
5.52418835831339e-31 0.376640767921761 0.303 0.202
9.84907542403695e-27 8 PRKCB Memory 1
7.70311152199321e-31 -0.59236780580043 0.159 0.281
1.37338775325617e-26 8 HSPA5 Memory 1
7.96906627960439e-31 -1.23193018695553 0.079 0.179
1.42080482699067e-26 8 MZB1 Memory 1
9.43511214368158e-31 -0.416297411962104 0.283 0.434

1.68218614409699e-26	8	DNAJA1	Memory 1
1.3022961990428e-30	-0.347630485975163	0.134	0.273
2.3218638932734e-26	8	TXNDC17	Memory 1
1.39741755499126e-30	-0.365228874984946	0.101	0.205
2.49145575879392e-26	8	NPM3	Memory 1
1.67432275764066e-30	-0.352625628628854	0.219	0.368
2.98515004459753e-26	8	ELOC	Memory 1
1.74384779779973e-30	-0.401894279207717	0.062	0.173
3.10910623869714e-26	8	CCDC88A	Memory 1
2.13183497758105e-30	-0.363770338667726	0.365	0.509
3.80084858152925e-26	8	EIF3I	Memory 1
2.18861066420369e-30	-0.474092475473024	0.139	0.274
3.90207395320876e-26	8	PIM1	Memory 1
4.04084981046123e-30	-0.379479639614813	0.198	0.343
7.20443112707133e-26	8	NDUFC1	Memory 1
4.53210170732044e-30	-0.370090772690843	0.224	0.373
8.0802841339816e-26	8	EWSR1	Memory 1
5.17896753283051e-30	-0.37019414339837	0.276	0.425
9.23358121428352e-26	8	EIF6	Memory 1
5.47297915995998e-30	-0.376528519882187	0.438	0.576
9.75777454429264e-26	8	RBM8A	Memory 1
7.74223624259991e-30	-0.345366481671733	0.338	0.491
1.38036329969314e-25	8	UBE2L3	Memory 1
8.41989917759978e-30	-0.363073112700709	0.151	0.281
1.50118382437427e-25	8	ATOX1	Memory 1
8.90758456824218e-30	-0.352433914448082	0.117	0.243
1.5881332526719e-25	8	HNRNPAB	Memory 1
1.27830318404215e-29	-0.360706281885917	0.153	0.289
2.27908674682875e-25	8	PRDX3	Memory 1
1.68493234476242e-29	-0.394529769051408	0.447	0.571
3.00406587747692e-25	8	UQCRQ	Memory 1
1.69710472701397e-29	-0.352096472459988	0.352	0.495
3.02576801779322e-25	8	CCT3	Memory 1
1.8581278700341e-29	-0.369996077257815	0.096	0.212
3.31285617948379e-25	8	GNL3	Memory 1
2.07539597769108e-29	-0.363921954563457	0.207	0.345
3.70022348862543e-25	8	STRAP	Memory 1
2.31715735334332e-29	-0.331706000073548	0.085	0.192
4.13125984527581e-25	8	YARS	Memory 1
5.6699355791375e-29	-0.354664176624609	0.066	0.169
1.01089281440442e-24	8	DEF8	Memory 1
6.77023510183783e-29	-0.328084557584252	0.103	0.222
1.20706521630667e-24	8	MRPL12	Memory 1
6.81905613610888e-29	-0.337913640939394	0.124	0.25
1.21576951850685e-24	8	AK2	Memory 1
7.55653568242907e-29	-0.470957976477267	0.174	0.308
1.34725474682028e-24	8	BCL7A	Memory 1
7.97602853016666e-29	0.364203789783269	0.274	0.17
1.42204612664341e-24	8	MARCH1	Memory 1
8.17247481773553e-29	-0.45806914583	0.089	0.175

1.45707053525407e-24 8 TNFRSF18 Memory 1
1.15850208549605e-28 -0.351417748878832 0.248 0.393
2.0654933682309e-24 8 NDUFS6 Memory 1
1.34143173527668e-28 -0.349006352726366 0.502 0.625
2.39163864082479e-24 8 ACTR3 Memory 1
1.53991970304645e-28 -0.341674891349042 0.087 0.192
2.74552283856151e-24 8 GPATCH4 Memory 1
1.55680537967778e-28 -0.371564936653833 0.261 0.404
2.77562831142752e-24 8 PSMA3 Memory 1
1.6212621273054e-28 -0.370285140191964 0.209 0.352
2.89054824677279e-24 8 RBM17 Memory 1
1.6970639293059e-28 -0.329626036613868 0.18 0.319
3.02569527955949e-24 8 STOML2 Memory 1
1.90997410366833e-28 -0.394864603264936 0.32 0.449
3.40529282943026e-24 8 C1QBP Memory 1
3.01403235969098e-28 -0.378037111564749 0.577 0.681
5.37371829409304e-24 8 HNRNPA3 Memory 1
3.33741989433719e-28 -0.355833643559102 0.187 0.328
5.95028592961377e-24 8 HSBP1 Memory 1
3.68715960240121e-28 -0.348314760484131 0.375 0.515
6.57383685512111e-24 8 PSMD8 Memory 1
4.49506165517318e-28 -0.384837072780022 0.502 0.62
8.01424542500826e-24 8 SERBP1 Memory 1
6.97089995731241e-28 0.370247236948671 0.312 0.226
1.24284175338923e-23 8 ATM Memory 1
1.0893285593367e-27 0.343845891452282 0.428 0.328
1.9421638884414e-23 8 RIPOR2 Memory 1
1.20282128443789e-27 -0.362698057715126 0.264 0.409
2.14451006802432e-23 8 BZW1 Memory 1
1.22292839417967e-27 -0.334901183503137 0.232 0.376
2.18035903398293e-23 8 PSMD7 Memory 1
1.23777329464905e-27 -0.355565113076616 0.211 0.343
2.20682600702979e-23 8 MIF Memory 1
1.24770396622568e-27 -0.336393212597834 0.306 0.451
2.22453140138376e-23 8 PSMB7 Memory 1
1.2513592405299e-27 -0.431056137890999 0.492 0.623
2.23104838994075e-23 8 CLEC2D Memory 1
1.36760879532816e-27 -0.387123035874285 0.267 0.404
2.43830972119057e-23 8 BLOC1S2 Memory 1
1.53365546038401e-27 -0.365725121377544 0.03 0.113
2.73435432031865e-23 8 CD9 Memory 1
2.86457204244925e-27 -0.323916037357207 0.197 0.337
5.10724549448277e-23 8 ACP1 Memory 1
3.01678221277644e-27 -0.330981628475318 0.27 0.418
5.37862100715912e-23 8 RNF181 Memory 1
3.88738549209099e-27 0.356746131958479 0.56 0.468
6.93081959384903e-23 8 GPSM3 Memory 1
3.92439912052114e-27 -0.319442758152651 0.122 0.244
6.99681119197714e-23 8 DDX1 Memory 1
4.87676607120575e-27 -0.332361939508564 0.128 0.246

8.69478622835273e-23	8	POLD2	Memory 1	
7.58797598740083e-27	-0.421781949044827		0.369	0.506
1.35286023879369e-22	8	FOXP1	Memory 1	
8.13161523844576e-27	-0.487008175830754		0.242	0.357
1.44978568086249e-22	8	TUBB4B	Memory 1	
1.08495591206179e-26	-0.357151572370761		0.172	0.283
1.93436789561496e-22	8	WARS	Memory 1	
1.2895743067864e-26	-0.378099800574338		0.482	0.599
2.29918203156948e-22	8	SRSF2	Memory 1	
1.39783265933565e-26	-0.369886478477741		0.54	0.649
2.49219584832953e-22	8	HNRNPC	Memory 1	
1.54951256343621e-26	-0.33556583155229		0.567	0.679
2.76262594935041e-22	8	ATP5ME	Memory 1	
1.88494096048534e-26	-0.309068618505191		0.057	0.149
3.36066123844932e-22	8	PRDX4	Memory 1	
1.89473685273941e-26	-0.325735461268966		0.139	0.265
3.37812633474909e-22	8	AK6	Memory 1	
2.18299779071714e-26	-0.400016648646617		0.802	0.847
3.8920667610696e-22	8	HNRNPA2B1		Memory 1
2.19330259957584e-26	-0.318281733313122		0.088	0.186
3.91043920478376e-22	8	TUBA1C	Memory 1	
3.88416158440041e-26	0.336838396937819		0.148	0.073
6.92507168882749e-22	8	CAPN2	Memory 1	
4.85136321311344e-26	0.394678474931651		0.378	0.295
8.64949547265995e-22	8	BLK	Memory 1	
7.41188943838409e-26	-0.35487915728796		0.314	0.451
1.3214657679695e-21	8	ATP5F1C	Memory 1	
8.50182843216903e-26	-0.322294052385025		0.141	0.267
1.51579099117142e-21	8	ATXN10	Memory 1	
9.77893555805369e-26	-0.324354173620632		0.057	0.148
1.74348642064539e-21	8	MID1IP1	Memory 1	
1.38451159285787e-25	-0.361090832959889		0.454	0.569
2.4684457189063e-21	8	SNRPB	Memory 1	
1.58049325933159e-25	-0.32663063038812		0.309	0.449
2.8178614320623e-21	8	CCT7	Memory 1	
1.83747369958375e-25	-0.425150661511289		0.131	0.247
3.27603185898787e-21	8	KPNA2	Memory 1	
3.14133569363264e-25	-0.318740449357487		0.374	0.502
5.60068740817764e-21	8	TUFM	Memory 1	
3.14491639720521e-25	-0.305703720000673		0.48	0.61
5.60707144457716e-21	8	NOP10	Memory 1	
3.18577985875546e-25	-0.324260205967403		0.173	0.298
5.6799269101751e-21	8	SSRP1	Memory 1	
3.67645535377113e-25	-0.370389417012532		0.119	0.22
6.55475225023855e-21	8	NFKBIE	Memory 1	
4.25661130817848e-25	-0.303741338672184		0.124	0.24
7.5891123013514e-21	8	EIF2S1	Memory 1	
4.53182565246313e-25	-0.345259027739106		0.292	0.425
8.07979195577652e-21	8	MAGOH	Memory 1	
4.75454992124226e-25	-0.319995658666908		0.39	0.528

8.47688705458283e-21 8 SELENOT Memory 1
5.75350850290743e-25 -0.314423243043681 0.219 0.353
1.02579303098336e-20 8 CYC1 Memory 1
7.03330798727969e-25 0.361520823956449 0.285 0.199
1.2539684810521e-20 8 BIN1 Memory 1
7.6709768655573e-25 0.371452127948785 0.55 0.478
1.36765846536021e-20 8 ISCU Memory 1
7.7820910540951e-25 -0.358339191101625 0.622 0.706
1.38746901403462e-20 8 TPI1 Memory 1
1.05847814410134e-24 -0.307671613345857 0.966 0.964
1.88716068311827e-20 8 EIF1 Memory 1
1.07906144644874e-24 -0.305405768014193 0.119 0.232
1.92385865287346e-20 8 MRPL3 Memory 1
1.16433422949922e-24 -0.322884090363299 0.438 0.571
2.07589149777417e-20 8 RAC1 Memory 1
1.29863452786613e-24 -0.313294988377142 0.258 0.394
2.31533549973252e-20 8 NOL7 Memory 1
1.31142765389591e-24 -0.388080748436895 0.105 0.199
2.33814436413102e-20 8 CLEC2B Memory 1
1.31513985849152e-24 0.331230222734156 0.2 0.123
2.34476285370453e-20 8 ZBTB20 Memory 1
1.73695061253001e-24 -0.300492079106653 0.112 0.229
3.09680924707975e-20 8 DAZAP1 Memory 1
1.78522004248622e-24 -0.400765463993921 0.15 0.26
3.18286881374869e-20 8 SRGN Memory 1
1.82607755595283e-24 -0.307441705324207 0.101 0.205
3.2557136745083e-20 8 TOMM40 Memory 1
1.87956020541189e-24 -0.351465347428914 0.292 0.425
3.35106789022887e-20 8 HNRNPD Memory 1
1.93162817645053e-24 -0.316642253834922 0.047 0.124
3.44389987579365e-20 8 ZNF318 Memory 1
2.39394113934703e-24 -0.339687759513778 0.458 0.59
4.26815765734181e-20 8 NDUFV2 Memory 1
2.77769804073933e-24 -0.328349220766314 0.092 0.189
4.95235783683415e-20 8 WDR43 Memory 1
3.48791164781964e-24 -0.312603513738743 0.174 0.299
6.21859767689763e-20 8 ILF3 Memory 1
3.65583448300599e-24 0.328055714429261 0.229 0.141
6.51798729975138e-20 8 SUN2 Memory 1
4.19713962576365e-24 -0.371088350023201 0.27 0.402
7.48308023877402e-20 8 NUCKS1 Memory 1
4.86744900448638e-24 -0.338368146009258 0.319 0.449
8.67817483009877e-20 8 ATP5MC1 Memory 1
5.52362896844875e-24 -0.487675905319373 0.251 0.364
9.84807808784729e-20 8 H2AFV Memory 1
7.19580100694516e-24 -0.308535670290033 0.501 0.631
1.28293936152825e-19 8 SELENOH Memory 1
8.69063861120103e-24 -0.404396009129217 0.083 0.179
1.54945395799103e-19 8 PDIA4 Memory 1
1.13267444307398e-23 -0.315966626692298 0.363 0.496

2.01944526455659e-19	8	SF3B6	Memory 1	
1.51191318522271e-23	-0.334155181185595		0.171	0.297
2.69559001793357e-19	8	HSPA4	Memory 1	
1.61104179562665e-23	0.334683095225787		0.163	0.088
2.87232641742276e-19	8	AHNAK	Memory 1	
1.6616202177883e-23	-0.335582306502388		0.242	0.373
2.96250268629477e-19	8	PFDN2	Memory 1	
1.72384258474981e-23	-0.305809591872218		0.303	0.439
3.07343894435044e-19	8	NDUFB7	Memory 1	
1.93803291938727e-23	-0.320902551056058		0.742	0.808
3.45531889197556e-19	8	HNRNPK	Memory 1	
2.37293770452473e-23	-0.313574682776645		0.511	0.622
4.23071063339715e-19	8	NDUFA13	Memory 1	
3.20633801367395e-23	-0.425487054551668		0.038	0.112
5.71658004457929e-19	8	VPREB3	Memory 1	
3.38391347199622e-23	-0.313837636736235		0.196	0.327
6.03317932922206e-19	8	RNASEH2C	Memory 1	
4.41904852129539e-23	-0.307682588862384		0.555	0.663
7.87872160861755e-19	8	UBL5	Memory 1	
4.54209951528883e-23	-0.337451001482304		0.043	0.121
8.09810922580845e-19	8	UBE2S	Memory 1	
4.62655387081409e-23	-2.05485969267397		0.609	0.444
8.24868289627444e-19	8	IGHG3	Memory 1	
5.66921085969509e-23	-0.311361002535678		0.19	0.321
1.01076360417504e-18	8	NDUFAF3	Memory 1	
8.84829959260244e-23	-1.38453244865673		0.237	0.305
1.57756333436509e-18	8	HMGB2	Memory 1	
9.45801169934446e-23	-0.350262158867992		0.351	0.481
1.68626890587612e-18	8	TNFAIP8	Memory 1	
1.06612069771177e-22	-0.387734802884258		0.129	0.221
1.90078659195031e-18	8	EIF4EBP1	Memory 1	
1.12888961005094e-22	-0.358912779377055		0.393	0.514
2.01269728575981e-18	8	TALDO1	Memory 1	
1.23751394586391e-22	-0.347010524878211		0.872	0.896
2.20636361408077e-18	8	PPIA	Memory 1	
1.49046509699826e-22	-0.563062197894743		0.116	0.212
2.6573502214382e-18	8	FOS	Memory 1	
1.62605976489589e-22	-0.313797380146202		0.552	0.661
2.89910195483288e-18	8	RBX1	Memory 1	
2.53490166805324e-22	-0.362582956152187		0.25	0.366
4.51947618397212e-18	8	HSPA9	Memory 1	
2.67276097883228e-22	0.330263618559183		0.367	0.281
4.76526554916007e-18	8	ACAP1	Memory 1	
2.71949364334159e-22	-0.363250605008031		0.639	0.71
4.84858521671372e-18	8	NDUFA4	Memory 1	
3.04774512107619e-22	-0.341623034066138		0.128	0.238
5.43382477636674e-18	8	ZCCHC7	Memory 1	
3.69153531189611e-22	-0.351663819371578		0.098	0.196
6.58163830757957e-18	8	MANF	Memory 1	
7.34258444914689e-22	-0.319412490707565		0.305	0.432

1.3091093814384e-17 8 MINOS1 Memory 1
8.23431996443174e-22 -0.344200206683022 0.905 0.919
1.46809690645854e-17 8 CFL1 Memory 1
8.52172818825034e-22 -0.315857701562491 0.186 0.306
1.51933891868315e-17 8 NOP58 Memory 1
8.90993501748551e-22 -0.318542897051639 0.121 0.221
1.58855231426749e-17 8 HMGCS1 Memory 1
1.07973761389053e-21 -0.352078024804629 0.156 0.267
1.92506419180543e-17 8 FKBP2 Memory 1
1.37287009536931e-21 0.316202139599213 0.199 0.12
2.44769009303394e-17 8 DDAH2 Memory 1
1.40663684179939e-21 0.349940034209569 0.238 0.168
2.50789282524413e-17 8 MFSD10 Memory 1
1.73784790842444e-21 -0.359962931116409 0.378 0.494
3.09840903592993e-17 8 CBX3 Memory 1
1.8541570588407e-21 -0.372569483967188 0.372 0.488
3.30577662020708e-17 8 RPS27L Memory 1
2.12306288767737e-21 -0.308474563064068 0.507 0.619
3.78520882243998e-17 8 UQCR10 Memory 1
2.46669577951094e-21 -0.324542685438774 0.295 0.419
4.39787190529005e-17 8 HDAC1 Memory 1
2.77771082935383e-21 -0.315259953206093 0.349 0.473
4.95238063765494e-17 8 PRDX5 Memory 1
3.12464270604172e-21 0.350046645882722 0.198 0.139
5.57092548060178e-17 8 MYO1G Memory 1
3.49054687138568e-21 -0.350671129236138 0.196 0.313
6.22329601699353e-17 8 CALR Memory 1
3.75052628435406e-21 -0.331066387925655 0.859 0.884
6.68681331237486e-17 8 H3F3B Memory 1
7.2602705539361e-21 -0.326373512162823 0.317 0.439
1.29443363706127e-16 8 EIF5B Memory 1
8.56000539904176e-21 -0.335221299484547 0.051 0.123
1.52616336259515e-16 8 LPP Memory 1
1.23221901055221e-20 0.322753959562016 0.3 0.193
2.19692327391353e-16 8 ACP5 Memory 1
2.01128629477225e-20 -0.312548078785913 0.14 0.244
3.58592233494945e-16 8 SLC38A1 Memory 1
2.8701369063507e-20 -0.342912801404861 0.357 0.472
5.11716709033266e-16 8 CYCS Memory 1
3.73816517167319e-20 -0.334676832061866 0.344 0.461
6.66477468457614e-16 8 COX17 Memory 1
4.00648252559275e-20 -0.323547240490077 0.296 0.427
7.14315769487932e-16 8 TMEM123 Memory 1
4.03107106109031e-20 -0.528626794836896 0.069 0.133
7.18699659481792e-16 8 MYC Memory 1
4.36722229403933e-20 -0.481187816572211 0.326 0.412
7.78632062804272e-16 8 TXN Memory 1
4.71647568254549e-20 -0.315348679905419 0.207 0.326
8.40900449441035e-16 8 NAA38 Memory 1
5.68042086431501e-20 -0.354406727937752 0.184 0.292

1.01276223589872e-15	8	RUBCNL	Memory 1	
9.13537110131282e-20	-0.373344391722088		0.146	0.245
1.62874531365306e-15	8	PHACTR1	Memory 1	
9.37862642419292e-20	0.307208695290108		0.134	0.084
1.67211530516935e-15	8	EBI3	Memory 1	
1.08468084126291e-19	-0.321674452333832		0.517	0.612
1.93387747188764e-15	8	POLR2L	Memory 1	
1.47585396761433e-19	-0.326240805891931		0.157	0.269
2.63130003885958e-15	8	FAM3C	Memory 1	
1.48201831859782e-19	-0.335715149774672		0.171	0.275
2.64229046022806e-15	8	SMS	Memory 1	
1.51968870277088e-19	-0.307215742847707		0.493	0.607
2.7094529881702e-15	8	LSM7	Memory 1	
2.00695897756555e-19	-0.307545128229172		0.811	0.862
3.57820716110162e-15	8	UBB	Memory 1	
2.00982257653729e-19	-0.318833685650848		0.216	0.326
3.58331267170833e-15	8	NOP56	Memory 1	
3.12136058115753e-19	-0.344667838675425		0.151	0.25
5.56507378014576e-15	8	CKS1B	Memory 1	
9.44249376183301e-19	-0.32894030523852		0.242	0.354
1.68350221279721e-14	8	PDIA6	Memory 1	
1.41558848379515e-18	-0.303435933774143		0.253	0.37
2.52385270775837e-14	8	PDIA3	Memory 1	
1.53301939158596e-18	-0.301481286495283		0.151	0.25
2.73322027325861e-14	8	DKC1	Memory 1	
1.78831599240711e-18	-0.31528755398731		0.14	0.24
3.18838858286264e-14	8	DSTN	Memory 1	
2.02660684723761e-18	-0.324106362355184		0.072	0.149
3.61323734793994e-14	8	SDF2L1	Memory 1	
2.33311862143051e-18	0.311919368828213		0.234	0.161
4.15971719014847e-14	8	MARCKS	Memory 1	
3.05906751870405e-18	-0.302114128803025		0.365	0.473
5.45401147909745e-14	8	SNRPF	Memory 1	
4.37784812488937e-18	-0.389990494938512		0.275	0.379
7.80526542186526e-14	8	ACADM	Memory 1	
6.50542728491376e-18	-0.506843199405701		0.225	0.319
1.15985263062727e-13	8	CD69	Memory 1	
6.65978875516187e-18	-0.32853375335413		0.223	0.333
1.18737373715781e-13	8	HNRNPH1	Memory 1	
9.26998530442657e-18	0.330935575607166		0.365	0.302
1.65274567992621e-13	8	CD99	Memory 1	
1.05398419101457e-17	-0.490771565352487		0.84	0.857
1.87914841415988e-13	8	CD79B	Memory 1	
1.32884793976189e-17	-0.418898694730399		0.829	0.852
2.36920299180148e-13	8	ATP5MG	Memory 1	
1.65528902183936e-17	-0.315886453680416		0.226	0.336
2.95121479703739e-13	8	BPTF	Memory 1	
4.89157876594528e-17	-0.333460821121648		0.173	0.278
8.72119578180384e-13	8	NCF1	Memory 1	
5.35521535776519e-17	-0.327714121376154		0.151	0.244

9.54781346135956e-13	8	MYDGF	Memory	1
5.39033959803957e-17	-1.28600086754519	0.345	0.425	
9.61043646934474e-13	8	HIST1H4C	Memory	1
6.88472216783577e-17	-0.351642487140651	0.389	0.496	
1.22747711530344e-12	8	C4orf3	Memory	1
7.11077334519024e-17	-0.319070813734142	0.255	0.365	
1.26777977971397e-12	8	TPD52	Memory	1
1.31220267104149e-16	-0.387396453313851	0.901	0.899	
2.33952614219987e-12	8	PFN1	Memory	1
2.70376629106104e-16	0.309228532579685	0.201	0.161	
4.82054492033273e-12	8	LYPLAL1	Memory	1
7.95203097528608e-16	-0.340803081350958	0.453	0.544	
1.41776760258375e-11	8	PARP1	Memory	1
5.30877407616124e-15	-0.310738744329369	0.118	0.196	
9.46501330038788e-11	8	DNMT1	Memory	1
9.10574015123398e-15	-0.321635600282292	0.312	0.412	
1.62346241156351e-10	8	PPIB	Memory	1
9.42586443784406e-15	-0.432670850142403	0.086	0.154	
1.68053737062322e-10	8	DUSP2	Memory	1
1.14139262745708e-14	-1.61023205157095	0.171	0.124	
2.03498891549323e-10	8	IGHG2	Memory	1
6.40167792630274e-14	-0.33458293375403	0.122	0.193	
1.14135515748052e-09	8	DAAM1	Memory	1
3.86977741284781e-12	-0.42579760980584	0.927	0.932	
6.89942614936636e-08	8	HMGB1	Memory	1
5.19468800388742e-12	-0.310288616177081	0.486	0.555	
9.26160924213087e-08	8	TCEA1	Memory	1
6.36929829347173e-12	-0.365794262635412	0.848	0.867	
1.13558219274307e-07	8	CD79A	Memory	1
8.88326899672215e-12	-0.304895035650034	0.095	0.154	
1.58379802942559e-07	8	NFKBID	Memory	1
9.22862539965234e-12	-0.375041509158289	0.995	0.99	
1.64537162250402e-07	8	ACTB	Memory	1
1.53572491206872e-11	-0.323792604973378	0.315	0.401	
2.73804394572732e-07	8	BASP1	Memory	1
3.71006563363494e-11	-0.428156689912998	0.479	0.536	
6.61467601820774e-07	8	ISG20	Memory	1
2.80315959231703e-10	-0.304660228718901	0.528	0.619	
4.99775323714202e-06	8	MEF2C	Memory	1
4.58291231471741e-10	-1.26381851291044	0.361	0.405	
8.17087436590967e-06	8	JCHAIN	Memory	1
5.28961766607813e-10	-0.329139607041094	0.554	0.607	
9.4308593368507e-06	8	DYNLL1	Memory	1
1.55626605456793e-09	-0.423882386856911	0.778	0.786	
2.77466674868917e-05	8	ACTG1	Memory	1
1.63595667577775e-09	-0.323621536040047	0.941	0.929	
2.91674715724415e-05	8	NPM1	Memory	1
1.66905223214434e-09	-0.30003511927883	0.176	0.243	
2.97575322469014e-05	8	RGS1	Memory	1
1.27955352814916e-07	-0.300752977330143	0.277	0.337	

0.00228131598533713 8 DUT Memory 1
1.73678123178851e-07 -0.69278847163109 0.148 0.214
0.00309650725815574 8 PLCG2 Memory 1
1.133179590316e-06 -0.38075319894035 0.446 0.501
0.020203458915744 8 SSR4 Memory 1
1.44268372773046e-06 -1.75921472194198 0.56 0.648
0.0257216081817064 8 IGLC2 Memory 1
8.58497564415188e-05 -0.356679306897098 0.275 0.314 1
8 HSP90B1 Memory 1
0.000172038520472277 -1.10154155830781 0.381 0.431 1
8 IGLC3 Memory 1
0.000447151849428913 -1.0986302809319 0.914 0.927 1 8
IGKC Memory 1
0.00440557608805478 -1.38915438923069 0.268 0.242 1
8 IGHG4 Memory 1
0 3.08267290432663 0.824 0.185 0 9 LGALS1
Memory LGALS1
0 2.14313246384017 0.714 0.248 0 9 S100A4
Memory LGALS1
0 1.51652260669439 0.854 0.452 0 9 S100A6
Memory LGALS1
0 0.615459714060329 1 0.998 0 9 B2M
Memory LGALS1
6.52166652510445e-322 1.3039649815301 0.792 0.425
1.16274792476087e-317 9 COTL1 Memory LGALS1
2.98288116981579e-298 1.22507136717837 0.889 0.559
5.31817883766458e-294 9 VIM Memory LGALS1
3.93215802945247e-260 -1.58394266496617 0.237 0.669
7.01064455071082e-256 9 TCL1A Memory LGALS1
8.81605088906097e-255 1.19086698531522 0.635 0.235
1.57181371301068e-250 9 TNFRSF13B Memory LGALS1
4.80203388400387e-241 1.16545405970551 0.606 0.225
8.5615462117905e-237 9 CAPG Memory LGALS1
7.3476314677521e-224 0.595867707348495 1 0.998
1.31000921438552e-219 9 TMSB4X Memory LGALS1
6.57154593127733e-204 1.06386786975665 0.652 0.292
1.17164092408743e-199 9 CD27 Memory LGALS1
9.64667547453537e-202 0.851794158832797 0.943 0.769
1.71990577035491e-197 9 HLA-DPB1 Memory LGALS1
2.04518536689899e-196 1.20745647836339 0.526 0.186
3.6463609906442e-192 9 ACP5 Memory LGALS1
4.08596632469301e-195 0.984229832699323 0.75 0.434
7.28486936029517e-191 9 ANXA2 Memory LGALS1
1.1740463499015e-193 0.745982086836826 0.961 0.851
2.09320723723938e-189 9 SH3BGRL3 Memory LGALS1
3.58808177685326e-191 0.792724747918777 0.185 0.014
6.39719099995167e-187 9 FUT7 Memory LGALS1
1.46440746272636e-178 -1.13854535862614 0.696 0.913
2.61089206529483e-174 9 IGHM Memory LGALS1
2.57114454486985e-173 0.997948504110509 0.619 0.326

4.58409360904845e-169	9	S100A11	Memory	LGALS1
2.33549775557581e-167	0.924391316959341	0.364	0.111
4.16395894841611e-163	9	PYCARD	Memory	LGALS1
9.97817686075744e-166	0.436271530119104	0.999	0.986
1.77900915250444e-161	9	HLA-B	Memory	LGALS1
5.26172946286185e-163	0.861009277562669	0.2	0.036
9.3811374593364e-159	9	GSN	Memory	LGALS1
5.76277285670665e-161	0.853259062612589	0.328	0.083
1.02744477262223e-156	9	ITGB7	Memory	LGALS1
3.78513673337491e-159	0.705405154439422	0.966	0.865
6.74852028193412e-155	9	CD52	Memory	LGALS1
4.92528262846606e-158	0.754032310890853	0.92	0.69
8.78128639829213e-154	9	EMP3	Memory	LGALS1
3.89366301838426e-157	0.921600921297879	0.748	0.507
6.94201179547729e-153	9	LSP1	Memory	LGALS1
2.05629296194988e-147	0.78519113307303	0.312	0.064
3.66616472186045e-143	9	MYO1F	Memory	LGALS1
2.7504538044002e-142	0.661577956939326	0.168	0.022
4.90378408786511e-138	9	ZBED2	Memory	LGALS1
2.39623429426976e-137	0.70574199032431	0.265	0.062
4.27224612325356e-133	9	THEMIS2	Memory	LGALS1
2.81287047048894e-136	0.802955290865642	0.706	0.417
5.01506676183474e-132	9	PLP2	Memory	LGALS1
1.89937027726663e-134	-1.04973707834447	0.299	0.616
3.38638726733867e-130	9	IGHD	Memory	LGALS1
2.29669181994311e-134	0.649380124587767	0.186	0.023
4.09477184577658e-130	9	ZBTB32	Memory	LGALS1
4.91972780795802e-129	0.482760661621196	0.976	0.902
8.77138270880835e-125	9	HLA-A	Memory	LGALS1
6.97059872457781e-126	1.12824055330417	0.296	0.088
1.24278804660498e-121	9	KLK1	Memory	LGALS1
4.42628665771537e-120	0.798655548482811	0.565	0.258
7.89162648204073e-116	9	GPR183	Memory	LGALS1
8.85305241713671e-120	0.759837643744319	0.271	0.069
1.5784107154513e-115	9	CAPN2	Memory	LGALS1
4.37542006187038e-113	0.825201721038615	0.53	0.297
7.8009364283087e-109	9	CD99	Memory	LGALS1
2.96554119681176e-108	0.59268995135435	0.935	0.81
5.2872633997957e-104	9	HLA-DPA1	Memory	LGALS1
5.4345103256139e-106	0.732339448617684	0.35	0.144
9.68918845953702e-102	9	CLECL1	Memory	LGALS1
1.66019811578706e-104	0.512716089101223	0.98	0.945
2.95996722063675e-100	9	HLA-DRA	Memory	LGALS1
5.49478105847941e-103	0.787451635161478	0.326	0.134
9.79664514916293e-99	9	ITGB1	Memory	LGALS1
1.26305041421976e-101	0.718602805773942	0.292	0.105
2.2518925835124e-97	9	CRIP1	Memory	LGALS1
9.36997986270008e-98	0.558315523146643	0.899	0.759
1.6705737097208e-93	9	MYL12A	Memory	LGALS1
1.04036011784399e-97	0.48513444658187	0.144	0.019

1.85485805410405e-93 9 CALHM2 Memory LGALS1
4.7640054548681e-97 0.697369207612806 0.574 0.341
8.49374532548434e-93 9 S100A10 Memory LGALS1
3.16531924685605e-96 0.508231375481194 0.924 0.789
5.64344768521964e-92 9 HLA-E Memory LGALS1
1.05736426477589e-91 -1.08336091658378 0.246 0.479
1.88517474766894e-87 9 CD83 Memory LGALS1
1.31673207336739e-87 0.637230506490436 0.272 0.085
2.34760161360673e-83 9 AHNAK Memory LGALS1
2.11976620463131e-85 -1.01490290324374 0.383 0.595
3.77933116623715e-81 9 MARCKSL1 Memory LGALS1
7.14361979987338e-85 0.82340016098362 0.358 0.152
1.27363597411943e-80 9 LMNA Memory LGALS1
2.17705769778567e-83 0.451843881899884 0.976 0.885
3.88147616938207e-79 9 GAPDH Memory LGALS1
1.03260450270892e-81 0.651560914647453 0.319 0.135
1.84103056787973e-77 9 MYO1G Memory LGALS1
8.37299590354652e-81 0.623923036201349 0.592 0.382
1.49282143964331e-76 9 ARL6IP5 Memory LGALS1
1.80000383258336e-76 0.628373360373611 0.312 0.134
3.20922683311288e-72 9 AIM2 Memory LGALS1
5.23158351196363e-75 0.536620542923546 0.516 0.266
9.32739024347995e-71 9 KLF2 Memory LGALS1
4.70651863841967e-74 0.601162695495606 0.489 0.278
8.39125208043843e-70 9 ACAP1 Memory LGALS1
1.53118342789752e-73 0.590555321755028 0.488 0.272
2.72994693359848e-69 9 RNASET2 Memory LGALS1
2.23632755399125e-73 0.388254034669009 0.123 0.019
3.98714839601101e-69 9 HMOX1 Memory LGALS1
1.59483011463102e-72 0.632561333241827 0.373 0.184
2.84342261137565e-68 9 S1PR4 Memory LGALS1
8.0512207290659e-72 0.657613159386537 0.587 0.413
1.43545214378516e-67 9 CTSH Memory LGALS1
2.18823063154054e-71 -1.22818147465486 0.057 0.211
3.90139639297363e-67 9 RGS13 Memory LGALS1
1.15941635697328e-70 0.493569056937825 0.849 0.708
2.06712342284767e-66 9 CLIC1 Memory LGALS1
2.64053989445947e-70 -0.764365442880025 0.154 0.353
4.7078185778318e-66 9 ABRACL Memory LGALS1
1.52025792119673e-68 0.536004746708856 0.252 0.086
2.71046784770165e-64 9 ANXA4 Memory LGALS1
9.91245971835916e-68 -0.628329307112828 0.436 0.639
1.76729244318625e-63 9 GSTP1 Memory LGALS1
1.95419595011324e-66 0.481030982735397 0.193 0.056
3.48413595945689e-62 9 FLNA Memory LGALS1
1.31987491091577e-63 -0.71657927450554 0.053 0.207
2.35320497867173e-59 9 HMCES Memory LGALS1
1.61797301064378e-62 0.56208335200625 0.483 0.291
2.88468408067679e-58 9 PDLIM1 Memory LGALS1
3.51114549960893e-62 0.594387409632637 0.45 0.261

6.26002131125275e-58 9 HHEX Memory LGALS1
4.40732836042503e-62 0.47106834596701 0.873 0.748
7.85782573380179e-58 9 HLA-C Memory LGALS1
5.91988568987545e-60 0.524598421032727 0.648 0.466
1.05545641964789e-55 9 GPSM3 Memory LGALS1
1.1299101612451e-59 -0.665399897897797 0.433 0.625
2.0145168264839e-55 9 CLEC2D Memory LGALS1
2.76831909082804e-59 0.362507613885372 0.92 0.81
4.93563610703731e-55 9 CORO1A Memory LGALS1
5.29971109103235e-59 -1.22048657202261 0.142 0.292
9.44885490420157e-55 9 MIR155HG Memory LGALS1
6.27519262459673e-59 0.575596951654443 0.551 0.344
1.11880409303935e-54 9 LY6E Memory LGALS1
2.976482242664e-58 0.322952142266454 0.995 0.985
5.30677019044565e-54 9 TMSB10 Memory LGALS1
3.01577659659924e-58 -0.802006679312371 0.142 0.304
5.37682809407679e-54 9 NME1 Memory LGALS1
6.49730683107848e-57 -0.500549383353373 0.011 0.122
1.15840483491298e-52 9 CD38 Memory LGALS1
8.78430818069011e-57 0.426683923167266 0.708 0.505
1.56615430553524e-52 9 BANK1 Memory LGALS1
2.87926139637146e-54 0.384288700909757 0.885 0.773
5.13343514359068e-50 9 ARHGDIB Memory LGALS1
4.85085412217936e-53 0.524110154095166 0.493 0.33
8.64858781443358e-49 9 VOPP1 Memory LGALS1
5.50713589035184e-52 0.437327279065709 0.167 0.06
9.8186725789083e-48 9 ARID3A Memory LGALS1
1.76947335481221e-51 0.479378190132472 0.271 0.112
3.15479404429469e-47 9 FCGR2B Memory LGALS1
3.54140886754438e-51 0.410193233225447 0.168 0.051
6.31397786994488e-47 9 TNFRSF1B Memory LGALS1
4.24434929903206e-51 0.528457899125341 0.372 0.214
7.56725036524427e-47 9 CCDC50 Memory LGALS1
1.50172599811603e-50 -0.342102024684808 0.006 0.106
2.67742728204108e-46 9 SEMA4A Memory LGALS1
1.76729942778442e-50 0.487888589805416 0.462 0.272
3.15091814979684e-46 9 IFITM2 Memory LGALS1
3.83249231432514e-50 0.488794206458678 0.528 0.356
6.8329505472103e-46 9 ANXA6 Memory LGALS1
9.41997995972531e-50 0.333252175902608 0.993 0.962
1.67948822701943e-45 9 FTL Memory LGALS1
4.01867574393352e-49 0.442163085818369 0.796 0.673
7.16489698385907e-45 9 TAGLN2 Memory LGALS1
8.5176151177333e-49 0.378305044796062 0.138 0.034
1.51860559934067e-44 9 RAB31 Memory LGALS1
1.02040535729235e-47 -0.507969868329166 0.653 0.775
1.81928071151654e-43 9 SEC62 Memory LGALS1
1.05126768711735e-47 0.470066000352135 0.265 0.136
1.87430515936153e-43 9 CORO1B Memory LGALS1
1.05270578151856e-47 0.394722255965053 0.823 0.693

1.87686913786944e-43 9 HLA-DQB1 Memory LGALS1
2.27148161449064e-47 0.490722738954979 0.748 0.616
4.04982457047537e-43 9 SUB1 Memory LGALS1
4.03990316968836e-47 0.489286583111088 0.298 0.158
7.20274336123737e-43 9 KIAA1551 Memory LGALS1
3.45690090352369e-46 0.523663761574152 0.423 0.28
6.16330862089239e-42 9 CD82 Memory LGALS1
1.03263390785676e-44 0.488348701606117 0.617 0.487
1.84108299431781e-40 9 RAC2 Memory LGALS1
1.51575373001683e-44 0.472252267480691 0.246 0.11
2.70243732524701e-40 9 CLN8 Memory LGALS1
1.98401659322864e-44 0.591894500703883 0.256 0.121
3.53730318406735e-40 9 LGALS3 Memory LGALS1
3.84748512245979e-44 -0.395800170278834 0.02 0.118
6.85968122483357e-40 9 PYCR1 Memory LGALS1
9.46343693237503e-44 0.470946236334067 0.364 0.214
1.68723617067314e-39 9 LY86 Memory LGALS1
3.4292592467773e-43 0.497497022836317 0.291 0.164
6.11402631107925e-39 9 SAMSN1 Memory LGALS1
4.74625807252769e-43 -0.595980756429612 0.081 0.202
8.46210351750963e-39 9 YBX3 Memory LGALS1
1.33560693263131e-42 -0.531099680743462 0.141 0.294
2.38125360018836e-38 9 RRAS2 Memory LGALS1
1.61372714885784e-42 0.326128472407969 0.117 0.039
2.87711413369864e-38 9 BHLHE41 Memory LGALS1
2.29696215512445e-42 0.44784119466411 0.401 0.249
4.09525382637138e-38 9 TBC1D10C Memory LGALS1
8.47756693358625e-42 0.442131940411941 0.303 0.166
1.51146540858909e-37 9 MFSD10 Memory LGALS1
9.37192375289918e-42 0.438204539608515 0.625 0.475
1.6709202859044e-37 9 ISCU Memory LGALS1
1.91967193044035e-41 -0.318269935771023 0.013 0.102
3.4225830847821e-37 9 FAM241A Memory LGALS1
8.47072369995237e-41 0.45102659758574 0.355 0.21
1.51024532846451e-36 9 YPEL3 Memory LGALS1
9.23798348382084e-41 0.44826332619197 0.383 0.237
1.64704007533042e-36 9 ARHGAP45 Memory LGALS1
9.79829706150296e-41 0.524536717689935 0.418 0.294
1.74693838309536e-36 9 TCF4 Memory LGALS1
1.13885776742731e-40 0.494425053500087 0.622 0.48
2.03046951354615e-36 9 NEAT1 Memory LGALS1
1.94754416967537e-40 0.448708201955592 0.364 0.223
3.47227650011422e-36 9 PCSK7 Memory LGALS1
2.14810626566283e-40 0.463117688374463 0.369 0.235
3.82985866105027e-36 9 NCF4 Memory LGALS1
7.6686199901917e-40 0.505470076584166 0.39 0.262
1.36723825805128e-35 9 GRN Memory LGALS1
8.82462765897007e-40 0.342209990045797 0.136 0.043
1.57334286531777e-35 9 HCK Memory LGALS1
1.6391791780605e-39 0.391795256223759 0.22 0.091

2.92249255656407e-35 9 CYSLTR1 Memory LGALS1
4.32209053214268e-39 0.361857890049002 0.77 0.649
7.70585520975719e-35 9 HLA-DQA1 Memory LGALS1
9.37664680291388e-39 0.380689701808671 0.133 0.046
1.67176235849152e-34 9 PLD4 Memory LGALS1
8.85798588254645e-38 0.450652706077778 0.236 0.125
1.57929030299921e-33 9 EEA1 Memory LGALS1
1.41787003973368e-37 0.42993853821346 0.271 0.156
2.52792049384118e-33 9 IL10RA Memory LGALS1
3.84927599314558e-37 0.340729558548188 0.13 0.059
6.86287416817925e-33 9 TESC Memory LGALS1
3.9427662403482e-37 -0.579195310955869 0.111 0.237
7.0295579299168e-33 9 IL4R Memory LGALS1
5.08192014111004e-37 0.403875530070131 0.217 0.103
9.06055541958508e-33 9 CARD19 Memory LGALS1
5.30446569818716e-37 0.368027426404037 0.171 0.079
9.45733189329789e-33 9 KCNN4 Memory LGALS1
7.08603150012946e-37 0.377486303608528 0.74 0.618
1.26336855615808e-32 9 ARPC1B Memory LGALS1
1.00759860445026e-36 -0.345632994811399 0.017 0.104
1.79644755187436e-32 9 MYBL2 Memory LGALS1
1.10883433860144e-36 0.309903669516163 0.116 0.033
1.9769407422925e-32 9 ARL4C Memory LGALS1
1.12924655902407e-36 0.369526499784926 0.487 0.326
2.01333369008402e-32 9 RIPOR2 Memory LGALS1
1.4796189804424e-36 0.401049790207901 0.549 0.377
2.63801268023076e-32 9 CD44 Memory LGALS1
1.60126788075238e-36 0.3692297343041 0.887 0.782
2.85490050459343e-32 9 ACTG1 Memory LGALS1
3.96899139422119e-36 0.418324265518345 0.236 0.116
7.07631475675697e-32 9 IL27RA Memory LGALS1
5.62726288572261e-36 -0.910528376135899 0.641 0.718
1.00328469989548e-31 9 HMGN2 Memory LGALS1
8.11587708147594e-36 -0.513685924882975 0.269 0.427
1.44697972485635e-31 9 TMEM123 Memory LGALS1
2.27732570456301e-35 0.41680401802869 0.267 0.144
4.06024399866539e-31 9 MVP Memory LGALS1
7.26516429461998e-35 -0.892027582465439 0.08 0.185
1.2953061420878e-30 9 XBP1 Memory LGALS1
1.5806379640327e-34 0.485924007884557 0.209 0.117
2.8181194260739e-30 9 NCBP3 Memory LGALS1
1.80995243276413e-34 0.388086565489277 0.326 0.178
3.22696419237516e-30 9 MBP Memory LGALS1
1.92058937539838e-34 -0.492561281439321 0.042 0.138
3.42421879739777e-30 9 NEIL1 Memory LGALS1
4.04091963142085e-34 0.319583909663889 0.119 0.042
7.20455561086024e-30 9 VSIR Memory LGALS1
8.70247348956281e-34 -0.768949689460335 0.628 0.698
1.55156399845415e-29 9 H2AFZ Memory LGALS1
1.39661979977592e-33 0.360141132531043 0.209 0.097

2.49003344102049e-29 9 CDKN2D Memory LGALS1
1.79257197538444e-33 -0.442676069920709 0.045 0.139
3.19597657491292e-29 9 PSAT1 Memory LGALS1
1.92750176640141e-33 0.357634308601119 0.174 0.08
3.43654289931707e-29 9 OAS1 Memory LGALS1
1.23174402670086e-32 0.360980617443685 0.453 0.298
2.19607642520496e-28 9 ZFP36L2 Memory LGALS1
1.72631550931825e-32 0.376310999175381 0.268 0.141
3.07784792156351e-28 9 SUN2 Memory LGALS1
4.28180315013225e-32 -0.92919634468567 0.322 0.432
7.63402683637079e-28 9 STMN1 Memory LGALS1
2.16283680143056e-31 0.322228763072232 0.205 0.095
3.85612173327054e-27 9 ARRB2 Memory LGALS1
2.41046272457769e-31 0.313644346271513 0.647 0.498
4.29761399164956e-27 9 TSC22D3 Memory LGALS1
3.10757068761854e-31 -0.500539205634174 0.262 0.389
5.5404877789551e-27 9 NHP2 Memory LGALS1
4.48192403117545e-31 0.367989042459826 0.423 0.292
7.9908223551827e-27 9 TWF2 Memory LGALS1
5.26124582931478e-31 0.362035721743964 0.583 0.441
9.38027518908532e-27 9 KLF6 Memory LGALS1
6.54121707810409e-31 -0.42853517457768 0.106 0.228
1.16623359285518e-26 9 SIAH2 Memory LGALS1
6.58094062709124e-31 -0.531091600170746 0.362 0.478
1.1733159044041e-26 9 SNRPD1 Memory LGALS1
1.13926505915365e-30 0.313038520568545 0.139 0.056
2.03119567396505e-26 9 TTC39C Memory LGALS1
1.28667912182267e-30 -0.475407748795854 0.038 0.125
2.29402020629764e-26 9 AL139020.1 Memory LGALS1
1.45842169483725e-30 0.392554853046299 0.46 0.323
2.60022003972534e-26 9 ZFP36 Memory LGALS1
1.71760773001354e-30 0.342639905192327 0.172 0.074
3.06232282184114e-26 9 TIMP1 Memory LGALS1
2.47942355207666e-30 0.373453789823328 0.33 0.207
4.42056425099747e-26 9 IDS Memory LGALS1
2.68381281143769e-30 -0.465148791436371 0.096 0.21
4.78496986151226e-26 9 EAF2 Memory LGALS1
2.72056171197793e-30 0.305946079071846 0.115 0.044
4.85048947628545e-26 9 HAGHL Memory LGALS1
3.26030811310861e-30 0.454573461275795 0.332 0.207
5.81280333486134e-26 9 PLAC8 Memory LGALS1
3.58741621086728e-30 -1.26658306725386 0.082 0.179
6.39600436235527e-26 9 MZB1 Memory LGALS1
5.10403971776682e-30 0.395788084273434 0.273 0.18
9.09999241280647e-26 9 TRABD Memory LGALS1
7.04827273411335e-30 0.305829077184248 0.627 0.498
1.25663654576507e-25 9 PSMB9 Memory LGALS1
1.02985561463181e-29 0.410364020785835 0.521 0.42
1.83612957532706e-25 9 GABARAPL2 Memory LGALS1
1.52138014792895e-29 0.364722995750516 0.467 0.334

2.71246866574253e-25 9 SPIB Memory LGALS1
2.25718351005781e-29 -0.571060355957073 0.354 0.465
4.02433248008206e-25 9 HSPD1 Memory LGALS1
3.620573274054e-29 0.306199250079302 0.839 0.75
6.45512009031087e-25 9 TOMM7 Memory LGALS1
4.23872213391463e-29 0.378551279201843 0.331 0.207
7.55721769255639e-25 9 FMNL1 Memory LGALS1
4.25756958792188e-29 0.382883347776557 0.418 0.294
7.59082081830592e-25 9 BLK Memory LGALS1
6.25314514199222e-29 -0.469800743386552 0.029 0.112
1.11487324736579e-24 9 VPREB3 Memory LGALS1
7.19365515264937e-29 -0.479847508346497 0.125 0.241
1.28255677716586e-24 9 GCHFR Memory LGALS1
9.14987207756288e-29 0.346797869293897 0.389 0.254
1.63133069270869e-24 9 CNN2 Memory LGALS1
9.91637272706647e-29 0.312388247803148 0.528 0.384
1.76799009350868e-24 9 EVI2B Memory LGALS1
1.83289863695059e-28 -0.852111411821011 0.614 0.667
3.26787497981921e-24 9 TUBA1B Memory LGALS1
2.1738833469126e-28 0.374600927193642 0.315 0.199
3.87581661921048e-24 9 PTPN1 Memory LGALS1
2.59046825484609e-28 -0.63454706663961 0.724 0.749
4.6185458515651e-24 9 RAN Memory LGALS1
2.60696288132619e-28 0.357388129977029 0.482 0.363
4.64795412111646e-24 9 CXXC5 Memory LGALS1
2.9082796764903e-28 0.367485726566202 0.319 0.204
5.18517183521455e-24 9 ARHGAP30 Memory LGALS1
3.16717421669294e-28 -0.427232157233489 0.169 0.282
5.64675491094185e-24 9 WARS Memory LGALS1
3.28653511589678e-28 -0.648638748687999 0.733 0.769
5.85956345813237e-24 9 HMGN1 Memory LGALS1
3.56522720113118e-28 0.380602131749975 0.47 0.341
6.35644357689677e-24 9 CAST Memory LGALS1
8.1661554571501e-28 0.36178361344 0.494 0.373
1.45594385645529e-23 9 ZFAND6 Memory LGALS1
1.14928219375321e-27 -0.446586132467493 0.271 0.393
2.04905522324261e-23 9 CCT5 Memory LGALS1
1.78053836481002e-27 0.3116685778469 0.164 0.08
3.17452185061979e-23 9 CDC42EP3 Memory LGALS1
1.89732250929751e-27 -0.330085447139829 0.032 0.114
3.38273630182653e-23 9 AC023590.1 Memory LGALS1
2.18845903071773e-27 -0.485842688700006 0.607 0.682
3.90180360586663e-23 9 SRSF3 Memory LGALS1
3.086046780824e-27 0.308146569875721 0.14 0.06
5.5021128055311e-23 9 KYNU Memory LGALS1
6.18824504638545e-27 0.332037821882039 0.267 0.163
1.10330220932006e-22 9 ARHGAP25 Memory LGALS1
9.29868750159149e-27 -0.469965486160953 0.559 0.643
1.65786299465875e-22 9 ERH Memory LGALS1
9.50959563850517e-27 0.386691472625399 0.35 0.233

1.69546580638909e-22 9 AC004687.1 Memory LGALS1
1.65502794217114e-26 0.307197393328148 0.619 0.494
2.95074931809693e-22 9 SAT1 Memory LGALS1
2.69655532213417e-26 0.352939747654453 0.336 0.207
4.807688483833e-22 9 LBH Memory LGALS1
2.83006755550226e-26 -0.669778605375007 0.839 0.848
5.04572744470497e-22 9 HSP90AB1 Memory LGALS1
2.9725651030572e-26 0.327947331055871 0.702 0.591
5.29978632224069e-22 9 TPM3 Memory LGALS1
3.73906729791865e-26 0.315574713914511 0.302 0.17
6.66638308545917e-22 9 MARCH1 Memory LGALS1
4.24099637416905e-26 -0.50146800945489 0.181 0.283
7.561272435506e-22 9 SRM Memory LGALS1
4.24857949836227e-26 0.349263187506287 0.494 0.355
7.57479238763009e-22 9 ARID5B Memory LGALS1
4.98188805811879e-26 0.32445672900685 0.229 0.119
8.88220821882e-22 9 DDAH2 Memory LGALS1
6.22348136830206e-26 -0.459931660169141 0.352 0.467
1.10958449315457e-21 9 PRMT1 Memory LGALS1
1.275058060522e-25 0.375905982835332 0.424 0.32
2.27330101610468e-21 9 AES Memory LGALS1
1.45765321653063e-25 -0.553753403490226 0.398 0.488
2.59884991975247e-21 9 RANBP1 Memory LGALS1
1.91440535572962e-25 -0.303272709887539 0.061 0.158
3.41319330873035e-21 9 PAICS Memory LGALS1
2.12952989537054e-25 0.346015750692506 0.222 0.123
3.79673885045614e-21 9 ZEB2 Memory LGALS1
2.31107903110791e-25 -0.48408855157637 0.487 0.574
4.12042280456229e-21 9 SNRPE Memory LGALS1
3.34022906158917e-25 0.348865512005849 0.194 0.108
5.95529439390733e-21 9 IVNS1ABP Memory LGALS1
5.69777353226584e-25 -0.368851968949376 0.14 0.254
1.01585604306768e-20 9 GARS Memory LGALS1
5.70307677873432e-25 0.32564774583444 0.496 0.348
1.01680155888054e-20 9 TXNIP Memory LGALS1
5.79741599195051e-25 -0.462785853163477 0.285 0.405
1.03362129720486e-20 9 RPL22L1 Memory LGALS1
6.23103797918424e-25 -0.666341097195996 0.105 0.193
1.11093176130876e-20 9 PTTG1 Memory LGALS1
6.88879387412813e-25 0.31772282043374 0.208 0.115
1.2282030598183e-20 9 HPCAL1 Memory LGALS1
1.35615730199861e-24 0.311629377405821 0.192 0.087
2.41789285373332e-20 9 CCR6 Memory LGALS1
2.14009871240664e-24 -0.463887111937639 0.154 0.273
3.81558199434981e-20 9 PIM1 Memory LGALS1
2.25331755358811e-24 0.340445653552508 0.453 0.339
4.01743986629224e-20 9 CMTM6 Memory LGALS1
2.26163087417918e-24 -0.377759652642671 0.07 0.162
4.03226168557406e-20 9 APLP2 Memory LGALS1
3.89296971926612e-24 0.30937388125106 0.252 0.155

6.94077571247957e-20	9	CTSZ	Memory	LGALS1
4.01790667720571e-24	0.384473323072927	0.441	0.341	
7.16352581479006e-20	9	RNH1	Memory	LGALS1
4.11990395726743e-24	-0.348806226359346	0.081	0.184	
7.3453767654121e-20	9	LCK	Memory	LGALS1
5.01660578953703e-24	0.319486013416971	0.241	0.144	
8.94410646216557e-20	9	SELENOW	Memory	LGALS1
5.17025932509585e-24	-0.34802544860947	0.101	0.202	
9.21805535071339e-20	9	EBNA1BP2	Memory	LGALS1
7.04444000740644e-24	-0.421173461515079	0.277	0.392	
1.25595320892049e-19	9	SHMT2	Memory	LGALS1
8.57818844144211e-24	-0.324687003638665	0.07	0.159	
1.52940521722471e-19	9	GAR1	Memory	LGALS1
8.68253286719401e-24	-0.555545644986573	0.48	0.562	
1.54800878489202e-19	9	NCL	Memory	LGALS1
1.10980423193544e-23	0.32248772020267	0.503	0.383	
1.9786699651177e-19	9	PSAP	Memory	LGALS1
1.44305516908205e-23	-0.502741357015911	0.428	0.515	
2.57282306095638e-19	9	PA2G4	Memory	LGALS1
1.52088358087431e-23	0.334229518563995	0.327	0.206	
2.71158333634081e-19	9	MGST3	Memory	LGALS1
1.66428850651906e-23	-0.355860918420472	0.148	0.256	
2.96725997827284e-19	9	RRP7A	Memory	LGALS1
1.96603388327437e-23	0.339680656968265	0.46	0.344	
3.50524181048987e-19	9	LITAF	Memory	LGALS1
2.41745032917008e-23	-0.41985826957663	0.348	0.453	
4.31007219187734e-19	9	ILF2	Memory	LGALS1
2.60117229790945e-23	-0.316045656280263	0.079	0.175	
4.63763008994276e-19	9	ASNS	Memory	LGALS1
3.30393715917961e-23	-0.361263566214818	0.107	0.211	
5.89058956110133e-19	9	GNL3	Memory	LGALS1
3.36513622919667e-23	0.324337146331893	0.189	0.102	
5.99970138303475e-19	9	SCIMP	Memory	LGALS1
3.81882939914853e-23	0.333512853599459	0.281	0.176	
6.80859093574191e-19	9	RHOF	Memory	LGALS1
6.26874699291917e-23	-0.513682490498032	0.147	0.238	
1.11765490136756e-18	9	UBE2J1	Memory	LGALS1
7.06372868678777e-23	0.332926241944225	0.403	0.287	
1.25939218756739e-18	9	YWHAH	Memory	LGALS1
7.49305186120406e-23	-0.436867925886645	0.094	0.174	
1.33593621633407e-18	9	TNFRSF18	Memory	LGALS1
7.6052564377872e-23	-0.328758334293674	0.09	0.182	
1.35594117029308e-18	9	ABCE1	Memory	LGALS1
7.92874391727393e-23	0.305912461958347	0.272	0.174	
1.41361575301077e-18	9	RGS19	Memory	LGALS1
8.11893762605314e-23	-0.427512455398453	0.325	0.432	
1.44752538934901e-18	9	CCT2	Memory	LGALS1
9.72449941852988e-23	-0.346622512001278	0.138	0.246	
1.73378100132969e-18	9	POLD2	Memory	LGALS1
1.01369730374917e-22	-0.442115386904134	0.092	0.178	

1.8073209228544e-18 9 PDIA4 Memory LGALS1
1.10942661287915e-22 -0.335658782876359 0.051 0.134
1.97799670810224e-18 9 PHGDH Memory LGALS1
1.15908120039961e-22 0.328985379974833 0.265 0.176
2.06652587219247e-18 9 ICAM3 Memory LGALS1
1.52235358629592e-22 0.467657981627092 0.605 0.485
2.714204209007e-18 9 LTB Memory LGALS1
1.66444689107856e-22 -0.376322299170414 0.295 0.415
2.96754236210397e-18 9 HNRNPR Memory LGALS1
2.04322131816797e-22 -0.45607116103685 0.225 0.33
3.64285928816167e-18 9 SLC3A2 Memory LGALS1
3.32551467398807e-22 0.304464747696518 0.356 0.241
5.92906011225333e-18 9 RASGRP2 Memory LGALS1
3.53741755995503e-22 -0.565799156898222 0.063 0.133
6.30686176764382e-18 9 MYC Memory LGALS1
6.16842768777741e-22 0.32572479928975 0.298 0.193
1.09976897245383e-17 9 TYMP Memory LGALS1
1.01939954439017e-21 0.302742569788082 0.309 0.202
1.81748744769324e-17 9 PRKCB Memory LGALS1
1.0586556755365e-21 -0.380029773118299 0.216 0.33
1.88747720391402e-17 9 PMAIP1 Memory LGALS1
2.08144644888022e-21 -0.464053989862221 0.826 0.857
3.71101087370854e-17 9 CD79B Memory LGALS1
2.10484730975321e-21 -0.309347942968846 0.08 0.166
3.752732268559e-17 9 MRTO4 Memory LGALS1
2.39187503573143e-21 -0.3820012619848 0.197 0.305
4.26447400120557e-17 9 NOP58 Memory LGALS1
2.42231294392117e-21 0.309752348189643 0.746 0.677
4.31874174771706e-17 9 HLA-DMA Memory LGALS1
2.66509040046091e-21 0.306956678548375 0.172 0.1
4.75158967498175e-17 9 RUNX3 Memory LGALS1
2.75650960269859e-21 -0.430556538748413 0.333 0.443
4.91458097065132e-17 9 RHOH Memory LGALS1
2.76357749942548e-21 -0.417497146613258 0.56 0.636
4.92718232372569e-17 9 PSMA4 Memory LGALS1
3.13331501897914e-21 -0.488460851572127 0.228 0.32
5.58638734733791e-17 9 DDX21 Memory LGALS1
3.66354535629281e-21 -0.422754934183938 0.103 0.186
6.53173501573445e-17 9 PLPP5 Memory LGALS1
4.1688571358436e-21 0.315044297476924 0.375 0.28
7.43265538749556e-17 9 MTPN Memory LGALS1
4.89496902795978e-21 0.340063182596961 0.172 0.11
8.7272402799495e-17 9 TRAC Memory LGALS1
5.81308400748137e-21 -0.437023973667705 0.198 0.307
1.03641474769385e-16 9 BCL7A Memory LGALS1
6.95929941000359e-21 0.330305878519438 0.299 0.216
1.24077349180954e-16 9 GNAI2 Memory LGALS1
1.00033286667792e-20 -0.416770883832436 0.299 0.403
1.78349346800007e-16 9 REL Memory LGALS1
1.18645154823189e-20 -0.361767030846608 0.079 0.172

2.11532446534265e-16 9 CCDC88A Memory LGALS1
1.21086143564936e-20 -0.309928932959723 0.056 0.131
2.15884485361924e-16 9 ICAM2 Memory LGALS1
1.91708159874923e-20 -0.315349017981571 0.122 0.221
3.41796478241e-16 9 MRPL12 Memory LGALS1
1.9673169758534e-20 -0.460065153442706 0.368 0.211
3.50752943624902e-16 9 IGHG1 Memory LGALS1
3.7278188842298e-20 -0.440605699546904 0.141 0.233
6.64632828869332e-16 9 CKS2 Memory LGALS1
5.32920747678802e-20 -0.474330465455124 0.414 0.5
9.50144401036535e-16 9 HSPE1 Memory LGALS1
6.26814637509261e-20 0.317513791180903 0.302 0.214
1.11754781721526e-15 9 C4orf48 Memory LGALS1
7.79315217975438e-20 -0.549905311362788 0.227 0.319
1.38944110212841e-15 9 CD69 Memory LGALS1
8.91343264225348e-20 0.30542354132917 0.271 0.171
1.58917590578737e-15 9 ASAH1 Memory LGALS1
9.20650825518688e-20 -0.382334484818221 0.304 0.404
1.64142835681727e-15 9 RPA3 Memory LGALS1
1.12451690577821e-19 -0.430869682646051 0.511 0.62
2.00490119131198e-15 9 MEF2C Memory LGALS1
1.25553810248154e-19 -0.385704145190449 0.322 0.429
2.23849888291433e-15 9 CD40 Memory LGALS1
1.4779505616671e-19 0.319814068712071 0.406 0.31
2.63503805639626e-15 9 LPXN Memory LGALS1
2.30495660655752e-19 -0.393616756364991 0.252 0.352
4.10950713383141e-15 9 TIMM13 Memory LGALS1
2.63389265699676e-19 -0.457903001020402 0.241 0.334
4.69596721815953e-15 9 GRHPR Memory LGALS1
2.82270470478569e-19 -0.310078877372552 0.134 0.231
5.03260021816241e-15 9 MRPL3 Memory LGALS1
3.95966720559669e-19 -0.390595464688841 0.269 0.365
7.05969066085835e-15 9 HSPA9 Memory LGALS1
7.23957285320521e-19 -0.355598596243202 0.156 0.249
1.29074344399796e-14 9 DKC1 Memory LGALS1
7.56734415811223e-19 -0.356341543757687 0.139 0.237
1.34918178994983e-14 9 ZCCHC7 Memory LGALS1
9.02595346507486e-19 -0.315462730348698 0.12 0.204
1.6092372432882e-14 9 NPM3 Memory LGALS1
1.02254657726042e-18 -0.303506590633088 0.073 0.145
1.82309829259761e-14 9 NOP16 Memory LGALS1
1.17466443714121e-18 -0.459737842884598 0.336 0.42
2.09430922497907e-14 9 METAP2 Memory LGALS1
1.22853675954958e-18 -0.365068311175253 0.616 0.68
2.19035818860095e-14 9 HNRNPA3 Memory LGALS1
1.45299506006642e-18 -1.36313084278112 0.335 0.405
2.59054489259243e-14 9 JCHAIN Memory LGALS1
1.45935038139688e-18 -0.457657362848611 0.464 0.532
2.60187579499251e-14 9 PSME2 Memory LGALS1
1.47690926894269e-18 -0.542415082539129 0.262 0.343

2.63318153559793e-14 9 LRMP Memory LGALS1
1.9153019109664e-18 -1.55897134468375 0.206 0.123
3.41479177706199e-14 9 IGHG2 Memory LGALS1
1.97434036208749e-18 -0.385056558337034 0.507 0.588
3.52005143156579e-14 9 EIF2S2 Memory LGALS1
2.89430313608947e-18 -0.366510730052547 0.178 0.278
5.16025306133391e-14 9 ITGAE Memory LGALS1
8.68229363224825e-18 -0.36030936354174 0.364 0.46
1.54796613169354e-13 9 SFPQ Memory LGALS1
9.6818618159949e-18 -0.311570289291061 0.107 0.187
1.72617914317373e-13 9 FKBP4 Memory LGALS1
1.10857607963381e-17 0.325437225524215 0.609 0.5
1.97648029237911e-13 9 HLA-DRB5 Memory LGALS1
1.33631732237586e-17 -0.318887890746555 0.147 0.239
2.38252015406392e-13 9 NIFK Memory LGALS1
1.81821494989676e-17 -0.300126610142314 0.104 0.189
3.24169543417093e-13 9 WDR43 Memory LGALS1
2.12686297139912e-17 -0.312707496762889 0.173 0.273
3.79198399170749e-13 9 TOP1 Memory LGALS1
2.42673265523999e-17 -0.307481541378226 0.92 0.927
4.32662165102738e-13 9 HNRNPA1 Memory LGALS1
2.6131531534324e-17 0.32305944359235 0.223 0.161
4.65899075725463e-13 9 LYPLAL1 Memory LGALS1
3.2184952511847e-17 -0.35084644341771 0.338 0.438
5.73825518333721e-13 9 EIF5B Memory LGALS1
3.69004668207236e-17 -0.355156115786968 0.088 0.157
6.57898422946681e-13 9 MCM7 Memory LGALS1
3.81778817082513e-17 -0.329271074167427 0.08 0.154
6.80673452976413e-13 9 CD72 Memory LGALS1
4.30980889878882e-17 -0.360406487352039 0.077 0.139
7.6839582856506e-13 9 FCER2 Memory LGALS1
6.39228370795353e-17 -0.50937259906371 0.197 0.28
1.13968026229103e-12 9 HSPA5 Memory LGALS1
6.58701558334845e-17 -0.38019111353976 0.221 0.306
1.17439900835519e-12 9 ODC1 Memory LGALS1
8.33530233016148e-17 -0.342926465355086 0.376 0.469
1.48610105244449e-12 9 PRDX6 Memory LGALS1
9.39674546346099e-17 -0.35716521151383 0.341 0.434
1.67534574868046e-12 9 SLIRP Memory LGALS1
1.42124439052937e-16 -0.584702234956614 0.334 0.406
2.53393662387481e-12 9 FABP5 Memory LGALS1
2.9694060212919e-16 -0.395971034045718 0.191 0.28
5.29415399536132e-12 9 PIM2 Memory LGALS1
4.36652812586327e-16 -0.300290504557109 0.148 0.241
7.78508299560163e-12 9 AMD1 Memory LGALS1
5.64676905277375e-16 -0.310912278983336 0.226 0.327
1.00676245441903e-11 9 IMP4 Memory LGALS1
7.70743673424036e-16 -0.330971603379301 0.332 0.423
1.37415889534771e-11 9 MAGOH Memory LGALS1
9.62933081405067e-16 -0.322123849307589 0.492 0.569

1.71681339083709e-11 9 UQCRQ Memory LGALS1
9.76512665967551e-16 -0.592723469681478 0.545 0.578
1.74102443215355e-11 9 TUBB Memory LGALS1
1.24658657471924e-15 -0.344769494870778 0.263 0.349
2.22253920406693e-11 9 H2AFY Memory LGALS1
2.29404464920475e-15 -0.334797765571977 0.138 0.221
4.09005220506716e-11 9 NANS Memory LGALS1
2.82010691166036e-15 -0.456919166811136 0.735 0.748
5.02796861279926e-11 9 HSP90AA1 Memory LGALS1
3.08584880158257e-15 -0.358199080442001 0.124 0.197
5.50175982834156e-11 9 FAM129C Memory LGALS1
3.29627526122292e-15 -0.358636487926524 0.313 0.396
5.87692916323434e-11 9 YWHAE Memory LGALS1
3.40362808517646e-15 -0.301066026924361 0.275 0.37
6.06832851306112e-11 9 EWSR1 Memory LGALS1
6.90940428993891e-15 -0.309007528453016 0.305 0.396
1.23187769085321e-10 9 PHB Memory LGALS1
7.51514188282256e-15 -0.35090507263913 0.455 0.532
1.33987464628843e-10 9 SRSF7 Memory LGALS1
1.09638001103174e-14 -0.377740671820439 0.6 0.647
1.9547359216685e-10 9 SNRPG Memory LGALS1
1.11562583035444e-14 -0.308947846147524 0.171 0.255
1.98904929293892e-10 9 SLC50A1 Memory LGALS1
1.15555968277166e-14 -0.319473994801046 0.242 0.332
2.0602473584136e-10 9 RFTN1 Memory LGALS1
1.1986082016039e-14 -0.329517246895043 0.535 0.596
2.1369985626396e-10 9 SRSF2 Memory LGALS1
1.45487137883955e-14 -0.508033368029828 0.294 0.394
2.59389018133304e-10 9 EIF5A Memory LGALS1
1.66876076793211e-14 -0.317488739469606 0.44 0.517
2.97523357314616e-10 9 SSBP1 Memory LGALS1
1.99533259329424e-14 -0.388328464152102 0.535 0.585
3.5574784805843e-10 9 ANP32B Memory LGALS1
3.67347740525205e-14 -0.544532697280689 0.729 0.751
6.54944286582387e-10 9 CXCR4 Memory LGALS1
4.06130414673133e-14 -0.356718508934741 0.209 0.291
7.24089916320728e-10 9 RUBCNL Memory LGALS1
4.14902169623601e-14 -0.326444671279439 0.339 0.422
7.39729078221919e-10 9 HNRNPD Memory LGALS1
4.56120815162179e-14 -0.341490652076106 0.37 0.444
8.13217801352649e-10 9 PPA1 Memory LGALS1
5.17551027832544e-14 -0.322454099728468 0.065 0.123
9.22741727522642e-10 9 LPP Memory LGALS1
7.33953521542454e-14 -0.352538230625682 0.557 0.609
1.30856573355804e-09 9 POMP Memory LGALS1
1.03631413206659e-13 -0.343199786317848 0.317 0.4
1.84764446606153e-09 9 NUCKS1 Memory LGALS1
1.50710826559458e-13 -0.326364273511509 0.831 0.867
2.68702332672857e-09 9 CD79A Memory LGALS1
3.08089216998934e-13 -0.309379997320601 0.224 0.305

5.492922649874e-09 9 ATP1B3 Memory LGALS1
7.31785946690245e-13 -0.313978501430269 0.387 0.459
1.30470116435404e-08 9 LSM5 Memory LGALS1
7.34448333572519e-13 -0.30395342870923 0.124 0.194
1.30944793392644e-08 9 MANF Memory LGALS1
7.72192991944787e-13 -0.317633859366762 0.349 0.431
1.37674288533836e-08 9 DNAJA1 Memory LGALS1
7.96825191948504e-13 -0.305226972723458 0.356 0.438
1.42065963472499e-08 9 ROMO1 Memory LGALS1
1.4922769656538e-12 -0.368607475784618 0.96 0.941
2.66058060206416e-08 9 YBX1 Memory LGALS1
1.72733910639863e-12 -2.1910792632157 0.594 0.445
3.07967289279812e-08 9 IGHG3 Memory LGALS1
1.76866860890692e-12 -0.324982114641605 0.281 0.363
3.15335926282015e-08 9 TPD52 Memory LGALS1
2.39107664201328e-12 -0.316892017785042 0.516 0.573
4.26305054504547e-08 9 RBM8A Memory LGALS1
2.63436843332682e-12 -0.331011016666275 0.511 0.561
4.69681547977838e-08 9 ATP5PF Memory LGALS1
5.98884513826886e-12 -0.346754989174854 0.169 0.222
1.06775119970196e-07 9 GBP2 Memory LGALS1
6.14327916904171e-12 -0.303819513912861 0.187 0.268
1.09528524304845e-07 9 FAM3C Memory LGALS1
1.24077707905749e-11 -0.489082700978345 0.584 0.6
2.21218145425159e-07 9 PRDX1 Memory LGALS1
1.40203890001346e-11 -0.358330293213747 0.491 0.536
2.49969515483399e-07 9 SRSF9 Memory LGALS1
1.41775717713777e-11 -0.317797823749819 0.389 0.454
2.52771927111893e-07 9 MRPL51 Memory LGALS1
2.21796231513267e-11 -0.314181404793345 0.208 0.28
3.95440501165005e-07 9 GADD45B Memory LGALS1
7.0633377047716e-11 0.314095754180168 0.205 0.159
1.25932247938373e-06 9 C12orf75 Memory LGALS1
1.05062799476851e-10 -0.302205760534157 0.431 0.504
1.87316465187277e-06 9 FOXP1 Memory LGALS1
1.74654331447137e-10 -0.49257721753116 0.721 0.715
3.113912075371e-06 9 ENO1 Memory LGALS1
2.09568950509544e-10 -0.386005211897247 0.575 0.597
3.73640481863466e-06 9 LDHA Memory LGALS1
2.41600979716541e-10 -0.353097172418239 0.295 0.355
4.3075038673662e-06 9 TUBB4B Memory LGALS1
2.71148687497797e-10 -0.333823367611028 0.602 0.638
4.83430994939822e-06 9 SET Memory LGALS1
3.01356606196319e-10 -1.17586751741481 0.287 0.303
5.37288693187417e-06 9 HMGB2 Memory LGALS1
4.56504929263572e-10 -0.343840650490116 0.318 0.377
8.13902638384023e-06 9 ACADM Memory LGALS1
6.58978814942538e-10 -0.30003035278448 0.752 0.76
1.17489332916105e-05 9 PSMA7 Memory LGALS1
6.99763583896739e-10 -0.417122220055164 0.496 0.535

1.2476084937295e-05 9 ISG20 Memory LGALS1
7.14446009626146e-10 -0.321357818655291 0.088 0.151
1.27378579056246e-05 9 BIK Memory LGALS1
9.62617306862667e-10 -0.35802639917841 0.098 0.153
1.71625039640545e-05 9 DUSP2 Memory LGALS1
1.04855260903998e-09 -0.311836937852273 0.277 0.349
1.86946444665738e-05 9 BIRC3 Memory LGALS1
2.94119503872202e-09 -0.320257517330798 0.234 0.298
5.24385663453749e-05 9 NFKBIA Memory LGALS1
1.59112669678321e-08 -0.316181477471439 0.572 0.599
0.000283681978769479 9 SEC61B Memory LGALS1
1.03810230194187e-07 -0.342516508601297 0.948 0.929
0.00185083259413217 9 NPM1 Memory LGALS1
2.21348340007541e-07 -0.38710130082373 0.942 0.932
0.00394641955399444 9 HMGB1 Memory LGALS1
3.93836179600569e-06 -0.311679648983205 0.404 0.43
0.0702170524609855 9 IRF8 Memory LGALS1
7.3864534598298e-06 -0.32179577138185 0.302 0.336
0.131693078735306 9 DUT Memory LGALS1
1.03001806267573e-05 -1.53852508134803 0.577 0.647
0.183641920394457 9 IGLC2 Memory LGALS1
3.85624405822758e-05 -0.322400049606777 0.864 0.851
0.687529753141395 9 ATP5MG Memory LGALS1
0.000111195047768837 -0.403175790107831 0.287 0.313 1
9 HSP90B1 Memory LGALS1
0.000133465003055907 -1.11320032615681 0.928 0.926 1
9 IGKC Memory LGALS1
0.000218460907813951 -0.305744240795103 0.552 0.543 1
9 CCR7 Memory LGALS1
0.000256500200001883 -0.304368136947543 0.173 0.21 1
9 FOS Memory LGALS1
0.000669583225006324 -0.306960424722957 0.947 0.933 1
9 SERF2 Memory LGALS1
0.00456205982050845 -0.375230733755767 0.497 0.499 1
9 SSR4 Memory LGALS1
0.00538754653992973 -1.10725808143919 0.404 0.43 1
9 IGLC3 Memory LGALS1
0 4.4199108849186 0.965 0.404 0 10 HIST1H4C DZ
1
0 2.80634383472283 0.994 0.279 0 10 HMGB2 DZ
1
0 2.74930040757588 0.993 0.409 0 10 STMN1 DZ
1
0 2.73523424631451 0.942 0.066 0 10 PCLAF DZ
1
0 2.54446607867781 0.984 0.655 0 10 TUBA1B DZ
1
0 2.47769158204973 0.868 0.039 0 10 TK1 DZ
1
0 2.45724866986668 0.798 0.019 0 10 RRM2 DZ

1<br>0	2.35754723068359	0.974	0.314	0	10	DUT	DZ
1<br>0	2.33040970584133	0.892	0.031	0	10	TYMS	DZ
1<br>0	2.29099883109967	0.986	0.563	0	10	TUBB	DZ
1<br>0	2.28432637548716	0.999	0.706	0	10	HMGN2	DZ
1<br>0	2.1852835565387	0.996	0.686	0	10	H2AFZ	DZ
1<br>0	2.14606467413439	0.804	0.035	0	10	MKI67	DZ
1<br>0	2.11408367110249	0.873	0.165	0	10	PCNA	DZ
1<br>0	2.08998908147668	0.831	0.056	0	10	NUSAP1	DZ
1<br>0	2.057025070838	0.902	0.129	0	10	MCM7	DZ
1<br>0	1.90041199748346	0.806	0.028	0	10	CDK1	DZ
1<br>0	1.89471912671013	0.867	0.074	0	10	CENPM	DZ
1<br>0	1.8555819718454	0.732	0.046	0	10	TOP2A	DZ
1<br>0	1.79178936648125	0.905	0.169	0	10	DNMT1	DZ
1<br>0	1.79069513182546	0.97	0.405	0	10	DEK	DZ
1<br>0	1.76134684841963	0.808	0.037	0	10	ZWINT	DZ
1<br>0	1.74801610947188	0.999	0.93	0	10	HMGB1	DZ
1<br>0	1.74491456357069	0.877	0.145	0	10	SMC4	DZ
1<br>0	1.74328356266117	0.969	0.339	0	10	H2AFV	DZ
1<br>0	1.70062019364345	0.845	0.091	0	10	FEN1	DZ
1<br>0	1.69133398444566	0.886	0.221	0	10	SLBP	DZ
1<br>0	1.66749511548085	0.762	0.023	0	10	ASF1B	DZ
1<br>0	1.66187614439689	0.799	0.08	0	10	RMI2	DZ
1<br>0	1.65106239687911	0.736	0.055	0	10	GINS2	DZ
1<br>0	1.6480170326103	0.93	0.182	0	10	RGS13	DZ
1<br>0	1.63692220913379	0.999	0.884	0	10	GAPDH	DZ

1<br>0	1.63605167645583	0.841	0.076	0	10	MYBL2	DZ
1<br>0	1.58839795483027	0.666	0.029	0	10	AURKB	DZ
1<br>0	1.58364537133788	0.661	0.035	0	10	UBE2C	DZ
1<br>0	1.51014039995222	0.923	0.263	0	10	CARHSP1	DZ
1<br>0	1.48863546348489	0.831	0.177	0	10	MCM3	DZ
1<br>0	1.47898341603261	0.959	0.325	0	10	H2AFY	DZ
1<br>0	1.4604850173818	0.795	0.093	0	10	LMNB1	DZ
1<br>0	1.43826252204188	0.724	0.049	0	10	DHFR	DZ
1<br>0	1.41877169190913	0.763	0.077	0	10	RRM1	DZ
1<br>0	1.4109171863419	0.96	0.422	0	10	HMGA1	DZ
1<br>0	1.40748731281484	0.875	0.216	0	10	GCHFR	DZ
1<br>0	1.39960629268357	0.807	0.207	0	10	MCM5	DZ
1<br>0	1.39600556734166	0.997	0.76	0	10	HMGN1	DZ
1<br>0	1.39439835636735	0.89	0.167	0	10	PTTG1	DZ
1<br>0	1.39385701745007	0.831	0.181	0	10	HMCES	DZ
1<br>0	1.39061579536485	0.851	0.216	0	10	CCND3	DZ
1<br>0	1.38914489840895	0.722	0.057	0	10	GMNN	DZ
1<br>0	1.37260831633725	0.677	0.02	0	10	CLSPN	DZ
1<br>0	1.36780632036442	0.684	0.022	0	10	CDT1	DZ
1<br>0	1.36666111997599	0.89	0.213	0	10	UBE2J1	DZ
1<br>0	1.36091002764956	0.707	0.052	0	10	CDCA7	DZ
1<br>0	1.34966733822197	0.842	0.145	0	10	CCDC167	DZ
1<br>0	1.34698105145718	0.806	0.119	0	10	SMC2	DZ
1<br>0	1.31491414804085	0.833	0.146	0	10	TFDP1	DZ
1<br>0	1.31350084152537	0.67	0.044	0	10	BIRC5	DZ

1<br>0	1.3017144767156	0.66	0.045	0	10	CDKN3	DZ
1<br>0	1.29102043566948	0.903	0.293	0	10	PSIP1	DZ
1<br>0	1.26634464880253	0.585	0.013	0	10	SPC25	DZ
1<br>0	1.25501646644433	0.776	0.14	0	10	C12orf75	DZ
1<br>0	1.25165243582616	0.986	0.646	0	10	SLC25A5	DZ
1<br>0	1.24517655457653	0.608	0.014	0	10	UHRF1	DZ
1<br>0	1.2394970531344	0.854	0.226	0	10	CKS1B	DZ
1<br>0	1.23629248766398	0.655	0.036	0	10	CENPU	DZ
1<br>0	1.23238093042563	0.682	0.113	0	10	PHGDH	DZ
1<br>0	1.21911264697799	0.835	0.186	0	10	EAF2	DZ
1<br>0	1.21839154705266	0.979	0.57	0	10	ANP32B	DZ
1<br>0	1.21644697770988	0.724	0.056	0	10	MAD2L1	DZ
1<br>0	1.21238935605912	0.793	0.14	0	10	DNAJC9	DZ
1<br>0	1.20332090036469	0.873	0.3	0	10	PRDX2	DZ
1<br>0	1.19809786644906	0.799	0.177	0	10	IDH2	DZ
1<br>0	1.19419925291594	0.987	0.644	0	10	TCL1A	DZ
1<br>0	1.1868663204727	0.68	0.065	0	10	TACC3	DZ
1<br>0	1.17960926823733	0.795	0.211	0	10	CKS2	DZ
1<br>0	1.17456024810994	0.65	0.049	0	10	CENPW	DZ
1<br>0	1.15562598823808	0.676	0.059	0	10	UBE2T	DZ
1<br>0	1.15135052746319	0.889	0.271	0	10	SIVA1	DZ
1<br>0	1.14290632503923	0.907	0.401	0	10	METAP2	DZ
1<br>0	1.13795216226611	0.957	0.469	0	10	RANBP1	DZ
1<br>0	1.13631618499314	0.97	0.575	0	10	MARCKSL1	DZ
1<br>0	1.13581836347748	0.746	0.114	0	10	NEIL1	DZ

1
0 1.13479515009969 0.684 0.074 0 10 KIF22 DZ
1
0 1.12468081841215 0.762 0.133 0 10 CD81 DZ
1
0 1.12424421405562 0.881 0.29 0 10 CALM3 DZ
1
0 1.12288078893133 0.618 0.054 0 10 HELLS DZ
1
0 1.12066545233806 0.834 0.218 0 10 HNRNPAB DZ
1
0 1.11230643199134 0.88 0.271 0 10 YWHAH DZ
1
0 1.11026208427746 0.773 0.14 0 10 SUSD3 DZ
1
0 1.10939883999436 0.967 0.473 0 10 CBX3 DZ
1
0 1.10844388260521 0.688 0.075 0 10 PXMP2 DZ
1
0 1.10464903618672 0.916 0.347 0 10 RBBP7 DZ
1
0 1.09437543746913 0.577 0.023 0 10 CCNA2 DZ
1
0 1.08666603462984 0.566 0.021 0 10 KIFC1 DZ
1
0 1.06398806005671 0.995 0.896 0 10 PFN1 DZ
1
0 1.04423021128654 0.631 0.057 0 10 EZH2 DZ
1
0 1.04373606515022 0.562 0.05 0 10 MCM6 DZ
1
0 1.04366844697364 0.799 0.173 0 10 CBX5 DZ
1
0 1.04170960099373 0.965 0.526 0 10 PARP1 DZ
1
0 1.0321026249588 0.779 0.17 0 10 USP1 DZ
1
0 1.02132288037074 0.561 0.036 0 10 ATAD2 DZ
1
0 1.01641991684094 0.568 0.032 0 10 SHCBP1 DZ
1
0 1.01093381129735 0.507 0.022 0 10 GTSE1 DZ
1
0 1.01037804174298 0.597 0.044 0 10 ORC6 DZ
1
0 0.998877121858072 0.921 0.384 0 10
RPA3 DZ 1
0 0.99676161416102 0.959 0.458 0 10 SNRPD1 DZ
1
0 0.992503737248191 0.516 0.02 0 10 PBK

DZ 1
0 0.981942015005227 0.575 0.042 0 10
WDR76 DZ 1
0 0.98057125530848 0.962 0.492 0 10 MZT2B DZ
1
0 0.977896126710836 0.729 0.134 0 10
DTYMK DZ 1
0 0.976805796876367 0.96 0.503 0 10
COX8A DZ 1
0 0.975963556719097 0.605 0.041 0 10
CENPK DZ 1
0 0.973151652659995 0.987 0.808 0 10
CORO1A DZ 1
0 0.97165019166962 0.574 0.028 0 10 CHAF1A DZ
1
0 0.969072650186732 0.681 0.095 0 10
SKA2 DZ 1
0 0.959695206654478 0.549 0.024 0 10
MND1 DZ 1
0 0.954420156464488 0.614 0.07 0 10
CDCA4 DZ 1
0 0.939487640214312 0.525 0.029 0 10
MCM4 DZ 1
0 0.938879664483908 0.996 0.938 0 10
H3F3A DZ 1
0 0.936021059442328 0.999 0.989 0 10
ACTB DZ 1
0 0.931997762819166 0.996 0.846 0 10
ATP5MG DZ 1
0 0.928478315798025 0.999 0.931 0 10
SERF2 DZ 1
0 0.923108937940091 0.598 0.065 0 10
RFC4 DZ 1
0 0.914374550281165 0.453 0.023 0 10
CDCA3 DZ 1
0 0.913525974663585 0.577 0.057 0 10
SAC3D1 DZ 1
0 0.900043313921595 0.568 0.055 0 10
TMEM106C DZ 1
0 0.897595916207681 1 0.999 0 10
PTMA DZ 1
0 0.887560447675132 0.967 0.552 0 10
SNRPB DZ 1
0 0.883248140706843 0.514 0.043 0 10
FBXO5 DZ 1
0 0.87391654042959 0.524 0.027 0 10 RAD51AP1 DZ
1
0 0.873483178273541 0.995 0.916 0 10
CFL1 DZ 1
0 0.850690380191643 0.461 0.018 0 10

NDC80 DZ 1
0 0.838039625173266 0.397 0.017 0 10
MXD3 DZ 1
0 0.827577713422341 0.497 0.04 0 10
MCM2 DZ 1
0 0.826406736899547 0.455 0.016 0 10
E2F1 DZ 1
0 0.815068696273201 0.484 0.024 0 10
BRCA1 DZ 1
0 0.794434836149794 0.488 0.035 0 10
RFC3 DZ 1
0 0.780353632050807 0.439 0.016 0 10
NCAPG DZ 1
0 0.772865494865466 0.413 0.007 0 10
ESCO2 DZ 1
0 0.771095600545781 0.425 0.011 0 10
CDCA5 DZ 1
0 0.769015523954284 0.423 0.006 0 10
CDC45 DZ 1
0 0.748366919637934 0.407 0.011 0 10
CDC6 DZ 1
0 0.719957821404014 0.434 0.021 0 10
TCF19 DZ 1
0 0.713627561364093 0.421 0.022 0 10
CDCA2 DZ 1
0 0.692732897651261 0.406 0.017 0 10
CHEK1 DZ 1
0 0.676541576980873 0.4 0.012 0 10
NCAPH DZ 1
0 0.67206173744696 0.367 0.004 0 10 PKMYT1 DZ
1
0 0.652874101125571 0.362 0.008 0 10
MELK DZ 1
0 0.488655427641103 0.282 0.005 0 10
MCM10 DZ 1
0 -1.23438819044572 0.993 0.998 0 10 B2M
DZ 1
0 -1.50110452804474 0.998 1 0 10
MALAT1 DZ 1
0 -2.29549055568735 0.27 0.712 0 10
EMP3 DZ 1
4.94065645841247e-324 0.937547105954559 0.967 0.566
8.80869639970358e-320 10 LDHB DZ 1
1.97626258336499e-323 0.412667123052863 0.24 0.003
3.52347855988143e-319 10 E2F8 DZ 1
4.57010722403153e-321 0.98029152330474 0.919 0.403
8.14804416972582e-317 10 HNRNPD DZ 1
1.58911620174331e-317 0.844075686140011 0.558 0.056
2.83323527608814e-313 10 HAUS8 DZ 1
2.01148304106007e-317 1.29081161696867 0.749 0.128

3.586273113906e-313 10 BIK DZ 1
2.17418132856382e-317 -2.15548032720698 0.129 0.557
3.87634789069644e-313 10 CCR7 DZ 1
2.2295043292569e-317 0.977057380120855 0.825 0.244
3.97498326863212e-313 10 HSPB11 DZ 1
1.50554604516843e-315 0.829426294888432 0.989 0.841
2.68423804393079e-311 10 HNRNPA2B1 DZ 1
8.19756446229283e-314 0.955298311985457 0.896 0.347
1.46154376798219e-309 10 VPS29 DZ 1
1.76007460983703e-312 0.885651319806469 0.476 0.03
3.13803702187844e-308 10 TPX2 DZ 1
1.94053739496987e-312 1.1551887075015 0.43 0.036
3.45978412149178e-308 10 HES6 DZ 1
1.60238572344138e-311 0.552945952760146 0.27 0.007
2.85689350632363e-307 10 SPC24 DZ 1
2.95131847741282e-309 0.984838528281142 0.828 0.265
5.26190571337932e-305 10 PRDX3 DZ 1
3.15246212051211e-309 0.952328061989772 0.744 0.141
5.62052471466104e-305 10 TMPO DZ 1
1.02627015185075e-308 0.89900776324106 0.529 0.045
1.8297370537347e-304 10 HMGB3 DZ 1
3.5571961022493e-308 0.617573325826994 0.325 0.011
6.34212493070028e-304 10 HJURP DZ 1
3.71127156540101e-308 0.910995292880534 0.566 0.067
6.61682607395346e-304 10 H2AFX DZ 1
9.97584989676003e-308 0.992924546680988 0.648 0.093
1.77859427809335e-303 10 AC023590.1 DZ 1
1.22931256665527e-305 1.08963782773441 0.78 0.199
2.19174137508969e-301 10 NANS DZ 1
1.16241947275464e-302 0.896702476173743 0.711 0.146
2.07247767797425e-298 10 SAE1 DZ 1
1.93914685952958e-302 0.400120616402317 0.224 0.003
3.45730493585529e-298 10 RAD54L DZ 1
2.48466238010699e-302 0.925385880043915 0.93 0.376
4.42990455749275e-298 10 YWHAE DZ 1
3.91579263792035e-302 0.61169640749047 0.358 0.015
6.98146669414819e-298 10 PAQR4 DZ 1
5.61904607925255e-302 1.10449400192854 0.894 0.321
1.00181972546994e-297 10 LRMP DZ 1
4.48439102570605e-301 0.94962221415722 0.88 0.321
7.99522075973131e-297 10 RBBP4 DZ 1
7.0540233817876e-301 0.816949175432639 0.369 0.02
1.25766182873891e-296 10 HIST1H1D DZ 1
3.35047455612503e-300 0.84206126746185 0.976 0.635
5.97356108611531e-296 10 SNRPG DZ 1
1.00877303969064e-299 1.01900147152876 0.81 0.244
1.79854145246444e-295 10 ANP32E DZ 1
1.27673077756007e-299 0.689139669279495 0.995 0.892
2.27628330331184e-295 10 PPIA DZ 1
3.02026271266788e-298 0.84618640753361 0.568 0.06

5.38482639041557e-294	10	LIG1	DZ 1		
2.27554357699525e-297	0.752710309887968			0.995	0.865
4.05706664342483e-293	10	ARPC2	DZ 1		
6.20049594135204e-296	0.924438722150073			0.991	0.799
1.10548642138365e-291	10	CALM2	DZ 1		
3.06854727561498e-295	0.940109789504396			0.819	0.235
5.47091293769395e-291	10	MPC2	DZ 1		
8.35259275954701e-295	0.960731641319583			0.901	0.373
1.48918376309964e-290	10	BUB3	DZ 1		
1.52993919191666e-293	0.629436136512582			0.369	0.016
2.72772858526821e-289	10	KIF2C	DZ 1		
1.51188299698578e-290	0.725201131503017			0.447	0.04
2.69553619532595e-286	10	CDKN2C	DZ 1		
3.46577681635651e-290	0.907443534432101			0.957	0.539
6.17913348588203e-286	10	TCEA1	DZ 1		
6.4551681903741e-288	0.965047114677673			0.916	0.38
1.1508919366618e-283	10	NUCKS1	DZ 1		
1.38466210422742e-287	0.457686425960941			0.275	0.007
2.46871406562708e-283	10	DSCC1	DZ 1		
1.44996912233482e-286	0.748600783664769			0.469	0.037
2.58514994821075e-282	10	RPL39L	DZ 1		
8.16468588297049e-285	1.11593353337233		0.825	0.337	
1.45568184607481e-280	10	TUBB4B	DZ 1		
2.42154966761108e-284	0.934767722980407			0.883	0.303
4.3173809023838e-280	10	NAA38	DZ 1		
3.3648760120515e-284	0.428752817215837			0.253	0.006
5.99923744188661e-280	10	SKA3	DZ 1		
3.50124372352396e-283	0.980351272610135			0.85	0.27
6.24236743467086e-279	10	DCK	DZ 1		
6.65278802025066e-283	0.887668377990703			0.956	0.521
1.18612557613049e-278	10	SRSF9	DZ 1		
1.14927444903132e-282	0.869190063285999			0.983	0.74
2.04904141517794e-278	10	HSP90AA1	DZ 1		
1.49541432078208e-280	0.709481438220377			0.44	0.034
2.66617419252237e-276	10	CENPN	DZ 1		
3.31896783257457e-279	0.867392389891552			0.732	0.155
5.9173877486972e-275	10	NUDT1	DZ 1		
5.57973806782803e-279	0.878244827476597			0.934	0.435
9.9481150011306e-275	10	MRPL51	DZ 1		
3.93206578592458e-278	0.974582124311611			0.904	0.357
7.01048008972493e-274	10	ACADM	DZ 1		
6.06022349752403e-278	0.861810921283803			0.631	0.106
1.08047724737356e-273	10	RFC2	DZ 1		
1.72723884889819e-277	1.05497538153513		0.741	0.172	
3.07949414370058e-273	10	DAAM1	DZ 1		
8.37407462030716e-277	0.815332056539674			0.465	0.027
1.49301376405456e-272	10	NUF2	DZ 1		
2.64810063352466e-275	0.727502254719434			0.988	0.792
4.72129861951112e-271	10	SUMO2	DZ 1		
4.37643081685103e-273	0.917697549308122			0.583	0.075

7.80273850336369e-269 10 GCSAM DZ 1
3.66306146461793e-272 0.804779953001551 0.968 0.545
6.53087228526731e-268 10 ATP5PF DZ 1
4.90918871049135e-272 1.03159006644949 0.62 0.102
8.75259255193503e-268 10 UBE2S DZ 1
3.19839211056285e-271 1.04384378064597 0.605 0.104
5.7024132939225e-267 10 LPP DZ 1
8.88370082067016e-270 0.809707775998679 0.533 0.052
1.58387501931728e-265 10 SGO1 DZ 1
8.40610324748478e-269 0.746097850414093 0.547 0.077
1.49872414799406e-264 10 TUBG1 DZ 1
9.47493429091344e-269 0.882319573088761 0.665 0.131
1.68928603472696e-264 10 SMARCA4 DZ 1
1.46086989907996e-268 0.536090907738028 0.338 0.015
2.60458494306966e-264 10 DONSON DZ 1
5.41998214075097e-268 0.749493455822259 0.971 0.694
9.66328615874491e-264 10 TPI1 DZ 1
8.34217281087936e-267 0.416063722230995 0.238 0.004
1.48732599045168e-262 10 CCNE2 DZ 1
3.67924810384836e-266 0.991285917924538 0.551 0.038
6.55973144435124e-262 10 CCNB2 DZ 1
1.60249137688214e-265 0.882312114870205 0.776 0.238
2.85708187584316e-261 10 EIF4A3 DZ 1
8.70978473406144e-265 0.842549247183394 0.609 0.096
1.55286752023581e-260 10 BRI3BP DZ 1
1.26318435127754e-263 0.816148813508613 0.707 0.151
2.25213137989273e-259 10 YEATS4 DZ 1
3.47492507269296e-263 0.783512791313452 0.973 0.629
6.19544391210427e-259 10 ERH DZ 1
2.20083541916924e-262 0.844945897977928 0.703 0.141
3.92386946883683e-258 10 VRK1 DZ 1
1.35059431899822e-260 0.770493873708586 0.976 0.667
2.40797461134192e-256 10 HNRNPA3 DZ 1
1.89306754026218e-260 0.812723451235657 0.935 0.483
3.37515011753344e-256 10 COX5A DZ 1
1.28463973664635e-259 0.802510699662848 0.967 0.572
2.29038418646677e-255 10 ATP5MC3 DZ 1
2.52469693598417e-259 0.773372077433907 0.952 0.575
4.50128216716618e-255 10 HNRNPF DZ 1
3.59967626230355e-259 0.912521298737928 0.92 0.38
6.417862808061e-255 10 BASP1 DZ 1
3.16820099712039e-258 0.509184734738567 0.318 0.015
5.64858555776595e-254 10 OIP5 DZ 1
1.69671068289961e-257 0.760666944531216 0.977 0.677
3.02506547654172e-253 10 PPP1CA DZ 1
1.3291974646461e-255 0.666513557620679 0.988 0.74
2.36982615971753e-251 10 RAN DZ 1
1.79530809041003e-254 0.821107000652547 0.911 0.4
3.20085479439204e-250 10 DDX39A DZ 1
9.3562664933198e-253 0.588990697633818 0.32 0.014

1.66812875309399e-248 10 KIF23 DZ 1
1.04592597667238e-251 0.880815427597786 0.856 0.332
1.86478142380919e-247 10 NASP DZ 1
1.26363300554366e-251 0.904009446817012 0.675 0.148
2.25293128558379e-247 10 DEF8 DZ 1
1.16871686041249e-250 0.816714467269225 0.962 0.543
2.08370529042943e-246 10 ATP5IF1 DZ 1
1.5251297284804e-250 0.630940160872591 0.41 0.033
2.7191537929077e-246 10 CTNNAL1 DZ 1
2.64132024551219e-250 0.976320198331398 0.512 0.038
4.70920986572369e-246 10 CENPF DZ 1
7.71051955211342e-249 0.899690405430996 0.841 0.27
1.3747085309463e-244 10 BCAS4 DZ 1
1.7584223600749e-248 0.833951507510606 0.852 0.313
3.13509122577754e-244 10 RALY DZ 1
7.0575551253516e-248 0.903956329169821 0.816 0.24
1.25829150329894e-243 10 SYNE2 DZ 1
3.47588680078889e-247 0.772887924793013 0.925 0.418
6.1971585771265e-243 10 LSM3 DZ 1
6.51219056993451e-247 0.862905890158167 0.748 0.197
1.16105845671362e-242 10 SNRNP25 DZ 1
4.23824736248265e-246 0.687497785429067 0.472 0.048
7.55637122257031e-242 10 TCTEX1D2 DZ 1
9.07238702131745e-246 0.918265405312461 0.875 0.313
1.61751588203069e-241 10 GRHPR DZ 1
1.20290438131538e-245 0.770426158715018 0.912 0.43
2.1446582214472e-241 10 PGAM1 DZ 1
5.92511825340564e-245 0.541672358352527 0.287 0.012
1.05638933339969e-240 10 CKAP2L DZ 1
3.82233542701309e-244 0.511416229543728 0.331 0.016
6.81484183282165e-240 10 POC1A DZ 1
9.87065190035901e-241 0.520591429624494 0.312 0.013
1.75983852731501e-236 10 C21orf58 DZ 1
2.18592908429829e-239 0.34001584713374 0.202 0.004
3.89729296439543e-235 10 DIAPH3 DZ 1
3.47117655402668e-239 0.860005249968707 0.623 0.104
6.18876067817418e-235 10 VNN2 DZ 1
8.21414344962036e-239 1.01073871921702 0.86 0.331
1.46449963563281e-234 10 S100A10 DZ 1
1.90847915646571e-235 0.777332450010987 0.965 0.621
3.40262748806271e-231 10 GSTP1 DZ 1
3.18936647015459e-234 -0.817715886470625 1 0.999
5.68632147963861e-230 10 RPL34 DZ 1
1.3168685747987e-233 -0.845615829444619 1 0.999
2.3478449820086e-229 10 RPS29 DZ 1
2.16050661848873e-232 0.767804894827357 0.987 0.779
3.85196725010356e-228 10 ACTG1 DZ 1
3.19567553115326e-232 0.790268600113254 0.712 0.172
5.69756990449314e-228 10 NUDT21 DZ 1
5.95298017106783e-230 0.78846814075921 0.791 0.257

1.06135683469968e-225	10	SMC3	DZ 1		
4.93884360432973e-229	0.850709585573225			0.753	0.222
8.80546426215947e-225	10	ZBTB8OS	DZ 1		
7.89928661961942e-229	0.757600365538191			0.838	0.302
1.40836381141195e-224	10	LSM4	DZ 1		
8.81401530506808e-229	0.679895635914178			0.488	0.059
1.57145078874059e-224	10	LRR1	DZ 1		
2.21409676237524e-228	0.754186735561319			0.928	0.432
3.94751311763882e-224	10	SEM1	DZ 1		
1.23568081967311e-227	0.798805571272011			0.527	0.085
2.2030953333952e-223	10	FAM241A	DZ 1		
2.84746886345494e-227	0.706704321994172			0.956	0.582
5.07675223665381e-223	10	SRSF2	DZ 1		
1.66371687086955e-226	0.511296801142776			0.312	0.016
2.96624080907331e-222	10	FANCI	DZ 1		
2.58767158355964e-226	0.754230516697052			0.529	0.083
4.61355966632848e-222	10	PHF19	DZ 1		
3.98160776891253e-226	0.758937395338018			0.611	0.11
7.09880849119414e-222	10	COMMD4	DZ 1		
5.08212721830242e-226	0.730157581201119			0.556	0.089
9.06092461751138e-222	10	BCL2L12	DZ 1		
1.26721003214464e-225	-0.772099954208237			0.999	1
2.25930876631068e-221	10	RPS27	DZ 1		
2.41062792319757e-224	0.758482855264602			0.59	0.096
4.29790852426895e-220	10	CKLF	DZ 1		
4.35318458679819e-223	0.621337339030939			0.439	0.044
7.76129279980249e-219	10	RFC5	DZ 1		
6.58833331015809e-221	0.340911260714205			0.194	0.004
1.17463394586809e-216	10	CCNE1	DZ 1		
1.40702249593159e-220	0.761024733707536			0.934	0.469
2.50858040799644e-216	10	NDUFB1	DZ 1		
3.43805498348435e-220	0.805808001031697			0.862	0.311
6.12970823005424e-216	10	RFTN1	DZ 1		
1.2704960317848e-219	0.824881535037874			0.829	0.256
2.26516737506912e-215	10	ITGAE	DZ 1		
3.15999994499623e-219	0.747052230969879			0.579	0.097
5.63396390193379e-215	10	WDR34	DZ 1		
3.6989879868488e-219	0.749833615721052			0.63	0.122
6.59492568175272e-215	10	RPA1	DZ 1		
9.20849294014902e-219	0.370347026862504			0.211	0.006
1.64178220629917e-214	10	SKA1	DZ 1		
1.01657179925602e-218	0.745911892310052			0.661	0.143
1.81244586089355e-214	10	TPGS2	DZ 1		
2.447748014728e-218	0.767851984527155			0.835	0.32
4.36408993545855e-214	10	PPM1G	DZ 1		
5.44565621838682e-218	0.617926761887243			0.419	0.037
9.70906047176186e-214	10	ATAD5	DZ 1		
7.99019754276441e-218	0.640035616028589			0.394	0.033
1.42457231989947e-213	10	MYBL1	DZ 1		
4.560450069321e-217	0.764238354635487			0.642	0.141

8.13082642859242e-213	10	SMC1A	DZ 1
4.58976076728727e-217	0.770211242282934	0.463	0.06
8.18308447199647e-213	10	S1PR2	DZ 1
3.6814575547945e-216	0.748867911705474	0.93	0.441
6.56367067444311e-212	10	COX17	DZ 1
1.21905262674859e-215	0.710280303705807	0.974	0.684
2.17344892823006e-211	10	EZR	DZ 1
3.88571702624437e-215	0.72865069432026 0.695	0.194
6.92784488609109e-211	10	C19orf48 DZ 1
8.95918210435855e-215	0.830832685224501	0.622	0.102
1.59733257738609e-210	10	CD38	DZ 1
9.24043634954158e-215	0.706750580236772	0.575	0.095
1.64747739675977e-210	10	CENPH	DZ 1
1.57525670270392e-213	0.613343854488954	0.341	0.027
2.80852517525082e-209	10	HIST2H2AC	DZ 1
1.8983894269934e-213	0.320862556890075	0.197	0.005
3.38463850938653e-209	10	DTL	DZ 1
4.87393937749832e-211	0.608393349842419	0.983	0.736
8.68974651614175e-207	10	CHCHD2	DZ 1
1.32164557365234e-210	-1.25632586602853	0.885	0.905
2.35636189326476e-206	10	HLA-A	DZ 1
1.58228253807366e-210	0.755648451458428	0.552	0.088
2.82105153713152e-206	10	SEMA4A	DZ 1
1.43136119108462e-209	0.814121526557221	0.872	0.365
2.55197386758476e-205	10	LSM8	DZ 1
3.34091750435983e-209	0.77051863023839 0.803	0.256
5.95652181852314e-205	10	POU2AF1	DZ 1
7.88428319618107e-209	0.819454332527529	0.959	0.593
1.40568885104712e-204	10	DYNLL1	DZ 1
2.82771910952781e-208	0.879694224109305	0.705	0.15
5.04154040037714e-204	10	CCDC88A	DZ 1
4.31809899515973e-208	0.677289885550367	0.376	0.042
7.69873869847028e-204	10	UNG	DZ 1
1.20802074049076e-206	0.781574386856708	0.54	0.074
2.15378017822097e-202	10	FAM111A	DZ 1
3.96855401512084e-206	0.72967459875871 0.661	0.161
7.07553495355894e-202	10	PMVK	DZ 1
1.07543992765033e-205	0.389099905495607	0.248	0.009
1.91740184700777e-201	10	TEDC2	DZ 1
2.29086050506554e-205	0.492831935753145	0.33	0.025
4.08437519448135e-201	10	ACOT7	DZ 1
1.27992672982201e-204	0.678646598717617	0.512	0.077
2.28198136659966e-200	10	MSH6	DZ 1
1.95278952712379e-203	0.794306533104321	0.528	0.082
3.481628447909e-199	10	STAG3	DZ 1
4.02125965783372e-203	0.734301549012725	0.855	0.363
7.16950384395175e-199	10	ANAPC11	DZ 1
1.26677776732262e-202	0.630831040034381	0.98	0.698
2.2585380813595e-198	10	NDUFA4	DZ 1
2.12754069712445e-201	0.670010316560243	0.557	0.096

3.79319230890319e-197 10 RAD51C DZ 1
1.00516259178879e-200 0.734390642208932 0.824 0.307
1.79210438490023e-196 10 IMP4 DZ 1
1.85386109227615e-200 0.715066248165806 0.894 0.424
3.30524894141916e-196 10 FDPS DZ 1
2.50689999910353e-200 0.752838154552062 0.691 0.173
4.46955200840168e-196 10 RFC1 DZ 1
4.24360470136875e-200 0.614688117792473 0.379 0.039
7.56592282207034e-196 10 UCHL1 DZ 1
7.80514228436215e-200 0.720012113015984 0.749 0.231
1.39157881787893e-195 10 HAT1 DZ 1
2.90159066773577e-199 0.627950020055931 0.455 0.06
5.17324600150611e-195 10 HIRIP3 DZ 1
4.59525315868342e-199 0.702861296348355 0.856 0.329
8.19287685661667e-195 10 LSM6 DZ 1
1.96527868589034e-198 0.455904266408393 0.268 0.014
3.50389536907388e-194 10 KIF11 DZ 1
4.79540268765127e-198 0.74039384971693 0.535 0.097
8.54972345181344e-194 10 CRIP1 DZ 1
1.85560515612607e-197 0.6883630524605 0.934 0.516
3.30835843285716e-193 10 SRSF7 DZ 1
3.21654844538544e-197 0.716198911745168 0.876 0.367
5.73478422327771e-193 10 SMARCB1 DZ 1
3.32153423988866e-196 0.523684075319235 0.313 0.021
5.92196339629749e-192 10 PRC1 DZ 1
5.1512539037836e-196 0.710259053020333 0.619 0.138
9.18417058505578e-192 10 PRKDC DZ 1
1.31443492937755e-195 0.568704092998583 0.409 0.041
2.34350603558723e-191 10 GGH DZ 1
1.75029231156148e-195 0.756050621301147 0.746 0.209
3.12059616228296e-191 10 UBE2G1 DZ 1
2.37657431296781e-195 0.662426446531625 0.961 0.642
4.23719434259031e-191 10 YWHAB DZ 1
1.77652666548129e-194 0.631360715210238 0.468 0.057
3.16736939188658e-190 10 CCDC34 DZ 1
1.90001109240103e-194 -0.898654443086012 0.981 0.986
3.3875297766418e-190 10 HLA-B DZ 1
3.07741875817059e-194 0.602035788093733 0.976 0.67
5.48672990394234e-190 10 SRSF3 DZ 1
5.71479031113108e-194 0.65084837070993 0.892 0.43
1.01888996457156e-189 10 YWHAQ DZ 1
5.9124437433763e-194 0.402666279416883 0.23 0.009
1.05412959500656e-189 10 KIF15 DZ 1
6.6638911146519e-194 0.629794601385062 0.474 0.055
1.18810514683129e-189 10 PRIM1 DZ 1
5.31485084338888e-193 0.744898611941574 0.766 0.239
9.47584756867803e-189 10 RAD21 DZ 1
1.26386621413911e-192 0.823624952645064 0.736 0.226
2.25334707318862e-188 10 KPNA2 DZ 1
4.21913907370235e-192 0.69145414305741 0.904 0.419

7.52230305450392e-188 10 ROMO1 DZ 1
6.08319294815553e-192 0.668202735775972 0.783 0.277
1.08457247072665e-187 10 SSRP1 DZ 1
7.54210745299402e-190 0.739101212254198 0.702 0.173
1.3446823377943e-185 10 LBR DZ 1
5.6025616296368e-189 0.712233059849681 0.577 0.106
9.98880712947945e-185 10 SSBP2 DZ 1
4.14221474201636e-188 0.694129119616948 0.878 0.399
7.38515466354098e-184 10 HDAC1 DZ 1
5.66525453853472e-188 0.648104974188168 0.433 0.05
1.01005823167535e-183 10 FANCA DZ 1
1.14109760764664e-187 0.586334114859547 0.37 0.028
2.0344629246732e-183 10 CDCA8 DZ 1
1.92675358988302e-187 0.708003803081628 0.778 0.254
3.43520897540243e-183 10 SMS DZ 1
3.96484738842693e-187 0.664264517064705 0.446 0.056
7.06892640882637e-183 10 SNTA1 DZ 1
1.99673772425691e-186 0.779522431536954 0.765 0.265
3.55998368857764e-182 10 JPT1 DZ 1
5.73973159536094e-186 0.629457470046082 0.942 0.501
1.0233367461369e-181 10 SRP9 DZ 1
5.0212880232033e-184 0.453535871961538 0.271 0.015
8.95245441656917e-180 10 SPAG5 DZ 1
2.28643605112373e-183 0.66884289827182 0.862 0.366
4.0764868355485e-179 10 SRSF10 DZ 1
2.31894197763777e-183 0.435583824329315 0.268 0.014
4.13444165193038e-179 10 NCAPG2 DZ 1
8.31970454444872e-183 0.806028053936115 0.72 0.194
1.48332012322976e-178 10 FAM111B DZ 1
7.29368693479782e-182 0.720475000332134 0.82 0.286
1.3003914436051e-177 10 CD27 DZ 1
3.93336287019295e-181 0.671944717067222 0.79 0.296
7.012792661267e-177 10 MZT2A DZ 1
5.74248257725352e-181 0.693223876935539 0.824 0.306
1.02382721869853e-176 10 RNASEH2C DZ 1
6.20752258880822e-181 0.685993841447744 0.85 0.33
1.10673920235862e-176 10 RBM17 DZ 1
1.01399113125156e-179 0.611184928714196 0.505 0.081
1.80784478790841e-175 10 TEX30 DZ 1
1.13098619608822e-179 0.617556523535566 0.954 0.625
2.01643528900569e-175 10 UQCRH DZ 1
1.75427399400176e-179 0.754288190378739 0.665 0.164
3.12769510390573e-175 10 LCK DZ 1
4.96389452924179e-179 0.57259170020297 0.432 0.054
8.8501275561852e-175 10 DSN1 DZ 1
1.57268803143654e-178 -1.45718469094427 0.369 0.583
2.80394549124821e-174 10 SARAF DZ 1
1.14920988477018e-177 0.754170080353612 0.528 0.08
2.04892630355676e-173 10 WDR66 DZ 1
1.15248949890806e-177 0.444564334761293 0.27 0.016

2.05477352760318e-173	10	CEP55	DZ 1		
5.21222859853503e-177	0.587689377600927			0.959	0.596
9.2928823683281e-173	10	POLR2L	DZ 1		
9.30477475562986e-177	0.551478183286898			0.98	0.754
1.65894829118125e-172	10	PKM	DZ 1		
7.09372473161527e-176	0.747688489584313			0.843	0.285
1.26474018239969e-171	10	BCL7A	DZ 1		
1.43916521051278e-175	0.577726459293062			0.955	0.614
2.56588765382324e-171	10	HNRNPA0	DZ 1		
3.50405096446776e-175	0.579609438066603			0.967	0.629
6.24737246454957e-171	10	ATP5MF	DZ 1		
1.04585088260292e-174	0.76541405171064		0.51	0.07	
1.86464753859275e-170	10	AICDA	DZ 1		
1.94342330449164e-174	0.719577965154205			0.66	0.172
3.46492940957814e-170	10	PRPSAP2	DZ 1		
1.0626974285999e-173	0.396965208521729			0.237	0.013
1.89468324545076e-169	10	BUB1B	DZ 1		
1.46015550467586e-173	0.626831250122137			0.452	0.066
2.6033112492866e-169	10	WEE1	DZ 1		
3.40989555367231e-173	0.324318235301237			0.191	0.007
6.07950278264236e-169	10	FOXM1	DZ 1		
7.11070859822035e-173	0.495731062943479			0.321	0.025
1.26776823597671e-168	10	CIP2A	DZ 1		
1.47932719233917e-172	0.69348912424238		0.836	0.296	
2.6374924512215e-168	10	HMGN3	DZ 1		
1.67314921132585e-171	0.675940575756579			0.867	0.358
2.98305772887286e-167	10	PTP4A2	DZ 1		
1.27961257908759e-170	0.643728668139174			0.71	0.199
2.28142126725526e-166	10	HAUS1	DZ 1		
5.04990016332279e-170	0.746944733442339			0.777	0.273
9.0034670011882e-166	10	TUBA4A	DZ 1		
1.07492996430243e-169	0.521312507796897			0.372	0.038
1.9164926333548e-165	10	POLA2	DZ 1		
1.3333999865858e-169	0.712387686598993			0.551	0.126
2.37731883608381e-165	10	AIM2	DZ 1		
1.3402121207768e-169	0.63702944090185		0.494	0.082	
2.38946419013295e-165	10	FAM76B	DZ 1		
6.93760750601213e-169	0.658706266299127			0.708	0.219
1.2369060422469e-164	10	PIN1	DZ 1		
2.8654950820205e-168	0.928212134088255			0.507	0.096
5.10889118173436e-164	10	VPREB3	DZ 1		
2.89405353953371e-167	0.760436530476432			0.528	0.102
5.15980805563465e-163	10	GMDS	DZ 1		
3.73464594013486e-167	0.722133007175177			0.643	0.152
6.65850024666645e-163	10	SEL1L3	DZ 1		
4.06887003207833e-167	0.676899408804298			0.818	0.28
7.25438838019245e-163	10	GGA2	DZ 1		
1.68788543775907e-166	0.561027982921301			0.978	0.714
3.00933094698064e-162	10	COX6C	DZ 1		
2.53666856322947e-166	0.641978814925633			0.755	0.223

4.52262638138182e-162 10 MCUB DZ 1
6.92906066075297e-166 0.583668773187704 0.377 0.038
1.23538222520565e-161 10 LINC01991 DZ 1
8.45166256232661e-165 0.623241655111159 0.6 0.132
1.50684691823721e-160 10 ARL6IP6 DZ 1
3.49134618224691e-164 0.625913696592926 0.663 0.182
6.22472110832802e-160 10 ETFB DZ 1
9.51213099736664e-164 0.361422652649462 0.201 0.009
1.6959178355205e-159 10 PLK4 DZ 1
1.08031386832038e-163 0.673995747121862 0.611 0.131
1.9260915958284e-159 10 SH2B2 DZ 1
1.11077926360344e-163 0.552727247412275 0.97 0.675
1.98040834907858e-159 10 COX6A1 DZ 1
3.4708226820667e-163 0.626819315986232 0.825 0.342
6.18812975985673e-159 10 GNAS DZ 1
6.59351645723607e-163 0.610581882905289 0.9 0.444
1.17555804916062e-158 10 RAP1B DZ 1
1.21595144870206e-162 0.61339334781081 0.724 0.217
2.1679198378909e-158 10 MRPL27 DZ 1
1.83036330975016e-162 -0.868050034676581 0.816 0.909
3.26335474495356e-158 10 IGHM DZ 1
3.20788467680397e-162 0.607437314184777 0.96 0.626
5.71933759027379e-158 10 SET DZ 1
5.51953237068864e-162 0.619099129552311 0.829 0.335
9.84077426370078e-158 10 MRPS6 DZ 1
2.84933672625807e-161 0.605522985475438 0.556 0.112
5.08008244924552e-157 10 SUZ12 DZ 1
3.14388871153901e-160 0.625828689761291 0.718 0.226
5.6052391838029e-156 10 CEP57 DZ 1
3.36914869215712e-160 0.527155442819417 0.969 0.647
6.00685520324693e-156 10 RBX1 DZ 1
1.0826241594568e-159 0.596810801327376 0.325 0.023
1.93021061389552e-155 10 CENPA DZ 1
1.31042509186905e-159 0.516242172950009 0.356 0.035
2.33635689629333e-155 10 NSD2 DZ 1
1.16413803841496e-158 0.573011263392317 0.436 0.066
2.07554170869004e-154 10 BTG3 DZ 1
1.37632114978095e-158 0.797248290430322 0.435 0.066
2.45384297794446e-154 10 SUGCT DZ 1
1.91389373962743e-158 0.574909846964057 0.95 0.611
3.41228114838174e-154 10 ARPC1B DZ 1
2.03757880729465e-158 0.538533782919937 0.373 0.043
3.63279925552562e-154 10 A4GALT DZ 1
2.67428481070366e-158 0.536756749985713 0.423 0.049
4.76798238900356e-154 10 BARD1 DZ 1
4.51432057249396e-158 0.552285342547719 0.96 0.634
8.04858214869949e-154 10 HNRNPC DZ 1
1.96658594882635e-157 0.559626093475084 0.954 0.596
3.5062260881625e-153 10 POMP DZ 1
4.18349497808233e-157 0.703678639252092 0.53 0.078

7.45875319642299e-153	10	CCNB1	DZ 1		
4.87115186557568e-157	0.555723816228926			0.944	0.553
8.68477666113488e-153	10	RAC1	DZ 1		
6.94968265466962e-157	-1.4400154369216		0.322	0.518	
1.23905892050105e-152	10	BANK1	DZ 1		
5.35476056860393e-156	0.510602309313876			0.296	0.034
9.54700261776395e-152	10	ACY3	DZ 1		
8.03213164421705e-156	0.643435580829119			0.522	0.098
1.43204875084746e-151	10	CKAP2	DZ 1		
9.10440804483782e-156	0.628792388399734			0.834	0.34
1.62322491031413e-151	10	RAB11A	DZ 1		
8.75266734878284e-155	0.582011902260046			0.879	0.42
1.56051306161449e-150	10	KHDRBS1	DZ 1		
1.32074058411804e-154	0.418324154009552			0.29	0.023
2.35474838742406e-150	10	C9orf40	DZ 1		
1.5458875963617e-154	0.437827136590788			0.289	0.019
2.75616299555328e-150	10	FANCB	DZ 1		
2.15549549159765e-154	-0.657554599998247			0.999	0.999
3.84303291196946e-150	10	RPL13	DZ 1		
4.14397517130219e-154	0.663798616246549			0.586	0.132
7.38829333291468e-150	10	SEC14L1	DZ 1		
1.42359086030727e-153	0.558947513307354			0.335	0.023
2.53812014484184e-149	10	ASPM	DZ 1		
1.012949870797e-152	0.629221451947111			0.711	0.216
1.80598832464397e-148	10	MTF2	DZ 1		
3.38705219270332e-152	-0.589742092740315			1	0.999
6.03877535437074e-148	10	RPL13A	DZ 1		
8.89264454884136e-152	0.580015043050137			0.826	0.352
1.58546959661293e-147	10	CACYBP	DZ 1		
1.99701485824667e-151	0.557802766619467			0.375	0.045
3.56047779076798e-147	10	MME	DZ 1		
3.28083157277351e-151	0.593291273244261			0.566	0.13
5.84939461109788e-147	10	MAD2L2	DZ 1		
2.47340762695883e-150	0.584184662731372			0.833	0.359
4.40983845810491e-146	10	LCP1	DZ 1		
3.06419441160365e-150	0.583939328267054			0.628	0.169
5.46315221644816e-146	10	BAZ1B	DZ 1		
8.30319518157814e-150	0.481371311762169			0.374	0.046
1.48037666892357e-145	10	SHMT1	DZ 1		
9.26618834302024e-150	0.540094744915137			0.961	0.623
1.65206871967708e-145	10	PSMA4	DZ 1		
1.29588384742255e-149	-1.31385585132919			0.104	0.359
2.31043131156966e-145	10	LY6E	DZ 1		
1.31517770783215e-149	0.607077962857787			0.341	0.046
2.34483033529394e-145	10	TUBB2A	DZ 1		
2.45142522507924e-149	0.595438822736863			0.63	0.161
4.37064603379377e-145	10	POLR3K	DZ 1		
2.55363854555372e-149	0.680200404500364			0.449	0.073
4.55288216286773e-145	10	HIST1H1C	DZ 1		
2.56520815070455e-149	0.62003165311961		0.503	0.104	

4.57350961189114e-145 10 ABI3 DZ 1
5.35139948875113e-149 0.359177198865747 0.22 0.013
9.54101014849439e-145 10 ASRGL1 DZ 1
9.66182708418995e-149 0.575210111039428 0.871 0.401
1.72260715084023e-144 10 PPP1CC DZ 1
1.13571367396934e-148 0.335140025966613 0.196 0.009
2.02486390931993e-144 10 CNTLN DZ 1
1.27444950056851e-148 0.32043926227703 0.207 0.012
2.2722160145636e-144 10 TRIP13 DZ 1
2.44211741014455e-148 0.472626522846588 0.328 0.034
4.35405113054673e-144 10 AL441992.1 DZ 1
3.20149518850686e-148 0.595823064289724 0.875 0.452
5.70794577158888e-144 10 PRDX6 DZ 1
3.76375985782057e-148 0.550103254661317 0.827 0.359
6.71040745050829e-144 10 PSMC3 DZ 1
1.47823286680351e-147 0.48004959657312 0.301 0.03
2.63554137822397e-143 10 RACGAP1 DZ 1
1.80739769642607e-147 0.60604879897239 0.78 0.287
3.22240935295804e-143 10 UGP2 DZ 1
2.99356401684781e-147 0.577183989891664 0.502 0.095
5.33722528563796e-143 10 OGG1 DZ 1
3.25262241376937e-147 -1.27667465550262 0.136 0.391
5.79910050150942e-143 10 CD44 DZ 1
3.28121483836482e-147 0.685924489912385 0.405 0.065
5.85007793532064e-143 10 RGCC DZ 1
9.25743118569144e-147 -1.38553332126516 0.113 0.361
1.65050740609693e-142 10 TXNIP DZ 1
1.13693844413436e-146 0.613543494710298 0.726 0.233
2.02704755204716e-142 10 SH3KBP1 DZ 1
1.14795971214229e-146 0.68082345237706 0.415 0.063
2.04669737077848e-142 10 H1FX DZ 1
2.51425499548299e-146 0.570896499143027 0.71 0.234
4.48266523144662e-142 10 SUPT16H DZ 1
4.71305072761641e-146 0.478472079143705 0.991 0.899
8.4028981422673e-142 10 MYL6 DZ 1
8.58640694837669e-146 0.567489657236292 0.854 0.396
1.53087049482608e-141 10 MDH1 DZ 1
1.00836677212277e-144 0.56445640447214 0.606 0.158
1.79781711801768e-140 10 MRPL37 DZ 1
1.26219127776127e-144 0.602476409901841 0.635 0.167
2.25036082912056e-140 10 RASGRP3 DZ 1
2.34569002000481e-144 0.556173492718501 0.406 0.059
4.18213073666658e-140 10 RPRD1B DZ 1
2.85914552452306e-144 0.486330626003991 0.988 0.865
5.09757055567217e-140 10 ARPC3 DZ 1
4.37851098721891e-144 0.546870139793733 0.946 0.587
7.8064472391126e-140 10 PRDX1 DZ 1
4.3858097499237e-144 0.471843014780583 0.335 0.037
7.81946020313897e-140 10 RBBP8 DZ 1
4.80663222516343e-144 0.531089696852124 0.868 0.41

8.56974459424388e-140 10 BLOC1S1 DZ 1
6.46834888042855e-144 0.489564124591005 0.271 0.019
1.15324192189161e-139 10 DLGAP5 DZ 1
6.71238339786131e-144 -1.89030061587415 0.249 0.472
1.19675083600469e-139 10 S100A6 DZ 1
7.04410872736382e-144 0.460434280386362 0.31 0.027
1.2558941450017e-139 10 KNL1 DZ 1
1.08967641876069e-143 0.534112727558473 0.409 0.07
1.94278408700844e-139 10 GPR160 DZ 1
2.00299064393107e-143 0.492446588096528 0.922 0.501
3.57113201906471e-139 10 UBE2I DZ 1
6.21775929168932e-143 0.534375578615544 0.484 0.082
1.10856430411529e-138 10 MSH2 DZ 1
2.81062695266874e-142 0.303114097169455 0.193 0.009
5.01106679391309e-138 10 MTFR2 DZ 1
3.10240566598891e-142 0.527456518317845 0.898 0.454
5.53127906189163e-138 10 SNRPF DZ 1
4.29520795811178e-142 0.544404846552376 0.856 0.385
7.65792626851748e-138 10 ENY2 DZ 1
1.09560279618898e-141 0.458505908798324 0.358 0.042
1.95335022532533e-137 10 ZWILCH DZ 1
1.13636641906926e-141 0.55428642038096 0.868 0.416
2.02602768855859e-137 10 CLTA DZ 1
2.06383388060318e-141 0.534392156728694 0.896 0.431
3.67960942572742e-137 10 ATP5F1C DZ 1
3.85290961023729e-141 0.59000506546675 0.528 0.11
6.86935254409206e-137 10 SPATS2 DZ 1
4.67286235607815e-141 0.545290666187799 0.845 0.39
8.33124629465174e-137 10 PSMB2 DZ 1
7.06339174576961e-141 -0.819616532218175 0.995 0.996
1.25933211435326e-136 10 RPS12 DZ 1
1.17853264540748e-140 0.350023041633369 0.209 0.012
2.101205853497e-136 10 KIF4A DZ 1
1.28450719220862e-140 0.615949344932386 0.732 0.245
2.29014787298874e-136 10 CCDC69 DZ 1
1.32567112324821e-140 0.503546040770941 0.944 0.554
2.36353904563923e-136 10 UQCRQ DZ 1
2.27085687817914e-140 -0.533670066628599 0.999 0.999
4.04871072810559e-136 10 RPLP1 DZ 1
2.66582331089033e-140 0.597182239017541 0.625 0.168
4.75289638098638e-136 10 TRABD DZ 1
2.78244058800876e-140 0.593805300001363 0.364 0.036
4.96081332436082e-136 10 HMMR DZ 1
1.10690346230055e-138 0.565754326069897 0.689 0.22
1.97349818293565e-134 10 UFD1 DZ 1
1.21864754476736e-138 0.494522544015664 0.38 0.045
2.17272670756572e-134 10 TIMELESS DZ 1
3.19410843209637e-138 0.6285650819918 0.701 0.223
5.69477592358462e-134 10 NCF4 DZ 1
4.96867424265337e-138 0.556865332030112 0.954 0.653

8.8586493072267e-134	10	DBI	DZ 1
5.76506785791826e-138	0.555263579523575	0.585	0.152
1.02785394838825e-133	10	CENPX	DZ 1
2.04231408351987e-137	0.564404141810035	0.473	0.099
3.64124177950758e-133	10	NFYB	DZ 1
3.17972584557477e-137	0.48482671443992	0.337	0.042
5.66913321007525e-133	10	ASB13	DZ 1
4.29527102617884e-137	-0.648434756915873	0.999	0.998
7.65803871257425e-133	10	RPL18A	DZ 1
6.58689484937373e-137	0.516335776820698	0.474	0.088
1.17437748269484e-132	10	HADH	DZ 1
1.14853404980328e-136	0.508246157221387	0.923	0.51
2.04772135739428e-132	10	TRAPPC1	DZ 1
2.19134904305743e-136	0.366525064333874	0.226	0.013
3.90695620886709e-132	10	CCNF	DZ 1
3.29136392568547e-136	0.437800605424263	0.293	0.033
5.86817274310462e-132	10	NDC1	DZ 1
7.45075408774046e-136	0.482669038383268	0.306	0.026
1.32839494630325e-131	10	HIST1H1E	DZ 1
7.78412010963416e-136	0.54991503201517	0.863	0.423
1.38783077434667e-131	10	COTL1	DZ 1
1.01654531641267e-135	0.456368019623901	0.988	0.801
1.81239864463215e-131	10	TMA7	DZ 1
2.27948773342117e-135	0.595547621997115	0.909	0.478
4.06409867991661e-131	10	C4orf3	DZ 1
2.87355682965703e-135	0.4187483417885	0.264	0.03
5.12326447159552e-131	10	KCNK12	DZ 1
4.57888743877795e-135	0.524255273855383	0.829	0.348
8.16369841459721e-131	10	PPP2CA	DZ 1
7.72407751386357e-135	0.488780861700833	0.327	0.047
1.37712577994674e-130	10	BFSP2	DZ 1
1.45371359624759e-134	0.529977955737972	0.517	0.119
2.59182597074983e-130	10	PAFAH1B3	DZ 1
2.92368134996378e-134	0.632892358435982	0.763	0.258
5.21263147885042e-130	10	NCF1	DZ 1
1.08335493676578e-133	0.387412497742008	0.265	0.023
1.93151351675971e-129	10	TEDC1	DZ 1
1.90753321745173e-133	0.544472750091351	0.497	0.097
3.40094097339469e-129	10	MAP4K2	DZ 1
2.46714560992385e-133	0.547341594841264	0.961	0.69
4.39867390793324e-129	10	CCNI	DZ 1
5.21023317150481e-133	0.46887597376507	0.888	0.442
9.28932472147593e-129	10	LSM5	DZ 1
8.13672124234511e-133	0.515772407531309	0.392	0.06
1.45069603029771e-128	10	MTA3	DZ 1
1.35004700771993e-132	0.544086558526354	0.78	0.31
2.40699881006385e-128	10	SNRNP70	DZ 1
3.38714578298611e-132	0.343795807318057	0.23	0.015
6.03894221648594e-128	10	GINS3	DZ 1
5.81861876300346e-132	0.461682977254056	0.337	0.045

1.03740153925589e-127 10 BORCS8-MEF2B DZ 1
6.84001150542175e-132 0.488122219702522 0.901 0.471
1.21950565130164e-127 10 UBE2L3 DZ 1
8.98276849431186e-132 0.517505887016492 0.652 0.198
1.60153779485086e-127 10 COPS3 DZ 1
1.58486796145683e-131 0.523533060362149 0.435 0.08
2.82566108848139e-127 10 XPNPEP1 DZ 1
3.39358575519069e-131 0.449659809859275 0.333 0.042
6.05042404292948e-127 10 TIPIN DZ 1
3.47087894846463e-131 0.552375714334099 0.657 0.21
6.18823007721759e-127 10 DAZAP1 DZ 1
1.10622590832139e-130 0.500904808552835 0.889 0.476
1.9722901719462e-126 10 CCT8 DZ 1
2.29794497178924e-130 0.524945215901378 0.568 0.141
4.09700609020304e-126 10 RPP30 DZ 1
4.07044342359177e-130 0.505013700494925 0.919 0.51
7.25719357992176e-126 10 FKBP1A DZ 1
4.55678936606305e-130 0.564427254411547 0.803 0.316
8.1242997607538e-126 10 BPTF DZ 1
6.16231282093426e-130 0.574572716957191 0.572 0.125
1.09867875284437e-125 10 SOCS1 DZ 1
6.68824766692432e-130 0.639967878991816 0.666 0.195
1.19244767653594e-125 10 ADA DZ 1
3.6085825732784e-129 -1.36773926091984 0.219 0.377
6.43374186989807e-125 10 SELL DZ 1
5.11599352426927e-129 0.503120146932551 0.833 0.397
9.12130485441969e-125 10 HNRNPR DZ 1
5.35404601576007e-129 0.523658617649597 0.734 0.27
9.54572864149862e-125 10 UQCRC1 DZ 1
5.35442154504234e-129 0.537076916720978 0.799 0.346
9.54639817265599e-125 10 TKT DZ 1
1.01983454417401e-128 0.422163725030106 0.287 0.031
1.81826300880785e-124 10 EEPD1 DZ 1
1.18777835416762e-128 0.458389976983208 0.322 0.039
2.11769002764544e-124 10 RCCD1 DZ 1
1.62895367390391e-128 0.617843119942058 0.549 0.124
2.90426150520328e-124 10 SIT1 DZ 1
1.89310448050831e-128 0.534457226977053 0.716 0.25
3.37521597829826e-124 10 RAB5IF DZ 1
2.60081350814521e-128 0.512600705945717 0.827 0.368
4.63699040367209e-124 10 SNRPD3 DZ 1
3.68438183382162e-128 -1.247604171021 0.772 0.794
6.56888437152057e-124 10 HLA-E DZ 1
5.4150870960281e-128 0.508871655113983 0.514 0.108
9.6545587835085e-124 10 CFAP20 DZ 1
1.24795506570838e-127 0.542618229905715 0.609 0.176
2.22497908665147e-123 10 MAP2K1 DZ 1
1.8582594891045e-127 0.623060956500499 0.499 0.093
3.31309084312442e-123 10 HRK DZ 1
2.94829388861476e-127 0.537548374724884 0.47 0.104

5.25651317401125e-123	10	GOT2	DZ 1
3.75725982523597e-127	0.469163322790747	0.423	0.072
6.69881854241321e-123	10	NCAPH2	DZ 1
8.07824216415478e-127	0.420714859802449	0.982	0.799
1.44026979544716e-122	10	HNRNPK	DZ 1
9.28801649669877e-127	-0.621060289378032	0.999	0.999
1.65596046119642e-122	10	RPL32	DZ 1
1.02471072589403e-126	0.478762257593322	0.903	0.477
1.82695675319647e-122	10	SF3B6	DZ 1
2.40315365810584e-126	0.433511619468158	0.35	0.043
4.2845826570369e-122	10	POLD1	DZ 1
3.49487152331429e-125	0.481940885641755	0.396	0.054
6.23100643891704e-121	10	ACYP1	DZ 1
6.64013179059889e-125	-1.01640326723534	0.967	0.963
1.18386909694588e-120	10	FTL	DZ 1
7.55452267127623e-125	0.500819111143372	0.911	0.521
1.34689584706184e-120	10	SOD1	DZ 1
1.20673663173559e-124	-0.653795314194666	0.997	0.996
2.15149074072138e-120	10	RPL35A	DZ 1
1.22371474552926e-124	-1.19955830712931	0.092	0.28
2.18176101980413e-120	10	KLF2	DZ 1
1.23851097400732e-124	0.508642659076498	0.52	0.112
2.20814121555765e-120	10	ARL3	DZ 1
1.3366666019979e-124	0.516236945472805	0.818	0.352
2.38314288470205e-120	10	EWSR1	DZ 1
2.64137231919253e-124	0.316158831691644	0.194	0.012
4.70930270788836e-120	10	TTK	DZ 1
9.83244808964216e-124	0.507996224834985	0.622	0.173
1.7530271699023e-119	10	GLRX5	DZ 1
1.22907892836655e-123	0.507099323250674	0.355	0.048
2.19132482138472e-119	10	SGO2	DZ 1
1.51331185917722e-123	0.484832157094154	0.604	0.17
2.69808371372706e-119	10	BID	DZ 1
1.75165609146737e-123	0.390890127709328	0.276	0.029
3.12302764547717e-119	10	FKBP5	DZ 1
5.16725386083064e-123	0.522757506449403	0.75	0.28
9.21269690847494e-119	10	LYPLA1	DZ 1
5.3939828553425e-123	0.456727060936931	0.345	0.043
9.61693203279015e-119	10	BRCA2	DZ 1
5.82662185251323e-123	0.508346855434817	0.766	0.322
1.03882841008458e-118	10	ACAT2	DZ 1
1.09349689151034e-122	0.488033406425687	0.852	0.413
1.94959560787379e-118	10	MINOS1	DZ 1
1.21711698905903e-122	0.539726675335424	0.665	0.233
2.16999787979334e-118	10	SNRPA1	DZ 1
2.64749242990397e-122	0.474367721114779	0.44	0.073
4.72021425327578e-118	10	MIS18A	DZ 1
2.99839643511978e-122	0.477238854979078	0.902	0.467
5.34584100417506e-118	10	TRA2B	DZ 1
3.98339777800447e-122	0.480703428741628	0.906	0.489

7.10199989840417e-118 10 GNG5 DZ 1
4.32910141517481e-122 0.479702894116898 0.885 0.482
7.71835491311517e-118 10 ARL6IP4 DZ 1
6.21822587553676e-122 0.531609478865098 0.69 0.229
1.10864749134945e-117 10 DCTN3 DZ 1
8.13413873603178e-122 -0.569613582771485 0.999 0.999
1.45023559524711e-117 10 RPS27A DZ 1
9.76217816758139e-122 0.480000808278665 0.379 0.06
1.74049874549809e-117 10 TERF2 DZ 1
2.44247412030072e-121 0.547396682487075 0.562 0.135
4.35468710908416e-117 10 PTPN18 DZ 1
6.68022251202392e-121 0.486845018264159 0.643 0.219
1.19101687166875e-116 10 CDK4 DZ 1
8.62547733066315e-121 0.485391529802454 0.575 0.157
1.53783635328393e-116 10 EED DZ 1
1.16426842524807e-120 0.507008452357671 0.621 0.177
2.07577417537478e-116 10 HNRNPUL1 DZ 1
2.41401353353329e-120 0.502780174085714 0.715 0.251
4.3039447289365e-116 10 PPP1R7 DZ 1
2.71287903249579e-120 0.524116227283829 0.522 0.121
4.83679202703674e-116 10 MAP4K1 DZ 1
7.11122404000346e-120 0.528409661247228 0.782 0.308
1.26786013409222e-115 10 HSBP1 DZ 1
1.72646339506687e-119 0.322170556496289 0.188 0.013
3.07811158706473e-115 10 DEPDC1B DZ 1
2.33447022406006e-119 0.511478626617326 0.475 0.104
4.16212696247669e-115 10 DBF4 DZ 1
4.98129257836365e-119 0.456708132382014 0.364 0.052
8.88114653796455e-115 10 DTX1 DZ 1
5.9516278464513e-119 0.521202249309489 0.562 0.139
1.0611157287438e-114 10 MYL6B DZ 1
1.05114678444677e-118 0.51816599373421 0.655 0.193
1.87408960199015e-114 10 MIS18BP1 DZ 1
1.73737117557505e-118 0.431397432027802 0.907 0.489
3.09755906893275e-114 10 NDUFA11 DZ 1
1.82331982405751e-118 0.57324441857886 0.625 0.193
3.25079691431213e-114 10 SNHG25 DZ 1
2.18573952041935e-118 -1.05865270368717 0.501 0.624
3.89695499095566e-114 10 SUB1 DZ 1
2.66787496188373e-118 0.572259947940303 0.699 0.249
4.7565542695425e-114 10 CD22 DZ 1
7.34515690009829e-118 0.426040137179657 0.327 0.04
1.30956802371852e-113 10 POLD3 DZ 1
8.58304705600437e-118 0.47745521609692 0.802 0.348
1.53027145961502e-113 10 ELOC DZ 1
1.08000643248547e-117 0.469776840937529 0.371 0.051
1.92554346847835e-113 10 DCAF12 DZ 1
2.31201537911388e-117 0.489554669310446 0.705 0.251
4.12209221942215e-113 10 MTCH2 DZ 1
2.5047763993949e-117 0.485532411096877 0.358 0.053

4.46576584248116e-113 10 PITPNC1 DZ 1
2.59267216794467e-117 0.502453147555709 0.673 0.225
4.62247520822855e-113 10 CPSF6 DZ 1
3.16371349844541e-117 -0.636800714066652 0.999 0.998
5.64058479637832e-113 10 RPLP2 DZ 1
3.27437828018099e-117 0.360360034919572 0.223 0.017
5.83788903573469e-113 10 ARHGAP11A DZ 1
4.91906911176894e-117 0.343767732954322 0.94 0.585
8.77020831937283e-113 10 LDHA DZ 1
7.57046165030698e-117 -1.04108808220331 0.043 0.217
1.34973760763323e-112 10 PLAC8 DZ 1
1.13431233195542e-116 0.543167289654748 0.613 0.166
2.02236545664332e-112 10 ZNF106 DZ 1
2.50719663597013e-116 0.575773565033042 0.896 0.475
4.47008088227115e-112 10 UCP2 DZ 1
3.57431603187307e-116 0.518312868336708 0.624 0.182
6.37264805322649e-112 10 UBE2E1 DZ 1
4.70707610235304e-116 0.502281890587489 0.662 0.226
8.39224598288523e-112 10 SRSF1 DZ 1
5.60158864172139e-116 0.436281503895249 0.938 0.618
9.98707238932506e-112 10 ATP5F1B DZ 1
5.61000272936705e-116 0.403563081274532 0.933 0.559
1.00020738661885e-111 10 SNRPE DZ 1
5.78763840839544e-116 0.384972904858025 0.22 0.006
1.03187805183282e-111 10 HIST1H1B DZ 1
7.30900907128578e-116 0.389495983704214 0.248 0.028
1.30312322731954e-111 10 NUGGC DZ 1
1.07090599296588e-115 0.476638641520918 0.677 0.236
1.90931829485887e-111 10 NAP1L4 DZ 1
1.10256177256749e-115 0.432962733275792 0.294 0.036
1.96575738431058e-111 10 ASB2 DZ 1
1.17711456338553e-115 0.587637202022255 0.647 0.195
2.09867755506005e-111 10 SYAP1 DZ 1
1.43043768336537e-115 0.451052469619597 0.899 0.488
2.55032734567213e-111 10 RTRAF DZ 1
2.55256486321733e-115 0.499892349786087 0.551 0.142
4.55096789463017e-111 10 ACTL6A DZ 1
3.58948402713203e-115 0.410246193804511 0.971 0.687
6.39969107197369e-111 10 ATP5MPL DZ 1
4.23019032435155e-115 0.519367969485494 0.621 0.183
7.54200632928637e-111 10 POLD4 DZ 1
5.15589348905535e-115 0.51207346230817 0.742 0.294
9.19244250163678e-111 10 FKBP3 DZ 1
6.37423627626386e-115 0.43109952758084 0.322 0.038
1.13646258569508e-110 10 FANCL DZ 1
1.16152964061272e-114 -0.481745489032161 1 0.999
2.07089119624843e-110 10 RPL10 DZ 1
1.68860928824085e-114 0.498628632151578 0.774 0.307
3.01062150000461e-110 10 GNB2 DZ 1
2.21785456081417e-114 0.561265379553002 0.484 0.099

3.95421289647558e-110	10	CPNE5	DZ 1		
2.68356512519254e-114	0.545799120219174			0.707	0.255
4.78452826170579e-110	10	TOP1	DZ 1		
3.19177005035201e-114	0.508445630404154			0.423	0.079
5.6906068227726e-110	10	ABHD3	DZ 1		
5.04618363523051e-114	0.508221280245801			0.97	0.717
8.99684080325247e-110	10	LIMD2	DZ 1		
1.13663920321673e-113	0.485637361656718			0.486	0.115
2.0265140354151e-109	10	PGP	DZ 1		
2.19108495600098e-113	0.623483918994234			0.63	0.223
3.90648536805414e-109	10	DSTN	DZ 1		
2.39172262961212e-113	0.601333207860554			0.367	0.037
4.26420227633546e-109	10	CDC20	DZ 1		
3.02744691559708e-113	0.395481401147695			0.967	0.708
5.39763510581804e-109	10	COX6B1	DZ 1		
3.63386454015132e-113	0.486335827849811			0.699	0.252
6.4788170886358e-109	10	PMF1	DZ 1		
4.8194887343783e-113	0.489153518983087			0.661	0.226
8.59266646452308e-109	10	SUMO3	DZ 1		
8.52830620394856e-113	0.469555709860993			0.86	0.404
1.52051171310199e-108	10	NDUFA12	DZ 1		
9.54973676003044e-113	0.395277421017		0.97	0.691	
1.70262256694583e-108	10	ELOB	DZ 1		
9.96361103777521e-113	0.640988626913068			0.623	0.171
1.77641221192494e-108	10	RGS2	DZ 1		
1.0076896865112e-112	0.50847706247748		0.756	0.291	
1.79660994208082e-108	10	TIMM8B	DZ 1		
1.02049136574158e-112	0.369677538791237			0.197	0.015
1.81943405598066e-108	10	BEX3	DZ 1		
1.1946812540537e-112	0.424719587918785			0.327	0.046
2.12999720785235e-108	10	GALK1	DZ 1		
1.6467629699253e-112	0.452474158707546			0.791	0.358
2.93601369907982e-108	10	DDT	DZ 1		
2.48019847434135e-112	0.471958883148701			0.473	0.096
4.42194585990319e-108	10	RABL6	DZ 1		
3.12327880448333e-112	0.427913032717875			0.954	0.624
5.56849378051332e-108	10	BRK1	DZ 1		
4.96402454999255e-112	0.477960303478709			0.789	0.331
8.85035937018172e-108	10	UBE2A	DZ 1		
6.1557764585663e-112	0.488841313002097			0.825	0.371
1.09751338479779e-107	10	NDUFB3	DZ 1		
6.96665661471459e-112	0.400331083215867			0.95	0.674
1.24208520783746e-107	10	SLC25A3	DZ 1		
7.02327371675944e-112	0.44340909535604		0.358	0.057	
1.25217947096104e-107	10	TEX9	DZ 1		
1.06443011864236e-111	0.417928078452297			0.907	0.53
1.89777245852747e-107	10	SUMO1	DZ 1		
2.42632596134422e-111	0.439175076611467			0.872	0.442
4.32589655648061e-107	10	PPP4C	DZ 1		
3.55081062989041e-111	-1.13521339591275			0.126	0.302

6.33074027203162e-107	10	LINC00926	DZ 1
4.9165681624767e-111	0.462491509220764	0.794	0.341
8.76574937687971e-107	10	AP2S1	DZ 1
5.78030494110724e-111	0.454617513193038	0.312	0.042
1.03057056795001e-106	10	CPM	DZ 1
6.80037362426484e-111	0.508769793458431	0.612	0.178
1.21243861347018e-106	10	CNTRL	DZ 1
1.51953584359529e-110	0.493260414516606	0.576	0.164
2.70918045554605e-106	10	HINT2	DZ 1
1.67353281979484e-110	0.514940456814001	0.713	0.294
2.98374166441223e-106	10	FERMT3	DZ 1
1.73244122030276e-110	0.444286182549108	0.936	0.603
3.0887694516778e-106	10	ARPC5	DZ 1
1.95345555683634e-110	0.37856614141634 0.9	0.499
3.4828159122835e-106	10	PA2G4	DZ 1
2.65245623882419e-110	0.454198006727361	0.689	0.239
4.72906422819966e-106	10	LUC7L2	DZ 1
2.73373163560958e-110	0.520238043145545	0.681	0.236
4.87397013312833e-106	10	TIFA	DZ 1
2.84415273781209e-110	0.449783022410363	0.848	0.413
5.07083991624517e-106	10	ANP32A	DZ 1
6.89421995059684e-110	0.492735266931052	0.381	0.067
1.22917047499191e-105	10	KLHL6	DZ 1
7.32113664171765e-110	0.47090902409957 0.706	0.258
1.30528545185184e-105	10	CAMTA1	DZ 1
4.57392863103505e-109	0.462177537348246	0.642	0.202
8.15485735627239e-105	10	HPRT1	DZ 1
8.536516523124e-109	0.478358849643511	0.512	0.129
1.52197553090778e-104	10	ANAPC15	DZ 1
1.19312174585858e-108	0.470157158526131	0.456	0.093
2.12721676069126e-104	10	MGME1	DZ 1
1.4411760703441e-108	0.402301960697443	0.942	0.593
2.56947281581649e-104	10	PSMB3	DZ 1
1.82749436957158e-108	0.450845054228829	0.785	0.335
3.25823971150917e-104	10	LSM14A	DZ 1
2.2581841086536e-108	0.489952580007683	0.597	0.168
4.0261164473185e-104	10	MED30	DZ 1
3.00981547800208e-108	0.453145190835583	0.671	0.234
5.36620001572991e-104	10	MEA1	DZ 1
3.66858372694856e-108	0.466791789807936	0.493	0.114
6.54071792677658e-104	10	CMC2	DZ 1
5.07708806133396e-108	0.394629119342949	0.948	0.631
9.05194030455232e-104	10	HNRNPM	DZ 1
1.73500133009042e-107	0.434719997831838	0.679	0.233
3.09333387141821e-103	10	UBA2	DZ 1
1.91873614078616e-107	0.422626886912236	0.728	0.289
3.42091466540765e-103	10	ODC1	DZ 1
2.22692279143775e-107	0.348387486841029	0.266	0.025
3.97038064485436e-103	10	PSMC3IP	DZ 1
2.31105711137157e-107	0.492122702062582	0.708	0.253

4.12038372386437e-103 10 TXNDC17 DZ 1
4.08276331718171e-107 0.483084848208531 0.652 0.202
7.27915871820326e-103 10 PNKD DZ 1
5.07618256564965e-107 0.514351428707078 0.681 0.238
9.05032589629677e-103 10 RGS10 DZ 1
5.74374432797263e-107 0.443228053719642 0.314 0.049
1.02405217623424e-102 10 MBD2 DZ 1
6.81797831637993e-107 0.449388020106491 0.779 0.33
1.21557735402738e-102 10 MRPS34 DZ 1
7.02414129531437e-107 0.55181464860648 0.429 0.086
1.2523341515416e-102 10 CCDC28B DZ 1
8.88155225614804e-107 0.468479467459336 0.674 0.235
1.58349195174863e-102 10 MRPL13 DZ 1
8.99346597767472e-107 0.363768373895499 0.997 0.96
1.60344504915963e-102 10 OAZ1 DZ 1
1.00179797237877e-106 0.382904808918124 0.931 0.583
1.78610560495411e-102 10 TBCA DZ 1
1.93037421858296e-106 0.522036555155769 0.779 0.313
3.44166419431156e-102 10 GYPC DZ 1
2.3530119575768e-106 0.372265608769619 0.944 0.589
4.19518501916368e-102 10 PSMA2 DZ 1
2.46182488863753e-106 0.528033773782991 0.786 0.346
4.38918759395185e-102 10 TPD52 DZ 1
3.16239977038255e-106 0.360276841199965 0.243 0.02
5.63824255061505e-102 10 NCAPD3 DZ 1
5.36054229306931e-106 0.482756558322362 0.56 0.148
9.55731085431327e-102 10 UBE2R2 DZ 1
5.78568373805087e-106 0.354803776806158 0.242 0.027
1.03152955365709e-101 10 PDGFD DZ 1
6.41439269649133e-106 -0.94199338293651 0.978 0.968
1.14362207385744e-101 10 MT-ND3 DZ 1
6.45278729401622e-106 0.321970070526986 0.203 0.017
1.15046744665015e-101 10 FAM81A DZ 1
9.64380365206523e-106 0.489143623713788 0.547 0.144
1.71939375312671e-101 10 SMIM20 DZ 1
1.38897533252212e-105 0.33231077753665 0.228 0.017
2.47640412035368e-101 10 C1orf112 DZ 1
3.18838181095188e-105 0.443687880083514 0.735 0.291
5.68456593074611e-101 10 ELAVL1 DZ 1
2.7327120802925e-104 0.44403732470124 0.349 0.051
4.87215236795349e-100 10 TESC DZ 1
3.30030903395369e-104 0.437859726348929 0.659 0.228
5.88412097663603e-100 10 PTBP1 DZ 1
4.25288094418737e-104 0.372562350448835 0.934 0.607
7.58246143539165e-100 10 NDUFA13 DZ 1
4.58262007075781e-104 0.398713620765301 0.93 0.561
8.17035332415409e-100 10 PARK7 DZ 1
4.76895276895901e-104 0.421127275307454 0.331 0.054
8.50256589177701e-100 10 FAM129A DZ 1
7.94585366771615e-104 0.468418882486294 0.78 0.335

1.41666625041711e-99 10 PDIA6 DZ 1
1.61825388638742e-103 0.32180446736776 0.205 0.017
2.88518485404013e-99 10 TCEAL9 DZ 1
3.49278647554441e-103 0.56711048439965 0.268 0.026
6.22728900724812e-99 10 PLK1 DZ 1
3.67772972422659e-103 0.418461572221862 0.813 0.375
6.55702432532359e-99 10 CCT5 DZ 1
4.17868635658047e-103 -0.619337715442508 0.999 0.999
7.45017990514732e-99 10 RPL39 DZ 1
4.39423368305441e-103 0.452838281779888 0.44 0.088
7.83447923351771e-99 10 TACC1 DZ 1
4.99204775181354e-103 0.415093289226003 0.879 0.464
8.90032193670836e-99 10 UBE2N DZ 1
5.50869428742718e-103 0.379550034557175 0.854 0.448
9.82145104505392e-99 10 HSPD1 DZ 1
6.12544760695256e-103 0.455656049429274 0.849 0.388
1.09210605384357e-98 10 MBD4 DZ 1
8.67813000312683e-103 0.333199978397297 0.243 0.024
1.54722379825748e-98 10 RMI1 DZ 1
9.10625434191965e-103 0.330569737771567 0.216 0.024
1.62355408662086e-98 10 SCARB1 DZ 1
1.08313890904287e-102 -1.07715191013763 0.471 0.611
1.93112836093253e-98 10 IGHD DZ 1
1.19595964661484e-102 0.336583741529068 0.972 0.752
2.1322764539496e-98 10 PSMA7 DZ 1
1.35656626312601e-102 0.45350012121582 0.457 0.105
2.41862199052736e-98 10 SLC25A1 DZ 1
2.3162402341708e-102 0.438394362883856 0.741 0.311
4.12962471350311e-98 10 AKR1B1 DZ 1
3.24183580073757e-102 0.437580237265299 0.817 0.373
5.77986904913502e-98 10 NDUFS6 DZ 1
4.40652763178209e-102 0.388834499743451 0.262 0.032
7.85639811470429e-98 10 SYBU DZ 1
4.73532905949676e-102 0.384136311140404 0.322 0.042
8.44261818017678e-98 10 CDK2 DZ 1
4.8461062796668e-102 0.461032330133004 0.526 0.143
8.64012288601794e-98 10 GNB1 DZ 1
4.84717279013608e-102 0.440138008373749 0.336 0.052
8.64202436753361e-98 10 CCDC18 DZ 1
7.03554917609314e-102 0.384880266036977 0.261 0.027
1.25436806260565e-97 10 FANCD2 DZ 1
7.6796767329634e-102 0.439116833949686 0.661 0.231
1.36920956472004e-97 10 AK2 DZ 1
9.2466971805205e-102 0.460437833461177 0.708 0.256
1.648593640315e-97 10 TXNL4A DZ 1
1.7952959344648e-101 0.447211119639365 0.386 0.073
3.20083312155729e-97 10 BPNT1 DZ 1
1.83477883191626e-101 -0.614451508438083 0.999 0.998
3.2712271794235e-97 10 RPL11 DZ 1
8.54660005598204e-101 0.484416968510507 0.489 0.115

1.52377332398104e-96 10 MSI2 DZ 1
9.80759941456569e-101 0.482052720438373 0.42 0.09
1.74859689962292e-96 10 PTPN7 DZ 1
1.29172217141074e-100 0.406714177624887 0.864 0.415
2.30301145940821e-96 10 IRF8 DZ 1
1.80539253148971e-100 0.449830615013149 0.575 0.174
3.218834344393e-96 10 SNRPA DZ 1
2.39305870946166e-100 -0.704354758804892 0.993 0.991
4.26658437309919e-96 10 RPL9 DZ 1
2.77261652757339e-100 0.467808131396502 0.484 0.144
4.94329800701059e-96 10 PAICS DZ 1
5.33907982724301e-100 0.440938590269416 0.544 0.156
9.51904542399157e-96 10 EXOSC3 DZ 1
8.03040494314141e-100 -0.64350895311246 0.999 0.995
1.43174089731268e-95 10 RPL31 DZ 1
8.15748022016663e-100 0.456612195438204 0.479 0.121
1.45439714845351e-95 10 PRKCD DZ 1
1.09870091614647e-99 0.340602191088012 0.271 0.038
1.95887386339753e-95 10 ALYREF DZ 1
1.18310416342493e-99 0.446124362803816 0.756 0.322
2.10935641297031e-95 10 SEPHS2 DZ 1
1.28837180866362e-99 -0.96372666352435 0.038 0.226
2.29703809766637e-95 10 GBP2 DZ 1
2.2553222926472e-99 0.407620441215261 0.331 0.063
4.0210141155607e-95 10 NEK6 DZ 1
9.40199687038724e-99 0.393382416884148 0.905 0.503
1.67628202202134e-94 10 SMDT1 DZ 1
9.45380182623237e-99 -0.607267707275664 0.998 0.995
1.68551832759897e-94 10 RPS25 DZ 1
1.03738033992559e-98 0.43251230198828 0.643 0.213
1.84954540805334e-94 10 PLEKHJ1 DZ 1
1.05564815531274e-98 0.442704075751859 0.558 0.156
1.88211509610709e-94 10 CYB5R3 DZ 1
1.09686833176721e-98 0.371009084516329 0.959 0.688
1.95560654870776e-94 10 RHOA DZ 1
1.17472543954376e-98 0.41395185072334 0.661 0.234
2.09441798616257e-94 10 EXOSC8 DZ 1
1.5391777167506e-98 0.468851507995542 0.517 0.129
2.74419995119465e-94 10 CUL3 DZ 1
2.0873949266738e-98 -1.10620608295658 0.079 0.275
3.72161641476672e-94 10 GPR183 DZ 1
2.6494331143909e-98 0.414698035176825 0.788 0.362
4.72367429964753e-94 10 NONO DZ 1
2.89153037895517e-98 0.407287315189737 0.378 0.073
5.15530951263916e-94 10 SEPT11 DZ 1
3.05157953149251e-98 0.416414927814567 0.837 0.379
5.44066114669799e-94 10 SYPL1 DZ 1
3.27275450271212e-98 -0.984912482871882 0.091 0.258
5.83499400288543e-94 10 FCMR DZ 1
3.64399321554386e-98 0.399068522088634 0.404 0.08

6.49687550399315e-94	10	CSE1L	DZ 1		
3.70684891987694e-98	0.468919272996455			0.445	0.101
6.6089409392486e-94	10	TRAC	DZ 1		
4.83992671363865e-98	0.35033150329235		0.974	0.728	
8.62910533774635e-94	10	HSPA8	DZ 1		
4.92753848003746e-98	0.373530979146112			0.925	0.567
8.78530835605878e-94	10	COPE	DZ 1		
5.23456335172471e-98	0.414768467091634			0.507	0.132
9.33270299978998e-94	10	POP7	DZ 1		
5.78451514833062e-98	0.532318759816952			0.301	0.044
1.03132120579587e-93	10	SERPINA9	DZ 1		
6.17705023703593e-98	0.426715275319608			0.768	0.335
1.10130628676114e-93	10	POLR2K	DZ 1		
1.39555954815747e-97	0.443211664585895			0.524	0.137
2.48814311840995e-93	10	AKR7A2	DZ 1		
1.69755433574073e-97	0.530333882676044			0.598	0.172
3.02656962519215e-93	10	P2RX5	DZ 1		
1.87985514451906e-97	0.410423678597483			0.624	0.221
3.35159373716303e-93	10	MTHFD2	DZ 1		
2.19415834876538e-97	0.442716397546678			0.732	0.288
3.91196492001379e-93	10	HNRNPH3	DZ 1		
2.67940142927481e-97	0.390308807652778			0.85	0.427
4.77710480825405e-93	10	AURKAIP1	DZ 1		
3.32426285053208e-97	0.409611595674318			0.414	0.083
5.92682823621364e-93	10	DERA	DZ 1		
5.16180320770113e-97	0.472886139483817			0.547	0.16
9.20297893901034e-93	10	CTCF	DZ 1		
8.11586582539739e-97	0.401520832130731			0.346	0.063
1.4469777180101e-92	10	ATPAF1	DZ 1		
8.30268052273163e-97	0.439080484984875			0.442	0.099
1.48028491039782e-92	10	PTS	DZ 1		
1.34527762009335e-96	0.441263375383722			0.36	0.065
2.39849546886443e-92	10	PAG1	DZ 1		
3.07823735356274e-96	0.374855965576124			0.942	0.616
5.48818937766701e-92	10	ATP5PO	DZ 1		
5.97466947718946e-96	0.422488101718652			0.558	0.149
1.06522382108811e-91	10	BRD7	DZ 1		
8.53644026333982e-96	0.376994636667551			0.947	0.603
1.52196193455086e-91	10	UQCR10	DZ 1		
8.72593097604466e-96	0.401552557301786			0.665	0.246
1.555746233719e-91	10	NAA50	DZ 1		
1.29232905391564e-95	0.465667185909056			0.51	0.129
2.3040934702262e-91	10	INO80C	DZ 1		
1.49531773905746e-95	0.434022363619675			0.695	0.267
2.66600199696555e-91	10	BABAM1	DZ 1		
1.51275585279401e-95	0.444436048780143			0.718	0.274
2.69709240994644e-91	10	RRAS2	DZ 1		
4.69488874362249e-95	0.434662589089033			0.285	0.034
8.37051714100453e-91	10	AURKA	DZ 1		
4.73266976578314e-95	0.340361080169457			0.932	0.605

8.43787692541476e-91	10	SERBP1	DZ	1
6.39907815918362e-95	0.44900771917163	0.341	0.065	
1.14089164500085e-90	10	DOK3	DZ	1
7.46506310829555e-95	0.435735199230837	0.257	0.027	
1.33094610157801e-90	10	CENPE	DZ	1
8.6846999048391e-95	0.402058910072974	0.43	0.09	
1.54839514603376e-90	10	EXOSC9	DZ	1
1.04483563421211e-94	0.359498336363671	0.934	0.591	
1.86283745223678e-90	10	TRIR	DZ	1
1.18637058545728e-94	0.411221821734188	0.364	0.065	
2.11518011681179e-90	10	CUX1	DZ	1
1.5227676428729e-94	0.449476865241149	0.682	0.235	
2.7149424304781e-90	10	PPP2R5C	DZ	1
2.1710014451195e-94	0.311083418261338	0.917	0.595	
3.87067847650355e-90	10	PPDPF	DZ	1
2.19277326582397e-94	0.38592408950991	0.841	0.416	
3.90949545563756e-90	10	KTN1	DZ	1
2.31889508885649e-94	0.476667191981282	0.45	0.106	
4.13435805392223e-90	10	BRWD1	DZ	1
2.43831682644762e-94	0.40063932265135	0.886	0.511	
4.34727506987347e-90	10	SELENOT	DZ	1
3.63746996993155e-94	0.4567549025488	0.853	0.443	
6.48524520939096e-90	10	CAPZA1	DZ	1
4.4848113667523e-94	0.371851960738719	0.887	0.497	
7.99597018578268e-90	10	PSMD8	DZ	1
5.64368528453701e-94	0.426413572919265	0.369	0.069	
1.0062126493801e-89	10	MYO1E	DZ	1
6.3521219188547e-94	0.342067437010035	0.283	0.039	
1.13251981691261e-89	10	PARP2	DZ	1
6.36305832089459e-94	0.379244732084925	0.875	0.48	
1.1344696680323e-89	10	THRAP3	DZ	1
9.02247528130681e-94	0.429395692755082	0.463	0.107	
1.60861711790419e-89	10	CBFB	DZ	1
1.33898738692168e-93	0.385976807675097	0.763	0.335	
2.38728061214267e-89	10	CYC1	DZ	1
1.65753648264876e-93	0.407417115854063	0.367	0.072	
2.95522179491447e-89	10	LRWD1	DZ	1
2.18502742299178e-93	0.401956562385389	0.871	0.456	
3.89568539245204e-89	10	MTDH	DZ	1
2.20677127169936e-93	-1.07480394253559	0.209	0.386	
3.93445250031279e-89	10	PDCD4	DZ	1
2.45165287443335e-93	0.37486742514197	0.344	0.058	
4.37105190982721e-89	10	RHNO1	DZ	1
2.94283420541116e-93	0.435176133017609	0.668	0.248	
5.24677910482755e-89	10	ATXN10	DZ	1
6.00593539076721e-93	0.392453675204004	0.407	0.083	
1.07079822081989e-88	10	MLH1	DZ	1
6.27241113896816e-93	0.510858923953167	0.394	0.093	
1.11830818196663e-88	10	ALDH2	DZ	1
1.17107284139008e-92	0.374876834160776	0.845	0.406	

2.08790576891437e-88 10 FAM49B DZ 1
2.31983319381621e-92 -0.479990286500809 0.999 0.999
4.13603060125492e-88 10 RPS19 DZ 1
2.68251151145166e-92 0.368013293828979 0.247 0.032
4.78264977376717e-88 10 TOX DZ 1
2.73113541887791e-92 0.426104947648118 0.643 0.23
4.86934133831742e-88 10 ETFA DZ 1
3.09023714266941e-92 0.504962534101653 0.98 0.856
5.50958380166529e-88 10 UBB DZ 1
3.11076890601717e-92 0.424609345167136 0.543 0.158
5.54618988253801e-88 10 COMMD7 DZ 1
4.0251012453343e-92 0.38821634244289 0.459 0.111
7.17635301030653e-88 10 GMPS DZ 1
5.76806168347569e-92 0.312579035429844 0.223 0.025
1.02838771754688e-87 10 SMCO4 DZ 1
8.02572229136864e-92 0.418640849030362 0.788 0.351
1.43090602732811e-87 10 TERF2IP DZ 1
1.49322364572828e-91 0.331447830279204 0.981 0.8
2.66226843796895e-87 10 HINT1 DZ 1
1.97210678264807e-91 0.331922366586215 0.94 0.609
3.51606918278325e-87 10 ACTR3 DZ 1
2.46747131788183e-91 0.391698176837274 0.382 0.08
4.39925461265151e-87 10 SFXN1 DZ 1
2.54189432378823e-91 0.419867861172754 0.453 0.107
4.53194338988204e-87 10 ELP6 DZ 1
3.39705858383986e-91 0.368120001574121 0.886 0.502
6.05661574912809e-87 10 DNAJC8 DZ 1
3.89489420636327e-91 0.420617283958192 0.509 0.139
6.94420688052508e-87 10 PBRM1 DZ 1
4.76972278050428e-91 0.355235738871555 0.964 0.698
8.50393874536108e-87 10 COX7A2 DZ 1
5.36032436023133e-91 0.340496657491809 0.159 0.014
9.55692230185644e-87 10 TUBB2B DZ 1
5.44565421158923e-91 0.422484021759815 0.6 0.196
9.70905689384243e-87 10 CISD2 DZ 1
8.16795013356026e-91 0.398947689260041 0.86 0.455
1.45626382931246e-86 10 PRDX5 DZ 1
1.53884864687864e-90 0.411249708347009 0.67 0.25
2.74361325251994e-86 10 MRPL18 DZ 1
1.79697941198232e-90 0.41576717691916 0.493 0.133
3.20383459362328e-86 10 FLOT1 DZ 1
1.95362604801542e-90 0.35815072140261 0.937 0.644
3.48311988100669e-86 10 ATP5F1A DZ 1
2.61049058741289e-90 0.39667683000938 0.415 0.089
4.65424366829844e-86 10 RMDN1 DZ 1
2.97572447078588e-90 0.39637740640684 0.546 0.159
5.30541915896415e-86 10 THOC3 DZ 1
6.1199784454362e-90 0.349833541274803 0.844 0.417
1.09113095703682e-85 10 SLIRP DZ 1
8.30948293431632e-90 -0.669901774866328 0.992 0.991

1.48149771235926e-85	10	RPL30	DZ 1		
8.79943886359557e-90	0.413274682858566			0.521	0.141
1.56885195499045e-85	10	MZT1	DZ 1		
3.08186245432562e-89	0.376094652715519			0.816	0.393
5.49465256981715e-85	10	BANF1	DZ 1		
3.12723426430504e-89	0.338633126202134			0.262	0.035
5.57554596982945e-85	10	TTF2	DZ 1		
3.82315504697745e-89	0.400166151166715			0.79	0.339
6.81630313325609e-85	10	DBNL	DZ 1		
4.50680532934236e-89	0.390085459459593			0.978	0.77
8.0351832216845e-85	10	ARHGDIB	DZ 1		
6.65988913733268e-89	0.435199477408474			0.48	0.116
1.18739163429504e-84	10	RABGAP1L	DZ 1		
7.60217499416878e-89	0.396737286698716			0.754	0.319
1.35539177971035e-84	10	BAX	DZ 1		
7.91769424824743e-89	-1.02242633530028			0.963	0.934
1.41164570752003e-84	10	BTG1	DZ 1		
1.40430396123654e-88	0.30167687199048	0.262		0.03	
2.50373353248863e-84	10	RNASEH2A	DZ 1		
2.01430848070502e-88	0.308385344182801			0.194	0.019
3.59131059024898e-84	10	BUB1	DZ 1		
3.05976285826386e-88	0.358425827606626			0.769	0.34
5.45525119999864e-84	10	KPNB1	DZ 1		
3.60897450625896e-88	0.43706599141335	0.591		0.182	
6.4344406472091e-84	10	USP34	DZ 1		
3.92292223435778e-88	0.369806194915636			0.319	0.049
6.99417805163648e-84	10	TOPBP1	DZ 1		
5.20617518155171e-88	0.340295435180802			0.951	0.65
9.28208973118855e-84	10	UBL5	DZ 1		
5.96473893056635e-88	-0.978790030922395			0.089	0.254
1.06345330393067e-83	10	TNFRSF13B		DZ 1	
6.20561014693491e-88	0.459365992775692			0.608	0.22
1.10639823309702e-83	10	CHCHD10	DZ 1		
6.6548449423595e-88	-1.06089517964947			0.242	0.351
1.18649230477328e-83	10	TRAF3IP3	DZ 1		
7.9868106417291e-88	0.345300410842433			0.923	0.573
1.42396846931388e-83	10	NDUFB2	DZ 1		
1.57009075346081e-87	0.416850256112765			0.425	0.096
2.79931480434528e-83	10	IMMP1L	DZ 1		
1.97977439756423e-87	0.34194648203833	0.772		0.359	
3.52973977341726e-83	10	ARHGDIA	DZ 1		
2.311374346122e-87	0.411277457419951			0.528	0.144
4.12094932170092e-83	10	MFAP1	DZ 1		
3.65200405802501e-87	0.386560592732104			0.763	0.331
6.51115803505279e-83	10	POLR2J	DZ 1		
9.39968352573162e-87	0.37944564705879	0.383		0.077	
1.67586957580269e-82	10	FAF1	DZ 1		
3.08259981578088e-86	0.316678410714884			0.234	0.026
5.49596721155573e-82	10	RAPGEF5	DZ 1		
3.66663884185146e-86	0.400468000339874			0.702	0.283

6.53725039113697e-82 10 MRPL57 DZ 1
4.29999813645086e-86 0.360076695275102 0.777 0.351
7.66646667747824e-82 10 PCMT1 DZ 1
5.16427548612908e-86 0.469334290996226 0.34 0.068
9.20738676421953e-82 10 LMO2 DZ 1
8.51464438548675e-86 0.396764378473762 0.421 0.094
1.51807594748843e-81 10 HMGXB4 DZ 1
1.01069292902454e-85 0.396102086877321 0.566 0.175
1.80196442315785e-81 10 CHRAC1 DZ 1
1.21820104499502e-85 0.425168890008393 0.537 0.16
2.17193064312162e-81 10 BLOC1S6 DZ 1
1.25208204419803e-85 0.415637221108667 0.558 0.174
2.23233707660067e-81 10 KIF20B DZ 1
1.34183925754177e-85 0.314127651063295 0.911 0.583
2.39236521227121e-81 10 HNRNPU DZ 1
1.39504513432127e-85 0.361981496221687 0.36 0.074
2.48722596998139e-81 10 THOP1 DZ 1
1.51083475033608e-85 0.388211327850344 0.663 0.246
2.69366727637419e-81 10 UBE2V2 DZ 1
2.37916975376013e-85 -1.18504821660927 0.182 0.32
4.24182175397893e-81 10 CD69 DZ 1
2.62222008621594e-85 0.375418363528748 0.848 0.409
4.67515619171441e-81 10 GABARAPL2 DZ 1
3.51203488261132e-85 0.345616919812633 0.814 0.389
6.26160699220773e-81 10 MDH2 DZ 1
5.11168915701681e-85 0.393067615892652 0.542 0.172
9.11363059804527e-81 10 LRRC59 DZ 1
2.11574937734512e-84 0.409747198606994 0.257 0.041
3.77216956486862e-80 10 LRRK1 DZ 1
2.26575337214059e-84 0.442111251663827 0.436 0.096
4.03961168718946e-80 10 E2F5 DZ 1
2.52682575203631e-84 0.381537796649597 0.479 0.124
4.50507763330553e-80 10 ACAA2 DZ 1
2.85439058355707e-84 -0.78101485140088 0.996 0.985
5.0890929714239e-80 10 TMSB10 DZ 1
3.0332934630225e-84 0.384355294819466 0.676 0.249
5.40805891522281e-80 10 THOC7 DZ 1
3.20429883032103e-84 0.379122102291751 0.474 0.125
5.71294438457936e-80 10 APIP DZ 1
5.08273030165893e-84 0.40930622563044 0.721 0.302
9.06199985482771e-80 10 NDUFAF3 DZ 1
6.63869795719372e-84 0.38044719671341 0.567 0.172
1.18361345878807e-79 10 SPTSSA DZ 1
7.23371909406314e-84 0.383072236676654 0.506 0.143
1.28969977728052e-79 10 ACTN4 DZ 1
8.91614154056983e-84 0.344841544630374 0.801 0.404
1.58965887526819e-79 10 IDI1 DZ 1
1.01079543109317e-83 0.388277685609772 0.605 0.216
1.80214717409602e-79 10 HIKESHI DZ 1
1.30079577642352e-83 0.404356998704422 0.591 0.201

2.31918878978549e-79	10	FAM192A	DZ 1	
3.38823412735326e-83	0.472254844265285		0.475	0.118
6.04088262565812e-79	10	STIM2	DZ 1	
3.5280654066277e-83	0.340882802606179		0.268	0.035
6.29018781347652e-79	10	REC8	DZ 1	
4.36014769597627e-83	0.367549825796238		0.352	0.073
7.77370732715609e-79	10	PDZD11	DZ 1	
4.75815366533893e-83	0.415713906803746		0.756	0.316
8.48331216993279e-79	10	CCM2	DZ 1	
5.99386620000848e-83	0.357468342594866		0.742	0.32
1.06864640479951e-78	10	NDUFS7	DZ 1	
1.07047839140495e-82	0.380978591881103		0.46	0.111
1.90855592403589e-78	10	NFATC2IP	DZ 1	
1.28430950638191e-82	0.358791973317584		0.825	0.398
2.2897954189283e-78	10	MOB1A	DZ 1	
1.56570068761716e-82	0.364826511918239		0.787	0.367
2.79148775595263e-78	10	SRSF11	DZ 1	
1.69405701773137e-82	0.361099083763035		0.562	0.178
3.02033425691326e-78	10	CHCHD3	DZ 1	
1.73770470351725e-82	0.427061984433729		0.768	0.324
3.0981537159009e-78	10	SPIB	DZ 1	
1.93080013003245e-82	0.312432994818392		0.909	0.565
3.44242355183486e-78	10	NDUFB11	DZ 1	
2.53638568625071e-82	0.358583250471578		0.735	0.319
4.52212204001638e-78	10	EIF3A	DZ 1	
3.05178685142264e-82	0.418088492904901		0.47	0.129
5.44103077740142e-78	10	CORO1B	DZ 1	
3.31465798428517e-82	0.323029178466602		0.863	0.464
5.90970372018203e-78	10	FUS	DZ 1	
3.36713156965872e-82	-1.18397038926001		0.402	0.497
6.00325887554453e-78	10	FXYD5	DZ 1	
1.15718550068125e-81	0.370838674742413		0.673	0.261
2.0631460291646e-77	10	SEPT2	DZ 1	
1.62766008886421e-81	0.369091615586999		0.568	0.185
2.90195517243599e-77	10	UQCC2	DZ 1	
2.83111910881e-81	0.373726834991577		0.783	0.368
5.04760225909734e-77	10	NDUFC2	DZ 1	
2.93886157881289e-81	0.369563966310284		0.394	0.094
5.23969630886551e-77	10	NABP2	DZ 1	
3.74809248969272e-81	0.511962545041593		0.369	0.075
6.68247409987316e-77	10	SH3TC1	DZ 1	
4.01693209803258e-81	0.423685553473074		0.424	0.093
7.16178823758228e-77	10	SORL1	DZ 1	
4.85902668506461e-81	0.341939734780589		0.68	0.268
8.66315867680169e-77	10	PSMD14	DZ 1	
1.07792522947043e-80	0.388521219783495		0.352	0.064
1.92183289162284e-76	10	NUCB2	DZ 1	
1.15539214101761e-80	0.418724461251098		0.611	0.218
2.0599486482203e-76	10	SEPT1	DZ 1	
1.95154299389978e-80	0.379673235108597		0.442	0.11

3.47940600382392e-76	10	DYNLT1	DZ 1		
2.32598934277909e-80	0.347482150221703			0.778	0.366
4.14700639924084e-76	10	VDAC2	DZ 1		
3.16998702190869e-80	0.339459406018215			0.809	0.388
5.65176986136099e-76	10	UQCRFS1	DZ 1		
3.74987780817752e-80	0.359409134613529			0.348	0.073
6.68565714419969e-76	10	MTHFD1	DZ 1		
4.67808497735395e-80	0.491966506450584			0.114	0.008
8.34055770612436e-76	10	IGLL1	DZ 1		
1.00386617866245e-79	0.39379409016218		0.486	0.123	
1.78979300993728e-75	10	MPST	DZ 1		
1.0920950746412e-79	0.30207283771391		0.908	0.544	
1.94709630857779e-75	10	SNU13	DZ 1		
1.76336750890648e-79	0.330096130660265			0.798	0.372
3.14390793162936e-75	10	SNRPC	DZ 1		
2.12313922077709e-79	0.326080196508475			0.263	0.037
3.78534491672347e-75	10	SASS6	DZ 1		
4.80684415824803e-79	0.401078918758718			0.73	0.323
8.57012244974042e-75	10	COX14	DZ 1		
6.9770200909566e-79	0.312519856120825			0.901	0.545
1.24393291201665e-74	10	PGK1	DZ 1		
8.16297299070901e-79	0.371941201797124			0.613	0.213
1.45537645451351e-74	10	SREK1	DZ 1		
8.49012204012725e-79	0.304215950823382			0.239	0.027
1.51370385853429e-74	10	MMS22L	DZ 1		
8.98126386441168e-79	0.345319649450801			0.734	0.324
1.60126953438596e-74	10	BUD31	DZ 1		
1.02889046244372e-78	0.364025516745947			0.377	0.089
1.83440880549091e-74	10	ECI1	DZ 1		
1.450683388784e-78	-0.793239754409606			0.058	0.2
2.58642341386299e-74	10	CLEC2B	DZ 1		
1.97694977122967e-78	0.329534959567433			0.7	0.29
3.52470374712538e-74	10	GLRX3	DZ 1		
2.43408092807153e-78	0.343385365671325			0.356	0.074
4.33972288665873e-74	10	GSTZ1	DZ 1		
2.73234355056515e-78	0.358234891780919			0.614	0.216
4.8714953163026e-74	10	ATG3	DZ 1		
3.07179944806487e-78	-0.900317583208127			0.152	0.283
5.47671123595485e-74	10	IFITM2	DZ 1		
1.23774591869091e-77	0.375552472238085			0.539	0.167
2.20677719843402e-73	10	CBX1	DZ 1		
1.27803403819195e-77	-0.528790459321193			1	0.999
2.27860688669243e-73	10	EEF1A1	DZ 1		
1.49559166215797e-77	0.361373719680379			0.651	0.242
2.66649037446145e-73	10	CYB5B	DZ 1		
1.67625959959193e-77	0.411111706621766			0.72	0.307
2.98860324011246e-73	10	AP3S1	DZ 1		
1.96039554090474e-77	0.361924139649997			0.499	0.135
3.49518920987906e-73	10	ANKRD13A	DZ 1		
2.28305058439853e-77	0.364529277913633			0.36	0.072

4.07045088692413e-73	10	VGLL4	DZ 1		
6.36546399311051e-77	0.355851537658016			0.663	0.256
1.13489857533167e-72	10	NDUFS3	DZ 1		
7.35493399905076e-77	0.317053844348632			0.192	0.023
1.31131118269076e-72	10	CAMK1	DZ 1		
1.03437072691247e-76	0.373873304661865			0.641	0.236
1.84417956901224e-72	10	KIF5B	DZ 1		
1.74946381287261e-76	-0.590424454420428			0.998	0.996
3.11911903197057e-72	10	RPL12	DZ 1		
3.32409913901511e-76	0.379305918455154			0.392	0.092
5.92653635495004e-72	10	LIMS1	DZ 1		
3.84506144898821e-76	0.429391012139685			0.542	0.165
6.85536005740108e-72	10	PLIN3	DZ 1		
3.90172142801471e-76	0.490641122418491			0.886	0.522
6.95637913400742e-72	10	ISG20	DZ 1		
4.20881006331726e-76	0.334479193887224			0.722	0.313
7.50388746188835e-72	10	PSMG2	DZ 1		
4.24889378410403e-76	0.358615959047137			0.622	0.234
7.57535272767908e-72	10	TPM4	DZ 1		
5.24813269826735e-76	0.333004850099444			0.837	0.463
9.35689578774086e-72	10	PRELID1	DZ 1		
5.26220571016359e-76	0.364232548336637			0.501	0.145
9.38198656065067e-72	10	VPS25	DZ 1		
5.26319923010838e-76	0.374783163396473			0.605	0.206
9.38375790736022e-72	10	GNAI2	DZ 1		
5.76423587803361e-76	0.366441401592893			0.634	0.239
1.02770561469461e-71	10	MCTS1	DZ 1		
7.24564350193932e-76	0.425138942856141			0.388	0.089
1.29182577996076e-71	10	MX1	DZ 1		
7.42178434667107e-76	0.386070040061162			0.477	0.135
1.32322993116799e-71	10	PTEN	DZ 1		
9.2741312875415e-76	0.374098787461321			0.318	0.061
1.65348486725577e-71	10	ST14	DZ 1		
9.37076043619271e-76	0.379082859744368			0.473	0.126
1.6707128781688e-71	10	CLIC4	DZ 1		
9.58809020450657e-76	0.368966860429539			0.555	0.189
1.70946060256148e-71	10	LAGE3	DZ 1		
1.88851936834725e-75	0.337034116923263			0.801	0.405
3.36704118182631e-71	10	ELF1	DZ 1		
2.69785019087173e-75	0.50248611521332		0.325	0.062	
4.80999710530521e-71	10	RASSF6	DZ 1		
2.98972704796235e-75	0.361507164651496			0.488	0.134
5.33038435381208e-71	10	C1orf35	DZ 1		
7.44583661644729e-75	0.327845484905549			0.337	0.068
1.32751821034639e-70	10	OXCT1	DZ 1		
9.54227219328183e-75	0.302665157451026			0.837	0.436
1.70129170934022e-70	10	CSNK2B	DZ 1		
1.0229360282305e-74	0.356092366917997			0.549	0.175
1.82379264473215e-70	10	TECR	DZ 1		
1.44270080968756e-74	0.339663423986452			0.483	0.135

2.57219127359195e-70 10 SF3A2 DZ 1
1.6597546062745e-74 0.373850206594696 0.695 0.269
2.95917648752681e-70 10 LAT2 DZ 1
1.68935762323487e-74 0.375003303334662 0.828 0.415
3.01195570646545e-70 10 DNAJA1 DZ 1
1.69347643151453e-74 0.399314385028232 0.508 0.135
3.01929912974725e-70 10 RBM38 DZ 1
1.96908117195525e-74 0.367152386331071 0.793 0.394
3.51067482147901e-70 10 LSM10 DZ 1
4.70297471259982e-74 0.363187529357804 0.512 0.154
8.38493361509423e-70 10 COX11 DZ 1
4.87391112390719e-74 0.307437159653056 0.212 0.028
8.68969614281412e-70 10 CTPS2 DZ 1
5.35715886002394e-74 -0.423614126352395 1 1
9.55127853153668e-70 10 RPL21 DZ 1
5.40933667543424e-74 0.37346495500995 0.623 0.237
9.64430635863171e-70 10 MRPL16 DZ 1
6.97044541010437e-74 0.303890700067613 0.885 0.505
1.24276071216751e-69 10 ATP5PB DZ 1
7.73156441436133e-74 0.356762592157082 0.449 0.123
1.37846061943648e-69 10 NUP37 DZ 1
9.99951520662581e-74 0.398895589658307 0.981 0.852
1.78281356618932e-69 10 CD79B DZ 1
1.05848410696651e-73 0.31802891194733 0.258 0.039
1.88717131431059e-69 10 CCDC102A DZ 1
1.20325503442242e-73 0.306634374830728 0.945 0.659
2.14528340087174e-69 10 GDI2 DZ 1
1.2683014219673e-73 0.359356878311932 0.482 0.14
2.26125460522551e-69 10 HTATSF1 DZ 1
1.29839518587775e-73 0.379413811663784 0.575 0.173
2.31490877690144e-69 10 CDCA7L DZ 1
1.38033822662072e-73 0.368265290137114 0.516 0.166
2.46100502424209e-69 10 LAP3 DZ 1
1.87461891075712e-73 0.354839134759926 0.421 0.106
3.34225805598887e-69 10 MAZ DZ 1
2.12707872476475e-73 0.301466489286827 0.276 0.049
3.79236865838307e-69 10 CTPS1 DZ 1
4.77682521973306e-73 -0.881218909110009 0.976 0.96
8.51660168426207e-69 10 MT-ND2 DZ 1
5.17948792002376e-73 0.342345360246665 0.65 0.251
9.23450901261035e-69 10 SYNCRIP DZ 1
5.64527595689334e-73 0.373027783291368 0.402 0.097
1.00649625035451e-68 10 TCF3 DZ 1
6.62067729010735e-73 0.351865282147501 0.234 0.04
1.18040055405324e-68 10 CCDC144A DZ 1
7.21986842627442e-73 0.327689715086578 0.652 0.255
1.28723034172047e-68 10 FIBP DZ 1
9.18645085555823e-73 0.304750228577317 0.681 0.275
1.63785232303748e-68 10 MRPL14 DZ 1
1.15689965026749e-72 0.402640460456699 0.428 0.107

2.06263638646191e-68	10	TBC1D1	DZ 1
1.41684779916104e-72	0.3033559429506	0.823	0.407
2.52609794112422e-68	10	NDUFA2	DZ 1
1.45515766779003e-72	0.301534797409854	0.833	0.432
2.59440060590284e-68	10	NDUFB10	DZ 1
1.76941484957712e-72	0.312085169381036	0.867	0.483
3.15468973531105e-68	10	ARGLU1	DZ 1
1.88892017167234e-72	0.376481079363073	0.457	0.12
3.36775577407462e-68	10	C16orf87	DZ 1
3.54389793046046e-72	0.329706639566772	0.262	0.045
6.31841562021796e-68	10	SPDL1	DZ 1
4.31478055467161e-72	0.360821188053166	0.653	0.246
7.69282225092402e-68	10	ILK	DZ 1
6.70556855573247e-72	0.303858257543812	0.211	0.024
1.19553581780154e-67	10	FGD6	DZ 1
1.07298877694447e-71	0.343154788724802	0.509	0.161
1.9130316904143e-67	10	CLTB	DZ 1
4.65953610009188e-71	0.332671468611449	0.667	0.272
8.30748691285382e-67	10	SRSF4	DZ 1
8.17215312772567e-71	0.358253138915167	0.394	0.095
1.45701318114221e-66	10	ACSF3	DZ 1
1.66801324135811e-70	0.328030139525979	0.426	0.117
2.97390080801737e-66	10	RCC1	DZ 1
1.76487519747272e-70	0.333382634156278	0.591	0.203
3.14659598957411e-66	10	PIH1D1	DZ 1
1.78242136552642e-70	0.357471666436492	0.521	0.164
3.17787905259705e-66	10	ZNF428	DZ 1
1.86089453672428e-70	0.336715514600825	0.527	0.17
3.31778886952571e-66	10	DHX15	DZ 1
2.04002036133851e-70	0.321741417076836	0.31	0.058
3.63715230223043e-66	10	PPP5C	DZ 1
2.69968806417609e-70	-0.422603898179092	0.999	0.999
4.81327384961955e-66	10	RPS8	DZ 1
2.72286811372886e-70	0.352463074394665	0.505	0.162
4.85460155996718e-66	10	DRG1	DZ 1
4.53522748732115e-70	0.33385755581845	0.625	0.232
8.08585708714488e-66	10	PFKL	DZ 1
7.33401178740651e-70	0.363561507660094	0.353	0.076
1.30758096157671e-65	10	TMEM131L	DZ 1
8.63160457885049e-70	0.301207959176961	0.762	0.353
1.53892878036325e-65	10	PSMA5	DZ 1
9.00787286427984e-70	0.332980234649839	0.85	0.472
1.60601365297245e-65	10	RPS27L	DZ 1
2.04648785130805e-69	0.332902734744859	0.755	0.352
3.64868319009712e-65	10	PDIA3	DZ 1
3.44233133035901e-69	0.315650445641772	0.604	0.226
6.13733252889709e-65	10	MRPS16	DZ 1
3.52471353632285e-69	0.342963742627967	0.574	0.207
6.28421176391e-65	10	NDUFV1	DZ 1
5.18014303435859e-69	0.330065108532737	0.541	0.178

9.23567701595793e-65	10	MRPS11	DZ 1		
6.27926065475452e-69	0.347711259376217			0.573	0.196
1.11952938213618e-64	10	PRPF38A	DZ 1		
7.18380924994311e-69	0.311710625694296			0.326	0.065
1.28080135117236e-64	10	LRRC42	DZ 1		
9.01086529752965e-69	0.307754687762175			0.349	0.081
1.60654717389656e-64	10	UBE2M	DZ 1		
9.46930171324711e-69	0.387687228325227			0.581	0.212
1.68828180245483e-64	10	SIAH2	DZ 1		
1.38865187377987e-68	0.302574518587179			0.599	0.241
2.47582742576212e-64	10	RRP7A	DZ 1		
1.40213783710868e-68	0.333414451667143			0.396	0.102
2.49987154978106e-64	10	CNP	DZ 1		
1.44692122070318e-68	0.359082129666339			0.585	0.216
2.57971584439171e-64	10	SDHA	DZ 1		
1.60851290279522e-68	0.338997830617886			0.591	0.206
2.8678176543936e-64	10	WDR33	DZ 1		
2.18542938148848e-68	0.336773642733511			0.433	0.114
3.89640204425581e-64	10	NTAN1	DZ 1		
2.18890601404963e-68	0.334494966413963			0.632	0.248
3.90260053244909e-64	10	BAZ1A	DZ 1		
4.25357063387501e-68	-0.48046914507202			0.999	0.998
7.58369108313576e-64	10	RPS14	DZ 1		
7.26643786988641e-68	0.368049208291529			0.644	0.252
1.29553320782205e-63	10	RBM6	DZ 1		
7.26707703641567e-68	0.326051114518088			0.386	0.093
1.29564716482255e-63	10	ASH2L	DZ 1		
8.55391228679807e-68	0.352632717602132			0.995	0.989
1.52507702161323e-63	10	CD74	DZ 1		
9.24339823922803e-68	0.312846841932541			0.669	0.282
1.64800547207197e-63	10	ILF3	DZ 1		
1.38928630938661e-67	0.329687190644698			0.44	0.129
2.47695856100539e-63	10	ITGB3BP	DZ 1		
1.72593286375436e-67	-0.565791903484278			0.993	0.985
3.07716570278764e-63	10	RPL27	DZ 1		
1.87066308475758e-67	0.310851610027234			0.709	0.313
3.3352052138143e-63	10	MRPS36	DZ 1		
2.22811580375924e-67	0.336514760619343			0.602	0.216
3.97250766652235e-63	10	HDDC2	DZ 1		
2.26303735198166e-67	0.31177213008011	0.323		0.065	
4.0347692948481e-63	10	ZNF672	DZ 1		
2.27548047579013e-67	-1.24254136178247			0.413	0.457
4.05695414028622e-63	10	JUNB	DZ 1		
3.44271064664932e-67	-1.00151584654991			0.391	0.45
6.13800881191108e-63	10	PNRC1	DZ 1		
4.64876128891697e-67	0.322902544392605			0.551	0.179
8.28827650201006e-63	10	NDUFA8	DZ 1		
4.68053695808144e-67	0.320284672287391			0.621	0.251
8.3449293425634e-63	10	CD2BP2	DZ 1		
4.72800697507791e-67	0.308082274810015			0.707	0.3

8.4295636358664e-63	10	ZNF207	DZ 1		
5.98359602718789e-67	0.36145827002837	0.55	0.211		
1.06681533568733e-62	10	MRPL4	DZ 1		
6.37407218587582e-67	0.33304050199775	0.377	0.09		
1.1364333300198e-62	10	PPP1R35	DZ 1		
7.27610440774926e-67	0.369800574931405		0.546	0.164	
1.29725665485762e-62	10	SYK	DZ 1		
8.52622569625082e-67	0.340707118389893		0.278	0.055	
1.52014077938456e-62	10	NINJ1	DZ 1		
1.04414559003442e-66	0.314436429401684		0.684	0.29	
1.86160717247237e-62	10	COPS6	DZ 1		
1.10550866281927e-66	0.3210316033085	0.483	0.145		
1.97101139494047e-62	10	DDB2	DZ 1		
1.28105691706378e-66	0.307170320059057		0.312	0.072	
2.28399637743302e-62	10	KEAP1	DZ 1		
1.76573260677537e-66	0.316766590955485		0.47	0.137	
3.1481246646198e-62	10	RTN3	DZ 1		
2.07594172623092e-66	0.313892815726102		0.282	0.047	
3.70119650369712e-62	10	BCL6	DZ 1		
2.29362544051246e-66	0.317644742894011		0.61	0.233	
4.08930479788966e-62	10	VDAC3	DZ 1		
2.6417401494824e-66	0.337353725796304		0.738	0.324	
4.70995851251217e-62	10	NDUFC1	DZ 1		
2.7153660322128e-66	0.304401849034828		0.666	0.275	
4.8412260988322e-62	10	TXN2	DZ 1		
2.90913608591866e-66	0.366872784252352		0.578	0.196	
5.18669872758437e-62	10	GLRX	DZ 1		
5.19453890493604e-66	0.342072118357773		0.602	0.206	
9.26134341361046e-62	10	LY86	DZ 1		
6.62807151171157e-66	0.319956885082604		0.578	0.21	
1.18171886982305e-61	10	PSMD9	DZ 1		
7.87420013539507e-66	0.487214724502177		0.646	0.256	
1.40389114213959e-61	10	PIM1	DZ 1		
8.29763865826217e-66	0.336846853761919		0.346	0.08	
1.47938599638156e-61	10	DESI2	DZ 1		
1.297511147926e-65	0.33743656537607	0.443	0.126		
2.31333262563726e-61	10	GTF2E2	DZ 1		
1.58449116630829e-65	0.305943896285406		0.47	0.142	
2.82498930041104e-61	10	MPC1	DZ 1		
1.63799677231027e-65	0.371503097913338		0.595	0.229	
2.92038444535197e-61	10	RGS1	DZ 1		
1.79183687077286e-65	0.352735109742069		0.388	0.094	
3.19466595690093e-61	10	OTULIN	DZ 1		
2.87407651204224e-65	0.326510635193952		0.507	0.166	
5.12419101332012e-61	10	SLC25A11	DZ 1		
3.14939238713904e-65	0.342267918804705		0.619	0.242	
5.6150516870302e-61	10	C12orf57	DZ 1		
3.34052314768285e-65	0.449862647380049		0.407	0.119	
5.95581872000375e-61	10	NFATC1	DZ 1		
3.43496891671727e-65	0.341263951620174		0.387	0.099	

6.12420608161522e-61	10	PHF6	DZ 1
5.49558413133285e-65	0.322264738641818	0.491	0.153
9.79807694775333e-61	10	SRRT	DZ 1
6.41035383715596e-65	0.336638466463403	0.581	0.196
1.14290198562654e-60	10	TCEAL8	DZ 1
1.30522886234963e-64	0.335836113011116	0.354	0.082
2.32709253868316e-60	10	RITA1	DZ 1
1.61197803980202e-64	0.315346862761695	0.5	0.158
2.87399564716303e-60	10	MTX1	DZ 1
2.66658732681821e-64	0.309391207581433	0.319	0.072
4.75425854498419e-60	10	BLMH	DZ 1
5.60483471309474e-64	0.324478472103646	0.322	0.07
9.99285980997661e-60	10	FAM45A	DZ 1
7.18026401940012e-64	0.323406491716335	0.602	0.215
1.28016927201885e-59	10	IAH1	DZ 1
7.39296180301477e-64	0.308482571370436	0.689	0.287
1.3180911598595e-59	10	POLE4	DZ 1
9.07202960908545e-64	0.350872878107982	0.331	0.066
1.61745215900384e-59	10	CDC25B	DZ 1
9.17652786446564e-64	0.35852413012428 0.491	0.152
1.63608315295558e-59	10	LYPLAL1	DZ 1
1.14223050673232e-63	-0.909027897563009	0.369	0.44
2.03648277045306e-59	10	IL2RG	DZ 1
1.33598769461753e-63	0.305369199852583	0.682	0.304
2.3819324607336e-59	10	WDR1	DZ 1
1.72542604308458e-63	0.411512377841784	0.283	0.056
3.0762620922155e-59	10	HOPX	DZ 1
2.53945489967386e-63	0.318191728740818	0.454	0.13
4.52759414062853e-59	10	MTHFS	DZ 1
3.93496921357153e-63	0.304403525359222	0.705	0.325
7.01565661087668e-59	10	HSD17B10 DZ 1
4.11408968411782e-63	0.320485726135559	0.499	0.158
7.33501049781365e-59	10	TARDBP	DZ 1
4.39664069833892e-63	0.336905864778743	0.619	0.227
7.83877070106847e-59	10	PTRHD1	DZ 1
4.42050784591473e-63	0.326991343363073	0.395	0.101
7.88132343848138e-59	10	TRNAU1AP DZ 1
5.20600283847971e-63	0.302619072426266	0.381	0.093
9.28178246072547e-59	10	HACD3	DZ 1
5.76097406008388e-63	-0.819651026384772	0.911	0.867
1.02712406517236e-58	10	CD52	DZ 1
7.21004270347144e-63	0.408937883177654	0.801	0.383
1.28547851360192e-58	10	CSTB	DZ 1
4.89982698423619e-62	0.310354235933626	0.403	0.101
8.7359015301947e-58	10	TMEM14A	DZ 1
8.34483372870441e-62	0.318248152091943	0.442	0.131
1.48780040549071e-57	10	CISD1	DZ 1
1.64921885699923e-61	0.322543317874487	0.518	0.175
2.94039230014393e-57	10	COPS8	DZ 1
1.77442075743133e-61	0.314679312037698	0.466	0.14

3.16361476842432e-57	10	ARPC1A	DZ 1
1.95146738438909e-61	0.307716601260806		0.404	0.113
3.47927119962732e-57	10	NUP62	DZ 1
2.03081158998763e-61	0.300921739273132		0.171	0.022
3.62073398378895e-57	10	HTR3A	DZ 1
2.07288831953892e-61	0.328479321211834		0.426	0.127
3.69575258490595e-57	10	OSBPL9	DZ 1
2.87145398668715e-61	-1.49320984854321		0.18	0.29
5.11951531286451e-57	10	MIR155HG	DZ 1
5.61471058786723e-61	0.31069729396489	0.563	0.205
1.00104675071085e-56	10	IFI27L2	DZ 1
5.62818320130433e-61	-0.78987409135384		0.961	0.916
1.00344878296055e-56	10	PFDN5	DZ 1
1.10419100911933e-60	0.317547237303719		0.478	0.155
1.96866215015885e-56	10	TOR3A	DZ 1
1.2767754629097e-60	0.301456905809298		0.329	0.075
2.27636297282171e-56	10	PARN	DZ 1
1.52507e-60	0.3414124	0.367	0.092	2.719048e-56	10
AC084033.3	DZ 1
1.599264e-60	0.4072743	0.372	0.092	2.851328e-56	10
CDKN2D	DZ 1
2.288683e-60	0.3142692	0.338	0.084	4.080492e-56	10
FAM208B	DZ 1
3.138261e-60	-0.7170486	0.081	0.187	5.595205e-56	10
PLPP5	DZ 1
3.277484e-60	0.3002076	0.572	0.213	5.843427e-56	10
NUDT5	DZ 1
4.289159e-60	0.3057224	0.65	0.257	7.647142e-56	10
SEPT9	DZ 1
4.603308e-60	-0.9235784	0.179	0.271	8.207238e-56	10
JUN	DZ 1
7.736058e-60	0.3479814	0.562	0.198	1.379262e-55	10
EHD1	DZ 1
1.596866e-59	-0.4253625	0.997	0.998	2.847053e-55	10
RPS28	DZ 1
1.997122e-59	0.3022209	0.594	0.228	3.560668e-55	10
PRKRA	DZ 1
2.475081e-59	0.3481681	0.341	0.086	4.412821e-55	10
RNGTT	DZ 1
3.958174e-59	0.3225346	0.509	0.178	7.057029e-55	10
RUVBL2	DZ 1
6.748797e-59	0.3008888	0.53	0.183	1.203243e-54	10
PAFAH1B1	DZ 1
1.12979e-58	0.3229298	0.448	0.136	2.014303e-54	10
P2RY8	DZ 1
1.188006e-58	0.3340271	0.568	0.216	2.118095e-54	10
ADI1	DZ 1
5.135696e-58	0.3375869	0.556	0.212	9.156432e-54	10
HSPH1	DZ 1
5.153777e-58	0.3107604	0.53	0.188	9.188668e-54	10

NUDCD2	DZ 1	5.715548e-58	0.3248465	0.338	0.08	1.019025e-53	10
MAP4K4	DZ 1	5.900955e-58	0.3014807	0.412	0.112	1.052081e-53	10
BAD	DZ 1	6.96063e-58	-0.4193372	0.997	0.997	1.241011e-53	10
RPL7	DZ 1	8.479078e-58	-0.9199386	0.082	0.201	1.511735e-53	10
ACP5	DZ 1	1.221464e-57	0.325214	0.656	0.297	2.177748e-53	10
CALR	DZ 1	4.861102e-57	0.3242228	0.761	0.387	8.666858e-53	10
NDUFS5	DZ 1	5.719707e-57	0.3463414	0.502	0.191	1.019767e-52	10
RELB	DZ 1	9.635481e-57	0.3315724	0.537	0.204	1.71791e-52	10
PHAX	DZ 1	4.506433e-56	0.3475012	0.672	0.317	8.03452e-52	10
HNRNPH1	DZ 1	5.296825e-56	-0.8136077	0.232	0.308	9.44371e-52	10
ARHGAP24	DZ 1	9.158032e-56	-0.499516	0.993	0.992	1.632786e-51	10
RPL36	DZ 1	1.827211e-55	0.3017089	0.855	0.49	3.257734e-51	10
PSMB9	DZ 1	6.867255e-55	-0.6036707	0.042	0.153	1.224363e-50	10
CELF2	DZ 1	2.381788e-54	0.3037617	0.498	0.164	4.246491e-50	10
EHMT1	DZ 1	7.896672e-54	0.3558713	0.841	0.486	1.407898e-49	10
SAT1	DZ 1	3.05413e-53	0.3368093	0.401	0.104	5.445209e-49	10
TNFRSF17	DZ 1	5.836538e-53	-0.7671165	0.212	0.281	1.040596e-48	10
RNASET2	DZ 1	6.159564e-53	0.3072857	0.337	0.08	1.098189e-48	10
VEZT	DZ 1	1.220603e-52	0.3403061	0.245	0.052	2.176213e-48	10
RGS16	DZ 1	1.470468e-52	-0.7849454	0.058	0.176	2.621698e-48	10
TNFRSF18	DZ 1	1.725753e-52	0.3117785	0.418	0.117	3.076845e-48	10
CCNG2	DZ 1	2.161156e-52	0.3034654	0.367	0.099	3.853125e-48	10
CITED2	DZ 1	2.781729e-52	-0.7914216	0.163	0.24	4.959544e-48	10
CD55	DZ 1	3.008998e-52	0.3212008	0.583	0.222	5.364743e-48	10
ZCCHC7	DZ 1	3.74177e-52	0.7766087	0.205	0.07	6.671201e-48	10

	IGHA2	DZ 1			
4.981073e-52	-0.8825607	0.911	0.853	8.880755e-48	10
	SH3BGRL3	DZ 1			
2.233714e-51	0.3721185	0.511	0.193	3.982488e-47	10
	UBALD2	DZ 1			
2.929894e-51	-0.7861907	0.237	0.332	5.223707e-47	10
	TRBC2	DZ 1			
3.562087e-51	0.4565544	0.809	0.478	6.350845e-47	10
	LTB	DZ 1			
3.468433e-50	0.3149385	0.444	0.138	6.183868e-46	10
	ALOX5	DZ 1			
4.241646e-50	-0.8607745	0.215	0.28	7.56243e-46	10
	ADAM28	DZ 1			
1.576094e-49	-0.6268043	0.073	0.175	2.810019e-45	10
	BCL2	DZ 1			
1.034932e-48	0.3193214	0.343	0.091	1.84518e-44	10
	SYVN1	DZ 1			
5.014213e-48	-0.7401424	0.047	0.162	8.93984e-44	10
	LMNA	DZ 1			
6.282047e-48	0.3140465	0.346	0.092	1.120026e-43	10
	SYNC	DZ 1			
1.276982e-47	-1.108273	0.521	0.572	2.276732e-43	10
	VIM	DZ 1			
1.754692e-47	-1.255959	0.205	0.265	3.128441e-43	10
	S100A4	DZ 1			
2.945714e-46	-0.7962568	0.152	0.24	5.251914e-42	10
	CAPG	DZ 1			
4.272179e-46	-0.4054924	0.997	0.997	7.616869e-42	10
	RPS3A	DZ 1			
4.641975e-46	0.3081702	0.377	0.116	8.276177e-42	10
	DLAT	DZ 1			
1.307111e-45	-0.4995896	0.031	0.115	2.330448e-41	10
	P2RY10	DZ 1			
1.978472e-45	-0.7319145	0.112	0.201	3.527418e-41	10
	YBX3	DZ 1			
3.350957e-45	-0.6197265	0.072	0.167	5.974421e-41	10
	MARCKS	DZ 1			
4.579397e-45	-0.5086366	0.99	0.981	8.164607e-41	10
	RPS11	DZ 1			
1.254557e-44	-0.3555848	0.999	0.998	2.236749e-40	10
	RPL26	DZ 1			
1.031641e-43	-0.9525097	0.393	0.428	1.839313e-39	10
	PLP2	DZ 1			
3.833329e-43	-0.6439822	0.046	0.14	6.834443e-39	10
	FCER2	DZ 1			
2.5735e-42	-0.4366073	0.993	0.992	4.588293e-38	10
	RPL37	DZ 1			
1.236794e-41	-0.6088957	0.065	0.161	2.20508e-37	10
	SLC2A3	DZ 1			
1.34619e-41	-0.8540744	0.804	0.75	2.400122e-37	10

HLA-C	DZ 1	1.805795e-41	-0.9382039	0.43	0.464	3.219552e-37	10
CD63	DZ 1	1.130679e-40	-0.4779724	0.028	0.103	2.015888e-36	10
RHOC	DZ 1	1.547541e-40	-0.6701204	0.123	0.198	2.75911e-36	10
CHPT1	DZ 1	7.612081e-40	-0.6266693	0.117	0.176	1.357158e-35	10
MARCH1	DZ 1	1.586833e-39	-0.8158085	0.295	0.328	2.829164e-35	10
ZFP36	DZ 1	4.472147e-39	-0.8586456	0.408	0.414	7.973391e-35	10
NPC2	DZ 1	5.248483e-39	-0.4067202	0.993	0.992	9.35752e-35	10
RPL18	DZ 1	1.184582e-38	-0.8562266	0.45	0.445	2.111991e-34	10
KLF6	DZ 1	1.060986e-37	-0.4691256	0.031	0.107	1.891631e-33	10
MTSS1	DZ 1	1.911473e-37	-0.3734682	0.999	0.997	3.407964e-33	10
RPS23	DZ 1	2.465096e-37	-0.7981925	0.514	0.501	4.395019e-33	10
FOXP1	DZ 1	1.683649e-36	-0.4747062	0.045	0.12	3.001779e-32	10
FCGR2B	DZ 1	3.887271e-36	-0.6347503	0.186	0.233	6.930615e-32	10
HSD17B11	DZ 1	4.273277e-35	-0.8673917	0.539	0.502	7.618826e-31	10
TSC22D3	DZ 1	1.117145e-34	-0.3943078	0.997	0.993	1.991757e-30	10
RPS5	DZ 1	1.248279e-34	-0.3716636	0.025	0.1	2.225556e-30	10
PARVB	DZ 1	2.3206e-34	-0.8138968	0.524	0.491	4.137398e-30	10
ANKRD12	DZ 1	2.507057e-33	-0.5488487	0.984	0.965	4.469832e-29	10
RPL38	DZ 1	4.782474e-33	-0.7838017	0.517	0.465	8.526674e-29	10
ITM2B	DZ 1	1.418518e-32	-0.7775783	0.636	0.568	2.529076e-28	10
SNX2	DZ 1	6.115277e-32	-0.3019106	0.999	0.998	1.090293e-27	10
RPL19	DZ 1	1.316913e-31	-0.7969671	0.712	0.646	2.347924e-27	10
ZFP36L1	DZ 1	2.179222e-31	-0.7736666	0.545	0.492	3.885334e-27	10
SP100	DZ 1	4.243736e-31	-0.7100181	0.488	0.454	7.566157e-27	10
CTSS	DZ 1	2.680089e-30	-0.7354165	0.533	0.474	4.778331e-26	10

N4BP2L2	DZ	1	3.450714e-30	-0.5590609	0.14	0.191	6.152278e-26	10
IFNGR1	DZ	1	2.126292e-29	-0.7128626	0.425	0.387	3.790967e-25	10
EVI2B	DZ	1	4.676584e-29	-0.4302878	0.997	0.984	8.337882e-25	10
RPL29	DZ	1	7.379491e-29	-0.6761326	0.3	0.328	1.315689e-24	10
PTPN6	DZ	1	1.081048e-28	-0.4256033	0.046	0.111	1.927401e-24	10
SNX9	DZ	1	1.207236e-28	-0.4236578	0.067	0.122	2.152382e-24	10
IL27RA	DZ	1	1.278464e-28	-0.688391	0.34	0.336	2.279374e-24	10
CD48	DZ	1	2.195205e-28	-0.3024499	0.997	0.994	3.913832e-24	10
RPS9	DZ	1	3.959602e-27	-0.4939065	0.11	0.159	7.059574e-23	10
TGIF1	DZ	1	4.62213e-27	-0.3022447	1	0.999	8.240796e-23	10
RPS6	DZ	1	1.382455e-26	-0.6095753	0.205	0.243	2.46478e-22	10
PHACTR1	DZ	1	1.510695e-26	-0.4419578	0.066	0.111	2.693419e-22	10
LINC02397	DZ	1	4.37672e-26	-0.4469467	0.062	0.107	7.803253e-22	10
C1orf162	DZ	1	1.260569e-25	-0.8917454	0.586	0.496	2.247469e-21	10
SSR4	DZ	1	2.404995e-25	-0.4656876	0.994	0.982	4.287866e-21	10
RPS21	DZ	1	2.89777e-25	-0.339952	0.998	0.994	5.166435e-21	10
RPL37A	DZ	1	6.387577e-25	-0.4201005	0.085	0.128	1.138841e-20	10
ZEB2	DZ	1	1.574029e-24	-0.7329771	0.358	0.337	2.806337e-20	10
HVCN1	DZ	1	1.708199e-24	-0.4681752	0.127	0.164	3.045549e-20	10
TNFRSF14	DZ	1	3.950889e-24	-0.5435303	0.969	0.933	7.04404e-20	10
MT-ATP6	DZ	1	5.597535e-24	-0.4486739	0.977	0.96	9.979845e-20	10
RPL23	DZ	1	2.224103e-23	-0.7028623	0.35	0.345	3.965353e-19	10
CAST	DZ	1	4.131257e-23	-0.6006317	0.162	0.212	7.365618e-19	10
MGST3	DZ	1	8.438221e-23	-0.3040387	0.999	0.998	1.50445e-18	10
RPL23A	DZ	1	1.357833e-22	-0.6902962	0.896	0.813	2.420881e-18	10

NOP53	DZ 1				
2.13336e-22	-0.9385849	0.855	0.747	3.803567e-18	10
CXCR4	DZ 1				
2.847814e-22	-0.4233493	0.101	0.149	5.077367e-18	10
MVP	DZ 1				
3.434611e-22	-0.5086397	0.147	0.175	6.123568e-18	10
ASAH1	DZ 1				
7.398533e-22	-0.7626827	0.377	0.359	1.319084e-17	10
ARID5B	DZ 1				
3.005879e-21	-0.6152489	0.367	0.347	5.359182e-17	10
LITAF	DZ 1				
3.246663e-21	-0.562122	0.31	0.284	5.788475e-17	10
SP110	DZ 1				
3.504936e-21	-0.4057512	0.993	0.986	6.24895e-17	10
RPL14	DZ 1				
7.910596e-21	-0.3644578	0.068	0.106	1.41038e-16	10
PDE4B	DZ 1				
1.321299e-20	-0.373825	0.077	0.112	2.355745e-16	10
SESN3	DZ 1				
1.440927e-20	-0.6856762	0.348	0.301	2.569028e-16	10
ZFP36L2	DZ 1				
4.33409e-20	-0.6117902	0.417	0.382	7.72725e-16	10
C19orf70	DZ 1				
4.464265e-20	-0.4448045	0.988	0.973	7.959337e-16	10
MT-ND4	DZ 1				
6.394491e-20	-0.5966351	0.226	0.211	1.140074e-15	10
LBH	DZ 1				
7.773755e-20	-0.6641765	0.853	0.754	1.385983e-15	10
MT-ND5	DZ 1				
1.201527e-19	-0.464441	0.995	0.975	2.142203e-15	10
RPL36A	DZ 1				
1.225802e-19	-0.4687424	0.19	0.191	2.185483e-15	10
S1PR4	DZ 1				
1.482823e-19	-0.4157724	0.139	0.16	2.643726e-15	10
ARHGAP15	DZ 1				
2.116987e-19	-0.5363906	0.226	0.229	3.774376e-15	10
NT5C3A	DZ 1				
2.963123e-19	-0.6198576	0.462	0.387	5.282952e-15	10
ARL6IP5	DZ 1				
8.453913e-19	-0.5751186	0.539	0.443	1.507248e-14	10
BTG2	DZ 1				
9.028433e-19	-0.3826882	0.99	0.98	1.609679e-14	10
RPL5	DZ 1				
3.305636e-18	-0.5936289	0.379	0.335	5.893618e-14	10
S100A11	DZ 1				
4.401719e-18	-0.6099923	0.459	0.384	7.847825e-14	10
PSAP	DZ 1				
8.085886e-18	-0.7523831	0.614	0.52	1.441633e-13	10
ID3	DZ 1				
8.178419e-18	-0.460534	0.984	0.968	1.45813e-13	10

RPS17	DZ 1				
1.728019e-17	-0.4643343	0.133	0.148	3.080884e-13	10
ARRDC2	DZ 1				
3.650538e-17	-0.5039334	0.243	0.241	6.508545e-13	10
HLA-F	DZ 1				
3.908678e-17	-0.3561387	0.083	0.118	6.968782e-13	10
SLAMF1	DZ 1				
3.992001e-17	-0.5658723	0.337	0.283	7.117339e-13	10
PPM1K	DZ 1				
4.632585e-17	-0.5354909	0.128	0.161	8.259436e-13	10
DDIT4	DZ 1				
4.804811e-17	-0.4448189	0.148	0.171	8.566498e-13	10
PARP14	DZ 1				
5.849888e-17	-0.3667496	0.089	0.121	1.042977e-12	10
LACTB	DZ 1				
8.05474e-17	-0.3580111	0.077	0.116	1.43608e-12	10
ENTPD1	DZ 1				
1.008186e-16	-0.3232596	0.998	0.993	1.797495e-12	10
RPL35	DZ 1				
1.125843e-16	-0.4714769	0.175	0.183	2.007265e-12	10
MBP	DZ 1				
3.017977e-16	-0.4798206	0.988	0.977	5.380752e-12	10
RPS10	DZ 1				
3.15695e-16	-0.6021521	0.37	0.318	5.628526e-12	10
TUBA1A	DZ 1				
5.921512e-16	-0.4090317	0.136	0.15	1.055746e-11	10
ESYT1	DZ 1				
7.510616e-16	-1.825501	0.735	0.441	1.339068e-11	10
IGHG3	DZ 1				
9.032575e-16	0.3563886	0.942	0.747	1.610418e-11	10
HLA-DRB1	DZ 1				
1.444287e-15	-0.6195861	0.461	0.208	2.575018e-11	10
IGHG1	DZ 1				
5.320513e-15	-0.5449003	0.452	0.404	9.485942e-11	10
SMIM14	DZ 1				
5.331078e-15	-0.5798484	0.385	0.34	9.504779e-11	10
PKIG	DZ 1				
6.364056e-15	-0.7222877	0.565	0.475	1.134648e-10	10
SQSTM1	DZ 1				
6.56867e-15	-0.3662487	0.101	0.115	1.171128e-10	10
CLN8	DZ 1				
7.962095e-15	-0.549864	0.409	0.36	1.419562e-10	10
ANXA6	DZ 1				
1.279859e-14	-0.612499	0.109	0.131	2.281861e-10	10
MYC	DZ 1				
3.252506e-14	-0.5891422	0.831	0.713	5.798893e-10	10
EIF4A2	DZ 1				
3.66917e-14	-0.3279992	0.995	0.981	6.541763e-10	10
TPT1	DZ 1				
8.941684e-14	-0.4682033	0.182	0.197	1.594213e-09	10

	TYMP	DZ 1			
2.166944e-13	-0.5976133	0.571	0.477	3.863445e-09	10
	ISCU	DZ 1			
2.257356e-13	-0.7413917	0.552	0.469	4.024641e-09	10
	CD83	DZ 1			
3.389433e-13	-0.3496143	0.105	0.124	6.04302e-09	10
	RAB13	DZ 1			
1.504518e-12	-0.4400129	0.228	0.21	2.682406e-08	10
	FMNL1	DZ 1			
2.522895e-12	-0.3178066	0.108	0.113	4.498069e-08	10
	PBXIP1	DZ 1			
2.545097e-12	-0.5428915	0.957	0.844	4.537653e-08	10
	CD37	DZ 1			
3.797127e-12	-0.3632673	0.163	0.158	6.769897e-08	10
	EVI2A	DZ 1			
4.699616e-12	-0.5182724	0.311	0.281	8.378945e-08	10
	MT-ND4L	DZ 1			
8.363459e-12	-0.3890476	0.171	0.157	1.491121e-07	10
	GLIPR1	DZ 1			
9.492723e-12	-0.3319893	0.117	0.129	1.692458e-07	10
	ICAM2	DZ 1			
2.574374e-11	-0.4810255	0.991	0.975	4.589852e-07	10
	FTH1	DZ 1			
3.843692e-11	-0.5172059	0.963	0.915	6.852919e-07	10
	EEF1B2	DZ 1			
3.938541e-11	-0.5334456	0.481	0.38	7.022024e-07	10
	LAPTM4A	DZ 1			
7.804386e-11	-0.3111751	0.114	0.113	1.391444e-06	10
	HBP1	DZ 1			
8.357376e-11	-0.3151092	0.118	0.117	1.490037e-06	10
	SAMD9	DZ 1			
1.372957e-10	-0.4842316	0.354	0.304	2.447845e-06	10
	TMEM147	DZ 1			
4.695986e-10	-0.3981565	0.213	0.203	8.372474e-06	10
	NDFIP1	DZ 1			
5.294998e-10	-0.5402767	0.774	0.622	9.440451e-06	10
	ATP6V0E1	DZ 1			
5.479016e-10	-0.5320358	0.874	0.748	9.768537e-06	10
	TOMM7	DZ 1			
7.505981e-10	-0.4092659	0.251	0.205	1.338241e-05	10
	C16orf74	DZ 1			
8.051038e-10	-0.432871	0.165	0.166	1.435419e-05	10
	CBX6	DZ 1			
1.146249e-09	-0.6037121	0.551	0.469	2.043648e-05	10
	SNHG8	DZ 1			
1.352442e-09	-0.4018843	0.268	0.205	2.41127e-05	10
	CCDC32	DZ 1			
1.491096e-09	-0.3760335	0.159	0.144	2.658475e-05	10
	SUN2	DZ 1			
3.729747e-09	-0.4516155	0.888	0.637	6.649766e-05	10

	IGLC2	DZ 1				
4.071919e-09	-0.3349492	0.148	0.133	7.259824e-05	10	
	ODF2L	DZ 1				
4.120626e-09	-0.3063362	0.121	0.119	7.346663e-05	10	
	LIPA	DZ 1				
5.620972e-09	-0.3133088	0.993	0.981	0.0001002163	10	
	RPSA	DZ 1				
1.00264e-08	-0.3787051	0.211	0.193	0.0001787607	10	
	ESF1	DZ 1				
1.192751e-08	-0.3437158	0.12	0.131	0.0002126556	10	
	RP9	DZ 1				
1.731207e-08	-0.3023017	0.989	0.97	0.0003086568	10	
	RPL22	DZ 1				
1.872215e-08	-0.3592071	0.215	0.188	0.0003337972	10	
	SYTL1	DZ 1				
2.907059e-08	-0.3431754	0.981	0.958	0.0005182995	10	
	MT-CYB	DZ 1				
4.225608e-08	-0.5640412	0.72	0.57	0.0007533836	10	
	APRT	DZ 1				
4.654297e-08	-0.4489826	0.428	0.348	0.0008298145	10	
	APOBEC3G	DZ 1				
5.350056e-08	-0.3799285	0.262	0.217	0.0009538615	10	
	CCDC50	DZ 1				
1.097328e-07	-0.435459	0.336	0.272	0.001956426	10	
	TNRC6B	DZ 1				
1.41944e-07	-0.3448725	0.197	0.17	0.002530719	10	
	TRIM44	DZ 1				
1.72055e-07	-0.3390021	0.197	0.176	0.003067568	10	
	STAT6	DZ 1				
1.877766e-07	-0.3416044	0.131	0.133	0.00334787	10	
	AC016831.5		DZ 1			
3.308483e-07	-0.4079486	0.341	0.241	0.005898694	10	
	RASGRP2	DZ 1				
4.33534e-07	-0.3224515	0.199	0.171	0.007729477	10	
	DHRS7	DZ 1				
7.056404e-07	-0.3686436	0.236	0.202	0.01258086	10	
	PTPN1	DZ 1				
7.141202e-07	-0.5892555	0.935	0.845	0.01273205	10	
	HSP90AB1	DZ 1				
7.166664e-07	-0.4096058	0.965	0.897	0.01277744	10	
	DDX5	DZ 1				
9.363376e-07	-0.3725518	0.26	0.209	0.01669396	10	
	IDS	DZ 1				
1.094415e-06	-0.3001557	0.163	0.139	0.01951233	10	
	RAB29	DZ 1				
1.374813e-06	-0.5264968	0.884	0.765	0.02451154	10	
	RPL17	DZ 1				
2.117795e-06	-0.3939853	0.297	0.235	0.03775816	10	
	AC004687.1		DZ 1			
2.448028e-06	-0.3456106	0.241	0.192	0.04364589	10	

HSPB1 DZ 1
2.480593e-06 -0.4166971 0.358 0.264 0.0442265 10
HHEX DZ 1
2.65937e-06 -0.3472372 0.228 0.177 0.04741391 10
HEXA DZ 1
3.047401e-06 -0.3410005 0.229 0.185 0.0543321 10
SRSF8 DZ 1
3.185305e-06 -0.3913021 0.293 0.23 0.05679081 10
EPB41L4A-AS1 DZ 1
3.378442e-06 -0.3530935 0.993 0.963 0.06023424 10
EIF1 DZ 1
3.514164e-06 -0.3406177 0.169 0.145 0.06265403 10
TSPYL1 DZ 1
3.741273e-06 -0.563395 0.403 0.309 0.06670316 10
HSP90B1 DZ 1
4.908951e-06 -0.3189622 0.22 0.178 0.08752168 10
DNPEP DZ 1
5.800834e-06 -0.4702654 0.434 0.326 0.1034231 10
SMC6 DZ 1
9.092124e-06 -0.4840134 0.64 0.487 0.1621035 10
CYTIP DZ 1
1.183204e-05 -0.4360886 0.448 0.33 0.2109534 10
BTK DZ 1
1.186842e-05 -0.3501367 0.963 0.879 0.211602 10
MS4A1 DZ 1
1.335687e-05 -0.359668 0.318 0.249 0.2381397 10
DNPH1 DZ 1
1.55807e-05 -0.3052555 0.2 0.154 0.2777883 10
LMBRD1 DZ 1
1.992654e-05 -0.3553229 0.226 0.189 0.3552702 10
RNF145 DZ 1
3.995732e-05 -0.3408713 0.255 0.183 0.7123991 10
ANKRD44 DZ 1
4.296061e-05 -0.3892037 0.444 0.317 0.7659448 10
TNFRSF13C DZ 1
5.770196e-05 -0.3968589 0.282 0.23 1 10
TARS DZ 1
9.302054e-05 -0.5291191 0.82 0.609 1 10
MEF2C DZ 1
0.0001023595 -0.4662332 0.612 0.475 1 10
RSL1D1 DZ 1
0.0001611685 -0.349197 0.301 0.203 1 10
PRKCB DZ 1
0.0002077513 -0.5027307 0.572 0.44 1 10
ANXA2 DZ 1
0.0002870032 -0.3222222 0.259 0.191 1 10
CYTH1 DZ 1
0.000288797 -0.3527322 0.145 0.125 1 10
LGALS3 DZ 1
0.000418605 -0.3139627 0.228 0.183 1 10

FKBP4 DZ 1
0.0005909818 -0.4266223 0.554 0.424 1 10
PPP1R15A DZ 1
0.0009107241 -0.4184765 0.488 0.357 1 10
TAPBP DZ 1
0.0009226015 -0.4499236 0.397 0.292 1 10
NFKBIA DZ 1
0.001028872 -0.3955955 0.453 0.315 1 10
LTA4H DZ 1
0.00136511 -0.4562493 0.64 0.47 1 10
TNFAIP8 DZ 1
0.001519494 -0.3351297 0.293 0.2 1 10
IRF1 DZ 1
0.00156351 -0.4204417 0.621 0.401 1 10 TXN
DZ 1
0.001755646 -0.3750588 0.373 0.274 1 10 MSN
DZ 1
0.001799227 -0.3678066 0.936 0.821 1 10
EIF3E DZ 1
0.001849508 -0.3317053 0.257 0.197 1 10
PPP3CA DZ 1
0.002617499 -0.7306721 0.524 0.426 1 10
IGLC3 DZ 1
0.002950407 -0.3018496 0.187 0.151 1 10
CEBPB DZ 1
0.003177383 -0.3093178 0.28 0.202 1 10
AP1S2 DZ 1
0.004267641 -1.411861 0.264 0.121 1 10
IGHG2 DZ 1
0.007078761 -0.3021649 0.297 0.199 1 10
BIN1 DZ 1
0.007097243 -0.3280904 0.327 0.237 1 10
ANKRD11 DZ 1
0.008134742 -0.3052417 0.295 0.202 1 10
RALGPS2 DZ 1
0.008342502 -0.3152891 0.348 0.176 1 10
XBP1 DZ 1
0 7.494194 0.983 0.435 0 11 IGHG3 ASC IgG
0 6.609033 0.683 0.11 0 11 IGHG2 ASC IgG
0 6.271728 0.88 0.225 0 11 IGHG4 ASC IgG
0 5.283512 0.922 0.196 0 11 IGHG1 ASC IgG
0 4.769338 0.978 0.387 0 11 JCHAIN ASC IgG
0 4.690234 0.995 0.152 0 11 MZB1 ASC IgG
0 3.862643 0.993 0.158 0 11 XBP1 ASC IgG
0 3.182545 0.459 0.012 0 11 IGHGP ASC IgG
0 3.166738 0.993 0.485 0 11 SSR4 ASC IgG
0 3.101241 0.985 0.293 0 11 HSP90B1 ASC IgG
0 2.667154 0.98 0.3 0 11 SEC11C ASC IgG
0 2.641669 0.955 0.257 0 11 HSPA5 ASC IgG
0 2.569728 0.882 0.031 0 11 DERL3 ASC IgG

0	2.514216	0.923	0.067	0	11	FKBP11	ASC	IgG
0	2.429963	0.952	0.153	0	11	PDIA4	ASC	IgG
0	2.272908	0.925	0.124	0	11	SDF2L1	ASC	IgG
0	2.119114	0.886	0.097	0	11	CD38	ASC	IgG
0	2.111755	0.964	0.392	0	11	PPIB	ASC	IgG
0	2.084044	0.941	0.22	0	11	MYDGF	ASC	IgG
0	2.021646	0.869	0.223	0	11	RGS1	ASC	IgG
0	1.939058	0.913	0.171	0	11	MANF	ASC	IgG
0	1.929741	0.951	0.35	0	11	SELENOK	ASC	IgG
0	1.92956	0.93	0.189	0	11	SELENOS	ASC	IgG
0	1.927287	0.8	0.048	0	11	ITM2C	ASC	IgG
0	1.85686	0.949	0.221	0	11	SSR3	ASC	IgG
0	1.818542	0.888	0.125	0	11	PRDX4	ASC	IgG
0	1.80038	0.942	0.243	0	11	FKBP2	ASC	IgG
0	1.765269	0.992	0.862	0	11	CD79A	ASC	IgG
0	1.756436	0.878	0.093	0	11	DNAJB9	ASC	IgG
0	1.754371	0.801	0.089	0	11	VPREB3	ASC	IgG
0	1.741574	0.975	0.626	0	11	HERPUD1	ASC	IgG
0	1.699382	0.833	0.099	0	11	LMAN1	ASC	IgG
0	1.694654	0.843	0.127	0	11	SPCS3	ASC	IgG
0	1.677574	0.876	0.142	0	11	HM13	ASC	IgG
0	1.672705	0.936	0.264	0	11	RPN2	ASC	IgG
0	1.640505	0.889	0.207	0	11	KDELR2	ASC	IgG
0	1.637621	0.766	0.095	0	11	TNFRSF17	ASC	IgG
0	1.592178	0.942	0.332	0	11	PDIA6	ASC	IgG
0	1.557725	0.826	0.094	0	11	CRELD2	ASC	IgG
0	1.497756	0.921	0.186	0	11	RGS13	ASC	IgG
0	1.488472	0.792	0.065	0	11	RRBP1	ASC	IgG
0	1.46944	0.971	0.516	0	11	SPCS2	ASC	IgG
0	1.453035	0.987	0.756	0	11	CYBA	ASC	IgG
0	1.411774	0.819	0.126	0	11	ERLEC1	ASC	IgG
0	1.367348	0.965	0.486	0	11	SPCS1	ASC	IgG
0	1.358029	0.594	0.014	0	11	PRDM1	ASC	IgG
0	1.357674	0.951	0.393	0	11	TMED9	ASC	IgG
0	1.332906	0.871	0.189	0	11	GLRX	ASC	IgG
0	1.332004	0.8	0.138	0	11	DNAJB11	ASC	IgG
0	1.309608	0.889	0.253	0	11	ARF4	ASC	IgG
0	1.299504	0.926	0.307	0	11	P4HB	ASC	IgG
0	1.287172	0.929	0.298	0	11	TRAM1	ASC	IgG
0	1.282715	0.884	0.292	0	11	CALR	ASC	IgG
0	1.255304	0.877	0.216	0	11	UBE2J1	ASC	IgG
0	1.237469	0.961	0.543	0	11	SEC61G	ASC	IgG
0	1.236412	0.725	0.064	0	11	GMPPB	ASC	IgG
0	1.229319	0.975	0.587	0	11	SEC61B	ASC	IgG
0	1.226884	0.945	0.469	0	11	RABAC1	ASC	IgG
0	1.225948	0.658	0.057	0	11	NUCB2	ASC	IgG
0	1.225402	0.936	0.456	0	11	MTDH	ASC	IgG
0	1.223204	0.894	0.35	0	11	PDIA3	ASC	IgG
0	1.198806	0.911	0.304	0	11	LMAN2	ASC	IgG
0	1.195425	0.875	0.199	0	11	NANS	ASC	IgG

0	1.167071	0.864	0.215	0	11	TMED10	ASC IgG
0	1.164408	0.636	0.023	0	11	SELENOM	ASC IgG
0	1.148657	0.946	0.545	0	11	SERP1	ASC IgG
0	1.145954	0.646	0.015	0	11	CHPF	ASC IgG
0	1.129845	0.918	0.346	0	11	REEP5	ASC IgG
0	1.126191	0.755	0.098	0	11	DNAJC1	ASC IgG
0	1.091559	0.69	0.08	0	11	PDXK	ASC IgG
0	1.063319	0.695	0.077	0	11	DNAJC3	ASC IgG
0	1.038103	0.727	0.089	0	11	TXNDC15	ASC IgG
0	1.034065	0.93	0.378	0	11	TMED2	ASC IgG
0	1.025211	0.629	0.065	0	11	CLPTM1L	ASC IgG
0	1.00757	0.518	0.013	0	11	BEX5	ASC IgG
0	1.000408	0.639	0.065	0	11	TXNDC11	ASC IgG
0	0.9758317	0.648	0.062	0	11	SEC61A1	ASC IgG
0	0.9437166	0.553	0.043	0	11	ANKRD28	ASC IgG
0	0.9282912	0.515	0.024	0	11	AC012236.1	ASC IgG
0	0.871283	0.514	0.011	0	11	ZBP1	ASC IgG
0	-1.019783	0.996	1	0	11	RPLP1	ASC IgG
0	-1.160622	0.991	0.996	0	11	RPL8	ASC IgG
0	-1.175595	0.993	0.998	0	11	RPL3	ASC IgG
0	-1.199542	0.988	0.997	0	11	RPS15A	ASC IgG
0	-1.290168	0.998	1	0	11	RPL41	ASC IgG
0	-1.292356	0.994	0.998	0	11	RPS28	ASC IgG
0	-1.305582	0.984	0.994	0	11	RPS9	ASC IgG
0	-1.311925	0.982	0.988	0	11	RACK1	ASC IgG
0	-1.335394	0.971	0.992	0	11	RPL18	ASC IgG
0	-1.35029	0.98	0.993	0	11	RPS7	ASC IgG
0	-1.352444	0.992	0.999	0	11	RPL15	ASC IgG
0	-1.369231	0.983	0.997	0	11	RPS13	ASC IgG
0	-1.403264	0.968	0.986	0	11	RPL27	ASC IgG
0	-1.405555	0.993	0.998	0	11	RPL18A	ASC IgG
0	-1.412387	0.983	0.993	0	11	RPL35	ASC IgG
0	-1.423582	0.989	0.997	0	11	RPL28	ASC

IgG 0	−1.431957	0.979	0.994	0	11	RPL6	ASC
IgG 0	−1.446797	0.993	0.998	0	11	RPS15	ASC
IgG 0	−1.449901	0.968	0.985	0	11	RPL29	ASC
IgG 0	−1.459963	0.965	0.986	0	11	TMSB10	ASC
IgG 0	−1.466052	0.995	0.999	0	11	RPS29	ASC
IgG 0	−1.469504	0.988	0.998	0	11	RPS14	ASC
IgG 0	−1.481285	0.994	0.999	0	11	RPS19	ASC
IgG 0	−1.489942	0.933	0.966	0	11	RPL38	ASC
IgG 0	−1.497826	0.985	0.995	0	11	RPS25	ASC
IgG 0	−1.500761	0.987	0.998	0	11	RPL19	ASC
IgG 0	−1.502502	0.982	0.996	0	11	RPS3	ASC
IgG 0	−1.526051	0.952	0.978	0	11	RPS10	ASC
IgG 0	−1.526919	0.985	0.997	0	11	RPL27A	ASC
IgG 0	−1.527854	0.99	0.998	0	11	RPL23A	ASC
IgG 0	−1.534517	0.982	0.995	0	11	RPL37A	ASC
IgG 0	−1.536488	0.987	0.996	0	11	RPL35A	ASC
IgG 0	−1.537471	0.978	0.993	0	11	RPL36	ASC
IgG 0	−1.547599	0.993	0.999	0	11	PTMA	ASC
IgG 0	−1.550069	0.966	0.993	0	11	RPL37	ASC
IgG 0	−1.557367	0.947	0.971	0	11	RPL22	ASC
IgG 0	−1.563061	0.97	0.976	0	11	RPS24	ASC
IgG 0	−1.564955	0.997	0.999	0	11	RPS18	ASC
IgG 0	−1.597803	0.997	0.999	0	11	RPL13	ASC
IgG 0	−1.602996	0.994	0.999	0	11	RPS8	ASC
IgG 0	−1.657411	0.974	0.991	0	11	RPL10A	ASC

IgG
0 -1.66282 0.994 1 0 11 RPL13A ASC IgG
0 -1.681711 0.973 0.992 0 11 RPL9 ASC
IgG
0 -1.68384 0.995 1 0 11 RPL21 ASC IgG
0 -1.685675 0.935 0.969 0 11 RPS17 ASC
IgG
0 -1.721914 0.904 0.962 0 11 RPL23 ASC
IgG
0 -1.723169 0.994 1 0 11 RPS2 ASC
IgG
0 -1.723833 0.971 0.991 0 11 RPS20 ASC
IgG
0 -1.736253 0.993 0.999 0 11 RPL34 ASC
IgG
0 -1.739912 0.987 0.998 0 11 RPL26 ASC
IgG
0 -1.750126 0.932 0.977 0 11 RPL36A ASC
IgG
0 -1.752353 0.966 0.987 0 11 RPL14 ASC
IgG
0 -1.752976 0.992 0.998 0 11 RPL7 ASC
IgG
0 -1.759994 0.937 0.97 0 11 MT-ND3 ASC
IgG
0 -1.765906 0.991 0.999 0 11 RPS6 ASC
IgG
0 -1.77372 0.946 0.982 0 11 RPL5 ASC IgG
0 -1.789574 0.947 0.984 0 11 RPS21 ASC
IgG
0 -1.790043 0.994 0.999 0 11 RPS27A ASC
IgG
0 -1.79248 0.991 0.999 0 11 RPLP2 ASC IgG
0 -1.811464 0.994 0.999 0 11 EEF1A1 ASC
IgG
0 -1.856049 0.949 0.982 0 11 RPSA ASC
IgG
0 -1.857638 0.983 0.998 0 11 RPS3A ASC
IgG
0 -1.868614 0.788 0.91 0 11 IGHM ASC
IgG
0 -1.871521 0.984 0.998 0 11 RPL11 ASC
IgG
0 -1.87196 0.998 1 0 11 RPS27 ASC IgG
0 -1.87216 0.952 0.992 0 11 RPL30 ASC IgG
0 -1.875366 0.492 0.785 0 11 NAP1L1 ASC
IgG
0 -1.893758 0.99 0.999 0 11 RPL32 ASC
IgG
0 -1.92022 0.983 0.997 0 11 RPL12 ASC IgG

0	-1.923743	0.43	0.734	0	11	LIMD2	ASC IgG
0	-1.924687	0.979	0.998	0	11	RPS23	ASC IgG
0	-1.937758	0.981	0.996	0	11	RPL31	ASC IgG
0	-1.986773	0.805	0.93	0	11	HNRNPA1	ASC IgG
0	-2.031394	0.99	0.999	0	11	RPL39	ASC IgG
0	-2.072262	0.212	0.66	0	11	ZFP36L1	ASC IgG
0	-2.078344	0.982	0.997	0	11	RPS12	ASC IgG
0	-2.098328	0.8	0.939	0	11	BTG1	ASC IgG
0	-2.100425	0.947	0.991	0	11	ACTB	ASC IgG
0	-2.135551	0.982	0.999	0	11	TMSB4X	ASC IgG
0	-2.253342	0.578	0.89	0	11	MS4A1	ASC IgG
0	-2.308583	0.61	0.876	0	11	CD52	ASC IgG
0	-2.353238	0.502	0.857	0	11	CD37	ASC IgG
0	-2.735483	0.292	0.764	0	11	CXCR4	ASC IgG
1.333977e-322	-1.163694	0.983	0.993	2.378348e-318	11	RPS5	ASC IgG
4.199558e-322	-1.164679	0.983	0.991	7.487392e-318	11	RPS16	ASC IgG
4.248965e-322	-0.7851164	0.991	0.999	7.575479e-318	11	RPL10	ASC IgG
5.147621e-319	-0.9158427	0.994	0.997	9.177693e-315	11	RPS4X	ASC IgG
5.121904e-318	1.066966	0.931	0.464	9.131843e-314	11	SSR2	ASC IgG
6.030881e-315	0.9657147	0.733	0.113	1.075246e-310	11	SRPRB	ASC IgG
1.416425e-309	1.500506	0.726	0.171	2.525344e-305	11	RGS2	ASC IgG
6.580102e-304	0.3824939	0.212	0.001	1.173166e-299	11	SDC1	ASC IgG
7.778211e-303	0.9844663	0.715	0.107	1.386777e-298	11	SPATS2	ASC IgG
2.766578e-302	-2.184847	0.112	0.556	4.932532e-298	11	CCR7	ASC IgG
6.147245e-302	-1.870487	0.646	0.819	1.095992e-297	11	CORO1A	ASC IgG

2.708982e-301	-1.289841	0.958	0.977	4.829844e-297	11	UBA52	ASC IgG
3.791876e-300	0.4524369	0.177	0.618	6.760535e-296	11	IGHD	ASC IgG
6.442231e-298	1.227816	0.575	0.071	1.148585e-293	11	HIST1H1C	ASC IgG
2.031454e-295	0.931345	0.758	0.137	3.62188e-291	11	ALG5	ASC IgG
5.490216e-295	-2.220395	0.373	0.663	9.788505e-291	11	TCL1A	ASC IgG
3.123069e-294	0.90295	0.534	0.052	5.568119e-290	11	QPCT	ASC IgG
2.231596e-293	0.7434122	0.5	0.031	3.978712e-289	11	SIL1	ASC IgG
8.685409e-286	4.564946	0.987	0.925	1.548522e-281	11	IGKC	ASC IgG
3.230345e-283	-1.730348	0.312	0.635	5.759382e-279	11	HLA-DMB	ASC IgG
2.501233e-280	-1.726994	0.911	0.932	4.459449e-276	11	HMGB1	ASC IgG
2.13796e-279	0.9790503	0.941	0.439	3.811769e-275	11	TMEM59	ASC IgG
2.773717e-275	-1.35044	0.951	0.958	4.94526e-271	11	PABPC1	ASC IgG
2.439628e-273	1.275317	0.822	0.262	4.349613e-269	11	PIM2	ASC IgG
8.765776e-272	0.7978618	0.301	0.006	1.56285e-267	11	IGLV6-57	ASC IgG
2.667589e-271	0.3067786	0.203	0.002	4.756044e-267	11	HID1	ASC IgG
2.406027e-270	-1.232272	0.971	0.982	4.289706e-266	11	MT-CO3	ASC IgG
2.170304e-268	-1.361019	0.941	0.958	3.869435e-264	11	RPL24	ASC IgG
1.341168e-266	-1.501361	0.888	0.924	2.391168e-262	11	BTF3	ASC IgG
4.06596e-266	0.7433611	0.424	0.029	7.249201e-262	11	DENND6B	ASC IgG
2.049716e-265	0.9401613	0.766	0.16	3.654439e-261	11	DERL1	ASC IgG
5.093532e-263	-1.800534	0.137	0.523	9.081258e-259	11	BANK1	ASC IgG
2.446554e-260	-1.227986	0.97	0.98	4.36196e-256	11	RPL7A	ASC IgG
6.792002e-259	-2.43931	0.194	0.497	1.210946e-254	11	LTB	ASC IgG
7.310482e-258	0.8259469	0.639	0.081	1.303386e-253	11	RPN1	ASC IgG
6.906242e-257	-1.516586	0.845	0.896	1.231314e-252	11	PPIA	ASC IgG

1.608257e-256	0.8334802	0.634	0.088	2.867361e-252	11	SLC35B1	ASC IgG
1.665683e-256	0.8444759	0.594	0.07	2.969746e-252	11	ST6GALNAC4	ASC IgG
6.751821e-253	0.5601642	0.369	0.018	1.203782e-248	11	KCNN3	ASC IgG
4.796257e-251	0.9202245	0.911	0.424	8.551247e-247	11	DAD1	ASC IgG
3.639192e-245	-1.845756	0.158	0.533	6.488316e-241	11	ID3	ASC IgG
1.852406e-243	0.7774187	0.494	0.04	3.302654e-239	11	MEI1	ASC IgG
1.949282e-241	-1.672203	0.926	0.93	3.475375e-237	11	NPM1	ASC IgG
1.002313e-239	0.7978015	0.636	0.072	1.787023e-235	11	TENT5C	ASC IgG
5.151077e-239	0.9121292	0.808	0.206	9.183855e-235	11	SSR1	ASC IgG
1.221434e-237	0.838582	0.959	0.594	2.177694e-233	11	TMEM258	ASC IgG
1.691206e-235	5.288669	0.914	0.637	3.015251e-231	11	IGLC2	ASC IgG
2.396637e-234	0.9688738	0.805	0.19	4.272963e-230	11	EAF2	ASC IgG
3.395107e-234	0.7914174	0.606	0.067	6.053136e-230	11	PLD3	ASC IgG
8.764867e-233	0.8338825	0.782	0.191	1.562688e-228	11	TMEM208	ASC IgG
2.625013e-232	-1.218335	0.952	0.964	4.680135e-228	11	RPL4	ASC IgG
7.577505e-232	0.6783647	0.967	0.746	1.350993e-227	11	HLA-C	ASC IgG
8.53807e-231	-1.534644	0.712	0.828	1.522253e-226	11	EIF3E	ASC IgG
1.572317e-227	0.8569311	0.951	0.644	2.803284e-223	11	TMBIM6	ASC IgG
5.32413e-224	-1.448231	0.902	0.935	9.492391e-220	11	MT-ATP6	ASC IgG
8.88109e-224	-1.61646	0.181	0.51	1.583409e-219	11	FOXP1	ASC IgG
2.56704e-221	0.7782316	0.541	0.05	4.576776e-217	11	CHST2	ASC IgG
3.309e-219	4.934097	0.709	0.421	5.899616e-215	11	IGLC3	ASC IgG
3.54386e-215	-1.644153	0.454	0.683	6.318348e-211	11	TAGLN2	ASC IgG
1.580863e-214	0.8181147	0.722	0.161	2.818521e-210	11	DERL2	ASC IgG
6.117841e-211	-1.132832	0.978	0.98	1.09075e-206	11	MT-CO1	ASC IgG

7.627553e-210	−1.526341	0.089	0.433	1.359916e-205	11	DEK	ASC IgG
7.642736e-210	−1.042205	0.978	0.982	1.362623e-205	11	RPS11	ASC IgG
2.8867e-209	0.8338603	0.885	0.374	5.146698e-205	11	CUTA	ASC IgG
8.994241e-209	−1.565832	0.112	0.438	1.603583e-204	11	IRF8	ASC IgG
1.999423e-207	−1.455278	0.953	0.946	3.564771e-203	11	HLA−DRA	ASC IgG
2.155617e-207	0.8884316	0.965	0.61	3.84325e-203	11	SUB1	ASC IgG
1.848209e-206	0.8650746	0.866	0.358	3.295171e-202	11	OSTC	ASC IgG
2.659313e-206	0.7559254	0.649	0.112	4.741289e-202	11	SAR1B	ASC IgG
1.080129e-204	0.9469356	0.568	0.094	1.925762e-200	11	CITED2	ASC IgG
1.214198e-204	0.669984	0.481	0.043	2.164794e-200	11	SEL1L	ASC IgG
4.378506e-204	0.722631	0.565	0.058	7.806438e-200	11	CHST12	ASC IgG
2.491715e-202	0.6647363	0.198	0.004	4.442478e-198	11	FRZB	ASC IgG
1.427815e-201	0.7953078	0.707	0.169	2.545651e-197	11	LRRC59	ASC IgG
7.095651e-201	0.5883716	0.432	0.03	1.265084e-196	11	SLC17A9	ASC IgG
1.407575e-200	0.8038238	0.766	0.215	2.509566e-196	11	DDOST	ASC IgG
2.719305e-200	0.5779111	0.414	0.025	4.848248e-196	11	TRIB1	ASC IgG
6.010146e-200	−1.325892	0.914	0.919	1.071549e-195	11	CFL1	ASC IgG
2.909897e-199	−1.201685	0.963	0.974	5.188056e-195	11	MT−ND4	ASC IgG
8.721333e-199	0.354279	0.245	0.006	1.554927e-194	11	TXNDC5	ASC IgG
1.051396e-197	0.7643971	0.616	0.118	1.874534e-193	11	CYTOR	ASC IgG
1.582639e-196	−1.571249	0.409	0.654	2.821688e-192	11	ZFAS1	ASC IgG
2.620029e-196	−0.9696108	0.986	0.988	4.67125e-192	11	RPLP0	ASC IgG
1.27856e-193	−1.802062	0.149	0.481	2.279545e-189	11	CD83	ASC IgG
1.633286e-193	0.5852776	0.426	0.029	2.911986e-189	11	CKAP4	ASC IgG
3.437294e-193	0.8025395	0.81	0.265	6.128352e-189	11	SRM	ASC IgG

4.938443e-193	-1.360049	0.742	0.82	8.804749e-189	11	SEPT7	ASC IgG
3.697295e-191	0.7645656	0.701	0.151	6.591907e-187	11	MLEC	ASC IgG
1.080178e-189	-1.380068	0.148	0.442	1.925849e-185	11	CYB561A3	ASC IgG
9.062365e-189	-1.390495	0.893	0.917	1.615729e-184	11	EEF1B2	ASC IgG
2.892661e-188	0.5678676	0.429	0.033	5.157326e-184	11	KCNK6	ASC IgG
3.209367e-188	0.62053	0.501	0.053	5.72198e-184	11	CALU	ASC IgG
2.81203e-187	0.739751	0.599	0.099	5.013569e-183	11	PREB	ASC IgG
4.173568e-186	0.451852	0.312	0.012	7.441055e-182	11	ELL2	ASC IgG
1.826504e-185	-1.363745	0.954	0.961	3.256475e-181	11	MT-ND2	ASC IgG
2.97782e-183	0.9127721	0.93	0.522	5.309156e-179	11	ISG20	ASC IgG
7.104866e-183	-1.803776	0.551	0.702	1.266727e-178	11	EMP3	ASC IgG
9.483555e-183	-0.7431852	0.991	0.993	1.690823e-178	11	FAU	ASC IgG
1.484638e-182	-1.18537	0.907	0.918	2.646961e-178	11	COX4I1	ASC IgG
2.381094e-180	-1.146193	0.941	0.944	4.245253e-176	11	NACA	ASC IgG
1.376241e-179	0.9440768	0.603	0.096	2.4537e-175	11	CD9	ASC IgG
9.682124e-179	0.7207148	0.735	0.187	1.726226e-174	11	SEC13	ASC IgG
1.090882e-177	-1.501561	0.558	0.7	1.944934e-173	11	HNRNPDL	ASC IgG
2.626559e-177	0.7729034	0.712	0.153	4.682892e-173	11	SEL1L3	ASC IgG
2.920409e-177	0.7292484	0.78	0.241	5.206797e-173	11	SRGN	ASC IgG
8.879302e-177	-1.889094	0.61	0.719	1.583091e-172	11	HMGN2	ASC IgG
1.961329e-176	0.599495	0.398	0.03	3.496853e-172	11	CPEB4	ASC IgG
1.01904e-174	-1.407797	0.737	0.818	1.816847e-170	11	NOP53	ASC IgG
2.165466e-174	0.6468541	0.526	0.061	3.860809e-170	11	EDEM2	ASC IgG
6.820211e-173	0.7084486	0.34	0.022	1.215975e-168	11	DHRS9	ASC IgG
4.394098e-172	0.5284735	0.375	0.026	7.834238e-168	11	SEC24D	ASC IgG

1.444791e-170	-1.577432	0.433	0.615	2.575918e-166	11	SYNGR2	ASC IgG
1.891476e-168	-1.363851	0.953	0.942	3.372312e-164	11	YBX1	ASC IgG
4.79049e-168	0.6793202	0.945	0.568	8.540965e-164	11	COPE	ASC IgG
6.551966e-168	0.6653983	0.7	0.142	1.16815e-163	11	MGAT1	ASC IgG
1.851011e-166	0.6552667	0.462	0.044	3.300167e-162	11	CCPG1	ASC IgG
6.400301e-165	0.8719744	0.995	0.962	1.14111e-160	11	FTL	ASC IgG
5.094975e-164	0.8790205	0.771	0.29	9.083831e-160	11	CD27	ASC IgG
5.412351e-163	0.674443	0.742	0.194	9.649681e-159	11	CISD2	ASC IgG
1.218279e-162	0.5927235	0.524	0.078	2.172069e-158	11	UBA5	ASC IgG
1.063163e-161	0.6961959	0.74	0.201	1.895513e-157	11	RAB1A	ASC IgG
1.191183e-161	0.5266549	0.386	0.032	2.12376e-157	11	BHLHE41	ASC IgG
2.392848e-161	-1.404659	0.404	0.575	4.266208e-157	11	SNX2	ASC IgG
3.055136e-160	-1.351569	0.116	0.408	5.447001e-156	11	REL	ASC IgG
3.364604e-159	-1.195093	0.92	0.917	5.998752e-155	11	PFDN5	ASC IgG
2.4332e-158	-1.358323	0.08	0.345	4.338152e-154	11	HVCN1	ASC IgG
2.869825e-158	0.5972233	0.479	0.057	5.116611e-154	11	THEMIS2	ASC IgG
2.363546e-157	0.6613372	0.891	0.394	4.213967e-153	11	GUK1	ASC IgG
2.8998e-157	0.6827214	0.769	0.221	5.170053e-153	11	LRPAP1	ASC IgG
8.992909e-157	0.6475596	0.681	0.17	1.603346e-152	11	KDELR1	ASC IgG
1.217154e-155	-1.3109	0.846	0.846	2.170063e-151	11	HNRNPA2B1	ASC IgG
6.742223e-155	0.631348	0.683	0.158	1.202071e-150	11	EMC7	ASC IgG
2.024557e-154	0.3123605	0.205	0.005	3.609583e-150	11	B9D1	ASC IgG
3.09185e-154	-1.717114	0.861	0.848	5.51246e-150	11	HSP90AB1	ASC IgG
1.573033e-153	0.4307488	0.305	0.012	2.80456e-149	11	ADA2	ASC IgG
1.554517e-152	-1.420477	0.187	0.412	2.771549e-148	11	SMIM14	ASC IgG

9.841736e-152	-1.364962	0.85	0.855	1.754683e-147	11	SH3BGRL3	ASC IgG
1.116103e-149	-0.9121107	0.975	0.979	1.989901e-145	11	MT-CO2	ASC IgG
3.667787e-149	0.653243	0.506	0.077	6.539297e-145	11	TPST2	ASC IgG
1.392188e-148	0.6361229	0.749	0.216	2.482132e-144	11	UFM1	ASC IgG
2.594554e-148	-1.330104	0.496	0.623	4.62583e-144	11	IFI16	ASC IgG
2.934416e-148	0.4267376	0.341	0.021	5.23177e-144	11	HSPA13	ASC IgG
6.358763e-148	-1.51731	0.189	0.377	1.133704e-143	11	SELL	ASC IgG
5.424924e-147	-1.379024	0.321	0.539	9.672097e-143	11	IMPDH2	ASC IgG
2.017005e-146	0.3437279	0.256	0.009	3.596119e-142	11	PARM1	ASC IgG
4.550875e-146	0.9308743	0.369	0.066	8.113756e-142	11	H1FX	ASC IgG
1.415167e-145	0.4222535	0.316	0.016	2.523102e-141	11	WIPI1	ASC IgG
3.975605e-144	-1.280964	0.931	0.899	7.088105e-140	11	PFN1	ASC IgG
8.381929e-142	-1.287433	0.819	0.805	1.494414e-137	11	CALM2	ASC IgG
1.623328e-141	-1.0636	0.055	0.295	2.894231e-137	11	RUBCNL	ASC IgG
1.415416e-140	-1.158145	0.107	0.345	2.523545e-136	11	SPIB	ASC IgG
1.599109e-140	0.7554112	0.671	0.154	2.851052e-136	11	CCDC88A	ASC IgG
1.046579e-139	-1.360436	0.317	0.494	1.865947e-135	11	UCP2	ASC IgG
2.695626e-139	-1.18409	0.77	0.776	4.806032e-135	11	ARHGDIB	ASC IgG
3.537951e-139	0.4575583	0.344	0.033	6.307813e-135	11	PKHD1L1	ASC IgG
1.174327e-138	0.5843907	0.876	0.373	2.093708e-134	11	PSAP	ASC IgG
1.764479e-138	0.5620748	0.515	0.089	3.14589e-134	11	HDLBP	ASC IgG
2.020887e-138	0.5591521	0.543	0.085	3.60304e-134	11	TMED5	ASC IgG
4.24684e-138	0.5432231	0.584	0.108	7.571692e-134	11	NEU1	ASC IgG
3.370454e-137	-1.280154	0.282	0.481	6.009183e-133	11	TNFAIP8	ASC IgG
4.407665e-137	0.4628611	0.323	0.023	7.858427e-133	11	GLCCI1	ASC IgG

1.235843e-136	-1.184085	0.078	0.327	2.203384e-132	11	TUBA1A	ASC IgG
1.31255e-136	-1.373655	0.096	0.322	2.340146e-132	11	CD69	ASC IgG
9.157773e-136	-1.230343	0.076	0.303	1.632739e-131	11	LINC00926	ASC IgG
1.269064e-135	0.5727882	0.488	0.079	2.262615e-131	11	ZBTB38	ASC IgG
1.920659e-135	-1.192553	0.915	0.855	3.424343e-131	11	CD79B	ASC IgG
3.698708e-135	-1.220511	0.653	0.704	6.594427e-131	11	COMMD6	ASC IgG
4.132375e-135	0.6279815	0.861	0.388	7.367611e-131	11	UBXN4	ASC IgG
9.064645e-135	-1.716441	0.373	0.576	1.616136e-130	11	VIM	ASC IgG
4.001763e-133	0.5716848	0.666	0.171	7.134743e-129	11	YIF1A	ASC IgG
4.532874e-133	-1.241794	0.727	0.765	8.081661e-129	11	MYL12A	ASC IgG
1.21283e-132	0.3473241	0.26	0.014	2.162354e-128	11	PDIA5	ASC IgG
1.549264e-131	0.4042054	0.282	0.02	2.762182e-127	11	MIR4435-2HG	ASC IgG
2.452644e-131	0.5077338	0.352	0.035	4.372818e-127	11	IRF4	ASC IgG
2.224334e-130	-1.935371	0.24	0.471	3.965765e-126	11	S100A6	ASC IgG
2.055904e-129	-1.153017	0.094	0.343	3.665471e-125	11	CD48	ASC IgG
7.091142e-129	0.3273544	0.155	0.005	1.26428e-124	11	CADM1	ASC IgG
2.160394e-128	-1.091368	0.075	0.299	3.851767e-124	11	BCL11A	ASC IgG
2.766436e-128	-1.080966	0.053	0.273	4.93228e-124	11	HHEX	ASC IgG
1.229937e-127	0.5400518	0.568	0.114	2.192854e-123	11	MGAT2	ASC IgG
1.298477e-127	-1.111283	0.086	0.312	2.315054e-123	11	ARHGAP24	ASC IgG
4.524554e-127	0.5326532	0.543	0.103	8.066827e-123	11	SURF4	ASC IgG
4.800924e-127	0.5015594	0.437	0.056	8.559568e-123	11	C1GALT1C1	ASC IgG
9.333026e-127	-1.222662	0.694	0.754	1.663985e-122	11	TOMM7	ASC IgG
1.21809e-126	-1.055123	0.961	0.959	2.171733e-122	11	MT-CYB	ASC IgG
1.2863e-126	0.4787253	0.366	0.033	2.293344e-122	11	FNDC3A	ASC IgG

1.605254e-126	-1.294878	0.23	0.443	2.862007e-122	11	COTL1	ASC IgG
9.038983e-125	0.5485373	0.333	0.038	1.61156e-120	11	CCDC144A	ASC IgG
3.336379e-124	-1.261413	0.466	0.599	5.94843e-120	11	TPM3	ASC IgG
3.545351e-124	0.4768383	0.431	0.067	6.321007e-120	11	SLAMF7	ASC IgG
1.210864e-123	-1.24374	0.728	0.77	2.158849e-119	11	RPL17	ASC IgG
3.100998e-123	0.5567716	0.6	0.129	5.528769e-119	11	ARMCX3	ASC IgG
9.768608e-123	-1.418205	0.739	0.787	1.741645e-118	11	ACTG1	ASC IgG
2.021622e-122	-1.179807	0.239	0.408	3.60435e-118	11	STX7	ASC IgG
7.087863e-122	0.5459018	0.803	0.292	1.263695e-117	11	CNPY2	ASC IgG
1.282891e-121	0.5670568	0.655	0.173	2.287266e-117	11	TECR	ASC IgG
2.588115e-121	0.5462469	0.614	0.141	4.61435e-117	11	ARFGAP3	ASC IgG
3.452128e-121	0.4673824	0.402	0.049	6.154799e-117	11	GMPPA	ASC IgG
4.039586e-121	0.6261176	0.608	0.155	7.202179e-117	11	CCDC167	ASC IgG
5.536558e-121	0.5465448	0.647	0.158	9.87113e-117	11	SRPRA	ASC IgG
1.255659e-120	0.5755882	0.382	0.069	2.238714e-116	11	HCST	ASC IgG
2.691531e-120	-1.077661	0.893	0.869	4.79873e-116	11	ARPC3	ASC IgG
7.544755e-120	-1.709526	0.632	0.667	1.345154e-115	11	TUBA1B	ASC IgG
1.681845e-119	0.6204941	0.597	0.153	2.998561e-115	11	TOR3A	ASC IgG
1.95281e-119	0.586722	0.799	0.287	3.481666e-115	11	CDK2AP2	ASC IgG
4.029945e-119	0.5396705	0.519	0.083	7.184989e-115	11	KLF13	ASC IgG
7.902191e-119	0.5761339	0.854	0.368	1.408882e-114	11	NDUFC2	ASC IgG
1.263415e-118	0.572196	0.994	0.986	2.252543e-114	11	HLA-B	ASC IgG
1.408205e-118	-1.118203	0.142	0.337	2.510688e-114	11	RIPOR2	ASC IgG
1.700157e-118	1.107566	0.821	0.488	3.031209e-114	11	SAT1	ASC IgG
3.112005e-118	-1.019761	0.092	0.295	5.548394e-114	11	DCK	ASC IgG

3.550152e-118	-0.9597909	0.055	0.262	6.329566e-114	11	LYSMD2	ASC IgG
4.230139e-116	0.6422377	0.435	0.06	7.541915e-112	11	RASSF6	ASC IgG
4.948384e-116	0.4629375	0.433	0.059	8.822475e-112	11	SLC38A10	ASC IgG
1.247631e-115	-0.9941362	0.093	0.304	2.224402e-111	11	BLK	ASC IgG
1.904446e-115	0.5654347	0.969	0.723	3.395437e-111	11	OST4	ASC IgG
6.377973e-115	-1.111656	0.116	0.335	1.137129e-110	11	TRBC2	ASC IgG
2.013502e-114	0.6617003	0.37	0.049	3.589874e-110	11	RGS16	ASC IgG
2.335112e-113	-1.580458	0.493	0.579	4.163272e-109	11	TUBB	ASC IgG
3.592731e-113	-1.507871	0.452	0.656	6.40548e-109	11	RPS4Y1	ASC IgG
1.15606e-112	0.5583092	0.892	0.456	2.061139e-108	11	PRDX5	ASC IgG
2.591364e-112	-1.193137	0.165	0.386	4.620143e-108	11	PDCD4	ASC IgG
9.65123e-112	0.5924917	0.95	0.623	1.720718e-107	11	GSTP1	ASC IgG
1.792706e-111	0.5329343	0.726	0.229	3.196216e-107	11	OS9	ASC IgG
2.060337e-111	-1.176121	0.173	0.352	3.673374e-107	11	TRAF3IP3	ASC IgG
5.751894e-111	0.5581207	0.819	0.371	1.025505e-106	11	ATP6V0B	ASC IgG
9.560507e-111	0.5432583	0.625	0.16	1.704543e-106	11	GORASP2	ASC IgG
9.829277e-111	0.5376851	0.705	0.227	1.752462e-106	11	MORF4L2	ASC IgG
4.7722e-110	0.5418032	0.648	0.159	8.508356e-106	11	SEC63	ASC IgG
7.072942e-110	0.5383963	0.695	0.217	1.261035e-105	11	CCNC	ASC IgG
9.213413e-110	-0.8387976	0.948	0.937	1.642659e-105	11	EEF2	ASC IgG
9.732095e-110	0.503989	0.506	0.087	1.735135e-105	11	SYVN1	ASC IgG
1.575166e-109	-0.7129422	0.998	1	2.808364e-105	11	MALAT1	ASC IgG
5.359829e-109	0.4239431	0.302	0.028	9.55604e-105	11	SLC44A1	ASC IgG
1.378365e-108	0.3462181	0.249	0.012	2.457487e-104	11	P2RX1	ASC IgG
2.491195e-108	0.5165335	0.629	0.158	4.441551e-104	11	TM9SF2	ASC IgG

3.496598e-108	-1.543315	0.314	0.431	6.234085e-104	11	STMN1	ASC IgG
1.060353e-107	0.3242351	0.227	0.012	1.890504e-103	11	FICD	ASC IgG
1.857513e-107	2.011854	0.182	0.037	3.31176e-103	11	IGLL5	ASC IgG
1.886631e-107	0.3261407	0.224	0.013	3.363674e-103	11	MIXL1	ASC IgG
2.448918e-107	0.3707199	0.291	0.02	4.366176e-103	11	ARSA	ASC IgG
3.055338e-107	-1.089521	0.138	0.335	5.447362e-103	11	SMC6	ASC IgG
3.180981e-107	-1.56073	0.245	0.408	5.671371e-103	11	FABP5	ASC IgG
4.155703e-107	0.4818896	0.413	0.072	7.409202e-103	11	GPR160	ASC IgG
5.980046e-107	-1.060257	0.253	0.421	1.066182e-102	11	ESD	ASC IgG
6.459065e-107	0.6183169	0.642	0.184	1.151587e-102	11	IDH2	ASC IgG
1.376218e-106	0.4189318	0.374	0.046	2.453659e-102	11	TMEM214	ASC IgG
1.341286e-104	0.5775172	0.698	0.188	2.39138e-100	11	HMCES	ASC IgG
1.551731e-104	0.5129276	0.864	0.419	2.766581e-100	11	ZNF706	ASC IgG
2.238682e-104	-0.9714509	0.937	0.898	3.991346e-100	11	DDX5	ASC IgG
2.392848e-104	-1.113673	0.171	0.351	4.266209e-100	11	BIRC3	ASC IgG
2.577994e-104	-1.161489	0.662	0.668	4.596306e-100	11	GDI2	ASC IgG
5.887959e-104	-1.078998	0.684	0.721	1.049764e-99	11	EIF3F	ASC IgG
1.199834e-103	0.3072643	0.222	0.012	2.139183e-99	11	MANEA	ASC IgG
1.441361e-103	-1.389974	0.795	0.747	2.569803e-99	11	RAN	ASC IgG
3.790394e-103	-1.149079	0.386	0.515	6.757894e-99	11	C6orf48	ASC IgG
4.308076e-103	0.5357679	0.67	0.194	7.680869e-99	11	GNL3	ASC IgG
4.979952e-102	0.5816827	0.694	0.223	8.878756e-98	11	DSTN	ASC IgG
8.944387e-101	0.4914376	0.514	0.105	1.594695e-96	11	PGRMC2	ASC IgG
1.768822e-100	0.5270336	0.481	0.092	3.153633e-96	11	SEMA4A	ASC IgG
2.212783e-100	-0.8534543	0.963	0.939	3.945171e-96	11	H3F3A	ASC IgG

3.505805e-100	-1.158905	0.383	0.497	6.250499e-96	11	SP100	ASC IgG
4.010069e-100	0.5174313	0.749	0.281	7.149553e-96	11	ACADVL	ASC IgG
4.673779e-100	-0.948654	0.935	0.901	8.33288e-96	11	COX7C	ASC IgG
5.236417e-100	-1.073707	0.248	0.398	9.336008e-96	11	SYPL1	ASC IgG
9.005752e-100	-1.332861	0.817	0.767	1.605636e-95	11	HMGN1	ASC IgG
1.222157e-99	0.471202	0.303	0.037	2.178983e-95	11	GADD45A	ASC IgG
1.660815e-99	-1.097009	0.337	0.463	2.961068e-95	11	SWAP70	ASC IgG
1.707761e-99	0.4487351	0.492	0.09	3.044768e-95	11	CTSD	ASC IgG
2.729795e-99	0.4772659	0.53	0.114	4.866951e-95	11	LY96	ASC IgG
2.755176e-99	-1.104166	0.537	0.603	4.912203e-95	11	UBXN1	ASC IgG
5.441505e-99	0.4399001	0.466	0.086	9.70166e-95	11	SEC23B	ASC IgG
6.005034e-99	0.4165146	0.375	0.035	1.070638e-94	11	VSIR	ASC IgG
6.81674e-99	-1.015185	0.093	0.284	1.215357e-94	11	IFITM2	ASC IgG
9.306474e-99	-0.9475481	0.912	0.895	1.659251e-94	11	SLC25A6	ASC IgG
1.310523e-98	-1.123192	0.697	0.718	2.336531e-94	11	EIF4A2	ASC IgG
2.287662e-98	-1.027461	0.059	0.216	4.078673e-94	11	PLAC8	ASC IgG
5.492083e-98	0.4780892	0.69	0.21	9.791836e-94	11	SRP54	ASC IgG
1.445189e-97	-1.00982	0.082	0.257	2.576627e-93	11	FCMR	ASC IgG
2.204355e-97	-1.17162	0.593	0.636	3.930145e-93	11	GPX4	ASC IgG
2.980112e-97	-0.9516458	0.124	0.293	5.313241e-93	11	BCAS4	ASC IgG
1.781146e-96	0.6796389	0.39	0.077	3.175605e-92	11	LINC01480	ASC IgG
2.845626e-96	0.4149425	0.911	0.472	5.073467e-92	11	RPS27L	ASC IgG
5.763816e-96	-1.067075	0.852	0.797	1.027631e-91	11	SUMO2	ASC IgG
3.793404e-95	0.4352649	0.369	0.053	6.763261e-91	11	STT3A	ASC IgG
5.939218e-95	-0.9489831	0.084	0.249	1.058903e-90	11	RASGRP2	ASC IgG

2.965303e-94	0.5396163	0.687	0.242	5.286839e-90	11	C12orf57	ASC IgG
3.828551e-94	0.4125689	0.376	0.054	6.825924e-90	11	LARP1B	ASC IgG
4.181749e-94	0.3970011	0.324	0.034	7.45564e-90	11	CREB3L2	ASC IgG
4.617333e-94	-1.238028	0.185	0.357	8.232242e-90	11	TXNIP	ASC IgG
5.450417e-94	0.3523907	0.281	0.029	9.717548e-90	11	ZFAT	ASC IgG
6.458025e-94	0.3021229	0.871	0.451	1.151401e-89	11	CD63	ASC IgG
4.680594e-93	-0.743053	0.979	0.961	8.345032e-89	11	OAZ1	ASC IgG
5.789581e-93	-0.8335607	0.055	0.224	1.032224e-88	11	LY86	ASC IgG
8.724753e-93	0.4009978	0.385	0.055	1.555536e-88	11	SLC39A7	ASC IgG
1.048608e-92	0.361614	0.291	0.027	1.869562e-88	11	MCEE	ASC IgG
2.556112e-92	-1.092528	0.545	0.596	4.557292e-88	11	ST13	ASC IgG
5.165886e-92	0.4525761	0.447	0.077	9.210257e-88	11	PNOC	ASC IgG
1.294026e-91	0.4852163	0.699	0.226	2.30712e-87	11	ZBTB8OS	ASC IgG
3.450559e-91	-0.8192981	0.041	0.199	6.152001e-87	11	FAM129C	ASC IgG
1.372987e-90	0.4348031	0.607	0.156	2.447898e-86	11	ARL1	ASC IgG
1.556478e-90	-1.045063	0.267	0.402	2.775045e-86	11	BLOC1S2	ASC IgG
4.622596e-90	-1.072341	0.369	0.472	8.241626e-86	11	SNRPF	ASC IgG
4.688481e-90	0.4588821	0.618	0.166	8.359093e-86	11	MESD	ASC IgG
7.148707e-90	-0.7652082	0.033	0.2	1.274543e-85	11	AFF3	ASC IgG
2.347903e-89	0.3064808	0.158	0.011	4.186077e-85	11	CAMP	ASC IgG
2.370327e-89	0.4556798	0.523	0.142	4.226057e-85	11	MPC1	ASC IgG
4.026765e-89	0.4497485	0.602	0.162	7.17932e-85	11	ERGIC2	ASC IgG
6.988059e-89	0.4896627	0.652	0.17	1.245901e-84	11	PLPP5	ASC IgG
3.177337e-88	-1.080856	0.45	0.535	5.664874e-84	11	RBMX	ASC IgG
5.311538e-88	0.3000981	0.23	0.022	9.469942e-84	11	SLCO3A1	ASC IgG

8.796179e-88	-1.136882	0.233	0.387	1.568271e-83	11	CD44	ASC IgG
1.431367e-87	0.4871395	0.852	0.44	2.551985e-83	11	VCP	ASC IgG
2.284692e-87	-1.069566	0.545	0.579	4.073377e-83	11	POU2F2	ASC IgG
3.243543e-87	-1.02812	0.713	0.697	5.782914e-83	11	MYL12B	ASC IgG
5.277425e-87	-0.9503372	0.134	0.29	9.409121e-83	11	SP110	ASC IgG
6.947278e-87	0.4211664	0.486	0.115	1.23863e-82	11	ISOC2	ASC IgG
7.172842e-87	0.3938424	0.402	0.056	1.278846e-82	11	IFNAR2	ASC IgG
3.037018e-86	0.3981302	0.33	0.05	5.414699e-82	11	SLC1A4	ASC IgG
1.456537e-85	-1.053305	0.67	0.677	2.59686e-81	11	EIF3L	ASC IgG
2.29061e-85	-1.0935	0.595	0.621	4.083928e-81	11	ACTR3	ASC IgG
6.241228e-85	-1.153037	0.192	0.355	1.112749e-80	11	LY6E	ASC IgG
1.333585e-84	-0.9887701	0.314	0.405	2.377649e-80	11	MBD4	ASC IgG
1.478459e-84	-1.227452	0.806	0.76	2.635945e-80	11	PKM	ASC IgG
1.662441e-84	0.3604326	0.322	0.035	2.963966e-80	11	TMEM39A	ASC IgG
1.863106e-84	-0.8771037	0.086	0.237	3.321732e-80	11	IL4R	ASC IgG
2.219846e-84	0.4236402	0.593	0.169	3.957764e-80	11	NUDT22	ASC IgG
8.595613e-84	0.4301501	0.918	0.557	1.532512e-79	11	UQCRQ	ASC IgG
8.652909e-84	0.5056301	0.677	0.214	1.542727e-79	11	UBE2G1	ASC IgG
1.377304e-83	-0.8952857	0.174	0.318	2.455596e-79	11	LYN	ASC IgG
1.39753e-83	0.4093044	0.743	0.307	2.491657e-79	11	PRDX2	ASC IgG
1.468955e-83	-1.147338	0.705	0.693	2.618999e-79	11	EZR	ASC IgG
7.915163e-83	-0.8017175	0.036	0.178	1.411194e-78	11	MARCH1	ASC IgG
1.121396e-82	-1.104091	0.658	0.641	1.999338e-78	11	HNRNPM	ASC IgG
5.629174e-82	0.388731	0.759	0.3	1.003625e-77	11	AKR1A1	ASC IgG
5.801176e-82	-0.9832765	0.867	0.803	1.034292e-77	11	HNRNPK	ASC IgG

9.227868e-82	−0.8340849	0.074	0.215	1.645237e-77	11	FAM111B	ASC IgG
2.283868e-81	−0.9819888	0.19	0.346	4.071909e-77	11	PKIG	ASC IgG
2.289132e-81	0.412376	0.516	0.113	4.081293e-77	11	B4GALT3	ASC IgG
2.375686e-81	0.3164839	0.241	0.021	4.23561e-77	11	LAX1	ASC IgG
4.133447e-81	−1.059365	0.386	0.474	7.369523e-77	11	GPSM3	ASC IgG
4.98096e-81	−1.017413	0.329	0.428	8.880553e-77	11	CD40	ASC IgG
1.496903e-80	0.4368082	0.633	0.215	2.668828e-76	11	TIMM17A	ASC IgG
2.457515e-80	−0.9517545	0.265	0.368	4.381503e-76	11	SNAP23	ASC IgG
3.310965e-80	−0.88998	0.061	0.216	5.903119e-76	11	LBH	ASC IgG
3.76896e-80	−0.822127	0.956	0.927	6.71968e-76	11	EEF1D	ASC IgG
4.567253e-80	0.3707445	0.726	0.262	8.142955e-76	11	AUP1	ASC IgG
1.303632e-79	−0.9447158	0.317	0.423	2.324246e-75	11	FAM49B	ASC IgG
2.689868e-79	−0.9563102	0.055	0.225	4.795766e-75	11	GBP2	ASC IgG
4.643398e-79	0.4029124	0.496	0.109	8.278714e-75	11	USO1	ASC IgG
1.06358e-78	0.3870477	0.416	0.069	1.896256e-74	11	COPG1	ASC IgG
1.826232e-78	0.3174126	0.325	0.04	3.255989e-74	11	TBL2	ASC IgG
2.487519e-78	−1.041627	0.757	0.717	4.434997e-74	11	ATP5MC2	ASC IgG
3.115036e-78	−1.060925	0.538	0.56	5.553798e-74	11	YWHAZ	ASC IgG
7.331864e-78	−0.9464924	0.287	0.387	1.307198e-73	11	EVL	ASC IgG
1.071314e-77	0.3176853	0.207	0.019	1.910046e-73	11	SPINK2	ASC IgG
1.161305e-77	0.3815275	0.435	0.075	2.07049e-73	11	GUSB	ASC IgG
1.949237e-77	0.3922779	0.831	0.347	3.475294e-73	11	TPD52	ASC IgG
2.19274e-77	0.4192056	0.312	0.065	3.909436e-73	11	SOCS3	ASC IgG
2.219176e-77	−1.019959	0.717	0.668	3.956568e-73	11	SNX3	ASC IgG
2.403805e-77	0.4376777	0.547	0.135	4.285744e-73	11	SEC14L1	ASC IgG

3.056177e-77	0.3219637	0.232	0.032	5.448857e-73	11	KCNK12	ASC IgG
3.945856e-77	-0.8100668	0.068	0.21	7.035067e-73	11	PRKCB	ASC IgG
5.472828e-77	0.4003957	0.637	0.204	9.757506e-73	11	CANX	ASC IgG
5.5045e-77	-0.992714	0.277	0.366	9.813972e-73	11	RCSD1	ASC IgG
6.13546e-77	0.5199458	0.696	0.266	1.093891e-72	11	STK17B	ASC IgG
8.447595e-77	0.3265426	0.278	0.032	1.506122e-72	11	NEDD9	ASC IgG
1.086462e-76	-0.7950174	0.944	0.903	1.937053e-72	11	SRP14	ASC IgG
1.257843e-76	0.3712143	0.458	0.087	2.242608e-72	11	IKBIP	ASC IgG
2.572797e-76	0.3196532	0.279	0.026	4.587041e-72	11	PGM3	ASC IgG
2.789122e-76	0.3483953	0.348	0.054	4.972726e-72	11	UAP1	ASC IgG
3.641658e-76	0.4200704	0.692	0.254	6.492712e-72	11	GRN	ASC IgG
5.769103e-76	-1.111468	0.575	0.605	1.028573e-71	11	CDC42	ASC IgG
1.663963e-75	-0.8680355	0.184	0.315	2.966679e-71	11	PNRC2	ASC IgG
1.842164e-75	0.4233517	0.665	0.246	3.284395e-71	11	CDV3	ASC IgG
2.42867e-75	-1.582931	0.144	0.291	4.330076e-71	11	MIR155HG	ASC IgG
2.779481e-75	-1.003862	0.574	0.587	4.955537e-71	11	PRR13	ASC IgG
4.009558e-75	-1.167272	0.816	0.746	7.14864e-71	11	HSP90AA1	ASC IgG
1.5418e-74	0.3637333	0.356	0.057	2.748875e-70	11	MAGT1	ASC IgG
2.443597e-74	0.4072986	0.569	0.15	4.35669e-70	11	ST6GAL1	ASC IgG
2.712573e-74	0.311543	0.274	0.029	4.836247e-70	11	ACP2	ASC IgG
3.578775e-74	0.3900001	0.393	0.08	6.380599e-70	11	ATF6	ASC IgG
4.123253e-74	-1.091858	0.565	0.572	7.351348e-70	11	SNRPE	ASC IgG
5.783706e-74	0.3243588	0.291	0.046	1.031177e-69	11	CDKN2C	ASC IgG
9.903556e-74	-0.9997709	0.447	0.508	1.765705e-69	11	FDFT1	ASC IgG
1.379415e-73	-0.8894842	0.169	0.314	2.459359e-69	11	SERPINB1	ASC IgG

1.635279e-73	0.4092603	0.664	0.215	2.915539e-69	11	ADI1	ASC IgG
1.822829e-73	-0.9583169	0.291	0.395	3.249922e-69	11	FNBP1	ASC IgG
3.015514e-73	0.3926731	0.599	0.196	5.37636e-69	11	SND1	ASC IgG
1.472683e-71	-1.112899	0.712	0.679	2.625647e-67	11	SRSF3	ASC IgG
1.497389e-71	-0.8122556	0.98	0.976	2.669695e-67	11	FTH1	ASC IgG
4.064771e-71	-1.036158	0.591	0.61	7.24708e-67	11	RSL24D1	ASC IgG
1.172006e-70	-0.9866727	0.412	0.478	2.08957e-66	11	FUS	ASC IgG
1.395252e-70	-1.100932	0.851	0.772	2.487594e-66	11	HLA-DPB1	ASC IgG
1.438922e-70	-0.9370586	0.615	0.603	2.565454e-66	11	LSM7	ASC IgG
1.554622e-70	-0.928847	0.241	0.369	2.771736e-66	11	LRRFIP1	ASC IgG
1.880959e-70	-0.9445515	0.277	0.377	3.353561e-66	11	LCP1	ASC IgG
2.028828e-70	0.3927631	0.587	0.171	3.617197e-66	11	TRABD	ASC IgG
2.794721e-70	-0.7705916	0.12	0.252	4.982708e-66	11	OFD1	ASC IgG
3.618172e-70	-0.8885913	0.215	0.323	6.450839e-66	11	LTA4H	ASC IgG
1.644401e-69	-0.9954055	0.501	0.525	2.931803e-65	11	SYF2	ASC IgG
4.81352e-69	0.3700372	0.211	0.028	8.582024e-65	11	GPR137B	ASC IgG
1.248225e-68	-0.9753202	0.571	0.572	2.22546e-64	11	EIF3D	ASC IgG
2.206784e-68	0.344157	0.359	0.061	3.934474e-64	11	MAGED1	ASC IgG
3.113903e-68	0.3432484	0.946	0.7	5.551778e-64	11	COX7A2	ASC IgG
3.658794e-68	-1.036884	0.606	0.606	6.523265e-64	11	PPDPF	ASC IgG
4.499285e-68	-1.377893	0.777	0.713	8.021775e-64	11	ENO1	ASC IgG
8.156375e-68	-1.034506	0.594	0.602	1.4542e-63	11	ARF6	ASC IgG
1.468253e-67	-1.026691	0.201	0.276	2.617748e-63	11	KLF2	ASC IgG
1.646327e-67	-0.8207916	0.877	0.829	2.935237e-63	11	UQCRB	ASC IgG
6.179529e-67	0.3173601	0.369	0.066	1.101748e-62	11	YIPF2	ASC IgG

2.015133e-66	-0.9664059	0.812	0.744	3.592781e-62	11	SNRPD2	ASC IgG
3.862601e-66	-0.9036743	0.105	0.241	6.886631e-62	11	CAPG	ASC IgG
5.74423e-66	-0.7953383	0.188	0.292	1.024139e-61	11	RRAS2	ASC IgG
5.958631e-66	-0.6742207	0.078	0.189	1.062364e-61	11	CDCA7L	ASC IgG
5.969528e-66	-0.8929227	0.341	0.417	1.064307e-61	11	HDAC1	ASC IgG
8.89085e-66	-1.018288	0.358	0.448	1.58515e-61	11	KLF6	ASC IgG
1.03554e-65	-0.7769574	0.054	0.18	1.846265e-61	11	BCL2A1	ASC IgG
1.489243e-65	0.3371	0.462	0.112	2.655171e-61	11	SLC52A2	ASC IgG
3.52671e-65	-0.6262326	0.05	0.17	6.287772e-61	11	SNX10	ASC IgG
3.617857e-65	0.3564119	0.445	0.097	6.450277e-61	11	SEC31A	ASC IgG
3.786732e-65	0.3351197	0.337	0.057	6.751365e-61	11	MXD4	ASC IgG
3.94999e-65	-0.8420507	0.215	0.317	7.042437e-61	11	SPI1	ASC IgG
4.327177e-65	-1.174564	0.804	0.734	7.714923e-61	11	HSPA8	ASC IgG
7.385335e-65	0.3581254	0.769	0.318	1.316731e-60	11	CCM2	ASC IgG
1.061761e-64	-0.880367	0.226	0.321	1.893014e-60	11	SNHG7	ASC IgG
4.222812e-64	0.312664	0.413	0.087	7.528851e-60	11	ALG3	ASC IgG
5.828401e-64	-1.033453	0.661	0.615	1.039146e-59	11	SERBP1	ASC IgG
7.445257e-64	0.3369594	0.544	0.148	1.327415e-59	11	YIPF3	ASC IgG
8.010176e-64	0.3405011	0.746	0.32	1.428134e-59	11	SRP19	ASC IgG
8.57172e-64	-0.7933524	0.143	0.27	1.528252e-59	11	ALOX5AP	ASC IgG
9.676994e-64	0.3414524	0.453	0.108	1.725311e-59	11	GOSR2	ASC IgG
3.483977e-63	-0.949462	0.387	0.447	6.211582e-59	11	FBL	ASC IgG
4.598336e-63	0.3312073	0.676	0.238	8.198373e-59	11	IER3IP1	ASC IgG
7.358179e-63	-0.8696498	0.873	0.804	1.31189e-58	11	HINT1	ASC IgG
7.599148e-63	-1.113685	0.454	0.495	1.354852e-58	11	FXYD5	ASC IgG

7.785849e-63 0.3106586 0.253 0.033 1.388139e-58 11
MAN1A1 ASC IgG
1.389197e-62 -0.9486267 0.602 0.593 2.476799e-58 11
HNRNPU ASC IgG
1.696084e-62 -0.9348613 0.437 0.454 3.023947e-58 11
PTPRC ASC IgG
4.824144e-62 0.3214079 0.732 0.306 8.600966e-58 11
ERGIC3 ASC IgG
7.475582e-62 0.4535613 0.949 0.738 1.332822e-57 11
CHCHD2 ASC IgG
7.639499e-62 0.3416661 0.546 0.159 1.362046e-57 11
SRP68 ASC IgG
1.146395e-61 -0.8843836 0.351 0.399 2.043908e-57 11
NUCKS1 ASC IgG
1.361815e-61 0.3480679 0.481 0.133 2.42798e-57 11
GPAA1 ASC IgG
4.190532e-61 -0.9391667 0.118 0.252 7.4713e-57 11
TNFRSF13B ASC IgG
4.795395e-61 -0.6814353 0.044 0.175 8.54971e-57 11
BCL2 ASC IgG
5.756986e-61 0.3337287 0.751 0.319 1.026413e-56 11
COPZ1 ASC IgG
7.023786e-61 -0.945236 0.66 0.624 1.252271e-56 11
HNRNPA0 ASC IgG
9.355286e-61 0.3579929 0.613 0.21 1.667954e-56 11
COPB2 ASC IgG
1.029186e-60 0.3598708 0.519 0.155 1.834936e-56 11
DEF8 ASC IgG
1.448304e-60 -0.9848067 0.817 0.756 2.58218e-56 11
MT-ND5 ASC IgG
2.925423e-60 0.3286721 0.899 0.524 5.215737e-56 11
SRSF9 ASC IgG
2.938641e-60 -0.8660361 0.407 0.428 5.239304e-56 11
ANP32A ASC IgG
5.396581e-60 0.321818 0.509 0.134 9.621564e-56 11 TFG
ASC IgG
6.518614e-60 0.3089853 0.552 0.168 1.162204e-55 11
SDF4 ASC IgG
8.444625e-60 -0.8243327 0.298 0.37 1.505592e-55 11
MEAF6 ASC IgG
9.021818e-60 -0.9151253 0.215 0.33 1.6085e-55 11
PMAIP1 ASC IgG
1.047815e-59 0.3217718 0.541 0.157 1.86815e-55 11
SAR1A ASC IgG
1.756305e-59 -0.6990435 0.063 0.187 3.131316e-55 11
MBP ASC IgG
1.912709e-59 -0.6314642 0.073 0.178 3.410168e-55 11
QRSL1 ASC IgG
2.457096e-59 0.3118679 0.503 0.134 4.380756e-55 11
WDR45 ASC IgG

2.830537e-59	-0.7132231	0.148	0.25	5.046565e-55	11	CSK	ASC IgG
5.385677e-59	-0.8005972	0.245	0.343	9.602124e-55	11	EIF2S3	ASC IgG
6.287156e-59	0.3768888	0.705	0.253	1.120937e-54	11	FAM3C	ASC IgG
7.23107e-59	0.3141155	0.469	0.109	1.289228e-54	11	ATP6AP1	ASC IgG
4.186316e-58	-0.983593	0.397	0.441	7.463782e-54	11	HMGA1	ASC IgG
1.25289e-57	-0.7634024	0.173	0.274	2.233777e-53	11	SMS	ASC IgG
3.823554e-57	-0.8900313	0.285	0.34	6.817015e-53	11	STK17A	ASC IgG
3.854725e-57	0.3147431	0.481	0.13	6.872589e-53	11	TMEM205	ASC IgG
4.01984e-57	-1.157134	0.626	0.587	7.166974e-53	11	MARCKSL1	ASC IgG
4.548268e-57	-0.8979249	0.752	0.676	8.109107e-53	11	MORF4L1	ASC IgG
4.600432e-57	-0.8304114	0.232	0.321	8.20211e-53	11	FCRLA	ASC IgG
8.127123e-57	0.329009	0.696	0.289	1.448985e-52	11	SRP72	ASC IgG
1.890517e-56	-0.6269479	0.085	0.204	3.370603e-52	11	NMI	ASC IgG
3.885258e-56	-0.6443458	0.037	0.151	6.927026e-52	11	ARRDC2	ASC IgG
6.094804e-56	-0.8648868	0.435	0.467	1.086643e-51	11	ENSA	ASC IgG
7.028475e-56	-0.9533188	0.075	0.201	1.253107e-51	11	ACP5	ASC IgG
7.351252e-56	-0.7680477	0.162	0.261	1.310655e-51	11	CNN2	ASC IgG
8.585572e-56	-0.9298137	0.498	0.489	1.530722e-51	11	CBX3	ASC IgG
9.00598e-56	-0.9118539	0.253	0.329	1.605676e-51	11	PTPN6	ASC IgG
1.03845e-55	0.3265777	0.587	0.196	1.851453e-51	11	MRPS31	ASC IgG
3.573976e-55	-0.6851947	0.099	0.205	6.372042e-51	11	BIN1	ASC IgG
5.557141e-55	-0.5462454	0.029	0.129	9.907827e-51	11	ZEB2	ASC IgG
6.057513e-55	-1.450304	0.173	0.266	1.079994e-50	11	S100A4	ASC IgG
8.449492e-55	0.3944023	0.821	0.475	1.50646e-50	11	NEAT1	ASC IgG
2.260107e-54	-0.6117032	0.066	0.18	4.029545e-50	11	STAT6	ASC IgG

2.91869e-54	-0.927198	0.737	0.661	5.203732e-50	11	DBI	ASC IgG
2.962158e-54	-0.7474746	0.124	0.24	5.281231e-50	11	AC004687.1	ASC IgG
3.178115e-54	0.3035924	0.444	0.113	5.666262e-50	11	SCYL1	ASC IgG
3.376182e-54	0.313118	0.624	0.234	6.019395e-50	11	MRPL55	ASC IgG
4.094436e-54	-0.8859416	0.344	0.349	7.299969e-50	11	S100A10	ASC IgG
4.132421e-54	-0.9156733	0.634	0.586	7.367693e-50	11	HNRNPF	ASC IgG
1.405423e-53	-0.8236052	0.224	0.299	2.505728e-49	11	PDLIM1	ASC IgG
1.507178e-53	-0.8327864	0.352	0.403	2.687149e-49	11	RPA3	ASC IgG
1.761821e-53	-0.681346	0.151	0.243	3.14115e-49	11	MCUB	ASC IgG
1.76521e-53	-0.9277692	0.599	0.564	3.147193e-49	11	SNRPB	ASC IgG
2.031577e-53	-0.6704037	0.139	0.251	3.622099e-49	11	HNRNPA1P48	ASC IgG
4.805875e-53	0.4199187	0.534	0.138	8.568395e-49	11	BIK	ASC IgG
4.912614e-53	-0.6201632	0.042	0.152	8.7587e-49	11	CELF2	ASC IgG
5.262711e-53	-0.8916858	0.278	0.347	9.382887e-49	11	CAST	ASC IgG
7.577689e-53	-0.6981945	0.148	0.244	1.351026e-48	11	ARHGAP45	ASC IgG
8.678067e-53	-0.5784002	0.032	0.131	1.547213e-48	11	CD24	ASC IgG
1.2529e-52	0.3183622	0.394	0.118	2.233796e-48	11	SLC1A5	ASC IgG
1.570267e-52	0.322047	0.552	0.177	2.799629e-48	11	REXO2	ASC IgG
2.113621e-52	-0.5450266	0.037	0.145	3.768374e-48	11	NUB1	ASC IgG
2.497346e-52	-0.8534629	0.671	0.612	4.452518e-48	11	ARPC5	ASC IgG
2.763945e-52	0.3046786	0.77	0.316	4.927838e-48	11	GYPC	ASC IgG
9.305551e-52	0.3108162	0.418	0.091	1.659087e-47	11	FBH1	ASC IgG
1.116452e-51	-0.6119816	0.11	0.22	1.990523e-47	11	HPRT1	ASC IgG
1.157331e-51	0.309439	0.515	0.167	2.063406e-47	11	LAP3	ASC IgG
2.297684e-51	-0.8365362	0.486	0.465	4.09654e-47	11	SF1	ASC IgG

2.898101e-51	-0.8440807	0.34	0.398	5.167024e-47	11	CIB1	ASC IgG
6.697819e-51	-0.7889952	0.298	0.35	1.194154e-46	11	NASP	ASC IgG
1.289522e-50	-0.7265827	0.288	0.347	2.299089e-46	11	LSM6	ASC IgG
2.046114e-50	0.3084609	0.306	0.065	3.648016e-46	11	FBXW7	ASC IgG
2.480518e-50	-0.8519366	0.679	0.62	4.422515e-46	11	ARPC1B	ASC IgG
2.748494e-50	-0.9168251	0.713	0.643	4.90029e-46	11	HNRNPC	ASC IgG
2.972406e-50	0.3555285	0.188	0.03	5.299502e-46	11	AREG	ASC IgG
3.202494e-50	-0.9696949	0.452	0.448	5.709726e-46	11	PNRC1	ASC IgG
4.624814e-50	-0.7598963	0.214	0.288	8.24558e-46	11	PLEKHF2	ASC IgG
5.137684e-50	-0.7963154	0.342	0.392	9.159977e-46	11	ARPC4	ASC IgG
7.568772e-50	-0.5584027	0.096	0.186	1.349436e-45	11	APBB1IP	ASC IgG
7.782671e-50	-0.7430796	0.104	0.198	1.387572e-45	11	CHPT1	ASC IgG
1.74076e-49	-0.8095091	0.531	0.515	3.1036e-45	11	CAPZB	ASC IgG
2.276262e-49	-0.6786662	0.118	0.22	4.058347e-45	11	NFKBIE	ASC IgG
2.421623e-49	-0.8588634	0.676	0.617	4.317511e-45	11	CLEC2D	ASC IgG
3.055657e-49	-0.6658189	0.143	0.235	5.44793e-45	11	ZNF581	ASC IgG
5.567637e-49	-0.5896491	0.038	0.125	9.926541e-45	11	AL139020.1	ASC IgG
7.370194e-49	-0.8857871	0.228	0.298	1.314032e-44	11	NFKBIA	ASC IgG
7.475755e-49	-0.4998539	0.017	0.114	1.332852e-44	11	SESN3	ASC IgG
1.223919e-48	-0.7730107	0.249	0.322	2.182125e-44	11	RHOG	ASC IgG
1.858819e-48	-0.866641	0.796	0.696	3.314088e-44	11	CCNI	ASC IgG
2.500662e-48	-0.5047394	0.071	0.177	4.458429e-44	11	NUDT1	ASC IgG
4.479305e-48	-0.7565374	0.335	0.357	7.986152e-44	11	RAB11A	ASC IgG
1.67166e-47	-1.103956	0.465	0.455	2.980403e-43	11	JUNB	ASC IgG
1.843247e-47	-0.7416146	0.936	0.868	3.286325e-43	11	ARPC2	ASC IgG

1.859524e-47	-0.6609577	0.101	0.192	3.315345e-43	11	IFNGR1	ASC IgG
3.089862e-47	-0.9887346	0.527	0.483	5.508915e-43	11	RANBP1	ASC IgG
3.406315e-47	-0.7073148	0.246	0.311	6.07312e-43	11	PGLS	ASC IgG
4.395984e-47	-0.8850708	0.298	0.336	7.8376e-43	11	DUT	ASC IgG
5.559515e-47	-0.7797214	0.926	0.88	9.91206e-43	11	MT-ND1	ASC IgG
1.262633e-46	-0.7587885	0.256	0.315	2.251148e-42	11	ATP2B1	ASC IgG
1.742332e-46	-0.7152059	0.211	0.292	3.106403e-42	11	DCTPP1	ASC IgG
2.023556e-46	-0.788004	0.279	0.338	3.607798e-42	11	ACAT2	ASC IgG
2.111228e-46	-0.7580157	0.334	0.367	3.764108e-42	11	HADHA	ASC IgG
3.152706e-46	-0.8707286	0.711	0.624	5.62096e-42	11	SELENOH	ASC IgG
3.268143e-46	-0.9119871	0.461	0.452	5.826773e-42	11	ARL6IP1	ASC IgG
4.246977e-46	-0.4862163	0.038	0.133	7.571935e-42	11	UVRAG	ASC IgG
5.030201e-46	-0.5271159	0.022	0.108	8.968346e-42	11	C1orf162	ASC IgG
5.284075e-46	-0.8008827	0.454	0.445	9.420977e-42	11	YWHAQ	ASC IgG
6.318312e-46	-1.640343	0.295	0.303	1.126492e-41	11	HMGB2	ASC IgG
8.604395e-46	0.300216	0.413	0.114	1.534078e-41	11	LMO4	ASC IgG
1.201356e-45	-0.4336557	0.019	0.107	2.141897e-41	11	TRAF5	ASC IgG
1.347652e-45	-0.7988124	0.458	0.439	2.402729e-41	11	RHOH	ASC IgG
2.319874e-45	-0.8753754	0.654	0.592	4.136103e-41	11	TBCA	ASC IgG
7.662173e-45	-0.6268015	0.057	0.155	1.366089e-40	11	NFKBID	ASC IgG
9.734693e-45	-0.7078249	0.231	0.291	1.735598e-40	11	PFDN4	ASC IgG
1.189812e-44	-0.6017076	0.067	0.16	2.121315e-40	11	TGIF1	ASC IgG
1.210059e-44	-0.7604031	0.268	0.298	2.157415e-40	11	GGA2	ASC IgG
1.829405e-44	-0.8515018	0.421	0.422	3.261646e-40	11	APEX1	ASC IgG
2.090582e-44	-0.8510643	0.424	0.428	3.727298e-40	11	PPP1R15A	ASC IgG

2.973901e-44	-0.6748713	0.96	0.904	5.302169e-40	11	ATP5F1E	ASC IgG
2.989437e-44	-0.918788	0.769	0.681	5.329868e-40	11	SLC25A3	ASC IgG
3.186041e-44	-0.8580689	0.67	0.594	5.680392e-40	11	XRCC6	ASC IgG
4.438549e-44	-0.7025494	0.252	0.298	7.913489e-40	11	IFT57	ASC IgG
6.818197e-44	-0.7976098	0.398	0.407	1.215616e-39	11	BANF1	ASC IgG
1.231795e-43	-0.7931479	0.446	0.435	2.196167e-39	11	KHDRBS1	ASC IgG
1.619243e-43	-0.8417541	0.622	0.567	2.886948e-39	11	PCBP2	ASC IgG
1.881707e-43	-0.5063207	0.032	0.115	3.354895e-39	11	P2RY10	ASC IgG
2.064427e-43	-0.5387596	0.107	0.195	3.680667e-39	11	GGCT	ASC IgG
2.117798e-43	-0.6374984	0.161	0.24	3.775822e-39	11	NT5C	ASC IgG
3.031061e-43	-0.8787809	0.434	0.417	5.404078e-39	11	METAP2	ASC IgG
3.906055e-43	-0.495886	0.046	0.127	6.964106e-39	11	AC025164.1	ASC IgG
4.007034e-43	-0.8107495	0.302	0.337	7.144141e-39	11	APOBEC3C	ASC IgG
4.307778e-43	-0.5219124	0.035	0.111	7.680338e-39	11	LINC02397	ASC IgG
4.789112e-43	-0.80116	0.786	0.695	8.538508e-39	11	RHOA	ASC IgG
6.978065e-43	-0.8199828	0.396	0.389	1.244119e-38	11	ARL6IP5	ASC IgG
8.15598e-43	-0.6172653	0.154	0.233	1.45413e-38	11	TAF15	ASC IgG
1.086902e-42	-0.9105319	0.647	0.582	1.937838e-38	11	ANP32B	ASC IgG
1.470689e-42	-0.7658985	0.302	0.344	2.622092e-38	11	CMTM6	ASC IgG
2.029582e-42	-0.9404875	0.331	0.361	3.618541e-38	11	ARID5B	ASC IgG
3.419847e-42	0.6945342	0.135	0.018	6.097245e-38	11	IGLV3-21	ASC IgG
7.511152e-42	-0.6654436	0.115	0.196	1.339163e-37	11	HSPB1	ASC IgG
1.572528e-41	-0.8043435	0.697	0.63	2.80366e-37	11	EIF3H	ASC IgG
2.427143e-41	0.3010818	0.667	0.317	4.327353e-37	11	SLC3A2	ASC IgG
2.73951e-41	-0.8072078	0.583	0.524	4.884272e-37	11	VAMP8	ASC IgG

2.896341e-41	-0.7158003	0.252	0.285	5.163887e-37	11	CD82	ASC IgG
3.606072e-41	-0.445722	0.023	0.106	6.429267e-37	11	RMI2	ASC IgG
3.957134e-41	-0.5667798	0.105	0.182	7.055175e-37	11	LYL1	ASC IgG
4.80012e-41	-0.6746719	0.209	0.271	8.558135e-37	11	MITD1	ASC IgG
6.054243e-41	-0.7306531	0.291	0.313	1.079411e-36	11	PSIP1	ASC IgG
2.102594e-40	-0.7889983	0.459	0.421	3.748715e-36	11	TMEM123	ASC IgG
2.905581e-40	-0.7806399	0.565	0.513	5.180361e-36	11	UBE2I	ASC IgG
3.001668e-40	-0.7761825	0.749	0.675	5.351674e-36	11	HNRNPA3	ASC IgG
3.213172e-40	-0.4563606	0.044	0.116	5.728765e-36	11	LGALS9	ASC IgG
3.35827e-40	-0.7577597	0.459	0.452	5.987459e-36	11	PCNP	ASC IgG
5.175796e-40	-0.5121265	0.041	0.123	9.227927e-36	11	LPP	ASC IgG
5.709492e-40	-0.6752188	0.124	0.201	1.017945e-35	11	PPP3CA	ASC IgG
9.078654e-40	-0.8964638	0.337	0.353	1.618633e-35	11	TUBB4B	ASC IgG
1.089862e-39	-0.4880366	0.025	0.103	1.943115e-35	11	RHOC	ASC IgG
2.377064e-39	-0.7892803	0.434	0.429	4.238068e-35	11	NSA2	ASC IgG
2.419047e-39	-0.6260006	0.154	0.221	4.312918e-35	11	CCDC50	ASC IgG
2.630626e-39	-0.3718337	0.029	0.113	4.690144e-35	11	CENPH	ASC IgG
4.374762e-39	-0.9130836	0.789	0.653	7.799763e-35	11	SLC25A5	ASC IgG
7.836409e-39	-0.76616	0.731	0.632	1.397153e-34	11	BRK1	ASC IgG
8.295833e-39	-0.7045797	0.133	0.2	1.479064e-34	11	YBX3	ASC IgG
8.667827e-39	-0.7993844	0.675	0.6	1.545387e-34	11	TRIR	ASC IgG
1.120129e-38	-0.689964	0.273	0.302	1.997078e-34	11	ARID4B	ASC IgG
1.165716e-38	-0.7076746	0.282	0.316	2.078355e-34	11	STRBP	ASC IgG
1.476379e-38	-0.5271553	0.082	0.15	2.632236e-34	11	ALOX5	ASC IgG
1.891471e-38	-0.7353193	0.847	0.731	3.372304e-34	11	SKP1	ASC IgG

2.314843e-38	-0.4868605	0.046	0.122	4.127133e-34	11	IL27RA	ASC IgG
2.673543e-38	-0.5677262	0.126	0.203	4.766659e-34	11	RELB	ASC IgG
3.558142e-38	-0.7425498	0.455	0.44	6.343812e-34	11	ACTR2	ASC IgG
4.454978e-38	-0.3957422	0.029	0.103	7.94278e-34	11	BACH2	ASC IgG
5.491153e-38	-0.6289367	0.09	0.166	9.790177e-34	11	MARCKS	ASC IgG
5.797815e-38	-0.6623288	0.131	0.197	1.033692e-33	11	CLEC2B	ASC IgG
6.772509e-38	-0.7627619	0.375	0.377	1.207471e-33	11	ZFAND6	ASC IgG
1.146582e-37	-0.582382	0.147	0.221	2.044241e-33	11	PCM1	ASC IgG
1.519263e-37	-0.6110557	0.215	0.259	2.708694e-33	11	ABI1	ASC IgG
3.302844e-37	-0.5315024	0.128	0.191	5.888641e-33	11	NUP88	ASC IgG
3.419354e-37	-0.7889209	0.534	0.477	6.096366e-33	11	CD47	ASC IgG
4.143518e-37	-0.8004049	0.538	0.488	7.387478e-33	11	CCT3	ASC IgG
4.19703e-37	0.3954806	0.215	0.044	7.482885e-33	11	PLD4	ASC IgG
5.088041e-37	-0.680891	0.315	0.334	9.071469e-33	11	BAX	ASC IgG
4.012189e-36	-0.6763317	0.227	0.263	7.153332e-32	11	DAPP1	ASC IgG
4.244421e-36	-0.5520418	0.139	0.2	7.567378e-32	11	MCM3	ASC IgG
5.282704e-36	-0.6050292	0.162	0.222	9.418533e-32	11	TRAF4	ASC IgG
7.958923e-36	-0.7725322	0.419	0.388	1.418996e-31	11	CCT5	ASC IgG
8.056778e-36	-0.6569713	0.221	0.279	1.436443e-31	11	MSN	ASC IgG
8.614062e-36	-0.428079	0.036	0.103	1.535801e-31	11	CDKN2D	ASC IgG
1.685611e-35	-0.8053615	0.884	0.798	3.005276e-31	11	LAPTM5	ASC IgG
1.846195e-35	-0.6798001	0.431	0.411	3.29158e-31	11	MOB1A	ASC IgG
2.160371e-35	-0.7011577	0.366	0.368	3.851725e-31	11	CACYBP	ASC IgG
2.51425e-35	-0.5325056	0.143	0.225	4.482656e-31	11	RPIA	ASC IgG
2.807357e-35	-0.6372936	0.295	0.3	5.005237e-31	11	ITSN2	ASC IgG

2.94161e-35	-0.5721011	0.12	0.188	5.244597e-31	11	KIF20B	ASC IgG
3.264234e-35	-0.4360499	0.063	0.13	5.819802e-31	11	NFATC1	ASC IgG
3.998434e-35	-0.7564053	0.833	0.72	7.128808e-31	11	SRSF5	ASC IgG
4.887843e-35	-0.4973353	0.089	0.156	8.714535e-31	11	TCOF1	ASC IgG
8.177623e-35	-0.6772348	0.392	0.379	1.457988e-30	11	POLR2G	ASC IgG
1.048445e-34	-0.6340264	0.256	0.284	1.869273e-30	11	MTPN	ASC IgG
1.309951e-34	-0.6777597	0.267	0.292	2.335512e-30	11	YWHAH	ASC IgG
1.643654e-34	-0.7206819	0.446	0.42	2.930471e-30	11	MAGOH	ASC IgG
1.692467e-34	-0.7698113	0.672	0.563	3.0175e-30	11	RAC1	ASC IgG
1.811251e-34	-0.6173122	0.169	0.228	3.229279e-30	11	MCM5	ASC IgG
1.885006e-34	-0.7708001	0.471	0.442	3.360778e-30	11	CCT6A	ASC IgG
1.893225e-34	-0.7659058	0.455	0.424	3.375431e-30	11	BLOC1S1	ASC IgG
2.710552e-34	-0.4997079	0.09	0.167	4.832642e-30	11	NFKB1	ASC IgG
2.908141e-34	-0.7288535	0.54	0.476	5.184924e-30	11	UBE2N	ASC IgG
4.011404e-34	-0.462047	0.04	0.107	7.151933e-30	11	MTSS1	ASC IgG
5.702044e-34	-0.5940548	0.205	0.24	1.016617e-29	11	SP140	ASC IgG
6.488886e-34	-0.42527	0.083	0.153	1.156904e-29	11	CLECL1	ASC IgG
7.806689e-34	-0.9111449	0.593	0.51	1.391855e-29	11	PA2G4	ASC IgG
1.40762e-33	-0.7237634	0.439	0.39	2.509645e-29	11	SH3BGRL	ASC IgG
1.697777e-33	-0.8422933	0.482	0.472	3.026967e-29	11	SNHG8	ASC IgG
1.764165e-33	-0.7437902	0.584	0.512	3.14533e-29	11	DNAJC8	ASC IgG
2.879114e-33	-0.7332341	0.492	0.434	5.133173e-29	11	DAZAP2	ASC IgG
3.422505e-33	-0.4117743	0.057	0.125	6.101984e-29	11	MAPK1	ASC IgG
4.488902e-33	-0.4422875	0.071	0.154	8.003263e-29	11	PEA15	ASC IgG
4.537155e-33	-0.7527159	0.716	0.612	8.089294e-29	11	PNISR	ASC IgG

5.519777e-33	-0.6836892	0.346	0.345	9.841211e-29	11	PNN	ASC IgG
7.927374e-33	-0.6262777	0.255	0.294	1.413371e-28	11	SSRP1	ASC IgG
9.193435e-33	-0.6375895	0.19	0.255	1.639098e-28	11	RRP7A	ASC IgG
1.029998e-32	-0.7143816	0.462	0.42	1.836383e-28	11	SCAF11	ASC IgG
1.073777e-32	-0.5936626	0.241	0.27	1.914436e-28	11	STMP1	ASC IgG
1.086136e-32	-0.6199836	0.261	0.284	1.936472e-28	11	LAT2	ASC IgG
1.117409e-32	0.3326936	0.106	0.011	1.992228e-28	11	IGLV3-1	ASC IgG
1.136317e-32	-0.737021	0.455	0.427	2.025939e-28	11	CCT4	ASC IgG
1.249273e-32	-0.6694893	0.351	0.355	2.227328e-28	11	KPNB1	ASC IgG
1.299916e-32	-0.6652782	0.285	0.298	2.31762e-28	11	RNASEH2B	ASC IgG
1.663718e-32	-0.5252749	0.143	0.193	2.966243e-28	11	CNTRL	ASC IgG
1.708064e-32	-0.8155241	0.394	0.385	3.045308e-28	11	NHP2	ASC IgG
1.811651e-32	-0.5880108	0.188	0.229	3.229992e-28	11	PCSK7	ASC IgG
2.056908e-32	-0.8074732	0.762	0.612	3.667261e-28	11	MEF2C	ASC IgG
4.930427e-32	-0.5212114	0.097	0.153	8.790457e-28	11	CD72	ASC IgG
4.94849e-32	-0.4879305	0.086	0.156	8.822662e-28	11	RILPL2	ASC IgG
5.024005e-32	-0.6175286	0.056	0.139	8.957299e-28	11	FCER2	ASC IgG
5.348118e-32	-0.5328933	0.127	0.185	9.53516e-28	11	INPP5D	ASC IgG
7.618553e-32	-0.5585783	0.125	0.186	1.358312e-27	11	FKBP4	ASC IgG
8.920874e-32	-0.566178	0.178	0.213	1.590503e-27	11	WASHC4	ASC IgG
8.926961e-32	-0.6345663	0.249	0.278	1.591588e-27	11	CCNG1	ASC IgG
1.014571e-31	-0.6993074	0.347	0.333	1.808879e-27	11	BTK	ASC IgG
1.616874e-31	-0.7947079	0.875	0.756	2.882725e-27	11	PSMA7	ASC IgG
2.072721e-31	-0.62789	0.291	0.303	3.695454e-27	11	HNRNPH3	ASC IgG
2.76061e-31	-0.4107295	0.032	0.107	4.921891e-27	11	SCIMP	ASC IgG

3.509957e-31	-0.5571162	0.107	0.172	6.257903e-27	11	PARP14	ASC IgG
3.559588e-31	-0.4604675	0.089	0.146	6.34639e-27	11	GNB5	ASC IgG
3.62156e-31	-0.5520891	0.201	0.236	6.456879e-27	11	KRAS	ASC IgG
3.694193e-31	-0.3845033	0.037	0.101	6.586376e-27	11	TRIM22	ASC IgG
4.320858e-31	-0.5366061	0.181	0.207	7.703658e-27	11	CAT	ASC IgG
5.004937e-31	-0.552209	0.181	0.221	8.923302e-27	11	NELFCD	ASC IgG
5.716607e-31	-0.7161082	0.094	0.174	1.019214e-26	11	TNFRSF18	ASC IgG
6.235884e-31	-0.3758439	0.055	0.129	1.111796e-26	11	RCC1	ASC IgG
7.73088e-31	-0.364348	0.058	0.116	1.378339e-26	11	PAX5	ASC IgG
7.744119e-31	-0.4672708	0.132	0.183	1.380699e-26	11	MED30	ASC IgG
8.750335e-31	-0.6632102	0.352	0.345	1.560097e-26	11	DNAJB6	ASC IgG
8.772304e-31	-0.5317628	0.165	0.205	1.564014e-26	11	ELOVL5	ASC IgG
8.792693e-31	-0.4559861	0.112	0.174	1.567649e-26	11	SNX8	ASC IgG
1.156328e-30	-0.642812	0.225	0.252	2.061617e-26	11	SDCBP	ASC IgG
1.268244e-30	-0.6012096	0.256	0.268	2.261152e-26	11	MBNL1	ASC IgG
1.365054e-30	-1.014578	0.845	0.692	2.433756e-26	11	H2AFZ	ASC IgG
1.685957e-30	-0.5777392	0.217	0.245	3.005893e-26	11	CCDC115	ASC IgG
1.945837e-30	-0.6165955	0.276	0.29	3.469233e-26	11	RPA2	ASC IgG
2.371496e-30	-0.7353193	0.447	0.416	4.228141e-26	11	DDX39A	ASC IgG
2.431416e-30	-0.5811158	0.247	0.274	4.334972e-26	11	OLA1	ASC IgG
2.566198e-30	-0.590877	0.238	0.254	4.575274e-26	11	PRMT2	ASC IgG
3.345533e-30	-0.4353061	0.077	0.129	5.964751e-26	11	CCNG2	ASC IgG
3.448802e-30	-0.5161782	0.101	0.156	6.148869e-26	11	MCM7	ASC IgG
3.596838e-30	-0.4543072	0.12	0.181	6.412803e-26	11	CBX1	ASC IgG
3.656403e-30	-0.5870752	0.271	0.29	6.519e-26	11	MED28	ASC IgG

4.775254e-30	0.3427089	0.341	0.125	8.5138e-26	11	PHGDH	ASC IgG
5.506267e-30	-0.6072908	0.227	0.252	9.817123e-26	11	DNPH1	ASC IgG
5.801548e-30	-0.5456744	0.113	0.181	1.034358e-25	11	RHOF	ASC IgG
5.961493e-30	-0.5515321	0.163	0.21	1.062875e-25	11	LARS	ASC IgG
7.098476e-30	-0.6954915	0.403	0.39	1.265587e-25	11	EIF4B	ASC IgG
7.456439e-30	-0.7650676	0.48	0.427	1.329408e-25	11	CCT2	ASC IgG
9.84248e-30	-0.4513481	0.093	0.141	1.754816e-25	11	ADD3	ASC IgG
1.001676e-29	-0.6209301	0.279	0.3	1.785888e-25	11	CAMLG	ASC IgG
1.181545e-29	-0.5385891	0.211	0.251	2.106576e-25	11	SUPT16H	ASC IgG
1.418959e-29	-0.630272	0.348	0.323	2.529862e-25	11	AES	ASC IgG
1.457033e-29	-0.4685231	0.089	0.141	2.597743e-25	11	RAB29	ASC IgG
1.661212e-29	-0.7012862	0.361	0.32	2.961775e-25	11	TNFRSF13C	ASC IgG
1.961414e-29	-0.3737042	0.044	0.109	3.497005e-25	11	UBAC2	ASC IgG
2.087792e-29	-0.4041681	0.047	0.111	3.722324e-25	11	LPAR5	ASC IgG
2.151943e-29	-0.6763438	0.458	0.404	3.836699e-25	11	CAPZA2	ASC IgG
2.220836e-29	-0.7013633	0.842	0.698	3.959529e-25	11	SON	ASC IgG
4.981058e-29	-0.5635757	0.198	0.223	8.880727e-25	11	GCA	ASC IgG
5.269394e-29	-0.5516676	0.148	0.186	9.394803e-25	11	ANKRD44	ASC IgG
5.41988e-29	-0.6156726	0.253	0.266	9.663104e-25	11	WSB1	ASC IgG
5.447625e-29	-0.5773049	0.17	0.203	9.71257e-25	11	TRIM38	ASC IgG
5.760804e-29	-0.5522773	0.196	0.223	1.027094e-24	11	CDC40	ASC IgG
6.833771e-29	-0.62647	0.333	0.321	1.218393e-24	11	PRKAR1A	ASC IgG
7.655657e-29	-0.6897542	0.467	0.418	1.364927e-24	11	TOMM22	ASC IgG
1.15269e-28	-0.6273907	0.301	0.309	2.055131e-24	11	DYNC1I2	ASC IgG
1.244632e-28	-0.5775867	0.108	0.168	2.219054e-24	11	CBX6	ASC IgG

1.27595e-28	-0.5912156	0.241	0.265	2.274891e-24	11	VASP
ASC IgG						
1.301647e-28	-0.4988858	0.15	0.198	2.320707e-24	11	ATIC
ASC IgG						
1.582052e-28	-0.6673701	0.401	0.375	2.82064e-24	11	NONO
ASC IgG						
1.684668e-28	-0.733934	0.393	0.375	3.003594e-24	11	TAF1D
ASC IgG						
1.737717e-28	-0.4180373	0.084	0.136	3.098176e-24	11	PLEKHA2
ASC IgG						
2.177495e-28	-0.7181379	0.635	0.528	3.882256e-24	11	PAIP2
ASC IgG						
2.391888e-28	-0.7633781	0.778	0.63	4.264498e-24	11	PSMA4
ASC IgG						
3.233328e-28	-0.6786833	0.466	0.421	5.7647e-24	11	GPBP1
ASC IgG						
4.038942e-28	-0.7497995	0.07	0.132	7.20103e-24	11	MYC
ASC IgG						
4.123036e-28	-0.595894	0.287	0.297	7.35096e-24	11	TWF2
ASC IgG						
4.428279e-28	-0.6747066	0.395	0.329	7.895179e-24	11	GRHPR
ASC IgG						
4.772262e-28	-0.6824425	0.39	0.35	8.508466e-24	11	APOBEC3G
ASC IgG						
6.833667e-28	-0.7152527	0.792	0.653	1.218374e-23	11	RBX1
ASC IgG						
1.126213e-27	-0.5126297	0.165	0.209	2.007925e-23	11	PITHD1
ASC IgG						
1.574811e-27	-0.6866871	0.528	0.457	2.807731e-23	11	RAP1B
ASC IgG						
1.66175e-27	-0.6675548	0.703	0.634	2.962734e-23	11	CD53
ASC IgG						
1.688267e-27	-0.8115251	0.708	0.591	3.010012e-23	11	SRSF2
ASC IgG						
2.008009e-27	-0.4934517	0.102	0.151	3.58008e-23	11	ESYT1
ASC IgG						
2.159455e-27	-0.5984697	0.309	0.3	3.850093e-23	11	POLE4
ASC IgG						
2.161638e-27	-0.6629719	0.465	0.416	3.853984e-23	11	ELF1
ASC IgG						
2.191418e-27	-0.5460919	0.15	0.203	3.907078e-23	11	NPM3
ASC IgG						
2.380638e-27	-0.6364887	0.363	0.341	4.24444e-23	11	PDCD10
ASC IgG						
2.441286e-27	-0.521596	0.17	0.195	4.35257e-23	11	ADK
ASC IgG						
3.109905e-27	-0.4958286	0.123	0.172	5.54465e-23	11	MFSD10
ASC IgG						
3.226713e-27	-0.7023483	0.464	0.418	5.752906e-23	11	EIF6
ASC IgG						

4.384592e-27	-0.4352045	0.083	0.135	7.81729e-23	11	TTF1	ASC IgG
5.087352e-27	-0.5087935	0.105	0.146	9.07024e-23	11	SUN2	ASC IgG
5.811787e-27	-0.6310357	0.381	0.36	1.036183e-22	11	TKT	ASC IgG
6.07943e-27	-0.7025342	0.638	0.558	1.083901e-22	11	UXT	ASC IgG
8.870844e-27	-0.4763467	0.111	0.17	1.581583e-22	11	TNIP2	ASC IgG
9.417403e-27	-0.5719037	0.207	0.236	1.679029e-22	11	CNPPD1	ASC IgG
9.976638e-27	-0.5678591	0.237	0.269	1.778735e-22	11	JUN	ASC IgG
1.02332e-26	-0.7409835	0.693	0.567	1.824477e-22	11	RBM8A	ASC IgG
1.985341e-26	-0.5216219	0.183	0.213	3.539664e-22	11	NR3C1	ASC IgG
2.06011e-26	-0.5191034	0.181	0.216	3.672969e-22	11	EMG1	ASC IgG
2.388982e-26	-0.6133988	0.377	0.345	4.259315e-22	11	H2AFY	ASC IgG
3.523627e-26	-0.6636697	0.654	0.539	6.282275e-22	11	SUMO1	ASC IgG
3.628831e-26	-0.7045967	0.759	0.62	6.469842e-22	11	LAMTOR5	ASC IgG
4.030361e-26	-0.5542191	0.194	0.223	7.18573e-22	11	SKAP2	ASC IgG
4.794854e-26	-0.3423444	0.053	0.105	8.548745e-22	11	SORL1	ASC IgG
4.93231e-26	-0.6623837	0.561	0.47	8.793816e-22	11	PSMB8	ASC IgG
5.163157e-26	-0.5589773	0.205	0.246	9.205393e-22	11	DENR	ASC IgG
5.90998e-26	-0.3976029	0.103	0.158	1.05369e-21	11	BCL7C	ASC IgG
5.93024e-26	-0.4511149	0.083	0.135	1.057303e-21	11	NFKB2	ASC IgG
7.974797e-26	-0.5479468	0.234	0.247	1.421826e-21	11	SNX6	ASC IgG
8.620537e-26	-0.6156959	0.388	0.349	1.536956e-21	11	LSM14A	ASC IgG
8.896066e-26	-0.694157	0.324	0.327	1.58608e-21	11	ZFP36	ASC IgG
1.013919e-25	-0.5295957	0.266	0.245	1.807715e-21	11	IKZF1	ASC IgG
1.227108e-25	-0.5010545	0.133	0.175	2.187811e-21	11	TRIM13	ASC IgG
1.799567e-25	-0.5821179	0.243	0.236	3.208448e-21	11	CCND3	ASC IgG

2.021816e-25	3.805822	0.228	0.145	3.604695e-21	11	IGHA1	ASC IgG
2.490502e-25	-0.7042458	0.824	0.683	4.440316e-21	11	PPP1CA	ASC IgG
2.497155e-25	-0.4560242	0.108	0.154	4.452178e-21	11	MAD1L1	ASC IgG
2.497384e-25	-0.6522707	0.466	0.402	4.452586e-21	11	BZW1	ASC IgG
2.747664e-25	-0.4544544	0.104	0.146	4.898809e-21	11	MID1IP1	ASC IgG
2.950731e-25	-0.5212631	0.211	0.222	5.260859e-21	11	THUMPD3-AS1	ASC IgG
3.155688e-25	-0.6619444	0.556	0.463	5.626276e-21	11	PRDX6	ASC IgG
3.171402e-25	-0.5283514	0.181	0.205	5.654293e-21	11	AP1S2	ASC IgG
3.715791e-25	-0.696465	0.829	0.693	6.624884e-21	11	CIRBP	ASC IgG
3.990219e-25	-0.4814815	0.131	0.184	7.114162e-21	11	TUBA1C	ASC IgG
4.55467e-25	-0.3512888	0.048	0.104	8.120521e-21	11	IRF5	ASC IgG
4.917841e-25	-0.6056824	0.36	0.334	8.768018e-21	11	TBCB	ASC IgG
7.127991e-25	-0.6010089	0.352	0.332	1.27085e-20	11	LSM2	ASC IgG
7.762124e-25	-0.6931278	0.596	0.5	1.383909e-20	11	XRCC5	ASC IgG
8.723398e-25	-0.509324	0.178	0.206	1.555295e-20	11	RECQL	ASC IgG
9.357914e-25	-0.617546	0.573	0.483	1.668423e-20	11	GMFG	ASC IgG
9.47972e-25	-0.3961658	0.072	0.121	1.690139e-20	11	NAB2	ASC IgG
1.05257e-24	-0.6711017	0.378	0.375	1.876627e-20	11	FAM107B	ASC IgG
1.333755e-24	-0.5425619	0.253	0.262	2.377952e-20	11	HDAC2	ASC IgG
1.406009e-24	-0.6739253	0.493	0.395	2.506773e-20	11	BASP1	ASC IgG
1.494596e-24	-0.7162016	0.9	0.79	2.664716e-20	11	HLA-E	ASC IgG
1.626801e-24	-0.6214352	0.371	0.351	2.900424e-20	11	MRPS6	ASC IgG
1.645852e-24	-0.5837046	0.235	0.246	2.934389e-20	11	CKS1B	ASC IgG
1.726787e-24	-0.6714445	0.937	0.803	3.078689e-20	11	TMA7	ASC IgG
1.822988e-24	-0.6550646	0.44	0.381	3.250206e-20	11	SRSF10	ASC IgG

1.925369e-24	-0.5482229	0.249	0.264	3.43274e-20	11	MRPL11	ASC IgG
2.420645e-24	-0.3431259	0.049	0.108	4.315768e-20	11	TP53	ASC IgG
2.652781e-24	-0.7398658	0.553	0.477	4.729644e-20	11	RSL1D1	ASC IgG
3.261481e-24	-0.5790365	0.211	0.233	5.814894e-20	11	EPB41L4A-AS1	ASC IgG
3.656502e-24	-0.4631398	0.169	0.202	6.519178e-20	11	SNRNP40	ASC IgG
3.656573e-24	-0.6547597	0.267	0.282	6.519304e-20	11	MT-ND4L	ASC IgG
5.399395e-24	-0.6430321	0.377	0.357	9.62658e-20	11	POLR1D	ASC IgG
5.498883e-24	-0.6581912	0.762	0.65	9.803959e-20	11	HLA-DQA1	ASC IgG
5.815678e-24	-0.4652049	0.159	0.173	1.036877e-19	11	TMEM156	ASC IgG
8.09022e-24	-0.735526	0.587	0.471	1.442405e-19	11	SNRPD1	ASC IgG
9.081856e-24	-0.5089815	0.184	0.208	1.619204e-19	11	ARHGAP30	ASC IgG
9.287286e-24	-0.7328515	0.614	0.506	1.65583e-19	11	TALDO1	ASC IgG
9.438296e-24	-0.3881487	0.078	0.127	1.682754e-19	11	MAP4	ASC IgG
1.211966e-23	-0.5973088	0.295	0.293	2.160815e-19	11	TNIP1	ASC IgG
1.437336e-23	-0.5928035	0.289	0.282	2.562627e-19	11	RTN4	ASC IgG
1.452171e-23	-0.4738268	0.152	0.183	2.589077e-19	11	TLR10	ASC IgG
1.551309e-23	-0.4312442	0.071	0.116	2.765829e-19	11	CLN8	ASC IgG
1.623878e-23	-0.4616866	0.152	0.188	2.895213e-19	11	CHRAC1	ASC IgG
2.023756e-23	-0.6328286	0.287	0.274	3.608154e-19	11	TNRC6B	ASC IgG
2.245756e-23	-0.5758002	0.291	0.291	4.003959e-19	11	VPS35	ASC IgG
2.476123e-23	-0.3891123	0.108	0.155	4.41468e-19	11	PPIH	ASC IgG
2.502548e-23	-0.3328308	0.075	0.124	4.461792e-19	11	GMPS	ASC IgG
3.550775e-23	-0.5246971	0.232	0.235	6.330678e-19	11	FNTA	ASC IgG
6.377229e-23	-0.5940425	0.403	0.352	1.136996e-18	11	EIF1B	ASC IgG
7.222413e-23	-0.6008912	0.359	0.324	1.287684e-18	11	AKR1B1	ASC IgG

7.233888e-23	-0.567468	0.348	0.309	1.28973e-18	11	CHURC1	ASC IgG
7.576826e-23	-0.4904377	0.204	0.219	1.350872e-18	11	CHD4	ASC IgG
7.621943e-23	-0.4936476	0.248	0.253	1.358916e-18	11	RGS10	ASC IgG
9.127955e-23	-0.4187382	0.082	0.122	1.627423e-18	11	ZNF318	ASC IgG
1.338384e-22	-0.6762762	0.646	0.53	2.386205e-18	11	SOD1	ASC IgG
1.367226e-22	-0.5670849	0.336	0.315	2.437627e-18	11	WDR1	ASC IgG
1.72713e-22	-0.5528669	0.317	0.29	3.079301e-18	11	G3BP1	ASC IgG
1.991446e-22	-0.3851683	0.103	0.143	3.550549e-18	11	NUP107	ASC IgG
2.725848e-22	-0.6846641	0.622	0.503	4.859914e-18	11	SRRM1	ASC IgG
2.85881e-22	-0.5941536	0.401	0.362	5.096973e-18	11	CPNE1	ASC IgG
3.261173e-22	-0.4689329	0.136	0.16	5.814345e-18	11	AIDA	ASC IgG
3.950849e-22	-0.7224594	0.551	0.454	7.043968e-18	11	LSM5	ASC IgG
4.194801e-22	-0.4554585	0.194	0.213	7.47891e-18	11	NSL1	ASC IgG
5.050471e-22	-0.6795379	0.565	0.454	9.004485e-18	11	SFPQ	ASC IgG
6.596617e-22	-0.5218886	0.234	0.242	1.176111e-17	11	CDC42SE2	ASC IgG
9.59273e-22	-0.6502199	0.511	0.425	1.710288e-17	11	RNPS1	ASC IgG
9.85023e-22	-0.4836149	0.21	0.224	1.756198e-17	11	JMJD1C	ASC IgG
1.015342e-21	-0.6220045	0.561	0.453	1.810253e-17	11	PPP4C	ASC IgG
1.166383e-21	-0.46813	0.115	0.152	2.079545e-17	11	CTNNB1	ASC IgG
1.385299e-21	-0.5430923	0.127	0.158	2.46985e-17	11	SLC2A3	ASC IgG
1.543465e-21	-0.4635083	0.239	0.234	2.751844e-17	11	ZCCHC7	ASC IgG
1.621906e-21	-0.6378259	0.636	0.512	2.891696e-17	11	SRP9	ASC IgG
1.688231e-21	-0.3306248	0.064	0.101	3.009946e-17	11	CENPM	ASC IgG
1.763084e-21	-0.4227821	0.135	0.162	3.143402e-17	11	DNAJC9	ASC IgG
1.929938e-21	-0.4991916	0.219	0.229	3.440886e-17	11	NOB1	ASC IgG

2.082091e-21	-0.8274342	0.49	0.425	3.712161e-17	11	PLP2	ASC IgG
2.215067e-21	-0.4810333	0.211	0.212	3.949243e-17	11	APPL1	ASC IgG
2.347916e-21	-0.5510158	0.305	0.295	4.186099e-17	11	CLNS1A	ASC IgG
2.731027e-21	-0.4605663	0.167	0.191	4.869148e-17	11	PHF14	ASC IgG
3.202932e-21	-0.4186169	0.127	0.152	5.710508e-17	11	SCAF4	ASC IgG
3.221382e-21	-0.4012505	0.114	0.148	5.743402e-17	11	ARL6IP6	ASC IgG
3.764298e-21	-0.5247772	0.294	0.282	6.711367e-17	11	GNAI3	ASC IgG
4.526669e-21	-0.3199535	0.076	0.126	8.070597e-17	11	NUP93	ASC IgG
4.610323e-21	-0.4351023	0.107	0.138	8.219745e-17	11	SMIM29	ASC IgG
4.739077e-21	-0.4875246	0.2	0.203	8.4493e-17	11	IFNGR2	ASC IgG
4.794322e-21	-0.4300444	0.173	0.191	8.547796e-17	11	USP1	ASC IgG
5.321527e-21	-0.4776895	0.259	0.259	9.487751e-17	11	ILK	ASC IgG
5.525e-21	-0.4704923	0.207	0.228	9.850522e-17	11	FUBP1	ASC IgG
9.007526e-21	-0.5403365	0.28	0.275	1.605952e-16	11	SMC3	ASC IgG
9.439935e-21	-0.6363018	0.394	0.346	1.683046e-16	11	LITAF	ASC IgG
9.55003e-21	-0.5021641	0.227	0.235	1.702675e-16	11	DNAJC19	ASC IgG
9.651299e-21	-0.3597461	0.088	0.128	1.72073e-16	11	TOB2	ASC IgG
1.088048e-20	-0.5488306	0.409	0.352	1.939881e-16	11	DBNL	ASC IgG
2.146999e-20	-0.4958111	0.233	0.244	3.827885e-16	11	GTF2H5	ASC IgG
2.345955e-20	-0.3876276	0.099	0.135	4.182603e-16	11	PLEKHO1	ASC IgG
2.630513e-20	-0.3813469	0.094	0.134	4.689942e-16	11	MCMBP	ASC IgG
2.641887e-20	-0.4725354	0.132	0.164	4.71022e-16	11	CD58	ASC IgG
2.860835e-20	-0.4201421	0.16	0.185	5.100583e-16	11	DHX9	ASC IgG
3.106074e-20	-0.5442532	0.302	0.288	5.537819e-16	11	ZMAT2	ASC IgG
3.621755e-20	-0.4921771	0.226	0.228	6.457227e-16	11	SINHCAF	ASC IgG

3.686711e-20	-0.4998439	0.276	0.256	6.573038e-16	11	RAD21	ASC IgG
4.069801e-20	-0.7094639	0.751	0.607	7.256048e-16	11	PTGES3	ASC IgG
4.386173e-20	-0.5136248	0.266	0.249	7.820108e-16	11	ROCK1	ASC IgG
5.566526e-20	-0.5029873	0.254	0.24	9.924559e-16	11	SLC38A1	ASC IgG
5.629713e-20	-0.4672201	0.226	0.228	1.003721e-15	11	IAH1	ASC IgG
5.785833e-20	-0.523015	0.283	0.275	1.031556e-15	11	SRI	ASC IgG
6.725405e-20	-0.4413177	0.181	0.185	1.199072e-15	11	OSBPL8	ASC IgG
6.796915e-20	-0.6682051	0.333	0.277	1.211822e-15	11	ADAM28	ASC IgG
8.392616e-20	-0.5498459	0.329	0.283	1.496319e-15	11	CARHSP1	ASC IgG
8.607728e-20	-0.4486257	0.159	0.183	1.534672e-15	11	ARL2	ASC IgG
9.041553e-20	-0.5202418	0.263	0.246	1.612018e-15	11	MKRN1	ASC IgG
1.014817e-19	-0.3552131	0.069	0.112	1.809318e-15	11	ZNF267	ASC IgG
1.022404e-19	-0.4890142	0.216	0.214	1.822844e-15	11	VPS4B	ASC IgG
1.173161e-19	-0.5519625	0.371	0.325	2.091628e-15	11	PSMG2	ASC IgG
1.213159e-19	-0.6715043	0.745	0.595	2.162941e-15	11	CALM1	ASC IgG
1.348414e-19	0.4057135	0.246	0.044	2.404088e-15	11	XIST	ASC IgG
1.362198e-19	-0.6506652	0.707	0.575	2.428663e-15	11	C11orf58	ASC IgG
1.447056e-19	-0.4943431	0.266	0.255	2.579956e-15	11	CYB5B	ASC IgG
1.486036e-19	-0.6165657	0.548	0.447	2.649454e-15	11	MRPS21	ASC IgG
1.560701e-19	-0.4840572	0.184	0.193	2.782574e-15	11	CYTH1	ASC IgG
1.666128e-19	-0.4242593	0.167	0.18	2.97054e-15	11	TSR2	ASC IgG
1.767702e-19	-0.3696826	0.138	0.172	3.151635e-15	11	EED	ASC IgG
1.872962e-19	-0.5408042	0.964	0.881	3.339305e-15	11	H3F3B	ASC IgG
1.974553e-19	-0.3706327	0.131	0.163	3.52043e-15	11	WDR77	ASC IgG
1.996049e-19	-0.4942404	0.204	0.216	3.558756e-15	11	LYRM4	ASC IgG

2.210867e-19	-0.3331508	0.086	0.118	3.941756e-15	11	KLHL5	ASC IgG
2.220455e-19	-0.4416352	0.205	0.215	3.95885e-15	11	RBM22	ASC IgG
2.227831e-19	-0.4901021	0.165	0.181	3.972e-15	11	CDC42SE1	ASC IgG
2.510822e-19	-0.4645822	0.181	0.192	4.476545e-15	11	ARID1B	ASC IgG
2.534799e-19	-0.3736019	0.159	0.188	4.519293e-15	11	SNRPA	ASC IgG
2.539834e-19	-0.4746402	0.18	0.189	4.52827e-15	11	CWF19L2	ASC IgG
2.709546e-19	-0.5673439	0.27	0.257	4.830849e-15	11	MDM4	ASC IgG
2.767288e-19	-0.4057626	0.085	0.124	4.933798e-15	11	RAB13	ASC IgG
3.043538e-19	-0.4226849	0.162	0.171	5.426323e-15	11	CCDC82	ASC IgG
3.273986e-19	-0.5965162	0.466	0.381	5.83719e-15	11	JAK1	ASC IgG
3.985379e-19	-0.4073369	0.987	0.964	7.105532e-15	11	EIF1	ASC IgG
5.665246e-19	-0.4465364	0.173	0.189	1.010057e-14	11	PHF11	ASC IgG
5.995113e-19	-0.3332858	0.094	0.124	1.068869e-14	11	RFC2	ASC IgG
6.428289e-19	-0.3684559	0.089	0.125	1.1461e-14	11	DYNLT3	ASC IgG
6.712245e-19	-0.4717745	0.205	0.206	1.196726e-14	11	ERICH1	ASC IgG
7.221568e-19	-0.450308	0.181	0.189	1.287533e-14	11	CELF1	ASC IgG
7.231319e-19	-0.5827571	0.459	0.385	1.289272e-14	11	RSRC2	ASC IgG
8.433063e-19	-0.3131784	0.115	0.139	1.503531e-14	11	RPA1	ASC IgG
1.239872e-18	-0.6189467	0.608	0.485	2.210567e-14	11	EIF3M	ASC IgG
1.413147e-18	-0.4820288	0.204	0.213	2.519499e-14	11	COX20	ASC IgG
1.488112e-18	-0.3726008	0.105	0.133	2.653155e-14	11	GGPS1	ASC IgG
1.75875e-18	-0.3907338	0.153	0.163	3.135675e-14	11	LYPLAL1	ASC IgG
1.892572e-18	-0.4949509	0.319	0.294	3.374267e-14	11	IDH3B	ASC IgG
2.44772e-18	-0.4645228	0.262	0.243	4.364041e-14	11	WAS	ASC IgG
2.479836e-18	-0.5718799	0.383	0.33	4.4213e-14	11	TSTD1	ASC IgG

2.922137e-18	-0.3165948	0.089	0.126	5.209878e-14	11	BZW2	ASC IgG
3.293753e-18	-0.4597135	0.245	0.238	5.872432e-14	11	CTNNBL1	ASC IgG
3.421751e-18	-0.5162928	0.333	0.296	6.10064e-14	11	WASF2	ASC IgG
3.447382e-18	-0.6883305	0.683	0.525	6.146337e-14	11	SRSF7	ASC IgG
3.754034e-18	-0.4265406	0.167	0.178	6.693067e-14	11	STAG2	ASC IgG
3.910255e-18	-0.4237054	0.14	0.164	6.971594e-14	11	MRTO4	ASC IgG
4.006421e-18	-0.4004964	0.13	0.143	7.143048e-14	11	ZMYM2	ASC IgG
4.285005e-18	-0.5170154	0.195	0.188	7.639735e-14	11	PCNA	ASC IgG
4.806873e-18	-0.4939809	0.265	0.253	8.570173e-14	11	PRDM2	ASC IgG
7.379327e-18	-0.5847663	0.484	0.412	1.31566e-13	11	HCLS1	ASC IgG
7.760315e-18	-0.5052165	0.278	0.249	1.383587e-13	11	WIPF1	ASC IgG
8.586866e-18	-0.3534119	0.115	0.117	1.530952e-13	11	CARD16	ASC IgG
8.708687e-18	-0.4652794	0.245	0.228	1.552672e-13	11	GTF2I	ASC IgG
9.215043e-18	-0.5458702	0.432	0.344	1.64295e-13	11	UBE2A	ASC IgG
1.032304e-17	-0.5272538	0.363	0.301	1.840495e-13	11	UGP2	ASC IgG
1.149239e-17	-0.5852911	0.507	0.416	2.048978e-13	11	NDUFA12	ASC IgG
1.157538e-17	-0.4702574	0.222	0.222	2.063774e-13	11	RNMT	ASC IgG
1.170434e-17	-0.4400405	0.208	0.214	2.086768e-13	11	MCRIP1	ASC IgG
1.198419e-17	-0.4316071	0.165	0.173	2.136661e-13	11	PHF20	ASC IgG
1.280771e-17	-0.3044279	0.066	0.102	2.283486e-13	11	CXCR5	ASC IgG
1.318839e-17	-0.5633598	0.456	0.387	2.351357e-13	11	PDCD6	ASC IgG
1.465428e-17	-0.6009038	0.446	0.386	2.612711e-13	11	SHMT2	ASC IgG
1.815482e-17	-0.3864383	0.137	0.157	3.236822e-13	11	DDB2	ASC IgG
1.828563e-17	-0.4328633	0.225	0.22	3.260145e-13	11	SMARCC1	ASC IgG
1.887791e-17	-0.5220885	0.313	0.287	3.365742e-13	11	WTAP	ASC IgG

2.148455e-17	-0.5510931	0.456	0.363	3.83048e-13	11	RBBP7	ASC IgG
2.194825e-17	-0.4782197	0.26	0.242	3.913153e-13	11	TMEM126B	ASC IgG
2.22267e-17	-0.4971862	0.214	0.211	3.962798e-13	11	FMNL1	ASC IgG
2.310943e-17	-0.5218	0.292	0.253	4.12018e-13	11	TBC1D10C	ASC IgG
2.313406e-17	-0.4328632	0.195	0.193	4.124572e-13	11	UTP6	ASC IgG
2.898727e-17	-0.3765396	0.143	0.155	5.16814e-13	11	SVBP	ASC IgG
2.958622e-17	-0.3806999	0.154	0.169	5.274928e-13	11	YEATS4	ASC IgG
3.038416e-17	-0.6105216	0.515	0.416	5.417192e-13	11	NUDC	ASC IgG
3.237203e-17	-0.3114237	0.082	0.105	5.771609e-13	11	LNPEP	ASC IgG
3.365362e-17	-0.5075645	0.297	0.275	6.000104e-13	11	NCBP2	ASC IgG
3.500494e-17	-0.4117184	0.15	0.16	6.241031e-13	11	ARHGAP15	ASC IgG
3.525211e-17	-0.428718	0.132	0.149	6.285098e-13	11	TRIM56	ASC IgG
3.805952e-17	-0.4443912	0.253	0.232	6.785632e-13	11	MTF2	ASC IgG
4.276663e-17	-0.6224162	0.38	0.321	7.624863e-13	11	NOP56	ASC IgG
4.644351e-17	-0.502512	0.305	0.253	8.280414e-13	11	ZCCHC10	ASC IgG
5.397649e-17	-0.3156674	0.067	0.102	9.623469e-13	11	CCNB1IP1	ASC IgG
5.562326e-17	-0.5527116	0.42	0.333	9.917071e-13	11	POLR2J3.1	ASC IgG
6.006981e-17	-0.5083236	0.321	0.276	1.070985e-12	11	TANK	ASC IgG
6.938203e-17	-0.6030329	0.511	0.414	1.237012e-12	11	IDI1	ASC IgG
7.554917e-17	-0.5633826	0.192	0.191	1.346966e-12	11	DAAM1	ASC IgG
7.746436e-17	-0.4408491	0.219	0.217	1.381112e-12	11	ITGB1BP1	ASC IgG
8.183175e-17	-0.6182914	0.837	0.698	1.458978e-12	11	EIF3K	ASC IgG
9.036583e-17	-0.5996321	0.568	0.425	1.611132e-12	11	DNAJA1	ASC IgG
9.422167e-17	-0.3860344	0.15	0.157	1.679878e-12	11	CREB1	ASC IgG
9.434157e-17	-0.310098	0.087	0.118	1.682016e-12	11	LYAR	ASC IgG

9.440314e-17	-0.4577466	0.239	0.225	1.683114e-12	11	PAK2	ASC IgG
9.801582e-17	-0.4360797	0.181	0.184	1.747524e-12	11	R3HDM4	ASC IgG
1.037367e-16	-0.3910446	0.169	0.177	1.849522e-12	11	POLR3K	ASC IgG
1.083481e-16	-0.3981743	0.175	0.166	1.931738e-12	11	ARHGAP25	ASC IgG
1.336112e-16	-0.4558967	0.26	0.247	2.382155e-12	11	UBA2	ASC IgG
1.508593e-16	-0.5753348	0.649	0.514	2.68967e-12	11	ATP5PB	ASC IgG
1.590696e-16	-0.4790748	0.272	0.259	2.836052e-12	11	NAA50	ASC IgG
1.709696e-16	-0.4009326	0.147	0.151	3.048217e-12	11	CHMP1B	ASC IgG
1.748354e-16	-0.3303764	0.101	0.128	3.11714e-12	11	AMFR	ASC IgG
1.803031e-16	-0.3118184	0.086	0.117	3.214624e-12	11	MYH9	ASC IgG
1.892239e-16	-0.4947238	0.306	0.275	3.373673e-12	11	GLO1	ASC IgG
2.102366e-16	-0.3815784	0.102	0.121	3.748308e-12	11	LACTB	ASC IgG
2.174764e-16	-0.6226596	0.681	0.528	3.877387e-12	11	SNRPB2	ASC IgG
2.187089e-16	-0.3495019	0.126	0.141	3.899361e-12	11	SMC2	ASC IgG
2.219309e-16	-0.5583569	0.543	0.446	3.956806e-12	11	HIGD2A	ASC IgG
2.373224e-16	-0.3520014	0.142	0.154	4.231221e-12	11	PRKDC	ASC IgG
2.561848e-16	-0.3445689	0.078	0.106	4.567519e-12	11	PDE7A	ASC IgG
2.758115e-16	-0.4123222	0.211	0.193	4.917443e-12	11	CBX5	ASC IgG
2.767895e-16	-0.3718441	0.123	0.151	4.93488e-12	11	HCFC1R1	ASC IgG
2.918705e-16	-0.4686313	0.297	0.261	5.203759e-12	11	PMPCB	ASC IgG
2.920155e-16	-0.3615121	0.097	0.13	5.206344e-12	11	EEA1	ASC IgG
3.4383e-16	-0.3704492	0.172	0.182	6.130145e-12	11	DHX15	ASC IgG
3.911203e-16	-0.4446685	0.219	0.204	6.973284e-12	11	SAFB	ASC IgG
4.201221e-16	-0.5086381	0.33	0.276	7.490357e-12	11	PPP1R12A	ASC IgG
5.26723e-16	-0.3439617	0.106	0.123	9.390944e-12	11	MAP3K8	ASC IgG

5.590253e-16	-0.6109557	0.525	0.422	9.966862e-12	11	STK4	ASC IgG
5.6494e-16	-0.4498914	0.217	0.206	1.007232e-11	11	IKZF3	ASC IgG
5.94873e-16	-0.4187798	0.16	0.166	1.060599e-11	11	HIF1A	ASC IgG
6.859639e-16	-0.3745243	0.154	0.163	1.223005e-11	11	PTDSS1	ASC IgG
7.459173e-16	-0.4353731	0.174	0.184	1.329896e-11	11	KANSL1	ASC IgG
8.086949e-16	-0.4214655	0.175	0.173	1.441822e-11	11	CDK13	ASC IgG
8.559647e-16	-0.3205306	0.082	0.111	1.526099e-11	11	SRFBP1	ASC IgG
8.635342e-16	-0.3957603	0.177	0.181	1.539595e-11	11	COPRS	ASC IgG
1.061916e-15	-0.5337139	0.464	0.381	1.893289e-11	11	SNRPD3	ASC IgG
1.074015e-15	-0.4750733	0.27	0.228	1.914861e-11	11	ATM	ASC IgG
1.096825e-15	-0.5330462	0.394	0.332	1.95553e-11	11	DARS	ASC IgG
1.10094e-15	-0.3160813	0.082	0.108	1.962865e-11	11	LCOR	ASC IgG
1.122352e-15	-0.4811923	0.271	0.242	2.001041e-11	11	APH1A	ASC IgG
1.192873e-15	-0.3992311	0.168	0.173	2.126774e-11	11	OSGEP	ASC IgG
1.206024e-15	-0.37351	0.143	0.142	2.150221e-11	11	SH3BP5	ASC IgG
1.37883e-15	-0.4274716	0.244	0.223	2.458316e-11	11	HSPH1	ASC IgG
1.38724e-15	-0.5966648	0.619	0.492	2.47331e-11	11	ARGLU1	ASC IgG
1.443341e-15	-0.444495	0.25	0.231	2.573332e-11	11	RTCB	ASC IgG
1.479549e-15	-0.5519336	0.468	0.364	2.637888e-11	11	CXXC5	ASC IgG
1.49312e-15	-0.3708535	0.15	0.161	2.662084e-11	11	TMPO	ASC IgG
1.704249e-15	-0.4115146	0.222	0.213	3.038505e-11	11	GLOD4	ASC IgG
2.036057e-15	-0.5337299	0.408	0.344	3.630086e-11	11	DDX46	ASC IgG
2.060928e-15	-0.4925649	0.317	0.273	3.674428e-11	11	SPG21	ASC IgG
2.151016e-15	-0.3102608	0.124	0.128	3.835047e-11	11	STAP1	ASC IgG
2.198387e-15	-0.3552305	0.166	0.153	3.919505e-11	11	CD81	ASC IgG

2.38355e-15	-0.4907307	0.255	0.224	4.249632e-11	11	PSMA3-AS1	ASC IgG
2.401017e-15	-0.4108859	0.199	0.194	4.280774e-11	11	RABEP1	ASC IgG
2.444257e-15	-0.6104886	0.483	0.39	4.357866e-11	11	PHB	ASC IgG
2.499764e-15	-0.3376056	0.093	0.117	4.456829e-11	11	SIAH1	ASC IgG
2.787045e-15	-0.6459067	0.618	0.5	4.969023e-11	11	TSC22D3	ASC IgG
3.134897e-15	-0.4306958	0.222	0.212	5.589208e-11	11	IQGAP1	ASC IgG
3.42915e-15	-0.5854158	0.516	0.417	6.113831e-11	11	KMT2E	ASC IgG
3.625674e-15	-0.4018507	0.22	0.207	6.464214e-11	11	PRPF38A	ASC IgG
3.718575e-15	-0.3657987	0.122	0.129	6.629848e-11	11	FBXL3	ASC IgG
3.829168e-15	-0.3605675	0.156	0.156	6.827024e-11	11	DYNC1LI1	ASC IgG
3.92741e-15	-0.7006386	0.871	0.698	7.002179e-11	11	TPI1	ASC IgG
4.223905e-15	-0.5278488	0.382	0.312	7.530801e-11	11	LARP7	ASC IgG
4.671146e-15	-0.4650024	0.209	0.211	8.328186e-11	11	C19orf48	ASC IgG
4.904233e-15	-0.4551935	0.268	0.242	8.743757e-11	11	PTBP1	ASC IgG
5.432949e-15	-0.3190276	0.093	0.12	9.686405e-11	11	UHMK1	ASC IgG
5.597456e-15	-0.4209698	0.226	0.209	9.979705e-11	11	CHORDC1	ASC IgG
5.83783e-15	-0.3589094	0.106	0.119	1.040827e-10	11	LIPA	ASC IgG
6.922959e-15	-0.3848311	0.151	0.153	1.234294e-10	11	SRPK2	ASC IgG
6.963755e-15	-0.5495892	0.349	0.283	1.241568e-10	11	ACAP1	ASC IgG
7.249561e-15	-0.314049	0.097	0.114	1.292524e-10	11	SETX	ASC IgG
7.328508e-15	-0.6250417	0.561	0.447	1.3066e-10	11	DRAP1	ASC IgG
7.61196e-15	-0.4971703	0.272	0.246	1.357136e-10	11	DKC1	ASC IgG
7.612909e-15	-0.3205491	0.125	0.141	1.357306e-10	11	MTHFS	ASC IgG
7.696262e-15	-0.4491246	0.265	0.234	1.372166e-10	11	AKIRIN1	ASC IgG
7.795538e-15	-0.406611	0.198	0.183	1.389866e-10	11	PPP4R3A	ASC IgG

7.893453e-15	-0.4006189	0.154	0.149	1.407324e-10	11	ARHGAP9	ASC IgG
8.313042e-15	-0.3471846	0.139	0.148	1.482132e-10	11	AP1G2	ASC IgG
8.397108e-15	-0.5650044	0.484	0.389	1.49712e-10	11	SSB	ASC IgG
9.141492e-15	-0.3597961	0.12	0.13	1.629837e-10	11	SIPA1	ASC IgG
9.855236e-15	-0.3747981	0.143	0.155	1.75709e-10	11	SMC5	ASC IgG
1.000363e-14	-0.4010224	0.222	0.205	1.783547e-10	11	GID8	ASC IgG
1.056473e-14	-0.597995	0.79	0.622	1.883585e-10	11	ATP5PO	ASC IgG
1.187315e-14	-0.3741238	0.18	0.181	2.116863e-10	11	UTP18	ASC IgG
1.239277e-14	-0.5701148	0.523	0.414	2.209507e-10	11	LAMTOR1	ASC IgG
1.342554e-14	-0.4278715	0.225	0.204	2.39364e-10	11	OGA	ASC IgG
1.448639e-14	-0.4450001	0.287	0.25	2.582779e-10	11	PFDN1	ASC IgG
1.490948e-14	-0.3003478	0.091	0.116	2.658212e-10	11	C1orf174	ASC IgG
1.651497e-14	-0.3291037	0.097	0.112	2.944454e-10	11	BRAF	ASC IgG
1.659081e-14	-0.4042661	0.19	0.189	2.957975e-10	11	ATP1A1	ASC IgG
1.696138e-14	-0.6050749	0.546	0.435	3.024045e-10	11	IL2RG	ASC IgG
1.738662e-14	-0.45847	0.264	0.229	3.09986e-10	11	TTC19	ASC IgG
1.755008e-14	-0.3451452	0.114	0.129	3.129004e-10	11	ERV3-1	ASC IgG
1.958529e-14	-0.3145674	0.118	0.133	3.491862e-10	11	AP1S1	ASC IgG
2.114141e-14	-0.3592395	0.199	0.18	3.769302e-10	11	ZNF106	ASC IgG
2.419475e-14	-0.6177746	0.681	0.541	4.313683e-10	11	CNBP	ASC IgG
2.583333e-14	-0.4607541	0.273	0.241	4.605824e-10	11	NCOA3	ASC IgG
2.772692e-14	-0.4999188	0.378	0.305	4.943433e-10	11	MED4	ASC IgG
2.888697e-14	-0.5523523	0.427	0.335	5.150258e-10	11	IER2	ASC IgG
3.051431e-14	-0.3664971	0.15	0.157	5.440396e-10	11	NAA15	ASC IgG
3.148705e-14	-0.4340943	0.266	0.231	5.613826e-10	11	COMMD8	ASC IgG

3.171715e-14	-0.5607008	0.485	0.385	5.65485e-10	11	PPIG	ASC IgG
3.693508e-14	-0.353823	0.149	0.16	6.585155e-10	11	WBP4	ASC IgG
4.077534e-14	-0.4393975	0.218	0.198	7.269835e-10	11	RSF1	ASC IgG
4.424144e-14	-0.4435663	0.278	0.247	7.887806e-10	11	EXOSC8	ASC IgG
4.473406e-14	-0.3779869	0.189	0.189	7.975636e-10	11	NCOA4	ASC IgG
4.674756e-14	-0.42612	0.225	0.195	8.334623e-10	11	RBM5	ASC IgG
5.120074e-14	-0.4345673	0.188	0.188	9.12858e-10	11	GPATCH4	ASC IgG
5.467549e-14	-0.6071547	0.702	0.561	9.748092e-10	11	PCBP1	ASC IgG
5.788659e-14	-0.3015986	0.097	0.111	1.03206e-09	11	IVNS1ABP	ASC IgG
5.869921e-14	-0.3863852	0.186	0.181	1.046548e-09	11	TADA3	ASC IgG
6.437006e-14	-0.3630916	0.161	0.163	1.147654e-09	11	NOL11	ASC IgG
6.662245e-14	-0.4527407	0.351	0.289	1.187812e-09	11	CDC123	ASC IgG
6.729646e-14	-0.363309	0.127	0.142	1.199829e-09	11	GLTP	ASC IgG
6.814299e-14	-0.3056502	0.106	0.118	1.214921e-09	11	DCLRE1C	ASC IgG
6.911543e-14	-0.5042571	0.411	0.333	1.232259e-09	11	ANXA11	ASC IgG
7.043898e-14	-0.4567112	0.302	0.258	1.255857e-09	11	RAB14	ASC IgG
7.046196e-14	-0.4965624	0.21	0.211	1.256266e-09	11	GABPB1-AS1	ASC IgG
7.212296e-14	-0.4028783	0.09	0.112	1.28588e-09	11	C1orf56	ASC IgG
7.315641e-14	-0.7501734	0.695	0.555	1.304306e-09	11	NCL	ASC IgG
8.032319e-14	-0.3727501	0.161	0.156	1.432082e-09	11	ACAP2	ASC IgG
8.664519e-14	-0.3837343	0.134	0.147	1.544797e-09	11	ARL8B	ASC IgG
8.959579e-14	-0.4363988	0.249	0.223	1.597403e-09	11	WBP11	ASC IgG
9.105899e-14	-0.5160502	0.53	0.406	1.623491e-09	11	SEPT6	ASC IgG
9.497595e-14	-0.4092802	0.191	0.18	1.693326e-09	11	BDP1	ASC IgG
9.877092e-14	-0.3232767	0.114	0.123	1.760987e-09	11	SMAD2	ASC IgG

1.027179e-13 -0.4793128 0.235 0.217 1.831357e-09 11
HMGCS1 ASC IgG
1.095485e-13 -0.3225076 0.115 0.133 1.95314e-09 11
SMARCD1 ASC IgG
1.259775e-13 -0.4592837 0.298 0.258 2.246053e-09 11
PWP1 ASC IgG
1.338829e-13 -0.5915136 0.517 0.398 2.386997e-09 11
CMPK1 ASC IgG
1.368079e-13 -0.3432251 0.163 0.156 2.439147e-09 11
TDP2 ASC IgG
1.409345e-13 -0.4634512 0.317 0.269 2.512722e-09 11
SEPT9 ASC IgG
1.527826e-13 -0.4637049 0.226 0.192 2.72396e-09 11
DNMT1 ASC IgG
1.580336e-13 -0.4779222 0.342 0.293 2.817582e-09 11
ILF3 ASC IgG
1.699461e-13 -0.3427776 0.128 0.133 3.02997e-09 11
WDR54 ASC IgG
1.822737e-13 -0.3717599 0.158 0.156 3.249758e-09 11
RRAGC ASC IgG
1.874578e-13 -0.4287808 0.307 0.261 3.342186e-09 11
ATXN10 ASC IgG
1.920716e-13 -0.6379554 0.441 0.337 3.424445e-09 11
LRMP ASC IgG
1.954849e-13 -0.3594704 0.149 0.15 3.4853e-09 11
ATF1 ASC IgG
1.992744e-13 -0.4622169 0.34 0.284 3.552863e-09 11
SNW1 ASC IgG
2.014393e-13 -0.5221957 0.395 0.318 3.591461e-09 11
LSM4 ASC IgG
2.065946e-13 -0.4516775 0.294 0.256 3.683374e-09 11
UPF3A ASC IgG
2.119644e-13 -0.4463015 0.282 0.238 3.779113e-09 11
FBXO7 ASC IgG
2.301778e-13 -0.628488 0.828 0.64 4.103839e-09 11
SNRPG ASC IgG
2.475588e-13 -0.3748271 0.143 0.15 4.413725e-09 11
PUM3 ASC IgG
2.491923e-13 -0.5491668 0.553 0.427 4.442849e-09 11
CLTA ASC IgG
2.824363e-13 -0.5285361 0.372 0.283 5.035557e-09 11
PPM1K ASC IgG
3.246195e-13 -0.432682 0.155 0.162 5.787641e-09 11
FAM215B ASC IgG
3.263177e-13 -0.4073249 0.205 0.188 5.817918e-09 11
LYRM2 ASC IgG
3.333368e-13 -0.4198664 0.23 0.205 5.943062e-09 11
UPF3B ASC IgG
3.357046e-13 -0.3111068 0.118 0.129 5.985278e-09 11
SMARCE1 ASC IgG

3.673367e-13	-0.3701971	0.184	0.174	6.549246e-09	11	VPS26A	ASC IgG
3.805057e-13	-0.3994428	0.249	0.214	6.784037e-09	11	SNRNP25	ASC IgG
3.939538e-13	-0.4219995	0.232	0.21	7.023802e-09	11	ARPP19	ASC IgG
4.02144e-13	-0.5162284	0.425	0.329	7.169825e-09	11	BPTF	ASC IgG
4.066103e-13	-0.5764197	0.527	0.394	7.249454e-09	11	ORAI2	ASC IgG
4.155976e-13	-0.4522759	0.323	0.274	7.40969e-09	11	MRPL47	ASC IgG
4.497873e-13	-0.4181236	0.275	0.229	8.019258e-09	11	OAZ2	ASC IgG
4.534583e-13	-0.6652489	0.722	0.571	8.084708e-09	11	APRT	ASC IgG
4.723797e-13	-0.3555017	0.121	0.117	8.422057e-09	11	SAMD9	ASC IgG
5.525048e-13	-0.4794106	0.329	0.268	9.850608e-09	11	CNOT7	ASC IgG
5.778715e-13	-0.4039718	0.209	0.191	1.030287e-08	11	ARFGAP2	ASC IgG
6.072425e-13	-0.5245469	0.458	0.363	1.082653e-08	11	LUC7L3	ASC IgG
6.393344e-13	-0.3597733	0.192	0.174	1.139869e-08	11	PPP2R3C	ASC IgG
6.579427e-13	-0.3239998	0.105	0.12	1.173046e-08	11	NCBP3	ASC IgG
6.585284e-13	-0.3222129	0.101	0.123	1.17409e-08	11	CCDC137	ASC IgG
6.81879e-13	-0.3830838	0.199	0.173	1.215722e-08	11	BLCAP	ASC IgG
6.990345e-13	-0.3576507	0.154	0.154	1.246309e-08	11	CFAP97	ASC IgG
7.472926e-13	-0.3944363	0.23	0.204	1.332348e-08	11	MTMR14	ASC IgG
7.596892e-13	-0.3451158	0.155	0.155	1.35445e-08	11	C15orf40	ASC IgG
7.741984e-13	-0.3865321	0.207	0.192	1.380318e-08	11	TFAM	ASC IgG
8.617752e-13	-0.3321779	0.138	0.139	1.536459e-08	11	GLUD1	ASC IgG
1.105916e-12	-0.3228223	0.143	0.14	1.971738e-08	11	ABHD14B	ASC IgG
1.170526e-12	-0.4432474	0.276	0.241	2.086931e-08	11	POLD2	ASC IgG
1.20312e-12	-0.4130775	0.307	0.264	2.145043e-08	11	MTCH2	ASC IgG
1.261455e-12	-0.341684	0.153	0.152	2.249049e-08	11	RBM14	ASC IgG

1.300798e-12	-0.3489425	0.119	0.129	2.319192e-08	11	ZC3H8	ASC IgG
1.35405e-12	-0.4432021	0.332	0.254	2.414136e-08	11	ATG12	ASC IgG
1.613876e-12	-0.4949947	0.413	0.322	2.877379e-08	11	CCDC12	ASC IgG
1.733737e-12	-0.5563915	0.527	0.407	3.091079e-08	11	HNRNPR	ASC IgG
1.833598e-12	-0.5042022	0.436	0.341	3.269121e-08	11	ATRX	ASC IgG
1.892162e-12	-0.4149021	0.258	0.222	3.373536e-08	11	MTCH1	ASC IgG
2.024712e-12	0.3816056	0.131	0.049	3.609859e-08	11	CCL3	ASC IgG
2.059342e-12	-0.3264544	0.11	0.119	3.671602e-08	11	RNF41	ASC IgG
2.201301e-12	-0.3179348	0.138	0.134	3.924699e-08	11	UTP3	ASC IgG
2.217411e-12	-0.4055206	0.189	0.171	3.953422e-08	11	TRIM44	ASC IgG
2.369126e-12	-0.4084561	0.298	0.246	4.223914e-08	11	HAT1	ASC IgG
2.479677e-12	-0.31568	0.125	0.13	4.421015e-08	11	TGS1	ASC IgG
2.63525e-12	-0.5222784	0.375	0.327	4.698387e-08	11	HNRNPH1	ASC IgG
2.685588e-12	-0.6266434	0.66	0.51	4.788135e-08	11	SSBP1	ASC IgG
2.88243e-12	-0.5359924	0.629	0.489	5.139084e-08	11	THRAP3	ASC IgG
2.983316e-12	-0.3129662	0.142	0.142	5.318953e-08	11	MRPL45	ASC IgG
3.002517e-12	-0.3294574	0.145	0.146	5.353187e-08	11	ANKRD13A	ASC IgG
3.116377e-12	-0.3646203	0.158	0.155	5.556188e-08	11	EEF1G	ASC IgG
3.33131e-12	-0.3741831	0.203	0.182	5.939393e-08	11	CCDC90B	ASC IgG
3.334451e-12	-0.4611043	0.363	0.302	5.944993e-08	11	GLRX3	ASC IgG
3.60266e-12	-0.4079911	0.257	0.221	6.423182e-08	11	FXR1	ASC IgG
3.61124e-12	-0.4292591	0.211	0.194	6.43848e-08	11	ESF1	ASC IgG
3.925746e-12	-0.3683391	0.158	0.147	6.999212e-08	11	TSPAN3	ASC IgG
4.176131e-12	-0.3414987	0.163	0.15	7.445623e-08	11	FADS3	ASC IgG
4.248028e-12	-0.5000129	0.423	0.326	7.573809e-08	11	SLTM	ASC IgG

4.34422e-12	-0.3430743	0.168	0.158	7.745309e-08	11	CTSZ	ASC IgG
4.676575e-12	-0.6108128	0.799	0.634	8.337865e-08	11	ANAPC16	ASC IgG
4.757062e-12	-0.3998053	0.23	0.206	8.481367e-08	11	BRIX1	ASC IgG
4.809967e-12	-0.3215491	0.132	0.13	8.575689e-08	11	CRLF3	ASC IgG
5.531033e-12	-0.5455485	0.633	0.49	9.861279e-08	11	AP2M1	ASC IgG
5.619411e-12	-0.5017001	0.558	0.412	1.001885e-07	11	PPP1CC	ASC IgG
5.929029e-12	-0.4919672	0.298	0.23	1.057087e-07	11	HSD17B11	ASC IgG
6.169902e-12	-0.5092396	0.472	0.36	1.100032e-07	11	NCOR1	ASC IgG
6.397735e-12	-0.3829296	0.188	0.176	1.140652e-07	11	RGS19	ASC IgG
6.68558e-12	-0.3482837	0.173	0.159	1.191972e-07	11	RBCK1	ASC IgG
7.310291e-12	-0.3841917	0.15	0.145	1.303352e-07	11	TSPYL1	ASC IgG
7.68975e-12	-0.3733184	0.19	0.177	1.371005e-07	11	POLR3GL	ASC IgG
7.847261e-12	-0.374062	0.151	0.145	1.399088e-07	11	PAFAH1B2	ASC IgG
8.739305e-12	-0.3395288	0.152	0.15	1.558131e-07	11	ADSS	ASC IgG
8.788454e-12	-0.3019949	0.109	0.113	1.566894e-07	11	SENP6	ASC IgG
9.042401e-12	-0.3595484	0.205	0.185	1.61217e-07	11	NECAP2	ASC IgG
9.075358e-12	-0.3149686	0.154	0.147	1.618046e-07	11	RBM38	ASC IgG
9.107986e-12	-0.3985882	0.236	0.211	1.623863e-07	11	PBDC1	ASC IgG
9.223113e-12	-0.5251223	0.242	0.209	1.644389e-07	11	MGST3	ASC IgG
9.266542e-12	-0.3418268	0.138	0.134	1.652132e-07	11	ZNF75A	ASC IgG
1.045508e-11	-0.3395134	0.165	0.154	1.864036e-07	11	PDCD6IP	ASC IgG
1.06913e-11	-0.5889318	0.821	0.648	1.906152e-07	11	UBE2D3	ASC IgG
1.122645e-11	-0.3347651	0.123	0.122	2.001564e-07	11	SP140L	ASC IgG
1.128175e-11	-0.435718	0.306	0.243	2.011424e-07	11	EPC1	ASC IgG
1.142826e-11	-0.4487317	0.335	0.258	2.037545e-07	11	MAT2B	ASC IgG

1.185257e-11	-0.6214426	0.581	0.441	2.113194e-07	11	C1QBP	ASC IgG
1.28284e-11	-0.3304864	0.154	0.148	2.287175e-07	11	UBE2D1	ASC IgG
1.283717e-11	-0.3629564	0.201	0.18	2.28874e-07	11	PLRG1	ASC IgG
1.297796e-11	-0.4396578	0.321	0.261	2.31384e-07	11	ANP32E	ASC IgG
1.35191e-11	-0.6477946	0.394	0.315	2.410321e-07	11	DDX21	ASC IgG
1.363352e-11	-0.4985801	0.449	0.327	2.43072e-07	11	TSPO	ASC IgG
1.37236e-11	-0.4450097	0.331	0.275	2.446781e-07	11	QARS	ASC IgG
1.432541e-11	-0.5312151	0.473	0.365	2.554078e-07	11	PFDN2	ASC IgG
1.48092e-11	-0.3157552	0.159	0.153	2.640333e-07	11	DTYMK	ASC IgG
1.531809e-11	-0.3542734	0.222	0.195	2.731062e-07	11	GRSF1	ASC IgG
1.694221e-11	-0.3046945	0.161	0.14	3.020627e-07	11	MKNK2	ASC IgG
1.797183e-11	-0.3890093	0.255	0.219	3.204198e-07	11	NUTF2	ASC IgG
1.902995e-11	-0.3775801	0.256	0.214	3.39285e-07	11	IMMT	ASC IgG
1.972618e-11	-0.5340372	0.646	0.477	3.516981e-07	11	TRA2B	ASC IgG
2.133914e-11	-0.4071293	0.215	0.19	3.804555e-07	11	AP2B1	ASC IgG
2.152693e-11	-0.3136544	0.105	0.117	3.838037e-07	11	VRK2	ASC IgG
2.244784e-11	-0.3066024	0.139	0.143	4.002225e-07	11	STK25	ASC IgG
2.246758e-11	-0.4268819	0.272	0.222	4.005745e-07	11	PHIP	ASC IgG
2.247272e-11	-0.4937508	0.461	0.35	4.006661e-07	11	RTF1	ASC IgG
2.452595e-11	-0.3079199	0.173	0.159	4.372731e-07	11	VRK1	ASC IgG
2.603457e-11	-0.3335003	0.15	0.146	4.641703e-07	11	PANK2	ASC IgG
2.861249e-11	-0.4626324	0.367	0.302	5.101321e-07	11	KARS	ASC IgG
2.970872e-11	-0.4319268	0.31	0.239	5.296768e-07	11	DRAM2	ASC IgG
2.973447e-11	-0.3237952	0.178	0.164	5.301358e-07	11	SAE1	ASC IgG
3.180869e-11	-0.4055845	0.24	0.205	5.671171e-07	11	CFDP1	ASC IgG

3.3098e-11	-0.3966436	0.094	0.134	5.901043e-07	11	SULF2	ASC IgG
3.31884e-11	-0.5370622	0.53	0.413	5.917159e-07	11	PRRC2C	ASC IgG
4.099545e-11	-0.3110338	0.125	0.123	7.309078e-07	11	HTATIP2	ASC IgG
4.512967e-11	-0.3909944	0.26	0.221	8.046168e-07	11	ADH5	ASC IgG
4.909228e-11	-0.3066526	0.166	0.155	8.752662e-07	11	RPP30	ASC IgG
5.03803e-11	-0.3020753	0.122	0.117	8.982303e-07	11	CRYZL1	ASC IgG
5.707594e-11	-0.366344	0.241	0.21	1.017607e-06	11	EEF1E1	ASC IgG
6.230753e-11	-0.3226885	0.173	0.163	1.110881e-06	11	PPID	ASC IgG
6.456191e-11	-0.5536939	0.959	0.886	1.151074e-06	11	GAPDH	ASC IgG
6.851764e-11	-0.3665459	0.123	0.123	1.221601e-06	11	DDAH2	ASC IgG
6.992571e-11	-0.305946	0.125	0.127	1.246706e-06	11	CHMP1A	ASC IgG
7.394553e-11	-0.3348754	0.161	0.151	1.318375e-06	11	TATDN1	ASC IgG
8.001869e-11	-0.4952291	0.527	0.377	1.426653e-06	11	LSM8	ASC IgG
8.692109e-11	-0.4965861	0.809	0.648	1.549716e-06	11	YWHAB	ASC IgG
9.356155e-11	-0.4197223	0.28	0.237	1.668109e-06	11	KRR1	ASC IgG
1.045403e-10	-0.3392242	0.093	0.109	1.863848e-06	11	SNX9	ASC IgG
1.12118e-10	-0.3620012	0.279	0.226	1.998951e-06	11	NSMCE1	ASC IgG
1.149328e-10	-0.3767294	0.273	0.226	2.049137e-06	11	DDX54	ASC IgG
1.262208e-10	-0.3421666	0.225	0.195	2.25039e-06	11	UBE2E1	ASC IgG
1.359876e-10	-0.3357578	0.183	0.167	2.424523e-06	11	MIF4GD	ASC IgG
1.382373e-10	-0.3509871	0.179	0.16	2.464633e-06	11	COX19	ASC IgG
1.384309e-10	-0.4428716	0.302	0.243	2.468085e-06	11	PFKL	ASC IgG
1.491387e-10	-0.3561922	0.118	0.131	2.658993e-06	11	RP9	ASC IgG
1.520835e-10	-0.5426478	0.581	0.449	2.711497e-06	11	VDAC1	ASC IgG
1.582594e-10	-0.3679851	0.216	0.189	2.821608e-06	11	OARD1	ASC IgG

1.610402e-10	-0.3903	0.283	0.232	2.871186e-06	11	PHF5A	ASC IgG
1.647898e-10	-0.4708615	0.428	0.332	2.938038e-06	11	TPR	ASC IgG
1.683633e-10	-0.3182822	0.154	0.145	3.00175e-06	11	C1orf35	ASC IgG
1.777494e-10	-0.3012046	0.143	0.143	3.169093e-06	11	NIP7	ASC IgG
1.850562e-10	-0.4027406	0.293	0.247	3.299367e-06	11	MEA1	ASC IgG
1.864023e-10	-0.3506123	0.221	0.188	3.323367e-06	11	PPP2R2A	ASC IgG
2.231259e-10	-0.4805484	0.462	0.344	3.978111e-06	11	RBM17	ASC IgG
2.25509e-10	-0.4864638	0.345	0.279	4.0206e-06	11	MSMO1	ASC IgG
2.308508e-10	-0.3052148	0.165	0.158	4.115839e-06	11	TRIAP1	ASC IgG
2.389036e-10	-0.3421438	0.245	0.204	4.259412e-06	11	SNAPIN	ASC IgG
2.623759e-10	-0.3414618	0.151	0.129	4.677899e-06	11	CLK4	ASC IgG
2.67102e-10	-0.4027454	0.268	0.22	4.762161e-06	11	POLE3	ASC IgG
2.718992e-10	-0.3559743	0.234	0.199	4.847691e-06	11	THYN1	ASC IgG
2.783204e-10	-0.3051735	0.181	0.159	4.962174e-06	11	TPGS2	ASC IgG
3.266035e-10	-0.4057699	0.293	0.239	5.823014e-06	11	SUMO3	ASC IgG
3.296808e-10	-0.3468778	0.201	0.178	5.877878e-06	11	DUSP11	ASC IgG
3.540901e-10	-0.3741154	0.287	0.239	6.313072e-06	11	SRSF1	ASC IgG
3.543142e-10	-0.323655	0.159	0.151	6.317068e-06	11	AFG3L2	ASC IgG
3.58414e-10	-0.3338435	0.192	0.171	6.390163e-06	11	THOC3	ASC IgG
3.727064e-10	-0.4167811	0.333	0.274	6.644982e-06	11	TMEM14B	ASC IgG
3.942464e-10	-0.3497767	0.167	0.153	7.02902e-06	11	RRP15	ASC IgG
4.183632e-10	-0.350586	0.2	0.182	7.458997e-06	11	AHCY	ASC IgG
4.719489e-10	-0.3225764	0.218	0.176	8.414377e-06	11	PLIN3	ASC IgG
5.861828e-10	-0.3611545	0.245	0.208	1.045105e-05	11	MRPS35	ASC IgG
5.87755e-10	-0.5192887	0.674	0.497	1.047908e-05	11	RTRAF	ASC IgG

6.815381e-10	-0.3375675	0.204	0.184	1.215114e-05	11	HDAC3	ASC IgG
7.021239e-10	-0.3624723	0.207	0.179	1.251817e-05	11	MICU2	ASC IgG
7.129424e-10	-0.3554614	0.187	0.168	1.271105e-05	11	TMEM154	ASC IgG
7.754372e-10	-0.3572079	0.222	0.189	1.382527e-05	11	LYPLA2	ASC IgG
7.860657e-10	-0.3419063	0.177	0.156	1.401477e-05	11	GAR1	ASC IgG
7.906937e-10	-0.3203733	0.179	0.155	1.409728e-05	11	COMMD2	ASC IgG
8.021305e-10	-0.4173056	0.345	0.269	1.430118e-05	11	XRN2	ASC IgG
8.172872e-10	-0.531094	0.86	0.691	1.457141e-05	11	ATP5MPL	ASC IgG
9.167701e-10	-0.3535479	0.191	0.162	1.634509e-05	11	KIAA1551	ASC IgG
1.122041e-09	-0.3845061	0.257	0.215	2.000487e-05	11	AATF	ASC IgG
1.130211e-09	-0.3662147	0.264	0.214	2.015053e-05	11	HAUS1	ASC IgG
1.207531e-09	-0.3378022	0.216	0.18	2.152906e-05	11	MRPL44	ASC IgG
1.215706e-09	-0.5066398	0.588	0.452	2.167482e-05	11	PSMD4	ASC IgG
1.231735e-09	-0.3363362	0.17	0.156	2.19606e-05	11	NAMPT	ASC IgG
1.242595e-09	-0.4817175	0.506	0.381	2.215423e-05	11	UQCRC2	ASC IgG
1.247834e-09	-0.5294926	0.646	0.471	2.224763e-05	11	N4BP2L2	ASC IgG
1.25216e-09	-0.3932637	0.308	0.252	2.232476e-05	11	DDX27	ASC IgG
1.2637e-09	-0.4397464	0.418	0.312	2.25305e-05	11	FAM133B	ASC IgG
1.276619e-09	-0.3456796	0.17	0.154	2.276085e-05	11	FAM162A	ASC IgG
1.323078e-09	-0.351958	0.153	0.133	2.358917e-05	11	ODF2L	ASC IgG
1.492283e-09	-0.4136533	0.322	0.259	2.660591e-05	11	BAZ1A	ASC IgG
1.596694e-09	-0.4117504	0.349	0.265	2.846746e-05	11	MIEN1	ASC IgG
1.61042e-09	-0.3126767	0.172	0.148	2.871218e-05	11	GIT2	ASC IgG
1.61763e-09	-0.615408	0.647	0.488	2.884073e-05	11	ANKRD12	ASC IgG
1.620132e-09	-0.3368884	0.2	0.169	2.888534e-05	11	ZFR	ASC IgG

1.672683e-09	−0.5353879	0.536	0.384	2.982227e-05	11	EVI2B	ASC IgG
1.71775e-09	−0.5024571	0.556	0.413	3.062576e-05	11	RAB7A	ASC IgG
1.730478e-09	−0.341816	0.196	0.164	3.08527e-05	11	C1orf131	ASC IgG
1.74622e-09	−0.4894666	0.507	0.387	3.113336e-05	11	IK	ASC IgG
1.876633e-09	−0.3899756	0.268	0.224	3.345848e-05	11	DCAF13	ASC IgG
2.167594e-09	−0.5578204	0.751	0.601	3.864603e-05	11	RBM3	ASC IgG
2.370463e-09	−0.409444	0.357	0.277	4.226298e-05	11	VBP1	ASC IgG
2.526158e-09	−0.405014	0.313	0.235	4.503888e-05	11	ZNF22	ASC IgG
2.75148e-09	−0.3396673	0.173	0.142	4.905613e-05	11	DUSP1	ASC IgG
2.883383e-09	−0.5399965	0.837	0.693	5.140783e-05	11	HLA−DQB1	ASC IgG
2.936879e-09	−0.4149145	0.359	0.28	5.236162e-05	11	GGNBP2	ASC IgG
3.074267e-09	−0.41518	0.301	0.239	5.481111e-05	11	RBM23	ASC IgG
3.22492e-09	−0.3343086	0.18	0.167	5.74971e-05	11	MMAB	ASC IgG
3.812825e-09	−0.4026133	0.325	0.245	6.797885e-05	11	OSTF1	ASC IgG
4.064252e-09	−0.4097467	0.22	0.181	7.246155e-05	11	CMSS1	ASC IgG
4.160615e-09	−0.3276063	0.176	0.149	7.41796e-05	11	CHD1	ASC IgG
4.595859e-09	−0.4102452	0.321	0.247	8.193956e-05	11	SH3KBP1	ASC IgG
4.649693e-09	−0.3645293	0.321	0.252	8.289938e-05	11	NDUFA10	ASC IgG
4.852751e-09	−0.4628444	0.409	0.303	8.65197e-05	11	EAPP	ASC IgG
4.912401e-09	−0.4449189	0.367	0.286	8.75832e-05	11	U2SURP	ASC IgG
5.439726e-09	−0.3909373	0.321	0.252	9.698488e-05	11	LUC7L2	ASC IgG
5.589937e-09	−0.3244617	0.177	0.151	9.9663e-05	11	LRCH4	ASC IgG
6.219513e-09	−0.4118048	0.287	0.235	0.0001108877	11	NIFK	ASC IgG
6.450763e-09	−0.4338587	0.376	0.278	0.0001150107	11	HP1BP3	ASC IgG
6.645494e-09	−0.4311606	0.307	0.239	0.0001184825	11	HLA−F	ASC IgG

6.753945e-09	-0.3416684	0.221	0.181	0.0001204161	11	
SBNO1	ASC IgG					
6.837192e-09	-0.3666516	0.247	0.192	0.0001219003	11	
KRCC1	ASC IgG					
7.085848e-09	-0.3089748	0.141	0.134	0.0001263336	11	
NDUFAF2	ASC IgG					
7.220454e-09	-0.354238	0.235	0.192	0.0001287335	11	
DCAF7	ASC IgG					
7.355151e-09	-0.3448642	0.232	0.196	0.000131135	11	
SF3B4	ASC IgG					
7.543523e-09	-0.3401346	0.154	0.142	0.0001344935	11	
NOP16	ASC IgG					
7.844561e-09	-0.4976208	0.538	0.392	0.0001398607	11	
ATP5PD	ASC IgG					
7.943505e-09	-0.4930669	0.504	0.384	0.0001416247	11	
EIF4H	ASC IgG					
8.140846e-09	-0.3498318	0.241	0.203	0.0001451431	11	
PXK	ASC IgG					
8.463172e-09	-0.3276046	0.199	0.157	0.0001508899	11	
ZNF638	ASC IgG					
8.671258e-09	-0.4339467	0.614	0.451	0.0001545999	11	
CAPZA1	ASC IgG					
9.061795e-09	-0.4699173	0.392	0.276	0.0001615627	11	
RNASET2	ASC IgG					
9.073607e-09	-0.3160579	0.202	0.172	0.0001617733	11	
BCL7B	ASC IgG					
9.180144e-09	-0.3324066	0.206	0.166	0.0001636728	11	
YAF2	ASC IgG					
9.333232e-09	-0.4755796	0.458	0.35	0.0001664022	11	
PHB2	ASC IgG					
9.756137e-09	-0.3642941	0.255	0.206	0.0001739422	11	
ITPA	ASC IgG					
1.008627e-08	-0.4879948	0.698	0.497	0.000179828	11	
PSMB9	ASC IgG					
1.068721e-08	-0.3132078	0.209	0.176	0.0001905422	11	
ZNRD1	ASC IgG					
1.087432e-08	-0.4354017	0.466	0.331	0.0001938783	11	
FAM96A	ASC IgG					
1.108661e-08	-0.4865132	0.507	0.376	0.0001976633	11	
RBM25	ASC IgG					
1.139081e-08	-0.3916729	0.384	0.281	0.0002030868	11	
DPY30	ASC IgG					
1.229719e-08	-0.4120519	0.329	0.244	0.0002192466	11	
PSMB8-AS1		ASC IgG				
1.236399e-08	-0.332887	0.173	0.151	0.0002204376	11	
PPAN	ASC IgG					
1.355331e-08	-0.4236781	0.285	0.232	0.0002416419	11	
CDK4	ASC IgG					
1.410737e-08	-0.4956116	0.489	0.358	0.0002515203	11	
ANXA6	ASC IgG					

1.432253e-08	-0.5987533	0.684	0.53	0.0002553565	11	ATP6V1F	ASC IgG
1.51713e-08	-0.4714343	0.331	0.24	0.0002704891	11	KPNA2	ASC IgG
1.537573e-08	-0.3101824	0.177	0.152	0.0002741339	11	PGD	ASC IgG
1.549875e-08	-0.3812816	0.313	0.244	0.0002763272	11	MRPL9	ASC IgG
1.769993e-08	-0.4136474	0.355	0.273	0.000315572	11	YY1	ASC IgG
1.863929e-08	-0.3242936	0.252	0.203	0.00033232	11	COPS4	ASC IgG
1.886747e-08	-0.4271436	0.447	0.333	0.0003363881	11	PPM1G	ASC IgG
1.9439e-08	-0.3553716	0.162	0.145	0.000346578	11	SERPINB9	ASC IgG
1.958667e-08	-0.3894305	0.327	0.247	0.0003492108	11	THOC2	ASC IgG
2.046423e-08	-0.4102102	0.31	0.24	0.0003648568	11	ABCF1	ASC IgG
2.169097e-08	-0.3170618	0.176	0.155	0.0003867284	11	PEX16	ASC IgG
2.216557e-08	-0.3617299	0.194	0.154	0.00039519	11	JUND	ASC IgG
2.324378e-08	-0.473696	0.477	0.358	0.0004144134	11	UPF2	ASC IgG
2.350956e-08	-0.4250601	0.339	0.257	0.0004191519	11	C9orf78	ASC IgG
2.443956e-08	-0.4563482	0.943	0.863	0.0004357329	11	UBC	ASC IgG
2.452325e-08	-0.3762944	0.368	0.29	0.0004372251	11	HSPA4	ASC IgG
2.654863e-08	-0.3001456	0.232	0.18	0.0004733355	11	PCGF5	ASC IgG
2.715404e-08	-0.3137697	0.143	0.128	0.0004841294	11	C19orf66	ASC IgG
2.865546e-08	-0.3347063	0.261	0.2	0.0005108982	11	NUBP1	ASC IgG
2.878929e-08	-0.3183316	0.194	0.161	0.0005132843	11	PDCL3	ASC IgG
3.069429e-08	-0.3563312	0.249	0.191	0.0005472486	11	MAX	ASC IgG
3.347575e-08	-0.304416	0.194	0.16	0.0005968391	11	NIPSNAP2	ASC IgG
3.372454e-08	-0.5391241	0.726	0.545	0.0006012749	11	PSMA1	ASC IgG
3.395895e-08	-0.3726456	0.264	0.209	0.0006054541	11	DNTTIP2	ASC IgG
3.537583e-08	-0.5910529	0.576	0.44	0.0006307158	11	ANXA2	ASC IgG

3.636008e-08	-0.3173911	0.197	0.163	0.0006482639	11	
RDH14	ASC IgG					
4.221828e-08	-0.3713889	0.287	0.218	0.0007527098	11	
BNIP2	ASC IgG					
4.508745e-08	-0.3558289	0.246	0.19	0.0008038641	11	
USP15	ASC IgG					
4.62688e-08	-0.3977535	0.346	0.263	0.0008249265	11	
PDCD2	ASC IgG					
5.438469e-08	-0.4615939	0.444	0.353	0.0009696247	11	
SQLE	ASC IgG					
6.156381e-08	-0.7168762	0.784	0.591	0.001097621	11	
LDHA	ASC IgG					
6.201376e-08	-0.3832933	0.352	0.264	0.001105643	11	
PMF1	ASC IgG					
6.678197e-08	-0.309727	0.215	0.183	0.001190656	11	
GNL1	ASC IgG					
6.911076e-08	-0.301952	0.195	0.156	0.001232176	11	
ZNF148	ASC IgG					
7.22032e-08	-0.4188967	0.329	0.252	0.001287311	11	
DTNBP1	ASC IgG					
7.355136e-08	-0.4506698	0.396	0.31	0.001311347	11	
LPXN	ASC IgG					
9.15141e-08	-0.4041195	0.418	0.304	0.001631605	11	
SHKBP1	ASC IgG					
9.353857e-08	-0.352383	0.226	0.195	0.001667699	11	
MRPL1	ASC IgG					
1.032688e-07	-0.3630425	0.306	0.231	0.001841179	11	
SMARCA5	ASC IgG					
1.082257e-07	-0.3713309	0.311	0.232	0.001929555	11	
MRFAP1	ASC IgG					
1.086683e-07	-0.4358532	0.306	0.204	0.001937448	11	
C16orf74	ASC IgG					
1.176571e-07	-0.3868446	0.273	0.215	0.002097708	11	
FAM204A	ASC IgG					
1.20895e-07	-0.3677632	0.394	0.299	0.002155436	11	
AHSA1	ASC IgG					
1.246921e-07	-0.4122952	0.343	0.255	0.002223136	11	
MRPS26	ASC IgG					
1.278756e-07	-0.3703073	0.325	0.255	0.002279895	11	
C1D	ASC IgG					
1.308358e-07	-0.3367442	0.202	0.156	0.002332672	11	
SHISA5	ASC IgG					
1.319248e-07	-0.3683242	0.245	0.193	0.002352088	11	
CHMP3	ASC IgG					
1.321906e-07	-0.3019761	0.197	0.162	0.002356827	11	
BLOC1S4	ASC IgG					
1.391425e-07	-0.4324385	0.438	0.317	0.002480771	11	
IGBP1	ASC IgG					
1.430203e-07	-0.4456456	0.655	0.486	0.002549909	11	
SF3B6	ASC IgG					

1.452675e-07	-0.3788357	0.227	0.185	0.002589974	11	WDR43	ASC IgG
1.605644e-07	-0.3090083	0.206	0.163	0.002862703	11	PPP1R18	ASC IgG
1.784629e-07	-0.4696465	0.706	0.531	0.003181816	11	CAP1	ASC IgG
1.796417e-07	-0.4980781	0.895	0.718	0.003202833	11	COX6C	ASC IgG
1.874157e-07	-0.3002608	0.183	0.149	0.003341435	11	NIPBL	ASC IgG
1.912644e-07	-0.4984632	0.616	0.439	0.003410053	11	CCT7	ASC IgG
2.172473e-07	-0.3476727	0.276	0.205	0.003873302	11	EBLN3P	ASC IgG
2.193099e-07	-0.4014267	0.411	0.302	0.003910077	11	ELAVL1	ASC IgG
2.278379e-07	-0.5003699	0.67	0.492	0.004062123	11	EIF4G2	ASC IgG
2.286898e-07	-0.3647071	0.298	0.235	0.00407731	11	TPRKB	ASC IgG
2.304384e-07	-0.3421907	0.307	0.238	0.004108487	11	CPSF6	ASC IgG
2.397813e-07	-0.4116988	0.373	0.278	0.00427506	11	SMCHD1	ASC IgG
2.444481e-07	-0.3313767	0.241	0.191	0.004358265	11	FYTTD1	ASC IgG
2.46555e-07	-0.6256496	0.679	0.492	0.004395829	11	HSPE1	ASC IgG
2.595398e-07	-0.4837264	0.702	0.518	0.004627335	11	TRAPPC1	ASC IgG
2.734849e-07	-0.4574776	0.532	0.387	0.004875962	11	LAMTOR4	ASC IgG
2.752273e-07	-0.3138048	0.253	0.192	0.004907028	11	UBE2E3	ASC IgG
2.798808e-07	-0.3066543	0.203	0.156	0.004989994	11	NSUN5	ASC IgG
2.94072e-07	-0.308149	0.184	0.144	0.00524301	11	PHF3	ASC IgG
2.972456e-07	-0.3501528	0.265	0.204	0.005299592	11	ARL2BP	ASC IgG
3.069991e-07	-0.3882891	0.432	0.322	0.005473487	11	SNRNP70	ASC IgG
3.246115e-07	-0.3711413	0.294	0.224	0.005787498	11	METTL26	ASC IgG
3.42605e-07	-0.3969455	0.296	0.222	0.006108304	11	MAGED2	ASC IgG
3.437055e-07	-0.3437985	0.207	0.168	0.006127925	11	CYSTM1	ASC IgG
3.504837e-07	-0.4277397	0.458	0.346	0.006248773	11	FKBP8	ASC IgG

3.708384e-07	-0.3404816	0.198	0.157	0.006611679	11	TPP1	ASC IgG
3.82856e-07	-0.3223252	0.2	0.161	0.006825939	11	FNIP1	ASC IgG
4.184727e-07	-0.3753077	0.29	0.223	0.007460949	11	RPF1	ASC IgG
4.364511e-07	-0.6150084	0.625	0.457	0.007781486	11	HSPD1	ASC IgG
4.381465e-07	-0.3026593	0.204	0.157	0.007811714	11	OSER1	ASC IgG
4.404228e-07	-0.4212399	0.436	0.303	0.007852299	11	CWC15	ASC IgG
5.23862e-07	-0.4004145	0.279	0.203	0.009339935	11	RALGPS2	ASC IgG
5.246876e-07	-0.3291027	0.271	0.205	0.009354655	11	HADHB	ASC IgG
5.587812e-07	-0.3031945	0.213	0.164	0.00996251	11	KATNBL1	ASC IgG
5.648859e-07	-0.3278102	0.201	0.165	0.01007135	11	C8orf33	ASC IgG
5.913021e-07	-0.4547543	0.763	0.58	0.01054233	11	NDUFV2	ASC IgG
6.174241e-07	-0.5011895	0.82	0.635	0.01100805	11	ATP5MF	ASC IgG
6.266021e-07	-0.4405319	0.523	0.388	0.01117169	11	MAF1	ASC IgG
6.374609e-07	-0.4209723	0.337	0.24	0.01136529	11	SLBP	ASC IgG
6.6062e-07	-0.4806327	0.614	0.446	0.01177819	11	NDUFB9	ASC IgG
6.706835e-07	-0.4190141	0.439	0.321	0.01195762	11	PUF60	ASC IgG
7.120354e-07	-0.3354596	0.325	0.237	0.01269488	11	PTRHD1	ASC IgG
7.839866e-07	-0.464103	0.497	0.371	0.0139777	11	UBE2B	ASC IgG
8.220569e-07	-0.4363819	0.509	0.36	0.01465645	11	PPP2CA	ASC IgG
8.243638e-07	-0.3571826	0.309	0.235	0.01469758	11	STIP1	ASC IgG
8.441365e-07	-0.3557197	0.332	0.245	0.01505011	11	SNRPA1	ASC IgG
8.694329e-07	-0.300956	0.22	0.174	0.01550112	11	CDC5L	ASC IgG
8.707535e-07	-0.4212316	0.424	0.313	0.01552466	11	TCP1	ASC IgG
8.769168e-07	-0.4057318	0.344	0.246	0.01563455	11	BBX	ASC IgG
9.07035e-07	-0.3592842	0.308	0.23	0.01617153	11	PIN4	ASC IgG

9.118953e-07	-0.3802872	0.667	0.519	0.01625818	11	FKBP1A	ASC IgG
9.120391e-07	-0.3721536	0.271	0.197	0.01626075	11	CLK1	ASC IgG
9.141401e-07	-0.4954081	0.603	0.439	0.0162982	11	RAD23A	ASC IgG
1.028964e-06	-0.3034365	0.198	0.162	0.0183454	11	KAT5	ASC IgG
1.037543e-06	-0.4441944	0.515	0.377	0.01849836	11	ANXA7	ASC IgG
1.053211e-06	-0.508226	0.708	0.51	0.0187777	11	VAMP2	ASC IgG
1.191759e-06	-0.3275465	0.278	0.205	0.02124788	11	ARHGEF1	ASC IgG
1.224949e-06	-0.372021	0.316	0.236	0.02183962	11	HNRNPAB	ASC IgG
1.428291e-06	-0.409103	0.346	0.234	0.025465	11	GCHFR	ASC IgG
1.498872e-06	-0.4668041	0.65	0.471	0.02672338	11	VAPA	ASC IgG
1.593807e-06	-0.3286897	0.295	0.225	0.02841598	11	PLEKHJ1	ASC IgG
1.668862e-06	-0.4393017	0.388	0.282	0.02975414	11	CNPY3	ASC IgG
1.770516e-06	-0.4225055	0.439	0.323	0.03156652	11	MRPS36	ASC IgG
1.843383e-06	-0.4373225	0.53	0.373	0.03286568	11	SUPT4H1	ASC IgG
1.855982e-06	-0.318782	0.294	0.211	0.0330903	11	RAMMET	ASC IgG
1.977582e-06	-0.4184168	0.448	0.333	0.03525831	11	SEPHS2	ASC IgG
2.069629e-06	-0.3484405	0.276	0.216	0.03689942	11	WDR46	ASC IgG
2.153007e-06	-0.339679	0.247	0.195	0.03838596	11	GTPBP4	ASC IgG
2.45312e-06	-0.4305672	0.443	0.319	0.04373667	11	NMT1	ASC IgG
2.612313e-06	-0.4246966	0.466	0.336	0.04657492	11	STRAP	ASC IgG
2.619144e-06	-0.3159776	0.253	0.186	0.04669672	11	NCKAP1L	ASC IgG
2.680132e-06	-0.4051209	0.455	0.322	0.04778408	11	RAB5C	ASC IgG
2.740698e-06	-0.3204558	0.247	0.19	0.0488639	11	CSNK2A1	ASC IgG
2.84207e-06	-0.4304852	0.514	0.38	0.05067126	11	CHMP4A	ASC IgG
2.966947e-06	-0.4249091	0.473	0.337	0.05289769	11	MED10	ASC IgG

3.241131e-06	-0.4167572	0.835	0.71	0.05778613	11	CLIC1	ASC IgG
3.582038e-06	-0.3609773	0.304	0.205	0.06386415	11	CCDC32	ASC IgG
3.771998e-06	-0.3625476	0.305	0.218	0.06725096	11	NEMF	ASC IgG
3.930092e-06	-0.3678927	0.302	0.228	0.0700696	11	CCDC85B	ASC IgG
4.036519e-06	-0.4343517	0.408	0.309	0.07196711	11	MZT2A	ASC IgG
4.200961e-06	-0.4004833	0.361	0.265	0.07489893	11	CEBPZ	ASC IgG
4.372488e-06	-0.3233833	0.273	0.202	0.07795709	11	FAAP20	ASC IgG
4.717351e-06	-0.3010978	0.298	0.222	0.08410566	11	DAZAP1	ASC IgG
4.870813e-06	-0.4041821	0.505	0.363	0.08684172	11	EWSR1	ASC IgG
4.899689e-06	-0.3992243	0.388	0.278	0.08735656	11	JPT1	ASC IgG
5.67288e-06	-0.3430067	0.358	0.26	0.1011418	11	THOC7	ASC IgG
6.206518e-06	-0.408342	0.461	0.325	0.110656	11	RFTN1	ASC IgG
6.30156e-06	-0.339822	0.351	0.265	0.1123505	11	FIBP	ASC IgG
6.535473e-06	-0.3450264	0.325	0.233	0.1165209	11	ABT1	ASC IgG
7.175566e-06	-0.3959631	0.441	0.315	0.1279332	11	GTF3C6	ASC IgG
7.291899e-06	-0.4210523	0.485	0.339	0.1300073	11	MRPS18C	ASC IgG
7.399008e-06	-0.4205603	0.174	0.124	0.1319169	11	LGALS3	ASC IgG
7.472752e-06	-0.317041	0.224	0.177	0.1332317	11	ABCE1	ASC IgG
7.963676e-06	-0.3355141	0.282	0.219	0.1419844	11	RNF187	ASC IgG
8.027388e-06	-0.3407956	0.27	0.197	0.1431203	11	FNBP4	ASC IgG
8.407777e-06	-0.4172043	0.464	0.328	0.1499023	11	PSMC6	ASC IgG
8.787686e-06	-0.4107479	0.301	0.23	0.1566756	11	TARS	ASC IgG
8.874256e-06	-0.4901485	0.795	0.595	0.1582191	11	PSMA2	ASC IgG
9.159659e-06	-0.3794167	0.397	0.275	0.1633076	11	CNIH1	ASC IgG
9.204219e-06	-0.3924909	0.458	0.318	0.164102	11	GNB2	ASC IgG

9.368192e-06	-0.4573338	0.75	0.549	0.1670255	11	NEDD8	ASC	IgG
9.746765e-06	-0.3438213	0.316	0.227	0.1737751	11	BTF3L4	ASC	IgG
9.937505e-06	-0.4750963	0.379	0.234	0.1771758	11	CD55	ASC	IgG
9.994513e-06	-0.3122229	0.261	0.191	0.1781922	11	TRMT10C	ASC	IgG
1.138791e-05	-0.3616074	0.315	0.231	0.2030351	11	RALA	ASC	IgG
1.140842e-05	-0.3126596	0.236	0.173	0.2034008	11	CRBN	ASC	IgG
1.14523e-05	-0.3075454	0.286	0.215	0.204183	11	SMAP2	ASC	IgG
1.168746e-05	-0.3127512	0.288	0.22	0.2083757	11	MRPL4	ASC	IgG
1.267276e-05	-0.3312897	0.305	0.219	0.2259426	11	CAPN1	ASC	IgG
1.333923e-05	-0.4726515	0.823	0.631	0.2378252	11	SET	ASC	IgG
1.402981e-05	-0.3850799	0.501	0.362	0.2501375	11	POLR2E	ASC	IgG
1.642887e-05	-0.3104252	0.256	0.201	0.2929104	11	MAP2K3	ASC	IgG
1.64334e-05	-0.3548147	0.335	0.245	0.2929912	11	RPL7L1	ASC	IgG
1.662567e-05	-0.3853007	0.321	0.249	0.2964191	11	GARS	ASC	IgG
1.790545e-05	-0.4222259	0.668	0.486	0.3192363	11	RAC2	ASC	IgG
1.877705e-05	-0.3069188	0.337	0.246	0.3347761	11	MRPL13	ASC	IgG
1.887203e-05	-0.338335	0.321	0.24	0.3364694	11	CEP57	ASC	IgG
1.943606e-05	-0.3082252	0.306	0.219	0.3465255	11	MPLKIP	ASC	IgG
1.952573e-05	-0.4042821	0.303	0.243	0.3481242	11	GSTO1	ASC	IgG
1.987445e-05	-0.3258774	0.343	0.248	0.3543416	11	MRPL28	ASC	IgG
2.080821e-05	-0.323595	0.208	0.169	0.3709895	11	DDIT3	ASC	IgG
2.208728e-05	-0.3120127	0.242	0.183	0.3937941	11	SCNM1	ASC	IgG
2.283551e-05	-0.4045555	0.356	0.241	0.4071343	11	GCC2	ASC	IgG
2.314625e-05	-0.3039807	0.268	0.193	0.4126745	11	FIP1L1	ASC	IgG
2.37631e-05	-0.4103031	0.337	0.253	0.4236723	11	NARS	ASC	IgG

2.455483e-05	-0.3264486	0.233	0.166	0.437788	11	BNIP3L	ASC IgG
2.489089e-05	-0.3989122	0.459	0.316	0.4437797	11	PRPF40A	ASC IgG
2.968275e-05	-0.4508875	0.595	0.421	0.5292137	11	TAF7	ASC IgG
3.321549e-05	-0.3698116	0.435	0.31	0.592199	11	ZNF207	ASC IgG
3.56286e-05	-0.4069641	0.471	0.349	0.6352223	11	CHMP4B	ASC IgG
4.222527e-05	-0.3772683	0.464	0.325	0.7528343	11	PPP2R1A	ASC IgG
5.086017e-05	-0.3261338	0.26	0.199	0.9067859	11	SRSF6	ASC IgG
5.129072e-05	-0.4656984	0.686	0.499	0.9144622	11	EIF3I	ASC IgG
5.183867e-05	-0.4695519	0.508	0.357	0.9242317	11	HSPA9	ASC IgG
5.410635e-05	-0.3328001	0.295	0.206	0.9646621	11	MIS18BP1	ASC IgG
5.523641e-05	-0.3123601	0.291	0.212	0.98481	11	TIAL1	ASC IgG
5.569204e-05	-0.3118619	0.276	0.2	0.9929334	11	NGDN	ASC IgG
6.0695e-05	-0.3041584	0.279	0.199	1	11	ZC3H13	ASC IgG
6.115913e-05	-0.3153287	0.337	0.244	1	11	PRPF31	ASC IgG
6.554781e-05	-0.3645772	0.391	0.277	1	11	DCTN2	ASC IgG
6.63391e-05	-0.3657405	0.957	0.858	1	11	UBB	ASC IgG
7.209862e-05	0.3941009	0.941	0.748	1	11	HLA-DRB1	ASC IgG
8.899774e-05	-0.4195857	0.497	0.354	1	11	C8orf59	ASC IgG
8.918825e-05	-0.4045146	0.405	0.285	1	11	AIMP1	ASC IgG
9.663501e-05	-0.3789972	0.326	0.226	1	11	NT5C3A	ASC IgG
0.0001019884	-0.3816514	0.27	0.216	1	11	EIF4EBP1	ASC IgG
0.0001067095	-0.3607597	0.839	0.671	1	11	ATP6V1G1	ASC IgG
0.0001174777	-0.3675296	0.273	0.188	1	11	RNF145	ASC IgG
0.0001237901	-0.3081236	0.306	0.21	1	11	WIPI2	ASC IgG
0.0001239736	-0.3951091	0.514	0.358	1	11	DNAJC7	ASC IgG

0.000127333 -0.3475134 0.349 0.245 1 11
PRPF4B ASC IgG
0.0001342094 -0.3676495 0.363 0.249 1 11
NFE2L2 ASC IgG
0.0001347543 -0.3052328 0.337 0.247 1 11
SHOC2 ASC IgG
0.0001368234 -0.3909781 0.405 0.27 1 11
RSRP1 ASC IgG
0.0001448731 -0.4198685 0.547 0.398 1 11
PSMC5 ASC IgG
0.0001450308 -0.3195435 0.331 0.235 1 11
METTL9 ASC IgG
0.0001480963 -0.3042312 0.203 0.132 1 11
NEIL1 ASC IgG
0.0001619348 -0.3262923 0.315 0.223 1 11
FAM32A ASC IgG
0.0001806472 -0.4820945 0.794 0.573 1 11
LDHB ASC IgG
0.0001840693 -0.3691517 0.345 0.242 1 11
LAMP1 ASC IgG
0.0001860466 -0.3163166 0.239 0.166 1 11
SAMSN1 ASC IgG
0.0001924566 -0.3925394 0.488 0.324 1 11
CCNDBP1 ASC IgG
0.0001933051 -0.3115026 0.287 0.201 1 11
SLU7 ASC IgG
0.0002082649 -0.3351485 0.447 0.302 1 11
OCIAD2 ASC IgG
0.0002129668 -0.3187408 0.243 0.161 1 11
TNFRSF14 ASC IgG
0.0002211777 -0.4130701 0.618 0.424 1 11
KTN1 ASC IgG
0.0002314185 -0.304553 0.265 0.182 1 11
CSNK1D ASC IgG
0.0002385756 -0.326705 0.329 0.237 1 11
MRPL15 ASC IgG
0.0002430947 -0.3322763 0.332 0.231 1 11
CNDP2 ASC IgG
0.0002478714 -0.4133349 0.603 0.414 1 11
HNRNPD ASC IgG
0.0002515677 -0.4721302 0.789 0.567 1 11
PARK7 ASC IgG
0.0002575692 -0.3302221 0.306 0.209 1 11
TINF2 ASC IgG
0.0002594575 -0.4057312 0.501 0.348 1 11
NDUFA5 ASC IgG
0.0002930249 -0.378229 0.389 0.283 1 11
ERG28 ASC IgG
0.000312642 -0.3663058 0.523 0.349 1 11
DGUOK ASC IgG

```
0.0003690318    -0.3608318    0.361   0.252   1       11
M6PR    ASC IgG
0.0003735576    -0.3227447    0.382   0.262   1       11      AIP
        ASC IgG
0.0003793986    -0.3348579    0.36    0.247   1       11
CHTOP   ASC IgG
0.0004304966    -0.3130142    0.31    0.229   1       11
TLN1    ASC IgG
0.0004323736    -0.3282027    0.313   0.213   1       11
VPS51   ASC IgG
0.0004675382    -0.4004871    0.622   0.429   1       11
LSM3    ASC IgG
0.0004730601    -0.4567006    0.804   0.599   1       11
NOP10   ASC IgG
0.0005046662    -0.4591343    0.542   0.379   1       11
C19orf70 ASC IgG
0.0005262116    -0.3937045    0.295   0.206   1       11
PIM3    ASC IgG
0.0005311124    -0.3043195    0.862   0.674   1       11
HLA-DMA ASC IgG
0.0005358706    -0.3343244    0.504   0.351   1       11
CD19    ASC IgG
0.0005752778    -0.4201832    0.513   0.368   1       11      DDT
        ASC IgG
0.0006188199    -0.3560312    0.326   0.232   1       11
EIF4A1  ASC IgG
0.0006380707    -0.3521295    0.407   0.284   1       11
PFDN6   ASC IgG
0.0006408443    -0.3973533    0.504   0.345   1       11
DDX17   ASC IgG
0.0006959882    -0.3331917    0.35    0.247   1       11
MRPL42  ASC IgG
0.0007014685    -0.3106042    0.323   0.208   1       11
ASH1L   ASC IgG
0.0007787505    -0.3102617    0.595   0.391   1       11
CSTB    ASC IgG
0.0007951384    -0.3716209    0.425   0.282   1       11
ATF6B   ASC IgG
0.0008355016    -0.3289452    0.394   0.285   1       11
TXN2    ASC IgG
0.000846841     -0.4054691    0.638   0.443   1       11
CSNK2B  ASC IgG
0.000940515     -0.5078351    0.776   0.58    1       11
EIF2S2  ASC IgG
0.0009744697    -0.3761075    0.534   0.382   1       11
VPS28   ASC IgG
0.001023419     -0.4188466    0.458   0.324   1       11
FRG1    ASC IgG
0.001066763     -0.3405968    0.481   0.298   1       11
BCL7A   ASC IgG
```

0.001071586 -0.4035532 0.664 0.472 1 11
GRB2 ASC IgG
0.0011319 -0.3513912 0.266 0.172 1 11
ASAH1 ASC IgG
0.001225035 -0.4467435 0.633 0.45 1 11
DDX18 ASC IgG
0.001226664 -0.4001857 0.433 0.298 1 11
ATP1B3 ASC IgG
0.00122964 -0.3007771 0.338 0.239 1 11
PPP1CB ASC IgG
0.001234929 -0.331334 0.341 0.237 1 11
ANKRD11 ASC IgG
0.001236306 -0.3978862 0.588 0.413 1 11
COX7A2L ASC IgG
0.001248031 -0.3416547 0.39 0.256 1 11
HNRNPH2 ASC IgG
0.001381276 -0.4397993 0.542 0.387 1 11
ARPC5L ASC IgG
0.001437684 -0.3021651 0.22 0.155 1 11
NOLC1 ASC IgG
0.001517665 -0.317024 0.443 0.306 1 11
SF3B1 ASC IgG
0.00167182 -0.3665699 0.567 0.378 1 11
SMARCB1 ASC IgG
0.002514058 -0.3236881 0.449 0.305 1 11
CALM3 ASC IgG
0.002592761 -0.3522346 0.514 0.356 1 11
SDHC ASC IgG
0.002597202 -0.315314 0.333 0.226 1 11
DEGS1 ASC IgG
0.003026232 -1.001076 0.616 0.417 1 11
HIST1H4C ASC IgG
0.003080076 -0.4265649 0.78 0.578 1 11
NDUFB2 ASC IgG
0.003080471 -0.3441893 0.513 0.351 1 11
AP2S1 ASC IgG
0.003144567 -0.3661674 0.505 0.339 1 11
PRPF38B ASC IgG
0.003807589 -0.328187 0.365 0.227 1 11
CIR1 ASC IgG
0.004030879 -0.4176974 0.816 0.621 1 11
ATP6V0E1 ASC IgG
0.004267489 -0.3341094 0.366 0.267 1 11
TIMM10 ASC IgG
0.00431049 -0.4165082 0.637 0.44 1 11
PGAM1 ASC IgG
0.004927038 -0.3354108 0.417 0.287 1 11
RAD23B ASC IgG
0.005657204 -0.3549113 0.518 0.353 1 11
SSNA1 ASC IgG

0.005804675	-0.3026888	0.841	0.569	1	11
SARAF	ASC IgG
0.005890458	-0.4002809	0.721	0.512	1	11
COX8A	ASC IgG
0.006920745	-0.3334119	0.31	0.222	1	11
TMEM109	ASC IgG
0.006974451	-0.3453182	0.602	0.405	1	11
MDH1	ASC IgG
0.00712363	-0.3064492	0.451	0.284	1	11
TUBA4A	ASC IgG
0.007208281	-0.3120811	0.444	0.294	1	11
SDHD	ASC IgG
0.007214844	-0.5182689	0.395	0.265	1	11
GPR183	ASC IgG
0.007807682	-0.3713931	0.549	0.364	1	11
CDC37	ASC IgG
0.007884328	-0.3120679	0.43	0.277	1	11
NDUFS2	ASC IgG
0.007922319	-0.3450385	0.534	0.341	1	11
ABRACL	ASC IgG
0.009100765	-0.3844922	0.897	0.711	1	11
COX6B1	ASC IgG
0.009517634	-0.4605256	0.638	0.473	1	11
SQSTM1	ASC IgG
0.009576882	-0.3937395	0.633	0.421	1	11
MINOS1	ASC IgG
0	3.000747	0.976	0.285	0	12	HMGB2	DZ 3
0	2.560714	0.995	0.709	0	12	HMGN2	DZ 3
0	2.365346	0.858	0.173	0	12	PTTG1	DZ 3
0	2.205517	0.975	0.414	0	12	STMN1	DZ 3
0	1.867187	0.973	0.689	0	12	H2AFZ	DZ 3
0	1.77418	0.988	0.762	0	12	HMGN1	DZ 3
0	1.726624	0.995	0.93	0	12	HMGB1	DZ 3
0	1.628168	0.91	0.345	0	12	H2AFV	DZ 3
0	1.536923	0.661	0.08	0	12	PCLAF	DZ 3
0	1.527815	0.998	0.885	0	12	GAPDH	DZ 3
0	1.477411	0.641	0.08	0	12	WDR66	DZ 3
0	1.46717	0.594	0.051	0	12	BIRC5	DZ 3
0	1.309225	0.83	0.296	0	12	CALM3	DZ 3
0	1.266783	0.613	0.088	0	12	MYBL2	DZ 3
0	1.246498	0.997	0.931	0	12	SERF2	DZ 3
0	1.231091	1	0.989	0	12	ACTB	DZ 3
0	1.198554	0.993	0.848	0	12	ATP5MG	DZ 3
0	1.181296	0.986	0.78	0	12	ACTG1	DZ 3
0	1.121723	0.973	0.842	0	12	HNRNPA2B1	DZ
3
0	1.00433	0.995	0.897	0	12	PFN1	DZ 3
0	0.9719783	0.991	0.866	0	12	ARPC2	DZ
3
0	0.9189811	1	0.917	0	12	CFL1	DZ

3

0	0.758067	0.998	0.999	0	12	PTMA	DZ 3
0	-2.326285	0.995	1	0	12	MALAT1	DZ 3
0	-2.399661	0.175	0.712	0	12	EMP3	DZ 3
3.45846e-323	1.302275	0.907	0.473	6.166087e-319	12	RPS27L	DZ 3
5.582942e-322	1.188048	0.937	0.542	9.953827e-318	12	TCEA1	DZ 3
6.844509e-318	1.344799	0.71	0.179	1.220307e-313	12	DNMT1	DZ 3
3.15241e-298	1.342472	0.672	0.147	5.620432e-294	12	C12orf75	DZ 3
8.122322e-296	1.062649	0.949	0.649	1.448129e-291	12	SLC25A5	DZ 3
4.028756e-288	1.128484	0.91	0.496	7.182868e-284	12	MZT2B	DZ 3
5.192192e-285	0.7485667	0.991	0.96	9.257158e-281	12	OAZ1	DZ 3
7.021598e-285	-1.278893	0.936	0.987	1.251881e-280	12	HLA-B	DZ 3
1.467698e-284	1.461092	0.818	0.19	2.616759e-280	12	RGS13	DZ 3
8.033939e-282	0.8761476	0.979	0.867	1.432371e-277	12	ARPC3	DZ 3
2.36608e-280	-1.091057	0.991	0.998	4.218483e-276	12	B2M	DZ 3
7.806971e-280	1.16548	0.773	0.271	1.391905e-275	12	PRDX3	DZ 3
2.880904e-279	1.095085	0.926	0.574	5.136364e-275	12	ANP32B	DZ 3
1.541758e-277	1.159443	0.881	0.44	2.7488e-273	12	MRPL51	DZ 3
8.140843e-277	1.234646	0.928	0.596	1.451431e-272	12	DYNLL1	DZ 3
7.984595e-273	1.120095	0.877	0.437	1.423573e-268	12	SEM1	DZ 3
7.870699e-268	1.115586	0.857	0.381	1.403267e-263	12	YWHAE	DZ 3
6.488431e-261	-1.606025	0.73	0.909	1.156822e-256	12	HLA-A	DZ 3
6.862254e-260	1.178849	0.804	0.301	1.223471e-255	12	HMGN3	DZ 3
1.521323e-253	0.9271439	0.961	0.632	2.712367e-249	12	ATP5MF	DZ 3
1.178575e-251	0.9902378	0.477	0.05	2.101281e-247	12	HMGB3	DZ 3
6.878696e-251	1.180056	0.555	0.072	1.226403e-246	12	AICDA	DZ 3

8.918648e-250 1.018614 0.918 0.576 1.590106e-245 12
ATP5MC3 DZ 3
1.142025e-249 1.071119 0.869 0.446 2.036117e-245 12
LSM5 DZ 3
1.810592e-247 -2.111463 0.106 0.555 3.228104e-243 12
CCR7 DZ 3
2.027884e-247 1.231384 0.683 0.231 3.615515e-243 12
SLBP DZ 3
1.285327e-246 1.025384 0.89 0.479 2.29161e-242 12
CBX3 DZ 3
1.147366e-245 1.201751 0.761 0.221 2.045638e-241 12
UBE2J1 DZ 3
1.22954e-241 1.108801 0.736 0.25 2.192148e-237 12
ANP32E DZ 3
1.628315e-240 1.20395 0.835 0.336 2.903122e-236 12
S100A10 DZ 3
6.408396e-240 0.7792626 0.355 0.023 1.142553e-235 12
TROAP DZ 3
3.382762e-236 0.9193507 0.972 0.81 6.031126e-232 12
CORO1A DZ 3
9.229361e-236 1.017656 0.882 0.508 1.645503e-231 12
COX8A DZ 3
1.575606e-226 1.068545 0.828 0.413 2.809148e-222 12 DEK
DZ 3
1.778748e-225 0.8197873 0.975 0.803 3.171329e-221 12
TMA7 DZ 3
1.32177e-223 0.9766956 0.936 0.687 2.356584e-219 12
EZR DZ 3
3.886663e-223 1.109808 0.658 0.148 6.929532e-219 12
SUSD3 DZ 3
3.335399e-222 0.9349991 0.93 0.57 5.946683e-218 12
LDHB DZ 3
3.706643e-222 -1.633411 0.521 0.8 6.608573e-218 12
HLA-E DZ 3
5.294985e-222 1.008789 0.9 0.548 9.440429e-218 12
ATP5IF1 DZ 3
7.088264e-222 1.120797 0.827 0.407 1.263767e-217 12
METAP2 DZ 3
2.555918e-220 0.9144725 0.919 0.634 4.556946e-216 12
HNRNPM DZ 3
3.476756e-220 1.154153 0.717 0.225 6.198709e-216 12
GCHFR DZ 3
7.598085e-217 1.09661 0.816 0.363 1.354663e-212 12
ACADM DZ 3
3.575741e-214 0.9646033 0.898 0.525 6.375189e-210 12
SRSF9 DZ 3
5.921929e-211 0.953478 0.905 0.531 1.055821e-206 12
PARP1 DZ 3
5.446721e-210 0.8305412 0.964 0.716 9.71096e-206 12
COX6C DZ 3

1.076334e-207	1.037697	0.761	0.276	1.918996e-203	12	BCAS4	DZ 3
1.249188e-205	1.000783	0.552	0.1	2.227177e-201	12	AC023590.1	DZ 3
5.587743e-205	1.033824	0.637	0.14	9.962387e-201	12	CD81	DZ 3
1.679761e-204	0.9684616	0.428	0.063	2.994846e-200	12	S1PR2	DZ 3
6.358092e-204	1.119774	0.67	0.225	1.133584e-199	12	CCND3	DZ 3
2.097547e-202	0.9310069	0.873	0.463	3.739716e-198	12	SNRPD1	DZ 3
4.81247e-196	0.7802682	0.388	0.045	8.580153e-192	12	A4GALT	DZ 3
1.433488e-195	1.025474	0.473	0.087	2.555766e-191	12	STAG3	DZ 3
8.010951e-195	0.8410981	0.936	0.678	1.428272e-190	12	COX6A1	DZ 3
2.472012e-193	0.8088277	0.969	0.771	4.40735e-189	12	ARHGDIB	DZ 3
9.380427e-193	1.004799	0.54	0.095	1.672436e-188	12	HRK	DZ 3
1.614308e-192	-1.401218	0.733	0.911	2.87815e-188	12	IGHM	DZ 3
6.208504e-192	1.031462	0.863	0.482	1.106914e-187	12	C4orf3	DZ 3
1.973772e-191	1.121857	0.468	0.068	3.519039e-187	12	SUGCT	DZ 3
6.048648e-191	0.8219731	0.936	0.637	1.078413e-186	12	HNRNPC	DZ 3
1.8833e-190	0.9057542	0.593	0.157	3.357736e-186	12	TFDP1	DZ 3
1.285131e-188	0.8222805	0.431	0.059	2.291259e-184	12	CENPW	DZ 3
3.084906e-188	0.9413625	0.414	0.068	5.500078e-184	12	GINS2	DZ 3
1.284902e-187	0.8471471	0.909	0.6	2.290851e-183	12	POMP	DZ 3
3.080414e-187	1.147001	0.493	0.109	5.492069e-183	12	UBE2S	DZ 3
4.568386e-187	0.9504206	0.844	0.446	8.144976e-183	12	COX17	DZ 3
1.854664e-186	0.5635903	0.999	0.998	3.30668e-182	12	TMSB4X	DZ 3
2.561831e-186	1.022094	0.452	0.056	4.567489e-182	12	TK1	DZ 3
9.862663e-182	0.8688321	0.511	0.104	1.758414e-177	12	SKA2	DZ 3
2.98356e-181	0.8807705	0.834	0.436	5.319388e-177	12	ATP5F1C	DZ 3

5.885952e-181	0.8443057	0.924	0.626	1.049406e-176	12	PSMA4	DZ 3
9.104231e-181	0.9197952	0.815	0.409	1.623193e-176	12	HNRNPD	DZ 3
8.124684e-180	0.9428885	0.761	0.301	1.44855e-175	12	PSIP1	DZ 3
9.05336e-179	0.9453595	0.815	0.386	1.614124e-174	12	NUCKS1	DZ 3
1.029101e-178	0.9609544	0.84	0.419	1.834785e-174	12	IRF8	DZ 3
1.250709e-178	0.923457	0.676	0.233	2.229889e-174	12	DCTN3	DZ 3
1.639842e-178	0.8376309	0.901	0.55	2.923673e-174	12	ATP5PF	DZ 3
2.088328e-177	1.078738	0.657	0.178	3.72328e-173	12	DAAM1	DZ 3
1.232432e-175	0.7004641	0.968	0.742	2.197304e-171	12	RAN	DZ 3
1.16292e-173	0.9286386	0.753	0.31	2.07337e-169	12	NAA38	DZ 3
1.647899e-173	0.8019124	0.936	0.652	2.938038e-169	12	UBL5	DZ 3
3.589257e-173	0.6881879	0.963	0.738	6.399286e-169	12	CHCHD2	DZ 3
1.345265e-172	0.8230981	0.436	0.063	2.398473e-168	12	CDCA7	DZ 3
2.030407e-172	0.8678884	0.822	0.404	3.620013e-168	12	HDAC1	DZ 3
6.931507e-172	0.90037	0.812	0.43	1.235818e-167	12	HMGA1	DZ 3
1.348637e-171	0.7306986	0.352	0.049	2.404484e-167	12	BFSP2	DZ 3
1.645548e-170	0.8198501	0.87	0.506	2.933847e-166	12	SRP9	DZ 3
5.66833e-170	0.7503299	0.955	0.701	1.010607e-165	12	NDUFA4	DZ 3
9.605056e-170	1.081114	0.382	0.039	1.712485e-165	12	CDC20	DZ 3
3.075541e-169	0.9334876	0.506	0.121	5.483383e-165	12	PHGDH	DZ 3
1.434186e-166	0.8830286	0.828	0.442	2.557011e-162	12	MAP1LC3B	DZ 3
1.091109e-165	0.9412211	0.723	0.263	1.945338e-161	12	ITGAE	DZ 3
5.910642e-165	0.7766041	0.925	0.606	1.053808e-160	12	UQCR10	DZ 3
2.14545e-163	1.006819	0.616	0.136	3.825123e-159	12	BIK	DZ 3
3.109456e-163	0.65174	0.979	0.9	5.543849e-159	12	MYL6	DZ 3

1.894311e-162	-1.539701	0.294	0.614	3.377368e-158	12	IGHD	DZ 3
4.451589e-162	0.8755	0.835	0.427	7.936738e-158	12	COTL1	DZ 3
3.026489e-161	0.8204868	0.798	0.401	5.395927e-157	12	MDH1	DZ 3
4.626881e-160	0.9060675	0.705	0.27	8.249267e-156	12	JPT1	DZ 3
1.168335e-159	0.9354709	0.771	0.291	2.083024e-155	12	BCL7A	DZ 3
1.762511e-157	0.9522977	0.411	0.056	3.142381e-153	12	CDKN3	DZ 3
4.892377e-157	-1.519916	0.214	0.52	8.72262e-153	12	BANK1	DZ 3
5.74265e-157	0.8312796	0.488	0.09	1.023857e-152	12	CENPM	DZ 3
2.809329e-156	0.7512923	0.356	0.041	5.008752e-152	12	LINC01991	DZ 3
1.08052e-153	0.8138419	0.785	0.391	1.92646e-149	12	RPA3	DZ 3
2.726063e-153	0.7958756	0.45	0.086	4.860297e-149	12	PXMP2	DZ 3
1.202433e-152	0.7524326	0.908	0.621	2.143817e-148	12	ATP5F1B	DZ 3
6.56918e-152	0.521982	0.99	0.925	1.171219e-147	12	HNRNPA1	DZ 3
8.08754e-152	-1.126567	0.753	0.863	1.441927e-147	12	UBB	DZ 3
1.748291e-151	0.6119114	0.269	0.025	3.117028e-147	12	PTMS	DZ 3
4.898406e-150	0.6832361	0.951	0.71	8.733368e-146	12	COX6B1	DZ 3
8.402469e-150	1.001374	0.383	0.046	1.498076e-145	12	CCNB2	DZ 3
9.153158e-150	0.8454182	0.939	0.648	1.631916e-145	12	TCL1A	DZ 3
9.189227e-150	0.8714412	0.739	0.327	1.638347e-145	12	NDUFC1	DZ 3
1.577845e-149	0.9292299	0.524	0.145	2.81314e-145	12	MCM7	DZ 3
2.583511e-149	0.8190415	0.938	0.743	4.606142e-145	12	HSP90AA1	DZ 3
1.075697e-147	-1.053502	0.909	0.962	1.917861e-143	12	MT-ND2	DZ 3
1.830911e-146	0.7851986	0.562	0.157	3.264331e-142	12	CCDC167	DZ 3
3.995691e-145	-1.369084	0.295	0.584	7.123917e-141	12	SARAF	DZ 3
9.528522e-145	0.7564865	0.89	0.545	1.69884e-140	12	SEC61G	DZ 3

4.072644e-143	0.6404654	0.303	0.038	7.261117e-139	12	ASB2	DZ 3
8.472068e-143	0.7785398	0.806	0.425	1.510485e-138	12	LSM3	DZ 3
1.011061e-142	0.782682	0.788	0.407	1.802621e-138	12	DDX39A	DZ 3
1.050547e-142	0.5642999	0.997	0.903	1.87302e-138	12	ATP5F1E	DZ 3
1.519063e-141	0.7148606	0.919	0.628	2.708337e-137	12	UQCRH	DZ 3
1.654717e-141	0.846847	0.706	0.309	2.950196e-137	12	PRDX2	DZ 3
2.180434e-141	0.7737282	0.501	0.132	3.887495e-137	12	ANAPC15	DZ 3
5.246315e-141	-1.331231	0.086	0.39	9.353655e-137	12	CD44	DZ 3
1.008907e-140	0.6944691	0.9	0.597	1.798781e-136	12	PSMB3	DZ 3
2.981037e-140	0.8323394	0.383	0.051	5.31489e-136	12	TYMS	DZ 3
1.564633e-139	0.7468593	0.928	0.656	2.789584e-135	12	DBI	DZ 3
8.062072e-139	0.7204002	0.612	0.214	1.437387e-134	12	DAZAP1	DZ 3
1.463875e-138	0.7095586	0.384	0.061	2.609942e-134	12	SNTA1	DZ 3
1.936853e-138	-0.9559652	0.921	0.97	3.453216e-134	12	MT-ND3	DZ 3
5.363198e-137	0.7180234	0.902	0.639	9.562046e-133	12	SNRPG	DZ 3
7.208309e-137	0.7573311	0.863	0.522	1.285169e-132	12	PAIP2	DZ 3
7.746425e-137	0.7397417	0.839	0.482	1.38111e-132	12	SF3B6	DZ 3
2.265747e-136	0.7473962	0.798	0.418	4.0396e-132	12	MINOS1	DZ 3
3.063393e-136	0.7130127	0.864	0.557	5.461724e-132	12	SNRPB	DZ 3
2.055962e-135	0.6559178	0.942	0.701	3.665575e-131	12	COX7A2	DZ 3
4.018407e-135	0.6650534	0.939	0.69	7.164418e-131	12	ATP5MPL	DZ 3
5.850144e-135	0.818091	0.767	0.371	1.043022e-130	12	LSM8	DZ 3
1.078586e-134	0.7466489	0.856	0.514	1.923011e-130	12	TRAPPC1	DZ 3
1.414266e-134	0.7386204	0.905	0.625	2.521494e-130	12	GSTP1	DZ 3
7.851977e-134	0.7722511	0.742	0.336	1.399929e-129	12	H2AFY	DZ 3

1.25171e-133	0.7443122	0.838	0.475	2.231675e-129	12	NDUFB1	DZ	3
2.300785e-133	0.7053337	0.821	0.42	4.102069e-129	12	SLIRP	DZ	3
1.877712e-131	0.722336	0.842	0.493	3.347772e-127	12	RTRAF	DZ	3
1.010461e-130	-1.329872	0.079	0.358	1.801552e-126	12	LY6E	DZ	3
1.660079e-130	0.7534967	0.703	0.301	2.959755e-126	12	MZT2A	DZ	3
8.326947e-129	0.7219806	0.42	0.091	1.484611e-124	12	FAM241A	DZ	3
1.154384e-128	0.7547116	0.655	0.239	2.05815e-124	12	SH3KBP1	DZ	3
1.276081e-128	0.6874012	0.896	0.607	2.275125e-124	12	ARPC5	DZ	3
1.317679e-128	0.7770163	0.464	0.094	2.34929e-124	12	RMI2	DZ	3
1.600636e-128	0.7755899	0.8	0.387	2.853775e-124	12	BASP1	DZ	3
1.658525e-128	0.7504975	0.522	0.147	2.956984e-124	12	SMIM20	DZ	3
3.447849e-128	0.8127448	0.511	0.137	6.14717e-124	12	SEC14L1	DZ	3
3.578399e-128	0.7980591	0.624	0.216	6.379927e-124	12	UBE2G1	DZ	3
6.677082e-128	-1.863702	0.196	0.472	1.190457e-123	12	S100A6	DZ	3
6.788652e-128	0.8206957	0.606	0.197	1.210349e-123	12	SNHG25	DZ	3
5.634778e-127	0.7319726	0.746	0.355	1.004625e-122	12	RBBP7	DZ	3
6.545923e-127	0.7944207	0.615	0.208	1.167073e-122	12	NANS	DZ	3
1.685116e-126	0.6323792	0.894	0.586	3.004393e-122	12	TBCA	DZ	3
2.356121e-126	0.7781651	0.515	0.13	4.200728e-122	12	SOCS1	DZ	3
4.373798e-125	0.6620926	0.913	0.681	7.798044e-121	12	PPP1CA	DZ	3
1.510949e-124	0.720355	0.445	0.108	2.693871e-120	12	LMNB1	DZ	3
1.986511e-124	0.6091393	0.937	0.694	3.541751e-120	12	ELOB	DZ	3
2.275809e-124	0.7024161	0.825	0.46	4.05754e-120	12	SNRPF	DZ	3
2.311663e-124	0.7127761	0.755	0.391	4.121463e-120	12	ENY2	DZ	3
4.531649e-124	0.8322734	0.551	0.123	8.079478e-120	12	NEIL1	DZ	3

6.930257e-124	0.8106542	0.739	0.292	1.235596e-119	12	CD27	DZ 3
8.345341e-124	0.602296	0.949	0.741	1.487891e-119	12	SNRPD2	DZ 3
1.966515e-123	0.8578666	0.436	0.107	3.5061e-119	12	GMDS	DZ 3
1.97646e-123	0.7186349	0.742	0.365	3.523831e-119	12	LCP1	DZ 3
6.553724e-123	0.7394911	0.763	0.376	1.168463e-118	12	NDUFB3	DZ 3
1.571219e-122	0.6342657	0.909	0.633	2.801326e-118	12	ERH	DZ 3
2.077622e-122	0.759623	0.716	0.313	3.704193e-118	12	HSBP1	DZ 3
1.066927e-121	0.4192919	0.222	0.016	1.902224e-117	12	AP002360.1	DZ 3
2.604278e-121	0.7039203	0.743	0.357	4.643168e-117	12	EWSR1	DZ 3
3.069542e-121	0.6591886	0.899	0.615	5.472686e-117	12	ARPC1B	DZ 3
3.122623e-121	0.6441812	0.899	0.6	5.567324e-117	12	POLR2L	DZ 3
3.652439e-121	0.7585217	0.612	0.229	6.511934e-117	12	ZBTB8OS	DZ 3
2.156591e-120	0.7536731	0.65	0.26	3.844986e-116	12	TOP1	DZ 3
2.752632e-120	0.6778134	0.856	0.507	4.907667e-116	12	SMDT1	DZ 3
2.648658e-119	0.7101452	0.662	0.271	4.722292e-115	12	BABAM1	DZ 3
2.79674e-119	0.6124428	0.916	0.632	4.986307e-115	12	ATP5MD	DZ 3
3.37913e-119	0.7006776	0.695	0.292	6.024651e-115	12	HNRNPH3	DZ 3
1.562719e-118	0.5671707	0.28	0.037	2.786172e-114	12	CDT1	DZ 3
2.21899e-118	0.7445928	0.548	0.168	3.956237e-114	12	PLIN3	DZ 3
2.621841e-118	0.7183671	0.712	0.336	4.67448e-114	12	LSM6	DZ 3
3.742298e-118	0.6139468	0.888	0.593	6.672142e-114	12	PSMA2	DZ 3
3.261063e-117	0.5271431	0.976	0.893	5.81415e-113	12	PPIA	DZ 3
3.703562e-117	0.6498538	0.367	0.063	6.60308e-113	12	DHFR	DZ 3
4.517783e-117	0.6448465	0.358	0.065	8.054756e-113	12	HELLS	DZ 3
1.242389e-116	0.6835941	0.808	0.425	2.215055e-112	12	ROMO1	DZ 3

1.642266e-116	-1.34374	0.09	0.36	2.927997e-112	12	TXNIP	DZ 3
4.324063e-116	0.8887503	0.663	0.327	7.709371e-112	12	DUT	DZ 3
5.301995e-115	0.6767378	0.983	0.798	9.452926e-111	12	RPS26	DZ 3
5.334796e-115	0.5362318	0.989	0.938	9.511407e-111	12	H3F3A	DZ 3
7.178344e-115	0.6833329	0.731	0.344	1.279827e-110	12	DGUOK	DZ 3
2.438713e-114	0.741882	0.62	0.244	4.347982e-110	12	MPC2	DZ 3
7.139829e-114	0.6627749	0.722	0.353	1.27296e-109	12	ELOC	DZ 3
1.869698e-113	0.7184547	0.676	0.274	3.333485e-109	12	CARHSP1	DZ 3
3.485234e-113	0.6671613	0.809	0.476	6.213823e-109	12	UBE2L3	DZ 3
7.934927e-113	0.6809752	0.356	0.071	1.414718e-108	12	GMNN	DZ 3
3.041425e-112	0.673131	0.842	0.526	5.422556e-108	12	SOD1	DZ 3
9.722906e-112	0.6541826	0.461	0.117	1.733497e-107	12	COMMD4	DZ 3
1.062742e-111	0.8136211	0.861	0.525	1.894763e-107	12	ISG20	DZ 3
3.472463e-111	0.6979983	0.682	0.299	6.191054e-107	12	FKBP3	DZ 3
3.527319e-111	0.7323638	0.399	0.093	6.288858e-107	12	AC084033.3	DZ 3
9.8362e-111	0.5505177	0.264	0.036	1.753696e-106	12	MND1	DZ 3
1.93742e-110	0.70053	0.5	0.137	3.454225e-106	12	SH2B2	DZ 3
2.020464e-110	0.5525115	0.948	0.755	3.602284e-106	12	PSMA7	DZ 3
6.328618e-110	0.64656	0.346	0.054	1.128329e-105	12	ZWINT	DZ 3
2.759306e-109	0.5477023	0.969	0.827	4.919566e-105	12	UQCRB	DZ 3
3.061585e-109	0.5729938	0.939	0.729	5.458499e-105	12	SKP1	DZ 3
3.576895e-109	0.6775675	0.769	0.407	6.377247e-105	12	PPP1CC	DZ 3
1.261754e-108	0.5789305	0.27	0.035	2.24958e-104	12	CLSPN	DZ 3
1.776186e-108	0.6792942	0.355	0.071	3.166762e-104	12	MAD2L1	DZ 3
2.450695e-108	-1.437656	0.161	0.377	4.369343e-104	12	SELL	DZ 3

8.835571e-108	0.6072353	0.878	0.546	1.575294e-103	12	NEDD8	DZ 3
3.51551e-107	0.6019719	0.83	0.468	6.267802e-103	12	UBE2N	DZ 3
3.613476e-107	-1.300809	0.263	0.5	6.442466e-103	12	FXYD5	DZ 3
4.936146e-107	0.7492197	0.505	0.156	8.800654e-103	12	DEF8	DZ 3
6.952281e-106	0.6014332	0.378	0.084	1.239522e-101	12	MSH6	DZ 3
7.434677e-106	0.8243391	0.4	0.085	1.325529e-101	12	CCNB1	DZ 3
7.705669e-106	0.5252308	0.25	0.034	1.373844e-101	12	SYBU	DZ 3
6.935786e-105	0.624798	0.824	0.489	1.236581e-100	12	COX5A	DZ 3
1.957297e-104	0.5364435	0.275	0.04	3.489665e-100	12	LINC00467	DZ 3
1.273042e-103	0.5650547	0.307	0.053	2.269706e-99	12	WDR76	DZ 3
3.834399e-103	0.7108922	0.718	0.321	6.836349e-99	12	GRHPR	DZ 3
5.193438e-103	0.5649029	0.323	0.06	9.259381e-99	12	MCM6	DZ 3
8.242051e-103	-0.5989844	0.999	0.999	1.469475e-98	12	RPL34	DZ 3
1.275054e-102	0.8919478	0.827	0.48	2.273293e-98	12	LTB	DZ 3
1.530262e-102	-0.9830451	0.761	0.868	2.728304e-98	12	UBC	DZ 3
1.582907e-102	0.6719226	0.751	0.365	2.822166e-98	12	PTP4A2	DZ 3
4.323875e-102	0.5506735	0.907	0.63	7.709037e-98	12	SET	DZ 3
4.565139e-102	0.5514703	0.312	0.055	8.139186e-98	12	DTX1	DZ 3
1.706128e-101	0.6285202	0.749	0.393	3.041856e-97	12	UQCRFS1	DZ 3
2.206135e-101	0.5399944	0.887	0.563	3.933317e-97	12	SNRPE	DZ 3
3.230836e-101	0.6096584	0.74	0.36	5.760258e-97	12	FAM96B	DZ 3
4.057014e-101	0.6832035	0.379	0.077	7.233251e-97	12	TACC3	DZ 3
4.674172e-101	0.6289952	0.518	0.165	8.333581e-97	12	NUDT1	DZ 3
1.529528e-100	0.6692769	0.737	0.374	2.726995e-96	12	SMARCB1	DZ 3
2.44694e-100	0.6551578	0.69	0.314	4.36265e-96	12	IMP4	DZ 3

2.581726e-100	0.6338734	0.714	0.335	4.602959e-96	12	POLR2J	DZ 3
6.383013e-100	0.5550082	0.902	0.651	1.138027e-95	12	RBX1	DZ 3
1.515294e-99	0.6916194	0.527	0.159	2.701618e-95	12	SEL1L3	DZ 3
4.675054e-99	0.7058467	0.265	0.043	8.335154e-95	12	HES6	DZ 3
9.184778e-99	0.5425182	0.887	0.598	1.637554e-94	12	RBM3	DZ 3
1.756636e-98	0.4842695	0.223	0.028	3.131907e-94	12	UHRF1	DZ 3
2.156861e-98	0.5729288	0.888	0.647	3.845468e-94	12	ATP5F1A	DZ 3
4.356771e-98	0.7512806	0.369	0.077	7.767687e-94	12	SH3TC1	DZ 3
1.486302e-97	0.6003335	0.804	0.463	2.649927e-93	12	NDUFB4	DZ 3
1.735589e-97	0.6642338	0.705	0.329	3.094381e-93	12	STK17A	DZ 3
1.874547e-97	0.5837338	0.387	0.096	3.34213e-93	12	BCL2L12	DZ 3
4.786587e-97	0.5594704	0.864	0.571	8.534005e-93	12	COPE	DZ 3
9.859572e-97	0.6287348	0.762	0.437	1.757863e-92	12	PGAM1	DZ 3
1.190378e-96	0.5125555	0.94	0.802	2.122326e-92	12	HNRNPK	DZ 3
1.397243e-96	0.4931626	0.26	0.039	2.491145e-92	12	MCM4	DZ 3
5.27419e-96	-1.201778	0.073	0.279	9.403353e-92	12	KLF2	DZ 3
4.243469e-95	0.5981555	0.819	0.51	7.56568e-91	12	ATP5PB	DZ 3
9.485273e-95	-0.5826573	1	0.999	1.691129e-90	12	RPS29	DZ 3
3.25126e-94	0.5063587	0.271	0.045	5.796671e-90	12	UCHL1	DZ 3
1.049159e-93	0.5880222	0.561	0.213	1.870545e-89	12	PSMD9	DZ 3
1.274227e-93	0.5594744	0.907	0.612	2.27182e-89	12	NDUFA1	DZ 3
1.420878e-93	0.5906705	0.747	0.395	2.533284e-89	12	PSMB2	DZ 3
1.786423e-93	0.6058661	0.697	0.357	3.185014e-89	12	PSMA5	DZ 3
1.975876e-93	0.5967525	0.779	0.457	3.522789e-89	12	PRDX6	DZ 3
5.332856e-93	0.6737573	0.636	0.264	9.507948e-89	12	POU2AF1	DZ 3

5.395603e-93	0.4952441	0.233	0.037	9.61982e-89	12	ACY3	DZ	3
2.422571e-92	0.5791251	0.279	0.05	4.319201e-88	12	TUBB2A	DZ	3
2.812607e-92	0.5127618	0.276	0.046	5.014597e-88	12	RPL39L	DZ	3
2.877602e-92	0.5221671	0.916	0.668	5.130476e-88	12	ATP5ME	DZ	3
3.585903e-92	0.602421	0.769	0.412	6.393306e-88	12	NDUFA2	DZ	3
4.016117e-92	0.4901658	0.893	0.591	7.160335e-88	12	PRDX1	DZ	3
1.80054e-91	0.6061887	0.792	0.45	3.210182e-87	12	RAP1B	DZ	3
2.124831e-91	0.5398688	0.844	0.535	3.788362e-87	12	SUMO1	DZ	3
5.130059e-91	0.4612439	0.289	0.049	9.146383e-87	12	MCM2	DZ	3
1.146696e-90	0.632971	0.585	0.249	2.044445e-86	12	CDV3	DZ	3
1.234937e-90	-1.034324	0.034	0.216	2.201769e-86	12	PLAC8	DZ	3
1.458891e-90	0.5648612	0.89	0.62	2.601057e-86	12	ATP5PO	DZ	3
2.679052e-90	0.6539243	0.549	0.201	4.776481e-86	12	SYAP1	DZ	3
4.137185e-90	0.6338409	0.64	0.294	7.376187e-86	12	UGP2	DZ	3
8.067431e-90	0.6131115	0.47	0.144	1.438342e-85	12	MYL6B	DZ	3
8.692496e-90	0.8139093	0.536	0.177	1.549785e-85	12	RGS2	DZ	3
1.075067e-89	0.4996603	0.899	0.625	1.916738e-85	12	COX7B	DZ	3
1.267916e-89	0.6942928	0.568	0.192	2.260568e-85	12	HMCES	DZ	3
3.249662e-89	0.5741986	0.818	0.511	5.793822e-85	12	PSMB6	DZ	3
7.180823e-89	0.6663841	0.324	0.07	1.280269e-84	12	RGCC	DZ	3
9.327443e-89	0.5305725	0.312	0.064	1.66299e-84	12	TERF2	DZ	3
1.489042e-88	0.5977848	0.609	0.24	2.654813e-84	12	PPP2R5C	DZ	3
1.752151e-88	0.6393409	0.755	0.41	3.123911e-84	12	NDUFA12	DZ	3
4.060118e-88	-1.060757	0.458	0.625	7.238784e-84	12	SUB1	DZ	3
4.578497e-88	0.5958407	0.646	0.311	8.163002e-84	12	LSM4	DZ	3

5.217998e-88	0.5084315	0.253	0.046	9.303169e-84	12	ASB13	DZ 3
8.838389e-88	0.5125132	0.241	0.032	1.575796e-83	12	SLC2A5	DZ 3
1.290179e-87	-1.107204	0.05	0.274	2.300261e-83	12	GPR183	DZ 3
1.396676e-87	0.5301515	0.89	0.625	2.490134e-83	12	EIF3H	DZ 3
1.600049e-87	0.575438	0.706	0.355	2.852728e-83	12	PPP2CA	DZ 3
2.354164e-87	0.524694	0.286	0.05	4.197239e-83	12	MME	DZ 3
1.284931e-86	0.5721327	0.406	0.109	2.290903e-82	12	C12orf49	DZ 3
1.507341e-86	0.6000406	0.675	0.327	2.687438e-82	12	SEPHS2	DZ 3
1.657783e-86	0.5420059	0.862	0.559	2.955661e-82	12	UQCRQ	DZ 3
2.484374e-86	0.6001793	0.532	0.187	4.42939e-82	12	UBE2E1	DZ 3
3.390089e-86	0.3766185	0.987	0.922	6.044189e-82	12	BTF3	DZ 3
6.408237e-86	0.7006823	0.551	0.228	1.142525e-81	12	DSTN	DZ 3
1.198784e-85	0.5917882	0.477	0.17	2.137313e-81	12	PMVK	DZ 3
4.124977e-85	0.5607222	0.76	0.427	7.354421e-81	12	KHDRBS1	DZ 3
4.168101e-85	0.6249254	0.433	0.112	7.431308e-81	12	VNN2	DZ 3
8.099723e-85	0.5937782	0.615	0.262	1.4441e-80	12	MRPL33	DZ 3
8.638134e-85	0.5610457	0.573	0.225	1.540093e-80	12	MRPL27	DZ 3
1.015959e-84	0.3983686	0.201	0.021	1.811354e-80	12	AL118516.1	DZ 3
5.475075e-84	-1.087561	0.101	0.302	9.761512e-80	12	LINC00926	DZ 3
5.489531e-84	-0.962	0.032	0.225	9.787285e-80	12	GBP2	DZ 3
6.911452e-84	0.5806748	0.568	0.24	1.232243e-79	12	MRPL13	DZ 3
1.040311e-83	0.6471325	0.353	0.053	1.85477e-79	12	MKI67	DZ 3
1.641929e-83	0.6224673	0.405	0.104	2.927396e-79	12	CPNE5	DZ 3
2.359646e-83	0.5404745	0.868	0.581	4.207012e-79	12	MARCKSL1	DZ 3
6.655439e-83	0.5592509	0.519	0.189	1.186598e-78	12	ETFB	DZ 3

6.865498e-83	0.6124605	0.646	0.297	1.22405e-78	12	TIMM8B	DZ 3
9.395284e-83	0.5396023	0.282	0.056	1.675085e-78	12	DCAF12	DZ 3
1.344806e-82	-0.5189571	1	1	2.397655e-78	12	RPS27	DZ 3
1.532474e-82	0.5654898	0.379	0.095	2.732248e-78	12	SEMA4A	DZ 3
1.856553e-82	0.494612	0.865	0.565	3.310049e-78	12	PARK7	DZ 3
2.195272e-82	0.5594552	0.406	0.118	3.913951e-78	12	ARL3	DZ 3
4.34334e-82	0.5384586	0.453	0.147	7.74374e-78	12	GNB1	DZ 3
7.824282e-82	0.7610058	0.446	0.181	1.394991e-77	12	PCNA	DZ 3
1.016639e-81	0.56744	0.724	0.394	1.812565e-77	12	MDH2	DZ 3
1.200303e-81	0.5267853	0.758	0.437	2.14002e-77	12	YWHAQ	DZ 3
1.645881e-81	0.5398585	0.573	0.231	2.934441e-77	12	CPSF6	DZ 3
1.709112e-81	0.5611761	0.488	0.169	3.047176e-77	12	HINT2	DZ 3
1.721337e-81	0.4828925	0.252	0.051	3.068972e-77	12	CENPU	DZ 3
7.079632e-81	0.455158	0.258	0.044	1.262228e-76	12	C6orf226	DZ 3
8.856057e-81	0.5440336	0.627	0.282	1.578946e-76	12	YWHAH	DZ 3
1.003389e-80	0.5801825	0.466	0.141	1.788942e-76	12	PTPN18	DZ 3
1.33694e-80	0.5913702	0.678	0.323	2.383631e-76	12	BPTF	DZ 3
1.697434e-80	-0.9591328	0.65	0.754	3.026355e-76	12	HLA-C	DZ 3
9.95468e-80	0.5734149	0.445	0.141	1.77482e-75	12	SMARCA4	DZ 3
1.043612e-79	0.301521	0.992	0.941	1.860655e-75	12	YBX1	DZ 3
2.295688e-79	0.5236627	0.359	0.091	4.092982e-75	12	RAB30-AS1	DZ 3
2.468381e-79	0.5608075	0.736	0.398	4.400876e-75	12	LSM10	DZ 3
3.432755e-79	0.5181414	0.791	0.482	6.120259e-75	12	CCT8	DZ 3
4.080866e-79	-0.9694366	0.067	0.253	7.275777e-75	12	TNFRSF13B	DZ 3
1.950498e-78	-1.146532	0.239	0.432	3.477542e-74	12	PLP2	DZ 3

2.937885e-78	0.9891661	0.793	0.662	5.237956e-74	12	TUBA1B	DZ 3
3.377356e-78	-0.5079567	0.999	0.999	6.021488e-74	12	RPL13	DZ 3
4.886727e-78	-1.033947	0.173	0.386	8.712545e-74	12	PDCD4	DZ 3
1.181399e-77	0.5733317	0.385	0.06	2.106316e-73	12	TOP2A	DZ 3
8.867675e-77	0.5251165	0.724	0.395	1.581018e-72	12	BZW1	DZ 3
1.138726e-76	-0.9470204	0.076	0.257	2.030235e-72	12	FCMR	DZ 3
1.852081e-76	0.6058591	0.651	0.264	3.302075e-72	12	NCF1	DZ 3
2.324611e-76	-0.6446503	0.947	0.975	4.14455e-72	12	MT-ND4	DZ 3
4.643269e-76	0.4504935	0.913	0.677	8.278484e-72	12	SLC25A3	DZ 3
5.310596e-76	0.4277889	0.251	0.038	9.468261e-72	12	AC025159.1	DZ 3
1.057966e-75	0.5451826	0.484	0.18	1.886248e-71	12	GLRX5	DZ 3
1.810228e-75	0.4704656	0.806	0.496	3.227455e-71	12	EIF3I	DZ 3
4.267883e-75	0.4675013	0.847	0.589	7.609209e-71	12	XRCC6	DZ 3
4.514955e-75	0.5535089	0.512	0.179	8.049714e-71	12	PRPSAP2	DZ 3
5.133115e-75	0.5157936	0.775	0.468	9.15183e-71	12	PRELID1	DZ 3
6.076873e-75	0.4768095	0.823	0.506	1.083446e-70	12	DNAJC8	DZ 3
1.504713e-74	0.5414184	0.685	0.349	2.682753e-70	12	GNAS	DZ 3
2.198789e-74	-0.8956083	0.9	0.936	3.920221e-70	12	BTG1	DZ 3
2.660377e-74	-1.291668	0.287	0.46	4.743186e-70	12	JUNB	DZ 3
2.6743e-74	0.3064017	0.163	0.012	4.76801e-70	12	AC104986.2	DZ 3
2.981947e-74	0.5445777	0.592	0.26	5.316514e-70	12	TXNDC17	DZ 3
3.907344e-74	0.4897187	0.726	0.402	6.966404e-70	12	HNRNPR	DZ 3
7.765598e-74	0.39448	0.216	0.035	1.384528e-69	12	EEPD1	DZ 3
9.770269e-74	0.5259278	0.668	0.329	1.741941e-69	12	HSD17B10	DZ 3
1.008704e-73	0.5551734	0.428	0.146	1.798419e-69	12	MZT1	DZ 3

1.643681e-73	0.4873896	0.714	0.381	2.930519e-69	12	CCT5	DZ 3
6.333953e-73	-0.5874376	0.996	0.996	1.12928e-68	12	RPS12	DZ 3
6.43659e-73	0.5595707	0.468	0.138	1.14758e-68	12	RBM38	DZ 3
7.705053e-73	0.4728322	0.817	0.508	1.373734e-68	12	CAPZB	DZ 3
7.81179e-73	0.5222127	0.791	0.461	1.392764e-68	12	MTDH	DZ 3
8.790482e-73	0.4476807	0.27	0.056	1.567255e-68	12	TCTEX1D2	DZ 3
1.212012e-72	-0.9314377	0.142	0.333	2.160897e-68	12	TRBC2	DZ 3
1.855696e-72	0.4921859	0.305	0.076	3.30852e-68	12	LRWD1	DZ 3
2.308847e-72	0.4997653	0.698	0.377	4.116443e-68	12	SNRPC	DZ 3
2.629685e-72	0.4031138	0.231	0.041	4.688465e-68	12	CHAF1A	DZ 3
2.909772e-72	0.5102116	0.685	0.358	5.187832e-68	12	RPS19BP1	DZ 3
3.080871e-72	0.4704042	0.337	0.088	5.492885e-68	12	ILF3-DT	DZ 3
4.554661e-72	0.5199341	0.633	0.291	8.120505e-68	12	GTF2A2	DZ 3
1.017784e-71	0.5433016	0.584	0.254	1.814608e-67	12	THOC7	DZ 3
1.036545e-71	0.4766693	0.276	0.063	1.848056e-67	12	FUCA1	DZ 3
1.150727e-71	0.537116	0.64	0.322	2.051631e-67	12	RALY	DZ 3
1.159464e-71	0.4944331	0.871	0.618	2.067209e-67	12	LAMTOR5	DZ 3
1.187652e-71	0.5200283	0.555	0.238	2.117466e-67	12	TPM4	DZ 3
2.145335e-71	0.42374	0.245	0.05	3.824917e-67	12	BORCS8-MEF2B	DZ 3
2.745085e-71	-1.006038	0.327	0.496	4.894213e-67	12	ANKRD12	DZ 3
3.188355e-71	0.479561	0.885	0.646	5.684519e-67	12	YWHAB	DZ 3
3.507843e-71	0.5657521	0.486	0.173	6.254134e-67	12	ZNF106	DZ 3
4.007656e-71	0.5216769	0.766	0.477	7.14525e-67	12	RANBP1	DZ 3
3.163158e-70	0.5311258	0.543	0.222	5.639595e-66	12	SEPT1	DZ 3
3.915988e-70	0.6272818	0.8	0.571	6.981816e-66	12	TUBB	DZ 3

4.305305e-70	0.4788055	0.823	0.549	7.675929e-66	12	PGK1	DZ 3
7.152564e-70	-0.825188	0.95	0.963	1.275231e-65	12	FTL	DZ 3
8.618699e-70	0.5338445	0.476	0.174	1.536628e-65	12	MED30	DZ 3
1.711917e-69	0.3775542	0.215	0.028	3.052177e-65	12	SNHG21	DZ 3
1.793617e-69	-1.017756	0.285	0.452	3.197839e-65	12	PNRC1	DZ 3
2.003934e-69	0.4831621	0.936	0.72	3.572814e-65	12	LIMD2	DZ 3
2.138709e-69	0.5764839	0.532	0.202	3.813103e-65	12	ADA	DZ 3
2.69128e-69	0.4528432	0.81	0.515	4.798284e-65	12	NDUFB8	DZ 3
2.985479e-69	0.4759878	0.677	0.341	5.322811e-65	12	LSM14A	DZ 3
3.961136e-69	0.5673168	0.936	0.854	7.062309e-65	12	CD79B	DZ 3
4.503165e-69	0.5009707	0.329	0.084	8.028693e-65	12	RITA1	DZ 3
6.450577e-69	0.4976594	0.442	0.152	1.150073e-64	12	TPGS2	DZ 3
9.052283e-69	0.4715202	0.373	0.104	1.613932e-64	12	CENPH	DZ 3
1.130538e-68	0.5249591	0.612	0.287	2.015635e-64	12	LYPLA1	DZ 3
1.235516e-68	0.5885305	0.611	0.281	2.202802e-64	12	TUBA4A	DZ 3
1.356035e-68	0.5391046	0.535	0.221	2.417675e-64	12	HDDC2	DZ 3
1.478627e-68	0.4324424	0.86	0.614	2.636243e-64	12	ACTR3	DZ 3
1.829186e-68	0.522859	0.772	0.431	3.261255e-64	12	RHOH	DZ 3
2.029597e-68	0.4586917	0.803	0.502	3.618569e-64	12	PSMD8	DZ 3
2.722468e-68	0.5400718	0.661	0.328	4.853889e-64	12	COX14	DZ 3
3.326433e-68	0.39472	0.191	0.026	5.930698e-64	12	RNF144B	DZ 3
4.776745e-68	0.5214084	0.641	0.307	8.516459e-64	12	NDUFAF3	DZ 3
4.933564e-68	0.4972585	0.539	0.213	8.796051e-64	12	MPLKIP	DZ 3
8.266758e-68	0.4299441	0.927	0.76	1.47388e-63	12	MYL12A	DZ 3
8.729731e-68	0.4519147	0.753	0.432	1.556424e-63	12	ACTR2	DZ 3

8.828448e-68	0.4896988	0.446	0.162	1.574024e-63	12
RPL26L1	DZ 3				
1.328372e-67	0.4848739	0.578	0.252	2.368354e-63	12
UBE2V2	DZ 3				
1.530082e-67	0.4968909	0.637	0.301	2.727984e-63	12
SCP2	DZ 3				
2.118321e-67	-1.039816	0.542	0.651	3.776755e-63	12
ZFP36L1	DZ 3				
2.261639e-67	0.5423828	0.673	0.32	4.032276e-63	12
RFTN1	DZ 3				
2.772641e-67	0.4814823	0.34	0.093	4.943342e-63	12
RRM1	DZ 3				
3.545828e-67	0.4397079	0.259	0.05	6.321857e-63	12
SNHG19	DZ 3				
4.076684e-67	0.3090218	0.154	0.019	7.26832e-63	12
CLDN23	DZ 3				
4.805536e-67	0.5332746	0.376	0.074	8.56779e-63	12
NUSAP1	DZ 3				
4.885539e-67	-1.097146	0.383	0.526	8.710428e-63	12
ID3	DZ 3				
1.137461e-66	0.3305455	0.185	0.026	2.027979e-62	12
E2F1	DZ 3				
1.638089e-66	0.4708784	0.683	0.34	2.920549e-62	12
POLR2K	DZ 3				
2.214008e-66	-0.9414459	0.156	0.332	3.947354e-62	12
PTPN6	DZ 3				
2.735933e-66	0.4341593	0.864	0.607	4.877895e-62	12
COX5B	DZ 3				
3.286515e-66	0.5001301	0.636	0.308	5.859528e-62	12
SCAND1	DZ 3				
4.375741e-66	0.5375769	0.622	0.292	7.801509e-62	12
CDK2AP2	DZ 3				
5.976166e-66	0.4690694	0.815	0.516	1.065491e-61	12
FKBP1A	DZ 3				
1.009945e-65	0.4853395	0.361	0.106	1.800631e-61	12
BRI3BP	DZ 3				
1.153904e-65	-0.9840589	0.2	0.351	2.057296e-61	12
TRAF3IP3	DZ 3				
2.197639e-65	0.5415626	0.693	0.357	3.918171e-61	12
SNAP23	DZ 3				
4.163404e-65	0.4038611	0.877	0.59	7.422933e-61	12
SEC61B	DZ 3				
4.484825e-65	0.5667401	0.479	0.161	7.995994e-61	12
SMC4	DZ 3				
5.130138e-65	0.4557129	0.709	0.379	9.146523e-61	12
NDUFS6	DZ 3				
5.65954e-65	0.5808707	0.258	0.058	1.009039e-60	12
HOPX	DZ 3				
6.444207e-65	0.4431093	0.281	0.065	1.148938e-60	12
MTA3	DZ 3				

6.463995e-65	0.4527165	0.765	0.448	1.152466e-60	12	PPP4C	DZ 3
1.021948e-64	0.4855942	0.229	0.04	1.822031e-60	12	TPX2	DZ 3
1.281483e-64	0.505374	0.595	0.261	2.284756e-60	12	SEPT9	DZ 3
1.299591e-64	0.5229888	0.35	0.109	2.31704e-60	12	FEN1	DZ 3
3.537957e-64	0.459834	0.632	0.3	6.307823e-60	12	MMADHC	DZ 3
3.642398e-64	0.4998334	0.514	0.213	6.494032e-60	12	TAF9	DZ 3
3.822581e-64	0.4854676	0.33	0.089	6.815279e-60	12	FAM76B	DZ 3
4.836367e-64	0.4510116	0.723	0.412	8.622758e-60	12	MAGOH	DZ 3
5.715199e-64	0.3329453	0.178	0.026	1.018963e-59	12	CHEK1	DZ 3
5.97266e-64	0.4894058	0.661	0.339	1.064866e-59	12	LAMTOR2	DZ 3
6.120725e-64	0.4554699	0.731	0.409	1.091264e-59	12	COX7A2L	DZ 3
6.15918e-64	0.4812045	0.504	0.214	1.09812e-59	12	MRPL4	DZ 3
6.193381e-64	-1.169915	0.151	0.32	1.104218e-59	12	CD69	DZ 3
7.77659e-64	0.626531	0.592	0.26	1.386488e-59	12	PIM1	DZ 3
8.730299e-64	0.4891486	0.654	0.331	1.556525e-59	12	RBBP4	DZ 3
1.612376e-63	-1.509	0.111	0.291	2.874706e-59	12	MIR155HG	DZ 3
1.840185e-63	0.5181446	0.479	0.183	3.280866e-59	12	LBR	DZ 3
2.50868e-63	0.4667027	0.688	0.371	4.472726e-59	12	ANAPC11	DZ 3
2.541104e-63	0.3850732	0.207	0.033	4.530534e-59	12	ARRDC1-AS1	DZ 3
3.320869e-63	0.3730321	0.157	0.022	5.920778e-59	12	IQCD	DZ 3
3.335813e-63	0.4580175	0.697	0.384	5.947422e-59	12	SSB	DZ 3
4.099679e-63	-0.8537098	0.123	0.283	7.309318e-59	12	IFITM2	DZ 3
5.215725e-63	0.4947938	0.456	0.174	9.299117e-59	12	MRPS33	DZ 3
6.922165e-63	0.4853959	0.612	0.325	1.234153e-58	12	EIF3A	DZ 3
8.649208e-63	0.5365495	0.695	0.391	1.542067e-58	12	NDUFS5	DZ 3

1.124037e-62	-0.463718	1	0.999	2.004045e-58	12	RPL32	DZ 3
1.566007e-62	0.4210987	0.282	0.07	2.792033e-58	12	EZH2	DZ 3
1.845111e-62	0.4998965	0.358	0.11	3.289649e-58	12	ABI3	DZ 3
1.907194e-62	0.3980264	0.933	0.795	3.400336e-58	12	SUMO2	DZ 3
2.036775e-62	0.4622711	0.242	0.053	3.631366e-58	12	RGS16	DZ 3
3.118868e-62	0.3873461	0.246	0.047	5.56063e-58	12	UNG	DZ 3
1.02255e-61	0.4475488	0.278	0.061	1.823104e-57	12	ZBED5-AS1	DZ 3
2.341974e-61	0.4856869	0.562	0.259	4.175506e-57	12	PPP1R7	DZ 3
3.029149e-61	0.4032258	0.753	0.444	5.40067e-57	12	PCNP	DZ 3
3.046627e-61	0.4249736	0.83	0.559	5.431831e-57	12	RAC1	DZ 3
3.440554e-61	0.5200794	0.544	0.231	6.134165e-57	12	NCF4	DZ 3
4.199003e-61	0.4447868	0.725	0.406	7.486402e-57	12	HCLS1	DZ 3
6.15353e-61	0.4673331	0.449	0.161	1.097113e-56	12	CYB5R3	DZ 3
6.26932e-61	0.4731678	0.412	0.146	1.117757e-56	12	DTYMK	DZ 3
9.931323e-61	0.4710471	0.532	0.24	1.770655e-56	12	SNRPA1	DZ 3
1.265988e-60	0.4820064	0.726	0.415	2.25713e-56	12	GABARAPL2	DZ 3
1.299681e-60	0.4966754	0.445	0.165	2.317201e-56	12	BLOC1S6	DZ 3
1.475185e-60	0.5212253	0.5	0.219	2.630108e-56	12	MCM5	DZ 3
1.75607e-60	0.396978	0.886	0.672	3.130898e-56	12	HNRNPA3	DZ 3
1.808799e-60	0.3026456	0.163	0.022	3.224907e-56	12	AC022167.3	DZ 3
2.656152e-60	0.4076495	0.753	0.442	4.735653e-56	12	MRPS21	DZ 3
5.42958e-60	0.4797813	0.554	0.231	9.680398e-56	12	PTRHD1	DZ 3
5.933211e-60	0.4477276	0.785	0.495	1.057832e-55	12	GNG5	DZ 3
9.344786e-60	0.4932218	0.575	0.283	1.666082e-55	12	SIVA1	DZ 3
1.164907e-59	0.6032387	0.264	0.048	2.076912e-55	12	CENPF	DZ 3

1.897831e-59	0.4897494	0.579	0.253	3.383642e-55	12	CCDC69	DZ 3
1.905679e-59	0.5300348	0.447	0.192	3.397635e-55	12	MCM3	DZ 3
2.971727e-59	0.5170418	0.554	0.248	5.298292e-55	12	RAD21	DZ 3
3.880042e-59	0.4888166	0.473	0.186	6.917726e-55	12	CBX5	DZ 3
3.956968e-59	0.4473089	0.64	0.328	7.054878e-55	12	PPM1G	DZ 3
6.327111e-59	0.4685065	0.511	0.215	1.128061e-54	12	RPIA	DZ 3
8.109911e-59	0.4744742	0.527	0.211	1.445916e-54	12	LY86	DZ 3
8.132906e-59	0.448757	0.61	0.295	1.450016e-54	12	SNF8	DZ 3
8.519221e-59	0.3564132	0.95	0.796	1.518892e-54	12	LAPTM5	DZ 3
1.193255e-58	0.4675012	0.706	0.433	2.127455e-54	12	FDPS	DZ 3
1.80814e-58	0.3782916	0.227	0.046	3.223734e-54	12	PEX5	DZ 3
2.181737e-58	0.4972635	0.627	0.312	3.889819e-54	12	AP3S1	DZ 3
2.33326e-58	0.5417848	0.402	0.131	4.15997e-54	12	SIT1	DZ 3
2.342088e-58	0.406653	0.72	0.4	4.175708e-54	12	GUK1	DZ 3
2.944336e-58	-0.9397996	0.052	0.201	5.249457e-54	12	ACP5	DZ 3
6.174663e-58	0.4086898	0.766	0.49	1.100881e-53	12	EIF4G2	DZ 3
6.596851e-58	0.4055091	0.738	0.436	1.176153e-53	12	CCT7	DZ 3
7.72277e-58	0.4582663	0.459	0.179	1.376893e-53	12	MRPL36	DZ 3
1.368657e-57	-0.6735915	0.031	0.175	2.440179e-53	12	BCL2	DZ 3
1.771257e-57	-0.7538767	0.053	0.199	3.157974e-53	12	CLEC2B	DZ 3
2.20133e-57	-0.8351535	0.168	0.309	3.924751e-53	12	ARHGAP24	DZ 3
3.362175e-57	0.499271	0.676	0.352	5.994422e-53	12	TPD52	DZ 3
3.610752e-57	0.4390022	0.341	0.105	6.43761e-53	12	CRIP1	DZ 3
4.842993e-57	0.4819225	0.476	0.208	8.634572e-53	12	PHAX	DZ 3
5.071755e-57	0.613192	0.347	0.103	9.042431e-53	12	VPREB3	DZ 3

6.31173e-57	0.4593095	0.599	0.281	1.125318e-52	12	PFDN4	DZ 3
9.541533e-57	0.4239225	0.27	0.064	1.70116e-52	12	ST14	DZ 3
1.068126e-56	0.330812	0.17	0.031	1.904362e-52	12	KANK1	DZ 3
1.315333e-56	0.3728076	0.873	0.65	2.345107e-52	12	PSMB1	DZ 3
2.91601e-56	-0.8947898	0.12	0.282	5.198954e-52	12	ADAM28	DZ 3
3.478181e-56	0.4280864	0.409	0.144	6.201248e-52	12	ARPC1A	DZ 3
4.040129e-56	-0.825698	0.141	0.283	7.203147e-52	12	RNASET2	DZ 3
4.813121e-56	0.4181346	0.416	0.159	8.581314e-52	12	HIGD1A	DZ 3
4.9261e-56	0.3957342	0.296	0.077	8.782744e-52	12	GSTZ1	DZ 3
7.492253e-56	0.3777651	0.755	0.454	1.335794e-51	12	HSPD1	DZ 3
9.588663e-56	0.4702919	0.643	0.343	1.709563e-51	12	MRPS6	DZ 3
1.195545e-55	0.4168846	0.273	0.074	2.131537e-51	12	FAM45A	DZ 3
1.29482e-55	0.4465094	0.718	0.423	2.308535e-51	12	CLTA	DZ 3
1.409972e-55	0.4467414	0.509	0.221	2.513839e-51	12	HIKESHI	DZ 3
1.423011e-55	-0.4324616	0.997	0.998	2.537087e-51	12	RPL18A	DZ 3
1.573881e-55	0.4340197	0.345	0.103	2.806073e-51	12	MAP4K2	DZ 3
1.865104e-55	-0.3572618	1	0.999	3.325293e-51	12	RPLP1	DZ 3
1.973407e-55	0.4210065	0.585	0.279	3.518387e-51	12	MRPS15	DZ 3
1.97555e-55	0.4517644	0.552	0.224	3.522209e-51	12	IL4R	DZ 3
2.939685e-55	0.4581537	0.542	0.257	5.241165e-51	12	SYNCRIP	DZ 3
3.045211e-55	0.4481924	0.506	0.231	5.429307e-51	12	HNRNPAB	DZ 3
3.439802e-55	0.3402214	0.179	0.033	6.132824e-51	12	MSRB2	DZ 3
3.71984e-55	0.3232304	0.978	0.902	6.632102e-51	12	SRP14	DZ 3
3.880269e-55	0.3935244	0.863	0.596	6.918132e-51	12	LSM7	DZ 3
4.264807e-55	0.3999792	0.32	0.096	7.603724e-51	12	AMZ2	DZ 3

4.837961e-55	0.4308232	0.759	0.44	8.6256e-51	12	HIGD2A	DZ 3
6.597066e-55	0.4102746	0.666	0.36	1.176191e-50	12	PFDN2	DZ 3
1.244018e-54	0.3018417	0.159	0.02	2.217961e-50	12	SNHG10	DZ 3
1.34847e-54	0.453079	0.514	0.24	2.404187e-50	12	HNRNPA1P48	DZ 3
1.674424e-54	0.4298523	0.377	0.135	2.98533e-50	12	RUVBL1	DZ 3
2.648296e-54	0.497425	0.558	0.263	4.721648e-50	12	TXNL4A	DZ 3
2.741721e-54	0.3173061	0.171	0.027	4.888214e-50	12	MIR3681HG	DZ 3
3.17514e-54	0.3493005	0.214	0.045	5.660956e-50	12	SHCBP1	DZ 3
3.260296e-54	0.4767494	0.496	0.208	5.812781e-50	12	SNRNP25	DZ 3
3.43001e-54	-1.178661	0.348	0.475	6.115365e-50	12	CD83	DZ 3
3.471654e-54	0.446734	0.561	0.267	6.189613e-50	12	SDHB	DZ 3
3.910278e-54	0.3475944	0.866	0.647	6.971634e-50	12	UBE2D3	DZ 3
4.427967e-54	0.4162861	0.635	0.332	7.894623e-50	12	STRAP	DZ 3
4.473723e-54	0.3980039	0.592	0.295	7.976202e-50	12	ODC1	DZ 3
4.993437e-54	0.3023857	0.159	0.025	8.902799e-50	12	LINC00877	DZ 3
6.776699e-54	0.4160945	0.692	0.387	1.208218e-49	12	NDUFAB1	DZ 3
7.22735e-54	0.4077589	0.809	0.525	1.288564e-49	12	SNRPB2	DZ 3
7.956044e-54	0.4986043	0.516	0.199	1.418483e-49	12	EAF2	DZ 3
1.289486e-53	0.4417993	0.569	0.267	2.299025e-49	12	RNF7	DZ 3
1.390656e-53	0.346238	0.894	0.692	2.479401e-49	12	RHOA	DZ 3
1.732644e-53	0.4367999	0.513	0.236	3.089132e-49	12	ETFA	DZ 3
2.650329e-53	0.3933365	0.755	0.461	4.725271e-49	12	PRDX5	DZ 3
2.712086e-53	0.4677426	0.528	0.233	4.835378e-49	12	RGS1	DZ 3
3.105616e-53	0.4610175	0.492	0.21	5.537003e-49	12	IFI27L2	DZ 3
4.169012e-53	0.3935753	0.738	0.448	7.432932e-49	12	PSMD4	DZ 3

4.299407e-53	0.43193	0.448	0.174	7.665412e-49	12	TADA3	DZ 3
4.681322e-53	0.4202527	0.421	0.166	8.346329e-49	12	MRPL37	DZ 3
6.059548e-53	-0.4193326	0.998	0.998	1.080357e-48	12	RPLP2	DZ 3
6.111432e-53	0.3892704	0.346	0.113	1.089607e-48	12	FMC1	DZ 3
8.042444e-53	0.4862848	0.569	0.285	1.433887e-48	12	HSPA4	DZ 3
9.198013e-53	-0.8922602	0.124	0.272	1.639914e-48	12	JUN	DZ 3
9.671379e-53	0.3698667	0.818	0.552	1.72431e-48	12	YWHAZ	DZ 3
1.08494e-52	0.4336044	0.555	0.267	1.93434e-48	12	SEPT2	DZ 3
1.235989e-52	0.460181	0.336	0.106	2.203645e-48	12	TRAC	DZ 3
1.246329e-52	0.4799263	0.386	0.111	2.22208e-48	12	CD38	DZ 3
1.743094e-52	0.4607908	0.39	0.134	3.107762e-48	12	AIM2	DZ 3
1.904361e-52	-0.9712485	0.314	0.466	3.395285e-48	12	CD63	DZ 3
2.696108e-52	0.4745051	0.267	0.069	4.806892e-48	12	H1FX	DZ 3
2.959628e-52	-0.8002119	0.037	0.175	5.276721e-48	12	TNFRSF18	DZ 3
3.247326e-52	0.4326247	0.389	0.134	5.789658e-48	12	CORO1B	DZ 3
3.476064e-52	0.4314364	0.476	0.203	6.197474e-48	12	ZCRB1	DZ 3
3.643139e-52	0.3385784	0.196	0.039	6.495352e-48	12	AL441992.1	DZ 3
3.854086e-52	0.391447	0.645	0.365	6.87145e-48	12	ARHGDIA	DZ 3
3.964961e-52	0.3923768	0.29	0.082	7.069128e-48	12	CSTF3	DZ 3
4.783757e-52	-0.9049966	0.291	0.417	8.52896e-48	12	NPC2	DZ 3
5.795522e-52	0.3735297	0.239	0.058	1.033284e-47	12	ORC6	DZ 3
6.216986e-52	0.4215846	0.474	0.2	1.108426e-47	12	MRPS14	DZ 3
9.108016e-52	-0.8839819	0.185	0.331	1.623868e-47	12	ZFP36	DZ 3
9.344676e-52	0.399421	0.282	0.077	1.666062e-47	12	BPNT1	DZ 3
1.143582e-51	0.4286055	0.567	0.251	2.038893e-47	12	SYNE2	DZ 3

1.610896e-51	0.4207388	0.538	0.234	2.872067e-47	12	NCOA3	DZ 3
2.384538e-51	0.3889457	0.73	0.442	4.251392e-47	12	ILF2	DZ 3
2.915583e-51	0.3461063	0.235	0.048	5.198192e-47	12	ATAD2	DZ 3
3.180113e-51	0.4511544	0.521	0.249	5.669823e-47	12	RBM42	DZ 3
3.410849e-51	0.4035052	0.269	0.074	6.081203e-47	12	MYO1E	DZ 3
4.067982e-51	0.3862466	0.241	0.056	7.252805e-47	12	TESC	DZ 3
4.094983e-51	0.3549446	0.21	0.041	7.300946e-47	12	MYBL1	DZ 3
4.109511e-51	-0.3128999	0.999	0.999	7.326848e-47	12	RPL10	DZ 3
5.010839e-51	-1.347608	0.157	0.266	8.933825e-47	12	S100A4	DZ 3
6.298302e-51	0.4129054	0.705	0.421	1.122924e-46	12	COPS9	DZ 3
6.480511e-51	0.3776919	0.286	0.076	1.15541e-46	12	SHLD1	DZ 3
6.921857e-51	0.4440383	0.468	0.19	1.234098e-46	12	POLD4	DZ 3
7.136771e-51	0.4152667	0.698	0.422	1.272415e-46	12	KTN1	DZ 3
7.977511e-51	0.4352202	0.53	0.236	1.42231e-46	12	WAS	DZ 3
1.002922e-50	0.3690846	0.724	0.445	1.788109e-46	12	VDAC1	DZ 3
1.749684e-50	0.4348945	0.223	0.048	3.119511e-46	12	SERPINA9	DZ 3
2.265147e-50	0.4549611	0.427	0.158	4.038531e-46	12	PPP1R18	DZ 3
2.297194e-50	0.4484515	0.464	0.19	4.095666e-46	12	IDH2	DZ 3
2.714907e-50	0.3821976	0.57	0.286	4.840407e-46	12	SSRP1	DZ 3
2.983898e-50	0.4043703	0.518	0.214	5.319991e-46	12	THUMPD3-AS1	DZ 3
4.180503e-50	0.361166	0.839	0.612	7.453418e-46	12	NDUFA13	DZ 3
4.955708e-50	0.4558779	0.507	0.226	8.835532e-46	12	CHCHD10	DZ 3
5.835856e-50	0.3844894	0.763	0.487	1.040475e-45	12	AP2M1	DZ 3
6.48455e-50	0.3872091	0.91	0.697	1.15613e-45	12	COMMD6	DZ 3
7.697092e-50	0.4009236	0.773	0.523	1.372314e-45	12	SRSF7	DZ 3

1.2331e-49	-0.3616985	1	0.999	2.198495e-45	12	RPL13A	DZ 3
1.395922e-49	0.4370587	0.772	0.478	2.48879e-45	12	GMFG	DZ 3
2.267543e-49	0.4298475	0.645	0.353	4.042802e-45	12	TKT	DZ 3
3.15672e-49	0.4278525	0.476	0.212	5.628116e-45	12	CHMP5	DZ 3
3.341343e-49	0.3864385	0.257	0.065	5.95728e-45	12	ZSCAN16-AS1	DZ 3
4.590026e-49	0.3470388	0.231	0.054	8.183557e-45	12	SLC15A4	DZ 3
4.614783e-49	0.341214	0.781	0.49	8.227697e-45	12	HSPE1	DZ 3
4.929301e-49	0.3881098	0.208	0.042	8.788451e-45	12	ASF1B	DZ 3
5.106209e-49	0.3639342	0.268	0.074	9.10386e-45	12	WEE1	DZ 3
6.981295e-49	0.3807817	0.353	0.117	1.244695e-44	12	RFC2	DZ 3
1.113324e-48	0.3539443	0.873	0.663	1.984945e-44	12	GDI2	DZ 3
1.534063e-48	0.407599	0.434	0.18	2.73508e-44	12	CLINT1	DZ 3
1.581491e-48	0.3779481	0.756	0.488	2.819641e-44	12	ARL6IP4	DZ 3
1.680192e-48	-0.77931	0.064	0.201	2.995614e-44	12	YBX3	DZ 3
1.719712e-48	0.3832228	0.273	0.07	3.066075e-44	12	DNASE1	DZ 3
2.11631e-48	0.4049575	0.324	0.111	3.77317e-44	12	GOT2	DZ 3
2.322778e-48	0.396761	0.574	0.295	4.141281e-44	12	AHSA1	DZ 3
2.332301e-48	0.402259	0.315	0.1	4.15826e-44	12	ACSF3	DZ 3
2.666423e-48	-0.8686149	0.363	0.47	4.753966e-44	12	ITM2B	DZ 3
5.779726e-48	0.3801866	0.689	0.418	1.030467e-43	12	BLOC1S1	DZ 3
6.520975e-48	0.3534573	0.73	0.438	1.162625e-43	12	PSMB7	DZ 3
7.300673e-48	-0.5765803	0.912	0.934	1.301637e-43	12	MT-ATP6	DZ 3
7.681544e-48	0.3788608	0.641	0.337	1.369542e-43	12	PNN	DZ 3
7.74769e-48	0.3601663	0.236	0.067	1.381336e-43	12	NEK6	DZ 3
7.897275e-48	-0.6914719	0.057	0.187	1.408005e-43	12	PLPP5	DZ 3

8.003003e-48	0.5023573	0.453	0.161	1.426855e-43	12	CCDC88A	DZ 3
9.531282e-48	-0.6138734	0.031	0.152	1.699332e-43	12	CELF2	DZ 3
9.721064e-48	0.3571168	0.245	0.063	1.733168e-43	12	MFHAS1	DZ 3
1.009163e-47	0.3249471	0.171	0.031	1.799236e-43	12	AL391069.3	DZ 3
1.053568e-47	0.3917063	0.604	0.317	1.878406e-43	12	NDUFB6	DZ 3
1.207297e-47	0.3873743	0.299	0.094	2.15249e-43	12	PCBD1	DZ 3
1.567257e-47	0.3200841	0.821	0.573	2.794263e-43	12	C11orf58	DZ 3
2.089095e-47	-0.911282	0.215	0.341	3.724647e-43	12	HVCN1	DZ 3
2.460235e-47	0.4012357	0.385	0.145	4.386352e-43	12	HDGF	DZ 3
3.060249e-47	0.3067494	0.832	0.562	5.456119e-43	12	PCBP2	DZ 3
4.079423e-47	0.3889856	0.437	0.18	7.273204e-43	12	SNRPA	DZ 3
6.690988e-47	0.3830822	0.566	0.256	1.192936e-42	12	CD22	DZ 3
8.783038e-47	0.3981661	0.299	0.049	1.565928e-42	12	UBE2C	DZ 3
9.550323e-47	0.3856809	0.709	0.433	1.702727e-42	12	AURKAIP1	DZ 3
9.761703e-47	0.3474594	0.28	0.077	1.740414e-42	12	RFC4	DZ 3
1.075077e-46	0.3075222	0.942	0.802	1.916756e-42	12	HINT1	DZ 3
1.928827e-46	0.3894566	0.495	0.229	3.438906e-42	12	UFD1	DZ 3
2.351395e-46	0.3628699	0.555	0.272	4.192303e-42	12	VBP1	DZ 3
2.443764e-46	0.3810675	0.612	0.326	4.356986e-42	12	BAX	DZ 3
2.611308e-46	0.3995066	0.439	0.193	4.6557e-42	12	NDUFA7	DZ 3
2.980123e-46	0.3974226	0.193	0.037	5.313262e-42	12	NUF2	DZ 3
3.077255e-46	-0.4900519	0.935	0.96	5.486438e-42	12	MT-CYB	DZ 3
3.428462e-46	0.4088186	0.808	0.508	6.112605e-42	12	LSP1	DZ 3
3.751768e-46	0.3648442	0.658	0.372	6.689027e-42	12	VDAC2	DZ 3
4.939466e-46	-0.4771896	0.99	0.991	8.806574e-42	12	RPL9	DZ 3

6.061885e-46	0.4257125	0.548	0.264	1.080773e-41	12	SMS	DZ 3
7.487282e-46	-0.6641822	0.045	0.167	1.334908e-41	12	MARCKS	DZ 3
8.034603e-46	0.3707667	0.285	0.086	1.432489e-41	12	XPNPEP1	DZ 3
8.500516e-46	-0.8094972	0.323	0.441	1.515557e-41	12	IL2RG	DZ 3
1.056636e-45	-0.6681545	0.023	0.14	1.883876e-41	12	FCER2	DZ 3
1.30665e-45	0.32605	0.79	0.529	2.329626e-41	12	CAP1	DZ 3
1.386675e-45	0.3498029	0.304	0.095	2.472304e-41	12	RMDN1	DZ 3
1.480058e-45	0.3871017	0.406	0.163	2.638796e-41	12	MTX1	DZ 3
1.755452e-45	0.3918456	0.485	0.208	3.129796e-41	12	DMAC1	DZ 3
2.272883e-45	0.3647509	0.321	0.101	4.052324e-41	12	IMMP1L	DZ 3
2.441453e-45	0.3854162	0.664	0.389	4.352866e-41	12	ATP5PD	DZ 3
2.463528e-45	0.4285165	0.5	0.226	4.392225e-41	12	ZNF581	DZ 3
4.272852e-45	0.4193693	0.497	0.235	7.618067e-41	12	PPP1CB	DZ 3
6.769096e-45	0.3615067	0.597	0.298	1.206862e-40	12	OCIAD2	DZ 3
8.099589e-45	0.3775538	0.246	0.066	1.444076e-40	12	RPRD1B	DZ 3
9.42658e-45	0.3851257	0.489	0.22	1.680665e-40	12	PLEKHJ1	DZ 3
1.020019e-44	0.4196602	0.606	0.342	1.818591e-40	12	NASP	DZ 3
1.182982e-44	0.3223932	0.967	0.937	2.109139e-40	12	EEF2	DZ 3
1.457351e-44	0.3873129	0.511	0.232	2.598312e-40	12	CDC26	DZ 3
1.580629e-44	0.3840968	0.392	0.155	2.818104e-40	12	DNAJC9	DZ 3
1.618609e-44	0.3908218	0.637	0.358	2.885818e-40	12	VPS29	DZ 3
2.174791e-44	0.4049479	0.357	0.125	3.877434e-40	12	KMT2A	DZ 3
2.609147e-44	0.446969	0.287	0.09	4.651848e-40	12	RNGTT	DZ 3
3.237893e-44	0.3613035	0.461	0.192	5.772839e-40	12	NUDCD2	DZ 3
4.027352e-44	0.3674	0.384	0.14	7.180367e-40	12	P2RY8	DZ 3

5.269581e-44	0.3899633	0.517	0.233	9.395136e-40	12	MCUB	DZ 3
5.345087e-44	0.4214637	0.362	0.124	9.529756e-40	12	STIM2	DZ 3
5.460181e-44	0.3679336	0.657	0.332	9.734956e-40	12	LRMP	DZ 3
5.765663e-44	0.3908547	0.406	0.155	1.02796e-39	12	UBE2R2	DZ 3
5.966538e-44	0.3815349	0.524	0.256	1.063774e-39	12	HSPB11	DZ 3
7.875243e-44	0.3662471	0.615	0.313	1.404077e-39	12	IGBP1	DZ 3
7.99899e-44	0.3900732	0.715	0.418	1.42614e-39	12	TAF7	DZ 3
8.947816e-44	0.3595439	0.581	0.302	1.595306e-39	12	PSMD6	DZ 3
9.646073e-44	-0.7888074	0.22	0.332	1.719798e-39	12	SMC6	DZ 3
1.07637e-43	0.3951885	0.443	0.183	1.919061e-39	12	NUDT21	DZ 3
1.24263e-43	0.3065096	0.898	0.715	2.215485e-39	12	EIF3F	DZ 3
1.435144e-43	0.3831633	0.54	0.265	2.558718e-39	12	POLR2F	DZ 3
1.724131e-43	0.3783328	0.488	0.212	3.073953e-39	12	GNAI2	DZ 3
1.862163e-43	0.3096766	0.184	0.037	3.32005e-39	12	SLC30A4	DZ 3
2.011881e-43	0.4168176	0.513	0.246	3.586982e-39	12	RGS10	DZ 3
2.220402e-43	0.3800538	0.441	0.184	3.958755e-39	12	NDUFA8	DZ 3
2.460022e-43	0.3747295	0.436	0.175	4.385972e-39	12	RASGRP3	DZ 3
2.69431e-43	0.3618424	0.597	0.281	4.803685e-39	12	DCK	DZ 3
3.053091e-43	0.3414332	0.294	0.089	5.443356e-39	12	TXLNG	DZ 3
3.288848e-43	0.3667802	0.312	0.105	5.863687e-39	12	PTS	DZ 3
3.519559e-43	0.3067285	0.191	0.043	6.275022e-39	12	CENPN	DZ 3
4.479119e-43	0.3335324	0.677	0.372	7.985821e-39	12	POLR2G	DZ 3
4.782607e-43	0.4402428	0.488	0.22	8.52691e-39	12	NSMCE1	DZ 3
5.994171e-43	0.3608689	0.262	0.073	1.068701e-38	12	KLHL6	DZ 3
8.988047e-43	0.3392562	0.761	0.496	1.602479e-38	12	NDUFA11	DZ 3

9.424348e-43	0.3392391	0.239	0.063	1.680267e-38	12	ST20	DZ 3
1.145261e-42	0.3475368	0.25	0.062	2.041886e-38	12	FAM200B	DZ 3
1.268183e-42	0.4320546	0.49	0.234	2.261043e-38	12	NSRP1	DZ 3
1.622707e-42	0.3441083	0.231	0.058	2.893125e-38	12	FANCA	DZ 3
1.910999e-42	0.340965	0.686	0.403	3.407121e-38	12	EID1	DZ 3
2.948699e-42	0.3974278	0.558	0.282	5.257236e-38	12	RRAS2	DZ 3
3.547036e-42	0.3273477	0.513	0.264	6.32401e-38	12	TIMM10	DZ 3
3.808355e-42	-0.8061099	0.501	0.572	6.789915e-38	12	SNX2	DZ 3
3.900077e-42	0.3484408	0.518	0.25	6.953447e-38	12	C1D	DZ 3
6.129291e-42	0.3539308	0.533	0.258	1.092791e-37	12	MTCH2	DZ 3
6.275156e-42	0.3852752	0.679	0.39	1.118798e-37	12	ORAI2	DZ 3
6.987102e-42	-0.7720818	0.136	0.241	1.24573e-37	12	CD55	DZ 3
8.155733e-42	0.3566877	0.538	0.269	1.454086e-37	12	TMEM14B	DZ 3
8.895742e-42	0.3452392	0.302	0.094	1.586022e-37	12	GON7	DZ 3
9.264846e-42	0.3264017	0.7	0.412	1.651829e-37	12	TOMM22	DZ 3
1.032002e-41	0.326739	0.684	0.4	1.839957e-37	12	BANF1	DZ 3
1.141661e-41	0.331616	0.265	0.078	2.035468e-37	12	EPN1	DZ 3
1.297726e-41	0.3376618	0.551	0.265	2.313716e-37	12	CAMTA1	DZ 3
1.458016e-41	-0.6432451	0.043	0.161	2.599497e-37	12	SLC2A3	DZ 3
1.507162e-41	-0.7742387	0.71	0.759	2.687119e-37	12	MT-ND5	DZ 3
2.008499e-41	0.3560249	0.596	0.319	3.580952e-37	12	MRPS36	DZ 3
2.019988e-41	0.386151	0.399	0.137	3.601436e-37	12	HSH2D	DZ 3
3.026912e-41	0.3508084	0.314	0.103	5.396681e-37	12	OGG1	DZ 3
4.156879e-41	0.3353151	0.557	0.297	7.411299e-37	12	GLRX3	DZ 3
5.968254e-41	0.3753826	0.347	0.115	1.06408e-36	12	LPP	DZ 3

6.406918e-41	0.3512906	0.688	0.415	1.142289e-36	12	ECH1	DZ 3
8.559033e-41	0.3595405	0.424	0.182	1.52599e-36	12	RUVBL2	DZ 3
9.02027e-41	0.3164415	0.821	0.578	1.608224e-36	12	NDUFB2	DZ 3
1.109587e-40	0.3602298	0.621	0.348	1.978283e-36	12	AP2S1	DZ 3
1.578507e-40	0.3229197	0.767	0.519	2.814321e-36	12	VAMP8	DZ 3
1.970078e-40	0.3450022	0.427	0.174	3.512453e-36	12	TAF12	DZ 3
2.120948e-40	-0.8078612	0.413	0.496	3.781438e-36	12	SP100	DZ 3
2.305859e-40	0.3521844	0.62	0.361	4.111115e-36	12	CACYBP	DZ 3
2.960781e-40	0.3701008	0.64	0.358	5.278777e-36	12	TERF2IP	DZ 3
3.044423e-40	0.3701342	0.354	0.131	5.427902e-36	12	CLIC4	DZ 3
3.123934e-40	0.3249666	0.313	0.111	5.569662e-36	12	CPSF3	DZ 3
3.302629e-40	0.337331	0.554	0.281	5.888256e-36	12	WTAP	DZ 3
5.474805e-40	0.3297547	0.158	0.033	9.761029e-36	12	PBK	DZ 3
9.799505e-40	0.3284722	0.245	0.077	1.747154e-35	12	PDZD11	DZ 3
9.872793e-40	0.3338528	0.611	0.314	1.76022e-35	12	GNB2	DZ 3
1.380426e-39	0.3662676	0.356	0.132	2.461162e-35	12	RPA1	DZ 3
1.537195e-39	0.3458475	0.495	0.24	2.740665e-35	12	MRPL9	DZ 3
1.569337e-39	0.3244139	0.275	0.088	2.797971e-35	12	RTCA	DZ 3
1.601079e-39	0.3622068	0.396	0.171	2.854564e-35	12	LAP3	DZ 3
1.6709e-39	0.3572715	0.492	0.228	2.979048e-35	12	PIN1	DZ 3
1.712544e-39	-0.3286963	0.999	0.999	3.053295e-35	12	RPS19	DZ 3
1.957484e-39	0.3610243	0.566	0.287	3.489998e-35	12	DYNLRB1	DZ 3
2.145863e-39	-0.404297	1	0.999	3.82586e-35	12	RPL39	DZ 3
3.049756e-39	0.3484935	0.343	0.125	5.43741e-35	12	MAGEF1	DZ 3
3.139329e-39	0.3696447	0.457	0.217	5.597109e-35	12	HSPH1	DZ 3

4.494616e-39	0.3246193	0.545	0.27	8.013451e-35	12	CCNG1	DZ 3
5.763477e-39	0.3186378	0.685	0.386	1.02757e-34	12	SYPL1	DZ 3
5.781144e-39	0.3228283	0.503	0.243	1.03072e-34	12	NAP1L4	DZ 3
5.872827e-39	0.4309054	0.626	0.33	1.047066e-34	12	SPIB	DZ 3
6.401697e-39	0.3645235	0.383	0.16	1.141359e-34	12	COX11	DZ 3
7.99459e-39	0.3702703	0.762	0.484	1.425355e-34	12	RAC2	DZ 3
1.072228e-38	-0.7713423	0.121	0.24	1.911675e-34	12	CAPG	DZ 3
1.749838e-38	0.3196232	0.236	0.068	3.119786e-34	12	HAUS8	DZ 3
1.768491e-38	0.3266092	0.459	0.222	3.153043e-34	12	DEGS1	DZ 3
1.888441e-38	-0.4072803	1	0.999	3.366902e-34	12	EEF1A1	DZ 3
1.904258e-38	0.382472	0.334	0.122	3.395102e-34	12	MSI2	DZ 3
1.93837e-38	0.3205669	0.609	0.342	3.455921e-34	12	CYC1	DZ 3
2.236307e-38	-0.7155783	0.044	0.161	3.987112e-34	12	LMNA	DZ 3
2.717386e-38	0.3246964	0.394	0.165	4.844827e-34	12	EED	DZ 3
3.044303e-38	0.3288818	0.43	0.184	5.427688e-34	12	MAP2K1	DZ 3
4.593553e-38	0.3328983	0.55	0.28	8.189846e-34	12	NDUFB5	DZ 3
4.751173e-38	0.3911843	0.214	0.04	8.470866e-34	12	RRM2	DZ 3
5.755657e-38	0.3245611	0.916	0.803	1.026176e-33	12	CALM2	DZ 3
6.033409e-38	0.3298534	0.706	0.438	1.075697e-33	12	NDUFB10	DZ 3
6.139139e-38	0.3876101	0.583	0.322	1.094547e-33	12	HNRNPH1	DZ 3
6.363933e-38	0.3329694	0.272	0.09	1.134626e-33	12	RNF8	DZ 3
7.924853e-38	-0.743787	0.852	0.855	1.412922e-33	12	SH3BGRL3	DZ 3
8.29813e-38	-0.6830705	0.117	0.245	1.479474e-33	12	PHACTR1	DZ 3
9.499289e-38	0.3629986	0.337	0.126	1.693628e-33	12	C16orf87	DZ 3
1.817661e-37	0.3096547	0.947	0.881	3.240709e-33	12	H3F3B	DZ 3

2.661615e-37	0.3469687	0.529	0.247	4.745393e-33	12	ZCCHC10	DZ 3
2.89687e-37	0.3297191	0.399	0.165	5.16483e-33	12	COMMD7	DZ 3
3.32476e-37	0.3403898	0.521	0.255	5.927715e-33	12	ATXN10	DZ 3
3.337523e-37	0.3281249	0.588	0.311	5.95047e-33	12	SMIM26	DZ 3
3.346761e-37	0.3349878	0.518	0.259	5.96694e-33	12	RAB5IF	DZ 3
3.678969e-37	0.3225545	0.219	0.063	6.559234e-33	12	TEX9	DZ 3
5.088018e-37	0.3436777	0.367	0.147	9.071428e-33	12	UBA1	DZ 3
5.17065e-37	0.3353904	0.363	0.136	9.218751e-33	12	CUL3	DZ 3
5.780594e-37	0.3289921	0.308	0.112	1.030622e-32	12	ACYP2	DZ 3
8.801176e-37	0.3568434	0.198	0.043	1.569162e-32	12	HMMR	DZ 3
1.521685e-36	0.3219808	0.457	0.207	2.713012e-32	12	FAM192A	DZ 3
2.006999e-36	0.3194158	0.276	0.093	3.578279e-32	12	PHF19	DZ 3
2.671742e-36	-0.694534	0.234	0.351	4.763449e-32	12	LITAF	DZ 3
3.094056e-36	0.3632325	0.342	0.123	5.516392e-32	12	ETS1	DZ 3
3.096734e-36	-0.4032706	0.999	0.998	5.521166e-32	12	RPL11	DZ 3
4.080598e-36	0.3551295	0.285	0.098	7.275298e-32	12	ALDH2	DZ 3
4.857026e-36	0.3147852	0.413	0.197	8.659592e-32	12	MRPL23	DZ 3
5.007567e-36	0.3660574	0.514	0.27	8.927991e-32	12	ATOX1	DZ 3
5.862838e-36	0.303294	0.234	0.077	1.045285e-31	12	GPR160	DZ 3
7.123773e-36	0.3161121	0.263	0.087	1.270097e-31	12	FUNDC1	DZ 3
8.61774e-36	-0.6947742	0.099	0.213	1.536457e-31	12	MGST3	DZ 3
1.287516e-35	0.3203878	0.613	0.339	2.295513e-31	12	UBE2A	DZ 3
1.473631e-35	0.3271365	0.514	0.256	2.627337e-31	12	TRAPPC3	DZ 3
1.566002e-35	0.3720111	0.493	0.24	2.792024e-31	12	CKS1B	DZ 3
1.665683e-35	0.4889804	0.748	0.491	2.969747e-31	12	SAT1	DZ 3

1.854368e-35	0.3059666	0.273	0.1	3.306152e-31	12	SLC25A4	DZ 3
2.034063e-35	0.3066785	0.385	0.159	3.62653e-31	12	SAE1	DZ 3
2.077014e-35	0.3680897	0.661	0.395	3.703109e-31	12	RPL22L1	DZ 3
2.900883e-35	0.3300177	0.498	0.245	5.171984e-31	12	MCTS1	DZ 3
2.963664e-35	-0.6967937	0.409	0.48	5.283917e-31	12	CD47	DZ 3
3.377051e-35	0.344624	0.43	0.189	6.020944e-31	12	USP34	DZ 3
3.450937e-35	0.3138299	0.215	0.059	6.152676e-31	12	PITPNC1	DZ 3
3.609076e-35	0.3218511	0.476	0.242	6.434622e-31	12	HAT1	DZ 3
3.850318e-35	0.3355715	0.585	0.319	6.864732e-31	12	SNRNP70	DZ 3
4.058061e-35	0.3084623	0.337	0.125	7.235117e-31	12	NCBP2-AS2	DZ 3
6.756036e-35	0.3628238	0.385	0.157	1.204534e-30	12	LYPLAL1	DZ 3
7.084077e-35	0.3684295	0.44	0.204	1.26302e-30	12	EHD1	DZ 3
7.691344e-35	0.3074113	0.359	0.147	1.37129e-30	12	BCCIP	DZ 3
7.980641e-35	0.3051974	0.319	0.116	1.422868e-30	12	CFAP20	DZ 3
9.102869e-35	0.309421	0.313	0.108	1.622951e-30	12	ZNF296	DZ 3
9.279489e-35	0.3188812	0.361	0.148	1.65444e-30	12	PPIH	DZ 3
1.210688e-34	0.317439	0.129	0.025	2.158535e-30	12	DLGAP5	DZ 3
1.242983e-34	0.3134423	0.627	0.349	2.216115e-30	12	RAB11A	DZ 3
1.45737e-34	0.3766945	0.385	0.153	2.598345e-30	12	KIAA0040	DZ 3
1.983213e-34	0.328095	0.36	0.145	3.53587e-30	12	COA6	DZ 3
2.532988e-34	0.319153	0.272	0.095	4.516065e-30	12	PPP1R35	DZ 3
3.346026e-34	0.3140173	0.261	0.088	5.96563e-30	12	FAM208B	DZ 3
3.516753e-34	0.3473662	0.453	0.221	6.270019e-30	12	ADI1	DZ 3
3.958099e-34	0.3063171	0.307	0.107	7.056896e-30	12	HPS1	DZ 3
4.50507e-34	0.302129	0.23	0.071	8.032089e-30	12	CUX1	DZ 3

4.811133e-34	0.3098803	0.603	0.326	8.577769e-30	12	DARS	DZ	3
4.87724e-34	0.3462737	0.474	0.237	8.695632e-30	12	KPNA2	DZ	3
5.290276e-34	0.3599333	0.315	0.119	9.432033e-30	12	DLAT	DZ	3
5.711329e-34	0.3235879	0.311	0.105	1.018273e-29	12	MOB3A	DZ	3
6.006085e-34	0.317496	0.348	0.141	1.070825e-29	12	ANKRD13A	DZ	3
6.618898e-34	0.3109937	0.451	0.209	1.180083e-29	12	RAB1A	DZ	3
6.899916e-34	0.3389149	0.408	0.177	1.230186e-29	12	TRABD	DZ	3
1.016858e-33	0.3414177	0.494	0.259	1.812956e-29	12	MRPL41	DZ	3
1.075949e-33	0.3217337	0.595	0.289	1.91831e-29	12	GGA2	DZ	3
1.101734e-33	0.3086402	0.344	0.134	1.964281e-29	12	C22orf39	DZ	3
1.129399e-33	0.310977	0.437	0.195	2.013606e-29	12	CHCHD1	DZ	3
1.218931e-33	0.3031716	0.513	0.259	2.173232e-29	12	PDCD2	DZ	3
1.314378e-33	-0.3437705	0.996	0.996	2.343404e-29	12	RPL35A	DZ	3
1.750748e-33	0.3035137	0.44	0.202	3.121409e-29	12	PRPF38A	DZ	3
2.009305e-33	0.3216474	0.615	0.351	3.58239e-29	12	POLR1D	DZ	3
2.059441e-33	0.5407182	0.402	0.201	3.671777e-29	12	LGALS1	DZ	3
2.509348e-33	-0.6600488	0.394	0.478	4.473916e-29	12	N4BP2L2	DZ	3
2.93843e-33	-0.6272756	0.142	0.244	5.238926e-29	12	HLA-F	DZ	3
3.33292e-33	0.321083	0.529	0.272	5.942263e-29	12	CNIH1	DZ	3
3.819138e-33	-0.5305221	0.066	0.16	6.809141e-29	12	TGIF1	DZ	3
5.286397e-33	0.349597	0.276	0.086	9.425117e-29	12	GCSAM	DZ	3
7.263443e-33	-0.5671448	0.866	0.868	1.294999e-28	12	CD52	DZ	3
8.330312e-33	-0.6891262	0.311	0.39	1.485211e-28	12	EVI2B	DZ	3
8.417394e-33	0.3072352	0.56	0.307	1.500737e-28	12	ZNF207	DZ	3
9.603103e-33	0.3243136	0.322	0.109	1.712137e-28	12	HDAC7	DZ	3

1.11757e-32	0.3175367	0.357	0.151	1.992515e-28	12	VPS25	DZ 3
1.215404e-32	0.3093622	0.412	0.18	2.166944e-28	12	COPS8	DZ 3
1.398605e-32	0.3237471	0.335	0.129	2.493573e-28	12	MAP4K1	DZ 3
3.534787e-32	-0.4727602	0.021	0.107	6.302171e-28	12	MTSS1	DZ 3
4.232915e-32	0.3010742	0.407	0.187	7.546864e-28	12	FYTTD1	DZ 3
4.682863e-32	-0.8078115	0.41	0.48	8.349076e-28	12	SQSTM1	DZ 3
6.222974e-32	0.3233568	0.49	0.244	1.109494e-27	12	COA3	DZ 3
7.149319e-32	0.3017847	0.217	0.045	1.274652e-27	12	AURKB	DZ 3
7.389785e-32	0.3209616	0.387	0.17	1.317525e-27	12	EIF4E2	DZ 3
8.822709e-32	0.306122	0.376	0.167	1.573001e-27	12	CLTB	DZ 3
8.958921e-32	0.3121876	0.308	0.112	1.597286e-27	12	BRWD1	DZ 3
9.59158e-32	0.3125184	0.33	0.131	1.710083e-27	12	GTF2E2	DZ 3
1.079669e-31	-0.7738638	0.24	0.304	1.924942e-27	12	ZFP36L2	DZ 3
1.096827e-31	0.3022074	0.377	0.157	1.955533e-27	12	BRD7	DZ 3
1.597508e-31	-0.82075	0.214	0.315	2.848196e-27	12	HSP90B1	DZ 3
3.391361e-31	0.3031289	0.421	0.197	6.046458e-27	12	URM1	DZ 3
3.617773e-31	0.3283071	0.478	0.223	6.450127e-27	12	ATM	DZ 3
8.23368e-31	-0.4382657	0.023	0.111	1.467983e-26	12	SNX9	DZ 3
1.185405e-30	0.3087856	0.543	0.298	2.113458e-26	12	CHMP2A	DZ 3
1.203828e-30	0.3045707	0.558	0.29	2.146305e-26	12	IFT57	DZ 3
1.618646e-30	-0.4518981	0.029	0.115	2.885884e-26	12	P2RY10	DZ 3
2.510731e-30	0.3143681	0.415	0.184	4.476382e-26	12	RFC1	DZ 3
3.4005e-30	-0.6632221	0.265	0.338	6.062752e-26	12	CD48	DZ 3
4.159138e-30	0.3032052	0.458	0.222	7.415327e-26	12	IAH1	DZ 3
8.586076e-30	-0.6509634	0.189	0.246	1.530811e-25	12	RASGRP2	DZ 3

1.095986e-29	0.311967	0.466	0.204	1.954033e-25	12	FAM111B	DZ 3
1.118649e-29	-0.4967735	0.067	0.152	1.994439e-25	12	ESYT1	DZ 3
1.67081e-29	0.3090822	0.464	0.232	2.978888e-25	12	TSG101	DZ 3
2.206803e-29	0.3212499	0.476	0.271	3.934509e-25	12	EIF1AY	DZ 3
2.970638e-29	0.417494	0.209	0.067	5.296351e-25	12	RASSF6	DZ 3
3.66705e-29	-0.4512587	0.026	0.102	6.537984e-25	12	RHOC	DZ 3
4.977025e-29	0.3154836	0.334	0.137	8.873538e-25	12	INO80C	DZ 3
5.196362e-29	0.3038593	0.245	0.092	9.264595e-25	12	RAD17	DZ 3
6.044984e-29	-0.6067574	0.218	0.308	1.07776e-24	12	TMEM147	DZ 3
8.504202e-29	-0.3599486	0.019	0.1	1.516214e-24	12	PARVB	DZ 3
1.386804e-28	-0.5026969	0.085	0.165	2.472533e-24	12	TNFRSF14	DZ 3
1.530271e-28	-0.4520071	0.038	0.12	2.72832e-24	12	FCGR2B	DZ 3
3.37153e-28	-0.5363442	0.081	0.173	6.0111e-24	12	PARP14	DZ 3
3.740529e-28	-0.7156661	0.266	0.346	6.66899e-24	12	INSIG1	DZ 3
5.633213e-28	-0.554827	0.123	0.192	1.004346e-23	12	S1PR4	DZ 3
6.186407e-28	-0.7550377	0.383	0.447	1.102974e-23	12	KLF6	DZ 3
1.175295e-26	-0.6892699	0.452	0.493	2.095433e-22	12	CYTIP	DZ 3
1.312885e-26	-0.4381568	0.052	0.122	2.340743e-22	12	IL27RA	DZ 3
1.58778e-26	-0.7268164	0.442	0.505	2.830853e-22	12	TSC22D3	DZ 3
4.173316e-26	-0.4627626	0.086	0.161	7.440605e-22	12	ARHGAP15	DZ 3
5.134227e-26	-0.6418876	0.152	0.213	9.153813e-22	12	LBH	DZ 3
5.763865e-26	-0.6592343	0.346	0.388	1.027639e-21	12	PSAP	DZ 3
1.198116e-25	-0.6102095	0.314	0.385	2.136121e-21	12	LAPTM4A	DZ 3
1.242522e-25	0.3163714	0.261	0.094	2.215292e-21	12	MX1	DZ 3
1.784106e-25	-0.6031595	0.709	0.717	3.180882e-21	12	EIF4A2	DZ 3

3.65922e-25	0.3058982	0.271	0.103	6.524023e-21	12	E2F5	DZ 3
4.22648e-25	-0.5234335	0.928	0.917	7.535392e-21	12	PFDN5	DZ 3
5.801535e-25	-0.5442631	0.14	0.208	1.034356e-20	12	C16orf74	DZ 3
6.106225e-25	-0.3872445	0.997	0.991	1.088679e-20	12	RPL30	DZ 3
7.057755e-25	-0.4798689	0.113	0.191	1.258327e-20	12	SYTL1	DZ 3
1.042879e-24	-0.6315069	0.307	0.363	1.859349e-20	12	ANXA6	DZ 3
1.465524e-24	-0.6674183	0.461	0.502	2.612883e-20	12	FOXP1	DZ 3
2.360205e-24	-0.5399406	0.164	0.233	4.20801e-20	12	HSD17B11	DZ 3
1.002911e-23	-0.6735021	0.27	0.347	1.78809e-19	12	CAST	DZ 3
1.290781e-23	-0.5159602	0.184	0.253	2.301334e-19	12	DNPH1	DZ 3
1.464962e-23	-0.5730457	0.123	0.198	2.611881e-19	12	CHPT1	DZ 3
1.957184e-23	-0.3992821	0.053	0.115	3.489463e-19	12	LGALS9	DZ 3
2.017487e-23	-0.3576419	0.986	0.985	3.596978e-19	12	RPL27	DZ 3
3.723976e-23	-0.5728303	0.082	0.162	6.639476e-19	12	DDIT4	DZ 3
1.399222e-22	-0.6140346	0.192	0.281	2.494673e-18	12	WARS	DZ 3
1.80726e-22	-0.5612211	0.164	0.23	3.222165e-18	12	NT5C3A	DZ 3
2.086326e-22	-0.513201	0.113	0.176	3.71971e-18	12	MARCH1	DZ 3
4.212071e-22	-0.4922486	0.127	0.196	7.509702e-18	12	ADK	DZ 3
5.279629e-22	-0.3970054	0.054	0.113	9.413051e-18	12	SESN3	DZ 3
6.048437e-22	-0.7348151	0.305	0.361	1.078376e-17	12	ARID5B	DZ 3
8.225279e-22	-0.3844953	0.047	0.116	1.466485e-17	12	PYCR1	DZ 3
1.035821e-21	-0.57154	0.277	0.335	1.846764e-17	12	BTK	DZ 3
1.1376e-21	-0.4363632	0.048	0.107	2.028226e-17	12	C1orf162	DZ 3
1.983746e-21	-0.4915245	0.114	0.191	3.536821e-17	12	IFNGR1	DZ 3
2.277938e-21	-0.3648735	0.039	0.103	4.061335e-17	12	PHF1	DZ 3

3.899517e-21	-0.5283329	0.164	0.207	6.952449e-17	12	PRKCB	DZ 3
6.610674e-21	-0.4879385	0.087	0.149	1.178617e-16	12	ARRDC2	DZ 3
7.752274e-21	-0.4971153	0.148	0.218	1.382153e-16	12	NFKBIE	DZ 3
1.178089e-20	-0.5419043	0.671	0.624	2.100414e-16	12	HLA-DMB	DZ 3
2.083589e-20	-0.5095216	0.169	0.226	3.714831e-16	12	TMEM109	DZ 3
2.603472e-20	0.3197538	0.663	0.389	4.641731e-16	12	CSTB	DZ 3
3.289834e-20	-0.5064611	0.147	0.205	5.865445e-16	12	IRF1	DZ 3
6.424151e-20	-0.3722812	0.045	0.106	1.145362e-15	12	PDE4B	DZ 3
1.995173e-19	-0.3763793	0.051	0.117	3.557194e-15	12	ENTPD1	DZ 3
2.189407e-19	-0.5106407	0.13	0.198	3.903493e-15	12	TYMP	DZ 3
2.227597e-19	-0.3004156	0.997	0.995	3.971582e-15	12	RPL31	DZ 3
1.723826e-18	-0.4537874	0.1	0.172	3.07341e-14	12	DDIT3	DZ 3
2.086081e-18	-0.3452575	0.053	0.11	3.719275e-14	12	HEXB	DZ 3
2.160936e-18	-0.5451133	0.228	0.283	3.852732e-14	12	MT-ND4L	DZ 3
3.370791e-18	-0.5241989	0.88	0.847	6.009784e-14	12	CD37	DZ 3
3.60445e-18	-0.5550645	0.321	0.362	6.426373e-14	12	TAPBP	DZ 3
4.826421e-18	-0.4458075	0.991	0.985	8.605025e-14	12	TMSB10	DZ 3
5.92277e-18	-0.564602	0.166	0.219	1.055971e-13	12	EIF4EBP1	DZ 3
8.45124e-18	-0.5482279	0.35	0.384	1.506772e-13	12	C19orf70	DZ 3
1.375786e-17	-0.4556375	0.116	0.176	2.452889e-13	12	ASAH1	DZ 3
1.813726e-17	-0.3794904	0.079	0.124	3.233692e-13	12	RNF213	DZ 3
2.155796e-17	-0.4310565	0.13	0.186	3.843569e-13	12	FKBP4	DZ 3
3.495052e-17	-0.5454105	0.445	0.45	6.231329e-13	12	DRAP1	DZ 3
4.358117e-17	-0.4424304	0.136	0.197	7.770087e-13	12	AARS	DZ 3
6.231675e-17	-0.4345028	0.093	0.146	1.111045e-12	12	SUN2	DZ 3

6.299792e-17	-0.4607832	0.152	0.206	1.12319e-12	12	AP1S2	DZ 3
7.584701e-17	-0.492871	0.137	0.201	1.352276e-12	12	PPP3CA	DZ 3
1.221485e-16	-0.4524134	0.179	0.22	2.177786e-12	12	CCDC50	DZ 3
1.680213e-16	-0.6404899	0.268	0.296	2.995652e-12	12	NFKBIA	DZ 3
1.755253e-16	-0.3233661	0.066	0.116	3.129441e-12	12	U2AF1L4	DZ 3
2.480029e-16	-0.5857565	0.652	0.618	4.421644e-12	12	CLEC2D	DZ 3
2.886005e-16	-0.6462619	0.658	0.615	5.145458e-12	12	MEF2C	DZ 3
4.539073e-16	-0.3757392	0.089	0.149	8.092712e-12	12	MVP	DZ 3
8.590261e-16	-0.6769586	0.089	0.132	1.531558e-11	12	MYC	DZ 3
9.348225e-16	-0.4639507	0.908	0.881	1.666695e-11	12	MS4A1	DZ 3
1.068434e-15	-0.4031725	0.118	0.154	1.904911e-11	12	SRPK2	DZ 3
1.164299e-15	-0.8166134	0.564	0.57	2.075828e-11	12	VIM	DZ 3
1.317747e-15	-0.4997339	0.225	0.264	2.349411e-11	12	TTC3	DZ 3
3.020534e-15	-0.3549316	0.07	0.118	5.38531e-11	12	SLAMF1	DZ 3
3.056911e-15	-0.6249857	0.712	0.696	5.450166e-11	12	HLA-DQB1	DZ 3
3.423641e-15	-0.3597467	0.071	0.122	6.10401e-11	12	LACTB	DZ 3
5.079905e-15	-0.3023386	0.056	0.106	9.056963e-11	12	SERTAD1	DZ 3
5.103372e-15	-0.5124846	0.253	0.284	9.098802e-11	12	GNG7	DZ 3
5.985325e-15	-0.4651104	0.263	0.286	1.067124e-10	12	CNPY3	DZ 3
6.105945e-15	-0.3054855	0.059	0.104	1.088629e-10	12	PRCP	DZ 3
7.874057e-15	-0.320048	0.055	0.101	1.403866e-10	12	PRNP	DZ 3
1.515384e-14	-0.524621	0.322	0.342	2.701778e-10	12	PKIG	DZ 3
2.382427e-14	-0.7752396	0.797	0.749	4.24763e-10	12	CXCR4	DZ 3
3.552895e-14	-0.4111008	0.169	0.196	6.334456e-10	12	AFF3	DZ 3
4.653112e-14	-0.3594592	0.113	0.159	8.296033e-10	12	EVI2A	DZ 3

6.560912e-14	-0.4514657	0.257	0.287	1.169745e-09	12	ERG28	DZ 3
8.692174e-14	-0.3556048	0.079	0.118	1.549728e-09	12	SAMD9	DZ 3
9.151399e-14	-0.5932465	0.691	0.652	1.631603e-09	12	HLA-DQA1	DZ 3
1.132178e-13	-0.3259713	0.087	0.133	2.018559e-09	12	DUSP22	DZ 3
1.979307e-13	-0.3382965	0.096	0.13	3.528906e-09	12	MUM1	DZ 3
3.436136e-13	-0.4656174	0.32	0.345	6.126286e-09	12	RNH1	DZ 3
3.662361e-13	-0.4276406	0.179	0.195	6.529624e-09	12	FAM129C	DZ 3
4.07979e-13	-0.32657	0.121	0.161	7.273858e-09	12	VKORC1	DZ 3
4.26808e-13	-0.4052919	0.181	0.203	7.609561e-09	12	BIN1	DZ 3
4.515052e-13	-0.3650233	0.099	0.134	8.049887e-09	12	ODF2L	DZ 3
5.414484e-13	-0.3667528	0.109	0.149	9.653484e-09	12	SVIP	DZ 3
5.483906e-13	-0.3662255	0.073	0.11	9.777256e-09	12	LINC02397	DZ 3
5.778811e-13	-0.373672	0.123	0.159	1.030304e-08	12	GLIPR1	DZ 3
5.988413e-13	-0.3253611	0.082	0.124	1.067674e-08	12	SLC38A2	DZ 3
6.398238e-13	-0.4553408	0.464	0.452	1.140742e-08	12	TMEM59	DZ 3
6.423499e-13	-0.3842544	0.13	0.172	1.145246e-08	12	TRIM44	DZ 3
6.855192e-13	-0.4499345	0.252	0.263	1.222212e-08	12	FKBP2	DZ 3
8.351681e-13	-0.3169039	0.079	0.114	1.489021e-08	12	PBXIP1	DZ 3
8.519495e-13	-0.4218948	0.18	0.212	1.518941e-08	12	FMNL1	DZ 3
9.938911e-13	-0.4615192	0.978	0.976	1.772008e-08	12	FTH1	DZ 3
1.016502e-12	-0.4032642	0.114	0.164	1.812321e-08	12	CD58	DZ 3
1.096409e-12	-0.4611667	0.175	0.209	1.954788e-08	12	PIM3	DZ 3
1.252116e-12	-0.4973932	0.302	0.321	2.232398e-08	12	TUBA1A	DZ 3
1.344855e-12	-0.4365602	0.118	0.167	2.397741e-08	12	CBX6	DZ 3
1.772324e-12	-0.4615931	0.191	0.233	3.159876e-08	12	TARS	DZ 3

1.819221e-12	-0.4172155	0.922	0.916	3.24349e-08	12	EEF1B2	DZ	3
3.469186e-12	-0.3057723	0.988	0.982	6.185212e-08	12	RPS21	DZ	3
3.62947e-12	-0.3512713	0.079	0.124	6.470981e-08	12	DDAH2	DZ	3
4.128099e-12	-0.5217701	0.488	0.475	7.359988e-08	12	TNFAIP8	DZ	3
5.174333e-12	-0.4964582	0.849	0.815	9.225319e-08	12	NOP53	DZ	3
6.483723e-12	-0.3536412	0.155	0.173	1.155983e-07	12	IRF7	DZ	3
8.941242e-12	-0.3213121	0.082	0.12	1.594134e-07	12	LIPA	DZ	3
1.489589e-11	-0.710591	0.554	0.498	2.655787e-07	12	SSR4	DZ	3
1.616885e-11	-0.4768777	0.342	0.351	2.882745e-07	12	APOBEC3G	DZ	3
1.985103e-11	-0.4188844	0.096	0.137	3.539239e-07	12	PSAT1	DZ	3
2.185987e-11	-0.5037176	0.403	0.389	3.897396e-07	12	ARL6IP5	DZ	3
3.01852e-11	-0.3338053	0.141	0.18	5.381719e-07	12	DNPEP	DZ	3
3.482042e-11	-0.4579996	0.257	0.281	6.208132e-07	12	MSMO1	DZ	3
4.468247e-11	-0.3056957	0.09	0.118	7.966438e-07	12	HPCAL1	DZ	3
5.063561e-11	-0.5534023	0.349	0.338	9.027823e-07	12	IER2	DZ	3
5.480655e-11	-0.4604482	0.092	0.126	9.77146e-07	12	LGALS3	DZ	3
6.289332e-11	-0.4727205	0.727	0.675	1.121325e-06	12	TAGLN2	DZ	3
7.666263e-11	-0.4235294	0.262	0.284	1.366818e-06	12	DPP7	DZ	3
1.087416e-10	-0.3325664	0.094	0.124	1.938754e-06	12	RAB13	DZ	3
1.150881e-10	-0.4892439	0.605	0.574	2.051906e-06	12	APRT	DZ	3
1.729425e-10	-0.3697167	0.153	0.183	3.083392e-06	12	CMSS1	DZ	3
1.764547e-10	-0.3214126	0.095	0.127	3.146011e-06	12	ZEB2	DZ	3
2.121936e-10	-0.5029396	0.237	0.256	3.7832e-06	12	SRGN	DZ	3
2.236711e-10	-0.4273633	0.652	0.636	3.987832e-06	12	CD53	DZ	3
2.440874e-10	-0.3322939	0.138	0.173	4.351834e-06	12	DHRS7	DZ	3

2.559837e-10	-0.3164162	0.098	0.137	4.563934e-06	12	CD46	DZ 3
3.171724e-10	-0.3051531	0.113	0.142	5.654866e-06	12	TM2D3	DZ 3
3.864214e-10	-0.3630121	0.168	0.204	6.889506e-06	12	NDFIP1	DZ 3
4.198364e-10	-0.3438607	0.13	0.155	7.485264e-06	12	RILPL2	DZ 3
5.175651e-10	-0.4417188	0.443	0.421	9.227668e-06	12	APEX1	DZ 3
6.663675e-10	-0.3106454	0.109	0.135	1.188067e-05	12	PLEKHO1	DZ 3
1.326163e-09	-0.3074891	0.142	0.159	2.364415e-05	12	TPP1	DZ 3
1.991239e-09	-0.4071101	0.389	0.368	3.55018e-05	12	MEAF6	DZ 3
3.443003e-09	-0.4089852	0.305	0.297	6.13853e-05	12	PDLIM1	DZ 3
5.773347e-09	-0.3343728	0.096	0.131	0.000102933	12	RP9	DZ 3
7.265904e-09	-0.3763293	0.17	0.184	0.0001295438	12	MBP	DZ 3
7.561524e-09	-0.3082015	0.124	0.148	0.0001348144	12	TSPAN3	DZ 3
7.970997e-09	-0.307975	0.1	0.115	0.0001421149	12	CLN8	DZ 3
8.9808e-09	-0.3904532	0.312	0.298	0.0001601187	12	MPHOSPH8	DZ 3
9.866342e-09	-0.4131238	0.47	0.455	0.000175907	12	CTSS	DZ 3
1.175632e-08	-0.3999997	0.659	0.626	0.0002096034	12	ATP6V0E1	DZ 3
1.681922e-08	-0.3941387	0.296	0.284	0.0002998698	12	ACAP1	DZ 3
2.178264e-08	-0.5645584	0.336	0.326	0.0003883627	12	SLC3A2	DZ 3
2.480589e-08	-0.3648776	0.214	0.207	0.0004422643	12	CCDC32	DZ 3
2.553458e-08	-0.3489936	0.234	0.241	0.0004552561	12	SLC38A1	DZ 3
3.616796e-08	-0.3572301	0.177	0.185	0.0006448386	12	ANKRD44	DZ 3
6.099288e-08	-0.344851	0.319	0.305	0.001087442	12	FIS1	DZ 3
6.81343e-08	-0.3472037	0.226	0.214	0.001214766	12	YPEL3	DZ 3
8.277306e-08	-0.3943953	0.431	0.383	0.001475761	12	EVL	DZ 3
1.296626e-07	-0.3683633	0.418	0.394	0.002311755	12	TCF25	DZ 3

1.772614e-07	-0.344797	0.227	0.233	0.003160393	12	SPINT2	DZ 3
3.288583e-07	-0.3732724	0.144	0.143	0.005863215	12	DUSP1	DZ 3
3.5523e-07	-0.8558287	0.835	0.64	0.006333396	12	IGLC2	DZ 3
3.607081e-07	-0.4513848	0.524	0.479	0.006431064	12	ISCU	DZ 3
3.733749e-07	-0.3642225	0.249	0.252	0.006656901	12	NFE2L2	DZ 3
4.185273e-07	-0.3692474	0.239	0.24	0.007461923	12	ANKRD11	DZ 3
5.903638e-07	-0.3787875	0.344	0.324	0.0105256	12	P4HB	DZ 3
6.326723e-07	-0.5174747	0.543	0.483	0.01127991	12	NEAT1	DZ 3
7.125436e-07	-0.3616168	0.153	0.152	0.01270394	12	NFKBID	DZ 3
9.872928e-07	-0.4019644	0.285	0.28	0.01760244	12	SRM	DZ 3
1.36635e-06	-0.4207969	0.676	0.618	0.02436065	12	IFI16	DZ 3
1.551783e-06	-0.3478871	0.24	0.235	0.02766674	12	CNPPD1	DZ 3
1.651615e-06	-0.3193202	0.285	0.274	0.02944664	12	SPG21	DZ 3
1.792464e-06	-0.3838777	0.31	0.28	0.03195783	12	SMCHD1	DZ 3
1.891561e-06	-0.3360696	0.194	0.204	0.03372464	12	PTPN1	DZ 3
1.968605e-06	-0.3455553	0.904	0.865	0.03509825	12	CD79A	DZ 3
2.086623e-06	-0.3289062	0.175	0.181	0.0372024	12	CDC42SE1	DZ 3
2.22634e-06	-0.3532079	0.315	0.285	0.03969342	12	SP110	DZ 3
3.490717e-06	-0.3238653	0.213	0.205	0.06223599	12	RALGPS2	DZ 3
6.449751e-06	-0.318792	0.22	0.221	0.1149926	12	RNF187	DZ 3
7.019506e-06	-0.4069805	0.704	0.651	0.1251508	12	TMBIM6	DZ 3
7.670997e-06	-0.3190277	0.395	0.206	0.1367662	12	PLCG2	DZ 3
1.086028e-05	-0.3927116	0.374	0.335	0.193628	12	S100A11	DZ 3
1.28353e-05	-0.3245832	0.912	0.899	0.2288406	12	DDX5	DZ 3
1.48999e-05	-0.3302102	0.265	0.248	0.2656503	12	BBX	DZ 3

1.67876e-05 -0.3430022 0.191 0.19 0.2993061 12
RNF145 DZ 3
1.925048e-05 -0.3417333 0.302 0.281 0.3432168 12
RTN4 DZ 3
2.676039e-05 -0.3497006 0.293 0.274 0.477111 12
TNRC6B DZ 3
3.60201e-05 -0.3243625 0.224 0.212 0.6422023 12
WASHC4 DZ 3
8.624007e-05 -0.3415856 0.693 0.613 1 12
PNISR DZ 3
0.0001259037 -0.3728606 0.658 0.601 1 12
ARF6 DZ 3
0.0001306232 -0.3340229 0.226 0.217 1 12
HMGCS1 DZ 3
0.0002943932 -0.5154259 0.867 0.772 1 12
HLA-DPB1 DZ 3
0.0003258006 -0.4276864 0.209 0.146 1 12
IGHA1 DZ 3
0.000360312 -0.3287355 0.445 0.407 1 12
TMBIM4 DZ 3
0.0003718775 -0.3054823 0.337 0.305 1 12
EAPP DZ 3
0.0003720698 -0.3390518 0.37 0.318 1 12
LTA4H DZ 3
0.0004621782 -0.3077593 0.322 0.284 1 12
PPM1K DZ 3
0.0006060694 -0.3689323 0.457 0.397 1 12
BLOC1S2 DZ 3
0.0006362125 -0.3595261 0.419 0.329 1 12
RIPOR2 DZ 3
0.001141281 -0.3055707 0.378 0.334 1 12
VOPP1 DZ 3
0.001360364 -0.4001418 0.776 0.694 1 12
HNRNPDL DZ 3
0.002795865 -2.41346 0.626 0.446 1 12 IGHG3 DZ
3
0.003917643 -0.3910859 0.677 0.608 1 12
SYNGR2 DZ 3
0.004952813 -0.384797 0.874 0.813 1 12
HLA-DPA1 DZ 3
0.005353021 -0.3222661 0.492 0.418 1 12
KMT2E DZ 3
0.007658328 -0.3004877 0.577 0.48 1 12
RABAC1 DZ 3
0 2.945458 0.799 0.194 0 13 FOS Naive IER2
0 2.014825 0.679 0.329 0 13 IER2 Naive IER2
0 2.002731 0.849 0.446 0 13 JUNB Naive IER2
3.011744e-309 1.976956 0.698 0.306 5.369639e-305 13
CD69 Naive IER2
2.866117e-185 1.534163 0.577 0.26 5.11e-181 13 JUN

Naive IER2
2.697418e-168 1.358758 0.58 0.321 4.809227e-164 13
ZFP36 Naive IER2
1.666575e-145 0.443256 1 1 2.971337e-141 13
MALAT1 Naive IER2
1.212167e-132 1.331309 0.375 0.137 2.161172e-128 13
DUSP1 Naive IER2
2.575835e-128 0.9125367 0.177 0.022 4.592456e-124 13
EGR1 Naive IER2
1.915227e-110 0.8818226 0.889 0.747 3.414658e-106 13
CXCR4 Naive IER2
3.904174e-107 0.4093619 0.997 0.904 6.960752e-103 13
IGHM Naive IER2
1.544826e-99 0.742316 0.153 0.028 2.75427e-95 13
FOSB Naive IER2
1.474143e-77 0.5309451 0.969 0.934 2.628249e-73 13
BTG1 Naive IER2
2.307434e-75 -1.268671 0.032 0.211 4.113925e-71 13
RGS13 Naive IER2
4.035532e-75 1.292104 0.289 0.148 7.19495e-71 13
DUSP2 Naive IER2
1.230724e-71 0.9341249 0.58 0.443 2.194258e-67 13
BTG2 Naive IER2
2.481428e-71 -0.9197776 0.341 0.606 4.424137e-67 13
PRDX1 Naive IER2
2.059671e-68 0.6078868 0.117 0.023 3.672188e-64 13
NR4A1 Naive IER2
2.458172e-68 0.4118685 0.996 0.985 4.382675e-64 13
TMSB10 Naive IER2
4.087088e-65 -0.9421152 0.487 0.721 7.286868e-61 13
ENO1 Naive IER2
1.163259e-61 -0.9309233 0.78 0.891 2.073975e-57 13
GAPDH Naive IER2
3.351103e-60 0.5993134 0.776 0.602 5.974682e-56 13
IGHD Naive IER2
2.738574e-58 0.9691755 0.426 0.292 4.882603e-54 13
NFKBIA Naive IER2
6.765787e-55 -0.985624 0.176 0.41 1.206272e-50 13
FABP5 Naive IER2
1.163641e-54 -0.8359815 0.095 0.309 2.074656e-50 13
CD27 Naive IER2
9.388842e-54 0.7111722 0.129 0.038 1.673937e-49 13
EGR2 Naive IER2
1.356476e-53 -0.6933234 0.562 0.766 2.418461e-49 13
PKM Naive IER2
1.638246e-52 -0.5835551 0.781 0.902 2.920828e-48 13
PFN1 Naive IER2
6.312355e-51 -0.7730785 0.097 0.304 1.12543e-46 13
NME1 Naive IER2
2.164468e-50 -0.5539492 0.317 0.56 3.859029e-46 13

	SEC61G	Naive IER2				
2.018933e-47	-0.5644573		0.168	0.399	3.599555e-43	13
	YWHAE	Naive IER2				
2.790661e-47	-0.6304577		0.021	0.152	4.97547e-43	13
	BIK	Naive IER2				
4.249088e-47	-0.8407349		0.04	0.194	7.575699e-43	13
	PTTG1	Naive IER2				
4.840719e-47	0.4104486		0.937	0.867	8.630518e-43	13
	CD52	Naive IER2				
2.660263e-46	-0.5706257		0.33	0.577	4.742982e-42	13
	SNRPE	Naive IER2				
3.20661e-46	-0.7987314		0.192	0.414	5.717065e-42	13
	TXN	Naive IER2				
8.987097e-46	-0.5175646		0.326	0.565	1.602309e-41	13
	ATP5PF	Naive IER2				
1.017321e-45	-0.6253739		0.213	0.445	1.813782e-41	13
	HMGA1	Naive IER2				
1.144874e-44	-0.5755464		0.192	0.403	2.041196e-40	13
	BASP1	Naive IER2				
1.53832e-44	-0.5567909		0.126	0.334	2.74267e-40	13
	RFTN1	Naive IER2				
1.834461e-44	-0.5334006		0.359	0.585	3.270661e-40	13
	LDHB	Naive IER2				
1.227373e-43	-0.6478686		0.24	0.467	2.188283e-39	13
	HSPD1	Naive IER2				
1.520318e-42	-0.6137317		0.106	0.308	2.710574e-38	13
	ODC1	Naive IER2				
2.824526e-42	-0.7819228		0.626	0.79	5.035847e-38	13
	ACTG1	Naive IER2				
6.806895e-41	-0.6217502		0.561	0.74	1.213601e-36	13
	HSPA8	Naive IER2				
6.0643e-40	-0.4469574		0.013	0.121	1.081204e-35	13
	CD38	Naive IER2				
6.743689e-40	-0.5888483		0.079	0.238	1.202332e-35	13
	UBE2J1	Naive IER2				
1.603063e-39	-0.7670743		0.375	0.582	2.858101e-35	13
	TUBB	Naive IER2				
2.871222e-39	-0.5071573		0.394	0.603	5.119102e-35	13
	SEC61B	Naive IER2				
4.951486e-39	-0.5513444		0.339	0.558	8.828005e-35	13
	TCEA1	Naive IER2				
5.228634e-39	-0.4668838		0.191	0.41	9.322131e-35	13
	PSMB2	Naive IER2				
7.117561e-39	-0.9223051		0.049	0.185	1.26899e-34	13
	XBP1	Naive IER2				
1.524212e-38	-0.4381134		0.02	0.134	2.717518e-34	13
	PHGDH	Naive IER2				
1.753944e-38	-0.4673919		0.219	0.45	3.127107e-34	13
	C1QBP	Naive IER2				
2.120248e-38	-0.4581395		0.231	0.454	3.780191e-34	13

	SEM1	Naive IER2				
5.82977e-38	-0.5106942	0.26	0.479	1.03939e-33	13	
	SNRPD1	Naive IER2				
5.94281e-38	-0.4182209	0.422	0.645	1.059544e-33	13	
	ATP5MD	Naive IER2				
8.930886e-38	-0.5988335	0.581	0.752	1.592288e-33	13	
	RAN	Naive IER2				
2.456377e-37	-0.4971628	0.643	0.807	4.379475e-33	13	
	RPS26	Naive IER2				
2.949067e-37	-0.3469671	0.01	0.103	5.257891e-33	13	
	MYBL2	Naive IER2				
3.409833e-37	-0.5531664	0.103	0.284	6.079391e-33	13	
	SRM	Naive IER2				
4.699937e-37	-0.4569422	0.331	0.54	8.379517e-33	13	
	SRSF9	Naive IER2				
5.609513e-37	-0.4968886	0.482	0.685	1.00012e-32	13	
	SRSF3	Naive IER2				
7.117154e-37	-0.4460034	0.404	0.613	1.268917e-32	13	
	POMP	Naive IER2				
1.125208e-36	-0.6737921	0.723	0.851	2.006133e-32	13	
	HSP90AB1	Naive IER2				
2.098385e-36	-0.4783918	0.247	0.395	3.74121e-32	13	
	EIF5A	Naive IER2				
2.202673e-36	-0.4623666	0.423	0.633	3.927146e-32	13	
	ATP5F1B	Naive IER2				
2.962191e-36	-0.5239513	0.23	0.447	5.281291e-32	13	
	PPA1	Naive IER2				
3.607608e-36	-0.4550583	0.219	0.436	6.432005e-32	13	
	SLIRP	Naive IER2				
3.880095e-36	-0.5710862	0.138	0.324	6.917821e-32	13	
	SEC11C	Naive IER2				
9.724428e-36	-0.4459524	0.236	0.458	1.733768e-31	13	
	VDAC1	Naive IER2				
1.058114e-35	0.7126009	0.504	0.426	1.886511e-31	13	
	PPP1R15A	Naive IER2				
1.087294e-35	0.4384049	0.895	0.846	1.938536e-31	13	
	CD37	Naive IER2				
1.320961e-35	-0.5000355	0.074	0.238	2.355142e-31	13	
	MTHFD2	Naive IER2				
1.759838e-35	-0.4785541	0.371	0.589	3.137615e-31	13	
	ANP32B	Naive IER2				
1.863139e-35	-0.4158828	0.399	0.613	3.32179e-31	13	
	POLR2L	Naive IER2				
4.775969e-35	-0.4981553	0.015	0.111	8.515076e-31	13	
	VPREB3	Naive IER2				
8.654691e-35	-0.5263932	0.185	0.39	1.543045e-30	13	
	NHP2	Naive IER2				
9.460395e-35	-0.4258012	0.568	0.748	1.686694e-30	13	
	CHCHD2	Naive IER2				
1.174182e-34	0.6816612	0.349	0.235	2.093448e-30	13	

CD55	Naive IER2	2.838217e-34	-0.5065491	0.887	0.943	5.060257e-30	13
YBX1	Naive IER2	3.305319e-34	-0.4342128	0.187	0.394	5.893053e-30	13
CCT5	Naive IER2	8.24436e-34	-0.5953828	0.05	0.194	1.469887e-29	13
DAAM1	Naive IER2	9.874669e-34	-0.42367	0.388	0.589	1.760555e-29	13
ATP5MC3	Naive IER2	1.82394e-33	-0.4174812	0.136	0.328	3.251902e-29	13
IMP4	Naive IER2	2.387422e-33	-0.3870412	0.749	0.873	4.256535e-29	13
ARPC2	Naive IER2	2.433113e-33	0.5718796	0.546	0.445	4.337997e-29	13
PNRC1	Naive IER2	2.434505e-33	-0.3948153	0.542	0.727	4.340479e-29	13
COX6C	Naive IER2	2.976925e-33	-0.5667594	0.403	0.602	5.307559e-29	13
LDHA	Naive IER2	3.131919e-33	-0.542485	0.296	0.503	5.583899e-29	13
HSPE1	Naive IER2	5.023602e-33	-0.4110142	0.106	0.286	8.95658e-29	13
PSMD14	Naive IER2	7.232574e-33	-0.3943348	0.394	0.61	1.289496e-28	13
RBM3	Naive IER2	9.565901e-33	-0.3963201	0.185	0.393	1.705505e-28	13
NDUFS6	Naive IER2	1.082768e-32	-0.4490156	0.041	0.164	1.930466e-28	13
SUSD3	Naive IER2	1.204417e-32	-0.4669999	0.448	0.642	2.147355e-28	13
SET	Naive IER2	1.215311e-32	0.5267438	0.655	0.574	2.166777e-28	13
SARAF	Naive IER2	1.427298e-32	0.6138799	0.251	0.155	2.54473e-28	13
SLC2A3	Naive IER2	1.859248e-32	-0.6743505	0.524	0.7	3.314853e-28	13
H2AFZ	Naive IER2	2.15325e-32	0.607727	0.423	0.294	3.83903e-28	13
LINC00926	Naive IER2	2.524923e-32	-0.3623146	0.303	0.525	4.501686e-28	13
PSMB6	Naive IER2	3.986789e-32	-0.3890847	0.293	0.509	7.108047e-28	13
EIF3I	Naive IER2	4.467018e-32	-0.3516983	0.281	0.508	7.964247e-28	13
NDUFA11	Naive IER2	7.294962e-32	-0.3887249	0.375	0.591	1.300619e-27	13
EIF2S2	Naive IER2	7.500699e-32	-0.5208885	0.864	0.931	1.3373e-27	13
NPM1	Naive IER2	8.8569e-32	-0.3710426	0.23	0.44	1.579097e-27	13

	ROMO1	Naive IER2				
3.930709e-31	-0.3517353		0.496	0.69	7.008061e-27	13
	COX6A1	Naive IER2				
5.327051e-31	-0.4173949		0.028	0.143	9.4976e-27	13
	AIM2	Naive IER2				
5.895554e-31	-0.4415298		0.186	0.379	1.051118e-26	13
	LCP1	Naive IER2				
5.979738e-31	-0.4398345		0.11	0.278	1.066128e-26	13
	POU2AF1	Naive IER2				
8.006345e-31	-0.3933348		0.446	0.639	1.427451e-26	13
	PSMA4	Naive IER2				
9.011369e-31	-0.3875316		0.536	0.711	1.606637e-26	13
	NDUFA4	Naive IER2				
1.035136e-30	-0.3891674		0.449	0.645	1.845544e-26	13
	ATP5MF	Naive IER2				
1.079644e-30	-1.050398		0.14	0.29	1.924897e-26	13
	MIR155HG	Naive IER2				
1.363349e-30	-0.4105533		0.602	0.764	2.430715e-26	13
	PSMA7	Naive IER2				
1.612306e-30	-0.3516419		0.014	0.114	2.87458e-26	13
	AC023590.1	Naive IER2				
1.632513e-30	-0.4042082		0.25	0.457	2.910608e-26	13
	MRPL51	Naive IER2				
1.870139e-30	-0.5400442		0.185	0.364	3.33427e-26	13
	H2AFV	Naive IER2				
2.39142e-30	-0.3943437		0.05	0.171	4.263663e-26	13
	CCDC88A	Naive IER2				
2.481856e-30	-0.380769		0.294	0.503	4.4249e-26	13
	COX5A	Naive IER2				
2.958075e-30	-0.3483303		0.462	0.661	5.273952e-26	13
	PSMB1	Naive IER2				
3.452508e-30	-0.3877226		0.126	0.308	6.155477e-26	13
	GLRX3	Naive IER2				
3.624525e-30	-0.3787171		0.459	0.645	6.462165e-26	13
	ERH	Naive IER2				
3.816508e-30	-0.3723411		0.367	0.572	6.804452e-26	13
	UQCRQ	Naive IER2				
4.148661e-30	-0.3703152		0.241	0.456	7.396648e-26	13
	NDUFB9	Naive IER2				
4.210906e-30	-0.3498769		0.369	0.578	7.507624e-26	13
	PARK7	Naive IER2				
4.394935e-30	-0.392298		0.096	0.257	7.83573e-26	13
	RGS10	Naive IER2				
1.155081e-29	-0.416991		0.024	0.135	2.059394e-25	13
	CYTOR	Naive IER2				
2.377239e-29	-0.4169972		0.08	0.221	4.238379e-25	13
	NANS	Naive IER2				
2.550659e-29	-0.4630311		0.141	0.312	4.547569e-25	13
	CALR	Naive IER2				
4.488908e-29	0.6092595		0.401	0.3	8.003273e-25	13

	ZFP36L2	Naive IER2				
7.236419e-29	-0.3502058	0.203	0.407	1.290181e-24	13	
	MDH2	Naive IER2				
9.990091e-29	-0.3217939	0.246	0.451	1.781133e-24	13	
	ATP5F1C	Naive IER2				
1.112096e-28	-0.4135643	0.272	0.468	1.982756e-24	13	
	PRMT1	Naive IER2				
1.192524e-28	-0.3350299	0.037	0.158	2.126151e-24	13	
	PAICS	Naive IER2				
1.243839e-28	-0.3611347	0.04	0.165	2.217641e-24	13	
	PPP1R14B	Naive IER2				
1.265259e-28	-0.3411362	0.405	0.606	2.25583e-24	13	
	PSMA2	Naive IER2				
1.283429e-28	0.6666726	0.509	0.368	2.288226e-24	13	
	SELL	Naive IER2				
1.701365e-28	-0.4217987	0.03	0.137	3.033364e-24	13	
	NEIL1	Naive IER2				
2.37476e-28	-0.3923389	0.169	0.353	4.233959e-24	13	
	TIMM13	Naive IER2				
3.118032e-28	-0.3520522	0.13	0.31	5.559139e-24	13	
	TIMM8B	Naive IER2				
3.261411e-28	-0.4181536	0.203	0.398	5.81477e-24	13	
	PHB	Naive IER2				
3.439177e-28	-0.338014	0.195	0.393	6.131708e-24	13	
	MRPL20	Naive IER2				
3.516992e-28	-0.3511139	0.433	0.637	6.270445e-24	13	
	COX7B	Naive IER2				
8.245163e-28	-0.5703731	0.633	0.771	1.47003e-23	13	
	HMGN1	Naive IER2				
9.287741e-28	-0.3602969	0.02	0.117	1.655911e-23	13	
	PYCR1	Naive IER2				
1.173377e-27	-0.3655135	0.186	0.379	2.092013e-23	13	
	PSMC3	Naive IER2				
1.19919e-27	-0.3527835	0.205	0.4	2.138035e-23	13	
	NDUFAB1	Naive IER2				
1.229522e-27	-0.414003	0.339	0.534	2.192115e-23	13	
	PSME2	Naive IER2				
1.319019e-27	-0.3712807	0.246	0.449	2.351679e-23	13	
	ATP5MC1	Naive IER2				
1.382929e-27	-0.5121345	0.376	0.564	2.465625e-23	13	
	NCL	Naive IER2				
1.550194e-27	-0.3722404	0.187	0.379	2.763841e-23	13	
	PSMC1	Naive IER2				
1.908024e-27	-0.3383471	0.022	0.118	3.401815e-23	13	
	GMDS	Naive IER2				
3.515582e-27	-0.3781515	0.429	0.621	6.267932e-23	13	
	SERBP1	Naive IER2				
7.007002e-27	-0.3668257	0.158	0.342	1.249278e-22	13	
	MIF	Naive IER2				
9.692573e-27	-0.3353088	0.19	0.39	1.728089e-22	13	

	SNRPC	Naive IER2				
1.096638e-26	-0.4647495		0.078	0.205	1.955196e-22	13
	HMCES	Naive IER2				
1.261457e-26	0.6178568		0.574	0.501	2.249052e-22	13
	TSC22D3	Naive IER2				
2.418194e-26	-0.4229478		0.032	0.139	4.311398e-22	13
	PSAT1	Naive IER2				
3.519092e-26	-0.4287805		0.321	0.517	6.27419e-22	13
	PA2G4	Naive IER2				
6.641638e-26	-0.5383296		0.136	0.28	1.184138e-21	13
	HSPA5	Naive IER2				
7.024478e-26	-0.3515308		0.2	0.394	1.252394e-21	13
	NOL7	Naive IER2				
9.004452e-26	-0.4902296		0.183	0.344	1.605404e-21	13
	LRMP	Naive IER2				
9.09212e-26	0.6169153		0.398	0.271	1.621034e-21	13
	KLF2	Naive IER2				
9.259852e-26	-0.4617865		0.247	0.422	1.650939e-21	13
	METAP2	Naive IER2				
1.140264e-25	-0.3416244		0.281	0.474	2.032977e-21	13
	MTDH	Naive IER2				
1.568067e-25	-0.346745		0.275	0.481	2.795707e-21	13
	PRELID1	Naive IER2				
1.686968e-25	-0.3313233		0.338	0.528	3.007696e-21	13
	FKBP1A	Naive IER2				
1.865137e-25	-0.3017659		0.326	0.537	3.325352e-21	13
	GHITM	Naive IER2				
2.399746e-25	-0.311382		0.213	0.406	4.278507e-21	13
	UQCRFS1	Naive IER2				
2.626192e-25	-0.3532248		0.319	0.519	4.682237e-21	13
	SSBP1	Naive IER2				
2.672107e-25	-0.3663016		0.181	0.354	4.7641e-21	13
	PDIA6	Naive IER2				
4.037495e-25	-0.3043137		0.024	0.128	7.19845e-21	13
	SLC1A5	Naive IER2				
4.050145e-25	-0.3083917		0.433	0.622	7.221003e-21	13
	NDUFA13	Naive IER2				
4.366502e-25	-0.3819666		0.494	0.661	7.785036e-21	13
	SLC25A5	Naive IER2				
4.809138e-25	-0.3119024		0.531	0.705	8.574212e-21	13
	ELOB	Naive IER2				
9.382025e-25	-0.4474001		0.881	0.934	1.672721e-20	13
	SERF2	Naive IER2				
1.076015e-24	-0.3739313		0.237	0.421	1.918428e-20	13
	DDX39A	Naive IER2				
1.172988e-24	-0.3770255		0.12	0.275	2.091321e-20	13
	SMS	Naive IER2				
1.282332e-24	-0.4467293		0.313	0.489	2.28627e-20	13
	RANBP1	Naive IER2				
1.314811e-24	-0.3365008		0.131	0.288	2.344177e-20	13

CD82	Naive IER2				
1.434866e-24	-0.3613148	0.049	0.166	2.558223e-20	13
MRTO4	Naive IER2				
1.440579e-24	-0.3156887	0.425	0.609	2.568408e-20	13
PSMB3	Naive IER2				
1.524074e-24	-0.3453064	0.085	0.232	2.717272e-20	13
MRPL3	Naive IER2				
2.13461e-24	-0.372958	0.374	0.545	3.805796e-20	13
PARP1	Naive IER2				
2.195524e-24	-0.3801164	0.541	0.707	3.9144e-20	13
TPI1	Naive IER2				
2.548688e-24	-0.3657754	0.103	0.254	4.544057e-20	13
GARS	Naive IER2				
3.420423e-24	-0.3574065	0.246	0.439	6.098272e-20	13
EIF5B	Naive IER2				
3.458963e-24	-1.121163	0.076	0.178	6.166985e-20	13
MZB1	Naive IER2				
4.671852e-24	-0.307862	0.548	0.721	8.329444e-20	13
COX6B1	Naive IER2				
4.93937e-24	-0.3356179	0.14	0.311	8.806402e-20	13
FERMT3	Naive IER2				
6.417166e-24	-0.3183707	0.936	0.977	1.144117e-19	13
RPS24	Naive IER2				
9.790227e-24	-0.419205	0.26	0.442	1.7455e-19	13
COTL1	Naive IER2				
9.905046e-24	-0.3001163	0.188	0.372	1.765971e-19	13
EWSR1	Naive IER2				
1.910391e-23	-0.4217828	0.45	0.608	3.406037e-19	13
DYNLL1	Naive IER2				
1.99033e-23	-0.338003	0.049	0.156	3.54856e-19	13
CD81	Naive IER2				
2.224909e-23	-0.7898438	0.58	0.719	3.966789e-19	13
HMGN2	Naive IER2				
2.44187e-23	-0.3498169	0.246	0.433	4.35361e-19	13
CCT2	Naive IER2				
2.791766e-23	0.5348552	0.679	0.647	4.97744e-19	13
ZFP36L1	Naive IER2				
3.60028e-23	-0.3267915	0.147	0.318	6.41894e-19	13
STOML2	Naive IER2				
4.226117e-23	-0.3640738	0.105	0.244	7.534744e-19	13
MYDGF	Naive IER2				
4.394911e-23	-0.3365196	0.247	0.432	7.835686e-19	13
CCT4	Naive IER2				
5.650699e-23	-0.3290509	0.231	0.415	1.007463e-18	13
HNRNPR	Naive IER2				
6.28747e-23	-0.3366251	0.476	0.645	1.120993e-18	13
HNRNPM	Naive IER2				
7.346147e-23	-0.3138788	0.042	0.148	1.309744e-18	13
PRDX4	Naive IER2				
8.736421e-23	0.5260741	0.276	0.193	1.557616e-18	13

CLEC2B	Naive IER2					
1.128363e-22	-0.3455253	0.259	0.447	2.011759e-18	13	
CCT6A	Naive IER2					
1.260557e-22	-0.4191801	0.763	0.854	2.247447e-18	13	
ATP5MG	Naive IER2					
1.350617e-22	-0.3455026	0.06	0.186	2.408014e-18	13	
TUBA1C	Naive IER2					
1.398467e-22	-0.4717218	0.194	0.357	2.493327e-18	13	
TUBB4B	Naive IER2					
1.561314e-22	-0.3322268	0.119	0.28	2.783667e-18	13	
ATOX1	Naive IER2					
2.849648e-22	-0.3203295	0.087	0.229	5.080637e-18	13	
DCAF13	Naive IER2					
3.170098e-22	-0.3073514	0.152	0.317	5.651967e-18	13	
ZNF207	Naive IER2					
3.19555e-22	-0.3097855	0.081	0.217	5.697346e-18	13	
EPRS	Naive IER2					
4.111752e-22	-0.3031537	0.301	0.494	7.330843e-18	13	
CCT3	Naive IER2					
4.511932e-22	-0.3377973	0.471	0.636	8.044324e-18	13	
GSTP1	Naive IER2					
4.788377e-22	-0.3398414	0.142	0.306	8.537197e-18	13	
ATP1B3	Naive IER2					
5.344538e-22	-0.3040643	0.046	0.159	9.528776e-18	13	
GAR1	Naive IER2					
5.927219e-22	-0.3182165	0.056	0.175	1.056764e-17	13	
ASNS	Naive IER2					
6.045456e-22	-0.3928447	0.248	0.412	1.077844e-17	13	
PPIB	Naive IER2					
9.113852e-22	-0.3076246	0.228	0.412	1.624909e-17	13	
BANF1	Naive IER2					
1.066993e-21	-0.3808988	0.134	0.285	1.902342e-17	13	
JPT1	Naive IER2					
1.179824e-21	-0.4348284	0.1	0.234	2.103509e-17	13	
CKS2	Naive IER2					
1.194437e-21	-0.3075801	0.185	0.35	2.129562e-17	13	
H2AFY	Naive IER2					
1.273279e-21	-0.3249772	0.242	0.424	2.270129e-17	13	
EIF6	Naive IER2					
1.596433e-21	-0.3616735	0.185	0.357	2.846281e-17	13	
ANXA5	Naive IER2					
1.672762e-21	-0.3019733	0.082	0.221	2.982368e-17	13	
MRPL12	Naive IER2					
1.747818e-21	0.4389483	0.599	0.51	3.116185e-17	13	
BANK1	Naive IER2					
1.826698e-21	-1.323233	0.192	0.305	3.256819e-17	13	
HMGB2	Naive IER2					
2.422561e-21	0.5345678	0.287	0.205	4.319185e-17	13	
C16orf74	Naive IER2					
3.837684e-21	-0.3369799	0.394	0.569	6.842206e-17	13	

SNRPB	Naive IER2	1.242778e-20	0.5008489	0.339	0.25	2.215749e-16	13
FCMR	Naive IER2	1.822524e-20	0.5713881	0.311	0.24	3.249379e-16	13
PHACTR1	Naive IER2	1.941466e-20	-0.4478423	0.165	0.321	3.461439e-16	13
DDX21	Naive IER2	2.01976e-20	-0.3587781	0.077	0.196	3.601029e-16	13
DNMT1	Naive IER2	2.020206e-20	-0.432806	0.187	0.339	3.601825e-16	13
DUT	Naive IER2	2.141534e-20	-0.4451964	0.629	0.75	3.81814e-16	13
HSP90AA1	Naive IER2	4.042682e-20	-0.3000332	0.184	0.358	7.207698e-16	13
PHB2	Naive IER2	5.9122e-20	-0.3137427	0.294	0.473	1.054086e-15	13
SNRPF	Naive IER2	1.085714e-19	-0.4195646	0.119	0.259	1.93572e-15	13
SRGN	Naive IER2	1.195037e-19	0.4278406	0.535	0.493	2.130632e-15	13
FXYD5	Naive IER2	2.295222e-19	-0.3311117	0.335	0.511	4.092152e-15	13
MZT2B	Naive IER2	2.822424e-19	-0.4257144	0.176	0.33	5.0321e-15	13
SLC3A2	Naive IER2	3.50408e-19	-0.3248017	0.165	0.323	6.247424e-15	13
PRDX2	Naive IER2	4.643086e-19	-0.3049199	0.189	0.335	8.278158e-15	13
GRHPR	Naive IER2	6.309529e-19	-0.3122453	0.152	0.305	1.124926e-14	13
NOP58	Naive IER2	6.479829e-19	-0.3222934	0.317	0.488	1.155289e-14	13
RPS27L	Naive IER2	8.644954e-19	-0.3098022	0.078	0.201	1.541309e-14	13
EBNA1BP2	Naive IER2	2.076504e-18	0.3589097	0.904	0.899	3.702198e-14	13
DDX5	Naive IER2	2.359719e-18	-0.3072693	0.859	0.92	4.207143e-14	13
CFL1	Naive IER2	2.584698e-18	-0.3488543	0.489	0.639	4.608258e-14	13
HERPUD1	Naive IER2	4.06721e-18	-0.6701823	0.549	0.669	7.251428e-14	13
TUBA1B	Naive IER2	5.849889e-18	-1.269211	0.116	0.219	1.042977e-13	13
IGHG1	Naive IER2	5.919453e-18	-0.3120279	0.034	0.121	1.055379e-13	13
UBE2S	Naive IER2	8.182532e-18	-0.3147925	0.241	0.396	1.458864e-13	13
FNBP1	Naive IER2	1.129834e-17	-0.3035334	0.088	0.214	2.014381e-13	13

C19orf48 Naive IER2
1.699617e-17 -0.3569983 0.121 0.246 3.030247e-13 13
KPNA2 Naive IER2
2.579125e-17 -0.4615336 0.46 0.591 4.598322e-13 13
MARCKSL1 Naive IER2
3.127198e-17 -0.3033303 0.076 0.189 5.575482e-13 13
WDR43 Naive IER2
3.68832e-17 -0.31239 0.064 0.165 6.575907e-13 13
CD58 Naive IER2
5.026471e-17 0.3980668 0.153 0.085 8.961695e-13 13
RNASE6 Naive IER2
8.062419e-17 0.5107941 0.658 0.615 1.437449e-12 13
MEF2C Naive IER2
2.158944e-16 -1.990231 0.085 0.127 3.849181e-12 13
IGHG2 Naive IER2
2.454615e-16 -0.3289707 0.119 0.24 4.376334e-12 13
GCHFR Naive IER2
2.688132e-16 -2.733392 0.359 0.453 4.792671e-12 13
IGHG3 Naive IER2
5.168547e-16 -0.3145741 0.23 0.351 9.215003e-12 13
S100A10 Naive IER2
1.17297e-15 -0.4630773 0.358 0.503 2.091289e-11 13
SSR4 Naive IER2
4.763051e-15 -0.4446434 0.066 0.161 8.492044e-11 13
LMNA Naive IER2
5.643101e-15 -0.4539737 0.193 0.315 1.006108e-10 13
HSP90B1 Naive IER2
6.878533e-15 0.4393883 0.201 0.154 1.226374e-10 13
JUND Naive IER2
7.412075e-15 0.411403 0.417 0.346 1.321499e-10 13
TRAF3IP3 Naive IER2
2.152871e-14 0.4210003 0.513 0.466 3.838354e-10 13
ITM2B Naive IER2
1.180342e-13 0.45593 0.299 0.209 2.104432e-09 13
PLAC8 Naive IER2
1.399125e-13 -0.4096221 0.08 0.174 2.494501e-09 13
TNFRSF18 Naive IER2
1.710161e-13 -1.296473 0.335 0.405 3.049046e-09 13
JCHAIN Naive IER2
2.857286e-13 -0.3444365 0.051 0.127 5.094255e-09 13
LGALS3 Naive IER2
1.093776e-12 0.4035101 0.393 0.336 1.950093e-08 13
HVCN1 Naive IER2
1.125846e-12 0.4207297 0.336 0.277 2.00727e-08 13
ADAM28 Naive IER2
4.053644e-12 0.3845 0.201 0.157 7.227242e-08 13
GLIPR1 Naive IER2
4.896993e-12 0.4206394 0.299 0.243 8.730849e-08 13
RASGRP2 Naive IER2
5.408645e-12 0.3770363 0.435 0.413 9.643073e-08 13

NPC2	Naive IER2	8.524951e-12	0.4157948	0.39	0.359	1.519913e-07	13
ARID5B	Naive IER2	6.01282e-11	0.4338502	0.323	0.284	1.072026e-06	13
PPM1K	Naive IER2	6.543025e-11	0.3844582	0.51	0.493	1.166556e-06	13
SP100	Naive IER2	8.246103e-11	0.3888195	0.428	0.419	1.470198e-06	13
AC114760.2	Naive IER2	8.567175e-11	0.4315035	0.461	0.445	1.527442e-06	13
KLF6	Naive IER2	1.739982e-10	-0.3699267	0.985	0.99	3.102214e-06	13
ACTB	Naive IER2	2.529821e-10	-0.9818338	0.072	0.15	4.510418e-06	13
IGHA1	Naive IER2	2.937416e-10	0.4181011	0.368	0.331	5.237119e-06	13
RIPOR2	Naive IER2	3.009401e-10	0.3652155	0.58	0.57	5.365461e-06	13
SNX2	Naive IER2	3.946699e-10	0.3225675	0.151	0.11	7.036569e-06	13
SESN3	Naive IER2	6.318469e-10	0.3015145	0.193	0.151	1.12652e-05	13
NFKBID	Naive IER2	2.483399e-09	0.3932503	0.374	0.346	4.427651e-05	13
BIRC3	Naive IER2	2.613059e-09	0.3261868	0.243	0.193	4.658823e-05	13
FAM129C	Naive IER2	4.69953e-09	0.3630669	0.498	0.491	8.378792e-05	13
CYTIP	Naive IER2	1.37945e-08	0.3593567	0.405	0.383	0.0002459422	13
EVL	Naive IER2	2.017594e-08	0.3266435	0.451	0.404	0.0003597168	13
SMIM14	Naive IER2	2.967299e-08	0.3319115	0.109	0.085	0.0005290397	13
AC103591.3	Naive IER2	5.255687e-08	0.3224456	0.255	0.231	0.0009370364	13
HSD17B11	Naive IER2	6.652654e-08	-1.024415	0.331	0.425	0.001186102	13
HIST1H4C	Naive IER2	7.321409e-08	0.3072221	0.533	0.523	0.001305334	13
ID3	Naive IER2	1.244992e-07	-0.6660738	0.135	0.208	0.002219696	13
LGALS1	Naive IER2	1.380691e-07	0.3092077	0.156	0.141	0.002461634	13
LY9	Naive IER2	1.772052e-07	-1.525225	0.192	0.244	0.003159392	13
IGHG4	Naive IER2	2.06773e-07	0.3056369	0.492	0.492	0.003686556	13
ANKRD12	Naive IER2	2.606681e-07	0.3120873	0.342	0.329	0.004647451	13

SMC6 Naive IER2
2.952121e-07 0.3661958 0.302 0.279 0.005263337 13
RNASET2 Naive IER2
1.264771e-06 0.307692 0.112 0.079 0.0225496 13
CALHM6 Naive IER2
2.516105e-06 0.3320656 0.224 0.207 0.04485963 13
CCDC32 Naive IER2
6.464904e-06 0.3061464 0.468 0.485 0.1152628 13
NEAT1 Naive IER2
1.024248e-05 0.317583 0.327 0.321 0.1826132 13
TNFRSF13C Naive IER2
1.119899e-05 0.3299092 0.149 0.122 0.1996669 13
AL139020.1 Naive IER2
5.505882e-05 0.3147771 0.61 0.619 0.9816438 13
CLEC2D Naive IER2
0.00012659 -1.247421 0.353 0.431 1 13
IGLC3 Naive IER2
0.0001521681 -1.20305 0.629 0.645 1 13 IGLC2
Naive IER2
0.0002082475 -0.460394 0.387 0.429 1 13
STMN1 Naive IER2
0.0002735892 -0.607489 0.909 0.927 1 13
IGKC Naive IER2
0.0005344564 -0.3382777 0.5 0.572 1 13 VIM
Naive IER2
0.001676269 -0.4105969 0.145 0.213 1 13
PLCG2 Naive IER2
0 5.298719 0.894 0.036 0 14 CCL4 Activated
Chemokine
0 5.083317 0.818 0.03 0 14 CCL4L2 Activated
Chemokine
0 3.87063 0.735 0.034 0 14 CCL3 Activated
Chemokine
0 3.110052 0.895 0.112 0 14 MYC Activated
Chemokine
0 2.435703 0.907 0.271 0 14 MIR155HG Activated
Chemokine
0 2.038543 0.35 0.008 0 14 CCL3L1 Activated
Chemokine
0 1.921057 0.675 0.139 0 14 DUSP2 Activated
Chemokine
0 1.919963 0.951 0.46 0 14 CD83 Activated
Chemokine
0 1.914527 0.841 0.305 0 14 DDX21 Activated
Chemokine
0 1.747822 0.721 0.195 0 14 PIM3 Activated
Chemokine
0 1.703639 0.65 0.14 0 14 NFKBID Activated
Chemokine
0 1.690858 0.812 0.286 0 14 NME1 Activated

Chemokine
0 1.554761 0.993 0.844 0 14 HSP90AB1 Activated
Chemokine
0 1.554049 0.904 0.55 0 14 NCL Activated
Chemokine
0 1.49589 0.867 0.451 0 14 HSPD1 Activated
Chemokine
0 1.490916 0.939 0.591 0 14 PRDX1 Activated
Chemokine
0 1.434279 0.893 0.488 0 14 HSPE1 Activated
Chemokine
0 1.423445 0.885 0.458 0 14 CYCS Activated
Chemokine
0 1.377079 0.628 0.131 0 14 NOP16 Activated
Chemokine
0 1.32482 0.962 0.709 0 14 ENO1 Activated
Chemokine
0 1.313673 0.46 0.045 0 14 CCND2 Activated
Chemokine
0 1.254839 0.961 0.743 0 14 RAN Activated
Chemokine
0 1.1892 0.992 0.928 0 14 NPM1 Activated
Chemokine
0 1.034924 0.993 0.941 0 14 YBX1 Activated
Chemokine
6.09789e-317 1.679033 0.827 0.393 1.087193e-312 14
FABP5 Activated Chemokine
2.136201e-301 1.325691 0.882 0.503 3.808633e-297 14
PA2G4 Activated Chemokine
9.399846e-295 1.436504 0.731 0.269 1.675899e-290 14 SRM
Activated Chemokine
5.465351e-291 1.277585 0.884 0.475 9.744174e-287 14
RANBP1 Activated Chemokine
5.852568e-289 1.49005 0.785 0.315 1.043454e-284 14
SLC3A2 Activated Chemokine
1.382832e-278 1.2949 0.796 0.351 2.465451e-274 14
HSPA9 Activated Chemokine
1.506088e-273 1.29018 0.611 0.145 2.685205e-269 14
NOLC1 Activated Chemokine
2.994331e-270 1.397554 0.73 0.292 5.338592e-266 14
ODC1 Activated Chemokine
8.601427e-268 -1.611467 0.491 0.865 1.533548e-263 14
CD79B Activated Chemokine
1.231398e-264 1.474442 0.74 0.285 2.19546e-260 14
NFKBIA Activated Chemokine
2.690657e-262 1.441787 0.637 0.166 4.797172e-258 14
BCL2A1 Activated Chemokine
8.505635e-259 1.256573 0.654 0.213 1.51647e-254 14
SIAH2 Activated Chemokine
4.626364e-254 0.7603474 0.996 0.963 8.248345e-250 14

EIF1 Activated Chemokine
6.852121e-245 1.218072 0.351 0.033 1.221665e-240 14
EGR2 Activated Chemokine
1.700964e-237 1.158488 0.817 0.419 3.032649e-233 14
CCT2 Activated Chemokine
5.913007e-237 1.116881 0.876 0.521 1.05423e-232 14
SRSF7 Activated Chemokine
3.832051e-229 1.284281 0.65 0.224 6.832163e-225 14
MTHFD2 Activated Chemokine
1.303636e-226 1.148129 0.516 0.105 2.324252e-222 14
PYCR1 Activated Chemokine
5.004363e-225 1.20302 0.664 0.236 8.922279e-221 14
DKC1 Activated Chemokine
7.749028e-225 1.171978 0.717 0.292 1.381574e-220 14
ATP1B3 Activated Chemokine
3.411501e-224 1.09427 0.517 0.109 6.082364e-220 14
PNO1 Activated Chemokine
1.933809e-222 -0.9728773 0.99 0.998 3.447789e-218 14
TMSB4X Activated Chemokine
2.734284e-221 -1.582128 0.473 0.808 4.874955e-217 14
LAPTM5 Activated Chemokine
2.775937e-220 1.145472 0.803 0.392 4.949217e-216 14
RPL22L1 Activated Chemokine
3.524021e-219 1.13084 0.562 0.139 6.282977e-215 14
RPF2 Activated Chemokine
3.899944e-216 1.060082 0.839 0.465 6.95321e-212 14
SNRPD1 Activated Chemokine
4.533016e-216 1.17313 0.794 0.433 8.081915e-212 14
PPA1 Activated Chemokine
8.265598e-216 1.30876 0.461 0.115 1.473673e-211 14
PLEK Activated Chemokine
8.073022e-215 1.130612 0.605 0.191 1.439339e-210 14
TOMM40 Activated Chemokine
3.595824e-213 1.160538 0.736 0.312 6.410995e-209 14
NOP56 Activated Chemokine
3.92612e-211 1.108971 0.816 0.425 6.999879e-207 14
EIF5B Activated Chemokine
4.18455e-211 1.094662 0.897 0.589 7.460635e-207 14
LDHA Activated Chemokine
1.380771e-209 1.086425 0.553 0.142 2.461776e-205 14
PPAN Activated Chemokine
1.47351e-209 1.083183 0.939 0.731 2.62712e-205 14
HSPA8 Activated Chemokine
3.731064e-209 1.260882 0.785 0.399 6.652114e-205 14 TXN
Activated Chemokine
6.475249e-208 1.010682 0.905 0.609 1.154472e-203 14
SERBP1 Activated Chemokine
7.152749e-207 1.086856 0.786 0.375 1.275264e-202 14
NHP2 Activated Chemokine
1.070381e-206 1.280807 0.675 0.246 1.908383e-202 14

SRGN	Activated Chemokine
7.857209e-204	1.001287	0.832	0.44	1.400862e-199	14
ILF2	Activated Chemokine
6.93548e-202	1.056035	0.569	0.173	1.236527e-197	14
ZNF593	Activated Chemokine
9.409131e-201	1.117471	0.632	0.225	1.677554e-196	14
EIF4A1	Activated Chemokine
1.733519e-200	1.005408	0.486	0.108	3.09069e-196	14
LYAR	Activated Chemokine
1.080095e-199	1.052442	0.57	0.153	1.925702e-195	14
MRTO4	Activated Chemokine
3.751557e-199	1.088084	0.638	0.206	6.688651e-195	14
NFKBIE	Activated Chemokine
2.196608e-198	0.9799984	0.878	0.564	3.916333e-194	14
SNRPE	Activated Chemokine
1.411375e-194	1.109952	0.735	0.355	2.516341e-190	14
EIF3J	Activated Chemokine
3.539669e-193	0.9849656	0.417	0.077	6.310876e-189	14
SLC43A3	Activated Chemokine
4.00707e-192	1.025503	0.54	0.147	7.144205e-188	14
GAR1	Activated Chemokine
1.238455e-190	1.068987	0.576	0.187	2.208041e-186	14
GTPBP4	Activated Chemokine
7.823552e-190	1.006515	0.824	0.455	1.394861e-185	14
PRMT1	Activated Chemokine
3.64616e-189	1.045683	0.652	0.25	6.500739e-185	14	AK6
Activated Chemokine
4.966144e-188	0.9573	0.881	0.521	8.854138e-184	14
PSME2	Activated Chemokine
8.755595e-186	0.9797215	0.379	0.055	1.561035e-181	14
PTGER4	Activated Chemokine
3.786866e-185	0.9906687	0.8	0.422	6.751603e-181	14
SLIRP	Activated Chemokine
4.425844e-185	1.075638	0.579	0.176	7.890837e-181	14
WDR43	Activated Chemokine
1.388565e-183	1.111591	0.521	0.145	2.475672e-179	14
RILPL2	Activated Chemokine
5.73906e-183	1.046177	0.575	0.189	1.023217e-178	14
EBNA1BP2	Activated Chemokine
2.385912e-181	0.9790941	0.481	0.112	4.253843e-177	14
PAK1IP1	Activated Chemokine
2.706253e-180	0.985285	0.517	0.127	4.824979e-176	14
IFRD2	Activated Chemokine
1.377734e-179	1.011251	0.627	0.227	2.456362e-175	14
NIFK	Activated Chemokine
2.887336e-177	1.138538	0.564	0.201	5.147832e-173	14
CFLAR	Activated Chemokine
3.21257e-177	0.8414402	0.937	0.69	5.727691e-173	14
HNRNPDL	Activated Chemokine
3.776367e-177	1.052581	0.679	0.292	6.732885e-173	14

NOP58	Activated Chemokine	2.541183e-176	1.009062	0.582	0.178	4.530675e-172	14
GPATCH4	Activated Chemokine	9.027639e-176	0.8519503	0.928	0.674	1.609538e-171	14
SRSF3	Activated Chemokine	9.491782e-175	0.9611166	0.805	0.434	1.69229e-170	14
CCT6A	Activated Chemokine	5.840708e-174	1.213765	0.39	0.069	1.04134e-169	14
CD200	Activated Chemokine	3.21488e-172	0.9198395	0.85	0.506	5.731809e-168	14
SSBP1	Activated Chemokine	2.659847e-169	0.98452	0.582	0.198	4.742241e-165	14
GNL3	Activated Chemokine	4.042626e-169	0.8753633	0.412	0.08	7.207597e-165	14
POLR1C	Activated Chemokine	5.738608e-169	0.9984722	0.54	0.159	1.023136e-164	14
SNHG15	Activated Chemokine	1.294979e-168	0.9373599	0.532	0.144	2.308818e-164	14
RRP15	Activated Chemokine	1.775782e-167	0.954728	0.76	0.38	3.166042e-163	14
CCT5	Activated Chemokine	4.504766e-167	0.9173791	0.883	0.587	8.031548e-163	14
SRSF2	Activated Chemokine	9.22578e-166	0.9644073	0.719	0.34	1.644864e-161	14
TIMM13	Activated Chemokine	5.730151e-164	0.9464242	0.797	0.447	1.021629e-159	14
DDX18	Activated Chemokine	1.427155e-162	1.057728	0.583	0.188	2.544475e-158	14
YBX3	Activated Chemokine	1.527417e-161	0.9753923	0.618	0.247	2.723231e-157	14
GSPT1	Activated Chemokine	9.95223e-160	-2.273527	0.148	0.497	1.774383e-155	14
LTB	Activated Chemokine	2.471065e-159	0.9341671	0.594	0.216	4.405661e-155	14
DCAF13	Activated Chemokine	6.80486e-159	0.9462866	0.786	0.436	1.213238e-154	14
C1QBP	Activated Chemokine	7.981555e-159	0.9688741	0.374	0.067	1.423032e-154	14
TRAF1	Activated Chemokine	1.421998e-158	0.9463402	0.449	0.118	2.53528e-154	14
SLC1A5	Activated Chemokine	1.667384e-158	0.9179535	0.474	0.134	2.972779e-154	14
NIP7	Activated Chemokine	1.181146e-157	1.13056	0.734	0.383	2.105865e-153	14
EIF5A	Activated Chemokine	1.803764e-156	0.7589665	0.301	0.042	3.215931e-152	14
RCN1	Activated Chemokine	2.330761e-156	0.9465051	0.463	0.108	4.155513e-152	14
SLAMF1	Activated Chemokine	1.733279e-154	0.770425	0.954	0.742	3.090263e-150	14

HSP90AA1	Activated Chemokine	3.58467e-154	0.9576919	0.528	0.17	6.391109e-150	14
ABCE1	Activated Chemokine	2.181892e-153	0.9664132	0.486	0.138	3.890095e-149	14
ARL8B	Activated Chemokine	2.681975e-153	-1.562297	0.472	0.757	4.781694e-149	14
CXCR4	Activated Chemokine	3.341433e-153	1.086716	0.562	0.162	5.957441e-149	14
TNFRSF18	Activated Chemokine	1.967594e-152	0.9144614	0.564	0.209	3.508024e-148	14
MRPL12	Activated Chemokine	6.878347e-151	0.9646463	0.765	0.391	1.226341e-146	14
REL	Activated Chemokine	7.285502e-151	0.878341	0.507	0.158	1.298932e-146	14
ETF1	Activated Chemokine	2.09228e-150	-0.895511	0.949	0.986	3.730326e-146	14
TMSB10	Activated Chemokine	9.629326e-150	-1.218303	0.5	0.783	1.716812e-145	14
ARHGDIB	Activated Chemokine	1.535606e-149	0.975632	0.533	0.176	2.737831e-145	14
FKBP4	Activated Chemokine	1.743222e-149	0.9331275	0.493	0.142	3.10799e-145	14
PUM3	Activated Chemokine	7.966591e-149	0.8648367	0.817	0.471	1.420363e-144	14
RSL1D1	Activated Chemokine	9.968825e-148	0.9131738	0.595	0.229	1.777342e-143	14
HNRNPAB	Activated Chemokine	2.663241e-146	0.9903444	0.619	0.232	4.748293e-142	14
PHACTR1	Activated Chemokine	5.569256e-146	0.9241191	0.66	0.287	9.929427e-142	14
PDCD5	Activated Chemokine	4.824892e-145	-1.152247	0.658	0.871	8.602299e-141	14
CD79A	Activated Chemokine	8.01411e-145	0.6925192	0.956	0.755	1.428836e-140	14
PSMA7	Activated Chemokine	1.226092e-144	0.7393487	0.913	0.639	2.185999e-140	14
SNRPG	Activated Chemokine	1.515643e-144	0.913131	0.572	0.22	2.702239e-140	14
MRPL3	Activated Chemokine	2.898071e-140	0.3496039	0.964	0.799	5.166971e-136	14
RPS26	Activated Chemokine	2.090825e-139	0.8490143	0.767	0.419	3.727731e-135	14
CCT4	Activated Chemokine	1.650601e-138	0.6410932	0.229	0.021	2.942856e-134	14
SEMA7A	Activated Chemokine	1.089962e-137	0.9019856	0.574	0.211	1.943293e-133	14
TRAF4	Activated Chemokine	1.939713e-136	-1.121059	0.631	0.853	3.458315e-132	14
CD37	Activated Chemokine	4.913048e-136	0.7916011	0.889	0.603	8.759474e-132	14

RSL24D1 Activated Chemokine
6.64178e-136 0.8673024 0.576 0.228 1.184163e-131 14
EIF2S1 Activated Chemokine
8.735197e-136 -1.256265 0.529 0.781 1.557398e-131 14
HLA-DPB1 Activated Chemokine
6.294719e-135 0.7492195 0.884 0.601 1.122286e-130 14
POMP Activated Chemokine
1.572743e-134 0.7357289 0.319 0.058 2.804043e-130 14
METTL1 Activated Chemokine
4.111275e-134 0.8070133 0.852 0.564 7.329993e-130 14
RBM8A Activated Chemokine
1.487784e-133 0.8941084 0.601 0.242 2.652571e-129 14
GARS Activated Chemokine
4.117978e-132 0.6848748 0.941 0.739 7.341943e-128 14
CHCHD2 Activated Chemokine
1.510581e-131 1.094636 0.319 0.059 2.693214e-127 14 NPW
Activated Chemokine
7.957063e-131 -1.227547 0.175 0.499 1.418665e-126 14
CYTIP Activated Chemokine
6.816245e-130 0.6430973 0.217 0.025 1.215268e-125 14
MFSD2A Activated Chemokine
1.293008e-129 0.8223504 0.464 0.147 2.305305e-125 14
PAICS Activated Chemokine
1.311089e-129 0.8333092 0.383 0.089 2.337541e-125 14
STAT5A Activated Chemokine
3.573957e-128 -1.45359 0.22 0.542 6.372008e-124 14
ISG20 Activated Chemokine
5.101631e-128 0.8467029 0.659 0.297 9.095698e-124 14
TMEM147 Activated Chemokine
1.341248e-127 0.6233491 0.979 0.881 2.391311e-123 14
H3F3B Activated Chemokine
3.690063e-127 0.8514978 0.571 0.225 6.579013e-123 14
CDK4 Activated Chemokine
2.673111e-126 0.9019088 0.499 0.174 4.765889e-122 14
CMSS1 Activated Chemokine
2.754536e-125 0.9148976 0.589 0.263 4.911063e-121 14
SMS Activated Chemokine
5.863999e-125 0.8331827 0.582 0.222 1.045492e-120 14
CKS2 Activated Chemokine
3.122506e-124 -1.095412 0.672 0.873 5.567117e-120 14
CD52 Activated Chemokine
4.298871e-124 0.8098836 0.462 0.154 7.664458e-120 14
DCUN1D5 Activated Chemokine
1.676764e-123 0.8333715 0.717 0.385 2.989503e-119 14
PHB Activated Chemokine
6.528631e-122 0.7447353 0.386 0.094 1.16399e-117 14
RRS1 Activated Chemokine
1.212341e-121 0.793866 0.857 0.579 2.161482e-117 14
EIF2S2 Activated Chemokine
6.576753e-121 0.8194094 0.691 0.36 1.172569e-116 14

PFDN2	Activated Chemokine	8.198273e-121	0.7212174	0.356	0.081	1.46167e-116	14
TSR1	Activated Chemokine	1.6741e-120	0.7615992	0.789	0.449	2.984752e-116	14
SFPQ	Activated Chemokine	1.411679e-119	0.8182648	0.485	0.158	2.516883e-115	14
C8orf33	Activated Chemokine	1.442937e-119	0.8176178	0.73	0.412	2.572613e-115	14
EIF6	Activated Chemokine	1.661558e-119	0.5936	0.925	0.659	2.962391e-115	14
TUBA1B	Activated Chemokine	3.199652e-117	0.8142784	0.534	0.193	5.70466e-113	14
NPM3	Activated Chemokine	4.257448e-117	0.6675515	0.897	0.65	7.590604e-113	14
PSMB1	Activated Chemokine	1.391756e-116	0.7174484	0.194	0.022	2.481362e-112	14
NR4A1	Activated Chemokine	2.017238e-115	0.7881208	0.609	0.273	3.596534e-111	14
PSMD14	Activated Chemokine	3.289563e-114	0.6304116	0.276	0.044	5.864962e-110	14
PLK3	Activated Chemokine	1.518357e-113	0.813135	0.476	0.165	2.707078e-109	14
ASNS	Activated Chemokine	2.952356e-113	0.7548022	0.738	0.41	5.263755e-109	14
NUDC	Activated Chemokine	5.879114e-113	0.7850334	0.63	0.309	1.048187e-108	14
TCP1	Activated Chemokine	1.668778e-112	0.7667071	0.662	0.332	2.975264e-108	14
STRAP	Activated Chemokine	4.261182e-112	0.7497056	0.449	0.134	7.597261e-108	14
TM2D3	Activated Chemokine	1.033354e-111	0.7738003	0.525	0.199	1.842366e-107	14
BRIX1	Activated Chemokine	1.565491e-110	0.7742381	0.564	0.244	2.791113e-106	14
NAA20	Activated Chemokine	2.504856e-110	0.6730069	0.318	0.071	4.465908e-106	14
RRP9	Activated Chemokine	2.662919e-110	0.7453602	0.445	0.144	4.747717e-106	14
GNL2	Activated Chemokine	2.91252e-110	0.7712259	0.601	0.267	5.192733e-106	14
NDUFAF8	Activated Chemokine	1.027818e-109	0.7536049	0.763	0.462	1.832496e-105	14
SNRPF	Activated Chemokine	1.476447e-109	0.7370671	0.74	0.421	2.632357e-105	14
DNAJA1	Activated Chemokine	1.740235e-109	0.7394924	0.665	0.331	3.102665e-105	14
EIF1AX	Activated Chemokine	6.849459e-108	0.7909156	0.467	0.157	1.22119e-103	14
NFKB1	Activated Chemokine	8.982287e-108	0.7318569	0.532	0.219	1.601452e-103	14

TIMM17A	Activated Chemokine	1.05299e-106	0.6341355	0.228	0.033	1.877376e-102	14
TNFAIP3	Activated Chemokine	1.156e-106	0.751939	0.624	0.297	2.061032e-102	14
PSMD11	Activated Chemokine	2.699587e-106	0.8822813	0.427	0.129	4.813094e-102	14
PSAT1	Activated Chemokine	5.069412e-106	0.6547758	0.272	0.058	9.038255e-102	14
IL21R	Activated Chemokine	1.255861e-104	0.7937047	0.437	0.15	2.239075e-100	14
NAMPT	Activated Chemokine	1.376786e-104	0.4157065	0.994	0.976	2.454673e-100	14
RPS24	Activated Chemokine	1.198644e-103	0.6805005	0.826	0.543	2.137063e-99	14
PSMA1	Activated Chemokine	3.840872e-102	-0.7979923	0.977	0.989	6.84789e-98	14
CD74	Activated Chemokine	3.899737e-102	0.7584676	0.508	0.186	6.952841e-98	14
ESF1	Activated Chemokine	4.664956e-102	0.5726046	0.153	0.012	8.317151e-98	14
EGR3	Activated Chemokine	1.897462e-101	0.6753249	0.834	0.566	3.382985e-97	14
PARK7	Activated Chemokine	2.38456e-101	0.7167874	0.509	0.203	4.251431e-97	14
EEF1E1	Activated Chemokine	2.152392e-100	0.5828736	0.256	0.051	3.8375e-96	14
CTPS1	Activated Chemokine	2.616828e-100	0.7142319	0.662	0.339	4.665542e-96	14
ABRACL	Activated Chemokine	3.289474e-100	0.571246	0.257	0.044	5.864804e-96	14
SLC38A5	Activated Chemokine	4.238774e-100	0.6376103	0.342	0.086	7.55731e-96	14
SLC25A33	Activated Chemokine	5.638905e-100	0.8325958	0.256	0.05	1.00536e-95	14
HES1	Activated Chemokine	1.664375e-99	0.6329371	0.882	0.647	2.967414e-95	14
UBE2D3	Activated Chemokine	5.603146e-99	0.9454186	0.53	0.212	9.989849e-95	14
GBP2	Activated Chemokine	8.540027e-99	-1.196069	0.065	0.309	1.522601e-94	14
CD27	Activated Chemokine	1.872532e-98	0.7096852	0.509	0.197	3.338537e-94	14
NIPA2	Activated Chemokine	2.831024e-98	0.5938349	0.269	0.052	5.047433e-94	14
WDR3	Activated Chemokine	5.863201e-98	0.7219861	0.547	0.23	1.04535e-93	14
AMD1	Activated Chemokine	7.325739e-98	0.680475	0.718	0.396	1.306106e-93	14
BZW1	Activated Chemokine	9.582361e-98	0.6849637	0.439	0.146	1.708439e-93	14

NDUFAF4	Activated Chemokine	1.719952e-97	0.6802637	0.774	0.468	3.066502e-93	14
PRELID1	Activated Chemokine	9.897736e-97	0.7003631	0.378	0.119	1.764667e-92	14
BZW2	Activated Chemokine	1.106681e-96	0.6990323	0.691	0.382	1.973102e-92	14
NOL7	Activated Chemokine	2.225908e-96	0.7239419	0.405	0.128	3.968571e-92	14
NDUFAF2	Activated Chemokine	1.079145e-95	0.6061044	0.306	0.076	1.924007e-91	14
YRDC	Activated Chemokine	1.093996e-95	0.6338427	0.31	0.07	1.950485e-91	14
MTHFD1L	Activated Chemokine	2.582415e-95	-0.4909961	1	1	4.604189e-91	14
RPS27	Activated Chemokine	2.847818e-95	0.5933811	0.883	0.614	5.077374e-91	14
SUB1	Activated Chemokine	8.552057e-95	0.6367608	0.35	0.1	1.524746e-90	14
AIMP2	Activated Chemokine	1.371131e-94	0.7672758	0.662	0.367	2.444589e-90	14
UBE2B	Activated Chemokine	7.862593e-94	0.6396183	0.854	0.599	1.401822e-89	14
NOP10	Activated Chemokine	1.493131e-93	0.7197517	0.574	0.26	2.662103e-89	14
CEBPZ	Activated Chemokine	3.946696e-92	0.680293	0.763	0.483	7.036564e-88	14
CCT3	Activated Chemokine	6.518945e-92	0.6629349	0.825	0.569	1.162263e-87	14
APRT	Activated Chemokine	9.148056e-92	0.7238407	0.877	0.631	1.631007e-87	14
SET	Activated Chemokine	2.326141e-91	0.6584386	0.345	0.098	4.147278e-87	14
RIOK1	Activated Chemokine	4.06204e-91	0.7186427	0.589	0.283	7.242212e-87	14
DCTPP1	Activated Chemokine	5.390475e-91	0.670025	0.722	0.424	9.610678e-87	14
ZNF706	Activated Chemokine	1.504049e-90	0.6345706	0.783	0.497	2.681569e-86	14
EIF3I	Activated Chemokine	2.564496e-90	0.6260745	0.837	0.558	4.57224e-86	14
SNRPB	Activated Chemokine	7.075581e-90	0.5839403	0.857	0.591	1.261505e-85	14
SEC61B	Activated Chemokine	1.328707e-89	0.6425433	0.433	0.155	2.368952e-85	14
POLR2H	Activated Chemokine	1.331868e-89	0.6508916	0.678	0.367	2.374587e-85	14
PSMC3	Activated Chemokine	2.15628e-89	0.6742233	0.65	0.341	3.844431e-85	14
ZC3H15	Activated Chemokine	3.149994e-89	0.6894137	0.521	0.228	5.616125e-85	14

BUD23	Activated Chemokine	4.625117e-89	0.7670578	0.577	0.269	8.246122e-85	14
HSPA5	Activated Chemokine	6.005309e-89	0.5631129	0.883	0.626	1.070687e-84	14
COX7B	Activated Chemokine	7.303558e-89	0.6928314	0.535	0.235	1.302151e-84	14
POLD2	Activated Chemokine	9.428831e-89	0.6729419	0.724	0.437	1.681066e-84	14
CCT7	Activated Chemokine	4.297475e-88	0.6232402	0.694	0.388	7.661968e-84	14
NDUFAB1	Activated Chemokine	5.70549e-88	0.648711	0.19	0.028	1.017232e-83	14
NR4A2	Activated Chemokine	1.009421e-87	0.5672336	0.869	0.628	1.799697e-83	14
PSMA4	Activated Chemokine	1.405348e-87	0.641864	0.448	0.174	2.505595e-83	14
FARSA	Activated Chemokine	1.678049e-87	0.5552615	0.245	0.058	2.991794e-83	14
BCL2L1	Activated Chemokine	1.829039e-87	0.64779	0.718	0.412	3.260994e-83	14
TOMM22	Activated Chemokine	2.142486e-87	0.689231	0.394	0.122	3.819838e-83	14
CCDC58	Activated Chemokine	2.51903e-87	0.5426819	0.278	0.061	4.491178e-83	14
WDR12	Activated Chemokine	7.501927e-87	0.6580045	0.748	0.445	1.337519e-82	14
VDAC1	Activated Chemokine	8.6903e-87	0.6525444	0.726	0.422	1.549394e-82	14
NSA2	Activated Chemokine	1.073747e-86	0.7254898	0.551	0.248	1.914384e-82	14
NARS	Activated Chemokine	1.249583e-86	0.5803785	0.877	0.639	2.227882e-82	14
HNRNPC	Activated Chemokine	1.763987e-86	0.652009	0.683	0.392	3.145012e-82	14
PSMA3	Activated Chemokine	4.675008e-86	0.6315685	0.755	0.47	8.335071e-82	14
FUS	Activated Chemokine	6.318859e-86	0.6506652	0.508	0.208	1.126589e-81	14
EMG1	Activated Chemokine	7.239603e-86	0.6711816	0.475	0.186	1.290749e-81	14
WDR74	Activated Chemokine	1.217928e-85	0.6282621	0.679	0.381	2.171443e-81	14
MRPL20	Activated Chemokine	1.719653e-85	0.6690912	0.478	0.196	3.065969e-81	14
MAP2K3	Activated Chemokine	1.787662e-85	0.6447141	0.465	0.172	3.187223e-81	14
DNPEP	Activated Chemokine	2.448456e-85	0.5989704	0.796	0.526	4.365351e-81	14
SNRPB2	Activated Chemokine	3.19645e-85	0.7898222	0.503	0.211	5.698951e-81	14

EIF4EBP1	Activated Chemokine	3.478192e-85	0.8779946	0.596	0.334	6.201269e-81	14
LRMP	Activated Chemokine	6.399549e-85	0.5753685	0.311	0.08	1.140976e-80	14
UTP4	Activated Chemokine	6.403364e-85	0.6468971	0.488	0.217	1.141656e-80	14
PSMB5	Activated Chemokine	1.463148e-84	0.6922805	0.594	0.271	2.608647e-80	14
WARS	Activated Chemokine	3.288278e-84	0.6390111	0.386	0.13	5.862671e-80	14
PES1	Activated Chemokine	3.676032e-84	0.4555442	0.976	0.893	6.553998e-80	14
PPIA	Activated Chemokine	1.195754e-83	0.6432711	0.749	0.483	2.13191e-79	14
CCT8	Activated Chemokine	1.424814e-83	0.5414772	0.223	0.045	2.540301e-79	14
CCDC86	Activated Chemokine	3.634116e-83	0.7978881	0.627	0.308	6.479265e-79	14
CD69	Activated Chemokine	1.094184e-82	0.6663163	0.652	0.368	1.950821e-78	14
PSMC1	Activated Chemokine	4.163798e-82	0.6146695	0.385	0.132	7.423636e-78	14
PSME3	Activated Chemokine	7.854772e-82	0.603256	0.802	0.55	1.400427e-77	14
SNU13	Activated Chemokine	2.231738e-81	-1.008072	0.267	0.521	3.978966e-77	14
LSP1	Activated Chemokine	3.837149e-81	0.5751474	0.785	0.496	6.841253e-77	14
XRCC5	Activated Chemokine	6.866803e-81	0.6311356	0.401	0.148	1.224282e-76	14
UTP11	Activated Chemokine	8.595066e-81	0.6455726	0.411	0.153	1.532414e-76	14
DNAJC2	Activated Chemokine	1.979418e-80	0.6077552	0.373	0.13	3.529104e-76	14
RRP1	Activated Chemokine	4.599421e-80	0.6520407	0.541	0.257	8.200308e-76	14
SYNCRIP	Activated Chemokine	6.099265e-80	-0.9868487	0.15	0.394	1.087438e-75	14
EVI2B	Activated Chemokine	7.995815e-80	0.6786193	0.53	0.246	1.425574e-75	14
RRP7A	Activated Chemokine	1.095198e-79	-1.079422	0.257	0.494	1.952629e-75	14
UCP2	Activated Chemokine	1.866036e-79	0.6346522	0.429	0.159	3.326955e-75	14
NXT1	Activated Chemokine	2.110797e-79	0.6212334	0.643	0.356	3.763341e-75	14
ELOC	Activated Chemokine	2.670492e-79	0.6214791	0.466	0.187	4.76122e-75	14
TRMT10C	Activated Chemokine	4.177324e-79	0.6523487	0.852	0.63	7.447752e-75	14

HERPUD1	Activated Chemokine	1.222573e-78	0.6278322	0.699	0.421	2.179725e-74	14
MINOS1	Activated Chemokine	1.521804e-78	0.6351861	0.648	0.364	2.713224e-74	14
PSMD7	Activated Chemokine	2.77748e-78	-2.304236	0.195	0.408	4.951969e-74	14
JCHAIN	Activated Chemokine	4.23044e-78	0.6103061	0.706	0.41	7.542452e-74	14
DDX39A	Activated Chemokine	7.635387e-78	0.5966504	0.334	0.1	1.361313e-73	14
CD320	Activated Chemokine	9.791491e-78	0.6261985	0.588	0.296	1.745725e-73	14
GLRX3	Activated Chemokine	1.032564e-77	0.5635055	0.842	0.587	1.840959e-73	14
HNRNPU	Activated Chemokine	1.219089e-77	0.6663796	0.517	0.225	2.173514e-73	14
TARS	Activated Chemokine	1.712345e-77	0.5841051	0.361	0.117	3.052939e-73	14
ABCF2.1	Activated Chemokine	2.179315e-77	-1.078008	0.158	0.357	3.8855e-73	14
TXNIP	Activated Chemokine	3.056732e-77	0.6413686	0.569	0.281	5.449848e-73	14
AIMP1	Activated Chemokine	5.447769e-77	0.4936002	0.17	0.019	9.712827e-73	14
SPRY1	Activated Chemokine	9.81945e-77	0.562799	0.849	0.605	1.75071e-72	14
PTGES3	Activated Chemokine	1.570683e-76	-0.6538552	1	1	2.800371e-72	14
MALAT1	Activated Chemokine	2.955914e-76	0.5544726	0.318	0.091	5.2701e-72	14
NFKBIB	Activated Chemokine	2.970674e-76	-1.014831	0.279	0.509	5.296414e-72	14
TSC22D3	Activated Chemokine	3.054394e-76	0.5890637	0.385	0.133	5.445679e-72	14
ZPR1	Activated Chemokine	4.098596e-76	0.5414414	0.859	0.634	7.307387e-72	14
ATP5MD	Activated Chemokine	1.383449e-75	0.6115687	0.748	0.491	2.466551e-71	14
TUFM	Activated Chemokine	1.695599e-75	0.6163568	0.584	0.307	3.023084e-71	14
STOML2	Activated Chemokine	1.88443e-75	0.6742963	0.421	0.156	3.35975e-71	14
CD58	Activated Chemokine	3.630886e-75	0.6249341	0.451	0.198	6.473508e-71	14
UBALD2	Activated Chemokine	3.701231e-75	0.6282154	0.361	0.103	6.598926e-71	14
SNX9	Activated Chemokine	4.78105e-75	0.6143762	0.391	0.148	8.524134e-71	14
GRPEL1	Activated Chemokine	5.750392e-75	0.6463506	0.429	0.166	1.025237e-70	14

TIMM44	Activated Chemokine	1.547427e-74	0.5152161	0.846	0.589	2.758907e-70	14
XRCC6	Activated Chemokine	3.37178e-74	0.5894218	0.692	0.414	6.011547e-70	14
MAGOH	Activated Chemokine	5.947309e-74	0.5621267	0.314	0.096	1.060346e-69	14
FARSB	Activated Chemokine	6.704537e-74	0.5108102	0.858	0.635	1.195352e-69	14
ERH	Activated Chemokine	1.441605e-73	0.7904934	0.606	0.338	2.570238e-69	14
INSIG1	Activated Chemokine	1.585766e-73	0.5458182	0.3	0.084	2.827261e-69	14
RRP1B	Activated Chemokine	1.872493e-73	0.598348	0.589	0.307	3.338468e-69	14
EIF4E	Activated Chemokine	2.214e-73	0.5829118	0.502	0.211	3.947341e-69	14
WDR46	Activated Chemokine	2.500054e-73	0.5790685	0.726	0.453	4.457346e-69	14
GADD45GIP1	Activated Chemokine	2.535794e-73	0.6332238	0.609	0.331	4.521067e-69	14
MIF	Activated Chemokine	4.588938e-73	0.5894809	0.783	0.539	8.181617e-69	14
CNBP	Activated Chemokine	4.731492e-73	0.6242619	0.352	0.123	8.435777e-69	14
NFATC1	Activated Chemokine	4.857694e-73	0.5274837	0.289	0.081	8.660782e-69	14
POLR3H	Activated Chemokine	8.832115e-73	0.6378858	0.494	0.239	1.574678e-68	14
AK2	Activated Chemokine	1.28718e-72	0.5448781	0.828	0.595	2.294913e-68	14
PSMA2	Activated Chemokine	1.972398e-72	0.5963956	0.684	0.414	3.516588e-68	14
ADRM1	Activated Chemokine	3.719307e-72	0.6117234	0.418	0.167	6.631153e-68	14
SNX8	Activated Chemokine	5.501954e-72	0.5914975	0.464	0.19	9.809434e-68	14
ATIC	Activated Chemokine	5.696735e-72	0.6076243	0.14	0.017	1.015671e-67	14
TNFRSF4	Activated Chemokine	5.719696e-72	0.4016213	0.13	0.011	1.019765e-67	14
RAB20	Activated Chemokine	1.013338e-71	0.5774661	0.62	0.337	1.80668e-67	14
PSMC4	Activated Chemokine	1.440645e-71	0.5725685	0.783	0.525	2.568526e-67	14
GHITM	Activated Chemokine	1.635392e-71	0.5941861	0.532	0.26	2.91574e-67	14
MRPS12	Activated Chemokine	1.794361e-71	0.4976835	0.198	0.039	3.199165e-67	14
CENPV	Activated Chemokine	2.886477e-71	0.5368816	0.74	0.45	5.1463e-67	14

LSM5	Activated Chemokine	3.162701e-71	0.5843758	0.312	0.108	5.638779e-67	14
DESI1	Activated Chemokine	4.831668e-71	0.5816016	0.622	0.347	8.614381e-67	14
PHB2	Activated Chemokine	4.965694e-71	0.5047599	0.194	0.033	8.853335e-67	14
KDM6B	Activated Chemokine	6.496698e-71	0.6917861	0.529	0.278	1.158296e-66	14
CD82	Activated Chemokine	6.571061e-71	0.4795012	0.252	0.068	1.171554e-66	14
BYSL	Activated Chemokine	6.908188e-71	0.4746296	0.942	0.756	1.231661e-66	14
PKM	Activated Chemokine	7.513704e-71	0.6183013	0.539	0.263	1.339618e-66	14
TIMM10	Activated Chemokine	7.537222e-71	0.6150799	0.445	0.183	1.343811e-66	14
ATP1A1	Activated Chemokine	7.893479e-71	-1.131123	0.082	0.279	1.407328e-66	14
KLF2	Activated Chemokine	2.761897e-70	0.5828631	0.415	0.166	4.924187e-66	14
RARS	Activated Chemokine	3.390439e-70	0.5545449	0.344	0.104	6.044814e-66	14
SRFBP1	Activated Chemokine	4.241291e-70	0.5810826	0.65	0.376	7.561798e-66	14
TXNL1	Activated Chemokine	6.034846e-70	0.529426	0.662	0.345	1.075953e-65	14
TUBB4B	Activated Chemokine	6.282167e-70	0.3961395	0.988	0.925	1.120047e-65	14
HNRNPA1	Activated Chemokine	7.97596e-70	0.5552169	0.353	0.128	1.422034e-65	14
EXOSC4	Activated Chemokine	1.025292e-69	0.5990444	0.463	0.204	1.827992e-65	14
DNTTIP2	Activated Chemokine	1.062114e-69	0.5576847	0.331	0.108	1.893643e-65	14
DIMT1	Activated Chemokine	1.355104e-69	0.5241951	0.804	0.553	2.416015e-65	14
ATP5PF	Activated Chemokine	1.930525e-69	0.5779081	0.55	0.28	3.441933e-65	14
MRPS15	Activated Chemokine	1.950747e-69	0.6000146	0.495	0.23	3.477987e-65	14
MRPL21	Activated Chemokine	2.71735e-69	0.5926351	0.505	0.235	4.844763e-65	14
ABCF1	Activated Chemokine	6.473864e-69	0.6638122	0.605	0.32	1.154225e-64	14
PMAIP1	Activated Chemokine	8.446452e-69	0.5735284	0.65	0.381	1.505918e-64	14
NDUFS6	Activated Chemokine	1.092851e-68	0.5367223	0.324	0.104	1.948444e-64	14
LTV1	Activated Chemokine	1.185732e-68	0.5638396	0.683	0.404	2.114042e-64	14

HNRNPR	Activated Chemokine	1.540975e-68	0.5466213	0.676	0.398	2.747405e-64	14
PSMB2	Activated Chemokine	5.452955e-68	0.58263	0.509	0.25	9.722074e-64	14
EIF4A3	Activated Chemokine	5.903415e-68	0.5392605	0.86	0.604	1.05252e-63	14
SYNGR2	Activated Chemokine	7.133077e-68	0.5198999	0.824	0.582	1.271756e-63	14
HNRNPF	Activated Chemokine	8.992927e-68	-0.9325409	0.189	0.409	1.603349e-63	14
STX7	Activated Chemokine	1.544722e-67	0.5522006	0.718	0.439	2.754085e-63	14
FBL	Activated Chemokine	3.776344e-67	0.4998883	0.27	0.072	6.732843e-63	14
DPH2	Activated Chemokine	1.256916e-66	0.583067	0.433	0.187	2.240955e-62	14
TFAM	Activated Chemokine	1.636964e-66	0.5496807	0.545	0.277	2.918544e-62	14
MRPS7	Activated Chemokine	3.177553e-66	0.5455052	0.666	0.387	5.665259e-62	14
YWHAE	Activated Chemokine	4.124993e-66	0.5033101	0.274	0.082	7.35445e-62	14
MRPS17	Activated Chemokine	6.390716e-66	0.5636081	0.383	0.14	1.139401e-61	14
PRDX4	Activated Chemokine	1.85994e-65	0.4457712	0.161	0.023	3.316086e-61	14
SERPINE2	Activated Chemokine	2.333578e-65	0.5782775	0.477	0.222	4.160537e-61	14
NOB1	Activated Chemokine	2.96544e-65	0.5359406	0.356	0.137	5.287084e-61	14
FAM207A	Activated Chemokine	3.461334e-65	0.5809179	0.525	0.27	6.171212e-61	14
ATOX1	Activated Chemokine	3.581804e-65	0.5153557	0.887	0.699	6.385999e-61	14
TPI1	Activated Chemokine	4.313821e-65	0.5646891	0.463	0.211	7.691111e-61	14
PSMD1	Activated Chemokine	4.562101e-65	0.3993301	0.175	0.033	8.13377e-61	14
SLC7A5	Activated Chemokine	6.766443e-65	0.5755322	0.35	0.125	1.206389e-60	14
SRPRB	Activated Chemokine	9.525584e-65	0.6156772	0.269	0.09	1.698316e-60	14
FCRL5	Activated Chemokine	1.001764e-64	0.6109758	0.57	0.302	1.786046e-60	14
CALR	Activated Chemokine	1.177156e-64	0.5111618	0.24	0.056	2.098752e-60	14
CD84	Activated Chemokine	3.088265e-64	0.5650504	0.573	0.312	5.506068e-60	14
GTF3C6	Activated Chemokine	3.369918e-64	0.572334	0.339	0.136	6.008227e-60	14

RUVBL1	Activated Chemokine	5.506553e-64	0.54323	0.686	0.415	9.817633e-60	14
APEX1	Activated Chemokine	5.917551e-64	0.5834904	0.603	0.347	1.05504e-59	14
RWDD1	Activated Chemokine	6.097505e-64	0.5465964	0.376	0.144	1.087124e-59	14
MAGOHB	Activated Chemokine	1.340545e-63	0.506741	0.337	0.117	2.390058e-59	14
HSPBP1	Activated Chemokine	1.572629e-63	0.5859958	0.525	0.269	2.803839e-59	14
NCBP2	Activated Chemokine	1.590104e-63	-0.8076437	0.707	0.817	2.834996e-59	14
CORO1A	Activated Chemokine	3.260275e-63	0.3661424	0.982	0.922	5.812745e-59	14
BTF3	Activated Chemokine	3.977107e-63	0.5729928	0.404	0.176	7.090784e-59	14
PCGF5	Activated Chemokine	6.534746e-63	0.5379612	0.7	0.437	1.16508e-58	14
RAD23A	Activated Chemokine	7.430594e-63	0.5197588	0.69	0.44	1.324801e-58	14
PSMB7	Activated Chemokine	7.494248e-63	0.5881504	0.415	0.177	1.336149e-58	14
TUBA1C	Activated Chemokine	1.244346e-62	0.5394585	0.822	0.6	2.218545e-58	14
RBM3	Activated Chemokine	1.984921e-62	-0.6824935	0.779	0.884	3.538915e-58	14
MS4A1	Activated Chemokine	2.602185e-62	0.5239266	0.338	0.125	4.639435e-58	14
TIMM50	Activated Chemokine	2.860811e-62	0.5618202	0.569	0.306	5.100541e-58	14
AKR1A1	Activated Chemokine	3.439773e-62	0.4331249	0.919	0.742	6.132771e-58	14
SNRPD2	Activated Chemokine	8.109062e-62	0.5514406	0.469	0.216	1.445765e-57	14
RPIA	Activated Chemokine	1.515268e-61	0.5409804	0.549	0.284	2.701571e-57	14
G3BP1	Activated Chemokine	3.169084e-61	0.4869344	0.751	0.491	5.650159e-57	14
COX5A	Activated Chemokine	3.249891e-61	0.3031436	0.996	0.986	5.79423e-57	14
RPL14	Activated Chemokine	7.86193e-61	0.5322401	0.407	0.181	1.401703e-56	14
MRPL36	Activated Chemokine	1.299542e-60	0.5280877	0.417	0.175	2.316953e-56	14
NOC2L	Activated Chemokine	1.629805e-60	0.5313685	0.578	0.317	2.90578e-56	14
IMP4	Activated Chemokine	1.869823e-60	0.5369661	0.45	0.201	3.333708e-56	14
TMEM208	Activated Chemokine	1.295561e-59	0.520773	0.462	0.216	2.309856e-55	14

MRPL4	Activated Chemokine	1.332546e-59	0.5319726	0.377	0.154	2.375796e-55	14
COA4	Activated Chemokine	1.45955e-59	0.532147	0.524	0.269	2.602233e-55	14
MRPL47	Activated Chemokine	1.614285e-59	0.4984154	0.319	0.121	2.878108e-55	14
ISOC2	Activated Chemokine	2.462558e-59	0.5365582	0.658	0.381	4.390495e-55	14
SHMT2	Activated Chemokine	2.497002e-59	0.5068699	0.327	0.119	4.451904e-55	14
TWISTNB	Activated Chemokine	3.260856e-59	-0.9496904	0.15	0.353	5.813781e-55	14
S100A10	Activated Chemokine	4.152303e-59	-0.7500333	0.053	0.223	7.403142e-55	14
LY86	Activated Chemokine	5.546957e-59	0.5775078	0.807	0.614	9.88967e-55	14
CLEC2D	Activated Chemokine	6.314262e-59	0.5331251	0.397	0.17	1.12577e-54	14
SSSCA1	Activated Chemokine	7.31498e-59	0.497102	0.286	0.096	1.304188e-54	14
BOP1	Activated Chemokine	8.161419e-59	0.544885	0.387	0.163	1.455099e-54	14
SMIM37	Activated Chemokine	8.228853e-59	0.54512	0.442	0.203	1.467122e-54	14
MRPS35	Activated Chemokine	1.138233e-58	0.5440779	0.374	0.143	2.029356e-54	14
TSEN15	Activated Chemokine	1.145994e-58	0.5381545	0.451	0.211	2.043194e-54	14
AATF	Activated Chemokine	1.349896e-58	-0.8069322	0.049	0.215	2.406729e-54	14
FAM111B	Activated Chemokine	1.420903e-58	0.5350131	0.594	0.341	2.533329e-54	14
RBM17	Activated Chemokine	2.206679e-58	0.4329734	0.837	0.577	3.934288e-54	14
ANP32B	Activated Chemokine	2.718268e-58	0.5396063	0.545	0.287	4.8464e-54	14
SSRP1	Activated Chemokine	2.991596e-58	-1.08656	0.33	0.468	5.333717e-54	14
S100A6	Activated Chemokine	3.154598e-58	0.5107086	0.726	0.483	5.624333e-54	14
EIF3M	Activated Chemokine	5.408706e-58	-0.803658	0.084	0.279	9.643182e-54	14
NCF1	Activated Chemokine	8.312497e-58	0.4980562	0.379	0.152	1.482035e-53	14
TRIAP1	Activated Chemokine	9.351575e-58	0.4969983	0.337	0.124	1.667292e-53	14
GRWD1	Activated Chemokine	9.454349e-58	-0.8795911	0.174	0.368	1.685616e-53	14
RCSD1	Activated Chemokine	1.407541e-57	0.4136952	0.888	0.678	2.509505e-53	14

SLC25A3	Activated Chemokine	2.703968e-57	0.5155861	0.217	0.067	4.820905e-53	14
BATF	Activated Chemokine	3.013444e-57	0.5106554	0.463	0.218	5.372669e-53	14
MRPS23	Activated Chemokine	3.158529e-57	0.5267654	0.504	0.256	5.631341e-53	14
HDAC2	Activated Chemokine	4.106493e-57	0.5020881	0.599	0.343	7.321466e-53	14
POLR2K	Activated Chemokine	4.294957e-57	0.5248689	0.596	0.342	7.657478e-53	14
CYC1	Activated Chemokine	5.849627e-57	0.577351	0.603	0.35	1.04293e-52	14
SARS	Activated Chemokine	6.245233e-57	0.5298035	0.628	0.377	1.113463e-52	14
ATP6V0B	Activated Chemokine	6.485407e-57	0.5448871	0.405	0.18	1.156283e-52	14
YIF1A	Activated Chemokine	6.606085e-57	-0.7849399	0.282	0.476	1.177799e-52	14
GPSM3	Activated Chemokine	6.775235e-57	0.435604	0.859	0.636	1.207957e-52	14
HNRNPM	Activated Chemokine	9.519214e-57	0.5117815	0.408	0.174	1.697181e-52	14
SDAD1	Activated Chemokine	1.019573e-56	0.5079439	0.59	0.331	1.817797e-52	14
BUD31	Activated Chemokine	1.324639e-56	0.5461943	0.687	0.444	2.361699e-52	14
EIF5	Activated Chemokine	1.686667e-56	0.498974	0.402	0.178	3.007159e-52	14
LRRC59	Activated Chemokine	1.769187e-56	0.4818737	0.279	0.088	3.154283e-52	14
DDX10	Activated Chemokine	2.816351e-56	0.5263497	0.461	0.228	5.021273e-52	14
PHF5A	Activated Chemokine	6.066899e-56	0.5003924	0.356	0.142	1.081667e-51	14
TIMM9	Activated Chemokine	6.362344e-56	0.4321629	0.83	0.599	1.134342e-51	14
PSMB3	Activated Chemokine	6.993031e-56	0.4969427	0.696	0.444	1.246787e-51	14
NDUFB9	Activated Chemokine	7.67489e-56	0.5173691	0.443	0.207	1.368356e-51	14
PRPF19	Activated Chemokine	1.126136e-55	0.4036407	0.206	0.047	2.007789e-51	14
TRMT61A	Activated Chemokine	1.521589e-55	0.5377807	0.44	0.208	2.712841e-51	14
EPRS	Activated Chemokine	2.386984e-55	0.397345	0.186	0.041	4.255753e-51	14
RCL1	Activated Chemokine	2.563653e-55	0.5172362	0.671	0.41	4.570737e-51	14
IDI1	Activated Chemokine	3.087823e-55	0.472743	0.322	0.124	5.505281e-51	14

EIF3B	Activated Chemokine	3.679911e-55	0.4531202	0.791	0.561	6.560914e-51	14
UQCRQ	Activated Chemokine	5.949691e-55	0.5091902	0.378	0.163	1.06077e-50	14
SAR1A	Activated Chemokine	6.272631e-55	0.4373769	0.249	0.071	1.118347e-50	14
PNPT1	Activated Chemokine	8.89302e-55	0.5125613	0.492	0.244	1.585536e-50	14
SUPT16H	Activated Chemokine	1.487298e-54	0.4773319	0.796	0.574	2.651703e-50	14
LDHB	Activated Chemokine	2.667068e-54	0.5146209	0.665	0.421	4.755116e-50	14
RNPS1	Activated Chemokine	3.23957e-54	0.5206468	0.504	0.265	5.77583e-50	14
ARF4	Activated Chemokine	3.969683e-54	0.4933998	0.402	0.176	7.077547e-50	14
TMEM126A	Activated Chemokine	4.079022e-54	0.6151657	0.588	0.33	7.272487e-50	14
CD48	Activated Chemokine	4.210736e-54	0.5210368	0.531	0.289	7.50732e-50	14
ILF3	Activated Chemokine	5.541159e-54	0.4435164	0.239	0.073	9.879333e-50	14
ATAD3A	Activated Chemokine	6.960094e-54	0.5377671	0.425	0.19	1.240915e-49	14
MRPL1	Activated Chemokine	8.092426e-54	-0.6068838	0.759	0.857	1.442799e-49	14
SH3BGRL3	Activated Chemokine	8.74147e-54	0.513939	0.408	0.187	1.558517e-49	14
CCDC124	Activated Chemokine	1.122572e-53	0.4539157	0.303	0.114	2.001433e-49	14
POLR2D	Activated Chemokine	1.257534e-53	0.5277179	0.601	0.348	2.242058e-49	14
KPNB1	Activated Chemokine	1.555678e-53	0.464479	0.281	0.088	2.773619e-49	14
PDCD11	Activated Chemokine	1.938162e-53	0.5072875	0.567	0.318	3.455548e-49	14
PUF60	Activated Chemokine	2.047921e-53	0.5095543	0.421	0.182	3.651238e-49	14
YARS	Activated Chemokine	2.299615e-53	0.3768198	0.159	0.031	4.099984e-49	14
CD3EAP	Activated Chemokine	2.399168e-53	0.4987435	0.438	0.208	4.277477e-49	14
PSMD12	Activated Chemokine	2.861086e-53	-1.225438	0.046	0.21	5.101031e-49	14
RGS13	Activated Chemokine	4.603891e-53	0.4721592	0.378	0.152	8.208277e-49	14
NAA15	Activated Chemokine	4.767286e-53	0.4475043	0.75	0.504	8.499594e-49	14
PSMD8	Activated Chemokine	4.965034e-53	0.4579768	0.268	0.091	8.852159e-49	14

NOC4L	Activated Chemokine					
5.493508e-53	0.4943481	0.639	0.377	9.794376e-49	14	
C19orf70	Activated Chemokine					
6.137802e-53	0.3977596	0.795	0.548	1.094309e-48	14	
SEC61G	Activated Chemokine					
2.381354e-52	0.3567343	0.114	0.017	4.245717e-48	14	
LTA	Activated Chemokine					
4.069348e-52	0.5035575	0.393	0.178	7.25524e-48	14	
TMA16	Activated Chemokine					
4.842035e-52	0.4873329	0.316	0.123	8.632864e-48	14	
TOMM5	Activated Chemokine					
7.598451e-52	0.4727321	0.654	0.396	1.354728e-47	14	
PSMC5	Activated Chemokine					
9.8889e-52	0.4962444	0.339	0.124	1.763092e-47	14	
ZC3H8	Activated Chemokine					
1.316543e-51	-0.8232783	0.269	0.458	2.347264e-47	14	
PTPRC	Activated Chemokine					
2.27304e-51	0.4668964	0.321	0.122	4.052603e-47	14	
GART	Activated Chemokine					
2.935838e-51	0.6095057	0.44	0.212	5.234306e-47	14	
HMGCS1	Activated Chemokine					
4.28951e-51	0.4620408	0.33	0.125	7.647768e-47	14	
TGS1	Activated Chemokine					
4.927778e-51	-0.7136004	0.113	0.289	8.785735e-47	14	
ACAP1	Activated Chemokine					
5.375862e-51	0.4974473	0.428	0.204	9.584624e-47	14	
CHORDC1	Activated Chemokine					
7.046591e-51	0.4848307	0.211	0.058	1.256337e-46	14	
TRIB3	Activated Chemokine					
8.604013e-51	-0.3920435	0.999	0.999	1.534009e-46	14	
RPL34	Activated Chemokine					
1.495193e-50	0.3785946	0.152	0.033	2.66578e-46	14	
SLC9B2	Activated Chemokine					
1.670638e-50	0.4771401	0.432	0.207	2.97858e-46	14	
COPS3	Activated Chemokine					
2.054435e-50	0.5966342	0.335	0.14	3.662853e-46	14	
SERPINB9	Activated Chemokine					
2.42019e-50	0.4704768	0.373	0.155	4.314957e-46	14	
MPHOSPH10		Activated Chemokine				
3.007512e-50	0.4244558	0.824	0.603	5.362093e-46	14	
POLR2L	Activated Chemokine					
3.727762e-50	0.4621577	0.534	0.294	6.646227e-46	14	
GTF2A2	Activated Chemokine					
3.858446e-50	0.4215946	0.254	0.076	6.879223e-46	14	
TIMM8A	Activated Chemokine					
4.428656e-50	0.4227933	0.711	0.472	7.895851e-46	14	
UBE2N	Activated Chemokine					
6.366738e-50	0.4969016	0.377	0.163	1.135126e-45	14	
MARS	Activated Chemokine					
8.223438e-50	0.4700695	0.628	0.376	1.466157e-45	14	

SRSF10 Activated Chemokine
9.318606e-50 -0.6302791 0.051 0.212 1.661414e-45 14
GLRX Activated Chemokine
1.085961e-49 0.4442157 0.483 0.237 1.93616e-45 14
KPNA2 Activated Chemokine
1.380036e-49 0.3470985 0.152 0.036 2.460466e-45 14
SLC16A1 Activated Chemokine
1.633828e-49 -0.8245149 0.239 0.424 2.912953e-45 14
AC114760.2 Activated Chemokine
1.765913e-49 0.3837587 0.171 0.04 3.148446e-45 14
MAK16 Activated Chemokine
1.777069e-49 0.4477797 0.269 0.09 3.168337e-45 14
NOP14 Activated Chemokine
1.961272e-49 -0.8295614 0.181 0.351 3.496751e-45 14
TRAF3IP3 Activated Chemokine
1.974026e-49 0.4104325 0.225 0.074 3.519491e-45 14
BTG3 Activated Chemokine
2.13283e-49 0.4756738 0.21 0.071 3.802623e-45 14
KLF10 Activated Chemokine
3.22146e-49 -0.6790235 0.089 0.258 5.743542e-45 14
TBC1D10C Activated Chemokine
3.408262e-49 -1.262307 0.119 0.267 6.076591e-45 14
S100A4 Activated Chemokine
4.21566e-49 0.5050158 0.439 0.207 7.5161e-45 14
COX20 Activated Chemokine
6.922828e-49 0.4893049 0.456 0.232 1.234271e-44 14
STIP1 Activated Chemokine
8.967039e-49 0.3983529 0.228 0.071 1.598733e-44 14
PPIL1 Activated Chemokine
1.001099e-48 0.4561294 0.651 0.401 1.78486e-44 14
BANF1 Activated Chemokine
1.306414e-48 0.3287691 0.136 0.025 2.329206e-44 14
UCK2 Activated Chemokine
1.91762e-48 0.3413204 0.599 0.305 3.418925e-44 14
HSP90B1 Activated Chemokine
2.063792e-48 0.3764099 0.217 0.058 3.679535e-44 14
SLC25A32 Activated Chemokine
2.112957e-48 0.5006008 0.355 0.153 3.767191e-44 14
TRMT1 Activated Chemokine
2.442613e-48 0.3914613 0.836 0.623 4.354935e-44 14
ATP5F1B Activated Chemokine
2.451885e-48 0.4653056 0.281 0.103 4.371466e-44 14
MAT2A Activated Chemokine
2.878454e-48 0.4611799 0.548 0.298 5.131996e-44 14
KARS Activated Chemokine
3.101929e-48 0.4171576 0.237 0.073 5.530429e-44 14
ALKBH2 Activated Chemokine
3.613508e-48 0.45692 0.721 0.491 6.442523e-44 14
EIF4G2 Activated Chemokine
3.907395e-48 -0.7233121 0.092 0.263 6.966495e-44 14

SYNE2	Activated Chemokine	4.323941e-48	-0.5814503	0.886	0.936	7.709154e-44	14
BTG1	Activated Chemokine	4.369481e-48	-0.8377394	0.228	0.41	7.790348e-44	14
SMIM14	Activated Chemokine	4.966883e-48	0.4231159	0.25	0.082	8.855456e-44	14
UTP14A	Activated Chemokine	8.874018e-48	-0.8117249	0.116	0.271	1.582149e-43	14
HHEX	Activated Chemokine	9.302779e-48	0.4259182	0.711	0.476	1.658593e-43	14
TRA2B	Activated Chemokine	1.011165e-47	-0.7604615	0.228	0.393	1.802807e-43	14
ARL6IP5	Activated Chemokine	1.021102e-47	0.3403449	0.165	0.036	1.820523e-43	14
ATAD3B	Activated Chemokine	1.040042e-47	-0.766252	0.332	0.498	1.85429e-43	14
SP100	Activated Chemokine	1.076876e-47	0.4743452	0.449	0.222	1.919962e-43	14
FUBP1	Activated Chemokine	1.195493e-47	0.4345994	0.315	0.129	2.131444e-43	14
EIF4G1	Activated Chemokine	1.726943e-47	0.4598581	0.623	0.368	3.078967e-43	14
LCP1	Activated Chemokine	1.926146e-47	0.3253659	0.162	0.035	3.434126e-43	14
TTLL12	Activated Chemokine	1.937767e-47	0.4579384	0.531	0.296	3.454845e-43	14
AHSA1	Activated Chemokine	2.058367e-47	0.4494058	0.303	0.116	3.669862e-43	14
TRAP1	Activated Chemokine	2.320284e-47	-0.7243894	0.451	0.63	4.136835e-43	14
HLA-DMB	Activated Chemokine	3.419343e-47	0.4636001	0.383	0.184	6.096346e-43	14
RUVBL2	Activated Chemokine	8.457735e-47	0.4222459	0.583	0.326	1.50793e-42	14
LSM2	Activated Chemokine	8.576724e-47	0.4790678	0.466	0.242	1.529144e-42	14
RPL7L1	Activated Chemokine	1.000959e-46	0.4684043	0.381	0.176	1.784609e-42	14
RNF126	Activated Chemokine	1.054191e-46	0.456323	0.328	0.141	1.879517e-42	14
ADSL	Activated Chemokine	1.095902e-46	-0.797036	0.128	0.282	1.953884e-42	14
ADAM28	Activated Chemokine	1.241359e-46	-0.7641302	0.282	0.452	2.213218e-42	14
PNRC1	Activated Chemokine	1.420874e-46	0.4154538	0.763	0.527	2.533277e-42	14
RBMX	Activated Chemokine	1.538208e-46	-0.7165003	0.061	0.21	2.74247e-42	14
C16orf74	Activated Chemokine	2.022506e-46	0.4456937	0.679	0.444	3.605926e-42	14

SF3B5	Activated Chemokine					
2.111366e-46	-0.729726	0.694	0.766	3.764354e-42	14	
MYL12A	Activated Chemokine					
2.119266e-46	-0.7240109	0.123	0.288	3.778438e-42	14	
GNG7	Activated Chemokine					
2.637611e-46	0.4283718	0.705	0.479	4.702597e-42	14	
GTF3A	Activated Chemokine					
2.902335e-46	0.4238145	0.255	0.096	5.174574e-42	14	
FAM241A	Activated Chemokine					
3.035775e-46	-0.3824851	0.997	0.997	5.412482e-42	14	
RPS15A	Activated Chemokine					
3.378642e-46	0.3917748	0.224	0.067	6.023781e-42	14	
NOL10	Activated Chemokine					
4.872158e-46	0.4171466	0.263	0.092	8.686571e-42	14	
TBL3	Activated Chemokine					
5.603027e-46	-0.5679466	0.721	0.817	9.989637e-42	14	
HLA-DPA1	Activated Chemokine					
6.442832e-46	0.4354673	0.317	0.128	1.148693e-41	14	
IDH3A	Activated Chemokine					
6.478008e-46	0.4502431	0.471	0.235	1.154964e-41	14	
CCDC59	Activated Chemokine					
8.048641e-46	0.5017585	0.618	0.385	1.434992e-41	14	
ARPC5L	Activated Chemokine					
1.000512e-45	0.4375436	0.344	0.152	1.783813e-41	14	
DNAJB11	Activated Chemokine					
1.015364e-45	-0.8740324	0.152	0.306	1.810293e-41	14	
ZFP36L2	Activated Chemokine					
1.250228e-45	0.4527749	0.381	0.167	2.229031e-41	14	
SBDS	Activated Chemokine					
1.315387e-45	0.4523781	0.387	0.184	2.345204e-41	14	
TSTA3	Activated Chemokine					
1.89017e-45	0.4207632	0.465	0.244	3.369985e-41	14	
MRPL13	Activated Chemokine					
2.202043e-45	0.4479633	0.362	0.157	3.926022e-41	14	
PPP1R14B	Activated Chemokine					
2.63647e-45	0.3598431	0.802	0.571	4.700563e-41	14	
TUBB	Activated Chemokine					
3.008223e-45	0.3996905	0.206	0.052	5.36336e-41	14	
GNPDA1	Activated Chemokine					
3.022349e-45	0.4296039	0.632	0.394	5.388546e-41	14	
ENY2	Activated Chemokine					
3.030742e-45	0.4424885	0.342	0.157	5.40351e-41	14	
EI24	Activated Chemokine					
3.036883e-45	0.4349168	0.578	0.338	5.414458e-41	14	
MRPS18C	Activated Chemokine					
3.143965e-45	0.442005	0.489	0.262	5.605376e-41	14	
PSMD3	Activated Chemokine					
3.400114e-45	0.4805653	0.72	0.504	6.062063e-41	14	
TALDO1	Activated Chemokine					
3.615157e-45	0.4564731	0.362	0.162	6.445463e-41	14	

PARL	Activated Chemokine	4.054335e-45	0.4732606	0.426	0.218	7.228474e-41	14
HSPH1	Activated Chemokine	5.248325e-45	0.3476536	0.806	0.579	9.357238e-41	14
ATP5MC3	Activated Chemokine	7.386753e-45	-0.7462075	0.682	0.764	1.316984e-40	14
CYBA	Activated Chemokine	7.493383e-45	-0.6056034	0.03	0.164	1.335995e-40	14
SUSD3	Activated Chemokine	9.116775e-45	0.4293791	0.666	0.435	1.62543e-40	14
AURKAIP1	Activated Chemokine	1.673449e-44	0.4391873	0.67	0.44	2.983592e-40	14
PGAM1	Activated Chemokine	2.478959e-44	0.4155701	0.268	0.091	4.419737e-40	14
SURF6	Activated Chemokine	3.178253e-44	0.4460295	0.528	0.296	5.666507e-40	14
POLR2I	Activated Chemokine	4.102617e-44	0.446482	0.439	0.219	7.314556e-40	14
RHEB	Activated Chemokine	4.109769e-44	0.3010789	0.15	0.029	7.327307e-40	14
RRP12	Activated Chemokine	4.191967e-44	0.4460426	0.383	0.18	7.473859e-40	14
USP14	Activated Chemokine	5.367497e-44	0.4926451	0.46	0.243	9.56971e-40	14
EXOSC8	Activated Chemokine	8.860175e-44	0.4345166	0.334	0.137	1.579681e-39	14
ARMC10	Activated Chemokine	9.670887e-44	0.4825106	0.238	0.082	1.724222e-39	14
LINC01480	Activated Chemokine	1.100692e-43	0.4610695	0.559	0.326	1.962423e-39	14
PDAP1	Activated Chemokine	1.757874e-43	0.4399472	0.61	0.38	3.134113e-39	14
SNRPC	Activated Chemokine	1.823504e-43	-0.7335971	0.139	0.293	3.251125e-39	14
DCK	Activated Chemokine	1.835166e-43	0.3888478	0.268	0.093	3.271918e-39	14
GFM1	Activated Chemokine	2.266837e-43	0.3399632	0.859	0.652	4.041544e-39	14
SLC25A5	Activated Chemokine	2.507909e-43	0.350972	0.83	0.612	4.471351e-39	14
NDUFA13	Activated Chemokine	2.933336e-43	0.4069596	0.28	0.107	5.229845e-39	14
PRMT5	Activated Chemokine	4.287874e-43	0.4698721	0.499	0.282	7.644851e-39	14
PFDN6	Activated Chemokine	5.364736e-43	0.4700129	0.43	0.226	9.564787e-39	14
UFM1	Activated Chemokine	6.256885e-43	0.4334749	0.338	0.148	1.11554e-38	14
DDX56	Activated Chemokine	8.23626e-43	0.4545903	0.372	0.165	1.468443e-38	14

	RABGGTB	Activated Chemokine			
9.617252e-43	0.4296329	0.359	0.149	1.71466e-38	14
	CFAP97	Activated Chemokine			
1.343956e-42	0.4021938	0.312	0.13	2.396138e-38	14
	EXOSC7	Activated Chemokine			
1.38689e-42	-0.7099141	0.091	0.241	2.472687e-38	14
	GCHFR	Activated Chemokine			
1.548027e-42	0.3423122	0.836	0.636	2.759976e-38	14
	ATP5MF	Activated Chemokine			
1.660114e-42	0.4488098	0.49	0.268	2.959818e-38	14
	NAA10	Activated Chemokine			
2.140177e-42	0.4218176	0.322	0.139	3.815722e-38	14
	MPHOSPH6	Activated Chemokine			
2.298914e-42	-0.7351049	0.308	0.471	4.098734e-38	14
	ITM2B	Activated Chemokine			
4.007919e-42	0.4144043	0.38	0.177	7.145719e-38	14
	DHX15	Activated Chemokine			
4.994545e-42	0.3030922	0.137	0.027	8.904774e-38	14
	TWNK	Activated Chemokine			
5.004425e-42	0.3760221	0.12	0.024	8.922388e-38	14
	EGR1	Activated Chemokine			
5.106851e-42	0.3791074	0.228	0.075	9.105006e-38	14
	FASTKD2	Activated Chemokine			
5.978175e-42	0.3865242	0.185	0.055	1.065849e-37	14
	BCAT1	Activated Chemokine			
6.006387e-42	0.3217383	0.917	0.773	1.070879e-37	14
	NAP1L1	Activated Chemokine			
6.081985e-42	-0.6456267	0.112	0.262	1.084357e-37	14
	CNN2	Activated Chemokine			
6.572462e-42	0.4450618	0.495	0.273	1.171804e-37	14
	PHPT1	Activated Chemokine			
6.941681e-42	0.4072423	0.76	0.527	1.237632e-37	14
	IMPDH2	Activated Chemokine			
8.07896e-42	-0.6898534	0.053	0.177	1.440398e-37	14
	MARCH1	Activated Chemokine			
8.092663e-42	0.4289627	0.484	0.246	1.442841e-37	14
	NFE2L2	Activated Chemokine			
9.688014e-42	0.4432662	0.529	0.302	1.727276e-37	14
	HAX1	Activated Chemokine			
1.025923e-41	0.4396674	0.304	0.127	1.829118e-37	14
	GOT1	Activated Chemokine			
1.200058e-41	0.4252541	0.28	0.112	2.139584e-37	14
	TXNRD1	Activated Chemokine			
1.403415e-41	0.4136818	0.307	0.127	2.502149e-37	14
	CCDC47	Activated Chemokine			
1.783043e-41	0.3902368	0.172	0.06	3.178987e-37	14
	NINJ1	Activated Chemokine			
2.189485e-41	0.3399873	0.152	0.036	3.903633e-37	14
	DUSP5	Activated Chemokine			
2.56536e-41	-0.7118089	0.137	0.302	4.57378e-37	14

TCF4	Activated Chemokine					
3.072109e-41	0.4279873	0.47	0.248	5.477263e-37	14	
DDX27	Activated Chemokine					
3.092601e-41	0.4049185	0.459	0.235	5.513799e-37	14	
MYDGF	Activated Chemokine					
3.503155e-41	0.3150925	0.143	0.034	6.245775e-37	14	
PPAT	Activated Chemokine					
3.623309e-41	0.3726613	0.733	0.501	6.459997e-37	14	
SRRM1	Activated Chemokine					
3.650001e-41	0.3802884	0.262	0.1	6.507587e-37	14	
MRPL17	Activated Chemokine					
4.038517e-41	0.4747775	0.632	0.377	7.200272e-37	14	
CD44	Activated Chemokine					
4.383467e-41	0.3669135	0.234	0.075	7.815283e-37	14	
NAT10	Activated Chemokine					
4.732902e-41	0.4262054	0.484	0.258	8.438292e-37	14	
MRPL11	Activated Chemokine					
5.199027e-41	-0.422351	0.008	0.114	9.269345e-37	14	
CPNE5	Activated Chemokine					
5.519239e-41	0.3222478	0.885	0.696	9.840251e-37	14	
ELOB	Activated Chemokine					
6.867643e-41	0.4042061	0.301	0.121	1.224432e-36	14	
ALG13	Activated Chemokine					
8.568017e-41	0.4988212	0.412	0.206	1.527592e-36	14	
C19orf48	Activated Chemokine					
9.511537e-41	0.3768611	0.217	0.067	1.695812e-36	14	
FAM210A	Activated Chemokine					
1.024347e-40	0.4362244	0.727	0.529	1.826309e-36	14	
SOD1	Activated Chemokine					
1.156119e-40	0.3623997	0.204	0.062	2.061245e-36	14	
PINX1.1	Activated Chemokine					
1.192183e-40	0.3082996	0.922	0.803	2.125544e-36	14	
CALM2	Activated Chemokine					
1.207582e-40	0.3711151	0.676	0.443	2.152998e-36	14	
SEM1	Activated Chemokine					
1.216475e-40	0.4454783	0.46	0.241	2.168852e-36	14	
PABPC4	Activated Chemokine					
1.279025e-40	0.3584435	0.833	0.639	2.280374e-36	14	
TRMT112	Activated Chemokine					
1.735404e-40	0.4253035	0.445	0.235	3.094051e-36	14	
SRSF1	Activated Chemokine					
1.793497e-40	0.3475179	0.182	0.053	3.197625e-36	14	
RABEPK	Activated Chemokine					
2.039872e-40	-0.6254651	0.079	0.218	3.636888e-36	14	
YPEL3	Activated Chemokine					
2.084713e-40	0.4417159	0.402	0.208	3.716835e-36	14	
SLC25A39	Activated Chemokine					
2.137717e-40	0.3101645	0.161	0.043	3.811335e-36	14	
PELO	Activated Chemokine					
2.632597e-40	0.4854142	0.4	0.196	4.693657e-36	14	

SRSF6 Activated Chemokine
2.703577e-40 0.4107855 0.482 0.267 4.820207e-36 14
POLR2F Activated Chemokine
2.938122e-40 0.4372305 0.709 0.472 5.238378e-36 14
SQSTM1 Activated Chemokine
3.654368e-40 0.4377081 0.442 0.234 6.515372e-36 14
MRPL15 Activated Chemokine
3.947733e-40 0.4280966 0.3 0.126 7.038413e-36 14
UBE2G2 Activated Chemokine
4.159465e-40 0.4106277 0.428 0.215 7.415911e-36 14
SF3A3 Activated Chemokine
5.393293e-40 0.4407065 0.25 0.094 9.615702e-36 14
PLGRKT Activated Chemokine
6.258724e-40 -0.7514271 0.251 0.401 1.115868e-35 14
ORAI2 Activated Chemokine
6.769408e-40 0.460644 0.552 0.322 1.206918e-35 14
PTPN6 Activated Chemokine
8.90491e-40 0.4439745 0.37 0.181 1.587656e-35 14
MFNG Activated Chemokine
9.413626e-40 0.3113683 0.872 0.673 1.678355e-35 14
HNRNPA3 Activated Chemokine
1.094275e-39 0.3762487 0.249 0.095 1.950982e-35 14
DDX49 Activated Chemokine
1.159475e-39 -0.7360254 0.154 0.292 2.067228e-35 14
BCAS4 Activated Chemokine
1.390805e-39 0.4163238 0.386 0.189 2.479666e-35 14
MRPL32 Activated Chemokine
1.469114e-39 0.3928558 0.619 0.396 2.619283e-35 14
UQCRFS1 Activated Chemokine
1.562032e-39 -0.4863727 0.027 0.143 2.784947e-35 14
AIM2 Activated Chemokine
2.033859e-39 -0.3836578 0.999 0.998 3.626168e-35 14
B2M Activated Chemokine
2.470559e-39 0.4105825 0.472 0.249 4.404759e-35 14
M6PR Activated Chemokine
2.770769e-39 0.4512129 0.439 0.248 4.940004e-35 14
RGS10 Activated Chemokine
3.208587e-39 0.3670196 0.236 0.084 5.720589e-35 14
RIOK2 Activated Chemokine
3.731806e-39 0.4038055 0.306 0.137 6.653437e-35 14
CISD1 Activated Chemokine
3.752533e-39 0.3660449 0.774 0.573 6.690391e-35 14
RBM39 Activated Chemokine
3.940202e-39 0.4022982 0.32 0.141 7.024985e-35 14
TYW3 Activated Chemokine
4.259997e-39 0.4350746 0.418 0.212 7.595148e-35 14
HPRT1 Activated Chemokine
4.469636e-39 0.3730407 0.195 0.068 7.968914e-35 14
PPIF Activated Chemokine
5.092702e-39 0.3995914 0.356 0.159 9.079779e-35 14

NOL11	Activated Chemokine	1.459803e-38	0.3211804	0.179	0.05	2.602682e-34	14
PMM2	Activated Chemokine	1.591784e-38	0.373386	0.594	0.361	2.837992e-34	14
PSMA5	Activated Chemokine	1.819491e-38	0.3923696	0.457	0.242	3.24397e-34	14
SNRPA1	Activated Chemokine	1.847413e-38	0.4157332	0.504	0.292	3.293753e-34	14
C1orf43	Activated Chemokine	1.935223e-38	0.3682115	0.236	0.092	3.450309e-34	14
PSMG1	Activated Chemokine	1.946215e-38	0.410702	0.724	0.496	3.469907e-34	14
FOXP1	Activated Chemokine	2.039168e-38	-0.6584704	0.216	0.37	3.635632e-34	14
CXXC5	Activated Chemokine	2.50941e-38	-0.9537328	0.227	0.375	4.474026e-34	14
SELL	Activated Chemokine	3.065846e-38	-0.6863903	0.152	0.297	5.466097e-34	14
BCL11A	Activated Chemokine	3.568749e-38	0.4014721	0.367	0.168	6.362723e-34	14
GTPBP6	Activated Chemokine	3.884623e-38	0.3861412	0.646	0.41	6.925894e-34	14
PRRC2C	Activated Chemokine	3.92513e-38	0.3946288	0.497	0.285	6.998115e-34	14
CDC123	Activated Chemokine	4.353423e-38	-0.6944458	0.155	0.301	7.761718e-34	14
GGA2	Activated Chemokine	5.025562e-38	0.5007105	0.634	0.424	8.960074e-34	14
IRF8	Activated Chemokine	5.066812e-38	0.4080426	0.417	0.216	9.03362e-34	14
MRPL50	Activated Chemokine	5.691839e-38	0.3499074	0.18	0.053	1.014798e-33	14
WDR36	Activated Chemokine	7.81772e-38	0.3823027	0.522	0.302	1.393821e-33	14
CWC15	Activated Chemokine	8.811235e-38	0.3221347	0.15	0.041	1.570955e-33	14
IRF4	Activated Chemokine	8.890746e-38	0.3926874	0.664	0.437	1.585131e-33	14
CD164	Activated Chemokine	1.027152e-37	0.3293076	0.174	0.05	1.831309e-33	14
EEF2KMT	Activated Chemokine	1.059715e-37	0.4056292	0.414	0.215	1.889367e-33	14
NUTF2	Activated Chemokine	1.101219e-37	0.4477907	0.217	0.074	1.963363e-33	14
SLAMF7	Activated Chemokine	1.583655e-37	0.3943396	0.503	0.301	2.823499e-33	14
TIMM8B	Activated Chemokine	1.665139e-37	0.399575	0.639	0.42	2.968777e-33	14
CD40	Activated Chemokine	1.721711e-37	0.3960624	0.424	0.22	3.069638e-33	14

C19orf24 Activated Chemokine
1.794289e-37 0.4056323 0.374 0.184 3.199037e-33 14
METTL5 Activated Chemokine
2.078781e-37 -0.6904276 0.585 0.672 3.706258e-33 14
SNX3 Activated Chemokine
2.1408e-37 0.3910167 0.276 0.093 3.816832e-33 14
PARVB Activated Chemokine
2.168256e-37 -0.4500935 0.017 0.122 3.865784e-33 14
PYCARD Activated Chemokine
2.269582e-37 -0.6355603 0.07 0.194 4.046437e-33 14
S1PR4 Activated Chemokine
2.321522e-37 0.3489263 0.216 0.081 4.139042e-33 14
TIMM23 Activated Chemokine
2.834641e-37 0.3503131 0.219 0.079 5.053882e-33 14
ARMC6 Activated Chemokine
2.848354e-37 0.4133764 0.321 0.148 5.07833e-33 14
BCCIP Activated Chemokine
3.682734e-37 0.418441 0.292 0.125 6.565947e-33 14
CEBPG Activated Chemokine
3.857471e-37 0.4079286 0.572 0.354 6.877484e-33 14
BSG Activated Chemokine
4.80511e-37 0.4110664 0.472 0.268 8.567031e-33 14
OLA1 Activated Chemokine
5.251534e-37 0.3553035 0.736 0.511 9.362959e-33 14
EIF3G Activated Chemokine
5.372853e-37 0.376213 0.27 0.11 9.579259e-33 14
DPH3 Activated Chemokine
5.791565e-37 -0.5593101 0.055 0.19 1.032578e-32 14
HLA-DOB Activated Chemokine
6.401766e-37 0.3770726 0.616 0.397 1.141371e-32 14
MDH2 Activated Chemokine
6.988286e-37 0.429555 0.35 0.169 1.245941e-32 14
PRELID3B Activated Chemokine
8.060938e-37 0.3605182 0.221 0.086 1.437185e-32 14
MTFP1 Activated Chemokine
9.75829e-37 -0.6092998 0.094 0.233 1.739806e-32 14
ATM Activated Chemokine
1.269503e-36 0.405624 0.536 0.327 2.263397e-32 14
PSMC6 Activated Chemokine
1.370664e-36 0.3530952 0.246 0.094 2.443758e-32 14
UMPS Activated Chemokine
1.417735e-36 0.3883581 0.445 0.232 2.52768e-32 14
NELFE Activated Chemokine
1.433121e-36 0.3425619 0.763 0.551 2.555112e-32 14
PGK1 Activated Chemokine
1.718127e-36 0.4748471 0.448 0.246 3.063249e-32 14
SDCBP Activated Chemokine
1.726419e-36 0.3822515 0.312 0.141 3.078033e-32 14
MRPL39 Activated Chemokine
1.855651e-36 -0.5725972 0.025 0.137 3.30844e-32 14

NEIL1 Activated Chemokine
2.208278e-36 0.4078951 0.406 0.21 3.937138e-32 14
TMEM70 Activated Chemokine
2.490291e-36 0.3848012 0.427 0.217 4.439939e-32 14
RNMT Activated Chemokine
2.525198e-36 0.3807887 0.275 0.114 4.502176e-32 14
TMX2 Activated Chemokine
3.357734e-36 0.3364977 0.194 0.061 5.986505e-32 14
WDR75 Activated Chemokine
3.428409e-36 0.3338602 0.193 0.061 6.11251e-32 14
METTL2B Activated Chemokine
3.961268e-36 -0.3359525 0.998 0.997 7.062545e-32 14
RPS4X Activated Chemokine
6.293401e-36 0.3493674 0.243 0.094 1.12205e-31 14
SEH1L Activated Chemokine
6.527118e-36 0.4142591 0.438 0.237 1.16372e-31 14
PTBP1 Activated Chemokine
6.562761e-36 0.3890317 0.353 0.162 1.170075e-31 14
PITPNB Activated Chemokine
6.823278e-36 -0.6929253 0.261 0.406 1.216522e-31 14
MBD4 Activated Chemokine
7.252387e-36 -0.6421182 0.137 0.277 1.293028e-31 14
RSRP1 Activated Chemokine
7.485591e-36 0.361982 0.239 0.09 1.334606e-31 14
NSUN2 Activated Chemokine
7.617873e-36 -0.4832673 0.03 0.142 1.358191e-31 14
SOCS1 Activated Chemokine
8.429901e-36 0.4178281 0.475 0.27 1.502967e-31 14
AUP1 Activated Chemokine
8.55352e-36 0.4115499 0.27 0.108 1.525007e-31 14
NUDT4 Activated Chemokine
1.343618e-35 -0.4857636 0.045 0.162 2.395537e-31 14
IL10RA Activated Chemokine
1.388324e-35 -0.5913618 0.773 0.817 2.475242e-31 14
NOP53 Activated Chemokine
1.739991e-35 -0.4542624 0.031 0.15 3.10223e-31 14
SH2B2 Activated Chemokine
1.80525e-35 0.3266977 0.203 0.067 3.218579e-31 14
EXOSC2 Activated Chemokine
2.374781e-35 -0.3824169 0.017 0.119 4.233997e-31 14
ABI3 Activated Chemokine
2.434726e-35 0.3073778 0.204 0.065 4.340872e-31 14
GTF2H2 Activated Chemokine
2.613248e-35 0.3253742 0.212 0.075 4.65916e-31 14
DOHH Activated Chemokine
2.738709e-35 -0.6341081 0.126 0.24 4.882844e-31 14
CAPG Activated Chemokine
2.740623e-35 -0.5257894 0.904 0.902 4.886257e-31 14
MYL6 Activated Chemokine
3.339249e-35 0.3292866 0.175 0.049 5.953547e-31 14

CHST11 Activated Chemokine
3.796519e-35 0.3422737 0.728 0.518 6.768814e-31 14
FKBP1A Activated Chemokine
4.523155e-35 0.3689135 0.473 0.266 8.064334e-31 14
MRPL54 Activated Chemokine
4.661121e-35 0.3896962 0.374 0.186 8.310312e-31 14
FAM136A Activated Chemokine
5.804913e-35 0.371504 0.314 0.142 1.034958e-30 14 ME2
Activated Chemokine
6.706863e-35 0.3675172 0.295 0.124 1.195767e-30 14
MTREX Activated Chemokine
6.896905e-35 0.3950126 0.323 0.146 1.229649e-30 14
ADSS Activated Chemokine
7.101789e-35 0.4035644 0.335 0.159 1.266178e-30 14
MRPL24 Activated Chemokine
7.379854e-35 0.3163393 0.673 0.451 1.315754e-30 14
COX17 Activated Chemokine
8.015409e-35 0.3287057 0.179 0.054 1.429067e-30 14
LINC00665 Activated Chemokine
9.385391e-35 0.3937769 0.261 0.11 1.673321e-30 14
NIN Activated Chemokine
1.10922e-34 0.3561569 0.583 0.367 1.977629e-30 14
OSTC Activated Chemokine
1.147398e-34 0.3796161 0.356 0.176 2.045695e-30 14
SRA1 Activated Chemokine
1.222088e-34 0.3949929 0.528 0.327 2.178861e-30 14
ACP1 Activated Chemokine
1.686573e-34 0.3772912 0.265 0.096 3.006991e-30 14
PRNP Activated Chemokine
2.202603e-34 -0.724022 0.74 0.787 3.927021e-30 14
ACTG1 Activated Chemokine
2.206105e-34 0.4023544 0.485 0.283 3.933265e-30 14
U2SURP Activated Chemokine
2.697908e-34 -0.6934231 0.305 0.443 4.810101e-30 14
RHOH Activated Chemokine
3.091094e-34 0.3350099 0.224 0.082 5.511112e-30 14
ZMPSTE24 Activated Chemokine
3.580279e-34 0.3278904 0.197 0.066 6.38328e-30 14
DUS3L Activated Chemokine
3.622374e-34 0.3603634 0.522 0.315 6.45833e-30 14
PRPF40A Activated Chemokine
3.756931e-34 -0.6680773 0.372 0.495 6.698233e-30 14
ANKRD12 Activated Chemokine
3.81446e-34 0.3029471 0.784 0.598 6.800801e-30 14
LSM7 Activated Chemokine
3.900097e-34 0.3929985 0.53 0.326 6.953483e-30 14
PSMD2 Activated Chemokine
3.917489e-34 0.3596631 0.657 0.446 6.984491e-30 14
VCP Activated Chemokine
3.976624e-34 0.3845462 0.615 0.392 7.089923e-30 14

ATF4	Activated Chemokine	4.46207e-34	0.3595947	0.231	0.093	7.955425e-30	14
ALG3	Activated Chemokine	4.856491e-34	-0.6872819	0.182	0.327	8.658639e-30	14
AES	Activated Chemokine	4.85774e-34	0.3726593	0.402	0.208	8.660864e-30	14
TMX1	Activated Chemokine	4.859794e-34	0.3133344	0.775	0.579	8.664527e-30	14
SAP18	Activated Chemokine	5.618392e-34	0.36561	0.343	0.168	1.001703e-29	14
EMC7	Activated Chemokine	5.969114e-34	0.3835416	0.359	0.179	1.064233e-29	14
YDJC	Activated Chemokine	6.136846e-34	0.4055878	0.393	0.216	1.094138e-29	14
TAF9	Activated Chemokine	6.680478e-34	0.4375223	0.376	0.186	1.191062e-29	14
RNF145	Activated Chemokine	6.752769e-34	-0.8294262	0.397	0.5	1.203951e-29	14
SAT1	Activated Chemokine	7.722e-34	0.3965079	0.535	0.327	1.376755e-29	14
EIF3A	Activated Chemokine	7.766404e-34	0.3678977	0.421	0.225	1.384672e-29	14
BTF3L4	Activated Chemokine	7.968851e-34	0.406888	0.3	0.135	1.420766e-29	14
STX4	Activated Chemokine	8.140198e-34	0.3698694	0.506	0.304	1.451316e-29	14
PSMD6	Activated Chemokine	1.127379e-33	0.3375398	0.699	0.485	2.010004e-29	14
MRPL52	Activated Chemokine	1.153417e-33	0.3690828	0.338	0.164	2.056427e-29	14
EXOSC3	Activated Chemokine	1.354774e-33	0.3812927	0.329	0.148	2.415426e-29	14
PEA15	Activated Chemokine	1.43334e-33	0.3383947	0.626	0.403	2.555502e-29	14
TMED9	Activated Chemokine	2.50464e-33	0.3548854	0.339	0.165	4.465522e-29	14
RPL26L1	Activated Chemokine	2.688471e-33	0.3660412	0.336	0.154	4.793275e-29	14
ASNSD1	Activated Chemokine	2.793131e-33	-0.6442414	0.077	0.193	4.979873e-29	14
DAAM1	Activated Chemokine	2.921409e-33	0.4157793	0.482	0.276	5.20858e-29	14
MSMO1	Activated Chemokine	3.246235e-33	0.3726948	0.292	0.138	5.787712e-29	14
ATG101	Activated Chemokine	3.500978e-33	0.3773987	0.328	0.166	6.241894e-29	14
POLDIP2	Activated Chemokine	3.949538e-33	0.3121751	0.704	0.486	7.041632e-29	14
SF3B6	Activated Chemokine	4.083237e-33	-0.5975177	0.136	0.266	7.280003e-29	14

DAPP1	Activated Chemokine	4.268649e-33	0.3705888	0.282	0.125	7.610574e-29	14
NOL8	Activated Chemokine	5.122066e-33	0.3737059	0.375	0.197	9.132132e-29	14
NUBP1	Activated Chemokine	6.289129e-33	0.417721	0.3	0.129	1.121289e-28	14
NFKB2	Activated Chemokine	6.571588e-33	0.3070619	0.201	0.069	1.171648e-28	14
TFB2M	Activated Chemokine	6.582403e-33	0.3592255	0.273	0.119	1.173577e-28	14
NUP62	Activated Chemokine	6.777913e-33	0.3146403	0.806	0.622	1.208434e-28	14
SELENOH	Activated Chemokine	6.922854e-33	-0.6152783	0.164	0.289	1.234276e-28	14
PLEKHF2	Activated Chemokine	7.610842e-33	0.3690665	0.26	0.107	1.356937e-28	14
C20orf27	Activated Chemokine	8.179513e-33	0.376641	0.441	0.257	1.458325e-28	14
PSMC2	Activated Chemokine	8.64113e-33	-0.5486269	0.016	0.111	1.540627e-28	14
VPREB3	Activated Chemokine	8.693593e-33	0.3471492	0.392	0.202	1.549981e-28	14
TRAPPC4	Activated Chemokine	1.079576e-32	0.34213	0.244	0.099	1.924775e-28	14
C12orf45	Activated Chemokine	1.330835e-32	0.3792194	0.644	0.439	2.372747e-28	14
ATP5MC1	Activated Chemokine	1.42382e-32	0.3632556	0.352	0.178	2.538529e-28	14
E2F4	Activated Chemokine	1.507418e-32	-0.6348656	0.374	0.505	2.687575e-28	14
PSMB9	Activated Chemokine	2.044369e-32	0.3182249	0.726	0.518	3.644905e-28	14
SELENOT	Activated Chemokine	2.604867e-32	-0.6768168	0.335	0.448	4.644217e-28	14
KLF6	Activated Chemokine	3.564137e-32	-0.6730246	0.398	0.515	6.3545e-28	14
BANK1	Activated Chemokine	3.881063e-32	0.3657531	0.359	0.179	6.919547e-28	14
GNL1	Activated Chemokine	4.038288e-32	-0.6166877	0.172	0.3	7.199863e-28	14
PDLIM1	Activated Chemokine	5.080694e-32	0.3519977	0.272	0.121	9.058369e-28	14
NUP93	Activated Chemokine	5.445208e-32	0.3213673	0.687	0.47	9.708262e-28	14
VAPA	Activated Chemokine	5.815247e-32	-0.7123423	0.338	0.44	1.0368e-27	14
COTL1	Activated Chemokine	7.328789e-32	0.3522538	0.453	0.252	1.30665e-27	14
MRPS26	Activated Chemokine	8.089448e-32	0.3229209	0.222	0.08	1.442268e-27	14

RBM28	Activated Chemokine	9.532429e-32	0.3192308	0.633	0.412	1.699537e-27	14
RAB7A	Activated Chemokine	9.554323e-32	0.360773	0.29	0.123	1.70344e-27	14
IFRD1	Activated Chemokine	1.17314e-31	0.3617401	0.272	0.109	2.091591e-27	14
NOC3L	Activated Chemokine	1.223213e-31	0.384298	0.589	0.387	2.180866e-27	14
SSB	Activated Chemokine	1.243282e-31	-0.7316954	0.673	0.726	2.216648e-27	14
LIMD2	Activated Chemokine	1.268087e-31	0.3508448	0.555	0.34	2.260872e-27	14
RNH1	Activated Chemokine	1.467007e-31	-0.7624319	0.102	0.214	2.615527e-27	14
PLAC8	Activated Chemokine	1.497406e-31	0.3527351	0.361	0.183	2.669725e-27	14
REXO2	Activated Chemokine	1.59269e-31	0.4141158	0.406	0.225	2.839607e-27	14
CCDC85B	Activated Chemokine	1.874762e-31	-0.972061	0.102	0.208	3.342514e-27	14
LGALS1	Activated Chemokine	2.09692e-31	-0.5408733	0.061	0.189	3.738599e-27	14
P2RX5	Activated Chemokine	2.984225e-31	0.3448121	0.553	0.348	5.320575e-27	14
PSMD13	Activated Chemokine	2.991817e-31	0.3482882	0.328	0.158	5.334111e-27	14
WDR77	Activated Chemokine	3.232766e-31	0.3349319	0.659	0.451	5.763698e-27	14
PSMD4	Activated Chemokine	3.423097e-31	0.3048791	0.167	0.052	6.103039e-27	14
MAPK13	Activated Chemokine	3.644511e-31	0.3940721	0.445	0.263	6.497798e-27	14
PPP1R2	Activated Chemokine	3.97452e-31	0.3533955	0.484	0.292	7.086172e-27	14
MRPL57	Activated Chemokine	4.188431e-31	0.3377792	0.283	0.139	7.467553e-27	14
WDR18	Activated Chemokine	4.595259e-31	0.3811928	0.364	0.166	8.192888e-27	14
BCL2	Activated Chemokine	4.957055e-31	-0.7152803	0.174	0.306	8.837933e-27	14
BCL7A	Activated Chemokine	5.498209e-31	0.3813063	0.416	0.235	9.802756e-27	14
DDX1	Activated Chemokine	6.101878e-31	0.3412169	0.579	0.374	1.087904e-26	14
VDAC2	Activated Chemokine	8.669636e-31	-0.5912782	0.617	0.705	1.545709e-26	14
COMMD6	Activated Chemokine	9.518159e-31	0.3759393	0.408	0.221	1.696993e-26	14
RPF1	Activated Chemokine	9.722105e-31	-0.5262861	0.101	0.22	1.733354e-26	14

PNKD	Activated Chemokine	1.022947e-30	-0.6828467	0.375	0.487	1.823812e-26	14
NEAT1	Activated Chemokine	1.389808e-30	-0.6135238	0.163	0.287	2.477888e-26	14
CARHSP1	Activated Chemokine	1.555511e-30	0.3390332	0.566	0.342	2.77332e-26	14
LITAF	Activated Chemokine	1.635016e-30	-0.6345391	0.187	0.324	2.915069e-26	14
TNFRSF13C	Activated Chemokine	2.463236e-30	0.3452739	0.324	0.168	4.391704e-26	14
CBWD1	Activated Chemokine	2.823442e-30	-0.4203701	0.017	0.106	5.033914e-26	14
RMI2	Activated Chemokine	2.937375e-30	-0.6428868	0.172	0.288	5.237046e-26	14
SP110	Activated Chemokine	3.737895e-30	0.3434426	0.395	0.203	6.664292e-26	14
PITHD1	Activated Chemokine	3.930706e-30	0.3164057	0.232	0.099	7.008056e-26	14
BOLA3	Activated Chemokine	4.920952e-30	0.3036362	0.417	0.221	8.773565e-26	14
KDELR2	Activated Chemokine	5.218598e-30	0.3617388	0.412	0.236	9.304238e-26	14
MORF4L2	Activated Chemokine	5.231123e-30	0.3922362	0.382	0.204	9.326569e-26	14
LARS	Activated Chemokine	5.680197e-30	0.3127012	0.214	0.081	1.012722e-25	14
MAPKAPK5	Activated Chemokine	6.171655e-30	-0.6343175	0.191	0.316	1.100344e-25	14
PSIP1	Activated Chemokine	8.168014e-30	-0.4928167	0.994	0.98	1.456275e-25	14
MT-CO1	Activated Chemokine	8.637638e-30	-0.4633582	0.026	0.121	1.540004e-25	14
CD38	Activated Chemokine	1.031905e-29	-0.5837968	0.141	0.27	1.839783e-25	14
ALOX5AP	Activated Chemokine	1.090923e-29	0.3627032	0.425	0.236	1.945007e-25	14
SLC38A1	Activated Chemokine	1.465877e-29	0.3106893	0.653	0.44	2.613512e-25	14
YWHAQ	Activated Chemokine	1.562431e-29	-0.5830364	0.126	0.234	2.785658e-25	14
HSD17B11	Activated Chemokine	1.861763e-29	0.3373471	0.174	0.06	3.319336e-25	14
ZC3H12A	Activated Chemokine	2.754453e-29	0.3247315	0.578	0.375	4.910915e-25	14
RBM25	Activated Chemokine	2.951747e-29	0.327158	0.273	0.123	5.26267e-25	14
BMS1	Activated Chemokine	2.972018e-29	0.3424273	0.289	0.133	5.29881e-25	14
QTRT1	Activated Chemokine	3.658596e-29	0.374249	0.401	0.224	6.522911e-25	14

SINHCAF Activated Chemokine
3.984191e-29 0.3106594 0.206 0.082 7.103414e-25 14
TRNT1 Activated Chemokine
4.129395e-29 -0.5175765 0.135 0.262 7.362299e-25 14
ILK Activated Chemokine
4.23129e-29 0.3089246 0.536 0.332 7.543967e-25 14
PPM1G Activated Chemokine
7.365094e-29 0.3541512 0.272 0.118 1.313123e-24 14
IARS Activated Chemokine
9.398175e-29 0.3273665 0.26 0.11 1.675601e-24 14
SPOUT1 Activated Chemokine
9.775794e-29 0.3169233 0.228 0.093 1.742926e-24 14
PRPF4 Activated Chemokine
9.78464e-29 0.349681 0.462 0.27 1.744503e-24 14 SRI
Activated Chemokine
1.013259e-28 -0.3947167 0.027 0.123 1.806539e-24 14
VNN2 Activated Chemokine
1.076455e-28 0.3101298 0.219 0.078 1.919212e-24 14
TMEM192 Activated Chemokine
1.091084e-28 -0.5088979 0.06 0.176 1.945294e-24 14
TMEM156 Activated Chemokine
1.144463e-28 0.3241197 0.441 0.266 2.040463e-24 14
TOP1 Activated Chemokine
1.205745e-28 0.345283 0.426 0.243 2.149722e-24 14
UBA2 Activated Chemokine
1.481501e-28 0.3392121 0.347 0.179 2.641368e-24 14
AHCY Activated Chemokine
1.539255e-28 0.3330577 0.56 0.364 2.744337e-24 14
CDC37 Activated Chemokine
1.583688e-28 -0.6424301 0.173 0.288 2.823557e-24 14
PPM1K Activated Chemokine
1.936875e-28 -0.6354951 0.092 0.205 3.453254e-24 14
HMCES Activated Chemokine
2.527182e-28 0.3263151 0.19 0.075 4.505712e-24 14
GLA Activated Chemokine
2.614004e-28 0.3328739 0.484 0.296 4.660508e-24 14
SRP72 Activated Chemokine
2.874805e-28 0.3130735 0.252 0.109 5.125489e-24 14
MCRIP2 Activated Chemokine
3.062088e-28 0.3258228 0.284 0.129 5.459396e-24 14
LSM12 Activated Chemokine
3.285438e-28 0.3032063 0.72 0.525 5.857607e-24 14
JTB Activated Chemokine
3.383134e-28 0.3472626 0.388 0.225 6.03179e-24 14
HDDC2 Activated Chemokine
3.401411e-28 0.3378549 0.275 0.13 6.064375e-24 14
MRPS2 Activated Chemokine
3.699706e-28 -3.265311 0.37 0.452 6.596205e-24 14
IGHG3 Activated Chemokine
3.850027e-28 -0.4833074 0.048 0.156 6.864213e-24 14

CD81	Activated Chemokine	4.717815e-28	-0.5071802	0.059	0.16	8.411393e-24	14
GLIPR1	Activated Chemokine	4.978951e-28	0.3266118	0.351	0.183	8.876972e-24	14
TUSC2	Activated Chemokine	1.114984e-27	-0.7273227	0.665	0.694	1.987905e-23	14
EZR	Activated Chemokine	1.34046e-27	0.3198757	0.345	0.177	2.389906e-23	14
UTP18	Activated Chemokine	1.518271e-27	-0.571162	0.147	0.259	2.706925e-23	14
LYSMD2	Activated Chemokine	1.614781e-27	-0.6349066	0.339	0.447	2.878992e-23	14
ANXA2	Activated Chemokine	1.686269e-27	0.3125882	0.514	0.318	3.006449e-23	14
MLF2	Activated Chemokine	1.710195e-27	-0.5483223	0.167	0.288	3.049107e-23	14
YPEL5	Activated Chemokine	1.714482e-27	-0.5933455	0.544	0.624	3.05675e-23	14
ARPC1B	Activated Chemokine	1.82439e-27	-0.5713212	0.125	0.239	3.252705e-23	14
CCND3	Activated Chemokine	2.072346e-27	-1.083635	0.34	0.43	3.694786e-23	14
STMN1	Activated Chemokine	2.699928e-27	0.3369698	0.359	0.188	4.813701e-23	14
UTP6	Activated Chemokine	2.98499e-27	0.3236271	0.238	0.101	5.321939e-23	14
LPCAT1	Activated Chemokine	3.796902e-27	0.4385275	0.449	0.273	6.769497e-23	14
GADD45B	Activated Chemokine	3.835915e-27	-0.5254328	0.13	0.243	6.839053e-23	14
MCUB	Activated Chemokine	3.919249e-27	0.3346715	0.476	0.286	6.987629e-23	14
PYURF	Activated Chemokine	3.929718e-27	-0.477704	0.082	0.182	7.006295e-23	14
LYL1	Activated Chemokine	4.371033e-27	0.3012903	0.267	0.117	7.793114e-23	14
SURF2	Activated Chemokine	4.851287e-27	-0.5067638	0.061	0.163	8.64936e-23	14
C12orf75	Activated Chemokine	5.053937e-27	0.342198	0.312	0.162	9.010665e-23	14
U2AF2	Activated Chemokine	5.150835e-27	-0.3945921	0.022	0.108	9.183423e-23	14
HRK	Activated Chemokine	5.883386e-27	-0.4725222	0.087	0.2	1.048949e-22	14
POLD4	Activated Chemokine	7.19352e-27	-0.5419999	0.183	0.304	1.282533e-22	14
CDK2AP2	Activated Chemokine	7.289334e-27	0.3072476	0.198	0.077	1.299615e-22	14
LRPPRC	Activated Chemokine	7.294941e-27	-0.5789276	0.197	0.314	1.300615e-22	14

IL16	Activated Chemokine	7.952768e-27	0.3264604	0.417	0.238	1.417899e-22	14
BCAS2	Activated Chemokine	1.121169e-26	-0.4777329	0.067	0.169	1.998932e-22	14
SNX10	Activated Chemokine	1.141188e-26	-0.3330509	0.916	0.947	2.034624e-22	14
HLA-DRA	Activated Chemokine	1.142093e-26	0.3502961	0.407	0.22	2.036237e-22	14
TMEM109	Activated Chemokine	1.154182e-26	-0.3580317	0.017	0.103	2.05779e-22	14
MYBL2	Activated Chemokine	1.16597e-26	0.3147704	0.54	0.345	2.078808e-22	14
PDIA6	Activated Chemokine	1.185337e-26	0.3165983	0.348	0.19	2.113337e-22	14
HARS	Activated Chemokine	1.413313e-26	0.3160024	0.217	0.09	2.519795e-22	14
PSMG4	Activated Chemokine	1.42392e-26	0.3185658	0.439	0.256	2.538706e-22	14
BAZ1A	Activated Chemokine	1.438099e-26	0.3238819	0.412	0.242	2.563987e-22	14
PRPF31	Activated Chemokine	1.470575e-26	0.3128037	0.461	0.278	2.621888e-22	14
IMP3	Activated Chemokine	1.502709e-26	-0.6239874	0.181	0.271	2.67918e-22	14
GPR183	Activated Chemokine	1.544717e-26	-0.4098556	0.05	0.152	2.754075e-22	14
PTPN18	Activated Chemokine	2.195813e-26	-0.4164455	0.988	0.99	3.914916e-22	14
ACTB	Activated Chemokine	2.371196e-26	0.3075938	0.318	0.158	4.227604e-22	14
CWC25	Activated Chemokine	2.433603e-26	0.3409078	0.658	0.471	4.338872e-22	14
TNFAIP8	Activated Chemokine	2.56443e-26	-1.534784	0.132	0.218	4.572121e-22	14
IGHG1	Activated Chemokine	3.053156e-26	0.3264011	0.37	0.203	5.443471e-22	14
FUNDC2	Activated Chemokine	3.10479e-26	-0.3739288	0.024	0.113	5.535529e-22	14
AC023590.1	Activated Chemokine	3.310004e-26	-1.342163	0.08	0.178	5.901406e-22	14
MZB1	Activated Chemokine	3.405186e-26	0.3151444	0.439	0.254	6.071106e-22	14
PWP1	Activated Chemokine	3.410929e-26	0.3371163	0.361	0.206	6.081345e-22	14
TMEM167A	Activated Chemokine	5.699681e-26	-0.5031047	0.122	0.242	1.016196e-21	14
NCF4	Activated Chemokine	5.847259e-26	0.305083	0.246	0.114	1.042508e-21	14
CRLS1	Activated Chemokine	8.602314e-26	0.3084274	0.672	0.479	1.533707e-21	14

WDR83OS	Activated Chemokine	9.285979e-26	0.3080565	0.298	0.151	1.655597e-21	14
ALG5	Activated Chemokine	1.213388e-25	0.303558	0.473	0.28	2.163349e-21	14
ATP6AP2	Activated Chemokine	1.736886e-25	0.3356582	0.377	0.199	3.096695e-21	14
NDFIP1	Activated Chemokine	1.843711e-25	-0.5609875	0.582	0.681	3.287152e-21	14
HLA-DMA	Activated Chemokine	2.029156e-25	0.3115047	0.412	0.23	3.617783e-21	14
DNAJC19	Activated Chemokine	2.099472e-25	0.3074588	0.414	0.248	3.743148e-21	14
MCTS1	Activated Chemokine	2.320176e-25	0.3288634	0.245	0.113	4.136642e-21	14
PTP4A1	Activated Chemokine	2.414584e-25	0.3192399	0.335	0.177	4.304962e-21	14
PLRG1	Activated Chemokine	2.435904e-25	-0.5132364	0.169	0.28	4.342972e-21	14
CCNG1	Activated Chemokine	2.958612e-25	0.3033059	0.474	0.299	5.27491e-21	14
SNF8	Activated Chemokine	3.131894e-25	0.3147572	0.383	0.212	5.583854e-21	14
CANX	Activated Chemokine	3.141583e-25	-0.5349404	0.491	0.619	5.601128e-21	14
MEF2C	Activated Chemokine	3.438691e-25	0.3201717	0.349	0.194	6.130842e-21	14
UQCC2	Activated Chemokine	3.77586e-25	0.312132	0.534	0.341	6.73198e-21	14
DNAJB6	Activated Chemokine	4.121178e-25	-0.4188585	0.033	0.115	7.347648e-21	14
PBXIP1	Activated Chemokine	4.233286e-25	0.3032801	0.403	0.229	7.547525e-21	14
SMARCA5	Activated Chemokine	4.264325e-25	-0.5172316	0.105	0.204	7.602865e-21	14
TRIM38	Activated Chemokine	4.361449e-25	-0.5011517	0.138	0.253	7.776028e-21	14
WIPF1	Activated Chemokine	4.481996e-25	-0.5129289	0.16	0.269	7.99095e-21	14
GRN	Activated Chemokine	4.587455e-25	0.3194845	0.378	0.219	8.178973e-21	14
LSM1	Activated Chemokine	4.948512e-25	-0.5387256	0.158	0.277	8.822702e-21	14
ITGAE	Activated Chemokine	5.293017e-25	0.3212267	0.595	0.418	9.43692e-21	14
TMEM123	Activated Chemokine	5.523805e-25	0.3070469	0.501	0.32	9.848392e-21	14
NDUFB6	Activated Chemokine	5.637554e-25	-0.4750544	0.06	0.147	1.00512e-20	14
SUN2	Activated Chemokine	8.33719e-25	0.3173582	0.298	0.147	1.486438e-20	14

AFG3L2 Activated Chemokine
1.152806e-24 -0.4120795 0.05 0.146 2.055337e-20 14
HSH2D Activated Chemokine
1.154526e-24 -0.6405603 0.201 0.299 2.058404e-20 14
LINC00926 Activated Chemokine
1.267243e-24 0.3153548 0.433 0.255 2.259367e-20 14
NAA50 Activated Chemokine
1.290682e-24 0.3160642 0.172 0.071 2.301157e-20 14
SPAG9 Activated Chemokine
1.365516e-24 0.324685 0.36 0.199 2.434578e-20 14
MRPL23 Activated Chemokine
1.429561e-24 -0.5513972 0.49 0.566 2.548763e-20 14
ERP29 Activated Chemokine
1.520336e-24 0.3105301 0.539 0.362 2.710608e-20 14
RPS19BP1 Activated Chemokine
1.797395e-24 0.3137493 0.434 0.255 3.204576e-20 14
RAB10 Activated Chemokine
1.824294e-24 -0.5037305 0.137 0.244 3.252533e-20 14
NCOA3 Activated Chemokine
2.215267e-24 0.308449 0.241 0.118 3.949599e-20 14
SNHG9 Activated Chemokine
2.253602e-24 0.3092034 0.437 0.269 4.017948e-20 14
CAMTA1 Activated Chemokine
2.319192e-24 -0.5581921 0.309 0.421 4.134887e-20 14
CTSH Activated Chemokine
2.499863e-24 -0.5086194 0.145 0.252 4.457005e-20 14
SH3KBP1 Activated Chemokine
3.809263e-24 -0.5660299 0.113 0.199 6.791535e-20 14
ACP5 Activated Chemokine
4.086995e-24 -0.5813795 0.155 0.251 7.286703e-20 14
TNFRSF13B Activated Chemokine
4.841054e-24 -0.4560533 0.067 0.171 8.631115e-20 14
SEL1L3 Activated Chemokine
5.276858e-24 -1.572495 0.235 0.304 9.408111e-20 14
HMGB2 Activated Chemokine
5.331221e-24 0.3146351 0.361 0.198 9.505035e-20 14
NGDN Activated Chemokine
6.660658e-24 -0.5881459 0.299 0.396 1.187529e-19 14
SYPL1 Activated Chemokine
7.350171e-24 0.3305975 0.609 0.436 1.310462e-19 14
FDPS Activated Chemokine
9.572115e-24 -0.5142586 0.167 0.281 1.706612e-19 14
STK17B Activated Chemokine
9.629961e-24 -0.4964027 0.15 0.264 1.716926e-19 14
CCDC69 Activated Chemokine
1.168976e-23 -0.4233586 0.061 0.147 2.084167e-19 14
GNB5 Activated Chemokine
1.553511e-23 -0.4408235 0.058 0.15 2.769755e-19 14
ALOX5 Activated Chemokine
2.746383e-23 -0.6072379 0.245 0.341 4.896526e-19 14

STK17A	Activated Chemokine	2.913908e-23	-0.4047559	0.993	0.978	5.195206e-19	14
MT-CO2	Activated Chemokine	3.532557e-23	-0.4796583	0.042	0.151	6.298196e-19	14
BIK	Activated Chemokine	3.888449e-23	-0.3936983	0.044	0.14	6.932715e-19	14
SIT1	Activated Chemokine	5.124077e-23	-1.163711	0.078	0.149	9.135716e-19	14
IGHA1	Activated Chemokine	5.79965e-23	-0.5301251	0.319	0.416	1.03402e-18	14
NPC2	Activated Chemokine	6.166698e-23	0.3175683	0.498	0.334	1.099461e-18	14
SPIB	Activated Chemokine	7.107075e-23	-0.5978189	0.087	0.188	1.26712e-18	14
RGS2	Activated Chemokine	8.294359e-23	-0.4970806	0.15	0.256	1.478801e-18	14
PRMT2	Activated Chemokine	8.844394e-23	-0.4251393	0.075	0.17	1.576867e-18	14
BNIP3L	Activated Chemokine	8.87231e-23	-0.3444043	0.036	0.113	1.581844e-18	14
CRIP1	Activated Chemokine	1.074039e-22	-0.5061539	0.21	0.321	1.914904e-18	14
FCRLA	Activated Chemokine	1.205998e-22	-0.6629414	0.939	0.932	2.150175e-18	14
HMGB1	Activated Chemokine	1.382374e-22	-0.4982954	0.101	0.209	2.464635e-18	14
EAF2	Activated Chemokine	1.455802e-22	0.3004475	0.375	0.217	2.59555e-18	14
POLE3	Activated Chemokine	1.680034e-22	0.3129219	0.373	0.222	2.995333e-18	14
BAG1	Activated Chemokine	1.883577e-22	-0.3972114	0.039	0.113	3.358229e-18	14
SESN3	Activated Chemokine	2.173517e-22	-0.4547697	0.063	0.161	3.875164e-18	14
KIAA0040	Activated Chemokine	2.422782e-22	-0.437542	0.948	0.937	4.319578e-18	14
EEF2	Activated Chemokine	3.18642e-22	-0.5100441	0.112	0.205	5.681068e-18	14
IRF1	Activated Chemokine	4.241842e-22	0.301256	0.327	0.164	7.56278e-18	14
TNIP2	Activated Chemokine	4.264189e-22	-0.3829021	0.057	0.149	7.602623e-18	14
SEC14L1	Activated Chemokine	5.320008e-22	-0.515894	0.288	0.394	9.485042e-18	14
SH3BGRL	Activated Chemokine	6.000448e-22	-0.4774957	0.117	0.207	1.06982e-17	14
RALGPS2	Activated Chemokine	6.090322e-22	-0.4684688	0.136	0.212	1.085843e-17	14
MGST3	Activated Chemokine	8.324601e-22	-0.5584874	0.503	0.572	1.484193e-17	14

SNX2	Activated Chemokine	9.956086e-22	0.3073695	0.536	0.351	1.775071e-17	14
SQLE	Activated Chemokine	1.035229e-21	-0.4340927	0.043	0.123	1.84571e-17	14
LPP	Activated Chemokine	1.359693e-21	-0.3565195	0.054	0.142	2.424196e-17	14
CORO1B	Activated Chemokine	3.407524e-21	-0.4916321	0.219	0.316	6.075274e-17	14
SPI1	Activated Chemokine	8.841423e-21	-0.6929245	0.538	0.589	1.576337e-16	14
MARCKSL1	Activated Chemokine	1.303302e-20	-0.3127044	0.04	0.126	2.323657e-16	14
SPATS2	Activated Chemokine	1.329195e-20	-0.3518876	0.061	0.148	2.369822e-16	14
P2RY8	Activated Chemokine	1.902022e-20	-0.3437535	0.062	0.149	3.391115e-16	14
ANKRD13A	Activated Chemokine	2.805376e-20	-0.463982	0.149	0.244	5.001704e-16	14
ARHGAP45	Activated Chemokine	2.971107e-20	-0.4727212	0.322	0.425	5.297186e-16	14
ECH1	Activated Chemokine	3.336257e-20	-0.4922599	0.203	0.3	5.948212e-16	14
BLK	Activated Chemokine	3.698449e-20	-0.5510355	0.564	0.621	6.593965e-16	14
IFI16	Activated Chemokine	4.56525e-20	-0.4368011	0.124	0.214	8.139385e-16	14
WASHC4	Activated Chemokine	4.85432e-20	-0.5386151	0.149	0.247	8.654768e-16	14
RASGRP2	Activated Chemokine	4.921144e-20	0.3436034	0.486	0.334	8.773908e-16	14
IER2	Activated Chemokine	5.446544e-20	0.3433952	0.306	0.16	9.710644e-16	14
MARCKS	Activated Chemokine	5.555985e-20	-0.4837122	0.183	0.285	9.905765e-16	14
LAT2	Activated Chemokine	1.015408e-19	-0.5255551	0.366	0.428	1.810372e-15	14
PLP2	Activated Chemokine	1.268373e-19	-0.4330978	0.117	0.211	2.261382e-15	14
MIS18BP1	Activated Chemokine	1.329442e-19	-0.4197994	0.068	0.171	2.370262e-15	14
CCDC88A	Activated Chemokine	3.512005e-19	-0.5096988	0.461	0.532	6.261553e-15	14
PAIP2	Activated Chemokine	3.924797e-19	-0.4756657	0.254	0.338	6.99752e-15	14
SEPHS2	Activated Chemokine	4.506286e-19	-0.3114439	0.037	0.105	8.034257e-15	14
TRIM8	Activated Chemokine	4.723272e-19	-0.3369023	0.031	0.1	8.421122e-15	14
MX1	Activated Chemokine	4.848394e-19	-0.3787038	0.068	0.143	8.644202e-15	14

MYO1G	Activated Chemokine					
5.841287e-19	-0.4229337	0.115	0.209	1.041443e-14	14	
CAT	Activated Chemokine					
1.027729e-18	-0.4347413	0.117	0.204	1.832339e-14	14	
BIN1	Activated Chemokine					
1.050652e-18	-0.4735973	0.235	0.333	1.873207e-14	14	
TSPO	Activated Chemokine					
1.074188e-18	-0.4835077	0.242	0.331	1.91517e-14	14	
CCNDBP1	Activated Chemokine					
1.230433e-18	-0.4818767	0.187	0.277	2.19374e-14	14	
TNRC6B	Activated Chemokine					
1.383706e-18	-0.4287397	0.107	0.213	2.467009e-14	14	
ADA	Activated Chemokine					
1.657145e-18	-0.4269181	0.163	0.248	2.954524e-14	14	
TPM4	Activated Chemokine					
3.122169e-18	-0.4306152	0.143	0.236	5.566514e-14	14	
CPNE3	Activated Chemokine					
3.82814e-18	-0.3412016	0.039	0.115	6.825191e-14	14	
TNFRSF17	Activated Chemokine					
4.048481e-18	-0.4870614	0.19	0.281	7.218037e-14	14	
RNASET2	Activated Chemokine					
5.585724e-18	-0.5657053	0.247	0.334	9.958787e-14	14	
RIPOR2	Activated Chemokine					
5.990515e-18	-0.9184258	0.672	0.655	1.068049e-13	14	
TCL1A	Activated Chemokine					
6.276192e-18	-0.3951749	0.104	0.185	1.118982e-13	14	
INPP5D	Activated Chemokine					
6.510598e-18	-0.3247062	0.039	0.109	1.160775e-13	14	
FLI1	Activated Chemokine					
7.318759e-18	-0.3613091	0.058	0.119	1.304862e-13	14	
FCGR2B	Activated Chemokine					
8.548432e-18	-0.3944103	0.119	0.211	1.5241e-13	14	
TCEAL8	Activated Chemokine					
9.578733e-18	-0.3921461	0.918	0.917	1.707792e-13	14	
PFDN5	Activated Chemokine					
9.995073e-18	-0.3075129	0.036	0.106	1.782022e-13	14	
SORL1	Activated Chemokine					
1.278375e-17	-0.3810247	0.067	0.142	2.279216e-13	14	
ADD3	Activated Chemokine					
1.651371e-17	-0.3574729	0.072	0.149	2.944228e-13	14	
SPSB3	Activated Chemokine					
2.1218e-17	-0.4889486	0.249	0.336	3.782957e-13	14	
FAM96A	Activated Chemokine					
2.182772e-17	-0.3812832	0.07	0.135	3.891665e-13	14	
ODF2L	Activated Chemokine					
2.456856e-17	-0.3373678	0.051	0.132	4.380329e-13	14	
STIM2	Activated Chemokine					
2.635712e-17	-0.3818469	0.092	0.168	4.69921e-13	14	
DOCK8	Activated Chemokine					
2.649872e-17	-1.469267	0.939	0.926	4.724457e-13	14	

IGKC	Activated Chemokine					
2.931341e-17	-0.3735113	0.078	0.147	5.226288e-13	14	
COBLL1	Activated Chemokine					
3.425601e-17	-0.5096707	0.569	0.616	6.107505e-13	14	
PNISR	Activated Chemokine					
5.335828e-17	-0.3146525	0.055	0.129	9.513248e-13	14	
SASH3	Activated Chemokine					
5.817449e-17	-0.3506703	0.055	0.129	1.037193e-12	14	
CCNG2	Activated Chemokine					
6.020674e-17	-0.4130286	0.085	0.149	1.073426e-12	14	
ARRDC2	Activated Chemokine					
7.010272e-17	-0.5052227	0.345	0.408	1.249861e-12	14	
LSM10	Activated Chemokine					
7.558472e-17	-0.3699837	0.088	0.166	1.3476e-12	14	
PPP1R18	Activated Chemokine					
9.843706e-17	-0.3557677	0.063	0.124	1.755034e-12	14	
DDAH2	Activated Chemokine					
1.364247e-16	-0.3241556	0.072	0.155	2.432316e-12	14	
MYL6B	Activated Chemokine					
1.414713e-16	-0.4816731	0.222	0.301	2.522291e-12	14	
ITSN2	Activated Chemokine					
1.829285e-16	-0.5257883	0.146	0.213	3.261432e-12	14	
LBH	Activated Chemokine					
1.911002e-16	0.3650644	0.756	0.65	3.407125e-12	14	
HLA-DQA1	Activated Chemokine					
2.168459e-16	-0.4243814	0.287	0.365	3.866145e-12	14	
TMEM256	Activated Chemokine					
2.190596e-16	-0.3701909	0.092	0.179	3.905614e-12	14	
SYK	Activated Chemokine					
3.990606e-16	-0.3503845	0.052	0.119	7.114852e-12	14	
SAMD9	Activated Chemokine					
4.764674e-16	-0.455674	0.117	0.196	8.494938e-12	14	
FAM129C	Activated Chemokine					
5.191624e-16	-0.3599098	0.064	0.136	9.256146e-12	14	
PLEKHA2	Activated Chemokine					
5.867918e-16	-0.3045299	0.059	0.137	1.046191e-11	14	
MPST	Activated Chemokine					
6.945164e-16	-0.3064699	0.057	0.123	1.238253e-11	14	
ING4	Activated Chemokine					
1.083906e-15	-0.4937552	0.457	0.495	1.932497e-11	14	
FXYD5	Activated Chemokine					
1.099737e-15	-0.4420935	0.304	0.379	1.960721e-11	14	
ZFAND6	Activated Chemokine					
1.114201e-15	-0.500486	0.514	0.558	1.986509e-11	14	
ATP5IF1	Activated Chemokine					
1.186444e-15	-0.6034428	0.552	0.571	2.11531e-11	14	
VIM	Activated Chemokine					
1.479083e-15	-0.3081367	0.05	0.111	2.637056e-11	14	
LPAR5	Activated Chemokine					
2.023934e-15	-0.4642866	0.415	0.487	3.608471e-11	14	

	GMFG	Activated Chemokine			
2.067401e-15	-0.3824212	0.051	0.107	3.685969e-11	14
	C1orf162	Activated Chemokine			
2.199685e-15	-0.3462139	0.074	0.142	3.921818e-11	14
	ARID4A	Activated Chemokine			
2.853649e-15	-0.3467426	0.105	0.184	5.08777e-11	14
	TLR10	Activated Chemokine			
2.90971e-15	-0.3247871	0.061	0.128	5.187722e-11	14
	ZBTB20	Activated Chemokine			
3.561409e-15	-0.4713735	0.306	0.386	6.349636e-11	14
	EVL	Activated Chemokine			
4.052011e-15	-0.4953498	0.298	0.361	7.22433e-11	14
	ARID5B	Activated Chemokine			
4.240724e-15	-0.4146152	0.246	0.332	7.560787e-11	14
	CCM2	Activated Chemokine			
5.920072e-15	-0.9305812	0.127	0.213	1.05549e-10	14
	PLCG2	Activated Chemokine			
6.612575e-15	-0.3020229	0.075	0.146	1.178956e-10	14
	FLOT1	Activated Chemokine			
7.000593e-15	-0.416181	0.246	0.322	1.248136e-10	14
	IGBP1	Activated Chemokine			
7.831375e-15	-0.3261821	0.115	0.199	1.396256e-10	14
	ETFB	Activated Chemokine			
1.127084e-14	-0.3739558	0.113	0.186	2.009479e-10	14
	APBB1IP	Activated Chemokine			
2.016657e-14	-0.3768724	0.174	0.249	3.595497e-10	14
	OSTF1	Activated Chemokine			
2.392363e-14	-0.4549248	0.481	0.524	4.265343e-10	14
	TRAPPC1	Activated Chemokine			
2.878006e-14	-0.3298448	0.993	0.982	5.131197e-10	14
	MT-CO3	Activated Chemokine			
3.217351e-14	-0.4369166	0.395	0.457	5.736215e-10	14
	CTSS	Activated Chemokine			
8.99706e-14	-0.3835166	0.159	0.235	1.604086e-09	14
	AKAP13	Activated Chemokine			
1.080479e-13	-0.4659158	0.302	0.367	1.926386e-09	14
	SNAP23	Activated Chemokine			
1.688779e-13	-0.3287852	0.073	0.142	3.010924e-09	14
	MKNK2	Activated Chemokine			
1.871153e-13	-0.431947	0.438	0.481	3.336078e-09	14
	ISCU	Activated Chemokine			
2.031331e-13	-0.4187494	0.107	0.171	3.62166e-09	14
	SMC4	Activated Chemokine			
2.756968e-13	-0.4051527	0.239	0.305	4.915398e-09	14
	UGP2	Activated Chemokine			
3.597892e-13	-2.136781	0.594	0.646	6.414682e-09	14
	IGLC2	Activated Chemokine			
3.623047e-13	-0.4093904	0.284	0.351	6.45953e-09	14
	DDX17	Activated Chemokine			
4.127611e-13	-0.3075918	0.074	0.143	7.359118e-09	14

SLC43A2	Activated Chemokine				
4.240949e-13	-0.3692048	0.209	0.288	7.561188e-09	14
GSTK1	Activated Chemokine				
4.835072e-13	-0.3859905	0.168	0.241	8.62045e-09	14
SP140	Activated Chemokine				
5.82851e-13	-0.3698193	0.133	0.195	1.039165e-08	14
CYTH1	Activated Chemokine				
5.852591e-13	-0.3659802	0.174	0.251	1.043458e-08	14
PPP2R5C	Activated Chemokine				
6.261456e-13	-0.3766175	0.178	0.25	1.116355e-08	14
SNRPN	Activated Chemokine				
6.424309e-13	-0.4591574	0.445	0.493	1.14539e-08	14
RAC2	Activated Chemokine				
7.820152e-13	-0.3335176	0.092	0.143	1.394255e-08	14
NUB1	Activated Chemokine				
9.684029e-13	-0.4661052	0.152	0.207	1.726565e-08	14
PRKCB	Activated Chemokine				
1.056785e-12	-0.3241411	0.067	0.124	1.884143e-08	14
RNF213	Activated Chemokine				
1.124221e-12	-0.3508123	0.153	0.213	2.004374e-08	14
IDS	Activated Chemokine				
1.16764e-12	-0.3461183	0.161	0.228	2.081785e-08	14
ALKBH7	Activated Chemokine				
1.226829e-12	-0.5256649	0.142	0.21	2.187314e-08	14
FOS	Activated Chemokine				
1.294805e-12	-0.4002869	0.066	0.124	2.308508e-08	14
AL139020.1	Activated Chemokine				
1.538299e-12	-0.4029464	0.402	0.454	2.742632e-08	14
TMEM59	Activated Chemokine				
1.629732e-12	-0.5339692	0.185	0.236	2.905648e-08	14
UBE2J1	Activated Chemokine				
1.703151e-12	-0.3855759	0.147	0.209	3.036548e-08	14
SNHG25	Activated Chemokine				
2.998702e-12	-0.4223158	0.173	0.242	5.346386e-08	14
RGS1	Activated Chemokine				
4.004658e-12	0.3001035	0.27	0.158	7.139904e-08	14
DDIT4	Activated Chemokine				
4.896679e-12	-0.392344	0.172	0.238	8.730289e-08	14
DSTN	Activated Chemokine				
5.50417e-12	-0.4024561	0.261	0.326	9.813385e-08	14
BLNK	Activated Chemokine				
6.335195e-12	-0.4192643	0.243	0.29	1.129502e-07	14
C7orf50	Activated Chemokine				
7.918673e-12	-0.3541805	0.148	0.208	1.41182e-07	14
ARHGEF1	Activated Chemokine				
8.748073e-12	-0.4120546	0.267	0.321	1.559694e-07	14
LTA4H	Activated Chemokine				
1.342532e-11	-0.3183975	0.129	0.191	2.393601e-07	14
MAP2K1	Activated Chemokine				
1.635896e-11	-0.4233857	0.485	0.516	2.916639e-07	14

VAMP2	Activated Chemokine	1.718244e-11	-0.3294651	0.106	0.167	3.063457e-07	14
DEF8	Activated Chemokine	2.003621e-11	-0.3433913	0.062	0.11	3.572255e-07	14
LINC02397	Activated Chemokine	4.584402e-11	-0.3859156	0.2	0.245	8.17353e-07	14
GCC2	Activated Chemokine	5.610266e-11	-0.3480391	0.119	0.17	1.000254e-06	14
TMEM154	Activated Chemokine	5.966686e-11	-0.4113703	0.249	0.315	1.0638e-06	14
HMGN3	Activated Chemokine	9.955372e-11	-0.3963635	0.441	0.483	1.774943e-06	14
RABAC1	Activated Chemokine	1.183578e-10	-0.4518924	0.71	0.699	2.110201e-06	14
CCNI	Activated Chemokine	1.247268e-10	-0.3263199	0.147	0.208	2.223754e-06	14
IKZF3	Activated Chemokine	1.251198e-10	-0.4105256	0.7	0.687	2.23076e-06	14
PPP1CA	Activated Chemokine	1.441709e-10	-0.3230171	0.285	0.343	2.570423e-06	14
PKIG	Activated Chemokine	1.559025e-10	-0.370654	0.182	0.227	2.779586e-06	14
MCM5	Activated Chemokine	1.61576e-10	-0.3478872	0.182	0.242	2.880739e-06	14
DRAM2	Activated Chemokine	1.696911e-10	-0.3282786	0.16	0.225	3.025422e-06	14
PHIP	Activated Chemokine	2.381127e-10	-0.3684666	0.233	0.29	4.245311e-06	14
AKAP9	Activated Chemokine	2.733129e-10	-0.4024821	0.349	0.388	4.872896e-06	14
PSAP	Activated Chemokine	2.76209e-10	-0.3039259	0.158	0.22	4.92453e-06	14
GNAI2	Activated Chemokine	2.883791e-10	-0.3370871	0.187	0.248	5.14151e-06	14
PSMB8-AS1	Activated Chemokine	3.67106e-10	-0.3958494	0.139	0.193	6.545133e-06	14
FAM30A	Activated Chemokine	3.848836e-10	-0.4456837	0.334	0.351	6.86209e-06	14
LY6E	Activated Chemokine	5.075686e-10	-0.4596116	0.468	0.493	9.04944e-06	14
C4orf3	Activated Chemokine	5.155901e-10	-0.3579446	0.215	0.271	9.192456e-06	14
SEPT9	Activated Chemokine	7.135724e-10	-0.3269878	0.156	0.206	1.272228e-05	14
OGA	Activated Chemokine	7.256147e-10	-0.3189257	0.118	0.174	1.293698e-05	14
CDK13	Activated Chemokine	1.041156e-09	-0.3639713	0.157	0.196	1.856277e-05	14
CLEC2B	Activated Chemokine	1.088061e-09	-0.3605409	0.216	0.269	1.939903e-05	14

MBNL1	Activated Chemokine	1.319297e-09	-0.3006529	0.051	0.112	2.352174e-05	14
CD9	Activated Chemokine	1.55598e-09	-0.3327877	0.229	0.287	2.774157e-05	14
ATF6B	Activated Chemokine	1.825068e-09	-0.3293986	0.139	0.191	3.253913e-05	14
LBR	Activated Chemokine	2.099591e-09	-0.3295326	0.136	0.186	3.743361e-05	14
ANKRD44	Activated Chemokine	2.670033e-09	-0.3287234	0.185	0.241	4.760401e-05	14
PTRHD1	Activated Chemokine	4.833213e-09	-0.3639448	0.287	0.333	8.617136e-05	14
BPTF	Activated Chemokine	5.081736e-09	-0.3455359	0.378	0.421	9.060227e-05	14
KMT2E	Activated Chemokine	5.320009e-09	-0.3041669	0.193	0.246	9.485043e-05	14
TMEM134	Activated Chemokine	7.051555e-09	-0.3319966	0.171	0.228	0.0001257222	14
UBE2G1	Activated Chemokine	9.229413e-09	-0.3174028	0.172	0.212	0.0001645512	14
FMNL1	Activated Chemokine	1.003289e-08	-0.3166175	0.148	0.193	0.0001788765	14
ARID1B	Activated Chemokine	1.226474e-08	-0.3006409	0.116	0.156	0.0002186681	14
LMBRD1	Activated Chemokine	1.260571e-08	-0.3390854	0.795	0.77	0.0002247473	14
SEC62	Activated Chemokine	1.300645e-08	-0.3268716	0.351	0.389	0.000231892	14
CUTA	Activated Chemokine	1.309221e-08	-0.3256236	0.181	0.223	0.000233421	14
GCA	Activated Chemokine	1.335737e-08	-1.499105	0.432	0.429	0.0002381485	14
IGLC3	Activated Chemokine	1.412795e-08	-0.3007991	0.181	0.225	0.0002518872	14
MAGED2	Activated Chemokine	1.437832e-08	-0.3345255	0.139	0.187	0.000256351	14
CDCA7L	Activated Chemokine	1.60603e-08	-0.3311504	0.858	0.83	0.0002863391	14
UQCRB	Activated Chemokine	2.073015e-08	-0.302015	0.163	0.209	0.0003695978	14
ARHGAP30	Activated Chemokine	2.580107e-08	-0.327713	0.264	0.307	0.0004600074	14
EAPP	Activated Chemokine	2.94396e-08	-0.3060941	0.192	0.234	0.0005248785	14
SPINT2	Activated Chemokine	3.047898e-08	-0.4402466	0.348	0.376	0.0005434098	14
ACADM	Activated Chemokine	3.302086e-08	-0.3359681	0.225	0.264	0.0005887289	14
TTC3	Activated Chemokine	4.627082e-08	-0.3199352	0.279	0.321	0.0008249624	14

TUBA1A	Activated Chemokine						
4.631169e-08	-0.3235486	0.159	0.2	0.0008256911	14		
CLK1	Activated Chemokine						
4.796127e-08	-0.3040913	0.18	0.225	0.0008551014	14		
JMJD1C	Activated Chemokine						
4.852798e-08	-0.4859249	0.973	0.932	0.0008652054	14		
SERF2	Activated Chemokine						
5.232826e-08	-0.3612119	0.333	0.362	0.0009329606	14		
ANXA6	Activated Chemokine						
5.275849e-08	-0.3229155	0.235	0.278	0.0009406312	14		
TANK	Activated Chemokine						
5.555574e-08	-0.3046693	0.197	0.248	0.0009905033	14		
CSK	Activated Chemokine						
5.885616e-08	-0.3619248	0.248	0.281	0.001049347	14		
SMCHD1	Activated Chemokine						
6.490995e-08	-0.3331633	0.29	0.337	0.00115728	14		
S100A11	Activated Chemokine						
6.723916e-08	-0.3681041	0.576	0.586	0.001198807	14		
NDUFV2	Activated Chemokine						
7.304456e-08	-0.3849892	0.251	0.289	0.001302311	14		
RUBCNL	Activated Chemokine						
8.21276e-08	-0.3441914	0.513	0.525	0.001464253	14		
SYF2	Activated Chemokine						
9.278577e-08	-0.3553429	0.107	0.144	0.001654278	14		
DUSP1	Activated Chemokine						
9.450957e-08	-0.3512835	0.46	0.477	0.001685011	14		
SSR2	Activated Chemokine						
1.046596e-07	-0.3070483	0.166	0.204	0.001865977	14		
IFNGR2	Activated Chemokine						
1.247972e-07	-0.3489657	0.265	0.292	0.002225009	14		
YWHAH	Activated Chemokine						
1.311265e-07	-0.3591976	0.22	0.265	0.002337854	14		
CD22	Activated Chemokine						
2.345529e-07	-1.326667	0.427	0.422	0.004181844	14		
HIST1H4C	Activated Chemokine						
2.576654e-07	-0.3563918	0.909	0.868	0.004593917	14		
ARPC3	Activated Chemokine						
2.606607e-07	-0.3314718	0.228	0.261	0.00464732	14		
MAT2B	Activated Chemokine						
2.869231e-07	-0.3388763	0.368	0.384	0.005115552	14		
LAPTM4A	Activated Chemokine						
3.020386e-07	-2.205244	0.076	0.127	0.005385046	14		
IGHG2	Activated Chemokine						
4.204982e-07	-0.3013331	0.207	0.247	0.007497062	14		
NBDY	Activated Chemokine						
6.974759e-07	-0.3505495	0.461	0.476	0.0124353	14		
N4BP2L2	Activated Chemokine						
7.676282e-07	-0.3370961	0.397	0.415	0.01368604	14		
HDAC1	Activated Chemokine						
8.061382e-07	-0.3193574	0.283	0.314	0.01437264	14		

ATP2B1 Activated Chemokine
1.213453e-06 -0.3042698 0.175 0.219 0.02163466 14
ISG15 Activated Chemokine
1.431493e-06 -0.4024395 0.613 0.576 0.02552209 14
SARAF Activated Chemokine
1.818966e-06 -0.4260714 0.348 0.36 0.03243035 14
H2AFV Activated Chemokine
1.8679e-06 -0.3925766 0.221 0.238 0.0333028 14
CD55 Activated Chemokine
2.317497e-06 -0.3666238 0.343 0.361 0.04131866 14
TPD52 Activated Chemokine
3.684747e-06 -0.3321725 0.636 0.613 0.06569536 14
ARPC5 Activated Chemokine
4.255155e-06 -0.3188221 0.361 0.384 0.07586515 14
JAK1 Activated Chemokine
5.607118e-06 -0.3158228 0.324 0.344 0.0999693 14
ATRX Activated Chemokine
6.185502e-06 -0.8127835 0.973 0.905 0.1102813 14
IGHM Activated Chemokine
1.615012e-05 -0.3221084 0.364 0.375 0.2879405 14
PTP4A2 Activated Chemokine
2.293452e-05 -0.3057127 0.313 0.336 0.4088995 14
POLR2J3.1 Activated Chemokine
4.768299e-05 -0.3041 0.574 0.559 0.8501401 14
YWHAZ Activated Chemokine
0.0002533444 -0.3065477 0.357 0.397 1 14
CSTB Activated Chemokine
0.0003151073 -0.310699 0.774 0.719 1 14
EIF3F Activated Chemokine
0.000445521 -0.3467786 0.259 0.268 1 14 JUN
Activated Chemokine
0.0007003605 -1.838351 0.255 0.243 1 14
IGHG4 Activated Chemokine
0.001396689 -0.4705575 0.672 0.604 1 14
IGHD Activated Chemokine
0.002801876 -0.3295761 0.721 0.676 1 14
TAGLN2 Activated Chemokine
0 1.634599 0.951 0.561 0 15 VIM Memory
LGALS3
9.095223e-309 1.748763 0.647 0.147 1.621587e-304 15
LMNA Memory LGALS3
5.557212e-306 1.927627 0.547 0.115 9.907953e-302 15
LGALS3 Memory LGALS3
4.387345e-294 -2.194833 0.6 0.914 7.822198e-290 15
IGHM Memory LGALS3
2.026863e-290 -2.471041 0.16 0.667 3.613693e-286 15
TCL1A Memory LGALS3
3.228636e-273 1.464055 0.851 0.398 5.756335e-269 15 TXN
Memory LGALS3
7.400816e-266 1.24134 0.913 0.646 1.319491e-261 15

HLA-DQA1	Memory	LGALS3	2.130993e-225	1.217198	0.834	0.435	3.799348e-221	15
ANXA2	Memory	LGALS3	6.340537e-213	0.9142659	0.976	0.756	1.130454e-208	15
PKM	Memory	LGALS3	2.488218e-206	1.303382	0.493	0.123	4.436244e-202	15
CYTOR	Memory	LGALS3	8.405913e-196	0.6655973	0.991	0.903	1.49869e-191	15
HLA-A	Memory	LGALS3	1.135094e-187	-1.357319	0.606	0.872	2.023759e-183	15
CD79A	Memory	LGALS3	4.597563e-187	0.4791063	1	0.998	8.196995e-183	15
B2M	Memory	LGALS3	3.639257e-185	-1.628796	0.212	0.616	6.488432e-181	15
IGHD	Memory	LGALS3	8.141396e-184	0.9685036	0.956	0.709	1.451529e-179	15
ENO1	Memory	LGALS3	1.044774e-179	0.85772	0.969	0.789	1.862728e-175	15
HLA-E	Memory	LGALS3	4.079095e-174	1.094891	0.671	0.238	7.272618e-170	15
TNFRSF13B	Memory	LGALS3	4.062543e-171	-1.488254	0.623	0.862	7.243108e-167	15
CD79B	Memory	LGALS3	1.469458e-166	0.9274189	0.256	0.023	2.619897e-162	15
ZBTB32	Memory	LGALS3	5.559114e-165	0.47709	1	0.986	9.911344e-161	15
HLA-B	Memory	LGALS3	7.07138e-163	0.7666456	0.991	0.945	1.260756e-158	15
HLA-DRA	Memory	LGALS3	3.556369e-144	0.9282642	0.389	0.068	6.340651e-140	15
CAPN2	Memory	LGALS3	6.055615e-140	1.015289	0.492	0.155	1.079656e-135	15
CD58	Memory	LGALS3	1.138296e-136	1.151417	0.818	0.456	2.029469e-132	15
S100A6	Memory	LGALS3	1.97133e-131	0.9268743	0.89	0.692	3.514684e-127	15
HLA-DQB1	Memory	LGALS3	5.603276e-129	0.7554587	0.947	0.692	9.990081e-125	15
EMP3	Memory	LGALS3	2.744343e-124	0.7632377	0.2	0.024	4.89289e-120	15
DUSP4	Memory	LGALS3	1.904108e-123	0.774736	0.96	0.811	3.394834e-119	15
HLA-DPA1	Memory	LGALS3	2.191596e-113	0.6483071	0.948	0.77	3.907397e-109	15
HLA-DPB1	Memory	LGALS3	1.317799e-111	0.595149	0.985	0.845	2.349503e-107	15
HSP90AB1	Memory	LGALS3	4.392007e-109	0.8427489	0.848	0.536	7.830509e-105	15
CCR7	Memory	LGALS3	1.575363e-108	0.3947827	0.878	0.75	2.808714e-104	15

HLA-DRB1	Memory	LGALS3	2.516221e-108	0.802632	0.644	0.328	4.486171e-104	15
VOPP1	Memory	LGALS3	7.638136e-108	1.003122	0.799	0.497	1.361803e-103	15
HLA-DRB5	Memory	LGALS3	1.375303e-104	0.6061828	0.981	0.886	2.452027e-100	15
GAPDH	Memory	LGALS3	7.051246e-104	-1.990061	0.207	0.496	1.257167e-99	15
LTB	Memory	LGALS3	3.43325e-101	0.3476943	1	0.999	6.121141e-97	15
EEF1A1	Memory	LGALS3	2.84925e-96	0.7412881	0.306	0.071	5.079928e-92	15
RDX	Memory	LGALS3	1.159204e-94	0.9058913	0.656	0.278	2.066744e-90	15
MIR155HG	Memory	LGALS3	2.277772e-94	-0.9874796	0.683	0.851	4.061039e-90	15
CD37	Memory	LGALS3	1.187987e-93	0.7987071	0.721	0.396	2.118062e-89	15
FABP5	Memory	LGALS3	9.15948e-92	0.6332839	0.931	0.731	1.633044e-87	15
HSPA8	Memory	LGALS3	1.188779e-85	0.6861521	0.683	0.385	2.119474e-81	15
FNBP1	Memory	LGALS3	3.213636e-85	-0.8731795	0.868	0.937	5.729592e-81	15
BTG1	Memory	LGALS3	5.621289e-83	0.4327576	0.986	0.897	1.00222e-78	15
PFN1	Memory	LGALS3	1.14265e-81	0.7380014	0.579	0.306	2.03723e-77	15
LPXN	Memory	LGALS3	1.613465e-81	0.5674408	0.84	0.57	2.876646e-77	15
TUBB	Memory	LGALS3	9.163109e-81	0.8083076	0.315	0.115	1.633691e-76	15
NCBP3	Memory	LGALS3	1.297267e-74	1.005723	0.451	0.191	2.312898e-70	15
ACP5	Memory	LGALS3	3.351725e-74	0.603646	0.276	0.074	5.975791e-70	15
CLIP1	Memory	LGALS3	9.039714e-72	0.7480376	0.733	0.456	1.611691e-67	15
CD63	Memory	LGALS3	1.057144e-70	0.5345544	0.609	0.312	1.884782e-66	15
SEC11C	Memory	LGALS3	1.16429e-70	-1.124607	0.6	0.754	2.075812e-66	15
CXCR4	Memory	LGALS3	3.037378e-70	1.269988	0.434	0.2	5.415341e-66	15
LGALS1	Memory	LGALS3	4.625606e-69	0.4873365	0.143	0.019	8.246993e-65	15
TFEC	Memory	LGALS3	4.126055e-68	0.408605	0.993	0.941	7.356344e-64	15
YBX1	Memory	LGALS3	5.63714e-68	0.5541876	0.788	0.551	1.005046e-63	15

PGK1	Memory	LGALS3	1.08639e-67	0.3782107	0.129	0.024	1.936926e-63	15
MIR4435-2HG	Memory	LGALS3	4.533727e-67	0.6673579	0.253	0.07	8.083182e-63	15
TRAF1	Memory	LGALS3	1.157434e-65	0.562525	0.298	0.103	2.063589e-61	15
CLDND1	Memory	LGALS3	8.994719e-65	0.6368694	0.585	0.33	1.603668e-60	15
S100A11	Memory	LGALS3	2.32551e-63	0.4893552	0.931	0.747	4.146153e-59	15
HLA-C	Memory	LGALS3	3.567979e-63	0.9526107	0.29	0.09	6.361349e-59	15
KLK1	Memory	LGALS3	3.778554e-62	0.5691796	0.265	0.082	6.736784e-58	15
EBI3	Memory	LGALS3	6.211914e-61	0.5743888	0.827	0.631	1.107522e-56	15
GPX4	Memory	LGALS3	8.577859e-61	0.5897628	0.673	0.422	1.529346e-56	15
PPP1R15A	Memory	LGALS3	1.346107e-60	-0.8322151	0.416	0.623	2.399973e-56	15
CLEC2D	Memory	LGALS3	1.095009e-59	0.5003775	0.887	0.709	1.952291e-55	15
CLIC1	Memory	LGALS3	2.685239e-59	0.6049668	0.394	0.163	4.787513e-55	15
TNIP2	Memory	LGALS3	6.777598e-59	0.5943827	0.671	0.376	1.208378e-54	15
CD44	Memory	LGALS3	1.6139e-58	-0.9856577	0.291	0.494	2.877422e-54	15
UCP2	Memory	LGALS3	1.284898e-57	0.6218782	0.407	0.174	2.290846e-53	15
RHOF	Memory	LGALS3	3.812491e-57	-2.187009	0.222	0.408	6.797291e-53	15
JCHAIN	Memory	LGALS3	4.861412e-57	0.4075785	0.932	0.744	8.667411e-53	15
RAN	Memory	LGALS3	6.263256e-57	-0.8205007	0.758	0.871	1.116676e-52	15
CD52	Memory	LGALS3	1.527244e-56	-1.042654	0.423	0.592	2.722924e-52	15
MARCKSL1	Memory	LGALS3	3.098608e-56	0.6842878	0.71	0.472	5.524508e-52	15
SQSTM1	Memory	LGALS3	8.331836e-56	0.6541473	0.358	0.163	1.485483e-51	15
SAMSN1	Memory	LGALS3	3.736425e-55	0.496771	0.942	0.863	6.661673e-51	15
UBC	Memory	LGALS3	2.965034e-54	0.5569831	0.406	0.184	5.286358e-50	15
ATP1A1	Memory	LGALS3	6.116234e-54	-0.9119634	0.106	0.308	1.090463e-49	15
BCL7A	Memory	LGALS3	1.321485e-53	0.6366246	0.539	0.262	2.356076e-49	15

GPR183	Memory LGALS3	1.773676e-53	0.4943084	0.192	0.064	3.162287e-49	15
TEX9	Memory LGALS3	2.470313e-53	0.5871535	0.387	0.178	4.404321e-49	15
TUBA1C	Memory LGALS3	2.47069e-53	0.4887018	0.241	0.079	4.404994e-49	15
KCNN4	Memory LGALS3	2.48417e-53	0.5512073	0.364	0.157	4.429027e-49	15
PPP1R14B	Memory LGALS3	1.19647e-52	0.3769132	0.106	0.011	2.133187e-48	15
TMEM273	Memory LGALS3	9.597661e-52	-0.733536	0.043	0.198	1.711167e-47	15
FAM129C	Memory LGALS3	9.861779e-52	0.3391662	0.105	0.018	1.758257e-47	15
NEDD4L	Memory LGALS3	3.817276e-51	0.3761262	0.926	0.756	6.805821e-47	15
PSMA7	Memory LGALS3	6.735117e-51	0.4829427	0.824	0.591	1.200804e-46	15
LDHA	Memory LGALS3	7.429425e-51	0.5152826	0.544	0.298	1.324592e-46	15
CD27	Memory LGALS3	5.125307e-50	0.4194015	0.206	0.068	9.13791e-46	15
NEK6	Memory LGALS3	1.179162e-49	0.4290166	0.885	0.699	2.102328e-45	15
TPI1	Memory LGALS3	2.611838e-49	0.5020248	0.614	0.375	4.656645e-45	15
ANXA7	Memory LGALS3	4.393938e-49	0.3974719	0.154	0.039	7.833952e-45	15
BHLHE41	Memory LGALS3	2.069389e-48	0.6608698	0.407	0.166	3.689515e-44	15
TNFRSF18	Memory LGALS3	2.628227e-48	0.4403323	0.831	0.6	4.685866e-44	15
RBM3	Memory LGALS3	2.922501e-48	0.3253507	0.996	0.928	5.210527e-44	15
NPM1	Memory LGALS3	7.663234e-48	0.4086939	0.821	0.603	1.366278e-43	15
POLR2L	Memory LGALS3	1.148345e-47	0.4341204	0.121	0.019	2.047384e-43	15
CCL22	Memory LGALS3	1.977276e-47	0.5760152	0.307	0.125	3.525285e-43	15
EEA1	Memory LGALS3	4.246539e-47	-1.070646	0.387	0.537	7.571154e-43	15
ISG20	Memory LGALS3	9.412377e-47	0.5455831	0.527	0.299	1.678133e-42	15
CD99	Memory LGALS3	2.689941e-46	0.4409915	0.818	0.606	4.795896e-42	15
PTGES3	Memory LGALS3	1.284911e-45	0.403109	0.823	0.623	2.290868e-41	15
ATP5F1B	Memory LGALS3	2.111811e-45	0.4845243	0.315	0.147	3.765148e-41	15

CLECL1	Memory LGALS3				
4.477216e-45	-1.295121	0.274	0.432	7.982428e-41	15
STMN1	Memory LGALS3				
5.947233e-45	0.5675268	0.407	0.191	1.060332e-40	15
TYMP	Memory LGALS3				
9.418731e-45	-1.120519	0.204	0.376	1.679266e-40	15
SELL	Memory LGALS3				
1.09275e-44	0.5584189	0.482	0.231	1.948264e-40	15
CAPG	Memory LGALS3				
2.438446e-44	0.4268681	0.204	0.057	4.347505e-40	15
FLNA	Memory LGALS3				
5.288694e-44	0.3412076	0.84	0.593	9.429213e-40	15
PRDX1	Memory LGALS3				
5.439449e-44	-0.4328758	0.973	0.976	9.697994e-40	15
FTH1	Memory LGALS3				
1.05775e-43	0.4299973	0.133	0.034	1.885863e-39	15
ATF5	Memory LGALS3				
2.724714e-43	-1.231198	0.054	0.21	4.857892e-39	15
RGS13	Memory LGALS3				
1.798765e-42	0.4211625	0.841	0.673	3.207018e-38	15
TAGLN2	Memory LGALS3				
5.037187e-42	0.4164123	0.592	0.347	8.9808e-38	15
TUBB4B	Memory LGALS3				
1.818452e-41	0.4842114	0.366	0.159	3.242118e-37	15
MARCKS	Memory LGALS3				
4.498651e-41	-0.6132026	0.015	0.125	8.020645e-37	15
AL139020.1		Memory LGALS3			
8.228989e-41	0.3794507	0.821	0.615	1.467146e-36	15
SUB1	Memory LGALS3				
1.97578e-40	0.4616569	0.39	0.188	3.522619e-36	15
CYTH1	Memory LGALS3				
2.23759e-40	0.6273123	0.352	0.162	3.989399e-36	15
CBX6	Memory LGALS3				
4.78968e-40	0.4250272	0.655	0.44	8.539521e-36	15
PGAM1	Memory LGALS3				
6.566776e-40	0.3167349	0.125	0.025	1.17079e-35	15
ATXN1	Memory LGALS3				
9.448781e-40	0.4823829	0.666	0.421	1.684623e-35	15
PLP2	Memory LGALS3				
4.046589e-39	0.4562669	0.648	0.432	7.214664e-35	15
COTL1	Memory LGALS3				
6.687377e-39	0.5232274	0.551	0.348	1.192292e-34	15
ANXA5	Memory LGALS3				
7.148273e-39	-0.6022556	0.017	0.137	1.274466e-34	15
NEIL1	Memory LGALS3				
7.619492e-39	0.4448427	0.607	0.378	1.358479e-34	15
C19orf70	Memory LGALS3				
7.985977e-39	0.3703679	0.596	0.393	1.42382e-34	15
BASP1	Memory LGALS3				
5.20826e-38	0.4292197	0.534	0.315	9.285806e-34	15

RHOG	Memory LGALS3	9.268135e-38	0.3946279	0.194	0.059	1.652416e-33	15
KYNU	Memory LGALS3	9.739334e-38	0.3513061	0.855	0.675	1.736426e-33	15
SRSF3	Memory LGALS3	1.454987e-37	0.4526562	0.544	0.34	2.594097e-33	15
RNH1	Memory LGALS3	2.728988e-37	-0.6930488	0.061	0.205	4.865513e-33	15
HMCES	Memory LGALS3	3.285797e-37	0.3934072	0.663	0.434	5.858248e-33	15
HMGA1	Memory LGALS3	6.048027e-36	0.4158944	0.756	0.571	1.078303e-31	15
APRT	Memory LGALS3	1.397952e-35	-0.696846	0.095	0.236	2.492408e-31	15
IL4R	Memory LGALS3	3.511638e-35	-0.61663	0.063	0.21	6.260899e-31	15
EAF2	Memory LGALS3	4.721838e-35	0.4172603	0.575	0.367	8.418565e-31	15
ARHGDIA	Memory LGALS3	5.242858e-35	0.4437979	0.563	0.351	9.347492e-31	15
SARS	Memory LGALS3	6.39315e-35	-0.7815351	0.475	0.62	1.139835e-30	15
MEF2C	Memory LGALS3	6.54035e-35	-0.544095	0.041	0.155	1.166079e-30	15
CD72	Memory LGALS3	1.232944e-34	0.4272791	0.646	0.44	2.198216e-30	15
C1QBP	Memory LGALS3	3.352731e-34	0.4329576	0.546	0.347	5.977584e-30	15
CHMP4B	Memory LGALS3	4.366562e-34	0.4113917	0.441	0.237	7.785144e-30	15
HLA-F	Memory LGALS3	4.429925e-34	0.3344931	0.836	0.674	7.898113e-30	15
MORF4L1	Memory LGALS3	2.449008e-33	0.440708	0.479	0.299	4.366337e-29	15
GLRX3	Memory LGALS3	2.457704e-33	-0.6114234	0.094	0.2	4.38184e-29	15
YBX3	Memory LGALS3	2.658535e-33	0.3346182	0.858	0.692	4.739903e-29	15
HNRNPDL	Memory LGALS3	3.63703e-33	-0.7980786	0.082	0.212	6.484461e-29	15
FOS	Memory LGALS3	5.628666e-33	0.3107949	0.999	0.998	1.003535e-28	15
TMSB4X	Memory LGALS3	6.727004e-33	0.3941723	0.244	0.098	1.199358e-28	15
RUNX3	Memory LGALS3	8.771211e-33	-0.3485781	0.007	0.105	1.563819e-28	15
SEMA4A	Memory LGALS3	1.227205e-32	0.3546571	0.712	0.51	2.187984e-28	15
UBE2I	Memory LGALS3	1.365173e-32	0.3961427	0.321	0.159	2.433968e-28	15

KIAA1551	Memory LGALS3	1.398446e-32	0.5468932	0.369	0.207	2.49329e-28	15
C19orf48	Memory LGALS3	1.882967e-32	0.3848009	0.596	0.414	3.357141e-28	15
CTSH	Memory LGALS3	2.08459e-32	-0.4794462	0.016	0.121	3.716616e-28	15
CD38	Memory LGALS3	2.41691e-32	0.4121518	0.4	0.212	4.309109e-28	15
C4orf48	Memory LGALS3	2.622284e-32	-0.7029684	0.133	0.255	4.675269e-28	15
FCMR	Memory LGALS3	3.017247e-32	0.3296434	0.808	0.622	5.379449e-28	15
ATP6V0E1	Memory LGALS3	3.570148e-32	0.4394546	0.368	0.186	6.365217e-28	15
RNF145	Memory LGALS3	6.582459e-32	-0.662286	0.08	0.214	1.173587e-27	15
FAM111B	Memory LGALS3	1.088762e-31	-0.6634561	0.127	0.278	1.941153e-27	15
NCF1	Memory LGALS3	1.584878e-31	0.3862022	0.772	0.591	2.825678e-27	15
TPM3	Memory LGALS3	1.872515e-31	0.4737177	0.41	0.227	3.338507e-27	15
TARS	Memory LGALS3	2.386113e-31	0.4051343	0.451	0.28	4.254201e-27	15
CD82	Memory LGALS3	2.507681e-31	0.3967038	0.247	0.087	4.470945e-27	15
AHNAK	Memory LGALS3	3.084003e-31	-0.6443787	0.203	0.35	5.498468e-27	15
ABRACL	Memory LGALS3	5.341219e-31	0.370017	0.655	0.455	9.52286e-27	15
GADD45GIP1	Memory LGALS3	6.67332e-31	0.4121612	0.308	0.144	1.189786e-26	15
MVP	Memory LGALS3	2.592768e-30	-0.5542335	0.016	0.111	4.622645e-26	15
VPREB3	Memory LGALS3	3.423274e-30	0.3370083	0.939	0.852	6.103355e-26	15
SH3BGRL3	Memory LGALS3	6.971267e-30	-0.6862221	0.226	0.367	1.242907e-25	15
RCSD1	Memory LGALS3	8.856348e-30	0.486897	0.4	0.206	1.578998e-25	15
MGST3	Memory LGALS3	1.00684e-29	0.4025024	0.558	0.34	1.795095e-25	15
CAST	Memory LGALS3	1.485283e-29	0.4017235	0.472	0.285	2.648111e-25	15
DCTPP1	Memory LGALS3	1.710051e-29	-0.3781266	0.01	0.103	3.04885e-25	15
MYBL2	Memory LGALS3	1.774048e-29	-0.6725868	0.305	0.443	3.162951e-25	15
RHOH	Memory LGALS3	5.187425e-29	0.3489483	0.812	0.649	9.248661e-25	15

TMBIM6	Memory LGALS3					
5.634061e-29	-0.5168193	0.718	0.772	1.004497e-24	15	
SEC62	Memory LGALS3					
5.948379e-29	0.4527398	0.419	0.246	1.060536e-24	15	
GARS	Memory LGALS3					
5.992398e-29	0.3985822	0.389	0.215	1.068385e-24	15	
CCDC50	Memory LGALS3					
1.519757e-28	0.3489686	0.685	0.493	2.709575e-24	15	
HSPE1	Memory LGALS3					
2.809745e-28	-0.5145925	0.056	0.139	5.009494e-24	15	
FCER2	Memory LGALS3					
3.16803e-28	0.3043773	0.892	0.754	5.648281e-24	15	
MT-ND5	Memory LGALS3					
4.289656e-28	-0.5446269	0.048	0.164	7.648027e-24	15	
SUSD3	Memory LGALS3					
9.20659e-28	0.3071771	0.512	0.394	1.641443e-23	15	
CSTB	Memory LGALS3					
1.094223e-27	0.3951758	0.313	0.161	1.95089e-23	15	
TES	Memory LGALS3					
1.137386e-27	0.3580594	0.583	0.388	2.027845e-23	15	
PHB	Memory LGALS3					
1.21623e-27	0.3311318	0.645	0.44	2.168416e-23	15	
KLF6	Memory LGALS3					
1.7366e-27	0.331759	0.786	0.628	3.096185e-23	15	
COX7B	Memory LGALS3					
1.907087e-27	0.3561153	0.23	0.092	3.400145e-23	15	
CYSLTR1	Memory LGALS3					
2.352794e-27	0.3173111	0.717	0.525	4.194797e-23	15	
PSME2	Memory LGALS3					
2.846346e-27	0.4221703	0.187	0.075	5.07475e-23	15	
SLAMF7	Memory LGALS3					
3.854457e-27	0.387598	0.346	0.206	6.872111e-23	15	
CFLAR	Memory LGALS3					
4.118458e-27	0.3617084	0.549	0.37	7.342798e-23	15	
PSMC3	Memory LGALS3					
4.171904e-27	-0.5350126	0.096	0.198	7.438087e-23	15	
CLEC2B	Memory LGALS3					
5.204851e-27	0.3758979	0.513	0.347	9.279729e-23	15	
MRPS6	Memory LGALS3					
6.19013e-27	0.3801298	0.297	0.156	1.103638e-22	15	
RBPJ	Memory LGALS3					
1.086457e-26	-0.6018515	0.096	0.209	1.937044e-22	15	
C16orf74	Memory LGALS3					
1.217505e-26	-0.6413296	0.128	0.247	2.17069e-22	15	
RASGRP2	Memory LGALS3					
2.353436e-26	0.4842588	0.69	0.53	4.195941e-22	15	
ATP6V1F	Memory LGALS3					
2.617293e-26	-0.6057637	0.173	0.281	4.666372e-22	15	
ADAM28	Memory LGALS3					
5.523927e-26	-0.4997242	0.061	0.16	9.848609e-22	15	

GLIPR1	Memory LGALS3				
8.149106e-26	-0.6011173	0.107	0.214	1.452904e-21	15
LBH	Memory LGALS3				
9.170973e-26	0.3667128	0.394	0.235	1.635093e-21	15
MRPL15	Memory LGALS3				
9.750754e-26	0.3591144	0.503	0.316	1.738462e-21	15
TUBA1A	Memory LGALS3				
1.26517e-25	0.350704 0.254	0.12	2.255671e-21	15	
HTATIP2 Memory LGALS3					
1.265938e-25	0.3417467	0.668	0.476	2.257041e-21	15
ISCU	Memory LGALS3				
1.905882e-25	0.3443467	0.326	0.167	3.397997e-21	15
MFSD10	Memory LGALS3				
2.427627e-25	-0.5684031	0.142	0.267	4.328216e-21	15
CD22	Memory LGALS3				
4.402167e-25	-0.4687814	0.036	0.123	7.848624e-21	15
LPP	Memory LGALS3				
5.500043e-25	-0.5331603	0.145	0.264	9.806026e-21	15
CCDC69	Memory LGALS3				
7.670503e-25	0.5047583	0.495	0.322	1.367574e-20	15
SLC3A2	Memory LGALS3				
9.480295e-25	0.3963804	0.246	0.123	1.690242e-20	15
SLC1A5	Memory LGALS3				
1.131033e-24	0.3694533	0.236	0.108	2.016519e-20	15
NUDT4	Memory LGALS3				
2.087356e-24	0.3113207	0.338	0.193	3.721548e-20	15
TAP1	Memory LGALS3				
2.807318e-24	0.3591328	0.316	0.165	5.005168e-20	15
CYSTM1	Memory LGALS3				
2.895577e-24	0.3197717	0.629	0.438	5.162524e-20	15
CCT6A	Memory LGALS3				
3.281242e-24	-0.5930016	0.315	0.436	5.850127e-20	15
CYB561A3	Memory LGALS3				
3.393176e-24	-0.4394148	0.048	0.136	6.049693e-20	15
PLEKHA2	Memory LGALS3				
4.011025e-24	0.3283719	0.99	0.962	7.151257e-20	15
FTL	Memory LGALS3				
4.931521e-24	-0.6511482	0.237	0.34	8.792408e-20	15
HVCN1	Memory LGALS3				
5.033063e-24	0.3587502	0.419	0.273	8.973448e-20	15
ATOX1	Memory LGALS3				
5.244238e-24	0.3155829	0.788	0.599	9.349952e-20	15
CDC42	Memory LGALS3				
7.32424e-24	-0.6309217	0.245	0.35	1.305839e-19	15
TRAF3IP3	Memory LGALS3				
7.651749e-24	-0.7120049	0.111	0.214	1.36423e-19	15
PLAC8	Memory LGALS3				
8.347875e-24	0.3690046	0.575	0.375	1.488343e-19	15
PDCD4	Memory LGALS3				
1.098979e-23	0.3346768	0.473	0.292	1.959369e-19	15

PDCD5	Memory LGALS3				
1.217563e-23	-0.6459196	0.127	0.237	2.170792e-19	15
UBE2J1	Memory LGALS3				
1.966612e-23	-0.4683609	0.988	0.985	3.506273e-19	15
TMSB10	Memory LGALS3				
2.118093e-23	-0.5990487	0.18	0.291	3.776348e-19	15
RUBCNL	Memory LGALS3				
2.352829e-23	0.3241797	0.632	0.459	4.194858e-19	15
PRMT1	Memory LGALS3				
2.784775e-23	0.3456367	0.248	0.114	4.964976e-19	15
VRK2	Memory LGALS3				
3.331276e-23	0.3897705	0.347	0.2	5.939332e-19	15
PTPN1	Memory LGALS3				
5.84402e-23	-1.346829	0.075	0.178	1.04193e-18	15
MZB1	Memory LGALS3				
6.78559e-23	0.3371708	0.649	0.48	1.209803e-18	15
RPS27L	Memory LGALS3				
7.817756e-23	0.3288656	0.256	0.117	1.393828e-18	15
LACTB	Memory LGALS3				
8.606335e-23	0.3276121	0.375	0.228	1.534423e-18	15
RTCB	Memory LGALS3				
1.03968e-22	0.3987317	0.449	0.305	1.853646e-18	15
CALR	Memory LGALS3				
1.434342e-22	0.3305065	0.473	0.298	2.557288e-18	15
ATP1B3	Memory LGALS3				
2.231415e-22	0.3030219	0.507	0.34	3.978389e-18	15
PSMC4	Memory LGALS3				
3.254143e-22	0.3489199	0.548	0.355	5.801811e-18	15
ARID5B	Memory LGALS3				
3.624441e-22	0.3058561	0.514	0.338	6.462016e-18	15
CMTM6	Memory LGALS3				
5.65836e-22	0.3289817	0.252	0.128	1.008829e-17	15
EMD	Memory LGALS3				
1.736912e-21	-0.6999043	0.222	0.318	3.096741e-17	15
CD69	Memory LGALS3				
3.822279e-21	-0.4687344	0.055	0.161	6.814741e-17	15
KIAA0040	Memory LGALS3				
3.888844e-21	-0.3790347	0.029	0.106	6.93342e-17	15
RMI2	Memory LGALS3				
5.202459e-21	0.3693457	0.268	0.149	9.275464e-17	15
PEA15	Memory LGALS3				
7.500256e-21	-1.579309	0.921	0.927	1.337221e-16	15
IGKC	Memory LGALS3				
1.156289e-20	0.3032597	0.75	0.582	2.061547e-16	15
EIF2S2	Memory LGALS3				
1.21402e-20	-0.5494759	0.179	0.28	2.164476e-16	15
PIM2	Memory LGALS3				
1.216196e-20	-0.4547776	0.098	0.197	2.168355e-16	15
AFF3	Memory LGALS3				
1.346316e-20	-0.4113617	0.038	0.107	2.400346e-16	15

C1orf162	Memory LGALS3	1.948453e-20	0.3092737	0.2	0.089	3.473897e-16	15
NFE2L1	Memory LGALS3	2.101062e-20	-0.5456561	0.043	0.151	3.745983e-16	15
BIK	Memory LGALS3	2.2542e-20	0.3015542	0.143	0.057	4.019013e-16	15
DUSP10	Memory LGALS3	2.787565e-20	0.3058445	0.559	0.381	4.969949e-16	15
NHP2	Memory LGALS3	2.982377e-20	0.3186199	0.316	0.182	5.317281e-16	15
YIF1A	Memory LGALS3	5.058427e-20	-0.4870634	0.12	0.222	9.01867e-16	15
LY86	Memory LGALS3	5.624054e-20	-0.4265422	0.081	0.183	1.002713e-15	15
LCK	Memory LGALS3	6.948727e-20	0.3133118	0.463	0.313	1.238889e-15	15
TCP1	Memory LGALS3	8.404303e-20	-0.5501822	0.385	0.488	1.498403e-15	15
GMFG	Memory LGALS3	8.723317e-20	-0.3490147	0.026	0.102	1.55528e-15	15
CENPM	Memory LGALS3	1.222823e-19	-0.5463488	0.191	0.3	2.180171e-15	15
GGA2	Memory LGALS3	1.228787e-19	-0.551482	0.283	0.386	2.190804e-15	15
EVL	Memory LGALS3	1.803954e-19	0.3150873	0.318	0.179	3.21627e-15	15
ARL2	Memory LGALS3	2.659507e-19	-0.3662164	0.054	0.149	4.741636e-15	15
SEC14L1	Memory LGALS3	3.05838e-19	-0.5378029	0.721	0.778	5.452786e-15	15
ARHGDIB	Memory LGALS3	5.617209e-19	-0.4758923	0.087	0.188	1.001492e-14	15
P2RX5	Memory LGALS3	6.469649e-19	-0.4446974	0.058	0.156	1.153474e-14	15
CD81	Memory LGALS3	6.825673e-19	-0.484709	0.96	0.939	1.216949e-14	15
H3F3A	Memory LGALS3	9.130914e-19	-0.490153	0.105	0.185	1.627951e-14	15
PLPP5	Memory LGALS3	1.289163e-18	0.3325824	0.266	0.142	2.298449e-14	15
SERPINB9	Memory LGALS3	1.729191e-18	0.3169032	0.354	0.225	3.082975e-14	15
SINHCAF	Memory LGALS3	1.966725e-18	-0.567367	0.309	0.406	3.506475e-14	15
STX7	Memory LGALS3	2.150069e-18	-0.3367099	0.032	0.113	3.833358e-14	15
AC023590.1	Memory LGALS3	2.544275e-18	-0.4998466	0.203	0.286	4.536188e-14	15
GNG7	Memory LGALS3	3.878929e-18	-0.3892891	0.051	0.127	6.915743e-14	15

AC025164.1	Memory LGALS3					
4.747057e-18	-0.4039698	0.068	0.156	8.463527e-14	15	
TSPAN13	Memory LGALS3					
4.7919e-18	-0.5058834	0.097	0.176	8.543478e-14	15	
MARCH1	Memory LGALS3					
4.924665e-18	-0.5085986	0.147	0.239	8.780184e-14	15	
CCND3	Memory LGALS3					
1.103036e-17	-0.3897376	0.08	0.167	1.966603e-13	15	
DEF8	Memory LGALS3					
1.149175e-17	0.3243795	0.296	0.174	2.048864e-13	15	
BCL2A1	Memory LGALS3					
1.164705e-17	-0.4985217	0.207	0.295	2.076553e-13	15	
BCL11A	Memory LGALS3					
1.382784e-17	-0.52795	0.217	0.324	2.465366e-13	15	
TNFRSF13C	Memory LGALS3					
2.143734e-17	-0.6005657	0.677	0.726	3.822062e-13	15	
LIMD2	Memory LGALS3					
2.746375e-17	0.3193564	0.547	0.394	4.896512e-13	15	
ATF4	Memory LGALS3					
4.084421e-17	-0.5667401	0.547	0.577	7.282114e-13	15	
SARAF	Memory LGALS3					
5.318352e-17	-0.4531435	0.104	0.188	9.482089e-13	15	
CDCA7L	Memory LGALS3					
6.201528e-17	-0.5495683	0.311	0.4	1.10567e-12	15	
ORAI2	Memory LGALS3					
6.279921e-17	-0.6029431	0.293	0.377	1.119647e-12	15	
ACADM	Memory LGALS3					
7.049917e-17	-0.4755193	0.163	0.261	1.25693e-12	15	
SYNE2	Memory LGALS3					
7.736207e-17	-0.3432799	0.05	0.129	1.379288e-12	15	
STAP1	Memory LGALS3					
1.033002e-16	0.3179894	0.313	0.185	1.84174e-12	15	
GPATCH4	Memory LGALS3					
1.069255e-16	0.3928463	0.247	0.133	1.906375e-12	15	
PSAT1	Memory LGALS3					
1.106454e-16	-0.4142869	0.066	0.148	1.972696e-12	15	
TAGAP	Memory LGALS3					
1.703758e-16	-0.494433	0.179	0.277	3.03763e-12	15	
ITGAE	Memory LGALS3					
1.948543e-16	-0.3875148	0.078	0.161	3.474058e-12	15	
APLP2	Memory LGALS3					
4.325334e-16	-0.5948082	0.238	0.298	7.711637e-12	15	
LINC00926	Memory LGALS3					
6.089767e-16	-0.5391397	0.093	0.188	1.085745e-11	15	
RGS2	Memory LGALS3					
6.878137e-16	-0.4283603	0.093	0.189	1.226303e-11	15	
HLA-DOB	Memory LGALS3					
7.402611e-16	-0.4454877	0.068	0.171	1.319812e-11	15	
CCDC88A	Memory LGALS3					
8.058651e-16	0.3151645	0.193	0.097	1.436777e-11	15	

SGK1 Memory LGALS3
8.960388e-16 -0.3962689 0.05 0.111 1.597548e-11 15
LINC02397 Memory LGALS3
9.748055e-16 -0.5545885 0.404 0.212 1.737981e-11 15
IGHG1 Memory LGALS3
9.90441e-16 -0.3311922 0.057 0.123 1.765857e-11 15
ZNF318 Memory LGALS3
1.020632e-15 0.311081 0.168 0.093 1.819685e-11 15
MT2A Memory LGALS3
1.615345e-15 -0.3245626 0.043 0.122 2.879998e-11 15
VNN2 Memory LGALS3
1.682208e-15 -0.456119 0.164 0.244 2.999208e-11 15
PHACTR1 Memory LGALS3
3.841607e-15 -0.5357578 0.263 0.348 6.849201e-11 15
BIRC3 Memory LGALS3
4.743905e-15 -0.5829714 0.957 0.933 8.457908e-11 15
SERF2 Memory LGALS3
5.751229e-15 -0.3709295 0.078 0.171 1.025387e-10 15
SEL1L3 Memory LGALS3
1.051871e-14 -0.3504861 0.081 0.151 1.87538e-10 15
ARHGAP9 Memory LGALS3
2.984406e-14 -0.3325601 0.055 0.115 5.320898e-10 15
LGALS9 Memory LGALS3
4.807521e-14 0.3033147 0.347 0.231 8.571329e-10 15
MTHFD2 Memory LGALS3
7.597117e-14 -0.3680663 0.113 0.159 1.35449e-09 15
SLC2A3 Memory LGALS3
8.012292e-14 -0.5010461 0.402 0.468 1.428511e-09 15
ITM2B Memory LGALS3
1.067476e-13 -0.5219448 0.241 0.325 1.903203e-09 15
AES Memory LGALS3
2.765563e-13 -0.4581428 0.341 0.424 4.930723e-09 15
TMEM123 Memory LGALS3
4.426338e-13 -0.4032836 0.092 0.175 7.891717e-09 15
TMEM156 Memory LGALS3
8.105549e-13 0.3103012 0.284 0.157 1.445138e-08 15
DDIT4 Memory LGALS3
9.673629e-13 -0.3858089 0.136 0.209 1.724711e-08 15
SNHG25 Memory LGALS3
1.135618e-12 -0.4247261 0.253 0.334 2.024694e-08 15
BPTF Memory LGALS3
1.516644e-12 -0.4749207 0.224 0.291 2.704025e-08 15
DCK Memory LGALS3
1.648568e-12 -0.460357 0.364 0.448 2.939232e-08 15
BTG2 Memory LGALS3
2.116436e-12 -0.3261171 0.067 0.129 3.773394e-08 15
CCNG2 Memory LGALS3
2.567923e-12 -0.4340748 0.672 0.7 4.57835e-08 15
CCNI Memory LGALS3
3.053806e-12 -0.9172164 0.132 0.213 5.44463e-08 15

PLCG2	Memory LGALS3				
3.808896e-12	-0.3241993	0.124	0.199	6.790881e-08	15
POLD4	Memory LGALS3				
5.128762e-12	-0.3107628	0.08	0.147	9.144069e-08	15
MID1IP1	Memory LGALS3				
1.304753e-11	-0.4748318	0.28	0.34	2.326244e-07	15
STK17A	Memory LGALS3				
1.488954e-11	-0.4381131	0.215	0.287	2.654656e-07	15
PPM1K	Memory LGALS3				
1.585014e-11	-0.3448747	0.111	0.17	2.825921e-07	15
TMEM154	Memory LGALS3				
3.82176e-11	-0.4557329	0.177	0.242	6.813816e-07	15
RGS1	Memory LGALS3				
4.914345e-11	-0.5179276	0.271	0.342	8.761785e-07	15
LRMP	Memory LGALS3				
5.683847e-11	-0.3775408	0.189	0.221	1.013373e-06	15
GBP2	Memory LGALS3				
6.849788e-11	-0.3460983	0.191	0.26	1.221249e-06	15
CNN2	Memory LGALS3				
7.427329e-11	-0.3184255	0.068	0.14	1.324218e-06	15
SIT1	Memory LGALS3				
8.86166e-11	-0.3286474	0.1	0.157	1.579945e-06	15
JUND	Memory LGALS3				
9.567832e-11	-0.3768705	0.165	0.236	1.705849e-06	15
ZCCHC7	Memory LGALS3				
1.266508e-10	-0.4742609	0.47	0.504	2.258056e-06	15
TSC22D3	Memory LGALS3				
1.306048e-10	-0.4795546	0.389	0.43	2.328553e-06	15
IRF8	Memory LGALS3				
1.60635e-10	-0.3039076	0.16	0.212	2.863962e-06	15
GABPB1-AS1	Memory LGALS3				
1.913384e-10	-0.4098314	0.088	0.144	3.411372e-06	15
DUSP1	Memory LGALS3				
1.939734e-10	-0.3602822	0.139	0.21	3.458352e-06	15
GLRX	Memory LGALS3				
1.98316e-10	-0.4505224	0.387	0.455	3.535776e-06	15
PTPRC	Memory LGALS3				
2.0262e-10	-0.3061757	0.08	0.148	3.612512e-06	15
RBM38	Memory LGALS3				
2.870157e-10	-0.481332	0.252	0.304	5.117203e-06	15
ZFP36L2	Memory LGALS3				
3.333516e-10	-0.3817169	0.715	0.723	5.943325e-06	15
SRSF5	Memory LGALS3				
4.021664e-10	-0.3193535	0.091	0.149	7.170224e-06	15
ALOX5	Memory LGALS3				
4.711274e-10	-1.420761	0.294	0.303	8.39973e-06	15
HMGB2	Memory LGALS3				
8.760603e-10	-0.3522353	0.135	0.186	1.561928e-05	15
ANKRD44	Memory LGALS3				
8.953157e-10	-2.196193	0.543	0.647	1.596258e-05	15

IGLC2	Memory LGALS3					
9.377024e-10	-0.5141122	0.432	0.456	1.67183e-05	15	
JUNB	Memory LGALS3					
1.109805e-09	-0.4639325	0.194	0.239	1.978672e-05	15	
CD55	Memory LGALS3					
1.313356e-09	-0.3769426	0.152	0.209	2.341582e-05	15	
CCDC32	Memory LGALS3					
1.601714e-09	-0.3637669	0.201	0.266	2.855696e-05	15	
RBM6	Memory LGALS3					
1.623955e-09	-0.3285527	0.068	0.12	2.895349e-05	15	
UBE2S	Memory LGALS3					
2.378695e-09	-1.803909	0.163	0.125	4.240975e-05	15	
IGHG2	Memory LGALS3					
2.807709e-09	-0.3798066	0.12	0.176	5.005864e-05	15	
QRSL1	Memory LGALS3					
3.738552e-09	-0.3316735	0.182	0.248	6.665464e-05	15	
CSK	Memory LGALS3					
5.202503e-09	-2.337613	0.628	0.446	9.275542e-05	15	
IGHG3	Memory LGALS3					
5.589075e-09	-0.3295665	0.189	0.252	9.964761e-05	15	
TIFA	Memory LGALS3					
7.358587e-09	-0.3363308	0.19	0.229	0.0001311962	15	
NT5C3A	Memory LGALS3					
7.520849e-09	-0.4655615	0.578	0.627	0.0001340892	15	
HLA-DMB	Memory LGALS3					
8.279021e-09	-0.3774808	0.301	0.362	0.0001476067	15	
TPD52	Memory LGALS3					
9.199307e-09	-0.3376182	0.608	0.627	0.0001640144	15	
SELENOH	Memory LGALS3					
1.005197e-08	-0.3841851	0.282	0.321	0.0001792166	15	
LTA4H	Memory LGALS3					
1.594649e-08	-0.3760662	0.176	0.239	0.00028431	15	
GCHFR	Memory LGALS3					
1.83126e-08	-0.3785102	0.466	0.502	0.0003264954	15	
FOXP1	Memory LGALS3					
2.45429e-08	-0.3624423	0.476	0.507	0.0004375754	15	
FDFT1	Memory LGALS3					
4.083421e-08	-0.3554378	0.304	0.359	0.0007280331	15	
TMEM243	Memory LGALS3					
4.20561e-08	-0.4370553	0.461	0.493	0.0007498183	15	
C4orf3	Memory LGALS3					
5.783511e-08	-0.3472626	0.29	0.328	0.001031142	15	
PTPN6	Memory LGALS3					
6.057686e-08	-0.4016224	0.351	0.353	0.001080025	15	
TXNIP	Memory LGALS3					
8.113454e-08	-0.3147498	0.215	0.264	0.001446548	15	
NSD3	Memory LGALS3					
9.342755e-08	-0.3315063	0.298	0.356	0.00166572	15	
CD19	Memory LGALS3					
1.484127e-07	-0.4188865	0.373	0.421	0.00264605	15	

AC114760.2	Memory LGALS3					
1.984478e-07	-0.3437657	0.229	0.284	0.003538126	15	
LAT2	Memory LGALS3					
2.222431e-07	-0.3541762	0.277	0.323	0.003962373	15	
NAA38	Memory LGALS3					
2.834058e-07	-0.360944	0.171	0.219	0.005052843	15	
NANS	Memory LGALS3					
3.122932e-07	-0.3667567	0.556	0.571	0.005567875	15	
SNX2	Memory LGALS3					
3.48103e-07	-0.3691413	0.216	0.258	0.006206329	15	
MDM4	Memory LGALS3					
4.159237e-07	-0.3452998	0.448	0.492	0.007415504	15	
RAC2	Memory LGALS3					
6.289254e-07	-0.3631386	0.601	0.62	0.01121311	15	
IFI16	Memory LGALS3					
6.493446e-07	-0.353627	0.515	0.493	0.01157717	15	
FXYD5	Memory LGALS3					
7.533875e-07	-0.339789	0.232	0.275	0.01343215	15	
RSRP1	Memory LGALS3					
1.04312e-06	-0.3301971	0.305	0.33	0.01859779	15	
SMC6	Memory LGALS3					
1.234684e-06	-0.3223182	0.682	0.675	0.02201319	15	
ATP6V1G1	Memory LGALS3					
1.594108e-06	-0.4059028	0.371	0.406	0.02842135	15	
SMIM14	Memory LGALS3					
2.484973e-06	-1.347361	0.413	0.422	0.04430458	15	
HIST1H4C	Memory LGALS3					
2.500575e-06	-0.3260029	0.188	0.231	0.04458276	15	
ATM	Memory LGALS3					
2.512766e-06	-0.3772254	0.289	0.332	0.04480011	15	
RIPOR2	Memory LGALS3					
2.6062e-06	-0.320111	0.157	0.194	0.04646594	15	
DNMT1	Memory LGALS3					
3.05675e-06	-0.3542481	0.349	0.389	0.05449879	15	
EVI2B	Memory LGALS3					
3.143602e-06	-0.304279	0.263	0.312	0.05604727	15	
IL16	Memory LGALS3					
4.300986e-06	-0.5742415	0.161	0.191	0.07668228	15	
PTTG1	Memory LGALS3					
4.772791e-06	-0.3276473	0.356	0.404	0.08509409	15	
MBD4	Memory LGALS3					
6.477597e-06	-0.3057082	0.172	0.215	0.1154891	15	
YPEL3	Memory LGALS3					
7.41686e-06	-0.3977766	0.784	0.762	0.1322352	15	
CYBA	Memory LGALS3					
9.470407e-06	-1.433143	0.451	0.428	0.1688479	15	
IGLC3	Memory LGALS3					
1.397973e-05	-0.3232343	0.153	0.193	0.2492445	15	
FAM30A	Memory LGALS3					
1.774047e-05	-0.3573468	0.173	0.207	0.3162948	15	

PRKCB Memory LGALS3
2.391991e-05 -0.3325246 0.821 0.8 0.4264681 15
LAPTM5 Memory LGALS3
4.410723e-05 -0.3030489 0.447 0.455 0.7863878 15
CTSS Memory LGALS3
6.70695e-05 -0.4865501 0.254 0.274 1 15
KLF2 Memory LGALS3
0.0001796233 -0.3224465 0.292 0.313 1 15
PSIP1 Memory LGALS3
0.0002432534 -0.3511016 0.292 0.332 1 15
GRHPR Memory LGALS3
0.0002505228 -0.421483 0.495 0.498 1 15
SAT1 Memory LGALS3
0.0006601566 -0.3121208 0.272 0.286 1 15
SP110 Memory LGALS3
0.001462821 -0.3128535 0.256 0.267 1 15
HHEX Memory LGALS3
0.002188118 -0.7429264 0.802 0.714 1 15
HMGN2 Memory LGALS3
0.002413521 -0.3466325 0.326 0.338 1 15
IER2 Memory LGALS3
0.002872321 -0.3788509 0.364 0.359 1 15
H2AFV Memory LGALS3
0.006982009 -0.3962154 0.901 0.85 1 15
ATP5MG Memory LGALS3
0 2.581614 0.959 0.189 0 16 RGS13 GC LMO2
0 2.183352 0.985 0.579 0 16 MARCKSL1 GC LMO2
0 2.002801 0.853 0.187 0 16 HMCES GC LMO2
0 1.692754 0.576 0.066 0 16 LMO2 GC LMO2
0 1.54388 0.884 0.387 0 16 BASP1 GC LMO2
0 1.507149 0.794 0.252 0 16 CD22 GC LMO2
0 1.46815 0.983 0.781 0 16 ACTG1 GC LMO2
0 1.396189 1 0.989 0 16 CD74 GC LMO2
0 1.382215 0.93 0.629 0 16 CD53 GC LMO2
0 1.329577 0.935 0.531 0 16 PARP1 GC LMO2
0 1.304886 0.987 0.763 0 16 HMGN1 GC LMO2
0 1.209011 0.99 0.81 0 16 CORO1A GC LMO2
0 1.199461 0.999 0.99 0 16 ACTB GC LMO2
0 1.174964 0.996 0.932 0 16 SERF2 GC LMO2
0 1.137996 0.995 0.867 0 16 ARPC2 GC LMO2
4.940656e-323 1.874887 0.585 0.098 8.808696e-319 16
VPREB3 GC LMO2
3.164805e-317 1.52353 0.859 0.328 5.642531e-313 16
LRMP GC LMO2
8.553794e-314 1.887483 0.64 0.137 1.525056e-309 16 BIK
GC LMO2
3.012054e-312 1.525741 0.708 0.207 5.370191e-308 16
NANS GC LMO2
3.126002e-310 1.439838 0.446 0.044 5.573349e-306 16
SERPINA9 GC LMO2

6.595776e-289 1.025733 0.996 0.945 1.175961e-284 16
HLA-DRA GC LMO2
7.716883e-288 1.279949 0.923 0.544 1.375843e-283 16
TCEA1 GC LMO2
9.472566e-280 1.027593 0.987 0.796 1.688864e-275 16
LAPTM5 GC LMO2
4.321647e-271 1.36353 0.649 0.169 7.705065e-267 16 LCK
GC LMO2
3.708859e-270 1.332432 0.568 0.101 6.612525e-266 16
AC023590.1 GC LMO2
1.701626e-258 1.397726 0.752 0.223 3.033829e-254 16
UBE2J1 GC LMO2
1.588123e-255 1.456434 0.62 0.123 2.831464e-251 16
NEIL1 GC LMO2
5.042804e-245 1.091001 0.349 0.039 8.990816e-241 16
CCDC144A GC LMO2
1.851681e-243 1.283715 0.548 0.111 3.301363e-239 16
VNN2 GC LMO2
1.846377e-240 0.9983265 0.29 0.023 3.291905e-236 16
CAMK1 GC LMO2
3.821673e-238 1.355758 0.715 0.226 6.813661e-234 16
GCHFR GC LMO2
1.858581e-233 1.10891 0.959 0.672 3.313663e-229 16
HLA-DMA GC LMO2
9.949966e-233 -1.99259 0.231 0.709 1.773979e-228 16
EMP3 GC LMO2
1.474034e-230 0.9206038 0.993 0.886 2.628055e-226 16
GAPDH GC LMO2
2.346833e-230 1.283049 0.613 0.178 4.184168e-226 16
PRPSAP2 GC LMO2
1.022625e-228 1.408814 0.488 0.107 1.823238e-224 16
GMDS GC LMO2
2.545914e-226 1.414979 0.643 0.18 4.539111e-222 16
DAAM1 GC LMO2
4.12744e-222 1.195997 0.784 0.318 7.358812e-218 16
RFTN1 GC LMO2
8.429295e-222 0.9365367 0.985 0.848 1.502859e-217 16
ATP5MG GC LMO2
3.921473e-218 1.051396 0.974 0.757 6.991594e-214 16
CYBA GC LMO2
7.90205e-218 1.085012 0.411 0.063 1.408856e-213 16
RPRD1B GC LMO2
1.167897e-216 1.347131 0.51 0.112 2.082243e-212 16 LPP
GC LMO2
5.041398e-215 0.833889 0.989 0.897 8.988309e-211 16
PFN1 GC LMO2
8.207915e-210 1.062056 0.937 0.691 1.463389e-205 16
HLA-DQB1 GC LMO2
1.163096e-206 1.030932 0.295 0.025 2.073684e-202 16
DHRS9 GC LMO2

3.618379e-205	1.008861	0.931	0.615	6.451208e-201	16	ARPC1B	GC LMO2
1.189198e-204	1.272946	0.769	0.321	2.120221e-200	16	GRHPR	GC LMO2
1.63087e-203	1.096687	0.936	0.646	2.907678e-199	16	HLA-DQA1	GC LMO2
7.655088e-201	1.197597	0.586	0.159	1.364826e-196	16	SEL1L3	GC LMO2
1.063334e-199	1.365848	0.956	0.749	1.895819e-195	16	HLA-DRB1	GC LMO2
2.121639e-197	1.178776	0.73	0.279	3.782671e-193	16	BCAS4	GC LMO2
3.269821e-194	1.061202	0.402	0.065	5.829764e-190	16	S1PR2	GC LMO2
2.601109e-193	-1.977412	0.117	0.553	4.637517e-189	16	CCR7	GC LMO2
1.848144e-192	1.193942	0.767	0.292	3.295055e-188	16	BCL7A	GC LMO2
1.256802e-191	1.125332	0.564	0.156	2.240753e-187	16	DEF8	GC LMO2
2.141382e-189	1.101447	0.443	0.095	3.817871e-185	16	SEMA4A	GC LMO2
7.153894e-184	-1.170246	0.715	0.911	1.275468e-179	16	IGHM	GC LMO2
1.486304e-183	0.9782756	0.951	0.72	2.649932e-179	16	LIMD2	GC LMO2
8.814098e-182	0.8748059	0.322	0.049	1.571466e-177	16	BORCS8-MEF2B	GC LMO2
2.071686e-181	1.185021	0.422	0.095	3.69361e-177	16	ALDH2	GC LMO2
2.492602e-181	1.305756	0.638	0.232	4.444061e-177	16	RGS1	GC LMO2
9.496593e-177	1.19062	0.563	0.152	1.693147e-172	16	SUSD3	GC LMO2
4.265919e-176	0.8477369	0.96	0.811	7.605707e-172	16	HLA-DPA1	GC LMO2
3.518108e-175	0.6779962	0.217	0.015	6.272434e-171	16	CAMK2B	GC LMO2
5.698635e-175	1.119471	0.644	0.197	1.01601e-170	16	EAF2	GC LMO2
3.347862e-174	-0.6806524	1	1	5.968903e-170	16	RPL41	GC LMO2
1.323588e-173	1.118706	0.78	0.366	2.359826e-169	16	ACADM	GC LMO2
4.774372e-171	0.8679987	0.987	0.853	8.512228e-167	16	CD79B	GC LMO2
1.883908e-170	0.8074455	0.254	0.022	3.35882e-166	16	HTR3A	GC LMO2
2.14393e-168	-0.8189858	0.992	0.999	3.822413e-164	16	RPS29	GC LMO2

8.106025e-164	1.167264	0.567	0.159	1.445223e-159	16	CCDC88A	GC LMO2
3.632265e-163	1.131841	0.76	0.409	6.475966e-159	16	METAP2	GC LMO2
4.427496e-160	0.9146173	0.846	0.393	7.893782e-156	16	JCHAIN	GC LMO2
9.44985e-160	0.7684856	0.277	0.034	1.684814e-155	16	TOX	GC LMO2
1.646509e-159	0.9547084	0.831	0.451	2.935561e-155	16	SWAP70	GC LMO2
1.418436e-158	0.8527262	0.946	0.688	2.528929e-154	16	EZR	GC LMO2
6.088079e-156	0.7944851	0.985	0.879	1.085444e-151	16	MS4A1	GC LMO2
4.404632e-155	-0.9903791	0.994	0.996	7.853018e-151	16	RPS12	GC LMO2
2.895472e-154	1.047467	0.497	0.11	5.162337e-150	16	CD38	GC LMO2
2.278336e-152	1.010584	0.898	0.598	4.062045e-148	16	DYNLL1	GC LMO2
2.382734e-152	-1.913921	0.201	0.579	4.248177e-148	16	VIM	GC LMO2
1.089416e-151	1.038692	0.736	0.357	1.942319e-147	16	SNAP23	GC LMO2
4.194838e-151	1.052219	0.695	0.265	7.478977e-147	16	NCF1	GC LMO2
4.91688e-149	0.8028258	0.642	0.501	8.766306e-145	16	HLA-DRB5	GC LMO2
1.705164e-147	0.7357103	0.99	0.863	3.040137e-143	16	CD79A	GC LMO2
2.565266e-147	0.8883528	0.965	0.648	4.573612e-143	16	TCL1A	GC LMO2
9.456036e-146	0.9512982	0.733	0.366	1.685917e-141	16	LCP1	GC LMO2
1.560336e-145	0.9812358	0.617	0.241	2.781923e-141	16	SH3KBP1	GC LMO2
3.66665e-145	1.01149	0.708	0.319	6.537271e-141	16	GYPC	GC LMO2
7.522073e-144	0.7773384	0.29	0.046	1.34111e-139	16	ASB13	GC LMO2
9.856347e-143	0.6232487	0.991	0.917	1.757288e-138	16	CFL1	GC LMO2
1.940317e-142	0.946333	0.653	0.299	3.459391e-138	16	FERMT3	GC LMO2
3.368115e-142	0.880324	0.865	0.55	6.005013e-138	16	ATP5IF1	GC LMO2
1.047536e-140	0.6847679	0.974	0.867	1.867652e-136	16	ARPC3	GC LMO2
3.181064e-136	0.7738338	0.889	0.614	5.67152e-132	16	ACTR3	GC LMO2

6.228832e-135	0.9483144	0.482	0.132	1.110538e-130	16	SOCS1	GC LMO2
7.423328e-134	0.7907256	0.908	0.67	1.323505e-129	16	ATP6V1G1	GC LMO2
8.881869e-134	0.9216052	0.468	0.133	1.583549e-129	16	AIM2	GC LMO2
2.368658e-133	0.868929	0.4	0.103	4.22308e-129	16	ITGB2	GC LMO2
2.895154e-133	0.7989834	0.905	0.647	5.16177e-129	16	YWHAB	GC LMO2
1.059863e-132	0.9690917	0.634	0.258	1.88963e-128	16	ALOX5AP	GC LMO2
2.90714e-132	0.565989	1	0.998	5.183141e-128	16	TMSB4X	GC LMO2
5.244121e-132	0.5432193	0.996	0.96	9.349743e-128	16	OAZ1	GC LMO2
8.702914e-132	1.022564	0.456	0.131	1.551643e-127	16	SIT1	GC LMO2
3.481689e-129	0.9557158	0.324	0.057	6.207503e-125	16	HOPX	GC LMO2
1.397102e-127	0.7416738	0.306	0.066	2.490894e-123	16	NEK6	GC LMO2
7.065169e-127	0.7310842	0.286	0.048	1.259649e-122	16	A4GALT	GC LMO2
4.182277e-126	0.8803618	0.709	0.357	7.456581e-122	16	TERF2IP	GC LMO2
9.913983e-125	-1.303748	0.255	0.584	1.767564e-120	16	SARAF	GC LMO2
2.988643e-124	-0.578565	1	0.999	5.328452e-120	16	RPLP1	GC LMO2
2.375131e-122	0.7923176	0.945	0.771	4.23462e-118	16	HLA-DPB1	GC LMO2
1.19916e-121	0.8331813	0.914	0.619	2.137982e-117	16	HLA-DMB	GC LMO2
9.168256e-121	0.6074069	0.981	0.9	1.634608e-116	16	MYL6	GC LMO2
9.186071e-121	-1.954723	0.148	0.472	1.637785e-116	16	S100A6	GC LMO2
1.743667e-119	-0.7032837	0.997	0.999	3.108785e-115	16	RPLP2	GC LMO2
2.600382e-119	0.5667841	0.996	0.938	4.636222e-115	16	H3F3A	GC LMO2
4.062046e-118	0.8473237	0.879	0.593	7.242222e-114	16	PRDX1	GC LMO2
9.587383e-118	0.8795571	0.642	0.275	1.709335e-113	16	LAT2	GC LMO2
1.462952e-116	-0.8460017	0.949	0.983	2.608296e-112	16	RPS21	GC LMO2
1.417458e-115	-0.7554766	1	0.998	2.527185e-111	16	B2M	GC LMO2

1.526295e-115	0.7881464	0.32	0.069	2.721231e-111	16	PAG1	GC LMO2
5.1859e-115	0.8473247	0.763	0.411	9.245941e-111	16	CTSH	GC LMO2
2.919115e-114	0.7226677	0.282	0.058	5.20449e-110	16	FAM129A	GC LMO2
5.304513e-114	0.866936	0.489	0.175	9.457417e-110	16	RASGRP3	GC LMO2
1.469797e-113	0.7623021	0.322	0.074	2.620502e-109	16	MYO1E	GC LMO2
8.218613e-113	0.9089034	0.414	0.099	1.465297e-108	16	HRK	GC LMO2
3.714798e-112	0.8335706	0.771	0.422	6.623113e-108	16	IRF8	GC LMO2
9.200743e-112	0.7942117	0.741	0.407	1.6404e-107	16	HDAC1	GC LMO2
2.305849e-111	0.7494829	0.869	0.577	4.111098e-107	16	ANP32B	GC LMO2
5.645206e-111	0.8630417	0.779	0.446	1.006484e-106	16	PTPRC	GC LMO2
1.835692e-109	0.9393062	0.855	0.526	3.272855e-105	16	ISG20	GC LMO2
2.744227e-109	0.8658028	0.599	0.253	4.892682e-105	16	CCDC69	GC LMO2
1.030994e-108	-0.6386458	0.996	0.999	1.83816e-104	16	RPL13	GC LMO2
3.079804e-108	0.8242511	0.62	0.287	5.490982e-104	16	LYPLA1	GC LMO2
3.331593e-108	-1.301645	0.073	0.357	5.939897e-104	16	LY6E	GC LMO2
5.164423e-108	0.9090315	0.493	0.145	9.207651e-104	16	CD81	GC LMO2
2.752608e-105	0.8134934	0.773	0.432	4.907625e-101	16	RHOH	GC LMO2
3.772634e-105	0.877999	0.519	0.178	6.726229e-101	16	P2RX5	GC LMO2
6.807477e-105	0.9066492	0.648	0.296	1.213705e-100	16	CD27	GC LMO2
1.506604e-104	0.7764175	0.394	0.098	2.686125e-100	16	SCIMP	GC LMO2
1.797594e-104	0.8155964	0.386	0.117	3.204931e-100	16	SPATS2	GC LMO2
2.715754e-104	0.7559411	0.396	0.126	4.841917e-100	16	PRKCD	GC LMO2
5.202393e-104	0.8235386	0.697	0.353	9.275346e-100	16	TPD52	GC LMO2
1.2788e-103	0.8312396	0.604	0.266	2.279972e-99	16	POU2AF1	GC LMO2
1.507309e-103	0.8482477	0.436	0.14	2.687381e-99	16	SEC14L1	GC LMO2

3.192942e-103	0.8432346	0.393	0.105	5.692697e-99	16	CPNE5 GC LMO2
1.164822e-102	0.7354581	0.321	0.072	2.076762e-98	16	KLHL6 GC LMO2
1.32991e-101	0.8030381	0.368	0.101	2.371097e-97	16	E2F5 GC LMO2
1.531416e-101	0.5905319	0.214	0.036	2.730362e-97	16	EEPD1 GC LMO2
2.701118e-101	0.8166574	0.599	0.251	4.815823e-97	16	SYNE2 GC LMO2
3.750604e-101	0.790687	0.44	0.151	6.686952e-97	16	SMIM20 GC LMO2
4.743998e-101	0.498226	0.154	0.013	8.458075e-97	16	BTNL9 GC LMO2
6.094686e-100	0.7455074	0.83	0.528	1.086622e-95	16	SRSF9 GC LMO2
8.356517e-100	-0.6845776	0.996	0.995	1.489883e-95	16	RPS25 GC LMO2
1.216592e-99	0.7975824	0.541	0.217	2.169062e-95	16	SIAH2 GC LMO2
1.995708e-99	0.6279841	0.199	0.029	3.558148e-95	16	GPR137B GC LMO2
2.084563e-99	0.6373493	0.246	0.052	3.716568e-95	16	BFSP2 GC LMO2
2.10199e-99	0.7656458	0.689	0.37	3.747637e-95	16	ZFAND6 GC LMO2
2.214616e-99	0.6455053	0.959	0.772	3.948439e-95	16	ARHGDIB GC LMO2
5.572835e-99	-0.5984438	0.998	0.999	9.935808e-95	16	RPS27A GC LMO2
1.061512e-98	0.8103441	0.355	0.107	1.892569e-94	16	PIP4K2A GC LMO2
2.792534e-97	0.7887157	0.609	0.27	4.978809e-93	16	STK17B GC LMO2
5.106562e-96	0.6746459	0.284	0.058	9.104489e-92	16	ZNF608 GC LMO2
8.830934e-96	-0.5596521	1	0.999	1.574467e-91	16	RPL13A GC LMO2
7.54352e-95	-1.198873	0.243	0.518	1.344934e-90	16	BANK1 GC LMO2
3.331297e-94	0.7918963	0.513	0.18	5.93937e-90	16	HLA-DOB GC LMO2
3.875258e-94	0.7822749	0.509	0.186	6.909197e-90	16	DNMT1 GC LMO2
3.936893e-94	0.6791445	0.308	0.078	7.019087e-90	16	BPNT1 GC LMO2
8.657374e-94	0.7840735	0.701	0.367	1.543523e-89	16	PTP4A2 GC LMO2
9.438725e-94	0.6640914	0.315	0.076	1.68283e-89	16	VGLL4 GC LMO2

1.161754e-93	0.7936284	0.517	0.227	2.071291e-89	16	CHCHD10	GC LMO2
2.771257e-93	0.7768186	0.643	0.289	4.940874e-89	16	GGA2	GC LMO2
4.75427e-93	0.7734089	0.478	0.198	8.476388e-89	16	MTMR14	GC LMO2
4.901351e-93	0.754398	0.402	0.122	8.738619e-89	16	RABGAP1L	GC LMO2
7.216741e-93	-1.290053	0.091	0.359	1.286673e-88	16	TXNIP	GC LMO2
8.26386e-93	0.7598893	0.708	0.386	1.473364e-88	16	SYPL1	GC LMO2
1.298883e-92	0.662637	0.277	0.058	2.315778e-88	16	PITPNC1	GC LMO2
2.496061e-92	1.040397	0.482	0.179	4.450228e-88	16	RGS2	GC LMO2
4.851258e-92	0.476821	0.155	0.018	8.649308e-88	16	SPRED2	GC LMO2
6.992491e-92	0.828273	0.515	0.212	1.246691e-87	16	LY86	GC LMO2
7.084026e-92	-0.6180858	0.999	0.999	1.263011e-87	16	RPL32	GC LMO2
3.994012e-91	0.6673	0.283	0.077	7.120924e-87	16	GPR160	GC LMO2
1.005116e-90	0.7556869	0.566	0.24	1.792022e-86	16	CSK	GC LMO2
1.116547e-90	0.7729825	0.555	0.243	1.990692e-86	16	WIPF1	GC LMO2
1.781642e-90	0.7404095	0.67	0.346	3.176489e-86	16	DBNL	GC LMO2
1.956313e-90	-0.601347	1	0.999	3.48791e-86	16	RPL34	GC LMO2
1.996072e-90	-0.9465735	0.321	0.613	3.558797e-86	16	IGHD	GC LMO2
2.057286e-90	0.5393502	0.185	0.03	3.667935e-86	16	PDGFD	GC LMO2
3.329146e-90	-1.178184	0.116	0.389	5.935534e-86	16	CD44	GC LMO2
4.520764e-90	0.5113351	0.169	0.025	8.060069e-86	16	LINC00877	GC LMO2
7.843263e-90	-0.9736702	0.841	0.918	1.398375e-85	16	EEF1B2	GC LMO2
4.549623e-89	0.6381603	0.911	0.694	8.111524e-85	16	CCNI	GC LMO2
4.759498e-89	0.5441398	0.21	0.039	8.485709e-85	16	ACY3	GC LMO2
1.487696e-88	0.8260065	0.536	0.232	2.652413e-84	16	NCF4	GC LMO2
9.790929e-88	0.6569327	0.795	0.516	1.745625e-83	16	SELENOT	GC LMO2

1.053089e-87	0.7413041	0.352	0.111	1.877552e-83	16	ABI3	GC LMO2
2.548054e-87	0.7610975	0.484	0.168	4.542925e-83	16	PDIA4	GC LMO2
4.594353e-87	0.7024225	0.325	0.09	8.191271e-83	16	FAM76B	GC LMO2
5.547781e-86	0.7609913	0.428	0.157	9.891138e-82	16	LYPLAL1	GC LMO2
6.937993e-86	0.7129859	0.696	0.376	1.236975e-81	16	SMARCB1	GC LMO2
2.812553e-85	0.9634881	0.264	0.066	5.014501e-81	16	RASSF6	GC LMO2
5.238501e-85	0.686963	0.755	0.452	9.339724e-81	16	RAP1B	GC LMO2
3.338743e-84	0.7691804	0.527	0.22	5.952645e-80	16	UBE2G1	GC LMO2
3.47623e-84	0.4255619	0.134	0.018	6.197771e-80	16	NLRP4	GC LMO2
4.438353e-84	-0.5810314	0.999	0.998	7.913139e-80	16	RPL18A	GC LMO2
4.581347e-84	0.7757423	0.56	0.283	8.168083e-80	16	C7orf50	GC LMO2
9.854143e-84	0.6189857	0.873	0.619	1.756895e-79	16	LAMTOR5	GC LMO2
1.142115e-83	0.7094227	0.374	0.129	2.036276e-79	16	RAB9A	GC LMO2
2.239102e-83	0.7116018	0.659	0.331	3.992095e-79	16	STK17A	GC LMO2
1.222202e-82	0.7614631	0.516	0.233	2.179064e-78	16	ZBTB8OS	GC LMO2
1.508311e-82	0.7354287	0.559	0.258	2.689167e-78	16	RBM6	GC LMO2
1.01966e-81	0.6669108	0.338	0.103	1.817952e-77	16	OGG1	GC LMO2
1.972985e-81	0.6129665	0.227	0.047	3.517635e-77	16	CPM	GC LMO2
2.250498e-81	0.6187784	0.857	0.578	4.012412e-77	16	ATP5MC3	GC LMO2
1.059304e-80	-0.8413986	0.953	0.969	1.888633e-76	16	MT-ND3	GC LMO2
1.315551e-80	0.6172713	0.253	0.063	2.345496e-76	16	TEX9	GC LMO2
2.114827e-80	0.7184785	0.327	0.092	3.770525e-76	16	STAG3	GC LMO2
2.118619e-80	0.6922177	0.332	0.103	3.777286e-76	16	PHF6	GC LMO2
2.504319e-80	0.5550016	0.907	0.692	4.46495e-76	16	RHOA	GC LMO2
4.618058e-80	0.6240365	0.245	0.061	8.233536e-76	16	QPCT	GC LMO2

9.632068e-80	0.7063386	0.658	0.348	1.717301e-75	16	CD19	GC LMO2
6.64999e-79	-0.6099198	0.991	0.993	1.185627e-74	16	RPS5	GC LMO2
7.380971e-79	-1.02274	0.038	0.215	1.315953e-74	16	PLAC8	GC LMO2
1.109473e-78	0.6495489	0.274	0.064	1.978079e-74	16	SNTA1	GC LMO2
1.336833e-78	-0.606417	0.998	0.999	2.38344e-74	16	RPL39	GC LMO2
1.828855e-78	0.5943355	0.287	0.086	3.260665e-74	16	ZBTB38	GC LMO2
2.095072e-78	0.7075745	0.411	0.141	3.735304e-74	16	SH2B2	GC LMO2
3.59683e-78	-1.15504	0.169	0.432	6.412789e-74	16	PLP2	GC LMO2
4.273519e-78	-1.296108	0.592	0.754	7.619257e-74	16	CXCR4	GC LMO2
1.073713e-77	0.7163833	0.524	0.258	1.914323e-73	16	VASP	GC LMO2
1.240067e-77	0.7694604	0.339	0.089	2.210915e-73	16	WDR66	GC LMO2
1.777549e-77	0.5745737	0.891	0.652	3.169193e-73	16	SLC25A5	GC LMO2
2.853648e-77	-0.6108491	0.995	0.998	5.087769e-73	16	RPL11	GC LMO2
1.384691e-76	0.636917	0.559	0.182	2.468766e-72	16	PTTG1	GC LMO2
2.887002e-76	0.6079351	0.791	0.53	5.147236e-72	16	CAP1	GC LMO2
4.729173e-76	0.6867614	0.709	0.419	8.431642e-72	16	CD40	GC LMO2
1.500864e-75	0.5570385	0.913	0.702	2.67589e-71	16	NDUFA4	GC LMO2
2.044414e-75	0.6971802	0.507	0.226	3.644985e-71	16	MTF2	GC LMO2
2.072785e-75	0.7361187	0.507	0.227	3.695568e-71	16	CPNE3	GC LMO2
4.369464e-75	0.3740198	0.113	0.012	7.790318e-71	16	RGS20	GC LMO2
5.696259e-75	0.7000833	0.508	0.226	1.015586e-70	16	ZNF581	GC LMO2
1.708001e-74	0.4952218	0.173	0.028	3.045195e-70	16	PTAFR	GC LMO2
7.085276e-74	0.7165425	0.49	0.202	1.263234e-69	16	GLRX	GC LMO2
8.730156e-74	-0.8885182	0.942	0.961	1.556499e-69	16	MT-ND2	GC LMO2
8.787707e-74	0.6857194	0.316	0.086	1.56676e-69	16	GCSAM	GC LMO2

9.339965e-74	0.831007	0.745	0.483	1.665222e-69	16	UCP2	GC	LMO2
1.166803e-73	-0.6602478	0.985	0.991	2.080293e-69	16	RPL30	GC	LMO2
3.755951e-73	0.8051339	0.495	0.228	6.696485e-69	16	ZCCHC7	GC	LMO2
4.134587e-73	-0.463603	0.998	0.999	7.371555e-69	16	RPS18	GC	LMO2
9.845623e-73	0.5338779	0.205	0.05	1.755376e-68	16	CDKN2C	GC	LMO2
1.372635e-72	-0.6709476	0.987	0.991	2.44727e-68	16	RPL9	GC	LMO2
1.540323e-72	-0.5855846	0.987	0.996	2.746241e-68	16	RPL31	GC	LMO2
1.971613e-72	0.5897492	0.256	0.071	3.515189e-68	16	HS2ST1	GC	LMO2
2.429575e-72	-0.5654728	0.997	0.997	4.33169e-68	16	RPS3A	GC	LMO2
3.911013e-72	0.654708	0.516	0.252	6.972945e-68	16	CDV3	GC	LMO2
4.119243e-72	-0.9959626	0.053	0.253	7.344198e-68	16	TNFRSF13B	GC	LMO2
4.250904e-72	0.7135777	0.446	0.175	7.578936e-68	16	ZNF106	GC	LMO2
4.954811e-72	0.6975725	0.696	0.396	8.833932e-68	16	MBD4	GC	LMO2
5.415271e-72	-0.5597506	0.995	0.996	9.654887e-68	16	RPL35A	GC	LMO2
6.127075e-72	0.663319	0.367	0.129	1.092396e-67	16	MAP4K1	GC	LMO2
6.806284e-72	0.608213	0.414	0.179	1.213492e-67	16	HSD17B12	GC	LMO2
8.69649e-72	0.7421628	0.492	0.235	1.550497e-67	16	NSRP1	GC	LMO2
3.089277e-71	-1.058661	0.087	0.278	5.507871e-67	16	KLF2	GC	LMO2
4.765004e-71	0.6333716	0.382	0.141	8.495525e-67	16	P2RY8	GC	LMO2
6.175456e-71	0.5333724	0.853	0.573	1.101022e-66	16	LDHB	GC	LMO2
7.320151e-71	0.6661015	0.355	0.122	1.30511e-66	16	AMFR	GC	LMO2
7.789936e-71	0.3683902	0.11	0.013	1.388868e-66	16	BAIAP2L1	GC	LMO2
3.049203e-70	0.6918414	0.581	0.293	5.436424e-66	16	ITSN2	GC	LMO2
4.238501e-70	0.7306781	0.62	0.34	7.556824e-66	16	ABRACL	GC	LMO2
1.194785e-69	-0.7419837	0.833	0.906	2.130182e-65	16	HLA-A	GC	LMO2

1.325865e-69	-0.9397848	0.063	0.257	2.363885e-65	16	FCMR	GC LMO2
1.640308e-69	-0.6119139	0.981	0.985	2.924505e-65	16	RPL27	GC LMO2
9.001252e-69	0.6136813	0.303	0.097	1.604833e-64	16	LIMS1	GC LMO2
9.753145e-69	0.6709243	0.558	0.283	1.738888e-64	16	RRAS2	GC LMO2
1.931291e-68	0.6449443	0.452	0.191	3.443299e-64	16	POLD4	GC LMO2
2.20869e-68	0.4761279	0.132	0.018	3.937874e-64	16	ETV7	GC LMO2
1.578933e-67	0.6115227	0.618	0.333	2.815079e-63	16	RBBP4	GC LMO2
1.581068e-67	0.6595073	0.601	0.317	2.818886e-63	16	IMP4	GC LMO2
1.703083e-67	-1.501831	0.079	0.268	3.036426e-63	16	S100A4	GC LMO2
2.854405e-67	0.6143362	0.688	0.414	5.089119e-63	16	FAM49B	GC LMO2
3.075289e-67	0.6603563	0.519	0.264	5.482934e-63	16	ERP44	GC LMO2
4.02292e-67	0.5666248	0.837	0.609	7.172463e-63	16	ARPC5	GC LMO2
6.038846e-67	-0.5540235	0.985	0.992	1.076666e-62	16	RPL37	GC LMO2
1.563968e-66	0.6452711	0.346	0.115	2.788398e-62	16	CD180	GC LMO2
1.879576e-66	0.683812	0.216	0.055	3.351097e-62	16	RGS16	GC LMO2
2.307037e-66	0.6299574	0.334	0.112	4.113216e-62	16	BRWD1	GC LMO2
1.170696e-65	-0.9433383	0.035	0.224	2.087233e-61	16	GBP2	GC LMO2
1.282761e-65	0.5839599	0.67	0.408	2.287034e-61	16	HCLS1	GC LMO2
1.998332e-65	-0.5875131	0.992	0.993	3.562826e-61	16	RPL35	GC LMO2
6.029607e-65	-1.12832	0.2	0.376	1.075019e-60	16	SELL	GC LMO2
7.82636e-65	-1.102955	0.267	0.499	1.395362e-60	16	FXYD5	GC LMO2
8.425132e-65	0.6754476	0.302	0.073	1.502117e-60	16	SUGCT	GC LMO2
9.660423e-65	0.8200792	0.619	0.342	1.722357e-60	16	S100A10	GC LMO2
9.917085e-65	0.7585803	0.42	0.192	1.768117e-60	16	IDH2	GC LMO2
3.073339e-64	0.5978985	0.675	0.412	5.479455e-60	16	ELF1	GC LMO2

4.037299e-64	0.6056688	0.396	0.156	7.1981e-60	16	UBE2R2	GC LMO2
8.445503e-64	0.5969158	0.563	0.307	1.505749e-59	16	LPXN	GC LMO2
8.548591e-64	-0.6865487	0.957	0.977	1.524128e-59	16	RPL36A	GC LMO2
1.977215e-63	0.6899034	0.58	0.312	3.525177e-59	16	FCRLA	GC LMO2
3.209109e-63	-0.4448368	0.998	0.999	5.72152e-59	16	RPS8	GC LMO2
5.591679e-63	0.5947679	0.595	0.33	9.969404e-59	16	SEPHS2	GC LMO2
1.01587e-62	0.6192242	0.579	0.299	1.811194e-58	16	TIMM8B	GC LMO2
3.083583e-62	0.5385203	0.233	0.057	5.49772e-58	16	DCAF12	GC LMO2
6.991904e-62	0.4469353	0.165	0.027	1.246587e-57	16	FGD6	GC LMO2
1.476564e-61	0.3781279	0.125	0.016	2.632565e-57	16	AL512631.1	GC LMO2
2.058144e-61	0.4189337	0.143	0.027	3.669466e-57	16	ZFAND4	GC LMO2
3.225278e-61	0.5902129	0.658	0.388	5.750347e-57	16	YWHAE	GC LMO2
5.22589e-61	0.6103244	0.335	0.119	9.317239e-57	16	PLEK	GC LMO2
6.733614e-61	0.6097401	0.281	0.09	1.200536e-56	16	RNGTT	GC LMO2
2.069764e-60	0.6944575	0.288	0.094	3.690183e-56	16	MX1	GC LMO2
3.779678e-60	0.6309976	0.74	0.487	6.738788e-56	16	C4orf3	GC LMO2
4.539704e-60	0.7311315	0.493	0.26	8.093839e-56	16	FAM3C	GC LMO2
6.087339e-60	-0.4692186	0.996	0.998	1.085312e-55	16	RPS28	GC LMO2
2.598901e-59	0.6381289	0.533	0.287	4.633581e-55	16	HSPA4	GC LMO2
3.538449e-59	-0.9359474	0.232	0.449	6.3087e-55	16	ANXA2	GC LMO2
3.560148e-59	0.6537729	0.503	0.251	6.347387e-55	16	LYSMD2	GC LMO2
4.170924e-59	-0.5015395	0.998	0.998	7.43634e-55	16	RPS14	GC LMO2
6.663368e-59	0.3970462	0.151	0.033	1.188012e-54	16	NDFIP2	GC LMO2
7.811983e-59	0.5260376	0.777	0.517	1.392798e-54	16	TRAPPC1	GC LMO2
9.867972e-59	0.5082506	0.858	0.635	1.759361e-54	16	ERH	GC LMO2

1.027891e-58	0.5583992	0.674	0.401	1.832627e-54	16	LSM10	GC LMO2
1.717515e-58	0.5730248	0.309	0.105	3.062157e-54	16	MAP4K2	GC LMO2
2.079268e-58	-0.9316291	0.309	0.506	3.707128e-54	16	FOXP1	GC LMO2
4.324214e-58	-0.4732358	1	1	7.709641e-54	16	RPS27	GC LMO2
7.064003e-58	0.5615672	0.695	0.431	1.259441e-53	16	COTL1	GC LMO2
7.228393e-58	-0.8721585	0.133	0.333	1.28875e-53	16	TRBC2	GC LMO2
2.268732e-57	0.4920785	0.234	0.067	4.044922e-53	16	TERF2	GC LMO2
2.304629e-57	-0.515913	1	0.999	4.108924e-53	16	EEF1A1	GC LMO2
3.943083e-57	0.551663	0.665	0.386	7.030123e-53	16	FNBP1	GC LMO2
4.752313e-57	-0.9654321	0.122	0.301	8.472899e-53	16	LINC00926	GC LMO2
7.375188e-57	0.4982134	0.842	0.623	1.314922e-52	16	ATP5F1B	GC LMO2
1.20246e-56	-0.7953826	0.509	0.681	2.143866e-52	16	TAGLN2	GC LMO2
1.238089e-56	0.5475724	0.312	0.106	2.207389e-52	16	MOB3A	GC LMO2
1.432362e-56	0.5769098	0.553	0.31	2.553759e-52	16	WDR1	GC LMO2
1.48655e-56	0.524271	0.211	0.053	2.650369e-52	16	MME	GC LMO2
2.568219e-56	0.5416459	0.666	0.377	4.578878e-52	16	EVL	GC LMO2
3.175693e-56	0.5309717	0.363	0.141	5.661944e-52	16	ANKRD13A	GC LMO2
4.752864e-56	0.4479466	0.879	0.693	8.473881e-52	16	CIRBP	GC LMO2
1.096549e-55	-0.9146447	0.171	0.351	1.955037e-51	16	TRAF3IP3	GC LMO2
1.29297e-55	0.5709269	0.346	0.141	2.305237e-51	16	DNAJC10	GC LMO2
2.247326e-55	0.5410643	0.766	0.485	4.006757e-51	16	RAC2	GC LMO2
3.447055e-55	0.5205564	0.758	0.511	6.145755e-51	16	SMDT1	GC LMO2
3.631353e-55	0.3492915	0.981	0.902	6.474339e-51	16	SRP14	GC LMO2
3.758402e-55	0.5416544	0.335	0.117	6.700855e-51	16	SSBP2	GC LMO2
4.190179e-55	-0.5263884	0.997	0.994	7.47067e-51	16	RPL37A	GC LMO2

4.744538e-55	0.519813	0.242	0.071	8.459036e-51	16	TMED8	GC LMO2
5.659388e-55	0.596214	0.598	0.356	1.009012e-50	16	REEP5	GC LMO2
5.663462e-55	-1.022515	0.341	0.527	1.009739e-50	16	ID3	GC LMO2
1.128104e-54	0.4902819	0.738	0.484	2.011296e-50	16	CCT8	GC LMO2
1.226372e-54	-0.6020293	0.981	0.987	2.186498e-50	16	RPL14	GC LMO2
1.334978e-54	0.5693087	0.474	0.251	2.380133e-50	16	ACO2	GC LMO2
1.588303e-54	-0.478563	0.998	0.997	2.831785e-50	16	RPS23	GC LMO2
1.986716e-54	0.6109727	0.496	0.231	3.542115e-50	16	CCND3	GC LMO2
2.011485e-54	-0.9879168	0.234	0.468	3.586276e-50	16	CD63	GC LMO2
2.360046e-54	0.5306158	0.226	0.07	4.207726e-50	16	DOK3	GC LMO2
2.774506e-54	0.450461	0.912	0.761	4.946667e-50	16	MYL12A	GC LMO2
2.956972e-54	0.5209311	0.391	0.172	5.271986e-50	16	LAP3	GC LMO2
3.436934e-54	0.5876691	0.648	0.375	6.127709e-50	16	LSM8	GC LMO2
4.603033e-54	-1.064894	0.132	0.32	8.206747e-50	16	CD69	GC LMO2
5.018184e-54	0.5014629	0.183	0.041	8.946921e-50	16	IZUMO4	GC LMO2
8.635467e-54	0.4298027	0.148	0.035	1.539617e-49	16	IGLC5	GC LMO2
3.502786e-53	0.4631089	0.849	0.63	6.245118e-49	16	HERPUD1	GC LMO2
4.188436e-53	0.5632633	0.389	0.175	7.467562e-49	16	TMC6	GC LMO2
4.34263e-53	0.4499672	0.136	0.025	7.742475e-49	16	FEZ1	GC LMO2
5.983069e-53	0.5330588	0.54	0.295	1.066721e-48	16	ATP6V0D1	GC LMO2
1.013596e-52	0.3433331	0.131	0.022	1.80714e-48	16	SERP2	GC LMO2
1.092923e-52	0.5765363	0.384	0.167	1.948572e-48	16	BLOC1S6	GC LMO2
1.156618e-52	-0.6227901	0.945	0.966	2.062134e-48	16	RPL38	GC LMO2
1.214314e-52	0.3596584	0.949	0.802	2.165e-48	16	HNRNPK	GC LMO2
2.825396e-52	0.4671834	0.869	0.683	5.037398e-48	16	PPP1CA	GC LMO2

5.730899e-52	0.5717269	0.586	0.307	1.021762e-47	16	PSIP1	GC LMO2
8.756752e-52	-0.549965	0.99	0.992	1.561241e-47	16	RPL36	GC LMO2
9.707385e-52	0.5715393	0.554	0.308	1.73073e-47	16	SPI1	GC LMO2
1.060946e-51	0.502539	0.691	0.434	1.89156e-47	16	HMGA1	GC LMO2
1.089932e-51	0.5316934	0.533	0.28	1.94324e-47	16	PLEKHF2	GC LMO2
1.566131e-51	0.6888664	0.677	0.39	2.792255e-47	16	CSTB	GC LMO2
3.897748e-51	0.5033237	0.742	0.511	6.949295e-47	16	CAPZB	GC LMO2
4.643606e-51	0.5487366	0.571	0.317	8.279085e-47	16	HSBP1	GC LMO2
1.019882e-50	0.5978835	0.528	0.269	1.818348e-46	16	ITGAE	GC LMO2
1.372784e-50	0.511432	0.297	0.113	2.447537e-46	16	ACYP2	GC LMO2
1.503557e-50	0.5426251	0.33	0.14	2.680692e-46	16	MDFIC	GC LMO2
2.46987e-50	-0.4675587	0.998	0.997	4.403531e-46	16	RPL7	GC LMO2
2.47568e-50	-0.3982374	0.998	0.999	4.41389e-46	16	RPS19	GC LMO2
2.575408e-50	0.4718281	0.828	0.603	4.591695e-46	16	POMP	GC LMO2
4.575697e-50	0.5761819	0.581	0.325	8.15801e-46	16	CCM2	GC LMO2
4.974811e-50	-0.8100419	0.114	0.282	8.86959e-46	16	IFITM2	GC LMO2
5.899172e-50	0.5255092	0.699	0.45	1.051763e-45	16	CAPZA1	GC LMO2
6.079199e-50	0.7854987	0.363	0.147	1.08386e-45	16	DUSP2	GC LMO2
6.740611e-50	0.5168789	0.608	0.358	1.201784e-45	16	PPP2CA	GC LMO2
7.228627e-50	0.5568154	0.484	0.247	1.288792e-45	16	RGS10	GC LMO2
7.30457e-50	-0.4289867	0.999	0.998	1.302332e-45	16	RPL26	GC LMO2
9.323156e-50	0.467377	0.834	0.609	1.662225e-45	16	UQCR10	GC LMO2
1.563126e-49	-0.7929316	0.042	0.201	2.786898e-45	16	YBX3	GC LMO2
1.796728e-49	0.5091263	0.622	0.385	3.203386e-45	16	ARPC4	GC LMO2
2.357746e-49	-0.4376652	0.998	0.998	4.203625e-45	16	RPL23A	GC LMO2

2.41377e-49	0.5111408	0.649	0.406	4.30351e-45	16	MOB1A	GC LMO2
2.875903e-49	0.3140438	0.115	0.022	5.127447e-45	16	AC090152.1	GC LMO2
4.745433e-49	0.4988937	0.588	0.345	8.460633e-45	16	LSM14A	GC LMO2
4.771471e-49	0.5330258	0.284	0.1	8.507056e-45	16	OTULIN	GC LMO2
7.205981e-49	0.5600159	0.354	0.145	1.284754e-44	16	PTPN18	GC LMO2
8.252979e-49	0.4820006	0.716	0.45	1.471424e-44	16	COX17	GC LMO2
1.540247e-48	0.4823489	0.235	0.078	2.746107e-44	16	RANBP2	GC LMO2
1.852555e-48	0.3178452	0.114	0.019	3.30292e-44	16	LOXL2	GC LMO2
1.978552e-48	0.4107469	0.822	0.613	3.527561e-44	16	NDUFA13	GC LMO2
4.677551e-48	-0.5220707	0.98	0.984	8.339606e-44	16	RPL29	GC LMO2
8.27996e-48	0.507216	0.682	0.43	1.476234e-43	16	ROMO1	GC LMO2
8.668751e-48	0.5746069	0.578	0.326	1.545552e-43	16	BPTF	GC LMO2
1.026454e-47	0.54287	0.538	0.291	1.830065e-43	16	PDHB	GC LMO2
1.051166e-47	0.4733596	0.766	0.518	1.874123e-43	16	FKBP1A	GC LMO2
1.134015e-47	0.4920431	0.26	0.085	2.021836e-43	16	MAP4K4	GC LMO2
1.399259e-47	0.5000599	0.332	0.13	2.494738e-43	16	CERS4	GC LMO2
1.411148e-47	0.4751689	0.199	0.066	2.515935e-43	16	EYA3	GC LMO2
3.286896e-47	0.439628	0.738	0.499	5.860206e-43	16	EIF3I	GC LMO2
3.820001e-47	0.422713	0.173	0.042	6.810679e-43	16	MYBL1	GC LMO2
4.625603e-47	0.4916947	0.66	0.424	8.246987e-43	16	KTN1	GC LMO2
5.645676e-47	0.4118415	0.796	0.574	1.006568e-42	16	COPE	GC LMO2
7.575341e-47	0.5389098	0.386	0.172	1.350608e-42	16	RGS19	GC LMO2
1.093949e-46	0.6169593	0.451	0.205	1.950402e-42	16	ADA	GC LMO2
1.240261e-46	0.4973064	0.693	0.473	2.211261e-42	16	GRB2	GC LMO2
2.536233e-46	0.5100423	0.37	0.173	4.52185e-42	16	TRAPPC6A	GC LMO2

2.632241e-46	0.5105538	0.249	0.079	4.693022e-42	16	GPR18	GC LMO2
3.479139e-46	-0.9452955	0.093	0.272	6.202956e-42	16	GPR183	GC LMO2
3.901464e-46	-0.8568118	0.115	0.272	6.95592e-42	16	JUN	GC LMO2
3.986556e-46	0.5488411	0.294	0.097	7.10763e-42	16	MYBL2	GC LMO2
4.118383e-46	0.4959068	0.696	0.463	7.342665e-42	16	PRDX5	GC LMO2
6.934278e-46	-0.7965785	0.151	0.324	1.236312e-41	16	TUBA1A	GC LMO2
8.737611e-46	0.5508366	0.396	0.171	1.557829e-41	16	SYK	GC LMO2
1.74277e-45	0.5664241	0.259	0.094	3.107185e-41	16	CCDC28B	GC LMO2
1.8555e-45	0.4469348	0.718	0.472	3.308171e-41	16	UBE2N	GC LMO2
2.07533e-45	0.4075181	0.847	0.643	3.700105e-41	16	UQCR11	GC LMO2
2.411542e-45	0.5057695	0.336	0.137	4.299538e-41	16	CUL3	GC LMO2
2.440536e-45	0.5201912	0.423	0.204	4.351232e-41	16	TCEAL8	GC LMO2
3.208182e-45	0.3314571	0.977	0.937	5.719867e-41	16	EEF2	GC LMO2
4.030827e-45	-0.5416807	0.996	0.996	7.186562e-41	16	RPL12	GC LMO2
4.971031e-45	0.4362899	0.227	0.075	8.862851e-41	16	WEE1	GC LMO2
6.926413e-45	0.4137695	0.184	0.06	1.23491e-40	16	BCAR3	GC LMO2
7.083657e-45	0.4301903	0.158	0.047	1.262945e-40	16	SHCBP1	GC LMO2
9.416064e-45	-0.7124068	0.055	0.198	1.67879e-40	16	CLEC2B	GC LMO2
9.950404e-45	0.491636	0.454	0.241	1.774058e-40	16	TPM4	GC LMO2
1.231026e-44	0.5345042	0.31	0.123	2.194796e-40	16	MSI2	GC LMO2
1.242432e-44	0.5262248	0.444	0.226	2.215133e-40	16	SEPT1	GC LMO2
1.265844e-44	0.4949859	0.676	0.441	2.256874e-40	16	ATP5F1C	GC LMO2
1.32048e-44	0.5338853	0.562	0.316	2.354284e-40	16	NAA38	GC LMO2
1.38747e-44	-0.6364785	0.959	0.968	2.47372e-40	16	RPS17	GC LMO2
1.952715e-44	0.4804726	0.651	0.41	3.481496e-40	16	PPP1CC	GC LMO2

2.502158e-44	0.3649451	0.139	0.031	4.461097e-40	16	RAPGEF5	GC LMO2
3.382288e-44	0.4246526	0.16	0.042	6.030281e-40	16	ASB2	GC LMO2
5.255966e-44	0.3457339	0.139	0.035	9.370862e-40	16	PRDM15	GC LMO2
5.80085e-44	0.3710864	0.908	0.719	1.034233e-39	16	COX6C	GC LMO2
6.639556e-44	0.4569586	0.709	0.48	1.183766e-39	16	UBE2L3	GC LMO2
1.14672e-43	-1.12103	0.555	0.668	2.044486e-39	16	TUBA1B	GC LMO2
1.689577e-43	0.3655501	0.704	0.458	3.012347e-39	16	PRMT1	GC LMO2
2.697194e-43	0.5258113	0.296	0.12	4.808827e-39	16	DLAT	GC LMO2
3.363889e-43	0.4826048	0.637	0.386	5.997478e-39	16	SH3BGRL	GC LMO2
3.960321e-43	0.4894532	0.593	0.315	7.060857e-39	16	TNFRSF13C	GC LMO2
4.349873e-43	0.4485336	0.247	0.085	7.755389e-39	16	CHIC2	GC LMO2
4.514176e-43	0.576783	0.545	0.325	8.048324e-39	16	TSPO	GC LMO2
4.722052e-43	0.4682185	0.276	0.105	8.418946e-39	16	RAB11FIP1	GC LMO2
4.723411e-43	-0.4028436	0.996	0.997	8.42137e-39	16	RPL27A	GC LMO2
6.389325e-43	0.3826796	0.147	0.037	1.139153e-38	16	SYBU	GC LMO2
6.394178e-43	0.4166421	0.796	0.579	1.140018e-38	16	NDUFB2	GC LMO2
6.754687e-43	-1.157216	0.74	0.444	1.204293e-38	16	IGHG3	GC LMO2
7.678942e-43	0.7009543	0.714	0.493	1.369079e-38	16	SAT1	GC LMO2
8.350929e-43	0.5004941	0.387	0.188	1.488887e-38	16	REPIN1	GC LMO2
8.422832e-43	0.4385433	0.855	0.666	1.501707e-38	16	SNX3	GC LMO2
1.171e-42	0.3861203	0.875	0.68	2.087775e-38	16	COX6A1	GC LMO2
1.183244e-42	0.4011455	0.842	0.64	2.109606e-38	16	HNRNPC	GC LMO2
1.796115e-42	0.3967255	0.153	0.038	3.202293e-38	16	SLC30A4	GC LMO2
2.340533e-42	-0.7135181	0.057	0.199	4.172937e-38	16	CHPT1	GC LMO2
3.989112e-42	-0.4777846	0.982	0.988	7.112188e-38	16	RPLP0	GC LMO2

1.006191e-41	0.325017	0.979	0.903	1.793937e-37	16	ATP5F1E	GC LMO2
2.131473e-41	0.4855434	0.491	0.276	3.800204e-37	16	BABAM1	GC LMO2
2.391624e-41	0.3555636	0.881	0.696	4.264027e-37	16	ELOB	GC LMO2
2.643554e-41	0.4275987	0.672	0.447	4.713193e-37	16	MAP1LC3B	GC LMO2
2.822429e-41	0.4421715	0.767	0.606	5.032109e-37	16	SYNGR2	GC LMO2
3.269776e-41	-0.3809393	0.992	0.994	5.829684e-37	16	RPS9	GC LMO2
3.518195e-41	-0.6474269	0.035	0.166	6.27259e-37	16	MARCKS	GC LMO2
4.433053e-41	-1.43974	0.139	0.29	7.90369e-37	16	MIR155HG	GC LMO2
4.862027e-41	0.3673065	0.141	0.037	8.668508e-37	16	MGST2	GC LMO2
4.939178e-41	0.3747212	0.139	0.032	8.806061e-37	16	MCTP2	GC LMO2
5.076308e-41	0.5120242	0.527	0.323	9.05055e-37	16	BST2	GC LMO2
1.367679e-40	0.5191592	0.39	0.191	2.438435e-36	16	USP34	GC LMO2
1.473902e-40	0.4177413	0.181	0.051	2.627819e-36	16	BCL6	GC LMO2
1.54628e-40	-0.8100518	0.088	0.241	2.756863e-36	16	CAPG	GC LMO2
1.546778e-40	0.4510762	0.814	0.63	2.75775e-36	16	BRK1	GC LMO2
1.989311e-40	0.3825835	0.861	0.67	3.546742e-36	16	ATP5ME	GC LMO2
2.322368e-40	0.3202985	0.112	0.028	4.14055e-36	16	SCARB1	GC LMO2
3.16038e-40	0.4848233	0.425	0.215	5.634642e-36	16	SNX5	GC LMO2
3.542053e-40	0.4754901	0.364	0.181	6.315127e-36	16	MVD	GC LMO2
3.983833e-40	0.4739036	0.571	0.344	7.102776e-36	16	PDIA6	GC LMO2
4.73176e-40	0.3906649	0.81	0.603	8.436254e-36	16	POLR2L	GC LMO2
5.439507e-40	-0.6132738	0.965	0.978	9.698098e-36	16	RPS10	GC LMO2
6.747801e-40	-0.3879803	0.999	0.999	1.203065e-35	16	RPS6	GC LMO2
7.376944e-40	0.4771431	0.298	0.122	1.315235e-35	16	COMMD4	GC LMO2
8.569764e-40	0.486128	0.475	0.227	1.527903e-35	16	IL4R	GC LMO2

1.125775e-39	0.5188761	0.389	0.186	2.007145e-35	16	LBR	GC LMO2
1.152897e-39	0.4723713	0.496	0.279	2.055501e-35	16	PRDX3	GC LMO2
1.157538e-39	0.4691579	0.374	0.178	2.063775e-35	16	TLR10	GC LMO2
1.512209e-39	0.4759537	0.269	0.108	2.696118e-35	16	TRAC	GC LMO2
1.908725e-39	0.4558762	0.532	0.328	3.403065e-35	16	EIF3A	GC LMO2
2.087513e-39	0.3434626	0.104	0.019	3.721827e-35	16	ANK1	GC LMO2
2.276567e-39	0.5348478	0.231	0.094	4.058891e-35	16	PDXK	GC LMO2
2.805319e-39	0.4816005	0.526	0.321	5.001604e-35	16	MRPS36	GC LMO2
3.122682e-39	0.4738656	0.316	0.123	5.567431e-35	16	STAP1	GC LMO2
5.012098e-39	0.469918	0.483	0.273	8.93607e-35	16	CNIH1	GC LMO2
5.632017e-39	0.4094401	0.787	0.573	1.004132e-34	16	POU2F2	GC LMO2
6.0117e-39	0.419693	0.661	0.425	1.071826e-34	16	CLTA	GC LMO2
7.74172e-39	-0.718567	0.06	0.214	1.380271e-34	16	MGST3	GC LMO2
1.337034e-38	0.3945265	0.208	0.067	2.383798e-34	16	MTA3	GC LMO2
1.53142e-38	0.4818593	0.448	0.241	2.730369e-34	16	NBDY	GC LMO2
2.03718e-38	0.499603	0.443	0.237	3.632088e-34	16	NCOA3	GC LMO2
2.777726e-38	0.3657414	0.141	0.032	4.952407e-34	16	AC012368.1	GC LMO2
3.798314e-38	0.3879964	0.833	0.649	6.772014e-34	16	ATP5F1A	GC LMO2
4.079513e-38	0.4575638	0.474	0.27	7.273364e-34	16	SEPT2	GC LMO2
4.283755e-38	0.3818805	0.194	0.065	7.637506e-34	16	MFHAS1	GC LMO2
5.265217e-38	0.387767	0.504	0.298	9.387355e-34	16	ODC1	GC LMO2
5.934012e-38	-0.6546867	0.474	0.624	1.057975e-33	16	SUB1	GC LMO2
6.456624e-38	0.3161734	0.122	0.026	1.151151e-33	16	LHFPL2	GC LMO2
1.270405e-37	0.4050618	0.189	0.059	2.265006e-33	16	PCED1B	GC LMO2
1.331474e-37	0.3248153	0.853	0.652	2.373885e-33	16	PSMB1	GC LMO2

1.81822e-37	-0.3317253	0.997	0.998	3.241705e-33	16	RPL19	GC LMO2
1.821294e-37	0.4148115	0.234	0.089	3.247186e-33	16	FAM208B	GC LMO2
3.670481e-37	0.4519618	0.369	0.186	6.544101e-33	16	MAP2K1	GC LMO2
4.836822e-37	0.4688599	0.325	0.162	8.623571e-33	16	TOR3A	GC LMO2
7.331495e-37	0.4472105	0.676	0.443	1.307132e-32	16	SEM1	GC LMO2
8.033875e-37	0.4388833	0.279	0.113	1.43236e-32	16	KLHL5	GC LMO2
8.056383e-37	0.4804734	0.539	0.319	1.436373e-32	16	BLNK	GC LMO2
8.855053e-37	-0.6057391	0.043	0.174	1.578767e-32	16	BCL2	GC LMO2
1.593143e-36	0.4035594	0.237	0.084	2.840414e-32	16	PTK2B	GC LMO2
1.620412e-36	0.3233526	0.816	0.635	2.889033e-32	16	ANAPC16	GC LMO2
1.65959e-36	0.4527534	0.405	0.211	2.958882e-32	16	DMAC1	GC LMO2
1.978568e-36	0.4736648	0.465	0.249	3.527589e-32	16	MPC2	GC LMO2
2.095595e-36	0.4328557	0.282	0.119	3.736237e-32	16	LMO4	GC LMO2
3.112017e-36	0.3751427	0.78	0.554	5.548416e-32	16	ATP5PF	GC LMO2
1.059177e-35	-0.4830187	0.974	0.982	1.888407e-31	16	RPSA	GC LMO2
1.490637e-35	0.4581681	0.419	0.222	2.657658e-31	16	CYBC1	GC LMO2
1.713841e-35	0.4399682	0.273	0.1	3.055606e-31	16	SORL1	GC LMO2
1.788989e-35	0.4130103	0.249	0.096	3.189588e-31	16	TACC1	GC LMO2
1.822799e-35	0.4001751	0.703	0.464	3.249868e-31	16	MTDH	GC LMO2
2.082107e-35	0.4469898	0.531	0.317	3.712189e-31	16	GNB2	GC LMO2
2.319906e-35	0.441604	0.513	0.311	4.136161e-31	16	SFT2D1	GC LMO2
3.369006e-35	0.4752552	0.414	0.205	6.006601e-31	16	SYAP1	GC LMO2
3.522476e-35	0.4697962	0.513	0.311	6.280223e-31	16	NDUFAF3	GC LMO2
4.669728e-35	0.4754468	0.495	0.279	8.325658e-31	16	CD82	GC LMO2
6.378716e-35	0.451288	0.332	0.167	1.137261e-30	16	COMMD7	GC LMO2

6.432853e-35	0.4675791	0.275	0.124	1.146913e-30	16	PRKD3	GC LMO2
7.237805e-35	0.3874366	0.778	0.549	1.290428e-30	16	SEC61G	GC LMO2
7.904009e-35	-0.7451566	0.35	0.495	1.409206e-30	16	ANKRD12	GC LMO2
8.298864e-35	0.4950721	0.557	0.308	1.479605e-30	16	HMGN3	GC LMO2
8.503415e-35	0.4014717	0.241	0.091	1.516074e-30	16	PDLIM2	GC LMO2
8.747542e-35	0.35618	0.767	0.551	1.559599e-30	16	SNU13	GC LMO2
9.209745e-35	-0.5364874	0.984	0.985	1.642005e-30	16	TMSB10	GC LMO2
1.163435e-34	0.3572826	0.754	0.552	2.074288e-30	16	PGK1	GC LMO2
1.312785e-34	0.4278454	0.502	0.299	2.340565e-30	16	CHMP2A	GC LMO2
1.36543e-34	0.355978	0.165	0.047	2.434426e-30	16	SYNE1	GC LMO2
1.539046e-34	0.4686912	0.348	0.171	2.743965e-30	16	EHMT1	GC LMO2
2.296962e-34	0.4594668	0.553	0.333	4.095253e-30	16	NDUFC1	GC LMO2
5.904421e-34	0.4098716	0.303	0.137	1.052699e-29	16	CORO1B	GC LMO2
7.421632e-34	0.4924349	0.512	0.284	1.323203e-29	16	TUBA4A	GC LMO2
7.738609e-34	0.4306718	0.657	0.461	1.379717e-29	16	PRDX6	GC LMO2
7.886637e-34	0.413794	0.463	0.261	1.406108e-29	16	GRN	GC LMO2
8.398292e-34	0.3638149	0.742	0.524	1.497331e-29	16	SPCS2	GC LMO2
1.022341e-33	0.3819429	0.205	0.073	1.822732e-29	16	RAB3GAP2	GC LMO2
1.196496e-33	0.4708656	0.398	0.205	2.133232e-29	16	EHD1	GC LMO2
1.269651e-33	0.4778029	0.654	0.414	2.263661e-29	16	AC114760.2	GC LMO2
1.572578e-33	0.4029138	0.447	0.259	2.80375e-29	16	CD2BP2	GC LMO2
1.684212e-33	-0.6191451	0.062	0.186	3.002782e-29	16	PLPP5	GC LMO2
1.740572e-33	0.4166869	0.396	0.202	3.103267e-29	16	ARHGEF1	GC LMO2
1.793711e-33	0.4440347	0.337	0.148	3.198008e-29	16	CD72	GC LMO2
2.013911e-33	0.3769006	0.708	0.493	3.590602e-29	16	COX5A	GC LMO2

2.03542e-33 0.374423 0.667 0.451 3.62895e-29 16
PPP4C GC LMO2
2.874295e-33 0.3906156 0.208 0.082 5.12458e-29 16
NCOA7 GC LMO2
3.125787e-33 0.3935122 0.482 0.273 5.572965e-29 16
TANK GC LMO2
3.782361e-33 0.4281539 0.557 0.359 6.743572e-29 16
CPNE1 GC LMO2
3.848365e-33 0.3004186 0.851 0.673 6.861251e-29 16
HNRNPA3 GC LMO2
3.9423e-33 -0.5698004 0.039 0.152 7.028727e-29 16
CELF2 GC LMO2
4.46018e-33 0.3581505 0.205 0.068 7.952054e-29 16
FOXN3 GC LMO2
4.488717e-33 0.4311203 0.338 0.165 8.002933e-29 16
CYB5R3 GC LMO2
4.502431e-33 0.4085415 0.41 0.217 8.027384e-29 16
CAPN1 GC LMO2
4.662812e-33 0.454776 0.387 0.213 8.313328e-29 16
IFI27L2 GC LMO2
4.700804e-33 0.3936808 0.639 0.417 8.381064e-29 16
ECH1 GC LMO2
6.24905e-33 0.4061629 0.303 0.142 1.114143e-28 16
PTEN GC LMO2
6.250682e-33 0.4146177 0.379 0.197 1.114434e-28 16
NUBP1 GC LMO2
6.511139e-33 0.4091076 0.262 0.109 1.160871e-28 16
ZNF296 GC LMO2
6.515136e-33 0.3752129 0.678 0.471 1.161584e-28 16
PRELID1 GC LMO2
8.81266e-33 0.3538696 0.197 0.073 1.571209e-28 16
EZH2 GC LMO2
1.120509e-32 0.3993865 0.521 0.33 1.997756e-28 16
NDUFS7 GC LMO2
1.206421e-32 0.444679 0.744 0.51 2.150928e-28 16
LSP1 GC LMO2
1.343379e-32 -0.6326986 0.165 0.3 2.39511e-28 16
PDLIM1 GC LMO2
1.403972e-32 0.3328744 0.157 0.052 2.503142e-28 16
KCTD9 GC LMO2
1.51244e-32 0.4283089 0.517 0.312 2.696529e-28 16
SCAND1 GC LMO2
1.63736e-32 0.4303461 0.197 0.06 2.919249e-28 16
SNX22 GC LMO2
1.768429e-32 0.3808559 0.565 0.387 3.152931e-28 16
ARPC5L GC LMO2
2.932635e-32 -0.7203503 0.035 0.161 5.228595e-28 16
LMNA GC LMO2
3.129547e-32 0.3973739 0.527 0.324 5.57967e-28 16
PPP2R1A GC LMO2

3.393266e-32	0.3486368	0.185	0.066	6.049853e-28	16	TRIM26	GC LMO2
3.427503e-32	0.3180705	0.147	0.041	6.110894e-28	16	GCH1	GC LMO2
3.863316e-32	0.3773033	0.293	0.149	6.887906e-28	16	PEA15	GC LMO2
6.095564e-32	0.5083744	0.269	0.128	1.086778e-27	16	PHGDH	GC LMO2
6.147848e-32	0.388187	0.359	0.188	1.0961e-27	16	FYTTD1	GC LMO2
6.927263e-32	0.4026268	0.439	0.239	1.235062e-27	16	WAS	GC LMO2
7.027607e-32	0.4061725	0.424	0.239	1.252952e-27	16	ETFA	GC LMO2
8.179588e-32	0.408555	0.227	0.085	1.458339e-27	16	VEZT	GC LMO2
8.823352e-32	-0.4924051	0.975	0.974	1.573115e-27	16	MT-ND4	GC LMO2
9.241635e-32	0.3068227	0.142	0.043	1.647691e-27	16	STK38L	GC LMO2
1.179863e-31	0.3017653	0.151	0.044	2.103578e-27	16	LIMK1	GC LMO2
1.249223e-31	0.4162179	0.637	0.417	2.227239e-27	16	TMEM123	GC LMO2
1.536264e-31	0.4076526	0.244	0.106	2.739005e-27	16	NADK	GC LMO2
1.687505e-31	0.3910603	0.441	0.258	3.008652e-27	16	MAPK1IP1L	GC LMO2
2.018627e-31	0.3951041	0.495	0.292	3.59901e-27	16	TWF2	GC LMO2
2.030929e-31	0.3999941	0.367	0.19	3.620943e-27	16	USF2	GC LMO2
2.794423e-31	0.427942	0.553	0.356	4.982178e-27	16	TKT	GC LMO2
3.191768e-31	0.3505889	0.208	0.08	5.690604e-27	16	PARN	GC LMO2
4.085724e-31	0.4078114	0.278	0.123	7.284437e-27	16	ZBTB20	GC LMO2
6.120412e-31	0.3486036	0.705	0.505	1.091208e-26	16	PSMD8	GC LMO2
7.414846e-31	0.3620301	0.735	0.541	1.321993e-26	16	UBE2D2	GC LMO2
8.425983e-31	-0.4772156	0.017	0.102	1.502268e-26	16	RHOC	GC LMO2
8.963136e-31	0.3777733	0.203	0.08	1.598038e-26	16	COMMD10	GC LMO2
9.861631e-31	0.4110862	0.456	0.266	1.75823e-26	16	ARF4	GC LMO2
1.14543e-30	0.4087296	0.632	0.415	2.042187e-26	16	HNRNPD	GC LMO2

1.158251e-30	0.3961827	0.406	0.23	2.065045e-26	16	CNDP2	GC LMO2
1.372272e-30	-0.3074656	0.997	0.998	2.446625e-26	16	RPS15	GC LMO2
2.349861e-30	0.3956723	0.531	0.34	4.189568e-26	16	SNX17	GC LMO2
2.502553e-30	-0.8051944	0.078	0.2	4.461802e-26	16	ACP5	GC LMO2
2.878555e-30	0.6650254	0.694	0.484	5.132175e-26	16	LTB	GC LMO2
3.485085e-30	0.3954543	0.372	0.201	6.213558e-26	16	PXK	GC LMO2
4.004811e-30	-0.4837048	0.948	0.959	7.140177e-26	16	MT-CYB	GC LMO2
4.64607e-30	0.3972156	0.501	0.301	8.283478e-26	16	OCIAD2	GC LMO2
4.693999e-30	0.4057084	0.445	0.262	8.368931e-26	16	PPP1R7	GC LMO2
4.728042e-30	0.3729558	0.259	0.112	8.429626e-26	16	EBF1	GC LMO2
5.399118e-30	-0.3349944	0.995	0.996	9.626087e-26	16	RPS3	GC LMO2
5.557748e-30	0.4320725	0.615	0.405	9.908908e-26	16	SEPT6	GC LMO2
5.595263e-30	0.4263684	0.273	0.118	9.975794e-26	16	EPS15	GC LMO2
6.067037e-30	0.4035105	0.542	0.341	1.081692e-25	16	LSM6	GC LMO2
6.294754e-30	0.4028086	0.391	0.213	1.122292e-25	16	SMAP2	GC LMO2
6.311181e-30	0.4090349	0.307	0.143	1.12522e-25	16	MGMT	GC LMO2
6.8809e-30	0.3888362	0.327	0.169	1.226796e-25	16	SNX8	GC LMO2
7.0319e-30	0.384864	0.36	0.189	1.253717e-25	16	RFXANK	GC LMO2
7.224673e-30	0.4086715	0.501	0.279	1.288087e-25	16	CARHSP1	GC LMO2
9.22978e-30	0.3796944	0.804	0.628	1.645578e-25	16	GSTP1	GC LMO2
1.210267e-29	0.3947079	0.456	0.268	2.157785e-25	16	NKTR	GC LMO2
1.294488e-29	0.3725619	0.602	0.407	2.307943e-25	16	MDH1	GC LMO2
1.356541e-29	0.3606944	0.175	0.059	2.418577e-25	16	FANCA	GC LMO2
1.413956e-29	-0.7227135	0.236	0.383	2.520942e-25	16	PDCD4	GC LMO2
1.552116e-29	0.3992685	0.316	0.152	2.767267e-25	16	ACAP2	GC LMO2

1.780155e-29	0.3899918	0.403	0.218	3.173838e-25	16	SKAP2	GC LMO2
1.813758e-29	0.3861189	0.306	0.144	3.23375e-25	16	ZNF580	GC LMO2
2.099659e-29	0.3985868	0.226	0.096	3.743482e-25	16	SYVN1	GC LMO2
2.390193e-29	0.3443086	0.163	0.061	4.261474e-25	16	IL21R	GC LMO2
2.570899e-29	0.3158393	0.826	0.653	4.583656e-25	16	RBX1	GC LMO2
2.728295e-29	0.3807062	0.654	0.428	4.864277e-25	16	CYB561A3	GC LMO2
3.353757e-29	0.396397	0.337	0.161	5.979414e-25	16	PPP1R18	GC LMO2
3.464092e-29	0.317221	0.88	0.699	6.17613e-25	16	COMMD6	GC LMO2
3.853318e-29	0.423935	0.361	0.192	6.87008e-25	16	UBE2E1	GC LMO2
4.787945e-29	0.3686286	0.694	0.498	8.536427e-25	16	GNG5	GC LMO2
4.814561e-29	0.3912441	0.427	0.245	8.58388e-25	16	THOC2	GC LMO2
4.900268e-29	0.3137708	0.81	0.628	8.736689e-25	16	EIF3H	GC LMO2
5.522894e-29	0.3714926	0.213	0.089	9.846768e-25	16	MSH6	GC LMO2
5.576909e-29	-0.4409647	0.981	0.98	9.943072e-25	16	RPL7A	GC LMO2
5.763585e-29	0.7527981	0.329	0.178	1.02759e-24	16	XBP1	GC LMO2
6.129218e-29	0.3898682	0.336	0.172	1.092778e-24	16	ZNF428	GC LMO2
6.37496e-29	0.381588	0.447	0.259	1.136592e-24	16	THOC7	GC LMO2
6.550331e-29	0.3809005	0.575	0.379	1.167859e-24	16	JAK1	GC LMO2
7.069391e-29	0.374983	0.281	0.133	1.260402e-24	16	GTF2E2	GC LMO2
8.075595e-29	0.3383103	0.206	0.082	1.439798e-24	16	CCDC88C	GC LMO2
8.648773e-29	0.4652201	0.343	0.173	1.54199e-24	16	BCL2A1	GC LMO2
9.970753e-29	0.3471254	0.172	0.06	1.777686e-24	16	DTX1	GC LMO2
1.387477e-28	0.3570892	0.6	0.381	2.473732e-24	16	NDUFB3	GC LMO2
1.394448e-28	0.3271395	0.759	0.562	2.486162e-24	16	RAC1	GC LMO2
1.489045e-28	0.374312	0.192	0.073	2.654819e-24	16	DNASE1	GC LMO2

2.006891e-28	0.3898017	0.231	0.098	3.578086e-24	16	PTPN7	GC LMO2
2.070473e-28	0.386831	0.413	0.238	3.691447e-24	16	CEP57	GC LMO2
2.367168e-28	0.4086508	0.29	0.138	4.220424e-24	16	INO80C	GC LMO2
2.534915e-28	0.3442439	0.187	0.072	4.5195e-24	16	CUX1	GC LMO2
3.086233e-28	0.3720147	0.241	0.111	5.502445e-24	16	NIN	GC LMO2
3.112132e-28	0.3318793	0.14	0.046	5.548619e-24	16	LRRK1	GC LMO2
3.333653e-28	-0.4553539	0.034	0.128	5.94357e-24	16	ZEB2	GC LMO2
3.666814e-28	0.3834766	0.454	0.249	6.537563e-24	16	TBC1D10C	GC LMO2
4.123759e-28	0.3963316	0.346	0.176	7.35225e-24	16	LYL1	GC LMO2
4.979548e-28	0.3623689	0.345	0.179	8.878037e-24	16	MAP2K2	GC LMO2
5.526765e-28	0.4392478	0.301	0.137	9.853669e-24	16	MKNK2	GC LMO2
5.764431e-28	-0.5729229	0.676	0.754	1.02774e-23	16	TOMM7	GC LMO2
5.956377e-28	0.3544365	0.527	0.324	1.061962e-23	16	CCNDBP1	GC LMO2
6.448036e-28	0.3806212	0.437	0.258	1.14962e-23	16	ATXN10	GC LMO2
7.243723e-28	-0.6624142	0.208	0.332	1.291483e-23	16	SMC6	GC LMO2
7.495236e-28	0.3329305	0.691	0.486	1.336326e-23	16	SF3B6	GC LMO2
8.879605e-28	0.4368723	0.263	0.11	1.583145e-23	16	TNFRSF17	GC LMO2
1.034279e-27	0.3956146	0.27	0.124	1.844015e-23	16	SASH3	GC LMO2
1.096109e-27	0.3523608	0.429	0.245	1.954254e-23	16	SHOC2	GC LMO2
1.503519e-27	0.3441549	0.621	0.436	2.680624e-23	16	ACTR2	GC LMO2
1.667759e-27	0.3663301	0.487	0.304	2.973448e-23	16	MMADHC	GC LMO2
1.856361e-27	-0.3263746	0.998	0.996	3.309705e-23	16	RPS13	GC LMO2
1.976294e-27	0.403804	0.243	0.11	3.523535e-23	16	BRI3BP	GC LMO2
2.156218e-27	0.3795189	0.331	0.161	3.844321e-23	16	SCPEP1	GC LMO2
2.188122e-27	0.3759462	0.396	0.23	3.901203e-23	16	MRPL27	GC LMO2

2.213188e-27	0.3869696	0.262	0.118	3.945892e-23	16	TFEB	GC LMO2
2.508159e-27	0.3355634	0.186	0.072	4.471797e-23	16	MPZL1	GC LMO2
2.515343e-27	0.3552751	0.518	0.323	4.484606e-23	16	PTPN6	GC LMO2
2.655021e-27	-0.6239266	0.128	0.24	4.733636e-23	16	CD55	GC LMO2
2.787424e-27	0.3751834	0.473	0.282	4.969698e-23	16	GSTK1	GC LMO2
3.388976e-27	0.3276921	0.707	0.515	6.042205e-23	16	PSMB6	GC LMO2
3.427604e-27	0.364353	0.477	0.299	6.111075e-23	16	COPS6	GC LMO2
5.246015e-27	-0.5974287	0.752	0.817	9.35312e-23	16	NOP53	GC LMO2
5.678334e-27	0.3821417	0.486	0.306	1.01239e-22	16	SCP2	GC LMO2
6.115746e-27	-0.5345864	0.098	0.193	1.090376e-22	16	S1PR4	GC LMO2
6.693695e-27	0.3525742	0.573	0.375	1.193419e-22	16	POLR2G	GC LMO2
7.223691e-27	-0.6882256	0.2	0.305	1.287912e-22	16	ZFP36L2	GC LMO2
7.983373e-27	0.3508044	0.337	0.181	1.423356e-22	16	NAPA	GC LMO2
8.456733e-27	0.3623935	0.256	0.116	1.507751e-22	16	SS18	GC LMO2
9.038208e-27	-0.6217141	0.109	0.214	1.611422e-22	16	LBH	GC LMO2
1.099862e-26	-0.4589121	0.029	0.114	1.960943e-22	16	P2RY10	GC LMO2
1.341253e-26	0.3457747	0.633	0.418	2.391319e-22	16	GABARAPL2	GC LMO2
1.495537e-26	0.3660336	0.333	0.178	2.666394e-22	16	TADA3	GC LMO2
1.515602e-26	0.3957205	0.389	0.225	2.702168e-22	16	NGLY1	GC LMO2
1.546804e-26	0.3002056	0.143	0.048	2.757796e-22	16	PEX5	GC LMO2
1.768972e-26	-0.4526831	0.02	0.107	3.1539e-22	16	MTSS1	GC LMO2
1.870684e-26	-0.6798452	0.287	0.417	3.335242e-22	16	NPC2	GC LMO2
2.146352e-26	0.3266983	0.643	0.441	3.826731e-22	16	NDUFB10	GC LMO2
2.153712e-26	0.3411855	0.277	0.139	3.839854e-22	16	FAM207A	GC LMO2
2.290354e-26	0.3158661	0.189	0.079	4.083472e-22	16	PDZD11	GC LMO2

3.483655e-26	0.3579961	0.237	0.107	6.211008e-22	16	PTS	GC LMO2
3.61511e-26	-0.492623	0.926	0.934	6.445381e-22	16	MT-ATP6	GC LMO2
4.506499e-26	0.3769832	0.291	0.152	8.034638e-22	16	SMAP1	GC LMO2
4.795824e-26	0.3700835	0.257	0.121	8.550475e-22	16	TLE4	GC LMO2
5.190827e-26	0.357109	0.562	0.384	9.254726e-22	16	CUTA	GC LMO2
1.036611e-25	0.3800121	0.538	0.361	1.848175e-21	16	PDIA3	GC LMO2
1.062191e-25	0.3761845	0.423	0.245	1.89378e-21	16	PPP2R5C	GC LMO2
1.094091e-25	0.3680295	0.343	0.179	1.950654e-21	16	INPP5D	GC LMO2
1.180293e-25	0.3506915	0.206	0.082	2.104344e-21	16	TMEM131L	GC LMO2
1.233546e-25	0.3277117	0.205	0.089	2.199289e-21	16	PPP6R1	GC LMO2
2.203386e-25	-0.5100229	0.109	0.221	3.928417e-21	16	CCDC50	GC LMO2
2.627382e-25	0.3536577	0.397	0.233	4.68436e-21	16	DCXR	GC LMO2
3.225708e-25	0.419796	0.337	0.186	5.751114e-21	16	RFC1	GC LMO2
3.682895e-25	0.3143937	0.216	0.102	6.566234e-21	16	SLC25A4	GC LMO2
4.732643e-25	0.3018542	0.829	0.665	8.437829e-21	16	GDI2	GC LMO2
6.219685e-25	-0.3901897	0.984	0.982	1.108908e-20	16	RPS11	GC LMO2
6.500159e-25	-0.4482066	0.98	0.981	1.158913e-20	16	RPL5	GC LMO2
1.224172e-24	0.389275	0.384	0.225	2.182576e-20	16	HDDC2	GC LMO2
1.23653e-24	0.3158309	0.198	0.085	2.20461e-20	16	FAF1	GC LMO2
1.238007e-24	0.3257487	0.347	0.181	2.207243e-20	16	NECAP2	GC LMO2
1.697823e-24	0.3284246	0.556	0.341	3.027049e-20	16	H2AFY	GC LMO2
1.769163e-24	0.3069429	0.678	0.491	3.154241e-20	16	ARGLU1	GC LMO2
1.792979e-24	0.3593595	0.318	0.17	3.196702e-20	16	AKIRIN2	GC LMO2
1.993654e-24	-0.4818245	0.947	0.963	3.554487e-20	16	FTL	GC LMO2
2.178958e-24	0.3530129	0.42	0.244	3.884864e-20	16	SNRPN	GC LMO2

2.306126e-24	0.3594894	0.411	0.24	4.111591e-20	16	CCDC115	GC	LMO2
2.319467e-24	-0.3560378	0.016	0.1	4.135379e-20	16	PARVB	GC	LMO2
2.424215e-24	0.3430203	0.524	0.342	4.322134e-20	16	UBE2A	GC	LMO2
2.86638e-24	0.3665246	0.45	0.266	5.110469e-20	16	SEPT9	GC	LMO2
3.176941e-24	0.34248	0.323	0.174	5.664169e-20	16	PMVK	GC	LMO2
3.251931e-24	0.3048584	0.204	0.087	5.797868e-20	16	SREBF2	GC	LMO2
3.738746e-24	0.3241875	0.463	0.28	6.665811e-20	16	RTF2	GC	LMO2
3.887187e-24	0.3505385	0.535	0.35	6.930466e-20	16	DGUOK	GC	LMO2
4.25039e-24	0.3344716	0.202	0.093	7.57802e-20	16	UBR5	GC	LMO2
4.286614e-24	0.3608528	0.492	0.284	7.642604e-20	16	DCK	GC	LMO2
4.936988e-24	0.3196881	0.309	0.169	8.802155e-20	16	CLTB	GC	LMO2
6.195127e-24	0.313241	0.188	0.076	1.104529e-19	16	FAM45A	GC	LMO2
6.529212e-24	0.3335849	0.302	0.161	1.164093e-19	16	WDR61	GC	LMO2
6.898055e-24	0.4233647	0.206	0.092	1.229854e-19	16	PXMP2	GC	LMO2
7.006079e-24	0.3012849	0.394	0.236	1.249114e-19	16	MRPL15	GC	LMO2
7.910319e-24	0.3284952	0.688	0.498	1.410331e-19	16	RTRAF	GC	LMO2
1.329112e-23	0.315836	0.253	0.118	2.369673e-19	16	NCK1	GC	LMO2
1.505155e-23	0.3153483	0.185	0.073	2.683541e-19	16	OTUD1	GC	LMO2
2.150339e-23	0.3361699	0.335	0.17	3.833839e-19	16	BLCAP	GC	LMO2
2.258919e-23	0.3003	0.711	0.527	4.027428e-19	16	PAIP2	GC	LMO2
2.519144e-23	0.3118804	0.27	0.145	4.491381e-19	16	UBQLN1	GC	LMO2
2.742765e-23	0.3121462	0.263	0.133	4.890076e-19	16	DPP3	GC	LMO2
2.798797e-23	0.3168468	0.486	0.299	4.989975e-19	16	UGP2	GC	LMO2
2.914455e-23	0.3332981	0.55	0.373	5.196182e-19	16	SUPT4H1	GC	LMO2
3.058029e-23	0.3129653	0.493	0.321	5.45216e-19	16	SPAG7	GC	LMO2

3.364419e-23	0.3490149	0.316	0.173	5.998423e-19	16	EIF4E2 GC LMO2
3.489741e-23	0.3111488	0.582	0.4	6.22186e-19	16	PSMB2 GC LMO2
3.621321e-23	0.3476284	0.286	0.152	6.456454e-19	16	GNB1 GC LMO2
4.218377e-23	-0.3540673	0.995	0.992	7.520944e-19	16	RPL18 GC LMO2
4.282076e-23	0.3528409	0.265	0.134	7.634513e-19	16	CLIC4 GC LMO2
5.369033e-23	-0.6281387	0.063	0.174	9.572449e-19	16	TNFRSF18 GC LMO2
5.642575e-23	0.3559516	0.403	0.244	1.006015e-18	16	OTUB1 GC LMO2
5.66347e-23	-0.483465	0.071	0.16	1.00974e-18	16	TGIF1 GC LMO2
5.882546e-23	0.3564707	0.497	0.316	1.048799e-18	16	AP3S1 GC LMO2
6.138363e-23	0.3010821	0.58	0.398	1.094409e-18	16	MDH2 GC LMO2
6.971139e-23	0.335639	0.366	0.208	1.242884e-18	16	NR3C1 GC LMO2
8.787768e-23	0.3311483	0.514	0.343	1.566771e-18	16	LAMTOR2 GC LMO2
9.731435e-23	0.345864	0.425	0.235	1.735018e-18	16	SP140 GC LMO2
1.006652e-22	0.3176453	0.189	0.08	1.794759e-18	16	N4BP3 GC LMO2
1.453043e-22	0.372775	0.422	0.265	2.590631e-18	16	TXNDC17 GC LMO2
1.730019e-22	0.3867902	0.325	0.174	3.08445e-18	16	PLIN3 GC LMO2
1.836053e-22	0.335935	0.287	0.151	3.273499e-18	16	ATP6V1H GC LMO2
2.502202e-22	0.3625066	0.286	0.14	4.461176e-18	16	RAB30 GC LMO2
2.55692e-22	0.3032042	0.302	0.158	4.558733e-18	16	RSU1 GC LMO2
2.62735e-22	0.313672	0.544	0.363	4.684303e-18	16	EWSR1 GC LMO2
2.781844e-22	0.3287936	0.297	0.159	4.959749e-18	16	ST6GAL1 GC LMO2
3.066104e-22	0.3086156	0.611	0.421	5.466558e-18	16	TAF7 GC LMO2
4.101211e-22	0.3179482	0.358	0.21	7.31205e-18	16	ACTR10 GC LMO2
4.730035e-22	0.3485457	0.531	0.352	8.433179e-18	16	RAB11A GC LMO2
8.74444e-22	0.3267777	0.573	0.396	1.559046e-17	16	RAP1A GC LMO2

8.827456e-22	0.3773955	0.347	0.169	1.573847e-17	16	TMEM156	GC LMO2
1.051113e-21	-0.484706	0.199	0.29	1.87403e-17	16	RUBCNL	GC LMO2
1.175188e-21	-0.4400732	0.04	0.111	2.095243e-17	16	LINC02397	GC LMO2
1.232016e-21	0.3282659	0.505	0.305	2.196561e-17	16	CALM3	GC LMO2
1.484951e-21	0.3111961	0.16	0.065	2.64752e-17	16	SYT17	GC LMO2
1.606338e-21	0.3002586	0.273	0.141	2.86394e-17	16	FLOT1	GC LMO2
2.179632e-21	0.3157198	0.472	0.303	3.886065e-17	16	SHKBP1	GC LMO2
2.527231e-21	0.3674096	0.296	0.143	4.505801e-17	16	RBM38	GC LMO2
2.569021e-21	0.3205887	0.325	0.184	4.580308e-17	16	DAXX	GC LMO2
2.722386e-21	0.3139368	0.333	0.191	4.853741e-17	16	PAFAH1B1	GC LMO2
2.928449e-21	0.3475512	0.615	0.414	5.221131e-17	16	NDUFA12	GC LMO2
3.277842e-21	0.3704778	0.314	0.157	5.844065e-17	16	C12orf75	GC LMO2
3.572201e-21	0.3224556	0.338	0.194	6.368876e-17	16	ETFB	GC LMO2
5.20042e-21	-0.8037011	0.371	0.458	9.271829e-17	16	JUNB	GC LMO2
5.696752e-21	0.3270769	0.437	0.285	1.015674e-16	16	ZNHIT1	GC LMO2
5.94745e-21	0.312297	0.384	0.234	1.060371e-16	16	TSG101	GC LMO2
9.404779e-21	0.3171895	0.225	0.109	1.676778e-16	16	TMEM14A	GC LMO2
1.101729e-20	0.3132237	0.283	0.154	1.964273e-16	16	VPS25	GC LMO2
1.188639e-20	-0.3707847	0.968	0.971	2.119224e-16	16	RPL22	GC LMO2
1.266026e-20	0.3240935	0.481	0.305	2.257198e-16	16	PGLS	GC LMO2
1.288664e-20	0.3185517	0.294	0.166	2.297559e-16	16	MTX1	GC LMO2
1.335147e-20	0.3250096	0.408	0.239	2.380434e-16	16	SLBP	GC LMO2
1.73462e-20	0.3181094	0.537	0.366	3.092654e-16	16	FAM96B	GC LMO2
2.118594e-20	0.3347206	0.38	0.225	3.777241e-16	16	GTF2I	GC LMO2
2.894376e-20	-0.8148777	0.305	0.411	5.160384e-16	16	TXN	GC LMO2

3.701083e-20	0.3462913	0.335	0.204	6.59866e-16	16	MRPS31 GC LMO2
3.729296e-20	0.404648	0.355	0.217	6.648961e-16	16	TRAF4 GC LMO2
3.9603e-20	0.3586117	0.948	0.8	7.060819e-16	16	RPS26 GC LMO2
4.06023e-20	0.344104	0.387	0.224	7.238984e-16	16	NSMCE1 GC LMO2
4.307028e-20	0.3126527	0.242	0.111	7.679001e-16	16	PAX5 GC LMO2
4.341544e-20	0.3252248	0.324	0.198	7.740539e-16	16	LAGE3 GC LMO2
4.794737e-20	0.3252878	0.471	0.305	8.548537e-16	16	FKBP3 GC LMO2
5.60479e-20	0.3245516	0.289	0.163	9.99278e-16	16	COX11 GC LMO2
7.687979e-20	0.3110678	0.457	0.323	1.37069e-15	16	PMAIP1 GC LMO2
8.775081e-20	0.3178924	0.217	0.114	1.564509e-15	16	TMEM18 GC LMO2
8.973255e-20	0.3344851	0.304	0.178	1.599842e-15	16	MCL1 GC LMO2
9.961153e-20	0.3362645	0.179	0.083	1.775974e-15	16	RRBP1 GC LMO2
1.154508e-19	0.3187543	0.19	0.1	2.058372e-15	16	AMZ2 GC LMO2
1.198319e-19	0.3150434	0.183	0.089	2.136483e-15	16	XPNPEP1 GC LMO2
1.355804e-19	0.3300221	0.424	0.271	2.417263e-15	16	RNF7 GC LMO2
1.389462e-19	0.3141219	0.241	0.101	2.477272e-15	16	RMI2 GC LMO2
1.460505e-19	0.3233565	0.287	0.14	2.603934e-15	16	HSH2D GC LMO2
1.883048e-19	0.3515337	0.418	0.267	3.357286e-15	16	SMS GC LMO2
1.976764e-19	0.3541744	0.392	0.236	3.524373e-15	16	PTRHD1 GC LMO2
2.12307e-19	0.339471	0.269	0.127	3.785222e-15	16	STIM2 GC LMO2
2.293236e-19	0.3213111	0.316	0.182	4.08861e-15	16	METTL23 GC LMO2
2.817457e-19	0.3078186	0.394	0.248	5.023244e-15	16	MCTS1 GC LMO2
3.140517e-19	0.3751481	0.526	0.334	5.599229e-15	16	SPIB GC LMO2
3.15564e-19	-0.4265978	0.041	0.107	5.626191e-15	16	C1orf162 GC LMO2
3.39858e-19	-0.3130761	0.991	0.991	6.059329e-15	16	RPS20 GC LMO2

3.513484e-19	0.3743015	0.209	0.081	6.264191e-15	16	AICDA	GC LMO2
4.28778e-19	0.3434867	0.321	0.175	7.644683e-15	16	MTERF4	GC LMO2
4.575704e-19	0.396319	0.27	0.145	8.158022e-15	16	ALOX5	GC LMO2
6.867414e-19	0.3114279	0.352	0.217	1.224391e-14	16	SMARCC1	GC LMO2
7.523268e-19	0.3785652	0.401	0.254	1.341323e-14	16	MDM4	GC LMO2
2.104042e-18	0.3054385	0.328	0.198	3.751296e-14	16	RELB	GC LMO2
3.440927e-18	0.3179071	0.172	0.071	6.134829e-14	16	CDCA7	GC LMO2
4.739028e-18	0.3301474	0.429	0.266	8.449212e-14	16	TOP1	GC LMO2
6.030828e-18	0.3172096	0.265	0.131	1.075236e-13	16	PLEKHA2	GC LMO2
6.816097e-18	-0.5122784	0.229	0.307	1.215242e-13	16	ARHGAP24	GC LMO2
7.328139e-18	-0.5622821	0.416	0.496	1.306534e-13	16	SP100	GC LMO2
9.806387e-18	-0.3762384	0.072	0.129	1.748381e-13	16	CD24	GC LMO2
1.287348e-17	-0.4375856	0.901	0.918	2.295213e-13	16	PFDN5	GC LMO2
1.614719e-17	0.3073152	0.28	0.15	2.878882e-13	16	MYL6B	GC LMO2
1.937801e-17	0.3051752	0.265	0.146	3.454905e-13	16	SMARCA4	GC LMO2
2.474537e-17	0.3155508	0.401	0.255	4.411852e-13	16	CNN2	GC LMO2
3.112931e-17	-0.6872694	0.057	0.132	5.550044e-13	16	MYC	GC LMO2
3.365344e-17	0.3001017	0.527	0.354	6.000071e-13	16	TMEM243	GC LMO2
4.415458e-17	0.3129728	0.355	0.216	7.87232e-13	16	GNAI2	GC LMO2
7.748111e-17	0.3532437	0.31	0.155	1.381411e-12	16	KIAA0040	GC LMO2
1.031389e-16	0.3039675	0.456	0.305	1.838864e-12	16	CALR	GC LMO2
1.177193e-15	-0.5923409	0.38	0.48	2.098818e-11	16	SQSTM1	GC LMO2
1.641978e-15	-0.529691	0.653	0.697	2.927482e-11	16	HNRNPDL	GC LMO2
5.623345e-15	-0.6008781	0.25	0.123	1.002586e-10	16	IGHG2	GC LMO2
7.806519e-15	0.3118876	0.335	0.201	1.391824e-10	16	UBALD2	GC LMO2

2.345156e-14	-0.3294157	0.04	0.116	4.181179e-10	16	PYCR1	GC LMO2
2.645612e-14	-0.4499266	0.373	0.452	4.716862e-10	16	DRAP1	GC LMO2
2.956841e-14	-0.4199347	0.121	0.203	5.271752e-10	16	NPM3	GC LMO2
3.087872e-14	-0.5497331	0.221	0.297	5.505367e-10	16	NFKBIA	GC LMO2
3.37467e-14	-0.5508596	0.385	0.449	6.016699e-10	16	PNRC1	GC LMO2
3.462032e-14	-0.3154621	0.046	0.11	6.172457e-10	16	HEXB	GC LMO2
8.190749e-14	-0.4085244	0.752	0.777	1.460329e-09	16	NAP1L1	GC LMO2
8.385149e-14	-0.4323598	0.177	0.248	1.494988e-09	16	CKS1B	GC LMO2
1.204756e-13	-0.436872	0.575	0.621	2.147959e-09	16	IFI16	GC LMO2
1.838504e-13	-0.4275004	0.12	0.191	3.277868e-09	16	IFNGR1	GC LMO2
2.827103e-13	-0.3296733	0.871	0.882	5.040443e-09	16	MT-ND1	GC LMO2
7.683961e-13	-0.5461785	0.299	0.354	1.369973e-08	16	TUBB4B	GC LMO2
1.216685e-12	-0.3540439	0.057	0.119	2.169227e-08	16	FCGR2B	GC LMO2
1.525238e-12	-0.4491141	0.975	0.976	2.719346e-08	16	FTH1	GC LMO2
1.665888e-12	-0.3906809	0.145	0.204	2.970112e-08	16	IRF1	GC LMO2
2.436925e-12	-0.4453673	0.092	0.168	4.344794e-08	16	CBX6	GC LMO2
2.488638e-12	-0.4109526	0.171	0.233	4.436993e-08	16	HSD17B11	GC LMO2
2.637678e-12	-0.367099	0.126	0.195	4.702715e-08	16	ADK	GC LMO2
3.15181e-12	-0.3966195	0.188	0.253	5.619362e-08	16	DNPH1	GC LMO2
5.621128e-12	-0.3233355	0.071	0.121	1.002191e-07	16	IL27RA	GC LMO2
5.667672e-12	-0.3696522	0.103	0.176	1.010489e-07	16	ASAH1	GC LMO2
5.685957e-12	-0.5231761	0.262	0.347	1.013749e-07	16	CAST	GC LMO2
9.972758e-12	-0.3566336	0.089	0.149	1.778043e-07	16	SVIP	GC LMO2
2.715381e-11	-0.4386952	0.199	0.284	4.841252e-07	16	MT-ND4L	GC LMO2
3.152466e-11	-0.4518031	0.256	0.329	5.620532e-07	16	ZFP36	GC LMO2

3.43536e-11	-0.3497395	0.093	0.151	6.124903e-07	16	ESYT1
GC LMO2						
3.473704e-11	-0.3105655	0.096	0.158	6.193267e-07	16	DDB2
GC LMO2						
4.565923e-11	-0.3283787	0.293	0.332	8.140584e-07	16	TSTD1
GC LMO2						
6.542395e-11	-0.4009718	0.099	0.149	1.166444e-06	16	ARRDC2
GC LMO2						
9.348815e-11	-0.4426295	0.735	0.769	1.6668e-06	16	RPL17
GC LMO2						
1.504578e-10	-0.3169095	0.092	0.151	2.682512e-06	16	CHD1
GC LMO2						
2.077817e-10	-0.4564947	0.427	0.481	3.704539e-06	16	ISCU
GC LMO2						
2.685356e-10	-0.3491724	0.118	0.186	4.787721e-06	16	FKBP4
GC LMO2						
2.804189e-10	-0.4311379	0.262	0.338	4.999589e-06	16	APOBEC3C
GC LMO2						
3.461968e-10	-0.3350097	0.111	0.161	6.172343e-06	16	AIDA
GC LMO2						
3.952654e-10	-0.3138471	0.053	0.11	7.047187e-06	16	SNX9
GC LMO2						
4.073977e-10	-0.3870587	0.285	0.349	7.263493e-06	16	LITAF
GC LMO2						
4.476656e-10	-0.3435547	0.136	0.175	7.98143e-06	16	MARCH1
GC LMO2						
5.068961e-10	-0.3507397	0.146	0.227	9.03745e-06	16	TMEM109
GC LMO2						
6.866708e-10	-0.3716998	0.127	0.184	1.224265e-05	16	MBP
GC LMO2						
7.106178e-10	-0.3534388	0.239	0.299	1.26696e-05	16	RNASEH2B
GC LMO2						
8.072054e-10	-0.3934646	0.133	0.201	1.439166e-05	16	PPP3CA
GC LMO2						
8.478419e-10	-0.3093044	0.069	0.124	1.511617e-05	16	RAB13
GC LMO2						
9.491797e-10	-0.3228593	0.139	0.2	1.692292e-05	16	NENF
GC LMO2						
1.008085e-09	-0.418597	0.532	0.571	1.797316e-05	16	SNX2
GC LMO2						
1.413036e-09	-0.656471	0.589	0.576	2.519302e-05	16	TUBB
GC LMO2						
1.960247e-09	-0.4119432	0.1	0.159	3.494925e-05	16	SLC2A3
GC LMO2						
2.86575e-09	-0.5051091	0.519	0.53	5.109346e-05	16	PSME2
GC LMO2						
4.046893e-09	-0.7195471	0.394	0.404	7.215206e-05	16	FABP5
GC LMO2						
4.888056e-09	-0.5222525	0.469	0.485	8.714915e-05	16	RANBP1
GC LMO2						

4.999489e-09	-0.3037121	0.968	0.96	8.91359e-05	16	RPL23	GC LMO2
9.881036e-09	-0.3255844	0.137	0.188	0.000176169	16	KIF20B	GC LMO2
1.912953e-08	-0.4592971	0.165	0.219	0.0003410603	16	EIF4EBP1	GC LMO2
2.057691e-08	-0.5033846	0.258	0.328	0.0003668658	16	SLC3A2	GC LMO2
2.110501e-08	-0.3342884	0.142	0.205	0.0003762813	16	NDFIP1	GC LMO2
3.11933e-08	-0.3250465	0.145	0.189	0.0005561453	16	PCNA	GC LMO2
3.188165e-08	-0.3141704	0.19	0.249	0.0005684179	16	EXOSC8	GC LMO2
3.237412e-08	-0.384318	0.285	0.343	0.0005771981	16	PKIG	GC LMO2
1.443501e-07	-0.3387359	0.312	0.359	0.002573619	16	C8orf59	GC LMO2
1.512003e-07	-0.3582542	0.443	0.476	0.00269575	16	N4BP2L2	GC LMO2
3.294475e-07	-0.3777126	0.302	0.337	0.005873719	16	CD48	GC LMO2
7.0524e-07	-0.3697251	0.09	0.138	0.01257372	16	FCER2	GC LMO2
1.128503e-06	-0.3879497	0.458	0.467	0.02012009	16	ITM2B	GC LMO2
2.089322e-06	-0.3459749	0.746	0.758	0.03725053	16	MT-ND5	GC LMO2
2.132043e-06	-0.50532	0.337	0.36	0.0380122	16	ARID5B	GC LMO2
2.166832e-06	-0.3571277	0.421	0.446	0.03863245	16	FBL	GC LMO2
2.808273e-06	-0.3143477	0.199	0.242	0.05006869	16	HLA-F	GC LMO2
3.402929e-06	-0.3248215	0.397	0.439	0.06067081	16	IL2RG	GC LMO2
3.661866e-06	0.3426176	0.175	0.109	0.06528741	16	CD9	GC LMO2
4.66416e-06	-0.3550532	0.948	0.926	0.0831573	16	HNRNPA1	GC LMO2
5.10203e-06	-0.3314576	0.437	0.457	0.09096409	16	LSM5	GC LMO2
5.633249e-06	-0.3954738	0.25	0.281	0.1004352	16	SRM	GC LMO2
9.98968e-06	-0.3140437	0.174	0.233	0.178106	16	TARS	GC LMO2
2.111751e-05	-0.3677949	0.636	0.648	0.3765041	16	ZFP36L1	GC LMO2
3.088079e-05	-0.9714623	0.362	0.24	0.5505736	16	IGHG4	GC LMO2

4.338318e-05	-0.3073876	0.147	0.198	0.7734787	16	TYMP	GC LMO2
4.828705e-05	-0.4034703	0.223	0.257	0.8609098	16	SRGN	GC LMO2
5.636614e-05	-0.3067814	0.206	0.257	1	16	NARS	GC LMO2
7.310974e-05	-0.3620086	0.117	0.161	1	16	DDIT4	GC LMO2
9.000649e-05	-0.3730586	0.425	0.446	1	16	KLF6	GC LMO2
0.0001004224	-0.3040564	0.31	0.32	1	16	LTA4H	GC LMO2
0.0001119412	-0.3529631	0.448	0.453	1	16	ARL6IP1	GC LMO2
0.0001594043	-0.3034811	0.363	0.384	1	16	LAPTM4A	GC LMO2
0.0002570859	-0.3059357	0.376	0.402	1	16	CMPK1	GC LMO2
0.0003289125	-0.3972408	0.529	0.512	1	16	PA2G4	GC LMO2
0.0004628302	-0.6035095	0.184	0.206	1	16	LGALS1	GC LMO2
0.001007032	-0.4591621	0.517	0.499	1	16	SSR4	GC LMO2
0.001359737	-0.3093743	0.466	0.468	1	16	CYCS	GC LMO2
0.00136348	-0.5320158	0.51	0.426	1	16	STMN1	GC LMO2
0.001379978	-0.3556197	0.435	0.473	1	16	SNHG8	GC LMO2
0	3.328184	0.933	0.058	0	17	IGHA2	Memory IgA
0	2.582398	0.949	0.133	0	17	IGHA1	Memory IgA
8.824468e-278	-2.509407	0.554	0.913	1.573314e-273	17	IGHM	Memory IgA
2.240009e-252	-2.60336	0.116	0.665	3.993711e-248	17	TCL1A	Memory IgA
2.020587e-198	-1.978451	0.127	0.615	3.602504e-194	17	IGHD	Memory IgA
1.060221e-194	0.5457432	1	0.998	1.890269e-190	17	B2M	Memory IgA
1.936318e-183	1.976083	0.596	0.199	3.452261e-179	17	LGALS1	Memory IgA
5.633453e-144	1.123452	0.695	0.296	1.004388e-139	17	CD27	Memory IgA
2.455447e-106	1.049612	0.585	0.242	4.377817e-102	17	TNFRSF13B	Memory IgA
6.169941e-103	0.547075	0.982	0.903	1.100039e-98	17	HLA-A	Memory IgA
4.719752e-101	0.4304007	0.999	0.986	8.414847e-97	17	HLA-B	Memory IgA

4.651283e-92	0.6994923	0.938	0.772	8.292773e-88	17	HLA-DPB1	Memory IgA
8.225887e-89	0.9520849	0.839	0.565	1.466593e-84	17	VIM	Memory IgA
5.069942e-82	1.009992	0.757	0.46	9.0392e-78	17	S100A6	Memory IgA
3.5454e-80	0.355022	0.999	0.998	6.321093e-76	17	RPS14	Memory IgA
2.893925e-79	0.8249781	0.71	0.432	5.15958e-75	17	COTL1	Memory IgA
1.703893e-71	0.700285	0.908	0.694	3.037872e-67	17	EMP3	Memory IgA
1.985961e-67	0.5208488	0.982	0.945	3.54077e-63	17	HLA-DRA	Memory IgA
1.661409e-64	0.8396284	0.55	0.263	2.962127e-60	17	GPR183	Memory IgA
5.395812e-61	0.3919015	1	0.998	9.620193e-57	17	TMSB4X	Memory IgA
7.585136e-59	1.080455	0.288	0.092	1.352354e-54	17	KLK1	Memory IgA
6.778252e-57	0.5452284	0.925	0.791	1.208495e-52	17	HLA-E	Memory IgA
1.643423e-55	0.7660236	0.489	0.233	2.930058e-51	17	CAPG	Memory IgA
6.045569e-55	0.7376312	0.659	0.44	1.077864e-50	17	ANXA2	Memory IgA
1.286924e-54	0.6924268	0.329	0.137	2.294457e-50	17	AIM2	Memory IgA
1.814451e-53	1.088245	0.48	0.259	3.234985e-49	17	S100A4	Memory IgA
1.363968e-52	0.5674162	0.928	0.812	2.431819e-48	17	HLA-DPA1	Memory IgA
5.857762e-52	0.6800992	0.257	0.073	1.04438e-47	17	CAPN2	Memory IgA
8.162121e-52	-0.8588277	0.118	0.351	1.455225e-47	17	ABRACL	Memory IgA
4.016141e-51	0.9991312	0.356	0.155	7.160378e-47	17	LMNA	Memory IgA
7.532786e-44	0.5460098	0.885	0.749	1.34302e-39	17	HLA-C	Memory IgA
4.773021e-43	0.598831	0.237	0.088	8.509819e-39	17	ITGB7	Memory IgA
9.488433e-43	0.5827497	0.307	0.148	1.691693e-38	17	CLECL1	Memory IgA
1.003209e-42	-0.7256007	0.048	0.201	1.78862e-38	17	YBX3	Memory IgA
1.993953e-42	0.572151	0.234	0.069	3.555018e-38	17	MYO1F	Memory IgA
1.75362e-41	-0.7512616	0.056	0.236	3.126529e-37	17	IL4R	Memory IgA

1.923557e-39	0.6210895	0.631	0.423	3.429509e-35	17	PLP2	Memory IgA
3.33446e-39	0.6079659	0.519	0.333	5.945008e-35	17	S100A11	Memory IgA
5.631629e-39	-0.6956571	0.406	0.622	1.004063e-34	17	CLEC2D	Memory IgA
6.925179e-38	-0.8378424	0.746	0.859	1.23469e-33	17	CD79B	Memory IgA
3.230489e-37	0.4919073	0.131	0.027	5.759639e-33	17	ZBTB32	Memory IgA
1.724379e-36	-1.239649	0.049	0.209	3.074395e-32	17	RGS13	Memory IgA
1.124494e-35	0.4940652	0.183	0.067	2.00486e-31	17	THEMIS2	Memory IgA
1.96262e-35	-0.613017	0.008	0.125	3.499156e-31	17	AL139020.1	Memory IgA
1.123381e-34	-0.8356651	0.413	0.591	2.002876e-30	17	MARCKSL1	Memory IgA
2.365405e-34	0.5816312	0.315	0.165	4.217281e-30	17	SAMSN1	Memory IgA
4.430535e-34	-0.705744	0.047	0.205	7.899201e-30	17	HMCES	Memory IgA
5.445361e-34	-0.5977045	0.452	0.636	9.708534e-30	17	GSTP1	Memory IgA
2.410313e-33	0.6143273	0.263	0.138	4.297347e-29	17	ITGB1	Memory IgA
2.53334e-33	0.4495751	0.927	0.853	4.516692e-29	17	SH3BGRL3	Memory IgA
5.210774e-33	0.4647093	0.16	0.065	9.290288e-29	17	TEX9	Memory IgA
2.294559e-31	0.595833	0.233	0.118	4.09097e-27	17	NCBP3	Memory IgA
2.418791e-31	0.4049478	0.461	0.27	4.312462e-27	17	KLF2	Memory IgA
2.478093e-31	0.5477144	0.546	0.356	4.418193e-27	17	ARID5B	Memory IgA
7.716085e-31	0.7147769	0.374	0.194	1.375701e-26	17	ACP5	Memory IgA
9.351629e-31	0.4617857	0.662	0.512	1.667302e-26	17	LSP1	Memory IgA
3.159359e-30	0.3955398	0.878	0.762	5.632821e-26	17	MYL12A	Memory IgA
7.441524e-30	-0.4913642	0.01	0.121	1.326749e-25	17	CD38	Memory IgA
9.96732e-30	-0.7482556	0.124	0.306	1.777073e-25	17	BCL7A	Memory IgA
1.76374e-28	0.4456353	0.229	0.093	3.144573e-24	17	CYSLTR1	Memory IgA
1.938372e-28	0.4777203	0.618	0.442	3.455923e-24	17	KLF6	Memory IgA

2.223842e-28	0.4794886	0.544	0.386	3.964888e-24	17	ARL6IP5	Memory IgA
3.358224e-28	0.5247335	0.417	0.282	5.987378e-24	17	CD82	Memory IgA
4.123407e-28	0.471721	0.562	0.416	7.351623e-24	17	CTSH	Memory IgA
1.044582e-27	−0.5825772	0.055	0.21	1.862386e-23	17	EAF2	Memory IgA
1.537033e-27	−0.5274924	0.033	0.154	2.740377e-23	17	CD72	Memory IgA
4.097386e-27	−0.5593695	0.128	0.292	7.305229e-23	17	RRAS2	Memory IgA
4.721685e-27	−0.7686449	0.331	0.475	8.418291e-23	17	CD83	Memory IgA
4.860238e-27	0.4838519	0.215	0.1	8.665318e-23	17	RUNX3	Memory IgA
6.63665e-27	0.5421929	0.462	0.333	1.183248e-22	17	VOPP1	Memory IgA
7.425587e-27	−0.7237128	0.449	0.619	1.323908e-22	17	MEF2C	Memory IgA
8.602403e-27	0.5143594	0.22	0.109	1.533722e-22	17	CRIP1	Memory IgA
1.376227e-26	0.3939391	0.765	0.651	2.453675e-22	17	HLA−DQA1	Memory IgA
5.618458e-26	0.5053987	0.452	0.301	1.001715e-21	17	CD99	Memory IgA
8.128341e-26	−0.6000002	0.787	0.868	1.449202e-21	17	CD79A	Memory IgA
9.024502e-25	0.3756694	0.681	0.509	1.608979e-20	17	BANK1	Memory IgA
1.059179e-24	0.4310997	0.498	0.364	1.888409e-20	17	CXXC5	Memory IgA
2.025302e-24	0.48431	0.294	0.168	3.61091e-20	17	MFSD10	Memory IgA
3.075597e-24	0.4809906	0.613	0.478	5.483482e-20	17	ISCU	Memory IgA
4.452833e-24	0.4977927	0.4	0.296	7.938956e-20	17	TCF4	Memory IgA
8.817746e-24	−0.7254672	0.192	0.343	1.572116e-19	17	LRMP	Memory IgA
1.816278e-23	0.3845887	0.805	0.695	3.238243e-19	17	HLA−DQB1	Memory IgA
6.472213e-23	0.453335	0.352	0.237	1.153931e-18	17	NCF4	Memory IgA
2.042367e-22	0.4311487	0.213	0.089	3.641336e-18	17	AHNAK	Memory IgA
2.890668e-22	−0.4945132	0.239	0.391	5.153773e-18	17	SHMT2	Memory IgA
5.441969e-22	0.338342	0.63	0.5	9.702487e-18	17	PSMB9	Memory IgA

8.853289e-22	0.4351716	0.226	0.109	1.578453e-17	17	SESN3	Memory IgA
1.51838e-21	-0.5248779	0.088	0.22	2.70712e-17	17	NANS	Memory IgA
2.995246e-21	0.4274608	0.51	0.374	5.340223e-17	17	ZFAND6	Memory IgA
4.089781e-21	0.4380795	0.24	0.115	7.29167e-17	17	FCGR2B	Memory IgA
5.490575e-21	0.3799688	0.814	0.711	9.789147e-17	17	CLIC1	Memory IgA
5.640079e-21	-0.5156591	0.029	0.136	1.00557e-16	17	NEIL1	Memory IgA
7.499114e-21	-0.435085	0.049	0.161	1.337017e-16	17	APLP2	Memory IgA
1.001625e-20	-0.5052552	0.088	0.197	1.785798e-16	17	CLEC2B	Memory IgA
1.142345e-20	-1.32817	0.06	0.178	2.036686e-16	17	MZB1	Memory IgA
1.798629e-20	-0.4128476	0.051	0.167	3.206776e-16	17	DEF8	Memory IgA
2.018999e-20	-0.3944299	0.665	0.773	3.599674e-16	17	SEC62	Memory IgA
3.057768e-20	-0.5174346	0.129	0.266	5.451695e-16	17	CD22	Memory IgA
3.790324e-20	0.4166098	0.161	0.081	6.757768e-16	17	KCNN4	Memory IgA
6.920466e-20	-0.348563	0.013	0.103	1.23385e-15	17	MYBL2	Memory IgA
7.431456e-20	0.4039596	0.207	0.117	1.324954e-15	17	PYCARD	Memory IgA
7.672264e-20	-0.3077386	0.013	0.104	1.367888e-15	17	SEMA4A	Memory IgA
1.445863e-19	-0.4816965	0.055	0.138	2.57783e-15	17	FCER2	Memory IgA
3.528059e-19	-0.5053528	0.081	0.185	6.290177e-15	17	PLPP5	Memory IgA
6.253868e-19	0.3349425	0.838	0.751	1.115002e-14	17	TOMM7	Memory IgA
1.650099e-18	0.3490772	0.143	0.061	2.941962e-14	17	KYNU	Memory IgA
1.8691e-18	0.403058	0.228	0.124	3.332419e-14	17	ZBTB20	Memory IgA
2.011231e-18	-0.4777298	0.121	0.244	3.585824e-14	17	PHACTR1	Memory IgA
2.488211e-18	0.3346911	0.638	0.5	4.436231e-14	17	TSC22D3	Memory IgA
3.386748e-18	-0.4787832	0.539	0.666	6.038233e-14	17	DBI	Memory IgA
3.799012e-18	-0.4854608	0.131	0.222	6.773259e-14	17	GBP2	Memory IgA

4.105072e-18	0.4863244	0.496	0.35	7.318932e-14	17	TXNIP	Memory IgA
5.803694e-18	0.3986013	0.4	0.282	1.034741e-13	17	ACAP1	Memory IgA
7.042418e-18	-0.9787351	0.312	0.43	1.255593e-13	17	STMN1	Memory IgA
1.697544e-17	0.4535461	0.317	0.194	3.02655e-13	17	TYMP	Memory IgA
5.643474e-17	-0.4922005	0.114	0.239	1.006175e-12	17	GCHFR	Memory IgA
5.941021e-17	-0.5155935	0.185	0.301	1.059225e-12	17	NME1	Memory IgA
8.212387e-17	0.3097148	0.46	0.346	1.464186e-12	17	S100A10	Memory IgA
8.53378e-17	-0.4684364	0.289	0.442	1.521488e-12	17	RHOH	Memory IgA
1.276476e-16	0.3425314	0.594	0.469	2.275829e-12	17	GPSM3	Memory IgA
1.503411e-16	-0.4088533	0.106	0.226	2.680432e-12	17	SIAH2	Memory IgA
1.542672e-16	-0.495574	0.119	0.232	2.750429e-12	17	CKS2	Memory IgA
1.814996e-16	0.3319849	0.435	0.33	3.235956e-12	17	RIPOR2	Memory IgA
3.009316e-16	0.3191286	0.139	0.059	5.365309e-12	17	FLNA	Memory IgA
4.481601e-16	0.3904419	0.319	0.217	7.990246e-12	17	CCDC50	Memory IgA
6.526692e-16	-0.701833	0.636	0.697	1.163644e-11	17	H2AFZ	Memory IgA
8.309438e-16	0.3267826	0.761	0.675	1.48149e-11	17	TAGLN2	Memory IgA
9.054587e-16	-0.8651724	0.665	0.717	1.614342e-11	17	HMGN2	Memory IgA
2.535489e-15	0.4244119	0.221	0.139	4.520524e-11	17	MYO1G	Memory IgA
2.760739e-15	0.3235166	0.395	0.277	4.922122e-11	17	RNASET2	Memory IgA
3.682798e-15	0.3322626	0.184	0.111	6.566061e-11	17	TRAC	Memory IgA
1.026307e-14	-0.4188883	0.51	0.61	1.829802e-10	17	POMP	Memory IgA
1.081854e-14	-0.3897062	0.313	0.442	1.928837e-10	17	FDPS	Memory IgA
1.819422e-14	-0.322358	0.03	0.113	3.243848e-10	17	AC023590.1	Memory IgA
3.279524e-14	-0.4661295	0.43	0.537	5.847064e-10	17	SRSF9	Memory IgA
3.389776e-14	-0.4394374	0.568	0.659	6.043632e-10	17	SLC25A5	Memory IgA

5.090995e-14	0.5173682	0.207	0.124	9.076736e-10	17	LGALS3	Memory IgA
5.750017e-14	0.329229	0.232	0.146	1.025171e-09	17	SELENOW	Memory IgA
5.818091e-14	-0.3391348	0.059	0.147	1.037307e-09	17	MID1IP1	Memory IgA
8.558938e-14	-0.3131682	0.048	0.148	1.525973e-09	17	SEC14L1	Memory IgA
1.043694e-13	-0.7879998	0.1	0.183	1.860802e-09	17	XBP1	Memory IgA
1.119022e-13	-0.4986689	0.05	0.15	1.995104e-09	17	BIK	Memory IgA
1.728423e-13	-0.3933341	0.289	0.403	3.081605e-09	17	RPA3	Memory IgA
1.735286e-13	-0.3950461	0.153	0.269	3.093841e-09	17	ALOX5AP	Memory IgA
1.797115e-13	0.3130659	0.421	0.303	3.204077e-09	17	ARHGAP24	Memory IgA
2.190022e-13	-0.474442	0.399	0.486	3.90459e-09	17	RANBP1	Memory IgA
2.419791e-13	-0.3920785	0.078	0.188	4.314245e-09	17	P2RX5	Memory IgA
2.458384e-13	-0.3678036	0.529	0.628	4.383053e-09	17	SELENOH	Memory IgA
2.761654e-13	-0.3826666	0.907	0.936	4.923754e-09	17	BTG1	Memory IgA
3.89453e-13	-0.3981845	0.234	0.351	6.943557e-09	17	TIMM13	Memory IgA
4.106151e-13	0.4763344	0.656	0.501	7.320857e-09	17	HLA-DRB5	Memory IgA
4.948528e-13	-0.3563993	0.07	0.137	8.822731e-09	17	PSAT1	Memory IgA
5.049358e-13	-0.363115	0.28	0.401	9.002501e-09	17	BLOC1S2	Memory IgA
5.272864e-13	-0.5130473	0.129	0.237	9.400989e-09	17	UBE2J1	Memory IgA
5.856426e-13	0.3210714	0.249	0.161	1.044142e-08	17	KIAA1551	Memory IgA
6.900593e-13	-0.3683849	0.41	0.516	1.230307e-08	17	SSBP1	Memory IgA
8.104443e-13	-0.3832554	0.407	0.526	1.444941e-08	17	FKBP1A	Memory IgA
1.419131e-12	-0.4011659	0.485	0.573	2.530169e-08	17	SNRPE	Memory IgA
1.838202e-12	0.3276311	0.337	0.24	3.27733e-08	17	HLA-F	Memory IgA
2.010184e-12	-0.3515308	0.201	0.325	3.583958e-08	17	RNASEH2C	Memory IgA
2.090349e-12	-0.4257476	0.375	0.476	3.726883e-08	17	SNRPD1	Memory IgA

2.126643e-12	0.316008	0.331	0.24	3.791591e-08	17	ARHGAP45	Memory IgA
3.16023e-12	0.3231832	0.239	0.146	5.634373e-08	17	MVP	Memory IgA
3.543673e-12	0.3362607	0.509	0.38	6.318014e-08	17	CD44	Memory IgA
4.017694e-12	-0.3515825	0.477	0.567	7.163146e-08	17	SNRPB	Memory IgA
4.535361e-12	0.3648104	0.207	0.128	8.086095e-08	17	EEA1	Memory IgA
4.857709e-12	-0.3686854	0.552	0.642	8.660809e-08	17	ERH	Memory IgA
7.089864e-12	-0.3897956	0.544	0.636	1.264052e-07	17	PSMA4	Memory IgA
7.983175e-12	-0.3483336	0.154	0.253	1.42332e-07	17	DNPH1	Memory IgA
8.193095e-12	-0.3314155	0.215	0.322	1.460747e-07	17	LSM4	Memory IgA
8.717664e-12	0.3011541	0.386	0.276	1.554272e-07	17	IFITM2	Memory IgA
1.28472e-11	-0.531635	0.541	0.6	2.290528e-07	17	PRDX1	Memory IgA
1.742965e-11	0.3521847	0.423	0.341	3.107533e-07	17	CMTM6	Memory IgA
2.177847e-11	0.3003744	0.172	0.088	3.882884e-07	17	ADGRE5	Memory IgA
3.341425e-11	-0.5338971	0.075	0.132	5.957427e-07	17	MYC	Memory IgA
3.562235e-11	-0.3773587	0.251	0.346	6.35111e-07	17	INSIG1	Memory IgA
3.768441e-11	-0.3336208	0.179	0.286	6.718753e-07	17	PRDX3	Memory IgA
3.79571e-11	-0.4094664	0.035	0.111	6.767372e-07	17	VPREB3	Memory IgA
5.336125e-11	-0.3699498	0.292	0.396	9.513777e-07	17	YWHAE	Memory IgA
5.76221e-11	-0.3130297	0.155	0.272	1.027344e-06	17	TOP1	Memory IgA
6.155771e-11	0.3269583	0.294	0.21	1.097512e-06	17	IDS	Memory IgA
6.195033e-11	-0.355557	0.24	0.328	1.104512e-06	17	SLC3A2	Memory IgA
6.607427e-11	-0.3211767	0.146	0.235	1.178038e-06	17	MTHFD2	Memory IgA
8.352267e-11	-0.3767602	0.162	0.277	1.489126e-06	17	ITGAE	Memory IgA
9.212623e-11	-0.3423917	0.151	0.252	1.642519e-06	17	TIFA	Memory IgA
1.07271e-10	-0.3474589	0.282	0.376	1.912535e-06	17	LCP1	Memory IgA

1.190913e-10	-0.397295	0.165	0.267	2.123278e-06	17
FAM3C	Memory IgA				
1.208664e-10	-0.3371913	0.146	0.243	2.154927e-06	17
NSRP1	Memory IgA				
1.267428e-10	-0.4294666	0.167	0.276	2.259697e-06	17
NCF1	Memory IgA				
1.602766e-10	-0.4298778	0.49	0.56	2.857571e-06	17
NCL	Memory IgA				
1.734452e-10	-0.3418116	0.227	0.34	3.092355e-06	17
NDUFC1	Memory IgA				
1.878292e-10	-0.337581	0.054	0.122	3.348807e-06	17
LPP	Memory IgA				
2.294347e-10	-0.3883251	0.254	0.363	4.090591e-06	17
TPD52	Memory IgA				
2.738877e-10	-0.3776065	0.407	0.503	4.883144e-06	17
FOXP1	Memory IgA				
2.918783e-10	-0.5052247	0.232	0.317	5.203899e-06	17
CD69	Memory IgA				
3.282896e-10	-0.3946048	0.332	0.431	5.853076e-06	17
IRF8	Memory IgA				
3.669888e-10	-0.4705061	0.227	0.333	6.543043e-06	17
GRHPR	Memory IgA				
3.925236e-10	-0.3138884	0.403	0.508	6.998304e-06	17
FDFT1	Memory IgA				
3.940671e-10	-0.3774952	0.072	0.17	7.025821e-06	17
CCDC88A	Memory IgA				
4.435789e-10	-0.347253	0.221	0.326	7.908568e-06	17
BLNK	Memory IgA				
4.739339e-10	-0.3016978	0.129	0.229	8.449767e-06	17
NSMCE1	Memory IgA				
5.266346e-10	-0.6902887	0.621	0.667	9.389369e-06	17
TUBA1B	Memory IgA				
5.528833e-10	-0.4581242	0.92	0.933	9.857357e-06	17
SERF2	Memory IgA				
5.618822e-10	-0.3654058	0.109	0.196	1.00178e-05	17
FAM129C	Memory IgA				
5.638904e-10	-0.3180877	0.399	0.499	1.00536e-05	17
COX5A	Memory IgA				
7.266124e-10	-0.3313757	0.192	0.294	1.295477e-05	17
HSPA4	Memory IgA				
7.632929e-10	-0.3390441	0.1	0.188	1.360875e-05	17
CDCA7L	Memory IgA				
8.249771e-10	0.3344154	0.438	0.343	1.470852e-05	17
CAST	Memory IgA				
8.493744e-10	-0.3881009	0.71	0.749	1.51435e-05	17
RAN	Memory IgA				
9.45094e-10	-0.3073739	0.158	0.254	1.685008e-05	17
SLC50A1	Memory IgA				
9.939831e-10	-0.3290709	0.295	0.409	1.772173e-05	17
LSM10	Memory IgA				

1.123024e-09	0.3067011	0.185	0.114	2.002239e-05	17	ENTPD1	Memory IgA
1.161389e-09	-0.3379373	0.153	0.219	2.07064e-05	17	EIF4EBP1	Memory IgA
1.316016e-09	-0.381027	0.441	0.514	2.346324e-05	17	PA2G4	Memory IgA
1.662367e-09	-0.332832	0.438	0.534	2.963834e-05	17	SNRPB2	Memory IgA
1.697508e-09	-0.3463374	0.53	0.598	3.026487e-05	17	LDHA	Memory IgA
1.717978e-09	-0.3269724	0.381	0.477	3.062984e-05	17	TNFAIP8	Memory IgA
1.872909e-09	-0.3175245	0.051	0.106	3.33921e-05	17	C1orf162	Memory IgA
1.96219e-09	-0.3096653	0.57	0.642	3.498388e-05	17	HNRNPM	Memory IgA
2.412303e-09	-0.4704518	0.728	0.769	4.300895e-05	17	HMGN1	Memory IgA
3.020666e-09	0.3295629	0.381	0.312	5.385545e-05	17	LPXN	Memory IgA
3.318264e-09	0.3044169	0.419	0.35	5.916133e-05	17	MRPS6	Memory IgA
3.319866e-09	-0.5709453	0.111	0.192	5.918989e-05	17	PTTG1	Memory IgA
3.437189e-09	-0.3105284	0.227	0.325	6.128165e-05	17	IMP4	Memory IgA
3.508281e-09	-0.3232631	0.105	0.192	6.254914e-05	17	LBR	Memory IgA
3.644976e-09	-1.283867	0.244	0.303	6.498628e-05	17	HMGB2	Memory IgA
5.645186e-09	-0.3007137	0.05	0.12	0.000100648	17	UBE2S	Memory IgA
6.271232e-09	-0.3262366	0.481	0.561	0.0001118098	17	ATP5PF	Memory IgA
6.299321e-09	-0.3574353	0.519	0.585	0.0001123106	17	ANP32B	Memory IgA
7.077573e-09	-0.3471125	0.076	0.162	0.000126186	17	SUSD3	Memory IgA
9.383538e-09	-0.6454705	0.25	0.287	0.0001672991	17	MIR155HG	Memory IgA
9.772685e-09	-0.3656435	0.1	0.177	0.0001742372	17	PDIA4	Memory IgA
1.367782e-08	-0.3095587	0.573	0.647	0.0002438619	17	SNRPG	Memory IgA
1.748009e-08	-0.4257912	0.078	0.153	0.0003116525	17	DUSP2	Memory IgA
2.122739e-08	-0.3890734	0.335	0.419	0.0003784632	17	METAP2	Memory IgA
2.391279e-08	-0.312112	0.317	0.424	0.0004263411	17	TMEM123	Memory IgA

2.517025e-08	-0.36674	0.803	0.848	0.0004487604	17	CD37	Memory IgA
2.698156e-08	-0.3095616	0.231	0.325	0.0004810543	17	HSBP1	Memory IgA
3.019217e-08	-0.3208066	0.149	0.236	0.0005382962	17	ZCCHC7	Memory IgA
3.17117e-08	-0.302362	0.449	0.531	0.0005653879	17	PSME2	Memory IgA
3.640575e-08	-1.275279	0.906	0.927	0.0006490781	17	IGKC	Memory IgA
4.816135e-08	-0.3363669	0.313	0.403	0.0008586687	17	RPL22L1	Memory IgA
5.201126e-08	-0.3058582	0.223	0.324	0.0009273088	17	NAA38	Memory IgA
5.282633e-08	0.3230418	0.557	0.461	0.0009418406	17	CD63	Memory IgA
5.888941e-08	-0.4306778	0.13	0.21	0.001049939	17	FOS	Memory IgA
6.228488e-08	-0.300054	0.35	0.43	0.001110477	17	CCT2	Memory IgA
7.864629e-08	-0.3584612	0.397	0.462	0.001402185	17	HSPD1	Memory IgA
1.490323e-07	-0.3630836	0.263	0.339	0.002657097	17	HVCN1	Memory IgA
1.934405e-07	-0.315887	0.481	0.556	0.003448851	17	SEC61G	Memory IgA
2.288828e-07	0.3092002	0.184	0.131	0.004080752	17	CYTOR	Memory IgA
2.359109e-07	-0.5456405	0.284	0.393	0.004206055	17	EIF5A	Memory IgA
2.670541e-07	-0.313834	0.25	0.319	0.004761307	17	DDX21	Memory IgA
3.228906e-07	-0.3301151	0.585	0.638	0.005756816	17	SET	Memory IgA
4.343115e-07	-0.3889625	0.925	0.932	0.00774334	17	HMGB1	Memory IgA
1.176575e-06	-0.3383912	0.413	0.494	0.02097716	17	C4orf3	Memory IgA
1.454205e-06	-2.899026	0.398	0.451	0.02592702	17	IGHG3	Memory IgA
1.634264e-06	-0.3797316	0.829	0.852	0.02913729	17	ATP5MG	Memory IgA
3.58253e-06	-0.3355927	0.174	0.271	0.06387293	17	PIM1	Memory IgA
6.740446e-06	-0.3911456	0.215	0.278	0.1201754	17	HSPA5	Memory IgA
1.279356e-05	-1.993756	0.535	0.647	0.2280963	17	IGLC2	Memory IgA
2.386268e-05	-0.3494899	0.845	0.848	0.4254477	17	HSP90AB1	Memory IgA

0.0001697784	-1.209283	0.378	0.423	1	17	HIST1H4C	Memory IgA
0.000809517	-0.3057969	0.723	0.751	1	17	CXCR4	Memory IgA
0.001632427	-0.36321	0.566	0.577	1	17	TUBB	Memory IgA
0.00291218	-0.3877127	0.507	0.534	1	17	ISG20	Memory IgA
0.003426467	-0.3021369	0.321	0.36	1	17	H2AFV	Memory IgA
0	3.860268	0.999	0.29	0	18	HMGB2	DZ 2
0	3.383629	0.883	0.041	0	18	UBE2C	DZ 2
0	3.264488	0.966	0.032	0	18	CDC20	DZ 2
0	3.251831	0.97	0.078	0	18	CCNB1	DZ 2
0	3.23978	0.988	0.176	0	18	PTTG1	DZ 2
0	3.238605	0.905	0.053	0	18	TOP2A	DZ 2
0	3.215331	0.952	0.038	0	18	CENPF	DZ 2
0	3.204885	0.966	0.039	0	18	CCNB2	DZ 2
0	2.834465	0.933	0.231	0	18	KPNA2	DZ 2
0	2.74143	0.904	0.019	0	18	PLK1	DZ 2
0	2.735905	0.974	0.217	0	18	CKS2	DZ 2
0	2.705582	0.955	0.049	0	18	BIRC5	DZ 2
0	2.697802	0.912	0.046	0	18	MKI67	DZ 2
0	2.680904	0.911	0.067	0	18	NUSAP1	DZ 2
0	2.657001	0.925	0.104	0	18	UBE2S	DZ 2
0	2.600889	0.99	0.66	0	18	TUBA1B	DZ 2
0	2.581186	0.918	0.05	0	18	CDKN3	DZ 2
0	2.560081	0.99	0.418	0	18	STMN1	DZ 2
0	2.559021	0.903	0.032	0	18	HMMR	DZ 2
0	2.555259	0.962	0.342	0	18	TUBB4B	DZ 2
0	2.505779	0.663	0.043	0	18	CDK1	DZ 2
0	2.471285	0.999	0.711	0	18	HMGN2	DZ 2
0	2.339582	0.877	0.027	0	18	AURKA	DZ 2
0	2.317225	0.856	0.036	0	18	AURKB	DZ 2
0	2.298707	0.972	0.444	0	18	ARL6IP1	DZ 2
0	2.217005	0.93	0.156	0	18	SMC4	DZ 2
0	2.211468	0.851	0.027	0	18	NUF2	DZ 2
0	2.188388	0.848	0.019	0	18	CENPA	DZ 2
0	2.187973	0.838	0.02	0	18	CENPE	DZ 2
0	2.161751	0.938	0.234	0	18	CKS1B	DZ 2
0	2.141114	0.806	0.02	0	18	ASPM	DZ 2
0	2.13207	0.851	0.031	0	18	TPX2	DZ 2
0	2.125271	0.876	0.071	0	18	TACC3	DZ 2
0	2.10653	0.942	0.269	0	18	JPT1	DZ 2
0	2.06793	0.994	0.691	0	18	H2AFZ	DZ 2
0	2.061773	0.947	0.194	0	18	RGS13	DZ 2
0	2.039766	0.975	0.57	0	18	TUBB	DZ 2
0	2.034578	1	0.931	0	18	HMGB1	DZ 2
0	1.942677	0.768	0.072	0	18	AICDA	DZ 2
0	1.923863	0.782	0.028	0	18	CCNA2	DZ 2

0	1.886015	0.991	0.802	0	18	CALM2	DZ 2
0	1.884098	0.964	0.349	0	18	H2AFV	DZ 2
0	1.868763	0.785	0.025	0	18	GTSE1	DZ 2
0	1.849803	0.842	0.065	0	18	MAD2L1	DZ 2
0	1.820661	0.785	0.048	0	18	HMGB3	DZ 2
0	1.791262	0.759	0.027	0	18	CDCA8	DZ 2
0	1.736836	0.741	0.015	0	18	DLGAP5	DZ 2
0	1.719314	0.707	0.025	0	18	CDCA3	DZ 2
0	1.714532	0.813	0.1	0	18	CKAP2	DZ 2
0	1.702175	0.969	0.387	0	18	NUCKS1	DZ 2
0	1.664051	0.593	0.019	0	18	PIF1	DZ 2
0	1.648273	0.72	0.027	0	18	KIFC1	DZ 2
0	1.605182	0.917	0.381	0	18	BUB3	DZ 2
0	1.544157	0.712	0.047	0	18	SGO2	DZ 2
0	1.480265	0.685	0.025	0	18	PBK	DZ 2
0	1.466599	0.755	0.083	0	18	KIF22	DZ 2
0	1.445123	0.662	0.021	0	18	TROAP	DZ 2
0	1.400042	0.71	0.05	0	18	ZWINT	DZ 2
0	1.364946	0.623	0.024	0	18	CDCA2	DZ 2
0	1.361597	0.703	0.058	0	18	CENPW	DZ 2
0	1.359892	0.617	0.009	0	18	DEPDC1	DZ 2
0	1.34816	0.628	0.017	0	18	KIF2C	DZ 2
0	1.341093	0.657	0.032	0	18	KNSTRN	DZ 2
0	1.333285	0.611	0.022	0	18	NDC80	DZ 2
0	1.322291	0.562	0.014	0	18	KIF23	DZ 2
0	1.295784	0.605	0.015	0	18	BUB1	DZ 2
0	1.285366	0.617	0.02	0	18	PRC1	DZ 2
0	1.269637	0.58	0.007	0	18	NEK2	DZ 2
0	1.242335	0.585	0.015	0	18	CEP55	DZ 2
0	1.209507	0.991	0.843	0	18	HNRNPA2B1	DZ 2
0	1.116435	0.518	0.012	0	18	CKAP2L	DZ 2
0	1.085137	0.482	0.013	0	18	HJURP	DZ 2
0	0.9269084	0.449	0.011	0	18	KIF4A	DZ 2
0	0.8712773	0.417	0.004	0	18	KIF20A	DZ 2
0	0.7202793	0.355	0.002	0	18	PSRC1	DZ 2
4.940656e-324	1.471386	0.798	0.172	8.808696e-320	18	TUBA1C	DZ 2
9.881313e-324	1.50023	0.887	0.245	1.761739e-319	18	RAD21	DZ 2
6.422853e-322	0.9611329	0.486	0.016	1.145131e-317	18	SPAG5	DZ 2
6.743996e-321	1.535756	0.915	0.278	1.202387e-316	18	DCK	DZ 2
6.201222e-317	1.134097	0.571	0.03	1.105616e-312	18	RACGAP1	DZ 2
2.756378e-314	1.312351	0.685	0.057	4.914346e-310	18		

SGO1	DZ 2	3.80328e-312	0.871056	0.437	0.011	6.780868e-308	18
TTK	DZ 2	4.991372e-308	1.483685	0.841	0.175	8.899117e-304	18
KIF20B	DZ 2	7.348135e-307	1.49735	0.865	0.252	1.310099e-302	18
ANP32E	DZ 2	1.388821e-305	1.589186	0.89	0.223	2.476129e-301	18
UBE2J1	DZ 2	7.836004e-302	1.392883	0.672	0.064	1.397081e-297	18
CDC25B	DZ 2	1.152151e-300	0.8759544	0.427	0.012	2.05417e-296	18
DEPDC1B	DZ 2	3.454788e-296	1.295659	0.991	0.764	6.159542e-292	18
HMGN1	DZ 2	9.836924e-294	1.156866	0.562	0.041	1.753825e-289	18
SHCBP1	DZ 2	1.016863e-293	1.076826	0.553	0.033	1.812965e-289	18
MND1	DZ 2	7.226309e-292	1.448172	0.808	0.166	1.288379e-287	18
PLIN3	DZ 2	5.976797e-290	1.347787	0.948	0.443	1.065603e-285	18
MRPL51	DZ 2	4.185145e-288	1.154991	0.997	0.886	7.461696e-284	18
GAPDH	DZ 2	6.56162e-285	1.452598	0.806	0.18	1.169871e-280	18
LBR	DZ 2	2.637588e-283	0.7899826	0.359	0.005	4.702555e-279	18
KIF14	DZ 2	5.403451e-281	0.9560786	0.486	0.022	9.633813e-277	18
NCAPG	DZ 2	4.189973e-278	1.053738	1	0.99	7.470303e-274	18
ACTB	DZ 2	2.770883e-274	1.399583	0.887	0.299	4.940207e-270	18
CALM3	DZ 2	8.36514e-270	1.325308	0.72	0.09	1.491421e-265	18
MYBL2	DZ 2	6.066244e-263	1.273344	0.925	0.408	1.081551e-258	18
DDX39A	DZ 2	2.201856e-260	0.858601	0.437	0.013	3.925688e-256	18
PRR11	DZ 2	1.021778e-259	1.224203	0.947	0.448	1.821729e-255	18
LSM5	DZ 2	3.630629e-259	0.8105835	0.413	0.018	6.473048e-255	18
NCAPH	DZ 2	1.165609e-258	0.7058656	0.338	0.007	2.078164e-254	18
FAM83D	DZ 2	3.432235e-256	1.037365	1	0.932	6.119332e-252	18
SERF2	DZ 2	7.018754e-251	1.497876	0.803	0.18	1.251374e-246	18

DAAM1 DZ 2
1.148958e-249 1.389946 0.769 0.143 2.048478e-245 18
CD81 DZ 2
1.204582e-247 0.8839238 0.437 0.017 2.147649e-243 18
ARHGAP11A DZ 2
1.461405e-247 1.235531 0.699 0.104 2.605539e-243 18
SKA2 DZ 2
6.889758e-247 1.444377 0.771 0.123 1.228375e-242 18
NEIL1 DZ 2
1.343124e-246 1.254053 0.699 0.106 2.394656e-242 18
LMNB1 DZ 2
9.105196e-244 1.305391 0.899 0.304 1.623365e-239 18
HMGN3 DZ 2
8.855673e-243 1.24108 0.63 0.09 1.578878e-238 18
FAM241A DZ 2
3.605208e-242 0.9876115 0.434 0.022 6.427725e-238 18
MXD3 DZ 2
2.630292e-239 0.6283941 0.26 0.003 4.689547e-235 18
FAM72D DZ 2
3.433228e-236 1.496371 0.79 0.137 6.121101e-232 18 BIK
DZ 2
7.771241e-236 1.190371 0.759 0.143 1.385535e-231 18
MZT1 DZ 2
1.485216e-230 1.305626 0.956 0.598 2.647991e-226 18
DYNLL1 DZ 2
2.366676e-229 1.182234 0.947 0.499 4.219547e-225 18
MZT2B DZ 2
5.775008e-229 1.282091 0.931 0.476 1.029626e-224 18
RPS27L DZ 2
1.74537e-226 0.939948 0.504 0.028 3.11182e-222 18
KNL1 DZ 2
3.763267e-226 0.7782422 0.382 0.014 6.709528e-222 18
CCNF DZ 2
3.316885e-225 0.8846557 0.995 0.938 5.913675e-221 18
H3F3A DZ 2
1.070123e-220 0.7742644 0.38 0.016 1.907922e-216 18
KIF11 DZ 2
7.310895e-219 1.191561 0.926 0.415 1.303459e-214 18 DEK
DZ 2
1.56798e-216 1.185386 0.975 0.744 2.795552e-212 18
HSP90AA1 DZ 2
1.590807e-214 1.172124 0.926 0.482 2.83625e-210 18
CBX3 DZ 2
2.269324e-212 1.154902 0.683 0.09 4.045978e-208 18
CENPM DZ 2
4.826472e-212 -2.278982 0.238 0.706 8.605117e-208 18
EMP3 DZ 2
7.936292e-212 1.132673 0.724 0.131 1.414962e-207 18
SMC2 DZ 2
1.586707e-211 1.362683 0.789 0.192 2.828939e-207 18

HMCES	DZ 2	2.148119e-209	1.246139	0.873	0.303	3.829882e-205	18
PSIP1	DZ 2	1.032758e-208	0.9627688	0.519	0.044	1.841304e-204	18
SPDL1	DZ 2	5.274261e-208	0.7042729	0.365	0.014	9.40348e-204	18
BUB1B	DZ 2	4.887267e-207	0.8965276	0.99	0.867	8.713508e-203	18
ARPC2	DZ 2	3.597448e-203	1.122287	0.99	0.782	6.41389e-199	18
ACTG1	DZ 2	2.440318e-202	0.8430239	0.426	0.026	4.350843e-198	18
PARPBP	DZ 2	1.653498e-201	1.228241	0.797	0.197	2.948021e-197	18
EAF2	DZ 2	3.881662e-201	1.326691	0.716	0.084	6.920615e-197	18
PCLAF	DZ 2	1.158658e-200	1.22141	0.903	0.366	2.065772e-196	18
ACADM	DZ 2	5.327362e-198	0.8983124	0.443	0.024	9.498154e-194	18
SPC25	DZ 2	2.312027e-197	1.12675	0.747	0.151	4.122114e-193	18
TMPO	DZ 2	8.563128e-196	1.105931	0.953	0.545	1.52672e-191	18
TCEA1	DZ 2	1.275709e-195	1.080641	0.699	0.144	2.274461e-191	18
DTYMK	DZ 2	2.892961e-193	1.129906	0.855	0.314	5.15786e-189	18
IMP4	DZ 2	5.114021e-193	1.050054	0.483	0.048	9.117787e-189	18
TUBB2A	DZ 2	1.3009e-190	1.082569	0.803	0.234	2.319375e-186	18
DCTN3	DZ 2	4.401822e-189	1.174452	0.832	0.265	7.848008e-185	18
ITGAE	DZ 2	3.795728e-188	-1.142027	0.994	0.998	6.767404e-184	18
B2M	DZ 2	1.645756e-187	1.086195	0.684	0.139	2.934218e-183	18
SMARCA4	DZ 2	5.053668e-185	2.693151	0.797	0.416	9.010185e-181	18
HIST1H4C	DZ 2	2.275518e-183	1.046828	0.619	0.092	4.057021e-179	18
AC084033.3	DZ 2	3.866821e-183	1.083953	0.912	0.449	6.894155e-179	18
COX17	DZ 2	3.060289e-181	0.6846239	0.352	0.019	5.456188e-177	18
OIP5	DZ 2	4.344211e-181	0.9392454	0.526	0.053	7.745294e-177	18
CCDC18	DZ 2	3.770847e-178	0.9012959	0.99	0.849	6.723042e-174	18

ATP5MG DZ 2
1.31006e-177 0.8961668 0.475 0.044 2.335706e-173 18
RPL39L DZ 2
4.199067e-177 1.062103 0.907 0.451 7.486516e-173 18
RAP1B DZ 2
1.832368e-176 0.9141118 0.966 0.622 3.266928e-172 18
ATP5F1B DZ 2
2.019793e-176 1.178222 0.712 0.151 3.60109e-172 18
C12orf75 DZ 2
3.100951e-174 1.078475 0.883 0.366 5.528686e-170 18
PTP4A2 DZ 2
1.588691e-172 1.15273 0.973 0.65 2.832477e-168 18
TCL1A DZ 2
8.112938e-171 1.184712 0.63 0.112 1.446456e-166 18 LPP
DZ 2
2.991881e-170 0.9441756 0.959 0.652 5.334224e-166 18
SLC25A5 DZ 2
2.036668e-169 0.9494732 0.981 0.689 3.631175e-165 18
EZR DZ 2
1.331194e-168 1.142816 0.724 0.151 2.373385e-164 18
SUSD3 DZ 2
5.548028e-168 1.066302 0.9 0.421 9.891579e-164 18
DNAJA1 DZ 2
1.559502e-167 0.8276687 0.981 0.744 2.780436e-163 18
RAN DZ 2
7.468644e-167 0.7302705 0.992 0.918 1.331585e-162 18
CFL1 DZ 2
2.930492e-166 0.6100509 0.999 0.999 5.224774e-162 18
PTMA DZ 2
7.907303e-165 1.12137 0.731 0.159 1.409793e-160 18
CCDC88A DZ 2
2.219061e-164 1.077861 0.727 0.195 3.956364e-160 18
UBALD2 DZ 2
1.661363e-163 0.7574158 0.389 0.029 2.962043e-159 18
NUGGC DZ 2
4.468947e-163 0.9382365 0.946 0.577 7.967686e-159 18
ANP32B DZ 2
6.886853e-162 0.5157085 0.266 0.008 1.227857e-157 18
ARHGEF39 DZ 2
1.109721e-161 0.7547665 0.991 0.881 1.978522e-157 18
H3F3B DZ 2
1.966415e-161 -2.095149 0.117 0.551 3.505922e-157 18
CCR7 DZ 2
1.115274e-160 0.98649 0.904 0.385 1.988421e-156 18
YWHAE DZ 2
9.680645e-160 0.9220072 0.974 0.732 1.725962e-155 18
HSPA8 DZ 2
5.52896e-157 1.04089 0.744 0.214 9.857582e-153 18
HSPH1 DZ 2
3.197953e-156 1.134072 0.852 0.41 5.70163e-152 18

METAP2	DZ 2	3.142e-153	0.8703699	0.975	0.811	5.601872e-149	18
CORO1A	DZ 2	4.52909e-152	0.9418654	0.937	0.533	8.074915e-148	18
PARP1	DZ 2	1.590928e-151	0.8624749	0.51	0.065	2.836466e-147	18
HAUS8	DZ 2	4.366997e-151	1.034944	0.545	0.083	7.785919e-147	18
GCSAM	DZ 2	6.995161e-151	0.9345876	0.939	0.55	1.247167e-146	18
ATP5IF1	DZ 2	1.547238e-150	1.061719	0.738	0.209	2.75857e-146	18
NANS	DZ 2	4.139915e-149	0.7705157	0.46	0.054	7.381055e-145	18
EMC9	DZ 2	5.567965e-149	0.9839152	0.804	0.295	9.927125e-145	18
UGP2	DZ 2	8.07502e-149	0.9398365	0.894	0.411	1.439695e-144	18
HNRNPD	DZ 2	1.375121e-148	0.873097	0.482	0.054	2.451704e-144	18
G2E3	DZ 2	1.690216e-147	0.8794205	0.955	0.628	3.013486e-143	18
PSMA4	DZ 2	2.410462e-147	1.006145	0.826	0.279	4.297613e-143	18
BCAS4	DZ 2	1.364682e-146	0.8683928	0.54	0.09	2.433091e-142	18
PHF19	DZ 2	1.860884e-146	0.4745467	0.222	0.005	3.31777e-142	18
SAPCD2	DZ 2	4.708078e-146	1.077748	0.861	0.34	8.394032e-142	18
S100A10	DZ 2	4.807955e-146	0.3903089	0.219	0.005	8.572104e-142	18
CDC25C	DZ 2	5.617244e-146	0.5588053	0.285	0.01	1.001499e-141	18
ECT2	DZ 2	8.040717e-145	0.9872095	0.641	0.138	1.433579e-140	18
SEC14L1	DZ 2	2.383633e-144	1.068439	0.839	0.331	4.24978e-140	18
LRMP	DZ 2	1.2341e-143	0.6058686	0.317	0.016	2.200276e-139	18
KIF18A	DZ 2	2.088822e-142	0.9861973	0.591	0.095	3.724161e-138	18
RMI2	DZ 2	1.451517e-141	0.977474	0.756	0.217	2.587909e-137	18
UBE2G1	DZ 2	2.612219e-141	0.725399	0.433	0.041	4.657325e-137	18
RCCD1	DZ 2	3.162088e-141	0.9294752	0.722	0.185	5.637686e-137	18
CBX5	DZ 2	9.358852e-141	1.122735	0.378	0.043	1.66859e-136	18

HES6 DZ 2
1.013496e-140 0.9935137 0.795 0.25 1.806963e-136 18
SYNE2 DZ 2
4.847886e-140 0.8989988 0.608 0.113 8.643296e-136 18
SSBP2 DZ 2
2.718185e-138 -0.8861703 0.995 0.999 4.846251e-134 18
RPS29 DZ 2
3.091483e-138 1.013893 0.707 0.184 5.511805e-134 18
DNMT1 DZ 2
9.760616e-137 0.9478903 0.503 0.067 1.74022e-132 18
H1FX DZ 2
1.810074e-136 0.9369586 0.825 0.338 3.227181e-132 18
H2AFY DZ 2
3.721094e-136 0.9208718 0.707 0.183 6.634338e-132 18
CNTRL DZ 2
9.302501e-136 0.8060639 0.557 0.096 1.658543e-131 18
BCL2L12 DZ 2
1.203905e-134 0.9408761 0.784 0.272 2.146443e-130 18
HP1BP3 DZ 2
4.913299e-133 0.3413116 0.146 0.001 8.759921e-129 18
FAM72C DZ 2
5.239355e-133 0.7903232 0.921 0.526 9.341246e-129 18
RBMX DZ 2
5.659375e-133 0.9133636 0.605 0.112 1.00901e-128 18
VNN2 DZ 2
1.243433e-130 0.8442521 0.877 0.428 2.216918e-126 18
KHDRBS1 DZ 2
1.80406e-130 0.5433842 0.28 0.013 3.216459e-126 18
INCENP DZ 2
2.332175e-130 0.8425735 0.554 0.109 4.158035e-126 18
DBF4 DZ 2
3.215716e-130 -0.8645686 1 0.999 5.7333e-126 18
RPL34 DZ 2
5.10064e-130 0.7849428 0.494 0.072 9.093931e-126 18
UBE2T DZ 2
1.641964e-129 0.9025168 0.775 0.275 2.927457e-125 18
PRDX3 DZ 2
2.149433e-129 0.8942767 0.47 0.077 3.832224e-125 18
H2AFX DZ 2
3.15829e-129 0.9182078 0.655 0.157 5.630915e-125 18
DEF8 DZ 2
6.228417e-129 0.9668373 0.851 0.373 1.110464e-124 18
LSM8 DZ 2
7.016005e-129 0.9080544 0.753 0.241 1.250884e-124 18
SH3KBP1 DZ 2
1.056384e-128 1.014414 0.552 0.124 1.883428e-124 18
PHGDH DZ 2
1.024601e-127 0.704482 0.399 0.034 1.826761e-123 18
POLH DZ 2
3.721563e-127 0.6781014 0.358 0.028 6.635175e-123 18

MYEF2	DZ 2					
4.496862e-127	0.9136137	0.763	0.261	8.017455e-123	18	
TOP1	DZ 2					
9.566761e-127	0.9019254	0.72	0.241	1.705658e-122	18	
KIF5B	DZ 2					
1.87574e-126	0.8286412	0.889	0.449	3.344256e-122	18	
SFPQ	DZ 2					
3.731952e-126	0.7775355	0.946	0.602	6.653697e-122	18	
POMP	DZ 2					
6.200866e-126	0.6817761	0.389	0.037	1.105552e-121	18	
CKAP5	DZ 2					
7.925869e-126	0.7368896	0.396	0.039	1.413103e-121	18	
MYBL1	DZ 2					
1.037272e-125	0.6286355	0.33	0.021	1.849353e-121	18	
NCAPD2	DZ 2					
2.089258e-125	0.9030534	0.573	0.087	3.724938e-121	18	
WDR66	DZ 2					
3.78641e-125	0.8433206	0.92	0.511	6.750791e-121	18	
COX8A	DZ 2					
2.353974e-124	0.7557358	0.981	0.773	4.196901e-120	18	
ARHGDIB	DZ 2					
3.268088e-124	0.6289548	0.36	0.029	5.826673e-120	18	
CIP2A	DZ 2					
3.438756e-124	0.8625549	0.62	0.119	6.130958e-120	18	
CCNG2	DZ 2					
5.240967e-124	0.8481067	0.877	0.433	9.34412e-120	18	
ACTR2	DZ 2					
5.883537e-124	0.7575398	0.949	0.683	1.048976e-119	18	
PPP1CA	DZ 2					
1.316252e-123	0.6587544	0.995	0.898	2.346746e-119	18	
PFN1	DZ 2					
7.597506e-123	0.888399	0.608	0.141	1.354559e-118	18	PTPN18
DZ 2						
4.451387e-122	0.7123025	0.408	0.039	7.936378e-118	18	
GPSM2	DZ 2					
5.529015e-121	1.028738	0.523	0.109	9.857681e-117	18	GMDS
DZ 2						
1.244406e-120	0.8080994	0.856	0.403	2.218652e-116	18	
MDH1	DZ 2					
2.408487e-120	0.8794893	0.574	0.103	4.294091e-116	18	
AC023590.1	DZ 2					
5.874766e-120	0.9089038	0.593	0.11	1.047412e-115	18	
CD38	DZ 2					
1.793422e-119	0.8914706	0.474	0.052	3.197493e-115	18	
TYMS	DZ 2					
4.205777e-119	0.8232156	0.778	0.283	7.498481e-115	18	
YWHAH	DZ 2					
6.589384e-119	0.9059778	0.835	0.294	1.174821e-114	18	
BCL7A	DZ 2					
4.267228e-118	0.6573301	0.39	0.041	7.60804e-114	18	

CENPN DZ 2
1.057116e-117 0.839495 0.821 0.357 1.884732e-113 18
VPS29 DZ 2
1.393049e-117 0.8090382 0.89 0.441 2.483667e-113 18
SEM1 DZ 2
2.890177e-117 0.8160871 0.816 0.314 5.152896e-113 18
HSBP1 DZ 2
1.050723e-116 0.8755383 0.812 0.321 1.873334e-112 18
RFTN1 DZ 2
1.149813e-116 0.6660753 0.979 0.868 2.050001e-112 18
ARPC3 DZ 2
4.986643e-116 0.8264718 0.826 0.368 8.890687e-112 18
FAM107B DZ 2
1.429054e-115 0.7493695 0.433 0.055 2.54786e-111 18
DCAF12 DZ 2
2.383577e-115 0.8967321 0.854 0.39 4.249679e-111 18
BASP1 DZ 2
2.793011e-115 -0.9803605 0.991 0.996 4.97966e-111 18
RPS12 DZ 2
8.871488e-115 0.7630337 0.955 0.695 1.581698e-110 18
CCNI DZ 2
1.506729e-114 0.7572403 0.579 0.126 2.686346e-110 18
NUP37 DZ 2
2.859754e-114 -0.7514079 0.999 1 5.098655e-110 18
RPS27 DZ 2
3.284139e-114 0.9091118 0.637 0.173 5.855291e-110 18
ZNF106 DZ 2
3.9057e-114 -0.6256576 1 1 6.963473e-110 18
RPL41 DZ 2
8.36616e-114 0.6331805 0.307 0.027 1.491603e-109 18
PTMS DZ 2
1.512712e-113 0.9109192 0.747 0.228 2.697015e-109 18
GCHFR DZ 2
3.775809e-113 0.7706576 0.644 0.169 6.73189e-109 18
PMVK DZ 2
3.879416e-113 0.8111092 0.839 0.357 6.916611e-109 18
RBBP7 DZ 2
1.470968e-112 0.6632872 0.398 0.05 2.622589e-108 18
CENPU DZ 2
2.214339e-112 -0.7790953 0.997 0.999 3.947944e-108 18
RPL13 DZ 2
1.918967e-111 0.7469055 0.39 0.044 3.421325e-107 18
LINC01991 DZ 2
3.246854e-111 0.842212 0.962 0.859 5.788816e-107 18 UBB
DZ 2
5.868506e-110 -1.223225 0.93 0.964 1.046296e-105 18
FTL DZ 2
5.658208e-109 0.6822138 0.407 0.056 1.008802e-104 18
ORC6 DZ 2
3.597788e-108 -0.9733123 0.997 1 6.414496e-104 18

MALAT1	DZ	2	5.758856e-108	0.720149	0.944	0.578	1.026747e-103	18
ATP5MC3	DZ	2	7.71367e-108	0.4036779	0.211	0.009	1.37527e-103	18
FOXM1	DZ	2	9.718324e-108	0.7804595	0.652	0.18	1.73268e-103	18
GLRX5	DZ	2	1.170949e-107	-0.788281	0.997	0.999	2.087686e-103	18
RPL32	DZ	2	3.77388e-107	0.7769254	0.547	0.119	6.728451e-103	18
COMMD4	DZ	2	1.142787e-106	0.7507345	0.87	0.432	2.037475e-102	18
HMGA1	DZ	2	1.193897e-106	0.7815646	0.444	0.066	2.128599e-102	18
CDCA7	DZ	2	1.555315e-106	0.7867752	0.812	0.338	2.772972e-102	18
LSM6	DZ	2	2.11561e-106	0.7173257	0.696	0.216	3.771921e-102	18
DAZAP1	DZ	2	4.507422e-106	0.735883	0.475	0.079	8.036282e-102	18
BRD8	DZ	2	6.182138e-106	0.7550955	0.583	0.144	1.102213e-101	18
HDGF	DZ	2	8.043328e-106	0.823872	0.495	0.09	1.434045e-101	18
STAG3	DZ	2	1.296073e-105	0.7724179	0.745	0.241	2.310768e-101	18
PPP2R5C	DZ	2	6.871416e-105	0.8035261	0.499	0.058	1.225105e-100	18
TK1	DZ	2	7.838496e-105	0.6792931	0.927	0.565	1.397525e-100	18
RBM8A	DZ	2	1.010458e-104	0.726422	0.843	0.381	1.801546e-100	18
CCT5	DZ	2	1.131184e-104	0.6719445	0.951	0.673	2.016787e-100	18
HNRNPA3	DZ	2	2.365551e-104	0.7601832	0.482	0.078	4.217541e-100	18
TMEM131L	DZ	2	3.806299e-104	0.4234472	0.231	0.011	6.78625e-100	18
PLK4	DZ	2	5.553661e-104	0.8017548	0.679	0.2	9.901622e-100	18
MIS18BP1	DZ	2	6.191807e-104	0.8511252	0.684	0.239	1.103937e-99	18
TPM4	DZ	2	7.83184e-104	0.6671816	0.426	0.053	1.396339e-99	18
CENPK	DZ	2	2.743733e-103	0.7734338	0.766	0.265	4.891802e-99	18
POU2AF1	DZ	2	3.280484e-103	0.7125783	0.896	0.467	5.848775e-99	18
SNRPD1	DZ	2	1.503935e-102	0.666027	0.439	0.069	2.681365e-98	18

EZH2 DZ 2
4.285113e-102 -0.7287315 0.999 0.999 7.639929e-98 18
RPS27A DZ 2
6.025483e-102 0.5876154 0.328 0.036 1.074283e-97 18
NDC1 DZ 2
9.048373e-101 0.7904976 0.797 0.324 1.613235e-96 18
BPTF DZ 2
1.086659e-100 0.8358724 0.785 0.295 1.937405e-96 18
CD27 DZ 2
1.178728e-100 0.6661898 0.952 0.635 2.101555e-96 18
ERH DZ 2
2.358113e-100 0.7280591 0.602 0.152 4.20428e-96 18
VRK1 DZ 2
3.445727e-100 -0.6719596 1 0.999 6.143386e-96 18
RPL13A DZ 2
5.65139e-100 0.8160915 0.88 0.486 1.007586e-95 18
C4orf3 DZ 2
8.625323e-100 0.8490275 0.703 0.228 1.537809e-95 18
CCND3 DZ 2
9.548993e-100 0.7506438 0.606 0.16 1.70249e-95 18
TFDP1 DZ 2
1.955976e-99 0.6807416 0.943 0.648 3.48731e-95 18
YWHAB DZ 2
2.137104e-99 0.5802954 0.342 0.035 3.810242e-95 18
REEP4 DZ 2
9.286143e-99 0.6870724 0.904 0.508 1.655626e-94 18
SRP9 DZ 2
1.007476e-98 0.7621068 0.799 0.343 1.796229e-94 18
MRPS6 DZ 2
1.416775e-98 0.4396489 0.215 0.012 2.525969e-94 18
SPC24 DZ 2
1.69339e-98 0.7779555 0.715 0.246 3.019145e-94 18
MPC2 DZ 2
1.876885e-98 0.8768726 0.47 0.071 3.346298e-94 18
SUGCT DZ 2
9.872131e-98 0.7319562 0.89 0.421 1.760102e-93 18
IRF8 DZ 2
6.377924e-97 0.6558118 0.392 0.05 1.13712e-92 18
MME DZ 2
1.592041e-96 0.7198859 0.891 0.517 2.83845e-92 18
TRAPPC1 DZ 2
2.05528e-96 0.4328803 0.232 0.016 3.664358e-92 18
MELK DZ 2
3.544899e-96 0.7534387 0.845 0.408 6.320201e-92 18
PPP1CC DZ 2
3.92862e-96 0.7402836 0.891 0.484 7.004336e-92 18
RAC2 DZ 2
5.059376e-96 0.3975943 0.19 0.011 9.020362e-92 18
SKA3 DZ 2
5.204746e-96 0.366236 0.197 0.01 9.279541e-92 18

SKA1 DZ 2
5.707634e-96 0.5129023 0.246 0.019 1.017614e-91 18
NPAS1 DZ 2
6.579469e-96 0.6885149 0.525 0.112 1.173053e-91 18
SS18 DZ 2
9.379115e-96 0.7826992 0.53 0.105 1.672202e-91 18
CPNE5 DZ 2
1.005876e-95 0.7500291 0.823 0.376 1.793376e-91 18
SMARCB1 DZ 2
1.346182e-95 0.6736091 0.872 0.442 2.400107e-91 18
ILF2 DZ 2
1.554032e-95 0.7151614 0.645 0.174 2.770684e-91 18
MED30 DZ 2
1.556304e-95 0.6067393 0.394 0.041 2.774735e-91 18
ASF1B DZ 2
4.939709e-95 -1.136172 0.791 0.908 8.807007e-91 18
IGHM DZ 2
1.264159e-94 -0.9037611 0.984 0.991 2.253869e-90 18
RPL9 DZ 2
2.738306e-94 -0.6099909 1 0.999 4.882125e-90 18
RPLP1 DZ 2
4.371282e-94 -0.6505659 0.996 0.999 7.793559e-90 18
RPS19 DZ 2
6.158748e-94 0.7544112 0.753 0.276 1.098043e-89 18
CARHSP1 DZ 2
8.648073e-94 0.7149719 0.804 0.359 1.541865e-89 18
EWSR1 DZ 2
8.772244e-94 0.7266885 0.618 0.175 1.564003e-89 18
TRABD DZ 2
4.569212e-93 0.6900258 0.939 0.582 8.146448e-89 18
MARCKSL1 DZ 2
6.461997e-93 0.6770993 0.92 0.528 1.152109e-88 18
SRSF9 DZ 2
7.801577e-93 0.5939709 0.97 0.718 1.390943e-88 18
COX6C DZ 2
9.708439e-93 0.45689 0.224 0.017 1.730918e-88 18
RGS9 DZ 2
1.270632e-92 0.4625966 0.241 0.017 2.26541e-88 18
ARHGAP11B DZ 2
1.795567e-92 0.4492766 0.273 0.022 3.201316e-88 18
POC1A DZ 2
3.07576e-92 0.8430115 0.408 0.065 5.483772e-88 18
RASSF6 DZ 2
4.323926e-92 0.6765531 0.841 0.428 7.709128e-88 18
LSM3 DZ 2
5.658684e-92 0.5835949 0.304 0.032 1.008887e-87 18
SCLT1 DZ 2
6.673152e-92 0.7618184 0.771 0.314 1.189756e-87 18
NAA38 DZ 2
8.750449e-92 0.6515769 0.413 0.062 1.560117e-87 18

RRM2B	DZ 2				
4.24321e-91	0.6624751	0.409	0.065	7.56522e-87	18
RPRD1B	DZ 2				
5.459555e-91	0.4055215	0.223	0.013	9.73384e-87	18
KIF15	DZ 2				
1.111455e-90	-1.128654	0.848	0.906	1.981613e-86	18
HLA-A	DZ 2				
4.284722e-90	0.6778009	0.427	0.071	7.639231e-86	18
KLHL6	DZ 2				
9.959162e-90	0.3562069	0.188	0.008	1.775619e-85	18
SMTN	DZ 2				
2.498462e-89	0.7193799	0.488	0.106	4.454508e-85	18
BRI3BP	DZ 2				
5.877822e-89	0.4089532	0.192	0.009	1.047957e-84	18
AC091057.6	DZ 2				
7.839123e-89	0.7494347	0.528	0.12	1.397637e-84	18
MSI2	DZ 2				
9.984219e-89	-1.880732	0.179	0.47	1.780086e-84	18
S100A6	DZ 2				
1.581448e-88	0.6961585	0.658	0.204	2.819563e-84	18
CCAR1	DZ 2				
2.276328e-88	0.7538911	0.566	0.132	4.058465e-84	18
SOCS1	DZ 2				
2.94256e-88	0.6217853	0.9	0.511	5.246291e-84	18
ATP5PB	DZ 2				
4.149554e-88	0.4437586	0.253	0.021	7.39824e-84	18
CDCA5	DZ 2				
4.181278e-88	0.6259863	0.426	0.073	7.454801e-84	18
WEE1	DZ 2				
1.536358e-87	-0.7256418	0.997	0.998	2.739174e-83	18
RPLP2	DZ 2				
1.741176e-87	0.6569184	0.835	0.393	3.104343e-83	18
ENY2	DZ 2				
3.340685e-87	-0.7650815	0.997	0.998	5.956107e-83	18
RPL11	DZ 2				
5.245824e-87	0.7124398	0.788	0.289	9.352779e-83	18
GGA2	DZ 2				
4.987713e-86	0.4803779	0.992	0.961	8.892594e-82	18
OAZ1	DZ 2				
1.099823e-85	0.4038247	0.189	0.01	1.960874e-81	18
TBC1D4	DZ 2				
3.080703e-85	0.5180943	0.288	0.027	5.492586e-81	18
KMT5A	DZ 2				
4.164882e-85	0.3583687	0.215	0.015	7.425569e-81	18
TRIP13	DZ 2				
6.437764e-85	0.6611474	0.675	0.232	1.147789e-80	18
MORF4L2	DZ 2				
7.356288e-85	0.6169366	0.933	0.615	1.311553e-80	18
ACTR3	DZ 2				
2.125378e-84	-1.098715	0.943	0.961	3.789337e-80	18

MT-ND2	DZ 2	3.447417e-84	0.624315	0.385	0.065	6.1464e-80	18
TERF2	DZ 2	4.395775e-84	0.6711677	0.615	0.182	7.837227e-80	18
MAP2K1	DZ 2	4.704778e-84	0.6538889	0.793	0.356	8.388148e-80	18
PPP2CA	DZ 2	5.082153e-84	0.729505	0.762	0.266	9.06097e-80	18
NCF1	DZ 2	5.621679e-84	-1.304987	0.082	0.355	1.002289e-79	18
LY6E	DZ 2	1.646226e-83	0.5859675	0.385	0.056	2.935057e-79	18
DTX1	DZ 2	1.981462e-83	0.7014771	0.663	0.225	3.532748e-79	18
MTF2	DZ 2	3.315623e-83	0.682006	0.742	0.312	5.911424e-79	18
PRDX2	DZ 2	4.175422e-83	0.5877696	0.94	0.636	7.444359e-79	18
HNRNPM	DZ 2	7.826888e-83	0.4565138	0.242	0.022	1.395456e-78	18
FANCI	DZ 2	1.076575e-82	0.578879	0.948	0.64	1.919425e-78	18
SNRPG	DZ 2	5.056583e-82	0.7201869	0.553	0.14	9.015381e-78	18
SH2B2	DZ 2	5.590876e-82	0.5536737	0.321	0.046	9.967973e-78	18
UCHL1	DZ 2	6.560701e-82	0.6728337	0.799	0.358	1.169707e-77	18
TERF2IP	DZ 2	1.051536e-81	0.6702392	0.522	0.13	1.874783e-77	18
CETN3	DZ 2	1.412957e-81	0.6733176	0.823	0.433	2.519161e-77	18
FDPS	DZ 2	3.603027e-81	0.7769709	0.605	0.179	6.423836e-77	18
P2RX5	DZ 2	3.860748e-81	0.5278759	0.262	0.025	6.883327e-77	18
TRIM59	DZ 2	1.039627e-80	0.625085	0.867	0.439	1.853552e-76	18
ATP5F1C	DZ 2	1.824306e-80	0.7819606	0.911	0.527	3.252555e-76	18
ISG20	DZ 2	3.962879e-80	0.6192306	0.845	0.424	7.065417e-76	18
ZNF706	DZ 2	4.506005e-80	0.6495285	0.833	0.422	8.033757e-76	18
KTN1	DZ 2	6.227847e-80	-0.6767346	1	0.998	1.110363e-75	18
RPL18A	DZ 2	7.848319e-80	0.6717256	0.516	0.122	1.399277e-75	18
RABGAP1L	DZ 2	9.897104e-80	0.662871	0.569	0.155	1.764555e-75	18

UBE2R2	DZ 2	1.651564e-79	-1.279662	0.13	0.387	2.944574e-75	18
CD44	DZ 2	5.4483e-79	-0.7107645	0.991	0.995	9.713773e-75	18
RPS25	DZ 2	6.988991e-79	0.6658522	0.17	0.009	1.246067e-74	18
IGLL1	DZ 2	7.18446e-79	0.573213	0.882	0.49	1.280917e-74	18
EIF4G2	DZ 2	7.646498e-79	0.5959189	0.462	0.098	1.363294e-74	18
CDC27	DZ 2	9.479936e-79	-0.9848906	0.955	0.969	1.690178e-74	18
MT-ND3	DZ 2	1.646851e-78	0.6432707	0.601	0.192	2.936171e-74	18
NUDCD2	DZ 2	1.651498e-78	0.5199342	0.319	0.048	2.944455e-74	18
CDKN2C	DZ 2	3.632796e-78	0.6848773	0.692	0.253	6.476912e-74	18
CCDC69	DZ 2	9.115399e-78	0.5345947	0.966	0.76	1.625185e-73	18
MYL12A	DZ 2	1.204733e-77	0.6823077	0.569	0.165	2.147918e-73	18
BLOC1S6	DZ 2	2.582057e-77	0.6577863	0.582	0.175	4.60355e-73	18
RASGRP3	DZ 2	1.096833e-76	0.6327243	0.446	0.095	1.955544e-72	18
PTPN7	DZ 2	1.364393e-76	0.6026192	0.802	0.367	2.432576e-72	18
LCP1	DZ 2	1.943479e-76	0.6291995	0.437	0.084	3.465029e-72	18
DCP2	DZ 2	2.750571e-76	0.6607255	0.773	0.33	4.903993e-72	18
NDUFC1	DZ 2	3.421325e-76	0.6567937	0.464	0.101	6.09988e-72	18
E2F5	DZ 2	7.517819e-76	0.5872158	0.464	0.103	1.340352e-71	18
AZIN1	DZ 2	7.661151e-76	-0.7059695	0.997	0.996	1.365907e-71	18
RPL35A	DZ 2	8.463371e-76	0.5317816	0.32	0.039	1.508934e-71	18
RAD51AP1	DZ 2	1.126125e-75	0.5303426	0.925	0.599	2.007768e-71	18
PSMB3	DZ 2	1.255639e-75	0.6965836	0.775	0.323	2.238678e-71	18
GRHPR	DZ 2	1.263755e-75	0.5384676	0.953	0.693	2.253149e-71	18
RHOA	DZ 2	3.222606e-75	0.4130456	0.236	0.019	5.745585e-71	18
C21orf58	DZ 2	3.686388e-75	0.676724	0.672	0.243	6.572462e-71	18

TIFA DZ 2
4.139185e-75 -0.7318295 0.994 0.995 7.379752e-71 18
RPL31 DZ 2
5.027647e-75 0.6346864 0.376 0.068 8.963792e-71 18
S1PR2 DZ 2
8.663614e-75 0.556648 0.358 0.047 1.544636e-70 18
APOLD1 DZ 2
9.175596e-75 0.6391338 0.624 0.189 1.635917e-70 18
UBE2E1 DZ 2
9.794865e-75 0.7502184 0.709 0.282 1.746327e-70 18
TUBA4A DZ 2
1.415085e-74 0.631532 0.447 0.089 2.522955e-70 18
PXMP2 DZ 2
2.071731e-74 0.744364 0.628 0.203 3.693689e-70 18 ADA
DZ 2
3.728968e-74 0.4101277 0.229 0.024 6.648376e-70 18
SLCO3A1 DZ 2
4.210669e-74 0.615297 0.742 0.303 7.507201e-70 18
PNRC2 DZ 2
4.672189e-74 0.6586348 0.596 0.19 8.330045e-70 18
POLD4 DZ 2
9.236658e-74 0.8415873 0.584 0.179 1.646804e-69 18
RGS2 DZ 2
1.500639e-73 -0.5303258 0.999 0.999 2.675489e-69 18
RPL10 DZ 2
2.138076e-73 0.6468157 0.933 0.659 3.811976e-69 18
DBI DZ 2
2.809524e-73 0.6086118 0.434 0.091 5.0091e-69 18
RAD17 DZ 2
2.866675e-73 0.526948 0.377 0.068 5.110994e-69 18
LRR1 DZ 2
2.962392e-73 0.6284653 0.734 0.309 5.281648e-69 18
NDUFAF3 DZ 2
4.049576e-73 0.7023769 0.614 0.203 7.21999e-69 18
SYAP1 DZ 2
5.213982e-73 0.5594796 0.876 0.478 9.296008e-69 18
UBE2L3 DZ 2
6.356866e-73 0.6821077 0.637 0.232 1.133366e-68 18
ZBTB8OS DZ 2
7.569891e-73 0.613712 0.519 0.135 1.349636e-68 18
ANAPC15 DZ 2
7.962703e-73 0.6310257 0.468 0.111 1.41967e-68 18
ABI3 DZ 2
8.760887e-73 0.6436563 0.692 0.295 1.561979e-68 18
AHSA1 DZ 2
9.912606e-73 0.4310191 0.259 0.025 1.767319e-68 18
BORA DZ 2
1.001755e-72 0.6016247 0.738 0.295 1.78603e-68 18
HNRNPH3 DZ 2
1.220901e-72 0.5792402 0.394 0.077 2.176745e-68 18

BPNT1	DZ	2				
1.518291e-72	0.594803	0.522	0.138	2.706962e-68	18	
FLOT1	DZ	2				
2.604306e-72	0.5893294	0.481	0.112	4.643217e-68	18	
CBFB	DZ	2				
5.468762e-72	0.6092142	0.763	0.343	9.750256e-68	18	
LSM14A	DZ	2				
6.83214e-72	0.5967148	0.723	0.292	1.218102e-67	18	
GTF2A2	DZ	2				
8.590449e-72	0.4824079	0.328	0.047	1.531591e-67	18	
ZWILCH	DZ	2				
1.091381e-71	-0.6806848	0.996	0.999	1.945823e-67	18	
RPL39	DZ	2				
1.157659e-71	0.5439795	0.842	0.439	2.063991e-67	18	
YWHAQ	DZ	2				
1.328954e-71	0.5924126	0.819	0.408	2.369392e-67	18	
HDAC1	DZ	2				
1.352153e-71	0.5996296	0.601	0.187	2.410753e-67	18	
DCAF7	DZ	2				
2.964658e-71	0.6736175	0.657	0.232	5.285689e-67	18	
NCF4	DZ	2				
4.360748e-71	0.5236906	0.312	0.052	7.774778e-67	18	
BFSP2	DZ	2				
5.477116e-71	0.5596642	0.916	0.574	9.765151e-67	18	
LDHB	DZ	2				
6.187579e-71	0.415024	0.253	0.027	1.103183e-66	18	
TEDC1	DZ	2				
1.067657e-70	0.5009921	0.307	0.038	1.903525e-66	18	
IGF2BP3	DZ	2				
1.204881e-70	0.3270558	0.144	0.007	2.148182e-66	18	
ACKR4	DZ	2				
1.359997e-70	0.5630614	0.811	0.395	2.424738e-66	18	
UQCRFS1	DZ	2				
1.760302e-70	0.4960701	0.962	0.702	3.138442e-66	18	
COX7A2	DZ	2				
6.193289e-70	0.6304045	0.593	0.193	1.104201e-65	18	
RALBP1	DZ	2				
9.917091e-70	0.6725979	0.468	0.1	1.768118e-65	18	
HRK	DZ	2				
1.033319e-69	0.5370958	0.926	0.609	1.842304e-65	18	
ARPC5	DZ	2				
1.187557e-69	0.6532468	0.592	0.19	2.117295e-65	18	
IDH2	DZ	2				
1.413911e-69	0.4543708	0.952	0.648	2.520863e-65	18	
UBE2D3	DZ	2				
2.083336e-69	0.5390041	0.905	0.553	3.71438e-65	18	
ATP5PF	DZ	2				
2.588265e-69	0.562588	0.479	0.125	4.614618e-65	18	
GOT1	DZ	2				
3.64166e-69	0.5040438	0.934	0.64	6.492715e-65	18	

HNRNPC	DZ 2					
3.656034e-69	0.543971	0.883	0.52	6.518342e-65	18	
VAMP8	DZ 2					
3.766205e-69	0.599879	0.453	0.097	6.714767e-65	18	
SEMA4A	DZ 2					
3.919484e-69	0.4824198	0.293	0.039	6.988048e-65	18	
CDT1	DZ 2					
4.148601e-69	-1.272915	0.324	0.515	7.396541e-65	18	
BANK1	DZ 2					
4.281502e-69	0.341938	0.167	0.01	7.63349e-65	18	
SPA17	DZ 2					
8.425906e-69	0.6337268	0.584	0.181	1.502255e-64	18	
PRPSAP2	DZ 2					
1.009224e-68	0.5919391	0.506	0.131	1.799346e-64	18	
CLIC4	DZ 2					
1.230133e-68	0.6362609	0.571	0.161	2.193204e-64	18	
SEL1L3	DZ 2					
1.262453e-68	0.5711265	0.574	0.182	2.250828e-64	18	
RUVBL2	DZ 2					
3.386882e-68	0.5631331	0.728	0.301	6.038473e-64	18	
MMADHC	DZ 2					
3.428034e-68	0.6158693	0.62	0.227	6.111842e-64	18	
MRPL27	DZ 2					
3.770364e-68	-0.7536554	0.975	0.986	6.722181e-64	18	
HLA-B	DZ 2					
6.65117e-68	-1.083619	0.448	0.623	1.185837e-63	18	
SUB1	DZ 2					
6.84129e-68	0.6418154	0.536	0.161	1.219734e-63	18	
CCDC167	DZ 2					
7.328751e-68	0.6249795	0.745	0.332	1.306643e-63	18	
RBBP4	DZ 2					
7.396917e-68	0.5880991	0.62	0.215	1.318796e-63	18	
FXR1	DZ 2					
7.483363e-68	0.5093099	0.949	0.68	1.334209e-63	18	
COX6A1	DZ 2					
8.122378e-68	0.5466486	0.549	0.158	1.448139e-63	18	
SAE1	DZ 2					
8.483012e-68	0.4932376	0.333	0.052	1.512436e-63	18	
RNF26	DZ 2					
2.863394e-67	0.5223694	0.43	0.085	5.105145e-63	18	
FOPNL	DZ 2					
3.242241e-67	0.6275088	0.728	0.313	5.780592e-63	18	
AP3S1	DZ 2					
4.135871e-67	0.5087323	0.892	0.559	7.373844e-63	18	
SNRPB	DZ 2					
8.110896e-67	0.5296726	0.873	0.484	1.446092e-62	18	
SF3B6	DZ 2					
1.112196e-66	-0.7515955	0.984	0.985	1.982934e-62	18	
RPL27	DZ 2					
1.313122e-66	0.4578808	0.972	0.802	2.341164e-62	18	

HNRNPK DZ 2
1.898915e-66 -0.7524355 0.994 0.996 3.385575e-62 18
RPL12 DZ 2
2.173362e-66 -0.516874 0.999 1 3.874888e-62 18
RPL21 DZ 2
2.40978e-66 0.533304 0.811 0.396 4.296396e-62 18
BZW1 DZ 2
2.54594e-66 0.5519125 0.499 0.135 4.539156e-62 18
RUVBL1 DZ 2
2.93095e-66 0.5361081 0.878 0.495 5.225591e-62 18
RTRAF DZ 2
5.120041e-66 0.5634952 0.635 0.229 9.128522e-62 18
MAPRE1 DZ 2
9.582045e-66 0.5388153 0.725 0.309 1.708383e-61 18
TCP1 DZ 2
1.710817e-65 0.5191427 0.312 0.04 3.050215e-61 18
DZIP3 DZ 2
2.246647e-65 0.4529765 0.966 0.795 4.005547e-61 18
SUMO2 DZ 2
2.777583e-65 -0.5858106 0.996 0.998 4.952152e-61 18
RPS28 DZ 2
3.076952e-65 0.6416211 0.449 0.095 5.485898e-61 18
CDKN2D DZ 2
3.244976e-65 0.5701303 0.812 0.416 5.785468e-61 18
GABARAPL2 DZ 2
3.59765e-65 0.4814815 0.955 0.703 6.414249e-61 18
NDUFA4 DZ 2
6.400301e-65 0.5842196 0.455 0.111 1.14111e-60 18
C12orf49 DZ 2
8.018643e-65 0.5337062 0.377 0.064 1.429644e-60 18
SNTA1 DZ 2
1.785554e-64 0.5417983 0.589 0.183 3.183464e-60 18
USP1 DZ 2
2.910989e-64 -1.210534 0.421 0.579 5.190003e-60 18
SARAF DZ 2
5.012831e-64 0.5299829 0.389 0.076 8.937376e-60 18
VGLL4 DZ 2
6.419209e-64 0.5717176 0.567 0.183 1.144481e-59 18
NUDT21 DZ 2
9.953261e-64 -1.279763 0.193 0.375 1.774567e-59 18
SELL DZ 2
1.052376e-63 0.52943 0.852 0.465 1.876281e-59 18
NDUFB4 DZ 2
1.664965e-63 0.5675739 0.788 0.379 2.968466e-59 18
NDUFB3 DZ 2
2.241967e-63 0.5374656 0.837 0.459 3.997204e-59 18
PRDX6 DZ 2
4.250112e-63 0.4457721 0.253 0.027 7.577524e-59 18
LMO7 DZ 2
4.487799e-63 0.5762527 0.828 0.449 8.001297e-59 18

CAPZA1 DZ 2
4.567269e-63 0.4930841 0.899 0.56 8.142983e-59 18
RAC1 DZ 2
5.017031e-63 0.716205 0.583 0.23 8.944865e-59 18
DSTN DZ 2
5.940944e-63 0.3814045 0.234 0.025 1.059211e-58 18
LINC00877 DZ 2
6.411989e-63 0.5640246 0.772 0.374 1.143194e-58 18
SRSF11 DZ 2
6.876825e-63 0.4778397 0.938 0.635 1.226069e-58 18
ATP5MF DZ 2
7.158861e-63 0.4708624 0.323 0.053 1.276353e-58 18
MBD2 DZ 2
7.403026e-63 0.5210763 0.636 0.241 1.319885e-58 18
EXOSC8 DZ 2
7.842856e-63 0.5839155 0.719 0.301 1.398303e-58 18
FKBP3 DZ 2
9.903619e-63 0.4724594 0.291 0.045 1.765716e-58 18
RFC3 DZ 2
1.255178e-62 0.5388785 0.519 0.132 2.237857e-58 18
GRK6 DZ 2
1.294153e-62 0.5536676 0.763 0.361 2.307345e-58 18
CACYBP DZ 2
1.297152e-62 0.5715578 0.944 0.721 2.312693e-58 18
LIMD2 DZ 2
1.33137e-62 0.5334547 0.802 0.405 2.373699e-58 18
MOB1A DZ 2
1.486633e-62 0.5311734 0.829 0.431 2.650518e-58 18
COTL1 DZ 2
2.162428e-62 0.5465096 0.745 0.342 3.855393e-58 18
POLR2K DZ 2
4.576504e-62 0.5185847 0.764 0.359 8.159449e-58 18
PSMA5 DZ 2
4.829073e-62 0.5595979 0.725 0.294 8.609753e-58 18
CDK2AP2 DZ 2
5.736948e-62 0.4182312 0.232 0.023 1.02284e-57 18
ALPK1 DZ 2
7.24345e-62 -0.735602 0.99 0.991 1.291435e-57 18
RPL30 DZ 2
1.24547e-61 -0.6228787 1 0.999 2.220549e-57 18
EEF1A1 DZ 2
1.272902e-61 -0.6007738 0.997 0.998 2.269457e-57 18
RPS14 DZ 2
1.323938e-61 -0.5053393 0.999 0.999 2.360449e-57 18
RPS18 DZ 2
1.330653e-61 0.5209547 0.781 0.346 2.372422e-57 18
EIF1B DZ 2
1.395383e-61 0.50408 0.295 0.047 2.487828e-57 18 CPM
DZ 2
1.416198e-61 -1.171961 0.088 0.277 2.52494e-57 18

KLF2	DZ 2	1.538894e-61	0.5404478	0.506	0.139	2.743695e-57	18
PTEN	DZ 2	1.659754e-61	0.5217054	0.345	0.066	2.959175e-57	18
CCDC34	DZ 2	1.769883e-61	0.3967494	0.246	0.034	3.155524e-57	18
KCNK12	DZ 2	2.285494e-61	0.4702195	0.369	0.077	4.074807e-57	18
PDZD11	DZ 2	2.678637e-61	0.4322673	0.97	0.797	4.775742e-57	18
LAPTM5	DZ 2	5.920209e-61	0.6013644	0.481	0.134	1.055514e-56	18
AIM2	DZ 2	6.268616e-61	0.437237	0.981	0.9	1.117632e-56	18
MYL6	DZ 2	1.481566e-60	0.4462908	0.355	0.062	2.641483e-56	18
RHNO1	DZ 2	1.550255e-60	0.5379688	0.64	0.243	2.76395e-56	18
THOC2	DZ 2	1.579914e-60	0.5371728	0.584	0.216	2.816829e-56	18
PSMD9	DZ 2	1.584426e-60	0.5255063	0.571	0.185	2.824873e-56	18
HNRNPUL1	DZ 2	1.783929e-60	0.4584305	0.302	0.047	3.180568e-56	18
ASB13	DZ 2	2.287242e-60	0.5593712	0.433	0.101	4.077924e-56	18
TCF3	DZ 2	2.419579e-60	0.5642755	0.627	0.223	4.313867e-56	18
OAZ2	DZ 2	3.916931e-60	0.5223293	0.358	0.07	6.983496e-56	18
PAG1	DZ 2	5.197167e-60	0.5460675	0.455	0.119	9.26603e-56	18
ARL3	DZ 2	8.548395e-60	0.3723509	0.237	0.028	1.524093e-55	18
C9orf40	DZ 2	1.035453e-59	0.5125847	0.751	0.339	1.846109e-55	18
UBE2A	DZ 2	1.464583e-59	0.5184948	0.547	0.167	2.611206e-55	18
NUDT1	DZ 2	1.761112e-59	0.5598109	0.5	0.15	3.139887e-55	18
ACTL6A	DZ 2	2.642284e-59	0.5583624	0.706	0.326	4.710928e-55	18
EIF3A	DZ 2	5.2759e-59	0.4964061	0.75	0.359	9.406403e-55	18
LRRFIP1	DZ 2	5.621686e-59	0.4960661	0.33	0.059	1.00229e-54	18
PITPNC1	DZ 2	5.943408e-59	0.5316733	0.728	0.324	1.05965e-54	18
RALY	DZ 2	6.861342e-59	0.5008508	0.382	0.084	1.223309e-54	18

DESI2	DZ 2	9.694642e-59	0.5938538	0.62	0.227	1.728458e-54	18
ZCCHC7	DZ 2	1.476623e-58	0.4828046	0.829	0.449	2.632671e-54	18
PPP4C	DZ 2	1.758205e-58	0.4760006	0.381	0.077	3.134703e-54	18
ODF2	DZ 2	2.178288e-58	0.5226662	0.797	0.411	3.88367e-54	18
ELF1	DZ 2	2.644875e-58	0.4761671	0.929	0.675	4.715547e-54	18
SRSF3	DZ 2	3.129662e-58	-1.057195	0.424	0.609	5.579874e-54	18
IGHD	DZ 2	5.231323e-58	0.517229	0.851	0.447	9.326926e-54	18
PTPRC	DZ 2	7.15207e-58	0.5174551	0.576	0.19	1.275143e-53	18
RNF5	DZ 2	7.245553e-58	0.5176574	0.53	0.171	1.29181e-53	18
TIPRL	DZ 2	7.500137e-58	0.4564552	0.246	0.036	1.337199e-53	18
SYBU	DZ 2	1.002098e-57	0.5487876	0.649	0.274	1.786641e-53	18
BABAM1	DZ 2	1.549922e-57	0.4616549	0.282	0.046	2.763357e-53	18
PEX5	DZ 2	1.673385e-57	0.4868149	0.439	0.099	2.983478e-53	18
MGME1	DZ 2	2.042371e-57	0.4018664	0.238	0.03	3.641343e-53	18
CTPS2	DZ 2	3.441658e-57	0.5615888	0.847	0.479	6.136131e-53	18
GMFG	DZ 2	3.550029e-57	0.5324962	0.671	0.264	6.329347e-53	18
SMS	DZ 2	5.860682e-57	0.6085195	0.347	0.072	1.044901e-52	18
RGCC	DZ 2	5.969856e-57	0.5120296	0.807	0.414	1.064366e-52	18
NDUFA2	DZ 2	8.608247e-57	0.493316	0.276	0.044	1.534764e-52	18
LRRK1	DZ 2	9.41672e-57	0.4483032	0.269	0.041	1.678907e-52	18
ASB2	DZ 2	1.069275e-56	0.4645318	0.894	0.589	1.90641e-52	18
ST13	DZ 2	1.109435e-56	0.4302083	0.915	0.594	1.978012e-52	18
PRDX1	DZ 2	2.128318e-56	0.5583654	0.758	0.354	3.794578e-52	18
TPD52	DZ 2	2.525681e-56	0.4409691	0.298	0.044	4.503037e-52	18
HYLS1	DZ 2	3.96774e-56	-1.086858	0.733	0.794	7.074083e-52	18

HLA-E	DZ 2	6.274274e-56	0.4977939	0.417	0.102	1.11864e-51	18
IMMP1L	DZ 2	7.089605e-56	0.4774038	0.391	0.079	1.264006e-51	18
MIS18A	DZ 2	7.812811e-56	0.4761601	0.754	0.356	1.392946e-51	18
ELOC	DZ 2	7.92209e-56	0.4200431	0.244	0.039	1.412429e-51	18
ACY3	DZ 2	8.137143e-56	0.5496314	0.386	0.09	1.450771e-51	18
RNGTT	DZ 2	1.128808e-55	0.5127354	0.635	0.242	2.012551e-51	18
MRPL13	DZ 2	1.237313e-55	0.5091088	0.512	0.154	2.206006e-51	18
TPGS2	DZ 2	1.387081e-55	0.5397422	0.421	0.107	2.473027e-51	18
TRAC	DZ 2	1.745074e-55	0.4950279	0.451	0.12	3.111292e-51	18
DNTTIP1	DZ 2	1.96349e-55	-1.21051	0.158	0.356	3.500706e-51	18
TXNIP	DZ 2	3.104964e-55	-1.099714	0.123	0.3	5.53584e-51	18
LINC00926	DZ 2	3.714652e-55	0.5178818	0.456	0.131	6.622852e-51	18
OSBPL9	DZ 2	4.800935e-55	0.5096788	0.65	0.257	8.559588e-51	18
MRPL18	DZ 2	7.092399e-55	0.4915883	0.759	0.347	1.264504e-50	18
DGUOK	DZ 2	7.79896e-55	0.471008	0.889	0.524	1.390477e-50	18
PAIP2	DZ 2	8.858336e-55	0.4897893	0.6	0.231	1.579353e-50	18
STIP1	DZ 2	1.219755e-54	0.4599748	0.79	0.419	2.1747e-50	18
BLOC1S1	DZ 2	1.912602e-54	0.5053238	0.341	0.039	3.409979e-50	18
RRM2	DZ 2	1.933805e-54	0.4442598	0.933	0.664	3.447781e-50	18
GDI2	DZ 2	2.173228e-54	0.3855286	0.168	0.016	3.874649e-50	18
TUBB2B	DZ 2	2.308894e-54	0.5590627	0.613	0.227	4.116528e-50	18
CPNE3	DZ 2	2.535178e-54	0.379929	0.209	0.022	4.519969e-50	18
AC011447.3	DZ 2	2.728134e-54	0.4093655	0.301	0.05	4.863989e-50	18
KCTD9	DZ 2	3.010552e-54	0.4982779	0.756	0.331	5.367513e-50	18
STK17A	DZ 2	3.226557e-54	0.4382948	0.931	0.655	5.752628e-50	18

UBL5	DZ 2					
3.405447e-54	0.5201037	0.728	0.328	6.071572e-50	18	
DUT	DZ 2					
4.05166e-54	0.505608	0.687	0.295	7.223705e-50	18	
ARID4B	DZ 2					
4.621877e-54	-0.9808251	0.049	0.214	8.240344e-50	18	
PLAC8	DZ 2					
5.491782e-54	0.5257594	0.681	0.305	9.791298e-50	18	
MZT2A	DZ 2					
7.437395e-54	0.5317134	0.473	0.136	1.326013e-49	18	
CUL3	DZ 2					
1.054066e-53	0.4813731	0.335	0.069	1.879295e-49	18	
DOK3	DZ 2					
1.116565e-53	0.4264248	0.329	0.068	1.990724e-49	18	
SEPHS1	DZ 2					
1.143326e-53	0.5034536	0.447	0.118	2.038436e-49	18	
SPATS2	DZ 2					
1.213216e-53	0.5626514	0.398	0.093	2.163043e-49	18	
MX1	DZ 2					
1.697038e-53	-1.2082	0.335	0.497	3.025649e-49	18	
FXYD5	DZ 2					
2.143515e-53	0.4719922	0.399	0.094	3.821673e-49	18	
PPP1R35	DZ 2					
2.300207e-53	0.476847	0.685	0.263	4.101039e-49	18	
SEPT9	DZ 2					
2.612312e-53	-0.9997436	0.071	0.251	4.657492e-49	18	
TNFRSF13B	DZ 2					
2.977288e-53	0.3087328	0.197	0.019	5.308207e-49	18	
NCAPG2	DZ 2					
4.678966e-53	0.6387946	0.846	0.492	8.342128e-49	18	
SAT1	DZ 2					
5.773176e-53	0.4264209	0.297	0.048	1.0293e-48	18	
BRCA2	DZ 2					
8.663201e-53	0.4757863	0.714	0.306	1.544562e-48	18	
ZNF207	DZ 2					
1.976259e-52	0.4692854	0.797	0.387	3.523473e-48	18	
SYPL1	DZ 2					
2.224131e-52	0.3785735	0.276	0.044	3.965403e-48	18	
RANGAP1	DZ 2					
2.390085e-52	-0.5483652	0.992	0.997	4.261282e-48	18	
RPL7	DZ 2					
3.013975e-52	0.5002364	0.6	0.213	5.373617e-48	18	
SNX5	DZ 2					
3.300407e-52	0.444871	0.345	0.07	5.884295e-48	18	
CUX1	DZ 2					
4.542525e-52	0.3523722	0.956	0.74	8.098867e-48	18	
CHCHD2	DZ 2					
4.860161e-52	0.4307111	0.892	0.526	8.665181e-48	18	
SNRPB2	DZ 2					
5.691966e-52	-0.6440995	0.994	0.992	1.014821e-47	18	

RPL36	DZ 2	5.81972e-52	0.4929128	0.556	0.191	1.037598e-47	18
ETFB	DZ 2	6.565751e-52	0.4834101	0.679	0.282	1.170608e-47	18
RRAS2	DZ 2	6.740294e-52	-0.589703	0.991	0.993	1.201727e-47	18
RPS5	DZ 2	7.140508e-52	0.3591879	0.222	0.031	1.273081e-47	18
KANK1	DZ 2	8.008748e-52	0.4835061	0.604	0.23	1.42788e-47	18
TPRKB	DZ 2	1.147815e-51	-0.4467649	0.999	0.999	2.04644e-47	18
RPS2	DZ 2	1.240342e-51	0.498021	0.74	0.35	2.211405e-47	18
RAB11A	DZ 2	1.304068e-51	0.4284449	0.256	0.038	2.325023e-47	18
MCOLN2	DZ 2	2.047081e-51	0.4395111	0.824	0.446	3.649741e-47	18
MAP1LC3B	DZ 2	2.35135e-51	0.5001276	0.639	0.253	4.192223e-47	18
ILK	DZ 2	3.493831e-51	0.4764458	0.746	0.379	6.229152e-47	18
SNRPC	DZ 2	3.65972e-51	-1.065541	0.073	0.272	6.524915e-47	18
GPR183	DZ 2	3.923434e-51	-0.9638993	0.085	0.255	6.99509e-47	18
FCMR	DZ 2	4.287678e-51	0.5128233	0.544	0.178	7.644501e-47	18
OSBPL8	DZ 2	4.311786e-51	0.4405696	0.352	0.077	7.687483e-47	18
LRWD1	DZ 2	5.059314e-51	0.4355882	0.925	0.627	9.020251e-47	18
GSTP1	DZ 2	5.280465e-51	0.5096335	0.528	0.18	9.414542e-47	18
COPS8	DZ 2	5.675082e-51	0.4636666	0.409	0.094	1.01181e-46	18
RRM1	DZ 2	6.394367e-51	0.5376275	0.61	0.235	1.140052e-46	18
NSRP1	DZ 2	1.076552e-50	0.4777669	0.494	0.149	1.919385e-46	18
GNB1	DZ 2	1.339568e-50	0.4784365	0.613	0.254	2.388317e-46	18
AK6	DZ 2	1.920227e-50	-1.06057	0.202	0.35	3.423572e-46	18
TRAF3IP3	DZ 2	2.170968e-50	0.4352977	0.869	0.51	3.870618e-46	18
SMDT1	DZ 2	2.413952e-50	0.4435795	0.864	0.528	4.303835e-46	18
SOD1	DZ 2	2.914251e-50	0.4646852	0.622	0.235	5.195818e-46	18

CEP57	DZ	2	2.993473e-50	0.433365	0.632	0.231	5.337062e-46	18
TSG101	DZ	2	3.662214e-50	0.3974023	0.22	0.03	6.529361e-46	18
RAPGEF5	DZ	2	3.826977e-50	0.4853543	0.61	0.245	6.823118e-46	18
MCTS1	DZ	2	4.346419e-50	0.4448274	0.768	0.398	7.74923e-46	18
PSMB2	DZ	2	6.084475e-50	0.4907428	0.791	0.396	1.084801e-45	18
MBD4	DZ	2	6.409086e-50	0.4739368	0.342	0.074	1.142676e-45	18
GMNN	DZ	2	6.72109e-50	0.47028	0.536	0.18	1.198303e-45	18
CLINT1	DZ	2	7.032695e-50	0.4610872	0.532	0.173	1.253859e-45	18
CBX1	DZ	2	8.588332e-50	0.471743	0.62	0.25	1.531214e-45	18
RBM42	DZ	2	1.25428e-49	0.4030869	0.277	0.045	2.236256e-45	18
PCBD2	DZ	2	2.038845e-49	0.4482196	0.723	0.339	3.635057e-45	18
DNAJB6	DZ	2	2.232473e-49	0.4019773	0.896	0.573	3.980277e-45	18
COPE	DZ	2	2.297878e-49	0.4591812	0.746	0.373	4.096886e-45	18
ANAPC11	DZ	2	2.54273e-49	0.4872296	0.422	0.112	4.533433e-45	18
BRWD1	DZ	2	2.794334e-49	0.4577777	0.802	0.424	4.982018e-45	18
CLTA	DZ	2	3.143009e-49	-0.8556301	0.917	0.917	5.603671e-45	18
PFDN5	DZ	2	4.958445e-49	-0.6983882	0.982	0.983	8.840411e-45	18
RPS21	DZ	2	5.878067e-49	0.456551	0.705	0.319	1.048001e-44	18
MRPS36	DZ	2	8.063891e-49	0.4684076	0.834	0.463	1.437711e-44	18
MTDH	DZ	2	1.263542e-48	0.4519638	0.39	0.1	2.252769e-44	18
ACSF3	DZ	2	1.381347e-48	0.4539152	0.562	0.203	2.462804e-44	18
CHORDC1	DZ	2	2.409702e-48	0.4442403	0.347	0.076	4.296258e-44	18
SNAPC1	DZ	2	3.338787e-48	0.4190483	0.311	0.056	5.952724e-44	18
XRCC4	DZ	2	3.488994e-48	0.4859781	0.676	0.286	6.220528e-44	18
HSPA4	DZ	2	3.834494e-48	0.4087266	0.931	0.692	6.836519e-44	18

ATP5MPL DZ 2
3.958888e-48 0.4825666 0.398 0.084 7.058301e-44 18
FAM111A DZ 2
4.463453e-48 0.4413028 0.834 0.471 7.95789e-44 18
UBE2N DZ 2
5.670351e-48 0.4314498 0.867 0.517 1.010967e-43 18
FKBP1A DZ 2
7.979374e-48 0.4104527 0.693 0.307 1.422643e-43 18
EIF4E DZ 2
8.762121e-48 0.4707904 0.681 0.276 1.562199e-43 18
MTPN DZ 2
1.143067e-47 0.4663332 0.411 0.108 2.037974e-43 18
WDR34 DZ 2
3.004415e-47 0.4419416 0.37 0.087 5.356572e-43 18
FUNDC1 DZ 2
4.736189e-47 0.6433588 0.277 0.059 8.444151e-43 18
HOPX DZ 2
5.019702e-47 0.4477846 0.374 0.09 8.949626e-43 18
FAM76B DZ 2
5.453501e-47 0.4671839 0.723 0.34 9.723046e-43 18
RBM17 DZ 2
5.759167e-47 0.3514107 0.196 0.023 1.026802e-42 18
IQCD DZ 2
5.775352e-47 -0.5542331 0.995 0.995 1.029687e-42 18
RPL37A DZ 2
6.566217e-47 0.4355347 0.598 0.229 1.170691e-42 18
PIN1 DZ 2
8.31641e-47 0.5631776 0.57 0.235 1.482733e-42 18
RGS1 DZ 2
1.351953e-46 0.4102801 0.284 0.053 2.410397e-42 18
MDM2 DZ 2
1.421057e-46 0.3875722 0.918 0.632 2.533603e-42 18
SET DZ 2
1.447359e-46 0.456289 0.588 0.239 2.580497e-42 18
VDAC3 DZ 2
1.536979e-46 0.4113959 0.667 0.27 2.740281e-42 18
CCNG1 DZ 2
1.538699e-46 0.360358 0.925 0.673 2.743346e-42 18
MORF4L1 DZ 2
1.626032e-46 0.3731337 0.93 0.648 2.899052e-42 18
ATP5F1A DZ 2
1.787786e-46 0.5247207 0.737 0.359 3.187444e-42 18
SNAP23 DZ 2
1.838553e-46 0.4531627 0.587 0.218 3.277957e-42 18
TXNDC9 DZ 2
2.952526e-46 -0.928227 0.885 0.868 5.264059e-42 18
CD52 DZ 2
3.006105e-46 0.3747313 0.902 0.595 5.359584e-42 18
PSMA2 DZ 2
3.059184e-46 0.4918341 0.595 0.258 5.454219e-42 18

SYNCRIP DZ 2
3.16281e-46 0.5041586 0.491 0.141 5.638974e-42 18
RBM38 DZ 2
3.460295e-46 0.3448027 0.177 0.023 6.16936e-42 18
AC022167.3 DZ 2
3.761501e-46 0.4361705 0.398 0.101 6.70638e-42 18
DNAJB1 DZ 2
4.313912e-46 0.4040864 0.913 0.617 7.691273e-42 18
ARPC1B DZ 2
4.638984e-46 0.4534547 0.68 0.317 8.270845e-42 18
MLF2 DZ 2
5.283375e-46 0.436355 0.474 0.141 9.419728e-42 18
ANKRD13A DZ 2
5.618276e-46 0.4468092 0.523 0.175 1.001682e-41 18
TADA3 DZ 2
5.750745e-46 0.3262578 0.183 0.022 1.0253e-41 18
SPINK2 DZ 2
6.019304e-46 0.4729977 0.278 0.054 1.073182e-41 18
RGS16 DZ 2
7.117301e-46 -0.489301 0.996 0.998 1.268944e-41 18
RPL26 DZ 2
7.374016e-46 0.4681996 0.381 0.104 1.314713e-41 18
PHF6 DZ 2
8.358684e-46 0.4128918 0.31 0.06 1.49027e-41 18
USP7 DZ 2
8.979527e-46 0.443783 0.395 0.106 1.60096e-41 18
CRIP1 DZ 2
1.032826e-45 0.4319563 0.434 0.118 1.841425e-41 18
PRPSAP1 DZ 2
1.070112e-45 0.3581739 0.908 0.62 1.907903e-41 18
HNRNPA0 DZ 2
1.161589e-45 0.4448519 0.729 0.323 2.070998e-41 18
CCM2 DZ 2
1.391292e-45 0.4965426 0.563 0.202 2.480535e-41 18
GLRX DZ 2
1.495507e-45 -0.4677759 0.999 0.999 2.666339e-41 18
RPS8 DZ 2
1.758987e-45 0.3963212 0.848 0.517 3.136098e-41 18
SELENOT DZ 2
1.783058e-45 0.4596046 0.553 0.189 3.179015e-41 18
USP34 DZ 2
1.893159e-45 0.4263285 0.525 0.171 3.375314e-41 18
RANGRF DZ 2
1.992038e-45 0.4663214 0.626 0.252 3.551605e-41 18
ABI1 DZ 2
3.233728e-45 0.621277 0.339 0.08 5.765414e-41 18
SH3TC1 DZ 2
3.935882e-45 -1.58825 0.122 0.29 7.017283e-41 18
MIR155HG DZ 2
4.80625e-45 0.3974318 0.773 0.401 8.569062e-41 18

BANF1	DZ 2	4.811057e-45	-0.9109509	0.057	0.223	8.577633e-41	18
GBP2	DZ 2	5.493239e-45	0.57738	0.626	0.283	9.793897e-41	18
RUBCNL	DZ 2	7.368715e-45	0.3660169	0.24	0.035	1.313768e-40	18
BRCA1	DZ 2	7.578457e-45	-0.6292589	0.982	0.982	1.351163e-40	18
RPS11	DZ 2	9.962671e-45	0.407095	0.81	0.429	1.776245e-40	18
ROMO1	DZ 2	1.101148e-44	0.4611319	0.624	0.257	1.963237e-40	18
HSPB11	DZ 2	1.357764e-44	0.4910472	0.768	0.356	2.420757e-40	18
RCSD1	DZ 2	1.584592e-44	0.3973606	0.755	0.384	2.825169e-40	18
IK	DZ 2	1.591251e-44	0.3111787	0.21	0.029	2.837041e-40	18
XKR6	DZ 2	1.68936e-44	0.4753116	0.517	0.196	3.01196e-40	18
RELB	DZ 2	1.742792e-44	0.4426901	0.703	0.323	3.107223e-40	18
SLTM	DZ 2	1.914231e-44	-0.5055015	0.995	0.997	3.412883e-40	18
RPS3A	DZ 2	2.149574e-44	0.4519897	0.597	0.252	3.832476e-40	18
CDV3	DZ 2	2.371946e-44	0.4244928	0.374	0.095	4.228943e-40	18
RMDN1	DZ 2	2.6157e-44	0.4696491	0.512	0.169	4.663531e-40	18
EHMT1	DZ 2	2.645183e-44	0.3912803	0.351	0.086	4.716097e-40	18
CSE1L	DZ 2	3.005206e-44	0.4660122	0.541	0.215	5.357982e-40	18
TAF9	DZ 2	3.378407e-44	0.4070877	0.496	0.15	6.023363e-40	18
CGGBP1	DZ 2	3.704367e-44	0.3753095	0.907	0.622	6.604515e-40	18
ATP5PO	DZ 2	3.922369e-44	0.4028484	0.788	0.413	6.993192e-40	18
FAM49B	DZ 2	4.470982e-44	0.4166264	0.459	0.137	7.971314e-40	18
DR1	DZ 2	4.472364e-44	0.4646177	0.469	0.148	7.973778e-40	18
RSRC1	DZ 2	4.587397e-44	0.3990999	0.769	0.408	8.178871e-40	18
HCLS1	DZ 2	4.763136e-44	0.3352328	0.905	0.606	8.492196e-40	18
PTGES3	DZ 2	4.813745e-44	0.4874891	0.405	0.113	8.582425e-40	18

TBC1D1 DZ 2
4.908388e-44 0.423165 0.835 0.475 8.751164e-40 18
TRA2B DZ 2
4.997743e-44 0.4068593 0.333 0.079 8.910475e-40 18
GSTZ1 DZ 2
5.320017e-44 0.3741487 0.864 0.54 9.485059e-40 18
UBE2D2 DZ 2
5.539216e-44 0.3401745 0.271 0.042 9.875868e-40 18
TRIOBP DZ 2
6.16854e-44 0.4841801 0.525 0.178 1.099789e-39 18
APBB1IP DZ 2
6.804268e-44 0.3811895 0.732 0.366 1.213133e-39 18
ARHGDIA DZ 2
6.958605e-44 0.3833508 0.821 0.472 1.24065e-39 18
GRB2 DZ 2
1.12014e-43 0.4616979 0.527 0.184 1.997097e-39 18
RFC1 DZ 2
1.220482e-43 0.4118672 0.754 0.395 2.175997e-39 18
RPA3 DZ 2
1.278493e-43 0.3347637 0.949 0.696 2.279424e-39 18
ELOB DZ 2
1.373727e-43 0.4214717 0.626 0.251 2.449218e-39 18
UPF3A DZ 2
1.802064e-43 0.3609457 0.207 0.027 3.2129e-39 18
SGPP1 DZ 2
2.024281e-43 0.4082997 0.473 0.146 3.60909e-39 18
PBRM1 DZ 2
2.038889e-43 0.4583006 0.63 0.259 3.635136e-39 18
RBM6 DZ 2
3.350783e-43 0.3968867 0.733 0.369 5.974111e-39 18
NONO DZ 2
5.400302e-43 0.4618339 0.803 0.454 9.628199e-39 18
SWAP70 DZ 2
5.77242e-43 0.4082491 0.711 0.33 1.029165e-38 18
PPM1G DZ 2
5.971421e-43 -0.7507563 0.992 0.985 1.064645e-38 18
TMSB10 DZ 2
6.190492e-43 0.3971683 0.839 0.496 1.103703e-38 18
GNG5 DZ 2
6.346082e-43 0.4173312 0.663 0.301 1.131443e-38 18
SHKBP1 DZ 2
6.717098e-43 0.4123754 0.729 0.354 1.197591e-38 18
TKT DZ 2
7.071866e-43 0.3675103 0.294 0.062 1.260843e-38 18
NIF3L1 DZ 2
7.8082e-43 -1.244884 0.345 0.458 1.392124e-38 18
JUNB DZ 2
9.918709e-43 0.4022734 0.719 0.339 1.768407e-38 18
POLR2J DZ 2
1.67642e-42 0.4156912 0.609 0.25 2.988889e-38 18

GSPT1	DZ 2	1.733151e-42	0.4116103	0.341	0.089	3.090034e-38	18
RTCA	DZ 2	1.802607e-42	0.3869501	0.315	0.066	3.213868e-38	18
MTA3	DZ 2	2.056388e-42	0.3886964	0.788	0.424	3.666334e-38	18
SLIRP	DZ 2	2.106665e-42	0.5167167	0.677	0.322	3.755973e-38	18
HNRNPH1	DZ 2	2.157215e-42	0.3374887	0.881	0.553	3.8461e-38	18
YWHAZ	DZ 2	3.476072e-42	0.4136511	0.698	0.33	6.19749e-38	18
SEPHS2	DZ 2	3.641478e-42	0.3748666	0.793	0.438	6.492391e-38	18
CCT7	DZ 2	4.14058e-42	-0.9518498	0.131	0.281	7.382239e-38	18
ADAM28	DZ 2	4.323496e-42	-0.8778343	0.133	0.281	7.70836e-38	18
IFITM2	DZ 2	4.783903e-42	0.3979715	0.642	0.276	8.52922e-38	18
GGNBP2	DZ 2	4.884267e-42	0.4267906	0.6	0.238	8.708159e-38	18
CCDC115	DZ 2	5.150198e-42	0.4346034	0.364	0.092	9.182288e-38	18
TOP2B	DZ 2	5.255336e-42	0.3219953	0.219	0.029	9.369738e-38	18
ZNF714	DZ 2	5.686699e-42	0.3977883	0.694	0.337	1.013882e-37	18
MRPS18C	DZ 2	6.116281e-42	0.3729837	0.321	0.077	1.090472e-37	18
GPR160	DZ 2	6.210328e-42	0.3981109	0.358	0.088	1.107239e-37	18
FAM208B	DZ 2	7.322966e-42	0.3336729	0.241	0.04	1.305612e-37	18
AL441992.1	DZ 2	7.598614e-42	0.5034848	0.505	0.175	1.354757e-37	18
LCK	DZ 2	1.138069e-41	0.4027112	0.304	0.059	2.029063e-37	18
ZNF608	DZ 2	1.1649e-41	0.431742	0.544	0.202	2.0769e-37	18
PRPF38A	DZ 2	1.260064e-41	0.3882912	0.725	0.369	2.246569e-37	18
PSMC1	DZ 2	1.289337e-41	0.4159811	0.291	0.058	2.298758e-37	18
FANCA	DZ 2	1.37349e-41	0.3976869	0.697	0.316	2.448795e-37	18
GNB2	DZ 2	1.61064e-41	0.4138277	0.589	0.234	2.87161e-37	18
SRSF1	DZ 2	1.95779e-41	0.4359719	0.578	0.213	3.490543e-37	18

LY86 DZ 2
2.171036e-41 0.4477461 0.675 0.293 3.87074e-37 18
ITSN2 DZ 2
2.540937e-41 0.4553188 0.793 0.433 4.530236e-37 18
RHOH DZ 2
2.663013e-41 0.3181302 0.927 0.679 4.747885e-37 18
SLC25A3 DZ 2
2.931749e-41 0.4147081 0.378 0.106 5.227014e-37 18
NFYB DZ 2
3.068089e-41 0.3675459 0.834 0.492 5.470095e-37 18
COX5A DZ 2
3.780014e-41 0.4233271 0.398 0.099 6.739386e-37 18
LNPEP DZ 2
5.081686e-41 0.409883 0.614 0.276 9.060138e-37 18
PSMD14 DZ 2
5.692658e-41 0.3810615 0.738 0.374 1.014944e-36 18
POLR2G DZ 2
6.209588e-41 0.3693216 0.304 0.057 1.107107e-36 18
DLEU2 DZ 2
7.319581e-41 0.3878658 0.873 0.549 1.305008e-36 18
SEC61G DZ 2
7.474071e-41 0.3817517 0.75 0.411 1.332552e-36 18
IDI1 DZ 2
7.641279e-41 -0.4239952 0.997 0.998 1.362364e-36 18
RPS15 DZ 2
8.557311e-41 -0.5627043 0.995 0.992 1.525683e-36 18
RPL37 DZ 2
9.626709e-41 0.3560901 0.744 0.381 1.716346e-36 18
EIF4H DZ 2
1.404579e-40 0.4281935 0.365 0.095 2.504224e-36 18
TACC1 DZ 2
1.697491e-40 -1.122987 0.171 0.318 3.026458e-36 18
CD69 DZ 2
1.834083e-40 0.4833617 0.584 0.247 3.269987e-36 18
RGS10 DZ 2
2.093317e-40 0.4574372 0.608 0.258 3.732175e-36 18
CD22 DZ 2
2.73903e-40 0.33662 0.22 0.036 4.883417e-36 18 TOX
DZ 2
3.413575e-40 0.3376851 0.88 0.579 6.086062e-36 18
SAP18 DZ 2
5.179893e-40 0.4501797 0.459 0.152 9.23523e-36 18
SMIM20 DZ 2
5.227082e-40 0.3087592 0.973 0.804 9.319364e-36 18
TMA7 DZ 2
8.573373e-40 0.3391433 0.834 0.484 1.528547e-35 18
CCT8 DZ 2
8.681357e-40 0.3840195 0.734 0.371 1.547799e-35 18
ZFAND6 DZ 2
1.135281e-39 0.4099127 0.495 0.17 2.024092e-35 18

EIF4E2 DZ 2
1.381409e-39 0.4103703 0.609 0.257 2.462914e-35 18
THOC7 DZ 2
1.413921e-39 0.3665678 0.333 0.075 2.52088e-35 18
SUGP2 DZ 2
1.508505e-39 0.4216314 0.82 0.478 2.689513e-35 18
NDUFB1 DZ 2
1.522934e-39 0.3603333 0.589 0.264 2.715238e-35 18
TIMM10 DZ 2
1.717917e-39 0.3881929 0.723 0.347 3.062875e-35 18
DBNL DZ 2
2.156807e-39 -0.4759034 0.999 0.998 3.845371e-35 18
RPL23A DZ 2
2.203087e-39 0.4100879 0.596 0.234 3.927884e-35 18
MCUB DZ 2
2.708278e-39 0.3550884 0.903 0.609 4.828589e-35 18
UQCR10 DZ 2
3.246229e-39 0.4166879 0.609 0.269 5.787702e-35 18
SEPT2 DZ 2
3.822085e-39 0.4151777 0.557 0.204 6.814395e-35 18
EHD1 DZ 2
4.021319e-39 0.4245605 0.631 0.265 7.169609e-35 18
TXNL4A DZ 2
4.146025e-39 0.3551566 0.746 0.376 7.391948e-35 18
SRSF10 DZ 2
5.539834e-39 0.3332637 0.236 0.042 9.87697e-35 18
H2AFJ DZ 2
8.606655e-39 0.3948809 0.383 0.109 1.53448e-34 18
NIN DZ 2
8.64438e-39 0.3525422 0.633 0.273 1.541206e-34 18
VBP1 DZ 2
1.239687e-38 0.3194539 0.271 0.052 2.210238e-34 18
GEMIN2 DZ 2
1.354995e-38 0.408459 0.487 0.167 2.41582e-34 18
CTCF DZ 2
1.51895e-38 0.412083 0.501 0.176 2.708135e-34 18 TDG
DZ 2
1.793752e-38 0.3346085 0.839 0.51 3.19808e-34 18
CAPZB DZ 2
1.825576e-38 0.4064638 0.584 0.238 3.254819e-34 18
ETFA DZ 2
2.383522e-38 0.3823132 0.25 0.05 4.249582e-34 18
A4GALT DZ 2
2.77046e-38 0.3904448 0.437 0.129 4.939454e-34 18
MAP4K1 DZ 2
3.028128e-38 0.3350392 0.337 0.088 5.39885e-34 18
TUBG1 DZ 2
3.22721e-38 0.358902 0.684 0.308 5.753792e-34 18
LARP7 DZ 2
3.447101e-38 -0.4434728 0.995 0.997 6.145836e-34 18

RPL27A	DZ 2					
4.652765e-38	0.3739029	0.671	0.31	8.295414e-34	18	
WDR1	DZ 2					
5.704089e-38	0.4033696	0.561	0.223	1.016982e-33	18	
HDDC2	DZ 2					
6.376447e-38	0.3637086	0.237	0.043	1.136857e-33	18	
LINC00467	DZ 2					
6.574828e-38	0.3939918	0.461	0.147	1.172226e-33	18	
UBA1	DZ 2					
6.879301e-38	0.3364903	0.26	0.052	1.226511e-33	18	
FZR1	DZ 2					
7.08204e-38	0.3461272	0.242	0.051	1.262657e-33	18	
BORCS8-MEF2B	DZ 2					
7.99612e-38	0.3994763	0.567	0.227	1.425628e-33	18	
CHCHD7	DZ 2					
1.363675e-37	0.3998961	0.58	0.241	2.431297e-33	18	
SNRPA1	DZ 2					
1.396999e-37	0.3143043	0.859	0.564	2.49071e-33	18	
PCBP2	DZ 2					
1.414143e-37	0.3609927	0.604	0.249	2.521276e-33	18	
CYB5B	DZ 2					
1.447504e-37	0.3631802	0.561	0.209	2.580755e-33	18	
PIH1D1	DZ 2					
1.767162e-37	-0.8021369	0.146	0.281	3.150674e-33	18	
RNASET2	DZ 2					
1.927967e-37	0.3903484	0.598	0.261	3.437373e-33	18	
PPP1R7	DZ 2					
2.282184e-37	0.3766468	0.65	0.289	4.068906e-33	18	
LYPLA1	DZ 2					
2.398953e-37	0.3191209	0.206	0.037	4.277093e-33	18	
EEPD1	DZ 2					
2.503824e-37	0.3815781	0.527	0.199	4.464067e-33	18	
UBE2K	DZ 2					
3.113099e-37	0.3961224	0.447	0.153	5.550345e-33	18	
SMC1A	DZ 2					
3.405288e-37	0.3612308	0.725	0.363	6.071287e-33	18	
FAM96B	DZ 2					
4.007301e-37	-0.9538726	0.382	0.468	7.144618e-33	18	
ITM2B	DZ 2					
4.206197e-37	0.3812358	0.72	0.351	7.499229e-33	18	
GNAS	DZ 2					
4.250562e-37	0.3334707	0.882	0.594	7.578327e-33	18	
CALM1	DZ 2					
4.472117e-37	0.3314025	0.845	0.53	7.973337e-33	18	
CAP1	DZ 2					
4.632925e-37	0.3545142	0.589	0.234	8.260043e-33	18	
CPSF6	DZ 2					
5.014047e-37	0.3727452	0.395	0.118	8.939545e-33	18	
CCNH	DZ 2					
5.593645e-37	0.3100946	0.801	0.455	9.972909e-33	18	

HSPD1	DZ 2	5.691945e-37	0.3969271	0.495	0.187	1.014817e-32	18
FYTTD1	DZ 2	6.036037e-37	0.4370703	0.422	0.137	1.076165e-32	18
INO80C	DZ 2	7.263136e-37	-0.5185841	0.994	0.992	1.294945e-32	18
RPL18	DZ 2	7.543236e-37	0.3704013	0.425	0.143	1.344884e-32	18
UBQLN1	DZ 2	9.084662e-37	0.3627681	0.631	0.282	1.619704e-32	18
WTAP	DZ 2	1.126776e-36	0.3277648	0.663	0.299	2.008928e-32	18
ELAVL1	DZ 2	1.193233e-36	-0.5455626	0.996	0.993	2.127415e-32	18
RPL35	DZ 2	1.22183e-36	-0.6731614	0.969	0.965	2.1784e-32	18
RPL38	DZ 2	1.423317e-36	0.4076884	0.674	0.316	2.537632e-32	18
RNASEH2C	DZ 2	1.790749e-36	0.323979	0.718	0.359	3.192726e-32	18
PCMT1	DZ 2	1.851732e-36	-1.005893	0.604	0.649	3.301453e-32	18
ZFP36L1	DZ 2	2.114502e-36	0.3919089	0.372	0.105	3.769946e-32	18
MAP4K2	DZ 2	2.159985e-36	0.3295458	0.82	0.487	3.851037e-32	18
THRAP3	DZ 2	2.227507e-36	0.3377892	0.729	0.359	3.971423e-32	18
HADHA	DZ 2	2.235711e-36	0.3994871	0.556	0.233	3.986049e-32	18
HNRNPAB	DZ 2	2.776806e-36	0.3525668	0.762	0.41	4.950767e-32	18
ESD	DZ 2	3.090784e-36	0.3422562	0.672	0.309	5.51056e-32	18
STRBP	DZ 2	3.929071e-36	0.3222725	0.812	0.484	7.005141e-32	18
CCT3	DZ 2	5.300246e-36	-0.9320813	0.246	0.382	9.449809e-32	18
PDCD4	DZ 2	5.682357e-36	0.3511066	0.398	0.112	1.013107e-31	18
HAGH	DZ 2	6.04438e-36	0.3589152	0.6	0.261	1.077653e-31	18
PMF1	DZ 2	6.135394e-36	0.3952739	0.561	0.225	1.093879e-31	18
SEPT1	DZ 2	6.277389e-36	0.3041984	0.902	0.653	1.119196e-31	18
RBX1	DZ 2	7.183776e-36	0.3715809	0.269	0.057	1.280795e-31	18
TESC	DZ 2	7.297601e-36	0.3750867	0.626	0.272	1.301089e-31	18

CNIH1 DZ 2
7.70007e-36 0.3915468 0.465 0.164 1.372845e-31 18
CYB5R3 DZ 2
8.241833e-36 -0.4016368 0.997 0.998 1.469436e-31 18
RPL19 DZ 2
9.093974e-36 0.3823362 0.338 0.084 1.621365e-31 18
MAP4K4 DZ 2
1.008933e-35 0.3537023 0.413 0.132 1.798826e-31 18
APIP DZ 2
1.019797e-35 0.3595674 0.665 0.304 1.818197e-31 18
SCP2 DZ 2
1.102605e-35 0.3827572 0.425 0.13 1.965834e-31 18
MPST DZ 2
1.175279e-35 0.4012455 0.468 0.157 2.095405e-31 18
LYPLAL1 DZ 2
1.325933e-35 -0.8134357 0.036 0.174 2.364005e-31 18
TNFRSF18 DZ 2
1.335966e-35 0.31416 0.878 0.561 2.381894e-31 18
UQCRQ DZ 2
1.674385e-35 0.3784501 0.61 0.262 2.985261e-31 18
TXNDC17 DZ 2
1.680812e-35 0.3169963 0.819 0.488 2.99672e-31 18
ARF1 DZ 2
2.061143e-35 0.3768143 0.725 0.386 3.674811e-31 18
SSB DZ 2
2.115131e-35 0.3479856 0.67 0.315 3.771067e-31 18
PRKAR1A DZ 2
2.163676e-35 0.3746002 0.326 0.077 3.857618e-31 18
SLC35E3 DZ 2
2.173898e-35 0.3665913 0.469 0.166 3.875843e-31 18
COMMD7 DZ 2
2.916861e-35 0.3558658 0.479 0.168 5.200471e-31 18
AKIRIN2 DZ 2
2.978413e-35 0.33315 0.6 0.251 5.310212e-31 18 C1D
DZ 2
3.563855e-35 0.4149473 0.223 0.042 6.353997e-31 18
EML6 DZ 2
3.925169e-35 0.3594478 0.391 0.106 6.998184e-31 18
MOB3A DZ 2
4.167964e-35 -0.8795482 0.872 0.854 7.431062e-31 18
SH3BGRL3 DZ 2
4.281343e-35 0.3136968 0.83 0.504 7.633207e-31 18
PSMD8 DZ 2
5.372972e-35 0.3963401 0.574 0.234 9.579471e-31 18
PTRHD1 DZ 2
5.688441e-35 0.3336457 0.244 0.05 1.014192e-30 18
HLTF DZ 2
6.058153e-35 -0.6871961 0.978 0.968 1.080108e-30 18
RPS17 DZ 2
6.521913e-35 0.3841931 0.553 0.222 1.162792e-30 18

IAH1	DZ 2	6.563116e-35	0.5023589	0.396	0.104	1.170138e-30	18
VPREB3	DZ 2	8.555689e-35	0.3730684	0.516	0.199	1.525394e-30	18
MTMR14	DZ 2	9.368717e-35	0.365414	0.449	0.151	1.670348e-30	18
MFAP1	DZ 2	9.614277e-35	0.3173613	0.771	0.435	1.71413e-30	18
AURKAIP1	DZ 2	1.046578e-34	0.3726075	0.348	0.087	1.865943e-30	18
CEP135	DZ 2	1.243328e-34	0.3383559	0.574	0.229	2.216729e-30	18
KRAS	DZ 2	1.288499e-34	0.4369789	0.495	0.171	2.297264e-30	18
SYK	DZ 2	1.292988e-34	-0.4364289	0.997	0.996	2.305268e-30	18
RPS13	DZ 2	1.300718e-34	0.3649867	0.601	0.256	2.319051e-30	18
ATXN10	DZ 2	1.423215e-34	0.3766621	0.671	0.312	2.537451e-30	18
FCRLA	DZ 2	1.602513e-34	-0.4660319	1	0.997	2.857121e-30	18
RPS23	DZ 2	1.805635e-34	0.3532624	0.449	0.143	3.219267e-30	18
SNX1	DZ 2	1.9709e-34	0.3399055	0.613	0.277	3.513917e-30	18
MRPL22	DZ 2	2.080939e-34	0.3280114	0.898	0.62	3.710106e-30	18
LAMTOR5	DZ 2	2.808243e-34	0.7535361	0.24	0.072	5.006816e-30	18
IGHA2	DZ 2	3.348906e-34	0.345042	0.574	0.244	5.970765e-30	18
NAP1L4	DZ 2	3.625364e-34	0.3897477	0.455	0.147	6.463662e-30	18
MYL6B	DZ 2	3.878247e-34	0.4034697	0.49	0.188	6.914526e-30	18
REPIN1	DZ 2	4.343433e-34	0.3821345	0.417	0.124	7.743908e-30	18
ETS1	DZ 2	4.576205e-34	0.3893639	0.552	0.227	8.158917e-30	18
ZNF581	DZ 2	4.577684e-34	0.3898871	0.376	0.116	8.161552e-30	18
CFAP20	DZ 2	4.700127e-34	0.3733837	0.469	0.172	8.379857e-30	18
HINT2	DZ 2	5.00086e-34	0.320702	0.338	0.091	8.916034e-30	18
TEX30	DZ 2	5.148454e-34	0.3865977	0.354	0.112	9.179179e-30	18
GOT2	DZ 2	6.785112e-34	0.3171388	0.825	0.509	1.209718e-29	18

DNAJC8	DZ 2	7.369685e-34	0.3501788	0.337	0.095	1.313941e-29	18
ARMT1	DZ 2	8.971024e-34	0.3902627	0.367	0.118	1.599444e-29	18
LMO4	DZ 2	9.778729e-34	0.3425916	0.44	0.146	1.74345e-29	18
ARPC1A	DZ 2	1.014842e-33	0.356514	0.447	0.157	1.809363e-29	18
PPP4R2	DZ 2	1.057972e-33	0.3627895	0.338	0.099	1.886259e-29	18
MRPL35	DZ 2	1.103276e-33	0.3459818	0.338	0.098	1.967031e-29	18
AMZ2	DZ 2	1.188625e-33	0.3443501	0.499	0.191	2.1192e-29	18
WAPL	DZ 2	1.215225e-33	0.36069	0.37	0.099	2.166625e-29	18
OTULIN	DZ 2	1.28214e-33	0.372089	0.403	0.128	2.285927e-29	18
PAPSS1	DZ 2	1.286048e-33	0.3785306	0.276	0.066	2.292895e-29	18
ZSCAN16-AS1	DZ 2	1.393188e-33	-0.7753351	0.982	0.976	2.483914e-29	18
FTH1	DZ 2	1.425273e-33	0.3342159	0.6	0.254	2.541119e-29	18
HNRNPH2	DZ 2	1.566285e-33	0.3028304	0.223	0.043	2.792529e-29	18
NSD2	DZ 2	1.574062e-33	0.3308787	0.255	0.051	2.806395e-29	18
BCL6	DZ 2	1.593406e-33	0.3634829	0.521	0.199	2.840883e-29	18
PXK	DZ 2	1.595674e-33	0.3426781	0.636	0.298	2.844927e-29	18
SNF8	DZ 2	1.815854e-33	0.3564298	0.301	0.075	3.237485e-29	18
FAM45A	DZ 2	1.886135e-33	0.3248387	0.778	0.44	3.36279e-29	18
PGAM1	DZ 2	1.937338e-33	0.3614253	0.538	0.211	3.454079e-29	18
IFI27L2	DZ 2	1.939109e-33	0.3515799	0.356	0.098	3.457237e-29	18
RASSF1	DZ 2	1.953043e-33	-0.9814257	0.317	0.428	3.482081e-29	18
PLP2	DZ 2	2.050659e-33	0.3773434	0.608	0.269	3.65612e-29	18
YY1	DZ 2	3.279314e-33	0.3289765	0.798	0.489	5.84669e-29	18
AP2M1	DZ 2	3.619829e-33	0.3543879	0.61	0.269	6.453793e-29	18
SDHB	DZ 2	4.745024e-33	0.3500989	0.523	0.208	8.459903e-29	18

FAM192A	DZ 2					
4.786143e-33	-0.8002306	0.197	0.307	8.533215e-29	18	
ARHGAP24	DZ 2					
6.493813e-33	0.3219564	0.294	0.067	1.157782e-28	18	
SPAG16	DZ 2					
7.078865e-33	0.3392961	0.301	0.067	1.262091e-28	18	
DHFR	DZ 2					
8.373938e-33	0.3498345	0.373	0.108	1.492989e-28	18	
HPS1	DZ 2					
8.525194e-33	0.3204967	0.728	0.382	1.519957e-28	18	
NDUFS6	DZ 2					
8.714205e-33	0.3507725	0.452	0.156	1.553656e-28	18	
DNAJC9	DZ 2					
1.128786e-32	-0.6121027	0.974	0.974	2.012513e-28	18	
MT-ND4	DZ 2					
1.209336e-32	0.4245716	0.434	0.133	2.156126e-28	18	
SIT1	DZ 2					
1.438851e-32	-0.5246085	0.982	0.984	2.565327e-28	18	
RPL29	DZ 2					
1.71426e-32	0.3232782	0.67	0.319	3.056354e-28	18	
SNRNP70	DZ 2					
1.941041e-32	0.3657061	0.276	0.066	3.460683e-28	18	
ST14	DZ 2					
2.137935e-32	0.3727105	0.308	0.089	3.811724e-28	18	
ATP6V1C1	DZ 2					
2.506802e-32	0.3374254	0.772	0.413	4.469377e-28	18	
NDUFA12	DZ 2					
3.02638e-32	-0.873601	0.063	0.199	5.395734e-28	18	
ACP5	DZ 2					
3.314421e-32	0.3205711	0.545	0.223	5.90928e-28	18	
HIKESHI	DZ 2					
3.352246e-32	0.3689112	0.536	0.216	5.97672e-28	18	
MPLKIP	DZ 2					
3.859795e-32	0.3014877	0.775	0.44	6.881628e-28	18	
PSMB7	DZ 2					
4.681443e-32	0.3478343	0.459	0.164	8.346545e-28	18	
TARDBP	DZ 2					
4.752211e-32	0.3533337	0.369	0.112	8.472717e-28	18	
CYTH2	DZ 2					
4.791528e-32	0.3035701	0.794	0.463	8.542815e-28	18	
SNRPF	DZ 2					
5.044004e-32	0.3430133	0.627	0.278	8.992954e-28	18	
DPY30	DZ 2					
5.209772e-32	-0.6671091	0.972	0.977	9.288502e-28	18	
RPS10	DZ 2					
5.236218e-32	0.3682998	0.518	0.202	9.335652e-28	18	
EBLN3P	DZ 2					
6.905398e-32	-0.7024147	0.069	0.185	1.231163e-27	18	
PLPP5	DZ 2					
7.995653e-32	0.3400646	0.667	0.314	1.425545e-27	18	

IGBP1	DZ 2	1.112645e-31	0.3243091	0.734	0.405	1.983734e-27	18
HNRNPR	DZ 2	1.178702e-31	0.316342	0.365	0.107	2.101507e-27	18
RAD51C	DZ 2	1.254672e-31	0.3161999	0.456	0.15	2.236956e-27	18
SVBP	DZ 2	1.298778e-31	0.3069256	0.676	0.323	2.315591e-27	18
PPP2R1A	DZ 2	1.416121e-31	0.3210829	0.539	0.222	2.524802e-27	18
FUBP1	DZ 2	1.588113e-31	0.3120379	0.317	0.084	2.831447e-27	18
CDCA4	DZ 2	1.861306e-31	0.3279665	0.364	0.114	3.318523e-27	18
XPO1	DZ 2	1.913595e-31	0.3406105	0.382	0.122	3.411748e-27	18
CMC2	DZ 2	2.044442e-31	0.3201712	0.242	0.056	3.645036e-27	18
TSC1	DZ 2	2.138594e-31	0.3178963	0.328	0.083	3.812899e-27	18
RFX5	DZ 2	2.168377e-31	0.3624834	0.636	0.3	3.865999e-27	18
TIMM8B	DZ 2	2.262929e-31	0.3063846	0.575	0.248	4.034577e-27	18
NOSIP	DZ 2	2.484902e-31	0.3540865	0.466	0.168	4.430331e-27	18
HMG20B	DZ 2	2.569503e-31	0.3199909	0.749	0.421	4.581167e-27	18
ANP32A	DZ 2	2.616914e-31	0.3247978	0.241	0.048	4.665696e-27	18
NNT	DZ 2	2.735567e-31	0.351827	0.361	0.114	4.877242e-27	18
POLR2D	DZ 2	2.775416e-31	0.3070786	0.709	0.373	4.948289e-27	18
VDAC2	DZ 2	2.972131e-31	0.3325331	0.281	0.063	5.299013e-27	18
FAM200B	DZ 2	3.059906e-31	0.3145554	0.37	0.114	5.455507e-27	18
PLEKHB2	DZ 2	3.453708e-31	0.3330089	0.448	0.147	6.157616e-27	18
VRK3	DZ 2	3.515317e-31	0.3055089	0.681	0.332	6.267458e-27	18
BUD31	DZ 2	3.996362e-31	0.3090999	0.496	0.191	7.125115e-27	18
TXNDC12	DZ 2	4.535104e-31	0.3251878	0.69	0.35	8.085637e-27	18
AP2S1	DZ 2	4.816041e-31	0.3056014	0.699	0.361	8.58652e-27	18
RPS19BP1	DZ 2	5.667362e-31	0.4109469	0.58	0.251	1.010434e-26	18

MDM4 DZ 2
6.641959e-31 0.3382193 0.299 0.078 1.184195e-26 18
SHLD1 DZ 2
7.997512e-31 0.3089205 0.667 0.336 1.425876e-26 18
PDCD10 DZ 2
1.282289e-30 0.311722 0.764 0.444 2.286192e-26 18
EIF5 DZ 2
1.315296e-30 0.3015063 0.285 0.069 2.345042e-26 18
KATNA1 DZ 2
1.344859e-30 0.3428373 0.472 0.18 2.397749e-26 18
MFNG DZ 2
1.619403e-30 0.3382257 0.557 0.223 2.887233e-26 18
NGLY1 DZ 2
1.632906e-30 0.3636004 0.427 0.148 2.911307e-26 18
TAF11 DZ 2
1.75538e-30 -0.6171435 0.978 0.976 3.129667e-26 18
RPL36A DZ 2
2.425804e-30 0.3184561 0.409 0.138 4.324966e-26 18
CEBPZOS DZ 2
2.500542e-30 0.3326058 0.516 0.213 4.458216e-26 18
COPS5 DZ 2
2.905643e-30 0.3213296 0.387 0.123 5.18047e-26 18
DDA1 DZ 2
3.160343e-30 0.3233882 0.421 0.143 5.634575e-26 18
MCRS1 DZ 2
4.141836e-30 0.3354989 0.308 0.088 7.384479e-26 18
MSH6 DZ 2
4.827139e-30 -0.5828341 0.99 0.981 8.606307e-26 18
RPL5 DZ 2
5.104349e-30 0.3405411 0.354 0.097 9.100545e-26 18
BACH2 DZ 2
5.734125e-30 0.351424 0.523 0.213 1.022337e-25 18
CHMP5 DZ 2
5.833952e-30 0.3220415 0.516 0.209 1.040135e-25 18
TIAL1 DZ 2
6.232049e-30 0.3117977 0.591 0.27 1.111112e-25 18
MRPL47 DZ 2
6.754358e-30 0.4157882 0.784 0.484 1.204234e-25 18
UCP2 DZ 2
9.53966e-30 0.3085454 0.391 0.127 1.700826e-25 18
CNIH4 DZ 2
1.133956e-29 0.3057129 0.29 0.075 2.021729e-25 18
TPP2 DZ 2
1.168965e-29 0.310979 0.228 0.043 2.084147e-25 18
FCHO1 DZ 2
1.345653e-29 0.311968 0.602 0.273 2.399165e-25 18
PPHLN1 DZ 2
1.384139e-29 0.3462977 0.443 0.165 2.467782e-25 18
RBBP6 DZ 2
1.483906e-29 0.3114739 0.286 0.071 2.645657e-25 18

TMED8	DZ 2	1.53814e-29	0.3244264	0.326	0.094	2.74235e-25	18
IFT27	DZ 2	1.670698e-29	-1.242518	0.17	0.265	2.978688e-25	18
S100A4	DZ 2	1.674229e-29	0.3401496	0.468	0.172	2.984983e-25	18
RRAGA	DZ 2	1.722112e-29	0.3314984	0.412	0.148	3.070353e-25	18
MPC1	DZ 2	1.924083e-29	-0.4048921	1	0.999	3.430448e-25	18
RPS6	DZ 2	2.032178e-29	0.3261812	0.418	0.141	3.623171e-25	18
P2RY8	DZ 2	2.10306e-29	0.3257301	0.63	0.308	3.749546e-25	18
SPI1	DZ 2	2.188173e-29	0.3213389	0.483	0.182	3.901294e-25	18
CHRAC1	DZ 2	2.519126e-29	0.312523	0.609	0.279	4.491349e-25	18
RTF2	DZ 2	2.54946e-29	-0.7412575	0.075	0.2	4.545432e-25	18
YBX3	DZ 2	2.972313e-29	0.3263714	0.535	0.234	5.299336e-25	18
PRKRA	DZ 2	3.545577e-29	0.3097894	0.263	0.064	6.32141e-25	18
TEX9	DZ 2	4.855618e-29	0.3554252	0.508	0.207	8.657081e-25	18
TRA2A	DZ 2	5.196011e-29	0.3172649	0.288	0.076	9.263969e-25	18
MYO1E	DZ 2	5.336726e-29	0.3285145	0.549	0.224	9.514849e-25	18
ATM	DZ 2	5.427275e-29	0.3298426	0.539	0.21	9.676289e-25	18
SNRNP25	DZ 2	6.972211e-29	0.3187407	0.513	0.209	1.243076e-24	18
MOB4	DZ 2	8.725786e-29	0.306983	0.422	0.143	1.55572e-24	18
C5orf15	DZ 2	9.882164e-29	0.3135178	0.465	0.175	1.761891e-24	18
TAF12	DZ 2	9.920891e-29	-0.7482305	0.924	0.916	1.768796e-24	18
EEF1B2	DZ 2	1.052832e-28	0.3671782	0.417	0.135	1.877094e-24	18
MKNK2	DZ 2	1.138362e-28	-0.6235427	0.982	0.96	2.029586e-24	18
RPL23	DZ 2	1.253454e-28	0.314389	0.347	0.111	2.234783e-24	18
ASXL1	DZ 2	1.344871e-28	0.3077284	0.536	0.228	2.397771e-24	18
PHF5A	DZ 2	1.395034e-28	-0.9876531	0.481	0.524	2.487205e-24	18

ID3	DZ 2					
1.39536e-28	0.3074136	0.329	0.105	2.487787e-24	18	
DAP	DZ 2					
1.464203e-28	0.3110974	0.222	0.036	2.610527e-24	18	
MILR1	DZ 2					
1.587025e-28	0.3069089	0.58	0.244	2.829506e-24	18	
WIPF1	DZ 2					
1.705133e-28	0.3038021	0.387	0.122	3.040081e-24	18	
SUZ12	DZ 2					
1.814662e-28	0.3112461	0.249	0.058	3.235361e-24	18	
BARD1	DZ 2					
1.849914e-28	0.312536 0.31	0.088	3.298212e-24	18		
XPNPEP1 DZ 2						
1.949313e-28	0.3692348	0.38	0.124	3.475431e-24	18	
NFATC1	DZ 2					
2.271964e-28	0.3083736	0.561	0.229	4.050684e-24	18	
MRFAP1	DZ 2					
2.405168e-28	-0.5357183	0.97	0.971	4.288174e-24	18	
RPL22	DZ 2					
2.577569e-28	0.3243513	0.435	0.161	4.595549e-24	18	
COX11	DZ 2					
2.738348e-28	0.3687148	0.662	0.322	4.882201e-24	18	
GYPC	DZ 2					
3.51093e-28	0.3150144	0.418	0.161	6.259637e-24	18	
HIGD1A	DZ 2					
3.675047e-28	-0.682991	0.085	0.197	6.552241e-24	18	
CLEC2B	DZ 2					
3.96794e-28	0.4379949	0.589	0.263	7.07444e-24	18	
PIM1	DZ 2					
5.024581e-28	0.3247277	0.513	0.21	8.958325e-24	18	
RAB1A	DZ 2					
7.39246e-28	0.344842 0.539	0.228	1.318002e-23	18		
AKAP13 DZ 2						
8.495194e-28	0.3400778	0.324	0.095	1.514608e-23	18	
SYVN1	DZ 2					
1.376438e-27	0.3214369	0.308	0.085	2.454051e-23	18	
VEZT	DZ 2					
1.385217e-27	0.3359471	0.652	0.292	2.469703e-23	18	
TCF4	DZ 2					
1.597732e-27	-0.3747232	0.997	0.998	2.848597e-23	18	
RPL3	DZ 2					
1.68806e-27	0.3137616	0.574	0.266	3.009642e-23	18	
MRPL33	DZ 2					
1.995862e-27	0.3110658	0.573	0.26	3.558422e-23	18	
MTCH2	DZ 2					
2.751e-27	-0.545588	0.992	0.987	4.904758e-23	18	
RPL14	DZ 2					
2.791828e-27	0.3102866	0.808	0.497	4.977549e-23	18	
PSMB9	DZ 2					
3.140591e-27	0.3104314	0.39	0.126	5.59936e-23	18	

KMT2A DZ 2
3.426458e-27 -0.6138925 0.063 0.173 6.109032e-23 18
BCL2 DZ 2
3.726199e-27 0.3051105 0.251 0.065 6.643441e-23 18
MFHAS1 DZ 2
4.136858e-27 0.5006031 0.325 0.106 7.375604e-23 18
CD9 DZ 2
4.966174e-27 0.3840857 0.39 0.144 8.854192e-23 18
ALOX5 DZ 2
5.132254e-27 0.3386636 0.72 0.394 9.150295e-23 18
NDUFS5 DZ 2
6.626691e-27 0.6129522 0.415 0.202 1.181473e-22 18
LGALS1 DZ 2
8.387466e-27 -0.7278595 0.043 0.16 1.495401e-22 18
LMNA DZ 2
9.435241e-27 0.3109222 0.486 0.202 1.682209e-22 18
MRPS14 DZ 2
1.098765e-26 0.3326395 0.411 0.125 1.958988e-22 18
STIM2 DZ 2
1.524492e-26 0.3292323 0.434 0.149 2.718016e-22 18
MCM7 DZ 2
1.578844e-26 0.3319376 0.37 0.12 2.814921e-22 18
DLAT DZ 2
2.345986e-26 0.319132 0.522 0.219 4.182658e-22 18
SIAH2 DZ 2
2.415219e-26 0.3449575 0.494 0.202 4.306094e-22 18
SNHG25 DZ 2
2.98408e-26 0.3321289 0.487 0.192 5.320316e-22 18
SREK1IP1 DZ 2
3.995206e-26 0.3030394 0.367 0.13 7.123052e-22 18
PPP4R3B DZ 2
4.59077e-26 0.311502 0.198 0.044 8.184884e-22 18
ALDH5A1 DZ 2
4.729485e-26 0.303373 0.394 0.136 8.432199e-22 18
CORO1B DZ 2
7.158781e-26 0.327376 0.58 0.302 1.276339e-21 18
FERMT3 DZ 2
7.520468e-26 0.3158146 0.276 0.072 1.340824e-21 18
DNASE1 DZ 2
7.861033e-26 0.3065712 0.381 0.132 1.401543e-21 18
GTF2E2 DZ 2
9.910015e-26 -0.7597492 0.22 0.329 1.766857e-21 18
PTPN6 DZ 2
1.294758e-25 -0.7533534 0.363 0.423 2.308423e-21 18
APEX1 DZ 2
1.305891e-25 -0.8439801 0.784 0.757 2.328273e-21 18
MT-ND5 DZ 2
2.232606e-25 0.3019671 0.548 0.255 3.980513e-21 18
UBE2V2 DZ 2
2.597762e-25 0.3074926 0.295 0.079 4.63155e-21 18

GPR18 DZ 2
4.60072e-25 -0.3610564 0.994 0.994 8.202624e-21 18
RPS9 DZ 2
7.977084e-25 0.306146 0.446 0.177 1.422234e-20 18
MCL1 DZ 2
8.228461e-25 0.3195555 0.433 0.179 1.467052e-20 18
LRRC59 DZ 2
1.272171e-24 0.3109081 0.294 0.087 2.268154e-20 18
RITA1 DZ 2
1.57852e-24 0.3326468 0.528 0.222 2.814344e-20 18
NSMCE1 DZ 2
1.664013e-24 0.324678 0.382 0.145 2.966768e-20 18
TBL1XR1 DZ 2
1.686045e-24 -0.5721735 0.053 0.151 3.006049e-20 18
CELF2 DZ 2
1.796093e-24 -0.6127094 0.949 0.934 3.202254e-20 18
MT-ATP6 DZ 2
2.047255e-24 -0.8912851 0.429 0.479 3.650051e-20 18
SQSTM1 DZ 2
2.060982e-24 -0.6364111 0.04 0.138 3.674524e-20 18
FCER2 DZ 2
2.474134e-24 0.3259909 0.455 0.186 4.411133e-20 18
NDUFA8 DZ 2
7.62034e-24 0.3104163 0.444 0.172 1.35863e-19 18
RGS19 DZ 2
1.561355e-23 0.3332395 0.788 0.484 2.783739e-19 18
LTB DZ 2
1.644222e-23 0.3029685 0.31 0.099 2.931483e-19 18
ALDH2 DZ 2
1.826495e-23 -0.5017395 0.984 0.981 3.256457e-19 18
RPSA DZ 2
4.165392e-23 0.4590026 0.562 0.205 7.426478e-19 18
PLCG2 DZ 2
4.181725e-23 -0.4842967 0.031 0.119 7.455597e-19 18
FCGR2B DZ 2
4.319336e-23 0.3416878 0.175 0.032 7.700944e-19 18
PEG10 DZ 2
6.512158e-23 -0.7371058 0.141 0.239 1.161053e-18 18
CAPG DZ 2
6.752995e-23 -0.7073064 0.937 0.935 1.203992e-18 18
BTG1 DZ 2
7.692578e-23 -0.6929743 0.199 0.268 1.37151e-18 18
HHEX DZ 2
9.327317e-23 0.3528014 0.429 0.154 1.662967e-18 18
KIAA0040 DZ 2
1.793361e-22 0.3102616 0.447 0.183 3.197383e-18 18
DECR1 DZ 2
1.880044e-22 0.3394329 0.205 0.05 3.35193e-18 18
SERPINA9 DZ 2
2.134973e-22 -0.6924754 0.304 0.385 3.806443e-18 18

	C19orf70	DZ 2			
2.416895e-22	-0.6539152	0.225	0.307	4.309081e-18	18
	TMEM147	DZ 2			
8.642134e-22	0.3014418	0.254	0.072	1.540806e-17	18
	ADAM17	DZ 2			
1.265312e-21	-0.7051316	0.251	0.33	2.255925e-17	18
	TRBC2	DZ 2			
2.541941e-21	-0.7314322	0.431	0.45	4.532026e-17	18
	DRAP1	DZ 2			
5.809349e-21	-0.6966765	0.197	0.281	1.035749e-16	18
	SRM	DZ 2			
5.983273e-21	-0.7683236	0.769	0.751	1.066758e-16	18
	HLA-C	DZ 2			
8.085066e-21	-0.599001	0.07	0.159	1.441486e-16	18
	SLC2A3	DZ 2			
1.458521e-20	-0.4406063	0.984	0.98	2.600397e-16	18
	RPL7A	DZ 2			
1.661101e-20	-0.9514244	0.517	0.499	2.961577e-16	18
	SSR4	DZ 2			
2.432857e-20	-0.7889842	0.591	0.575	4.337541e-16	18
	APRT	DZ 2			
3.292296e-20	-0.5701038	0.075	0.165	5.869834e-16	18
	MARCKS	DZ 2			
6.752705e-20	-0.5002545	0.962	0.959	1.20394e-15	18
	MT-CYB	DZ 2			
9.057763e-20	-0.3649266	0.996	0.993	1.614909e-15	18
	RPL6	DZ 2			
1.333082e-19	-0.6792758	0.161	0.213	2.376752e-15	18
	LBH	DZ 2			
1.654015e-19	-0.5925551	0.184	0.252	2.948944e-15	18
	DNPH1	DZ 2			
1.836355e-19	-0.4726871	0.067	0.149	3.274038e-15	18
	MVP	DZ 2			
1.98813e-19	-0.5765981	0.142	0.226	3.544636e-15	18
	TMEM109	DZ 2			
2.033469e-19	-0.7929647	0.435	0.448	3.625471e-15	18
	PNRC1	DZ 2			
2.044975e-19	-0.4560914	0.032	0.102	3.645986e-15	18
	RHOC	DZ 2			
3.019395e-19	-0.6009553	0.149	0.208	5.38328e-15	18
	C16orf74	DZ 2			
7.701689e-19	-0.666481	0.637	0.627	1.373134e-14	18
	ATP6V0E1	DZ 2			
4.402364e-18	-0.7197768	0.42	0.438	7.848974e-14	18
	IL2RG	DZ 2			
1.030698e-17	-0.446236	0.035	0.106	1.837631e-13	18
	MTSS1	DZ 2			
1.639508e-17	-0.4029306	0.039	0.116	2.923079e-13	18
	PYCR1	DZ 2			
5.361746e-17	-0.584951	0.13	0.197	9.559458e-13	18

CHPT1	DZ 2				
1.242248e-16	-0.5499005	0.088	0.167	2.214805e-12	18
CBX6	DZ 2				
1.599776e-16	-0.5998078	0.124	0.212	2.852241e-12	18
MGST3	DZ 2				
1.791251e-16	-0.4752574	0.088	0.151	3.193622e-12	18
ESYT1	DZ 2				
2.053347e-16	-0.633367	0.148	0.219	3.660913e-12	18
EIF4EBP1	DZ 2				
5.152795e-16	-0.529869	0.152	0.191	9.186918e-12	18
S1PR4	DZ 2				
5.871484e-16	-0.4184292	0.06	0.121	1.046827e-11	18
IL27RA	DZ 2				
1.511724e-15	-0.4071767	0.079	0.147	2.695253e-11	18
PRDX4	DZ 2				
1.545829e-15	-0.5219514	0.118	0.175	2.756058e-11	18
ASAH1	DZ 2				
1.615125e-15	-0.8913777	0.461	0.472	2.879605e-11	18
CD83	DZ 2				
1.85485e-15	-0.4459742	0.051	0.106	3.307012e-11	18
C1orf162	DZ 2				
2.533637e-15	-0.633275	0.206	0.238	4.517222e-11	18
CD55	DZ 2				
3.004332e-15	-0.7175341	0.395	0.414	5.356424e-11	18
NPC2	DZ 2				
9.644542e-15	-0.7033821	0.308	0.328	1.719525e-10	18
ZFP36	DZ 2				
1.052766e-14	-0.7210186	0.462	0.472	1.876977e-10	18
SNHG8	DZ 2				
1.910643e-14	-0.674574	0.513	0.501	3.406485e-10	18
FOXP1	DZ 2				
1.994912e-14	-0.540339	0.262	0.286	3.556729e-10	18
SP110	DZ 2				
2.264651e-14	-0.5634239	0.089	0.161	4.037645e-10	18
DDIT4	DZ 2				
4.805609e-14	-0.4159811	0.058	0.11	8.567921e-10	18
LINC02397	DZ 2				
7.070212e-14	-0.3783211	0.052	0.105	1.260548e-09	18
PDE4B	DZ 2				
7.972414e-14	-0.3864536	0.062	0.12	1.421402e-09	18
LIPA	DZ 2				
1.473456e-13	-0.6134368	0.404	0.389	2.627025e-09	18
ARL6IP5	DZ 2				
1.479105e-13	-0.7810602	0.456	0.463	2.637096e-09	18
CD63	DZ 2				
2.044742e-13	-0.4188071	0.097	0.151	3.645571e-09	18
CYB5A	DZ 2				
2.699072e-13	-0.6156026	0.392	0.387	4.812175e-09	18
PSAP	DZ 2				
6.186625e-13	-0.5471169	0.317	0.345	1.103013e-08	18

RNH1	DZ 2				
8.364411e-13	-0.3403116	0.995	0.99	1.491291e-08	18
RPL10A	DZ 2				
8.406506e-13	-0.5924099	0.329	0.348	1.498796e-08	18
LITAF	DZ 2				
8.737354e-13	-0.4305487	0.131	0.18	1.557783e-08	18
DNPEP	DZ 2				
1.009417e-12	-0.3536768	0.088	0.14	1.79969e-08	18
TMEM205	DZ 2				
1.204663e-12	-0.596876	0.324	0.337	2.147793e-08	18
CD48	DZ 2				
1.275802e-12	-0.4080264	0.066	0.113	2.274628e-08	18
P2RY10	DZ 2				
1.441116e-12	-0.3910786	0.053	0.11	2.569366e-08	18
SNX9	DZ 2				
1.73682e-12	-0.5949286	0.833	0.751	3.096577e-08	18
TOMM7	DZ 2				
2.618531e-12	-0.3041325	0.053	0.106	4.668578e-08	18
CD320	DZ 2				
4.354667e-12	-0.6314603	0.816	0.768	7.763935e-08	18
RPL17	DZ 2				
4.67499e-12	-0.8352385	0.404	0.409	8.33504e-08	18
TXN	DZ 2				
5.022784e-12	-0.3879935	0.073	0.115	8.955122e-08	18
CLN8	DZ 2				
8.427684e-12	-0.4948109	0.131	0.171	1.502572e-07	18
PARP14	DZ 2				
1.311713e-11	-0.3306068	0.99	0.982	2.338653e-07	18
TPT1	DZ 2				
1.44583e-11	-0.3143228	0.056	0.11	2.577771e-07	18
HEXB	DZ 2				
1.624226e-11	-0.6247404	0.631	0.569	2.895832e-07	18
SNX2	DZ 2				
2.786704e-11	-0.3358287	0.087	0.138	4.968414e-07	18
QTRT1	DZ 2				
3.603484e-11	-0.6112291	0.342	0.329	6.424651e-07	18
SMC6	DZ 2				
3.649213e-11	-0.5261541	0.286	0.285	6.506182e-07	18
CNPY3	DZ 2				
4.780449e-11	-0.3565799	0.067	0.118	8.523063e-07	18
SLAMF1	DZ 2				
5.743537e-11	-0.6357628	0.903	0.847	1.024015e-06	18
HSP90AB1	DZ 2				
7.26758e-11	-0.7079728	0.325	0.327	1.295737e-06	18
SLC3A2	DZ 2				
8.05886e-11	-0.5707387	0.367	0.362	1.436814e-06	18
ANXA6	DZ 2				
8.348469e-11	-0.439721	0.104	0.153	1.488449e-06	18
CEBPB	DZ 2				
9.524606e-11	-0.3961967	0.106	0.149	1.698142e-06	18

SVIP DZ 2
1.265532e-10 -0.50724 0.083 0.126 2.256317e-06 18
LGALS3 DZ 2
1.501607e-10 -0.6331523 0.532 0.491 2.677216e-06 18
ANKRD12 DZ 2
1.753952e-10 -0.3656772 0.122 0.164 3.12712e-06 18
MRPL24 DZ 2
1.803877e-10 -0.3174983 0.08 0.131 3.216133e-06 18
SRPRB DZ 2
1.963092e-10 -0.5893881 0.521 0.475 3.499996e-06 18
N4BP2L2 DZ 2
2.123382e-10 -0.6118039 0.078 0.132 3.785778e-06 18
MYC DZ 2
2.156834e-10 -0.6860391 0.549 0.502 3.84542e-06 18
TSC22D3 DZ 2
2.728787e-10 -0.4975309 0.159 0.197 4.865154e-06 18
TYMP DZ 2
3.437212e-10 -0.6494673 0.355 0.337 6.128206e-06 18
HVCN1 DZ 2
3.52097e-10 -0.5024349 0.202 0.229 6.277538e-06 18
NT5C3A DZ 2
1.001231e-09 -0.3378816 0.115 0.156 1.785094e-05 18
FAM162A DZ 2
1.245802e-09 -0.4301978 0.188 0.217 2.221141e-05 18
C4orf48 DZ 2
1.488387e-09 -0.3084284 0.078 0.115 2.653645e-05 18
U2AF1L4 DZ 2
2.487858e-09 -0.4797098 0.231 0.242 4.435603e-05 18
HLA-F DZ 2
2.615925e-09 -0.5966482 0.338 0.302 4.663933e-05 18
ZFP36L2 DZ 2
3.223698e-09 -0.4071224 0.146 0.163 5.747531e-05 18
TNFRSF14 DZ 2
4.00593e-09 -0.462556 0.176 0.189 7.142173e-05 18
IFNGR1 DZ 2
4.41907e-09 -0.4094808 0.218 0.23 7.878759e-05 18
CCDC85B DZ 2
5.347834e-09 -0.5533882 0.272 0.268 9.534653e-05 18
JUN DZ 2
6.00172e-09 -0.3310566 0.089 0.114 0.0001070047 18
LGALS9 DZ 2
7.181009e-09 -0.5007448 0.374 0.361 0.0001280302 18
TAPBP DZ 2
7.586232e-09 -0.5368416 0.415 0.383 0.0001352549 18
LAPTM4A DZ 2
1.122802e-08 -0.4846267 0.268 0.263 0.0002001844 18
TTC3 DZ 2
1.299107e-08 -0.5172285 0.273 0.282 0.0002316179 18
MT-ND4L DZ 2
1.304354e-08 -0.3486998 0.1 0.129 0.0002325532 18

ICAM2 DZ 2
1.819621e-08 -0.6484817 0.378 0.359 0.0003244202 18
ARID5B DZ 2
2.065176e-08 -0.3933634 0.973 0.944 0.0003682002 18
NACA DZ 2
2.372337e-08 -0.3391015 0.109 0.13 0.000422964 18
MUM1 DZ 2
2.780205e-08 -0.4368251 0.135 0.148 0.0004956827 18
ARRDC2 DZ 2
2.993616e-08 -0.5316198 0.36 0.341 0.0005337318 18
PKIG DZ 2
3.682471e-08 -0.3481874 0.093 0.123 0.0006565478 18
RAB13 DZ 2
5.755067e-08 -0.5710994 0.563 0.49 0.001026071 18
CYTIP DZ 2
5.885457e-08 -0.4077567 0.176 0.202 0.001049318 18
NPM3 DZ 2
7.532526e-08 -0.45883 0.288 0.286 0.001342974 18
ERG28 DZ 2
1.06975e-07 -0.3784235 0.093 0.134 0.001907258 18
AC016831.5 DZ 2
1.311807e-07 -0.3133442 0.113 0.117 0.00233882 18
CARD16 DZ 2
2.008295e-07 -0.3327705 0.109 0.137 0.003580589 18
IFRD2 DZ 2
2.284216e-07 -0.3330895 0.152 0.163 0.004072528 18
HM13 DZ 2
2.414571e-07 -0.3281736 0.109 0.127 0.004304939 18
ZEB2 DZ 2
2.650332e-07 -0.3890248 0.148 0.158 0.004725277 18
TGIF1 DZ 2
3.221119e-07 -0.4139925 0.179 0.183 0.005742933 18
MBP DZ 2
3.283361e-07 -0.3745745 0.157 0.158 0.005853904 18
GLIPR1 DZ 2
3.504032e-07 -0.4245414 0.215 0.217 0.006247338 18
NFKBIE DZ 2
3.522651e-07 -0.4667468 0.242 0.242 0.006280535 18
PHACTR1 DZ 2
3.664226e-07 -0.4227185 0.184 0.174 0.006532949 18
MARCH1 DZ 2
5.349119e-07 -0.3241419 0.15 0.16 0.009536944 18
VKORC1 DZ 2
5.688356e-07 -0.3276464 0.931 0.711 0.01014177 18
ENO1 DZ 2
6.36932e-07 -0.4185358 0.321 0.298 0.01135586 18
MPHOSPH8 DZ 2
6.421236e-07 -0.5174765 0.808 0.715 0.01144842 18
EIF4A2 DZ 2
6.895158e-07 -0.4890836 0.497 0.443 0.01229338 18

ATP5MC1	DZ 2				
7.190724e-07	-0.3028995	0.988	0.976	0.01282034	18
UBA52	DZ 2				
8.209814e-07	-0.3369677	0.135	0.147	0.01463728	18
TSPAN3	DZ 2				
9.654156e-07	-0.3544653	0.202	0.208	0.01721239	18
GNL3	DZ 2				
9.859403e-07	-0.3132955	0.113	0.123	0.01757833	18
RNF213	DZ 2				
9.871415e-07	-0.463059	0.229	0.232	0.01759975	18
TARS	DZ 2				
1.147804e-06	-0.5608574	0.37	0.345	0.0204642	18
CAST	DZ 2				
1.343387e-06	-0.4632993	0.92	0.846	0.02395125	18
CD37	DZ 2				
1.458459e-06	-0.3183348	0.119	0.138	0.02600287	18
SMIM29	DZ 2				
1.473248e-06	-0.3447266	0.097	0.131	0.02626653	18
RP9	DZ 2				
1.565419e-06	-0.3356967	0.911	0.881	0.02790986	18
MT-ND1	DZ 2				
2.534743e-06	-0.4773749	0.351	0.322	0.04519194	18
NOP56	DZ 2				
2.625796e-06	-0.5353348	0.747	0.651	0.04681531	18
HLA-DQA1	DZ 2				
2.745824e-06	-0.3219679	0.167	0.185	0.0489553	18
YIF1A	DZ 2				
3.970314e-06	-0.9933814	0.966	0.926	0.07078672	18
IGKC	DZ 2				
5.630232e-06	-0.3162088	0.111	0.12	0.1003814	18
LACTB	DZ 2				
6.478444e-06	-0.8264108	0.881	0.641	0.1155042	18
IGLC2	DZ 2				
7.140185e-06	-0.4529789	0.528	0.459	0.1273024	18
GADD45GIP1	DZ 2				
1.198885e-05	-0.5116026	0.895	0.814	0.2137492	18
NOP53	DZ 2				
1.478211e-05	-0.4015351	0.367	0.324	0.2635502	18
P4HB	DZ 2				
1.607619e-05	-0.4681821	0.394	0.337	0.2866224	18
IER2	DZ 2				
2.150697e-05	-0.3034783	0.981	0.957	0.3834478	18
RPL24	DZ 2				
3.118695e-05	-0.3136269	0.228	0.225	0.5560321	18
C19orf24	DZ 2				
4.200527e-05	-0.3603378	0.201	0.196	0.748912	18
AARS	DZ 2				
4.457062e-05	-0.3715984	0.225	0.204	0.7946495	18
AP1S2	DZ 2				
5.220529e-05	-0.4138938	0.312	0.243	0.930768	18

RASGRP2 DZ 2
5.496677e-05 −0.3401444 0.237 0.233 0.9800025 18
CDK4 DZ 2
6.568704e-05 −0.4018904 0.212 0.199 1 18
PPP3CA DZ 2
6.670439e-05 −0.4405212 0.391 0.318 1 18
LTA4H DZ 2
9.296005e-05 −0.320993 0.175 0.171 1 18
TRIM44 DZ 2
0.0001642129 −0.3088494 0.168 0.155 1 18
LMBRD1 DZ 2
0.00018608 −0.510663 0.626 0.531 1 18
IMPDH2 DZ 2
0.0002035011 −0.3882147 0.334 0.29 1 18
PYURF DZ 2
0.0002984736 −0.3990355 0.361 0.296 1 18
PDLIM1 DZ 2
0.0003733282 −0.3508098 0.264 0.229 1 18
TTC19 DZ 2
0.0004662365 −0.3065049 0.212 0.189 1 18
SYTL1 DZ 2
0.0007048264 −0.3281651 0.123 0.136 1 18
PSAT1 DZ 2
0.000783987 −0.4839485 0.652 0.651 1 18
RPS4Y1 DZ 2
0.0007983759 −0.4839601 0.584 0.478 1 18
ISCU DZ 2
0.0008058492 −0.418297 0.554 0.478 1 18
RSL1D1 DZ 2
0.0008099755 −0.4248046 0.29 0.279 1 18
WARS DZ 2
0.0008115495 −0.4190514 0.499 0.406 1 18
PPIB DZ 2
0.0009705813 −0.3258652 0.218 0.203 1 18
NDFIP1 DZ 2
0.000992151 −0.8807615 0.544 0.427 1 18
IGLC3 DZ 2
0.00110159 −0.329708 0.308 0.256 1 18
MRPS26 DZ 2
0.001102575 −0.4046647 0.409 0.335 1 18
S100A11 DZ 2
0.00129289 −0.453957 0.291 0.255 1 18
SRGN DZ 2
0.001666568 −0.3315932 0.306 0.252 1 18
SLC50A1 DZ 2
0.001916348 −0.3627723 0.933 0.747 1 18
CXCR4 DZ 2
0.003322671 −0.4758004 0.626 0.492 1 18
SP100 DZ 2
0.004173038 −0.378714 0.652 0.4 1 18

FABP5 DZ 2
0.004310741 -0.3863923 0.373 0.318 1 18
SNHG7 DZ 2
0.004441736 -0.4522409 0.797 0.695 1 18
HLA-DQB1 DZ 2
0.005032343 -0.4354322 0.361 0.295 1 18
NFKBIA DZ 2
0.005830825 -0.4177609 0.499 0.397 1 18
BLOC1S2 DZ 2
0.007535123 -0.3322744 0.266 0.202 1 18
IRF1 DZ 2
0.007718462 -0.3053134 0.218 0.187 1 18
GPATCH4 DZ 2
0.007724927 -0.3009747 0.237 0.184 1 18
ANKRD44 DZ 2
0.008452393 -0.3993049 0.576 0.431 1 18
CYB561A3 DZ 2
0.009602653 -0.3076045 0.345 0.289 1 18
DCTPP1 DZ 2
1.31751e-298 2.102598 0.789 0.138 2.348988e-294 19 BIK
LZ
3.00384e-226 1.922664 0.698 0.143 5.355546e-222 19
DUSP2 LZ
2.546294e-212 1.532765 0.639 0.084 4.539788e-208 19
FCRL2 LZ
2.374113e-211 1.506478 0.723 0.168 4.232807e-207 19
BCL2A1 LZ
4.289765e-200 1.326358 0.948 0.593 7.648222e-196 19
PRDX1 LZ
6.400608e-200 1.347489 0.772 0.211 1.141164e-195 19
TRAF4 LZ
6.111142e-187 1.464274 0.748 0.159 1.089555e-182 19
CCDC88A LZ
4.652112e-186 1.418215 0.933 0.583 8.29425e-182 19
MARCKSL1 LZ
8.586839e-176 1.298456 0.597 0.121 1.530948e-171 19
NFATC1 LZ
1.629386e-175 1.603894 0.85 0.196 2.905032e-171 19
RGS13 LZ
3.20242e-175 1.300992 0.887 0.367 5.709594e-171 19
ACADM LZ
7.150632e-175 0.7982722 0.994 0.846 1.274886e-170 19
HSP90AB1 LZ
1.034273e-151 1.272075 0.822 0.33 1.844006e-147 19
SPIB LZ
3.075554e-150 1.145712 0.943 0.63 5.483405e-146 19
HERPUD1 LZ
2.756297e-144 1.112672 0.744 0.263 4.914203e-140 19 SMS
LZ
6.788654e-143 1.328446 0.796 0.26 1.210349e-138 19

PIM1	LZ	3.121506e-135	1.11375	0.527	0.088	5.565334e-131	19
FCRL5	LZ	4.08593e-130	1.175438	0.552	0.109	7.284805e-126	19
GMDS	LZ	1.386246e-129	1.0646	0.884	0.39	2.471538e-125	19
BASP1	LZ	1.536807e-125	1.026023	0.506	0.096	2.739974e-121	19
SEMA4A	LZ	1.977744e-125	0.7498874	0.994	0.868	3.526119e-121	19
ARPC2	LZ	7.993238e-125	0.9189109	0.717	0.292	1.425114e-120	19
NME1	LZ	3.31235e-123	0.7780513	0.999	0.886	5.905588e-119	19
GAPDH	LZ	1.045162e-120	1.001411	0.677	0.196	1.86342e-116	19
UBALD2	LZ	3.521849e-119	0.8068958	0.916	0.605	6.279105e-115	19
SYNGR2	LZ	3.57773e-117	0.945239	0.834	0.397	6.378735e-113	19
FABP5	LZ	3.024126e-116	1.144612	0.759	0.34	5.391714e-112	19
ABRACL	LZ	9.829557e-115	0.7879464	0.999	0.932	1.752512e-110	19
SERF2	LZ	1.556925e-113	0.9473275	0.698	0.245	2.775841e-109	19
RGS10	LZ	4.853268e-112	0.8224153	0.947	0.592	8.652892e-108	19
SEC61B	LZ	4.298842e-109	-1.782388	0.309	0.704	7.664405e-105	19
EMP3	LZ	2.087885e-108	0.7328602	0.972	0.758	3.722491e-104	19
PKM	LZ	9.442997e-107	0.9848055	0.829	0.466	1.683592e-102	19
CD83	LZ	8.437765e-105	0.9699239	0.478	0.098	1.504369e-100	19
SORL1	LZ	3.414024e-103	1.032113	0.642	0.203	6.086864e-99	19
SYAP1	LZ	5.482312e-103	0.9717736	0.613	0.173	9.774414e-99	19
LCK	LZ	8.705462e-103	0.7708205	0.936	0.602	1.552097e-98	19
POMP	LZ	4.543463e-102	0.7913534	0.943	0.617	8.100541e-98	19
ARPC1B	LZ	2.736651e-97	0.8214255	0.608	0.21	4.879175e-93	19
NFKBIE	LZ	1.281207e-95	0.4677641	0.996	0.941	2.284264e-91	19
YBX1	LZ	6.443339e-95	1.128431	0.503	0.146	1.148783e-90	19

NFKBID	LZ				
5.978657e-94	0.7751673	0.396	0.058	1.065935e-89	19
PITPNC1	LZ				
1.771314e-93	-1.665512	0.547	0.754	3.158075e-89	19
CXCR4	LZ				
2.976023e-93	0.6838619	0.818	0.439	5.305951e-89	19
C1QBP	LZ				
2.730987e-92	0.7343139	0.827	0.458	4.869077e-88	19
PRMT1	LZ				
1.436247e-91	0.9273354	0.442	0.092	2.560684e-87	19
CCDC28B	LZ				
4.822217e-91	0.7751431	0.815	0.387	8.597531e-87	19
YWHAE	LZ				
1.902026e-90	0.8149838	0.488	0.146	3.391122e-86	19
PPAN	LZ				
8.050903e-90	0.7339328	0.259	0.021	1.435395e-85	19
ID2	LZ				
1.907002e-89	0.5868644	0.965	0.745	3.399993e-85	19
RAN	LZ				
3.661529e-89	0.7602542	0.92	0.632	6.52814e-85	19
SET	LZ				
1.359558e-88	0.702533	0.982	0.783	2.423956e-84	19
ACTG1	LZ				
5.053495e-87	0.9303465	0.799	0.324	9.009876e-83	19
GRHPR	LZ				
1.687586e-86	0.7275095	0.924	0.636	3.008797e-82	19
ERH	LZ				
1.017722e-85	0.7965989	0.537	0.162	1.814496e-81	19
SAMSN1	LZ				
4.484471e-85	0.7591878	0.912	0.579	7.995363e-81	19
ATP5MC3	LZ				
2.833818e-84	0.7544253	0.738	0.343	5.052415e-80	19
TIMM13	LZ				
6.568327e-84	0.8128504	0.773	0.322	1.171067e-79	19
RFTN1	LZ				
9.207755e-84	0.7712329	0.783	0.386	1.641651e-79	19
FNBP1	LZ				
1.313894e-82	0.3652362	0.999	0.928	2.342542e-78	19
NPM1	LZ				
5.324702e-81	0.6223546	0.243	0.024	9.493411e-77	19
FEZ1	LZ				
2.023554e-79	0.9060633	0.523	0.132	3.607795e-75	19
SIT1	LZ				
2.03466e-79	0.8346375	0.782	0.359	3.627596e-75	19
SNAP23	LZ				
2.22947e-79	0.6316505	0.86	0.498	3.974922e-75	19
EIF3I	LZ				
5.21251e-79	0.6224116	0.958	0.732	9.293384e-75	19
HSPA8	LZ				
6.94483e-79	0.4364232	0.957	0.711	1.238194e-74	19

ENO1	LZ	4.302203e-78	0.6933673	0.761	0.368	7.670398e-74	19
LCP1	LZ	5.511791e-78	0.6284102	0.31	0.038	9.826972e-74	19
MCOLN2	LZ	1.151603e-77	0.514048	0.997	0.902	2.053193e-73	19
SRP14	LZ	1.111716e-76	0.6112107	0.255	0.034	1.982079e-72	19
CD70	LZ	2.602692e-76	0.8736467	0.589	0.218	4.640339e-72	19
SIAH2	LZ	2.667592e-76	0.7194177	0.901	0.535	4.756049e-72	19
PARP1	LZ	1.377267e-75	0.5716339	0.892	0.566	2.455529e-71	19
SNRPE	LZ	2.528188e-75	0.7087606	0.524	0.167	4.507506e-71	19
SNX8	LZ	7.207735e-75	0.7007435	0.481	0.139	1.285067e-70	19
MDFIC	LZ	7.635703e-75	0.5794958	0.881	0.554	1.36137e-70	19
NCL	LZ	1.286233e-74	0.7730375	0.462	0.149	2.293225e-70	19
RILPL2	LZ	2.224558e-74	0.7554117	0.71	0.299	3.966165e-70	19
TIMM8B	LZ	1.377744e-72	0.6418283	0.906	0.574	2.45638e-68	19
LDHB	LZ	1.897327e-72	0.9156384	0.719	0.334	3.382744e-68	19
LRMP	LZ	7.049107e-72	0.6109745	0.79	0.456	1.256785e-67	19
HSPD1	LZ	1.445604e-71	0.7349484	0.808	0.412	2.577367e-67	19
CTSH	LZ	1.652758e-71	0.5444642	0.943	0.679	2.946701e-67	19
SLC25A3	LZ	1.669477e-71	0.6038581	0.94	0.629	2.97651e-67	19
PSMA4	LZ	3.511989e-70	0.5820592	0.836	0.484	6.261525e-66	19
CCT3	LZ	7.408038e-70	0.7614169	0.554	0.204	1.320779e-65	19
CFLAR	LZ	2.297271e-69	0.7378475	0.359	0.073	4.095804e-65	19
LMO2	LZ	6.278153e-69	0.691629	0.415	0.117	1.119332e-64	19
MAP3K8	LZ	1.812372e-68	0.6417805	0.62	0.274	3.231278e-64	19
SRM	LZ	3.272193e-68	0.5129621	0.892	0.601	5.833994e-64	19
RBM3	LZ	4.291054e-68	0.6601797	0.336	0.073	7.650519e-64	19

HCST	LZ					
5.069907e-68	0.8024578	0.745	0.322	9.039137e-64	19	
GYPC	LZ					
1.543707e-66	0.69857	0.309	0.057	2.752275e-62	19	
TESC	LZ					
2.644445e-66	0.7210286	0.713	0.318	4.714782e-62	19	
BLNK	LZ					
3.371731e-66	0.6418045	0.337	0.07	6.01146e-62	19	
DOK3	LZ					
3.517485e-66	0.6236833	0.666	0.307	6.271324e-62	19	
AKR1A1	LZ					
6.217212e-66	0.5890447	0.905	0.554	1.108467e-61	19	
ATP5PF	LZ					
6.42361e-66	0.5877937	0.754	0.383	1.145265e-61	19	
CCT5	LZ					
3.320778e-65	-0.8953836	0.997	0.985	5.920616e-61	19	
TMSB10	LZ					
9.078562e-65	0.6789143	0.722	0.341	1.618617e-60	19	
RBM17	LZ					
3.088574e-64	0.7192919	0.396	0.099	5.506618e-60	19	
OTULIN	LZ					
5.721088e-64	-1.280097	0.203	0.374	1.020013e-59	19	
SELL	LZ					
1.659803e-63	0.4092767	0.999	0.898	2.959263e-59	19	
PFN1	LZ					
6.64823e-63	0.5590161	0.848	0.468	1.185313e-58	19	
SNRPD1	LZ					
5.57978e-62	0.5731682	0.933	0.653	9.94819e-58	19	
SLC25A5	LZ					
5.763191e-62	0.4568669	0.945	0.676	1.027519e-57	19	
SRSF3	LZ					
1.129634e-61	0.5551491	0.887	0.568	2.014025e-57	19	
PARK7	LZ					
2.284009e-61	0.5035737	0.917	0.6	4.07216e-57	19	
PSMB3	LZ					
3.124732e-61	0.5551924	0.508	0.192	5.571084e-57	19	
ATIC	LZ					
3.419856e-61	0.3608984	0.992	0.922	6.097261e-57	19	
BTF3	LZ					
1.044437e-60	0.583419	0.27	0.039	1.862127e-56	19	
IGF2BP3	LZ					
1.293378e-60	0.6172764	0.4	0.11	2.305964e-56	19	
NIN	LZ					
1.830459e-60	0.5091027	0.794	0.436	3.263525e-56	19	
PPA1	LZ					
2.071009e-60	0.4833996	0.716	0.38	3.692402e-56	19	
NHP2	LZ					
9.559706e-60	0.4998724	0.947	0.696	1.7044e-55	19	
ELOB	LZ					
2.067088e-59	0.6241964	0.778	0.41	3.68541e-55	19	

PPP1CC	LZ				
4.350552e-59	0.7211272	0.59	0.231	7.756599e-55	19
DSTN	LZ				
1.902793e-58	-0.6663338	0.997	0.998	3.392491e-54	19
B2M	LZ				
2.140016e-58	0.5176427	0.371	0.109	3.815435e-54	19
MCRIP2	LZ				
3.268262e-58	0.4532224	0.899	0.616	5.826985e-54	19
ACTR3	LZ				
3.688497e-58	0.4961481	0.908	0.603	6.57622e-54	19
POLR2L	LZ				
4.941028e-58	0.5860771	0.786	0.425	8.809359e-54	19
CLTA	LZ				
6.154031e-58	0.6571041	0.483	0.147	1.097202e-53	19
CD72	LZ				
9.256503e-58	0.564559	0.787	0.432	1.650342e-53	19
COTL1	LZ				
2.264518e-57	0.498919	0.117	0.004	4.03741e-53	19
RSPH1	LZ				
4.392761e-57	0.5508994	0.873	0.518	7.831854e-53	19
FKBP1A	LZ				
6.670266e-57	0.7393701	0.287	0.049	1.189242e-52	19
SERPINA9	LZ				
8.808132e-57	0.5823512	0.31	0.063	1.570402e-52	19
MYO1C	LZ				
1.428246e-56	0.4442311	0.864	0.592	2.546419e-52	19
LDHA	LZ				
2.65327e-56	0.4859254	0.773	0.423	4.730515e-52	19
CCT2	LZ				
2.678766e-56	0.6538459	0.572	0.204	4.775972e-52	19
EHD1	LZ				
3.874821e-56	0.4892787	0.958	0.719	6.908418e-52	19
COX6C	LZ				
4.509005e-56	-0.9698502	0.045	0.214	8.039105e-52	19
PLAC8	LZ				
4.813928e-56	0.5400291	0.253	0.033	8.582753e-52	19
PASK	LZ				
9.877927e-56	0.5233281	0.842	0.518	1.761136e-51	19
NDUFB8	LZ				
1.297433e-55	0.4894265	0.905	0.624	2.313194e-51	19
ATP5F1B	LZ				
2.716825e-55	0.9332983	0.751	0.385	4.843827e-51	19
EIF5A	LZ				
3.120428e-55	0.5734098	0.815	0.443	5.563411e-51	19
SEM1	LZ				
3.226031e-55	0.5534111	0.586	0.27	5.75169e-51	19
NDUFAF8	LZ				
6.793142e-55	0.5615155	0.308	0.067	1.211149e-50	19
BATF	LZ				
1.712874e-54	0.4610957	0.892	0.612	3.053883e-50	19

SERBP1 LZ
1.71941e-54 0.5139663 0.779 0.422 3.065537e-50 19
MINOS1 LZ
2.715114e-54 0.501282 0.955 0.744 4.840777e-50 19
HSP90AA1 LZ
3.812002e-54 0.5141164 0.61 0.298 6.796419e-50 19
ODC1 LZ
4.290147e-54 0.6166891 0.736 0.352 7.648903e-50 19
TMEM243 LZ
7.360347e-54 0.4610474 0.917 0.635 1.312276e-49 19
ATP5MD LZ
8.897834e-54 0.7535104 0.698 0.292 1.586395e-49 19
TCF4 LZ
1.756616e-53 0.549826 0.464 0.179 3.131871e-49 19 BID
LZ
1.824586e-53 0.5017605 0.8 0.47 3.253054e-49 19
PRELID1 LZ
1.951285e-53 0.5262636 0.818 0.492 3.478946e-49 19
COX5A LZ
4.223399e-53 0.5531934 0.294 0.059 7.529899e-49 19
PTPRE LZ
4.347925e-53 0.5940531 0.787 0.43 7.751915e-49 19
ROMO1 LZ
1.311013e-52 0.6844397 0.313 0.069 2.337405e-48 19
LINC01857 LZ
1.357188e-52 0.5699103 0.32 0.057 2.41973e-48 19
DCAF12 LZ
2.620073e-52 0.6360101 0.717 0.396 4.671329e-48 19
RPL22L1 LZ
2.895587e-52 0.5723408 0.565 0.229 5.162543e-48 19
MRPL27 LZ
4.850316e-52 0.4255457 0.211 0.031 8.647628e-48 19
PDGFD LZ
8.066349e-52 0.6474006 0.435 0.141 1.438149e-47 19
TYW3 LZ
8.851857e-52 0.5979437 0.691 0.314 1.578198e-47 19
AP3S1 LZ
9.885949e-52 0.3969046 0.922 0.7 1.762566e-47 19
TPI1 LZ
1.269496e-51 0.6389177 0.42 0.119 2.263385e-47 19
DLAT LZ
1.780779e-51 0.5407041 0.424 0.137 3.17495e-47 19
FAM207A LZ
2.36508e-51 0.6102532 0.905 0.6 4.216702e-47 19
DYNLL1 LZ
2.576986e-51 0.5313413 0.874 0.573 4.594508e-47 19
POU2F2 LZ
3.690146e-51 -0.6431011 1 0.999 6.579161e-47 19
RPS29 LZ
9.209809e-51 0.6527313 0.552 0.236 1.642017e-46 19

RGS1	LZ	1.017639e-50	0.5903672	0.74	0.378	1.814348e-46	19
SMARCB1	LZ	1.312914e-50	0.5751877	0.582	0.259	2.340793e-46	19
VASP	LZ	1.830826e-50	0.6730488	0.471	0.135	3.26418e-46	19
MKNK2	LZ	2.911285e-50	0.3941763	0.801	0.493	5.19053e-46	19
TUFM	LZ	4.952035e-50	0.6349626	0.684	0.282	8.828984e-46	19
BCAS4	LZ	5.031277e-50	0.5113588	0.4	0.131	8.970264e-46	19
RRP1	LZ	5.100897e-50	0.4787121	0.747	0.429	9.09439e-46	19
EIF5B	LZ	3.089421e-49	0.463568	0.793	0.493	5.508129e-45	19
HSPE1	LZ	3.158744e-49	0.5622458	0.666	0.278	5.631725e-45	19
CD82	LZ	4.157111e-49	0.394715	0.82	0.512	7.411714e-45	19
EIF3G	LZ	4.190139e-49	0.5196788	0.508	0.213	7.4706e-45	19
MRPL12	LZ	4.556784e-49	0.4467633	0.677	0.357	8.124291e-45	19
HSPA9	LZ	6.001609e-49	0.8493261	0.536	0.18	1.070027e-44	19
RGS2	LZ	6.326084e-49	-1.34506	0.303	0.547	1.127877e-44	19
CCR7	LZ	6.562393e-49	0.4175788	0.817	0.509	1.170009e-44	19
SSBP1	LZ	8.371061e-49	0.6126592	0.434	0.137	1.492477e-44	19
INO80C	LZ	9.139071e-49	-1.135714	0.124	0.354	1.629405e-44	19
LY6E	LZ	1.210655e-48	0.6568949	0.899	0.649	2.158477e-44	19
HLA-DQA1	LZ	1.279241e-48	0.4072676	0.923	0.652	2.28076e-44	19
PSMB1	LZ	4.899597e-48	0.4952335	0.733	0.397	8.735492e-44	19
MDH2	LZ	5.208102e-48	0.8464547	0.789	0.39	9.285526e-44	19
CSTB	LZ	7.199306e-48	0.5129691	0.41	0.151	1.283564e-43	19
PAICS	LZ	8.981596e-48	0.4187404	0.945	0.692	1.601329e-43	19
ATP5MPL	LZ	1.724685e-47	0.7448431	0.919	0.651	3.074941e-43	19
TCL1A	LZ	2.266641e-47	0.53717	0.285	0.062	4.041195e-43	19

QPCT	LZ	2.794234e-47	0.5189655	0.483	0.178	4.981839e-43	19
ZNF593	LZ	2.801471e-47	0.5123676	0.442	0.157	4.994743e-43	19
WDR77	LZ	3.814135e-47	-0.9366216	0.978	0.976	6.800222e-43	19
FTH1	LZ	4.467183e-47	0.4723717	0.808	0.485	7.96454e-43	19
CCT8	LZ	9.354104e-47	0.4901689	0.808	0.46	1.667743e-42	19
PRDX6	LZ	2.315799e-46	0.436126	0.993	0.849	4.128838e-42	19
ATP5MG	LZ	2.662051e-46	0.5176367	0.701	0.355	4.746171e-42	19
TKT	LZ	2.91772e-46	0.6052512	0.958	0.721	5.202002e-42	19
LIMD2	LZ	3.073165e-46	0.5145854	0.684	0.318	5.479146e-42	19
IMP4	LZ	3.508966e-46	0.5865198	0.508	0.173	6.256135e-42	19
MTERF4	LZ	3.717992e-46	0.3893522	0.773	0.475	6.628808e-42	19
RSL1D1	LZ	3.999838e-46	0.426784	0.985	0.868	7.131311e-42	19
ARPC3	LZ	4.132905e-46	0.4377463	0.947	0.703	7.368557e-42	19
NDUFA4	LZ	4.148304e-46	0.4217124	0.173	0.016	7.396011e-42	19
ZNF804A	LZ	4.801638e-46	-0.9827884	0.123	0.276	8.56084e-42	19
KLF2	LZ	4.987449e-46	0.4878136	0.751	0.425	8.892123e-42	19
SLIRP	LZ	6.925483e-46	0.514885	0.607	0.266	1.234744e-41	19
MRPL54	LZ	7.575456e-46	0.4571779	0.811	0.48	1.350628e-41	19
UBE2L3	LZ	9.419163e-46	0.421922	0.985	0.811	1.679343e-41	19
CORO1A	LZ	1.004325e-45	0.5519818	0.551	0.224	1.790611e-41	19
NGLY1	LZ	1.045235e-45	0.4185444	0.923	0.649	1.86355e-41	19
ATP5F1A	LZ	1.372239e-45	0.4799997	0.59	0.283	2.446566e-41	19
MRPL14	LZ	1.586769e-45	0.6477323	0.982	0.8	2.82905e-41	19
RPS26	LZ	2.46442e-45	-1.196838	0.131	0.356	4.393815e-41	19
TXNIP	LZ	6.031276e-45	0.440911	0.871	0.579	1.075316e-40	19

NDUFB2	LZ	6.256162e-45	0.3278976	0.99	0.894	1.115411e-40	19
SLC25A6	LZ	6.762749e-45	0.4522796	0.712	0.384	1.205731e-40	19
NOL7	LZ	8.899149e-45	0.4757851	0.47	0.194	1.586629e-40	19
EBNA1BP2	LZ	1.308471e-44	0.4118585	0.699	0.388	2.332873e-40	19
PHB	LZ	1.331722e-44	0.5650905	0.834	0.486	2.374327e-40	19
RAC2	LZ	1.472928e-44	0.5438253	0.681	0.317	2.626083e-40	19
HSBP1	LZ	3.346433e-44	0.5021545	0.538	0.241	5.966356e-40	19
AK2	LZ	3.993879e-44	0.6400195	0.243	0.052	7.120687e-40	19
HES1	LZ	5.585247e-44	0.7381189	0.548	0.205	9.957937e-40	19
ADA	LZ	6.212092e-44	0.4497919	0.853	0.528	1.107554e-39	19
SNRPB2	LZ	1.35108e-43	0.5149379	0.548	0.247	2.40884e-39	19
DNPH1	LZ	1.541228e-43	0.6544861	0.415	0.092	2.747855e-39	19
FCRL3	LZ	1.862387e-43	0.5562585	0.303	0.074	3.320449e-39	19
KLHL6	LZ	3.144491e-43	0.4328759	0.245	0.046	5.606313e-39	19
PLK3	LZ	3.899778e-43	0.4330893	0.515	0.221	6.952914e-39	19
DCAF13	LZ	6.475623e-43	0.4062494	0.931	0.643	1.154539e-38	19
UQCR11	LZ	2.09727e-42	0.4458323	0.929	0.695	3.739223e-38	19
CCNI	LZ	2.384712e-42	0.4142604	0.757	0.444	4.251703e-38	19
ILF2	LZ	2.452179e-42	0.4461179	0.552	0.255	4.37199e-38	19
AK6	LZ	2.483371e-42	0.4937899	0.745	0.409	4.427601e-38	19
HDAC1	LZ	2.714729e-42	0.4448814	0.571	0.249	4.84009e-38	19
DTNBP1	LZ	3.618345e-42	0.441082	0.73	0.399	6.451147e-38	19
PSMB2	LZ	4.304407e-42	0.4676944	0.745	0.415	7.674327e-38	19
FAM49B	LZ	4.788534e-42	0.399053	0.924	0.671	8.537477e-38	19
ATP5ME	LZ	5.402241e-42	0.5047004	0.569	0.261	9.631656e-38	19

MTCH2	LZ					
7.001534e-42	0.4319717	0.171	0.018	1.248303e-37	19	
BACE2	LZ					
9.951355e-42	0.4338621	0.407	0.159	1.774227e-37	19	
MRTO4	LZ					
1.11628e-41	0.4544098	0.143	0.019	1.990216e-37	19	
CCL22	LZ					
1.206207e-41	0.528837	0.572	0.263	2.150547e-37	19	
PPP1R2	LZ					
1.55064e-41	0.4277134	0.529	0.217	2.764636e-37	19	
MRPL4	LZ					
1.583307e-41	0.4507765	0.394	0.13	2.822878e-37	19	
EXOSC7	LZ					
1.753628e-41	0.4843471	0.547	0.252	3.126543e-37	19	
GSPT1	LZ					
2.348156e-41	0.4769449	0.745	0.426	4.186527e-37	19	
ZNF706	LZ					
2.612875e-41	0.4986753	0.448	0.183	4.658495e-37	19	
PDHA1	LZ					
3.678333e-41	0.4780828	0.524	0.242	6.558099e-37	19	
DKC1	LZ					
3.726941e-41	0.3801002	0.877	0.613	6.644763e-37	19	
NDUFA13	LZ					
3.739338e-41	0.4371154	0.701	0.36	6.666866e-37	19	
POLR2E	LZ					
4.868745e-41	0.370932	0.759	0.455	8.680486e-37	19	
GADD45GIP1	LZ					
6.668452e-41	0.7072856	0.425	0.105	1.188918e-36	19	
CD9	LZ					
7.487926e-41	0.5589039	0.669	0.33	1.335022e-36	19	
ANXA11	LZ					
1.053212e-40	0.3847755	0.997	0.938	1.877772e-36	19	
H3F3A	LZ					
1.333177e-40	0.400437	0.859	0.551	2.376921e-36	19	
SNU13	LZ					
1.472833e-40	0.4000634	0.87	0.562	2.625914e-36	19	
UQCRQ	LZ					
2.677701e-40	0.6070952	0.382	0.107	4.774074e-36	19	
AC023590.1	LZ					
2.863919e-40	0.3276654	0.877	0.59	5.106082e-36	19	
SRSF2	LZ					
2.996918e-40	0.4072612	0.871	0.549	5.343206e-36	19	
SEC61G	LZ					
4.660148e-40	0.4666881	0.533	0.233	8.308579e-36	19	
AMD1	LZ					
5.827814e-40	0.5085939	0.343	0.104	1.039041e-35	19	
PHF6	LZ					
6.025296e-40	0.4062159	0.585	0.285	1.07425e-35	19	
DCTPP1	LZ					
6.351524e-40	0.5355259	0.306	0.074	1.132413e-35	19	

CRYZ	LZ	7.114472e-40	0.4588563	0.703	0.383	1.268439e-35	19
MRPL20	LZ	8.272728e-40	-0.7068716	0.999	1	1.474945e-35	19
MALAT1	LZ	8.502056e-40	0.5376033	0.498	0.183	1.515832e-35	19
PRPSAP2	LZ	1.302554e-39	0.6280658	0.592	0.231	2.322323e-35	19
GCHFR	LZ	1.376669e-39	0.4068376	0.299	0.098	2.454464e-35	19
BOP1	LZ	1.504354e-39	0.3416455	0.989	0.937	2.682112e-35	19
EEF2	LZ	1.575386e-39	0.3104417	0.994	0.918	2.808756e-35	19
CFL1	LZ	1.718451e-39	0.3524091	0.89	0.637	3.063827e-35	19
HNRNPM	LZ	1.763114e-39	0.3689165	0.818	0.505	3.143455e-35	19
PSMD8	LZ	1.803076e-39	0.4735824	0.578	0.258	3.214705e-35	19
THOC7	LZ	2.186174e-39	0.4464973	0.863	0.529	3.89773e-35	19
SRSF9	LZ	2.188282e-39	0.4537434	0.243	0.06	3.901488e-35	19
BCL2L1	LZ	3.261074e-39	0.568878	0.947	0.675	5.81417e-35	19
HLA-DMA	LZ	4.250102e-39	0.4594575	0.368	0.125	7.577507e-35	19
EIF3B	LZ	4.625738e-39	0.3507416	0.743	0.448	8.247228e-35	19
VDAC1	LZ	4.830688e-39	-1.066415	0.379	0.514	8.612633e-35	19
BANK1	LZ	5.362384e-39	0.3929607	0.199	0.031	9.560595e-35	19
CTPS2	LZ	5.396418e-39	0.4104583	0.817	0.518	9.621273e-35	19
SELENOT	LZ	8.765185e-39	0.4268464	0.484	0.198	1.562745e-34	19
MAP2K3	LZ	1.053852e-38	0.389277	0.793	0.498	1.878913e-34	19
NDUFA11	LZ	1.167381e-38	0.5613991	0.249	0.055	2.081323e-34	19
RGS16	LZ	1.341581e-38	0.3486036	0.92	0.641	2.391904e-34	19
SNRPG	LZ	1.979442e-38	0.4529718	0.786	0.446	3.529146e-34	19
MRPL51	LZ	2.140979e-38	0.4137807	0.829	0.529	3.817151e-34	19
SOD1	LZ	2.155152e-38	0.4106577	0.761	0.441	3.842421e-34	19

PSMB7 LZ
2.235037e-38 0.5087026 0.962 0.759 3.984848e-34 19
CYBA LZ
2.261749e-38 0.4103543 0.572 0.313 4.032472e-34 19
DDX21 LZ
2.601028e-38 0.5821796 0.582 0.272 4.637374e-34 19
PIM2 LZ
2.6029e-38 0.3400802 0.18 0.033 4.64071e-34 19
NDFIP2 LZ
3.179989e-38 0.3494685 0.726 0.415 5.669602e-34 19
EIF6 LZ
4.25264e-38 0.3149691 0.999 0.99 7.582033e-34 19
ACTB LZ
4.394817e-38 0.44829 0.544 0.235 7.83552e-34 19
PRKRA LZ
6.830549e-38 0.3891202 0.747 0.439 1.217819e-33 19
CCT7 LZ
8.616587e-38 0.4056474 0.592 0.297 1.536251e-33 19
NOP58 LZ
9.819613e-38 0.5661151 0.427 0.147 1.750739e-33 19
CLECL1 LZ
1.073311e-37 0.404253 0.799 0.472 1.913606e-33 19
UBE2N LZ
1.113646e-37 0.3937561 0.759 0.435 1.98552e-33 19
AURKAIP1 LZ
1.124358e-37 -0.8451735 0.253 0.408 2.004617e-33 19
SMIM14 LZ
1.155507e-37 0.3656617 0.954 0.731 2.060153e-33 19
SKP1 LZ
1.173925e-37 0.4545594 0.399 0.137 2.09299e-33 19
CISD1 LZ
1.314339e-37 0.3637217 0.916 0.636 2.343335e-33 19
ATP5MF LZ
2.144346e-37 0.4473658 0.234 0.041 3.823154e-33 19
IZUMO4 LZ
2.361608e-37 0.4308046 0.375 0.129 4.21051e-33 19
PRKCD LZ
3.688497e-37 0.4393657 0.424 0.158 6.576221e-33 19
EI24 LZ
3.737493e-37 0.3900653 0.621 0.32 6.663577e-33 19
PUF60 LZ
4.011873e-37 0.4109558 0.832 0.511 7.152769e-33 19
SMDT1 LZ
4.203164e-37 0.4587493 0.768 0.441 7.493822e-33 19
ATP5F1C LZ
5.107965e-37 0.4620714 0.352 0.105 9.106992e-33 19
NADK LZ
8.09384e-37 0.4237502 0.586 0.282 1.443051e-32 19
MRPS15 LZ
8.501458e-37 0.3922704 0.807 0.493 1.515725e-32 19

C19orf53 LZ
1.053702e-36 0.4079977 0.759 0.412 1.878645e-32 19
METAP2 LZ
1.683249e-36 0.4552268 0.488 0.2 3.001065e-32 19
MTMR14 LZ
1.683422e-36 0.3658309 0.899 0.641 3.001374e-32 19
HNRNPC LZ
2.732329e-36 0.4368744 0.621 0.299 4.871469e-32 19
CHMP2A LZ
2.931087e-36 0.4070674 0.508 0.232 5.225836e-32 19
NIFK LZ
3.370496e-36 0.331434 0.92 0.694 6.009258e-32 19
RHOA LZ
3.370535e-36 0.483223 0.39 0.134 6.009327e-32 19
RPA1 LZ
7.859936e-36 0.3963001 0.748 0.422 1.401348e-31 19
ANP32A LZ
7.969921e-36 0.3644491 0.449 0.192 1.420957e-31 19
GTPBP4 LZ
8.502975e-36 0.5091361 0.4 0.138 1.515995e-31 19
WDR18 LZ
9.220142e-36 0.552018 0.466 0.173 1.643859e-31 19
PLIN3 LZ
1.047038e-35 0.3717599 0.618 0.315 1.866764e-31 19
RHOG LZ
1.066429e-35 0.3427169 0.737 0.415 1.901335e-31 19
ADRM1 LZ
1.132058e-35 0.4242108 0.48 0.191 2.018347e-31 19
GRSF1 LZ
1.252083e-35 0.4693053 0.196 0.038 2.232339e-31 19
EGR2 LZ
1.737891e-35 0.501289 0.371 0.127 3.098485e-31 19
PHGDH LZ
2.174062e-35 0.3894761 0.312 0.101 3.876136e-31 19
LPCAT1 LZ
2.650515e-35 0.4609527 0.434 0.164 4.725603e-31 19
CFAP298 LZ
3.791303e-35 0.400816 0.811 0.497 6.759514e-31 19
RTRAF LZ
4.273898e-35 0.445489 0.344 0.113 7.619933e-31 19
EHD4 LZ
4.968291e-35 0.4077937 0.571 0.271 8.857965e-31 19
ATOX1 LZ
6.399974e-35 0.3958533 0.695 0.39 1.141051e-30 19
NDUFAB1 LZ
7.430218e-35 0.4282817 0.218 0.048 1.324734e-30 19
UCHL1 LZ
9.519404e-35 0.3629585 0.484 0.23 1.697215e-30 19
MTHFD2 LZ
1.02926e-34 0.397958 0.607 0.313 1.835068e-30 19

GTF3C6 LZ
1.574067e-34 0.4801021 0.769 0.423 2.806404e-30 19
DNAJA1 LZ
1.731668e-34 0.3517122 0.592 0.299 3.08739e-30 19
GLRX3 LZ
1.900995e-34 0.5039313 0.533 0.222 3.389285e-30 19
NSMCE1 LZ
3.316083e-34 0.4955916 0.583 0.265 5.912245e-30 19
TOP1 LZ
4.753045e-34 0.4123929 0.403 0.158 8.474204e-30 19
DCUN1D5 LZ
4.795976e-34 0.3043754 0.965 0.803 8.550746e-30 19
HNRNPK LZ
5.210926e-34 0.3817325 0.578 0.29 9.290561e-30 19
ILF3 LZ
5.671013e-34 0.4131956 0.273 0.083 1.011085e-29 19
EBI3 LZ
7.363139e-34 0.4220329 0.509 0.211 1.312774e-29 19
DMAC1 LZ
7.692157e-34 -1.120781 0.316 0.497 1.371435e-29 19
FXYD5 LZ
8.143576e-34 0.3907052 0.305 0.087 1.451918e-29 19
MAP1S LZ
8.413751e-34 0.3418996 0.996 0.961 1.500088e-29 19
OAZ1 LZ
9.244641e-34 0.3809922 0.414 0.186 1.648227e-29 19
ATP1A1 LZ
1.069758e-33 0.3965961 0.343 0.121 1.907271e-29 19
BZW2 LZ
1.139967e-33 0.4206266 0.632 0.332 2.032448e-29 19
PPM1G LZ
1.222041e-33 0.4152572 0.695 0.383 2.178777e-29 19
NDUFS6 LZ
1.436854e-33 0.4208304 0.769 0.455 2.561767e-29 19
SWAP70 LZ
1.844711e-33 0.4147034 0.262 0.059 3.288935e-29 19
DTX1 LZ
2.151217e-33 0.400603 0.894 0.665 3.835405e-29 19
GDI2 LZ
2.727059e-33 0.3481816 0.569 0.298 4.862073e-29 19
ATP1B3 LZ
2.821274e-33 0.3473559 0.635 0.335 5.030049e-29 19
STRAP LZ
3.05205e-33 0.397913 0.497 0.229 5.441501e-29 19
RALA LZ
4.676051e-33 0.5024289 0.862 0.694 8.336932e-29 19
HLA-DQB1 LZ
5.137151e-33 0.3962974 0.29 0.076 9.159027e-29 19
MYO1E LZ
5.885829e-33 0.410516 0.813 0.525 1.049384e-28 19

SRSF7 LZ
7.351369e-33 0.4242282 0.459 0.197 1.310676e-28 19
RELB LZ
8.732795e-33 0.4495964 0.547 0.242 1.55697e-28 19
TRAPPC2L LZ
9.150679e-33 0.4648833 0.559 0.263 1.631474e-28 19
TXNDC17 LZ
1.180401e-32 0.3868472 0.34 0.122 2.104537e-28 19
ISOC2 LZ
1.408192e-32 0.3237404 0.828 0.552 2.510666e-28 19
PGK1 LZ
1.539001e-32 -0.8916905 0.164 0.299 2.743885e-28 19
LINC00926 LZ
1.671621e-32 0.4979059 0.71 0.376 2.980333e-28 19
LSM8 LZ
1.972025e-32 0.3543839 0.389 0.153 3.515924e-28 19
GAR1 LZ
2.278133e-32 0.5246114 0.467 0.176 4.061683e-28 19
ZNF106 LZ
2.566819e-32 0.3364741 0.859 0.597 4.576382e-28 19
PSMA2 LZ
2.701432e-32 0.3238711 0.719 0.44 4.816383e-28 19
ATP5MC1 LZ
3.066694e-32 0.424222 0.347 0.108 5.467609e-28 19
TRAC LZ
5.019715e-32 0.3592561 0.329 0.117 8.94965e-28 19
TRAP1 LZ
6.226901e-32 -0.8637085 0.147 0.27 1.110194e-27 19
JUN LZ
7.664821e-32 -0.9844022 0.077 0.271 1.366561e-27 19
GPR183 LZ
8.320023e-32 0.4081303 0.555 0.262 1.483377e-27 19
PPP1R7 LZ
8.505563e-32 0.3763386 0.476 0.219 1.516457e-27 19
MRPS23 LZ
9.42502e-32 0.4363607 0.611 0.311 1.680387e-27 19
NDUFAF3 LZ
9.846594e-32 0.3671921 0.327 0.114 1.755549e-27 19
LYAR LZ
1.025834e-31 0.4356876 0.618 0.309 1.82896e-27 19
LYN LZ
1.117695e-31 0.3252615 0.248 0.074 1.992739e-27 19
RRP9 LZ
1.183753e-31 0.3275796 0.783 0.484 2.110513e-27 19
EIF3M LZ
1.349927e-31 0.3482389 0.754 0.464 2.406785e-27 19
SNRPF LZ
1.462079e-31 0.408978 0.382 0.137 2.606741e-27 19
TM2D3 LZ
1.676016e-31 0.3343741 0.476 0.247 2.988169e-27 19

GARS	LZ	1.710799e-31	-0.9636837	0.383	0.525	3.050184e-27	19
ID3	LZ	2.082389e-31	0.3455081	0.744	0.441	3.712692e-27	19
PGAM1	LZ	2.290275e-31	0.3390484	0.467	0.224	4.083331e-27	19
MRPL3	LZ	2.573905e-31	0.3356649	0.227	0.07	4.589015e-27	19
BYSL	LZ	2.584497e-31	0.3325758	0.719	0.423	4.6079e-27	19
PPP1R15A	LZ	2.60754e-31	0.3469497	0.628	0.344	4.648983e-27	19
CYC1	LZ	2.704384e-31	0.4389637	0.576	0.265	4.821646e-27	19
SEPT9	LZ	3.777964e-31	0.4427736	0.552	0.261	6.735733e-27	19
AIP	LZ	3.800363e-31	0.3466062	0.359	0.153	6.775667e-27	19
NAMPT	LZ	5.248061e-31	0.4042513	0.421	0.205	9.356767e-27	19
PIM3	LZ	6.897338e-31	0.318917	0.801	0.515	1.229726e-26	19
PSMB6	LZ	9.092502e-31	0.4017959	0.417	0.169	1.621102e-26	19
MRPL37	LZ	1.35886e-30	0.338641	0.731	0.435	2.422712e-26	19
HMGA1	LZ	1.553739e-30	0.3789123	0.176	0.027	2.770162e-26	19
FGD6	LZ	1.820067e-30	0.3584684	0.578	0.286	3.244998e-26	19
G3BP1	LZ	2.272912e-30	0.3539859	0.691	0.403	4.052375e-26	19
BANF1	LZ	2.385333e-30	0.3292652	0.429	0.197	4.25281e-26	19
TOMM40	LZ	2.415539e-30	0.3918836	0.575	0.28	4.306664e-26	19
RTF2	LZ	2.44958e-30	0.3994811	0.738	0.414	4.367356e-26	19
HNRNPD	LZ	2.491838e-30	0.434169	0.566	0.256	4.442699e-26	19
CCDC69	LZ	3.55454e-30	0.411145	0.543	0.258	6.33739e-26	19
ATXN10	LZ	3.619841e-30	0.3757321	0.31	0.091	6.453815e-26	19
PDLIM2	LZ	3.73227e-30	0.3170059	0.603	0.34	6.654264e-26	19
INSIG1	LZ	4.498879e-30	0.3317291	0.28	0.085	8.021051e-26	19
POLR1C	LZ	5.324782e-30	0.3892481	0.628	0.34	9.493554e-26	19

MRPS34 LZ
5.837561e-30 0.303539 0.796 0.51 1.040779e-25 19
DNAJC8 LZ
6.163331e-30 0.3622613 0.68 0.387 1.09886e-25 19
SSB LZ
6.453157e-30 -0.3796735 1 1 1.150533e-25 19
RPL41 LZ
7.384042e-30 0.3768753 0.389 0.156 1.316501e-25 19
COA4 LZ
7.680373e-30 -0.7123023 0.063 0.197 1.369334e-25 19
CLEC2B LZ
8.026779e-30 0.4908453 0.671 0.308 1.431094e-25 19
HMGN3 LZ
1.0747e-29 0.3866696 0.652 0.341 1.916083e-25 19
LSM6 LZ
1.67212e-29 0.4021136 0.72 0.421 2.981223e-25 19
CD40 LZ
1.740425e-29 0.3425001 0.365 0.142 3.103003e-25 19
ADSL LZ
1.953264e-29 0.3112066 0.284 0.098 3.482475e-25 19
RRS1 LZ
2.891211e-29 0.4340158 0.215 0.043 5.15474e-25 19
EML6 LZ
2.933872e-29 0.3878633 0.596 0.315 5.2308e-25 19
PRDX2 LZ
3.561331e-29 0.3866548 0.519 0.26 6.349497e-25 19
SYNCRIP LZ
3.892213e-29 0.3303853 0.385 0.164 6.939426e-25 19
SNHG15 LZ
5.349632e-29 0.4665919 0.355 0.114 9.537858e-25 19
TBC1D1 LZ
6.559943e-29 0.3215485 0.733 0.444 1.169572e-24 19
CSNK2B LZ
7.523575e-29 0.3074264 0.159 0.029 1.341378e-24 19
MIR3681HG LZ
7.543028e-29 0.365844 0.559 0.279 1.344846e-24 19
MRPS7 LZ
7.841665e-29 0.3299389 0.754 0.473 1.39809e-24 19
GRB2 LZ
8.66471e-29 0.3804205 0.636 0.342 1.544831e-24 19
UBE2A LZ
9.148158e-29 0.3172149 0.102 0.009 1.631025e-24 19
RARRES2 LZ
1.047603e-28 0.3223549 0.376 0.163 1.867772e-24 19
ETF1 LZ
1.274396e-28 0.3607541 0.632 0.333 2.272121e-24 19
HSD17B10 LZ
1.358247e-28 0.418779 0.313 0.085 2.421619e-24 19
VEZT LZ
1.390471e-28 0.3322217 0.631 0.349 2.479071e-24 19

PHB2 LZ
1.406552e-28 0.3071676 0.839 0.623 2.507741e-24 19
SELENOH LZ
1.496347e-28 0.3657142 0.369 0.14 2.667837e-24 19
MPHOSPH6 LZ
1.588665e-28 0.4276181 0.541 0.235 2.832432e-24 19
PTRHD1 LZ
1.798905e-28 0.3730198 0.533 0.244 3.207267e-24 19
MRPL13 LZ
1.867157e-28 0.3547366 0.657 0.362 3.328954e-24 19
EWSR1 LZ
2.488498e-28 0.3085044 0.257 0.082 4.436744e-24 19
EXOSC5 LZ
2.516501e-28 0.3884359 0.32 0.102 4.48667e-24 19
ACSF3 LZ
2.595256e-28 0.300819 0.916 0.683 4.627082e-24 19
PPP1CA LZ
2.705783e-28 0.3302372 0.621 0.328 4.824141e-24 19
LSM2 LZ
2.967912e-28 0.3397753 0.523 0.274 5.29149e-24 19
PHPT1 LZ
3.624862e-28 0.3584745 0.526 0.253 6.462766e-24 19
CDV3 LZ
4.64106e-28 0.4037858 0.6 0.355 8.274546e-24 19
BSG LZ
4.932442e-28 0.3870941 0.628 0.345 8.79405e-24 19
PDIA6 LZ
5.195793e-28 0.3576404 0.866 0.615 9.26358e-24 19
CLEC2D LZ
6.695679e-28 0.3114385 0.645 0.37 1.193773e-23 19
PSMC3 LZ
7.688654e-28 0.3230538 0.797 0.513 1.37081e-23 19
ATP5PB LZ
7.752107e-28 0.3223764 0.537 0.277 1.382123e-23 19
PSMD14 LZ
7.835314e-28 0.3263712 0.887 0.61 1.396958e-23 19
UQCR10 LZ
8.816768e-28 0.3402631 0.401 0.177 1.571941e-23 19
FARSA LZ
8.911214e-28 0.3428086 0.185 0.038 1.58878e-23 19
TESPA1 LZ
1.069138e-27 0.3519004 0.383 0.163 1.906167e-23 19
PARL LZ
1.130632e-27 0.3677111 0.737 0.424 2.015805e-23 19
IRF8 LZ
1.662861e-27 -0.8699431 0.333 0.45 2.964715e-23 19
PNRC1 LZ
1.792531e-27 0.3665646 0.327 0.132 3.195903e-23 19
NDUFAF2 LZ
1.97632e-27 0.5625493 0.976 0.855 3.523581e-23 19

CD79B LZ
2.497808e-27 0.3636989 0.599 0.331 4.453342e-23 19
VOPP1 LZ
2.768449e-27 0.3332066 0.467 0.225 4.935868e-23 19
NOB1 LZ
2.892576e-27 0.3364166 0.422 0.186 5.157175e-23 19
CHCHD3 LZ
3.294447e-27 0.3687857 0.273 0.079 5.873669e-23 19
COMMD10 LZ
3.795884e-27 0.3183741 0.656 0.381 6.767682e-23 19
SNRPC LZ
3.858762e-27 0.5079823 0.519 0.197 6.879788e-23 19
HMCES LZ
4.128905e-27 0.3111196 0.462 0.231 7.361425e-23 19
EIF4A1 LZ
4.613899e-27 0.3370861 0.61 0.328 8.22612e-23 19
STUB1 LZ
4.974574e-27 -0.8072437 0.246 0.349 8.869167e-23 19
TRAF3IP3 LZ
5.326136e-27 0.3566977 0.207 0.052 9.495968e-23 19
BORCS8-MEF2B LZ
6.805477e-27 -0.8319748 0.067 0.222 1.213349e-22 19
GBP2 LZ
7.100823e-27 0.3095186 0.357 0.146 1.266006e-22 19
RPF2 LZ
1.086194e-26 0.3162352 0.895 0.63 1.936576e-22 19
BRK1 LZ
1.284075e-26 0.3891269 0.442 0.204 2.289378e-22 19
GNL3 LZ
1.524943e-26 0.4428453 0.548 0.222 2.718821e-22 19
UBE2G1 LZ
1.573429e-26 0.3846389 0.45 0.189 2.805266e-22 19
REPIN1 LZ
1.699764e-26 0.3035301 0.547 0.271 3.03051e-22 19
MRPL47 LZ
2.460252e-26 0.3466613 0.413 0.179 4.386383e-22 19
TMA16 LZ
2.652859e-26 0.3331837 0.652 0.375 4.729782e-22 19
VDAC2 LZ
2.686993e-26 0.3411445 0.614 0.344 4.790639e-22 19
ZC3H15 LZ
2.812721e-26 0.310733 0.664 0.38 5.014801e-22 19
UQCRC2 LZ
2.819181e-26 0.3146005 0.73 0.445 5.026318e-22 19
SF3B5 LZ
2.825305e-26 0.3255044 0.688 0.397 5.037237e-22 19
UQCRFS1 LZ
2.93017e-26 0.3353056 0.523 0.247 5.224199e-22 19
MCTS1 LZ
3.058839e-26 0.3363873 0.336 0.13 5.453604e-22 19

EXOSC4	LZ					
3.391896e-26	0.3561065	0.459	0.197	6.047412e-22	19	
NUBP1	LZ					
3.449554e-26	0.346824	0.439	0.207	6.15021e-22	19	
EEF1E1	LZ					
3.491311e-26	0.3328332	0.558	0.286	6.224659e-22	19	
CDC123	LZ					
3.579159e-26	0.4256711	0.698	0.394	6.381283e-22	19	
NDUFS5	LZ					
4.496866e-26	0.3557463	0.347	0.131	8.017462e-22	19	
PMPCA	LZ					
4.790353e-26	0.5174344	0.355	0.102	8.540721e-22	19	
HRK	LZ					
5.221131e-26	0.3562537	0.513	0.263	9.308755e-22	19	
PSMD3	LZ					
5.981039e-26	0.3801817	0.649	0.36	1.066359e-21	19	
TERF2IP	LZ					
6.932709e-26	0.3695405	0.467	0.216	1.236033e-21	19	
SMARCC1	LZ					
7.599508e-26	0.5723093	0.292	0.081	1.354916e-21	19	
SH3TC1	LZ					
8.206276e-26	0.3187107	0.642	0.362	1.463097e-21	19	
RPS19BP1	LZ					
9.724858e-26	0.3279745	0.214	0.061	1.733845e-21	19	
IL21R	LZ					
1.007968e-25	0.3026587	0.326	0.133	1.797106e-21	19	
PES1	LZ					
1.128181e-25	0.3379949	0.436	0.204	2.011434e-21	19	
ITPA	LZ					
1.195618e-25	-0.4286436	1	0.999	2.131668e-21	19	
RPL34	LZ					
1.861648e-25	0.525977	0.276	0.067	3.319133e-21	19	
RASSF6	LZ					
2.210764e-25	0.3273887	0.564	0.312	3.94157e-21	19	
WDR1	LZ					
2.352122e-25	-0.7774238	0.39	0.468	4.193599e-21	19	
ITM2B	LZ					
3.999128e-25	0.3825826	0.379	0.145	7.130045e-21	19	
SMARCA4	LZ					
4.276723e-25	0.3425547	0.372	0.145	7.62497e-21	19	
METTL21A	LZ					
4.877935e-25	0.3762014	0.597	0.305	8.696871e-21	19	
PGLS	LZ					
5.782027e-25	0.3308062	0.345	0.132	1.030878e-20	19	
DPP3	LZ					
7.664554e-25	0.4200759	0.594	0.284	1.366513e-20	19	
RUBCNL	LZ					
7.68201e-25	0.3284398	0.506	0.236	1.369625e-20	19	
SLC38A1	LZ					
9.987019e-25	0.3144928	0.51	0.242	1.780586e-20	19	

PRPF31 LZ
1.28294e-24 0.4736341 0.407 0.156 2.287353e-20 19
C12orf75 LZ
1.731924e-24 0.3039042 0.743 0.48 3.087847e-20 19
GTF3A LZ
1.855926e-24 0.3207524 0.456 0.221 3.308931e-20 19
DAZAP1 LZ
2.268734e-24 0.3091445 0.462 0.219 4.044925e-20 19
RPIA LZ
2.801017e-24 0.3462514 0.42 0.183 4.993934e-20 19
MRPL36 LZ
4.748657e-24 0.3349275 0.375 0.154 8.466381e-20 19
CHCHD5 LZ
5.704029e-24 0.3093939 0.239 0.067 1.016971e-19 19
SH3BP1 LZ
5.795473e-24 0.3461157 0.724 0.405 1.033275e-19 19
SEPT6 LZ
6.196479e-24 0.358712 0.467 0.219 1.10477e-19 19
HSPH1 LZ
6.546689e-24 0.5353471 0.414 0.13 1.167209e-19 19
NEIL1 LZ
7.093485e-24 0.3262434 0.267 0.085 1.264697e-19 19
TSPAN33 LZ
7.377669e-24 0.3669682 0.641 0.333 1.315365e-19 19
NDUFC1 LZ
9.04236e-24 -0.5990407 0.117 0.208 1.612162e-19 19
C16orf74 LZ
1.093641e-23 0.3257348 0.284 0.101 1.949852e-19 19
UCHL3 LZ
1.367657e-23 0.3257632 0.337 0.129 2.438396e-19 19
ERCC1 LZ
1.47349e-23 1.005507 0.933 0.751 2.627085e-19 19
HLA-DRB1 LZ
1.485935e-23 0.3252803 0.519 0.269 2.649274e-19 19
CAMTA1 LZ
1.514204e-23 0.320036 0.699 0.406 2.699675e-19 19
EID1 LZ
1.669837e-23 0.3248324 0.502 0.226 2.977153e-19 19
CKS2 LZ
1.968856e-23 0.3252719 0.666 0.377 3.510274e-19 19
NDUFC2 LZ
2.350385e-23 0.3125185 0.238 0.072 4.190501e-19 19
RNF121 LZ
2.43809e-23 0.3882117 0.372 0.162 4.346871e-19 19
TOR3A LZ
2.787566e-23 -0.6409973 0.083 0.185 4.969952e-19 19
PLPP5 LZ
3.206529e-23 0.3427186 0.203 0.065 5.71692e-19 19
TEX9 LZ
3.25462e-23 0.324168 0.417 0.185 5.802662e-19 19

RUVBL2 LZ
3.756634e-23 0.3215245 0.737 0.451 6.697702e-19 19
CAPZA1 LZ
4.92758e-23 0.3298682 0.396 0.174 8.785383e-19 19
LAP3 LZ
5.466681e-23 0.3994345 0.621 0.326 9.746545e-19 19
CCM2 LZ
5.489837e-23 0.3327645 0.464 0.219 9.78783e-19 19
PSMB5 LZ
5.810044e-23 0.3922551 0.445 0.18 1.035873e-18 19
APBB1IP LZ
7.093715e-23 0.3573125 0.726 0.429 1.264738e-18 19
CYB561A3 LZ
9.837198e-23 0.3862071 0.476 0.229 1.753874e-18 19
ZNF581 LZ
1.110646e-22 -0.7466483 0.408 0.493 1.980171e-18 19
CYTIP LZ
1.179208e-22 0.3105128 0.343 0.138 2.102409e-18 19
RUVBL1 LZ
1.228179e-22 -0.3874647 1 0.999 2.18972e-18 19
RPLP1 LZ
1.273857e-22 0.3371212 0.344 0.133 2.27116e-18 19
GTF2E2 LZ
1.328256e-22 0.3767429 0.565 0.304 2.368147e-18 19
CALR LZ
1.932229e-22 0.3203447 0.401 0.176 3.444972e-18 19
TMC6 LZ
2.356636e-22 0.3917931 0.45 0.172 4.201646e-18 19
SYK LZ
2.619232e-22 0.3009081 0.415 0.209 4.669828e-18 19
PRPF19 LZ
3.495029e-22 0.3364755 0.722 0.417 6.231287e-18 19
TMEM123 LZ
3.541981e-22 0.3425672 0.358 0.144 6.314998e-18 19
ZNF580 LZ
4.800131e-22 0.3586162 0.471 0.225 8.558154e-18 19
HDDC2 LZ
5.263851e-22 0.3808743 0.576 0.292 9.384919e-18 19
PDHB LZ
6.206175e-22 0.3206966 0.607 0.334 1.106499e-17 19
EIF1AX LZ
8.982971e-22 -1.115384 0.434 0.572 1.601574e-17 19
VIM LZ
9.855229e-22 0.3159349 0.562 0.304 1.757089e-17 19
MMADHC LZ
1.024158e-21 0.3615387 0.408 0.179 1.825972e-17 19
RASGRP3 LZ
1.092793e-21 0.3267711 0.641 0.341 1.948341e-17 19
H2AFY LZ
1.510901e-21 0.322778 0.421 0.189 2.693785e-17 19

						RFXANK	LZ
1.588413e-21	0.3393923	0.653	0.35	2.831981e-17	19	CD19	LZ
1.603289e-21	0.3937157	0.52	0.241	2.858504e-17	19	IKZF1	LZ
1.827567e-21	0.3109428	0.643	0.353	3.258369e-17	19	GNAS	LZ
2.034274e-21	-0.3945765	1	1	3.626907e-17	19	RPL21	LZ
2.071557e-21	0.3304589	0.375	0.153	3.693379e-17	19	MFAP1	LZ
2.181615e-21	0.3664618	0.516	0.235	3.889602e-17	19	SP140	LZ
2.589661e-21	0.3203399	0.583	0.304	4.617107e-17	19	FKBP3	LZ
2.738918e-21	0.4009999	0.4	0.143	4.883217e-17	19	RBM38	LZ
3.389558e-21	0.326863	0.329	0.126	6.043243e-17	19	DNAJB2	LZ
4.24136e-21	0.3467865	0.544	0.285	7.561921e-17	19	RRAS2	LZ
4.591748e-21	0.3232034	0.334	0.142	8.186628e-17	19	MRPL39	LZ
6.082435e-21	0.3183305	0.357	0.158	1.084437e-16	19	DCPS	LZ
6.313746e-21	0.3447396	0.66	0.396	1.125678e-16	19	RAP1A	LZ
6.972698e-21	0.325308	0.393	0.172	1.243162e-16	19	EIF4E2	LZ
7.119485e-21	0.3553474	0.608	0.317	1.269333e-16	19	NAA38	LZ
7.506816e-21	0.305585	0.529	0.274	1.33839e-16	19	CNIH1	LZ
9.867105e-21	0.3128003	0.204	0.06	1.759206e-16	19	NINJ1	LZ
1.230501e-20	0.327147	0.399	0.183	2.19386e-16	19	COPS8	LZ
1.238598e-20	-1.401201	0.341	0.467	2.208296e-16	19	S100A6	LZ
1.84668e-20	0.3615471	0.299	0.121	3.292445e-16	19	PLEK	LZ
2.303986e-20	-0.7451186	0.231	0.322	4.107776e-16	19	TUBA1A	LZ
2.921e-20	-0.6562026	0.084	0.198	5.207851e-16	19	CHPT1	LZ
2.967227e-20	0.3446215	0.636	0.362	5.290268e-16	19	CXXC5	LZ
3.067852e-20	-0.6503621	0.032	0.138	5.469673e-16	19	FCER2	LZ
3.219471e-20	0.3980242	0.292	0.092	5.739996e-16	19		

PXMP2 LZ
3.49998e-20 -0.72202 0.071 0.212 6.240114e-16 19
MGST3 LZ
3.865481e-20 0.3161705 0.215 0.061 6.891766e-16 19
ZNF608 LZ
6.012608e-20 0.3063854 0.501 0.245 1.071988e-15 19
SH3KBP1 LZ
7.309234e-20 0.3317991 0.403 0.162 1.303163e-15 19
ARHGAP25 LZ
7.943303e-20 -0.6884593 0.179 0.253 1.416212e-15 19
FCMR LZ
8.180657e-20 0.3471017 0.993 0.945 1.458529e-15 19
HLA-DRA LZ
1.161064e-19 0.307916 0.571 0.322 2.07006e-15 19
SNRNP70 LZ
1.162589e-19 0.4053305 0.508 0.273 2.072779e-15 19
HSPA5 LZ
2.799609e-19 0.4283238 0.152 0.031 4.991424e-15 19
GNG11 LZ
2.896783e-19 0.3004452 0.455 0.216 5.164674e-15 19
SNX5 LZ
4.594617e-19 -0.6580167 0.185 0.285 8.191743e-15 19
GNG7 LZ
4.970604e-19 0.3186749 0.449 0.209 8.86209e-15 19
STARD7 LZ
5.240035e-19 -0.6343789 0.22 0.298 9.342458e-15 19
PDLIM1 LZ
6.096516e-19 0.3049965 0.413 0.184 1.086948e-14 19
GLRX5 LZ
8.871522e-19 0.4039683 0.316 0.124 1.581704e-14 19
MSI2 LZ
1.638075e-18 0.3078229 0.401 0.179 2.920525e-14 19
TRABD LZ
1.787105e-18 -0.7664039 0.531 0.577 3.18623e-14 19
SARAF LZ
3.410366e-18 -0.5333399 0.045 0.151 6.080342e-14 19
CELF2 LZ
3.790701e-18 -0.9132801 0.309 0.428 6.758441e-14 19
PLP2 LZ
3.795899e-18 0.3171193 0.529 0.267 6.767709e-14 19
TXNL4A LZ
4.263995e-18 0.3617481 0.309 0.104 7.602277e-14 19
E2F5 LZ
5.29838e-18 -0.3537493 0.906 0.905 9.446482e-14 19
HLA-A LZ
6.111454e-18 -0.5054195 0.123 0.192 1.089611e-13 19
S1PR4 LZ
6.177852e-18 0.3219082 0.376 0.169 1.101449e-13 19
CBWD1 LZ
7.58614e-18 0.3111063 0.343 0.132 1.352533e-13 19

	MPST	LZ			
8.128956e-18	0.5148242	0.757	0.493	1.449312e-13	19
	SAT1	LZ			
9.133845e-18	0.3160808	0.248	0.082	1.628473e-13	19
	TMEM131L	LZ			
3.761185e-17	0.3117988	0.58	0.326	6.705817e-13	19
	TSPO	LZ			
3.969733e-17	0.3878379	0.586	0.269	7.077636e-13	19
	NCF1	LZ			
5.862826e-17	0.3558593	0.538	0.23	1.045283e-12	19
	UBE2J1	LZ			
8.827658e-17	0.3002233	0.359	0.154	1.573883e-12	19
	SMIM20	LZ			
1.936242e-16	-0.6475981	0.952	0.935	3.452127e-12	19
	BTG1	LZ			
2.009858e-16	0.3035514	0.406	0.193	3.583377e-12	19
	UBE2E1	LZ			
4.023855e-16	0.3145907	0.306	0.123	7.174132e-12	19
	COMMD4	LZ			
5.629466e-16	0.3117542	0.26	0.098	1.003678e-11	19
	PTPN7	LZ			
6.846649e-16	-0.7499264	0.238	0.317	1.220689e-11	19
	CD69	LZ			
6.995232e-16	0.3069236	0.252	0.097	1.24718e-11	19
	FAM241A	LZ			
9.078361e-16	0.3120705	0.473	0.214	1.618581e-11	19
	NANS	LZ			
1.124055e-15	0.3512313	0.344	0.127	2.004078e-11	19
	STIM2	LZ			
1.87258e-15	-0.4216255	1	0.998	3.338622e-11	19
	RPL11	LZ			
2.098733e-15	-0.4149361	1	0.998	3.741831e-11	19
	RPLP2	LZ			
3.035786e-15	-0.5565759	0.989	0.968	5.412503e-11	19
	MT-ND3	LZ			
3.057233e-15	-0.6018322	0.285	0.363	5.45074e-11	19
	ANXA6	LZ			
3.929976e-15	0.3156576	0.33	0.154	7.006753e-11	19
	DNAJB11	LZ			
7.28006e-15	-0.3638112	1	1	1.297962e-10	19
	RPS27	LZ			
8.354998e-15	-0.5102236	0.124	0.175	1.489613e-10	19
	MARCH1	LZ			
8.966375e-15	-0.7445365	0.341	0.415	1.598615e-10	19
	NPC2	LZ			
9.670495e-15	0.3537501	0.997	0.989	1.724152e-10	19
	CD74	LZ			
2.256297e-14	-0.5724102	0.357	0.389	4.022751e-10	19
	EVI2B	LZ			
3.25588e-14	0.4475982	0.368	0.155	5.804908e-10	19

KIAA0040	LZ				
6.287747e-14	0.3228767	0.41	0.224	1.121042e-09	19
ADI1	LZ				
7.534256e-14	-0.3654876	0.993	0.985	1.343283e-09	19
RPL27	LZ				
9.262905e-14	-0.8713109	0.404	0.457	1.651483e-09	19
JUNB	LZ				
1.221657e-13	-0.4807261	0.083	0.146	2.178092e-09	19
SUN2	LZ				
1.306323e-13	-0.5844189	0.656	0.615	2.329043e-09	19
MEF2C	LZ				
2.134054e-13	-0.4471949	0.102	0.159	3.804804e-09	19
GLIPR1	LZ				
3.308443e-13	0.3165177	0.173	0.045	5.898623e-09	19
COL9A3	LZ				
4.108178e-13	-0.3119527	1	0.999	7.324471e-09	19
RPL13A	LZ				
4.891651e-13	0.3873215	0.39	0.157	8.721324e-09	19
SUSD3	LZ				
1.377451e-12	-0.6452014	0.572	0.57	2.455857e-08	19
SNX2	LZ				
1.661054e-12	-0.5711487	0.206	0.238	2.961494e-08	19
CD55	LZ				
2.07411e-12	-0.6598939	0.221	0.279	3.697931e-08	19
ADAM28	LZ				
2.9104e-12	-0.8024294	0.621	0.606	5.188952e-08	19
IGHD	LZ				
5.072653e-12	-0.4217921	0.199	0.211	9.044032e-08	19
FAM111B	LZ				
5.749643e-12	-0.5381439	0.157	0.23	1.025104e-07	19
NT5C3A	LZ				
8.56936e-12	0.3165067	0.175	0.061	1.527831e-07	19
HOPX	LZ				
9.592305e-12	-0.9509283	0.71	0.665	1.710212e-07	19
TUBA1B	LZ				
1.165734e-11	0.4120947	0.372	0.15	2.078387e-07	19
CD81	LZ				
1.653577e-11	-0.3902039	0.063	0.11	2.948162e-07	19
LINC02397	LZ				
2.269988e-11	-0.7104377	0.418	0.446	4.047162e-07	19
KLF6	LZ				
2.801979e-11	-0.3617908	0.997	0.995	4.995648e-07	19
RPL31	LZ				
3.64071e-11	-0.3834357	0.039	0.101	6.491022e-07	19
RHOC	LZ				
3.856923e-11	-0.406946	0.05	0.119	6.876508e-07	19
FCGR2B	LZ				
4.122625e-11	-0.5214203	0.273	0.285	7.350228e-07	19
PPM1K	LZ				
9.00318e-11	-0.4745205	0.143	0.194	1.605177e-06	19

HSPB1	LZ				
9.717025e-11	-0.5383987	0.183	0.212	1.732448e-06	19
LBH	LZ				
1.404239e-10	-0.3470347	1	0.997	2.503618e-06	19
RPS3A	LZ				
1.898878e-10	-0.3403278	0.999	0.996	3.385509e-06	19
RPL35A	LZ				
3.569024e-10	-0.9321112	0.137	0.207	6.363212e-06	19
LGALS1	LZ				
4.872026e-10	-0.3663875	1	0.999	8.686335e-06	19
RPL39	LZ				
5.237668e-10	-0.5284919	0.366	0.389	9.338238e-06	19
ARL6IP5	LZ				
9.648983e-10	-0.6109617	0.483	0.503	1.720317e-05	19
TSC22D3	LZ				
1.169252e-09	-0.456788	0.257	0.267	2.08466e-05	19
HHEX	LZ				
1.254486e-09	-0.6389277	0.154	0.239	2.236623e-05	19
CAPG	LZ				
1.734722e-09	-0.3706171	0.066	0.113	3.092836e-05	19
P2RY10	LZ				
3.784988e-09	-0.4441411	0.357	0.331	6.748254e-05	19
RIPOR2	LZ				
3.793079e-09	-0.5104654	0.252	0.279	6.76268e-05	19
IFITM2	LZ				
4.354742e-09	0.3133402	0.765	0.484	7.764069e-05	19
LTB	LZ				
5.069733e-09	-0.5762064	0.986	0.963	9.038826e-05	19
FTL	LZ				
6.654976e-09	-0.3712298	0.067	0.106	0.0001186516	19
C1orf162	LZ				
1.022189e-08	-0.3098025	1	0.999	0.0001822461	19
RPL13	LZ				
1.38144e-08	-0.4074838	0.175	0.204	0.0002462969	19
IRF1	LZ				
2.82811e-08	-0.3893036	0.124	0.143	0.0005042237	19
DUSP1	LZ				
3.177458e-08	-0.5264503	0.197	0.249	0.0005665089	19
TNFRSF13B		LZ			
3.655336e-08	-0.360661	0.101	0.149	0.0006517099	19
SVIP	LZ				
4.205596e-08	-0.4055767	0.303	0.318	0.0007498157	19
FCRLA	LZ				
5.878193e-08	-0.4471415	0.229	0.246	0.001048023	19
CKS1B	LZ				
6.44329e-08	-0.34494	0.092	0.151	0.001148774	19
CYB5A	LZ				
6.797073e-08	-0.3728256	0.057	0.106	0.00121185	19
MTSS1	LZ				
9.050589e-08	-0.8819483	0.25	0.264	0.001613629	19

S100A4	LZ	9.720699e-08	-0.6329884	0.129	0.198	0.001733103	19
ACP5	LZ	1.252761e-07	-0.3850664	0.071	0.123	0.002233548	19
DDAH2	LZ	1.2819e-07	-0.3951328	0.164	0.205	0.002285499	19
AP1S2	LZ	1.282865e-07	-0.4423333	0.113	0.171	0.002287221	19
PARP14	LZ	1.342418e-07	-0.4175398	0.108	0.148	0.002393397	19
ARRDC2	LZ	4.183808e-07	-0.3004403	0.092	0.113	0.007459311	19
PBXIP1	LZ	5.933114e-07	-0.5036502	0.396	0.387	0.01057815	19
PSAP	LZ	8.010041e-07	-0.4642913	0.677	0.619	0.0142811	19
IFI16	LZ	1.088456e-06	-0.5222841	0.2	0.209	0.01940608	19
FOS	LZ	1.396183e-06	-0.399047	1	0.996	0.02489255	19
RPS12	LZ	1.756267e-06	-0.4604295	0.259	0.275	0.03131248	19
TNRC6B	LZ	2.172396e-06	-0.3206708	0.213	0.214	0.03873165	19
YPEL3	LZ	2.264953e-06	0.3166142	0.183	0.047	0.04038185	19
XIST	LZ	2.922395e-06	-0.3034409	1	0.995	0.05210339	19
RPS25	LZ	3.00575e-06	-0.4722756	0.322	0.33	0.05358952	19
SMC6	LZ	3.284863e-06	-0.3564016	0.999	0.991	0.05856582	19
RPL30	LZ	3.637705e-06	-0.3864636	0.166	0.187	0.06485664	19
KIF20B	LZ	5.529801e-06	-0.3738928	0.999	0.991	0.09859082	19
RPL9	LZ	6.068801e-06	-0.3279323	0.074	0.124	0.1082006	19
RAB13	LZ	8.23473e-06	-0.491475	0.442	0.284	0.146817	19
MIR155HG	LZ	9.092184e-06	-1.124879	0.432	0.3	0.1621045	19
HMGB2	LZ	1.112436e-05	-0.3985951	0.994	0.982	0.1983363	19
RPS21	LZ	1.526817e-05	-0.4869048	0.351	0.302	0.2722162	19
ZFP36L2	LZ	1.780203e-05	-0.5613083	0.292	0.346	0.3173925	19
CAST	LZ	1.801917e-05	-0.4061041	0.639	0.598	0.3212638	19

CALM1 LZ
1.888554e-05 -0.3022114 0.129 0.151 0.3367103 19
TMEM19 LZ
2.019879e-05 -0.3883508 0.234 0.232 0.3601242 19
HSD17B11 LZ
2.321905e-05 -0.3469651 0.129 0.165 0.4139725 19
TES LZ
2.851079e-05 -0.3048344 0.112 0.134 0.5083189 19
ODF2L LZ
5.520905e-05 -0.4375435 0.109 0.158 0.9843222 19
SLC2A3 LZ
7.653792e-05 -0.3015876 0.565 0.652 1 19
RPS4Y1 LZ
8.792113e-05 -0.3041587 0.145 0.16 1 19
AIDA LZ
9.898438e-05 -0.3769399 0.299 0.284 1 19
ACAP1 LZ
0.0001325541 -0.5983169 0.361 0.36 1 19
ARID5B LZ
0.0001814914 -0.3250525 0.429 0.208 1 19
PLCG2 LZ
0.0001923842 -0.3126822 0.397 0.322 1 19 AES
LZ
0.0002171631 -0.3143786 0.087 0.112 1 19
C1orf56 LZ
0.0002552327 -0.4562175 0.548 0.493 1 19
SP100 LZ
0.0002597377 -0.3238884 0.197 0.218 1 19 EBP
LZ
0.0003056432 -0.4236506 0.401 0.401 1 19
CMPK1 LZ
0.0003442273 -0.4135524 0.348 0.337 1 19
HVCN1 LZ
0.0004552031 -0.3293556 0.197 0.199 1 19
CLK1 LZ
0.001379637 -0.6116434 0.684 0.399 1 19
JCHAIN LZ
0.001464869 -0.3364875 0.989 0.965 1 19
RPL38 LZ
0.001519217 -0.3041963 0.215 0.205 1 19
RALGPS2 LZ
0.001606203 -0.5882269 0.694 0.418 1 19
HIST1H4C LZ
0.002377423 -0.6242795 0.666 0.575 1 19
TUBB LZ
0.002541151 -0.3798787 0.966 0.916 1 19
PFDN5 LZ
0.002915007 -0.4596885 0.55 0.491 1 19
ANKRD12 LZ
0.003061051 -1.708747 0.344 0.241 1 19

IGHG4 LZ
0.003187934 -0.4147901 0.478 0.437 1 19
IL2RG LZ
0.003282974 -0.4183436 0.18 0.198 1 19
YBX3 LZ
0.00348674 -0.3207047 0.183 0.189 1 19
IFNGR1 LZ
0.003728104 -0.5999926 0.979 0.926 1 19
IGKC LZ
0.003802331 -0.4640587 0.122 0.161 1 19
DDIT4 LZ
0.00406321 -0.3035723 0.478 0.406 1 19
LSM10 LZ
0.004585779 -0.3444653 0.403 0.383 1 19
LAPTM4A LZ
0.004810422 -0.4760941 0.985 0.977 1 19
RPS10 LZ
0.005439567 -0.3186968 0.529 0.452 1 19
TMEM59 LZ
0.006063952 -0.3326056 0.364 0.319 1 19
LTA4H LZ
0.007214543 -0.4471186 0.098 0.159 1 19
LMNA LZ
1.261577e-273 1.859697 0.729 0.189 2.249266e-269 20
ACP5 B LY9
7.447929e-195 1.678745 0.523 0.136 1.327891e-190 20 LY9
B LY9
2.477637e-142 0.5446066 1 1 4.41738e-138 20
MALAT1 B LY9
2.689054e-123 0.7192384 0.999 0.963 4.794314e-119 20
FTL B LY9
2.219532e-99 1.093991 0.687 0.409 3.957204e-95 20
NPC2 B LY9
5.801046e-88 1.494165 0.31 0.092 1.034268e-83 20
KLK1 B LY9
5.483541e-75 0.330964 1 0.999 9.776606e-71 20
RPL34 B LY9
6.894677e-74 0.3075007 1 1 1.229252e-69 20
RPS27 B LY9
1.855569e-73 0.6677346 0.926 0.748 3.308295e-69 20
CXCR4 B LY9
1.6489e-66 0.9877323 0.384 0.171 2.939824e-62 20
ASAH1 B LY9
5.347113e-59 -1.098496 0.329 0.603 9.533368e-55 20
PRDX1 B LY9
3.078381e-51 -0.8285904 0.078 0.333 5.488446e-47 20
RFTN1 B LY9
4.163495e-48 -0.9390686 0.075 0.303 7.423095e-44 20
NME1 B LY9
6.278082e-48 0.7712172 0.207 0.075 1.119319e-43 20

SLAMF7	B LY9				
6.180154e-47	-1.174217	0.164	0.408	1.10186e-42	20
FABP5	B LY9				
1.229666e-46	-1.444229	0.084	0.29	2.192371e-42	20
MIR155HG	B LY9				
2.542251e-44	-1.292274	0.027	0.209	4.532578e-40	20
RGS13	B LY9				
2.7942e-42	-0.7827551	0.275	0.533	4.98178e-38	20
PSME2	B LY9				
8.269025e-42	-0.8468889	0.154	0.402	1.474284e-37	20
BASP1	B LY9				
4.097025e-41	-1.035305	0.442	0.659	7.304586e-37	20
TCL1A	B LY9				
8.830935e-40	0.7595441	0.637	0.482	1.574467e-35	20
NEAT1	B LY9				
5.042591e-39	-0.6786521	0.397	0.638	8.990436e-35	20
PSMA4	B LY9				
1.722537e-38	-0.6188478	0.734	0.872	3.071111e-34	20
ARPC2	B LY9				
2.298048e-38	0.7065949	0.544	0.348	4.097189e-34	20
LY6E	B LY9				
2.804485e-38	-0.6799448	0.154	0.397	5.000116e-34	20
YWHAE	B LY9				
2.941941e-37	0.5767417	0.154	0.044	5.245187e-33	20
RENBP	B LY9				
7.089804e-37	-0.7271425	0.01	0.151	1.264041e-32	20
BIK	B LY9				
6.415442e-36	-0.8758331	0.548	0.718	1.143809e-31	20
ENO1	B LY9				
1.667261e-35	0.6548119	0.221	0.08	2.97256e-31	20
FGR	B LY9				
2.229886e-35	-0.7369821	0.037	0.205	3.975664e-31	20
HMCES	B LY9				
4.438438e-34	-0.6304953	0.057	0.237	7.913292e-30	20
MTHFD2	B LY9				
3.335329e-33	-0.7216358	0.558	0.739	5.946558e-29	20
HSPA8	B LY9				
3.451877e-33	-0.6584909	0.246	0.478	6.154351e-29	20
SNRPD1	B LY9				
6.667764e-33	-0.636755	0.169	0.388	1.188796e-28	20
NHP2	B LY9				
7.267534e-33	-0.610291	0.388	0.611	1.295729e-28	20
POMP	B LY9				
7.616224e-33	-0.5618948	0.422	0.649	1.357897e-28	20
SNRPG	B LY9				
8.989118e-33	-0.5627401	0.582	0.762	1.60267e-28	20
PSMA7	B LY9				
1.098054e-32	-0.7232561	0.618	0.763	1.95772e-28	20
PKM	B LY9				
1.472012e-32	-0.7269879	0.123	0.334	2.62445e-28	20

GRHPR B LY9
1.521115e-32 -0.5726528 0.387 0.612 2.711996e-28 20
POLR2L B LY9
2.134753e-32 -0.9476439 0.159 0.395 3.806051e-28 20
EIF5A B LY9
6.697906e-32 -0.5934861 0.377 0.601 1.19417e-27 20
SEC61B B LY9
1.199209e-31 -0.5824824 0.358 0.588 2.138069e-27 20
ATP5MC3 B LY9
6.90442e-31 -0.5522816 0.344 0.576 1.230989e-26 20
PARK7 B LY9
7.55208e-31 -0.6993669 0.059 0.237 1.34646e-26 20
UBE2J1 B LY9
1.43504e-30 -0.7616259 0.136 0.343 2.558533e-26 20
LRMP B LY9
2.487626e-30 0.4186767 0.873 0.773 4.435189e-26 20
HLA-DPB1 B LY9
3.060378e-30 -0.670954 0.283 0.501 5.456349e-26 20
HSPE1 B LY9
3.373328e-30 -0.6896322 0.249 0.465 6.014306e-26 20
HSPD1 B LY9
6.285215e-30 -1.833473 0.217 0.406 1.120591e-25 20
JCHAIN B LY9
6.341626e-30 -0.6462273 0.586 0.751 1.130648e-25 20
RAN B LY9
2.953866e-29 -0.6237004 0.186 0.405 5.266447e-25 20
RPL22L1 B LY9
4.328315e-29 -0.4720914 0.01 0.133 7.716952e-25 20
PHGDH B LY9
4.365865e-28 -0.358141 0.003 0.104 7.7839e-24 20
SEMA4A B LY9
5.614794e-28 0.6055271 0.602 0.476 1.001062e-23 20
SQSTM1 B LY9
9.134774e-28 -0.5536668 0.101 0.292 1.628639e-23 20
RRAS2 B LY9
9.379212e-28 0.6677422 0.38 0.264 1.67222e-23 20
GRN B LY9
1.157527e-27 0.7291276 0.412 0.266 2.063754e-23 20
GPR183 B LY9
1.837867e-27 -0.4864339 0.008 0.12 3.276733e-23 20
CD38 B LY9
2.596117e-27 -0.68616 0.619 0.77 4.628616e-23 20
HMGN1 B LY9
2.597163e-27 -0.510051 0.588 0.746 4.630482e-23 20
CHCHD2 B LY9
3.196755e-27 -0.5442069 0.027 0.163 5.699495e-23 20
SUSD3 B LY9
3.828966e-27 -0.5721616 0.507 0.682 6.826664e-23 20
SRSF3 B LY9
4.135304e-27 -0.5874939 0.395 0.608 7.372834e-23 20

DYNLL1	B LY9					
5.328584e-27	-0.5548447	0.016	0.136	9.500333e-23	20	
NEIL1	B LY9					
6.669601e-27	-0.7021894	0.025	0.154	1.189123e-22	20	
DUSP2	B LY9					
8.956226e-27	0.6549036	0.584	0.461	1.596806e-22	20	
CD63	B LY9					
1.464436e-26	-0.6189611	0.108	0.283	2.610944e-22	20	
SRM	B LY9					
1.990229e-26	-0.4766682	0.525	0.71	3.548379e-22	20	
NDUFA4	B LY9					
2.452467e-26	-0.5099037	0.353	0.562	4.372503e-22	20	
ATP5PF	B LY9					
2.855916e-26	-0.4578183	0.351	0.57	5.091812e-22	20	
UQCRQ	B LY9					
2.892041e-26	-0.5209074	0.205	0.432	5.156219e-22	20	
DNAJA1	B LY9					
3.323112e-26	-0.5948297	0.846	0.9	5.924776e-22	20	
PFN1	B LY9					
4.410739e-26	-0.461474	0.299	0.527	7.863906e-22	20	
FKBP1A	B LY9					
7.711485e-26	-0.4637256	0.438	0.642	1.374881e-21	20	
ATP5MD	B LY9					
8.031805e-26	0.4542216	0.865	0.815	1.431991e-21	20	
NOP53	B LY9					
1.039417e-25	-0.5008928	0.129	0.326	1.853177e-21	20	
IMP4	B LY9					
1.225929e-25	-0.9597405	0.047	0.184	2.185709e-21	20	
XBP1	B LY9					
1.532747e-25	-0.4522976	0.018	0.134	2.732735e-21	20	
CYTOR	B LY9					
2.496698e-25	-0.4012189	0.013	0.116	4.451363e-21	20	
PYCR1	B LY9					
2.504726e-25	-0.5262914	0.452	0.643	4.465675e-21	20	
ERH	B LY9					
3.371293e-25	-0.9312821	0.52	0.668	6.010679e-21	20	
TUBA1B	B LY9					
5.424229e-25	0.639324	0.618	0.533	9.670859e-21	20	
ATP6V1F	B LY9					
6.113482e-25	-0.4242122	0.516	0.703	1.089973e-20	20	
ELOB	B LY9					
6.789748e-25	-0.5223656	0.387	0.582	1.210544e-20	20	
LDHB	B LY9					
8.336461e-25	-0.437472	0.285	0.501	1.486308e-20	20	
COX5A	B LY9					
1.021887e-24	-0.329166	0.006	0.101	1.821922e-20	20	
FAM241A	B LY9					
1.168109e-24	0.3027987	0.936	0.867	2.082621e-20	20	
CD52	B LY9					
2.258844e-24	-0.4673957	0.441	0.631	4.027293e-20	20	

	ATP5F1B	B LY9			
2.554109e-24	-0.5372211	0.385	0.574	4.55372e-20	20
	SNRPE	B LY9			
2.625892e-24	-0.5295867	0.218	0.429	4.681703e-20	20
	CD40	B LY9			
4.153934e-24	-0.5582219	0.103	0.287	7.406049e-20	20
	CD82	B LY9			
4.271551e-24	-0.5853853	0.055	0.193	7.615749e-20	20
	DAAM1	B LY9			
5.514186e-24	-0.4547046	0.198	0.408	9.831242e-20	20
	PSMB2	B LY9			
5.896677e-24	-0.4934589	0.244	0.445	1.051319e-19	20
	PPA1	B LY9			
7.048934e-24	-0.4949418	0.242	0.452	1.256755e-19	20
	SEM1	B LY9			
8.097626e-24	-0.5123711	0.195	0.392	1.443726e-19	20
	CCT5	B LY9			
8.938428e-24	-0.4533665	0.212	0.423	1.593632e-19	20
	TOMM22	B LY9			
9.961032e-24	-0.4722739	0.456	0.643	1.775952e-19	20
	ATP5MF	B LY9			
1.123609e-23	-0.4066467	0.831	0.906	2.003283e-19	20
	ATP5F1E	B LY9			
1.838096e-23	-0.5897841	0.3	0.488	3.277141e-19	20
	RANBP1	B LY9			
1.853517e-23	-0.4223092	0.181	0.389	3.304635e-19	20
	SNRPC	B LY9			
2.227797e-23	-0.5205595	0.195	0.395	3.97194e-19	20
	FNBP1	B LY9			
3.608536e-23	-0.5738551	0.61	0.75	6.433659e-19	20
	HSP90AA1	B LY9			
4.09034e-23	-0.5717072	0.982	0.99	7.292668e-19	20
	ACTB	B LY9			
4.308339e-23	-0.4848298	0.55	0.706	7.681338e-19	20
	TPI1	B LY9			
4.85581e-23	-0.4616663	0.227	0.434	8.657424e-19	20
	SLIRP	B LY9			
6.373385e-23	-0.5244021	0.275	0.471	1.136311e-18	20
	CYCS	B LY9			
6.833969e-23	-0.7421204	0.856	0.888	1.218428e-18	20
	GAPDH	B LY9			
7.025725e-23	-0.5748763	0.373	0.562	1.252616e-18	20
	NCL	B LY9			
8.795752e-23	-0.5369903	0.363	0.555	1.568195e-18	20
	TCEA1	B LY9			
1.149895e-22	-0.3837966	0.033	0.17	2.050149e-18	20
	CCDC167	B LY9			
1.2291e-22	-0.4973384	0.238	0.438	2.191362e-18	20
	EIF5B	B LY9			
1.348911e-22	-0.5689818	0.763	0.853	2.404973e-18	20

	ATP5MG	B LY9				
1.360671e-22	-0.7258669		0.411	0.579	2.425941e-18	20
	TUBB	B LY9				
1.462309e-22	-0.3595251		0.018	0.127	2.60715e-18	20
	SLC1A5	B LY9				
1.574208e-22	-0.3728742		0.008	0.102	2.806655e-18	20
	MYBL2	B LY9				
1.671741e-22	-0.4190186		0.106	0.284	2.980546e-18	20
	PSMD14	B LY9				
2.275475e-22	-0.4005613		0.084	0.251	4.056945e-18	20
	MRPL13	B LY9				
2.308374e-22	-0.5152027		0.133	0.306	4.115599e-18	20
	ODC1	B LY9				
2.514592e-22	-0.427216		0.237	0.449	4.483266e-18	20
	ATP5F1C	B LY9				
2.780523e-22	-0.4166502		0.445	0.635	4.957395e-18	20
	COX7B	B LY9				
2.822439e-22	-0.4236964		0.508	0.688	5.032127e-18	20
	COX6A1	B LY9				
2.826256e-22	-0.4861666		0.258	0.46	5.038932e-18	20
	COX17	B LY9				
3.259923e-22	-0.4815942		0.102	0.274	5.812116e-18	20
	SMS	B LY9				
4.674628e-22	0.5930158		0.21	0.12	8.334395e-18	20
	ZNF318	B LY9				
8.894963e-22	-0.4349576		0.373	0.574	1.585883e-17	20
	RBM8A	B LY9				
9.524732e-22	-0.4206043		0.3	0.506	1.698164e-17	20
	XRCC5	B LY9				
1.078502e-21	-0.3944682		0.467	0.66	1.922861e-17	20
	RBX1	B LY9				
1.234692e-21	-0.4658426		0.255	0.452	2.201333e-17	20
	ILF2	B LY9				
1.404997e-21	-0.4100592		0.313	0.517	2.504968e-17	20
	SSBP1	B LY9				
1.452312e-21	-0.4767781		0.268	0.466	2.589327e-17	20
	PRMT1	B LY9				
1.512426e-21	-0.6250999		0.241	0.411	2.696505e-17	20
	TXN	B LY9				
1.563378e-21	-0.4141066		0.317	0.526	2.787348e-17	20
	SELENOT	B LY9				
1.686981e-21	-0.4394187		0.28	0.479	3.007718e-17	20
	PRELID1	B LY9				
1.942242e-21	-0.4130782		0.125	0.306	3.462823e-17	20
	GLRX3	B LY9				
2.738133e-21	0.5287328		0.605	0.492	4.881817e-17	20
	FXYD5	B LY9				
2.901828e-21	-0.4977557		0.49	0.66	5.17367e-17	20
	SLC25A5	B LY9				
3.004501e-21	-0.4800101		0.367	0.557	5.356724e-17	20

SEC61G	B LY9				
3.061434e-21	-0.4494854	0.428	0.608	5.458231e-17	20
RBM3	B LY9				
3.541742e-21	0.5560411	0.45	0.335	6.314573e-17	20
CD48	B LY9				
3.841122e-21	-0.5628205	0.101	0.271	6.848336e-17	20
PIM1	B LY9				
3.958811e-21	-0.4381588	0.258	0.46	7.058163e-17	20
SFPQ	B LY9				
4.73157e-21	-0.3978217	0.475	0.656	8.435917e-17	20
UBE2D3	B LY9				
5.163126e-21	-0.4269146	0.195	0.391	9.205337e-17	20
NDUFS6	B LY9				
6.483158e-21	-0.4030733	0.076	0.228	1.155882e-16	20
DCAF13	B LY9				
7.126798e-21	-0.3819539	0.316	0.522	1.270637e-16	20
PSMB6	B LY9				
7.477696e-21	-0.7211371	0.058	0.192	1.333198e-16	20
PTTG1	B LY9				
9.52594e-21	-0.4157918	0.186	0.377	1.69838e-16	20
PSMC3	B LY9				
9.868031e-21	-0.4692256	0.244	0.431	1.759371e-16	20
CCT2	B LY9				
1.316239e-20	-0.530367	0.235	0.42	2.346723e-16	20
METAP2	B LY9				
1.330554e-20	-0.4119398	0.237	0.438	2.372245e-16	20
ROMO1	B LY9				
1.458084e-20	-0.3971404	0.101	0.273	2.599618e-16	20
TOP1	B LY9				
1.654876e-20	-0.4722132	0.351	0.538	2.950478e-16	20
SRSF9	B LY9				
1.893516e-20	-0.3826888	0.154	0.341	3.37595e-16	20
HSD17B10	B LY9				
1.89481e-20	-0.3923329	0.353	0.553	3.378257e-16	20
PSMA1	B LY9				
1.899035e-20	-0.4550218	0.101	0.265	3.385789e-16	20
ANP32E	B LY9				
2.292794e-20	-0.4173832	0.865	0.92	4.087823e-16	20
CFL1	B LY9				
2.613145e-20	-0.4352941	0.221	0.414	4.658976e-16	20
HNRNPR	B LY9				
3.391591e-20	-0.3510425	0.52	0.686	6.046868e-16	20
SLC25A3	B LY9				
3.413061e-20	-0.539004	0.343	0.515	6.085147e-16	20
PA2G4	B LY9				
3.437024e-20	-0.415063	0.208	0.402	6.12787e-16	20
PSMA3	B LY9				
3.481377e-20	-0.4011365	0.16	0.349	6.206947e-16	20
LSM6	B LY9				
3.845393e-20	-0.4011993	0.14	0.326	6.855952e-16	20

HSBP1 B LY9
4.1727e-20 0.5565495 0.269 0.178 7.439507e-16 20
HEXA B LY9
4.718156e-20 -0.3966736 0.034 0.144 8.412001e-16 20
NOP16 B LY9
5.267777e-20 -0.3812546 0.205 0.405 9.39192e-16 20
UQCRFS1 B LY9
5.41417e-20 -0.4015553 0.058 0.185 9.652924e-16 20
TUBA1C B LY9
5.465125e-20 0.5629334 0.388 0.277 9.743771e-16 20
IFITM2 B LY9
7.005942e-20 -0.4103018 0.047 0.184 1.249089e-15 20
RASGRP3 B LY9
8.126283e-20 -0.3790577 0.071 0.22 1.448835e-15 20
MRPL12 B LY9
8.145171e-20 -0.3776567 0.201 0.398 1.452202e-15 20
NDUFAB1 B LY9
8.708145e-20 -0.4090243 0.059 0.206 1.552575e-15 20
UBALD2 B LY9
9.587514e-20 -0.3528091 0.035 0.156 1.709358e-15 20
PAICS B LY9
9.950321e-20 -0.3814833 0.407 0.597 1.774043e-15 20
SRSF2 B LY9
1.049895e-19 -0.419565 0.232 0.423 1.871858e-15 20
EIF6 B LY9
1.204069e-19 -0.4221635 0.585 0.725 2.146734e-15 20
COX6C B LY9
1.240623e-19 -0.4218482 0.256 0.445 2.211907e-15 20
CCT6A B LY9
1.393757e-19 -0.3775126 0.156 0.343 2.48493e-15 20
STRAP B LY9
1.452219e-19 -0.3863834 0.232 0.43 2.589161e-15 20
MINOS1 B LY9
1.505223e-19 -0.5163509 0.435 0.599 2.683662e-15 20
LDHA B LY9
1.726438e-19 -0.389725 0.129 0.309 3.078066e-15 20
TIMM8B B LY9
1.924092e-19 -0.4762909 0.065 0.209 3.430463e-15 20
EAF2 B LY9
2.190309e-19 -0.5452688 0.16 0.32 3.905102e-15 20
DDX21 B LY9
2.197324e-19 0.5095459 0.208 0.113 3.917609e-15 20
CLN8 B LY9
2.257675e-19 -1.29039 0.054 0.177 4.025208e-15 20
MZB1 B LY9
2.300481e-19 -0.399938 0.314 0.507 4.101527e-15 20
EIF3I B LY9
2.5916e-19 -0.4473365 0.269 0.455 4.620563e-15 20
MRPL51 B LY9
2.705365e-19 -0.4449761 0.262 0.442 4.823394e-15 20

HMGA1 B LY9
3.186883e-19 -0.4347837 0.079 0.226 5.681894e-15 20
SIAH2 B LY9
3.70624e-19 -0.4015531 0.21 0.406 6.607856e-15 20
BZW1 B LY9
3.941839e-19 0.3027902 0.986 0.968 7.027904e-15 20
MT-ND3 B LY9
5.686339e-19 -0.4182118 0.499 0.659 1.013817e-14 20
PSMB1 B LY9
6.864804e-19 -0.3559445 0.017 0.113 1.223926e-14 20
AC023590.1 B LY9
7.458487e-19 -0.4016898 0.297 0.488 1.329774e-14 20
UBE2L3 B LY9
7.966279e-19 -0.3640379 0.319 0.513 1.420308e-14 20
PSMD8 B LY9
8.946825e-19 -0.3469849 0.047 0.164 1.595129e-14 20
PPP1R14B B LY9
9.805771e-19 -0.448164 0.176 0.352 1.748271e-14 20
TIMM13 B LY9
1.00486e-18 -0.3603976 0.078 0.229 1.791565e-14 20
TIMM17A B LY9
1.012824e-18 -0.4432873 0.078 0.222 1.805764e-14 20
TRAF4 B LY9
1.017504e-18 -0.3866425 0.254 0.447 1.814108e-14 20
CCT7 B LY9
1.238152e-18 0.4149628 0.133 0.06 2.207502e-14 20
AMDHD2 B LY9
1.275474e-18 0.5042597 0.266 0.156 2.274043e-14 20
SLC2A3 B LY9
1.29226e-18 -0.3524876 0.093 0.254 2.30397e-14 20
MCTS1 B LY9
1.321332e-18 -0.4262055 0.446 0.619 2.355803e-14 20
SERBP1 B LY9
1.34094e-18 -0.3997114 0.435 0.607 2.390761e-14 20
PSMB3 B LY9
1.684117e-18 -1.11318 0.36 0.491 3.002612e-14 20 LTB
B LY9
1.789769e-18 -0.3593821 0.075 0.222 3.190979e-14 20
NUTF2 B LY9
2.560012e-18 -0.5527335 0.142 0.298 4.564245e-14 20
NFKBIA B LY9
2.602415e-18 -0.4358116 0.044 0.17 4.639845e-14 20
CCDC88A B LY9
3.181825e-18 -0.4816548 0.166 0.329 5.672876e-14 20
PMAIP1 B LY9
3.49233e-18 -0.4052859 0.273 0.455 6.226476e-14 20
VDAC1 B LY9
3.659836e-18 -0.3632211 0.31 0.501 6.525121e-14 20
TUFM B LY9
3.927691e-18 -0.4881183 0.418 0.619 7.00268e-14 20

MEF2C	B LY9	6.458274e-18	-0.4155403	0.034	0.138	1.151446e-13	20
PSAT1	B LY9	7.346987e-18	-0.3889304	0.099	0.248	1.309894e-13	20
AK2	B LY9	7.449378e-18	0.4043819	0.765	0.696	1.32815e-13	20
HLA-DQB1	B LY9	8.084793e-18	-0.3729987	0.106	0.267	1.441438e-13	20
SYNCRIP	B LY9	8.42479e-18	-0.495116	0.096	0.245	1.502056e-13	20
KPNA2	B LY9	9.355794e-18	-0.3191489	0.678	0.809	1.668045e-13	20
TMA7	B LY9	1.037365e-17	-0.3964117	0.484	0.647	1.849519e-13	20
HNRNPC	B LY9	1.095435e-17	-0.5022307	0.194	0.355	1.953052e-13	20
TUBB4B	B LY9	1.251033e-17	-0.4927635	0.933	0.942	2.230466e-13	20
YBX1	B LY9	1.287544e-17	-0.3310256	0.068	0.205	2.295563e-13	20
MRPL23	B LY9	1.384197e-17	-0.4090358	0.164	0.338	2.467884e-13	20
VOPP1	B LY9	1.810929e-17	-0.4344772	0.425	0.586	3.228706e-13	20
ANP32B	B LY9	1.827174e-17	-0.4306972	0.079	0.22	3.257669e-13	20
NANS	B LY9	1.979398e-17	-0.3600123	0.261	0.454	3.529069e-13	20
VCP	B LY9	1.987044e-17	-0.3368466	0.47	0.65	3.5427e-13	20
UQCR11	B LY9	2.127673e-17	-0.3415009	0.421	0.604	3.793427e-13	20
PSMA2	B LY9	2.273488e-17	-0.3470457	0.443	0.62	4.053401e-13	20
NDUFA13	B LY9	2.644177e-17	-0.4223523	0.35	0.532	4.714303e-13	20
SRSF7	B LY9	2.687399e-17	-0.3454316	0.065	0.198	4.791363e-13	20
GTPBP4	B LY9	2.821794e-17	-0.3351576	0.38	0.581	5.030977e-13	20
POU2F2	B LY9	2.899489e-17	-0.3730967	0.296	0.487	5.1695e-13	20
GTF3A	B LY9	2.972937e-17	-0.3576683	0.323	0.505	5.30045e-13	20
NDUFA11	B LY9	2.978307e-17	-0.3717464	0.027	0.125	5.310024e-13	20
PLEK	B LY9	3.082922e-17	0.5338661	0.462	0.385	5.496541e-13	20
PSAP	B LY9	3.294589e-17	-0.4043554	0.025	0.12	5.873923e-13	20

UBE2S	B LY9
3.84135e-17	-0.410547	0.143	0.304	6.848742e-13	20
NOP58	B LY9
4.671423e-17	-0.3684752	0.191	0.37	8.328679e-13	20
EWSR1	B LY9
5.319782e-17	-0.3571906	0.133	0.292	9.48464e-13	20
DCTPP1	B LY9
5.594366e-17	0.5139425	0.38	0.282	9.974195e-13	20
GNG7	B LY9
6.004885e-17	-0.332763	0.106	0.264	1.070611e-12	20
PSMC2	B LY9
6.439465e-17	-0.3886054	0.174	0.35	1.148092e-12	20
RBM17	B LY9
6.695696e-17	0.511768 0.61	0.521	1.193776e-12	20	ID3
B LY9
7.376434e-17	-0.3091686	0.565	0.719	1.315144e-12	20
COX6B1	B LY9
8.32479e-17	0.4572814	0.157	0.089	1.484227e-12	20
AC073111.5	B LY9
8.890866e-17	-0.3798142	0.265	0.459	1.585152e-12	20
CAPZA1	B LY9
1.014827e-16	-0.3583006	0.19	0.363	1.809335e-12	20
TKT	B LY9
1.102221e-16	-0.3437794	0.084	0.225	1.96515e-12	20
PSMB5	B LY9
1.130209e-16	-0.3774983	0.2	0.374	2.015049e-12	20
PSMD7	B LY9
1.463719e-16	-0.3524005	0.14	0.304	2.609665e-12	20
AHSA1	B LY9
1.587671e-16	0.4862101	0.174	0.089	2.830659e-12	20
CCR6	B LY9
1.725581e-16	0.4600266	0.246	0.146	3.076538e-12	20
ARRDC2	B LY9
1.73769e-16	0.4586332	0.697	0.634	3.098127e-12	20
GPX4	B LY9
1.988757e-16	-0.3653924	0.173	0.34	3.545755e-12	20
BUD31	B LY9
2.353969e-16	-0.3468634	0.249	0.43	4.196891e-12	20
CCT4	B LY9
2.747522e-16	-0.3416252	0.827	0.896	4.898556e-12	20
PPIA	B LY9
2.878711e-16	-0.441476	0.092	0.233	5.132453e-12	20
CKS2	B LY9
3.085624e-16	-0.3520855	0.071	0.2	5.501359e-12	20
EBNA1BP2 B LY9
3.098578e-16	-0.425534	0.373	0.543	5.524455e-12	20
PARP1	B LY9
3.337235e-16	-0.3203036	0.339	0.526	5.949957e-12	20
NDUFB8	B LY9
3.630692e-16	-0.3517776	0.153	0.316	6.473161e-12	20

	STOML2	B LY9			
3.825664e-16	-0.3336269	0.295	0.48	6.820777e-12	20
	UBE2N	B LY9			
3.877332e-16	-0.3478175	0.479	0.644	6.912895e-12	20
	HNRNPM	B LY9			
3.970543e-16	-0.3181936	0.018	0.113	7.07908e-12	20
	CPNE5	B LY9			
4.03176e-16	-0.3367772	0.176	0.352	7.188226e-12	20
	POLR2K	B LY9			
4.569986e-16	-0.3658899	0.18	0.349	8.147827e-12	20
	H2AFY	B LY9			
4.584839e-16	-0.3568867	0.159	0.322	8.17431e-12	20
	RHOG	B LY9			
5.446612e-16	-0.344106	0.414	0.588	9.710764e-12	20
	EIF2S2	B LY9			
6.470778e-16	-0.3455209	0.095	0.238	1.153675e-11	20
	BUD23	B LY9			
7.293946e-16	-0.3341286	0.266	0.447	1.300438e-11	20
	ATP5MC1	B LY9			
8.650801e-16	-0.4129229	0.205	0.364	1.542351e-11	20
	HSPA9	B LY9			
9.626559e-16	-0.3106577	0.055	0.178	1.716319e-11	20
	SSSCA1	B LY9			
9.667799e-16	-0.3664989	0.099	0.24	1.723672e-11	20
	HNRNPAB	B LY9			
1.148993e-15	-0.319175	0.187	0.356	2.048539e-11	20
	PHB2	B LY9			
1.20895e-15	-0.3300045	0.161	0.334	2.155437e-11	20
	COPZ1	B LY9			
1.212546e-15	-0.3527773	0.357	0.536	2.161848e-11	20
	SOD1	B LY9			
1.257582e-15	0.4363044	0.652	0.542	2.242143e-11	20
	CCR7	B LY9			
1.573692e-15	-0.3376037	0.225	0.403	2.805736e-11	20
	ENY2	B LY9			
1.576855e-15	0.3936635	0.122	0.057	2.811374e-11	20
	CRIP3	B LY9			
1.736916e-15	-0.3689719	0.232	0.395	3.096747e-11	20
	PHB	B LY9			
1.826973e-15	-0.3837432	0.211	0.377	3.257311e-11	20
	LCP1	B LY9			
1.837393e-15	-0.3401032	0.399	0.568	3.275888e-11	20
	SNRPB	B LY9			
2.026302e-15	-0.3112739	0.04	0.138	3.612694e-11	20
	IFRD2	B LY9			
2.041135e-15	-0.3273267	0.215	0.393	3.639139e-11	20
	ARPC4	B LY9			
2.225913e-15	-0.4438737	0.592	0.727	3.96858e-11	20
	LIMD2	B LY9			
2.264352e-15	-0.4281565	0.269	0.426	4.037114e-11	20

	DEK	B LY9			
4.323017e-15	-0.4644916	0.069	0.179	7.707506e-11	20
	BCL2A1	B LY9			
6.284236e-15	-0.312276	0.048	0.158	1.120417e-10	20
	GAR1	B LY9			
6.816724e-15	-0.3338058	0.344	0.51	1.215354e-10	20
	MZT2B	B LY9			
7.112225e-15	-0.3159764	0.276	0.452	1.268039e-10	20
	SF3B5	B LY9			
9.423822e-15	-0.3379894	0.072	0.19	1.680173e-10	20
	GPATCH4	B LY9			
1.062179e-14	-0.3582001	0.057	0.167	1.893758e-10	20
	DEF8	B LY9			
1.083937e-14	-0.3355354	0.067	0.184	1.932551e-10	20
	CMSS1	B LY9			
1.322011e-14	-0.4546398	0.024	0.11	2.357013e-10	20
	VPREB3	B LY9			
1.375806e-14	-0.317897	0.178	0.34	2.452924e-10	20
	MIF	B LY9			
1.711527e-14	-0.316057	0.127	0.275	3.051481e-10	20
	NAA10	B LY9			
1.793319e-14	-0.3340762	0.129	0.277	3.197309e-10	20
	NDUFAF8	B LY9			
1.969222e-14	-0.3299056	0.057	0.164	3.510926e-10	20
	CD58	B LY9			
2.025839e-14	-0.506245	0.194	0.351	3.611869e-10	20
	S100A10	B LY9			
2.119883e-14	-0.3208175	0.163	0.319	3.779539e-10	20
	TCP1	B LY9			
2.476283e-14	-0.4173074	0.7	0.807	4.414966e-10	20
	CALM2	B LY9			
2.720282e-14	-0.328515	0.106	0.244	4.84999e-10	20
	POLD2	B LY9			
2.822988e-14	-0.3399746	0.071	0.188	5.033105e-10	20
	WDR43	B LY9			
3.096098e-14	-0.5974097	0.722	0.787	5.520032e-10	20
	ACTG1	B LY9			
3.125031e-14	0.4688172	0.279	0.194	5.571618e-10	20
	CLEC2B	B LY9			
3.405355e-14	-0.432916	0.497	0.639	6.071408e-10	20
	SET	B LY9			
3.474641e-14	-0.410639	0.059	0.171	6.194938e-10	20
	SMC4	B LY9			
3.739041e-14	-0.308842	0.203	0.369	6.666336e-10	20
	PSMA5	B LY9			
4.199134e-14	-0.3064949	0.05	0.15	7.486637e-10	20
	TSEN15	B LY9			
5.158378e-14	-0.3751427	0.293	0.447	9.196872e-10	20
	C1QBP	B LY9			
5.434513e-14	-0.348455	0.255	0.422	9.689193e-10	20

HNRNPD	B LY9				
5.832001e-14	-0.3039554	0.252	0.421	1.039787e-09	20
NDUFA12	B LY9				
5.892175e-14	-0.3347265	0.071	0.186	1.050516e-09	20
FKBP4	B LY9				
5.949512e-14	-1.381992	0.194	0.304	1.060738e-09	20
HMGB2	B LY9				
6.039359e-14	-0.3466154	0.119	0.253	1.076757e-09	20
GARS	B LY9				
6.10712e-14	0.4393949	0.358	0.278	1.088838e-09	20
RNASET2	B LY9				
6.377971e-14	-0.3212218	0.176	0.335	1.137128e-09	20
EIF3A	B LY9				
6.779251e-14	-0.336178	0.079	0.212	1.208673e-09	20
EHD1	B LY9				
7.171833e-14	-0.3244067	0.065	0.174	1.278666e-09	20
ASNS	B LY9				
7.249767e-14	-0.301155	0.241	0.41	1.292561e-09	20
BANF1	B LY9				
7.354741e-14	-0.3626799	0.207	0.368	1.311277e-09	20
PDIA3	B LY9				
7.619709e-14	-0.3149271	0.091	0.225	1.358518e-09	20
HSPH1	B LY9				
8.116593e-14	-0.3138626	0.079	0.203	1.447107e-09	20
TOMM40	B LY9				
9.092499e-14	-0.3094269	0.22	0.39	1.621102e-09	20
MRPL20	B LY9				
9.672736e-14	-0.3930183	0.5	0.634	1.724552e-09	20
GSTP1	B LY9				
1.174818e-13	-0.3798936	0.164	0.312	2.094583e-09	20
CALM3	B LY9				
1.447873e-13	-0.3273499	0.059	0.166	2.581414e-09	20
NFKB1	B LY9				
1.567626e-13	-0.3769768	0.275	0.442	2.79492e-09	20
RHOH	B LY9				
1.627744e-13	-0.3011201	0.5	0.655	2.902105e-09	20
YWHAB	B LY9				
1.815771e-13	-0.3058092	0.293	0.459	3.237338e-09	20
LSM5	B LY9				
1.962293e-13	0.4875463	0.397	0.328	3.498571e-09	20
SMC6	B LY9				
2.324162e-13	0.4903542	0.29	0.209	4.143749e-09	20
MGST3	B LY9				
2.370692e-13	-0.4443421	0.898	0.934	4.226706e-09	20
SERF2	B LY9				
2.374761e-13	-0.3127719	0.286	0.448	4.233961e-09	20
PGAM1	B LY9				
2.515269e-13	-0.3148659	0.055	0.164	4.484473e-09	20
HM13	B LY9				
2.589215e-13	-0.4243077	0.171	0.321	4.616311e-09	20

	SEC11C	B LY9				
2.655601e-13		-0.3188642	0.047	0.142	4.734672e-09	20
	AIM2	B LY9				
2.765168e-13		-0.3123973	0.33	0.492	4.930017e-09	20
	CCT3	B LY9				
3.272608e-13		-0.304903	0.096	0.225	5.834733e-09	20
	RPIA	B LY9				
3.410981e-13		0.4025973	0.823	0.817	6.081437e-09	20
	SEPT7	B LY9				
3.707936e-13		-0.3145317	0.364	0.535	6.610879e-09	20
	SNRPB2	B LY9				
4.146692e-13		-0.3031515	0.095	0.227	7.393137e-09	20
	DAZAP1	B LY9				
4.6995e-13		0.3488024	0.596	0.511	8.378738e-09	20
	BANK1	B LY9				
4.700677e-13		0.3922215	0.531	0.454	8.380837e-09	20
	JUNB	B LY9				
4.880669e-13		-0.3590906	0.19	0.349	8.701745e-09	20
	ABRACL	B LY9				
5.425262e-13		-0.305249	0.261	0.43	9.672699e-09	20
	ANP32A	B LY9				
5.790198e-13		-0.361758	0.329	0.495	1.032334e-08	20
	C4orf3	B LY9				
5.851239e-13		0.4616224	0.239	0.169	1.043217e-08	20
	DDIT3	B LY9				
6.229421e-13		-0.4121465	0.256	0.41	1.110643e-08	20
	PPIB	B LY9				
7.18046e-13		0.4204217	0.156	0.098	1.280204e-08	20
	MX1	B LY9				
7.712552e-13		-0.463394	0.164	0.305	1.375071e-08	20
	BCL7A	B LY9				
8.019593e-13		0.4430858	0.419	0.336	1.429813e-08	20
	HVCN1	B LY9				
8.128927e-13		-0.3308091	0.069	0.182	1.449306e-08	20
	LCK	B LY9				
1.004817e-12		0.4123889	0.343	0.251	1.791488e-08	20
	FCMR	B LY9				
1.037801e-12		0.4261975	0.279	0.195	1.850296e-08	20
	CHPT1	B LY9				
1.178324e-12		-0.4291563	0.085	0.173	2.100834e-08	20
	TNFRSF18	B LY9				
1.351478e-12		-0.3029868	0.057	0.158	2.40955e-08	20
	NOLC1	B LY9				
1.494044e-12		0.3855923	0.17	0.108	2.663731e-08	20
	HEXB	B LY9				
1.665573e-12		-0.3640287	0.132	0.258	2.96955e-08	20
	SRGN	B LY9				
1.67025e-12		-0.3069093	0.118	0.255	2.977889e-08	20
	RGS10	B LY9				
2.148686e-12		0.3968611	0.204	0.136	3.830892e-08	20

SMIM29	B LY9	3.123125e-12	-0.31064	0.167	0.309	5.568219e-08	20
FERMT3	B LY9	3.534329e-12	-0.3322029	0.102	0.229	6.301355e-08	20
UBE2G1	B LY9	3.640598e-12	0.3183206	0.103	0.049	6.490821e-08	20
PARP15	B LY9	3.72724e-12	-0.3572475	0.149	0.283	6.645296e-08	20
JPT1	B LY9	3.740732e-12	-0.331399	0.305	0.461	6.669351e-08	20
RAP1B	B LY9	5.164695e-12	0.4085028	0.551	0.491	9.208135e-08	20
ANKRD12	B LY9	5.861536e-12	0.4267088	0.212	0.151	1.045053e-07	20
CD72	B LY9	6.572987e-12	-0.3386509	0.245	0.4	1.171898e-07	20
NUCKS1	B LY9	6.665461e-12	-0.3493912	0.089	0.213	1.188385e-07	20
ADA	B LY9	7.75795e-12	0.4217218	0.139	0.093	1.383165e-07	20
HSD17B4	B LY9	8.323985e-12	0.4450412	0.326	0.263	1.484083e-07	20
CD22	B LY9	1.021786e-11	-0.3001637	0.186	0.341	1.821742e-07	20
EIF1AX	B LY9	1.171222e-11	-0.3066638	0.059	0.17	2.088172e-07	20
SEL1L3	B LY9	2.501762e-11	-0.3471077	0.188	0.331	4.460391e-07	20
HNRNPH1	B LY9	3.349898e-11	-0.3612763	0.071	0.165	5.972533e-07	20
MARCKS	B LY9	3.64726e-11	-0.5017113	0.154	0.279	6.5027e-07	20
HSPA5	B LY9	3.737508e-11	-0.5943231	0.61	0.697	6.663604e-07	20
H2AFZ	B LY9	3.993832e-11	-0.3137137	0.157	0.295	7.120603e-07	20
HSPA4	B LY9	4.322708e-11	0.4465301	0.305	0.25	7.706957e-07	20
SDCBP	B LY9	4.392914e-11	-0.3102943	0.091	0.211	7.832126e-07	20
CFLAR	B LY9	5.48066e-11	-0.3092487	0.211	0.363	9.771468e-07	20
TPD52	B LY9	5.838933e-11	-0.3179761	0.176	0.31	1.041023e-06	20
CALR	B LY9	6.56372e-11	-0.4922469	0.347	0.474	1.170246e-06	20
CD83	B LY9	7.993707e-11	0.3841551	0.373	0.302	1.425198e-06	20
ZFP36L2	B LY9	1.047077e-10	-0.3184538	0.348	0.492	1.866834e-06	20

CBX3	B LY9	1.211782e-10	0.3157427	0.106	0.061	2.160487e-06	20
ST3GAL5	B LY9	1.329627e-10	-0.3741511	0.737	0.815	2.370591e-06	20
CORO1A	B LY9	1.964688e-10	-0.5235057	0.806	0.849	3.502843e-06	20
HSP90AB1	B LY9	2.124178e-10	-0.3653442	0.171	0.291	3.787196e-06	20
BCAS4	B LY9	4.394204e-10	-0.3219101	0.068	0.162	7.834427e-06	20
C12orf75	B LY9	4.501253e-10	-0.3182187	0.092	0.195	8.025285e-06	20
DNMT1	B LY9	4.704324e-10	-0.3044084	0.061	0.155	8.387338e-06	20
CD81	B LY9	4.900086e-10	0.4415404	0.208	0.136	8.736364e-06	20
FCER2	B LY9	5.251875e-10	0.4291124	0.197	0.157	9.363568e-06	20
MGAT1	B LY9	6.923176e-10	-1.282526	0.123	0.218	1.234333e-05	20
IGHG1	B LY9	1.286164e-09	-0.3913751	0.088	0.16	2.293101e-05	20
LMNA	B LY9	1.456447e-09	-0.5036778	0.062	0.132	2.596699e-05	20
MYC	B LY9	1.957598e-09	-0.3098835	0.082	0.187	3.490202e-05	20
P2RX5	B LY9	2.589011e-09	0.3618236	0.49	0.455	4.615947e-05	20
CTSS	B LY9	2.735401e-09	0.303948	0.154	0.097	4.876947e-05	20
CREBRF	B LY9	3.956237e-09	0.3669345	0.289	0.231	7.053575e-05	20
HSD17B11	B LY9	4.133771e-09	-0.3006706	0.238	0.377	7.3701e-05	20
ACADM	B LY9	4.159633e-09	-0.3080586	0.048	0.122	7.416209e-05	20
LPP	B LY9	4.927944e-09	-0.3663354	0.235	0.361	8.786032e-05	20
H2AFV	B LY9	5.740723e-09	-1.653014	0.327	0.431	0.0001023514	20
IGLC3	B LY9	5.800442e-09	0.3550848	0.163	0.115	0.0001034161	20
SIAH1	B LY9	5.90976e-09	-0.3465504	0.081	0.177	0.0001053651	20
PDIA4	B LY9	9.882401e-09	0.3163111	0.118	0.072	0.0001761933	20
SOCS3	B LY9	3.422054e-08	-0.9241636	0.084	0.149	0.0006101179	20
IGHA1	B LY9	3.94089e-08	0.3251977	0.214	0.162	0.0007026212	20

TNFRSF14 B LY9
5.408795e-08 -0.3212714 0.218 0.337 0.0009643341 20
DUT B LY9
5.839931e-08 0.3373948 0.201 0.158 0.001041201 20
TPP1 B LY9
6.815165e-08 -0.3445187 0.922 0.93 0.001215076 20
NPM1 B LY9
7.718389e-08 -0.4161308 0.17 0.27 0.001376112 20
JUN B LY9
1.01813e-07 0.3031592 0.637 0.62 0.001815224 20
IFI16 B LY9
1.225704e-07 0.3403461 0.214 0.179 0.002185307 20
LYL1 B LY9
1.362363e-07 0.3446048 0.299 0.263 0.002428958 20
TTC3 B LY9
2.189363e-07 0.3183557 0.414 0.383 0.003903415 20
LAPTM4A B LY9
2.40778e-07 0.3083547 0.211 0.167 0.004292831 20
BNIP3L B LY9
2.943786e-07 -0.3283392 0.137 0.234 0.005248477 20
IL4R B LY9
2.988087e-07 -0.4083593 0.378 0.501 0.005327461 20
SSR4 B LY9
3.368404e-07 -2.802518 0.341 0.452 0.006005527 20
IGHG3 B LY9
4.152691e-07 -0.4660048 0.218 0.317 0.007403833 20
CD69 B LY9
4.30555e-07 0.3645097 0.276 0.241 0.007676364 20
PHACTR1 B LY9
7.426243e-07 0.3583825 0.29 0.265 0.01324025 20
WSB1 B LY9
8.545042e-07 0.3115557 0.149 0.111 0.01523496 20
SESN3 B LY9
1.346257e-06 -0.3198437 0.061 0.123 0.02400241 20
AL139020.1 B LY9
1.589319e-06 -2.24386 0.047 0.127 0.02833597 20
IGHG2 B LY9
1.730315e-06 0.3734457 0.348 0.331 0.03084978 20
MPG B LY9
2.58577e-06 0.3048583 0.2 0.157 0.0461017 20
TGIF1 B LY9
3.302413e-06 0.3291938 0.279 0.251 0.05887872 20
DNPH1 B LY9
5.379554e-06 0.3222608 0.466 0.426 0.09591206 20
PLP2 B LY9
5.718143e-06 -0.3622478 0.501 0.59 0.1019488 20
MARCKSL1 B LY9
6.946379e-06 0.3049724 0.397 0.359 0.123847 20
ARID5B B LY9
8.371041e-06 -0.4477923 0.228 0.314 0.1492473 20

HSP90B1 B LY9
9.193343e-06 -1.823572 0.149 0.245 0.1639081 20
IGHG4 B LY9
9.880237e-06 0.3031873 0.31 0.278 0.1761547 20
ADAM28 B LY9
1.499347e-05 -2.024671 0.54 0.646 0.2673186 20
IGLC2 B LY9
5.407275e-05 -0.6805466 0.127 0.213 0.9640631 20
PLCG2 B LY9
9.829104e-05 0.302645 0.222 0.202 1 20 BIN1 B
LY9
0.0001235058 -1.130021 0.356 0.423 1 20
HIST1H4C B LY9
0.0008464105 0.3049321 0.174 0.169 1 20
CREM B LY9
0.001094031 -0.5499794 0.687 0.716 1 20
HMGN2 B LY9
0.004546433 0.3435775 0.13 0.125 1 20
LGALS3 B LY9
0 5.20417 0.931 0.202 0 21 PLCG2 B PLCG2
0 -1.058405 0.572 0.987 0 21 MT-CO3 B
PLCG2
0 -1.279438 0.499 0.985 0 21 MT-CO2 B
PLCG2
0 -1.42721 0.53 0.986 0 21 MT-CO1 B PLCG2
6.860818e-318 -1.156266 0.458 0.975 1.223215e-313 21
MT-ND3 B PLCG2
2.578808e-314 -1.322005 0.444 0.966 4.597756e-310 21
MT-CYB B PLCG2
9.186407e-307 -1.428282 0.439 0.967 1.637844e-302 21
MT-ND2 B PLCG2
5.54311e-289 -1.283963 0.636 0.991 9.882811e-285 21
HLA-B B PLCG2
2.381678e-279 -1.069669 0.549 0.979 4.246294e-275 21
MT-ND4 B PLCG2
1.196793e-254 2.438168 0.377 0.027 2.133763e-250 21
AC007952.4 B PLCG2
8.134362e-229 2.482734 0.244 0.002 1.450275e-224 21
HIST1H3B B PLCG2
8.194732e-223 2.370197 0.42 0.082 1.461039e-218 21
ZNF277 B PLCG2
3.887656e-213 1.737873 0.23 0.002 6.931302e-209 21
HIST1H4B B PLCG2
4.624778e-211 -0.9080054 0.458 0.94 8.245516e-207 21
MT-ATP6 B PLCG2
4.936642e-211 -1.046345 0.917 0.999 8.801539e-207 21
B2M B PLCG2
2.250128e-208 -1.342896 0.549 0.968 4.011754e-204 21
FTL B PLCG2
1.265065e-198 -1.330451 0.364 0.912 2.255485e-194 21

1.487212e-197	2.106003	0.228	0.005	2.651551e-193	21	B PLCG2	HLA-A
5.702934e-187	-1.025944	0.38	0.888	1.016776e-182	21	B PLCG2	HIST1H2AG
9.304405e-176	1.774707	0.183	0.001	1.658882e-171	21	B PLCG2	MT-ND1
1.046037e-174	1.887693	0.219	0.009	1.864979e-170	21	B PLCG2	HIST1H4D
2.9285e-167	1.860761	0.2	0.004	5.221223e-163	21	B PLCG2	HIST1H4J
7.36107e-164	1.586585	0.176	0.005	1.312405e-159	21	B PLCG2	HIST1H3H
7.502485e-164	2.584786	0.675	0.297	1.337618e-159	21	B PLCG2	HIST1H2AJ
4.973143e-151	1.597429	0.162	0.002	8.866617e-147	21	B PLCG2	HMGB2
2.347726e-147	1.911541	0.188	0.011	4.185762e-143	21	B PLCG2	HIST1H2AI
8.366831e-146	1.065946	0.94	0.958	1.491722e-141	21	B PLCG2	HIST1H1B
1.050186e-143	1.398246	0.162	0.002	1.872376e-139	21	B PLCG2	PABPC1
1.903584e-143	-0.8241935	0.964	1	3.393901e-139	21	B PLCG2	HIST2H2BF
6.812852e-143	-0.8356579	0.547	0.942	1.214663e-138	21	B PLCG2	EEF1A1
1.393639e-139	1.857007	0.316	0.062	2.484718e-135	21	B PLCG2	EEF2
2.707948e-137	1.482885	0.15	0.002	4.828e-133	21	B PLCG2	TMEM107
5.708815e-135	1.365571	0.168	0.003	1.017825e-130	21	B PLCG2	HIST1H3C
1.231468e-133	1.341118	0.161	0.003	2.195585e-129	21	B PLCG2	HIST1H3G
1.905736e-133	-0.9066804	0.689	0.98	3.397737e-129	21	B PLCG2	HIST1H4E
2.210291e-133	-0.5895183	0.755	0.987	3.940728e-129	21	B PLCG2	FTH1
6.78496e-133	-0.6268164	0.841	0.995	1.209691e-128	21	B PLCG2	RPL29
1.910964e-132	-1.282219	0.259	0.8	3.407058e-128	21	B PLCG2	RPS5
2.86365e-132	1.705718	0.187	0.012	5.105602e-128	21	B PLCG2	HLA-E
2.084199e-128	0.7963415	0.998	0.998	3.715918e-124	21	B PLCG2	HIST1H2BJ
1.794362e-127	1.328953	0.145	0.001	3.199169e-123	21	B PLCG2	TMSB4X
1.140709e-122	-0.6211919	0.921	0.999	2.033771e-118	21	B PLCG2	HIST1H3J

RPL3	B PLCG2					
2.334731e-122	-0.6271057	0.941	0.999	4.162592e-118	21	
RPL18A	B PLCG2					
7.368806e-121	2.026971	0.394	0.091	1.313784e-116	21	
PCLAF	B PLCG2					
1.0198e-120	1.596046	0.15	0.002	1.818202e-116	21	
RMRP	B PLCG2					
2.552505e-120	-1.688456	0.168	0.705	4.550861e-116	21	
EMP3	B PLCG2					
2.443871e-116	-0.5280525	0.807	0.99	4.357178e-112	21	
RACK1	B PLCG2					
8.335226e-116	1.292003	0.13	0.001	1.486087e-111	21	
HIST1H4F	B PLCG2					
6.914395e-115	1.346954	0.152	0.005	1.232768e-110	21	
Z93241.1	B PLCG2					
8.870801e-115	1.431166	0.145	0.006	1.581575e-110	21	
HIST1H2BH	B PLCG2					
8.336987e-114	-0.8460535	0.558	0.94	1.486401e-109	21	
BTG1	B PLCG2					
1.190382e-111	-1.026984	0.249	0.764	2.122332e-107	21	
MT-ND5	B PLCG2					
4.23791e-111	1.563647	0.166	0.015	7.555769e-107	21	
HIST1H2BC	B PLCG2					
7.854417e-111	1.152092	0.131	0.003	1.400364e-106	21	
HIST1H2AH	B PLCG2					
3.673579e-109	1.080702	0.133	0.001	6.549624e-105	21	
HIST1H3A	B PLCG2					
5.239281e-109	0.9305676	0.981	0.999	9.341114e-105	21	
PTMA	B PLCG2					
8.88797e-109	1.126979	0.116	0	1.584636e-104	21	
HIST1H4L	B PLCG2					
4.144939e-106	1.450089	0.898	0.932	7.390011e-102	21	
HMGB1	B PLCG2					
1.87569e-105	-0.9766501	0.288	0.758	3.344167e-101	21	
HLA-C	B PLCG2					
3.815394e-104	1.718236	0.813	0.715	6.802466e-100	21	
HMGN2	B PLCG2					
1.741894e-103	-0.4950568	0.839	0.994	3.105622e-99	21	
RPL18	B PLCG2					
2.356001e-103	-0.8910925	0.627	0.946	4.200514e-99	21	
YBX1	B PLCG2					
1.386547e-102	1.078007	0.111	0.001	2.472074e-98	21	
HIST1H4I	B PLCG2					
1.938428e-102	0.9490224	0.111	0	3.456023e-98	21	
HIST1H4A	B PLCG2					
1.0221e-98	0.9170919	0.112	0.001	1.822302e-94	21	
HIST1H2AL	B PLCG2					
2.312044e-98	-0.4239122	0.876	0.996	4.122143e-94	21	
RPS9	B PLCG2					
4.931066e-98	-0.5863619	0.959	0.999	8.791598e-94	21	

RPL13	B PLCG2	6.531869e-97	0.9519676	0.102	0.001	1.164567e-92	21
HIST1H3F	B PLCG2	1.305195e-96	-0.5615267	0.791	0.984	2.327032e-92	21
RPSA	B PLCG2	1.068058e-93	-0.3949556	0.967	0.999	1.90424e-89	21
RPL10	B PLCG2	9.174316e-93	1.77576	0.358	0.106	1.635689e-88	21
VPREB3	B PLCG2	1.625865e-92	-1.181157	0.459	0.853	2.898755e-88	21
HSP90AB1	B PLCG2	3.27846e-92	1.302935	0.874	0.851	5.845167e-88	21
ATP5MG	B PLCG2	1.778327e-91	-0.6381643	0.461	0.87	3.170579e-87	21
UBC	B PLCG2	2.845708e-87	-0.4947473	0.839	0.993	5.073612e-83	21
RPL9	B PLCG2	1.101762e-86	1.38267	0.145	0.014	1.964332e-82	21
HIST1H4H	B PLCG2	8.78622e-86	1.847739	0.542	0.202	1.566495e-81	21
RGS13	B PLCG2	1.6198e-85	1.356055	0.225	0.047	2.887941e-81	21
LINC01991	B PLCG2	4.60637e-84	-0.4440551	0.976	1	8.212697e-80	21
RPLP1	B PLCG2	3.089732e-81	-1.237144	0.314	0.72	5.508683e-77	21
ENO1	B PLCG2	1.41288e-80	-1.215807	0.687	0.909	2.519025e-76	21
IGHM	B PLCG2	2.095445e-80	-0.9495102	0.382	0.766	3.735969e-76	21
PKM	B PLCG2	1.900048e-78	1.363942	0.178	0.033	3.387595e-74	21
HIST1H1E	B PLCG2	2.394219e-77	0.9799301	0.109	0.007	4.268653e-73	21
HIST1H2AE	B PLCG2	2.822969e-77	-0.5513639	0.554	0.87	5.033072e-73	21
CD79A	B PLCG2	4.884715e-77	-0.6448342	0.399	0.821	8.708959e-73	21
NOP53	B PLCG2	2.337549e-75	-1.498645	0.159	0.575	4.167616e-71	21
VIM	B PLCG2	1.327901e-74	-0.7070465	0.313	0.719	2.367514e-70	21
PSME1	B PLCG2	3.521771e-73	1.138706	0.929	0.933	6.278965e-69	21
SERF2	B PLCG2	5.48371e-71	-0.5122617	0.58	0.899	9.776906e-67	21
SLC25A6	B PLCG2	5.560537e-71	1.080958	0.131	0.013	9.913882e-67	21
AL031777.3	B PLCG2	1.020569e-70	-1.007217	0.173	0.582	1.819573e-66	21

SARAF	B PLCG2	4.174209e-70	1.344849	0.235	0.071	7.442197e-66	21
CDCA7	B PLCG2	1.251376e-68	-0.3581083	0.755	0.967	2.231079e-64	21
RPL4	B PLCG2	2.469378e-68	-0.5987044	0.489	0.859	4.402654e-64	21
SH3BGRL3	B PLCG2	2.660609e-68	-1.428117	0.143	0.548	4.743601e-64	21
CCR7	B PLCG2	6.094565e-68	-0.8510551	0.216	0.626	1.0866e-63	21
SUB1	B PLCG2	1.159729e-67	1.327364	0.793	0.768	2.067681e-63	21
HMGN1	B PLCG2	1.346996e-67	1.574082	0.378	0.205	2.401559e-63	21
SNHG25	B PLCG2	3.646037e-67	1.139426	0.884	0.94	6.500519e-63	21
H3F3A	B PLCG2	3.516032e-66	-0.3504492	0.582	0.922	6.268734e-62	21
PFDN5	B PLCG2	4.375679e-65	-0.3560074	0.908	0.997	7.801398e-61	21
RPS3	B PLCG2	4.447725e-65	1.709827	0.415	0.187	7.929849e-61	21
PTTG1	B PLCG2	1.91918e-63	0.8755391	0.105	0.01	3.421706e-59	21
HIST2H2BE	B PLCG2	3.17386e-63	-0.3234675	0.63	0.932	5.658676e-59	21
EEF1D	B PLCG2	4.230252e-62	-0.3271853	0.934	0.998	7.542117e-58	21
RPS4X	B PLCG2	4.917827e-62	-0.7029299	0.311	0.681	8.767995e-58	21
TAGLN2	B PLCG2	5.412959e-62	-0.3810272	0.499	0.757	9.650764e-58	21
HLA-DRB1	B PLCG2	6.547736e-62	-0.4000333	0.983	1	1.167396e-57	21
RPS2	B PLCG2	8.662891e-61	-0.3823928	0.934	0.998	1.544507e-56	21
RPL7	B PLCG2	3.189786e-60	-0.8716266	0.192	0.575	5.687069e-56	21
SNX2	B PLCG2	7.718272e-60	-0.7461191	0.473	0.819	1.376091e-55	21
HLA-DPA1	B PLCG2	5.055313e-59	0.8089122	0.109	0.011	9.013117e-55	21
HIST1H2BG	B PLCG2	7.462019e-59	-0.6566656	0.237	0.632	1.330403e-54	21
ATP6V0E1	B PLCG2	3.087598e-57	-0.3689035	0.617	0.86	5.504879e-53	21
CD79B	B PLCG2	9.943516e-57	-0.9794369	0.235	0.611	1.772829e-52	21
IGHD	B PLCG2	2.390411e-56	-0.4018871	0.63	0.905	4.261863e-52	21

MYL6 B PLCG2
3.30451e-56 -0.3944565 0.964 0.999 5.89161e-52 21
RPL32 B PLCG2
3.876301e-56 -1.003005 0.121 0.482 6.911056e-52 21
SQSTM1 B PLCG2
9.135105e-56 -0.4393236 0.57 0.904 1.628698e-51 21
DDX5 B PLCG2
1.619492e-55 -0.3389536 0.946 0.998 2.887392e-51 21
RPS14 B PLCG2
1.964193e-55 -0.716227 0.152 0.51 3.501959e-51 21
FDFT1 B PLCG2
3.107737e-55 -0.9806131 0.164 0.516 5.540784e-51 21
BANK1 B PLCG2
4.651795e-55 1.180356 0.8 0.807 8.293686e-51 21
TMA7 B PLCG2
5.158632e-55 -0.7246744 0.273 0.64 9.197324e-51 21
GPX4 B PLCG2
1.794579e-54 -0.6929835 0.157 0.498 3.199555e-50 21
AP2M1 B PLCG2
1.933617e-54 -0.6923222 0.202 0.58 3.447446e-50 21
APRT B PLCG2
1.981422e-54 1.405504 0.212 0.112 3.532678e-50 21
CSKMT B PLCG2
2.442727e-54 -0.4092783 0.971 0.999 4.355138e-50 21
RPL39 B PLCG2
4.649885e-54 -0.4898287 0.321 0.682 8.29028e-50 21
EIF3L B PLCG2
1.218191e-53 -0.8383255 0.105 0.442 2.171912e-49 21
IL2RG B PLCG2
7.383676e-53 -0.6801655 0.238 0.6 1.316436e-48 21
XRCC6 B PLCG2
8.287599e-53 -0.6030185 0.271 0.658 1.477596e-48 21
TMBIM6 B PLCG2
1.29333e-52 -0.9307826 0.104 0.432 2.305877e-48 21
PPP1R15A B PLCG2
1.388608e-52 -1.029992 0.138 0.498 2.475749e-48 21
FXYD5 B PLCG2
3.464485e-52 -0.9653778 0.097 0.467 6.17683e-48 21
CD63 B PLCG2
4.636444e-52 -0.7058098 0.173 0.521 8.266316e-48 21
EIF3G B PLCG2
5.409078e-52 1.518303 0.598 0.426 9.643845e-48 21
STMN1 B PLCG2
1.44603e-51 -0.3232889 0.857 0.988 2.578128e-47 21
RPL14 B PLCG2
2.881832e-50 1.433161 0.456 0.334 5.138019e-46 21 DUT
B PLCG2
5.328968e-50 -0.6444851 0.325 0.702 9.501017e-46 21
HLA-DQB1 B PLCG2
7.957464e-50 1.509522 0.494 0.358 1.418736e-45 21

H2AFV	B PLCG2	9.050461e-50	-0.3218338	0.767	0.967	1.613607e-45	21
EIF1	B PLCG2	1.771877e-49	-0.4093991	0.775	0.98	3.159079e-45	21
RPS10	B PLCG2	2.348197e-49	-0.8061191	0.152	0.508	4.186601e-45	21
TSC22D3	B PLCG2	4.365234e-49	-0.6689384	0.181	0.514	7.782776e-45	21
TALDO1	B PLCG2	4.66902e-49	-1.016032	0.081	0.387	8.324396e-45	21
CD44	B PLCG2	5.792541e-49	-0.9965712	0.081	0.384	1.032752e-44	21
PDCD4	B PLCG2	2.491089e-48	-0.4304814	0.351	0.691	4.441362e-44	21
PPP1CA	B PLCG2	2.886095e-48	-0.7168533	0.247	0.615	5.145619e-44	21
SYNGR2	B PLCG2	6.896129e-48	-0.8568292	0.136	0.448	1.229511e-43	21
ANXA2	B PLCG2	1.176522e-47	-0.3139394	0.484	0.829	2.097622e-43	21
EIF3E	B PLCG2	1.927731e-47	-0.7913821	0.199	0.537	3.436951e-43	21
IMPDH2	B PLCG2	2.196827e-47	-0.610711	0.193	0.536	3.916724e-43	21
GHITM	B PLCG2	2.357998e-47	1.009817	0.147	0.044	4.204075e-43	21
MYBL1	B PLCG2	2.473012e-46	1.331907	0.2	0.084	4.409132e-42	21
HIST1H1C	B PLCG2	2.312308e-45	-0.3994767	0.953	0.998	4.122614e-41	21
RPL11	B PLCG2	7.021201e-45	-0.5364957	0.188	0.514	1.25181e-40	21
PSMD8	B PLCG2	1.007284e-44	-0.4295119	0.952	0.997	1.795887e-40	21
RPS12	B PLCG2	1.31812e-44	-0.5479341	0.192	0.528	2.350076e-40	21
SF3B2	B PLCG2	1.614966e-44	-0.3144237	0.86	0.987	2.879324e-40	21
TMSB10	B PLCG2	2.62026e-44	-1.364017	0.143	0.469	4.671661e-40	21
S100A6	B PLCG2	3.989279e-44	-0.7505276	0.23	0.563	7.112485e-40	21
NCL	B PLCG2	1.071126e-43	-0.5499967	0.316	0.657	1.90971e-39	21
HLA-DQA1	B PLCG2	1.472082e-43	-0.6677573	0.145	0.467	2.624574e-39	21
PRMT1	B PLCG2	2.411884e-43	-0.3025985	0.402	0.738	4.300148e-39	21
SKP1	B PLCG2	3.211523e-43	-0.6920592	0.126	0.423	5.725825e-39	21

EIF6	B PLCG2	6.379026e-43	-0.3325684	0.344	0.629	1.137317e-38	21
HLA-DMB	B PLCG2	9.690873e-43	1.621495	0.53	0.421	1.727786e-38	21
HIST1H4C	B PLCG2	1.933065e-42	-0.6325165	0.104	0.395	3.446461e-38	21
MAF1	B PLCG2	9.900944e-42	-0.3601274	0.66	0.92	1.765239e-37	21
EEF1B2	B PLCG2	1.129737e-41	-0.9749624	0.059	0.33	2.014208e-37	21
SLC3A2	B PLCG2	1.904641e-41	-0.615103	0.18	0.505	3.395784e-37	21
FOXP1	B PLCG2	2.741501e-41	-0.5393911	0.247	0.576	4.887823e-37	21
EIF3D	B PLCG2	3.467768e-41	1.070198	0.181	0.082	6.182684e-37	21
H2AFX	B PLCG2	6.729869e-41	0.9607887	0.192	0.07	1.199868e-36	21
DHFR	B PLCG2	1.613978e-40	-0.5408554	0.297	0.653	2.877562e-36	21
ZFP36L1	B PLCG2	1.777948e-40	-0.5388843	0.478	0.779	3.169903e-36	21
HLA-DPB1	B PLCG2	2.113641e-40	-0.6810407	0.105	0.39	3.768411e-36	21
PSAP	B PLCG2	2.190042e-40	-0.618687	0.221	0.549	3.904625e-36	21
CNBP	B PLCG2	2.410656e-40	-0.6046042	0.059	0.288	4.297958e-36	21
UQCRC1	B PLCG2	3.688568e-40	-0.5987239	0.097	0.366	6.576347e-36	21
CPNE1	B PLCG2	5.361948e-40	-0.6618306	0.185	0.496	9.559817e-36	21
CYTIP	B PLCG2	5.767832e-40	1.296729	0.547	0.452	1.028347e-35	21
PTPRC	B PLCG2	6.274026e-40	1.041496	0.185	0.076	1.118596e-35	21
KLHL6	B PLCG2	1.24014e-39	-0.5535746	0.104	0.338	2.211046e-35	21
ANXA11	B PLCG2	1.425999e-39	1.312011	0.694	0.696	2.542414e-35	21
H2AFZ	B PLCG2	1.586066e-39	-0.7485663	0.152	0.452	2.827798e-35	21
PNRC1	B PLCG2	1.593928e-39	-0.5456303	0.15	0.455	2.841814e-35	21
VCP	B PLCG2	1.801223e-39	-0.3197572	0.332	0.657	3.2114e-35	21
ATP5F1A	B PLCG2	1.836685e-39	-0.3087852	0.404	0.724	3.274625e-35	21
EIF3F	B PLCG2	2.716885e-39	-0.7658476	0.092	0.365	4.843934e-35	21

ANXA6 B PLCG2
4.900699e-39 -0.3773477 0.25 0.519 8.737456e-35 21
LSP1 B PLCG2
6.732493e-39 -0.6798355 0.081 0.354 1.200336e-34 21
APOBEC3G B PLCG2
8.450346e-39 -0.9142404 0.135 0.375 1.506612e-34 21
SELL B PLCG2
1.043686e-38 -0.9223419 0.088 0.363 1.860787e-34 21
ARID5B B PLCG2
1.39325e-38 -0.5460387 0.437 0.74 2.484025e-34 21
HSPA8 B PLCG2
1.653313e-38 -0.5157876 0.259 0.607 2.947692e-34 21
ARF6 B PLCG2
3.099789e-38 -0.5671096 0.155 0.448 5.526613e-34 21
CCT7 B PLCG2
3.554626e-38 -0.8399105 0.124 0.43 6.337542e-34 21
PLP2 B PLCG2
4.920273e-38 -0.3210076 0.988 1 8.772354e-34 21
RPL13A B PLCG2
5.247693e-38 -0.6827111 0.117 0.391 9.356111e-34 21
SHMT2 B PLCG2
8.186939e-38 -0.4993218 0.155 0.464 1.459649e-33 21
GADD45GIP1 B PLCG2
8.769574e-38 -0.389002 0.328 0.632 1.563527e-33 21
ATP5F1B B PLCG2
1.652584e-37 -0.7393977 0.192 0.503 2.946393e-33 21
SSR4 B PLCG2
2.304531e-37 -0.7444653 0.078 0.332 4.108748e-33 21
TRBC2 B PLCG2
2.428879e-37 -0.5832309 0.079 0.328 4.330448e-33 21
RAB5C B PLCG2
2.9165e-37 -0.7290423 0.09 0.351 5.199829e-33 21
LITAF B PLCG2
3.446266e-37 -0.7290713 0.057 0.289 6.144347e-33 21
ERG28 B PLCG2
4.215326e-37 -0.4657567 0.794 0.948 7.515504e-33 21
HLA-DRA B PLCG2
4.961885e-37 -0.8866173 0.1 0.354 8.846544e-33 21
LY6E B PLCG2
6.50538e-37 -0.6821766 0.086 0.339 1.159844e-32 21
S100A11 B PLCG2
8.011449e-37 1.13707 0.242 0.11 1.428361e-32 21
AC023590.1 B PLCG2
1.085107e-36 -0.5035031 0.19 0.488 1.934638e-32 21
GTF3A B PLCG2
1.541471e-36 -0.6087644 0.169 0.471 2.748289e-32 21
ITM2B B PLCG2
2.455612e-36 -0.5453731 0.123 0.405 4.37811e-32 21
PSMC5 B PLCG2
2.609735e-36 -0.9310607 0.085 0.356 4.652896e-32 21

TXNIP	B PLCG2	2.755774e-36	-0.633324	0.226	0.533	4.913269e-32	21
PSME2	B PLCG2	3.015406e-36	-0.3712469	0.275	0.605	5.376168e-32	21
UBXN1	B PLCG2	3.67461e-36	0.8708064	0.105	0.034	6.551463e-32	21
SLC20A1	B PLCG2	3.908594e-36	-0.5764604	0.138	0.422	6.968633e-32	21
NUDC	B PLCG2	4.059676e-36	-0.3765164	0.389	0.707	7.237996e-32	21
TPI1	B PLCG2	4.77334e-36	-0.5913165	0.095	0.353	8.510387e-32	21
FKBP8	B PLCG2	6.986287e-36	-0.5480202	0.09	0.347	1.245585e-31	21
PSMC4	B PLCG2	7.827218e-36	-0.5621046	0.107	0.363	1.395515e-31	21
BSG	B PLCG2	1.173769e-35	-0.6511339	0.086	0.348	2.092712e-31	21
RNH1	B PLCG2	1.187818e-35	-0.8275773	0.054	0.251	2.117761e-31	21
TNFRSF13B	B PLCG2	1.432282e-35	-0.61522	0.19	0.496	2.553615e-31	21
ANKRD12	B PLCG2	3.848397e-35	0.7236972	0.126	0.04	6.861306e-31	21
MND1	B PLCG2	5.485814e-35	-0.7302817	0.064	0.282	9.780657e-31	21
RNASET2	B PLCG2	6.593011e-35	-0.3387651	0.273	0.583	1.175468e-30	21
COPE	B PLCG2	9.714692e-35	-0.6922762	0.09	0.359	1.732032e-30	21
SARS	B PLCG2	1.050308e-34	0.7935537	0.836	0.785	1.872593e-30	21
ACTG1	B PLCG2	1.369281e-34	-0.5012522	0.204	0.507	2.44129e-30	21
XRCC5	B PLCG2	1.390601e-34	-0.3143323	0.477	0.763	2.479303e-30	21
PSMA7	B PLCG2	1.438406e-34	-0.5780618	0.081	0.327	2.564535e-30	21
PUF60	B PLCG2	2.321297e-34	-0.6854062	0.152	0.449	4.138641e-30	21
KLF6	B PLCG2	2.419143e-34	-0.6667887	0.136	0.417	4.313091e-30	21
NPC2	B PLCG2	2.649208e-34	-0.4144368	0.235	0.554	4.723273e-30	21
PSMA1	B PLCG2	3.351881e-34	-0.646634	0.095	0.339	5.976068e-30	21
ACAT2	B PLCG2	6.33138e-34	-0.8303267	0.079	0.349	1.128822e-29	21
CAST	B PLCG2	6.755642e-34	-0.3267883	0.302	0.606	1.204463e-29	21

TRIR B PLCG2
8.484633e-34 -0.6331607 0.1 0.365 1.512725e-29 21
HSPA9 B PLCG2
1.254115e-33 1.228897 0.674 0.486 2.235962e-29 21 LTB
B PLCG2
1.608198e-33 -0.3916729 0.199 0.481 2.867256e-29 21
SSR2 B PLCG2
1.631105e-33 -0.8535931 0.078 0.276 2.908097e-29 21
KLF2 B PLCG2
2.571363e-33 -0.5768767 0.126 0.396 4.584482e-29 21
PHB B PLCG2
2.999926e-33 -0.4980503 0.105 0.356 5.348568e-29 21
PSMD13 B PLCG2
3.835501e-33 -1.208998 0.074 0.266 6.838314e-29 21
S100A4 B PLCG2
5.48188e-33 -0.6574033 0.1 0.359 9.773643e-29 21
SQLE B PLCG2
6.428867e-33 -0.5283325 0.183 0.484 1.146203e-28 21
ISCU B PLCG2
7.975072e-33 -0.485035 0.2 0.501 1.421876e-28 21
TUFM B PLCG2
1.186848e-32 -0.4127504 0.168 0.454 2.116032e-28 21
UFC1 B PLCG2
1.234942e-32 -0.4367974 0.176 0.422 2.201779e-28 21
CTSH B PLCG2
1.826534e-32 -0.5752364 0.088 0.31 3.256527e-28 21
SHKBP1 B PLCG2
6.3291e-32 -0.4626486 0.095 0.321 1.128415e-27 21
ERGIC3 B PLCG2
7.367768e-32 -0.6603204 0.088 0.33 1.313599e-27 21
PTPN6 B PLCG2
3.778596e-31 -0.3359114 0.288 0.598 6.736858e-27 21
ATP5F1D B PLCG2
6.092541e-31 1.160598 0.202 0.066 1.086239e-26 21
TOP2A B PLCG2
1.039301e-30 -0.3178415 0.313 0.621 1.85297e-26 21
NDUFA13 B PLCG2
1.245862e-30 -0.5537712 0.043 0.234 2.221248e-26 21
RTCB B PLCG2
1.498138e-30 -0.3187477 0.294 0.598 2.67103e-26 21
ST13 B PLCG2
1.538566e-30 -0.4380471 0.18 0.456 2.743108e-26 21
TMEM59 B PLCG2
2.18042e-30 -0.6350323 0.155 0.394 3.887471e-26 21
EIF5A B PLCG2
4.645154e-30 -0.4244069 0.211 0.493 8.281845e-26 21
CCT3 B PLCG2
1.031496e-29 -0.7966931 0.093 0.299 1.839053e-25 21
LINC00926 B PLCG2
1.128729e-29 -0.6254884 0.079 0.309 2.012411e-25 21

TMEM147 B PLCG2
1.234231e-29 -0.49537 0.211 0.497 2.20051e-25 21
SP100 B PLCG2
1.323239e-29 -0.357685 0.275 0.56 2.359202e-25 21
PGK1 B PLCG2
1.49443e-29 -0.3307831 0.207 0.497 2.66442e-25 21
THRAP3 B PLCG2
1.500626e-29 -0.3641489 0.325 0.62 2.675466e-25 21
SERBP1 B PLCG2
1.605189e-29 1.031569 0.204 0.099 2.861892e-25 21
CENPM B PLCG2
1.832177e-29 -0.3563254 0.116 0.357 3.266588e-25 21
DBNL B PLCG2
2.367209e-29 1.120493 0.199 0.076 4.220498e-25 21
SUGCT B PLCG2
2.904026e-29 -0.4329634 0.124 0.378 5.177588e-25 21
PSMC1 B PLCG2
3.074378e-29 0.8428523 0.135 0.058 5.481309e-25 21
CENPK B PLCG2
3.413704e-29 -0.4142726 0.152 0.42 6.086294e-25 21
RAB7A B PLCG2
3.675764e-29 -0.426301 0.176 0.453 6.55352e-25 21
DRAP1 B PLCG2
5.214696e-29 0.8760193 0.159 0.048 9.297281e-25 21
AURKB B PLCG2
6.880357e-29 -0.7493798 0.185 0.476 1.226699e-24 21
CD83 B PLCG2
6.896487e-29 -0.5866822 0.043 0.221 1.229575e-24 21
CCDC50 B PLCG2
1.284202e-28 -0.4570308 0.066 0.27 2.289604e-24 21
PSMD3 B PLCG2
1.716277e-28 1.162446 0.528 0.484 3.05995e-24 21
NDUFB1 B PLCG2
1.730368e-28 -0.4555077 0.126 0.386 3.085072e-24 21
TXNL1 B PLCG2
2.782807e-28 1.143598 0.204 0.093 4.961466e-24 21
WDR66 B PLCG2
5.034273e-28 -0.693532 0.052 0.282 8.975606e-24 21
WARS B PLCG2
5.657181e-28 1.197048 0.404 0.337 1.008619e-23 21
NDUFC1 B PLCG2
6.429479e-28 -0.3784786 0.145 0.37 1.146312e-23 21
CXXC5 B PLCG2
6.757269e-28 0.7615842 0.116 0.043 1.204754e-23 21
RAD51AP1 B PLCG2
8.034378e-28 -0.3286481 0.292 0.591 1.432449e-23 21
PRR13 B PLCG2
1.270398e-27 -0.3324093 0.199 0.442 2.264992e-23 21
RHOH B PLCG2
1.404724e-27 1.010207 0.159 0.052 2.504482e-23 21

CDK1	B PLCG2	1.435275e-27	-0.4517161	0.088	0.304	2.558952e-23	21
ATP6V0D1	B PLCG2	1.478065e-27	-0.4995409	0.123	0.384	2.635242e-23	21
ANXA7	B PLCG2	2.15118e-27	-0.3705756	0.238	0.507	3.835339e-23	21
EIF3I	B PLCG2	2.925995e-27	-0.4520106	0.048	0.239	5.216757e-23	21
LRPAP1	B PLCG2	3.097946e-27	-0.7713585	0.041	0.222	5.523327e-23	21
GBP2	B PLCG2	3.297237e-27	-0.3934136	0.138	0.39	5.878645e-23	21
VPS28	B PLCG2	3.713888e-27	-0.3824861	0.244	0.538	6.62149e-23	21
ATP6V1F	B PLCG2	4.519079e-27	-0.4358051	0.081	0.308	8.057065e-23	21
PSMD11	B PLCG2	5.411992e-27	-0.3170813	0.193	0.453	9.649041e-23	21
SF3B5	B PLCG2	5.566711e-27	-0.4421378	0.06	0.258	9.924889e-23	21
ACO2	B PLCG2	7.251766e-27	1.170299	0.435	0.339	1.292917e-22	21
LRMP	B PLCG2	7.990545e-27	-0.3999854	0.155	0.419	1.424634e-22	21
PRRC2C	B PLCG2	8.355264e-27	-0.6683667	0.1	0.339	1.48966e-22	21
APOBEC3C	B PLCG2	9.260167e-27	-0.5828009	0.074	0.282	1.650995e-22	21
SRM	B PLCG2	9.45989e-27	-0.3681921	0.152	0.391	1.686604e-22	21
CUTA	B PLCG2	1.135379e-26	0.888725	0.14	0.06	2.024267e-22	21
DCAF12	B PLCG2	1.255081e-26	-0.4882415	0.143	0.392	2.237684e-22	21
ARL6IP5	B PLCG2	1.399695e-26	1.07481	0.594	0.583	2.495517e-22	21
ANP32B	B PLCG2	1.470411e-26	-0.323885	0.19	0.459	2.621597e-22	21
PSMD4	B PLCG2	1.580175e-26	-0.4539592	0.13	0.387	2.817295e-22	21
C19orf70	B PLCG2	1.674827e-26	-0.5094757	0.288	0.608	2.986049e-22	21
CDC42	B PLCG2	1.808135e-26	-0.3531461	0.114	0.347	3.223724e-22	21
SNX17	B PLCG2	1.818651e-26	-0.6111316	0.107	0.323	3.242472e-22	21
TUBA1A	B PLCG2	2.187667e-26	-0.3163535	0.225	0.51	3.900392e-22	21
SRRM1	B PLCG2	2.746838e-26	-0.4434978	0.035	0.195	4.897337e-22	21

HARS	B PLCG2	3.749512e-26	-0.5147152	0.107	0.345	6.685004e-22	21
PKIG	B PLCG2	3.966394e-26	-0.4660146	0.311	0.6	7.071684e-22	21
LDHA	B PLCG2	4.419017e-26	-0.4415837	0.2	0.453	7.878665e-22	21
ILF2	B PLCG2	4.772356e-26	-0.3050275	0.361	0.657	8.508633e-22	21
UBE2D3	B PLCG2	4.936966e-26	-0.5958981	0.1	0.33	8.802117e-22	21
ZFP36	B PLCG2	5.111509e-26	-0.4439457	0.135	0.377	9.113309e-22	21
PSMC3	B PLCG2	5.461492e-26	-0.393454	0.169	0.424	9.737294e-22	21
ADRM1	B PLCG2	5.993114e-26	0.8431613	0.147	0.061	1.068512e-21	21
ZWINT	B PLCG2	6.433851e-26	-0.32374	0.216	0.472	1.147091e-21	21
PRDX5	B PLCG2	6.903559e-26	-0.4532618	0.086	0.307	1.230836e-21	21
KARS	B PLCG2	8.961684e-26	-0.4360901	0.171	0.425	1.597779e-21	21
APEX1	B PLCG2	9.85978e-26	-0.4584038	0.048	0.227	1.7579e-21	21
C19orf24	B PLCG2	9.893442e-26	-0.8225275	0.035	0.199	1.763902e-21	21
ACP5	B PLCG2	1.221674e-25	-0.5512304	0.052	0.226	2.178123e-21	21
MAGED2	B PLCG2	1.245741e-25	1.019543	0.214	0.102	2.221031e-21	21
RMI2	B PLCG2	1.493312e-25	-0.4273612	0.155	0.388	2.662427e-21	21
UQCRC2	B PLCG2	1.655211e-25	-0.5966895	0.174	0.459	2.951075e-21	21
JUNB	B PLCG2	1.67523e-25	-0.5089587	0.041	0.193	2.986768e-21	21
S1PR4	B PLCG2	1.698453e-25	-0.6479152	0.06	0.243	3.028172e-21	21
RGS1	B PLCG2	1.824819e-25	-0.3447758	0.278	0.568	3.253469e-21	21
PCBP1	B PLCG2	1.886004e-25	0.8881065	0.851	0.906	3.362557e-21	21
ATP5F1E	B PLCG2	1.903139e-25	-0.3525923	0.149	0.34	3.393106e-21	21
SPIB	B PLCG2	1.907405e-25	-0.5213921	0.48	0.754	3.400713e-21	21
CXCR4	B PLCG2	1.988819e-25	1.095782	0.168	0.054	3.545866e-21	21
UBE2C	B PLCG2	2.895819e-25	-0.414893	0.166	0.423	5.162955e-21	21

KMT2E B PLCG2
5.733237e-25 -0.4422678 0.085 0.296 1.022179e-20 21
TNIP1 B PLCG2
5.907899e-25 -0.3525097 0.218 0.482 1.053319e-20 21
CD47 B PLCG2
6.318805e-25 0.8892432 0.104 0.032 1.12658e-20 21
ASPM B PLCG2
6.637209e-25 -0.8552085 0.067 0.271 1.183348e-20 21
GPR183 B PLCG2
7.115401e-25 1.142754 0.439 0.38 1.268605e-20 21
LSM8 B PLCG2
7.217451e-25 -0.5577235 0.029 0.198 1.286799e-20 21
AARS B PLCG2
7.451627e-25 0.7522282 0.128 0.055 1.328551e-20 21
SNHG19 B PLCG2
7.606101e-25 -0.4390217 0.062 0.239 1.356092e-20 21
UBE2L6 B PLCG2
8.664946e-25 -0.4545383 0.086 0.288 1.544873e-20 21
CNPY3 B PLCG2
9.475585e-25 0.5745346 0.955 0.976 1.689402e-20 21
RPS24 B PLCG2
1.085556e-24 -0.7558501 0.021 0.174 1.935437e-20 21
TNFRSF18 B PLCG2
1.38549e-24 0.8550863 0.143 0.06 2.470191e-20 21
HMGB3 B PLCG2
1.542164e-24 0.8484366 0.154 0.078 2.749523e-20 21
GMNN B PLCG2
1.665894e-24 0.8075949 0.145 0.079 2.970122e-20 21
UBE2T B PLCG2
2.289753e-24 -0.469088 0.105 0.327 4.082401e-20 21
P4HB B PLCG2
2.432077e-24 -0.6353172 0.064 0.258 4.336151e-20 21
SRGN B PLCG2
2.518988e-24 -0.450806 0.09 0.294 4.491103e-20 21
VPS35 B PLCG2
2.547903e-24 -0.4157601 0.069 0.286 4.542657e-20 21
DPP7 B PLCG2
2.597242e-24 0.8839968 0.154 0.077 4.630623e-20 21
MAD2L1 B PLCG2
2.64307e-24 -0.5520113 0.071 0.283 4.712329e-20 21
MSMO1 B PLCG2
2.867276e-24 -0.4622928 0.081 0.269 5.112067e-20 21
PMF1 B PLCG2
3.33891e-24 -0.6813885 0.098 0.315 5.952943e-20 21
HSP90B1 B PLCG2
4.65396e-24 -0.3001915 0.244 0.519 8.297545e-20 21
VAMP2 B PLCG2
5.564874e-24 -0.4112371 0.04 0.209 9.921613e-20 21
SND1 B PLCG2
6.173074e-24 0.7336499 0.119 0.055 1.100597e-19 21

NSUN6 B PLCG2
6.174243e-24 1.066558 0.263 0.187 1.100806e-19 21
PCNA B PLCG2
6.183609e-24 0.8743075 0.152 0.084 1.102476e-19 21
TMEM131L B PLCG2
6.217185e-24 -0.5325004 0.06 0.24 1.108462e-19 21
CD55 B PLCG2
7.839452e-24 -0.734534 0.014 0.16 1.397696e-19 21
LMNA B PLCG2
1.135389e-23 -0.4696038 0.083 0.296 2.024285e-19 21
ACADVL B PLCG2
1.418499e-23 -0.357993 0.114 0.334 2.529043e-19 21
COPZ1 B PLCG2
1.9586e-23 -0.3862446 0.192 0.446 3.491989e-19 21
CCT6A B PLCG2
1.960609e-23 -0.4125126 0.083 0.276 3.495569e-19 21
NAA10 B PLCG2
2.19879e-23 -0.4812315 0.038 0.203 3.920222e-19 21
TOMM40 B PLCG2
2.70377e-23 -0.4224756 0.086 0.292 4.820551e-19 21
C7orf50 B PLCG2
2.71927e-23 -0.4183594 0.142 0.396 4.848187e-19 21
TMED2 B PLCG2
3.172486e-23 -0.610235 0.126 0.34 5.656224e-19 21
HVCN1 B PLCG2
3.196303e-23 -0.326397 0.168 0.387 5.698689e-19 21
EVL B PLCG2
3.850098e-23 -0.8154413 0.102 0.318 6.86434e-19 21
CD69 B PLCG2
4.350771e-23 -0.3364761 0.197 0.458 7.75699e-19 21
CTSS B PLCG2
5.325597e-23 1.126621 0.482 0.456 9.495006e-19 21
COX17 B PLCG2
6.191215e-23 -0.4487374 0.071 0.257 1.103832e-18 21
PQBP1 B PLCG2
6.54078e-23 1.03945 0.202 0.12 1.166156e-18 21 LPP
B PLCG2
7.347364e-23 -1.064762 0.079 0.289 1.309961e-18 21
MIR155HG B PLCG2
8.282977e-23 -0.3145222 0.145 0.369 1.476772e-18 21
POLR2E B PLCG2
1.03161e-22 0.9325783 0.164 0.098 1.839257e-18 21
SYVN1 B PLCG2
1.097336e-22 -0.3354998 0.149 0.393 1.95644e-18 21
IK B PLCG2
1.100023e-22 -0.3182465 0.133 0.376 1.961231e-18 21
ARHGDIA B PLCG2
1.306308e-22 -0.3292537 0.111 0.334 2.329017e-18 21
PSMD2 B PLCG2
1.326713e-22 -0.4217322 0.059 0.239 2.365397e-18 21

STIP1	B PLCG2	1.454145e-22	-0.3528106	0.069	0.26	2.592595e-18	21
NSFL1C	B PLCG2	1.495376e-22	-0.4051027	0.124	0.364	2.666106e-18	21
TAPBP	B PLCG2	2.017035e-22	1.073847	0.301	0.242	3.596172e-18	21
SLBP	B PLCG2	2.066307e-22	0.8635446	0.187	0.113	3.684019e-18	21
SKA2	B PLCG2	3.066589e-22	-0.3259122	0.083	0.278	5.467421e-18	21
ASNA1	B PLCG2	3.121334e-22	-0.3948705	0.048	0.218	5.565026e-18	21
VPS51	B PLCG2	3.157292e-22	-0.413653	0.085	0.276	5.629136e-18	21
ATRAID	B PLCG2	3.342587e-22	-0.3353979	0.363	0.639	5.959498e-18	21
HERPUD1	B PLCG2	4.391579e-22	-0.4197359	0.097	0.297	7.829747e-18	21
YTHDF2	B PLCG2	4.91643e-22	-0.4194994	0.093	0.28	8.765503e-18	21
PIM2	B PLCG2	5.405893e-22	1.042382	0.489	0.474	9.638166e-18	21
SNRPD1	B PLCG2	6.689632e-22	0.8459816	0.187	0.115	1.192694e-17	21
LMNB1	B PLCG2	6.768184e-22	-0.365241	0.25	0.516	1.2067e-17	21
PA2G4	B PLCG2	6.936025e-22	-0.3491401	0.13	0.351	1.236624e-17	21
CYC1	B PLCG2	7.279839e-22	-0.403365	0.116	0.336	1.297922e-17	21
BTK	B PLCG2	7.641859e-22	-0.4139491	0.145	0.386	1.362467e-17	21
LAPTM4A	B PLCG2	8.509279e-22	-0.3347505	0.195	0.449	1.517119e-17	21
FBL	B PLCG2	1.158983e-21	-0.3424817	0.238	0.475	2.066351e-17	21
GPSM3	B PLCG2	1.369339e-21	-0.3049712	0.157	0.386	2.441395e-17	21
ATP6V0B	B PLCG2	1.567615e-21	-0.3947348	0.123	0.356	2.794902e-17	21
ANXA5	B PLCG2	2.076345e-21	-0.3473534	0.233	0.5	3.701916e-17	21
EIF4G2	B PLCG2	3.087987e-21	0.8034637	0.841	0.9	5.505572e-17	21
PFN1	B PLCG2	5.350184e-21	-0.4153279	0.13	0.339	9.538842e-17	21
CD48	B PLCG2	6.300334e-21	0.8690674	0.168	0.063	1.123287e-16	21
BIRC5	B PLCG2	6.506789e-21	-0.3137181	0.128	0.33	1.160095e-16	21

BST2	B PLCG2	7.614801e-21	-0.4498232	0.145	0.35	1.357643e-16	21
TRAF3IP3	B PLCG2	7.775643e-21	-0.6880256	0.06	0.24	1.386319e-16	21
CAPG	B PLCG2	7.824896e-21	1.020123	0.211	0.139	1.395101e-16	21
MKNK2	B PLCG2	8.085938e-21	0.7569377	0.142	0.068	1.441642e-16	21
CENPW	B PLCG2	8.490029e-21	-0.3681437	0.114	0.318	1.513687e-16	21
TRAM1	B PLCG2	8.546489e-21	-0.4401887	0.054	0.212	1.523753e-16	21
STX5	B PLCG2	8.58154e-21	-0.5237831	0.059	0.219	1.530003e-16	21
NFKBIE	B PLCG2	9.638533e-21	-0.3746821	0.064	0.232	1.718454e-16	21
DDOST	B PLCG2	1.007581e-20	-0.3546172	0.214	0.443	1.796416e-16	21
FDPS	B PLCG2	1.035143e-20	-0.4273186	0.036	0.186	1.845556e-16	21
PEPD	B PLCG2	1.048805e-20	-0.3674156	0.105	0.3	1.869914e-16	21
PDLIM1	B PLCG2	1.084798e-20	-0.6298026	0.052	0.22	1.934087e-16	21
EIF4EBP1	B PLCG2	1.174342e-20	0.7016015	0.128	0.052	2.093735e-16	21
SERPINA9	B PLCG2	1.204756e-20	-0.3711917	0.218	0.483	2.14796e-16	21
RSL1D1	B PLCG2	1.390885e-20	-0.3951763	0.059	0.22	2.479808e-16	21
PSMD1	B PLCG2	2.288918e-20	-0.3745934	0.209	0.459	4.080912e-16	21
DDX18	B PLCG2	2.303894e-20	0.9681106	0.63	0.646	4.107613e-16	21
SNRPG	B PLCG2	2.562545e-20	0.961114	0.174	0.08	4.568762e-16	21
NUSAP1	B PLCG2	2.596189e-20	-0.5932137	0.052	0.197	4.628746e-16	21
CLEC2B	B PLCG2	2.772479e-20	-0.3621202	0.114	0.308	4.943052e-16	21
FIS1	B PLCG2	2.845372e-20	-0.4614412	0.038	0.186	5.073014e-16	21
FKBP4	B PLCG2	3.457284e-20	-0.4246782	0.088	0.266	6.163991e-16	21
MRPL11	B PLCG2	3.518475e-20	0.6436127	0.126	0.06	6.273089e-16	21
DLEU2	B PLCG2	3.823712e-20	-0.3084753	0.164	0.392	6.817296e-16	21
CCT5	B PLCG2	3.854456e-20	-0.5064568	0.092	0.254	6.872109e-16	21

FCMR	B PLCG2	5.451327e-20	-0.4114805	0.069	0.224	9.719171e-16	21
CAPN1	B PLCG2	6.09361e-20	-0.343256	0.114	0.323	1.08643e-15	21
RHOG	B PLCG2	6.420805e-20	-0.390537	0.059	0.22	1.144765e-15	21
MRPL12	B PLCG2	6.924825e-20	-0.3431565	0.086	0.288	1.234627e-15	21
ATP6AP2	B PLCG2	7.9113e-20	-0.3659862	0.074	0.247	1.410506e-15	21
VDAC3	B PLCG2	1.009488e-19	-0.3619636	0.135	0.357	1.799815e-15	21
KPNB1	B PLCG2	1.518236e-19	1.291227	0.827	0.803	2.706863e-15	21
RPS26	B PLCG2	1.595565e-19	-0.3036561	0.178	0.446	2.844733e-15	21
CD164	B PLCG2	2.218559e-19	0.7676183	0.133	0.072	3.955469e-15	21
HELLS	B PLCG2	2.498272e-19	-0.3993236	0.054	0.22	4.454169e-15	21
WDR46	B PLCG2	2.551287e-19	-0.437378	0.073	0.234	4.548689e-15	21
HSD17B11	B PLCG2	2.721671e-19	-0.3862301	0.067	0.24	4.852467e-15	21
NELFE	B PLCG2	3.216711e-19	-0.3500609	0.088	0.274	5.735073e-15	21
BCAP31	B PLCG2	3.563451e-19	-0.3930517	0.081	0.253	6.353276e-15	21
DNPH1	B PLCG2	3.716248e-19	-0.4041122	0.064	0.209	6.625699e-15	21
CCDC32	B PLCG2	3.744509e-19	-0.3976312	0.168	0.404	6.676086e-15	21
CMPK1	B PLCG2	3.778722e-19	-0.3957671	0.048	0.199	6.737083e-15	21
TSSC4	B PLCG2	4.00907e-19	0.8039221	0.145	0.058	7.147772e-15	21
TYMS	B PLCG2	4.182964e-19	0.5965121	0.107	0.055	7.457807e-15	21
CENPU	B PLCG2	4.25617e-19	-0.3159905	0.067	0.23	7.588326e-15	21
DDX54	B PLCG2	4.422853e-19	-0.3433648	0.204	0.431	7.885504e-15	21
CCT2	B PLCG2	4.698242e-19	-0.4053842	0.04	0.187	8.376496e-15	21
YIF1A	B PLCG2	5.929948e-19	-0.3785682	0.076	0.244	1.05725e-14	21
ABCF1	B PLCG2	7.40158e-19	0.7984022	0.133	0.074	1.319628e-14	21
DNASE1	B PLCG2	1.072275e-18	-0.6232096	0.041	0.212	1.911759e-14	21

MGST3 B PLCG2
1.073038e-18 0.8322094 0.102 0.045 1.91312e-14 21
EML6 B PLCG2
1.146025e-18 -0.3963093 0.104 0.281 2.043247e-14 21
IFITM2 B PLCG2
1.289253e-18 -0.3119289 0.1 0.285 2.298609e-14 21
RPN2 B PLCG2
1.34199e-18 -0.3383383 0.109 0.282 2.392634e-14 21
DCTN2 B PLCG2
1.444733e-18 -0.5547053 0.052 0.198 2.575815e-14 21
CHPT1 B PLCG2
1.467462e-18 -0.4362085 0.092 0.28 2.616338e-14 21
MSN B PLCG2
1.496473e-18 -0.5398814 0.102 0.279 2.668061e-14 21
HSPA5 B PLCG2
1.632072e-18 -0.4128913 0.128 0.347 2.909821e-14 21
INSIG1 B PLCG2
1.670627e-18 -0.6093544 0.169 0.412 2.97856e-14 21
TXN B PLCG2
1.754203e-18 -0.3590893 0.1 0.269 3.127569e-14 21
HHEX B PLCG2
1.787252e-18 -0.3521194 0.093 0.277 3.186492e-14 21
SPG21 B PLCG2
1.793468e-18 -0.3723415 0.064 0.248 3.197575e-14 21
LAMP1 B PLCG2
1.840849e-18 -0.5283233 0.079 0.244 3.282049e-14 21
PHACTR1 B PLCG2
2.246805e-18 -0.3493966 0.159 0.377 4.005829e-14 21
TAX1BP1 B PLCG2
2.488642e-18 -0.3400267 0.066 0.232 4.436999e-14 21
PSMF1 B PLCG2
3.364722e-18 -0.323029 0.054 0.205 5.998963e-14 21
MAP2K3 B PLCG2
3.567481e-18 -0.3887774 0.066 0.222 6.360462e-14 21
C14orf119 B PLCG2
4.04383e-18 -0.3883703 0.078 0.25 7.209745e-14 21
OSTF1 B PLCG2
4.34479e-18 -0.3111197 0.109 0.295 7.746326e-14 21
HSPA4 B PLCG2
4.458672e-18 -0.3446096 0.214 0.448 7.949365e-14 21
C1QBP B PLCG2
5.061714e-18 0.769039 0.14 0.092 9.02453e-14 21
FAM208B B PLCG2
5.467538e-18 -0.3552073 0.06 0.225 9.748073e-14 21
TMEM9B B PLCG2
5.720621e-18 -0.3974287 0.043 0.196 1.019929e-13 21
FAM50A B PLCG2
6.294409e-18 1.000484 0.382 0.374 1.12223e-13 21
LCP1 B PLCG2
6.577432e-18 -0.4525505 0.05 0.205 1.17269e-13 21

NDFIP1 B PLCG2
6.849569e-18 -0.4651012 0.074 0.213 1.22121e-13 21
PLAC8 B PLCG2
6.931667e-18 0.7616165 0.149 0.09 1.235847e-13 21
HIST1H2AC B PLCG2
7.367906e-18 0.795337 0.155 0.064 1.313624e-13 21
CDKN3 B PLCG2
8.844042e-18 0.9715867 0.339 0.233 1.576804e-13 21
UBE2J1 B PLCG2
9.752361e-18 -0.327145 0.083 0.255 1.738748e-13 21
NOSIP B PLCG2
1.010712e-17 0.6013556 0.111 0.046 1.801998e-13 21
ASF1B B PLCG2
1.214936e-17 -0.4612854 0.052 0.203 2.166109e-13 21
NPM3 B PLCG2
1.264403e-17 -0.3068151 0.107 0.287 2.254304e-13 21
PPM1K B PLCG2
1.339943e-17 0.6985024 0.124 0.043 2.388984e-13 21
RRM2 B PLCG2
1.796511e-17 -0.3742375 0.029 0.17 3.202999e-13 21
MARS B PLCG2
1.873207e-17 -0.3885269 0.131 0.332 3.339741e-13 21
SMC6 B PLCG2
1.908639e-17 -0.3004443 0.128 0.3 3.402912e-13 21
BLK B PLCG2
2.021823e-17 -0.356534 0.043 0.169 3.604708e-13 21
HMOX2 B PLCG2
2.136378e-17 -0.4054041 0.092 0.255 3.808949e-13 21
SLC50A1 B PLCG2
2.463295e-17 -0.3444708 0.124 0.322 4.391808e-13 21
LTA4H B PLCG2
2.482016e-17 -0.4621784 0.04 0.185 4.425186e-13 21
PLPP5 B PLCG2
2.578094e-17 -0.3085916 0.038 0.19 4.596484e-13 21
YARS B PLCG2
2.69771e-17 -0.8681655 0.067 0.208 4.809747e-13 21
LGALS1 B PLCG2
2.940624e-17 1.039762 0.373 0.313 5.242838e-13 21
HMGN3 B PLCG2
3.476763e-17 -0.3808528 0.057 0.213 6.19872e-13 21
FMNL1 B PLCG2
3.65506e-17 1.021325 0.468 0.448 6.516606e-13 21
SEM1 B PLCG2
3.773248e-17 0.7318995 0.152 0.095 6.727324e-13 21
FAM76B B PLCG2
3.978852e-17 -0.3998699 0.043 0.184 7.093896e-13 21
CMSS1 B PLCG2
4.271801e-17 -0.4407614 0.071 0.209 7.616193e-13 21
PITHD1 B PLCG2
4.870207e-17 -0.3429448 0.145 0.348 8.683092e-13 21

DNAJB6 B PLCG2
4.886616e-17 -0.3095128 0.117 0.303 8.712347e-13 21
SRP72 B PLCG2
5.043935e-17 -0.3343587 0.078 0.208 8.992831e-13 21
CAT B PLCG2
5.521378e-17 -0.4027346 0.076 0.253 9.844065e-13 21
GARS B PLCG2
8.184926e-17 -0.3220031 0.016 0.124 1.459291e-12 21
ABCF2.1 B PLCG2
8.513457e-17 -0.5411715 0.254 0.507 1.517864e-12 21
HLA-DRB5 B PLCG2
8.681682e-17 -0.3833963 0.098 0.27 1.547857e-12 21
JUN B PLCG2
8.712208e-17 -0.3748547 0.04 0.181 1.5533e-12 21
DNPEP B PLCG2
9.219907e-17 -0.3275958 0.066 0.216 1.643817e-12 21
RBM22 B PLCG2
1.026015e-16 -0.308382 0.067 0.217 1.829282e-12 21
IMMT B PLCG2
1.386878e-16 0.8494534 0.176 0.083 2.472666e-12 21
AICDA B PLCG2
1.837221e-16 -0.5261403 0.021 0.138 3.275581e-12 21
PSAT1 B PLCG2
2.020611e-16 -0.3343492 0.048 0.191 3.602547e-12 21
RPS6KB2 B PLCG2
2.086089e-16 1.0658 0.278 0.277 3.719287e-12 21
GADD45B B PLCG2
2.22628e-16 -0.3477058 0.074 0.227 3.969234e-12 21
ORMDL2 B PLCG2
2.316732e-16 -0.3272698 0.124 0.325 4.130501e-12 21
NOP56 B PLCG2
2.384348e-16 -0.4450384 0.04 0.173 4.251055e-12 21
BCL2 B PLCG2
2.756435e-16 1.006722 0.394 0.386 4.914447e-12 21
NDUFB3 B PLCG2
2.853406e-16 0.7779304 0.157 0.09 5.087338e-12 21
GCSAM B PLCG2
2.946266e-16 -0.3132956 0.057 0.214 5.252898e-12 21
TINF2 B PLCG2
3.110291e-16 -0.3224484 0.071 0.214 5.545339e-12 21
IDH3G B PLCG2
3.112881e-16 -0.3311611 0.047 0.174 5.549955e-12 21
GTPBP6 B PLCG2
3.273235e-16 -0.3551867 0.052 0.182 5.835851e-12 21
FARSA B PLCG2
3.281172e-16 0.9520411 0.313 0.263 5.850002e-12 21
CD22 B PLCG2
3.465984e-16 -0.3145808 0.104 0.268 6.179503e-12 21
GRN B PLCG2
3.630541e-16 0.8260895 0.25 0.197 6.472891e-12 21

POLD4 B PLCG2
4.162688e-16 -0.3343517 0.135 0.311 7.421657e-12 21
CALR B PLCG2
4.332942e-16 -0.3130529 0.054 0.179 7.725202e-12 21
RRAGA B PLCG2
5.496149e-16 -0.3061389 0.054 0.191 9.799084e-12 21
NUP88 B PLCG2
5.775597e-16 -0.345387 0.064 0.208 1.029731e-11 21
STXBP2 B PLCG2
5.979269e-16 -0.3145754 0.036 0.142 1.066044e-11 21
SLC43A2 B PLCG2
6.105094e-16 -0.3246271 0.093 0.255 1.088477e-11 21
PRMT2 B PLCG2
6.435841e-16 -0.4389071 0.064 0.234 1.147446e-11 21
TARS B PLCG2
6.566416e-16 -0.3312939 0.052 0.193 1.170726e-11 21
SAT2 B PLCG2
7.01504e-16 -0.3401439 0.102 0.278 1.250712e-11 21
GLO1 B PLCG2
7.15827e-16 0.7249699 0.154 0.065 1.276248e-11 21
TK1 B PLCG2
7.45079e-16 -0.3402088 0.022 0.126 1.328401e-11 21
RPP38 B PLCG2
8.041059e-16 -0.3076881 0.083 0.241 1.43364e-11 21
FBXO7 B PLCG2
8.692355e-16 -0.4236698 0.043 0.159 1.54976e-11 21
TGIF1 B PLCG2
9.003692e-16 -0.3225362 0.067 0.219 1.605268e-11 21
EBP B PLCG2
1.067589e-15 1.098215 0.28 0.189 1.903405e-11 21
DAAM1 B PLCG2
1.167296e-15 -0.3474716 0.076 0.208 2.081172e-11 21
C16orf74 B PLCG2
1.272535e-15 -0.3396375 0.078 0.23 2.268802e-11 21
NOB1 B PLCG2
1.668894e-15 -0.3996564 0.076 0.258 2.975472e-11 21
NARS B PLCG2
2.007544e-15 -0.3257881 0.022 0.132 3.57925e-11 21
SRPRB B PLCG2
2.093411e-15 -0.3331412 0.047 0.181 3.732342e-11 21
SDF4 B PLCG2
2.343767e-15 -0.3536617 0.111 0.246 4.178703e-11 21
RASGRP2 B PLCG2
2.383874e-15 -0.3128529 0.048 0.161 4.250209e-11 21
YIPF3 B PLCG2
2.674071e-15 -0.3035259 0.062 0.217 4.7676e-11 21
SCAMP2 B PLCG2
2.867029e-15 -0.3432707 0.029 0.147 5.111626e-11 21
PRDX4 B PLCG2
2.967922e-15 0.7776158 0.145 0.084 5.291508e-11 21

TACC3	B PLCG2				
3.707145e-15	-0.3053595	0.076	0.223	6.609469e-11	21
COPB2	B PLCG2				
4.065875e-15	-0.3463879	0.038	0.155	7.249048e-11	21
PGD	B PLCG2				
4.12297e-15	-0.3183741	0.05	0.186	7.350843e-11	21
CSNK1D	B PLCG2				
4.695563e-15	0.8522569	0.142	0.1	8.371719e-11	21
AC084033.3	B PLCG2				
5.753686e-15	-0.3144584	0.111	0.291	1.025825e-10	21
TMCO1	B PLCG2				
7.527669e-15	-0.3982259	0.052	0.184	1.342108e-10	21
ARL2	B PLCG2				
8.752279e-15	-0.3263196	0.026	0.132	1.560444e-10	21
SLC39A1	B PLCG2				
9.576447e-15	-0.3243114	0.093	0.284	1.707385e-10	21
MT-ND4L	B PLCG2				
1.118008e-14	-0.3516739	0.047	0.169	1.993297e-10	21
GOLGA4	B PLCG2				
1.19455e-14	-0.4796792	0.036	0.168	2.129762e-10	21
CBX6	B PLCG2				
1.19713e-14	-0.3754687	0.088	0.254	2.134364e-10	21
NFE2L2	B PLCG2				
1.216589e-14	-0.3563041	0.035	0.17	2.169056e-10	21
TNIP2	B PLCG2				
1.278558e-14	-0.3128293	0.019	0.108	2.279541e-10	21
LEPROTL1	B PLCG2				
1.410924e-14	-0.3971893	0.143	0.321	2.515537e-10	21
SEC11C	B PLCG2				
1.449154e-14	-0.3007647	0.036	0.15	2.583697e-10	21
MRPS30	B PLCG2				
1.452048e-14	-0.3539425	0.031	0.141	2.588856e-10	21
RAB29	B PLCG2				
1.590906e-14	0.9825059	0.446	0.435	2.836427e-10	21
ROMO1	B PLCG2				
1.683497e-14	0.8794648	0.472	0.469	3.001507e-10	21
SNRPF	B PLCG2				
1.722669e-14	-0.3246778	0.043	0.164	3.071346e-10	21
KIAA1551	B PLCG2				
1.847141e-14	0.6156929	0.1	0.051	3.293267e-10	21
APOLD1	B PLCG2				
2.299545e-14	-0.5308655	0.022	0.127	4.099858e-10	21
LGALS3	B PLCG2				
2.665792e-14	-0.3582176	0.045	0.174	4.752841e-10	21
TIMM44	B PLCG2				
2.948662e-14	0.960897	0.385	0.346	5.25717e-10	21
BIRC3	B PLCG2				
3.015128e-14	-0.3848841	0.124	0.32	5.375672e-10	21
DDX21	B PLCG2				
3.054024e-14	-0.3561198	0.045	0.174	5.445019e-10	21

SBDS	B PLCG2	3.251021e-14	-0.3135393	0.142	0.305	5.796246e-10	21
ZFP36L2	B PLCG2	3.516722e-14	-0.312554	0.057	0.198	6.269963e-10	21
ATIC	B PLCG2	3.973906e-14	-0.5252739	0.052	0.2	7.085077e-10	21
YBX3	B PLCG2	3.982904e-14	-0.3257426	0.062	0.212	7.101119e-10	21
DNTTIP2	B PLCG2	4.432506e-14	-0.3055225	0.078	0.238	7.902716e-10	21
EIF2S1	B PLCG2	4.779908e-14	-0.3172431	0.074	0.232	8.522098e-10	21
TTC19	B PLCG2	5.749572e-14	-0.3238214	0.085	0.228	1.025091e-09	21
ALKBH7	B PLCG2	5.809775e-14	-0.5076871	0.031	0.138	1.035825e-09	21
FCER2	B PLCG2	5.913067e-14	-0.3019782	0.029	0.125	1.054241e-09	21
SART1	B PLCG2	6.771337e-14	-0.4538018	0.029	0.143	1.207262e-09	21
LY9	B PLCG2	8.686547e-14	-0.467706	0.052	0.165	1.548725e-09	21
MARCKS	B PLCG2	9.285841e-14	0.8240865	0.264	0.24	1.655573e-09	21
SRSF1	B PLCG2	9.488541e-14	-0.3034453	0.105	0.25	1.691712e-09	21
SNRPN	B PLCG2	1.124539e-13	-0.3277138	0.035	0.15	2.00494e-09	21
PIP4P1	B PLCG2	1.228857e-13	-0.3050031	0.055	0.186	2.190929e-09	21
MVD	B PLCG2	1.392292e-13	-0.3035395	0.062	0.195	2.482318e-09	21
HSPB1	B PLCG2	1.405727e-13	-0.3274284	0.136	0.329	2.506271e-09	21
PMAIP1	B PLCG2	1.412545e-13	-0.3009284	0.036	0.135	2.518427e-09	21
UNC45A	B PLCG2	1.438609e-13	-0.318342	0.047	0.16	2.564895e-09	21
EVI2A	B PLCG2	1.634143e-13	-0.3238729	0.038	0.147	2.913514e-09	21
SLC39A3	B PLCG2	1.635146e-13	-0.3458172	0.076	0.211	2.915303e-09	21
SELENOS	B PLCG2	1.666561e-13	-0.3223564	0.095	0.242	2.971312e-09	21
MYDGF	B PLCG2	2.638864e-13	0.8187796	0.225	0.16	4.704831e-09	21
C12orf75	B PLCG2	3.172805e-13	-0.325578	0.059	0.207	5.656795e-09	21
MT-ND6	B PLCG2	3.392856e-13	-0.3733271	0.054	0.171	6.049122e-09	21

CYSTM1 B PLCG2
4.040598e-13 0.8409703 0.477 0.49 7.203981e-09 21
CBX3 B PLCG2
5.383674e-13 -0.3116242 0.021 0.13 9.598552e-09 21
EEA1 B PLCG2
5.711245e-13 -0.3077559 0.09 0.234 1.018258e-08 21
EPB41L4A-AS1 B PLCG2
5.724767e-13 -0.3818907 0.021 0.119 1.020669e-08 21
FCGR2B B PLCG2
6.969848e-13 -0.3136902 0.026 0.119 1.242654e-08 21
HPCAL1 B PLCG2
6.9869e-13 -0.331061 0.126 0.286 1.245694e-08 21
GNG7 B PLCG2
1.095422e-12 -0.3250656 0.043 0.174 1.953027e-08 21
ASNS B PLCG2
1.197654e-12 -0.3785012 0.028 0.124 2.135297e-08 21
RAB13 B PLCG2
1.451544e-12 0.7929197 0.23 0.193 2.587958e-08 21
CBX5 B PLCG2
1.57153e-12 -0.3187966 0.057 0.19 2.80188e-08 21
GPATCH4 B PLCG2
2.104169e-12 0.7412443 0.195 0.161 3.751523e-08 21
TMPO B PLCG2
2.201581e-12 0.5997385 0.1 0.046 3.925199e-08 21
SOX4 B PLCG2
2.301861e-12 1.081813 0.207 0.133 4.103988e-08 21
NEIL1 B PLCG2
3.048507e-12 -0.324841 0.017 0.102 5.435183e-08 21
RHOC B PLCG2
3.334908e-12 -0.3358472 0.022 0.118 5.945808e-08 21
SLAMF1 B PLCG2
3.835569e-12 0.6268047 0.1 0.065 6.838437e-08 21
UHRF2 B PLCG2
4.016815e-12 -0.5171532 0.076 0.21 7.16158e-08 21
FOS B PLCG2
5.30676e-12 -0.4450642 0.13 0.301 9.461422e-08 21
NME1 B PLCG2
5.596627e-12 -0.3088964 0.029 0.124 9.978226e-08 21
HTATIP2 B PLCG2
6.079455e-12 -0.360616 0.069 0.196 1.083906e-07 21
ADK B PLCG2
6.281284e-12 -0.3010046 0.033 0.127 1.11989e-07 21
ZEB2 B PLCG2
6.628271e-12 0.6843682 0.157 0.124 1.181754e-07 21
TLE4 B PLCG2
7.622235e-12 0.8188971 0.173 0.129 1.358968e-07 21
STIM2 B PLCG2
8.2059e-12 -0.3740937 0.029 0.113 1.46303e-07 21
P2RY10 B PLCG2
8.575951e-12 -0.3830243 0.057 0.187 1.529006e-07 21

WDR43	B PLCG2					
9.201071e-12	-0.3471363	0.079	0.227	1.640459e-07	21	
TMEM109	B PLCG2					
9.285944e-12	0.659711	0.152	0.12	1.655591e-07	21	
SNHG9	B PLCG2					
9.94272e-12	-0.6662378	0.073	0.183	1.772687e-07	21	
XBP1	B PLCG2					
1.234882e-11	-0.408579	0.036	0.159	2.201671e-07	21	
SLC2A3	B PLCG2					
1.239086e-11	0.489943	0.102	0.062	2.209166e-07	21	
ORC6	B PLCG2					
1.265466e-11	-0.3285419	0.029	0.127	2.2562e-07	21	
MAP4	B PLCG2					
1.299873e-11	0.7639629	0.202	0.167	2.317544e-07	21	
CCDC167	B PLCG2					
1.935189e-11	0.667159	0.109	0.062	3.450248e-07	21	
HOPX	B PLCG2					
2.431941e-11	0.8937274	0.454	0.452	4.335908e-07	21	
MRPL51	B PLCG2					
2.561439e-11	-0.3016211	0.019	0.116	4.566789e-07	21	
PYCR1	B PLCG2					
2.774575e-11	0.9849945	0.339	0.321	4.94679e-07	21	
NAA38	B PLCG2					
3.548385e-11	-0.3386947	0.059	0.176	6.326416e-07	21	
ASAH1	B PLCG2					
4.894118e-11	-0.3397537	0.038	0.151	8.725723e-07	21	
CELF2	B PLCG2					
7.136541e-11	0.5956661	0.105	0.067	1.272374e-06	21	
FAM200B	B PLCG2					
9.537729e-11	0.5340523	0.107	0.067	1.700482e-06	21	
SGO1	B PLCG2					
1.070524e-10	-0.3017387	0.047	0.155	1.908638e-06	21	
RILPL2	B PLCG2					
1.54975e-10	0.5386693	0.109	0.074	2.76305e-06	21	
CUX1	B PLCG2					
1.559044e-10	-0.3355107	0.059	0.181	2.779619e-06	21	
RHOF	B PLCG2					
1.893572e-10	-0.3037729	0.045	0.149	3.376049e-06	21	
MVP	B PLCG2					
1.973927e-10	0.5762356	0.104	0.07	3.519315e-06	21	
NUP210	B PLCG2					
2.081853e-10	0.6318656	0.105	0.074	3.711736e-06	21	
PAG1	B PLCG2					
2.402091e-10	0.5150567	0.107	0.075	4.282688e-06	21	
EZH2	B PLCG2					
2.74036e-10	0.5541216	0.104	0.078	4.885789e-06	21	
WEE1	B PLCG2					
2.832328e-10	0.9389085	0.375	0.348	5.049757e-06	21	
S100A10	B PLCG2					
2.932688e-10	-0.3149559	0.026	0.116	5.228689e-06	21	

ENTPD1	B PLCG2	4.34369e-10	-0.3062513	0.064	0.161	7.744364e-06	21
AIDA	B PLCG2	4.705849e-10	-0.3431996	0.079	0.219	8.390059e-06	21
HMGCS1	B PLCG2	6.020483e-10	0.8264845	0.269	0.201	1.073392e-05	21
HMCES	B PLCG2	6.384559e-10	0.8436392	0.268	0.268	1.138303e-05	21
TXNDC17	B PLCG2	7.805551e-10	-0.339652	0.126	0.28	1.391652e-05	21
ADAM28	B PLCG2	8.53862e-10	0.6989095	0.131	0.108	1.522351e-05	21
PHF6	B PLCG2	9.287117e-10	-0.5984945	0.038	0.132	1.6558e-05	21
MYC	B PLCG2	9.919615e-10	0.7886712	0.237	0.238	1.768568e-05	21
AMD1	B PLCG2	1.210274e-09	-0.3331615	0.047	0.147	2.157798e-05	21
TSPYL1	B PLCG2	1.239223e-09	-0.3083249	0.052	0.149	2.209411e-05	21
ARRDC2	B PLCG2	1.798089e-09	0.7381493	0.25	0.233	3.205812e-05	21
CHCHD10	B PLCG2	1.855126e-09	0.8393702	0.282	0.268	3.307504e-05	21
MBNL1	B PLCG2	2.041575e-09	0.7732267	0.527	0.595	3.639924e-05	21
TBCA	B PLCG2	2.532682e-09	-0.3521008	0.033	0.134	4.515519e-05	21
AC016831.5	B PLCG2	2.980961e-09	0.937944	0.484	0.507	5.314755e-05	21
MZT2B	B PLCG2	3.022269e-09	-0.3535932	0.078	0.201	5.388403e-05	21
PPP3CA	B PLCG2	3.897241e-09	0.7375697	0.145	0.121	6.948391e-05	21
EPS15	B PLCG2	4.932771e-09	0.731143	0.152	0.145	8.794637e-05	21
DNAJC10	B PLCG2	5.670807e-09	0.7922791	0.418	0.457	0.0001011048	21
LSM5	B PLCG2	6.336684e-09	0.7722502	0.309	0.289	0.0001129767	21
DCK	B PLCG2	6.88079e-09	0.6759752	0.155	0.128	0.0001226776	21
RABGAP1L	B PLCG2	8.552293e-09	0.5816885	0.105	0.047	0.0001524788	21
CDC20	B PLCG2	9.96262e-09	0.797599	0.219	0.169	0.0001776236	21
SMC4	B PLCG2	1.093167e-08	0.8337808	0.238	0.237	0.0001949007	21
AC004687.1	B PLCG2	1.323852e-08	0.6764199	0.242	0.222	0.0002360296	21

THUMPD3-AS1 B PLCG2
1.529624e-08 -0.316131 0.022 0.107 0.0002727167 21
AC058791.1 B PLCG2
1.547395e-08 -0.3058176 0.028 0.11 0.0002758851 21
SNX9 B PLCG2
1.636513e-08 0.7955985 0.195 0.168 0.000291774 21
SEL1L3 B PLCG2
1.663554e-08 0.8062343 0.701 0.807 0.0002965951 21
CALM2 B PLCG2
1.832522e-08 -0.3218614 0.024 0.106 0.0003267203 21
MTSS1 B PLCG2
2.251884e-08 0.7664887 0.211 0.195 0.0004014883 21
USP34 B PLCG2
2.309872e-08 0.5755961 0.128 0.1 0.0004118271 21
PTPN7 B PLCG2
2.421716e-08 0.567812 0.105 0.052 0.0004317677 21
CENPF B PLCG2
2.497506e-08 0.6667956 0.105 0.054 0.0004452804 21
CCNB2 B PLCG2
2.791478e-08 0.8365732 0.558 0.621 0.0004976926 21
NDUFA1 B PLCG2
3.066652e-08 -0.3120017 0.064 0.164 0.0005467533 21
FAM215B B PLCG2
3.854546e-08 0.5984274 0.13 0.107 0.000687227 21
IMMP1L B PLCG2
5.39606e-08 0.7938687 0.539 0.615 0.0009620635 21
ARPC5 B PLCG2
6.689943e-08 0.4823049 0.1 0.075 0.00119275 21
EPM2AIP1 B PLCG2
7.011078e-08 0.6281258 0.188 0.168 0.001250005 21
TFDP1 B PLCG2
9.705576e-08 0.7323212 0.187 0.165 0.001730407 21
DEF8 B PLCG2
1.060711e-07 0.7899454 0.525 0.557 0.001891142 21
ATP5IF1 B PLCG2
1.138965e-07 0.699171 0.283 0.274 0.002030661 21
RSRP1 B PLCG2
1.430678e-07 0.6593807 0.261 0.263 0.002550755 21
HSPB11 B PLCG2
1.455358e-07 0.5925508 0.109 0.072 0.002594758 21
LINC01857 B PLCG2
1.682681e-07 0.5952963 0.138 0.127 0.003000051 21
PRKD3 B PLCG2
1.852342e-07 0.8107288 0.644 0.724 0.003302541 21
COX6C B PLCG2
2.112097e-07 0.5208357 0.116 0.089 0.003765658 21
DCP2 B PLCG2
2.131468e-07 0.3377396 0.964 0.996 0.003800194 21
RPL31 B PLCG2
2.141207e-07 0.6042083 0.145 0.136 0.003817559 21

CETN3 B PLCG2
2.157647e-07 0.5801114 0.123 0.097 0.003846869 21
STAG3 B PLCG2
2.488655e-07 0.7425656 0.168 0.155 0.004437023 21
JUND B PLCG2
2.916446e-07 0.4640803 0.105 0.082 0.005199731 21
RFC4 B PLCG2
3.557515e-07 0.4938968 0.119 0.099 0.006342694 21
GON7 B PLCG2
4.524258e-07 0.4772796 0.105 0.077 0.008066299 21
GINS2 B PLCG2
5.365512e-07 0.6481135 0.166 0.143 0.009566171 21
RAB30 B PLCG2
5.550505e-07 0.8494362 0.466 0.493 0.009895995 21
C4orf3 B PLCG2
5.5867e-07 0.7572764 0.37 0.401 0.009960527 21
ENY2 B PLCG2
6.069992e-07 0.6312678 0.183 0.174 0.01082219 21
NUDT1 B PLCG2
7.174155e-07 0.6244211 0.589 0.588 0.0127908 21
MARCKSL1 B PLCG2
1.292273e-06 0.5367066 0.131 0.115 0.02303994 21
FEN1 B PLCG2
1.432828e-06 0.8460493 0.249 0.259 0.0255459 21
SYNE2 B PLCG2
2.061903e-06 0.4415926 0.117 0.1 0.03676166 21
SYNC B PLCG2
2.214177e-06 0.6832097 0.744 0.831 0.03947657 21
UQCRB B PLCG2
2.33659e-06 0.7984672 0.58 0.66 0.04165906 21
UBL5 B PLCG2
2.663388e-06 0.5860262 0.183 0.154 0.04748554 21
MCM7 B PLCG2
2.804314e-06 0.5397257 0.173 0.159 0.04999811 21
VRK1 B PLCG2
3.013347e-06 0.763465 0.226 0.23 0.05372497 21
CKS2 B PLCG2
3.626649e-06 0.6948073 0.444 0.485 0.06465953 21
RPS27L B PLCG2
3.711503e-06 0.6932746 0.66 0.747 0.06617239 21
SNRPD2 B PLCG2
3.877112e-06 0.5262272 0.145 0.139 0.06912503 21
ITGB3BP B PLCG2
4.166002e-06 0.6220803 0.164 0.169 0.07427564 21
TARDBP B PLCG2
4.311198e-06 0.4936851 0.123 0.111 0.07686435 21
RAD51C B PLCG2
4.44177e-06 0.6905426 0.244 0.236 0.07919232 21
DSTN B PLCG2
4.872224e-06 0.6967526 0.249 0.271 0.08686688 21

MRPL33 B PLCG2
5.962015e-06 0.5416379 0.131 0.117 0.1062968 21
BRWD1 B PLCG2
6.222835e-06 0.609623 0.169 0.163 0.1109469 21
LYPLAL1 B PLCG2
6.379181e-06 0.5787055 0.104 0.07 0.1137344 21
RASSF6 B PLCG2
6.437196e-06 0.7935898 0.195 0.16 0.1147688 21
SUSD3 B PLCG2
6.626677e-06 0.5560833 0.123 0.104 0.118147 21
LNPEP B PLCG2
9.548623e-06 0.7489819 0.237 0.206 0.1702424 21
EAF2 B PLCG2
9.772147e-06 0.5062188 0.112 0.104 0.1742276 21
BCL2L12 B PLCG2
1.22305e-05 0.652645 0.278 0.291 0.2180575 21
SIVA1 B PLCG2
1.495289e-05 0.6373761 0.168 0.146 0.266595 21
SEC14L1 B PLCG2
1.866888e-05 0.5992224 0.152 0.12 0.3328475 21
VNN2 B PLCG2
1.871909e-05 0.6443763 0.244 0.257 0.3337426 21
MDM4 B PLCG2
1.88352e-05 0.6073046 0.188 0.19 0.3358127 21
LBR B PLCG2
1.933824e-05 0.5971989 0.214 0.193 0.3447814 21
DNMT1 B PLCG2
2.054792e-05 0.5345616 0.105 0.06 0.3663489 21
MKI67 B PLCG2
2.150528e-05 0.4136419 0.105 0.091 0.3834177 21
MSH6 B PLCG2
2.693155e-05 0.5288697 0.112 0.107 0.4801625 21
MAT2A B PLCG2
3.050805e-05 0.5169096 0.131 0.136 0.543928 21
PUS7L B PLCG2
3.895703e-05 0.772247 0.193 0.153 0.6945648 21
CD81 B PLCG2
3.903799e-05 0.4825067 0.114 0.115 0.6960082 21
EBF1 B PLCG2
4.021544e-05 0.6367424 0.364 0.402 0.7170011 21
RPA3 B PLCG2
4.870195e-05 0.4157201 0.119 0.111 0.8683071 21
NFYB B PLCG2
5.217066e-05 0.4584545 0.104 0.098 0.9301507 21
RAB30-AS1 B PLCG2
5.372886e-05 0.6514377 0.321 0.349 0.9579318 21
NASP B PLCG2
5.492227e-05 0.5376941 0.105 0.107 0.9792091 21
TCF3 B PLCG2
5.51655e-05 0.6403995 0.199 0.148 0.9835456 21

BIK B PLCG2
5.9092e-05 0.5063723 0.121 0.108 1 21
OGG1 B PLCG2
5.957092e-05 0.6344155 0.307 0.313 1 21
PSIP1 B PLCG2
6.294464e-05 0.6652344 0.594 0.676 1 21
ATP5ME B PLCG2
6.74143e-05 0.6182993 0.155 0.157 1 21
SMIM20 B PLCG2
7.23015e-05 0.4647614 0.155 0.153 1 21
MZT1 B PLCG2
7.790702e-05 0.523523 0.126 0.107 1 21 E2F5 B
PLCG2
9.097833e-05 0.6935701 0.508 0.56 1 21
ATP5PF B PLCG2
9.320365e-05 0.7157791 0.375 0.428 1 21
MINOS1 B PLCG2
9.461079e-05 0.5487251 0.178 0.182 1 21
RASGRP3 B PLCG2
9.461522e-05 -0.7857552 0.071 0.149 1 21
IGHA1 B PLCG2
9.698805e-05 0.742564 0.269 0.275 1 21 ITGAE B
PLCG2
0.0001083214 0.5330264 0.136 0.125 1 21
AC025164.1 B PLCG2
0.000109204 0.5136203 0.123 0.112 1 21
BRI3BP B PLCG2
0.000115263 0.4649409 0.112 0.119 1 21
FMC1 B PLCG2
0.0001244367 0.5464547 0.154 0.158 1 21
ANKRD49 B PLCG2
0.0001582468 0.5835238 0.173 0.182 1 21
MED30 B PLCG2
0.0001623137 0.6969254 0.306 0.297 1 21
GGA2 B PLCG2
0.0001689426 0.645537 0.472 0.53 1 21 SRSF7 B
PLCG2
0.0001968094 0.4351764 0.185 0.211 1 21
GABPB1-AS1 B PLCG2
0.0002561591 0.6821809 0.611 0.697 1 21
ATP5MPL B PLCG2
0.0002802167 0.5563618 0.13 0.126 1 21
COMMD4 B PLCG2
0.0002877979 0.5537636 0.112 0.095 1 21
RNGTT B PLCG2
0.0002923774 0.6897912 0.278 0.312 1 21
MZT2A B PLCG2
0.0003529258 0.5436727 0.29 0.329 1 21
HNRNPH1 B PLCG2
0.0004007597 0.4947631 0.109 0.101 1 21

BACH2 B PLCG2
0.0004039222 0.7007228 0.28 0.321 1 21
AP3S1 B PLCG2
0.0004112966 0.7962527 0.2 0.168 1 21
CCDC88A B PLCG2
0.0004313115 0.6437601 0.261 0.281 1 21
JPT1 B PLCG2
0.0004780221 0.5835341 0.335 0.383 1 21
SRSF10 B PLCG2
0.00048107 0.6130962 0.309 0.346 1 21
LSM6 B PLCG2
0.0005281253 0.3924742 0.33 0.397 1 21
CSTB B PLCG2
0.0005680171 0.4939534 0.155 0.152 1 21
CD72 B PLCG2
0.0005695965 0.5421552 0.143 0.118 1 21
CD38 B PLCG2
0.0005998663 0.7375624 0.615 0.708 1 21
NDUFA4 B PLCG2
0.000663416 0.7028023 0.487 0.572 1 21
SNRPE B PLCG2
0.0006936912 0.4955489 0.114 0.112 1 21
CKLF B PLCG2
0.0008080735 0.539126 0.228 0.263 1 21 ANP32E B
PLCG2
0.000846089 0.6147379 0.207 0.237 1 21
ATP6V1E1 B PLCG2
0.0008577985 0.5992766 0.328 0.38 1 21
ANAPC11 B PLCG2
0.0008695652 0.6507399 0.133 0.134 1 21
PLEKHA2 B PLCG2
0.0009162 0.5431675 0.204 0.233 1 21
CHCHD7 B PLCG2
0.0009270319 0.4780782 0.119 0.12 1 21
CD180 B PLCG2
0.0009456746 0.7022013 0.449 0.519 1 21
COX8A B PLCG2
0.0009731904 0.3939719 0.121 0.123 1 21
NUP62 B PLCG2
0.001004543 0.580914 0.126 0.116 1 21 GMDS B
PLCG2
0.001015238 0.6190047 0.295 0.31 1 21
CALM3 B PLCG2
0.001035991 0.6209115 0.361 0.394 1 21
SYPL1 B PLCG2
0.001076902 0.5375338 0.112 0.114 1 21
PAX5 B PLCG2
0.001188719 0.6926707 0.399 0.478 1 21 FUS
B PLCG2
0.001216537 0.482017 0.686 0.655 1 21 TCL1A B

PLCG2
0.00139379 0.5560212 0.124 0.112 1 21
CPNE5 B PLCG2
0.001505546 0.4095033 0.107 0.111 1 21
ZNF644 B PLCG2
0.001559948 0.4382424 0.114 0.093 1 21
CCNB1 B PLCG2
0.001691928 0.4183122 0.145 0.141 1 21
SMC2 B PLCG2
0.001932743 0.3418604 0.396 0.556 1 21
SEC61G B PLCG2
0.001985701 0.7222397 0.585 0.664 1 21 DBI
B PLCG2
0.001993111 0.6070449 0.187 0.209 1 21
MIS18BP1 B PLCG2
0.002061325 0.5463874 0.309 0.363 1 21
ELOC B PLCG2
0.002602705 0.4196625 0.117 0.122 1 21
NFATC2IP B PLCG2
0.002835775 0.4315816 0.131 0.142 1 21
ANAPC15 B PLCG2
0.002957783 0.4409271 0.085 0.101 1 21
SMG1 B PLCG2
0.002963109 0.6238155 0.261 0.237 1 21
GCHFR B PLCG2
0.003008729 0.5612887 0.396 0.43 1 21
IRF8 B PLCG2
0.003209103 0.4648463 0.164 0.187 1 21
KIF20B B PLCG2
0.003876655 0.3559828 0.128 0.134 1 21
NUP37 B PLCG2
0.003879406 0.3434584 0.459 0.626 1 21
LAMTOR5 B PLCG2
0.004045405 0.3493897 0.1 0.099 1 21
HERC4 B PLCG2
0.004258476 0.304016 0.466 0.624 1 21 ARPC1B B
PLCG2
0.004352917 0.380225 0.09 0.102 1 21 BOLA3 B
PLCG2
0.00459502 0.564971 0.15 0.182 1 21 MCL1 B
PLCG2
0.004962217 0.534379 0.373 0.424 1 21 DEK B
PLCG2
0.005011071 0.5290662 0.109 0.101 1 21
MYBL2 B PLCG2
0.005255716 0.5026131 0.285 0.318 1 21
SMIM26 B PLCG2
0.005293589 0.3808697 0.478 0.65 1 21
UQCR11 B PLCG2
0.005507312 0.4266464 0.104 0.117 1 21

ACYP2	B PLCG2
0.005840218	-1.683611	0.427	0.451	1	21
IGHG3	B PLCG2
0.007026856	0.3069014	0.432	0.623	1	21
ACTR3	B PLCG2
0.007522057	0.5619035	0.117	0.129	1	21
ETS1	B PLCG2
0.007640056	0.3352228	0.102	0.099	1	21
RRM1	B PLCG2
0.008144491	0.5264299	0.111	0.114	1	21
HDAC7	B PLCG2
0.008162702	0.5158951	0.264	0.311	1	21
CHURC1	B PLCG2
0.00832441	0.6371634	0.775	0.897	1	21
PPIA	B PLCG2
0.008658265	0.3827335	0.09	0.103	1	21
MRPL35	B PLCG2
0.009221373	0.5373797	0.206	0.234	1	21
ZCCHC7	B PLCG2
0	2.175821	0.547	0.015	0	22	IFI44L	Naive IFN1
1.214861e-238	2.053225	0.565	0.093	2.165975e-234	22	MX1
	Naive IFN1
5.285282e-218	1.692904	0.353	0.011	9.423129e-214	22
IFIT3	Naive IFN1
1.462961e-192	2.159296	0.623	0.213	2.608313e-188	22
ISG15	Naive IFN1
4.57532e-150	1.324339	0.24	0.006	8.157339e-146	22
IFIT1	Naive IFN1
7.356768e-140	1.491519	0.391	0.056	1.311638e-135	22	MX2
	Naive IFN1
1.55548e-137	1.538383	0.733	0.346	2.773265e-133	22
LY6E	Naive IFN1
2.737739e-105	1.274231	0.305	0.035	4.881115e-101	22
XAF1	Naive IFN1
2.265355e-96	1.237015	0.267	0.04	4.038901e-92	22
IFI6	Naive IFN1
1.20275e-81	1.134807	0.283	0.06	2.144383e-77	22
PLSCR1	Naive IFN1
3.60167e-80	0.4234197	1	0.998	6.421417e-76	22
	B2M	Naive IFN1
1.811746e-72	1.17665	0.315	0.081	3.230162e-68	22
OAS1	Naive IFN1
1.941179e-70	0.7944895	0.168	0.012	3.460928e-66	22
	IFI44	Naive IFN1
8.86178e-65	0.3854182	1	1	1.579967e-60	22
	MALAT1	Naive IFN1
1.864516e-63	1.175325	0.405	0.17	3.324246e-59	22
IRF7	Naive IFN1
2.561246e-58	1.059085	0.267	0.067	4.566446e-54	22
SAMD9L	Naive IFN1

5.171741e-58	1.105418	0.313	0.122	9.220697e-54	22	EIF2AK2	Naive IFN1
3.940873e-57	1.063224	0.782	0.531	7.026183e-53	22	ISG20	Naive IFN1
4.028637e-54	0.8096357	0.156	0.018	7.182657e-50	22	HERC5	Naive IFN1
8.14949e-54	0.9132776	0.246	0.059	1.452973e-49	22	EPSTI1	Naive IFN1
5.476873e-49	0.8153478	0.884	0.749	9.764716e-45	22	CXCR4	Naive IFN1
1.082338e-48	0.4228712	0.998	0.905	1.9297e-44	22	IGHM	Naive IFN1
2.177115e-43	-1.273808	0.03	0.208	3.881578e-39	22	RGS13	Naive IFN1
4.211431e-41	-1.037121	0.311	0.602	7.50856e-37	22	PRDX1	Naive IFN1
1.00816e-40	0.6828208	0.132	0.019	1.797448e-36	22	IFIT2	Naive IFN1
1.310213e-40	0.8674279	0.747	0.618	2.335979e-36	22	IFI16	Naive IFN1
3.188297e-39	-1.025611	0.77	0.889	5.684415e-35	22	GAPDH	Naive IFN1
5.792159e-38	0.4666514	0.996	0.985	1.032684e-33	22	TMSB10	Naive IFN1
8.224428e-38	0.7137199	0.192	0.054	1.466333e-33	22	STAT1	Naive IFN1
2.047997e-37	0.8773565	0.605	0.369	3.651375e-33	22	SELL	Naive IFN1
5.692421e-35	0.4774575	0.972	0.935	1.014902e-30	22	BTG1	Naive IFN1
5.119359e-34	-0.7971572	0.529	0.764	9.127305e-30	22	PKM	Naive IFN1
3.741169e-33	0.803892	0.25	0.097	6.670131e-29	22	TRIM22	Naive IFN1
8.718748e-33	0.929475	0.457	0.283	1.554466e-28	22	SP110	Naive IFN1
6.307312e-32	0.5890658	0.772	0.604	1.124531e-27	22	IGHD	Naive IFN1
8.798184e-32	-0.9107296	0.068	0.302	1.568628e-27	22	NME1	Naive IFN1
7.955764e-31	-1.009945	0.499	0.718	1.418433e-26	22	ENO1	Naive IFN1
9.824076e-31	0.7741577	0.261	0.115	1.751534e-26	22	SAMD9	Naive IFN1
6.258012e-30	0.9051837	0.363	0.235	1.115741e-25	22	UBE2L6	Naive IFN1
7.917275e-29	-1.068834	0.172	0.406	1.411571e-24	22	FABP5	Naive IFN1
4.227342e-28	-0.6504569	0.152	0.396	7.536928e-24	22	YWHAE	Naive IFN1

4.13626e-27	-0.5628057	0.385	0.631	7.374539e-23	22
ATP5F1B	Naive IFN1				
1.54077e-26	-0.5446088	0.357	0.611	2.747039e-22	22
POLR2L	Naive IFN1				
1.347952e-25	-0.8214898	0.369	0.599	2.403264e-21	22
LDHA	Naive IFN1				
5.812236e-25	-0.6012986	0.782	0.901	1.036264e-20	22
PFN1	Naive IFN1				
1.173194e-24	-0.6344861	0.242	0.477	2.091687e-20	22
SNRPD1	Naive IFN1				
2.319111e-24	-0.7266311	0.02	0.153	4.134743e-20	22
DUSP2	Naive IFN1				
3.960609e-24	-0.5265304	0.218	0.451	7.06137e-20	22
SEM1	Naive IFN1				
4.517879e-24	-0.5662419	0.367	0.6	8.054926e-20	22
SEC61B	Naive IFN1				
8.3831e-24	-0.5546005	0.337	0.562	1.494623e-19	22
ATP5PF	Naive IFN1				
9.309959e-24	-0.6416944	0.076	0.236	1.659873e-19	22
UBE2J1	Naive IFN1				
5.278989e-23	-0.6216082	0.359	0.586	9.41191e-19	22
ANP32B	Naive IFN1				
5.627347e-23	-0.6524159	0.184	0.393	1.0033e-18	22
EIF5A	Naive IFN1				
6.013502e-23	-0.5849797	0.218	0.452	1.072147e-18	22
ILF2	Naive IFN1				
6.015346e-23	0.4994519	0.918	0.847	1.072476e-18	22
CD37	Naive IFN1				
6.129888e-23	-0.621465	0.206	0.447	1.092898e-18	22
C1QBP	Naive IFN1				
2.670985e-22	0.7066941	0.341	0.227	4.7621e-18	22
NT5C3A	Naive IFN1				
4.767045e-22	0.4473124	0.954	0.867	8.499164e-18	22
CD52	Naive IFN1				
9.740517e-22	-0.6877651	0.545	0.738	1.736637e-17	22
HSPA8	Naive IFN1				
1.199726e-21	-0.5911499	0.222	0.442	2.138992e-17	22
HMGA1	Naive IFN1				
1.411472e-21	-0.6231224	0.16	0.387	2.516513e-17	22
NHP2	Naive IFN1				
1.863409e-21	0.7093877	0.417	0.284	3.322272e-17	22
PPM1K	Naive IFN1				
1.882186e-21	0.6656814	0.238	0.121	3.35575e-17	22
RNF213	Naive IFN1				
3.408551e-21	-0.7489234	0.054	0.192	6.077106e-17	22
PTTG1	Naive IFN1				
4.463311e-21	-0.6255306	0.876	0.943	7.957638e-17	22
YBX1	Naive IFN1				
7.528956e-21	-0.6413857	0.138	0.305	1.342338e-16	22
CD27	Naive IFN1				

2.600988e-20	-0.514779	0.379	0.582	4.637301e-16	22	LDHB	Naive IFN1
2.866089e-20	-0.6797827	0.337	0.561	5.10995e-16	22	NCL	Naive IFN1
2.979752e-20	-0.5180606	0.345	0.557	5.3126e-16	22	SEC61G	Naive IFN1
3.080128e-20	-0.5493829	0.569	0.75	5.49156e-16	22	HSP90AA1	Naive IFN1
3.589009e-20	-0.470954	0.381	0.587	6.398844e-16	22	ATP5MC3	Naive IFN1
4.560769e-20	-0.4773425	0.327	0.537	8.131395e-16	22	SRSF9	Naive IFN1
4.626496e-20	-0.5140399	0.375	0.608	8.248579e-16	22	RBM3	Naive IFN1
7.162113e-20	-0.8265789	0.19	0.411	1.276933e-15	22	TXN	Naive IFN1
1.122776e-19	-0.5118795	0.485	0.682	2.001798e-15	22	SRSF3	Naive IFN1
2.086072e-19	-0.459921	0.433	0.636	3.719257e-15	22	PSMA4	Naive IFN1
2.356345e-19	-0.3294634	0.01	0.104	4.201128e-15	22	SEMA4A	Naive IFN1
3.443647e-19	-0.5564447	0.064	0.236	6.139679e-15	22	MTHFD2	Naive IFN1
6.427786e-19	-0.4804254	0.144	0.351	1.14601e-14	22	TIMM13	Naive IFN1
6.545388e-19	-0.6522796	0.589	0.75	1.166977e-14	22	RAN	Naive IFN1
7.231775e-19	-0.4568468	0.064	0.22	1.289353e-14	22	NANS	Naive IFN1
7.235672e-19	-0.5099889	0.427	0.643	1.290048e-14	22	HNRNPM	Naive IFN1
8.623153e-19	-0.5955995	0.607	0.77	1.537422e-14	22	HMGN1	Naive IFN1
9.149868e-19	-0.816674	0.393	0.579	1.63133e-14	22	TUBB	Naive IFN1
1.203871e-18	-0.6119903	0.289	0.5	2.146381e-14	22	HSPE1	Naive IFN1
1.458733e-18	-0.3566128	0.527	0.725	2.600776e-14	22	COX6C	Naive IFN1
1.533557e-18	-0.5180298	0.347	0.555	2.734179e-14	22	TCEA1	Naive IFN1
1.629248e-18	-0.5097597	0.513	0.705	2.904786e-14	22	TPI1	Naive IFN1
1.665651e-18	-0.4511947	0.136	0.325	2.969689e-14	22	IMP4	Naive IFN1
1.938982e-18	0.6181359	0.194	0.102	3.457011e-14	22	IFI35	Naive IFN1
2.065711e-18	-0.5279468	0.062	0.192	3.682956e-14	22	DAAM1	Naive IFN1

3.759744e-18	-0.7548006	0.655	0.787	6.703247e-14	22
ACTG1	Naive IFN1				
3.876515e-18	-0.5026418	0.222	0.445	6.911439e-14	22
CCT6A	Naive IFN1				
4.102694e-18	-0.8979608	0.503	0.668	7.314693e-14	22
TUBA1B	Naive IFN1				
4.991602e-18	-0.3980755	0.359	0.575	8.899527e-14	22
PARK7	Naive IFN1				
5.522735e-18	-0.4808464	0.228	0.444	9.846484e-14	22
PPA1	Naive IFN1				
7.409382e-18	-0.3047861	0.934	0.977	1.321019e-13	22
RPS24	Naive IFN1				
9.159786e-18	-0.4364873	0.19	0.397	1.633098e-13	22
NDUFAB1	Naive IFN1				
1.016535e-17	-0.5153407	0.19	0.391	1.812381e-13	22
CCT5	Naive IFN1				
1.207665e-17	-0.3975212	0.433	0.642	2.153145e-13	22
ERH	Naive IFN1				
1.886077e-17	-0.4640987	0.415	0.61	3.362688e-13	22
POMP	Naive IFN1				
1.969212e-17	-0.460718	0.345	0.578	3.510907e-13	22
APRT	Naive IFN1				
2.48108e-17	-0.3930504	0.497	0.687	4.423518e-13	22
COX6A1	Naive IFN1				
2.883871e-17	-0.4089546	0.766	0.871	5.141654e-13	22
ARPC2	Naive IFN1				
3.017459e-17	-0.4183241	0.222	0.422	5.379827e-13	22
HNRNPD	Naive IFN1				
3.487305e-17	-0.5961891	0.162	0.355	6.217516e-13	22
TUBB4B	Naive IFN1				
3.546348e-17	-0.4404175	0.353	0.567	6.322784e-13	22
SNRPB	Naive IFN1				
4.005905e-17	-0.3181546	0.014	0.102	7.142127e-13	22
MYBL2	Naive IFN1				
4.436734e-17	-0.4051946	0.517	0.702	7.910252e-13	22
ELOB	Naive IFN1				
7.360654e-17	-0.4358859	0.206	0.419	1.312331e-12	22
DDX39A	Naive IFN1				
7.417529e-17	0.6637627	0.357	0.21	1.322471e-12	22
PLAC8	Naive IFN1				
7.937113e-17	0.6125769	0.555	0.466	1.415108e-12	22
ITM2B	Naive IFN1				
7.963631e-17	-0.5039432	0.042	0.15	1.419836e-12	22
BIK	Naive IFN1				
8.428573e-17	-0.4658546	0.068	0.235	1.50273e-12	22
CDK4	Naive IFN1				
9.275869e-17	-0.3793566	0.371	0.586	1.653795e-12	22
NDUFB2	Naive IFN1				
1.040513e-16	-0.5722698	0.11	0.282	1.85513e-12	22
SRM	Naive IFN1				

1.081322e-16	-0.4723388	0.609	0.761	1.927889e-12	22	PSMA7	Naive IFN1
1.311477e-16	-0.4502881	0.226	0.433	2.338232e-12	22	SLIRP	Naive IFN1
1.439073e-16	-0.3873723	0.447	0.643	2.565723e-12	22	ATP5MF	Naive IFN1
1.501359e-16	-0.4370694	0.104	0.284	2.676772e-12	22	PSMD14	Naive IFN1
1.840769e-16	-0.4067698	0.062	0.219	3.281908e-12	22	MRPL12	Naive IFN1
2.016779e-16	-0.795438	0.749	0.849	3.595715e-12	22	HSP90AB1	Naive IFN1
2.24277e-16	-0.5174205	0.18	0.35	3.998635e-12	22	S100A10	Naive IFN1
2.353184e-16	-0.4269675	0.443	0.641	4.195492e-12	22	ATP5MD	Naive IFN1
2.39978e-16	-0.5819933	0.287	0.487	4.278568e-12	22	RANBP1	Naive IFN1
2.52826e-16	0.4975427	0.174	0.091	4.507635e-12	22	PARP9	Naive IFN1
3.087272e-16	-0.460476	0.224	0.4	5.504297e-12	22	BASP1	Naive IFN1
3.689889e-16	-0.4263105	0.05	0.2	6.578703e-12	22	EBNA1BP2	Naive IFN1
3.723591e-16	-0.4373613	0.112	0.283	6.63879e-12	22	JPT1	Naive IFN1
4.066925e-16	-0.4621211	0.218	0.43	7.250921e-12	22	CCT4	Naive IFN1
4.720657e-16	-0.3879677	0.429	0.634	8.41646e-12	22	COX7B	Naive IFN1
5.239929e-16	-0.5987013	0.864	0.93	9.34227e-12	22	NPM1	Naive IFN1
5.46628e-16	-0.4458197	0.449	0.634	9.745831e-12	22	GSTP1	Naive IFN1
7.033158e-16	-0.3253961	0.435	0.638	1.253942e-11	22	UQCRH	Naive IFN1
8.172745e-16	-0.4177471	0.842	0.928	1.457119e-11	22	HNRNPA1	Naive IFN1
9.061249e-16	-0.4227924	0.174	0.376	1.61553e-11	22	PSMC3	Naive IFN1
1.059595e-15	0.4950572	0.617	0.511	1.889151e-11	22	BANK1	Naive IFN1
1.082267e-15	0.3485095	0.974	0.969	1.929573e-11	22	MT-ND3	Naive IFN1
1.088554e-15	-0.5834381	0.267	0.463	1.940782e-11	22	HSPD1	Naive IFN1
1.123516e-15	-0.4084526	0.026	0.132	2.003116e-11	22	PHGDH	Naive IFN1
1.21374e-15	-0.3697451	0.309	0.521	2.163977e-11	22	PSMB6	Naive IFN1

1.334144e-15	-0.5240951	0.124	0.305	2.378645e-11	22	
ODC1	Naive IFN1					
1.387474e-15	-0.4230709	0.12	0.285	2.473728e-11	22	
PRDX3	Naive IFN1					
1.726927e-15	-0.5376109	0.086	0.232	3.078938e-11	22	
CKS2	Naive IFN1					
1.792999e-15	-0.3941024	0.489	0.68	3.196738e-11	22	
HNRNPA3	Naive IFN1					
2.27116e-15	-0.4540541	0.253	0.466	4.049251e-11	22	
PRMT1	Naive IFN1					
2.29696e-15	-0.4409796	0.022	0.133	4.095249e-11	22	
CYTOR	Naive IFN1					
2.671555e-15	-0.4001512	0.281	0.492	4.763115e-11	22	
CCT3	Naive IFN1					
2.992397e-15	-0.4496104	0.212	0.403	5.335145e-11	22	
RPL22L1	Naive IFN1					
3.203002e-15	-0.4165551	0.369	0.574	5.710632e-11	22	
SNRPE	Naive IFN1					
3.367191e-15	-0.385035	0.415	0.607	6.003365e-11	22	
PSMB3	Naive IFN1					
3.834642e-15	-0.387848	0.17	0.333	6.836783e-11	22	
GRHPR	Naive IFN1					
3.991171e-15	-0.4239542	0.184	0.377	7.115859e-11	22	
PSMC1	Naive IFN1					
4.227403e-15	-0.3463822	0.499	0.685	7.537037e-11	22	
SLC25A3	Naive IFN1					
4.391083e-15	-0.409202	0.094	0.262	7.828862e-11	22	
AK6	Naive IFN1					
4.613782e-15	-0.3611465	0.297	0.505	8.225912e-11	22	
NDUFA11	Naive IFN1					
4.733073e-15	-0.3428263	0.325	0.526	8.438595e-11	22	
FKBP1A	Naive IFN1					
5.032971e-15	-0.4010917	0.094	0.264	8.973284e-11	22	
ANP32E	Naive IFN1					
5.140947e-15	-0.5585698	0.178	0.342	9.165794e-11	22	
LRMP	Naive IFN1					
5.641128e-15	-0.4906461	0.255	0.439	1.005757e-10	22	
COTL1	Naive IFN1					
6.576388e-15	-0.3763646	0.545	0.709	1.172504e-10	22	
NDUFA4	Naive IFN1					
6.964194e-15	-0.3887466	0.084	0.235	1.241646e-10	22	
CHCHD10	Naive IFN1					
7.194661e-15	-0.4094949	0.359	0.542	1.282736e-10	22	
PARP1	Naive IFN1					
7.616437e-15	-0.3601648	0.373	0.569	1.357935e-10	22	
UQCRQ	Naive IFN1					
7.649115e-15	-0.3912893	0.014	0.116	1.363761e-10	22	
PYCR1	Naive IFN1					
9.305079e-15	-0.3528835	0.038	0.135	1.659003e-10	22	
NEIL1	Naive IFN1					

9.522918e-15	-0.3602733	0.246	0.459	1.697841e-10	22	SFPQ	Naive IFN1
1.087014e-14	-0.3214239	0.112	0.273	1.938037e-10	22	TXNL4A	Naive IFN1
1.125625e-14	0.614954	0.377	0.277	2.006876e-10	22	IFITM2	Naive IFN1
1.206796e-14	-0.354651	0.351	0.559	2.151596e-10	22	PGK1	Naive IFN1
1.394432e-14	-0.3741325	0.056	0.205	2.486132e-10	22	MRPL23	Naive IFN1
1.498686e-14	-0.3409917	0.226	0.436	2.672008e-10	22	NDUFB7	Naive IFN1
1.531045e-14	-1.05901	0.128	0.288	2.7297e-10	22	MIR155HG	Naive IFN1
1.685284e-14	-0.3540697	0.034	0.156	3.004693e-10	22	PAICS	Naive IFN1
1.74366e-14	-0.4040761	0.17	0.351	3.108771e-10	22	PDIA6	Naive IFN1
1.794652e-14	-0.3738885	0.1	0.254	3.199686e-10	22	RGS10	Naive IFN1
2.211036e-14	-0.3545396	0.8	0.896	3.942055e-10	22	PPIA	Naive IFN1
2.279983e-14	-0.4551456	0.024	0.11	4.064981e-10	22	VPREB3	Naive IFN1
2.284326e-14	-0.3062862	0.086	0.228	4.072726e-10	22	UBE2G1	Naive IFN1
2.318039e-14	-0.3597021	0.07	0.226	4.132831e-10	22	DAZAP1	Naive IFN1
2.445357e-14	0.5216752	0.25	0.17	4.359828e-10	22	PARP14	Naive IFN1
2.792736e-14	-0.4655595	0.144	0.31	4.979169e-10	22	CALR	Naive IFN1
3.011758e-14	0.4967426	0.112	0.045	5.369663e-10	22	IRF9	Naive IFN1
4.077433e-14	-1.147362	0.072	0.177	7.269656e-10	22	MZB1	Naive IFN1
4.233275e-14	-0.4185431	0.246	0.446	7.547505e-10	22	ATP5MC1	Naive IFN1
5.265887e-14	-0.6099858	0.044	0.173	9.388549e-10	22	TNFRSF18	Naive IFN1
5.300279e-14	-0.3940766	0.076	0.23	9.449867e-10	22	MRPL3	Naive IFN1
5.362225e-14	-0.3553265	0.269	0.458	9.560311e-10	22	COX17	Naive IFN1
5.410859e-14	-0.4188615	0.12	0.298	9.647021e-10	22	PDCD5	Naive IFN1
5.565484e-14	-0.3566212	0.327	0.509	9.922701e-10	22	MZT2B	Naive IFN1
6.053766e-14	-0.3598426	0.269	0.454	1.079326e-09	22	MRPL51	Naive IFN1

6.475555e-14	-0.4443869	0.152	0.34	1.154527e-09	22	MIF	Naive IFN1
6.603132e-14	-0.4160622	0.188	0.377	1.177272e-09	22	LCP1	Naive IFN1
6.970157e-14	0.4826902	0.681	0.618	1.242709e-09	22	CLEC2D	Naive IFN1
7.598495e-14	-0.4436441	0.214	0.394	1.354736e-09	22	FNBP1	Naive IFN1
9.581714e-14	-0.4042583	0.082	0.221	1.708324e-09	22	TRAF4	Naive IFN1
1.006248e-13	-0.3402406	0.465	0.648	1.79404e-09	22	SNRPG	Naive IFN1
1.014566e-13	-0.3707499	0.172	0.331	1.80887e-09	22	RFTN1	Naive IFN1
1.022109e-13	-0.3479435	0.431	0.62	1.822317e-09	22	NDUFA13	Naive IFN1
1.104315e-13	-0.3725217	0.122	0.275	1.968884e-09	22	POU2AF1	Naive IFN1
1.175803e-13	-0.3576413	0.05	0.166	2.09634e-09	22	DEF8	Naive IFN1
1.211747e-13	-0.3199142	0.028	0.119	2.160424e-09	22	CD38	Naive IFN1
1.222704e-13	-0.4598817	0.447	0.637	2.17996e-09	22	HERPUD1	Naive IFN1
1.288598e-13	0.5288996	0.559	0.493	2.297441e-09	22	SP100	Naive IFN1
1.354314e-13	-0.4799223	0.246	0.425	2.414606e-09	22	DEK	Naive IFN1
1.528307e-13	-0.4195843	0.13	0.304	2.724818e-09	22	ATP1B3	Naive IFN1
1.612082e-13	-0.406832	0.174	0.366	2.87418e-09	22	EIF3J	Naive IFN1
1.695469e-13	-0.3880352	0.75	0.852	3.022852e-09	22	ATP5MG	Naive IFN1
1.843852e-13	-0.4039018	0.064	0.203	3.287403e-09	22	TOMM40	Naive IFN1
2.054284e-13	0.5588523	0.244	0.166	3.662583e-09	22	ADAR	Naive IFN1
2.223407e-13	-0.3588414	0.076	0.212	3.964112e-09	22	CLPP	Naive IFN1
2.483623e-13	-0.321293	0.407	0.603	4.428052e-09	22	PSMA2	Naive IFN1
2.48742e-13	-0.3139632	0.323	0.525	4.434822e-09	22	NDUFB8	Naive IFN1
2.52345e-13	-0.3380504	0.086	0.242	4.49906e-09	22	SRSF1	Naive IFN1
3.394105e-13	-0.4216141	0.134	0.295	6.051351e-09	22	SSRP1	Naive IFN1
3.39499e-13	-0.3384841	0.142	0.318	6.052927e-09	22	NDUFAF3	Naive IFN1

3.495319e-13	-0.3197115	0.144	0.308	6.231805e-09	22	TIMM8B	Naive IFN1
3.905919e-13	-0.327434	0.062	0.189	6.963864e-09	22	PRPSAP2	Naive IFN1
4.197298e-13	-0.4731127	0.463	0.639	7.483362e-09	22	SET	Naive IFN1
4.775943e-13	-0.5142202	0.429	0.59	8.515029e-09	22	MARCKSL1	Naive IFN1
4.941063e-13	-0.3587372	0.082	0.223	8.809421e-09	22	MRPL4	Naive IFN1
5.197126e-13	-0.4104739	0.122	0.273	9.265956e-09	22	SMS	Naive IFN1
6.09122e-13	-0.7748523	0.585	0.717	1.086004e-08	22	HMGN2	Naive IFN1
6.869948e-13	-0.387626	0.323	0.516	1.224843e-08	22	SSBP1	Naive IFN1
7.045093e-13	-0.3405286	0.036	0.141	1.25607e-08	22	AIM2	Naive IFN1
8.26525e-13	-0.4173355	0.064	0.203	1.473611e-08	22	NPM3	Naive IFN1
8.916455e-13	-0.4513058	0.261	0.446	1.589715e-08	22	ANXA2	Naive IFN1
8.957429e-13	-2.979395	0.291	0.452	1.59702e-08	22	IGHG3	Naive IFN1
1.02159e-12	-0.3716417	0.03	0.119	1.821393e-08	22	UBE2S	Naive IFN1
1.069146e-12	-0.3360833	0.852	0.92	1.90618e-08	22	CFL1	Naive IFN1
1.096207e-12	-0.3718037	0.142	0.309	1.954427e-08	22	FERMT3	Naive IFN1
1.187482e-12	-0.4010553	0.04	0.158	2.117162e-08	22	NOLC1	Naive IFN1
1.510642e-12	-1.27443	0.192	0.304	2.693324e-08	22	HMGB2	Naive IFN1
1.527836e-12	-0.4490137	0.102	0.244	2.723979e-08	22	KPNA2	Naive IFN1
1.588726e-12	-0.8149134	0.07	0.183	2.83254e-08	22	XBP1	Naive IFN1
1.617504e-12	-0.3522986	0.052	0.184	2.883847e-08	22	TUBA1C	Naive IFN1
1.729661e-12	-0.4392636	0.463	0.606	3.083812e-08	22	DYNLL1	Naive IFN1
1.801496e-12	-0.3153556	0.497	0.661	3.211886e-08	22	UBL5	Naive IFN1
1.844022e-12	-0.3695897	0.14	0.305	3.287706e-08	22	GLRX3	Naive IFN1
1.908632e-12	-0.3322194	0.261	0.437	3.402899e-08	22	ROMO1	Naive IFN1
2.10916e-12	-1.21423	0.287	0.424	3.760421e-08	22	HIST1H4C	Naive IFN1

2.252974e-12	-0.3163066	0.489	0.68	4.016828e-08	22	MORF4L1	Naive IFN1
2.255835e-12	-0.3834634	0.501	0.659	4.021927e-08	22	SLC25A5	Naive IFN1
2.411489e-12	-0.3087777	0.032	0.117	4.299443e-08	22	GMDS	Naive IFN1
2.526391e-12	-0.3719733	0.208	0.395	4.504302e-08	22	PHB	Naive IFN1
2.547966e-12	-0.3754578	0.116	0.276	4.542769e-08	22	NDUFAF8	Naive IFN1
2.622553e-12	-0.3319475	0.024	0.127	4.675749e-08	22	SLC1A5	Naive IFN1
2.72306e-12	0.5255356	0.363	0.251	4.854943e-08	22	FCMR	Naive IFN1
2.804394e-12	-0.3735183	0.066	0.17	4.999954e-08	22	SMC4	Naive IFN1
2.854217e-12	-0.3314363	0.216	0.401	5.088784e-08	22	PSMA3	Naive IFN1
3.220879e-12	-0.317176	0.062	0.162	5.742505e-08	22	SUSD3	Naive IFN1
3.503782e-12	-0.3171147	0.12	0.278	6.246892e-08	22	ATOX1	Naive IFN1
3.51634e-12	-0.3006601	0.251	0.442	6.269283e-08	22	AURKAIP1	Naive IFN1
3.516909e-12	-0.3175151	0.106	0.25	6.270298e-08	22	MRPL13	Naive IFN1
3.61428e-12	-0.3429352	0.194	0.369	6.443899e-08	22	EWSR1	Naive IFN1
3.681344e-12	-0.6025265	0.044	0.16	6.563468e-08	22	LMNA	Naive IFN1
3.724913e-12	-0.4385115	0.218	0.361	6.641148e-08	22	H2AFV	Naive IFN1
3.760244e-12	-0.3680083	0.485	0.647	6.70414e-08	22	HNRNPC	Naive IFN1
3.832029e-12	-0.6352497	0.571	0.697	6.832125e-08	22	H2AFZ	Naive IFN1
4.160266e-12	-0.3192993	0.305	0.492	7.417338e-08	22	CCT8	Naive IFN1
4.217917e-12	-0.3036526	0.299	0.487	7.520125e-08	22	UBE2L3	Naive IFN1
5.024412e-12	-0.3933872	0.894	0.934	8.958024e-08	22	SERF2	Naive IFN1
5.034457e-12	-0.3685892	0.074	0.194	8.975934e-08	22	DNMT1	Naive IFN1
5.50088e-12	-0.305462	0.285	0.471	9.807519e-08	22	SNRPF	Naive IFN1
5.797082e-12	-0.3259749	0.315	0.506	1.033562e-07	22	EIF3I	Naive IFN1
6.183918e-12	-0.3553643	0.192	0.348	1.102531e-07	22	H2AFY	Naive IFN1

6.276252e-12	-0.3105977	0.403	0.598	1.118993e-07	22	XRCC6	Naive IFN1
6.339463e-12	-0.3373218	0.403	0.597	1.130263e-07	22	SRSF2	Naive IFN1
8.250628e-12	-0.3084645	0.07	0.179	1.471005e-07	22	PLIN3	Naive IFN1
1.18622e-11	-0.388011	0.281	0.47	2.114912e-07	22	CYCS	Naive IFN1
1.20256e-11	-0.3333342	0.086	0.225	2.144044e-07	22	HSPH1	Naive IFN1
1.233588e-11	-0.3090754	0.088	0.226	2.199365e-07	22	SIAH2	Naive IFN1
1.338933e-11	-0.317076	0.198	0.368	2.387184e-07	22	RPS19BP1	Naive IFN1
1.580429e-11	-0.3511182	0.108	0.238	2.817747e-07	22	DSTN	Naive IFN1
1.585962e-11	-0.3160588	0.469	0.655	2.827612e-07	22	UBE2D3	Naive IFN1
1.808427e-11	-0.5200499	0.156	0.319	3.224244e-07	22	DDX21	Naive IFN1
2.006853e-11	-0.3089627	0.208	0.387	3.578018e-07	22	SNRPC	Naive IFN1
2.508781e-11	-0.4168527	0.18	0.355	4.472906e-07	22	ANXA5	Naive IFN1
2.945219e-11	-0.3951574	0.329	0.514	5.25103e-07	22	PA2G4	Naive IFN1
3.382645e-11	-0.4378816	0.244	0.41	6.030917e-07	22	PPIB	Naive IFN1
3.429959e-11	0.4257123	0.635	0.576	6.115274e-07	22	SARAF	Naive IFN1
3.432638e-11	-0.410925	0.106	0.252	6.12005e-07	22	GARS	Naive IFN1
3.654983e-11	-0.3055099	0.226	0.413	6.516469e-07	22	HNRNPR	Naive IFN1
3.750647e-11	-0.345361	0.281	0.458	6.687029e-07	22	LSM5	Naive IFN1
5.524078e-11	0.3931624	0.13	0.068	9.848878e-07	22	STAT2	Naive IFN1
5.566492e-11	-0.3137206	0.162	0.333	9.924498e-07	22	PDAP1	Naive IFN1
6.226786e-11	-0.3389365	0.068	0.198	1.110174e-06	22	GTPBP4	Naive IFN1
6.231053e-11	-0.5351173	0.986	0.99	1.110934e-06	22	ACTB	Naive IFN1
6.74659e-11	-0.3200588	0.196	0.374	1.20285e-06	22	ARHGDIA	Naive IFN1
7.372664e-11	-0.3134009	0.05	0.163	1.314472e-06	22	PPP1R14B	Naive IFN1
7.538082e-11	-0.3064787	0.257	0.429	1.343965e-06	22	MINOS1	Naive IFN1

8.23585e-11	-0.3315913	0.07	0.198	1.46837e-06	22	ATIC	Naive IFN1
8.756814e-11	0.5002401	0.675	0.615	1.561252e-06	22	MEF2C	Naive IFN1
9.050991e-11	0.4677743	0.22	0.148	1.613701e-06	22	TRIM56	Naive IFN1
9.517286e-11	-0.3575233	0.144	0.286	1.696837e-06	22	CD82	Naive IFN1
1.040695e-10	-0.3004901	0.265	0.446	1.855455e-06	22	CCT7	Naive IFN1
1.128674e-10	-0.3220979	0.251	0.43	2.012313e-06	22	CCT2	Naive IFN1
1.240415e-10	-0.4316994	0.034	0.137	2.211536e-06	22	PSAT1	Naive IFN1
1.246947e-10	-0.360893	0.144	0.303	2.223181e-06	22	NOP58	Naive IFN1
1.248677e-10	-0.3357267	0.064	0.161	2.226266e-06	22	C12orf75	Naive IFN1
1.435994e-10	-0.3088443	0.156	0.315	2.560233e-06	22	STOML2	Naive IFN1
1.501462e-10	-0.313484	0.082	0.214	2.676957e-06	22	PRPF19	Naive IFN1
1.51713e-10	0.5170297	0.415	0.337	2.704891e-06	22	HVCN1	Naive IFN1
1.524121e-10	-0.3347066	0.112	0.244	2.717355e-06	22	SLBP	Naive IFN1
1.819584e-10	-0.3342153	0.176	0.321	3.244136e-06	22	PRDX2	Naive IFN1
2.024071e-10	-0.3157225	0.066	0.184	3.608715e-06	22	TMA16	Naive IFN1
2.265803e-10	-0.38841	0.07	0.177	4.0397e-06	22	PDIA4	Naive IFN1
2.409333e-10	-0.3050225	0.439	0.622	4.295601e-06	22	ACTR3	Naive IFN1
2.560763e-10	-0.3295664	0.244	0.377	4.565584e-06	22	ACADM	Naive IFN1
2.576293e-10	-0.3519697	0.066	0.189	4.593273e-06	22	GPATCH4	Naive IFN1
2.6344e-10	0.5277464	0.385	0.273	4.696872e-06	22	KLF2	Naive IFN1
2.82911e-10	-0.3046767	0.106	0.253	5.04402e-06	22	NAA20	Naive IFN1
2.956446e-10	-0.3764139	0.074	0.189	5.271047e-06	22	PCNA	Naive IFN1
3.441292e-10	-0.3410085	0.216	0.393	6.13548e-06	22	ARPC5L	Naive IFN1
4.018659e-10	-0.3685498	0.427	0.612	7.164866e-06	22	SYNGR2	Naive IFN1
4.02324e-10	-0.3149423	0.13	0.275	7.173034e-06	22	NAA10	Naive IFN1

4.219454e-10	-0.3267332	0.04	0.144	7.522865e-06	22	NOP16	Naive IFN1
4.234984e-10	-0.3614338	0.194	0.363	7.550554e-06	22	HSPA9	Naive IFN1
4.332999e-10	-0.3161225	0.098	0.235	7.725303e-06	22	RALA	Naive IFN1
4.431168e-10	-0.3422045	0.255	0.431	7.900329e-06	22	NSA2	Naive IFN1
4.600265e-10	-0.3247128	0.619	0.745	8.201812e-06	22	CHCHD2	Naive IFN1
4.801946e-10	-0.3409911	0.07	0.189	8.56139e-06	22	YARS	Naive IFN1
5.122307e-10	-0.3783221	0.19	0.337	9.132562e-06	22	DUT	Naive IFN1
6.77585e-10	0.3885322	0.525	0.494	1.208066e-05	22	FXYD5	Naive IFN1
6.80112e-10	-0.470404	0.16	0.328	1.212572e-05	22	SLC3A2	Naive IFN1
6.927928e-10	-0.3510293	0.122	0.272	1.23518e-05	22	TIMM10	Naive IFN1
8.282369e-10	-0.3112937	0.118	0.258	1.476664e-05	22	GSPT1	Naive IFN1
9.070811e-10	-0.3988481	0.084	0.209	1.617235e-05	22	PIM3	Naive IFN1
9.54801e-10	-0.4932168	0.146	0.278	1.702315e-05	22	HSPA5	Naive IFN1
1.020406e-09	0.4548028	0.349	0.277	1.819281e-05	22	ADAM28	Naive IFN1
1.197616e-09	0.4526602	0.505	0.447	2.13523e-05	22	PNRC1	Naive IFN1
1.241482e-09	-0.3087797	0.126	0.259	2.213439e-05	22	CDV3	Naive IFN1
1.263728e-09	-0.3056661	0.036	0.138	2.2531e-05	22	IFRD2	Naive IFN1
1.585934e-09	0.5080492	0.379	0.302	2.827562e-05	22	ZFP36L2	Naive IFN1
1.688959e-09	0.4896523	0.261	0.194	3.011246e-05	22	CLEC2B	Naive IFN1
1.859369e-09	0.5743999	0.365	0.327	3.315069e-05	22	BST2	Naive IFN1
2.68614e-09	-0.3093863	0.142	0.292	4.789119e-05	22	DCTPP1	Naive IFN1
2.909562e-09	-0.3062385	0.048	0.151	5.187457e-05	22	RPF2	Naive IFN1
3.313172e-09	-0.3059408	0.291	0.462	5.907055e-05	22	GADD45GIP1	Naive IFN1
4.026605e-09	-0.3366617	0.108	0.203	7.179033e-05	22	HMCES	Naive IFN1
4.8631e-09	0.4746193	0.489	0.455	8.670421e-05	22	JUNB	Naive IFN1

5.005726e-09	-0.3707972	0.283	0.419	8.924708e-05	22	METAP2	Naive IFN1
9.746371e-09	0.3949738	0.144	0.086	0.0001737681	22	RNASE6	Naive IFN1
1.044262e-08	0.3497075	0.419	0.352	0.0001861814	22	TXNIP	Naive IFN1
1.077681e-08	0.4670608	0.265	0.202	0.0001921397	22	IRF1	Naive IFN1
1.199921e-08	-0.3067758	0.118	0.243	0.000213934	22	SSR3	Naive IFN1
1.256481e-08	0.440127	0.417	0.347	0.0002240179	22	TRAF3IP3	Naive IFN1
1.412056e-08	-0.3079046	0.114	0.238	0.0002517555	22	NIFK	Naive IFN1
1.546026e-08	0.4838262	0.473	0.449	0.0002756409	22	DRAP1	Naive IFN1
1.625186e-08	0.5256724	0.269	0.205	0.0002897544	22	PRKCB	Naive IFN1
1.682633e-08	-0.3988498	0.182	0.321	0.0002999967	22	SEC11C	Naive IFN1
2.267312e-08	-1.294647	0.116	0.217	0.0004042391	22	IGHG1	Naive IFN1
3.431653e-08	0.4164586	0.164	0.105	0.0006118294	22	C1orf162	Naive IFN1
3.507507e-08	0.4784645	0.301	0.237	0.0006253534	22	CD55	Naive IFN1
4.302172e-08	-1.049633	0.058	0.149	0.0007670343	22	IGHA1	Naive IFN1
6.164218e-08	-0.3181614	0.569	0.695	0.001099018	22	EZR	Naive IFN1
8.510814e-08	-0.3163077	0.034	0.13	0.001517393	22	CCDC58	Naive IFN1
1.041553e-07	0.3805067	0.172	0.113	0.001856985	22	LGALS9	Naive IFN1
1.342813e-07	0.3974047	0.445	0.413	0.002394102	22	NPC2	Naive IFN1
1.528764e-07	0.4625747	0.253	0.218	0.002725633	22	CHMP5	Naive IFN1
1.561784e-07	0.3651532	0.142	0.096	0.002784505	22	UNC93B1	Naive IFN1
1.901436e-07	-0.300861	0.074	0.187	0.003390071	22	WDR43	Naive IFN1
2.347018e-07	-1.768152	0.134	0.244	0.004184498	22	IGHG4	Naive IFN1
2.406305e-07	0.3742252	0.184	0.142	0.004290201	22	NUB1	Naive IFN1
2.693392e-07	-0.5135341	0.192	0.314	0.004802048	22	HSP90B1	Naive IFN1
2.74245e-07	-0.405871	0.064	0.161	0.004889515	22	DDIT4	Naive IFN1

2.888e-07	0.350112	0.591	0.57	0.005149015	22	SNX2	Naive IFN1
2.907919e-07	0.385607	0.357	0.296	0.005184529	22	PDLIM1	Naive IFN1
3.12225e-07	0.3232114	0.275	0.219	0.00556666	22	GBP2	Naive IFN1
3.619367e-07	0.4108403	0.202	0.157	0.006452969	22	SHISA5	Naive IFN1
5.037756e-07	-0.4060343	0.046	0.126	0.008981816	22	LGALS3	Naive IFN1
5.549273e-07	0.3410981	0.102	0.056	0.009893799	22	TNFSF10	Naive IFN1
6.674191e-07	0.4276026	0.15	0.095	0.01189941	22	MT2A	Naive IFN1
6.743209e-07	-0.302687	0.064	0.152	0.01202247	22	CTNNB1	Naive IFN1
6.746587e-07	-0.4269623	0.89	0.932	0.01202849	22	HMGB1	Naive IFN1
6.795749e-07	-0.3852877	0.136	0.257	0.01211614	22	SRGN	Naive IFN1
8.196491e-07	-0.6654037	0.114	0.207	0.01461352	22	LGALS1	Naive IFN1
8.874133e-07	-0.327325	0.088	0.178	0.01582169	22	BCL2A1	Naive IFN1
1.091696e-06	0.32556	0.928	0.934	0.01946384	22	MT-ATP6	Naive IFN1
1.187326e-06	0.3996691	0.383	0.359	0.02116883	22	ARID5B	Naive IFN1
1.215967e-06	0.4188422	0.325	0.279	0.02167947	22	RNASET2	Naive IFN1
1.462949e-06	0.3864095	0.367	0.329	0.02608293	22	SMC6	Naive IFN1
1.514891e-06	0.3554095	0.168	0.128	0.02700899	22	C19orf66	Naive IFN1
1.931851e-06	0.3653326	0.17	0.117	0.03444298	22	CARD16	Naive IFN1
2.273761e-06	0.3813116	0.226	0.188	0.04053888	22	PHF11	Naive IFN1
2.807273e-06	0.3128408	0.22	0.196	0.05005087	22	TYMP	Naive IFN1
3.549247e-06	0.3979736	0.267	0.211	0.06327952	22	LBH	Naive IFN1
3.897989e-06	0.3799192	0.301	0.256	0.06949724	22	LYSMD2	Naive IFN1
3.961688e-06	0.3974381	0.549	0.488	0.07063293	22	UCP2	Naive IFN1
4.371335e-06	0.321569	0.523	0.491	0.07793654	22	CYTIP	Naive IFN1
5.292655e-06	0.3633654	0.238	0.173	0.09436275	22	MARCH1	Naive IFN1

6.185285e-06	-0.5728578	0.457	0.571	0.1102775	22	VIM	Naive IFN1
8.32097e-06	0.4171732	0.323	0.268	0.1483546	22	JUN	Naive IFN1
9.24702e-06	0.4359181	0.527	0.502	0.1648651	22	PSMB9	Naive IFN1
9.825512e-06	0.3405392	0.162	0.109	0.1751791	22	LINC02397	Naive IFN1
1.097008e-05	-2.029209	0.072	0.126	0.1955856	22	IGHG2	Naive IFN1
1.129793e-05	-0.3528056	0.116	0.219	0.2014308	22	EIF4EBP1	Naive IFN1
1.220912e-05	-0.3995494	0.371	0.501	0.2176764	22	SSR4	Naive IFN1
1.249534e-05	0.3905237	0.259	0.206	0.2227794	22	C16orf74	Naive IFN1
1.41412e-05	0.3283035	0.373	0.336	0.2521235	22	CD48	Naive IFN1
1.510572e-05	-0.3551694	0.144	0.242	0.2693199	22	RGS1	Naive IFN1
2.158158e-05	-1.131977	0.371	0.404	0.384778	22	JCHAIN	Naive IFN1
2.187647e-05	0.3339065	0.23	0.195	0.3900356	22	CHPT1	Naive IFN1
3.75671e-05	0.3655729	0.228	0.186	0.6697838	22	CDCA7L	Naive IFN1
4.034327e-05	-0.4621649	0.058	0.131	0.7192802	22	MYC	Naive IFN1
5.087496e-05	0.3372102	0.493	0.492	0.9070497	22	ANKRD12	Naive IFN1
5.12962e-05	0.3661196	0.186	0.159	0.9145599	22	AIDA	Naive IFN1
9.038648e-05	0.3395257	0.218	0.201	1	22	NMI	Naive IFN1
0.0001100697	0.3347856	0.457	0.455	1	22	CTSS	Naive IFN1
0.0002617102	0.3679612	0.184	0.177	1	22	LAP3	Naive IFN1
0.0002949907	0.3122449	0.148	0.122	1	22	ZC3HAV1	Naive IFN1
0.0003416338	-0.5143228	0.136	0.212	1	22	PLCG2	Naive IFN1
0.0003707151	0.3812086	0.433	0.422	1	22	TMEM123	Naive IFN1
0.0003858281	-0.3719764	0.367	0.473	1	22	CD83	Naive IFN1
0.0004042581	0.3970493	0.399	0.363	1	22	RCSD1	Naive IFN1
0.0005939612	0.3125635	0.473	0.476	1	22	N4BP2L2	Naive IFN1

0.0008020495	0.3074769	0.154	0.122	1	22
AL139020.1	Naive IFN1
0.0008241087	0.3046701	0.475	0.472	1	22
GPSM3	Naive IFN1
0.000884767	0.3740536	0.16	0.144	1	22
HSH2D	Naive IFN1
0.0009569697	0.3720921	0.224	0.207	1	22
CCDC32	Naive IFN1
0.001666532	0.3335397	0.397	0.384	1	22	EVL
Naive IFN1
0.003024793	-0.4305117	0.395	0.428	1	22
STMN1	Naive IFN1
0.004187886	0.3548205	0.403	0.403	1	22
STX7	Naive IFN1
0.005185881	0.3492803	0.337	0.323	1	22	AES
Naive IFN1
0.007313198	0.3650378	0.515	0.497	1	22
SAT1	Naive IFN1
0.0086371	0.322102 0.208	0.226	1	22	BAG1
Naive IFN1
0	4.270956 0.997	0.906	0	23	IGHM	ASC IgM
1.177773e-266	3.534745 0.938	0.17	2.099851e-262	23
MZB1	ASC IgM
1.727266e-244	2.587461 0.911	0.308	3.079543e-240	23
HSP90B1	ASC IgM
6.511188e-228	3.225788 0.973	0.399	1.16088e-223	23
JCHAIN	ASC IgM
3.312784e-222	2.56983 0.926	0.176	5.906362e-218	23
XBP1	ASC IgM
1.711882e-175	1.886325 0.97	0.633	3.052115e-171	23
HERPUD1	ASC IgM
1.230135e-172	2.005618 0.881	0.236	2.193208e-168	23
RGS1	ASC IgM
1.813651e-169	2.019751 0.884	0.315	3.233559e-165	23
SEC11C	ASC IgM
4.049659e-164	-1.337277	1	1	7.220137e-160	23
RPL21	ASC IgM
1.041388e-161	-1.868718	0.967	0.998	1.856691e-157	23
TMSB4X	ASC IgM
1.940343e-154	-1.280239	0.997	1	3.459438e-150	23
RPS27	ASC IgM
4.041849e-154	2.036995 0.914	0.496	7.206213e-150	23
SSR4	ASC IgM
1.354298e-152	1.819619 0.884	0.404	2.414577e-148	23
PPIB	ASC IgM
1.093025e-150	1.826258 0.682	0.087	1.948754e-146	23
FKBP11	ASC IgM
1.447263e-148	1.070818 1	0.986	2.580325e-144	23
HLA-B	ASC IgM
2.838303e-147	1.786085 0.742	0.066	5.06041e-143	23

RASSF6	ASC IgM
8.605834e-143	1.835625	0.763	0.171	1.534334e-138	23
PDIA4	ASC IgM
7.980444e-142	1.784292	0.733	0.142	1.422833e-137	23
SDF2L1	ASC IgM
3.657468e-140	-1.728962	0.976	0.996	6.520899e-136	23
RPS12	ASC IgM
5.168938e-134	1.81246	0.843	0.273	9.215699e-130	23
HSPA5	ASC IgM
1.33874e-133	1.64795	0.813	0.236	2.38684e-129	23
MYDGF	ASC IgM
1.911724e-131	-1.441959	0.988	0.999	3.408414e-127	23
RPL39	ASC IgM
4.823261e-126	1.342242	0.961	0.595	8.599392e-122	23
SEC61B	ASC IgM
5.402459e-122	1.319957	0.561	0.026	9.632044e-118	23
PRDM1	ASC IgM
6.358548e-122	-1.17531	0.982	0.999	1.133666e-117	23
RPS27A	ASC IgM
6.179782e-121	-1.257802	0.994	0.999	1.101793e-116	23
RPL34	ASC IgM
1.192749e-118	-1.386627	0.982	0.995	2.126552e-114	23
RPL31	ASC IgM
2.325975e-118	-1.301441	0.994	0.999	4.146982e-114	23
RPL32	ASC IgM
1.963141e-117	-1.088786	0.991	0.999	3.500084e-113	23
RPS18	ASC IgM
5.952239e-117	-1.215623	0.988	0.999	1.061225e-112	23
RPS6	ASC IgM
1.673382e-116	1.71898	0.588	0.082	2.983472e-112	23
LINC01480	ASC IgM
3.248518e-116	-1.228189	0.988	0.999	5.791783e-112	23
RPS19	ASC IgM
8.065632e-116	1.877939	0.816	0.144	1.438022e-111	23	BIK
ASC IgM
1.37687e-115	-1.323451	0.979	0.998	2.454821e-111	23
RPS3A	ASC IgM
3.329207e-114	1.44402	0.852	0.346	5.935643e-110	23
PDIA6	ASC IgM
1.408596e-113	1.370299	0.935	0.551	2.511385e-109	23
SEC61G	ASC IgM
6.611728e-112	-1.120159	0.997	0.999	1.178805e-107	23
RPL13A	ASC IgM
8.634896e-111	-1.109924	0.988	0.999	1.539516e-106	23
RPS8	ASC IgM
1.465793e-110	-1.331285	0.988	0.998	2.613362e-106	23
RPL11	ASC IgM
3.487096e-110	-1.260932	0.991	0.999	6.217144e-106	23
RPS29	ASC IgM
4.371252e-110	1.509459	0.721	0.188	7.793506e-106	23

MANF ASC IgM
6.421991e-109 1.970378 0.896 0.201 1.144977e-104 23
RGS13 ASC IgM
6.558501e-109 1.543817 0.718 0.114 1.169315e-104 23
CD38 ASC IgM
1.311577e-108 −1.278756 0.994 0.999 2.33841e-104 23
RPLP2 ASC IgM
1.35457e-106 1.526123 0.825 0.273 2.415063e-102 23
PIM2 ASC IgM
1.501887e-105 1.515086 0.632 0.081 2.677714e-101 23
HIST1H1C ASC IgM
8.395754e-105 1.484818 0.84 0.363 1.496879e-100 23
SELENOK ASC IgM
2.96858e-104 1.28795 0.78 0.252 5.292682e-100 23
SRGN ASC IgM
6.059907e-104 −1.200953 0.994 0.997 1.080421e-99 23
RPS23 ASC IgM
1.625713e-103 1.607377 0.721 0.106 2.898484e-99 23 CD9
ASC IgM
6.775884e-101 −1.016903 1 0.999 1.208072e-96 23
RPS2 ASC IgM
1.1406e-100 1.307685 0.887 0.526 2.033576e-96 23
SPCS2 ASC IgM
4.147022e-100 1.409025 0.807 0.305 7.393725e-96 23
CALR ASC IgM
5.138937e-100 1.546533 0.819 0.164 9.162212e-96 23
CCDC88A ASC IgM
4.411092e-99 −1.105531 0.985 0.998 7.864537e-95 23
RPL26 ASC IgM
2.105055e-98 1.82938 0.739 0.182 3.753102e-94 23
RGS2 ASC IgM
2.765881e-97 1.295242 0.644 0.115 4.931289e-93 23
LMAN1 ASC IgM
9.036719e-97 1.186438 0.976 0.761 1.611157e-92 23
CYBA ASC IgM
3.430392e-95 1.358947 0.76 0.237 6.116046e-91 23
SSR3 ASC IgM
4.751439e-95 1.40471 0.908 0.481 8.471341e-91 23
NEAT1 ASC IgM
8.469756e-95 −1.129964 0.994 0.999 1.510073e-90 23
RPL13 ASC IgM
9.325037e-95 −1.314344 0.982 0.996 1.662561e-90 23
RPL12 ASC IgM
8.769543e-93 1.209339 0.953 0.618 1.563522e-88 23
SUB1 ASC IgM
2.240808e-91 1.270876 0.671 0.159 3.995136e-87 23
HM13 ASC IgM
2.652365e-91 −1.144952 0.997 0.997 4.728902e-87 23
RPL7 ASC IgM
1.051065e-90 −0.9273536 0.997 1 1.873944e-86 23

RPL41	ASC IgM	9.659565e-90	-1.087056	0.982	0.998	1.722204e-85	23
RPL23A	ASC IgM	1.013592e-89	-1.167679	0.988	0.998	1.807134e-85	23
RPL18A	ASC IgM	3.011327e-88	1.15545	0.813	0.321	5.368895e-84	23
P4HB	ASC IgM	1.136866e-87	1.032227	0.596	0.077	2.026918e-83	23
TXNDC11	ASC IgM	2.679035e-87	-1.301431	0.973	0.991	4.776452e-83	23
RPL30	ASC IgM	2.357679e-86	1.186489	0.804	0.266	4.203506e-82	23
ARF4	ASC IgM	5.217544e-86	1.285935	0.763	0.259	9.302359e-82	23
FKBP2	ASC IgM	2.011504e-85	1.051636	0.561	0.079	3.586311e-81	23
GMPPB	ASC IgM	3.044466e-85	1.126698	0.623	0.093	5.427978e-81	23
PDXK	ASC IgM	4.85829e-84	-1.485028	0.926	0.976	8.661845e-80	23
RPL36A	ASC IgM	4.120319e-83	1.169025	0.638	0.111	7.346117e-79	23
DNAJB9	ASC IgM	1.371149e-82	1.233071	0.78	0.279	2.444622e-78	23
RPN2	ASC IgM	2.173133e-82	-1.008508	0.994	0.999	3.874479e-78	23
EEF1A1	ASC IgM	6.59234e-82	1.259407	0.724	0.222	1.175348e-77	23
KDELR2	ASC IgM	1.888983e-79	-1.055511	0.973	0.996	3.367868e-75	23
RPL35A	ASC IgM	2.693698e-79	-1.023398	0.991	0.997	4.802595e-75	23
RPL27A	ASC IgM	9.727037e-78	1.392445	0.51	0.051	1.734233e-73	23
DERL3	ASC IgM	2.108208e-77	-0.9349258	0.976	0.997	3.758724e-73	23
RPS15A	ASC IgM	3.003656e-77	-1.090197	0.985	0.995	5.355218e-73	23
RPS25	ASC IgM	1.129552e-76	1.298774	0.825	0.23	2.013879e-72	23
UBE2J1	ASC IgM	1.162278e-76	-1.525904	0.828	0.936	2.072226e-72	23
BTG1	ASC IgM	1.348326e-76	0.7349896	0.967	0.75	2.40393e-72	23
HLA-C	ASC IgM	2.067565e-75	1.038604	0.884	0.358	3.686262e-71	23
REEP5	ASC IgM	2.813735e-75	-0.9046495	0.997	0.998	5.016609e-71	23
RPL19	ASC IgM	1.243781e-74	-1.846317	0.386	0.753	2.217538e-70	23

CXCR4	ASC IgM	1.625963e-74	-1.291802	0.688	0.883	2.898929e-70	23
MS4A1	ASC IgM	4.490294e-74	0.930637	0.27	0.011	8.005746e-70	23
ADM	ASC IgM	4.974033e-74	-1.142063	0.961	0.99	8.868203e-70	23
ACTB	ASC IgM	6.601697e-74	-0.9146636	0.991	0.998	1.177017e-69	23
RPS15	ASC IgM	1.38831e-73	1.080288	0.804	0.312	2.475217e-69	23
TRAM1	ASC IgM	2.834234e-73	-1.366971	0.973	0.983	5.053155e-69	23
RPS21	ASC IgM	5.925471e-73	-0.8303389	0.994	0.999	1.056452e-68	23
PTMA	ASC IgM	4.862626e-72	1.34642	0.567	0.11	8.669576e-68	23
TNFRSF17	ASC IgM	7.847823e-72	-1.0129	0.991	0.998	1.399188e-67	23
RPS14	ASC IgM	9.609337e-72	-0.948884	0.985	0.996	1.713249e-67	23
RPS3	ASC IgM	3.066723e-71	-1.247611	0.976	0.991	5.46766e-67	23
RPL9	ASC IgM	4.709578e-71	-1.806732	0.237	0.651	8.396707e-67	23
ZFP36L1	ASC IgM	7.566888e-71	-1.091327	0.991	0.992	1.3491e-66	23
RPL37	ASC IgM	1.65388e-70	1.082498	0.635	0.153	2.948702e-66	23
DNAJB11	ASC IgM	2.411923e-70	1.007599	0.947	0.63	4.300217e-66	23
GSTP1	ASC IgM	2.77696e-70	-1.098982	0.985	0.991	4.951042e-66	23
RPS20	ASC IgM	3.11741e-70	1.044551	0.721	0.229	5.558031e-66	23
TMED10	ASC IgM	1.405309e-68	-1.22328	0.955	0.987	2.505526e-64	23
RPL14	ASC IgM	1.525311e-68	-1.113068	0.991	0.993	2.719477e-64	23
RPL35	ASC IgM	1.693185e-68	-1.415748	0.7	0.849	3.01878e-64	23
CD37	ASC IgM	1.824034e-68	-1.161551	0.982	0.992	3.25207e-64	23
RPL36	ASC IgM	4.202349e-68	0.6868138	0.386	0.03	7.492368e-64	23
CHPF	ASC IgM	4.836628e-68	1.095685	0.703	0.206	8.623224e-64	23
SELENOS	ASC IgM	5.543389e-68	1.025358	0.884	0.496	9.883307e-64	23
SPCS1	ASC IgM	6.630291e-68	1.019191	0.57	0.111	1.182115e-63	23

CRELD2	ASC IgM	1.064767e-67	1.050292	0.831	0.405	1.898372e-63	23
TMED9	ASC IgM	2.758116e-67	-1.225575	0.97	0.981	4.917444e-63	23
RPSA	ASC IgM	8.260938e-67	-1.034734	0.982	0.995	1.472843e-62	23
RPL37A	ASC IgM	5.349173e-66	0.8221164	0.442	0.036	9.53704e-62	23
MAN1A1	ASC IgM	2.451795e-63	-0.9175671	0.997	0.996	4.371306e-59	23
RPS13	ASC IgM	2.85078e-63	0.9828896	0.878	0.474	5.082655e-59	23
SSR2	ASC IgM	8.574836e-63	3.048968	0.976	0.926	1.528808e-58	23
IGKC	ASC IgM	1.377413e-62	-0.8163548	0.997	0.999	2.45579e-58	23
RPLP1	ASC IgM	2.871904e-61	0.97238	0.866	0.45	5.120318e-57	23
TMEM59	ASC IgM	3.342401e-61	0.8449251	0.49	0.078	5.959167e-57	23
CLPTM1L	ASC IgM	5.465428e-61	0.9900029	0.611	0.143	9.744312e-57	23
SPCS3	ASC IgM	5.754005e-61	1.076196	0.748	0.232	1.025882e-56	23
DSTN	ASC IgM	9.002043e-61	0.9153262	0.849	0.434	1.604974e-56	23
DAD1	ASC IgM	3.276963e-60	0.8798515	0.843	0.385	5.842496e-56	23
CUTA	ASC IgM	8.26827e-60	-1.288205	0.935	0.968	1.47415e-55	23
RPS17	ASC IgM	9.971201e-60	0.7504467	0.43	0.054	1.777765e-55	23
ANKRD28	ASC IgM	1.910539e-59	0.8224248	0.487	0.06	3.406301e-55	23
CHST2	ASC IgM	3.008184e-59	0.9709451	0.872	0.479	5.363292e-55	23
RABAC1	ASC IgM	7.442233e-59	-0.7271728	0.997	0.998	1.326876e-54	23
RPL15	ASC IgM	7.546799e-58	0.9618266	0.881	0.466	1.345519e-53	23
MTDH	ASC IgM	8.847519e-58	1.043988	0.982	0.865	1.577424e-53	23
CD79A	ASC IgM	2.33682e-56	0.9040481	0.617	0.142	4.166317e-52	23
ERLEC1	ASC IgM	3.219191e-56	-1.996552	0.154	0.546	5.739495e-52	23
CCR7	ASC IgM	7.753725e-56	0.8783813	0.466	0.071	1.382412e-51	23
DOK3	ASC IgM	4.697198e-55	-1.06214	0.964	0.976	8.374634e-51	23

RPS24	ASC IgM	5.448632e-55	0.9706634	0.73	0.204	9.714367e-51	23
GLRX	ASC IgM	9.773285e-55	1.202568	0.629	0.105	1.742479e-50	23
VPREB3	ASC IgM	1.762767e-54	0.9414163	0.745	0.317	3.142837e-50	23
LMAN2	ASC IgM	2.232013e-54	-0.878702	0.976	0.99	3.979456e-50	23
RPL10A	ASC IgM	2.732475e-53	-1.137794	0.935	0.961	4.871729e-49	23
RPL23	ASC IgM	2.818453e-53	-0.6947456	1	0.996	5.025019e-49	23
RPL8	ASC IgM	3.904356e-53	-1.103223	0.961	0.981	6.961077e-49	23
RPL5	ASC IgM	2.18807e-52	-0.7012139	0.991	0.997	3.90111e-48	23
RPL28	ASC IgM	2.270534e-52	0.7224183	0.478	0.075	4.048135e-48	23
SEC61A1	ASC IgM	2.631347e-52	0.8928903	0.804	0.369	4.691429e-48	23
OSTC	ASC IgM	7.548292e-52	0.7887122	0.51	0.091	1.345785e-47	23
DNAJC3	ASC IgM	1.905473e-51	0.9033832	0.872	0.554	3.397268e-47	23
SERP1	ASC IgM	5.870569e-51	0.9893146	0.745	0.362	1.046664e-46	23
PDIA3	ASC IgM	8.343212e-51	-0.8760099	0.988	0.993	1.487511e-46	23
RPL6	ASC IgM	1.347012e-50	0.9062904	0.537	0.143	2.401587e-46	23
PRDX4	ASC IgM	3.270809e-50	0.6131809	0.353	0.034	5.831526e-46	23
TRIB1	ASC IgM	3.559642e-50	0.8027136	0.65	0.174	6.346485e-46	23
DERL1	ASC IgM	4.897052e-50	0.7548655	0.786	0.39	8.730954e-46	23
TMED2	ASC IgM	5.256533e-50	-0.7983063	0.973	0.993	9.371873e-46	23
RPS7	ASC IgM	1.079938e-49	0.6074827	0.356	0.039	1.925422e-45	23
BHLHE41	ASC IgM	5.542157e-49	-0.9221919	0.979	0.993	9.881112e-45	23
RPS5	ASC IgM	5.965274e-49	-1.037699	0.769	0.869	1.063549e-44	23
CD52	ASC IgM	7.724598e-49	-0.917251	0.982	0.985	1.377219e-44	23
RPL27	ASC IgM	2.568695e-48	0.7337988	0.911	0.576	4.579726e-44	23
COPE	ASC IgM	3.734155e-48	0.7735462	0.442	0.075	6.657625e-44	23

HCST	ASC IgM	4.437282e-48	-0.7022095	0.982	0.994	7.911229e-44	23
RPS9	ASC IgM	1.512492e-47	-1.4499	0.136	0.408	2.696621e-43	23
SMIM14	ASC IgM	2.09599e-47	-0.9327261	0.97	0.984	3.73694e-43	23
RPL29	ASC IgM	2.970615e-47	0.9839077	0.754	0.3	5.29631e-43	23
CD27	ASC IgM	4.25798e-47	0.8515742	0.475	0.082	7.591553e-43	23
RRBP1	ASC IgM	6.365411e-47	0.8913868	0.537	0.099	1.134889e-42	23
SEMA4A	ASC IgM	8.056662e-47	1.247953	0.861	0.495	1.436422e-42	23
SAT1	ASC IgM	1.289109e-46	0.7022125	0.917	0.602	2.298352e-42	23
TMEM258	ASC IgM	1.522588e-46	0.6899786	0.507	0.129	2.714621e-42	23
CYTOR	ASC IgM	1.614991e-46	0.9205388	0.706	0.214	2.879367e-42	23
NANS	ASC IgM	1.998156e-46	0.7850279	0.659	0.199	3.562513e-42	23
SEC13	ASC IgM	2.446571e-46	0.7691787	0.875	0.466	4.361991e-42	23
PRDX5	ASC IgM	5.787219e-46	-0.7600417	0.994	0.998	1.031803e-41	23
RPS28	ASC IgM	1.333638e-45	0.965393	0.742	0.198	2.377742e-41	23
HMCES	ASC IgM	2.275975e-45	-0.7169361	0.991	0.998	4.057835e-41	23
RPL3	ASC IgM	3.601149e-45	-0.8334323	0.979	0.992	6.420489e-41	23
RPL18	ASC IgM	6.508053e-45	0.337634	0.142	0.003	1.160321e-40	23
CTHRC1	ASC IgM	8.308203e-45	-0.9496122	0.944	0.971	1.48127e-40	23
RPL22	ASC IgM	8.424692e-45	-0.8261729	0.988	0.991	1.502038e-40	23
RPS16	ASC IgM	1.278438e-44	-1.599329	0.297	0.49	2.279326e-40	23
LTB	ASC IgM	2.201204e-44	0.6977872	0.519	0.127	3.924526e-40	23
SRPRB	ASC IgM	4.580031e-44	-1.534637	0.226	0.525	8.165738e-40	23
ID3	ASC IgM	5.422397e-44	0.6827957	0.439	0.063	9.667592e-40	23
QPCT	ASC IgM	1.187438e-43	0.4976536	0.3	0.026	2.117084e-39	23
KCNN3	ASC IgM	1.192209e-43	0.671688	0.487	0.082	2.125589e-39	23

ST6GALNAC4	ASC IgM	1.255494e-42	-1.474036	0.264	0.514	2.238421e-38	23
BANK1	ASC IgM	2.342447e-42	0.9097854	0.65	0.2	4.176348e-38	23
UBALD2	ASC IgM	3.134519e-42	-1.057647	0.958	0.965	5.588535e-38	23
RPL38	ASC IgM	5.526484e-42	-1.354464	0.332	0.572	9.853168e-38	23
SNX2	ASC IgM	7.28891e-42	-1.19349	0.964	0.977	1.29954e-37	23
RPS10	ASC IgM	7.989299e-42	-1.102111	0.89	0.932	1.424412e-37	23
HMGB1	ASC IgM	2.250014e-41	0.6902469	0.632	0.204	4.01155e-37	23
TMEM208	ASC IgM	3.549542e-41	-1.327823	0.134	0.426	6.328479e-37	23
DEK	ASC IgM	1.003891e-40	0.7431247	0.656	0.22	1.789837e-36	23
SSR1	ASC IgM	2.214923e-40	0.3820384	0.142	0.003	3.948987e-36	23
LINC01485	ASC IgM	2.2657e-40	0.6597297	0.769	0.303	4.039516e-36	23
CNPY2	ASC IgM	3.85165e-40	0.6607266	0.496	0.114	6.867108e-36	23
DNAJC1	ASC IgM	3.925712e-40	0.5887233	0.454	0.088	6.999152e-36	23
UBA5	ASC IgM	4.257091e-40	0.563154	0.356	0.038	7.589967e-36	23
CKAP4	ASC IgM	5.015879e-40	-0.5596834	0.988	0.997	8.94281e-36	23
RPS4X	ASC IgM	1.179797e-39	0.5210996	0.982	0.864	2.10346e-35	23
RPL36AL	ASC IgM	1.254643e-39	-1.168052	0.647	0.778	2.236903e-35	23
NAP1L1	ASC IgM	1.923812e-39	0.7029849	0.846	0.428	3.429965e-35	23
ZNF706	ASC IgM	2.584223e-39	0.7432178	0.22	0.027	4.607411e-35	23
DUSP4	ASC IgM	7.605475e-39	0.6111608	0.564	0.151	1.35598e-34	23
ALG5	ASC IgM	1.360956e-38	0.7229871	0.359	0.043	2.426448e-34	23
EML6	ASC IgM	2.117239e-38	0.760664	0.766	0.312	3.774825e-34	23
NDUFAF3	ASC IgM	2.198095e-38	-1.305843	0.875	0.917	3.918984e-34	23
EEF1B2	ASC IgM	2.427839e-38	-0.9956865	0.964	0.969	4.328594e-34	23
MT-ND3	ASC IgM	4.187839e-38	0.6238497	0.718	0.291	7.466498e-34	23

ACADVL	ASC IgM	5.381051e-38	-1.073935	0.727	0.815	9.593876e-34	23
CORO1A	ASC IgM	5.468006e-38	-0.9740581	0.932	0.946	9.748909e-34	23
HLA-DRA	ASC IgM	6.233394e-38	0.6204726	0.522	0.11	1.111352e-33	23
PREB	ASC IgM	7.47535e-38	0.5026003	0.323	0.04	1.33278e-33	23
IQGAP2	ASC IgM	2.044433e-37	0.5439184	0.32	0.033	3.645019e-33	23
SEC24D	ASC IgM	2.052489e-37	0.5941712	0.323	0.036	3.659383e-33	23
AC012236.1	ASC IgM	2.151238e-37	0.5670498	0.49	0.113	3.835442e-33	23
SURF4	ASC IgM	2.429681e-37	0.5334451	0.341	0.038	4.331878e-33	23
CPEB4	ASC IgM	6.242411e-37	0.8119301	0.362	0.045	1.112959e-32	23
COL9A3	ASC IgM	7.36341e-37	0.6594361	0.591	0.181	1.312822e-32	23
LRRC59	ASC IgM	1.405395e-36	0.496214	0.338	0.042	2.505679e-32	23
SIL1	ASC IgM	3.358473e-36	0.6427238	0.62	0.204	5.987822e-32	23
SND1	ASC IgM	5.852107e-36	-1.121147	0.175	0.395	1.043372e-31	23
SYPL1	ASC IgM	8.218344e-36	0.605081	0.243	0.026	1.465249e-31	23
FEZ1	ASC IgM	9.990773e-36	0.5996775	0.896	0.651	1.781255e-31	23
TMBIM6	ASC IgM	1.054194e-35	0.6789161	0.315	0.042	1.879522e-31	23
GADD45A	ASC IgM	1.390313e-35	0.6099489	0.448	0.101	2.47879e-31	23
SLC35B1	ASC IgM	1.614151e-35	0.465252	0.291	0.023	2.87787e-31	23
ZBP1	ASC IgM	1.661494e-35	0.5866839	0.507	0.124	2.962278e-31	23
SAR1B	ASC IgM	7.291339e-35	0.6940554	0.522	0.12	1.299973e-30	23
SPATS2	ASC IgM	7.434969e-35	-1.381529	0.409	0.611	1.325581e-30	23
SYNGR2	ASC IgM	1.286734e-34	-0.83707	0.961	0.976	2.294119e-30	23
UBA52	ASC IgM	2.906637e-34	0.7361467	0.917	0.597	5.182243e-30	23
PRDX1	ASC IgM	4.12757e-34	-0.4312464	0.994	0.999	7.359045e-30	23
RPL10	ASC IgM	9.62786e-34	0.4033196	0.237	0.018	1.716551e-29	23

ELL2 ASC IgM
1.02534e-33 0.7081728 0.582 0.165 1.828078e-29 23
CCDC167 ASC IgM
1.064479e-33 0.5365088 0.226 0.017 1.897859e-29 23
ZNF804A ASC IgM
1.129255e-33 0.5761024 0.478 0.104 2.013348e-29 23
TXNDC15 ASC IgM
3.149676e-33 -1.21372 0.101 0.339 5.615557e-29 23
HVCN1 ASC IgM
3.161293e-33 0.7000798 0.534 0.124 5.636269e-29 23
MSI2 ASC IgM
3.45231e-33 0.6797598 0.62 0.19 6.155124e-29 23
REPIN1 ASC IgM
8.788052e-33 0.4578891 0.332 0.042 1.566822e-28 23
KCNK6 ASC IgM
9.681806e-33 0.6230168 0.893 0.564 1.726169e-28 23
UQCRQ ASC IgM
1.23852e-32 0.5209011 0.323 0.04 2.208157e-28 23
CREB3L2 ASC IgM
1.2448e-32 0.5763868 0.383 0.061 2.219354e-28 23
ITPR2 ASC IgM
1.284477e-32 0.5034415 0.472 0.095 2.290094e-28 23
TMED5 ASC IgM
1.488623e-32 0.573964 0.807 0.398 2.654065e-28 23
UBXN4 ASC IgM
1.633058e-32 0.3522713 0.231 0.019 2.911579e-28 23
NT5DC2 ASC IgM
2.045442e-32 -0.923017 0.045 0.258 3.646818e-28 23
LYSMD2 ASC IgM
2.066822e-32 0.6373871 0.558 0.167 3.684938e-28 23
SRP68 ASC IgM
2.195263e-32 0.5365711 0.445 0.094 3.913934e-28 23
RPN1 ASC IgM
4.382949e-32 0.5878252 0.659 0.233 7.814359e-28 23
LRPAP1 ASC IgM
4.7176e-32 0.591792 0.641 0.227 8.411009e-28 23
UFM1 ASC IgM
5.386373e-32 0.604056 0.57 0.17 9.603364e-28 23
GORASP2 ASC IgM
6.510291e-32 0.7916083 0.979 0.963 1.16072e-27 23
FTL ASC IgM
1.348788e-31 -0.9904182 0.071 0.295 2.404754e-27 23
BCL11A ASC IgM
1.461589e-31 0.6139542 0.552 0.163 2.605866e-27 23
MLEC ASC IgM
1.893351e-31 0.5413666 0.341 0.051 3.375656e-27 23
MEI1 ASC IgM
2.875837e-31 1.190846 0.504 0.149 5.12733e-27 23
DUSP2 ASC IgM
3.284169e-31 3.278607 0.917 0.643 5.855346e-27 23

IGLC2	ASC IgM	3.618854e-31	-0.6575091	0.982	0.988	6.452054e-27	23
RACK1	ASC IgM	7.85658e-31	0.5409391	0.415	0.07	1.40075e-26	23
CHST12	ASC IgM	1.03119e-30	-0.8395427	0.979	0.98	1.838509e-26	23
RPL7A	ASC IgM	1.344779e-30	0.6127374	0.941	0.728	2.397607e-26	23
OST4	ASC IgM	1.378807e-30	0.7651988	0.84	0.372	2.458276e-26	23
ACADM	ASC IgM	3.198574e-30	0.6085567	0.617	0.219	5.702738e-26	23
COPB2	ASC IgM	3.440432e-30	-1.49222	0.493	0.699	6.133946e-26	23
EMP3	ASC IgM	5.535762e-30	-1.195592	0.243	0.373	9.869711e-26	23
SELL	ASC IgM	5.838359e-30	-1.119377	0.086	0.298	1.040921e-25	23
LINC00926	ASC IgM	6.000729e-30	0.3162679	0.131	0.005	1.06987e-25	23
IL17RB	ASC IgM	7.420224e-30	0.5429469	0.439	0.098	1.322952e-25	23
CTSD	ASC IgM	8.461742e-30	0.6442194	0.543	0.156	1.508644e-25	23
IL10RA	ASC IgM	1.286683e-29	0.596634	0.783	0.378	2.294027e-25	23
NDUFC2	ASC IgM	1.545502e-29	0.5757549	0.614	0.206	2.755475e-25	23
CISD2	ASC IgM	1.547157e-29	-1.031498	0.202	0.431	2.758425e-25	23
IRF8	ASC IgM	3.66518e-29	0.4517201	0.365	0.056	6.53465e-25	23
GMPPA	ASC IgM	6.321157e-29	-0.7789404	0.516	0.626	1.126999e-24	23
HLA-DMB	ASC IgM	8.49556e-29	0.3932389	0.252	0.04	1.514673e-24	23
PKHD1L1	ASC IgM	1.243179e-28	0.6412416	0.614	0.165	2.216465e-24	23
SEL1L3	ASC IgM	2.689801e-28	0.4692596	0.368	0.063	4.795646e-24	23
CALU	ASC IgM	2.743882e-28	-1.365586	0.632	0.717	4.892067e-24	23
HMGN2	ASC IgM	3.03451e-28	0.5858471	0.789	0.405	5.410227e-24	23
GUK1	ASC IgM	3.132206e-28	0.4792682	0.288	0.039	5.584411e-24	23
SELENOM	ASC IgM	3.359557e-28	-0.9625033	0.267	0.435	5.989753e-24	23
CYB561A3	ASC IgM	4.035549e-28	0.5685232	0.733	0.328	7.194981e-24	23

COPZ1	ASC IgM	5.981576e-28	0.8094714	0.309	0.073	1.066455e-23	23
H1FX	ASC IgM	5.998143e-28	0.5819582	0.599	0.227	1.069409e-23	23
DDOST	ASC IgM	7.544776e-28	0.5332204	0.95	0.705	1.345158e-23	23
COX7A2	ASC IgM	9.39753e-28	0.4485528	0.472	0.118	1.675486e-23	23
NEU1	ASC IgM	1.263766e-27	2.222715	0.543	0.428	2.253168e-23	23
IGLC3	ASC IgM	1.607416e-27	0.4277775	0.243	0.034	2.865862e-23	23
SLCO4A1	ASC IgM	2.183099e-27	1.283998	0.234	0.05	3.892247e-23	23
CCL3	ASC IgM	3.051611e-27	0.309268	0.136	0.008	5.440717e-23	23
CADM1	ASC IgM	3.165039e-27	0.5295492	0.95	0.705	5.642948e-23	23
NDUFA4	ASC IgM	5.01522e-27	0.4540642	0.418	0.099	8.941635e-23	23
HDLBP	ASC IgM	5.091046e-27	0.4799005	0.418	0.076	9.076826e-23	23
COPG1	ASC IgM	5.219045e-27	-0.7226655	0.97	0.982	9.305035e-23	23
RPS11	ASC IgM	7.528105e-27	0.5219209	0.57	0.176	1.342186e-22	23
MESD	ASC IgM	7.853617e-27	0.4989259	0.558	0.169	1.400221e-22	23
SRPRA	ASC IgM	8.992236e-27	0.5722813	0.662	0.236	1.603226e-22	23
ZBTB8OS	ASC IgM	9.146061e-27	0.5441536	0.608	0.237	1.630651e-22	23
MORF4L2	ASC IgM	9.854602e-27	-0.7965824	0.039	0.196	1.756977e-22	23
FAM129C	ASC IgM	1.160181e-26	0.3765317	0.383	0.093	2.068487e-22	23
KLK1	ASC IgM	1.390145e-26	0.5597204	0.421	0.087	2.47849e-22	23
ABHD3	ASC IgM	1.52632e-26	-0.978972	0.264	0.404	2.721276e-22	23
STX7	ASC IgM	1.989252e-26	0.5163489	0.516	0.14	3.546638e-22	23
USP48	ASC IgM	2.091595e-26	-0.7944429	0.953	0.957	3.729106e-22	23
PABPC1	ASC IgM	2.362693e-26	0.4294115	0.291	0.039	4.212446e-22	23
DENND6B	ASC IgM	2.781891e-26	-0.9210503	0.92	0.927	4.959833e-22	23
HNRNPA1	ASC IgM	3.18891e-26	0.3000897	0.187	0.015	5.685508e-22	23

ZNF215 ASC IgM
4.255304e-26 0.4610859 0.418 0.094 7.586782e-22 23
SEC23B ASC IgM
4.598961e-26 0.5493425 0.801 0.449 8.199487e-22 23
VCP ASC IgM
4.694952e-26 -0.6870419 0.982 0.985 8.37063e-22 23
TMSB10 ASC IgM
4.840163e-26 0.6831284 0.659 0.223 8.629527e-22 23
UBE2G1 ASC IgM
5.031944e-26 0.3630902 0.157 0.01 8.971453e-22 23
IGHV1-69D ASC IgM
6.923779e-26 0.3896567 0.223 0.019 1.234441e-21 23
AEBP1 ASC IgM
8.414517e-26 -0.7953381 0.973 0.957 1.500224e-21 23
RPL24 ASC IgM
9.857036e-26 0.7891745 0.976 0.904 1.757411e-21 23
HLA-A ASC IgM
1.09587e-25 0.5235147 0.481 0.139 1.953827e-21 23
ARMCX3 ASC IgM
1.211335e-25 0.3726975 0.228 0.025 2.159689e-21 23
MIR4435-2HG ASC IgM
1.563573e-25 0.44191 0.371 0.066 2.787694e-21 23
THEMIS2 ASC IgM
1.96546e-25 0.375016 0.249 0.025 3.504219e-21 23
LAX1 ASC IgM
2.173109e-25 -0.854771 0.181 0.339 3.874436e-21 23
SPIB ASC IgM
2.304478e-25 0.499268 0.893 0.557 4.108654e-21 23
ATP5PF ASC IgM
2.331114e-25 0.4683881 0.318 0.053 4.156144e-21 23
SEL1L ASC IgM
2.470945e-25 0.4355921 0.264 0.042 4.405448e-21 23
IRF4 ASC IgM
2.477794e-25 0.506352 0.546 0.159 4.417659e-21 23
ST6GAL1 ASC IgM
4.014277e-25 0.6770774 0.436 0.122 7.157055e-21 23
DLAT ASC IgM
4.979622e-25 -0.8804192 0.092 0.268 8.878168e-21 23
HHEX ASC IgM
5.076104e-25 0.5094131 0.442 0.108 9.050186e-21 23
STT3B ASC IgM
5.229283e-25 0.4119578 0.19 0.026 9.323288e-21 23
BEX5 ASC IgM
6.927482e-25 0.4734108 0.54 0.17 1.235101e-20 23
EMC7 ASC IgM
7.05621e-25 -0.8984339 0.062 0.212 1.258052e-20 23
PLAC8 ASC IgM
8.624027e-25 0.4540257 0.383 0.072 1.537578e-20 23
EDEM2 ASC IgM
1.005879e-24 0.4398879 0.65 0.277 1.793382e-20 23

SRM	ASC IgM	1.182215e-24	0.611154	0.763	0.355	2.107771e-20	23
TMEM243	ASC IgM	1.246497e-24	0.5128153	0.641	0.24	2.222379e-20	23
OS9	ASC IgM	1.39666e-24	-1.519402	0.626	0.666	2.490105e-20	23
TUBA1B	ASC IgM	1.427249e-24	0.524302	0.579	0.213	2.544642e-20	23
RAB1A	ASC IgM	1.842233e-24	0.3771049	0.341	0.061	3.284516e-20	23
LARP1B	ASC IgM	2.209783e-24	0.3210451	0.243	0.028	3.939823e-20	23
HSPA13	ASC IgM	2.718396e-24	-0.6845967	0.694	0.725	4.846628e-20	23
LIMD2	ASC IgM	3.149521e-24	0.5828937	0.54	0.184	5.615281e-20	23
REXO2	ASC IgM	3.403901e-24	-0.5703476	0.537	0.656	6.068815e-20	23
TCL1A	ASC IgM	3.588511e-24	0.7219261	0.632	0.204	6.397956e-20	23
EAF2	ASC IgM	4.514323e-24	0.3187725	0.315	0.075	8.048586e-20	23
SLAMF7	ASC IgM	4.527106e-24	0.3825877	0.297	0.041	8.071378e-20	23
FNDC3A	ASC IgM	8.678342e-24	0.4079944	0.261	0.039	1.547262e-19	23
SLC17A9	ASC IgM	8.868649e-24	-0.9869266	0.407	0.489	1.581191e-19	23
UCP2	ASC IgM	8.951658e-24	0.4605958	0.525	0.166	1.595991e-19	23
ARL1	ASC IgM	9.002945e-24	0.555217	0.694	0.298	1.605135e-19	23
CDK2AP2	ASC IgM	9.008337e-24	0.5630023	0.614	0.224	1.606096e-19	23
ADI1	ASC IgM	9.461484e-24	-0.7771479	0.033	0.175	1.686888e-19	23
MARCH1	ASC IgM	9.686928e-24	0.4764119	0.739	0.381	1.727082e-19	23
ATP6V0B	ASC IgM	1.004757e-23	0.4760658	0.513	0.174	1.791381e-19	23
DERL2	ASC IgM	1.068001e-23	0.6284857	0.718	0.32	1.904138e-19	23
HSBP1	ASC IgM	1.242964e-23	0.574074	0.531	0.173	2.21608e-19	23
EHMT1	ASC IgM	1.587579e-23	0.3862937	0.332	0.063	2.830495e-19	23
SLC39A7	ASC IgM	1.872301e-23	0.4502114	0.439	0.121	3.338126e-19	23
PIGT	ASC IgM	2.321575e-23	-0.943168	0.089	0.253	4.139135e-19	23

FCMR ASC IgM
2.670337e-23 0.753007 0.872 0.531 4.760944e-19 23
ISG20 ASC IgM
2.708845e-23 0.4742036 0.585 0.224 4.8296e-19 23
TIMM17A ASC IgM
2.990026e-23 0.4741142 0.953 0.742 5.330916e-19 23
CHCHD2 ASC IgM
3.03278e-23 0.6484069 0.635 0.342 5.407144e-19 23
INSIG1 ASC IgM
3.313467e-23 0.4612544 0.697 0.31 5.90758e-19 23
AKR1A1 ASC IgM
4.235083e-23 0.5680105 0.261 0.045 7.55073e-19 23
ALDH5A1 ASC IgM
5.67006e-23 -1.013323 0.326 0.477 1.010915e-18 23
TNFAIP8 ASC IgM
5.736406e-23 -1.194079 0.136 0.354 1.022744e-18 23
TXNIP ASC IgM
6.497034e-23 0.368455 0.208 0.021 1.158356e-18 23 SPN
ASC IgM
6.541718e-23 0.4523975 0.742 0.384 1.166323e-18 23
PSAP ASC IgM
6.610206e-23 -1.033099 0.294 0.438 1.178534e-18 23
COTL1 ASC IgM
7.554462e-23 -0.7892001 0.929 0.923 1.346885e-18 23
BTF3 ASC IgM
8.806616e-23 0.3952606 0.234 0.036 1.570132e-18 23
PRDM15 ASC IgM
9.674869e-23 0.5531879 0.329 0.072 1.724932e-18 23
NUCB2 ASC IgM
9.938927e-23 0.4357646 0.54 0.171 1.772011e-18 23
ERGIC2 ASC IgM
1.208945e-22 0.3617927 0.255 0.029 2.155427e-18 23
GLCCI1 ASC IgM
2.162398e-22 0.4059515 0.24 0.035 3.855339e-18 23
ZFAT ASC IgM
3.558971e-22 0.3272782 0.184 0.017 6.345289e-18 23
TRAM2 ASC IgM
3.862598e-22 0.471952 0.855 0.432 6.886626e-18 23
ROMO1 ASC IgM
6.890624e-22 -0.9274904 0.549 0.62 1.228529e-17 23
IFI16 ASC IgM
1.013761e-21 0.4401487 0.593 0.205 1.807434e-17 23
GNL3 ASC IgM
1.770708e-21 0.3634271 0.377 0.085 3.156995e-17 23
TENT5C ASC IgM
1.803049e-21 0.4078455 0.309 0.064 3.214656e-17 23
MAGT1 ASC IgM
2.39546e-21 0.4812299 0.668 0.316 4.270865e-17 23
PRDX2 ASC IgM
3.5916e-21 0.3617425 0.303 0.062 6.403464e-17 23

SLC39A8	ASC IgM	3.700614e-21	0.3836545	0.846	0.491	6.597825e-17	23
ARF1	ASC IgM	4.712546e-21	0.384319	0.499	0.152	8.401998e-17	23
ARFGAP3	ASC IgM	6.291214e-21	0.4276835	0.537	0.178	1.121661e-16	23
NUDT22	ASC IgM	6.466558e-21	0.4010209	0.552	0.214	1.152923e-16	23
CANX	ASC IgM	6.740456e-21	-0.8831445	0.151	0.307	1.201756e-16	23
ARHGAP24	ASC IgM	6.85894e-21	0.4944459	0.54	0.18	1.22288e-16	23
TRABD	ASC IgM	8.243807e-21	0.4393095	0.534	0.182	1.469788e-16	23
NAPA	ASC IgM	8.79202e-21	-0.9577494	0.148	0.321	1.567529e-16	23
TUBA1A	ASC IgM	1.22785e-20	-0.9776887	0.519	0.648	2.189134e-16	23
ZFAS1	ASC IgM	1.348153e-20	-0.8258986	0.166	0.319	2.403623e-16	23
FCRLA	ASC IgM	1.60341e-20	0.5241356	0.412	0.11	2.85872e-16	23
BRI3BP	ASC IgM	1.723059e-20	0.3703382	0.855	0.507	3.072041e-16	23
PSMD8	ASC IgM	1.820739e-20	0.3639793	0.504	0.183	3.246196e-16	23
YIF1A	ASC IgM	1.945374e-20	0.4849292	0.412	0.098	3.468407e-16	23
PTPN7	ASC IgM	2.09034e-20	0.4342143	0.579	0.22	3.726867e-16	23
SRP54	ASC IgM	2.163951e-20	0.4419047	0.528	0.17	3.858109e-16	23
SEC63	ASC IgM	2.26728e-20	-0.8373232	0.279	0.417	4.042334e-16	23
ESD	ASC IgM	3.077692e-20	0.5369683	0.754	0.325	5.487216e-16	23
GYPC	ASC IgM	3.33815e-20	-0.712001	0.074	0.22	5.951588e-16	23
LY86	ASC IgM	6.896465e-20	0.3907141	0.493	0.165	1.229571e-15	23
SAR1A	ASC IgM	6.897774e-20	-1.228046	0.38	0.572	1.229804e-15	23
VIM	ASC IgM	7.571409e-20	0.4173717	0.579	0.227	1.349906e-15	23
CCNC	ASC IgM	8.118527e-20	-0.6848501	0.988	0.988	1.447452e-15	23
RPLP0	ASC IgM	1.017706e-19	0.3741523	0.392	0.118	1.814469e-15	23
CTSA	ASC IgM	1.024162e-19	0.4719577	0.881	0.489	1.825978e-15	23

C4orf3	ASC IgM	1.152362e-19	0.3843589	0.439	0.132	2.054547e-15	23
AP3D1	ASC IgM	1.274443e-19	0.459607	0.519	0.171	2.272204e-15	23
AKIRIN2	ASC IgM	1.526705e-19	0.3935446	0.199	0.032	2.721962e-15	23
GPR137B	ASC IgM	2.038065e-19	0.4189308	0.46	0.137	3.633666e-15	23
GOLGB1	ASC IgM	2.956577e-19	0.4981974	0.596	0.239	5.271281e-15	23
PHACTR1	ASC IgM	2.988468e-19	0.3515531	0.22	0.031	5.32814e-15	23
ESR2	ASC IgM	3.555487e-19	0.3199216	0.846	0.529	6.339078e-15	23
GHITM	ASC IgM	3.615393e-19	0.4182644	0.359	0.102	6.445885e-15	23
SLC25A4	ASC IgM	3.660145e-19	0.4869215	0.442	0.129	6.525673e-15	23
C16orf87	ASC IgM	6.765477e-19	-1.071079	0.154	0.317	1.206217e-14	23
CD69	ASC IgM	7.545417e-19	0.4033192	0.439	0.122	1.345272e-14	23
B4GALT3	ASC IgM	1.032282e-18	0.3384197	0.276	0.046	1.840455e-14	23
FUT8	ASC IgM	1.124451e-18	0.7282473	0.392	0.09	2.004784e-14	23
FCRL2	ASC IgM	1.220731e-18	0.3473206	0.703	0.357	2.176441e-14	23
BSG	ASC IgM	1.347189e-18	0.3836389	0.3	0.061	2.401903e-14	23
BCL2L1	ASC IgM	1.351393e-18	-0.7800731	0.914	0.917	2.409399e-14	23
PFDN5	ASC IgM	1.561162e-18	0.3176419	0.226	0.027	2.783395e-14	23
RNF103	ASC IgM	1.602483e-18	0.4117322	0.789	0.447	2.857067e-14	23
SF3B5	ASC IgM	2.020309e-18	0.3582004	0.457	0.142	3.602009e-14	23
WDR45	ASC IgM	2.554287e-18	0.3863199	0.439	0.14	4.554038e-14	23
GPAA1	ASC IgM	2.830747e-18	0.5742421	0.362	0.081	5.04694e-14	23
OAS1	ASC IgM	2.942315e-18	0.3572125	0.297	0.056	5.245853e-14	23
MBTPS1	ASC IgM	3.304419e-18	0.3774598	0.484	0.165	5.891448e-14	23
GOLGA4	ASC IgM	3.38474e-18	0.364725	0.409	0.124	6.034653e-14	23
MGAT2	ASC IgM	3.702473e-18	0.4865062	0.703	0.321	6.601138e-14	23

BLNK	ASC IgM						
4.166958e-18	0.3430283	0.849	0.5	7.429269e-14	23		
NDUFA11	ASC IgM						
4.69329e-18	0.319846	0.864	0.574	8.367666e-14	23		
NDUFB11	ASC IgM						
4.734952e-18	0.4709682	0.908	0.582	8.441946e-14	23		
ATP5MC3	ASC IgM						
5.695469e-18	-0.5950936	0.97	0.964	1.015445e-13	23		
RPL4	ASC IgM						
5.705379e-18	0.3298017	0.359	0.08	1.017212e-13	23		
PLD3	ASC IgM						
5.759001e-18	0.3043387	0.119	0.013	1.026772e-13	23		
CHDH	ASC IgM						
6.539638e-18	-0.8034031	0.234	0.332	1.165952e-13	23		
RIPOR2	ASC IgM						
6.644855e-18	-0.9095538	0.401	0.502	1.184711e-13	23		
FOXP1	ASC IgM						
6.653117e-18	0.3361613	0.285	0.06	1.186184e-13	23		
STT3A	ASC IgM						
1.011561e-17	0.3271049	0.819	0.437	1.803512e-13	23		
AURKAIP1	ASC IgM						
1.057398e-17	-1.109549	0.288	0.473	1.885235e-13	23		
CD83	ASC IgM						
1.256562e-17	0.3316959	0.228	0.036	2.240324e-13	23		
PIK3CG	ASC IgM						
1.333431e-17	0.345652	0.733	0.363	2.377374e-13	23		
PSMA5	ASC IgM						
1.427905e-17	0.3877508	0.49	0.182	2.545812e-13	23		
KDELR1	ASC IgM						
1.497694e-17	-0.7557626	0.81	0.825	2.670238e-13	23		
EIF3E	ASC IgM						
1.590865e-17	0.3146362	0.315	0.068	2.836353e-13	23		
SLC38A10	ASC IgM						
1.843304e-17	0.3499741	0.409	0.123	3.286426e-13	23		
ISOC2	ASC IgM						
1.90782e-17	0.3701153	0.522	0.184	3.401452e-13	23		
TECR	ASC IgM						
1.937064e-17	-0.8940417	0.181	0.338	3.453592e-13	23		
CD48	ASC IgM						
2.167556e-17	0.3828417	0.599	0.242	3.864535e-13	23		
MRPL55	ASC IgM						
2.195452e-17	0.3733103	0.409	0.114	3.914272e-13	23		
PGRMC2	ASC IgM						
2.294985e-17	-0.9314729	0.941	0.929	4.091728e-13	23		
NPM1	ASC IgM						
3.011534e-17	0.3672484	0.671	0.329	5.369265e-13	23		
SRP19	ASC IgM						
3.124277e-17	-1.147389	0.519	0.577	5.570274e-13	23		
TUBB	ASC IgM						
3.570193e-17	0.3506792	0.712	0.364	6.365298e-13	23		

RPS19BP1 ASC IgM					
3.583473e-17	0.3537363	0.412	0.117	6.388974e-13	23
USO1 ASC IgM					
3.937993e-17	0.3942925	0.573	0.255	7.021048e-13	23
CDV3 ASC IgM					
5.499645e-17	-0.7205712	0.045	0.178	9.805318e-13	23
BCL2A1 ASC IgM					
6.229169e-17	0.3785948	0.905	0.618	1.110599e-12	23
NDUFA1 ASC IgM					
6.651412e-17	0.3250854	0.142	0.011	1.18588e-12	23
HIST1H2BG ASC IgM					
6.968771e-17	-0.8576897	0.154	0.269	1.242462e-12	23
JUN ASC IgM					
7.305905e-17	0.3990183	0.757	0.362	1.30257e-12	23
TERF2IP ASC IgM					
1.041254e-16	-0.9627212	0.665	0.697	1.856452e-12	23
HNRNPDL ASC IgM					
1.089002e-16	-0.7783472	0.887	0.845	1.941582e-12	23
HNRNPA2B1 ASC IgM					
1.164655e-16	0.4000282	0.685	0.332	2.076463e-12	23
VOPP1 ASC IgM					
1.198631e-16	0.3708422	0.638	0.298	2.13704e-12	23
SRP72 ASC IgM					
1.270068e-16	-0.7313436	0.178	0.29	2.264404e-12	23
DCK ASC IgM					
1.278681e-16	-0.5410443	0.03	0.129	2.279759e-12	23
CD24 ASC IgM					
1.493384e-16	0.4185249	0.421	0.142	2.662555e-12	23
MDFIC ASC IgM					
2.239505e-16	0.3920239	0.546	0.215	3.992813e-12	23
IFI27L2 ASC IgM					
2.61993e-16	0.392669	0.549	0.205	4.671073e-12	23
MRPS31 ASC IgM					
2.648084e-16	0.3553988	0.38	0.105	4.721268e-12	23
SEC31A ASC IgM					
2.820754e-16	0.3467905	0.84	0.495	5.029122e-12	23
COX5A ASC IgM					
2.846521e-16	0.4831192	0.941	0.721	5.075062e-12	23
COX6C ASC IgM					
2.938532e-16	0.3247178	0.463	0.155	5.23911e-12	23
MGAT1 ASC IgM					
2.97591e-16	0.3355076	0.344	0.085	5.30575e-12	23
PNOC ASC IgM					
3.106587e-16	0.4060503	0.3	0.063	5.538734e-12	23
AC243960.1 ASC IgM					
3.579668e-16	0.3822363	0.469	0.153	6.38219e-12	23
GEMIN7 ASC IgM					
3.637836e-16	0.3773963	0.451	0.153	6.485897e-12	23
SMAP1 ASC IgM					
3.687162e-16	0.3402199	0.214	0.034	6.573841e-12	23

SLC44A1	ASC IgM	4.070109e-16	0.3021514	0.599	0.272	7.256597e-12	23
AUP1	ASC IgM	4.118999e-16	0.5055804	0.377	0.093	7.343763e-12	23
FCRL5	ASC IgM	4.304813e-16	-0.5526078	0.053	0.168	7.675051e-12	23
SNX10	ASC IgM	4.365538e-16	0.3287756	0.234	0.049	7.783318e-12	23
STX11	ASC IgM	4.407996e-16	0.3413318	0.415	0.117	7.859017e-12	23
ATP6AP1	ASC IgM	4.937558e-16	-0.9365367	0.522	0.596	8.803172e-12	23
TPM3	ASC IgM	6.436845e-16	0.4326307	0.332	0.067	1.147625e-11	23
ITM2C	ASC IgM	6.511817e-16	-0.4353861	0.012	0.105	1.160992e-11	23
RMI2	ASC IgM	7.123099e-16	0.4137122	0.507	0.176	1.269977e-11	23
ICAM3	ASC IgM	7.542223e-16	0.3136081	0.849	0.52	1.344703e-11	23
SELENOT	ASC IgM	7.683843e-16	0.3367362	0.424	0.134	1.369952e-11	23
CD46	ASC IgM	7.750497e-16	0.414221	0.626	0.325	1.381836e-11	23
BST2	ASC IgM	8.650639e-16	0.3137428	0.552	0.207	1.542322e-11	23
STX5	ASC IgM	9.413355e-16	0.4245029	0.914	0.602	1.678307e-11	23
DYNLL1	ASC IgM	1.133855e-15	0.3063274	0.214	0.04	2.02155e-11	23
ELL	ASC IgM	1.285784e-15	0.4163196	0.861	0.532	2.292424e-11	23
SRSF9	ASC IgM	1.364594e-15	0.4136624	0.861	0.554	2.432935e-11	23
ATP5IF1	ASC IgM	1.369424e-15	0.3264542	0.739	0.393	2.441546e-11	23
NDUFAB1	ASC IgM	2.629138e-15	-0.8765593	0.267	0.348	4.68749e-11	23
TRAF3IP3	ASC IgM	2.717954e-15	-0.5816143	0.071	0.196	4.845841e-11	23
AFF3	ASC IgM	3.422213e-15	0.3447635	0.356	0.107	6.101464e-11	23
DAP	ASC IgM	3.555776e-15	-0.6469497	0.953	0.944	6.339593e-11	23
NACA	ASC IgM	3.710172e-15	0.4799955	0.558	0.25	6.614865e-11	23
RGS10	ASC IgM	4.16388e-15	0.4044699	0.632	0.271	7.423781e-11	23
POU2AF1	ASC IgM	4.288936e-15	0.4346571	0.614	0.276	7.646744e-11	23

STK17B	ASC IgM					
4.671615e-15	0.377021	0.341	0.087	8.329022e-11	23	
TPST2	ASC IgM					
4.802776e-15	0.4502618	0.341	0.106	8.56287e-11	23	
CITED2	ASC IgM					
4.84678e-15	0.4424097	0.49	0.18	8.641324e-11	23	
RASGRP3	ASC IgM					
5.155387e-15	0.3716624	0.32	0.072	9.191539e-11	23	
GPX7	ASC IgM					
5.782779e-15	0.3516104	0.798	0.47	1.031012e-10	23	
NDUFB4	ASC IgM					
5.957621e-15	-0.822937	0.205	0.33	1.062184e-10	23	
SMC6	ASC IgM					
6.728847e-15	0.3453532	0.371	0.096	1.199686e-10	23	
IFT27	ASC IgM					
6.778328e-15	-0.3662148	0.994	0.993	1.208508e-10	23	
FAU	ASC IgM					
6.80447e-15	0.3339484	0.605	0.259	1.213169e-10	23	
PSMC2	ASC IgM					
8.295658e-15	0.3187514	0.309	0.063	1.479033e-10	23	
MXD4	ASC IgM					
9.118273e-15	0.3916844	0.401	0.13	1.625697e-10	23	
PAPSS1	ASC IgM					
1.166376e-14	0.4052993	0.772	0.465	2.079531e-10	23	
ITM2B	ASC IgM					
1.255409e-14	0.3485012	0.611	0.245	2.238269e-10	23	
SNRPN	ASC IgM					
2.000116e-14	0.3114858	0.318	0.088	3.566007e-10	23	
UBE2M	ASC IgM					
2.313728e-14	-0.6928128	0.202	0.286	4.125145e-10	23	
SP110	ASC IgM					
2.523903e-14	0.3186869	0.326	0.081	4.499866e-10	23	
ALG2	ASC IgM					
2.831798e-14	-0.8294291	0.415	0.477	5.048814e-10	23	
FUS	ASC IgM					
3.047589e-14	-0.8180033	0.816	0.815	5.433546e-10	23	
NOP53	ASC IgM					
3.185513e-14	0.3456392	0.365	0.101	5.679452e-10	23	
IFI35	ASC IgM					
3.809882e-14	0.3431067	0.691	0.313	6.792639e-10	23	
SCAND1	ASC IgM					
3.879947e-14	-0.640552	0.932	0.895	6.917557e-10	23	
PPIA	ASC IgM					
4.052638e-14	0.3405809	0.205	0.037	7.225449e-10	23	
PDE4DIP	ASC IgM					
4.076797e-14	0.3593568	0.291	0.069	7.268521e-10	23	
UBE2H	ASC IgM					
4.306894e-14	-0.9623693	0.22	0.352	7.678762e-10	23	
LY6E	ASC IgM					
4.335966e-14	-0.8243398	0.398	0.472	7.730593e-10	23	

GPSM3	ASC IgM	4.446848e-14	0.300726	0.573	0.248	7.928285e-10	23
IER3IP1	ASC IgM	4.499314e-14	0.3101751	0.739	0.399	8.021827e-10	23
UQCRFS1	ASC IgM	8.495103e-14	0.4942296	0.264	0.076	1.514592e-09	23
CRYZ	ASC IgM	8.666328e-14	-0.6070485	0.151	0.241	1.54512e-09	23
MCUB	ASC IgM	9.687121e-14	0.333939	0.641	0.316	1.727117e-09	23
ERGIC3	ASC IgM	1.189799e-13	-0.8130149	0.976	0.961	2.121292e-09	23
MT-ND2	ASC IgM	1.316844e-13	-0.7508101	0.685	0.668	2.3478e-09	23
GDI2	ASC IgM	1.42774e-13	0.314064	0.92	0.645	2.545517e-09	23
UQCR11	ASC IgM	2.258208e-13	-0.7873767	0.181	0.279	4.026159e-09	23
IFITM2	ASC IgM	2.411055e-13	0.5320661	0.427	0.136	4.29867e-09	23
SIT1	ASC IgM	2.667939e-13	-0.6634974	0.214	0.298	4.756669e-09	23
BLK	ASC IgM	3.156651e-13	0.4412926	0.525	0.207	5.627993e-09	23
EHD1	ASC IgM	3.75981e-13	-0.8291393	0.828	0.774	6.703366e-09	23
HLA-DPB1	ASC IgM	3.91231e-13	-0.504536	0.104	0.175	6.975257e-09	23
QRSL1	ASC IgM	4.421932e-13	-0.7272724	0.214	0.28	7.883862e-09	23
RNASET2	ASC IgM	4.53352e-13	-0.6932829	0.875	0.805	8.082814e-09	23
CALM2	ASC IgM	4.638145e-13	0.5595554	0.481	0.158	8.269348e-09	23
SUSD3	ASC IgM	4.751519e-13	-0.814726	0.315	0.399	8.471484e-09	23
BLOC1S2	ASC IgM	4.962836e-13	0.3142096	0.273	0.065	8.84824e-09	23
C1GALT1C1	ASC IgM	5.207957e-13	0.4426246	0.774	0.444	9.285267e-09	23
BTG2	ASC IgM	5.464176e-13	-0.8777393	0.231	0.384	9.742079e-09	23
CD44	ASC IgM	5.480544e-13	0.3020051	0.178	0.034	9.771262e-09	23
NDFIP2	ASC IgM	6.767303e-13	0.3076938	0.721	0.396	1.206543e-08	23
PSMA3	ASC IgM	7.382645e-13	0.4573515	0.377	0.116	1.316252e-08	23
TBC1D1	ASC IgM	8.461066e-13	-0.8019412	0.297	0.401	1.508524e-08	23

REL	ASC IgM	8.825081e-13	0.3573568	0.377	0.13	1.573424e-08	23
DUSP22	ASC IgM	1.032102e-12	0.6578213	0.436	0.151	1.840134e-08	23
CD81	ASC IgM	1.138192e-12	0.3453702	0.288	0.068	2.029282e-08	23
MAGED1	ASC IgM	1.154163e-12	-0.6248574	0.148	0.242	2.057757e-08	23
ARHGAP45	ASC IgM	1.183358e-12	0.3250029	0.579	0.262	2.109809e-08	23
EMC6	ASC IgM	1.263685e-12	-0.5765671	0.148	0.212	2.253024e-08	23
FAM111B	ASC IgM	1.354279e-12	0.4378694	0.694	0.295	2.414544e-08	23
TCF4	ASC IgM	1.626381e-12	0.3486529	0.335	0.116	2.899675e-08	23
GOSR2	ASC IgM	1.781833e-12	0.3151218	0.243	0.061	3.176831e-08	23
NINJ1	ASC IgM	1.862221e-12	0.3356181	0.445	0.163	3.320154e-08	23
DEF8	ASC IgM	2.000617e-12	0.3789269	0.605	0.267	3.5669e-08	23
TOP1	ASC IgM	2.094643e-12	-0.6163982	0.777	0.702	3.734539e-08	23
COMMD6	ASC IgM	2.331261e-12	0.38987	0.682	0.328	4.156406e-08	23
CCM2	ASC IgM	2.96952e-12	0.312695	0.309	0.09	5.294357e-08	23
HIST1H2AC	ASC IgM	2.984085e-12	-0.7182453	0.228	0.274	5.320326e-08	23
KLF2	ASC IgM	3.111565e-12	-0.8136413	0.46	0.494	5.54761e-08	23
SP100	ASC IgM	3.378581e-12	-0.5732472	0.151	0.233	6.023672e-08	23
IL4R	ASC IgM	3.626631e-12	-0.3842355	0.03	0.102	6.465921e-08	23
BACH2	ASC IgM	3.653726e-12	0.3370703	0.754	0.396	6.514229e-08	23
NDUFS5	ASC IgM	3.809636e-12	0.3391859	0.659	0.303	6.7922e-08	23
TIMM8B	ASC IgM	3.828136e-12	-0.5459205	0.187	0.284	6.825183e-08	23
LAT2	ASC IgM	3.968642e-12	-0.6600379	0.97	0.934	7.075691e-08	23
MT-ATP6	ASC IgM	4.073456e-12	-0.6532023	0.774	0.764	7.262565e-08	23
MYL12A	ASC IgM	4.098828e-12	0.4160441	0.834	0.513	7.3078e-08	23
LSP1	ASC IgM	4.578781e-12	-0.63122	0.383	0.46	8.163509e-08	23

SWAP70	ASC IgM	4.645669e-12	0.3016386	0.344	0.105	8.282764e-08	23
MCUR1	ASC IgM	4.886059e-12	0.6146999	0.519	0.208	8.711355e-08	23
ADA	ASC IgM	5.952615e-12	-0.8159645	0.401	0.44	1.061292e-07	23
HMGA1	ASC IgM	7.122882e-12	0.3625893	0.353	0.097	1.269939e-07	23
SYVN1	ASC IgM	1.025839e-11	0.3614573	0.57	0.287	1.828968e-07	23
C7orf50	ASC IgM	1.027994e-11	-1.117244	0.371	0.404	1.832811e-07	23
FABP5	ASC IgM	1.179503e-11	0.3205794	0.267	0.084	2.102936e-07	23
DUS1L	ASC IgM	1.26208e-11	0.3261013	0.231	0.061	2.250163e-07	23
ORC6	ASC IgM	1.27482e-11	-0.5510918	0.16	0.268	2.272877e-07	23
ALOX5AP	ASC IgM	1.309849e-11	-0.6521653	0.742	0.72	2.335329e-07	23
EIF3F	ASC IgM	1.372077e-11	0.3163047	0.32	0.093	2.446276e-07	23
KLF13	ASC IgM	1.372201e-11	0.3034766	0.202	0.047	2.446496e-07	23
LRRK1	ASC IgM	1.424474e-11	0.305127	0.739	0.397	2.539694e-07	23
RAP1A	ASC IgM	1.44327e-11	-0.844393	0.763	0.769	2.573205e-07	23
RPL17	ASC IgM	1.60478e-11	-0.5431604	0.979	0.974	2.861162e-07	23
MT-ND4	ASC IgM	1.644293e-11	0.4153247	0.659	0.333	2.931611e-07	23
ANXA11	ASC IgM	1.801871e-11	-0.7502973	0.724	0.717	3.212556e-07	23
EIF4A2	ASC IgM	2.085131e-11	-0.6565261	0.223	0.312	3.717581e-07	23
PNRC2	ASC IgM	2.454868e-11	0.4588306	0.585	0.263	4.376784e-07	23
FAM3C	ASC IgM	2.625928e-11	-0.6958981	0.371	0.426	4.681768e-07	23
CD40	ASC IgM	2.948164e-11	-0.5948335	0.148	0.206	5.256281e-07	23
PRKCB	ASC IgM	3.254027e-11	-1.102978	0.454	0.652	5.801605e-07	23
RPS4Y1	ASC IgM	3.563852e-11	-0.6154192	0.116	0.212	6.353992e-07	23
LBH	ASC IgM	3.62715e-11	0.7415929	0.157	0.04	6.466845e-07	23
IGLL5	ASC IgM	3.847423e-11	0.3077436	0.285	0.083	6.859571e-07	23

RBM33	ASC IgM	4.414301e-11	-0.8393876	0.377	0.446	7.870256e-07	23
KLF6	ASC IgM	4.799948e-11	-0.3878358	0.027	0.105	8.557828e-07	23
TRAF5	ASC IgM	5.038118e-11	0.3286524	0.73	0.372	8.98246e-07	23
PTP4A2	ASC IgM	5.252232e-11	0.3222344	0.593	0.243	9.364204e-07	23
NBDY	ASC IgM	5.70758e-11	0.3998318	0.433	0.144	1.017604e-06	23
SEC14L1	ASC IgM	5.800345e-11	0.3423267	0.733	0.416	1.034143e-06	23
CTSH	ASC IgM	6.184874e-11	-0.5238685	0.95	0.917	1.102701e-06	23
COX4I1	ASC IgM	6.489794e-11	0.3049526	0.401	0.129	1.157065e-06	23
MAGEF1	ASC IgM	6.588375e-11	-0.6773495	0.089	0.197	1.174641e-06	23
CHPT1	ASC IgM	7.328192e-11	-0.5959605	0.074	0.184	1.306543e-06	23
MBP	ASC IgM	9.028851e-11	0.3274369	0.644	0.318	1.609754e-06	23
AP3S1	ASC IgM	9.406228e-11	-0.8049538	0.104	0.221	1.677036e-06	23
GBP2	ASC IgM	1.071547e-10	0.3274847	0.481	0.207	1.910461e-06	23
CFLAR	ASC IgM	1.386991e-10	-0.5668344	0.415	0.403	2.472865e-06	23
MBD4	ASC IgM	1.587056e-10	-0.6648052	0.042	0.137	2.829562e-06	23
FCER2	ASC IgM	1.712069e-10	0.3192026	0.344	0.119	3.052448e-06	23
SNHG9	ASC IgM	1.79175e-10	-0.6621962	0.228	0.328	3.194511e-06	23
PTPN6	ASC IgM	1.837096e-10	-0.8033909	0.976	0.942	3.275359e-06	23
YBX1	ASC IgM	1.958198e-10	-0.4950288	0.098	0.148	3.491271e-06	23
ALOX5	ASC IgM	2.094747e-10	0.3290001	0.35	0.131	3.734725e-06	23
PRKCD	ASC IgM	2.407201e-10	-0.7204956	0.7	0.677	4.291799e-06	23
TAGLN2	ASC IgM	2.415022e-10	-0.5924122	0.961	0.928	4.305743e-06	23
EEF1D	ASC IgM	2.480391e-10	-0.6796003	0.451	0.469	4.422289e-06	23
SNRPF	ASC IgM	2.696903e-10	-0.771235	0.442	0.533	4.808308e-06	23
IMPDH2	ASC IgM	2.84955e-10	-0.4836502	0.033	0.106	5.080462e-06	23

C1orf162	ASC IgM					
3.4453e-10	0.3413625	0.825	0.489	6.142626e-06	23	
RAC2	ASC IgM					
3.517235e-10	-0.5758783	0.226	0.289	6.270878e-06	23	
RRAS2	ASC IgM					
3.562579e-10	-0.4711824	0.056	0.127	6.351722e-06	23	
ZEB2	ASC IgM					
3.953707e-10	-0.7947645	0.896	0.854	7.049065e-06	23	
SH3BGRL3	ASC IgM					
4.102484e-10	-0.5367716	0.187	0.245	7.314319e-06	23	
RASGRP2	ASC IgM					
4.447834e-10	0.4282839	0.635	0.272	7.930044e-06	23	
NCF1	ASC IgM					
5.477417e-10	-0.7173497	0.211	0.327	9.765687e-06	23	
PMAIP1	ASC IgM					
5.633726e-10	-0.5958198	0.297	0.339	1.004437e-05	23	
STK17A	ASC IgM					
5.787051e-10	0.5498617	0.611	0.266	1.031773e-05	23	
PIM1	ASC IgM					
6.266998e-10	0.4021741	0.145	0.03	1.117343e-05	23	
DHRS9	ASC IgM					
6.565575e-10	-0.6589983	0.484	0.49	1.170576e-05	23	
CBX3	ASC IgM					
7.725472e-10	0.3747136	0.496	0.185	1.377374e-05	23	
HLA-DOB	ASC IgM					
7.94937e-10	-0.5636885	0.113	0.187	1.417293e-05	23	
KIF20B	ASC IgM					
8.073595e-10	-0.611948	0.22	0.286	1.439441e-05	23	
PLEKHF2	ASC IgM					
8.415257e-10	-0.3365751	0.036	0.111	1.500356e-05	23	
CENPH	ASC IgM					
8.543828e-10	-0.5079606	0.101	0.155	1.523279e-05	23	
MCM7	ASC IgM					
1.035899e-09	-0.703275	0.288	0.366	1.846905e-05	23	
LRRFIP1	ASC IgM					
1.066675e-09	-0.6202614	0.157	0.254	1.901775e-05	23	
RRP7A	ASC IgM					
1.095506e-09	0.3060441	0.49	0.224	1.953177e-05	23	
ALKBH7	ASC IgM					
1.156275e-09	-0.7123441	0.706	0.615	2.061522e-05	23	
MEF2C	ASC IgM					
1.221393e-09	-0.4039191	0.086	0.159	2.177622e-05	23	
CTSZ	ASC IgM					
1.483268e-09	-0.7696654	0.131	0.238	2.644519e-05	23	
CAPG	ASC IgM					
1.49045e-09	-0.6540991	0.249	0.297	2.657324e-05	23	
PDLIM1	ASC IgM					
1.867353e-09	-0.6105846	0.282	0.341	3.329304e-05	23	
EIF2S3	ASC IgM					
2.228833e-09	-0.4262075	0.22	0.289	3.973786e-05	23	

BCAS4 ASC IgM
2.268516e-09 -0.7819473 0.039 0.131 4.044536e-05 23
MYC ASC IgM
2.521633e-09 0.3027431 0.407 0.141 4.49582e-05 23
RAB30 ASC IgM
3.020915e-09 0.3092755 0.306 0.115 5.385989e-05 23
GOT2 ASC IgM
3.035198e-09 0.4593724 0.326 0.105 5.411455e-05 23
HRK ASC IgM
3.274121e-09 -0.4587126 0.068 0.121 5.83743e-05 23
LPP ASC IgM
3.692132e-09 -0.4463252 0.024 0.101 6.582702e-05 23
RHOC ASC IgM
3.740864e-09 -0.4212318 0.053 0.131 6.669586e-05 23
UVRAG ASC IgM
4.138713e-09 -0.6479508 0.374 0.401 7.378912e-05 23
RPA3 ASC IgM
4.454348e-09 0.3134782 0.237 0.068 7.941658e-05 23
ST14 ASC IgM
4.580817e-09 0.4990047 0.282 0.048 8.167139e-05 23
XIST ASC IgM
5.638193e-09 0.3469235 0.398 0.142 0.0001005233 23
HSH2D ASC IgM
6.384421e-09 -0.9410339 0.43 0.456 0.0001138278 23
JUNB ASC IgM
6.602914e-09 -0.4610695 0.071 0.146 0.0001177234 23
COBLL1 ASC IgM
6.628886e-09 -0.6284068 0.769 0.669 0.0001181864 23
SNX3 ASC IgM
6.665009e-09 -0.7168858 0.315 0.335 0.0001188304 23
DUT ASC IgM
7.180265e-09 0.3131534 0.273 0.101 0.0001280169 23
AMZ2 ASC IgM
7.467751e-09 -0.4469779 0.047 0.11 0.0001331425 23
LINC02397 ASC IgM
7.537533e-09 -0.7503221 0.866 0.767 0.0001343867 23
HMGN1 ASC IgM
7.608147e-09 -0.5396497 0.362 0.365 0.0001356457 23
SNAP23 ASC IgM
8.160747e-09 -0.6628454 0.365 0.397 0.000145498 23
CIB1 ASC IgM
8.515532e-09 -0.548415 0.11 0.19 0.0001518234 23
IFNGR1 ASC IgM
8.630266e-09 -0.5335367 0.065 0.148 0.000153869 23
ARRDC2 ASC IgM
8.933291e-09 -0.3554201 0.039 0.1 0.0001592717 23
TRIM22 ASC IgM
9.515367e-09 0.3017181 0.383 0.133 0.0001696495 23
MPST ASC IgM
1.218239e-08 -0.6741989 0.273 0.319 0.0002171998 23

SNHG7	ASC IgM					
1.322191e-08	-0.5945099	0.878	0.805	0.0002357334	23	
HNRNPK	ASC IgM					
1.327414e-08	-0.648181	0.217	0.311	0.0002366646	23	
SERPINB1	ASC IgM					
1.609671e-08	-0.3767296	0.062	0.125	0.0002869883	23	
AC025164.1	ASC IgM					
1.781207e-08	-0.5305592	0.065	0.15	0.0003175713	23	
CELF2	ASC IgM					
1.814336e-08	-0.3729574	0.047	0.114	0.000323478	23	
LGALS9	ASC IgM					
2.050776e-08	-0.4903533	0.926	0.899	0.0003656328	23	
DDX5	ASC IgM					
2.275866e-08	-0.4978223	0.092	0.178	0.0004057641	23	
STAT6	ASC IgM					
2.46996e-08	-0.6444009	0.261	0.342	0.0004403692	23	
PKIG	ASC IgM					
2.58437e-08	-0.3651426	0.05	0.101	0.0004607673	23	
CDKN2D	ASC IgM					
2.585492e-08	-0.610169	0.318	0.375	0.0004609674	23	
LCP1	ASC IgM					
2.672121e-08	-0.5076514	0.202	0.249	0.0004764125	23	
OFD1	ASC IgM					
2.723109e-08	-0.7563312	0.448	0.448	0.0004855031	23	
PNRC1	ASC IgM					
2.926981e-08	-0.4933999	0.958	0.919	0.0005218515	23	
CFL1	ASC IgM					
3.554407e-08	-0.5743164	0.401	0.42	0.0006337153	23	
FAM49B	ASC IgM					
3.768258e-08	-0.8195962	0.095	0.198	0.0006718427	23	
ACP5	ASC IgM					
4.018582e-08	-0.6004531	0.496	0.465	0.000716473	23	
SF1	ASC IgM					
4.908864e-08	-0.4464329	0.955	0.905	0.0008752013	23	
ATP5F1E	ASC IgM					
5.747932e-08	-1.353131	0.398	0.466	0.001024799	23	
S100A6	ASC IgM					
6.060661e-08	-0.4511218	0.128	0.18	0.001080555	23	
LYL1	ASC IgM					
6.180308e-08	-1.349383	0.371	0.302	0.001101887	23	
HMGB2	ASC IgM					
6.746042e-08	-0.5478015	0.315	0.347	0.001202752	23	
BIRC3	ASC IgM					
8.032294e-08	-0.5596269	0.291	0.32	0.001432078	23	
LTA4H	ASC IgM					
9.295358e-08	-0.6603586	0.724	0.697	0.001657269	23	
HLA-DQB1	ASC IgM					
1.011165e-07	-0.4980239	0.273	0.314	0.001802807	23	
LYN	ASC IgM					
1.081792e-07	-0.386716	0.074	0.141	0.001928726	23	

	AIM2	ASC IgM				
1.094596e-07	-0.4153836		0.258	0.289	0.001951556	23
	RUBCNL	ASC IgM				
1.123778e-07	-0.5756516		0.807	0.786	0.002003584	23
	ACTG1	ASC IgM				
1.227931e-07	0.4310618		0.451	0.156	0.002189277	23
	KIAA0040	ASC IgM				
1.327734e-07	-0.4573951		0.139	0.199	0.002367217	23
	MCM3	ASC IgM				
1.446847e-07	-0.3313029		0.062	0.117	0.002579583	23
	KLHL5	ASC IgM				
1.494817e-07	-0.3771944		0.045	0.106	0.002665109	23
	PDE7A	ASC IgM				
1.854277e-07	-0.3375784		0.142	0.173	0.00330599	23
	TMEM156	ASC IgM				
1.970406e-07	-0.4513267		0.205	0.259	0.003513038	23
	CNN2	ASC IgM				
2.028683e-07	-0.4772033		0.166	0.232	0.003616939	23
	TAF15	ASC IgM				
2.133785e-07	-0.4711598		0.136	0.201	0.003804325	23
	NMI	ASC IgM				
2.260986e-07	-0.3840538		0.092	0.14	0.004031112	23
	ADD3	ASC IgM				
2.280148e-07	0.3360058		0.496	0.208	0.004065276	23
	SYAP1	ASC IgM				
2.474282e-07	-0.5990101		0.973	0.976	0.004411397	23
	FTH1	ASC IgM				
2.478363e-07	-0.4319355		0.056	0.113	0.004418673	23
	P2RY10	ASC IgM				
2.61633e-07	-0.6132114		0.513	0.511	0.004664654	23
	C6orf48	ASC IgM				
2.702427e-07	0.3160577		0.318	0.106	0.004818158	23
	E2F5	ASC IgM				
3.166735e-07	-0.3907459		0.065	0.12	0.005645971	23
	IL27RA	ASC IgM				
3.231115e-07	0.4772093		0.76	0.394	0.005760755	23
	CSTB	ASC IgM				
3.49785e-07	0.3813072		0.439	0.184	0.006236317	23
	P2RX5	ASC IgM				
3.773244e-07	-0.6012246		0.638	0.601	0.006727317	23
	UBXN1	ASC IgM				
4.210159e-07	-0.3905319		0.056	0.112	0.007506293	23
	SESN3	ASC IgM				
4.21691e-07	-0.8876874		0.374	0.427	0.007518328	23
	PLP2	ASC IgM				
4.377606e-07	-0.6182371		0.804	0.752	0.007804834	23
	TOMM7	ASC IgM				
5.705547e-07	-0.3400752		0.994	0.982	0.01017242	23
	MT-CO3	ASC IgM				
5.848843e-07	-0.5316526		0.249	0.29	0.0104279	23

RPA2	ASC IgM				
6.083026e-07	-0.5294575	0.288	0.315	0.01084543	23
STRBP	ASC IgM				
6.092431e-07	-0.5101802	0.125	0.185	0.01086219	23
FKBP4	ASC IgM				
6.958906e-07	-0.4882895	0.157	0.188	0.01240703	23
PCNA	ASC IgM				
7.052733e-07	-0.4131023	0.101	0.152	0.01257432	23
SCAF4	ASC IgM				
8.351513e-07	-0.7408979	0.647	0.61	0.01488991	23
RSL24D1	ASC IgM				
9.800262e-07	-0.7593319	0.837	0.747	0.01747289	23
RAN	ASC IgM				
1.19823e-06	-0.5455532	0.967	0.899	0.02136325	23
PFN1	ASC IgM				
1.285392e-06	0.3115229	0.409	0.178	0.02291726	23
LCK	ASC IgM				
1.334364e-06	-0.7443108	0.641	0.602	0.02379038	23
ARF6	ASC IgM				
1.351356e-06	-0.3625342	0.089	0.128	0.02409333	23
CCNG2	ASC IgM				
1.456244e-06	-0.631869	0.255	0.296	0.02596337	23
NFKBIA	ASC IgM				
1.633144e-06	-0.5336377	0.733	0.677	0.02911733	23
EIF3L	ASC IgM				
1.723363e-06	-0.5887749	0.401	0.389	0.03072584	23
ARL6IP5	ASC IgM				
1.851204e-06	-0.3414705	0.05	0.105	0.03300511	23
SCIMP	ASC IgM				
1.996764e-06	-0.6734603	0.665	0.587	0.03560031	23
HNRNPF	ASC IgM				
2.189484e-06	-0.5255226	0.377	0.368	0.03903632	23
MEAF6	ASC IgM				
2.285422e-06	0.3175157	0.208	0.057	0.04074678	23
RGS16	ASC IgM				
2.434744e-06	-0.5025027	0.466	0.397	0.04340905	23
NUCKS1	ASC IgM				
2.750154e-06	-0.8050032	0.804	0.695	0.04903249	23
H2AFZ	ASC IgM				
2.755182e-06	-0.6257187	0.789	0.735	0.04912214	23
HSPA8	ASC IgM				
2.855063e-06	-0.3387117	0.122	0.174	0.05090292	23
NUDT1	ASC IgM				
3.025457e-06	-0.6650808	0.347	0.353	0.05394087	23
TUBB4B	ASC IgM				
3.043497e-06	-0.6075251	0.478	0.456	0.0542625	23
LSM5	ASC IgM				
3.294525e-06	-0.3337372	0.107	0.141	0.05873809	23
SMC2	ASC IgM				
3.30169e-06	-0.3413805	0.122	0.157	0.05886583	23

BCL7C	ASC IgM	3.369004e-06	-0.3776383	0.166	0.186	0.06006597	23
CDCA7L	ASC IgM	3.492838e-06	-0.3725188	0.157	0.22	0.06227381	23
NELFCD	ASC IgM	3.823457e-06	-0.4148369	0.243	0.247	0.06816842	23
CSK	ASC IgM	4.106501e-06	-0.4658897	0.294	0.314	0.0732148	23
SPI1	ASC IgM	5.319571e-06	-0.400494	0.166	0.19	0.09484263	23
NUP88	ASC IgM	5.778028e-06	-0.6226443	0.635	0.593	0.1030165	23
HNRNPU	ASC IgM	6.289111e-06	-0.4224103	0.217	0.222	0.1121286	23
THUMPD3-AS1	ASC IgM	6.530433e-06	-0.4572263	0.172	0.194	0.1164311	23
ADK	ASC IgM	6.683507e-06	-0.48281	0.353	0.349	0.1191603	23
NASP	ASC IgM	6.808389e-06	-0.4405996	0.214	0.228	0.1213868	23
PCSK7	ASC IgM	6.8976e-06	-0.4769655	0.217	0.226	0.1229773	23
MCM5	ASC IgM	7.14467e-06	-0.8635768	0.487	0.494	0.1273823	23
FXYD5	ASC IgM	7.551689e-06	-0.7090647	0.338	0.36	0.1346391	23
ARID5B	ASC IgM	7.851316e-06	-0.4216404	0.326	0.299	0.1399811	23
ITSN2	ASC IgM	7.973395e-06	-0.3827737	0.065	0.117	0.1421577	23
SLAMF1	ASC IgM	9.727612e-06	1.247869	0.24	0.147	0.1734336	23
IGHA1	ASC IgM	1.036976e-05	-1.208876	0.231	0.287	0.1848825	23
MIR155HG	ASC IgM	1.047533e-05	-0.3685926	0.104	0.151	0.1867646	23
ATF1	ASC IgM	1.126103e-05	0.3249591	0.134	0.055	0.200773	23
HES1	ASC IgM	1.131148e-05	-0.4223584	0.202	0.235	0.2016724	23
FNTA	ASC IgM	1.142644e-05	-0.6295014	0.674	0.583	0.203722	23
ANP32B	ASC IgM	1.234478e-05	-0.4955161	0.267	0.277	0.2200951	23
CCNG1	ASC IgM	1.287419e-05	-0.4920475	0.16	0.194	0.229534	23
HSPB1	ASC IgM	1.333879e-05	-0.3037759	0.053	0.107	0.2378174	23
TP53	ASC IgM	1.460594e-05	-0.3501918	0.077	0.121	0.2604093	23

NBEAL1	ASC IgM					
1.525967e-05	-0.5725757	0.436	0.422	0.2720647	23	
APEX1	ASC IgM					
1.664859e-05	-0.3867041	0.062	0.129	0.2968277	23	
EEA1	ASC IgM					
1.981454e-05	-0.5742776	0.721	0.625	0.3532735	23	
HNRNPA0	ASC IgM					
1.990926e-05	-0.4466444	0.107	0.169	0.3549621	23	
TNIP2	ASC IgM					
2.175969e-05	-0.4124009	0.071	0.133	0.3879536	23	
SULF2	ASC IgM					
2.180362e-05	-0.4297973	0.11	0.145	0.3887368	23	
SUN2	ASC IgM					
2.239813e-05	-1.373387	0.24	0.216	0.3993362	23	
IGHG1	ASC IgM					
2.32184e-05	-0.5931845	0.76	0.679	0.4139609	23	
SRSF3	ASC IgM					
2.370934e-05	-0.4171396	0.077	0.118	0.4227138	23	
FCGR2B	ASC IgM					
2.510637e-05	-0.4285463	0.142	0.181	0.4476215	23	
CDC42SE1	ASC IgM					
2.558419e-05	-0.5281114	0.404	0.377	0.4561406	23	
ZFAND6	ASC IgM					
2.597769e-05	-0.513642	0.445	0.421	0.4631563	23	
SCAF11	ASC IgM					
2.796818e-05	-0.385751	0.953	0.901	0.4986447	23	
COX7C	ASC IgM					
2.912932e-05	-1.158577	0.537	0.45	0.5193467	23	
IGHG3	ASC IgM					
2.926097e-05	-0.5237649	0.407	0.367	0.5216938	23	
CACYBP	ASC IgM					
3.084609e-05	-0.3035564	0.089	0.124	0.5499549	23	
MAPK1	ASC IgM					
3.136057e-05	-0.4390833	0.237	0.253	0.5591277	23	
SLC50A1	ASC IgM					
3.166306e-05	-0.4542915	0.19	0.237	0.5645207	23	
AC004687.1	ASC IgM					
3.173559e-05	-0.4093079	0.211	0.222	0.5658138	23	
CDC40	ASC IgM					
3.274139e-05	-0.5217446	0.089	0.158	0.5837462	23	
SLC2A3	ASC IgM					
3.335965e-05	-0.3503528	0.116	0.145	0.5947693	23	
GNB5	ASC IgM					
3.382912e-05	0.3359202	0.374	0.133	0.6031394	23	
NEIL1	ASC IgM					
3.477464e-05	-0.4697145	0.226	0.26	0.6199971	23	
NAA50	ASC IgM					
3.615757e-05	-0.4391557	0.16	0.209	0.6446533	23	
LARS	ASC IgM					
3.680967e-05	-0.567665	0.674	0.62	0.6562797	23	

ACTR3 ASC IgM
3.706173e-05 -0.3837437 0.116 0.155 0.6607736 23
TCOF1 ASC IgM
4.176748e-05 -0.324414 0.125 0.161 0.7446725 23
DNAJC9 ASC IgM
4.862105e-05 -0.4857892 0.122 0.171 0.8668648 23
PARP14 ASC IgM
4.877485e-05 -0.5319643 0.166 0.199 0.8696068 23
PPP3CA ASC IgM
4.986523e-05 -0.3230595 0.223 0.234 0.8890472 23
ZCCHC7 ASC IgM
5.047624e-05 -0.3566427 0.255 0.239 0.8999408 23
SP140 ASC IgM
5.249586e-05 -0.465162 0.783 0.718 0.9359487 23
ATP5MC2 ASC IgM
5.256076e-05 -0.656351 0.623 0.604 0.9371057 23
CDC42 ASC IgM
5.268435e-05 -0.3847093 0.335 0.309 0.9393093 23
PGLS ASC IgM
5.273889e-05 -0.3117206 0.389 0.363 0.9402817 23
RCSD1 ASC IgM
5.610629e-05 -0.6032807 0.703 0.615 1 23
SERBP1 ASC IgM
5.723468e-05 -0.3887023 0.068 0.123 1 23
RAB13 ASC IgM
5.893958e-05 -0.5141255 0.338 0.343 1 23
CMTM6 ASC IgM
6.693259e-05 -0.5141645 0.659 0.586 1 23
PRR13 ASC IgM
6.790018e-05 -0.5810037 0.469 0.442 1 23
CCT6A ASC IgM
8.222563e-05 -0.3896122 0.136 0.16 1 23
AIDA ASC IgM
8.69344e-05 -0.3675009 0.181 0.192 1 23
CNTRL ASC IgM
8.771694e-05 -0.4878629 0.273 0.282 1 23
RTN4 ASC IgM
8.922445e-05 -0.4250853 0.979 0.959 1 23 MT-
CYB ASC IgM
9.33445e-05 -0.3361988 0.148 0.179 1 23
CBX1 ASC IgM
9.379171e-05 -0.5724099 0.555 0.533 1 23
RBMX ASC IgM
9.431516e-05 -0.3707125 0.098 0.142 1 23
NUB1 ASC IgM
9.745869e-05 -0.3620495 0.131 0.143 1 23
DUSP1 ASC IgM
0.0001004379 -0.5239977 0.593 0.478 1 23
CD47 ASC IgM
0.0001059281 -0.4352638 0.252 0.246 1 23

CKS1B	ASC IgM
0.0001068155	-0.3659562	0.104	0.095	1	23
PCLAF	ASC IgM
0.0001076737	-0.4242545	0.217	0.248	1	23
HNRNPA1P48	ASC IgM
0.0001125321	-0.40776 0.178	0.185	1	23	ANKRD44	ASC
IgM
0.0001163596	-0.5477672	0.591	0.524	1	23
SYF2	ASC IgM
0.0001201903	-0.330789	0.101	0.134	1	23
TTF1	ASC IgM
0.0001204476	-0.300377	0.053	0.102	1	23
PAXX	ASC IgM
0.0001343401	-0.3987385	0.119	0.15	1	23
ESYT1	ASC IgM
0.000136447	-0.3604096	0.089	0.114	1	23
CLN8	ASC IgM
0.0001405985	-0.3417753	0.172	0.173	1	23
BLCAP	ASC IgM
0.0001411948	-0.3789547	0.172	0.193	1	23
UTP6	ASC IgM
0.0001462724	-0.4523223	0.869	0.817	1	23
SEPT7	ASC IgM
0.0001537413	-0.5501785	0.709	0.635	1	23
GPX4	ASC IgM
0.0001612673	-0.3784957	0.119	0.158	1	23
TGIF1	ASC IgM
0.0001783567	-0.5540455	0.733	0.64	1	23
HNRNPM	ASC IgM
0.0001791111	-0.3637744	0.178	0.202	1	23
BIN1	ASC IgM
0.0001795569	-0.4121884	0.777	0.662	1	23	DBI
ASC IgM
0.0001949321	-0.3487747	0.226	0.233	1	23
ZNF581	ASC IgM
0.0002100632	-0.5472876	0.166	0.198	1	23
YBX3	ASC IgM
0.0002147282	-0.3282357	0.427	0.33	1	23
GRHPR	ASC IgM
0.0002183556	-0.5142081	0.828	0.722	1	23
SRSF5	ASC IgM
0.0002198222	-0.3450427	0.101	0.152	1	23
PEA15	ASC IgM
0.0002227006	-0.5513391	0.475	0.445	1	23	FBL
ASC IgM
0.000241423	-0.4091991	0.205	0.219	1	23
PCM1	ASC IgM
0.0002820033	-0.3243369	0.211	0.235	1	23
KRAS	ASC IgM
0.0002881956	-0.5467576	0.65	0.564	1	23

SNRPB ASC IgM
0.0002932529 -0.3202859 0.125 0.147 1 23
ARL6IP6 ASC IgM
0.0002945458 -0.4724399 0.659 0.594 1 23
ST13 ASC IgM
0.0002960065 -0.3861933 0.73 0.652 1 23
HLA-DQA1 ASC IgM
0.0003248931 -0.3999219 0.22 0.219 1 23
CCDC50 ASC IgM
0.0003513409 -0.5317291 0.662 0.568 1 23
PCBP2 ASC IgM
0.0003797016 -0.4573858 0.445 0.407 1 23
BANF1 ASC IgM
0.0004033937 -0.3188776 0.157 0.161 1 23
TMPO ASC IgM
0.0004150583 -0.406729 0.893 0.798 1 23
SUMO2 ASC IgM
0.0004447984 -0.4338899 0.125 0.18 1 23
RHOF ASC IgM
0.0004991233 -0.3715878 0.386 0.346 1 23
LSM6 ASC IgM
0.0005036017 -0.3563525 0.184 0.193 1 23
TFAM ASC IgM
0.000514175 -0.4664936 0.35 0.336 1 23
ACAT2 ASC IgM
0.0005184261 -0.3499465 0.175 0.188 1 23
CELF1 ASC IgM
0.0005303168 -0.3624928 0.208 0.212 1 23
NR3C1 ASC IgM
0.0005481476 -0.3044485 0.139 0.154 1 23
PPIH ASC IgM
0.0005627615 -0.4799451 0.3 0.237 1 23
CD55 ASC IgM
0.0005775741 -0.3347557 0.122 0.133 1 23
ODF2L ASC IgM
0.0005883223 -0.3846848 0.211 0.238 1 23
NT5C ASC IgM
0.000589706 -0.3930857 0.196 0.208 1 23
PITHD1 ASC IgM
0.0005934752 -0.3252505 0.116 0.15 1 23
ADSS ASC IgM
0.0006171748 -0.3515333 0.178 0.193 1 23
GGCT ASC IgM
0.0006310537 -0.4192156 0.472 0.397 1 23
ORAI2 ASC IgM
0.000655628 -0.4075712 0.338 0.301 1 23
ARID4B ASC IgM
0.000697033 -0.6504 0.347 0.345 1 23 CAST ASC
IgM
0.000720377 -0.4071819 0.184 0.195 1 23

CLEC2B	ASC IgM	0.0007205183	−0.3450851	0.335	0.296	1	23	
IFT57	ASC IgM	0.0007504885	−0.3217203	0.599	0.407	1	23	TXN
	ASC IgM	0.0008113222	−0.3579721	0.653	0.588	1	23	
MARCKSL1	ASC IgM	0.0008192869	−0.4454395	0.282	0.29	1	23	
DCTPP1	ASC IgM	0.0008369003	−0.4525243	0.555	0.465	1	23	
PRDX6	ASC IgM	0.0008653597	−0.4278096	0.166	0.211	1	23	
C19orf48	ASC IgM	0.0009023926	−0.3715596	0.145	0.183	1	23	
TUBA1C	ASC IgM	0.000963737	−0.307238	0.151	0.162	1	23	
WDR77	ASC IgM	0.0009690042	−0.4557402	0.439	0.365	1	23	
HADHA	ASC IgM	0.001029403	−0.4520252	0.255	0.246	1	23	
DKC1	ASC IgM	0.001047416	−0.4169746	0.214	0.211	1	23	
FMNL1	ASC IgM	0.001056453	−0.4191438	0.315	0.299	1	23	
CAMLG	ASC IgM	0.001181139	−0.4070784	0.445	0.379	1	23	
POLR2G	ASC IgM	0.001187241	−0.3407457	0.264	0.229	1	23	ATM
	ASC IgM	0.001258884	−0.4331863	0.65	0.571	1	23	
EIF3D	ASC IgM	0.001313092	−0.3629141	0.344	0.302	1	23	
HNRNPH3	ASC IgM	0.001328132	−0.4836242	0.501	0.44	1	23	
ACTR2	ASC IgM	0.001346703	−0.3820205	0.35	0.313	1	23	
ATP2B1	ASC IgM	0.001355812	−0.3625895	0.478	0.347	1	23	
S100A10	ASC IgM	0.001386066	−0.5290321	0.685	0.571	1	23	
SNRPE	ASC IgM	0.001473966	−0.4829368	0.496	0.42	1	23	
MAGOH	ASC IgM	0.001521143	−0.3804135	0.736	0.602	1	23	
LSM7	ASC IgM	0.001609205	−0.3587402	0.917	0.83	1	23	
UQCRB	ASC IgM	0.001647819	−0.3186164	0.211	0.19	1	23	
USP1	ASC IgM	0.001668675	−0.3490931	0.139	0.165	1	23	

NFKB1	ASC IgM	0.001689374	-0.3552766	0.294	0.236	1	23	
CCND3	ASC IgM	0.001733462	-0.5970845	0.19	0.21	1	23	
MGST3	ASC IgM	0.001739252	-0.3167073	0.107	0.133	1	23	
MCMBP	ASC IgM	0.001741624	-0.3248902	0.288	0.262	1	23	
ATXN10	ASC IgM	0.001826959	-0.3275018	0.276	0.264	1	23	
CD22	ASC IgM	0.001860105	-0.5372936	0.706	0.61	1	23	
PTGES3	ASC IgM	0.001860511	-0.4854675	0.377	0.333	1	23	BTK
	ASC IgM	0.001909721	-0.3836662	0.478	0.414	1	23	
HDAC1	ASC IgM	0.001941368	-0.3684594	0.341	0.283	1	23	
MTPN	ASC IgM	0.001950271	-0.4390965	0.178	0.202	1	23	
NPM3	ASC IgM	0.001985662	-0.5040763	0.326	0.283	1	23	
GNG7	ASC IgM	0.002029653	-0.3045315	0.116	0.122	1	23	
SP140L	ASC IgM	0.002276304	-0.5039203	0.644	0.529	1	23	
SRSF7	ASC IgM	0.002280596	0.3894417	0.371	0.217	1	23	
ISG15	ASC IgM	0.002312417	-0.3824362	0.217	0.205	1	23	
RECQL	ASC IgM	0.002481831	-0.4319604	0.852	0.745	1	23	
SNRPD2	ASC IgM	0.002499933	-0.4630397	0.626	0.559	1	23	
YWHAZ	ASC IgM	0.002505235	-0.3085513	0.196	0.189	1	23	
OARD1	ASC IgM	0.00260854	-0.3523089	0.116	0.143	1	23	
NOP16	ASC IgM	0.00277242	-0.3470858	0.169	0.171	1	23	
MFSD10	ASC IgM	0.002821604	-0.5736345	0.647	0.605	1	23	
PPDPF	ASC IgM	0.002840378	-0.3292883	0.297	0.26	1	23	
MAT2B	ASC IgM	0.002861665	-0.4991898	0.671	0.594	1	23	
SRSF2	ASC IgM	0.002896635	-0.5206697	0.353	0.336	1	23	
APOBEC3C	ASC IgM	0.003061878	-0.3843381	0.211	0.235	1	23	

CNPPD1	ASC IgM	0.003091529	-0.3479015	0.347	0.299	1	23	
POLE4	ASC IgM	0.003323594	-0.3484689	0.677	0.577	1	23	
POU2F2	ASC IgM	0.003330416	-0.3737366	0.234	0.222	1	23	GCA
	ASC IgM	0.003344985	-0.3610185	0.22	0.217	1	23	
ITGB1BP1	ASC IgM	0.003539118	-0.3442552	0.217	0.217	1	23	
HPRT1	ASC IgM	0.003686077	-0.4104912	0.318	0.285	1	23	
CNPY3	ASC IgM	0.004046416	-0.3758639	0.727	0.631	1	23	
EIF3H	ASC IgM	0.00407383	-0.4756896	0.475	0.429	1	23	
NSA2	ASC IgM	0.004138862	-0.3808027	0.611	0.514	1	23	
SRP9	ASC IgM	0.004144078	-0.531976	0.567	0.452	1	23	
ARL6IP1	ASC IgM	0.004153509	-0.6193495	0.92	0.848	1	23	
HSP90AB1	ASC IgM	0.004170621	-0.3983152	0.383	0.324	1	23	
AKR1B1	ASC IgM	0.004184357	-0.3642569	0.148	0.184	1	23	
KANSL1	ASC IgM	0.004246393	-0.4724984	0.528	0.451	1	23	
PCNP	ASC IgM	0.004331625	-0.3378365	0.134	0.151	1	23	
HCFC1R1	ASC IgM	0.004519178	-0.3715889	0.208	0.189	1	23	
CWF19L2	ASC IgM	0.004603989	-0.3446625	0.211	0.204	1	23	
AP1S2	ASC IgM	0.004608614	-0.4995001	0.537	0.449	1	23	
DRAP1	ASC IgM	0.004683183	-0.3634345	0.546	0.421	1	23	
TMEM123	ASC IgM	0.004687753	-0.3138221	0.136	0.147	1	23	
TSPAN3	ASC IgM	0.005113542	-0.3446776	0.128	0.163	1	23	
FAM215B	ASC IgM	0.005290425	-0.3583733	0.312	0.289	1	23	
PFDN4	ASC IgM	0.005491704	-0.3450236	0.401	0.32	1	23	
TNFRSF13C	ASC IgM	0.00551081	-0.332958	0.252	0.236	1	23	
TPRKB	ASC IgM	0.0055274	-0.3008146	0.128	0.151	1	23	

CTNNB1	ASC IgM
0.005951125	-1.691197	0.273	0.125	1	23
IGHG2	ASC IgM
0.006324488	-0.4355254	0.528	0.445	1	23
YWHAQ	ASC IgM
0.00687435	-0.3604107	0.329	0.253	1	23
PRMT2	ASC IgM
0.00703687	-0.3369263	0.546	0.458	1	23
RAP1B	ASC IgM
0.007125975	-0.4886732	0.243	0.248	1	23
TNFRSF13B	ASC IgM
0.00717598	-0.3265819	0.941	0.895	1	23
SLC25A6	ASC IgM
0.007744853	-0.4907134	0.412	0.391	1	23
ARPC5L	ASC IgM
0.00794115	-0.3212071	0.226	0.207	1	23
ARHGAP30	ASC IgM
0.008220243	-0.360943	0.356	0.311	1	23
MZT2A	ASC IgM
0.008263147	-0.4973279	0.279	0.282	1	23	MT-
ND4L	ASC IgM
0.008492003	-0.3107619	0.395	0.297	1	23
GGA2	ASC IgM
0.008719391	-0.3756258	0.703	0.565	1	23
RAC1	ASC IgM
0.008835453	-0.3094922	0.323	0.254	1	23
ZCCHC10	ASC IgM
0.009170721	-0.3755177	0.436	0.344	1	23	PNN
ASC IgM
0.009406538	-0.3301983	0.392	0.333	1	23
DARS	ASC IgM
0.009455744	-0.362042	0.282	0.264	1	23
MRPL11	ASC IgM
0.009551164	-0.3069954	0.166	0.156	1	23
DDB2	ASC IgM
0.009714137	-0.3014084	0.208	0.173	1	23
CDK13	ASC IgM
0	3.466277	1	0.068	0	24	IGHA2	GC IgA
3.296026e-218	2.306851	0.95	0.142	5.876484e-214	24
IGHA1	GC IgA
4.859058e-209	1.417603	1	0.933	8.663215e-205	24
SERF2	GC IgA
9.509469e-163	1.327696	1	0.813	1.695443e-158	24
CORO1A	GC IgA
4.462341e-157	1.340538	0.994	0.85	7.955907e-153	24
ATP5MG	GC IgA
1.0005e-147	1.523805	0.988	0.784	1.783792e-143	24
ACTG1	GC IgA
7.444464e-146	1.18831	1	0.99	1.327274e-141	24
ACTB	GC IgA

1.166327e-137	1.732708	0.892	0.201	2.079445e-133	24	RGS13	GC IgA
6.464703e-127	1.637297	0.941	0.586	1.152592e-122	24	MARCKSL1	GC IgA
6.988778e-122	1.315136	0.981	0.766	1.246029e-117	24	HMGN1	GC IgA
2.202481e-113	1.622314	0.923	0.486	3.926803e-109	24	LTB	GC IgA
1.61587e-111	1.16181	0.981	0.723	2.880935e-107	24	LIMD2	GC IgA
7.939084e-109	1.353054	0.848	0.285	1.415459e-104	24	BCAS4	GC IgA
8.859023e-109	0.9891474	0.991	0.869	1.579475e-104	24	ARPC2	GC IgA
4.877062e-108	1.464179	0.7	0.13	8.695314e-104	24	NEIL1	GC IgA
3.448359e-104	1.205433	0.969	0.692	6.148079e-100	24	EZR	GC IgA
1.971424e-103	1.224072	0.972	0.653	3.514851e-99	24	TCL1A	GC IgA
2.771639e-102	1.539444	0.567	0.075	4.941556e-98	24	SUGCT	GC IgA
2.461569e-99	1.054299	0.991	0.887	4.388731e-95	24	GAPDH	GC IgA
5.547064e-99	1.283062	0.87	0.394	9.889861e-95	24	BASP1	GC IgA
1.059741e-98	1.256541	0.449	0.05	1.889412e-94	24	SERPINA9	GC IgA
2.36297e-97	0.8541608	1	0.989	4.212939e-93	24	CD74	GC IgA
1.000857e-96	1.433105	0.858	0.414	1.784427e-92	24	METAP2	GC IgA
6.27109e-96	1.266697	0.848	0.372	1.118073e-91	24	ACADM	GC IgA
6.60324e-94	1.200183	0.95	0.55	1.177292e-89	24	TCEA1	GC IgA
1.481473e-93	0.9155987	0.988	0.856	2.641318e-89	24	CD79B	GC IgA
1.589468e-92	1.381437	0.625	0.117	2.833863e-88	24	LPP	GC IgA
9.50314e-91	1.309068	0.904	0.531	1.694315e-86	24	ISG20	GC IgA
2.100674e-89	-2.054888	0.241	0.701	3.745292e-85	24	EMP3	GC IgA
4.65705e-89	1.271127	0.78	0.231	8.303055e-85	24	UBE2J1	GC IgA
6.392651e-87	1.206474	0.901	0.426	1.139746e-82	24	IRF8	GC IgA
2.022018e-86	1.375738	0.684	0.199	3.605056e-82	24	HMCES	GC IgA

3.582623e-86	1.293821	0.647	0.162	6.387459e-82	24	DEF8	GC IgA
9.080858e-86	1.353571	0.842	0.3	1.619026e-81	24	CD27	GC IgA
1.005931e-85	0.6982521	1	0.998	1.793475e-81	24	TMSB4X	GC IgA
5.876991e-84	-2.255469	0.656	0.908	1.047809e-79	24	IGHM	GC IgA
1.695973e-83	0.9389434	0.991	0.799	3.02375e-79	24	LAPTM5	GC IgA
2.508815e-81	1.215193	0.483	0.1	4.472966e-77	24	SEMA4A	GC IgA
2.736822e-80	1.261449	0.628	0.157	4.879479e-76	24	SUSD3	GC IgA
1.122335e-79	1.425439	0.666	0.187	2.001011e-75	24	DAAM1	GC IgA
6.846042e-78	-2.050251	0.08	0.547	1.220581e-73	24	CCR7	GC IgA
3.121102e-76	1.148706	0.752	0.271	5.564613e-72	24	NCF1	GC IgA
7.376883e-76	1.21487	0.44	0.075	1.315224e-71	24	LMO2	GC IgA
8.457344e-75	0.8143049	0.997	0.899	1.50786e-70	24	PFN1	GC IgA
1.246079e-74	1.032459	0.901	0.538	2.221634e-70	24	PARP1	GC IgA
3.631602e-74	1.094182	0.789	0.3	6.474783e-70	24	BCL7A	GC IgA
1.675212e-72	1.22921	0.712	0.261	2.986736e-68	24	CD22	GC IgA
8.235018e-70	1.1744	0.563	0.137	1.468221e-65	24	SOCS1	GC IgA
2.453534e-69	1.05277	0.548	0.108	4.374406e-65	24	AC023590.1	GC IgA
5.443618e-69	-1.07802	0.997	0.998	9.705426e-65	24	B2M	GC IgA
2.545899e-68	1.07959	0.78	0.326	4.539083e-64	24	RFTN1	GC IgA
1.062138e-67	1.151522	0.669	0.23	1.893685e-63	24	ZNF581	GC IgA
2.424954e-67	1.082176	0.913	0.602	4.32345e-63	24	DYNLL1	GC IgA
3.818796e-67	1.136933	0.591	0.165	6.808531e-63	24	SEL1L3	GC IgA
2.008817e-66	1.100885	0.511	0.118	3.58152e-62	24	VNN2	GC IgA
2.320734e-66	0.624138	0.91	0.4	4.137637e-62	24	JCHAIN	GC IgA
3.497788e-66	1.144869	0.793	0.337	6.236207e-62	24	LRMP	GC IgA

3.32525e-65	-1.655675	0.703	0.906	5.928589e-61	24	HLA-A	GC IgA
3.328614e-65	0.9367205	0.92	0.62	5.934585e-61	24	ARPC1B	GC IgA
2.045467e-64	1.046006	0.576	0.15	3.646864e-60	24	CD81	GC IgA
8.069398e-64	1.061001	0.495	0.104	1.438693e-59	24	HRK	GC IgA
3.684161e-63	1.055626	0.474	0.109	6.56849e-59	24	CPNE5	GC IgA
1.788082e-62	1.130625	0.567	0.177	3.187971e-58	24	LCK	GC IgA
1.680782e-61	1.132519	0.458	0.092	2.996666e-57	24	WDR66	GC IgA
1.925672e-61	1.164756	0.579	0.145	3.433281e-57	24	BIK	GC IgA
2.070599e-61	-1.900163	0.195	0.573	3.691671e-57	24	VIM	GC IgA
5.040337e-61	1.00741	0.759	0.294	8.986417e-57	24	GGA2	GC IgA
3.522905e-60	1.080973	0.625	0.215	6.280987e-56	24	NANS	GC IgA
1.21119e-59	1.007242	0.752	0.328	2.159431e-55	24	GRHPR	GC IgA
2.327397e-59	1.016722	0.402	0.07	4.149517e-55	24	S1PR2	GC IgA
4.435043e-59	0.581134	0.994	0.961	7.907238e-55	24	OAZ1	GC IgA
1.136228e-57	-1.812768	0.217	0.609	2.025781e-53	24	IGHD	GC IgA
1.885858e-57	0.6604298	0.985	0.869	3.362296e-53	24	ARPC3	GC IgA
4.643094e-57	0.9700701	0.616	0.204	8.278172e-53	24	EAF2	GC IgA
1.87026e-56	0.9668097	0.557	0.166	3.334486e-52	24	CCDC88A	GC IgA
9.363528e-56	1.00018	0.675	0.258	1.669423e-51	24	CCDC69	GC IgA
2.545714e-55	1.004103	0.548	0.137	4.538753e-51	24	AIM2	GC IgA
5.364629e-55	0.8363566	0.334	0.054	9.564596e-51	24	BFSP2	GC IgA
6.477314e-55	1.035208	0.663	0.234	1.15484e-50	24	GCHFR	GC IgA
1.047807e-53	1.038806	0.477	0.136	1.868135e-49	24	SIT1	GC IgA
1.328539e-53	1.010488	0.824	0.49	2.368652e-49	24	C4orf3	GC IgA
1.802425e-53	0.9595303	0.768	0.381	3.213544e-49	24	SMARCB1	GC IgA

2.343924e-53	0.6605757	0.991	0.918	4.178982e-49	24	CFL1	GC IgA
4.770289e-53	0.9617785	0.461	0.114	8.504948e-49	24	ABI3	GC IgA
1.06856e-52	0.7290981	0.291	0.041	1.905135e-48	24	ACY3	GC IgA
2.04924e-52	0.9299754	0.759	0.336	3.653589e-48	24	STK17A	GC IgA
2.954919e-52	1.001825	0.427	0.095	5.268325e-48	24	STAG3	GC IgA
3.239537e-52	0.9397989	0.492	0.116	5.775771e-48	24	CD38	GC IgA
6.344148e-52	0.9653045	0.746	0.358	1.131098e-47	24	TPD52	GC IgA
6.774661e-51	0.7732107	0.941	0.651	1.207854e-46	24	YWHAB	GC IgA
9.463612e-51	0.9349446	0.536	0.18	1.687267e-46	24	RASGRP3	GC IgA
1.453122e-50	0.9545238	0.489	0.144	2.590772e-46	24	SEC14L1	GC IgA
8.520291e-50	0.9525089	0.684	0.271	1.519083e-45	24	POU2AF1	GC IgA
1.288522e-49	0.8906065	0.848	0.532	2.297306e-45	24	SRSF9	GC IgA
2.880207e-49	0.9414181	0.824	0.435	5.135122e-45	24	COTL1	GC IgA
3.154115e-49	0.8175512	0.907	0.618	5.623471e-45	24	ACTR3	GC IgA
2.801361e-48	0.8551067	0.876	0.554	4.994546e-44	24	ATP5IF1	GC IgA
1.326191e-47	0.8571542	0.641	0.23	2.364465e-43	24	IL4R	GC IgA
3.075776e-47	0.9130681	0.412	0.106	5.4838e-43	24	OGG1	GC IgA
3.749711e-47	-0.8152629	0.994	0.996	6.68536e-43	24	RPS12	GC IgA
1.194812e-46	0.8818959	0.545	0.185	2.13023e-42	24	PRPSAP2	GC IgA
3.715964e-46	0.7027088	0.276	0.038	6.625191e-42	24	SYBU	GC IgA
1.824963e-45	0.6424893	0.978	0.901	3.253727e-41	24	MYL6	GC IgA
2.984183e-45	-0.9857209	0.969	0.986	5.3205e-41	24	HLA-B	GC IgA
1.03749e-44	0.7169783	0.932	0.655	1.849742e-40	24	SLC25A5	GC IgA
1.709436e-44	0.8589613	0.638	0.247	3.047754e-40	24	SH3KBP1	GC IgA
8.228627e-44	0.9292065	0.619	0.256	1.467082e-39	24	SYNE2	GC IgA

1.030361e-43	0.8783542	0.48	0.155	1.837031e-39	24	SMIM20	GC IgA
1.639011e-43	-0.6524226	0.994	0.999	2.922193e-39	24	RPS29	GC IgA
2.143605e-43	0.8999004	0.734	0.372	3.821834e-39	24	LCP1	GC IgA
5.052812e-43	0.8751499	0.409	0.122	9.008659e-39	24	DLAT	GC IgA
7.48856e-43	0.6676098	0.954	0.761	1.335135e-38	24	CYBA	GC IgA
1.040977e-42	0.6498816	0.932	0.677	1.855958e-38	24	HLA-DMA	GC IgA
2.15055e-42	0.9353569	0.508	0.158	3.834216e-38	24	C12orf75	GC IgA
2.256734e-42	0.6380097	0.245	0.038	4.02353e-38	24	EEPD1	GC IgA
6.117768e-42	0.9858765	0.619	0.238	1.090737e-37	24	RGS1	GC IgA
7.502641e-42	0.7949243	0.337	0.077	1.337646e-37	24	MYO1E	GC IgA
7.844125e-42	0.7562028	0.344	0.069	1.398529e-37	24	RPRD1B	GC IgA
8.480989e-42	-1.737201	0.183	0.467	1.512076e-37	24	S100A6	GC IgA
8.910127e-42	0.7337271	0.91	0.622	1.588586e-37	24	LAMTOR5	GC IgA
9.580402e-42	0.8007113	0.752	0.372	1.70809e-37	24	PTP4A2	GC IgA
1.632177e-41	0.851189	0.737	0.404	2.910008e-37	24	LSM10	GC IgA
6.110171e-41	0.7146498	0.331	0.067	1.089382e-36	24	SNTA1	GC IgA
1.794073e-40	0.8134503	0.759	0.412	3.198653e-36	24	HDAC1	GC IgA
1.949283e-40	0.6937678	0.291	0.064	3.475376e-36	24	QPCT	GC IgA
2.402418e-40	0.9009946	0.604	0.234	4.283271e-36	24	CCND3	GC IgA
5.505252e-40	-1.232441	0.093	0.385	9.815314e-36	24	CD44	GC IgA
8.899472e-40	0.8422224	0.628	0.28	1.586687e-35	24	LAT2	GC IgA
1.058777e-38	0.7932425	0.789	0.457	1.887693e-34	24	RAP1B	GC IgA
1.490395e-38	0.6815705	0.892	0.577	2.657225e-34	24	LDHB	GC IgA
4.845778e-38	0.6177318	0.207	0.026	8.639538e-34	24	HTR3A	GC IgA
4.981165e-38	0.6244478	0.272	0.053	8.88092e-34	24	BORCS8-MEF2B	GC IgA

7.382608e-38	0.5457131	0.981	0.865	1.316245e-33	24	CD79A	GC IgA
7.469423e-38	-1.237477	0.077	0.353	1.331723e-33	24	LY6E	GC IgA
8.49571e-38	0.7812241	0.322	0.08	1.5147e-33	24	GPR160	GC IgA
1.118388e-37	0.7816576	0.44	0.132	1.993974e-33	24	MAP4K1	GC IgA
1.807086e-37	0.7663108	0.52	0.183	3.221853e-33	24	P2RX5	GC IgA
2.586014e-37	-1.268898	0.303	0.578	4.610605e-33	24	SARAF	GC IgA
3.812403e-37	0.7862164	0.372	0.082	6.797133e-33	24	AICDA	GC IgA
8.954006e-37	0.8570724	0.625	0.305	1.59641e-32	24	FERMT3	GC IgA
9.355224e-37	0.8374843	0.545	0.19	1.667943e-32	24	DNMT1	GC IgA
9.727923e-37	0.5832915	0.211	0.032	1.734391e-32	24	RAPGEF5	GC IgA
1.154767e-36	0.8159753	0.474	0.16	2.058834e-32	24	LYPLAL1	GC IgA
2.21924e-36	0.8610865	0.362	0.114	3.956683e-32	24	GMDS	GC IgA
4.168502e-36	0.525622	0.988	0.937	7.432022e-32	24	EEF2	GC IgA
7.433864e-36	0.6605037	0.241	0.045	1.325384e-31	24	CCDC144A	GC IgA
1.726016e-35	-0.8802045	0.935	0.961	3.077314e-31	24	MT-ND2	GC IgA
3.696664e-35	0.6355138	0.26	0.052	6.590782e-31	24	A4GALT	GC IgA
3.782016e-35	0.5706441	0.238	0.043	6.742956e-31	24	ASB2	GC IgA
7.710904e-35	0.6380708	0.201	0.027	1.374777e-30	24	CAMK1	GC IgA
1.447328e-34	0.7194701	0.307	0.071	2.580441e-30	24	CDCA7	GC IgA
1.587629e-34	0.6894833	0.814	0.514	2.830584e-30	24	SMDT1	GC IgA
2.603179e-34	0.7036461	0.31	0.076	4.641208e-30	24	KLHL6	GC IgA
3.419016e-34	0.7607968	0.69	0.363	6.095764e-30	24	SNAP23	GC IgA
5.338381e-34	0.7173609	0.762	0.437	9.517799e-30	24	RHOH	GC IgA
6.026783e-34	-0.4664057	1	1	1.074515e-29	24	RPL41	GC IgA
8.124148e-34	0.7006607	0.752	0.417	1.448454e-29	24	FAM49B	GC IgA

1.463599e-33	0.7297591	0.774	0.437	2.60945e-29	24	HMGA1	GC IgA
1.971331e-33	0.7046835	0.743	0.4	3.514685e-29	24	MBD4	GC IgA
2.007043e-33	0.7320256	0.517	0.193	3.578356e-29	24	USP34	GC IgA
4.609471e-33	-1.183571	0.238	0.496	8.218225e-29	24	FXYD5	GC IgA
6.948298e-33	0.765192	0.567	0.237	1.238812e-28	24	NCF4	GC IgA
7.289141e-33	0.8142998	0.337	0.101	1.299581e-28	24	ALDH2	GC IgA
8.760634e-33	0.6925355	0.548	0.224	1.561933e-28	24	UBE2G1	GC IgA
2.244496e-32	0.854463	0.508	0.184	4.001712e-28	24	RGS2	GC IgA
2.395824e-32	0.694439	0.56	0.217	4.271515e-28	24	LY86	GC IgA
3.023056e-32	0.5502459	0.207	0.038	5.389807e-28	24	TOX	GC IgA
3.088475e-32	0.6695473	0.743	0.414	5.506442e-28	24	PPP1CC	GC IgA
5.024873e-32	0.7275508	0.591	0.24	8.958846e-28	24	SLBP	GC IgA
6.140875e-32	-0.7962466	0.932	0.969	1.094857e-27	24	MT-ND3	GC IgA
8.547001e-32	0.7255048	0.724	0.391	1.523845e-27	24	SYPL1	GC IgA
3.059521e-31	-1.506949	0.096	0.374	5.454821e-27	24	SELL	GC IgA
3.795349e-31	0.5922763	0.229	0.049	6.766728e-27	24	UCHL1	GC IgA
4.024259e-31	0.5687593	0.251	0.05	7.174851e-27	24	ASB13	GC IgA
4.125418e-31	0.5448429	0.263	0.062	7.355207e-27	24	PITPNC1	GC IgA
1.810792e-30	0.6687801	0.337	0.106	3.22846e-26	24	E2F5	GC IgA
2.139417e-30	0.4682924	0.954	0.775	3.814367e-26	24	ARHGDIB	GC IgA
2.630445e-30	0.7439988	0.554	0.256	4.689821e-26	24	CDV3	GC IgA
3.051306e-30	-1.108612	0.393	0.622	5.440173e-26	24	SUB1	GC IgA
3.175596e-30	0.6780496	0.796	0.487	5.661769e-26	24	UCP2	GC IgA
4.661816e-30	0.4857987	0.994	0.946	8.311552e-26	24	HLA-DRA	GC IgA
5.016768e-30	-0.9086865	0.969	0.986	8.944395e-26	24	TMSB10	GC IgA

6.115301e-30	0.6989473	0.523	0.195	1.090297e-25	24	POLD4	GC IgA
7.030065e-30	-1.197361	0.111	0.354	1.25339e-25	24	TXNIP	GC IgA
7.568996e-30	0.6255859	0.245	0.06	1.349476e-25	24	DCAF12	GC IgA
8.297794e-30	0.6640557	0.678	0.352	1.479414e-25	24	CD19	GC IgA
1.068403e-29	0.6528975	0.616	0.286	1.904856e-25	24	RRAS2	GC IgA
1.343622e-29	0.678503	0.437	0.16	2.395543e-25	24	UBE2R2	GC IgA
1.842107e-29	0.714303	0.322	0.094	3.284293e-25	24	FAM76B	GC IgA
2.188016e-29	0.6405147	0.672	0.351	3.901014e-25	24	DBNL	GC IgA
2.426553e-29	0.6373447	0.291	0.073	4.326302e-25	24	PAG1	GC IgA
2.933235e-29	0.6950172	0.721	0.416	5.229665e-25	24	CTSH	GC IgA
5.31315e-29	0.6837927	0.768	0.458	9.472814e-25	24	SWAP70	GC IgA
6.464015e-29	0.6559927	0.678	0.363	1.152469e-24	24	TERF2IP	GC IgA
1.024036e-28	0.7318036	0.554	0.24	1.825754e-24	24	NCOA3	GC IgA
1.522911e-28	0.6943534	0.347	0.122	2.715198e-24	24	SPATS2	GC IgA
2.497483e-28	0.4209021	0.167	0.02	4.452762e-24	24	LOXL2	GC IgA
2.748285e-28	0.7293754	0.35	0.116	4.899917e-24	24	BRWD1	GC IgA
2.893471e-28	0.6100961	0.399	0.126	5.158769e-24	24	RABGAP1L	GC IgA
3.845593e-28	-0.5926049	0.991	0.991	6.856308e-24	24	RPL9	GC IgA
4.135131e-28	-0.9720806	0.065	0.28	7.372524e-24	24	IFITM2	GC IgA
6.751618e-28	-0.9497788	0.031	0.221	1.203746e-23	24	GBP2	GC IgA
1.238328e-27	0.4843825	0.18	0.03	2.207815e-23	24	MIR3681HG	GC IgA
1.555754e-27	0.760842	0.554	0.263	2.773755e-23	24	RBM6	GC IgA
1.651603e-27	0.6746375	0.365	0.126	2.944643e-23	24	MSI2	GC IgA
1.862228e-27	0.5312852	0.245	0.055	3.320167e-23	24	MME	GC IgA
2.027293e-27	0.7185698	0.635	0.334	3.61446e-23	24	SEPHS2	GC IgA

2.05422e-27	-0.505388	0.988	0.995	3.662469e-23	24	RPS25	GC IgA
2.094933e-27	0.6041343	0.811	0.513	3.735056e-23	24	LSP1	GC IgA
2.799843e-27	0.5713469	0.279	0.069	4.99184e-23	24	TERF2	GC IgA
2.868581e-27	0.5534875	0.882	0.606	5.114394e-23	24	POMP	GC IgA
2.87484e-27	0.596378	0.3	0.081	5.125552e-23	24	BPNT1	GC IgA
8.21844e-27	0.6311968	0.7	0.39	1.465266e-22	24	FNBP1	GC IgA
8.779439e-27	0.4361409	0.988	0.939	1.565286e-22	24	H3F3A	GC IgA
1.197705e-26	-0.4676681	1	0.998	2.135389e-22	24	RPLP2	GC IgA
1.501405e-26	0.6029854	0.793	0.521	2.676855e-22	24	FKBP1A	GC IgA
1.559528e-26	0.6979504	0.347	0.097	2.780482e-22	24	MX1	GC IgA
1.636466e-26	0.5435907	0.851	0.582	2.917656e-22	24	ATP5MC3	GC IgA
2.034206e-26	0.5896664	0.755	0.451	3.626786e-22	24	PTPRC	GC IgA
2.075793e-26	0.4632132	0.142	0.02	3.700931e-22	24	NLRP4	GC IgA
2.810828e-26	0.6424856	0.393	0.144	5.011425e-22	24	P2RY8	GC IgA
2.885357e-26	0.563205	0.811	0.521	5.144302e-22	24	TRAPPC1	GC IgA
3.3787e-26	0.6799872	0.368	0.102	6.023885e-22	24	RMI2	GC IgA
3.662039e-26	-1.640992	0.099	0.288	6.52905e-22	24	MIR155HG	GC IgA
5.21114e-26	0.6602399	0.437	0.176	9.290941e-22	24	PLIN3	GC IgA
7.954308e-26	0.6595213	0.502	0.222	1.418174e-21	24	SIAH2	GC IgA
1.127386e-25	0.668167	0.604	0.326	2.010016e-21	24	GYPC	GC IgA
1.228751e-25	0.5874264	0.313	0.093	2.190741e-21	24	PXMP2	GC IgA
1.372934e-25	0.5770409	0.325	0.099	2.447803e-21	24	MYBL2	GC IgA
1.449314e-25	0.5328039	0.251	0.061	2.583982e-21	24	FANCA	GC IgA
1.572859e-25	0.6251438	0.554	0.247	2.80425e-21	24	PPP2R5C	GC IgA
1.695034e-25	0.6549472	0.582	0.304	3.022077e-21	24	TIMM8B	GC IgA

1.707011e-25	0.6129811	0.65	0.337	3.043431e-21	24	RBBP4	GC IgA
2.064942e-25	0.6103451	0.7	0.391	3.681585e-21	24	YWHAE	GC IgA
2.218389e-25	-0.4651048	0.997	0.999	3.955167e-21	24	RPL13	GC IgA
2.473069e-25	-1.024506	0.217	0.428	4.409235e-21	24	PLP2	GC IgA
2.8425e-25	0.6634517	0.563	0.276	5.067894e-21	24	STK17B	GC IgA
2.96165e-25	0.6358634	0.396	0.145	5.280325e-21	24	ANKRD13A	GC IgA
3.501065e-25	0.6078875	0.412	0.147	6.242049e-21	24	PTPN18	GC IgA
3.870908e-25	0.636989	0.576	0.265	6.901442e-21	24	ALOX5AP	GC IgA
5.344579e-25	0.69249	0.508	0.231	9.528849e-21	24	CHCHD10	GC IgA
5.371073e-25	0.5459645	0.811	0.521	9.576087e-21	24	SELENOT	GC IgA
6.011902e-25	0.5954666	0.337	0.108	1.071862e-20	24	ITGB2	GC IgA
6.431687e-25	0.4866237	0.916	0.699	1.146706e-20	24	ELOB	GC IgA
8.509731e-25	0.616523	0.533	0.245	1.5172e-20	24	CSK	GC IgA
9.627365e-25	0.5735324	0.842	0.582	1.716463e-20	24	ANP32B	GC IgA
1.054447e-24	-1.375154	0.511	0.667	1.879973e-20	24	TUBA1B	GC IgA
1.088635e-24	0.3799507	0.13	0.015	1.940928e-20	24	AC104170.1	GC IgA
1.107951e-24	0.4282816	0.167	0.033	1.975366e-20	24	PDGFD	GC IgA
1.978689e-24	0.4776885	0.214	0.044	3.527805e-20	24	MYBL1	GC IgA
2.026778e-24	0.488703	0.916	0.685	3.613543e-20	24	PPP1CA	GC IgA
2.729514e-24	0.5753521	0.269	0.066	4.86645e-20	24	MFHAS1	GC IgA
3.05099e-24	0.5005483	0.91	0.683	5.43961e-20	24	COX6A1	GC IgA
3.11754e-24	0.6170653	0.545	0.26	5.558263e-20	24	ATXN10	GC IgA
3.204346e-24	-0.7294988	0.867	0.917	5.713029e-20	24	EEF1B2	GC IgA
4.165161e-24	0.4258553	0.155	0.029	7.426065e-20	24	SCARB1	GC IgA
4.272641e-24	0.5365068	0.759	0.446	7.617691e-20	24	SEM1	GC IgA

4.848192e-24	0.4175259	0.167	0.028	8.643841e-20	24	LINC00877	GC IgA
5.046607e-24	0.5164063	0.879	0.633	8.997595e-20	24	BRK1	GC IgA
5.923981e-24	0.5186857	0.195	0.057	1.056187e-19	24	RGS16	GC IgA
7.104075e-24	0.5490043	0.536	0.188	1.266585e-19	24	PTTG1	GC IgA
7.127735e-24	-1.038561	0.08	0.27	1.270804e-19	24	GPR183	GC IgA
7.134892e-24	0.3531549	0.149	0.02	1.27208e-19	24	SPRED2	GC IgA
7.158198e-24	0.5676879	0.368	0.14	1.276235e-19	24	CUL3	GC IgA
8.069498e-24	0.3983391	0.142	0.019	1.438711e-19	24	CAMK2B	GC IgA
1.091445e-23	0.3956948	0.152	0.029	1.945937e-19	24	FGD6	GC IgA
1.108501e-23	0.6286628	0.43	0.17	1.976347e-19	24	BLOC1S6	GC IgA
1.141501e-23	0.6674631	0.347	0.103	2.035182e-19	24	SCIMP	GC IgA
1.316992e-23	-0.6629146	1	1	2.348065e-19	24	MALAT1	GC IgA
1.430912e-23	0.5091413	0.232	0.053	2.551172e-19	24	BCL6	GC IgA
1.458496e-23	-0.3659172	1	0.999	2.600353e-19	24	RPLP1	GC IgA
1.685731e-23	0.5980662	0.653	0.358	3.00549e-19	24	TKT	GC IgA
1.703054e-23	0.4557533	0.189	0.037	3.036375e-19	24	SLC2A5	GC IgA
1.866832e-23	0.6619505	0.824	0.635	3.328375e-19	24	CD53	GC IgA
2.154455e-23	0.5514098	0.867	0.674	3.841177e-19	24	ATP6V1G1	GC IgA
2.258115e-23	-0.4813415	1	0.999	4.025994e-19	24	RPL34	GC IgA
2.267951e-23	0.5945127	0.718	0.454	4.04353e-19	24	CAPZA1	GC IgA
4.517377e-23	0.5411496	0.461	0.185	8.054032e-19	24	HLA-DOB	GC IgA
5.555644e-23	0.4343279	0.164	0.031	9.905157e-19	24	SMCO4	GC IgA
6.462075e-23	0.4562813	0.211	0.04	1.152123e-18	24	SLC30A4	GC IgA
7.298843e-23	0.5659398	0.43	0.176	1.301311e-18	24	TRAPPC6A	GC IgA
8.006511e-23	0.5911471	0.319	0.109	1.427481e-18	24	MOB3A	GC IgA

9.626832e-23	0.5992961	0.381	0.145	1.716368e-18	24	
SH2B2	GC IgA					
9.875934e-23	-0.9620328	0.272	0.464	1.76078e-18	24	
CD63	GC IgA					
1.108174e-22	0.5933726	0.672	0.41	1.975764e-18	24	
MOB1A	GC IgA					
1.208594e-22	-0.464517	0.991	0.996	2.154803e-18	24	
RPL35A	GC IgA					
1.533844e-22	0.4422796	0.981	0.905	2.734691e-18	24	
ATP5F1E	GC IgA					
1.541776e-22	0.5739832	0.616	0.328	2.748832e-18	24	
CCM2	GC IgA					
2.884404e-22	-0.586485	0.994	0.983	5.142603e-18	24	
RPS21	GC IgA					
2.940538e-22	0.5160116	0.842	0.592	5.242686e-18	24	
TBCA	GC IgA					
3.096662e-22	-0.9262331	0.139	0.33	5.521039e-18	24	
TRBC2	GC IgA					
3.202113e-22	0.5292911	0.322	0.108	5.709048e-18	24	
MAP4K2	GC IgA					
3.297674e-22	0.4251044	0.954	0.806	5.879424e-18	24	
TMA7	GC IgA					
3.424425e-22	-1.392046	0.087	0.265	6.105408e-18	24	
S100A4	GC IgA					
3.437582e-22	0.5242138	0.588	0.287	6.128865e-18	24	
DCK	GC IgA					
4.328627e-22	0.5442009	0.582	0.265	7.71751e-18	24	
HHEX	GC IgA					
5.244285e-22	0.5292709	0.771	0.473	9.350035e-18	24	
PRELID1	GC IgA					
5.344155e-22	-1.236276	0.111	0.317	9.528093e-18	24	
CD69	GC IgA					
7.706205e-22	0.5276027	0.641	0.344	1.373939e-17	24	
LSM6	GC IgA					
1.070726e-21	-1.062071	0.347	0.524	1.908997e-17	24	
ID3	GC IgA					
1.152131e-21	0.4740799	0.895	0.698	2.054134e-17	24	
CCNI	GC IgA					
1.236622e-21	0.5754181	0.375	0.144	2.204773e-17	24	
DNAJC10	GC IgA					
1.551748e-21	0.5597118	0.498	0.228	2.766611e-17	24	
ATM	GC IgA					
1.774203e-21	0.4068944	0.161	0.031	3.163227e-17	24	
XKR6	GC IgA					
1.792127e-21	0.6117067	0.536	0.264	3.195183e-17	24	
GRN	GC IgA					
2.274945e-21	0.5755369	0.291	0.104	4.056e-17	24	
ACSF3	GC IgA					
2.63167e-21	0.5274454	0.467	0.195	4.692004e-17	24	
ETFB	GC IgA					

3.515853e-21	0.4939107	0.836	0.616	6.268414e-17	24	NDUFA13	GC IgA
4.002819e-21	0.5698788	0.548	0.268	7.136625e-17	24	SEPT9	GC IgA
6.029432e-21	0.5375654	0.7	0.444	1.074987e-16	24	ATP5F1C	GC IgA
6.210064e-21	0.3885331	0.167	0.04	1.107192e-16	24	DENND6B	GC IgA
7.301437e-21	0.5581923	0.598	0.311	1.301773e-16	24	PSIP1	GC IgA
7.488557e-21	0.558028	0.557	0.284	1.335135e-16	24	PLEKHF2	GC IgA
8.145022e-21	0.5343255	0.709	0.433	1.452176e-16	24	ROMO1	GC IgA
8.151577e-21	0.5854784	0.362	0.142	1.453345e-16	24	HSH2D	GC IgA
9.003506e-21	0.6809638	0.542	0.282	1.605235e-16	24	GNG7	GC IgA
1.053438e-20	0.371353	0.152	0.025	1.878174e-16	24	IQCD	GC IgA
1.237257e-20	0.4564738	0.214	0.061	2.205905e-16	24	DTX1	GC IgA
1.275377e-20	0.4770252	0.885	0.696	2.27387e-16	24	RHOA	GC IgA
1.414085e-20	0.5181756	0.854	0.63	2.521171e-16	24	EIF3H	GC IgA
1.544331e-20	-1.025853	0.322	0.513	2.753388e-16	24	BANK1	GC IgA
1.662422e-20	0.5266249	0.635	0.336	2.963932e-16	24	SPIB	GC IgA
1.935069e-20	0.5699205	0.517	0.261	3.450035e-16	24	CD2BP2	GC IgA
1.979839e-20	-0.917032	0.074	0.254	3.529854e-16	24	FCMR	GC IgA
2.086426e-20	0.496166	0.567	0.272	3.719888e-16	24	ITGAE	GC IgA
2.190188e-20	0.4302321	0.929	0.763	3.904886e-16	24	MYL12A	GC IgA
2.239046e-20	0.5387241	0.56	0.298	3.991995e-16	24	ITSN2	GC IgA
2.285801e-20	0.4801948	0.272	0.074	4.075354e-16	24	DNASE1	GC IgA
2.340508e-20	0.4910187	0.282	0.087	4.172892e-16	24	MAP4K4	GC IgA
2.498895e-20	0.5394979	0.452	0.203	4.455279e-16	24	MTMR14	GC IgA
2.85118e-20	0.4508427	0.898	0.696	5.083369e-16	24	CIRBP	GC IgA
3.056942e-20	-0.4067749	0.994	0.999	5.450222e-16	24	RPL32	GC IgA

3.680967e-20	0.5300062	0.616	0.348	6.562796e-16	24	LSM14A	GC IgA
3.767822e-20	0.5522706	0.359	0.125	6.71765e-16	24	AMFR	GC IgA
3.785379e-20	0.521973	0.245	0.066	6.748953e-16	24	TEX9	GC IgA
3.792123e-20	0.4421506	0.26	0.073	6.760976e-16	24	DOK3	GC IgA
4.037181e-20	0.5722476	0.514	0.248	7.197891e-16	24	WIPF1	GC IgA
4.189387e-20	0.5252055	0.245	0.077	7.469258e-16	24	FAM45A	GC IgA
4.606388e-20	0.5329164	0.483	0.229	8.212729e-16	24	SEPT1	GC IgA
4.746861e-20	-0.9963856	0.115	0.298	8.463178e-16	24	LINC00926	GC IgA
4.91154e-20	0.4609787	0.18	0.052	8.756784e-16	24	CDKN2C	GC IgA
5.057953e-20	0.4815932	0.867	0.634	9.017825e-16	24	UQCRH	GC IgA
5.387152e-20	0.570117	0.492	0.237	9.604753e-16	24	ZBTB8OS	GC IgA
5.773309e-20	0.4442444	0.873	0.652	1.029323e-15	24	ATP5F1A	GC IgA
6.097219e-20	0.5820094	0.582	0.321	1.087073e-15	24	HSBP1	GC IgA
7.992701e-20	0.567909	0.279	0.098	1.425019e-15	24	FAM241A	GC IgA
8.008792e-20	0.4560879	0.858	0.606	1.427888e-15	24	POLR2L	GC IgA
8.812522e-20	0.5301833	0.31	0.116	1.571184e-15	24	ACYP2	GC IgA
9.315968e-20	-0.8013526	0.077	0.249	1.660944e-15	24	TNFRSF13B	GC IgA
1.071196e-19	0.6535091	0.307	0.108	1.909835e-15	24	VPREB3	GC IgA
1.166598e-19	0.5743627	0.467	0.231	2.079928e-15	24	MTF2	GC IgA
1.176208e-19	0.3750971	0.146	0.023	2.097062e-15	24	MAP3K7CL	GC IgA
1.232609e-19	0.5615962	0.585	0.312	2.197619e-15	24	SPI1	GC IgA
1.382484e-19	0.5322324	0.406	0.167	2.464831e-15	24	CYB5R3	GC IgA
1.524233e-19	0.5052416	0.331	0.125	2.717555e-15	24	COMMD4	GC IgA
1.536384e-19	0.5030525	0.604	0.307	2.739218e-15	24	PGLS	GC IgA
1.909878e-19	0.7124954	0.768	0.502	3.405121e-15	24	HLA-DRB5	GC IgA

2.150929e-19	0.4584778	0.211	0.062	3.834892e-15	24	FAM129A	GC IgA
2.15573e-19	0.5615809	0.536	0.282	3.843451e-15	24	PRDX3	GC IgA
2.356662e-19	0.4999826	0.201	0.048	4.201692e-15	24	LINC01991	GC IgA
2.80302e-19	0.3640842	0.124	0.02	4.997505e-15	24	ANK1	GC IgA
3.084587e-19	0.5108026	0.266	0.086	5.499511e-15	24	PTK2B	GC IgA
3.797929e-19	0.3984391	0.854	0.624	6.771328e-15	24	HLA-DMB	GC IgA
3.932062e-19	0.5287205	0.415	0.179	7.010473e-15	24	ZNF106	GC IgA
4.698129e-19	0.5472057	0.477	0.205	8.376294e-15	24	SNHG25	GC IgA
5.325439e-19	0.4441149	0.743	0.476	9.494726e-15	24	GRB2	GC IgA
5.35813e-19	0.4921346	0.728	0.476	9.55301e-15	24	UBE2N	GC IgA
6.514205e-19	0.5141402	0.158	0.033	1.161418e-14	24	AC012368.1	GC IgA
6.982969e-19	0.5120718	0.743	0.514	1.244994e-14	24	CAPZB	GC IgA
7.052399e-19	0.3475079	0.981	0.903	1.257372e-14	24	SRP14	GC IgA
7.723415e-19	-0.426004	0.994	0.993	1.377008e-14	24	RPS5	GC IgA
8.517929e-19	0.5137445	0.656	0.375	1.518661e-14	24	ZFAND6	GC IgA
8.63065e-19	0.4516381	0.251	0.077	1.538759e-14	24	WEE1	GC IgA
8.857939e-19	0.5324849	0.548	0.293	1.579282e-14	24	LYPLA1	GC IgA
8.910501e-19	0.5257918	0.3	0.102	1.588653e-14	24	SORL1	GC IgA
9.268078e-19	0.4270281	0.186	0.049	1.652406e-14	24	PEX5	GC IgA
9.459058e-19	0.5429746	0.591	0.335	1.686455e-14	24	COX14	GC IgA
9.744299e-19	0.532705	0.263	0.09	1.737311e-14	24	GCSAM	GC IgA
1.027228e-18	0.4911987	0.693	0.423	1.831445e-14	24	CD40	GC IgA
1.245691e-18	0.5270653	0.56	0.279	2.220943e-14	24	JPT1	GC IgA
1.271148e-18	0.4033523	0.895	0.673	2.26633e-14	24	ATP5ME	GC IgA
1.561989e-18	0.3518254	0.161	0.036	2.78487e-14	24	PRDM15	GC IgA

1.658783e-18	-0.7502333	0.062	0.211	2.957445e-14	24	MGST3	GC IgA
1.682782e-18	0.475652	0.276	0.086	3.000232e-14	24	DENND2D	GC IgA
1.727243e-18	0.5199239	0.421	0.173	3.079502e-14	24	EHMT1	GC IgA
1.738363e-18	0.5042094	0.594	0.321	3.099327e-14	24	IMP4	GC IgA
1.886296e-18	0.5321484	0.356	0.145	3.363078e-14	24	RBM38	GC IgA
1.971236e-18	0.4068608	0.907	0.706	3.514517e-14	24	NDUFA4	GC IgA
1.972522e-18	0.4785331	0.176	0.05	3.516809e-14	24	CPM	GC IgA
2.467531e-18	0.4314601	0.238	0.069	4.39936e-14	24	MTA3	GC IgA
2.475581e-18	0.4478172	0.839	0.613	4.413714e-14	24	UQCR10	GC IgA
3.119087e-18	0.4838673	0.297	0.111	5.56102e-14	24	BRI3BP	GC IgA
3.514268e-18	0.4799216	0.257	0.082	6.265589e-14	24	PARN	GC IgA
4.382269e-18	-0.3839316	1	0.997	7.813147e-14	24	RPS3A	GC IgA
4.7727e-18	0.4970435	0.245	0.074	8.509247e-14	24	TMED8	GC IgA
4.963136e-18	-0.3424981	1	0.999	8.848776e-14	24	RPL13A	GC IgA
5.594947e-18	0.4833	0.266	0.091	9.975232e-14	24	FAM208B	GC IgA
5.996866e-18	0.470228	0.449	0.209	1.069181e-13	24	ADA	GC IgA
6.016359e-18	-0.5331613	0.985	0.985	1.072657e-13	24	RPL27	GC IgA
6.234933e-18	-0.4145652	1	0.999	1.111626e-13	24	RPL39	GC IgA
7.16298e-18	0.4128245	0.155	0.033	1.277088e-13	24	MCTP2	GC IgA
7.677059e-18	-0.7044308	0.028	0.164	1.368743e-13	24	MARCKS	GC IgA
8.425836e-18	0.4742878	0.325	0.119	1.502242e-13	24	CD180	GC IgA
8.667165e-18	0.431459	0.257	0.08	1.545269e-13	24	VGLL4	GC IgA
9.830606e-18	0.4743013	0.728	0.488	1.752699e-13	24	CCT8	GC IgA
1.017317e-17	0.507688	0.303	0.115	1.813775e-13	24	C12orf49	GC IgA
1.254293e-17	-0.4025923	0.997	0.999	2.23628e-13	24	EEF1A1	GC IgA

1.270439e-17	0.4946197	0.378	0.164	2.265065e-13	24	TOR3A	GC IgA
1.385125e-17	0.4772495	0.533	0.252	2.469539e-13	24	TBC1D10C	GC IgA
1.427194e-17	-0.4268915	1	0.998	2.544545e-13	24	RPL11	GC IgA
1.469942e-17	0.318503	0.133	0.025	2.62076e-13	24	AC022167.3	GC IgA
1.7133e-17	0.5817524	0.48	0.244	3.054643e-13	24	TPM4	GC IgA
1.726857e-17	-0.9474329	0.059	0.212	3.078813e-13	24	PLAC8	GC IgA
1.942808e-17	0.4946307	0.508	0.254	3.463832e-13	24	LYSMD2	GC IgA
2.170148e-17	0.4471986	0.223	0.069	3.869157e-13	24	ST14	GC IgA
2.172667e-17	-1.059543	0.102	0.275	3.873649e-13	24	KLF2	GC IgA
2.234583e-17	0.3676775	0.904	0.701	3.984038e-13	24	COMMD6	GC IgA
2.301655e-17	0.5434848	0.563	0.32	4.10362e-13	24	NAA38	GC IgA
2.501783e-17	0.5113836	0.307	0.115	4.460429e-13	24	KLHL5	GC IgA
2.522758e-17	0.4817221	0.632	0.384	4.497826e-13	24	NDUFB3	GC IgA
2.563834e-17	0.5463988	0.356	0.146	4.571059e-13	24	ALOX5	GC IgA
2.782683e-17	-0.3589296	1	0.999	4.961245e-13	24	RPS27A	GC IgA
3.527868e-17	0.4513735	0.789	0.565	6.289836e-13	24	RAC1	GC IgA
3.656895e-17	0.4095649	0.235	0.071	6.519879e-13	24	NEK6	GC IgA
3.82848e-17	0.4215102	0.799	0.552	6.825797e-13	24	SEC61G	GC IgA
4.442519e-17	-0.7369007	0.04	0.173	7.920567e-13	24	TNFRSF18	GC IgA
4.696648e-17	0.4927292	0.331	0.129	8.373654e-13	24	KMT2A	GC IgA
4.871434e-17	0.5614329	0.254	0.083	8.685279e-13	24	SH3TC1	GC IgA
5.029205e-17	0.3185251	0.988	0.923	8.96657e-13	24	BTF3	GC IgA
5.236513e-17	-0.777353	0.214	0.381	9.336179e-13	24	PDCD4	GC IgA
6.44047e-17	0.4136803	0.864	0.643	1.148271e-12	24	HNRNPC	GC IgA
6.615259e-17	0.3167333	0.118	0.023	1.179435e-12	24	AC090152.1	GC IgA

8.452457e-17	0.4676551	0.399	0.175	1.506989e-12	24	SYK	GC IgA
8.711957e-17	0.5222054	0.464	0.235	1.553255e-12	24	DSTN	GC IgA
8.735684e-17	0.3471754	0.966	0.881	1.557485e-12	24	MS4A1	GC IgA
8.907456e-17	0.420564	0.207	0.057	1.58811e-12	24	SLC15A4	GC IgA
8.908715e-17	0.5451318	0.675	0.45	1.588335e-12	24	MAP1LC3B	GC IgA
9.954129e-17	0.4104386	0.861	0.668	1.774722e-12	24	SNX3	GC IgA
1.113605e-16	-0.4638719	0.994	0.991	1.985446e-12	24	RPL30	GC IgA
1.169793e-16	0.5358001	0.272	0.107	2.085624e-12	24	PHF6	GC IgA
1.292226e-16	0.4539188	0.229	0.074	2.303909e-12	24	CUX1	GC IgA
1.380852e-16	0.4986444	0.65	0.412	2.461921e-12	24	HCLS1	GC IgA
1.438663e-16	-0.4129435	0.994	0.993	2.564992e-12	24	RPL35	GC IgA
1.555853e-16	0.4973399	0.644	0.389	2.773931e-12	24	ARPC4	GC IgA
1.602657e-16	0.358478	0.136	0.034	2.857378e-12	24	NUGGC	GC IgA
1.68933e-16	0.4526598	0.588	0.33	3.011907e-12	24	BPTF	GC IgA
1.850777e-16	-1.099809	0.307	0.457	3.29975e-12	24	JUNB	GC IgA
1.879647e-16	0.437938	0.743	0.483	3.351223e-12	24	UBE2L3	GC IgA
1.893386e-16	0.4042274	0.675	0.421	3.375719e-12	24	GABARAPL2	GC IgA
1.925615e-16	0.4681657	0.653	0.401	3.433179e-12	24	STX7	GC IgA
1.958616e-16	-0.4214998	0.997	0.995	3.492016e-12	24	RPL31	GC IgA
2.000183e-16	0.4434244	0.749	0.49	3.566126e-12	24	RAC2	GC IgA
2.400232e-16	0.5005208	0.402	0.188	4.279374e-12	24	RFC1	GC IgA
2.592699e-16	0.3383336	0.864	0.634	4.622523e-12	24	HERPUD1	GC IgA
2.687325e-16	0.5090547	0.452	0.233	4.791232e-12	24	ZCCHC7	GC IgA
2.845819e-16	-0.3565587	0.994	0.995	5.07381e-12	24	RPL37A	GC IgA
2.875724e-16	-0.3193725	0.994	0.998	5.127127e-12	24	RPL18A	GC IgA

3.182303e-16	0.4147653	0.217	0.074	5.673728e-12	24	EZH2 GC IgA
3.289197e-16	0.4557507	0.548	0.282	5.864309e-12	24	CARHSP1 GC IgA
3.513818e-16	-0.7518025	0.087	0.238	6.264787e-12	24	CAPG GC IgA
3.543965e-16	0.4620462	0.399	0.189	6.318534e-12	24	LBR GC IgA
3.863147e-16	0.4355923	0.241	0.081	6.887604e-12	24	GPR18 GC IgA
3.972074e-16	0.4301159	0.508	0.209	7.081811e-12	24	PLCG2 GC IgA
4.22401e-16	0.4472037	0.768	0.532	7.530987e-12	24	SOD1 GC IgA
4.288482e-16	0.4895316	0.3	0.12	7.645934e-12	24	SSBP2 GC IgA
4.30685e-16	0.4749869	0.536	0.299	7.678683e-12	24	ATP6V0D1 GC IgA
4.366595e-16	0.4677248	0.56	0.314	7.785203e-12	24	NDUFAF3 GC IgA
4.750722e-16	-0.7992744	0.167	0.321	8.470063e-12	24	TUBA1A GC IgA
5.089923e-16	0.501279	0.393	0.194	9.074823e-12	24	GRSF1 GC IgA
5.142601e-16	0.4752445	0.498	0.252	9.168742e-12	24	MPC2 GC IgA
5.455361e-16	0.4446647	0.3	0.111	9.726362e-12	24	PIP4K2A GC IgA
5.608452e-16	-0.7126176	0.037	0.159	9.999308e-12	24	LMNA GC IgA
5.873255e-16	0.4634915	0.303	0.124	1.047143e-11	24	ARL3 GC IgA
6.797465e-16	0.4013134	0.811	0.577	1.21192e-11	24	COPE GC IgA
7.707112e-16	0.4519415	0.498	0.261	1.374101e-11	24	THOC7 GC IgA
8.220344e-16	0.4743992	0.567	0.347	1.465605e-11	24	S100A10 GC IgA
9.063545e-16	0.5982604	0.743	0.496	1.615939e-11	24	SAT1 GC IgA
1.102874e-15	0.4810485	0.294	0.111	1.966313e-11	24	TRAC GC IgA
1.107943e-15	0.3597238	0.941	0.804	1.975351e-11	24	HNRNPK GC IgA
1.221551e-15	-0.5435074	0.978	0.974	2.177903e-11	24	MT-ND4 GC IgA
1.513557e-15	0.4995112	0.198	0.068	2.698521e-11	24	EYA3 GC IgA
1.719229e-15	-0.8538762	0.121	0.279	3.065213e-11	24	ADAM28 GC IgA

1.780772e-15	0.3302849	0.139	0.036	3.174938e-11	24	MSRB2	GC IgA
2.137416e-15	0.3168321	0.136	0.033	3.810799e-11	24	KANK1	GC IgA
2.18003e-15	0.4290982	0.26	0.103	3.886776e-11	24	OTULIN	GC IgA
2.415765e-15	0.3988742	0.158	0.036	4.307068e-11	24	IGLC5	GC IgA
2.612661e-15	0.4588893	0.381	0.176	4.658113e-11	24	PMVK	GC IgA
3.008272e-15	0.4475478	0.384	0.176	5.363449e-11	24	LAP3	GC IgA
3.324659e-15	0.497928	0.492	0.263	5.927535e-11	24	VASP	GC IgA
3.835449e-15	0.3758545	0.851	0.626	6.838223e-11	24	ATP5F1B	GC IgA
3.925991e-15	-0.6067344	0.978	0.976	6.999649e-11	24	FTH1	GC IgA
4.021737e-15	0.4222495	0.563	0.314	7.170355e-11	24	SCAND1	GC IgA
4.201464e-15	0.4107222	0.232	0.087	7.490791e-11	24	VEZT	GC IgA
4.522024e-15	0.4390773	0.437	0.205	8.062316e-11	24	HADHB	GC IgA
5.625163e-15	0.4712222	0.656	0.429	1.00291e-10	24	CLTA	GC IgA
5.983389e-15	0.4302015	0.808	0.604	1.066778e-10	24	RBM3	GC IgA
6.117372e-15	0.3810591	0.217	0.072	1.090666e-10	24	ACSL5	GC IgA
6.526989e-15	0.4496351	0.591	0.344	1.163697e-10	24	H2AFY	GC IgA
6.680501e-15	0.3939814	0.167	0.045	1.191066e-10	24	FCHO1	GC IgA
6.757423e-15	0.3646015	0.867	0.667	1.204781e-10	24	GDI2	GC IgA
7.051232e-15	0.4514852	0.226	0.075	1.257164e-10	24	RGCC	GC IgA
7.398788e-15	0.4038967	0.248	0.087	1.31913e-10	24	CHIC2	GC IgA
7.416502e-15	0.5158752	0.272	0.093	1.322288e-10	24	RNGTT	GC IgA
7.534157e-15	0.3793992	0.57	0.312	1.343265e-10	24	HMGN3	GC IgA
8.403386e-15	0.4163538	0.669	0.418	1.49824e-10	24	HNRNPD	GC IgA
8.712237e-15	-0.4738981	0.963	0.968	1.553305e-10	24	RPS17	GC IgA
9.420807e-15	0.3698237	0.805	0.557	1.679636e-10	24	ATP5PF	GC IgA

1.017846e-14	0.4458731	0.601	0.364	1.814717e-10	24	PDIA3	GC IgA
1.085965e-14	0.4541882	0.625	0.387	1.936167e-10	24	CUTA	GC IgA
1.149526e-14	0.4661568	0.579	0.336	2.049491e-10	24	NDUFC1	GC IgA
1.276151e-14	0.4245084	0.307	0.128	2.275249e-10	24	ETS1	GC IgA
1.394821e-14	0.421296	0.752	0.534	2.486826e-10	24	CAP1	GC IgA
1.76002e-14	0.4580446	0.322	0.14	3.13794e-10	24	INO80C	GC IgA
1.902332e-14	0.4469147	0.325	0.133	3.391667e-10	24	CERS4	GC IgA
1.953609e-14	0.4189479	0.734	0.502	3.483089e-10	24	EIF3I	GC IgA
2.273937e-14	-0.4318324	0.988	0.984	4.054202e-10	24	RPL29	GC IgA
2.277971e-14	0.4992068	0.467	0.249	4.061395e-10	24	TIFA	GC IgA
2.318154e-14	0.4095101	0.17	0.048	4.133037e-10	24	MYL5	GC IgA
2.37808e-14	0.4642321	0.44	0.242	4.23988e-10	24	ETFA	GC IgA
2.775661e-14	0.3209645	0.155	0.042	4.948727e-10	24	IKBKE	GC IgA
2.824358e-14	0.4433391	0.61	0.365	5.035547e-10	24	RPS19BP1	GC IgA
2.835516e-14	0.3518621	0.901	0.721	5.055442e-10	24	COX6C	GC IgA
2.893234e-14	-0.7479319	0.802	0.644	5.158347e-10	24	IGLC2	GC IgA
3.272433e-14	0.4782923	0.449	0.242	5.83442e-10	24	WAS	GC IgA
3.484516e-14	0.3594045	0.867	0.662	6.212543e-10	24	DBI	GC IgA
3.619245e-14	0.3588525	0.18	0.06	6.452751e-10	24	TESC	GC IgA
3.988232e-14	-0.7347989	0.706	0.794	7.110619e-10	24	HLA-E	GC IgA
4.041726e-14	0.3592242	0.854	0.639	7.205994e-10	24	ATP5MF	GC IgA
4.056308e-14	0.3365718	0.105	0.022	7.231992e-10	24	FAM81A	GC IgA
4.643263e-14	0.3895606	0.254	0.096	8.278474e-10	24	CCDC28B	GC IgA
5.080609e-14	0.4448567	0.579	0.364	9.058217e-10	24	RBBP7	GC IgA
5.173425e-14	0.4371124	0.378	0.171	9.223699e-10	24	CDK13	GC IgA

5.278985e-14	0.4057378	0.7	0.455	9.411902e-10	24	COX17	GC IgA
5.778623e-14	0.3680016	0.885	0.706	1.030271e-09	24	COX7A2	GC IgA
5.888684e-14	-0.6768063	0.068	0.196	1.049893e-09	24	CLEC2B	GC IgA
6.234886e-14	0.4276567	0.279	0.108	1.111618e-09	24	RAB11FIP1	GC IgA
6.294627e-14	0.4128803	0.186	0.062	1.122269e-09	24	ZNF608	GC IgA
7.626624e-14	0.4445638	0.198	0.062	1.359751e-09	24	HOPX	GC IgA
7.707838e-14	0.341817	0.124	0.03	1.37423e-09	24	RNF144B	GC IgA
7.782101e-14	0.4075874	0.322	0.139	1.387471e-09	24	MKNK2	GC IgA
8.586486e-14	0.415748	0.254	0.098	1.530885e-09	24	SYVN1	GC IgA
8.951039e-14	-0.8429342	0.204	0.348	1.595881e-09	24	TRAF3IP3	GC IgA
9.41778e-14	-0.4733632	0.957	0.965	1.679096e-09	24	RPL38	GC IgA
9.866214e-14	0.3287686	0.892	0.729	1.759047e-09	24	OST4	GC IgA
9.88212e-14	0.4176933	0.56	0.332	1.761883e-09	24	NDUFS7	GC IgA
1.075652e-13	0.3011036	0.858	0.597	1.91778e-09	24	PRDX1	GC IgA
1.136589e-13	0.3763895	0.402	0.208	2.026424e-09	24	SYAP1	GC IgA
1.161322e-13	0.3902927	0.622	0.382	2.07052e-09	24	EVL	GC IgA
1.183321e-13	0.3335986	0.13	0.031	2.109743e-09	24	PTAFR	GC IgA
1.187088e-13	0.3832827	0.533	0.291	2.116458e-09	24	BCL11A	GC IgA
1.415274e-13	0.4163817	0.372	0.169	2.523292e-09	24	COMMD7	GC IgA
1.428993e-13	0.3326954	0.842	0.646	2.547751e-09	24	UQCR11	GC IgA
1.510834e-13	0.3611587	0.656	0.403	2.693665e-09	24	PSMB2	GC IgA
1.704856e-13	0.4032601	0.409	0.213	3.039587e-09	24	PHAX	GC IgA
1.80552e-13	0.3613224	0.728	0.467	3.219062e-09	24	MTDH	GC IgA
1.906437e-13	0.3716949	0.879	0.682	3.398987e-09	24	SLC25A3	GC IgA
2.066092e-13	0.4087031	0.455	0.238	3.683636e-09	24	PTRHD1	GC IgA

2.168673e-13	0.488909	0.48	0.257	3.866526e-09	24	CNN2	GC IgA
2.242947e-13	0.3696734	0.786	0.555	3.998951e-09	24	PGK1	GC IgA
2.278509e-13	0.4534552	0.443	0.215	4.062353e-09	24	IFI27L2	GC IgA
2.382901e-13	0.4291045	0.539	0.314	4.248474e-09	24	WDR1	GC IgA
2.721604e-13	0.5003492	0.288	0.13	4.852347e-09	24	PHGDH	GC IgA
3.03678e-13	0.3246816	0.139	0.042	5.414275e-09	24	IGF2BP3	GC IgA
3.353631e-13	0.4672767	0.539	0.317	5.979188e-09	24	FCRLA	GC IgA
3.49524e-13	0.4254509	0.545	0.311	6.231664e-09	24	LPXN	GC IgA
3.568948e-13	0.4178791	0.22	0.089	6.363078e-09	24	ZBTB38	GC IgA
3.69232e-13	0.3709679	0.759	0.544	6.583038e-09	24	UBE2D2	GC IgA
3.710853e-13	0.4206661	0.601	0.395	6.61608e-09	24	CSTB	GC IgA
4.005699e-13	0.4575306	0.489	0.277	7.14176e-09	24	CNIH1	GC IgA
4.426314e-13	0.3484048	0.728	0.483	7.891675e-09	24	NDUFB1	GC IgA
4.44327e-13	0.4013487	0.269	0.103	7.921906e-09	24	RHBDF2	GC IgA
4.651281e-13	0.3918396	0.644	0.416	8.292769e-09	24	COX7A2L	GC IgA
4.916635e-13	0.4788964	0.406	0.221	8.765868e-09	24	RPIA	GC IgA
4.919866e-13	-0.354902	0.994	0.992	8.77163e-09	24	RPL37	GC IgA
4.932203e-13	0.3172918	0.858	0.651	8.793625e-09	24	UBE2D3	GC IgA
5.064696e-13	0.3847949	0.56	0.331	9.029847e-09	24	EIF3A	GC IgA
5.210489e-13	0.3101696	0.115	0.029	9.28978e-09	24	ZFAND4	GC IgA
5.659035e-13	0.3429343	0.777	0.553	1.008949e-08	24	NEDD8	GC IgA
6.119549e-13	0.4255021	0.356	0.163	1.091054e-08	24	PPP1R18	GC IgA
6.284688e-13	0.3774297	0.604	0.38	1.120497e-08	24	LSM8	GC IgA
6.588111e-13	0.3555548	0.161	0.049	1.174594e-08	24	GNA13	GC IgA
7.274563e-13	0.4237059	0.297	0.128	1.296982e-08	24	DNAJB2	GC IgA

7.537556e-13	0.3729122	0.421	0.205	1.343871e-08	24	ARHGEF1	GC IgA
8.286961e-13	0.3018303	0.118	0.027	1.477482e-08	24	B3GLCT	GC IgA
9.260956e-13	0.388382	0.644	0.428	1.651136e-08	24	KTN1	GC IgA
9.357764e-13	0.3304495	0.851	0.655	1.668396e-08	24	PSMB1	GC IgA
9.955987e-13	-0.4993255	0.015	0.113	1.775053e-08	24	P2RY10	GC IgA
1.082041e-12	0.3765582	0.622	0.39	1.929172e-08	24	SH3BGRL	GC IgA
1.09661e-12	0.4075758	0.378	0.178	1.955145e-08	24	TMC6	GC IgA
1.114347e-12	0.3110266	0.149	0.038	1.98677e-08	24	INPP5B	GC IgA
1.204691e-12	0.4104776	0.461	0.238	2.147844e-08	24	SP140	GC IgA
1.332784e-12	0.5229396	0.356	0.131	2.37622e-08	24	SULF2	GC IgA
1.35301e-12	0.4188106	0.362	0.183	2.412281e-08	24	HSD17B12	GC IgA
1.441009e-12	0.3325482	0.721	0.491	2.569175e-08	24	THRAP3	GC IgA
1.441492e-12	-0.4557914	0.019	0.109	2.570036e-08	24	SNX9	GC IgA
1.452077e-12	-0.7416965	0.09	0.198	2.588907e-08	24	ACP5	GC IgA
1.479143e-12	0.3743288	0.793	0.582	2.637164e-08	24	NDUFB2	GC IgA
1.520409e-12	0.4135831	0.375	0.18	2.710738e-08	24	TADA3	GC IgA
1.598009e-12	0.3803202	0.282	0.111	2.849091e-08	24	HPS1	GC IgA
1.728616e-12	0.4044774	0.461	0.251	3.081949e-08	24	RGS10	GC IgA
1.742717e-12	0.360215	0.251	0.093	3.10709e-08	24	PDLIM2	GC IgA
1.80269e-12	0.3572517	0.211	0.074	3.214017e-08	24	HS2ST1	GC IgA
1.80273e-12	0.3716786	0.731	0.496	3.214087e-08	24	COX5A	GC IgA
1.986519e-12	0.4136992	0.418	0.225	3.541764e-08	24	PLEKHJ1	GC IgA
2.054544e-12	-0.6587754	0.062	0.184	3.663047e-08	24	PLPP5	GC IgA
2.055286e-12	0.3745988	0.557	0.322	3.664369e-08	24	BLNK	GC IgA
2.060018e-12	0.3514305	0.697	0.467	3.672805e-08	24	PRDX5	GC IgA

2.12193e-12	0.3700336	0.557	0.325	3.783188e-08	24	PMAIP1	GC IgA
2.238965e-12	0.4130797	0.706	0.5	3.99185e-08	24	RTRAF	GC IgA
2.367156e-12	0.3792174	0.542	0.314	4.220403e-08	24	SFT2D1	GC IgA
2.429569e-12	-0.569986	0.059	0.172	4.331678e-08	24	BCL2	GC IgA
2.719235e-12	0.4238951	0.412	0.227	4.848123e-08	24	NGLY1	GC IgA
3.117104e-12	0.3692786	0.186	0.067	5.557485e-08	24	ST20	GC IgA
3.291077e-12	-0.6712772	0.195	0.33	5.867662e-08	24	SMC6	GC IgA
3.515343e-12	0.4069454	0.316	0.146	6.267504e-08	24	ZNF580	GC IgA
3.585685e-12	0.3721787	0.359	0.174	6.392917e-08	24	PDIA4	GC IgA
3.814941e-12	-0.3547183	0.988	0.992	6.801659e-08	24	RPL36	GC IgA
3.884898e-12	0.3189	0.721	0.489	6.926385e-08	24	SF3B6	GC IgA
4.458833e-12	0.339179	0.214	0.082	7.949654e-08	24	TFPT	GC IgA
4.706859e-12	0.4240471	0.536	0.301	8.391859e-08	24	ODC1	GC IgA
4.920553e-12	0.4734291	0.526	0.317	8.772854e-08	24	PRDX2	GC IgA
5.089175e-12	0.3491159	0.26	0.109	9.07349e-08	24	PTS	GC IgA
5.105674e-12	-0.6712638	0.074	0.199	9.102907e-08	24	YBX3	GC IgA
6.305963e-12	0.3587084	0.607	0.381	1.12429e-07	24	JAK1	GC IgA
6.327431e-12	0.3844704	0.529	0.296	1.128118e-07	24	BLK	GC IgA
6.458643e-12	0.335689	0.142	0.047	1.151511e-07	24	LRRK1	GC IgA
6.577827e-12	0.3663885	0.502	0.295	1.172761e-07	24	PDHB	GC IgA
6.651404e-12	0.3714842	0.402	0.207	1.185879e-07	24	GLRX	GC IgA
6.692857e-12	0.3846853	0.328	0.147	1.19327e-07	24	SMARCA4	GC IgA
7.067694e-12	0.4063569	0.594	0.396	1.260099e-07	24	ORAI2	GC IgA
8.335297e-12	-0.7131226	0.115	0.243	1.4861e-07	24	PHACTR1	GC IgA
8.780746e-12	0.4169167	0.474	0.279	1.565519e-07	24	BABAM1	GC IgA

1.05947e-11	0.3810008	0.536	0.308	1.888929e-07	24	CALM3	GC IgA
1.181172e-11	0.3599186	0.867	0.715	2.105911e-07	24	COX6B1	GC IgA
1.271261e-11	0.3558989	0.437	0.226	2.266531e-07	24	NSMCE1	GC IgA
1.274911e-11	-0.6963868	0.368	0.479	2.273039e-07	24	SQSTM1	GC IgA
1.32922e-11	0.3223008	0.173	0.062	2.369866e-07	24	SNX22	GC IgA
1.407063e-11	0.3673041	0.539	0.32	2.508653e-07	24	GNB2	GC IgA
1.412682e-11	0.3621	0.322	0.139	2.518671e-07	24	ITGB1	GC IgA
1.435319e-11	0.360274	0.232	0.094	2.55903e-07	24	UBR5	GC IgA
1.441143e-11	0.4047434	0.601	0.374	2.569414e-07	24	FAM107B	GC IgA
1.446109e-11	0.3395308	0.294	0.126	2.578268e-07	24	STAP1	GC IgA
1.479257e-11	0.4173546	0.567	0.356	2.637368e-07	24	GNAS	GC IgA
1.502349e-11	0.3047777	0.149	0.046	2.678538e-07	24	CCDC102A	GC IgA
1.536636e-11	-0.7129227	0.189	0.306	2.739668e-07	24	ARHGAP24	GC IgA
1.605443e-11	0.3531972	0.511	0.302	2.862345e-07	24	UGP2	GC IgA
1.614043e-11	0.3788801	0.495	0.284	2.877678e-07	24	GSTK1	GC IgA
1.692141e-11	0.3433384	0.632	0.421	3.016918e-07	24	ECH1	GC IgA
1.779563e-11	0.3479444	0.74	0.518	3.172784e-07	24	PSMB6	GC IgA
1.868352e-11	0.3922228	0.276	0.135	3.331085e-07	24	GTF2E2	GC IgA
1.887514e-11	0.3728192	0.313	0.15	3.365248e-07	24	ARPC1A	GC IgA
1.956807e-11	0.3992496	0.539	0.343	3.488792e-07	24	SNX17	GC IgA
1.982603e-11	0.3825721	0.474	0.265	3.534783e-07	24	PPP1R7	GC IgA
2.15423e-11	0.3445109	0.214	0.08	3.840777e-07	24	RANBP2	GC IgA
2.181161e-11	0.4012794	0.368	0.175	3.888792e-07	24	RGS19	GC IgA
2.194073e-11	0.3655207	0.362	0.181	3.911814e-07	24	TRABD	GC IgA
2.222522e-11	0.3381479	0.316	0.133	3.962535e-07	24	PLEKHA2	GC IgA

2.233648e-11	0.3420301	0.585	0.365	3.982371e-07	24	EWSR1	GC IgA
2.302847e-11	0.3798724	0.294	0.136	4.105747e-07	24	CLIC4	GC IgA
2.347662e-11	0.4261735	0.406	0.227	4.185647e-07	24	IAH1	GC IgA
2.396902e-11	0.3766306	0.384	0.207	4.273437e-07	24	TCEAL8	GC IgA
2.668947e-11	-0.7375581	0.241	0.354	4.758466e-07	24	TUBB4B	GC IgA
2.712773e-11	0.3762347	0.232	0.1	4.836604e-07	24	PTPN7	GC IgA
2.752669e-11	0.3727652	0.567	0.362	4.907734e-07	24	PPP2CA	GC IgA
2.911669e-11	0.4147244	0.402	0.214	5.191215e-07	24	DMAC1	GC IgA
2.966924e-11	0.4724876	0.449	0.256	5.289729e-07	24	MDM4	GC IgA
3.738953e-11	0.3794867	0.539	0.332	6.66618e-07	24	BAX	GC IgA
3.873412e-11	0.3579623	0.217	0.09	6.905907e-07	24	RITA1	GC IgA
4.173703e-11	0.3757246	0.402	0.22	7.441295e-07	24	MPLKIP	GC IgA
4.248065e-11	0.3622014	0.356	0.177	7.573874e-07	24	MTERF4	GC IgA
4.388128e-11	0.3672791	0.368	0.19	7.823594e-07	24	NDUFA8	GC IgA
5.055038e-11	0.372871	0.526	0.319	9.012627e-07	24	IGBP1	GC IgA
5.715463e-11	-0.6124915	0.207	0.346	1.01901e-06	24	CAST	GC IgA
5.826275e-11	0.3874375	0.43	0.25	1.038767e-06	24	MCTS1	GC IgA
6.617173e-11	-0.5257115	0.05	0.15	1.179776e-06	24	CELF2	GC IgA
6.692804e-11	0.4116285	0.57	0.362	1.19326e-06	24	CPNE1	GC IgA
6.939563e-11	0.3855008	0.604	0.404	1.237255e-06	24	SMIM14	GC IgA
7.134376e-11	0.3478582	0.421	0.227	1.271988e-06	24	DEGS1	GC IgA
7.231675e-11	0.3386458	0.709	0.516	1.289335e-06	24	ATP5PB	GC IgA
7.59885e-11	0.3959808	0.313	0.151	1.354799e-06	24	PEA15	GC IgA
7.696645e-11	-0.4098251	0.923	0.934	1.372235e-06	24	MT-ATP6	GC IgA
8.390524e-11	0.3557225	0.297	0.141	1.495947e-06	24	FAM207A	GC IgA

9.10538e-11	0.3630299	0.251	0.114	1.623398e-06	24	HIBCH	GC IgA
9.539691e-11	0.3722202	0.294	0.14	1.700832e-06	24	CISD1	GC IgA
1.02072e-10	-0.588128	0.09	0.197	1.819842e-06	24	CHPT1	GC IgA
1.025034e-10	0.3500243	0.288	0.136	1.827532e-06	24	OSBPL9	GC IgA
1.064372e-10	0.353402	0.297	0.125	1.89767e-06	24	ZBTB20	GC IgA
1.107482e-10	0.3275334	0.567	0.353	1.974529e-06	24	DGUOK	GC IgA
1.123401e-10	0.3907121	0.146	0.048	2.002911e-06	24	HES6	GC IgA
1.140866e-10	0.3271656	0.827	0.696	2.03405e-06	24	HLA-DQB1	GC IgA
1.143407e-10	0.3436347	0.61	0.409	2.038581e-06	24	EID1	GC IgA
1.15377e-10	0.33884	0.344	0.167	2.057056e-06	24	TFDP1	GC IgA
1.188586e-10	0.3132977	0.56	0.344	2.11913e-06	24	POLR2J	GC IgA
1.209467e-10	0.3799248	0.786	0.609	2.156359e-06	24	SYNGR2	GC IgA
1.222248e-10	0.3379767	0.285	0.13	2.179147e-06	24	C16orf87	GC IgA
1.254557e-10	-0.7242731	0.124	0.269	2.23675e-06	24	JUN	GC IgA
1.265868e-10	0.3028652	0.282	0.129	2.256916e-06	24	STIM2	GC IgA
1.280749e-10	0.3066099	0.693	0.47	2.283448e-06	24	GPSM3	GC IgA
1.416467e-10	-0.6636426	0.328	0.445	2.525418e-06	24	ANXA2	GC IgA
1.634988e-10	0.3305849	0.269	0.13	2.915021e-06	24	MAGEF1	GC IgA
1.690392e-10	0.3151274	0.514	0.309	3.0138e-06	24	SCP2	GC IgA
1.757459e-10	0.316721	0.387	0.208	3.133373e-06	24	EHD1	GC IgA
1.76893e-10	0.3899978	0.412	0.22	3.153826e-06	24	SKAP2	GC IgA
1.835546e-10	0.3724155	0.269	0.132	3.272596e-06	24	PRKCD	GC IgA
2.028671e-10	0.3386734	0.489	0.286	3.616918e-06	24	NDUFB5	GC IgA
2.042232e-10	0.3232554	0.56	0.343	3.641095e-06	24	MRPS34	GC IgA
2.10425e-10	0.3094911	0.811	0.612	3.751667e-06	24	COX5B	GC IgA

2.105761e-10	-0.4609837	0.022	0.105	3.754361e-06	24	MTSS1	GC IgA
2.157727e-10	0.3232877	0.539	0.323	3.847012e-06	24	SPAG7	GC IgA
2.175698e-10	-0.670194	0.118	0.239	3.879052e-06	24	CD55	GC IgA
2.297521e-10	0.3677228	0.56	0.376	4.09625e-06	24	SUPT4H1	GC IgA
2.40577e-10	0.3127088	0.214	0.091	4.289247e-06	24	XPNPEP1	GC IgA
2.520922e-10	0.3287394	0.235	0.098	4.494552e-06	24	TACC1	GC IgA
2.59244e-10	0.3816263	0.365	0.195	4.622061e-06	24	UBE2E1	GC IgA
2.872359e-10	0.3346522	0.489	0.288	5.121128e-06	24	TUBA4A	GC IgA
3.023748e-10	-0.4884338	0.053	0.149	5.39104e-06	24	SVIP	GC IgA
3.109707e-10	0.331854	0.204	0.091	5.544297e-06	24	MSH6	GC IgA
3.151776e-10	0.352104	0.387	0.2	5.619301e-06	24	LAGE3	GC IgA
3.403877e-10	0.4033259	0.303	0.165	6.068772e-06	24	COX11	GC IgA
3.594345e-10	0.3445629	0.328	0.155	6.408357e-06	24	ACAP2	GC IgA
3.610975e-10	0.3450009	0.399	0.215	6.438007e-06	24	SMAP2	GC IgA
3.643049e-10	0.339625	0.272	0.122	6.495193e-06	24	PLEK	GC IgA
3.892149e-10	0.3113156	0.7	0.487	6.939313e-06	24	EIF3M	GC IgA
3.905237e-10	0.3222933	0.319	0.159	6.962648e-06	24	TPGS2	GC IgA
4.529288e-10	0.360622	0.495	0.295	8.075267e-06	24	TWF2	GC IgA
4.612896e-10	0.3319414	0.22	0.09	8.224333e-06	24	PPP6R1	GC IgA
4.709419e-10	-0.7952193	0.322	0.409	8.396424e-06	24	TXN	GC IgA
4.919261e-10	-0.5468659	0.084	0.191	8.770551e-06	24	S1PR4	GC IgA
6.039286e-10	-0.7354691	0.35	0.448	1.076744e-05	24	PNRC1	GC IgA
6.149605e-10	0.3179151	0.458	0.256	1.096413e-05	24	UPF3A	GC IgA
6.866683e-10	0.3509095	0.226	0.098	1.224261e-05	24	EPS15L1	GC IgA
7.111925e-10	0.345761	0.563	0.359	1.267985e-05	24	SDHC	GC IgA

7.541614e-10	0.3076977	0.638	0.424	1.344594e-05	24	TAF7	GC IgA
7.990833e-10	0.3315206	0.43	0.245	1.424686e-05	24	NBDY	GC IgA
8.010254e-10	0.3437779	0.467	0.276	1.428148e-05	24	CCNG1	GC IgA
8.120168e-10	0.358148	0.18	0.08	1.447745e-05	24	CLPTM1L	GC IgA
8.648761e-10	0.3757936	0.226	0.108	1.541988e-05	24	DAP	GC IgA
9.049482e-10	0.4164009	0.467	0.288	1.613432e-05	24	C7orf50	GC IgA
9.345867e-10	0.3242894	0.319	0.16	1.666275e-05	24	RSU1	GC IgA
9.52673e-10	0.4113087	0.402	0.233	1.698521e-05	24	CPNE3	GC IgA
9.851726e-10	0.3427043	0.167	0.059	1.756464e-05	24	ZNF101	GC IgA
1.019792e-09	0.3658442	0.35	0.188	1.818187e-05	24	NUDT21	GC IgA
1.036797e-09	0.3492401	0.427	0.255	1.848506e-05	24	ACO2	GC IgA
1.052084e-09	-0.5475581	0.121	0.242	1.87576e-05	24	HLA-F	GC IgA
1.123546e-09	0.3322466	0.257	0.12	2.00317e-05	24	TFEB	GC IgA
1.224942e-09	0.3357006	0.452	0.273	2.183949e-05	24	RNF7	GC IgA
1.267162e-09	0.3466197	0.706	0.506	2.259222e-05	24	MZT2B	GC IgA
1.307897e-09	0.3671042	0.458	0.273	2.331849e-05	24	SEPT2	GC IgA
1.396339e-09	0.3337252	0.325	0.165	2.489533e-05	24	DOCK8	GC IgA
1.423128e-09	0.3906047	0.337	0.186	2.537295e-05	24	SNRPA	GC IgA
1.427202e-09	-0.4747356	0.102	0.202	2.544559e-05	24	NPM3	GC IgA
1.478808e-09	0.3003109	0.266	0.115	2.636566e-05	24	LMNB1	GC IgA
1.512949e-09	0.3300343	0.226	0.103	2.697436e-05	24	SLC25A4	GC IgA
1.535035e-09	0.3570905	0.375	0.204	2.736814e-05	24	ARL2BP	GC IgA
1.54528e-09	0.3048725	0.347	0.176	2.75508e-05	24	RRAGA	GC IgA
1.553791e-09	0.3100935	0.619	0.4	2.770254e-05	24	UQCRFS1	GC IgA
1.71879e-09	-0.4662991	0.115	0.226	3.064431e-05	24	TMEM109	GC IgA

1.878893e-09	0.308973	0.201	0.082	3.349877e-05	24	GSTZ1	GC IgA
1.929387e-09	0.3171595	0.69	0.493	3.439904e-05	24	ARL6IP4	GC IgA
1.945977e-09	-0.315126	0.981	0.981	3.469483e-05	24	RPL5	GC IgA
1.986964e-09	0.3217488	0.56	0.358	3.542558e-05	24	H2AFV	GC IgA
2.019193e-09	0.3157416	0.223	0.099	3.600019e-05	24	RMDN1	GC IgA
2.088385e-09	0.3415058	0.576	0.378	3.723381e-05	24	POLR2G	GC IgA
2.106912e-09	0.3289081	0.223	0.094	3.756414e-05	24	STK40	GC IgA
2.132614e-09	-0.3467736	0.981	0.981	3.802238e-05	24	RPSA	GC IgA
2.202866e-09	0.3484825	0.322	0.171	3.927489e-05	24	CLTB	GC IgA
2.519311e-09	0.330746	0.421	0.253	4.49168e-05	24	ZCCHC10	GC IgA
3.065268e-09	-0.5669279	0.616	0.677	5.465066e-05	24	TAGLN2	GC IgA
3.263831e-09	0.3490187	0.291	0.139	5.819084e-05	24	CORO1B	GC IgA
3.270192e-09	-0.5934742	0.198	0.298	5.830425e-05	24	PDLIM1	GC IgA
3.383921e-09	-0.6067836	0.313	0.414	6.033193e-05	24	NPC2	GC IgA
3.388514e-09	0.3051069	0.167	0.055	6.041381e-05	24	TUBB2A	GC IgA
3.516687e-09	0.3103792	0.303	0.153	6.269901e-05	24	ATP6V1H	GC IgA
3.545546e-09	0.3292173	0.186	0.073	6.321355e-05	24	DOCK2	GC IgA
3.777959e-09	0.3579342	0.396	0.227	6.735723e-05	24	GTF2I	GC IgA
3.785987e-09	-0.5495774	0.223	0.349	6.750036e-05	24	LITAF	GC IgA
3.852259e-09	0.3638424	0.446	0.269	6.868192e-05	24	TOP1	GC IgA
3.91997e-09	0.3613531	0.502	0.322	6.988914e-05	24	MLF2	GC IgA
4.145434e-09	-0.6569914	0.582	0.648	7.390894e-05	24	ZFP36L1	GC IgA
4.168517e-09	0.3613381	0.418	0.242	7.432048e-05	24	GTF2H5	GC IgA
4.201106e-09	0.3195066	0.474	0.281	7.490153e-05	24	GGNBP2	GC IgA
4.236633e-09	0.3578809	0.226	0.113	7.553493e-05	24	PAX5	GC IgA

4.449e-09	0.3492745	0.31	0.151	7.932123e-05	24	CD72	GC IgA
4.56385e-09	0.3440396	0.347	0.189	8.136888e-05	24	MRPS11	GC IgA
4.664651e-09	0.3270427	0.319	0.172	8.316607e-05	24	MRPL37	GC IgA
4.677989e-09	0.3237708	0.31	0.152	8.340387e-05	24	MYL6B	GC IgA
5.025144e-09	-0.3649893	0.978	0.959	8.95933e-05	24	MT-CYB	GC IgA
5.249005e-09	0.3190471	0.167	0.069	9.358451e-05	24	UBAP1	GC IgA
5.600638e-09	0.3114931	0.746	0.554	9.985377e-05	24	SNU13	GC IgA
5.779395e-09	-0.4111007	0.985	0.977	0.0001030408	24	RPS10	GC IgA
6.408109e-09	0.3326361	0.372	0.212	0.0001142502	24	ACTR10	GC IgA
6.552092e-09	0.3218024	0.303	0.154	0.0001168173	24	GNB1	GC IgA
7.226809e-09	0.3202976	0.372	0.204	0.0001288468	24	OGA	GC IgA
7.314195e-09	0.3668345	0.384	0.226	0.0001304048	24	CYBC1	GC IgA
7.840703e-09	0.31598	0.195	0.086	0.0001397919	24	PPP3CB	GC IgA
7.86206e-09	0.3417494	0.334	0.196	0.0001401727	24	IDH2	GC IgA
8.396879e-09	0.3131437	0.22	0.108	0.000149708	24	NADK	GC IgA
9.608641e-09	0.3508098	0.341	0.191	0.0001713125	24	RFXANK	GC IgA
9.91016e-09	0.3242015	0.372	0.211	0.0001766882	24	NR3C1	GC IgA
1.006885e-08	0.3246747	0.523	0.326	0.0001795175	24	BST2	GC IgA
1.103069e-08	0.3412142	0.291	0.143	0.0001966662	24	FLOT1	GC IgA
1.158978e-08	0.3539833	0.805	0.646	0.0002066342	24	ZFAS1	GC IgA
1.244501e-08	0.30697	0.167	0.071	0.0002218821	24	USP6NL	GC IgA
1.291665e-08	0.3120642	0.17	0.073	0.000230291	24	FDX2	GC IgA
1.315016e-08	0.3021468	0.483	0.306	0.0002344542	24	SHKBP1	GC IgA
1.409976e-08	0.3290865	0.316	0.17	0.0002513846	24	EED	GC IgA
1.450602e-08	0.3016798	0.536	0.348	0.0002586278	24	TIMM13	GC IgA

1.703494e-08	-0.4492093	0.071	0.158	0.0003037159	24	TGIF1	GC IgA
1.7797e-08	-0.6917256	0.201	0.303	0.0003173027	24	ZFP36L2	GC IgA
2.017411e-08	0.3091139	0.415	0.243	0.0003596843	24	DCTN3	GC IgA
2.328053e-08	0.3127444	0.402	0.248	0.0004150686	24	THOC2	GC IgA
2.425819e-08	0.3846334	0.492	0.325	0.0004324993	24	MRPS36	GC IgA
2.583796e-08	0.3145407	0.573	0.384	0.000460665	24	UQCRC2	GC IgA
2.683234e-08	0.3136422	0.489	0.3	0.0004783937	24	CDK2AP2	GC IgA
2.716813e-08	0.3393789	0.449	0.283	0.0004843806	24	RTF2	GC IgA
2.736248e-08	0.3271925	0.356	0.203	0.0004878457	24	PXK	GC IgA
2.74539e-08	0.302237	0.474	0.302	0.0004894756	24	COPS6	GC IgA
2.773936e-08	0.3271957	0.337	0.182	0.0004945651	24	INPP5D	GC IgA
2.790122e-08	0.3011293	0.415	0.249	0.0004974508	24	COA3	GC IgA
2.828499e-08	-0.4172173	0.028	0.101	0.0005042932	24	RHOC	GC IgA
3.025649e-08	0.3092396	0.406	0.247	0.000539443	24	SNRPN	GC IgA
3.115135e-08	-0.6592678	0.477	0.53	0.0005553975	24	PSME2	GC IgA
3.696216e-08	0.3229252	0.248	0.133	0.0006589984	24	CIAPIN1	GC IgA
3.748866e-08	-0.4729918	0.071	0.158	0.0006683853	24	SLC2A3	GC IgA
4.003152e-08	0.3172932	0.372	0.205	0.0007137221	24	IKZF3	GC IgA
4.233127e-08	0.432822	0.498	0.274	0.0007547242	24	EIF1AY	GC IgA
4.822108e-08	0.3172055	0.279	0.144	0.0008597337	24	SUDS3	GC IgA
4.958151e-08	-0.3472581	0.969	0.976	0.0008839888	24	RPL36A	GC IgA
4.970268e-08	0.3260202	0.446	0.27	0.000886149	24	TXNL4A	GC IgA
5.117097e-08	0.3022848	0.412	0.246	0.0009123271	24	TRAPPC2L	GC IgA
6.578458e-08	0.3258256	0.291	0.16	0.001172873	24	DCPS	GC IgA
6.737584e-08	0.3097869	0.257	0.133	0.001201244	24	RAB9A	GC IgA

6.858285e-08	0.3086333	0.322	0.182	0.001222764	24	MAP2K2	GC IgA
8.784816e-08	-0.5097212	0.133	0.229	0.001566245	24	NT5C3A	GC IgA
1.08541e-07	0.3279962	0.551	0.368	0.001935177	24	FAM96B	GC IgA
1.110192e-07	-0.6482608	0.372	0.468	0.001979361	24	ITM2B	GC IgA
1.171353e-07	0.3021691	0.508	0.336	0.002088405	24	BUD31	GC IgA
1.194363e-07	-0.5997909	0.421	0.493	0.002129429	24	ANKRD12	GC IgA
1.202263e-07	0.3282666	0.52	0.357	0.002143515	24	TMEM243	GC IgA
1.328244e-07	-0.4384682	0.121	0.22	0.002368126	24	CCDC50	GC IgA
1.401124e-07	-0.4589158	0.087	0.167	0.002498063	24	CBX6	GC IgA
1.432526e-07	0.3170425	0.378	0.228	0.002554051	24	HIKESHI	GC IgA
1.4398e-07	-0.444288	0.105	0.185	0.00256702	24	FKBP4	GC IgA
1.521369e-07	0.3127293	0.334	0.177	0.002712448	24	PHYKPL	GC IgA
1.878182e-07	0.3043801	0.365	0.213	0.00334861	24	TIAL1	GC IgA
2.294445e-07	0.3324993	0.461	0.302	0.004090765	24	GLRX3	GC IgA
3.364035e-07	0.30074	0.325	0.181	0.005997739	24	NDUFS4	GC IgA
3.513791e-07	-0.7580136	0.446	0.5	0.006264738	24	SSR4	GC IgA
3.562314e-07	0.3193134	0.492	0.311	0.006351249	24	MZT2A	GC IgA
3.667913e-07	-0.3900596	0.402	0.174	0.006539523	24	MZB1	GC IgA
4.297108e-07	-0.4840464	0.173	0.252	0.007661315	24	DNPH1	GC IgA
5.048094e-07	-0.3955794	0.056	0.12	0.009000248	24	IL27RA	GC IgA
5.621108e-07	-0.4407041	0.966	0.963	0.01002187	24	FTL	GC IgA
5.869794e-07	0.3113223	0.192	0.1	0.01046526	24	SYNC	GC IgA
7.263017e-07	-0.666616	0.598	0.597	0.01294923	24	LDHA	GC IgA
8.017545e-07	-0.3637893	0.062	0.123	0.01429448	24	RAB13	GC IgA
8.067176e-07	-0.4172395	0.043	0.106	0.01438297	24	C1orf162	GC IgA

9.554596e-07	-0.4771725	0.158	0.232	0.01703489	24	HSD17B11	GC IgA
1.118009e-06	-0.580088	0.433	0.502	0.01993298	24	FOXP1	GC IgA
1.186325e-06	-0.5565798	0.251	0.327	0.02115099	24	SLC3A2	GC IgA
1.415985e-06	0.3247958	0.433	0.303	0.0252456	24	CHMP2A	GC IgA
1.446605e-06	-0.4404628	0.13	0.219	0.02579153	24	EIF4EBP1	GC IgA
1.844842e-06	-0.3181607	0.046	0.115	0.03289169	24	PYCR1	GC IgA
3.407891e-06	-0.3292282	0.056	0.116	0.06075929	24	ENTPD1	GC IgA
3.841795e-06	-0.4492655	0.74	0.753	0.06849537	24	TOMM7	GC IgA
4.27912e-06	-0.3540854	0.043	0.105	0.07629243	24	PDE4B	GC IgA
4.292365e-06	-0.4451354	0.604	0.635	0.07652858	24	GPX4	GC IgA
5.875571e-06	-0.4273367	0.214	0.298	0.1047555	24	RNASEH2B	GC IgA
6.371501e-06	0.3277878	0.155	0.066	0.1135975	24	RASGEF1B	GC IgA
7.239297e-06	-0.5633489	0.266	0.328	0.1290694	24	ZFP36	GC IgA
7.304019e-06	-0.4408152	0.102	0.161	0.1302234	24	DDIT4	GC IgA
7.719976e-06	0.3604	0.932	0.802	0.1376395	24	RPS26	GC IgA
8.017002e-06	-0.4058352	0.133	0.208	0.1429351	24	GNL3	GC IgA
8.871022e-06	-0.3053753	0.046	0.109	0.1581614	24	HEXB	GC IgA
9.085087e-06	-0.3569686	0.901	0.917	0.161978	24	PFDN5	GC IgA
9.687183e-06	-0.4443317	0.737	0.757	0.1727128	24	MT-ND5	GC IgA
1.449295e-05	-0.7556094	0.755	0.751	0.2583947	24	CXCR4	GC IgA
1.645529e-05	-0.426797	0.121	0.194	0.2933814	24	ADK	GC IgA
1.729528e-05	-0.4782629	0.297	0.337	0.3083575	24	CD48	GC IgA
1.787585e-05	-0.4636748	0.133	0.194	0.3187085	24	HSPB1	GC IgA
1.945427e-05	-0.4163681	0.235	0.307	0.3468501	24	TMEM147	GC IgA
2.525107e-05	-0.5179143	0.226	0.296	0.4502013	24	NFKBIA	GC IgA

2.766013e-05	-0.3760668	0.217	0.282	0.4931525	24	MT-ND4L	GC IgA
2.990361e-05	-0.3710108	0.099	0.16	0.5331515	24	AIDA	GC IgA
3.286966e-05	-0.3961558	0.087	0.137	0.5860332	24	FCER2	GC IgA
3.426947e-05	-0.7120848	0.263	0.313	0.6109903	24	HSP90B1	GC IgA
3.447255e-05	-0.3430454	0.084	0.15	0.6146111	24	CHD1	GC IgA
3.703216e-05	-0.4970116	0.585	0.62	0.6602464	24	IFI16	GC IgA
3.904244e-05	-0.3689549	0.214	0.294	0.6960876	24	ACADVL	GC IgA
3.91061e-05	-0.7385884	0.625	0.576	0.6972227	24	TUBB	GC IgA
3.928436e-05	-0.3016755	0.087	0.137	0.7004008	24	IFRD2	GC IgA
4.871345e-05	-0.4876358	0.402	0.453	0.8685121	24	ARL6IP1	GC IgA
4.942373e-05	-0.4366028	0.136	0.204	0.8811757	24	IRF1	GC IgA
5.362302e-05	-0.4091996	0.186	0.252	0.9560448	24	NFE2L2	GC IgA
5.441426e-05	-0.4077507	0.313	0.384	0.9701519	24	LAPTM4A	GC IgA
5.796205e-05	-0.3380312	0.062	0.112	1	24	SESN3	GC IgA
6.855075e-05	0.3447941	0.341	0.205	1	24	LGALS1	GC IgA
6.916389e-05	-0.4338272	0.412	0.45	1	24	DRAP1	GC IgA
7.078419e-05	-0.3737447	0.167	0.232	1	24	TARS	GC IgA
7.942476e-05	-0.344514	0.099	0.15	1	24	ESYT1	GC IgA
8.08072e-05	-0.4035699	0.152	0.213	1	24	APPL1	GC IgA
8.525976e-05	-0.3788231	0.873	0.854	1	24	SH3BGRL3	GC IgA
8.716153e-05	-0.3695963	0.808	0.751	1	24	HLA-C	GC IgA
9.371417e-05	-0.5435197	0.573	0.3	1	24	HMGB2	GC IgA
0.0001045074	-0.3299971	0.068	0.123	1	24	RNF213	GC IgA
0.0001069038	-0.5826475	0.093	0.131	1	24	MYC	GC IgA
0.0001315408	-0.3652682	0.142	0.197	1	24	TYMP	GC IgA

0.000155123 -0.3383865 0.084 0.134 1 24
ODF2L GC IgA
0.0001608001 -0.4561466 0.402 0.438 1 24
IL2RG GC IgA
0.0001626065 -0.3164355 0.077 0.121 1 24
LACTB GC IgA
0.0001640013 -0.3906443 0.158 0.208 1 24
PIM3 GC IgA
0.0002362214 -0.3199642 0.065 0.117 1 24
SAMD9 GC IgA
0.0002557112 -0.3027393 0.149 0.204 1 24
NDFIP1 GC IgA
0.0002730326 -0.4771691 0.17 0.212 1 24 LBH
GC IgA
0.0002785637 -3.086757 0.402 0.451 1 24
IGHG3 GC IgA
0.0002967966 -0.3802926 0.303 0.331 1 24
TSTD1 GC IgA
0.0003004546 -0.3513117 0.121 0.163 1 24
CD58 GC IgA
0.000336908 -0.3317484 0.084 0.137 1 24
PSAT1 GC IgA
0.0003636213 -0.3622607 0.508 0.533 1 24
IMPDH2 GC IgA
0.000442537 -0.3226756 0.235 0.291 1 24
PYURF GC IgA
0.00045891 -0.3331777 0.127 0.172 1 24
TRIM44 GC IgA
0.0005298926 -0.3852314 0.204 0.246 1 24
CKS1B GC IgA
0.000676496 -0.4004834 0.291 0.336 1 24
S100A11 GC IgA
0.000778519 -0.3135402 0.127 0.171 1 24
PARP14 GC IgA
0.0008141565 -0.3515619 0.288 0.325 1 24
AKR1B1 GC IgA
0.0009550802 -0.4922281 0.489 0.485 1 24
RANBP1 GC IgA
0.001145394 -0.4005041 0.241 0.281 1 24
SMCHD1 GC IgA
0.001215868 -0.311059 0.152 0.204 1 24
PTPN1 GC IgA
0.001367181 -0.3604213 0.161 0.189 1 24
IFNGR1 GC IgA
0.001429991 -0.333354 0.238 0.279 1 24
WARS GC IgA
0.0014398 -0.4377422 0.477 0.494 1 24
SP100 GC IgA
0.001477798 -0.3002266 0.201 0.247 1 24
PABPC4 GC IgA

0.001621538	−0.338069	0.709	0.714	1	24
PSME1	GC IgA				
0.001648381	−0.4011238	0.387	0.393	1	24
TMED2	GC IgA				
0.00174315	−0.3086538	0.115	0.148	1	24
ARRDC2	GC IgA				
0.001822105	−0.3118494	0.139	0.187	1	24
KIF20B	GC IgA				
0.001908151	−0.3105045	0.189	0.235	1	24
EIF4A1	GC IgA				
0.002177464	−0.5230632	0.461	0.472	1	24
CD83	GC IgA				
0.002379407	−0.3289925	0.176	0.211	1	24
FMNL1	GC IgA				
0.002690149	−2.066047	0.17	0.244	1	24
IGHG4	GC IgA				
0.002804636	−0.3993045	0.294	0.336	1	24
APOBEC3C	GC IgA				
0.004288789	−0.4097692	0.889	0.868	1	24
CD52	GC IgA				
0.004394376	−0.3677032	0.09	0.126	1	24
LGALS3	GC IgA				
0.004631871	−0.4754515	0.356	0.36	1	24
ARID5B	GC IgA				
0.004658641	−0.3074322	0.279	0.296	1	24
PDCD5	GC IgA				
0.005408635	−0.350939	0.167	0.188	1	24
PCNA	GC IgA				
0.006432337	−0.3470623	0.836	0.815	1	24
NOP53	GC IgA				
0.006926568	−0.3393764	0.604	0.599	1	24
CALM1	GC IgA				
0.006959726	−0.337919	0.331	0.342	1	24
PKIG	GC IgA				
0.007191506	−0.7030806	0.588	0.421	1	24
HIST1H4C	GC IgA				
0.007683048	−0.3041836	0.077	0.112	1	24
C1orf56	GC IgA				
0.007840741	−0.3168369	0.167	0.208	1	24
CCDC32	GC IgA				
0.008164896	−0.3192911	0.167	0.199	1	24
PPP3CA	GC IgA				
0.009001075	−0.3064014	0.276	0.285	1	24
CNPY3	GC IgA				
0.009231296	−0.3073085	0.254	0.276	1	24
GLO1	GC IgA				
