# Supplementary material for: Distinct stage-specific transcriptional states of B cells derived from human tonsillar tissue
Source: JCI Insight. 2023 Apr 10;8(7):e155199. doi: 10.1172/jci.insight.155199 (PMC10132144; doi:10.1172/jci.insight.155199)
Supplement: Supplemental table 5 [file jciinsight-8-155199-s230.pdf]

| Donor Code | Sex | Age |
| --- | --- | --- |
| Donor 1 | Male | 33 |
| Donor 2 | Female | 33 |
| Donor 3 | Male | 30 |
