# Supplementary material for: Distinct stage-specific transcriptional states of B cells derived from human tonsillar tissue
Source: JCI Insight. 2023 Apr 10;8(7):e155199. doi: 10.1172/jci.insight.155199 (PMC10132144; doi:10.1172/jci.insight.155199)
Supplement: Supplemental table 6 [file jciinsight-8-155199-s231.pdf]

gene_name	set
CNTN1	CD40L_up_only
PRDM11	CD40L_up_only
TBPL1	CD40L_up_only
PIAS1	CD40L_up_only
ARID1B	CD40L_up_only
MSH4	CD40L_up_only
STAG3	CD40L_up_only
TP53BP1	CD40L_up_only
FGFR3	CD40L_up_only
MEF2A	CD40L_up_only
RAB27A	CD40L_up_only
SPTB	CD40L_up_only
SLC44A1	CD40L_up_only
RPS6KA2	CD40L_up_only
PICALM	CD40L_up_only
MARK3	CD40L_up_only
BAZ2A	CD40L_up_only
ICAM3	CD40L_up_only
TULP3	CD40L_up_only
RBFOX1	CD40L_up_only
DLGAP4	CD40L_up_only
TCF7	CD40L_up_only
COL19A1	CD40L_up_only
ZNF264	CD40L_up_only
NCOA1	CD40L_up_only
RAD54L	CD40L_up_only
PIGS	CD40L_up_only
CPXM1	CD40L_up_only
SNAP23	CD40L_up_only
CYP26A1	CD40L_up_only
JAK2	CD40L_up_only
CRYBB2P1	CD40L_up_only
DCAF11	CD40L_up_only
HCK	CD40L_up_only
SMAD7	CD40L_up_only
KLF5	CD40L_up_only
SYT17	CD40L_up_only
HERC1	CD40L_up_only
CD276	CD40L_up_only
DMXL2	CD40L_up_only
IKBKB	CD40L_up_only
FCER2	CD40L_up_only
PLIN3	CD40L_up_only
CCL24	CD40L_up_only
FKTN	CD40L_up_only
PALD1	CD40L_up_only
PPP3CB	CD40L_up_only
MED13	CD40L_up_only
COL1A1	CD40L_up_only

SGCA	CD40L_up_only
GALNT7	CD40L_up_only
NRXN2	CD40L_up_only
CD69	CD40L_up_only
KCNA1	CD40L_up_only
ALDH2	CD40L_up_only
STX2	CD40L_up_only
WWC1	CD40L_up_only
C3orf52	CD40L_up_only
RNF19B	CD40L_up_only
MYCL	CD40L_up_only
TNFSF4	CD40L_up_only
NSL1	CD40L_up_only
STX12	CD40L_up_only
SLC5A9	CD40L_up_only
MYB	CD40L_up_only
WDR35	CD40L_up_only
KLHL29	CD40L_up_only
ATAD2B	CD40L_up_only
PLEKHG1	CD40L_up_only
ENOX1	CD40L_up_only
NACA3P	CD40L_up_only
TMEM131L	CD40L_up_only
ECHDC2	CD40L_up_only
A1BG	CD40L_up_only
DPPA4	CD40L_up_only
ADGRB2	CD40L_up_only
PIK3CA	CD40L_up_only
MTERF4	CD40L_up_only
KIAA1191	CD40L_up_only
MED13L	CD40L_up_only
MED20	CD40L_up_only
TTF1	CD40L_up_only
SH2D3A	CD40L_up_only
KDM5C	CD40L_up_only
ST3GAL3	CD40L_up_only
ASL	CD40L_up_only
SPECC1	CD40L_up_only
SMO	CD40L_up_only
TPH1	CD40L_up_only
ZNF337	CD40L_up_only
ABCB7	CD40L_up_only
PIAS3	CD40L_up_only
CLUHP3	CD40L_up_only
FLOT2	CD40L_up_only
FAM83F	CD40L_up_only
RRAS2	CD40L_up_only
KATNBL1	CD40L_up_only
DOCK2	CD40L_up_only
DDB2	CD40L_up_only

KCNH3	CD40L_up_only
NHSL1	CD40L_up_only
FBXL8	CD40L_up_only
TM6SF1	CD40L_up_only
TANK	CD40L_up_only
KIF13A	CD40L_up_only
FCHSD2	CD40L_up_only
PRCP	CD40L_up_only
FXYD2	CD40L_up_only
KYAT3	CD40L_up_only
TMOD3	CD40L_up_only
BBS7	CD40L_up_only
CCDC65	CD40L_up_only
TCF12	CD40L_up_only
ZFHX3	CD40L_up_only
CTTNBP2NL	CD40L_up_only
PI4KB	CD40L_up_only
FANCD2	CD40L_up_only
GRIP2	CD40L_up_only
ZNF660	CD40L_up_only
NXPE3	CD40L_up_only
OTULINL	CD40L_up_only
GNA12	CD40L_up_only
TMEM168	CD40L_up_only
ZCCHC7	CD40L_up_only
PROSER2	CD40L_up_only
CCDC82	CD40L_up_only
PLCB3	CD40L_up_only
ADAM8	CD40L_up_only
BICD1	CD40L_up_only
RABGAP1L	CD40L_up_only
ATG10	CD40L_up_only
CAMK4	CD40L_up_only
RASGRP3	CD40L_up_only
STK32B	CD40L_up_only
CHST9	CD40L_up_only
JPH3	CD40L_up_only
LRGUK	CD40L_up_only
BACH1	CD40L_up_only
SAMD8	CD40L_up_only
ST3GAL2	CD40L_up_only
APPL1	CD40L_up_only
CCDC24	CD40L_up_only
RSPRY1	CD40L_up_only
TCAF2P1	CD40L_up_only
ICOSLG	CD40L_up_only
CFAP410	CD40L_up_only
PMF1	CD40L_up_only
IER2	CD40L_up_only
VCAM1	CD40L_up_only

SLC30A7	CD40L_up_only
VANGL2	CD40L_up_only
CAPN2	CD40L_up_only
ARHGAP25	CD40L_up_only
DNAJC5G	CD40L_up_only
MST1R	CD40L_up_only
IL15	CD40L_up_only
CMYA5	CD40L_up_only
ACSL6	CD40L_up_only
USP49	CD40L_up_only
TAGAP	CD40L_up_only
NOS3	CD40L_up_only
MYORG	CD40L_up_only
ANKS6	CD40L_up_only
CKB	CD40L_up_only
PLEKHF1	CD40L_up_only
MSS51	CD40L_up_only
RNF169	CD40L_up_only
MAPK7	CD40L_up_only
NDST2	CD40L_up_only
ZSCAN21	CD40L_up_only
NOD2	CD40L_up_only
SNX20	CD40L_up_only
GNG8	CD40L_up_only
DHRS13	CD40L_up_only
SLC43A2	CD40L_up_only
RILP	CD40L_up_only
SDHAF2	CD40L_up_only
PKIG	CD40L_up_only
CXXC4	CD40L_up_only
PARM1	CD40L_up_only
PTPN9	CD40L_up_only
ROBO1	CD40L_up_only
TCAF2	CD40L_up_only
DCLK2	CD40L_up_only
SPATA24	CD40L_up_only
PAQR8	CD40L_up_only
ATF7IP	CD40L_up_only
MOB3A	CD40L_up_only
TMCC1	CD40L_up_only
IQCK	CD40L_up_only
CEP135	CD40L_up_only
TRAF6	CD40L_up_only
TP53I11	CD40L_up_only
KCNE5	CD40L_up_only
SYNE3	CD40L_up_only
ANO6	CD40L_up_only
OR2T35	CD40L_up_only
HIC1	CD40L_up_only
TBL1XR1	CD40L_up_only

JUN	CD40L_up_only
CASC2	CD40L_up_only
PARP10	CD40L_up_only
GPC5	CD40L_up_only
PIK3CD-AS1	CD40L_up_only
TIGD2	CD40L_up_only
MAP6D1	CD40L_up_only
PJA1	CD40L_up_only
DHTKD1	CD40L_up_only
PHLDA2	CD40L_up_only
IBA57	CD40L_up_only
TMEM30B	CD40L_up_only
ALG12	CD40L_up_only
RGS7	CD40L_up_only
LINC01547	CD40L_up_only
LHFPL6	CD40L_up_only
MAATS1	CD40L_up_only
MED12	CD40L_up_only
NUTM2A	CD40L_up_only
RAD51D	CD40L_up_only
SPATA12	CD40L_up_only
PPARA	CD40L_up_only
TSPYL4	CD40L_up_only
TCTEX1D4	CD40L_up_only
HBA2	CD40L_up_only
CFAP54	CD40L_up_only
BNIP5	CD40L_up_only
TDRD7	CD40L_up_only
OR8A1	CD40L_up_only
SEMA4A	CD40L_up_only
LCOR	CD40L_up_only
ZNF140	CD40L_up_only
MRTFA	CD40L_up_only
OR2T4	CD40L_up_only
FBXL22	CD40L_up_only
TXNRD3	CD40L_up_only
ADAMTSL2	CD40L_up_only
ZNF71	CD40L_up_only
HYLS1	CD40L_up_only
ZXDB	CD40L_up_only
SMURF1	CD40L_up_only
L1CAM	CD40L_up_only
RNU5F-1	CD40L_up_only
RNU1-87P	CD40L_up_only
RNY1P11	CD40L_up_only
RNU6-302P	CD40L_up_only
SNHG5	CD40L_up_only
SYS1	CD40L_up_only
HLA-G	CD40L_up_only
INSYN2B	CD40L_up_only

TCTN1	CD40L_up_only
ACOT6	CD40L_up_only
HACD2	CD40L_up_only
MIR181B2	CD40L_up_only
IGKC	CD40L_up_only
IGKV4-1	CD40L_up_only
IGLV1-51	CD40L_up_only
IGLV1-47	CD40L_up_only
IGLV7-46	CD40L_up_only
IGLV1-44	CD40L_up_only
IGLV1-40	CD40L_up_only
IGLV3-25	CD40L_up_only
IGLV2-23	CD40L_up_only
IGLV3-19	CD40L_up_only
IGLV3-1	CD40L_up_only
IGLJ1	CD40L_up_only
IGLC3	CD40L_up_only
IGHE	CD40L_up_only
PDCL3P5	CD40L_up_only
SUPT4H1	CD40L_up_only
RPL7AP50	CD40L_up_only
GNL3LP1	CD40L_up_only
RAET1K	CD40L_up_only
ZGLP1	CD40L_up_only
LINC01465	CD40L_up_only
PGAM1P6	CD40L_up_only
TNPO1P1	CD40L_up_only
NUTM2B-AS1	CD40L_up_only
KCNMA1-AS3	CD40L_up_only
MROCKI	CD40L_up_only
EEF1A1P11	CD40L_up_only
ARL17B	CD40L_up_only
LINC02768	CD40L_up_only
LINC01362	CD40L_up_only
HNRNPA1P68	CD40L_up_only
LINC01712	CD40L_up_only
PRDX3P2	CD40L_up_only
TRAF3IP2-AS1	CD40L_up_only
C4BPAP2	CD40L_up_only
ATP11A-AS1	CD40L_up_only
TNF	CD40L_up_only
HNRNPA1P49	CD40L_up_only
MAP3K5-AS1	CD40L_up_only
USP27X-AS1	CD40L_up_only
ERICH3-AS1	CD40L_up_only
SETP9	CD40L_up_only
C12orf75	CD40L_up_only
LINC01055	CD40L_up_only
ASH1L-AS1	CD40L_up_only
CHCHD4P3	CD40L_up_only

TMSB4XP1	CD40L_up_only
NONOP2	CD40L_up_only
ZEB2-AS1	CD40L_up_only
RNU7-45P	CD40L_up_only
IGKV1-17	CD40L_up_only
RPS6P25	CD40L_up_only
RN7SL328P	CD40L_up_only
RPL36A	CD40L_up_only
IGKV1-33	CD40L_up_only
LINC00971	CD40L_up_only
RPSAP26	CD40L_up_only
APOBEC3C	CD40L_up_only
LINC02709	CD40L_up_only
FRG1-DT	CD40L_up_only
LRP4-AS1	CD40L_up_only
LINC01596	CD40L_up_only
CTBP2P4	CD40L_up_only
LINC02615	CD40L_up_only
IGKV1D-39	CD40L_up_only
RNU6-367P	CD40L_up_only
IGLL5	CD40L_up_only
SYS1-DBNDD2	CD40L_up_only
LINC00273	CD40L_up_only
DENND11	CD40L_up_only
MGAM	CD40L_up_only
SPESP1	CD40L_up_only
LINC02316	CD40L_up_only
LINC02490	CD40L_up_only
SPDYE6	CD40L_up_only
FBXL19-AS1	CD40L_up_only
ADAMTS7P3	CD40L_up_only
LINC01686	CD40L_up_only
IKBKE	CD40L_up_only
DLGAP1-AS4	CD40L_up_only
MIR4298	CD40L_up_only
RN7SL405P	CD40L_up_only
MIR4540	CD40L_up_only
TSPOAP1-AS1	CD40L_up_only
MIR4476	CD40L_up_only
LINC02132	CD40L_up_only
SNORD11B	CD40L_up_only
LINC01215	CD40L_up_only
RNA5SP108	CD40L_up_only
RN7SL726P	CD40L_up_only
BMS1P14	CD40L_up_only
RN7SL575P	CD40L_up_only
ARRDC3-AS1	CD40L_up_only
PRR33	CD40L_up_only
TPTEP2-CSNK1E	CD40L_up_only
FUCA2	BCR_up_only

LASP1	BCR_up_only
MATK	BCR_up_only
RHBDF1	BCR_up_only
RHOBTB2	BCR_up_only
CD22	BCR_up_only
XYLT2	BCR_up_only
AKAP11	BCR_up_only
SH2D2A	BCR_up_only
POU2F2	BCR_up_only
ATP6V0A1	BCR_up_only
THAP3	BCR_up_only
TNFRSF17	BCR_up_only
USP13	BCR_up_only
ANKS1A	BCR_up_only
NTHL1	BCR_up_only
KDM4A	BCR_up_only
SYT1	BCR_up_only
GBA2	BCR_up_only
NDST1	BCR_up_only
LMCD1	BCR_up_only
PTPN18	BCR_up_only
CIC	BCR_up_only
TDRD3	BCR_up_only
NOA1	BCR_up_only
ZNF213	BCR_up_only
GNAS	BCR_up_only
ERP29	BCR_up_only
NAT14	BCR_up_only
WHRN	BCR_up_only
NEFH	BCR_up_only
KIAA0930	BCR_up_only
IL2RB	BCR_up_only
PYGB	BCR_up_only
ABHD12	BCR_up_only
CTSZ	BCR_up_only
UBL4A	BCR_up_only
ACP5	BCR_up_only
HCFC1R1	BCR_up_only
MPG	BCR_up_only
NME4	BCR_up_only
METRN	BCR_up_only
CLUAP1	BCR_up_only
HOMER2	BCR_up_only
CCDC61	BCR_up_only
DNASE2	BCR_up_only
USF2	BCR_up_only
FKBP8	BCR_up_only
ERF	BCR_up_only
PIK3CG	BCR_up_only
PLEKHA8	BCR_up_only

IMPDH1	BCR_up_only
RPA3	BCR_up_only
ALDOC	BCR_up_only
CCDC34	BCR_up_only
KRT18	BCR_up_only
ACAD10	BCR_up_only
OAS2	BCR_up_only
RASAL1	BCR_up_only
ENO2	BCR_up_only
PTPN6	BCR_up_only
MAN1A1	BCR_up_only
RNF130	BCR_up_only
DBN1	BCR_up_only
ACTR8	BCR_up_only
BCL6	BCR_up_only
RAP1A	BCR_up_only
CD48	BCR_up_only
ID3	BCR_up_only
LRMP	BCR_up_only
PHF19	BCR_up_only
KDSR	BCR_up_only
LGALSL	BCR_up_only
TRIM25	BCR_up_only
CRY2	BCR_up_only
SEPTIN7	BCR_up_only
AGO2	BCR_up_only
USP22	BCR_up_only
DEK	BCR_up_only
MBOAT7	BCR_up_only
AHDC1	BCR_up_only
RAC2	BCR_up_only
THEM6	BCR_up_only
ANGPTL6	BCR_up_only
NFATC1	BCR_up_only
KCNC3	BCR_up_only
MGAT1	BCR_up_only
NDFIP1	BCR_up_only
C19orf57	BCR_up_only
FCRLA	BCR_up_only
TBC1D14	BCR_up_only
PRMT7	BCR_up_only
HMGB1P5	BCR_up_only
CD180	BCR_up_only
GSTM1	BCR_up_only
TAOK3	BCR_up_only
SPRY2	BCR_up_only
TTYH3	BCR_up_only
SLC31A1	BCR_up_only
ATP6V1G1	BCR_up_only
HINT2	BCR_up_only

ZSCAN9	BCR_up_only
MYOF	BCR_up_only
SLC39A8	BCR_up_only
RAB20	BCR_up_only
ITGAX	BCR_up_only
ZCCHC14	BCR_up_only
RANBP10	BCR_up_only
EVI5L	BCR_up_only
SH3BGRL3	BCR_up_only
LMO4	BCR_up_only
CD53	BCR_up_only
FCRL5	BCR_up_only
OCIAD2	BCR_up_only
ANKRD31	BCR_up_only
FAM50B	BCR_up_only
DOK3	BCR_up_only
CRIP3	BCR_up_only
PLPP5	BCR_up_only
ADM	BCR_up_only
ENDOD1	BCR_up_only
SLX4IP	BCR_up_only
GRIK4	BCR_up_only
NUDT22	BCR_up_only
QDPR	BCR_up_only
RNF144A	BCR_up_only
RMND5A	BCR_up_only
ENAH	BCR_up_only
PIK3AP1	BCR_up_only
CCNB2	BCR_up_only
H2BC5	BCR_up_only
CD1C	BCR_up_only
SELENON	BCR_up_only
GFI1	BCR_up_only
H3−3A	BCR_up_only
KBTBD8	BCR_up_only
IFI16	BCR_up_only
MTMR14	BCR_up_only
FYCO1	BCR_up_only
TEX264	BCR_up_only
DUSP7	BCR_up_only
GFM2	BCR_up_only
PEX2	BCR_up_only
HECTD2	BCR_up_only
SKA3	BCR_up_only
ARF6	BCR_up_only
PIP4P1	BCR_up_only
TPP1	BCR_up_only
ANKRD11	BCR_up_only
KLK1	BCR_up_only
KLK4	BCR_up_only

VWCE	BCR_up_only
PNOC	BCR_up_only
BMI1	BCR_up_only
SLC33A1	BCR_up_only
TM2D2	BCR_up_only
IRF2BP1	BCR_up_only
RNF139	BCR_up_only
MFN1	BCR_up_only
TADA3	BCR_up_only
GAA	BCR_up_only
LDHAL6B	BCR_up_only
QARS1	BCR_up_only
CXCR6	BCR_up_only
CERS6	BCR_up_only
VEGFB	BCR_up_only
DDIT3	BCR_up_only
LONRF3	BCR_up_only
RPS6KB2	BCR_up_only
MTHFR	BCR_up_only
DEAF1	BCR_up_only
HASPIN	BCR_up_only
GBA	BCR_up_only
CD151	BCR_up_only
DMAP1	BCR_up_only
COX14	BCR_up_only
DPM3	BCR_up_only
MAGED1	BCR_up_only
SEPHS2	BCR_up_only
H2BC4	BCR_up_only
D2HGDH	BCR_up_only
IDH2	BCR_up_only
PAPPA	BCR_up_only
BEGAIN	BCR_up_only
ZNRF3	BCR_up_only
CLECL1	BCR_up_only
KNTC1	BCR_up_only
RNLS	BCR_up_only
BRI3BP	BCR_up_only
IFNLR1	BCR_up_only
ROR1	BCR_up_only
ARHGAP30	BCR_up_only
GCNT1	BCR_up_only
TRPV2	BCR_up_only
FOCAD	BCR_up_only
HMGB1	BCR_up_only
TECPR2	BCR_up_only
GM2A	BCR_up_only
SERTAD1	BCR_up_only
ZNF398	BCR_up_only
H4C11	BCR_up_only

SERPINA1	BCR_up_only
PELI1	BCR_up_only
MPEG1	BCR_up_only
CARD11	BCR_up_only
ZNF627	BCR_up_only
F5	BCR_up_only
BHLHB9	BCR_up_only
ARMCX6	BCR_up_only
RNU6-212P	BCR_up_only
RNU6-979P	BCR_up_only
MDP1	BCR_up_only
CPNE1	BCR_up_only
BCAR3-AS1	BCR_up_only
LINC01567	BCR_up_only
KCNQ5-AS1	BCR_up_only
KNOP1P4	BCR_up_only
GPX1	BCR_up_only
RNASEH1-AS1	BCR_up_only
NFS1	BCR_up_only
RN7SL211P	BCR_up_only
NEAT1	BCR_up_only
FAM86EP	BCR_up_only
MIR2116	BCR_up_only
SOX5-AS1	BCR_up_only
CENPUP2	BCR_up_only
NSFP1	BCR_up_only
DYNLL2	BCR_up_only
LINC01480	BCR_up_only
PHACTR2P1	BCR_up_only
H2AC14	BCR_up_only
GLIDR	BCR_up_only
DPM1	COSTIM_up
GCLC	COSTIM_up
NFYA	COSTIM_up
LAS1L	COSTIM_up
ENPP4	COSTIM_up
ANKIB1	COSTIM_up
CYP51A1	COSTIM_up
LAP3	COSTIM_up
MAD1L1	COSTIM_up
SNX11	COSTIM_up
M6PR	COSTIM_up
ALS2	COSTIM_up
CFLAR	COSTIM_up
ARF5	COSTIM_up
POLDIP2	COSTIM_up
AK2	COSTIM_up
FKBP4	COSTIM_up
KDM1A	COSTIM_up
RECQL	COSTIM_up

NDUFAB1	COSTIM_up
ARX	COSTIM_up
SLC25A13	COSTIM_up
ST7	COSTIM_up
HCCS	COSTIM_up
DVL2	COSTIM_up
UPF1	COSTIM_up
SLC25A5	COSTIM_up
POLR2J	COSTIM_up
DHX33	COSTIM_up
LIG3	COSTIM_up
RPAP3	COSTIM_up
CIAPIN1	COSTIM_up
GCFC2	COSTIM_up
IBTK	COSTIM_up
MYCBP2	COSTIM_up
LAMP2	COSTIM_up
REX1BD	COSTIM_up
CRLF1	COSTIM_up
AP2B1	COSTIM_up
ZNF263	COSTIM_up
RALA	COSTIM_up
BAIAP2L1	COSTIM_up
AGK	COSTIM_up
GGCT	COSTIM_up
DBF4	COSTIM_up
IFRD1	COSTIM_up
COX10	COSTIM_up
GTF2IRD1	COSTIM_up
PAF1	COSTIM_up
ELAC2	COSTIM_up
PNPLA4	COSTIM_up
ADIPOR2	COSTIM_up
PRSS21	COSTIM_up
CCDC124	COSTIM_up
PAFAH1B1	COSTIM_up
KIAA0100	COSTIM_up
NA	COSTIM_up
RPUSD1	COSTIM_up
TSR3	COSTIM_up
PIGQ	COSTIM_up
TEAD3	COSTIM_up
DNAJC11	COSTIM_up
PSMB1	COSTIM_up
JARID2	COSTIM_up
NADK	COSTIM_up
CYB561	COSTIM_up
SPAG9	COSTIM_up
CELSR3	COSTIM_up
SS18L2	COSTIM_up

MPND	COSTIM_up
CRY1	COSTIM_up
NFIX	COSTIM_up
IL32	COSTIM_up
PKD1	COSTIM_up
MED24	COSTIM_up
RPS20	COSTIM_up
CSDE1	COSTIM_up
UBE3C	COSTIM_up
VTA1	COSTIM_up
BAZ1B	COSTIM_up
RANBP9	COSTIM_up
SPRTN	COSTIM_up
EEF1AKNMT	COSTIM_up
ZNF207	COSTIM_up
UQCRC1	COSTIM_up
STARD3NL	COSTIM_up
SCMH1	COSTIM_up
ELOA	COSTIM_up
LYPLA2	COSTIM_up
CLCN6	COSTIM_up
MRC2	COSTIM_up
NME1-NME2	COSTIM_up
BTBD7	COSTIM_up
APBA3	COSTIM_up
ABHD5	COSTIM_up
UTP18	COSTIM_up
PTBP1	COSTIM_up
LARS2	COSTIM_up
WIZ	COSTIM_up
PPP5C	COSTIM_up
ZBTB32	COSTIM_up
ERCC1	COSTIM_up
MBTPS2	COSTIM_up
PRICKLE3	COSTIM_up
MAP4K5	COSTIM_up
PSMC4	COSTIM_up
SLC25A39	COSTIM_up
MVP	COSTIM_up
POLR3B	COSTIM_up
RNF14	COSTIM_up
DNASE1L1	COSTIM_up
DDX11	COSTIM_up
POLA2	COSTIM_up
ZC3H3	COSTIM_up
MDH1	COSTIM_up
SLC30A9	COSTIM_up
ZMYND11	COSTIM_up
BID	COSTIM_up
MATR3	COSTIM_up

NUDCD3	COSTIM_up
GLT8D1	COSTIM_up
ATP2C1	COSTIM_up
IGF1	COSTIM_up
SLC38A5	COSTIM_up
RALBP1	COSTIM_up
RUFY3	COSTIM_up
AGPS	COSTIM_up
CXorf56	COSTIM_up
TTC27	COSTIM_up
CD74	COSTIM_up
NCDN	COSTIM_up
ZFP64	COSTIM_up
MNAT1	COSTIM_up
RUNX3	COSTIM_up
AQR	COSTIM_up
RTF2	COSTIM_up
GABRA1	COSTIM_up
RNF10	COSTIM_up
RNH1	COSTIM_up
NDUFS1	COSTIM_up
ALAS1	COSTIM_up
BIRC3	COSTIM_up
GLRX2	COSTIM_up
SNAPC1	COSTIM_up
STRAP	COSTIM_up
GCLM	COSTIM_up
RRAGD	COSTIM_up
HSF2	COSTIM_up
PHF20	COSTIM_up
TYMP	COSTIM_up
NCAPH2	COSTIM_up
TOMM34	COSTIM_up
KPNA6	COSTIM_up
VIM	COSTIM_up
RTEL1-TNFRSF6B	COSTIM_up
FAS	COSTIM_up
CD44	COSTIM_up
SLAMF7	COSTIM_up
MIPEP	COSTIM_up
IFNGR1	COSTIM_up
B4GALT7	COSTIM_up
VRK2	COSTIM_up
TNFRSF1B	COSTIM_up
VEZT	COSTIM_up
BRD9	COSTIM_up
SNX1	COSTIM_up
ARNTL2	COSTIM_up
BCLAF1	COSTIM_up
SLC39A9	COSTIM_up

BAK1	COSTIM_up
GRN	COSTIM_up
ARHGAP31	COSTIM_up
SARS1	COSTIM_up
RANBP3	COSTIM_up
EIPR1	COSTIM_up
ALG1	COSTIM_up
ABCF2	COSTIM_up
CHPF2	COSTIM_up
UBA6	COSTIM_up
MAP2K3	COSTIM_up
GABARAPL2	COSTIM_up
FAM136A	COSTIM_up
NSMAF	COSTIM_up
RPL26L1	COSTIM_up
NSUN2	COSTIM_up
METTL1	COSTIM_up
PI4K2B	COSTIM_up
EDC4	COSTIM_up
TRIO	COSTIM_up
CLEC16A	COSTIM_up
MTREX	COSTIM_up
PHLPP2	COSTIM_up
SPDL1	COSTIM_up
CTNS	COSTIM_up
PHF23	COSTIM_up
INPP4A	COSTIM_up
PSMA4	COSTIM_up
LSG1	COSTIM_up
TDP1	COSTIM_up
AIFM2	COSTIM_up
AP2S1	COSTIM_up
ZPBP	COSTIM_up
DCUN1D1	COSTIM_up
JADE2	COSTIM_up
CUL7	COSTIM_up
HSPA5	COSTIM_up
GEMIN8	COSTIM_up
ATP6V1H	COSTIM_up
POLR2B	COSTIM_up
TPR	COSTIM_up
DTNBP1	COSTIM_up
MAP4	COSTIM_up
HDAC9	COSTIM_up
NOP16	COSTIM_up
ZNF800	COSTIM_up
MRPS10	COSTIM_up
CELF2	COSTIM_up
FAM120A	COSTIM_up
R3HDM1	COSTIM_up

ERCC8	COSTIM_up
PER3	COSTIM_up
TNFRSF9	COSTIM_up
RCN1	COSTIM_up
RFC2	COSTIM_up
CLPTM1L	COSTIM_up
NEDD4L	COSTIM_up
PTCD2	COSTIM_up
MCUR1	COSTIM_up
HERPUD1	COSTIM_up
HEBP2	COSTIM_up
MPHOSPH9	COSTIM_up
SIKE1	COSTIM_up
RRP12	COSTIM_up
MSMO1	COSTIM_up
MRTO4	COSTIM_up
USE1	COSTIM_up
TRAPPC3	COSTIM_up
THRAP3	COSTIM_up
PHPT1	COSTIM_up
LY75	COSTIM_up
CHRDL2	COSTIM_up
RELT	COSTIM_up
NOP58	COSTIM_up
SZRD1	COSTIM_up
CUL1	COSTIM_up
TAB2	COSTIM_up
EIF2AK2	COSTIM_up
MRPL43	COSTIM_up
HPF1	COSTIM_up
ZFR	COSTIM_up
ZNF280C	COSTIM_up
TRAF1	COSTIM_up
SOAT1	COSTIM_up
GDI2	COSTIM_up
PRDM1	COSTIM_up
ATG5	COSTIM_up
TMCC3	COSTIM_up
PITHD1	COSTIM_up
SEC61A1	COSTIM_up
RASGRF1	COSTIM_up
POLR3E	COSTIM_up
RIOK2	COSTIM_up
NDC1	COSTIM_up
ALDH18A1	COSTIM_up
TARBP1	COSTIM_up
GATB	COSTIM_up
CDK17	COSTIM_up
DNAJC25	COSTIM_up
PSD	COSTIM_up

CTDP1	COSTIM_up
YBX3	COSTIM_up
WNK1	COSTIM_up
CCAR1	COSTIM_up
OGFR	COSTIM_up
PIGV	COSTIM_up
SNRNP40	COSTIM_up
QSER1	COSTIM_up
MPC1	COSTIM_up
BCAT1	COSTIM_up
MRPS35	COSTIM_up
SFSWAP	COSTIM_up
GPBP1	COSTIM_up
DGAT2	COSTIM_up
CS	COSTIM_up
MRPS24	COSTIM_up
ELMO2	COSTIM_up
WAPL	COSTIM_up
VMP1	COSTIM_up
POLD1	COSTIM_up
EIF4B	COSTIM_up
RPL18	COSTIM_up
ISOC2	COSTIM_up
U2AF2	COSTIM_up
EPN1	COSTIM_up
AHRR	COSTIM_up
RNF4	COSTIM_up
INTS13	COSTIM_up
TAF2	COSTIM_up
TNPO3	COSTIM_up
RFXANK	COSTIM_up
CTSA	COSTIM_up
SUGP2	COSTIM_up
DDX20	COSTIM_up
BCAS1	COSTIM_up
SBNO2	COSTIM_up
PMS1	COSTIM_up
TAF11	COSTIM_up
AP3D1	COSTIM_up
UHRF1BP1	COSTIM_up
GNAI3	COSTIM_up
IPO5	COSTIM_up
WDR3	COSTIM_up
WDR18	COSTIM_up
MCM10	COSTIM_up
KARS1	COSTIM_up
PDIA5	COSTIM_up
TBC1D22B	COSTIM_up
NDUFB4	COSTIM_up
SPEN	COSTIM_up

ZC3H15	COSTIM_up
SEC61A2	COSTIM_up
MTHFD2	COSTIM_up
SLC9A7	COSTIM_up
YBX1	COSTIM_up
ELAVL1	COSTIM_up
SMARCD1	COSTIM_up
NFYC	COSTIM_up
SLC9A3	COSTIM_up
CD84	COSTIM_up
ELOVL1	COSTIM_up
SPI1	COSTIM_up
ZNRD1	COSTIM_up
GOLGA5	COSTIM_up
LRRC40	COSTIM_up
ISOC1	COSTIM_up
TRMT11	COSTIM_up
MSANTD3	COSTIM_up
ARFGEF1	COSTIM_up
FECH	COSTIM_up
PFKP	COSTIM_up
IDI1	COSTIM_up
KLF6	COSTIM_up
PLPP1	COSTIM_up
TRAM1	COSTIM_up
PHKA1	COSTIM_up
STOML1	COSTIM_up
PKM	COSTIM_up
DHX29	COSTIM_up
DNTTIP2	COSTIM_up
RRP15	COSTIM_up
RHOA	COSTIM_up
IARS2	COSTIM_up
NAV3	COSTIM_up
IDH3G	COSTIM_up
HYAL2	COSTIM_up
HDAC4	COSTIM_up
COASY	COSTIM_up
TFE3	COSTIM_up
ACSL4	COSTIM_up
GPKOW	COSTIM_up
FTSJ1	COSTIM_up
POLR1A	COSTIM_up
TTC7A	COSTIM_up
SRBD1	COSTIM_up
KIF2A	COSTIM_up
PSME4	COSTIM_up
NUP133	COSTIM_up
NUCKS1	COSTIM_up
VPS35	COSTIM_up

DNAJA2	COSTIM_up
KCNAB2	COSTIM_up
FUNDC1	COSTIM_up
ATP1B3	COSTIM_up
MAPK6	COSTIM_up
HDHD5	COSTIM_up
UFD1	COSTIM_up
PHRF1	COSTIM_up
ELP1	COSTIM_up
EXOC5	COSTIM_up
RNF126	COSTIM_up
JMJD6	COSTIM_up
ASNS	COSTIM_up
ST6GALNAC2	COSTIM_up
PABPC1	COSTIM_up
CFAP20	COSTIM_up
CSNK2A2	COSTIM_up
EIF2B3	COSTIM_up
TCOF1	COSTIM_up
CDC42	COSTIM_up
SLC12A3	COSTIM_up
RAD18	COSTIM_up
NCK2	COSTIM_up
RPL31	COSTIM_up
WDR1	COSTIM_up
ING3	COSTIM_up
BUD23	COSTIM_up
SEL1L	COSTIM_up
TRIP13	COSTIM_up
ATP6AP1	COSTIM_up
TCF3	COSTIM_up
DAZAP1	COSTIM_up
MBD3	COSTIM_up
FAM50A	COSTIM_up
CPSF1	COSTIM_up
PDCD2	COSTIM_up
RDH11	COSTIM_up
ZFYVE26	COSTIM_up
TFRC	COSTIM_up
SREBF1	COSTIM_up
SMC1A	COSTIM_up
HSD17B10	COSTIM_up
HMMR	COSTIM_up
CHFR	COSTIM_up
TRNT1	COSTIM_up
STK10	COSTIM_up
FBXW11	COSTIM_up
EVC	COSTIM_up
DERL2	COSTIM_up
NDE1	COSTIM_up

AP1M1	COSTIM_up
PVR	COSTIM_up
SCARB1	COSTIM_up
MCM2	COSTIM_up
TP63	COSTIM_up
NLE1	COSTIM_up
SMARCE1	COSTIM_up
FERMT2	COSTIM_up
NSF	COSTIM_up
MRPS34	COSTIM_up
CLNS1A	COSTIM_up
EED	COSTIM_up
TSG101	COSTIM_up
MGLL	COSTIM_up
BCS1L	COSTIM_up
SLC24A1	COSTIM_up
SCARF1	COSTIM_up
LMAN1	COSTIM_up
ENO1	COSTIM_up
MYDGF	COSTIM_up
TUBE1	COSTIM_up
TIPIN	COSTIM_up
SRI	COSTIM_up
EIF4G3	COSTIM_up
NUP37	COSTIM_up
TIMM21	COSTIM_up
RASAL2	COSTIM_up
VPS9D1	COSTIM_up
SLC25A3	COSTIM_up
FNDC3B	COSTIM_up
FSCN1	COSTIM_up
ACTB	COSTIM_up
DLG1	COSTIM_up
RAB7A	COSTIM_up
SART3	COSTIM_up
EXOSC7	COSTIM_up
MKRN2	COSTIM_up
MCM6	COSTIM_up
REXO2	COSTIM_up
RBM7	COSTIM_up
PTPN23	COSTIM_up
MLH1	COSTIM_up
SPAG5	COSTIM_up
TRAF4	COSTIM_up
XAB2	COSTIM_up
MAP2K7	COSTIM_up
TM9SF3	COSTIM_up
NFKB2	COSTIM_up
UBE2T	COSTIM_up
GTF3C1	COSTIM_up

SNRPA	COSTIM_up
EXOSC5	COSTIM_up
DYNC1I2	COSTIM_up
SIRT6	COSTIM_up
CAPZB	COSTIM_up
SLC25A43	COSTIM_up
UBE2A	COSTIM_up
ARAF	COSTIM_up
LAMP3	COSTIM_up
UBE2K	COSTIM_up
TIGAR	COSTIM_up
SYNJ2	COSTIM_up
PMS2P1	COSTIM_up
GNB1	COSTIM_up
MLLT10	COSTIM_up
NRDC	COSTIM_up
VDAC3	COSTIM_up
SDF4	COSTIM_up
THOC1	COSTIM_up
XRCC5	COSTIM_up
REXO1	COSTIM_up
SAR1A	COSTIM_up
SENP1	COSTIM_up
FDFT1	COSTIM_up
PAFAH1B3	COSTIM_up
PGM1	COSTIM_up
DDX1	COSTIM_up
EPB41L2	COSTIM_up
KEAP1	COSTIM_up
RIF1	COSTIM_up
RAB21	COSTIM_up
SRCAP	COSTIM_up
PUM3	COSTIM_up
CNOT4	COSTIM_up
CPOX	COSTIM_up
CLDND1	COSTIM_up
HSP90AA1	COSTIM_up
PCNP	COSTIM_up
UBA5	COSTIM_up
JMJD4	COSTIM_up
IL12RB2	COSTIM_up
WDR70	COSTIM_up
BZW1	COSTIM_up
ME2	COSTIM_up
C5orf22	COSTIM_up
MRPL22	COSTIM_up
GEMIN5	COSTIM_up
NFE2L1	COSTIM_up
GSK3B	COSTIM_up
ITGB5	COSTIM_up

XPO1	COSTIM_up
PALB2	COSTIM_up
LYRM2	COSTIM_up
TNPO1	COSTIM_up
PLOD1	COSTIM_up
DIS3	COSTIM_up
PIBF1	COSTIM_up
NUFIP1	COSTIM_up
CYLD	COSTIM_up
SLC27A5	COSTIM_up
ZNF324	COSTIM_up
RPS5	COSTIM_up
CHMP2B	COSTIM_up
PPIE	COSTIM_up
ZMPSTE24	COSTIM_up
STARD7	COSTIM_up
GSTP1	COSTIM_up
WBP11	COSTIM_up
EIF3I	COSTIM_up
TXLNA	COSTIM_up
RAB10	COSTIM_up
HADHA	COSTIM_up
CAD	COSTIM_up
CD59	COSTIM_up
CD82	COSTIM_up
AK6	COSTIM_up
MECOM	COSTIM_up
PREP	COSTIM_up
SEH1L	COSTIM_up
WDFY1	COSTIM_up
AKR1B1	COSTIM_up
RRN3	COSTIM_up
CTTN	COSTIM_up
MTIF2	COSTIM_up
EPS15	COSTIM_up
CHERP	COSTIM_up
POMGNT1	COSTIM_up
MAST2	COSTIM_up
DNAJA1	COSTIM_up
CHMP5	COSTIM_up
NFX1	COSTIM_up
DIMT1	COSTIM_up
IPO11	COSTIM_up
EIF2AK1	COSTIM_up
EPDR1	COSTIM_up
SEPHS1	COSTIM_up
MRPL28	COSTIM_up
ITPKC	COSTIM_up
RBM22	COSTIM_up
TMED2	COSTIM_up

TXLNG	COSTIM_up
LAT2	COSTIM_up
HUWE1	COSTIM_up
ZW10	COSTIM_up
ALG9	COSTIM_up
ACOX3	COSTIM_up
MTMR2	COSTIM_up
PPP1R15A	COSTIM_up
TRIP6	COSTIM_up
ACHE	COSTIM_up
FTL	COSTIM_up
SRRT	COSTIM_up
BAX	COSTIM_up
ATXN7L3	COSTIM_up
PSMC5	COSTIM_up
OGFOD1	COSTIM_up
SH3BP2	COSTIM_up
NOP14	COSTIM_up
ADD1	COSTIM_up
L2HGDH	COSTIM_up
RTRAF	COSTIM_up
SF3B2	COSTIM_up
KLHL42	COSTIM_up
DNM1L	COSTIM_up
ERGIC2	COSTIM_up
PIR	COSTIM_up
METTL2A	COSTIM_up
CNOT3	COSTIM_up
DDX18	COSTIM_up
KHSRP	COSTIM_up
TPX2	COSTIM_up
PDRG1	COSTIM_up
ANKRD10	COSTIM_up
COQ9	COSTIM_up
KIF9	COSTIM_up
CRLS1	COSTIM_up
ATRN	COSTIM_up
FKBP1A	COSTIM_up
NSFL1C	COSTIM_up
ZNF343	COSTIM_up
XRN2	COSTIM_up
DYNLL1	COSTIM_up
TESC	COSTIM_up
SNX5	COSTIM_up
RPL6	COSTIM_up
MAPKAPK5	COSTIM_up
P2RX7	COSTIM_up
ESF1	COSTIM_up
RBBP9	COSTIM_up
ANAPC5	COSTIM_up

SLC23A2	COSTIM_up
TMEM230	COSTIM_up
KDM2B	COSTIM_up
GCN1	COSTIM_up
RPLP0	COSTIM_up
TRMT6	COSTIM_up
PEBP1	COSTIM_up
BRAP	COSTIM_up
FUS	COSTIM_up
CDIP1	COSTIM_up
KCNH4	COSTIM_up
GANAB	COSTIM_up
MLF2	COSTIM_up
DDX24	COSTIM_up
DHX32	COSTIM_up
GPATCH2L	COSTIM_up
SLC9A1	COSTIM_up
SPTLC1	COSTIM_up
CCNK	COSTIM_up
RGS1	COSTIM_up
MRPS33	COSTIM_up
NDUFB2	COSTIM_up
NUDC	COSTIM_up
MAEA	COSTIM_up
ICAM1	COSTIM_up
STRN4	COSTIM_up
IRAK3	COSTIM_up
MUL1	COSTIM_up
TFAP4	COSTIM_up
PDCD7	COSTIM_up
DNAJB11	COSTIM_up
RAB11FIP3	COSTIM_up
GOLGA3	COSTIM_up
PABPC4	COSTIM_up
CERS4	COSTIM_up
EFNB1	COSTIM_up
AARS1	COSTIM_up
RBM27	COSTIM_up
PUS7	COSTIM_up
DLD	COSTIM_up
WDR7	COSTIM_up
TXNL1	COSTIM_up
CMTM6	COSTIM_up
MAP3K20	COSTIM_up
FH	COSTIM_up
CDV3	COSTIM_up
ALKBH5	COSTIM_up
SPAG7	COSTIM_up
ZC3HC1	COSTIM_up
ESR1	COSTIM_up

TMEM101	COSTIM_up
PSME1	COSTIM_up
PPP2R3C	COSTIM_up
JPH4	COSTIM_up
RNF31	COSTIM_up
SCFD1	COSTIM_up
HECTD1	COSTIM_up
HNRNPC	COSTIM_up
SUPT16H	COSTIM_up
TOX4	COSTIM_up
TGM1	COSTIM_up
TINF2	COSTIM_up
TRPM7	COSTIM_up
WDR76	COSTIM_up
TBX15	COSTIM_up
PHGDH	COSTIM_up
MYL6	COSTIM_up
AGO1	COSTIM_up
CLSPN	COSTIM_up
RFFL	COSTIM_up
NUP50	COSTIM_up
CDC45	COSTIM_up
COMT	COSTIM_up
ECHDC1	COSTIM_up
LRRFIP2	COSTIM_up
XYLB	COSTIM_up
HDAC6	COSTIM_up
CDC6	COSTIM_up
CDC23	COSTIM_up
AAAS	COSTIM_up
CBX5	COSTIM_up
MSH2	COSTIM_up
DHPS	COSTIM_up
ARCN1	COSTIM_up
EPB41L4B	COSTIM_up
TMEM38B	COSTIM_up
PSMD5	COSTIM_up
NUP188	COSTIM_up
NANS	COSTIM_up
BTAF1	COSTIM_up
WAC	COSTIM_up
NUBP2	COSTIM_up
HIVEP1	COSTIM_up
FKBP5	COSTIM_up
SRPK1	COSTIM_up
BRPF3	COSTIM_up
MRPS18A	COSTIM_up
HSP90AB1	COSTIM_up
CDC5L	COSTIM_up
HNRNPH3	COSTIM_up

ABL1	COSTIM_up
ACOT7	COSTIM_up
SH3GLB1	COSTIM_up
CDC7	COSTIM_up
SCD	COSTIM_up
TMED1	COSTIM_up
WASHC2A	COSTIM_up
PSMD8	COSTIM_up
FBXL19	COSTIM_up
SETD1A	COSTIM_up
BCL7C	COSTIM_up
ATP5F1D	COSTIM_up
IGFALS	COSTIM_up
HNRNPM	COSTIM_up
NDUFB7	COSTIM_up
TIMM13	COSTIM_up
CDC34	COSTIM_up
MTAP	COSTIM_up
CEP170B	COSTIM_up
POLR2E	COSTIM_up
POLRMT	COSTIM_up
IZUMO4	COSTIM_up
GADD45B	COSTIM_up
MKNK2	COSTIM_up
TRMT2A	COSTIM_up
RANBP1	COSTIM_up
ZDHHC8	COSTIM_up
MED15	COSTIM_up
SNAP29	COSTIM_up
CRKL	COSTIM_up
LZTR1	COSTIM_up
SMARCB1	COSTIM_up
BCL2L13	COSTIM_up
DDT	COSTIM_up
SF3A1	COSTIM_up
SPECC1L	COSTIM_up
SNRPD3	COSTIM_up
PES1	COSTIM_up
PPM1F	COSTIM_up
MFNG	COSTIM_up
GGA1	COSTIM_up
HIRA	COSTIM_up
SH3BP1	COSTIM_up
LGALS1	COSTIM_up
SRRD	COSTIM_up
TFIP11	COSTIM_up
ANKRD54	COSTIM_up
EIF3L	COSTIM_up
SNU13	COSTIM_up
MICALL1	COSTIM_up

POLR2F	COSTIM_up
TCF20	COSTIM_up
TOMM22	COSTIM_up
XBP1	COSTIM_up
RTCB	COSTIM_up
POLDIP3	COSTIM_up
CYB5R3	COSTIM_up
RHBDD3	COSTIM_up
AP1B1	COSTIM_up
HMGXB4	COSTIM_up
MCAT	COSTIM_up
THOC5	COSTIM_up
TTLL12	COSTIM_up
RPL3	COSTIM_up
ZMAT5	COSTIM_up
ASCC2	COSTIM_up
MIEF1	COSTIM_up
MYH9	COSTIM_up
SAMM50	COSTIM_up
TXN2	COSTIM_up
FOXRED2	COSTIM_up
EIF3D	COSTIM_up
CSF2RB	COSTIM_up
SLC25A17	COSTIM_up
ST13	COSTIM_up
RBX1	COSTIM_up
L3MBTL2	COSTIM_up
RANGAP1	COSTIM_up
ZC3H7B	COSTIM_up
PHF5A	COSTIM_up
ACO2	COSTIM_up
POLR3H	COSTIM_up
TRMU	COSTIM_up
DESI1	COSTIM_up
BRD1	COSTIM_up
ZBED4	COSTIM_up
KHNYN	COSTIM_up
FKBP3	COSTIM_up
PRMT5	COSTIM_up
TRIM9	COSTIM_up
PSMC6	COSTIM_up
GNPNAT1	COSTIM_up
DDHD1	COSTIM_up
CNIH1	COSTIM_up
PSMA3	COSTIM_up
VTI1B	COSTIM_up
TIMM9	COSTIM_up
AHSA1	COSTIM_up
SNW1	COSTIM_up
ITPK1	COSTIM_up

DHRS7	COSTIM_up
PPM1A	COSTIM_up
CEP128	COSTIM_up
ERH	COSTIM_up
SUSD6	COSTIM_up
EIF5	COSTIM_up
ZFYVE21	COSTIM_up
MTHFD1	COSTIM_up
TELO2	COSTIM_up
GSKIP	COSTIM_up
PSMC1	COSTIM_up
PPP4R3A	COSTIM_up
PSMB5	COSTIM_up
YY1	COSTIM_up
ACIN1	COSTIM_up
CCNB1IP1	COSTIM_up
APEX1	COSTIM_up
PABPN1	COSTIM_up
CINP	COSTIM_up
SRP54	COSTIM_up
PCK2	COSTIM_up
PRORP	COSTIM_up
PSMA6	COSTIM_up
NFKBIA	COSTIM_up
EMC9	COSTIM_up
PSME2	COSTIM_up
BRMS1L	COSTIM_up
TM9SF1	COSTIM_up
SEC23A	COSTIM_up
PNN	COSTIM_up
RABGGTA	COSTIM_up
PLTP	COSTIM_up
GSS	COSTIM_up
TRPC4AP	COSTIM_up
GINS1	COSTIM_up
CD40	COSTIM_up
UQCC1	COSTIM_up
RAB5IF	COSTIM_up
NFATC2	COSTIM_up
PFDN4	COSTIM_up
CSTF1	COSTIM_up
RAE1	COSTIM_up
TPD52L2	COSTIM_up
DNAJC5	COSTIM_up
NELFCD	COSTIM_up
PRPF6	COSTIM_up
PRELID3B	COSTIM_up
MTG2	COSTIM_up
PSMA7	COSTIM_up
MRGBP	COSTIM_up

TCFL5	COSTIM_up
GID8	COSTIM_up
SLC17A9	COSTIM_up
ARFGAP1	COSTIM_up
C20orf27	COSTIM_up
ARFRP1	COSTIM_up
NDUFAF5	COSTIM_up
TRIB3	COSTIM_up
RASSF2	COSTIM_up
CSNK2A1	COSTIM_up
HM13	COSTIM_up
SIRPB1	COSTIM_up
SEC23B	COSTIM_up
TM9SF4	COSTIM_up
CRNKL1	COSTIM_up
POFUT1	COSTIM_up
KIF3B	COSTIM_up
NOP56	COSTIM_up
MANBAL	COSTIM_up
IDH3B	COSTIM_up
MAPRE1	COSTIM_up
JAG1	COSTIM_up
CDK5RAP1	COSTIM_up
TTI1	COSTIM_up
E2F1	COSTIM_up
RPRD1B	COSTIM_up
CHMP4B	COSTIM_up
ASIP	COSTIM_up
ACTR5	COSTIM_up
AHCY	COSTIM_up
DHX35	COSTIM_up
DNTTIP1	COSTIM_up
PIGU	COSTIM_up
APMAP	COSTIM_up
ADNP2	COSTIM_up
RBFA	COSTIM_up
USP14	COSTIM_up
VAPA	COSTIM_up
RNMT	COSTIM_up
MIB1	COSTIM_up
RBBP8	COSTIM_up
RIOK3	COSTIM_up
CSTF2	COSTIM_up
PSMD10	COSTIM_up
PGRMC1	COSTIM_up
POLA1	COSTIM_up
NKAP	COSTIM_up
NXT2	COSTIM_up
MCTS2P	COSTIM_up
PRPS2	COSTIM_up

SUV39H1	COSTIM_up
XIAP	COSTIM_up
CCDC22	COSTIM_up
NAA10	COSTIM_up
RENBP	COSTIM_up
PIM2	COSTIM_up
PQBP1	COSTIM_up
EMD	COSTIM_up
PGK1	COSTIM_up
MAGT1	COSTIM_up
SMS	COSTIM_up
EEA1	COSTIM_up
JADE3	COSTIM_up
CDK16	COSTIM_up
HTATSF1	COSTIM_up
RBM3	COSTIM_up
PBDC1	COSTIM_up
GLA	COSTIM_up
ARMCX3	COSTIM_up
NDFIP2	COSTIM_up
DNAJC3	COSTIM_up
ARHGEF7	COSTIM_up
MRPS31	COSTIM_up
SLC25A15	COSTIM_up
KPNA3	COSTIM_up
VWA8	COSTIM_up
INTS6	COSTIM_up
MGRN1	COSTIM_up
TRADD	COSTIM_up
HSF4	COSTIM_up
LYRM1	COSTIM_up
NUTF2	COSTIM_up
NUP93	COSTIM_up
CCL22	COSTIM_up
DHODH	COSTIM_up
CCL17	COSTIM_up
ACD	COSTIM_up
POLR2C	COSTIM_up
USB1	COSTIM_up
CYB5B	COSTIM_up
NME3	COSTIM_up
NDRG4	COSTIM_up
PSMD7	COSTIM_up
SETD6	COSTIM_up
SLC38A7	COSTIM_up
VAC14	COSTIM_up
MON1B	COSTIM_up
CMC2	COSTIM_up
AXIN1	COSTIM_up
NPRL3	COSTIM_up

TAF1C	COSTIM_up
USP10	COSTIM_up
ABCC1	COSTIM_up
NOMO3	COSTIM_up
CLCN7	COSTIM_up
HAGHL	COSTIM_up
ANTKMT	COSTIM_up
SLC7A5	COSTIM_up
STUB1	COSTIM_up
NUBP1	COSTIM_up
UBE2I	COSTIM_up
EEF2K	COSTIM_up
CAPN15	COSTIM_up
PIEZO1	COSTIM_up
GSPT1	COSTIM_up
UBFD1	COSTIM_up
EARS2	COSTIM_up
ELOB	COSTIM_up
USP31	COSTIM_up
HMOX2	COSTIM_up
DNAJA3	COSTIM_up
BFAR	COSTIM_up
RRN3P2	COSTIM_up
RPGRIP1L	COSTIM_up
MAZ	COSTIM_up
STX4	COSTIM_up
BCKDK	COSTIM_up
KAT8	COSTIM_up
NOMO1	COSTIM_up
IL21R	COSTIM_up
VPS35L	COSTIM_up
RNF40	COSTIM_up
KNOP1	COSTIM_up
AAGAB	COSTIM_up
LACTB	COSTIM_up
CSK	COSTIM_up
TRIP4	COSTIM_up
RAB11A	COSTIM_up
CTSH	COSTIM_up
FAH	COSTIM_up
RPAP1	COSTIM_up
EHD4	COSTIM_up
ZNF106	COSTIM_up
ATP8B4	COSTIM_up
GABPB1	COSTIM_up
DNAJC17	COSTIM_up
EIF3J	COSTIM_up
VPS18	COSTIM_up
CSPP1	COSTIM_up
BRF2	COSTIM_up

CA2	COSTIM_up
INTS9	COSTIM_up
RIPK2	COSTIM_up
NBN	COSTIM_up
TRPA1	COSTIM_up
IMPAD1	COSTIM_up
LAPTM4B	COSTIM_up
POP1	COSTIM_up
JPH1	COSTIM_up
STK3	COSTIM_up
GDAP1	COSTIM_up
RAB2A	COSTIM_up
EIF3E	COSTIM_up
EMC2	COSTIM_up
IL7	COSTIM_up
ARMC1	COSTIM_up
CHRAC1	COSTIM_up
SNX16	COSTIM_up
UBR5	COSTIM_up
TSTA3	COSTIM_up
PYCR3	COSTIM_up
EEF1D	COSTIM_up
SQLE	COSTIM_up
SLC39A14	COSTIM_up
MTMR9	COSTIM_up
LEPROTL1	COSTIM_up
DCTN6	COSTIM_up
R3HCC1	COSTIM_up
GSR	COSTIM_up
TNFRSF10A	COSTIM_up
UBXN8	COSTIM_up
PPP2CB	COSTIM_up
KLHDC4	COSTIM_up
MCM4	COSTIM_up
MAN2B1	COSTIM_up
KCNN4	COSTIM_up
HNRNPL	COSTIM_up
NFKBIB	COSTIM_up
SARS2	COSTIM_up
SNRNP70	COSTIM_up
CLPTM1	COSTIM_up
RELB	COSTIM_up
PPP1R37	COSTIM_up
ERCC2	COSTIM_up
DOT1L	COSTIM_up
PLEKHJ1	COSTIM_up
RNASEH2A	COSTIM_up
SF3A2	COSTIM_up
OAZ1	COSTIM_up
TRMT1	COSTIM_up

RSPH6A	COSTIM_up
TBC1D17	COSTIM_up
IL4I1	COSTIM_up
PTOV1	COSTIM_up
SGTA	COSTIM_up
C19orf53	COSTIM_up
TIMM44	COSTIM_up
FAM32A	COSTIM_up
PPP6R1	COSTIM_up
MED26	COSTIM_up
AKAP8	COSTIM_up
ILVBL	COSTIM_up
POP4	COSTIM_up
CCNE1	COSTIM_up
URI1	COSTIM_up
PDCD5	COSTIM_up
ANKRD27	COSTIM_up
RPS16	COSTIM_up
TIMM50	COSTIM_up
FBL	COSTIM_up
GPI	COSTIM_up
PIAS4	COSTIM_up
EBI3	COSTIM_up
YJU2	COSTIM_up
TBCB	COSTIM_up
POLR2I	COSTIM_up
SLC1A5	COSTIM_up
CACTIN	COSTIM_up
CCDC9	COSTIM_up
HNRNPUL1	COSTIM_up
TGFB1	COSTIM_up
DMAC2	COSTIM_up
MRPL4	COSTIM_up
RPS19	COSTIM_up
BABAM1	COSTIM_up
TYK2	COSTIM_up
CDC37	COSTIM_up
NAPA	COSTIM_up
MEGF8	COSTIM_up
GRWD1	COSTIM_up
PLA2G4C	COSTIM_up
TMEM205	COSTIM_up
BCAT2	COSTIM_up
MIER2	COSTIM_up
PPP2R1A	COSTIM_up
TNPO2	COSTIM_up
WDR83OS	COSTIM_up
GCDH	COSTIM_up
PRPF31	COSTIM_up
TFPT	COSTIM_up

JAK3	COSTIM_up
RPL18A	COSTIM_up
KCNN1	COSTIM_up
PIK3R2	COSTIM_up
ELL	COSTIM_up
COPE	COSTIM_up
DDX49	COSTIM_up
ARMC6	COSTIM_up
TMEM147	COSTIM_up
KXD1	COSTIM_up
SUGP1	COSTIM_up
PBX4	COSTIM_up
GSK3A	COSTIM_up
ATP13A1	COSTIM_up
ZNF574	COSTIM_up
SMG9	COSTIM_up
CDK6	COSTIM_up
PMPCB	COSTIM_up
DNAJC2	COSTIM_up
TFPI2	COSTIM_up
NAMPT	COSTIM_up
TWISTNB	COSTIM_up
WDR91	COSTIM_up
CBLL1	COSTIM_up
MTPN	COSTIM_up
STEAP1B	COSTIM_up
TTC26	COSTIM_up
OGDH	COSTIM_up
TFEC	COSTIM_up
CAV1	COSTIM_up
MET	COSTIM_up
DNAJB6	COSTIM_up
SSBP1	COSTIM_up
GARS1	COSTIM_up
CHCHD2	COSTIM_up
HSPB1	COSTIM_up
PDAP1	COSTIM_up
BUD31	COSTIM_up
PTCD1	COSTIM_up
EIF3B	COSTIM_up
SNX8	COSTIM_up
TAF6	COSTIM_up
AIMP2	COSTIM_up
RBM28	COSTIM_up
USP42	COSTIM_up
LSM5	COSTIM_up
AP1S1	COSTIM_up
ZNHIT1	COSTIM_up
PHF14	COSTIM_up
EZH2	COSTIM_up

MEST	COSTIM_up
MEOX2	COSTIM_up
AHR	COSTIM_up
CHCHD3	COSTIM_up
PSMA2	COSTIM_up
MRPL32	COSTIM_up
BLVRA	COSTIM_up
URGCP	COSTIM_up
TMEM248	COSTIM_up
RHEB	COSTIM_up
POLD2	COSTIM_up
BCL7B	COSTIM_up
YKT6	COSTIM_up
TBL2	COSTIM_up
CLIP2	COSTIM_up
EIF4H	COSTIM_up
FSD1L	COSTIM_up
TGFBR1	COSTIM_up
SEC61B	COSTIM_up
OGN	COSTIM_up
ECM2	COSTIM_up
SUSD1	COSTIM_up
CDC37L1	COSTIM_up
PLGRKT	COSTIM_up
NCS1	COSTIM_up
FUBP3	COSTIM_up
CREB3	COSTIM_up
EDF1	COSTIM_up
PIP5K1B	COSTIM_up
BAG1	COSTIM_up
RAPGEF1	COSTIM_up
EXOSC3	COSTIM_up
ZFAND5	COSTIM_up
DVL1	COSTIM_up
CCNJ	COSTIM_up
RASSF4	COSTIM_up
ERLIN1	COSTIM_up
EIF3A	COSTIM_up
TRDMT1	COSTIM_up
DDX50	COSTIM_up
MAPK8	COSTIM_up
SEC23IP	COSTIM_up
NSMCE4A	COSTIM_up
MICU1	COSTIM_up
CCSER2	COSTIM_up
TWNK	COSTIM_up
NPM3	COSTIM_up
GBF1	COSTIM_up
ARHGAP21	COSTIM_up
FBXL15	COSTIM_up

CUEDC2	COSTIM_up
LARP4B	COSTIM_up
GTPBP4	COSTIM_up
BCCIP	COSTIM_up
MTPAP	COSTIM_up
PITRM1	COSTIM_up
STN1	COSTIM_up
MAP3K8	COSTIM_up
GLRX3	COSTIM_up
TASOR2	COSTIM_up
XPNPEP1	COSTIM_up
SMC3	COSTIM_up
TFAM	COSTIM_up
CCDC6	COSTIM_up
CUL2	COSTIM_up
CCNY	COSTIM_up
UBE2S	COSTIM_up
RPL28	COSTIM_up
ZMIZ1	COSTIM_up
PPIF	COSTIM_up
NUFIP2	COSTIM_up
RPL19	COSTIM_up
UBTF	COSTIM_up
PSMD3	COSTIM_up
CASC3	COSTIM_up
RAD51C	COSTIM_up
MTMR4	COSTIM_up
DHX40	COSTIM_up
KPNB1	COSTIM_up
PNPO	COSTIM_up
CBX1	COSTIM_up
INTS2	COSTIM_up
CAMTA2	COSTIM_up
ENO3	COSTIM_up
PFN1	COSTIM_up
RNF167	COSTIM_up
SLC25A11	COSTIM_up
C1QBP	COSTIM_up
BLMH	COSTIM_up
GOSR1	COSTIM_up
CCDC47	COSTIM_up
DRG2	COSTIM_up
FTSJ3	COSTIM_up
SMARCD2	COSTIM_up
SYNGR2	COSTIM_up
UTP6	COSTIM_up
C17orf75	COSTIM_up
CYTH1	COSTIM_up
PSMD11	COSTIM_up
CCL2	COSTIM_up

PEX12	COSTIM_up
KAT2A	COSTIM_up
RAB5C	COSTIM_up
MLX	COSTIM_up
CNTNAP1	COSTIM_up
PPP1R9B	COSTIM_up
MRPL27	COSTIM_up
LRRC59	COSTIM_up
EFTUD2	COSTIM_up
SLC16A6	COSTIM_up
YWHAE	COSTIM_up
MMD	COSTIM_up
RANGRF	COSTIM_up
DHRS7B	COSTIM_up
NAT9	COSTIM_up
TMEM104	COSTIM_up
TMEM97	COSTIM_up
UNC119	COSTIM_up
SUPT6H	COSTIM_up
RAB34	COSTIM_up
TMEM33	COSTIM_up
SLAIN2	COSTIM_up
OCIAD1	COSTIM_up
USP46	COSTIM_up
CRACD	COSTIM_up
LAMTOR3	COSTIM_up
NFKB1	COSTIM_up
UBE2D3	COSTIM_up
NDUFC1	COSTIM_up
ZNF330	COSTIM_up
GRPEL1	COSTIM_up
GAR1	COSTIM_up
CLCN3	COSTIM_up
DHX15	COSTIM_up
FBXW7	COSTIM_up
NSD2	COSTIM_up
SH3D19	COSTIM_up
MFSD10	COSTIM_up
UFSP2	COSTIM_up
UGDH	COSTIM_up
HTATIP2	COSTIM_up
CTSC	COSTIM_up
ZPR1	COSTIM_up
MTCH2	COSTIM_up
HSPA8	COSTIM_up
LPXN	COSTIM_up
DTX4	COSTIM_up
EHD1	COSTIM_up
OSBP	COSTIM_up
PUS3	COSTIM_up

DCPS	COSTIM_up
FOXRED1	COSTIM_up
CCND1	COSTIM_up
CCDC86	COSTIM_up
PRPF19	COSTIM_up
TMEM109	COSTIM_up
CHORDC1	COSTIM_up
ANAPC15	COSTIM_up
PANX1	COSTIM_up
CEP164	COSTIM_up
EIF4G2	COSTIM_up
UBE4A	COSTIM_up
DDX6	COSTIM_up
NECTIN1	COSTIM_up
PDHX	COSTIM_up
COMMD9	COSTIM_up
AMBRA1	COSTIM_up
PTPMT1	COSTIM_up
CARS1	COSTIM_up
SLC35F2	COSTIM_up
C11orf58	COSTIM_up
RPS13	COSTIM_up
AIP	COSTIM_up
NUP98	COSTIM_up
NDUFS8	COSTIM_up
CHKA	COSTIM_up
HPS5	COSTIM_up
GTF2H1	COSTIM_up
POU2AF1	COSTIM_up
PSMD9	COSTIM_up
PRPF40B	COSTIM_up
COQ5	COSTIM_up
KCTD10	COSTIM_up
MLEC	COSTIM_up
MVK	COSTIM_up
CAMKK2	COSTIM_up
ATP5F1B	COSTIM_up
PTGES3	COSTIM_up
BCL7A	COSTIM_up
RSRC2	COSTIM_up
CYP27B1	COSTIM_up
METAP2	COSTIM_up
WNT5B	COSTIM_up
MAGOHB	COSTIM_up
PRR4	COSTIM_up
ARPC3	COSTIM_up
GPN3	COSTIM_up
VPS29	COSTIM_up
SH2B3	COSTIM_up
NAA25	COSTIM_up

OGFOD2	COSTIM_up
CDK2AP1	COSTIM_up
OAS3	COSTIM_up
GTF2H3	COSTIM_up
EIF2B1	COSTIM_up
DDX55	COSTIM_up
SLC38A1	COSTIM_up
C12orf49	COSTIM_up
VDR	COSTIM_up
COPZ1	COSTIM_up
CAND1	COSTIM_up
NUP107	COSTIM_up
CNOT2	COSTIM_up
CPSF6	COSTIM_up
KRR1	COSTIM_up
MRPL51	COSTIM_up
GAPDH	COSTIM_up
NOP2	COSTIM_up
UHRF1BP1L	COSTIM_up
COPS7A	COSTIM_up
USP5	COSTIM_up
TPI1	COSTIM_up
SPSB2	COSTIM_up
ATN1	COSTIM_up
NT5DC3	COSTIM_up
GOLT1B	COSTIM_up
LDHB	COSTIM_up
CMAS	COSTIM_up
AICDA	COSTIM_up
RAB35	COSTIM_up
COX6A1	COSTIM_up
SRSF9	COSTIM_up
DSE	COSTIM_up
RWDD1	COSTIM_up
PAK1IP1	COSTIM_up
RNGTT	COSTIM_up
HDDC2	COSTIM_up
HINT3	COSTIM_up
UST	COSTIM_up
ULBP1	COSTIM_up
MTRF1L	COSTIM_up
KCTD20	COSTIM_up
SRSF3	COSTIM_up
SOD2	COSTIM_up
MRPL18	COSTIM_up
MCM3	COSTIM_up
RNF8	COSTIM_up
PHACTR1	COSTIM_up
CD83	COSTIM_up
MDN1	COSTIM_up

BAG2	COSTIM_up
CCNC	COSTIM_up
E2F3	COSTIM_up
PTP4A1	COSTIM_up
ASCC3	COSTIM_up
ACOT13	COSTIM_up
SMAP1	COSTIM_up
RPS12	COSTIM_up
C6orf62	COSTIM_up
GMNN	COSTIM_up
SNX3	COSTIM_up
HBS1L	COSTIM_up
PEX7	COSTIM_up
ZBTB24	COSTIM_up
ARFGEF3	COSTIM_up
EPM2A	COSTIM_up
SLC39A7	COSTIM_up
CUTA	COSTIM_up
QKI	COSTIM_up
TFEB	COSTIM_up
BYSL	COSTIM_up
TBP	COSTIM_up
PPP2R5D	COSTIM_up
MRPL2	COSTIM_up
SRF	COSTIM_up
DNPH1	COSTIM_up
DUSP22	COSTIM_up
EXOC2	COSTIM_up
COX7A2	COSTIM_up
TMEM30A	COSTIM_up
GMDS	COSTIM_up
VEGFA	COSTIM_up
PRPF4B	COSTIM_up
SLC29A1	COSTIM_up
FBRSL1	COSTIM_up
NUDT12	COSTIM_up
HMGCS1	COSTIM_up
DAP	COSTIM_up
MRPS30	COSTIM_up
HSPA9	COSTIM_up
MRPS27	COSTIM_up
PFDN1	COSTIM_up
SLC4A9	COSTIM_up
LOX	COSTIM_up
IK	COSTIM_up
HMGCR	COSTIM_up
THG1L	COSTIM_up
ARSB	COSTIM_up
CLINT1	COSTIM_up
CNOT6	COSTIM_up

TTC1	COSTIM_up
POLR3G	COSTIM_up
DROSHA	COSTIM_up
LMNB1	COSTIM_up
GOLPH3	COSTIM_up
SUB1	COSTIM_up
TARS1	COSTIM_up
RAD1	COSTIM_up
BRIX1	COSTIM_up
PRLR	COSTIM_up
RAD50	COSTIM_up
ST8SIA4	COSTIM_up
GNPDA1	COSTIM_up
SKP1	COSTIM_up
NUP155	COSTIM_up
PPP2CA	COSTIM_up
TRAPPC13	COSTIM_up
SEC24A	COSTIM_up
TXNDC15	COSTIM_up
RARS1	COSTIM_up
MACROH2A1	COSTIM_up
TCERG1	COSTIM_up
CSNK1A1	COSTIM_up
HMGXB3	COSTIM_up
ATP6V0E1	COSTIM_up
BNIP1	COSTIM_up
SELENOK	COSTIM_up
TIMMDC1	COSTIM_up
CD86	COSTIM_up
NIT2	COSTIM_up
FAM162A	COSTIM_up
KPNA1	COSTIM_up
PCCB	COSTIM_up
UBE3A	COSTIM_up
ARMC8	COSTIM_up
RNF7	COSTIM_up
COL7A1	COSTIM_up
PRKAR2A	COSTIM_up
HES1	COSTIM_up
USP4	COSTIM_up
TFG	COSTIM_up
TUSC2	COSTIM_up
RPL24	COSTIM_up
C3orf14	COSTIM_up
FXR1	COSTIM_up
GBE1	COSTIM_up
UMPS	COSTIM_up
NCBP2	COSTIM_up
SNX4	COSTIM_up
PLXNA1	COSTIM_up

ATP6V1A	COSTIM_up
PODXL2	COSTIM_up
KLHL18	COSTIM_up
SCAP	COSTIM_up
MRPL3	COSTIM_up
MAPKAPK3	COSTIM_up
COMMD2	COSTIM_up
RRP9	COSTIM_up
EIF1B	COSTIM_up
ABHD14A-ACY1	COSTIM_up
SSR3	COSTIM_up
FOXP1	COSTIM_up
EIF4G1	COSTIM_up
SPCS1	COSTIM_up
NEK4	COSTIM_up
INO80D	COSTIM_up
EEF1B2	COSTIM_up
DGUOK	COSTIM_up
MOB1A	COSTIM_up
IL1A	COSTIM_up
FAHD2A	COSTIM_up
NCL	COSTIM_up
ACTR1B	COSTIM_up
ACTR3	COSTIM_up
SF3B6	COSTIM_up
MPV17	COSTIM_up
GTF3C2	COSTIM_up
EIF2B4	COSTIM_up
NRBP1	COSTIM_up
PSMD14	COSTIM_up
SNX17	COSTIM_up
PPM1G	COSTIM_up
IFIH1	COSTIM_up
RPS15	COSTIM_up
INO80B	COSTIM_up
MOGS	COSTIM_up
NDUFS7	COSTIM_up
PCGF1	COSTIM_up
AUP1	COSTIM_up
HTRA2	COSTIM_up
LOXL3	COSTIM_up
POLE4	COSTIM_up
MRPL19	COSTIM_up
LANCL1	COSTIM_up
WDR75	COSTIM_up
FANCL	COSTIM_up
FN1	COSTIM_up
STAT1	COSTIM_up
GLS	COSTIM_up
CCT4	COSTIM_up

TXNDC9	COSTIM_up
COQ10B	COSTIM_up
SF3B1	COSTIM_up
CHST10	COSTIM_up
PDCL3	COSTIM_up
HSPE1	COSTIM_up
PLCD4	COSTIM_up
ZNF142	COSTIM_up
IL1R1	COSTIM_up
CNPPD1	COSTIM_up
UXS1	COSTIM_up
ABCB6	COSTIM_up
STK16	COSTIM_up
HDLBP	COSTIM_up
PPP1R7	COSTIM_up
PASK	COSTIM_up
STK25	COSTIM_up
ID2	COSTIM_up
TAF1B	COSTIM_up
ODC1	COSTIM_up
NOL10	COSTIM_up
PLEKHB2	COSTIM_up
GORASP2	COSTIM_up
CEBPZ	COSTIM_up
DCAF17	COSTIM_up
RAB3GAP1	COSTIM_up
SLC25A12	COSTIM_up
DARS1	COSTIM_up
SRSF7	COSTIM_up
KYNU	COSTIM_up
COX7A2L	COSTIM_up
PNO1	COSTIM_up
PLEK	COSTIM_up
ATF2	COSTIM_up
THADA	COSTIM_up
AAK1	COSTIM_up
ARID3A	COSTIM_up
SUMO1	COSTIM_up
GRIN3B	COSTIM_up
NFE2L2	COSTIM_up
MSH6	COSTIM_up
SPR	COSTIM_up
FARSB	COSTIM_up
BCL9	COSTIM_up
DHCR24	COSTIM_up
MARK1	COSTIM_up
CACYBP	COSTIM_up
SCP2	COSTIM_up
CEP104	COSTIM_up
FAM20B	COSTIM_up

LRRC42	COSTIM_up
MRPL37	COSTIM_up
ICMT	COSTIM_up
RPL22	COSTIM_up
CHD5	COSTIM_up
PARK7	COSTIM_up
AMPD2	COSTIM_up
SRSF4	COSTIM_up
MECR	COSTIM_up
EDEM3	COSTIM_up
WDR77	COSTIM_up
ATP5PB	COSTIM_up
HDAC1	COSTIM_up
CAPZA1	COSTIM_up
SCAMP3	COSTIM_up
DLGAP3	COSTIM_up
SFPQ	COSTIM_up
ARHGEF2	COSTIM_up
LAMTOR2	COSTIM_up
MEF2D	COSTIM_up
SRM	COSTIM_up
FBXO6	COSTIM_up
MAD2L2	COSTIM_up
IVNS1ABP	COSTIM_up
MFN2	COSTIM_up
MIIP	COSTIM_up
SMG7	COSTIM_up
NCF2	COSTIM_up
RO60	COSTIM_up
UCHL5	COSTIM_up
BCAS2	COSTIM_up
AGMAT	COSTIM_up
PLEKHM2	COSTIM_up
CRYZ	COSTIM_up
PHTF1	COSTIM_up
ZBTB17	COSTIM_up
CD58	COSTIM_up
TTF2	COSTIM_up
KIF21B	COSTIM_up
ADPRHL2	COSTIM_up
MAP7D1	COSTIM_up
WARS2	COSTIM_up
MRPS15	COSTIM_up
GNPAT	COSTIM_up
TSNAX	COSTIM_up
C1orf109	COSTIM_up
RRAGC	COSTIM_up
NID1	COSTIM_up
MTR	COSTIM_up
RIMS3	COSTIM_up

ETV3	COSTIM_up
ACADM	COSTIM_up
SLAMF1	COSTIM_up
ADGRL2	COSTIM_up
SDHB	COSTIM_up
RPF1	COSTIM_up
UAP1	COSTIM_up
KLHL12	COSTIM_up
ZNHIT6	COSTIM_up
RBBP5	COSTIM_up
GBP1	COSTIM_up
PINK1-AS	COSTIM_up
CDK18	COSTIM_up
RAB29	COSTIM_up
ECE1	COSTIM_up
GALE	COSTIM_up
PRPF3	COSTIM_up
APH1A	COSTIM_up
EBNA1BP2	COSTIM_up
CDC20	COSTIM_up
IPO13	COSTIM_up
ATP6V0B	COSTIM_up
B4GALT2	COSTIM_up
ERI3	COSTIM_up
PTCH2	COSTIM_up
AKR1A1	COSTIM_up
PRDX1	COSTIM_up
PIK3R3	COSTIM_up
SLC19A2	COSTIM_up
FAAH	COSTIM_up
NSUN4	COSTIM_up
DR1	COSTIM_up
PRRC2C	COSTIM_up
DPH5	COSTIM_up
PRDX6	COSTIM_up
DARS2	COSTIM_up
UTP25	COSTIM_up
RCAN3	COSTIM_up
MTFR1L	COSTIM_up
RPS6KA1	COSTIM_up
DHDDS	COSTIM_up
ARID1A	COSTIM_up
RPA2	COSTIM_up
PPP1R8	COSTIM_up
CD3EAP	COSTIM_up
RCN2	COSTIM_up
CTSD	COSTIM_up
STK11	COSTIM_up
KPTN	COSTIM_up
RPS25	COSTIM_up

CAMSAP2	COSTIM_up
ATF6	COSTIM_up
MREG	COSTIM_up
FASTKD2	COSTIM_up
NRP2	COSTIM_up
KLF7	COSTIM_up
B4GALT6	COSTIM_up
ATP10B	COSTIM_up
SPCS2	COSTIM_up
UBE3D	COSTIM_up
ANKRD13C	COSTIM_up
ZC2HC1B	COSTIM_up
PLAGL1	COSTIM_up
FBXO30	COSTIM_up
TNFAIP3	COSTIM_up
SGK1	COSTIM_up
MED28	COSTIM_up
RXYLT1	COSTIM_up
VAMP8	COSTIM_up
DCLRE1B	COSTIM_up
MYL12B	COSTIM_up
RPN2	COSTIM_up
TGIF2	COSTIM_up
CCNI	COSTIM_up
RARRES1	COSTIM_up
MFSD1	COSTIM_up
RAB3GAP2	COSTIM_up
EEF2KMT	COSTIM_up
UBN1	COSTIM_up
UCHL3	COSTIM_up
LDAH	COSTIM_up
CCND2	COSTIM_up
ELL2	COSTIM_up
CYP20A1	COSTIM_up
NDUFB3	COSTIM_up
GTF3C3	COSTIM_up
TJP2	COSTIM_up
CPSF3	COSTIM_up
SENP5	COSTIM_up
HEATR1	COSTIM_up
RAD23B	COSTIM_up
ABITRAM	COSTIM_up
SET	COSTIM_up
GLE1	COSTIM_up
RAB14	COSTIM_up
FBXW2	COSTIM_up
NEK6	COSTIM_up
PPP6C	COSTIM_up
NDUFA8	COSTIM_up
RBM18	COSTIM_up

MAPKAP1	COSTIM_up
ALG2	COSTIM_up
ZBTB45	COSTIM_up
DCAF4	COSTIM_up
FCF1	COSTIM_up
NEK9	COSTIM_up
ACOT2	COSTIM_up
AREL1	COSTIM_up
MLH3	COSTIM_up
SLIRP	COSTIM_up
RBM25	COSTIM_up
EIF2B2	COSTIM_up
NRDE2	COSTIM_up
GPR75	COSTIM_up
SUPT7L	COSTIM_up
DNMT3A	COSTIM_up
TMEM214	COSTIM_up
FAM98A	COSTIM_up
IDE	COSTIM_up
GPAM	COSTIM_up
SMNDC1	COSTIM_up
HELLS	COSTIM_up
TCTN3	COSTIM_up
KCNIP2	COSTIM_up
GOT1	COSTIM_up
PANK3	COSTIM_up
RCL1	COSTIM_up
CAAP1	COSTIM_up
CD274	COSTIM_up
NUP43	COSTIM_up
MTHFD1L	COSTIM_up
PCMT1	COSTIM_up
CYSTM1	COSTIM_up
WDR55	COSTIM_up
CENPL	COSTIM_up
ACAT2	COSTIM_up
TCP1	COSTIM_up
MSANTD2	COSTIM_up
PDZD11	COSTIM_up
NUDCD1	COSTIM_up
ENY2	COSTIM_up
KIAA1217	COSTIM_up
TAF12	COSTIM_up
TNFSF11	COSTIM_up
DNAJC15	COSTIM_up
PROSER1	COSTIM_up
UFM1	COSTIM_up
WBP4	COSTIM_up
HSPH1	COSTIM_up
ALG5	COSTIM_up

EXOSC8	COSTIM_up
ETF1	COSTIM_up
SIL1	COSTIM_up
SERP1	COSTIM_up
UTP20	COSTIM_up
TMPO	COSTIM_up
ARL1	COSTIM_up
MTERF2	COSTIM_up
SOCS2	COSTIM_up
DUSP4	COSTIM_up
TNFRSF10B	COSTIM_up
SORBS3	COSTIM_up
PPP3CC	COSTIM_up
PDLIM2	COSTIM_up
UBIAD1	COSTIM_up
TARDBP	COSTIM_up
TNFRSF8	COSTIM_up
ZNF706	COSTIM_up
LYPLA1	COSTIM_up
COPS5	COSTIM_up
AKAP1	COSTIM_up
COIL	COSTIM_up
SLC35B1	COSTIM_up
PYROXD1	COSTIM_up
LHX4	COSTIM_up
RNF2	COSTIM_up
SEC22A	COSTIM_up
B4GALT4	COSTIM_up
NAA50	COSTIM_up
CD80	COSTIM_up
DESI2	COSTIM_up
ZCCHC17	COSTIM_up
KHDRBS1	COSTIM_up
TMEM39B	COSTIM_up
ZNF639	COSTIM_up
PDS5A	COSTIM_up
LIAS	COSTIM_up
GTDC1	COSTIM_up
ZRANB3	COSTIM_up
RPL21	COSTIM_up
GTF3A	COSTIM_up
RASL11A	COSTIM_up
FYTTD1	COSTIM_up
MRPS2	COSTIM_up
COPA	COSTIM_up
RBBP6	COSTIM_up
ZC3H7A	COSTIM_up
SHLD2	COSTIM_up
PRXL2A	COSTIM_up
RPL5	COSTIM_up

TRMT13	COSTIM_up
RPAP2	COSTIM_up
PMS2	COSTIM_up
ZMIZ2	COSTIM_up
CBX3	COSTIM_up
HNRNPA2B1	COSTIM_up
INHBA	COSTIM_up
ARL4A	COSTIM_up
CCZ1	COSTIM_up
MRM2	COSTIM_up
SMU1	COSTIM_up
SLC25A51	COSTIM_up
CLTA	COSTIM_up
DCAF10	COSTIM_up
PLAU	COSTIM_up
SRGN	COSTIM_up
CISD1	COSTIM_up
ECD	COSTIM_up
P4HA1	COSTIM_up
SLC25A16	COSTIM_up
ZWINT	COSTIM_up
VPS26A	COSTIM_up
RBM19	COSTIM_up
DDX54	COSTIM_up
CDKN2C	COSTIM_up
RNF11	COSTIM_up
ACOT9	COSTIM_up
PRDX4	COSTIM_up
DDX39A	COSTIM_up
PKN1	COSTIM_up
TRIR	COSTIM_up
ADGRE5	COSTIM_up
GIPC1	COSTIM_up
SPRYD7	COSTIM_up
EBPL	COSTIM_up
ZC3H13	COSTIM_up
NLN	COSTIM_up
OPTN	COSTIM_up
ATF1	COSTIM_up
TSFM	COSTIM_up
SPATS2	COSTIM_up
ORMDL2	COSTIM_up
ATG101	COSTIM_up
IKZF4	COSTIM_up
TUBA1B	COSTIM_up
EEF1AKMT3	COSTIM_up
KBTBD4	COSTIM_up
ATPAF1	COSTIM_up
AMD1	COSTIM_up
NDUFAF4	COSTIM_up

MORF4L2	COSTIM_up
RAB9A	COSTIM_up
NMI	COSTIM_up
SLC36A1	COSTIM_up
BATF3	COSTIM_up
G0S2	COSTIM_up
RAP2C	COSTIM_up
EXOSC9	COSTIM_up
PLA2G12A	COSTIM_up
CKS2	COSTIM_up
ACSL3	COSTIM_up
CHPF	COSTIM_up
DNPEP	COSTIM_up
ENKD1	COSTIM_up
TTPAL	COSTIM_up
PREX1	COSTIM_up
NCOA5	COSTIM_up
VAPB	COSTIM_up
ATP5F1E	COSTIM_up
TOX2	COSTIM_up
SRSF6	COSTIM_up
ARFGEF2	COSTIM_up
ZNFX1	COSTIM_up
CSE1L	COSTIM_up
RAB22A	COSTIM_up
STAU1	COSTIM_up
MOCS3	COSTIM_up
DDX27	COSTIM_up
KCNK15	COSTIM_up
MTRR	COSTIM_up
FASTKD3	COSTIM_up
PEPD	COSTIM_up
VAMP7	COSTIM_up
STAMBP	COSTIM_up
SNRNP27	COSTIM_up
MPHOSPH10	COSTIM_up
ZNF576	COSTIM_up
BTN2A2	COSTIM_up
MRS2	COSTIM_up
WRNIP1	COSTIM_up
RRP36	COSTIM_up
SNRPC	COSTIM_up
XPO5	COSTIM_up
ABCC10	COSTIM_up
PEX6	COSTIM_up
AARS2	COSTIM_up
RPS10	COSTIM_up
MAD2L1BP	COSTIM_up
MEA1	COSTIM_up
CDKN1A	COSTIM_up

GLO1	COSTIM_up
CPNE5	COSTIM_up
RREB1	COSTIM_up
SSR1	COSTIM_up
RIOK1	COSTIM_up
RPP40	COSTIM_up
ATXN1	COSTIM_up
NUP153	COSTIM_up
EEF1E1	COSTIM_up
RUNX2	COSTIM_up
AHNAK	COSTIM_up
EMC3	COSTIM_up
CNOT1	COSTIM_up
GOT2	COSTIM_up
RAP2A	COSTIM_up
ABCC4	COSTIM_up
HROB	COSTIM_up
UPF3B	COSTIM_up
NDUFA1	COSTIM_up
FAM193A	COSTIM_up
MRPS7	COSTIM_up
GGA3	COSTIM_up
NUP85	COSTIM_up
SLC25A19	COSTIM_up
NT5C	COSTIM_up
MSTO1	COSTIM_up
C1orf61	COSTIM_up
GTF3C4	COSTIM_up
DDX31	COSTIM_up
PPP1R12C	COSTIM_up
PPDPF	COSTIM_up
IL1B	COSTIM_up
CHCHD5	COSTIM_up
POLR1B	COSTIM_up
GTF2F1	COSTIM_up
CLPP	COSTIM_up
MED1	COSTIM_up
RPL23	COSTIM_up
CD70	COSTIM_up
TRIP10	COSTIM_up
TNFSF14	COSTIM_up
OPA3	COSTIM_up
SNRPD2	COSTIM_up
EML2	COSTIM_up
VASP	COSTIM_up
SYMPK	COSTIM_up
GZF1	COSTIM_up
CENPB	COSTIM_up
PSMF1	COSTIM_up
DTD1	COSTIM_up

RBCK1	COSTIM_up
STK35	COSTIM_up
SNRPB	COSTIM_up
DSTN	COSTIM_up
SNRPB2	COSTIM_up
ITPA	COSTIM_up
MCM8	COSTIM_up
MRPS26	COSTIM_up
NCLN	COSTIM_up
HNRNPR	COSTIM_up
ARMCX5	COSTIM_up
RALY	COSTIM_up
DYNLRB1	COSTIM_up
EIF2S2	COSTIM_up
ERGIC3	COSTIM_up
ROMO1	COSTIM_up
PLAGL2	COSTIM_up
PSMB2	COSTIM_up
AGO3	COSTIM_up
UROD	COSTIM_up
PCID2	COSTIM_up
CAPNS1	COSTIM_up
PDCD2L	COSTIM_up
RBM42	COSTIM_up
KIRREL2	COSTIM_up
UBA2	COSTIM_up
FFAR1	COSTIM_up
COX6B1	COSTIM_up
CCR7	COSTIM_up
PRDX5	COSTIM_up
BCL2L12	COSTIM_up
PRMT1	COSTIM_up
SCAF1	COSTIM_up
SBDS	COSTIM_up
STAT5A	COSTIM_up
BECN1	COSTIM_up
TRAP1	COSTIM_up
NSRP1	COSTIM_up
DNAJC8	COSTIM_up
ZNF384	COSTIM_up
EMG1	COSTIM_up
UXT	COSTIM_up
ELK1	COSTIM_up
TIMM17B	COSTIM_up
PCNX4	COSTIM_up
KTN1	COSTIM_up
TRMT5	COSTIM_up
NUP214	COSTIM_up
MAP2K2	COSTIM_up
HNRNPH2	COSTIM_up

TIMM8A	COSTIM_up
CANX	COSTIM_up
INTS11	COSTIM_up
IPPK	COSTIM_up
OMD	COSTIM_up
HIVEP3	COSTIM_up
COX7C	COSTIM_up
TRAF2	COSTIM_up
ABHD8	COSTIM_up
RAP1B	COSTIM_up
RAB3IP	COSTIM_up
YEATS4	COSTIM_up
TRPV5	COSTIM_up
PIN1	COSTIM_up
EMC1	COSTIM_up
UBR4	COSTIM_up
ADGRE2	COSTIM_up
SIN3B	COSTIM_up
SLC35E1	COSTIM_up
UQCR11	COSTIM_up
GFER	COSTIM_up
WDR24	COSTIM_up
CHTF18	COSTIM_up
TUBBP1	COSTIM_up
MACF1	COSTIM_up
SMARCA4	COSTIM_up
TICAM1	COSTIM_up
EMC6	COSTIM_up
METTL16	COSTIM_up
AAMP	COSTIM_up
RNF6	COSTIM_up
ECHS1	COSTIM_up
GNG11	COSTIM_up
SEM1	COSTIM_up
POR	COSTIM_up
STYXL1	COSTIM_up
MTERF1	COSTIM_up
ZFP36	COSTIM_up
SRD5A3	COSTIM_up
PAICS	COSTIM_up
PPAT	COSTIM_up
TUBGCP6	COSTIM_up
ADM2	COSTIM_up
DGCR8	COSTIM_up
SDF2L1	COSTIM_up
YWHAH	COSTIM_up
MGAT3	COSTIM_up
ATF4	COSTIM_up
A4GALT	COSTIM_up
EMC4	COSTIM_up

ATP6V1F	COSTIM_up
LSM8	COSTIM_up
STRIP2	COSTIM_up
IFT22	COSTIM_up
LRRC4	COSTIM_up
CALU	COSTIM_up
IRF5	COSTIM_up
NDUFA5	COSTIM_up
MRPS12	COSTIM_up
MTX2	COSTIM_up
EIF2S2P4	COSTIM_up
HAT1	COSTIM_up
PSMG2	COSTIM_up
INO80	COSTIM_up
ICE2	COSTIM_up
KNSTRN	COSTIM_up
DUT	COSTIM_up
CHAC1	COSTIM_up
CLN6	COSTIM_up
ARPP19	COSTIM_up
CALML4	COSTIM_up
ANAPC13	COSTIM_up
COPB1	COSTIM_up
PSMA1	COSTIM_up
SUMF2	COSTIM_up
SPCS3	COSTIM_up
DCTD	COSTIM_up
USP6	COSTIM_up
PLD2	COSTIM_up
CD68	COSTIM_up
TXNDC17	COSTIM_up
KIF1C	COSTIM_up
MPDU1	COSTIM_up
CCNT1	COSTIM_up
PUS7L	COSTIM_up
KRI1	COSTIM_up
ILF3	COSTIM_up
NGDN	COSTIM_up
DTD2	COSTIM_up
SNX6	COSTIM_up
EGLN3	COSTIM_up
NEDD8	COSTIM_up
DAD1	COSTIM_up
MAP7D3	COSTIM_up
TTI2	COSTIM_up
CHRNA10	COSTIM_up
KLF16	COSTIM_up
DOHH	COSTIM_up
ABHD17A	COSTIM_up
CBFA2T3	COSTIM_up

GALNT8	COSTIM_up
CRACR2A	COSTIM_up
GNL3L	COSTIM_up
ECSIT	COSTIM_up
LDLR	COSTIM_up
ELOF1	COSTIM_up
TOMM40	COSTIM_up
APOC1	COSTIM_up
GADD45G	COSTIM_up
XPO7	COSTIM_up
RPL36	COSTIM_up
KIF1A	COSTIM_up
GTPBP3	COSTIM_up
BST2	COSTIM_up
NSUN5	COSTIM_up
DDA1	COSTIM_up
PGLS	COSTIM_up
LSM7	COSTIM_up
SNX9	COSTIM_up
MLLT1	COSTIM_up
ACTN4	COSTIM_up
STK33	COSTIM_up
NDUFA10	COSTIM_up
ARPC1B	COSTIM_up
UBAC1	COSTIM_up
ZBTB46	COSTIM_up
SAMD10	COSTIM_up
LSP1	COSTIM_up
COL5A1	COSTIM_up
ATXN10	COSTIM_up
TUBGCP2	COSTIM_up
CEP85	COSTIM_up
ADRM1	COSTIM_up
ASS1	COSTIM_up
EXOSC2	COSTIM_up
UCK1	COSTIM_up
CHMP2A	COSTIM_up
UBE2M	COSTIM_up
TRIM28	COSTIM_up
METTL26	COSTIM_up
YIPF2	COSTIM_up
EIF2S3	COSTIM_up
TMEM160	COSTIM_up
ZC3H4	COSTIM_up
LRRC47	COSTIM_up
SESN2	COSTIM_up
ATP5IF1	COSTIM_up
CLIP1	COSTIM_up
ZNF317	COSTIM_up
PPAN	COSTIM_up

EIF3G	COSTIM_up
DNMT1	COSTIM_up
ZNF426	COSTIM_up
DKC1	COSTIM_up
PLXNA3	COSTIM_up
NOL11	COSTIM_up
UBE4B	COSTIM_up
CASZ1	COSTIM_up
UBA1	COSTIM_up
AKAP12	COSTIM_up
LATS1	COSTIM_up
AAR2	COSTIM_up
RBM39	COSTIM_up
ZNF341	COSTIM_up
ATP6V1E1	COSTIM_up
COX4I1	COSTIM_up
EMC8	COSTIM_up
CHMP1A	COSTIM_up
COX7B	COSTIM_up
CAP1	COSTIM_up
PPT1	COSTIM_up
RLIM	COSTIM_up
TRAF3	COSTIM_up
MRPS25	COSTIM_up
RFTN1	COSTIM_up
SLC6A6	COSTIM_up
NR1H2	COSTIM_up
KIF3A	COSTIM_up
TUBG1	COSTIM_up
PSME3	COSTIM_up
RPL27	COSTIM_up
PSMC3IP	COSTIM_up
AOC3	COSTIM_up
ACLY	COSTIM_up
VPS25	COSTIM_up
AOC2	COSTIM_up
NDUFA2	COSTIM_up
DIAPH1	COSTIM_up
UBE2D2	COSTIM_up
C1orf159	COSTIM_up
PPFIA1	COSTIM_up
KREMEN2	COSTIM_up
THOC6	COSTIM_up
TRAF7	COSTIM_up
NINJ1	COSTIM_up
MAP1B	COSTIM_up
IL13RA1	COSTIM_up
WDR44	COSTIM_up
ZCCHC9	COSTIM_up
RARA	COSTIM_up

KHDRBS3	COSTIM_up
CHD1L	COSTIM_up
PEX11B	COSTIM_up
PDHA1	COSTIM_up
MCCC2	COSTIM_up
SELENOS	COSTIM_up
SNRPA1	COSTIM_up
THAP1	COSTIM_up
C19orf12	COSTIM_up
ACTR10	COSTIM_up
GCH1	COSTIM_up
LGALS3	COSTIM_up
DNAJB1	COSTIM_up
ZSWIM4	COSTIM_up
FBXW9	COSTIM_up
TRIM21	COSTIM_up
LRRC41	COSTIM_up
DHX30	COSTIM_up
HSD17B7	COSTIM_up
ENOSF1	COSTIM_up
EMILIN2	COSTIM_up
SLX1A	COSTIM_up
ARFIP2	COSTIM_up
RRP8	COSTIM_up
PTCD3	COSTIM_up
IMMT	COSTIM_up
MRPL35	COSTIM_up
ILKAP	COSTIM_up
PER2	COSTIM_up
PTPRE	COSTIM_up
RAN	COSTIM_up
CLUH	COSTIM_up
MYBBP1A	COSTIM_up
RPA1	COSTIM_up
UBE2G1	COSTIM_up
EEFSEC	COSTIM_up
COQ3	COSTIM_up
SEC61G	COSTIM_up
LANCL2	COSTIM_up
FIGNL1	COSTIM_up
GRSF1	COSTIM_up
ANKRD17	COSTIM_up
UTP3	COSTIM_up
WBP2	COSTIM_up
H3-3B	COSTIM_up
ZRANB2	COSTIM_up
EIF5A	COSTIM_up
KDM6B	COSTIM_up
GPS2	COSTIM_up
RIDA	COSTIM_up

MATN2	COSTIM_up
ERAL1	COSTIM_up
NIP7	COSTIM_up
VPS4A	COSTIM_up
PCNA	COSTIM_up
NXT1	COSTIM_up
POLR3F	COSTIM_up
DAP3	COSTIM_up
KHDC4	COSTIM_up
MMACHC	COSTIM_up
DPH2	COSTIM_up
TOE1	COSTIM_up
NASP	COSTIM_up
MUTYH	COSTIM_up
CTNNBL1	COSTIM_up
ZBED3	COSTIM_up
DCTN4	COSTIM_up
MTUS2	COSTIM_up
XPO4	COSTIM_up
POMP	COSTIM_up
CDK8	COSTIM_up
PEMT	COSTIM_up
SCO1	COSTIM_up
SPART	COSTIM_up
RFC3	COSTIM_up
BEX2	COSTIM_up
TCEAL4	COSTIM_up
FAM104A	COSTIM_up
SLC39A11	COSTIM_up
SRRM1	COSTIM_up
ZNF414	COSTIM_up
HSPBP1	COSTIM_up
CNDP2	COSTIM_up
MACROD1	COSTIM_up
WDR74	COSTIM_up
MYH11	COSTIM_up
MED10	COSTIM_up
MORC2	COSTIM_up
AGAP3	COSTIM_up
KRBA1	COSTIM_up
ACTR3B	COSTIM_up
C12orf29	COSTIM_up
ATP13A3	COSTIM_up
KRAS	COSTIM_up
IPO8	COSTIM_up
LARS1	COSTIM_up
CCDC59	COSTIM_up
SWAP70	COSTIM_up
AMPD3	COSTIM_up
DPF2	COSTIM_up

MEN1	COSTIM_up
ERG28	COSTIM_up
COX16	COSTIM_up
MED6	COSTIM_up
EIF2S1	COSTIM_up
ELP3	COSTIM_up
ADAMDEC1	COSTIM_up
CTIF	COSTIM_up
MBD2	COSTIM_up
IER3IP1	COSTIM_up
MRPS36	COSTIM_up
CCNB1	COSTIM_up
CDK7	COSTIM_up
THUMPD3	COSTIM_up
BHLHE40	COSTIM_up
ARL8B	COSTIM_up
EDEM1	COSTIM_up
MEIS2	COSTIM_up
DPH6	COSTIM_up
EMC7	COSTIM_up
PRPF38B	COSTIM_up
LAMTOR5	COSTIM_up
TRIM45	COSTIM_up
NAPG	COSTIM_up
FKBP11	COSTIM_up
ARF3	COSTIM_up
TMEM106C	COSTIM_up
SLC38A2	COSTIM_up
YWHAQ	COSTIM_up
LDHA	COSTIM_up
CDC73	COSTIM_up
TIMM17A	COSTIM_up
NARS1	COSTIM_up
RBM17	COSTIM_up
IL2RA	COSTIM_up
ECHDC3	COSTIM_up
IL15RA	COSTIM_up
TMEM241	COSTIM_up
SOX5	COSTIM_up
PRH2	COSTIM_up
RBMX2	COSTIM_up
PUM1	COSTIM_up
YARS1	COSTIM_up
GNL2	COSTIM_up
BTF3L4	COSTIM_up
PRPF38A	COSTIM_up
RNF138	COSTIM_up
TPGS2	COSTIM_up
SLC43A3	COSTIM_up
TIMM10	COSTIM_up

DHX34	COSTIM_up
FADS2	COSTIM_up
TMEM258	COSTIM_up
TMEM165	COSTIM_up
CLOCK	COSTIM_up
STT3A	COSTIM_up
KLB	COSTIM_up
TMED7	COSTIM_up
WDR36	COSTIM_up
RFK	COSTIM_up
UBQLN1	COSTIM_up
C9orf40	COSTIM_up
AGTPBP1	COSTIM_up
PSAT1	COSTIM_up
P2RX4	COSTIM_up
DTX1	COSTIM_up
TRAFD1	COSTIM_up
HILPDA	COSTIM_up
RINT1	COSTIM_up
KCP	COSTIM_up
TES	COSTIM_up
MDFIC	COSTIM_up
SYNCRIP	COSTIM_up
ORC3	COSTIM_up
LMO2	COSTIM_up
NAT10	COSTIM_up
CAPRIN1	COSTIM_up
ATP5MC2	COSTIM_up
DNAJC14	COSTIM_up
CD63	COSTIM_up
FAM186B	COSTIM_up
BLOC1S1	COSTIM_up
CDK4	COSTIM_up
TSPAN31	COSTIM_up
B4GALNT1	COSTIM_up
TFCP2	COSTIM_up
ESPL1	COSTIM_up
HNRNPA1	COSTIM_up
ACVR1B	COSTIM_up
LTV1	COSTIM_up
CD164	COSTIM_up
SMPD2	COSTIM_up
MICAL1	COSTIM_up
STX11	COSTIM_up
TEC	COSTIM_up
CCT7	COSTIM_up
SMYD5	COSTIM_up
EMX1	COSTIM_up
GNS	COSTIM_up
MDM2	COSTIM_up

MPHOSPH6	COSTIM_up
DYNC1LI2	COSTIM_up
FHOD1	COSTIM_up
CCDC102A	COSTIM_up
SLC9A5	COSTIM_up
URB2	COSTIM_up
COG2	COSTIM_up
NTPCR	COSTIM_up
TAF5L	COSTIM_up
STX6	COSTIM_up
DHX9	COSTIM_up
KIAA1614	COSTIM_up
NIBAN1	COSTIM_up
GPR55	COSTIM_up
MRPL44	COSTIM_up
DOCK10	COSTIM_up
TTLL4	COSTIM_up
USP37	COSTIM_up
HTR2B	COSTIM_up
SERPINE2	COSTIM_up
EIF4E2	COSTIM_up
ARMC9	COSTIM_up
CAB39	COSTIM_up
COX5B	COSTIM_up
TGFBRAP1	COSTIM_up
MRPS9	COSTIM_up
C2orf49	COSTIM_up
ISCU	COSTIM_up
ALDH1L2	COSTIM_up
SCYL2	COSTIM_up
PLXNC1	COSTIM_up
PWP1	COSTIM_up
DRAM1	COSTIM_up
SLC41A2	COSTIM_up
VILL	COSTIM_up
TBC1D4	COSTIM_up
LRCH1	COSTIM_up
SUCLA2	COSTIM_up
MED4	COSTIM_up
PHF11	COSTIM_up
NUDT15	COSTIM_up
LCP1	COSTIM_up
GPNMB	COSTIM_up
RAC1	COSTIM_up
KDELR2	COSTIM_up
IL6	COSTIM_up
BZW2	COSTIM_up
TBRG4	COSTIM_up
DDX56	COSTIM_up
MYO1G	COSTIM_up

TTC5	COSTIM_up
ZFHX2	COSTIM_up
MTHFS	COSTIM_up
ADAMTS7	COSTIM_up
IREB2	COSTIM_up
ALPK3	COSTIM_up
RSAD1	COSTIM_up
NMT1	COSTIM_up
SRSF1	COSTIM_up
TACO1	COSTIM_up
TEX2	COSTIM_up
DCAF7	COSTIM_up
ACTL6A	COSTIM_up
MRPL47	COSTIM_up
TRA2B	COSTIM_up
EPRS1	COSTIM_up
IL10	COSTIM_up
RPS6KC1	COSTIM_up
CBWD2	COSTIM_up
IL36G	COSTIM_up
SMPD4	COSTIM_up
WDR33	COSTIM_up
IMP4	COSTIM_up
HS6ST1	COSTIM_up
UGGT1	COSTIM_up
GYPC	COSTIM_up
ABI1	COSTIM_up
YME1L1	COSTIM_up
TXN	COSTIM_up
ODF2	COSTIM_up
ECPAS	COSTIM_up
C9orf78	COSTIM_up
SMC2	COSTIM_up
TOR1A	COSTIM_up
SLC2A8	COSTIM_up
PRPF4	COSTIM_up
FPGS	COSTIM_up
TEX10	COSTIM_up
MRPL50	COSTIM_up
DPM2	COSTIM_up
WDR38	COSTIM_up
TSTD2	COSTIM_up
PSMB7	COSTIM_up
RABEPK	COSTIM_up
ANP32B	COSTIM_up
RPL35	COSTIM_up
ARPC5L	COSTIM_up
DERL1	COSTIM_up
MYC	COSTIM_up
DMAC1	COSTIM_up

POLR1E	COSTIM_up
PLAA	COSTIM_up
UBAP2	COSTIM_up
APTX	COSTIM_up
TLN1	COSTIM_up
DNAJB5	COSTIM_up
DCTN3	COSTIM_up
GRHPR	COSTIM_up
ALDH1B1	COSTIM_up
DENND4C	COSTIM_up
RPS6	COSTIM_up
FOXP4	COSTIM_up
PPIL1	COSTIM_up
PIM1	COSTIM_up
CMTR1	COSTIM_up
TMEM14B	COSTIM_up
TMEM63B	COSTIM_up
TJAP1	COSTIM_up
IRF4	COSTIM_up
TUBB2A	COSTIM_up
TUBB2B	COSTIM_up
UQCC2	COSTIM_up
HMGA1	COSTIM_up
IER3	COSTIM_up
MDC1	COSTIM_up
ATAT1	COSTIM_up
MTCH1	COSTIM_up
VARS2	COSTIM_up
CPEB2	COSTIM_up
THAP12	COSTIM_up
LRRC32	COSTIM_up
NARS2	COSTIM_up
RNF121	COSTIM_up
MRPL15	COSTIM_up
GGH	COSTIM_up
SLCO5A1	COSTIM_up
TGS1	COSTIM_up
SDCBP	COSTIM_up
BUD13	COSTIM_up
DCUN1D5	COSTIM_up
SLC37A4	COSTIM_up
RDX	COSTIM_up
C11orf1	COSTIM_up
CASP1	COSTIM_up
ALKBH8	COSTIM_up
SQOR	COSTIM_up
SLTM	COSTIM_up
NDUFAF1	COSTIM_up
HAUS2	COSTIM_up
RPLP1	COSTIM_up

TUBGCP4	COSTIM_up
RMDN3	COSTIM_up
RSL24D1	COSTIM_up
BCAR3	COSTIM_up
GTF2B	COSTIM_up
RABGGTB	COSTIM_up
IFI44L	COSTIM_up
RTCA	COSTIM_up
SELENOI	COSTIM_up
HADHB	COSTIM_up
ADCY3	COSTIM_up
PNPT1	COSTIM_up
THUMPD2	COSTIM_up
RAB1A	COSTIM_up
ACTR2	COSTIM_up
PREB	COSTIM_up
SLC5A6	COSTIM_up
ATRAID	COSTIM_up
CENPO	COSTIM_up
LRPPRC	COSTIM_up
ACTR1A	COSTIM_up
ATAD1	COSTIM_up
DUSP5	COSTIM_up
DBR1	COSTIM_up
ANXA7	COSTIM_up
ZNF365	COSTIM_up
RPS24	COSTIM_up
ATIC	COSTIM_up
ASNSD1	COSTIM_up
METTL5	COSTIM_up
SSB	COSTIM_up
PPIG	COSTIM_up
FASTKD1	COSTIM_up
OLA1	COSTIM_up
WDR12	COSTIM_up
COX17	COSTIM_up
PARP9	COSTIM_up
SPPL2A	COSTIM_up
SHF	COSTIM_up
INTS14	COSTIM_up
PPCDC	COSTIM_up
SEMA7A	COSTIM_up
AP1AR	COSTIM_up
COPS4	COSTIM_up
HNRNPD	COSTIM_up
SEC31A	COSTIM_up
LARP1B	COSTIM_up
NUP54	COSTIM_up
CXCL9	COSTIM_up
BMP2K	COSTIM_up

G3BP2	COSTIM_up
SEPTIN11	COSTIM_up
USO1	COSTIM_up
PPA2	COSTIM_up
GSTCD	COSTIM_up
INTS12	COSTIM_up
SEC24B	COSTIM_up
RNF185	COSTIM_up
KIF21A	COSTIM_up
CPNE8	COSTIM_up
YARS2	COSTIM_up
SINHCAF	COSTIM_up
AEBP2	COSTIM_up
ETNK1	COSTIM_up
ZCRB1	COSTIM_up
NDUFA9	COSTIM_up
PEX5	COSTIM_up
AMIGO2	COSTIM_up
LLPH	COSTIM_up
SNRPF	COSTIM_up
ASCL1	COSTIM_up
SLC15A4	COSTIM_up
TDG	COSTIM_up
RITA1	COSTIM_up
MMAB	COSTIM_up
GLTP	COSTIM_up
NUP58	COSTIM_up
MTMR6	COSTIM_up
SLC7A1	COSTIM_up
TARBP2	COSTIM_up
NABP2	COSTIM_up
KANSL2	COSTIM_up
C12orf10	COSTIM_up
TMBIM6	COSTIM_up
ANKRD52	COSTIM_up
ESD	COSTIM_up
SBNO1	COSTIM_up
SETD1B	COSTIM_up
VPS33A	COSTIM_up
RHOF	COSTIM_up
DENR	COSTIM_up
RBM26	COSTIM_up
ZIC5	COSTIM_up
CUL4A	COSTIM_up
TMX1	COSTIM_up
NAA30	COSTIM_up
WDR89	COSTIM_up
WARS1	COSTIM_up
NIPA2	COSTIM_up
MFAP1	COSTIM_up

SORD	COSTIM_up
SERF2	COSTIM_up
ZSCAN29	COSTIM_up
BNIP2	COSTIM_up
GTF2A2	COSTIM_up
SRP14	COSTIM_up
BAHD1	COSTIM_up
ANP32A	COSTIM_up
ETFA	COSTIM_up
BCL2A1	COSTIM_up
WDR61	COSTIM_up
MAN2C1	COSTIM_up
DNAJA4	COSTIM_up
TLNRD1	COSTIM_up
PML	COSTIM_up
CYP1A1	COSTIM_up
CELF6	COSTIM_up
SCAMP2	COSTIM_up
HAPLN3	COSTIM_up
POLG	COSTIM_up
ABHD2	COSTIM_up
ZNF710	COSTIM_up
UNC45A	COSTIM_up
SEC11A	COSTIM_up
GLYR1	COSTIM_up
PMM2	COSTIM_up
ARMC5	COSTIM_up
UQCRC2	COSTIM_up
ARHGAP17	COSTIM_up
DHX38	COSTIM_up
TXNL4B	COSTIM_up
CPNE2	COSTIM_up
NLRC5	COSTIM_up
KATNB1	COSTIM_up
GCSH	COSTIM_up
MAP1LC3B	COSTIM_up
RHOT2	COSTIM_up
RPS2	COSTIM_up
NDUFB10	COSTIM_up
COPS3	COSTIM_up
MYOCD	COSTIM_up
KSR1	COSTIM_up
UTP4	COSTIM_up
GFOD2	COSTIM_up
NOB1	COSTIM_up
C17orf80	COSTIM_up
VPS53	COSTIM_up
NPEPPS	COSTIM_up
G6PC3	COSTIM_up
CLTC	COSTIM_up

PTRH2	COSTIM_up
TAF4B	COSTIM_up
AFG3L2	COSTIM_up
SLC39A6	COSTIM_up
C18orf21	COSTIM_up
GALNT1	COSTIM_up
PELP1	COSTIM_up
WRAP53	COSTIM_up
PIK3R5	COSTIM_up
TP53	COSTIM_up
ARHGDIA	COSTIM_up
EIF4A3	COSTIM_up
ANAPC11	COSTIM_up
RPTOR	COSTIM_up
FOXK2	COSTIM_up
TRIM65	COSTIM_up
RNF157	COSTIM_up
CEP131	COSTIM_up
WDR45B	COSTIM_up
DYM	COSTIM_up
MBD1	COSTIM_up
PMAIP1	COSTIM_up
NT5C3B	COSTIM_up
MIEN1	COSTIM_up
TXNL4A	COSTIM_up
SAMD1	COSTIM_up
BRD4	COSTIM_up
SLC39A3	COSTIM_up
NFIC	COSTIM_up
PRDM15	COSTIM_up
FEM1A	COSTIM_up
DUS3L	COSTIM_up
DPP9	COSTIM_up
CCDC97	COSTIM_up
HUNK	COSTIM_up
SOD1	COSTIM_up
SCYL1	COSTIM_up
APP	COSTIM_up
URB1	COSTIM_up
AKT1	COSTIM_up
EMP3	COSTIM_up
SAE1	COSTIM_up
GEMIN7	COSTIM_up
CAPN10	COSTIM_up
ERVK3-1	COSTIM_up
ZNF787	COSTIM_up
CARM1	COSTIM_up
PSMB6	COSTIM_up
ACP4	COSTIM_up
ZNF473	COSTIM_up

RPL13A	COSTIM_up
CTU1	COSTIM_up
NOSIP	COSTIM_up
ZNF614	COSTIM_up
EFHD2	COSTIM_up
PEX14	COSTIM_up
PGD	COSTIM_up
RPL11	COSTIM_up
ZNF593	COSTIM_up
C1orf216	COSTIM_up
GPN2	COSTIM_up
SERBP1	COSTIM_up
RPS8	COSTIM_up
IGSF3	COSTIM_up
STRIP1	COSTIM_up
PSMA5	COSTIM_up
ITGA10	COSTIM_up
TIPRL	COSTIM_up
POGK	COSTIM_up
UCK2	COSTIM_up
TMCO1	COSTIM_up
UFC1	COSTIM_up
SDHC	COSTIM_up
PFDN2	COSTIM_up
PRCC	COSTIM_up
MRPL24	COSTIM_up
ISG20L2	COSTIM_up
CRABP2	COSTIM_up
HDGF	COSTIM_up
ABL2	COSTIM_up
RGS16	COSTIM_up
RGL1	COSTIM_up
SF3B4	COSTIM_up
TARS2	COSTIM_up
SETDB1	COSTIM_up
MCL1	COSTIM_up
RFX5	COSTIM_up
PIP5K1A	COSTIM_up
ANP32E	COSTIM_up
CERS2	COSTIM_up
ENSA	COSTIM_up
MRPL9	COSTIM_up
DTL	COSTIM_up
EIF2D	COSTIM_up
INTS7	COSTIM_up
TAF1A	COSTIM_up
DUSP10	COSTIM_up
HHIPL2	COSTIM_up
TP53BP2	COSTIM_up
ADAM15	COSTIM_up

JTB	COSTIM_up
RAB13	COSTIM_up
TPM3	COSTIM_up
NUP210L	COSTIM_up
SNAPIN	COSTIM_up
UBAP2L	COSTIM_up
SLC39A1	COSTIM_up
HAX1	COSTIM_up
C1orf43	COSTIM_up
ILF2	COSTIM_up
RIT1	COSTIM_up
INTS3	COSTIM_up
FLG	COSTIM_up
GALNT2	COSTIM_up
SCCPDH	COSTIM_up
ACP1	COSTIM_up
SNAP47	COSTIM_up
SRP9	COSTIM_up
SDE2	COSTIM_up
ARF1	COSTIM_up
ITPKB	COSTIM_up
GUK1	COSTIM_up
C1orf35	COSTIM_up
PARP1	COSTIM_up
PYCR2	COSTIM_up
ARL8A	COSTIM_up
PDIA6	COSTIM_up
EML4	COSTIM_up
CALM2	COSTIM_up
CHAC2	COSTIM_up
RPS27A	COSTIM_up
VPS54	COSTIM_up
SNRPG	COSTIM_up
CIAO1	COSTIM_up
SNRNP200	COSTIM_up
MRPS5	COSTIM_up
TPRKB	COSTIM_up
TEX261	COSTIM_up
TMEM177	COSTIM_up
NT5DC4	COSTIM_up
SLC20A1	COSTIM_up
ZC3H8	COSTIM_up
UBXN4	COSTIM_up
SPOPL	COSTIM_up
POLR2D	COSTIM_up
AMMECR1L	COSTIM_up
LNPK	COSTIM_up
CDCA7	COSTIM_up
HSPD1	COSTIM_up
METTL21A	COSTIM_up

COPS7B	COSTIM_up
DIS3L2	COSTIM_up
TAMM41	COSTIM_up
RETREG2	COSTIM_up
CNOT9	COSTIM_up
GMPPA	COSTIM_up
EAF1	COSTIM_up
DYNC1LI1	COSTIM_up
GOLGA4	COSTIM_up
IQSEC1	COSTIM_up
CAND2	COSTIM_up
RPL32	COSTIM_up
SHQ1	COSTIM_up
SLC25A26	COSTIM_up
UBA3	COSTIM_up
LRIG1	COSTIM_up
NFKBIZ	COSTIM_up
PLA1A	COSTIM_up
RABL3	COSTIM_up
ADPRH	COSTIM_up
ATG3	COSTIM_up
NR1I2	COSTIM_up
SRPRB	COSTIM_up
EIF2A	COSTIM_up
DCAF1	COSTIM_up
MANF	COSTIM_up
EIF2B5	COSTIM_up
FIP1L1	COSTIM_up
LYAR	COSTIM_up
ENOPH1	COSTIM_up
TRMT10A	COSTIM_up
PYURF	COSTIM_up
CISD2	COSTIM_up
ANK2	COSTIM_up
TIFA	COSTIM_up
SPATA5	COSTIM_up
METTL14	COSTIM_up
SETD7	COSTIM_up
NAF1	COSTIM_up
RPS3A	COSTIM_up
NDUFS6	COSTIM_up
RPL37	COSTIM_up
PPIP5K2	COSTIM_up
BDP1	COSTIM_up
GTF2H2	COSTIM_up
SLC30A5	COSTIM_up
BTF3	COSTIM_up
TNFAIP8	COSTIM_up
FEM1C	COSTIM_up
YIPF5	COSTIM_up

DDX46	COSTIM_up
RNF145	COSTIM_up
GLRA1	COSTIM_up
TNIP1	COSTIM_up
G3BP1	COSTIM_up
NHP2	COSTIM_up
RMND5B	COSTIM_up
BOD1	COSTIM_up
TBC1D7	COSTIM_up
FARS2	COSTIM_up
CDKAL1	COSTIM_up
ZMAT2	COSTIM_up
TRIM41	COSTIM_up
HIGD2A	COSTIM_up
ABT1	COSTIM_up
RPL7L1	COSTIM_up
NFKBIE	COSTIM_up
PM20D2	COSTIM_up
ABRACL	COSTIM_up
AIG1	COSTIM_up
DYNLT1	COSTIM_up
WTAP	COSTIM_up
ZMYM4	COSTIM_up
ARMT1	COSTIM_up
CCZ1B	COSTIM_up
CREB5	COSTIM_up
CDCA5	COSTIM_up
PURB	COSTIM_up
MDH2	COSTIM_up
POMZP3	COSTIM_up
NIPSNAP2	COSTIM_up
CCT6A	COSTIM_up
PSPH	COSTIM_up
GIGYF1	COSTIM_up
MEPCE	COSTIM_up
NOM1	COSTIM_up
NLGN4X	COSTIM_up
LUC7L2	COSTIM_up
SH3KBP1	COSTIM_up
CHST7	COSTIM_up
NDUFB11	COSTIM_up
NONO	COSTIM_up
EBP	COSTIM_up
SNX12	COSTIM_up
IL2RG	COSTIM_up
PRPS1	COSTIM_up
RBMX	COSTIM_up
NSDHL	COSTIM_up
CETN2	COSTIM_up
RPL10	COSTIM_up

ATP6V1B2	COSTIM_up
CCDC25	COSTIM_up
CHRNA6	COSTIM_up
BIN3	COSTIM_up
SLC25A37	COSTIM_up
PLPBP	COSTIM_up
TACC1	COSTIM_up
GOLGA7	COSTIM_up
MRPS28	COSTIM_up
LACTB2	COSTIM_up
RPL7	COSTIM_up
MTDH	COSTIM_up
EBAG9	COSTIM_up
POLR2K	COSTIM_up
EIF3H	COSTIM_up
NDUFB9	COSTIM_up
TATDN1	COSTIM_up
ZNF7	COSTIM_up
ARHGAP39	COSTIM_up
SLC39A4	COSTIM_up
VLDLR	COSTIM_up
PLIN2	COSTIM_up
HAUS6	COSTIM_up
SIGMAR1	COSTIM_up
CBWD5	COSTIM_up
CEP78	COSTIM_up
NTRK2	COSTIM_up
MFSD14B	COSTIM_up
INIP	COSTIM_up
UGCG	COSTIM_up
STOM	COSTIM_up
MRRF	COSTIM_up
NR6A1	COSTIM_up
POLE3	COSTIM_up
SURF4	COSTIM_up
GBGT1	COSTIM_up
SURF2	COSTIM_up
SURF6	COSTIM_up
MED22	COSTIM_up
REXO4	COSTIM_up
RPL7A	COSTIM_up
GTF3C5	COSTIM_up
ASB6	COSTIM_up
PTGES2	COSTIM_up
NTMT1	COSTIM_up
CIZ1	COSTIM_up
SLC25A25	COSTIM_up
LRSAM1	COSTIM_up
PAXX	COSTIM_up
INPP5E	COSTIM_up

SEC16A	COSTIM_up
DPH7	COSTIM_up
PDSS1	COSTIM_up
FAM171A1	COSTIM_up
RSU1	COSTIM_up
NRBF2	COSTIM_up
POLR3A	COSTIM_up
GLUD1	COSTIM_up
RPP30	COSTIM_up
DNAJB12	COSTIM_up
EIF4EBP2	COSTIM_up
TCF7L2	COSTIM_up
MKI67	COSTIM_up
FUOM	COSTIM_up
MTG1	COSTIM_up
GSTO1	COSTIM_up
PPRC1	COSTIM_up
PDCD11	COSTIM_up
ADAM12	COSTIM_up
RGS10	COSTIM_up
LIN7C	COSTIM_up
PGAP2	COSTIM_up
HSD17B12	COSTIM_up
APIP	COSTIM_up
DGKZ	COSTIM_up
EIF3M	COSTIM_up
TNKS1BP1	COSTIM_up
SSRP1	COSTIM_up
SLC43A1	COSTIM_up
ARFGAP2	COSTIM_up
CELF1	COSTIM_up
HIKESHI	COSTIM_up
SERPINH1	COSTIM_up
PAK1	COSTIM_up
RPS3	COSTIM_up
ZC3H12C	COSTIM_up
AASDHPPT	COSTIM_up
GLB1L2	COSTIM_up
LAMTOR1	COSTIM_up
HYOU1	COSTIM_up
KAT14	COSTIM_up
MTA2	COSTIM_up
TMEM138	COSTIM_up
FADS1	COSTIM_up
EML3	COSTIM_up
ZP1	COSTIM_up
CPSF7	COSTIM_up
B3GAT3	COSTIM_up
EI24	COSTIM_up
CHEK1	COSTIM_up

FEZ1	COSTIM_up
ESAM	COSTIM_up
COMMD7	COSTIM_up
YTHDF1	COSTIM_up
CABLES2	COSTIM_up
FERMT3	COSTIM_up
MRPL49	COSTIM_up
FAU	COSTIM_up
VPS51	COSTIM_up
PPP4C	COSTIM_up
ALDOA	COSTIM_up
HIRIP3	COSTIM_up
TAOK2	COSTIM_up
HMGA2	COSTIM_up
CWC15	COSTIM_up
EEF1AKMT1	COSTIM_up
SAP18	COSTIM_up
PDCD4	COSTIM_up
WDR17	COSTIM_up
CD226	COSTIM_up
MTMR12	COSTIM_up
CCT5	COSTIM_up
ATPSCKMT	COSTIM_up
DLAT	COSTIM_up
NKAPD1	COSTIM_up
TIMM8B	COSTIM_up
SEC24D	COSTIM_up
RILPL2	COSTIM_up
DHX37	COSTIM_up
UBC	COSTIM_up
SLC7A11	COSTIM_up
NOCT	COSTIM_up
DCP1B	COSTIM_up
UEVLD	COSTIM_up
C12orf45	COSTIM_up
PLBD2	COSTIM_up
TWF1	COSTIM_up
EIF4E	COSTIM_up
TEX30	COSTIM_up
SRFBP1	COSTIM_up
FAM177A1	COSTIM_up
MBIP	COSTIM_up
TMEM18	COSTIM_up
NDUFC2	COSTIM_up
ANKRD50	COSTIM_up
UPF2	COSTIM_up
CDC123	COSTIM_up
SCLT1	COSTIM_up
VTI1A	COSTIM_up
TEX9	COSTIM_up

QTRT2	COSTIM_up
MMAA	COSTIM_up
POU4F2	COSTIM_up
CENPU	COSTIM_up
ACSL1	COSTIM_up
SLC25A4	COSTIM_up
SAV1	COSTIM_up
NBAS	COSTIM_up
GUF1	COSTIM_up
SACS	COSTIM_up
FBXO4	COSTIM_up
CACUL1	COSTIM_up
BAG3	COSTIM_up
AP1S3	COSTIM_up
MZT2B	COSTIM_up
FAM168B	COSTIM_up
GEMIN6	COSTIM_up
OBI1	COSTIM_up
ARL14EP	COSTIM_up
ATP5F1A	COSTIM_up
UHMK1	COSTIM_up
HOMER1	COSTIM_up
SUV39H2	COSTIM_up
RPP38	COSTIM_up
USP12	COSTIM_up
CCDC50	COSTIM_up
PFKM	COSTIM_up
TMEM123	COSTIM_up
SPEF2	COSTIM_up
SPARCL1	COSTIM_up
NADK2	COSTIM_up
GPD1L	COSTIM_up
SLC30A6	COSTIM_up
PELO	COSTIM_up
GPR180	COSTIM_up
HNRNPDL	COSTIM_up
CNTNAP4	COSTIM_up
SREK1IP1	COSTIM_up
CWC27	COSTIM_up
SRP19	COSTIM_up
CENPH	COSTIM_up
TEKT5	COSTIM_up
ANAPC1	COSTIM_up
CAST	COSTIM_up
SCOC	COSTIM_up
CETN3	COSTIM_up
SMARCA5	COSTIM_up
HNRNPU	COSTIM_up
RANBP2	COSTIM_up
AHCTF1	COSTIM_up

ADGRF1	COSTIM_up
FAM49B	COSTIM_up
ASAP1	COSTIM_up
LPCAT1	COSTIM_up
TMEM251	COSTIM_up
UBP1	COSTIM_up
RPIA	COSTIM_up
GTF2E1	COSTIM_up
CFDP1	COSTIM_up
JAZF1	COSTIM_up
TRIP12	COSTIM_up
CEBPG	COSTIM_up
KCTD15	COSTIM_up
MCOLN2	COSTIM_up
SREK1	COSTIM_up
ZUP1	COSTIM_up
NUS1	COSTIM_up
PPP2R5E	COSTIM_up
CABYR	COSTIM_up
IMPACT	COSTIM_up
OTULIN	COSTIM_up
ROBO3	COSTIM_up
GPR15	COSTIM_up
TOMM70	COSTIM_up
CC2D1B	COSTIM_up
ABCA6	COSTIM_up
ABCA5	COSTIM_up
UCHL1	COSTIM_up
NEIL2	COSTIM_up
LONRF1	COSTIM_up
SH3RF1	COSTIM_up
BUB3	COSTIM_up
DIPK1A	COSTIM_up
ATP5MC3	COSTIM_up
ELOC	COSTIM_up
C21orf91	COSTIM_up
L3MBTL4	COSTIM_up
RABGEF1	COSTIM_up
MRPL39	COSTIM_up
ATP5PF	COSTIM_up
GABPA	COSTIM_up
TSEN2	COSTIM_up
DPH3	COSTIM_up
OXNAD1	COSTIM_up
PLCL2	COSTIM_up
CXXC1	COSTIM_up
USP43	COSTIM_up
EPHB1	COSTIM_up
ANKRD40	COSTIM_up
VOPP1	COSTIM_up

APOOL	COSTIM_up
FBXL18	COSTIM_up
KLF10	COSTIM_up
AZIN1	COSTIM_up
OTUD6B	COSTIM_up
GTF3C6	COSTIM_up
MMS19	COSTIM_up
PI4K2A	COSTIM_up
TRMT44	COSTIM_up
HSPA13	COSTIM_up
SAMSN1	COSTIM_up
C16orf87	COSTIM_up
DBI	COSTIM_up
SLC16A1	COSTIM_up
HEATR3	COSTIM_up
NIFK	COSTIM_up
OXA1L	COSTIM_up
LARP1	COSTIM_up
MIER3	COSTIM_up
NUP205	COSTIM_up
ZKSCAN2	COSTIM_up
PDIA4	COSTIM_up
RNF20	COSTIM_up
PPARGC1B	COSTIM_up
ELMO1	COSTIM_up
RRAGA	COSTIM_up
RMND1	COSTIM_up
VBP1	COSTIM_up
RAB39B	COSTIM_up
CLIC2	COSTIM_up
GRIP1	COSTIM_up
CARNMT1	COSTIM_up
KCNMA1	COSTIM_up
BATF	COSTIM_up
N6AMT1	COSTIM_up
USP16	COSTIM_up
CCT8	COSTIM_up
TSPAN7	COSTIM_up
TIAM1	COSTIM_up
SCAF4	COSTIM_up
SFXN2	COSTIM_up
ATP5MPL	COSTIM_up
UQCRB	COSTIM_up
MTERF3	COSTIM_up
RPL30	COSTIM_up
SUPV3L1	COSTIM_up
EEF1A1	COSTIM_up
HK1	COSTIM_up
CD109	COSTIM_up
UBE2L6	COSTIM_up

ZDHHC5	COSTIM_up
MED19	COSTIM_up
ZFAND3	COSTIM_up
NPTN	COSTIM_up
UTP14A	COSTIM_up
AIFM1	COSTIM_up
MAPK13	COSTIM_up
ZNF689	COSTIM_up
PRR14	COSTIM_up
FBRS	COSTIM_up
MFSD14A	COSTIM_up
SASS6	COSTIM_up
MALSU1	COSTIM_up
LHFPL4	COSTIM_up
B3GNT7	COSTIM_up
EIF4A2	COSTIM_up
BRPF1	COSTIM_up
RPUSD3	COSTIM_up
TATDN2	COSTIM_up
SEC13	COSTIM_up
ZFYVE9	COSTIM_up
SMG1	COSTIM_up
NECAP2	COSTIM_up
LRP8	COSTIM_up
PAXIP1	COSTIM_up
ZFHX2-AS1	COSTIM_up
FAM81A	COSTIM_up
MYO1E	COSTIM_up
VPS26C	COSTIM_up
ETS2	COSTIM_up
SLC35B2	COSTIM_up
TMEM164	COSTIM_up
C2CD2	COSTIM_up
TAB3	COSTIM_up
SLC38A10	COSTIM_up
TMEM268	COSTIM_up
ACAN	COSTIM_up
PSMG3	COSTIM_up
GAREM2	COSTIM_up
PRXL2B	COSTIM_up
TNFRSF14	COSTIM_up
C12orf43	COSTIM_up
PEX10	COSTIM_up
RER1	COSTIM_up
BABAM2	COSTIM_up
WDR66	COSTIM_up
MRPL17	COSTIM_up
DUSP2	COSTIM_up
GALNT14	COSTIM_up
FANCC	COSTIM_up

FAIM	COSTIM_up
RNF207	COSTIM_up
GPR153	COSTIM_up
MITD1	COSTIM_up
EIF5B	COSTIM_up
CNOT11	COSTIM_up
TSPAN33	COSTIM_up
B4GALT5	COSTIM_up
SPATA2	COSTIM_up
FAM86C1	COSTIM_up
TSR2	COSTIM_up
ZC3H18	COSTIM_up
TMED4	COSTIM_up
PPP1R15B	COSTIM_up
COPG2	COSTIM_up
GPAT4	COSTIM_up
TAGLN2	COSTIM_up
SLC45A3	COSTIM_up
DUSP23	COSTIM_up
NIT1	COSTIM_up
DEDD	COSTIM_up
ZNF276	COSTIM_up
NDUFS2	COSTIM_up
TOMM40L	COSTIM_up
CCAR2	COSTIM_up
MIS18A	COSTIM_up
ALG8	COSTIM_up
FBXW5	COSTIM_up
CFAP298	COSTIM_up
PAXBP1	COSTIM_up
MRPL10	COSTIM_up
IFNGR2	COSTIM_up
GART	COSTIM_up
SON	COSTIM_up
CSRP1	COSTIM_up
ATP5MC1	COSTIM_up
UBE2Z	COSTIM_up
SNF8	COSTIM_up
CBR1	COSTIM_up
CHAF1B	COSTIM_up
PTMS	COSTIM_up
ADIPOR1	COSTIM_up
CYB5R1	COSTIM_up
PSMD4	COSTIM_up
PSMB4	COSTIM_up
HK2	COSTIM_up
ALDH4A1	COSTIM_up
THEM4	COSTIM_up
MED8	COSTIM_up
GPBP1L1	COSTIM_up

NAE1	COSTIM_up
TMEM69	COSTIM_up
EFCAB14	COSTIM_up
CHCHD6	COSTIM_up
CTBP1	COSTIM_up
ATP6V0D1	COSTIM_up
CARMIL2	COSTIM_up
PIP	COSTIM_up
ZYX	COSTIM_up
CCDC117	COSTIM_up
GNE	COSTIM_up
PTGIR	COSTIM_up
CALM3	COSTIM_up
DFFA	COSTIM_up
CCDC28B	COSTIM_up
ATAD3B	COSTIM_up
SSU72	COSTIM_up
UBE2J2	COSTIM_up
CCDC58	COSTIM_up
VMA21	COSTIM_up
FAM86C2P	COSTIM_up
WDR4	COSTIM_up
NDUFV3	COSTIM_up
CBS	COSTIM_up
U2AF1	COSTIM_up
HSF2BP	COSTIM_up
RRP1B	COSTIM_up
PDXK	COSTIM_up
G6PD	COSTIM_up
CSTB	COSTIM_up
RRP1	COSTIM_up
AGPAT3	COSTIM_up
TRAPPC10	COSTIM_up
ITGB2	COSTIM_up
FAM207A	COSTIM_up
RALGDS	COSTIM_up
SPATC1L	COSTIM_up
LSS	COSTIM_up
VAV2	COSTIM_up
MCM3AP	COSTIM_up
PCNT	COSTIM_up
SLC2A6	COSTIM_up
C19orf47	COSTIM_up
CFAP157	COSTIM_up
TOR2A	COSTIM_up
ST6GALNAC6	COSTIM_up
SHKBP1	COSTIM_up
ZDHHC12	COSTIM_up
PKN3	COSTIM_up
TAOK1	COSTIM_up

MED27	COSTIM_up
TLCD1	COSTIM_up
SAFB	COSTIM_up
CHTOP	COSTIM_up
CXCR5	COSTIM_up
ZBTB7B	COSTIM_up
FLAD1	COSTIM_up
SHC1	COSTIM_up
NLRX1	COSTIM_up
ADAR	COSTIM_up
IL6R	COSTIM_up
UBE2Q1	COSTIM_up
CHRNB2	COSTIM_up
CRTC2	COSTIM_up
FDPS	COSTIM_up
RUSC1	COSTIM_up
FAM189B	COSTIM_up
SLC25A44	COSTIM_up
LMNA	COSTIM_up
NBEAL2	COSTIM_up
CCDC12	COSTIM_up
UBQLN4	COSTIM_up
GPATCH4	COSTIM_up
NACC1	COSTIM_up
CPSF4	COSTIM_up
VPS28	COSTIM_up
TONSL	COSTIM_up
PWWP3A	COSTIM_up
LRRC14	COSTIM_up
PPP1R16A	COSTIM_up
SQSTM1	COSTIM_up
MGAT4B	COSTIM_up
RPL8	COSTIM_up
PSMC2	COSTIM_up
MFSD12	COSTIM_up
YDJC	COSTIM_up
DVL3	COSTIM_up
AP2M1	COSTIM_up
ABCF3	COSTIM_up
PCYT1A	COSTIM_up
IKZF3	COSTIM_up
GRIN2C	COSTIM_up
SAP30BP	COSTIM_up
SRSF2	COSTIM_up
LSM12	COSTIM_up
EMC10	COSTIM_up
JOSD2	COSTIM_up
FMNL3	COSTIM_up
LARP4	COSTIM_up
RAVER1	COSTIM_up

ZNF653	COSTIM_up
SENP3	COSTIM_up
EIF4A1	COSTIM_up
RPL26	COSTIM_up
POLR3K	COSTIM_up
SNRNP25	COSTIM_up
JMJD8	COSTIM_up
CCDC78	COSTIM_up
TEDC2	COSTIM_up
TBC1D24	COSTIM_up
NTN3	COSTIM_up
FLYWCH2	COSTIM_up
CLPB	COSTIM_up
UBXN1	COSTIM_up
LBHD1	COSTIM_up
TAF6L	COSTIM_up
RPL29	COSTIM_up
CMPK1	COSTIM_up
COA7	COSTIM_up
CZIB	COSTIM_up
MAGOH	COSTIM_up
PARS2	COSTIM_up
NOL9	COSTIM_up
KLHL21	COSTIM_up
GMEB1	COSTIM_up
AK4	COSTIM_up
DHRS3	COSTIM_up
PEF1	COSTIM_up
RBBP4	COSTIM_up
TMCO4	COSTIM_up
ALPL	COSTIM_up
WNT4	COSTIM_up
TTLL10	COSTIM_up
FAAP20	COSTIM_up
USP1	COSTIM_up
FUBP1	COSTIM_up
TYW3	COSTIM_up
NTNG1	COSTIM_up
HENMT1	COSTIM_up
C1orf52	COSTIM_up
ATXN7L2	COSTIM_up
ZNF326	COSTIM_up
AGL	COSTIM_up
ZNF281	COSTIM_up
ARPC5	COSTIM_up
PEA15	COSTIM_up
PEX19	COSTIM_up
NCSTN	COSTIM_up
FLVCR1	COSTIM_up
ATF3	COSTIM_up

IER5	COSTIM_up
BPNT1	COSTIM_up
C1orf115	COSTIM_up
TFB2M	COSTIM_up
MRPL55	COSTIM_up
REL	COSTIM_up
MEMO1	COSTIM_up
DPY30	COSTIM_up
TYW5	COSTIM_up
MAIP1	COSTIM_up
NUP35	COSTIM_up
CCDC138	COSTIM_up
FBXO41	COSTIM_up
WDCP	COSTIM_up
SGCB	COSTIM_up
SPATA18	COSTIM_up
SGPP2	COSTIM_up
SMARCAD1	COSTIM_up
PDLIM5	COSTIM_up
PACRGL	COSTIM_up
SCNM1	COSTIM_up
VPS72	COSTIM_up
ERCC3	COSTIM_up
IWS1	COSTIM_up
BOLA3	COSTIM_up
CDC42EP3	COSTIM_up
S100A11	COSTIM_up
GNPDA2	COSTIM_up
PAQR3	COSTIM_up
MRPS18C	COSTIM_up
CGGBP1	COSTIM_up
PMVK	COSTIM_up
HIPK1	COSTIM_up
YY1AP1	COSTIM_up
EOGT	COSTIM_up
NAXE	COSTIM_up
POGLUT1	COSTIM_up
ATP1A1	COSTIM_up
LRRC58	COSTIM_up
TMEM183A	COSTIM_up
KRTCAP2	COSTIM_up
ARPC2	COSTIM_up
CCT3	COSTIM_up
RNF25	COSTIM_up
CIP2A	COSTIM_up
CWC22	COSTIM_up
FCRL4	COSTIM_up
STT3B	COSTIM_up
CHCHD4	COSTIM_up
SUCLG1	COSTIM_up

EIF5A2	COSTIM_up
RPL22L1	COSTIM_up
SNHG16	COSTIM_up
PPP4R2	COSTIM_up
NEPRO	COSTIM_up
COX18	COSTIM_up
THOC7	COSTIM_up
PSMD6	COSTIM_up
GMPS	COSTIM_up
RPL9	COSTIM_up
ABHD6	COSTIM_up
DNASE1L3	COSTIM_up
CRELD1	COSTIM_up
PRRT3	COSTIM_up
U2SURP	COSTIM_up
MTHFD2L	COSTIM_up
CXCL1	COSTIM_up
RCHY1	COSTIM_up
ZNF513	COSTIM_up
SLC4A1AP	COSTIM_up
KIAA1143	COSTIM_up
WDR43	COSTIM_up
ZDHHC3	COSTIM_up
ELP6	COSTIM_up
DTX3L	COSTIM_up
SMIM12	COSTIM_up
TPRA1	COSTIM_up
YEATS2	COSTIM_up
ZC3H12A	COSTIM_up
SNIP1	COSTIM_up
POLR2H	COSTIM_up
KLF15	COSTIM_up
RPN1	COSTIM_up
RFC4	COSTIM_up
RPL39L	COSTIM_up
BAP1	COSTIM_up
TKT	COSTIM_up
PRKCD	COSTIM_up
RFT1	COSTIM_up
GNL3	COSTIM_up
PBRM1	COSTIM_up
UVSSA	COSTIM_up
SLBP	COSTIM_up
LRPAP1	COSTIM_up
PIGX	COSTIM_up
MELTF	COSTIM_up
EXO5	COSTIM_up
AIMP1	COSTIM_up
METAP1	COSTIM_up
H2AZ1	COSTIM_up

SLC9B2	COSTIM_up
PGRMC2	COSTIM_up
CDC25A	COSTIM_up
CCDC51	COSTIM_up
ATRIP	COSTIM_up
BSN	COSTIM_up
APEH	COSTIM_up
MON1A	COSTIM_up
RAD54L2	COSTIM_up
WDR82	COSTIM_up
SAP30	COSTIM_up
ANXA5	COSTIM_up
NAA15	COSTIM_up
ICE1	COSTIM_up
ANAPC10	COSTIM_up
ABCE1	COSTIM_up
OTUD4	COSTIM_up
LSM6	COSTIM_up
TMEM184C	COSTIM_up
TMEM161B	COSTIM_up
NDUFAF2	COSTIM_up
SLC25A46	COSTIM_up
ANKRD33B	COSTIM_up
PRRC1	COSTIM_up
AGGF1	COSTIM_up
WDR41	COSTIM_up
NDUFS4	COSTIM_up
GRPEL2	COSTIM_up
RHOBTB3	COSTIM_up
CASP3	COSTIM_up
ERAP1	COSTIM_up
CFAP97	COSTIM_up
UBLCP1	COSTIM_up
UTP15	COSTIM_up
NSA2	COSTIM_up
LINC01600	COSTIM_up
SEPTIN8	COSTIM_up
UQCRQ	COSTIM_up
CGAS	COSTIM_up
SFXN1	COSTIM_up
PDSS2	COSTIM_up
RPS14	COSTIM_up
PTTG1	COSTIM_up
RELL2	COSTIM_up
MIOS	COSTIM_up
FABP5	COSTIM_up
BRI3	COSTIM_up
RAD21	COSTIM_up
TNFRSF11B	COSTIM_up
ORC5	COSTIM_up

DNAAF5	COSTIM_up
OSGIN2	COSTIM_up
GPR146	COSTIM_up
GPER1	COSTIM_up
INTS1	COSTIM_up
SLC4A2	COSTIM_up
FASTK	COSTIM_up
TMUB1	COSTIM_up
PHAX	COSTIM_up
FOXK1	COSTIM_up
COX6C	COSTIM_up
YWHAZ	COSTIM_up
BAALC	COSTIM_up
FZD6	COSTIM_up
CTHRC1	COSTIM_up
SLC25A32	COSTIM_up
DCAF13	COSTIM_up
VIRMA	COSTIM_up
GEM	COSTIM_up
WASHC5	COSTIM_up
RPP25L	COSTIM_up
FAM219A	COSTIM_up
NUDT2	COSTIM_up
UBAP1	COSTIM_up
METTL2B	COSTIM_up
FXN	COSTIM_up
ZMAT4	COSTIM_up
KDM1B	COSTIM_up
C9orf64	COSTIM_up
HNRNPK	COSTIM_up
ZHX1	COSTIM_up
DYNLT3	COSTIM_up
MID1IP1	COSTIM_up
PIGA	COSTIM_up
WNK2	COSTIM_up
NLGN4Y	COSTIM_up
HDX	COSTIM_up
NDUFB6	COSTIM_up
NOL6	COSTIM_up
VCP	COSTIM_up
STOML2	COSTIM_up
WRN	COSTIM_up
CFL2	COSTIM_up
SUGT1	COSTIM_up
GTF2A1	COSTIM_up
SLC16A9	COSTIM_up
REEP3	COSTIM_up
PKNOX2	COSTIM_up
LRR1	COSTIM_up
RPL36AL	COSTIM_up

DNAAF2	COSTIM_up
ZNF22	COSTIM_up
EML5	COSTIM_up
RPUSD4	COSTIM_up
AKR1E2	COSTIM_up
NUDT5	COSTIM_up
ATP5F1C	COSTIM_up
VDAC2	COSTIM_up
COMTD1	COSTIM_up
PDZD8	COSTIM_up
ABRAXAS2	COSTIM_up
FAM204A	COSTIM_up
PRDX3	COSTIM_up
ENOX2	COSTIM_up
GHITM	COSTIM_up
SNAPC4	COSTIM_up
PMPCA	COSTIM_up
ENTR1	COSTIM_up
HPRT1	COSTIM_up
DIPK1B	COSTIM_up
ZMYND19	COSTIM_up
DDX21	COSTIM_up
BMS1	COSTIM_up
FUNDC2	COSTIM_up
NSMF	COSTIM_up
TRUB1	COSTIM_up
E2F7	COSTIM_up
PACSIN3	COSTIM_up
SLC39A13	COSTIM_up
PSMC3	COSTIM_up
RAPSN	COSTIM_up
TC2N	COSTIM_up
CPSF2	COSTIM_up
SMCO2	COSTIM_up
IFI27L1	COSTIM_up
ARL5B	COSTIM_up
TAF1D	COSTIM_up
ABTB2	COSTIM_up
SPRED1	COSTIM_up
GPT2	COSTIM_up
RAB8B	COSTIM_up
RPUSD2	COSTIM_up
HIF1AN	COSTIM_up
NDUFB8	COSTIM_up
BRD7	COSTIM_up
TRMT61A	COSTIM_up
BAG5	COSTIM_up
API5	COSTIM_up
HPS6	COSTIM_up
NOLC1	COSTIM_up

ALKBH3	COSTIM_up
COPS2	COSTIM_up
CCT2	COSTIM_up
PCBD1	COSTIM_up
ZNF202	COSTIM_up
STXBP4	COSTIM_up
CUL5	COSTIM_up
MYRFL	COSTIM_up
TRIM44	COSTIM_up
TAF10	COSTIM_up
NETO1	COSTIM_up
CYB5A	COSTIM_up
USP54	COSTIM_up
CYB5R2	COSTIM_up
SERPINB8	COSTIM_up
IDH3A	COSTIM_up
RPL27A	COSTIM_up
DENND2B	COSTIM_up
ATMIN	COSTIM_up
LEO1	COSTIM_up
TMX3	COSTIM_up
WEE1	COSTIM_up
HDGFL3	COSTIM_up
MCM7	COSTIM_up
TMED3	COSTIM_up
SEC11C	COSTIM_up
TMEM135	COSTIM_up
CENPV	COSTIM_up
CIAO2B	COSTIM_up
HSP90B1	COSTIM_up
TVP23A	COSTIM_up
PLEKHA7	COSTIM_up
B2M	COSTIM_up
SAAL1	COSTIM_up
PPIB	COSTIM_up
SCNN1G	COSTIM_up
DCTN5	COSTIM_up
CLPX	COSTIM_up
ZBTB39	COSTIM_up
STAT6	COSTIM_up
PATL1	COSTIM_up
STX3	COSTIM_up
MRPL16	COSTIM_up
YWHAB	COSTIM_up
MAP1A	COSTIM_up
RCCD1	COSTIM_up
MARS1	COSTIM_up
MBD6	COSTIM_up
PDIA3	COSTIM_up
NUDT21	COSTIM_up

PBX3	COSTIM_up
PHB	COSTIM_up
SNRPD1	COSTIM_up
SAMD14	COSTIM_up
GOLGA2	COSTIM_up
TRUB2	COSTIM_up
COQ4	COSTIM_up
SLC27A4	COSTIM_up
URM1	COSTIM_up
DOLPP1	COSTIM_up
ENDOG	COSTIM_up
C15orf39	COSTIM_up
COQ7	COSTIM_up
CRK	COSTIM_up
FBXO22	COSTIM_up
TBC1D2B	COSTIM_up
RNF214	COSTIM_up
CDK12	COSTIM_up
ATP5MG	COSTIM_up
RRM1	COSTIM_up
PRRT2	COSTIM_up
IRGQ	COSTIM_up
ZNF668	COSTIM_up
VKORC1	COSTIM_up
SMG8	COSTIM_up
RAB8A	COSTIM_up
GPX4	COSTIM_up
KLHL26	COSTIM_up
GATAD2A	COSTIM_up
MVD	COSTIM_up
TRAPPC2L	COSTIM_up
RSKR	COSTIM_up
RPL13	COSTIM_up
KMT2D	COSTIM_up
CORO6	COSTIM_up
RHEBL1	COSTIM_up
TUBA1A	COSTIM_up
TUBA1C	COSTIM_up
NFKBID	COSTIM_up
ZNF526	COSTIM_up
ZNF146	COSTIM_up
SPINT2	COSTIM_up
YIF1B	COSTIM_up
DAPK3	COSTIM_up
EEF2	COSTIM_up
CHAF1A	COSTIM_up
UBXN6	COSTIM_up
HDGFL2	COSTIM_up
ZNF444	COSTIM_up
GLOD4	COSTIM_up

MFSD3	COSTIM_up
GPT	COSTIM_up
SRR	COSTIM_up
TSR1	COSTIM_up
TRPV3	COSTIM_up
C19orf48	COSTIM_up
OTUB1	COSTIM_up
CD320	COSTIM_up
SPRYD3	COSTIM_up
NDUFV1	COSTIM_up
CDK2AP2	COSTIM_up
PRDX2	COSTIM_up
ZNF232	COSTIM_up
MIS12	COSTIM_up
MRPL58	COSTIM_up
ATP5PD	COSTIM_up
TMEM88	COSTIM_up
SRP68	COSTIM_up
FAM234A	COSTIM_up
ZNF598	COSTIM_up
RAB26	COSTIM_up
MLST8	COSTIM_up
E4F1	COSTIM_up
ECI1	COSTIM_up
CASKIN1	COSTIM_up
KCTD5	COSTIM_up
DDB1	COSTIM_up
VPS37C	COSTIM_up
BEST1	COSTIM_up
FTH1	COSTIM_up
POLR2G	COSTIM_up
SLC3A2	COSTIM_up
RPSA	COSTIM_up
FADD	COSTIM_up
SAC3D1	COSTIM_up
SF1	COSTIM_up
CCDC88B	COSTIM_up
COPS6	COSTIM_up
NUDT16L1	COSTIM_up
RNF187	COSTIM_up
HOOK3	COSTIM_up
MAPK1IP1L	COSTIM_up
DDIT4	COSTIM_up
RBPJ	COSTIM_up
ZCCHC4	COSTIM_up
UBTD2	COSTIM_up
NKIRAS2	COSTIM_up
DNAJC7	COSTIM_up
KCNV2	COSTIM_up
IRF2BP2	COSTIM_up

NT5DC2	COSTIM_up
SMIM4	COSTIM_up
COA6	COSTIM_up
MGAT2	COSTIM_up
MMADHC	COSTIM_up
PDHB	COSTIM_up
MPLKIP	COSTIM_up
IRF2	COSTIM_up
ARF4	COSTIM_up
SEPTIN2	COSTIM_up
FILIP1L	COSTIM_up
MFSD2A	COSTIM_up
DTYMK	COSTIM_up
TAP1	COSTIM_up
ING5	COSTIM_up
STIP1	COSTIM_up
SCNN1B	COSTIM_up
ATXN2L	COSTIM_up
POLR3D	COSTIM_up
FEN1	COSTIM_up
SLC20A2	COSTIM_up
STAT3	COSTIM_up
ZSWIM1	COSTIM_up
NDUFS5	COSTIM_up
TMEM208	COSTIM_up
AHCYL1	COSTIM_up
DNAJC21	COSTIM_up
SEMA4C	COSTIM_up
PPIP5K1	COSTIM_up
ZBTB5	COSTIM_up
CHTF8	COSTIM_up
LCMT2	COSTIM_up
ZNF507	COSTIM_up
STX18	COSTIM_up
GFM1	COSTIM_up
DDX19A	COSTIM_up
USP39	COSTIM_up
TNIP2	COSTIM_up
TMEM150A	COSTIM_up
RNF181	COSTIM_up
MAT2A	COSTIM_up
LETM1	COSTIM_up
MFF	COSTIM_up
PXDC1	COSTIM_up
E2F6	COSTIM_up
FEM1B	COSTIM_up
COMMD8	COSTIM_up
ATP5ME	COSTIM_up
UQCRFS1	COSTIM_up
SLC49A3	COSTIM_up

MAP2K1	COSTIM_up
HNRNPH1	COSTIM_up
ASMTL	COSTIM_up
SLC25A6	COSTIM_up
CSNK1G1	COSTIM_up
ZNF354A	COSTIM_up
ATF5	COSTIM_up
UBE2V2	COSTIM_up
ZBTB43	COSTIM_up
XPO6	COSTIM_up
APEX2	COSTIM_up
NSMCE1	COSTIM_up
NPIPB12	COSTIM_up
CD2BP2	COSTIM_up
TBC1D10B	COSTIM_up
LMAN2	COSTIM_up
PRELID1	COSTIM_up
SLC50A1	COSTIM_up
CXCL10	COSTIM_up
NPIPB3	COSTIM_up
NMD3	COSTIM_up
GPRIN1	COSTIM_up
MRPL1	COSTIM_up
PGM2	COSTIM_up
MINAR1	COSTIM_up
SNUPN	COSTIM_up
SIN3A	COSTIM_up
ARL13B	COSTIM_up
NPR1	COSTIM_up
CXCL8	COSTIM_up
PCBP1	COSTIM_up
HINT1	COSTIM_up
NFU1	COSTIM_up
RAMAC	COSTIM_up
APLF	COSTIM_up
BOLA2B	COSTIM_up
RGPD8	COSTIM_up
HIC2	COSTIM_up
BUB1	COSTIM_up
SPNS1	COSTIM_up
CHRNA5	COSTIM_up
CENPX	COSTIM_up
ASPSCR1	COSTIM_up
FASN	COSTIM_up
CNBP	COSTIM_up
DUS1L	COSTIM_up
GPS1	COSTIM_up
DCXR	COSTIM_up
ZNF32	COSTIM_up
NRG4	COSTIM_up

LIMS1	COSTIM_up
TMEM266	COSTIM_up
UGP2	COSTIM_up
HNRNPF	COSTIM_up
CSGALNACT2	COSTIM_up
AVEN	COSTIM_up
SYAP1	COSTIM_up
TOR1AIP2	COSTIM_up
GUSB	COSTIM_up
ZNF764	COSTIM_up
ZNF768	COSTIM_up
PUSL1	COSTIM_up
SF3B5	COSTIM_up
ZNF35	COSTIM_up
CHD3	COSTIM_up
ALCAM	COSTIM_up
YWHAG	COSTIM_up
UBE2E3	COSTIM_up
CNTROB	COSTIM_up
TRAPPC1	COSTIM_up
TMEM192	COSTIM_up
UBE2E1	COSTIM_up
HNRNPA3	COSTIM_up
USP38	COSTIM_up
NANP	COSTIM_up
USP47	COSTIM_up
MRAP	COSTIM_up
GON7	COSTIM_up
FAXDC2	COSTIM_up
ELP5	COSTIM_up
UBB	COSTIM_up
NFRKB	COSTIM_up
TMED10	COSTIM_up
SETMAR	COSTIM_up
GPRC5C	COSTIM_up
HARS1	COSTIM_up
RIOX1	COSTIM_up
NUDT9	COSTIM_up
PA2G4	COSTIM_up
ELOVL6	COSTIM_up
TMC7	COSTIM_up
ARL6IP1	COSTIM_up
SERPINB9	COSTIM_up
NUDCD2	COSTIM_up
HSPA4	COSTIM_up
COMMD5	COSTIM_up
ZNF16	COSTIM_up
ARMC10	COSTIM_up
RNF34	COSTIM_up
ATF7	COSTIM_up

SOCS6	COSTIM_up
ZNF296	COSTIM_up
HOXB9	COSTIM_up
KIF5B	COSTIM_up
CDCA4	COSTIM_up
USP32	COSTIM_up
RIOX2	COSTIM_up
TRIAP1	COSTIM_up
LSM3	COSTIM_up
KIAA0232	COSTIM_up
TMEM43	COSTIM_up
RPS9	COSTIM_up
TSEN34	COSTIM_up
MSANTD4	COSTIM_up
NDUFA3	COSTIM_up
DNAJC24	COSTIM_up
TRMT61B	COSTIM_up
JAGN1	COSTIM_up
ZNF672	COSTIM_up
ZNF692	COSTIM_up
GRIK1	COSTIM_up
TMEM126A	COSTIM_up
NETO2	COSTIM_up
SCAND1	COSTIM_up
JUNB	COSTIM_up
NPTX1	COSTIM_up
FAM98B	COSTIM_up
CANT1	COSTIM_up
ZDHHC16	COSTIM_up
EXOSC1	COSTIM_up
PGAM1	COSTIM_up
CLCN5	COSTIM_up
MRPL36	COSTIM_up
ZNF581	COSTIM_up
POLR1C	COSTIM_up
ASXL1	COSTIM_up
DLK2	COSTIM_up
ZNF562	COSTIM_up
ZNF318	COSTIM_up
HOPX	COSTIM_up
RSL1D1	COSTIM_up
PPID	COSTIM_up
ETFDH	COSTIM_up
PTGER4	COSTIM_up
TBCA	COSTIM_up
MAP6	COSTIM_up
BCL2L1	COSTIM_up
PLRG1	COSTIM_up
PIK3CD	COSTIM_up
SLC25A33	COSTIM_up

RGS19	COSTIM_up
HDAC3	COSTIM_up
GPHN	COSTIM_up
CAMTA1	COSTIM_up
SLFNL1	COSTIM_up
BCL2	COSTIM_up
CTPS1	COSTIM_up
COL8A2	COSTIM_up
EXOSC10	COSTIM_up
RPS21	COSTIM_up
MRM3	COSTIM_up
RPS7	COSTIM_up
RNASEH1	COSTIM_up
PRNP	COSTIM_up
PPIH	COSTIM_up
ZNF57	COSTIM_up
SYNPO	COSTIM_up
THOP1	COSTIM_up
USP19	COSTIM_up
SERF1A	COSTIM_up
SMN1	COSTIM_up
NME6	COSTIM_up
CYCS	COSTIM_up
FRMD3	COSTIM_up
MRPL13	COSTIM_up
CEBPB	COSTIM_up
PAIP1	COSTIM_up
MACROD2	COSTIM_up
BSG	COSTIM_up
COPRS	COSTIM_up
POP7	COSTIM_up
ALG14	COSTIM_up
SUCLG2	COSTIM_up
GNB2	COSTIM_up
MCRIP2	COSTIM_up
CLP1	COSTIM_up
COPS9	COSTIM_up
ZNF24	COSTIM_up
MANEA	COSTIM_up
FIBP	COSTIM_up
PPP1CA	COSTIM_up
HCFC1	COSTIM_up
RASGRP1	COSTIM_up
CHCHD1	COSTIM_up
MRPL52	COSTIM_up
EFEMP2	COSTIM_up
ZMAT3	COSTIM_up
MUS81	COSTIM_up
CFL1	COSTIM_up
CBWD1	COSTIM_up

RPL38	COSTIM_up
METAP1D	COSTIM_up
DHCR7	COSTIM_up
RNASEH2C	COSTIM_up
MYEOV	COSTIM_up
MYD88	COSTIM_up
OXSR1	COSTIM_up
KAT5	COSTIM_up
RELA	COSTIM_up
FAM222B	COSTIM_up
TRMT112	COSTIM_up
MRPL57	COSTIM_up
NOC3L	COSTIM_up
ESRRA	COSTIM_up
COMMD1	COSTIM_up
MTX1	COSTIM_up
PARP14	COSTIM_up
CKS1B	COSTIM_up
IQCB1	COSTIM_up
GPR137	COSTIM_up
MZT2A	COSTIM_up
ZBTB21	COSTIM_up
FAM86B3P	COSTIM_up
MAP3K11	COSTIM_up
DAG1	COSTIM_up
NAA20	COSTIM_up
MICOS10	COSTIM_up
RNF26	COSTIM_up
PPP1R14B	COSTIM_up
ZNRD2	COSTIM_up
SMARCC1	COSTIM_up
FKBP2	COSTIM_up
GMPPB	COSTIM_up
ZNF622	COSTIM_up
NABP1	COSTIM_up
CEP83	COSTIM_up
NUDT4	COSTIM_up
SLC19A1	COSTIM_up
UQCRH	COSTIM_up
TAS1R1	COSTIM_up
EIF1AX	COSTIM_up
PSMD1	COSTIM_up
HEG1	COSTIM_up
TOMM20	COSTIM_up
AGFG1	COSTIM_up
CNP	COSTIM_up
EIF1	COSTIM_up
PLK3	COSTIM_up
GOLIM4	COSTIM_up
ATP5MD	COSTIM_up

RBM4	COSTIM_up
XXYLT1	COSTIM_up
NRROS	COSTIM_up
FBXO45	COSTIM_up
GNG5	COSTIM_up
FAM3C2	COSTIM_up
C16orf91	COSTIM_up
RGMB	COSTIM_up
TRMT10C	COSTIM_up
CTU2	COSTIM_up
MGA	COSTIM_up
C12orf66	COSTIM_up
PRPF8	COSTIM_up
PITPNA	COSTIM_up
DDX23	COSTIM_up
ZNHIT2	COSTIM_up
LIG4	COSTIM_up
ATP2A2	COSTIM_up
RPL4	COSTIM_up
SNAPC5	COSTIM_up
DENND4A	COSTIM_up
MRPL11	COSTIM_up
AKIRIN1	COSTIM_up
SLC29A2	COSTIM_up
TMEM167A	COSTIM_up
LARP7	COSTIM_up
BRMS1	COSTIM_up
RPL15	COSTIM_up
FAM241A	COSTIM_up
SRP72	COSTIM_up
PDE12	COSTIM_up
YIF1A	COSTIM_up
NDUFA11	COSTIM_up
RSRC1	COSTIM_up
RAB1B	COSTIM_up
PTDSS2	COSTIM_up
MICOS13	COSTIM_up
KCTD13	COSTIM_up
DHX36	COSTIM_up
FBXW8	COSTIM_up
KLC2	COSTIM_up
SLC22A1	COSTIM_up
ATR	COSTIM_up
SNHG29	COSTIM_up
MRPS22	COSTIM_up
MARCKSL1	COSTIM_up
PSMD2	COSTIM_up
CSRP2	COSTIM_up
PARL	COSTIM_up
DCTN2	COSTIM_up

ZNF408	COSTIM_up
CKAP5	COSTIM_up
ARHGAP1	COSTIM_up
MED16	COSTIM_up
CENPS	COSTIM_up
LSM1	COSTIM_up
BANF1	COSTIM_up
PTPN2	COSTIM_up
EIF1AD	COSTIM_up
EIF3F	COSTIM_up
CLTB	COSTIM_up
LPL	COSTIM_up
RFESD	COSTIM_up
SART1	COSTIM_up
DRAP1	COSTIM_up
MRPL48	COSTIM_up
FOSL1	COSTIM_up
ERCC4	COSTIM_up
CCDC85B	COSTIM_up
TMEM70	COSTIM_up
ZNF77	COSTIM_up
GPR156	COSTIM_up
MTLN	COSTIM_up
B3GNTL1	COSTIM_up
AURKAIP1	COSTIM_up
TTLL11	COSTIM_up
TOMM5	COSTIM_up
RUVBL1	COSTIM_up
CTDNEP1	COSTIM_up
SWI5	COSTIM_up
UBE2O	COSTIM_up
TUBB6	COSTIM_up
B3GALT6	COSTIM_up
TPRN	COSTIM_up
SLC35A4	COSTIM_up
SSNA1	COSTIM_up
CSTF3	COSTIM_up
CHMP6	COSTIM_up
UFSP1	COSTIM_up
TMEM39A	COSTIM_up
CCDC57	COSTIM_up
SPHK1	COSTIM_up
ENTHD1	COSTIM_up
SMIM19	COSTIM_up
ANAPC2	COSTIM_up
ZBTB8OS	COSTIM_up
COX8A	COSTIM_up
ZSCAN2	COSTIM_up
CDC26	COSTIM_up
KCMF1	COSTIM_up

SPRYD4	COSTIM_up
CLK2	COSTIM_up
LMNB2	COSTIM_up
MEX3C	COSTIM_up
MYO1D	COSTIM_up
RBIS	COSTIM_up
RUFY1	COSTIM_up
BASP1	COSTIM_up
WSB2	COSTIM_up
TYMS	COSTIM_up
PNMA1	COSTIM_up
ANKLE2	COSTIM_up
THAP4	COSTIM_up
DPP7	COSTIM_up
SEC24C	COSTIM_up
SMCR8	COSTIM_up
MTX3	COSTIM_up
FBXO46	COSTIM_up
WDR73	COSTIM_up
RHOG	COSTIM_up
NUDT4B	COSTIM_up
FAM210A	COSTIM_up
TALDO1	COSTIM_up
NAP1L4P1	COSTIM_up
RPS6KA3	COSTIM_up
PUS1	COSTIM_up
GATD1	COSTIM_up
CCDC71	COSTIM_up
TIMM22	COSTIM_up
ZFAS1	COSTIM_up
PAWR	COSTIM_up
ARIH2	COSTIM_up
SLC25A22	COSTIM_up
ATOX1	COSTIM_up
RPLP2	COSTIM_up
CSTF2T	COSTIM_up
ACAD9	COSTIM_up
POLR2L	COSTIM_up
FLII	COSTIM_up
HNRNPA0	COSTIM_up
CHID1	COSTIM_up
GRB2	COSTIM_up
UBE2N	COSTIM_up
MAMDC4	COSTIM_up
RIC8A	COSTIM_up
IMP3	COSTIM_up
ASB8	COSTIM_up
IMPDH2	COSTIM_up
NDUFAF3	COSTIM_up
C2orf69	COSTIM_up

STAP2	COSTIM_up
DDX10	COSTIM_up
ZNF366	COSTIM_up
PLEC	COSTIM_up
GALNT11	COSTIM_up
WDR6	COSTIM_up
GEN1	COSTIM_up
TMPRSS9	COSTIM_up
TMEM11	COSTIM_up
ZFAND2A	COSTIM_up
BEND3	COSTIM_up
NT5DC1	COSTIM_up
RPS3AP5	COSTIM_up
TIMM23B-AGAP6	COSTIM_up
KLHL11	COSTIM_up
EPM2AIP1	COSTIM_up
GTPBP6	COSTIM_up
SUZ12	COSTIM_up
RPP25	COSTIM_up
GRINA	COSTIM_up
COX5A	COSTIM_up
CPNE7	COSTIM_up
MPI	COSTIM_up
EXOSC4	COSTIM_up
PFAS	COSTIM_up
ZBTB7A	COSTIM_up
TUFM	COSTIM_up
EIF3K	COSTIM_up
MRFAP1L1	COSTIM_up
MRFAP1	COSTIM_up
C3orf38	COSTIM_up
RRS1	COSTIM_up
RCC2	COSTIM_up
CYC1	COSTIM_up
FARSA	COSTIM_up
SPTY2D1	COSTIM_up
SAMD4B	COSTIM_up
EDC3	COSTIM_up
RPS2P28	COSTIM_up
CALR	COSTIM_up
RAD23A	COSTIM_up
GADD45GIP1	COSTIM_up
PTPN11	COSTIM_up
FAM156B	COSTIM_up
HLA-DQB1	COSTIM_up
PACS2	COSTIM_up
GEMIN4	COSTIM_up
IL6-AS1	COSTIM_up
FJX1	COSTIM_up
OR9A2	COSTIM_up

SHARPIN	COSTIM_up
GCC1	COSTIM_up
LSMEM2	COSTIM_up
ZFPM1	COSTIM_up
PLD6	COSTIM_up
LACC1	COSTIM_up
MAF1	COSTIM_up
SERTAD2	COSTIM_up
C14orf119	COSTIM_up
FIZ1	COSTIM_up
PUF60	COSTIM_up
DCTPP1	COSTIM_up
ZNF771	COSTIM_up
SOCS4	COSTIM_up
TMEM86B	COSTIM_up
TRNAU1AP	COSTIM_up
EXOC3	COSTIM_up
FAHD1	COSTIM_up
RCC1	COSTIM_up
PRKRA	COSTIM_up
CCDC43	COSTIM_up
HCLS1	COSTIM_up
PAK2	COSTIM_up
MCFD2	COSTIM_up
TSPYL5	COSTIM_up
MB21D2	COSTIM_up
SSTR2	COSTIM_up
PCGF5	COSTIM_up
YOD1	COSTIM_up
LINC02363	COSTIM_up
SMG1P3	COSTIM_up
GPR157	COSTIM_up
SLC36A4	COSTIM_up
PPA1	COSTIM_up
PSMG4	COSTIM_up
SSR4	COSTIM_up
SCRIB	COSTIM_up
KCTD2	COSTIM_up
FAM83H	COSTIM_up
ST20	COSTIM_up
PITPNB	COSTIM_up
MRPL14	COSTIM_up
NQO1	COSTIM_up
AEN	COSTIM_up
TRAPPC5	COSTIM_up
HIGD1A	COSTIM_up
NPM1	COSTIM_up
POLR2A	COSTIM_up
LRRC75A	COSTIM_up
HSP90AA6P	COSTIM_up

OGFOD3	COSTIM_up
WASHC1	COSTIM_up
ZBTB2	COSTIM_up
C6orf223	COSTIM_up
MRPS23	COSTIM_up
C5orf30	COSTIM_up
SIAH2	COSTIM_up
COPG1	COSTIM_up
SLC35C1	COSTIM_up
TIGIT	COSTIM_up
C5orf24	COSTIM_up
ADO	COSTIM_up
COA4	COSTIM_up
PRKAG1	COSTIM_up
MRPS11	COSTIM_up
SNRPE	COSTIM_up
OGFRP1	COSTIM_up
TMEM259	COSTIM_up
TNRC18	COSTIM_up
NOP10	COSTIM_up
MRPL41	COSTIM_up
MRPS16	COSTIM_up
ARL6IP4	COSTIM_up
SHMT2	COSTIM_up
ATP6AP2	COSTIM_up
CYB5D1	COSTIM_up
BACE2	COSTIM_up
UBE2E2	COSTIM_up
AP1S2	COSTIM_up
C8orf33	COSTIM_up
FBXL6	COSTIM_up
YBEY	COSTIM_up
NPLOC4	COSTIM_up
EXOC7	COSTIM_up
KPNA2	COSTIM_up
WASH6P	COSTIM_up
GLRX5	COSTIM_up
FAM104B	COSTIM_up
MFSD5	COSTIM_up
ADI1	COSTIM_up
RWDD4	COSTIM_up
SKA2	COSTIM_up
ANXA2	COSTIM_up
PAQR7	COSTIM_up
NGRN	COSTIM_up
RPS17	COSTIM_up
OR2T29	COSTIM_up
CRIP2	COSTIM_up
DDX28	COSTIM_up
ACBD3	COSTIM_up

RRP7BP	COSTIM_up
RBM10	COSTIM_up
RPL35A	COSTIM_up
CEP63	COSTIM_up
SRPRA	COSTIM_up
EWSR1	COSTIM_up
HMGN4	COSTIM_up
MTA1	COSTIM_up
PYCR1	COSTIM_up
NAA38	COSTIM_up
SPNS2	COSTIM_up
AP2A2	COSTIM_up
SLC25A10	COSTIM_up
GAS6	COSTIM_up
NEB	COSTIM_up
PTGDR2	COSTIM_up
HSP90AB3P	COSTIM_up
RUVBL2	COSTIM_up
DDX41	COSTIM_up
DAZAP2	COSTIM_up
OR2T34	COSTIM_up
BOLA2	COSTIM_up
SF3A3	COSTIM_up
TRIM61	COSTIM_up
GTF2H2C	COSTIM_up
GPR132	COSTIM_up
EP400	COSTIM_up
UTP11	COSTIM_up
PSMG1	COSTIM_up
PRR14L	COSTIM_up
SETD3	COSTIM_up
SFXN4	COSTIM_up
MRPL54	COSTIM_up
NDUFB1	COSTIM_up
ALYREF	COSTIM_up
UPP1	COSTIM_up
TMEM50A	COSTIM_up
TBK1	COSTIM_up
CBX6	COSTIM_up
MACC1	COSTIM_up
TBL3	COSTIM_up
TUBA8	COSTIM_up
TOB2	COSTIM_up
KMT5A	COSTIM_up
NPW	COSTIM_up
COA3	COSTIM_up
ST6GALNAC3	COSTIM_up
ACTG1	COSTIM_up
DENND5A	COSTIM_up
OR2T10	COSTIM_up

DIABLO	COSTIM_up
VPS33B	COSTIM_up
UQCR10	COSTIM_up
EIF3C	COSTIM_up
NR2C2AP	COSTIM_up
CRELD2	COSTIM_up
SCFD2	COSTIM_up
PGP	COSTIM_up
IRAK1	COSTIM_up
CMSS1	COSTIM_up
TSSC4	COSTIM_up
RPL23AP82	COSTIM_up
SRPK3	COSTIM_up
CSF1	COSTIM_up
MAML2	COSTIM_up
SS18L1	COSTIM_up
KCND2	COSTIM_up
COPB2	COSTIM_up
THAP7	COSTIM_up
PTP4A3	COSTIM_up
TMEM255B	COSTIM_up
HDDC3	COSTIM_up
ZFP1	COSTIM_up
SOCS3	COSTIM_up
XPOT	COSTIM_up
SNN	COSTIM_up
EIF4ENIF1	COSTIM_up
ATL3	COSTIM_up
NDUFA12	COSTIM_up
UBE2G2	COSTIM_up
APOO	COSTIM_up
PRR16	COSTIM_up
TMED9	COSTIM_up
SUMO3	COSTIM_up
IMMP2L	COSTIM_up
OR6C70	COSTIM_up
NOC4L	COSTIM_up
NDUFA6	COSTIM_up
DGAT1	COSTIM_up
AP3M1	COSTIM_up
NELFA	COSTIM_up
INTS5	COSTIM_up
RPS27L	COSTIM_up
MANEAL	COSTIM_up
ANO9	COSTIM_up
FAF1	COSTIM_up
HSF1	COSTIM_up
C6orf120	COSTIM_up
DDX51	COSTIM_up
NOMO2	COSTIM_up

TNFAIP2	COSTIM_up
PRMT3	COSTIM_up
PRPF39	COSTIM_up
PPIL6	COSTIM_up
UBALD2	COSTIM_up
CCDC137	COSTIM_up
ATP6V0A2	COSTIM_up
TEDC1	COSTIM_up
HGS	COSTIM_up
MAPK11	COSTIM_up
MRPL30	COSTIM_up
LINC00158	COSTIM_up
FAAP100	COSTIM_up
BRCC3	COSTIM_up
SP1	COSTIM_up
MRPL40	COSTIM_up
P4HB	COSTIM_up
PSMD13	COSTIM_up
ZFP36L1	COSTIM_up
UBE2L3	COSTIM_up
MOSMO	COSTIM_up
DRG1	COSTIM_up
YTHDF3	COSTIM_up
ZNF696	COSTIM_up
KCNQ5	COSTIM_up
MORF4L1	COSTIM_up
SLC52A2	COSTIM_up
PCYT2	COSTIM_up
NAT8L	COSTIM_up
BCAP31	COSTIM_up
GNB1L	COSTIM_up
TRIM69	COSTIM_up
ATP6V0C	COSTIM_up
LAMP1	COSTIM_up
ZNF267	COSTIM_up
LRCH3	COSTIM_up
NDUFA13	COSTIM_up
C15orf41	COSTIM_up
C2orf76	COSTIM_up
ZNRF1	COSTIM_up
ZNF749	COSTIM_up
KDM4D	COSTIM_up
GPAT2	COSTIM_up
TOR3A	COSTIM_up
PPP1CC	COSTIM_up
CA5BP1	COSTIM_up
KRT10	COSTIM_up
NKRF	COSTIM_up
KPNA4	COSTIM_up
RPS23	COSTIM_up

INSIG1	COSTIM_up
NF2	COSTIM_up
MIR22HG	COSTIM_up
HPDL	COSTIM_up
KATNA1	COSTIM_up
ZFP91	COSTIM_up
C17orf58	COSTIM_up
HYAL3	COSTIM_up
LILRB4	COSTIM_up
TNFRSF4	COSTIM_up
TNFRSF18	COSTIM_up
RTN4RL2	COSTIM_up
EMID1	COSTIM_up
PTRH1	COSTIM_up
TMEM216	COSTIM_up
RPS19BP1	COSTIM_up
NAP1L1	COSTIM_up
CMC1	COSTIM_up
RNF220	COSTIM_up
MT1X	COSTIM_up
FNBP1	COSTIM_up
CHP1	COSTIM_up
PTMA	COSTIM_up
HSPA14	COSTIM_up
USP7	COSTIM_up
NANOS3	COSTIM_up
TET3	COSTIM_up
ZNF286A	COSTIM_up
TMEM203	COSTIM_up
TCEA1	COSTIM_up
NHEJ1	COSTIM_up
FANCA	COSTIM_up
MCRS1	COSTIM_up
CARD9	COSTIM_up
ZFP69B	COSTIM_up
PLSCR3	COSTIM_up
EIF4EBP1	COSTIM_up
PMS2CL	COSTIM_up
MORN2	COSTIM_up
UBQLN2	COSTIM_up
NWD1	COSTIM_up
C11orf95	COSTIM_up
MAPK12	COSTIM_up
AGRN	COSTIM_up
TMPPE	COSTIM_up
LINC00265	COSTIM_up
PRKAR1B	COSTIM_up
NUTM2B	COSTIM_up
NCR3LG1	COSTIM_up
TUBB4B	COSTIM_up

IL17REL	COSTIM_up
HES4	COSTIM_up
CENPP	COSTIM_up
GTF2F2	COSTIM_up
H2AX	COSTIM_up
C19orf54	COSTIM_up
C22orf34	COSTIM_up
FAM83G	COSTIM_up
SRSF10	COSTIM_up
OR2G6	COSTIM_up
NDOR1	COSTIM_up
NPIPP1	COSTIM_up
SUMO2	COSTIM_up
RTL6	COSTIM_up
PARVB	COSTIM_up
UROS	COSTIM_up
ZDHHC9	COSTIM_up
ZBED6CL	COSTIM_up
SMIM15	COSTIM_up
TMEM120B	COSTIM_up
RBM34	COSTIM_up
MTF1	COSTIM_up
TMEM201	COSTIM_up
ZDHHC11	COSTIM_up
SLX4	COSTIM_up
RPL14	COSTIM_up
ASTL	COSTIM_up
NOC2L	COSTIM_up
NELFB	COSTIM_up
ADAT2	COSTIM_up
NDUFA4	COSTIM_up
ALKBH2	COSTIM_up
LITAF	COSTIM_up
TMEM120A	COSTIM_up
ARID2	COSTIM_up
SF3B3	COSTIM_up
JPT1	COSTIM_up
PNRC2	COSTIM_up
RRP7A	COSTIM_up
SLC25A51P4	COSTIM_up
S100A14	COSTIM_up
RPS2P46	COSTIM_up
SH2D5	COSTIM_up
PAX5	COSTIM_up
HLA-DRB1	COSTIM_up
ZNF79	COSTIM_up
STK40	COSTIM_up
EEF1A1P5	COSTIM_up
TUBB	COSTIM_up
SUPT5H	COSTIM_up

XPNPEP3	COSTIM_up
OR2T2	COSTIM_up
PPIA	COSTIM_up
SUPT3H	COSTIM_up
NIF3L1	COSTIM_up
IARS1	COSTIM_up
POM121	COSTIM_up
WDR5	COSTIM_up
LONP1	COSTIM_up
FUT4	COSTIM_up
PTPN1	COSTIM_up
THEM5	COSTIM_up
XRCC6	COSTIM_up
TSC22D2	COSTIM_up
YRDC	COSTIM_up
ZNF777	COSTIM_up
PIK3R4	COSTIM_up
IPO4	COSTIM_up
NCOR2	COSTIM_up
PRPF40A	COSTIM_up
ANAPC7	COSTIM_up
SLC6A9	COSTIM_up
NACA	COSTIM_up
OR2T3	COSTIM_up
HDAC2	COSTIM_up
SDHAF3	COSTIM_up
RABL6	COSTIM_up
ZNF502	COSTIM_up
TRAPPC4	COSTIM_up
ZNF512B	COSTIM_up
VKORC1L1	COSTIM_up
HLA-DQA1	COSTIM_up
COL27A1	COSTIM_up
SNHG17	COSTIM_up
ZNF239	COSTIM_up
ILRUN	COSTIM_up
ADA	COSTIM_up
ARID5A	COSTIM_up
PPTC7	COSTIM_up
NHLRC2	COSTIM_up
CBWD3	COSTIM_up
ARHGEF12	COSTIM_up
FLNA	COSTIM_up
NOP9	COSTIM_up
AP2A1	COSTIM_up
LAGE3	COSTIM_up
METTL9	COSTIM_up
CXorf40B	COSTIM_up
ANXA6	COSTIM_up
GMFB	COSTIM_up

MAFG	COSTIM_up
IGF2R	COSTIM_up
DYNC1H1	COSTIM_up
PCBP2	COSTIM_up
ZGPAT	COSTIM_up
SRC	COSTIM_up
ZNF682	COSTIM_up
PCNX3	COSTIM_up
ACSL5	COSTIM_up
LRRC8B	COSTIM_up
ABCB8	COSTIM_up
SND1	COSTIM_up
PSMD12	COSTIM_up
NOL4L	COSTIM_up
C1D	COSTIM_up
TBC1D9B	COSTIM_up
GTF2E2	COSTIM_up
ZNF165	COSTIM_up
ZNF720	COSTIM_up
SVIL	COSTIM_up
MRPL21	COSTIM_up
HTT	COSTIM_up
MAP3K5	COSTIM_up
HNRNPAB	COSTIM_up
PDGFA	COSTIM_up
ZNF628	COSTIM_up
RPF2	COSTIM_up
ENTPD6	COSTIM_up
FAR1	COSTIM_up
CXorf40A	COSTIM_up
CDC42SE1	COSTIM_up
SERPINB2	COSTIM_up
DPP4	COSTIM_up
NMB	COSTIM_up
FAM114A1	COSTIM_up
RPE	COSTIM_up
ZNF460	COSTIM_up
PTMAP2	COSTIM_up
RPL37A	COSTIM_up
MCMBP	COSTIM_up
EME2	COSTIM_up
TAF13	COSTIM_up
ATAD3A	COSTIM_up
FAM118B	COSTIM_up
SLC9A8	COSTIM_up
GPAA1	COSTIM_up
SGTB	COSTIM_up
FAM49A	COSTIM_up
MYO1C	COSTIM_up
KIF13B	COSTIM_up

ADH5	COSTIM_up
SPG7	COSTIM_up
HRNR	COSTIM_up
HES5	COSTIM_up
ERO1A	COSTIM_up
S100A6	COSTIM_up
RPL12	COSTIM_up
ZNF121	COSTIM_up
MPZL1	COSTIM_up
VPS13A	COSTIM_up
GOLGA6L9	COSTIM_up
C1orf122	COSTIM_up
SNHG12	COSTIM_up
NOL8	COSTIM_up
MRPL42	COSTIM_up
RPS4X	COSTIM_up
MAK16	COSTIM_up
AVPR1B	COSTIM_up
SIRPA	COSTIM_up
CD2AP	COSTIM_up
ZNF544	COSTIM_up
ZNF770	COSTIM_up
MAN1A2	COSTIM_up
DDRGK1	COSTIM_up
TFDP1	COSTIM_up
CACNA1E	COSTIM_up
DDX42	COSTIM_up
RPL23A	COSTIM_up
UBL5	COSTIM_up
ZNF485	COSTIM_up
SDAD1	COSTIM_up
PIM3	COSTIM_up
GET3	COSTIM_up
SPRED2	COSTIM_up
BZW1P2	COSTIM_up
TXNRD1	COSTIM_up
YTHDF2	COSTIM_up
TMA16	COSTIM_up
HLA-DRB5	COSTIM_up
MAFK	COSTIM_up
GPN1	COSTIM_up
ZNF511	COSTIM_up
WDHD1	COSTIM_up
DDX39B	COSTIM_up
BAZ1A	COSTIM_up
COPS8	COSTIM_up
PPIAP22	COSTIM_up
KLHL9	COSTIM_up
C6orf89	COSTIM_up
CALM1	COSTIM_up

FAN1	COSTIM_up
EIF1AY	COSTIM_up
MT-ND6	COSTIM_up
IPO9	COSTIM_up
ANKRD13B	COSTIM_up
UNC13B	COSTIM_up
MT-CYB	COSTIM_up
CTR9	COSTIM_up
MSRB1	COSTIM_up
GPATCH3	COSTIM_up
CDC42BPB	COSTIM_up
OXCT2	COSTIM_up
RPL10A	COSTIM_up
COLGALT2	COSTIM_up
MT-ND2	COSTIM_up
FAM169A	COSTIM_up
MT-ND5	COSTIM_up
CNOT7	COSTIM_up
MTOR	COSTIM_up
ALPK2	COSTIM_up
PNP	COSTIM_up
FOXJ3	COSTIM_up
SFT2D1	COSTIM_up
INPP5F	COSTIM_up
HMGN2	COSTIM_up
UBE2J1	COSTIM_up
OPA1	COSTIM_up
RYR3	COSTIM_up
KTI12	COSTIM_up
SELENOT	COSTIM_up
TOX	COSTIM_up
RUSC2	COSTIM_up
OSTC	COSTIM_up
TSEN15	COSTIM_up
GRK5	COSTIM_up
TYW1	COSTIM_up
DCAF12	COSTIM_up
ITPRIPL1	COSTIM_up
MT-ND4	COSTIM_up
MT-ND1	COSTIM_up
PRMT6	COSTIM_up
CAPZA2	COSTIM_up
MT-ATP6	COSTIM_up
TOP1	COSTIM_up
C1orf174	COSTIM_up
SPOUT1	COSTIM_up
RPL39	COSTIM_up
DCLRE1A	COSTIM_up
APRT	COSTIM_up
CCDC167	COSTIM_up

MT-CO3	COSTIM_up
SMG5	COSTIM_up
RNVU1-22	COSTIM_up
RN7SKP90	COSTIM_up
SNORD1B	COSTIM_up
SNORA73B	COSTIM_up
RNU6-1214P	COSTIM_up
SNORA33	COSTIM_up
SNORD14E	COSTIM_up
SNORA74A	COSTIM_up
RNY1	COSTIM_up
RNU6-777P	COSTIM_up
SNORA65	COSTIM_up
SNORD14B	COSTIM_up
RNVU1-6	COSTIM_up
RNU6-619P	COSTIM_up
RNY3	COSTIM_up
VTRNA1-3	COSTIM_up
E2F3P2	COSTIM_up
OR2T5	COSTIM_up
COX20	COSTIM_up
CENPW	COSTIM_up
PRR9	COSTIM_up
EEF1AKMT2	COSTIM_up
GDI1	COSTIM_up
LINC00963	COSTIM_up
TRAF3IP1	COSTIM_up
ASAH2B	COSTIM_up
AGAP6	COSTIM_up
TIMM23B	COSTIM_up
ZDHHC18	COSTIM_up
AGAP9	COSTIM_up
BMS1P1	COSTIM_up
MACO1	COSTIM_up
PTPN20	COSTIM_up
RPL12P16	COSTIM_up
DAXX	COSTIM_up
PFDN6	COSTIM_up
RING1	COSTIM_up
HSD17B8	COSTIM_up
RXRB	COSTIM_up
BRD2	COSTIM_up
PSMB8	COSTIM_up
TAP2	COSTIM_up
NBDY	COSTIM_up
HLA-DRA	COSTIM_up
RNF5	COSTIM_up
AGPAT1	COSTIM_up
FKBPL	COSTIM_up
MRPL38	COSTIM_up

STK19	COSTIM_up
SKIV2L	COSTIM_up
NELFE	COSTIM_up
SDHD	COSTIM_up
NEU1	COSTIM_up
SNHG32	COSTIM_up
HSPA1B	COSTIM_up
HSPA1A	COSTIM_up
LSM2	COSTIM_up
VARS1	COSTIM_up
MSH5	COSTIM_up
CSNK2B	COSTIM_up
GPANK1	COSTIM_up
BAG6	COSTIM_up
PRRC2A	COSTIM_up
LST1	COSTIM_up
NFKBIL1	COSTIM_up
ZNF814	COSTIM_up
MICB	COSTIM_up
ZNF551	COSTIM_up
ZNF805	COSTIM_up
HLA-C	COSTIM_up
DHX16	COSTIM_up
MRPS18B	COSTIM_up
ABCF1	COSTIM_up
PRR3	COSTIM_up
LILRB3	COSTIM_up
GNL1	COSTIM_up
HLA-E	COSTIM_up
PPP1R11	COSTIM_up
RACK1	COSTIM_up
HLA-F	COSTIM_up
ZFP57	COSTIM_up
AKT1S1	COSTIM_up
TRIM27	COSTIM_up
MRPL53	COSTIM_up
DCTN1	COSTIM_up
FAM216A	COSTIM_up
MZT1	COSTIM_up
UQCC3	COSTIM_up
ZNF783	COSTIM_up
C12orf73	COSTIM_up
SLC35B4	COSTIM_up
PSENEN	COSTIM_up
ZBTB10	COSTIM_up
C4orf46	COSTIM_up
PSMB10	COSTIM_up
E2F4	COSTIM_up
TMEM170B	COSTIM_up
SARNP	COSTIM_up

IPO7	COSTIM_up
PRR13	COSTIM_up
NAP1L4	COSTIM_up
TMSB4X	COSTIM_up
SMN2	COSTIM_up
SERF1B	COSTIM_up
HMGN1	COSTIM_up
EIF3CL	COSTIM_up
DPF3	COSTIM_up
RNPS1	COSTIM_up
HSP90AB2P	COSTIM_up
DNAJC19	COSTIM_up
JPT2	COSTIM_up
HCP5	COSTIM_up
HLA-H	COSTIM_up
HLA-A	COSTIM_up
ANKRD28	COSTIM_up
THUMPD3-AS1	COSTIM_up
RNU6-431P	COSTIM_up
SNORD58A	COSTIM_up
SNORD24	COSTIM_up
RNU6-608P	COSTIM_up
SNORD21	COSTIM_up
SNORD101	COSTIM_up
RNVU1-30	COSTIM_up
SNORA75	COSTIM_up
SNORD51	COSTIM_up
RNVU1-15	COSTIM_up
SNORD20	COSTIM_up
RNU6-1127P	COSTIM_up
MIR181A2	COSTIM_up
SNORD12C	COSTIM_up
SNORD83B	COSTIM_up
SNORD83A	COSTIM_up
MT-TF	COSTIM_up
MT-TV	COSTIM_up
MT-TQ	COSTIM_up
MT-TA	COSTIM_up
MT-TN	COSTIM_up
MT-TC	COSTIM_up
MT-TY	COSTIM_up
MT-TE	COSTIM_up
GPX3	COSTIM_up
SELENOH	COSTIM_up
STK38L	COSTIM_up
TSN	COSTIM_up
IGLV2-14	COSTIM_up
IGLC1	COSTIM_up
IGLC2	COSTIM_up
PRR22	COSTIM_up

TAS2R15P	COSTIM_up
TAS2R13	COSTIM_up
SNORD19	COSTIM_up
SNORA26	COSTIM_up
LINC02693	COSTIM_up
RNF208	COSTIM_up
MT-ND4L	COSTIM_up
NUP62	COSTIM_up
AURKAP1	COSTIM_up
DENND1B	COSTIM_up
SCAF8	COSTIM_up
CRIP1	COSTIM_up
KLHL23	COSTIM_up
RPL22P1	COSTIM_up
TRIM59	COSTIM_up
BTF3L4P2	COSTIM_up
DNLZ	COSTIM_up
NPM1P32	COSTIM_up
RBMS2P1	COSTIM_up
NRAS	COSTIM_up
ANKRD39	COSTIM_up
QTRT1	COSTIM_up
CHUK	COSTIM_up
RPS3P6	COSTIM_up
COG8	COSTIM_up
HAUS7	COSTIM_up
SYNJ2BP	COSTIM_up
ARL2	COSTIM_up
FIRRE	COSTIM_up
RBMXL1	COSTIM_up
SRA1	COSTIM_up
YBX1P6	COSTIM_up
DNAJC9	COSTIM_up
RPLP0P6	COSTIM_up
VDAC1	COSTIM_up
ZBTB9	COSTIM_up
TMX2	COSTIM_up
NDUFS3	COSTIM_up
ADAT3	COSTIM_up
PPP1CB	COSTIM_up
LAT	COSTIM_up
RPL17P50	COSTIM_up
CLIC1	COSTIM_up
RPS29	COSTIM_up
CENPBD1P1	COSTIM_up
GTF2H4	COSTIM_up
DDX47	COSTIM_up
TMX2P1	COSTIM_up
RPL21P75	COSTIM_up
YBX1P10	COSTIM_up

RPL13AP7	COSTIM_up
ITGA1	COSTIM_up
NUDT19	COSTIM_up
PGAM1P7	COSTIM_up
REPIN1	COSTIM_up
MRPL23	COSTIM_up
TSPAN4	COSTIM_up
LYRM4	COSTIM_up
MYCBP	COSTIM_up
ALG3	COSTIM_up
ST7-OT4	COSTIM_up
ZNF788P	COSTIM_up
SH3D21	COSTIM_up
SMIM30	COSTIM_up
TTC41P	COSTIM_up
EEF1A1P12	COSTIM_up
HNRNPA1P10	COSTIM_up
FIS1	COSTIM_up
SOGA3	COSTIM_up
RPS3AP26	COSTIM_up
FAM187A	COSTIM_up
PPME1	COSTIM_up
NUTM2D	COSTIM_up
B3GNT10	COSTIM_up
IFRD2	COSTIM_up
ZBED1	COSTIM_up
TOMM6	COSTIM_up
HNRNPUL2	COSTIM_up
CYCSP55	COSTIM_up
LRRC69	COSTIM_up
DNAJA1P3	COSTIM_up
PHB2	COSTIM_up
NEURL4	COSTIM_up
UBXN2B	COSTIM_up
CBWD6	COSTIM_up
PEX26	COSTIM_up
FASTKD5	COSTIM_up
HOMEZ	COSTIM_up
DDX3X	COSTIM_up
VPS16	COSTIM_up
MYL5	COSTIM_up
MIR17HG	COSTIM_up
MCM3AP-AS1	COSTIM_up
AATBC	COSTIM_up
GCGR	COSTIM_up
KRT19P2	COSTIM_up
IFI30	COSTIM_up
MTCO2P33	COSTIM_up
HMGB1P17	COSTIM_up
PAM16	COSTIM_up

TATDN2P2	COSTIM_up
NEPNP	COSTIM_up
MORF4L1P1	COSTIM_up
ZNF579	COSTIM_up
ARL4AP5	COSTIM_up
RNASEK	COSTIM_up
PPIAP9	COSTIM_up
ZSCAN12P1	COSTIM_up
ASS1P1	COSTIM_up
RPL21P16	COSTIM_up
HSPE1P26	COSTIM_up
MIR663B	COSTIM_up
PPP3R1	COSTIM_up
AP4M1	COSTIM_up
HMSD	COSTIM_up
PPP2R2A	COSTIM_up
TRIM16	COSTIM_up
UBA52	COSTIM_up
BTBD19	COSTIM_up
CYTOR	COSTIM_up
SNORD19C	COSTIM_up
SNORD12B	COSTIM_up
RNA5SP44	COSTIM_up
RNU2-63P	COSTIM_up
MIR1913	COSTIM_up
NUTM2A-AS1	COSTIM_up
EXOSC6	COSTIM_up
HLA-DQB1-AS1	COSTIM_up
SAMSN1-AS1	COSTIM_up
LINC00571	COSTIM_up
AFG3L1P	COSTIM_up
MIR181A2HG	COSTIM_up
RPS24P8	COSTIM_up
MIR3681HG	COSTIM_up
C5orf66	COSTIM_up
DTNB-AS1	COSTIM_up
MDC1-AS1	COSTIM_up
HSP90AA2P	COSTIM_up
ADM5	COSTIM_up
SMIM13	COSTIM_up
HNRNPA1P48	COSTIM_up
SVIL-AS1	COSTIM_up
RPS27AP16	COSTIM_up
LINC00240	COSTIM_up
MRPL20-AS1	COSTIM_up
NDUFAF8	COSTIM_up
VPS26BP1	COSTIM_up
DYNC1I2P1	COSTIM_up
SLC9A3-AS1	COSTIM_up
GOLGA2P7	COSTIM_up

NPM1P39	COSTIM_up
LINC00618	COSTIM_up
RPSAP58	COSTIM_up
RBMS1P1	COSTIM_up
C2CD4D	COSTIM_up
MTND2P28	COSTIM_up
MCRIP1	COSTIM_up
FGD5-AS1	COSTIM_up
NPM1P18	COSTIM_up
OR6V1	COSTIM_up
RUSC1-AS1	COSTIM_up
NOL7	COSTIM_up
MTND1P23	COSTIM_up
YBX1P8	COSTIM_up
UQCRFS1P1	COSTIM_up
GTF2H2B	COSTIM_up
CFLAR-AS1	COSTIM_up
SLC16A1-AS1	COSTIM_up
PLCL2-AS1	COSTIM_up
TMEM185B	COSTIM_up
TEX41	COSTIM_up
NAALADL2-AS2	COSTIM_up
LINC01276	COSTIM_up
DANCR	COSTIM_up
LTA	COSTIM_up
LINC01692	COSTIM_up
FUNDC2P4	COSTIM_up
WDR46	COSTIM_up
RPS28P7	COSTIM_up
LINC01630	COSTIM_up
LINC02672	COSTIM_up
MIR663AHG	COSTIM_up
SCAMP4	COSTIM_up
RCC2-AS1	COSTIM_up
MYL12BP2	COSTIM_up
RPL23AP89	COSTIM_up
RAPGEF4-AS1	COSTIM_up
PABPC4-AS1	COSTIM_up
MELTF-AS1	COSTIM_up
NACA4P	COSTIM_up
GAPDHP1	COSTIM_up
MT-ATP8	COSTIM_up
PSMD8P1	COSTIM_up
FAM174C	COSTIM_up
LINC02474	COSTIM_up
OST4	COSTIM_up
MIR34AHG	COSTIM_up
SNHG26	COSTIM_up
SAPCD1	COSTIM_up
HCG22	COSTIM_up

LINC01128	COSTIM_up
RASA4CP	COSTIM_up
AKR7A2P1	COSTIM_up
RPL41	COSTIM_up
ST13P13	COSTIM_up
RALGAPA1P1	COSTIM_up
RPL4P4	COSTIM_up
NAMPTP1	COSTIM_up
PET100	COSTIM_up
RPL4P6	COSTIM_up
TPRG1-AS2	COSTIM_up
ACBD6	COSTIM_up
EXOSC10-AS1	COSTIM_up
HLA-K	COSTIM_up
YY2	COSTIM_up
LINC02541	COSTIM_up
HSBP1	COSTIM_up
ECE1-AS1	COSTIM_up
HLA-DPA1	COSTIM_up
RPS18	COSTIM_up
PRH1	COSTIM_up
MTND5P15	COSTIM_up
TAPBP	COSTIM_up
FAM242A	COSTIM_up
LSM12P1	COSTIM_up
TMA7	COSTIM_up
MCTS1	COSTIM_up
IMPDH1P10	COSTIM_up
USP12-AS1	COSTIM_up
SMIM26	COSTIM_up
AJM1	COSTIM_up
EMSLR	COSTIM_up
EEF1B2P3	COSTIM_up
MRTFA-AS1	COSTIM_up
MRAP-AS1	COSTIM_up
HLA-DQB2	COSTIM_up
LINC00665	COSTIM_up
PET117	COSTIM_up
SNHG15	COSTIM_up
VPS13A-AS1	COSTIM_up
SNHG7	COSTIM_up
NPIPA9	COSTIM_up
EEF1A1P14	COSTIM_up
LINC00456	COSTIM_up
LINC01762	COSTIM_up
KLF4P1	COSTIM_up
SNRPEP4	COSTIM_up
EEF1A1P6	COSTIM_up
HNRNPLP1	COSTIM_up
FAM224A	COSTIM_up

KRT8P39	COSTIM_up
GAS6-AS1	COSTIM_up
YEATS2-AS1	COSTIM_up
TXNP6	COSTIM_up
NUDT9P1	COSTIM_up
TPT1P1	COSTIM_up
TRIM26	COSTIM_up
HSPA8P1	COSTIM_up
LINC01283	COSTIM_up
GAPDHP49	COSTIM_up
C2CD4D-AS1	COSTIM_up
RPSAP9	COSTIM_up
YWHAEP5	COSTIM_up
GAS5	COSTIM_up
EIF5AP4	COSTIM_up
HLA-B	COSTIM_up
FOXP4-AS1	COSTIM_up
LINC02621	COSTIM_up
SLC25A25-AS1	COSTIM_up
HSPA8P3	COSTIM_up
NXT1-AS1	COSTIM_up
RPL23AP42	COSTIM_up
HNRNPUL2-BSCL2	COSTIM_up
LINC00163	COSTIM_up
EIF3EP1	COSTIM_up
MIR155HG	COSTIM_up
SNHG20	COSTIM_up
FABP5P7	COSTIM_up
COX20P2	COSTIM_up
HGH1	COSTIM_up
SNORA71B	COSTIM_up
L3MBTL2-AS1	COSTIM_up
RPL6P27	COSTIM_up
NUS1P1	COSTIM_up
H3P6	COSTIM_up
BHLHE40-AS1	COSTIM_up
CPB2-AS1	COSTIM_up
ASMTL-AS1	COSTIM_up
TAS2R39	COSTIM_up
KCNMA1-AS1	COSTIM_up
RPL13AP5	COSTIM_up
BCL2L1-AS1	COSTIM_up
EIF1AXP1	COSTIM_up
MKLN1-AS	COSTIM_up
LINC00106	COSTIM_up
PRMT5-AS1	COSTIM_up
CDKN2AIPNL	COSTIM_up
ZNF295-AS1	COSTIM_up
RSL24D1P8	COSTIM_up
SMG1P1	COSTIM_up

TTN-AS1	COSTIM_up
SRRM1P3	COSTIM_up
RNF223	COSTIM_up
WAKMAR2	COSTIM_up
HLA-DQA2	COSTIM_up
KLF7-IT1	COSTIM_up
CELF2-AS2	COSTIM_up
TNFRSF14-AS1	COSTIM_up
LRIG2-DT	COSTIM_up
TMEM250	COSTIM_up
RNVU1-2	COSTIM_up
NME1	COSTIM_up
APOBEC3G	COSTIM_up
MRPS17	COSTIM_up
GET4	COSTIM_up
ADSL	COSTIM_up
LILRA4	COSTIM_up
RN7SL254P	COSTIM_up
RPS2P5	COSTIM_up
RPL23AP7	COSTIM_up
RPL13P5	COSTIM_up
SETP14	COSTIM_up
L1TD1	COSTIM_up
ISY1	COSTIM_up
RN7SL288P	COSTIM_up
TMEM189	COSTIM_up
RDH14	COSTIM_up
MIF	COSTIM_up
TPM3P9	COSTIM_up
ARHGAP31-AS1	COSTIM_up
RN7SL443P	COSTIM_up
RN7SL302P	COSTIM_up
CRCP	COSTIM_up
PDXP	COSTIM_up
RPP21	COSTIM_up
ATP5MF	COSTIM_up
PSMC1P1	COSTIM_up
CBX5P1	COSTIM_up
ARPC4	COSTIM_up
ARPC1A	COSTIM_up
ATP5PO	COSTIM_up
PLEKHO2	COSTIM_up
PISD	COSTIM_up
PWP2	COSTIM_up
RPL7AP6	COSTIM_up
MTFP1	COSTIM_up
SNHG3	COSTIM_up
ARFGAP3	COSTIM_up
EIF6	COSTIM_up
MRPL20	COSTIM_up

DECR2	COSTIM_up
GNG10	COSTIM_up
CNTF	COSTIM_up
RN7SL40P	COSTIM_up
AP5Z1	COSTIM_up
RN7SL846P	COSTIM_up
RPS26P21	COSTIM_up
EIF4EBP3	COSTIM_up
MRPL33	COSTIM_up
PPAN-P2RY11	COSTIM_up
TICAM2	COSTIM_up
C4orf48	COSTIM_up
NME2	COSTIM_up
TTC4	COSTIM_up
HLA-L	COSTIM_up
LINC02044	COSTIM_up
MRPS6	COSTIM_up
ACY1	COSTIM_up
FAM86DP	COSTIM_up
DDOST	COSTIM_up
TMEM199	COSTIM_up
ATP1B3-AS1	COSTIM_up
P2RY11	COSTIM_up
TMEM141	COSTIM_up
TMEM225B	COSTIM_up
SIAH2-AS1	COSTIM_up
RPL7P23	COSTIM_up
RBM12	COSTIM_up
RN7SL396P	COSTIM_up
UBE2V1	COSTIM_up
SMARCA5-AS1	COSTIM_up
EEF1A1P4	COSTIM_up
ARNTL2-AS1	COSTIM_up
SNHG6	COSTIM_up
RRS1-AS1	COSTIM_up
SOCS2-AS1	COSTIM_up
PGAM5	COSTIM_up
SNHG10	COSTIM_up
ZCCHC3	COSTIM_up
DNAJC3-DT	COSTIM_up
SPTY2D1OS	COSTIM_up
MARS2	COSTIM_up
MTND4P12	COSTIM_up
NRAV	COSTIM_up
ANK2-AS1	COSTIM_up
CDK11B	COSTIM_up
MTATP6P1	COSTIM_up
LY75-CD302	COSTIM_up
ATP5MF-PTCD1	COSTIM_up
LINC02362	COSTIM_up

HAUS5	COSTIM_up
EEF1A1P9	COSTIM_up
IL20RB-AS1	COSTIM_up
SERBP1P5	COSTIM_up
NOP14-AS1	COSTIM_up
PVT1	COSTIM_up
PDCD6	COSTIM_up
LINC02223	COSTIM_up
YJEFN3	COSTIM_up
ANKRD33B-AS1	COSTIM_up
ARPC4-TTLL3	COSTIM_up
EEF1A1P13	COSTIM_up
PKD1P6	COSTIM_up
MRPS31P4	COSTIM_up
LINC02260	COSTIM_up
CHCHD10	COSTIM_up
ZBED3-AS1	COSTIM_up
LINC01181	COSTIM_up
TMED7-TICAM2	COSTIM_up
HSPD1P11	COSTIM_up
CENPS-CORT	COSTIM_up
LINC02201	COSTIM_up
SNORA74D	COSTIM_up
RNU7-13P	COSTIM_up
RNU6-786P	COSTIM_up
RNU6-1003P	COSTIM_up
KLF3P1	COSTIM_up
C8orf88	COSTIM_up
SHLD3	COSTIM_up
LINC01845	COSTIM_up
SERPINE3	COSTIM_up
LINC02099	COSTIM_up
MIR3142HG	COSTIM_up
MTCO1P4	COSTIM_up
ALG11	COSTIM_up
PRKDC	COSTIM_up
MTND1P5	COSTIM_up
SLC2A3P1	COSTIM_up
THAP12P7	COSTIM_up
LYN	COSTIM_up
PINX1	COSTIM_up
LINC02055	COSTIM_up
LINC02365	COSTIM_up
CHKB-CPT1B	COSTIM_up
LINC02732	COSTIM_up
SLC25A1P1	COSTIM_up
CHMP4A	COSTIM_up
PHBP2	COSTIM_up
BBOX1-AS1	COSTIM_up
PKD1P5	COSTIM_up

MEX3A	COSTIM_up
EEF1G	COSTIM_up
MPV17L2	COSTIM_up
BORCS8	COSTIM_up
DPP3	COSTIM_up
ANKHD1-EIF4EBP3	COSTIM_up
BRK1	COSTIM_up
LINC02739	COSTIM_up
ZFP91-CNTF	COSTIM_up
EID3	COSTIM_up
MSH5-SAPCD1	COSTIM_up
HTD2	COSTIM_up
NECTIN1-AS1	COSTIM_up
EID1	COSTIM_up
UVRAG-DT	COSTIM_up
POLR2M	COSTIM_up
FDXACB1	COSTIM_up
RAB44	COSTIM_up
SNHG1	COSTIM_up
GOLGA2P10	COSTIM_up
CCND2-AS1	COSTIM_up
COA8	COSTIM_up
LINC00944	COSTIM_up
SMLR1	COSTIM_up
HMBS	COSTIM_up
HSPA8P5	COSTIM_up
SMIM10L1	COSTIM_up
PPP1R14B-AS1	COSTIM_up
LSM14A	COSTIM_up
GATC	COSTIM_up
LINC02388	COSTIM_up
ZNF271P	COSTIM_up
HSPD1P4	COSTIM_up
GIHCG	COSTIM_up
CPNE8-AS1	COSTIM_up
CNPY2	COSTIM_up
LINC02416	COSTIM_up
CLEC5A	COSTIM_up
NENFP2	COSTIM_up
CHURC1	COSTIM_up
C17orf49	COSTIM_up
RTEL1	COSTIM_up
PDF	COSTIM_up
LINC00641	COSTIM_up
BCL2L2-PABPN1	COSTIM_up
HSPE1P2	COSTIM_up
EEF1A1P33	COSTIM_up
NDUFC2-KCTD14	COSTIM_up
LINC00924	COSTIM_up
CSPG4P12	COSTIM_up

ZHX1-C8orf76	COSTIM_up
INAFM2	COSTIM_up
TGIF2-RAB5IF	COSTIM_up
UBE2Q2P2	COSTIM_up
THTPA	COSTIM_up
MRPL46	COSTIM_up
TYRO3P	COSTIM_up
EHD4-AS1	COSTIM_up
IL21R-AS1	COSTIM_up
RBM15B	COSTIM_up
NORAD	COSTIM_up
LINC02166	COSTIM_up
SNHG19	COSTIM_up
ATP2A1-AS1	COSTIM_up
SLC7A5P1	COSTIM_up
LINC01963	COSTIM_up
LINC01311	COSTIM_up
TMEM178B	COSTIM_up
ZNF865	COSTIM_up
BOP1	COSTIM_up
VPS9D1-AS1	COSTIM_up
TMEM249	COSTIM_up
GAN	COSTIM_up
LINC02605	COSTIM_up
LINC00662	COSTIM_up
LINC01978	COSTIM_up
CORO7	COSTIM_up
MIR193BHG	COSTIM_up
LINC01979	COSTIM_up
OVCA2	COSTIM_up
MRPL12	COSTIM_up
CCNQ	COSTIM_up
MIR378H	COSTIM_up
RN7SL471P	COSTIM_up
AK4P1	COSTIM_up
UBBP4	COSTIM_up
RN7SL4P	COSTIM_up
LINC00667	COSTIM_up
SNORD43	COSTIM_up
MIR4439	COSTIM_up
SINHCAFP1	COSTIM_up
RHEX	COSTIM_up
MIR3681	COSTIM_up
MIR4670	COSTIM_up
RNU4ATAC	COSTIM_up
RN7SL753P	COSTIM_up
MIR548AC	COSTIM_up
MIR3648-2	COSTIM_up
OTUD7B	COSTIM_up
MIR4511	COSTIM_up

SNORD3C	COSTIM_up
AGAP12P	COSTIM_up
MIR3142	COSTIM_up
RBM8A	COSTIM_up
TIMM23	COSTIM_up
RNF115	COSTIM_up
RPL17	COSTIM_up
MAFG-DT	COSTIM_up
RN7SL5P	COSTIM_up
SNHG25	COSTIM_up
NCOA4	COSTIM_up
MRPS21	COSTIM_up
AARSD1	COSTIM_up
UPK3BL1	COSTIM_up
MIR497HG	COSTIM_up
S1PR2	COSTIM_up
FDX2	COSTIM_up
SMIM22	COSTIM_up
NDUFV2P1	COSTIM_up
NDUFA7	COSTIM_up
ZNF587B	COSTIM_up
PTOV1-AS2	COSTIM_up
SPIB	COSTIM_up
SNHG8	COSTIM_up
COMMD3-BMI1	COSTIM_up
FMC1-LUC7L2	COSTIM_up
MIR222HG	COSTIM_up
GAS5-AS1	COSTIM_up
TERC	COSTIM_up
NCBP2AS2	COSTIM_up
TAF15	COSTIM_up
RNVU1-31	COSTIM_up
RPS10-NUDT3	COSTIM_up
HNRNPA3P9	COSTIM_up
NUDT4P2	COSTIM_up
ARMCX5-GPRASP2	COSTIM_up
BNIP3P4	COSTIM_up
SRXN1	COSTIM_up
NBPF19	COSTIM_up
SNORA51	COSTIM_up
LINC02343	COSTIM_up
OR2AS2P	COSTIM_up
SNORD14A	COSTIM_up
GTF2H5	COSTIM_up
NUDT3	COSTIM_up
KMT2B	COSTIM_up
DCP1A	COSTIM_up
ATP6V1FNB	COSTIM_up
CWC25	COSTIM_up
EPOP	COSTIM_up

CYFIP1	COSTIM_up
TAF9	COSTIM_up
NOL12	COSTIM_up
RN7SL2	COSTIM_up
SNORD1C	COSTIM_up
RNVU1-27	COSTIM_up
SOCS7	COSTIM_up
H2BC6	COSTIM_up
RNVU1-25	COSTIM_up
NPEPPSP1	COSTIM_up
RCC1L	COSTIM_up
SPDYE10P	COSTIM_up
RN7SKP236	COSTIM_up
RNA5-8SN5	COSTIM_up
TBC1D3I	COSTIM_up
RNU11	COSTIM_up
MLLT6	COSTIM_up
HMGB1P24	COSTIM_up
PPP4R3B	COSTIM_up
SNORD91B	COSTIM_up
LENG9	COSTIM_up
RNA5-8SN3	COSTIM_up
RNVU1-26	COSTIM_up
MIR1299	COSTIM_up
HNF1B	COSTIM_up
RNVU1-19	COSTIM_up
AATF	COSTIM_up
MIR3648-1	COSTIM_up
TUBGCP5	COSTIM_up
IKBKGP1	COSTIM_up
U2AF1L5	COSTIM_up
SNORD27	COSTIM_up
ORAI1	COSTIM_up
RN7SL1	COSTIM_up
TADA2A	COSTIM_up
PIP4K2B	COSTIM_up
RNA5-8SN4	COSTIM_up
CSPG4P10	COSTIM_up
MIR6821	COSTIM_up
LINC02227	COSTIM_up
MIR6797	COSTIM_up
PIGW	COSTIM_up
GPR179	COSTIM_up
SEC22B4P	COSTIM_up
MARCKS	COSTIM_up
GPIHBP1	COSTIM_up
CCL3	COSTIM_up
PSMB3	COSTIM_up
SENP3-EIF4A1	COSTIM_up
DDX52	COSTIM_up

RNA5-8SN1	COSTIM_up
RNA5-8SN2	COSTIM_up
MYO19	COSTIM_up
GGNBP2	COSTIM_up
PMS2P2	COSTIM_up
DHRS11	COSTIM_up
ACACA	COSTIM_up
C11orf98	COSTIM_up
MRM1	COSTIM_up
GOLGA6L10	COSTIM_up
RN7SL3	COSTIM_up
MRPL45	COSTIM_up
SND1-IT1	COSTIM_up
OR2T11	COSTIM_up
WASH9P	COSTIM_up
BCLAF1P2	COSTIM_up
TMEM75	COSTIM_up
ERICD	COSTIM_up
LINC01943	COSTIM_up
JAKMIP2-AS1	COSTIM_up
PAGR1	COSTIM_up
SLFNL1-AS1	COSTIM_up
ABALON	COSTIM_up
SNHG4	COSTIM_up
WASH5P	COSTIM_up
PCBP2-OT1	COSTIM_up
HYMAI	COSTIM_up
MIR4521	COSTIM_up
SCO2	COSTIM_up
SMIM38	COSTIM_up
EEF1AKMT4	COSTIM_up
TBCE	COSTIM_up
GGT1	COSTIM_up
DERPC	COSTIM_up
RNVU1-8	COSTIM_up
