# Supplementary material for: Distinct stage-specific transcriptional states of B cells derived from human tonsillar tissue
Source: JCI Insight. 2023 Apr 10;8(7):e155199. doi: 10.1172/jci.insight.155199 (PMC10132144; doi:10.1172/jci.insight.155199)
Supplement: Supplemental table 7 [file jciinsight-8-155199-s232.pdf]

feature	group	avgExpr	logFC	statistic	auc	pval	padj	pct_in	pct_out
ATF1-regulon	Activated	0.115282891346733	0.00944753984421344	55666838	0.681446360306627	1.08353600431113e-156	6.41092135884088e-156	100	100
ATF3-regulon	Activated	0.0709182646823648	0.0207555023509353	65221267.5	0.798407039977013	0	0	100	100
ATF4-regulon	Activated	0.125604397215999	0.0175803622398163	59208270.5	0.724798854791703	2.1157655784312e-239	2.31106701644023e-238	100	100
ATF5-regulon	Activated	0.125307174692447	0.00717050244946621	46137207.5	0.564789257934619	1.68806183996941e-21	3.28362714076242e-21	100	99.9977010437262
ATF6-regulon	Activated	0.132908195633545	-0.00365710853031054	42714479	0.522889880092415	0.000767297673447804	0.000972823835978466	100	100
ATF6B-regulon	Activated	0.0575383321125472	0.000252261160736872	43519060.0332031	0.532739169837379	1.49182100783425e-06	2.09741171398479e-06	99.8402555910543	99.9241344429629
BACH1-regulon	Activated	0.056524256874996	0.0138131113678839	55149965	0.675119052393238	4.30066068736415e-146	2.34882237540658e-145	100	99.5448066577774
BATF-regulon	Activated	0.0397888194025127	0.0213803455259567	55509184	0.679516436704935	1.2793530506638e-172	8.65086348544092e-172	75.2928647497338	50.6988827072509
BATF3-regulon	Activated	0.0981003034457006	0.0155815710564632	54203387	0.663531504833121	1.17828147642704e-127	5.7695161949186e-127	100	100
BCL11A-regulon	Activated	0.191107722119286	-0.0139708274568562	25518116	0.312380366746937	2.15107878202696e-167	1.38842357749013e-166	100	100
BHLHE40-regulon	Activated	0.0646026815849241	0.00933798108191049	52916910	0.647783079006093	1.29912276399642e-104	5.12431756909698e-104	100	100
BRF2-regulon	Activated	0.0511085794683546	0.00990623879018914	53647892	0.656731405177406	2.02184454312048e-117	8.7000583370639e-117	99.9467518636848	99.7195273345901
CEBPB-regulon	Activated	0.158725820343702	0.0163087790675349	59950323.5	0.733882706761248	5.79206665817656e-259	7.47703150419156e-258	100	100
CEBPG-regulon	Activated	0.0850318970096619	0.0177038830397894	58511073.5	0.716264108160923				

1.00190530559868e-221	9.48470355966755e-221	100	100
CLOCK-regulon	Activated	0.0177756049539532
0.00227503585698379	43344964.5	0.530607976981645
1.68912215585164e-06	2.35152300128365e-06	55.4313099041534
50.9057887718976
CREB1-regulon	Activated	0.0439780625454643
0.00105033700306615	42571888.5	0.521144356532422
0.00188501684287281	0.00228779821955504	100
99.9931031311784
CREB3-regulon	Activated
0.236162543467567	-0.00379451563503488	41888336
0.512776639235393	0.0603953584510415	0.0686091272003832
100	100
CREB3L2-regulon	Activated
0.122996387577075	-0.00394404970098702	41313636.5
0.505741447429725	0.398741450943733	0.416332985544192
100	100
CREB3L4-regulon	Activated
0.0108892589098173	-0.000221242377143858	40530302.5
0.496152253532913	0.491688489588395	0.50594032986632
30.5644302449414 31.3807531380753
CREB5-regulon	Activated	0.0551936085274399
0.0167109192596535	57699203 0.70632558430826
5.28413587637412e-202	4.68967059028203e-201	99.8935037273695
99.6114763897191
CREM-regulon	Activated	0.155234552482291
0.00546101218668354	49282065 0.603287074122023
4.72335805326702e-52	1.28984008377676e-51	100	100
CTCF-regulon	Activated
0.361445373524502	-0.00980049638608332	32044556
0.392273871453627	1.82842685569735e-56
5.09091399037303e-56	100	100
CUX1-regulon	Activated
0.0683453848486769	-0.00871330263150961	28435156.5
0.348089358006545	1.99048705856917e-110
8.0756903519092e-110	100	100
DDIT3-regulon	Activated	0.0974298942230949
0.0130623867688133	55092057 0.674410170817593
6.27507942571583e-145	3.30022695722832e-144	100	100
E2F1-regulon	Activated	0.41527431284209 -0.0233886124210815
31319513.5	0.383398253752967
7.72594600016931e-66	2.43796518227565e-65	100	100
E2F2-regulon	Activated
0.0495272455601046	-0.00458344365810631	33795814
0.413711920262109	7.3905748767054e-37
1.69268005240672e-36	100	100
E2F3-regulon	Activated	0.0248268011384617
0.00387174051199218	51701268 0.632901780802378
5.67006041714376e-85	2.11881205061688e-84	100	100
E2F4-regulon	Activated	0.418412345176259

0.0206077260724427	53888284	0.659674167140046
8.5094944496894e-122	3.77608816204967e-121	100	100
E2F6-regulon	Activated	0.0854968420534152
0.0136610183308621	57427005	0.70299346876071
1.34304586404819e-195	1.12183830996966e-194	100	100
E2F7-regulon	Activated
0.0302316933268387	-0.00035711397848185	40435057.5
0.494986310560054	0.461179530057777	0.478010899767915
100	100
E2F8-regulon	Activated
0.0695306705002889	-0.00549329411434434	35441722
0.43386032560174	2.45023348575551e-22	4.90046697151102e-22	100
100
EGR1-regulon	Activated	0.0511347992905428
0.00383031852564597	49997166.5	0.612040998934939
6.26380781743759e-61	1.89246959590668e-60	100	100
EGR2-regulon	Activated	0.0632183228894073
0.0179117364758025	61623846.5	0.75436916150185
6.37579262914455e-306	9.05362553338526e-305	100
99.9954020874523
EGR3-regulon	Activated	0.0593242589395023
0.0186239136132887	63618907	0.778791721955463	0	0
100	99.9954020874523
ELF1-regulon	Activated
0.691134449677632	-0.0165971033328927	30793202
0.37695540431247	4.18626726071662e-73	1.38244174656223e-72	100
100
ELF2-regulon	Activated
0.0316981619578316	-0.000999256825570102	40339064.5
0.493811210934943	0.363022442551117	0.381845828461175
99.8935037273695	99.519518138765
ELF4-regulon	Activated
0.0175355866101296	-0.000950869407638989	38988456.5
0.477277724592481	0.000838678948570836	0.00105391513891202
98.2960596379127	98.3378546140052
ELK1-regulon	Activated
0.065378644018588	-0.000705527333793948	39574255.5
0.484448791079521	0.0222718283585051	0.0259229477615387
100	100
ELK3-regulon	Activated
0.0574172019745134	-0.0078115549904933	30329679
0.371281181155257	7.96135365850284e-80
2.89874928078821e-79	100	100
ELK4-regulon	Activated	0.0504158886906491
0.00565281116247896	46125137	0.564641496743439
2.07672510000595e-21	3.98506708379519e-21	97.6038338658147
96.6918019219274
ESRRA-regulon	Activated	0.100648533006368
0.00929761263421881	53993654	0.660964055439171
9.68915997676309e-124	4.43826037645277e-123	100	100

ETS1-regulon	Activated
0.217706609122993	-0.0263217902565514	21922787
0.268368097518444	4.81021531691578e-254	5.692088125017e-253
	100	100
ETV2-regulon	Activated
0.0284306898171057	-0.00017873327595111	40743639.5
0.498763821342256	0.855824943688811	0.855824943688811
	100	100
ETV3-regulon	Activated	0.0143041216496818
0.00299195347813 49	47802535.5	0.585175393470406
4.33907071934158e-36	9.78012765311911e-36	90.7348242811502
84.0728309347556
ETV5-regulon	Activated
0.0330524360826381	-0.000428959675870526	39961317
0.489187009736557	0.111996921779761	0.125224904667135
	100	100
ETV6-regulon	Activated
0.456820152625032	-0.00287367681333139	37218795.5
0.455614395207281	6.85713359691804e-11
1.11921031122111e-10	100	100
ETV7-regulon	Activated
0.0546225331907406	-0.00986169747093458	26222114
0.320998367912427	1.49419476610115e-152
8.48702627145453e-152	100	100
FLI1-regulon	Activated
0.127386255722948	-0.0172595915067291	24762522
0.303130752440309	4.25489418773373e-184
3.02097487329095e-183	100	100
FOS-regulon	Activated
0.0834198513787681	-0.0029810515740029	37568395
0.45989402227789 3.75285363534454e-09	5.73016361525725e-09	100
	100
FOSB-regulon	Activated	0.0431168168461675
0.00171428492976881	43166727 0.5284260801826
2.94037639839194e-05	4.01474469780438e-05	99.8935037273695
99.2988183364752
FOSL1-regulon	Activated	0.0800514098839791
0.0200891430090245	61688154.5	0.755156388765209
8.34811266481193e-308	1.31714666489255e-306	100	100
FOXO1-regulon	Activated
0.0151194768139147	-0.00164561625059504	38988922
0.477283423017111	0.000406712523270511
0.000529845672517547	48.2428115015974 52.5196560761414
FOXO3-regulon	Activated
0.0176141979664484	-0.00222166387037508	39696635
0.485946901406016	0.0249902734261344	0.0288505595651307
	45.2076677316294 46.8274403420847
GABPA-regulon	Activated	0.027026844139647
0.00160535895529126	45301525 0.554559239157606
1.06557809999751e-15	1.89140112749557e-15	100	100

GTF2B-regulon	Activated	0.0818986744085045
0.00296438225071007	44450992.5	0.544147433902069
8.65553533715009e-11	1.39668865667649e-10	100	100
HINFP-regulon	Activated	0.0169572952200816
0.000758126076717047	41616402.5	0.509447761568218
0.154310578456234	0.16726795527317 64.0575079872204
63.1983079681824
HIVEP3-regulon	Activated	0.097560826928012
0.0207308582457828	54176618 0.663203811752744
3.75285535115237e-127	1.77635153287879e-126	99.7337593184239
99.6919398593039
HOXB2-regulon	Activated	0.037715959250703
0.000639710766900267	42198396.5	0.516572249095609
0.014645262593813	0.0171870023828219	85.7827476038339
83.6406271552715
IKZF1-regulon	Activated
0.142111937320416	-0.00182784574091008	37055612.5
0.453616788276312	9.27316803507534e-12
1.53115100114035e-11	100	100
IRF1-regulon	Activated
0.1331898512155	-0.00234984692778456	39333575.5
0.481502503560934	0.0065530719059396	0.0078859000901985
100	100
IRF2-regulon	Activated	0.0872564206242086
0.00233253711307431	43983402.5	0.538423424508617
1.62860117098913e-08	2.46022730085592e-08	100	100
IRF3-regulon	Activated
0.138668363575549	-0.00707080844919553	29946738.5
0.3665934097762	1.32227290055223e-85	5.07466896968695e-85	100
100
IRF4-regulon	Activated	0.0630460032045203
0.0048337083303709	49721487.5	0.608666270678181
2.0136903167632e-57	5.83559234653826e-57	100
99.9954020874523
IRF5-regulon	Activated	0.069281976452826
0.0231972987076649	54265152 0.664287601927128
9.03515695987619e-130	4.58211531536578e-129	94.8349307774228
79.8588440847855
IRF7-regulon	Activated
0.0997826357949905	-0.00839970224588014	30333702
0.371330428764893	9.13305686334821e-80
3.24223518648862e-79	100	100
IRF8-regulon	Activated
0.074539996242139	-0.0075452057154005	32041447.5
0.392235818708275	1.67285775297316e-56
4.75091601844377e-56	100	100
IRF9-regulon	Activated
0.0337073533469014	-0.000217885526767464	41083582.5
0.502925238235771	0.667233767212025	0.676765678172196
100	100

JUN-regulon	Activated	0.092549856731376
0.00432872873097362	46159039	0.565056508541075
1.1557288606467e-21	2.279354141831e-21	100	100
JUNB-regulon	Activated	0.163841686637132
0.0042344492168018	45015028	0.551052082205584
6.2097519623585e-14	1.08862318352458e-13	100	100
JUND-regulon	Activated	0.0421441255525095
0.00384559753950761	48425051.5	0.592795931616162
2.3460037603949e-42	5.74366437889785e-42	100	100
KLF11-regulon	Activated
0.0188775905533662	-0.00319096336320084	38996454
0.477375626098339	0.000188577282720957
0.000250261440620336	37.5399361022364	41.0018851441446
KLF12-regulon	Activated
0.0414917213917226	-0.00579786259406592	35042032
0.428967514988877	1.62305648015668e-25
3.38932382620954e-25	98.2960596379127	98.6160283231413
KLF13-regulon	Activated
0.0622839857137959	-0.00418116091186255	35163487
0.430454308035951	1.58449724696965e-24
3.26084940680711e-24	100	100
KLF2-regulon	Activated
0.0989998299982755	-0.0102737417520557	30382247.5
0.371924699168473	4.76812568488204e-79
1.65139962744695e-78	100	100
KLF3-regulon	Activated
0.0651017011280778	-0.0072266074732425	37246249
0.455950467603789	9.52185597383298e-11
1.51921746998234e-10	99.8935037273695	99.8620626235689
KLF4-regulon	Activated	0.0162112248397282
0.000324879162001496	41376268	0.506508151795358
0.306103959985708	0.324378823268437	50.4259850905218
49.7861970665318
KLF6-regulon	Activated	0.0714591005028526
0.00216899274558612	42574702.5	0.521178804151988
0.0018529786615933	0.00226830146505387	100	100
KLF7-regulon	Activated	0.0495598808813143
0.0037148931424759	44924335.5	0.549941868723868
2.12865702931238e-13	3.64179877304046e-13	99.5740149094782
97.4688491424893
KLF8-regulon	Activated
0.0214028467489597	-0.00386378960554287	37999648
0.465173211787833	2.63733687715361e-07
3.86084367583312e-07	76.6773162939297	77.6771345809003
MAFB-regulon	Activated	0.0568119828119019
0.00107350723337552	42919975.5	0.525405468313552
0.000188410400405463	0.000250261440620336	100
99.9977010437262
MAFF-regulon	Activated
0.0118473349202659	-0.00056290302572801	40738139

0.498696486896121	0.81002603725495 0.815770902767396
28.4877529286475 28.5484390086901
MAFG-regulon	Activated	0.0383591266222019
0.0135109590106561	49646538 0.607748775346727
1.15894879954993e-60	3.42855686533521e-60	71.2460063897764
58.0555427835763
MAX-regulon	Activated
0.197449272652884	-0.000230476152899478	39094252
0.478572821655688	0.001636377214428	0.00203829442498926
100	100
MAZ-regulon	Activated
0.132190123818292	-0.00429129536019204	36187865.5
0.442994251483096	5.354059251272e-17
9.62375207190662e-17	100	100
MEF2A-regulon	Activated
0.0387789075703292	-0.00206069473109018	37489418.5
0.458927230370745	1.57169011024603e-09
2.42586951798843e-09	100	99.9977010437262
MLX-regulon	Activated
0.0488271449369439	-0.00427525503674941	35730488
0.437395258548359	3.52726173236845e-20
6.50482033761454e-20	100	99.9885052186307
MXD4-regulon	Activated
0.0674454845520272	-0.0257910734965062	36877889.5
0.451441189736069	9.17081153215123e-13
1.55030385424461e-12	86.4217252396166 88.6983309577452
MXI1-regulon	Activated	0.072320843473168
0.00739745416385036	48607743.5	0.595032358238007
2.45229196145457e-44	6.21831175940267e-44	100	100
MYBL1-regulon	Activated
0.017466725230504	-0.012959870564486	32682346
0.400081386479718	3.33781986627958e-49
8.77723001873519e-49	72.8966986155485 80.3692123775806
MYC-regulon	Activated	0.356776915202617
0.0437039542867505	62201929 0.761445766348382	0	0
100	100
NFATC1-regulon	Activated	0.0875586073907065
0.0386399780101956	62788936.5	0.768631626704735	0
0	99.2012779552716 94.6388339693779
NFE2L1-regulon	Activated	0.228984307360897
0.048316880567486	55997142 0.685489781249536
1.1624505558319e-163	7.17686864904915e-163	99.9467518636848
99.9356292243322
NFE2L2-regulon	Activated	0.130452463586805
0.0204169415202104	61877004 0.757468192507694	0	0
100	100
NFKB1-regulon	Activated	0.102565322368217
0.0478780806240203	69885765.5	0.855507556172267	0
0	100	99.9287323555106
NFKB2-regulon	Activated	0.109374159000838

0.037551739334129 71229722 0.871959618086318 0 0
100 100
NFYA-regulon Activated 0.0241415723205907
0.00333102926393507 47908720.5 0.586475258603201
5.20015599385355e-37 1.21052811660197e-36 99.7337593184239
98.9861602832314
NFYB-regulon Activated
0.103354367973859 -0.00549808064866812 38898668
0.476178577439155 0.000463086371472151
0.000597802406809504 100 100
NFYC-regulon Activated
0.0791995786927037 -0.00452232799092103 34318241
0.420107217542618 7.71187201632284e-32
1.65922094896643e-31 100 100
NR1H3-regulon Activated
0.00829763936265615 -0.000246970386209914 40399665
0.49455305278624 0.270265403849575 0.288554040200298
20.9797657082002 22.1527426548347
NR2C2-regulon Activated 0.0229344795002537
0.00119788866167183 42828956 0.524291251856854
0.000324009107756254 0.000426011975012853 79.5527156549521
75.8241758241758
NR3C1-regulon Activated
0.223307094074902 -0.00226420093275928 35690876
0.436910347707466 1.81236130404655e-20 3.3862540154554e-20
100 100
NRF1-regulon Activated
0.0265691230558906 -0.000166952660188548 41658386.5
0.509961709279621 0.143150004599075 0.157575974054796
100 99.9655156558922
PAX5-regulon Activated
0.092749227632403 -0.00784305603677896 30518569.5
0.373593486799804 4.74419708124693e-77
1.60399044175492e-76 100 100
POU2F1-regulon Activated 0.00572140382326439
0.000877750880915879 42398274 0.519019052251236
3.45096188746981e-05 4.66701512400679e-05 21.7784877529286
18.1985378638098
POU6F1-regulon Activated
0.0249839670777476 -0.00627799334762588 37131509.5
0.454545882442002 8.84127831829937e-12
1.47701355435119e-11 60.0638977635783 65.5823256241666
REL-regulon Activated 0.286621770018938
0.0238989043227965 63122215.5 0.772711466150917 0
0 100 100
RELA-regulon Activated 0.0961540178191518
0.00426612028270307 49246519 0.602851937275855
1.24980713585049e-51 3.34853987341073e-51 100 100
RELB-regulon Activated 0.115670526351618
0.0109396108759855 57228748.5 0.700566509098799

5.34283519924145e-191	4.21490332384603e-190	100	100
REST-regulon	Activated
0.215318331494598	-0.00344262217306118	33895730.5
0.414935049466243	7.21063286472429e-36	1.5998591668607e-35
	100	100
RFX5-regulon	Activated
0.0599030366584162	-0.00568054133414616	35166291.5
0.430488639361138	1.669155081208e-24
3.38600030759337e-24	100	100
RXRA-regulon	Activated	0.0202799330594858
0.00599803820377144	48384120.5	0.592294874218692
7.70292510079661e-44	1.91897432335635e-43	75.3993610223642
63.7822428617408
SETDB1-regulon	Activated
0.0587749659025093	-0.000983255520312878	40049643
0.49026825367609 0.152588000435805	0.166673046629879
92.7582534611289 95.1515012184468
SOX5-regulon	Activated	0.00920346994709279
0.00146061776918862	41860037.5	0.512430222759804
0.0124484923216702	0.0147307159139765	24.6006389776358
22.4033288886845
SP1-regulon	Activated	0.0150981130492121
0.00219508858749111	43847509 0.536759882365909
2.30830110969578e-08	3.45030271133474e-08	64.2172523961661
59.6326267874385
SP2-regulon	Activated	0.0284566244660685
0.000225813264241703	41631761.5	0.509635778977218
0.156700587028322	0.168571843621377	100	100
SP3-regulon	Activated
0.0276009595719513	-0.000697821851121497	38921855
0.476462421417439	0.000541123050635782
0.000692247506218749	100	100
SP4-regulon	Activated	0.00935284615438201
0.000745788160713887	41520204 0.508270146312041
0.129349200721127	0.14349676955	30.3514376996805
28.9576532254357
SPI1-regulon	Activated
0.396682789280343	-0.0228039889039222	22975592
0.281256024354932	8.61838801098459e-227
8.74150783971294e-226	100	100
SPIB-regulon	Activated	0.566015606611142
0.00163192798713641	42333968 0.518231849470905
0.00736887438309282	0.00879311060839647	100	100
SREBF1-regulon	Activated	0.0499320103889161
0.00939079185702722	48920537.5	0.598861430275937
7.73456489937214e-48	1.99692402856517e-47	97.8168264110756
96.8550278173709
SREBF2-regulon	Activated
0.0952788187917446	-0.00386372520912667	33582095.5
0.411095682315287	5.07885774701315e-39

1.22236915267096e-38	100	100
SRF-regulon	Activated	0.0244925949758523
0.0038841751855187	48028743.5	0.58794452180265
3.20879993981574e-38	7.59415985756392e-38	99.1480298189563
97.8826612717826
STAT1-regulon	Activated
0.0784054350529892	-0.00142192514545501	38130728
0.466777829404322	1.04505484154613e-06	1.4839778749955e-06
	100	100
STAT2-regulon	Activated
0.0631996106838971	-0.00316576720656135	37798822.5
0.462714803677214	4.24992181000303e-08
6.28634267729615e-08	100	99.9609177433445
STAT5A-regulon	Activated	0.0834972757265512
0.0124027671166473	56959446	0.697269838854183
7.71524937039817e-185	5.76613373998179e-184	100	100
TBP-regulon	Activated
0.0182560738695231	-0.00275939592847584	39222949.5
0.480148273865774	0.00180372483170516	0.00222720805306202
	46.9116080937167	50.0137937376431
TCF12-regulon	Activated
0.00878763620453667	-0.000267133086166773	40585895.5
0.496832796004331	0.541674420277284	0.553365235103412
	24.6006389776358	25.2471377994391
TFDP1-regulon	Activated
0.522509943267325	-0.00589743940327492	39728290
0.486334406522357	0.0445844723722771	0.0510564119101883
	100	100
TFDP2-regulon	Activated
0.0244901528779202	-0.00274667907039674	35653546
0.436453372000847	9.63189093188567e-21
1.82363801643702e-20	100	99.9954020874523
TFEC-regulon	Activated	0.101429485180837
0.0103148456474631	50727131	0.620976869366058
9.90997156584515e-71	3.19821809625002e-70	100	100
THAP1-regulon	Activated	0.0394547425780819
0.00238848291936467	47405312.5	0.580312782671853
3.70914263353272e-32	8.1030500609484e-32	100	100
THAP11-regulon	Activated
0.176887856787648	-0.00165849410793933	37271165.5
0.456255483280026	1.28005144132691e-10
2.01963671853801e-10	100	100
TP53-regulon	Activated	0.105274722718269
0.00721671235640681	50145729.5	0.613859634935537
7.2893592240268e-63	2.25019349959088e-62	100	100
USF2-regulon	Activated
0.118467941708074	-0.00711263782483419	28271648
0.346087766462865	2.64112303405781e-113
1.10305726716532e-112	100	100
XBP1-regulon	Activated

0.189259607059289	-0.00629061240878687	38207855.5
0.467721986752626	2.09344416994875e-06
2.88610749643419e-06	100	100
YBX1-regulon	Activated	0.0957192218667629
0.000558578035462123	43656974.5	0.53442745167283
4.19014265598192e-07	6.07143119540238e-07	100	100
YY1-regulon	Activated
0.637217763076378	-0.00235086562703679	39884439
0.488245906645923	0.0840593306054255	0.0947335313172256
100	100
YY2-regulon	Activated	0.0568571862592235
0.00610792098271007	44933375.5	0.550052532007763
1.84193021045367e-13	3.18968402298075e-13	93.1309904153355
90.4179502505862
ZBTB33-regulon	Activated
0.359546250907185	-0.00655088783544988	34704971
0.424841378137861	2.27330161498174e-28
4.81804222876727e-28	100	100
ZNF143-regulon	Activated	0.0251831686475474
0.00193241048383334	45892028	0.561787889725115
1.07069890414353e-19	1.94922108190232e-19	100
99.9931031311784
ZNF274-regulon	Activated	0.0671588580662753
0.00189578930021538	44395599.5	0.543469339733393
1.66889039456349e-10	2.60420259371446e-10	100	100
ZNF76-regulon	Activated	0.0153758829808857
0.000854126506457418	43581646	0.533505316807682
8.45432670245764e-07	1.21264079974645e-06	99.6272630457934
99.4528484068233
ATF1-regulon	Activated Chemokine	0.134621820018288
0.0290943484519726	42752092	0.885654670828108	0	0
100	100
ATF3-regulon	Activated Chemokine	0.0855995664622852
0.0354288396067979	44198787.5	0.915624493751416	0
0	100	100
ATF4-regulon	Activated Chemokine	0.143052994488331
0.0351456023695265	41874382	0.867471982571998	0	0
100	100
ATF5-regulon	Activated Chemokine	0.141985315860068
0.024131550584132	34141568.5	0.707278596130987
2.71863076396726e-121	5.36174400671321e-121	100
99.9977419500519
ATF6-regulon	Activated Chemokine
0.135042948327873	-0.00140474119571918	28036517.5
0.580806026466003	6.86040605028125e-20	9.2778824679994e-20
100	100
ATF6B-regulon	Activated Chemokine
0.0542706701222256	-0.00310031555126004	21018226.609375
0.435414729391876	2.92761204063209e-13
3.64667464710313e-13	100	99.9187102018697

BACH1-regulon	Activated Chemokine	0.0511839040496024
0.00809553512340592	29660890.5	0.614456626175066
2.97630662612162e-38	4.35706743205433e-38	100
99.5529061102832
BATF-regulon	Activated Chemokine	0.112234269792366
0.0952284475133916	45667750.5	0.946055611419849	0
	0	98.4403669724771 50.5667705369643
BATF3-regulon	Activated Chemokine	0.152870322440903
0.071422380882002	43890393.5	0.90923578681854 0	0
	100	100
BCL11A-regulon	Activated Chemokine
0.165252519467043	-0.0402138086137048	5796177
0.120073919025915	0	0	100	100
BHLHE40-regulon	Activated Chemokine	0.0766892812503233
0.0215559103896964	39085385.5	0.809694978884126
3.109608222725e-268	1.05134373244512e-267	100	100
BRF2-regulon	Activated Chemokine	0.0562345718393626
0.0149821298993476	36090212 0.747646801213298
2.82423049890515e-172	6.91449535938848e-172	100
99.722259856388
CEBPB-regulon	Activated Chemokine	0.182183880609407
0.0400540173981899	43473685.5	0.900603241151034	0
	0	100	100
CEBPG-regulon	Activated Chemokine	0.115936337635515
0.0490539538974721	42810005.5	0.886854410054413	0
	0	100	100
CLOCK-regulon	Activated Chemokine	0.017798935215093
0.00225845964044082	25752027 0.533480396604722
5.68441906727381e-05	6.16173669887696e-05	55.6880733944954
50.9799936774601
CREB1-regulon	Activated Chemokine
0.0306982722466979	-0.0125749944821716	12859424.5
0.266396539673109	1.62314214755906e-153
3.77846204841617e-153	100	99.9932258501558
CREB3-regulon	Activated Chemokine	0.246305964985769
0.00666608078664127	33214700 0.688077537706327
3.30634778761677e-100	5.94305551698205e-100	100	100
CREB3L2-regulon	Activated Chemokine
0.120834112907937	-0.0060893656975184	21254875
0.440317150365825	1.54891869498878e-11
1.86395300583396e-11	100	100
CREB3L4-regulon	Activated Chemokine
0.00801021209876415	-0.00316721377002557	22648773.5
0.469193227756033	2.3151398152148e-05
2.52884502892694e-05	26.1467889908257 31.474958226076
CREB5-regulon	Activated Chemokine	0.0994612340940955
0.0617707479489901	45254998 0.937505008106192	0	0
	100	99.6138734588809
CREM-regulon	Activated Chemokine	0.163232256864617
0.0135583918653484	36499604 0.756127788225575

3.91709115298402e-184	1.03004989578469e-183	100	100
CTCF-regulon	Activated Chemokine
0.353790167829334	-0.0174697332210133	13714874.5
0.284118088554504	2.11386110966356e-131
4.4801235458541e-131	100	100
CUX1-regulon	Activated Chemokine
0.0548476117736833	-0.022388253373895	6307072
0.130657647725149	0	0	100	100
DDIT3-regulon	Activated Chemokine	0.115675348151254
0.0315244866800791	40199849.5	0.83278227592376 0	0
100	100
E2F1-regulon	Activated Chemokine	0.451904967309995
0.0145597867055828	31845687.5	0.659717000050133
8.54959220110562e-73	1.42828481477294e-72	100	100
E2F2-regulon	Activated Chemokine
0.0465026998457231	-0.00760087654283702	14876598.5
0.308184426333088	3.7536270038921e-104
7.0133557177984e-104	100	100
E2F3-regulon	Activated Chemokine	0.0222341521472386
0.00114638775606057	26412363.5	0.54715996357289
9.91100381737485e-08	1.15357585415347e-07	100	100
E2F4-regulon	Activated Chemokine	0.496968018570164
0.100730187974098	43931755 0.910092633909613	0	0
100	100
E2F6-regulon	Activated Chemokine	0.138993177001454
0.0682309684766749	44765767.5	0.927370082371176	0
0	100	100
E2F7-regulon	Activated Chemokine
0.0211948154299558	-0.0096100599576607	10100644
0.209245492290106	1.11024991367138e-236
3.42729321176817e-236	100	100
E2F8-regulon	Activated Chemokine	0.0769434591719516
0.00219968819445462	30965485 0.641482677028009
1.6162786744635e-57	2.52210518432766e-57	100	100
EGR1-regulon	Activated Chemokine	0.0481931606747768
0.00074812359209573	24116524.5	0.499599237566328
0.963885078129431	0.963885078129431	100	100
EGR2-regulon	Activated Chemokine	0.077569427192542
0.0322973495480173	41641208 0.862641537263832	0	0
100	99.9954839001039
EGR3-regulon	Activated Chemokine	0.076272964645415
0.0356583901111485	43626963 0.903778546205295	0	0
100	99.9954839001039
ELF1-regulon	Activated Chemokine
0.67342207241756 -0.0344501113518308	10476525.5
0.217032273955735	2.76027304564098e-224
8.16580776002125e-224	100	100
ELF2-regulon	Activated Chemokine
0.019813443978972	-0.0131587101207465	13407405
0.277748533614077	3.77238087818007e-139

8.50282674129476e-139	98.5321100917431 99.5596802601274
ELF4-regulon	Activated Chemokine
0.00993472525102016	-0.00872188959319946	12390526
0.256682812759598	2.2396253146776e-166
5.39028465566473e-166	92.8440366972477 98.4713001851601
ELK1-regulon	Activated Chemokine	0.0742363039371977
0.0083826976584353	31856297.5	0.659936797389114
5.45235783349812e-73	9.21708109948491e-73	100	100
ELK3-regulon	Activated Chemokine
0.0388667920299352	-0.0266795470492178	6379214
0.132152145333895	0	0	100	100
ELK4-regulon	Activated Chemokine	0.0460516462131142
0.00108056978017621	24852379 0.514843239543468
0.0935257386240356	0.0955442797454176	97.9816513761468
96.6987309759292
ESRRA-regulon	Activated Chemokine	0.146961996991936
0.0565855416197526	43890031.5	0.909228287606786	0
0	100	100
ETS1-regulon	Activated Chemokine
0.192836396862672	-0.0513357716683338	6266805
0.129823474355803	0	0	100	100
ETV2-regulon	Activated Chemokine
0.0212480060554418	-0.00753502231871423	13510038.5
0.279874694800726	1.54179294514279e-136
3.42085309703556e-136	100	100
ETV3-regulon	Activated Chemokine	0.0140719072790281
0.00270789956095395	27933956.5	0.57868136719331
5.24918771321079e-19	6.96621173155077e-19	91.3761467889908
84.1755859639615
ETV5-regulon	Activated Chemokine
0.0302136451277632	-0.00332998840151338	18670385
0.38677671449175 1.80604142863881e-37	2.6169171721093e-37	100
100
ETV6-regulon	Activated Chemokine
0.455647168208499	-0.00402439901799412	19971587.5
0.413732496487593	1.90192317526199e-22
2.59685664314618e-22	100	100
ETV7-regulon	Activated Chemokine
0.0429407456715799	-0.0216555323817945	6114326.5
0.126664721429143	0	0	100	100
FLI1-regulon	Activated Chemokine
0.100064456465498	-0.0449467478314987	6110084
0.126576833567632	0	0	100	100
FOS-regulon	Activated Chemokine
0.0680430694136747	-0.0186832552006064	9965529
0.206446442576961	3.21849934421382e-241
1.01561534861858e-240	100	100
FOSB-regulon	Activated Chemokine
0.0315977030165401	-0.0101188489197058	17429972.5
0.361080261453182	1.61614663297453e-55

2.46766475142348e-55	99.2660550458716 99.3248430655286
FOSL1-regulon	Activated Chemokine	0.134073981251242
0.0750839032869862	46090237 0.954807864808685	0	0
	100	100
FOXO1-regulon	Activated Chemokine
0.00688204796604814	-0.0100565096660158	17797921.5
0.368702713015939	1.31041706017838e-55
2.02260024505794e-55	30.8256880733945 52.8722395339385
FOXO3-regulon	Activated Chemokine
0.0121637610604061	-0.00776671995850038	20866989
0.432281682823118	1.01315560113014e-16
1.33211199407852e-16	36.5137614678899 47.0125999187102
GABPA-regulon	Activated Chemokine
0.0193512422248321	-0.00628772543578963	14283924
0.295906549049195	1.18344315672396e-117
2.30204011307948e-117	100	100
GTF2B-regulon	Activated Chemokine	0.0936634903052057
0.0149660160156731	34470012.5	0.71408265995798
2.97269244928321e-129	6.20768129115023e-129	100	100
HINFP-regulon	Activated Chemokine
0.0125866293082585	-0.00373360358419918	21670088
0.44891872553175 3.21181693183963e-09	3.76923970513411e-09
58.4403669724771 63.3518493429075
HIVEP3-regulon	Activated Chemokine	0.10913014125861З
0.032216052003803	35390939 0.733160623586388
6.08330572999438e-153	1.39327324783742e-152	99.8165137614679
99.6906471571151
HOXB2-regulon	Activated Chemokine
0.0282040826185365	-0.00911766190494869	20767082
0.430212003959252	2.74452557982439e-15	3.5110147057213e-15
	83.1192660550459 83.7442984238811
IKZF1-regulon	Activated Chemokine
0.141443034322317	-0.00248068862574777	20794322
0.430776309285723	5.23183540683764e-15
6.63321989081201e-15	100	100
IRF1-regulon	Activated Chemokine
0.112431367426499	-0.0235774421278614	9502831.5
0.196861175917835	4.35924793809482e-257
1.43956559816154e-256	100	100
IRF2-regulon	Activated Chemokine
0.0757924741205907	-0.0094550724202606	16718065
0.346332346834815	1.59838282901127e-67
2.57920865590455e-67	100	100
IRF3-regulon	Activated Chemokine
0.130260322056068	-0.0155599809852115	11044297
0.228794259332686	3.37074799208904e-206
9.57292429753288e-206	100	100
IRF4-regulon	Activated Chemokine	0.074382103888998
0.0163628132956992	37390053.5	0.774574388658872
2.66637034291918e-211	7.72703242233722e-211	100

99.9954839001039
IRF5-regulon	Activated Chemokine	0.054651638930039
0.00779410883240807	27583843.5	0.571428407179853
5.47356326358845e-16	7.06587257663237e-16	91.8348623853211
80.1991600054193
IRF7-regulon	Activated Chemokine
0.0815278361408023	-0.0269543430745549	7115592.5
0.14740700252363 0	0	100	100
IRF8-regulon	Activated Chemokine
0.062171553359975	-0.0200838148044431	9932033
0.205752537613104	2.37679925479184e-242
7.67057941319185e-242	100	100
IRF9-regulon	Activated Chemokine
0.0255584990193618	-0.00856342859164902	13992806
0.28987573267506 1.36741584076887e-124	2.73483168153773e-124	100
100
JUN-regulon	Activated Chemokine
0.0772447345513765	-0.0114301174569789	13908517.5
0.288129607509487	1.22960187208489e-126
2.53048501211674e-126	100	100
JUNB-regulon	Activated Chemokine
0.153156546141079	-0.00678902730245365	19171272.5
0.397153127274882	3.25714500037659e-31
4.57935237676709e-31	100	100
JUND-regulon	Activated Chemokine
0.0363559394587748	-0.00215347808372406	21245598.5
0.440124977885612	1.33351845409116e-11
1.61845829470893e-11	100	100
KLF11-regulon	Activated Chemokine
0.013506160190834	-0.00863782117379691	21493541.5
0.445261378603713	3.79938019981524e-12	4.6509654170152e-12
33.302752293578	41.0445739059748
KLF12-regulon	Activated Chemokine
0.0243230120290333	-0.0232859771295233	10302796.5
0.213433294511447	5.66158431997208e-230
1.71052122007667e-229	92.1100917431193 98.7625886284605
KLF13-regulon	Activated Chemokine
0.0519230500186359	-0.0147227104603663	8942224
0.185247600355819	5.3884549623111e-277
1.91290151162044e-276	100	100
KLF2-regulon	Activated Chemokine
0.0763096570839817	-0.0333395769518565	6767228
0.140190264531587	0	0	100	100
KLF3-regulon	Activated Chemokine
0.0431223661829778	-0.029618328012012	11856297
0.245615695643041	1.1565978171791e-181
2.93280160784701e-181	99.2660550458716 99.8780653028045
KLF4-regulon	Activated Chemokine
0.0118447012767195	-0.00415489724532231	22412851
0.464305844371883	1.5973459680094e-05

1.77205568326043e-05	46.2385321100917 49.9006458022851
KLF6-regulon	Activated Chemokine
0.0489382843852774	-0.0209447162919114	12512833.5
0.259216541603845	5.6959128394163e-163
1.34803270532852e-162	100	100
KLF7-regulon	Activated Chemokine
0.0365306092419973	-0.00970116533428697	18859219.5
0.390688620298336	4.83549160576084e-35
6.93575563654586e-35	98.4403669724771 97.5342094567132
KLF8-regulon	Activated Chemokine
0.0106093057469204	-0.0148542391900473	15024029
0.311238604616283	4.82701370800994e-102
8.78764034022323e-102	58.6238532110092 78.1036896536151
MAFB-regulon	Activated Chemokine
0.0502144661104132	-0.0057054938402517	18401291.5
0.381202158861479	4.47431953327875e-41
6.61826430964148e-41	100	99.9977419500519
MAFF-regulon	Activated Chemokine
0.00700724941399277	-0.00551210034199902	21966053.5
0.455049962980411	1.86121090341323e-10
2.22094074188805e-10	20.9174311926606 28.7336855891252
MAFG-regulon	Activated Chemokine	0.0347960025101
0.00961973028349333	28690546.5	0.594354926919974
1.95584587547489e-28	2.6964088768683e-28	73.1192660550459
58.2441403603848
MAX-regulon	Activated Chemokine
0.19574306425941 -0.001974578055393	20307576 0.420692852588285
3.23255429453123e-19	4.33040292286259e-19	100	100
MAZ-regulon	Activated Chemokine	0.141010152516692
0.00482217532947141	31058573.5	0.643411103473792
4.78360874071561e-59	7.54747156868462e-59	100	100
MEF2A-regulon	Activated Chemokine
0.0235399083255473	-0.0176381007348638	8567505.5
0.177484911461654	1.01876729181299e-290
3.70935783172935e-290	100	99.9977419500519
MLX-regulon	Activated Chemokine
0.0488532415779865	-0.00417244461917029	21477827.5
0.444935846522209	4.92738244024689e-10
5.83073588762548e-10	100	99.9887097502597
MXD4-regulon	Activated Chemokine
0.0820539718226196	-0.0103641195086625	25442114.5
0.527060232342982	0.00221593295103984	0.00236588330111021
93.1192660550459 88.4929774646615
MXI1-regulon	Activated Chemokine
0.0540671551949068	-0.0114371335883849	17045360
0.353112607915107	7.44789105258448e-62
1.18831520164831e-61	100	100
MYBL1-regulon	Activated Chemokine
0.0102020302460963	-0.0201727690337013	13991146
0.289841344024475	1.30500032353736e-125

2.64728637060436e-125	57.9816513761468 80.6033509461229
MYC-regulon	Activated Chemokine	0.465864781854003
0.154699129312613	45626374.5	0.945198463945986	0
	0	100	100
NFATC1-regulon	Activated Chemokine	0.114186113035978
0.0652353217641544	42212202.5	0.87447029048466 0	0
	100	94.7003567718918
NFE2L1-regulon	Activated Chemokine	0.319062654513512
0.139752579851908	42733053.5	0.885260268223188	0
	0	100	99.9345165515061
NFE2L2-regulon	Activated Chemokine	0.165488461725779
0.0559519842342995	45474679.5	0.94205594204808 0	0
	100	100
NFKB1-regulon	Activated Chemokine	0.114074382798328
0.0588184951691351	43549901 0.902182125608068	0	0
	100	99.93000045161
NFKB2-regulon	Activated Chemokine	0.102564440964836
0.0299062410683808	40159367.5	0.831943648602681
7.77486287080266e-308	2.9838662909567e-307	100	100
NFYA-regulon	Activated Chemokine
0.0193049450999793	-0.00168391107437605	21980539
0.455350045388876	4.54095104729324e-07
5.15852038972512e-07	99.4495412844037 99.0064580228515
NFYB-regulon	Activated Chemokine	0.114681930752429
0.00620611427052872	33348893.5	0.690857497575186
3.91665466376821e-103	7.22292158772839e-103	100	100
NFYC-regulon	Activated Chemokine
0.083332565401795	-0.000207149393428044	25520011.5
0.528673950845774	0.00119649897390842	0.00128714283556815
	100	100
NR1H3-regulon	Activated Chemokine
0.00398929922331403	-0.00465695614917009	21878451
0.453235184809994	3.43978227211362e-13
4.24738332730551e-13	13.5779816513761 22.3140495867769
NR2C2-regulon	Activated Chemokine
0.0112386675614195	-0.0108071038597105	16533120
0.342501016122477	8.29429626243763e-72
1.36952333635598e-71	57.9816513761468 76.4214424423068
NR3C1-regulon	Activated Chemokine
0.202424774802416	-0.0236202034837524	7469512.5
0.154738828556833	0	0	100	100
NRF1-regulon	Activated Chemokine
0.0260733780158601	-0.000671928685121871	24353079
0.504499713496965	0.611174861411469	0.615509434896656
	100	99.966129250779
PAX5-regulon	Activated Chemokine
0.0727419638078563	-0.0282031986359221	7274494
0.15069881466879 0	0	100	100
POU2F1-regulon	Activated Chemokine
0.00256230367941107	-0.00237472158942802	22761667

0.471531935662564 1.88606519990096e-06
2.10882880618848e-06 13.1192660550459 18.4753646750666
POU6F1-regulon Activated Chemokine
0.0131881011774799 -0.0182524808229593 16393377
0.339606092508785 1.68217199272936e-76
2.87793280683818e-76 43.2110091743119 65.8989296843246
REL-regulon Activated Chemokine 0.321311786284839
0.0590174935264992 45772523 0.948226084247222 0 0
100 100
RELA-regulon Activated Chemokine
0.0855427829805686 -0.0066821950950308 15589593
0.322954859302772 5.14723246739847e-89
8.91350012647052e-89 100 100
RELB-regulon Activated Chemokine 0.128310526552144
0.0236960629314615 41664315.5 0.863120233494794 0
0 100 100
REST-regulon Activated Chemokine
0.202966755668602 -0.0160369481092035 9020858.5
0.186876596948857 3.70624473132868e-274
1.28362622402115e-273 100 100
RFX5-regulon Activated Chemokine
0.0536715000941009 -0.0119643767803608 14516167.5
0.300717718068584 2.90476860082419e-112
5.57401542320318e-112 100 100
RXRA-regulon Activated Chemokine 0.0336073304880645
0.0195467337647753 36733383 0.760970766746755
4.0201516523832e-200 1.11933634242826e-199 90.8256880733945
63.6092670369869
SETDB1-regulon Activated Chemokine 0.0658326973100108
0.00626568151840808 27648048 0.572758471105454
2.01655024936091e-16 2.62706546247017e-16 97.9816513761468
94.9803549654518
SOX5-regulon Activated Chemokine
0.00529372671357056 -0.00257134438062002 22525975
0.466649327328992 2.54326470328403e-07
2.93612673062059e-07 16.1467889908257 22.6504990290385
SP1-regulon Activated Chemokine 0.0140827584391982
0.00111568515094381 24882842.5 0.515474323071843
0.0706110029771499 0.072657698715618 62.0183486238532
59.7683240753286
SP2-regulon Activated Chemokine
0.0275217334344335 -0.000736105994749627 23122641.5
0.479009903102726 0.01771334402252 0.018494815082337 100
100
SP3-regulon Activated Chemokine
0.0178497461055524 -0.0106766227957232 8308772.5
0.172124984514749 2.17584619727447e-300
8.13079368455198e-300 100 100
SP4-regulon Activated Chemokine 0.0095017807040875
0.00088511826631823 24972643 0.517334635130203

0.0145352988899057	0.0152889810545675	32.3853211009174
28.9323939845549
SPI1-regulon	Activated Chemokine
0.371020478921496	-0.0486921578870497	7354159
0.152349159156061	0	0	100	100
SPIB-regulon	Activated Chemokine	0.591894424559912
0.0281186570660672	36834152.5	0.763058313207686
4.04676296218803e-194	1.10507757813596e-193	100	100
SREBF1-regulon	Activated Chemokine	0.0489114541737106
0.00817802252124377	29183769 0.604572551144831
3.25519865297483e-32	4.62238208722426e-32	98.6238532110092
96.8522783723976
SREBF2-regulon	Activated Chemokine
0.0858048457151089	-0.0135021298259498	11493294.5
0.238095716044211	1.94243957485848e-192
5.20427206848875e-192	100	100
SRF-regulon	Activated Chemokine
0.0186942228231885	-0.0021260236322897	22041143
0.45660552116	9.44185286314365e-07	1.06408183060825e-06
98.8990825688073 97.91130379804
STAT1-regulon	Activated Chemokine
0.0690630312930436	-0.0109689701708959	12108167
0.250833448307436	2.26634034067287e-174
5.64597067325522e-174	100	100
STAT2-regulon	Activated Chemokine
0.0461293524935018	-0.020599841430664	11847569
0.245434885918759	6.42355884235869e-182
1.65844610111806e-181	100	99.9616131508829
STAT5A-regulon	Activated Chemokine	0.107371222731043
0.036643629664358	42548551.5	0.881438114723024	0
0	100	100
TBP-regulon	Activated Chemokine
0.0179479561725272	-0.00302599811939958	22431571
0.464693648913422	1.9847554239067e-05
2.18476953639342e-05	43.4862385321101 50.0429029490132
TCF12-regulon	Activated Chemokine
0.00533665187505766	-0.00379830239616407	22475681.5
0.465607444438506	3.50035488627164e-07
4.00847091814978e-07	19.5412844036697 25.3601589667163
TFDP1-regulon	Activated Chemokine	0.551086236168037
0.0234871301878486	34569044 0.716134201916069
1.05303154988824e-131	2.26561333460803e-131	100	100
TFDP2-regulon	Activated Chemokine
0.0210058675661015	-0.00626784935449581	15417833
0.319396669769932	1.49754818149531e-92
2.62533137990536e-92	100	99.9954839001039
TFEC-regulon	Activated Chemokine	0.100454522722095
0.00913235016586424	30667776.5	0.635315331496234
9.11626594885523e-53	1.36264185761836e-52	100	100
THAP1-regulon	Activated Chemokine	0.0372521001316256

8.91280850861612e-05	24389506.5	0.505254347574792
0.552740113994476	0.560636401337254	100	100
THAP11-regulon	Activated Chemokine	0.179495461598913
0.00104280131889944	25138243 0.520765213766896
0.0189685729210634	0.0196608566043139	100	100
TP53-regulon	Activated Chemokine	0.129760876204049
0.0321771271880439	41432494.5	0.858317817008461	0
0	100	100
USF2-regulon	Activated Chemokine
0.103402593171441	-0.0224222276860152	6721470
0.139242339306601	0	0	100	100
XBP1-regulon	Activated Chemokine
0.181137407268426	-0.0145007902332031	14863753
0.307918318254117	1.95286056246702e-104
3.69741599827088e-104	100	100
YBX1-regulon	Activated Chemokine	0.0967403518691899
0.00159490182849339	27463305.5	0.568931335394166
6.79826530086367e-15	8.54295285595258e-15	100	100
YY1-regulon	Activated Chemokine	0.663217421722516
0.0243305459441425	33074946 0.685182386216034
3.32351633624377e-97	5.8992414968327e-97	100	100
YY2-regulon	Activated Chemokine
0.033470990162198	-0.0179625544685162	16557244.5
0.343000780580936	1.8497666009142e-70
3.01915927965306e-70	82.2018348623853 90.7352210630899
ZBTB33-regulon	Activated Chemokine	0.365877954652536
5.32193036772433e-05	22836055.5	0.4730729718879
0.00234747910846377	0.00248762711493922	100	100
ZNF143-regulon	Activated Chemokine
0.0171032697001461	-0.00638074116902623	13541523.5
0.280526939778844	9.64100080584629e-136
2.10618786835411e-135	100	99.9932258501558
ZNF274-regulon	Activated Chemokine
0.0600095346133211	-0.0054632312247772	19296146.5
0.399740023873181	9.55405520628247e-30
1.33007435224717e-29	100	100
ZNF76-regulon	Activated Chemokine
0.0110399926768242	-0.00360367980082213	17480406.5
0.362125054949335	1.03128916251632e-54
1.55790490507785e-54	98.7155963302752 99.478390461997
ATF1-regulon	Activated NME1	0.125560304367744
0.021461872969762	156920744.5	0.853267789968554	0
0	100	100
ATF3-regulon	Activated NME1	0.0673125520358079
0.0180837640778633	146192473.5	0.794932047836297	0
0	100	100
ATF4-regulon	Activated NME1	0.132435097715441
0.0262901010976888	149416627	0.812463613469829	0
0	100	100
ATF5-regulon	Activated NME1	0.138915290986944

0.0227361205696601	126061245.5	0.685467053391837	0
	0	100	99.9975536365193
ATF6-regulon	Activated NME1
0.13402365912673 -0.00265336583860609	102607122
0.557933576615001	2.22782662866752e-37
2.82456590420347e-37	100	100
ATF6B-regulon	Activated NME1
0.0543039480388969	-0.00332193056624079	82519289.2597656
0.448704547004338	1.13026248128184e-29
1.33747726951685e-29	100	99.9119309146953
BACH1-regulon	Activated NME1	0.0453110009091249
0.00225138869224373	98889241 0.537717332329746
8.95714880993797e-17	1.03407734228552e-16	99.9333185152256
99.5229591212662
BATF-regulon	Activated NME1	0.0297817756494759
0.011642797963409	115782260	0.629574333352494
4.16900187846297e-202	7.04759841359216e-202	70.5712380529006
49.6416077500795
BATF3-regulon	Activated NME1	0.138082012545245
0.060962820410377	168422975	0.915811992328261	0
	0	100	100
BCL11A-regulon	Activated NME1
0.175095202557603	-0.0326415133289934	25948597
0.141097355136335	0	0	100	100
BHLHE40-regulon	Activated NME1	0.0635444099144618
0.0087619777816877	117686945.5	0.639931197209778
4.21491545991816e-209	7.2110601844383e-209	100	100
BRF2-regulon	Activated NME1	0.0549484006427983
0.0148038579276171	138318147	0.752114833378423	0
	0	100	99.6990972918756
CEBPB-regulon	Activated NME1	0.172415111854923
0.0325504455682356	163053385	0.886614462027624	0
	0	100	100
CEBPG-regulon	Activated NME1	0.10003425288193 0.0354925851829828
	153276291	0.83345081297487 0	0	100	100
CLOCK-regulon	Activated NME1	0.0214136809416	0.00645939882385069
	107537590.5	0.58474335230087 5.17213585710454e-88
6.92871029914004e-88	62.3249611024672 49.8568877363799
CREB1-regulon	Activated NME1
0.0314563221185427	-0.012782223124315	48168057
0.26191726068104 0	0	99.9777728384085 99.9951072730386
CREB3-regulon	Activated NME1	0.248606123569203
0.00977532719130753	136691646.5	0.743270620387719	0
	0	100	100
CREB3L2-regulon	Activated NME1
0.122463229189436	-0.00478877776967536	89782625.5
0.488199458153599	0.00925769825682471	0.0095955704559789
	100	100
CREB3L4-regulon	Activated NME1
0.00799194684792451	-0.00345162397048344	86537616

0.47055448652595 2.89116042148142e-15 3.31084499879324e-15
27.3394087575017 31.7880470680334
CREB5-regulon Activated NME1 0.0606771924482186
0.0238695273612463 143048127.5 0.777834441201398 0
0 100 99.5816718448027
CREM-regulon Activated NME1 0.160118160410013
0.0112322749082542 131892044 0.717172437952047 0
0 100 100
CTCF-regulon Activated NME1
0.367551057630833 -0.00365120898776761 87471379
0.47563188973292 7.70385876439825e-08 8.16379063092949e-08 100
100
CUX1-regulon Activated NME1
0.0592464930622106 -0.0193723255514861 29111489.5
0.158295809693649 0 0 100 100
DDIT3-regulon Activated NME1 0.102935270665034
0.0200112449812679 134135448 0.729371107918761 0
0 100 100
E2F1-regulon Activated NME1 0.46427414545475 0.0295045757159242
139157910.5 0.75668110757005 0 0 100 100
E2F2-regulon Activated NME1
0.0492451347428163 -0.00519049080621324 72475513
0.394090794059081 1.18943920780746e-120
1.74124090215113e-120 100 100
E2F3-regulon Activated NME1 0.0218497291816754
0.000815259233297554 98170380 0.533808474143284
8.93062841742104e-14 1.00646764704269e-13 100 100
E2F4-regulon Activated NME1 0.481203175197123
0.0916307843463519 170976624.5 0.929697644427109 0
0 100 100
E2F6-regulon Activated NME1 0.121593549838473
0.0546065314922971 171108525.5 0.930414865563953 0
0 100 100
E2F7-regulon Activated NME1
0.0232551032303709 -0.00812445864552989 46018602.5
0.250229447851086 0 0 100 100
E2F8-regulon Activated NME1 0.0792306930286648
0.00492210570398595 131072726.5 0.712717340350164 0
0 100 100
EGR1-regulon Activated NME1
0.0397946147189648 -0.00851239131764498 46518345.5
0.252946836215008 0 0 100 100
EGR2-regulon Activated NME1
0.0447954513976569 -0.00139030514923336 89808042.5
0.488337664911964 0.0101139362155182 0.0104070937869825
100 99.9951072730386
EGR3-regulon Activated NME1 0.0434948626963249
0.00224645425105589 102461653 0.55714257850615
2.06571931650691e-36 2.55071428646941e-36 100
99.9951072730386

ELF1-regulon	Activated NME1	0.69501593111025 -0.0133526114755896
	73662748 0.400546469424701	1.26917604460216e-106
1.76689214052457e-106	100	100
ELF2-regulon	Activated NME1
0.0185836030137128	-0.0156213004879757	43499426.5
0.236531247878158	0	0	98.4885530117804
99.6501700222619
ELF4-regulon	Activated NME1
0.0116615329213865	-0.00753240153812388	53896269
0.293064823798237	0	0	95.7768392976217
98.6178046334124
ELK1-regulon	Activated NME1	0.0710593533708843
0.00555517380444821	112480778.5	0.61162229117921
8.47113454074302e-134	1.2662116892479e-133	100	100
ELK3-regulon	Activated NME1
0.044727844263274	-0.0223983979861946	28808402.5
0.156647752418098	0	0	100	100
ELK4-regulon	Activated NME1
0.0394516145483027	-0.00615575809226711	81369746.5
0.442453825895253	6.63714595908607e-37
8.26732215956336e-37	96.8659702156035 96.7145338454388
ESRRA-regulon	Activated NME1	0.136930518031419
0.0501690169743369	170400876.5	0.926566973430714	0
	0	100	100
ETS1-regulon	Activated NME1
0.209945223307933	-0.0366251406738177	35390097.5
0.192436190491032	0	0	100	100
ETV2-regulon	Activated NME1
0.022477997754931	-0.00679805012722454	55554291.5
0.302080439922166	0	0	100	100
ETV3-regulon	Activated NME1
0.0113163426508015	-0.000125118296695009	93710984.5
0.509560191642427	0.0346543273261153	0.0354022624482617
	87.2860635696821 84.0252464711207
ETV5-regulon	Activated NME1
0.0291772702934311	-0.00475813809311046	61704420.5
0.335522206952856	4.39168524795104e-288
7.9951192975519e-288	100	100
ETV6-regulon	Activated NME1	0.463240966709196
0.00406956640303235	93421181 0.507984364349751
0.0782719788932824	0.0793901500203293	100	100
ETV7-regulon	Activated NME1
0.0454291797426329	-0.0206992128251107	22727791
0.123583991773868	0	0	100	100
FLI1-regulon	Activated NME1
0.111571063444102	-0.0359221047582902	26979423.5
0.146702548078152	0	0	100	100
FOS-regulon	Activated NME1
0.0672849374380364	-0.0210829472891998	28569293
0.155347577382123	0	0	100	100

FOSB-regulon	Activated NME1
0.0287232753790156	-0.0141535184024856	57324812.5
0.3117077746992	0	0	99.4443209602134	99.3101254984466
FOSL1-regulon	Activated NME1	0.0950227126254206
0.0379963151846319	161249709	0.876806844562877	0
0	100	100
FOXO1-regulon	Activated NME1
0.00805631327128275	-0.00959168051909024	70467407
0.383171573824037	6.7175142588685e-164
1.07178317388688e-163	36.2747277172705	54.1111138292928
FOXO3-regulon	Activated NME1
0.0102574321028243	-0.0105305805920538	74666238.5
0.406003020908175	4.53822607450841e-112
6.44428102580194e-112	33.4074238719715	48.2300560217237
GABPA-regulon	Activated NME1
0.0203566566163269	-0.00569602772270255	57570674
0.313044664218886	0	0	100	100
GTF2B-regulon	Activated NME1	0.0922025559241314
0.0145924022505192	130435286.5	0.709251214684175	0
0	100	100
HINFP-regulon	Activated NME1
0.0127751273896565	-0.00383572863439903	82639365
0.449357467444049	2.17951610694516e-30
2.62280751852722e-30	58.7463880862414	63.7277686718693
HIVEP3-regulon	Activated NME1
0.0713767835819882	-0.00700580470421297	80858357.5
0.439673111572015	2.19504640706641e-40
2.80807738561649e-40	99.1775950211158	99.75047092497
HOXB2-regulon	Activated NME1
0.0297688601241197	-0.00814104339920279	81673324.5
0.44410455301848	4.67355907715251e-35	5.7210809392729e-35
84.885530117804	83.602025588962
IKZF1-regulon	Activated NME1	0.146775737064165
0.00323206064863499	105317613.5	0.572672068324958
8.34993518445712e-58	1.10812223943263e-57	100	100
IRF1-regulon	Activated NME1
0.117658886374804	-0.0197408494330837	42570027.5
0.231477574233823	0	0	100	100
IRF2-regulon	Activated NME1
0.0783455278964084	-0.00740954495369411	69581696.5
0.378355459528282	1.5892218506857e-158
2.47988464612494e-158	100	100
IRF3-regulon	Activated NME1
0.135260391385977	-0.0113072429972145	51257070
0.278713990164401	0	0	100	100
IRF4-regulon	Activated NME1	0.0670456662777295
0.00958351534027485	126839339.5	0.689697995259231	0
0	100	99.9951072730386
IRF5-regulon	Activated NME1	0.0575822431214909
0.0116972625080937	109609835	0.596011330224525

3.11213415596466e-100	4.29051504997069e-100	92.1315847966215
79.1961249602466
IRF7-regulon	Activated NME1
0.0858382001939905	-0.0244174709034975	26970337.5
0.146653142302234	0	0	100	100
IRF8-regulon	Activated NME1
0.0622654618103084	-0.021654491097371	31127629
0.169258712660461	0	0	100	100
IRF9-regulon	Activated NME1
0.026391788124676	-0.00835258634848519	54731255
0.297605119991356	0	0	100	100
JUN-regulon	Activated NME1
0.0768098857346486	-0.0128660587031243	47409085.5
0.257790298777325	0	0	100	100
JUNB-regulon	Activated NME1
0.148531600256583	-0.0124891848122751	58293931.5
0.316977428689062	0	0	100	100
JUND-regulon	Activated NME1
0.0300732578020233	-0.00930723617859822	51220109.5
0.278513014797813	0	0	100	100
KLF11-regulon	Activated NME1
0.0177670365301458	-0.00462834904054012	87245298
0.474402558099053	2.31900250955e-10
2.47592749139925e-10	37.9862191598133 41.1747437434254
KLF12-regulon	Activated NME1
0.0282875502207906	-0.0208270640743912	44319078.5
0.240988164347754	0	0	94.5098910869082
99.0532573329745
KLF13-regulon	Activated NME1
0.0541965115867716	-0.0134268503885327	37813647.5
0.205614417238346	0	0	100	100
KLF2-regulon	Activated NME1
0.0820776927432949	-0.0297171054336894	28892574.5
0.157105443698152	0	0	100	100
KLF3-regulon	Activated NME1
0.0497449729330298	-0.0247368904383786	53808719.5
0.29258876711997 0	0	99.6888197377195 99.8825745529271
KLF4-regulon	Activated NME1
0.0121793398595902	-0.0041299317237077	85045939.5
0.462443388693993	7.9008163870898e-19
9.19603218825206e-19	45.9879973327406 50.2336277124055
KLF6-regulon	Activated NME1
0.0537440258707877	-0.0173567624839658	54121043
0.294287048525971	0	0	100	100
KLF7-regulon	Activated NME1
0.034669541640992	-0.0125761090945385	65892602
0.358295743899032	2.20109900237031e-214
3.81165924800712e-214	98.0440097799511 97.5022628862196
KLF8-regulon	Activated NME1	0.01382301480101 -0.0125256152538608
	65130048.5	0.354149304613704

1.57602305131193e-229	2.76290460847276e-229	68.3485218937542
78.6579249944957
MAFB-regulon	Activated NME1
0.0500811116616035	-0.0063293439263785	68274288
0.371246332147223	2.38316019648901e-177
3.84555395342544e-177	100	99.9975536365193
MAFF-regulon	Activated NME1
0.00635428541054495	-0.00669662084708313	81443025
0.44285228298865 2.52083316482106e-56	3.31442879078324e-56
19.4932207157146 29.5422853927637
MAFG-regulon	Activated NME1	0.0272455854726445
0.00204055218932905	97573636 0.530563635892743
2.69307673573505e-12	3.01115666515257e-12	63.8586352522783
58.0228490349096
MAX-regulon	Activated NME1
0.19692233011101 -0.000830192931940504	81337331 0.442277564291767
4.04891495725059e-37	5.08801702592552e-37	100	100
MAZ-regulon	Activated NME1	0.14719940565314 0.0120947820769731
144332561	0.784818640374036	0	0	100
100
MEF2A-regulon	Activated NME1
0.0263292250589928	-0.016012742937284	36081464.5
0.196195548082834	0	0	100	99.9975536365193
MLX-regulon	Activated NME1
0.0505451181650139	-0.00264232429952349	86126346
0.468318176437705	2.81159412343183e-12
3.11911223068218e-12	100	99.9877681825966
MXD4-regulon	Activated NME1
0.0792544943902249	-0.0143360443411446	94163095.5
0.512018577594009	0.00799133338904392	0.00834389221503115
91.2869526561458 88.3088289258018
MXI1-regulon	Activated NME1
0.0539614040302117	-0.012508341192955	63130015.5
0.343273981894507	9.09414179824595e-262
1.63464320930497e-261	100	100
MYBL1-regulon	Activated NME1
0.0105031220941494	-0.0215208774061021	51634314
0.280765281440035	0	0	59.1687041564792
82.3592729407735
MYC-regulon	Activated NME1	0.417445912460915
0.113852558239538	171598660	0.933080007020775	0
0	100	100
NFATC1-regulon	Activated NME1	0.0616868537530113
0.0123982941611037	114516722.5	0.622692882533559
2.9531799797406e-161	4.65946174581294e-161	98.2885085574572
94.4467548988429
NFE2L1-regulon	Activated NME1	0.294378966770086
0.124007039075174	162276789	0.882391665642545	0
0	100	99.9290554590601
NFE2L2-regulon	Activated NME1	0.144849264324918

0.0377073990884539 164626239 0.89516696833136 0 0
100 100
NFKB1-regulon Activated NME1 0.0831406743037153
0.0293854226192917 145001397.5 0.78845548675801 0 0
100 99.9241627320987
NFKB2-regulon Activated NME1 0.0822195998805535
0.00981628502370291 117119089 0.636843436809977
4.56904854682901e-200 7.54424294941534e-200 100 100
NFYA-regulon Activated NME1
0.0172480742374773 -0.00410759737986414 73523242.5
0.399787898274323 3.1665462603918e-108
4.45197593045184e-108 99.0886863747499 99.0092227903222
NFYB-regulon Activated NME1 0.116934534771769
0.00922421255390991 136595844.5 0.742749690149496 0
0 100 100
NFYC-regulon Activated NME1 0.0862007900454493
0.00295948194322243 112581096.5 0.612167777490958
4.33161773417315e-135 6.54350764098497e-135 100 100
NR1H3-regulon Activated NME1
0.00599887648154437 -0.0028145750019005 87778604.5
0.477302450398703 5.46733235908925e-12
5.92642133580666e-12 18.5596799288731 22.4943122049074
NR2C2-regulon Activated NME1
0.0139808152573328 -0.00866442517017826 70665065.5
0.384246356077976 9.99415885377467e-146
1.54257669264783e-145 66.5925761280284 77.011522371994
NR3C1-regulon Activated NME1
0.208261967731519 -0.0191104010844849 33299385
0.181067791494336 0 0 100 100
NRF1-regulon Activated NME1 0.0270649788531771
0.00037277309599015 97751687.5 0.531531803679543
3.5570323692316e-12 3.91549299558827e-12 100
99.9633045477897
PAX5-regulon Activated NME1
0.0812507915909873 -0.0211099220907379 37285835
0.202744398957285 0 0 100 100
POU2F1-regulon Activated NME1
0.0026946494226805 -0.00242585316590786 86174842.5
0.468581879630728 1.01285692695447e-24 1.1886420134507e-24
12.8695265614581 18.9495315213934
POU6F1-regulon Activated NME1
0.0163200281911418 -0.01629804157726 67270929.5
0.365790498423205 8.80191117404107e-201
1.47043692554568e-200 50.6334741053567 66.9740930107395
REL-regulon Activated NME1 0.295277384428737
0.0350395503508582 160924291 0.87503736087504 0 0
100 100
RELA-regulon Activated NME1
0.0885434903885212 -0.00390849604020052 72650877
0.3950443483721 1.59483572029879e-118 2.31088441104519e-118 100

100
RELB-regulon Activated NME1 0.119271011179693
0.0156378108335334 144283518.5 0.784551968266897 0
0 100 100
REST-regulon Activated NME1 0.2092533671981 -0.0103958460552805
48314637.5 0.262714302650768 0 0 100
100
RFX5-regulon Activated NME1
0.0528102752338925 -0.0139181771987151 47515994
0.258371621404964 0 0 100 100
RXRA-regulon Activated NME1 0.0152720230959841
0.000823537521926652 95646779.5 0.520086215634636
5.77886764341386e-06 6.07851263233162e-06 68.2596132473883
63.823176847616
SETDB1-regulon Activated NME1 0.0685320657939079
0.00978468371087743 112233947 0.610280127215034
1.15493301021098e-130 1.70833841093707e-130 98.6219159813292
94.6595885216626
SOX5-regulon Activated NME1
0.00465433935726055 -0.00349554512121881 84979450
0.462081847274458 2.67668642478123e-30
3.19402917915071e-30 16.0924649922205 23.198864887345
SP1-regulon Activated NME1
0.00838885482524338 -0.00511185600195994 75897475.5
0.412697960301083 3.38152570386647e-88
4.57311095189561e-88 49.4332073794176 60.9658243021748
SP2-regulon Activated NME1
0.0264633932775172 -0.00197231775922203 80067188
0.435371070736646 4.31268483703153e-46
5.61836006292181e-46 100 100
SP3-regulon Activated NME1
0.0204790921076675 -0.00864827929983677 39973308.5
0.21735772864324 0 0 100 100
SP4-regulon Activated NME1
0.00539499145206512 -0.00359985619193185 84157843.5
0.457614303071092 1.96775871389753e-31
2.38821997755085e-31 22.3605245610136 29.7477799251413
SPI1-regulon Activated NME1
0.391227429196005 -0.0303219494710899 41717771
0.226843368459702 0 0 100 100
SPIB-regulon Activated NME1 0.585871119980678
0.0237774146400309 133983635 0.728545613855428 0
0 100 100
SREBF1-regulon Activated NME1
0.0402041520102126 -0.000805603092083176 90570690.5
0.492484618047813 0.0974383157465199 0.098129367631247
97.5550122249389 96.8221738385889
SREBF2-regulon Activated NME1
0.0884086959094003 -0.0117377257395564 45337685
0.246526910164133 0 0 100 100

SRF-regulon	Activated NME1
0.0193766162167961	-0.00154582772589084	86171537.5
0.468563908456459	4.12963260341748e-12
4.51082945911755e-12	98.6441431429207 97.8569855909191
STAT1-regulon	Activated NME1
0.071996030172313	-0.0086279339443346	54059675.5
0.293953358348374	0	0	100	100
STAT2-regulon	Activated NME1
0.0502784559674177	-0.0177120350898054	50578067.5
0.275021865427138	0	0	99.9777728384085
99.960858184309
STAT5A-regulon	Activated NME1	0.0993409502014644
0.0307854817382289	161832065.5	0.879973449751452	0
	0	100	100
TBP-regulon	Activated NME1
0.0143036083803525	-0.00732380747445865	81070723.5
0.440827866910845	2.84512126905432e-44
3.67279291096103e-44	42.1871527006001 50.7326858624654
TCF12-regulon	Activated NME1
0.00633868656860472	-0.00300274711114148	87128313
0.473766443780786	3.33715986984049e-14	3.7910136121388e-14
	21.2047121582574 25.6623529123957
TFDP1-regulon	Activated NME1	0.559362147011918
0.0346326486276138	146633964.5	0.797332686777065	0
	0	100	100
TFDP2-regulon	Activated NME1
0.020822191442834	-0.00699445811293859	55264165
0.30050285629385 0	0	100	99.9951072730386
TFEC-regulon	Activated NME1	0.0982928509377953
0.00749436701717561	112713282	0.612886545616933
8.42474638842718e-137	1.2863591259749e-136	100	100
THAP1-regulon	Activated NME1
0.0370581291523171	-0.000118758734275526	91697766.5
0.498613174541161	0.759728124485584	0.759728124485584
	100	100
THAP11-regulon	Activated NME1	0.182966441779073
0.00498277016081733	115762243	0.629465489480982
2.71480080828008e-179	4.43105419282495e-179	100	100
TP53-regulon	Activated NME1	0.12766579761856 0.0325349210743099
	161935482	0.880535784378926	0	0	100
	100
USF2-regulon	Activated NME1
0.110297540629443	-0.0166383452662519	33900029.5
0.184333838993058	0	0	100	100
XBP1-regulon	Activated NME1
0.183088041252005	-0.0135447813838775	60699817
0.330059603452147	2.16848259591494e-307
3.99901985220678e-307	100	100
YBX1-regulon	Activated NME1	0.0972298575547724
0.00227129271633567	108766684.5	0.591426638977754

2.09322836448793e-90	2.85806180535853e-90	100	100
YY1-regulon	Activated NME1	0.678621725628686
0.0434593598289669	149055720	0.810501155802071	0
	0	100	100
YY2-regulon	Activated NME1
0.0317199653001101	-0.0214043151731979	58081539
0.315822529254584	0	0	80.6845965770171
91.6138659882085
ZBTB33-regulon	Activated NME1	0.36964566467101 0.00424004892435642
	97648236 0.530969278737062	8.50951931298156e-12
9.15417986699532e-12	100	100
ZNF143-regulon	Activated NME1
0.0182368788340467	-0.00565449654699762	56253675
0.305883387807017	0	0	100	99.9926609095579
ZNF274-regulon	Activated NME1
0.0602218850897296	-0.00568312358546303	72678783.5
0.395196091965062	3.46318592640025e-118
4.96739799544279e-118	100	100
ZNF76-regulon	Activated NME1
0.0110106659667809	-0.00393676865851541	64791382
0.352307781257999	1.06433826566503e-232
1.88920042155543e-232	98.7330517892865 99.540083665631
ATF1-regulon	ASC IgG 0.0847176905544194	-0.022124486274079
	5178834 0.0929527078831718	0	0	100	100
ATF3-regulon	ASC IgG 0.0364414161242288	-0.0149978155852696
	11656076.5	0.209210002477083
6.54080008344923e-273	1.57422646076236e-272	100	100
ATF4-regulon	ASC IgG
0.104486713306767	-0.00438703847749566	24211702.5
0.434565639647218	1.97962680272254e-15
2.21344099202048e-15	100	100
ATF5-regulon	ASC IgG 0.133629712777597	0.0156313559199938
	34813455.5	0.624852034163539
7.03132788681047e-52	9.60046692237583e-52	100
99.9977330945526
ATF6-regulon	ASC IgG 0.292295085039482	0.160344174887955
	55374345.5	0.993890779562219	0	0	100
	100
ATF6B-regulon	ASC IgG
0.0551223003404527	-0.00223646094622511	22992161.4628906
0.412676611774541	2.96826881882186e-26
3.63357045062676e-26	99.9208234362629 99.9206583093419
BACH1-regulon	ASC IgG
0.0432286792234352	-5.57072345341927e-05	28239839
0.506864963278375	0.40468203931185 0.413416184045199
99.7624703087886 99.5579534377621
BATF-regulon	ASC IgG
0.0111166300690657	-0.00841083112702434	24263592.5
0.435496991378526	9.46668167610478e-17	1.0754150384055e-16
	44.2596991290578 51.9302699884388

BATF3-regulon ASC IgG 0.0714860928637652 -0.0120118613912421
22821012 0.409604722227891 5.18510424913175e-28
6.40247655110181e-28 100 100
BCL11A-regulon ASC IgG 0.156428977228396 -0.0494476861897257
3282841.5 0.0589223379193566 0 0 100
100
BHLHE40-regulon ASC IgG 0.0363634207056072 -0.0198399836909514
9564921.5 0.171676743088303 0 0 100
100
BRF2-regulon ASC IgG
0.0342813105866521 -0.00754091940880204 20720241.5
0.371898878283852 1.60872600818277e-54
2.23959895256817e-54 99.5249406175772 99.7347720626573
CEBPB-regulon ASC IgG 0.15643781935699 0.013727901958755
40097203 0.719687790222903 1.15663227003695e-156
1.97881665476201e-156 100 100
CEBPG-regulon ASC IgG 0.0698811652596658 0.00187255232192179
30044628 0.539258360075369 1.88577789522186e-06
2.05984970093465e-06 100 100
CLOCK-regulon ASC IgG
0.0117346513849727 -0.00397059368981877 25410530.5
0.456082897950181 1.40390826721697e-08
1.54538739492101e-08 46.4766429136975 51.2252623943055
CREB1-regulon ASC IgG 0.0270840997037513 -0.0163419603959422
10905729.5 0.195742340547387
1.43298646577717e-298 3.69971051164289e-298 99.8416468725257
99.9977330945526
CREB3-regulon ASC IgG 0.394451895950807 0.159079722920381
54738328 0.982475169622591 0 0 100 100
CREB3L2-regulon ASC IgG 0.269949157897225 0.147271113552776
55228244.5 0.99126847431466 0 0 100 100
CREB3L4-regulon ASC IgG 0.0152292312779765 0.0042460722520932
31599663 0.567169027631639 3.64435195464457e-23
4.3124831463294e-23 44.8139350752177 30.9613946002312
CREB5-regulon ASC IgG 0.0233107992268065 -0.0163177020123123
14880953.5 0.26709196002586 7.84193790856983e-176
1.39194397877114e-175 98.4164687252573 99.6576972774466
CREM-regulon ASC IgG 0.162799097408037 0.0131660031883354
41501694.5 0.744896415972232
3.51697182618747e-194 6.65879999091494e-194 100 100
CTCF-regulon ASC IgG 0.3104388206223 -0.0621307853986845
1888529 0.0338964107491954 0 0 100 100
CUX1-regulon ASC IgG 0.0615769825247789 -0.0155540149551062
11946675.5 0.214425841401085
2.76449413002606e-263 6.54263610772833e-263 100 100
DDIT3-regulon ASC IgG 0.0909397904209529 0.00620435584847656
32105221.5 0.576243083986478
2.15014190653068e-20 2.50262418628981e-20 100 100
E2F1-regulon ASC IgG 0.396900502503576 -0.0419624120103513
13689599 0.2457088404233 3.35323579009254e-209

6.43458759720461e-209	100	100
E2F2-regulon	ASC IgG	0.0430439745712076	-0.011188437345737
	10878627.5	0.195255898176566
1.61344782646644e-299	4.24277021033767e-299	100	100
E2F3-regulon	ASC IgG	0.0146682793928053	-0.0066316077786654
	14217450	0.255183015461318	4.6854482141173e-194
8.75439008427179e-194	100	100
E2F4-regulon	ASC IgG	0.375014912875276	-0.0243195207623676
	21050729	0.377830659075926	9.47514483434862e-50
1.26931185516746e-49	100	100
E2F6-regulon	ASC IgG
0.0658209493982128	-0.00676866977459317	27879129.5
0.500390740550984	0.96217199099831	0.96217199099831	100	100
E2F7-regulon	ASC IgG	0.0194339945253931	-0.0114589831716364
	8914532	0.160003176180427	0	0	100	100
E2F8-regulon	ASC IgG	0.0521631815850432	-0.0232814472922707
	4112762.5	0.0738182400237898	0	0	100
	100
EGR1-regulon	ASC IgG	0.0480334510794343
0.000586775299827473	28848210	0.517784357846263
0.0308721418923045	0.0322341481522591	100	100
EGR2-regulon	ASC IgG	0.0355674332288905	-0.0107805421533597
	17697704	0.317648627106959	1.4763295415485e-108
2.32931994333207e-108	100	99.9954661891053
EGR3-regulon	ASC IgG	0.0313809306619997	-0.0103791052426524
	17006958	0.305250718396336	1.52285844118594e-123
2.45733975736823e-123	100	99.9954661891053
ELF1-regulon	ASC IgG	0.633251188440448	-0.0759062331438033
	3413438	0.0612663594336714	0	0	100	100
ELF2-regulon	ASC IgG	0.0166672395155743	-0.0164465987382068
	12189310	0.218780785738146	2.21570512642361e-255
5.07467948309923e-255	98.2581155977831	99.5715548704463
ELF4-regulon	ASC IgG
0.0103313115227792	-0.00834815369294973	14845199
0.266450217580744	8.60391186684116e-177
1.5465259304955e-176	95.1702296120348	98.4267676195226
ELK1-regulon	ASC IgG
0.0583471760622128	-0.00792847727322665	18616746.5
0.33414413343806	3.8646451371244e-90	5.90085601582435e-90	100
	100
ELK3-regulon	ASC IgG	0.0903068403131027	0.0261286516508818
	47704769.5	0.85623279370753	0	0	100	100
ELK4-regulon	ASC IgG	0.0225993631599332	-0.023038938238983
	13463117	0.241643810498263	6.9781722318117e-216
1.35739788618803e-215	86.6983372921615	97.0167524312561
ESRRA-regulon	ASC IgG
0.0912762009804512	-0.000472681151695992	32438014
0.582216236251681	1.87128266017612e-23
2.23295914071436e-23	100	100
ETS1-regulon	ASC IgG	0.209189310656621	-0.0347159832201295

11116778 0.199530361088243 3.09485768097508e-291
7.70999632804318e-291 100 100
ETV2-regulon ASC IgG
0.0279973025588627 -0.000622037047295691 27159481.5
0.487474082028485 0.128402455682121 0.133088676692417
100 100
ETV3-regulon ASC IgG
0.00821938690496731 -0.00330156472098872 23025945
0.413282978237762 5.28898907689825e-26
6.41911494803036e-26 81.1559778305622 84.4399610092263
ETV5-regulon ASC IgG
0.0252940188502073 -0.00840352794685916 12378291.5
0.222172735000243 2.6164156165954e-249
5.80517214932105e-249 100 100
ETV6-regulon ASC IgG 0.410593163130779 -0.05038412869942
5455963 0.09792677945661 0 0 100 100
ETV7-regulon ASC IgG 0.122408163831764 0.0600021911766684
54772479.5 0.983088140496589 0 0 100
100
FLI1-regulon ASC IgG 0.113544209740141 -0.0312573252754418
9134891.5 0.16395831593443 0 0 100 100
FOS-regulon ASC IgG 0.151949323059974 0.0675520487303158
54698366 0.981757908534009 0 0 100 100
FOSB-regulon ASC IgG 0.0634919908058085 0.0226489211676321
42900128 0.769996309233831 1.43030506831396e-235
2.98681352500857e-235 100 99.3040600276562
FOSL1-regulon ASC IgG
0.0512856891256164 -0.00978024170935817 22299605.5
0.40024621680314 9.53264094323523e-34 1.21949100354901e-33 100
100
FOXO1-regulon ASC IgG 0.0046522231018446 -0.0123896157619585
18443351.5 0.331031939692633
1.35901570528044e-104 2.12066186977827e-104 24.0696753760887
53.1521320245732
FOXO3-regulon ASC IgG 0.0661643817485583 0.0477495342475788
44636497.5 0.801161673273449 0 0
85.9857482185273 45.6373404665291
GABPA-regulon ASC IgG 0.0156579665264377 -0.0101114022338024
10201971.5 0.183110884935092 0 0 100
100
GTF2B-regulon ASC IgG 0.113869550417463 0.0358092890740571
49252935.5 0.884020172479018 0 0 100
100
HINFP-regulon ASC IgG 0.0213037315741593 0.00521843592790493
32871483.5 0.589996397540837
3.80855280617745e-29 4.74398682874736e-29 77.4346793349169
62.8272844739646
HIVEP3-regulon ASC IgG 0.064510670153598 -0.0135545766966823
21254100.5 0.381480888380681
6.31729126516773e-47 8.30606814494275e-47 99.6832937450515

99.6939677646045
HOXB2-regulon	ASC IgG	0.104076327989539	0.0688911257641067
	50265361.5	0.902191779877773	0	0
99.3665874901029 83.2815723256183
IKZF1-regulon	ASC IgG	0.122477360735168	-0.021999097344781
	4672351.5	0.0838620670419248	0	0	100
	100
IRF1-regulon	ASC IgG	0.244097919412466	0.11176639223904
54977130.5	0.986761335007361	0	0	100	100
IRF2-regulon	ASC IgG	0.116970459097048	0.0328647996982315
	48515877 0.870791020232912	0	0	100	100
IRF3-regulon	ASC IgG	0.181406736588479	0.0369897850500218
	52450798 0.941417258157579	0	0	100	100
IRF4-regulon	ASC IgG	0.122092558516174	0.0655034375076755
	54730770 0.982339514267316	0	0	100
99.9954661891053
IRF5-regulon	ASC IgG	0.00654835290567023	-0.0416558566157308
	8838532.5	0.158639093199052	0	0
29.9287410926366 81.9259628680888
IRF7-regulon	ASC IgG	0.182165630891415	0.0764591058691104
	54676816 0.981371116670264	0	0	100	100
IRF8-regulon	ASC IgG	0.0513582781860765	-0.0312854486595875
	4545087.5	0.0815778591650081	0	0	100
	100
IRF9-regulon	ASC IgG
0.0333873907564144	-0.000543971327029907	27736383.5
0.497828652783836	0.792116318094936	0.803432265496292
	100	100
JUN-regulon	ASC IgG	0.101813791151963	0.0137975501690764
	41015267 0.736165733870075	9.98655723739792e-181
1.8180655483468e-180	100	100
JUNB-regulon	ASC IgG	0.151244654593863	-0.0087822830903117
	20981088.5	0.376580711104367
9.77397552895808e-51	1.32181383344005e-50	100	100
JUND-regulon	ASC IgG	0.027386929295166	-0.0113877255140143
	12736946 0.228610073399096	5.48745336535343e-238
1.16301250429879e-237	100	100
KLF11-regulon	ASC IgG	0.00676577804035995	-0.0156050626564205
	21313553.5	0.382547985210156
1.11969977399573e-57	1.58997367907393e-57	21.4568487727633
41.4140956180718
KLF12-regulon	ASC IgG
0.0415136529852907	-0.00569447235796546	23949441.5
0.429858427536896	1.68030408432061e-17
1.92421919333489e-17	98.9707046714173 98.5922517171809
KLF13-regulon	ASC IgG	0.135521398633612	0.0712114050575879
	55195307.5	0.990677301989085	0	0	100
	100
KLF2-regulon	ASC IgG	0.0805989998717862	-0.0290581752896085
	8693193 0.156030455793917	0	0	100	100

KLF3-regulon ASC IgG 0.0429234640621619 -0.0299390804745564
13162395.5 0.236246287089772
6.69925092963526e-225 1.35899090286887e-224 99.6041171813143
99.8707863894997
KLF4-regulon ASC IgG 0.0269497732915539 0.0113663538716203
34591687.5 0.620871613836911
1.57521034375733e-55 2.21465216647071e-55 67.8543151227237
49.2961258585904
KLF6-regulon ASC IgG 0.0420483324755483 -0.0281140743898501
10125834 0.181744325049903 0 0 100 100
KLF7-regulon ASC IgG 0.0240748395251268 -0.0225516019011844
13206067 0.237030128429796 1.39638736675287e-223
2.79277473350574e-223 94.6159936658749 97.6401514292839
KLF8-regulon ASC IgG 0.0150440627083541 -0.0103507651914044
21491809.5 0.38574742699501 3.30263203267936e-44
4.30251145541714e-44 70.8630245447348 77.8296647246843
MAFB-regulon ASC IgG 0.0607275841287688 0.00508624992613332
33280056 0.597329693074464 3.29796255085948e-32
4.18134537698255e-32 100 99.9977330945526
MAFF-regulon ASC IgG
0.005388472017359 -0.00719884203965776 24187634
0.434133644288864 1.10621159030477e-23
1.33120377816337e-23 16.8646080760095 28.8803753995421
MAFG-regulon ASC IgG
0.0205880805223671 -0.00495725280032153 25766690
0.462475454646016 2.29477570748088e-06
2.48746679742202e-06 55.1068883610451 58.7015165597443
MAX-regulon ASC IgG 0.167080799000072 -0.0314652168786717
2486214.5 0.0446240157829747 0 0 100
100
MAZ-regulon ASC IgG 0.116019114059117 -0.0208654705961951
6898219 0.123813224293566 0 0 100 100
MEF2A-regulon ASC IgG 0.0259752872143907 -0.0152021666330565
11550081.5 0.207307542913391
1.82510992165678e-276 4.46837256681488e-276 100
99.9977330945526
MLX-regulon ASC IgG 0.0894311505876856 0.0375508878747655
50660350.5 0.909281270897193 0 0 100
99.9886654727631
MXD4-regulon ASC IgG 0.631014412280549 0.554272970545154
55488007 0.99593084190194 0 0 99.9208234362629
88.2800988370775
MXI1-regulon ASC IgG 0.0369295389424644 -0.0291102707402816
7742715 0.138970726927654 0 0 100 100
MYBL1-regulon ASC IgG
0.0243640092458474 -0.00568443067426361 27772815
0.498482546416504 0.853286714808313 0.859338393636741
85.9065716547902 79.8925486817945
MYC-regulon ASC IgG 0.28474219364003 -0.0310024918291402
20089681.5 0.360581222710645 3.04195582540268e-64

4.40773191027735e-64	100	100
NFATC1-regulon	ASC IgG
0.0436860496236848	-0.00702739368777646	25904304.5
0.464945439283289	2.08750391061267e-05
2.24564814626515e-05	95.5661124307205 94.8065196200666
NFE2L1-regulon	ASC IgG	0.16727566516061 -0.0158321510111689
26151928 0.469389929077808	0.000202770300675668
0.00021649159921763	99.8416468725257 99.9387935529209
NFE2L2-regulon	ASC IgG	0.0903218763543448	-0.021147267336477
12569222 0.225599666041572	3.00470479237883e-243
6.56412431565837e-243	100	100
NFKB1-regulon	ASC IgG	0.0224869521399837	-0.0351605054404075
6120180	0.109848530331814	0	0
98.9707046714173 99.9591957019473
NFKB2-regulon	ASC IgG	0.0460042570729513	-0.0281560336482183
6205179	0.111374141544176	0	0	100	100
NFYA-regulon	ASC IgG	0.0106213290787583	-0.0106227517153387
12217295 0.219283076703662	1.77464553830362e-254
3.99999470538276e-254	96.9121140142518 99.0773694829189
NFYB-regulon	ASC IgG	0.0942174523063235	-0.0148199441748659
15514095.5	0.278455958828402
2.74863528755248e-159	4.75983183942014e-159	100	100
NFYC-regulon	ASC IgG
0.0770358677830578	-0.00668494024857011	20248735
0.363436006919464	1.02990322734236e-61
1.47723493214763e-61	100	100
NR1H3-regulon	ASC IgG
0.0034531342174493	-0.00522673550491385	24807540
0.44526007570818 5.70179837302075e-20	6.58256397535729e-20
12.0348376880443 22.3924920091583
NR2C2-regulon	ASC IgG	0.00803675855302943	-0.0141430694964261
15884796 0.285109505802228	4.60422004153336e-152
7.60231681276438e-152	49.4061757719715 76.7392832044975
NR3C1-regulon	ASC IgG	0.181739424861183	-0.0449904284545342
1740990	0.0312482954459485	0	0	100	100
NRF1-regulon	ASC IgG	0.0164247159629372	-0.0105994768676521
11969451.5	0.214834638221903
1.54414353695612e-262	3.59456364340605e-262	99.8416468725257
99.9705302291841
PAX5-regulon	ASC IgG	0.0698509716605271	-0.0312875686184946
5388927	0.0967235785574006	0	0	100	100
POU2F1-regulon	ASC IgG
0.00304674522223774	-0.00188572304903557	26788996
0.480824393999008	0.00056393580607591
0.000597603615393875	14.9643705463183 18.4435427198332
POU6F1-regulon	ASC IgG	0.0877868152319009	0.0584104885908424
46708973 0.838359662192678	0	0
94.9326999208234 64.5070614104686
REL-regulon	ASC IgG	0.213245786073431	-0.0519110944002524
2019530	0.0362476924634584	0	0	100	100

RELA-regulon	ASC	IgG	0.0817688246289306	-0.0105904115825824	13264477	0.238078504892038	8.13897504842065e-222	1.60518674566074e-221	100	100
RELB-regulon	ASC	IgG	0.080521792477155	-0.0253679811938433	4676059.5	0.0839286203704985	0	0	100	100
REST-regulon	ASC	IgG	0.199023543599544	-0.0201559511892134	7909987	0.141973021527758	0	0	100	100
RFX5-regulon	ASC	IgG	0.0511385661415943	-0.0146167524567361	13152664	0.236071620499423	3.39520183867272e-225	6.98722697234095e-225	100	100
RXRA-regulon	ASC	IgG	0.0110424720876027	-0.00358752211984039	25066422	0.449906639572211	4.84091836975781e-10	5.37039381645007e-10	60.5700712589074	64.3687801781788
SETDB1-regulon	ASC	IgG	0.0474201860357993	-0.0126494262429307	19570440	0.351261575958052	7.15070554952211e-73	1.05770852920015e-72	92.6365795724465	95.1216194772516
SOX5-regulon	ASC	IgG	0.00365880864049794	-0.00426315597950448	25120069	0.450869526955704	3.41326318441723e-16	3.84669343005751e-16	13.5391923990499	22.7506630698434
SP1-regulon	ASC	IgG	0.00582872572224317	-0.00737029347289584	20278546	0.363971071989073	2.29498728420522e-65	3.35967210677465e-65	40.3008709422011	60.3812934962483
SP2-regulon	ASC	IgG	0.0371669018364665	0.00918232655514619	43006128.5	0.771898867514705	7.14343529323558e-239	1.53692092672644e-238	100	100
SP3-regulon	ASC	IgG	0.0279613313081796	-0.000317403644662272	27196259	0.488134185869267	0.149780699989062	0.154122169553963	100	100
SP4-regulon	ASC	IgG	0.00358874504794808	-0.00519374239772433	23699079.5	0.425364785560527	1.25624550597371e-29	1.5786447951174e-29	15.2810768012668	29.4085643687802
SPI1-regulon	ASC	IgG	0.356654219090561	-0.0636606966262636	4491822.5	0.0806218281384494	0	0	100	100
SPIB-regulon	ASC	IgG	0.504051129714633	-0.0621294062990844	4178871	0.0750047936165666	0	0	100	100
SREBF1-regulon	ASC	IgG	0.0312453240751917	-0.00996183469667878	19215311	0.344887515272221	4.42771688711324e-79	6.61827155757978e-79	97.070467141726	96.8898057262032
SREBF2-regulon	ASC	IgG	0.0816198911670267	-0.0178598558031826	7337276.5	0.13169368223862	0	0	100	100
SRF-regulon	ASC	IgG	0.0121007174242836	-0.0089166455140273	15624252	0.280433111400957	1.70592638152059e-156			

2.88382793066576e-156	95.0910530482977 98.0164577335479
STAT1-regulon	ASC IgG	0.109533088362021	0.0306167682219218
52271225 0.93819417809502 0	0	100	100
STAT2-regulon	ASC IgG	0.0854600725952632	0.0197761697853046
40015510 0.718221517010613	1.31947096064979e-154
2.20429266367377e-154	99.9208234362629 99.963729512842
STAT5A-regulon	ASC IgG
0.0632993615009093	-0.00854634669740899	21074751.5
0.378261828799675	2.0633549804522e-49
2.73828417966554e-49	100	100
TBP-regulon	ASC IgG
0.0117227734572639	-0.00944128133664718	22409156.5
0.402212501511495	6.26032083981787e-37
8.08150508412853e-37	33.3333333333333 50.3593045134087
TCF12-regulon	ASC IgG
0.00736636131777228	-0.00172537628916426	26987103
0.484380133012966	0.0129324132519346	0.0136029828279609
22.6444972288203 25.2941309817967
TFDP1-regulon	ASC IgG	0.468100919955296	-0.0617820293557516
5483059.5	0.0984131231102503	0	0	100
100
TFDP2-regulon	ASC IgG	0.0319005047993006	0.0049141314936739
37718607 0.676995373520595	2.18701658593122e-102
3.37561255654602e-102	100	99.9954661891053
TFEC-regulon	ASC IgG	0.0679797423209247	-0.0242364014244788
10764078 0.193199897499259	1.52093170515879e-303
4.07494909684053e-303	100	100
THAP1-regulon	ASC IgG
0.0317305103669565	-0.00559020086562922	17013723.5
0.305372149503258	2.15876130288414e-123
3.44431578662414e-123	100	100
THAP11-regulon	ASC IgG	0.16852786111093 -0.0102347231096028
15424464 0.276847200826769	1.40310585132488e-161
2.45976581343373e-161	100	100
TP53-regulon	ASC IgG	0.0819996218705949	-0.016825389839392
14540567.5	0.260982515230849	4.556986811734e-185
8.40379386060036e-185	100	100
USF2-regulon	ASC IgG	0.109780481936975	-0.0159496677777073
10052440 0.180427007089455	0	0	100	100
XBP1-regulon	ASC IgG	0.361186663236969	0.170646590405572
55201996.5	0.990797360029762	0	0	100
100
YBX1-regulon	ASC IgG
0.0897666926364585	-0.00557216555137399	19111791
0.343029478439979	6.14002388237816e-81
9.27535522657127e-81	100	100
YY1-regulon	ASC IgG	0.564783464851753	-0.0768262568695265
4261580.5	0.0764893115587642	0	0	100
100
YY2-regulon	ASC IgG	0.0174146500309007	-0.0345490485115602

        10949029.5   0.196519513990549
2.6169589664851e-297   6.63586023644435e-297   63.341250989707
91.3086845147689
ZBTB33-regulon ASC IgG 0.32742991412107 -0.0394954189711232
7210825.5   0.129424066555913   0   0   100   100
ZNF143-regulon ASC IgG
0.0210995595493503   -0.00229505713086858   23424603
0.420438322591199   4.57186533605947e-22
5.36532956793756e-22   100   99.9931992836579
ZNF274-regulon ASC IgG 0.0763114882872051   0.011284038534801
        39056028.5   0.70100018811905 1.79597149267580e-131
2.93135576965475e-131   100   100
ZNF76-regulon   ASC IgG
0.0114221815108206   -0.00322468123065279   20858337.5
0.374377505161607   1.68637430554937e-52
2.32490438240787e-52   98.9707046714173 99.4740779362093
ATF1-regulon   ASC IgM 0.0883769994901334   -0.0179829183761072
        2351210.5   0.154907652405172
5.79849061046156e-106   5.14616041678463e-105   100   100
ATF3-regulon   ASC IgM 0.0388696781865659   -0.0122430298091073
        3972826.5   0.261746545674263
1.75788049075795e-51   6.40048794070845e-51   100   100
ATF4-regulon   ASC IgM
0.101778632767131   -0.00702518463829781   6023742
0.396869498462361   6.41008648941579e-11
9.68332214358556e-11   100   100
ATF5-regulon   ASC IgM 0.130627032523787   0.0122848283599058
        9316137.5   0.61378638348578 5.63833764541614e-13
8.89604384054547e-13   100   99.997779702036
ATF6-regulon   ASC IgM 0.240636297073781   0.105002185308303
        14527897 0.957159054305919   1.90360167212425e-184
9.01038124805479e-183   100   100
ATF6B-regulon   ASC IgM
0.0560371892002155   -0.00126874497258444   5847940.828125
0.385286976682523   3.6429695486528e-13   5.8784281353261e-13
        99.7032640949555 99.9222895712605
BACH1-regulon   ASC IgM 0.044418905609399   0.00114457024473204
        7849084.5   0.517130751765878   0.277780097799305
        0.29003510211398 99.406528189911 99.5648215990586
BATF-regulon   ASC IgM
0.0109265075047833   -0.00842944992080871   6481559.5
0.427032443955759   9.23392850785314e-07
1.18127734064428e-06   41.839762611276 51.7906703079553
BATF3-regulon   ASC IgM
0.0733692853235033   -0.00986761474497187   6740777.5
0.444110817772635   0.000398815271180797
0.000488204900928217   100   100
BCL11A-regulon ASC IgM 0.169761013322454   -0.0349992520056701
        2392987.5   0.157660097154178
2.6058816665854e-104   2.1766776273831e-103   100   100

BHLHE40-regulon	ASC IgM	0.040413682858243	-0.0153515065858686
	3549706	0.233869584704796	8.74933138319376e-64
4.28415536694315e-63	100	100
BRF2-regulon	ASC IgM	0.0438948441058869	0.00229958744839617
	8195320.5	0.539942237993146	0.0113885367587101
	0.0130417114494906	100	99.7269033504296
CEBPB-regulon	ASC IgM	0.144305344826815	0.00122240224750411
	8180746.5	0.538982041478987	0.0135221966334391
	0.0153612153755868	100	100
CEBPG-regulon	ASC IgM
0.0658504568512045	-0.00222681507505507	7576586
0.499177402663817	0.958438091229891	0.965235524501025
	100	100
CLOCK-regulon	ASC IgM
0.0134906014521102	-0.00211986965058164	7171404.5
0.472482338583844	0.0635572864099739	0.0683722323501234
	48.0712166172107 51.1156997269034
CREB1-regulon	ASC IgM
0.0338374669758388	-0.00920207166251366	4998127
0.329297661775884	2.92695480969795e-27
6.02358815908853e-27	100	99.993339106108
CREB3-regulon	ASC IgM	0.350278924403021	0.111305558743188
	14312065 0.942939132936091	2.81312170448034e-173
9.98658205090522e-172	100	100
CREB3L2-regulon	ASC IgM	0.228701458056705	0.102686893674153
	14616861.5	0.9630204103361	3.74170211306982e-189
2.65660850027957e-187	100	100
CREB3L4-regulon	ASC IgM	0.0111623636330126
6.14755877951234e-05	7988029.5	0.526285033682974
0.0428883283393441	0.0464896383525715	38.2789317507418
31.2950998023935
CREB5-regulon	ASC IgM	0.0235329305766098	-0.0157584172992397
	4140053.5	0.272764164891581
5.43809364047124e-47	1.71602065988204e-46	99.1097922848665
99.6269899420502
CREM-regulon	ASC IgM	0.166762635206882	0.0168885052911347
	11887325 0.783187047321929	5.60648085505232e-72
3.46139252790187e-71	100	100
CTCF-regulon	ASC IgM	0.333543863450318	-0.0375754548930483
	3036957	0.200087520587993	1.67313972252743e-80
1.07993563908589e-79	100	100
CUX1-regulon	ASC IgM
0.0709733777576467	-0.00576752218361842	6107048.5
0.402358081617758	6.16637884201076e-10
8.93495709760743e-10	100	100
DDIT3-regulon	ASC IgM	0.0877383359117061	0.00285138545998496
	8079347.5	0.532301448207465	0.0407109720147036
	0.0448136281092087	100	100
E2F1-regulon	ASC IgM	0.423006842296007	-0.0147979885563905
	6788881.5	0.447280111934642

0.000837556684453436	0.000991108743269899	100	100
E2F2-regulon	ASC IgM	0.0453474934963445	-0.0086376487463195
	4159590	0.274051311810674	1.76325131983911e-46
5.44308016124248e-46	100	100
E2F3-regulon	ASC IgM	0.01499394342857	-0.0061716140796663
4186103	0.27579809993884	8.61007487419767e-46
2.60134177050228e-45	100	100
E2F4-regulon	ASC IgM	0.386979989901663	-0.0117649078407812
	7334680.5	0.483239649277253	0.288302531567107
	0.298824521770287	100	100
E2F6-regulon	ASC IgM
0.0640566424153489	-0.00840701444128555	7070641
0.465843614729417	0.0304657707588075	0.034064090139769
	100	100
E2F7-regulon	ASC IgM
0.0213152715562028	-0.00932803343991598	3401616
0.224112791663644	2.07539405443113e-68
1.13348444511239e-67	100	100
E2F8-regulon	ASC IgM	0.0579595716416163	-0.0169630206975483
	2738374.5	0.180415647684964
3.75110025952277e-91	2.95920131584574e-90	100	100
EGR1-regulon	ASC IgM	0.0545127511899376	0.00710249207610044
	10349223	0.681850408182345	1.03210439086818e-30
2.15527681622473e-30	100	100
EGR2-regulon	ASC IgM	0.0479048834921213	0.00187086940503135
	7944652	0.523427141251733	0.137748327441219
0.145972108183978	100	99.995559404072
EGR3-regulon	ASC IgM
0.0363389714222303	-0.00517057243795163	6152998.5
0.405385461185864	2.04341015829819e-09
2.87291329186478e-09	100	99.995559404072
ELF1-regulon	ASC IgM	0.671177353201987	-0.0361356604376875
	4588427	0.302304899881362	5.44883385754927e-36
1.31141425046101e-35	100	100
ELF2-regulon	ASC IgM	0.0192682534055096	-0.0134879816130201
	4135685.5	0.272476382651027	4.1766975265087e-47
	1.3479342017369e-46	99.406528189911	99.5359577255268
ELF4-regulon	ASC IgM
0.0114909674669331	-0.00700818303548404	4624030
0.304650575501891	3.50624007164434e-35
8.03042080924994e-35	97.3293768545994	98.3436577188659
ELK1-regulon	ASC IgM
0.0615332257154975	-0.00455557913999875	6149063.5
0.405126206809357	1.84674384320244e-09
2.62237625734747e-09	100	100
ELK3-regulon	ASC IgM	0.0803655363879577	0.0155757588616082
	11147231	0.734426536895851	6.73918791092528e-50
2.33406020329607e-49	100	100
ELK4-regulon	ASC IgM	0.0282214086605778	-0.0169011466928441
	4681284.5	0.308422743151122

6.67385080207399e-34	1.48076064671017e-33	91.0979228486647
96.7716867603632
ESRRA-regulon	ASC IgM
0.0915384133155673	-0.00019878853275021	8789388.5
0.57908194039284 5.43600457779273e-07	7.08176743161989e-07	100
	100
ETS1-regulon	ASC IgM 0.243817830232737
0.000885400130914288	7597188 0.500534749211415
0.972975202426225	0.972975202426225	100	100
ETV2-regulon	ASC IgM
0.0249087468700281	-0.00372091350171883	5967055
0.39313472010377 1.28368136701618e-11	1.96002961415374e-11	100
	100
ETV3-regulon	ASC IgM 0.00689875243911196	-0.0045642006718539
	5485856 0.361431302893905	1.41879413393012e-18
2.37022078844796e-18	74.4807121661721 84.4223894846688
ETV5-regulon	ASC IgM
0.0309152980494518	-0.00256741187359575	6273934
0.413353201376479	4.0302617288432e-08
5.45044919519747e-08	100	100
ETV6-regulon	ASC IgM 0.417622232237861	-0.0422665696288673
	2093921 0.137956336292259	1.97139463668228e-116
2.3328169867407e-115	100	100
ETV7-regulon	ASC IgM 0.100323116803205	0.0365182523015273
	13569942 0.894044943442686	1.47569849693006e-137
2.32832429515632e-136	100	100
FLI1-regulon	ASC IgM 0.130915780752945	-0.013113123817104
	5158741 0.339879588695402	3.50735462719498e-24
6.46810853326867e-24	100	100
FOS-regulon	ASC IgM 0.126664271208513	0.0406889365400767
	12955673 0.853574314064639	3.86935789652744e-111
3.92463443790641e-110	100	100
FOSB-regulon	ASC IgM 0.0533263521385505	0.0119415581896191
	9724162.5	0.640668789324228
5.00774265643878e-19	8.5674633399314e-19	99.7032640949555
99.32058882302
FOSL1-regulon	ASC IgM 0.0502892603207204	-0.0105830448272803
	5859879.5	0.386073546678273
5.28245975827217e-13	8.42819422106346e-13	100	100
FOXO1-regulon	ASC IgM 0.00546997483744364	-0.0113110152834818
	5320754.5	0.350553720570428
1.16742929290606e-23	2.09841721003367e-23	27.5964391691395
52.5278092319989
FOXO3-regulon	ASC IgM 0.034460233243096	0.0148264340167319
	9154499.5	0.603136991132578
1.32976268004591e-12	2.07501429193977e-12	61.1275964391691
46.6529008192899
GABPA-regulon	ASC IgM
0.017817495113766	-0.00772782516399374	3864275
0.254594715572254	1.64944600664831e-54

6.33030629578541e-54	100	100
GTF2B-regulon	ASC IgM	0.103646686133706	0.0247736956678461	11858642	0.781297290452462	4.80118951949396e-71	2.84070379903392e-70	100	100
HINFP-regulon	ASC IgM	0.0199406344367142	0.00373784871731076	8406288	0.553841665610872	0.000466644003674435	0.000566354260869827	70.6231454005935	63.1785785652435
HIVEP3-regulon	ASC IgM	0.0708789841036898	-0.00685993090480215	6637662.5	0.437317167192324	7.14876048511714e-05	8.9045963937424e-05	99.406528189911	99.6958191789338
HOXB2-regulon	ASC IgM	0.0692247443216763	0.0323623697311023	10628250.5	0.700233915308348	4.99074003503483e-37	1.24330716662271e-36	94.0652818991098	83.6519460911654
IKZF1-regulon	ASC IgM	0.136577177821397	-0.00734147918616296	5428795.5	0.357671916781915	1.92926760843862e-19	3.38217284442326e-19	100	100
IRF1-regulon	ASC IgM	0.203133814791234	0.0681978651734658	13378547	0.881435034575705	5.08444035944958e-129	7.21990531041841e-128	100	100
IRF2-regulon	ASC IgM	0.0964644172568767	0.0115296244265737	9712430	0.639895802800119	7.78055244672991e-19	1.31528386599482e-18	100	100
IRF3-regulon	ASC IgM	0.173587778324251	0.0283518137106509	12920969	0.851287868351221	9.88882895016098e-110	9.36142473948573e-109	100	100
IRF4-regulon	ASC IgM	0.105593606001575	0.0475342851633832	13700133	0.902622474962846	1.59814353840387e-143	3.24194832076213e-142	100	99.995559404072
IRF5-regulon	ASC IgM	0.0140518507933039	-0.0332397718142159	3606518	0.237612598589959	1.6632430376879e-62	7.87268371172271e-62	51.6320474777448	80.6945092031351
IRF7-regulon	ASC IgM	0.157065827781561	0.0495994997955399	13649754	0.899303294217217	3.3666142361844e-141	5.97574026922731e-140	100	100
IRF8-regulon	ASC IgM	0.0633824587057562	-0.018528070815502	3834882.5	0.252658213853961	2.41138643107818e-55	9.51157981147505e-55	100	100
IRF9-regulon	ASC IgM	0.0371221440590664	0.00322991093745128	8808062	0.580312229236475	3.6149154432083e-07	4.75294437903314e-07	100	100
JUN-regulon	ASC IgM	0.0917934710307148	0.00341857689528387	8312209.5	0.547643377717551	0.00254069506079102	0.00298164213745723	100	100
JUNB-regulon	ASC IgM	0.153718568559394	-0.00610929491913675	6240613.5	0.411157906471167	1.81674164392074e-08	2.48055109073794e-08	100	100

JUND-regulon	ASC IgM	0.0291957331869404	-0.0093312562491743
	4242855.5	0.279537193713355
2.46265745068983e-44	6.99394715995912e-44	100	100
KLF11-regulon	ASC IgM	0.0096501930540702	-0.0123782258331793
	6347433.5	0.418195658059092
5.90361146850906e-09	8.21875322086555e-09	28.7833827893175
40.9489553498079
KLF12-regulon	ASC IgM
0.0463529054168533	-0.000701932564940375	7419048.5
0.488798168524305	0.477894620830668	0.491746638825759
	99.406528189911	98.5967716867604
KLF13-regulon	ASC IgM	0.115147936746221	0.0492213971159021
	14305654.5	0.942516782191339
5.96426123509651e-173	1.69385019076741e-171	100	100
KLF2-regulon	ASC IgM	0.0884709864552091	-0.0205298524243679
	3787557	0.249540210551449	1.05685305143909e-56
4.41391568542207e-56	100	100
KLF3-regulon	ASC IgM	0.0458911573205367	-0.0263336354644217
	4089145	0.269410098455391	2.45885123683676e-48
8.31325894359094e-48	100	99.8623415262328
KLF4-regulon	ASC IgM	0.0240144908552611	0.00817541669463265
	9163557	0.603733737388032	2.04319565012957e-12
3.15362806867825e-12	67.0623145400593	49.6836075401319
KLF6-regulon	ASC IgM	0.0454366350260414	-0.0241223949694327
	3382456.5	0.222850483092695
5.08937229187836e-69	2.89076346178691e-68	100	100
KLF7-regulon	ASC IgM	0.024340434847789	-0.0218203592732038
	3767774.5	0.248236856116061
2.82119905537786e-57	1.21397050261714e-56	95.2522255192878
97.5732143253625
KLF8-regulon	ASC IgM	0.011818712115448	-0.0133874378514649
	5090392	0.335376468649689	9.74859151467178e-26
1.89630136312794e-25	62.9080118694362	77.7459535069606
MAFB-regulon	ASC IgM	0.0577463778755895	0.0019781639922568
	8405870.5	0.553814158952119
0.000651049622179684	0.0007768827424329	100
99.997779702036
MAFF-regulon	ASC IgM
0.00523446822683142	-0.00720599025054735	6655270
0.438477223465348	1.00483762694359e-06
1.27399056273206e-06	17.8041543026706	28.6263016496814
MAFG-regulon	ASC IgM	0.0287759302441775	0.0033937826371874
	8235655.5	0.542599677707609	0.00510917823358186
	0.00594674843580839	64.6884272997033	58.555918204223
MAX-regulon	ASC IgM	0.17413391791103	-0.0237124001080216
1824134.5	0.120181665174719	6.03639617202229e-128
7.79243869479241e-127	100	100
MAZ-regulon	ASC IgM	0.122369266262283	-0.0140388107648729
	3688036	0.242983347831154	1.2961654646039e-59
5.7517342491798e-59	100	100

MEF2A-regulon	ASC IgM
0.0313360084625726	-0.00948877837710699	4816090
0.317304297370238	5.5354759312489e-31
1.17319042124977e-30	100	99.997779702036
MLX-regulon	ASC IgM	0.0803302248409666	0.0276098206029531
	12258035	0.807610983767909	1.36794911462402e-84
9.24994163221956e-84	100	99.9888985101801
MXD4-regulon	ASC IgM	0.488432437987334	0.399228310666966
	14210495.5	0.936247306406324
2.17530038355292e-168	5.14821090774191e-167	99.406528189911
88.5232798241524
MXI1-regulon	ASC IgM	0.0394806967837534	-0.0259415181278384
	2745311.5	0.18087268646764	6.74847008534574e-91
5.04359343220576e-90	100	100
MYBL1-regulon	ASC IgM
0.0267606245729378	-0.00315301124029694	7700713.5
0.507355445260991	0.639883423867199	0.653693857475844
	85.1632047477745	80.0217589200471
MYC-regulon	ASC IgM	0.290815225271631	-0.0242466093550776
	6390218.5	0.421014514094379
5.61129235706792e-07	7.24366831548768e-07	100	100
NFATC1-regulon	ASC IgM	0.073265988567623	0.0229183572408636
	10123153	0.666955964244111	3.75897611573651e-26
7.51795223147302e-26	99.1097922848665	94.795621572415
NFE2L1-regulon	ASC IgM	0.182994892962146
0.000330202783516115	8130826	0.535693068644827
0.0237383126213707	0.0267527015256718	100
99.9356113590444
NFE2L2-regulon	ASC IgM	0.0977781040631353	-0.0132004621661211
	5167645.5	0.340466254666332	5.1285644955177e-24
	9.33661741491685e-24	100	100
NFKB1-regulon	ASC IgM	0.0329402511424648	-0.0239060920747822
	3636778.5	0.239606287804773
3.83758089872803e-61	1.75785963748187e-60	100
99.9311707631164
NFKB2-regulon	ASC IgM	0.0564503379027167	-0.0170529041184844
	3876022	0.255368657417446	3.54213330541696e-54
1.32363928781371e-53	100	100
NFYA-regulon	ASC IgM
0.0135093891144812	-0.00749467870596104	4607733
0.303576860489455	1.49959156349494e-35
3.49085249207018e-35	97.9228486646884	99.0252891938098
NFYB-regulon	ASC IgM
0.100116459956316	-0.00857210031882985	6126802.5
0.403659558353087	1.03664820300971e-09
1.48690954371089e-09	100	100
NFYC-regulon	ASC IgM
0.0811865795211023	-0.00236572910943111	7205268
0.474713408616588	0.109148049072878	0.116534007280817
	100	100

NR1H3-regulon	ASC IgM
0.00334727473259459	-0.00522592560476547	6904757
0.454914478009596	8.37451352987671e-05
0.000103407036629782	14.2433234421365 22.1630142765159
NR2C2-regulon	ASC IgM	0.00750301479974394	-0.0143900260051273
	4237281.5	0.279169955112427
4.45636618024241e-45	1.29143672978454e-44	45.6973293768546
76.2050667199538
NR3C1-regulon	ASC IgM	0.197902611070766	-0.0277813013825538
	2809190	0.185081271141009	1.447988004479e-88
1.02807148318009e-87	100	100
NRF1-regulon	ASC IgM
0.0186750240011935	-0.00811440630384374	4357621
0.287098428312344	1.8299568203856e-41
4.90290317914633e-41	100	99.9666955305402
PAX5-regulon	ASC IgM	0.0845665924721093	-0.0158185684522591
	4370266.5	0.287931567122539
3.73875947270664e-41	9.83155268748784e-41	100	100
POU2F1-regulon	ASC IgM
0.00223648464179098	-0.00266327593775303	7027231.5
0.462983614003373	0.000511907972200862
0.000616024847902732	11.2759643916914 18.3996092275583
POU6F1-regulon	ASC IgM	0.0688078395982239	0.038088587332181
	11658724.5	0.768125883383758
1.91158501871348e-67	1.00535212095302e-66	91.9881305637982
65.1546437531917
REL-regulon	ASC IgM	0.228236421154679	-0.0357410033489111
	2109167.5	0.138960839939379
8.49289538184216e-116	9.27685495555067e-115	100	100
RELA-regulon	ASC IgM
0.084304222306261	-0.00781830460569541	4592829.5
0.302594955127251	6.86781483370494e-36
1.62538284397684e-35	100	100
RELB-regulon	ASC IgM	0.0921144377012664	-0.0131670304561542
	4275231.5	0.281670260979884
1.62697884043623e-43	4.53001951650871e-43	100	100
REST-regulon	ASC IgM
0.209774644822874	-0.00891000034858566	5098082
0.335883118244439	2.54121139401329e-25
4.87637862094442e-25	100	100
RFX5-regulon	ASC IgM
0.0555546079603429	-0.00986714819504123	5132300
0.338137544230543	1.12584140386564e-24
2.13159305798561e-24	100	100
RXRA-regulon	ASC IgM	0.0208998936001903	0.00641741591249043
	8946631	0.589441738689641	6.61132535698276e-09
9.1146427251607e-09	72.700296735905	64.1999156286774
SETDB1-regulon	ASC IgM
0.050616814362891	-0.00916880776403309	6025000.5
0.396952413743895	6.6210425189906e-11

9.89671618628068e-11	94.9554896142433 95.0531761362375
SOX5-regulon	ASC IgM
0.00454420622079757	-0.00328348318391183	7124174.5
0.469370627223633	0.00794278829015314	0.00916972306668086
	17.2106824925816 22.5338040365017
SP1-regulon	ASC IgM
0.00788955005443789	-0.00514251628748833	6098160.5
0.401772502736336	1.23144517659349e-10
1.82151265704453e-10	45.1038575667656 59.9325029418948
SP2-regulon	ASC IgM 0.0346553183932771	0.00646316217076322
	10394521 0.684834831243848	1.12959070640295e-31
2.46772123552645e-31	100	100
SP3-regulon	ASC IgM 0.0284616034699164
0.000193137561535075	7571714.5	0.498856447722228
0.942245496714174	0.955706146667234	100	100
SP4-regulon	ASC IgM
0.00329253923549348	-0.00538538141026118	6357137
0.418834965515874	1.39759262720263e-10
2.04596034085333e-10	13.353115727003	29.1325295854704
SPI1-regulon	ASC IgM 0.388484950934774	-0.0302829330604887
	4348704.5	0.286510971730863
1.10391782833999e-41	3.01454483892845e-41	100	100
SPIB-regulon	ASC IgM 0.541226858170966	-0.0233981359629862
	5144975 0.338972626625009	1.94402319287945e-24
3.63225386038003e-24	100	100
SREBF1-regulon	ASC IgM
0.0322515082654652	-0.00874330691526768	5573670
0.367216859137511	4.00937922506902e-17
6.62013779023024e-17	95.5489614243323 96.9049046382025
SREBF2-regulon	ASC IgM 0.0886277381579272	-0.0104323753632783
	4126109.5	0.271845475431349
2.33921862072395e-47	7.72486149169306e-47	100	100
SRF-regulon	ASC IgM
0.0143209051577684	-0.00649651957116463	5194726.5
0.342250465027243	1.61438142576413e-23
2.86552703073133e-23	97.0326409495549 97.9417837873843
STAT1-regulon	ASC IgM 0.0974498738656476	0.0178136627275513
	11677955.5	0.769392902675907
2.59364790326231e-65	1.31535000808303e-64	100	100
STAT2-regulon	ASC IgM 0.0839384328888943	0.0178365473128387
	10639215 0.700956302757195	3.94570418911227e-37
1.00051784795347e-36	100	99.9622549346122
STAT5A-regulon	ASC IgM
0.0647882401828743	-0.00687061508448136	6354863
0.418685144816464	2.58115961568267e-07	3.4577798625183e-07
	100	100
TBP-regulon	ASC IgM 0.0130968296489564	-0.007862831542647
	6649244 0.438080205200333	2.71835358119575e-05
3.41598414628138e-05	41.2462908011869 49.9500432958103
TCF12-regulon	ASC IgM

0.00618225174628441	-0.00288287218099285	7218235.5
0.475567762143235	0.0424280787518719	0.0463445167905063
	21.0682492581602 25.2514487444215
TFDP1-regulon	ASC IgM 0.494473095327819	-0.0339422907095336
	4447411 0.29301417176001 2.75144947670653e-39
7.10374228531505e-39	100	100
TFDP2-regulon	ASC IgM 0.0327047723533541	0.00562338255985192
	10377348 0.683703401661191	2.62413719982296e-31
5.64587094507364e-31	100	99.995559404072
TFEC-regulon	ASC IgM 0.0760661139992432	-0.015591224822102
	4567656 0.300936418901838	1.82012604259126e-36
4.45617065599929e-36	100	100
THAP1-regulon	ASC IgM 0.0323520493565443	-0.0048490769687152
	5048930.5	0.332644810369753
2.89145149103998e-26	5.86551588182396e-26	100	100
THAP11-regulon	ASC IgM
0.170646647681681	-0.00788965743243683	5059379.5
0.333333234507014	4.6057413929361e-26	9.0835455249573e-26
	100	100
TP53-regulon	ASC IgM
0.0887570977225554	-0.00967142249442791	5645496.5
0.371949091532475	4.95133039198289e-16
8.08148178921346e-16	100	100
USF2-regulon	ASC IgM 0.116635405730208	-0.0087155281564042
	4657587 0.306861452023479	1.98837619387303e-34
4.48173681793604e-34	100	100
XBP1-regulon	ASC IgM 0.31343516079359 0.119029304995454
14621954 0.96335592568867 2.00378089580392e-189
2.65660850027957e-187	100	100
YBX1-regulon	ASC IgM
0.0903219826432203	-0.00489815714427357	5433603.5
0.357988688076005	2.3165585786298e-19
4.01160144104185e-19	100	100
YY1-regulon	ASC IgM 0.601242099314868	-0.0385152794663985
	4213401.5	0.277596640115988
4.35088013889441e-45	1.28713537442293e-44	100	100
YY2-regulon	ASC IgM 0.0239146588756049	-0.0272900769478265
	4009414.5	0.264157117244185
1.59982379083085e-50	5.67937445744951e-50	73.8872403560831
90.6547658695797
ZBTB33-regulon	ASC IgM 0.340884865441482	-0.0251277680667447
	3822355 0.25183284938085 1.0576911114357e-55
4.29120393782484e-55	100	100
ZNF143-regulon	ASC IgM
0.0209621163896133	-0.00238634241502332	6359361.5
0.418981524946761	2.85255115380889e-07
3.78562863402676e-07	100	99.993339106108
ZNF274-regulon	ASC IgM 0.0663696593516593	0.00103582144599904
	7890410 0.519853449792903	0.208458068535507
0.219267005422534	100	100

ZNF76-regulon	ASC IgM
0.0135130737396063	-0.00105184478683829	7085816.5
0.466843440597443	0.0356727869012441	0.0395744979685676
99.406528189911	99.4604675947512
ATF1-regulon	B EIF5A	0.107262018183195	0.00109059984857016
52288738	0.530829976607995	6.49429599793748e-07
9.31505082532447e-07	100	100
ATF3-regulon	B EIF5A	0.0542854781748648	0.00343684197485872
56468968	0.573267248532898	2.89170442610029e-32
8.05141232365178e-32	100	100
ATF4-regulon	B EIF5A
0.107832511986311	-0.000967892027727277	49032719
0.497775201225862	0.719536710938601	0.729815806809153
100	100
ATF5-regulon	B EIF5A
0.111860418892572	-0.00692173301527618	42956717
0.436092243807189	6.05289589874564e-25
1.50791441688049e-24	99.9562554680665 100
ATF6-regulon	B EIF5A	0.126519312455805	-0.0104195606851842
36280357	0.368314512728146	3.03907341214035e-100
2.53852014425841e-99	100	100
ATF6B-regulon	B EIF5A	0.0598402121205192	0.00267864848483335
54591216.5039062 0.554204505371129
2.14925056535399e-18	4.62414515576162e-18	99.912510936133
99.9210953817591
BACH1-regulon	B EIF5A	0.0488358619319509	0.00584762379541885
57106277	0.579737145005865	6.68819581988693e-38
2.06461697048683e-37	99.9562554680665 99.542817359016
BATF-regulon	B EIF5A	0.019925807688535
0.000666007197832712	51337751	0.521175652822928
0.000285631110435311	0.000365401961097425	54.5494313210849
51.5664887444883
BATF3-regulon	B EIF5A	0.084689298919873	0.00160662419239002
50803369	0.515750660837852	0.0110179219561149
0.0134874561876579	100	100
BCL11A-regulon	B EIF5A	0.202597659634682	-0.0020036121610521
46503991.5	0.472103815550557
6.71912078920116e-06	9.35407011829966e-06	100	100
BHLHE40-regulon	B EIF5A	0.0593791452417265	0.00392574416319359
55734543.5	0.565811445331923
2.35819675365048e-26	5.97971319675658e-26	100	100
BRF2-regulon	B EIF5A	0.0430970430021434	0.00156347403585166
51885004.5	0.526731314973421
1.60044451187924e-05	2.16441067320811e-05	99.737532808399
99.7284752842887
CEBPB-regulon	B EIF5A	0.144010128447368
0.000966814490405171	51699161	0.524844650568598
6.07483732464121e-05	7.98728611202826e-05	100	100
CEBPG-regulon	B EIF5A
0.0678546723445331	-0.000216993314925196	49476425.5

0.502279664711208	0.712922960663134	0.728309787152267
	100	100
CLOCK-regulon	B EIF5A	0.0186026601781033	0.00316750911019067
	52660263	0.534601660810036	2.79736645354448e-09
4.4136226267035e-09	55.3805774278215	50.8656300765839
CREB1-regulon	B EIF5A
0.0425780059794145	-0.000414049844086904	48241670
0.48974455183123	0.0978815717113085	0.110310977642903	100
	99.9930378278023
CREB3-regulon	B EIF5A	0.224772158144463	-0.0158251094340668
	33047224.5	0.335492078777923
2.4805212720017e-155	4.40292525780301e-154	100	100
CREB3L2-regulon	B EIF5A
0.120252825292387	-0.00687050722031783	43081024
0.437354195891445	4.94136204532159e-24
1.16945568405944e-23	100	100
CREB3L4-regulon	B EIF5A	0.0162254092414996	0.00539590523391537
	54424555.5	0.55251257972539 9
6.67991241203961e-25	1.63542683191315e-24	39.9825021872266
30.888837317243
CREB5-regulon	B EIF5A	0.0416421946039159	0.00259880732797826
	54433933	0.552607779156406	2.05204351252734e-17
4.34910714595346e-17	99.5625546806649	99.6263634253887
CREM-regulon	B EIF5A	0.140300565143333	-0.0102135414573265
	32471890	0.329651341157199	2.07145892921127e-166
4.90245279913334e-165	100	100
CTCF-regulon	B EIF5A	0.356074553286215	-0.0155490445795856
	34826137	0.353551418453756	1.62278373437998e-123
1.64596635915684e-122	100	100
CUX1-regulon	B EIF5A
0.0691235711986506	-0.00797633451030395	36904135.5
0.374647048934386	5.14351546595012e-91
3.31990543711326e-90	100	100
DDIT3-regulon	B EIF5A
0.0842149316422637	-0.000729970814526679	48832742
0.495745054959335	0.492246394451911	0.510211591329718
	100	100
E2F1-regulon	B EIF5A	0.4068888909496	-0.03244035198157
32993912.5	0.334950860749044	2.42216745938266e-156
4.9135397033191e-155	100	100
E2F2-regulon	B EIF5A
0.0498402637834519	-0.00429721785420516	41835192
0.424706635504398	5.58258933056317e-34	1.6515160102916e-33
	100	100
E2F3-regulon	B EIF5A	0.0242526316534494	0.00330377011461408
	60928262.5	0.618537555020754	1.3734259084794e-81
	8.12610329183648e-81	100	100
E2F4-regulon	B EIF5A
0.390735856019437	-0.00834192395639022	44734443
0.454139538255096	1.34348637604895e-13

2.61335706025961e-13	100	100
E2F6-regulon	B EIF5A	0.0753573814560314	0.00311299167530632
	52433262 0.53229716963031 1.8610258826325e-07
2.72438840550324e-07	100	100
E2F7-regulon	B EIF5A
0.0300757104907388	-0.000524753330398454	48094450.5
0.488249994365696	0.0579031093658666	0.0657779322396245
	100	100
E2F8-regulon	B EIF5A
0.0700533725808572	-0.00499487532554845	44211890
0.448834633080937	1.48127266695356e-16	3.0932458633442e-16
	100	100
EGR1-regulon	B EIF5A	0.0487058205929684	0.00130874583075925
	53331978 0.54142084351315 2.30522758056812e-11
4.14357362583131e-11	100	100
EGR2-regulon	B EIF5A
0.0449182651119381	-0.00118957318370819	48589229
0.493272935626607	0.277595674438493	0.298625649774742
	100	99.9953585518682
EGR3-regulon	B EIF5A	0.0439386374797361	0.00259839951247336
	55453031.5	0.562953563996656	2.9723909844465e-24
	7.15389016595599e-24	100	99.9953585518682
ELF1-regulon	B EIF5A	0.678765573354329	-0.0297793207895184
	30324630 0.307852574937764	3.68248669362946e-211
1.30728277623846e-209	100	100
ELF2-regulon	B EIF5A	0.0340516400716322	0.00146961590840498
	52061573 0.528523820516866	4.15055920165276e-06
5.89379406634692e-06	99.6062992125984 99.5312137386865
ELF4-regulon	B EIF5A	0.0217757077564474	0.00350519423629235
	57695561.5	0.585719501614863
1.56696722196207e-43	5.17463594229334e-43	98.8188976377953
98.3105128800186
ELK1-regulon	B EIF5A
0.0646720755876952	-0.00145626078174889	46591829
0.47299553296149 1.30971327179285e-05	1.80562412227752e-05	100
	100
ELK3-regulon	B EIF5A
0.0593299758282606	-0.00587126926407152	40207101.5
0.408178425509529	1.090274545863e-49	4.3005273753485e-49
	100	100
ELK4-regulon	B EIF5A	0.0738695996333791	0.0304043065138739
	76211260 0.773688999016687	0	0
99.5188101487314 96.5815734509167
ESRRA-regulon	B EIF5A
0.0909157915412883	-0.000863432869719599	47710695.5
0.484354152441318	0.0115628123553439	0.0140334987560584
	100	100
ETS1-regulon	B EIF5A	0.221347016752729	-0.0227374819209238
	30881504.5	0.313505908506621
4.86903937253463e-199	1.38280718179983e-197	100	100

ETV2-regulon	B EIF5A	0.0301901744485001	0.0016724027523052
	53994149.5	0.548143141570056
7.83479152057849e-15	1.58934342274592e-14	100	100
ETV3-regulon	B EIF5A	0.0140775002751472	0.00278894935595978
	56520565.5	0.573791061131283
7.99819822125138e-33	2.27148829483539e-32	88.4951881014873
84.1285681132513
ETV5-regulon	B EIF5A	0.0343917201942661
0.000977314176565623	52777563 0.535792478539393
7.61137565733789e-09	1.17479928624128e-08	100	100
ETV6-regulon	B EIF5A
0.456070823522664	-0.00368996870214311	46836764
0.475482088294312	7.58493925316257e-05	9.8812970087072e-05
	100	100
ETV7-regulon	B EIF5A
0.0558639548683323	-0.00864779221509191	34887732
0.354176724660404	1.76157429090712e-122
1.66762366205874e-121	100	100
FLI1-regulon	B EIF5A	0.132325495700467	-0.0122217395715895
	36601844 0.371578216217983	1.97478575352188e-95
1.47589251052688e-94	100	100
FOS-regulon	B EIF5A
0.0803422721987433	-0.00625012797026644	40381130
0.409945145230019	7.30282286416764e-48
2.80270499111299e-47	100	100
FOSB-regulon	B EIF5A	0.0421724718330355
0.000736072558811869	50672090 0.514417929715156
0.0199636846993428	0.023623693560889	99.6062992125984
99.3084242283592
FOSL1-regulon	B EIF5A	0.0632864043502608	0.00262493969618349
	53676055.5	0.544913883472851
4.19779398388395e-13	8.05522629339894e-13	100	100
FOXO1-regulon	B EIF5A	0.0224708063314083	0.00608013257176618
	55836074 0.566842172693138	3.16558618481914e-30
8.48138185366638e-30	60.673665791776	51.9006730099791
FOXO3-regulon	B EIF5A
0.019607533512805	-0.000143614325414217	49471113.5
0.502225737824777	0.69664978117215 0.716842528452502
46.6754155730534 46.7649106521235
GABPA-regulon	B EIF5A	0.0267052552520852	0.00128190964203426
	53169358 0.539769941730131	1.37350570440281e-10
2.37850987835608e-10	100	100
GTF2B-regulon	B EIF5A
0.0716839911569304	-0.00776413943100575	39226359
0.398222026899689	1.22986573039476e-60
5.13649805047224e-60	100	100
HINFP-regulon	B EIF5A	0.0165991427778977
0.000388151377779148	49125872.5	0.498720886130821
0.832282094458174	0.838184804347948	61.3298337707786
63.3348804827106

HIVEP3-regulon B EIF5A
0.0692580022612606 -0.00887718960555701 41854961.5
0.424907333467744 8.29283891705076e-34 2.4032308698392e-33
99.4313210848644 99.7075887676955
HOXB2-regulon B EIF5A 0.0371321318818938
3.09674692861722e-05 50203756.5 0.509663455418038
0.118044335116536 0.130955434269907 83.7707786526684
83.7270828498492
IKZF1-regulon B EIF5A
0.139079609334253 -0.00503835109626907 39059508
0.396528172432844 1.30666520725345e-62 5.7983268571872e-62
100 100
IRF1-regulon B EIF5A
0.130245650073889 -0.00547249275160103 43779477.5
0.444444825140649 3.06050365132549e-19
6.68602336135723e-19 100 100
IRF2-regulon B EIF5A
0.0838655558726579 -0.00121613320078573 47595145
0.483181095458914 0.00663673438967081 0.00826680950292329
100 100
IRF3-regulon B EIF5A
0.137033614406387 -0.00885923425906285 33369159.5
0.338760330318422 2.66286662384142e-149
4.20141178428313e-148 100 100
IRF4-regulon B EIF5A 0.0584877749957086
7.94263576568421e-05 51745127 0.525311292748884
4.4038799633472e-05 5.84440144668508e-05 100
99.9953585518682
IRF5-regulon B EIF5A 0.0527272932557965 0.00598400544064977
53915410.5 0.547343791210364
1.72275868303595e-14 3.44551736607191e-14 86.6141732283465
80.15316778835
IRF7-regulon B EIF5A
0.0998140532492855 -0.00844615107647262 38288554
0.388701525444618 3.77063522509592e-72
1.98307482208748e-71 100 100
IRF8-regulon B EIF5A
0.0732676393195593 -0.00895650559126174 36665317
0.372222587690579 1.70420392692514e-94
1.20998478811685e-93 100 100
IRF9-regulon B EIF5A 0.0380308805833437 0.00433294934410479
60063081 0.609754319988256 3.26023933795346e-70
1.65340709281925e-69 100 100
JUN-regulon B EIF5A 0.0891783387635406
0.000819332630108915 49723651.5 0.504789478044184
0.439514662178695 0.458905014921872 100 100
JUNB-regulon B EIF5A
0.158898636810618 -0.000930743952087448 47597079.5
0.483200734307144 0.00670052075295063 0.00827368649494773
100 100

JUND-regulon	B EIF5A	0.0404270390561339	0.0020738288446035
	54127603.5	0.549497953072645
1.36162096421126e-15	2.80217647707244e-15	100	100
KLF11-regulon	B EIF5A	0.0246484770339974	0.0028558649471481
	50993950.5	0.517685424939195	0.00135023365956178
	0.00171190338980155	43.0008748906387 40.7449524251566
KLF12-regulon	B EIF5A
0.0415195398326602	-0.00582346571224631	42232906
0.428744187784139	1.31022344856509e-30
3.57791787877389e-30	97.7252843394576 98.6493385936412
KLF13-regulon	B EIF5A
0.0632851021664498	-0.00316652302595906	45448469
0.461388257948378	4.60897818840792e-10
7.79136788992768e-10	100	100
KLF2-regulon	B EIF5A
0.106032476146417	-0.00296527888403024	45996552
0.466952341098927	9.61589333223278e-08	1.4223508887261e-07
	100	100
KLF3-regulon	B EIF5A	0.0731532391396434	0.00118365321284979
	50524139 0.512915946135649	0.0371045655930983
0.043187281264098	99.6937882764654 99.872360176375
KLF4-regulon	B EIF5A	0.0173758566328049	0.00155437284825685
	50400020.5	0.511655907684317	0.0441402378666704
	0.0505476917505419	50.3937007874016 49.7818519378046
KLF6-regulon	B EIF5A	0.0723052245291627	0.00308054235937233
	52513921 0.5331160116357	9.04825314897677e-08
1.35247573384705e-07	100	100
KLF7-regulon	B EIF5A	0.0489235173456834	0.00307994407330307
	53046275.5	0.538520420645957
5.06177800412963e-10	8.4561467833695e-10	98.9938757655293
97.4796936644233
KLF8-regulon	B EIF5A	0.0269807976282167	0.00197349683999223
	52029721 0.528200462236256	4.71287727412354e-06
6.62602547451032e-06	80.6211723534558 77.4773729403574
MAFB-regulon	B EIF5A
0.0556815259363517	-0.000106757798737554	49792213.5
0.505485512529778	0.375966740604046	0.401408098990786
	100	99.9976792759341
MAFF-regulon	B EIF5A	0.014395923295826	0.00211556255085397
	50799541.5	0.515711804445192	0.00146346507034661
	0.00183904460167451	31.1023622047244 28.4103040148526
MAFG-regulon	B EIF5A	0.0271285690758412	0.00181252995641345
	50558990.5	0.513269755036712	0.0262838640668385
	0.030845526425546	59.7550306211724 58.5402645625435
MAX-regulon	B EIF5A	0.19432072309693 -0.00352718302041763
41370908.5	0.419993276397424	3.80429422505037e-38
1.20046617768256e-37	100	100
MAZ-regulon	B EIF5A
0.129436207390541	-0.00723194424235907	38851698
0.394418506343008	4.09950016520081e-65

1.94043007819505e-64	100	100
MEF2A-regulon	B EIF5A	0.0464670166025497	0.00601577015625621
	59837644	0.607465706378255	2.15976180366767e-67
1.05753853834762e-66	100	99.9976792759341
MLX-regulon	B EIF5A	0.0530349123526026	0.00011526132894879
	49970714	0.507297631541706	0.238866398420329
0.260915604428359	99.9562554680665	99.9907171037364
MXD4-regulon	B EIF5A	0.0704977753933591	-0.0228210574549721
	45520719	0.462121732636751	9.47459242604127e-10
1.52885468692939e-09	83.4645669291339	88.8767695521003
MXI1-regulon	B EIF5A	0.0681339026071762	0.00305843194439363
	53010188	0.538154063997976	7.36682172462019e-10
1.21638219173961e-09	100	100
MYBL1-regulon	B EIF5A	0.0200265611407815	-0.0103869421106813
	42635059	0.432826804342657	1.36080190447254e-27
3.51334309882002e-27	75.4593175853018	80.304014852634
MYC-regulon	B EIF5A
0.313460290425754	-0.00149688007217058	48540836
0.49278165478793	0.244007580023308	0.264496766132136	100
	100
NFATC1-regulon	B EIF5A	0.0581158482072139	0.00800109358963407
	57812457.5	0.586906217977104
1.05829652097026e-44	3.57805014232803e-44	96.8066491688539
94.7226734741239
NFE2L1-regulon	B EIF5A	0.182852564820755
0.000195259257902675	50026393	0.507862879115047
0.204421472159017	0.225022085632406	99.8687664041995
99.9396611742864
NFE2L2-regulon	B EIF5A
0.110648802473326	-0.000244019597101922	50222506.5
0.509853803520557	0.111747637813561	0.124946177712801
	100	100
NFKB1-regulon	B EIF5A	0.0630347110225679	0.00670363728390446
	57388218	0.582599381505717	1.52010908634357e-40
4.90580659683608e-40	99.912510936133	99.9326990020887
NFKB2-regulon	B EIF5A	0.0755823345534526	0.00232276007034984
	52854109	0.536569565785015	3.58540654999231e-09
5.59481022086712e-09	100	100
NFYA-regulon	B EIF5A	0.0248142495783574	0.00407093325994591
	59302029	0.602028197101958	6.31350623503008e-61
2.71672086477052e-60	99.6937882764654	98.9812021350661
NFYB-regulon	B EIF5A	0.0981996095059098	-0.0109783669597918
	36318918	0.368705980097812	1.16509093143101e-99
9.19127290351129e-99	100	100
NFYC-regulon	B EIF5A
0.0759616691492137	-0.00797483421662767	34130735
0.346491767723743	1.62780730712566e-135
2.10135125101676e-134	100	100
NR1H3-regulon	B EIF5A	0.010982813555525	0.00257831854992472
	51461091.5	0.522427793096993

6.20647339286823e-07	8.99305328354376e-07	26.0279965004374
21.8960315618473
NR2C2-regulon	B EIF5A	0.024789638365988	0.00316280922944907
	53086482	0.5389285929651	2.49951059242492e-10
4.27627113402818e-10	79.2650918635171	75.8041308888373
NR3C1-regulon	B EIF5A
0.219262662778599	-0.00654463510499431	38007382
0.385847095754943	8.41950434516381e-76
4.78227846805305e-75	100	100
NRF1-regulon	B EIF5A	0.0277209626883117	0.00104441328261449
	53001035	0.538061143668251	8.09745722297972e-10
1.32165393754382e-09	100	99.9651891390114
PAX5-regulon	B EIF5A	0.0953657775376852	-0.0051619559755333
	41592870	0.422246607083142	4.01198268762025e-36
1.21213093966399e-35	100	100
POU2F1-regulon	B EIF5A	0.0058186165643322	0.00098843196000863
	52177427	0.529699958600557	1.2319479568126e-12
2.33248813156519e-12	24.0157480314961	18.045950336505
POU6F1-regulon	B EIF5A
0.0273390783755196	-0.00385738278921623	45898623.5
0.465958180877193	1.99733807869994e-08
3.01725539548289e-08	59.8862642169729	65.6440009282896
REL-regulon	B EIF5A
0.262160022395524	-0.00163429380197327	48731890.5
0.494721220737405	0.394222167605431	0.41775781940277	100
	100
RELA-regulon	B EIF5A
0.0919078515234028	-0.000164918583868426	48768123.5
0.495089054486662	0.428000591884298	0.450193215167188
	100	100
RELB-regulon	B EIF5A
0.100125642407282	-0.00532637410012937	40540803.5
0.41156613444322	3.22038716921757e-46	1.14323744507224e-45	100
	100
REST-regulon	B EIF5A
0.213889838404005	-0.00497949597963646	40415939
0.410298522675383	1.67686266088571e-47
6.26617099594136e-47	100	100
RFX5-regulon	B EIF5A
0.0562797355565817	-0.00954985142143264	34398736.5
0.349212491830259	7.98798096528382e-131
8.72533305438694e-130	100	100
RXRA-regulon	B EIF5A	0.016962445221413	0.00256134457292306
	53320958.5	0.541308974664312
8.80707136299535e-12	1.60333863275044e-11	69.4663167104112
63.9870039452309
SETDB1-regulon	B EIF5A
0.0594907067007116	-0.000238853426858733	49254394
0.500025623392574	0.996700748593361	0.996700748593361
	92.3884514435696	95.1937804595034

SOX5-regulon B EIF5A 0.0118915465958711 0.004305131532586
54224006.5 0.550476626572758
7.63788358182686e-29 2.00848049744336e-28 31.6272965879265
22.0097470410768
SP1-regulon B EIF5A 0.0181143807500735 0.00539215889006672
57759285.5 0.586366421214057
4.18397011371074e-47 1.52339424653058e-46 70.3412073490814
59.264330471107
SP2-regulon B EIF5A
0.0277983157096507 -0.000465281800028503 47761630
0.484871234330798 0.0146159771944897 0.0175887183187927
100 100
SP3-regulon B EIF5A 0.0296165572923499 0.0014180994959555
53574962.5 0.543887597567361
1.40674980833534e-12 2.62840095767919e-12 100 100
SP4-regulon B EIF5A 0.0109735081871126 0.00245949068953154
52490201 0.532875208596141 3.57958671400193e-11
6.35376641735342e-11 34.7331583552056 28.7119981434207
SPI1-regulon B EIF5A 0.395361686213455 -0.0244110996079357
29456529.5 0.299039706512666
8.91407048639531e-231 4.21932669689378e-229 100 100
SPIB-regulon B EIF5A 0.54360217005885 -0.0219551286473042
33412072 0.33919597367572 1.66002869670064e-148
2.35724074931491e-147 100 100
SREBF1-regulon B EIF5A
0.0372939532028227 -0.00382881918494328 43692645.5
0.443563315463961 8.32018391895043e-20
1.84604080701713e-19 94.5319335083115 97.0201902993734
SREBF2-regulon B EIF5A
0.0944990948881601 -0.00472139872350062 39054942
0.396481818862918 1.15265049847586e-62
5.27988292850231e-62 100 100
SRF-regulon B EIF5A 0.0231183301147838 0.00247378068888025
55161545.5 0.559994427622748
3.55955859995453e-22 8.15253743860553e-22 98.9063867016623
97.8834996518914
STAT1-regulon B EIF5A
0.0785666412230692 -0.00126563028088776 46615188.5
0.473232676241532 1.55887057980338e-05
2.12845790703924e-05 100 100
STAT2-regulon B EIF5A 0.0681057939895882 0.00197072248150459
51534971 0.523177810304462 0.000183391817937078
0.000236742164973319 99.9562554680665 99.9628684149455
STAT5A-regulon B EIF5A 0.0745104412722433 0.00305660162082272
53441008.5 0.542527710115372
6.70048167149167e-12 1.23567324331405e-11 100 100
TBP-regulon B EIF5A 0.0639999140864883 0.0453851076720392
80243141.5 0.81462025198231 0 0
88.1014873140857 47.8579716871664
TCF12-regulon B EIF5A 0.0114629884274931 0.00254762189572501

51246906.5	0.520253408652301	1.8235742102636e-05
2.4429013005418e-05	28.3902012248469 25.0522162914829
TFDP1-regulon	B EIF5A	0.508380097489148	-0.0208327384821119
38258787.5	0.388399338948958
1.56399277378684e-72	8.54180668760502e-72	100	100
TFDP2-regulon	B EIF5A
0.0270422316812533	-8.52150945480044e-05	50504303
0.512714573071033	0.0401589372226185	0.0463623502895271
100	99.9953585518682
TFEC-regulon	B EIF5A	0.0940809889907786	0.00267416553384214
53288912.5	0.540983646915335
3.72325422309098e-11	6.52718641578913e-11	100	100
THAP1-regulon	B EIF5A	0.0382507813314205	0.00114326489900245
52917087.5	0.537208917143654
1.90793502381519e-09	3.04412104923323e-09	100	100
THAP11-regulon	B EIF5A
0.175761753088677	-0.00286004312257296	43521888
0.441829802604449	6.08184545434812e-21
1.37082865796418e-20	100	100
TP53-regulon	B EIF5A	0.108066947739947	0.0102254016398844
61617135 0.625530918927545	2.87132892932012e-91
1.94156527601646e-90	100	100
USF2-regulon	B EIF5A
0.120859351169776	-0.00466170629775849	40190536.5
0.40801025930589 7.27612683659699e-50	2.95202860227649e-49	100
100
XBP1-regulon	B EIF5A	0.18279372915476 -0.0131590794697917
34334771.5	0.348563125623453	6.17239904143782e-132
7.30400553236809e-131	100	100
YBX1-regulon	B EIF5A
0.0930438981737781	-0.00225338734059748	44692673.5
0.453715498518127	8.00478142211145e-14
1.57872078047198e-13	100	100
YY1-regulon	B EIF5A	0.614927210739505	-0.0258462301202783
35334527.5	0.358712547361146
4.22688458311727e-115	3.75136006751657e-114	100	100
YY2-regulon	B EIF5A	0.0527885915509142	0.00188131337715656
50722064 0.514925260705837	0.0159553426616987
0.0190391483862287	89.8075240594926 90.5685773961476
ZBTB33-regulon	B EIF5A
0.358980300572642	-0.00720889026332328	43201160
0.438573804405802	3.61547851383747e-23
8.41635981909707e-23	100	100
ZNF143-regulon	B EIF5A	0.0268536454520769	0.00370980614314494
61373174 0.623054251544155	8.89669087654833e-88
5.49273958465157e-87	100	99.9930378278023
ZNF274-regulon	B EIF5A	0.0670056624074452	0.00175241672698287
52737361.5	0.535384356979745
1.12329806742188e-08	1.71514328574093e-08	100	100
ZNF76-regulon	B EIF5A	0.0169065490555792	0.0024740844414755

57945199.5 0.588253801327747
4.88355652529221e-46 1.69137811363779e-45 99.6062992125984
99.4523091204456
ATF1-regulon B LY9
0.102701708080722 -0.00358036005674849 14004290.5
0.444058776003567 3.24291997582238e-07
5.29304179961814e-07 100 100
ATF3-regulon B LY9
0.0488824181950674 -0.00217317503798128 15552173.5
0.493140236458613 0.531015290010309 0.558549416158992
100 100
ATF4-regulon B LY9
0.106801062011555 -0.00198140903356452 15770592.5
0.500066033506019 0.995189606918874 0.998566325283668
100 100
ATF5-regulon B LY9
0.114664005179128 -0.00382901151308394 14843864
0.47068061598718 0.00741626471487056 0.00957372354101473 100
99.9977613610925
ATF6-regulon B LY9
0.134217647025864 -0.00223101053220523 17766041.5
0.563339259701773 7.27853285602432e-09
1.29193958194432e-08 100 100
ATF6B-regulon B LY9 0.0600382989038765 0.00278512086619856
18373790.7460938 0.582610238573389
4.52885151133774e-14 9.45730756779352e-14 100
99.9194089993284
BACH1-regulon B LY9 0.0598419350358669 0.0168208122343383
22535328.5 0.714567467059348
1.70333602858726e-85 4.83747432118782e-84 100
99.5567494963062
BATF-regulon B LY9
0.0152140554905628 -0.00414377026148587 14961737.5
0.474418239262936 0.0131398393924507 0.016084975808
47.3087818696884 51.7864338482203
BATF3-regulon B LY9 0.0683099708976349 -0.0150884027733824
12205815.5 0.387031352359862
5.92218680752682e-25 1.55731579012742e-24 100 100
BCL11A-regulon B LY9 0.215236117320346 0.0109054624563451
19480616 0.617706301990486 5.9672467126873e-27
1.80287028340765e-26 100 100
BHLHE40-regulon B LY9 0.0703562279879648 0.0149374618913212
23145553 0.733916933178848 3.0073275798302e-101
2.13520258167944e-99 100 100
BRF2-regulon B LY9
0.0406474774494405 -0.000980107237846355 15310210
0.4854678723608 0.184467345885802 0.203057078416929
99.7167138810198 99.7291246921872
CEBPB-regulon B LY9
0.139968445430035 -0.00317294318788722 15304438

0.485284849361164	0.17900039059508 0.198578558316417	100
	100
CEBPG-regulon	B LY9
0.0593882090642635	-0.00880959211429685	13002714
0.412300020737533	1.15572362942319e-15
2.52481162120143e-15	100	100
CLOCK-regulon	B LY9	0.0199754363325252	0.00444994534007528
	17429381 0.552664170552576	3.08549498403229e-07
5.09465450851844e-07	59.2067988668555 50.9648533691516
CREB1-regulon	B LY9	0.0446100380586315	0.00166474322464401
	16834625 0.533805191486069	0.0020204433364789
0.00273240908361909	100	99.9932840832774
CREB3-regulon	B LY9
0.233324727519268	-0.00657762654046978	14446882
0.458092806485838	0.00012965412874725	0.00019178006543864
	100	100
CREB3L2-regulon	B LY9	0.12638590639485 -0.000397480815270967
18892331 0.599052510351327	1.4853080583381e-19
3.5748092251527e-19	100	100
CREB3L4-regulon	B LY9	0.012459950444924	0.00138007831152889
	16429742 0.520966851021434	0.019905225849577
0.0237524543751255	35.6940509915014 31.2782628162077
CREB5-regulon	B LY9	0.03593733511178 -0.00328813737525351
15262445 0.483953303133904	0.142800665036167
0.162221555481086	99.4334277620397 99.6261473024401
CREM-regulon	B LY9	0.143104869528493	-0.0070036576139498
	11550469 0.36625112328305 2.60324220634596e-34
9.99082144057097e-34	100	100
CTCF-regulon	B LY9	0.36206955228623 -0.00890931847570314
12506142.5	0.396554351045216	3.48586115796417e-21
8.53434973156746e-21	100	100
CUX1-regulon	B LY9
0.0745368612676811	-0.00219536162696822	15295160.5
0.484990671280926	0.170467058232787	0.190600962748471
	100	100
DDIT3-regulon	B LY9	0.092531316772253	0.00774367247057173
	19409158 0.615440456961374	5.50530634745973e-26
1.56350700267856e-25	100	100
E2F1-regulon	B LY9	0.405139320587518	-0.033070142637915
	9257328 0.293538451001395	2.68290545687322e-79
5.44246535537139e-78	100	100
E2F2-regulon	B LY9	0.0525081185162547	-0.001435203514261
	16253222.5	0.515369635431629	0.160435276494957
	0.180808010018126	100	100
E2F3-regulon	B LY9	0.0231010916359227	0.00201717426468389
	18028590.5	0.571664364610226
5.96360483377033e-11	1.09978167064336e-10	100	100
E2F4-regulon	B LY9	0.369850368047542	-0.0292624447166676
	9976535 0.316343617754626	3.90054657283921e-63
3.95626866673691e-62	100	100

E2F6-regulon	B LY9	0.0591009716952773	-0.0135104552947174
	11763898.5	0.373018709440524
4.29839096965804e-31	1.41946864579405e-30	100	100
E2F7-regulon	B LY9	0.032562093879538	0.0020194876123412
	18073748.5	0.573096269083128
2.46535755430843e-11	4.73082125286212e-11	100	100
E2F8-regulon	B LY9
0.067797535459356	-0.00710969426915428	12072904
0.382816892655045	1.00054418065684e-26
2.95994320110981e-26	100	100
EGR1-regulon	B LY9	0.0494848027632652	0.00205374866368488
	18029787.5	0.571702320003602
5.82685644238445e-11	1.08870212476131e-10	100	100
EGR2-regulon	B LY9
0.0452738234829196	-0.000786319495059987	15863135
0.503000442020204	0.784077129958236	0.812693083606346
	100	99.9955227221849
EGR3-regulon	B LY9	0.0420418985365079
0.000579776330972809	16642490 0.52771282765461 0.0113791686861001
	0.0141740522230369	100	99.9955227221849
ELF1-regulon	B LY9	0.692754808480787	-0.0145156794206889
	12224329.5	0.387618408460914
1.03344776008239e-24	2.66817421694e-24	100	100
ELF2-regulon	B LY9	0.0403836715391173	0.00784974276610189
	20149682 0.638921559487865	6.99715987882354e-37
3.20515065417078e-36	100	99.5276471905082
ELF4-regulon	B LY9	0.0219197952122911	0.00352757850992159
	18799344.5	0.596104023144863
1.68512672025639e-18	3.79822213137155e-18	99.4334277620397
98.3187821804343
ELK1-regulon	B LY9
0.0643232141892095	-0.00175912719729679	14553498
0.461473468323894	0.000434189063572426
0.000610444029973113	100	100
ELK3-regulon	B LY9	0.0719651066774835	0.00717122688214672
	19556652.5	0.620117325606541
5.35404044905845e-28	1.68949720836956e-27	100	100
ELK4-regulon	B LY9	0.0470795040653397	0.00211538372929196
	16527450.5	0.52406506702282 0.0279672168174376
0.0330945399006345	97.8753541076487 96.7114394448175
ESRRA-regulon	B LY9	0.0791357039174231	-0.0127991622649816
	11509177.5	0.364941820755417
5.94229746797532e-35	2.41087497272141e-34	100	100
ETS1-regulon	B LY9	0.232809954126705	-0.0102891392364237
	12966048.5	0.411137402963248
4.84779717385496e-16	1.07560499794907e-15	100	100
ETV2-regulon	B LY9	0.0328461564879497	0.00431120831920522
	19706907.5	0.624881726301344
3.96373105850978e-30	1.27920411433725e-29	100	100
ETV3-regulon	B LY9	0.0128610096498295	0.0014545858324131

17001731 0.539103916603408 0.000346265735643484
0.000491697344613747 87.1104815864023 84.3049026192075
ETV5-regulon B LY9 0.0335286890591028
6.60749290137611e-05 16065721 0.509424194169265
0.389430850600538 0.412680453621466 100 100
ETV6-regulon B LY9 0.470810220991784 0.0114128978773999
19398557 0.615104312328812 7.62733806739703e-26
2.12369020700074e-25 100 100
ETV7-regulon B LY9 0.0648811696138257
0.000817814411085693 17186380.5 0.544958924464011
4.02900974499779e-05 6.08637642329454e-05 100 100
FLI1-regulon B LY9 0.149656728165037 0.00581569836965617
17237412 0.546577070376339 2.10375341435198e-05
3.21218263266647e-05 100 100
FOS-regulon B LY9 0.0963145056572438 0.0101956134133453
22695874.5 0.71965818266913 1.65162688458171e-89
5.86327544026507e-88 100 100
FOSB-regulon B LY9 0.0537079256062185 0.0124278065137936
21138033 0.670260950463931 1.61999230597619e-54
1.27799393027011e-53 100 99.3127378553839
FOSL1-regulon B LY9
0.0532933869210775 -0.00761886069751678 14154401.5
0.448818610636008 2.95291895947797e-06
4.65904991384302e-06 100 100
FOXO1-regulon B LY9 0.0222287414158795 0.00561918457377148
17940431.5 0.568868951473538
2.73588621201431e-11 5.17994456141376e-11 61.7563739376771
52.1938661293933
FOXO3-regulon B LY9 0.0234560357467139 0.00377079239050264
16915078.5 0.536356272723295
0.000314365646494648 0.000450908301032727 52.1246458923513
46.6756212222968
GABPA-regulon B LY9 0.0275002512779848 0.00204412863547071
18018739 0.571351985698078 7.2149509199042e-11
1.31349106490564e-10 100 100
GTF2B-regulon B LY9 0.0731556557181761 -0.0059945940665089
13185971 0.41811087414093 7.52276873334736e-14
1.54816400019612e-13 100 100
HINFP-regulon B LY9 0.0183652646263222 0.00216845730608043
16465573.5 0.522103023684546 0.038392803717011
0.0450560175852526 65.1558073654391 63.2034922766958
HIVEP3-regulon B LY9 0.0819739725585603 0.00435374452891921
17317852 0.549127723545218 7.23959880054229e-06
1.11741633660544e-05 99.8583569405099 99.6910678307589
HOXB2-regulon B LY9 0.0597920023843358 0.0230478771473103
22055324.5 0.699347132354293
2.29089327258886e-74 3.25306844707618e-73 94.4759206798867
83.5594358629953
IKZF1-regulon B LY9
0.138714123073008 -0.00523140482042786 11577880

0.367120292278725	6.88654830109788e-34	2.5733943651471e-33
	100	100
IRF1-regulon	B LY9	0.145401377178331	0.01011633222352
21714607	0.688543400739829	1.93049851656265e-66
2.28442324459913e-65	100	100
IRF2-regulon	B LY9	0.0951040483151798	0.0102429964954111
	20721717	0.657060083673093	1.17353413789962e-46
6.94341031590606e-46	100	100
IRF3-regulon	B LY9	0.151846307502967	0.00650092585017725
	20430484.5	0.647825460363725
1.56476056667363e-41	8.22948149880207e-41	100	100
IRF4-regulon	B LY9	0.0552400067885167	-0.0032224814958161
	14822039	0.469988572160591	0.00612990183548173
0.0079857436755817	100	99.9955227221849
IRF5-regulon	B LY9	0.0644634193535156	0.0176939613218648
	19861031.5	0.629768808213331
1.25226358688282e-32	4.44553573343401e-32	93.4844192634561
80.2731139467204
IRF7-regulon	B LY9	0.115835612817275	0.00812737042918966
	20828938	0.660459929314818	1.27428203707562e-48
8.6165737745113e-48	100	100
IRF8-regulon	B LY9
0.077434573187421	-0.00440691817191347	13933608.5
0.441817536977178	1.0758018080254e-07
1.79722184399537e-07	100	100
IRF9-regulon	B LY9	0.0377022988716599	0.00384591590526329
	18849743	0.597702097408062	4.55850744999595e-19
1.06116075065479e-18	100	100
JUN-regulon	B LY9	0.0965095963134052	0.00823747897054061
	20244426	0.64192577485127	2.03119159999463e-38
9.61430690664125e-38	100	100
JUNB-regulon	B LY9	0.171973117231321	0.0123832967690701
	20918472.5	0.663298957859684
2.71087111997638e-50	1.92471849518323e-49	100	100
JUND-regulon	B LY9	0.0441978468921254	0.00583088115692668
	20425061	0.647653487869177	1.93629754742134e-41
9.81979470477965e-41	100	100
KLF11-regulon	B LY9	0.065851639879911	0.0446092218014056
	24623049.5	0.780766524547976
2.79917950197073e-182	3.97483489279843e-180	82.0113314447592
40.2081934184016
KLF12-regulon	B LY9	0.0505515515926049	0.00355727396638814
	17083907.5	0.541709632045133
0.000139485891561528	0.000204195841255021	99.2917847025496
98.5918961271547
KLF13-regulon	B LY9	0.0705395775898452	0.00431460934908026
	20790211	0.659231943918607	6.59980600701702e-48
4.25987478634735e-47	100	100
KLF2-regulon	B LY9	0.121228318719712	0.0125756141816202
	20749061	0.65792712818142	3.73704732921285e-47

2.30722052499228e-46	100	100
KLF3-regulon	B LY9	0.0926851398629044	0.0209823851390282
	21787945	0.690868858249765	4.81405712100931e-68
6.21451010166657e-67	100	99.8612043877323
KLF4-regulon	B LY9	0.0186953608090408	0.00283975257515711
	16547993.5	0.524716460210889	0.0157356461042987
	0.0189850583081635	52.1246458923513	49.7761361092456
KLF6-regulon	B LY9	0.0850350555383821	0.0159026053758606
	21649647	0.686483599274757	4.89077238341096e-65
5.34222829572582e-64	100	100
KLF7-regulon	B LY9	0.0615379838903264	0.0157848404756738
	21380542.5	0.677950627548196
2.18994182368416e-59	1.82924552331265e-58	99.7167138810198
97.5218267293486
KLF8-regulon	B LY9	0.0349759777521817	0.0100252355527267
	19918400.5	0.631587908432693
1.26984494053044e-33	4.623537988598e-33	92.2096317280453
77.4054175061563
MAFB-regulon	B LY9	0.0574803508966381	0.00172427328968783
	16951410	0.537508299769604	0.000613910983155078
0.000854660388313933	100	99.9977613610925
MAFF-regulon	B LY9	0.0156154083362493	0.00327949280301193
	16686939.5	0.529122266466521
0.000846784365888873	0.00116741145588563	33.7110481586402
28.4642937094247
MAFG-regulon	B LY9	0.0394213349701077	0.0142354703613184
	19851630.5	0.629470714100445
1.36359570750426e-34	5.37862751293347e-34	74.6458923512748
58.3478844862324
MAX-regulon	B LY9	0.201486605323548	0.00387671268334888
	18090634	0.573631687458105	1.76426561741672e-11
3.43185914620786e-11	100	100
MAZ-regulon	B LY9
0.129938027234084	-0.00646639579544714	12168814.5
0.38585809629445	1.92981632364288e-25	5.26988303764017e-25	100
	100
MEF2A-regulon	B LY9	0.0440037370981556	0.00330077826822682
	17870375	0.56664754628053	1.15410676308239e-09
2.07447038427468e-09	100	99.9977613610925
MLX-regulon	B LY9	0.0574792341258559	0.00462574782734605
	18286510	0.579842673784651	3.06477289336926e-13
6.12954578673852e-13	100	99.9888068054623
MXD4-regulon	B LY9	0.0664689315693134	-0.0261063842197145
	14152857	0.44876963644631	2.84080259993193e-06
4.53251650775657e-06	86.5439093484419	88.6366689053056
MXI1-regulon	B LY9	0.0809277970249861	0.0159463529345525
	22162635.5	0.702749831784994
1.53862182367978e-76	2.73105373703161e-75	100	100
MYBL1-regulon	B LY9
0.02421134792803 6	-0.0057686243629012 9	15809068

0.501286044147481	0.906136505701988	0.932401331954219
	84.8441926345609 79.9843295276472
MYC-regulon	B LY9	0.287886137608992	-0.0274222816324883
	12271920 0.389127444508073	4.26754130666697e-24
1.08212654561913e-23	100	100
NFATC1-regulon	B LY9
0.0419662883614995	-0.00868670930785328	14175883.5
0.449499778355723	3.98466786419813e-06
6.21783337050698e-06	93.7677053824363 94.8444145959257
NFE2L1-regulon	B LY9	0.158270259248782	-0.0247824709248002
	13787074 0.437171108747751	9.59371007968311e-09
1.66134979428659e-08	100	99.9350794716812
NFE2L2-regulon	B LY9
0.107043403599118	-0.00389776995198539	15245482
0.483415427329532	0.129883471142663	0.148737523405308
	100	100
NFKB1-regulon	B LY9
0.0482209390804344	-0.00858137419034707	13798368
0.437529227555425	1.16305544483112e-08
1.98980570079542e-08	100	99.9306021938661
NFKB2-regulon	B LY9
0.0716604738596836	-0.00174324198363757	15471177
0.490571937361235	0.389236252469384	0.412680453621466
	100	100
NFYA-regulon	B LY9	0.0240164383042496	0.0031165218221883
	18405557.5	0.583617523152156
2.23628646839572e-14	4.73959221659989e-14	99.8583569405099
99.0038056861428
NFYB-regulon	B LY9	0.0960069199428106	-0.0128174012516817
	10056001 0.318863386585036	1.82704975445711e-61
1.62150665708068e-60	100	100
NFYC-regulon	B LY9
0.0784537811257311	-0.00516126110024694	12667600.5
0.401673985049951	2.7203702552204e-19	6.4382096040216e-19
	100	100
NR1H3-regulon	B LY9	0.0135235967910218	0.00506806192993417
	17158537.5	0.544076057281252
2.97496975798687e-08	5.02911554326352e-08	29.8866855524079
21.9811954331766
NR2C2-regulon	B LY9	0.025880181419748	0.00415871796069785
	17749884 0.562826925308732	7.56999215992878e-09
1.32708504532085e-08	83.1444759206799 75.8652339377658
NR3C1-regulon	B LY9	0.228884570868929	0.0034608322102665
	17018565.5	0.539637717831298
0.000294764784603758	0.000427108157282996	100	100
NRF1-regulon	B LY9
0.0253682005187395	-0.00138247521510297	14934859.5
0.473565971039749	0.0157763160588965	0.0189850583081635
	100	99.9664204163868
PAX5-regulon	B LY9

0.0991539155588365	-0.00113136628918666	14904161.5
0.472592575328931	0.0123165215575055	0.0152082266188329
100	100
POU2F1-regulon	B LY9	0.00536587919107498
0.000493577839889243	16387211.5	0.519618261332237
0.00794685373443063	0.0101662453179203	22.2379603399433
18.2852025968211
POU6F1-regulon	B LY9	0.0330699883487345	0.00210054055422592
16458351.5	0.521874022973635	0.0413012007375378
0.0480718893830358	67.2804532577904 65.3234833221401
REL-regulon	B LY9
0.256830237585625	-0.00699050896487607	13230497.5
0.419522754527853	1.98992881480518e-13
4.03671273860479e-13	100	100
RELA-regulon	B LY9	0.0966544454257976	0.00466252749353435
19513610.5	0.618752516883333
2.10850175821715e-27	6.50885325362686e-27	100	100
RELB-regulon	B LY9
0.0975715372900946	-0.00773244982699933	11034156.5
0.349879490833313	8.90141807522195e-43
4.86154371800584e-42	100	100
REST-regulon	B LY9	0.223656066877849	0.00511721292739156
19045370.5	0.603905204106158
2.33268586053855e-21	5.81125249467498e-21	100	100
RFX5-regulon	B LY9
0.062659109453694	-0.00273186988569875	15400530.5
0.488331823996053	0.286614227422774	0.313070925338722
100	100
RXRA-regulon	B LY9
0.0133681762576946	-0.00118032708418239	15435057.5
0.489426632573401	0.322948874197244	0.350066718595486
64.3059490084986 64.2623684799642
SETDB1-regulon	B LY9	0.0598804651214988
0.000165513408065754	15975565 0.506565458626084
0.548761471316347	0.572971536227362	94.6175637393768
95.0593239310499
SOX5-regulon	B LY9	0.01007459150816 0.00230718521780054
16525146 0.52399199417066 0.00272501778244646	0.00361637873932148
26.7705382436261 22.4266845757779
SP1-regulon	B LY9	0.0158687907031102	0.00292035443478778
17426516.5	0.552573340791235
6.87409443446114e-07	1.10922887465168e-06	67.4220963172804
59.7022610252966
SP2-regulon	B LY9	0.0285792875727116
0.000344490407320888	16090380 0.51020610063982 0.35130731955052
0.37792151042556 100	100
SP3-regulon	B LY9	0.0320239236145409	0.00381335485821492
19826220.5	0.628664994346327
7.04934473617026e-32	2.44148037203946e-31	100	100
SP4-regulon	B LY9	0.0107409889140256	0.00213630309550755

16494117 0.523008102858165 0.00874853415573243
0.011091891518875 32.8611898016997 28.9545556301769
SPI1-regulon B LY9 0.417098725297297 -0.0014670785009519
14871009.5 0.471541366305377 0.00935111212072518
0.0117509550543626 100 100
SPIB-regulon B LY9 0.543666579225551 -0.0211131377448872
9088678.5 0.288190783403124
2.32672786058816e-83 5.50658927005865e-82 100 100
SREBF1-regulon B LY9
0.0408346258340751 -9.67597350235022e-05 15767889
0.499980308856068 0.998566325283668 0.998566325283668
95.6090651558074 96.9151555854041
SREBF2-regulon B LY9 0.106219286986685 0.00735102676666725
21039967.5 0.667151414432943
1.31091093160434e-52 9.7973343309377e-52 100 100
SRF-regulon B LY9 0.0264879798855211 0.00580918822209148
20112460.5 0.637741311639464
2.75187005154692e-36 1.22114233537394e-35 99.4334277620397
97.9113498992612
STAT1-regulon B LY9 0.0852750976774545 0.00559361789278565
20072221 0.636465366734079 1.19550426547206e-35
4.99298840285389e-35 100 100
STAT2-regulon B LY9 0.0727481816783633 0.00661677670682546
18490815.5 0.58632094915753 3.19256165475648e-15
6.86884477235484e-15 100 99.9619431385718
STAT5A-regulon B LY9
0.0643579059448316 -0.00736450591393296 12721387.5
0.403379504468082 1.10708081017684e-18
2.53557217814696e-18 100 100
TBP-regulon B LY9
0.0208812273245853 -2.03546209970491e-05 15735226
0.498944605419282 0.917894413855948 0.937705084658595
47.8753541076487 49.9171703604209
TCF12-regulon B LY9 0.0112667708812332 0.00225819252535703
16510915.5 0.523540762570465 0.00482621798338741
0.00634558290408345 29.6033994334278 25.1511081262592
TFDP1-regulon B LY9 0.501967610458996 -0.0266097092606765
9450503 0.299663791949905 8.99984549411758e-75
1.41997562240522e-73 100 100
TFDP2-regulon B LY9
0.0263923914300598 -0.000742311834838371 15772065
0.500112724664537 0.991787538788378 0.998566325283668
100 99.9955227221849
TFEC-regulon B LY9 0.101196742555401 0.00980779553618874
20089707.5 0.63701984207766 6.32687120393725e-36
2.72247185139118e-35 100 100
THAP1-regulon B LY9 0.0381256090392047
0.000975676439812716 16827735.5 0.533586733939985
0.00216030616524992 0.00289399505156121 100 100
THAP11-regulon B LY9 0.179499234331596 0.00103766936930905

16340398.5 0.51813387885095 0.097711729662483
0.112805411480265 100 100
TP53-regulon B LY9
0.0886814393239899 -0.00982816819386267 11751997.5
0.372641343411648 2.87220551782131e-31
9.71079008406251e-31 100 100
USF2-regulon B LY9 0.136308913834113 0.0111969203287787
23005570.5 0.729478260786847
1.62725580002886e-97 7.70234412013661e-96 100 100
XBP1-regulon B LY9 0.196349902989977 0.0010767900065701
19345929.5 0.613435559225317
3.79559792601602e-25 1.01693378395146e-24 100 100
YBX1-regulon B LY9
0.0931361625274997 -0.00207996134300149 14420220
0.457247387356193 9.44901415631102e-05
0.000141237895810123 100 100
YY1-regulon B LY9 0.616325541521492 -0.0235116050664732
9991108 0.316805709607312 7.92887656602622e-63
7.50600314917149e-62 100 100
YY2-regulon B LY9 0.0681126186732421 0.0173809905557293
20598009.5 0.653137471454183
1.76885100759828e-44 1.00470737231582e-43 98.5835694050991
90.402955003358
ZBTB33-regulon B LY9 0.372324368327533 0.00660105970448471
18228624.5 0.578007195987446 1.0491691157704e-12
2.06919464499162e-12 100 100
ZNF143-regulon B LY9 0.0274598525303932 0.00419437651877764
20323322.5 0.644427485539217
1.00667655039002e-39 4.92924379846146e-39 100
99.9932840832774
ZNF274-regulon B LY9 0.0709108734550072 0.00565736497111861
19416008.5 0.615657677865569
4.45723072113761e-26 1.29168727020723e-25 100 100
ZNF76-regulon B LY9 0.015402915677565
0.000859176861929859 16865076 0.534770755131588
0.00149635334020971 0.00204309782990172 99.7167138810198
99.4560107454668
ATF1-regulon B PLCG2 0.0871437567811967 -0.0193292470902561
4564108 0.175965860654914 1.20973008876125e-158
6.36228417052213e-158 100 100
ATF3-regulon B PLCG2 0.0392293014639523 -0.0119448970631449
7151722 0.275729434293554 5.20315747354451e-77
1.21122682171036e-76 100 100
ATF4-regulon B PLCG2 0.0823730539372226 -0.0267195311273126
4037541 0.155664453381582 7.01756249553629e-179
5.86172867274208e-178 100 100
ATF5-regulon B PLCG2 0.0857556408142917 -0.0331001604060155
5433261 0.209475421709517 6.35564462475142e-128
2.654416284455e-127 100 99.997767707659
ATF6-regulon B PLCG2 0.107592430001563 -0.0291940328184539

2800020 0.107952732308476 2.87952395120581e-231
1.36297467023742e-229 100 100
ATF6B-regulon B PLCG2 0.0590832703108211 0.00180985270564116
13989026.5625 0.539336733222521 0.00112153066651685
0.00131617648467266 98.7910189982729 99.9352635221108
BACH1-regulon B PLCG2 0.0254820614996661 -0.0180308489200338
6500542.5 0.250623682817398
9.09738697871396e-95 2.53299794309291e-94 96.3730569948187
99.6048842556421
BATF-regulon B PLCG2
0.0154053981599447 -0.00393820685793029 12272585
0.473160578580874 0.0182941728915585 0.0206172424650898
47.1502590673575 51.7757885572695
BATF3-regulon B PLCG2 0.0555968900114848 -0.0279230240903841
6240676.5 0.240604738404832
2.23676944877867e-102 7.38654097038537e-102 100 100
BCL11A-regulon B PLCG2 0.183901824237746 -0.0208647426164945
7119117.5 0.27447239153652 7.44207012543889e-78
1.7612899296872e-77 100 100
BHLHE40-regulon B PLCG2 0.03368961020649 -0.0222454187951622
4368874.5 0.168438775218687 5.28073418974588e-166
3.26027936932137e-165 100 100
BRF2-regulon B PLCG2 0.0261619620884002 -0.0156500688737452
6423242.5 0.247643437602205
5.32867666703709e-97 1.54422874840667e-96 97.4093264248705
99.7589124271715
CEBPB-regulon B PLCG2 0.111183330114296 -0.03232111000223
2628032.5 0.10132187947603 4.39103816659669e-239
3.11763709828365e-237 100 100
CEBPG-regulon B PLCG2 0.0492519139772856 -0.0190519232451358
6945203.5 0.267767263899326
1.93817514819295e-82 4.66476052615929e-82 100 100
CLOCK-regulon B PLCG2 0.016429862775358
0.000845929696433781 12951552.5 0.499337676163625
0.953451850417856 0.960213920278975 49.2227979274611
51.117262316673
CREB1-regulon B PLCG2
0.0397454591921645 -0.00326742975645598 11472244
0.442304014081871 1.76700956129561e-06
2.22048989118563e-06 100 99.993303122977
CREB3-regulon B PLCG2 0.191937389423649 -0.0484812472038214
2164634.5 0.0834559070021613
8.51230318611639e-261 1.20874705242853e-258 100 100
CREB3L2-regulon B PLCG2 0.100136289027772 -0.026985246906415
3025892.5 0.116661082080387
3.36009827371012e-221 9.54267909733674e-220 100 100
CREB3L4-regulon B PLCG2 0.01424521012266 0.00318449988420979
13557681.5 0.52270653841511 0.0222228585715469
0.0246534837278098 34.3696027633851 31.3079000825948
CREB5-regulon B PLCG2

0.0299142887281885	-0.00937970956212293	9995386
0.385364829243323	2.22097668950167e-21
3.62504241274984e-21	99.6545768566494 99.6227425943702
CREM-regulon	B PLCG2	0.121751591970336	-0.0286130702523405
	3747660.5	0.144488321776112
1.52214021689717e-190	1.96494464363089e-189	100	100
CTCF-regulon	B PLCG2	0.356574827899285	-0.0144498040067788
	11068593 0.426741543689142	1.30093598901681e-09
1.82903871723156e-09	100	100
CUX1-regulon	B PLCG2	0.0832787973714758	0.0066657876368756
	16482541 0.635472366746123	3.25814255623567e-29
5.78320303731832e-29	100	100
DDIT3-regulon	B PLCG2	0.0529203972400767	-0.0324011705121235
	3202361	0.123464696605061	1.7156459154964e-213
2.70690800000544e-212	100	100
E2F1-regulon	B PLCG2	0.423188920423986	-0.0146934979899566
	11400196 0.439526255902514	5.48705940983733e-07
6.95680746604376e-07	100	100
E2F2-regulon	B PLCG2	0.0676633429977206	0.0139199706473415
	18421601 0.710231413149389	6.75930047755103e-68
1.49971979345663e-67	100	100
E2F3-regulon	B PLCG2	0.0230154963542859	0.00192475395459239
	14724587 0.567695730303307	2.06369388637942e-08
2.73873394267176e-08	100	100
E2F4-regulon	B PLCG2	0.334761713566725	-0.0647216597364801
	4656064.5	0.179511176555702
3.09680697576457e-155	1.57052353770918e-154	100	100
E2F6-regulon	B PLCG2	0.0523732545613102	-0.0202868256956028
	6612416	0.254936884150929	1.38965638974017e-91
3.65428161746489e-91	100	100
E2F7-regulon	B PLCG2	0.0355421121515557	0.00503229726888751
	16071313 0.61961777063547 3.89015997490562e-23
6.5762228147214e-23	100	100
E2F8-regulon	B PLCG2	0.0898299497072593	0.0152276443938152
	17490246 0.674323699276217	3.00421051419671e-47
6.00842102839342e-47	100	100
EGR1-regulon	B PLCG2
0.0464093280594815	-0.00106729886715823	12240753
0.471933318998855	0.0200993723158602	0.0224733139279697
	100	100
EGR2-regulon	B PLCG2
0.038025777963995	-0.00812581657948196	9628235
0.37120958977368 1.46100202036288e-26	2.56126280112998e-26
99.8272884283247 99.997767707659
EGR3-regulon	B PLCG2
0.0333081966887322	-0.00826845204679139	9234087
0.356013500626488	8.76893337810527e-33
1.63840597327756e-32	99.8272884283247 99.997767707659
ELF1-regulon	B PLCG2	0.675748464401392	-0.0317006779174678
	10369716.5	0.399796869107823

1.05127821350582e-16	1.62262506867203e-16	100	100
ELF2-regulon	B PLCG2
0.0281367354457422	-0.00457773875061314	11178118.5
0.430964219592333	1.08065345671261e-08
1.44766783823765e-08	99.6545768566494 99.53345090073
ELF4-regulon	B PLCG2	0.0210822396810723	0.00266919687152296
14807475 0.570891416789684	4.32504659760378e-09
5.90535208518978e-09	98.272884283247	98.3369422059513
ELK1-regulon	B PLCG2
0.0570031017400558	-0.00916886475424796	8606901.5
0.331832820349469	4.30309118645338e-44
8.48665206217194e-44	100	100
ELK3-regulon	B PLCG2	0.0542660808122168	-0.010776888917491
8345424.5	0.321751765004927
2.55398976977029e-49	5.25603691749828e-49	100	100
ELK4-regulon	B PLCG2
0.0355514284218947	-0.00956768911332207	10262721
0.395671735512452	5.58979629683813e-18
8.91855139495522e-18	90.846286701209	96.8055896600219
ESRRA-regulon	B PLCG2	0.066729282514555	-0.0253296505559138
4511187	0.173925530033527	1.27146399945912e-160
6.94414953550751e-160	100	100
ETS1-regulon	B PLCG2	0.236930869136543	-0.0060857916870467
12204826 0.470548179673548	0.0147196068469383
0.0167214733781219	100	100
ETV2-regulon	B PLCG2	0.0300881692822462	0.00150535188010127
14095186.5	0.543429652314106
0.000322090650698727	0.000387600613552706	100	100
ETV3-regulon	B PLCG2	0.0142733925428102	0.00288109996843053
14960752.5	0.576800919195528
1.85488237855113e-10	2.63393297754261e-10	89.9827288428325
84.2757327499609
ETV5-regulon	B PLCG2	0.0383433171665207	0.00494274464989262
16829952 0.648866544889144	6.30999250966856e-35
1.21083640050397e-34	100	100
ETV6-regulon	B PLCG2	0.39425897994034 -0.0661601215654362
3030899	0.116854104042481	5.58382561790212e-221
1.32150539623683e-219	100	100
ETV7-regulon	B PLCG2
0.0581250532929533	-0.00602794304155881	10334755
0.398448953932002	4.08334869459637e-17
6.44261682925205e-17	100	100
FLI1-regulon	B PLCG2	0.117110462170063	-0.0271677147937451
5795874	0.223455701893435	4.29124342195258e-116
1.6035699103086e-115	100	100
FOS-regulon	B PLCG2	0.0701481356069804	-0.0163378609332459
6525715	0.251594190225929	4.78821192232419e-94
1.30755017878853e-93	100	100
FOSB-regulon	B PLCG2	0.0283117530760479	-0.0133318438234226
8287125.5	0.319504089509448

1.58781938954373e-50	3.31574048992955e-50	97.5820379965458
99.3459383440855
FOSL1-regulon	B PLCG2	0.042606254419732	-0.0184225245382309
	6407101.5	0.247021133100026	1.8078991701306e-97
	5.46216345018182e-97	100	100
FOXO1-regulon	B PLCG2	0.0248145027555498	0.00822243635643568
	14739114.5	0.568255827487831
2.14781356363041e-09	2.99009339250508e-09	59.9309153713299
52.2445699488805
FOXO3-regulon	B PLCG2
0.0164715484778633	-0.00331465942423846	12150192.5
0.468441824861591	0.00456228695052983	0.00531020284405931
	41.6234887737478 46.8267964372614
GABPA-regulon	B PLCG2	0.0343967205371258	0.00902393944347293
	19477266 0.750931808558146	6.26767961102019e-96
1.78002100952973e-95	100	100
GTF2B-regulon	B PLCG2	0.0603703611436132	-0.0189281434820706
	6795219	0.261984720710734	1.68498983288684e-86
4.19769396964792e-86	100	100
HINFP-regulon	B PLCG2	0.0216684324777642	0.00550817091076739
	14510600.5	0.559445636606788
4.40888102471558e-07	5.64019013972623e-07	69.0846286701209
63.1582472040538
HIVEP3-regulon	B PLCG2	0.0537528105587042	-0.0242445181328805
	7447080	0.287116746923167	1.4232094364342e-69
3.20786888847074e-69	97.2366148531952 99.7254280420564
HOXB2-regulon	B PLCG2	0.0223990975595251	-0.0148936709413634
	9298294	0.358488954760148	7.4655441720916e-32
1.37676269147663e-31	65.2849740932643 83.9676764069022
IKZF1-regulon	B PLCG2	0.125822098621396	-0.0182752272438964
	5053641.5	0.194839468301121
6.2546919600239e-141	2.8650524462045e-140	100	100
IRF1-regulon	B PLCG2	0.103232134955301	-0.0326266262179958
	3998918.5	0.154175391016461
2.05882698748979e-180	1.82720895139718e-179	100	100
IRF2-regulon	B PLCG2	0.0606036592687004	-0.0247323481465954
	4695364	0.181026340162876	8.63132931140933e-154
4.22637504213836e-153	100	100
IRF3-regulon	B PLCG2	0.117555230454498	-0.0282517926295051
	3040740	0.117233516631908	1.51423556482669e-220
3.07173500293414e-219	100	100
IRF4-regulon	B PLCG2	0.0387608876573633	-0.0199054570197407
	5245347.5	0.202230553543344
2.78144692166477e-134	1.19686503901939e-133	99.6545768566494 100
IRF5-regulon	B PLCG2	0.0183506996259275	-0.0290649266270362
	6938604.5	0.267512844259286
3.20480912774375e-83	7.84625682999333e-83	51.1226252158895
80.8580931758823
IRF7-regulon	B PLCG2	0.0816200201753387	-0.0265534989987859
	4411473	0.170081129368744	2.20277581061877e-164

1.25117666043146e-163   100   100
IRF8-regulon   B PLCG2 0.0867656421934963   0.0050572483085987
        15390579.5   0.593372586208605
1.04927152211533e-14   1.56838480147766e-14   100   100
IRF9-regulon   B PLCG2
0.0304236215199486   -0.00353774136712598   10864444
0.418870727642098   1.82794761696189e-11
2.64865879192436e-11   100   100
JUN-regulon   B PLCG2 0.0690590303366398   -0.019591238166566
        5205003.5   0.200675119999207
1.14476061648371e-135   5.07987523564648e-135   100   100
JUNB-regulon   B PLCG2 0.124530261141009   -0.0357078637247948
        3992693.5   0.153935390674099
1.16409350024142e-180   1.10200851356188e-179   100   100
JUND-regulon   B PLCG2 0.041527774830558   0.00310976787316639
        14420487.5   0.555971395506183
3.55967570080689e-06   4.43398201328578e-06   100   100
KLF11-regulon   B PLCG2
0.0132597287853594   -0.00878890595257688   11166092
0.430500546641744   1.02561937950465e-10
1.47109042312789e-10   28.3246977547496 41.0206040583075
KLF12-regulon   B PLCG2 0.0509233377968183   0.0039237805853281
        14003389.5   0.539890485819681
0.000954033376063235   0.00112893949500816   98.1001727115717
98.6092818715539
KLF13-regulon   B PLCG2 0.0547163028434213   -0.0117254127568881
        6418076 0.247444246956613   3.77130001861026e-97
1.11567625550554e-96   100   100
KLF2-regulon   B PLCG2 0.0809047894876136   -0.0283047475711034
        4858751.5   0.187325626257279
7.43291695948149e-148   3.51824736082124e-147   100   100
KLF3-regulon   B PLCG2 0.0514038591921044   -0.0208919402539864
        8275499.5   0.319055857544741
9.08747938657039e-51   1.92600309387014e-50   98.6183074265976
99.8794562135857
KLF4-regulon   B PLCG2 0.0184791363053017   0.00261268264505664
        13494666.5   0.520277040973514   0.0723758056518028
        0.07845316337829 52.3316062176166 49.780119204411
KLF6-regulon   B PLCG2 0.0383773375094813   -0.0314032464481932
        4159598 0.160370272142653   4.42723495260694e-174
3.30877559615887e-173   100   100
KLF7-regulon   B PLCG2 0.0306470474446675   -0.0155501106795069
        8354057 0.322084584756805   3.83606312468641e-49
7.78172805293529e-49   92.9188255613126 97.6159117798067
KLF8-regulon   B PLCG2
0.0155287174285447   -0.00970180173460396   10007537.5
0.385833321477895   1.93169677627715e-21
3.18954583989948e-21   64.9395509499137 77.7998526687055
MAFB-regulon   B PLCG2 0.0643496683898074   0.00867748818939677
        16272825.5   0.627386938344741

5.06791606228267e-26	8.67041061257998e-26	100
99.997767707659
MAFF-regulon	B PLCG2	0.0143624332362135	0.00200102563500503
	13372647	0.515572667997637	0.105604215065898
0.112750364957575	31.2607944732297	28.5108377793156
MAFG-regulon	B PLCG2
0.0166537024894986	-0.00886679086546033	10441506.5
0.402564680285038	5.66095635057854e-17
8.83358023936432e-17	42.1416234887737	58.8142063084582
MAX-regulon	B PLCG2	0.170829333795342	-0.0271877937651774
	3910251	0.150756880115839	5.89209042276848e-184
5.97626314309374e-183	100	100
MAZ-regulon	B PLCG2
0.133351557033652	-0.00299041391158913	12191937.5
0.470051272940611	0.0131250107428836	0.0150302542378183
	100	100
MEF2A-regulon	B PLCG2	0.0413321229188857	0.0005852758051463
	13055725.5	0.503353990326656	0.781183786282921
	0.798043868001258	100	99.997767707659
MLX-regulon	B PLCG2
0.044020750117586	-0.00901980076593771	9337810
0.36001246536718	4.43030260628667e-31	7.96332873535072e-31
99.6545768566494	99.993303122977
MXD4-regulon	B PLCG2	0.0539508300265227	-0.0387122699890688
	9636367.5	0.371523132389625
1.77450227208371e-26	3.07291856873032e-26	73.4024179620035
88.800589325178
MXI1-regulon	B PLCG2	0.0465402053235162	-0.0189309055396441
	6776377.5	0.261258300397383
5.12201326117624e-87	1.29879621979826e-86	100	100
MYBL1-regulon	B PLCG2	0.0650921965831417	0.0356569622582601
	19940145	0.768777771364917	1.23448056055551e-110
4.17371999044958e-110	96.0276338514681	79.8535616224301
MYC-regulon	B PLCG2	0.2483351413056	-0.0674067310039951
4120177.5	0.158850443468584	1.26796994756828e-175
1.00028740308164e-174	100	100
NFATC1-regulon	B PLCG2
0.0412370013881167	-0.00940079537661053	11111650.5
0.428401594249985	3.02554330379562e-09
4.17113737028134e-09	91.0189982728843	94.8768890773936
NFE2L1-regulon	B PLCG2	0.0784473183581414	-0.105566862559201
	2980756	0.114920877188336	3.40937268723625e-223
1.21032730396887e-221	96.7184801381693	99.97767707659
NFE2L2-regulon	B PLCG2	0.0800438785930261	-0.0312352128587094
	3753254	0.144703975095791	2.5765140153374e-190
3.04887491814925e-189	100	100
NFKB1-regulon	B PLCG2	0.0315428614794441	-0.0254506871956903
	5851900	0.225615743528964	2.56133073395778e-114
9.32587087748731e-114	99.4818652849741	99.9374958144519
NFKB2-regulon	B PLCG2	0.046686432001988	-0.0270351305387389

4179971 0.161155738323366 2.7598970603997e-173
1.95952691288379e-172 100 100
NFYA-regulon B PLCG2
0.0189532691245406 -0.00202092405521179 11669846.5
0.449922434588148 3.36218622171533e-05
4.15156907377023e-05 98.4455958549223 99.0244882469808
NFYB-regulon B PLCG2 0.120089166406677 0.0116124450662268
15864707 0.611652226742454 2.3074674472976e-20
3.72341338086658e-20 100 100
NFYC-regulon B PLCG2
0.081947516502174 -0.00160773705619925 12321536
0.475047848742955 0.0387773733316485 0.0426851706441402
100 100
NR1H3-regulon B PLCG2
0.00838620372562142 -0.000150099813055492 12759570
0.491935930665231 0.357734890799902 0.379092197713328
20.2072538860104 22.1287139763823
NR2C2-regulon B PLCG2
0.0213297989606998 -0.00046226807950079 12688153.5
0.489182519508558 0.366961623474718 0.385989263210444
73.4024179620035 76.0117865035605
NR3C1-regulon B PLCG2 0.206330098628041 -0.0193949674659004
6749693.5 0.260229518206927
9.42656569005466e-88 2.43376786906866e-87 100 100
NRF1-regulon B PLCG2 0.0281586460473722 0.00144795607850055
13477072 0.519598697837179 0.104553640048636
0.112474370355351 100 99.9665156148849
PAX5-regulon B PLCG2
0.098029866218124 -0.00226673650070623 11812064.5
0.455405546024297 0.000221343659219032
0.000268639312898312 100 100
POU2F1-regulon B PLCG2 0.0580989483610636 0.0539068211881433
18874708 0.727700623611492 8.9716894266911e-172
6.06657094566732e-171 57.5129533678757 17.8404803893118
POU6F1-regulon B PLCG2
0.0309527924028667 -4.99751474971125e-05 12816596.5
0.494134545849762 0.619750298290346 0.637714075052385
61.8307426597582 65.3994687144228
REL-regulon B PLCG2 0.222929812646891 -0.0413092777971962
3605773 0.139017952526814 2.17742530620489e-196
3.09194393481094e-195 100 100
RELA-regulon B PLCG2 0.0805747984543741 -0.0116381667932928
6280705 0.242148008076195 3.47664351175672e-101
1.09707417482101e-100 100 100
RELB-regulon B PLCG2 0.0951186677189491 -0.0101951010878487
8828646 0.34038201808712 6.76245029439224e-40
1.3154355367174e-39 100 100
REST-regulon B PLCG2 0.192551186362723 -0.0264042046741469
4403148 0.169760165055464 1.06402558940851e-164
6.29548473733369e-164 100 100

RFX5-regulon	B PLCG2	0.0720661769286686	0.00680452856107068
	15313685.5	0.590407994027789
7.01389921776197e-14	1.03747259262729e-13	100	100
RXRA-regulon	B PLCG2
0.0143354119174499	-0.000197243689384846	12903448.5
0.497483061469813	0.831032050946325	0.842903937388415
	64.9395509499137 64.2543027434873
SETDB1-regulon	B PLCG2	0.0548193077266681	-0.0049615285400705
	11879973 0.458023708795267	0.000507624982396652
0.000605737373952307	90.3281519861831 95.1135120655401
SOX5-regulon	B PLCG2	0.0132126301672144	0.00547924204461027
	14568023 0.561659519282977	2.83819158926214e-12
4.15487840902292e-12	34.1968911917098 22.3430140411188
SP1-regulon	B PLCG2	0.0146950547196828	0.00172316870812707
	13586153.5	0.523804255643661	0.0414807701165103
	0.0453097642811113	62.0034542314335 59.7941826461593
SP2-regulon	B PLCG2	0.0305858026500608	0.0023759630029873
	14803307.5	0.570730741861685
4.68656423380618e-09	6.33802020190931e-09	100	100
SP3-regulon	B PLCG2	0.0286892658879458
0.000424785867680442	13230240.5	0.510082289081241
0.403708695118379	0.42151937284419 100	100
SP4-regulon	B PLCG2	0.0142347920578061	0.00566920710602286
	14361094.5	0.553681541637283
2.90037708651616e-08	3.77847290169995e-08	37.6511226252159
28.9037212313325
SPI1-regulon	B PLCG2	0.379040979258869	-0.0400125606821262
	6596437.5	0.254320844717928
4.91514210742091e-92	1.31688713066749e-91	100	100
SPIB-regulon	B PLCG2	0.512573880151348	-0.052547853120859
	5903774 0.227615707827709	1.09743613673179e-112
3.80087637599789e-112	100	100
SREBF1-regulon	B PLCG2
0.0326778230862634	-0.00835871462035849	9946894.5
0.383495274769163	4.95917297991049e-22
8.28473603702694e-22	88.7737478411054 96.9997990936893
SREBF2-regulon	B PLCG2
0.0904959109572208	-0.00859641371176516	8160166
0.314609258430557	3.32470875480937e-53
7.15316126034743e-53	100	100
SRF-regulon	B PLCG2	0.0207944022384556
2.55521255347058e-05	12955097 0.499474331780252
0.965275410826659	0.965275410826659	97.5820379965458
97.9395941692524
STAT1-regulon	B PLCG2	0.0616514931191323	-0.018351179078235
	4332435.5	0.167033896106184
2.13963767546109e-167	1.38103886325216e-166	100	100
STAT2-regulon	B PLCG2	0.0534774701773057	-0.0129217668499698
	9137459 0.352288078444681	2.05782016880362e-34
3.89613951960152e-34	99.4818652849741 99.9687479072259

STAT5A-regulon B PLCG2 0.0534170957867485 -0.0184258472868398
5769739 0.222448085998234 6.30116425706609e-117
2.41828466081996e-116 100 100
TBP-regulon B PLCG2 0.0260107839080181 0.00517555904658587
14074151 0.542618643928282 0.000159846754417938
0.000195674475235751 55.440414507772 49.8136035895261
TCF12-regulon B PLCG2
0.00878301961355565 -0.000264063160801775 12829066
0.494615298342787 0.558781765527765 0.579175260620019
24.006908462867 25.2360649150613
TFDP1-regulon B PLCG2 0.506480880185866 -0.0219626668202766
10499422 0.404797570217257 3.15326362720295e-15
4.76344079854063e-15 100 100
TFDP2-regulon B PLCG2 0.0368623262394333 0.00986505105805966
18602134 0.71719173151206 2.42500850312631e-72
5.55405173296672e-72 100 99.995535415318
TFEC-regulon B PLCG2 0.0571498320672161 -0.0348362252764764
3144328.5 0.121227295823034
5.18551792642427e-216 9.20429431940308e-215 100 100
THAP1-regulon B PLCG2
0.0349301192302052 -0.00226388104129132 11221494.5
0.432636549688765 2.41827937354937e-08
3.17958954670381e-08 100 100
THAP11-regulon B PLCG2 0.159861634806353 -0.0188566873971558
5437870.5 0.209653137625681
9.06126595770114e-128 3.67628504569589e-127 100 100
TP53-regulon B PLCG2 0.0758662726958416 -0.0227811075326184
5880560 0.226720708960626 2.04905498093908e-113
7.27414518233375e-113 100 100
USF2-regulon B PLCG2 0.11132446223991 -0.0141421980144215
6278068 0.242046340461286 2.90324918171836e-101
9.36957690463652e-101 100 100
XBP1-regulon B PLCG2 0.162757049964002 -0.0329533024379572
3793007.5 0.146236642342391
1.07541050676363e-188 1.17467916892642e-187 100 100
YBX1-regulon B PLCG2
0.0935089045252694 -0.00169650495124844 12124349
0.467445447536638 0.00701409666350907 0.00809757500990479
100 100
YY1-regulon B PLCG2 0.575416899524749 -0.0648823344769417
6293614 0.242645705171705 8.39305213573009e-101
2.59089870276885e-100 100 100
YY2-regulon B PLCG2 0.0393232723545075 -0.0118297325810763
10381356 0.4002456215552 1.39471028927565e-16
2.12955764599078e-16 82.2107081174439 90.6377659218251
ZBTB33-regulon B PLCG2 0.326915080321346 -0.039413856186116
5690045.5 0.219375561133331
1.73864052396675e-119 6.85797095564663e-119 100 100
ZNF143-regulon B PLCG2 0.025200806564276 0.00189424134060901
14618571.5 0.563608379894364

1.37873233423515e-07	1.77981810419447e-07	100
99.993303122977
ZNF274-regulon	B PLCG2
0.0577573738599807	-0.00768218193995263	9326296
0.359568551480922	2.88182648617596e-31
5.24640206457675e-31	100	100
ZNF76-regulon	B PLCG2	0.0205104344242471	0.00603027437265563
	17840571.5	0.687830243844589
1.44543701741842e-54	3.15772394574485e-54	100
99.4530883764538
ATF1-regulon	DZ 1
0.100154478665602	-0.00627603110877981	24188400.5
0.373298524300733	8.8653589598573e-62
1.18762355877334e-61	100	100
ATF3-regulon	DZ 1	0.0319933866222915	-0.0196681645920935
	7991621.5	0.123334344191961	0	0	100
	100
ATF4-regulon	DZ 1	0.0881129055656642	-0.0213326498500696
	12119862.5	0.187045306529375	0	0	100
	100
ATF5-regulon	DZ 1
0.11341039581603 6	-0.00519192990316961	29597215
0.456772521312912	1.52624061915114e-08
1.61735946208554e-08	100	99.997722095672
ATF6-regulon	DZ 1	0.11794917693632 -0.0190855885935479
10946814 0.168941700464841	0	0	100	100
ATF6B-regulon	DZ 1
0.0509597244498521	-0.00654984157933301	11891315.5214844
0.183518151031298	0	0	100	99.9179954441913
BACH1-regulon	DZ 1	0.015803014810626	-0.0284037440007435
	7084763 0.109338836725497	0	0
96.3414634146341 99.6719817767654
BATF-regulon	DZ 1	0.00855574024396562	-0.0110986316676576
	23881518.5	0.36856242785093 1.59796028652145e-74
2.2920238453136e-74	28.6585365853659 52.4920273348519
BATF3-regulon	DZ 1	0.0867151391165874	0.00367093305731661
	39055436.5	0.602740838997228
3.11899605385239e-41	3.7218272239247e-41	100	100
BCL11A-regulon	DZ 1	0.193465100367571	-0.0114062564310747
	20818268 0.32128741720219 4.92637644926441e-121
7.86006130107356e-121	100	100
BHLHE40-regulon	DZ 1	0.0315236440259714	-0.0249387450381343
	6679003.5	0.103076768153786	0	0	100
	100
BRF2-regulon	DZ 1	0.0312721106255504	-0.0106878823534267
	19968847 0.3081783401547	3.87258887951561e-139
6.4695014222496e-139	99.8644986449865 99.7243735763098
CEBPB-regulon	DZ 1	0.116901410866619	-0.0270711875296699
	6997999.5	0.107999819434413	0	0	100
	100

CEBPG-regulon DZ 1
0.0645524026309115 -0.00362628775547649 32204688
0.497013537789136 0.695845597421511 0.700780672580529
100 100
CLOCK-regulon DZ 1 0.004421870212973 -0.0115485092915635
22117584 0.341339704057633 3.02527352827325e-108
4.72075649466814e-108 25.6775067750677 51.9476082004556
CREB1-regulon DZ 1
0.0398414777501404 -0.00323494561989168 28516096
0.440087659190943 4.41035779765276e-15
4.85481245943172e-15 100 99.9931662870159
CREB3-regulon DZ 1 0.223339994342704 -0.0170134357672554
17815185 0.274940968942719 9.15453877213363e-191
1.85706357948996e-190 100 100
CREB3L2-regulon DZ 1 0.106143636820433 -0.0213273050621158
7532371 0.11624675136273 0 0 100 100
CREB3L4-regulon DZ 1 0.00217036802402656 -0.0092312527051172
24975004.5 0.38543814934163 2.77830025472808e-74
3.94518636171388e-74 10.4336043360434 32.0501138952164
CREB5-regulon DZ 1 0.0213646392831573 -0.0184084679378624
15182611 0.234312569834127 5.0235006891168e-265
1.13228110770569e-264 98.7127371273713 99.6537585421412
CREM-regulon DZ 1 0.156752302061446 0.00697978389521944
41211572.5 0.636016391342729
6.47545282204776e-71 9.0148460855959e-71 100 100
CTCF-regulon DZ 1 0.445277894086636 0.0769403749709687
62741065.5 0.968280112784044 0 0 100
100
CUX1-regulon DZ 1 0.103880474316327 0.0280963321598793
57706696.5 0.890584916754634 0 0 100
100
DDIT3-regulon DZ 1 0.0644568728709937 -0.0211388637176105
13865305 0.213982644097512 8.6051031212722e-307
2.07105871732314e-306 100 100
E2F1-regulon DZ 1 0.587277235119036 0.154611543010171
63639538.5 0.982146207196696 0 0 100
100
E2F2-regulon DZ 1 0.106355701470562 0.0541976624863925
64241482 0.991435974838108 0 0 100 100
E2F3-regulon DZ 1
0.0156820587023468 -0.00561591945308678 18476855.5
0.285152500756215 4.97248628429961e-174
9.29069805750717e-174 100 100
E2F4-regulon DZ 1 0.41157770740461 0.0133545864504041
42251852.5 0.652070986968412 3.57559473805864e-88
5.28890055004507e-88 100 100
E2F6-regulon DZ 1
0.0643134147419047 -0.00835973171597405 28808741
0.44604036644011 4.12263303165674e-13
4.50318377304043e-13 100 100

E2F7-regulon DZ 1 0.0481064095140929 0.0181218537101787
56807063.5 0.876700920112846 0 0 100
100
E2F8-regulon DZ 1 0.148144522382903 0.0758140053676476
64401204.5 0.993900965177078 0 0 100
100
EGR1-regulon DZ 1
0.0403002141373164 -0.00740362051672551 18137641
0.279917418251631 1.63682309136159e-182
3.18395724621022e-182 100 100
EGR2-regulon DZ 1 0.0336194507883291 -0.0128463259051041
17617363.5 0.271887998407319
6.42431490213443e-196 1.32210538565665e-195 99.9322493224932
99.997722095672
EGR3-regulon DZ 1 0.0241170547043478 -0.0179375650347048
11432483.5 0.176437016562649 0 0
99.9322493224932 99.997722095672
ELF1-regulon DZ 1 0.793936448880426 0.0898132736913855
61677024.5 0.951858814687236 0 0 100
100
ELF2-regulon DZ 1 0.0146905691577894 -0.0185695262936985
11753598 0.181392762560883 0 0
98.1707317073171 99.5808656036446
ELF4-regulon DZ 1
0.013123690100021 -0.00550239484137061 22413766.5
0.345910675593089 1.76987338125882e-90
2.64549494882897e-90 96.6124661246612 98.3940774487472
ELK1-regulon DZ 1 0.0671315233120055 0.00111274767318324
34235316.5 0.528352138390404
0.000206142963113165 0.000213666428920215 100 100
ELK3-regulon DZ 1 0.051202626008023 -0.0141635448298849
16171940.5 0.249580848627393
1.11630972681412e-235 2.43870740319393e-235 100 100
ELK4-regulon DZ 1 0.0300254343668978 -0.0154749721059005
21414015 0.330481554530807 4.21531194118024e-109
6.65082550719548e-109 92.8184281842818 96.8610478359909
ESRRA-regulon DZ 1
0.0851728520299066 -0.00678352951309054 29219724.5
0.450946726978659 1.35205589331463e-10
1.46558730420364e-10 100 100
ETS1-regulon DZ 1 0.286344332707468 0.0448646950521562
55646545 0.858790688988894 0 0 100 100
ETV2-regulon DZ 1
0.0201595307417107 -0.00872634747704877 15463272.5
0.238644006457149 1.59393248667408e-256
3.53653770480811e-256 100 100
ETV3-regulon DZ 1
0.00360290778513733 -0.00808927747331163 14969018
0.231016198430777 1.26055723174294e-272
2.88708269205642e-272 53.1842818428184 85.3963553530752

ETV5-regulon DZ 1 0.0399961979135958 0.00675219245695948
45953942 0.709205171892266 4.08272229086706e-165
7.43264827311696e-165 100 100
ETV6-regulon DZ 1 0.428016185680409 -0.0326197721664119
9100553.5 0.140448443123383 0 0 100
100
ETV7-regulon DZ 1
0.0628720042774972 -0.00124455846373682 32538083.5
0.502158815921872 0.777488430423106 0.777488430423106
100 100
FLI1-regulon DZ 1 0.135540365927297 -0.0086732757796624
23314783.5 0.359816031446191
3.27445425775445e-75 4.7446173938891e-75 100 100
FOS-regulon DZ 1 0.0697588967027501 -0.0170740150511383
13819633 0.213277790124143 0 0 100 100
FOSB-regulon DZ 1 0.0142091501326966 -0.0281810095424914
7860341.5 0.121308305708342 0 0
93.0216802168022 99.5353075170843
FOSL1-regulon DZ 1 0.0468025778976903 -0.014461536619665
19286069.5 0.297641064935706
1.28729569942001e-154 2.31387328250179e-154 100 100
FOXO1-regulon DZ 1 0.00778543792366176 -0.0092111700308087
25174193.5 0.388512224444568
7.04876606327528e-54 9.01734036923504e-54 36.3143631436314
52.8815489749431
FOXO3-regulon DZ 1 0.00844205284498133 -0.0116818494775687
25510504.5 0.393702497360965
1.60555978870538e-51 2.03562044639433e-51 30.6233062330623
47.3029612756264
GABPA-regulon DZ 1
0.0207150368566727 -0.00493336363177631 21678593.5
0.334564782920039 5.34827132673494e-104
8.07930349357832e-104 100 100
GTF2B-regulon DZ 1 0.0814340480522025 0.00245698892022382
35504613 0.547941135618645 3.48250524224827e-10
3.74633139696405e-10 100 100
HINFP-regulon DZ 1
0.00926733855640657 -0.00719732358737763 25872606.5
0.399290801649474 1.13756193813953e-41
1.36893046793062e-41 52.3712737127371 63.5990888382688
HIVEP3-regulon DZ 1
0.0714230805095051 -0.00647552413464939 28114946.5
0.433896736547092 5.01249461065181e-18
5.60452153316974e-18 99.8644986449865 99.6879271070615
HOXB2-regulon DZ 1 0.00706025644738404 -0.0310525519695848
11234926 0.173388120327673 0 0
43.4959349593496 85.0820045558087
IKZF1-regulon DZ 1 0.147195119084679 0.00344297999310009
37570707.5 0.579827081442796
1.47192420650027e-25 1.6721058985843e-25 100 100

IRF1-regulon	DZ 1	0.097578517413465	-0.0391369823295193
	4070792.5	0.0628243621559222	0	0	100
	100
IRF2-regulon	DZ 1	0.0593997201409923	-0.0264821171248728
	7323907.5	0.113029543307962	0	0	100
	100
IRF3-regulon	DZ 1
0.136825773800253	-0.00891060090129692	19830216.5
0.306038861726886	3.25136468602691e-142
5.49635458828359e-142	100	100
IRF4-regulon	DZ 1	0.0427511082668811	-0.0161878019985386
	14550253	0.224553416547833	1.08426454537113e-284
2.56609275737833e-284	100	99.995444191344
IRF5-regulon	DZ 1	0.00654146972694152	-0.0418650827792193
	10075190	0.15548996549191	0	0	28.3875338753388
82.2300683371298
IRF7-regulon	DZ 1	0.087429486542663	-0.0210912699035286
	11406416	0.176034717978159	0	0	100	100
IRF8-regulon	DZ 1	0.0998468776693617	0.018681633107817
	50945235.5	0.786235585618954
2.94952965901243e-307	7.22126226861665e-307	100	100
IRF9-regulon	DZ 1	0.0203639179248936	-0.0140079569556346
	9922481.5	0.15313322190739	0	0	100	100
JUN-regulon	DZ 1	0.0578384096046849	-0.0315894210132311
	3204485.5	0.0494546842108512	0	0	100
	100
JUNB-regulon	DZ 1	0.125737517242686	-0.0351896291209551
	5485115.5	0.0846515469995247	0	0	100
	100
JUND-regulon	DZ 1	0.0252491069942707	-0.0136526779117344
	11216451	0.173102996462766	0	0	100	100
KLF11-regulon	DZ 1	0.00216901520397043	-0.0204320920142754
	20928698.5	0.322991686266521
3.13380893383943e-149	5.56251085756499e-149	8.26558265582656
41.9544419134396
KLF12-regulon	DZ 1	0.0590944854731514	0.0124498313403698
	43322064	0.668587514121155	6.3364586371326e-108
9.78018615731336e-108	99.9322493224932	98.5580865603645
KLF13-regulon	DZ 1	0.0483310595494028	-0.018564922892476
	6925643	0.106883144742609	0	0	100	100
KLF2-regulon	DZ 1	0.0816344902944426	-0.0281288581579053
	10516771	0.162304865702416	0	0	100	100
KLF3-regulon	DZ 1	0.0371176574772931	-0.0360853516302758
	11232863.5	0.173356289855609	0	0
99.8644986449865	99.8633257403189
KLF4-regulon	DZ 1
0.00878578094637766	-0.00735319700036295	27063269
0.417666243803668	9.19161562450032e-31
1.06114586884475e-30	38.4823848238482	50.1936218678815
KLF6-regulon	DZ 1	0.0303710489921622	-0.0403203776515166

4404504.5 0.0679745248192801 0 0 100
100
KLF7-regulon DZ 1 0.0139195496472474 -0.0331577505276903
7655833.5 0.118152142711632 0 0
84.9593495934959 97.9794988610478
KLF8-regulon DZ 1 0.00374288714397114 -0.0220821286400424
12127431 0.187162110858011 0 0
32.7913279132791 79.1435079726651
MAFB-regulon DZ 1
0.0472351729015308 -0.00883512320855654 19743538.5
0.304701163953553 3.72796967476301e-144
6.37797221465479e-144 100 99.997722095672
MAFF-regulon DZ 1 0.00249756586680362 -0.0102218741772898
25648745.5 0.395835964652357
1.27716881238861e-65 1.72721877484935e-65 9.28184281842818
29.1936218678815
MAFG-regulon DZ 1 0.00767810007907073 -0.0183253431748428
20760720 0.320399281441562 2.10984631854933e-131
3.44365720958626e-131 32.5880758807588 59.4760820045558
MAX-regulon DZ 1 0.177946849903244 -0.0203864962730413
6666588.5 0.102885168003161 0 0 100
100
MAZ-regulon DZ 1 0.189699477107949 0.0551909259520239
63905131.5 0.986245092319944 0 0 100
100
MEF2A-regulon DZ 1
0.0342039559643471 -0.00677059461243739 23462286
0.362092431060985 7.54614865363462e-73
1.06094367209516e-72 100 99.997722095672
MLX-regulon DZ 1
0.0446884996162847 -0.00851390008334302 23761450.5
0.366709423671685 3.56005383134482e-68
4.86084273125927e-68 100 99.9886104783599
MXD4-regulon DZ 1
0.0859676143345284 -0.00641002172984624 35040061
0.540771724972375 9.23959985924199e-08
9.71869022231379e-08 94.1734417344173 88.4168564920273
MXI1-regulon DZ 1 0.0398259970991896 -0.0262576694270269
10091167 0.155736537832349 0 0 100 100
MYBL1-regulon DZ 1 0.0930687926388676 0.0653027553737562
56983022.5 0.879416487644375 0 0
99.3224932249322 79.4123006833713
MYC-regulon DZ 1 0.301415674536074 -0.0139188394742591
31173357 0.481097051688057 0.0133443926451628
0.0136324011195188 100 100
NFATC1-regulon DZ 1
0.0459574319134062 -0.00471373974875824 27181920
0.419497379484045 5.72676634762659e-26
6.55807114002399e-26 91.7344173441735 94.9316628701595
NFE2L1-regulon DZ 1 0.146756166020572 -0.0371183706271997

24592095 0.37952872381799 5.00505909527426e-56
6.52035221586188e-56 100 99.9339407744875
NFE2L2-regulon DZ 1 0.0953381453272577 -0.0160649472232144
18473746.5 0.285104519695539
4.16641408282852e-174 7.88841066348867e-174 100 100
NFKB1-regulon DZ 1 0.0304133223472561 -0.0271382323910593
12572714.5 0.19403415158869 0 0
99.9322493224932 99.9316628701595
NFKB2-regulon DZ 1 0.0454750656158627 -0.0288396287899933
8068080.5 0.124514332586378 0 0 100
100
NFYA-regulon DZ 1 0.00872372616496585 -0.0126356964973801
10793522.5 0.166575959466884 0 0
95.1219512195122 99.1480637813212
NFYB-regulon DZ 1 0.186337110323165 0.0803250433892105
63890066 0.986012587119037 0 0 100 100
NFYC-regulon DZ 1 0.115295687980621 0.0328288116651352
60986065.5 0.941195274737485 0 0 100
100
NR1H3-regulon DZ 1
0.00209190247570834 -0.00665909418773354 27707376.5
0.427606726608268 6.38615365023043e-39
7.49449436638612e-39 8.73983739837398 22.5535307517084
NR2C2-regulon DZ 1 0.0082259656857937 -0.0140161221375748
18767793 0.289642526436654 3.10619437824578e-169
5.72830651572598e-169 51.3550135501355 76.8063781321185
NR3C1-regulon DZ 1 0.227473955919822 0.0020634922756709
33637596 0.519127544122822 0.0122852302165461
0.0126413238460112 100 100
NRF1-regulon DZ 1 0.0314366593987196 0.00486576816126033
39180003 0.604663268329722 1.0056344023074e-42
1.23103521661768e-42 100 99.9658314350797
PAX5-regulon DZ 1 0.101309676354502 0.0010770312010509
32798572 0.506178923520442 0.418609507870239
0.424589643696957 100 100
POU2F1-regulon DZ 1
0.000823278029160382 -0.00419309677027755 27828831.5
0.429481136297696 1.40988321292355e-42
1.71114030970209e-42 4.94579945799458 18.7972665148064
POU6F1-regulon DZ 1 0.0169472440908694 -0.0145274372867338
24720509 0.381510531449278 1.55845393306055e-56
2.04907831939443e-56 50.7452574525745 65.8451025056948
REL-regulon DZ 1 0.254017509505922 -0.0100204187882824
23660107.5 0.365145401596385
9.68126119124302e-70 1.33469814481215e-69 100 100
RELA-regulon DZ 1 0.0769127011794402 -0.0156611909746363
8086267 0.124795004043435 0 0 100 100
RELB-regulon DZ 1 0.119056564500284 0.0143393180163436
49935751 0.770656255594447 5.98073487943765e-275
1.39223664406581e-274 100 100

REST-regulon	DZ 1
0.209783084900046	-0.00913244940223693	17976830
0.277435629139891	1.32282361644259e-186
2.64564723288517e-186	100	100
RFX5-regulon	DZ 1	0.0978833570793956	0.0336287661154823
	57088623.5	0.88104622324697 0	0	100	100
RXRA-regulon	DZ 1
0.00520511055741407	-0.00963855307668243	19824951
0.305957599496268	4.83351372190387e-149
8.47356726555987e-149	31.7073170731707 65.3576309794989
SETDB1-regulon	DZ 1	0.0514862493347628	-0.008508028510265
	24601566.5	0.379674897062182
6.67116963495933e-56	8.61187352876568e-56	98.0352303523035
94.9521640091116
SOX5-regulon	DZ 1
0.00193756093111926	-0.00606295981286215	27551873.5
0.425206855627782	6.66128526199946e-41
7.88252089336603e-41	8.60433604336043 22.9612756264237
SP1-regulon	DZ 1	0.00311196986938232	-0.0102141519259514
	18932783 0.292188809872153	4.14712165674022e-174
7.88841066348867e-174	25.7452574525745 60.9681093394077
SP2-regulon	DZ 1	0.0310972721847919	0.00295317668001769
	39183608.5	0.604718911853128
9.09623127871176e-43	1.12318681876267e-42	100	100
SP3-regulon	DZ 1
0.0217587471630331	-0.00673007027670056	16633123.5
0.256698265644388	1.36669408995615e-222
2.89657553393692e-222	100	100
SP4-regulon	DZ 1
0.00203076952032542	-0.00682929967954941	26035141
0.401799189461143	6.78435160929408e-58
9.00353204224075e-58	11.1111111111111 29.6173120728929
SPI1-regulon	DZ 1	0.443385256390348	0.0256775225111792
	48153603.5	0.743152451370755
2.54773413088777e-222	5.32026833214798e-222	100	100
SPIB-regulon	DZ 1	0.603267150967071	0.0401209949949686
	55174603.5	0.85150723651314 0	0	100	100
SREBF1-regulon	DZ 1
0.0359973251006631	-0.00509839670712534	27677140
0.427140088029582	1.46043829755566e-21
1.64589077978495e-21	98.3062330623306 96.8473804100228
SREBF2-regulon	DZ 1
0.0952753419660433	-0.00383193812277023	25470161.5
0.393079885610929	1.64623123953417e-44
2.06871536295444e-44	100	100
SRF-regulon	DZ 1	0.00766382296823556	-0.0135459796445996
	10395469.5	0.160432824971758	0	0
88.2791327913279 98.2596810933941
STAT1-regulon	DZ 1	0.0579517472723943	-0.0225502832963389
	4283380.5	0.0661052234383392	0	0	100

100
STAT2-regulon	DZ 1	0.0500711949373756	-0.0167065953272247
18030493.5	0.278263815582347
3.10641473966191e-185	6.12654018099988e-185	99.7967479674797
99.9681093394077
STAT5A-regulon	DZ 1	0.0624143975128842	-0.0095025311567979
22964198	0.354405460797205	5.56417188102675e-81
8.14548873304947e-81	100	100
TBP-regulon	DZ 1
0.0154953891459289	-0.00558763175659797	29515695
0.455514426727411	4.71192963625771e-10
5.03078201765861e-10	43.8346883468835	50.0888382687927
TCF12-regulon	DZ 1
0.00236675514713284	-0.00690144997364097	27129414
0.418687056688335	2.96116561721591e-44
3.68846945302332e-44	10.2981029810298	25.7220956719818
TFDP1-regulon	DZ 1	0.639143828973834	0.114711899285989
63669157.5	0.982603315924959	0	0	100
100
TFDP2-regulon	DZ 1	0.0376399978736046	0.0108704400984067
48452332.5	0.747762722929052
9.47571440414423e-231	2.03871431119467e-230	100
99.995444191344
TFEC-regulon	DZ 1	0.0696450863293575	-0.022632658913699
13259217	0.204628914569328	0	0	100	100
THAP1-regulon	DZ 1
0.0316736816933035	-0.00567606352348928	19588563.5
0.302309441573915	1.1719477703558e-147
2.02947052915272e-147	100	100
THAP11-regulon	DZ 1
0.177302526843309	-0.00121469494900839	30264682
0.467073510256743	1.63135592831693e-05
1.70332751338973e-05	100	100
TP53-regulon	DZ 1	0.101049133179728	0.00278296590382934
36341618	0.560858597082554	1.6317091499172e-15
1.81017733818939e-15	100	100
USF2-regulon	DZ 1
0.117436416018758	-0.00811371366248316	20045788
0.309365767233982	1.90775516241839e-137
3.15001433794665e-137	100	100
XBP1-regulon	DZ 1	0.168087695979404	-0.0281167584446077
7899840	0.121917884326907	0	0	100	100
YBX1-regulon	DZ 1	0.115145574227304	0.0206329656514457
60029642.5	0.926434840515831	0	0	100
100
YY1-regulon	DZ 1	0.709059985801249	0.0719283541074913
60270629.5	0.930153982320005	0	0	100
100
YY2-regulon	DZ 1	0.0302610904061583	-0.0214383165950516
20494851	0.316296136822416	6.96311342429026e-128

1.12359330255593e-127 85.9756097560976 90.6833712984055
ZBTB33-regulon DZ 1 0.355401757999115 -0.0107747387075849
21557759 0.332699949379904 2.59738644074275e-106
3.96590187726312e-106 100 100
ZNF143-regulon DZ 1 0.0133893757944393 -0.0102756071361469
10146494 0.156590396997364 0 0
99.9322493224932 99.995444191344
ZNF274-regulon DZ 1 0.0462897470254072 -0.0196923403100151
7024998 0.10841648610108 0 0 100 100
ZNF76-regulon DZ 1
0.0122475498590906 -0.00238720840448039 26456448
0.408301201918625 3.40134291056914e-33
3.95894010902309e-33 99.390243902439 99.4624145785877
ATF1-regulon DZ 2
0.0981965434243653 -0.00816879730924991 11462715.5
0.332886670983294 2.98853941065816e-57
4.76823141925234e-57 100 100
ATF3-regulon DZ 2 0.0348125749906271 -0.0164897528067283
6296581 0.182857882817266 3.44405379599685e-201
9.78111278063105e-201 100 100
ATF4-regulon DZ 2 0.0843339003169048 -0.0248403611915022
4906248 0.142481470794459 4.17309713477447e-255
1.60156700848101e-254 100 100
ATF5-regulon DZ 2 0.10513679693765 -0.0135267811284812
12778772.5 0.371106046972715 9.0711069068004e-35
1.25057978715112e-34 100 99.9977580486055
ATF6-regulon DZ 2 0.112719569276413 -0.0241044753033959
3400934.5 0.0987659306328622 0 0 100
100
ATF6B-regulon DZ 2
0.0509705518422259 -0.00643544842562033 7338480.28515625
0.213115493636931 4.9612221754179e-165
1.13627991759571e-164 99.6113989637306 99.9260156039817
BACH1-regulon DZ 2 0.0153476150057449 -0.0284187199170236
3864324 0.112223142235437 9.82747074516303e-300
5.5820033832526e-299 95.8549222797927 99.627836068514
BATF-regulon DZ 2 0.00676438314838548 -0.012745819876675
12224892 0.355020902421447 7.92183374549646e-49
1.17177124152135e-48 26.6839378238342 52.1500313873195
BATF3-regulon DZ 2
0.0751442997818233 -0.00815811237642501 16234426
0.471461062299299 0.00646205930860601 0.00674715016045627
100 100
BCL11A-regulon DZ 2
0.197498057838109 -0.00712346827954977 12818516
0.372260230848972 3.52553752902501e-34
4.81371470309185e-34 100 100
BHLHE40-regulon DZ 2 0.0298950624691208 -0.0262018972266556
3255091.5 0.0945305301506452 0 0 100
100

BRF2-regulon DZ 2 0.0290911407616952 -0.0127379096910081
9598361.5 0.27874429986762 5.96226434209706e-99
1.09953446308803e-98 99.6113989637306 99.7309658326607
CEBPB-regulon DZ 2 0.112283803517421 -0.0313414421045129
2551244 0.0740902207706458 0 0 100 100
CEBPG-regulon DZ 2
0.0608544205568581 -0.00733103907850567 15253683
0.442979480220413 5.29054916622275e-08
5.73479375269947e-08 100 100
CLOCK-regulon DZ 2 0.00571359931904858 -0.010052149089372
12715057.5 0.369255711051728
3.14019325006572e-40 4.45907441509332e-40 31.3471502590674
51.434848892476
CREB1-regulon DZ 2 0.0539420719695411 0.0111607580169106
24267476.5 0.704747445337043
5.18821473539855e-85 9.09538879539005e-85 100
99.9932741458165
CREB3-regulon DZ 2 0.21615198000279 -0.0240573304831512
6045325.5 0.175561216773235 1.86332835222185e-210
6.01346877307961e-210 100 100
CREB3L2-regulon DZ 2 0.104674767405743 -0.0224849814094668
3505578.5 0.101804878323606 0 0 100
100
CREB3L4-regulon DZ 2
0.00315788626782094 -0.00808094264918463 13867239
0.402716007951144 1.51142354633591e-29 2.0058144259785e-29
13.4715025906736 31.656353690252
CREB5-regulon DZ 2 0.0211843703984597 -0.0183013098238767
8078288.5 0.234600131705932
1.64648744983574e-141 3.38842344748805e-141 98.4455958549223
99.6435297282755
CREM-regulon DZ 2 0.151123012929533 0.00114289950117516
18032772.5 0.523686521411449 0.0238015979769111
0.0246702694359225 100 100
CTCF-regulon DZ 2 0.458040636883276 0.0887096376640182
33464803.5 0.971845374006281 0 0 100
100
CUX1-regulon DZ 2 0.117410493343205 0.0414170730244439
32664064 0.948591241381265 0 0 100 100
DDIT3-regulon DZ 2 0.062194197701264 -0.0231070591360586
6405413.5 0.18601846798749 3.0600929765338e-197
8.35640774361154e-197 100 100
E2F1-regulon DZ 2 0.525404532219088 0.0892276695219814
31257310.5 0.9077379645544 0 0 100 100
E2F2-regulon DZ 2 0.0755368336835231 0.0219899658875422
31975434.5 0.928592875217864 0 0 100
100
E2F3-regulon DZ 2
0.0121348647696793 -0.00913586978475541 5960650
0.17310217072068 1.25862218285177e-213

4.25534166583219e-213	100	100
E2F4-regulon	DZ 2
0.397581041849418	-0.00109511144874069	18900428
0.548883949626024	3.08894473944214e-06
3.29797107519387e-06	100	100
E2F6-regulon	DZ 2	0.0578498023321957	-0.0148032709854322
11678925.5	0.339165586928936	3.6618390234664e-53
5.59119506808848e-53	100	100
E2F7-regulon	DZ 2
0.0283752145262518	-0.00223686947115581	14904507
0.432839122446789	1.46558147132975e-10
1.65168705499067e-10	100	100
E2F8-regulon	DZ 2	0.115747818146269	0.041659985297243
32863975	0.954396820982621	0	0	100	100
EGR1-regulon	DZ 2
0.0455664847201391	-0.00192934819115074	14993159
0.435413649325347	7.12722561902176e-10
7.84547316202395e-10	100	100
EGR2-regulon	DZ 2
0.0369271819056188	-0.00927858712348516	11813431
0.343071737101113	1.06824065194323e-50
1.59673865869408e-50	100	99.995516097211
EGR3-regulon	DZ 2	0.0246683872614503	-0.0170935754397018
6521661.5	0.189394405367115
4.5694290496737e-193	1.17974350009757e-192	100
99.995516097211
ELF1-regulon	DZ 2	0.80935244073909	0.104078530213222
33164665.5	0.9631291200213	0	0	100	100
ELF2-regulon	DZ 2	0.0161812947889455	-0.0167599102783163
7281152.5	0.21145064768001	6.65404931332613e-167
1.54897541392182e-166	98.7046632124352	99.5493677697067
ELF4-regulon	DZ 2
0.0157136368941402	-0.00278077544180375	14797366
0.429727659825578	2.00241356304525e-11
2.27474180761941e-11	98.4455958549223	98.3342301138911
ELK1-regulon	DZ 2
0.0638685385707478	-0.00222427523913923	15271868.5
0.44350760207384	7.01205106840966e-08	7.5432670584407e-08	100
100
ELK3-regulon	DZ 2	0.0560995806489089	-0.0089582863475721
11575188	0.336152964742584	4.18351746700393e-55
6.52812615730283e-55	100	100
ELK4-regulon	DZ 2	0.0345470246506516	-0.0106308760365307
13207956	0.383569888246274	1.11291358965976e-28
1.46327527529339e-28	95.0777202072539	96.758138283562
ESRRA-regulon	DZ 2	0.079403259543375	-0.0125459145882327
12249227	0.355727610804672	4.00445572422793e-43
5.80237462082007e-43	100	100
ETS1-regulon	DZ 2	0.328032598471426	0.0865663810441887
33252544	0.965681183824681	0	0	100	100

ETV2-regulon DZ 2
0.0227658039435869 -0.00593723436091178 11193686
0.325073833383748 1.48455607495402e-62
2.45124375166827e-62 100 100
ETV3-regulon DZ 2
0.00468892137198908 -0.00685679151110653 9529327
0.276739481298408 4.25709271120287e-101
8.06009553321077e-101 62.0466321243523 84.7345529548919
ETV5-regulon DZ 2 0.0345321886918574 0.00108704076797862
18474613 0.536517932358584 0.000492557541232163
0.000518097561888646 100 100
ETV6-regulon DZ 2 0.423668060404163 -0.0365283053232991
4563810.5 0.132536804594304
2.2502016102974e-269 9.98526964569473e-269 100 100
ETV7-regulon DZ 2
0.0637683901852811 -0.000313014743216836 17906463.5
0.52001840433001 0.0560969435941359 0.0573076689954482 100
100
FLI1-regulon DZ 2
0.141357367407792 -0.00261870118258911 14974540
0.434872938275942 5.13782660906967e-10
5.69977639443667e-10 100 100
FOS-regulon DZ 2
0.0817168940434581 -0.00463956536214254 14659789
0.425732310771171 1.36958632139266e-12
1.59410866916195e-12 100 100
FOSB-regulon DZ 2 0.01886068456029 -0.0230041765924333
6475160 0.188043963621376 9.89613592924033e-195
2.60231722583727e-194 97.1502590673575 99.3610438525693
FOSL1-regulon DZ 2 0.0422364004634463 -0.0188784935176116
7954976 0.231019035445135 2.67124897614806e-145
5.7472326456519e-145 100 100
FOXO1-regulon DZ 2
0.00757734483485858 -0.00927748175836326 13355669
0.387859595064083 9.11017979452646e-30
1.22042031209694e-29 35.880829015544 52.6275670343467
FOXO3-regulon DZ 2 0.0119865987089939 -0.0078915765164521
15267080.5 0.443368554622067
4.49873486083213e-09 4.91400269413971e-09 40.2849740932642
46.8724778046812
GABPA-regulon DZ 2 0.0339403681447792 0.00859873484651914
26100305.5 0.757974304565264
8.19700531284239e-134 1.66282107774803e-133 100 100
GTF2B-regulon DZ 2 0.0977126663042179 0.0189785758093731
26637605 0.773577923260676 3.07283551390401e-150
6.71296373806721e-150 100 100
HINFP-regulon DZ 2
0.00877232752447907 -0.00758730441374362 13203166.5
0.383430797233269 3.68721972916575e-30
4.98652572896702e-30 48.7046632124352 63.48533763788

HIVEP3-regulon DZ 2 0.0610783466615522 -0.0168970978521168
11525733.5 0.334716765451924 4.8202908921617e-56
7.60534785207735e-56 99.7409326424871 99.6928526589544
HOXB2-regulon DZ 2 0.00903432378350951 -0.0285542048251444
6946774.5 0.201740035978093
6.0135857232593e-179 1.47229167707383e-178 46.3730569948187
84.3758407317729
IKZF1-regulon DZ 2 0.146409362371901 0.00258928179306997
19491784 0.566057413471131 2.90780165155445e-10
3.25124279150182e-10 100 100
IRF1-regulon DZ 2 0.098187800410397 -0.0378994417316186
2618046.5 0.0760302202270016 0 0 100
100
IRF2-regulon DZ 2 0.0599196013779982 -0.0255352616530252
4376857.5 0.127107535953698
2.4979447986967e-277 1.14421987553204e-276 100 100
IRF3-regulon DZ 2 0.129026172596753 -0.016704557523472
6182783 0.179553095449512 2.32296732983852e-205
6.87211168410563e-205 100 100
IRF4-regulon DZ 2 0.0401313362697548 -0.018597419107646
6239575.5 0.181202396285935
2.8380671812935e-203 8.22460285191177e-203 100
99.995516097211
IRF5-regulon DZ 2 0.00645572696241281 -0.0412915388236701
5586452 0.162235153519074 1.29072130146447e-229
4.69954935405013e-229 30.9585492227979 81.3357546408394
IRF7-regulon DZ 2 0.0879896723753979 -0.0201884978122753
6466553 0.187794009273547 4.86065173650261e-195
1.30228782374221e-194 100 100
IRF8-regulon DZ 2 0.107671458490842 0.0263467821896839
29577757 0.858962351711759 3.74255152588817e-257
1.47622865743367e-256 100 100
IRF9-regulon DZ 2 0.0207672826263845 -0.013376518555679
5863293 0.170274843493207 2.661795304131e-217
9.21890080942931e-217 100 100
JUN-regulon DZ 2 0.066067449561108 -0.0227193674427896
4566808.5 0.132623868976179
3.01186254347147e-269 1.29601357931197e-268 100 100
JUNB-regulon DZ 2 0.126811653861981 -0.0335414916862353
3642999.5 0.105795697009911 0 0 100
100
JUND-regulon DZ 2
0.0294764532560818 -0.00913668048491617 9473707.5
0.275124245345221 3.75743101028542e-102
7.30897538987028e-102 100 100
KLF11-regulon DZ 2 0.00352086127841632 -0.0187343617602151
11705459.5 0.339936156077919
6.19101963441421e-66 1.03426445657273e-65 11.139896373057
41.372971033988
KLF12-regulon DZ 2 0.0605757750438429 0.0137602589768167

23446169 0.680896001102157 9.04867316512474e-67
1.52965665410442e-66 100 98.5786028158909
KLF13-regulon DZ 2 0.0501958398896208 -0.0163748507367493
4823802.5 0.140087185772507
1.66320745700926e-258 6.74787025415187e-258 100 100
KLF2-regulon DZ 2 0.0854627979208336 -0.0237903234301167
7186853 0.208712112763882 4.7766598675849e-170
1.13047616866176e-169 100 100
KLF3-regulon DZ 2 0.0402920715181797 -0.0322864479654255
7184232 0.20863599677159 3.9021642100159e-170
9.39164945461453e-170 99.8704663212435 99.8632409649359
KLF4-regulon DZ 2
0.00917319466558759 -0.00684301998459923 14570506.5
0.423139473654864 4.25238519128714e-15
5.07427476607373e-15 39.119170984456 49.9977580486055
KLF6-regulon DZ 2 0.0352313991392065 -0.0347395150800219
4144999.5 0.120374189238355
2.3469152809075e-287 1.14917920651333e-286 100 100
KLF7-regulon DZ 2 0.0172886430022728 -0.0292070053826556
5522339 0.160373259351261 2.01192554621633e-230
7.51824809375578e-230 87.4352331606218 97.7311451887723
KLF8-regulon DZ 2 0.0040157022022307 -0.0214560620395991
6704785 0.194712462183043 1.13957773686811e-188
2.83894804623283e-188 32.3834196891192 78.418975876603
MAFB-regulon DZ 2
0.0501561824051495 -0.00572410953321297 13310735.5
0.386554689325942 2.60160070621541e-27
3.32817387641971e-27 99.8704663212435 100
MAFF-regulon DZ 2
0.00314343827533907 -0.00940348780318624 14088627
0.409145297268815 1.4598937347557e-27
1.88459009395735e-27 11.9170984455959 28.8337368845843
MAFG-regulon DZ 2 0.00678192440831677 -0.018947794650967
10507778 0.305154501815168 6.47820184745717e-83
1.12183495407185e-82 26.9430051813472 59.1494036409291
MAX-regulon DZ 2 0.178982723226633 -0.0190109272366695
4237531.5 0.12306139450306 2.47562792425051e-283
1.17179721747857e-282 100 100
MAZ-regulon DZ 2 0.159905878462459 0.024010566701554
30305707.5 0.880102632004472
4.50440817358027e-288 2.28437843088714e-287 100 100
MEF2A-regulon DZ 2
0.0389018681823161 -0.00188450898312804 15897997
0.461690887873157 0.000256483950972076
0.000271796425656976 100 99.9977580486055
MLX-regulon DZ 2 0.0371484270836447 -0.0160500974105424
8054761.5 0.233916888306214
3.14336366152761e-142 6.56408294024883e-142 99.8704663212435
99.991032194422
MXD4-regulon DZ 2 0.0738367568453174 -0.018649666921592

16447425 0.477646728168156 0.0327879668662792
0.0337383427174756 90.5440414507772 88.5705317908708
MXI1-regulon DZ 2 0.044502459114904 -0.0210858341723035
7539502 0.218953329309437 1.92687379493681e-158
4.3431123631909e-158 100 100
MYBL1-regulon DZ 2 0.165024084052308 0.137472743791682
33664240 0.97763717373799 0 0 100
79.7148237826204
MYC-regulon DZ 2 0.285379371289825 -0.0300130111429866
12136410 0.352451312482488 5.03880526303347e-45
7.37639533351292e-45 100 100
NFATC1-regulon DZ 2
0.049524209601617 -0.00101083020783418 16693928
0.484805377709567 0.147040025712313 0.149140597508204
96.6321243523316 94.79643081338
NFE2L1-regulon DZ 2 0.125825117227384 -0.0578258393994659
9590291.5 0.278509940440761
3.71352616127399e-99 6.93843045922245e-99 100
99.9349834095597
NFE2L2-regulon DZ 2 0.0923168341379362 -0.018884992368975
8594203.5 0.249582726960987
3.33811152153192e-126 6.67622304306383e-126 100 100
NFKB1-regulon DZ 2 0.0282988399804644 -0.0288609800431138
6139625.5 0.178299766209773
5.92793876100712e-207 1.79099426396385e-206 99.6113989637306
99.9372253609542
NFKB2-regulon DZ 2 0.0445277999060951 -0.0293481040428464
4104028.5 0.119184357754108
3.80197127091715e-289 1.99955526100087e-288 100 100
NFYA-regulon DZ 2 0.00907716437620279 -0.0120767075590059
6325984 0.183711770082192 4.05094243622169e-200
1.12790946263427e-199 92.4870466321243 99.1301228589364
NFYB-regulon DZ 2 0.178978834445513 0.0715716141727647
33453224 0.971509095817518 0 0 100 100
NFYC-regulon DZ 2 0.136365170038765 0.0537448132447719
33910380 0.984785281461315 0 0 100 100
NR1H3-regulon DZ 2
0.0024517152801522 -0.00618795105880769 14807577.5
0.430024210170978 3.73819195414101e-20
4.61585441293934e-20 8.80829015544041 22.3343197919469
NR2C2-regulon DZ 2 0.00828778398944933 -0.0137320127004945
10203183.5 0.296308827410632 2.5949262234124e-85
4.60599404655701e-85 52.3316062176166 76.3877679131916
NR3C1-regulon DZ 2 0.241824128972994 0.0166294673877381
26389517.5 0.766373258538118
1.5540076563219e-142 3.29356846563747e-142 100 100
NRF1-regulon DZ 2 0.0679466347988666 0.0419308552155949
33935598 0.985517632889636 0 0 100
99.9663707290826
PAX5-regulon DZ 2 0.104943571279181 0.00475682194037315

19690041.5 0.571814974074678
7.22973127892619e-12 8.27920840006063e-12 100 100
POU2F1-regulon DZ 2
0.00117791636383529 -0.00376613934043978 15213268.5
0.441805809953149 1.91466848509798e-16
2.32378568276849e-16 7.25388601036269 18.538696081069
POU6F1-regulon DZ 2
0.0212304266378903 -0.00994083054510801 14769576
0.428920615405203 4.2549710692397e-12 4.9122430230247e-12
58.0310880829015 65.4806743789795
REL-regulon DZ 2
0.255680727830987 -0.00817025805056643 13703480
0.397960312116806 2.09089951661634e-22
2.60445378385544e-22 100 100
RELA-regulon DZ 2 0.0788777248012285 -0.0134149711219682
6054720.5 0.175834055288148
4.17420689114807e-210 1.31719417454006e-209 100 100
RELB-regulon DZ 2 0.13248489084559 0.027773737717741
31413769.5 0.912281662394181 0 0 100 100
REST-regulon DZ 2 0.214817595639985 -0.0038666614972358
13917363 0.40417165007158 5.98798751753021e-20
7.33012265076974e-20 100 100
RFX5-regulon DZ 2 0.15761601576808 0.0938644954068566
34237124.5 0.994274210054815 0 0 100 100
RXRA-regulon DZ 2 0.00380482116233411 -0.0109109499519663
9872204.5 0.286696925459879
2.05204369690654e-96 3.68848360709782e-96 29.6632124352332
64.8618957940992
SETDB1-regulon DZ 2
0.0503717037454073 -0.00950757941581987 12751190.5
0.370305043043143 3.47762383939513e-35
4.84139789405988e-35 97.279792746114 95.0139000986459
SOX5-regulon DZ 2
0.00266776113277242 -0.00522442766196831 15239983
0.442581620970354 6.62179236570626e-14
7.77102905727512e-14 12.0466321243523 22.67509640391
SP1-regulon DZ 2
0.00438811319492802 -0.00875470787394194 11470276
0.333106234111767 6.11969062056792e-61
9.87495531955278e-61 34.3264248704663 60.2636534839925
SP2-regulon DZ 2
0.0279360964383461 -0.000309323253922161 16909516
0.491066230264439 0.393927967731083 0.393927967731083
100 100
SP3-regulon DZ 2
0.0221322005751347 -0.00624393021073518 9447651
0.274367543188348 7.9313556279185e-103
1.56423958217281e-102 100 100
SP4-regulon DZ 2 0.0035220858949613 -0.0052043826588813
15023241.5 0.43628726982826 3.29685808489947e-14

3.90128206713104e-14	18.0051813471503 29.2059008160703
SPI1-regulon	DZ 2	0.45488920353578 0.0369753014059703
28417436.5	0.825265691568822	1.61007207337017e-211
5.31698219578055e-211	100	100
SPIB-regulon	DZ 2	0.597959537338163	0.0340882746895745
	27832140.5	0.808268215100019
3.33820001213697e-190	8.46472145934733e-190	100	100
SREBF1-regulon	DZ 2
0.0330128190451332	-0.00805408847230126	13255074
0.384938233658265	4.76452215283718e-28
6.20699216241174e-28	95.8549222797927 96.9128329297821
SREBF2-regulon	DZ 2
0.0930457394111704	-0.00603964956325727	11640727.5
0.3380562856418 7.1239387499216e-54	1.09956445922703e-53	100
	100
SRF-regulon	DZ 2	0.0076912808545724	-0.0133042457480832
	5820742.5	0.169039142031919
6.41604075941348e-219	2.27769446959178e-218	86.139896373057
98.1391803425702
STAT1-regulon	DZ 2	0.0592102161210909	-0.020914114299857
	3086318.5	0.0896292236389496	0	0	100
	100
STAT2-regulon	DZ 2	0.0533281183958122	-0.0131296156401926
	11216934 0.325748974394359	4.35931989296919e-62
7.11521177932902e-62	99.8704663212435 99.9641287776881
STAT5A-regulon	DZ 2	0.0579132499397872	-0.013931602324013
	9479382.5	0.275289052005373
5.26893010281115e-102	1.01106496567457e-101	100	100
TBP-regulon	DZ 2
0.019899209973225	-0.00101939871435887	16764453
0.486853481622736	0.179660180787204	0.180934366466546
	48.3160621761658 49.9125638956147
TCF12-regulon	DZ 2
0.00208335770009006	-0.00708082450900932	14248563
0.413789970043812	4.04511190282725e-27
5.12862401965598e-27	8.93782383419689 25.5021971123666
TFDP1-regulon	DZ 2	0.610453979971549	0.0837149535989418
	32213729.5	0.935513157699093	0	0	100
	100
TFDP2-regulon	DZ 2	0.0680408621428374	0.0416259065868703
	33668326 0.9777558345333	0	0	100
99.995516097211
TFEC-regulon	DZ 2	0.0669058699585809	-0.0250620663696472
	6069406.5	0.176260548787883
1.47079665277472e-209	4.54028531943502e-209	100	100
THAP1-regulon	DZ 2
0.0347670827738959	-0.00243953504726525	14322830.5
0.415946759230218	1.04979744733797e-15
1.26331557222027e-15	100	100
THAP11-regulon	DZ 2	0.167907625239301	-0.0107530302987386

7623103 0.221381171000254 9.45029303265332e-156
2.09678376661996e-155 100 100
TP53-regulon DZ 2 0.088121857475824 -0.0104119778307377
12389364 0.359797304361281 8.0187452633551e-41
1.15016346201659e-40 100 100
USF2-regulon DZ 2
0.119005119672567 -0.00638979761428962 11806302.5
0.342864719607387 7.93488541819651e-51
1.19867418019564e-50 100 100
XBP1-regulon DZ 2 0.167955239693469 -0.02780773096917
4084918 0.118629373141097 5.5327728083547e-290
3.02174514917834e-289 100 100
YBX1-regulon DZ 2 0.128546137088777 0.0339398065756577
33896477 0.984381526924558 0 0 100 100
YY1-regulon DZ 2 0.705465645454182 0.0671365338922607
31152836 0.904703939282845 0 0 100 100
YY2-regulon DZ 2 0.0378482552266465 -0.0133814659588444
13458088.5 0.390833941448129
1.97301648956641e-25 2.4793658541454e-25 90.4145077720207
90.5322392610528
ZBTB33-regulon DZ 2 0.352006284588407 -0.0140589191719858
10329439 0.299975390808139 3.20036545737849e-81
5.47532403551501e-81 100 100
ZNF143-regulon DZ 2
0.017177682853995 -0.00625954919160726 9654695.5
0.280380285487535 1.60268799047529e-97
2.91771403394219e-97 100 99.9932741458165
ZNF274-regulon DZ 2 0.047104807874297 -0.0185523616386521
4697337.5 0.136414538322965
9.16853436005297e-264 3.82921140919859e-263 100 100
ZNF76-regulon DZ 2
0.0112381015925816 -0.00337644994838587 12728303.5
0.369640385768975 1.59016946559714e-35
2.23568380311678e-35 99.2227979274611 99.464173616716
ATF1-regulon DZ 3
0.102201553162691 -0.00413086592240695 22365190.5
0.434226514035357 1.63755623653564e-14
1.97061852193271e-14 100 100
ATF3-regulon DZ 3 0.0383292350273652 -0.0130270061379358
12597162 0.244577471495209 2.9102142610343e-195
6.88750708444783e-195 100 100
ATF4-regulon DZ 3 0.087990156650212 -0.0213085698854496
9473727 0.183935095483879 7.37938698280565e-298
2.61968237889601e-297 100 100
ATF5-regulon DZ 3
0.111517219240823 -0.00709847103720826 22757667
0.441846556549003 1.14509033969352e-11
1.32197421330471e-11 100 99.9977381194725
ATF6-regulon DZ 3 0.119871977104282 -0.0169778643060616
10878310.5 0.211205482332432

4.94181562835024e-249	1.37595650828575e-248	100	100
ATF6B-regulon	DZ 3
0.052767828010656	-0.00464801832479883	13855485.7597656
0.269008184022826	4.2822411741877e-160
9.80771365701055e-160	100	99.9185723010111
BACH1-regulon	DZ 3	0.0175896061464413	-0.0263702696880569
	6953531.5	0.135004785382	0	0
96.824034334764	99.6358372350772
BATF-regulon	DZ 3	0.00868782063396714	-0.0108849980872059
	20772470	0.403303393995416	4.54423177668891e-33
6.51798901302853e-33	39.1416309012876	52.0481328176246
BATF3-regulon	DZ 3
0.0806851187707392	-0.00254380679009775	27886805.5
0.541430234624964	1.32984160506187e-06
1.44150769403653e-06	100	100
BCL11A-regulon	DZ 3
0.196171223806185	-0.00854858745953746	18424635
0.357719511864825	6.38224934253535e-62
1.02986296209093e-61	100	100
BHLHE40-regulon	DZ 3	0.0317646384074757	-0.0245159699196796
	5804082	0.112687897473324	0	0	100	100
BRF2-regulon	DZ 3
0.0330392381557943	-0.00879900605060021	17820299
0.345986157097019	3.07757797357786e-72
5.20257228866733e-72	99.9141630901288	99.7240505756486
CEBPB-regulon	DZ 3	0.120606187341229	-0.0230783559575807
	7573656.5	0.14704468806095	0	0	100	100
CEBPG-regulon	DZ 3
0.0614930271384601	-0.00674077163626462	23368621.5
0.453708411370638	6.56815833107595e-08
7.28655064853738e-08	100	100
CLOCK-regulon	DZ 3	0.0057448572048595	-0.0101094229978013
	18658788	0.362265658741639	1.33043566716189e-65
2.19676586903475e-65	29.0128755364807	51.6749225305919
CREB1-regulon	DZ 3
0.0397165042087179	-0.00334045626945401	22637216.5
0.439507975944075	1.66615149629249e-12
1.93929108584863e-12	100	99.9932143584176
CREB3-regulon	DZ 3	0.227521384436132	-0.0126021821108583
	18216720	0.353682783196422	2.22433410520965e-65
3.63052233264104e-65	100	100
CREB3L2-regulon	DZ 3	0.108678039980314	-0.0185760922495335
	8618896.5	0.167338318983983	0	0	100
	100
CREB3L4-regulon	DZ 3	0.00405419050688691	-0.0072328530178547
	21269479	0.412952964631275	4.73690280323934e-35
6.93443503154625e-35	15.450643776824	31.7658501277963
CREB5-regulon	DZ 3	0.024860728286126	-0.0146907603961333
	15154342.5	0.294225855857246
1.91536752531418e-127	3.8854598370659e-127	99.5708154506438

99.6245278324399
CREM-regulon DZ 3 0.153214664104979 0.00329982703597478
29630874 0.5752918189917 1.53386443749528e-18
1.96224099211108e-18 100 100
CTCF-regulon DZ 3 0.416879481229578 0.047252405044529
46285968.5 0.898655200388539 0 0 100
100
CUX1-regulon DZ 3 0.0975449622475231 0.0213962313945543
43312776 0.840929825108097 0 0 100 100
DDIT3-regulon DZ 3 0.0585647996407548 -0.0270374981814124
7219250.5 0.140163795097699 0 0 100
100
E2F1-regulon DZ 3 0.548581428130687 0.113808459438674
48141692.5 0.934684607941841 0 0 100
100
E2F2-regulon DZ 3 0.0745811520764111 0.0212045741974536
45371870 0.880907718866307 0 0 100 100
E2F3-regulon DZ 3
0.0156765968559845 -0.00558202032822258 14983729
0.290913346386228 1.62468906147778e-131
3.39273304014477e-131 100 100
E2F4-regulon DZ 3 0.421848658666791 0.0238022444560282
37001770.5 0.718399864170677
2.60887267406407e-143 5.69938338026305e-143 100 100
E2F6-regulon DZ 3 0.0664021994816857 -0.0061570994996485
26006565.5 0.504924841981435 0.565448453102871
0.565448453102871 100 100
E2F7-regulon DZ 3
0.0303723823323619 -0.00020695843842897 25245770
0.490153781665235 0.250501568847095 0.255908077527248
100 100
E2F8-regulon DZ 3 0.10453535189943 0.0305223839266624
46626428 0.90526531810049 0 0 100 100
EGR1-regulon DZ 3
0.0402653458908202 -0.00738732718638489 14645277
0.284342204855898 8.75463989576765e-140
1.88357403818031e-139 100 100
EGR2-regulon DZ 3 0.0306920330784365 -0.0157605169129437
12153831.5 0.235970084154576
1.67973750811338e-208 4.18460923073861e-208 100
99.9954762389451
EGR3-regulon DZ 3 0.0233748937069909 -0.0185731017719483
8456995 0.164194955462796 0 0 100
99.9954762389451
ELF1-regulon DZ 3 0.779489256281213 0.0743535976039891
46660478.5 0.905926418211225 0 0 100
100
ELF2-regulon DZ 3 0.0158225301317256 -0.017277110744838
10483096 0.203532280772569 2.51117053313044e-262
7.75187425444615e-262 98.7124463519313 99.5566714166158

ELF4-regulon	DZ 3
0.0134068442257676	-0.00517307303445507	18494121.5
0.359068611961581	8.68736861713786e-61
1.37067371514842e-60	96.824034334764	98.3759697812762
ELK1-regulon	DZ 3	0.069359414566686	0.00339151831953875
30098197	0.58436502752165	7.12706325063279e-23
9.54757529801751e-23	100	100
ELK3-regulon	DZ 3
0.0559910824294859	-0.00914927550726393	17460868
0.339007702334193	9.27413589515845e-79
1.60600889891768e-78	100	100
ELK4-regulon	DZ 3	0.0325982869776861	-0.0127254645988985
18626129	0.361631574997891	1.15429343598242e-58
1.80120514186268e-58	92.7896995708155	96.8333672615412
ESRRA-regulon	DZ 3
0.0901662127099427	-0.00161087085583585	28924174.5
0.561571047851587	6.67968545877744e-13
7.83896971195368e-13	100	100
ETS1-regulon	DZ 3	0.299877327087883	0.0584386977402767
46136031	0.895744121319117	0	0	100	100
ETV2-regulon	DZ 3
0.0217718482748041	-0.00701015887133963	15145947.5
0.294062864552284	1.21181127785145e-127
2.49387248485371e-127	100	100
ETV3-regulon	DZ 3	0.006240087247341	-0.0053257023320311
16976648	0.329606433758984	2.49334838666323e-88
4.4817148215972e-88	69.4420600858369	84.7413539616837
ETV5-regulon	DZ 3
0.0322726475996158	-0.00122237837159614	23596653.5
0.458135717297163	1.02958316132201e-06
1.12462160698251e-06	100	100
ETV6-regulon	DZ 3	0.431011434520693	-0.0293161336936235
8355246	0.162219469782198	0	0	100	100
ETV7-regulon	DZ 3
0.0591961649507477	-0.00500850471259705	21617499
0.41970987159411	7.21867403948665e-21	9.49121957043615e-21	100
100
FLI1-regulon	DZ 3	0.13494133162114	-0.00922708347251436
18484461	0.358881050615353	6.05516925961663e-61
9.66105657152316e-61	100	100
FOS-regulon	DZ 3	0.0628157224499075	-0.0240800419724041
6521958.5	0.12662567323709	0	0	100	100
FOSB-regulon	DZ 3	0.0169924072373697	-0.0251261732268831
8204824	0.159298984007922	0	0
96.0515021459228	99.4096491823302
FOSL1-regulon	DZ 3	0.0473458702639151	-0.0138021990724814
16554516	0.321410621305575	1.77094451000267e-96
3.3088700055313e-96	100	100
FOXO1-regulon	DZ 3
0.0101346566166213	-0.00673525179120056	22138380

0.429822923877624 4.20668331298966e-18
5.33347348611189e-18 44.2918454935622 52.554794055778
FOXO3-regulon DZ 3 0.00723891504308656 -0.0128345156853281
19642930.5 0.381373064381177
5.05825272015243e-51 7.6411902793792e-51 28.2403433476395
47.2484223383321
GABPA-regulon DZ 3
0.0243217120494693 -0.00119694578015976 23724768.5
0.460623106342459 4.315057356944e-06
4.60705371944397e-06 100 100
GTF2B-regulon DZ 3 0.0735829201918458 -0.005618306851094
21964743.5 0.426451722004593
9.18883813301925e-18 1.15470355299888e-17 100 100
HINFP-regulon DZ 3 0.0179135086008619 0.00172731009141002
27809605 0.539931365031308 1.74754805032992e-06
1.87993805414279e-06 71.4163090128755 63.0182533758567
HIVEP3-regulon DZ 3 0.0561181932157559 -0.0221381573669963
14871602.5 0.288736378601135
3.16013799130697e-134 6.69760589202372e-134 99.8283261802575
99.6901223677365
HOXB2-regulon DZ 3 0.0106425183612599 -0.027157456625291
11507978 0.223430655354158 1.49853460146457e-229
3.86894388014489e-229 55.6223175965665 84.4699282983873
IKZF1-regulon DZ 3 0.146774420383883 0.00298697603423981
29917400 0.580854802511134 3.85783704244368e-21
5.11974635539255e-21 100 100
IRF1-regulon DZ 3 0.0975417205515296 -0.0388994417378725
3607572 0.0700420331180081 0 0 100 100
IRF2-regulon DZ 3 0.0581638653640674 -0.0275642507099967
5470094 0.106203425768527 0 0 100 100
IRF3-regulon DZ 3 0.13322290938401 -0.012545722904218
12436898.5 0.241465910208391 5.35406338729942e-200
1.28860508643477e-199 100 100
IRF4-regulon DZ 3 0.0388986710152653 -0.0200278822117835
8603015.5 0.167029984866757 0 0 100
99.9954762389451
IRF5-regulon DZ 3 0.007591635697023 -0.0404927462158583
8686871 0.16865806317209 0 0 32.1888412017167
81.7511479043677
IRF7-regulon DZ 3 0.0834384967974421 -0.0250390604022539
7099641.5 0.137841552453835 0 0 100
100
IRF8-regulon DZ 3 0.104282712374728 0.0231029411011707
42710180.5 0.829230262641218 0 0 100
100
IRF9-regulon DZ 3 0.0229342641123181 -0.0112713415609641
11440207 0.222114862176242 9.89726329180431e-231
2.60261368043743e-230 100 100
JUN-regulon DZ 3 0.058222656491571 -0.0309728347185429
3197106.5 0.0620727290695235 0 0 100

100
JUNB-regulon DZ 3 0.12475910255445 -0.0359462862930801
4581921.5 0.0889593048862541 0 0 100 100
JUND-regulon DZ 3 0.0242350329085473 -0.014597434711699
8540816 0.165822363940848 0 0 100 100
KLF11-regulon DZ 3 0.00373189642443036 -0.0186842989565289
17931292 0.348141117658268 4.0218649618585e-88
7.13881030729884e-88 14.0772532188841 41.5643165727986
KLF12-regulon DZ 3 0.0522195647149752 0.00530617248013459
29723074 0.577081908130179 2.33895119579695e-19
3.01937336184697e-19 99.3991416309013 98.581800909276
KLF13-regulon DZ 3 0.0520865842183607 -0.0145798427450423
9244892 0.179492199084705 3.13969251383774e-306
1.14317009478195e-305 100 100
KLF2-regulon DZ 3 0.083888753476351 -0.0256173220915557
9884271 0.191905923632118 3.92566095377418e-283
1.29638105915333e-282 100 100
KLF3-regulon DZ 3 0.0381943846877661 -0.0347264111344843
9619910 0.186773279871409 1.42734615603639e-292
4.82578938469445e-292 99.8283261802575 99.8642871683518
KLF4-regulon DZ 3 0.00884200324985244 -0.0072437675029622
21329619 0.414120599780821 7.89677269067277e-27
1.08868128356848e-26 37.0815450643777 50.1481531745493
KLF6-regulon DZ 3 0.0319661000647982 -0.0383996639168906
4448481.5 0.0863685294563342 0 0 100
100
KLF7-regulon DZ 3 0.0151846434303559 -0.0316260738880204
7026578 0.136423003888008 0 0
84.549356223176 97.8987129899799
KLF8-regulon DZ 3 0.00500966844314483 -0.020626630907841
10969184.5 0.212969826804993
8.75882660393125e-249 2.39183341876584e-248 38.5407725321888
78.6659428648979
MAFB-regulon DZ 3
0.0496235237692292 -0.00632168690784988 18910789
0.367158329598318 3.27488717807696e-54
5.00036536867664e-54 100 99.9977381194725
MAFF-regulon DZ 3
0.00453633302590841 -0.00805747840518162 21842189
0.424072291643186 1.01762249412683e-28
1.43071677392089e-28 14.8497854077253 28.9068331410735
MAFG-regulon DZ 3 0.00840873147770427 -0.0174465502936193
16855539 0.327255068189873 3.79787063544257e-97
7.19063506977127e-97 32.618025751073 59.2861505055303
MAX-regulon DZ 3 0.185195263965061 -0.01280367216117
10192334 0.19788705411224 2.49608658434103e-272
7.87653988836502e-272 100 100
MAZ-regulon DZ 3 0.161447435494662 0.025806179662286
44127985.5 0.856757348660535 0 0 100
100

MEF2A-regulon	DZ 3
0.0356155003844409	-0.00527422731627487	20351522.5
0.395130578945309	1.92136811694925e-34
2.78402318986524e-34	100	99.9977381194725
MLX-regulon	DZ 3
0.047411650504319	-0.00565910110522973	21378175
0.41506332828633 3.66257671691929e-23	4.95319898859562e-23	100
99.9886905973626
MXD4-regulon	DZ 3	0.0730612175657893	-0.019611422806961
24877578.5	0.483005239311328	0.0471562587275604
0.0485231068066201	90.2145922746781 88.5616701725815
MXI1-regulon	DZ 3	0.0414089321444141	-0.0244483143840757
9509440.5	0.18462848321107 1.45857985790776e-296
5.05166682494881e-296	100	100
MYBL1-regulon	DZ 3	0.059692935960181	0.0305880456957728
40791553 0.791979565802424	1.58889833179712e-256
4.60456251255493e-256	98.4549356223176 79.575218836941
MYC-regulon	DZ 3
0.305866064344224	-0.00925326649443287	28033624
0.544280757425157	2.36713152625363e-07
2.60567966455826e-07	100	100
NFATC1-regulon	DZ 3	0.0376575059033336	-0.0131992177875085
20196101 0.392113026461187	2.33230082968061e-36
3.44986164390257e-36	92.1888412017167 94.8971975300265
NFE2L1-regulon	DZ 3	0.142676570048627	-0.0410443603935005
18734572 0.363737026586221	6.04141068642251e-57
9.32478605947823e-57	99.9141630901288 99.9366673452308
NFE2L2-regulon	DZ 3	0.0908177968658546	-0.0205914029287675
11491149.5	0.223103925255818
4.16878839008887e-229	1.05708562748682e-228	100	100
NFKB1-regulon	DZ 3	0.0303571752295727	-0.027004956539764
10113494.5	0.19635636286893 4.49356390127957e-275
1.45019562268568e-274	99.7424892703863 99.9366673452308
NFKB2-regulon	DZ 3	0.0433902979078611	-0.0307764611386077
5073670 0.0985067414232742	0	0	100	100
NFYA-regulon	DZ 3	0.0101385632748824	-0.0110946919892202
10703386.5	0.207809283281897
7.12141772981014e-255	2.02248263526608e-254	94.8497854077253
99.1269141163964
NFYB-regulon	DZ 3	0.15092999665460	0.0434198777444947
47582819.5	0.923833930207686	0	0	100
100
NFYC-regulon	DZ 3	0.101403302592859	0.0183394167230867
43499083 0.844547028330685	0	0	100	100
NR1H3-regulon	DZ 3
0.00388652873696773	-0.00477033483998545	23071303
0.447935888404057	5.88997771939126e-17	7.2728420535092e-17
12.4463519313305 22.3586890140463
NR2C2-regulon	DZ 3	0.0103584359042042	-0.0117288637038054
16951282.5	0.329113955385426

1.02972919632302e-89 1.87463520356241e-89 57.5107296137339
76.465133111669
NR3C1-regulon DZ 3 0.227502302655148 0.0020780704431948
27112328 0.526393534399951 0.00206766373850693
0.0021588841975587 100 100
NRF1-regulon DZ 3
0.0242752297938228 -0.00251859961259579 22218640
0.431381194531142 1.1624137844523e-15 1.4229548051054e-15
100 99.9660717920879
PAX5-regulon DZ 3 0.101140625736504 0.00089594962529374
26189738.5 0.508481197705541 0.322260622097767
0.326864345270592 100 100
POU2F1-regulon DZ 3
0.00184212740890347 -0.00311790366107896 23577755
0.457768797561984 2.834376210514e-13
3.38219682263015e-13 10.5579399141631 18.5519440863134
POU6F1-regulon DZ 3
0.022062920957328 -0.00917476518050238 22398007.5
0.434863665393898 8.15708947560738e-15
9.90005731227562e-15 59.2274678111588 65.5153694781842
REL-regulon DZ 3
0.259490489231246 -0.00433273295761871 24285717.5
0.471514090205155 0.000885663523752306 0.0009315868175765
100 100
RELA-regulon DZ 3 0.0788953964655497 -0.0135160820267361
9133020.5 0.177320182196903 0 0 100
100
RELB-regulon DZ 3 0.12060657011971 0.0158292984982144
40853794.5 0.79318800217024 1.33251550788434e-256
3.94202504415783e-256 100 100
REST-regulon DZ 3 0.215293732315126 -0.0034123496788479
21318138 0.413897692910985 9.29622477532435e-24
1.26929222893852e-23 100 100
RFX5-regulon DZ 3 0.0745443289413349 0.00943817356940649
35628105.5 0.691729768764944
6.68132012598891e-111 1.33626402519778e-110 100 100
RXRA-regulon DZ 3
0.00520363121221489 -0.00957226949931276 16345160.5
0.317345924921293 1.46672094526972e-105
2.85307361956576e-105 35.6223175965665 65.0177557621406
SETDB1-regulon DZ 3
0.0572025644201879 -0.00258123404321058 24276182.5
0.471328965477005 0.000818838251411087
0.000867724117166973 98.7124463519313 94.9560064237407
SOX5-regulon DZ 3
0.00409226983404218 -0.00380882279535902 23677723.5
0.459709714330314 1.25729328322758e-10
1.41694957316124e-10 15.2789699570815 22.684399810002
SP1-regulon DZ 3
0.0045431355275226 -0.00867342282792732 17114671.5

0.332286199140815	4.27003981351406e-91
7.87461887687008e-91	33.8197424892704 60.5075659903644
SP2-regulon	DZ 3
0.0275302955150851	-0.000728567033359044	24701114
0.479579131016566	0.0171591825270763	0.0177854300645608
	100	100
SP3-regulon	DZ 3
0.0221755186510876	-0.00625497414908498	14460743
0.280759424930952	2.12107930297903e-144
4.70614470348473e-144	100	100
SP4-regulon	DZ 3
0.00315904505657937	-0.00562325270179729	21685297.5
0.421026198692322	1.31511401820807e-30
1.86746190585545e-30	14.5922746781116 29.3953993350071
SPI1-regulon	DZ 3	0.44987105757585 0.0321536032397844
40890571.5	0.793902038051432	7.6513066144941e-258
2.31167136012375e-257	100	100
SPIB-regulon	DZ 3	0.605002113705772	0.0416194465067605
	44053224.5	0.855305842650971	0	0	100
	100
SREBF1-regulon	DZ 3	0.0301571211193062	-0.011056630958378
	17537827 0.340501883136885	2.42867054938055e-77
4.1550749158077e-77	93.5622317596567 96.9826513763543
SREBF2-regulon	DZ 3
0.0934772714732791	-0.00565043383477599	18076719
0.350964624091474	9.22063919231034e-68
1.54038913565655e-67	100	100
SRF-regulon	DZ 3	0.0104927179755997	-0.0105472521893594
	12159739 0.236084779941838	2.52749663165221e-208
6.18800899473472e-208	92.4463519313305 98.0796634321775
STAT1-regulon	DZ 3	0.0582538001121084	-0.0220816422848253
	3840989.5	0.0745739000538095	0	0	100
	100
STAT2-regulon	DZ 3	0.0466402029181027	-0.0201104754185993
	12740606.5	0.247362487128881
4.50491016614989e-191	1.04868400589063e-190	99.3991416309013
99.9773811947253
STAT5A-regulon	DZ 3
0.0662227010459486	-0.00552703030166926	22564539.5
0.43809693138532 5.02629764570959e-13	5.94778554742302e-13	100
	100
TBP-regulon	DZ 3
0.0195397331035661	-0.00139740975533029	25467299.5
0.494454839710817	0.488819232907473	0.492286035977739
	50.0429184549356 49.8812512723078
TCF12-regulon	DZ 3
0.0050938472391887	-0.00405394863436049	23576822
0.457750683102481	1.01770919138871e-10
1.16544117078384e-10	17.6824034334764 25.4190133677139
TFDP1-regulon	DZ 3	0.607661672031014	0.0815932239763286

47451574 0.921285761617402 0 0 100 100
TFDP2-regulon DZ 3 0.0297593835592599 0.00270569688974129
30584434 0.593805456723673 6.80416642401186e-28
9.47246698244788e-28 100 99.9954762389451
TFEC-regulon DZ 3 0.0664392066541368 -0.0257638081575671
8775500.5 0.170378830040841 0 0 100
100
THAP1-regulon DZ 3
0.0320234058936334 -0.00527719579667975 16344169.5
0.317326684375347 7.49364616210956e-101
1.43796993921562e-100 100 100
THAP11-regulon DZ 3
0.176737739311898 -0.00178582043579159 22910803
0.444819735402692 1.19488583103388e-10
1.35739030405448e-10 100 100
TP53-regulon DZ 3 0.100269693776923 0.00196341093475501
28376327 0.550934433325635 2.77372926870939e-09
3.1013350878483e-09 100 100
USF2-regulon DZ 3
0.120343591129504 -0.00507285634701307 19294627
0.37461065318547 1.70596830200842e-48 2.54997367247574e-48 100
100
XBP1-regulon DZ 3 0.173930360225965 -0.0219223488015727
11054086.5 0.214618223204506
3.12408931089269e-243 8.3702015499389e-243 100 100
YBX1-regulon DZ 3 0.10119097184219 0.0061655052876327
35475500.5 0.688766899426793 1.46558978926188e-107
2.89046875104427e-107 100 100
YY1-regulon DZ 3 0.703064312838044 0.0652687134183909
45576233.5 0.884875494155369 0 0 100
100
YY2-regulon DZ 3 0.031585765112337 -0.0199279287746308
16983183.5 0.329733322344283 6.0707854531806e-88
1.06426115352055e-87 83.519313304721 90.7149804347334
ZBTB33-regulon DZ 3
0.362199406125476 -0.00372217203594616 21771113.5
0.422692340660953 1.83967776782285e-19
2.39664443147564e-19 100 100
ZNF143-regulon DZ 3
0.0169093447734217 -0.00659060054933265 13976206
0.271352001710875 7.016236454656e-157
1.58143742311294e-156 100 99.9932143584176
ZNF274-regulon DZ 3 0.0493604333210905 -0.0164022138892298
8956165.5 0.173886492233935 0 0 100
100
ZNF76-regulon DZ 3
0.0127424841462317 -0.00186243945496316 22044211
0.427994606045939 4.32958029229043e-17
5.39298597811614e-17 99.5708154506438 99.4571486734071
ATF1-regulon GC

0.0985494361680518	-0.00798377736625101	25932830
0.34079900010261 6.2733935646445e-113	1.46036374783528e-112	100
	100
ATF3-regulon	GC	0.0394036682042259	-0.0120824965284893
	20091966.5	0.264040680993749
1.21926051639583e-245	4.3283748332052e-245	100	100
ATF4-regulon	GC	0.0929541263103047	-0.0164289534265963
	19446482 0.255557978867459	1.83578952332886e-263
6.86005558717627e-263	100	100
ATF5-regulon	GC
0.114730504747721	-0.00385094563313607	35989311.5
0.47295730445082 0.000124965296336649	0.000144268878697595	100
	99.9977081041437
ATF6-regulon	GC
0.129512016128611	-0.00717780409028504	32882853
0.43213345488792 6.13230707465809e-22	8.13820191216308e-22	100
	100
ATF6B-regulon	GC
0.0533480312292308	-0.00410630355958089	22270952.5898438
0.292676054790448	3.81201735654806e-190
1.12772180131214e-189	99.7706422018349 99.9266593325999
BACH1-regulon	GC	0.0235749954580695	-0.0204955759024167
	15694374.5	0.206249265384298	0	0
99.2545871559633 99.5759992665933
BATF-regulon	GC
0.0110327732140161	-0.00859076095895571	32914821
0.432553565706341	3.10432742830336e-24
4.27975237688425e-24	43.9220183486239 52.0283278327833
BATF3-regulon	GC	0.0656586440739552	-0.0182046561660772
	26301352.5	0.345641977113422
2.79581563706204e-106	6.40331968488404e-106	100	100
BCL11A-regulon	GC	0.222666611623756	0.0188923981810711
	55464263 0.728889418232725	2.88410971435465e-231
9.98886779117952e-231	100	100
BHLHE40-regulon	GC	0.0347326273009331	-0.021754677348951
	11044567.5	0.145143339950394	0	0	100
	100
BRF2-regulon	GC
0.0324887507160043	-0.00948826032661809	25603599
0.336472376452095	4.83466596320364e-119
1.16359757080494e-118	99.6559633027523 99.7318481848185
CEBPB-regulon	GC	0.121473310345662	-0.0224828250318899
	11502232 0.151157785885622	0	0	100	100
CEBPG-regulon	GC	0.055259106435326	-0.0133133168462666
	25842269.5	0.339608889811955
1.3560146223017e-114	3.20923460611403e-114	100	100
CLOCK-regulon	GC
0.00840148417154636	-0.00748076170964913	30977293.5
0.407091345244043	1.08791105087297e-44
1.73576819352766e-44	38.0733944954128 51.6134946828016

CREB1-regulon	GC	0.049934433274808	0.00724156209144833
	48627990	0.639049820979804	1.3132734387263e-86
2.6265468774526e-86	100	99.9931243124312
CREB3-regulon	GC
0.234125992431642	-0.00590081547714755	36382060
0.478118649976618	0.00190919618596731	0.00211801451880749
	100	100
CREB3L2-regulon	GC
0.118459891259132	-0.00864976005721702	28555118
0.375260072356624	4.57093882422867e-70
8.54043832947988e-70	100	100
CREB3L4-regulon	GC
0.00685402302125478	-0.00441708985607851	34618931
0.454948305658165	7.81566382631795e-15
9.99841678682116e-15	23.9105504587156	31.6442060872754
CREB5-regulon	GC	0.0281167466515633	-0.0114995443372609
	26753437.5	0.351583099465336
2.10756853293285e-98	4.53446563146159e-98	99.3119266055046
99.6355885588559
CREM-regulon	GC	0.153221148554264	0.00335035963188063
	43136976	0.566889085697561	2.34078722013397e-21
3.07770171536133e-21	100	100
CTCF-regulon	GC	0.391797805482221	0.0217952411237536
	60912951	0.800493921955269	0	0	100	100
CUX1-regulon	GC	0.106421715368862	0.0309117238682282
	70351815.5	0.924535747845618	0	0	100
	100
DDIT3-regulon	GC	0.0659984132522742	-0.0196655477828686
	17529351.5	0.230363807715825	0	0	100
	100
E2F1-regulon	GC	0.49151912016819	0.0559755801736544
64128130.5	0.842746539920621	0	0	100	100
E2F2-regulon	GC	0.0518747593518903	-0.0021280217827337
	37386454	0.49131799886793	0.218100270934528
0.226060134837248	100	100
E2F3-regulon	GC
0.0165883025842265	-0.00470794695650761	24631602.5
0.323698782698415	4.82708027850596e-138
1.26934333249601e-137	100	100
E2F4-regulon	GC	0.38909022035839	-0.00994971275752898
37727798.5	0.49580381334674	0.551673397448067
0.563580017536875	100	100
E2F6-regulon	GC	0.0568048124377904	-0.0162198056582956
	24607358.5	0.323380177634545
1.55442889998792e-138	4.16469629808084e-138	100	100
E2F7-regulon	GC
0.0273185854286948	-0.00338556398565688	30053984
0.394957576797435	3.25045235480627e-50
5.43016746332342e-50	100	100
E2F8-regulon	GC	0.0770861675216768	0.00238107181556531

47891012 0.629364747445693 3.22903971770373e-75
6.19626540424229e-75 100 100
EGR1-regulon GC 0.0477740748286372
0.000323500230223747 38922800.5 0.511508057222962
0.102576483259851 0.107895263873324 100 100
EGR2-regulon GC
0.037383984162875 -0.00901022737510709 26769314
0.351791742152044 3.9339905445836e-98
8.33771130344584e-98 100 99.9954162082875
EGR3-regulon GC 0.0303801558082533 -0.0115343011856602
21888991 0.287656466573645 2.47681009359226e-199
7.64580507152392e-199 100 99.9954162082875
ELF1-regulon GC 0.767161251415614 0.0625195121674119
68268269.5 0.897154609980302 0 0 100
100
ELF2-regulon GC 0.0204106349492354 -0.0127348849225502
21629171 0.284242014845598 9.99145183087459e-206
3.1528581332982e-205 99.4266055045871 99.5393289328933
ELF4-regulon GC
0.0170310047126887 -0.00147269952493033 35373978
0.464870834847246 6.25078838752527e-07
7.45892395822344e-07 99.197247706422 98.3017051705171
ELK1-regulon GC 0.0666276703818246
0.000595590215732841 39417795 0.518013079260908
0.0106106232001773 0.0114144582910998 100 100
ELK3-regulon GC 0.0697160463367214 0.00500287272650975
44769427.5 0.588342117970398
4.99624692256072e-36 7.71159851090894e-36 100 100
ELK4-regulon GC 0.0452112276103101
0.000222755747250773 38277595 0.503029021604378
0.66741946696168 0.676954030775418 96.8463302752293
96.7248808214155
ESRRA-regulon GC 0.0798060016555008 -0.0124065628028446
28629116 0.37623252481976 5.24048270819891e-69
9.54036595595186e-69 100 100
ETS1-regulon GC 0.305192487666072 0.0647417948338664
70966476.5 0.932613379720044 0 0 100
100
ETV2-regulon GC
0.0240093541011047 -0.00477624380011414 27555244
0.362120123518468 3.44946040907735e-85
6.70990928889018e-85 100 100
ETV3-regulon GC
0.00858165907948748 -0.00296120879964128 31721585
0.416872529903984 3.27229027196359e-32
4.84026269394615e-32 80.2178899082569 84.5136596993033
ETV5-regulon GC 0.0356388091369937 0.00226210982153971
43871117.5 0.576536883069997
1.84241600105186e-27 2.61623072149365e-27 100 100
ETV6-regulon GC 0.433012499339347 -0.0276241123376765

13866055 0.182222213285931 0 0 100 100
ETV7-regulon GC 0.0778705929255131 0.0143458892999249
59605777 0.783315557998843 0 0 100 100
FLI1-regulon GC 0.159046633452398 0.0157192791641869
51536038.5 0.677266244758077
1.55016573510439e-139 4.23314489201583e-139 100 100
FOS-regulon GC
0.0797841361618993 -0.00675293351316959 28754384
0.377878747354858 3.11851696749423e-67
5.60543556182508e-67 100 100
FOSB-regulon GC 0.0230341915448879 -0.0191763210440911
18223367 0.239484285058858 5.878878605078e-299
2.87862331696923e-298 98.4518348623853 99.3582691602494
FOSL1-regulon GC 0.0418222250845915 -0.0197297842604159
16123100.5 0.211883412992484 0 0 100
100
FOXO1-regulon GC 0.0188152923330231 0.00220297738647499
41168851.5 0.541024771556858 7.1516365139438e-10
8.90817881561421e-10 61.2385321100917 51.9870737073707
FOXO3-regulon GC
0.0119611767983185 -0.00809381700491925 32758855
0.430503922190766 1.03223683016815e-26 1.4512636622166e-26
36.6399082568807 47.1649248258159
GABPA-regulon GC 0.0282496097041646 0.00287206898819245
45181869.5 0.593762267687969
2.29112306117863e-40 3.61488305208184e-40 100 100
GTF2B-regulon GC
0.0715335617338276 -0.00782413489147306 29796650
0.391575795098623 2.20711713552516e-53
3.73107896719729e-53 100 100
HINFP-regulon GC
0.0158662528256795 -0.000378854318008524 38255829.5
0.502742988007707 0.689779540388542 0.694671593866475
66.0550458715596 63.1211037770444
HIVEP3-regulon GC
0.0730387450278824 -0.00483505492127843 34940301.5
0.45917162972509 6.96527320569315e-09 8.60059821920372e-09
99.9426605504587 99.6837183718372
HOXB2-regulon GC 0.0157675050048806 -0.0221880024046528
21609365 0.283981732223299 4.21649534122391e-207
1.36077804194044e-206 66.6857798165138 84.4105243857719
IKZF1-regulon GC 0.150986563683643 0.00740711889942378
50914473 0.669097876674135 3.74693240979635e-127
9.33446319633476e-127 100 100
IRF1-regulon GC 0.117309638845359 -0.0188575852166362
18020872.5 0.236823182389913
4.73575055087827e-305 2.40170206508826e-304 100 100
IRF2-regulon GC 0.0681390401587379 -0.0175561412875113
17423162 0.228968307285622 0 0 100 100
IRF3-regulon GC 0.141425412897294 -0.0041818425902922

31365216.5 0.412189275956456
1.28903510146845e-35 1.96820413342495e-35 100 100
IRF4-regulon GC 0.0441279310024704 -0.0148553767713087
19056684 0.250435407646269 1.57366940830416e-274
6.2072515549775e-274 100 99.9954162082875
IRF5-regulon GC 0.0153657090960545 -0.0329452798036958
18309876.5 0.240621158708952
1.40916653308716e-298 6.67005492327925e-298 51.6628440366973
81.6304547121379
IRF7-regulon GC
0.103815340516548 -0.00418001097464955 32824370
0.431364894421399 2.11061317106083e-22
2.82742519142111e-22 100 100
IRF8-regulon GC 0.122561953452043 0.0424193933843997
72479566 0.952497803775026 0 0 100 100
IRF9-regulon GC
0.0289770405781744 -0.00513660287947117 28891691
0.379683181668702 2.58196248555513e-65
4.52640336973862e-65 100 100
JUN-regulon GC 0.0710369975304691 -0.0180573078648803
13898582.5 0.182649676832171 0 0 100
100
JUNB-regulon GC 0.139579320329036 -0.0210107045646251
16079728.5 0.211313435314288 0 0 100
100
JUND-regulon GC
0.0335692510258475 -0.00508383078356053 28332848.5
0.372339094455126 2.68177377825922e-73
5.07749168683745e-73 100 100
KLF11-regulon GC 0.00668552756174421 -0.0158605513082221
28531993 0.374956172748391 2.86753366889015e-88
5.81699687117715e-88 19.4380733944954 41.714796479648
KLF12-regulon GC 0.067881729064028 0.0216647772511426
57487256.5 0.755474799080634
1.38970771237349e-287 6.36575790829146e-287 99.9426605504587
98.5492299229923
KLF13-regulon GC 0.0608757276434215 -0.0056328670596239
29136918 0.382905857959649 5.84671975009978e-62
1.00028217411346e-61 100 100
KLF2-regulon GC 0.0977317454750389 -0.0115609603515151
26573483.5 0.349218215136689
1.67915645529628e-101 3.66831102541648e-101 100 100
KLF3-regulon GC 0.0444731579441697 -0.0286574932935487
18935592 0.248844064452317 5.15608538011291e-278
2.09189749707438e-277 99.8853211009174 99.8624862486249
KLF4-regulon GC
0.0124695455159058 -0.00356735531740527 35444677
0.465799933156542 2.09562250515756e-07 2.5218508112913e-07
46.1009174311927 49.9610377704437
KLF6-regulon GC 0.0390902163489567 -0.0315003584735899

11276527.5	0.148191666572047	0	0	100
100
KLF7-regulon	GC	0.0223224423632007	-0.0246226528301821
16232429	0.213320164919779	0	0
93.2912844036697	97.7264393105977
KLF8-regulon	GC	0.0105421740169446	-0.0151467043947061
23440303	0.308043195613522	4.26953995044052e-165
1.18877386855403e-164	58.4862385321101	78.4011734506784
MAFB-regulon	GC
0.0543811269119016	-0.00145780848187222	36754008.5
0.483006650125066	0.015925735642407	0.0170034170016677
100	99.9977081041437
MAFF-regulon	GC
0.0088726410945011	-0.00365476858386099	36334061.5
0.477487872664369	6.14999642349561e-05
7.15819255849489e-05	25.4587155963303	28.6693252658599
MAFG-regulon	GC	0.0132980940131743	-0.0125932737582902
28917179	0.380018134888795	8.81097722065353e-70
1.62488151341922e-69	42.6032110091743	59.2409240924092
MAX-regulon	GC
0.191531426967319	-0.00638415423375158	24929592.5
0.327614849477111	4.57242246110124e-132
1.15943569549353e-131	100	100
MAZ-regulon	GC
0.132871836906671	-0.00356915457644205	34631119
0.455108475536009	1.91330165667211e-10
2.40432597564105e-10	100	100
MEF2A-regulon	GC	0.0508738384095479	0.0105240072045157
52821428.5	0.694158331998146
5.43098804940968e-167	1.54240060603235e-166	100
99.9977081041437
MLX-regulon	GC
0.0512691930654562	-0.00172246671500624	36959296.5
0.485704463866685	0.042569371050137	0.0451108260382048
99.9426605504587	99.990832416575
MXD4-regulon	GC
0.0880158931614854	-0.00431924399267161	43288146.5
0.568875708647891	1.40378664356283e-22
1.89845431796117e-22	93.348623853211	88.4144664466447
MXI1-regulon	GC	0.0491511486120662	-0.0167210674000319
21268385.5	0.279500714430197
8.95001284185575e-215	2.95558563614771e-214	100	100
MYBL1-regulon	GC	0.0698986534224423	0.0416075981807706
63330639.5	0.832266228462487	0	0
98.5091743119266	79.3225155848918
MYC-regulon	GC	0.281127378138613	-0.035103565966519
24681210	0.324350704852595	4.87415120999705e-137
1.25841722149015e-136	100	100
NFATC1-regulon	GC
0.0492651902083437	-0.00130272127290523	37591966.5

0.494018762899799	0.396140741912029	0.407623082257306
	95.4701834862385 94.8019801980198
NFE2L1-regulon	GC	0.121983610070254	-0.0631090935399632
	19314099 0.25381825381506 3.39612229436977e-267
1.3033766643257e-266	99.9426605504587 99.9358269160249
NFE2L2-regulon	GC	0.0925498187803862	-0.0190634004512732
	18787899.5	0.246903148003065
2.70594975357218e-282	1.20076520314765e-281	100	100
NFKB1-regulon	GC	0.0362660761773483	-0.0212182307520452
	20743586 0.272604006864754	2.75724956953498e-228
9.32212949699921e-228	99.9426605504587 99.9312431243124
NFKB2-regulon	GC	0.0570320587536715	-0.0169978360666368
	18810774 0.247203755639325	1.25128852249598e-281
5.38433243013423e-281	100	100
NFYA-regulon	GC
0.013278126829703	-0.00797686541235505	22096899
0.290388711319526	2.7356662283037e-194
8.2652043493431e-194	98.3371559633027 99.0442794279428
NFYB-regulon	GC	0.11956591776749 0.0113783409282183
57221756.5	0.751985703038003	7.70191972197299e-280
3.21668411917696e-279	100	100
NFYC-regulon	GC	0.0862052532055902	0.00277725668655245
	45873442.5	0.60285064666157 3.25158712109038e-48
5.36889966505621e-48	100	100
NR1H3-regulon	GC
0.00531869479501389	-0.00334422686470237	35921788
0.472069937307186	4.87060886384926e-08
5.91133725356064e-08	17.3738532110092 22.293270993766
NR2C2-regulon	GC
0.015258495313771	-0.00678858859186915	31704652.5
0.416650009682734	1.09441778126647e-32
1.65326941425361e-32	72.1903669724771 76.1299046571324
NR3C1-regulon	GC	0.243725961601728	0.0189777759608569
	61819773 0.812411018194709	0	0	100	100
NRF1-regulon	GC
0.0234880202705743	-0.00337069643379616	31936907.5
0.419702213077768	4.65001518419797e-30	6.7377771036338e-30
	100	99.9656215621562
PAX5-regulon	GC	0.124776131609699	0.0254880716764291
	65148315.5	0.856153407891439	0	0	100
	100
POU2F1-regulon	GC
0.0021342296622248	-0.00285550070849957	35170720.5
0.462199705133931	1.95348456477282e-15
2.52177098361583e-15	11.4105504587156 18.6239457279061
POU6F1-regulon	GC	0.0319397820480431
0.000975130763134779	39718970.5	0.521971008621313
0.00145442224301473	0.00162620439770151	69.954128440367
65.1700586725339
REL-regulon	GC	0.255602964282134	-0.008433140559227

30453162 0.400203416270526 1.69612401446323e-45
2.76838632245723e-45 100 100
RELA-regulon GC 0.0869690933324948 -0.005299033586577
27551887.5 0.362076013722358
3.05111504713636e-85 6.01747689851893e-85 100 100
RELB-regulon GC 0.111548962573668 0.00661970837677298
49586423.5 0.651645175149205
1.20681082979385e-102 2.6776115286051e-102 100 100
REST-regulon GC 0.224852844309542 0.00648356422366794
49049030 0.644582962214417 1.75298904794401e-93
3.60760064939202e-93 100 100
RFX5-regulon GC 0.077308384004388 0.0124379550380026
58313026 0.766326735406721 0 0 100 100
RXRA-regulon GC
0.00912364691034414 -0.00562259292527969 31113216.5
0.408877591577009 5.75200941124751e-40
8.97566303733128e-40 53.3256880733945 64.7002200220022
SETDB1-regulon GC
0.0573766455480963 -0.00243444795160461 36580921
0.480732002624957 0.00626737586964763 0.00679364407244247
96.8463302752293 94.9807480748075
SOX5-regulon GC
0.00606612075149156 -0.00180661911843223 36756387
0.483037907431798 0.000996661797063218 0.00112322202526172
19.6674311926606 22.6072607260726
SP1-regulon GC
0.00837326244971853 -0.00480530017931637 32258951.5
0.423934388015445 6.52320891165955e-29
9.35652187328946e-29 51.5481651376147 60.1530986431977
SP2-regulon GC
0.0270592292597059 -0.00122813026026597 35091118
0.461153600547364 3.57603323686637e-08
4.37755792788814e-08 100 100
SP3-regulon GC
0.0275500341024207 -0.000748639736558109 35995534
0.473039078085943 0.000130997908561719
0.000150013733998097 100 100
SP4-regulon GC
0.00540573529705937 -0.00336138175758937 35033879.5
0.460401394807868 2.40130596628664e-12
3.04451292154199e-12 22.3623853211009 29.2812614594793
SPI1-regulon GC 0.467991529923278 0.0514250436851625
68545448.5 0.900797186823996 0 0 100
100
SPIB-regulon GC 0.604008599581292 0.0411385142234595
65116690 0.855737798072621 0 0 100 100
SREBF1-regulon GC 0.0419225995978384 0.00103239915981449
40243035 0.528858057107316 4.24437934780482e-05
4.98100716849822e-05 97.0756880733945 96.8876054272094
SREBF2-regulon GC 0.103966344873147 0.00518291309871516

47173615.5 0.619937006243629 6.4871995985388e-65
1.12339310121038e-64 100 100
SRF-regulon GC
0.0141404368707139 -0.00689369439766841 25349851
0.333137720547666 7.24937187364127e-124
1.77484621733976e-123 96.4449541284404 97.9945911257792
STAT1-regulon GC 0.0678051239010194 -0.0124415708381184
16538407.5 0.217341213407465 0 0 100
100
STAT2-regulon GC
0.0607201677559597 -0.00573459259830156 32618217.5
0.428655719762534 4.47511312868234e-24
6.11025061800858e-24 100 99.9610377704437
STAT5A-regulon GC
0.0620202521970721 -0.00997079793806651 26868057.5
0.353089390193798 1.8709346251458e-96
3.90695171721623e-96 100 100
TBP-regulon GC 0.0227505136147737 0.00192316404894683
39444524 0.518364341212409 0.00533074335600507
0.00582281197348246 54.1284403669725 49.7158049138247
TCF12-regulon GC
0.00797280747500268 -0.00111371065762323 37450985
0.492166039759557 0.145143408195045 0.151546793850709
24.0825688073394 25.2658599193253
TFDP1-regulon GC 0.559656024713245 0.0327515074378071
59094551.5 0.776597234575331 0 0 100
100
TFDP2-regulon GC 0.0274703100328577
0.000361032381241379 40435447.5 0.531386666117873
8.49179408296803e-06 1.00486229981788e-05 100
99.9954162082875
TFEC-regulon GC 0.0735231810797982 -0.0187385702905068
19734170 0.259338660834738 1.92619346442335e-255
7.01331979354144e-255 100 100
THAP1-regulon GC
0.0347717839946424 -0.00248899199339078 31818945
0.418151996535663 3.63831739610522e-31
5.32619660048393e-31 100 100
THAP11-regulon GC
0.175184342316797 -0.00342500571453272 30507920.5
0.400923030830415 7.2098408612742e-45
1.16340613897834e-44 100 100
TP53-regulon GC
0.0930149390075797 -0.00555526658363763 33340430.5
0.438146757503541 1.72016896030467e-18
2.24095405837856e-18 100 100
USF2-regulon GC 0.131046333739658 0.00599036479465845
47260186 0.621074681531609 4.07234485916882e-66
7.22841212502465e-66 100 100
XBP1-regulon GC

0.191696407179173 -0.00373709241651948 39627290
0.520766179733417 0.00322108260062438 0.00354568782394312
100 100
YBX1-regulon GC
0.0921185024409748 -0.00318778000728891 31701459.5
0.41660804853899 2.74216922647727e-32 4.09882137010287e-32 100
100
YY1-regulon GC 0.663658470259106 0.0251539140098695
53060580 0.697301166469858 2.24865824638702e-172
6.51651981606034e-172 100 100
YY2-regulon GC
0.0416518212008376 -0.00972397089254216 32321730
0.42475939824487 1.29179254726822e-26 1.79837785992242e-26
89.6788990825688 90.5642647598093
ZBTB33-regulon GC 0.366070106989523
0.000253849801196304 38158059 0.501458126747308
0.836132078097772 0.836132078097772 100 100
ZNF143-regulon GC
0.0191116921203667 -0.00438768169397612 26440705.5
0.347473299150443 8.0808798599862e-104
1.82140466685403e-103 100 99.9931243124312
ZNF274-regulon GC 0.0523715383925523 -0.0134884115908986
17977665.5 0.23625537307649 2.32831126265795e-306
1.22451925665715e-305 100 100
ZNF76-regulon GC 0.0153474322723595
0.000821915461766664 40031199.5 0.526074198709053
0.000216631306924042 0.000246093164665712 99.2545871559633
99.4682801613495
ATF1-regulon GC IgA
0.100135795771469 -0.00613423125384775 5556944
0.381864936645996 2.32758950938674e-13
4.13147137916146e-13 100 100
ATF3-regulon GC IgA 0.0410165287352449 -0.0100769833102241
4460690 0.306531990289524 3.47819837256313e-33
1.02896701854992e-32 100 100
ATF4-regulon GC IgA 0.0963364360474433 -0.0125042152415343
4647677 0.319381459153818 3.86901532596717e-29
1.01740773386544e-28 100 100
ATF5-regulon GC IgA 0.124502661675215 0.00611273240132641
8294268.5 0.56996980989504 1.42116236592761e-05
1.99806986100714e-05 100 99.9977803919828
ATF6-regulon GC IgA 0.131109836839473 -0.0053421356067575
6924907.5 0.475869356208536 0.134409094724549
0.144591601900651 100 100
ATF6B-regulon GC IgA
0.0544749755858022 -0.00284176436170396 4975829.40625
0.341931605029481 1.0558435421776e-22
2.45786529490524e-22 99.3808049535604 99.9245333274144
BACH1-regulon GC IgA 0.0240652872605643 -0.0193553256596506
3253184.5 0.223554006121033

6.36256117120622e-66	3.34623587522697e-65	99.0712074303406
99.5671764366413
BATF-regulon	GC IgA
0.0117059667080055	-0.00764178310385315	6395442.5
0.439485307947248	6.74828497896301e-05
9.12625206678806e-05	43.9628482972136 51.7723570017535
BATF3-regulon	GC IgA	0.0707905612034447	-0.0124617602861201
5988689 0.41153381167375 4.06542038129556e-08
6.34384279279088e-08	100	100
BCL11A-regulon	GC IgA	0.222153854592353	0.0177800868838821
10433830.5	0.716997332141113
2.63874454428786e-41	8.92146964973516e-41	100	100
BHLHE40-regulon	GC IgA	0.0368622365698634	-0.0189236439727088
2726569 0.187365771266714	8.63513643918374e-84
8.75849553117208e-83	100	100
BRF2-regulon	GC IgA	0.034056133766371	-0.0076103744762293
5318075.5	0.365450248173479
7.01525579642386e-17	1.46495047513557e-16	99.6904024767802
99.7292078218987
CEBPB-regulon	GC IgA	0.123358536079352	-0.0198749610214533
2810390 0.193125825867697	8.43821439336232e-81
7.48891527410906e-80	100	100
CEBPG-regulon	GC IgA
0.0600021057616057	-0.00811640297443093	6169895
0.423986018805921	2.41099989770917e-06
3.49348964770105e-06	100	100
CLOCK-regulon	GC IgA
0.00717636787717903	-0.00847871333515244	5699296.5
0.391647188976396	8.48773181429921e-13
1.46982672881767e-12	34.3653250773994 51.213015781413
CREB1-regulon	GC IgA	0.0480688154354793	0.00513416558490427
8793749 0.604293367859348	9.81176366046844e-11
1.62008190672851e-10	100	99.9933411759483
CREB3-regulon	GC IgA
0.236684963792564	-0.00313738255889021	7656119.5
0.526117158607623	0.105195265861387	0.115796339165247
100	100
CREB3L2-regulon	GC IgA
0.118933952915572	-0.00789948081357236	5702213
0.391847606523833	1.95671254734562e-11
3.26886096144798e-11	100	100
CREB3L4-regulon	GC IgA
0.00627149133004565	-0.00486448011499175	6525156.5
0.44839906133258 9.93788830132178e-05	0.000131885994279224
22.2910216718266 31.4118926597563
CREB5-regulon	GC IgA	0.0275188572481153	-0.01173901733723
5005104.5	0.343943345982808
3.63316122305572e-22	8.32111118828892e-22	99.6904024767802
99.6226666370719
CREM-regulon	GC IgA	0.162280976724702	0.0123694682822461

10432777.5 0.716924971545381
2.80400703710508e-41 9.25974416904467e-41 100 100
CTCF-regulon GC IgA 0.387448212458509 0.0167270287809396
10845138.5 0.745261806888742
2.82160640789272e-52 1.29247777393795e-51 100 100
CUX1-regulon GC IgA 0.108018178887492 0.0315446577240111
13381438.5 0.919552575126688
2.45810299044538e-149 1.74525312321622e-147 100 100
DDIT3-regulon GC IgA 0.0736679828750185 -0.0113207286897759
5034012.5 0.345929860798967 1.2038112884162e-21
2.71335242785874e-21 100 100
E2F1-regulon GC IgA 0.491479255011999 0.0541699241517675
12144147 0.834527741286338 1.16666683748652e-95
1.84074101025651e-94 100 100
E2F2-regulon GC IgA
0.0536721320480051 -0.000250643974104597 7942528
0.545798725257813 0.00449559980119561 0.00545619804931433
100 100
E2F3-regulon GC IgA
0.0165451776716999 -0.00460288943327068 4778855.5
0.328395850803584 1.83119670505551e-26 4.6433916449622e-26
100 100
E2F4-regulon GC IgA
0.391299837939386 -0.00741043348237092 7404843.5
0.508849845166879 0.583007623822376 0.599906395527372
100 100
E2F6-regulon GC IgA 0.0597966421358273 -0.0126949436464749
5555012 0.38173217247605 2.18880776692605e-13
3.934312694981e-13 100 100
E2F7-regulon GC IgA 0.0276471857247401 -0.0029478250236128
5999904.5 0.412304524172734
5.32282300062461e-08 8.2156615879206e-08 100 100
E2F8-regulon GC IgA 0.078508550662298 0.0037385519908902
9658261.5 0.663701382595896
3.14193299829536e-24 7.56194043657527e-24 100 100
EGR1-regulon GC IgA 0.0482051960873074
0.000747508940104759 7647366.5 0.525515665450509
0.113453655165198 0.12392630025737 100 100
EGR2-regulon GC IgA
0.0399018396533873 -0.00619013229070625 5827896.5
0.400484389936613 6.68217764433554e-10
1.07826048351778e-09 100 99.9955607839656
EGR3-regulon GC IgA
0.0322128652744241 -0.00932465328825514 4744846.5
0.326058802845139 3.82051924611579e-27
9.86388605360804e-27 100 99.9955607839656
ELF1-regulon GC IgA 0.770984716390965 0.064398484043461
13097858.5 0.900065378794662
5.73178446156821e-136 1.62782678708537e-134 100 100
ELF2-regulon GC IgA 0.0194686597756224 -0.0132819471382277

4034612 0.277252543083244 1.9821196643735e-43
7.03652480852594e-43 98.7616099071207 99.5405411404346
ELF4-regulon GC IgA
0.0171788468608671 -0.00127734757632158 6910595
0.474885822470253 0.11924362729399 0.129256450959898
99.0712074303406 98.3308547710474
ELK1-regulon GC IgA 0.0675934245089185 0.00154948286604377
7834174.5 0.538352833700714 0.0173497441356447
0.0203608567542277 100 100
ELK3-regulon GC IgA 0.0717174936579628 0.00686087533464415
9037854.5 0.621067935192119 5.8932930844467e-14
1.07288156152748e-13 100 100
ELK4-regulon GC IgA
0.0404884939621782 -0.00454086261189238 6522096
0.448188748319059 0.00130827457477948 0.00164402645680253
95.9752321981424 96.7349566066633
ESRRA-regulon GC IgA
0.0828150957820609 -0.00898458466976501 6329647
0.434963938928757 5.47184003459783e-05 7.5437017952708e-05
100 100
ETS1-regulon GC IgA 0.300785151086187 0.0582608635949304
13245922.5 0.910240116920429
7.2069833713152e-143 3.41130546242253e-141 100 100
ETV2-regulon GC IgA 0.0230756396034937 -0.0055660066705919
4930883.5 0.338842989120691
1.56526475790503e-23 3.7044599270419e-23 100 100
ETV3-regulon GC IgA
0.0081859634150173 -0.00326634294757168 5977101.5
0.410737535887385 2.88906501583035e-08
4.55830258053233e-08 78.328173374613 84.3917164228797
ETV5-regulon GC IgA 0.0373616218253227 0.00392592556054069
9144809.5 0.628417723906738
1.63397440744649e-15 3.26794881489298e-15 100 100
ETV6-regulon GC IgA 0.430585627797673 -0.0291971010275124
2612440.5 0.179523030288579
5.97413645457018e-88 7.06939480457472e-87 100 100
ETV7-regulon GC IgA 0.0799291142880545 0.0159666905040952
11769813.5 0.8088040992518 8.52330788495905e-82
8.0687314644279e-81 100 100
FLI1-regulon GC IgA 0.159988440807136 0.016172042683515
9954865.5 0.684083568860315
3.33776086916132e-30 9.29337340040994e-30 100 100
FOS-regulon GC IgA
0.0821165973907679 -0.00419075831410726 6231950
0.428250346221056 8.54800710195376e-06
1.21381700847743e-05 100 100
FOSB-regulon GC IgA 0.0237829829896494 -0.0178173280180739
3754382 0.257995553774677 6.06156766735582e-51
2.60831093565008e-50 98.1424148606811 99.3318979868155
FOSL1-regulon GC IgA 0.0439935203852778 -0.0169206323948799

3882830.5 0.266822343879953
2.01187136322617e-47 8.16244953080333e-47 100 100
FOXO1-regulon GC IgA 0.0195965682957369 0.00292037128295072
7975340.5 0.5480535515137 0.0015954995909253
0.00198737668343326 60.9907120743034 52.2806472376978
FOXO3-regulon GC IgA
0.0102824980517333 -0.00952924667311141 6175780
0.424390427263548 3.5769627961103e-07
5.40349698986875e-07 37.1517027863777 46.8292899473953
GABPA-regulon GC IgA 0.0264580688118478
0.000977097075955991 7698359 0.529019794299373
0.071823226267901 0.0809436359527139 100 100
GTF2B-regulon GC IgA
0.0725707404429984 -0.00653274215873349 5910761
0.40617871527851 5.87811643319789e-09 9.37856779229327e-09 100
100
HINFP-regulon GC IgA 0.0139786374352648 -0.002268053349584
6807277.5 0.467785997352001 0.0403664627105097
0.0462261105233257 59.4427244582043 63.2610480989057
HIVEP3-regulon GC IgA 0.0819425270585242 0.00428506197361292
7894466.5 0.542496010374846 0.00838322991296524
0.00992015539700886 100 99.6914744856059
HOXB2-regulon GC IgA 0.0162596636349412 -0.0209924917524289
4310486 0.296210194542802 8.73929068652577e-37
2.75773172774813e-36 67.4922600619195 83.8456928506426
IKZF1-regulon GC IgA 0.14802271668237 0.00418839789336758
8757405 0.601795862169626 2.70352633879491e-10
4.41265218515952e-10 100 100
IRF1-regulon GC IgA 0.114921733341629 -0.0206678302956414
3092574 0.212517091153529 3.85137306211543e-71
2.4858862491836e-70 100 100
IRF2-regulon GC IgA 0.0655571191794102 -0.0196028411651758
2960597.5 0.20344786212922 1.39946335582696e-75
9.93618982637142e-75 100 100
IRF3-regulon GC IgA 0.145846453974942 0.00040279235655219
7471925 0.513459586194973 0.403739383833419
0.421551415473128 100 100
IRF4-regulon GC IgA 0.0449401861577442 -0.0135687503464816
3975200 0.273169838701841 5.69491601499359e-45
2.12810019507655e-44 100 99.9955607839656
IRF5-regulon GC IgA 0.0129192892802467 -0.0343701239419562
3254817 0.223666189095897 2.37548999760196e-66
1.2973829986903e-65 44.2724458204334 80.7382416265287
IRF7-regulon GC IgA
0.104020416848523 -0.00384162420274352 6344060.5
0.435954413237 7.09559711754922e-05 9.50542255369801e-05 100
100
IRF8-regulon GC IgA 0.12449778849456 0.0430311726863328
13810728 0.949052711842172 8.88010946495592e-171
1.26097554402374e-168 100 100

IRF9-regulon	GC IgA
0.026529893324485	-0.00743928285842231	4598924.5
0.316031259777356	3.62274531199296e-30
9.89288142890385e-30	100	100
JUN-regulon	GC IgA	0.0701125621287226	-0.0184188319878833
2624746	0.180368645968329	1.69724374743228e-87
1.85391240104141e-86	100	100
JUNB-regulon	GC IgA	0.13856970194258 -0.0213648703852694
3069633	0.210940619713184	6.66388235377548e-72
4.50605378207676e-71	100	100
JUND-regulon	GC IgA
0.0325950458223893	-0.00590467316406083	5087310.5
0.34959242018293 1.05360565433921e-20	2.33768754556512e-20	100
100
KLF11-regulon	GC IgA	0.00710624974898678	-0.0149365610439267
5650467.5	0.388291732633577
7.18467512444376e-15	1.39756694201509e-14	21.9814241486068
40.993940470113
KLF12-regulon	GC IgA	0.0729699454331223	0.0261061520206395
11605196.5	0.797491863556091	4.7631216424234e-76
3.5598067011796e-75	100	98.5927685170799
KLF13-regulon	GC IgA
0.0605386955053201	-0.00579465131831392	5464494
0.375511909983694	1.13980480659313e-14
2.15803043381633e-14	100	100
KLF2-regulon	GC IgA	0.0957844760879672	-0.0131575503909856
4801901.5	0.329979537687948
5.23364960196363e-26	1.30382147978743e-25	100	100
KLF3-regulon	GC IgA	0.0402944070883117	-0.0319623276190161
3135947	0.215497619281426	1.0345361438633e-69
6.12100551785786e-69	99.6904024767802 99.8646039109493
KLF4-regulon	GC IgA	0.0137113419221679	-0.0022041394241999
7020362.5	0.482428882006806	0.243525316397873
0.256152555025911	49.5356037151703 49.8146627305618
KLF6-regulon	GC IgA	0.0384202987894396	-0.0311815377466611
2228309	0.153126084249311	1.04996573391935e-102
1.86368917770685e-101	100	100
KLF7-regulon	GC IgA	0.0221441262952655	-0.0240256333248648
3173472	0.21807628153673 1.73089952303009e-68
9.83150929081091e-68	93.4984520123839 97.5850664772601
KLF8-regulon	GC IgA	0.00966833877050595	-0.0155490678599032
4407818.5	0.30289873935198 9.50384115493208e-35
2.87137328510714e-34	59.4427244582043 77.7661864914656
MAFB-regulon	GC IgA
0.0531164210985506	-0.00268560119565091	6631643
0.455716655423172	0.00601229491942056	0.00717433511393042
100	99.9977803919828
MAFF-regulon	GC IgA
0.00655271103008841	-0.00587605729815579	6630552.5
0.455641717883148	0.000554945948495646

0.000722957107214511 21.3622291021672 28.5974296939161
MAFG-regulon GC IgA 0.0140465218844671 -0.0114422803705399
5700392 0.391722470108992 3.19777524388943e-12
5.40576291228927e-12 44.5820433436532 58.7019732315273
MAX-regulon GC IgA 0.19038869429279 -0.00733371929430823
4491268.5 0.308633299384097 1.66643908339937e-32
4.82927244576961e-32 100 100
MAZ-regulon GC IgA
0.133267671063713 -0.00305790916852136 6770695.5
0.465272136655837 0.0312138127565604 0.036035458629525
100 100
MEF2A-regulon GC IgA 0.0469204539363036 0.00621034578140185
9095474.5 0.6250274959956 8.76071859455959e-15
1.68111086544252e-14 100 99.9977803919828
MLX-regulon GC IgA
0.0518128588163732 -0.00112057555757757 7235289.5
0.497198346165256 0.862024136639712 0.868137782998859
100 99.9889019599139
MXD4-regulon GC IgA
0.0852596499386107 -0.00695901596718113 8030050
0.551813107080831 0.00129719967792836 0.00164402645680253
96.5944272445821 88.5468226311233
MXI1-regulon GC IgA 0.0469620754834901 -0.0183984417259775
3725063 0.255980795649074 9.13569123799129e-52
4.05396298685864e-51 100 100
MYBL1-regulon GC IgA 0.0741742447121461 0.0446015127557539
12397419.5 0.851932251241211
1.70674078738332e-106 4.03928653014051e-105 99.0712074303406
79.9236454842075
MYC-regulon GC IgA 0.284430362324914 -0.0306697130005455
5145810.5 0.353612453279141
1.07453599603886e-19 2.31188047632603e-19 100 100
NFATC1-regulon GC IgA
0.0442165836321719 -0.00634643434576557 6504920
0.447008439114606 0.00101063238641603 0.0012928810709106
94.7368421052632 94.8283133198677
NFE2L1-regulon GC IgA 0.129402710323609 -0.0536463027154565
4316960 0.296655078205449 1.75728688563568e-36
5.42466821217971e-36 100 99.9356313675005
NFE2L2-regulon GC IgA 0.0938504541778775 -0.0171521687055998
3999926.5 0.274869007049764
2.51461821546433e-44 9.15578939989578e-44 100 100
NFKB1-regulon GC IgA 0.0374295725974377 -0.0193771564738256
4259341.5 0.292695620479739
7.54812908206207e-38 2.4359871128473e-37 100
99.931192151466
NFKB2-regulon GC IgA 0.0563362645706944 -0.0171624961735348
3604406 0.247689425849253 3.21684031122775e-55
1.52263774731447e-54 100 100
NFYA-regulon GC IgA

0.0122178399491299	-0.00879315848366938	3945122
0.271102923223759	9.21302864584712e-46
3.63402796586192e-45	95.6656346749226 99.0411293365592
NFYB-regulon	GC IgA	0.120262559311752	0.0117210968879422
	11115500.5	0.763840681896568
3.27316386513815e-60	1.60272161672282e-59	100	100
NFYC-regulon	GC IgA	0.0872283734736143	0.00372011562003682
	9155761.5	0.629170329077161
1.11887169193193e-15	2.26971114649048e-15	100	100
NR1H3-regulon	GC IgA
0.0053520384019669	-0.00320516518437383	6739110.5
0.463101662376455	0.00162125209576149	0.00200189389215767
	14.8606811145511 22.1561272279315
NR2C2-regulon	GC IgA
0.015373046339851	-0.00645909995074758	6114032
0.420147196432355	6.08973405863564e-07	9.1025498560659e-07
	70.5882352941177 76.017135373893
NR3C1-regulon	GC IgA	0.246401581081594	0.0210740068417757
	12138806 0.834160715700579	1.87288814984142e-95
2.65950117277481e-94	100	100
NRF1-regulon	GC IgA
0.0236628013905351	-0.00308834836082069	6134604
0.421560873711932	1.1390322209627e-06
1.66744923068766e-06	99.6904024767802 99.9689254877589
PAX5-regulon	GC IgA	0.123760512560315	0.0236612614787281
	12097756 0.831339820681785	6.99766400427904e-94
9.03334807825112e-93	100	100
POU2F1-regulon	GC IgA
0.00196802226487165	-0.00293283541108361	6643352
0.456521280509045	6.44298640434614e-05
8.79715451362646e-05	9.90712074303406 18.4072092868399
POU6F1-regulon	GC IgA
0.0303129552674957	-0.000694115520320962	7379712
0.507122845820598	0.651739070441125	0.665805381313955
	68.7306501547988 65.3297227709587
REL-regulon	GC IgA
0.254241904281009	-0.00953797188847061	5473877.5
0.376156730164177	1.55893482411366e-14
2.91274664505447e-14	100	100
RELA-regulon	GC IgA
0.0850365156993805	-0.00707833165670926	4581605.5
0.314841123825334	1.5458480439719e-30
4.39020844488018e-30	100	100
RELB-regulon	GC IgA	0.110450480887947	0.00530456153694712
	9066411.5	0.6230303298097	2.30838387224383e-14
4.25701960855355e-14	100	100
REST-regulon	GC IgA	0.223863022236646	0.00528215008606181
	8957952.5	0.615577188449325
7.51000880389453e-13	1.31656944463336e-12	100	100
RFX5-regulon	GC IgA	0.0804406251778126	0.0152003513573668

11621669.5	0.798623863644875
1.29587433153977e-76	1.08243620634498e-75	100	100
RXRA-regulon	GC IgA	0.00738170211854422	-0.007199686178246
5469125.5	0.375830179783439
3.14801205772017e-15	6.20857933605923e-15	46.4396284829721
64.3908285796728
SETDB1-regulon	GC IgA	0.0585361836629925	-0.0011898126988854
7239917	0.497516341090944	0.877545977692294
0.877545977692294	97.2136222910217	95.0369564734868
SOX5-regulon	GC IgA
0.00601723402917066	-0.00179887442095905	7054255
0.484757924258316	0.195864626678777	0.207558037226763
19.8142414860681	22.5134841187046
SP1-regulon	GC IgA
0.00800688203454129	-0.00502274510338967	6162171.5
0.423455271359449	9.09171532576818e-07
1.34481622526988e-06	52.3219814241486	59.8761458726389
SP2-regulon	GC IgA
0.0272949305833515	-0.000952003113093052	6745261
0.463524315599673	0.0236503549152862	0.0275274622784479
100	100
SP3-regulon	GC IgA	0.0264793771005087	-0.0018033600671268
6298145.5	0.432799202645333
3.06220400649167e-05	4.26306832276291e-05	100	100
SP4-regulon	GC IgA	0.00525463227449797	-0.0034075479901202
6744428.5	0.463467107436381	0.00468740369981878
0.00564077394384972	22.9102167182663	29.0591081614987
SPI1-regulon	GC IgA	0.471237213206883	0.0530720180842084
13178477	0.90560536235307	1.05312580125262e-139
3.73859659444679e-138	100	100
SPIB-regulon	GC IgA	0.604757804325571	0.0405955556139305
12384402.5	0.851037742338418
3.86920840413366e-105	7.848965619814e-104	100	100
SREBF1-regulon	GC IgA	0.0420404387538209	0.00111852062421793
7721010	0.530576337370523	0.0578509424570826
0.0657186706312459	96.9040247678019	96.8947683839034
SREBF2-regulon	GC IgA	0.109627789852606	0.0107214745235847
10689207	0.734546425850421	5.83568067469486e-48
2.43725487001962e-47	100	100
SRF-regulon	GC IgA
0.0139251166671818	-0.00689312683982291	4838683.5
0.332507142087005	2.74081470278316e-25
6.71027047922775e-25	96.9040247678019	97.9424233680332
STAT1-regulon	GC IgA	0.066557520402032	-0.0133057038416718
2932558	0.201521029342874	1.53133456106208e-76
1.20805282039342e-75	100	100
STAT2-regulon	GC IgA
0.0603999186929685	-0.00587626497084281	6221297
0.427518287886458	6.91164265574093e-06
9.91366926379001e-06	100	99.9622666637072

STAT5A-regulon	GC IgA
0.0618464086384306	-0.00983140259140643	5141882.5
0.353342526954322	9.21166496923115e-20	2.0123945009705e-19
	100	100
TBP-regulon	GC IgA	0.0224453829016061	0.00155518794626231
	7572577	0.520376242112918	0.176382866259535
0.188318548938752	54.7987616099071	49.8501764588374
TCF12-regulon	GC IgA
0.00643147081377833	-0.00263097054032213	6974836
0.479300368558009	0.0922892431436646	0.102383379112503
	21.9814241486068	25.2436019798904
TFDP1-regulon	GC IgA	0.562142221709896	0.0342225256352835
	11416632	0.784533991235228	9.99242092389966e-70
6.12100551785786e-69	100	100
TFDP2-regulon	GC IgA
0.0260961290974675	-0.00103438770483516	7143430.5
0.490885932145002	0.571809974210361	0.592678951371323
	100	99.9955607839656
TFEC-regulon	GC IgA	0.0744439044212331	-0.0172202196651597
	4084548	0.280684070821576	3.72781574444034e-42
1.29109716026958e-41	100	100
THAP1-regulon	GC IgA
0.0346587533118928	-0.00252432865549623	6022768
0.413875669928208	9.15675416397506e-08
1.39812805514458e-07	100	100
THAP11-regulon	GC IgA
0.173434054545058	-0.00507981504598848	5206232
0.357764529000897	1.10880719187932e-18
2.35000927234124e-18	100	100
TP53-regulon	GC IgA
0.0936956182575227	-0.00469449070942808	6585365
0.452536500010754	0.00323592150499357	0.00396121425611282
	100	100
USF2-regulon	GC IgA	0.136351818089212	0.0111449460538902
	10590553	0.727767069524377	2.49920644405985e-45
9.59154905558105e-45	100	100
XBP1-regulon	GC IgA
0.193165229511701	-0.00213986935735175	8054300.5
0.553479565415868	0.000907958454447972	0.00117209182301466
	100	100
YBX1-regulon	GC IgA
0.0936468194262508	-0.00154796141775562	6859924
0.47140378662379	0.0760697410220217	0.0850543561033629	100
	100
YY1-regulon	GC IgA	0.662646057621229	0.0233408727548067
	9930365.5	0.682399965255919
1.10406553201068e-29	2.95806236878334e-29	100	100
YY2-regulon	GC IgA	0.0352918752780998	-0.0158228132238375
	5358136	0.36820314622221	2.8508796705921e-16
5.86702772788519e-16	85.4489164086687	90.5666659267973

ZBTB33-regulon	GC IgA
0.361735389694494	-0.00411995111210073	6407533.5
0.440316183505646	0.000213505756810976
0.000280720532103321	100	100
ZNF143-regulon	GC IgA	0.0201490219186016	-0.0032045246875205
	5603625.5	0.385072819979001
1.00741285696981e-12	1.72352561071943e-12	100
99.9933411759483
ZNF274-regulon	GC IgA	0.0514454344320656	-0.0139957221016388
	3253923	0.223604754743965	6.71672429555646e-66
3.40633874988935e-65	100	100
ZNF76-regulon	GC IgA	0.0146461331464729
8.96647568768491e-05	7231156	0.496914298185714
0.84819733795579 0.86031444278373 99.3808049535604 99.4606352518145
ATF1-regulon	GC LMO2	0.09760007663517 -0.00882405627287154
14516251.5	0.321774618340929	2.10162062016886e-84
5.32910942971389e-84	100	100
ATF3-regulon	GC LMO2	0.0403738713268834	-0.010892029839206
	13113657 0.290683994847351	1.09393695364661e-115
3.6125359864609e-115	100	100
ATF4-regulon	GC LMO2	0.0942282176687774	-0.0148563972683602
	12888928.5	0.285702548547813
3.64825249621825e-121	1.26354110844632e-120	100	100
ATF5-regulon	GC LMO2	0.121242399138479	0.00287335737978862
	24453496 0.54204863717754 4.37374041332076e-06
5.17559282242956e-06	100	99.997745666043
ATF6-regulon	GC LMO2	0.137155195634148
0.000758244441396189	25756841.5	0.570939256827446
9.30756673365628e-15	1.30858859027643e-14	100	100
ATF6B-regulon	GC LMO2
0.0535259697213253	-0.00385698730571815	14973569.2128906
0.331911755502401	2.70111259799466e-75
6.39263314858736e-75	99.4100294985251 99.932369981289
BACH1-regulon	GC LMO2	0.025210522798456	-0.0184866493648834
	10678134.5	0.236696963629392
6.90818006456673e-182	3.63319099692028e-181	98.6234021632252
99.585202551906
BATF-regulon	GC LMO2	0.0147608567876183	-0.004636411143745
	21551425 0.477719854473322	0.00978637402697048
0.0109422449750379	50.4424778761062 51.7459816497216
BATF3-regulon	GC LMO2	0.0710043440516161	-0.0124380413366384
	18749305 0.415606636502038	3.0295549948214e-20
4.67605227461564e-20	100	100
BCL11A-regulon	GC LMO2	0.218541786699304	0.0143633774806592
	29935401.5	0.663563344334793
2.19792405786322e-71	4.95405105105679e-71	100	100
BHLHE40-regulon	GC LMO2	0.0381100356105423	-0.0179432990516605
	9081987.5	0.201315956918326
1.87872190260892e-233	1.48210283428037e-232	100	100
BRF2-regulon	GC LMO2

0.0350785166720309	-0.00668361673054707	17386457
0.385397054155197	5.98430125553259e-36
1.04909972627855e-35	99.9016715830875 99.724971257242
CEBPB-regulon	GC LMO2	0.123311337620291	-0.0202341868882947
8428561.5	0.186831783661612
1.97187995509168e-256	1.75004346014386e-255	100	100
CEBPG-regulon	GC LMO2
0.0583759993849718	-0.00990677215792012	17771890
0.393940758187261	4.94567527310983e-31
8.16611498583251e-31	100	100
CLOCK-regulon	GC LMO2
0.00752047646213164	-0.00825936564705727	17932686
0.397505044155353	1.00599473422823e-32
1.70061014595724e-32	36.3815142576205 51.4303748957371
CREB1-regulon	GC LMO2	0.0491254991903914	0.00629539989989825
28134078 0.623634290906569	1.48075486737506e-41
2.76667356799024e-41	100	99.9932369981289
CREB3-regulon	GC LMO2	0.243685590050746	0.00397465949786044
27709848 0.614230592827986	9.98049376684667e-36
1.72832940840516e-35	100	100
CREB3L2-regulon	GC LMO2
0.125581585859321	-0.00122302839645405	23382670.5
0.518312174181412	0.0454834892604822	0.048929208143852
100	100
CREB3L4-regulon	GC LMO2
0.00666997204126869	-0.00453296877450498	20652709.5
0.457798469327193	2.08755866109067e-08
2.69484845340796e-08	24.4837758112094 31.5043170495277
CREB5-regulon	GC LMO2	0.0277177058066056	-0.011719267699997
15537458.5	0.344411212414274
9.05588105631814e-65	1.91930613432414e-64	99.6066863323501
99.6235262291756
CREM-regulon	GC LMO2	0.172059741708094	0.0225659481594807
38500686 0.853425799595297	0	0	100	100
CTCF-regulon	GC LMO2	0.381635388443419	0.0110426322766279
30845495 0.6837369400194	1.38415269399917e-89
3.70848457637514e-89	100	100
CUX1-regulon	GC LMO2	0.105858953748294	0.0298294475647242
41116126.5	0.911400984764892	0	0	100
100
DDIT3-regulon	GC LMO2
0.0752821300272596	-0.00984668834915983	16661972.5
0.369337762024483	3.28714647150106e-46
6.39417532812534e-46	100	100
E2F1-regulon	GC LMO2	0.477401836735614	0.0406172515408446
35719628 0.791779452634859	7.0096820500685e-223
4.97687425554864e-222	100	100
E2F2-regulon	GC LMO2
0.0524307200706666	-0.00152443862235499	22955752.5
0.508848892526856	0.3337822417862	0.343457089374206	100

100
E2F3-regulon GC LMO2
0.0164825310922512 -0.00473898484695796 14450314.5
0.320313025242356 9.20056096090556e-86
2.37541755717926e-85 100 100
E2F4-regulon GC LMO2 0.38722422961289 -0.0116954183616473
21184845 0.469594055633903 0.000896622529256829
0.00101856319323576 100 100
E2F6-regulon GC LMO2 0.0603115461522054 -0.0123668480231173
17815903.5 0.394916383827555
1.70544756873486e-30 2.78360407770517e-30 100 100
E2F7-regulon GC LMO2
0.0270569308818937 -0.00359773134060343 17638471
0.390983324733836 1.0830049346452e-32
1.80925530258374e-32 100 100
E2F8-regulon GC LMO2 0.0749746308186783
0.000182101400425766 26169019.5 0.58007580414054
2.20424584791031e-18 3.36562269250821e-18 100 100
EGR1-regulon GC LMO2 0.0555117417286169 0.00823326349328438
32757530.5 0.726120091983032
1.12146773032043e-134 4.30401128933788e-134 100 100
EGR2-regulon GC LMO2 0.0496014366963987 0.00363499820398333
25084359 0.556032667493522 9.34605346011766e-10
1.24031737508103e-09 100 99.9954913320859
EGR3-regulon GC LMO2
0.0410635963167681 -0.000416890209841432 22058672.5
0.488963760706064 0.228034381034781 0.236356803700284
100 99.9954913320859
ELF1-regulon GC LMO2 0.766876008659559 0.0612030969947427
40339737.5 0.894191151072007 0 0 100
100
ELF2-regulon GC LMO2 0.0200753787335096 -0.0128691152863113
12768375 0.283030298314882 3.72514876576632e-124
1.35633621727902e-123 99.4100294985251 99.5378615388084
ELF4-regulon GC LMO2
0.0176760070891273 -0.000788773368166192 21764391
0.482440567211703 0.0551184479503139 0.0584091015592879
99.3117010816126 98.3137582001398
ELK1-regulon GC LMO2 0.0675035113923368 0.00148175012930545
24175936.5 0.535896112045319
8.82663985869917e-05 0.000102736299994695 100 100
ELK3-regulon GC LMO2 0.0745176153229987 0.00983253313024177
29945822.5 0.663794341524235
1.39798611591625e-71 3.20183916871142e-71 100 100
ELK4-regulon GC LMO2
0.0400061295636517 -0.00510532812284149 20259097.5
0.449073465418683 2.65825304025298e-08
3.40064803347678e-08 96.5585054080629 96.73347009626
ESRRA-regulon GC LMO2
0.0823186297229144 -0.00963299752083575 18977347.5

0.420661542612132		4.48423024756698e-18
6.70274415952117e-18	100	100
ETS1-regulon	GC LMO2	0.297727807069742	0.0560449208830714
	40772106.5	0.903775262366679	0	0	100
	100
ETV2-regulon	GC LMO2
0.0246069232276469	-0.00408669656215426	17228548.5
0.381896773981608	4.50771600455564e-38
8.10247686894812e-38	100	100
ETV3-regulon	GC LMO2	0.00875300954207445	-0.0027373985832774
	19068922.5	0.422691440666362
2.66687288341854e-17	3.94474947338992e-17	81.2192723697148
84.4202980229491
ETV5-regulon	GC LMO2	0.0426388994108642	0.00938561446381948
	35110323 0.778273287918147	6.48551244094894e-203
4.00409898528152e-202	100	100
ETV6-regulon	GC LMO2	0.425738680585403	-0.0346119630581505
	6619872.5	0.146739462811946	0	0	100
	100
ETV7-regulon	GC LMO2	0.0884900196600399	0.0249736682395954
	40311900 0.893574090879982	0	0	100	100
FLI1-regulon	GC LMO2	0.164097687322568	0.0206285130166297
	33464205.5	0.741784609673159
1.07867100007607e-153	4.94100909712264e-153	100	100
FOS-regulon	GC LMO2	0.0900314691191925	0.00384000958595826
	26613313.5	0.589924251054067
9.05104618297207e-23	1.4440995033506e-22	100	100
FOSB-regulon	GC LMO2	0.0277556745446962	-0.0140323097273716
	14098477 0.312514016160671	3.36499287697998e-93
9.18901901021455e-93	98.4267453294002 99.3439888185036
FOSL1-regulon	GC LMO2	0.0459530249152167	-0.0151809277426456
	13450060 0.29814087494713 9.94762340718154e-108
3.13902783071062e-107	100	100
FOXO1-regulon	GC LMO2	0.0193484463409232	0.00271225017672072
	24473406.5	0.542489983453366
8.89715072173965e-07	1.08913396766123e-06	60.7669616519174
52.1495074280304
FOXO3-regulon	GC LMO2
0.0174207971073357	-0.00237637666351076	22276683
0.493796292398685	0.462123811813039	0.468725580553225
	47.6892822025565 46.7391059311526
GABPA-regulon	GC LMO2	0.0260190897046156
0.000543340440345257	23521222.5	0.521383388325117
0.0195115580682405	0.0214778391138772	100	100
GTF2B-regulon	GC LMO2
0.0758660885937696	-0.00326404821542468	20604551
0.456730963507432	2.28893812165105e-06
2.75448485825804e-06	100	100
HINFP-regulon	GC LMO2
0.0147581964078022	-0.00150610562954419	21905162.5

0.485560979921953	0.10570087988112 0.111181666245327
62.8318584070796 63.2430848305868
HIVEP3-regulon	GC LMO2	0.104374429458476	0.0272982912238191
33447571.5	0.741415891963805
3.12669466169722e-153	1.38747075612814e-152	100
99.6866475799725
HOXB2-regulon	GC LMO2	0.0187647837163147	-0.0187583670080429
14659571 0.324951511315903	7.82422633283157e-82
1.91558644700359e-81	72.5663716814159 83.9852115692419
IKZF1-regulon	GC LMO2	0.148889839568131	0.00514092874848859
28033904 0.621413783042146	3.87171818925061e-40
7.04851260094341e-40	100	100
IRF1-regulon	GC LMO2	0.121171271664457	-0.0145983612654226
13327908 0.295433191549692	1.38375373292103e-110
4.46575068351787e-110	100	100
IRF2-regulon	GC LMO2	0.0698413716668099	-0.0155270534084722
11803302.5	0.261638010136434
1.97862771614786e-149	8.51409502099987e-149	100	100
IRF3-regulon	GC LMO2	0.148797513768423	0.00342781156538072
26164652 0.579978991912837	2.4205539247643e-18
3.65658146081416e-18	100	100
IRF4-regulon	GC LMO2
0.0508071480101109	-0.00777956329824939	17065710.5
0.378287224002304	2.50335805059379e-40
4.61658237901712e-40	100	99.9954913320859
IRF5-regulon	GC LMO2	0.0127830655996316	-0.0350471940235024
9924465 0.219990741935885	5.94992263572927e-207
3.84040461033435e-206	45.3294001966568 81.2845194887171
IRF7-regulon	GC LMO2	0.111512256200636	0.00376187486722571
25981467.5	0.575918431059819	1.1114643797193e-16
1.62709218474372e-16	100	100
IRF8-regulon	GC LMO2	0.123623940795929	0.0428105166902839
42474729 0.941516459198118	0	0	100	100
IRF9-regulon	GC LMO2
0.028147093005099	-0.00590139448393695	16223812
0.359625273393409	4.63784448508536e-53
9.40819881260174e-53	100	100
JUN-regulon	GC LMO2	0.0727627738434575	-0.0159960240488356
9623020.5	0.21330876973814 3.00952878734549e-215
2.03501470382409e-214	100	100
JUNB-regulon	GC LMO2	0.143350812085881	-0.0168084008116757
12122262 0.26870822873789 8.15974917095086e-141
3.21856772854173e-140	100	100
JUND-regulon	GC LMO2
0.0341649532146134	-0.00439115224037666	17562717
0.389304123017209	1.18112621039901e-33	2.0207219503212e-33
100	100
KLF11-regulon	GC LMO2	0.00655190026736678	-0.0157373035187903
17110942 0.37928984845046 1.37074457413398e-49
2.74148914826795e-49	20.353982300885	41.3287044342749

KLF12-regulon	GC LMO2	0.0801716511114696	0.0338814009298352
	38760476	0.859184436947288	0	0	100
98.5707522712415
KLF13-regulon	GC LMO2
0.0635214216994063	-0.00283419919432673	20412863.5
0.452481920829077	2.10084944046159e-07
2.61684754864514e-07	100	100
KLF2-regulon	GC LMO2	0.0960276319453406	-0.0131146706999909
	14939621.5	0.331159253221841
6.07817657412694e-76	1.46288317546784e-75	100	100
KLF3-regulon	GC LMO2	0.0393584402446586	-0.0334198060758163
	9120250.5	0.202164114049082
3.85291217071915e-232	2.87954488548484e-231	99.3117010816126
99.8760116323632
KLF4-regulon	GC LMO2	0.0171917526553403	0.00132158127888259
	23333150	0.517214477576504	0.0442512290859626
0.047966981146616	52.9006882989184	49.7418787619198
KLF6-regulon	GC LMO2	0.0392735434065387	-0.0307965687313698
	7038335.5	0.156015326633595	0	0	100
	100
KLF7-regulon	GC LMO2	0.0248022326674809	-0.0216824685732204
	11151353.5	0.247186576813393
7.58198676571737e-168	3.58880706910622e-167	94.3952802359882
97.6284406772019
KLF8-regulon	GC LMO2	0.0092438316627107	-0.0162265738365504
	13142901.5	0.291332243317424
2.90933605688483e-116	9.83632666851539e-116	56.6371681415929
78.1171802790865
MAFB-regulon	GC LMO2
0.0542667253544368	-0.00155094084069908	21491765
0.476397400551232	0.00993723974698045	0.0110241253443064
	100	99.997745666043
MAFF-regulon	GC LMO2
0.00846922120899032	-0.00400753947509998	21337948
0.472987814648884	0.000213910311250819
0.000244961808045293	24.385447394297	28.6413129240966
MAFG-regulon	GC LMO2
0.0176894275154879	-0.00789487075048431	19419153.5
0.43045483925147	3.25233519807904e-15	4.61831598127223e-15
51.4257620452311	58.7659775919205
MAX-regulon	GC LMO2	0.187024586818481	-0.0108896908433622
	11074183.5	0.245475987320136
4.27608439653886e-170	2.09380684244316e-169	100	100
MAZ-regulon	GC LMO2
0.131254455165593	-0.00516512244104914	18736239.5
0.415317019979761	2.25459727871092e-20	3.5181627865599e-20
	100	100
MEF2A-regulon	GC LMO2	0.0490805140841151	0.00851709005515435
	29866845.5	0.662043697149363
4.24516256309668e-70	9.41895443687075e-70	100

99.997745666043
MLX-regulon	GC LMO2
0.051921919811932	-0.00102654565840226	22183424
0.491729065943436	0.366315845976909	0.374221943372094
	100	99.9887283302148
MXD4-regulon	GC LMO2	0.0962012991639772	0.00412461346921295
	25101762	0.556418431248234	6.96432623869834e-10
9.32956911221852e-10	94.1986234021632	88.4758448116504
MXI1-regulon	GC LMO2	0.0457441721708173	-0.0199321124415858
	10636772.5	0.235780112487496
3.84498388908887e-183	2.09995273942546e-182	100	100
MYBL1-regulon	GC LMO2	0.0759973771609955	0.047164237520773
	38929138.5	0.862923095757789	0	0
99.1150442477876	79.6230753623842
MYC-regulon	GC LMO2	0.283646763358627	-0.0319511072501136
	15446249.5	0.342389427302307
2.04615049292413e-66	4.47005184608041e-66	100	100
NFATC1-regulon	GC LMO2	0.0601124801621753	0.00981461021135867
	26468486	0.586713930983643	2.74461624838576e-21
4.33039452523087e-21	97.6401179941003	94.7631822178137
NFE2L1-regulon	GC LMO2	0.134087871880066	-0.049693027017148
	14445318.5	0.320202281363798	7.2512896268208e-86
	1.9068206055714e-85	100	99.9346243152461
NFE2L2-regulon	GC LMO2	0.0957629053687166	-0.0154642185679991
	13599698	0.301457827008707	2.78939099697582e-104
8.61072872979492e-104	100	100
NFKB1-regulon	GC LMO2	0.041497853290657	-0.0155187611506063
	15533987.5	0.344334272461817	7.846643890039e-65
	1.68821732179627e-64	99.9016715830875	99.932369981289
NFKB2-regulon	GC LMO2	0.0617016391154155	-0.0119426205452106
	14537307	0.322241345269466	5.6768903361182e-84
1.41424285566453e-83	100	100
NFYA-regulon	GC LMO2
0.0135707360720142	-0.00754681473190917	13737288.5
0.304507728054087	3.67629326982557e-101
1.11070988152177e-100	98.2300884955752	99.0351450663901
NFYB-regulon	GC LMO2	0.119537818391017	0.0111631176102522
	34197037	0.758028925653817	9.35852506171676e-175
4.74610913844207e-174	100	100
NFYC-regulon	GC LMO2	0.0898006749006348	0.00640959260988752
	31044770.5	0.688154182167429
7.50291921780315e-94	2.13082905785609e-93	100	100
NR1H3-regulon	GC LMO2
0.00574841346295159	-0.00284984765852144	21402103.5
0.474409918111818	0.000118603889451274
0.000136924815464073	17.6007866273353	22.2074438107261
NR2C2-regulon	GC LMO2
0.0176502536272551	-0.00423073721697476	20355479
0.451209906798032	8.03037513264297e-08
1.00912678657991e-07	74.1396263520157	76.0206496990464

NR3C1-regulon GC LMO2 0.245436528500378 0.0204165333825655
36556908 0.810339027222313 7.39106412630616e-252
6.17371238785574e-251 100 100
NRF1-regulon GC LMO2
0.0236962255643267 -0.00310247533510866 19252450.5
0.426759615715195 1.24704930963802e-15
1.80694899967958e-15 100 99.9661849906445
PAX5-regulon GC LMO2 0.125840963217374 0.0261595920200148
38516350 0.853773015791 0 0 100 100
POU2F1-regulon GC LMO2
0.00209255625705391 -0.00285133071859798 20907191
0.463439435766589 3.29356275033818e-09
4.33042509766686e-09 11.6027531956736 18.5013187853649
POU6F1-regulon GC LMO2 0.0346773826615945 0.00375951376040052
24311869.5 0.538909272102165
1.41764238946031e-05 1.6636794983749e-05 72.4680432645035
65.1908293694628
REL-regulon GC LMO2 0.252298082125985 -0.0116755815414598
15778603.5 0.349756555207475
1.61098483159887e-60 3.36411538363294e-60 100 100
RELA-regulon GC LMO2
0.0847483649568798 -0.00748382975769142 14085555
0.312227580532423 1.76968990436558e-93
4.92737189058652e-93 100 100
RELB-regulon GC LMO2 0.110790987420588 0.0057358649958113
28427391 0.630136016137041 7.48316833948737e-46
1.43595933000974e-45 100 100
REST-regulon GC LMO2 0.223217474897985 0.00470444220537672
27255554 0.604160480825272 5.44525461947278e-30
8.78666086324016e-30 100 100
RFX5-regulon GC LMO2 0.088415771959057 0.0235961516276109
40108377 0.88906269648532 0 0 100 100
RXRA-regulon GC LMO2
0.0111386846883405 -0.00346920851014316 20250793.5
0.448889394728622 1.10019538911805e-08
1.43328206655746e-08 58.7020648967552 64.3905408147163
SETDB1-regulon GC LMO2
0.0554192897316084 -0.00439678104901747 20617645
0.457021211775213 2.67037850292931e-06
3.18650207912573e-06 95.771878072763 95.0359566266147
SOX5-regulon GC LMO2
0.00566886629332943 -0.00218337256631123 21635945.5
0.479593378890386 0.00229608329126768 0.00258764942349215
18.9773844641101 22.5749002457224
SP1-regulon GC LMO2
0.00916873290731819 -0.00391283820509683 19830425.5
0.439571303707484 8.76804835306091e-12
1.20879889915985e-11 54.1789577187807 59.9517572533195
SP2-regulon GC LMO2
0.0279637627443042 -0.000282731083180172 22255946.5

0.493336636586492	0.46673107506606 0.470041224534613	100
	100
SP3-regulon	GC LMO2
0.026570544352808	-0.00173831637016003	19730035
0.43734599679388 7.7327555732531e-12	1.07652087392347e-11	100
	100
SP4-regulon	GC LMO2 0.00481700456687575	-0.0039085203557328
	20385973 0.451885852320999	5.4814508082638e-11
7.48428860359095e-11	20.6489675516224 29.2071507473117
SPI1-regulon	GC LMO2 0.465477200783423	0.0480102641407813
	39748397 0.881083196604765	0	0	100	100
SPIB-regulon	GC LMO2 0.600064806004783	0.0364300835291677
	36967086.5	0.819431252600824
1.05270294869936e-266	1.06774156225221e-265	100	100
SREBF1-regulon	GC LMO2
0.0372248879086462	-0.00378993497301015	20435602
0.452985953992125	2.81829031318292e-07
3.47997586497369e-07	95.771878072763	96.9205798146938
SREBF2-regulon	GC LMO2 0.113079002624021	0.0144195500896496
	36686820 0.81321872272896 1.63248731563654e-256
1.54542132546926e-255	100	100
SRF-regulon	GC LMO2
0.0153690404636159	-0.00552394231897802	16624101.5
0.368498294165223	8.78061389113948e-47
1.73173218408584e-46	96.6568338249754 97.9643364367998
STAT1-regulon	GC LMO2
0.0705463575970419	-0.00943358503613859	12538968
0.277945146003368	5.99204690547885e-130
2.23913331731052e-129	100	100
STAT2-regulon	GC LMO2
0.0659372628224737	-0.000303903120245169	22639259
0.501833336536394	0.841288254389557	0.841288254389557
	100	99.9616763227305
STAT5A-regulon	GC LMO2 0.061038518717166	-0.0108116277196947
	15058841 0.333801933331875	1.21426096120612e-73
2.82664027034867e-73	100	100
TBP-regulon	GC LMO2 0.0185790920419254	-0.0023754126897792
	21790994 0.483030262848468	0.0474255447327335
0.0506347921206628	48.2792527040315 49.9222254784824
TCF12-regulon	GC LMO2
0.00649506309827131	-0.00260708204089315	21838519
0.484083726184829	0.0226602993303505	0.0247520192685367
	23.2055063913471 25.2665749904191
TFDP1-regulon	GC LMO2 0.553179665297578	0.0255899025604971
	33081806 0.7333081477459	3.02700514783447e-143
1.22809923140713e-142	100	100
TFDP2-regulon	GC LMO2
0.0260097235197388	-0.00113895734261267	21937550
0.486278897729558	0.133953232311727	0.139862933737245
	100	99.9954913320859

TFEC-regulon GC LMO2 0.0736807098733097 -0.0182703233251654
11945409 0.26478801513609 1.46791615594175e-145
6.13070865128612e-145 100 100
THAP1-regulon GC LMO2
0.0354413513501653 -0.00176328164141257 19951606
0.442257452341507 2.84572675471906e-10
3.84850665876293e-10 100 100
THAP11-regulon GC LMO2
0.170622786935084 -0.00803500949834585 12866657
0.28520886714443 1.02874921641429e-121 3.65205971827072e-121 100
100
TP53-regulon GC LMO2
0.0922175762840769 -0.00627986479674787 19253534.5
0.426783644210863 1.2739124661904e-15
1.82722798180845e-15 100 100
USF2-regulon GC LMO2 0.138587206516026 0.0136059478306773
34799246.5 0.771377807906497
4.38051158414607e-193 2.48813057979497e-192 100 100
XBP1-regulon GC LMO2 0.204057822194491 0.00896897476087172
31135452.5 0.690164285529195
7.95393366096608e-96 2.30501750991262e-95 100 100
YBX1-regulon GC LMO2
0.0931649885591326 -0.00206505699363146 20300030
0.449980796044998 4.67174416467399e-08
5.92310420878309e-08 100 100
YY1-regulon GC LMO2 0.65336631722129 0.0142135494450886
27762140 0.615389723912363 2.01977962135897e-36
3.58510882791217e-36 100 100
YY2-regulon GC LMO2 0.0293983418047986 -0.0220990143839596
13975715.5 0.309792822276047
6.00274645520822e-96 1.7758124929991e-95 82.3992133726647
90.7166527649406
ZBTB33-regulon GC LMO2 0.354234697959273 -0.0118570649841255
15925970 0.353023156044043 5.39241522854687e-58
1.10974342384588e-57 100 100
ZNF143-regulon GC LMO2
0.0197590427920553 -0.00365357973788914 16805697
0.37252363243557 4.54928634717199e-44 8.61331548397898e-44 100
99.9932369981289
ZNF274-regulon GC LMO2 0.051474226437309 -0.0141852341535023
10197387 0.226040469882996 9.77348717204298e-197
5.7826465767921e-196 100 100
ZNF76-regulon GC LMO2 0.0156999774094529 0.00116907287289054
24562611.5 0.544467346881459
1.19196747311936e-06 1.44666137763204e-06 99.6066863323501
99.4567055163552
ATF1-regulon LZ 0.109902710534213 0.00373520529308961
19307738 0.60464005218489 6.85382173070407e-22
1.1186697537471e-21 100 100
ATF3-regulon LZ

0.0485571259497651	-0.00250411298156023	15194822
0.475840202877215	0.0264083569463439	0.0288460514336987
	100	100
ATF4-regulon	LZ
0.104299969181777	-0.00452294238313813	14266392.5
0.4467655561563	9.98385900720266e-07	1.20144743984981e-06	100
	100
ATF5-regulon	LZ	0.128356172404225	0.0100815887623772
	19393127.5	0.607314105030233
6.10772405835631e-23	1.00848467010069e-22	100
99.9977609099662
ATF6-regulon	LZ	0.13178333697426 -0.00470474224435602
15418370.5	0.482840835302715	0.114833936838121
0.121689694261292	100	100
ATF6B-regulon	LZ
0.0521918348986017	-0.00518639980318974	8454008.32226562
0.264745255666209	1.15834273322327e-103
5.67188510750705e-103	99.8601398601399 99.9216318488166
BACH1-regulon	LZ	0.0309532653604492	-0.0125269607192822
	10198708.5	0.319382189651552
7.22916415259545e-62	1.93687039560105e-61	99.7202797202797
99.5611383533732
BATF-regulon	LZ	0.0449438244122143	0.0260611221649377
	22678643 0.710203126176794	1.94234072985727e-93
7.66145510110369e-93	80.6993006993007 51.2527708739168
BATF3-regulon	LZ	0.0983537040421334	0.0154332747425667
	21749465.5	0.681105055129372
3.42566442565601e-62	9.35469900852218e-62	100	100
BCL11A-regulon	LZ
0.198470895000458	-0.00612596493724438	12443555
0.389681678121256	3.76012652126142e-24
6.67422457523903e-24	100	100
BHLHE40-regulon	LZ	0.0425707298265197	-0.0132898578600661
	8747716.5	0.273943004667798
7.53085129535692e-96	3.24054813315358e-95	100	100
BRF2-regulon	LZ	0.0440496655412964	0.00247635065362301
	17681048.5	0.553698734037284	8.0305962981405e-07
	9.74653567808505e-07	100	99.7245919258413
CEBPB-regulon	LZ
0.137791751988585	-0.00538512379485878	13887803.5
0.43490968403308 2.21164158250617e-09	2.96276513882902e-09	100
	100
CEBPG-regulon	LZ
0.0676233117637773	-0.000444424881943933	16735076
0.5240747117015	0.0269435598852913	0.0292059962115371	100
	100
CLOCK-regulon	LZ
0.00803392197703036	-0.00768184987021716	12909940
0.404286964910328	7.97390447877138e-21
1.28669822271084e-20	37.4825174825175 51.3109872147959

CREB1-regulon	LZ	0.046206817176478	0.00328742144368133
	18119286	0.567422555277731	5.80017759663732e-10
7.91947325694711e-10	100	99.9932827298986
CREB3-regulon	LZ	0.244678456228939	0.00495654410710142
	20842665	0.65270774097267	9.78995793200431e-45
2.01474496571683e-44	100	100
CREB3L2-regulon	LZ	0.12285954332926	-0.00398037926787048
15810193.5	0.495111142635829	0.653245452169407
0.658135303585789	100	100
CREB3L4-regulon	LZ
0.0041247290259148	-0.00708830767186056	13508116.5
0.423019427002768	7.88435629474671e-18
1.25795347624049e-17	17.9020979020979	31.5622131165894
CREB5-regulon	LZ
0.0369591281431482	-0.00225064857395069	15555658
0.487140123037214	0.23730417928255	0.245964915752716
99.7202797202797	99.6215937842861
CREM-regulon	LZ	0.169615753061679	0.0199302403794519
	26264103	0.822485192647079	5.34921729910649e-193
1.51917771294624e-191	100	100
CTCF-regulon	LZ	0.376033156397335	0.00527604047907204
	19454487.5	0.609235651386521
1.03583424734022e-23	1.75105313240847e-23	100	100
CUX1-regulon	LZ	0.0849083813500176	0.00834175883610822
	21659762	0.678295905299331	2.47078555999295e-60
6.15529034243858e-60	100	100
DDIT3-regulon	LZ
0.0783754609791892	-0.00663725091703334	13141741.5
0.411546047826024	4.34854129030035e-16
6.71187894807229e-16	100	100
E2F1-regulon	LZ	0.487465147677132	0.0505670150328627
	26410291	0.827063207945857	1.85925841343786e-198
6.60036736770442e-197	100	100
E2F2-regulon	LZ
0.0476904067020347	-0.00633033367510999	11071026.5
0.346699651751039	4.53504961685499e-45
9.47025067049131e-45	100	100
E2F3-regulon	LZ
0.0170829586628162	-0.00409689947346119	10995116
0.344322442743884	2.00947095121389e-46
4.32340719806625e-46	100	100
E2F4-regulon	LZ	0.441164721243537	0.0431877182581645
	25104109	0.786158884889321	2.10544107895528e-152
1.99315088807766e-151	100	100
E2F6-regulon	LZ	0.0885520792444252	0.0164094269751037
	23180434.5	0.725917200955825
9.83954139206558e-96	4.10945552256857e-95	100	100
E2F7-regulon	LZ
0.0227067939519801	-0.00799318371441721	7935846
0.248518513125217	3.69123704901578e-118

2.27893765634887e-117	100	100
E2F8-regulon	LZ	0.0750866389185429
0.000294671314143041	18258787.5	0.571791176513417
4.18939453904228e-11	5.83229435827455e-11	100	100
EGR1-regulon	LZ	0.055953959479275	0.00862688717118879
22693069 0.710654889992567	1.74379148320953e-83
5.62769069581257e-83	100	100
EGR2-regulon	LZ	0.0656901235588109	0.0199566767515128
23342953.5	0.731006636944704
5.23648253092988e-100	2.39864683674852e-99	100
99.9955218199324
EGR3-regulon	LZ	0.058014750441935	0.0168084623444441
22708965 0.711152688246797	7.16863479639645e-84
2.36731660718208e-83	100	99.9955218199324
ELF1-regulon	LZ	0.732088133012172	0.0254444265514419
24098199 0.754657863128341	4.10851525683387e-121
2.65185984759277e-120	100	100
ELF2-regulon	LZ	0.0184489087926076	-0.014434602562053
8186010 0.256352635072323	4.93953690804311e-111
2.59783052200786e-110	98.7412587412587 99.5477038131703
ELF4-regulon	LZ
0.0106237814744039	-0.00794856781827925	8864812.5
0.277609976508344	7.92393224562792e-93
3.04107669967342e-92	95.3846153846154 98.383376995589
ELK1-regulon	LZ	0.0709576259826713	0.00498114367427677
19470343 0.609732181344998	6.51605518069636e-24
1.11479498272155e-23	100	100
ELK3-regulon	LZ
0.0581295608492649	-0.00688437389838458	11971715
0.37490556285478 1.38971427878346e-30	2.56284970892534e-30	100
100
ELK4-regulon	LZ	0.0479551478824206	0.00300547244869868
17126984.5	0.536347696547871
0.000837137988998219	0.000950988755501977	98.4615384615385
96.7018203801975
ESRRA-regulon	LZ	0.110090123933903	0.0186482430821312
23948747 0.74997763258662 8.96648067417185e-117
5.30516773221835e-116	100	100
ETS1-regulon	LZ	0.270643036861273	0.0281475585515932
23939200.5	0.749678674922176
1.68676588357608e-116	9.58083021871214e-116	100	100
ETV2-regulon	LZ
0.0208775938286336	-0.00784809618258055	8587802
0.268935131056445	4.67287689831713e-100
2.21182839853677e-99	100	100
ETV3-regulon	LZ
0.007125754426834	-0.00437219479780463	11809975.5
0.369840537644662	4.31286152461694e-33
8.27603157426495e-33	74.2657342657343 84.5099751461006
ETV5-regulon	LZ	0.038031191259167	0.00464067322516646

20786467.5 0.650947863180012
9.45583459332668e-44 1.91818358893198e-43 100 100
ETV6-regulon LZ 0.444836029699554 -0.0149748271622394
9110959 0.285318286648306 1.23763168107382e-86
4.28643167591421e-86 100 100
ETV7-regulon LZ
0.0610855501668822 -0.00303840617723092 14668160.5
0.459347300557753 0.000187147611288704
0.000216056591894276 100 100
FLI1-regulon LZ 0.144559953033389
0.000638498416938416 15493074 0.485180245964823
0.173244002505578 0.182227024857719 100 100
FOS-regulon LZ 0.0726373861944332 -0.0138585101446532
8282060 0.259360531544316 2.34550181577115e-108
1.18950449228394e-107 100 100
FOSB-regulon LZ 0.0255582443700753 -0.0161700324399381
9031597 0.28283299065861 1.31674169578161e-88
4.79429027694841e-88 98.4615384615385 99.3372293499922
FOSL1-regulon LZ 0.0681124415344892 0.0074359041396318
20586541.5 0.644686991654144
2.44345271955422e-40 4.88690543910845e-40 100 100
FOXO1-regulon LZ
0.00782698748190721 -0.00901200178226094 12654240
0.39627947789431 5.91807865864038e-24 1.02483801161821e-23
38.4615384615385 52.5648776337297
FOXO3-regulon LZ 0.00578912325661902 -0.0141781985416088
11353621.5 0.355549381095159
4.77172163082765e-47 1.04243764858081e-46 21.3986013986014
47.1664315622131
GABPA-regulon LZ
0.0234688925217079 -0.00205135820466057 13848058.5
0.433665031817783 1.08875235611803e-09 1.4724079482739e-09
100 100
GTF2B-regulon LZ 0.0820802600068792 0.00307168059947692
17725670.5 0.555096114114049
4.12636530613371e-07 5.18534401301758e-07 100 100
HINFP-regulon LZ
0.0122286468887057 -0.00406596759139901 14368221.5
0.449954427471724 2.38595241548371e-06 2.8471028823419e-06
58.7412587412587 63.3057925259175
HIVEP3-regulon LZ 0.115834848862897 0.0387575936788788
24526695 0.76807662009516 5.35641329183168e-134
4.47418051435352e-133 100 99.6887664853004
HOXB2-regulon LZ 0.0175061632394759 -0.0199102923102217
9945043.5 0.31143843058265 1.51291686538972e-67
4.57094031670936e-67 71.6083916083916 83.9233335572423
IKZF1-regulon LZ 0.161592936688541 0.0180126327957263
27484277 0.860696093946581 6.46265233077325e-241
3.05898876989934e-239 100 100
IRF1-regulon LZ 0.11277666332701 -0.02302864815595

6353256.5	0.198958228131332	1.88806621003517e-168
2.43732183477268e-167	100	100
IRF2-regulon	LZ	0.0686422929417581	-0.0166403340358337
	7588753	0.237648967990877	2.02895942094274e-128
1.60062354318816e-127	100	100
IRF3-regulon	LZ
0.144148697016116	-0.00131860942934439	15128026
0.473748423046468	0.0158488996292853	0.0175823730262384
	100	100
IRF4-regulon	LZ	0.0608776734146125	0.00250479193049014
	18320911.5	0.573736648251326	1.2354992400917e-11
	1.73703853557446e-11	100	99.9955218199324
IRF5-regulon	LZ
0.0380104377838089	-0.00917895343721174	14005566.5
0.438597543608627	1.48178692951658e-08
1.91285221810322e-08	80.5594405594406 80.4773739952083
IRF7-regulon	LZ	0.097529220630825	-0.0104704600161319
	10499393.5	0.328798424432199
9.05659785805688e-56	2.14339482640679e-55	100	100
IRF8-regulon	LZ	0.0944604998770516	0.0128906969107118
	22736051 0.712000911920305	1.56866469377418e-84
5.30358063133175e-84	100	100
IRF9-regulon	LZ	0.0273691156207345	-0.0066519213565724
	10888478 0.34098297305122 2.32736598991635e-48
5.1638432901269e-48	100	100
JUN-regulon	LZ	0.069835362171845	-0.0188621361544387
	5696405.5	0.178388318651636
5.7698752916363e-192	1.17046041630336e-190	100	100
JUNB-regulon	LZ	0.147596082178817	-0.0123815068150752
	10251122 0.321023567910113	8.81764275824056e-61
2.31871346605585e-60	100	100
JUND-regulon	LZ
0.0311820603162496	-0.00739210654456967	10254739.5
0.321136853339446	1.0470325532472e-60
2.65497540287683e-60	100	100
KLF11-regulon	LZ	0.0114077913062685	-0.0106972556594974
	13813799.5	0.43259217887417 3.51446651582187e-12
5.09239025761945e-12	31.4685314685315 41.0089339692349
KLF12-regulon	LZ	0.0523538515193747	0.005389144657658
	18354495.5	0.574788362932381
6.30178932984639e-12	9.03893014988068e-12	99.7202797202797
98.5848950986319
KLF13-regulon	LZ
0.058621217127666	-0.00779368855843865	10481536.5
0.328239215610748	4.01674302956046e-56
9.66741542707773e-56	100	100
KLF2-regulon	LZ	0.0877619381130509	-0.0214240120220074
	7678421.5	0.240457021762859
9.98325300132363e-126	7.46116803256819e-125	100	100
KLF3-regulon	LZ	0.037557651479706	-0.0350234380939885

5939066 0.18598746140897 4.25377509449971e-183
6.71151181576621e-182 99.8601398601399 99.8634155079376
KLF4-regulon LZ 0.00951555518618337 -0.0064864448268178
13745220 0.430444547056356 7.99697506829912e-12
1.13557045969847e-11 40.8391608391608 49.9563377443407
KLF6-regulon LZ 0.0408946791434642 -0.0289412314358411
5696140 0.178380004268363 5.64089460845727e-192
1.17046041630336e-190 100 100
KLF7-regulon LZ 0.0195295001710399 -0.0268929968697072
6132510.5 0.192045358640374
3.49783140500642e-176 4.96692059510912e-175 87.4125874125874
97.7183672555473
KLF8-regulon LZ 0.00833627910550987 -0.0170389309162599
8880364.5 0.278097002077656
1.87596718468394e-93 7.61106686357483e-93 54.965034965035
77.9987013277804
MAFB-regulon LZ
0.0517425650682268 -0.00410502411275241 13363300
0.418484361521911 6.84302359380963e-14
1.03373335140528e-13 100 99.9977609099662
MAFF-regulon LZ
0.00540429677485515 -0.00709443260785198 14016194
0.438930353809107 1.90129004623119e-12
2.78333182025597e-12 17.9020979020979 28.7163296836166
MAFG-regulon LZ
0.0179153802669913 -0.00761191508404458 14011004.5
0.438767839714975 5.28910103551868e-09
7.01918081349208e-09 53.1468531468531 58.6887888762007
MAX-regulon LZ
0.191403627238729 -0.00636690754823133 10253907
0.321110782815626 1.00645844014105e-60
2.59849270000053e-60 100 100
MAZ-regulon LZ 0.13644883146698 0.000147340021254855
17086429 0.535077662759533 0.00126658071278513
0.00142741635885309 100 100
MEF2A-regulon LZ
0.0396675509843516 -0.00110416282345398 15166449.5
0.474951691241071 0.0213462938197969 0.0234974707163655
100 99.9977609099662
MLX-regulon LZ
0.0509093465510898 -0.00204838814950491 15310971.5
0.479477534176265 0.059308108035034 0.0633214386539461
100 99.9888045498309
MXD4-regulon LZ
0.0908091908091908 -0.00138171066088917 17799491
0.557407872797139 1.29621288293472e-07
1.65821828267324e-07 93.986013986014 88.517946306621
MXI1-regulon LZ 0.0426122920603618 -0.0229793503882208
6423809 0.201167646307701 5.12347196492311e-166
5.59640783860832e-165 100 100

MYBL1-regulon	LZ
0.0242345522893156	-0.00574621099378379	15810936
0.495134394724641	0.653500547926735	0.658135303585789
	85.4545454545455	79.973578737601
MYC-regulon	LZ	0.352582824562713	0.0383046404399546
	24120750	0.75536406899341	8.95394905195546e-122
6.05457507322703e-121	100	100
NFATC1-regulon	LZ	0.143937437154911	0.094915195424823
	28895407	0.904886962749527	4.79106864604513e-303
3.40165873869204e-301	100	94.7448556906473
NFE2L1-regulon	LZ	0.190872504755435	0.00833672591072895
	18433047.5	0.577248293007009
1.25936344468128e-12	1.86280842859106e-12	100
99.9350663890195
NFE2L2-regulon	LZ	0.121440302636867	0.0107288306796024
	21349064	0.668566103966117	4.03323696167544e-54
9.38884669767069e-54	100	100
NFKB1-regulon	LZ	0.0660492731931427	0.009530653223516
	20318810.5	0.6363027425095	5.41597187102785e-36
1.05351781600816e-35	100	99.9305882089519
NFKB2-regulon	LZ	0.0825581734777225	0.00932857299341804
	20439994.5	0.64009773393128	6.28682595600077e-38
1.23990178576682e-37	100	100
NFYA-regulon	LZ
0.0141514759579542	-0.00690574554148693	10329793
0.32348722458214	3.60786922041534e-59	8.83305912584445e-59
97.3426573426573	99.043908555563
NFYB-regulon	LZ	0.114943921138376	0.00642018880762529
	21999908.5	0.688947914225002
1.56490540821513e-67	4.62951183263642e-67	100	100
NFYC-regulon	LZ	0.0873276654000135	0.00385364948124337
	20036179	0.627451870133404	1.10460168155466e-31
2.06386103658897e-31	100	100
NR1H3-regulon	LZ	0.00398187261131423	-0.0046253991170876
	14689349	0.460010838448401	4.18405313491993e-07
5.21171530840903e-07	15.1048951048951	22.2162513154654
NR2C2-regulon	LZ	0.0103927541285172	-0.011575817143873
	10721967	0.335768523811783	3.66354177062766e-52
8.25750684808139e-52	59.1608391608392	76.2477329213408
NR3C1-regulon	LZ	0.231474820821199	0.00609324817327958
	18829978.5	0.589678562184776
1.70814528602897e-16	2.66545747929795e-16	100	100
NRF1-regulon	LZ
0.0231461139604565	-0.00364041484801726	13101073
0.410272475335954	1.6450726281337e-16
2.59555903549984e-16	100	99.9664136494928
PAX5-regulon	LZ	0.114786598083132	0.0147513595773599
	22839900.5	0.71525305710165	4.37527818319649e-87
1.55322375503475e-86	100	100
POU2F1-regulon	LZ

0.00152517166091827 -0.00340851800856538 14245208.5
0.446102159187401 2.16987473354358e-13
3.24339170698093e-13 7.97202797202797 18.5127963995432
POU6F1-regulon LZ
0.0256219755371819 -0.00546628787487383 14658646
0.459049345003533 0.00012108395959008
0.000140933789030992 60.8391608391608 65.4262107879358
REL-regulon LZ 0.280701358904919 0.0172613682076824
23316657 0.730183137209402 2.61226935257087e-99
1.15919452520332e-98 100 100
RELA-regulon LZ
0.0858405176886717 -0.00632358616832687 10680505
0.334470102119729 2.97565507890636e-52
6.81521001943069e-52 100 100
RELB-regulon LZ 0.123678663979269 0.0187910805148989
26233102 0.821514367050741 7.51730508161064e-192
1.33432165198589e-190 100 100
REST-regulon LZ 0.219627070477499 0.00102474554542656
16224338 0.508080468824742 0.457754177845515
0.467633764417721 100 100
RFX5-regulon LZ 0.0732003059473891 0.00797753530605549
22233525.5 0.69626385123799 1.02403816633463e-72
3.16116129607645e-72 100 100
RXRA-regulon LZ 0.0295172064402833 0.0152270030387747
22744473.5 0.712264670463099
1.06774311482411e-88 3.98998742907955e-88 86.4335664335664
63.9081077450124
SETDB1-regulon LZ 0.0598416580589754 0.00012611841806582
16044117.5 0.502436693643787 0.822810672584136
0.822810672584136 98.4615384615385 94.9978728644679
SOX5-regulon LZ
0.00488735561585962 -0.00296263075546606 15044366.5
0.47112854678516 0.000284215888238903 0.000325473033305841
17.3426573426573 22.5767448109088
SP1-regulon LZ
0.00918961237867844 -0.00386516564894309 13760930.5
0.430936536202876 5.27533483442215e-11
7.27279171347519e-11 49.9300699300699 59.9807438257092
SP2-regulon LZ
0.0251094652202786 -0.00318081262329494 12523674.5
0.392190695938933 3.87770656715615e-23
6.47805097101381e-23 100 100
SP3-regulon LZ
0.0231442945375912 -0.00520766412483396 10133616.5
0.317343772190283 3.13599244319933e-63
8.90621853868609e-63 100 100
SP4-regulon LZ
0.00312971370065774 -0.00559639427700813 13745905
0.430465998478358 1.55114137116868e-15
2.36840940544035e-15 16.9230769230769 29.2089294910548

SPI1-regulon LZ 0.435360796089156 0.0170870631560839
20924719 0.655277339485038 3.40720435768758e-46
7.22123908644233e-46 100 100
SPIB-regulon LZ 0.62005276861915 0.0564917011902971
29540589.5 0.925091462130489 0 0 100 100
SREBF1-regulon LZ
0.0377487441119787 -0.00323206436007574 14544574.5
0.455477088237215 4.28636656476268e-05
5.07220043496917e-05 97.3426573426573 96.8876648530037
SREBF2-regulon LZ 0.100704302705985 0.00174923174460868
16906711 0.529449623840703 0.00680457715293497
0.00760826736784855 100 100
SRF-regulon LZ
0.0135702524134703 -0.00731417487049001 10118454.5
0.316868959839337 1.50045256111178e-63
4.34825027914026e-63 96.5034965034965 97.957949889165
STAT1-regulon LZ 0.0681874704119705 -0.0117664464191995
7398154.5 0.231680195937602
3.0853279210263e-134 2.73822852991084e-133 100 100
STAT2-regulon LZ
0.057885070049972 -0.00848295241387322 12389562.5
0.387990851986284 7.57505993994168e-25
1.36159305249585e-24 100 99.9619354694252
STAT5A-regulon LZ 0.0768940024635359 0.00537080308227182
19562686.5 0.612624005268595
4.20517871886223e-25 7.65558177023637e-25 100 100
TBP-regulon LZ 0.0141077689975372 -0.0069022566885666
14358712.5 0.4496566441552 7.49263576551991e-07
9.17201964399851e-07 44.6153846153846 49.9697722845436
TCF12-regulon LZ
0.0047669547772154 -0.00434522727722649 14622253
0.457909663834296 3.96230500161736e-07
5.02363669847915e-07 17.9020979020979 25.3375428225969
TFDP1-regulon LZ 0.5645440224595 0.0369631570779982
25531427 0.799540751673485 8.53613425951078e-167
1.01010922070878e-165 100 100
TFDP2-regulon LZ
0.0247037775422055 -0.00245810916543534 14220739.5
0.445335889340726 5.07818235462248e-07
6.27045125527298e-07 100 99.9955218199324
TFEC-regulon LZ
0.0869235916149632 -0.00469188487655316 14601692.5
0.457265792356811 8.59971921661573e-05
0.000100922324690862 100 100
THAP1-regulon LZ
0.0334174618615228 -0.00380764917418012 11857312.5
0.371322940510823 2.90852567104171e-32
5.50680860383897e-32 100 100
THAP11-regulon LZ
0.178315362631696 -0.000164946407998912 15219688.5

0.476618920811841 0.0316666420233802 0.0340656300554544
100 100
TP53-regulon LZ 0.110309367008396 0.0121440311096432
21797245 0.682601315300986 3.41415654754751e-63
9.50608293630875e-63 100 100
USF2-regulon LZ
0.120997807324993 -0.00435705286203447 12445204.5
0.389733333771757 3.94726500004525e-24
6.91989666674599e-24 100 100
XBP1-regulon LZ 0.190481136886898 -0.0048857150780281
15606649 0.488736954364683 0.300663494558757
0.309378378459011 100 100
YBX1-regulon LZ
0.0943352008170597 -0.000862146207312828 15522218.5
0.486092933510143 0.201255479498162 0.210134397711317
100 100
YY1-regulon LZ 0.680269540467937 0.0414513669168742
25370657.5 0.79450610292956 2.6481348182336e-161
2.68596531563694e-160 100 100
YY2-regulon LZ 0.0280839856322263 -0.0232849780512014
9530035.5 0.298442063075636
1.18383787393395e-76 3.73566617996934e-76 80.4195804195804
90.6921027294508
ZBTB33-regulon LZ 0.371872310032725 0.00614309441826505
17939794.5 0.561801609420337
1.35300063213324e-08 1.76262467672404e-08 100 100
ZNF143-regulon LZ
0.016118340391835 -0.00732786224458525 8089315
0.25332453981611 9.24150835442538e-114 5.04728533203232e-113
99.7202797202797 99.9977609099662
ZNF274-regulon LZ 0.0526195053445954 -0.0129256986211276
7802541.5 0.244343956797776
4.7632560704762e-122 3.3819118100381e-121 100 100
ZNF76-regulon LZ 0.012818683959738 -0.0017662539577195
13942462.5 0.436621382245081
5.74033389088378e-09 7.54747604171756e-09 99.8601398601399
99.4536620317503
ATF1-regulon Memory 1
0.103659971137685 -0.00266573284336021 34217637
0.463205883581229 2.70855166940717e-07
3.77072879466488e-07 100 100
ATF3-regulon Memory 1
0.048778415527101 -0.00233020382301172 36198073
0.490015145929067 0.162843524549776 0.176517408290597
100 100
ATF4-regulon Memory 1 0.106588975310731 -0.002246381770995
36547098 0.49473991501575 0.46222036400622 0.482612438888848
100 100
ATF5-regulon Memory 1 0.11852293249536 9.29550557832726e-05
37825273.5 0.512042641438658 0.0923389924764069

0.102438569778514	100	99.9977108847431
ATF6-regulon	Memory 1
0.134416593991111	-0.00207466688745661	41569722.5
0.562731437031698	1.820192733147e-18
3.31368420649839e-18	100	100
ATF6B-regulon	Memory 1 0.0603693768791612	0.00319181282590388
47259622.453125	0.639755900622684
5.53503701901032e-85	3.57261480317939e-84	100
99.9175918507497
BACH1-regulon	Memory 1 0.0442928205332679	0.00104908006954242
38376200.5	0.519500568116171	0.00641899373798467
0.00779057359652841	99.8817267888823 99.5513334096372
BATF-regulon	Memory 1
0.0153898574122718	-0.00405459607602397	35819576.5
0.48489141965554 0.0249849318987579	0.0286117768518035
50.7983441750443 51.7523177291977
BATF3-regulon	Memory 1 0.066520165208724	-0.0172877000074857
26443700 0.357969705028344	1.07120491377956e-87
7.24338560746181e-87	100	100
BCL11A-regulon	Memory 1 0.220122549376242	0.0162269371384526
51070898 0.691349330562389	1.42333155212301e-157
2.88732972002097e-156	100	100
BHLHE40-regulon	Memory 1 0.0597299828033509	0.00423669271347783
42101294 0.569927347326267	1.46061304350124e-22
2.88065350246077e-22	100	100
BRF2-regulon	Memory 1
0.0390994374302159	-0.00261016949128843	33869865.5
0.458498083187477	6.60469794345402e-09
9.47340513101486e-09	99.8817267888823 99.7230170539087
CEBPB-regulon	Memory 1 0.13918657191303 -0.00405662504087309
34578979 0.468097388520189	8.23453733384326e-06
1.11362314419595e-05	100	100
CEBPG-regulon	Memory 1
0.0588506504215955	-0.00956659587307642	29774762.5
0.403062466652322	8.04738216953371e-42
2.38068389182039e-41	100	100
CLOCK-regulon	Memory 1 0.0165715258445797	0.00101460950074744
38123794 0.516083728553166	0.016744072800982
0.0194890027683561	53.9917208752218 50.9808858876044
CREB1-regulon	Memory 1 0.0474696750120323	0.00467260996319931
43558026 0.589647202125155	5.12305316643335e-36
1.37259160308214e-35	100	99.9931326542291
CREB3-regulon	Memory 1
0.233898418238886	-0.00613003977267668	34319996.5
0.464591529312419	7.45942592594412e-07
1.02838687522725e-06	100	100
CREB3L2-regulon	Memory 1
0.124659283409104	-0.00219990185691583	40999152.5
0.555007602069192	1.49040024935194e-14
2.54984139045753e-14	100	100

CREB3L4-regulon	Memory 1
0.0102554650159618	-0.000878622699979249	36183738.5
0.4898210990772	0.0836613061172534	0.0935425627452755
29.4500295683028	31.4203960169394
CREB5-regulon	Memory 1
0.035923746161122	-0.00337639255528517	35607032.5
0.482014200772194	0.0119419344478051	0.0141312890965693
	99.8225901833235	99.6154286368319
CREM-regulon	Memory 1
0.145884209258168	-0.00427464956029369	30374464.5
0.411180662972992	2.18905440470717e-35
5.75640232348923e-35	100	100
CTCF-regulon	Memory 1
0.366139309685066	-0.00488291048598155	33636095
0.455333520099508	4.29295006614673e-10
6.62607510209605e-10	100	100
CUX1-regulon	Memory 1	0.0776680747091794	0.00100755722203866
	40947254.5	0.554305056217002
3.19511071790667e-14	5.33771437579702e-14	100	100
DDIT3-regulon	Memory 1
0.0806538429488696	-0.00441896309830397	33315187.5
0.450989379033153	7.3766276735597e-12	1.1769450894893e-11
	100	100
E2F1-regulon	Memory 1	0.416856052588706	-0.0216455268478831
	28674609.5	0.388169639820371
4.51877292723919e-55	1.64529680940504e-54	100	100
E2F2-regulon	Memory 1
0.0518823504078892	-0.00211755510283239	36573782
0.495101137674038	0.493528255407165	0.511540235531514
	100	100
E2F3-regulon	Memory 1	0.0213781923119917
0.000273066148122555	37986423.5	0.514224137143318
0.0468015326654761	0.0531665411079808	100	100
E2F4-regulon	Memory 1	0.37582257950443	-0.0237188586110978
26666985	0.360992325372216	4.39204461157618e-84
2.71161015149486e-83	100	100
E2F6-regulon	Memory 1	0.0595013789981437	-0.0133991794950249
	27889158	0.377536943118735	1.11736534701135e-65
5.28886264252038e-65	100	100
E2F7-regulon	Memory 1	0.0339640130976732	0.00352120860902794
	45754366	0.619379167846364	1.67099869318228e-62
7.19035801308738e-62	100	100
E2F8-regulon	Memory 1
0.0679080518765645	-0.00715520773848849	28544321
0.386405917802893	9.15583923697586e-57
3.42139255697519e-56	100	100
EGR1-regulon	Memory 1
0.0466755921666811	-0.000817896010616839	35385176.5
0.479010924873633	0.0033503400587518	0.00410127834778238
	100	100

EGR2-regulon	Memory 1
0.0427975352968383	-0.00337619193260746	33729214.5
0.456594083483126	1.30484987078271e-09
1.97115618777814e-09	100	99.9954217694861
EGR3-regulon	Memory 1
0.0387353431698709	-0.00284169961341336	34062263.5
0.461102584649377	5.4291599355235e-08
7.70940710844337e-08	100	99.9954217694861
ELF1-regulon	Memory 1 0.704460994408036	-0.0026836558793023
38336043 0.518956954006585	0.00805888748431649
0.00969798324383849	100	100
ELF2-regulon	Memory 1 0.0403358875621291	0.00797710343646459
47458545 0.642448725205792	3.33916959501722e-88
2.49558990785497e-87	99.9408633944412 99.5192857960398
ELF4-regulon	Memory 1 0.0226571501834917	0.00437301479252373
45511406.5	0.616090212800405
3.31129651136744e-59	1.30612251281716e-58	99.8225901833235
98.2785853267712
ELK1-regulon	Memory 1 0.0662568127722245
0.000209654512982563	37042926.5	0.501451970510618
0.839182348682212	0.839182348682212	100	100
ELK3-regulon	Memory 1 0.0775980321246631	0.0131838921807649
52620982 0.712332896109161	1.49667802349722e-193
7.08427597788682e-192	100	100
ELK4-regulon	Memory 1 0.0459637141884334	0.00100410002822406
38174206.5	0.516766165116686	0.0191073696294636
0.0220589145315759	97.2797161442933 96.7082522605013
ESRRA-regulon	Memory 1 0.0816779417800863	-0.0104471098345089
30116498.5	0.40768856417716 4.37021476327304e-38
1.19340480073995e-37	100	100
ETS1-regulon	Memory 1 0.247696289872888	0.00494143346978934
43125422.5	0.583791026654656
1.11446763277675e-31	2.51197466435395e-31	100	100
ETV2-regulon	Memory 1 0.0310386223711019	0.00253091467397989
42598347.5	0.576655985708123
8.73783060876838e-27	1.8799574946138e-26	100	100
ETV3-regulon	Memory 1 0.0122613340966449
0.000864495163845442	39000927 0.527957522359654
9.03645207776226e-05	0.000116652381367476	86.6942637492608
84.2577543779329
ETV5-regulon	Memory 1 0.0338515013721027	0.00040287280745633
38307827.5	0.518574999355298	0.00942591821530002
0.0112477343409462	100	100
ETV6-regulon	Memory 1 0.477039082085373	0.0181402071458427
51635580 0.698993459370945	3.0114912779024e-170
7.12719602436901e-169	100	100
ETV7-regulon	Memory 1 0.0714374071872933	0.00764627688856276
50581736.5	0.684727526583891
5.40209444526252e-147	9.58871764034097e-146	100	100
FLI1-regulon	Memory 1 0.162231140000031	0.0190079834633682

52697401 0.713367383979185 2.01792905670376e-195
1.43272963025967e-193 100 100
FOS-regulon Memory 1 0.0915616855662657 0.00548870520146308
46484766.5 0.62926663637526 5.74993394510488e-73
2.91603792930319e-72 100 100
FOSB-regulon Memory 1 0.0448344635572429 0.00349108167647573
41356114 0.559839807957985 6.07567061368227e-17
1.0784315339286e-16 99.9408633944412 99.2995307313723
FOSL1-regulon Memory 1
0.0525085459649091 -0.00860587044947174 31938206.5
0.432349117556898 3.21506284638348e-21
6.16944492143857e-21 100 100
FOXO1-regulon Memory 1 0.018151246546934 0.00151055437191484
38422982.5 0.520133858417477 0.00288264702103629
0.00355944240858394 55.4109994086339 52.22387547213
FOXO3-regulon Memory 1
0.0142332826623249 -0.00572394066323638 33412174
0.452302290191452 4.66010397271809e-13
7.60614671409159e-13 39.4441159077469 47.043607645645
GABPA-regulon Memory 1 0.0266262262309067 0.00118236157972437
40103947.5 0.542889166684208
2.04020705136536e-09 2.98669485869981e-09 100 100
GTF2B-regulon Memory 1
0.0731778659803316 -0.00610668888852897 30627796
0.414610024307805 7.78835603079818e-33
1.87448568876838e-32 100 100
HINFP-regulon Memory 1 0.0164929797910091
0.000272592221024333 37238545 0.504100068043985
0.556629433430131 0.572763619906367 63.4535777646363
63.225363397047
HIVEP3-regulon Memory 1 0.0865147514481661 0.00916845944835923
43845201.5 0.593534711400572
4.68224870033548e-39 1.30368493225027e-38 99.9408633944412
99.6841020945405
HOXB2-regulon Memory 1
0.0362306423476039 -0.000905839552624368 37420165.5
0.506558673943012 0.358263570293966 0.382506969787543
86.0437610881135 83.6396932585556
IKZF1-regulon Memory 1 0.145087493052685 0.00127071495637665
38679284.5 0.523603431561106
0.000970209432936968 0.00121920123431017 100 100
IRF1-regulon Memory 1 0.143199466110083 0.00805728840086628
48296838.5 0.653796746735388
1.69666492369627e-102 1.50579011978044e-101 100 100
IRF2-regulon Memory 1 0.0911379421937043 0.00635432349964388
44557986 0.603183711246047 3.76941727340411e-47
1.1636027235291e-46 100 100
IRF3-regulon Memory 1 0.153150103737944 0.00800177213427275
50114373.5 0.67840081000296 3.11442689591509e-137
3.68540516016619e-136 100 100

IRF4-regulon	Memory 1
0.0566107888321154	-0.00187129775455584	36668999
0.496390095021296	0.613873727225154	0.622643351899799
	100	99.9954217694861
IRF5-regulon	Memory 1	0.0527730079461207	0.00594998613312915
	40956340.5	0.55442805385878	2.24410066999436e-14
3.79359875165713e-14	90.1833234772324	80.1030101865629
IRF7-regulon	Memory 1	0.116591721953794	0.00909600195148359
	50162470.5	0.679051901525808
3.19543769927212e-138	4.12501957542401e-137	100	100
IRF8-regulon	Memory 1	0.0845732297900485	0.00290870200896071
	42548402.5	0.575979877715761
2.41524435717286e-26	5.11887610027682e-26	100	100
IRF9-regulon	Memory 1	0.0373097614266101	0.0035249006382404
	44024086	0.59595627992915	5.16357059372498e-41
1.49638168226316e-40	100	100
JUN-regulon	Memory 1	0.0947879839543121	0.00663496173075061
	45093997.5	0.610439726045292
9.36725722344743e-54	3.24426957495008e-53	100	100
JUNB-regulon	Memory 1	0.169893114144564	0.0105019948863383
	47197925	0.63892069907766	5.56517659633219e-84
3.16102030671668e-83	100	100
JUND-regulon	Memory 1	0.0411760358385883	0.00282357245998895
	42457105	0.574743978838341	1.51414172515531e-25
3.16188419076549e-25	100	100
KLF11-regulon	Memory 1	0.0262094903411879	0.00443840586928437
	39680941	0.537162906288346	5.46451663256939e-09
7.91797307984545e-09	47.2501478415139	40.6111937736065
KLF12-regulon	Memory 1	0.0505012800621773	0.00358526512793576
	40256768	0.544957905525871	3.30550088775916e-10
5.15803435232748e-10	99.7043169722058	98.5601465033764
KLF13-regulon	Memory 1	0.0703627481726634	0.00422821993795067
	48346055.5	0.654462999754912
2.27247126813289e-103	2.15127280049914e-102	100	100
KLF2-regulon	Memory 1	0.129613668831075	0.0215691045472405
	56040051	0.758617006177024	4.20334400247474e-286
5.96874848351413e-284	100	100
KLF3-regulon	Memory 1	0.0858192326404102	0.0143238122475104
	47018590	0.636493032107786	3.88489440524527e-81
2.12175002132626e-80	100	99.8580748540689
KLF4-regulon	Memory 1	0.0159265554818161
2.77998627474102e-05	37195023	0.503510908527645
0.599569455114542	0.612509803066655	50.7983441750443
49.7745221471901
KLF6-regulon	Memory 1	0.0866963248383531	0.0179867491792418
	52496631.5	0.710649557098163
1.5813116680079e-190	5.61365642142805e-189	100	100
KLF7-regulon	Memory 1	0.0556480042541682	0.0100227791708375
	45494594	0.615862621137143	5.54901273838323e-59
2.12962110500113e-58	99.8817267888823	97.4659494105528

KLF8-regulon Memory 1 0.0394442362508596 0.0148925024556783
50303383 0.680959441168892 1.03017982446413e-142
1.62539483415452e-141 93.4949733885275 77.0218610507039
MAFB-regulon Memory 1 0.0576496840036503 0.00193903964551091
40147898 0.543484126826732 1.21894438390277e-09
1.86118389800208e-09 100 99.9977108847431
MAFF-regulon Memory 1 0.0149550571803947 0.00266752545410287
38760452 0.524702200115918 1.47693659665013e-05
1.9785377049464e-05 32.8208160851567 28.3804509557056
MAFG-regulon Memory 1 0.0262352007456857
0.000859893200703211 37665785 0.509883637543575
0.151799721311518 0.165812003278735 60.3784742755766
58.532677120293
MAX-regulon Memory 1 0.206670866991404 0.00934906290502149
51694748 0.699794419581019 1.32457212244692e-171
3.76178482774925e-170 100 100
MAZ-regulon Memory 1
0.130727189148534 -0.00579248916453165 29744261.5
0.402649573071882 3.66042083989048e-42
1.10591438141372e-41 100 100
MEF2A-regulon Memory 1 0.0458004467541717 0.00524146188836162
44580326.5 0.603486135725041
2.04116841620308e-47 6.44102033557417e-47 100
99.9977108847431
MLX-regulon Memory 1 0.055588352927253 0.00276597299255444
40593921.5 0.549521969516322
4.46465897918846e-12 7.20433608005411e-12 100
99.9885544237152
MXD4-regulon Memory 1
0.0834186211632023 -0.00908923127119934 39733549.5
0.537875070214989 1.1728313367671e-07
1.64893118634582e-07 92.3122412773507 88.460569989699
MXI1-regulon Memory 1 0.0701652801464298 0.00512678538869384
42244846 0.571870618014417 9.63732126026535e-24
1.92746425205307e-23 100 100
MYBL1-regulon Memory 1 0.0233818874214 -0.00676026210918884
36087334 0.488516066482351 0.107071336319794
0.11786147098768 83.6191602602011 79.9221700812636
MYC-regulon Memory 1 0.289328526557362 -0.0265423710564726
28969810.5 0.392165790695403
2.47980303077996e-51 8.18911698536639e-51 100 100
NFATC1-regulon Memory 1 0.0381182426580962 -0.0128795748339118
30004592.5 0.40617368699239 2.70492731289071e-39
7.68199356860962e-39 92.9036073329391 94.9021403227652
NFE2L1-regulon Memory 1 0.159592173089926 -0.0239681770345664
32474540 0.439609491286437 3.15396044184793e-17
5.66914408534692e-17 100 99.9336156575484
NFE2L2-regulon Memory 1 0.103629506734009 -0.0075317012225333
31509326.5 0.426543347294319
9.92856479997555e-25 2.04326985738627e-24 100 100

NFKB1-regulon	Memory 1
0.0518270696449834	-0.00502914512042642	35033861
0.474255149172544	0.000320267179657026
0.000406053031350873	100	99.9290374270344
NFKB2-regulon	Memory 1	0.0778292263661702	0.00462499014135852
	43317870.5	0.586396204969086	1.4228611765617e-33
	3.60796941199573e-33	100	100
NFYA-regulon	Memory 1	0.0233305179753796	0.00247432091002284
	42016850.5	0.568784231393679
6.98492177459192e-22	1.35871081094802e-21	99.7043169722058
98.9905001716836
NFYB-regulon	Memory 1	0.0963982388746582	-0.0126999387904567
	23971183.5	0.324499124051298
7.14582086766003e-133	7.24790402291232e-132	100	100
NFYC-regulon	Memory 1
0.0789857478242881	-0.00472507754227552	30427218
0.411894789771973	7.58029642018676e-35
1.95709471212095e-34	100	100
NR1H3-regulon	Memory 1	0.0105974931816127	0.00214296552660217
	38541302.5	0.521735562244814
2.87136074493117e-05	3.7753076461132e-05	26.0792430514488
21.9503261989241
NR2C2-regulon	Memory 1	0.0270865161105466	0.00550551846707224
	42712770.5	0.578204935649261
3.52896280662189e-28	7.70942643908167e-28	84.6836191602602
75.6415245507611
NR3C1-regulon	Memory 1	0.235481247242272	0.0103908930284868
	49940197	0.676042973908621	1.10740038718067e-133
1.20962196138196e-132	100	100
NRF1-regulon	Memory 1	0.0272978986033293
0.000590747680615712	39033905	0.528403947214437
7.18758979599594e-05	9.36364909203141e-05	100
99.9656632711457
PAX5-regulon	Memory 1	0.105897963333955	0.00584822661567609
	43265377	0.585685597803262	4.73619954976827e-33
1.15955230356396e-32	100	100
POU2F1-regulon	Memory 1	0.00521557210429092
0.000348581612405605	37616972	0.509222853492495
0.0561602232985761	0.0632916802253794	19.9881726788882
18.2831635572851
POU6F1-regulon	Memory 1	0.0375813773036258	0.00683392312634582
	41624101.5	0.563467568306434	1.2900828943739e-19
	2.41041803948808e-19	73.4476641040804	65.0406317958109
REL-regulon	Memory 1
0.258701857025966	-0.00520406161694126	32694332.5
0.442584833481079	1.01644537963234e-15
1.76018590131454e-15	100	100
RELA-regulon	Memory 1	0.0975211783166015	0.00566794036372426
	47441275	0.64221494034188	6.40719182574827e-88
4.54910619628127e-87	100	100

RELB-regulon	Memory 1
0.0989254659114843	-0.00650046168359579	27992574
0.378936890743886	3.15769800127072e-64
1.40122848806388e-63	100	100
REST-regulon	Memory 1	0.226756089757294	0.00845261622168492
	50349555	0.68158447386933	4.20751479867747e-142
5.97467101412201e-141	100	100
RFX5-regulon	Memory 1	0.066849954403977	0.00155960061050424
	43175321.5	0.584466511942691
3.64331301211193e-32	8.481154880654e-32	100	100
RXRA-regulon	Memory 1	0.0146905003794419
0.000166569039669783	37390587	0.506158268291753
0.378277158559726	0.400860869518516	65.1685393258427
64.2279958795925
SETDB1-regulon	Memory 1
0.0595799209303088	-0.000142932569100099	36826728.5
0.498525287244369	0.836688678838646	0.839182348682212
	94.2637492607924	95.0829804280646
SOX5-regulon	Memory 1	0.00983836277643753	0.00211383423940743
	38379481.5	0.519544983179199
0.000186327571487963	0.000238365001362979	25.7835600236546
22.3669451756896
SP1-regulon	Memory 1	0.0149580488322778	0.00204020623717699
	40177574	0.543885852340424	2.25304272687351e-10
3.55480074684487e-10	66.7060910703726	59.5559116401511
SP2-regulon	Memory 1
0.0277405441658109	-0.000518952371214479	35646446
0.482547743316132	0.0147164483759533	0.0172705427221931
	100	100
SP3-regulon	Memory 1	0.0318775157288466	0.00374726238514814
	46509728	0.629604541463884	2.44042635597069e-73
1.28348349091792e-72	100	100
SP4-regulon	Memory 1	0.00992666879436827	0.00133863041150851
	38298524	0.518449057405014	0.00129345896535656
0.00161115064105817	32.2885866351271	28.8886345427492
SPI1-regulon	Memory 1	0.430555300711557	0.0124773072990722
	45654717	0.618030214290834	3.85844630120132e-61
1.56542678505882e-60	100	100
SPIB-regulon	Memory 1
0.560154508292411	-0.00446303293410211	33044402.5
0.447323748785642	1.80492754169869e-13	2.9802291967583e-13
	100	100
SREBF1-regulon	Memory 1	0.0449700828356721	0.004196594701636
	41681973.5	0.564250984498926
2.69714548867391e-19	4.97395661547656e-19	97.043169722058
96.8890923658006
SREBF2-regulon	Memory 1	0.104814035637188	0.00605712900153968
	47202369	0.638980857730539	4.72468170006405e-84
2.79543667253789e-83	100	100
SRF-regulon	Memory 1	0.0233233747937771	0.00265306895359621

41883133 0.566974090829684 7.90308397153185e-21
1.49631723194336e-20 98.9355410999409 97.89630307886
STAT1-regulon Memory 1 0.0847148069594519 0.0051377626107508
46257722 0.626193123489646 1.2610463956444e-69
6.17477890281053e-69 100 100
STAT2-regulon Memory 1 0.0684685427999388 0.00232067120857277
39397758 0.53332944368746 3.18644434804149e-06
4.35072209059511e-06 100 99.9610850406318
STAT5A-regulon Memory 1
0.065260264580662 -0.00659327123076443 30903580
0.41834332627128 3.59815036149544e-30 7.98339611456801e-30 100
100
TBP-regulon Memory 1 0.0210789548422794 0.00018456776749335
37332178.5 0.505367589471613 0.422312607190593
0.444210297933809 50.7983441750443 49.8500629506696
TCF12-regulon Memory 1 0.0119639675674613 0.00303329419576482
39368219.5 0.532929579518226
1.60055610246168e-09 2.39241017420588e-09 31.0467179183915
24.9948494906719
TFDP1-regulon Memory 1 0.50888577998557 -0.0200237347427396
26240755 0.355222428293735 4.77652454073332e-91
3.98980285167136e-90 100 100
TFDP2-regulon Memory 1
0.0267061476368906 -0.000433147946001515 37530607
0.508053726117174 0.260309484995818 0.280029900525804
99.9408633944412 99.9977108847431
TFEC-regulon Memory 1 0.0964921778073385 0.00514226614612524
43191605.5 0.584686949274709
2.52466430974802e-32 5.97503886640365e-32 100 100
THAP1-regulon Memory 1 0.0380743197695079
0.000944401159772727 39154393.5 0.530035006136007
2.69329633896548e-05 3.57428112273924e-05 100 100
THAP11-regulon Memory 1 0.181387203039689 0.00302211648907955
42277062 0.57230672763664 5.18383907059086e-24
1.05157878289129e-23 100 100
TP53-regulon Memory 1
0.0893924363105866 -0.00931125269442681 27924274.5
0.378012316956232 3.48995959546867e-65
1.59862665340823e-64 100 100
USF2-regulon Memory 1 0.132144529767724 0.00712380312899275
47585691.5 0.644169913810276
2.66534492292548e-90 2.10266099475232e-89 100 100
XBP1-regulon Memory 1 0.196043664567344
0.000782976649378458 44852348 0.607168504535623
1.00981192950643e-50 3.25893849977075e-50 100 100
YBX1-regulon Memory 1 0.0919794684400941 -0.0033283283530272
30567541.5 0.413794356092251
1.96623233921421e-33 4.89833319593715e-33 100 100
YY1-regulon Memory 1 0.62694461856428 -0.0130116094409021
28962104.5 0.392061474183457 1.98846543735104e-51

6.72290695485353e-51   100   100
YY2-regulon   Memory 1 0.057467541112343   0.00671575617994892
        41339656 0.559617015179163   7.67497120050232e-17
1.34548877835967e-16   94.9733885274985 90.3582465377132
ZBTB33-regulon   Memory 1 0.374936316393466   0.00946295278560327
        45675851 0.618316306318276   1.98849535386458e-61
8.30489236025797e-61   100   100
ZNF143-regulon   Memory 1 0.026475878848795   0.0032668816109869
        45142857 0.611101139569225   2.2256178686998e-54
7.90094343388431e-54   100   99.9931326542291
ZNF274-regulon   Memory 1 0.0692346246600355   0.00404379141442672
        43143211 0.584031830479306   7.48864707062285e-32
1.71514174843298e-31   100   100
ZNF76-regulon   Memory 1 0.0155047091922372
0.000984283230952711   40105980 0.542916680739559
1.99247370471693e-09   2.94720068822712e-09   99.4677705499704
99.4597687993591
ATF1-regulon   Memory 2
0.102651772960789   -0.00375742540161711   42098469.5
0.441677534847002   2.05328957290996e-20
3.31326271992289e-20   100   100
ATF3-regulon   Memory 2
0.0476156593851464   -0.00358034138031871   43891563
0.460489836724869   3.54593820990759e-10
4.75021911138564e-10   100   100
ATF4-regulon   Memory 2
0.104523728537579   -0.00444416756033325   43602622
0.457458402325663   1.43732432124041e-11
2.02079261006078e-11   100   100
ATF5-regulon   Memory 2
0.110903594435036   -0.00791499124845711   40931457
0.429433783227109   3.92354943465326e-29
7.14287204770209e-29   100   99.9976834692365
ATF6-regulon   Memory 2
0.134552624544514   -0.00195652568076635   53987318.5
0.566409801384345   5.44264089011137e-26
9.66068757994769e-26   100   100
ATF6B-regulon   Memory 2 0.058634011702825   0.00140591208900521
        54936558.1015625 0.576368781180446
7.73365995945389e-34   1.44497330821375e-33   99.9547101449275
99.9189214232765
BACH1-regulon   Memory 2 0.0484740919327763   0.00545678358650052
        55122534.5   0.578319958935296
1.69960604638016e-35   3.30608299432852e-35   100
99.5413269088213
BATF-regulon   Memory 2 0.0131116106642568   -0.0064979325893957
        43027154 0.451420860090942   2.67438354422803e-16
4.04002620511043e-16   44.8822463768116 52.0663454410675
BATF3-regulon   Memory 2 0.0677935936544815   -0.0161561824887027
        35323119 0.370593712985867   8.51080123120205e-94

3.55451110244321e-93	100	100
BCL11A-regulon	Memory 2	0.220550175851185	0.0168707776088836
	66651848.5	0.699280151704228
1.08811390142725e-219	1.71680193336299e-218	100	100
BHLHE40-regulon	Memory 2	0.0609151899141922	0.00553326257991596
	56664004.5	0.594492344243522
7.12148008988388e-51	1.77412311011142e-50	100	100
BRF2-regulon	Memory 2
0.0380448110394954	-0.00374999956755624	41951181
0.440132252503868	2.00299634047046e-21
3.26925839479086e-21	99.5923913043478	99.7359154929577
CEBPB-regulon	Memory 2
0.138022782621967	-0.00532852501347786	42347283
0.444287970205386	9.14559542591144e-19
1.42711489063673e-18	100	100
CEBPG-regulon	Memory 2	0.0578478585645441	-0.0107352535216111
	36464054	0.382563871621222	1.39485340174582e-77
4.71593292971207e-77	100	100
CLOCK-regulon	Memory 2	0.0160611384984345
0.000490267786820431	48786186	0.511841941595224
0.0454204110703588	0.0499976617983795	54.2119565217391
50.933561897702
CREB1-regulon	Memory 2	0.0466293742104347	0.00384528996175575
	54217777	0.568827664631477	8.52703063704889e-28
1.53270677273537e-27	100	99.9930504077094
CREB3-regulon	Memory 2
0.234550972215748	-0.00551752451388232	45575643.5
0.478158424978983	0.000525002170762806	0.0006061000670595
	100	100
CREB3L2-regulon	Memory 2
0.125756835396691	-0.00107255822902649	55637332.5
0.583720979786758	2.57897295013001e-40
5.54869937755245e-40	100	100
CREB3L4-regulon	Memory 2	0.0127502492519033	0.00173324445981956
	49386103	0.518135991350947	0.000463607030853458
0.000544067755216455	34.2391304347826	31.1990363232024
CREB5-regulon	Memory 2
0.0331844616391291	-0.00629622598331132	41721961
0.437727383021911	4.75552144531569e-23
8.13595235222684e-23	99.5471014492754	99.6270385470719
CREM-regulon	Memory 2
0.143014429651403	-0.00734241070881217	33611205
0.352633108613063	4.62583964877336e-121
2.43284900046599e-120	100	100
CTCF-regulon	Memory 2
0.368095006073021	-0.00288566218681074	46521908.5
0.488086196640896	0.0585593020440193	0.0634764953454256
	100	100
CUX1-regulon	Memory 2
0.0766135224197752	-8.88673887891184e-05	50636381.5

0.531253331062126	6.98009152258002e-07
9.01066360187602e-07	100	100
DDIT3-regulon	Memory 2 0.08025601068728 -0.0048900676346205242245997 0.443225324666822	1.99039795728837e-19
3.14040566594388e-19	100	100
E2F1-regulon	Memory 2 0.41708960037541 -0.021659270199691736979552 0.387972236546664	9.06910470238214e-71
2.92684742667787e-70	100	100
E2F2-regulon	Memory 2
0.0523295860836938	-0.00167280459233509	48820710.5
0.512204156569614	0.0526738430545602	0.0575360439519042
100	100
E2F3-regulon	Memory 2 0.0215263662171904
0.000432089367194011	49563146 0.519993444050075
0.00150225697144196	0.00170656391955806	100	100
E2F4-regulon	Memory 2 0.372762884982345	-0.0272191215034274
31698531 0.332566223823202	1.08092748589358e-155
9.0289237056993e-155	100	100
E2F6-regulon	Memory 2 0.0583099974843684	-0.0148119737724525
33141784 0.347708162111494	3.743137008351e-129
2.12610182074337e-128	100	100
E2F7-regulon	Memory 2 0.0322795040573547	0.00179271024536439
53982404.5	0.566358245985016
5.93752866618066e-26	1.0409000871576e-25	100	100
E2F8-regulon	Memory 2
0.0679203178213638	-0.00722800849111968	36701420
0.385054205141221	2.09562757364116e-74
6.92044454551267e-74	100	100
EGR1-regulon	Memory 2 0.0488416856670487	0.00144919550242961
52439117 0.550166792313281	1.65622624428459e-15
2.47562238619381e-15	100	100
EGR2-regulon	Memory 2
0.0429081621289933	-0.00330034148999467	43632862.5
0.457775671567304	2.03177862207667e-11
2.82855455230281e-11	100	99.9953669384729
EGR3-regulon	Memory 2
0.0376190385457217	-0.00404913561520936	41840186.5
0.438967749904989	3.33342713992091e-22
5.56878416316199e-22	100	99.9953669384729
ELF1-regulon	Memory 2 0.701076818941126	-0.0062730692428149
45472550 0.477076815992254	0.00027328930652933
0.000328873572264109	100	100
ELF2-regulon	Memory 2 0.0503189491539399	0.0185663261307441
74853503 0.785328090839564	0	0	100
99.5112120088955
ELF4-regulon	Memory 2 0.0232452574962272	0.00504357646020397
60915546 0.639097537527798	4.52462716044273e-108
2.00780330244646e-107	99.411231884058	98.2811341734618
ELK1-regulon	Memory 2
0.0647745324955556	-0.00134593199672182	45027983.5

0.472412631328829 1.18737838539562e-05
1.49210381173609e-05 100 100
ELK3-regulon Memory 2 0.0752402969862147 0.0108634576601761
64743138 0.679254850110388 3.73933314897977e-178
4.8271391559557e-177 100 100
ELK4-regulon Memory 2 0.0490705031683292 0.0042818237232376
51854819.5 0.544036615076855
2.71825907669397e-12 3.93870192745453e-12 98.4148550724638
96.6433469236471
ESRRA-regulon Memory 2 0.0814755004516001 -0.0107850252696884
38290399 0.401725032750373 6.99226135953887e-55
1.80527475100822e-54 100 100
ETS1-regulon Memory 2 0.249014085809825 0.00638581425042123
56824564.5 0.596176864983522
1.22171014493656e-52 3.0979078675177e-52 100 100
ETV2-regulon Memory 2 0.0326539805526621 0.00425920821857136
59594422 0.625236919826549 5.69219352425444e-88
2.07254225754905e-87 100 100
ETV3-regulon Memory 2 0.0122154726791133
0.000826641575951458 50011231 0.524694543176776
8.5600824089299e-05 0.000105698408875482 85.9148550724638
84.2684395848777
ETV5-regulon Memory 2 0.0337348402995698
0.000285069629505058 48732310.5 0.511276704941462
0.0733994420033792 0.078960005791514 100 100
ETV6-regulon Memory 2 0.480545876921689 0.0220436265673373
70797445 0.742773819391847 0 0 100 100
ETV7-regulon Memory 2 0.0665561049771942 0.0026068763155904
56022029.5 0.587757041540097
4.00927067114382e-44 9.03676881432417e-44 100 100
FLI1-regulon Memory 2 0.16590506588172 0.0230974754078194
72356200 0.759127550869673 0 0 100 100
FOS-regulon Memory 2 0.0907628763971774 0.00471477302328956
58157541.5 0.610161838840298
1.71412635226219e-68 5.40902093380512e-68 100 100
FOSB-regulon Memory 2 0.0512601996187911 0.0102872985212577
60809254 0.637982371368754 2.24269174275493e-106
9.6503705294303e-106 100 99.2888250555967
FOSL1-regulon Memory 2
0.0519249137558606 -0.00932242275998324 39972071.5
0.419368357390002 1.60945669473671e-37
3.26489786646589e-37 100 100
FOXO1-regulon Memory 2 0.0205483068455114 0.00404831300605419
52773859 0.553678749472905 1.8022360668207e-19
2.87547776953414e-19 61.0960144927536 51.8949221645663
FOXO3-regulon Memory 2
0.0184943388961257 -0.00131348823685454 46805347
0.491059901372863 0.123476946399493 0.129879454731318
45.4710144927536 46.8263528539659
GABPA-regulon Memory 2 0.0264493261958532 0.00101057379585975

51055433.5	0.535649829474799
1.51439617473184e-08	1.9911505260363e-08	100	100
GTF2B-regulon	Memory 2
0.0727940243522081	-0.00658330017172716	38731909
0.406357150039347	5.3854490019173e-50
1.31850647977975e-49	100	100
HINFP-regulon	Memory 2	0.0170827226415701
0.000895764519146205	49074353.5	0.514865260792683
0.0154778314801381	0.0173059218124378	65.036231884058
63.1416790214974
HIVEP3-regulon	Memory 2
0.0736188279286228	-0.00427727192877184	44550020
0.467398060895886	2.26659363652053e-07
2.95281005858637e-07	99.6829710144928	99.6942179392142
HOXB2-regulon	Memory 2	0.0437838639337602	0.00702287300400839
55199401	0.579126406453116	2.40878857208773e-36
4.81757714417545e-36	91.4402173913043	83.3348776871757
IKZF1-regulon	Memory 2	0.148118072914542	0.00447152461733086
57029882	0.598330960567946	6.08298884034676e-55
1.59960076912822e-54	100	100
IRF1-regulon	Memory 2	0.144462747250747	0.00948168293251053
64471281	0.676402653082396	1.35531112891876e-172
1.6037848358872e-171	100	100
IRF2-regulon	Memory 2	0.0937139134309851	0.00913815541477009
61333185.5	0.643479216648336
7.33940212424465e-115	3.47398367214247e-114	100	100
IRF3-regulon	Memory 2	0.152152576367605	0.00704905517348989
62697573.5	0.657793740087598
1.6589938232197e-138	1.12179582331998e-137	100	100
IRF4-regulon	Memory 2
0.0548330565732627	-0.00376237076967828	43781241
0.459332389682776	1.07102725225648e-10
1.46236413288866e-10	100	99.9953669384729
IRF5-regulon	Memory 2	0.0544871312376547	0.00782304491063734
53784956.5	0.564286713529413
1.25113793477898e-24	2.16660471632458e-24	90.8967391304348
79.9457931801334
IRF7-regulon	Memory 2	0.118211097641975	0.0109071450258862
67573109	0.708945587797859	2.6283433399875e-241
4.66530942847781e-240	100	100
IRF8-regulon	Memory 2	0.0819802556336336
0.000217935784376552	50410996	0.528888691368271
4.50746930919778e-06	5.71482715987576e-06	100	100
IRF9-regulon	Memory 2	0.0389256339023564	0.00526563921981543
59643469.5	0.625751503352927
1.11324667075404e-88	4.16002703281772e-88	100	100
JUN-regulon	Memory 2	0.097125686030694	0.00917169830952898
62722504.5	0.658055304528113
5.84708603592965e-139	4.15143108551005e-138	100	100
JUNB-regulon	Memory 2	0.171950249784595	0.0127901277198784

63691709.5 0.668223751985838
3.80548426777236e-157 3.6025251068245e-156 100 100
JUND-regulon Memory 2 0.0413632611358769 0.00305419055792216
55044124 0.577497312488585 8.63933250926179e-35
1.63571362175357e-34 100 100
KLF11-regulon Memory 2 0.0311771479601429 0.00971331061243029
54731326 0.574215581556655 5.85704401564272e-40
1.24134365704667e-39 52.8985507246377 40.2427724240178
KLF12-regulon Memory 2 0.0471629839220571
0.000119157279924453 47912289.5 0.502673426530052
0.671241425135885 0.676002002619118 99.411231884058
98.5614343958488
KLF13-regulon Memory 2 0.0713186929486918 0.00528369945669099
65033188 0.682297919621083 3.49506389078142e-184
4.96299072490961e-183 100 100
KLF2-regulon Memory 2 0.13396230937478 0.026398495308037
77285154 0.81083984060254 0 0 100 100
KLF3-regulon Memory 2 0.090492261534873 0.0194074105158366
64047340.5 0.671954866804517
4.20489380403053e-164 4.26496371551668e-163 100
99.8563750926612
KLF4-regulon Memory 2 0.0171781703411169 0.00134376651361553
48897296.5 0.513007661212076 0.027138280557366
0.0301065299933279 50.9963768115942 49.7521312083024
KLF6-regulon Memory 2 0.0966588079587203 0.0286742209962231
77399747.5 0.812042102233203 0 0 100
100
KLF7-regulon Memory 2 0.0598166638579935 0.0145246989960436
63317925.5 0.664302184345825
5.32045236446343e-150 4.19724575418782e-149 99.8188405797101
97.440233506301
KLF8-regulon Memory 2 0.0344586673391022 0.00983028620358606
59853793 0.627958119557831 9.09065091443759e-93
3.68820694242897e-92 90.5797101449275 76.9736842105263
MAFB-regulon Memory 2 0.0577060853443626 0.00202154869695846
52090995.5 0.54651446367109 1.52632043558136e-13
2.25768231096409e-13 100 99.9976834692365
MAFF-regulon Memory 2 0.0153716782330775 0.00313740377939722
50912356 0.534148726982413 1.02691523873458e-11
1.4582196390031e-11 34.8731884057971 28.2222942920682
MAFG-regulon Memory 2 0.0270545347934932 0.00173143384511752
49270115 0.516919099275765 0.0053225903023919
0.00599847478523531 61.231884057971 58.4669199406968
MAX-regulon Memory 2 0.207025198807899 0.00983348715509463
67862543.5 0.71198219976922 2.61396293786077e-248
5.30261053108899e-247 100 100
MAZ-regulon Memory 2
0.131077839872096 -0.00549327654559409 39207567
0.411347532240065 5.42133180147648e-45
1.24165986420913e-44 100 100

MEF2A-regulon	Memory 2	0.0447838950079873	0.00423568871752717
55581032	0.583130301162428	8.99341426512904e-40
1.87803650830636e-39	100	99.9976834692365
MLX-regulon	Memory 2	0.0550962930907693	0.00228187136396072
51595668	0.541317718237342	5.38858023916457e-11
7.42891644622688e-11	100	99.9884173461824
MXD4-regulon	Memory 2	0.0753958877186663	-0.0176311763542812
47541586.5	0.498784183307079	0.846825330116003
0.846825330116003	89.9909420289855	88.5331727205337
MXI1-regulon	Memory 2	0.072396216802355	0.00753323298286582
57348182.5	0.601670421167115
1.30024531990497e-58	3.55066991204818e-58	100	100
MYBL1-regulon	Memory 2
0.0218764898545213	-0.00842362329120189	45042374
0.472563609752527	1.22385978907508e-05
1.52445693025141e-05	83.4692028985507	79.8855633802817
MYC-regulon	Memory 2	0.284426677841506	-0.0320128281813543
32865257.5	0.344806974864298
4.81244563309241e-134	2.84736366624634e-133	100	100
NFATC1-regulon	Memory 2	0.0395998017239955	-0.0114764872693937
39900111.5	0.418613386585004
3.37380713045659e-38	6.94319728296864e-38	93.4329710144928
94.8989992587102
NFE2L1-regulon	Memory 2	0.161514774632954	-0.0222342903571772
41936438	0.439977575814344	1.58155475236042e-21
2.6114043585486e-21	100	99.9328206078577
NFE2L2-regulon	Memory 2
0.102560196031835	-0.00874590924592733	38975273
0.408910411781808	2.11242702409558e-47
4.99941062369286e-47	100	100
NFKB1-regulon	Memory 2	0.0499270868640804	-0.0070865414949056
43329501.5	0.45459294924414	5.63542399707548e-13
8.24979595448162e-13	99.9547101449275	99.9305040770941
NFKB2-regulon	Memory 2	0.073637549090267
0.000274303795725184	49763178	0.522092086630193
0.000452445615216561	0.000535393978006263	100	100
NFYA-regulon	Memory 2	0.0270650354185051	0.0064294887984991
62831258.5	0.659196300844493
6.05879210788809e-141	4.52814989115847e-140	100
98.9668272794663
NFYB-regulon	Memory 2	0.0968609062496662	-0.0123657068025561
31646102	0.332016163173741	1.05323376585774e-156
9.34744967198741e-156	100	100
NFYC-regulon	Memory 2
0.078828271558169	-0.00494719828312014	38882853.5
0.407940789431718	2.22957950721941e-48
5.36610661059588e-48	100	100
NR1H3-regulon	Memory 2	0.0112858333946394	0.00289217880225892
50624785	0.531131665985137	1.00183292564915e-11
1.43697247921394e-11	27.7173913043478	21.8170867309118

NR2C2-regulon	Memory 2 0.0290463369513252	0.0076315188265665
	57442629.5	0.602661315102908
1.54087135913859e-60	4.46538230607509e-60	85.6884057971015
75.4818383988139
NR3C1-regulon	Memory 2 0.231572015375916	0.00640615402107275
	57044498 0.598484304832619	4.15197415910994e-55
1.11241571810115e-54	100	100
NRF1-regulon	Memory 2
0.0253599542435728	-0.00143924552540338	45349603.5
0.475786918575958	0.000120958354947781	0.00014680415728705
	99.9094202898551 99.9698851000741
PAX5-regulon	Memory 2 0.108256598326853	0.00839754450388108
	59661170 0.625937208755009	6.16778491158249e-89
2.36709583093166e-88	100	100
POU2F1-regulon	Memory 2
0.00396730250071042	-0.000959360957054524	47340369.5
0.496673108258869	0.433902953526466	0.440101567148272
	17.8894927536232 18.3700889547813
POU6F1-regulon	Memory 2 0.0365093360813738	0.00578889430303725
	52505868.5	0.550867117962111
1.59400450143014e-16	2.43385633551699e-16	71.5126811594203
65.0389177168273
REL-regulon	Memory 2
0.254975000304526	-0.00918386958691914	36040201.5
0.378117008598358	2.01155337320002e-83
7.14101447486006e-83	100	100
RELA-regulon	Memory 2 0.0965630000336543	0.00472863410448215
	58977561.5	0.618765106760174
2.63948679361396e-79	9.14163718763861e-79	100	100
RELB-regulon	Memory 2
0.0967295325847011	-0.00888656734812816	31056095
0.325826084522486	2.59528686660333e-168
2.83485180813595e-167	100	100
REST-regulon	Memory 2 0.225671996097605	0.00741430471048563
	62660174 0.657401361952224	7.90413741860617e-138
5.10176142473671e-137	100	100
RFX5-regulon	Memory 2
0.0622171305241673	-0.00329150907385947	45650083
0.478939409543167	0.000826774212914088
0.000946789824466133	100	100
RXRA-regulon	Memory 2
0.014367427727935	-0.00017103354653444	48268822.5
0.506414004712629	0.297235326908995	0.31034865015498
66.8025362318841 64.1331541882876
SETDB1-regulon	Memory 2 0.0604809234106772
0.000802443459693734	48641664.5	0.510325689327373
0.1011189522699 0.107961588137789	94.429347826087
95.0843217197924
SOX5-regulon	Memory 2 0.00905364928606905	0.00131429969907231
	49191598.5	0.516095340726424

0.000473092865951921	0.000550649073485023	25.3623188405797
22.3475722757598
SP1-regulon	Memory 2	0.014664161950072	0.00175572179169999
	51328138	0.538510918078072	2.57144275591812e-10
3.47757020324164e-10	65.625	59.5255744996294
SP2-regulon	Memory 2
0.0280680360326866	-0.00018092483749883	47036421
0.493484222159329	0.300915087216365	0.311897389669517
	100	100
SP3-regulon	Memory 2	0.0322363694793131	0.00416935009906336
	61154626	0.64160585353751	6.23073776177067e-112
2.85407987797237e-111	100	100
SP4-regulon	Memory 2	0.00957597167458425
0.000986027544000867	49378415	0.518055332435594
0.000347958410740497	0.000415210876681937	32.6086956521739
28.8315418828762
SPI1-regulon	Memory 2	0.434149470913799	0.0164047497325999
	62572816.5	0.656484847748534
2.98531619336879e-136	1.84310825851465e-135	100	100
SPIB-regulon	Memory 2
0.559280476491772	-0.00543522194014223	41759735
0.438123690236864	8.89649583693979e-23
1.50393143910173e-22	100	100
SREBF1-regulon	Memory 2	0.0409935562856367
6.69331636064599e-05	48233563.5	0.506044083706328
0.337256911591642	0.347032474246472	96.9655797101449
96.8912157153447
SREBF2-regulon	Memory 2	0.102837598332076	0.00405214189389147
	56251751	0.590167172526482	1.75504251122437e-46
4.08550879662065e-46	100	100
SRF-regulon	Memory 2	0.0237963483026543	0.00318200896906649
	55205428.5	0.579189644175839
2.98905848071873e-36	5.89508755919527e-36	99.320652173913
97.8641586360267
STAT1-regulon	Memory 2	0.0893113114159406	0.010030905899469
	70087100.5	0.735321215737167
1.70338178723144e-305	4.03133689644773e-304	100	100
STAT2-regulon	Memory 2	0.0727106171545652	0.00680751685651472
	55978999.5	0.587305590821099
1.09107841681604e-43	2.42083023731058e-43	100
99.96061897702
STAT5A-regulon	Memory 2
0.0643858850562288	-0.00759133846869496	37951643
0.398170962572249	8.64336823741101e-59
2.40658488178895e-58	100	100
TBP-regulon	Memory 2	0.0196289443100463	-0.0013373988831463
	47178724	0.494977198958434	0.39370328528415
0.402200478491722	49.6829710144928	49.8957561156412
TCF12-regulon	Memory 2	0.0111993642133376	0.00226591028412874
	49900158.5	0.523529222238225

9.71355120918517e-07	1.24263447901288e-06	29.2119565217391
25.0162157153447
TFDP1-regulon	Memory 2 0.50834893415244 -0.020827853242232
33305679 0.349427672118236	2.68287701871131e-126
1.46526360252695e-125	100	100
TFDP2-regulon	Memory 2
0.026160653384405	-0.00101173126440569	46728060
0.490249042164889	0.1215971243315	0.128856654142336	100
99.9953669384729
TFEC-regulon	Memory 2 0.101935640674514	0.0109257428570806
61453177.5	0.644738116826675
7.51477874304239e-117	3.81106636254292e-116	100	100
THAP1-regulon	Memory 2 0.0381277511902077	0.00101187613070668
51175497 0.536909479797837	4.63112132380055e-09
6.14597409326802e-09	100	100
THAP11-regulon	Memory 2 0.181442551064343	0.00311648977928428
55062067 0.577685562087725	5.9640778879649e-35
1.14445818931218e-34	100	100
TP53-regulon	Memory 2
0.0892288140632645	-0.00959475993908734	35621500
0.373724187468441	2.09668346905665e-89
8.27025146127902e-89	100	100
USF2-regulon	Memory 2 0.13209679517886 0.0071589449357341
61335157.5	0.643499905953887	6.80933210056584e-115
3.33422468372534e-114	100	100
XBP1-regulon	Memory 2 0.196092870896818
0.000844077125053438	58151350 0.610096880505957
2.05404245143039e-68	6.34073974137206e-68	100	100
YBX1-regulon	Memory 2
0.0920212501742367	-0.00332427112985402	39611761.5
0.415588152682543	5.91745343007109e-41
1.29273598010784e-40	100	100
YY1-regulon	Memory 2 0.626967829193125	-0.0131430446842875
37388030 0.392257797476123	1.34738227706752e-65
3.98600590299142e-65	100	100
YY2-regulon	Memory 2 0.0563147928157428	0.00558447697402103
52751842.5	0.553447762608978
2.08047087499308e-17	3.2111615679241e-17	94.2028985507246
90.3423832468495
ZBTB33-regulon	Memory 2 0.373889585789909	0.00847601567300094
57936066 0.60783822104538 1.03704367204485e-65
3.13319577511422e-65	100	100
ZNF143-regulon	Memory 2 0.0253115510454914	0.00208213192445545
54457833 0.571346220378622	9.61858704991561e-30
1.77381735206236e-29	100	99.9930504077094
ZNF274-regulon	Memory 2 0.0702456759101156	0.00515498724663789
57370151 0.601900904437399	7.18180418602977e-59
2.03963238883246e-58	100	100
ZNF76-regulon	Memory 2 0.0151196276328832
0.000591293367932833	50013085.5	0.524713999727052

8.71978499225595e-05	0.000106742195594857	99.6829710144928
99.4486656782802
ATF1-regulon	Memory 3	0.112318504998996	0.00657854627992387
92691059	0.657474534324634	4.54478725166107e-203
2.68899912389946e-202	100	100
ATF3-regulon	Memory 3	0.0582126545599008	0.00776500026996162
94234885.5	0.668425176383492
5.4458488345634e-232	3.86655267254001e-231	100	100
ATF4-regulon	Memory 3	0.121611797920629	0.0138869234687617
97940013	0.694706319397253	0	0	100	100
ATF5-regulon	Memory 3	0.124101617050706	0.0061207284927834
77791318.5	0.551787965927617
1.53070617686947e-23	2.55717973077018e-23	100
99.9976202375003
ATF6-regulon	Memory 3	0.134351057934091	-0.0022275906594765
79717556.5	0.565451122285	1.30765947524198e-36
2.61531895048397e-36	100	100
ATF6B-regulon	Memory 3
0.0569216490947047	-0.000404791714806604	75836528.5371094
0.537922285306211	2.42789787386975e-13
3.62906840094216e-13	100	99.9143285500107
BACH1-regulon	Memory 3	0.0552891618714063	0.0129649234286983
95117008	0.674682231661119	2.18705092350689e-249
1.72534017298877e-248	99.9403874813711	99.5335665500583
BATF-regulon	Memory 3
0.0164374044230455	-0.00308397078651715	70188225.5
0.497857844904813	0.660611209317124	0.672710486350415
53.4128166915052	51.5813521810523
BATF3-regulon	Memory 3	0.0978673467170506	0.0158776928986653
96663057	0.685648638316567	2.10250131797365e-281
2.13253705108756e-280	100	100
BCL11A-regulon	Memory 3
0.195838875009165	-0.00935299633962036	49760116.5
0.352957553584289	2.54369734447902e-177
1.29001793898579e-176	100	100
BHLHE40-regulon	Memory 3	0.0632230574827012	0.00817642792504884
89756324	0.636657925384054	1.94133291056299e-153
8.35361434242258e-153	100	100
BRF2-regulon	Memory 3	0.0501857492939443	0.0092579242022229
92764025	0.657992095429115	2.15893810394905e-204
1.33290961200333e-203	100	99.7072892125366
CEBPB-regulon	Memory 3	0.156381738640658	0.0143507822403635
102685331	0.728365722752136	0	0	100
100
CEBPG-regulon	Memory 3	0.0761159417064469	0.0086983440809613
87179942	0.618383179427248	1.21099507482221e-115
4.09431668154174e-115	100	100
CLOCK-regulon	Memory 3	0.0174340704546422	0.00198619833574517
74302584	0.527041737806847	2.752352898984e-08
3.61883436718267e-08	55.3502235469449	50.7531948311558

CREB1-regulon	Memory 3
0.0391484048091994	-0.00412800719833852	59684969.5
0.423356340423217	1.48974838823394e-49
3.46793887097081e-49	100	99.9928607125009
CREB3-regulon	Memory 3	0.243323884626271	0.00380522061170877
	90580513	0.642504047812869	1.14163502556925e-166
5.22942495583333e-166	100	100
CREB3L2-regulon	Memory 3
0.124183018446414	-0.00280130676486505	76441835
0.542215834102678	3.59996419364741e-16
5.67994350553258e-16	100	100
CREB3L4-regulon	Memory 3
0.0104739296212114	-0.000677508453411031	70198717.5
0.49793226656844	0.627362968602152	0.645547402474678
31.3263785394933	31.3486114085814
CREB5-regulon	Memory 3	0.0429023182929491	0.00402565337036617
	81639586.5	0.579084430533296
1.20902119291796e-52	2.96001740335087e-52	99.7615499254843
99.6120987125485
CREM-regulon	Memory 3	0.152745908403394	0.00296562181700477
	78507564.5	0.556868429031528
4.74121992022858e-28	8.31176825521553e-28	100	100
CTCF-regulon	Memory 3	0.374253634986006	0.00368591133660179
	82982243.5	0.58860814075256	1.27063544746764e-65
3.40434402906424e-65	100	100
CUX1-regulon	Memory 3
0.0696476141968901	-0.00761336662527975	52194801
0.37022721341054	1.45039477079709e-138	5.56637993116722e-138	100
	100
DDIT3-regulon	Memory 3	0.0904236891952161	0.0059559301379027
	80983545.5	0.574431012440696
7.79715672433584e-47	1.78580041105756e-46	100	100
E2F1-regulon	Memory 3	0.440169242212463	0.00267186530586727
	82387246	0.584387715304224	1.08623832409536e-59
2.70606740388668e-59	100	100
E2F2-regulon	Memory 3
0.0528832555107985	-0.00112059029336811	75638296.5
0.536516189424981	1.77908533624103e-12
2.60443420356934e-12	100	100
E2F3-regulon	Memory 3	0.0217143522114026
0.000646878614892644	74040082	0.525179763393443
1.16279075123453e-06	1.50105715159367e-06	100	100
E2F4-regulon	Memory 3	0.419010342123782	0.0219778105754248
	96501147.5	0.684500184795119
5.84706409392674e-278	5.53522067558398e-277	100	100
E2F6-regulon	Memory 3	0.0814081794164875	0.00972608504182688
	96154732	0.68204299666929	1.15968320460805e-270
9.68676559143199e-270	100	100
E2F7-regulon	Memory 3
0.0296539122061545	-0.000993577972160774	67225268.5

0.476841052186986	7.76066196081354e-06
9.83941070031717e-06	100	100
E2F8-regulon	Memory 3
0.0741174356391858	-0.000733401214706067	79868012
0.566518330501912	9.31866713332874e-38
1.91775468540968e-37	100	100
EGR1-regulon	Memory 3
0.0443016755928644	-0.00341373660020712	55961922
0.396948087591291	4.2395524975505e-88
1.28088607372802e-87	100	100
EGR2-regulon	Memory 3
0.0428027202653895	-0.00350428765243505	64143645.5
0.454982539955627	3.55272119312738e-18	5.7328001070919e-18
100	99.9952404750006
EGR3-regulon	Memory 3
0.0394302433320419	-0.00220384707297054	67033895
0.475483605156474	2.2036866265559e-06
2.81913063937781e-06	100	99.9952404750006
ELF1-regulon	Memory 3
0.703949338734892	-0.00334243342404739	71818204
0.509419578763595	0.0689428686363551	0.0747319644760491
100	100
ELF2-regulon	Memory 3 0.0344151140105099	0.0018994965123077
76573238 0.543147899473016	7.99714623576512e-17
1.27594917469511e-16	99.9403874813711 99.5026296375622
ELF4-regulon	Memory 3 0.0187382488042308
0.000314392380620473	72729210.5	0.515881513504833
0.00216570371656994	0.00248008006252365	98.956780923994
98.2865710002142
ELK1-regulon	Memory 3 0.0661450817096798
9.73048881657834e-05	70808168 0.502255209773582
0.663235690768014	0.672710486350415	100	100
ELK3-regulon	Memory 3
0.0621887058817043	-0.00293365835377036	62809515
0.445519309751128	7.02513420209015e-26
1.20189042975518e-25	100	100
ELK4-regulon	Memory 3 0.046318132141083	0.00142657668195421
73556559 0.521750046841599	2.67249699353488e-05
3.27150494036166e-05	97.7049180327869 96.6516741629185
ESRRA-regulon	Memory 3 0.100167400846117	0.00910486902881155
95113556 0.674657745997486	2.56570220525843e-249
1.91752480603525e-248	100	100
ETS1-regulon	Memory 3 0.230310747650951	-0.0136365113332594
53975849.5	0.382860514246461
2.87815971167128e-113	9.50462044319352e-113	100	100
ETV2-regulon	Memory 3 0.029680789698241	0.001164893557247
76123171.5	0.539955495958642
1.21104180013412e-14	1.86921669151136e-14	100	100
ETV3-regulon	Memory 3
0.0111604722953626	-0.000290027221208437	70245489

0.498264025321808	0.736993565567364	0.736993565567364
	85.3651266766021	84.2673901144666
ETV5-regulon	Memory 3
0.0314034666631489	-0.00222466205848885	60030155.5
0.425804807482002	1.50331549389483e-46
3.38842539893754e-46	100	100
ETV6-regulon	Memory 3	0.483512332868005	0.0258486275119316
	111538394.5	0.791162111797696	0	0	100
	100
ETV7-regulon	Memory 3
0.0564385241133953	-0.00824734567202911	49171247
0.34878059515413	2.02947767621928e-187	1.06735492601162e-186	100
	100
FLI1-regulon	Memory 3
0.135689020476303	-0.00890058441996813	53252764
0.377731537325511	3.1595125661267e-123
1.09427020582925e-122	100	100
FOS-regulon	Memory 3
0.0782235976118692	-0.00869696085987438	48563117.5
0.344467022042169	3.82712106296971e-198
2.1738047637668e-197	100	100
FOSB-regulon	Memory 3	0.046549495651811	0.00548128791223351
	82570120	0.58568487383588	1.75108502124902e-61
4.52098314577021e-61	100	99.2694129125913
FOSL1-regulon	Memory 3	0.0689489272625316	0.00880634185706542
	96176110.5	0.682194638256771
4.1382533465883e-271	3.67269984509712e-270	100	100
FOXO1-regulon	Memory 3	0.0138234643824534	-0.0031029455690815
	66014454.5	0.468252528338059
8.50501399019194e-11	1.1957544421854e-10	48.5543964232489
52.6451060184194
FOXO3-regulon	Memory 3
0.0130525323586143	-0.00722562703540262	62165878
0.44095387548579	3.6713166946312e-35	7.24065236996708e-35
38.628912071535	47.4096285190738
GABPA-regulon	Memory 3
0.0237764346550689	-0.00184813966671324	62219519.5
0.441334364398242	9.58493170199122e-30
1.70132537710344e-29	100	100
GTF2B-regulon	Memory 3	0.0841578463408511	0.00550812414610659
	82449831	0.58483164208826	2.66377448837566e-60
6.75457102409542e-60	100	100
HINFP-regulon	Memory 3	0.0146738010274089	-0.0016810372052468
	67567140.5	0.479266012441228
4.01165761534458e-05	4.86884941349513e-05	61.2518628912072
63.392113467076
HIVEP3-regulon	Memory 3	0.0608204749646278	-0.0182142104832217
	47980780.5	0.340336399822231
1.07428832704887e-208	6.93404283822453e-208	99.2548435171386
99.7287070750339

HOXB2-regulon	Memory 3	0.0445867849377067	0.00808159551919539	84041336	0.596120476416394	3.23222023253339e-77	9.36684230652532e-77	93.204172876304	82.9727993146284
IKZF1-regulon	Memory 3	0.146968818641144	0.00335256687778099	81323032	0.576839051909713	8.49635198328e-50	2.0108033027096e-49	100	100
IRF1-regulon	Memory 3	0.133673675202171	-0.0019099889209109	68744151	0.487614761918594	0.0167831987153596	0.0186188610748521	100	100
IRF2-regulon	Memory 3	0.0924418800081111	0.00801399548674393	88947743.5	0.630922516883635	5.38418830952748e-141	2.12376316653584e-140	100	100
IRF3-regulon	Memory 3	0.145004432647623	-0.000477393581987534	69358638	0.491973429933958	0.121184094303775	0.130364707508606	100	100
IRF4-regulon	Memory 3	0.0577719774452538	-0.000691500628209774	72765856	0.516141446699119	0.0018288947119728	0.00212871351721424	100	99.9952404750006
IRF5-regulon	Memory 3	0.065146331658197	0.0195468237544304	91025983.5	0.64566385106361	2.4393472735347e-175	1.19443900979975e-174	93.919523099851	79.4055353275743
IRF7-regulon	Memory 3	0.102651214751946	-0.00559733489304098	57940161	0.410980096496355	3.23512151640351e-66	8.83437029479421e-66	100	100
IRF8-regulon	Memory 3	0.0708466040499732	-0.0117986892820306	44128741	0.313013183281328	1.91709019998416e-285	2.09405237229039e-284	100	100
IRF9-regulon	Memory 3	0.0311706404735021	-0.0029647906519763	61562100.5	0.436671171901098	2.20433656562991e-34	4.22994313945198e-34	100	100
JUN-regulon	Memory 3	0.0907889644969754	0.00257939592850552	74978255.5	0.531834398605112	7.90637769802297e-10	1.07952464723006e-09	100	100
JUNB-regulon	Memory 3	0.164264562352458	0.00483992481119117	78915021	0.559758592068667	8.43013782534371e-31	1.53471739897283e-30	100	100
JUND-regulon	Memory 3	0.0351213497216986	-0.00360271469801695	58293962	0.413489671316496	1.22592713120666e-62	3.22373430798788e-62	100	100
KLF11-regulon	Memory 3	0.0277530400955549	0.00628095176349551	77999759	0.553266472292205	7.49951824178568e-31	1.38302803939424e-30	50.1639344262295	40.1156564574855
KLF12-regulon	Memory 3	0.0418650283337243	-0.00559854004703382	60864207	0.431720886416489	1.0876844902181e-39	2.30524175538762e-39	98.4798807749628	98.6125984626734

KLF13-regulon	Memory 3
0.0642305808011599	-0.00222611167416222	68229600
0.483964958121323	0.00196050222069559	0.00226334402714451
	100	100
KLF2-regulon	Memory 3	0.108885204726454
3.97787995264215e-05	70206417.5	0.497986884068433
0.697494329367944	0.702441097661333	100	100
KLF3-regulon	Memory 3
0.0637535302653351	-0.00893642638059339	62023240.5
0.439942121764325	4.29917336706765e-31
8.03266602794219e-31	99.9403874813711 99.8572142500178
KLF4-regulon	Memory 3
0.0133039319128551	-0.00280311582449381	66263328
0.470017833323066	5.87082225418432e-10
8.09375495237062e-10	45.8122205663189 50.1320768187335
KLF6-regulon	Memory 3	0.0919138109022553	0.0243330664556009
	109061171.5	0.77359071865671 0	0	100	100
KLF7-regulon	Memory 3	0.0526294881187278	0.00716015611042795
	83105543.5	0.589482730070633	6.8842723987577e-67
	1.91679741298744e-66	99.8211624441133 97.3751219628281
KLF8-regulon	Memory 3
0.0211769772425511	-0.00424350146318983	65754849
0.46641109932579 6.93078846985817e-11	9.8417196271986e-11
79.2846497764531 77.504105090312
MAFB-regulon	Memory 3
0.0543944148230213	-0.00149934907370309	67242678.5
0.476964544482425	8.67384958819344e-06
1.08042687852936e-05	100	99.9976202375003
MAFF-regulon	Memory 3
0.00973869509776975	-0.00285968435779026	66947952.5
0.474873999378141	1.14799925637617e-09
1.55253232767063e-09	24.3517138599106 28.8807976963899
MAFG-regulon	Memory 3	0.0340753205593024	0.00936002739428835
	83435074.5	0.591820153368068
1.47362666726068e-75	4.18509973502033e-75	70.521609538003
57.6497465552938
MAX-regulon	Memory 3	0.206172062253408	0.00918064894254103
	98889088 0.701438280930502	0	0	100	100
MAZ-regulon	Memory 3	0.139307240384566	0.00324322400070517
	86231061.5	0.611652597517862
4.39495675231168e-103	1.356704040931e-102	100	100
MEF2A-regulon	Memory 3	0.0370396152368518	-0.0040112855081228
	59546949 0.422377335922203	8.80979356437215e-51
2.12032319684889e-50	100	99.9976202375003
MLX-regulon	Memory 3
0.0501103937106586	-0.00303982168992756	64856679.5
0.46004021975954 1.20321122021855e-14	1.86921669151136e-14	100
	99.9881011875015
MXD4-regulon	Memory 3	0.0650572240347398	-0.0292765480160743
	61048256.5	0.433026382983372

2.63171761089849e-38	5.49564559922919e-38	86.3189269746647
88.7865591014017
MXI1-regulon	Memory 3	0.0651444333549318	-9.191414544929e-05
71217553	0.505159052011855	0.319180530021108
0.338236084052219	100	100
MYBL1-regulon	Memory 3	0.0153515441132921	-0.0156994576090197
51956960.5	0.368540167500523
2.93860074239907e-143	1.19223230120191e-142	70.9388971684054
80.7881773399015
MYC-regulon	Memory 3	0.338783594605951	0.0258101827222286
100482645.5	0.712741674014316	0	0	100
100
NFATC1-regulon	Memory 3
0.0443039755605785	-0.00670998811157271	65839808
0.467013728959805	1.89462233015268e-10
2.63761147923217e-10	95.618479880775	94.7645225006544
NFE2L1-regulon	Memory 3	0.246287759657233	0.0687001528360357
106222129.5	0.753452877563773	0	0	100
99.9309868875086
NFE2L2-regulon	Memory 3	0.121927267197322	0.0119287216124538
96987454.5	0.687949648765143
2.27945914868784e-288	2.69735999261394e-287	100	100
NFKB1-regulon	Memory 3	0.0718596676528152	0.016403725818461
97566824	0.692059221968038	5.07542695675693e-301
7.20710627859484e-300	100	99.9262273625092
NFKB2-regulon	Memory 3	0.0854426599175957	0.0130294342151479
97233178.5	0.689692613774016
1.05936645652541e-293	1.3675457893328e-292	100	100
NFYA-regulon	Memory 3	0.0271287280953456	0.00667376529254244
94065574.5	0.667224222676824
1.00539377520285e-228	6.79837695613355e-228	99.8509687034277
98.9505247376312
NFYB-regulon	Memory 3
0.107913613550512	-0.000768072743856055	80320360
0.569726917110602	2.57049721249147e-41
5.53046369960286e-41	100	100
NFYC-regulon	Memory 3	0.083721495335875
0.000201667419896204	76052221	0.539452231162114
2.58240721313287e-14	3.9010832368603e-14	100	100
NR1H3-regulon	Memory 3	0.00886155387611659
0.000353286859032919	71477246.5	0.507001105224125
0.0626692465233423	0.0684541000485739	23.4873323397914
21.9937650222508
NR2C2-regulon	Memory 3	0.0314710519311139	0.0104581345429217
90802844	0.64408108201949	1.06773713114148e-172
5.05395575406969e-172	89.2399403874814	74.919683015635
NR3C1-regulon	Memory 3
0.225458442456475	-2.06712836653689e-05	66329902
0.470490054809371	1.21238830509262e-08
1.60896391890796e-08	100	100

NRF1-regulon	Memory 3 0.02675255993512 2.52617743278297e-05
73038877.5	0.518078037838649	0.000481888935140516
0.000565522551983085	100	99.9643035625045
PAX5-regulon	Memory 3
0.0939533409390099	-0.00681848138043396	54104482
0.383772927956574	1.53569587463888e-111
4.9561094136073e-111	100	100
POU2F1-regulon	Memory 3
0.0035052700433607	-0.00148446916015399	68501780
0.485895580348354	5.46590678656152e-05
6.57761664145538e-05	15.8569299552906 18.5454891601818
POU6F1-regulon	Memory 3
0.0239647572214828	-0.00759924373701015	62948988.5
0.446508620645323	5.07013631724853e-26
8.77999215913769e-26	61.1624441132638 65.6885842792889
REL-regulon	Memory 3 0.271397747468979	0.00829940489293668
89612302 0.635636351152364	3.49419621221571e-151
1.45934077098421e-150	100	100
RELA-regulon	Memory 3 0.0966675185225917	0.00497056966698337
88346830.5	0.626660131718258
4.30467113361778e-132	1.60858763414138e-131	100	100
RELB-regulon	Memory 3 0.106424670966103	0.00134007424646036
76081024.5	0.539656539624588	1.901095001406e-14
2.90274720644787e-14	100	100
REST-regulon	Memory 3 0.220268251003001	0.00178149897264457
73752548.5	0.52314023599938 7.89287180766407e-06
9.91847607688759e-06	100	100
RFX5-regulon	Memory 3 0.0541675886649839	-0.012073579284231
40461358.5	0.286999772415261	0	0	100
100
RXRA-regulon	Memory 3
0.0128857299349103	-0.00177570013072244	67683730
0.480093002962716	8.33310289860763e-05
9.94370261850658e-05	63.2488822652757 64.344018466957
SETDB1-regulon	Memory 3 0.0617825602176684	0.00222990768953839
74216144.5	0.526428606717151
3.33735577507454e-07	4.34774789046408e-07	95.8271236959762
94.9905999381262
SOX5-regulon	Memory 3 0.00818592548802169
0.000413170899058953	71631308 0.50809389145467 0.0325313567459177
0.0358097105265141	23.9940387481371 22.3745270222032
SP1-regulon	Memory 3 0.01174432752535 -0.00134931126227555
68658086.5	0.487004290772079	0.00946310656154532
0.0105807963129089	59.9403874813711 59.8129506675234
SP2-regulon	Memory 3 0.0291475930608751
0.000979886635870904	75562394 0.535977799192094
3.73581986810883e-12	5.35844869971166e-12	100	100
SP3-regulon	Memory 3
0.0263242039903747	-0.00210104272029949	60226951
0.427200713744327	7.02095751938914e-45

1.55777494961447e-44	100	100
SP4-regulon	Memory 3
0.00802631558360923	-0.000660440202866496	70060045
0.496948637312881	0.462254808646567	0.48264840314568
28.822652757079	29.0307227338712
SPI1-regulon	Memory 3	0.419517133888741	0.0010519338332699
	70070303.5	0.497021402718554	0.565205675183198
	0.58583361953295	100	100
SPIB-regulon	Memory 3	0.566883441711856	0.00262641272493414
	73236051	0.519476625323702	0.000169455389002465
0.000200522210319583	100	100
SREBF1-regulon	Memory 3	0.0440122522209264	0.00332847189658183
	77469580	0.549505816249494	1.18836120564545e-21
1.93962403680062e-21	97.973174366617	96.8087384878989
SREBF2-regulon	Memory 3	0.0968067288683867	-0.0023496316486728
	61165619.5	0.433858860080995
2.38171545197659e-37	4.8314799168668e-37	100	100
SRF-regulon	Memory 3	0.023344628091218	0.00278107867167673
	80331460.5	0.569805654975365
2.09041656587823e-41	4.56675619007244e-41	99.3144560357675
97.8248970752719
STAT1-regulon	Memory 3	0.0812343997218916	0.001582927603796
	75835317	0.537913691653215	2.46816748311942e-13
3.65083106878081e-13	100	100
STAT2-regulon	Memory 3
0.064978137711991	-0.00135651458852046	68319304
0.484601244903061	0.00294622937843916	0.00334691657390688
	100	99.9595440375051
STAT5A-regulon	Memory 3	0.0780030506791472	0.00690582347609345
	90385365	0.641119827567587	1.73835289281217e-163
7.71394096185401e-163	100	100
TBP-regulon	Memory 3
0.0171184883655577	-0.00408479769459981	66511031.5
0.471774839285346	5.57407842206256e-09
7.46716165974419e-09	46.7064083457526	50.1392161062326
TCF12-regulon	Memory 3	0.00926865788952966
0.000242904381051377	71187066	0.504942802177791
0.210862354896403	0.225131236054806	26.1400894187779
25.1469503343566
TFDP1-regulon	Memory 3	0.536974479190212	0.00951467035228215
	86369843.5	0.612637003476829
7.10682137856909e-105	2.24259696834847e-104	100	100
TFDP2-regulon	Memory 3
0.0234843015099297	-0.00392938192518155	56530537
0.400981377170332	1.74863373821613e-81
5.17304147555604e-81	100	99.9952404750006
TFEC-regulon	Memory 3	0.119890613550329	0.0306124870152405
	116810856	0.828560639841884	0	0	100
	100
THAP1-regulon	Memory 3	0.0386016568970594	0.00155123900346138

78066884.5	0.553742605668282	3.1560834743138e-25
5.33528396848286e-25	100	100
THAP11-regulon	Memory 3	0.182912162220483	0.00478850352474133
87754720	0.622460184285829	1.31548527079311e-123
4.66997271131553e-123	100	100
TP53-regulon	Memory 3	0.105314357508374	0.00751317251647379
87985393.5	0.624096393361761	7.036514728892e-127
2.56201305513504e-126	100	100
USF2-regulon	Memory 3
0.123360359187739	-0.00207960744740295	61595172
0.436905754063569	3.8455959774688e-34
7.28099505067427e-34	100	100
XBP1-regulon	Memory 3
0.189885536106624	-0.00583581785549053	67303944
0.477399111813052	1.27747343644474e-05
1.57740198239263e-05	100	100
YBX1-regulon	Memory 3	0.0964794724883297	0.00139916136195592
78817583.5	0.559067450165344
3.94170500893069e-30	7.08509001605264e-30	100	100
YY1-regulon	Memory 3	0.658095421087987	0.0201110553506564
92061774.5	0.653010904951328
7.75981978197145e-192	4.2380554193844e-191	100	100
YY2-regulon	Memory 3
0.0463231415130338	-0.00505248488844114	65357150
0.463590148010233	2.01788031175627e-12
2.92386739050398e-12	90.521609538003	90.5309250136836
ZBTB33-regulon	Memory 3	0.371477814287913	0.00610304611735879
79519939	0.564049385427221	3.93882091715618e-35
7.66181603063256e-35	100	100
ZNF143-regulon	Memory 3	0.0236233273983958
0.000315952373914945	72650141.5	0.515320662711721
0.00309426822227553	0.00348719117113592	100
99.9928607125009
ZNF274-regulon	Memory 3
0.0651867440610479	-0.000167145049390346	71203359
0.50505837138914	0.328716095880484	0.345760634185398	100
100
ZNF76-regulon	Memory 3
0.0132431299571716	-0.00141888594812511	63370398.5
0.449497758394949	1.82106518484559e-22
3.00687507265202e-22	99.4634873323398	99.4597939125675
ATF1-regulon	Memory IgA	0.106316782578359
9.21200895805108e-05	19641820.5	0.526884979625202
0.0075984762546704	0.0110100370220734	100	100
ATF3-regulon	Memory IgA	0.051277164388243
0.000260182633532662	20046410.5	0.537737965167279
0.00017896006277143	0.00032167504953852	100	100
ATF4-regulon	Memory IgA	0.108901157921124
0.000152325163638209	19506287.5	0.523249354203234
0.0209753409984477	0.0280990417149016	100	100

ATF5-regulon	Memory IgA	0.122153426884688
0.00378989324012713	20327604	0.545280882664068
6.92633902327541e-06	1.53678147078923e-05	100
99.9977547767125
ATF6-regulon	Memory IgA	0.137191772245964
0.000792444049342866	23360574	0.626639244362458
2.93436367318274e-36	2.97628315422821e-35	100	100
ATF6B-regulon	Memory IgA	0.0594977516043622
0.0022426070372108	22601493.3867188	0.606277171841605
4.91729080412276e-26	2.79302117674173e-25	100
99.9191719616516
BACH1-regulon	Memory IgA	0.0477844922854887
0.00458625385093355	21202337	0.568745290094249
8.74842528860427e-12	2.70060084996045e-11	100
99.5554457890837
BATF-regulon	Memory IgA
0.0152686118590692	-0.00410037647462289	17999926.5
0.482841746120612	0.0705346883223296	0.0894279084086678
50.179211469534	51.745661105997
BATF3-regulon	Memory IgA
0.0750768585472359	-0.00823872691121702	16606021
0.44545071757685	6.08792574439539e-08	1.51664115035815e-07	100
100
BCL11A-regulon	Memory IgA	0.211326155966397
0.00695409868942229	21194364.5	0.56853143056427
1.01409750205518e-11	3.06386904876247e-11	100	100
BHLHE40-regulon	Memory IgA	0.0622241712459117
0.00669651801697203	22877560	0.613682562391523
1.51200648740717e-29	1.2629701247754e-28	100	100
BRF2-regulon	Memory IgA	0.0419074443239423
0.000300654854909044	18930391.5	0.507801144999497
0.438590990227366	0.486561879783485	99.5221027479092
99.7328184287927
CEBPB-regulon	Memory IgA
0.142335334096877	-0.000770907116376479	19243383.5
0.516197046160637	0.107790494264422	0.130822651158529
100	100
CEBPG-regulon	Memory IgA
0.0618244549276307	-0.00635347420125029	16538863
0.44364922766599	2.20533673919645e-08	5.592103874391e-08	100
100
CLOCK-regulon	Memory IgA
0.0148980353475552	-0.000709784430562754	18588599
0.49863268047766	0.885127115360791	0.891404612632853
51.8518518518518	51.0788297896226
CREB1-regulon	Memory IgA	0.0447769116722757
0.00183964922736404	19822461.5	0.531730611403808
0.00162973125376806	0.00262979361403482	100
99.9932643301376
CREB3-regulon	Memory IgA

0.239529386668522	-0.000275712611559037	20836362
0.558928138449964	4.886967352541e-09
1.26172611647422e-08	100	100
CREB3L2-regulon	Memory IgA
0.126056466714423	-0.000734280584593572	22012602
0.590480365924721	2.61577726359417e-19
1.09247168067757e-18	100	100
CREB3L4-regulon	Memory IgA
0.010503305500968	-0.000609277774556058	18421661
0.494154626891503	0.480386831400736	0.524730231222343
30.2270011947431 31.3680145490469
CREB5-regulon	Memory IgA
0.0381754792536638	-0.00101760407666217	19328755
0.518487106852215	0.0664191455494744	0.0857410788002306
99.7610513739546 99.62055726442
CREM-regulon	Memory IgA
0.148158371625168	-0.00187578693900725	17222391.5
0.461984641116884	0.000160299812477496
0.000295617835997461	100	100
CTCF-regulon	Memory IgA
0.367143939166948	-0.00376577554974733	17767721.5
0.476612928038609	0.0202274428479501	0.0275641543572578
100	100
CUX1-regulon	Memory IgA
0.0760374030576295	-0.000673077967982508	19258649.5
0.516606551282576	0.0991755315392714	0.121404529987729
100	100
DDIT3-regulon	Memory IgA
0.0817214718258304	-0.00324654067285823	17334824
0.46500060368877 0.000510681536985432	0.000873696123517244	100
100
E2F1-regulon	Memory IgA
0.424956911216472	-0.0129773968714213	16968154
0.45516480891205 8.5198425902858e-06	1.8330570421524e-05	100
100
E2F2-regulon	Memory IgA
0.0521925244824719	-0.00176094963647519	18879029.5
0.506423377275599	0.523619457197971	0.559052352797834
100	100
E2F3-regulon	Memory IgA
0.021037102312448	-7.96696185042384e-05	18644795
0.500140118564421	0.988900948488648	0.988900948488648
100	100
E2F4-regulon	Memory IgA
0.388306625013098	-0.0105454161392146	16974079.5
0.455323758381463	9.16864640096912e-06
1.94320565513077e-05	100	100
E2F6-regulon	Memory IgA
0.0666008830297811	-0.00590933925998749	17759082.5
0.476381189878748	0.0190213994870075	0.0262236769626705

100	100
E2F7-regulon	Memory IgA	0.0325388597366733
0.00200175664421414	21313359	0.571723416495921
1.0682088161056e-12	3.37079226415546e-12	100	100
E2F8-regulon	Memory IgA
0.0694744872133794	-0.00542213970256533	16172489.5
0.433821386398287	5.00201019469079e-11
1.42057089529219e-10	100	100
EGR1-regulon	Memory IgA
0.0459639561845478	-0.00152722291809606	16991983.5
0.455804026932701	1.14282817422901e-05
2.38649412853704e-05	100	100
EGR2-regulon	Memory IgA
0.0432758361223441	-0.00282416682722567	17382869.5
0.466289407457677	0.000816544151015251	0.00138034844576388
100	99.9955095534251
EGR3-regulon	Memory IgA
0.0369081757634262	-0.00464871676164255	15935524
0.427464869565269	5.9329190936168e-13
2.00589169355616e-12	100	99.9955095534251
ELF1-regulon	Memory IgA
0.703175550910986	-0.00394179912457548	18889759
0.506711192368344	0.505185343756885	0.547605487125783
100	100
ELF2-regulon	Memory IgA	0.0418496081197217
0.00936631598471628	24516661	0.657650874645911
3.16115538798225e-55	5.61105081366849e-54	100
99.5262578863468
ELF4-regulon	Memory IgA	0.022128435122568
0.00375051475351767	22396411.5	0.600775921807001
1.43336768109682e-23	7.26922181127673e-23	99.6415770609319
98.3115920878331
ELK1-regulon	Memory IgA	0.0677307722272363
0.00170729341992992	20032007	0.537351596306814
0.000208375049121076	0.00036986571218991	100	100
ELK3-regulon	Memory IgA	0.0767948948506576
0.0121128712582556	25739460	0.690452031045885
9.43763498862769e-80	2.68028833677026e-78	100	100
ELK4-regulon	Memory IgA	0.0481960826907064
0.00325916753613933	19949160.5	0.535129267858974
0.000486563536296804	0.000842585636026173	97.9689366786141
96.7062574373021
ESRRA-regulon	Memory IgA
0.0887019251754153	-0.00309081305314605	19001034.5
0.50969611881904	0.335684687700437	0.384413110108564	100
100
ETS1-regulon	Memory IgA
0.242534012940636	-0.000412603716969634	19547477.5
0.524354261577311	0.0156003170788473	0.0217180884823168
100	100

ETV2-regulon	Memory IgA	0.0306865443806063
0.00212369196964644	21051744	0.564705685428445
1.32191337006688e-10	3.68062154018621e-10	100	100
ETV3-regulon	Memory IgA	0.0117719766505414
0.000349365449565377	19312700	0.518056437080649
0.0724515548496549	0.0910453167137256	86.4994026284349
84.3081344439704
ETV5-regulon	Memory IgA
0.0329881913499068	-0.000484385751216503	17982679.5
0.482379101365072	0.080191079316331	0.0998871338852544
100	100
ETV6-regulon	Memory IgA	0.478299365946858
0.0190763508310668	26718597	0.716717039337519
1.0598006391293e-102	5.01638969187867e-101	100	100
ETV7-regulon	Memory IgA	0.0691992614363216
0.00521945944634526	23671360	0.634975970343524
5.90393699887737e-41	7.62144594400533e-40	100	100
FLI1-regulon	Memory IgA	0.156761236582049
0.0130708237747341	23762632.5	0.637424323300565
2.16612797822831e-42	3.0759017290842e-41	100	100
FOS-regulon	Memory IgA	0.08837161783687	0.00213344649491135
20894189	0.560479327542481	1.91339409478736e-09
5.0315178048112e-09	100	100
FOSB-regulon	Memory IgA	0.047419319077031
0.00605757442023276	21985125	0.589743304989602
5.07494194914138e-19	2.00178265771688e-18	99.8805256869773
99.3129616740385
FOSL1-regulon	Memory IgA
0.0575707482673738	-0.00328352587414474	19126118.5
0.513051453462865	0.195017506748757	0.234682084392572
100	100
FOXO1-regulon	Memory IgA
0.0163442865912668	-0.000359326594922584	18712917.5
0.501967480851156	0.836114400359682	0.854160034899819
52.9271206690562	52.331664384023
FOXO3-regulon	Memory IgA
0.0120390897027962	-0.00784961601304603	16168791
0.433722175426619	9.2247698555481e-13
3.03407216657055e-12	37.037037037037	46.9431284941287
GABPA-regulon	Memory IgA
0.024614889597078	-0.000889443981372546	17602147
0.47217144986407	0.00572558095781532	0.00846908850010183	100
100
GTF2B-regulon	Memory IgA
0.0782117011839087	-0.000861164325599462	18456349.5
0.495085133797201	0.625552709812226	0.648383100681286
100	100
HINFP-regulon	Memory IgA	0.016864190006283
0.000645551671652193	19108656	0.512583027995037
0.199975475173158	0.238626197265449	65.4719235364397

63.1918094254474
HIVEP3-regulon Memory IgA 0.0817573445775812
0.00414585098098103 20496128.5 0.549801493559012
7.6225450440862e-07 1.77442851845941e-06 99.8805256869773
99.6901591863311
HOXB2-regulon Memory IgA 0.0380267661687676
0.000941406714401474 19690782 0.528198354774411
0.00501818040521813 0.00750085913201025 89.3667861409797
83.6233413412964
IKZF1-regulon Memory IgA 0.145595703780099
0.00176411129204629 20123058 0.539794007603662
7.77774135966173e-05 0.00014924855041513 100 100
IRF1-regulon Memory IgA 0.139647825541106
0.00428441144466499 22010927 0.590435434634321
2.72403824641265e-19 1.1051812314017e-18 100 100
IRF2-regulon Memory IgA 0.0897453557289104
0.00481372771403608 21581505.5 0.578916352771307
4.6658385233861e-15 1.74355018505481e-14 100 100
IRF3-regulon Memory IgA 0.15316987175109 0.00786848400391205
24981618 0.670123183894007 5.19325465194339e-64
1.0534888008228e-62 100 100
IRF4-regulon Memory IgA 0.0586269772720634
0.000218660595058526 19835258.5 0.532073886462465
0.00144946005987533 0.00239329451746857 100
99.9955095534251
IRF5-regulon Memory IgA 0.0515194146698812
0.00455874841651373 20129333.5 0.539962345700919
6.81356469269085e-05 0.000132537833748233 88.2915173237754
80.331844001886
IRF7-regulon Memory IgA 0.112747078012137
0.0050046988426759 22486641 0.603196296653064
1.22880194106507e-24 6.71114906273999e-24 100 100
IRF8-regulon Memory IgA
0.0810405562231054 -0.000746131446870413 18956462
0.508500477063005 0.398663038832217 0.452881212113398
100 100
IRF9-regulon Memory IgA
0.0337536380825649 -0.000165638395066461 18834779
0.505236373057181 0.60312046203949 0.638998829054352 100
100
JUN-regulon Memory IgA 0.0930687595961783
0.00475620868751349 21375879 0.573400493675512
3.1473760498154e-13 1.11731849768447e-12 100 100
JUNB-regulon Memory IgA 0.16696672640469 0.00731924552695704
22078875 0.592258116019459 5.17480023567008e-20
2.22673222262167e-19 100 100
JUND-regulon Memory IgA
0.0379960328093972 -0.000470330598755196 18331428
0.491734158159162 0.411808931560305 0.460447781744593
100 100

KLF11-regulon	Memory IgA	0.0314278763963097
0.00966975563072584	21367062.5	0.573163994140101
3.43586152564429e-16	1.31862793686889e-15	53.1660692951016
40.6273153865152
KLF12-regulon	Memory IgA	0.0475830347479184
0.000543434018142816	18905387.5	0.507130421426265
0.47895797061861 0.524730231222343	99.6415770609319
98.5832641056153
KLF13-regulon	Memory IgA	0.0705481193018115
0.00433600189374703	24488640 0.656899221100657
1.01872228705953e-54	1.60731738624949e-53	100	100
KLF2-regulon	Memory IgA	0.126827473315417
0.0183169789269556	26728435.5	0.716980953666236
6.02102743312049e-103	4.27492947751555e-101	100	100
KLF3-regulon	Memory IgA	0.0776798950279206
0.00575686823970373	20953055 0.562058387447372
7.19323119470424e-10	1.92724307480755e-09	100
99.8607961561777
KLF4-regulon	Memory IgA	0.0165499307292395
0.000662356860980425	19020757.5	0.51022518141042
0.277376566068516	0.325516300675448	51.9713261648746
49.7721098363232
KLF6-regulon	Memory IgA	0.093988193302715
0.02507076856988	28950012 0.776573967915518
5.12892523372085e-166	7.2830738318836e-164	100	100
KLF7-regulon	Memory IgA	0.0535010189328942
0.00764326774601822	22153771 0.594267175079642
7.98429853349516e-21	3.6573238443752e-20	100
97.5100473742114
KLF8-regulon	Memory IgA	0.0335894955966641
0.00864218455402076	22705625 0.609070466024393
1.27666271779707e-27	7.88200460552971e-27	88.5304659498208
77.4310155144929
MAFB-regulon	Memory IgA	0.0567118182108428
0.000946369444749705	19598480.5	0.525722399251506
0.0106500120784982	0.0152757749004721	100
99.9977547767125
MAFF-regulon	Memory IgA	0.013632740139663
0.00126921106046931	19134142.5	0.51326669446237
0.0983716114854646	0.121404529987729	30.8243727598566
28.5031096342531
MAFG-regulon	Memory IgA	0.0273083179226617
0.00193668918240611	19519677 0.523608522867599
0.0150177277302743	0.0211140330465242	62.962962962963
58.5194997642516
MAX-regulon	Memory IgA	0.205905669040675
0.00839022409110121	25462073 0.683011221583071
8.72837298572647e-74	2.06571493995527e-72	100	100
MAZ-regulon	Memory IgA
0.133068741337269	-0.0032958669264358	17215819

0.461808336098284 0.000149408472799539
0.000279157936020191 100 100
MEF2A-regulon Memory IgA 0.0426664664399351
0.0019480853152132 20053024 0.537915369996569
0.000166804282923541 0.000303669335578755 100
99.9977547767125
MLX-regulon Memory IgA 0.0538603014730844
0.000952411760375985 19502905.5 0.523158633233602
0.0214810743561519 0.0285075940053605 100
99.9887738835627
MXD4-regulon Memory IgA
0.0899127970189214 -0.00229873469214022 21317974.5
0.571847225672543 9.40135037528904e-13
3.03407216657055e-12 93.5483870967742 88.511192438088
MXI1-regulon Memory IgA 0.0666881662625049
0.0014860257326863 19555925 0.524580862816508
0.0146615191455369 0.0208193571866623 100 100
MYBL1-regulon Memory IgA
0.0210248437962526 -0.00903197785703773 17005550.5
0.456167956972616 1.24565537830473e-05
2.56352266259815e-05 79.3309438470729 80.0736433238286
MYC-regulon Memory IgA 0.30155762351411 -0.0135745296502531
17570016.5 0.471309560415592 0.0043901893146875
0.00663198811367687 100 100
NFATC1-regulon Memory IgA
0.036726571997613 -0.014050442859281 14481638.5
0.388464898455418 1.65712204275871e-28
1.17655665035869e-27 91.9952210274791 94.8808909046005
NFE2L1-regulon Memory IgA 0.190357401384261
0.00783477817931941 20524205.5 0.550554649284722
5.17867271720433e-07 1.24639241668308e-06 100
99.9348885246638
NFE2L2-regulon Memory IgA
0.107487806574224 -0.00345647981222656 17959037.5
0.481744912966481 0.0699017141301159 0.0894238144727608
100 100
NFKB1-regulon Memory IgA
0.0553931179070064 -0.00129965196004114 19038865.5
0.510710922190459 0.287561339236175 0.334702542389647
100 99.9303980780889
NFKB2-regulon Memory IgA 0.0807779701582164
0.00754046773913054 23306706.5 0.625194267475516
1.78418769317708e-35 1.68903101620764e-34 100 100
NFYA-regulon Memory IgA 0.0251329847575693
0.00426321744230872 22787929 0.611278242099074
2.22100782122783e-28 1.50182433625882e-27 100
98.9986304137947
NFYB-regulon Memory IgA
0.0998705538261479 -0.00891885904089237 14862777
0.398688805694916 8.36828261747132e-24

4.40109678400344e-23	100	100
NFYC-regulon	Memory IgA
0.0806820697238969	-0.00290627786698537	17040605
0.457108281700574	2.05601553940659e-05
4.11203107881318e-05	100	100
NR1H3-regulon	Memory IgA	0.0117576740177399
0.00328385941870543	19860498.5	0.53275094065333
7.53569802323965e-06	1.64626018353851e-05	27.9569892473118
21.9942073239184
NR2C2-regulon	Memory IgA	0.0308628871739341
0.00924729310506123	23388057 0.627376466245482
3.74042085199146e-37	4.08569046909837e-36	89.4862604540024
75.7246458160264
NR3C1-regulon	Memory IgA	0.232461312093888
0.0071149685591588	22830740 0.612426632232399
6.19768474883046e-29	4.88928463518848e-28	100	100
NRF1-regulon	Memory IgA	0.0273257532039983
0.000607798621155724	19751124 0.529817007864156
0.00307109440117413	0.00484550449963029	100
99.9663216506882
PAX5-regulon	Memory IgA	0.102514693159256
0.00228924116524128	19738043.5	0.529466128016945
0.00343691278381531	0.00530480016632362	100	100
POU2F1-regulon	Memory IgA
0.00443172214203206	-0.000456682641045352	18474381.5
0.495568835903765	0.514512933737306	0.553491186293162
17.3237753882915 18.3659264913896
POU6F1-regulon	Memory IgA	0.0350855814485713
0.00416018992468319	20414285.5	0.547606083648436
1.37722140633978e-06	3.15428128548788e-06	73.4767025089606
65.2012842677204
REL-regulon	Memory IgA
0.260678901014787	-0.00309008023753005	17615216
0.472522021227795	0.00636639339090594	0.00931987486091385
100	100
RELA-regulon	Memory IgA	0.0967823237776879
0.00480652262582351	23200348.5	0.622341251246039
5.93155341679303e-34	5.26425365740382e-33	100	100
RELB-regulon	Memory IgA
0.100080639141134	-0.00519893864207364	15097441.5
0.404983599006018	3.94060188467237e-21
1.86521822541159e-20	100	100
REST-regulon	Memory IgA	0.224566814534639
0.00606012679092696	23458067 0.629254460060952
1.0619832885566e-37	1.25668022479198e-36	100	100
RFX5-regulon	Memory IgA
0.0649145646594927	-0.00044206408711106	20139410
0.540232644296571	6.477632223713e-05
0.000127753302189895	100	100
RXRA-regulon	Memory IgA	0.0145360900438558

6.06311570586562e-06	18772297.5	0.503560328626653
0.717458102947442	0.738253989989397	66.1887694145759
64.2268573609645
SETDB1-regulon	Memory IgA
0.0586926859678198	-0.00104410029275558	18328188.5
0.491647259702295	0.40688150678263 0.458548999707409
94.9820788530466 95.0537730977346
SOX5-regulon	Memory IgA	0.00855247785998533
0.000763253211333401	19195053 0.5149005973662	0.0429892236352841
0.0565228681130587	25.5675029868578 22.4365163115472
SP1-regulon	Memory IgA
0.012938817116608	-5.60912651644055e-05	18821586.5
0.50488248884906 0.616152732523105	0.643335941310889
61.6487455197133 59.7880509216642
SP2-regulon	Memory IgA
0.0279917758810621	-0.000253048879078215	18274958
0.490219370118031	0.331489596097165	0.382695306063394
100	100
SP3-regulon	Memory IgA	0.0302602179940825
0.00202772076792346	21121829 0.566585691092738
3.8097292641483e-11	1.10404399083481e-10	100	100
SP4-regulon	Memory IgA	0.00986225093156292
0.0012473347983586	19194150.5	0.514876388118686
0.0653281112794405	0.0851063468044087	31.5412186379928
28.9678708547565
SPI1-regulon	Memory IgA	0.429622508421086
0.0112877430204441	22663954.5	0.607952669405517
8.32325844418475e-27	4.92459457947598e-26	100	100
SPIB-regulon	Memory IgA	0.564557985599573
0.000108771920418471	18569662 0.498124702061955
0.852290214623843	0.864465789118469	100	100
SREBF1-regulon	Memory IgA	0.0430221475353781
0.0021315864153849	19995758 0.536379229533254
0.000303670185976488	0.000532360079119275	97.0131421744325
96.892610970161
SREBF2-regulon	Memory IgA	0.103979697070484
0.00509097046699537	22802774.5	0.611676467455274
1.42883438492575e-28	1.06786569820766e-27	100	100
SRF-regulon	Memory IgA	0.0227237617781819
0.00199131720609354	20512051 0.550228609064323
6.12537630613176e-07	1.44967239245118e-06	98.9247311827957
97.9164327892409
STAT1-regulon	Memory IgA	0.0835864183757783
0.00388965624010132	22254215 0.596961550323193
6.13104776614601e-22	3.00209925100943e-21	100	100
STAT2-regulon	Memory IgA
0.0637147590625279	-0.0025669451294878	17551512.5
0.470813197073763	0.00375601204460711	0.00573498613262591
100	99.9618312041133
STAT5A-regulon	Memory IgA

0.0674679552955786	-0.00421767169976621	17040196
0.457097310418322	2.04602069447695e-05
4.11203107881318e-05	100	100
TBP-regulon	Memory IgA
0.0206404104906995	-0.000265756877665457	18819880
0.504836712582153	0.607498886777025	0.638998829054352
	52.3297491039427 49.8394665349469
TCF12-regulon	Memory IgA	0.0115046432675501
0.00250717702483704	19725719.5	0.529135541018204
0.000149118183306647	0.000279157936020191	30.5854241338112
25.119558140057
TFDP1-regulon	Memory IgA
0.517266814138158	-0.0111012606149384	16154724.5
0.433344846473536	3.63619438890917e-11
1.07570750671896e-10	100	100
TFDP2-regulon	Memory IgA
0.0254022330856314	-0.00175326106780611	17438790.5
0.467789468765417	0.00138296125393617	0.00231035880069336
	100	99.9955095534251
TFEC-regulon	Memory IgA	0.107973559036128
0.016740812573947	26020049 0.697978733041154
5.0052064566923e-86	1.77684829212577e-84	100	100
THAP1-regulon	Memory IgA	0.0380313773075011
0.000882543557526046	19822555 0.531733119508675
0.00162834256189765	0.00262979361403482	100	100
THAP11-regulon	Memory IgA	0.181360908451057
0.00293738107213157	21370253.5	0.573249591601395
3.51710608577045e-13	1.21811966873025e-12	100	100
TP53-regulon	Memory IgA
0.0938341615891465	-0.00460752032412269	16688777
0.447670618393776	2.03857482725711e-07	4.990993542595e-07
	100	100
USF2-regulon	Memory IgA	0.129815485552065
0.00461439703092376	22129165.5	0.593607141129827
1.48239783571958e-20	6.57814039600563e-20	100	100
XBP1-regulon	Memory IgA	0.196723978294932
0.00146106222930681	22784177 0.611177596008578
2.48253194975136e-28	1.60236153120315e-27	100	100
YBX1-regulon	Memory IgA
0.093584192170145	-0.00162962976553586	17538256.5
0.470457609500304	0.00335418690212426	0.00523400593518291
	100	100
YY1-regulon	Memory IgA
0.637119468799092	-0.00239606084939026	17768795.5
0.476641737713767	0.0203819451233244	0.0275641543572578
	100	100
YY2-regulon	Memory IgA	0.0535203722075922
0.00256564073057542	19805822.5	0.531284276036067
0.00188654059402039	0.00300998611630219	93.7873357228196
90.4690271447495

ZBTB33-regulon   Memory IgA   0.3699737956224   0.00422572913471841
20428480.5   0.54798685956917 1.89213303239896e-06
4.26480778731194e-06   100   100
ZNF143-regulon   Memory IgA   0.0255060550815819
0.00221619897295699   21527731 0.577473870576907
1.44419899001234e-14   5.25836555337828e-14   100
99.9932643301376
ZNF274-regulon   Memory IgA   0.0682573900015716
0.0029706555620192   20987349.5   0.562978325440582
4.02333676805797e-10   1.09868042512352e-09   100   100
ZNF76-regulon   Memory IgA   0.0148122777941818
0.000259966452457627   19049550 0.51099753017391 0.274857690484904
0.325248267073803   99.2831541218638 99.463391634298
ATF1-regulon   Memory LGALS1
0.102954513387177   -0.00338231626530371   29188850
0.448707802818064   1.7265889330351e-11
2.95392323483113e-11   100   100
ATF3-regulon   Memory LGALS1
0.0483138178616031   -0.0027993925747915   30907499
0.47512786447193 0.00110545913106615   0.001414190960463   100
100
ATF4-regulon   Memory LGALS1
0.10391494719045 -0.00499999744046492   29093177.5
0.447237070080559   4.5051901908782e-12
7.89798774203339e-12   100   100
ATF5-regulon   Memory LGALS1   0.119396447361146
0.000995519938073536   33755646 0.518911219502117
0.0131241223445973   0.0156607174196035   100
99.9977217842985
ATF6-regulon   Memory LGALS1   0.137803457198732
0.00143642597918742   42065220 0.646650773883126
1.89907182756937e-82   1.03718538274942e-81   100   100
ATF6B-regulon   Memory LGALS1   0.0596676770004826
0.00245122362064725   39657228.65625   0.609633744947111
6.85272326908272e-47   2.3168731052613e-46   99.9325236167341
99.9202624504488
BACH1-regulon   Memory LGALS1   0.0482587993902809
0.0051439677280216   37047139 0.569509944426909
7.73165871710107e-20   1.66347808761871e-19   99.9325236167341
99.5511915068119
BATF-regulon   Memory LGALS1
0.012416580517517   -0.00710895434358909   29123201.5
0.447698616289876   3.29205576336804e-13
5.99322972305465e-13   45.27665317139   51.9342051305418
BATF3-regulon   Memory LGALS1
0.0684312134487346   -0.0152298138672322   24856940
0.382115188922497   6.29159230500963e-54
2.35106870345097e-53   100   100
BCL11A-regulon   Memory LGALS1   0.22015782252895 0.0161861371365232
43885732 0.674636732203646   4.11908309297987e-116

3.44064587766554e-115	100	100
BHLHE40-regulon	Memory	LGALS1	0.0652187686290279
0.00989062438634428	43157061.5	0.663435189866988
6.16140717394403e-102	4.37459909350026e-101	100	100
BRF2-regulon	Memory	LGALS1
0.0405402400738156	-0.00110829255640754	31354733
0.482003002940405	0.0182515749031449	0.0212436363626769
	99.6626180836707	99.7311705472274
CEBPB-regulon	Memory	LGALS1
0.137101524405049	-0.0061927548328406	27846031.5
0.428065223932001	3.9128924592733e-21
8.81953538439379e-21	100	100
CEBPG-regulon	Memory	LGALS1
0.0584983991530165	-0.00988518918937403	25645408.5
0.394235980533892	9.37019320596144e-44
2.71544374540107e-43	100	100
CLOCK-regulon	Memory	LGALS1
0.0150413313637476	-0.000572080209643432	32194190.5
0.494907626808222	0.477222102985555	0.491054627709774
	51.0121457489879	51.0958217524035
CREB1-regulon	Memory	LGALS1	0.0463706495811815
0.00351422946853953	36694134.5	0.564083356069373
4.27374587104829e-17	8.66959876698368e-17	100
99.9931653528956
CREB3-regulon	Memory	LGALS1	0.240748040468755
0.000980035331324736	38444174.5	0.590985978243378
7.90162733326931e-33	2.04005651149862e-32	100	100
CREB3L2-regulon	Memory	LGALS1
0.126463170727607	-0.00032463483972664	39278708.5
0.603814915235311	3.20389719294506e-42
9.09906802796397e-42	100	100
CREB3L4-regulon	Memory	LGALS1
0.00963719728021788	-0.00151358156982066	31534168.5
0.484761388726503	0.0150919444282312	0.0177112075108168
	28.7449392712551	31.4348202487812
CREB5-regulon	Memory	LGALS1
0.0330994220141944	-0.00627999820632929	28445767
0.43728470323581	1.94053972552169e-16	3.88107945104339e-16
99.7300944669366	99.6195379778557
CREM-regulon	Memory	LGALS1
0.147387968050106	-0.0026997655055003	28994410
0.445718759221624	1.08324102229992e-12
1.92275281458237e-12	100	100
CTCF-regulon	Memory	LGALS1
0.368367927116255	-0.00255579805947209	32108842
0.493595600541041	0.400910533663938	0.430234932797147
	100	100
CUX1-regulon	Memory	LGALS1	0.0777689290291464
0.00110701924790589	36084174	0.554706692180223
7.21509951310146e-13	1.29689130488659e-12	100	100

DDIT3-regulon	Memory LGALS1
0.0808571029003349	-0.00418779968541756	29515806.5
0.453733966326804	1.29332279514997e-09
2.08695269217382e-09	100	100
E2F1-regulon	Memory LGALS1
0.425783256061492	-0.0123138482610062	30131505.5
0.463198845740939	1.387330269794e-06
2.05209269073696e-06	100	100
E2F2-regulon	Memory LGALS1
0.052906057190023	-0.00104920207137947	34448249
0.529558311468919	0.000105817822404814
0.000141755950768713	100	100
E2F3-regulon	Memory LGALS1
0.0201515804726428	-0.000996260175458154	30228957
0.464696926290406	3.65085125294863e-06
5.29000895835413e-06	100	100
E2F4-regulon	Memory LGALS1
0.379286932954361	-0.020024600999721	25820243
0.396923637099731	1.20098343282279e-41
3.34391465609483e-41	100	100
E2F6-regulon	Memory LGALS1
0.0603757003710466	-0.0124315384704817	25597100
0.39349335446632 2.39816847101522e-44	7.09458172675337e-44	100
100
E2F7-regulon	Memory LGALS1	0.0326401172627778
0.00213584773958107	37503350 0.576523082506396
1.05110636873029e-23	2.44683777638855e-23	100	100
E2F8-regulon	Memory LGALS1
0.0688088971604364	-0.00618987776130898	26693567.5
0.410348883984832	6.37941161382271e-32
1.61763651636219e-31	100	100
EGR1-regulon	Memory LGALS1	0.0479948867806114
0.000549836542076423	33910823 0.521296689663425
0.00521797312627233	0.00638751882698854	100	100
EGR2-regulon	Memory LGALS1	0.0469750620932189
0.000958456994956267	34757635.5	0.534314378824658
6.77452231251483e-06	9.61982168377106e-06	100
99.9954435685971
EGR3-regulon	Memory LGALS1
0.0368784638968594	-0.00474774232250722	27373970
0.420808422843229	2.84989119839196e-25	6.859060172401e-25
100	99.9954435685971
ELF1-regulon	Memory LGALS1
0.706980368054765	-6.64420762142015e-05	35315852.5
0.542895611849108	1.84263710956221e-08
2.90727188397594e-08	100	100
ELF2-regulon	Memory LGALS1	0.0480662870403589
0.0159305228583886	48551217.5	0.746357260685739
4.73420407567224e-229	1.1204282979091e-227	100
99.5192964869914

ELF4-regulon Memory LGALS1 0.0247976291804093
0.00656494113490518 43476077.5 0.668339287439308
4.99424896351887e-108 3.73254396220884e-107 99.7975708502024
98.2867817925001
ELK1-regulon Memory LGALS1 0.0685965686891213
0.00262740971168615 36229402 0.556939220587052
8.13527299366549e-14 1.52001153302697e-13 100 100
ELK3-regulon Memory LGALS1 0.0833652420146409
0.0190830465640942 51697940 0.794730490157032 0 0
100 100
ELK4-regulon Memory LGALS1 0.0480814623972519
0.00318856910867811 34888491.5 0.536325972575202
1.89251703467871e-06 2.77048885489048e-06 97.9082321187584
96.6897525857748
ESRRA-regulon Memory LGALS1
0.0853328927165883 -0.0066190126082336 31107770.5
0.478206553242885 0.00425764024457069 0.00530337644499156
100 100
ETS1-regulon Memory LGALS1 0.255244673740465
0.0127211461134305 41161598 0.632759776389286
6.61020195510423e-68 3.23671957801655e-67 100 100
ETV2-regulon Memory LGALS1 0.0308595321291157
0.00233372690373274 37320405 0.573710746666288
4.12935452026995e-22 9.45755390126343e-22 100 100
ETV3-regulon Memory LGALS1
0.0107019591752015 -0.000751645445911308 31528397
0.484672665906524 0.0439897737035819 0.0495757767135606
84.7503373819163 84.3349888367431
ETV5-regulon Memory LGALS1 0.0339963866978697
0.000550731652064818 34005597 0.522753610141768
0.00284182565191814 0.00357114373957854 100 100
ETV6-regulon Memory LGALS1 0.479934027791853
0.0210465212909147 47904075.5 0.736409021377534
4.30247660237645e-211 7.6368959692182e-210 100 100
ETV7-regulon Memory LGALS1 0.073092837418391
0.00932119214569536 45829661.5 0.704519935371233
1.66427792089774e-158 1.96939553972899e-157 100 100
FLI1-regulon Memory LGALS1 0.171283805552733
0.028275789623106 49984232.5 0.768386392085411
1.90353512207259e-271 6.75754968335769e-270 100 100
FOS-regulon Memory LGALS1 0.0899930378206607
0.00384096064658816 38539410 0.592449993165353
7.72123814123802e-34 2.10849195395346e-33 100 100
FOSB-regulon Memory LGALS1 0.0489775296215195
0.00775740804596195 39487247.5 0.607020696774901
9.28466245776947e-45 2.86613493261579e-44 99.9325236167341
99.3028659953524
FOSL1-regulon Memory LGALS1
0.0524337916790822 -0.00864217207497278 27852494
0.428164569201709 4.43036308481529e-21

9.82986809443392e-21	100	100
FOXO1-regulon	Memory LGALS1	0.0174414156564089
0.000769564889247537	33588688.5	0.516344652714148
0.0231995871512221	0.026783263215232	55.7354925775978
52.2280949560304
FOXO3-regulon	Memory LGALS1
0.0140035155506281	-0.00593421106150809	29145257
0.448037666130656	1.4011356756659e-13
2.58391254473452e-13	38.19163292847‌5	47.0497106666059
GABPA-regulon	Memory LGALS1
0.0250854194546905	-0.00041609747727081	31841291.5
0.489482660257409	0.16775698275743 0.186105402746524	100
100
GTF2B-regulon	Memory LGALS1
0.0738828922411545	-0.0053487818946618	27864518.5
0.428349416736812	5.57970907601423e-21
1.21895182891388e-20	100	100
HINFP-regulon	Memory LGALS1
0.0159927492932066	-0.000245825577364592	32407693
0.498189710126721	0.807567658461818	0.813295088663675
63.697705802969	63.2182074998861
HIVEP3-regulon	Memory LGALS1	0.0813979846607209
0.00383527911000275	35593511 0.547163938126736
6.17126888945298e-10	1.01897695616549e-09	99.6626180836707
99.694719096004
HOXB2-regulon	Memory LGALS1	0.0373788718628672
0.000285470944279193	33737654 0.51863463612222 0.0143099229640163
0.0169334088407526	88.191632928475	83.5786212238575
IKZF1-regulon	Memory LGALS1	0.148931401942145
0.0052383558718149	39851483.5	0.612619942215103
2.24800510790082e-49	7.78577378833943e-49	100	100
IRF1-regulon	Memory LGALS1	0.142048480816739
0.00682907766713153	41270684.5	0.634436716855666
1.3835856850875e-69	7.01675597437233e-69	100	100
IRF2-regulon	Memory LGALS1	0.092034635471168
0.00725103604684318	40051634.5	0.6156967785907
5.19998274448279e-52	1.89332705055527e-51	100	100
IRF3-regulon	Memory LGALS1	0.157530702256785
0.0124921732828323	48832415.5	0.750679998194645
4.38236816365838e-237	1.24459255847898e-235	100	100
IRF4-regulon	Memory LGALS1	0.0586231579044048
0.000217925379697274	34792549.5	0.534851096928578
4.85290682964664e-06	6.96073504858407e-06	100
99.9954435685971
IRF5-regulon	Memory LGALS1	0.0484189457202728
0.00142058633675839	33634101.5	0.517042767489118
0.024853027069415	0.0284607245472333	87.0445344129555
80.256982731125
IRF7-regulon	Memory LGALS1	0.119072123292072
0.0116168391209796	45905780.5	0.705690080452067

2.66488580006055e-160	3.4401253055327e-159	100	100
IRF8-regulon	Memory LGALS1	0.085888836894163
0.00425487846215314	38022878.5	0.584509573640386
1.49642566382585e-28	3.72793761865388e-28	100	100
IRF9-regulon	Memory LGALS1	0.0358851777323952
0.00203543479001313	36231138	0.556965907378264
7.92174975286216e-14	1.4998512865419e-13	100	100
JUN-regulon	Memory LGALS1	0.0938341851755963
0.00561736751776623	38461997.5	0.591259963658001
5.12746490397368e-33	1.37377361578163e-32	100	100
JUNB-regulon	Memory LGALS1	0.168600268996879
0.00911549426318667	40307865.5	0.619635709005015
1.73338839553521e-55	6.65246357205407e-55	100	100
JUND-regulon	Memory LGALS1
0.0383195522283965	-0.000142799411797671	32512407.5
0.499799441692651	0.979014785427983	0.979014785427983
100	100
KLF11-regulon	Memory LGALS1	0.0412383077090531
0.0199535102128751	41413099.5	0.636626002207379
4.79609658237884e-90	2.72418285879118e-89	61.4035087719298
40.1649428167859
KLF12-regulon	Memory LGALS1	0.04970827626533	0.00274841588436076
35173114.5	0.540701361155482
9.37845184573991e-08	1.41674485329262e-07	99.6626180836707
98.56700232378
KLF13-regulon	Memory LGALS1	0.0739910996742154
0.00795894349395805	48433705	0.744550790897492
9.79909304375735e-226	1.98781601744792e-224	100	100
KLF2-regulon	Memory LGALS1	0.142116608477133
0.0343914822418943	55446392.5	0.852353859534136	0
0	100	100
KLF3-regulon	Memory LGALS1	0.0837510937403041
0.0121176440356604	40360027.5	0.620437573292597
3.28776526881683e-56	1.3731254946235e-55	100
99.8587506265093
KLF4-regulon	Memory LGALS1
0.0147068061407709	-0.00123326443466407	32330918
0.497009480636304	0.674738364944126	0.684377484443327
50.7422402159244	49.7812912926596
KLF6-regulon	Memory LGALS1	0.100637751263646
0.0323132387882003	54616510	0.83959642807753	0	0	100
100
KLF7-regulon	Memory LGALS1	0.0596474634038472
0.0141095495159673	43041324	0.661656006400403
8.98670630422181e-100	6.0767252152357e-99	99.7975708502024
97.4802934341824
KLF8-regulon	Memory LGALS1	0.0378676803778019
0.0131918068638183	42569079.5	0.654396392130299
3.43558738994175e-92	2.03272253904887e-91	91.9028340080972
77.1540529457329

MAFB-regulon	Memory LGALS1
0.0553985161580099	-0.000397367413533446	32110658
0.4936235171383	0.402966521563525	0.430234932797147	100
	99.9977217842985
MAFF-regulon	Memory LGALS1	0.0141150851009254
0.00178649192109364	33760158	0.518980580563149
0.00178628732393613	0.0022647571428476	31.9838056680162
28.429853738552
MAFG-regulon	Memory LGALS1	0.0258782800807734
0.000486827411073459	32910694	0.505922131017756
0.420325857316691	0.445419938350523	59.7165991902834
58.563812821798
MAX-regulon	Memory LGALS1	0.206048087409006
0.00866074102781583	44800044.5	0.68869207021676
3.19885750551539e-135	3.2445554698799e-134	100	100
MAZ-regulon	Memory LGALS1
0.133386248683197	-0.00301607061802767	30398486
0.467303023656488	1.79871866580634e-05
2.45594279369712e-05	100	100
MEF2A-regulon	Memory LGALS1	0.0437811326187523
0.0031290122655554	36536290.5	0.561656887248984
6.12128480593962e-16	1.20725339228254e-15	99.9325236167341	100
MLX-regulon	Memory LGALS1	0.0552501831990037
0.0024032154870985	35538552	0.546319076745247
1.23847481962666e-09	2.02141867111477e-09	100
99.9886089214927
MXD4-regulon	Memory LGALS1
0.0906179378719424	-0.00160356479488211	37837516
0.581660074598805	8.3415236204032e-27
2.04223509327113e-26	94.6018893387315	88.4016038638538
MXI1-regulon	Memory LGALS1	0.0666928144338967
0.00151266723317968	34326728	0.527690220711446
0.000281416506686393	0.000373468635041755	100	100
MYBL1-regulon	Memory LGALS1
0.0233498309818603	-0.00676121208457801	31653920
0.486602277711481	0.0776909094684345	0.0868670011379347
	83.2658569500675	79.951701827129
MYC-regulon	Memory LGALS1
0.287699600696218	-0.0280999138715381	24730550
0.380172249094509	1.16525061552086e-55
4.59626631677671e-55	100	100
NFATC1-regulon	Memory LGALS1
0.0347537466200124	-0.0162963411592365	24274666.5
0.373164145533526	3.76832752419276e-62
1.67219533886054e-61	91.025641025641	94.9560304369618
NFE2L1-regulon	Memory LGALS1
0.17670726830041	-0.00616109840716575	33583641	0.516267059638891
	0.0328774395257968	0.0373487713013052	100
99.9339317446576
NFE2L2-regulon	Memory LGALS1

0.101414770995262 -0.00978535145550029 25527359.5
0.392421263358845 3.29758492318544e-45 1.0405712424274e-44
100 100
NFKB1-regulon Memory LGALS1
0.0500181244147822 -0.0068752199241682 29382987.5
0.45169219621039 2.35773790638014e-10 3.93880920830565e-10 100
99.9293753132547
NFKB2-regulon Memory LGALS1 0.0767109144012846
0.00344689867878525 37026923.5 0.569199180125203
1.12512385009819e-19 2.34952333402857e-19 100 100
NFYA-regulon Memory LGALS1 0.0261100246837371
0.00533589113210867 41486264.5 0.63775073669994
5.76548993499083e-73 3.03222063247666e-72 99.8650472334683
98.9884722285506
NFYB-regulon Memory LGALS1
0.0984982901437867 -0.0104685127733432 23905367
0.367487061056857 1.1631016385524e-67
5.50534775581469e-67 100 100
NFYC-regulon Memory LGALS1
0.0806673792715307 -0.00296417059015422 29859707
0.459020602756229 7.66540796218371e-08
1.17041712970977e-07 100 100
NR1H3-regulon Memory LGALS1 0.010396006067927
0.00192447190411015 33788312 0.519413380056125
0.000454341469450256 0.000586513533290331 25.5735492577598
21.9870597348157
NR2C2-regulon Memory LGALS1 0.0310766801095698
0.00960418863292775 40503016.5 0.622635682502695
5.25418727972759e-59 2.26089270824642e-58 87.1794871794872
75.6003098373354
NR3C1-regulon Memory LGALS1 0.232007464325929
0.00675034830130378 39484295 0.606975309245491
1.00982655152018e-44 3.05096532586948e-44 100 100
NRF1-regulon Memory LGALS1
0.0265072681376985 -0.000229389793155493 33131988
0.509323989758913 0.221356915560918 0.243664201625197
99.9325236167341 99.9681049801795
PAX5-regulon Memory LGALS1 0.109864075173176
0.00992040075004096 41025122.5 0.630661796450251
7.79009984702058e-66 3.56836831702233e-65 100 100
POU2F1-regulon Memory LGALS1
0.00400609221161733 -0.000903393900579079 32356728
0.497406246812112 0.614256307705971 0.627513638088114
18.0161943319838 18.3578621223857
POU6F1-regulon Memory LGALS1 0.0377014959889615
0.00692555787215131 36945643 0.567949689495495
8.74287686254783e-20 1.85296793206238e-19 74.1565452091768
65.0567275709664
REL-regulon Memory LGALS1
0.255581191371714 -0.00840531180715892 25437687

0.391042766074841	2.49852476005039e-46
8.06342081652626e-46	100	100
RELA-regulon	Memory LGALS1	0.0963189152283828
0.00439809734583965	39917294.5	0.613631626786824
3.11099901751124e-50	1.10440465121649e-49	100	100
RELB-regulon	Memory LGALS1
0.0973337347332984	-0.0081149829723709	22343722
0.34348055525989 1.18602003105067e-93	7.32238453953022e-93	100
100
REST-regulon	Memory LGALS1	0.226966790522296
0.00863018402417204	44463330 0.683515901115477
5.17564413489789e-128	4.89960978103667e-127	100	100
RFX5-regulon	Memory LGALS1	0.0662115291219795
0.000892194082880604	36788641 0.565536164383747
8.27710548446234e-18	1.70340431709225e-17	100	100
RXRA-regulon	Memory LGALS1
0.0135583315070495	-0.00100461854138841	32013191.5
0.492125206000199	0.290392391211618	0.317197842708075
64.8448043184885 64.2434045655443
SETDB1-regulon	Memory LGALS1
0.0579354155162841	-0.00184228110784895	31116288.5
0.478337496841704	0.00449136985020383	0.00554586538025169
94.4669365721997 95.0722194377364
SOX5-regulon	Memory LGALS1	0.00995515513752415
0.00222450491924136	34129934 0.524664990072083
9.63116870183073e-06	1.35408510461382e-05	26.9905533063428
22.3424613842439
SP1-regulon	Memory LGALS1	0.0138904970959481
0.000926896201835788	34436861 0.529383248578175
6.73975550952278e-05	9.11471697478318e-05	66.5991902834008
59.5935663188591
SP2-regulon	Memory LGALS1
0.0275762901473711	-0.000686281147246223	31208726.5
0.479758506983484	0.0079341066228993	0.00962942855086924
100	100
SP3-regulon	Memory LGALS1	0.0304744821673545
0.00227901550118312	37517810 0.576745369949333
7.81884142822662e-24	1.85045913801363e-23	100	100
SP4-regulon	Memory LGALS1	0.0100469680964162
0.00145661754810398	33928521.5	0.521568761192388
0.000415895024990052	0.000546824940264698	32.8609986504723
28.8854968788445
SPI1-regulon	Memory LGALS1	0.443358223181473
0.0256530865119349	46708182.5	0.718025065845353
7.52640886390175e-180	1.18750006519339e-178	100	100
SPIB-regulon	Memory LGALS1	0.568241557430711
0.00391831111252638	34681098 0.533137800320942
1.3843239876523e-05	1.90848549754006e-05	100	100
SREBF1-regulon	Memory LGALS1	0.0412665940319033
0.000348082461866177	33044302 0.507976030096306

0.295493532580949	0.320305966614464	97.3009446693657
96.8811227046977
SREBF2-regulon	Memory LGALS1	0.106412048105643
0.00768025457239831	43889689 0.674697561485229
3.42980385404682e-116	3.04395092046655e-115	100	100
SRF-regulon	Memory LGALS1	0.0225573687935001
0.00184856766230648	35910556 0.552037736352581
8.77614840817198e-12	1.51977204141515e-11	99.1228070175439
97.8949286918485
STAT1-regulon	Memory LGALS1	0.0862647182082246
0.00671554044914924	42937333.5	0.660057404579195
7.59435764675112e-98	4.901812662903e-97	100	100
STAT2-regulon	Memory LGALS1	0.0683810846424522
0.0022192103793774	34688094.5	0.533245354546012
1.29801195935325e-05	1.80703625713884e-05	100
99.9612703330751
STAT5A-regulon	Memory LGALS1
0.0648588975085012	-0.00697679605372739	26595630.5
0.408843340049919	6.03644183785867e-33
1.58736063143691e-32	100	100
TBP-regulon	Memory LGALS1
0.0200010287927934	-0.000930631282146287	32194905
0.494918610513477	0.475945706657387	0.491054627709774
49.4601889338731 49.8997585091356
TCF12-regulon	Memory LGALS1	0.0107098853386129
0.0017224272503249	33855159.5	0.520440998302437
0.00044006422596482	0.000573294679697289	28.8798920377868
25.0968241673122
TFDP1-regulon	Memory LGALS1
0.512633428211799	-0.0160542116536025	25434529
0.390994219481149	2.28015862883212e-46
7.52982616963166e-46	100	100
TFDP2-regulon	Memory LGALS1
0.0252506382591421	-0.00193573749363908	30028105
0.461609313739326	4.77104905097461e-07	7.1314627919831e-07
100	99.9954435685971
TFEC-regulon	Memory LGALS1	0.108767593888254
0.0178076542735024	46460368.5	0.714215526399724
1.08467049356038e-173	1.54023210085574e-172	100	100
THAP1-regulon	Memory LGALS1	0.037705907045275
0.000559052920442005	33762456 0.51901590674184 0.0126272007012109
0.0151954449116266	100	100
THAP11-regulon	Memory LGALS1	0.180549702125715
0.00214194918104899	35846126.5	0.551047288994029
2.15184278429564e-11	3.63763899249976e-11	100	100
TP53-regulon	Memory LGALS1
0.0895365826191069	-0.00911790429353938	24725969.5
0.380101835012049	1.00718217491098e-55	4.0862819667817e-55
100	100
USF2-regulon	Memory LGALS1	0.13430548824662 0.00932380260692688

44845391 0.689389162715454 3.3027011960933e-136
3.60756592188652e-135 100 100
XBP1-regulon Memory LGALS1 0.199462295140579
0.0043133028618759 43632682.5 0.67074670963855
4.39972176966885e-111 3.47089161829432e-110 100 100
YBX1-regulon Memory LGALS1
0.0933154015064696 -0.0019314422308775 29838988
0.458702098362716 6.07406684360398e-08
9.47821419551391e-08 100 100
YY1-regulon Memory LGALS1
0.634630709119997 -0.00500405780675262 29859284
0.459014100156757 7.62920953966469e-08
1.17041712970977e-07 100 100
YY2-regulon Memory LGALS1
0.0494977438940639 -0.00155510342770226 32160516.5
0.494389970698026 0.461657803646841 0.485595615687789
92.2402159244265 90.4725019364834
ZBTB33-regulon Memory LGALS1 0.370601291279154
0.00493650596555556 36503235.5 0.561148746763074
1.05545210993397e-15 2.05307122754279e-15 100 100
ZNF143-regulon Memory LGALS1 0.0245692039333794
0.00128028268910775 35522473.5 0.546071908788729
1.51497168249817e-09 2.4171458305027e-09 100
99.9931653528956
ZNF274-regulon Memory LGALS1 0.0679052881997217
0.00265031796409605 36374725 0.559173209388561
8.41812711967344e-15 1.61537033918058e-14 100 100
ZNF76-regulon Memory LGALS1
0.0142695057156398 -0.000297311250054947 32166548.5
0.494482698073945 0.469283647252034 0.489987337571977
99.5951417004049 99.4555064473504
ATF1-regulon Memory LGALS3 0.115685850153189
0.00969034308489279 33767642.5 0.705213051550037
5.52819796870115e-118 3.89845921261373e-117 100 100
ATF3-regulon Memory LGALS3 0.0632927128723501
0.0125703985174344 34454988.5 0.719567780937222
8.84934179433916e-135 7.39180314585977e-134 100 100
ATF4-regulon Memory LGALS3 0.124477922685581
0.0161100731101773 33892061.5 0.7078114533384
6.11202623961066e-121 4.56793540013007e-120 100 100
ATF5-regulon Memory LGALS3 0.136830094381634
0.0188456150663379 31802714.5 0.664176936252497
3.30661105769303e-76 1.42284475815882e-75 100
99.9977424088498
ATF6-regulon Memory LGALS3 0.139745021411501
0.00341236933399028 33000433 0.689190430110794
1.42539162536442e-100 8.43356711673951e-100 100 100
ATF6B-regulon Memory LGALS3 0.0576706622730563
0.000383281845196835 27177260.7695312 0.567577644783826
2.84935358067933e-14 4.70474660995889e-14 100

99.9187267185913
BACH1-regulon Memory LGALS3 0.0569507521208443
0.0140014757095795 32453276.5 0.677763458120066
5.13665236613444e-89 2.60501655711104e-88 99.9074930619797
99.5552545434022
BATF-regulon Memory LGALS3 0.0199176169976122
0.000639498574631414 25436332.5 0.531219603576601
0.000192053670106193 0.000241341780133446 58.6493987049029
51.5475787334914
BATF3-regulon Memory LGALS3 0.106564298434571
0.0239717668908233 35547679 0.742387840167141
8.26991086690125e-164 9.78606119249982e-163 100 100
BCL11A-regulon Memory LGALS3
0.187336875052239 -0.0175823232697294 12967347.5
0.270813773895668 1.14793757636154e-146
1.16433668459528e-145 100 100
BHLHE40-regulon Memory LGALS3 0.0653902294802307
0.00997673043003442 31719576 0.662440648168829
1.21149372996889e-74 4.91520313301663e-74 100 100
BRF2-regulon Memory LGALS3 0.055094868464969
0.013811568448042 34691671 0.724510725594181
7.97228926664702e-141 7.07540672414923e-140 100
99.7223162885201
CEBPB-regulon Memory LGALS3 0.159825403868229
0.0171417535588342 35898157.5 0.749707332858634
1.00144032148954e-173 1.42204525651515e-172 100 100
CEBPG-regulon Memory LGALS3 0.0795752580877738
0.0117955312505964 30806682 0.643375510190017
1.47541519274656e-58 4.36476994520859e-58 100 100
CLOCK-regulon Memory LGALS3
0.0152742752755288 -0.000328272396006447 24075633
0.502802368152552 0.737184609301198 0.7477158180055
52.7289546716004 51.0531662715882
CREB1-regulon Memory LGALS3
0.0369985130112086 -0.00611844408414072 18447191
0.385256384351865 3.85474701913135e-38
8.82861414059117e-38 100 99.9932272265493
CREB3-regulon Memory LGALS3 0.250898835266822
0.0113696836119256 35661695.5 0.744768993186398
5.2925952248048e-167 6.83225929020256e-166 100 100
CREB3L2-regulon Memory LGALS3
0.126254121606252 -0.000535846813084356 28369073.5
0.592467800871271 2.34586516410067e-25
4.16391066627869e-25 100 100
CREB3L4-regulon Memory LGALS3
0.00766338060711226 -0.00352186600710122 22184357.5
0.463304432616282 5.14731454463748e-07 7.4583537279441e-07
24.9768732654949 31.5024269104865
CREB5-regulon Memory LGALS3 0.0485965387281629
0.00965217127767055 30329120.5 0.633401979976357

6.16998160343838e-51	1.68487959170817e-50	100
99.6139519133085
CREM-regulon	Memory	LGALS3	0.157953031582674
0.008147574574001	31364223	0.655019355032731
3.81612871543723e-68	1.29021494664783e-67	100	100
CTCF-regulon	Memory	LGALS3
0.369318645358519	-0.00155874046870941	24296235.5
0.507409493515377	0.404405648646571	0.416127551505892
100	100
CUX1-regulon	Memory	LGALS3
0.0682475361648872	-0.0086567608023715	16772388.5
0.350279332525738	1.08786410652928e-63
3.43281562504794e-63	100	100
DDIT3-regulon	Memory	LGALS3	0.0937402989629915
0.00904771701427669	29393289.5	0.61385781916486
1.39995933015165e-37	3.05837269048514e-37	100	100
E2F1-regulon	Memory	LGALS3	0.448813307173294
0.0113897176584378	30646930.5	0.640039214420933
6.0120839262722e-56	1.74227738271562e-55	100	100
E2F2-regulon	Memory	LGALS3
0.0517782920664081	-0.00219499144305852	23471322
0.490181765325593	0.269232788912123	0.283193007596455
100	100
E2F3-regulon	Memory	LGALS3
0.0201169795065286	-0.00102268647883414	22330532.5
0.46635719289738	0.000153228993126266	0.000194272473427944	100
100
E2F4-regulon	Memory	LGALS3	0.434801157292788
0.037025705097612	36165305.5	0.755286527683842
1.75378804397798e-181	3.11297377806091e-180	100	100
E2F6-regulon	Memory	LGALS3	0.0920538978141368
0.0201322934316366	36544193.5	0.763199332454731
8.90741218058904e-193	2.10808754940607e-191	100	100
E2F7-regulon	Memory	LGALS3
0.0294461980118892	-0.00115535340889829	22515595
0.470222090790459	0.000805594339818416
0.000977729882514659	100	100
E2F8-regulon	Memory	LGALS3	0.0748344210435566
3.87329781140755e-05	27884276	0.582343151975251
1.93332328780846e-20	3.30761333576869e-20	100	100
EGR1-regulon	Memory	LGALS3
0.0412418856880405	-0.00637294621060153	14734365.5
0.307716680455515	7.99298572569249e-104
4.93479988281884e-103	100	100
EGR2-regulon	Memory	LGALS3
0.043163518648033	-0.00295478234636292	22039048
0.460269747683385	7.7931636479606e-06
1.04398984717963e-05	100	99.9954848176995
EGR3-regulon	Memory	LGALS3
0.0386043244415571	-0.00293678188120398	22003256

0.459522257373954	5.24098049693335e-06
7.08780219585272e-06	100	99.9954848176995
ELF1-regulon	Memory LGALS3
0.701563633702398	-0.00561476795273452	23110918
0.482654985668682	0.0509608753474342	0.0569798763727217
	100	100
ELF2-regulon	Memory LGALS3
0.0302029156616123	-0.00251301432071104	22510914.5
0.470124341897039	0.000774179045980584
0.000947701935596921	99.5374653098982 99.53493622305
ELF4-regulon	Memory LGALS3
0.0181033070910471	-0.000352184963345065	23538437.5
0.49158342451934 0.343584478759388	0.356124058276154
98.9824236817761 98.3203521842194
ELK1-regulon	Memory LGALS3	0.0729544126750265
0.00706781918025029	31160493.5	0.650764610201618
1.48261472154063e-64	4.78480205588113e-64	100	100
ELK3-regulon	Memory LGALS3
0.064266033995984	-0.000655026914329546	23420756.5
0.489125741039676	0.221078994408154	0.236039227112465
	100	100
ELK4-regulon	Memory LGALS3
0.0443479061795435	-0.000664968833466929	23861158.5
0.498323221684904	0.850337619319782	0.856368382577369
	97.6873265494912 96.7061745117959
ESRRA-regulon	Memory LGALS3	0.11270229917121 0.0214782537049131
	37417629 0.781440407895137	4.0112186284557e-220
1.42398261310177e-218	100	100
ETS1-regulon	Memory LGALS3
0.224496175941787	-0.0188929190339744	16071981.5
0.335651833499207	2.3134608224519e-76
1.02659823996303e-75	100	100
ETV2-regulon	Memory LGALS3
0.0268781180358574	-0.00176597894081145	21780820
0.454876840675569	3.82141365388745e-07
5.59423442115482e-07	100	100
ETV3-regulon	Memory LGALS3
0.0100891408557057	-0.00137261472555079	22314394.5
0.466020162314747	0.000127595212910123
0.000163294017251578	84.6438482886217 84.3413477819167
ETV5-regulon	Memory LGALS3
0.0308289471744005	-0.00269899358124404	19510128.5
0.407455073466214	2.14146546582358e-25
3.84921640692339e-25	100	100
ETV6-regulon	Memory LGALS3	0.479313544905519
0.0202203628873032	34909021 0.729049924821797
1.70574346941966e-146	1.61477048438395e-145	100	100
ETV7-regulon	Memory LGALS3
0.0618751627206914	-0.00225462916897309	22866728
0.477555252246131	0.0115476258150079	0.0135517592209184

100 100
FLI1-regulon Memory LGALS3
0.130278928619714 -0.0139857723444195 15943016
0.332958481311541 7.99887861017785e-79
3.78613587548418e-78 100 100
FOS-regulon Memory LGALS3
0.0792398403993435 -0.00720943581822664 17631216
0.368215330338736 9.43720763554007e-50
2.52845940423904e-49 100 100
FOSB-regulon Memory LGALS3 0.0418188991894955
0.000353847032078872 24881920.5 0.519641105659965
0.0270922943488691 0.0307768463803152 99.8149861239593
99.311434699176
FOSL1-regulon Memory LGALS3 0.0762135424658417
0.015796150144286 35279958 0.736796678646936
1.97020929821516e-156 2.15207477189656e-155 100 100
FOXO1-regulon Memory LGALS3
0.0110794483059016 -0.00575463030247177 20865041
0.435751451536086 1.91802576791109e-14
3.20423128286323e-14 43.8482886216466 52.5499492041991
FOXO3-regulon Memory LGALS3
0.00883264581272571 -0.0111775515421807 18892192
0.39454991182133 6.02507151215078e-38 1.33681274175845e-37
29.5097132284921 47.181397448922
GABPA-regulon Memory LGALS3
0.0211613788350332 -0.00443213567727493 16913960.5
0.353235962445462 2.85442672934264e-61
8.81149120797076e-61 100 100
GTF2B-regulon Memory LGALS3 0.0868812422649747
0.00801520935086528 29741156.5 0.621122772547483
2.66397771673326e-42 6.52215234096764e-42 100 100
HINFP-regulon Memory LGALS3
0.0144172046190336 -0.00185759528226915 22755825
0.475239122446544 0.00426002818803756 0.00508339498068348
60.4995374653099 63.3005982616548
HIVEP3-regulon Memory LGALS3 0.0932419395615455
0.0159335599691676 31514465 0.658157051698733
7.43017124519291e-71 2.70534440209588e-70 100
99.6861948301163
HOXB2-regulon Memory LGALS3 0.0404355748703764
0.00341418709328205 26678705.5 0.557165674715365
1.14332443734843e-10 1.78408868245579e-10 91.0268270120259
83.5511908793318
IKZF1-regulon Memory LGALS3 0.146285735266372
0.00248070033993883 26526749.5 0.553992182385798
1.23475875271874e-09 1.9058232922398e-09 100 100
IRF1-regulon Memory LGALS3
0.127950297190288 -0.00767498916610815 18748703
0.391553246728294 2.98255125670512e-34
6.13800403553808e-34 100 100

IRF2-regulon	Memory LGALS3	0.0892341821655639
0.00431659571159368	27288527.5	0.56990137083399
3.66445243929826e-15	6.19466959976611e-15	100	100
IRF3-regulon	Memory LGALS3	0.149917734643051
0.00458032364004654	28681400.5	0.598990526784147
8.07835537242808e-29	1.50937692484841e-28	100	100
IRF4-regulon	Memory LGALS3	0.0625085625041471
0.00419617870491797	28761035.5	0.600653646777205
9.70992594557222e-30	1.88878011544007e-29	100
99.9954848176995
IRF5-regulon	Memory LGALS3	0.0680285598064488
0.0214959039494298	31397536 0.655715073200983
3.04740404426948e-69	1.05544237630797e-68	93.154486586494
80.1693193362682
IRF7-regulon	Memory LGALS3
0.100662006882684	-0.00734773463858426	18297856
0.38213762973187 3.79981327045803e-40	9.14531329500067e-40	100
100
IRF8-regulon	Memory LGALS3
0.070679126129378	-0.0113645377682613	15327673
0.320107483058407	4.09031326272574e-91
2.32329793322822e-90	100	100
IRF9-regulon	Memory LGALS3
0.0270434442752362	-0.0070405039548304	15876383
0.331566898785046	4.12843418668982e-80	2.0215091534826e-79
100	100
JUN-regulon	Memory LGALS3
0.0844391100827837	-0.00405784394596755	19551272
0.408314326023938	5.88795882354897e-25
1.03221006536291e-24	100	100
JUNB-regulon	Memory LGALS3
0.157978829787301	-0.00184767852772305	22250299.5
0.464681584102214	7.05773820045158e-05
9.27961874503818e-05	100	100
JUND-regulon	Memory LGALS3
0.0297969543649141	-0.00887209477446265	13575202
0.283508380184615	4.37915602556936e-131
3.4546675312825e-130	100	100
KLF11-regulon	Memory LGALS3	0.034037231303346
0.0123960567625821	28185290.5	0.588629624420161
4.13875736481594e-29	7.94193980816031e-29	54.6716003700278
40.5215035557061
KLF12-regulon	Memory LGALS3
0.0399734055829159	-0.00724891128247188	19470861
0.406634999826138	8.08470273892432e-26
1.47183049862468e-25	98.8899167437558 98.595778304549
KLF13-regulon	Memory LGALS3
0.0643224344896424	-0.00201773305545525	23387337
0.488427798695129	0.192847172656156	0.207456806948289
100	100

KLF2-regulon	Memory LGALS3
0.106306531524656	-0.00260386793701071	22276600
0.465230851225683	9.13369788165462e-05
0.000118989458641739	100	100
KLF3-regulon	Memory LGALS3
0.0545269249928294	-0.0179294280118328	17056365.5
0.356209988974142	6.92427297223922e-59
2.09201438735738e-58	100	99.8600293486849
KLF4-regulon	Memory LGALS3
0.0138751245336043	-0.00207407819622841	23157111
0.483619693420793	0.04859714860773 0.0547682150976005
48.7511563367253 49.8385822327577
KLF6-regulon	Memory LGALS3	0.0939067706780598
0.0251254617522905	36482989.5	0.761921130708576
6.27284782746987e-191	1.27249198785817e-189	100	100
KLF7-regulon	Memory LGALS3	0.0501772122350502
0.0042804481187163	26730066.5	0.558238312449571
5.61952548502257e-11	8.86636243192451e-11	99.722479185939
97.5031041878316
KLF8-regulon	Memory LGALS3
0.0202817978951189	-0.00494267599173229	21827453
0.455850737512843	5.8508886179425e-07
8.39218367422056e-07	79.0934320074006 77.600180607292
MAFB-regulon	Memory LGALS3
0.0520817254619758	-0.00379150558466821	20717894
0.432678391730492	3.57450115828369e-14
5.83424326984235e-14	100	99.9977424088498
MAFF-regulon	Memory LGALS3
0.00800286755855251	-0.004491064608155	22296671
0.465650019699101	1.23462659869743e-06
1.73581165361421e-06	22.8492136910268 28.6849531549836
MAFG-regulon	Memory LGALS3	0.0414905905958775
0.0164757422805062	30962600 0.646631746054619
1.07599180292186e-65	3.55327525616055e-65	77.0582793709528
58.1510328479512
MAX-regulon	Memory LGALS3	0.203588557247816
0.00606278194109516	30041120.5	0.627387306051566
1.32791200328151e-46	3.36720543689239e-46	100	100
MAZ-regulon	Memory LGALS3	0.139757596472387
0.00353807142058016	29463676 0.61532779085308
1.63778645990531e-38	3.81255208699269e-38	100	100
MEF2A-regulon	Memory LGALS3
0.0344038246109813	-0.00650547158316579	17803568
0.371814778534172	3.62873454513379e-47
9.36873282561816e-47	100	99.9977424088498
MLX-regulon	Memory LGALS3
0.0523623685629928	-0.000576831162274048	23877685
0.49866836581205 0.880886296599154	0.880886296599154	100
99.9887120442488
MXD4-regulon	Memory LGALS3	0.101243472236514

0.00929579804086815 28902530 0.603608658164883
1.8666848500302e-31 3.7867035529184e-31 93.9870490286771
88.4727395868608
MXI1-regulon Memory LGALS3
0.057264673781639 -0.00815925713294184 19117843.5
0.399262481936399 8.71799156489664e-30 1.7193816697435e-29
100 100
MYBL1-regulon Memory LGALS3
0.0149872849134535 -0.0152666335622642 17674407
0.369117343468894 1.78290208957615e-49
4.68837216147803e-49 70.3977798334875 80.2957444406818
MYC-regulon Memory LGALS3 0.358857795266535
0.0450492520001228 37023014 0.773199155982528
1.52995840635432e-207 4.34508187404626e-206 100 100
NFATC1-regulon Memory LGALS3
0.0459964951898975 -0.00463168844647067 22889705.5
0.478035120892335 0.0134416937371375 0.0156452500874879
95.7446808510638 94.8052827632916
NFE2L1-regulon Memory LGALS3 0.284024204490351
0.103830636484267 40090110 0.837253261315967 0 0
100 99.934529856643
NFE2L2-regulon Memory LGALS3 0.125308407377581
0.0147799848221324 33766870 0.705196918440291
5.76532700456961e-118 3.89845921261373e-117 100 100
NFKB1-regulon Memory LGALS3 0.0814371814952032
0.0253728463873968 36004503.5 0.751928292973931
8.57716477693151e-177 1.35328599813808e-175 100
99.9300146743425
NFKB2-regulon Memory LGALS3 0.0967605108757047
0.0239545921713486 37846794 0.790403211835876
3.13873280611868e-234 1.48566686156284e-232 100 100
NFYA-regulon Memory LGALS3 0.0271783790344958
0.00638201269607865 31609819 0.660148451759235
1.32614563664769e-72 5.08953190281006e-72 99.8149861239593
98.9976295292922
NFYB-regulon Memory LGALS3 0.109371692364043
0.000765020870825278 28445672.5 0.594067516176706
3.48717261850872e-26 6.43088976400309e-26 100 100
NFYC-regulon Memory LGALS3 0.0861043566470529
0.00263232828778572 28690094.5 0.599172094753252
6.42105344253503e-29 1.21571945178663e-28 100 100
NR1H3-regulon Memory LGALS3
0.00761249141062906 -0.000944395334248558 23510412
0.490998132005176 0.163042448034333 0.176733035273857
20.3515263644773 22.1469691838808
NR2C2-regulon Memory LGALS3 0.0336334482236256
0.0121364074003189 31567027.5 0.659254781900718
8.44671029797554e-73 3.33175795086813e-72 89.5467160037003
75.6473642623321
NR3C1-regulon Memory LGALS3

0.224376736757318 -0.00112771432025874 21765768.5
0.454562500868003 3.17053406761454e-07
4.68974830834651e-07 100 100
NRF1-regulon Memory LGALS3 0.0275901319517371
0.0008819774883181 25972608 0.542419333668108
1.81152770766096e-06 2.49744596590151e-06 100
99.9661361327464
PAX5-regulon Memory LGALS3
0.0904777070946132 -0.0100288919158046 16455277.5
0.343656696196001 2.78267434666981e-69
9.87849393067782e-69 100 100
POU2F1-regulon Memory LGALS3
0.00293901378953346 -0.00198833553115466 22611853.5
0.472232380686256 3.66978168321014e-06
5.01066345207539e-06 13.0434782608696 18.4761259735862
POU6F1-regulon Memory LGALS3
0.0236318135715141 -0.00755018562216687 21207636
0.44290630297103 5.28835276139053e-11 8.43759654064556e-11
59.2969472710453 65.5017496331414
REL-regulon Memory LGALS3 0.276394843447163
0.0129923810839181 32458666.5 0.677876024413311
3.98420995751513e-89 2.09539931098944e-88 100 100
RELA-regulon Memory LGALS3 0.0969042541531773
0.00495790549610019 29910768 0.624664987361353
1.04517443002408e-44 2.6037678783056e-44 100 100
RELB-regulon Memory LGALS3 0.110403685041711
0.00534739818501576 29426202 0.614545173177186
5.15053708916554e-38 1.16091470898652e-37 100 100
REST-regulon Memory LGALS3
0.217931359696468 -0.000703881060972023 22452870.5
0.46891213448978 0.000468281602533023 0.000583298136488502 100
100
RFX5-regulon Memory LGALS3
0.0644553624923836 -0.000914908001141926 25420787.5
0.530894957374653 0.000507880547123963
0.000627122066883502 100 100
RXRA-regulon Memory LGALS3 0.0171399505349551
0.00267350307607055 26239383.5 0.547990749097355
3.23985715134969e-08 4.89425229246442e-08 71.4153561517114
64.0884975730895
SETDB1-regulon Memory LGALS3
0.058447095402557 -0.00130143583764314 23176881.5
0.48403258616673 0.072351348065614 0.0802647767602905
95.2821461609621 95.0468450163675
SOX5-regulon Memory LGALS3 0.00793741040941297
0.000137379707470529 24439694.5 0.510405532079879
0.109215949148356 0.120222207589664 24.7918593894542
22.4381984422621
SP1-regulon Memory LGALS3
0.00975065115035066 -0.0033223720000914 21737905.5

0.453980602050064	8.55261389901951e-08	1.2783907091166e-07
	55.4116558741906 59.9300146743425
SP2-regulon	Memory LGALS3
0.0280070301983931	-0.000238816212259966	23646858.5
0.493847719524895	0.488744941444506	0.499293393418129
	100	100
SP3-regulon	Memory LGALS3
0.0237569885676153	-0.00462304736816426	16333539.5
0.341114285174278	1.71317395573664e-71
6.40186057143692e-71	100	100
SP4-regulon	Memory LGALS3
0.00630874457633906	-0.00238602234542373	22489035.5
0.469667414637315	2.05446572136572e-05
2.72648721900871e-05	23.5892691951896 29.1477593407834
SPI1-regulon	Memory LGALS3
0.415157464297454	-0.00346813535989104	21864107
0.456616230075479	1.05061121324965e-06
1.49186792281451e-06	100	100
SPIB-regulon	Memory LGALS3	0.573591710449433
0.0093635600259796	28268492.5	0.590367238655892
2.72979255418064e-24	4.72720174016647e-24	100	100
SREBF1-regulon	Memory LGALS3	0.048677024064476
0.00793620961144995	29248819 0.610840656146626
1.04892997722584e-35	2.19041259950101e-35	99.0749306197965
96.8416299808105
SREBF2-regulon	Memory LGALS3
0.0983165656403765	-0.000682323470889951	22923730.5
0.478745708671124	0.0167698776780656	0.0193603465876854
	100	100
SRF-regulon	Memory LGALS3	0.021879574761413
0.00113749739178909	25571580.5	0.534044161281393
0.000127645323344543	0.000163294017251578	98.7974098057354
97.9139857771758
STAT1-regulon	Memory LGALS3
0.0788837778917422	-0.000906323858804542	22934505
0.478970726394049	0.0179623339901688	0.0205697695693868
	100	100
STAT2-regulon	Memory LGALS3
0.0537440510786676	-0.0127951239326764	15996933
0.334084499276829	8.64637242414836e-78
3.96059640073893e-77	100	99.9616209504459
STAT5A-regulon	Memory LGALS3	0.08153654947251 0.0101710271935106
	32530017 0.679366128551751	1.35834464861614e-90
7.41865154244198e-90	100	100
TBP-regulon	Memory LGALS3
0.0186080660402272	-0.00234916372861436	22857365.5
0.477359723132864	0.00643163266819118	0.00761076532402623
	46.3459759481961 49.971780110622
TCF12-regulon	Memory LGALS3	0.00957388908621255
0.000543114477409245	24881738 0.519637294278051

0.00376776271051324	0.00453408732960067	29.6947271045328
25.1111863641495
TFDP1-regulon	Memory LGALS3	0.540642823329645
0.0127840782603946	30607825.5	0.639222534477082
2.56244557112503e-55	7.27734542199509e-55	100	100
TFDP2-regulon	Memory LGALS3
0.0223213335926375	-0.00491900643623675	17510825.5
0.365701060890324	1.3401734668611e-51
3.73146337831913e-51	99.9074930619797 99.9977424088498
TFEC-regulon	Memory LGALS3	0.131604239891987
0.0410404069476579	41719330 0.871278355245647	0	0
100	100
THAP1-regulon	Memory LGALS3	0.0372866152948135
0.000124467466326619	24345431 0.508436906331582
0.342421204868959	0.356124058276154	100	100
THAP11-regulon	Memory LGALS3	0.182689680036914
0.00431476137377865	29359862.5	0.61315972018818
3.84170041017932e-37	8.26547664008279e-37	100	100
TP53-regulon	Memory LGALS3	0.109011236406762
0.0109145637026304	31795991 0.664036520765923
4.43076837885903e-76	1.85049738175877e-75	100	100
USF2-regulon	Memory LGALS3
0.120285783648163	-0.0051224545486207	18362147
0.383480301264157	2.81836633262855e-39
6.67013365388756e-39	100	100
XBP1-regulon	Memory LGALS3
0.193360211665938	-0.0019767473513069	26476352.5
0.552939677101813	2.56554049121511e-09
3.91727687905963e-09	100	100
YBX1-regulon	Memory LGALS3	0.0965789696536433
0.00142925709366783	27037863 0.564666422111696
3.41778006371225e-13	5.51505419371749e-13	100	100
YY1-regulon	Memory LGALS3	0.665094237744678
0.0262482212778856	33471291.5	0.699023972965712
4.31620134306049e-111	2.7859117759754e-110	100	100
YY2-regulon	Memory LGALS3
0.0391018691841594	-0.0121906066303688	19107653.5
0.399049671077741	6.27322020684229e-30
1.25464404136846e-29	86.8640148011101 90.6197087707416
ZBTB33-regulon	Memory LGALS3	0.368196082680812
0.00242790941401261	24431648 0.510237486684963
0.249317369737237	0.264201988826027	100	100
ZNF143-regulon	Memory LGALS3
0.0228633572488197	-0.000478784756809687	23321650.5
0.487055983143876	0.145234835029843	0.158641127494136
100	99.9932272265493
ZNF274-regulon	Memory LGALS3
0.0626963511716302	-0.00270973405284228	21897131.5
0.457305923127664	1.55290709240591e-06
2.16189026589842e-06	100	100

ZNF76-regulon	Memory LGALS3
0.0116782396322569	-0.00294912450185108	18593536.5
0.388312705403464	3.16568367617834e-36	6.7093594330944e-36
	99.3524514338575 99.4626933062422
ATF1-regulon	Naive 1	0.0994937483497286	-0.0076685341756237
	77407532 0.350859097800407	3.4929913243409e-284
8.70183803607732e-284	100	100
ATF3-regulon	Naive 1	0.0472146595641573	-0.0043363608761183
	98865570 0.44812027715498 5.03404072686619e-36
6.8079407925238e-36	100	100
ATF4-regulon	Naive 1	0.100615083535986	-0.0092676464891993
	84786916 0.384307057523019	7.57246978229657e-172
1.62922834710017e-171	100	100
ATF5-regulon	Naive 1	0.104883315570558	-0.0154337696663472
	79303336 0.359452061093003	1.277143390204e-252
3.07380273574522e-252	100	99.997489833827
ATF6-regulon	Naive 1
0.130336464396129	-0.00692233002360701	99983994.5
0.453189672869959	1.21440988667672e-29
1.58207526521187e-29	100	100
ATF6B-regulon	Naive 1	0.0603519042839175	0.00348013220587956
	129952517.0625	0.589025663464387
1.38860981788416e-102	2.34741183499465e-102	99.9277717587577
99.919674682464
BACH1-regulon	Naive 1	0.0465746590538568	0.00374942937768467
	122576137	0.555591319455568	4.1555646046626e-41
	5.67394397944317e-41	99.9277717587577 99.5130277624379
BATF-regulon	Naive 1
0.0139177459823071	-0.00612288663655771	101015870.5
0.457866776932673	3.32316725021758e-27	4.1760154825743e-27
	46.4969302997472 52.4423916863296
BATF3-regulon	Naive 1	0.0582674717802224	-0.0283570307705884
	50659151.5	0.229618794597716	0	0	100
	100
BCL11A-regulon	Naive 1	0.222128861398929	0.0200791245352361
	164088930.5	0.743753128755787	0	0	100
	100
BHLHE40-regulon	Naive 1	0.0578154410036894	0.0024651255707324
	119337457.5	0.540911608863133	4.9832117767633e-23
	6.1001385543137e-23	100	100
BRF2-regulon	Naive 1
0.034858185078847	-0.00769306493605722	81954186
0.371467362645366	1.27544999755072e-211
2.87482380400321e-211	99.2777175875767 99.7916562076409
CEBPB-regulon	Naive 1	0.133391567450104	-0.0110489429042689
	75490017.5	0.342167729013592	0	0	100
	100
CEBPG-regulon	Naive 1	0.0554359809430864	-0.0143797575925541
	73488941 0.333097605250706	0	0	100	100
CLOCK-regulon	Naive 1	0.0187798840378498	0.00362793507249291

118355922.5 0.536462681534465
7.07503329756486e-21 8.51402312079839e-21 54.9656915854099
50.5547467242331
CREB1-regulon Naive 1 0.0492961263910224 0.00720417751933879
138655441 0.628472729687049
1.99961178805767e-211 4.43663865475295e-211 100
99.992469501481
CREB3-regulon Naive 1 0.224781223646112 -0.0171065969504268
62224358 0.282039506298813 0 0 100 100
CREB3L2-regulon Naive 1 0.123236992003311 -0.004032346188278
113297014.5 0.513532562838325 0.00108034482308597
0.00113636270280154 100 100
CREB3L4-regulon Naive 1 0.0158198948768453 0.00537449010288813
121167513 0.549206559045173
2.45377281810306e-47 3.44985881357064e-47 38.4976525821596
30.3529293639239
CREB5-regulon Naive 1 0.0335759235329488 -0.0063766380931176
97058434 0.439929212407397 1.05032201162358e-47
1.49145725650548e-47 99.4763452509931 99.6435564034339
CREM-regulon Naive 1 0.13766876480289 -0.014044933823086
51721588.5 0.23443442012741 0 0 100 100
CTCF-regulon Naive 1 0.359900051749704 -0.0124610299878272
77332371.5 0.350518423649729
1.79197568762747e-285 4.54393835076966e-285 100 100
CUX1-regulon Naive 1 0.0770654149720365
0.000418415809331815 120613697 0.546696320350217
1.6613733006394e-29 2.14468189718904e-29 100 100
DDIT3-regulon Naive 1
0.0811873789364411 -0.00423798067115229 100991866
0.457757973603132 1.91553108279682e-24
2.36526446745346e-24 100 100
E2F1-regulon Naive 1 0.391633917757274 -0.0524640903273461
32171056.5 0.145819244810388 0 0 100
100
E2F2-regulon Naive 1
0.0513125982131878 -0.00297099428319698 104433276.5
0.473356587226298 1.23001521192886e-10
1.35397023328604e-10 100 100
E2F3-regulon Naive 1 0.0234027207777438 0.00260539932583722
130919986.5 0.593410836912246
1.00287736237646e-112 1.8026403222463e-112 100 100
E2F4-regulon Naive 1 0.353657985015137 -0.0512550574318989
30952360.5 0.140295356268728 0 0 100
100
E2F6-regulon Naive 1 0.0536957696693749 -0.0213057504177513
52954546 0.24002295066054 0 0 100 100
E2F7-regulon Naive 1 0.0354598892540277 0.00556506035835446
150103984.5 0.680364652084713 0 0 100
100
E2F8-regulon Naive 1 0.0654653223913124 -0.0106284588377597

66721051.5 0.302421319072471 0 0 100
100
EGR1-regulon Naive 1 0.0500650007017968 0.00296370336024933
131650042 0.596719902676987
1.03580837460171e-120 1.88570242555695e-120 100 100
EGR2-regulon Naive 1
0.0447484746515752 -0.00148007230321334 108906103.5
0.493630222172279 0.123905107212704 0.127496559595681
100 99.994979667654
EGR3-regulon Naive 1 0.0418686382301406
0.000452752373182229 116594639 0.528479448846195
6.0245906198417e-12 6.68353021888689e-12 100
99.994979667654
ELF1-regulon Naive 1 0.687322608464808 -0.0224636504756074
69210981.5 0.313707231060805 0 0 100
100
ELF2-regulon Naive 1 0.0442620588940406 0.0132193813439121
158037844 0.71632583976662 0 0 100
99.4703549374969
ELF4-regulon Naive 1 0.0225365206809891 0.00465790117239422
137129262 0.621555136874221
1.72441414729292e-189 3.76718167562453e-189 99.3318887685085
98.1977006877855
ELK1-regulon Naive 1
0.0614904884425879 -0.00519900535624154 86118789.5
0.390343936913441 1.37313760747579e-154
2.8674344156112e-154 100 100
ELK3-regulon Naive 1 0.0720072214241673 0.00808900305163454
140401932 0.636388913561462
5.14683585070378e-238 1.19811588655727e-237 100 100
ELK4-regulon Naive 1 0.0473067964898951 0.00263085024961334
117235869.5 0.531385904444238
3.42268784155488e-14 3.88817338800635e-14 96.7858432647165
96.7217229780612
ESRRA-regulon Naive 1 0.0720043505784193 -0.0224742925721011
43291899 0.196225822381294 0 0 100 100
ETS1-regulon Naive 1
0.238948848802039 -0.00454484072752417 106279732
0.481725872412378 1.01467267567113e-05
1.09154181776742e-05 100 100
ETV2-regulon Naive 1 0.0338065500335476 0.00592802081289096
145972188.5 0.661636781819384 0 0 100
100
ETV3-regulon Naive 1 0.0139037129649336 0.00281866697023079
127592860.5 0.578330231750616 3.8915024259872e-80
6.20891398303575e-80 88.5698808234019 83.7617350268588
ETV5-regulon Naive 1 0.0343605232181816 0.00102155916163972
118707690 0.53805711071334 3.83805670849403e-20
4.5417004383846e-20 100 100
ETV6-regulon Naive 1

0.457139641586075 -0.00277378540413681 97438973.5
0.44165405419214 4.18361788389123e-45 5.76770620885976e-45 100
100
ETV7-regulon Naive 1 0.0667795491446797 0.00307928709612
132125174.5 0.598873498793262 4.66766911664367e-126
8.72117124425529e-126 100 100
FLI1-regulon Naive 1 0.158577439885158 0.0166818982173994
150938382 0.68414666071479 0 0 100 100
FOS-regulon Naive 1 0.0978402481285551 0.013170092413082
171823050 0.778808970479956 0 0 100
100
FOSB-regulon Naive 1 0.0510859895881914 0.0109487712289061
144004631.5 0.652718589286248
7.08863431476598e-298 1.93574244749379e-297 99.9638858793788
99.2343993172348
FOSL1-regulon Naive 1 0.0493429568356075 -0.0130425529122035
78568941 0.356123326014236 1.26244018336379e-264
3.09080182823549e-264 100 100
FOXO1-regulon Naive 1 0.0242738808682469 0.00863018280789686
131614353 0.596558137923378
1.12179806554858e-134 2.15263953118782e-134 64.192849404117
50.6953160299212
FOXO3-regulon Naive 1 0.0285933396905618 0.0100796124263215
132265350.5 0.599508863642425
5.47349415445458e-150 1.11033738561793e-149 61.2314915131817
44.7487323660826
GABPA-regulon Naive 1 0.0296483891654215 0.00473882049617964
143443792 0.650176515719288
4.10214908163135e-288 1.05910030834846e-287 100 100
GTF2B-regulon Naive 1 0.0696707457804081 -0.0106910434832852
76985897 0.348947985640145 1.85908126517254e-291
4.88869517878705e-291 100 100
HINFP-regulon Naive 1 0.0194669297771729 0.00368628309020568
120209399 0.544863790260994
1.04426524086844e-28 1.33590688471457e-28 66.5583243048032
62.7717254882273
HIVEP3-regulon Naive 1
0.0766273283909141 -0.00120808167187847 108962178
0.493884386695695 0.139623347227344 0.142636800764624
99.8916576381365 99.6661478989909
HOXB2-regulon Naive 1 0.0424942912525993 0.00614106460807957
124705682 0.56524374239324 3.45724424506867e-56
5.11384044583073e-56 88.1545684362586 83.1141121542246
IKZF1-regulon Naive 1
0.138099911529778 -0.00656552327712498 73825918.5
0.334624996947279 0 0 100 100
IRF1-regulon Naive 1 0.149349487525028 0.0158403036175607
172170828 0.780385316762574 0 0 100
100
IRF2-regulon Naive 1 0.0900292861270492 0.00570516201620068

130488696.5	0.591455962284667
3.88105659680692e-108	6.80382761415534e-108	100	100
IRF3-regulon	Naive 1	0.146481022987133	0.00117830231278998
116949100.5	0.53008608890927 3.67171457483785e-13
4.1053816506061e-13	100	100
IRF4-regulon	Naive 1	0.0552074463067239	-0.0036504270505531
102303098.5	0.463701295138775
1.82241117705306e-18	2.1211671077175e-18	100
99.994979667654
IRF5-regulon	Naive 1	0.0515533448302869	0.00513534089767739
120611296.5	0.546685439790632	1.0589565709822e-29
1.39233178777289e-29	87.5406283856988 79.4969626989307
IRF7-regulon	Naive 1	0.118415842049717	0.0120520638265773
161846513	0.733589097419123	0	0	100
100
IRF8-regulon	Naive 1	0.0841484707000638	0.00270577787023209
126938504	0.575364280953608
4.80948972213702e-74	7.42334283199409e-74	100	100
IRF9-regulon	Naive 1	0.043424841370145	0.0108304420879404
164488023	0.745562064280161	0	0	100
100
JUN-regulon	Naive 1	0.103617744186319	0.0173328857747727
177013491	0.802335278571606	0	0	100
100
JUNB-regulon	Naive 1	0.172318224164827	0.0142783633437699
151605668.5	0.687171218316812	0	0	100
100
JUND-regulon	Naive 1	0.0476704440389509	0.0104934492046354
165661359.5	0.750880355345252	0	0	100
100
KLF11-regulon	Naive 1	0.0271044928369587	0.00588642486188523
120149517.5	0.544592370044872
1.13102073391237e-33	1.51514098316563e-33	47.7609245214879
39.8990913198454
KLF12-regulon	Naive 1	0.0497890609114082	0.00312025329907564
119047274.5	0.539596318955983
1.12958580697511e-21	1.37095029564501e-21	99.4221740700614
98.4888799638536
KLF13-regulon	Naive 1	0.0708781995398091	0.00522362842574867
151096918	0.6848652445075	0	0	100	100
KLF2-regulon	Naive 1	0.131527237544162	0.0258315283415644
178960003	0.811158082070595	0	0	100
100
KLF3-regulon	Naive 1	0.112243181356895	0.0458042279981848
186938844.5	0.847323156164191	0	0	100
99.844369697274
KLF4-regulon	Naive 1	0.0214004139328155	0.00626528033728269
123637415.5	0.560401693942446
6.25732321329554e-55	9.1602051163708e-55	56.0671722643554
48.9432200411667

KLF6-regulon	Naive 1	0.0807844361043295	0.0129899411290758
	144275732.5	0.653947387696625
1.18315202897658e-302	3.29426643362106e-302	100	100
KLF7-regulon	Naive 1	0.0613758611132589	0.017514743232997
	152004298.5	0.68897805750342 0	0
99.783315276273	97.2463477082183
KLF8-regulon	Naive 1	0.0415582765348026	0.0187385325995408
	157057988	0.711884522710622	0	0
93.1022029613579 75.4857171544756
MAFB-regulon	Naive 1	0.0602032133855988	0.0050347882963361
	130632100	0.592105956171973
1.18633197159836e-109	2.10573924958709e-109	100
99.997489833827
MAFF-regulon	Naive 1	0.0204373536110149	0.0091695248832348
	126205845	0.572043414506976
1.08977685881451e-105	1.8871745603861e-105	39.4185626579993
27.034489683217
MAFG-regulon	Naive 1	0.0265017800268877	0.00124656699474225
	111967991.5	0.507508603687477	0.0598842675395348
	0.0620698247490069	58.974358974359	58.5496259852402
MAX-regulon	Naive 1	0.201556532689019	0.00442657210956376
	128429075.5	0.582120478421536
1.46210659635637e-87	2.41417600793726e-87	100	100
MAZ-regulon	Naive 1	0.125682996290055	-0.0120972484637506
	58728904 0.266195933907914	0	0	100	100
MEF2A-regulon	Naive 1	0.0487408300644686	0.00909674420606227
	148739420	0.674179596742031	0	0	100
	99.997489833827
MLX-regulon	Naive 1	0.0551449199680047	0.00252799629049878
	120181148.5	0.544735741417602
3.23505521427361e-27	4.10158786095404e-27	100
99.987449169135
MXD4-regulon	Naive 1	0.0628867628255257	-0.0333529964358842
	92839121 0.420804660645205	1.09845476034119e-81
1.77250654509601e-81	82.6110509209101 89.4372207440133
MXI1-regulon	Naive 1	0.0857964788295568	0.0234259975689206
	173219782	0.785139828947178	0	0	100
	100
MYBL1-regulon	Naive 1
0.0247812843291018	-0.00581914293277874	110162675
0.499325786045982	0.870123290546145	0.876294377713139
	82.3040808956302 79.7479793162307
MYC-regulon	Naive 1	0.270596930300293	-0.0504409958171291
	41856068.5	0.189717745185082	0	0	100
	100
NFATC1-regulon	Naive 1	0.0373883528332971	-0.0149546590518981
	85994381.5	0.389780042451089
3.49413372250688e-156	7.40547744173099e-156	90.9895269050199
95.3612129122948
NFE2L1-regulon	Naive 1	0.129935794782367	-0.0600616907434827

60841546 0.275771741932581 0 0
99.9819429396894 99.929715347156
NFE2L2-regulon Naive 1 0.0990156112873432 -0.013514295087461
74474447 0.33756453162212 0 0 100 100
NFKB1-regulon Naive 1 0.0464194858588007 -0.0116740982596429
88900754.5 0.402953533225236
1.62984304848687e-121 3.00568458292385e-121 99.9819429396894
99.92469501481
NFKB2-regulon Naive 1
0.0709733435073124 -0.00273733235298666 104921509
0.475569560693361 3.61197890635603e-09
3.91527484505768e-09 100 100
NFYA-regulon Naive 1 0.0243868905394688 0.00391647849848029
132590534 0.6009827975928 2.08463559929945e-131
3.94691006800696e-131 99.7472011556519 98.9156082132637
NFYB-regulon Naive 1 0.0937450417396988 -0.0169483482920082
54131097.5 0.245355814105995 0 0 100
100
NFYC-regulon Naive 1
0.0755887230109528 -0.00905061525087129 67674957.5
0.306745014582443 0 0 100 100
NR1H3-regulon Naive 1 0.0107835990155287 0.00256188029894533
115469525.5 0.523379734421337
7.45058991103023e-15 8.53212715617978e-15 25.8757674250632
21.5798985892866
NR2C2-regulon Naive 1 0.0254960649075528 0.00422562027328195
123626067 0.560350255479437
8.73687229205504e-49 1.25316754088062e-48 81.1303719754424
75.2623123650786
NR3C1-regulon Naive 1 0.229405266704297 0.00447368013098776
123278708.5 0.558775810631831
9.56189080659947e-46 1.33116519072267e-45 100 100
NRF1-regulon Naive 1
0.0260322675912731 -0.000793776303412937 108197722
0.490419396461048 0.0206597133629479 0.0215711713054309
99.9277717587577 99.972388172097
PAX5-regulon Naive 1 0.105642143422252 0.00612158480326205
130115970 0.589766533877154
2.99130070930986e-104 5.1176469966506e-104 100 100
POU2F1-regulon Naive 1 0.00673911006745123 0.00211757232678099
121218610.5 0.549438164707912
4.85625135897421e-70 7.41492142983159e-70 26.9772481040087
17.1469451277675
POU6F1-regulon Naive 1 0.0383048533989226 0.00831789705995796
125078562 0.566933866558261
2.88134883630719e-61 4.30685826058548e-61 71.9212712170459
64.4409859932728
REL-regulon Naive 1 0.251352511014118 -0.014077598181879
68234594.5 0.309281637671211 0 0 100
100

RELA-regulon Naive 1 0.0971152311856447 0.0057528921576134
140839487.5 0.63837218733342 6.33112951509298e-245
1.49836731857201e-244 100 100
RELB-regulon Naive 1 0.0949756123100796 -0.0116271204795729
60798545 0.275576834645464 0 0 100 100
REST-regulon Naive 1 0.220612927761584 0.00227171095253151
118617884.5 0.537650056310579
9.53023020231813e-20 1.11842370969353e-19 100 100
RFX5-regulon Naive 1
0.0638986312395765 -0.00165139031171137 113865539
0.516109469606874 9.97658653552381e-05
0.000106516938950705 100 100
RXRA-regulon Naive 1 0.0153582536933928
0.000943233668151345 115906090 0.525358516364697
3.61103679953095e-10 3.94436327333381e-10 67.8945467677862
63.7582207942166
SETDB1-regulon Naive 1
0.0572728029401434 -0.00278457239828518 103582709.5
0.469501288361599 1.74163031980833e-13
1.96278972549828e-13 91.6215240158902 95.5293940458858
SOX5-regulon Naive 1 0.00994298148782617 0.00243712102506362
115760459.5 0.524698428327757
3.33005786249604e-16 3.84445704450763e-16 26.489707475623
21.9388523520257
SP1-regulon Naive 1 0.0183397988669252 0.00608907824296489
132433277.5 0.600270013290192
1.95385722725924e-138 3.80065378453167e-138 71.0906464427591
58.2559365429991
SP2-regulon Naive 1
0.0280741182478414 -0.000189120357203816 108976312
0.493948450777835 0.143817764905092 0.145872304403736
100 100
SP3-regulon Naive 1 0.0348153284503518 0.00745532776108779
164853254 0.747217518417993 0 0 100
100
SP4-regulon Naive 1 0.0123160437892243 0.00418942590725782
121208224 0.549391086627457
4.07929115925914e-50 5.9108096389265e-50 36.7280606717226
27.9431698378433
SPI1-regulon Naive 1
0.413173482078442 -0.00611592532095689 93029783
0.421668859458633 7.73302441454239e-80 1.2200994076278e-79
100 100
SPIB-regulon Naive 1 0.536906751711274 -0.0313735076146372
43532592 0.197316792815888 0 0 100 100
SREBF1-regulon Naive 1 0.04193858647236 0.0011489296791837
113701616 0.515366468578385 0.000205832138753365
0.000218120624649089 96.677500902853 96.9250464380742
SREBF2-regulon Naive 1 0.102489438655468 0.00399429609392338
129572876.5 0.587304896223711

1.02381184259678e-98 1.71036801939697e-98 100 100
SRF-regulon Naive 1 0.0248375341711046 0.00463391267491998
133971131 0.607240522200865
6.07811824104271e-148 1.21562364820854e-147 99.0249187432286
97.7835232692404
STAT1-regulon Naive 1 0.0869391694988802 0.00816747412620478
153221200 0.694493812254546 0 0 100
100
STAT2-regulon Naive 1 0.0774921832513154 0.0128228131650225
146000284 0.661764128106335 0 0 100
99.957327175059
STAT5A-regulon Naive 1 0.0624296851688848 -0.0104540244265462
76730924 0.34779228936057 6.69477745837647e-296
1.79369509262162e-295 100 100
TBP-regulon Naive 1
0.0204181707776482 -0.000550250885932017 110226943.5
0.499617090875685 0.921198731390781 0.921198731390781
49.6749729144095 49.914654350118
TCF12-regulon Naive 1 0.0123994716799855 0.00382225241106188
117742883 0.533684005088793
1.46001786750128e-26 1.81861874723844e-26 30.3358613217768
24.5092625131784
TFDP1-regulon Naive 1 0.489047995734102 -0.044552842774014
34993905.5 0.158614152848107 0 0 100
100
TFDP2-regulon Naive 1 0.028609361289723 0.00169280974672033
125866229 0.570504063486735
4.92876394458814e-65 7.44557957586719e-65 100
99.994979667654
TFEC-regulon Naive 1
0.0833258248866372 -0.00935781247894625 87288637.5
0.395646415925995 3.43561012826534e-140
6.77578664185664e-140 100 100
THAP1-regulon Naive 1 0.0381833850263545 0.00115982503537684
118736387 0.538187183372543
2.86429583771624e-20 3.4178992349219e-20 100 100
THAP11-regulon Naive 1
0.176174197804569 -0.00262373027570073 93304832
0.422915552661446 2.23876842367627e-77
3.49346281496737e-77 100 100
TP53-regulon Naive 1 0.0839797873624619 -0.0163754815157924
60445998.5 0.273978874553897 0 0 100
100
USF2-regulon Naive 1 0.132136105431948 0.00780212553416056
144925527.5 0.656892662937479 0 0 100
100
XBP1-regulon Naive 1
0.194389562042917 -0.00102545863799194 127981479.5
0.580091694856404 2.21384658536177e-83
3.61340477151001e-83 100 100

YBX1-regulon	Naive 1
0.0908695779973771	-0.00491391166300309	81252018.5
0.368284702648471	3.99402478873459e-222
9.14760516129535e-222	100	100
YY1-regulon	Naive 1	0.59927370717949 -0.045785617586778
35253959 0.159792877114756	0	0	100	100
YY2-regulon	Naive 1	0.0774843771438548	0.0301637070256276
164104112.5	0.743821943025991	0	0
99.1513181654027 89.3317937647472
ZBTB33-regulon	Naive 1
0.363108948570616	-0.00309477257619711	99794221.5
0.452329503557664	1.11415998333977e-30
1.47860483770325e-30	100	100
ZNF143-regulon	Naive 1	0.0279620259769666	0.00527509968887765
149469414	0.677488383750506	0	0	100
99.992469501481
ZNF274-regulon	Naive 1	0.0745978069296128	0.0105430188916002
151866756	0.688354629314814	0	0	100
100
ZNF76-regulon	Naive 1	0.0171588932608143	0.0029634687795554
134150746.5	0.608054651403188
3.64015012827504e-150	7.4913234523921e-150	99.7110870350307
99.4251719463828
ATF1-regulon	Naive 2
0.103720419761196	-0.00301977486447313	134129971.5
0.461349047087692	8.45304579141804e-27
1.25034635664725e-26	100	100
ATF3-regulon	Naive 2
0.0469071697560528	-0.00495829510331563	126897038.5
0.436470888165552	1.87509820215598e-69
4.51294821535846e-69	100	100
ATF4-regulon	Naive 2
0.106893656967002	-0.00223895771692879	145690737.5
0.501113078333339	0.757598802803502	0.762971843958136
100	100
ATF5-regulon	Naive 2
0.114005683445814	-0.00533565134139753	132948950.5
0.4572868460237	2.32359120211151e-32	3.70730281685208e-32	100
99.9973443101846
ATF6-regulon	Naive 2	0.13080361948783 -0.00676069988154043
134287494.5	0.46189085802772 4.24025379611435e-26
6.14404121477794e-26	100	100
ATF6B-regulon	Naive 2	0.058965355797645	0.00201103393187438
167738692.273438 0.576948499836861
5.0417947935419e-101	1.46109155241418e-100	100
99.9043951666445
BACH1-regulon	Naive 2	0.043329962450787
5.67896557510661e-05	146751929.5	0.504763119502379
0.186593287277441	0.196268494765901	99.9740966196089
99.4794847961758

BATF-regulon	Naive 2
0.0122704387114778	-0.00846293380821503	126151954
0.433908119977125	2.71719663413585e-84
7.28003626504323e-84	43.9062297629841 53.3182844243792
BATF3-regulon	Naive 2	0.0718771948485954	-0.013600653243612
119595212.5	0.411355767142059
2.09795119607805e-133	7.26607487422153e-133	100	100
BCL11A-regulon	Naive 2	0.217521276288572	0.0156908347169611
196708398.5	0.676591750428583	0	0	100
100
BHLHE40-regulon	Naive 2	0.0570073552639765	0.00163425770712054
153164027	0.526817959583056
1.03683548428993e-13	1.31455927472474e-13	100	100
BRF2-regulon	Naive 2
0.0408113581965403	-0.000965214115899532	141296216.5
0.485997828154099	0.000103369948261595
0.000122321105442888	99.8445797176532 99.7052184304873
CEBPB-regulon	Naive 2	0.140674244103109	-0.0029135321103049
143887280.5	0.494909966835521	0.158135142139292
0.168836016419394	100	100
CEBPG-regulon	Naive 2
0.0605459218634649	-0.00905569257121433	121798438.5
0.418933910969659	6.79513886493721e-112
2.01022858087726e-111	100	100
CLOCK-regulon	Naive 2	0.0156613984524288
8.03419331804472e-05	146731129	0.504691574785365
0.166243383492385	0.176168361611333	51.9362776842378
50.9201965210463
CREB1-regulon	Naive 2	0.0457501433941445	0.00334875845303644
164073174	0.564340703506025
3.42711990386211e-71	8.39053493704172e-71	100
99.9920329305537
CREB3-regulon	Naive 2
0.233373661506881	-0.00774404857099528	130258523
0.448032939909334	4.50950508364073e-47
8.20961181893568e-47	100	100
CREB3L2-regulon	Naive 2
0.123835908209614	-0.00354439478612216	155399492
0.534506991616794	1.08837077662625e-21
1.48604471423969e-21	100	100
CREB3L4-regulon	Naive 2	0.013612133761585	0.00302561594350836
154658603.5	0.531958655852232	4.5382803507399e-27
6.78353484005333e-27	35.9668436730993 30.3996813172222
CREB5-regulon	Naive 2
0.035344756791143	-0.00461479034470321	136899086
0.470873602424317	6.6810687320533e-16
8.62465236319608e-16	99.8057246470664 99.585712388793
CREM-regulon	Naive 2
0.147441492588279	-0.00308258604844766	127130253
0.437273045104369	9.31896573533807e-68

2.16933300724263e-67	100	100
CTCF-regulon	Naive 2
0.368959164263314	-0.00226679652735329	146178039
0.502789184576823	0.439292374531925	0.458672920467157
100	100
CUX1-regulon	Naive 2
0.0754837631925271	-0.00146328992523855	147364525.5
0.506870184595207	0.0567834039789187	0.0615514760687516
100	100
DDIT3-regulon	Naive 2	0.086994736432066	0.00251445969311143
159923554.5	0.55006780848717 8.04512846829415e-44
1.41038054629354e-43	100	100
E2F1-regulon	Naive 2	0.419827668164519	-0.0215308674009209
116270686	0.399920834921912
1.73780165466659e-169	7.47781318068653e-169	100	100
E2F2-regulon	Naive 2
0.0516545010868104	-0.00273122521472591	141831838.5
0.487840136003238	0.000747045742759246
0.000855487866708169	100	100
E2F3-regulon	Naive 2	0.0224790631393359	0.00164339421968682
163633549.5	0.56282858550672 5.69675793177348e-68
1.34823271051972e-67	100	100
E2F4-regulon	Naive 2	0.382550184012292	-0.0194100798373943
119791827.5	0.412032037642073
2.08529590607442e-131	7.05028615863255e-131	100	100
E2F6-regulon	Naive 2	0.0619574974259661	-0.012585163363678
119145231	0.409808025545528
4.93350581823523e-138	1.79630211843437e-137	100	100
E2F7-regulon	Naive 2	0.0317955215877111	0.00147195664133161
162100858	0.557556790134689
2.44955475986103e-57	5.04111269420675e-57	100	100
E2F8-regulon	Naive 2
0.0691021116734863	-0.00686213232534184	119158405
0.409853338403485	6.75774620964786e-138
2.39899990442499e-137	100	100
EGR1-regulon	Naive 2	0.0489318712830744	0.00177004737648825
162840040	0.560099256277868	2.3782645771809e-62
5.36053285650298e-62	100	100
EGR2-regulon	Naive 2	0.0477948596127311	0.00210515585133435
161671251	0.556079127999554
1.59846616796075e-54	3.15253049792259e-54	100
99.9946886203691
EGR3-regulon	Naive 2	0.0437438201785657	0.00273868025852006
166751178.5	0.573551879877382
1.86839587782614e-92	5.20220028728063e-92	100
99.9946886203691
ELF1-regulon	Naive 2	0.706165187440606	-0.0010597807473488
158322128.5	0.544559596185183
4.54638841461433e-35	7.33621766903676e-35	100	100
ELF2-regulon	Naive 2	0.0370072416108785	0.00524337073265197

174591362.5 0.600518719405802
5.83422406683674e-171 2.5889369296588e-170 99.9870483098044
99.4423051387598
ELF4-regulon Naive 2 0.0196351628382664 0.00143166786441255
159045512.5 0.54704772404614 6.74201717612985e-39
1.1397219512029e-38 99.0545266157233 98.188819545877
ELK1-regulon Naive 2 0.0660828377610898 3.3580329156721e-05
144876397 0.498312099480675 0.63976808587306
0.653576030172478 100 100
ELK3-regulon Naive 2 0.0666631441903325 0.00211809467556849
155242473.5 0.533966915938406 4.5803227306163e-21
6.13590403535391e-21 100 100
ELK4-regulon Naive 2
0.0444250554091248 -0.000689259621217339 144819698.5
0.498117081181232 0.601595704874556 0.619033261537587
97.3319518197125 96.606028415881
ESRRA-regulon Naive 2 0.081454390094295 -0.0123894801331825
112638476.5 0.387427606354814
6.82921261015043e-214 4.4079463210971e-213 100 100
ETS1-regulon Naive 2 0.243545441367119
0.000730782613994452 157127399 0.540450243814579
3.39924651102975e-29 5.24666309311113e-29 100 100
ETV2-regulon Naive 2 0.030928141825087 0.00280307641154799
169896216 0.584369447624945
4.90918080947135e-121 1.5491192776554e-120 100 100
ETV3-regulon Naive 2 0.0124678827564206 0.00125183437043077
156300038 0.53760448007752 1.51116812640771e-25
2.16753408030197e-25 86.8281310711048 83.8401274731111
ETV5-regulon Naive 2 0.0340154424617778
0.000664944615026516 153527724 0.52806891984572
7.08297894039355e-15 9.06110819401698e-15 100 100
ETV6-regulon Naive 2 0.471916799009759 0.0148725596156444
188926749 0.649826244244938 0 0 100
100
ETV7-regulon Naive 2
0.0632592730567659 -0.000984289162925242 148832751.5
0.511920246549551 0.000948873375558587 0.00107792015463455
100 100
FLI1-regulon Naive 2 0.155468688462096 0.0139028218460203
188316601.5 0.647727600932336 0 0 100
100
FOS-regulon Naive 2 0.0900700877034922 0.0045702122455107
177603105.5 0.610877811766625
1.45453125469705e-207 8.26173752667927e-207 100 100
FOSB-regulon Naive 2 0.0438871744631174 0.00290861009432487
160432911.5 0.55181977610447 8.13843427232374e-47
1.46285780591136e-46 99.909338168631 99.2032930553711
FOSL1-regulon Naive 2 0.0522992438953064 -0.0102362167386924
120767122 0.415386628589741
1.00370255645644e-121 3.23922188674577e-121 100 100

FOXO1-regulon Naive 2 0.0204876308338786 0.00456790176979148
162264038 0.558118058706223
2.70063024672882e-65 6.18531443605634e-65 59.8756637741225
50.7980347895366
FOXO3-regulon Naive 2 0.0227318636252183 0.00360061776387648
159484956 0.548559219483786
2.34944123463478e-48 4.38974546471234e-48 54.5395674135475
45.1653166910105
GABPA-regulon Naive 2 0.0268982126610997 0.00169945881998928
162510515 0.558965832904692
4.34036298158848e-60 9.33835671796309e-60 100 100
GTF2B-regulon Naive 2
0.0753309003994177 -0.00449009731230737 128075938
0.440525792187783 4.25172327480711e-61
9.28838007727092e-61 100 100
HINFP-regulon Naive 2 0.0172095407962896 0.00117973345773619
149238141 0.513314610966637
0.000152352372424764 0.000178793693258814 63.4114751975133
63.1974505377772
HIVEP3-regulon Naive 2 0.0811796257417061 0.00420760820462743
158627433.5 0.545609713241393
1.16631222441287e-36 1.92577134728637e-36 99.909338168631
99.6494489443633
HOXB2-regulon Naive 2 0.040942886255577 0.00462757080631503
162166522.5 0.557782647593418 5.1332159123778e-58
1.07193626405536e-57 89.2371454474809 82.5999203293055
IKZF1-regulon Naive 2 0.144891727188101 0.00123829799490008
151837372.5 0.522254842312957 6.7921792500959e-10
8.46043380275104e-10 100 100
IRF1-regulon Naive 2 0.140672992475455 0.0063030507541352
182683614.5 0.628352563752764
2.01575738499684e-277 2.38531290557959e-276 100 100
IRF2-regulon Naive 2 0.0898018268270522 0.00576181254029308
172762907.5 0.594229625607756
1.74709082325419e-150 7.08819705434557e-150 100 100
IRF3-regulon Naive 2 0.149504761866843 0.00489035673729007
178008666 0.612272764349698
9.12632098126681e-213 5.63451121452125e-212 100 100
IRF4-regulon Naive 2 0.0551735136207373 -0.0039029462859982
134180696 0.461523517412835
1.42430345182199e-26 2.08506278514147e-26 100
99.9946886203691
IRF5-regulon Naive 2 0.0535044391439135 0.00778421368362221
165164684 0.568095025472661
4.24180242261243e-80 1.09515626183812e-79 90.0531019298018
78.5154693931749
IRF7-regulon Naive 2 0.112440462969942 0.00555016113661727
179256615.5 0.616565170485329
3.50552383906211e-229 2.37040183403247e-228 100 100
IRF8-regulon Naive 2 0.082112653755049

0.000409389167369892	155499162.5	0.53484981499686
4.32119361009675e-22	5.95737371489066e-22	100	100
IRF9-regulon	Naive 2	0.0369984459968633	0.00371422216017368
174922236	0.601656781035314
8.31220155063289e-175	3.80752458125765e-174	100	100
JUN-regulon	Naive 2	0.0946104115950572	0.00748348904087875
181232823.5	0.623362470652108
1.96052289782761e-256	1.73996407182201e-255	100	100
JUNB-regulon	Naive 2	0.169659892614883	0.0119027217229819
191107759.5	0.657327976367972	0	0	100
100
JUND-regulon	Naive 2	0.0435860797480566	0.00617994729386931
190962695	0.656829017275587	0	0	100
100
KLF11-regulon	Naive 2	0.0234255679221186	0.00179440968561947
152014633	0.522864541710092
1.08852960654502e-12	1.36788676220701e-12	45.1107369511721
39.9867215509229
KLF12-regulon	Naive 2	0.0492081463697743	0.00260111732007424
156076197.5	0.536834565641396
1.72426482727674e-24	2.44845605473297e-24	99.4689807019816
98.4251759394503
KLF13-regulon	Naive 2	0.0670147018529606	0.00087076983631755
167328016.5	0.575535952927184
2.09631436980837e-97	5.95353281025576e-97	100	100
KLF2-regulon	Naive 2	0.117313261742195	0.0102005858473289
182143409.5	0.626494492367265
1.6459575970787e-269	1.55817319190117e-268	100	100
KLF3-regulon	Naive 2	0.0858493533869234	0.0166538971458312
193426239.5	0.665302544070701	0	0	100
99.8353472314434
KLF4-regulon	Naive 2
0.0154579100487158	-0.000532487536004168	143416547.5
0.493290849060769	0.0465498220736236	0.0508467287265734
48.5688382333895 50.0677200902935
KLF6-regulon	Naive 2	0.0735220815293188	0.00499154623567749
161820597.5	0.55659281531858 1.71190739871143e-55
3.42381479742287e-55	100	100
KLF7-regulon	Naive 2	0.0540366845459538	0.00968609387858452
179323772	0.616796159778283
4.37115455802107e-230	3.10351973619496e-229	99.6503043647196
97.1265436197052
KLF8-regulon	Naive 2	0.0320692230677985	0.00839013070194468
179216952	0.616428745212703
3.26073048067559e-231	2.4369669908207e-230	89.3407589690455
75.2356924711194
MAFB-regulon	Naive 2	0.0576349617537632	0.00223181277661038
159594814.5	0.548937085174225
6.08591068665848e-42	1.04120399699458e-41	100
99.9973443101846

MAFF-regulon	Naive 2	0.0164347232987904	0.00487776335814122
	158674949	0.545773145995473	4.239150443594e-57
	8.59941947129069e-57	35.5005828260588	27.1199043951666
MAFG-regulon	Naive 2
0.0240803434458146	-0.00159910690124104	141947943
0.488239485230249	0.000716463586958764
0.000827136823968654	57.6997798212667	58.7863497543487
MAX-regulon	Naive 2	0.202980406054011	0.00639902954483396
	182178637	0.626615659719905
5.05798172520678e-270	5.13023860699545e-269	100	100
MAZ-regulon	Naive 2
0.133462889411605	-0.00342344320848351	134796248
0.463640749866231	6.65334621199506e-24
9.35420952577523e-24	100	100
MEF2A-regulon	Naive 2	0.0441211423416171	0.00405718086758732
	169132187.5	0.581741520275965
9.83323572080058e-114	2.97089249436954e-113	100
99.9973443101846
MLX-regulon	Naive 2
0.0515410663064074	-0.00166825514613594	139752500
0.480688111553969	8.56375892147751e-08
1.05743805813027e-07	100	99.9867215509229
MXD4-regulon	Naive 2	0.0649035206340803	-0.0328563077862643
	125258704.5	0.430835728318288
4.30213193743368e-82	1.13130136132515e-81	86.128739800544
89.1116717567388
MXI1-regulon	Naive 2	0.0748649478045778	0.0116110938616708
	190667403	0.655813340605496	0	0	100
	100
MYBL1-regulon	Naive 2
0.0217929192944696	-0.00975761699555188	133458273.5
0.459038696695716	4.0347123844671e-30
6.30805108019565e-30	80.4688511850796	79.9760987916611
MYC-regulon	Naive 2	0.296047276450633	-0.0226964144056556
	130243621.5	0.447981685199083
3.67048065543062e-47	6.76893835157335e-47	100	100
NFATC1-regulon	Naive 2
0.0423661891108812	-0.00982311534664348	129632725.5
0.445880467370451	6.56894677747454e-51
1.24372058986851e-50	94.5602901178604	94.8824857256672
NFE2L1-regulon	Naive 2	0.15520597074449	-0.0330919700273945
118011593.5	0.405908803212748	4.76632782919054e-150
1.88005153262516e-149	99.9870483098044	99.925640685168
NFE2L2-regulon	Naive 2
0.107685524362912	-0.00385012646334537	142926187
0.491604221181298	0.019912456291405	0.0220903811982774
	100	100
NFKB1-regulon	Naive 2
0.0502262103106653	-0.00776361167812641	132166741.5
0.454596385623703	2.40950970908554e-36

3.93276297344996e-36	99.9740966196089 99.9229849953525
NFKB2-regulon	Naive 2
0.0702947639260835	-0.00371374514290607	133410957
0.458875948415504	4.04248343871693e-30
6.30805108019565e-30	100	100
NFYA-regulon	Naive 2	0.021841998671934	0.00107681999843847
155355862.5	0.534356925020755
1.62608112673379e-21	2.19908114282094e-21	99.6762077451107
98.8819545877042
NFYB-regulon	Naive 2	0.0994389162886248	-0.0110695272267946
108452282	0.373028909166551
1.57748567291831e-271	1.72309973503385e-270	100	100
NFYC-regulon	Naive 2
0.0806741316813329	-0.00344716256903579	130966255
0.450467231664876	6.30744106466066e-43
1.09226418436806e-42	100	100
NR1H3-regulon	Naive 2	0.010570361609291	0.00245344115798548
152462462.5	0.524404881358064
1.17921126564742e-20	1.55044444186976e-20	25.8515736303588
21.3358119771611
NR2C2-regulon	Naive 2	0.0232606531858727	0.00177682162167171
154563894	0.531632896164919
1.02228552982361e-18	1.33178481866929e-18	79.7694599145188
75.2011685035188
NR3C1-regulon	Naive 2	0.230787921903469	0.00639919881492265
172381109	0.592916404019884
2.24658219188475e-146	8.62201814182797e-146	100	100
NRF1-regulon	Naive 2
0.0261684297943912	-0.000675712767140558	144613284.5
0.497407106362475	0.47216501240003 0.489397312122658
99.9611449294133 99.9681317222148
PAX5-regulon	Naive 2	0.1061628368661	0.00710393523039279
176287986	0.606354369903884	3.85708556985707e-191
1.9560933961418e-190	100	100
POU2F1-regulon	Naive 2	0.00507733604635108
0.000237822025570598	148843951	0.511958767982122
8.9846509476983e-07	1.09044481587449e-06	20.2305400854812
17.9604302217501
POU6F1-regulon	Naive 2	0.0313450670716712
0.000413255044197213	149178482	0.513109409828574
0.000204797714194798	0.000238371109964437	67.4783059189224
64.9183375381755
REL-regulon	Naive 2
0.261550452254701	-0.00260474238844849	142375243
0.489709212283912	0.00432481235863122	0.00487399488036217
100	100
RELA-regulon	Naive 2	0.0948918628116588	0.00340714790697165
170313800.5	0.585805757563724
4.00827964286268e-125	1.32366444020116e-124	100	100
RELB-regulon	Naive 2	0.10021632977293 -0.00598588315153176

113839576.5	0.391558870488103	1.24317857654401e-198
6.5381984396018e-198	100	100
REST-regulon	Naive 2	0.225131562291883	0.00784857207207001
	193207994.5	0.664551875732703	0	0	100
	100
RFX5-regulon	Naive 2
0.0637968641570769	-0.00186976152897249	150096166
0.516265845591535	6.47613423311576e-06	7.7933140771393e-06
	100	100
RXRA-regulon	Naive 2	0.0134087367347309	-0.0013513408217881
	141331065	0.486117691910779
8.13881540656305e-05	9.71186376245338e-05	63.7093640720114
64.3765768158279
SETDB1-regulon	Naive 2	0.0623584485443716	0.00318243154254615
	155539217	0.534987585140251
2.95618646537695e-22	4.11547527532869e-22	95.4410050511592
94.9727791793918
SOX5-regulon	Naive 2	0.00832043103480123
0.000623162343381356	146823168.5	0.505008150828323
0.057486962550783	0.0618420354712969	23.1576220696801
22.3582525561014
SP1-regulon	Naive 2	0.015315250728299	0.00279736562753777
	160679583	0.552668219298754
1.57436187463167e-51	3.02107278645538e-51	66.0665716876052
58.5420262913292
SP2-regulon	Naive 2
0.0277133449170487	-0.000634832756561864	139836664
0.480977599285643	1.33075860073946e-07
1.62903208021555e-07	100	100
SP3-regulon	Naive 2	0.0307291306752935	0.00296348525043611
	175636874	0.604114826441762
2.91689818393762e-183	1.42827428316946e-182	100	100
SP4-regulon	Naive 2	0.0106411998693472	0.0024140388151638
	154589894	0.531722324911456
5.03315435916113e-28	7.60327573405191e-28	33.7262012692656
28.049395830567
SPI1-regulon	Naive 2	0.425128113790775	0.00793539066903631
	164388093	0.56542388856105 1.51719675629859e-73
3.77968314727018e-73	100	100
SPIB-regulon	Naive 2
0.556267625719468	-0.00986160609098696	113826122.5
0.391512594551337	8.44796138866131e-199
4.61388660457656e-198	100	100
SREBF1-regulon	Naive 2
0.0407759231875681	-0.000185525126846296	145723655
0.501226300285806	0.733830146599973	0.744313434408544
	97.0340629452143 96.8662860177931
SREBF2-regulon	Naive 2	0.104564882316525	0.00672686494101357
	189918633.5	0.653237897612031	0	0	100
	100

SRF-regulon	Naive 2	0.0231686667508819	0.00289149608507328
	166314093.5	0.572048496658916
8.59253422799264e-89	2.34642280841337e-88	99.0027198549411
97.7161067587306
STAT1-regulon	Naive 2	0.0834923897378786	0.00448744538054802
	177628807	0.61096621380236 6.83841445900181e-208
4.04606188824274e-207	100	100
STAT2-regulon	Naive 2	0.0739738131030225	0.00932640199227804
	180951321.5	0.622394225613353
1.85552083796145e-252	1.54990564112074e-251	100
99.9548532731377
STAT5A-regulon	Naive 2
0.0668673772932277	-0.00571246068969752	127998107
0.440258087235025	1.24108469060338e-61
2.75365665727625e-61	100	100
TBP-regulon	Naive 2
0.0190191852258313	-0.00226799264886471	142905947
0.491534604341687	0.0120525427487768	0.0134760714198921
	49.7085869705997 49.9216571504448
TCF12-regulon	Naive 2	0.00960083571226177
0.000671358010369187	147056428.5	0.505810464267446
0.0346704085420747	0.0381643256819737	26.0199456029012
25.0564334085779
TFDP1-regulon	Naive 2	0.515282114766877	-0.0155224210649317
	116670433	0.401295791581216
6.44385289492237e-165	2.69125620905581e-164	100	100
TFDP2-regulon	Naive 2
0.0264447820484241	-0.000817468940733833	145252406
0.499605407694391	0.912875194438426	0.912875194438426
	100	99.9946886203691
TFEC-regulon	Naive 2
0.0870584846891417	-0.00540229351580983	130672420
0.449456566444157	1.26631284578035e-44
2.24770530126012e-44	100	100
THAP1-regulon	Naive 2	0.038537992229535	0.00165438231870561
	161330975.5	0.554908727559468
2.40724189346661e-52	4.68258012153779e-52	100	100
THAP11-regulon	Naive 2	0.180927080027543	0.00295160316097004
	164662117	0.566366412516475
1.26194446034978e-75	3.19993059588695e-75	100	100
TP53-regulon	Naive 2
0.0938250885915372	-0.00546078990985582	128466022.5
0.441867514029264	1.87437053284861e-58
3.97254650245526e-58	100	100
USF2-regulon	Naive 2	0.132644079252153	0.00886657541084916
	197964310	0.680911542398057	0	0	100
	100
XBP1-regulon	Naive 2
0.192521415235118	-0.00333611076274126	157102383
0.540364199602142	4.45026983744113e-29

6.79503566577033e-29	100	100
YBX1-regulon	Naive 2
0.0936940840004509	-0.00179513022003032	135493046.5
0.46603743511407 4.63324726584135e-21	6.14879543691095e-21	100
	100
YY1-regulon	Naive 2 0.63104953981379 -0.0101486456462108
121109868	0.416565526480531	2.06641854131142e-118
6.37894419274394e-118	100	100
YY2-regulon	Naive 2 0.0619972438752862	0.0132497039249072
	179778567.5	0.618360459451192
1.98455546048057e-236	1.56559375215689e-235	96.6196088589561
89.2816359049263
ZBTB33-regulon	Naive 2 0.375064521165253	0.0111328246491484
	185199534.5	0.637006239598426	0	0	100
	100
ZNF143-regulon	Naive 2 0.0254888053140338	0.00260057258277177
	172068844.5	0.591842349295923
4.67300963770085e-143	1.74622991724611e-142	100
99.9920329305537
ZNF274-regulon	Naive 2 0.0695084872441402	0.00502137343129905
	175087055	0.602223687057447
9.71909412935524e-177	4.60037122122815e-176	100	100
ZNF76-regulon	Naive 2 0.0154917004363083	0.00112622832652083
	159019669	0.546958833591865
9.31705396992956e-39	1.55649607497647e-38	99.6891594353063
99.4130925507901
ATF1-regulon	Naive IER2
0.103055148934322	-0.00325087998287522	22164572
0.450302030199444	1.42732701211367e-08
2.15617484808661e-08	100	100
ATF3-regulon	Naive IER2
0.0503477747759822	-0.000690938657397333	25673826.5
0.521597087276862	0.013738581410263	0.0157328916149787
	100	100
ATF4-regulon	Naive IER2
0.105604591682537	-0.00322611104235529	23495478.5
0.477341122086968	0.00973317393864056	0.0112907812238142
	100	100
ATF5-regulon	Naive IER2
0.110153875163709	-0.00848756560340466	20796800.5
0.422513978018742	9.53440328197904e-19
2.02072427767317e-18	100	99.9977408277607
ATF6-regulon	Naive IER2
0.133380814033377	-0.00310932980165976	26371827.5
0.535777882980079	4.46642981273591e-05
5.76575484916818e-05	100	100
ATF6B-regulon	Naive IER2	0.0592273764232438
0.0019793721898586	27043317.4121094 0.549420071544843
1.71245350690566e-08	2.55966734716424e-08	100
99.9186697993855

BACH1-regulon	Naive IER2	0.047220389180006
0.00403647247768725	27493238.5	0.558560802045152
2.36919956844817e-11	3.91193417115861e-11	99.910071942446
99.5549430688596
BATF-regulon	Naive IER2
0.0192677048821272	-2.62927613624633e-05	25363519
0.51529278790956 0.0640094753914308	0.0699180423506398
53.8669064748201 51.6627507681186
BATF3-regulon	Naive IER2
0.0658370524007427	-0.0177618403578975	17078665.5
0.3469752426416	2.95541550002954e-68	1.31146562813811e-67	100
100
BCL11A-regulon	Naive IER2	0.21787280638548 0.013708417923018
32365672 0.657550608708768	3.05495478708351e-72
1.60667992505873e-71	100	100
BHLHE40-regulon	Naive IER2	0.0597481379441849
0.00419988543418495	27967089.5	0.568187699749833
7.27627654017927e-15	1.37764169160728e-14	100	100
BRF2-regulon	Naive IER2
0.0375496626185022	-0.00416473513652753	21208560
0.430879406361049	3.11976258832696e-15
6.06857928140312e-15	99.5503597122302 99.7334176757636
CEBPB-regulon	Naive IER2
0.13867501102544 -0.00452797423969484	22419482 0.455480857497266
3.78984753601596e-07	5.27606225602221e-07	100	100
CEBPG-regulon	Naive IER2
0.0603961181518532	-0.00785716602627801	21113938.5
0.428957047853494	5.25969040431801e-16	1.0373278297405e-15
100	100
CLOCK-regulon	Naive IER2	0.0172560505988654
0.00170305917428646	25658155.5	0.521278710584758
0.00978004289105034	0.0112907812238142	54.3165467625899
51.0121091632026
CREB1-regulon	Naive IER2	0.0466525334514206
0.00377381960030827	27958576 0.568014736954337
8.50286880108007e-15	1.58869390757022e-14	100
99.9932224832821
CREB3-regulon	Naive IER2
0.230526481564216	-0.00950650158185443	19898669
0.404267271615565	9.02640072590797e-28
2.28883732692666e-27	100	100
CREB3L2-regulon	Naive IER2
0.124740065879022	-0.00208831391795469	27288570.5
0.554402706147029	5.40589704195306e-10
8.52930422174816e-10	100	100
CREB3L4-regulon	Naive IER2	0.0141071784018296
0.00308134632994188	26376158.5	0.535865872862888
6.51325439502506e-07	8.97943809799572e-07	37.5
31.1923911078981
CREB5-regulon	Naive IER2

0.0362216117589015	-0.00302687871436812	24024026
0.488079250136851	0.173815275991042	0.181483596990646
	99.8201438848921 99.6181998915597
CREM-regulon	Naive IER2
0.143948307588687	-0.00620326993032255	18641965.5
0.378735709922935	1.56446118597845e-43
4.72666996614767e-43	100	100
CTCF-regulon	Naive IER2
0.362930969261824	-0.00810797938788016	20384888.5
0.414145451441124	1.18052848152772e-22
2.74811548158911e-22	100	100
CUX1-regulon	Naive IER2
0.075872999638201	-0.000845793176756746	25370643
0.515437521210214	0.0781914794404933	0.084757176187405
	100	100
DDIT3-regulon	Naive IER2	0.0858271019984974
0.000942061261668667	25779729 0.523748633932182
0.00673850176611877	0.00797389375657388	100	100
E2F1-regulon	Naive IER2
0.402057130850105	-0.0365330903931071	13093854.5
0.26601863841477 5.36428904314043e-157	5.85945418558417e-156	100
	100
E2F2-regulon	Naive IER2
0.0511010271406896	-0.0028908078628158	23052798
0.468347493521539	0.0003047162171481
0.000376258285522002	100	100
E2F3-regulon	Naive IER2	0.0231879748128689
0.00212474212544142	28501761.5	0.579050254961402
1.8998313275993e-19	4.21525075811094e-19	100	100
E2F4-regulon	Naive IER2
0.367257865130643	-0.0321884786248593	14357584.5
0.291692952569085	7.52444597904968e-125
5.34235664512528e-124	100	100
E2F6-regulon	Naive IER2
0.0592206313372646	-0.013511710692667	18101952
0.367764635210321	1.9771111757914e-51
7.79860519339942e-51	100	100
E2F7-regulon	Naive IER2	0.0325742627531492
0.00205048541596473	28125300.5	0.571401961432842
3.75220193462664e-16	7.61160963881404e-16	100	100
E2F8-regulon	Naive IER2
0.067091122984974	-0.00789906505152452	18174849.5
0.369245642479329	2.5222509083381e-50
9.67998997254082e-50	100	100
EGR1-regulon	Naive IER2	0.0606741752149668
0.0135430579517263	41320952.5	0.83948874810327 0	0
	100	100
EGR2-regulon	Naive IER2	0.0667659619323777
0.0212385320449638	37869722 0.769372523849708
2.06870496487783e-207	3.67195131265815e-206	100

99.9954816555214
EGR3-regulon	Naive IER2	0.0568364240646317
0.0157512877071368	36032118.5	0.732039225162433
1.95590860248605e-154	1.98385015395014e-153	100
99.9954816555214
ELF1-regulon	Naive IER2
0.692926260975185	-0.0144730609826585	19275539.5
0.391607587551863	3.96384622006873e-35
1.10365914362698e-34	100	100
ELF2-regulon	Naive IER2	0.0481236591274094
0.0158561740052438	36593197 0.743438262673794
8.92786596443728e-170	1.15250633359099e-168	100
99.5233146575095
ELF4-regulon	Naive IER2	0.0206016721892196
0.0022086974006711	27697737.5	0.562715464489063
8.3508062921323e-13	1.41168392081284e-12	99.3705035971223
98.3101391650099
ELK1-regulon	Naive IER2
0.0628419692345574	-0.00329371911633701	21217211
0.431055162647399	3.66254336097876e-15
7.02812374674303e-15	100	100
ELK3-regulon	Naive IER2	0.0699270212480735
0.00514771688913307	28894101.5	0.587021150971867
3.13530928042837e-23	7.54599860713269e-23	100	100
ELK4-regulon	Naive IER2	0.0481331402410539
0.00321489215381972	26424254.5	0.536843005488976
2.62797015276727e-05	3.45529408974956e-05	97.7517985611511
96.7038677028737
ESRRA-regulon	Naive IER2
0.0773548911058601	-0.014742109626654	15917374
0.323382099489395	2.66578885609705e-90
1.64583485898166e-89	100	100
ETS1-regulon	Naive IER2
0.238886774803557	-0.00415403114626378	23763445.5
0.482785219276233	0.0495247369609796	0.0545156019260395
100	100
ETV2-regulon	Naive IER2	0.0340794792635027
0.00561505805806952	32277114.5	0.655751448226924
1.21121498963708e-70	6.14259030458803e-70	100	100
ETV3-regulon	Naive IER2	0.0126336070573483
0.00123481224239713	26294118 0.534199113689349
9.25625280530369e-05	0.000116317513128595	86.4208633093525
84.2964937646846
ETV5-regulon	Naive IER2	0.0353274624161882
0.00191064311698332	27823410 0.565268664338365
9.58125985564984e-14	1.72220113861048e-13	100	100
ETV6-regulon	Naive IER2	0.465246485063484
0.00581407163159442	26977527 0.54808345398505
4.11424366155636e-08	5.90123838324245e-08	100	100
ETV7-regulon	Naive IER2

0.0639435433427844 -0.000135865713256203 25857254.5
0.525323665024243 0.00386229569507605 0.00464784736187118
100 100
FLI1-regulon Naive IER2 0.155232406797076
0.0115847921511826 30654875 0.622793548551724
1.36155214702777e-44 4.29645344173208e-44 100 100
FOS-regulon Naive IER2 0.100109304383603
0.0141792616614273 38454105.5 0.781245032665355
6.59659209009127e-226 1.33816582398994e-224 100 100
FOSB-regulon Naive IER2 0.0812888778825248
0.0408156381587934 44119566 0.896346211481926 0 0
100 99.3064341225375
FOSL1-regulon Naive IER2
0.0546585353870082 -0.00628929899168629 23187572.5
0.47108561230719 0.000970772068685802 0.0011883589116671 100
100
FOXO1-regulon Naive IER2 0.0218600484345053
0.00529276979458557 27612009 0.56097377881176 1.7497743943235e-13
3.03009712187729e-13 60.6115107913669 52.1349177661305
FOXO3-regulon Naive IER2 0.0596865074362217
0.0409460324296688 40175259 0.81621249855348 0 0
87.589928057554 45.7346828122176
GABPA-regulon Naive IER2 0.0277183653089999
0.00228647136313525 28461833 0.578239055692009
4.40287714767232e-19 9.47285689347681e-19 100 100
GTF2B-regulon Naive IER2
0.0747438623805913 -0.0044214723350172 21775033
0.442388040137202 4.93144363924324e-11
8.04902295140851e-11 100 100
HINFP-regulon Naive IER2 0.0188493312520496
0.00268457426060951 26303140 0.534382407321928
5.72224054260631e-05 7.32034375720808e-05 65.4676258992806
63.1777516717875
HIVEP3-regulon Naive IER2 0.085461796561606
0.00796912321681326 27890680.5 0.566635351803502
2.90515667626258e-14 5.28887497473445e-14 100
99.6859750587385
HOXB2-regulon Naive IER2 0.0432154199205294
0.00626625850109962 27802108.5 0.56483589673535
1.23325606450476e-13 2.18902951449595e-13 88.8489208633094
83.6006687149828
IKZF1-regulon Naive IER2
0.141277489147638 -0.00265162557215975 21111794.5
0.428913489712477 5.04897310162851e-16 1.0097946203257e-15
100 100
IRF1-regulon Naive IER2 0.148640416380036
0.0135295319727374 36296476 0.737409990677258
1.42850886965279e-161 1.69040216242247e-160 100 100
IRF2-regulon Naive IER2 0.0916636744080671
0.00681014474608863 29873439.5 0.606917672756788

3.17314088735094e-34	8.5016227547893e-34	100	100
IRF3-regulon	Naive	IER2	0.147435108625449
0.00203853690270112	27022447	0.548996062051497
2.27074079140446e-08	3.32417724102509e-08	100	100
IRF4-regulon	Naive	IER2
0.0551430441569553	-0.0033514373935441	22921749.5
0.465685073258942	9.03985860945043e-05
0.000114612493084104	100	99.9954816555214
IRF5-regulon	Naive	IER2	0.0543034694057786
0.00744106640654218	27795096	0.564693428701824
1.27706971527721e-13	2.23881357493042e-13	91.5467625899281
80.2006144948491
IRF7-regulon	Naive	IER2	0.115915911771861
0.00828423284012281	32737313.5	0.665100987843378
3.79262595773914e-79	2.15421154399583e-78	100	100
IRF8-regulon	Naive	IER2
0.081419818168316	-0.000361977193346366	25502060.5
0.518107438186447	0.0388388532436958	0.0434261193748409
100	100
IRF9-regulon	Naive	IER2	0.0397234188333224
0.00595308609408338	31889584.5	0.647878273605587
7.28674628180972e-64	3.13550900611206e-63	100	100
JUN-regulon	Naive	IER2	0.108420914747428
0.0205235896185669	41126695	0.835542155016272	0	0
100	100
JUNB-regulon	Naive	IER2	0.19002292615431	0.0310001354681884
42266646.5	0.858701748388024	0	0	100
100
JUND-regulon	Naive	IER2	0.0557838765558418
0.0177614572406631	42414844.5	0.86171258298801	0	0
100	100
KLF11-regulon	Naive	IER2	0.0267940977167504
0.00497964270452773	26224411	0.532782925566288
2.67216101261237e-05	3.48116388799043e-05	46.1330935251799
40.7260979577083
KLF12-regulon	Naive	IER2	0.0478559998742906
0.000826632778518133	25200663	0.511984157026448
0.171536826086622	0.180431328180003	99.1007194244604
98.5902765226821
KLF13-regulon	Naive	IER2	0.0693002797116579
0.00308375246481983	30730779	0.624335636768012
1.12586644392118e-45	3.63347806901836e-45	100	100
KLF2-regulon	Naive	IER2	0.126382155020419
0.0179742716761676	35321689	0.717605928360511
4.60275708812888e-136	3.84465592067236e-135	100	100
KLF3-regulon	Naive	IER2	0.104326315924057
0.0331084662278886	37843417.5	0.768838113812222
1.34753420970925e-206	2.1261095308746e-205	100
99.8599313211639
KLF4-regulon	Naive	IER2	0.0275097409339083

0.0119016144062501	30430544 0.61823597330341 3.2241367892692e-47
	1.17391647199033e-46	65.4676258992806 49.4193927345021
KLF6-regulon	Naive IER2	0.0839047219660382
0.0148897380706899	33090589.5	0.672278248023306
5.20243692327453e-86	3.07810851293743e-85	100	100
KLF7-regulon	Naive IER2	0.0663345266048588
0.0208466642477628	35660268 0.724484599921725
1.12706682558234e-144	1.06695659488461e-143	99.6402877697842
97.5036146755829
KLF8-regulon	Naive IER2	0.0354203084418912
0.0105726825753786	31114659.5	0.632134667063024
6.47014070177305e-52	2.62502851329078e-51	91.4568345323741
77.2885414784023
MAFB-regulon	Naive IER2	0.0645967690749586
0.00903528553926054	32143030 0.653027347686283
2.93988538480613e-68	1.31146562813811e-67	100
99.9977408277607
MAFF-regulon	Naive IER2	0.0178305071678698
0.00558031962981552	26734643.5	0.543148960634493
6.53583448480844e-10	1.01987746905802e-09	35.6115107913669
28.3684258087837
MAFG-regulon	Naive IER2	0.0275761705099298
0.00222330287581463	25701611.5	0.522161575592228
0.00871079780039383	0.0102225891541812	61.6007194244604
58.5261160310862
MAX-regulon	Naive IER2	0.202249989142664
0.00469483236357809	29142814.5	0.592074078176461
8.20966271061004e-26	2.00995190501142e-25	100	100
MAZ-regulon	Naive IER2
0.128087414369775	-0.00842281107702753	17000232.5
0.345381774509906	1.20371756729377e-69
5.89406532950742e-69	100	100
MEF2A-regulon	Naive IER2	0.0465653324354139
0.00595700151963501	30241190.5	0.614389011337469
6.29432281081574e-39	1.86207049819966e-38	100
99.9977408277607
MLX-regulon	Naive IER2	0.0533181725596847
0.000402580558037227	25141140.5	0.510774880231365
0.218954420966362	0.226945458227908	100
99.9887041388035
MXD4-regulon	Naive IER2
0.0614971320515581	-0.0314425392646916	20677856
0.420097466216436	7.3144700253822e-20
1.64865832318139e-19	82.0143884892086 88.7696547984818
MXI1-regulon	Naive IER2	0.0869836362415831
0.0223005906804348	37820094 0.768364266656438
7.07622618924483e-206	1.00482411887277e-204	100	100
MYBL1-regulon	Naive IER2
0.0227542438382437	-0.00731524501782968	23443922
0.476293684914711	0.00661844938084628	0.00789764547966531

80.3057553956834 80.0537682992951
MYC-regulon Naive IER2
0.289370578094755 -0.0261520727688696 19053166
0.387089781455154 5.6835113401434e-38
1.64705838836809e-37 100 100
NFATC1-regulon Naive IER2 0.0507722434485079
0.000260792365043526 25552055.5 0.519123151460758
0.0291150846338687 0.0330747361440749 94.7841726618705
94.8287547442617
NFE2L1-regulon Naive IER2
0.146138546454548 -0.0374462673043997 18400024
0.373820354524261 5.48814552929585e-47
1.90077235404881e-46 100 99.9344840050605
NFE2L2-regulon Naive IER2
0.109298928489174 -0.00162133305494308 25056234.5
0.50904990470844 0.30183358882125 0.308347982824587 100 100
NFKB1-regulon Naive IER2
0.0540640981190951 -0.00267013375529857 24681452
0.501435712084589 0.86988833954662 0.876057760394468 100
99.929965660582
NFKB2-regulon Naive IER2 0.0763897039216764
0.00308880634113266 27774067.5 0.564266207447922
2.26440308701245e-13 3.87403901633456e-13 100 100
NFYA-regulon Naive IER2 0.0239235021933432
0.00304983645465081 28388919 0.576757713204098
1.99896097449925e-18 4.17430085851314e-18 99.6402877697842
99.0014458702332
NFYB-regulon Naive IER2
0.0968741682570224 -0.0120459302007714 16578118.5
0.336805981069112 2.25687788082004e-77 1.2326025349094e-76
100 100
NFYC-regulon Naive IER2
0.0784988935769003 -0.00516235565614892 19922621.5
0.404753897722234 1.66102879431846e-27
4.13800155777582e-27 100 100
NR1H3-regulon Naive IER2 0.0100651458528609
0.00156921328032193 25559654.5 0.519277535002542
0.00245624671110854 0.0029810857519437 25.8093525179856
22.0111151274173
NR2C2-regulon Naive IER2 0.0239199301151334
0.00218736598830165 26432085.5 0.537002102411691
2.12663170828402e-05 2.82225890258253e-05 79.7661870503597
75.883336345563
NR3C1-regulon Naive IER2 0.229573267146124
0.00419857349328157 27361360.5 0.555881529414098
1.82289945432903e-10 2.9414968467582e-10 100 100
NRF1-regulon Naive IER2
0.0258494283323058 -0.000901838392153083 24105082
0.48972600791588 0.241130248443284 0.248119531006858
99.910071942446 99.9683715886499

PAX5-regulon	Naive IER2	0.104001102070847
0.00382721405727014	27180518 0.55220747945291
2.57871099444891e-09	3.98018436099723e-09	100	100
POU2F1-regulon	Naive IER2	0.00615810519005609
0.00131023335075047	26178373.5	0.531847614037814
7.31898944685654e-08	1.03929650145363e-07	24.4604316546763
18.1931140430146
POU6F1-regulon	Naive IER2	0.0358436258342111
0.00496312400818403	26708449.5	0.542616795547838
6.81539601300414e-07	9.3056368639095e-07	70.863309352518
65.2155250316284
REL-regulon	Naive IER2
0.259127121005556	-0.00470004187145828	22238234.5
0.451798579435747	3.81225538413528e-08
5.52388025048173e-08	100	100
RELA-regulon	Naive IER2	0.0959969144231611
0.00403124381801338	29590525 0.601169897716383
8.050359705362e-31	2.11694644103964e-30	100	100
RELB-regulon	Naive IER2
0.0991879882215737	-0.00614631430293847	19128416.5
0.388618592971276	5.36644258671907e-37
1.52406969462822e-36	100	100
REST-regulon	Naive IER2	0.221790015629368
0.00325121904969131	27566182 0.560042743863828
7.36845295282047e-12	1.23096508153001e-11	100	100
RFX5-regulon	Naive IER2
0.063192895409136	-0.00220973152629739	24634614.5
0.500484147518421	0.955950769454464	0.955950769454464
	100	100
RXRA-regulon	Naive IER2	0.0216323278373638
0.00728061023017895	29766082 0.604736565889165
2.10047206837806e-34	5.73590449441702e-34	74.5503597122302
64.0046087113681
SETDB1-regulon	Naive IER2
0.0585052755758041	-0.00124270551434897	24003446.5
0.487661150900353	0.159180032385588	0.168683317901146
	93.1654676258993 95.0998554129767
SOX5-regulon	Naive IER2	0.0104693384631511
0.00273301105884754	26077807 0.529804475143904
3.29520383914366e-06	4.41432967130566e-06	27.9676258992806
22.3567684800289
SP1-regulon	Naive IER2	0.0216039772585879
0.00882640650965402	31282092 0.635536275479887
1.48407857193023e-57	6.19821050629682e-57	76.0791366906475
59.4139707211278
SP2-regulon	Naive IER2	0.0286816494135892
0.000452583530333603	25299285.5	0.513987801038764
0.110516105563665	0.11799463902286 100	100
SP3-regulon	Naive IER2	0.0327413104834819
0.0045837409199695	32185507.5	0.653890333197024

5.21543072218602e-69	2.46863720850138e-68	100	100
SP4-regulon	Naive	IER2	0.0131900052113683
0.00466643825195517	27245065	0.553518835482852
2.56238359280934e-14	4.7254346776484e-14	38.1294964028777
28.7863726730526
SPI1-regulon	Naive	IER2
0.414567363186047	-0.00407548987433565	21927624.5
0.445488134388567	4.99381625513562e-10
7.96766189021638e-10	100	100
SPIB-regulon	Naive	IER2
0.546106911182564	-0.0188051545315081	15577006.5
0.316467092230788	2.34358227948331e-97
1.51267583493922e-96	100	100
SREBF1-regulon	Naive	IER2	0.0443351681889132
0.00349083572496019	26812017	0.544720903649392
3.35518122437569e-07	4.71718548377572e-07	97.4820143884892
96.8800831375384
SREBF2-regulon	Naive	IER2	0.102252191998654
0.00335169581353077	28281536.5	0.574576098429046
1.76285585211604e-17	3.62790624638374e-17	100	100
SRF-regulon	Naive	IER2	0.0243354934373112
0.00365591028390685	28882347	0.586782343057417
4.11695523667599e-23	9.74346072679985e-23	99.4604316546763
97.8967106452196
STAT1-regulon	Naive	IER2	0.0848338804934895
0.0051926224546163	30761107.5	0.624951799585093
4.12286647590441e-46	1.39392152280578e-45	100	100
STAT2-regulon	Naive	IER2	0.0755877613745902
0.00958838297013112	30825053	0.626250935362319
4.87880254147115e-47	1.73197490222226e-46	100
99.961594071932
STAT5A-regulon	Naive	IER2
0.0650905027676279	-0.00668105380770329	20398702
0.414426090611335	1.61976941972643e-22
3.70979447743794e-22	100	100
TBP-regulon	Naive	IER2	0.0220016186033649
0.00112799642476875	25295032	0.513901385668982
0.0898176467802267	0.0966220139605469	52.2482014388489
49.8260437375746
TCF12-regulon	Naive	IER2	0.0108309514916284
0.00183213716530342	25282894.5	0.513654796612737
0.041114013459255	0.045610858681361	27.2482014388489
25.1694379179469
TFDP1-regulon	Naive	IER2
0.499516469172808	-0.0293664987275548	13770273
0.279760957635482	2.5067150042572e-139
2.22470956627826e-138	100	100
TFDP2-regulon	Naive	IER2	0.0280831915877154
0.000984155934654604	27033823	0.549227180247488
1.95016267593001e-08	2.88461562481314e-08	100

99.9954816555214
TFEC-regulon	Naive	IER2
0.0888336877439296	-0.00277588443258096	23689081.5
0.481274418157504	0.0326456318029633	0.0367911088573078
	100	100
THAP1-regulon	Naive	IER2	0.0386968777963919
0.00157024574581064	27172301.5	0.552040550597657
2.89675206625526e-09	4.4229977785833e-09	100	100
THAP11-regulon	Naive	IER2
0.177075670273541	-0.00143726170186309	22532403.5
0.457775004242043	1.45382226685438e-06
1.96612154184116e-06	100	100
TP53-regulon	Naive	IER2
0.0893143079156647	-0.00926954699087314	18641733
0.378730986383855	1.55277501606972e-43
4.72666996614767e-43	100	100
USF2-regulon	Naive	IER2	0.130396218937907
0.00523838757175746	29548202.5	0.600310061231694
2.50690640260178e-30	6.4723765303537e-30	100	100
XBP1-regulon	Naive	IER2	0.19743348320845	0.00219746849780697
	30991194	0.629626305281457	1.72083037225277e-49
6.43047139104983e-49	100	100
YBX1-regulon	Naive	IER2
0.093659895655519	-0.00156214889695626	22948879.5
0.466236254399697	0.00011709477913947
0.000145854900331621	100	100
YY1-regulon	Naive	IER2
0.611500406505434	-0.0286736111695289	14029690.5
0.285031360642554	7.81416081555664e-133
6.16450464338358e-132	100	100
YY2-regulon	Naive	IER2	0.0732363679350018
0.0227928812268669	33699624	0.684651573879158
1.32114003105357e-98	8.93342306712415e-98	97.2122302158273
90.3623712271824
ZBTB33-regulon	Naive	IER2	0.367184343696422
0.00139245389793075	24926414	0.506412432858701
0.464413840274691	0.471048323707186	100	100
ZNF143-regulon	Naive	IER2	0.0269108958518955
0.00367010077829127	30755191	0.624831598213206
5.01737813770457e-46	1.65690161756756e-45	100
99.9932224832821
ZNF274-regulon	Naive	IER2	0.0765686593239556
0.0115091764153549	35024755	0.711573328992689
9.84628276799009e-129	7.35880080555049e-128	100	100
ZNF76-regulon	Naive	IER2	0.0165330195335459
0.00202555175744165	28475578.5	0.578518313354016
3.29980078673495e-19	7.20879556486711e-19	99.4604316546763
99.4600578348093
ATF1-regulon	Naive	IFN1
0.100754807787662	-0.00553264025477078	8939001.5

0.397600409209436 2.88634897902108e-15
7.73323688718856e-15 100 100
ATF3-regulon Naive IFN1
0.0467041703345378 -0.0043658140137273 9972831.5
0.443584430025742 1.36116497645155e-05
2.32874008019422e-05 100 100
ATF4-regulon Naive IFN1
0.102179385949706 -0.00664563152115363 9424195.5
0.419181492168866 4.61484928954184e-10
1.04017237954752e-09 100 100
ATF5-regulon Naive IFN1
0.107104488343467 -0.0114554335426059 8853066
0.393778059479926 2.60242078891155e-16
7.24595592206744e-16 100 99.9977715877437
ATF6-regulon Naive IFN1
0.130940290607503 -0.00553476468664346 10643529
0.473416576318116 0.0403872280829009 0.0526145540162562
100 100
ATF6B-regulon Naive IFN1 0.0590564901942148
0.0017796277901611 12488801.2675781 0.555492970274632
1.87553610527742e-05 3.17054913034992e-05 100
99.9197771587744
BACH1-regulon Naive IFN1 0.0442678811284809
0.000996042616297363 11505492 0.511756075592548
0.364684958011719 0.401436155330729 100
99.5587743732591
BATF-regulon Naive IFN1
0.0144858507620027 -0.00486117516086274 10482089
0.466235840297122 0.00571618305677411 0.00803661380259331
47.3053892215569 51.7660167130919
BATF3-regulon Naive IFN1
0.063417372681198 -0.0199666961238113 7236049.5
0.32185431921672 6.15600614953281e-43 4.85640485129811e-42 100
100
BCL11A-regulon Naive IFN1 0.222854178132567
0.0185587551050765 16229260 0.721865906070867
1.30674675684262e-65 2.06175599412946e-64 100 100
BHLHE40-regulon Naive IFN1
0.0556002388140931 -5.15062299338634e-05 11167230
0.496710423164812 0.799767740385563 0.828956344049269
100 100
BRF2-regulon Naive IFN1
0.0363400207513596 -0.00533117650020536 9315694
0.414355422859017 4.00762309178994e-11
9.48470798390286e-11 99.6007984031936 99.7303621169916
CEBPB-regulon Naive IFN1
0.135059669499613 -0.00812202760605582 8768673.5
0.390024341289566 2.25403424924166e-17
6.95810572591989e-17 100 100
CEBPG-regulon Naive IFN1

0.0601017723832119	-0.00804781795175567	9503401
0.422704496299879	2.52202334519271e-09
5.26657816202008e-09	100	100
CLOCK-regulon	Naive IFN1
0.0152469692551477	-0.000351640418092854	11218928.5
0.499009935560634	0.935252230215393	0.948612976361328
51.2974051896208 51.0908077994429
CREB1-regulon	Naive IFN1	0.0471525717155813
0.00422805765042012	13044073 0.580191060775385
6.2778874932528e-10	1.39290628756547e-09	100
99.9933147632312
CREB3-regulon	Naive IFN1
0.228212119695222	-0.0117172650737974	8331806.5
0.370592808811347	1.89881809697505e-23
7.09558341501202e-23	100	100
CREB3L2-regulon	Naive IFN1
0.124463006764658	-0.00234003257131425	12277168.5
0.54607969576168 0.000380769544533253	0.000575205056635339	100
100
CREB3L4-regulon	Naive IFN1	0.0139720558882236
0.00290276088626535	12059460.5	0.536396199244964
0.00064429079441482	0.000963045187441099	38.12375249501
31.2713091922006
CREB5-regulon	Naive IFN1
0.0344483469548989	-0.00477872809268432	10267712.5
0.456700526523555	0.000841740884785347	0.00124507505874499
99.6007984031936 99.6233983286908
CREM-regulon	Naive IFN1
0.141866227124249	-0.0082241342250727	7692902
0.342174792476329	4.52245101357607e-34
2.37847423676964e-33	100	100
CTCF-regulon	Naive IFN1
0.361908947225608	-0.0090310166359987	8988058.5
0.39978242956983 1.09700815919778e-14	2.78169926082295e-14	100
100
CUX1-regulon	Naive IFN1
0.0753347976478039	-0.00137848785943662	11279008.5
0.501682251096692	0.896794583119493	0.916149861891857
100	100
DDIT3-regulon	Naive IFN1
0.0829792609609404	-0.00195040078515167	10906450
0.485111114817718	0.250953857501529	0.28971908752209 100
100
E2F1-regulon	Naive IFN1
0.401420669303438	-0.0366792377114666	6086120
0.270706275471341	5.96369710293208e-70
2.11711247154089e-68	100	100
E2F2-regulon	Naive IFN1
0.0511866958044371	-0.0027648226842 10513472.5
0.467631755986634	0.0125667548227153	0.017158453700246

100	100
E2F3-regulon	Naive IFN1	0.0231124116336311
0.00201940569625499	12808120	0.569696039675524
7.69869653664279e-08	1.49755466877161e-07	100	100
E2F4-regulon	Naive IFN1
0.364608364363712	-0.0344292939198002	6250654.5
0.278024652644572	1.13049020994773e-65
2.00662012265722e-64	100	100
E2F6-regulon	Naive IFN1
0.0565521084159405	-0.0160260558925507	7302116.5
0.324792932241367	1.37096865987794e-41	9.7338774851334e-41
100	100
E2F7-regulon	Naive IFN1	0.0327911936991595
0.00224191966704019	12983485	0.577496149761758
2.29393575303604e-09	4.86177428255399e-09	100	100
E2F8-regulon	Naive IFN1
0.0657123089441472	-0.00918572214723648	7410290.5
0.329604434584869	1.97618745309857e-39
1.33627913495237e-38	100	100
EGR1-regulon	Naive IFN1	0.0496192612667627
0.00218032628103635	12840227.5	0.5711241583685
4.15417333053745e-08	8.30834666107489e-08	100	100
EGR2-regulon	Naive IFN1	0.046402240825963
0.000358287989161127	11765928	0.523340083065068
0.0719108337216335	0.0919940395357834	100
99.9955431754875
EGR3-regulon	Naive IFN1	0.041561644779481
9.15122947231645e-05	11666747	0.51892858294553	0.144420767116333
0.176790939056201	100	99.9955431754875
ELF1-regulon	Naive IFN1
0.690688976814782	-0.0165382635632055	8392255.5
0.373281537204143	1.50143307152712e-22
5.33008740392126e-22	100	100
ELF2-regulon	Naive IFN1	0.0413629395665324
0.00880408413089159	14520719	0.64587122134561	2.3773154689615e-29
1.08896385997591e-28	100	99.5298050139276
ELF4-regulon	Naive IFN1	0.0236278332487367
0.00523857082963682	14197987.5	0.631516354477674
3.63902746351832e-24	1.43539416616556e-23	99.4011976047904
98.3242339832869
ELK1-regulon	Naive IFN1
0.0634367749937314	-0.00264742678188884	9857102
0.438436864432694	2.06509502177836e-06
3.85846701437536e-06	100	100
ELK3-regulon	Naive IFN1	0.0678429933665993
0.00297033295221864	12321892	0.548068965133799
0.000210182873785762	0.000320924387930948	100	100
ELK4-regulon	Naive IFN1	0.0464802688994306
0.00149979490360286	11612294.5	0.516506574594544
0.203091446826558	0.238338722722076	96.6067864271457

96.7309192200557
ESRRA-regulon	Naive IFN1
0.0761455902937064	-0.0157641888393648	6887836
0.30636603116886 2.08590664505122e-50	1.97465829064849e-49	100
100
ETS1-regulon	Naive IFN1
0.24044581941792 -0.00252102119747913	11121150.5
0.494660839880128	0.680570376318431	0.721201443561322
100	100
ETV2-regulon	Naive IFN1	0.0324514916820296
0.00389244268268452	13852918.5	0.616167931546378
3.32544936178796e-19	1.07321320312248e-18	100	100
ETV3-regulon	Naive IFN1	0.0127034064237626
0.00128857816485525	12208422 0.543021900488716
0.000888423961134752	0.00130057940702201	87.4251497005988
84.3142061281337
ETV5-regulon	Naive IFN1
0.0332003253893166	-0.000266256552672373	11076448.5
0.49267252681267 0.572075262676176	0.615414297727401	100
100
ETV6-regulon	Naive IFN1	0.466828347571107
0.00733443253140509	12800513.5	0.569357707982364
8.89494664390718e-08	1.70686813977678e-07	100	100
ETV7-regulon	Naive IFN1	0.0644485089110703
0.000376587358105099	12112320.5	0.538747374332116
0.00281092455237525	0.00399151286437285	100	100
FLI1-regulon	Naive IFN1	0.156950679966518
0.0131645148326803	14394616.5	0.640262272113155
2.91649966263653e-27	1.18326557741253e-26	100	100
FOS-regulon	Naive IFN1	0.0935195298294603
0.00732285744874943	14909916 0.663182426233883
2.63534268431649e-36	1.55924442155393e-35	100	100
FOSB-regulon	Naive IFN1	0.0476746606561959
0.00627041084343027	13365391.5	0.594483078411422
3.20939283930012e-13	7.99532952948452e-13	99.4011976047904
99.3225626740947
FOSL1-regulon	Naive IFN1
0.0510675691147821	-0.0098347234793657	9171648.5
0.407948381787956	1.26737528735081e-12	3.1028843242037e-12
100	100
FOXO1-regulon	Naive IFN1	0.0237886760620389
0.0071708650565905	12961080.5	0.576499613586198
4.20047159798328e-10	9.62043495021976e-10	62.4750499001996
52.2295264623955
FOXO3-regulon	Naive IFN1	0.0235981239491461
0.00389724101074796	12245577.5	0.544674550620208
0.00018525865734411	0.000285942710248518	54.4910179640719
46.6740947075209
GABPA-regulon	Naive IFN1	0.0284095897498511
0.00295428119545145	13510132 0.600921032586637

7.15468146828808e-15	1.88141623795724e-14	100	100
GTF2B-regulon	Naive	IFN1
0.0712170650086452	-0.00792744309719504	8775040.5
0.390307540907044	2.71873134880081e-17
8.21403939424926e-17	100	100
HINFP-regulon	Naive	IFN1	0.0183841701759869
0.0021776678793136	11873045.5	0.528104593042328
0.0262155608599108	0.0344686077972902	65.4690618762475
63.2089136490251
HIVEP3-regulon	Naive	IFN1	0.0788060081579512
0.00113052290729318	11584194.5	0.515256706642426
0.239436150085392	0.278687977968243	100
99.6902506963788
HOXB2-regulon	Naive	IFN1	0.0421326568895146
0.00508608826735026	12557190	0.558534852300969
6.09121706546569e-06	1.09487699151409e-05	89.0219560878243
83.6701949860724
IKZF1-regulon	Naive	IFN1
0.141675358931559	-0.00221321036235919	9865231
0.438798436553078	2.36928224236357e-06
4.31330869763625e-06	100	100
IRF1-regulon	Naive	IFN1	0.147737741590395
0.0124325667295036	16263840.5	0.723404022039487
1.69435012421749e-66	4.00996196064806e-65	100	100
IRF2-regulon	Naive	IFN1	0.099607226426529
0.0147496571238016	15947484	0.709332710623322
1.31359510850403e-58	1.86530505407573e-57	100	100
IRF3-regulon	Naive	IFN1	0.146015659048124
0.000575484199229759	11617274.5	0.516728081441574
0.197102561383761	0.234471503648409	100	100
IRF4-regulon	Naive	IFN1	0.0622722675004607
0.00390301088907113	13287350.5	0.591011870409599
2.25628683238479e-12	5.43038525760408e-12	100
99.9955431754875
IRF5-regulon	Naive	IFN1	0.0521436195911608
0.00515578870709942	12341987.5	0.54896279863671
0.00015095376657815	0.000238171498378859	90.0199600798403
80.3721448467967
IRF7-regulon	Naive	IFN1	0.126589627327793
0.0189643186518363	18199952	0.809520880245081
6.87125681511335e-126	3.25239489248699e-124	100	100
IRF8-regulon	Naive	IFN1	0.0818898662693873
0.000118247223352144	11763769.5	0.523244074525045
0.0730882484441049	0.0926654578487759	100	100
IRF9-regulon	Naive	IFN1	0.0569242555372054
0.023264904047775	19873437.5	0.883956321340606
1.26123811767236e-192	1.79095812709475e-190	100	100
JUN-regulon	Naive	IFN1	0.0978800288016969
0.00958558061733203	14958211	0.665330553377924
3.19264291842473e-37	1.97110997572309e-36	100	100

JUNB-regulon	Naive IFN1	0.170639218711156
0.0109779362099912	14438749.5	0.642225276466565
5.53358832696875e-28	2.31108688949871e-27	100	100
JUND-regulon	Naive IFN1	0.0446409224475277
0.00625226646644794	14738050.5	0.655537971410939
3.86338534023052e-33	1.89172661487149e-32	100	100
KLF11-regulon	Naive IFN1	0.0252976280940657
0.00339866516042946	11685416.5	0.519758988985817
0.0871299132461617	0.109441279188305	43.5129740518962
40.8289693593315
KLF12-regulon	Naive IFN1	0.0474200432827046
0.000374553916624373	11345387 0.504634719419101
0.720816234673969	0.752616950909585	99.4011976047904
98.5938718662953
KLF13-regulon	Naive IFN1	0.0685803894799454
0.00231383799328355	13662016 0.607676724545338
1.01833417834643e-16	2.95109088418761e-16	100	100
KLF2-regulon	Naive IFN1	0.123027850668578
0.0143377882385458	15186841.5	0.675499874902007
1.00863338055317e-41	7.53820737044998e-41	100	100
KLF3-regulon	Naive IFN1	0.0958553469517124
0.0240921328361479	15718110 0.699130318749687
3.31258563827298e-53	4.27624691486148e-52	100
99.8618384401114
KLF4-regulon	Naive IFN1	0.0162782810157839
0.000382714982360911	11414752.5	0.50772004737044
0.524201870367065	0.568218821313917	52.2954091816367
49.7849582172702
KLF6-regulon	Naive IFN1	0.0753998869706235
0.00608721945869813	12832166.5	0.570765610839602
4.85549713223121e-08	9.57611934412267e-08	100	100
KLF7-regulon	Naive IFN1	0.0575431334604247
0.0116732811351785	14146529 0.629227517110626
2.18147336803281e-23	7.94280046822201e-23	99.8003992015968
97.5309192200557
KLF8-regulon	Naive IFN1	0.0368891845937754
0.0119140043628848	14535325 0.646520885804992
6.50127634302419e-30	3.07727080236478e-29	92.4151696606786
77.4707520891365
MAFB-regulon	Naive IFN1	0.0583707154161183
0.00261670124314848	12521222 0.556935021322258
1.133045132684e-05	1.98632603507566e-05	100
99.9977715877437
MAFF-regulon	Naive IFN1	0.0170152644513567
0.00467999586829322	12128744.5	0.53947790213445
0.000133753887655506	0.000213405079180694	35.5289421157685
28.4679665738162
MAFG-regulon	Naive IFN1
0.0247864119939077	-0.000627873068032494	10985030
0.488606297154994	0.36205210136131 0.401436155330729

56.4870259481038 58.625069637883
MAX-regulon Naive IFN1 0.200993640427255
0.00336053438286421 12536629.5 0.557620335929812
8.87421450222239e-06 1.57517307414447e-05 100 100
MAZ-regulon Naive IFN1
0.127416159815879 -0.00898687813150084 7576213
0.336984549007834 3.10167020741339e-36
1.76174867781081e-35 100 100
MEF2A-regulon Naive IFN1 0.0455508157711157
0.00485005029762512 13159185 0.585311160408987
4.76541439509784e-11 1.10932597394081e-10 100
99.9977715877437
MLX-regulon Naive IFN1 0.0532337202938423
0.000311704062531441 11531831 0.512927615521047
0.318857691928818 0.362222338031137 100
99.9888579387187
MXD4-regulon Naive IFN1
0.0665118098078265 -0.0259437669112842 10070327
0.447920960307797 5.85369347710381e-05 9.4457326562357e-05
85.2295409181637 88.641782729805
MXI1-regulon Naive IFN1 0.0793851300468729
0.0143136162403922 15370638 0.683675012092806
1.56045965654465e-45 1.3034427719373e-44 100 100
MYBL1-regulon Naive IFN1
0.0228070954497166 -0.00716220191079708 10745509
0.477952573960714 0.0878613086441325 0.109441279188305
82.0359281437126 80.0378830083565
MYC-regulon Naive IFN1
0.279350390128833 -0.0359280534414024 6932123
0.308335885332399 2.01176064694486e-49
1.78543757416356e-48 100 100
NFATC1-regulon Naive IFN1
0.044578558057086 -0.00600559231364613 10555757
0.469512540378852 0.0187251574269215 0.0253235462345034
93.4131736526946 94.8434540389972
NFE2L1-regulon Naive IFN1
0.138867398952806 -0.0442887390529477 7708908.5
0.342886750176527 8.84692786640522e-34
4.48665627510551e-33 100 99.9353760445682
NFE2L2-regulon Naive IFN1
0.101472353501049 -0.00951321120902889 8889571.5
0.395401798075159 7.30848006641353e-16
1.99577724890523e-15 100 100
NFKB1-regulon Naive IFN1
0.050179046584365 -0.00656220387709086 10283921.5
0.45742149127928 0.00102672393042913 0.00148770202164221 100
99.9309192200557
NFKB2-regulon Naive IFN1
0.0716851332340194 -0.00171034374683517 10969399.5
0.487911063666539 0.35126420510914 0.392752103350377 100

100
NFYA-regulon Naive IFN1 0.0231449868851658
0.00222110418283695 12623511 0.5614847630644
2.12763965666133e-06 3.92369910708973e-06 99.2015968063872
99.0150417827298
NFYB-regulon Naive IFN1
0.0952108317228007 -0.0135638242447641 6806279
0.302738433995519 3.02401845933643e-52
3.30315862481364e-51 100 100
NFYC-regulon Naive IFN1
0.0771026680628007 -0.00650388055022973 8288042.5
0.368646217314674 4.13801955050394e-24
1.58810480046367e-23 100 100
NR1H3-regulon Naive IFN1 0.015085902644795
0.0066246577566791 12522457.5 0.556989975480793
1.437423551684e-09 3.09263855059285e-09 31.936127744511
21.9944289693593
NR2C2-regulon Naive IFN1 0.0232614160862448
0.00149171778342806 11772961.5 0.523652928127033
0.0662722243456105 0.0855514168825154 77.8443113772455
75.9576601671309
NR3C1-regulon Naive IFN1 0.227545995113998
0.00209150220338442 11616401 0.51668922878477 0.198144932660627
0.234471503648409 100 100
NRF1-regulon Naive IFN1
0.0257875500565671 -0.0009521284285278 10846818.5
0.482458748241678 0.176198886318914 0.213848221002442
99.8003992015968 99.9688022284123
PAX5-regulon Naive IFN1 0.105982221835035
0.00577834191533831 13041672.5 0.580084288248016
6.61392570825917e-10 1.44488838549662e-09 100 100
POU2F1-regulon Naive IFN1 0.00611040362713486
0.00124415961935388 11983777 0.533029851161187
0.000161165289373144 0.000251488693307544 24.750499001996
18.275208913649
POU6F1-regulon Naive IFN1 0.0372461892011825
0.00631377017131926 12397089 0.551413674044668
5.13380663647458e-05 8.37931657907345e-05 71.2574850299401
65.2880222841226
REL-regulon Naive IFN1
0.253873846022881 -0.00994797234567257 8409510.5
0.374049027293602 2.6881459148731e-22
9.31016390029221e-22 100 100
RELA-regulon Naive IFN1 0.0947140104918501
0.00267912930525634 12685294.5 0.564232849065101
7.31528140391184e-07 1.38502661247398e-06 100 100
RELB-regulon Naive IFN1
0.0965733548738974 -0.00870645257023787 7491761.5
0.333228206539567 7.62921827627088e-38
4.92431361468393e-37 100 100

REST-regulon	Naive IFN1	0.220639063526179
0.00204315002520852	11971916.5	0.532502304583034
0.0122052581955417	0.0168266666385139	100	100
RFX5-regulon	Naive IFN1
0.0631984905351351	-0.00217398711627172	11244739
0.50015796818619 0.990283012569109	0.990283012569109	100
100
RXRA-regulon	Naive IFN1	0.0154786072949745
0.00095905755759744	11875506.5	0.528214056566533
0.0259531323961909	0.0344424747687768	70.6586826347305
64.191643454039
SETDB1-regulon	Naive IFN1
0.0583490630510343	-0.00138374186988515	11123661.5
0.494772527368661	0.686876126334569	0.722491925477843
92.6147704590818 95.0796657381616
SOX5-regulon	Naive IFN1	0.00995689720497441
0.00217763714713767	11787561 0.524302303471052
0.0103676792747617	0.0144334358530997	26.9461077844311
22.4445682451253
SP1-regulon	Naive IFN1	0.0155591962956732
0.00259396271354003	12441678.5	0.55339698319239
2.06575197931837e-05	3.45102095368481e-05	69.061876247505
59.7192200557103
SP2-regulon	Naive IFN1	0.0286306251853698
0.000394827454195019	11527619 0.512740268766089
0.325921407983944	0.367308253442223	100	100
SP3-regulon	Naive IFN1	0.0324622882160987
0.00423919317385509	14461180.5	0.643222991343219
2.35686032050893e-28	1.01416413791596e-27	100	100
SP4-regulon	Naive IFN1	0.0113643342403459
0.00275684850815446	12205421 0.542888418149773
3.68694800296185e-05	6.08775135372771e-05	37.125748502994
28.924791086351
SPI1-regulon	Naive IFN1
0.417681424355669	-0.000871172014814847	10753221
0.478295598218605	0.0942171695684866	0.116337722423696
100	100
SPIB-regulon	Naive IFN1
0.544374764535341	-0.020300596021105	6859456
0.305103709016507	4.82284553335453e-51
4.89174332668816e-50	100	100
SREBF1-regulon	Naive IFN1
0.0404196761874689	-0.000515900000621732	11106527.5
0.494010419272875	0.644193242832584	0.687785266783662
97.2055888223553 96.891364902507
SREBF2-regulon	Naive IFN1	0.101845274167912
0.0028945996657916	12519003 0.556836321785399
1.17337671073101e-05	2.03194503565614e-05	100	100
SRF-regulon	Naive IFN1	0.022570302729831
0.00182123496967643	12125153 0.539318154776797

0.00243167091843246	0.00348785121633747	98.8023952095808
97.925348189415
STAT1-regulon	Naive IFN1	0.10094247672537 0.021410359954294
	19499801 0.867337236390728	1.73677273768196e-176
1.23310864375419e-174	100	100
STAT2-regulon	Naive IFN1	0.0824905219472696
0.0164376567695997	15695421.5	0.698121150456747
1.09628725817242e-52	1.29727325550403e-51	100
99.9621169916435
STAT5A-regulon	Naive IFN1
0.0628625948206457	-0.00884286833975363	8427916.5
0.374867713041883	4.98439420701723e-22
1.68519994618202e-21	100	100
TBP-regulon	Naive IFN1
0.0202489311935767	-0.000659616939511717	11231056
0.499549358108296	0.970354829321867	0.977236778465994
	50.0998003992016 49.883008356546
TCF12-regulon	Naive IFN1	0.0102192495780039
0.00118866035548445	11479705.5	0.510609110469868
0.28352276148453 0.324679291377446	26.9461077844311
25.2011142061281
TFDP1-regulon	Naive IFN1
0.497849032248096	-0.0306527090266784	6226154
0.276934887884398	2.66078733606486e-66
5.39759716744585e-65	100	100
TFDP2-regulon	Naive IFN1
0.0269199868786721	-0.000205435065983271	11631837
0.517375811051991	0.180311984525167	0.216985608496387
	100	99.9955431754875
TFEC-regulon	Naive IFN1
0.0849574459362756	-0.00665760661365732	9565140
0.425450602972328	9.01563997839502e-09	1.8553925752639e-08
	100	100
THAP1-regulon	Naive IFN1
0.0371382098658062	-2.72035281492847e-05	11308848
0.503009490767768	0.816498319936261	0.840164937905428
	100	100
THAP11-regulon	Naive IFN1
0.17763114224884 -0.000856019066743319	10579299 0.470559671742865
	0.0232043173925685	0.0310850289598559	100	100
TP53-regulon	Naive IFN1
0.0876867162577296	-0.0107890992105733	7974475
0.354698958628704	3.90935778191876e-29
1.73477751572645e-28	100	100
USF2-regulon	Naive IFN1	0.130818728555648
0.00559429044882201	13693459.5	0.609075308992044
4.08376008798775e-17	1.20811235936304e-16	100	100
XBP1-regulon	Naive IFN1	0.195954575124487
0.000672129523542786	13495949.5	0.60029020510511
1.04978983017553e-14	2.710366470635e-14	100	100

YBX1-regulon   Naive IFN1
0.0910038569115036   -0.00422657098213518   8742689
0.388868569268149   1.04378505650107e-17
3.29372173384782e-17   100   100
YY1-regulon   Naive IFN1
0.609010231162041   -0.0308011793022795   6165905
0.274255055348912   7.36325581534074e-68
2.09116465155677e-66   100   100
YY2-regulon   Naive IFN1   0.0707218609643046
0.0199399627035918   14844763.5   0.660284489516788
4.07708575581619e-35   2.22671606663807e-34   98.0039920159681
90.4467966573816
ZBTB33-regulon   Naive IFN1   0.367037834314061
0.00122534974047944   11456149.5   0.509561356395843
0.460972831208879   0.503524169474314   100   100
ZNF143-regulon   Naive IFN1   0.0268325813162046
0.00354094129200605   13884347.5   0.617565871043428
1.24432325701212e-19   4.1091605231563e-19   100
99.9933147632312
ZNF274-regulon   Naive IFN1   0.0709673433102925
0.00568862104542152   13642336 0.60680137218599
1.79358826911853e-16   5.09379068429663e-16   100   100
ZNF76-regulon   Naive IFN1   0.0164997822877186
0.00196436433926987   12913560.5   0.574385957889235
9.71233407546092e-09   1.97021634102207e-08   99.6007984031936
99.458495821727
