# Supplementary material for: Distinct stage-specific transcriptional states of B cells derived from human tonsillar tissue
Source: JCI Insight. 2023 Apr 10;8(7):e155199. doi: 10.1172/jci.insight.155199 (PMC10132144; doi:10.1172/jci.insight.155199)
Supplement: Supplemental table 9 [file jciinsight-8-155199-s234.pdf]

gene	waldStat	df	pvalue	meanLogFC
AL669831.5	1.73940096115019	2	0.419077052169076	0.623181363715432
LINC00115	0.779324906570484	2	0.677285451397376	0.654162571461824
NOC2L	4.41776065950463	2	0.109823545857215	0.331839125148629
ISG15	8.45015879161615	2	0.0146241735654222	0.460082609627823
C1orf159	0.180743605812028	2	0.913591446162555	0.424203432400884
TNFRSF18	27.3781339160999	2	1.13478544738754e-06	2.34621054023136
SDF4	1.53048104386959	2	0.465222021415801	0.246686230198593
B3GALT6	0.142193081865845	2	0.931371972268088	0.167828335412873
UBE2J2	1.92636163788977	2	0.38167690795773	0.132234614252536
ACAP3	5.47996003144414	2	0.0645716372978207	1.47711759565409
PUSL1	0.930693264681093	2	0.627917410980056	0.364298047856842
INTS11	16.1623779285488	2	0.000309303066415079	0.603330895256006
CPTP	10.9585044766929	2	0.00417244852869891	0.718162602080155
TAS1R3	1.59002851132354	2	0.451574797379132	1.07201857567995
DVL1	0.0384132906809357	2	0.980976627047298	0.188628688642065
AURKAIP1	23.0775911741689	2	9.74461642411217e-06	0.231946704577756
CCNL2	1.92072376053316	2	0.382754349663754	0.13795362693411
MRPL20	2.41283017861078	2	0.299268208491615	0.138448827096095
ATAD3B	0.0471505989049534	2	0.976700426892446	0.0858307234033682
ATAD3A	4.38165312608299	2	0.111824280589789	0.615947283598747
SSU72	0.243586863968168	2	0.885331230730994	0.0421837595569917
AL645728.1	0.466842618474085	2	0.791819902200973	0.481195363574131
AL691432.2	1.11136541409841	2	0.573680471770882	0.909530638650177
MIB2	5.57424907923668	2	0.0615980823494279	0.598048641990712
CDK11B	1.30346977361732	2	0.521140871610368	0.20751082335141

SLC35E2B	3.4851318452042	2	0.175070606169579	0.723321588635796
CDK11A	0.00210515310548891	2	0.998947977211645	0.0101775869834333
SLC35E2A	1.60602406665659	2	0.447977606490702	0.695787890925631
NADK	9.69722289893165	2	0.00783925519738782	0.414659438830642
GNB1	7.21698014739803	2	0.0270927240143983	0.217059446670497
PRKCZ	1.01761803467523	2	0.601211184687979	0.329060435895135
FAAP20	1.10665774737957	2	0.575032410498019	0.19270134573102
MORN1	0.00470101815510081	2	0.997652251205797	0.0621931542150357
RER1	11.1285992716751	2	0.00383226358565891	0.231050180557001
PEX10	0.00783984448277221	2	0.996087750624849	0.0274749357407188
PANK4	0.440840177824683	2	0.802181739515724	0.0963491176079054
AL139246.5	6.01686693219447	2	0.0493689563595505	1.91358894002851
TNFRSF14-AS1	0.473499654005278	2	0.789188697025197	0.241423580711263
TNFRSF14	0.589809924895027	2	0.744602349288193	0.136394340019794
AL139246.3	0.0340133005238877	2	0.983137146493618	0.127095743732825
FAM213B	3.07350338443875	2	0.215078609473856	1.29186401618246
TPRG1L	2.34017593790394	2	0.310339639811793	0.531787824552472
WRAP73	15.4357068816907	2	0.000444814400269444	0.653850971018505
SMIM1	0.191305361136746	2	0.90877959807513	0.377951012744528
LRRC47	0.26724857109794	2	0.874918722466221	0.0962582730364654
CEP104	1.84747651758123	2	0.39703205487725	0.13476550990656
DFFB	2.16797008633856	2	0.338244915721944	1.01296628614639
C1orf174	3.12146503500365	2	0.209982199214687	0.599043699906528
LINC01134	1.63379059881647	2	0.441801187819724	0.661219048181836
KCNAB2	2.3588906180215	2	0.307449230629447	0.255071147165406
RPL22	54.958642463494	2	1.16384679671455e-12	

0.313660550464476
ICMT	1.47104296457877	2	0.479255470558306
0.594237214657511
ACOT7	2.13972726787556	2	0.343055295328922
0.584113752349751
NOL9	17.3328715896379	2	0.000172272024130504
1.58285855914773
ZBTB48	4.55091035828186	2	0.102750128074344
0.798007270975348
PHF13	4.11417486256084	2	0.127825729465566
1.17104942343094
THAP3	8.65873037554116	2	0.0131759090639918
0.695723237359492
DNAJC11	3.35703684416541	2	0.186650308259979
0.647900427451885
CAMTA1	3.59805903518622	2	0.165459385751902
0.196867160353168
VAMP3	2.3953019699507	2	0.301902553267854
0.62765561376817
PARK7	0.0749263786464791	2	0.963229874211775
0.0181198749615941
RERE	1.26322096494514	2	0.531734761555087
0.256930525852795
ENO1	24.5919703907351	2	4.57005556098178e-06
0.167585274937489
SLC2A5	80.313452797267	2	0	2.1584724629917
GPR157	0.00551114095467685	2	0.997248222622139
0.0755394238236158
H6PD	0.119045610932255	2	0.942214045715706
0.067195441470261
SPSB1	2.66678470170433	2	0.263581581725719
0.337026682647423
SLC25A33	3.26335558719511	2	0.195601120357732
0.404824496575265
PIK3CD	0.356699271959373	2	0.836649849454775
0.147619674276405
PIK3CD-AS1	0.71815444010536	2	0.698320424929499
0.764868952353039
PIK3CD-AS2	3.83885305570337	2	0.146691061251828
0.409325908027109
CLSTN1	0.982409154246218	2	0.61188888272886
0.291392512077255
CTNNBIP1	1.03257257637076	2	0.596732530744264
0.300169337351292
LZIC	8.1714219323056	2	0.0168111828841387
0.76655025521383
NMNAT1	1.81906286554418	2	0.402712877888513
0.605679729246459
UBE4B	0.177422223985335	2	0.9151098996677
0.107242534940832

PGD	0.883176470497904	2	0.643014351595808	0.201526176573235
CENPS	1.9726952844788	2	0.372936303417196	0.491303675164538
DFFA	10.1241358236962	2	0.00633245101349145	0.293632224016342
PEX14	0.0781168010820011	2	0.961694544024698	0.111724330123803
CASZ1	0.446511690978769	2	0.799910169703664	0.342461359001362
TARDBP	6.38684626201462	2	0.041031175178425	0.396497645604223
SRM	7.30173817692852	2	0.0259685500014593	0.259895436412863
EXOSC10	2.77154566629602	2	0.25013041599781	0.360385872555977
AL109811.2	2.29301035197408	2	0.317745295086921	1.68304871781109
MTOR	3.26593689156293	2	0.195348830189018	2.21233486775629
UBIAD1	1.87633196121511	2	0.391344911748803	0.413558912842575
FBXO2	4.92477012053979	2	0.0852314262659276	4.38724786641993
FBXO44	0.158667634896863	2	0.923731515274305	0.161362958427209
FBXO6	1.24132105425108	2	0.537589228027302	0.777364233112098
MAD2L2	2.40234838571678	2	0.300840759370921	0.383691055779195
AGTRAP	0.0325163169614791	2	0.98387329203264	0.0749394104180268
MTHFR	0.0737965842475602	2	0.963774153786683	0.0800990401591109
CLCN6	2.00807847252785	2	0.366396486210891	0.406553446460174
NPPA-AS1	2.21136313192008	2	0.330985217090628	4.1387450345145
KIAA2013	0.47632458347122	2	0.788074782690539	0.149928276196512
PLOD1	0.453779688788759	2	0.797008572416743	0.225715761968995
MFN2	2.32125321942079	2	0.313289808928302	0.726971474250491
MIIP	2.7514798260872	2	0.25265258568 4018	0.275923061791141
TNFRSF1B	4.36092884783154	2	0.112989043639198	1.94787992000054
VPS13D	0.0419081004279559	2	0.979263960505119	0.113014682176817
PRDM2	22.9801870195436	2	1.02309459937278e-05	

0.310267176005116
EFHD2 2.19657042807952 2 0.33344237604103 0.378595037181512
CASP9 1.40618146108373 2 0.495052861828832
0.408434876580998
DNAJC16 0.0328887636876505 2 0.983690088898229
0.0799123110781106
AGMAT 10.6310912919608 2 0.00491459639383618
0.60453236617521
DDI2 2.01909044937121 2 0.364384655042169
0.773463412040443
PLEKHM2 2.00006805881746 2 0.367866922664576
0.722832438106316
UQCRHL 0.77783693852122 2 0.677789528442715
0.368618112549229
AL450998.2 3.80984463598784 2 0.148834203912616
0.994093155204605
SPEN 0.691374643765031 2 0.707733745368573
0.1377401175545
ZBTB17 0.178743915646251 2 0.914505352884205
0.161910656794
ARHGEF19 1.43978188278451 2 0.486805343378092
0.52536560979239
FBXO42 3.47330864527506 2 0.176108618693256
0.521984640843691
SZRD1 0.331300717987984 2 0.847342448068099
0.0966331213699599
NECAP2 0.685832971651288 2 0.709697478884335
0.047481546394673
AL137802.2 0.000791906771333579 2 0.99960412499353
0.0266933315990441
NBPF1 2.29676869945676 2 0.317148757146722
0.867954937165591
CROCC 0.00155791165047605 2 0.999221347482091
0.0373017899188054
BX284668.5 1.77732841009952 2 0.411204671172765
0.516941327874022
ATP13A2 3.48613386554182 2 0.1749829159843 0.851842777108657
SDHB 1.00563748670641 2 0.604823412722991
0.109757005036309
PADI2 3.50067030625477 2 0.173715712328556
1.69782698132172
PADI4 2.61092691019451 2 0.271046887870158
0.969517429616259
RCC2 3.36567674929802 2 0.185845726909701
0.495878627830819
ALDH4A1 2.89602694828259 2 0.235036731184017
1.26454910637439
UBR4 0.607179365615497 2 0.738163685571282
0.12076305222442
AL035413.1 4.69016002476105 2 0.0958395334744874

4.35239108308254
EMC1 0.0128566595598862 2 0.993592287729564
0.0518456490575677
MRTO4 0.556039113284662 2 0.757282011476077
0.164978735621102
AKR7A2 3.27372797356121 2 0.194589321121716
0.282903932758881
PQLC2 1.29334414922584 2 0.52378600021553 0.450385869940432
CAPZB 0.997897498165244 2 0.607168610889703
0.0691994757965824
MINOS1 0.708850962243655 2 0.701576396329573
0.0349391373993521
TMCO4 3.84904994664198 2 0.145945068193619
1.65220905886174
OTUD3 0.822829921717667 2 0.662711875046994
0.535288918044518
MUL1 3.30846680208273 2 0.191238602840636
0.451287312953125
PINK1 2.46497884548081 2 0.291565842841559
0.241348827209378
DDOST 2.01558116158937 2 0.365024581608521
0.129235328630959
HP1BP3 1.00316517727599 2 0.60557153033579 0.0701359747850312
EIF4G3 0.724800606116256 2 0.696003699660187
0.224353582451527
ECE1 2.10032722181624 2 0.349880500161642
0.301312764810094
USP48 0.500441217303175 2 0.77862899183081
0.123909109889761
CDC42 2.52283958936141 2 0.283251581783824
0.0847778742909905
ZBTB40 4.63922052059177 2 0.0983118941872403
2.898349482467
KDM1A 0.263980541771245 2 0.876349521142624
0.0407886767575355
LUZP1 1.73194804315103 2 0.420641638995427
0.795890092007143
LINC01355 4.53981580320782 2 0.103321695407348
3.20939663696398
HNRNPR 2.01567778259576 2 0.365006947513265
0.151425781563361
ASAP3 1.19357454109807 2 0.550577654751397
0.890258123890539
ID3 0.0591633493453533 2 0.970851580417903
0.034607814761721
RPL11 20.2811394634566 2 3.9446322600778e-05
0.164216153246403
ELOA 0.302530149141333 2 0.859619805105372
0.140944907892576
ELOA-AS1 2.280349942213 2 0.319763067623504

0.583763320103433
PITHD1 2.30465622714985 2 0.315900460455914
0.186369887253219
LYPLA2 2.7629944520379 2 0.251202164943658
0.191027049195548
GALE 0.85877068478418 2 0.650909057995045
0.341919862575851
HMGCL 0.70556550388331 2 0.70272984348921 0.167727894040224
FUCA1 8.0206320650364 2 0.018127665399696
0.596075634461003
CNR2 9.37543151617928 2 0.00920769475500616
0.592100297743351
PNRC2 2.6996674320859 2 0.2592833717265 0.12057136409193
SRSF10 0.0418777506895139 2 0.979278820820404
0.0216215361362811
IFNLR1 0.269680386183494 2 0.873855548683754
0.227571873496492
STPG1 1.85009420908492 2 0.396512741083838
0.512127014839221
NIPAL3 3.15863276561117 2 0.206115954464032
0.547230298083088
RCAN3 0.471434078483279 2 0.790004182491822
0.177898604491529
AL445686.2 1.63329579749109 2 0.441910503248165
1.0597146980771
SRRM1 0.255154229690278 2 0.880225534761135
0.0426541973079602
CLIC4 14.243264725586 2 0.000807447627308555
0.521309855091179
RUNX3 1.72370120749387 2 0.422379701144704
0.15736019471375
SYF2 4.50084743844819 2 0.105354574344459
0.107252830411888
RSRP1 6.74826293101051 2 0.0342478508377582
0.297982769034815
TMEM50A 6.8742248345626 2 0.0321574085676782
0.211038425280438
MACO1 0.568287728513849 2 0.752658356281822
0.202590496343431
MTFR1L 2.98574409941303 2 0.224726302693287
0.259630154424711
STMN1 20.0963162941646 2 4.32653641554426e-05
0.482273036495648
PAFAH2 7.07398991930065 2 0.0291006444311275
1.07515323332999
PDIK1L 4.75570604417287 2 0.0927494950154815
1.18117036137013
ZNF593 2.62437957070438 2 0.269229854820701
0.255146698197713
CEP85 0.458890011616792 2 0.794974686425943

0.493081768112431
SH3BGRL3 15.1376363709908 2 0.000516302261700075
0.252148187332548
UBXN11 1.71911046009453 2 0.423350333958333
0.387867299477383
CD52 5.00765476983976 2 0.0817714281981643
0.0977765458318958
DHDDS 1.89231368340766 2 0.388230190146024
0.518648459935588
HMGN2 88.1609054104135 2 0 0.734986130384536
RPS6KA1 0.463314120449732 2 0.793218102705515
0.130010449733179
ARID1A 2.82798668770657 2 0.243170279137164
0.276799023462642
PIGV 4.47704016020954 2 0.106616171076165
1.00709500661063
ZDHHC18 1.01599805387045 2 0.601698357253302
0.860259356188335
GPN2 0.693864503130658 2 0.706853214834458
0.175336337791805
GPATCH3 0.267127529320133 2 0.874971674927372
0.164155356528586
NUDC 2.33951268984259 2 0.310442572960635
0.158495922061906
KDF1 1.63552380542983 2 0.441418487298184
0.666079097747838
WDTC1 1.27089928589917 2 0.529697260124339
0.284668682012249
TMEM222 3.11962879584643 2 0.210175076511609
0.557831725075857
SYTL1 9.6405978090403 2 0.00806437630028389
0.585205360435194
WASF2 6.79924142549123 2 0.0333859304170824
0.281474355062
AHDC1 11.4323261370577 2 0.00329231910125038
1.8563149387929
FGR 1.13566649782998 2 0.566752120034078
1.05443884119893
IFI6 1.05012601778037 2 0.591518092294071
0.934308295746934
FAM76A 3.2212301521599 2 0.199764705787897
0.61390581896562
STX12 1.21648909173808 2 0.544305534292518
0.245709468208647
PPP1R8 2.06717625772177 2 0.355728269017316
0.134306141386425
THEMIS2 26.6019941357742 2 1.67282445806549e-06
1.14407720183369
RPA2 0.137893983809824 2 0.933376155258889
0.052769983259952

XKR8	0.924062924310034	2	0.630002518393205	0.460042075642778
EYA3	1.50493814896103	2	0.471201683206799	0.14849028981669
PTAFR	10.7848521158055	2	0.00455091913875461	0.651786489403448
DNAJC8	2.67798947460843	2	0.262109024611822	0.123185404283774
ATP5IF1	7.02164031860648	2	0.0298724042569126	0.157639437753085
AL353622.1	0.0161458762688508	2	0.991959560518629	0.0776757267021052
SESN2	1.84323775972931	2	0.39787440856531	0.722819373700761
MED18	3.74432520216037	2	0.153790713964061	0.502921235338563
PHACTR4	0.57972651703018	2	0.748365893245822	0.250887389926322
RCC1	0.487860471138573	2	0.783542295738329	0.129628882493552
TRNAU1AP	0.614487463180313	2	0.735471321465414	0.143072304053335
SNHG12	0.0176020349537318	2	0.99123759810893	0.037587051499505
TAF12	1.15208870130723	2	0.562117514364806	0.0845351409332562
GMEB1	2.69562944783935	2	0.259807391629733	0.543082586410517
YTHDF2	0.628447620586422	2	0.730355548776403	0.0906207480578285
EPB41	3.46864232667541	2	0.17651998786303	0.55341811016395
SRSF4	6.88577868812868	2	0.0319721731337733	0.312195596793511
MECR	0.0587860109620444	2	0.971034767481131	0.0966511232015433
LAPTM5	10.3781503172987	2	0.00557716242091988	0.100907124130972
PUM1	0.687780585017793	2	0.709006707129974	0.215254122380131
SNRNP40	0.24853152283705	2	0.883145103796554	0.0858714832879004
ZCCHC17	1.78980289253642	2	0.408647870428966	0.246875148497561
SERINC2	8.42292445736777	2	0.0148246754163227	1.5032476053747
PEF1	1.11779118777331	2	0.571840259114727	0.173704165143475
PTP4A2	12.1241351853395	2	0.00232957928363986	0.261462296283679
KHDRBS1	3.90586867136732	2	0.141857203615793	0.165345982371395

TMEM39B	0.44114892101803	2	0.802057914997513	0.155236179408745
KPNA6	0.833397410292817	2	0.659219509471208	0.242357693395525
TXLNA	1.05143026964067	2	0.591132473757522	0.424195918233836
CCDC28B	36.4252892141801	2	1.23125766249288e-08	1.00898896520511
IQCC	3.04355321154765	2	0.21832366710997	4.54125650832876
TMEM234	0.525856198289275	2	0.768797172498273	0.266589346857986
EIF3I	3.18521406136601	2	0.203394663806861	0.126862467303268
FAM167B	0.609117940343931	2	0.737448539485158	0.46050988251958
LCK	44.6436207937987	2	2.02190597597962e-10	0.777932534039811
HDAC1	16.3728631230973	2	0.000278405586974784	0.284872599876175
MARCKSL1	315.297315994821	2	0	1.03663978382898
FAM229A	0.951382575805822	2	0.621455303407144	0.810910254700599
BSDC1	1.28737439599661	2	0.525351772454587	0.254855652183052
ZBTB8A	0.342603679445043	2	0.84256721483145	0.329696320278107
ZBTB8OS	38.505137628417	2	4.35226776840381e-09	0.671498029399645
RBBP4	2.14514745203197	2	0.342126842555179	0.102263694913737
SYNC	8.76211281022898	2	0.0125121337548043	0.737819396043209
YARS	5.35727727232555	2	0.0686565571161916	0.412606865576774
S100PBP	0.0162998615259353	2	0.991883189884682	0.0419423360273436
RNF19B	5.69188929355015	2	0.0580793763223341	0.967248201334294
AK2	8.86108744094848	2	0.0119080132816473	0.181035884198382
TRIM62	6.74841339499688e-05	2	0.999966258502282	0.00613830799262475
ZNF362	2.71597476223	2	0.257177857448734	2.46798070062172
PHC2	0.149596588655091	2	0.927930636326302	0.286967667052925
SMIM12	0.202477655714963	2	0.903717174273864	0.0781590740615165
ZMYM6	5.52515944448187	2	0.0631287035984636	0.611767157880285

ZMYM1 1.1562637777001 2 0.560945296533128
1.16042621606696
SFPQ 1.19856555683979 2 0.549205396832815
0.106907430848385
ZMYM4 1.04694176522923 2 0.592460613898898
0.315715279774624
KIAA0319L 2.82218351300954 2 0.24387688358415
0.567495138638186
NCDN 0.0480931493963818 2 0.976240240604494
0.143619570594368
AC004865.2 0.974512397239743 2 0.614309627503223
0.391976863220137
PSMB2 6.72537361027068 2 0.034642057335977
0.170069656483566
C1orf216 2.79748613597605 2 0.246907114437851
0.71396987576888
CLSPN 1.78178035934647 2 0.410290358006165
0.534832049580216
AGO4 0.639106623263694 2 0.726473471857325
0.426333803147474
AGO1 1.2170692496966 2 0.544147665597028
0.431616446881666
AGO3 7.99706168330475 2 0.0183425672386611
0.797218621565768
ADPRHL2 3.47423885144056 2 0.176026729076836
0.33356449299362
TRAPPC3 3.88934773503127 2 0.143033863721532
0.235583082559502
MAP7D1 2.06779834862112 2 0.355617638564359
0.264015214033484
THRAP3 25.9425688613889 2 2.32617695294923e-06
0.417562088520523
SH3D21 3.34451548064411 2 0.187822532071756
1.32774349104816
STK40 23.9553092261952 2 6.28305259797735e-06
0.812901017345632
LSM10 86.9574097632724 2 0 0.671412907541106
MRPS15 2.24489095912082 2 0.325482858896494
0.107767681519822
LINC01137 2.68351894470864 2 0.261385363431201
1.3211138495845
ZC3H12A 0.776587062222054 2 0.67821323735857
0.302316433527309
MEAF6 0.851273069328499 2 0.653353770417113
0.0488017294951629
SNIP1 0.491865590244852 2 0.781974775678689
0.350462669568468
GNL2 0.0140042586296025 2 0.993022328473842
0.0251166493126159
C1orf109 3.5232395344621 2 0.171766416760951

0.387626591014416
CDCA8	1.57612372261269	2	0.454725262399086
1.89274321569539
YRDC	7.98024708543853	2	0.0184974287560042
0.64976539601639
C1orf122	0.000241603784257024	2	0.999879205404126
0.00304299292401753
MTF1	0.0602812268794162	2	0.970309085457429
0.0967565487523048
INPP5B	7.42340116230728	2	0.0244359326310423
0.617606321125996
SF3A3	0.113469776198206	2	0.944844525693839
0.0535059215051529
FHL3	0.0779014548271432	2	0.961798098258788
0.292930752072705
UTP11	6.47098206693513	2	0.0393408825065593
0.221899492130389
POU3F1	2.68448391488943	2	0.261259279309799
1.50610168921501
RRAGC	1.9666828333557	2	0.374059120941117
0.249578713271889
AL139260.1	3.14393635738955	2	0.207636114952482
0.915357262742237
MYCBP	1.14115272269966	2	0.565199585607909
0.325667114906087
AKIRIN1	4.6583383380615	2	0.0973766169993521
0.241620507608463
NDUFS5	10.1945805351491	2	0.00611328952074253
0.170631090391094
MACF1	3.50284355715539	2	0.17352705093512	0.926049228499671
PABPC4	0.443519011987363	2	0.801108002842625
0.121843387268642
PPIE	0.0494727127117519	2	0.975567080186576
0.0203855398642498
TRIT1	0.435246911825408	2	0.804428287360863
0.267529175163317
MFSD2A	0.242737311246747	2	0.885707378392868
0.16786444229957
CAP1	45.1892036780398	2	1.53917878442655e-10
0.469492311053148
PPT1	0.663652485090836	2	0.717612002457049
0.181879615366743
RLF	3.9482707081719	2	0.138881343529417
0.88893396535408
AL050341.2	5.30480334810842e-05	2	0.999973476335018
0.00345491134856794
ZMPSTE24	2.13230001723626	2	0.34433164263068	0.348588319468956
SMAP2	1.94798835015539	2	0.377571933896552
0.143755063625468
AL603839.3	2.58106932770492	2	0.275123645090585

2.84296146414718
EXO5 0.448530811840379 2 0.799103019550116
0.335548333309452
ZNF684 0.250550148003476 2 0.882254184014395
0.496558161284418
NFYC-AS1 3.46998197143585 2 0.176401790414671
1.46075198582749
NFYC 0.102586701926505 2 0.949999946253085
0.0542975213336806
CTPS1 0.411560441814252 2 0.814011959434846
0.263359012891411
SCMH1 0.468203477933036 2 0.791281307657775
0.369554756358564
HIVEP3 10.9126738655718 2 0.00426916537751998
2.64530138961356
FOXJ3 1.95583296449567 2 0.376093881375909
0.541373522425386
PPCS 2.24538495847105 2 0.32540247466394 0.266376001517774
CCDC30 0.187785104870012 2 0.910380575161812
0.400741391764728
PPIH 1.94592730307518 2 0.37796123121809 0.265139159481149
YBX1 1.13584021355383 2 0.566702895294498
0.0464375738453198
P3H1 1.37224005813685 2 0.503525949928386
0.404154716112326
C1orf50 0.610378122630573 2 0.736984026050042
0.350036516332875
SVBP 1.36964300374642 2 0.504180216767573
0.168687679516263
ERMAP 1.56549609781393 2 0.457148018471499
0.606669216727164
ZNF691 1.8968522794899 2 0.387350179017436
0.479738458078737
SLC2A1 14.5620349989647 2 0.000688484674376211
1.08308288749463
EBNA1BP2 7.40781457407275 2 0.0246271130360105
0.345792130411843
CDC20 2.00395222037355 2 0.367153188674052
1.06228417090862
ELOVL1 0.125402894897589 2 0.939223839508524
0.0690786115136189
MED8 0.112605534826353 2 0.945252900785683
0.0633744696270744
SZT2 1.11614664662174 2 0.572310659905707
0.535968504291379
HYI 15.6060842761235 2 0.00040849040265134
2.70384494612159
KDM4A 4.06592919215047 2 0.130946740950825
1.11073470181722
ST3GAL3 1.43671460689714 2 0.487552499310784

0.789580609599908
IPO13	0.597550003524455	2	0.741726277767173
0.394484315899979
DPH2	0.814653900343551	2	0.665426593392773
0.262122923000374
ATP6V0B	7.25890325440869	2	0.0265307291506746
0.163942158452998
CCDC24	2.01636361739331	2	0.364881801738846
0.989688412829854
DMAP1	0.012747985185241	2	0.993646278206657
0.0192963168498661
ERI3	3.51285604950385	2	0.172660502686998
0.306184706868551
RNF220	3.95321573367111	2	0.138538381800202
0.78214017434662
TMEM53	10.3396532442985	2	0.00568555448298746
1.59263075582803
ARMH1	0.482341289024312	2	0.785707538268405
0.464879703544023
RPS8	109.003532451581	2	0	0.286174458353229
PLK3	0.00261280467913022	2	0.998694450632489
0.0210366625271913
PTCH2	3.67887199638527	2	0.158907024686792
0.680249289254295
EIF2B3	0.000453843877959515	2	0.999773103805856
0.00576824651096365
HECTD3	0.268228031684509	2	0.874490353164941
0.283616017301693
UROD	0.00255250068787938	2	0.998724563717178
0.00751442758930234
MUTYH	0.00365294467254181	2	0.998175194649271
0.0220668884001519
TOE1	0.656007305022047	2	0.720360388576236
0.123512393471986
TESK2	3.09765988931884	2	0.212496461045597
1.16338822736104
CCDC163	3.03122216927516	2	0.219673904475302
2.64626620775925
PRDX1	258.740871253534	2	0	0.540461448346851
AKR1A1	41.6129947993607	2	9.20138076843102e-10
1.05458365388707
NASP	0.0338466002097994	2	0.983219094544319
0.0219737956235179
GPBP1L1	1.99427167355968	2	0.368934618314741
0.292622495048296
TMEM69	0.599906586893548	2	0.740852822555434
0.168198035161068
IPP	1.10733546527727	2	0.574837588630274
0.566963884051
MAST2	2.34448966355446	2	0.30967100111966	0.63537088663982

POMGNT1	4.21355036736277	2	0.12162956768766	0.837804654738284
LRRC41	5.57623398237601	2	0.0615369795617059	0.436055040562399
UQCRH	28.5408172161731	2	6.34512653086006e-07	0.346856241168228
NSUN4	2.45422318451431	2	0.293138058283956	0.533425322989667
FAAH	2.70607608275745	2	0.258453873150735	3.11913809664945
MKNK1	3.45151863911083	2	0.178037812841719	0.577470704165315
MOB3C	2.18353800355772	2	0.335622251893663	1.35220673163258
ATPAF1	0.740395616467085	2	0.690597711212233	0.210551247991463
EFCAB14	0.905284314325332	2	0.63594566151428	0.129023941419344
CMPK1	6.47785366779757	2	0.0392059470239132	0.298563134284076
BEND5	2.690981143299	2	0.260411925811418	0.596721749944971
FAF1	3.23012734168022	2	0.198878007307883	0.337310802348211
CDKN2C	6.87273987854807	2	0.0321812936022696	0.263625522256231
RNF11	9.68333237828227	2	0.00789389037362842	0.71201809735987
EPS15	1.26307686002764	2	0.53177307573236	0.156914623650859
AC104170.1	7.28590914010206	2	0.0261748940483821	0.634174922822184
OSBPL9	6.61155235105139	2	0.0366707378581788	0.390767526158515
NRDC	0.925297137600758	2	0.62961385958681	0.178268386041575
TXNDC12	1.2053979067501	2	0.547332416147286	0.209177273753752
KTI12	0.00421467435656488	2	0.997894881697795	0.0164497838161151
BTF3L4	0.30930131265372	2	0.856714412972418	0.077593930206428
CC2D1B	0.227851014708356	2	0.892324425013998	0.289137416566286
ORC1	0.0473129639075288	2	0.976621139127215	0.170436790239934
PRPF38A	4.26847945341723	2	0.118334522762814	0.218209233319476
TUT4	9.35067272732937	2	0.00932238889740611	0.449543790515365
GPX7	11.0274999736511	2	0.00403096294730509	0.929993255331849

SHISAL2A	3.34315422912209	2	0.187950412439982	0.278932778562717
COA7	0.456526645508542	2	0.795914649801369	0.277747597483579
ZYG11B	1.81671545429819	2	0.403185821751885	0.821929907996196
ECHDC2	13.9756787326824	2	0.000923038727234426	1.92489755432787
SCP2	2.54451566331672	2	0.28019826652589	0.087568344231656
CPT2	2.67701781995202	2	0.262236395276517	0.266728705794736
C1orf123	0.174467812924938	2	0.916462704011257	0.081314800199014
MAGOH	0.405789388658194	2	0.816364204679596	0.0595976578580843
AL606760.1	4.99663335241219	2	0.0822232906176517	2.0496118738576
LRP8	2.139982077515	2	0.343011591214984	1.24960061485504
AC119428.2	0.803016261810412	2	0.669309877582431	0.292260800303044
NDC1	1.36663850378923	2	0.504938190678017	0.28821032696812
YIPF1	2.7823465930106	2	0.248783236821764	0.412027302887779
HSPB11	0.0744889787893968	2	0.963440555553591	0.0313093462044551
LRRC42	0.00975802286581996	2	0.995132871609683	0.0232820435478604
TMEM59	20.9381687353665	2	2.84010521006683e-05	0.221190751020889
TCEANC2	4.54251187402348	2	0.103182507939438	0.940159579326328
CYB5RL	1.93555165346443	2	0.379927122810369	1.50878919787627
MRPL37	0.000984995835517486	2	0.999507623339434	0.004551092070147
SSBP3	6.70337390236514	2	0.0350252184017477	0.835450731547358
TTC4	1.01220237157372	2	0.602841369439305	0.927139905082559
PARS2	0.0799595136036152	2	0.960808888800222	0.192494141883075
DHCR24	2.51680391207424	2	0.28410768048733	0.903264365467699
USP24	2.56009221082772	2	0.278024481723897	0.562641465555163
OMA1	3.08087615338333	2	0.214287206631371	0.537974918788326
MYSM1	0.0256304263875595	2	0.987266551998675	0.0600245985810852

JUN	7.87609140371344	2	0.0194862595186587	0.528270490991269
FGGY	0.0187123791239419	2	0.990687443394153	0.080214947188977
NFIA	1.59710853727322	2	0.449979042899538	0.867168812197312
TM2D1	2.82713888112682	2	0.243273381669696	0.216114195844479
PATJ	1.60349784137984	2	0.448543810181409	1.21470041764525
L1TD1	5.80749072835399	2	0.0548175235038099	1.51081083523297
USP1	0.0376816444163991	2	0.981335556638058	0.0326285005488883
DOCK7	3.51086580376506	2	0.172832406620445	1.20918126325382
ATG4C	0.0904762220122988	2	0.955769875409697	0.0897955272166844
FOXD3-AS1	3.48870553281676	2	0.174758061658553	5.86864199355438
ALG6	1.84001291819763	2	0.398516467018961	0.331799610862633
ITGB3BP	11.4070657102528	2	0.00333416550229115	0.750263303090002
EFCAB7	0.196798586751245	2	0.906286957118893	0.242992834699326
PGM1	0.61339969899638	2	0.735871439945089	0.193533440265051
JAK1	2.91311313665791	2	0.233037342893535	0.0925846080517836
LEPROT	0.269602556633613	2	0.873889555237436	0.140535184474913
PDE4B	2.60045574073623	2	0.272469698189067	0.599861940067154
WDR78	1.86872859212048	2	0.392835513255544	1.78877945551708
MIER1	1.7115232940474	2	0.424959398728951	0.285694795756845
SLC35D1	5.8056450779779	2	0.054868133843902	0.99155649690869
SERBP1	3.72139063324044	2	0.155564426223219	0.120355937916277
GADD45A	7.97944614462953	2	0.0185048379122552	0.696393228925736
DEPDC1	0.0132386309246707	2	0.993402543948222	0.11591939671372
LRRC7	0.326289265090541	2	0.84946831876426	0.253969333498193
LRRC40	2.8080177410872	2	0.245610367526676	0.429247306217838

SRSF11	0.333836680737896	2	0.846268714506678	0.0562544201293218
ANKRD13C	0.410488287943572	2	0.814448449457053	0.260494239311295
HHLA3	0.128126845272508	2	0.93794551066733	0.0939643756192009
ZRANB2	6.48397584922156	2	0.0390861175616198	0.323460853124823
LRRIQ3	0.0144636217167755	2	0.992794275763374	0.0997762832991691
FPGT	0.870229456997273	2	0.64719041163425	0.582629858095505
CRYZ	8.52038110509891	2	0.0141196116258115	0.644233713895409
TYW3	0.271778628396196	2	0.872939249122377	0.0700093397748733
ACADM	0.240720263285785	2	0.886601086110748	0.010839576768293
RABGGTB	0.0464655271109388	2	0.977035039154363	0.0380680571718209
PIGK	0.997942170602297	2	0.607155049190387	0.437324783074442
AC118549.1	0.061905373535636	2	0.969521443184033	0.0836100658549486
USP33	0.190978229172602	2	0.908928255659906	0.0480633605241247
MIGA1	0.247266587764833	2	0.883703841077348	0.280825185623396
FUBP1	0.316059309660305	2	0.853824461573659	0.0418402179449728
DNAJB4	0.763583235053092	2	0.682637288031805	0.398243167903723
AC103591.3	5.50103675729463	2	0.0638947309562387	0.631921512796861
PRKACB	4.18275690778655	2	0.123516756260699	0.443918383426558
RPF1	4.36351693293949	2	0.112842925570486	0.206616760603956
GNG5	12.2958759407548	2	0.00213788561151218	0.26394880105374
CTBS	6.34675516297762	2	0.0418619666489001	0.712160603001645
SSX2IP	0.0187438253133539	2	0.990671866844113	0.0924118864059655
MCOLN2	1.63830366522498	2	0.440805372737182	0.275703889523871
C1orf52	0.032791939201072	2	0.983737712694912	0.0412298766065499
BCL10	3.47992853272455	2	0.175526672711468	0.170695687528703

ZNHIT6	1.81173522289965	2	0.404191052146923	0.386878048994685
ODF2L	0.362110118550111	2	0.834389416554641	0.148575640533846
SH3GLB1	2.32346049578284	2	0.312944241059466	0.204179515905102
SELENOF	2.40458645581438	2	0.300504296309167	0.069584195763541
HS2ST1	18.6006431640394	2	9.13948358159944e-05	0.907214511923433
LMO4	63.6335084655659	2	1.52100554373646e-14	1.06736218874305
PKN2	2.49393673298011	2	0.287374692339001	0.426822449759953
GTF2B	0.106378061329441	2	0.948200756521101	0.049341495175922
KYAT3	0.820995502015487	2	0.663319999753574	0.22061004513611
RBMXL1	0.98794169439413	2	0.610198571830098	0.359278675658851
GBP3	1.82244056232204	2	0.402033330880942	0.870840997182409
GBP1	3.72024152135775	2	0.155653832370524	0.998152127851887
GBP2	0.774923360069036	2	0.678777644488962	0.566551040347286
GBP4	2.56121367285234	2	0.277868628474772	0.744897558514079
LRRC8B	0.938852777878131	2	0.625360879338698	0.519432801172289
AC099568.2	1.58420364553744	2	0.452891895721961	0.792886762587158
LRRC8D	3.53815492186068	2	0.170490200168106	0.692750640985624
ZNF326	3.97056099332664	2	0.137342084717026	0.266065314551759
ZNF644	1.5809519043591	2	0.453628838259548	0.201597697677849
CDC7	2.40444389218891	2	0.300525717563608	1.24274011423503
GLMN	0.0118804518654656	2	0.994077382326402	0.0313161723639015
RPAP2	0.838265013115042	2	0.657617050922761	0.238801587879862
GFI1	0.124275770763084	2	0.939753299614933	0.25041454986949
EVI5	11.5959565503346	2	0.00303368181952901	0.697948892360458
RPL5	13.7077930731164	2	0.00105533551748849	0.163062821187784

FAM69A	0.190840802716732	2	0.908990713200119	0.203357336278034
MTF2	12.7690497872141	2	0.00168747003298386	0.395858103717655
TMED5	0.939248285369501	2	0.625237224109449	0.241531862275547
CCDC18	0.470014536402354	2	0.790565103621637	0.183036675912723
DR1	1.55466139266045	2	0.459631270720658	0.225353230048527
BCAR3	40.4646717284427	2	1.63383495621616e-09	2.0358232500287
DNTTIP2	0.17133074093713	2	0.917901336728212	0.0680925545589435
GCLM	0.0461526973989851	2	0.977187873901798	0.0782247276746934
ABCD3	7.10231743089377	2	0.0286913752462793	1.34350250530604
ALG14	0.746662816985439	2	0.688437041151572	0.266369605991598
RWDD3	4.82439009715058	2	0.0896183618553699	1.98371589567931
PTBP2	0.0184502840827911	2	0.990817279034409	0.0604879273072507
DPYD	5.87910913870061	2	0.0528892819995318	2.59628695413049
SNX7	4.49869825239012	2	0.105467848486527	1.06837201302966
AGL	1.33723309742099	2	0.512416991593341	0.315503942997179
SLC35A3	2.5758692322007	2	0.275839910464133	0.401369401523485
MFSD14A	0.0105191213377574	2	0.994754246603002	0.0442505778503135
SASS6	0.176965904509469	2	0.915318714723276	0.177563948917031
TRMT13	8.22722137147924	2	0.0163486380346822	0.566108238352687
DBT	0.527108273235347	2	0.768316027282306	0.260836274386465
RTCA	2.36238019591373	2	0.306913264320368	0.217164797181803
CDC14A	0.301043092722432	2	0.860259194352385	0.505138251770295
EXTL2	4.48625977419415	2	0.106125822180468	1.4032596984294
SLC30A7	3.96782905102338	2	0.137529818232747	0.908256810128276
DPH5	12.1730862349635	2	0.0022732537187593	1.39013914798861

AC093157.1	0.233688791490075	2	0.8897236271748
0.446184505014211
RNPC3	0.00871238729987364	2	0.995653280799117
0.0313349602084209
PRMT6	0.00227288957004563	2	0.9988642007238
0.0151619674475299
VAV3	5.66743970241443 2	0.0587937424177704
1.56738796712645
SLC25A24 3.47226588039129 2	0.176200462575721
0.974810703161425
FAM102B	1.34636301292319 2	0.510083160648461
0.987142700304395
HENMT1	5.59696529272421 2	0.0609024030337735
0.796771174052932
PRPF38B	2.53740632127547 2	0.28119605153202 0.0944352446773233
STXBP3	1.87971810339398 2	0.390682897569394
0.312822417586031
GPSM2	2.8666582212531	2	0.238513561025009
0.571341552549789
CLCC1	0.203259223326865	2	0.903364085232231
0.242707877050285
WDR47	4.54964932923701 2	0.102814933950653
2.57139917605392
TAF13	2.0624699232017	2	0.356566342810615
0.663715702075426
AL356488.3	1.34073334100793 2	0.511520983739146
0.695129863896171
TMEM167B 6.89524754422246 2	0.0318211609408253
0.4804389104622
KIAA1324 2.03907196870646 2	0.360762300693412
1.85791040532484
SARS	2.54160975779134 2	0.280605677273953
0.17421268831221
PSMA5	8.99394683905796 2	0.0111426697418434
0.210591256346102
ATXN7L2	2.46959665267944 2	0.290893421993768
1.09421721667354
GNAI3	0.00432764575828764	2	0.997838516497946
0.00813106756069407
AMPD2	1.34616716608726 2	0.510133112180671
0.543127906994256
GSTM4	0.499478887162159	2	0.779003731053046
0.229152139277625
AHCYL1	2.56336071426303 2	0.277570490805851
0.429442938333792
STRIP1	2.87016547087459 2	0.238095664250864
0.35445676653384
RBM15	2.38136758482517 2	0.304013310981062
0.363513607686494
SLC16A4	1.52399625539018 2	0.466732902730988

0.515580862787207
LAMTOR5 31.9264057821652 2 1.16753274825854e-07
0.331615263519664
CD53 196.36050833672 2 0 1.14126621479079
LRIF1 0.506560917370364 2 0.776250145197007
0.14295216641361
DRAM2 2.07552772572646 2 0.354245939449692
0.171402685907698
CEPT1 2.6766087378549 2 0.262290038869749
0.320850884794477
AL355816.2 1.53301859835725 2 0.464632132599587
0.408181505505226
DENND2D 16.4885240889376 2 0.000262761956379309
0.630063828552735
CHI3L2 7.15586467664847 2 0.0279333953967431
0.692275224841138
WDR77 1.65268007895115 2 0.43764813357033 0.149168208520104
ATP5PB 10.6748277641125 2 0.00480828944883616
0.238806884066541
C1orf162 8.4906524167862 2 0.0143310580050277
1.39251491127453
RAP1A 9.27951660517015 2 0.00966003215020117
0.162967312642205
DDX20 1.04070666232641 2 0.594310522439898
0.352023918541442
CTTNBP2NL 1.37318871068313 2 0.503287170975169
0.634101319789421
WNT2B 0.263291569782321 2 0.876651463283344
0.475049846471242
ST7L 0.0439989948482049 2 0.978240726691298
0.125193918793287
CAPZA1 2.83476538660373 2 0.242347485241344
0.11352678872706
MOV10 3.37669293104383 2 0.184824885781338
14.0824043957195
RHOC 0.419005390083767 2 0.810987453784992
0.43850882582529
SLC16A1 0.204586913534078 2 0.902764590415341
0.161275797445612
SLC16A1-AS1 2.55029066434887 2 0.27939036086204
0.693769235586272
LRIG2 3.83355356433019 2 0.147080270683924
0.863704328836942
PHTF1 4.35835009553573 2 0.113134822979046
0.877059857872631
RSBN1 0.775158642505167 2 0.678697796956842
0.228170281519223
PTPN22 5.47840200696921 2 0.0646219589915317
0.941932808465541
AP4B1 0.00264556656775855 2 0.998678091208298

0.0253222608498444
HIPK1-AS1 0.11043394025683 2 0.946279811226163
0.308721020438269
HIPK1 0.431151964569616 2 0.806077020357455
0.380728077429036
TRIM33 5.81953416511569 2 0.054488419694634
0.721971222070172
BCAS2 0.933243804533846 2 0.62711715716785
0.137507443595696
NRAS 0.0661108033186404 2 0.967484957832201
0.0695931871508113
CSDE1 6.04337992333711 2 0.0487188158217812
0.198393456545938
SIKE1 1.21929451820838 2 0.543542564954298
0.269627830976367
VANGL1 1.28350263942697 2 0.526369774588963
1.16413302440332
ATP1A1 3.29705226894951 2 0.192333173055762
0.327683364642891
CD58 4.09988099683302 2 0.128742563746312
0.483133050924802
TTF2 4.62833518465505 2 0.0988484319586418
0.389200569127607
MAN1A2 4.05593171374792 2 0.131602948296378
0.323675905569117
TENT5C 0.352692099506507 2 0.838327829997508
0.398328564022962
GDAP2 0.254620397347874 2 0.880460512548959
0.168612068809343
WDR3 0.153991728966528 2 0.925893682647398
0.126070443791071
WARS2 1.70048740377367 2 0.427310782814536
0.490412143161765
AL359915.2 1.76962018098195 2 0.412792559067933
1.13472427777725
PHGDH 8.45476552612238 2 0.0145905274874406
0.618583664676961
NOTCH2 2.19903130907954 2 0.333032347346816
1.48123631072654
SEC22B 0.364607005367694 2 0.833348378560903
0.121688094973589
LINC00623 5.17368656505837 2 0.0752572313324376
0.447228143946704
SRGAP2C 4.56669892233441 2 0.101942182854706
1.09895370105686
AL592494.3 2.74048073595032 2 0.25404588771761
2.06726742614127
AC239800.3 1.71361540306313 2 0.42451510045487
0.6625707827677
AC245595.1 1.46617710626402 2 0.48042288470858

0.36328207580749
NBPF15 8.92573858759108 2 0.01152923502032 0.915512278672084
SRGAP2B 7.12885656102565 2 0.0283131680477192
1.70879168163435
AC245014.3 5.24008485610659 2 0.0727997740092184
0.540007362124224
NBPF20 1.27716802941529 2 0.528039591224295
0.558414020379768
GPR89A 3.0364399656091 2 0.219101544567582
0.567243491590063
RNF115 4.25741652141313 2 0.118990899842492
0.391993219673883
POLR3C 0.191791445270199 2 0.908558753241794
0.129128285163159
NUDT17 2.7976189123835 2 0.246890723262132
0.649824706592435
PIAS3 0.0865215964253897 2 0.95766160106226
0.123367117942585
ITGA10 0.0631692514407601 2 0.968908958365631
0.219511646302959
PEX11B 1.6430229015787 2 0.439766466560199
0.262625813450696
RBM8A 10.674744993243 2 0.00480848844610304
0.227753566824209
LIX1L 2.89433645386782 2 0.235235479308499
0.622508813698137
POLR3GL 0.207966418600448 2 0.901240429751645
0.0779141901644304
TXNIP 0.179432054564372 2 0.914190753647229
0.125559972140625
NBPF12 1.64744389472246 2 0.438795437916577
0.542908886289809
PRKAB2 0.0241924154779543 2 0.987976657289103
0.0912825211176262
AC242426.2 2.65493365389628 2 0.265148077258716
1.32777459720573
CHD1L 1.70102115073923 2 0.427196760113189
0.345042293735019
BCL9 2.31597798609422 2 0.314117238089537
1.64622185112252
ACP6 1.13659900746652 2 0.566487930721902
0.558723298914233
GPR89B 0.159663754276715 2 0.923271556395229
0.174272912557399
NBPF11 2.7497240509394 2 0.25287448363598 1.3506227126808
NBPF14 1.04803286501282 2 0.592137485224512
1.05855188829068
PDE4DIP 4.09281970979396 2 0.129197911206461
0.672181235520811
NBPF9 0.10842278341196 2 0.947231848379703

0.256672754223678
AC245297.3 0.0867206415135494 2 0.95756629688589
0.075804885675605
NBPF19 2.89732647916323 2 0.234884062043825
2.23910536045325
HIST2H2BE 18.2834130586742 2 0.000107104396670943
1.27710043798838
HIST2H2AC 1.33244499738543 2 0.513645213126441
0.335649758638277
BOLA1 4.07394266528248 2 0.130423121558653
0.321392768916958
SF3B4 0.185826618929017 2 0.91127249557264
0.063989997233997
VPS45 1.18495701156901 2 0.552955082560747
0.176942180130174
PLEKHO1 0.534466674567577 2 0.765494432233015
0.154361905929716
ANP32E 0.743801660319375 2 0.689422609064266
0.141582185597565
APH1A 0.00524920247442061 2 0.997378840017318
0.010749206359052
MRPS21 8.0151527808455 2 0.0181773968069401
0.243539460506358
PRPF3 0.0482714453382909 2 0.976153214647031
0.0643586093202011
RPRD2 2.52633051054675 2 0.282757608540836
0.604923404717159
TARS2 0.441285796417556 2 0.802003025876998
0.184490792773813
MCL1 0.0044488537698968 2 0.997778045319116
0.00584579084816335
ENSA 0.972058003205868 2 0.615063969213849
0.0491277992556764
GOLPH3L 0.306653048629924 2 0.857849567332944
0.149704998431553
CTSS 2.13290577924622 2 0.344227366909079
0.146251302537852
ARNT 3.42852760155546 2 0.180096258645513
0.925427241547681
SETDB1 0.00138296327530261 2 0.999308757380681
0.0164592327630261
CERS2 3.94623026665381 2 0.139023105461066
0.403144254467346
MINDY1 2.07309247864881 2 0.354677540354486
2.37579338693936
PRUNE1 1.65518355279731 2 0.437100655962675
0.311131246955707
C1orf56 3.51528651261823 2 0.172450807635281
0.735788058377025
CDC42SE1 0.809507640414718 2 0.667141027284021

0.188129246641267
MLLT11	5.89026888320504 2	0.052594988386414
3.27678835584133
GABPB2	4.40547456527106 2	0.110500273530801
1.04643130044128
TNFAIP8L2	2.79497430053914 2	0.247217404266147
0.342914186352586
SCNM1	0.0904105309417728	2	0.955801268698405
0.0445888983357018
LYSMD1	2.04421871419685 2	0.3598351183259	1.6595439521082
VPS72	1.77723145909915 2	0.411224605008025
0.308258790598015
PIP5K1A	0.0148084254092939	2	0.992623130950542
0.0371060556265516
PSMD4	1.06059176765049 2	0.588430836751196
0.089137597143469
AL391069.2	2.3397364883635	2	0.31040783660983
2.2340158370492
ZNF687	3.53820772605751 2	0.170485698928484
1.51434781390411
PI4KB	0.0522493057100674	2	0.974213643527528
0.0543520621694459
RFX5	0.814432976695196	2	0.66550010168799
0.17354661637686
AL391069.3	0.0565183647249325	2	0.972136373544619
0.0571856778235231
PSMB4	1.66354264843564 2	0.435277585328613
0.146814019605129
POGZ	0.0236985244066078	2	0.988220663840894
0.0433784611273356
SNX27	1.23693192795386 2	0.53877029702746 0.334504846839473
MRPL9	0.755284842431104	2	0.685475568368259
0.104152681968009
TDRKH	0.208527203230771	2	0.900987764285413
0.291460971163226
TDRKH-AS1	0.345764000538902	2	0.841236874712733
0.540651684066172
THEM4	7.11177826818265 2	0.0285559735357563
0.910247098614951
S100A10	6.48151821699909 2	0.0391341767345008
0.231581609681992
S100A11	36.095174855164	2	1.45221984437782e-08
0.675930900827796
S100A6	0.0117518292061197	2	0.994141314820427
0.0272482048100811
S100A4	1.89664500948444 2	0.387390324134479
0.533640819450527
S100A13	0.517237296882303	2	0.772117415086561
0.305457728290404
CHTOP	0.0545395957448562	2	0.973098666155

0.0289983291597271
SNAPIN 0.340985972302912 2 0.843249004028671
0.0762118107348351
ILF2 6.80792835110687 2 0.0332412343385114
0.259236411957599
INTS3 0.868947104498501 2 0.647605507815705
0.474047053920274
SLC27A3 0.370147743489558 2 0.831042890996211
0.278148942165367
GATAD2B 3.24273022578384 2 0.197628729329722
0.533814758364272
DENND4B 1.52419108764283 2 0.466687437634127
0.379519938341958
CRTC2 3.0685990127977 2 0.215606669376198
0.650960284112995
SLC39A1 1.03743612185684 2 0.595283175806264
0.135363342453223
CREB3L4 0.0185023615704642 2 0.990791479732984
0.0575853099298091
JTB 0.516761051150718 2 0.772301295790401
0.0616141007655667
AL358472.2 3.22548959030531 2 0.199339715799206
3.94369790851997
RAB13 0.998576415086128 2 0.606962537346504
0.37600113434026
RPS27 63.2155324132402 2 1.87627691161651e-14
0.136259479448273
TPM3 5.68871713912086 2 0.0581715677898259
0.121888577719628
C1orf43 0.203448100668774 2 0.903278776756907
0.0470414462105246
UBAP2L 1.14580054388733 2 0.563887637318986
0.266893415833779
HAX1 3.43807258527996 2 0.179238798455103
0.130140226708726
UBE2Q1 1.08211381882434 2 0.582132665628148
0.211683582500582
ADAR 1.84661349100289 2 0.397203416454896
0.254551836060262
KCNN3 52.7166961588891 2 3.57036622489204e-12
1.01022707625007
PMVK 1.08558882058478 2 0.581122087803379
0.13672210912158
PBXIP1 0.129598404876738 2 0.937255643131519
0.0848258621974294
PYGO2 2.1693779536062 2 0.338006897533632
0.388090842812961
AL451085.2 5.8410018070666 2 0.0539066784907921
2.40445862854125
SHC1 0.00375179911067599 2 0.998125858844534

0.0221275849198509
CKS1B 0.982772241264296 2 0.611777808356669
0.133366487812796
FLAD1 6.46491382658414 2 0.0394604287390015
0.621629403487693
ZBTB7B 0.0011782808172444 2 0.999411033100513
0.0155800392052331
ADAM15 1.2574425607423 2 0.533273272209453
0.984506056519596
EFNA4 5.30674543314749 2 0.0704133279282489
0.774410162759875
EFNA3 1.81328468721685 2 0.403878033609025
1.15718083220908
SLC50A1 7.04607750398126 2 0.0295096263478274
0.262216352087078
DPM3 0.0189379969293918 2 0.990575691334153
0.0322910233869133
KRTCAP2 0.841657682366076 2 0.656502457977528
0.194670862496365
MTX1 0.50107730222416 2 0.778381394125816
0.0854382706471303
GBA 4.06871354808823 2 0.130764566621944
0.915206066941679
FAM189B 0.8848636736051 2 0.642472132430438
0.344353312335848
SCAMP3 1.43033801995223 2 0.489109440435541
0.144629671827638
CLK2 3.92940699032715 2 0.14019744967557 1.07923502006058
FDPS 6.93615064556971 2 0.0311769785726612
0.243365362081178
RUSC1 3.35787640483824 2 0.186571972573826
1.03474784544691
ASH1L 0.00209494766002945 2 0.998953074579199
0.00703191627130792
ASH1L-AS1 0.159257343593429 2 0.923459189170621
0.220430394283738
MSTO1 2.25134204315387 2 0.324434691622028
0.783711772747331
YY1AP1 0.45738606676212 2 0.795572710291115
0.165372601562583
DAP3 2.78851628527764 2 0.248016961344215
0.215159518132119
GON4L 1.74560206207016 2 0.417779694922345
0.295457920702018
SYT11 0.000717559751631944 2 0.999641284477987
0.00869055322531972
RIT1 1.43775516807035 2 0.487298901187252
0.249784139522098
KHDC4 0.0121503132597924 2 0.993943259820995
0.0319649736114717

ARHGEF2	1.11480357020909	2	0.572695117454237	0.239252244448642
SSR2	10.554556945061	2	0.00510630885919172	0.191035025299241
UBQLN4	0.412477620654336	2	0.813638747744468	0.27950342441716
LAMTOR2	1.64668993588943	2	0.438960885948025	0.116120128410642
RAB25	2.00716218086115	2	0.366564387693147	2.03211224116531
LMNA	0.781109237150534	2	0.676681470291752	0.489724169652144
SEMA4A	34.2288841663753	2	3.69225972018938e-08	0.845283472615644
SLC25A44	2.94344777144133	2	0.229529461373966	0.913692235954122
PMF1	0.602578438856977	2	0.739863758826221	0.071966413617703
SMG5	3.42154873596829	2	0.180725790151602	0.710729706383204
TMEM79	1.60864717100691	2	0.447390445616715	0.490645498203788
GLMP	0.0441252974075693	2	0.978178951488182	0.0896534950031737
CCT3	0.173596201798973	2	0.916862190598847	0.0331537400510146
TSACC	0.750610662025474	2	0.687079460093144	0.672598993809865
C1orf61	0.0171590897694688	2	0.991457154381152	0.132819150043031
MEF2D	0.0808949010455361	2	0.960359629581934	0.138774760072097
TTC24	0.0521931749743004	2	0.974240985575511	0.1789119829386
NAXE	0.0254100815162223	2	0.987375327551287	0.0242519835337752
GPATCH4	0.00301278640861188	2	0.998494740836429	0.00889497430367096
ISG20L2	4.70272367101173	2	0.0952393734997796	0.382739581502624
RRNAD1	6.3014328660925	2	0.0428214371821155	0.581361770576156
MRPL24	2.18969409552971	2	0.334590779442609	0.329820321655872
HDGF	0.63063573787982	2	0.729556933918504	0.145689066051212
PRCC	1.11378609833726	2	0.572986542163635	0.0923241637491618
ETV3	0.0240319198605958	2	0.988055943432104	0.0941756322465621

FCRL5	32.2377568118068	2	9.99217550923248e-08	2.23840904233134
FCRL4	1.3281523930089	2	0.514748834900618	0.982661585039773
FCRL3	5.88791886961372	2	0.0526568241768083	0.408649690417527
FCRL2	2.8263473596558	2	0.243369678776207	0.250272376190382
FCRL1	1.56574208737108	2	0.457091795109851	0.210371838010994
CD1C	7.25551095734036	2	0.0265757673930105	1.07661514248618
PYHIN1	5.65238897856231	2	0.0592378555686681	2.95025658458568
IFI16	16.8463119755864	2	0.000219720123415978	0.331786220125743
AIM2	23.2374231087015	2	8.99617065142078e-06	0.544917741676543
DUSP23	10.8740070741672	2	0.00435250587256375	0.503828004674505
TAGLN2	85.712322263342	2	0	0.876224796926044
PIGM	0.0209899833751583	2	0.98955988858057	0.108673096408624
IGSF8	2.8708045613702	2	0.23801959406741	0.297734822538013
AL121987.2	5.2966120341996	2	0.0707709964340819	2.14624590741528
PEA15	2.56368301262963	2	0.277525764151878	0.226387621559833
DCAF8	8.53782526397794	2	0.013996994765888	0.73399367487334
PEX19	2.06662198074911	2	0.355826868673586	0.404555526865759
COPA	2.08249115371424	2	0.353014701058789	0.317028375147951
NCSTN	0.793068719483995	2	0.672647164321149	0.283020260123061
SLAMF6	2.34436888113338	2	0.309689703090999	0.35888093126714
CD84	3.12938592389476	2	0.209152221006293	0.792963208768877
SLAMF1	38.2408513307986	2	4.96711993758225e-09	2.61013946831211
CD48	15.4652457906959	2	0.000438293011446156	0.577072677341318
SLAMF7	5.40262486253578	2	0.0671173679780787	1.90948114745936
LY9	5.88936630155324	2	0.0526187293788042	0.229782057514773
F11R	0.044807914378395	2	0.977845147680063	0.165914221982312

TSTD1	0.00827708652494957	2	0.995870008706061	0.013307189338596
USF1	3.06852196669214	2	0.215614975363287	1.57410717584585
ARHGAP30	0.970329067764639	2	0.615595902047603	0.0717651915178197
PFDN2	4.32317716227295	2	0.115142063165858	0.159546213013013
NIT1	2.07848165979281	2	0.353723116066982	0.312689140965284
DEDD	0.99457190170443	2	0.608179049626507	0.31851518280739
UFC1	0.626933613505356	2	0.730908639832206	0.0791951402681983
USP21	5.37940415678001	2	0.0679011655832703	1.8097474303212
PPOX	0.111113573019548	2	0.945958304474684	0.126604702135885
B4GALT3	0.241601001873076	2	0.886210740171204	0.110049738629289
NDUFS2	6.4940337591538	2	0.0388900486606599	0.187689992124608
TOMM40L	0.00232473459515774	2	0.998838307989619	0.028434073850689
MPZ	1.94364005489331	2	0.378393724045021	4.54808386162489
SDHC	0.207707066983097	2	0.901357306411058	0.0426373693200555
FCGR2B	1.06386511686196	2	0.587468554629616	0.446157308718851
FCRLA	24.1493116004551	2	5.7022153528985e-06	0.61088279886364
FCRLB	8.3761060815894	2	0.0151758027975798	1.45821282298638
DUSP12	1.90153454459931	2	0.386444401589486	0.311485462868673
ATF6	6.59985941634418	2	0.0368857600776166	0.32807440772951
UHMK1	5.68536133950911	2	0.0582692557845428	0.496185506914693
UAP1	0.115325558161079	2	0.943968219600722	0.13312999066232
HSD17B7	0.086678812416239	2	0.957586324162227	0.0639674907253298
RGS5.1	0.00686411842931335	2	0.996573823568625	0.0677538088115224
NUF2	2.24271566235578	2	0.325837062391021	1.04256208707987
MGST3	0.592935935291182	2	0.743439440998353	0.362120247242194

ALDH9A1 0.197875555023955 2 0.90579906734174
0.0856164109447561
TMCO1 17.7834785748555 2 0.000137520262589508
0.27471635956635
UCK2 0.101901377200721 2 0.950325531259093
0.370654600462315
POGK 1.7797151180917 2 0.410714251115776
0.464974065185504
TADA1 0.00150533525122595 2 0.999247615557612
0.0268662031957895
POU2F1 0.436377699295436 2 0.803973597198518
0.175591386724911
CD247 0.451154769062478 2 0.798055300922941
0.360510246576727
CREG1 0.160472448318753 2 0.922898309757478
0.149848410414398
AL031733.2 0.130988579387938 2 0.936604395042083
0.2942701362701
RCSD1 69.885483221009 2 6.66133814775094e-16
0.812442146238738
MPZL1 8.23506598432174 2 0.0162846392601912
0.548674913290885
MPC2 0.0184379462673579 2 0.99082339131362
0.0136486530564248
DCAF6 1.59375956771857 2 0.450733157157205
0.246690390805538
TIPRL 0.458808299566429 2 0.795007166595251
0.102580222747267
SFT2D2 0.0579829481207343 2 0.971424746740046
0.0657207821184202
TBX19 1.51025514788821 2 0.46995065744722 1.34915068532464
ATP1B1 0.513726523417689 2 0.773473970043735
0.708262860949585
NME7 0.622543945922695 2 0.732514624603399
0.237825461017835
BLZF1 5.33014615078528 2 0.0695942677190083
0.879031169175567
C1orf112 2.31881553939164 2 0.313671891885279
0.796659016583189
SELL 21.5468834888887 2 2.09485370529228e-05
0.70516420902616
METTL18 0.48096834067932 2 0.786247091374009
0.18840981132936
SCYL3 3.5961337572514 2 0.165618740092194
0.790072450457523
KIFAP3 0.0352565917962439 2 0.982526173500759
0.0747631155979297
GORAB 0.675897204230033 2 0.713231945569716
0.33482709995236
FMO4 4.38142340842415 2 0.111837125333411

2.94462911265554
PRRC2C	2.55077891088261 2	0.279322163499035
0.153636113692794
VAMP4	5.35890205875829 2	0.0686008036448683
0.376636298484744
METTL13	2.02883200208528 2	0.36261413428523 0.461441374778916
PIGC	2.51403017252098 2	0.284501974196522
0.550810209058207
SUCO	0.691382995843964	2	0.707730789850691
0.40676985196454
AL645568.1	0.00533378052189003	2	0.997336662731705
0.0398853061285722
PRDX6	5.70932142260996 2	0.0575753524735251
0.195128955715439
KLHL20	0.236191844982632	2	0.888610810763599
0.197635370931377
CENPL	3.51662924321546 2	0.172335069003115
1.04375577441731
DARS2	3.63953850308113 2	0.162063142440148
1.27158124656539
ZBTB37	1.24465607931776 2	0.536693538246292
0.26937376803134
RC3H1	6.67328933585025 2	0.035556060184895
0.716156947361848
AL121983.2	0.0844742776559049	2	0.958642422275366
0.0508563725558224
RABGAP1L 5.46248582562561 2	0.0651382781329237
0.316106699531508
CACYBP	0.0777185968389692	2	0.96188603851151
0.0322573251542164
MRPS14	5.27154958893829 2	0.0716634234688825
0.301521018948164
KIAA0040 36.9844468464996 2	9.3095654518649e-09
0.496412936776027
COP1	0.0216142919884163	2	0.989251041406178
0.0309888171918355
C1orf220 0.142366361358144	2	0.931291281932385
0.279667167970082
AL137796.1	0.350331333060271	2	0.839317962356359
0.552640305037656
RALGPS2	3.11112820483605 2	0.211070283794694
0.289895922304599
FAM20B	2.22865255743843 2	0.328136276838426
1.08952279758607
TOR3A	9.22899047397756 2	0.00990718292456116
0.354525902373717
ABL2	3.79774153142376 2	0.149737612634507
1.22103077342558
SOAT1	0.799935493860906	2	0.67034166626336
0.310719146043898

TOR1AIP2	0.3564695738683	2	0.836745943409477	0.135770703636592
TOR1AIP1	0.895551270634579	2	0.639048047760909	0.240823818146033
CEP350	1.8691443585177	2	0.39275385784021	0.266068185697168
QSOX1	0.263228509224111	2	0.876679104784429	0.123284745935808
ACBD6	0.24381615615392	2	0.885229736782543	0.0701813653065109
XPR1	0.0591482127626641	2	0.970858928133321	0.160032287496938
STX6	0.705401364319212	2	0.70278751874097	0.260775046519381
MR1	0.601573356338877	2	0.74023566433221	0.247821565708254
IER5	3.12279165912092	2	0.209842961673925	0.62561676986281
GLUL	12.4806357973709	2	0.00194923576485295	0.77949735355544
RNASEL	1.83199032527136	2	0.400118245169346	0.439316515680515
RGS16	1.49478916345809	2	0.473598869841758	0.236353031320737
DHX9	0.00104782673847911	2	0.999476223849405	0.00515094478013654
SMG7	5.08040405130853	2	0.0788504683639905	0.72160235152309
NCF2	21.005217822639	2	2.74647028262187e-05	2.45890749934706
ARPC5	18.3361817183444	2	0.00010431547267431	0.26831643073346
TSEN15	12.7841587507153	2	0.00167477010251038	0.909335625256723
C1orf21	3.92738070759263	2	0.140339561488943	1.52516794669461
EDEM3	0.228802056853962	2	0.891900206816467	0.246009248458951
FAM129A	12.7667949844491	2	0.00168937356185095	0.684927279348857
RNF2	0.769210356870354	2	0.680719345832246	0.28107304025161
TRMT1L	2.44046474917874	2	0.2951615709055	0.305171142004128
SWT1	3.25167061176924	2	0.196747262400196	2.31667394495793
IVNS1ABP	2.66408215570972	2	0.263937993150026	0.489597343721465
TPR	0.0495330352461057	2	0.975537656290954	0.0261316152880711
ODR4	0.0658754894004822	2	0.967598795867082	0.0989061210102643

AL390957.1	1.91750144098236	2	0.383371525126424	1.05086021443321
AL136987.1	2.41025073037629	2	0.299654430924287	0.905856952973372
RGS1	69.693139639911	2	7.7715611723761e-16	0.435183762249578
RGS13	306.830537399696	2	0	0.943644954557975
RGS2	27.3224285108441	2	1.16683657225281e-06	0.383308614680701
UCHL5	0.0868584403159141	2	0.957500323414235	0.0720398999463679
TROVE2	0.605109795611092	2	0.738927921624091	0.116642084515184
GLRX2	0.54029097869065	2	0.76326843883288	0.247062747287455
CDC73	2.1198097690462	2	0.346488765206917	0.267477587358841
ASPM	0.988460461091683	2	0.610040317006339	0.632975294791443
ZBTB41	0.0194008051440653	2	0.990346494570109	0.122401083848401
DENND1B	7.62338164522679	2	0.0221107619568804	0.989481330874888
C1orf53	0.859111613934773	2	0.650798110515493	0.844295493757506
NEK7	1.95400907883977	2	0.37643701392852	0.465912853388316
AL157402.1	3.63451761252968	2	0.162470504206222	2.33547037199182
PTPRC	17.0897929091234	2	0.000194535390616313	0.238083668487718
MIR181A1HG	1.17953876917954	2	0.554455135890335	0.364404579703001
ZNF281	4.10984178934915	2	0.128102968803766	0.465237408898803
DDX59	0.0248928011850685	2	0.987630735497444	0.0615029544109552
KIF21B	0.000396144683405694	2	0.999801947273328	0.0187071926017122
TMEM9	4.12407697773459	2	0.127194421029712	0.495883011940651
CSRP1	0.590605185834165	2	0.744306331563137	0.219223939366175
IPO9	2.58018992655858	2	0.275244643714657	0.287951637390983
TIMM17A	0.085692134988901	2	0.958058855116939	0.0387959118051764
RNPEP	2.4151328982843	2	0.298923841379211	0.252505826319088
ARL8A	0.183405866927913	2	0.912376145712327	0.130623601359674
PTPN7	6.82552314945313	2	0.0329500805070659	

0.220186700440384
UBE2T	2.3551159982511	2	0.308030030503885
0.445245174473873
PPP1R12B	1.53694154979322	2	0.4637216611794	0.697523996894178
KDM5B	0.478458949865739	2	0.787234211125969
0.291457009394971
RABIF	2.63187304794481	2	0.268223008299149
0.184531989578623
KLHL12	0.146383975590301	2	0.929422375143194
0.11251779893401
ADIPOR1	0.471370423939852	2	0.790029326569724
0.134244866959567
CYB5R1	0.0803605190723313	2	0.960616263302437
0.0903491846868767
TMEM183A	0.142985218505751	2	0.93100315837835
0.0205363752556314
LINC01353	6.08168842742426	2	0.0477945236656596
1.1900575271527
LINC01136	2.23052864584636	2	0.327828614828669
1.01785424195083
BTG2	9.24218351123849	2	0.00984204508550912
0.152150193756076
PRELP	9.31992395566733	2	0.00946682234396123
2.25756209873179
ATP2B4	6.90385928592603	2	0.0316844376990143
1.74718854297709
LAX1	1.50045991511739	2	0.472257940970264
0.428112051335842
SNRPE	13.5514492411079	2	0.00114114330439097
0.267867240968189
PPP1R15B	0.669907850683577	2	0.715371046069692
0.327353248470821
PIK3C2B	0.590854890975702	2	0.744213408805146
0.340749736640168
MDM4	2.12150170178232	2	0.346195771313605
0.104214690061037
RBBP5	0.582268588616465	2	0.747415297657625
0.207447499916881
DSTYK	4.2511397203437	2	0.119364927563583
1.03678430564954
TMCC2	0.00696133955268548	2	0.996525380732726
0.0743186911396957
NUAK2	12.2298140133024	2	0.00220968124552046
1.43225484729378
ELK4	1.01038711658706	2	0.603388773222108
0.192220025639577
SLC45A3	0.122846699257866	2	0.940425026904142
0.205047158482355
NUCKS1	2.08425537554071	2	0.352703440241918
0.143282195683684

RAB29	0.841612509889455	2	0.656517286065951
0.23002204431032
SLC41A1	5.46310209702262	2	0.0651182097961361
0.542535295557903
RHEX	9.00169784043072	2	0.0110995698883116
2.05137169553812
SRGAP2	1.94460875106124	2	0.378210494146903
0.736105990495743
IKBKE	2.65062886719619	2	0.265719394846508
0.337248855683241
RASSF5	5.84014231130697	2	0.0539298497501247
0.39885521853172
EIF2D	1.5812503098812	2	0.453561160633358
0.254502742415367
MAPKAPK2	0.156528774566862	2	0.924719910036487
0.126244118827233
FCMR	9.41034552543742	2	0.00904835086516687
1.21917792435169
YOD1	0.375707702756388	2	0.828735816979947
0.616251664005177
CD55	7.2106595476663	2	0.0271784805833292
0.350834953318334
CR2	6.43696799471448	2	0.0400156761714155
0.75861061428744
CR1	8.92698519482208	2	0.0115220510455792
0.81923748670385
CD46	19.5123572925325	2	5.79355920552471e-05
0.461848243310729
MIR29B2CHG	2.15619693851913	2	0.340241891323609
0.583652202517182
TRAF3IP3	0.0763576152926791	2	0.962540815845234
0.0555776161036082
C1orf74	1.44372618166102	2	0.48584623655546	1.30156544013684
UTP25	2.62975141003716	2	0.268507695324203
0.464204757298873
RCOR3	0.251676818025877	2	0.881757319307544
0.178720560643411
TRAF5	23.6704859541459	2	7.24468126411804e-06
0.908066589186203
LINC00467	2.66397939814195	2	0.263951554311511
0.358885333425842
LPGAT1	1.64822747090387	2	0.43862355676244	0.276887104920594
INTS7	2.31136967185551	2	0.314841848047001
0.331894662840236
DTL	0.673506665094146	2	0.714084959698499
0.750104673812754
PPP2R5A	2.12781630217337	2	0.345104451056549
0.349168830943542
TMEM206	0.106039684308583	2	0.948361194766456
0.0902574174570821

NENF	0.65554333364159	2	0.72052752126364	0.16736492845736
NSL1	3.1184128534149	2	0.210302895759673	0.279589983026722
TATDN3	6.57314824409894	2	0.0373816954048054	0.574494113663501
FLVCR1-DT	4.06453758278687	2	0.131037886012148	1.37160257980676
FLVCR1	6.38935793931756	2	0.0409796789849438	1.49411043782848
ANGEL2	2.15139970088051	2	0.341058981479416	0.553410851976696
RPS6KC1	1.53412941385252	2	0.464374143964417	0.415484981689414
SMYD2	0.795451460275605	2	0.671846269578627	0.23147852545574
CENPF	0.0357787100495393	2	0.98226970955358	0.0958724828639382
KCTD3	2.88526375048349	2	0.236305014232281	2.60841383679807
GPATCH2	1.97874426972488	2	0.371810064328083	0.420098990156418
RRP15	0.031013337909718	2	0.984612940390585	0.0360357085977368
LYPLAL1-DT	0.00874512874594277	2	0.995636981368435	0.0964084495118382
LYPLAL1	15.2053781803785	2	0.000499107482213601	0.305473655893005
AC096642.1	1.74313400013527	2	0.418295566238572	0.627463321975476
ZC3H11B	6.48745217825327	2	0.0390182384686169	1.23755927535368
EPRS	0.00117473877695912	2	0.999412803079151	0.00550227160963788
BPNT1	10.0283545620869	2	0.00664309518992678	0.55657448462475
IARS2	0.343464409674626	2	0.842204681311805	0.112445547812549
RAB3GAP2	12.5537439265858	2	0.00187926983486264	0.829572766505599
C1orf115	5.18696445480036	2	0.0747592575617225	3.08076238735275
MARC2	0.472742592134372	2	0.789487485907781	0.59034073361411
DUSP10	10.5755537348887	2	0.0050529812292921	1.03907478974146
TAF1A	1.61273919336326	2	0.446476015550039	0.520361340969949
MIA3	0.940154399826887	2	0.624954020024092	0.141676478375027
AIDA	4.82976449209875	2	0.089377862898576	

0.528305883346169
BROX 2.72206642229625 2 0.256395729126285
0.453524221773545
AL392172.1 1.35907370742847 2 0.506851684488107
0.573009939476968
TP53BP2 1.11119178920226 2 0.573730276538856
0.669453017855593
FBXO28 0.9676138657322 2 0.616432203223019
0.332845980953384
DEGS1 11.6767896385695 2 0.0029135155933746
0.47600772517749
NVL 9.52903500978071 2 0.00852700148652796
0.436703947480126
CNIH4 0.367286293253824 2 0.832232735901915
0.122991561280893
WDR26 0.0153799430941815 2 0.992339520637666
0.0351134119378347
CNIH3 0.22470581924729 2 0.893728796345834
0.350546131422414
LBR 0.474441419518596 2 0.788817169155916
0.0828840740876563
SRP9 12.2592479772178 2 0.00217739953383678
0.259013211677378
TMEM63A 4.53569720462238 2 0.103534684930943
0.599129164203568
PYCR2 3.50815664366098 2 0.173066680586398
0.30580323145336
SDE2 7.48247398503483 2 0.0237247376496587
1.66561299127626
H3F3A 9.21712566502391 2 0.00996613101998611
0.0695016060958362
ACBD3 1.60296803672937 2 0.448662646218971
0.272433876078846
MIXL1 8.53678459835812 2 0.0140042797566503
0.925517433780208
LIN9 0.0642761252893638 2 0.968372876729153
0.131520846449202
PARP1 44.9154651523617 2 1.76494263648408e-10
0.355012033540167
ITPKB 4.51665959579161 2 0.104524916781796
0.462748624885084
ITPKB-AS1 0.457694432684617 2 0.795450055990598
0.216475629297106
PSEN2 1.48168141319148 2 0.476712971267191
0.778137837006484
COQ8A 0.414864572399302 2 0.812668268766934
0.221808328986367
ZNF678 1.92824034389533 2 0.381318546948245
1.01337517666897
SNAP47 0.539623639262205 2 0.763523160888894

0.160595615866566
JMJD4 1.7405420006407 2 0.418838028626338
0.358556038448924
ARF1 8.09432583663673 2 0.0174718735224193
0.158007200869604
C1orf35 0.0122517255517228 2 0.99389286206673
0.0227153298501018
MRPL55 6.92159524643181 2 0.0314047029057051
0.232567129496532
GUK1 15.3365406467862 2 0.000467425620048978
0.205024041501163
IBA57 0.104440091397248 2 0.94911999409078
0.161697915940733
TRIM11 0.89292875265725 2 0.639886554887583
0.280049963119307
HIST3H2A 0.0382841573781421 2 0.981039967468023
0.098885104878461
RNF187 0.0279528896168487 2 0.986120772252998
0.0275531284228767
RAB4A 11.9939565840234 2 0.00248625356972143
0.516564093319176
NUP133 0.893606985253013 2 0.6396695957173
0.331534586167931
ABCB10 3.90458086789336 2 0.141948575129586
2.37285907767758
TAF5L 5.18117699259949 2 0.0749759040575484
1.88014394727441
URB2 0.589054904986675 2 0.744883497151671
0.858496981208334
GALNT2 2.91061922616649 2 0.233328111280608
0.274353185499706
COG2 0.120849299428416 2 0.941364698445158
0.086617944227859
C1orf198 0.0823318095921394 2 0.959669902900364
0.278427749740193
TTC13 4.42913637426975 2 0.109200658318166
0.669939694353394
ARV1 7.86160730423904 2 0.019627892215783
0.855370611889043
FAM89A 0.28750991646578 2 0.866099952721549
0.33571226281797
C1orf131 0.0327429448770792 2 0.9837618117722
0.0296071903167202
GNPAT 0.920827072862857 2 0.631022640692163
0.156583073369995
EXOC8 0.26469285566157 2 0.876037458749172
0.294650484796232
SPRTN 2.15954054144655 2 0.339673549641279
0.509366066504056
EGLN1 0.201328385427135 2 0.904236631157157

0.11049358976783
AL445524.1 5.60198439424394 2 0.0607497569785862
0.965600817276086
TSNAX 1.55085739408649 2 0.460506320978528
0.22269728436506
MAP10 5.05835106555073 2 0.0797247236186709
1.22500276418093
NTPCR 0.507992265619338 2 0.775694801800113
0.169237359842261
PCNX2 0.0824186933909479 2 0.959628213922512
0.213905386482062
AL355472.1 0.557256184540066 2 0.756821318580113
0.442155049178703
COA6 1.04904031705026 2 0.591839285278386
0.181216831770465
TARBP1 2.7177337420855 2 0.256951771548177
0.591164582718548
IRF2BP2 0.553148457176808 2 0.758377323764113
0.176463592276625
TOMM20 3.40104695832384 2 0.182587918060723
0.223555133017408
RBM34 0.9276476640186 2 0.628874332236576
0.352194075322264
ARID4B 10.9456867855629 2 0.00419927497841299
0.263238299271935
GGPS1 0.0704331997664215 2 0.965396288886477
0.0610095408153112
TBCE 0.0245643305643806 2 0.987792952658322
0.067729728660828
TBCE.1 0.0464265609615825 2 0.977054074986432
0.119009838156063
B3GALNT2 0.0958100223499657 2 0.953224328436164
0.287881629661589
LYST 0.0298277347160344 2 0.985196793551364
0.0725809916670937
GPR137B 206.590743237355 2 0 2.3721241622984
ERO1B 5.77699375694449 2 0.0556598132282935
0.875216002096405
LGALS8 17.6505266784928 2 0.00014697275342046
1.18181174940214
HEATR1 0.573076758019592 2 0.750858260781549
0.363905783129035
ACTN2 3.12989051713624 2 0.209099459263798
3.67007673231015
MTR 1.85201294000381 2 0.396132522869086
0.605452809859912
FH 5.20266176530759 2 0.074174794546769
0.282796068965872
KMO 20.7659843676726 2 3.09544996832667e-05
2.25904669582122

OPN3 1.08582641225729 2 0.581053057019359
0.427088364069496
CEP170 7.43699744476881 2 0.0242703770825026
0.781165434486907
SDCCAG8 7.29174343560248 2 0.026098649277796
0.5476313501083
ZBTB18 2.18175724098643 2 0.335921216742397
0.977376619425466
ADSS 1.50807823342201 2 0.470462457125019
0.278260842360357
DESI2 3.46676934768845 2 0.176685374406346
0.370644976285092
COX20 0.443625718104831 2 0.801065262420473
0.143584440254179
HNRNPU 1.24141756777402 2 0.537563286338091
0.0868998087376291
EFCAB2 0.796380060756664 2 0.67153440359939
0.427631579017356
SMYD3 6.304593927892 2 0.0427538100349534
0.652530501818845
TFB2M 0.601585503316798 2 0.740231168532727
0.217340361216701
CNST 0.597503235330911 2 0.741743622569027
0.290145597908812
SCCPDH 6.34943036781461 2 0.0418060094136772
0.637084098597807
AHCTF1 0.686701916130696 2 0.709389202004894
0.451332754087142
ZNF670 4.21966118114188 2 0.121258507027892
0.692722525014478
ZNF669 2.07513492783451 2 0.354315519811347
0.39722879290627
ZNF124 1.29757564137881 2 0.522678973547277
0.761083253943112
AL390728.6 1.69585314881564 2 0.428302064390842
0.763586353541926
ZNF496 3.1813783529913 2 0.203785119413309
2.4356029329166
SH3BP5L 1.54634417623175 2 0.461546677050476
0.414726028458268
ZNF672 2.86999166408542 2 0.238116356471422
0.435876047469446
ZNF692 0.0564269770919155 2 0.972180795180582
0.102916321423386
PGBD2 8.74245402965096 2 0.0126357268280085
1.38906764938356
SH3YL1 1.76761268102953 2 0.413207107606013
0.492889767758157
ACP1 1.5590334516073 2 0.458627600641831
0.130772460414183

TMEM18	1.10770445723708	2	0.574731543188853	0.188818785366249
PXDN	1.31775687724454	2	0.517431340181686	1.18742845063211
EIPR1	1.28022186738065	2	0.527233932792954	0.192606649664611
TRAPPC12	0.840753385226215	2	0.656799361742307	0.194751546621457
ADI1	19.7321068735919	2	5.19071798753545e-05	0.602626857972303
AC231981.1	2.60740064114376	2	0.271525201537919	1.54589219005794
RNASEH1	7.92046893336033	2	0.0190586451504429	0.364506550348409
RNASEH1-AS1	6.62010537171955	2	0.0365142499184105	0.853084817351843
RPS7	23.8941496524833	2	6.47815489718262e-06	0.188777484136941
LINC00487	7.89813573621767	2	0.0192726580644813	1.34837564183872
CMPK2	3.16295477805285	2	0.205671017533111	0.611877895319816
RSAD2	2.81265986582944	2	0.245040951625199	2.50978854292285
LINC01814	3.25273724155618	2	0.196642362129834	2.40558660715079
ID2	1.38698078235256	2	0.499828424136845	0.279902429842796
KIDINS220	0.0785989400115306	2	0.961462736777649	0.104627167793547
MBOAT2	8.59057820089118	2	0.0136326299281386	2.76468730037562
ITGB1BP1	1.5167408024728	2	0.468429156944228	0.139298325923598
CPSF3	1.40483832007341	2	0.495385436390463	0.208333109095011
IAH1	9.75155207264003	2	0.00762917140689523	0.368016240503607
ADAM17	1.63907205551526	2	0.440636049981599	0.152152759711114
YWHAQ	6.71128611643205	2	0.0348869286138436	0.236766957577329
TAF1B	3.06178721626507	2	0.216342255712671	0.284013794635257
AC010969.2	0.0105522480521086	2	0.994737770269566	0.0621824566922591
CYS1	3.51481328807683	2	0.172491616440202	1.08270101424996
RRM2	1.63988563355944	2	0.440456840526419	0.45905278098278

HPCAL1 3.49510084045384 2 0.174200138373529
0.47788927428272
ODC1 22.5023040046865 2 1.2992321844485e-05
0.614179697869775
NOL10 0.901519055140791 2 0.637144039332544
0.131083883446614
RN7SL832P 3.32915096515036 2 0.189270989762822
2.17029750462012
ATP6V1C2 7.62470935497421 2 0.0220960884908588
1.68445793253704
PDIA6 58.7453703081758 2 1.7519319328585e-13
0.676692813580663
PQLC3 7.30392862470277 2 0.0259401241943366
1.44495909741769
ROCK2 5.21671231536192 2 0.073655522365637
0.41643463021469
E2F6 0.68157952725533 2 0.711208414367053
0.174522366047054
GREB1 2.86720928527626 2 0.238447851956641
2.44164750067237
LPIN1 21.7319939031965 2 1.90966629736167e-05
1.12625598934199
MIR3681HG 29.7647266617973 2 3.44089743697928e-07
1.50874668577838
TRIB2 8.79070135651682 2 0.0123345541069669
2.52373972242326
NBAS 0.504743032112484 2 0.776956032801766
0.265731694977745
DDX1 10.4613043245802 2 0.00535003507356002
0.452601921093564
FAM49A 6.5537017825582 2 0.0377469390524068
0.557131585757956
SMC6 0.715219094406617 2 0.699346083338719
0.0969431727741237
GEN1 8.73465787460015 2 0.0126850779956217
0.992594953342086
RDH14 0.335385658885444 2 0.845613542378392
0.0956405620792566
LINC00954 3.16710989906863 2 0.205244167106794
0.362180468439533
TTC32 0.122791584133403 2 0.940450943082437
0.137464072613512
LAPTM4A 23.5782087227545 2 7.58677194001578e-06
0.640424241542901
PUM2 0.000118526587628561 2 0.99994073846222
0.00327791308830355
RHOB 1.53943679102901 2 0.46314347322855 0.7498190974209
HS1BP3 0.137148493981182 2 0.933724131323035
0.173935198640393
LDAH 1.95775393842127 2 0.375732821530454

0.499935657673177
ATAD2B 3.01359437678609 2 0.221618645259013
0.459323170327414
UBXN2A 2.25970430092684 2 0.323081020270359
0.396817277074512
WDCP 3.17854843428798 2 0.204073671170113
1.53460900329411
FKBP1B 0.0615616651937115 2 0.969688073805555
0.137254273980192
SF3B6 0.205479482056253 2 0.90236179067545
0.032905921641892
FAM228B 0.000510393818328464 2 0.999744835650797
0.0010600328698478
TP53I3 0.432513005923066 2 0.805528654886252
0.274129249617657
ITSN2 12.4585014100034 2 0.0019709281501008
0.240588996431828
NCOA1 0.0150439176218679 2 0.992506260322528
0.046736792937239
PTRHD1 2.25973702683289 2 0.323075733754053
0.166867011374301
CENPO 0.179959682018183 2 0.913949609387388
0.329374579968487
ADCY3 3.92629664242346 2 0.140415650723736
1.1937025611868
DNAJC27 1.63081157922497 2 0.442459745358951
1.1229058824284
DNAJC27-AS1 0.0254815560923874 2 0.987340042065268
0.165573778769277
POMC 2.52450692784851 2 0.283015542055105
0.432566387048714
DTNB 0.931881804491483 2 0.627544369414429
0.704574404312329
ASXL2 0.0192621017550245 2 0.990415179159339
0.0509575702885802
RAB10 1.07695053777893 2 0.583637464496417
0.14990546893417
HADHA 9.92332885182764 2 0.00700126504238674
0.295195835335615
HADHB 0.0618488902239541 2 0.969548824461606
0.0344138441772141
SELENOI 0.0660321302140878 2 0.967523016103357
0.181310415249873
SLC35F6 1.85847479599231 2 0.39485471257961 0.719871523362776
TMEM214 1.55501251007964 2 0.459550585530582
0.414921089734365
AGBL5 4.56267253991585 2 0.102147618682501
1.46464808188688
OST4 13.4329264502477 2 0.00121081302306292
0.187160512947002

KHK	1.4824645985806	2	0.476526330496208	0.979449510189501
PREB	7.59105570810879	2	0.0224710412553994	0.317217673136013
SLC5A6	2.17902109885543	2	0.336381095340886	0.277510084157908
ATRAID	0.0325916407607098	2	0.983836238193245	0.023410921537572
CAD	2.88277940260681	2	0.236598728546826	0.93596112508559
UCN	0.929006958794589	2	0.628447064651391	1.01980828970682
MPV17	0.558908241840605	2	0.756196420615174	0.155564490387096
GTF3C2	4.43197796604073	2	0.109045616639505	0.676491810232299
AC074117.1	0.0689234040808275	2	0.966125339607188	0.138790824754134
EIF2B4	0.0287347307241328	2	0.985735352713869	0.0398246011003788
SNX17	0.123483530221464	2	0.940125628685148	0.0313674532491282
ZNF513	0.941392564543873	2	0.624567241751582	0.482844103243818
PPM1G	5.29371946600478	2	0.0708734254536585	0.232468633447146
NRBP1	1.73707974878481	2	0.419563717945873	0.221005868870968
KRTCAP3	0.0150363789794016	2	0.992510001404499	0.097973375154172
IFT172	3.91443919400488	2	0.141250609064695	2.2704933846067
ZNF512	0.0246586088946223	2	0.987746390020692	0.065465525082876
GPN1	6.92499608423575	2	0.0313513471317129	0.257940930099616
SUPT7L	0.188989907163457	2	0.909832326008919	0.115546139000195
SLC4A1AP	0.650359473995049	2	0.722397500414963	0.205654418708121
MRPL33	1.54030530514802	2	0.462942393569027	0.0670371094379
RBKS	2.40338002128051	2	0.300685620372894	0.920653120896137
BABAM2	0.0606261970509234	2	0.97014173604467	0.0563265782007021
PPP1CB	5.12005752944888	2	0.0773025168257242	0.31081111913529
TRMT61B	0.0304277549224378	2	0.984901268892065	0.0537995608197627

WDR43 0.415208448010813 2 0.812528552379604
0.128278120160347
CLIP4 1.50099115279947e-06 2 0.999999249504705
0.000414833932332318
YPEL5 7.20049043900752 2 0.0273170229590892
0.268940334083293
LBH 9.72209787929603 2 0.00774235836363102
0.572203495431736
LCLAT1 0.00261226665127777 2 0.998694719295241
0.0386600301302354
GALNT14 8.02223715640029 2 0.0181131229563738
1.22740363815929
EHD3 5.57114968830074 2 0.0616936146223267
0.70132113357341
MEMO1 0.364019379817279 2 0.83359326293404
0.206660291629204
DPY30 6.24270869112553 2 0.0440974048339881
0.295614932875518
SPAST 2.15168022404336 2 0.341011147362038
0.444231651698705
SLC30A6 0.0030913261416874 2 0.998455530851108
0.0249835342966792
YIPF4 1.63335375985572 2 0.441897696344883
0.183866686676008
AL133245.1 0.382923461213547 2 0.825751225500171
0.579521363927029
BIRC6 0.344286218142253 2 0.841858686932662
0.114880640188062
TTC27 2.74633910419039 2 0.253302829346231
0.581070348748433
LTBP1 19.0170503664891 2 7.42164166771531e-05
1.04953694045421
RASGRP3 0.199331181119518 2 0.905140054809911
0.0436915058702289
FAM98A 0.50380290710987 2 0.77732133654902 0.0775062535135817
FEZ2 1.89452935527547 2 0.387800332940345
0.225768925203104
STRN 0.709105500230859 2 0.701487113089292
0.213281108066759
HEATR5B 0.989744273482532 2 0.609648854002099
0.436926040996095
GPATCH11 1.32556158093098 2 0.515416075730487
0.322637469461907
EIF2AK2 0.371900587373485 2 0.830314865847037
0.131058666467671
CEBPZOS 0.310052117083092 2 0.856392860843901
0.0970594685178
CEBPZ 0.611072585715135 2 0.736728166373886
0.0513028264285257
NDUFAF7 0.355604842101776 2 0.837107802030389

0.201329576445466
PRKD3 3.69186874361077 2 0.157877735445848
0.270182356154446
QPCT 12.4879716157209 2 0.00194209924116806
0.696284742278205
CDC42EP3 8.06022755187691 2 0.0177723077776283
0.597697023096096
RMDN2 1.29861086662466 2 0.522408498319567
0.554159236612054
ATL2 4.60338438073706 2 0.100089330138174
0.39708464044465
HNRNPLL 22.1247170840292 2 1.56920154974483e-05
3.20346709254356
GALM 1.0442410493944 2 0.593261188188895
0.326672936924079
SRSF7 2.2155265122851 2 0.330296925067584
0.127794288099476
GEMIN6 0.507007261962487 2 0.776076926999255
0.156696460619266
DHX57 1.01578734309182 2 0.601761752757449
0.448560004749486
MORN2 2.473125624902 2 0.290380597159998
0.793552395864728
SOS1 10.1943374399576 2 0.00611403262154619
0.985905714808433
MAP4K3 0.0037401394755065 2 0.998131677753179
0.0636774178940911
AC007388.1 0.0676108024553183 2 0.966759616568271
0.114107134063061
THUMPD2 0.00328543254363954 2 0.998358632248042
0.0163850782208327
EML4 2.43305318695011 2 0.296257404285883
0.485297007348359
COX7A2L 5.9962163793851 2 0.0498813452058093
0.202319655669298
MTA3 4.48083641644302 2 0.10641399186587 0.350630758672129
HAAO 1.37522499752634 2 0.502775013222127
0.814985667570027
ZFP36L2 20.4611808996864 2 3.60504774634673e-05
0.541289919498664
AC010883.1 0.918136253067177 2 0.631872196170736
0.784147155847349
THADA 0.517602366798944 2 0.771976489528691
0.342997716287486
DYNC2LI1 2.3159248921745 2 0.314125577057934
0.458016355273199
LRPPRC 0.798927024459618 2 0.67067976102517
0.246717504866547
PPM1B 0.684426396045543 2 0.710196776019176
0.158747927016546

PREPL 0.00594051137588715 2 0.99703415115725
0.027952445970478
CAMKMT 0.0425118712988855 2 0.978968379595998
0.0844189263958793
SRBD1 0.634621774249235 2 0.728104361664699
0.478763410036861
PRKCE 0.803384299725614 2 0.669186723208164
0.300201271246459
ATP6V1E2 0.107566345493395 2 0.947637557876065
0.161133012889947
RHOQ 3.34233234253185 2 0.188027665273969
0.361449233032494
PIGF 4.29343354530167 2 0.11686723025896 0.395422756352647
CRIPT 3.05483894545395 2 0.217095165100264
0.218964593120905
SOCS5 0.113583663131613 2 0.944790724502878
0.328604707389093
MCFD2 2.02742166804789 2 0.362869927991284
0.378134593864418
TTC7A 2.62390086714311 2 0.26929430317846 0.532564812208662
CALM2 19.2992367667912 2 6.44501575922662e-05
0.22455063468256
EPCAM 3.3504469383326 2 0.187266326555841
1.69573116030876
MSH2 1.21838768593212 2 0.543789071805877
0.308246891308076
KCNK12 7.98805396967545 2 0.0184253658517037
0.696393694387066
MSH6 0.0818971306768546 2 0.959878499703913
0.0523302889486256
FBXO11 3.61558611049633 2 0.164015711281729
0.455118921850118
AC079807.1 1.47352750896354 2 0.478660474464253
1.62984320551207
FOXN2 0.118035497622736 2 0.942690037380963
0.136901155260334
AC093635.1 0.170475750804879 2 0.918293818906974
0.320701429097934
PPP1R21 6.54503927348504 2 0.0379107852270203
0.850250833501674
ASB3 0.257867723360234 2 0.879032101329365
0.201992344493657
CHAC2 0.527988433506905 2 0.767977981049871
0.132440680422938
ERLEC1 4.70581326982377 2 0.0950923613535409
0.442368420584569
PSME4 8.73202348540142 2 0.0127017977209987
0.94912899767113
ACYP2 8.47254370087155 2 0.0144614057511824
0.231435239250357

SPTBN1	21.6988181436257	2	1.94160780083719e-05
1.83826973731614
EML6	0.282270796401745	2	0.868371727767893
0.133440358220926
RTN4	0.0895658931886114	2	0.956205006863559
0.0443826874409615
CLHC1	3.33310361760739	2	0.188897297931573
0.907236860541876
RPS27A	63.7155294636426	2	1.46549439250521e-14
0.264620479510922
MTIF2	7.28899287944576	2	0.0261345668707006
0.774657301775306
CCDC88A	8.64743038666511	2	0.0132505635771152
0.339911417324209
CFAP36	2.9936645739511	2	0.223838093135177
0.750957412525363
PPP4R3B	1.25778684671956	2	0.533181480855463
0.210782854940829
AC015982.1	6.16387819264697	2	0.0458702235731987
1.80578095727248
PNPT1	1.6021169498662	2	0.448853612290366
0.395450358757522
CCDC85A	3.29629315014727	2	0.192406188775768
1.17038235557226
VRK2	2.39124093020765	2	0.302516195196756
0.394068800306308
FANCL	1.61739403037372	2	0.445438087324515
0.341772342590118
AC007381.1	2.3736412523645	2	0.30519003641714
0.69623322472806
BCL11A	19.9276129808706	2	4.7073210853843e-05
0.361707244247937
PAPOLG	0.740350067670877	2	0.690613439338537
0.459142728082506
REL	11.612505317171	2	0.00300868353829342
0.364401991088051
PUS10	5.45156705524053	2	0.0654948655724535
0.641771230497421
PEX13	3.56144642232858	2	0.168516230368911
0.528530247150156
KIAA1841	0.00365504060809013	2	0.998174148594382
0.048108316088975
C2orf74	0.00492092021390589	2	0.997542564343994
0.0187397236523586
USP34	15.0666861172665	2	0.000534946905187517
0.436208635435677
AC016727.1	1.80501160424618	2	0.405552151992989
0.741444539258289
XPO1	3.22566908380787	2	0.199321826510077
0.455742811062688

CCT4	3.5911131687484	2	0.166035014129951
0.173547211144737
COMMD1	2.04256601556512	2	0.360132590720881
0.227319799105509
B3GNT2	0.000687616532661139	2	0.999656250828959
0.00854530895119502
EHBP1	0.359374208018094	2	0.835531605004156
0.217718577895837
WDPCP	0.654572876558495	2	0.720877226618576
0.508771749051663
MDH1	12.1828043893781	2	0.00226223459649166
0.297620697512478
UGP2	14.6048612904903	2	0.000673898774020021
0.277632360046979
VPS54	1.19993130166155	2	0.548830487641552
0.326392291959808
PELI1	1.93751182488112	2	0.379554944080409
0.32746820390841
AC012368.1	3.64622721458845	2	0.161522050942833
0.444304952595038
LGALSL	0.287994646996606	2	0.865890065612333
0.5345876310084
AC008074.3	1.5631757303401	2	0.457678701953539
0.347489157280383
AFTPH	4.07948941153441	2	0.130061910696092
0.346160920682792
SERTAD2	0.319267012261736	2	0.852456151676499
0.189266161389563
LINC02245	1.19379535858562	2	0.550516869519858
0.404598667044457
SLC1A4	3.86803716688548	2	0.144566078768167
0.38296707566282
LINC02576	2.5522720654177	2	0.279113705746038
0.517153633113746
CEP68	0.0675266348449707	2	0.966800302347724
0.110641122538304
RAB1A	0.0887330979243642	2	0.956603251272502
0.0377929668249515
ACTR2	6.95794224595457	2	0.0308391243818625
0.215217442070042
SPRED2	9.51232474401074	2	0.00859854417520522
0.657489594765053
AC007389.5	0.793401745121324	2	0.672535169270416
1.16448063098414
ETAA1	2.90868474534045	2	0.233553904840057
0.329223844907536
C1D	2.815369406379	2	0.244709202200704
0.142487724379801
WDR92	2.95402074023965	2	0.228319259133348
0.535429553964951

PNO1	1.45175085060951 2	0.48390076450362 0.210141912375312
PPP3R1	4.48747156358395 2	0.106061540583722
0.680808853962236
AC017083.1	0.120453584216445	2	0.941550973038066
0.284724382854925
PLEK	36.9306258654473 2	9.56349177627658e-09
1.32880938899606
FBXO48	0.138680080821461	2	0.933009365243553
0.230856173685228
APLF	3.2587759604556	2	0.196049523602215
0.977702274040526
ARHGAP25 2.83186844982882 2	0.242698772265151
0.279561954246068
GFPT1	1.69762966211331 2	0.427921791149558
0.345544690543448
NFU1	0.888280509152509	2	0.641375458675489
0.136163947530451
AAK1	1.38049363635335 2	0.501452286251778
0.710941614384744
ANXA4	0.0703845039569606	2	0.965419794549502
0.122634755580925
GMCL1	0.0400070496028646	2	0.980195218307157
0.0447071175022758
SNRNP27 1.06799018954191 2	0.586258128090564
0.130339566701387
MXD1	1.35824318481169 2	0.507062204089064
1.06233404957758
PCBP1	0.561737745427735	2	0.755127346788193
0.0598929367003341
LINC01816	3.44434940450037 2	0.178677155481361
1.876241673054
C2orf42 0.0304093673098402	2	0.98491032392518
0.0694969289902705
TIA1	0.777463742900732	2	0.677916014285152
0.181383880558378
PCYOX1	0.494368688175438	2	0.780996708134683
0.351982847994329
SNRPG	4.86598461031937 2	0.0877737932572278
0.134033742093735
FAM136A 0.00117621039925762	2	0.999412067700338
0.00660126556152945
TEX261	0.736226323821485	2	0.69203886481732
0.341994428097735
NAGK	6.58912498488164 2	0.0370842661452795
0.371863791655542
MCEE	2.24173963678536 2	0.325996113849779
0.5018517143855
MPHOSPH10	3.74040476130928 2	0.154092473323638
0.381299023438135
PAIP2B	1.43652120064418 2	0.487599649441536

0.657969449362361
ZNF638 1.24572696119952 2 0.536406247473693
0.115186033024291
SFXN5 0.226626256119582 2 0.892871033364488
0.23498584101682
SMYD5 4.05352442324198 2 0.131761446929299
1.01021583356704
PRADC1 0.0572426771456105 2 0.971784372063252
0.0786547031931389
CCT7 0.529953690437005 2 0.767223714667659
0.051422742471857
ALMS1 1.23977434338719 2 0.538005136378716
1.330945693636
TPRKB 0.148611982119741 2 0.928387572076994
0.0580264370141913
DUSP11 0.0817087122540682 2 0.959968933360178
0.0440105823703562
STAMBP 1.09256560696324 2 0.579098437166855
0.223037618024526
DGUOK 0.292889205463109 2 0.86377358170637
0.047562164023527
BOLA3 13.519463309936 2 0.00115954028903564
0.87839006360448
MOB1A 4.35047957903198 2 0.1135809148925 0.096167701866979
MTHFD2 0.0758516815216069 2 0.962784337597791
0.0331764278243191
DCTN1 4.9841451645895 2 0.0827383067973473
0.375796509446813
C2orf81 0.297520580184816 2 0.861775666307095
0.715525247677787
WDR54 1.58458906796033 2 0.452804626785204
0.195834151471411
RTKN 4.0223720218942 2 0.133829856832323
1.10989718703902
INO80B 0.0356988841508288 2 0.98230891561716
0.0694948685162178
WBP1 4.25857881877322 2 0.118921768527843
1.93754982298981
MOGS 0.110720542293831 2 0.946144218080978
0.131644499141928
MRPL53 0.730386673142593 2 0.694062450251055
0.222772064795392
CCDC142 0.755776749013158 2 0.685306994127845
0.420307906672412
TTC31 0.399098218275244 2 0.819099994530835
0.296624379308673
PCGF1 0.702400374992572 2 0.703842839215656
0.303808135880385
AUP1 1.79250038518226 2 0.408097079638381
0.183234063948383

HTRA2	2.62880564400125	2	0.26863469807987	0.366819692348959
DOK1	2.72454433888425	2	0.256078262215878	0.367973748025731
SEMA4F	1.60941576710987	2	0.447218547372409	1.57536666373111
POLE4	0.296703903130458	2	0.862127634369603	0.0608840926578963
MRPL19	0.0876723957348214	2	0.957110721410854	0.0436074450653518
GCFC2	0.0118435019609176	2	0.994095748028249	0.0480254174966717
SUCLG1	0.399296396703881	2	0.819018834577033	0.0748766827005125
TRABD2A	0.707140987004433	2	0.702176491963259	0.546588792777361
TMSB10	56.4372898771545	2	5.55666623824891e-13	0.215358561884377
KCMF1	0.60560725447497	2	0.738744151357386	0.216097970173036
TGOLN2	4.12887949095873	2	0.12688936099549	0.197488908048683
RETSAT	11.9390308038044	2	0.00255547949948742	1.45282642277428
ELMOD3	2.51179238692242	2	0.284820479560281	0.580444932066307
CAPG	0.27920900448758	2	0.869702132631058	0.0521065435009842
MAT2A	0.426888595878312	2	0.807797154863672	0.155165929108627
GGCX	2.43451573772503	2	0.296040837732323	0.42038553326222
VAMP8	2.69080554467925	2	0.260434790802538	0.103498950091351
VAMP5	1.28949476291705	2	0.524795098335121	0.492193286597356
RNF181	0.0816811403112941	2	0.959982167555649	0.0264850601269896
TMEM150A	1.6139134585104	2	0.446213951878579	1.22634390997772
USP39	0.235288400282241	2	0.889012306802986	0.0719281319817229
C2orf68	1.52215418260668	2	0.467162978747057	0.337670599856594
ST3GAL5	0.729116233808626	2	0.69450347242772	0.174853674992556
POLR1A	2.69393302671137	2	0.260027856491011	1.27333098688834
PTCD3	0.675091062590905	2	0.713519486500342	0.18198273143154
IMMT	1.4159849397744	2	0.492632179362047	0.109695824933745

MRPL35	0.247370000838611	2	0.883658148993391
0.0309047272510415
KDM3A	3.14322646735194 2	0.207709827438374
0.501464267325595
CHMP3	0.116519599088763	2	0.943404819453847
0.0139047525723989
RNF103	1.90725054106135 2	0.385341520939537
0.405742732248045
RMND5A	2.7930605443389	2	0.247454074400433
0.435052357481013
PLGLB1	1.42792127338917 2	0.489700824449467
1.32029440438555
CYTOR	15.6540840989566 2	0.000398803378009127
0.698098159595305
KRCC1	6.87442990979021 2	0.0321541113927827
0.371677026892637
THNSL2	0.903108347956883	2	0.636637936223422
0.8669986443034
EIF2AK3	0.186744450421594	2	0.910854394219247
0.133049421994872
AC062029.1	9.23152025340286 2	0.00989465935293676
1.59395917033062
RPIA	45.3613954771884 2	1.41220590776925e-10
0.787135249464887
IGKC	14.8949299254605 2	0.000582917458222809
0.349353815511611
IGKV4-1	0.999070642118265	2	0.606812567230363
0.568211936628946
IGKV1-5	1.30815800796455 2	0.5199206870299	1.00167933181278
IGKV3-11 1.51593344938667 2	0.46861828897849 1.37085313686609
IGKV3-20 0.315145500641042	2	0.854214666957098
0.252058594400701
IGKV1-39 0.000732213493351902	2	0.999633960262221
0.0256914930611643
MRPS5	2.22000515572729 2	0.329558111518219
0.189632782230518
ZNF514	1.12410621104124 2	0.570037514435213
0.668831554458934
ZNF2	3.82913404269815 2	0.147405642267178
0.865723708628442
FAHD2A	1.38305197254278 2	0.500811254567231
0.447256230511592
LINC00342	0.806357735305917	2	0.668192570597141
0.465071095474379
ANKRD36C 4.46139248012559 2	0.107453590591013
0.888898121050142
GPAT2	1.46785037089979 2	0.480021115486973
1.34328701073281
DUSP2	49.6326967085305 2	1.66877622831407e-11
1.17701931720497

STARD7	3.04201237024918	2	0.218491932980757	0.233216099626204
TMEM127	0.253800300763979	2	0.880821617908515	0.0893434175998921
CIAO1	0.012307749687012	2	0.993865021462585	0.0201953152379688
SNRNP200	0.210299571651537	2	0.900189676833434	0.0866234709170928
ITPRIPL1	3.67616388710374	2	0.159122339221944	1.48416261550607
NCAPH	1.91948326027926	2	0.382991826737704	1.47099286438848
ARID5A	9.51677425687731	2	0.00857943577238707	0.520430582354364
KANSL3	0.00019375385734231	2	0.999903127763747	0.00901707492900102
LMAN2L	0.610115291831628	2	0.737080883464369	0.344190449361654
CNNM3	0.448822068425997	2	0.798986656014718	0.532238343719734
ANKRD39	0.931814421134696	2	0.627565512793677	0.209642349970899
FAHD2B	0.00053012426421176	2	0.999734972993757	0.00947958510213532
ANKRD36	0.0152055143045974	2	0.992426070702537	0.0423692661010035
AC092683.1	0.000986290683313532	2	0.999506976234521	0.0319662456980322
ANKRD36B	0.0446917684556224	2	0.977901935692474	0.116497996012571
COX5B	0.535673811111896	2	0.765032543485923	0.0474313832418592
ACTR1B	0.761578941204679	2	0.683321733790678	0.0753237479218443
ZAP70	0.647965474846788	2	0.723262727650321	0.459274125603731
TMEM131	0.0553218628755058	2	0.972718129029567	0.0556455793800309
INPP4A	0.0314938487596873	2	0.984376410205114	0.146798574762331
COA5	1.88408636664807	2	0.389830525881497	0.280179439629215
UNC50	0.0169614147451692	2	0.991555152382554	0.0262498494788974
MGAT4A	5.68484425270187	2	0.0582843228639229	0.715703572713345
TSGA10	3.63818694226095	2	0.162172698550774	0.656550438020522
C2orf15	0.025272531514092	2	0.987443236625687	0.0171229768015527

LIPT1	1.4281463905368	2	0.48964570752507 0.504767539266829
MITD1	3.17978959168647 2	0.203947066684794
0.225693533647692
MRPL30	1.09408063551028 2	0.578659927944395
0.196277962314272
LYG2	0.09327368560281 2	0.954433944218538
0.306498673476622
TXNDC9	0.495006405316738	2	0.780747720338701
0.0881149814539618
EIF5B	2.89265708378789 2	0.235433085973412
0.0926726469143933
REV1	0.0939986288670759	2	0.954088051680917
0.074261205554686
AFF3	51.1290195805479 2	7.89723841876366e-12
1.13178182862643
CHST10	2.3580955405353	2	0.307571477907562
0.730557986386604
PDCL3	0.114483590454575	2	0.944365698639415
0.0583927438877417
RPL31	78.6972870010307 2	0	0.250247701626962
CNOT11	0.0259018258342484	2	0.987132589290258
0.0363329760946024
RNF149	3.20453878033278 2	0.201438855525862
0.553128059204649
MAP4K4	5.11290057269077 2	0.0775796377507277
0.218891756829377
MFSD9	0.0160321703540138	2	0.992015957956449
0.0625631970230719
TMEM182	4.1531930192391	2	0.125356135375597
2.48527283906132
LINC01102	3.25759406149732 2	0.196165413205151
1.2877013256944
MRPS9	1.01950838826323 2	0.600643202292094
0.169244893057298
TGFBRAP1 3.06611713756481 2	0.21587438988045 0.67760647312875
AC012360.3	0.13607107543702 2	0.934227272681434
0.070953694650082
C2orf49	0.334684655210567	2	0.84590998342729
0.130702735193485
NCK2	0.631489134671862	2	0.72924569955132
0.304920489845306
UXS1	0.00010527630688562	2	0.99994736323192
0.00196540831672723
GCC2	2.30765215094174 2	0.315427607849252
0.200208077583792
LIMS1	32.4131540318528 2	9.15320167438338e-08
0.816728068541753
RANBP2	18.9417975346506 2	7.70621154371209e-05
0.877969999704486
CCDC138	7.20544564674876 2	0.0272494259711304

0.751466933034486
RGPD5	0.93042719950877 2	0.628000950013718
0.951289133233751
SMIM37	1.94162867595399 2	0.37877446204869 0.24686188578206
BUB1	4.20861026455039 2	0.121930370318404
0.655266054899765
MIR4435-2HG	0.000349417470057213	2	0.999825306525654
	0.00769570222182683
BCL2L11	4.87574660517734 2	0.0873464134663331
0.49916303919768
ANAPC1	0.154764439588243	2	0.925536027801192
0.191558134003933
TMEM87B	0.000500834424163623	2	0.999749614139691
0.0103935321155765
ZC3H8	0.281021281275836	2	0.868914419079871
0.124131218225379
ZC3H6	0.70010987330221 2	0.704649377578358
0.287297154468911
TTL	0.00971346024435846	2	0.995155044721435
0.0940934026257392
POLR1B	2.40278888466877 2	0.300774506647643
0.999082798092891
CHCHD5	0.00342023419994002	2	0.998291344317094
0.00868854131029325
SLC20A1	0.788304158377493	2	0.674251508820505
0.442593571611604
PSD4	0.129292056431084	2	0.937399217531914
0.114628884538511
AC016745.1	3.37809709855095 2	0.184695168773208
2.86437279576439
AC016745.2	0.966312453601102	2	0.616833449929331
	0.439172700860529
CBWD2	0.733079534823008	2	0.693128572005326
0.253761164239652
RABL2A	0.429476921046583	2	0.806752410190826
0.340423449531611
SLC35F5	0.0203786741086594	2	0.989862398374797
0.0453084733851849
AC104653.1	0.861166800386469	2	0.650129698272496
	0.285213126267208
AC110769.2	2.00734729753201 2	0.366530460673733
0.642482958403882
ACTR3	9.21960718002875 2	0.00995377313630685
0.196705667004415
DDX18	1.02364345462492 2	0.599402635439991
0.0482333522130769
CCDC93	0.000333740827589354	2	0.999833143508298
0.00360057566306152
INSIG2	4.24330103044147 2	0.119833677886826
0.589995335485551

C2orf76	1.12936486007825	2	0.568540669529896	0.337756470388827
DBI	9.6407444405399	2	0.00806378507616212	0.125408456947784
TMEM177	0.142486253106334	2	0.931235456535721	0.196333590345871
PTPN4	2.51273885346033	2	0.284685724921378	0.556268294636806
EPB41L5	0.0545497158301323	2	0.973093742246716	0.188057959343997
TMEM185B	1.86566851702086	2	0.393437026393222	0.706724445324184
RALB	0.0568665953823907	2	0.97196712443523	0.0625286565942223
CLASP1	0.380659282301233	2	0.826686579108049	0.475797195518534
NIFK-AS1	0.178537380602791	2	0.914599796462	0.167249898107564
NIFK	0.272366486778076	2	0.872682704499966	0.0728818665195041
TSN	2.13717088238727	2	0.343494066475561	0.221653860671028
GYPC	27.4819803025502	2	1.07736732879449e-06	0.395305453503244
BIN1	9.65582087224193	2	0.00800322706014556	0.539955003734101
ERCC3	0.0639749028480393	2	0.968518735533884	0.0587196632891704
MAP3K2	1.86396754426171	2	0.393771781557566	0.256360667439154
IWS1	1.74115752032257	2	0.418709146934594	0.275855333639773
MYO7B	15.7704631182799	2	0.0003762594751332	3.85583217083739
LIMS2	13.7517119868979	2	0.00103241352109151	1.77341024841829
WDR33	9.45804733807004	2	0.0088350928048776	0.218104228203551
SFT2D3	0.594318413503674	2	0.74292572415465	0.447221451981243
POLR2D	1.49485895807073	2	0.473582342805299	0.117815729106424
AMMECR1L	0.130326085971153	2	0.93691469355485	0.255709625269483
AC012306.2	1.23355752502855	2	0.539680078336999	0.683106356712734
SAP130	0.00399577397635522	2	0.998004107459583	0.045914393023254
UGGT1	0.453207275260818	2	0.79723671430743	0.147336640518546

SMPD4	3.94442318513601 2	0.139148775268627
0.724451182217136
MZT2B	23.6204064169624 2	7.42837665368423e-06
0.384670797185143
CCDC115	3.48878775591704 2	0.174750877231421
0.224357295293282
IMP4	4.73204993889372 2	0.093853054538991
0.189971496382106
PTPN18	0.236358521822601	2	0.888536758428393
0.0590467749986621
FAM168B	0.904266830704793	2	0.636269275972475
0.220371381170916
PLEKHB2	2.34190757986137 2	0.310071057529984
0.324689811371971
MZT2A	28.0974571366449 2	7.91980894643274e-07
0.532902848393145
MGAT5	2.6153731911802 2	0.270444981866981
0.630056100205579
TMEM163	4.81548987078344 2	0.0900180624056739
1.0511255593595
CCNT2-AS1	0.531169304763152	2	0.766757532287051
0.886749640645551
CCNT2	0.193959464262759	2	0.907574400545578
0.143692262261679
RAB3GAP1 0.0222805659573848	2	0.988921540184915
0.0290247309026242
ZRANB3	4.08639127033256 2	0.12961384978272 1.00330027404743
R3HDM1	0.988454206970146	2	0.610042224642464
0.240199372563107
UBXN4	6.28620303401357 2	0.0431487635305572
0.140870510820924
MCM6	4.09704522051596 2	0.128925235776597
0.663070804894073
DARS	15.2654506799989 2	0.000484339069357298
0.445858099452819
DARS-AS1 4.60142760275549 2	0.100187304357509
3.54484733275766
CXCR4	244.279204390417 2	0	1.14623639071168
SPOPL	0.00432907098307633	2	0.997837805426105
0.030689001513015
KYNU	16.82651134442	2	0.000221906225672863
1.31321435566314
ARHGAP15 2.07481473026043 2	0.354372249837382
0.399523638431372
GTDC1	0.841992465335038	2	0.656392574253579
0.281654724743223
ZEB2	19.4622066525641 2	5.94067128949494e-05
1.86229989497322
ZEB2-AS1 1.65588621353007 2	0.43694711620224 2.41719644294755
ORC4	0.0912391883503825	2	0.95540533482622

0.0195914353693363
MBD5	0.248973926581908	2	0.882949772050638
0.153026250487592
EPC2	0.150750399303967	2	0.927395462588628
0.148833489125812
KIF5C	2.18386928156435	2	0.335566664362227
1.52076076862241
LYPD6B	0.138587493229893	2	0.933052558788361
0.237110236863308
MMADHC	0.128385237040834	2	0.93782433979534
0.036044967938818
RBM43	3.29663482509729	2	0.192373321395871
3.65947199051316
NMI	2.61517494577157	2	0.270471790433599
0.278454974747597
RIF1	1.55808919590624	2	0.458844182628286
0.328282211159697
ARL5A	0.467819808057854	2	0.791433117618808
0.107120147086469
CACNB4	0.86311663763365	2	0.649496183584713
0.525952756842911
STAM2	3.29003433081909	2	0.19300924968251	0.67186216650208
FMNL2	7.83493018159343	2	0.0198914539190423
1.92722763303732
PRPF40A	1.15575352467828	2	0.56108842680683	0.113872554549014
ARL6IP6	4.38315489055875	2	0.111740345240456
0.404785800577243
NR4A2	0.0968966125169832	2	0.952706587000967
0.213348107104065
GPD2	3.8386847799562	2	0.146703404045036
1.9546815946002
CYTIP	43.0523536494887	2	4.48022841048612e-10
0.309914497884152
DAPL1	2.26018809979263	2	0.323002876606612
0.453627162932436
WDSUB1	8.61519910353396	2	0.0134658348686942
0.55413244481716
BAZ2B	0.00243821430418169	2	0.998781635657148
0.00994875652777569
MARCH7	1.34235399097167	2	0.511106653401054
0.201020082212174
CD302	0.214118345447781	2	0.898472506351772
0.52067823286839
LY75	3.3358574949778	2	0.188637376923233
0.709994000795967
RBMS1	4.21803578648923	2	0.121357093547421
0.964247453585687
TANK	4.66352513190692	2	0.097124407960911
0.121545261710589
LINC01806	4.76126396593308	2	0.0924921056003849

2.32192812817922
PSMD14 0.622886815903527 2 0.732389056729421
0.0992413031172304
IFIH1 1.73853021250613 2 0.419259547280503
0.217983288540241
GCA 4.27198016129473 2 0.118127576632128
0.280833316235413
COBLL1 9.11812819509534 2 0.0104718549934069
0.55634808159823
AC019197.1 1.8222123362858 2 0.402079210735409
2.217093000957
GALNT3 1.32401018346009 2 0.515816038433887
0.837732587274057
TTC21B 3.47181958282551 2 0.1762397858818 0.417524524873647
STK39 0.00813599028930912 2 0.995940267939068
0.0379270193032513
DHRS9 164.827513258215 2 0 3.18733763812228
BBS5 1.1337371610229 2 0.567299111687277
1.17428560479946
FASTKD1 0.00938856449070119 2 0.995316718677006
0.0468557998894064
PPIG 10.6204681727997 2 0.00494077001523574
0.36881641724226
PHOSPHO2 1.07282868001879 2 0.584841540133431
0.609691343882138
KLHL23 3.2051856197283 2 0.201373716766237
1.64561920511694
SSB 0.0246787628453789 2 0.98773643657479
0.0154176389716449
METTL5 0.40128896146239 2 0.81820326687919 0.091370128861696
UBR3 2.14446650514919 2 0.342243347490947
0.705484955329308
GORASP2 18.2987609465408 2 0.000106285629141945
0.357053531556252
TLK1 0.684766569187137 2 0.710075991357168
0.161886877772018
METTL8 0.678544912852713 2 0.712288355109548
0.330084241855359
DCAF17 3.17143551426481 2 0.204800743155909
0.501247658908739
DYNC1I2 2.01318620134216 2 0.3654619531092 0.176987066708414
SLC25A12 0.479098570251133 2 0.78698248585551
0.310580571543908
HAT1 1.74628267226322 2 0.417637546551213
0.186337067702215
AC078883.3 0.177830896319218 2 0.914922928721585
0.116482498611667
AC078883.1 0.507499351107022 2 0.775886000972825
0.725555296942693
PDK1 1.02251550121588 2 0.599740779906846

0.476013944455416
MAP3K20 2.27624606427729 2 0.320419875556882
0.510890940698593
CDCA7 17.7804509821056 2 0.000137728597913278
0.601496846748972
SP3 0.203817815262345 2 0.903111814516561
0.0870876237874583
OLA1 3.77030004446117 2 0.151806283403765
0.2369895568311
CIR1 1.490894311289 2 0.474522067272111
0.172923778824332
SCRN3 0.291615250680562 2 0.86432396122014
0.181772369413132
WIPF1 18.4808065841285 2 9.70384474950903e-05
0.388651879701382
CHRNA1 1.16495578654748 2 0.558512715625469
0.286793485963879
ATF2 1.76669642995683 2 0.41339645170218 0.583691587782733
ATP5MC3 7.22310127116327 2 0.0270099318159335
0.122061111314706
LNPK 0.0850661567663195 2 0.958358764038232
0.137056541637277
MTX2 3.90521914380896 2 0.1419032811791 0.430168141931846
LINC01116 1.53925476651738 2 0.463185626879035
0.542031176134463
HNRNPA3 2.91634081247648 2 0.23266156170182 0.0689037639866356
NFE2L2 2.45035703490342 2 0.293705264127978
0.183547696102954
AGPS 0.820788720881173 2 0.663388584329993
0.231069060480789
TTC30B 0.0796586624065385 2 0.960953429923471
0.228061968283121
TTC30A 0.17646613054336 2 0.915547469535666
0.311277880592386
RBM45 3.09570690439217 2 0.212704063582952
0.275203283253969
AC009948.1 1.4416570329905 2 0.486349140703689
1.02370331265789
PRKRA 7.60166863324012 2 0.0223521153332082
0.344298746362991
FKBP7 0.501296786020479 2 0.778295977761095
0.670062315196691
PLEKHA3 0.284816449067446 2 0.867267144486558
0.0347883394885502
TTN-AS1 1.28947925000303 2 0.524799168901529
0.641850031181932
TTN 4.8883409636728 2 0.0867981056522865
0.618383129779818
CCDC141 4.48577224807352 2 0.106151694888938
0.972739472079176

SESTD1 0.0143298706448031 2 0.992860671632795
0.06050619576551
CWC22 1.76960574756061 2 0.412795538083148
0.329987366784383
UBE2E3 6.77108980448161 2 0.0338591873709821
0.223526553069439
ITGA4 2.52450839351144 2 0.283015334652486
0.515568952353076
SSFA2 0.0987489006615146 2 0.951824651905327
0.206933006767633
DNAJC10 29.7890793844258 2 3.39925387393691e-07
0.911909801088954
FRZB 76.9964648457826 2 0 4.86785176996001
NUP35 3.54914286579442 2 0.169556100097109
0.878182005296559
AC096667.1 3.35747800236061 2 0.186609141643831
2.36989937868131
ZNF804A 0.171743816761319 2 0.917711774879144
0.198841792382851
LINC01473 1.77244237269452 2 0.412210479979608
0.914905338493411
ZC3H15 0.226925975081752 2 0.89273723820024
0.0469087539332051
ITGAV 5.01643783333891 2 0.0814131137255729
0.853988854612725
WDR75 0.413190965601467 2 0.813348596947198
0.232386196996512
ASNSD1 0.0744934691207395 2 0.963438392472358
0.0549414700681037
ANKAR 0.00438766867576003 2 0.997808570357848
0.0551332147535162
OSGEPL1 4.28918041147497 2 0.11711602068507 2.1778071834352
ORMDL1 5.18499916837735 2 0.0748327553437969
0.163388889071244
PMS1 0.034999685229165 2 0.982652390320218
0.0786581081799288
C2orf88 5.97719777425381 2 0.0503579444816694
0.565505054265693
HIBCH 0.0575666116352417 2 0.971626987571864
0.0391240294854376
INPP1 2.31378754815432 2 0.314461453708811
0.637007767696709
MFSD6 1.6318491546421 2 0.442230262213188
0.796929636443374
NEMP2 0.232919974702434 2 0.890065710150838
0.234322570711414
NAB1 3.35480710005659 2 0.186858515513264
0.761772337605252
GLS 2.42344816212016 2 0.297683606082738
0.315735091723319

STAT1	1.12325292344355 2	0.570280769293754
0.408701350309302
NABP1	4.64445684865266 2	0.0980548341815854
1.21470253886088
SLC39A10 0.293910019546056	2	0.863332818082026
0.171989262492282
STK17B	2.54331128258978 2	0.280367050036654
0.155534490740363
AC114760.2	5.74473787909328 2	0.056564769218499
0.213055266134784
HECW2	2.16159790103295 2	0.339324313981108
0.940002038299538
GTF3C3	3.7609582432775 2	0.152517014046665
0.666600836897405
ANKRD44	7.64235088428612 2	0.0219020411754813
0.439762157406999
SF3B1	0.271416633283381	2	0.873097263293018
0.0567685332509498
COQ10B	4.59526425222989 2	0.100496525309558
0.396385409984336
HSPD1	2.84887646200322 2	0.240643611364291
0.153911356034077
HSPE1	0.314134946413453	2	0.854646391139568
0.0186538209079561
MOB4	9.10104030816724 2	0.010561709238625
0.312829488303007
MARS2	0.092744933482438	2	0.954686307062295
0.250317084867516
C2orf69	0.681826536657375	2	0.711120582208426
0.324377034737473
TYW5	0.497579848587674	2	0.779743761401459
0.31187979019799
MAIP1	1.5527789132137 2	0.460064097595734
0.297359300292635
SPATS2L	1.22830133360554 2	0.541100274624918
0.878915595056641
KCTD18	0.772768434548282	2	0.67950939627033
0.338381069884928
SGO2	0.980203154306791	2	0.612564168499607
0.393610289962214
BZW1	0.169064368431456	2	0.918942079470769
0.0400002765371778
CLK1	0.054060877223662	2	0.973331614210244
0.0423563097901363
PPIL3	2.04172834387614 2	0.360283458750907
0.288027819207381
NIF3L1	0.0536613291959655	2	0.973526079997579
0.0615159510201944
ORC2	11.439149240969 2	0.00328110632094836
0.452990995229316

AC005037.1	1.23092745398396 2	0.540390243654653
1.11231574475423
FAM126B	10.6608052788712 2	0.00484211999123729
0.981805200390139
NDUFB3	1.94218047789789 2	0.378669972221546
0.115263509100784
CFLAR	2.82082203724196 2	0.244042956337381
0.181733274313363
CASP8	0.671811793553144	2	0.714690357318093
0.256595069695338
TRAK2	0.737597583845918	2	0.691564544824573
0.507336223348997
STRADB	1.12472929393586 2	0.569859951783444
0.331749594989702
SUMO1	13.1862697459146 2	0.00136973926748707
0.283752422249319
NOP58	0.0143457495233816	2	0.992852788907062
0.015454007766004
BMPR2	0.823304126876665	2	0.66255476297853
0.703981215483269
FAM117B	4.32512912730964 2	0.115029741346064
1.18754402849631
ICA1L	0.482655250362972	2	0.785584207053712
0.360258220960152
WDR12	0.456847374489451	2	0.795787023587738
0.111792144736033
CARF	4.72166933260559 2	0.0943414466983156
1.09489460277089
NBEAL1	0.353348686694357	2	0.838052657512464
0.0909248447651416
CYP20A1	1.81866091784777 2	0.402793820778734
0.436334824283928
ABI2	11.3694752110895 2	0.00339742459806114
0.615133170598885
RAPH1	1.82732845517744 2	0.401051982627853
1.15327070908533
INO80D	0.074917656705712	2	0.963234074837888
0.0957318160824775
NDUFS1	1.60239941756318 2	0.448790223443724
0.264374257896101
EEF1B2	60.5243868720314 2	7.19424519957101e-14
0.453630445716989
ZDBF2	3.62450676562274 2	0.163285776568003
1.46418562169691
ADAM23	1.68691607848643 2	0.430220229729483
1.25291834541414
FASTKD2	0.49744234318577 2	0.779797372734047
0.220543618858333
KLF7	0.903851675910351	2	0.636401364801567
0.472534274711743

CREB1	2.12093272567131	2	0.346294273886148	0.17821944005413
METTL21A	0.569567518846255	2	0.752176887898909	0.118812610532203
LINC01857	2.07267284933855	2	0.354751964707684	0.206235098218678
CCNYL1	4.358388510307	2	0.113132649975741	1.68715453336019
IDH1	5.94364722245094	2	0.0512098382935271	0.474524129814928
PIKFYVE	3.74771931494229	2	0.153529943783494	0.725773068167025
MAP2	11.0967750546222	2	0.00389373071865495	1.31533498342178
RPE	0.227608444915497	2	0.89243265705279	0.130398199452165
LANCL1	10.0613204207322	2	0.00653449500477588	1.12829620341445
IKZF2	0.475412680400972	2	0.788434188527095	0.502229990465349
AC079610.2	2.59851856789497	2	0.27273373649008	1.19576003007823
SPAG16	7.63901805739914	2	0.0219385694584939	0.626001472520848
BARD1	5.69479831223865	2	0.0579949607332791	0.314106605297538
ATIC	3.45726533582056	2	0.1775269824334	0.307143583181726
MREG	3.61729848785029	2	0.16387534298633	1.02089462872846
XRCC5	0.153156657457985	2	0.926280357084141	0.03437800956267
SMARCAL1	2.27468494505886	2	0.320670080006772	0.385201311571522
RPL37A	18.6772851117675	2	8.79587541779969e-05	0.181459586524217
ARPC2	77.635688112572	2	0	0.319256757972908
AAMP	0.939092251906406	2	0.625286004976954	0.126181103414277
PNKD	5.71511488943674	2	0.057408813352399	0.227506334542255
TMBIM1	6.14317871692737	2	0.0463474336023336	0.657771942869177
SLC11A1	2.0031673397185	2	0.36729730266789	1.6458144113083
CTDSP1	0.847887730832185	2	0.654460618752263	0.162820120677638
USP37	2.54567205675496	2	0.280036303635199	0.881320980991292
CNOT9	0.710094914106518	2	0.701140168372703	0.213993582639242
ZNF142	2.113363738025	2	0.347607305439419	1.56296367180234

BCS1L	4.37819395013325 2	0.112017857877013
1.22566022415336
RNF25	0.749827166180708	2	0.68734867477285
0.28973283166156
TTLL4	2.0422499684615	2	0.360189504648741
2.06515607938671
WNT6	8.43486401763439e-05	2	0.999957826569236
0.00539380260547586
WNT10A	12.6107228448333 2	0.00182648591648316
2.60370116398615
CNPPD1	1.15842200788038 2	0.560340298488864
0.157365147096
RETREG2	3.38777002402442 2	0.183804054130196
0.421052665929995
ZFAND2B	0.705629057356703	2	0.702707513382796
0.162812667778596
ANKZF1	5.01732969512208 2	0.081376817196656
0.454021578618318
GLB1L	1.83686937465195 2	0.399143336471633
1.55245953870087
STK16	1.40867678982035 2	0.49443558716857 0.223585021527009
TUBA4A	8.08526384039988 2	0.0175512181679495
0.285877868894486
DNAJB2	1.70653389281516 2	0.426020868677146
0.206446662485322
DNPEP	3.69680093628189 2	0.157488873423679
0.260369888821103
GMPPA	0.0495285164804788	2	0.975539860406459
0.0743279370572074
CHPF	2.91759109289321 2	0.232516161057289
0.730087867291835
STK11IP	2.47454622524857 2	0.290174413006504
0.63269577134449
SGPP2	4.17763739236637 2	0.123833334238462
9.62937669504538
FARSB	1.69337543141974 2	0.428832998937197
0.205428571402148
ACSL3	0.252392466656399	2	0.881441861541131
0.158251214747778
AP1S3	4.3795482547452	2	0.111942030402513
0.657597566068259
WDFY1	2.97272034050471 2	0.226194468357442
0.737269605246612
MRPL44	1.02098881687903 2	0.600198762110597
0.147281254028959
CUL3	1.80874771050993 2	0.404795266196864
0.166908971420095
DOCK10	16.34467315518	2	0.000282357494869934
1.66701349590375
IRS1	2.6469750140872	2	0.266205288375746

0.83756780191275
RHBDD1	0.223273105859735	2	0.894369254322309
0.0419030523835688
COL4A4	3.81821777381372 2	0.148212401778288
1.59034396546895
MFF	0.265162551220212	2	0.875831747453748
0.0737756014361558
AGFG1	0.329895311501472	2	0.847938087608913
0.106726600492112
PID1	3.02899037276585 2	0.219919175024937
1.87704666130287
DNER	5.16221651114059 2	0.0756900735775857
1.20288015167788
TRIP12	0.467392661389521	2	0.791602164679837
0.183364944430622
SP110	29.598919945657	2	3.73831762767729e-07
0.653087120337765
SP140	12.4651871934813 2	0.00196435055089617
0.374592284904071
SP140L	6.11156590239247 2	0.0470858404193725
0.476976438775223
SP100	13.099389780858	2	0.00143055200683739
0.396522231005633
CAB39	6.58932468039976 2	0.0370805635492596
0.608023683139107
ITM2C	1.36559449517043 2	0.505201839396428
0.996382875009953
PSMD1	7.57633404395435 2	0.0226370570743772
0.318517904925253
ARMC9	3.80612401549597 2	0.149111339406174
0.91454673065052
B3GNT7	0.00154940607409259	2	0.999225596967875
0.0312365165536308
NCL	0.159638004211102	2	0.923283443623331
0.0315039137940536
PTMA	44.7690071974453 2	1.89903759384435e-10
0.267772991509027
PDE6D	5.65839253512247 2	0.0590603032800875
0.332347540904869
COPS7B	2.53473649434606 2	0.281571674583788
0.333285741122265
DIS3L2	1.68844240268953 2	0.429892027206627
0.522242781693307
TIGD1	0.224550264500916	2	0.893798310927251
0.238682965734241
EIF4E2	1.90779106420901 2	0.385237392005285
0.0949634834130612
GIGYF2	2.51766158122933 2	0.28398587141003 0.186487519198552
INPP5D	7.08133007868625 2	0.0289940384927587
0.351859109653725

ATG16L1 3.82680967063427 2 0.147577054632885
1.11225998540191
DGKD 2.63043714722958 2 0.268415648248563
0.187296769368252
USP40 0.0377123027513544 2 0.981320513696256
0.0868196161225139
ARL4C 1.08594980132145 2 0.581017210328675
0.523427875131515
IQCA1 0.256041756130235 2 0.87983500970026
0.33199682658982
COPS8 1.7534417869644 2 0.416145261455033
0.181871300307758
LRRFIP1 17.7458639911233 2 0.00014013112093525
0.541479624921863
RAMP1 14.9281442677159 2 0.000573316788873068
2.07551768351387
UBE2F 2.93817301442609 2 0.23013561642105 0.308156532500102
SCLY 0.158062501748653 2 0.924011047840747
0.185987793004768
ILKAP 3.14524618457139 2 0.207500175757905
0.226051617415785
HES6 19.9037690620626 2 4.76377744277334e-05
1.13235501749616
PER2 1.49660920349586 2 0.473168081432048
0.571425733087682
TRAF3IP1 3.06229949352728 2 0.21628684919962 0.748207474180504
ASB1 0.998882723607 2 0.606869585566147
0.246008845793467
AC062017.1 0.138297576535602 2 0.933187822348638
0.237785189257588
NDUFA10 0.309602743519585 2 0.856585302618473
0.0688063181505159
COPS9 4.3913880693993 2 0.11128130161275 0.146930748239315
ANKMY1 0.957663085527058 2 0.61950683631278
0.77758043551883
DUSP28 7.7950403105874 2 0.0202921705343597
1.10816047424464
RNPEPL1 0.0357069987782283 2 0.982304930089825
0.0499285134113746
CAPN10-DT 0.283447795624114 2 0.867860841686613
0.246734444629864
CAPN10 0.937812440618307 2 0.625686257068812
0.309371902959603
SNED1 0.971120732935168 2 0.615352277350695
0.63865716671509
MTERF4 3.12643746046442 2 0.209460787236976
0.23161780415885
PASK 0.24241544151813 2 0.885849931060166
0.0428965431978184
PPP1R7 0.815549411441675 2 0.665128711637169

0.0549668457318065
HDLBP	0.024452747962917	2	0.987848064449369
0.01829109359132
SEPT2	4.60887318010447	2	0.0998150215905145
0.125730573583198
FARP2	0.016079930413657	2	0.991992268868639
0.0645766977629392
STK25	4.82259556499674	2	0.0896988094573864
0.292956848432774
THAP4	0.0269690120858629	2	0.986606002630358
0.0427940868688575
ATG4B	3.62327650731479	2	0.163386249308287
0.230546853810447
DTYMK	0.889126749067504	2	0.641104137323462
0.102124218863196
ING5	7.08960049513249	2	0.0288743896632729
0.89855147422876
D2HGDH	1.05710437677938	2	0.589457775989814
0.388884356538429
CHL1	3.83103868393072	2	0.147265331656038
3.78439475646082
TRNT1	2.3935946538058	2	0.302160384853894
0.355882204534487
CRBN	0.0840042294844917	2	0.958867752812195
0.0482743572469411
SUMF1	0.0944781550684933	2	0.953859323992622
0.179411457536083
SETMAR	1.31764744801561	2	0.517459652012504
0.404744646018438
ITPR1	0.5170364981626	2	0.77219493907238	0.19627868968298
ARL8B	5.20751030714578	2	0.0739951925393423
0.410650028978085
AC026202.2	1.03829765802385	2	0.595026802036264
0.618095394875664
AC026202.3	1.47212147894801	2	0.478997098273355
1.84973999614362
EDEM1	0.0280125479200601	2	0.986091357545691
0.0409645746484822
AC069277.1	0.115871721280992	2	0.943710474481085
0.341001050961277
LMCD1	2.98188050493426	2	0.22516084793596	0.727726327191256
RAD18	0.460228174045928	2	0.794442961700547
0.279363704723989
THUMPD3-AS1	13.3245472451779	2	0.00127823683590178
0.565213826252934
THUMPD3	0.175558315203802	2	0.915963137884524
0.107489798726472
SETD5	2.88636628371445	2	0.23617478306621	0.25746132900473
MTMR14	13.4412806462061	2	0.00120576588696075
0.485356505135112

BRPF1	0.19265026912526 2	0.908168691030967
0.245135358873699
OGG1	5.60712328124852 2	0.060593864274767
0.219275721288021
CAMK1	84.8030952307791 2	0	3.27341800434212
TADA3	6.95839331603244 2	0.0308321698630176
0.237931299037437
ARPC4	6.15290786444694 2	0.0461225205894983
0.205613367910099
TTLL3	2.08253594459105 2	0.353006795228323
0.705371718116196
RPUSD3	0.00324271978302234	2	0.998379953802352
0.0100562251639012
JAGN1	0.0437712589243312	2	0.978352123311262
0.0374816535669443
CRELD1	1.26638638551339 2	0.530893845118632
0.654272998292325
PRRT3	4.3525721940828	2	0.113462136476716
1.70534009458536
EMC3	2.16519569315649 2	0.338714453511285
0.191709286647849
FANCD2	0.327888914120158	2	0.848789164816553
0.234552434103509
BRK1	44.5064498613405 2	2.16544560061038e-10
0.391284401928322
VHL	1.02022644549132 2	0.600427592902925
0.231858577420287
IRAK2	1.31284028945376 2	0.518704903241317
0.727949720222126
TATDN2	0.239027077220324	2	0.887351994225001
0.274031896422731
LINC00852	0.292870654595	2	0.863781593618422
0.502512401523829
GHRL	2.30918268565624 2	0.31518631373642 0.795842012830305
SEC13	15.0161688289957 2	0.000548631032416314
0.285240134909049
ATG7	1.54099490690364 2	0.462782798141253
0.392942841874724
VGLL4	22.0730515596018 2	1.61026648699547e-05
0.941109404746443
TAMM41	0.00879696048872253	2	0.995611178902823
0.0277085071739338
TSEN2	0.4288520431022	2	0.807004510465603
0.314574168891534
MKRN2	1.70768350999694 2	0.425776058588142
0.328158872903704
RAF1	0.942648037801484	2	0.624175301047421
0.202591438970057
RPL32	47.5308805854242 2	4.77310413415921e-11
0.158754699215518

IQSEC1 1.29122860163075 2 0.524340340453682
0.336663033074978
NUP210 0.379560093971854 2 0.827141046102719
0.144538369620172
CHCHD4 0.1143354735547 2 0.944435639489054
0.105147833189745
TMEM43 0.302258232425076 2 0.859736685547945
0.196308204328611
XPC 5.7868657732652 2 0.0553857528745039
0.513875829899979
LSM3 1.80157705629952 2 0.406249194483598
0.0788385354414757
SLC6A6 0.8183887315215 2 0.664185124928509
0.178940007907375
CCDC174 6.45742393074594 2 0.0396084830442917
0.436308008228758
FGD5-AS1 1.1844910609708 2 0.553083922444172
0.378569562902573
NR2C2 3.16315898966739 2 0.20565001839992 0.825082725067498
MRPS25 0.452828317406455 2 0.797387788176972
0.131947215248113
RBSN 1.6749895915193 2 0.432793402313015
1.44201099402768
CAPN7 0.850448911167954 2 0.653623059318269
0.23133411958736
SH3BP5 0.195572216605344 2 0.906842849167743
0.0755980933603952
METTL6 2.94551425340481 2 0.229292424607097
0.623152549876084
EAF1 0.641625885767577 2 0.725558959263708
0.304919883758086
EAF1-AS1 0.0159825259014329 2 0.992040582306655
0.106548692032722
HACL1 1.24049338973723 2 0.537811745830182
0.297035952939638
BTD 0.176125558109806 2 0.915703387925533
0.447331435662441
ANKRD28 12.1991625679432 2 0.00224380704079019
0.574168661669122
DPH3 3.14976519913367 2 0.20703185688414 0.346110563173939
OXNAD1 1.25428939443926 2 0.534114684969879
0.784941098996723
RFTN1 5.22982969738608 2 0.073174019294085
0.12338391433604
PLCL2 6.69617353527616 2 0.0351515428758822
0.721856236286655
TBC1D5 1.08460923256525 2 0.581406787637374
0.305383655325236
SATB1 4.12821250866317 2 0.126931684531 1.04898136829355
KCNH8 0.999512811078732 2 0.606678425218243

0.920501179900545
RAB5A	0.119116591977283	2	0.942180606640292
0.0586294708545279
KAT2B	1.28816234240721	2	0.525144838698737
0.635856386035568
SGO1	4.48779435027187	2	0.106044424338283
0.458833598179278
UBE2E2	0.519969392511887	2	0.771063385869982
0.107676992586263
UBE2E1	12.082639697016	2	0.00237841769006364
0.28374858682363
NKIRAS1	0.136230294733836	2	0.934152902137068
0.271166709552359
RPL15	43.476383607188	2	3.62429308786716e-10
0.194217722638288
NR1D2	0.00614122201105595	2	0.99693409849886
0.0746994680122801
TOP2B	1.03901229284665	2	0.594814226580363
0.227757667807125
NGLY1	0.656463000701506	2	0.720196274715077
0.0751844274890909
OXSM	1.2206458866306	2	0.543175425874098
0.430243382143293
SLC4A7	1.28985304408692	2	0.524701094654423
0.357863816891791
CMC1	1.98472599397775	2	0.370699692999945
0.125852987697783
AZI2	0.403155471947304	2	0.817440030593508
0.200751896934
TGFBR2	0.187662893991527	2	0.910436206066393
0.206972348923612
STT3B	0.0866876531839286	2	0.957582091272465
0.0620306340692687
OSBPL10	6.99195106794569	2	0.0303191566377383
0.379570917680257
OSBPL10-AS1	3.98702177573994	2	0.136216344626075
0.576923320950166
ZNF860	5.21054319427381	2	0.0738830680425707
1.59895589738984
GPD1L	1.73664855860177	2	0.419654183575664
1.15670607294149
CMTM7	0.355966160347538	2	0.83695658452894
0.118013840807979
CMTM6	0.508868629610135	2	0.775354980761208
0.0394999619262671
DYNC1LI1	0.340511599655735	2	0.84344903488116
0.115053853095027
CNOT10	2.23621334302787	2	0.326898134628286
0.459758409447921
GLB1	0.216728334402512	2	0.897300769414325

0.112995578246433
CRTAP	1.43628105394402 2	0.487658200680093
0.308481374224936
UBP1	5.74266351732158 2	0.0566234675510771
0.592747296407335
CLASP2	1.72898745984281 2	0.421264772398804
0.656568373047994
PDCD6IP	0.193111421478625	2	0.907959313106114
0.0428636122978366
TRANK1	1.5982830779567	2	0.449714861133888
0.297067578804363
EPM2AIP1	0.708041188602412	2	0.701860512879748
0.225387470347189
MLH1	0.00165928755939007	2	0.99917070027955
0.0100797283203011
LRRFIP2	1.4248468672853	2	0.49045417292779 0.281439777534169
GOLGA4	5.38728943140326 2	0.0676339829625228
0.240358213655645
VILL	0.00145255510366933	2	0.999273986123869
0.0225241183364932
PLCD1	1.12372491947086 2	0.570146200044638
1.00679877192832
ACAA1	0.329577708077449	2	0.848072752321116
0.0945707329790155
MYD88	0.194936976802221	2	0.907130926251313
0.126570505034207
OXSR1	2.76617525743364 2	0.250802969867426
0.463610007074731
EXOG	1.08860272093365 2	0.580247025275209
0.131561665602205
WDR48	0.0180739248331743	2	0.99100374820253
0.0393020657180936
GORASP1	0.288281541661091	2	0.86576586490075
0.20843585069591
TTC21A	2.3075214245879	2	0.315448225873612
0.440042195555204
CSRNP1	0.932855952525357	2	0.627238783285185
0.236310001751097
SLC25A38	3.67110605578788 2	0.159525255452976
0.352764659818133
RPSA	3.99093095915287 2	0.135950357321555
0.0910556604076596
EIF1B	6.56086400732851 2	0.0376120047730778
0.175125138187132
ENTPD3-AS1	1.22440823692651 2	0.542154578259595
0.353299941076506
RPL14	47.8113355175632 2	4.14859258057731e-11
0.268246624099555
ZNF620	0.0211088404701339	2	0.989501082221145
0.100307874389218

ZNF621	0.0196337392886592	2	0.990231158529883
0.0872706429053014
CTNNB1	1.98152884655219 2	0.371292757687026
0.27078945905727
ULK4	5.7678816399125	2	0.0559139811566673
11.7401762153883
TRAK1	2.30422614617459 2	0.315968399149497
0.510916831835063
SEC22C	3.77294042642971 2	0.151606002350769
0.680447140945239
SS18L2	3.90434151913058 2	0.141965563754034
0.21850074133828
NKTR	14.4257490764458 2	0.000737035470859349
0.430479832160005
HIGD1A	1.95699821848386 2	0.375874822749236
0.221436610034634
SNRK	1.04591651749538 2	0.592764401207454
0.370830252246403
ABHD5	5.29804068395132 2	0.0707204610023464
0.77868717077909
AC006058.1	10.2096127312592 2	0.00606751368061342
1.55786492387103
TCAIM	2.94672442962623 2	0.22915372445423 0.713851085017821
ZNF445	0.167134590510072	2	0.91982918444884
0.33162435107703
ZNF852	3.7783773420256	2	0.151194427509344
1.05099661552662
ZKSCAN7	0.243580558201823	2	0.885334022081334
0.489214470638306
ZNF660	0.129131040531267	2	0.937474688659135
0.213596317390039
ZNF197	0.132785707357994	2	0.935763174066517
0.23225359045834
ZNF35	3.11053478967206 2	0.211132919239976
1.11301696110424
KIAA1143 0.169938743392076	2	0.918540417305726
0.0809786585473527
TMEM42	1.39949086839957 2	0.496711733518303
0.360869746979738
ZDHHC3	7.04286818370275 2	0.0295570172819545
0.296760542758284
EXOSC7	0.474157739917421	2	0.788929062761185
0.115663051958981
LARS2	1.10032311360454 2	0.576856607743013
0.593319377609853
LIMD1	1.35321123542348 2	0.508339565994182
1.14590799711024
SACM1L	2.74103439796289 2	0.253975569672426
0.476152206406774
LZTFL1	0.0300645586184846	2	0.985080141383341

0.0942254783833484
CCRL2	0.514912950777227	2	0.773015270770553
0.61558862786596
LRRC2	14.6364687558017 2	0.000663332372172643
7.08209326546204
CCDC12	0.167095628396557	2	0.919847103867933
0.0429900807500146
SETD2	2.07944672308738 2	0.353552474642358
0.315478422815264
KIF9-AS1 4.52140083547928 2	0.10427742141452 1.46855117439889
KIF9	1.63720171350536 2	0.441048312777337
0.646815402437304
KLHL18	0.581538006013568	2	0.747688371837204
0.188562886463795
AC099778.1	0.766861878292992	2	0.681519142717251
0.710159261125039
PTPN23	6.50733847120092 2	0.0386321968232182
2.74486499101442
SCAP	1.88357840557658 2	0.3899295478216 0.590471301059006
ELP6	6.89477446050087 2	0.0318286888677467
0.419119870227307
SMARCC1 0.462459669033954	2	0.793557058271064
0.035692738015106
DHX30	0.579749365430974	2	0.748357343812723
0.189037879685727
MAP4	1.01251266407121 2	0.602747848117175
0.173698733101693
CAMP	0.283580403192858	2	0.8678033011361
0.199237429045259
ZNF589	4.82942278709021 2	0.0893931346348482
1.78685960594326
NME6	0.0160804089405797	2	0.991992031521163
0.0280536751004676
CCDC51	0.380530951000056	2	0.826739625692097
0.18936322408799
TMA7	22.2780945690076 2	1.45336009201857e-05
0.230082224350084
ATRIP	0.00745334724168256	2	0.996280261809261
0.0561272751132991
SHISA5	1.15276206776336 2	0.561928290681525
0.146228021251384
UQCRC1	0.939304095334424	2	0.625219777119107
0.113377428111668
SLC26A6 1.00840257857164 2	0.603987794347275
0.915679792831019
NCKIPSD 1.96221254577524 2	0.374896131933964
1.38572646276719
IP6K2	7.52010285151292 2	0.0232825430217227
0.367118388294308
PRKAR2A 7.55304281211359 2	0.0229022205458148

0.80310251080308
SLC25A20 7.37429949635873 2 0.0250432800684691
0.859227896269257
ARIH2OS 2.44992096975619 2 0.293769308424218
1.00288873680718
ARIH2 1.9726544756111 2 0.372943913048964
0.248300421034134
P4HTM 0.601301802578597 2 0.740336178044846
0.286605022976089
WDR6 0.77772546096873 2 0.677827308654484
0.125787679798642
DALRD3 2.60969897373184 2 0.271213353145479
0.319337361331121
NDUFAF3 19.9567272288548 2 4.63929238113669e-05
0.263545533355535
IMPDH2 20.235306691436 2 4.0360727163069e-05
0.444641216468762
QRICH1 0.028641373380323 2 0.985781366604909
0.0464147133077703
QARS 0.418600204579179 2 0.811151770609467
0.0698120872370289
USP19 0.000221804533592065 2 0.999889103882633
0.00807486318040697
CCDC71 0.701445446700108 2 0.704178979176967
0.229480101463288
C3orf62 2.05531463167343 2 0.357844295544595
0.708032224925824
USP4 1.97863783682767 2 0.371829851265753
0.290428524425113
RHOA 4.0701220398295 2 0.130672508635408
0.104092766440396
TCTA 0.507937522900576 2 0.775716033911878
0.198960877957527
AMT 0.72683080664615 2 0.695297544589994
0.840386236639348
DAG1 0.489165151660658 2 0.783031326233699
0.711084058905484
APEH 7.72181512254761 2 0.0210488876964465
0.566024592874962
RNF123 1.33395549230712 2 0.513257430337868
0.275847118492754
GMPPB 18.4859399243257 2 9.67897011748819e-05
0.981172857566772
IP6K1 0.00398761365185545 2 0.998008179486575
0.0250970815864007
INKA1 19.3582664842306 2 6.25757182731723e-05
1.12936097089204
UBA7 0.789043850834445 2 0.674002185551401
0.154658642969715
MON1A 0.0567277750160494 2 0.972034591192785

0.10579711831868
RBM6 5.6991137441263 2 0.0578699589894692
0.219517529729174
RBM5 1.36405769642909 2 0.505590185355171
0.177002912701886
GNAI2 1.55320583349577 2 0.459965902729296
0.156959964637352
IFRD2 0.981505519283457 2 0.61216540728735
0.167691941927724
HYAL3 1.33971365402916 2 0.511781845876168
0.76561240822486
NAA80 3.85519576368327 2 0.145497280707374
1.37299790996121
HYAL2 0.299602076561959 2 0.860879241400644
0.477918160611681
TUSC2 7.34587643048097 2 0.0254017244573517
0.430450306892346
RASSF1 4.12503386113524 2 0.127133580470133
0.471426612520489
RASSF1-AS1 1.06752140377881 2 0.586395558928315
0.641219425607439
NPRL2 1.45694184618501 2 0.482646429655687
0.171289808692873
CYB561D2 1.32804295222104 2 0.514777002930315
0.263082651787394
TMEM115 1.0959887507421 2 0.578108116305022
0.334757338204785
CYB561D2.1 1.25316930086738 2 0.534413897961338
0.683282770553725
HEMK1 0.764095178108249 2 0.682462574684181
0.292769563028946
MAPKAPK3 1.92791744692351 2 0.381380115220217
0.66660228330473
MANF 4.67709598631327 2 0.0964676082145044
0.377478831277916
RBM15B 0.299174997241069 2 0.861063092890631
0.156257842404273
DCAF1 0.0505109977535816 2 0.975060753272533
0.103729343175198
RAD54L2 5.23369943084956 2 0.0730325742013549
0.652514256295879
TEX264 0.958721343571188 2 0.61917912397502
0.164385596915643
RRP9 0.00195062867493192 2 0.999025161126974
0.0127744230946371
PARP3 0.611733054573609 2 0.736484913535803
0.47421196502916
PCBP4 0.0468130272737057 2 0.976865293983841
0.178605657256338
ABHD14B 1.08439372568768 2 0.581469439593494

0.181701997158374
ABHD14A 4.2198614636967 2 0.121246364654088
0.775439758122407
RPL29 33.1892260510722 2 6.2094213992836e-08
0.194351206364248
POC1A 0.27229877143425 2 0.872712252004861
0.385817110996754
ALAS1 8.57910894130934 2 0.0137110326041516
0.661888395956488
TWF2 6.30679156538526 2 0.0427068571480775
0.142125562932305
PPM1M 3.06911014062627 2 0.215551575132176
0.65521173617684
WDR82 0.0015885966449747 2 0.99920601704892
0.0111571695749173
GLYCTK 2.36515860814853 2 0.306487194553948
0.563545647436612
BAP1 1.9330235666591 2 0.380407670836248
0.42153222422918
PHF7 0.341210338034988 2 0.843154411244559
0.236362998835881
NISCH 1.10704380830679 2 0.574921422437611
0.419882861725951
NT5DC2 1.62256015824939 2 0.444288977016233
2.06271384632948
SMIM4 0.113378197000427 2 0.94488779073622
0.070497467474508
PBRM1 1.06463058317545 2 0.587243753957293
0.139238894994886
GNL3 9.35966996173511 2 0.00928054522823307
0.328627923504571
GLT8D1 3.49220865833264 2 0.174452229866082
0.611524611093784
SPCS1 54.9575540838982 2 1.16440190822686e-12
0.336113921357359
NEK4 0.0266822128975276 2 0.986747491675232
0.0830988393837951
STIMATE 1.28673876679173 2 0.525518763453975
0.948194878000548
SFMBT1 5.14425850404754 2 0.0763727553309983
0.587231129490942
RFT1 0.330778826595196 2 0.847563587284702
0.172791090768551
PRKCD 68.6488244854253 2 1.22124532708767e-15
1.05704336547563
TKT 9.25798042572134 2 0.0097646143076241
0.260861224423507
DCP1A 0.58144123421644 2 0.747724550286177
0.105784891818298
CACNA1D 5.23180933170224 2 0.073101626228122

2.4041736971944
CHDH	0.236699531411502	2	0.888385271566021
0.186334902739088
IL17RB	2.7719166917737	2	0.250084017923121
1.53183519218632
ACTR8	0.701135476362817	2	0.70428812493258
0.213692392424539
SELENOK	20.7669058327672	2	3.09402412226323e-05
0.267684319002179
CCDC66	6.93233417951517	2	0.0312365283121837
0.46828040181077
FAM208A	0.982866981290171	2	0.61174882912035
0.288765688644897
APPL1	2.61578181243397	2	0.270389732727383
0.284701076790931
PDE12	1.40055948252707	2	0.496446407819447
0.294742050977994
ARF4	6.68545806723845	2	0.0353403809132307
0.28752537343109
ARF4-AS1	2.40919583039757	2	0.299812525340474
1.18940068438915
DENND6A	1.49174423553342	2	0.474320456208933
0.419449436694527
SLMAP	4.73051668014973	2	0.0939250326339878
0.382052259539132
FLNB	0.49619914768846	2	0.780282243707086
0.645492748642381
ABHD6	4.43119288808155	2	0.109088429696937
1.1080618965047
RPP14	0.0364178386524018	2	0.981955861370038
0.0823889008605197
PXK	14.8232208056305	2	0.000604196910247823
0.476159384522751
PDHB	7.28528018277687	2	0.0261831267884988
0.13514094488188
KCTD6	2.16189136498536	2	0.339274527906637
0.584387123825803
C3orf67	31.3222694839806	2	1.57926714816625e-07
1.36868871809028
FHIT	0.0188030634406566	2	0.99064252450558
0.0720901579499128
C3orf14	0.0260229680238019	2	0.987072799399423
0.0703518033694491
CADPS	5.79992822041127	2	0.0550251948638978
1.00707400487443
THOC7	0.438957149596809	2	0.802937360603562
0.065745078537463
ATXN7	0.230739881769596	2	0.891036452113872
0.299695698794275
ATXN7.1	0.132048442910938	2	0.936108190114178

0.355232832412183
PSMD6 2.68735625855644 2 0.260884335398001
0.147788726180909
SLC25A26 3.09435487780007 2 0.21284790297125 0.274445805487289
LRIG1 4.59594651957597 2 0.100462248407587
0.772049718437322
KBTBD8 3.05352548734896 2 0.21723778462841 0.438444148761463
SUCLG2 0.526001033514989 2 0.768741500058126
0.144672880619076
TMF1 0.119393877136 2 0.942049989345534
0.0639017350349896
UBA3 0.049983914784696 2 0.975317756094838
0.0337699472917953
ARL6IP5 6.43164287988339 2 0.0401223621725187
0.168449605142069
LMOD3 1.16618710402432 2 0.558168968217983
0.886607219583693
FOXP1 53.0887339305596 2 2.96429547574917e-12
0.960203300302255
AC097634.1 6.90432409708204 2 0.0316770749145663
2.61925351781158
LINC00877 2.82624456039406 2 0.243382188209348
0.36083791949361
RYBP 13.0190193262465 2 0.00148920975852496
0.946765220770866
SHQ1 6.41325210682258 2 0.0404930042883302
0.539629315972513
PPP4R2 2.2179956846863 2 0.329889396658033
0.247127720983096
EBLN2 0.115370775348116 2 0.943946878048203
0.157479530198205
ZNF717 0.0104014122722978 2 0.994812794122243
0.0687076789434795
GBE1 0.209794517740746 2 0.900417027696705
0.113084998215895
CHMP2B 0.870615634076439 2 0.647065458646718
0.112222375148379
CGGBP1 0.000419727695009135 2 0.999790158172372
0.00320978554406458
ZNF654 5.27515206210955 2 0.0715344568732227
0.979494889875371
C3orf38 2.19992914325367 2 0.332882876987956
0.294262957308005
ARL13B 0.820389107653279 2 0.663521146999767
0.506489650877678
DHFR2 0.604159320046239 2 0.739279171547943
0.69564913238893
NSUN3 0.0423783012708651 2 0.979033762196209
0.073343455819682
MTRNR2L12 1.65046303626327 2 0.438133544862209

0.756214720925531
ARL6 1.85729323787115 2 0.395088053395421
0.671059256762332
RIOX2 0.445409847010956 2 0.800350979215981
0.27760182952734
CLDND1 2.44514889967107 2 0.294471089192353
0.290765046762151
CPOX 1.46621820106133 2 0.480413013369461
0.672982494402395
CMSS1 2.97309585720496 2 0.226152002444045
0.193247953583446
TBC1D23 0.698741158487484 2 0.705131774646674
0.127900422921034
NIT2 4.51223075886144 2 0.104756635153611
0.528133863381471
TOMM70 0.0289414380413921 2 0.985633478623346
0.0661568703992733
TFG 1.18205590282347 2 0.553757755993689
0.223839109185071
SENP7 0.451767158822554 2 0.797810977882952
0.263047009383559
TRMT10C 0.0676151737545412 2 0.966757503572799
0.0397807127324931
PCNP 5.83399818544791 2 0.0540957803865172
0.227771016137152
ZBTB11 0.425056488594217 2 0.808537479426156
0.272513732941977
ZBTB11-AS1 1.24622085649603 2 0.536273799566893
0.707482914025899
RPL24 43.0846209705831 2 4.40852576666373e-10
0.189152124222192
CEP97 0.0762940503950788 2 0.96257140823558
0.146144860283436
NXPE3 1.72325495865431 2 0.422473954885213
0.330800504406822
NFKBIZ 4.58292394922434 2 0.101118521026806
0.70955176423034
ALCAM 13.3882169264419 2 0.00123818527003716
1.48119901813741
CBLB 0.61858326294249 2 0.733966691025745
0.272105990865093
DUBR 1.09430778405382 2 0.578594210796437
0.484997600649005
BBX 12.0490015469851 2 0.00241875877449993
0.443852275025339
CD47 0.765449605288455 2 0.682000558213442
0.0459916570522766
LINC01215 3.80830002144711 2 0.14894919404852
0.506629280258805
IFT57 54.5394099329926 2 1.43518530393294e-12

0.888063457846023
CIP2A	1.00890699064783 2	0.603835484186182
0.630815931396745
DZIP3	0.753606232356216	2	0.68605113296918
0.284644520604165
CD96	0.0481074389959641	2	0.976233265588344
0.143640271793794
ZBED2	32.2460178187642 2	9.95098792255789e-08
1.80524125758392
ABHD10	0.804809834931761	2	0.668709918537102
0.445522843772263
C3orf52	3.32478665356663 2	0.189684459512999
1.23219634363443
GCSAM	0.00337135756926296	2	0.998315741173874
0.000526473676038933
CD200	0.259191127625575	2	0.878450636312767
0.47413739363463
BTLA	4.76947210425605 2	0.0921132894776054
0.528102389674198
ATG3	0.229619833570708	2	0.891535593753167
0.0651071874857882
SLC35A5	2.39414372521247 2	0.302077442425968
0.545619164021077
GTPBP8	11.3755142210698 2	0.00338718152981399
0.427718431243892
NEPRO	0.1173215622502	2	0.9430266073312	0.0843671053299564
SPICE1	0.164286337708374	2	0.92114007068698
0.278099451983599
SIDT1	0.722902060480515	2	0.696664710743701
0.654051783333955
USF3	2.4715836012538	2	0.290604570365797
0.531894550664673
NAA50	22.5932139014422 2	1.2414977507591e-05
0.596579391591562
ATP6V1A	0.562415034863249	2	0.754871670195176
0.146144021415931
GRAMD1C	12.8308432923619 2	0.0016361298947557
1.51532217436072
ZDHHC23	1.8764708058456	2	0.391317744621982
1.25378726881046
CCDC191	0.120080212343469	2	0.941726763771538
0.132927426784467
QTRT2	2.018019127642	2	0.364579893927785
0.345183659040344
AC093010.2	7.62765779013616 2	0.0220635380478477
1.00192274982278
TIGIT	2.84930860171513 2	0.240591621150808
3.06524871551277
ZBTB20	4.66676377504586 2	0.0969672595833962
0.348448202007117

ZBTB20-AS5 2.28385794125705 2 0.319202694946101
1.24866371006981
LSAMP 3.34053496480337 2 0.18819671959516 3.91322620783522
B4GALT4 2.81733632898155 2 0.244468658472386
0.283273682577631
TMEM39A 1.27784266511659 2 0.527861504081997
0.412090013055866
POGLUT1 0.042079692856642 2 0.979179946968538
0.0779896568119661
TIMMDC1 0.584895625939905 2 0.746434198203378
0.125496629585851
CD80 1.54115912236596 2 0.462744801655601
0.543691911792408
ADPRH 0.335337721650512 2 0.845633810808816
0.243615620479132
COX17 10.9058449641845 2 0.00428376714652845
0.135475595380196
GSK3B 0.0027597145366953 2 0.998621094296968
0.0139592381286753
LRRC58 0.00399913705072011 2 0.998002429279979
0.0167068202249029
NDUFB4 1.35290147467008 2 0.508418303914975
0.0451721557485096
RABL3 3.77700999408738 2 0.151297830546662
0.701370240961084
GTF2E1 0.0111481892889446 2 0.994441411796246
0.040496924816245
POLQ 0.72054180792541 2 0.69748734838817 0.647782986016577
HCLS1 15.3728689822155 2 0.000459011868422787
0.378342085260891
GOLGB1 0.0017627696967225 2 0.999119003457174
0.00261724100479377
IQCB1 0.0294948339801728 2 0.985360793568688
0.0301434577540352
EAF2 2.13683986965996 2 0.343550921634258
0.0667109193879532
SLC15A2 2.34506380029373 2 0.309582117128747
0.473316560051774
ILDR1 0.70181361713095 2 0.704049362168544
0.625717523001064
CD86 8.67038275867454 2 0.0130993668849342
0.540228011492539
CCDC58 0.0621041226399209 2 0.969425102211824
0.0596902316597769
FAM162A 5.5984147756943 2 0.0608582805263931
0.813442406392193
WDR5B 1.13182230639999 2 0.567842519445774
0.51952620281625
AC083798.2 1.44793763692252 2 0.484824253097881
0.768284796936731

KPNA1 0.857179204867425 2 0.651427218474919
0.200065297342267
PARP9 7.17827783243991 2 0.0276221051324723
1.34027295492039
DTX3L 0.167315981806595 2 0.919745763727672
0.29161986581091
PARP15 4.54523545386395 2 0.103042090671291
1.64900793053692
PARP14 7.64601709837681 2 0.0218619291655314
0.684362721207818
HSPBAP1 1.29777793569252 2 0.522626108728763
0.46989137058758
DIRC2 0.0978269754055926 2 0.952263508638733
0.243986291159158
PDIA5 0.0619342015809641 2 0.969507468580693
0.137675025806932
SEC22A 2.5749516491505 2 0.275966492512502
0.343147604427866
HACD2 12.3018594119528 2 0.00213149918104083
0.808550257619378
MYLK-AS1 0.0475906503148362 2 0.976485551332443
0.10931036355167
CCDC14 3.39364981309435 2 0.183264483125503
0.705083363514007
UMPS 0.176808423535511 2 0.915390790202206
0.0984673557899582
HEG1 0.82612521652798 2 0.661620858597736
0.953021886138294
SLC12A8 6.25334167538757 2 0.0438635834337552
0.796965631203089
ZNF148 3.24865923306619 2 0.197043725793012
0.297218357952054
SNX4 5.52668105160652 2 0.0630806933213912
0.490564359863034
OSBPL11 0.0602609125563682 2 0.970318941093591
0.0714670474704838
ALG1L 1.02887018335969 2 0.597838223031007
0.54025309021401
SLC41A3 0.642793361549131 2 0.725135546600224
0.224813100576153
ZXDC 6.5925485103386 2 0.0370208409805823
0.934895723411965
CHCHD6 6.14318801015215 2 0.0463472182442747
1.68400987011337
TPRA1 0.260746421571976 2 0.877767777380336
0.14863236853639
MCM2 12.7068440653612 2 0.00174077992629296
1.28908935780565
PODXL2 0.286260962531553 2 0.866640981105542
0.328432434132965

ABTB1	4.93706876233936 2	0.0847089190538447
0.480730073988254
MGLL	14.0032201250223 2	0.000910414959889527
3.13076443169447
SEC61A1	4.81191820979382 2	0.0901789630344645
0.669597406185727
RUVBL1	2.15819599523263 2	0.339901979808975
0.248473367739543
EEFSEC	0.229159852970172	2	0.89174066187301
0.153426867576579
RPN1	0.78348970655272 2	0.675876539647442
0.105039075442833
RAB7A	7.46559902557715 2	0.023925761517464
0.244008750410183
ACAD9	0.00172409648840794	2	0.999138323212638
0.0124551930610424
ISY1	3.41617063411269 2	0.181212425005001
0.281819135174976
CNBP	5.46182503805717 2	0.0651598029707742
0.185857295785166
COPG1	2.6503199316276	2	0.265760443102908
0.233749188467461
HMCES	130.918142497174 2	0	0.854020115434918
H1FX	9.2962341689287	2	0.00957962257972023
0.601253004800114
EFCAB12	1.87707654165007 2	0.391199244983336
1.91122862606035
MBD4	29.8714091205968 2	3.26216501700394e-07
0.286489179797702
IFT122	3.41858238319479 2	0.180994037255743
1.79739238487472
TMCC1	0.368363862878287	2	0.831784462315914
0.200527172098855
PIK3R4	1.3773205217905	2	0.502248500480543
1.00065566330815
ATP2C1	0.420307464274874	2	0.81045964269949
0.113928573433081
ASTE1	0.460588707500805	2	0.794299762975076
0.326426457344665
NUDT16	0.334467854392962	2	0.846001685385479
0.166410175787685
MRPL3	9.11572860281542 2	0.0104844266247955
0.456994027151632
DNAJC13	0.178471913431278	2	0.914629735082867
0.211392064961872
ACKR4	0.497842718262669	2	0.779641282641677
0.501434641549748
UBA5	5.09730205939512 2	0.0781870669251284
0.725240032511002
NPHP3	1.24286637440085 2	0.537174014724144

0.655358354429015
BFSP2	10.3766126262784 2	0.00558145204602467
0.504412404812172
BFSP2-AS1	3.07774181425401 2	0.2146232943041
0.988137832466523
CDV3	1.01656856936678 2	0.601526742613202
0.103317582062402
TOPBP1	0.673712552646995	2	0.714011452879659
0.25294898394091
SRPRB	10.2211138651389 2	0.00603272216837147
0.431167822621902
RYK	2.44483223070794 2	0.294517717810958
0.450307875811254
ANAPC13	4.22640008227177 2	0.120850620046294
0.349081015203225
CEP63	0.0224119120545998	2	0.988856596825075
0.0316737130924506
MSL2	3.8153489394936	2	0.148425152741069
0.740649998946842
PCCB	0.24583365503643 2	0.884337212022791
0.0654381167827477
STAG1	1.53389469368919 2	0.464428646150002
0.312180489120494
SLC35G2	0.0931305770920805	2	0.954502240472126
0.250834723759256
AC096992.2	0.00105436902078924	2	0.999472954426943
0.0256544316136274
NCK1-DT	0.143964436409923	2	0.930547442467986
0.127619412888773
NCK1	9.26519491964903 2	0.00972945438569639
0.486545980026919
DBR1	5.05105900979837 2	0.0800159327390912
1.15016843446498
ARMC8	1.38475357227612 2	0.500385345625943
0.295416186681397
FAIM	0.062587922402823	2	0.969190626755459
0.0894347785204164
PIK3CB	0.405350795880755	2	0.816543250032845
0.598715146304199
MRPS22	2.82163262819463 2	0.243944066872226
0.344179023410045
COPB2	4.83941097895507 2	0.0889478096681542
0.172700443369159
NMNAT3	16.0786809338918 2	0.000322521593640213
3.55564453948642
SLC25A36 0.166556142570442	2	0.920095259572915
0.0891694071598569
ZBTB38	48.0847329458701 2	3.61853880193053e-11
0.592727943876489
RASA2	0.395822863557259	2	0.82044251505431

0.218186977635635
RNF7 6.47855337257904 2 0.0391922331286751
0.281374871029788
ATP1B3 0.445693028666837 2 0.800237664880515
0.0867185386496466
TFDP2 1.02114337178907 2 0.600152382069844
0.280059893425122
GK5 0.889660889555359 2 0.640932940346951
0.928919167699888
XRN1 0.0386649783371729 2 0.980853184960631
0.0527587223705654
ATR 0.235706168118811 2 0.888826625822498
0.231854497410124
PLS1 0.964858148948484 2 0.617282144922426
1.22982753100752
U2SURP 2.37091105657031 2 0.305606935183142
0.139282335857859
AC026304.1 0.508710014290279 2 0.775416474788787
0.568956488241946
CHST2 71.9409545664792 2 2.22044604925031e-16
1.5845176964625
C3orf58 0.0656902697780434 2 0.967688409158374
0.1233079420325
PLSCR1 0.261039987844897 2 0.877638945328306
0.159522625944136
GYG1 0.0984015789769858 2 0.951989960929502
0.0598535544853399
HLTF 3.78754326313845 2 0.150503094797029
0.611153935331169
HPS3 2.84476178731968 2 0.24113920607996 0.50569131947274
COMMD2 4.3906765639166 2 0.111320897283576
0.345932269283776
RNF13 13.97353696487 2 0.000924027724008014
0.608678545097564
PFN2 0.208501805712674 2 0.900999205784584
0.201114602050584
TSC22D2 0.704627654633063 2 0.703059448091449
0.151276700395503
SERP1 2.64774220075114 2 0.266103193384922
0.127382295124992
EIF2A 3.80654404386688 2 0.149080027197806
0.255801745732439
SELENOT 6.17994994997438 2 0.0455030931058614
0.113598517558215
ERICH6-AS1 1.88618497201767 2 0.389421690197775
1.16299072058447
SIAH2 28.8804235958375 2 5.35421367131583e-07
0.681666238159954
MED12L 9.7283376942646 2 0.00771824056404113
1.08299410181572

P2RY12	1.48264166293135	2	0.476484144450984
1.63018335915616
IGSF10	10.9285945054233	2	0.00423531635855046
1.42120334707424
MBNL1	1.49425332663324	2	0.473725772698139
0.152368961844829
MBNL1-AS1	1.05844668037721	2	0.589062293072454
0.951110922252046
RAP2B	0.915214715848073	2	0.632795889727175
0.221775453490456
ARHGEF26	1.26412844057145	2	0.531493548115168
0.418320910572013
DHX36	2.22727225106645	2	0.328362819300833
0.213003814418154
MME	10.6510061371688	2	0.00486590251570962
0.506222559715778
SLC33A1	1.70234921040483	2	0.42691318288229	0.418045105553053
GMPS	0.860107944634006	2	0.650473986187696
0.196759409927213
SSR3	1.93581002493988	2	0.379878044814884
0.193174158512663
TIPARP-AS1	1.5695725979876	2	0.456217185440665
0.730720529542833
TIPARP	7.0851156805962	2	0.0289392104546619
0.521673846872115
LINC00886	2.9245172636826	2	0.231712330406353
1.25691533417329
LEKR1	0.876826490280957	2	0.645059160207555
0.584990345186465
CCNL1	2.10797302057547	2	0.348545495634499
0.133072583481457
RSRC1	1.98079777853771	2	0.371428502624786
0.255935463820454
AC106707.1	3.10040167396657	2	0.212205350864249
0.558463740833895
GFM1	0.303616951417164	2	0.859152813618447
0.0538205388345139
LXN	0.809602512883381	2	0.667109381376506
0.324910693564207
MFSD1	1.56282718225687	2	0.457758470421274
0.32651917041908
IFT80	4.76086260132505	2	0.0925106689918498
1.12224145681014
SMC4	3.27529059808392	2	0.194437345477143
0.168502831793996
TRIM59	3.68835390811785	2	0.158155436526822
0.71756337591558
KPNA4	4.11668082019099	2	0.127665666833009
0.53249582472923
B3GALNT1	22.1080514524632	2	1.58233204822222e-05

1.28718416000613
NMD3	3.46729340426644 2	0.176639083904975
0.182748825526657
PDCD10	7.63142180470886 2	0.022022053357968
0.322664227518502
SERPINI1 2.59278885031885 2	0.273516200421031
0.758363764797868
GOLIM4	1.91077753368724 2	0.384662571426574
0.540205830142751
MYNN	0.329715552143239	2	0.848014303437214
0.155389630679127
LRRC34	2.4613091265809	2	0.292101316292609
1.03902086154147
SEC62	2.17970355433638 2	0.336266332361053
0.0813384428290505
GPR160	16.6213058006648 2	0.000245883453554696
0.693331848101943
PHC3	6.59097241043464 2	0.0370500267509515
0.801250603433265
PRKCI	0.558482736754306	2	0.756357320442097
0.167191693431475
SKIL	0.604376824396869	2	0.739198777701454
0.161542041398307
RPL22L1	0.0929860028072148	2	0.954571241205438
0.0331493487427957
EIF5A2	5.22807101844711 2	0.0732383923961478
1.37202675078086
TNIK	3.30512153060758 2	0.191558743026471
8.40644277976036
FNDC3B	1.85035914963709 2	0.396460218410479
1.73326807387123
TNFSF10	3.16993410361031 2	0.204954545887863
0.509875075589185
NCEH1	1.73129431313372 2	0.420779154501677
1.27309605489928
TBL1XR1	3.99482519109006 2	0.135685903754311
0.353321926790386
LINC00501	2.02846112794499 2	0.362681382622861
1.62692093315181
ZMAT3	8.37084289163769 2	0.015215791958536
1.21598010987544
PIK3CA	2.87227263388175 2	0.23784494316367 0.548265156890771
KCNMB3	0.726355311001625	2	0.695462869719111
0.245206788853582
ZNF639	0.481200686913549	2	0.786155755904048
0.0720332858665473
MFN1	1.39110424479774 2	0.498798973858416
0.389218672283988
GNB4	10.6472033881358 2	0.00487516321999637
0.857303784075847

ACTL6A	0.0112561596826697	2	0.994387728129937
0.0176390954304927
MRPL47	4.75742197107478 2	0.0926699534654143
0.230282300822729
NDUFB5	1.45644948542505 2	0.482765262363676
0.130859144923945
USP13	2.49495745634326 2	0.287228064727515
1.09672515680985
TTC14	0.111244107087214	2	0.945896566596767
0.0552117233523742
FXR1	0.103129120594466	2	0.949742332335608
0.0367376401332286
DNAJC19	1.66312693726587 2	0.435368069609204
0.140672061194892
ATP11B	0.45157219654269 2	0.797888753197278
0.163647476288782
DCUN1D1	4.75635472143222 2	0.0927194176492547
0.484905958983256
MCCC1	3.99536787235655 2	0.1356490916498	0.578388912293276
KLHL6	27.7830072421225 2	9.26822635860525e-07
1.00901829381831
KLHL24	2.22408751297742 2	0.328886110615531
0.240790210231547
YEATS2	0.740507686584587	2	0.690559014613105
0.523658386900651
PARL	4.36227348276855 2	0.112913104661821
0.247162364454583
ABCC5	0.0594945658153901	2	0.9706908127138
0.138632011246875
EIF2B5	1.06781097278929 2	0.586310664083318
0.125561011187166
DVL3	3.49576255290204 2	0.174142512706939
0.337507740540357
AP2M1	4.91948999309465 2	0.0854567399528123
0.181620531633567
ABCF3	3.90079104138496 2	0.142217810374168
0.547390536898022
ALG3	1.29652165987021 2	0.522954493125561
0.202341670505341
EEF1AKMT4	1.66426622054092 2	0.435120136452336
0.53545022490038
PSMD2	0.000148886782973942	2	0.999925559379354
0.00140892965486248
EIF4G1	1.71098591792009 2	0.425073595587907
0.262954142747382
POLR2H	0.287314222104578	2	0.866184702306228
0.104480467944679
MAGEF1	1.55499353655087 2	0.45955494519939 0.192605169415117
VPS8	0.0828580457278972	2	0.959417429626308
0.0874685906906495

MAP3K13	4.78256731317775	2	0.0915121382902836	0.723227904180782
TMEM41A	0.179382980828961	2	0.914213185300009	0.205369511909279
SENP2	1.4353690378684	2	0.48788062744965	0.326861807186518
TRA2B	0.0888216719012274	2	0.956560887133477	0.0252419359107811
ETV5	0.714307793477207	2	0.699664813315798	0.592456670763014
DGKG	4.90013061296098	2	0.0862879511529617	3.1150115077205
TBCCD1	0.000155013887194846	2	0.999922496059988	0.0055617098150128
DNAJB11	0.00216989641141566	2	0.998915640137803	0.00782599718782578
EIF4A2	9.56516558689031	2	0.00837434181640584	0.227461022248552
RFC4	2.42504471026396	2	0.297446067801313	0.339722718700636
ST6GAL1	11.4707246582378	2	0.00322971193763655	0.314322498559631
RPL39L	1.94907923310141	2	0.377366046659584	0.295110866577631
RTP4	7.89731785863453	2	0.0192805410136926	2.25980987412601
BCL6	3.28045855325657	2	0.193935572299001	0.317180325352078
LINC01991	63.884847151617	2	1.34336985979644e-14	2.17785636187971
LPP-AS2	3.70256000563262	2	0.15703603105223	1.21675681442922
LPP	14.6631187616781	2	0.000654552094890071	0.278972161472567
CCDC50	2.6989554114316	2	0.259375695717664	0.27955720774959
OPA1	0.491404852917928	2	0.782154938913809	0.233861522521457
HES1	38.3078008453805	2	4.8035989630435e-09	3.37684125598845
ATP13A3	1.08882699455612	2	0.580181961872137	0.426014136176077
TMEM44-AS1	4.18331531884886	2	0.123482274513133	1.00628664364476
TMEM44	3.38837024215139	2	0.183748901144016	3.5150450941464
LSG1	0.498253395288906	2	0.779481208695169	0.175200281500768
FAM43A	0.0192004590082757	2	0.990445705585802	0.0303947756777899
XXYLT1	2.12659509615279	2	0.345315237220012	0.953605812022626

XXYLT1-AS2 2.49085589974126 2 0.287817710219532
1.06071326814149
ACAP2 1.45346274702375 2 0.483486747725557
0.158733242339979
PPP1R2 11.3190912067032 2 0.0034840997018013
0.427448805339781
MUC20-OT1 1.82056945296706 2 0.402409631041395
0.297626804825318
TNK2 1.13653349000307 2 0.566506488452013
0.332915892304577
TFRC 0.000127372392740979 2 0.999936315831552
0.00315451119981553
PCYT1A 1.57916026408121 2 0.454035390179979
0.478550906895954
TCTEX1D2 5.98339516507837 2 0.0502021420669128
0.551461906469814
UBXN7 1.65291722402252 2 0.437596243597817
0.414954982190397
RNF168 2.94506807698619 2 0.229343582749688
0.247393076619673
WDR53 0.674163500578041 2 0.713850480034224
0.1932002301138
FBXO45 0.864832876992886 2 0.648939077193907
0.631116932177159
PIGX 1.54131448678413 2 0.462708856013352
0.333249421511332
CEP19 1.68945241327996 2 0.429674984265047
0.683740128101067
PAK2 9.47120101464811 2 0.00877717649011267
0.430010377623163
SENP5 0.000433549491511678 2 0.999783248748192
0.00465106678510153
NCBP2 2.46194438837858 2 0.292008550622364
0.193909180875483
NCBP2-AS2 0.0403366880363871 2 0.980033676611956
0.0335828955842474
PIGZ 3.56510106904791 2 0.168208577900553
1.60537446243276
MELTF 6.21584716245383 2 0.0446936618166659
1.0981501893172
MELTF-AS1 3.52292619464272 2 0.171793329498107
6.1517258312494
DLG1 11.1538149479319 2 0.00378425033435981
0.705548485991089
BDH1 0.661822335755043 2 0.718268971563926
0.356400922819525
AC024560.1 2.64007618474211 2 0.267125126342614
2.39970294208496
RUBCN 1.85242669125364 2 0.39605058118203 0.245263411993821
FYTTD1 0.00269673490824827 2 0.998652541184833

0.00638020218372494
LRCH3	5.89886867280566 2	0.0523693209900599
0.651659119304747
IQCG	1.20387244418961 2	0.54775004295004 0.645179111562573
RPL35A	29.0671519925726 2	4.87694819639728e-07
0.144995996955149
ZNF595	1.95798567317256 2	0.375689288876535
0.202198788327874
ZNF718	0.0313841616575322	2	0.984430398383484
0.0679241091707189
ZNF141	7.59455765908192 2	0.0224317294400601
0.672774466436863
ZNF721	0.00362215701203738	2	0.998190560507049
0.0162538578746256
PIGG	0.281113323855407	2	0.86887443143774
0.227501859770804
PDE6B	0.172795602640463	2	0.917229283616666
0.190117901422254
AC107464.3	0.0850349678690192	2	0.958373709231297
	0.204744871202566
ATP5ME	24.2968993777234 2	5.29657756276336e-06
0.156346157154656
MYL5	0.767934968271716	2	0.681153575116574
0.141659308805112
PCGF3	1.93421104162943 2	0.380181875580614
0.535045768564713
AC139887.2	0.454529292228821	2	0.796709908206348
	0.454855948291758
CPLX1	4.28772132865067 2	0.117201492846135
3.93027971714423
GAK	0.00279378320838426	2	0.99860408359475
0.0173465721821642
TMEM175	0.13732433480719 2	0.933642041520515
0.179968074218565
DGKQ	0.339791561208078	2	0.843752747415932
0.543723871760791
IDUA	3.37417145467824 2	0.185058048519674
1.94776327286633
CTBP1	2.24030570868205 2	0.326229925151523
0.329497892471461
CTBP1-DT	0.396086094287826	2	0.820334539318786
0.397641814895628
MAEA	0.482261785961838	2	0.785738771966945
0.158528404538002
UVSSA	1.12789813421853 2	0.568957769105039
0.905962505670295
AC147067.1	0.214946075701816	2	0.898100736850332
	0.345839479948259
SLBP	33.7605351059699 2	4.66651803865403e-08
0.607715232251709

AC016773.1 0.243078730806455 2 0.88555619238612
0.381805418464419
TMEM129 0.0784003140282796 2 0.96155822726002
0.0557509954678864
TACC3 0.3322802339492 2 0.846927556948261
0.141342042813826
LETM1 5.31012951379106 2 0.0702942864780716
0.663610804263554
NSD2 0.0316812366898021 2 0.984284184396701
0.0621863714128544
NELFA 2.47109276141416 2 0.290675899268564
0.323788493763075
C4orf48 7.11793944625835 2 0.0284681396761914
0.437501494786849
HAUS3 5.95057692282252 2 0.0510327109129244
0.640984756549089
MXD4 14.2171128691025 2 0.000818075085056602
0.804188670755059
ZFYVE28 0.69926035596419 2 0.704948747085493
0.804495601470292
RNF4 4.67485740198127 2 0.0965756441032345
0.66918134847249
FAM193A 1.9261441335819 2 0.381718418400712
0.466911569276831
TNIP2 0.665293632274079 2 0.717023390531531
0.159395312584944
SH3BP2 3.95960622128068 2 0.138096424350947
1.20579579839374
ADD1 2.41772634735772 2 0.298536470710292
0.322002279301783
MFSD10 0.17896280722554 2 0.914405269600672
0.0615866347478739
NOP14-AS1 2.32546596049866 2 0.312630599018525
0.671746337375429
NOP14 0.549075690071092 2 0.759923244382521
0.18376050945762
HTT 3.30829099001982 2 0.19125541460619 0.312544775543868
DOK7 2.15586258618763 2 0.340298776413212
0.502076915151529
LRPAP1 4.24657061015399 2 0.119637935048576
0.236211368740114
TMEM128 3.32440895390757 2 0.189720284773533
0.630901815777743
LYAR 0.0536579282485403 2 0.973527735454494
0.0654558613981551
ZBTB49 0.159909368173486 2 0.923158179194753
0.256110335740504
NSG1 0.0168761156731396 2 0.991597442651559
0.0690182237601102
STX18 0.00559287569351513 2 0.997207468543384

0.0215441543973195
EVC2	1.7915002198559	2	0.408301212950312
0.691006695106188
MAN2B2	0.00311946025184627	2	0.998441485620949
0.0405250125341052
MRFAP1	0.684016700967502	2	0.710342272982803
0.106300829689789
LINC02482	4.96891833704031	2	0.0833706317835436
2.53026234376127
AC093323.1	1.26686463579037	2	0.530766910231662
0.45376194084418
MRFAP1L1	0.0362269633074515	2	0.982049581424062
0.0407984671188689
BLOC1S4	3.17983814221584	2	0.203942115875859
0.289856462695347
KIAA0232	1.18903631967815	2	0.551828394901737
0.511999238456279
TBC1D14	2.88758815207746	2	0.236030539884456
0.728050413187707
TADA2B	0.497473561906902	2	0.779785200690686
0.605297061643518
GRPEL1	0.0618068061350475	2	0.969569225965714
0.0460165201911399
ABLIM2	2.66853199193731	2	0.263351405524844
0.823714968626631
SH3TC1	103.292260012602	2	0	1.75596926104888
ACOX3	0.999622351902021	2	0.606645198101097
0.322201611700124
TRMT44	3.00111447227812	2	0.223005858595356
1.00981418052626
WDR1	1.80987648876866	2	0.404566868607626
0.124340333266714
ZNF518B	0.834631216470469	2	0.658812960333204
0.478643087117272
RAB28	0.0783913864651254	2	0.961562519455499
0.0713086283069432
BOD1L1	4.42310496982924	2	0.109530472045948
0.329446977416575
CPEB2	0.458284840158569	2	0.795215270817927
0.675745558258899
FBXL5	0.486795667914095	2	0.783959565987658
0.116178708873594
FAM200B	3.62225074719108	2	0.163470068350659
0.437490004767271
CD38	29.4895397416229	2	3.94846059514897e-07
0.420456983408782
TAPT1	0.398921980611601	2	0.819172175845718
0.291595925630936
QDPR	4.65479396635771	2	0.0975493394652539
0.879580585635

LAP3	8.22163607354546	2	0.0163943578517862	0.344423440065338
MED28	1.07830277655945	2	0.583242989260995	0.113164042987437
FAM184B	2.64817879340792	2	0.266045110374717	0.58140429087807
DCAF16	0.340301306425959	2	0.843537725354678	0.224987725556715
NCAPG	0.219354911180627	2	0.896123128192891	0.264738554649121
LCORL	0.001410691889906	2	0.999294902753022	0.0129756514934944
PACRGL	1.73822496510235	2	0.419323541108024	1.37371606449461
DHX15	0.128995466295016	2	0.937538239520535	0.0575438702884087
CCDC149	3.12455122314666	2	0.209658426797851	7.65046796314544
SEPSECS	4.61825199159861	2	0.0993480442335309	1.97337708286459
SEPSECS-AS1	0.323971195463036	2	0.850453452910991	0.386496696928085
PI4K2B	2.69538326205112	2	0.259839374041846	0.706087832570197
ZCCHC4	0.00967196571129003	2	0.995175691682574	0.0492148661432402
ANAPC4	2.85639072584429	2	0.23974117791289	0.665156095754226
SEL1L3	1.00238461097578	2	0.605807920826873	0.0910348744721638
SMIM20	3.57032516767928	2	0.167769782127341	0.202557601388303
RBPJ	0.0523976272996006	2	0.974141397748387	0.0426441214638371
TBC1D19	0.0744337599765441	2	0.963467155942666	0.203408861203078
STIM2	4.3827442229912	2	0.111763291664102	0.293297300467441
AC106047.1	2.67949434021907	2	0.261911879361712	1.39773959825287
ARAP2	6.29651027717993	2	0.0429269631600415	1.2482836571887
PGM2	3.71083904157469	2	0.156387321179015	0.485267624747822
TBC1D1	0.428461953462347	2	0.807161927866252	0.0710747748828691
KLF3	1.35953456781108	2	0.506734904012877	1.17182639529488
TLR10	8.85491379391511	2	0.0119448280081313	0.453059985647289
TLR1	6.83110452484249	2	0.0328582553101736	

1.15961965310026
TLR6	1.48125725867193 2	0.476814081969098
0.514870315714946
TMEM156	58.4354462962145 2	2.04614103438416e-13
0.883750564251815
KLHL5	6.59329256076398 2	0.0370070708559183
0.278405222727947
WDR19	2.21151555185064 2	0.330959993679875
0.828533770103414
RFC1	7.762365866541	2	0.0206264110765174
0.251098575640944
RPL9	0.657182855368696	2	0.719937103034537
0.0344909578198271
LIAS	3.95730847057897 2	0.138255171101579
0.602043358093837
UGDH	1.44402895099399 2	0.48577269245184 0.337136254945419
SMIM14	160.787012621735 2	0	1.28868428024952
UBE2K	5.88513737581017 2	0.0527301074393127
0.224222580900913
PDS5A	0.0139208470428561	2	0.993063744121518
0.0288496098775094
N4BP2	0.158610437384012	2	0.923757933224671
0.281990120801384
RHOH	7.88648898611085 2	0.0193852174001911
0.190043202654654
APBB2	4.18689380896623 2	0.123261532003737
1.61815349173076
UCHL1	17.8035236814119 2	0.00013614884247648
1.02229854374316
TMEM33	3.51713611965402 2	0.172291398244261
0.472929433466776
SLC30A9	0.764953321858559	2	0.682169812000146
0.245715298671338
ATP8A1	11.4655595753391 2	0.00323806358217726
0.777852390842304
GUF1	0.390011288032682	2	0.822830013976868
0.301162262467969
GNPDA2	2.87788830609309 2	0.237178050245856
0.516312365816774
COMMD8	2.74651510545153 2	0.253280539518298
0.138484323037584
ATP10D	2.70639528517686 2	0.258412626891493
0.544514466066501
NFXL1	3.79787522221455 2	0.149727603699115
0.959465425556336
TEC	6.86203441895987 2	0.0323540132196756
2.35559408278855
SLAIN2	0.227765664660047	2	0.892362505792932
0.139481770555528
FRYL	0.186155385725485	2	0.911122709814583

0.133595835655668
OCIAD1	3.38929016051193 2	0.183664403584274
0.216603477385573
OCIAD2	0.449135227380493	2	0.79886156089571
0.0273818291839284
DCUN1D4	0.539315463408473	2	0.763640819654535
0.4011765608968
SGCB	4.61463528139846 2	0.0995278633153768
0.734129979628001
USP46	0.773967307400723	2	0.679102195643884
0.552329334599875
USP46-AS1	0.00176889141144208	2	0.999115945301099
	0.0350442847340957
SCFD2	1.54525403228182 2	0.461798321785289
0.484065817955981
FIP1L1	0.0142153641132966	2	0.992917517775982
0.0172246712690188
LNX1	0.660398572645788	2	0.718780476039743
0.801527484241545
CHIC2	0.263486562696247	2	0.876565997038078
0.0790565188431147
GSX2	0.635272202919679	2	0.727867610188322
0.470729423478619
SRD5A3	0.399862066002821	2	0.818787220427844
0.543785697847921
TMEM165	0.0529793590860008	2	0.973858094444259
0.0086712878187356
CLOCK	1.63443983978182 2	0.441657793380534
0.703653447567678
EXOC1	0.16612146251873 2	0.920295254833121
0.109472810099026
CEP135	9.13183200173477 2	0.0104003481142873
0.560937189305827
AASDH	0.391597746961957	2	0.822177579764624
0.219643956100072
PPAT	0.543854893808262	2	0.761909537980311
0.293094620902985
PAICS	2.07278075858711 2	0.354732824715067
0.193080279853696
SRP72	0.342508500331712	2	0.842607313185789
0.0717372887497484
HOPX	30.5809614841794 2	2.28785548395116e-07
0.947377298529713
SPINK2	6.35865250961492 2	0.0416136826970518
1.11106299673571
REST	0.672611208162491	2	0.714404747445672
0.117693318941404
NOA1	7.9234262773758	2	0.0190304844906742
0.506405746408819
POLR2B	1.47256139361601 2	0.47889175093501 0.203234243253901

CENPC	0.520592895627947	2	0.770823043123657
0.145222815590002
STAP1	0.985937989542793	2	0.610810207082398
0.129432923479214
UBA6	5.11851953657901 2	0.0773619850480696
0.579718120136456
UBA6-AS1 0.198154958919067	2	0.905672534286601
0.222168013066254
YTHDC1	0.0457336434854053	2	0.977392642554756
0.0300251190318306
UGT2B17	2.3182187760384	2	0.313765499795036
0.948243005864234
FDCSP	0.66361367314273 2	0.717625928552073
0.833796131645096
ENAM	7.73725872669026 2	0.0208869782725983
1.56123401209872
JCHAIN	98.3729956725651 2	0	0.533020106759406
UTP3	0.186587945465584	2	0.910925673621555
0.0725903968586389
RUFY3	3.66058337917241 2	0.160366783631522
0.440979693415357
GRSF1	0.967096882044597	2	0.616591566515977
0.128693707924507
MOB1B	1.70713452761101 2	0.425892946408005
0.402997666499812
DCK	21.0006012153836 2	2.75281729252841e-05
0.295677220877771
COX18	1.57191175281233 2	0.455683916036155
0.732195948177713
ANKRD17	3.07662276982999 2	0.214743414406276
0.21735917646969
RASSF6	247.177804681314 2	0	1.08882927634702
MTHFD2L	2.51151265317781 2	0.28486031929599 0.475664037651891
AREG	1.74887124921081 2	0.417097352747207
0.411276482462525
PARM1	1.36995404101531 2	0.504101813445494
0.540800945966062
RCHY1	0.687510425197144	2	0.709102486161158
0.182033453901307
THAP6	0.40882494324287 2	0.815126085459757
0.243719383352044
G3BP2	1.89305739297837 2	0.388085851730127
0.218011345345598
USO1	1.5738187213178	2	0.455249635670635
0.272360949210065
NAAA	0.430083075401268	2	0.806507938996222
0.183217311292336
SDAD1	1.63945134024169 2	0.440552494642827
0.169868949593297
NUP54	3.74594114539301 2	0.153666505617491

0.504670738444527
SCARB2 0.00101046758272865 2 0.999494893817736
0.0199557989521467
SHROOM3 3.3935334159279 2 0.183275149169148
2.00796560994985
SEPT11 6.34674520658323 2 0.0418621750465437
0.558533287596043
CCNI 25.9372158940701 2 2.33241126679218e-06
0.303786526071498
CCNG2 10.8036073509625 2 0.00450844183866517
0.427507166743093
CNOT6L 0.904954991205782 2 0.636050385940644
0.222621389997397
MRPL1 0.112458315473459 2 0.945322483106806
0.0490034717836933
AC098818.2 1.72596745500501 2 0.421901363730389
0.847201447811124
BMP2K 0.82029025302873 2 0.663553943877225
0.287722255859167
PAQR3 0.572892858401016 2 0.750927305229694
0.407792065813793
BMP3 5.50770888065625 2 0.0636819293496937
5.88111728610696
RASGEF1B 0.0788504514199059 2 0.961341834956342
0.0847750880175031
AC124016.2 1.40958690035721 2 0.494210642834665
1.54372555798564
HNRNPD 1.28474326179533 2 0.526043362779686
0.0897278919692091
AC124016.1 2.19957265605169 2 0.332942216518948
0.972263208129719
HNRNPDL 7.19800347695949 2 0.0273510122869339
0.209299279161605
ENOPH1 0.614390392117556 2 0.735507018823103
0.130580633330714
SEC31A 0.0108362276819009 2 0.994596537664728
0.0239792628720955
THAP9-AS1 0.0508324755909786 2 0.974904035657034
0.0784630631963844
THAP9 5.15589389656832 2 0.0759297317756862
2.73777260170798
LIN54 0.802981975913228 2 0.669321351625604
0.681588695616324
COPS4 2.439127150902 2 0.295359040736267
0.142799592141184
PLAC8 7.69924307604492 2 0.0212877915336888
1.15422897811437
COQ2 8.56957691356266 2 0.0137765355457341
0.693403064508936
HELQ 4.74882404475759 2 0.0930691957302959

0.689698610440231
MRPS18C 1.46984293259765 2 0.479543117792045
0.123286875987075
ABRAXAS1 2.39387465416493 2 0.302118085306798
0.37612461548203
ARHGAP24 13.1176910688646 2 0.00141752124555006
0.628183204907527
MAPK10 4.08969921332002 2 0.129399649359514
0.56566915225638
AFF1 4.69695434098292 2 0.0955145038271361
1.72771183391247
KLHL8 0.0175724645465323 2 0.991252253866977
0.0801443330761759
HSD17B11 7.11610695915088 2 0.0284942353788407
0.258988386130138
NUDT9 0.155502579482731 2 0.925194503295331
0.114449706786153
PKD2 2.40023380840047 2 0.301159003101812
1.42026741868232
PPM1K 4.40393607582906 2 0.110585307984843
0.273253660787944
AC107067.1 1.68118795259386 2 0.431454173751381
1.34512018173461
PYURF 16.5494850748855 2 0.000254873672519418
0.346777542015543
HERC3 0.531734028402343 2 0.766541059798267
0.399492656609505
NAP1L5 0.0726591659127312 2 0.964322416869602
0.125793554845956
TIGD2 2.65655691802079 2 0.264932961887043
1.93389992533298
GPRIN3 13.8502987414024 2 0.000982756312185251
2.39306349026335
CCSER1 0.138589547258279 2 0.933051600530633
0.372353245130228
SMARCAD1 1.79005465591093 2 0.408596432383167
0.394760114674384
PDLIM5 0.458732299963069 2 0.795037377283913
0.147464505300056
RAP1GDS1 0.580421183453045 2 0.748106006053134
0.194414440257115
EIF4E 3.87371842963338 2 0.144156002542653
0.227112983651823
METAP1 1.1240674486063 2 0.570048562563308
0.248829587753182
ADH5 1.01206069165264 2 0.602884076210783
0.069146354211162
TRMT10A 1.95841186825008 2 0.375609238943263
0.456254203843711
DAPP1 0.0240650003928516 2 0.988039600859006

0.0184995804791654
LAMTOR3 0.867007315930951 2 0.648233921393984
0.188797972378062
DNAJB14 1.78171687150918 2 0.410303382436632
0.2925378138542
H2AFZ 2.40544233128826 2 0.300375726692522
0.0926515835547269
PPP3CA 1.20865760943846 2 0.546441072249456
0.268664348965393
AP001816.1 7.47998868172014 2 0.0237542375594789
1.35536624500427
BANK1 17.4380126087242 2 0.000163449529335224
0.518175847421316
SLC39A8 9.25762461748058 2 0.0097663516272769
0.836173033693847
AC098487.1 2.76950066558745 2 0.25038630523791
1.26208277904443
NFKB1 1.98106789126257 2 0.371378342229636
0.197597928616873
MANBA 0.706844323992495 2 0.702280654584876
0.265250088914993
UBE2D3 16.1408179974155 2 0.000312655379265614
0.150089225207644
AC018797.2 0.270639551782346 2 0.873436563070599
0.481993397910258
CISD2 0.207239639117217 2 0.901567990791102
0.0612714819861243
SLC9B1 0.11583468081667 2 0.943727952380012
0.316060134335514
SLC9B2 3.91426422468806 2 0.14126296686653 0.595093777785915
BDH2 10.6955658644656 2 0.00475868965008053
0.952012467095269
CENPE 6.20011232857012 2 0.0450466723083638
1.76823028228481
TET2 1.08872358012742 2 0.580211962240811
0.636610617692629
PPA2 0.996669978732581 2 0.607541380907996
0.177475786602913
INTS12 7.12059230049562 2 0.028430403796188
0.400180784862455
GSTCD 0.176067006296796 2 0.915730196364723
0.224799552783687
TBCK 1.97016169865572 2 0.373409035846647
0.602343098645097
AIMP1 8.36952715323049 2 0.0152258052528316
0.198444411735833
PAPSS1 3.32605955211502 2 0.189563773385788
0.19872743099799
CYP2U1 0.16405465111034 2 0.921246784772562
0.194975589923397

HADH 2.80999238641079 2 0.245367990516691
0.224376524217871
RPL34 54.4623564893574 2 1.4915846335839e-12
0.155731925759586
OSTC 0.229697027388193 2 0.891501183899276
0.0460112095165329
SEC24B 0.411548961184977 2 0.814016632133023
0.324888196634529
MCUB 47.7022376039749 2 4.38117320200604e-11
0.806584894795094
CASP6 6.38482071874342 2 0.041072751438896
0.492898336174559
PLA2G12A 0.856858810850935 2 0.651531583525669
0.443048084203152
GAR1 0.101695945592687 2 0.950423149723508
0.0618735247988778
FAM241A 59.7507990123473 2 1.06026298851702e-13
1.28738451077966
AC109347.1 0.0298449862908357 2 0.985188295489927
0.174695284858681
AP1AR 4.17463337175078 2 0.124019472939066
1.2143464073952
TIFA 22.6762567475613 2 1.19100452677934e-05
0.52881398390796
ALPK1 6.00092059703356 2 0.0497641567276409
0.823720479712765
ZGRF1 0.195097566561584 2 0.907058091206914
0.327023638378449
LARP7 5.42069091515097 2 0.0665138250409136
0.241775243211536
CAMK2D 2.12592755800902 2 0.345430512002756
0.1264526143746
UGT8 0.315562016176049 2 0.854036788640502
0.209025315975679
SNHG8 0.0100477270380353 2 0.994988734976774
0.00969208916669051
METTL14 0.270035209846242 2 0.873700530121976
0.195211328836668
SEC24D 2.20880316467293 2 0.331409144000023
0.475907441489341
USP53 23.8281190035589 2 6.69560309296546e-06
1.39047312605605
C4orf3 179.534830871299 2 0 0.940618838207383
MAD2L1 0.634940266049365 2 0.727988423261888
0.239103347935526
ANXA5 3.07080069012374 2 0.215369451811633
0.211202783707394
EXOSC9 4.76430630248764 2 0.0923515165003963
0.48572420729534
CCNA2 1.6956208627435 2 0.428351811581794

1.47292045554283
BBS7 2.04744763718118 2 0.35925464708415 0.637404869179358
KIAA1109 1.025507185334 2 0.598844333062327
0.37420383227369
NUDT6 4.13175645921566 2 0.126706963882755
16.3201055542446
SPATA5 0.640209552036228 2 0.726072958054727
0.762819347513283
MFSD8 1.44903476975811 2 0.484558367728658
0.406605414520236
ABHD18 0.0963700862367037 2 0.952957432546437
0.139132542920097
LARP1B 2.72393341409654 2 0.256156496443068
0.670981904628416
PGRMC2 1.79135799364585 2 0.40833024954978 0.285954666377552
JADE1 10.1826988024513 2 0.00614971585158341
0.490749796100128
SCLT1 0.404982316012622 2 0.81669370376676
0.28152761293238
C4orf33 1.21288012323546 2 0.545288611764202
0.455013057941448
ELF2 2.03771181348073 2 0.36100773050388 0.372688576128231
NDUFC1 3.94379790879776 2 0.139192285288054
0.101348793227687
NAA15 6.29015881250329 2 0.0430635043995951
0.464912811116298
AC097376.2 0.246925501093602 2 0.883854563730109
0.113355660000644
RAB33B 7.30195731139829 2 0.0259657048551118
2.22735006288422
SETD7 2.76234798301998 2 0.251283375276377
0.798931649229095
MGST2 5.89132482707458 2 0.0525672270378877
0.491642973177036
MAML3 0.31684809880924 2 0.853487784234689
0.0830374138914356
SCOC 2.04311770024026 2 0.360033264605024
0.147041140832418
SCOC-AS1 0.339018459641763 2 0.844078963746764
0.140210842068238
ELMOD2 0.595323215426713 2 0.742552571300523
0.30887989798229
ZNF330 2.17222977228994 2 0.337525273798318
0.210451438991624
USP38 0.74512280917736 2 0.688967344502567
0.462556513833533
SMARCA5 0.631229912783042 2 0.729340223900709
0.118780884266422
ANAPC10 1.57171159386604 2 0.455729522924512
0.278307380787535

ABCE1	3.23418463368493	2	0.198474963188546	0.352118019202037
OTUD4	0.802569105952152	2	0.669459537228481	0.35494576619386
MMAA	2.78218581069997	2	0.248803237597527	1.57776996962398
ZNF827	0.522696782872262	2	0.770012607080878	0.207496647455939
AC108206.1	2.59913213708332	2	0.272650078814513	0.453935434854682
LSM6	2.8018798722372	2	0.24636528744607	0.0891755252837596
SLC10A7	7.26707369555016	2	0.0264225663550579	1.11651504344794
TMEM184C	1.28834209597337	2	0.525097642490937	0.378481388773536
PRMT9	0.170913643170355	2	0.918092783989409	0.0878215857010487
ARHGAP10	1.21986363459569	2	0.543387917467988	0.578770441310141
LRBA	1.01745188886707	2	0.601261131121625	0.179555719746555
RPS3A	56.0676642991029	2	6.68465283126807e-13	0.273132861676087
AC095055.1	2.01946991448702	2	0.364315525967731	1.23710409134084
GATB	2.74239531386205	2	0.253802808761977	0.387617532631136
FBXW7	1.5890064547534	2	0.451805623847821	0.291544920108967
TMEM154	54.7771388894152	2	1.27442500996722e-12	0.787924512120615
AC106882.1	3.60759289484161	2	0.164672529405201	1.66212874918241
ARFIP1	1.91361803305553	2	0.384116642300603	0.469173036454871
MND1	11.4286569621968	2	0.00329836469237776	2.51729490754132
TMEM131L	6.32949429350484	2	0.0422248171465474	0.53021401822838
PLRG1	0.395661187409547	2	0.82050884072772	0.102650957112784
MAP9	9.4782595640645	2	0.00874625402215568	1.52541038995177
CTSO	0.695323238890643	2	0.706337846772991	0.572846927088867
C4orf46	0.215508280361825	2	0.897848314120595	0.298519661523768
ETFDH	0.760623666021875	2	0.683648191895884	0.31938391151855
PPID	0.35916079103758	2	0.835620768077432	

0.0722404759739022
RAPGEF2 1.61572333760583 2 0.445810337877461
1.0035893641596
NAF1 0.11547429347565 2 0.943898021505944
0.139774946356487
TMA16 2.8332175064989 2 0.242535120266539
0.263843959513548
MARCH1 53.356212161646 2 2.59325894091944e-12
1.49335560538735
TRIM61 0.10149283213252 2 0.950519676492178
0.104774637970957
TMEM192 1.67464874930564 2 0.432867165728908
0.433335258042092
KLHL2 0.336178224184666 2 0.845278506792051
0.248909184542008
MSMO1 1.57674154962061 2 0.454584813319364
0.0832231074689872
DDX60L 0.509446786278801 2 0.775130874828498
0.668025279904033
PALLD 0.020228607948765 2 0.989936673585785
0.0960216600868815
CBR4 2.58597331043524 2 0.274449870670578
0.28577222327174
SH3RF1 0.288288080521647 2 0.865763034344244
0.204960521490256
NEK1 0.00361913802586392 2 0.998192067269937
0.0255459725431258
CLCN3 0.745646920111009 2 0.688786820498161
0.307321300911955
HPF1 1.90823182454872 2 0.385152502677706
0.278141049730713
GALNT7 0.0625418097018985 2 0.969212973011825
0.120234864773079
HMGB2 22.6505651158685 2 1.20640264053007e-05
0.539452779703788
AC097534.2 3.18633562647285 2 0.203280635603441
2.44834368726638
SAP30 0.00466613862947147 2 0.997669650176147
0.0276572036552855
SCRG1 1.67764516142518 2 0.432219127087064
1.94622794303796
FBXO8 1.06722971285024 2 0.586481088297744
0.319729440814518
CEP44 0.883722284765646 2 0.642838892335207
0.436884603912765
GLRA3 10.2583173889793 2 0.00592154020305402
1.99724387123405
GPM6A 8.13788760410788 2 0.0170954351388802
3.96313133888005
SPCS3 0.0460741893731518 2 0.977226233200095

0.0389657585273802
AGA 0.0265637589831274 2 0.986805935457395
0.0493319588638691
DCTD 0.309710137815531 2 0.856539307665637
0.103008277260558
CDKN2AIP 0.878728402744881 2 0.644446028755964
0.285811713527549
ING2 0.544482783989912 2 0.761670377764762
0.20068591641942
RWDD4 0.398192552341628 2 0.819470994005528
0.109562727929835
TRAPPC11 0.448764193030178 2 0.799009777183738
0.12579070330351
IRF2 8.08013016042804 2 0.017596327205618
0.244286886718839
CASP3 0.100139943621022 2 0.951162867584252
0.0593775433958209
PRIMPOL 0.348337094983073 2 0.8401552796592
0.255572666412951
CENPU 2.47814474989478 2 0.289652782533807
0.517170565048417
ACSL1 2.00577879179534 2 0.36681802598607 0.581519020392359
SLC25A4 18.4089460601751 2 0.000100588458845308
0.364666065766252
CFAP97 1.05956837938889 2 0.588732010404569
0.230421322523238
SNX25 6.81408851780908 2 0.033139006082649
1.45397166145186
LRP2BP 2.269478604912 2 0.321505926215399
1.21257811355599
ANKRD37 2.77056715407015 2 0.250252823774803
0.480004004880841
UFSP2 0.554328411176397 2 0.757930030545256
0.175540294269787
CCDC110 1.67593160020052 2 0.432589602740947
3.13253172512525
AC106897.1 0.109378120493856 2 0.94677949357118
0.233266200038504
CYP4V2 0.190476382779841 2 0.909156355459666
0.357030224711594
FRG1 4.47911936201342 2 0.106505390402374
0.192924111904302
CCDC127 7.76166875262665 2 0.0206336018087168
0.472796776709203
SDHA 12.924441374647 2 0.00156132464389935
0.44293172455439
PDCD6 0.648618756921018 2 0.723026518942683
0.0777382447093897
EXOC3-AS1 6.10653514885493 2 0.047204428132364
1.56461727950525

EXOC3	4.94627513516728	2	0.0843198841954507	0.547668168907537
SLC9A3	0.0330387999081296	2	0.983616297094594	0.105429137183274
BRD9	1.6178872782114	2	0.445328245183223	0.284202024200279
CLPTM1L	1.40997657272123	2	0.494114362099696	0.252310339217517
LPCAT1	12.2601449980945	2	0.00217642316638933	0.89949536691802
MRPL36	0.185631827134515	2	0.911361254097274	0.0456119417919113
NDUFS6	7.80921329122141	2	0.0201488785825281	0.239220742009907
ICE1	0.149392493282666	2	0.928025334332478	0.109480568484916
MED10	4.42463248399172	2	0.109446849310104	0.227053824772486
NSUN2	1.08969963103175	2	0.579928873118556	0.238395400145273
SRD5A1	0.0332879309162165	2	0.983493780065617	0.0611688141813509
TENT4A	0.938821643518763	2	0.625370614519649	0.888358937423029
MTRR	0.468068081641813	2	0.791334877748238	0.22576622504946
FASTKD3	0.397567382978845	2	0.819727188124191	0.259879993808205
MIR4458HG	0.774927181346896	2	0.678776347591209	0.404754929524002
FAM173B	0.784041427559706	2	0.675690117719322	0.495007100778968
CCT5	1.31132266998707	2	0.519098650941531	0.049204528737073
MARCH6	3.01219151851429	2	0.221774149565106	0.342013793234256
ANKRD33B	0.521422826904417	2	0.77050324440493	0.158112915666314
DAP	0.130214697687162	2	0.936966875667936	0.0286451935588629
TRIO	10.817751040002	2	0.00447667130948326	3.17739066285384
OTULINL	0.627864759644238	2	0.730568427656237	0.556125692031483
AC010491.1	3.88184769634253	2	0.14357125045295	2.70866806410498
OTULIN	5.41659221457219	2	0.0666502749365707	0.388169971765185
ANKH	2.64870672943574	2	0.265974892243399	0.625706671435141

ZNF622	1.759384883062	2	0.414910501298493
0.126272591347688
BASP1	32.7828626254006 2	7.60837360891387e-08
0.358318977663287
DROSHA	2.73610852519418 2	0.2546018662914 8 0.935061410913263
C5orf22	2.12979603033808 2	0.344763013572017
0.391529401281339
GOLPH3	1.08943317450864 2	0.580006141181176
0.248841759413363
AC025181.2	0.251275518940056	2	0.881934261261688
0.317918270830895
MTMR12	6.87551456702077 2	0.0321366780258097
0.650387850254253
ZFR	2.03364305032395 2	0.361742905540192
0.223342127091049
SUB1	139.806243126786 2	0	0.630047375798525
TARS	4.99307010008602 2	0.0823699123572111
0.487031726638449
RAD1	2.06038359085877 2	0.356938494831769
0.426150198275906
BRIX1	1.95495799291236 2	0.376258453101612
0.243450169535021
DNAJC21	0.0597051106304909	2	0.970588631133504
0.0578757716047357
LMBRD2	2.80161045102958 2	0.24639847769819 0.836400054564204
SKP2	0.316785009747238	2	0.853514707531197
0.37668107311187
NADK2	0.500211126624845	2	0.778718574620201
0.31748015122255
NIPBL	9.18033387787398 2	0.0101511636074506
0.53078818523423
CPLANE1	2.29265665698119 2	0.317801492515905
0.909696710847347
NUP155	1.53435900891374 2	0.464320838019163
0.396563788275421
WDR70	0.220660115767887	2	0.895538506967382
0.109925006376583
RICTOR	4.47624984335136 2	0.106658309680006
0.439679476662568
TTC33	0.555628838590151	2	0.757437374233842
0.0999532512558942
PTGER4	1.0768126271841	2	0.583677710778945
0.53581566147025
PRKAA1	2.36819995298156 2	0.306021482118946
0.404689597514469
RPL37	64.2402663294101 2	1.12132525487141e-14
0.266692291001077
CARD6	0.0035898016053507	2	0.99820670906844
0.0498576137746733
OXCT1	1.782673181052	2	0.410107240813358

0.351080717002728
C5orf51 0.376996930877253 2 0.828201774364119
0.270127765209488
FBXO4 0.0955170301737415 2 0.953363982300496
0.105813968006937
CCDC152 0.925372946309708 2 0.629589994932183
0.227446818084794
ANXA2R 0.141648528221334 2 0.931625597795644
0.133196897724913
AC025171.2 0.393501893669335 2 0.821395178909533
0.576057423534971
ZNF131 0.950128601441342 2 0.621845070093305
0.247232418501522
HMGCS1 2.8486915860374 2 0.240665857002481
0.278395296349323
CCL28 1.68493038005282 2 0.430647585662521
0.858542013197592
TMEM267 0.509705600282049 2 0.775030573956078
0.254052056807122
C5orf34 3.20731702708738 2 0.201159226367221
2.4934277017659
PAIP1 0.348187376735503 2 0.840218175301405
0.110954907472412
NNT-AS1 3.61096054396498 2 0.164395483069725
0.433846966640416
NNT 3.07184631276269 2 0.215256883652875
0.54579330048712
MRPS30 3.08162823173974 2 0.214206641395082
0.306770800331937
EMB 5.39456744686463 2 0.0673883096503143
0.301380502091044
PARP8 0.54078159687399 2 0.763081225109002
0.192235567306956
PELO 3.26989953309839 2 0.194962164675511
0.717698203418437
MOCS2 1.48564255865271 2 0.47576974093244 0.153228435893836
NDUFS4 0.225937212406771 2 0.893178699946321
0.0707997876078997
ARL15 2.06563238872596 2 0.35600297395352 0.897716159404593
DHX29 0.383340716623103 2 0.825578968886653
0.133668681952203
MTREX 1.75048947577745 2 0.416760010231221
0.273509607597979
PLPP1 4.35662690191523 2 0.113232341586481
0.682803398009973
SLC38A9 11.7590840556813 2 0.00279606550201605
1.04967192839581
IL6ST 0.884271079517446 2 0.642662523228741
0.926115168438332
MAP3K1 1.32735438177762 2 0.514954263557256

0.391024705923493
SETD9 1.82121382872809 2 0.402280000419101
0.670866081708108
MIER3 0.830341762749973 2 0.660227450490423
0.553906053502441
GPBP1 0.480057427620992 2 0.786605274307497
0.0682237618125173
GAPT 2.62376371553018 2 0.269312770885688
0.896690067570412
PDE4D 6.63325953634272 2 0.0362748807294661
0.77403869573666
DEPDC1B 3.68674079524533 2 0.158283049268603
1.47517755129494
ERCC8 0.219710425779476 2 0.895963849922528
0.207215378237506
NDUFAF2 3.77338062022915 2 0.151572638011502
0.306589584740009
SMIM15 0.204059065133458 2 0.903002883282179
0.07714766832849
ZSWIM6 0.962086380869562 2 0.618138219467922
0.704608480589713
KIF2A 15.8199128174706 2 0.000367070581317241
0.682229245189508
DIMT1 0.580942973345679 2 0.747910854434988
0.12837685621532
IPO11 0.0299000785768389 2 0.985161157726028
0.174865585175932
SREK1IP1 1.50015462036711 2 0.472330035407715
0.190518267668041
CWC27 0.000924380049257316 2 0.999537916768727
0.00508244773189739
CENPK 0.0412117074789908 2 0.979604996134011
0.081718456068699
PPWD1 0.179088169729538 2 0.914347955329823
0.094120319280796
TRIM23 0.0039556959796778 2 0.998024106662616
0.0454839387246929
TRAPPC13 7.10879277597445 2 0.0285986321853459
0.427051797092718
SHLD3 0.0316239105740662 2 0.98431239739557
0.114184937403088
SGTB 0.1482505369547 2 0.928555367838385
0.147276757387019
NLN 3.726050343881 2 0.15520240550884 1.62052227802997
ERBIN 2.89702517552516 2 0.234919450420628
0.192588792866553
SREK1 8.54818728902001 2 0.0139246636966347
0.3704408525851
MAST4 1.483411671491 2 0.476300731325785
0.584529311638567

CD180	4.05824997597221	2	0.131450491600277	0.307760376746986
PIK3R1	1.26586463630929	2	0.531032359905919	0.345902610454857
SLC30A5	0.00691459839705261	2	0.996548670378828	0.0194979245358417
AC010273.1	0.020143545282839	2	0.98997877780743	0.13597368162743
CCNB1	5.02364049786019	2	0.0811204453669903	0.332081222369004
CENPH	7.54042476577714	2	0.0230471679432992	0.748532966156651
MRPS36	18.6005779300879	2	9.13978168877172e-05	0.456472480574355
CDK7	0.0399693785205396	2	0.980213680988415	0.0581408931474552
CCDC125	1.17773229033419	2	0.55495616786903	0.386811096271233
AK6	0.606971420639449	2	0.738240438276256	0.0814830926101192
TAF9	7.35283448277842	2	0.0253135047420715	0.277459180897076
RAD17	2.42915909501113	2	0.296834792989598	0.200271695399016
OCLN	0.0426317428579779	2	0.978909706121353	0.161852944544982
GTF2H2C	1.02690689639142	2	0.598425375167096	0.525850882977855
SMN2	1.00639031018065	2	0.604595792933623	0.988670897349882
SERF1A	1.65385425948929	2	0.437391270018257	1.05127658081961
SMN1	1.24458615302825	2	0.536712303068195	0.697403139438362
NAIP	0.0670331241419614	2	0.967038894931953	0.126175020119576
GTF2H2	0.451148846983239	2	0.798057663999804	0.238240053659751
BDP1	1.51940312736821e-05	2	0.99999240301322	0.0006674018511991
MCCC2	0.772013129465502	2	0.679766063183135	0.232997938838695
MAP1B	1.43809090445589	2	0.487217106066962	0.687038445887371
MRPS27	5.74422626656772	2	0.0565792406915855	0.303277272611337
PTCD2	0.0438550705336419	2	0.978311125537327	0.138960321913797
TNPO1	8.24733361236773	2	0.0161850580301277	0.296893912516852
BTF3	76.9718991512543	2	0	0.260242786099252

ANKRA2	2.31145696523805 2	0.314828106541948
0.420598265422089
UTP15	1.62225469894968 2	0.444356838298209
0.599820390885746
HEXB	0.0990632999698173	2	0.951675037159236
0.113719262697131
GFM2	0.221125693141553	2	0.895330059997326
0.0738087147562431
NSA2	1.07139469546167 2	0.585261017364886
0.0512267977603073
HMGCR	2.92589611247286 2	0.231552637327596
0.510671211401431
COL4A3BP 1.00674577313056 2	0.604488346780159
0.279670025685138
POLK	0.161149131789465	2	0.922586107560292
0.120002406042815
POC5	0.0471334219202828	2	0.976708815312598
0.0740570488430899
IQGAP2	23.2641499985135 2	8.87675052985148e-06
3.32905556150275
S100Z	2.25279033475761 2	0.324199838646269
0.574716579980772
AGGF1	0.00323355298484983	2	0.998384529786606
0.0260654851389197
PDE8B	4.08272049006593 2	0.129851960209673
3.00713019483101
WDR41	3.72844236143329 2	0.155016893029164
0.516510774289077
TBCA	42.0923905858785 2	7.24024951104241e-10
0.461200425595765
AP3B1	16.2562205175974 2	0.000295125384772388
0.634776133096426
SCAMP1-AS1	0.902888228084819	2	0.636708008409958
0.428352325462363
SCAMP1	1.67841496748906 2	0.432052796647185
0.443028636910481
LHFPL2	0.104111278241466	2	0.949276048488849
0.0765788932739288
ARSB	0.294194185940109	2	0.863210161709046
0.441550558667125
JMY	2.37087750883222 2	0.305612061436846
0.852794972092618
HOMER1	3.03324747396023 2	0.219451563777256
1.25627572868001
TENT2	0.0964853649684195	2	0.952902506267308
0.0719328540320422
MTX3	0.0158199101815564	2	0.992121246282638
0.0727526730478331
SERINC5	1.76241320113602 2	0.414282736201437
0.99070407825485

ZFYVE16	1.95838002545744	2	0.375615219214424
0.420283356620968
ANKRD34B	2.67843856583592	2	0.262050175787393
0.983695241091549
DHFR	3.02955008656785	2	0.219857637737362
0.388090529921313
MSH3	0.520124859606787	2	0.771003450707206
0.256198126870367
CKMT2-AS1	2.18635802852336	2	0.335149353803435
0.869605647447171
ZCCHC9	2.1316019910896	2	0.3444518398495	0.30554326139584
SSBP2	1.55549896097949	2	0.45943882472474	0.155835830598348
ATG10	0.290203848104248	2	0.864934131026444
0.226196803111619
RPS23	66.3814232200633	2	3.88578058618805e-15
0.289272176366289
ATP6AP1L	1.76117366910508	2	0.414539574143625
0.899523135217733
TMEM167A	0.356234048763167	2	0.836844486549855
0.0863925137535353
XRCC4	0.429276361930857	2	0.806833315022307
0.0773827884546986
COX7C	12.9658859481806	2	0.0015293033499657
0.167569982287453
RASA1	0.83310245083016	2	0.659316738156805
0.523669075837356
CCNH	1.1453210051401	2	0.564022856514638
0.0949781574340652
TMEM161B	1.6573577378693	2	0.436625745284796
0.741004107734062
TMEM161B-AS1	1.01034911299789	2	0.603400238800564
0.265174369728999
MEF2C	32.0033744128654	2	1.12345464708952e-07
0.400359530560066
MEF2C-AS1	0.0450091939423442	2	0.977746742509416
0.0964454056027667
CETN3	1.63489733367603	2	0.44155677706266	0.216105022847696
MBLAC2	0.905444623164735	2	0.635894689701655
0.766774387047692
LYSMD3	1.3848210420113	2	0.500368465477292
0.307579314465589
ARRDC3	0.230245549820527	2	0.89125671322631
0.125693841430952
ARRDC3-AS1	0.314143831068885	2	0.85464259452865
0.388746674205459
FAM172A	1.44900237964605	2	0.484566215242119
0.467534294702223
POU5F2	0.0323219755574711	2	0.983968900336198
0.161465451886377
SLF1	0.472865950799879	2	0.789438792348141

0.195740074521253
TTC37 0.16951769704135 2 0.918733811707364
0.0452397700525119
ARSK 0.476429616541371 2 0.788033396820303
0.256641297888268
RFESD 0.0229488274860135 2 0.988591166271963
0.095674088068826
RHOBTB3 1.3347627456053 2 0.513050307764047
1.28463349602542
GLRX 53.1606450869808 2 2.85960144452702e-12
0.419956446014899
ELL2 0.117572661599753 2 0.942908218079365
0.234710281104391
CAST 1.05385804215913 2 0.590415341516789
0.154993471313003
ERAP1 0.43582690983991 2 0.804195037778832
0.366411636376549
AC009126.1 6.48336479159917 2 0.0390980613211412
1.71711581827263
ERAP2 2.84172058699857 2 0.241506161320834
0.451622081718112
LNPEP 4.93242144833129 2 0.0849059823920429
0.342537665348379
RIOK2 0.441125167613101 2 0.802067440857296
0.175381850858382
CHD1 0.670937170050004 2 0.715002968159297
0.235659183936122
FAM174A 2.58352928304448 2 0.274785457175123
0.917495645112102
ST8SIA4 12.7899043482555 2 0.00166996572932254
0.480728545065433
GIN1 0.00648454918768217 2 0.99676297590238
0.0360300124838754
PPIP5K2 8.37222942486678 2 0.0152052470136087
0.861178059789637
C5orf30 2.17785634043616 2 0.33657705375284 1.35041316303895
FBXL17 0.0272395963283141 2 0.986472531640438
0.0909494468857488
FER 5.59073734596832 2 0.0610923470823411
1.43431103592115
AC008467.1 2.94769192453095 2 0.229042898731817
1.72002893692183
PJA2 1.57613766855051 2 0.454722091625024
0.211705848810397
MAN2A1 0.307632934233957 2 0.857429373056203
0.0735429108072343
SLC25A46 3.81160571560298 2 0.148703207153737
0.396100503094753
WDR36 1.06128133286696 2 0.588227991003389
0.174484548446119

CAMK4	3.61383626020746	2	0.164159275546446	1.04766614672583
STARD4	1.95493300597174	2	0.376263153904788	0.504623104034033
STARD4-AS1	2.5907166274345	2	0.273799740550128	2.23706727314298
NREP	3.61397061243036	2	0.164148248335044	2.03235425989196
EPB41L4A-AS1	7.39179095120279	2	0.0248252133333007	0.316410839585195
APC	5.99864930503796	2	0.049820703295427	1.03284489886757
SRP19	5.31016439748765	2	0.0702930604264832	0.19087470538931
REEP5	81.7533211795643	2	0	0.424095389969624
DCP2	3.21955266248452	2	0.199932327689805	0.354830895841088
YTHDC2	0.00410065955572255	2	0.997951770712427	0.0163154759674782
PGGT1B	0.0730255779869133	2	0.964145763363618	0.0579356212946347
AC008494.3	1.43265193812431	2	0.488543888047932	1.16661010901574
CCDC112	6.67059868394317	2	0.0356039268663925	0.98687763159556
FEM1C	0.634434016242703	2	0.728172718585036	0.710051105275983
AC010226.1	0.530273804842893	2	0.767100924812819	0.326478297615518
TMED7	1.07842237651349	2	0.583208112386467	0.322255827677998
ATG12	0.230338048897788	2	0.891215493967715	0.0605335578643699
AP3S1	7.26650368504649	2	0.0264300979984641	0.219302955461502
AC034236.2	0.865960770226545	2	0.648573213370324	0.831496025674901
COMMD10	0.358183531879397	2	0.836029176873801	0.101831144844268
DMXL1	0.928943508287864	2	0.628467002610009	0.271301572033753
TNFAIP8	49.4637240255008	2	1.81589188130715e-11	0.718350248830523
HSD17B4	4.51003500583732	2	0.104871708159245	0.548685099772308
SRFBP1	0.047037626959795	2	0.976755598324169	0.0620803797218239
SNX2	51.6456589694283	2	6.09945427498815e-12	0.649553410978651
SNX24	6.34122528152814	2	0.0419778726681008	

3.32531816656878
CEP120	2.57302948895932 2	0.276231845908014
0.877565143423286
CSNK1G3	0.00591929585159706	2	0.997044727514472
0.0208280995644723
ZNF608	37.4731537270849 2	7.29135185562058e-09
0.73639702208716
GRAMD2B	0.0279310063255513	2	0.986131562096084
0.147634782089052
ALDH7A1	1.81591292822643 2	0.403347637781971
0.845650493752706
PHAX	22.7337630876526 2	1.15724701454933e-05
0.62371991872849
LMNB1	6.92663143748845 2	0.0313257223457808
0.438005045495555
MARCH3	0.352513195617193	2	0.838402823406264
0.2150551060104
C5orf63	3.07007138120306 2	0.215448001563745
0.860641927380781
AC011416.3	0.0563437212174405	2	0.972221265904058
0.182125998983296
PRRC1	0.490518860151098	2	0.782501507481661
0.301432770486608
LINC01184	3.43069536216551 2	0.179901161607777
0.413345589294692
SLC12A2	4.70910525165193 2	0.0949359689363133
0.696881197671634
ISOC1	3.92149738878974 2	0.140753000479602
0.322935061956656
HINT1	14.3577738742382 2	0.00076251609834721
0.215434590169412
LYRM7	1.02679825175826 2	0.598457883902741
0.136875702043779
CDC42SE2	1.10216679775237 2	0.576325082080382
0.0633515893613701
RAPGEF6	0.164196675863358	2	0.921181367171781
0.19467517023642
FNIP1	0.817250292496661	2	0.664563299683491
0.153145595126885
P4HA2	12.4823987041673 2	0.00194751836138085
0.724920607874682
AC116366.3	1.00278539931632 2	0.605686532614389
0.579445941896671
IRF1	10.893610660795 2	0.00431005191197398
0.500739463840315
RAD50	0.978289215894293	2	0.613150654125297
0.551767558810563
KIF3A	21.041380578815 2	2.69725658160747e-05
1.17375111705139
UQCRQ	18.0676182134115 2	0.00011930717278863

0.161048242732516
LEAP2	0.620494260454655	2	0.733265721706451
0.306615654651494
AFF4	3.98049840715052	2	0.136661364700987
0.456206107605769
ZCCHC10	23.6035538802748	2	7.49123460574985e-06
0.537512155019472
HSPA4	4.81528467300687	2	0.0900272986326076
0.13199122804377
C5orf15	0.00419590140835972	2	0.99790424845622
0.0101805855079509
VDAC1	1.11588182985643	2	0.57238644365165	0.089686526459926
TCF7	0.390510927921569	2	0.822624480302764
0.25980293857786
SKP1	13.1216962750717	2	0.00141468535363509
0.194945700062677
PPP2CA	1.96869532090332	2	0.373682915588524
0.112013572388886
CDKL3	0.0352911410290975	2	0.982509200884581
0.0260541378966589
UBE2B	2.69770170453918	2	0.259538337237261
0.185782860336133
CDKN2AIPNL	4.32224144480329	2	0.115195945989648
0.46581041071314
JADE2	2.15405292307859	2	0.340606828830831
0.255266778743611
SAR1B	13.7069412993694	2	0.0010557850667543
0.326664990409301
SEC24A	1.1832056894694	2	0.553439495848809
0.649493902295681
CAMLG	22.6292745355769	2	1.2193137459704e-05
0.562522022883655
DDX46	2.79666793033627	2	0.247008145499255
0.117302792168716
C5orf24	0.764290779280711	2	0.682395832708051
0.200647387919204
TXNDC15	0.0461075354439425	2	0.977209940008349
0.0522357338671824
PCBD2	0.0337533748248551	2	0.983264926101763
0.062116441381146
PITX1	0.79403621889258	2	0.672321850145977
0.81353918721284
H2AFY	11.929282753697	2	0.00256796537410708
0.308222954117244
SMAD5	12.1101143607093	2	0.00234596797355602
0.730384822187709
KLHL3	0.990499980944457	2	0.609418539423389
0.676083940354913
HNRNPA0	2.40805489795994	2	0.299983607051687
0.065146143296878

AC106791.1	2.42478830158526 2	0.297484204122497
0.469647593655765
FAM13B	1.62025796802586 2	0.444800690344741
0.20217122609107
BRD8	0.105020614226786	2	0.948844541157122
0.104263728328317
CDC23	0.239780567312519	2	0.887017751723089
0.141315848036891
FAM53C	0.0456228198725395	2	0.977446803147228
0.0738330016329557
KDM3B	2.36795814896132 2	0.306058482967967
0.447212455943886
EGR1	21.5693133367667 2	2.07149132882556e-05
1.74050332919676
ETF1	0.509584892304487	2	0.775077351554236
0.130723796381279
HSPA9	4.7574692061035	2	0.0926677648573022
0.265084889502976
CTNNA1	7.81077503546329 2	0.020133151026379
0.601230299004136
SIL1	5.48770914082975 2	0.0643219350124681
0.41346438061923
MATR3.1	0.0195212963381503	2	0.990286832351476
0.0964860432686356
PAIP2	4.69305200426503 2	0.0957010506377587
0.115439454243785
MZB1	70.9802843056648 2	3.33066907387547e-16
0.547342961221433
SPATA24	8.4154142474116	2	0.0148804482797187
2.19300717996854
DNAJC18	0.149079415290482	2	0.928170617857635
0.0461882142385323
UBE2D2	1.01119489326823 2	0.603145120739274
0.035350999216369
CXXC5	6.14426919389095 2	0.046322170085948
0.267079294283237
PURA	2.30704992983336 2	0.315522600732021
0.414347019699139
IGIP	3.64875919776922 2	0.161317694768571
1.93710973692353
CYSTM1	0.543174172882402	2	0.762168906000175
0.144558515597442
PFDN1	1.5771075083179	2	0.454501641295934
0.158712911750623
HBEGF	6.71178737795766 2	0.0348781859719464
2.30468259703467
ANKHD1	0.0631798278612423	2	0.968903834584902
0.0529744318218163
SRA1	2.17126165085482 2	0.337688696074508
0.223006783243545

APBB3	0.000229429796274279	2	0.999885291681365
0.0109224169942244
SLC35A4	1.4255834160321	2	0.490273584479663
0.306758134223382
NDUFA2	5.59038673087433	2	0.0611030579706707
0.2261839656047
TMCO6	1.1965942438033	2	0.54974699158141	0.847678693071834
IK	0.0450214994842109	2	0.977740726676186
0.0206783257874218
WDR55	5.54844511448019	2	0.0623979686863432
0.630898916521072
DND1	1.56675893086056	2	0.456859458769303
1.21602026215703
HARS	0.247222170061803	2	0.883723467342676
0.0721818076800433
HARS2	1.4946050051803	2	0.473642480425655
0.53426130162041
ZMAT2	0.00825376996659977	2	0.995881618904322
0.0118294784206673
TAF7	4.99942184755337	2	0.0821087308753412
0.18684460005141
PCDHGA10	0.925997017006355	2	0.629393571245975
0.944636649314045
DIAPH1	2.75114314382584	2	0.252695121086089
0.376695599767879
HDAC3	1.47535776729186	2	0.478222638673047
0.213705622897707
RELL2	1.02099642324364	2	0.600196479449626
0.307281163368972
FCHSD1	0.00669733167107163	2	0.996656934692727
0.0485422650776771
DELE1	1.25325850307502	2	0.534390063043127
0.327115055761989
RNF14	4.01241560635304	2	0.134497750743528
0.61831535633247
GNPDA1	0.871810679929302	2	0.646678937689342
0.752614861398627
NDFIP1	7.85347482235571	2	0.019707866441752
0.477975352697609
ARHGAP26	0.500973559781298	2	0.778421770766652
0.519545613600366
NR3C1	10.8330489012518	2	0.00444256018433031
0.403555076294013
YIPF5	0.0692778443679576	2	0.965954137906444
0.0587728949243252
LARS	3.83846881971137	2	0.146719245951854
0.255563780307619
RBM27	0.985107967588594	2	0.611063752631716
0.510106771061599
TCERG1	6.34574048132838	2	0.0418832103220156

0.384519773690539
JAKMIP2 6.53332988051035 2 0.0381333923809152
3.06436167325907
FBXO38 0.334892950661215 2 0.845821888414215
0.110459847940884
GRPEL2 0.631173964242217 2 0.729360626946734
0.797669073845524
PCYOX1L 2.24671924399175 2 0.325185457157645
0.907840391671255
CSNK1A1 4.24221032871049 2 0.119899047109738
0.172819236974164
ARHGEF37 0.635350516935608 2 0.727839109628515
0.580329763519859
PDE6A 0.491796079404829 2 0.782001954012751
0.584578781710338
SLC26A2 1.02869223860158 2 0.597891416486338
0.665997421694281
TIGD6 4.15137184318316 2 0.125470335158289
3.03890337853222
HMGXB3 0.483373615497884 2 0.785302089570143
0.193642523496617
TCOF1 2.13285577588375 2 0.344235973279565
0.219380885410188
CD74 154.154516279597 2 0 0.552592865938353
RPS14 81.1634895352614 2 0 0.27103391221781
RBM22 3.42572322580194 2 0.18034896456522 0.257967475479043
DCTN4 0.271702860698467 2 0.872972320047382
0.307868352663138
TNIP1 6.82941247221449 2 0.0328860660214361
0.216636251516957
ANXA6 97.7683421881875 2 0 0.872966498340973
CCDC69 11.6094561228985 2 0.0030132740670642
0.272261526683575
GM2A 2.42029434316837 2 0.298153396493114
0.433147665641149
ATOX1 0.421575300612867 2 0.809946040414854
0.031768515724408
G3BP1 4.02179559736691 2 0.133868433797214
0.237412214997514
FAM114A2 0.66017574041163 2 0.71886056423089 0.290485285097527
MFAP3 0.240355803487807 2 0.886762666059016
0.205760141175225
GALNT10 5.59393293799875 2 0.0609948119150137
2.41575185617764
SAP30L 0.83259909747512 2 0.65948269368559 0.285442779898514
LARP1 2.58394150638091 2 0.274728826522463
0.347185706050254
CNOT8 3.94939746398001 2 0.138803122885147
0.435026544101102
GEMIN5 1.16235867696137 2 0.559238446087731

0.464433280178527
MRPL22	0.118986723829268	2	0.942241788251896
0.0415530206556432
MED7	1.01549750263019 2	0.601848966528921
0.132680621235343
CYFIP2	0.372995727223836	2	0.829860334853181
0.113767508140075
ADAM19	0.904457304954102	2	0.63620868240155
0.247617186282164
THG1L	0.507275708932792	2	0.775972766239933
0.128410085731266
CLINT1	17.0459090634439 2	0.000198851044877713
0.589593375645873
EBF1	7.19513066975919 2	0.027390327609026
0.427633033920696
RNF145	1.54877259056999 2	0.460986603857569
0.278125594871011
UBLCP1	0.858104409464723	2	0.651125936438504
0.179185390499858
TTC1	11.8234512491566 2	0.00270751070685926
0.229348988461402
PWWP2A	0.800104877526534	2	0.670284896203047
0.227802446488414
ZBED8	2.21804033097745 2	0.329882032571203
4.21241254217159
SLU7	5.56856924955076 2	0.0617732642909492
0.191055310605175
PTTG1	47.2915804431355 2	5.37977440373538e-11
0.347953849050421
MIR3142HG	2.1498799877177	2	0.341318235876747
0.508036175525419
CCNG1	4.31325835907101 2	0.115714517239763
0.19634420622091
NUDCD2	0.174846236208867	2	0.916289315002358
0.0576046804632444
HMMR	3.36694355508835 2	0.185728048960934
0.911203657843605
MAT2B	5.23704332379694 2	0.0729105696674004
0.339516935127118
RARS	3.23490598313306 2	0.198403391193845
0.318877973497764
PANK3	8.25455633963798 2	0.0161267133155264
1.25557637766309
SPDL1	1.07786719055004 2	0.583370029337817
0.482670749897323
DOCK2	0.0313696961790025	2	0.984437518537629
0.038437840344183
NPM1	7.61366761254758 2	0.0222184155148969
0.120802967335337
FBXW11	3.3029486960903	2	0.191766968840516

0.349034617712735
STK10 0.00108783207708288 2 0.999456231856972
0.00964741601246487
UBTD2 0.381364762834541 2 0.826395024888175
0.366861982919435
DUSP1 2.58638568309588 2 0.274393288692304
0.367670331055842
ERGIC1 2.87448289492275 2 0.23758223864596 0.318227591546081
RPL26L1 0.506672420048966 2 0.776206869418132
0.118775166923482
ATP6V0E1 13.5391796978068 2 0.00114816547566909
0.164287507217619
CREBRF 2.21005465849044 2 0.331201830622248
0.363677629722925
BNIP1 0.219065184696082 2 0.896252952897903
0.109228482714323
BOD1 2.43464478685487 2 0.296021736442327
0.461994416341283
CPEB4 35.4574868176472 2 1.99758177776843e-08
0.865512096529025
SFXN1 0.049235789781375 2 0.975682654137679
0.0442716313433116
THOC3 4.07233506965235 2 0.130527997522685
0.339225963442835
SIMC1 1.72905040113856 2 0.421251515132098
0.758490476561789
KIAA1191 1.24178310353037 2 0.537465046014723
0.329196617448681
ARL10 1.45869465554644 2 0.482223621368249
0.613259244243752
NOP16 3.66662240092872 2 0.159883284716325
0.383364969704467
HIGD2A 12.6765016259382 2 0.00176739103207835
0.287979053370451
CLTB 0.411043047510562 2 0.814222569251224
0.0929406388822587
FAF2 1.50727063603454 2 0.47065246761107 0.282388937816423
RNF44 0.885353023157357 2 0.642314954934735
0.1445049161245
TSPAN17 7.1696278016349 2 0.0277418298816836
0.677521065339555
UIMC1 0.914458256334644 2 0.633035277231482
0.174609558194065
ZNF346 0.33661231421738 2 0.845095063213196
0.178222957219335
NSD1 5.21894126259667 2 0.0735734809541725
0.324434820873598
RAB24 1.49782929861131 2 0.472879514428323
0.526510279495096
MXD3 0.556300458637434 2 0.757183061874003

0.462505503319944
PRELID1 3.97945218791245 2 0.136732872276932
0.125596092589141
LMAN2 8.44275386185129 2 0.0146784194141898
0.187530456029485
RGS14 3.12247373494437 2 0.209876321400717
0.620116315087662
GRK6 8.32943677115756 2 0.0155340889076416
0.461054803730502
PRR7-AS1 0.299086576421534 2 0.861101161684315
0.445137723779603
PRR7 0.0173738167883156 2 0.991350713775635
0.0565523456324425
DBN1 0.787830458008646 2 0.674411224328294
0.986264842729951
PDLIM7 6.02488231275311 2 0.0491714968156881
0.90228884152706
DOK3 13.3536174670591 2 0.00125979189604375
0.66831641055136
DDX41 8.75690158466175 2 0.012544778041182
0.681714235400352
FAM193B 2.57509696671183 2 0.275946441852089
0.925955941746711
TMED9 8.53576493044315 2 0.0140114214344006
0.171347687771138
B4GALT7 2.22346325600329 2 0.328988781362052
0.42374110245967
AC106795.2 1.56533724879942 2 0.457184328669549
1.16874521320741
N4BP3 7.26135714180975 2 0.0264981974010662
0.27443377650347
RMND5B 1.36948179534185 2 0.504220857449635
0.230182315446533
NHP2 0.74417106634528 2 0.689295282390346
0.0912500316381815
HNRNPAB 0.020623237287737 2 0.989741363827598
0.0207372721658451
PHYKPL 0.88018139521004 2 0.643978011171063
0.0634449236691597
COL23A1 0.462827269632373 2 0.793411215649684
0.445723067954415
CLK4 0.00297277179165319 2 0.99851471822857
0.0111685330678025
ZNF354A 0.0591808876536487 2 0.970843066908068
0.0752224829182322
ZNF354B 0.038307461751136 2 0.98102853627396
0.147735871255799
ZNF879 1.09235770279886 2 0.579158638784178
0.542353875192981
RUFY1 3.06767743517109 2 0.215706041410523

0.195870620437495
HNRNPH1 0.699545017787383 2 0.704848418227809
0.0634139858449595
CANX 0.254998212392993 2 0.880294202643912
0.0859961168814977
MAML1 0.0186552612983424 2 0.990715736754473
0.0802404296747503
MGAT4B 3.75854424055366 2 0.152701213432427
0.534426087715574
SQSTM1 12.6940123986074 2 0.0017519843847813
0.286616236867753
MRNIP 3.12406526584856 2 0.20970937550863 0.599848996694637
TBC1D9B 1.38907433015923 2 0.499305490530689
0.533722982398515
MAPK9 0.00112120850080391 2 0.999439552858801
0.0110455787216994
CNOT6 0.175489503921426 2 0.915994652725729
0.252883291684972
MGAT1 8.62734325667287 2 0.0133843170300432
0.558361842286491
LINC00847 1.81757086578897 2 0.403013413732041
0.503520445805655
ZFP62 3.53990803615902 2 0.170340821243432
0.655512012857864
BTNL9 50.1677825744539 2 1.27704513630533e-11
1.77820984647185
AC008443.5 1.0630738963071 2 0.587701009205173
0.667655346537499
TRIM41 0.618467789830376 2 0.734009068958112
0.295694202897246
RACK1 59.7126292024342 2 1.08024700296028e-13
0.183114355086419
TRIM52 0.165002142185437 2 0.920810451582906
0.139861048580123
DUSP22 46.5903463320324 2 7.63896723654511e-11
0.685925520897026
IRF4 8.05025858814995 2 0.0178611146677168
1.77393552796674
EXOC2 2.23616369048483 2 0.326906250390878
0.543494144154025
GMDS 83.394327908539 2 0 1.65914453502244
GMDS-DT 5.41732198755957 2 0.0666259595878868
2.0559660156686
WRNIP1 6.70791964822806 2 0.0349457009317085
0.966332190039339
SERPINB1 1.16093809129959 2 0.559635810252499
0.126013434282497
SERPINB9P1 1.38538363778712 2 0.500227732679624
1.15958367930579
SERPINB9 46.1751141832496 2 9.40159061713075e-11

0.880382391990481
SERPINB6 0.499993566667681 2 0.778803288217558
0.543882791599723
NQO2 1.45300705826967 2 0.48359692001299 0.583194420203086
RIPK1 2.2840311775111 2 0.319175047403929
0.759376386329947
BPHL 10.3618353859419 2 0.00562284400159452
1.58440311276092
TUBB2A 6.66256056366131 2 0.0357473091276849
0.429237132773276
TUBB2B 4.17213254689092 2 0.124174645424166
0.448899832030491
PSMG4 4.96081845221824 2 0.0837089626889372
0.700320074635347
PXDC1 1.67941419167463 2 0.431836991759113
1.66587401206745
FAM50B 1.52087079897391 2 0.467462849609487
0.186362721195934
PRPF4B 3.02590265910766 2 0.220258960968082
0.239750757825199
ECI2 5.26516970124128 2 0.0718923907679939
0.466850299547669
CDYL 0.27431804830794 2 0.871831572829516
0.255546670486314
RPP40 0.576145170363222 2 0.749707172632756
0.330185358986612
LYRM4 4.72912653668746 2 0.0939903399630273
0.357991874290611
FARS2 3.01019783381294 2 0.221995333654195
0.467131245330541
LY86-AS1 0.129988908708753 2 0.93707266003589
0.202615663454418
LY86 17.999868439145 2 0.000123417922303304
0.297655064953318
RREB1 1.90027991969849 2 0.386686899026873
0.539240717937231
SSR1 0.55082841555587 2 0.759257567693797
0.123577235204834
RIOK1 1.67016143592198 2 0.433839461382917
0.303942833306765
SNRNP48 0.285355540980139 2 0.867033407637515
0.134353804254857
TXNDC5 1.14168916155253 2 0.565048008428118
0.859786649206032
BLOC1S5 5.07406134809825 2 0.0791009278610508
0.674487824751105
EEF1E1 2.68075536924618 2 0.261746792170906
0.250554822226929
SLC35B3 0.0304899491323656 2 0.984870641790137
0.0490414050344365

GCNT2	4.75098676558913 2	0.0929686087812797
0.416864154929506
C6orf52	2.26990351310493 2	0.321437628219688
0.538032647787914
PAK1IP1	1.00792022084212 2	0.604133481005409
0.334732339867821
TMEM14C	0.000943404555508775	2	0.999528408956275
0.00629584349465036
TMEM14B	0.0430365987824779	2	0.978711567479394
0.027927839910267
SMIM13	9.35910416105724 2	0.00928317106903187
1.58324347297775
NEDD9	4.98824529356066 2	0.0825688616790394
0.792543557334316
HIVEP1	0.57575354042227 2	0.749853990894769
0.497204113616555
PHACTR1	75.1391870445864 2	0	1.07589156442295
TBC1D7	0.0184456592034203	2	0.990819570242255
0.0414740490316138
GFOD1	9.4090781477158 2	0.00905408652143758
0.74358438259323
SIRT5	0.0581900031232694	2	0.971324182769005
0.0923793237079188
NOL7	0.248780519371094	2	0.883035160605612
0.0452721103381681
RANBP9	3.61883520673715 2	0.163749476180668
0.840906538897247
MCUR1	0.0475076883398845	2	0.976526057757485
0.0454313081854104
CD83	47.1978612201617 2	5.63786795026999e-11
0.831716281675294
JARID2	0.851176858963303	2	0.653385200875517
0.397930767831677
DTNBP1	13.1625809030679 2	0.00138605949765613
0.242436827636391
MYLIP	1.02009378011789 2	0.600467422199317
0.66807280848685
FAM8A1	0.389686752600675	2	0.822963543557372
0.584979381839843
NUP153	0.655929298336206	2	0.720388485587433
0.261546805524293
AL138724.1	2.93799429267987 2	0.230156182459553
0.624103445072857
KIF13A	0.176512285448272	2	0.915526341276261
0.26723527929839
TPMT	1.72474219305271 2	0.422159912764179
0.475704593165787
KDM1B	2.8091991973689 2	0.24546532141652 0.608604916481396
DEK	48.2735417953949 2	3.29255511744009e-11
0.634868129212578

RNF144B	7.59728405937803	2	0.0224011713363043	0.696638587905244
E2F3	0.00286622338625171	2	0.998567914721056	0.0273421157054174
CDKAL1	1.18967343516142	2	0.551652633691021	0.210288023550111
SOX4	18.4099807860011	2	0.000100536431566822	0.738390399767609
MRS2	2.15650627369307	2	0.340189271000727	0.337209629803176
GPLD1	0.011978358031755	2	0.994028720364739	0.0704762830790057
ALDH5A1	3.04574370379811	2	0.218084679858342	0.336196077373919
TDP2	0.150484079401717	2	0.927518962745555	0.0603288691304494
ACOT13	1.71426962908861	2	0.42437625875111	0.224862286805917
C6orf62	1.67107344568161	2	0.433641673571	0.212332614406866
GMNN	0.254735609174636	2	0.880409794277787	0.0826808527857537
RIPOR2	9.7503128938836	2	0.00763389982515106	0.264355604534801
TRIM38	13.7832779187362	2	0.00101624688797708	0.666472552321123
HIST1H1C	24.9036674580536	2	3.91054530679558e-06	0.571692117044477
HIST1H4C	1.67346136832303	2	0.433124231150298	0.121858078461691
HIST1H2BC	19.8736510596877	2	4.83605804383336e-05	0.985738947714102
HIST1H2AC	59.8186207056229	2	1.02473585172902e-13	1.17047906331066
HIST1H1E	5.71606325945416	2	0.0573815974069463	1.06484452013607
HIST1H2BD	34.8859470499808	2	2.65835422563754e-08	1.43173030424276
HIST1H2BE	0.011266311248039	2	0.994382680846734	0.107768252692721
AL031777.3	10.8661801238592	2	0.00436957266961502	2.34076420411851
HIST1H2BG	10.4953585457772	2	0.00525971059931063	2.29070424791916
HIST1H2AE	7.10548791256371	2	0.0286459285381334	0.818144440864026
HIST1H1D	0.203318639308407	2	0.903337248498959	0.269879406457708
HIST1H2BH	1.50181723993035	2	0.471937545991877	1.09646141956512
HIST1H4H	13.8220010875804	2	0.000996759995371965	1.35873300794915

BTN3A2	24.4103995220274	2	5.00436632799861e-06	0.899309944413335
BTN2A2	9.83256698093449	2	0.00732630860808337	1.15999167129044
BTN3A1	3.09470217319026	2	0.212810945632365	1.2265166075053
BTN3A3	1.91012484710522	2	0.384788123961561	0.730598353158637
BTN2A1	0.947014571554506	2	0.622814046319406	0.290352894159692
HCG11	2.42438159270633	2	0.297544705007429	0.94125940372346
HMGN4	0.152327754006945	2	0.926664335141138	0.277890331248896
ABT1	6.41088591981209	2	0.0405409396491273	0.190731192260139
ZNF322	1.70838041899091	2	0.425627720851796	0.388322581110827
HIST1H2BJ	0.738519661383624	2	0.691245780245306	0.579036317223122
ZNF184	0.842996594314143	2	0.656063105565025	0.500183271319858
HIST1H3H	3.0880403052311	2	0.213520986735918	0.72366954730687
HIST1H4J	3.10312371280704	2	0.211916731713331	1.12316799058052
HIST1H2BN	0.0947721837351424	2	0.953719103307498	0.27945770894663
AL121944.1	5.83464601556075	2	0.0540782607863455	0.894045233412665
ZNF165	2.55539579536582	2	0.278678108086168	1.23456357761711
ZSCAN16-AS1	5.5129531871945	2	0.0635151643077669	0.627736173979857
ZSCAN16	0.653482526105922	2	0.721270338171325	0.17811579227347
ZKSCAN4	0.938643839413222	2	0.625426213722427	0.449539931888973
NKAPL	2.08572902021171	2	0.352443656188439	1.62257947670418
ZSCAN26	0.962564982212661	2	0.617990316274266	0.452595947552086
PGBD1	10.3002513824578	2	0.00579867583834348	1.51872992689615
ZKSCAN3	2.78493605712647	2	0.248461337621148	1.27561685640665
ZSCAN12	1.9657382974875	2	0.374235818790476	1.28105438885772
TRIM27	4.023324728525	2	0.133766121717743	0.272756552644051

HLA-F	26.2210335888634	2	2.02383406333606e-06	1.14264417013599
HLA-A	74.3075175861974	2	1.11022302462516e-16	0.677590496305534
ZNRD1	0.0810293140491015	2	0.960295089339563	0.0464762481751576
PPP1R11	4.78396705226504	2	0.0914481141387106	0.275692983693317
TRIM26	16.040100578433	2	0.000328803487128493	0.945857933509467
HCG18	0.939649869968925	2	0.625111693892527	0.40668912934364
TRIM39	0.209810604986818	2	0.900409785110688	0.22613660841938
RPP21	6.7397914090869	2	0.0343932242134439	1.14083827987078
HLA-E	110.867521077523	2	0	0.743934732979416
GNL1	0.478202313339336	2	0.787335234134083	0.113135470377326
PRR3	2.1069872098716	2	0.348717337922203	0.591180556478298
ABCF1	0.264354305790763	2	0.876185762485111	0.076528241556318
PPP1R10	1.4074039517003	2	0.494750355551874	0.19716185109387
MRPS18B	0.120105214216063	2	0.941714991378838	0.0421859305389199
ATAT1	0.493302309022132	2	0.781413238473811	0.336721839404789
C6orf136	3.00231183090452	2	0.222872389557655	0.642414623365596
DHX16	1.51903166025403	2	0.467892911828613	0.337308469896144
PPP1R18	4.05845638582666	2	0.13143692596189	0.199182300670073
NRM	0.037120064332236	2	0.981611144579666	0.0873485304931426
MDC1	1.2710485288965	2	0.529657734795687	0.494257187973658
TUBB	6.26547575101637	2	0.0435982670704262	0.239716992076706
FLOT1	1.96770981531842	2	0.373867094262223	0.136999988398449
IER3-AS1	2.56649679286636	2	0.277135590426838	2.7695059927299
VARS2	2.98543286321493	2	0.224761276894558	1.00725203927162
CCHCR1	0.403102243992582	2	0.817461786213477	0.36779421743186
TCF19	3.15641980467914	2	0.206344143961711	7.77261497122652

AL662844.4 2.62166671206047 2 0.269595293880222
0.567555534774255
HLA-C 36.2310597229114 2 1.35682970414663e-08
0.298108952180079
HLA-B 164.350094470606 2 0 0.466387242518364
MICA 0.909453683785261 2 0.634621296226312
0.495439929414246
MICB 0.953766008911515 2 0.620715145951762
0.590632214661252
DDX39B 0.0391764640523375 2 0.980602370837568
0.0643059539752866
NFKBIL1 2.39461306420408 2 0.302006562381877
0.263033267856149
LTA 5.40785355404035 2 0.0669421291394675
1.32808514081397
TNF 5.33705444686828 2 0.0693542935072908
9.21449787604274
LTB 853.133167161002 2 0 1.76973834248432
LST1 6.60981737766463 2 0.0367025630372713
0.891722377181831
NCR3 7.54315509410895 2 0.0230157262419225
1.54784110747073
AIF1 1.60774734804809 2 0.44759177700123 0.434817483470344
PRRC2A 0.391952799825996 2 0.822031634467359
0.182679425455049
BAG6 3.33264832383577 2 0.188940304708179
0.243167622350381
APOM 1.42970507785775 2 0.48926425390807 0.408118862286823
C6orf47 6.18694266087353 2 0.0453442759207617
0.980792092051686
GPANK1 2.47316509849211 2 0.290374866034221
0.310736168465543
CSNK2B 0.0177263762593513 2 0.991175974135987
0.0116282106657952
ABHD16A 3.99850799501556 2 0.135436281362936
2.74253827075802
DDAH2 6.67732896453466 2 0.035484316023915
0.936108231605822
CLIC1 4.98203579644364 2 0.0828256156052591
0.169249727864329
VARS 0.58977848605051 2 0.744614054098932
0.259981523827292
LSM2 1.32032900475051 2 0.516766318212404
0.150270769418521
HSPA1A 0.741374418711151 2 0.690259814607725
0.484703793025208
HSPA1B 0.0519774493904347 2 0.97434607559578
0.142491448389239
C6orf48 13.4306300869413 2 0.00121220405478728
0.221188320070199

NEU1	7.10880064077514	2	0.0285985197242958	0.766718252743552
EHMT2	0.246830247428901	2	0.883896659925684	0.133711737708293
NELFE	0.170130628523281	2	0.918452294408916	0.0637131371203452
SKIV2L	2.11357978180556	2	0.347569758269205	0.516329070098157
DXO	3.71515456794587	2	0.156050238178113	0.537078628125905
STK19	0.00645817606280535	2	0.99677611986626	0.0268696899238134
ATF6B	2.02557072380748	2	0.363205909439651	0.192522399232118
FKBPL	0.306699400311775	2	0.857829686178216	0.329780350855941
AGPAT1	0.428298397178703	2	0.80722793876795	0.261624155474686
RNF5	0.116426980615764	2	0.943448508822348	0.0510925750325238
AGER	1.2647325992658	2	0.531333019138591	1.05441003518122
PBX2	1.88048212538371	2	0.390533680910034	0.436109127623186
GPSM3	10.3444240324071	2	0.00567200835797477	0.242358563492394
NOTCH4	2.46192349736532	2	0.292011600815547	0.919292601107013
AL662796.1	3.95158015037102	2	0.13865172367075	0.809565235296418
HLA-DRA	119.118557553461	2	0	0.554372495430469
HLA-DRB5	40.5845882587398	2	1.53875201469589e-09	0.786048740358064
HLA-DRB1	74.8068715383073	2	1.11022302462516e-16	0.806956116289738
HLA-DQA1	114.660405898259	2	0	0.883774112268367
HLA-DQB1	162.108188579244	2	0	1.08497396833966
HLA-DQA2	4.03127271764629	2	0.133235590739456	1.21457132391104
HLA-DOB	7.08400798651735	2	0.0289552427900165	0.272047777397632
TAP2	0.840684728265304	2	0.656821909053365	0.191578913041551
PSMB8	0.491086713954974	2	0.782279365790373	0.0400848972571061
PSMB8-AS1	0.00964108648633502	2	0.995191056928217	0.0148409851767049
PSMB9	11.093352448561	2	0.00390039977659606	0.267173742886096
TAP1	6.88727917349272	2	0.0319481952405956	

0.416725237314187
HLA-DMB 24.797445675604 2 4.123852173743e-06
0.304891829131264
HLA-DMA 21.7305867969739 2 1.91101032176455e-05
0.248812268082112
BRD2 0.167490233317802 2 0.919665633673791
0.0574852757766966
HLA-DOA 5.15942207554093 2 0.0757959030118266
0.566937917947855
HLA-DPA1 156.731589895465 2 0 0.855614127143738
HLA-DPB1 127.933002533932 2 0 0.828322640639359
RXRB 0.189510748676471 2 0.909595417635553
0.104730672175871
SLC39A7 1.04796483852153 2 0.59215762608478 0.186395679078084
HSD17B8 0.0115751810304855 2 0.994229125323054
0.0657353916988235
RING1 0.417192598551444 2 0.81172286261489
0.131884474747692
VPS52 1.17938479860277 2 0.554497822421964
0.563110707114493
RPS18 53.8282972208216 2 2.04802841352603e-12
0.202033382500593
B3GALT4 1.37898590146298 2 0.501830457333219
0.397090125703711
WDR46 0.296955076099688 2 0.862019369589152
0.0805386876373291
PFDN6 2.83780612297151 2 0.2419793077889 0.197269363479864
RGL2 0.0611580087044046 2 0.969883803998629
0.0794596643534984
TAPBP 2.55918925980898 2 0.278150031307549
0.191903551416164
ZBTB22 0.000274163741835465 2 0.999862927524373
0.00733740454904272
DAXX 1.33371764102963 2 0.513318473435335
0.0817711554380136
KIFC1 1.15815808464457 2 0.56041424678029 0.893042236246437
PHF1 0.000320682612724892 2 0.999839671547618
0.00536702662994465
CUTA 3.34143022538911 2 0.188112495894397
0.0950718158964513
SYNGAP1 0.00174852319030056 2 0.999126120460171
0.0315349306685327
BAK1 0.412716528443029 2 0.813541561232228
0.135936991405252
ITPR3 1.59144624728419 2 0.451254803894514
0.73985205564344
UQCC2 1.20040632462722 2 0.548700149577615
0.167565298277554
LEMD2 0.000165047732465794 2 0.999917479538768
0.00318866183477672

HMGA1 27.0065232510522 2 1.36649481563555e-06
0.289825556722775
SMIM29 3.2486991779667 2 0.197039790386301
0.381530480492865
NUDT3 1.24043302952538 2 0.537827977290572
0.37055169877518
RPS10 27.784444966145 2 9.26156617619966e-07
0.237910354283332
PACSIN1 0.0621120056425676 2 0.96942128122903
0.211971142746408
C6orf106 4.21841289729486 2 0.12133421316894 0.494254599293445
AL451165.2 2.7310612458344 2 0.255245201095009
0.662771475344247
SNRPC 1.14399248669001 2 0.564397638362584
0.0524643574601179
UHRF1BP1 0.300641672959739 2 0.860431874201907
0.478791338134623
TAF11 4.99195010854429 2 0.0824160520775961
0.222712609907519
ANKS1A 1.62840386353724 2 0.442992724745334
0.490363637869477
ZNF76 0.0386971934613949 2 0.980837385934275
0.056343390995377
DEF6 0.626081167730063 2 0.731220236223321
0.179460889505767
PPARD 0.841871753382612 2 0.656432192663768
0.639591014572917
FANCE 0.344345327127143 2 0.841833806594124
0.380396844279576
RPL10A 18.1765374071669 2 0.00011298350723421
0.112565189414488
FKBP5 8.95056834549256 2 0.0113869857936718
0.848966274894436
SRPK1 0.0524965776583705 2 0.974093203120215
0.0454339296319167
MAPK14 2.04631686332101 2 0.359457822397104
0.479015035667827
MAPK13 0.676920824204293 2 0.712866999736261
0.202148260847633
ETV7 2.05951289621877 2 0.357093920878601
0.359806823089466
Z84484.1 2.25651177907517 2 0.323597153711123
1.0789127001445
KCTD20 0.74712725176282 2 0.688277192660115
0.251478371898362
STK38 0.173795436538657 2 0.916770859748057
0.118551051759376
SRSF3 26.4325498159227 2 1.82072659604682e-06
0.366582321724723
CDKN1A 0.413918265172029 2 0.813052876677125

0.636940722640546
CPNE5	25.1247472526285	2	3.50130911586088e-06
0.590295996657777
PPIL1	1.21775159687766	2	0.543962048449811
0.331011305779262
C6orf89	0.137147868243163	2	0.933724423456425
0.12481036128062
MTCH1	1.89861207213067	2	0.3870095009229	0.0982999356924926
FGD2	2.70454175440007	2	0.258652225778847
0.490481615419417
PIM1	21.0494145202114	2	2.68644351337777e-05
0.580282960529903
TBC1D22B	0.867773857851052	2	0.6479855197622
0.461378682527995
RNF8	1.97133348402769	2	0.373190322301254
0.259329920527455
CMTR1	1.28112395544375	2	0.526996180696701
0.500741153908772
CCDC167	3.04367544303268	2	0.218310324504672
0.190420781727716
ZFAND3	1.68177471611177	2	0.431327611533407
0.30482489395445
GLO1	1.65585318319081	2	0.436954332517572
0.095690887518256
SAYSD1	1.0262331227393	2	0.598627010754649
0.258530658128182
OARD1	4.13498257565534	2	0.126502742927579
0.250340038310488
NFYA	1.60855619853429	2	0.447410796187079
0.695977798831711
FOXP4	0.0344877412701224	2	0.982903953993001
0.11202526916206
TFEB	1.17066786613587	2	0.556919856800915
0.170165359292593
AL365205.1	1.02815403311414	2	0.598052332357443
0.700843016893669
USP49	0.162748966569563	2	0.921848409976936
0.182366568153407
MED20	0.62521090686635	2	0.731538481634601
0.322319049279886
BYSL	0.0710920201814402	2	0.965078329866975
0.0639575114504075
CCND3	0.212047136887562	2	0.899403450286471
0.0462965335596617
TAF8	0.000457793646710929	2	0.999771129371524
0.00261503308358975
GUCA1A	0.129429533268153	2	0.937334784406706
0.360031786293961
MRPS10	0.480110435832136	2	0.786584426314544
0.0966484878735651

TRERF1	0.817629120390984	2	0.664437434046489	0.443626926190305
UBR2	0.393845920306562	2	0.821253900150178	0.240753784479845
TBCC	6.07220272733441	2	0.0480217443352188	0.273520928261054
BICRAL	1.74476250775293	2	0.417955106109834	0.489185261443488
RPL7L1	0.055632299544171	2	0.972567157058916	0.0318220909265138
C6orf226	10.9119042205176	2	0.00427080856467643	0.811639690791754
CNPY3	3.78295768702118	2	0.150848562386209	0.287098593255718
GNMT	0.0337920433454121	2	0.983245915585537	0.191132899295425
PEX6	0.85746113067388	2	0.651335397874762	0.382185856419339
PPP2R5D	0.276720872515063	2	0.870784772770694	0.323003171610808
MEA1	1.0109361006133	2	0.603223170552397	0.14056321133077
KLHDC3	4.68698937712785	2	0.0959915906675746	0.259083821303568
RRP36	0.939364809724723	2	0.625200797488407	0.163430512099192
CUL7	0.337560891286716	2	0.844694339350888	0.585173394161182
KLC4	1.52963237405687	2	0.465419473248584	0.476870759736115
MRPL2	0.444711819723997	2	0.800630361378556	0.0560689075456494
SRF	0.299278108829318	2	0.861018701243419	0.230745265834439
CUL9	0.826479201626683	2	0.661503766997745	0.583635868987107
DNPH1	7.25336090905156	2	0.0266043523466186	0.696060903768928
CRIP3	0.072423783126839	2	0.964435915996897	0.0884722399272636
ZNF318	9.49388643463264	2	0.00867818201679449	0.781319964796403
ABCC10	1.19578909058216	2	0.549968351416107	0.879875840390296
TJAP1	0.094735184264501	2	0.953736747021681	0.11880269580222
POLR1C	3.69672673355909	2	0.157494716583682	0.55867091604204
YIPF3	0.191140290655805	2	0.90885460751321	0.0709873379851305

XPO5	1.99938396474268	2	0.367992771977592	0.896817245489549
POLH	0.00458766870197746	2	0.99770879447661	0.024547877718256
GTPBP2	2.10770030594576	2	0.348593025602844	1.74233607055431
MAD2L1BP	0.0140912964540887	2	0.9929791141626	0.029583057560963
MRPS18A	5.77448375394405	2	0.055729710228643	0.200901790208017
MRPL14	3.31670404085873	2	0.190452583591407	0.205657651782874
TMEM63B	5.12988128665325	2	0.0769237472440678	2.38927082894101
SLC29A1	4.25444693893043	2	0.119167707717288	2.51240879206385
HSP90AB1	48.8489568486966	2	2.46935805137127e-11	0.402343282856868
SLC35B2	4.80056999275903	2	0.0906921026850072	0.653803078987097
NFKBIE	11.1966261642321	2	0.00370410697336898	0.830759746348544
CDC5L	0.503568290448996	2	0.777412528165888	0.12253472112943
SUPT3H	2.9766874116505	2	0.225746248260274	1.87062329152607
ENPP4	2.10126755590032	2	0.349716036547528	0.655918648743698
ENPP5	0.127699200141626	2	0.93814608602567	0.265461120228387
CYP39A1	2.62237945466062	2	0.269499234972184	1.31112149198228
AL355353.1	0.329742949264609	2	0.848002686941381	0.528987314458143
CD2AP	4.72490262081089	2	0.094189053370953	0.624134928626527
MUT	0.771894207438133	2	0.679806483964039	0.279235776405868
CENPQ	0.690948355138102	2	0.707884610869336	0.318910137764169
MCM3	0.646869766485774	2	0.72365907872065	0.125994288542753
PAQR8	0.514395850623941	2	0.773215159767601	0.454512539830643
EFHC1	2.19981529816755	2	0.332901826067165	0.508961102405396
TRAM2	0.382094236317178	2	0.826093663221933	0.362681799461153
TRAM2-AS1	0.664979464529409	2	0.717136032189133	0.491249087366702

TMEM14A	6.04522114540316	2	0.0486739853812851	0.491671036744018
GSTA4	7.08486477065128	2	0.0289428412502585	1.40704073833613
FBXO9	2.27791827213104	2	0.320152083207399	0.291661570255819
ELOVL5	0.77745350583075	2	0.677919484230873	0.123428167010969
GCLC	0.0980988205296939	2	0.952134083339	0.140395446827527
LRRC1	0.242166874354721	2	0.885960034504335	0.307258164367979
KIAA1586	2.74953915214728	2	0.252897862809949	0.706338780401146
ZNF451	4.0006896316617	2	0.1352886255331	0.4449250992463
BAG2	0.0672208847617237	2	0.966948113282201	0.0749855496297538
PRIM2	0.00180860901801294	2	0.999096104251092	0.0185628373149158
AL021368.2	0.521430250244881	2	0.770500384556281	0.343346840650351
KHDRBS2	0.855454121428583	2	0.651989344021537	1.0935194427757
PTP4A1	5.36271656242853	2	0.0684700893284098	0.279605326081782
PHF3	16.9418357149644	2	0.000209472549153267	0.735469525490159
LMBRD1	3.15292440986818	2	0.206705086403961	0.373640576083826
COL19A1	1.50977600418087	2	0.470063257884768	2.25301774004268
FAM135A	0.786214974142106	2	0.674956194622501	0.463821434860989
SDHAF4	2.09616844893628	2	0.350608793868902	0.721763344418233
SMAP1	50.5464372086596	2	1.05676578598946e-11	0.504466710901986
B3GAT2	23.969673892228	2	6.23808729305342e-06	1.13805667489382
OGFRL1	0.0666122209744424	2	0.967242431215464	0.0714655657131332
KCNQ5	0.132878465449321	2	0.935719775269942	0.298807515585495
KHDC1	0.00155547914497123	2	0.999222562788545	0.0193102188097881
AC019205.1	0.642874692387448	2	0.725106059258836	0.341565713319625
DDX43	4.32250544946471	2	0.115180740859868	3.9868076177389
CGAS	10.7051514301138	2	0.00473593685222395	

1.24993357568082
MTO1	6.03383145405635	2	0.0489519669962654
0.798091057858092
EEF1A1	10.4317039493856	2	0.00542980544845051
0.115905320587383
SLC17A5	1.05906581988942	2	0.588879965425076
0.336802416831014
COX7A2	7.1767810457243	2	0.0276427850698808
0.145613685972204
TMEM30A	0.923520576367586	2	0.630173381843812
0.297770850676107
SENP6	2.95609895469047	2	0.228082134161954
0.314523519251343
MYO6	16.7974209540577	2	0.000225157482801719
1.50732309059792
IRAK1BP1	0.0178259456203379	2	0.991126629985103
0.12782072939582 1
PHIP	1.46714889823678	2	0.480189505860651
0.139987699931958
HMGN3	3.00548716167629	2	0.222518823525829
0.0946934753568473
HMGN3-AS1	3.38824240997698	2	0.183760646030145
1.30134386875943
TTK	1.91995009321995	2	0.382902440569844
1.46406749178016
BCKDHB	9.12527747878192	2	0.0104344886877396
1.04508041160949
IBTK	2.32201267792804	2	0.313170866207433
0.444694275465941
UBE3D	3.27261466530174	2	0.19469767022461	0.830509391084826
DOPEY1	0.723024586768613	2	0.696622032180498
0.51291221205672
PGM3	1.23118848134244	2	0.540319719937985
0.562386543899465
RWDD2A	1.31950643428217	2	0.516978900281536
0.423890850862161
CYB5R4	0.0532089122428458	2	0.973746324758703
0.0386709320306224
CEP162	1.73040354497917	2	0.420966604577681
0.540891743291241
SNX14	1.48632778505617	2	0.475606763858855
0.27152101764641
SYNCRIP	1.23423925491569	2	0.539496151666407
0.13793247285362
ZNF292	8.89959024545352	2	0.0116809598884705
0.563050848995218
SMIM8	0.501548202845767	2	0.778198145558458
0.187925939362733
SLC35A1	0.0425942077896351	2	0.978928078015111
0.0608501603845566

RARS2 0.000251188084683204 2 0.99987441384426
0.00370853968422305
ORC3 0.327136015076216 2 0.849108751242207
0.195026880513818
AKIRIN2 0.00195142472281468 2 0.999024763491121
0.0052126066871596
CNR1 10.8419971624909 2 0.00442272798878041
0.860286716750133
RNGTT 0.0204090754656332 2 0.989847351909092
0.0211738392568792
PNRC1 0.460085711294383 2 0.794499552981196
0.086596820802562
PM20D2 2.65045969374731 2 0.265741872130364
0.665380510241734
UBE2J1 9.23846836542983 2 0.00986034439266981
0.199539673615663
LYRM2 0.206084004064967 2 0.902089083110814
0.0589222774429121
MDN1 1.57459226505618 2 0.45507359196467 0.644476136022091
CASP8AP2 5.64169736693029 2 0.0595553775908627
0.95253848254225
BACH2 49.2416547104629 2 2.02913241764691e-11
1.26179804528962
AL132996.1 5.56218150724395 2 0.0619708755422744
1.35495692356115
MAP3K7 3.85464559745391 2 0.145537310057975
0.361971972149525
MANEA-DT 0.965583225735331 2 0.617058397006372
0.425474922630666
MANEA 3.40289084036376 2 0.182419660343077
0.879199826718906
UFL1 6.13568603363981 2 0.0465213925743304
0.452631288619222
NDUFAF4 1.12808630583945 2 0.568904240770382
0.203877427498169
MMS22L 4.87609343632962 2 0.0873312675510248
1.12160235969447
FBXL4 0.0475084695862106 2 0.976525676303862
0.102110676841052
COQ3 2.17222469068199 2 0.337526131384964
0.699582709013545
PNISR 5.9403223676278 2 0.0512950417350939
0.177284557187652
USP45 2.94968898195056 2 0.22881430696837 1.83422164429084
TSTD3 1.54664858909568 2 0.461476432023585
0.598787913724658
CCNC 1.35109074837974 2 0.508878815543341
0.153279836798018
ASCC3 1.52536175533117 2 0.466414349613858
0.378134154245344

PREP	1.7226084197618	2	0.42261054988397	0.539694124980269
ATG5	0.00465354066397602	2	0.997675934499853	0.0124269996070138
PRDM1	90.8529000020077	2	0	2.93741439892695
CRYBG1	5.69174477517132	2	0.0580835732426203	0.735701654190937
RTN4IP1	19.7191179222691	2	5.22453868413431e-05	1.01098415313191
QRSL1	4.19224408983001	2	0.122932230755702	0.303506716676453
CD24	31.9962937973991	2	1.12743907099144e-07	1.58515204153158
C6orf203	0.0409654998117812	2	0.979725596687496	0.0512837734375049
PDSS2	0.0821919378426981	2	0.959737020601406	0.109308047650234
SOBP	0.571256560056243	2	0.751541927174924	0.25356299762502
SEC63	3.23853851937177	2	0.198043364491132	0.330199144761342
OSTM1	0.601781634778928	2	0.740158580781272	0.264915932469331
SNX3	21.022116298745	2	2.72336246061045e-05	0.235807402744193
AFG1L	0.443733110138941	2	0.8010222495613	0.434918622683615
FOXO3	1.1512695979257	2	0.562347777692372	0.385675698776378
CEP57L1	0.15516451193814	2	0.925350905630596	0.229229004733613
CD164	2.88664082651335	2	0.236142365248294	0.166974635728119
SMPD2	0.375715160561571	2	0.828732726710573	0.200791385633119
MICAL1	6.65923106083423	2	0.0358068690735821	0.934339724263372
ZBTB24	0.218585676933776	2	0.896467858783085	0.140659051146759
AK9	3.26311700786808	2	0.195624454941323	0.77476544037791
FIG4	0.583667989591916	2	0.746892513726735	0.240580381479646
WASF1	0.33671202706408	2	0.845052930846246	0.259444076444135
CDC40	4.60824754956262	2	0.0998462501376643	0.319825855484569
CDK19	2.54108799659162	2	0.280678891401021	0.508348581392025
AMD1	1.30022885746848	2	0.521986043141251	0.0906079986014172

GTF3C6	0.558483943589016	2	0.756356864043101	0.0832028317638164
RPF2	2.84213929944146	2	0.241455605795685	0.349724604274981
MFSD4B	0.725603667385305	2	0.695724288952765	0.343579749283774
AL080317.1	0.643216618064354	2	0.724982103664967	0.827234712248619
REV3L	0.619008942385362	2	0.733810490383124	0.255987688295945
TRAF3IP2-AS1	0.469701356427482	2	0.790688907894283	0.109615425160574
TRAF3IP2	3.3435651263554	2	0.187911802254087	0.681578559851402
FYN	1.07681618932012	2	0.583676671210171	0.363353693249153
TUBE1	5.82670532704107	2	0.0542933968982436	1.10993082795713
FAM229B	2.87130996830613	2	0.237959453289757	6.25974957461784
MARCKS	6.93187932543877	2	0.0312436331511869	1.66427339951552
HDAC2	0.270039044232394	2	0.873698855070975	0.0733719339542697
NT5DC1	1.36112581085903	2	0.506331895158812	0.226840116319329
TSPYL4	1.917192116901	2	0.383430822734266	0.793054390671283
DSE	0.147466337602777	2	0.928919525485608	0.284956362553072
TSPYL1	0.472924727857814	2	0.789415592244234	0.0777336256348316
CALHM6	4.72894708951226	2	0.0939987734918662	0.717692549219427
RWDD1	0.000833998959308059	2	0.999583087452545	0.00234966156532992
ZUP1	1.17655165973167	2	0.555283863698865	0.329653171483765
KPNA5	0.0808235389060771	2	0.960393896852185	0.108056768046457
GOPC	0.330756276467876	2	0.84757314367198	0.0418739072227341
NUS1	1.1975948605375	2	0.549472017353422	0.235957345601632
CEP85L	0.0058620407901178	2	0.997073270851618	0.0220917868440079
MCM9	3.36907573447652	2	0.185530151708888	2.50333834242501
ASF1A	3.81362833782306	2	0.148552897965731	0.232401988165883

FAM184A	10.1898759564254	2	0.00612768667314378	1.76193900089551
MAN1A1	3.2623636727908	2	0.195698154202451	0.997286071693953
HSF2	0.603570356209808	2	0.739496907954621	0.262958081797825
SERINC1	0.902511635669305	2	0.636827909401487	0.208477108506389
PKIB	0.684967843148587	2	0.710004535048946	0.935082227970071
SMPDL3A	3.6983922315091	2	0.157363617613721	0.722227805524737
HDDC2	1.13889638213832	2	0.565837586802364	0.128501286049366
NCOA7	7.05811023718149	2	0.0293326186230919	0.474921233728849
HINT3	1.56938582388666	2	0.456259792207417	0.239343809565456
TRMT11	1.08347184990428	2	0.581737522671147	0.30955369305371
CENPW	3.43682981828017	2	0.179350209097787	0.733698746454907
RNF146	5.06138356234578	2	0.0796039327318767	0.570870356861015
ECHDC1	1.38604861012417	2	0.500061441523681	0.101942691816843
ARHGAP18	3.55911695306857	2	0.168712621407659	1.31750653107488
L3MBTL3	1.28943799346078	2	0.524809994712729	0.718889284321708
EPB41L2	7.44521786700086	2	0.0241708254381164	1.30733262188656
AKAP7	1.79992661076802	2	0.406584578931861	0.864364643455905
MED23	3.82516719164384	2	0.147698300517877	0.594768748486236
LINC01013	11.7210360098653	2	0.00284976710685758	1.2488011968961
STX7	33.8476123095881	2	4.46770381801187e-08	0.376643454738431
VNN2	15.1591424018433	2	0.000510780198141103	0.404342643771462
RPS12	30.2346999553071	2	2.72030953429869e-07	0.156439970897081
TBPL1	2.39001067304343	2	0.302702338800228	0.202219531523444
SGK1	17.8776500496072	2	0.000131195100644077	0.891990052596758
HBS1L	0.525743450841648	2	0.768840513679384	0.149743043366986

MYB	0.500980062163017	2	0.77841923997302
0.408033993090667
AHI1	3.99858540828268 2	0.135431039181877
0.764372748581807
BCLAF1	3.83089304246069 2	0.147276056016205
0.209752989600228
PEX7	2.03519980803591 2	0.361461442068038
0.810604528656836
IFNGR1	0.286903364957946	2	0.866362659671989
0.124996773588885
AL357060.1	7.96185872384231 2	0.0186682816830808
1.31359226718378
TNFAIP3	9.95842374641326 2	0.00687948232582891
0.948436743568894
HEBP2	3.46730096685442 2	0.176638415981931
0.366582558090274
CCDC28A	0.545013262820339	2	0.761468379549306
0.0542935723945616
REPS1	1.01251128209245 2	0.602748264609681
0.162071116693794
ABRACL	73.5357946385566 2	1.11022302462516e-16
0.861937664107468
HECA	0.321068623226885	2	0.851688600260579
0.252623357658808
TXLNB	5.896321570047	2	0.0524360584988441
1.10762707729834
AL158850.1	0.0215211825189227	2	0.989297096798048
0.0746745524465779
CITED2	1.00428416146832 2	0.605232812614684
0.19584379553149
VTA1	0.156493983099909	2	0.924735996357499
0.0975438235895653
HIVEP2	1.31088638233933 2	0.519211901458213
0.727354849994645
AIG1	0.0894467888354566	2	0.956261952648578
0.103800182381691
ADAT2	1.48332894200718 2	0.476320433790099
0.848829206228729
PEX3	0.160267811314698	2	0.922992744161167
0.115442546123645
FUCA2	0.000544866232965069	2	0.999727603990049
0.00992297758593954
PHACTR2	1.3918495586547	2	0.498613127595489
1.39420888474921
LTV1	1.50433291641545 2	0.471344298081606
0.258238709633423
PLAGL1	1.00109426496164 2	0.606198897855211
0.294851300465402
SF3B5	0.677135071752301	2	0.712790638822994
0.0685039628103566

STX11 4.74628126733074 2 0.0931875981070882
0.574935310620229
UTRN 3.10666312578038 2 0.211542032949792
0.421488265145475
EPM2A 2.66541168741105 2 0.263762594491465
1.39643172236697
FBXO30 3.20089139850874 2 0.201806552917298
0.869947757553352
SHPRH 3.61545489688512 2 0.164026472181606
0.558134580316709
STXBP5 2.88213668913049 2 0.236674773360587
1.23645755460228
TAB2 1.71805287443199 2 0.423574257779486
0.554394241668408
SUMO4 4.27946635612598 2 0.117686240103421
1.44443587938375
ZC3H12D 0.640750428854726 2 0.725876626587933
0.186401079688203
PPIL4 1.44527326424502 2 0.485470559749415
0.260400930021195
GINM1 2.3319457150796 2 0.311619353281079
0.2661540950244
KATNA1 2.89291104862289 2 0.23540319200904 0.506270415849076
LATS1 0.0741488386031035 2 0.963604421912604
0.16121067535056
NUP43 2.29576870239115 2 0.317307370709946
0.624594808550979
PCMT1 7.49228087588209 2 0.0236086894439449
0.282235580915516
PLEKHG1 12.7787208823848 2 0.00167932988823061
0.93315855128916
MTHFD1L 1.07418262736443 2 0.584445751792249
0.384731423911887
ZBTB2 3.26820404216338 2 0.195127513043712
0.552596449219221
RMND1 0.273538345222059 2 0.872171523974302
0.134806749875104
ARMT1 5.75494035500823 2 0.0562769536028203
0.32887807838671
SYNE1 0.00278246271482308 2 0.998609735956296
0.0106299424856923
FBXO5 0.00679719341816792 2 0.996607171983708
0.0330193291813292
AL080276.2 0.0232644768765937 2 0.98843515448396
0.11578216261711
MTRF1L 0.447003108290964 2 0.799713648995276
0.158368322432396
RGS17 1.91056245574383 2 0.384703939868278
1.08808724272793
SCAF8 10.2177586162096 2 0.00604285130473747

1.65640681357794
TIAM2 0.512641687021016 2 0.773893630206392
0.265937466528272
TFB1M 0.944271947951113 2 0.623668704438837
0.216957881028406
ARID1B 7.78596943949397 2 0.0203844133885603
0.280853672706881
TMEM242 3.33616037748804 2 0.188608811605147
0.338625357233828
ZDHHC14 5.64562327392732 2 0.0594385878180131
1.82705500307179
SNX9 7.48856734146363 2 0.0236525660060325
1.79717688532635
SYNJ2 18.7099913553019 2 8.65320512780654e-05
1.00535806060539
SERAC1 1.08018493438398 2 0.582694369770681
0.794127387055112
GTF2H5 3.01611314901926 2 0.221339717489743
0.2143877270694
TULP4 0.110884754344832 2 0.946066537128762
0.13177265597632
TMEM181 0.74327756580108 2 0.689603294042333
0.604131198633094
DYNLT1 3.31952405719804 2 0.190184233125246
0.349067924718045
EZR 177.01850892953 2 0 0.630633725320091
AL627422.2 2.10792048139869 2 0.348554651901469
5.09281959878702
C6orf99 2.87123452458961 2 0.237968429731828
0.938297163999613
RSPH3 4.49528075512389 2 0.105648220589871
1.62533709294872
TAGAP 28.7172861333414 2 5.80925644588781e-07
0.714847659886029
AL356417.1 13.4172434984807 2 0.00122034490736123
1.90971557382841
SOD2 0.479725054480689 2 0.786736008402922
0.281527582518723
WTAP 2.44508593615265 2 0.294480359806204
0.192502711167583
ACAT2 1.65992182329122 2 0.436066331103696
0.170883579853182
TCP1 0.529919775193255 2 0.767236725067618
0.0884627666864241
MRPL18 9.21018065243528 2 0.0100007986295932
0.204886645080746
IGF2R 0.411218048477933 2 0.814151327499476
0.494154353144388
MAP3K4 1.56176281562905 2 0.4580021466755 0.60665934581988
QKI 1.55935450905592 2 0.458553983647145

0.75710155709762
TBXT 2.54851591386842 2 0.27963839498402 7.60004549083257
SFT2D1 3.41730064343764 2 0.181110068058774
0.103546672567562
MPC1 5.71670459379832 2 0.0573631999622622
0.38371867033887
RNASET2 0.0802134076973761 2 0.960686924690828
0.0463022872041895
FGFR1OP 1.51721576446317 2 0.46831792712987 0.316205985069332
CCR6 0.92670456184836 2 0.62917094853972 0.689366988356011
AFDN 1.38450959464362 2 0.500446390765262
0.94458541601432
WDR27 0.371473073742377 2 0.830492370279383
0.424275262735242
C6orf120 1.26339979924907 2 0.53168721747277 0.452563882087558
PHF10 13.6407802448232 2 0.00109129510054717
0.759101865180044
TCTE3 2.53232002560181 2 0.281912084765466
1.73922826586566
ERMARD 1.95824046246042 2 0.375641431121826
0.792833195463457
FAM120B 0.949424650645771 2 0.622063982782892
0.548352022545165
PSMB1 2.85366583408204 2 0.240068034905194
0.0772828847713067
TBP 1.41784718588846 2 0.492173691668565
0.411332957803499
PDCD2 10.8490734994522 2 0.00440710728265281
0.394139106281738
PRKAR1B 6.43782762490854 2 0.0399984805254181
0.85449795582368
AC147651.4 0.273246247805129 2 0.872298912801198
0.235561591773678
DNAAF5 0.790271711206293 2 0.673588522257497
0.493450338048037
SUN1 0.995712911574507 2 0.607832179432295
0.377144249240068
COX19 0.48253728629179 2 0.785630543775875
0.0523718599733645
C7orf50 59.8551886589672 2 1.00586206031039e-13
0.820447216058889
GPR146 7.99024472619796 2 0.0184051941563294
4.29059999236232
GPER1 41.8952107933704 2 7.99043720078885e-10
2.65932160292332
ZFAND2A 4.66400304042785 2 0.097101202442473
0.548882857791183
AC091729.3 1.93846667158449 2 0.379373778936535
0.904548406553095
INTS1 0.151218755535008 2 0.927178312293773

0.194289954089337
MAFK	0.578944106126635	2	0.748658715336245
0.780180056992558
PSMG3	1.75081240676914 2	0.416692723301924
0.238849514212695
PSMG3-AS1	3.73305180752709 2	0.154660033412395
1.40662783645323
MAD1L1	36.1926246555295 2	1.38315678910317e-08
0.98364108129589
MRM2	5.93792544749246 2	0.0513565536467054
0.506152055910607
NUDT1	1.79005307014118 2	0.408596756353227
0.238273120897035
SNX8	2.64786289647796 2	0.2660871351103	0.185097792605529
EIF3B	0.350790049304612	2	0.839125480039144
0.0712283705168527
CHST12	0.0218336786448746	2	0.989142533118418
0.0538273271310567
BRAT1	0.328216008734448	2	0.848650358985283
0.148246266418528
IQCE	1.00158538582329 2	0.606050057668067
0.492126884511527
TTYH3	0.458759241594538	2	0.795026667554036
0.549049163839354
GNA12	0.794610442907739	2	0.672128846178046
0.444325759178181
CARD11	1.7696373864331	2	0.412789007942101
0.24576248107238
FOXK1	0.906681597886443	2	0.635501518471492
0.374830315649364
AP5Z1	5.93711290554181 2	0.0513774225627751
0.69543502380769
RBAK-RBAKDN	0.0850917308225	2	0.958346509556145
0.134529464489305
RBAK	2.99640116685107 2	0.223532025710206
1.16584355531253
WIPI2	0.144245904857548	2	0.930416491810786
0.0557379416539642
FBXL18	0.103648379804048	2	0.949495783116334
0.248513958954321
ACTB	29.6074773241125 2	3.72235669843235e-07
0.18605727265771
RNF216	0.0698979498578088	2	0.965654687599607
0.0917119496877657
CCZ1	3.06790257576982 2	0.215681760683537
0.218398933046869
PMS2	1.95809848641801 2	0.37566809811021 0.467413197694636
AIMP2	0.776042055358326	2	0.678398077976993
0.198943985656905
EIF2AK1	0.962638382073141	2	0.617967636488945

0.137794801757192
USP42	4.03819790656139	2	0.132775047718411
1.14779745052276
FAM220A	0.0733294205317241	2	0.963999300238177
0.130334033394209
RAC1	15.9502536788842	2	0.000343911280477727
0.283418596368542
DAGLB	0.829657034046835	2	0.66045352753155
0.955626583276672
KDELR2	7.45153841565427	2	0.0240945595730312
0.235086079159997
ZDHHC4	1.02830925393678	2	0.598005919071038
0.187636748266983
C7orf26	0.174727269009144	2	0.916343820810414
0.114107251589877
ZNF316	0.26139165235201	2	0.877484641660918
0.351201165895308
ZNF12	1.34321675021861	2	0.510886219954026
0.618016821354227
CCZ1B	0.631540127409227	2	0.729227106671131
0.128408803806431
C1GALT1	0.233607178841613	2	0.889759934266381
0.139051443477994
AC004982.2	3.21320479022398	2	0.200567908240743
2.36023426609231
MIOS	3.13087858072024	2	0.208996182996212
0.239361091545984
RPA3	6.64557969407485	2	0.036052111445937
0.272659034312994
UMAD1	2.15937111395631	2	0.339702325878641
0.385015872337054
AC006042.4	0.197265439823777	2	0.906075430382566
0.104190906629866
GLCCI1	3.03503124727238	2	0.219255925112594
0.573811349263558
ICA1	0.147111474885315	2	0.929084359562102
0.272431407857577
NDUFA4	36.0436329882854	2	1.49013131833797e-08
0.187660153007843
PHF14	9.51689476629869	2	0.00857891883654072
0.451820524930325
TMEM106B	0.310729237903902	2	0.856102969201152
0.127826840901642
ARL4A	2.5436239926962	2	0.280323216658502
0.884190931288749
ANKMY2	3.19961213351814	2	0.201935676237643
0.705898594651278
BZW2	2.75762608405723	2	0.251877343519519
0.21650392184932
TSPAN13	2.73010234019781	2	0.255367608467965

0.238501892144152
AHR	2.24639328352798 2	0.325238460277971
1.04341638803989
AC073332.1	0.552036692941197	2	0.758799009349822
	0.625659238863318
SNX13	0.0486169909417051	2	0.975984576489704
0.0777404155583007
HDAC9	21.6204163554769 2	2.01923209315336e-05
0.594579635781086
TWISTNB	1.87694544759569 2	0.391224887771279
0.244110015966717
MACC1	3.51251043573842 2	0.172690342188397
1.89574614060554
AC099342.1	2.66299453394837 2	0.264081564536828
5.44950610201704
AC004130.1	9.15168162424526 2	0.0102976371587882
1.77758285949543
SP4	1.29996701220153 2	0.522054387402465
0.916899398500969
CDCA7L	50.8767107598335 2	8.95905571951516e-12
1.09857221788326
RAPGEF5	1.86186568104269 2	0.394185826297983
0.182534170634531
STEAP1B	0.871301417545464	2	0.646843623284189
0.601156232789815
TOMM7	8.46257895386391 2	0.0145336376697998
0.231964586831176
FAM126A	5.81477811827745 2	0.0546181486208163
0.597017111016925
KLHL7	1.68544526114778 2	0.430536733781794
0.423987129642145
NUPL2	1.97194067638635 2	0.373077040342096
0.215058607854512
MALSU1	0.912461793589973	2	0.633667508409008
0.215453222794924
IGF2BP3	7.86522679633449 2	0.0195924028384866
0.511997026545845
TRA2A	0.114408717263348	2	0.944401053137958
0.0470647496375122
CCDC126	0.0177842995948844	2	0.99114726844241
0.0268034630220032
FAM221A	0.715424138772757	2	0.69927438852675
0.397535738578081
MPP6	1.20558328554843 2	0.547281686585573
0.308575516089557
GSDME	0.347876003174417	2	0.840348996347361
0.345323636642192
OSBPL3	0.558413044804901	2	0.756383676909357
0.306162332815372
CYCS	46.1989981251107 2	9.28997989646518e-11

0.376308266084574
C7orf31 4.11836968323314 2 0.127557907423901
1.42093645088877
NFE2L3 2.6194335430066 2 0.269896487936923
1.52896477044717
HNRNPA2B1 7.23854466772738 2 0.0268021724317686
0.112303276780549
CBX3 20.3340051762121 2 3.84173034915225e-05
0.335602892863804
SNX10 18.9016264868897 2 7.86255975128158e-05
0.63483609933562
AC004540.1 7.99453996441589 2 0.0183657092241286
1.28669553769038
SKAP2 8.46782291037736 2 0.0144955807019124
0.332683643981721
HOXA1 0.00185673669347997 2 0.999072062453829
0.0442082379044926
HOTAIRM1 0.00112408759439114 2 0.999438114119833
0.011110442167711
HOXA-AS2 0.97899615408248 2 0.612933962618172
1.15945927779904
HOXA7 2.58919030681189 2 0.274008773398003
0.765141382958177
HIBADH 4.66978305785781 2 0.0968209842327487
0.715932551180421
TAX1BP1 1.29804231568898 2 0.522557027350411
0.106394163755753
JAZF1 3.9769459861565 2 0.13690431975729 0.576910518712389
CHN2 0.279596608595813 2 0.869533598902903
0.571276242874646
PRR15 8.62685385503811 2 0.0133875925841095
4.77585756614532
SCRN1 2.22989912319142 2 0.327931818840118
0.355010248513617
FKBP14 2.40847576379149 2 0.299920487268044
1.28288352382229
PLEKHA8 0.0586628101697403 2 0.971094585449871
0.122034790074593
MTURN 4.45608474033427 2 0.107739137174411
1.67100379451184
ZNRF2 0.00194871786244102 2 0.999026115602308
0.0369856375376865
GGCT 15.1292169210667 2 0.000518480333544291
0.812192443016258
GARS 3.47368008361159 2 0.176075914984015
0.272095158679294
LSM5 2.02923748304666 2 0.362540625173228
0.165442407444792
AVL9 0.100487974436221 2 0.950997364990574
0.149491618606987

KBTBD2	0.133759440408062	2	0.935307693189898	0.188742899232228
NT5C3A	9.99771665119066	2	0.00674564393357491	0.565560447002995
RP9	5.01635277893914	2	0.081416576070955	0.75379384028336
BBS9	0.883639571417145	2	0.642865478563628	0.37299712343103
HERPUD2	0.210614659622161	2	0.900047868535024	0.143523487756778
AC018647.2	3.212615829961	2	0.200626980202067	5.43873773531286
SEPT7	8.11114694018531	2	0.0173255416540362	0.122152802226368
EEPD1	3.69081363001297	2	0.157961046892405	0.332485721426844
ELMO1	0.0585248503921575	2	0.971161573756778	0.0511606899318349
STARD3NL	0.0809881093173809	2	0.960314873894131	0.0767055667671385
TRG-AS1	0.477940452349868	2	0.787438327074668	0.353501255146615
VPS41	0.644087913122521	2	0.724666335789618	0.0885786024802366
YAE1D1	1.64661999620734	2	0.438976236608835	0.626592452199805
RALA	10.4345500266754	2	0.00542208412063994	0.456597561201393
CDK13	60.8041452528418	2	6.26165785888588e-14	1.03418783399405
MPLKIP	17.8135166498639	2	0.000135470273573746	0.493263929690529
SUGCT	241.663424882503	2	0	1.82187902831963
LINC01450	1.07529270605171	2	0.584121451413841	0.924749108685663
PSMA2	4.62016754484343	2	0.0992529365525092	0.129508588803093
MRPL32	4.70887494459791	2	0.0949469017774419	0.307286438250144
STK17A	76.3765606376049	2	0	0.656132829140978
COA1	12.6957499567304	2	0.00175046295841907	0.636728825857273
BLVRA	5.80076877631381	2	0.0550020738466993	1.94902685422692
MRPS24	7.85956315093481	2	0.019647963681688	0.805372497075291
URGCP	1.74539572301353	2	0.417822799279899	0.643649417192878
UBE2D4	5.21354849268361	2	0.0737721310796646	0.766979743311579

AC004951.1	5.76106971463786 2	0.0561047467732561
0.633272660498651
LINC00957	2.78120254150505 2	0.248925587950459
1.61420444989386
DBNL	0.309957074537031	2	0.856433558689862
0.0425664010641468
POLM	8.1186752751829	2	0.0172604480018071
0.414623997972819
AEBP1	6.17303268744815 2	0.0456607439972433
0.860774983641878
POLD2	1.45212528596607 2	0.483810178205916
0.116929688283101
YKT6	0.0440745540170431	2	0.978203769861293
0.0292722099787434
CAMK2B	65.8064299147122 2	5.10702591327572e-15
1.90576605083971
NUDCD3	10.3524481622015 2	0.00564929748156406
0.731355708200687
DDX56	1.33220027385447 2	0.513708067506957
0.206477335649786
TMED4	7.3203701381303	2	0.025727750874935
0.501399565010161
OGDH	2.28788645491311 2	0.318560385843195
0.586732626134444
ZMIZ2	0.643364886795561	2	0.724928359568805
0.321642234149005
PPIA	34.079668629192	2	3.97826752651653e-08
0.290429726621173
H2AFV	23.1786949427498 2	9.26425166702938e-06
0.422247282804275
PURB	0.423587639973375	2	0.80913150711442
0.282543012322601
MYO1G	3.03098090056231 2	0.219700406293875
0.299846401673325
SNHG15	6.26195327676966 2	0.043675121616578
0.492244441492729
CCM2	2.1447570640083	2	0.342193630184194
0.126872734425769
TBRG4	3.79425837777458 2	0.149998619406142
0.488361845708773
TNS3	0.0573907658134025	2	0.971712419600601
0.135753568761567
HUS1	2.41214118057486 2	0.299371323856098
0.213012446494795
UPP1	2.91623063973233 2	0.232674378536188
1.07735858684858
SPATA48	7.26595881154815 2	0.0264372995093767
1.16471611675822
AC020743.1	2.26746008280701 2	0.321830573424209
0.687342130577407

IKZF1	5.81661579228523	2	0.0545679864937179	0.275431946608542
FIGNL1	0.246428816157439	2	0.884074089611562	0.104712914459704
SEC61G	56.4280050122655	2	5.58220136781529e-13	0.372682572556127
LANCL2	0.283982667569678	2	0.867628775510982	0.29313289434414
VOPP1	7.21664678849672	2	0.0270972401911203	0.230099162956744
NIPSNAP2	2.15033451379259	2	0.341240675671343	0.282064897209224
MRPS17	1.5172527508304	2	0.468309266520535	0.343625091158627
PSPH	3.97031892226873	2	0.137358708994942	0.729116268341468
CCT6A	0.711706597009872	2	0.700575388153812	0.0384263136982559
SUMF2	4.35452532456243	2	0.113351387383857	0.304080846151678
PHKG1	0.94839235444682	2	0.622385142801294	0.489493567399341
CHCHD2	3.01629739133823	2	0.221319328357478	0.0844598722780681
ZNF736	0.0289725037077196	2	0.985618169061863	0.136442125327726
ZNF680	0.34275066501642	2	0.842505294495079	0.169992303157527
ZNF107	0.485097401202211	2	0.784625534913047	0.282840603437501
ZNF138	1.99516755530532	2	0.36876939442783	0.348087265932
ZNF273	0.0483874911372043	2	0.976096577050358	0.105344442938805
ZNF117	0.466602678865902	2	0.791914902377978	0.301664248424141
ERV3-1	0.428861733155591	2	0.807000600516678	0.119682703022244
ZNF92	3.20895297384394	2	0.200994750752765	0.392594551694759
VKORC1L1	0.132691959751855	2	0.935807037873282	0.129210830564495
GUSB	4.05681561729029	2	0.131544798990826	0.407701982099713
ASL	0.419114465494924	2	0.810943225596073	0.177587704012137
CRCP	1.95327743313875	2	0.376574748381662	0.387119849703581
AC008267.5	3.55028127325722	2	0.169459615594526	1.23442972999931
KCTD7	4.00105304092513	2	0.13526404519647	0.901576269974099

RABGEF1	1.08009367751984	2	0.582720957807729	0.415977103705948
AC027644.3	9.06417883224954	2	0.0107581742703214	0.710785154543012
TMEM248	4.66194280354163	2	0.0972012797188311	0.362202446192021
SBDS	0.571170321475896	2	0.75157433382803	0.0750152328219122
TYW1	1.06735076079796	2	0.586445593205851	0.38714276592036
AC006480.2	2.44375203566524	2	0.29467682906427	1.33767265330457
AUTS2	0.718977238121308	2	0.698033195686399	0.441502083987814
TYW1B	2.69029159344934	2	0.260501724792894	1.67885682621692
POM121	0.211643645676244	2	0.899584919284952	0.183160429882304
NSUN5	5.83988567717211	2	0.0539367703142952	0.254176253780088
BAZ1B	2.08764836406862	2	0.352105588148384	0.169969491301984
BCL7B	0.362136710791887	2	0.834378322485845	0.0940894219837075
TBL2	1.47776543569684	2	0.477647284289335	0.334201800660414
DNAJC30	0.230356354659289	2	0.891207336815907	0.106804200018892
BUD23	14.2172919134539	2	0.00081800185247316	0.538430127959383
STX1A	0.0631232481034728	2	0.968931245144752	0.262946772318127
ABHD11	0.346559524304917	2	0.840902329288512	0.217219247459272
LIMK1	20.3552819947042	2	3.80107707598221e-05	0.491118405123562
EIF4H	0.441688675182227	2	0.80184148715351	0.0661242562470181
LAT2	3.99723265122508	2	0.13552267281493	0.179069153140919
RFC2	2.67705860681463	2	0.262231047431134	0.315522278653362
CLIP2	2.002680119319	2	0.367386790936846	0.757761380532794
GTF2I	0.389881967041678	2	0.822883220293441	0.0720082663145466
NCF1	6.81466978083119	2	0.0331293762426714	0.192839257215306
GTF2IRD2	1.42814160822725	2	0.489646878345144	0.239333359368704
RCC1L	0.00522813297095802	2	0.997389347211125	

0.0148546449876014
GTF2IRD2B 2.50287477710175 2 0.286093273975408
0.378995559091456
TRIM73 2.50510009304491 2 0.285775127040687
1.01241088362415
POM121C 3.68134489337822e-05 2 0.999981593444936
0.00221265198703944
HIP1 7.5104666128238 2 0.0233949917708584
0.74291236779219
RHBDD2 2.83126374063297 2 0.24277216444953 0.456307574941964
POR 0.774723244926933 2 0.678845564729264
0.22189014438176
TMEM120A 1.6935097013907 2 0.428804210206417
0.454328785681341
STYXL1 0.501148507561659 2 0.778353682164193
0.179983629527119
MDH2 0.416808169146204 2 0.811878902679654
0.058447787262892
HSPB1 13.5417645680788 2 0.00114668250479466
0.929950963072934
YWHAG 2.67991179978244 2 0.261857216257419
0.299454844636468
DTX2 0.0767196348937177 2 0.962366602291822
0.110714096141992
CCDC146 3.00791010610305 2 0.222249411379788
12.8944350366278
GSAP 3.38149732386213 2 0.184381432948041
0.917692150218111
PTPN12 1.48001780439757 2 0.477109668177021
0.43531203411731
APTR 1.20594233526633 2 0.547183444736674
0.384971981109436
RSBN1L 5.19231958479009 2 0.0745593525394105
0.328008521921075
TMEM60 6.89605098168607 2 0.0318083803516804
0.343915446997373
PHTF2 7.66683808269656 2 0.0216355163028704
0.793074278462344
KIAA1324L 0.345628826308823 2 0.841293733407596
0.477864464527946
AC005076.1 1.52978523690807 2 0.465383901934143
1.10850636706336
DMTF1 1.46402730982644 2 0.480939568052875
0.122631838072815
TMEM243 0.384646342548686 2 0.825040196112015
0.0571539337926966
TP53TG1 8.64755241529098 2 0.0132497551277463
0.694739378310446
CROT 0.462722347640395 2 0.7934528398841
0.372653260082846

ABCB4	2.79870362886855 2	0.246756856348562
0.927808862625621
SLC25A40 2.19986875017933 2	0.332892929049892
0.596933897430141
DBF4	0.435887898147346	2	0.804170514905635
0.14615200123013
SRI	16.8392989221175 2	0.000220491930292721
0.664497947516075
STEAP4	0.405476169802352	2	0.816492065022473
0.431506191233017
GTPBP10	2.20486732731949 2	0.332061972390042
0.636274021408749
CLDN12	1.82539343018347 2	0.401440193201996
1.33488039592381
CDK14	4.08150683961648 2	0.129930781567584
0.595878889331457
MTERF1	0.0123554768211073	2	0.993841304580995
0.0412148539131291
AKAP9	0.245426034829287	2	0.88451746724986
0.0655269758090758
CYP51A1	0.261601812035123	2	0.877392440558127
0.175470205750841
KRIT1	0.656735272464119	2	0.720098236833893
0.213632454258361
ANKIB1	2.07440729881704 2	0.354444448389746
0.442972640505025
GATAD1	0.0386714705103092	2	0.98085000103145
0.0805858954141716
PEX1	3.46350369080748 2	0.176974106971917
1.2935352337108
RBM48	2.32914954077978 2	0.312055328989568
0.488898452306821
FAM133B	0.68406993759807 2	0.71032336511987 0.101725067524888
CDK6	0.124141169314219	2	0.939816547821086
0.113717818124347
SAMD9	0.114633638296753	2	0.944294851279418
0.102944990758
SAMD9L	8.13138215847155 2	0.0171511323855883
1.05338429964831
VPS50	2.22508689085175 2	0.328721810917257
0.494657973752798
GNG11	1.52277749736367 2	0.467017406643259
0.492323861101832
BET1	8.68675368485965 2	0.0129925801464563
0.33283876444986
AC003092.1	3.62191104581112 2	0.163497836212689
2.95401820424686
CASD1	4.82441800647815 2	0.0896171112699867
1.05772698398104
SGCE	29.3854759473204 2	4.15934528041895e-07

2.24299035892966
PEG10	45.6958361531516	2	1.19474541371289e-10
2.05791697403238
SLC25A13	0.10254913221583	2	0.95001779203226	0.125114345012442
SEM1	12.273933733637	2	0.00216146971119513
0.255987034472845
SDHAF3	4.46796612954963	2	0.107100989260881
0.587458116637394
ASNS	8.1475837684905	2	0.0170127556446633
0.544798209914651
TECPR1	0.22878802700407	2	0.891906463451422
0.307853204497355
BRI3	13.6578392593042	2	0.0010820264756799
0.533674659172714
BAIAP2L1	21.33332904405	2	2.33091511326888e-05
0.609161575448726
NPTX2	3.77752359946242	2	0.1512589818456	0.521242932897682
TRRAP	1.84099813491147	2	0.398320202821599
0.486893349388287
SMURF1	2.48520830137277	2	0.288631598222182
1.26820997818823
ARPC1A	1.04519684132102	2	0.592977738796927
0.155112033410978
ARPC1B	11.1268326297109	2	0.00383565020000654
0.174133711453492
PDAP1	2.78463636294964	2	0.248498571618808
0.190914424379771
BUD31	0.816827757398226	2	0.664703715175211
0.0923684679747519
PTCD1	0.00159932093649952	2	0.999200659174975
0.0257479441845013
CPSF4	1.8059207166525	2	0.405367847638114
0.325493348550302
ATP5MF	26.5736274109539	2	1.69671979111108e-06
0.323587823471795
ZNF789	1.39126146671662	2	0.498759764333683
0.527693236538523
ZNF394	0.237552927282539	2	0.888006280267852
0.119811210575861
ZKSCAN5	0.627874030381586	2	0.730565041210082
0.328039650354635
FAM200A	1.02334121435379	2	0.599493224092228
0.43027814847103
ZNF655	3.07779303147597	2	0.214617798170023
0.171821172562729
TMEM225B	2.7948673481552	2	0.247230624865007
2.76251891740576
ZSCAN25	2.36218570757155	2	0.306943111297552
1.82252400894981
CYP3A5	0.34631461302206	2	0.841005308827708

0.515942005384013
TRIM4	1.10639687849616 2	0.57510741942121 0.156904428415317
ZKSCAN1	0.00124841226726258	2	0.999375988642488
0.00987625059129972
ZSCAN21	2.25216271541184 2	0.324301591656289
0.653316871442141
ZNF3	1.35569744665649 2	0.507708038833407
0.412887507527616
COPS6	0.0431235438837078	2	0.978669021316041
0.0208944267723831
MCM7	2.25235380846739 2	0.324270607245504
0.34359000507816
AP4M1	0.333975724054736	2	0.846209882547247
0.223186857423267
TAF6	0.196952894291839	2	0.90621703636054
0.101137071645422
CNPY4	0.926773086004265	2	0.62914939220492
0.528712164762552
MBLAC1	1.67415430386051 2	0.432974193557438
0.960248186896061
LAMTOR4	0.881277559596614	2	0.643625154996198
0.0511829513224925
C7orf43	0.693069331720821	2	0.707134305443253
0.229022359163261
STAG3	28.1217048334252 2	7.82437009627657e-07
0.725603530459957
CASTOR3	1.51263073160325 2	0.469392785266475
1.36896382938461
PILRB	5.4896683352291 2	0.064258956276985
0.661523726571149
ZCWPW1	2.26896055935657 2	0.321589214359756
0.487543871283513
MEPCE	1.86830520934265 2	0.392918681953699
0.473632368833757
PPP1R35	1.75464607772252 2	0.415894756936468
0.230128860667487
TSC22D4	0.864863284289368	2	0.648929211027449
0.129319889748579
AGFG2	1.16367423758252 2	0.558870711006686
0.653530393662942
SAP25	0.446077585297514	2	0.800083811322267
0.274434672772722
LRCH4	4.2097571966822 2	0.121860467434006
0.179683813216867
MOSPD3	0.279818877481837	2	0.869436969140256
0.190520599660774
GNB2	1.14595438170853 2	0.563844265364305
0.102709003832499
GIGYF1	0.310043096952099	2	0.856396723240504
0.165061322800743

POP7	0.110427842372586	2	0.946282696382937	0.0724098293085632
SLC12A9	0.354607649591744	2	0.837525284914579	0.196850930089071
TRIP6	0.780221458989562	2	0.676981908483065	0.339912042453832
SRRT	0.741381168505209	2	0.690257485055859	0.144632555540677
TRIM56	0.303637055770683	2	0.859144177305908	0.121048522588563
AP1S1	1.103180578955	2	0.576033022340394	0.170959610710121
PLOD3	0.297242446330071	2	0.861895519134682	0.282079544551079
ZNHIT1	5.07735641254994	2	0.0789707138286668	0.17433232968863
CLDN15	2.92303591500732	2	0.231884017357411	0.415829164120982
FIS1	1.89332960734383	2	0.388033034052705	0.163282110299938
IFT22	0.169385691894595	2	0.918794452504388	0.131983011730462
CUX1	0.0620751002799706	2	0.969439169816023	0.0407280411073088
SH2B2	5.93261842449034	2	0.0514930098164025	0.199286833486253
PRKRIP1	0.459552527654061	2	0.794711388298669	0.144009535623684
ORAI2	136.002426802678	2	0	1.07573067352261
ALKBH4	2.58678196736645	2	0.274338925206201	0.440308446028238
LRWD1	8.31255901971982	2	0.0156657338396967	0.625892242060703
POLR2J	17.4296403131873	2	0.000164135187349368	0.423900796470387
POLR2J3	2.54601891797052	2	0.279987740980106	1.0863400855583
POLR2J3.1	0.098689131351122	2	0.951853097281895	0.032972366827056
RASA4	0.00046043242095971	2	0.999769810287238	0.0123469299531877
UPK3BL1	0.0213902044718123	2	0.989361887020425	0.072476937150014
POLR2J2	0.078874980153681	2	0.961330044779673	0.101035148026204
FAM185A	0.0393421826405644	2	0.980521122183464	0.0974930773222887
ARMC10	3.68656154105292	2	0.158297236354451	0.370727483424439
NAPEPLD	0.15045312503246	2	0.927533318238879	

0.0804371315228883
PMPCB	1.5595911583584	2	0.458499728616873
0.150299189931571
DNAJC2	1.76744808944526	2	0.413241114211531
0.244269731927811
PSMC2	0.163746035248085	2	0.921388951426417
0.0162925583630993
ORC5	5.3129670078305	2	0.0701946273808676
0.768167407064175
AC007384.1	5.06325058529821	2	0.0795296562215011
0.864993591595809
KMT2E-AS1	0.18191138624896	2	0.913058164757516
0.161516299422016
KMT2E	0.48903199419627	2	0.783083461202189
0.070686563523714
AC005070.3	1.69577738832889	2	0.428318288884569
0.468501490523799
SRPK2	0.504783009545659	2	0.77694050260304
0.159886194043588
PUS7	1.4690501480368	2	0.479733242661595
0.889517307549328
RINT1	0.952529764032588	2	0.621098942516404
0.340805550206189
ATXN7L1	2.20270918522024	2	0.332420484246224
1.26819512009008
SYPL1	25.6929788425673	2	2.63536353573013e-06
0.280873881349091
NAMPT	1.34321239015042	2	0.510887333704616
0.220936080575284
CCDC71L	2.14712082259968	2	0.341789437518083
0.531547412408473
PIK3CG	13.0970637895452	2	0.0014322167004357
1.35208557731091
PRKAR2B	7.48682883583321	2	0.0236731350041548
0.629650713311972
HBP1	0.214141663796071	2	0.898462030965423
0.109134830754257
COG5	0.512585456778173	2	0.773915388625641
0.292230354269764
DUS4L	1.35635362806966	2	0.507541491866958
0.699537771814942
BCAP29	1.62224084296112	2	0.444359916810508
0.516975512999121
AC002467.1	1.27100312196185	2	0.529669759999271
0.715143688991243
CBLL1	1.57404907876071	2	0.455197203619243
0.408447837976681
DLD	2.24772158944486	2	0.325022523907706
0.163579391832452
LAMB1	2.87646738874873	2	0.237346615320672

1.45601575510464
PNPLA8 5.03082953612987 2 0.0808293798076475
0.34666214006987
THAP5 1.39599900375381 2 0.497579716087932
0.462637087166817
DNAJB9 1.90107321991984 2 0.386533550040535
0.305190174369147
IMMP2L 1.36245300085141 2 0.50599600730585 0.644039588142656
ZNF277 3.75012622745193 2 0.153345288347002
0.472467785190007
IFRD1 0.83242133566411 2 0.659541311709543
0.180182548863274
TMEM168 1.78983426904102 2 0.408641459508362
0.472860007388069
BMT2 0.0243836862464672 2 0.987882176279783
0.149775188473854
SMIM30 0.766535377378549 2 0.681630410110884
0.133206481135045
MDFIC 46.1929638505173 2 9.31805743675795e-11
1.00645566611377
LINC01393 0.91393848963682 2 0.633199813938557
0.872111812625847
TFEC 1.01930650351215 2 0.600703835703971
0.591833316794579
TES 2.29381900235767 2 0.317616848628362
0.203836074677585
MET 0.59543914835235 2 0.742509529402013
0.563387103243955
CAPZA2 0.0403914544640455 2 0.98000684050766
0.0192574366283241
ST7 1.66701623517269 2 0.434522254221843
1.24383530758592
LSM8 1.58249472200991 2 0.453279039906372
0.104347877158538
ANKRD7 2.86570781322387 2 0.238626930561443
1.72627218039368
ING3 1.61097513097048 2 0.446869995049757
0.226996331173037
FAM3C 21.1631823741504 2 2.53789317428144e-05
0.291222278988603
NDUFA5 0.516826588414279 2 0.772275988948253
0.0810430146227284
WASL 0.0499280842338667 2 0.975344982738634
0.130830873801797
AC006333.2 0.662888847359906 2 0.717886052573094
0.440169208649833
POT1 0.0561703663126218 2 0.97230553921881
0.108536773675834
ZNF800 0.0357830523328505 2 0.982267576909212
0.0445739003745302

GCC1 0.548669946553689 2 0.760077426986707
0.52097833499153
ARF5 0.0558781328810954 2 0.972447619690832
0.0661937728507294
SND1 8.87105113793904 2 0.0118488368898979
0.242092018072554
SND1-IT1 6.42886081941894 2 0.0401782124269795
1.1808472307308
RBM28 2.80774708604857 2 0.245643607617529
0.340870540760729
IMPDH1 2.12077383142438 2 0.346321787062987
0.498861081177428
HILPDA 3.21172556237705 2 0.200716305930012
0.57835116782671
METTL2B 0.147586472743864 2 0.928863729222272
0.132475381939096
AC090114.2 0.661076694972146 2 0.718536806807188
0.283534662848387
CALU 9.30831392029715 2 0.00952193723176598
0.420077405079783
CCDC136 0.305810485542207 2 0.858211039658533
0.383342625269328
KCP 3.59027430937429 2 0.166104668750554
2.20525704176612
ATP6V1F 0.654325333556247 2 0.720966456196966
0.054603713155712
IRF5 2.25936988888073 2 0.32313504587946 0.410563051847113
TNPO3 6.70403177361197 2 0.0350136992543304
0.51261232136171
TSPAN33 0.580672459918348 2 0.748012021240864
0.125052918373789
SMKR1 0.639562375352556 2 0.726307944816714
0.791304423561201
AC078846.1 3.3352711853104 2 0.188692684988612
3.22696409200213
NRF1 2.83894649446886 2 0.241841373963813
1.55136040015619
UBE2H 6.77786856928828 2 0.0337446199032737
0.269845079409811
ZC3HC1 0.0176843357146116 2 0.991196809143908
0.0324598687192015
KLHDC10 3.84651930091668 2 0.146129852706475
0.848227928855628
TMEM209 1.57417029122768 2 0.455169616667218
0.723888361910301
CEP41 1.92709626419835 2 0.381536738753321
0.574924578798579
MEST 1.23495374640679 2 0.539303453383854
0.616369206757182
COPG2 0.0402553122703525 2 0.980073552918779

0.0836782662067521

AC016831.7	0.00187380504440935	2	0.999063536233929	0.0228127574617096
LINC00513	0.813200156688821	2	0.665910449065914	0.280984296708666
AC016831.1	0.896794823049782	2	0.638650826393492	0.763163395625249
AC016831.5	1.05380178184034	2	0.590431950228058	0.250894949437862
AC058791.1	0.756153302434114	2	0.685177978926983	0.243085399176343
MKLN1	0.608868960791681	2	0.737540350003359	0.233489880863778
CHCHD3	1.24168663972851	2	0.537490969600745	0.163246503159573
EXOC4	1.88225133438348	2	0.390188365814707	0.424077198676256
SLC35B4	7.85365598754386	2	0.0197060813329372	0.975470133080033
AKR1B1	1.80631357522639	2	0.40528822934079	0.183143009078485
BPGM	3.20862621401286	2	0.201027591940888	0.401618474318878
AGBL3	0.0727232153559411	2	0.964291535207155	0.251319742932614
CYREN	7.83774348243359	2	0.0198634932670729	0.406779607673269
TMEM140	1.440817980964	2	0.48655321962495	0.487877231612402
WDR91	2.26714540307254	2	0.321881214187704	0.626395967104301
CNOT4	8.02855158065246	2	0.018056026165703	0.507933769734459
NUP205	2.42353878995328	2	0.297670117178271	0.863800952810588
STMP1	6.09036873264474	2	0.0475875376394028	0.276733630981327
MTPN	4.02588070275908	2	0.133595279527803	0.232124910865609
CREB3L2	36.3640194996775	2	1.2695607787272e-08	1.57135758999451
TRIM24	0.63702049692751	2	0.72723162491044	0.314907605282326
ZC3HAV1	0.60259437311627	2	0.739857864259215	0.171520336028325
TTC26	2.40993273624934	2	0.299702078886704	1.38174027649325
UBN2	3.16143262001125	2	0.205827609011428	0.893494168725193
FMC1	0.500404205345772	2	0.778643401255679	0.134701231636412
LUC7L2	0.0438220283529737	2	0.978327288437321	0.0274601423580697

AC083880.1	1.74474476959769	2	0.41795881300255	2.41336815395001
HIPK2	2.67115284387635	2	0.263006529020851	1.05467802207926
PARP12	2.65969211380019	2	0.264517978883298	0.703345275710355
KDM7A	0.518181535134639	2	0.771752969724833	0.289007210091525
SLC37A3	0.164766059724657	2	0.920919151596983	0.331215679393191
MKRN1	0.566775662625294	2	0.753227605953595	0.0923403842119061
ADCK2	2.03471593129623	2	0.361548904039868	0.314782527402197
NDUFB2	1.41238967035784	2	0.493518548511368	0.0514012423028537
BRAF	0.347526352062024	2	0.840495923670998	0.138892661359518
MRPS33	6.06169026042043	2	0.0482748223704049	0.342746666189994
AGK	5.51214764389653	2	0.0635407515677964	0.93109477201292
KIAA1147	0.00603460875731653	2	0.99698724310933	0.0692320258719436
SSBP1	3.39296098714908	2	0.183327612661606	0.127957682802863
TAS2R4	0.194851906995874	2	0.907169511798037	0.361254783428338
TRBC2	9.41388185638245	2	0.00903236601967439	0.540565231421244
PIP	28.3556440871639	2	6.96065519667499e-07	1.19945872387406
GSTK1	8.1741146029708	2	0.016788564623937	0.29618205706897
CASP2	0.291802706672076	2	0.864242953663968	0.150972503564001
AC093673.1	1.02661475771086	2	0.598512793151242	1.0526301401624
ZYX	1.84620226909076	2	0.397285094225741	0.313689282349543
TCAF1	0.961052332186774	2	0.618457894606526	0.652858499858216
OR2A25	3.91895122429819	2	0.140932304735868	1.61132713196917
TPK1	0.0259227831621155	2	0.987122245513792	0.0986698167765668
CNTNAP2	1.89181909665986	2	0.388326208771489	1.345335334451
CUL1	0.169590799877825	2	0.918700231297273	0.0855908001462158

EZH2 2.23974592562593 2 0.32632124692327 0.276126825700553
PDIA4 52.5681249915169 2 3.84570153499908e-12
0.851736153725814
ZNF786 1.71782774454151 2 0.423621940076244
0.791670324717069
ZNF425 2.43448347977816 2 0.296045612605637
3.11692305485846
ZNF398 0.548941215557184 2 0.759974341254757
0.386737359808049
ZNF282 2.22353941210836 2 0.328976254348456
1.2320970846141
ZNF212 0.638478521225522 2 0.72670165742058
0.393297236985516
ZNF783 2.21789402542281 2 0.32990616524075 1.50205774700759
ZNF746 1.0957475513098 2 0.578177840184009
0.451725040915041
KRBA1 0.0217864918226627 2 0.989165870640149
0.0918503397552806
ATP6V0E2 0.00929432583457394 2 0.995363618436934
0.0279031777464111
ACTR3C 3.86409285258818 2 0.144851467116699
2.56004250930408
LRRC61 1.55068622674203 2 0.460545734487114
0.182882529702552
RARRES2 2.60200823781627 2 0.272258276052327
1.57921340829555
REPIN1 10.5649412767672 2 0.00507986476716149
0.221557856353212
ZNF775 0.243373124353231 2 0.885425850965101
0.0464685228161002
AC073111.5 5.68070964961349 2 0.0584049387663456
0.289338548029297
GIMAP2 3.79199230358377 2 0.150168669724656
1.10787944528034
ABCB8 1.00246717765109 2 0.60578291157016 0.211296501961003
CDK5 1.27641486117423 2 0.528238479996185
0.368619189160875
SLC4A2 3.02254953165065 2 0.220628548885196
0.714172550963119
FASTK 0.591940072729183 2 0.743809714929391
0.153449444849395
TMUB1 3.37475123244991 2 0.185004410023151
0.313389394555783
ABCF2.1 1.63089780024076 2 0.442440671105758
0.281177193897186
CHPF2 0.596847752734504 2 0.741986762428132
0.274594988573387
NUB1 0.0724833781136278 2 0.964407178652213
0.0474142832786187
RHEB 2.09796161162386 2 0.350294585443104

0.243658370342343
KMT2C 0.053271509886511 2 0.973715848122918
0.0525903424339539
LINC01003 1.99131724653961 2 0.369480016252658
0.562428047148603
XRCC2 0.657668938947183 2 0.719762149494245
0.728708071132324
ACTR3B 1.44656367423318 2 0.485157432746202
0.608672859064414
PAXIP1-AS2 1.62814748894656 2 0.443049514424353
0.73531143531421
PAXIP1 0.0248176966477831 2 0.987667823968517
0.125530688403517
PAXIP1-AS1 0.162501205372836 2 0.921962616183384
0.108057269576826
AC144652.1 0.0204127637051136 2 0.989845526513733
0.0874682878090481
INSIG1 8.51945022666369 2 0.0141261849764286
0.377500435031322
AC009403.1 1.62704645717357 2 0.443293487369879
0.405092536050985
RBM33 4.30733501450719 2 0.11605773371457 0.27338789795371
LINC01006 0.642529714624746 2 0.725231142779306
0.352933812255409
RNF32 2.27126690491818 2 0.321218580174857
2.35505936327376
LMBR1 1.89975000892832 2 0.386789367377284
0.515201687287512
NOM1 3.7414781794542 2 0.154009792684917
0.616596036673572
UBE3C 1.47807035311413 2 0.477574468352037
0.611959238426956
DNAJB6 1.49232528514958 2 0.474182674364917
0.138908651086023
PTPRN2 1.75932282401763 2 0.414923375972845
0.500190644485119
NCAPG2 3.87298424520057 2 0.144208930803317
2.6482998368072
ESYT2 1.1601829389956 2 0.559847155285163
0.680413871871305
WDR60 0.767039274144003 2 0.681458696063886
0.302087016283583
PLCXD1 0.781077914797882 2 0.676692068002561
0.371358103801167
GTPBP6 0.421578070037854 2 0.80994491887323
0.117819734297721
LINC00685 5.52856712475347 2 0.0630212339610913
0.610262363546983
PPP2R3B 3.49491059549064 2 0.174216709511117
1.00747448311254

SLC25A6	22.8721082720142	2	1.07990311519757e-05	0.225800064415845
ASMTL	4.96730938772094	2	0.0834377283292921	0.486153858569683
P2RY8	0.972676507941122	2	0.61487378863354	0.0535472483122765
AKAP17A	5.95661856826581e-05	2	0.999970217350671	0.000812982683034779
DHRSX	2.57740556943467	2	0.275628100264991	0.489519881991197
ZBED1	0.011277835519295	2	0.994376951095369	0.0385683440209686
CD99	6.1318972639955	2	0.046609605522583	0.270560118133702
PRKX	0.00910882519316768	2	0.995455943013261	0.03601761704371
BX890604.1	1.0993751410522	2	0.577130094667807	0.474839409741402
PUDP	3.44282612207506	2	0.178813295204967	0.475099260640911
STS	4.16654377442021	2	0.124522122610744	0.962073014096423
PNPLA4	0.818409099967939	2	0.664178360753382	0.260877230493128
TBL1X	0.0371555606116129	2	0.981593722962554	0.0776634624921117
GPR143	7.02005657888694	2	0.0298960686818185	2.01189011337983
WWC3	2.87709703565881	2	0.237271904800125	0.52435135960749
CLCN4	0.271295210056964	2	0.873150272045468	0.562151660489635
HCCS	1.80231452723562	2	0.406099423611339	0.264832875718541
MSL3	7.92975442267275	2	0.0189703658152941	0.444172996639916
PRPS2	0.523646027619512	2	0.769647228584848	0.10619314793023
TMSB4X	144.102383684738	2	0	0.269397484058249
TCEANC	0.674880971561368	2	0.713594442458924	0.380769449739658
RAB9A	40.7125789429729	2	1.44336376184384e-09	0.852198301139152
TRAPPC2	1.55485554320867	2	0.459586654054685	0.322053722603092
OFD1	5.32796240370114	2	0.0696702973584329	0.197290182163793
GPM6B	0.0180382221745457	2	0.991021439094694	0.0352359048651144
GEMIN8	0.00619045691634459	2	0.996909556822979	

0.0254196092277633
FANCB 6.43851076532237 2 0.0399848205691985
2.11256924819217
MOSPD2 1.67916675860281 2 0.431890420440749
0.310628740778098
PIGA 0.0265620926682604 2 0.986806757622438
0.110133516291318
VEGFD 3.86305986994319 2 0.144926300966369
1.36949047201664
CA5B 0.370612863229065 2 0.830849646241079
0.523905205801026
ZRSR2 4.06352847088194 2 0.131104018639936
0.267560590353667
AP1S2 1.42332467736178 2 0.490827597215425
0.222037043049565
CTPS2 2.74422105943175 2 0.253571224804569
0.387801598619254
SYAP1 2.81369302722636 2 0.244914400888906
0.187128329424252
TXLNG 5.33243819078502 2 0.0695145569801006
0.425747066817504
RBBP7 42.492033757375 2 5.92887294814659e-10
0.639261678333445
PHKA2 1.36972193504755 2 0.50416031935995 0.90135876465238
PDHA1 4.62679929952923 2 0.0989243710315107
0.320337988731789
SH3KBP1 8.35974723296941 2 0.0153004411680756
0.260837319843803
BCLAF3 2.93180636626042 2 0.23086937995505 1.24729770950883
EIF1AX 1.09128182885669 2 0.57947027344049 0.118905961291688
RPS6KA3 0.24310520868934 2 0.88554446863715 0.292829717493841
CNKSR2 1.01879230232862 2 0.600858296870785
0.868142022445349
KLHL34 0.762677359183392 2 0.682946550388337
0.860630063772279
MBTPS2 2.11123054027213 2 0.347978260795957
1.01151812426929
SMS 11.9428962996115 2 0.00255054517178399
0.507257797375897
PRDX4 5.02634243402424 2 0.0810109282282361
0.319024448943117
ACOT9 0.47481645986615 2 0.788669263891321
0.188025798572735
SAT1 14.4756194438733 2 0.000718884594165248
0.375664856801182
APOO 2.40488184968243 2 0.300459916023437
0.7221536684568
KLHL15 0.199236869656884 2 0.905182738357519
0.444963158780308
EIF2S3 1.42695666544658 2 0.489937066067401

0.115409986247683
ZFX 0.538372367252514 2 0.764000997929323
0.180479283788063
PDK3 0.0174128718713891 2 0.991331355322407
0.0362181687702315
POLA1 4.13474972036914 2 0.126517472201214
2.6620301583624
CXorf21 0.850964966235281 2 0.653454428328982
0.283477731567506
GK 0.392440973773035 2 0.821831011739258
0.121523654171177
TAB3 2.25857752377838 2 0.323263091709399
1.02044323956543
DMD 7.05163310230162 2 0.0294277682778161
0.480561343175057
CYBB 5.02425371219773 2 0.0810955770695009
0.209552680323808
DYNLT3 1.11387325509713 2 0.572961572882468
0.258461461580975
RPGR 1.67864633478812 2 0.432002818093786
0.375747933453453
MID1IP1 45.9778202485654 2 1.03763109216004e-10
1.21880893675287
BCOR 0.518574491203816 2 0.771601352113407
0.328387980022496
ATP6AP2 0.000368219444189593 2 0.99981590722506
0.00243998115171198
CXorf38 0.498333742788404 2 0.779449894641162
0.154761084074063
MED14 1.78517240868318 2 0.409595085204373
0.663625143845996
MED14OS 0.482498281221567 2 0.785645865712548
0.154751491488617
USP9X 0.127873671801489 2 0.938064249642842
0.0304272854673341
DDX3X 0.571831874178482 2 0.751325771923627
0.131187343951656
GPR82 7.48410997899248 2 0.023705338821111
1.47116807463615
FUNDC1 0.0288433801230995 2 0.985681804391574
0.0342930139358541
KDM6A 1.04808752652768 2 0.59212130187969 0.483258818577644
KRBOX4 1.97091239011815 2 0.373268904659513
0.305280580840123
SLC9A7 8.67984680660397 2 0.0130375267966957
0.869960919027881
RP2 1.04891581033376 2 0.591876130408294
0.356228413472063
NDUFB11 2.23816017654144 2 0.326580081330597
0.103203280232452

RBM10	1.81054099959406	2	0.404432471404067
0.301034658814592
UBA1	1.41674744060816	2	0.492444398936256
0.0899426175499934
CDK16	1.57230416134175	2	0.455594517678925
0.325022995376952
USP11	0.0203331747274481	2	0.989884917694255
0.0232106135886701
LINC01560	0.30336830576038	2	0.859259632566136
0.37458137506358
ARAF	0.881871432240581	2	0.643434067681724
0.199900332498834
TIMP1	9.09081246818286	2	0.0106158593162224
1.56018049374381
ELK1	1.88534573256723	2	0.38958513351003	0.583727221913068
UXT	17.8875931703627	2	0.000130544474932548
0.354415578839448
ZNF81	0.422221073054327	2	0.809684562214995
0.643172634275477
ZNF182	2.32607317334994	2	0.312535696767007
1.68199804484418
FTSJ1	0.222579142539977	2	0.894679637896308
0.20042990955498
EBP	11.1461665331538	2	0.0037987497992602
0.606911826268563
TBC1D25	0.0298546786794663	2	0.985183521087578
0.0682176608728172
RBM3	31.2138449344617	2	1.66724603345436e-07
0.368381531908608
WDR13	0.440909739603295	2	0.802153839406636
0.148915146638101
WAS	11.5326787728347	2	0.00313119866894318
0.378336261020785
SUV39H1	0.000297902742203347	2	0.999851059721603
0.00998522340523755
HDAC6	1.6320229579582	2	0.442191833339953
0.347104214600205
TIMM17B	0.042540963171544	2	0.978954139687846
0.0298624994257512
PQBP1	0.0642313392812576	2	0.968394561749702
0.0329547728640867
SLC35A2	0.257727107564237	2	0.87909390640135
0.17315813529485
PIM2	39.3273417472282	2	2.88520218827415e-09
0.732658999422163
OTUD5	2.96505186620244	2	0.227063416403279
0.468755009061313
GRIPAP1	0.653127937040956	2	0.721398226795367
0.182441992405026
TFE3	1.71776198521835	2	0.423635868851256

0.8449401121021
PRAF2 0.0619854260140298 2 0.969482637663466
0.0844353294244739
WDR45 6.2120075921308 2 0.0447795464590884
0.433451855683654
GPKOW 0.313404183021061 2 0.854958720343823
0.14337573370879
PLP2 3.50286529658589 2 0.17352516475574 0.389226249822444
PRICKLE3 0.532227964674089 2 0.766351771956653
0.720691517000086
CCDC22 0.241803604085289 2 0.886120970589936
0.0379377464391546
GSPT2 5.91511103719966 2 0.0519457424998546
0.639226058461334
MAGED1 2.40194416277496 2 0.300901568884249
0.22427569256975
TSPYL2 9.66536457000704 2 0.00796512784418724
0.510288170264621
KANTR 2.41795705821275 2 0.298502034894303
1.25805013687882
KDM5C 0.00122857127234595 2 0.999385902998621
0.0144482981763651
SMC1A 2.99818258272265 2 0.223333012605437
0.315602603521486
HSD17B10 3.81892785941106 2 0.148159789372743
0.144174352681481
HUWE1 0.00452152358695114 2 0.997741791803747
0.018922584370387
PHF8 0.46758962559232 2 0.791524209873827
0.407460915779089
FAM120C 0.347169104939093 2 0.840646069455669
0.289942532885957
TSR2 0.166143431836963 2 0.920285145758982
0.0714299887444313
GNL3L 0.670859477924757 2 0.715030743748857
0.318156319842497
MAGED2 4.25840899634719 2 0.118931866748183
0.318393850727163
TRO 1.69117796013688 2 0.429304431980067
1.62890552896336
APEX2 0.0646042707307563 2 0.968214006190137
0.0764471332775744
FAM104B 0.0496747622155149 2 0.975468528742508
0.0300207105786369
MAGEH1 0.317574782860887 2 0.853177732584958
0.169071113383623
USP51 2.42156535639411 2 0.297963978232647
2.75532700761809
RRAGB 4.56711656219056 2 0.101920897517893
1.42274571584323

AL445472.1	4.65805419847442	2	0.0973904522579875	0.642391005539786
UBQLN2	0.00461287940246131	2	0.997696218087082	0.0149525275104005
NBDY	34.1717585509136	2	3.79924159910061e-08	0.307957697293682
SPIN3	1.232641873296	2	0.539927214404698	0.977136415980367
SPIN2B	3.58690500690007	2	0.166384733025332	0.804907281411557
ZXDB	0.000684064709554571	2	0.99965802613162	0.0231314627621538
ZXDA	0.274155121610172	2	0.871902598042038	0.456112966215337
ARHGEF9	0.181053500767688	2	0.91344989843872	0.175662992726421
MTMR8	0.00360113340652456	2	0.998201053344484	0.0611027291909246
ZC4H2	0.258996283753572	2	0.878536220843276	0.346260426976992
LAS1L	3.96591939852547	2	0.137661198025665	0.43009959052434
MSN	1.41862452456402	2	0.491982436015765	0.164214379811689
YIPF6	0.0823834052653049	2	0.959645145812376	0.0716559534754602
PJA1	0.481495353875801	2	0.786039937372006	0.260782630963207
IGBP1	3.14038562408221	2	0.208005072607714	0.174185626714852
PDZD11	0.740588087441022	2	0.690531254402998	0.171786694460884
SNX12	0.163096679000634	2	0.921688154832159	0.127441243515625
IL2RG	5.4103108358311	2	0.0668599318078247	0.279278433411984
MED12	0.513149516691103	2	0.773697152078348	0.215653690875362
ZMYM3	3.74745864744195	2	0.153549955220885	1.06306451294567
NONO	0.784498697801917	2	0.6755356488866	0.0902830240952454
TAF1	0.855739694928724	2	0.651896255228105	0.504504331104851
OGT	0.000354990051772952	2	0.999822520725424	0.00496405153019355
GCNA	0.369192211451755	2	0.831440029911964	0.610478858536812
NHSL2	0.762428056960737	2	0.683031685740818	0.548287652156069

PIN4 10.8781652677293 2 0.00434346599226398
0.482974689563387
RPS4X 6.95831883229232 2 0.030833318132063
0.101660727453739
HDAC8 3.37687883115618 2 0.184807707096215
0.370746466154413
CHIC1 3.18661290663822 2 0.203252454712859
5.86396378680674
JPX 0.713758010190802 2 0.699857171763675
0.13826032858685
FTX 1.52328042763362 2 0.466899982812681
0.365437850968018
RLIM 0.139526954748795 2 0.932614378222816
0.162750548257494
ABCB7 0.232444433800472 2 0.890277366637976
0.112961212545553
UPRT 0.10984531449817 2 0.94655835454949 0.0905008252178775
PBDC1 10.5982742148873 2 0.00499590297500097
0.500448782848519
ATRX 0.613055028484198 2 0.735998267466254
0.0880546138831013
MAGT1 1.77096481103373 2 0.412515125699656
0.54478750141179
COX7B 7.78657731485581 2 0.0203782187386695
0.118916778404845
ATP7A 0.691733427344902 2 0.707606795132449
0.760046331681691
PGK1 25.6025928283025 2 2.75719578635147e-06
0.210369444719585
TAF9B 2.12937658377726 2 0.344835325984722
0.432827413326739
CYSLTR1 17.1151998603426 2 0.000192079745663154
1.71909040613268
P2RY10 0.0135693701193582 2 0.993238278952121
0.075028389210336
GPR174 7.06395029955587 2 0.0292470913937701
1.62332253500179
BRWD3 0.0681895504455051 2 0.96647990194875
0.0775518286588306
HMGN5 3.67179338338435 2 0.159470441817044
1.04086095119851
SH3BGRL 13.4119987458594 2 0.00122354931067647
0.354652257303352
HDX 1.93291503410558 2 0.380428314704322
1.37495478983249
APOOL 1.89217910882742 2 0.388256313982352
0.536167053163646
ZNF711 3.4664598429918 2 0.176712718998715
1.42835754954949
CHM 1.99322007881528 2 0.369128654174867

0.53100135554332
DIAPH2 4.02379160627622 2 0.133734899149117
0.720951235209178
SYTL4 3.90859282615382 2 0.141664114656583
15.1617190572795
CSTF2 0.951296525396413 2 0.621482042224005
0.238681533707145
XKRX 3.92385360424004 2 0.140587275922258
3.49488988360742
TRMT2B 0.264660850746146 2 0.876051477613727
0.235906642904567
TIMM8A 0.810031629631386 2 0.666966262826547
0.242754270541203
BTK 9.60637671676697 2 0.00820354945187951
0.267838416135509
RPL36A 50.2508481026955 2 1.22508669875288e-11
0.303781953939041
GLA 12.3990815742342 2 0.00203036279100122
1.59122193888154
HNRNPH2 1.7454012462207 2 0.417821645420551
0.0955259503798676
ARMCX6 1.98687958030086 2 0.370300740938507
0.235191358938567
ARMCX3 2.98518172070325 2 0.22478950222247 0.354706465260229
ARMCX2 2.70647784674719 2 0.258401959635536
1.7757339111327
ZMAT1 0.679817140534873 2 0.711835402707942
0.583994058155368
BEX5 7.54154814441503 2 0.0230342262301847
1.10994883885198
ARMCX5 1.06116174158472 2 0.588263165524865
0.443131822468569
BEX1 0.0248639944653631 2 0.987644960800775
0.104873055361186
BEX4 1.51038402959092 2 0.469920374402502
0.0937892747049714
TCEAL8 0.216123007413502 2 0.897572390703668
0.0162611738542521
BEX2 1.96459177614903 2 0.374450414970305
0.203117960177048
TCEAL9 0.303293534255903 2 0.859291757234367
0.257339952699265
BEX3 0.754618465208674 2 0.685703999074047
0.318536415998627
TCEAL4 0.214516038583576 2 0.898293865939139
0.0734558923489876
TCEAL3 7.62404756254904 2 0.0221034012126625
0.65952298446185
TCEAL1 22.0486261446942 2 1.63005277667994e-05
1.4763536377662

MORF4L2 0.526381715346351 2 0.768595191021823
0.116874203881278
TMSB15B 0.416440891945264 2 0.812028008675482
0.651460115973283
SLC25A53 0.228469139599221 2 0.892048683657928
0.208558160741662
ZCCHC18 9.67892941181769 2 0.00791128778395056
5.89372486265836
FAM199X 0.170291717272837 2 0.918378321222202
0.180404082618757
CXorf57 0.332087080889602 2 0.847009354222487
0.530051886443755
MORC4 2.57000498383326 2 0.276649894242642
0.844636002272316
RBM41 5.24113611003223 2 0.0727615185400456
0.635347490903525
PRPS1 0.442862250997247 2 0.801371114283162
0.122396277973798
TSC22D3 32.723646225735 2 7.83701193940445e-08
0.471608917009564
PSMD10 0.082568194471408 2 0.959556483876064
0.0720255434779617
ATG4A 2.00384863301407 2 0.36717220538119 0.372307605461112
NXT2 0.0291743879869182 2 0.985518683676268
0.0689404071198136
ACSL4 2.43514826229215 2 0.295947225984667
0.602570611921546
TMEM164 0.831845300070266 2 0.659731298703624
0.735427683735929
AMMECR1 0.0593110362904353 2 0.970779892012776
0.121060439069352
ALG13 1.43033626219004 2 0.489109870304769
0.123563302675641
WDR44 0.237388182891051 2 0.888079430307715
0.36410733999602
DOCK11 4.73573880023479 2 0.0936801086290753
0.960000722320934
PGRMC1 2.98782313480552 2 0.224492817102053
0.588297313412438
SLC25A43 2.06324911262705 2 0.356427453505764
1.04593235435552
SLC25A5 22.0527745183282 2 1.62667524672422e-05
0.250003151168276
CXorf56 0.112156996328281 2 0.945464915717218
0.126444827234917
UBE2A 5.99782657877209 2 0.0498412019119181
0.158401845602529
NKRF 0.0116217561813264 2 0.994205972406901
0.045206174854084
SEPT6 37.4127446133037 2 7.51494366735272e-09

0.308220913148382
SOWAHD 0.735128873978288 2 0.692418707994338
0.627082107138946
RPL39 33.1249369938595 2 6.41226297615205e-08
0.154657290194609
UPF3B 4.02014538593996 2 0.133978934988107
0.322793854789307
RNF113A 2.37028854265881 2 0.305702072272715
0.157653977165902
NDUFA1 13.8585919359514 2 0.000978689654761888
0.225605076783224
NKAP 0.306363095152349 2 0.857973944581273
0.0929402391744796
ZBTB33 1.17695921674712 2 0.555170720310244
0.820409520714762
LAMP2 8.77134619651999 2 0.0124545022086076
0.44165869772402
CUL4B 0.591994996873658 2 0.743789288653744
0.258300735157728
MCTS1 3.01046835343403 2 0.221965308638058
0.194401530887165
C1GALT1C1 4.12453362313648 2 0.127165382971107
0.466120997172176
THOC2 4.43173995785285 2 0.109058594286493
0.241269670374844
XIAP 0.0712799723144297 2 0.964987639863086
0.0794340246773794
AL121601.1 1.32490341599569 2 0.515585718036177
0.476554004734896
STAG2 3.07532892072033 2 0.214882382140017
0.202971441942531
SASH3 3.57531739820537 2 0.16735153163138 0.22678382892928
ZDHHC9 0.405844595494588 2 0.816341670548064
0.547763464787177
UTP14A 0.267548720925937 2 0.874787428966368
0.141836511486349
ELF4 0.0564610534696312 2 0.972164231121701
0.137124335179056
AIFM1 0.389088064817872 2 0.823209929542212
0.114670301869748
RAB33A 4.12716708499382 2 0.126998050568345
0.670839026654107
ZNF280C 0.216260441241077 2 0.897510714418204
0.463230023839071
SLC25A14 7.23010157309703 2 0.0269155582342121
0.636629122164525
RBMX2 6.35613815758498 2 0.0416660313196547
0.428669989424232
ENOX2 1.54997452415831e-06 2 0.999999225013038
0.000161562845434695

STK26	0.847026341787914	2	0.654742552064921	0.348800925344128
RAP2C	0.795324340680424	2	0.671888973348641	0.134274071296609
RAP2C-AS1	2.36706925582914	2	0.306194539842511	1.21677390248068
MBNL3	0.365702025155358	2	0.832892236961046	0.209808563665116
PHF6	23.7614250537107	2	6.92264575508084e-06	0.953150345326262
HPRT1	0.424811175705906	2	0.80863665784066	0.124337872146429
FAM122B	0.615133493649456	2	0.735233791388972	0.281608063014524
FAM122C	1.29130395041369	2	0.524320586622541	0.744377202376861
MOSPD1	0.532880482656398	2	0.76610178358336	0.205871451004329
RTL8C	0.505799284743745	2	0.776545810208963	0.324344472741239
RTL8A	3.27989821446622	2	0.193989914723181	1.16476214323626
ZNF75D	0.663093102261487	2	0.71781274044424	0.624683488120255
INTS6L	0.251053993625811	2	0.882031952054019	0.374840255947503
MMGT1	0.0993889948146389	2	0.951520071950647	0.129229927879927
SLC9A6	1.14374330487172	2	0.564467961558191	1.06511644928566
MAP7D3	0.0635574595426661	2	0.968720907463119	0.0789649529713703
HTATSF1	7.13247203740981	2	0.0282620314871831	0.503431554683213
ARHGEF6	0.702179946161127	2	0.703920417117963	0.342669939342004
AL683813.1	4.3606844861669	2	0.113002849577986	1.42122063558982
RBMX	1.06268657294203	2	0.587814835392973	0.0924164546857957
ATP11C	0.395219441279686	2	0.820690089045873	0.261404066976648
LDOC1	0.0144521021378932	2	0.992799994065829	0.121985304932955
FMR1	2.09821335210554	2	0.350250496554068	0.370420291551602
AFF2	0.567840684807524	2	0.752826610675982	0.291606001484945
IDS	4.80880458359188	2	0.0903194641641708	0.395129433201329

LINC00893 1.12565291901446 2 0.569596844069962
0.878784532564901
CXorf40A 0.0793449362953821 2 0.961104179837994
0.188854615556533
TMEM185A 4.82271691528325 2 0.0896933671343815
0.960638993603195
CXorf40B 1.71559574008709 2 0.424094967005322
0.689160961425566
MTM1 0.600316629264813 2 0.740700947600698
0.165175453613302
MTMR1 0.452150763729704 2 0.797657970453974
0.321818256451968
HMGB3 4.63153826011292 2 0.0986902491670791
0.870790123471278
VMA21 7.04939589553882 2 0.0294607046968792
0.260523055515127
CETN2 6.58403294799146 2 0.0371788036669773
0.516017161994142
NSDHL 1.56515190274597 2 0.457226699288335
0.355176043479995
ZNF185 1.36240263722582 2 0.506008749363014
0.938210042900274
ZNF275 8.37173866659975 2 0.0152089785217442
2.34306959009731
HAUS7 0.263312055212643 2 0.876642484038096
0.214337619509929
CCNQ 1.93466869141229 2 0.380094890456772
0.252487703857775
BCAP31 0.171349393039481 2 0.917892776373289
0.0581078929015435
IDH3G 0.772645534415942 2 0.679551153450672
0.062953273065046
SSR4 31.8710270576045 2 1.20031271921839e-07
0.328418289997376
ARHGAP4 0.379748205216771 2 0.827063252495287
0.167515464684371
NAA10 4.91620968022894e-05 2 0.99997541925371
0.000373114676281883
RENBP 0.099727330420017 2 0.951359119005273
0.159826787325224
HCFC1 0.0291801484914515 2 0.985515845137934
0.0564853453998885
TMEM187 0.270630575482691 2 0.873440483193556
0.310100580938559
IRAK1 0.150619228040127 2 0.92745628840071
0.145207696584062
MECP2 3.03663701213938 2 0.21907995903137 0.347858785893036
FLNA 1.1707681499571 2 0.556891932475331
0.618797119205716
EMD 2.03796931292849 2 0.360961253850252

0.310991100561486
RPL10	15.7814027508918	2	0.000374207023305373	0.135987601122471
AC245140.2	0.47953505853655	2	0.786810750278382	0.487265152801688
DNASE1L1	0.437863993647879	2	0.803376348439355	0.230610402691061
TAZ	1.17264596022976	2	0.556369309164798	0.358920659871596
AC244090.1	0.393225279825472	2	0.821508791404933	0.216813055759766
ATP6AP1	12.5847290594516	2	0.00185037949272482	0.674046325707995
GDI1	0.00487631499197333	2	0.997564812395823	0.0160159542843206
FAM50A	2.08351252697	2	0.352834467196941	0.241634238952134
PLXNA3	3.01878433257234	2	0.221044295308874	0.846829063913485
LAGE3	1.42404943864869	2	0.490649763018702	0.172957889327601
UBL4A	0.0166798391371931	2	0.991694761082361	0.0396803753244612
SLC10A3	1.37374938111678	2	0.503146101631243	0.370341120366474
FAM3A	0.947238663254312	2	0.622744266499597	0.253411054276503
G6PD	2.35914603024737	2	0.307409969990242	0.353892165171111
IKBKG	0.595998552186594	2	0.742301877104884	0.164096640441847
DKC1	0.0399028641954244	2	0.980246280656228	0.0316954797664412
MPP1	3.16499288075483	2	0.205461534959713	0.772571897768399
F8	0.267206678737464	2	0.874937048863409	0.557615341126546
F8A1	0.3770061748806	2	0.828197946422979	0.251866398095371
FUNDC2	0.125321968655191	2	0.939261844205464	0.0537060150872042
BRCC3	0.069448074829928	2	0.965871923995753	0.0704763252857916
VBP1	0.0217039997899745	2	0.989206670633229	0.0193892472656616
RAB39B	0.649519201861235	2	0.722701069425211	0.34862638623344
F8A3	0.623051966655936	2	0.732328581924433	0.386509283634057
TMLHE	0.501467086693042	2	0.778229708418331

0.382974184607288
VAMP7	2.1400971556376	2	0.342991855217811
0.339255986918282
ZNF596	1.62472140843585	2	0.443809126514965
1.08572929572247
FBXO25	0.836858513467992	2	0.658079682651544
0.185521709807151
ERICH1	0.327964490324481	2	0.848757091290882
0.0788016387858271
CLN8	4.01003785432036	2	0.134657746982639
0.37747191900714
AC100810.1	1.62046517027498	2	0.444754610879999
0.435791625528709
ARHGEF10	3.04731807627039	2	0.217913074151732
1.34988181881786
MYOM2	2.85167377718977	2	0.240307268618981
1.44934254163901
AC246817.2	0.352280826581496	2	0.838500238493017
0.520636184440012
AC016065.1	0.746414957904438	2	0.688522364124654
0.328718861474193
MCPH1	0.125509531687887	2	0.939173762935729
0.106335582121241
ANGPT2	5.8972138468344	2	0.0524126699775801
1.31122700047764
AGPAT5	5.93157541408305	2	0.0515198706924075
0.754560887003402
PRAG1	0.459601284926399	2	0.794692014555028
0.371417079959652
AC103957.1	1.87501173378388	2	0.391603329175643
1.57388778676183
AC103957.2	1.2723684807697	2	0.529308288761759
0.763430874874436
CLDN23	11.7540926852142	2	0.00280305231621947
1.18553670649156
MFHAS1	11.3327228522945	2	0.00346043343977442
0.580171850883857
ERI1	3.26990610749851	2	0.194961523796925
0.47670605634226
PPP1R3B	0.850985335297998	2	0.653447773235756
0.792614505980382
TNKS	7.00488056743605	2	0.0301237830786104
0.39550015015223
MSRA	2.77703105934246	2	0.249445324104537
0.273279868130096
PINX1.1	0.37329749342398	2	0.829735132398936
0.193558895012373
XKR6	5.10125669381353	2	0.0780326190387135
0.522863861733403
MTMR9	2.4143876914035	2	0.2990352421838	0.364087411843684

FAM167A-AS1	5.68332845942527	2	0.0583285130999877	2.7629380110897
FAM167A	0.15768180409101	2	0.924186949002281	0.294746724151408
AF131216.5	1.48844073626339	2	0.475104562244515	1.24725341705165
BLK	71.4902581765205	2	3.33066907387547e-16	0.796456107676014
NEIL2	4.17038308355526	2	0.124283312439171	1.50184943730705
FDFT1	2.18408743571054	2	0.335530063728831	0.0857907240170532
AC069185.1	0.746250494851822	2	0.688578984697519	0.638993113085346
CTSB	0.0598637364393859	2	0.970511653982826	0.0787596027027382
LONRF1	1.56080849584986	2	0.458220739076857	0.50049256530379
ZDHHC2	0.452109667608624	2	0.797674360946637	0.356685346168593
CNOT7	3.90908081225053	2	0.14162955381388	0.243152556704483
VPS37A	2.538138683523	2	0.281093101696166	0.431097981397837
PCM1	2.10904952293032	2	0.348357941091342	0.243055489122338
ASAH1	1.43235296406556	2	0.48861692448134	0.316950227707042
NAT1	16.5148466933654	2	0.000259326325144693	1.56616559342216
INTS10	1.24890489801737	2	0.535554591698358	0.229424155631242
ATP6V1B2	0.111882593596541	2	0.945594643694261	0.0625738574398136
DOK2	0.645950670525038	2	0.723991711213193	0.463089564262147
XPO7	0.277534097933904	2	0.870430772590279	0.303137537700749
FAM160B2	0.185922816331442	2	0.911228665603242	0.217453692575712
NUDT18	0.00559539554440384	2	0.997206212137112	0.0228735959322145
REEP4	0.711314122863625	2	0.700712880507593	0.333926220428606
POLR3D	0.50349811870873	2	0.777439804839401	0.118654797827354
PPP3CC	3.02241547707933	2	0.220643337513583	0.276989498875869
PDLIM2	5.28319181551858	2	0.071247474380043	0.457988604048749
C8orf58	0.452562343373153	2	0.797493837451467	0.347725278977732

CCAR2	1.46742247776294 2	0.480123825344222
0.452621940318717
BIN3	0.000547494497102582	2	0.999726290216808
0.00530651514827018
EGR3	0.162959267838156	2	0.92175148212800З
0.454716538225416
PEBP4	10.3616652719586 2	0.00562332228412998
3.6875159592278
RHOBTB2	1.56642499551279 2	0.456935745898992
0.277162324685357
TNFRSF10B	8.88798396017354e-05	2	0.999955561067638
	0.00566272957925421
TNFRSF10A	1.64871422488575 2	0.438516818870339
0.592977541564702
CHMP7	13.9881816359648 2	0.000917286394217731
0.603347260037849
R3HCC1	0.420957734883942	2	0.810196176490153
0.154145375290884
LOXL2	3.22859076482536 2	0.199030861689976
0.245614397926301
ENTPD4	1.98619833070891 2	0.370426896037397
0.216465381522078
SLC25A37 3.85490165334964 2	0.145518678407542
0.573864669411525
ADAM28	60.1393344591789 2	8.72635297355373e-14
1.02225037272077
DOCK5	0.261025952422287	2	0.877645104366665
0.355551723332105
KCTD9	5.47532145400945 2	0.0647215713706586
0.321068066665065
CDCA2	2.64887198542339 2	0.265952916179577
0.352373737580623
PPP2R2A	0.0356922105276695	2	0.982312193402394
0.026078175311648
BNIP3L	2.82542016762716 2	0.243482530145995
0.301184076226387
DPYSL2	1.58051172834487 2	0.45372868751394 0.346685461195241
TRIM35	0.416311136642754	2	0.812080692854433
0.454630486248568
PTK2B	0.0766192030524263	2	0.962414929630146
0.0415812476735938
CCDC25	0.0236459208222748	2	0.98824665615723
0.0256677007797976
PBK	2.08089519718731 2	0.353296511541284
2.81784592352768
NUGGC	0.21755430190329 2	0.896930275286638
0.106778396800274
ELP3	0.534163108324667	2	0.765610630185617
0.193753744978956
PNOC	0.229816092136956	2	0.891448112296789

0.10045677847813
ZNF395 0.292922298667385 2 0.863759289306821
0.250827931792834
FBXO16 6.54913398307281 2 0.037833247799542
1.80863743363098
FZD3 13.004330933346 2 0.00150018706796529
1.68271041474003
INTS9 1.76107481304137 2 0.414560064525296
0.498883341304475
HMBOX1 0.794467664782412 2 0.672176830539123
0.273717351672034
DUSP4 6.00431927765567 2 0.0496796623027924
26.6552416356497
LINC02099 12.4042990977227 2 0.00202507296117804
1.96348981078403
SARAF 13.7723757326554 2 0.00102180167037857
0.387041181239971
AC044849.1 1.40918131898991 2 0.494310874311454
0.907909305374098
LEPROTL1 3.99134808556077 2 0.135922006036075
0.778274362674729
AC026979.2 0.135104662077282 2 0.934678806623432
0.212585976905896
DCTN6 4.94054356918707 2 0.0845618732637677
0.234589559148093
GTF2E2 7.13541815118656 2 0.0282204305548139
0.451524937782094
GSR 2.84812974068741 2 0.240733474996079
0.790223327283234
UBXN8 1.58442950440347 2 0.452840753784729
0.807647023435464
PPP2CB 1.71564112065649 2 0.424085344278954
0.424902261035304
WRN 0.579408918463195 2 0.74848474264981
0.27936844948792
AC090204.1 2.39958981574038 2 0.301255990809576
0.741867744363834
FUT10 1.0396516684368 2 0.594624102123641
0.895958834392057
TTI2 0.199810037775431 2 0.904923364581946
0.140216660661884
MAK16 0.201541675711404 2 0.904140203854947
0.169637405868635
RNF122 0.085342125790709 2 0.958226534494693
0.271928150649822
ERLIN2 0.937891364461832 2 0.625661566773854
0.669272516440896
PLPBP 0.0491518210743269 2 0.975723618403081
0.0452159412753486
BRF2 1.35212057249936 2 0.508616855153308

0.251108589767834
RAB11FIP1 3.11379835705688 2 0.210788676926311
0.291179393643116
EIF4EBP1 0.00206544337235393 2 0.998967811387342
0.00946342624808507
ASH2L 3.58828117269879 2 0.166270285914888
0.295871391663765
LSM1 6.03039562748523 2 0.0490361345062647
0.319214346936264
BAG4 0.10564449744566 2 0.948548603223925
0.135413772768146
DDHD2 0.13272413128292 2 0.935791984821757
0.184106612474051
PLPP5 2.19689073712915 2 0.333388978011823
0.542807828649498
NSD3 1.50879706142579 2 0.470293396713726
0.132334291726187
AC087623.3 0.0162151430087536 2 0.991925206211098
0.0829688265378991
FGFR1 0.587230047986709 2 0.745563460245319
0.373851722878689
TACC1 5.19663216460792 2 0.0743987541704623
0.349939254654712
PLEKHA2 31.0741803196269 2 1.78783517990055e-07
0.725211254301032
AC108863.1 2.92824194159387 2 0.231281205082286
1.43453221943407
TM2D2 2.94170109165079 2 0.229730006168863
0.343676226894241
GOLGA7 1.39522035054263 2 0.497773474825076
0.234920510543255
GINS4 0.10142790914822 2 0.950550532280004
0.196827371542873
GPAT4 6.33820168988245 2 0.0420413826356081
0.341594141291376
NKX6-3 18.3852543590771 2 0.000101787100142081
1.4602569166122
ANK1 22.7706123937606 2 1.13612036306554e-05
0.9841171466101
KAT6A 2.56151529050398 2 0.277826726592845
0.518493372986352
AP3M2 1.54724877154231 2 0.461337967773558
0.668031808592642
IKBKB 6.56770834070632 2 0.0374835102135436
0.751436574122225
POLB 0.816167425576781 2 0.6649232139162
0.459698883026083
VDAC3 7.60590919313663 2 0.0223047727987155
0.206090996947083
SLC20A2 0.477769779827837 2 0.787505526984534

0.320863373083515
SMIM19 0.292915293637075 2 0.863762314642121
0.0930164018390803
THAP1 0.438620484998885 2 0.803072532271954
0.165840667535325
RNF170 1.59758594905288 2 0.449871643070693
0.313982574777505
HOOK3 1.94779945606734 2 0.377607596133685
0.332660103915821
FNTA 3.41572564801322 2 0.181252747995716
0.236922119358352
HGSNAT 6.87999035784207 2 0.0320648399146134
0.888174049388211
SPIDR 0.454588616699005 2 0.796686276360239
0.260524449057155
CEBPD 0.119564995992187 2 0.941969391535211
0.229878561945875
PRKDC 0.576422698986144 2 0.74960314725082
0.139437511555434
MCM4 1.62314084554132 2 0.444159999259627
0.480533902538202
UBE2V2 5.61910116568175 2 0.0602320556299059
0.271661975157448
AC026904.2 7.8564651413874 2 0.0196784220552173
1.44035911092907
AC022915.2 2.9603325996937 2 0.227599835418656
0.593885361239767
PCMTD1 0.00102098991543764 2 0.999489635322662
0.00825656926346864
AC064807.1 1.37482861534577 2 0.502874668625247
0.535933334107344
RB1CC1 5.41670502679399 2 0.0666465155597982
0.433510455264639
ATP6V1H 3.00634219967531 2 0.222423712833285
0.239453042113134
RGS20 15.6209914698228 2 0.000405456998801346
1.02318317262705
TCEA1 8.53293005009617 2 0.0140312958681122
0.111954424394883
LYPLA1 14.1090510000547 2 0.000863492366996121
0.391188505733035
MRPL15 1.17535914046917 2 0.555615055779095
0.130977891821704
TMEM68 0.50158354095482 2 0.778184395654466
0.235778391751058
TGS1 2.0993124173825 2 0.350058075350276
0.350696051600056
LYN 7.46356449981217 2 0.0239501126902633
0.189566480112425
RPS20 84.5747216677115 2 0 0.31933388382504

PLAG1	0.432864212380842	2	0.805387213872606	0.402402890920742
CHCHD7	2.06427122276001	2	0.356245345987266	0.179024716633596
SDR16C5	2.1671556599162	2	0.338382681568373	1.44302349752055
IMPAD1	0.11313200938095	2	0.945004107732962	0.164086145063962
UBXN2B	3.81460239416084	2	0.1484805661351	0.859927590096723
SDCBP	0.0399151852194392	2	0.980240241855847	0.0302285229741351
NSMAF	3.60421739077099	2	0.164950690469288	0.650826972834863
TOX	18.236634098199	2	0.000109639039276077	0.691214252008691
AC090152.1	0.556511600518062	2	0.75710312956551	0.183151134193246
CA8	4.79614747307408	2	0.0908928683804581	2.98080310985817
RAB2A	2.20231763683856	2	0.33248556996839	0.161072903190601
CHD7	1.55388587331308	2	0.459809531751194	0.141860267828774
AC022182.1	0.228924259826054	2	0.891845712053298	0.392817778579464
AC022182.2	1.88820182850719	2	0.389029184306534	0.728074190821348
ASPH	0.12481249503123	2	0.939501139250606	0.0457758024448089
GGH	3.60033499569681	2	0.16527120333211	0.691651183607565
YTHDF3-AS1	0.494560443762931	2	0.780921831482946	0.665157640529536
YTHDF3	0.101117538788444	2	0.950698055081729	0.117387752997564
ARMC1	0.563182974350019	2	0.75458187795297	0.14347921041363
MTFR1	0.886519997037326	2	0.641940281866225	0.39744163531864
PDE7A	15.7413458494329	2	0.000381777368313685	1.31445050578268
DNAJC5B	4.33521162604455	2	0.114451306976252	1.36215976532049
TRIM55	2.93207292564311	2	0.230838611805778	0.786375792667684
RRS1	0.20519717180319	2	0.902489172658288	0.107166468969993
MYBL1	0.674072730274911	2	0.713882878981666	0.156411614193943
VCPIP1	0.0282412391653558	2	0.985978608761713	0.0406702762419968
SGK3	0.00220282742896404	2	0.998899192618975	

0.0197890318926799
C8orf44 4.2101740603424 2 0.12183507048062 0.880891085474481
COPS5 2.06876422144099 2 0.355445939321927
0.147216301560762
CSPP1 2.41722960597026 2 0.298610627629448
0.393128557010934
ARFGEF1 1.09737223038556 2 0.577708354181341
0.313693571293913
SLCO5A1 6.82995477059826 2 0.0328771502000236
2.58762031599636
AC079089.1 3.29130038368736 2 0.19288710838884
1.13009395829534
NCOA2 3.50186779948649 2 0.173611731765789
0.743166712333358
TRAM1 14.0374183544618 2 0.000894980008123714
0.229535304218047
LACTB2-AS1 1.05127466093754 2 0.591178468225596
0.716281780718702
LACTB2 0.0223743071553949 2 0.988875189926198
0.0430695640050926
TERF1 0.0235909355728998 2 0.98827382602513
0.0272588709709138
C8orf89 2.00160728948843 2 0.367583915556748
0.484345960604953
RPL7 73.6485440278471 2 1.11022302462516e-16
0.230403882318143
STAU2 0.668368546633419 2 0.715921844778464
0.204417035908699
UBE2W 0.110151746147276 2 0.946413337940414
0.128540014352694
ELOC 5.05466821586782 2 0.079871665955333
0.220158124545915
TMEM70 4.2912505062351 2 0.116994862767605
0.320550612518112
LY96 2.12793993677804 2 0.345083118289735
0.197149961098988
GDAP1 0.495441029090212 2 0.780578073012387
0.354328757672304
PEX2 2.8728141742409 2 0.237780550563889
0.311343704552771
PKIA 0.369364186591086 2 0.831368539478157
0.324806240053546
ZC2HC1A 4.08094796037623 2 0.12996709444922 0.707560093091821
IL7 12.856787107202 2 0.00161504323203965
1.36742917628551
MRPS28 1.36784334189594 2 0.504634097896027
0.237507025651739
AC009686.2 0.579601266031018 2 0.748412761501315
0.69668498990665
TPD52 36.2252436840439 2 1.3607811433225e-08

0.502240927990545
ZBTB10	4.27436177289914	2	0.117986993348782
3.72796261622642
PAG1	33.2987616783036	2	5.87848987265716e-08
1.1776349039985
AC079209.1	0.33785996691595	2	0.844568035049232
0.380454119151847
FABP5	3.92666917545326	2	0.140389498425562
0.145822286021043
IMPA1	1.98864132631538	2	0.369974696634141
0.368948751549584
ZFAND1	2.12834800420738	2	0.345012716881615
0.23126828122916
SNX16	1.46274695120451	2	0.481247554186824
0.639957811131059
LRRCC1	0.324578914050742	2	0.850195073982742
0.368891061552844
E2F5	10.1682118897646	2	0.00619442277071469
0.211241925289435
C8orf59	1.08543584031831	2	0.581166539609341
0.131895091061947
CA13	2.39521525214161	2	0.301915643715634
2.09922376255658
WWP1	1.78750067433948	2	0.409118539554474
0.510427663641636
RMDN1	0.7458892543978	2	0.688703367222713
0.157973081966612
CPNE3	2.88668554910608	2	0.236137084857919
0.180051158663719
AF117829.1	0.0792322813402024	2	0.961158317936862
0.15593929739472
RIPK2	0.538928864911624	2	0.763788445118527
0.170373266352334
OSGIN2	6.72070883975785	2	0.0347229502598191
1.60015301604968
NBN	1.53979437159472	2	0.463060675077942
0.232882994840442
DECR1	0.605394869627186	2	0.738822604554949
0.116315995255469
TMEM64	2.11502317220135	2	0.347319009336762
0.591366103343754
PIP4P2	22.4256073464584	2	1.35002321923761e-05
1.04181869906148
OTUD6B-AS1	1.7966577919279	2	0.407249647947202
0.217389703276433
OTUD6B	0.0684966391856945	2	0.966331515793217
0.118965264809589
LRRC69	6.05670618752954	2	0.0483952750109981
2.38723377638504
TRIQK	2.60128370357163	2	0.272356924141922

0.576038432537338
FAM92A 2.53807550514426 2 0.281101981339636
0.533073321104564
RBM12B 2.07638497912413 2 0.354094132718624
0.765220103934846
TMEM67 1.77724189179027 2 0.411222459923977
1.5189331385377
PDP1 0.480573436384057 2 0.786402352878624
0.309400912042996
VIRMA 0.627851600434061 2 0.730573234523795
0.261764540838902
DPY19L4 0.339016006839243 2 0.844079998926903
0.501504114455206
INTS8 4.62114515380316 2 0.0992044331277939
0.611999001689025
CCNE2 2.24062001681474 2 0.326178660820526
1.44153541203368
NDUFAF6 0.501252651145738 2 0.778313152948346
0.120624945159223
TP53INP1 1.24545549738458 2 0.536479059858213
0.272665382527017
MIR3150BHG 0.178274306859122 2 0.914720107970733
0.299732884921522
PLEKHF2 4.03328780305518 2 0.13310141779587 0.159304670601318
UQCRB 46.0184744957231 2 1.01675223795894e-10
0.364914185822939
MTERF3 1.61646365737695 2 0.445645347312139
0.433750518752868
PTDSS1 6.78436169756932 2 0.0336352434752253
0.316533872087912
CPQ 3.36676516827858 2 0.185744615416802
1.33903288862382
TSPYL5 4.58062763631487 2 0.101234687585346
1.06420923625814
MTDH 24.6141833003869 2 4.51957927050106e-06
0.392666994522413
RPL30 42.0758590636438 2 7.30034366291932e-10
0.232521116693577
RIDA 0.265363473116247 2 0.875743764985571
0.16232409135989
POP1 6.52059828250801 2 0.0383769161814609
0.942066812098938
STK3 0.852560484563067 2 0.652933336950944
0.539205167635713
AC104986.2 5.62128153091376 2 0.0601664274697713
0.899830184487367
VPS13B 0.462120610906706 2 0.793691600660186
0.415527625324513
COX6C 17.6429425002489 2 0.000147531145266555
0.240658054823088

POLR2K 18.1165194890652 2 0.000116425410347953
0.261348056329164
SPAG1 0.447551350314289 2 0.799494460724201
0.129066788741499
RNF19A 1.9151699140523 2 0.383818706247012
0.187219719640592
ANKRD46 0.951660654841388 2 0.621368902568139
0.27837752384555
PABPC1 23.2239333622098 2 9.05705377574062e-06
0.194150028526512
YWHAZ 3.56081290365962 2 0.168569617912947
0.142088982748459
ZNF706 35.2782792934377 2 2.1848367226518e-08
0.312963888252589
NCALD 1.30477028766184 2 0.520802106253229
0.618600258014128
RRM2B 10.2536322881625 2 0.0059354279695204
0.663164005903609
UBR5-AS1 4.37482554729645 2 0.112206677472618
0.807776078715315
UBR5 0.0187516889233136 2 0.990667971723191
0.024415219263123
AP002852.1 1.29045500692811 2 0.524543193137472
0.758509640886667
KLF10 0.69587691599733 2 0.70614233228954 0.211327640414348
GASAL1 1.56296337895818 2 0.457727298885817
1.23610867473263
AZIN1 2.51158602005011 2 0.284849869832318
0.333026472161995
AZIN1-AS1 0.144665035058832 2 0.930221529414411
0.0749286920286646
AP003354.2 0.0405422117016065 2 0.979932971729726
0.121391168377188
ATP6V1C1 0.00104080405051051 2 0.999479733360393
0.0063045759551202
SLC25A32 2.25552548938214 2 0.323756773334385
0.304421048712887
DCAF13 4.50978730824353 2 0.104884697198451
0.297527983694631
OXR1 1.23995589174101 2 0.537956301621787
0.215793008939255
EIF3E 32.2709828781476 2 9.82754645439954e-08
0.314780614316671
EMC2 1.71415480071683 2 0.424400624667984
0.338686903774362
NUDCD1 3.20023388721618 2 0.201872908867875
0.426824478666206
ENY2 2.57175973017968 2 0.276407275496215
0.0745992509401546
PKHD1L1 0.528408038794419 2 0.767816874140018

0.225234273712935
EBAG9	1.79272684385413 2	0.40805087369304 0.159649173359944
SYBU	41.5633933413222 2	9.43243483320089e-10
1.26084818234306
TRPS1	2.06381569763885 2	0.356326494580418
1.52685212515132
EIF3H	30.4667044174866 2	2.42236283587438e-07
0.346710810898803
UTP23	0.157585084378666	2	0.924231643630915
0.0919527164232461
RAD21	10.3895720481509 2	0.00554540277073579
0.30453801064019
MED30	10.0586087833338 2	0.00654336060400751
0.487495985004793
EXT1	3.45054571739379 2	0.178124442338225
0.862927559861935
SAMD12	2.88799109118843 2	0.235982991706421
1.78260961583261
AC023590.1	2.84224591756905 2	0.241442734366477
0.156773279336305
TAF2	0.478841043195212	2	0.787083827021215
0.209499638936173
DSCC1	0.17711852194402 2	0.91524887059115 0.326807904579415
MRPL13	7.0149934384723 2	0.0299718485591774
0.251443801315794
SNTB1	0.0588458413343054	2	0.971005719229816
0.226409331135586
AC016405.3	1.48028311924769 2	0.47704638023487
1.24305903689008
ZHX2	0.507172247102742	2	0.776012909059469
0.241334490934916
DERL1	6.25819871772812 2	0.0437571890355967
0.288021658097478
TBC1D31	2.73243864411369 2	0.255069474462952
1.47967092079614
C8orf76	0.637544608143466	2	0.727041074753292
0.205722928725689
ZHX1-C8orf76	0.382269345730466	2	0.826021337999842
	0.390767846448964
ZHX1	0.273340476235074	2	0.872257816090828
0.248142374873552
ATAD2	0.808298501896094	2	0.66754448218667
0.275809532992902
WDYHV1	0.252499294069856	2	0.881394781721406
0.230379883688103
FBXO32	2.54154607947275 2	0.280614611665041
1.76997758011556
FAM91A1	4.370864412821	2	0.112429130560806
0.77370511592688
TMEM65	1.46460424634666 2	0.480800852260975

1.34142074455421
TRMT12	0.554026374085281	2	0.758044500679443
0.386234197714137
RNF139	0.146204313830197	2	0.929505869723233
0.0716494070253159
TATDN1	2.39113406821069	2	0.302532359370952
0.272181725772604
NDUFB9	7.23037216071817	2	0.0269119169721007
0.235708400113777
MTSS1	1.58087574548219	2	0.453646112519871
1.04689088149086
SQLE	1.53838559689278	2	0.463386964063625
0.127293936419303
WASHC5	7.88735732497892	2	0.0193768027581461
1.7160463678216
NSMCE2	2.76415818644068	2	0.251056041159401
0.33769260596786
TRIB1	1.24847758998808	2	0.535669027311315
0.458187945711165
MYC	6.56379152614294	2	0.037556990121197
0.786901926244053
PVT1	5.52724036894591	2	0.0630630547251217
0.553585920464707
FAM49B	9.12060380171648	2	0.0104589009155824
0.15824020801257
ASAP1	3.07072743197692	2	0.215377340739574
0.337469818734918
EFR3A	5.24696461080437	2	0.0725497819331477
0.254274715712873
PHF20L1	0.00145185405332475	2	0.999274336394616
0.00688855396667897
SLA	7.73951284000007	2	0.0208634507256651
7.01110873239213
NDRG1	1.52364333575299	2	0.466815269601342
16.2947492183047
ST3GAL1	2.68415839028524	2	0.261301805932315
0.498323572110445
ZFAT	3.76497840872509	2	0.152210750142656
0.441516489463541
TRAPPC9	1.84784697756251	2	0.396958519444135
0.717848926064192
AC107375.1	0.914350073389714	2	0.633069519967861
	0.990999927523433
CHRAC1	1.99120398342761	2	0.369500941073386
0.14015193813015
AGO2	0.665694020926943	2	0.716879860884218
0.286817285412608
PTK2	6.4610751471947	2	0.039536239436218
0.581330246947972
DENND3	3.98226755969144	2	0.136540530751985

0.429567343156317
PTP4A3 1.67453641229529 2 0.432891479913385
0.953896938628225
TSNARE1 0.00237121266607663 2 0.998815096220472
0.0459877347162013
JRK 0.137776235643314 2 0.933431108542008
0.185399518626285
THEM6 1.59097152654547 2 0.451361926614284
0.368104151276054
LY6E 4.53102722250603 2 0.103776719959313
0.444961352011221
ZFP41 0.612187002094171 2 0.736317769754931
0.772126732625318
GLI4 0.139435834044156 2 0.932656869430416
0.131183071554378
MINCR 4.00029178222704 2 0.135315540461626
0.799077353989581
ZNF696 0.102745555319334 2 0.949924493892266
0.192365399382373
TOP1MT 0.15386209209493 2 0.925953699572704
0.137902969767839
ZC3H3 3.1026585964751 2 0.21196602041082 0.674002761157131
GSDMD 2.91315139197122 2 0.233032885477882
0.295840266140686
NAPRT 0.990856662411932 2 0.609309864964745
0.281424400102489
EEF1D 2.30241217692172 2 0.316255107630402
0.0532555339846392
TIGD5 2.62148524230589 2 0.269619756685929
1.98945128274445
PYCR3 0.171444448968025 2 0.917849151834909
0.166422510907326
TSTA3 1.09763868819584 2 0.57763139185674 0.161170008783671
ZNF623 1.80046763013021 2 0.406474608741748
0.626412826071689
ZNF707 0.337942817912775 2 0.844533049122097
0.344583027214302
SCRIB 0.612689383737684 2 0.736132836716986
0.715981624342199
PUF60 2.99076079681436 2 0.224163317141143
0.187561712216636
PLEC 0.700041621899089 2 0.704673424643031
0.206680697937973
PARP10 3.04792918702886 2 0.217846499811311
0.699009776771661
GRINA 13.5075181541582 2 0.00116648645634143
0.392693344216888
EXOSC4 4.27879856857665 2 0.117725541367214
0.368700126885384
GPAA1 0.474467493515912 2 0.788806885414575

0.121452952195743
CYC1	4.44191484242458	2	0.108505173923	0.126287467536499
SHARPIN	1.18045338958325	2	0.554201635868711
0.180170195326812
MAF1	0.100204392779773	2	0.951132217254775
0.031928401551746
HGH1	0.231354036194165	2	0.890762877130585
0.138072505794736
MROH1	0.0659398533011479	2	0.967567657151746
0.11134954630359
BOP1	1.71293818283712	2	0.424658869900528
0.270528061459292
HSF1	2.63386038723357	2	0.26795661561296	0.247761613568156
DGAT1	0.0777709266814035	2	0.961860871168345
0.172725951941214
SLC52A2	3.05502773196129	2	0.217074673748412
0.35973763407914
FBXL6	1.02784761760633	2	0.598143965631284
0.47643644128354
ADCK5	0.409374439581953	2	0.814902161822545
0.183931156023849
CPSF1	1.04003858233374	2	0.594509079085721
0.329169772667357
SLC39A4	7.5585375108524	2	0.0228393864977038
0.851055283933784
VPS28	1.70216222272569	2	0.426953098500824
0.12951103958574
CYHR1	0.14268959250609	2	0.931140782919139
0.148218060873718
PPP1R16A	4.27579260277413	2	0.117902613878076
0.894233376388993
MFSD3	2.97336188608203	2	0.226121922962964
0.64507793100263
RECQL4	0.0224430342098558	2	0.988841209270532
0.025089896831941
LRRC14	0.0797819331240256	2	0.960894203039335
0.113693241396472
C8orf82	0.0100892104525199	2	0.994968097425753
0.0267037187946589
ZNF251	2.54644677360409	2	0.27992785022033	1.23009637027981
ZNF34	0.201193400693886	2	0.904297662286927
0.244743383773525
RPL8	66.151496487366	2	4.32986979603811e-15
0.243880867431342
ZNF7	1.08399120308832	2	0.581586478665956
0.407771461517362
COMMD5	1.36054002818527	2	0.506480217104537
0.229632518197719
ZNF250	0.127649517305328	2	0.938169391194343
0.19291173319895

ZNF16	0.659733205059219	2	0.719019642436237
0.21629353230502
C8orf33	0.483511940164024	2	0.785247778123639
0.132554627723285
WASHC1	0.34519297862188	2	0.841477091349777
0.218486988819299
CBWD1	3.91935422268256	2	0.14090390985118	0.216423607445441
C9orf66	4.11035679403513	2	0.128069986235881
1.10254177472557
DOCK8	0.396403302261186	2	0.820204441307743
0.0942061569379509
KANK1	9.14836437169384	2	0.0103147312628036
0.703764437122021
SMARCA2	7.39982003515497	2	0.0247257512532385
0.329484595262533
PUM3	0.128363289637689	2	0.93783463125624
0.0715721403670535
RFX3	0.19641611243044	2	0.906460289436343
0.267916535141162
RFX3-AS1	0.324026724518288	2	0.850429840800394
0.569232152751199
SLC1A1	0.969175432078244	2	0.615951091177969
0.402720025050412
SPATA6L	0.0772466038721836	2	0.962113067021935
0.242225159279904
PLPP6	2.48940143515213	2	0.288027096680421
1.29579132361345
CDC37L1-DT	4.24305733923949	2	0.11984827998291
1.19961285027729
CDC37L1	0.445734491627283	2	0.800221074941159
0.233059276295153
AK3	1.09114045099995	2	0.579511237020963
0.113147122458825
RCL1	0.648776520558999	2	0.72296948754509
0.231881718341472
JAK2	1.36855700113443	2	0.504454061626062
0.474023145539737
RLN2	0.496470457172086	2	0.780176401899908
0.446128146572665
PLGRKT	6.24468130293595	2	0.044053932745109
0.864689033801941
RIC1	1.04566471195168	2	0.592839036586922
0.473268965927634
KIAA2026	0.00512292204190204	2	0.99744181672113
0.0147421921037955
RANBP6	7.55140978390131	2	0.0229209281684319
0.764430660342441
UHRF2	4.04708861783899	2	0.132186125365812
0.23069494411759
AL354707.1	1.00675844082206	2	0.604484518056339

0.463919239479554
KDM4C 1.52087905682855 2 0.467460919493341
0.451051097612915
DMAC1 0.825188113806594 2 0.661930934588886
0.080924642846435
AL159169.2 0.219382290861915 2 0.89611086049404
0.472789350994978
ZDHHC21 0.00239832765316236 2 0.998801554883048
0.011861473289668
SNAPC3 2.21155836907812 2 0.330952908361052
0.398458616656848
PSIP1 4.07732148345217 2 0.130202969567966
0.180034302497573
CCDC171 0.825432691284679 2 0.661849992838795
0.404178475493863
CNTLN 0.427892443923081 2 0.807391803802659
0.325921782584736
RRAGA 0.0246367118422797 2 0.987757204447094
0.0207263997605802
HAUS6 0.697830472506797 2 0.705452924568662
0.321124764317233
PLIN2 7.19791087556784 2 0.0273522786871518
0.386463351217435
DENND4C 0.00724944198863825 2 0.996381840376717
0.0587227962189078
RPS6 73.8130662016702 2 1.11022302462516e-16
0.279730420267884
FOCAD 3.78734594443546 2 0.150517944067257
7.63659745122253
HACD4 6.84924332854845 2 0.032561597874762
0.587385280063322
KLHL9 1.20834329124647 2 0.546526957183008
0.325957688686988
MTAP 0.870920220442962 2 0.646966922491625
0.162508882368714
CDKN2A 5.26930430638707 2 0.0717439209624702
1.08700206299648
TUSC1 1.03227972507279 2 0.596819914089774
0.598271747545996
CAAP1 0.176983758761858 2 0.915310543594074
0.0998842448490695
PLAA 1.03457751815271 2 0.596134623494876
0.300478357144733
IFT74 3.21699116948008 2 0.200188554364859
0.789946083667731
C9orf72 1.1028920720819 2 0.576116123077073
0.468272906471838
ACO1 0.795896722004175 2 0.671696712511592
0.625638891289125
DDX58 0.0269587390011762 2 0.986611070386882

0.079722509161307
TOPORS	0.838447690535531	2	0.657556987772582
0.192868688529496
SMIM27	1.6935381842873	2	0.42879810345691	0.177391985079411
NDUFB6	2.5530856337114	2	0.279000189805179
0.0986418758073277
APTX	0.00991423147900607	2	0.995055150481995
0.025837240619471
DNAJA1	1.52614746400626	2	0.466231152700726
0.118396923805683
SMU1	7.61637715676042	2	0.0221883350060266
0.589769902462743
B4GALT1	3.33425402019016	2	0.188788675204825
0.667217655898749
B4GALT1-AS1	1.89269018005159	2	0.388157113342701
1.45058039186138
BAG1	4.89131309757163	2	0.0866692136509968
0.323892954124345
CHMP5	0.807092774756878	2	0.667947041768134
0.070446844697268
NFX1	2.96843448032133	2	0.226679707020835
0.314115384486404
AQP3	0.279499447963711	2	0.869575842146042
0.435980556748731
NOL6	0.350778879214266	2	0.839130166605943
0.553014867267796
UBE2R2	11.042481067804	2	0.00400088163298629
0.303922622597736
UBAP2	0.37064410675434	2	0.830836667006498
0.131893499772924
DCAF12	4.51407898780777	2	0.104659872747562
0.337677084969831
UBAP1	0.870587139525868	2	0.647074677632108
0.180200119459397
NUDT2	0.00441060119285192	2	0.997797129292394
0.0228883662022893
FAM219A	0.895259661594236	2	0.639141230647957
0.661903311912887
RPP25L	3.68198747086668	2	0.158659681994105
0.389891289415233
DCTN3	4.34449523564816	2	0.113921277448443
0.238611213534491
SIGMAR1	0.695830434340704	2	0.706158743812958
0.252335759755799
GALT	1.82597822762618	2	0.401322829762099
0.358609183661781
IL11RA	3.69561876854771	2	0.157581990073169
2.43059975299924
AL162231.1	0.299797717193443	2	0.860795034040097
0.326110541699401

DNAJB5	0.568734973392838	2	0.752490063801773
0.744618818841364
VCP	23.6323037498963 2	7.38431889091284e-06
0.276538477930721
FANCG	0.0531964247006651	2	0.973752404626836
0.147774091669616
PIGO	2.50649577138209 2	0.285575771530761
0.815304157347466
STOML2	0.845479865289429	2	0.655249019834476
0.102581808890196
FAM214B	1.25466183320928 2	0.534015231722068
1.02096876080306
TESK1	1.66168325094519 2	0.435682450525689
0.608816966506613
CD72	14.1044038527199 2	0.000865501087923826
0.685294839451415
SIT1	0.00720690952227961	2	0.99640302989058
0.00658305737226898
CCDC107	3.82987474113083 2	0.147351060810804
0.280920516179109
TLN1	0.273346379730102	2	0.872255241409787
0.0641796144701738
CREB3	0.549904114735874	2	0.759608539984748
0.172553369767219
GBA2	5.51723305772694 2	0.0633793912922694
0.934214635390799
RGP1	0.10595713535664 2	0.948400338685619
0.0997480892516889
MSMP	6.17045872753748 2	0.045719546290083
1.63107333607811
AL133410.1	0.147646634842881	2	0.928835788446693
0.312982765566849
HINT2	2.8067813228249 2	0.245762253042254
0.256053355852367
RECK	1.65784848906779 2	0.436518621124266
1.17089137173833
GLIPR2	4.10718446367087 2	0.128273287581058
2.25769498623612
CLTA	3.66336972516144 2	0.160143520517531
0.0890316767799801
GNE	0.045294823033965	2	0.977607116023051
0.186259447644066
RNF38	0.113914122142306	2	0.944634630094868
0.179046632055939
PAX5	2.89710615243611 2	0.234909939087471
0.211500459784598
AL161781.2	0.498277328366661	2	0.77947188105879
0.213119449020905
EBLN3P	3.05376202245349 2	0.217212093966578
0.233242249179793

ZCCHC7	18.6212403414766	2	9.04584281061771e-05	0.256638302190424
GRHPR	27.2068793714234	2	1.23623549674878e-06	0.275215360715562
ZBTB5	3.46809687096173	2	0.176568136346427	1.10327682924142
POLR1E	0.00293730248175729	2	0.998532426699584	0.0199497584788272
FBXO10	0.65704689080159	2	0.719986047666481	0.362742426810726
TOMM5	3.88595824095184	2	0.14327647546335	0.447163219580748
TRMT10B	1.3584722720578	2	0.507004126673356	0.239644594988741
EXOSC3	7.21387696865926	2	0.0271347934257525	0.347214938431325
DCAF10	0.0290819473177499	2	0.985564235732274	0.0516542380381388
SLC25A51	0.717576869665129	2	0.698522118668807	0.601170268818502
ALDH1B1	0.00565527397116844	2	0.997176357014463	0.0625639572817722
CBWD6	2.6765067190002	2	0.262303418475671	0.881646582199083
AL845472.1	3.59430963134625	2	0.165769863715937	1.24602626787105
AL590399.3	0.700144355749368	2	0.704637228665626	0.769760920870447
AL590399.1	0.0094126628824073	2	0.995304725983178	0.0488153667922624
LINC01410	2.80807144783235	2	0.245603772148523	0.684851212823427
AL512625.1	0.159177381775285	2	0.923496110646567	0.142406206774187
CBWD5	4.2866245925678	2	0.117265780024127	0.350665038102417
CBWD3	0.540530287846264	2	0.7631771157338	0.18796699028869
AL161457.2	0.19050988643037	2	0.909141125558826	0.297726227506996
FAM122A	3.94792428705128	2	0.138905401328218	0.477148899934906
FXN	0.24021324998766	2	0.886825873872536	0.175232917441339
PTAR1	0.890275832314492	2	0.640735902104935	0.28893335965129
SMC5	3.0289454906239	2	0.219924110302129	0.310420170350937
ABHD17B	4.9869100422557	2	0.0826240051747504	0.719511403694659
C9orf85	0.526435984091369	2	0.768574335956547	

0.222282795117667
ZFAND5 1.88598086828674 2 0.389461433435613
0.266401945812794
C9orf40 4.12784328800339 2 0.126955119594265
1.01529337550061
CARNMT1 0.0633302918438531 2 0.9688309447619
0.135998080268892
NMRK1 3.94754980389107 2 0.138931412630171
0.914956194710861
OSTF1 0.590604410804033 2 0.74430661999311
0.0732210368996217
RFK 0.564782036608202 2 0.753978807371076
0.0923767442058329
GCNT1 5.60270082335234 2 0.0607279994286406
0.910590705667509
VPS13A 2.46388911789883 2 0.291724749799404
0.293210623876227
CEP78 1.65553313887638 2 0.437024260487329
0.690488957188242
PSAT1 2.49500817729611 2 0.287220780579317
0.520445490204168
TLE4 7.6021882757418 2 0.0223463085330411
0.508958455901694
TLE1 0.865324957088015 2 0.648779431832869
0.469072075503619
IDNK 1.03310321655141 2 0.596574226616871
0.447683380461762
UBQLN1 7.86014885413285 2 0.019642210586549
0.413672882691955
GKAP1 0.912854177963406 2 0.633543199989451
0.312534407778223
KIF27 0.0475715536276763 2 0.976494875196501
0.135275802726353
C9orf64 0.141417656431026 2 0.931733147037876
0.16636146068917
HNRNPK 19.0020676709044 2 7.47744853999421e-05
0.200683534285019
RMI1 1.54397155806516 2 0.462094538968358
0.321443449979859
AGTPBP1 0.321595513465802 2 0.85146425660796
0.385097320789595
NAA35 4.00884891806788 2 0.134737820519396
0.738558027528994
ISCA1 0.32960874611871 2 0.8480595911647 0.146179253392812
TUT7 3.70840488461339 2 0.15657777269598 0.360938305729885
SPIN1 0.305098395714863 2 0.858516655737357
0.279576497861865
CKS2 1.38810537412656 2 0.499547451671983
0.162854553983296
SECISBP2 0.484334928880495 2 0.784924719566069

0.111585962050111
SEMA4D	3.3946582827706	2	0.183172098082356
0.332071923698455
GADD45G	12.5853009960502	2	0.00184985041850116
9.06575254987798
SYK	2.97618538414079	2	0.225802920786188
0.189158479847103
AUH	0.0999738895154847	2	0.951241843112357
0.113987531865593
SPTLC1	1.56021492118336	2	0.458356753370709
0.16187719495938
IARS	14.2755579931578	2	0.00079451475829917
1.14558386703971
NOL8	0.15087675099551	2	0.92733687544658	0.0728150477803775
CENPP	2.89977700202537	2	0.23459644390152	1.01316149469004
IPPK	4.12441945113755	2	0.127172642541299
1.12339214442115
BICD2	0.146015027791907	2	0.92959384512812
0.120919594121616
FGD3	0.83324004832861	2	0.659271379550202
0.234576352585215
SUSD3	26.7567403449083	2	1.5482734169181e-06
0.405080636938085
CARD19	0.941261383192558	2	0.624608208882478
0.122071667913156
NINJ1	0.148893479788436	2	0.928256911803757
0.103278469551599
WNK2	4.5298211217225	2	0.103839321424962
1.92862426198984
FAM120AOS	4.31623181791394	2	0.115542608884762
0.376114898756266
FAM120A	1.19684294417183	2	0.549678634691891
0.239792345265122
AL158152.1	1.84584914822519	2	0.39735524524668
1.49472140692802
ZNF169	5.33086277913369	2	0.0695693355734783
1.45534645326521
AL691447.2	2.66845501316783	2	0.263361541953484
1.3254497594695
MFSD14B	0.137025416024742	2	0.93378159352008
0.126350758631134
C9orf3	1.90911455064862	2	0.384982548103023
0.366772822517708
FANCC	3.15518330540653	2	0.206471755597527
4.50257195648215
AL161729.1	0.0588125852590267	2	0.971021865283702
0.253576210324679
LINC00476	1.79376381311608	2	0.407839360424301
0.375875381639136
ERCC6L2	0.290714813298379	2	0.864713183633668

0.217642973442956
SLC35D2 3.05375483049448 2 0.21721287505822 1.23383617701186
HABP4 4.59925250365553 2 0.100296322285732
0.914655387533997
AAED1 4.80921863166459 2 0.0903007677995037
0.936963236987245
ZNF510 0.146556484079765 2 0.929342211975445
0.349890491089153
ZNF782 0.0095555851642201 2 0.995233602913208
0.0861096532393756
MFSD14C 0.20964436882882 2 0.900484628552716
0.145495735513079
TDRD7 2.67999722897311 2 0.261846031371271
0.927864297056818
TSTD2 0.251918247611398 2 0.881650884579731
0.250767626215418
NCBP1 0.161652313856098 2 0.922354022364668
0.148817471609191
XPA 9.10411331968219 2 0.0105454935724935
0.525004223194511
TRMO 10.8898666227091 2 0.00431812796813624
1.00609895706892
ANP32B 46.8552527833089 2 6.69131416941582e-11
0.493765311708933
NANS 68.3105226556534 2 1.4432899320127e-15
0.758380239165309
TRIM14 5.35056384977202 2 0.0688874045830788
0.77159792929566
CORO2A 0.137076941005196 2 0.933757537290778
0.286819878471178
TBC1D2 0.782180511807895 2 0.676319111491751
0.507716411545051
TGFBR1 5.7526101674458 2 0.05634255974272 2.32717446766842
ALG2 8.73422908956318 2 0.0126877978729906
0.412477185958901
SEC61B 25.0904568960729 2 3.56185725591818e-06
0.209081378013664
STX17 0.736574629385849 2 0.691918354817514
0.278255335035218
ERP44 45.7768903624239 2 1.14729337141739e-10
0.814911868425651
INVS 0.00110025681464532 2 0.999450022885565
0.0221765595221462
TEX10 1.08690971709029 2 0.580738413448328
0.291626707538409
MSANTD3 0.616258243133003 2 0.734820430718091
0.491638321313856
MRPL50 3.34201842655005 2 0.188057180034772
0.278281174474748
ZNF189 3.06751527495245 2 0.215723531588985

0.414162633676377
RNF20	0.262309435890138	2	0.877082063558333
0.132313588733852
SMC2	0.424716981749897	2	0.808674743080391
0.171671298317056
NIPSNAP3A	11.572788623039	2	0.00306902820984067
0.906534126028453
NIPSNAP3B	1.79551400941635	2	0.407482617069932
1.18285791472249
ABCA1	0.613851567732868	2	0.735705200076553
0.539854408401493
SLC44A1	0.451969806981664	2	0.797730144515212
0.250364792556589
FSD1L	0.604192734154254	2	0.739266820474073
0.524735292479107
FKTN	0.173681926431366	2	0.916822892603932
0.340860644134474
TMEM38B	5.54919195589481	2	0.0623746723426807
1.53034937413539
RAD23B	1.1441591704644	2	0.564350602358334
0.0762994994843117
KLF4	1.15286021295005	2	0.561900716079613
1.06125288174803
ELP1	1.2808679907987	2	0.527063631208012
0.78395801897396
FAM206A	8.11153778633268	2	0.017322156174244
1.35506241807324
CTNNAL1	0.0497970760092937	2	0.975408873938446
0.114886383036321
TMEM245	1.93521187755815	2	0.37999167333463	0.564146806604974
AKAP2	1.01564903304357	2	0.601803369044949
0.928938330967549
TXN	14.5517256241361	2	0.000692042760193856
0.556799708110784
ECPAS	5.23793136497378	2	0.0728782030596067
2.14666892608459
DNAJC25	3.35487450315365	2	0.186852218198049
1.18159820094838
GNG10	0.3757120515882	2	0.828734014965564
0.307553499054042
UGCG	12.1459836520297	2	0.00230426891584024
0.695701234563884
SUSD1	3.97247289045565	2	0.137210855482487
4.7798444849231
PTBP3	10.5202502777185	2	0.00519465462487623
0.825191883010205
HSDL2	2.86568100997435	2	0.238630128571453
0.358013591224557
INIP	0.131159961496252	2	0.936524139862769
0.0483497090790484

SNX30	0.00319120213758069	2	0.998405671225813
0.0324155059606248
FKBP15	0.0550085786905777	2	0.972870509566967
0.0692013039428845
SLC31A1	0.0421072395448825	2	0.979166460479051
0.0851470648599597
CDC26	0.415560015036565	2	0.812385735809122
0.0780592025007578
PRPF4	0.465986909353992	2	0.79215875844229
0.102751875095649
HDHD3	0.349867600854417	2	0.839512594304811
0.204824940754356
ALAD	1.00566763649008 2	0.604814295144186
0.383375746644435
POLE3	0.934325873258703	2	0.6267779570043
0.150908491941461
RGS3	2.25702123129064 2	0.323514735565157
0.40521473618757
AKNA	1.86857187052664 2	0.39286629736554 0.184693657589893
ATP6V1G1 48.5834217259766 2	2.8199664825479e-11
0.481287107490596
TMEM268	0.582912416167758	2	0.74717473309978
0.501224813422987
TNFSF8	0.832813701553281	2	0.659411933644236
0.488498797677449
TRIM32	3.36201674078266 2	0.186186136761956
0.808688704310949
CDK5RAP2 3.89485233317749 2	0.142640733005808
0.663195843924367
MEGF9	0.258884656175257	2	0.878585256647098
0.226214130504247
FBXW2	0.000287973617084783	2	0.999856023557061
0.00318191229596674
B3GNT10	1.97128060726264 2	0.373200188980178
1.75365701166499
PSMD5	7.20021666771328 2	0.027320762523395
0.444907719841945
PHF19	13.6121526311873 2	0.00110702801815044
1.07960639895806
TRAF1	2.22453718233613 2	0.328812173924383
0.597387133633149
CNTRL	3.95229466656502 2	0.138602198067044
0.221390711799592
RAB14	0.0934912953244261	2	0.954330102815414
0.0360007244084854
GSN	7.55248989730545 2	0.0229085529095285
0.670837434809243
STOM	0.185522783023136	2	0.911410944740929
0.319034473246328
AL359644.1	0.199306379229893	2	0.905151279471376

0.445030020071845
TTLL11 0.311890900255004 2 0.85560586228873
0.365798509929727
NDUFA8 2.47730640219991 2 0.289774222855606
0.178875168171581
RBM18 0.350297659241128 2 0.839332093995959
0.144596967555703
MRRF 0.583201818775253 2 0.747066623763743
0.105407910515386
PTGS1 3.07871232996632 2 0.214519171929568
1.30788774371454
PDCL 0.000769769653875213 2 0.999615189231726
0.00650817173665824
RC3H2 5.6875299028468 2 0.058206109738875
0.668546975619678
ZBTB6 1.73667472714533 2 0.419648692742195
0.994888997695704
ZBTB26 1.12691110492925 2 0.569238627394411
0.299919841712361
RABGAP1 3.06799213006424 2 0.215672103285805
0.44215125724957
STRBP 4.46588637009464 2 0.107212419335305
0.150445602575108
CRB2 3.93769908671986 2 0.139617387605783
1.89076840315354
DENND1A 0.785480855448997 2 0.675203989077105
0.734504533068719
NEK6 5.82634800063656 2 0.0543030979969837
0.357178470138453
PSMB7 13.2959650742832 2 0.00129663538220959
0.31544940432782
NR6A1 0.2304905798151 2 0.891147527601082
0.319496001410155
RPL35 25.722291466925 2 2.59702049509869e-06
0.192667130467918
ARPC5L 21.702465509783 2 1.93807015029801e-05
0.555834485009155
GOLGA1 0.0289939642295713 2 0.985607593178477
0.0925907207866512
SCAI 0.228059553886022 2 0.892231387563669
0.198559968021742
PPP6C 4.72812460934761 2 0.0940374375047662
0.289253328102252
RABEPK 0.00118587476772176 2 0.999407238368771
0.0162997693100817
HSPA5 11.028467281165 2 0.00402901382831899
0.252179077110633
GAPVD1 1.13831211260204 2 0.566002911782 0.180480387209029
MAPKAP1 2.64265484913523 2 0.266780935252719
0.356326004521582

PBX3 1.36096914710316 2 0.506371558640458
0.720772720505822
ZBTB43 5.56946777932357 2 0.0617455179655764
0.664561968903404
RALGPS1 5.23553581270254 2 0.0729655471309181
1.17601628052989
SLC2A8 2.90076807939582 2 0.234480221086979
0.590861200911062
RPL12 52.4218162249351 2 4.13757916817303e-12
0.18795693956035
LRSAM1 3.02732993746815 2 0.220101831617234
0.976695145080707
PTRH1 2.29409609413045 2 0.317572847168718
0.549086368797253
TOR2A 1.79141226415983 2 0.408319169553851
0.16353109125427
SH2D3C 0.0218721768468187 2 0.989123493197174
0.0526547853666895
CDK9 0.839824742169378 2 0.657104398637934
0.193719576043879
FPGS 0.33530837701191 2 0.845646218309123
0.123478969820979
ENG 0.838863368109937 2 0.657420336126975
0.673221066260311
ST6GALNAC4 7.50505913602743 2 0.023458331296603
0.32228322946497
DPM2 0.54863981115352 2 0.7600888796917 0.15546251310496
FAM102A 3.49597802757886 2 0.174123752066745
0.375476128353966
NAIF1 0.303964911455528 2 0.859003351197568
0.132763355958455
SLC25A25 1.93466801160643 2 0.380095019652161
0.861651193476137
PTGES2 0.440234280024767 2 0.802424796406326
0.144857566273276
C9orf16 2.43146335528539 2 0.296492997613032
0.187919477529983
CIZ1 0.225704725179773 2 0.89328253230071
0.134808416038625
GOLGA2 2.18339320951115 2 0.335646550825223
0.409811084122574
SWI5 2.91365326251108 2 0.232974416644115
0.404760284570457
TRUB2 0.144792799449867 2 0.93016210671884
0.0738778089921891
COQ4 0.23188891062064 2 0.890524685841096
0.0999432195198631
SLC27A4 11.603584680997 2 0.00302213319647648
1.07525737231331
URM1 8.11062138051553 2 0.0173300950552562

0.276233476796571
ODF2	7.20341013042092 2	0.0272771734145729
0.686660790869839
GLE1	2.93590092060272 2	0.230397209840418
0.41684180812832
AL356481.1	0.416497178456168	2	0.812005155885377
	0.708497627747187
SPTAN1	1.55424836392229 2	0.459726200984436
0.308911927804916
WDR34	0.770530849998977	2	0.680270051561721
0.158749883154687
HMGA1P4	4.6416303090682	2	0.0981935100868683
0.942869290055755
SET	8.33187338481452 2	0.015515175144774
0.19343571104019
PKN3	2.09980100848101 2	0.349972568165425
2.1890862945447
ZDHHC12	13.3869540469451 2	0.00123896735632678
0.653926692184398
AL441992.1	0.034219711885457	2	0.98303568639083
0.0606256405533047
ZER1	3.11373759296444 2	0.210795081214923
1.77252818276437
TBC1D13	4.2973135250215	2	0.116640728793613
1.2942510320371
ENDOG	5.13189903626832 2	0.0768461799477967
1.14008199814866
SPOUT1	4.09323019791616 2	0.129171396823532
0.495151704509377
KYAT1	0.143121455790982	2	0.930939741866879
0.220269443783939
LRRC8A	0.0641727244188462	2	0.968422943322594
0.188352404657764
PHYHD1	6.01935802204686 2	0.0493075033856101
1.52057693856189
DOLK	1.15196750714524 2	0.562151578077424
0.613897725005801
NUP188	0.00393434367790707	2	0.998034761775443
0.0331192320037115
SH3GLB2	0.978649596905853	2	0.61304018015229
0.361501284727203
MIGA2	0.000253497263473367	2	0.999873259400532
0.00740890002581513
DOLPP1	4.2369307816803	2	0.120215971559129
1.72153954663751
PTPA	1.09525292698507 2	0.578320848278987
0.284248357029006
NTMT1	4.61269805699597 2	0.0996243139222247
0.206026690402274
ASB6	1.19463985359546 2	0.550284464215021

0.397547437576584
TOR1B	0.101711966056064	2	0.950415536644368
0.210077468846786
TOR1A	5.29041242422349	2	0.070990713085289
0.259398114815385
C9orf78	1.13294768116349	2	0.567523091502726
0.136515250039158
USP20	0.0155834260974111	2	0.992238563660569
0.099746010984616
FNBP1	22.4895664857441	2	1.30753308689169e-05
0.308964599117724
GPR107	0.37438793333812	2	0.829282867548785
0.28973191547058
FUBP3	2.89729124500841	2	0.234888200050978
0.424589772793936
EXOSC2	0.000741033011835135	2	0.999629552126846
0.00947140125300901
ABL1	1.36111408199098	2	0.506334864517515
1.10099477142305
NUP214	1.83679483269115	2	0.399158213212338
0.337716124562456
AL157938.3	3.36258753690054	2	0.186133007181824
0.599134030323688
FAM78A	0.241064238650588	2	0.886448614756707
0.162101627709434
PRRC2B	6.87959664653808	2	0.0320711526809123
0.563860598520616
POMT1	2.99050865849866	2	0.224191579003193
1.17875068321866
UCK1	0.815940362631396	2	0.664998707913294
0.275880503376131
RAPGEF1	7.36752806775481	2	0.0251282131584208
0.641132111697851
MED27	0.00117116700243577	2	0.999414587919339
0.00972228202580518
SETX	1.80642372337244	2	0.405265909081889
0.263942788303579
TTF1	0.764179512565605	2	0.682433797735456
0.204048222892586
DDX31	0.00195673359675554	2	0.999022111646374
0.027236136960073
GTF3C4	1.0285566753406	2	0.597931943914894
0.750893120658025
SPACA9	0.0700764107908355	2	0.965568525625536
0.23940733519534
TSC1	4.17040772177785	2	0.124281781388643
0.368703196257263
GFI1B	2.62123691599428	2	0.269653235604167
1.80942128731312
GTF3C5	2.41095861528356	2	0.299548389267193

0.371129269543186
RALGDS	1.7371242368371	2	0.419554385263359
0.815551637251235
GBGT1	0.458687098456272	2	0.795055345930671
0.401873002447045
SURF6	0.273587965871593	2	0.87214988538397
0.0477723377368382
MED22	0.260829655195808	2	0.877731248243957
0.286237909784902
RPL7A	17.5417166068176	2	0.000155190323086085
0.188891553251046
SURF1	0.158418719507044	2	0.923846487923878
0.0633008964604578
SURF2	0.178965940272325	2	0.914403837164549
0.0881364303536385
SURF4	8.16441598083349	2	0.0168701753140778
0.319443297850329
REXO4	2.59237871564749	2	0.273572295410946
0.289979626107852
CACFD1	1.92797672018081	2	0.381368812566855
0.900938055434577
SLC2A6	1.36231560878582	2	0.506030768418125
0.249916009608291
VAV2	1.67051724838194	2	0.433762285505163
0.726281687051119
BRD3OS	0.925045043291434	2	0.629693225624173
0.191975278231292
BRD3	0.469163099463975	2	0.790901733437027
0.209998641093412
WDR5	1.45202591441415	2	0.483834217287233
0.420632351248615
FCN1	12.6984705791123	2	0.00174808340290233
0.970874850607523
MRPS2	0.687051738095195	2	0.709265132893616
0.169982060825592
UBAC1	1.55640213076326	2	0.459231395932128
0.296051121000911
TMEM250	1.29533370772511	2	0.523265207850934
0.682205055593224
QSOX2	1.61678089763691	2	0.445574664595224
0.459959971999773
SNAPC4	1.57195526851812	2	0.455674001440404
0.662996063108311
ENTR1	0.41429986241994	2	0.812897762105346
0.253938670952896
PMPCA	0.760111088775179	2	0.683823425604083
0.0641730213106432
INPP5E	0.0976742687997152	2	0.952336219878722
0.160056397526014
SEC16A	0.196371144360517	2	0.906480670550306

0.241724707782723
AGPAT2	1.29215544421096 2	0.524097406271438
0.306324241532581
SNHG7	14.923774478548	2	0.00057457079505685
0.402678184719661
TMEM141	5.15164101313654 2	0.0760913637143538
0.619389368428452
RABL6	2.96382261772477 2	0.227203017979614
0.367718119157879
PHPT1	2.70439950630608 2	0.258670622826139
0.147459501495718
EDF1	4.7337623763772	2	0.0937727301872399
0.0850948728047616
TRAF2	0.559661698835548	2	0.755911593528451
0.129949279682577
FBXW5	1.05496554296608 2	0.590088489288836
0.0724785962304211
PAXX	4.35864963122718 2	0.113117880289099
0.623438173521571
UAP1L1	2.38862759483474 2	0.302911741701288
0.737919668041246
MAN1B1-DT	2.49232628385971 2	0.287606186689211
0.983298986487565
MAN1B1	0.489195326723126	2	0.783019512313229
0.226958119382126
DPP7	0.000970900527956974	2	0.999514667547936
0.00266584184520761
ANAPC2	2.76931549186417 2	0.250409488793339
0.536279420853096
SSNA1	3.04425744517928 2	0.218246805208469
0.182814450689458
TPRN	0.0674834259194339	2	0.966821189774491
0.242229738421355
TMEM203	0.958014166111495	2	0.619398097446008
0.196009959908653
TUBB4B	2.16118407788923 2	0.33939453137243 0.226543160384686
NELFB	0.727095296441649	2	0.695205601116966
0.219446275432437
TOR4A	1.85053671117272 2	0.39642502193029 0.604733998302646
EXD3	0.00577625768761864	2	0.997116037788078
0.0472690070091969
NOXA1	0.0260890850393612	2	0.987040168784968
0.144831977737956
NSMF	1.86655522015323 2	0.393262634132772
0.669661253858338
PNPLA7	2.42412320628261e-05	2	0.999987879457423
0.00463738051178051
MRPL41	10.3167343992342 2	0.00575108239305899
0.458525183699874
DPH7	3.50615388134636 2	0.173240073100804

0.381975731749676
ZMYND19 0.522445757894531 2 0.770109259345057
0.386175210308072
ARRDC1 1.72872619350315 2 0.421319807145963
0.227065382201712
ARRDC1-AS1 1.51351024425651 2 0.469186412199736
0.440910616488699
EHMT1 6.90155665155336 2 0.0317209375440942
0.24655835465566
BET1L 0.744721262001788 2 0.689105684835268
0.386238288059752
RIC8A 15.4325606092271 2 0.000445514704611627
0.615421544226205
SIRT3 1.78607322381607 2 0.40941064201924 0.719744637235535
PSMD13 3.57956261263858 2 0.166996686793964
0.118022634698391
IFITM2 7.01669463998365 2 0.029946365321711
0.426426384597412
SIGIRR 0.0728938955326156 2 0.964209245993692
0.0890881641085464
ANO9 1.15759032371318 2 0.56057336002115 1.05929319296257
PTDSS2 0.847182002714971 2 0.654691595131629
0.417015407394103
RNH1 5.72658687065701 2 0.0570804595554982
0.295456971139016
HRAS 0.0955052770109744 2 0.953369584837988
0.0939724778806474
LRRC56 0.818550398964894 2 0.66413143854283
0.773183964142898
LMNTD2 2.24932001903197 2 0.324762864873852
2.28099083996175
RASSF7 0.207155705918829 2 0.901605827327548
0.131583722755797
PHRF1 0.0578350598556557 2 0.971496580556083
0.113495340953527
IRF7 3.69751303679602 2 0.1574328094512 0.29165288645669
DEAF1 4.88603528439705 2 0.0868982276501901
0.786858651646464
EPS8L2 0.014651247251759 2 0.992701143353388
0.05464760457467
TMEM80 0.016403194697108 2 0.991831943990822
0.0309724269096413
TALDO1 12.4796770365473 2 0.00195017041430501
0.18158458383687
GATD1 2.11626724487587 2 0.347103031472339
0.368116783131551
AP006621.3 2.0276125326214 2 0.362835300136594
0.899889672641483
CEND1 1.13372943370384 2 0.56730130354213 0.821705779811664
SLC25A22 0.0103786103395047 2 0.994824136014134

0.0508136288099344
PANO1 0.00392377527552926 2 0.998040035605852
0.0436709475923537
PIDD1 0.153408383758718 2 0.926163779856818
0.215407285295325
RPLP2 58.7488691885381 2 1.74971148680925e-13
0.227137407709659
PNPLA2 4.7992426004136 2 0.0907523146654751
0.314169152283642
CRACR2B 0.00163123480383066 2 0.999184715123547
0.0414346939414267
CD151 0.389110121672448 2 0.823200850881423
0.375859057084281
POLR2L 10.9116976975885 2 0.00427124959739422
0.204777580487688
TSPAN4 1.46814387829783 2 0.479950675781439
0.972933952562784
CHID1 0.467779901297368 2 0.791448909542291
0.159051355608655
AP2A2 0.194099540675722 2 0.907510837888249
0.118195779689051
TOLLIP 2.59626249379181 2 0.273041563837742
0.167768745973999
MOB2 0.0413093509613825 2 0.979557171279872
0.0488582186426626
CTSD 13.3369059163009 2 0.00127036253559376
0.978636532286192
LSP1 41.9415723765296 2 7.80734366045976e-10
0.451410502066698
MRPL23 2.50068131565259 2 0.2864072133811 0.156772521229493
CD81 23.759979109903 2 6.92765244314497e-06
0.481702967714878
TSSC4 10.4734167717383 2 0.00531773198110785
0.529777716892065
KCNQ1OT1 1.50245174692754 2 0.471787845901987
0.363626235682187
NAP1L4 0.0743999593993019 2 0.963483438953271
0.0320834503031258
CARS 2.29196817398061 2 0.317910911810734
0.274560605504935
ZNF195 0.61562682933245 2 0.735052455222474
0.0954980886073006
NUP98 0.140262133778689 2 0.932271621956557
0.13561128504478
PGAP2 0.915514803778218 2 0.63270094964557
0.436966622967573
RHOG 3.08111316108886 2 0.214261814276363
0.197368888749833
STIM1 1.59519765681153 2 0.45040917643088 0.371636437253471
RRM1 0.555862659611059 2 0.757348827019934

0.186539303180379
TRIM21 0.0971083052861036 2 0.952605751789802
0.0799887245563486
TRIM68 1.46910600224206 2 0.479719845289167
0.785122100777567
TRIM5 0.0491456671521543 2 0.975726620671304
0.0798956681576042
TRIM22 9.19281443713999 2 0.01008801474614 0.606901689375154
FAM160A2 0.30471197574498 2 0.858682545752772
0.223661522052935
SMPD1 2.69005866270884 2 0.260532065989538
0.411866653761248
APBB1 5.38895786404957 2 0.0675775851172078
0.975304127344784
TRIM3 2.07042232726283 2 0.35515137795161 1.04625886353287
ARFIP2 1.55557504904495 2 0.459421346151536
0.249469580362947
TIMM10B 2.28659344264604 2 0.318766403675263
0.184862893705365
DNHD1 0.194309428307295 2 0.907415605235179
0.296023310047733
RRP8 0.56428635888062 2 0.754165695780166
0.178747158971681
ILK 3.52386729062919 2 0.171712511507523
0.121158556522782
TAF10 6.21197142383582 2 0.0447803562663335
0.470862374757289
TPP1 2.47424621113623 2 0.290217944480908
0.180736783154611
MRPL17 0.186086606185569 2 0.911154043653756
0.108760139179087
ZNF215 1.61281117517686 2 0.446459946762543
0.590126141591382
PPFIBP2 2.88699163644799 2 0.236100948336906
0.750177931266426
EIF3F 21.8961409491699 2 1.75919266437452e-05
0.270335808644565
RIC3 1.12155459714949 2 0.570765236373184
0.317688051276174
STK33 0.0155703558222504 2 0.992245048097283
0.109729058472765
TRIM66 0.626653080651722 2 0.731011168965974
0.479972233232247
RPL27A 69.0813418472308 2 9.99200722162641e-16
0.291810122903939
AKIP1 0.484249699433224 2 0.784958169628796
0.158632366813684
TMEM9B 3.49613192777361 2 0.174110353742576
0.209057703782756
DENND5A 2.09311966721146 2 0.351143666284291

1.4346437338352
TMEM41B 0.422305076442026 2 0.809650554806087
0.195125599291673
IPO7 0.133219438423719 2 0.935560261290329
0.120182191543924
AC132192.1 0.0219597023413356 2 0.989080207382893
0.122773222326002
AC132192.2 6.4958472030923 2 0.0388548021809476
0.904635595100303
ZNF143 2.88351181814964 2 0.236512100116696
0.27821587956237
WEE1 2.79539949540159 2 0.247164852067475
0.270593380750777
SWAP70 38.2326545368121 2 4.98751884236981e-09
0.451742272392278
AC026250.1 0.162290527635815 2 0.922059739797538
0.401539103941038
SBF2-AS1 0.0243208697659601 2 0.987913204407784
0.0631813977443452
SBF2 3.29505800662443 2 0.1925250501036 0.944343971745354
AMPD3 0.338571051632824 2 0.844267808712943
0.22141518253682
MTRNR2L8 0.297970518340392 2 0.861581815236252
0.33225311258683
RNF141 8.45089290456643 2 0.0146188066528617
1.00878277764871
CTR9 5.33356607372732 2 0.0694753658903499
0.512658122693806
EIF4G2 0.248176263844003 2 0.883301990350126
0.0428596240513296
ZBED5 0.236889501639692 2 0.888300892197097
0.091902320775709
ZBED5-AS1 31.0544608826869 2 1.80554991846549e-07
1.38265814337919
USP47 6.3322008974267 2 0.042167712867085
0.485449175211304
ARNTL 0.590034424761266 2 0.744518772415122
0.793636930101103
BTBD10 11.8435243626528 2 0.00268047253404968
0.818351266622013
FAR1 1.74226618596758 2 0.418477107030973
0.424136019696924
SPON1 3.40893132998479 2 0.181869539478611
0.534839371916175
RRAS2 28.4571364176068 2 6.61624138587058e-07
0.392525282609007
COPB1 5.87120015965334 2 0.0530988461977276
0.237806662523395
PSMA1 13.7109289749539 2 0.00105368209977197
0.19441707751178

CYP2R1 0.281568874275045 2 0.868676545919376
0.181487876717321
C11orf58 7.56025293796897 2 0.0228198052449952
0.14855618746216
RPS13 54.4485590648614 2 1.50190970771291e-12
0.236097172324265
PIK3C2A 4.19517645932993 2 0.122752121462704
0.836067596149878
NUCB2 0.141973318058371 2 0.931474318816402
0.140477746881064
KCNC1 7.30232285729991 2 0.0259609594602938
0.944323822440096
SERGEF 2.46685698107822 2 0.291292171265538
0.349876928319217
SAAL1 0.355843522401885 2 0.837007907420521
0.13009603189263
HPS5 0.268445859601484 2 0.874395114145442
0.183976949443847
GTF2H1 0.0625224770129074 2 0.969222341803592
0.0374832569151465
LDHA 4.84761447996538 2 0.0885837151677866
0.179956313105871
TSG101 3.22326973662453 2 0.199561091132612
0.208282041591506
UEVLD 4.72265389743102 2 0.0942950154928865
0.899273683179766
SPTY2D1OS 1.33016943517235 2 0.514229961540168
1.0861434549214
SPTY2D1 0.766333504119386 2 0.681699215059512
0.280553905350578
IGSF22 0.305642338716785 2 0.858283195422611
0.329213694150114
ZDHHC13 3.32179010333872 2 0.189968872029084
0.627954843882603
HTATIP2 1.175542237 2 0.555564192512757
0.32235135758122
PRMT3 0.429338692863452 2 0.806808170077649
0.359196002084522
FANCF 2.48153674099814 2 0.289161949046456
0.431720890259477
SVIP 0.887188235072973 2 0.641725833237424
0.303006367379167
CCDC34 1.35066506279545 2 0.508987138258752
0.369586457027456
LIN7C 0.201865441217207 2 0.90399385099606
0.193676915299938
KIF18A 4.0182283964417 2 0.134107414657495
1.13406494207689
METTL15 2.87134512847495 2 0.237955269979254
0.694589363975318

ARL14EP 4.07023326693134 2 0.130665241675268
0.292227162031626
DNAJC24 1.47540004417697 2 0.478212529898113
0.509703282793779
IMMP1L 2.41077533515485 2 0.29957584115869 0.323663988873363
ELP4 0.0163130206103467 2 0.991876663768841
0.029801260871341
EIF3M 7.08495324516544 2 0.0289415609266686
0.15689239400463
PRRG4 2.64482429808848 2 0.266491708336471
2.15959977456056
QSER1 2.39333412029454 2 0.302199748870764
0.887129521135321
TCP11L1 8.02175014149351 2 0.0181175341738782
4.61341218311532
LINC00294 1.01954095690779 2 0.60063342130425
0.791906053551896
CSTF3 1.93105592684062 2 0.380782107635431
0.294706419487316
CSTF3-DT 0.274340209363688 2 0.871821912528993
0.52097927529985
HIPK3 1.53248605574125 2 0.464755867278039
0.341542363510551
CD59 4.69229690640263 2 0.0957371892887667
0.40405838299228
FBXO3 4.84950401780346 2 0.0885000635489102
0.593235671982201
LMO2 136.847201652074 2 0 2.27346253588196
CAPRIN1 1.20011179137429 2 0.548780960747833
0.235920546822638
NAT10 3.37867989612542 2 0.184641356665791
0.588626802140118
CAT 17.7854729589207 2 0.000137383196831098
0.284287971723592
APIP 1.64519306143181 2 0.439289543591526
0.141710087881887
PDHX 0.0942494219886744 2 0.953968419821405
0.0792221680949158
CD44 0.545639195503436 2 0.76123010286459
0.187534462469082
SLC1A2 0.0827374490058562 2 0.959475282669053
0.224626178940786
TRIM44 2.10550632793901 2 0.348975638141087
0.312022088373191
COMMD9 8.19029746591166 2 0.0166532692087483
0.297183677630308
TRAF6 0.0572833419564384 2 0.971764613550294
0.11776204575831
API5 1.02107573093908 2 0.600172679821703
0.228707079607004

TTC17	3.90094235058014	2	0.142207051349944	0.396791076060415
HSD17B12	27.9357844851156	2	8.58660480718143e-07	0.553954849885825
ALKBH3	9.96510938658997	2	0.0068565238485212	0.723700093967372
ACCS	6.57089762018148	2	0.037423785151288	10.2606155923873
EXT2	1.90485932227082	2	0.385802514411506	0.682348797375343
CD82	37.5437002710838	2	7.03864511120145e-09	0.614900263705326
AC010768.1	0.000532126142893237	2	0.999733972320193	0.0235709219610337
PRDM11	1.28988104965485	2	0.52469374742979	1.1437438243682
AC103736.1	2.20025509784788	2	0.332828629057122	1.70171072643529
SLC35C1	0.0969055416488047	2	0.95270233358911	0.181737140696734
CRY2	3.50462536154354	2	0.173372524149058	1.79779267092176
PEX16	1.13800899189343	2	0.566088701884872	0.156742786478632
LARGE2	0.11797743190256	2	0.942717406766229	0.312792847793889
PHF21A	1.70324870434487	2	0.426721223141719	0.320183534073498
DGKZ	4.26768006907938	2	0.118381829598331	0.484318779710012
HARBI1	0.0400699078086931	2	0.980164412134872	0.132880692586071
ATG13	1.12282465819085	2	0.570402898088053	0.392699722589567
ARHGAP1	1.08014511882267	2	0.582705970037852	0.346482099795968
ZNF408	0.0743393442636783	2	0.963512640235441	0.102739461365973
CKAP5	0.0372882536506159	2	0.981528599795842	0.0778545530993595
C11orf49	5.1309567197773	2	0.07688239519	0.501718251627983
ARFGAP2	0.00404442749008636	2	0.997979829551613	0.00951152214827353
DDB2	5.94008482429884	2	0.0513011344943969	0.588672555717817
ACP2	10.1795896497154	2	0.00615928348940098	0.954519150890789
NR1H3	1.8823848503504	2	0.390162318495678	0.765421684892609
MADD	0.0274421239886812	2	0.986372642711167	0.083674199029499

SPI1	1.99872191359443	2	0.368114607160378	0.107467943065267
SLC39A13	3.35012561597962	2	0.187296415401172	0.737579051673666
PSMC3	11.7240260102388	2	0.00284550988756549	0.236993078491058
CELF1	3.97272362592578	2	0.13719365474654	0.203504505790543
NDUFS3	0.146264619279454	2	0.929477843011245	0.0469983691155587
PTPMT1	1.48336508499572	2	0.476311826045888	0.258660815440693
KBTBD4	1.20012781347597	2	0.548776564453263	0.358899398647841
MTCH2	1.73854741805892	2	0.419255940499879	0.126432425070729
FNBP4	4.08036522126165	2	0.130004968421385	0.23658598438562
NUP160	2.17023879938813	2	0.337861442933369	0.644039165579184
PTPRJ	2.89103077406771	2	0.23562460738923	2.77987147998805
SSRP1	0.626729942747715	2	0.730983075980479	0.103770027501528
SLC43A3	1.66755253247575	2	0.434405753285797	0.745643596280471
SLC43A1	0.0281951053593326	2	0.986001352496965	0.0497209867735964
TIMM10	0.0140490575259768	2	0.993000085570762	0.0162937254358645
UBE2L6	0.0827568540951708	2	0.959465973362437	0.042174186709095
CLP1	3.19017877478737	2	0.202890391848631	0.635958000041297
ZDHHC5	2.61185388646785	2	0.270921289961968	0.813979612551375
MED19	1.3207936483861	2	0.516646276066727	0.238169448620259
TMX2	3.72134673405355	2	0.155567840836603	0.276391733896977
SELENOH	2.08685088153183	2	0.352246015172375	0.059678604036317
CTNND1	0.887583297536822	2	0.64159908486182	0.659113113323531
LPXN	13.6880455907195	2	0.00106580723972882	0.345003076801408
ZFP91	0.0539607331156011	2	0.973380352143614	0.0240950315083968
FAM111B	36.9083097705574	2	9.67079916147640e-09	0.962331806653496
FAM111A-DT	0.0469229728766255	2	0.97681594438003	0.0984596092766461

FAM111A 2.13583052290565 2 0.343724346395853
0.438640144092987
MPEG1 29.5686515643833 2 3.79532432703833e-07
4.00671254791328
OSBP 0.399724600417301 2 0.818843499894281
0.268576791614992
PATL1 1.52946098488903 2 0.46545935888566 0.469338064127012
STX3 10.3415890492886 2 0.00568005408297245
4.28559247116606
MRPL16 0.0474936073243885 2 0.976532933020964
0.00419433730111809
MS4A7 8.22781019358363 2 0.0163438255234195
1.17965398593337
MS4A1 160.383590979543 2 0 0.76239180426691
CCDC86 0.269531443416307 2 0.873920628338781
0.200541605244076
PRPF19 0.540564446992715 2 0.763164081105681
0.102459827311998
TMEM109 2.34679111749258 2 0.309314859297436
0.336849357021446
SLC15A3 0.998723643006068 2 0.606917858075106
0.476864539830606
VPS37C 2.26313637645188 2 0.322527076469399
0.824859751046779
DDB1 8.88474533536744 2 0.0117679838543066
0.401453512758476
TKFC 0.928809795136311 2 0.628509021166385
0.28523092899001
CYB561A3 1.67953797608612 2 0.43181026524226 0.0669497994584
TMEM138 3.02763940412845 2 0.220067777162605
0.410496250347534
TMEM216 4.84011942576844 2 0.0889163078516659
0.384090745988871
CPSF7 1.29435260132607 2 0.523521960243045
0.179565441065664
SDHAF2 0.667392018653242 2 0.716271488987379
0.128629837942612
TMEM258 13.472084688045 2 0.0011873369413602
0.169777642739797
FEN1 0.478844343711284 2 0.787082528130876
0.184472768025593
FADS2 0.85925303736493 2 0.650752093091933
0.411778112049004
FADS1 1.09601733855335 2 0.578099852941225
0.401887379819772
FADS3 0.126576318049186 2 0.938672947632741
0.0582789296769253
FTH1 48.8853824417881 2 2.42479369916282e-11
0.187347837981345
INCENP 0.0381382752382275 2 0.981111528182747

0.187925573239988
ASRGL1	1.70631466617907 2	0.426067568797547
0.599860289962193
EEF1G	3.0432967021945	2	0.218351669936993
0.400319473353404
TUT1	0.287455576909901	2	0.866123484784617
0.21318157638758
MTA2	0.11144292770436 2	0.945802539400917
0.12464415336528
EML3	6.56728137294778 2	0.0374915131929356
0.768396617448141
ROM1	1.41745695274264 2	0.492269732281815
1.44921479287941
B3GAT3	0.000876273133886588	2	0.999561959400866
0.00737304089577023
GANAB	1.29800882909313 2	0.522565776751648
0.403573813067624
INTS5	1.21794582623931 2	0.543909224314138
0.328550938739638
C11orf98 1.03561195286599 2	0.595826372044081
0.868434890184584
CSKMT	0.596602232838359	2	0.742077854275659
0.172840821456776
UQCC3	0.919271098409477	2	0.63151375926359
0.15670449380466
UBXN1	9.41775197505712 2	0.00901490475520983
0.215945479232008
HNRNPUL2 1.1635206875582	2	0.558913619959466
0.31448499257451
TTC9C	3.11929389723644 2	0.21021027312884 0.350085918122252
ZBTB3	0.00316403757775682	2	0.998419231943198
0.037900144862936
POLR2G	1.89539898302824 2	0.387631748628373
0.0816255324001556
AP001160.2	2.93241661668893 2	0.230798946632064
2.06484002435687
TAF6L	0.510356201496008	2	0.774778497042552
0.306038647702036
TMEM223	0.481533631516348	2	0.786024893638877
0.109958262518261
AP001160.3	4.52667898539821 2	0.10400258829494
0.921135147667168
TMEM179B 4.78010300937462 2	0.091624964640821
0.342720395188398
NXF1	0.546591517801739	2	0.760867720946983
0.153422646189213
STX5	0.672941924493867	2	0.714286624553637
0.119557039515857
WDR74	0.44433549277409 2	0.800781024943752
0.1238277580637

SLC3A2 14.747085378312 2 0.00062764069989274
0.743160837559592
ATL3 1.26242162835226 2 0.531947321555314
0.465701595779442
RTN3 4.24915337817886 2 0.119483536247503
0.317103040899194
SPINDOC 2.49333969696208 2 0.287460491665684
0.831963142016367
MARK2 3.67973551825907 2 0.158838429650327
0.462048840963285
NAA40 0.122831062663999 2 0.940432379454988
0.162510847477344
COX8A 15.8291580614345 2 0.00036537766864142
0.322606316619464
OTUB1 0.88294475938793 2 0.643088852695977
0.0555501450353024
MACROD1 1.86447282903195 2 0.393672310681289
1.24686627999422
STIP1 12.7786273264205 2 0.00167940844573145
0.64312379698999
FERMT3 43.490615847349 2 3.59859364529314e-10
0.768894420299806
TRPT1 0.485781177575604 2 0.784357326562926
0.0582773469954361
NUDT22 0.031710277314005 2 0.984269892386909
0.0268861603029106
DNAJC4 4.00536656586745 2 0.134972627152807
0.372765450431255
VEGFB 1.51947782627604 2 0.467788544510735
0.366931015596143
FKBP2 5.09741793931985 2 0.0781825369006492
0.170673335851761
PPP1R14B 3.14397125865878 2 0.207632491602121
0.352226482117488
PLCB3 1.48576340541153 2 0.475740994185368
1.03730580586658
BAD 0.0398392341455065 2 0.980277467712219
0.03436493818209
GPR137 0.462959310170979 2 0.793358836156631
0.209708413887564
ESRRA 0.963162401328939 2 0.617805744228047
0.308521073781329
TRMT112 8.97602485035642 2 0.0112429678567172
0.157545335913819
PRDX5 6.28740754892769 2 0.0431227846897185
0.0973806390259071
AP003774.4 0.0942834519286757 2 0.953952188215451
0.0929581123560742
CCDC88B 3.72935120092713 2 0.154946466294727
0.431079636476207

RPS6KA4	1.78688069319105	2	0.409245382104452	0.538545722832254
RASGRP2	1.75732746897661	2	0.415337542265633	0.191105269449783
SF1	0.878810616598762	2	0.644419538104621	0.0826168882484859
AP001462.1	1.69215441828719	2	0.429094884232096	0.966163725982555
MAP4K2	10.0179450231552	2	0.0066777611047204	0.306359807455634
MEN1	11.0442715274783	2	0.00399730152712141	0.729605882899273
EHD1	6.64356389897043	2	0.0360884665988725	0.170372454775715
ATG2A	1.26903986419253	2	0.530189954412176	0.361530817242657
PPP2R5B	1.12343593204166	2	0.570228588539114	0.288993460455216
ARL2	1.17281964552085	2	0.556320994679999	0.106629078941821
SNX15	14.6102705938686	2	0.00067207857517404	0.796962517107849
SAC3D1	5.3999206838286	2	0.0672081780345832	0.630802926492329
NAALADL1	9.49099899654528	2	0.0086907199218651	1.31116410424659
CDCA5	0.338754752158279	2	0.844190266054111	0.633293255118188
ZFPL1	0.94052545430446	2	0.624838084785293	0.270074524882651
VPS51	0.849686212249209	2	0.65387236565197	0.14236569727221
TM7SF2	3.23165722535532	2	0.198725935370034	0.373127700924076
ZNHIT2	1.73016972907763	2	0.421015821797651	0.315959569675082
FAU	10.3398808503024	2	0.00568490748663553	0.103487588356547
SYVN1	0.950674936259892	2	0.621675225485599	0.0829314045171005
MRPL49	0.612986115974693	2	0.736023627646961	0.13023877240556
AP003068.2	2.70059205599668	2	0.259163529628268	3.70214373197454
CAPN1	0.746216893358123	2	0.688590553435909	0.0936034833660436
POLA2	8.73921709220296	2	0.0126561939148869	1.22644198440336
DPF2	0.330670203611465	2	0.847609620977647	0.118841978889214

SLC25A45 6.24930113639492 2 0.0439522892680406
0.7082043525897
FRMD8 0.472207465437381 2 0.789698752085388
0.0827835317173001
NEAT1 63.0097791427263 2 2.07611705604904e-14
0.459882820965579
MALAT1 497.14873088953 2 0 0.756832359626639
SCYL1 1.51731935506554 2 0.468293671089964
0.274120113789484
LTBP3 7.88782548511817 2 0.0193722675656273
0.905537548267413
SSSCA1 10.2246471556879 2 0.00602207389688891
0.341752274480665
FAM89B 0.0124962241966915 2 0.993771366764231
0.0436378043936152
EHBP1L1 3.28664659087119 2 0.193336459302281
0.702756859574384
MAP3K11 2.01415581397487 2 0.365284817787575
0.266121048746179
SIPA1 8.56526156941432 2 0.0138062928835035
0.614998418435017
RELA 1.01378214028399 2 0.602365382485028
0.243626429073824
KAT5 0.526047008099162 2 0.768723828975831
0.0824587690295209
RNASEH2C 4.70955934310269 2 0.0949144165771594
0.226281105221432
AP5B1 1.40872574935857 2 0.494423483647703
0.690034346784991
CFL1 12.1264697441649 2 0.00232686160015838
0.121554698120435
MUS81 2.67089564227439 2 0.263040354046062
0.24156763104176
FIBP 1.36742545059206 2 0.5047395500131 0.138263871862972
CCDC85B 4.61562407490841 2 0.0994786692244259
0.312488168792053
C11orf68 0.0430768374566797 2 0.978691876649527
0.0839067617141975
DRAP1 9.89533516921395 2 0.00709994966350647
0.397878390572561
SART1 1.71620107790039 2 0.423966626068729
0.322017300013621
EIF1AD 0.308284578043775 2 0.85715004929192
0.164499448602236
BANF1 0.49938883005283 2 0.779038809254888
0.0727749937098018
SF3B2 2.27351646124282 2 0.320857483645359
0.125166344069711
AP006287.2 0.699762314699747 2 0.704771841695363
0.946572221662071

PACS1	4.39220798275581	2	0.111235690449927	0.468540333737784
AP000759.1	6.34840740035762e-05	2	0.999968258466771	0.00812830803678162
KLC2	3.50065573521552	2	0.173716977942398	0.449470394819233
RAB1B	5.68140350604733	2	0.0583846799594591	0.513458489734302
YIF1A	0.00341391952824995	2	0.998294496263115	0.0115145426448202
BRMS1	0.000137234369552887	2	0.999931385169329	0.000653122848828124
B4GAT1	3.1733261551098	2	0.20460723231015	1.14879174710981
SLC29A2	0.266070551685212	2	0.875434209884676	0.556353308488527
MRPL11	0.644442237332629	2	0.724537963747774	0.119164726048374
AP002748.3	0.692899388336011	2	0.707194394394838	0.67196862128041
DPP3	2.07177410641776	2	0.354911415939876	0.220094450676446
ZDHHC24	0.0246693319819453	2	0.987741094189492	0.0372605484914525
CCS	0.0932802971402292	2	0.954430789085884	0.0503056853008091
RBM14	11.5409212691987	2	0.00311832077684326	0.651391559524437
RBM4	0.051106547863457	2	0.97477044772818	0.0406781576235061
RBM4B	0.180857153156916	2	0.913539579693382	0.0799750541361527
AP001157.1	2.45587631698042	2	0.292895860373452	1.0030318276184
C11orf80	2.10732979424801	2	0.348657610481893	0.654903172892036
RCE1	0.0988195092361312	2	0.951791049007516	0.120701950512145
KDM2A	0.924162964705911	2	0.629971006330659	0.350906136587207
GRK2	4.54639495597758	2	0.102982369223787	0.535892495264397
ANKRD13D	0.0973261805640441	2	0.952501982820608	0.0853298782685912
RAD9A	0.219445184943249	2	0.896082680902453	0.152159051632577
POLD4	5.70932903697376	2	0.0575751332741025	0.25919666043521
CLCF1	5.29530859722815	2	0.0708171342335062	3.86050710984324
PPP1CA	0.555484615829164	2	0.75749199605794	

0.039194101051242
TBC1D10C 4.07668506303484 2 0.130244408074809
0.13425628786463
RPS6KB2 1.69870662259772 2 0.427691425748988
0.121501809930954
CORO1B 2.27663973698495 2 0.320356811483738
0.154234614323758
TMEM134 7.67463377865435 2 0.0215513484926064
0.334750237357137
AIP 0.594715251870564 2 0.742778328062693
0.0847542191579519
PITPNM1 0.177141258105168 2 0.915238466027383
0.14264200122132
CDK2AP2 3.34296601044943 2 0.187968101160884
0.108597841739982
GSTP1 60.4719226846764 2 7.39408534400354e-14
0.325594272487828
NDUFV1 4.80112645037147 2 0.0906668730395137
0.279307208618172
NUDT8 2.99977019815886 2 0.223155799482201
0.870357787412349
ACY3 5.07228657770652 2 0.0791711520067393
0.205690208181129
UNC93B1 0.0692157994018205 2 0.965984104667151
0.0559226086738263
NDUFS8 7.369691798416 2 0.0251010425159233
0.178117571737678
TCIRG1 12.6745667702506 2 0.00176910168270572
1.28074835855113
AP002807.1 0.906463216400462 2 0.635570913143025
0.790430822159706
CHKA 0.916422668412749 2 0.632413811412954
0.424537388781554
KMT5B 0.0942144375519905 2 0.953985106991243
0.0840455157593343
C11orf24 3.06847247330869 2 0.215620311186635
0.667644872781773
PPP6R3 0.522603391651663 2 0.770048564129016
0.292374706463014
TESMIN 1.3455427570165 2 0.510292402916294
1.72572316821023
CPT1A 0.909527935082791 2 0.634597735936318
0.700931973827569
MRPL21 1.9249487504594e-05 2 0.999990375302565
0.000556670543373819
IGHMBP2 0.208636383076622 2 0.900938580775278
0.314488420132384
TPCN2 5.83821090195714 2 0.0539819552133411
0.958087653066826
ORAOV1 0.57250943506059 2 0.751071280558022

0.288458680663607
FADD 0.0624531615902438 2 0.969255933413831
0.056288653442756
PPFIA1 1.78437934065717 2 0.40975753579368 0.446421229795979
AP000487.1 1.35169853767351 2 0.508724193490983
0.606212285721119
CTTN 0.053604960234938 2 0.973553518711081
0.126530659020037
SHANK2 2.09104274254336 2 0.351508505158495
0.82316857435113
DHCR7 0.0270566355243954 2 0.986562778672009
0.0491562359145681
AP002387.2 0.443708473085723 2 0.801032117035972
0.355099846494101
NADSYN1 0.0208401618133907 2 0.989634020061143
0.0339647102602528
FAM86C1 0.462097159984939 2 0.793700907114564
0.168598660173942
AP002495.2 0.78282285128391 2 0.676101933137327
0.584155826430735
RNF121 0.762554994458 2 0.682988335950141
0.181452582819794
NUMA1 2.61962679357599 2 0.269870410371833
0.337926583994934
LRTOMT 0.478110239666164 2 0.787371481391944
0.579169855047137
LAMTOR1 2.56129289798158 2 0.277857621603776
0.147188446269643
ANAPC15 9.23671189784903 2 0.009869007884029
0.468672449757349
FOLR2 4.57445731591521 2 0.101547495096855
0.677160726704101
INPPL1 1.69971048269014 2 0.427476808437844
0.715252732717872
CLPB 7.35881429584642 2 0.0252379327617219
0.518839357868578
ARAP1 2.80746924535203 2 0.245677734883481
0.779356600358553
STARD10 8.94329284632929 2 0.0114284842311775
0.943452998342391
ATG16L2 5.32384643905002 2 0.069813825237275
1.08838218223737
FCHSD2 1.74036396029173 2 0.418875315320337
0.373197152489867
AP002761.2 1.44170323811831 2 0.486337904921373
1.55358070140385
RELT 3.16672506444132 2 0.20528366343784 0.493263322644908
FAM168A 3.85507816795727 2 0.145505835888063
1.43654860991984
AP000763.3 4.73906620983027 2 0.0935243821602527

1.24199965107074
PLEKHB1 0.222640231782385 2 0.894652310663021
0.309943337065393
RAB6A 1.45524121357122 2 0.483057006320566
0.159050990511767
MRPL48 0.214743580144856 2 0.89819167215821
0.0727482722393431
COA4 2.45150634760689 2 0.293536533018255
0.268715476985076
PAAF1 1.92907512363476 2 0.381159421660565
0.365843305595456
UCP2 55.9346586651101 2 7.14428516346288e-13
0.397228079196676
C2CD3 0.0248817077666455 2 0.987636213613135
0.115220288036755
PPME1 0.512108353820255 2 0.774100029308465
0.310888349340666
P4HA3 2.73059813265595 2 0.255304311646645
0.911501932891517
PGM2L1 1.03408209077869 2 0.596282312491954
0.400251294058529
LIPT2 6.7742603506979 2 0.0338055538349127
1.77655348051907
POLD3 0.425342553151012 2 0.808421840738533
0.265710771811245
RNF169 0.0476858714121454 2 0.976439061426304
0.085932999152192
XRRA1 2.96988339845651 2 0.226515546322678
0.524338637042301
SPCS2 11.8401741033193 2 0.00268496643599592
0.230940276222689
NEU3 0.654694976815365 2 0.720833218314674
0.621704560802529
RPS3 46.9751909032432 2 6.30183683014707e-11
0.277709360104363
GDPD5 0.105390813287042 2 0.948668926731965
0.114399328690843
SERPINH1 2.4975577950821 2 0.286854862261499
1.49799115861916
UVRAG 8.57479190427127 2 0.0137406600861737
0.353336161099964
THAP12 2.1313064526894 2 0.344502742983213
0.487808507216524
GVQW3 1.2090524590151 2 0.546333201884932
0.71144054711823
AP002360.1 2.47412798785757 2 0.290235100246417
0.774745798297265
EMSY 2.11734273382769 2 0.346916428911337
0.488789860250591
ACER3 0.746394820103777 2 0.688529296822616

0.272616890378385
PAK1 4.15942239257081 2 0.124966297718496
1.74688477419066
CLNS1A 7.67450594495253 2 0.0215527260309585
0.407674596159265
RSF1 5.54960971811921 2 0.0623616448123985
0.267366565869575
AAMDC 1.53293676347008 2 0.464651144547616
0.59159595819531
INTS4 1.9566850927948 2 0.375933675387622
0.503198847707081
NDUFC2 2.45039112197126 2 0.293700258395004
0.0738487380313794
ALG8 0.010089149815152 2 0.994968127591877
0.0253627848611118
KCTD21-AS1 1.01951980021347 2 0.600639775046703
0.880254746756229
KCTD21 2.04752595593171 2 0.359240579172057
1.41673011358189
NARS2 0.394449638462963 2 0.821006034617023
0.198536054883851
PRCP 8.36698875329336 2 0.0152451421129554
0.967702681167075
RAB30 5.92941553495843 2 0.0515755390928571
0.254733220311905
RAB30-AS1 11.1526281498419 2 0.00378649657128582
0.632230308537832
PCF11 0.812414495690116 2 0.666172090387126
0.171775168002372
ANKRD42 0.140384448055629 2 0.932214608635284
0.104831828193574
CCDC90B 4.46582491607363 2 0.107215713703053
0.203869139340968
DLG2 3.5010627622855 2 0.173681627783368
4.80816401329161
TMEM126B 1.40654626143256 2 0.494962572335149
0.171377330651738
TMEM126A 1.47398909637463 2 0.478550015386773
0.151342334923478
CREBZF 2.32976726121367 2 0.311958962395627
0.384290311236349
SYTL2 0.00766113795087843 2 0.996176758295041
0.0437474646399868
PICALM 0.152468540187952 2 0.926599106670572
0.115259357637424
EED 0.014527823641309 2 0.992762406623307
0.0169454059705216
HIKESHI 0.381337642398364 2 0.826406231060919
0.073905859198141
TMEM135 0.548129498274203 2 0.760282846008892

0.520297337172399
CTSC	4.90538054688982 2	0.0860617451532845
0.641665668153525
CHORDC1	0.393382053046557	2	0.821444398639042
0.0795123518124438
FAT3	1.46619204915426 2	0.480419295268773
1.39734944059399
SLC36A4	0.000668086173655703	2	0.999666012699352
0.00696284366905617
SMCO4	19.5430732462509 2	5.70526162891882e-05
1.31017441141935
CEP295	2.4539906123249 2	0.293172148146013
0.653324283016854
TAF1D	5.55710824112964 2	0.0621282724584684
0.271812551264794
C11orf54	0.55108594077537 2	0.759159810001811
0.268209429718237
MED17	5.54883979943886 2	0.0623856561314321
0.731665509768844
PANX1	0.309085014668849	2	0.85680707078332
0.352043434060059
MRE11	0.193558354021533	2	0.907756437492555
0.121288311723723
ANKRD49	0.558514754697142	2	0.756345212036294
0.103285665372183
CWC15	0.000385666303289972	2	0.999807185439472
0.00244283934584872
KDM4D	0.920471946456346	2	0.631134697041819
0.512164729258046
SRSF8	5.68434899733427 2	0.0582987574629249
0.45918833366292
ENDOD1	3.99492553707756 2	0.13567909615709 2.00439730167393
SESN3	2.85079473780064 2	0.240412911610674
0.366677522181127
FAM76B	2.76803301074224 2	0.250570113008281
0.220407933416513
CEP57	2.15290401929051 2	0.34080254727881 0.173570931384015
MTMR2	0.540916305106728	2	0.763029830178217
0.231407880578531
CCDC82	0.0687710490807356	2	0.96619893942358
0.0445385341957778
JRKL	7.0858135149297 2	0.028929114828713
2.41660149412347
CEP126	0.836068948964007	2	0.658339532119256
1.04874270927001
BIRC3	18.3316357152628 2	0.000104552851583506
0.502479109106177
BIRC2	3.12464234326691 2	0.209648874964914
0.45115941897008
TMEM123	8.53327980469274 2	0.01402884232754 0.20396150220952

AP001830.1 4.14400779619628 2 0.12593317144421
2.43711089057402
DCUN1D5 0.38714641296728 2 0.824009511148248
0.113995071935267
PDGFD 10.5783496950753 2 0.00504592219746436
0.66296204970119
CASP4 3.12015729667186 2 0.210119544998334
0.261969863137292
CASP1 15.9203825013996 2 0.000349086348066852
1.3141473051707
CARD16 4.93262695149864 2 0.0848972586160874
0.612098129242417
MSANTD4 0.145394339080933 2 0.929882384101756
0.215266946674129
KBTBD3 0.47270923070321 2 0.789500655233825
0.206136990654103
AASDHPPT 0.639097149592099 2 0.726476913051022
0.14469587413476
AP000766.1 3.27366299066292 2 0.194595643713461
2.05201656575027
CWF19L2 2.54476682237001 2 0.280163081569515
0.324376130162913
ALKBH8 1.28718637974777 2 0.525401162110855
0.793449607249453
SLC35F2 0.166244526610847 2 0.920238628925285
0.253283508195215
CUL5 4.73258274831219 2 0.0938280549734347
0.533241153161637
ACAT1 2.35321743931297 2 0.308322575919407
0.336785193732494
NPAT 5.34367434621905 2 0.0691251137819253
0.525928255954303
ATM 23.1254658121383 2 9.51412609828672e-06
0.532956424567644
DDX10 1.3121091896818 2 0.518894550420194
0.31437448460166
RDX 5.64602467423548 2 0.059426659681309
0.979639974607757
FDX1 4.7162963290056 2 0.0945952359146389
0.173397275192278
COLCA1 3.80759269258029 2 0.149001881397111
1.9187011459757
POU2AF1 43.4770488586815 2 3.62308738566242e-10
0.53579028354352
SIK2 0.0734908326921651 2 0.963921502772726
0.236534509108038
PPP2R1B 0.248844429915032 2 0.883006943427743
0.236901501440842
ALG9 1.1237520524756 2 0.57013846520733 1.19491656258042
FDXACB1 3.59911475865172 2 0.16537206912142 1.09870803628764

C11orf1 0.173100553986055 2 0.917089439126251
0.16117205462139
DLAT 10.5338879345127 2 0.00515935365880238
0.420734931956363
NKAPD1 0.35794056578182 2 0.836130746416447
0.167354905219664
TIMM8B 14.8688687609915 2 0.000590562916302773
0.332529010553708
SDHD 6.16940761019587 2 0.0457435809093093
0.29110584891438
BCO2 0.253987641314954 2 0.880739114969006
0.335915966950103
PTS 4.2047132470121 2 0.122168184330297
0.329823451036222
AP002884.1 0.057980665001744 2 0.971425855679823
0.251715693761508
ZW10 0.455977149830174 2 0.796133355674684
0.268632494607465
USP28 8.76518543588351 2 0.0124929259615879
1.14104643264846
HTR3A 29.2650541644223 2 4.41747633805889e-07
0.513259925948972
C11orf71 0.990608989529338 2 0.609385324402287
0.37219088476081
RBM7 0.0465690171215397 2 0.976984483779089
0.0462389050662956
REXO2 11.348901274167 2 0.00343255417639421
0.727988481354572
CADM1 4.54068963349445 2 0.103276562454346
1.05792775622699
BUD13 1.27776195857073 2 0.527882805451129
0.424998144551171
ZPR1 2.76965973127801 2 0.25036639209452 0.201146436534633
SIK3 0.434179044418547 2 0.804857913421188
0.3476225646428
PAFAH1B2 1.07098056217431 2 0.585382217947292
0.240783276310187
SIDT2 1.38807574694066 2 0.4995548518194 0.6021468611503
PCSK7 2.45265938764594 2 0.293367352103285
0.147179294214898
RNF214 1.7963748701692 2 0.40730726191548 0.464303477303031
BACE1-AS 0.0541654431884353 2 0.973280726860885
0.191961504719473
CEP164 0.720450344001553 2 0.69751924658241
0.35140743368392
IL10RA 28.0270774008188 2 8.20346766294833e-07
0.528420270978803
JAML 12.7917421405195 2 0.00166843190909205
2.11206281301077
MPZL3 0.704445739447709 2 0.703123399594749

0.584412442653905
UBE4A	0.848073799221413	2	0.654399734367899
0.336220666994658
ATP5MG	269.244359645799 2	0	0.63247238361374
AP001267.2	3.55863010512528 2	0.168753695102988
3.03414145146475
KMT2A	60.0158532821908 2	9.28146448586631e-14
1.24769384085167
TMEM25	0.229863551356844	2	0.891426958831782
0.35446600012352
IFT46	0.61632036389314 2	0.734797607270685
0.48506730927789
ARCN1	0.220645017616976	2	0.895545267480662
0.13560662988366
DDX6	0.192202946233067	2	0.908371836070712
0.0671336957933067
AP004609.3	3.3990780134808	2	0.182767759340421
1.01093839777456
CXCR5	2.97893698087396 2	0.225492475100902
0.319361846242719
AP004609.1	0.343882224046768	2	0.842028757078294
0.218405074785669
BCL9L	1.2919543993552	2	0.52415009246319 1.11943943176506
AP003392.4	0.474134390146013	2	0.788938273471588
0.97629959225069
CCDC84	0.280577284160678	2	0.869107338240661
0.189222142984807
RPS25	43.875069428907	2	2.96927038512251e-10
0.242289192122137
TRAPPC4	8.10887768545487 2	0.0173452108442047
0.497421129478218
SLC37A4	0.301562155902813	2	0.86003595888528
0.347804579083914
HYOU1	0.027352233831953	2	0.986416976303171
0.116072379282212
VPS11	0.369923372832456	2	0.831136127045738
0.226312223888541
HMBS	0.74289367262377 2	0.689735673746659
0.234269146251291
H2AFX	1.58590702173489 2	0.452506337295434
0.393638182339123
DPAGT1	3.89619140950378 2	0.142545261555898
0.585277300422242
C2CD2L	2.32707987864338 2	0.312378420682831
1.79653667210056
HINFP	2.79027762079385 2	0.247798636952633
0.622644873589315
NLRX1	2.62427730215088 2	0.269243622046593
1.56196660343244
CBL	5.56700803153845 2	0.0618215038830747

0.767426433698218
RNF26 2.25487865700983 2 0.323861498449245
0.429120746972406
ARHGEF12 2.31659097618296 2 0.314020977465166
0.389354895941578
TBCEL 1.76818049165043 2 0.41308981256498 0.556960446680531
SC5D 2.93015770843181 2 0.231059770721901
0.29374959663215
AP000977.1 4.28879065890587 2 0.11713884604403
2.35471221411346
SORL1 0.781121234335754 2 0.676677411167459
0.128244807451727
HSPA8 53.0985488662855 2 2.94986257642904e-12
0.32339227021123
GRAMD1B 2.36459420873074 2 0.30657369735599 1.59364934564758
ZNF202 2.16664636529485 2 0.3384688607804 1.09996173440821
TBRG1 0.40190366217149 2 0.817951830456536
0.134295197263863
SPA17 1.15946641790709 2 0.560047762364372
1.07369824498165
NRGN 1.55677480130289 2 0.459145832898013
0.951688683025113
MSANTD2 1.79413939467374 2 0.407762779144023
0.691804413988613
TMEM218 0.0225589093034413 2 0.988783919896318
0.0468610217194296
FEZ1 65.5431577583086 2 5.88418203051333e-15
3.87124201850332
EI24 0.0946529229397139 2 0.953775975652579
0.0489037845259876
STT3A 0.650160707325754 2 0.722469298255191
0.248566996062836
CHEK1 4.67407451914144 2 0.0966134552104253
1.38822179952259
HYLS1 0.516681372061735 2 0.772332064535137
0.283302813229372
PUS3 0.219348227816584 2 0.89612612275644
0.18163011972671
RPUSD4 1.72157991719886 2 0.422827933790997
0.266215870522268
FAM118B 5.12915528514911 2 0.0769516756908862
0.438729710184228
SRPRA 6.73657130929769 2 0.034448643622499
0.542907834250469
FOXRED1 4.07219414181723 2 0.130537195360795
0.577784667148334
TIRAP 2.9054960762733 2 0.23392656488945 1.24156300173614
DCPS 2.43950399442146 2 0.295303393908761
0.228929742499346
ST3GAL4 1.41273261139083 2 0.49343393188579 1.13472266904326

ETS1	0.603359366095994	2	0.73957492533816
0.122683936426263
FLI1	0.00904793551783589	2	0.995486249969193
0.0127102428373047
KCNJ1	6.80893030116789 2	0.0332245854808036
1.80645505350261
NFRKB	0.27462202619181	2	0.871699074140639
0.199107245963529
PRDM10	1.07908362026136 2	0.583015322899423
1.21109946582775
APLP2	6.70804393944843 2	0.0349435292772807
0.423166676325412
ST14	47.6806245951967 2	4.42877956530197e-11
1.23426848503112
ZBTB44	3.68934236392542 2	0.158077291009393
0.404319249325844
AP003486.1	2.17968693980459 2	0.336269125826491
2.44352372240018
SNX19	1.19342949280057 2	0.550617586375116
0.496668869716577
NCAPD3	2.10487788877925 2	0.349085310349167
0.694215346196886
VPS26B	0.948155554009026	2	0.6224588377011
0.484138041976488
THYN1	19.0662993703873 2	7.24111919776504e-05
0.531309785057333
ACAD8	3.1545601893557	2	0.206536093551989
0.296093602211941
LARP4B	11.5939197151721 2	0.0030367729481926
1.10752729334666
GTPBP4	0.677805172251613	2	0.7125518581455
0.0598590318952213
IDI1	4.34098117346624 2	0.114121616624383
0.143418077004845
WDR37	1.5263853407695	2	0.466175703219564
0.66800157534253
PFKP	15.1652834779734 2	0.000509214233507249
0.908891106734987
PITRM1	3.97829580414014 2	0.13681195297396 0.674037531595998
KLF6	0.625590093234382	2	0.731399800071592
0.0897249328535814
NET1	1.44545693644479 2	0.485425978073733
0.224977518891191
ASB13	10.7427678945627 2	0.00464769469290627
0.417345328177395
FAM208B	5.32078153661043 2	0.0699208935364379
0.262118014512641
GDI2	22.9309183160285 2	1.04861086963615e-05
0.201750464423418
ANKRD16	0.167352319137818	2	0.919729053326246

0.190210040218003
FBH1 13.0460083941556 2 0.00146924855336794
0.683654369567388
IL15RA 5.04261433918087 2 0.0803545011087459
2.44412629529967
IL2RA 4.71270893117614 2 0.0947650635509281
1.3685522654797
RBM17 13.6541912131815 2 0.00108400191800306
0.392166186788206
PFKFB3 0.0797186175425967 2 0.960924623308431
0.0884190749682478
SFMBT2 1.99793246081639 2 0.368259940391551
1.02776527415413
KIN 0.0526607895564753 2 0.974013227556587
0.0506381981103741
ATP5F1C 19.8963685307443 2 4.78143733785297e-05
0.339854251008613
TAF3 2.08026236688696 2 0.353408317597672
0.323377677248284
CELF2 0.48714423314044 2 0.783822947371347
0.402471587074457
USP6NL 2.03703265435178 2 0.361130342168805
0.279053671392699
AL512631.1 2.97195038156564 2 0.226281565348083
0.370007902152341
ECHDC3 0.945672222196398 2 0.623232203649663
0.892021881654831
UPF2 4.0619765452735 2 0.131205789962158
0.237576754200271
DHTKD1 8.41885602623012 2 0.014854862695164
0.566553430770535
SEC61A2 0.180815240935548 2 0.913558724130524
0.368397410234976
NUDT5 5.37594087028267 2 0.0680188480408391
0.182580692113231
CDC123 0.425361297190045 2 0.808414264228768
0.0774696903931591
CAMK1D 0.770078502726757 2 0.680423928113573
0.531491892082211
OPTN 0.0519666899543776 2 0.974351317317029
0.0819479161195632
PHYH 0.00698668827144169 2 0.996512750491962
0.0417789788780291
SEPHS1 0.0316870215254255 2 0.984281337439711
0.0416722961808743
PRPF18 0.966597555764753 2 0.616745525920717
0.201784860200754
FAM107B 4.51957470341643 2 0.10437267706659 0.217983095728242
HSPA14 0.324841026819701 2 0.850083657791304
0.357618404946038

HSPA14.1 0.664366368033377 2 0.717355902682125
0.235119222611826
SUV39H2 1.00849403287954 2 0.603960176335871
0.325068638093677
DCLRE1C 1.6179745730443 2 0.44530880818004 0.206642285317662
MEIG1 0.120547684877931 2 0.941506673795529
0.367045924497099
RPP38 2.2241518669833 2 0.328875528216438
0.322674244165887
NMT2 1.78790072170471 2 0.409036714341327
2.86063381084253
MINDY3 3.16978288835913 2 0.204970042600259
0.589150572971909
PTER 0.284903030453834 2 0.867229600703343
0.175401036462613
RSU1 2.61382258638913 2 0.270654739811695
0.245444952145879
TRDMT1 0.00236251726396 5 2 0.998819438779361
0.0338842744080253
VIM 9.86392188290581 2 0.00721234641646051
0.571642771788069
HACD1 0.331970994748649 2 0.847058518672942
0.24359453003845
STAM-AS1 4.14413926912295 2 0.125924893314993
4.26919066208026
STAM 9.46288044087212 2 0.00881376812545109
2.36748766008191
NSUN6 0.253707628598915 2 0.880862432677289
0.167821487093958
ARL5B 7.37980298277055 2 0.0249744621207718
2.14433250859574
MLLT10 1.33232012804918 2 0.513677283395994
0.372128799368261
DNAJC1 0.956918559362236 2 0.619737498768135
0.248108771382689
COMMD3 8.81852972359426 2 0.0121641173511734
0.452720733235519
BMI1 9.66028940230699 2 0.00798536569072439
1.23703028751985
PIP4K2A 46.1273160656904 2 9.62898649703448e-11
1.53000014247818
MSRB2 0.493919867989702 2 0.781171991345411
0.171574335472061
OTUD1 2.93687366787762 2 0.230285177958295
0.394434448578311
KIAA1217 9.64461528906484 2 0.00804819332407303
2.0819628190725
THNSL1 0.00539663200707215 2 0.997305321178933
0.0747870882954434
APBB1IP 7.10870823672917 2 0.0285998410642855

0.319764628295328
PDSS1	5.00122873877384	2	0.0820345836019277
1.50860681051037
ABI1	10.8446621486585	2	0.00441683865894082
0.255985828053492
ANKRD26	3.16495548269629	2	0.205465376926889
0.377078178316142
YME1L1	0.128263775805577	2	0.937881296176203
0.0707232414730391
MASTL	0.00410571774229606	2	0.997949246802494
0.0537702614259222
ACBD5	0.190239383607472	2	0.909264096495073
0.171714377689214
RAB18	0.819771955558376	2	0.663725925325927
0.126810155867972
WAC-AS1	4.82513035438858	2	0.0895851976727443
0.463038023340999
WAC	1.32769921714512	2	0.5148654839897	0.158949860274549
MTPAP	0.160762583493753	2	0.922764436836719
0.181255914725855
MAP3K8	0.213652897319634	2	0.898681626857652
0.0836889053914293
ZNF438	0.0564355257190512	2	0.9721766397839
0.177291008267421
ZEB1-AS1	3.29729483368114	2	0.192309847848006
0.447657246509827
ZEB1	10.6982708287968	2	0.00475225795755252
1.01365733609512
ARHGAP12	3.04725927162548	2	0.217919481396399
0.838787102319489
KIF5B	1.46310467268446	2	0.481161485590546
0.152337041425052
EPC1	1.87678071161397	2	0.391257113506425
0.0885070728135991
CCDC7	0.0316740395792605	2	0.984287726404114
0.10739374271963
ITGB1	24.49105753587	2	4.8065606657044e-06
0.727138203561973
CUL2	0.9548715653191	2	0.620372122964866
0.264283205420558
CREM	57.744894403647	2	2.88991053309928e-13
1.10732690091249
CCNY	0.885276224175388	2	0.642339619975616
0.474161183732316
ZNF248	2.06094162073655	2	0.356838917551866
0.631349741724968
ZNF33A	5.2692831595919	2	0.0717446795434813
0.563547325146447
ZNF37A	1.04344454458434	2	0.593497502937237
0.332042697889073

AL117339.5 0.145844985311484 2 0.929672883709657
0.108880436616904
ZNF33B 6.03743119490881 2 0.0488639390417831
0.825160369120805
BMS1 4.44423523417831 2 0.1083793596663 0.429141126592335
CSGALNACT2 0.0456338219994487 2 0.97744142616513
0.0738775687729002
HNRNPF 2.27894181207597 2 0.319988280902717
0.107753153620994
ZNF487 5.9005373574531 2 0.0523256452718771
3.86608176748605
ZNF485 0.31162613283299 2 0.855719138065707
0.60545745720761
ZNF32 8.30382677885357 2 0.0157342818558669
0.897623018622281
ZNF22 0.359400069806764 2 0.835520800903112
0.0525739693175983
OR13A1 2.3097551220187 2 0.315096114591886
1.81859121053581
ALOX5 32.202347001259 2 1.0170661413067e-07
0.678090867668437
MARCH8 0.0320303579833797 2 0.984112382108244
0.0927622796804268
ZFAND4 1.42565362334368 2 0.490256374386581
0.167761310340314
WASHC2C 3.12324159367618 2 0.209795759183814
0.357751046171805
AGAP4 8.76303706282439 2 0.0125063529046002
0.92457410772823
TIMM23 0.808577763825435 2 0.667451278813852
0.230337714878124
NCOA4 4.04231526322952 2 0.132501987772106
0.29184026159616
SYT15 1.13043605555482 2 0.568236241965807
0.3864729308232
PTPN20 0.136707867552147 2 0.933929865750054
0.296709309299099
MAPK8 2.51309921223162 2 0.284634435043188
0.49068593852046
WDFY4 0.420587257739355 2 0.810346269974286
0.159687649108373
ERCC6 0.0629500435361923 2 0.969015160436848
0.127098541936821
PARG 0.716125820395397 2 0.699029097564501
0.343235782952926
TIMM23B 0.752111237105487 2 0.686564146276183
0.473446255037295
AGAP6 3.79324072891225 2 0.150074961789124
1.40417463728339
WASHC2A 0.109568136374178 2 0.946689546274631

0.129222804091927
SGMS1	5.16576215621509 2	0.0755560073822009
0.539004888121027
SGMS1-AS1	8.49909791199322 2	0.0142706691573479
1.16809332832002
CSTF2T	1.32381846711063 2	0.515865485987767
0.366254077676405
ZWINT	3.54081917236886 2	0.170263237072086
0.824689672438999
IPMK	1.41582165456255 2	0.492672400778802
0.810020121605195
CISD1	1.42983070279357 2	0.489233522977973
0.184544547137853
UBE2D1	3.78594050631051 2	0.150623753068444
0.283864861917455
TFAM	1.04881566317257 2	0.591905768507448
0.0724410206975251
SLC16A9	1.86532820778618 2	0.393503977215729
42.2992795816703
CCDC6	1.17805872228251 2	0.554865597548936
0.342401917581308
CDK1	0.327037771221037	2	0.849150462125252
0.0926465814004879
ARID5B	0.000618375575108934	2	0.999690860006064
0.00287533359402002
RTKN2	0.244853712967916	2	0.884770617810669
0.492315847780492
ADO	0.0827239475351683	2	0.959481759854618
0.124163121201453
EGR2	25.9341432047228 2	2.33599740850909e-06
5.07829150289196
NRBF2	1.54493732109657 2	0.461871455922656
0.137181431428761
JMJD1C	0.706085663826545	2	0.702547101296283
0.104853663325314
REEP3	6.69536416614899 2	0.0351657710414354
0.898960046536738
SIRT1	0.0276033988877666	2	0.986293107343768
0.0902149469747205
HERC4	0.583528435246836	2	0.746944631592832
0.137102650750367
PBLD	0.154041575411502	2	0.925870606680711
0.227111194830102
HNRNPH3	5.24583498307001 2	0.0725907706304468
0.27184099854431
RUFY2	2.21624335830187 2	0.330178560263644
0.63887887032766
DNA2	1.69775953803775 2	0.42789400368269 1.18883794331658
SLC25A16 2.96180304622078 2	0.22743256022472 0.759690749085547
CCAR1	0.235012816792605	2	0.889134813799937

0.0644295835569494
DDX50	2.80145157095876	2	0.246418052379479
0.2057049694569
DDX21	0.0164849108922586	2	0.991791420452339
0.00916742046462278
KIF1BP	0.0226678528355103	2	0.988730060556895
0.0717377549379648
SRGN	3.46430075996678	2	0.176903590723101
0.334713869387645
VPS26A	0.00112826521282293	2	0.999436026486469
0.00596132155139425
SUPV3L1	2.06943852739813	2	0.355326119864643
0.488677019813188
HK1	3.48400519765023	2	0.175169255387802
0.38107802437199
AIFM2	5.49427638788795	2	0.064111072379439
1.03129613573908
TYSND1	0.545659688501624	2	0.761222302960992
0.193736066080548
SAR1A	0.643719767278325	2	0.724799739517223
0.142619136299658
PPA1	1.67238797491102	2	0.433356749888885
0.101327295479115
LRRC20	1.37042892104106	2	0.503982133713358
1.41810586485981
EIF4EBP2	1.37572342257237	2	0.502649731004171
0.165381609629838
PALD1	1.93303854703553	2	0.380404821521866
0.488976249735732
SGPL1	1.43422146848971	2	0.488160646211465
0.411270934479627
PCBD1	1.1481637571224	2	0.563221737448508
0.125652189465997
SLC29A3	0.000300287383467306	2	0.999849867579266
0.0101119234601496
VSIR	0.172036100464244	2	0.917577668580764
0.339280918789963
PSAP	19.3596024680726	2	6.25339321569385e-05
0.2913938442067
SPOCK2	1.15976553550179	2	0.559964008557789
1.07385953309802
ASCC1	0.0924969833540962	2	0.954804671695456
0.0634164327431971
ANAPC16	1.59799565636012	2	0.449779494659743
0.0800112638447845
DDIT4	5.27693488151847	2	0.0714707187767621
0.72244260204115
DNAJB12	1.5209623980327	2	0.467441440521232
0.139340189938318
MICU1	0.158636003169667	2	0.923746125001484

0.103701203668731
MCU	0.591380139068876	2	0.744017986130671
0.420349084464
P4HA1	1.16103119695388 2	0.559609758229761
0.433542760148626
NUDT13	1.51548491275113 2	0.468723396999595
1.17390695693771
ECD	4.30679522768842 2	0.116089061159353
0.619050529005614
FAM149B1 0.00361402556790225	2	0.998194618880689
0.0375696597428174
DNAJC9	2.69422286259613 2	0.259990176519404
0.236850077026173
MRPS16	0.0533592772649991	2	0.973673118816794
0.0340242099739775
AC016394.1	1.46127380715491 2	0.481602158251926
0.879622201679124
CFAP70	2.21619179882016 2	0.330187072291081
1.39106907514453
ANXA7	3.01331535493673 2	0.221649565637954
0.108192253591754
PPP3CB	1.48486054502357 2	0.475955806517396
0.239490475147396
AC073389.1	1.96093093561784 2	0.375136444267751
1.62211402105118
SEC24C	2.02197185147568 2	0.363860063666642
0.620177737108976
FUT11	2.66108656817137 2	0.264333614036904
0.738373741361007
CHCHD1	0.152410972921305	2	0.926625777943342
0.0517419125250085
ZSWIM8	0.0998145568255708	2	0.951317628091884
0.118676746899049
NDST2	0.326367169056944	2	0.849435230933
0.396551121441672
CAMK2G	6.81559112564448 2	0.0331141179679899
0.988493284221768
VCL	1.06453774382916 2	0.587271014253111
0.401099454991331
AP3M1	0.0594533189620789	2	0.970710831891013
0.0698983372564423
ADK	13.9820141069518 2	0.000920119455444235
1.06955023810935
KAT6B	11.6385049133427 2	0.00296982438759574
0.748382198799078
SAMD8	1.15284386004218 2	0.561905310453718
0.443202690802139
VDAC2	1.28941353084096 2	0.524816413865676
0.106756149407848
COMTD1	4.23409062470269 2	0.120386808946891

0.42017298894128
KCNMA1 8.38635004181255 2 0.0150982713642043
2.71164324327566
POLR3A 0.230741521111281 2 0.891035721757572
0.235149989873759
RPS24 111.273169765849 2 0 0.373867263832385
ZMIZ1 2.74049437638543 2 0.254044155075294
0.667914366080494
PPIF 0.0396858939950104 2 0.980352628540689
0.0595178177527871
EIF5AL1 0.476172537501924 2 0.78813469676504
0.289763890254557
NUTM2B-AS1 4.41458394817921 2 0.109998123317022
0.398208391315042
AL135925.1 2.05086931478903 2 0.358640545757406
0.758661759979719
TMEM254 3.3219709513422e-06 2 0.999998339015904
0.00116294166872376
ANXA11 2.00234931925335 2 0.367447561749735
0.14550217382435
FAM213A 5.02911729185471 2 0.0808986092592722
0.594724010023724
TSPAN14 3.43666042963614 2 0.179365399685419
0.663719947464307
GHITM 5.31025726086298 2 0.0702897966768277
0.108291439934025
CCSER2 1.02386970183157 2 0.599334832689143
0.146303801439578
WAPL 0.433995084574206 2 0.804931947594199
0.0865127016410207
BMPR1A 0.283611072399324 2 0.867789993818824
0.383096752394485
GLUD1 2.44108319440929 2 0.295070314382633
0.304688442184034
SHLD2 2.54930531005889 2 0.279528044021256
0.290792109517656
NUTM2A-AS1 2.39815344457717 2 0.301472426229644
0.459046466215047
MINPP1 1.55386698518024 2 0.459813874243458
0.598901420313491
ATAD1 0.595577920963224 2 0.742458011196365
0.169034964664946
PTEN 5.907461897834 2 0.0521447930104141
0.392693374619652
RNLS 1.32616169354225 2 0.51526144508705 0.425194662619077
STAMBPL1 2.93135931544403 2 0.230920990895408
0.469878329721341
ACTA2 0.258337906238837 2 0.878825472696735
0.109962185279964
FAS 0.296813625080181 2 0.862080338504487

0.166495204261081
LIPA 2.8775406642186 2 0.237219280340066
0.423247452453938
IFIT2 0.0309564021407105 2 0.984640970637031
0.122798576688113
IFIT5 2.91418696370742 2 0.232912255575904
1.05729638972477
PANK1 3.29267786499987 2 0.192754304934044
1.19606554787981
KIF20B 0.00166528417490477 2 0.99916770446278
0.00883556082305185
RPP30 2.80752393332325 2 0.245671017166879
0.247941045839167
PCGF5 6.76721103780079 2 0.0339249170317919
0.463596437364198
TNKS2-AS1 3.25398132079132 2 0.196520080825947
0.689614726579866
TNKS2 4.73389972241742 2 0.0937662907517645
0.723562737010148
FGFBP3 2.22678052442345 2 0.328443561599623
2.10433216404734
BTAF1 0.140085286730774 2 0.932354060343348
0.168162192547599
CPEB3 1.52534415168933 2 0.466418454927506
1.76596190166743
MARCH5 5.97774978507188 2 0.0503440473345367
0.395750147014765
IDE 4.74117335565444 2 0.0934258992931394
0.727288287420578
KIF11 8.65784094515031 2 0.0131817698940696
0.800096243599671
HHEX 54.1020072469228 2 1.78601577971449e-12
0.722180235579638
EXOC6 0.298270893768754 2 0.861452425949426
0.138038997201718
CEP55 1.64805410788172 2 0.438661578963019
1.35217933177313
FRA10AC1 0.14467823635867 2 0.930215389368012
0.0871598090388057
PLCE1 4.27594971580068 2 0.11789335222361 1.2110215051339
NOC3L 0.26556759070297 2 0.875654392194339
0.131512987803447
TBC1D12 2.17517872673421 2 0.337027967193936
0.966048597186158
HELLS 2.00809483542631 2 0.366393488568903
0.237635851967175
PDLIM1 6.27302914823473 2 0.0434339200956605
0.411637040450994
ALDH18A1 3.55319435516804 2 0.16921297039215 0.392675536346936
TCTN3 1.5354611162916 2 0.464065042793451

0.296696561519456
ENTPD1 8.31709308602815 2 0.0156302593278383
1.13009377357783
ENTPD1-AS1 1.71752825937344 2 0.423685379069845
0.784649577813117
CCNJ 2.41814037586307 2 0.298474675802347
0.982656492961737
ZNF518A 0.000219598994255266 2 0.999890206530617
0.00297997944125603
BLNK 3.32822824041952 2 0.189358332421123
0.105627147236134
TM9SF3 2.19909728506318 2 0.333021361459669
0.335869204777864
PIK3AP1 0.0042565800753835 2 0.997873973165681
0.0129441609997789
LCOR 3.22902289838897 2 0.198987862377739
0.386959333595469
ARHGAP19 0.272813038323916 2 0.872487877345506
0.519119979554399
FRAT1 3.31963730806865 2 0.190173464165158
0.980799462198693
FRAT2 0.477674063221029 2 0.787543216564849
0.210280410598765
RRP12 1.05527017324991 2 0.589998616721498
0.300013502631656
PGAM1 2.62445892855372 2 0.26921917228151 0.141976390644454
EXOSC1 0.420233735020048 2 0.810489520542966
0.102730593953022
ZDHHC16 0.339674038939613 2 0.843802328741095
0.144826773248938
MMS19 1.35100800008033 2 0.508899870407199
0.416635696779726
UBTD1 2.74481633418536 2 0.253495763760955
2.34948123511805
PI4K2A 0.642097694301656 2 0.725387816996618
0.435714674652858
ZFYVE27 6.20079555283577 2 0.0450312864467078
0.764447389828027
R3HCC1L 0.00794028146822018 2 0.996037729855387
0.0394682034241002
HPS1 6.28069582032106 2 0.0432677419960028
0.411478014600272
GOT1 2.55203427974885 2 0.279146892338425
0.179100240841813
SLC25A28 0.0234790626309563 2 0.98832910812144
0.0887523698442381
CUTC 4.17264880262232 2 0.12414259662449 0.426062981390886
COX15 1.69419620470431 2 0.428657047709147
0.405123213114356
DNMBP 3.35064257602212 2 0.187248009276016

1.46144903180528
ERLIN1	2.34586431608869	2	0.309458229236655
0.871336195826498
CHUK	0.0412603853235311	2	0.979581153894303
0.0895391133920162
CWF19L1	1.74819195881053	2	0.417239041921719
0.239897985340343
BLOC1S2	8.88865942751672	2	0.0117449758888658
0.225289295112947
SCD	2.42442332608911	2	0.297538496298672
1.71222087570516
NDUFB8	6.26621174429803	2	0.0435822260063082
0.143323548692322
HIF1AN	0.00845402837090788	2	0.995781907064498
0.0210998149452826
AL138762.1	0.583108728062811	2	0.747101397055128
0.682336222822015
SLF2	5.9985221177077	2	0.0498238716772924
0.779990304738988
MRPL43	4.23696660658948	2	0.120213818215283
0.302800610980893
TWNK	0.0552657700365828	2	0.972745410672806
0.116908297537548
LZTS2	0.00720972809306272	2	0.996401625675335
0.0518253345657838
BTRC	1.12075637705346	2	0.570993079978389
0.196595859323116
DPCD	0.183984613698176	2	0.912112166534645
0.228541157801453
POLL	0.253672914993852	2	0.880877721765275
0.17944420102641
FBXW4	1.09547577016679	2	0.578256414439771
0.337811761209379
NPM3	3.52520504362244	2	0.171597695447562
0.532886390479794
OGA	3.68099261533509	2	0.158738623357426
0.251523486350484
ARMH3	0.00124504125530004	2	0.999377673098114
0.0158964824961406
HPS6	0.0850120887603031	2	0.958384672662147
0.0913284846588603
LDB1	0.454345123771459	2	0.796783276001684
0.372331205173956
PPRC1	2.64024449933099	2	0.267102646760628
1.84884062990987
NOLC1	0.538993344648091	2	0.763763821076638
0.188511498962824
GBF1	0.250118026963183	2	0.882444824906538
0.231882012066435
NFKB2	4.97838226464799	2	0.0829770568970926

0.552594216637988
FBXL15 0.542505520898557 2 0.762423761475717
0.124264017335342
CUEDC2 1.17632226932413 2 0.555347555747279
0.182037529456018
C10orf95 1.39832007151462 2 0.497002592919478
0.700498259264337
MFSD13A 1.85726176785588 2 0.395094270157868
0.530276820205663
ACTR1A 4.71607878383977 2 0.0946055258424013
0.238215620798431
SUFU 0.0218955478845679 2 0.989111934843457
0.102127995278942
TRIM8 4.69728045293807 2 0.095498930886007
0.399374289460793
ARL3 2.19020680537723 2 0.334505016442207
0.241999098047871
SFXN2 1.0965422379609 2 0.577948150714041
0.845176673806336
WBP1L 1.95966575523463 2 0.375373826977978
0.533054384413819
BORCS7 0.242274338554147 2 0.885912431290341
0.0930940169303264
NT5C2 24.4400823508876 2 4.93064288664691e-06
1.21927365596883
INA 1.31413780095076 2 0.518368499587265
1.43531769332741
PCGF6 1.025432774258 2 0.598866613802397
0.426785616478342
TAF5 0.3262873232097 2 0.849469143547787
0.12207142958157
ATP5MD 5.11872200127918 2 0.0773541539089047
0.102322451805343
PDCD11 0.078614461293459 2 0.961455275239502
0.0958579493123247
CALHM2 0.682208049177615 2 0.710984944442957
0.63345850921522
STN1 0.0313588839410578 2 0.984442840538361
0.0580692681145263
SLK 2.08207211317879 2 0.353088672542458
0.971747550739405
SFR1 6.42170814136874 2 0.0403221605863238
0.964539678696789
GSTO1 2.75931717581607 2 0.251664459683796
0.234526244753883
ITPRIP 0.0832268789310741 2 0.959240513438074
0.103600951088986
XPNPEP1 16.4090463306864 2 0.000273414071803924
0.552660141259434
ADD3 3.63466959103152 2 0.162458158663381

0.406592867217787
MXI1 0.0344233564944347 2 0.982935596527623
0.11192726515262
SMNDC1 0.771721054487419 2 0.679865341761159
0.185165661350694
DUSP5 8.39822859216573e-05 2 0.999958009738655
0.00811537534773716
SMC3 1.19041491016571 2 0.551448153278176
0.13661154725167
PDCD4-AS1 7.46440661609964 2 0.0239400304230308
0.913356741072534
PDCD4 18.3557226569857 2 0.000103301224457897
0.403150266272761
BBIP1 2.18639189931986 2 0.335143677963717
0.236952515863614
SHOC2 4.51260589061148 2 0.104736988226279
0.146406344254487
GPAM 1.60497088073035 2 0.448213570468949
1.04219062374931
ACSL5 0.2664918349715 2 0.875249826404343
0.101930467294503
AL157786.1 1.64501821502593 2 0.439327949369199
1.23377208790321
ZDHHC6 0.59066131366158 2 0.744285443707575
0.151769343905523
VTI1A 1.47052067277966 2 0.479380642502641
0.228929478379463
CASP7 0.57604192529959 2 0.749745875414087
0.234163481788277
NHLRC2 0.0194002751762562 2 0.990346756996025
0.0664327717091472
CCDC186 0.00921026055370763 2 0.995405457077296
0.0202493490494554
ABLIM1 2.06163253591162 2 0.356715666130565
0.408649451707415
FAM160B1 2.68540953095496 2 0.261138394392113
0.772813285239543
TRUB1 0.0212790661568987 2 0.989416866554475
0.0781757826641977
HSPA12A 0.12543326791394 2 0.939209576086312
0.143588443500001
PDZD8 0.0176195523738709 2 0.991228916184219
0.0626079867944333
RAB11FIP2 0.825003291125405 2 0.661992107340416
0.220589415350334
FAM204A 1.53042180159331 2 0.46523580202566 0.199262560588897
CACUL1 2.09917501451654 2 0.350082125667814
0.316926376565932
EIF3A 0.0163333390273163 2 0.991866587138208
0.00939941186483631

FAM45A	7.61665817447233	2	0.0221852175674783
0.349155139160516
SFXN4	7.34815897984568	2	0.0253727506490222
0.682679304180866
PRDX3	13.8059079887744	2	0.00100481282923082
0.408462622938125
GRK5	8.99795444322314	2	0.0111203643922254
1.14440994087048
RGS10	18.1883673524883	2	0.000112317185458122
0.547995233164061
TIAL1	3.00313132194017	2	0.222781087301609
0.119077498265502
BAG3	1.47921784351218	2	0.477300540883443
0.66662823602291
INPP5F	8.33947032517994	2	0.0154563530024096
0.749042199825898
MCMBP	2.24556223401892	2	0.325373632991202
0.37109417476833
SEC23IP	1.35230960127762	2	0.5085687858136	0.308820885802686
WDR11	1.45359703448616	2	0.48345428571115	0.303415313948362
ATE1	0.169459299918181	2	0.918760637804785
0.174265059747006
NSMCE4A	7.75868234584902	2	0.0206644349872355
0.283523795364683
PLEKHA1	4.86182311900999	2	0.087956618336426
1.62428128248728
FAM24B	0.269628732652934	2	0.873878117837341
0.315322670381741
C10orf88	1.47747761581986	2	0.477716027426927
0.526180737692841
PSTK	0.766120597897147	2	0.681771787924538
0.478072886821393
IKZF5	0.228610501001268	2	0.891985635259798
0.193944536667446
ACADSB	1.16954836708768	2	0.557231679688933
0.302039151232861
BUB3	3.79267817855309	2	0.150117180088154
0.113983361467384
CHST15	8.98993328243506	2	0.0111650530614814
1.4039770394848
OAT	0.56836035977443	2	0.752631023515526
0.118677558425642
LHPP	9.25714291702477	2	0.00976870413858222
0.542371640806399
FAM53B	0.628991705957906	2	0.73015688791471
0.327470869748515
EEF1AKMT2	2.46325491400779	2	0.291817270953667
0.567471152380731
ABRAXAS2	1.27800653900749	2	0.527818254494627
0.142833698381943

ZRANB1	0.0707634938915178	2	0.965236869689341	0.134842295647737
AL731577.1	0.38365040944988	2	0.825451140841464	0.448586660410703
AL158835.1	1.94146806321966	2	0.378804881271123	1.69944628800624
EDRF1	1.87261930997838	2	0.392072050028	0.563329850330548
UROS	3.70560281488404	2	0.156797297359506	0.250358833863337
BCCIP	2.23508637012127	2	0.327082389206363	0.197223523049562
DHX32	5.95233957049581	2	0.0509877543818893	0.935979855899988
PTPRE	0.0544886406647832	2	0.973123458631051	0.059372618210152
MKI67	10.1897438621406	2	0.00612809140270365	1.24888313137758
MGMT	25.2242587939423	2	3.3313617722186e-06	0.762332750684918
C10orf143	3.62493361622063	2	0.163250930970924	0.77162185946975
GLRX3	4.46332038539898	2	0.107350060326193	0.227920778401133
PPP2R2D	1.88196003893058	2	0.390245200001858	0.49013987518494
BNIP3	0.189592622230477	2	0.909558182492927	0.0948771708729661
STK32C	0.965437662546079	2	0.617103309134852	0.394771895079129
LRRC27	4.55435855352755	2	0.102573129448084	2.61164823211282
ADAM8	2.94899779257513	2	0.228893397643203	1.17804444057101
TUBGCP2	0.333419952940802	2	0.846445064727122	0.124380652163512
ZNF511	0.200424721160591	2	0.904645286638082	0.0895593535215511
FUOM	2.32662916166535	2	0.312448825744609	0.406792895317628
ECHS1	3.61019085315627	2	0.16445876209142	0.139471836261124
PAOX	10.7446390073976	2	0.00464334854566006	0.790626792417658
MTG1	8.87668188113943	2	0.0118155249258051	0.545597417927366
KDM5A	0.277282262611918	2	0.870540382097961	0.0821528334519154
CCDC77	2.88267140101963	2	0.236611505410907	0.785415690001926
B4GALNT3	1.34755535512971	2	0.509779154436687	0.438531046477337

NINJ2	6.07428327778596	2	0.0479718144791873	0.48301385025576
AC021054.1	0.495763751705978	2	0.780452128075214	0.460214207952369
WNK1	1.75961191968278	2	0.414863404032675	0.245910385443059
RAD52	1.54571915037452	2	0.461690938894884	0.549629760329749
AC004803.1	0.319144022458665	2	0.852508574995477	0.326785513454915
ERC1	0.00692775405504256	2	0.996542115273648	0.0303708527563466
ADIPOR2	0.841889439536755	2	0.656426387808963	0.246918064732649
DCP1B	0.510430395911967	2	0.774749755456628	0.279743140243819
FKBP4	0.0561423021398728	2	0.972319182789843	0.053570446518403
ITFG2-AS1	3.50611889310662	2	0.173243103809919	1.64521737241539
ITFG2	1.68653482566814	2	0.430302248884279	0.197990204222112
RHNO1	0.094103574867844	2	0.954037989131692	0.0829777284131646
TULP3	0.396086843314433	2	0.820334232092645	0.395316131056404
CRACR2A	0.861553239396903	2	0.650004092668987	0.429666654399373
PARP11	1.39142100025916	2	0.498719981464337	0.415318675688213
TIGAR	3.87181971593685	2	0.144292923013796	0.245104167817325
C12orf4	6.56310893242127	2	0.0375698103916624	0.901660539814298
RAD51AP1	1.3434726158536	2	0.510820865021095	0.875860969790967
DYRK4	1.38521209833804	2	0.500270638914464	0.231592104361096
NDUFA9	0.00757182930375509	2	0.996221242887538	0.0110942021659468
CD9	28.4069667005882	2	6.78430801537289e-07	0.590472578769796
CD27	77.7462164018402	2	0	0.704001177695909
TAPBPL	1.77757379439155	2	0.411154222684086	0.439099277102678
VAMP1	0.718149385114632	2	0.698322189933366	0.209060868028843
MRPL51	2.34708042191071	2	0.309270119455677	0.078515984196336
NCAPD2	5.26164189754584	2	0.0720193137958799

1.38154729214747
GAPDH 17.4780980866924 2 0.000160206164611987
0.18352627541877
IFFO1 0.283803255796562 2 0.867706610410564
0.218276115590977
NOP2 7.80933291151731 2 0.0201476735111572
0.417052553161019
CHD4 0.217321550796856 2 0.897034662117541
0.0660914957094259
LPAR5 8.30919145972499 2 0.0156921337085292
0.721235139552408
ACRBP 0.183712393687309 2 0.912236322575836
0.305615434135662
ING4 5.42673267994197 2 0.0663131977860338
0.426847252431146
ZNF384 0.0259076079830445 2 0.987129735420627
0.115539639794018
COPS7A 0.00394839443715574 2 0.998027750226993
0.0139690366418117
MLF2 5.87517578035711 2 0.0529934005992302
0.258480105879918
PTMS 2.35047519051834 2 0.308745614478462
0.323321512786865
GNB3 0.874025783804392 2 0.64596310366379
0.828647647184015
USP5 1.40766926619138 2 0.494684727685581
0.239827388580932
TPI1 44.9171143025892 2 1.76348824432182e-10
0.331712968284531
SPSB2 1.7280613974462 2 0.42145987629728 0.436969012594247
LRRC23 2.51466365206108 2 0.284411875376328
0.256384748706882
ENO2 48.6341039577884 2 2.74940070710272e-11
1.06265830986275
ATN1 1.26750245575581 2 0.53059767035307 0.626533785941258
C12orf57 9.93720090259913 2 0.00695287211162743
0.213483004192645
PTPN6 91.6056403832454 2 0 1.87474034179023
PHB2 7.28355610720354 2 0.0262057073644201
0.274242737850883
EMG1 3.1716097937214 2 0.204782897652333
0.289999508275522
LPCAT3 0.0952185915048319 2 0.953506253253952
0.089967582293466
C1RL 4.75145237124944 2 0.0929469679451613
1.52674482929558
C1RL-AS1 0.696829011998269 2 0.705806254645099
0.865222352126522
CLSTN3 0.436225927903062 2 0.804034609609613
0.269261719484467

PEX5 4.48833345924283 2 0.106015843440271
0.438491204798566
CD163L1 1.15729638612203 2 0.560655752867143
0.519405600295357
SLC2A3 11.0519121514431 2 0.00398205972096499
0.562311600033635
NECAP1 3.21254748507461 2 0.200633836233295
0.485477027503232
LINC00937 1.29465443981116 2 0.523442956667081
1.12059518296398
AICDA 166.598184386865 2 0 1.84112042556624
RIMKLB 0.480227414249025 2 0.786538420959495
0.332076426354479
PHC1 1.27860704732181 2 0.527659798659193
0.740342551913903
M6PR 0.960433183929083 2 0.618649382808844
0.139980309471808
KLRG1 1.23868949803588 2 0.538297041725189
0.458328404754762
CLEC2D 22.2260958871644 2 1.49164199221286e-05
0.274679809133171
CLECL1 10.517764832964 2 0.00520111415128821
0.633345477774434
CD69 4.08649216950052 2 0.129607310982861
0.515840528991515
CLEC2B 0.0418052369490072 2 0.979314327049221
0.0785500896400181
OLR1 14.8492358774353 2 0.000596388690189964
3.30492337598533
TMEM52B 41.1652704788254 2 1.15099940689589e-09
2.948972878055
GABARAPL1 7.9074480822756 2 0.0191831298250392
0.802692366184598
MAGOHB 1.99945507713403 2 0.367979687787198
0.177477034923935
YBX3 1.38320044871883 2 0.500774076677255
0.351282191232463
PRH1 16.0601976931718 2 0.000325516031181805
0.520694369338775
SMIM10L1 0.042837935123151 2 0.978808789518646
0.0464267030724187
ETV6 2.2197310999524 2 0.329603273264169
0.87163718096006
MANSC1 3.08237941182672 2 0.214126202620272
2.05314723849167
BORCS5 6.53953815113378 2 0.0380152047012463
1.36261853953537
CREBL2 1.10426858529475 2 0.575719743770283
0.352092015985194
CDKN1B 4.12961642167246 2 0.126842615274427

0.43680237567218
APOLD1 0.0186354373134455 2 0.990725556770042
0.0428866605604834
DDX47 4.33888094813176 2 0.114241520124679
1.0573192193634
GPRC5D-AS1 0.218903372411343 2 0.896325468200336
0.383772659121162
ATF7IP 1.85939799818949 2 0.394672489270975
0.315195170505506
GUCY2C 15.972526033372 2 0.000340102669545539
2.95302129257388
H2AFJ 0.594270954519365 2 0.742943353613961
0.313095978047582
WBP11 0.205322904287693 2 0.902432438338686
0.0796531462224867
C12orf60 0.992676559329542 2 0.608755676571862
1.16154549800527
ARHGDIB 25.6371298684302 2 2.70999183982212e-06
0.149700268309169
EPS8 3.11671232331584 2 0.210481785002611
1.54189910123014
STRAP 0.556411552225574 2 0.757141003950495
0.0797736940975284
DERA 4.38078801727432 2 0.111872661137725
0.355200083683472
AEBP2 0.0051582175896823 2 0.997424214248802
0.0209787868371109
PYROXD1 0.92935231289079 2 0.628338555636157
0.214361975655399
RECQL 6.53411654800538 2 0.0381183961802338
0.348584363770132
GOLT1B 7.5289491580334 2 0.0231797881833755
0.350752845212638
LDHB 22.9901010080545 2 1.01803567420511e-05
0.306401484471034
CMAS 1.48580093865415 2 0.475732066218062
0.150974099529365
C2CD5 1.05343874143431 2 0.590539135283362
0.826245289127996
ETNK1 3.80666712092638 2 0.149070853314394
0.230322523579912
SOX5 15.6113640231512 2 0.000407413461775974
2.8648494490403
BCAT1 5.68288135706887 2 0.0583415539654099
0.6828326956582
C12orf77 0.00497677274985531 2 0.997514707092002
0.00216878372505075
LRMP 126.061815852255 2 0 0.980278515166476
ETFRF1 0.208099035713126 2 0.901180671781088
0.13884988816152

KRAS 3.43589030641545 2 0.179434479714242
0.149286792294648
AC087239.1 0.00745660569128664 2 0.99627863864607
0.0603112858622194
BHLHE41 41.423471547719 2 1.01159680632179e-09
1.88398054429543
SSPN 53.437860774294 2 2.48956411041945e-12
1.33899210210456
ITPR2 1.86786845073114 2 0.393004496632416
0.404764885553142
INTS13 5.5980816529927 2 0.060868418008038
0.662478845373931
FGFR1OP2 1.45662232988813 2 0.482723542515179
0.183899809203281
TM7SF3 3.62059035861798 2 0.163605836616648
0.553419009523507
MED21 1.45615947565847 2 0.482835270759857
0.265917699983687
AC092747.4 0.0741020070400705 2 0.963626985727389
0.22408822260188
STK38L 1.30850545610085 2 0.519830372138196
0.255397806155299
ARNTL2 0.0220706973571398 2 0.989025317419411
0.111393157071687
PPFIBP1 0.955709003654984 2 0.620112415641735
0.801805267587286
AC009509.1 0.532935007474583 2 0.766080898087825
0.3482427830429
MRPS35 2.52147411555546 2 0.283445034122544
0.23166283239641
KLHL42 5.55185570567758 2 0.0622916523811028
0.869898494210824
CCDC91 0.0331165363926217 2 0.983578066401066
0.0331832995857938
ERGIC2 1.29158613246448 2 0.524246614911833
0.213787744463652
OVCH1-AS1 0.517264343184117 2 0.772106973696839
0.671706009205193
IPO8 4.33831130708795 2 0.114274063088297
0.97019324142615
CAPRIN2 2.45607440533897 2 0.292866852179919
0.722076626454354
DDX11 0.0811221552925767 2 0.960250512879106
0.148619826088108
SINHCAF 12.5229510925886 2 0.00190842774457434
0.446484961945942
DENND5B 0.234577278430979 2 0.889328461044407
0.0633548708284893
AMN1 3.52197676133244 2 0.171874902013245
0.489528474896578

AC023157.3	1.12764209382157	2	0.569030611854137	0.491308244589958
LINC02422	0.0294283006267936	2	0.985393573792872	0.150459307461632
KIAA1551	0.748565657497682	2	0.687782359693541	0.150962775469655
AC016957.2	0.543058888414612	2	0.762212840384748	0.742280527680967
BICD1	4.21248772460084	2	0.121694209248731	0.480070649522382
DNM1L	0.676582534761018	2	0.71298758762461	0.0732975498452931
YARS2	0.110319705758954	2	0.946333861669297	0.0726216879166169
ALG10	1.94610565901533	2	0.377927526905588	0.794492078646749
ALG10B	0.0778458086699917	2	0.961824858815129	0.168345427760743
SLC2A13	1.21411337866592	2	0.544952475339559	1.16544061341236
AC079630.1	2.91286282026429	2	0.233066511252452	8.61666190333485
LRRK2	4.7385857935904	2	0.0935468501746513	3.3920729069974
GXYLT1	0.229233204737216	2	0.891707957096105	0.163489620244428
YAF2	7.43446696087487	2	0.0243011044163129	0.461943177437793
PPHLN1	0.370495971818628	2	0.830898207253662	0.0665085173593047
ZCRB1	8.74740660740706	2	0.0126044758274202	0.453823439516928
PRICKLE1	1.14048533197796	2	0.565388221559302	0.6911377673725
PUS7L	4.31721673023485	2	0.115485723223219	0.439705824018703
IRAK4	0.479026720569876	2	0.787010758583736	0.116524101729443
TWF1	0.134386833636945	2	0.935014336348012	0.12234306061988[illegible]
ANO6	2.02042939239988	2	0.364140791534301	2.19544612026899
AC008124.1	0.207157247385253	2	0.90160513243026	0.146241898146082
ARID2	0.27723310294467	2	0.870561780098695	0.222679987682871
SCAF11	1.52901028739581	2	0.465564261388162	0.105438243796579
SLC38A1	6.23804325311894	2	0.0442003917613206	0.420168079900957

SLC38A2	4.79813516591581	2	0.0908025797025073	0.413771841636154
AMIGO2	1.63091880860419	2	0.442436023652961	0.92159876841974
PCED1B	18.6462600818722	2	8.9333853718121e-05	0.814110127460244
AC008083.2	0.264097506659601	2	0.876298271579288	0.188446688812936
LINC02156	0.390269464837176	2	0.822723803020492	0.489191130359347
RPAP3	2.26374925367492	2	0.322428256861754	0.202212890844263
AC004241.1	0.536354305187021	2	0.764772287707503	0.558698541339171
SLC48A1	2.38670888136035	2	0.303202481560787	0.355263131416547
HDAC7	14.6842964561813	2	0.000647657708957361	0.590463862782096
VDR	9.54888056047948	2	0.00844280837186839	0.588996709221117
TMEM106C	0.721012847753093	2	0.697323095571136	0.269784972342765
SENP1	0.589698068437959	2	0.744643994743118	0.362904795472137
PFKM	0.451732615639836	2	0.797824757467141	0.200982731719644
ASB8	2.41368419631971	2	0.299140445596615	0.324075643127428
ZNF641	0.475439969254273	2	0.788423430868032	0.256252405756244
KANSL2	0.0386517647380507	2	0.98085966528243	0.0604256625930629
CCNT1	1.02796608547754	2	0.598108536259462	0.485257387704909
DDX23	0.0606639466197194	2	0.970123425001376	0.0654902119727058
RND1	1.70853648460479	2	0.425594509221842	0.753515301185238
FKBP11	11.4512024141687	2	0.00326139191451913	0.966725893115207
ARF3	0.275263090354736	2	0.871419711396748	0.0765043953794184
DDN-AS1	0.214447148127978	2	0.898324808408889	0.305169274400767
PRKAG1	0.522325336032591	2	0.770155629736499	0.116011336445439
KMT2D	0.40988411621638	2	0.814694519985604	0.391732282199678
RHEBL1	2.36108406993083	2	0.307112227912065	2.20343543963404

LMBR1L	4.77314646007892	2	0.0919442163331055
0.697472881362454
AC011603.2	0.801497104517177	2	0.669818464204729
	0.693740232516119
TUBA1B	9.03428015992467	2	0.0109202099794561
0.323553306441639
TUBA1A	3.76493901415408	2	0.152213748310786
0.42185743031068
TUBA1C	1.94338366645953	2	0.3784422350415	0.186367246928465
TROAP	2.1806577586681	2	0.336105937231054
1.05694074623471
SPATS2	1.7191165785423	2	0.423349038836862
0.160298796327044
MCRS1	6.12909159220387	2	0.0466750370344062
0.315805124027378
PRPF40B	3.51358603035835	2	0.172597494755699
1.55071747118435
FMNL3	0.0521866882190209	2	0.974244145412064
0.0727079800065184
TMBIM6	23.2048092799297	2	9.1440730746184e-06
0.18341063942158
NCKAP5L	1.67474297483836	2	0.432846772639656
0.50175579960071
BCDIN3D	0.388168764407983	2	0.823588405131566
0.17024839737093
RACGAP1	0.0068744069430883	2	0.996568696950056
0.0521811534443776
SMARCD1	0.104854681087949	2	0.948923266799366
0.0627589057053739
COX14	46.7098561248906	2	7.19587722741721e-11
0.67908625193337
AC074032.1	8.6571414801001	2	0.0131863807939838
0.842777638039063
CERS5	2.90051733063198	2	0.234509620742714
0.225746754433335
LIMA1	2.56434185412932	2	0.277434356463171
0.636709396643069
LARP4	0.712429702576009	2	0.70032213895671
0.160412576824654
DIP2B	0.673931491800007	2	0.713933294626346
0.48536749269777
ATF1	3.69533905815527	2	0.157604030274494
0.356083237429334
METTL7A	0.15019570985349	2	0.927652706499359
0.0853353929599283
SLC11A2	2.87078171289077	2	0.238022313275841
1.77011927648929
LETMD1	0.670046259773772	2	0.715321540854886
0.167438372030706
TFCP2	0.253217351576212	2	0.881078392451688

0.239020248014672
DAZAP2 0.673254429891005 2 0.714175024060165
0.0721137718123068
SMAGP 6.08001790627702 2 0.0478344612237241
1.30713429645659
BIN2 0.791733396163461 2 0.673096415000692
0.231143989885127
GALNT6 4.5491652353934 2 0.102839823000968
1.46986678444501
SLC4A8 3.96259658001797 2 0.137890099710984
1.02347582428118
NR4A1 0.92789848290042 2 0.628795470403281
0.479964808148125
ATG101 5.61748823412861 2 0.0602806503137646
0.335108917861628
KRT8 0.000541078500396489 2 0.999729497342245
0.0174523434724149
EIF4B 4.0501681166852 2 0.131982748470447
0.210110672021026
SPRYD3 0.0105079556151819 2 0.994759800193479
0.0577182056679323
CSAD 0.2383711576865 2 0.887643057704142
0.227775244510424
ZNF740 3.80245320404532 2 0.149385270522701
1.59331046814467
ITGB7 7.90626560152802 2 0.0191944750194254
0.763019325902371
MFSD5 0.151357016022278 2 0.927114218446588
0.158854086063116
PFDN5 1.50362700287047 2 0.471510691606922
0.0609014855958851
C12orf10 5.94695846524932 2 0.0511251243358705
0.421505654520438
AAAS 9.51780531878526 2 0.00857501394757043
0.785723151879247
SP1 0.741149431336664 2 0.690337468847151
0.48575291096354
PRR13 0.714507850640049 2 0.699594830337259
0.0328036340517175
PCBP2 4.76155019862436 2 0.0924788694154108
0.103642581582544
MAP3K12 0.0209583793218976 2 0.989575525755831
0.0752659117484098
AC023509.4 1.9982294544875 2 0.368205259015848
1.14783275820568
TARBP2 0.00189497949551361 2 0.999052958978922
0.00093709261117925
ATF7 0.0284672575077256 2 0.985867190432096
0.0472017508944402
ATP5MC2 14.7714210048633 2 0.000620049960058711

0.259759174683195
CALCOCO1 8.38926480114977 2 0.0150762834767287
0.65463994807805
SMUG1 0.0187627187289763 2 0.990662508300654
0.0306484245158539
CBX5 9.56993848079397 2 0.00835438072142514
0.459203566898854
HNRNPA1 124.86018975696 2 0 0.56584760964982
COPZ1 5.40927088314795 2 0.0668947064307712
0.140593218221916
ZNF385A 0.447653694451667 2 0.799453549985493
0.316366182671319
GTSF1 0.301210941734606 2 0.860187000553849
0.0669980456117736
NCKAP1L 1.01397775683834 2 0.602306469045896
0.133881080956098
TESPA1 1.45573463931256 2 0.482937844639803
0.425396994651594
BLOC1S1 2.75624094393015 2 0.252051846648171
0.0918716196962496
RDH5 2.00140337591226 2 0.367621395142747
0.934459214963286
CD63 1.8164344612969 2 0.403242471928434
0.24055498383754
AC009779.2 0.0293269674662873 2 0.985443501580284
0.122865293692119
GDF11 0.0116198827167768 2 0.994206903712159
0.0449216770029721
SARNP 1.58399782741919 2 0.452938504799043
0.376905593564632
ORMDL2 2.29282380689245 2 0.317774933380124
0.108542791604871
DNAJC14 2.34810765533038 2 0.309111313940547
0.416216949959149
PYM1 0.265603204829922 2 0.875638799499825
0.0813723223310401
DGKA 1.68316724948563 2 0.431027397013157
0.43296668282354
CDK2 2.92819861442454 2 0.231286215516525
0.401806055757273
RAB5B 2.27588919736814 2 0.320477054283339
0.228278636872822
IKZF4 0.65586672585563 2 0.720411024187266
1.08545325237938
RPS26 8.43158400421657 2 0.0147606266889783
0.206055535143882
PA2G4 0.805140189087172 2 0.668599472108785
0.0443987671063198
RPL41 100.312102206419 2 0 0.297278440431922
ZC3H10 1.55966946755702 2 0.458481776595168

0.592277640345558
ESYT1 1.86774021348335 2 0.393029696347802
0.404848223359135
MYL6B 1.45273923201737 2 0.483661684324644
0.173851297729165
MYL6 3.17152959973999 2 0.204791108994901
0.0761565507973012
SMARCC2 1.59212289635416 2 0.451102159146016
0.297027375950224
RNF41 0.00485340761381685 2 0.997576238258453
0.0145908642292569
NABP2 2.15038114428274 2 0.34123271965411 0.285312151411862
ANKRD52 0.192506412096208 2 0.908234016605209
0.299257562474351
COQ10A 1.11162664664105 2 0.573605544660188
0.311475339090414
CS 4.27781985456956 2 0.117783165283562
0.580403786994793
AC073896.2 4.76938304302156 2 0.092117391430573
0.503997656185752
AC073896.3 2.2005845672101 2 0.332773805154874
0.826243219940867
CNPY2 7.51974768192859 2 0.0232866780144313
0.311661514849562
PAN2 0.468806573376939 2 0.791042734554423
0.366171576301047
IL23A 0.00555551650522331 2 0.997226096148153
0.0520349818242834
STAT2 9.70032186204304 2 0.00782711782179724
0.888321377033438
TIMELESS 9.26721161217491 2 0.00971964867133468
1.00555573328107
SPRYD4 0.831115652768426 2 0.65997202819388
0.160157551123022
BAZ2A 2.34931957970305 2 0.308924060912787
0.363478217240066
ATP5F1B 12.6989118558322 2 0.00174769775119366
0.121636636037422
PTGES3 1.09529044235241 2 0.578310000421197
0.0410171555732777
NACA 58.4866823594469 2 1.99396055222678e-13
0.326125156359505
PRIM1 1.9106068465202 2 0.384695401309755
0.544330350289769
HSD17B6 2.25207104159327 2 0.324316456979615
1.2227322401031
ZBTB39 4.09189128618843 2 0.129257900324487
1.41615919505133
NEMP1 2.56825748658471 2 0.276891722340241
1.71321582270374

NAB2	19.9761282899873	2	4.59450635887926e-05	1.70927451082327
STAT6	3.90448842315021	2	0.141955136481009	0.364485469261974
NXPH4	5.18243833780874	2	0.0749286337164736	5.02215440010221
SHMT2	7.25898631161157	2	0.0265296273894751	0.326470251262803
NDUFA4L2	0.355220946804496	2	0.837268498326792	0.282333197302894
STAC3	1.06061285962094	2	0.588424631201009	0.268733451942444
R3HDM2	2.14285094168181	2	0.34251991710443	0.423100365709321
ARHGAP9	4.1486238672266	2	0.125642848378833	0.466546302933986
MARS	2.55605137849318	2	0.278586774723325	0.330449579103892
DDIT3	4.08842423319521	2	0.129482166649241	0.484035512837513
MBD6	1.71052526862922	2	0.425171511788918	0.51149687592409
DCTN2	10.2470869865619	2	0.00595488437225589	0.321267387555356
PIP4K2C	0.61155748991004	2	0.7365495667365	0.257123628505488
DTX3	2.2216834834435	2	0.329281674266186	0.28714786524391
OS9	13.10043509563	2	0.0014298045136234	0.472532303891823
AGAP2	0.00402677055883326	2	0.997988640221126	0.0311148204258561
TSPAN31	0.00281514773993836	2	0.998593416297498	0.0127036588948148
CDK4	0.281177501492598	2	0.868846550731059	0.0873053196542844
MARCH9	1.4105647342486	2	0.493969073935017	0.239424181612264
METTL1	1.2577739863714	2	0.533184909316227	0.273210171547326
TSFM	0.050896815293733	2	0.974872673643558	0.0498150129587145
AVIL	0.0305770227400006	2	0.984827764603595	0.092846045142944
CTDSP2	2.12066712953924	2	0.346340264149639	0.19469143900697
AC084033.3	33.5891470157058	2	5.08404516263994e-08	1.30057909015752
ATP23	0.124211488089323	2	0.939783505027739	0.179336368980286
SLC16A7	1.17677927203561	2	0.555220672574924	0.368629558769534

FAM19A2 2.133900139271 2 0.344056266479916
1.51016380897321
USP15 2.30770915839941 2 0.315418617114386
0.208751561490962
MON2 0.0314353247272667 2 0.984405215465034
0.0883428812895657
AC048341.1 0.0541581425252895 2 0.973284279664736
0.154267722244909
DPY19L2 0.309246333567429 2 0.856737963983938
0.75471375158337
RXYLT1 0.114770125684201 2 0.944230411309638
0.0832461361188325
SRGAP1 7.39605041880901 2 0.0247723984980281
0.950705723628159
C12orf66 3.89496655547897 2 0.142632586862024
1.23080277959437
XPOT 2.5299437934672 2 0.282247228098108
0.915012787480221
TBK1 2.84784775033523 2 0.240767419647737
0.330142472385768
RASSF3 5.91867456624773 2 0.0518532698254234
0.580314866125937
GNS 1.05012636354771 2 0.59151799003026 0.609525259145576
LEMD3 0.0563586494342083 2 0.972214009166239
0.0934775671513609
LLPH 0.01190126683779 2 0.994067036533638
0.0135110592752281
TMBIM4 5.23663567646122 2 0.0729254320817405
0.187159376929558
CAND1 0.37857098527951 2 0.827550213471377
0.119099091470242
DYRK2 1.8468844377478 2 0.397149609613296
0.342878859206202
IFNG-AS1 4.8559457701187 2 0.0882154743635668
1.01837870436179
MDM1 0.914984333138551 2 0.632868786541461
0.174033960326659
RAP1B 5.2906569540423 2 0.0709820339427734
0.143183085825255
AC090061.1 0.0884266598944841 2 0.956749832309565
0.195621200933769
NUP107 0.105414335097595 2 0.948657769592188
0.076700855742265
SLC35E3 27.5377528140373 2 1.04773862474872e-06
1.16008055902966
MDM2 10.3021900490961 2 0.00579305771200489
0.852809262190046
CPM 16.1329454628299 2 0.00031388849976266
0.737995391019411
CPSF6 3.61904154832118 2 0.16373258288898 0.220825393176282

AC020656.1 0.667727032251805 2 0.71615151869104
3 0.52159147769486
YEATS4 1.56788773355315 2 0.456601679428054
0.244371196071747
FRS2 0.792568910850888 2 0.672815282756917
0.63422038125841
CCT2 4.87277937969169 2 0.0874760978953401
0.20206194712081
RAB3IP 1.6089023146064 2 0.447333374852767
0.261446200448568
MYRFL 0.0106171504785915 2 0.994705490345828
0.0814431713947605
AC025159.1 2.08298833853433 2 0.352926955190402
0.337672006250422
CNOT2 0.316925600400439 2 0.853454711544816
0.0850598497091955
KCNMB4 3.47340868724818 2 0.176099809786724
0.608954753281483
AC025569.1 4.29163103219632 2 0.116972605093772
0.52531177345123
AC083809.1 0.0127281920069402 2 0.99365611196429
3 0.100808888517158
ZFC3H1 10.5531597125652 2 0.0051098774559224
0.626988593331205
THAP2 1.81925872217651 2 0.40267344282542 0.378232049251688
TMEM19 6.80432492937683 2 0.0333011794172197
0.530929340126562
RAB21 2.91493371343611 2 0.232825308227062
0.383311300889638
TBC1D15 6.2646375755076 2 0.0436165423994838
0.483389273864323
ATXN7L3B 0.0609016542281524 2 0.970008128993575
0.0495941316493995
GLIPR1 2.80569848298378 2 0.245895349649099
0.28797007936976
AC121761.1 0.425265267898987 2 0.80845308088498
2 0.716688966949905
KRR1 3.48057693192382 2 0.175469776257851
0.246497198016167
PHLDA1 0.706182986647002 2 0.702512915195361
0.578923602558947
NAP1L1 66.6995941180522 2 3.33066907387547e-15
0.467865627769855
OSBPL8 14.7132628285765 2 0.000638345162225162
0.416571139801482
ZDHHC17 1.02366822708903 2 0.599395211145831
0.450044785847899
PAWR 2.4351170381747 2 0.295951846366206
0.318517488768796
PPP1R12A 0.209339014215155 2 0.900622122616493

0.0501285614545973
CCDC59	2.7988487330319	2	0.24673895427439	0.229988091127136
METTL25	2.85119745174839	2	0.24036450766771	0.720349892210276
C12orf29	0.104936583202244	2	0.948884408184095
0.0228657216943313
CEP290	0.978755077622006	2	0.613007849046258
0.339202252765103
TMTC3	1.41113542182118	2	0.493828143036984
1.37985021432068
DUSP6	0.893187598761104	2	0.639803744175686
0.635656579346581
POC1B	0.000372265601999301	2	0.999813884520635
0.006356532226674
POC1B-AS1	0.739859715073527	2	0.690782782143982
0.48111992058986
ATP2B1	5.55191937868912	2	0.0622896692641203
0.243726931708774
ATP2B1-AS1	4.51675652224055	2	0.104519851290037
0.323830170907147
AC126178.1	2.01699146944997	2	0.364767273821604
0.98059809123958
BTG1	9.24492611401651	2	0.00982855792500714
0.093365614223474
AC025164.1	2.42179978974947	2	0.297929053931944
0.160798558302572
LINC02397	39.5948333835057	2	2.52401055611529e-09
2.05971035654589
PLEKHG7	0.144707704892442	2	0.930201683427178
0.294921515276914
EEA1	3.03960404156591	2	0.218755191646925
0.42306437516966
LINC02413	0.370952398197478	2	0.830708606959164
0.244579463694268
NUDT4	1.14209268859496	2	0.564934013852643
0.279974859055587
UBE2N	10.6290846940026	2	0.00491952967774645
0.198099162117683
MRPL42	10.093242168371	2	0.00643102667355333
0.346564455151579
CRADD	0.267905766145951	2	0.874631273570397
0.237256623085093
CEP83	0.311247703065612	2	0.855881068182198
0.365429547857605
NDUFA12	5.83610437552001	2	0.0540388423745517
0.196054281080491
NR2C1	0.00198904208970023	2	0.999005973327303
0.0187978716301818
FGD6	9.1781903370516	2	0.010162049156607
0.846056440725025
VEZT	0.972992977897832	2	0.614776501790199

0.156730166408322
METAP2 2.71146659046612 2 0.257758212265429
0.134202883310693
USP44 1.8520334491725 2 0.396128460715546
0.753266293755285
SNRPF 28.3629431648861 2 6.93529831341522e-07
0.453290439251802
LTA4H 0.0566646472546943 2 0.97206527286085
0.0328132831541472
ELK3 1.67121805435966 2 0.433610320529914
0.21545076691238
CDK17 1.13930294730439 2 0.565722573566629
0.373560728921167
NEDD1 0.387604266110504 2 0.823820895066326
0.15089742772252
LINC02453 2.22959975323478 2 0.327980908981249
1.6164802393914
TMPO-AS1 1.01603626081032 2 0.601686862836616
0.696764727567167
TMPO 0.0356010548670552 2 0.982356966081166
0.0385164786475238
SLC25A3 5.63458710431422 2 0.0597674815836728
0.14657187607404
IKBIP 3.25606444958133 2 0.196315499067888
0.500145840081385
APAF1 0.113874303466954 2 0.944653437331918
0.119173997147028
UHRF1BP1L 0.310787512893525 2 0.856078024868738
0.257050041669329
ACTR6 1.00529617576499 2 0.604926637954909
0.100185650300699
SCYL2 3.40598654478185 2 0.182137520080886
0.366622562118189
UTP20 0.000951643877471368 2 0.99952429124657
0.013200707744022
ARL1 8.24668248166535 2 0.0161903281820722
0.521065432969423
CHPT1 16.3863209878991 2 0.000276538503384605
1.41867718153241
GNPTAB 5.61949084602745 2 0.0602203211489869
0.709081662771206
WASHC3 3.05030170446866 2 0.217588230718578
0.273703724748787
NUP37 2.95448563087845 2 0.228266193557895
0.223985881894745
PARPBP 0.822276289128768 2 0.662895349885773
0.427859476127877
HSP90B1 210.691610766965 2 0 0.966450807849151
C12orf73 2.68451701873765 2 0.261254955001823
0.352241435559271

TDG	0.788434620602045	2	0.674207528079103
0.124136650105973
HCFC2	0.65622117721372	2	0.720283360167318
0.145236617416484
NFYB	0.479440600279646	2	0.786847911541914
0.130202447785854
TXNRD1	0.893530752718666	2	0.639693977999191
0.182530898970308
EID3	1.52684698286314	2	0.466068112473328
0.736577691198677
CHST11	14.2358919518384	2	0.00081042968477496
1.50419001439597
SLC41A2	0.00873429857561075	2	0.995642372842082
0.0949893827757071
C12orf45	0.947832332808406	2	0.622559441776653
0.207280961725361
WASHC4	8.35838554183714	2	0.0153108619526748
0.404056942540235
APPL2	1.01696574828286	2	0.601407297604045
0.611826654105889
C12orf75	0.571081062663643	2	0.751607876892707
0.102384373156424
CKAP4	9.45162180802486	2	0.00886352352817432
0.770334083786371
TCP11L2	1.49484303665833	2	0.473586112870198
0.496066102867322
POLR3B	1.41035832901225	2	0.494020055467411
1.12447342015962
RFX4	3.63096557140394	2	0.162759311550529
1.77205539993314
RIC8B	12.8969427650936	2	0.00158294003003023
0.636252361367738
TMEM263	1.89412339962597	2	0.38787905579759	0.341912106546975
MTERF2	0.497443221687778	2	0.779797030207343
0.461031498686168
PWP1	1.25382265046065	2	0.534239346922167
0.128723344550049
PRDM4	9.4179958333072	2	0.00901380564276888
1.3807085792923
FICD	0.235153104429198	2	0.889072448676407
0.379071099730383
SART3	0.879841326979656	2	0.644087518712141
0.177118055686705
ISCU	6.85868149562291	2	0.0324082989739388
0.191931954871063
CORO1C	29.123545619879	2	4.74135443684354e-07
2.03392746856001
USP30	3.83493006601476	2	0.146979077390921
1.23798896846069
USP30-AS1	1.08405948212933	2	0.581566623921359

0.327963225826205
ALKBH2 2.50892843128511 2 0.285228628329558
0.479049338883861
UNG 0.0144725582116973 2 0.992789839722783
0.0435247465596954
KCTD10 0.689687498705445 2 0.708331022002235
0.262029717316732
UBE3B 1.66271287700963 2 0.435458213247338
0.463877023955394
MMAB 0.900023982925178 2 0.637620505573491
0.0909323220284859
MVK 2.98810872646623 2 0.224460762752494
0.34889784725299
GLTP 0.0182199907304185 2 0.990931374919553
0.0290651062234486
TCHP 3.3015044388726 2 0.19190549926732 0.608757894875306
GIT2 2.33411202903227 2 0.311282003339066
0.199576611001494
ANKRD13A 2.75890804829976 2 0.251715946377439
0.132463116375407
C12orf76 0.132387804030146 2 0.935949364227843
0.149787490886384
IFT81 3.98755189915699 2 0.13618024367374 1.04149275131126
ATP2A2 7.53735478000441 2 0.023082572347946
1.04696644515015
ANAPC7 1.29472989155166 2 0.523423209698503
0.192351227368636
ARPC3 8.23310396178054 2 0.0163006225134371
0.104643044866625
GPN3 0.0510917869746859 2 0.974777641993806
0.0467410435958736
FAM216A 0.379035583042646 2 0.827357996809068
0.183718663925773
VPS29 2.61604803375766 2 0.270353743366441
0.151262111077051
PPTC7 0.0532493110633933 2 0.973726655855838
0.0797296070708267
TCTN1 12.1025701836491 2 0.00235483388342717
1.16590976518546
HVCN1 16.8560003604737 2 0.000218658330694899
0.488716372066856
PPP1CC 19.0370443249582 2 7.34781729526679e-05
0.259199577701936
SH2B3 0.104960428016096 2 0.948873095265494
0.221120104059093
ATXN2 1.82689155248296 2 0.401139602543767
0.221856201975602
BRAP 0.403031787359156 2 0.817490584523437
0.196314463826223
ACAD10 2.11034843791573 2 0.34813177086828 0.643064612102035

ALDH2	87.8478302440993	2	0	2.00447653645077
MAPKAPK5-AS1	7.71757482512622	2	0.0210935618097051	0.568018153118735
MAPKAPK5	1.91450670196849	2	0.383946003954178	0.371715304192281
TMEM116	1.919122544091	2	0.383060908643194	0.826339411739402
ERP29	11.5024349491505	2	0.00317890819968381	0.247397213957784
NAA25	0.000201706434851723	2	0.999899151868089	0.0028464329702181
TRAFD1	0.108487665718677	2	0.947201119584485	0.0671139476716842
HECTD4	0.0244326222576736	2	0.98785800506887	0.065927346306919
RPL6	10.5183010091574	2	0.00519971998138336	0.12093692718863 4
PTPN11	3.18391938546737	2	0.203526371516533	0.559683593025211
OAS1	11.2188804134938	2	0.00366311937339547	0.938509901920783
OAS2	1.63995283065146	2	0.440442042065601	0.56129709281314
DTX1	1.46551921121124	2	0.48058094462341	0.218697209445669
RASAL1	7.68440409281638	2	0.021446323510546	1.26969964899858
CFAP73	11.1788674377971	2	0.00373714353935806	2.09622305487893
DDX54	17.7232551144327	2	0.000141724212087357	0.602423084587852
RITA1	15.1711594585752	2	0.000507720362591724	0.671940554584686
IQCD	6.53124748919382	2	0.038173117380657	0.568982760461185
TPCN1	8.50565393429266	2	0.0142239663327458	0.92455218531913
SLC8B1	1.3378587952688	2	0.512256707562581	0.602435450120454
PLBD2	2.95947665558116	2	0.227697262634803	5.67963456298757
RBM19	1.68347456968775	2	0.430961170388098	0.355699333433224
MED13L	0.233832480735327	2	0.88965970761273	0.0727959747729466
C12orf49	16.0256247230106	2	0.000331191976428724	0.629453847759339
RNFT2	4.26657938860602	2	0.118446997813151	0.571470382971874
HRK	44.0464573869695	2	2.72541877954779e-10	0.704230425693218

FBXW8	0.0487380139924186	2	0.975925519961074	0.0970009894901316
TESC	8.70880666992662	2	0.0128501045053321	0.636629089477076
FBXO21	1.74023260670769	2	0.41890282661072	0.278404599547126
RFC5	1.75048624357037	2	0.416760683759093	0.593714747919336
WSB2	0.175341540455391	2	0.916062422104389	0.180502287876139
VSIG10	1.50889313396236	2	0.470270806116537	1.04734207658727
PEBP1	6.08974959372348	2	0.0476022715682322	0.226824949480122
TAOK3	10.9743599506399	2	0.00413950122556317	0.40974123873691
SUDS3	0.747269046237691	2	0.68822839743831	0.0773896149510324
PRKAB1	2.36220054491222	2	0.306940834196244	0.464917269042748
BICDL1	3.40028921566084	2	0.182657108494662	0.926233010342173
RAB35	5.2218987063288	2	0.073464766638051	0.566059621280579
AC004812.2	0.00717273270430185	2	0.996420056978555	0.0485382347784436
GCN1	0.477713942047166	2	0.7875275135719	0.125574036897341
RPLP0	18.4959243044102	2	9.63077126826528e-05	0.201991549573759
PXN-AS1	0.0675673393079596	2	0.966780626004391	0.126902413101593
COX6A1	16.7221142148284	2	0.000233797054492113	0.239932747177723
TRIAP1	0.000360861908910307	2	0.999819585322231	0.00238070758473571
GATC	0.551553441629559	2	0.758982376810341	0.115917491402921
SRSF9	129.948395669032	2	0	0.681644539571583
DYNLL1	47.8221428321636	2	4.12623268886136e-11	0.456562661025045
NRAV	5.69933505278712	2	0.0578635557821842	1.21941805895433
COQ5	1.08701849671419	2	0.580706828054198	0.209621128679988
RNF10	0.47227047096676	2	0.789673874783279	0.118086429791936
POP5	0.480916262144301	2	0.786267564938907	0.141810065778511
MLEC	2.88663582917111	2	0.236142955291139	0.215503026338214

UNC119B 0.955027720135514 2 0.620323687808251
0.533277254342136
ACADS 4.13804526141332 2 0.126309172102504
1.00564799326934
SPPL3 0.415409953453283 2 0.812446692040766
0.245310439559416
C12orf43 1.20832453262127 2 0.546532083254222
0.298418664384345
P2RX4 1.37722604402039 2 0.50227222670012 0.295367119230686
CAMKK2 12.0668304304732 2 0.00239729271144995
1.49278160589501
ANAPC5 0.0591286992537788 2 0.970868400611691
0.0303001233148304
RNF34 0.335944678465624 2 0.845377218143672
0.104938125915248
KDM2B 3.22418032655907 2 0.199470252652877
0.462463873322333
ORAI1 2.28830272719801 2 0.31849408881299 0.332168249952833
TMEM120B 1.19503518017983 2 0.550175703925498
0.429057562754193
RHOF 0.401380467871404 2 0.818165832314174
0.0626187658369467
LINC01089 6.5303579535719 2 0.0381900993307444
1.76159867663014
SETD1B 4.68414938381698 2 0.096127995229363
1.18873248599702
PSMD9 3.92075633316992 2 0.140805163043852
0.256479415375944
WDR66 32.3240026199657 2 9.57044241634009e-08
0.643305864369836
AC069503.1 4.96889022991015 2 0.0833718034463762
0.547837255182061
BCL7A 2.49797901498724 2 0.286794454134064
0.0737555661781516
MLXIP 4.26261321057922 2 0.11868212181204 0.762138055179676
VPS33A 0.246883196570259 2 0.883873259450848
0.117791185442502
CLIP1 5.76688971504657 2 0.0559417192687607
0.334422498129144
ZCCHC8 0.247509829072713 2 0.883596370973732
0.154387995719644
RSRC2 1.06044448652588 2 0.588474170724441
0.0975817301861485
KNTC1 2.90600633545228 2 0.233866890913601
1.18792706014746
DENR 1.64419981240868 2 0.439507759727804
0.177053523679632
HIP1R 1.08878547319038 2 0.580194006970885
0.243869502358193
VPS37B 1.35656752224861 2 0.507487214684059

0.499362891635058
OGFOD2 2.85275496595075 2 0.240177394967621
0.538889221986716
ARL6IP4 14.8304128775093 2 0.000602028098343976
0.304163008446589
MPHOSPH9 14.5028703446258 2 0.000709155897252622
1.41816217902423
C12orf65 0.125276933412997 2 0.939282994385907
0.0229158029402915
SBNO1 7.72354190347275 2 0.0210307221306781
0.476306706551572
AC137767.1 4.89136656208596 2 0.0866668968182562
0.843124391747544
KMT5A 0.0405149022751807 2 0.979946352524778
0.0894906449831557
RILPL2 2.40591647439656 2 0.300304524592496
0.32910775191899
SNRNP35 0.00509669037943542 2 0.997454899085456
0.012997719833193
TMED2 6.69204743398221 2 0.0352241371462347
0.304239740891849
DDX55 0.00176039119380051 2 0.999120191661615
0.0142464604750903
EIF2B1 0.524503761007353 2 0.769317223291638
0.0548694844906836
GTF2H3 1.20884923092648 2 0.546388719831768
0.320524031410682
ATP6V0A2 0.159875340828377 2 0.923173885639342
0.139211590393096
CCDC92 7.374392648766 2 0.0250421136747203
1.39843817426365
ZNF664 8.6981899270866 2 0.0129184990039269
1.16238052938458
NCOR2 0.00212285076613637 2 0.998939137729602
0.0223675501828632
SCARB1 0.0651804613127757 2 0.967935108470753
0.0607629956719027
UBC 18.1484688370974 2 0.000114580328894887
0.247478949404326
DHX37 0.0489114611605186 2 0.975840887872069
0.102210261166139
BRI3BP 0.579081408595038 2 0.748607320755613
0.0554438585910449
AACS 3.88698910127873 2 0.14320264547492 0.701648248593833
SLC15A4 7.80125266001757 2 0.0202292372987444
0.419928002026298
PIWIL1 1.72916896452398 2 0.421226543369414
0.965785368812321
STX2 1.47722306687077 2 0.477776832352677
0.653246457187553

RAN	7.31612952513364	2	0.0257823594653185
0.155632587647059
SFSWAP	4.13641136116076	2	0.126412402557929
0.465130180311931
PUS1	0.280361011968464	2	0.869201325196916
0.149795514186002
EP400	0.912699459667496	2	0.633592212247343
0.327896845538111
DDX51	1.75136004354806	2	0.416578640791187
0.644837826666905
NOC4L	1.81603669941026	2	0.403322677147019
0.198320566174207
FBRSL1	1.79486391854749	2	0.407615088962746
0.903159629796349
POLE	0.116764393272926	2	0.943289356513598
0.225153340381388
PXMP2	19.3613189402259	2	6.24802863039564e-05
0.656720341461334
PGAM5	0.425822684277276	2	0.808227789787496
0.258001374620388
ANKLE2	2.64507511945968	2	0.266458289524202
0.306633487927523
GOLGA3	0.965722815015297	2	0.617015331140605
0.305168448610899
CHFR	0.364511971106797	2	0.833387977825331
0.154837962856936
ZNF605	0.597654903313644	2	0.74168737532231
0.348188226272745
ZNF26	0.687021345645878	2	0.709275911127812
0.367114110866044
ZNF84	1.40252290872217	2	0.495959279028141
0.330183670255288
ZNF140	1.25328450488938	2	0.534383115532681
0.239280136495432
ZNF891	0.196931850502841	2	0.906226571530753
0.283054197326362
ZNF10	0.669965214782012	2	0.715350528056391
0.432669760984291
ZNF268	0.432494914129428	2	0.805535941648307
0.424673489246239
MPHOSPH8	5.89296781016361	2	0.0525240612379551
0.360379723636347
PSPC1	0.0435154672466134	2	0.978477258478736
0.0353569344897103
ZMYM5	0.0795738727272244	2	0.960994170253634
0.0378088896888559
AL355001.2	5.55851394635473	2	0.0620846207820025
0.962192511899981
ZMYM2	1.63290021396515	2	0.441997918150368
0.224744471953585

CRYL1	1.29129563225695	2	0.524322767317486	1.1191259841683
IFT88	1.2709531507075	2	0.529682994295752	0.688142265199186
EEF1AKMT1	4.25689310740133	2	0.119022044669851	0.599257389594819
XPO4	2.49033430059539	2	0.287892782744455	0.792389224822837
SAP18	8.42857108757657	2	0.0147828797152822	0.146242663474271
MRPL57	6.6810242350125	2	0.0354188144813131	0.333298689056982
ZDHHC20	2.26582385286043	2	0.322093975567118	0.856490359533837
MICU2	14.3556871507163	2	0.000763312093669644	0.572142985771357
MIPEP	0.686077367349883	2	0.709610760677261	0.423481012032435
SPATA13	0.0398353469501297	2	0.980279372979091	0.16031619084934
PARP4	0.106078695626085	2	0.948342696537029	0.137613639995939
CENPJ	0.545212472852686	2	0.761392537256245	0.541559775477869
MTMR6	0.608813087729578	2	0.737560954610058	0.274487116855753
NUP58	0.313272501177481	2	0.855015013467238	0.162040737437266
RNF6	0.0162817268269132	2	0.991892183677014	0.0345964980335927
CDK8	0.0321346377909799	2	0.984061071920978	0.102358861101075
USP12	0.97775233179603	2	0.613315271637459	0.204554473033047
USP12-AS2	0.161749333106639	2	0.922309280401894	0.266292093175738
RPL21	12.444523760156	2	0.00198475086789374	0.112378838251431
RASL11A	2.16397167526636	2	0.338921813233185	0.784625500077784
GTF3A	10.1686257130436	2	0.00619314120513348	0.237897946046481
MTIF3	0.399043513090259	2	0.81912239934561	0.0910681863033308
POLR1D	22.9695495707209	2	1.02855065435836e-05	0.438885809689647
PAN3	1.9273500594404	2	0.381488325720654	0.311540058084706
POMP	1.74875997531124	2	0.417120559417245	0.0791682545898991

SLC46A3 1.4767110763135 2 0.477899156622548
0.255281050106505
SLC7A1 1.41755008000398 2 0.492246810949464
0.908406126487857
UBL3 1.18931616516026 2 0.55175118696183 0.325498072223931
KATNAL1 0.140373369531848 2 0.932219772430442
0.0946261461646931
LINC00426 0.938480912613702 2 0.625477165143405
0.145111048367219
UBE2L5 0.984236143746875 2 0.611330180671253
1.14406704077342
HMGB1 1.7724296034834 2 0.41221311178933 0.0565031319696051
USPL1 1.28084734081335 2 0.527069073164238
0.470385672017411
ALOX5AP 22.7403990021784 2 1.15341368134292e-05
0.304738533566108
HSPH1 1.97517765122695 2 0.372473708219926
0.207443201199424
B3GLCT 0.0592869083635128 2 0.970791603536565
0.0687120429478143
BRCA2 0.661813230754329 2 0.718272241491119
0.423304372548555
N4BP2L1 0.0715797046714756 2 0.964843031689386
0.0147713167479085
N4BP2L2 0.411373566521052 2 0.814088022350154
0.0675007560956745
PDS5B 0.847021439383985 2 0.654744156973118
0.204054116192866
RFC3 0.0288344736768704 2 0.985686193862343
0.0697133540495598
SPART 1.20147242722813 2 0.548407742190314
0.934838782092324
RFXAP 0.233515129312208 2 0.889800886200392
0.179336967609556
SMAD9 6.23688479545923 2 0.0442260013187007
4.28966018702247
ALG5 0.820211040028718 2 0.663580225446964
0.150902906477221
EXOSC8 1.39567788925245 2 0.497659612532945
0.221219530957644
SUPT20H 1.49384500470298 2 0.473822498882619
0.316093956931326
UFM1 2.30711876065981 2 0.315511742078188
0.136482401842771
PROSER1 0.491119095080627 2 0.782266700349684
0.502065536692884
NHLRC3 6.2938380144153 2 0.0429843575573562
0.275449151431035
LHFPL6 17.6719861874264 2 0.000145404201997379
2.11384196305631

COG6 2.45354007339218 2 0.293238198318642
0.673548253360738
FOXO1 2.90342541874291 2 0.234168881207905
0.508265883896569
MRPS31 46.5295557269431 2 7.87472309582427e-11
0.422491600597321
SLC25A15 0.774332303342461 2 0.678978272179401
0.674115104804457
ELF1 8.02010409919699 2 0.0181324514254224
0.232782719301797
WBP4 0.736432928869769 2 0.691967379148168
0.142828746847885
KBTBD6 0.601758858546984 2 0.740167009841023
0.544662778969618
KBTBD7 2.42231562027104 2 0.297852223390615
0.442282498479392
MTRF1 1.17388047876787 2 0.55602599102075 0.376179787593999
NAA16 1.11630843149249 2 0.572264366175066
0.448583076222301
RGCC 28.812890343186 2 5.53809444969744e-07
1.00055514366269
VWA8 0.00507564270799284 2 0.997465396192188
0.063441615187288
AKAP11 2.64882563852927 2 0.265959079296811
0.778920685949703
EPSTI1 1.4919266678249 2 0.474277192498278
0.462960066475478
DNAJC15 7.64561754670151 2 0.0218662970870311
0.222582333997699
CCDC122 0.0166106232903881 2 0.991729082172579
0.0273533735373101
LACC1 0.00124316144638973 2 0.999378612418083
0.0201300602026865
SERP2 0.467063045399118 2 0.791732637796806
0.139474903912214
TSC22D1 12.172574317574 2 0.00227383565228634
0.453915307558301
NUFIP1 1.0766558746903 2 0.583723459040081
0.191999798925666
GPALPP1 0.2163203128269 2 0.897483847125471
0.122437381370344
GTF2F2 0.140165112529739 2 0.932316848132084
0.0690422886477108
TPT1 13.2726380661229 2 0.0013118472335778
0.15029814503963
COG3 1.38830757555557 2 0.499496949620629
0.614541789750628
ZC3H13 1.44250102083735 2 0.48614394761981 0.0882843037094038
CPB2-AS1 0.494120581956314 2 0.781093599214581
0.778335477640282

LCP1	31.6034058541219 2	1.37216903284276e-07
0.522488436294039
RUBCNL	184.124790772105 2	0	2.1700626234592
LRCH1	1.14509885944961 2	0.564085507617449
0.266312346268584
ESD	15.4485421409113 2	0.000441968886695565
0.246936194570249
SUCLA2	3.36936221383313 2	0.185503578332867
0.30409367442519
LINC00562	2.14979073591125 2	0.341333467851178
1.17967696517171
NUDT15	0.0146141912508751	2	0.992719536291002
0.0546700771321159
MED4	10.7508790702821 2	0.00462888372927528
0.287403118743176
ITM2B	1.22451271043372 2	0.542126258604145
0.0809422689989069
RB1	2.81868728037581 2	0.244303581593864
0.349604450010186
RCBTB2	0.0696234439099014	2	0.965787235673393
0.0763484960595035
FNDC3A	0.543105030005518	2	0.762195255731062
0.480239776766549
CDADC1	0.568890844255879	2	0.752431420449163
0.180776533865831
CAB39L	1.57850311146189 2	0.454184599964996
0.497739054795541
SETDB2	1.56505747369352 2	0.457248287539955
0.169238073323064
PHF11	1.07033114276789 2	0.585572328097181
0.172512306340726
RCBTB1	1.62339072964008 2	0.444104508465704
0.689753451073705
ARL11	0.997569642525419	2	0.607268150874869
0.271631602253157
EBPL	0.786193241941007	2	0.674963528804225
0.207128542029631
KPNA3	2.97309714363701 2	0.226151856979501
0.363733784304801
SPRYD7	1.33503163361498 2	0.512981335862519
0.401788208488563
DLEU2	3.44943299683053 2	0.178223571276274
0.53091301799369
TRIM13	10.5554516361737 2	0.00510402508547025
0.572171008736103
RNASEH2B 2.31104522603959 2	0.314892926750095
0.232668448548772
INTS6	2.44384788350047 2	0.294662707334578
0.172043910551705
INTS6-AS1	0.803619291626268	2	0.669108101097155

0.21119778229111
WDFY2 4.03199589462823 2 0.133187422992234
0.892970880744853
DHRS12 5.5702834875793 2 0.0617203399360274
0.807896435064545
AL162377.1 1.54026423671944 2 0.462951899824944
0.711816632559725
ALG11 2.54357106827481 2 0.280330634728673
1.23656983796417
UTP14C 0.684428206307491 2 0.710196133198368
0.580152782937597
NEK5 0.279587201578479 2 0.869537688771341
0.436053114592496
NEK3 2.24029690311067 2 0.32623136147513 1.43841561942943
VPS36 17.2376222076399 2 0.000180674930796987
1.12015143710909
AL359513.1 0.190724241265361 2 0.909043691382315
0.311954689536824
CKAP2 3.07087161334865 2 0.215361814599013
0.47725666818707
HNRNPA1L2 1.61887872192989 2 0.445107540946025
0.706564327196561
SUGT1 0.0124483701628197 2 0.993795145033024
0.0163927235003257
TDRD3 4.61190957665882 2 0.0996635975716271
0.393994945379329
PCDH9 3.84694174950023 2 0.146098989791444
1.58919378161596
MZT1 0.864739701765478 2 0.648969310421194
0.141436557688788
BORA 0.190322989425768 2 0.909226087405106
0.246844103389675
DIS3 3.59858879004042 2 0.165415565099321
0.440294653938631
PIBF1 0.00735273828121201 2 0.996330380430585
0.0317633161205115
KLF5 2.13196852108568 2 0.344388719667783
4.92883330743908
KLF12 1.27993909162518 2 0.527308482549872
0.252583382662632
TBC1D4 2.60430508796531 2 0.271945787290673
0.608270580526731
COMMD6 23.4658588419557 2 8.0251562319944e-06
0.244674459310381
UCHL3 0.460213431201211 2 0.794448817896742
0.121697371550982
LMO7 3.95390545695219 2 0.138490613463783
1.02141589060951
CLN5 3.63988334186666 2 0.162035202020343
0.494378529990299

FBXL3	0.0454103033800288	2	0.977550670448619
0.0520402511516205
MYCBP2	3.45906443351894 2	0.177367360045484
0.213089733608195
SLAIN1	3.01780574170853 2	0.221152477737243
1.2109106094605
POU4F1	7.35206138452601 2	0.0253232915466292
1.89891331246074
RNF219	3.80590096134669 2	0.149127970285033
0.433501082001423
RBM26	0.537256831750612	2	0.764427251911973
0.136140609483114
RBM26-AS1	0.71363158492622 2	0.699901412976017
0.509374389257189
NDFIP2	5.21013107789955 2	0.0738982938222756
0.476281515381411
TGDS	1.18141990170801 2	0.553933879271107
0.198657583010919
GPR180	3.80158309850268 2	0.14945027513783 1.06608974221424
DNAJC3-DT	3.22228245756613 2	0.199659626694209
1.29009303885425
DNAJC3	0.423860745871322	2	0.809021025364492
0.170989584644854
UGGT2	1.61166639580188e-05	2	0.999991941700489
0.00251896830653761
MBNL2	3.27094391740814 2	0.194860383539588
0.447192720479091
RAP2A	9.84646326215687 2	0.00727558082169033
1.19787438470044
IPO5	5.17425760871789 2	0.0752357468173399
0.491343871934518
STK24	0.150340200123684	2	0.927585690525073
0.102177407857374
DOCK9	2.30922166801364 2	0.315180170443525
1.13289463114085
UBAC2-AS1	4.9342953861404	2	0.0848264653850299
1.20203318892264
UBAC2	1.58246315315216 2	0.453286194713604
0.180324385460741
GPR18	24.5402170701592 2	4.68985667778643e-06
0.765273492286244
GPR183	22.789618018348	2	1.12537516019362e-05
0.812184498783396
TM9SF2	14.4400010058533 2	0.00073180205083756
0.574647792001106
CLYBL	1.56760969183097 2	0.456665160999232
0.954980197351669
AL355338.1	0.741636607354269	2	0.690169331396677
0.736512091197744
PCCA	2.4710639336956	2	0.290680089060268

3.09080888980095
GGACT	0.0464371885329656	2	0.977048883144262
0.20142700839785
TMTC4	0.411903343838231	2	0.813872408224069
0.338275635022203
TPP2	4.11490103125286 2	0.127779326368809
0.47819695900798
TEX30	2.22112351655169 2	0.329373880591541
0.290226419308532
BIVM	1.53089254900111 2	0.465126310637954
0.726064678843272
EFNB2	0.621860730485813	2	0.732764899998603
0.44796112492974
ARGLU1	8.58570085282139 2	0.0136659160392628
0.218844931637439
LIG4	0.0208610205092798	2	0.989623698877432
0.0872754818270344
ABHD13	0.649853816305439	2	0.722580166431129
0.250318122141936
IRS2	0.319724545376421	2	0.852261160521973
0.330058786560785
NAXD	2.85453685465832 2	0.239963505569592
0.470765113149196
CARS2	1.6795520393491	2	0.431807228922278
0.220142923299563
ING1	1.09078538600397 2	0.57961412823148 0.400095951604051
ANKRD10	0.222747695571482	2	0.894604240590867
0.0908760682223831
ARHGEF7	0.359229155755473	2	0.835592205076572
0.124330915924368
TUBGCP3	2.50204811173188 2	0.286211550118495
0.566830790556203
PCID2	1.17628335773259 2	0.555358360581014
0.205006865177567
CUL4A	1.71678346421387 2	0.423843187861585
0.302008579609354
LAMP1	1.02472862065136 2	0.599077497966932
0.0822882252506654
DCUN1D2	0.216534778023765	2	0.897387612760315
0.498713941994331
TMCO3	0.0476241595348825	2	0.976469190834894
0.0560861162552361
TFDP1	3.39676552827423 2	0.182979205428208
0.268332777692915
TMEM255B	0.495706767627051	2	0.780474365064833
0.716274468794924
GAS6-AS1	2.58904707678168 2	0.274028397243122
1.17090280709752
RASA3	0.315287881239377	2	0.854153857323957
0.35212880920752

CDC16 0.684830769458198 2 0.710053198187442
0.168341930156344
UPF3A 0.000399563331647054 2 0.999800238289205
0.00209333637279781
CHAMP1 1.75718948351643 2 0.415366198525108
0.294041349022394
OR11G2 0.647987340551093 2 0.723254820369076
0.879131067827023
TTC5 0.249588510397869 2 0.882678490413994
0.151416481076413
CCNB1IP1 1.14663871463647 2 0.563651369768879
0.141691260780306
AL355075.4 0.673820116339524 2 0.713973053058085
0.809534589346912
PARP2 2.43807692757729 2 0.295514177941752
0.585305388319992
TEP1 0.574454896491955 2 0.750341045672288
0.448801029520623
OSGEP 1.12796122342291 2 0.568939821841618
0.140087594406187
APEX1 6.95505336808913 2 0.0308837018006998
0.229086066136584
PIP4P1 1.93426602814122 2 0.380171423286706
0.295317546333568
PNP 0.351571687458383 2 0.838797597869121
0.223299865315759
RNASE6 5.46151667349362 2 0.065169850232411
1.72997899704395
METTL17 0.713331259738111 2 0.700006519879137
0.20191907212404
ZNF219 0.226119007342952 2 0.89309751595371
0.473456039304664
HNRNPC 3.81078374224107 2 0.148764334751747
0.114997552795288
SUPT16H 3.20007309856029 2 0.201889138957109
0.268949464000468
CHD8 2.40902385435114 2 0.299838306735293
0.325969135486439
RAB2B 5.21764469385414 2 0.073621192955816
1.18287323999602
TOX4 1.9454386599308 2 0.378053586582073
0.17428743541811
METTL3 1.44485385371217 2 0.485572376157834
0.29198158055302
TRAC 1.66651249486106 2 0.434631711193614
0.19678439169097
DAD1 53.8792433390126 2 1.99651406518342e-12
0.340195649046361
ABHD4 2.83133950485818 2 0.242762967901252
1.54916127023978

OXA1L	2.1683625491698	2	0.338178547955239
0.137128260691267
SLC7A7	0.0590748793901377	2	0.970894526965686
0.141402589217646
MRPL52	4.32444704589318	2	0.115068977860765
0.131927308518532
LRP10	3.0519172472591	2	0.217412540138333
0.249012497089309
RBM23	0.531644618158317	2	0.766575328875869
0.102688700364697
PRMT5	2.72844110677092	2	0.255579809188163
0.392688168244689
HAUS4	7.96380737087829	2	0.0186501015952782
1.1181486571819
C14orf93	1.9623286052104	2	0.374874377448515
0.541628593035748
PSMB5	0.42033896165905	2	0.810446879120633
0.0985954917574127
ACIN1	0.376166644890303	2	0.828545667905488
0.0950183099790005
C14orf119	2.26987828830738	2	0.321441682344803
0.216741333318549
HOMEZ	0.361317458638523	2	0.834720175615752
0.407759722602749
PPP1R3E	1.1041883449734	2	0.57574284220226	0.471734113429636
BCL2L2	4.91811918001321	2	0.0855153326389357
2.93932295297926
PABPN1	0.459773515794081	2	0.794623582254005
0.082686698047856
NGDN	2.47701442917713	2	0.289816529071485
0.260136817444162
THTPA	0.842285702839741	2	0.656296341848198
0.371313702567891
AP1G2	0.0411671093202054	2	0.979626840667149
0.0321254903106188
DHRS4-AS1	0.0939372825352016	2	0.954117317030873
0.0961486538925516
DHRS4	0.0234912801899518	2	0.988323070655288
0.0686753100382384
DHRS4L2	1.56076998468341	2	0.458229562469385
0.149676884695063
NRL	1.47344672840926	2	0.478679808083915
0.694780287985274
PCK2	6.92828259777296	2	0.0312998711240662
1.1106108877646
DCAF11	7.20647059195832	2	0.0272354649644465
0.481356081763733
PSME1	8.23963207547234	2	0.0162475030944926
0.12530288368408
EMC9	0.00870256521843 3	2	0.995658170504929

0.0302275099942669
AL136295.2 0.401986264318588 2 0.81791804886544
0.348608094646939
PSME2 3.80802881246884 2 0.148969393597429
0.194541258119372
AL136295.5 0.556246017837751 2 0.757203672980221
0.485903625005857
IRF9 0.0424834593358384 2 0.978982286901494
0.0734347893322922
REC8 0.112098587955404 2 0.945492527654077
0.112411309823527
IPO4 3.05173013667415 2 0.217432881183605
0.52316384382922
TM9SF1 2.97619360781965 2 0.225801992322744
0.786385972162673
CHMP4A 0.350604969136633 2 0.839203136374651
0.0607257925925224
MDP1 2.91116155943399 2 0.233264849059803
0.824142697822417
NEDD8 7.36212447780041 2 0.0251961962353315
0.181343843610779
GMPR2 2.2873206008521 2 0.318650527938414
0.182816786814927
TINF2 0.358317570022293 2 0.835973148852157
0.0893732143709187
RABGGTA 3.9954862356354 2 0.135641063951718
0.746462857149037
DHRS1 0.969819517439549 2 0.615752760574589
0.217548057124513
NOP9 0.916963557294327 2 0.632242801738729
0.5139979752849
LTB4R 0.0067326793524006 2 0.996639320092525
0.0500775205860171
RIPK3 2.39111713879759 2 0.302534920229435
0.767464146266419
NFATC4 1.68094405630695 2 0.431506791995139
1.09693553935065
CBLN3 1.46555985288854 2 0.480571178914799
1.40047276698013
KHNYN 3.02006944860881 2 0.22090230714724 0.720260892850495
SDR39U1 7.44337983402478 2 0.0241930490355857
0.475105911030032
STXBP6 4.06713190230101 2 0.130868019135818
0.309843179817122
G2E3 3.51883849100413 2 0.172144808670463
0.461580449936041
SCFD1 0.367297089087432 2 0.83222824359097
0.0833193428079075
COCH 1.54285608677689 2 0.462352337448718
0.406541458899347

STRN3 0.214660392725912 2 0.898229032058635
0.148001216889722
AP4S1 1.37441690695415 2 0.502978198141376
0.292411539349169
HECTD1 1.27970520290699 2 0.527370151908283
0.234716161161979
HEATR5A 1.67652100048996 2 0.432462137305378
1.04084250793273
AL139353.1 0.153514725376619 2 0.926114536288595
0.3629880046749
DTD2 2.46495537512335 2 0.291569264438912
0.719947445071195
NUBPL 4.0027860931799 2 0.135146886135237
0.56423926354216
ARHGAP5 0.634406108626936 2 0.728182879438148
0.276814375096166
SPTSSA 0.0696049583116785 2 0.965796162292051
0.0424743687100046
EAPP 1.48181079079966 2 0.4766821342726 0.142886220879501
SNX6 1.03252442070756 2 0.596746898942628
0.141982560252592
CFL2 0.00109825515543667 2 0.999451023165236
0.0108228592057043
BAZ1A 12.7146199032714 2 0.00173402505472631
0.335230998526948
AL121603.2 0.00875001531704808 2 0.995634548745955
0.0388661773345134
SRP54 1.42949854202093 2 0.489314781818032
0.165408977845678
FAM177A1 6.76416949772164 2 0.0339765482788851
0.261380013607638
PPP2R3C 0.00347626019547527 2 0.998263379575584
0.00948433843935215
KIAA0391 0.45081561821038 2 0.798190642965624
0.632644746826916
PSMA6 0.642726607333399 2 0.725159749931496
0.247056827987695
NFKBIA 1.07286723332568 2 0.5848302664544 0.202401068342103
RALGAPA1 0.21377807047058 2 0.898625383212253
0.17881648457012
BRMS1L 0.399516241863884 2 0.818928810861577
0.247227508478309
MBIP 0.750903356691885 2 0.686978915203876
0.231065490476986
SEC23A 0.0453131132725588 2 0.97759817573023
0.0602175711932229
GEMIN2 1.41470205600573 2 0.492948275643322
0.187846043641831
TRAPPC6B 2.27597792301237 2 0.320462837332143
0.36909291527816

PNN	7.8413529191385	2	0.0198276775845608	0.284192333035617
MIA2	0.0445971382326624	2	0.97794820632622	0.0744768040141668
AL132639.2	0.733965234136098	2	0.692821688211979	0.653984068476996
FBXO33	2.30320590244606	2	0.316129622656641	0.649026040914147
C14orf28	3.07280778503097	2	0.215153426760506	1.1477173765932
KLHL28	0.0790794819989509	2	0.96123175292094	0.050360092946995
TOGARAM1	0.626103553400054	2	0.731212051841674	0.0931436568471013
PRPF39	0.141066631685088	2	0.931896692085157	0.151429609414795
FKBP3	2.7505950700178	2	0.252764378363825	0.16471699498754
FANCM	1.50794544756665	2	0.470493693541839	0.55098921951124
MIS18BP1	1.45987785673441	2	0.481938421957803	0.161964397900648
RPS29	7.28123204972341	2	0.0262361768493112	0.0930451678691387
LRR1	0.66677311507414	2	0.716493174780214	0.237478220878335
RPL36AL	28.9203290521352	2	5.24844123495072e-07	0.159168483734287
MGAT2	6.11717968120498	2	0.0469538609852039	0.27623790685894
DNAAF2	3.19086039586704	2	0.202821256446364	0.200908941643608
POLE2	0.00767640414679344	2	0.996169154409283	0.0890883274271996
KLHDC2	0.0546158506492443	2	0.973061565089415	0.0351152654727958
NEMF	3.62342049898772	2	0.16337448660204	0.299644794567817
AL627171.1	1.00729850492775	2	0.604321309897721	0.462639837383716
ARF6	2.7066042096094	2	0.258385633945671	0.135461599527195
LINC01588	1.2864366312561	2	0.525598158397371	0.902214104414095
VCPKMT	0.294783621767432	2	0.862955795696087	0.0484650269206955
SOS2	3.69583139444017	2	0.157565237958031	1.09050186361495
L2HGDH	1.47341750340412	2	0.478686802844945	0.904345311289994
ATP5S	1.13623300847153	2	0.566591607214646	

0.140023940709754
CDKL1	2.52572723551193 2	0.282842911708576
1.94204153139403
MAP4K5	10.2541795206264 2	0.00593380416224465
1.39607619018054
ATL1	0.442750360798591	2	0.801415948323859
0.626514071431289
SAV1	2.70606335375056 2	0.258455518086537
0.430970594236582
NIN	11.9192131603635 2	0.00258092716021074
0.692191173807429
TMX1	9.13292449048013 2	0.0103946685340165
0.436867327849703
GNG2	21.0781703646515 2	2.64809438850211e-05
0.49295614029679
RTRAF	11.6290793149457 2	0.00298385360606779
0.240898715113339
TXNDC16	0.00212116982371995	2	0.998939977309539
0.0252900400702581
ERO1A	0.0139241980660662	2	0.993062080233085
0.0675374010937958
AL133453.1	0.0778839667722154	2	0.961806508284543
0.264326402743654
PSMC6	2.31047506792649 2	0.314982708925467
0.0959818269704789
STYX	1.82740449912206 2	0.401036734130365
0.422118461362426
GNPNAT1	3.58742356125459 2	0.166341598853509
0.702011085809447
DDHD1	0.57941052280336 2	0.748484142237983
0.1461022721552
CDKN3	2.30645825231114 2	0.315615958356045
0.451116075146706
CNIH1	5.67228248642473 2	0.0586515519388451
0.236827693112986
GMFB	0.336639977884855	2	0.84508337407963
0.0661518521185683
CGRRF1	1.44228657931728 2	0.486196075137888
0.348574811296056
SAMD4A	1.38751997891638 2	0.499693689415389
0.761144815635861
GCH1	23.0904771035899 2	9.68203402895718e-06
0.919944738971902
WDHD1	0.801867399002359	2	0.669694460642858
0.505590980079556
SOCS4	5.12661219549604 2	0.0770495854311242
0.753200856134635
MAPK1IP1L	13.1886562010122 2	0.00136810583158253
0.492690303839613
LGALS3	41.0349875865326 2	1.22847310102259e-09

2.67803186849264
FBXO34 3.54983505117038 2 0.169497428124219
0.479007432063728
ATG14 0.143813930108823 2 0.930617471729697
0.228414605793159
KTN1 1.21156784644446 2 0.545646513962857
0.0858190557333478
TMEM260 0.418985757384904 2 0.810995414760297
0.276922988670981
EXOC5 3.90701966246657 2 0.141775588913131
0.662601214481153
AP5M1 0.0586973610418782 2 0.97107780951235
0.0467576934841647
NAA30 2.86881640998978 2 0.238256321202517
0.66058474730731
ACTR10 5.04108941111367 2 0.0804157918888174
0.239877351456577
PSMA3 3.38069863376233 2 0.18445507946479 0.100310973253914
PSMA3-AS1 2.74955922728494 2 0.252895324342985
0.239295627148635
ARID4A 2.60142408094776 2 0.272337808437591
0.301570015697588
TIMM9 4.62114103691976 2 0.0992046373345461
0.427539243256545
KIAA0586 2.75295898166596 2 0.252465798523548
0.699583856094958
DAAM1 5.65661346169691 2 0.0591128629615403
0.124684128373328
JKAMP 0.0363293799289835 2 0.981999293611496
0.0444188386584902
PCNX4 21.0788232345714 2 2.64723009899548e-05
0.680713967145936
DHRS7 3.58733645740772 2 0.166348843507844
0.441838324744874
PPM1A 0.750172721433723 2 0.687229926559052
0.184358797701535
MNAT1 0.0592311253684213 2 0.970818680745803
0.0515912847205361
TRMT5 3.77118773708 2 0.151738919695803
0.818487100536428
SLC38A6 1.0562519773912 2 0.589709056257602
0.894665379198565
PRKCH 1.63763502266315 2 0.440952767991342
0.791387457135433
HIF1A 4.46940252403289 2 0.107024097240942
0.319631214966824
SNAPC1 0.655399866520237 2 0.72057920912216
0.180224095919349
PPP2R5E 0.141406904463964 2 0.931738156033394
0.0242365646858945

WDR89 2.08982062320636 2 0.351723363468071
0.6615304579728
SGPP1 0.500113059896657 2 0.778756758747738
0.244685156957945
SYNE2 12.2095925293776 2 0.00223213608868478
0.289058628348155
ESR2 5.21013966477431 2 0.0738979765452595
0.565526925487424
MTHFD1 1.2117761552871 2 0.545589685425474
0.362908982165964
ZBTB25 0.000904683479838152 2 0.999547760551182
0.00738158274916908
AKAP5 0.00260754390376475 2 0.998697077589526
0.0370503023489641
ZBTB1 3.66314587898349 2 0.160161445278106
0.439771602667864
HSPA2 1.40435562724196 2 0.495505010318728
1.08027772574733
CHURC1 2.84927625493521 2 0.240595512364385
0.174530261591262
MAX 0.0711399993843061 2 0.965055178300189
0.0429202684934878
FUT8 3.56065875246042 2 0.168582611018041
0.365707851960139
GPHN 3.98154703162168 2 0.136589730256392
1.24468288608096
FAM71D 1.18784158968841 2 0.55215813634622 0.734009465157084
MPP5 1.26420674482885 2 0.531472739418726
0.735747612431407
ATP6V1D 0.464045033830685 2 0.792928268806969
0.110775069067287
EIF2S1 7.00983792637652 2 0.0300492083375488
0.407584316003782
TMEM229B 2.03734477128241 2 0.361073989119128
1.17641862227445
PIGH 0.108606197663404 2 0.947144984452578
0.0995151145304723
VTI1B 0.454401172779804 2 0.796760946858324
0.117723350331166
RDH11 2.0702050084284 2 0.355189970590049
0.267935011696898
ZFYVE26 0.212949747922027 2 0.898997636127111
0.344164377279731
RAD51B 1.78634572605748 2 0.409354863160488
0.426341505266885
AL121820.2 0.639074668870863 2 0.7264850789594
0.730974956322423
AL121820.1 0.94486557427393 2 0.623483618828282
0.88729239348042
ZFP36L1 39.4275419550242 2 2.74421441126549e-09

0.537080253011952
ACTN1 2.7883367745411 2 0.248039223196963
1.58421846912056
DCAF5 0.154345341975657 2 0.925729993092886
0.104817029553386
EXD2 1.10005904267693 2 0.576932778301264
1.04110410319283
ERH 20.2082137982963 2 4.091119159777e-05
0.18426605087685
SLC39A9 0.206941157486168 2 0.901702551574029
0.193038644076505
SUSD6 3.2191919974863 2 0.199968385237187
0.621732893913923
SRSF5 2.67379926152926 2 0.26265874660536 0.0634060905530394
COX16 3.73103579150374 2 0.154816010565063
0.493074170846393
SYNJ2BP 4.99874964174301 2 0.082136332496562
0.608209785422996
MED6 0.364304331887346 2 0.833474504331435
0.0939082506591337
TTC9 0.438487461299023 2 0.80312594788807
0.125483087336676
MAP3K9 2.9694650795318 2 0.226562929147672
1.27504556466864
AC004816.1 2.42210515209071 2 0.297883569247645
0.763126106783452
PCNX1 1.30752700120424 2 0.520084749643865
0.632143361227907
SIPA1L1 1.05766552995535 2 0.589292411138101
0.189310671707707
DCAF4 0.399281822801598 2 0.81902480274901
0.229293478927877
ZFYVE1 0.642276381234057 2 0.725323011229737
0.448877015756069
RBM25 1.38741853498147 2 0.499719035505238
0.114088903862237
PSEN1 2.35405651536832 2 0.308193250004484
0.537263622844632
AC004846.1 0.0896373118826227 2 0.95617086201681
0.184838226199923
NUMB 1.15638812307784 2 0.56091042213986 0.223228749106926
RIOX1 1.34712045227389 2 0.509890018695098
0.41104092757718
ACOT2 0.947685614713699 2 0.622605113819431
0.427271741406615
DNAL1 2.02522397972883 2 0.363268884647778
0.400321904829384
PNMA1 7.84173183671315 2 0.0198239214126412
1.01142333965507
ELMSAN1 0.550638940389467 2 0.759329501328144

0.177012856348225
AC005520.2 1.67025889663999 2 0.433818320745313
2.01366827605952
PTGR2 0.0138117504502571 2 0.993117915534343
0.121454897462709
ZNF410 0.49354073908278 2 0.781320087823479
0.777829625035595
COQ6 1.31357313769287 2 0.518514872071965
0.408882316220639
ENTPD5 1.47273805914793 2 0.47884945097032 1.03506249484328
ALDH6A1 3.24476193164461 2 0.197428069543577
0.673603839742855
LIN52 4.02278511017392 2 0.133802217914083
1.15616089373167
ABCD4 1.29552791706744 2 0.52321439882193 0.208001919959373
NPC2 3.08792541840699 2 0.213533252462231
0.249465483941599
ISCA2 0.402254770647878 2 0.817808248149671
0.10392905696236
FCF1 6.95312083714774 2 0.0309135580775787
0.533451003218696
YLPM1 0.433663778466493 2 0.805065298074117
0.207940322876346
DLST 0.0763780139969825 2 0.962530998602558
0.0686132641003602
EIF2B2 2.39401501547107 2 0.302096883206264
0.317195044595901
MLH3 0.397276739147742 2 0.819846321105506
0.161931541336764
ACYP1 4.43079931848485 2 0.1091098987539 0.649001384952291
NEK9 4.07561075630079 2 0.130314388090461
1.09976922924776
TMED10 34.9303924453624 2 2.59993000462089e-08
0.504639784370596
FOS 6.26180179570736 2 0.0436784297187643
0.261421191159387
JDP2 0.302342672373018 2 0.859700388253701
0.396060709373886
BATF 0.006882895237959 2 0.996564467374552
0.0245263301834064
TTLL5 0.682506608375106 2 0.710878816817279
0.46050874376158
ERG28 1.38799855071258 2 0.499574134066669
0.165850814899091
IFT43 0.000919131986552746 2 0.999540539591
0.00976926549202538
GPATCH2L 1.40608961216174 2 0.49507559738673 0.312341872200334
ANGEL1 1.9399016455884 2 0.379101680806373
4.47116478445064
IRF2BPL 2.33374613758468 2 0.311338956259959

2.96951345832827
CIPC	3.55193700712024 2	0.169319383637258
2.11385012618387
POMT2	1.07088744247464 2	0.585409473889969
0.580284272670093
GSTZ1	0.0191183785942788	2	0.990486354516695
0.0250539299636291
TMED8	5.98222006409345 2	0.0502316470271851
0.370917040333965
SAMD15	1.32435814153201 2	0.515726305062877
0.339821747703729
VIPAS39	0.676250635856839	2	0.713105917342228
0.290475395596362
AHSA1	2.68675351092373 2	0.260962970954541
0.179710339514401
SPTLC2	0.151316925862755	2	0.927132802711306
0.0359763118512813
ALKBH1	1.57093728444175 2	0.455905994915552
0.606599168661437
SLIRP	0.372801382642045	2	0.829940978201128
0.0508369212353085
SNW1	1.26605717986897 2	0.53098123893627 0.104019475457678
ADCK1	2.93630808708071 2	0.230350309604396
1.36052466337927
CEP128	5.94949735590666 2	0.0510602649620293
2.35030048262373
GTF2A1	0.675614966297911	2	0.713332603226621
0.262155888614377
SEL1L	0.169445887505306	2	0.918766799223949
0.165511688030927
GALC	0.0216448442720994	2	0.989235929582378
0.101557804464226
GPR65	14.7887325944967 2	0.000614706095888984
1.33485651273219
ZC3H14	2.89089242535788 2	0.235640907133204
0.428535328810607
TTC8	0.462576855836333	2	0.79351056242617
0.303138633372614
FOXN3	47.6150278852875 2	4.57643922757711e-11
1.38966437392199
EFCAB11	2.60758178157436 2	0.271500610555579
0.850724314969017
TDP1	3.31674424129343 2	0.190448755491554
0.712955058687193
PSMC1	0.130350549595504	2	0.936903233460383
0.036914170485235
NRDE2	0.848405828995627	2	0.654291103287313
0.281198507850707
CALM1	6.18664887207872 2	0.0453509372300914
0.143159415652524

RPS6KA5 4.81487140964034 2 0.0900459030469283
0.778577886495653
DGLUCY 1.77606400686855 2 0.411464717622407
0.514122449869972
GPR68 1.37094014861605 2 0.503853325394622
1.15346274288851
CCDC88C 12.4229558921647 2 0.00200627011276422
0.312524808286921
PPP4R3A 0.947510463109012 2 0.622659641349428
0.154258288603437
FBLN5 1.31074360611188 2 0.519248968339534
0.750525959571484
TRIP11 10.7935770260394 2 0.00453110919967281
0.673315716188785
ATXN3 0.395837512282962 2 0.820436505857638
0.130589425607079
NDUFB1 33.6917978798763 2 4.82968768311665e-08
0.448403421723811
CPSF2 0.0478774300731219 2 0.976345543225365
0.0568342334590346
RIN3 0.100519962146374 2 0.950982154998174
0.260449784281778
LGMN 4.39629871033838 2 0.111008405515808
0.71727244371336
GOLGA5 0.386413140218121 2 0.824311678397402
0.242334108551859
ITPK1 1.32708132565838 2 0.515024574063221
0.879196813837401
MOAP1 3.77787201811175 2 0.151232633415661
0.250707211028979
TMEM251 1.9937624820884 2 0.369028559453294
0.412719481977568
GON7 0.0726280195502084 2 0.964337434554331
0.0539825991353084
UBR7 1.40896240095554 2 0.494364984055285
0.428474178269532
BTBD7 1.53436918517743 2 0.464318475499531
0.170981622226432
ASB2 31.1687958906715 2 1.70522608922141e-07
0.977701947177126
DDX24 0.957823478170079 2 0.619457156135467
0.0962554372276105
IFI27L1 4.69673056835916 2 0.0955251911905741
0.888640527949276
IFI27L2 1.81763773498106 2 0.402999939366609
0.157417447234785
SERPINA9 56.307069220847 2 5.93081139754759e-13
1.35128387455637
DICER1 0.832178011424596 2 0.659621557784947
0.212100679005038

DICER1-AS1	2.14016791998988	2	0.342979719634268	1.72280764733676
SYNE3	0.885451377089597	2	0.642283368610624	0.314645506125837
AL133467.1	5.54192786234868	2	0.0626016319846018	0.662170075070293
SNHG10	4.43347902208304	2	0.10896380555323	0.585379255411669
GLRX5	2.22683656303102	2	0.328434358968619	0.195663130418162
TCL1B	9.94631237004114	2	0.00692126872066745	1.25364020445833
TCL1A	175.917704471741	2	0	0.638293374570733
AL139020.1	8.4936084859164	2	0.014309891851948	0.571097146524379
ATG2B	0.295685962133177	2	0.862566543588007	0.391497270378205
GSKIP	0.360778815255201	2	0.834945014141099	0.0451753525855174
AL163051.1	2.98878017324143	2	0.224385418672932	2.91860863453823
PAPOLA	0.781400347702992	2	0.676582982901278	0.0805088105993968
AL137786.1	0.180331357141607	2	0.913779779001373	0.391635089585439
VRK1	2.60082733865383	2	0.272419078305545	0.263735526041973
SETD3	0.0368611852728388	2	0.981738212088169	0.0635210993127555
CCNK	0.0512704446745786	2	0.974690570117167	0.0592558337306462
EVL	3.75025581736001	2	0.153335352667986	0.171604913846618
DEGS2	1.72216685310903	2	0.422703865547832	1.1637212902657
YY1	4.73926188163725	2	0.0935152325654126	0.222563201821412
SLC25A29	0.481913479573039	2	0.785875622800206	0.686348932440642
WARS	8.18482373511903	2	0.0166989093920116	0.575620736077171
WDR25	0.79552350205695	2	0.671822069513459	0.542682085946252
PPP2R5C	24.7983975242496	2	4.12188999920904e-06	0.524689131889605
AL118558.3	1.13566059984618	2	0.566753791383953	0.669090154162865
DYNC1H1	8.65909401835643	2	0.0131735136194371	0.635971417296013
HSP90AA1	35.0339780042065	2	2.46870011100242e-08	0.380352234622701

WDR20	1.12552158507682	2	0.569634248996283	0.289403301677191
MOK	4.68746022212651	2	0.0959689947472834	1.63018049501408
ZNF839	0.332117467751717	2	0.84699648534202	0.285828569094463
CINP	0.602913423400496	2	0.739739847741826	0.182854179759322
TECPR2	0.376481443838524	2	0.828415265515987	0.563697497385378
RCOR1	2.3136206326858	2	0.314487699044423	0.367284077092867
TRAF3	12.8404857624853	2	0.00162826071277256	0.492115104535104
AMN	7.40150827836988	2	0.0247048885189263	1.11737446085097
CDC42BPB	0.821896458888783	2	0.663021255691019	0.296937150982378
EIF5	0.00936707442968544	2	0.99532741344297	0.00839455734631632
MARK3	0.227566917000651	2	0.892451187678868	0.0949724122014582
CKB	3.69598085943644	2	0.157553463154165	1.20650514876687
TRMT61A	0.193439883822616	2	0.907810210128012	0.172037181646185
BAG5	0.211935002005177	2	0.899453878950218	0.137230750366629
KLC1	2.13821000903753	2	0.343315645910626	0.248087042151696
APOPT1	1.28977723061135	2	0.524720984738215	0.194747177361785
AL049840.1	0.861073944265856	2	0.650159883234044	0.562842246745278
XRCC3	0.0982144759784803	2	0.952079025183603	0.221982312764781
ZFYVE21	0.632389668065778	2	0.728917418411491	0.191503467984104
PPP1R13B	0.340333911381134	2	0.843523973711909	0.4503568941811
ATP5MPL	29.8575338417863	2	3.28487542944345e-07	0.325420805941095
INF2	2.29152255088927	2	0.317981753924308	0.625815676282559
SIVA1	3.07056106631863	2	0.215395257181271	0.123524383914603
AKT1	2.24516370234883	2	0.325438475300094	1.11491572769273
PLD4	43.6617434813898	2	3.3034885937866e-10	3.1801491386802

CDCA4	0.0839244373613459	2	0.958906008622225
0.0677901354441554
GPR132	0.331885948782474	2	0.847094538893844
0.406781894389316
NUDT14	0.0493864772166293	2	0.975609145348539
0.0755773141121613
BRF1	2.61150679037957 2	0.270968311902122
1.23439648495697
BTBD6	0.0269234402638858	2	0.986628483603038
0.166025683551065
PACS2	0.67097979113258 2	0.714987731221377
0.683320073527355
MTA1	0.0526492069838812	2	0.974018868362379
0.0512633451440678
CRIP1	11.9386169621204 2	0.00255600833616909
0.821078309909561
TEDC1	0.256842932388844	2	0.879482628824138
0.140488806155228
TMEM121	3.07470883055107 2	0.214949015695542
3.88343891456723
IGHA2	9.40577894904826 2	0.0090690344621982
0.903023378199432
IGHE	0.342329030274961	2	0.842682927969543
1.27446643298204
IGHG4	74.3949260670656 2	1.11022302462516e-16
0.727415713185999
IGHG2	22.769351245258	2	1.13683699723977e-05
0.682877196742431
IGHGP	15.1905491331477 2	0.000502821879654336
0.943781086499395
IGHA1	24.023945485566	2	6.07108790284805e-06
0.727877794814374
IGHG1	34.7412577661505 2	2.85779931941121e-08
0.483315078610664
IGHG3	203.330274283503 2	0	0.912276764616527
IGHD	1.54544847281003 2	0.46175342781282 0.237305234185678
IGHM	46.3626958004355 2	8.55989723547168e-11
0.874935185193054
FAM30A	1.12614258983299 2	0.569457403664199
0.194763780864171
IGHV1-2	5.43288986846342 2	0.0661093602833959
1.3996650440231
IGHV3-7	1.36949001765553 2	0.50421878452287 6.65920718440571
IGHV3-21 0.0379431203116941	2	0.981207267227759
0.20274082350988
IGHV3-23 0.915943835873158	2	0.632565239695124
0.620578926956322
IGHV3-30 0.0020753021574848	2	0.998962887094977
0.0500976107408437
IGHV3-33 1.60401560788363 2	0.448427704730739

1.15512415345569
IGHV1-46 0.848708995499038 2 0.654191931230916
1.15174656651806
IGHV5-51 0.0549866439803439 2 0.972881179441839
0.218954563478554
IGHV1-69 4.54395221712869 2 0.103108225583724
1.1183335101686
IGHV1-69D 7.00276474597605 2 0.0301556682147974
0.986596981992524
NIPA1 0.205366547064078 2 0.902412746224987
0.243943966610726
NIPA2 16.0090847652028 2 0.000333942283904265
0.347551357066884
TUBGCP5 2.44471293277644 2 0.294535286012177
0.633405803618419
NDN 0.599491406336234 2 0.74100663236347
0.395164671309295
SNRPN 3.5722460300477 2 0.167608728149828
0.213447341727358
SNURF 6.19900499183763 2 0.0450716201315984
1.51426292261757
PWAR6 2.07622911434629 2 0.354121729195604
1.28227195176058
UBE3A 4.02355216788985 2 0.133750910741772
0.296502852891763
HERC2 0.0381676824877364 2 0.98109710239305
0.0358280300025911
NSMCE3 0.0849655344695676 2 0.958406981381131
0.0402799817127645
ARHGAP11B 1.39660003320318 2 0.49743020852228
0.428298390368239
AC091057.6 0.549247847729722 2 0.759857833894545
0.536207652370628
FAN1 1.55086058094964 2 0.4605055871938 1.10456481798584
MTMR10 2.03845721266266 2 0.360873208140158
1.12489294398698
LINC02352 4.00415632483905 2 0.135054326574858
3.23958056848583
AC012236.1 3.32961281183744 2 0.189227287719137
0.393104466274423
KLF13 1.62765257637384 2 0.443159163377969
0.309687130249278
LINC02256 3.66049961182741 2 0.160373500522031
1.72005403849613
AC123768.3 1.06718892427257 2 0.586493049284425
0.812280279065661
ARHGAP11A 0.266452398837363 2 0.875267084809273
0.276271405482112
AVEN 0.201436056428453 2 0.904187952435738
0.271407068772117

EMC7	4.10737666039923 2	0.12826096132023 0.199434076783115
PGBD4	2.32601732606378 2	0.312544424024101
0.892597543491954
KATNBL1	2.40632088164194 2	0.30024380806847 0.150244328774959
EMC4	11.9457808625711 2	0.00254686921923575
0.340525202947514
SLC12A6	0.862265036793034	2	0.649772798219806
0.79843298941739
NOP10	7.67133786199317 2	0.0215868934971891
0.224280130820987
LPCAT4	0.422866645553903	2	0.809423249348015
0.324343052161338
GOLGA8A	1.11188872375051 2	0.573530385143135
1.04763252471637
GOLGA8B	1.57850666957311 2	0.454183791946055
0.609846916868778
AQR	2.61560919221234 2	0.270413071102333
0.370706353338131
ZNF770	0.0540641191549578	2	0.973330036474412
0.0681731050864252
DPH6	4.90798267399516 2	0.08594984616302 1.78890013632839
C15orf41	0.212302904942473	2	0.899288438305196
0.398617953118956
FAM98B	0.326326149666055	2	0.849452652769544
0.193759691886195
RASGRP1	4.62704453489685 2	0.098912241897901
0.908935314005131
C15orf53	1.54855433335056 2	0.461036913429803
1.27800425420661
EIF2AK4	4.34200729562239 2	0.114063080282369
0.594183366779773
SRP14	0.455599192865944	2	0.796283821964798
0.0257839968902485
SRP14-AS1	8.6123091761015	2	0.0134853065760556
1.20624029407033
BMF	2.18858211566926 2	0.334776860271465
0.521550774970215
BUB1B	0.706925231340918	2	0.702252245326703
0.448137923355193
PLCB2	3.53559167531304 2	0.170708844456476
1.55058330069064
KNSTRN	8.06965628333404 2	0.0176887198058721
1.23236864740942
IVD	0.65594187128142 2	0.720383956899187
0.21103469370144
BAHD1	3.13906848768393e-05	2	0.999984304780733
0.0052025848755594
CHST14	0.0938217349761253	2	0.954172441586762
0.2361608909603
CCDC32	7.51061776719541 2	0.0233932237100317

0.479860239420844
RPUSD2 7.37103442076657 2 0.025084197560307
0.940654144423125
KNL1 1.35439977086036 2 0.508037565943434
0.441531792338466
RMDN3 0.113298891321469 2 0.944925258962968
0.0951871062919374
GCHFR 26.8845798117961 2 1.45240478055531e-06
0.460891830109519
DNAJC17 1.335988354963 2 0.512736004448072
0.34126123768174
C15orf62 0.00943763310946427 2 0.995292299568251
0.0483032725234233
ZFYVE19 0.152283956286368 2 0.926684628256145
0.135702683389094
SPINT1-AS1 0.00313215137492861 2 0.998435149969158
0.0318823625357348
SPINT1 0.133395398734314 2 0.935477954174
0.144721177980537
VPS18 4.91194399042015 2 0.0857797773740248
0.85216101862086
CHAC1 1.74309312996667 2 0.418304114231069
0.447120412197031
INO80 0.23749687161493 2 0.888031169509086
0.113965781298374
CHP1 0.703435102452637 2 0.703478790639887
0.100187606735321
OIP5 3.60593775136077 2 0.164808864142568
1.47075205380263
NUSAP1 1.53143937828603 2 0.464999155677839
0.286463152524545
NDUFAF1 7.59862594701454 2 0.0223861464498655
0.713738914653017
RTF1 10.5633569743439 2 0.00508389038242929
0.374339237165767
RPAP1 0.295105219806894 2 0.862817044405902
0.351121914924837
MGA 3.89740877933187 2 0.142458522806582
0.501905515964661
EHD4 11.2826609858343 2 0.00354814448324126
0.837013020743654
VPS39 5.90755640611487 2 0.0521423290112597
0.941330010944055
TMEM87A 11.4973513625887 2 0.00318699860489924
0.500775005199828
GANC 5.42275657445309 2 0.0664451630543776
2.18362466960432
ZNF106 4.29502663822193 2 0.116774177145876
0.172455996589036
SNAP23 32.9825694272381 2 6.88535044535143e-08

0.340506138666954
LRRC57	2.35813639313254 2	0.307565195424871
0.579835492789963
HAUS2	0.0138243935141161	2	0.993111637527573
0.0385967971337535
CDAN1	0.0973502623084151	2	0.952490513935024
0.303456140595729
TTBK2	0.524107475327507	2	0.769469673093982
0.432987248688003
UBR1	0.791894704363149	2	0.673042129204447
0.336840369913733
TMEM62	0.0910354834822693	2	0.9555026501409
0.166994240705452
CCNDBP1	13.3921801249792 2	0.00123573411241984
0.20232681423308
LCMT2	0.382947966942007	2	0.825741107744503
0.36765178317778
ADAL	1.75577295124745 2	0.415660492543876
0.463190313772992
ZSCAN29	3.4157256583197	2	0.181252747061677
1.12462219725446
TUBGCP4	2.93292332783751 2	0.230740479839151
0.464315155845449
TP53BP1	1.69717249220809 2	0.428019618812448
0.459218359376166
CATSPER2 0.50206024645692 2	0.777998935366301
0.481547345762197
PDIA3	3.53133094134698 2	0.171072904595577
0.181546696457601
ELL3	1.69697568258951 2	0.428061740073841
0.486874018531062
SERF2	194.395798602127 2	0	0.408795719508685
HYPK	0.512144304094554	2	0.774086114879328
0.343624569136309
MFAP1	6.53661801075297 2	0.0380707501086418
0.35493324595246
WDR76	4.84047076963883 2	0.0889006891237214
0.500734028076873
CASC4	1.01286157141861 2	0.602642705712264
0.298926275513802
CTDSPL2	0.000229286324552303	2	0.999885363409
0.00459290076795096
EIF3J-DT 0.724061835725745	2	0.696260840611791
0.322242771853355
EIF3J	1.76421587984413 2	0.413909495101309
0.140225398418488
SPG11	0.903567657357684	2	0.63649174611618
0.173424796336824
B2M	133.895873427191 2	0	0.230924141861395
TRIM69	1.94904864516389 2	0.377371818128246

0.578636400907785
SORD	0.694145079114332	2	0.70675405877179
0.379494281333255
DUOX1	0.000645293208295282	2	0.99967740544067
0.0131972074313131
SHF	0.363020091583831	2	0.834009866971817
0.657437410936834
GATM	0.0492426585156253	2	0.975679303291001
0.0641389467695593
SPATA5L1	0.976421104145367	2	0.613723638653837
0.478648147885617
SLC30A4	22.8712582483768	2	1.08036218433583e-05
0.842826226911816
AC090527.3	2.84020908362431	2	0.241688748996486
2.42106194529249
BLOC1S6	37.5495344935936	2	7.01814251158339e-09
0.636405099997917
SQOR	3.1361948188919	2	0.208441383940969
0.329768241097778
MYEF2	0.0153952900321248	2	0.99233190598036
0.0477652259693943
DUT	3.97996382902093	2	0.136697897671566
0.245217335478834
CEP152	1.83488965647395	2	0.399538627740106
0.751784293181828
EID1	10.21925139751	2	0.00603834265983749
0.291508361289191
SECISBP2L	2.43208900743366	2	0.296400261378492
0.27063232092733
COPS2	0.0311509297462074	2	0.984545205369208
0.0319701395798682
GALK2	1.90527337512493	2	0.385722651362565
0.524702382805162
FAM227B	4.28846609533802	2	0.117157857087465
2.02097528563905
DTWD1	2.99172158693733	2	0.224055656052621
0.392434152882749
GABPB1	1.74443910636094	2	0.418022695205842
0.381602598814691
GABPB1-AS1	13.9860180749553	2	0.0009182792336766
0.604266430466466
USP8	2.49000500724429	2	0.287940187236439
0.180309935033196
TRPM7	0.755172539532081	2	0.685514059895695
0.310134902934071
SPPL2A	0.945834129910216	2	0.623181752641157
0.177375869857508
AP4E1	0.549940013996275	2	0.759594905414726
0.432131973947597
DMXL2	7.59581568064191	2	0.022417624077106

1.63380558921313
LYSMD2 25.3026506776467 2 3.20331182246392e-06
0.447651583358898
TMOD2 3.18272075812085 2 0.203648384212138
0.914432724977944
TMOD3 4.59335577088637 2 0.100592468950762
0.518326335878406
LEO1 0.555008798739651 2 0.75767223131529
0.158992412207126
MAPK6 0.624397645948044 2 0.731836007950808
0.394899774908822
GNB5 5.50328765632395 2 0.0638228611128209
0.280391950649956
CERNA1 2.613738490678 2 0.270666120502367
1.8861882629279
MYO5A 0.12708724949746 2 0.938433179496021
0.160966768190753
ARPP19 1.79972764776196 2 0.406625028588847
0.187942885891642
FAM214A 7.12897519320393 2 0.028311488671127
0.593158780487673
RSL24D1 14.4141640746431 2 0.000741317138298059
0.189077440344992
RAB27A 0.668635383918146 2 0.715826333829508
0.279472213660766
PIGBOS1 0.157883990836837 2 0.92409352454878
0.0866507660964771
PIGB 0.0623257176453034 2 0.969317698281606
0.103552671728106
CCPG1 1.24542243979313 2 0.536487927284287
0.539337139454462
RFX7 3.2162534448673 2 0.200262409997177
0.946984916206048
TEX9 0.300587987115748 2 0.860454971017581
0.0836097368112365
AC084782.1 0.721506429002209 2 0.69715102400256
0.463427522072507
ZNF280D 3.50178393219483 2 0.173619012091305
0.50717374145258
TCF12 1.82996880440364 2 0.400522873317041
0.619385817668212
LINC00926 8.37261809879626 2 0.0152022923591645
0.718515048973277
POLR2M 2.03279867015564 2 0.361895662051799
0.405432452566717
ADAM10 1.85719160235604 2 0.395108131394486
0.426823668115912
MINDY2 0.256561042331826 2 0.879606596264423
0.232564572410493
RNF111 1.27111007578388 2 0.529641435654002

0.388972967544584
SLTM 3.33633756165097 2 0.188592103098077
0.0942055316350795
CCNB2 0.928559179525294 2 0.628587783187214
0.372157772153963
MYO1E 4.49867310043413 2 0.105469174856209
0.215175017042182
FAM81A 8.39294520094414 2 0.0150485656124522
0.8915282636032
GTF2A2 4.08422183441745 2 0.129754520533378
0.130730781883001
BNIP2 3.35336942828785 2 0.18699288440832 0.280323331540965
ANXA2 37.4776623550883 2 7.27493343344321e-09
0.99804939051332
ICE2 0.576939037972179 2 0.749409647565263
0.241890014217107
VPS13C 1.80960038524605 2 0.404622723631757
0.244771105341674
TPM1 1.97213743806909 2 0.37304033851438 0.9134373280656
LACTB 1.13487664152461 2 0.566975990605357
0.314198533401989
RPS27L 0.581912922331117 2 0.747548224687956
0.0682197215916756
RAB8B 0.011451035736643 2 0.994290841671937
0.0242446964083893
APH1B 2.08526864951916 2 0.352524792891364
0.576465050992053
USP3 1.77652626169027 2 0.411369627836914
0.324155661708774
USP3-AS1 2.35213507219338 2 0.308489480187458
1.06089099593853
FBXL22 2.44022192734073 2 0.295197408918597
1.33668347239465
HERC1 2.69384430961001 2 0.26003939120569 0.698182589782211
DAPK2 0.377860222798435 2 0.827844361557107
0.445022327564057
FAM96A 0.588251033970062 2 0.745182952455557
0.0801021699186195
SNX1 0.101214431394531 2 0.950651998391297
0.0512245856857106
SNX22 21.9782431134132 2 1.68843811325647e-05
1.28368172828887
PPIB 46.0097638639046 2 1.02119090961139e-10
0.366325252513452
CSNK1G1 0.260742402347442 2 0.877769541355001
0.264190860268836
PCLAF 40.3913481676508 2 1.69484581924451e-09
1.35937863668262
TRIP4 5.03808504721976 2 0.0805366818162436
0.458843597541174

ZNF609 0.603866833646581 2 0.739387294005452
0.289266476551981
OAZ2 11.5725401429884 2 0.00306940952967028
0.368123251654631
PIF1 4.57591374287123 2 0.101473573760791
0.906559251811888
PLEKHO2 0.218143494817647 2 0.896666081722357
0.214323253366332
ANKDD1A 4.16055859693096 2 0.124895324254325
1.42850195081277
AC103691.1 5.43404839556112 2 0.0660710766299787
1.20731793508746
SPG21 0.770694659054331 2 0.680214336646138
0.102322481027455
MTFMT 2.11393898080147 2 0.347507340520382
0.324631707422983
PDCD7 1.16645739522601 2 0.558093539234462
0.171818563842359
CLPX 2.64010173345435 2 0.267121714012917
0.342571926613279
PARP16 0.396601762593291 2 0.820123056322825
0.209209427320842
DPP8 0.564596050399826 2 0.754048925461064
0.205423654380604
HACD3 0.131785489552562 2 0.936231274601568
0.088265946614847
INTS14 1.34523380531454 2 0.510371236858296
0.232963478818454
DENND4A 23.6897608322741 2 7.17519645554443e-06
1.26781763733786
RAB11A 16.2534048805302 2 0.000295541160358459
0.379155104574239
MEGF11 2.69427494354127 2 0.259983406340494
3.20244941155142
DIS3L 0.0940557400535081 2 0.954060807519609
0.0931223893354344
TIPIN 1.73929375304112 2 0.419099517000332
0.509446566448263
MAP2K1 2.37307043501222 2 0.30527715273269 0.119953558080247
AC116913.1 0.168357130138226 2 0.919267092446718
0.292552426712366
SNAPC5 3.87637632641288 2 0.143964553896063
0.56429812997726
RPL4 41.8208476246312 2 8.29312729599962e-10
0.278784417717634
ZWILCH 1.77767266715994 2 0.411133897208384
0.544528696928891
SMAD3 1.93407647190976 2 0.380207456925434
0.596903361429507
AAGAB 0.49787439177744 2 0.779628935749603

0.193833099936244
C15orf61 0.0631057465030269 2 0.968939724105607
0.0964191793636021
MAP2K5 1.37274713511107 2 0.503398302903221
0.500035419597158
PIAS1 0.0424680228565631 2 0.978989842950544
0.0295560992393911
CLN6 0.237359754005419 2 0.888092053951712
0.256042520543274
FEM1B 0.471333451123597 2 0.790043931509284
0.317756390759727
ANP32A 0.427959277370122 2 0.807364823864772
0.0615989605837344
GLCE 1.18466808229113 2 0.553034970787474
0.904570690167511
KIF23 2.61821550115747 2 0.270060910608883
1.25888625166971
RPLP1 41.1565319859937 2 1.15603937533848e-09
0.195916236255676
TLE3 0.283521843496937 2 0.867828710656808
0.191508607099927
UACA 2.488255784938 2 0.288192133096903
7.50243467528139
MYO9A 1.15490788161488 2 0.561325717237089
0.242994008275695
SENP8 2.03500294562074 2 0.361497022905379
1.05924871248618
PKM 82.5559257484526 2 0 0.53797674195678
PARP6 2.99402498740568 2 0.223797759639263
0.900908232715352
HEXA 0.876650797154411 2 0.645115828926926
0.166191525370376
ARIH1 0.283812213417996 2 0.867702724125601
0.186778921240354
BBS4 0.0328119054795333 2 0.983727891953381
0.106548102726978
ADPGK 2.71697257114546 2 0.257049582270413
0.201409421578062
STOML1 1.71948802072158 2 0.423270421292764
0.649565552726244
PML 1.94026926468868 2 0.37903200470073 0.427742558722886
SEMA7A 6.04475362445006 2 0.0486853647652766
2.07558360194768
UBL7 1.32791752241737 2 0.514809288131891
0.173543720956901
UBL7-AS1 1.95896342477887 2 0.375505668361169
0.470948809949803
ARID3B 4.22939941928384 2 0.12066952000643 1.50815198859225
CLK3 1.70297287723254 2 0.426780077841431
0.238859045103095

EDC3	0.173291039800464	2	0.917002097021327	0.0594122730922505
CSK	8.20077503379514	2	0.0165662544541983	0.324368770354395
ULK3	9.21545894066156	2	0.00997443987833413	1.58972430601786
SCAMP2	4.65406787583128	2	0.097584760720247	0.343167635261167
MPI	0.373974509049584	2	0.829454308107353	0.209167634737569
FAM219B	0.16041303110862	2	0.922925728186158	0.119865816651268
COX5A	2.48985599559725	2	0.287961641256428	0.081319990029655
RPP25	12.5780210038393	2	0.00185659613656564	1.35212602114173
SCAMP5	17.9324827237452	2	0.000127647070772952	2.69227839081855
PPCDC	3.11363195931999	2	0.210806215035278	0.394924551260816
C15orf39	2.94155849423011	2	0.229746386205959	1.22003043490246
COMMD4	0.575402488687903	2	0.749985621218707	0.105105275871591
AC068338.3	2.61479630542641	2	0.270523001047091	1.08368294277596
NEIL1	49.1865670363925	2	2.08579820082377e-11	0.538773183632349
MAN2C1	0.571850389556463	2	0.751318816415496	0.344514301441937
AC068338.2	0.650239089213393	2	0.722440984556336	0.244281639903485
SIN3A	0.40243026408087	2	0.817736491309433	0.215142778189432
PTPN9	0.199228954156204	2	0.905186320851899	0.131586842617798
SNUPN	0.0750598996035139	2	0.963165570670941	0.0695545351792774
IMP3	1.07195674949552	2	0.58509656631573	0.130742385376123
UBE2Q2	1.97949104215092	2	0.371671261491346	0.588550796006585
FBXO22	0.511935972459697	2	0.774166752392008	0.187722837058334
ETFA	0.0141175066378429	2	0.992966101165346	0.0132531343581061
ISL2	3.14662736562808	2	0.20735692757051	1.1439010637637
SCAPER	0.613231703232209	2	0.735933254183627	0.376193943647835
RCN2	5.88474381866875	2	0.0527404846154574	0.202758522183977

PSTPIP1 8.50311443753106 2 0.0142420386621928
0.852030341579146
TSPAN3 0.0187797210818218 2 0.990654086539693
0.0299586588707822
HMG20A 0.0473976926462871 2 0.976579766064912
0.0701114823995039
TBC1D2B 0.63467571609883 2 0.728084724281537
0.380835871606321
CIB2 0.00420915072513354 2 0.997897637703368
0.0452723623433669
IDH3A 0.33682404592457 2 0.845005601238528
0.114396309485378
DNAJA4 0.013290421722268 2 0.993376819726249
0.117020438690504
WDR61 15.3939273804556 2 0.000454204195948549
0.543790703764264
IREB2 0.890642411785227 2 0.640618472553142
0.254001785955826
PSMA4 30.0547744557848 2 2.9763818631956e-07
0.321542705653289
CHRNA5 0.290739331143574 2 0.864702583246656
0.171925047642479
ADAMTS7 0.297035320841285 2 0.861984784022202
0.409149477094429
MORF4L1 9.62030167262262 2 0.00814663079726186
0.160631039442508
CTSH 12.7501016329667 2 0.00170353322603511
0.31232217627530З
RASGRF1 6.59036501586611 2 0.0370612804522306
0.949112815398595
AC011944.1 2.06140806252922 2 0.356755704963503
0.553673104243692
TMED3 0.0691228528039222 2 0.966028998178394
0.0561559975270351
MTHFS 3.18782235823079 2 0.203129579866875
0.282123727622518
ST20 0.00216564673453519 2 0.998917762674409
0.0051125306371084
ST20-AS1 0.0120251332864458 2 0.994005472663311
0.0683807943772013
BCL2A1 35.2395315565702 2 2.2275781552672e-08
1.20710516857237
ZFAND6 2.58309763035817 2 0.27484476951585 0.121487232363319
FAH 5.91749419959865 2 0.051883881793045
1.18137402751186
MESD 0.860027210709407 2 0.650500244376551
0.169414507825834
TLNRD1 10.4617650481195 2 0.00534880277195571
0.832232801427965
IL16 1.93555241169604 2 0.37992697877402 0.126102918877035

STARD5	0.0624655477653686	2	0.969249930745553	0.1586876034904
EFL1	0.346554241566147	2	0.840904550425114	0.256527624817632
RPS17	23.4428472870198	2	8.11802513367432e-06	0.21882191789403
SNHG21	0.0212992528116568	2	0.989406880096525	0.0643825012735458
WHAMM	2.48726388247208	2	0.288335097789469	0.31388399400646
HOMER2	1.3385301928624	2	0.512084772463018	1.16941676970194
RAMMET	2.27917788487826	2	0.319950512866675	0.200053635050712
C15orf40	1.03972006033489	2	0.594603768735807	0.180781718544251
BTBD1	1.8208646282142	2	0.402350244742698	0.341513281121796
TM6SF1	2.29216339242699	2	0.317879882287997	1.50305437221641
ZSCAN2	0.00128995237504995	2	0.999355231764906	0.0300747312488415
WDR73	2.64550855979217	2	0.266400548896405	0.421422720872356
SEC11A	1.78053389144318	2	0.410546144586469	0.13656703102043
ZNF592	0.648554579983069	2	0.723049720128947	0.401545518521634
AKAP13	3.74230865761716	2	0.153945855075679	0.23915793400371
KLHL25	0.222920718514181	2	0.894526850409355	0.466890211873879
MRPL46	0.0399239296876962	2	0.980235956025377	0.0371260150836752
MRPS11	0.125136487221244	2	0.939348956061667	0.0529510860609355
DET1	0.953470802771429	2	0.620806772174916	0.640190875230766
AEN	0.366171628455781	2	0.832696695446993	0.233825464973179
ISG20	88.2207668797293	2	0	0.620280665079417
MFGE8	7.10298744821565	2	0.0286817649969258	2.07960070560457
ABHD2	5.51501645288344	2	0.0634496737650511	1.68128796132957
FANCI	4.47034226378816	2	0.106973821653884	2.3272178003041
POLG	0.000255826539845924	2	0.999872094910631	0.00450393767460234
AC124068.2	0.429429799122906	2	0.806771418277499	

0.662393553419314
PEX11A 2.06872982427586 2 0.35545205254083 0.519553347994489
AP3S2 0.19564337638411 2 0.906810584373474
0.232777627082957
ZNF710 0.311407975413447 2 0.855812483896143
0.438914150991678
IDH2 32.184913470343 2 1.0259704197324e-07
0.731914139304182
SEMA4B 2.91212695735691 2 0.2331522795302 0.904051625551345
CIB1 5.14861154433064 2 0.0762067092576162
0.241992857024666
GDPGP1 0.892070741853695 2 0.640161128568739
0.93016950445346
NGRN 0.398268333386279 2 0.819439944409781
0.139688987627694
IQGAP1 6.15667207618515 2 0.0460357947619943
0.29444617593366
CRTC3 0.735577105038789 2 0.692263543596465
0.23803890870713
BLM 1.99927917590954 2 0.368012053249293
0.519330228467385
FURIN 2.77504885421239 2 0.249692672558691
1.21286080105124
FES 1.16737892505422 2 0.557836448546526
0.414981850612295
MAN2A2 2.8337180190622 2 0.242474431923309
1.17285203188511
HDDC3 0.0040064834152007 2 0.997998763441913
0.0129889437538236
UNC45A 0.197939700142689 2 0.905770016513248
0.0831779602616225
RCCD1 3.71345570770167 2 0.15618284826451 0.658416811538927
PRC1 2.88642027405642 2 0.236168407573613
0.714035891328476
VPS33B 0.431244145258567 2 0.806039868846083
0.243427761786036
SLCO3A1 6.28370898806222 2 0.0432026045935958
0.378304005542677
CHD2 3.26621163112659 2 0.19532199700591 0.63755406339356
MCTP2 9.35435538753605 2 0.00930523909616243
0.712178904900469
LRRC28 0.0321089767922982 2 0.984073697996912
0.0714317613463502
MEF2A 1.55065606817302 2 0.460552679239634
0.209473424037988
LINS1 0.22752155177784 2 0.892471431031942
0.101540327723855
ASB7 0.336960171982299 2 0.844948089555132
0.291509147080413
AC015712.2 1.31834714287273 2 0.517278651746949

0.335224256352134
LRRK1 13.0475128804658 2 0.00146814373679804
0.840696970944994
SELENOS 10.9148058075142 2 0.00426461699580849
0.271303258720257
SNRPA1 4.31573200745959 2 0.115571487194954
0.272083460374442
TM2D3 0.504119910566852 2 0.777198139537312
0.184042162574209
TARSL2 0.0413220819872608 2 0.979550935915869
0.0690955678727835
POLR3K 0.849577565211826 2 0.653907887264468
0.158893508942263
SNRNP25 0.588229166037275 2 0.74519110030546
0.115366698377871
MPG 0.636868274220888 2 0.72728697760004
0.0929015383621417
NPRL3 0.189685254577146 2 0.90951605621406
0.115527604357666
LUC7L 0.841866234859053 2 0.656434003934527
0.175572748989246
FAM234A 1.59869750258138 2 0.449621684331644
0.432816067711544
AXIN1 2.09479918731277 2 0.350848913639375
1.30598248571713
MRPL28 3.75088508437237 2 0.153287115817217
0.227792091226063
TMEM8A 1.10678076888042 2 0.574997040910747
0.43501418480539
NME4 1.94008562354285 2 0.379066809234416
0.537391968555323
CAPN15 3.47473296974271 2 0.17598324543432 1.7731156225097
PIGQ 0.0230541866894782 2 0.988539089054764
0.076596425068507
RAB40C 2.31054071213681 2 0.314972370699534
0.820922569309471
METTL26 1.43898816160855 2 0.486998575573481
0.168569136303123
MCRIP2 1.61244103298924 2 0.446542581239623
0.253107993669576
RHOT2 3.2006044069607 2 0.201835513382615
0.312444024845164
STUB1 5.85018196638159 2 0.0536598095499368
0.247459775235601
JMJD8 1.07862137467293 2 0.583150086602804
0.427098117819184
WDR24 0.61314781626518 2 0.735964122435299
0.319040628689774
METRN 0.266196164860755 2 0.875379228575747
0.299063076562266

FAM173A 0.984905865370174 2 0.611125504421708
0.0677310930762665
HAGHL 1.59626095467443 2 0.450169780516446
0.445699893748271
NARFL 1.51280920418906 2 0.469350900263261
0.190049057765989
RPUSD1 0.101552473373475 2 0.950491331828279
0.0752748534299786
CHTF18 1.12398423782104 2 0.570072280150958
0.904405324702157
LMF1 0.367741826358362 2 0.832043202706454
0.411307609632163
UBE2I 2.31657935923529 2 0.314022801453095
0.117219726526585
AL031714.1 1.17491796106987 2 0.555737632256427
1.17163992865945
TSR3 0.161818757999185 2 0.922277265346215
0.0701489875376883
GNPTG 1.98309200509344 2 0.371002676339835
0.485486083157929
UNKL 1.61875862137958 2 0.445134270578884
0.214374195185699
C16orf91 14.4263759117978 2 0.000736804507111
0.420023878337069
CLCN7 0.201713802790304 2 0.904062393697201
0.150881679250536
TELO2 1.09396497743247 2 0.578693392259477
0.263591765428621
AL031708.1 1.74589532929652 2 0.417718438867373
1.37755751501421
JPT2 2.16910277749649 2 0.338053406444653
0.319452057050305
MAPK8IP3 2.08999780559822 2 0.351692205254856
0.861548711639427
NME3 3.19891795434437 2 0.202005778173207
0.450359069450803
MRPS34 0.201569415805894 2 0.904127663474571
0.0432392000719833
EME2 1.05581512932293 2 0.589837876956891
0.87678431932088
SPSB3 4.78770025078822 2 0.0912775763687383
0.20459606859119
NUBP2 6.976915337619 2 0.0305479509138787
0.423843409753703
HAGH 1.74940695087349 2 0.416985647835318
0.299552253313348
FAHD1 0.249749466565001 2 0.882607456999036
0.147399641464097
MSRB1 0.376368662108977 2 0.828461981886376
0.145831486778538

NDUFB10 1.80095125909591 2 0.406376329177633
0.0988554570007652
RPS2 57.7071657106719 2 2.94431146130592e-13
0.239214168022202
SNHG9 0.0130999514300683 2 0.993471428617783
0.0220461327263054
TBL3 0.120940440106759 2 0.941321801113996
0.0944919363876755
GFER 0.767491416826584 2 0.681304655195201
0.192734756632551
SYNGR3 0.000238866621158264 2 0.999880573821295
0.0122241912706801
ZNF598 0.0918412009672274 2 0.955117795071148
0.147981448890276
NTHL1 3.74453721484578 2 0.153774412037001
0.528269564793843
TSC2 2.05851311106365 2 0.357272474104138
0.434031885734537
PKD1 4.36643377908783 2 0.112678472794087
1.7082930816558
SNHG19 6.03811518773363 2 0.0488472306072097
0.602251377796653
TRAF7 1.74455509096714 2 0.417998453809913
0.279335682538253
MLST8 3.63559612417589 2 0.162382914659455
0.479781454171426
PGP 3.24529557478018 2 0.197375398503732
0.407659125932998
E4F1 0.209234564712976 2 0.900669158610883
0.158080542273484
ECI1 9.99644987988199 2 0.00674991788105728
0.9826120274295
RNPS1 0.892451988842556 2 0.640039110447543
0.066874910874246
CCNF 6.10333009657926 2 0.0472801351072687
2.06283687990021
TEDC2 1.00118521720302 2 0.606171330907795
0.559886202077227
TBC1D24 0.999824469119583 2 0.606583894479035
0.669056502548011
AMDHD2 2.35464126926764 2 0.308103154573697
0.530827892549369
PDPK1 0.96875800504631 2 0.616079661912631
0.295764030066763
KCTD5 0.278970770728664 2 0.869805735005428
0.138112067021987
SRRM2-AS1 0.787902048553416 2 0.674387084026877
0.609139762148508
SRRM2 1.84671322543612 2 0.397183609519951
0.132070438725551

ELOB	9.05884721881682	2	0.0107868917441576	0.15874956207244
FLYWCH2	0.417315329280376	2	0.811673052473904	0.295069041352359
FLYWCH1	0.205023884205959	2	0.902567371136074	0.249675581028478
KREMEN2	2.20792073139972	2	0.331555399490683	0.700500054478608
PKMYT1	3.50664535636185	2	0.173197506747288	1.73845249988272
PAQR4	0.685215926524255	2	0.709916470350008	0.465862298391317
TNFRSF12A	0.0389647330257976	2	0.980706188306118	0.17608476375846
HCFC1R1	7.05161682918882	2	0.0294280077194866	0.558203769211063
THOC6	1.61247201028195	2	0.446535664953062	0.225783354878583
MMP25-AS1	5.68061793584794	2	0.0584076170961856	1.80801069440333
ZNF213-AS1	2.34026981465751	2	0.310325073314716	0.740044652173404
ZNF213	1.96291248648017	2	0.374764952358352	0.941895554655813
ZNF200	0.277242832677021	2	0.870557544942439	0.333722852632494
LINC00921	3.15466015790783	2	0.206525770252874	0.951487902672585
ZNF263	1.18826653594783	2	0.552040830041554	0.377598426934118
ZNF75A	0.614566152076979	2	0.735442385321248	0.174184887457627
ZSCAN32	0.668856651451277	2	0.715747143646624	0.36593466695439
ZNF174	0.736086046403955	2	0.692087405231975	0.360156554894467
NAA60	4.29435183186954	2	0.11681358377173	0.512383432655453
CLUAP1	2.13558641667989	2	0.343766301582631	0.579637117244032
NLRC3	0.0993163849585847	2	0.951554617445458	0.120269411018609
SLX4	3.35476825465805	2	0.186862144845261	0.691637244227887
DNASE1	0.1369456765102	2	0.933818823907736	0.0641079621766051
TRAP1	0.0975376236467369	2	0.95240128816574	0.0737208342992874
CREBBP	3.41213244537488	2	0.181578679618791	0.458298882099995
LINC01569	2.34824638674989	2	0.309089872958505	

1.54658553600763
TFAP4 3.29426545055835 2 0.192601358670472
1.33704694552119
CORO7 4.77188790734114 2 0.0920020928639567
0.576224150853414
DNAJA3 0.184398515320113 2 0.911923423713
0.0280256460484764
NMRAL1 0.0631362417123522 2 0.968924950208386
0.0652476711973184
HMOX2 0.48720571297613 2 0.783798853088669
0.124841806720715
CDIP1 0.143075574407128 2 0.930961098513667
0.260864322428562
UBALD1 0.344006485884609 2 0.841976442683047
0.160653433372753
MGRN1 0.0103100126485413 2 0.994858257918624
0.0350586106491934
NUDT16L1 1.83503020260628 2 0.399510551922181
0.194770560289272
ANKS3 0.675378932294964 2 0.713416793569356
0.346480323188312
ZNF500 0.886232980976635 2 0.642032412062229
0.541866054255119
ROGDI 0.00465701331304133 2 0.997674202212157
0.0188479422914873
GLYR1 0.0919970514294733 2 0.955043370196043
0.0731291588706105
UBN1 3.24637692074731 2 0.197268711802049
0.372396684084523
NAGPA 1.08501678491121 2 0.581288322857804
0.319849568138645
ALG1 3.70092417056928 2 0.157164526117151
0.523548395332636
EEF2KMT 5.46107011377278 2 0.0651844029720811
1.07556596886354
METTL22 0.581283984963198 2 0.747783342160973
0.305452199803993
ABAT 2.10805344862622 2 0.348531479498917
1.16803051612418
TMEM186 0.280050695608676 2 0.869336199355639
0.190600958663431
PMM2 4.25845884226762 2 0.118928902650937
0.600099253011229
CARHSP1 10.1864053743594 2 0.00613832922417168
0.219542481361989
USP7 2.93095142383233 2 0.230968091065761
0.277698857810941
AC022167.3 0.491006608590193 2 0.782310698804833
0.0665290196217939
C16orf72 2.37368602200366 2 0.3051832048697 0.415754553889948

ATF7IP2 0.365550876693796 2 0.832955184529755
0.140543599292243
NUBP1 5.28804006622816 2 0.0710749707406315
0.240844995815305
TVP23A 0.0461839400566051 2 0.977172609047939
0.155758786497642
AC133065.1 4.70499947120483 2 0.0951310622428603
1.90069135051966
CIITA 6.34369406427559 2 0.0419260875123423
0.643008001061755
DEXI 21.0454183186519 2 2.691816664524e-05
0.603784940621595
CLEC16A 5.31611839075372 2 0.0700841093998383
0.582423261748245
RMI2 33.5791754105137 2 5.10945650233907e-08
0.856855045518959
SOCS1 5.93490802944816 2 0.0514340942209126
0.258844263285584
AC009121.2 5.5992309402821 2 0.0608334504063839
1.27271623083785
LITAF 31.4982183619911 2 1.44626803533221e-07
0.546599805707599
SNN 3.63245224716287 2 0.162638371344306
0.421800882884043
TXNDC11 4.8854793956263 2 0.0869223838815533
0.342772013632687
ZC3H7A 2.71286652277522 2 0.257577853370711
0.185156074554387
AC010654.1 0.186807711228994 2 0.910825583982688
0.431125574714992
RSL1D1 1.13097296905967 2 0.568083715583965
0.105892035061202
GSPT1 3.93400538839657 2 0.139875478114795
0.172404356582002
TNFRSF17 7.03732131163902 2 0.0296391055593537
0.230110973530435
SNX29 23.8233767726088 2 6.71149797792925e-06
0.907104114048554
CPPED1 1.16414626853945 2 0.558738824432677
0.238612672804929
ERCC4 13.2559776030965 2 0.0013228208677426
0.875475182690712
MKL2 1.56848998016252 2 0.456464206720577
1.01082076900923
MIR193BHG 5.73576467684476 2 0.0568191229408469
0.589009934680901
PARN 4.58498158322162 2 0.101014542070326
0.356787253890899
BFAR 10.0490970067106 2 0.0065745542140897
0.570787706335181

NOMO1	8.88032318078787	2	0.0117940325633956
2.10221820266254
PDXDC1	2.4289270063302	2	0.296869240986086
0.339528940624615
NTAN1	1.07638637346621	2	0.583802121433236
0.23929268956729
RRN3	9.28747637094625	2	0.00962166275704013
0.501056310327789
MARF1	1.95719363850375	2	0.375838097810794
0.414155424516216
NDE1	2.95304871216706	2	0.228430252468018
0.341672454731569
AC026401.3	0.177059344456934	2	0.915275952055911
	0.198836961184133
MYH11	3.91999524853027	2	0.140858755563704
1.43212508970594
FOPNL	0.0125233823918522	2	0.99375787233749
0.0250998011139023
ABCC1	0.195848225212131	2	0.906717709587155
0.343346659480645
NOMO3	3.66661551768734	2	0.159883834974893
2.66080753228005
XYLT1	0.28083967009806	2	0.868993324947881
0.397361334913208
AC109446.3	0.0228174912882765	2	0.988656087306008
	0.102038017897633
RPS15A	78.5853827920415	2	0	0.277178739523972
ARL6IP1	23.0531754506371	2	9.86430644756009e-06
0.632858607127098
SMG1	3.42217647449783	2	0.180669074781794
0.379113838461897
COQ7	2.11805896751562	2	0.346792214537652
0.344596721781208
ITPRIPL2	4.46849540700201	2	0.107072649941516
0.86211247625966
SYT17	11.9927512014329	2	0.00248775246474586
0.57030832816855
TMC5	4.5391180040504	2	0.103357750592802
0.68674172405102
GDE1	0.938488933089284	2	0.62547465683627
0.262880998078072
CCP110	0.721973014225846	2	0.69698840278924
0.359311177146212
VPS35L	1.83070434186397	2	0.400375600611328
0.39159122087977
KNOP1	1.89178030958874	2	0.388333739862655
0.208904806302194
THUMPD1	0.8885737943601	2	0.641281412603967
0.108839765332507
REXO5	1.66911367088397	2	0.434066801837436

1.45793612195806
LYRM1 0.00155788545427544 2 0.999221360569993
0.00681020591402523
AC008551.1 1.33314146929812 2 0.513466374535246
0.901193414977517
TMEM159 7.23891890202533 2 0.0267971577548576
0.799138455628045
CRYM 16.858336342881 2 0.000218403088777297
2.70529385912975
METTL9 2.41115056022182 2 0.299519642248135
0.226429612683327
NPIPB4 2.60724094868875 2 0.271546882666502
1.54582903632344
UQCRC2 1.49642450467267 2 0.473211780243702
0.112081194110863
MOSMO 0.767359711843462 2 0.681349522281546
0.54949020908781
EEF2K 1.81707731226118 2 0.403112880350609
1.07038995224179
POLR3E 0.0908743094363497 2 0.955579654357633
0.0721853351772121
CDR2 0.460685915069552 2 0.794261157938851
0.39159349867199
NPIPB5 0.136291837073148 2 0.934124157601886
0.141134502067607
COG7 2.13515156442723 2 0.34384105348416 0.681124265119849
AC008915.2 0.777852395152792 2 0.677784290291444
0.481915783671133
GGA2 26.3636084862005 2 1.88458250738321e-06
0.306724780651122
EARS2 1.5725910283123 2 0.45552917485567 0.68654025221167
UBFD1 2.74735340350847 2 0.253174399472031
0.376060648943961
NDUFAB1 0.0276280822560928 2 0.986280934900859
0.0142978572158696
PALB2 1.31518453925588 2 0.518097272487062
0.837525300169642
DCTN5 0.586509284198254 2 0.745832196238273
0.184475648495703
PRKCB 38.7790739871865 2 3.79516862381024e-09
1.04682469744057
RBBP6 0.172934847163712 2 0.917165426262486
0.0662132122448907
TNRC6A 0.0503434850338993 2 0.975142424232035
0.0544354890049883
AC008731.1 0.682915413309519 2 0.710733526282614
0.579538206045038
ARHGAP17 0.129661538705463 2 0.937226057329865
0.0832979926513406
LCMT1 1.0082997812238 2 0.604018839316798

0.177511418659038
ZKSCAN2 0.0801541659838818 2 0.960715381482058
0.104482696156579
AC008741.2 3.78251836282137 2 0.150881701737786
1.10265050912223
KDM8 1.10811650903575 2 0.574613145802674
0.471931445197921
NSMCE1 17.0360509584487 2 0.000199833611689404
0.390966547430903
AC106739.1 1.39428508511186 2 0.49800630442188
1.12836723486511
IL4R 60.4281205386687 2 7.54951656745106e-14
0.723942790419448
IL21R 12.2008101201194 2 0.0022419594073263
0.768947850426384
GTF3C1 0.0035014889045655 2 0.998250787206808
0.030348013455031
XPO6 1.49802485517223 2 0.47283327934297 0.500914094698009
CLN3 2.96428335133199 2 0.227150683974832
3.06357515001544
SGF29 4.8519682894993 2 0.0883910865990587
0.476739134335198
SULT1A1 0.549323189046637 2 0.75982921008875
0.434536376276014
EIF3C 0.063505118719336 2 0.968746259619792
0.243742070760902
ATXN2L 1.3233659814721 2 0.515982210053171
0.229793262697579
TUFM 2.00396168734051 2 0.367151450764613
0.114116599503725
SH2B1 1.36923786605032 2 0.504282358318292
0.212641664985538
ATP2A1-AS1 0.315839409783735 2 0.853918344681642
0.129852536180124
RABEP2 5.17386507803265 2 0.0752505144361046
0.422663481072147
CD19 12.9901092042655 2 0.00151089271307636
0.17610746388565
NFATC2IP 2.11261052010636 2 0.347738242119388
0.198674093748099
SPNS1 0.521893009710039 2 0.770322127006738
0.178512687683344
LAT 2.18513990949307 2 0.335353541881398
1.79115228435413
NPIPB11 0.476197251206667 2 0.788124957961114
0.356948459346562
BOLA2-SMG1P6 3.39846676499087 2 0.182823626135562
0.471881989037599
SPN 0.305890152371089 2 0.858176854863365
0.222308032728443

C16orf54	0.0545401026058748	2	0.973098419542141	0.076560216532519
KIF22	0.177922626315395	2	0.914880966745508	0.140241715467562
MAZ	0.0893175781545413	2	0.956323734273286	0.0735348689810692
AC009133.1	0.0232647105488028	2	0.988435038999054	0.133936240205276
PAGR1	0.791985977888659	2	0.673011414441339	0.230066411175201
MVP	3.03876926880334	2	0.218846516142228	0.373585290262106
CDIPT	3.1955403522876	2	0.202347213966536	0.208353697959159
KCTD13	1.19136049252801	2	0.551187495077749	0.578154474213052
TMEM219	5.15610941598514	2	0.0759215500507684	0.350143626800144
TAOK2	5.28979310173825	2	0.0710126995617215	1.24241630648446
HIRIP3	1.58474367725162	2	0.452769624236931	0.31029084221388
INO80E	1.00646046508132	2	0.604574585626675	0.220363561794036
ALDOA	5.52934174500698	2	0.0629968299252479	0.286523805529953
PPP4C	0.499583690721126	2	0.778962910940847	0.0550826839746073
YPEL3	11.3646181125263	2	0.00340568543799624	0.441819344527891
AC012645.1	1.22937001888996	2	0.54081121890875	1.32395128253584
MAPK3	0.0353634000648829	2	0.982473703942075	0.0533375947047871
CORO1A	14.8243179115973	2	0.000603865567118111	0.112972705546205
AC012645.3	3.29211097055443	2	0.192808948350342	0.909429243827275
BOLA2B	0.0576196503984788	2	0.971601220966649	0.0999842724563539
SLX1A	1.34982356661947	2	0.509201338683035	1.05902647203544
SULT1A3	0.0913535443685428	2	0.955350708213046	0.171207318007805
CD2BP2	4.66821544547289	2	0.0968969028686117	0.223424243625453
TBC1D10B	1.13127364521016	2	0.567998317391046	0.363521114189698
MYLPF	5.8951442495195	2	0.0524669346097483	0.625346150830173

ZNF48 0.18542646534893 2 0.911454838289073
0.0770530795170696
SEPT1 0.0355622727506313 2 0.982376015206971
0.0193956854328923
ZNF771 0.173596966858031 2 0.916861839872052
0.154401928943146
DCTPP1 2.13230695984041 2 0.34433044735361 0.173768381814208
SEPHS2 10.7551010103219 2 0.00461912260084663
0.221397179236479
ITGAL 1.39717268535696 2 0.497287801670518
0.281032665039515
ZNF768 0.076760155170728 2 0.962347104808678
0.205569980213696
ZNF747 0.364209145543853 2 0.833514172970636
0.304613607168333
AC002310.1 0.11369406458741 2 0.944738572806592
0.247918124199615
ZNF764 0.0100976776077594 2 0.99496388516
0.0527462793218717
ZNF688 0.729023545515286 2 0.694535659344341
0.266513094731686
ZNF785 3.28323230597743 2 0.193666794062505
1.27097888077603
ZNF689 0.112944690783378 2 0.945092620299989
0.164340798626963
PRR14 0.018461296542148 2 0.990811823381921
0.0302114232118995
FBRS 9.60010459612036 2 0.00822931666046789
1.19939136681955
AC093249.6 0.369531905945739 2 0.831298824103892
0.31316404554059
PHKG2 0.37487429219126 2 0.829081227534936
0.152250770832874
CCDC189 7.15007003918435 2 0.0280144447026152
0.853495735166753
RNF40 0.130659282781614 2 0.936758618062392
0.13135019113415
BCL7C 8.5444546812825 2 0.0139506756158854
0.597825132705487
MIR762HG 1.12604396648389 2 0.569485485254729
0.940035432105579
FBXL19 0.511892528626873 2 0.774183568960136
0.427675004281913
ORAI3 5.1046842230205 2 0.0778990040233318
0.503349516036226
SETD1A 2.37615703626957 2 0.304806381675031
1.03414039679022
STX4 2.30149115942718 2 0.316400779412825
0.214756184250172
ZNF668 1.65327001189646 2 0.437519061081071

0.576513274295906
ZNF646 0.0299312341138337 2 0.985145811233112
0.148568118202848
VKORC1 6.41993264759904 2 0.0403579723523146
0.381328018397352
BCKDK 2.29704061387018 2 0.317105641418603
0.254304858727818
KAT8 0.423440004605416 2 0.809191237532863
0.165362570260512
FUS 5.54362808784295 2 0.0625484361536908
0.172432538568993
PYCARD 1.51640693319584 2 0.46850736052346 0.199978808441027
ARMC5 0.00361367579420895 2 0.998194793451813
0.0354280369004345
C16orf58 0.0054525088982318 2 0.997277458407714
0.0349183253022419
ZNF720 3.32592492905533 2 0.18957653364283 0.434795438888008
ZNF267 10.5151908491127 2 0.0052078122524819
0.661642709508426
SHCBP1 1.94036683025593 2 0.379013514915452
0.295204761910002
VPS35 0.0309206864659574 2 0.984658554352362
0.0218258794105599
ORC6 17.6211307632882 2 0.00014914890604234
0.539557653564541
C16orf87 0.202583928531666 2 0.903669155264848
0.0655332136083733
GPT2 8.30049147486768 2 0.0157605430535446
0.727328135807351
DNAJA2 2.1249562610296 2 0.345598310551468
0.259625504363812
NETO2 4.40907309751485 2 0.110301632889454
3.46740791205245
ITFG1 0.245097451857992 2 0.884662797876601
0.110303246001268
PHKB 1.23272111960855 2 0.539905821208142
0.172853510827449
LONP2 0.536105643991393 2 0.764867378214432
0.0688199755087887
SIAH1 4.74911362466307 2 0.0930557212213546
0.566893559572499
N4BP1 2.41606185428886 2 0.29878503007043 0.464675797013721
CNEP1R1 2.89429236469089 2 0.235240665034991
0.858452345053258
HEATR3 3.10160593027542 2 0.212077614508661
0.968636414409344
TENT4B 4.41284801321599 2 0.110093639557598
0.957531397512538
ADCY7 2.01906849399391 2 0.364388655165417
0.768090725105524

BRD7 1.84944765445553 2 0.396640945379673
0.12483333797796
SNX20 0.542584295532556 2 0.762393732240725
0.194514955819857
AC007728.2 5.94620684938726e-05 2 0.999970269407716
0.00720491778111718
CYLD 0.891162734815828 2 0.640451829958528
0.240222401413805
HNRNPA1P48 14.104134010514 2 0.00086561787016326
0.511177162537209
CHD9 1.10152918694989 2 0.576508846920474
0.133940213842296
RBL2 0.0401368883079428 2 0.980131586733705
0.0640758047551971
AKTIP 1.78597427994194 2 0.409430896857775
0.436438057358347
RPGRIP1L 1.11614849608752 2 0.572310130671459
1.00526391334762
FTO 0.000438361930513699 2 0.999780843053136
0.0107028452099175
CRNDE 2.26356597307952 2 0.322457805637107
3.86410190466683
AMFR 15.5263527039532 2 0.000425104153512046
0.399942443078995
AC092140.1 1.72702758337301 2 0.421677788188224
0.300740083537188
NUDT21 0.158940099341771 2 0.923605681848506
0.0575366630476862
OGFOD1 0.136351704133867 2 0.934096196386546
0.119668524056616
BBS2 0.0968554408108787 2 0.952726199480638
0.143457251773848
MT2A 20.773793471707 2 3.08338718810752e-05
1.69307500643913
MT1F 0.461217664762771 2 0.794050011945765
0.36291222865788
MT1X 3.93226550963572 2 0.139997214245412
0.970096012988669
NUP93 0.938678616024316 2 0.625415338714875
0.222190087735535
HERPUD1 39.595066919959 2 2.52371590292455e-09
0.27532452253358
NLRC5 2.31031557312234 2 0.31500782897982 0.777517175383079
CPNE2 2.70042886894287 2 0.259184676557411
18.4924507689071
FAM192A 0.0628620419370744 2 0.969057798816746
0.0325959200965961
RSPRY1 2.8915296229397 2 0.23556584418322 0.399988917167827
ARL2BP 6.94532800633717 2 0.0310342451122252
0.350057085154738

CIAPIN1 7.53598342244248 2 0.0230984050054535
0.302858629793761
COQ9 0.00389563206694144 2 0.998054079729114
0.015289518208427
POLR2C 0.192913549422203 2 0.908049147438183
0.064961167921642
DOK4 0.828194677337292 2 0.660936613444672
0.740763278190177
CCDC102A 4.49097071796884 2 0.105876139964961
0.311626862587411
ADGRG5 12.0149700439022 2 0.00246026792602272
1.05371725187408
KATNB1 2.68280354882508 2 0.26147887716155 0.614328249520547
USB1 0.801788951496367 2 0.669720729088136
0.215399958192152
CFAP20 0.073285021036805 2 0.964020701016739
0.0489102605285524
CSNK2A2 0.934351422577926 2 0.62676995018039
0.337434571573861
GINS3 4.5490246585044 2 0.102847051706204
0.715397159912318
AC009118.2 1.21861441900299 2 0.543727427817048
0.585100698748466
NDRG4 0.129374988122842 2 0.937360348286311
0.292396407131006
SETD6 2.07066121506959 2 0.355108959818081
0.746382959680089
CNOT1 0.45050886239893 2 0.798313077163921
0.15591049096007
SLC38A7 0.142176694897534 2 0.931379603480848
0.194519831852245
GOT2 0.848409239972155 2 0.654289987402467
0.163817600280586
TK2 0.373282742614249 2 0.829741252054037
0.525756570249089
CKLF 6.47432032565577 2 0.0392752722557371
0.459401413314795
CMTM3 8.09126257790408 2 0.0174986544609915
1.68420758857191
DYNC1LI2 0.397614973837737 2 0.819707682595794
0.0625590161052395
NAE1 2.48384875924256 2 0.288827868332942
0.195369367988636
FAM96B 6.93625813946012 2 0.0311753029503323
0.237715405766755
CES2 2.38564909157977 2 0.303363189581908
0.488579883209298
CES4A 0.075893362359977 2 0.962764272977689
0.234800996849352
CBFB 1.97210802115754 2 0.373045825402052

0.276535980628776
C16orf70 0.879655076880288 2 0.64414750218726
0.52654805575847
TRADD 1.99178789300745 2 0.369393079249914
0.258320341439411
KIAA0895L 1.44484366526163 2 0.485574849779204
1.33004952806298
E2F4 0.0330562809919008 2 0.983607699792723
0.028266594810108
ELMO3 0.93469200589356 2 0.626663225573917
0.803751996946649
LRRC29 0.605862090793078 2 0.738650027934252
0.696719505397756
TMEM208 2.92385045309977 2 0.231789597403294
0.234646430554516
FHOD1 4.56836942010945 2 0.101857071309495
0.902659806652354
ATP6V0D1 2.2821067974169 2 0.319482302252899
0.140646142866175
AC009061.2 1.35105028494289 2 0.5088891111404
0.605612337731456
AC027682.6 0.0482140247699196 2 0.976181240685548
0.211686153690046
RIPOR1 1.41635025742426 2 0.492542203964693
1.21004305558851
AC027682.4 0.591811134000586 2 0.743857669414652
0.329012187519095
AC027682.1 0.215194237381514 2 0.897989306669952
0.401272310799044
CTCF 2.77803180858358 2 0.249320539217337
0.274372906859718
CARMIL2 2.55936139033357 2 0.278126093282278
1.03109612847565
ACD 0.217922483312363 2 0.896765173957616
0.101138459181172
PARD6A 0.000809762965853583 2 0.99959520047052
0.00882451656365171
ENKD1 0.496071487827352 2 0.780332050658084
0.722396483431692
C16orf86 1.68667526943846 2 0.430272033310083
1.07156713976485
GFOD2 0.0207441878322352 2 0.989681510758998
0.0464597144117182
RANBP10 0.517805843638142 2 0.771897953855794
0.439387942176647
CENPT 0.449317624944649 2 0.79878870901636
0.240941162089062
THAP11 0.62485694450247 2 0.731667961637139
0.194851037507006
NUTF2 2.85752104118948 2 0.239605724626593

0.244133897339997
PSKH1	0.642202952557308	2	0.725349641473055
0.521379427177018
AC040162.1	0.519678067786801	2	0.771175708964863
	0.80041980438652
PSMB10	0.0511072242790162	2	0.974770118053287
0.0471299474774701
DPEP2	0.0142686823035967	2	0.992891047846233
0.0665829810438307
DUS2	4.83802719200903 2	0.0890093733724096
0.367675373886868
DDX28	4.1859436046795	2	0.123320107735413
0.648771891695708
NFATC3	1.19963449559188 2	0.548911941795402
0.263344179973392
PLA2G15	2.44100753903624 2	0.295081476421105
0.587748296831377
SLC7A6	4.96288239356565 2	0.0836226220524807
1.20564260302223
SLC7A6OS 0.441345465222986	2	0.80197909895267
0.154774395761423
PRMT7	1.6477673145811	2	0.43872448607429 0.452283990047415
ZFP90	0.154183219568683	2	0.925805036921761
0.249278812706184
TANGO6	0.15272079000004 2	0.926482246814927
0.241046502180905
CHTF8	0.86701490949154 2	0.648231460196877
0.152637765832649
UTP4	1.17382289616594 2	0.556041999962861
0.256916578481224
SNTB2	1.3752664883071	2	0.502764583066394
0.534129001673298
VPS4A	1.46984101029768 2	0.479543578705128
0.277157462563631
COG8	3.71954226281099 2	0.155708263021555
0.735089077252463
COG8.1	0.183506718859291	2	0.912330139424073
0.200222381511009
NIP7	2.76523427780257 2	0.250920997873766
0.307354551596996
TERF2	2.45068722895807 2	0.293656778264507
0.162186660002908
CYB5B	1.9252127712209	2	0.381896218880368
0.165241899106468
NFAT5	0.865197536995215	2	0.648820766917291
0.216521165948276
NOB1	0.264415035991921	2	0.876159157420242
0.0706869673396981
WWP2	5.05017016174157 2	0.0800515016455081
0.829231054173494

PDPR	1.56695898889235 2	0.456813761852788
0.889084611636149
EXOSC6	0.462939633798602	2	0.79336664140697
0.156511701668397
AARS	0.00495808155566268	2	0.997524029506114
0.0161561943817229
DDX19B	4.56558704603894 2	0.101998872159432
0.391247939573725
DDX19A	0.108675889911193	2	0.947111980696133
0.10760743695732
ST3GAL2	0.775302744026771	2	0.67864889802584
0.235818853727732
COG4	1.49059463629247 2	0.47459317379862 0.265537530145262
SF3B3	2.75644792750542 2	0.252025762701745
0.447846067055438
VAC14	0.317715147290048	2	0.853117856783387
0.18523787428581
CMTR2	2.42776825563799 2	0.297041289540863
0.340271576174062
PHLPP2	1.21666705205243 2	0.544257104055222
0.801916442885267
AP1G1	0.936722815570421	2	0.6260272316529
0.290907802499482
ATXN1L	0.196154805830354	2	0.906578729201621
0.169933817114337
IST1	0.12079497443598 2	0.941390268607493
0.0517357550724369
ZNF821	3.87428979420814 2	0.144114825608148
0.311540076554044
DHODH	1.90777055868078 2	0.385241341773643
0.773809535871804
TXNL4B	0.468729789982239	2	0.791073104610655
0.306797619589922
DHX38	0.91971742424845 2	0.631372844533433
0.141229345289446
ZFHX3	1.59392535788177 2	0.450695795143949
1.72285051799483
PSMD7	0.466005508045063	2	0.792151391918528
0.0754238614611432
GLG1	2.99461047196699 2	0.223732254161281
0.284271823329974
RFWD3	0.505051582907456	2	0.776836176846626
0.190396001561997
MLKL	2.38459859904867 2	0.303522571818139
147.484153999174
WDR59	1.09328485408823 2	0.5788902171665 0.38605789885533
ZFP1	0.756621585340077	2	0.685017569139469
0.46576239324485
BCAR1	4.15271255309578 2	0.125386253682612
2.69394023479031

CFDP1 0.89409102562508 2 0.639514801495163
0.126138755425723
TMEM170A 4.33973025627153 2 0.1141930172974 0.456583155242868
GABARAPL2 1.21167554628421 2 0.545617131732921
0.0883326695601783
ADAT1 1.42174213821091 2 0.491216127855851
0.254806922451154
KARS 0.347898534765162 2 0.840339529200855
0.0807067218555313
TERF2IP 18.1395400839728 2 0.000115093002161504
0.316445002826233
MON1B 2.07494088347571 2 0.354349897942968
0.521854350664576
SYCE1L 3.62297667877192 2 0.163410745074901
1.04395209428366
NUDT7 2.01015468332841 2 0.366016325397548
0.522880344368852
WWOX 0.903776397221377 2 0.636425318982575
0.414476913515363
CMC2 0.0266548117956872 2 0.986761010752096
0.0305848287685704
CENPN 9.06074716954497 2 0.0107766493285372
1.73504433995267
ATMIN 2.10596204184124 2 0.348896130674677
0.477172135185067
GCSH 2.78774575275115 2 0.248112532321088
0.403784116686915
GAN 2.41829120507784 2 0.298452167300602
1.08272397129405
CMIP 0.827134282206707 2 0.66128713334212
0.423816259158055
AC099524.1 1.24296733127321 2 0.537146899704294
0.426121179749258
PLCG2 6.13179351825159 2 0.0466120233593919
0.287869942009354
SDR42E1 0.914528797382112 2 0.633012950139458
0.544972118150997
MPHOSPH6 5.36570561309334 2 0.0683678354749165
0.483000106566515
HSBP1 16.0032143983592 2 0.000334923905675133
0.346797996053934
MLYCD 8.02858658619992 2 0.0180557101379282
0.971385397532547
MBTPS1 0.432304387043403 2 0.805612683511425
0.121999640547107
AC040169.1 0.713240700037187 2 0.70003821678729
0.530643940168141
HSDL1 1.87957784684185 2 0.390710296448196
0.353192610731569
TAF1C 0.848353910376021 2 0.654308088453225

0.404766995242679
TLDC1 1.92733168229578 2 0.381491831069825
0.458554039437185
COTL1 19.4066851338914 2 6.10789928399136e-05
0.236159836438859
KLHL36 1.03797217025068 2 0.595123646890889
0.275729266966927
USP10 2.1612468914986 2 0.339383872242057
0.235037054277873
ZDHHC7 4.7695342722312 2 0.0921104262737596
0.888793310034635
GSE1 1.72012738700048 2 0.423135130501895
0.430871289339553
GINS2 7.68163518254818 2 0.0214760355459819
0.748051917105998
C16orf74 63.5525768963246 2 1.58761892521397e-14
1.11876603405559
AC018695.2 3.11346945085847 2 0.210823344628036
1.2461904446815
EMC8 4.01074495675409 2 0.134610146987347
0.26417844406503
COX4I1 59.6659507389465 2 1.10578213252666e-13
0.348670021658502
IRF8 81.3187752649304 2 0 0.502863883242274
LINC02132 0.273744882886432 2 0.87208146049001
0.185403907894474
AC092723.3 0.0020872582350227 2 0.998956915273959
0.0466042056104942
MTHFSD 1.5155698697336 2 0.468703486759765
0.317136058250041
C16orf95 0.0983891761030183 2 0.95199586465356
0.174068436470474
FBXO31 4.90802982475166 2 0.0859478198867716
0.959970899423979
MAP1LC3B 22.8008521201419 2 1.11907159094926e-05
0.333335778556602
KLHDC4 0.448930699076405 2 0.798943259973208
0.181260226758035
AC126696.1 1.52473354610282 2 0.466560875524217
0.922074991427521
SLC7A5 9.41956627007823 2 0.00900673061493928
1.43313945923422
BANP 1.71067675378217 2 0.425139309422732
0.306559069220691
ZC3H18 0.99690834206308 2 0.607468977429181
0.119882455031094
IL17C 10.7317192335256 2 0.00467344114501689
2.87381863312947
CYBA 154.163257343649 2 0 0.332861874300009
MVD 22.1245615870629 2 1.56932355752648e-05

0.614776814977539
SNAI3-AS1 0.352361613801665 2 0.838466369125386
0.437183536193522
SNAI3 0.826142887285397 2 0.661615012952713
0.592158448860405
RNF166 7.24267934620407 2 0.0267468204843681
0.32967339639524
CTU2 1.55149529782411 2 0.460359465048083
0.433110863907686
PIEZO1 0.966439899113823 2 0.616794144854013
0.903069136218447
CDT1 10.5441361061376 2 0.00513298430503051
1.22314911156442
APRT 3.73129461971815 2 0.154795976485649
0.152038772824239
GALNS 0.486898616337642 2 0.783919213325506
0.164838243271489
TRAPPC2L 0.0594108681503591 2 0.970731435841055
0.0284007285295075
CBFA2T3 7.14453697614766 2 0.0280920548528169
0.811779164223355
ACSF3 8.26365414691059 2 0.016053521049178
0.456447217909242
AC009113.1 1.94357476251139 2 0.378406077360438
1.08329605391961
ZNF778 0.0169611249496862 2 0.991555296056666
0.126454996563879
ANKRD11 6.98701293764008 2 0.0303941091040386
0.483742502229761
AC137932.3 0.102900893451891 2 0.949850717008918
0.173412850901308
SPG7 0.828792613944077 2 0.660739043881691
0.18094922146272
RPL13 26.2214763139331 2 2.02338611188235e-06
0.128204624261883
CHMP1A 6.81797672063523 2 0.0330746430784712
0.554375203459698
SPATA33 3.17319886352895 2 0.204620255113596
0.883299426065963
CDK10 1.174913707913 2 0.555738814077348
0.276017799426835
SPATA2L 6.28064764161291 2 0.043268784300514
1.25315984028221
ZNF276 0.0665278556415334 2 0.967283232940877
0.118887460875298
FANCA 8.83809388821205 2 0.012045707047909
0.478409010127724
SPIRE2 4.40326986531296 2 0.110622150668301
3.25718148680564
TCF25 5.85256154837803 2 0.0535960035569929

0.23155040903702
TUBB3 2.7037932549622 2 0.258749044417692
1.38065499188069
DEF8 0.826121482258249 2 0.661622093934262
0.0630194745851376
CENPBD1 1.80499530168244 2 0.405555457776366
0.700021788659849
DBNDD1 12.0492545492479 2 0.00241845281813069
1.23493437086782
GAS8 3.55265975043165 2 0.169258207465583
1.42846222233418
FAM157C 1.5599796120489 2 0.458410684308712
1.54296945414013
VPS53 0.0542523052823757 2 0.973238457177836
0.0715119931478155
FAM57A 1.31951643777916 2 0.516976314489566
1.24607217173035
GEMIN4 1.34260989093282 2 0.511041261498216
0.416605708607811
GLOD4 0.456086809526612 2 0.796089705000321
0.099534275484839
MRM3 1.22411619237215 2 0.542233750686025
0.262348375582699
TIMM22 5.1764221740393 2 0.0751543645203625
0.450516274441274
ABR 8.68076076106359 2 0.0130315703049108
0.745698761630749
YWHAE 19.9411068808734 2 4.67566792659024e-05
0.329670856910647
CRK 0.332385317215651 2 0.846883059160199
0.155258491079533
MYO1C 6.70201650793413 2 0.0350489979886064
0.621835558153231
INPP5K 1.03509712726671 2 0.595979765120433
0.130942387079867
PITPNA-AS1 2.23601620781762 2 0.32693035778259
0.367552220468677
PITPNA 0.583569491108678 2 0.746929298522411
0.175177429249799
SLC43A2 6.3754839538634 2 0.0412649430133132
0.289925787104744
RILP 12.3992227439163 2 0.00203021948322402
1.46685530697703
PRPF8 3.68099648914659 2 0.158738315895971
0.362518885414431
MIR22HG 0.37145322422741 2 0.830500612755652
0.448650098654999
WDR81 0.12342036313965 2 0.940155321650302
0.274243975913796
SERPINF1 0.868894583134934 2 0.647622514601171

0.282656740987069
SMYD4 0.0991868635927825 2 0.951616242767729
0.100736759790503
RPA1 1.24176802352917 2 0.537469098516771
0.0946431342902245
SMG6 0.00356528601627945 2 0.998218944956176
0.0207267117646175
SRR 2.51969019633927 2 0.283697968430921
0.84836684152085
TSR1 0.21327571402761 2 0.898851126687525
0.138205704135461
SGSM2 1.2328069890939 2 0.539882640988261
0.744785076858258
MNT 1.18029029041901 2 0.554246832623392
0.76763769075353
METTL16 0.0221746985023506 2 0.988973888873752
0.064481751244828
PAFAH1B1 0.0117738716220566 2 0.994130358242624
0.0140314395803371
CLUH 0.00515848407535179 2 0.997424081349181
0.0501347095256921
OR3A3 1.29899188434069 2 0.522308984352581
1.2452587673967
TRPV3 3.86049037103986 2 0.145112614609615
1.59841483906503
TRPV1 0.873961560169247 2 0.645983847046185
1.07686459170918
SHPK 0.0272971345488853 2 0.986444152111611
0.123807770561221
CTNS 1.55168687331838 2 0.460315370363953
0.586983582281547
TAX1BP3 0.134595540597101 2 0.934916769438904
0.0836374278537787
P2RX5-TAX1BP3 0.0589473897695031 2 0.970956418425754
0.0909354509548284
EMC6 1.20627701342873 2 0.547091887222573
0.0953321503049118
P2RX5 8.45868119160727 2 0.0145619896202972
0.168772411638983
ITGAE 4.32563114806232 2 0.115000871310889
0.186289511937892
HASPIN 0.543683941948753 2 0.761974665689845
0.596759392584939
NCBP3 7.14739994191212 2 0.0280518703257615
0.527444179214122
P2RX1 1.69586740505131 2 0.42829901141414 1.18334538726307
ATP2A3 0.0130648043635661 2 0.993488887574375
0.0299163867901192
ZZEF1 4.82319066821201 2 0.0896721234028656
0.781857094431081

CYB5D2	0.654695823285436	2	0.720832913232866
0.251070332570169
ANKFY1	1.46296308014538	2	0.48119555123462	0.332514139855576
UBE2G1	19.5861633335474	2	5.58365621898815e-05
0.419915464548346
SPNS3	5.23374412738462	2	0.0730309420680858
0.758806267877839
MYBBP1A	0.907789970361323	2	0.635149429846429
0.49440227531639
PELP1	2.60934830786931	2	0.271260909946699
0.50381763484829
ARRB2	2.1405511949511	2	0.342913998162446
0.189115742446512
MED11	0.679283372503397	2	0.712025405551995
0.0651367789887546
CXCL16	3.35981183736656	2	0.186391511173329
3.19196107189408
PSMB6	0.319910044538314	2	0.852182117322146
0.0395989203488498
MINK1	0.190835293869774	2	0.90899321694893
0.357022427794629
SLC25A11	4.16592684679742	2	0.124560539104032
0.167458051489391
RNF167	0.0574280339950461	2	0.971694312791824
0.0430919542913281
PFN1	6.72918889797271	2	0.0345760356212941
0.113677519587871
SPAG7	1.5692774533903	2	0.456284515427303
0.122974524396832
CAMTA2	2.71440798794146	2	0.257379406211098
1.17921185874411
AC004771.4	0.000707452583645542	2	0.999646336261946
0.0235911657759148
KIF1C	0.0502631501539351	2	0.975181593993503
0.118848496426842
ZFP3	2.60145692999075	2	0.272333335456135
1.29387555404647
ZNF232	1.45003054800611	2	0.484317171436911
0.500681228741006
ZNF594	1.60773265364711	2	0.447595065559836
1.20147769829123
AC087500.1	0.0139196286019186	2	0.993064349116462
0.0613533065027535
SCIMP	14.7317073403941	2	0.000632485242154424
0.627296209279874
RABEP1	1.16682652254779	2	0.557990544952535
0.149820163046744
NUP88	11.9929564365829	2	0.00248749719071872
0.385641332910206
RPAIN	1.08365370761098	2	0.581684628350105

0.156204979487751
AC004148.2	0.892302218386808	2	0.640087041716827
	0.216793746915435
C1QBP	3.01855489684969 2	0.221069654492238
0.1566358853917
DHX33	3.49556908128848 2	0.174159359338224
1.27776431075131
DERL2	4.76515940338478 2	0.092312132319874
0.357599043214511
MIS12	0.980537735520547	2	0.612461700839285
0.304413149494819
NLRP1	1.73961358611056 2	0.419032501416463
0.3556764603431
KIAA0753 0.000604033596713398	2	0.999698028804126
0.0217364282612746
TXNDC17	10.918875045978	2	0.00425594894513359
0.227752863564945
MED31	0.00260312073358782	2	0.998699286295533
0.0101659857298156
C17orf100	0.953601344273484	2	0.620766252973027
	0.483355226138016
XAF1	3.91744536578941 2	0.141038456748455
1.9051297281979
ALOX12-AS1	2.05659726217125 2	0.357614878113438
0.530469086253398
RNASEK	1.06563633187366 2	0.58694851837618 0.43922689664177
C17orf49 2.60109576894441 2	0.272382517992899
0.84439791804191
ACADVL	30.1410656224066 2	2.85069504446511e-07
0.708872214372541
DVL2	10.5920320154029 2	0.00501152004502725
0.805277039960655
PHF23	1.70875241630378 2	0.425548562029521
0.258176163132113
GABARAP	0.538509548416553	2	0.763948596453355
0.10924449783528
CTDNEP1	0.190629224943074	2	0.909086879402375
0.0685126583533905
ELP5	2.71550416825932 2	0.25723837774313 0.219586273778296
EIF5A	0.214298181674326	2	0.89839172103126
0.0595697167289417
GPS2	0.0131418073957152	2	0.993450637482348
0.0314369298148768
ACAP1	0.0508453272135255	2	0.974897771127819
0.0263538340403714
KCTD11	0.443187345487343	2	0.801240864202457
0.407086583001683
TMEM256	0.594256713800172	2	0.742948643656632
0.044236028836891
TMEM102	0.817717781648394	2	0.664407979770171

0.308367896393807
CHRNB1	0.968609393077321	2	0.616125442019284
0.144718144931654
ZBTB4	3.2376418228598	2	0.198132176796144
2.38953523658692
POLR2A	0.03549455873261	2	0.982409276083623
0.0546925523389256
SENP3	1.85694064208942	2	0.395157712726157
0.870437279669584
EIF4A1	0.239895635266368	2	0.886966719532286
0.0510677113809776
CD68	0.403208812402964	2	0.817418229572375
0.377228005502391
AC016876.1	2.27850102975419	2	0.320058811263264
0.722489704207821
MPDU1	3.02711379715316	2	0.220125619342191
0.337548356409346
FXR2	0.327198466543179	2	0.8490822376126
0.229430692459511
SAT2	8.52923591061131	2	0.0140572365999527
0.248215856503628
TP53	1.44414048148325	2	0.485745603974116
0.15512807210555
WRAP53	1.02701485391398	2	0.598393073778428
0.407503126770424
KDM6B	0.435176206216332	2	0.804456726659573
0.286972758598397
NAA38	3.34017299779974	2	0.188230783178883
0.158427125468843
CHD3	0.746716236439835	2	0.688418653431576
0.19182727202542
TRAPPC1	11.0561947602429	2	0.00397354204169054
0.214983485970917
CNTROB	3.52874470590339	2	0.171294265091974
0.611137161940648
PER1	1.94474099040577	2	0.378185487819697
1.89044170143046
VAMP2	2.28094102252562	2	0.31966857875983	0.121021290302045
TMEM107	1.78452562725384	2	0.409727565872056
0.443936012697554
BORCS6	0.0860838533349859	2	0.95787122887661
0.0662552306038166
AURKB	5.17119427658853	2	0.0753510711542313
0.564664117699756
LINC00324	4.27256912367399	2	0.118092795404294
0.555666748979225
CTC1	1.87305575262811	2	0.391986500880463
1.33694619041378
PFAS	0.00547977163086014	2	0.997263864246016
0.0533617183888301

SLC25A35 5.01403664711031 2 0.0815109164482152
3.24223013240657
RANGRF 0.581020074921885 2 0.74788202243787
0.112802131791839
KRBA2 1.10190925933704 2 0.576399299782933
1.12749969960205
RPL26 44.6349896060471 2 2.03065009252157e-10
0.183642181702522
NDEL1 6.10182949402338 2 0.0473156227646134
0.812069777611618
MYH10 2.96183883423431 2 0.22742849058136 1.0716899999076
STX8 1.57433287044958 2 0.455132617610002
0.236798055797742
GAS7 0.792897660317234 2 0.672704698013256
0.761165513082845
MYH3 1.33591097594114 2 0.512755842337076
1.27324736085153
SCO1 0.000247156127195131 2 0.999876429571857
0.00179955406385421
ADPRM 2.99069093737429 2 0.224171147239804
0.562800471348181
ZNF18 2.20417114191811 2 0.332177580858873
1.31057740748873
MAP2K4 0.130914197872462 2 0.936639228716977
0.148711317042067
ARHGAP44 0.023410667576628 2 0.98836290711088
0.0586963533224304
ELAC2 3.96168859050876 2 0.137952715305465
0.381256090189619
COX10 0.00118897764436086 2 0.999405687851288
0.0167176225960877
TVP23C 3.10545028806798 2 0.211670354931954
1.56571628324876
AC005838.2 0.410017086744221 2 0.814640356605982
0.633988411537919
TRIM16 0.522802008444143 2 0.76997209563813
0.473253815618169
ZNF286A 0.83205517695366 2 0.659662071161578
0.830061778590213
ZSWIM7 2.29662268231796 2 0.317171912569031
0.299357184102389
TTC19 0.62999701059184 2 0.72978996508828 0.113593844837451
NCOR1 2.1487171100789 2 0.341516749254954
0.151214576979632
PIGL 0.237539144737152 2 0.888012399782368
0.295398531892937
CENPV 2.40295649851335 2 0.300749300718168
1.18753591727988
UBB 39.6424792557454 2 2.46459186392656e-09
0.39294571450044

TRPV2	6.22441351914045	2	0.0445026402739906	0.637199343612833
LRRC75A	0.987201990137064	2	0.610424296810577	0.325769971492729
ZNF287	0.659216814727846	2	0.71920531380064	0.394944467582614
CCDC144A	14.5777428282953	2	0.00068309855324955	0.696926799230995
AC098850.3	6.26151080261339	2	0.0436847852418083	1.42441353255909
TNFRSF13B	0.866629654252353	2	0.648356339507216	0.465170700627432
AC104024.1	0.83501395983676	2	0.65868689425123	0.95888873028789
MPRIP	1.58699507658152	2	0.452260228389788	0.524381425655546
FLCN	4.72878054994482	2	0.0940066010753008	0.8208226618661
COPS3	6.09695459494534	2	0.0474310928766262	0.349442098617452
MED9	4.26223265592246	2	0.118704706477711	1.44022982150398
PEMT	2.28372610215664	2	0.319223737337721	0.746029936101498
RAI1	0.500807055886238	2	0.77848657859272	0.669701907296135
SMCR5	0.441140833369072	2	0.802061158385501	0.177261400989419
SREBF1	2.1959617498426	2	0.333543871043477	1.75423513361842
TOM1L2	0.061105973620215	2	0.969909038319592	0.140152275925658
ATPAF2	0.0356692561551787	2	0.982323467647086	0.0411914229029395
GID4	0.0302273011457915	2	0.984999987428595	0.093765358894271
DRG2	1.42417619974706	2	0.490618666352736	0.212097745015964
ALKBH5	1.63921039819923	2	0.440605571648819	0.193885763602386
LLGL1	0.568051320237367	2	0.752747328872576	0.490930803726433
FLII	0.857311685203335	2	0.651384069255678	0.103422006004481
MIEF2	0.0159331751375657	2	0.992065061588934	0.0561914115511809
TOP3A	0.273782585864068	2	0.872065020611067	0.337753703598674
SMCR8	0.728950101304385	2	0.694561164624363	0.536339335413376

SHMT1	5.65462185139821	2	0.0591717571735831	0.406179971824771
TRIM16L	2.36822097403575	2	0.30601826568877	1.55531975543961
TVP23B	2.90393617691948	2	0.234109087007937	1.01659811840398
PRPSAP2	30.65122065504	2	2.20887938939818e-07	0.646293398670873
FAM83G	0.735619455412931	2	0.692248884941628	0.892088585171649
GRAP	0.953448254233058	2	0.620813771357033	0.197971963687574
AC007952.4	4.45394178895931	2	0.107854638908083	0.457164822550027
GRAPL	2.26325952592102	2	0.322507217561683	1.63300892873424
AC007952.5	6.20291112558029	2	0.0449836781497223	2.8870717903931
EPN2	11.1082522075401	2	0.00387145023748148	3.38857476323324
MAPK7	0.33059901040576	2	0.847639793537716	0.235623248372838
ALDH3A2	0.77252764391629	2	0.679591210943769	0.518980903441348
AKAP10	4.35897704068949	2	0.113099363872571	0.275584607921027
SPECC1	1.22380576328101	2	0.542317919783195	0.567849953543141
AC107926.1	1.10177398379192	2	0.576438287466181	0.895496395388521
USP22	0.180538755476749	2	0.913685025711951	0.0806372536502131
DHRS7B	1.61384187779881	2	0.446229922320469	0.356865412756397
TMEM11	1.30779530551858	2	0.520014983832511	0.160739906653271
MAP2K3	0.728840591246051	2	0.694599196382396	0.119509414466241
MTRNR2L1	0.0122552907308689	2	0.993891090365257	0.0720621082529412
WSB1	0.210554300745471	2	0.900075031884065	0.0670074613097611
LGALS9	30.0501238326164	2	2.98331093162574e-07	1.95111872694195
LYRM9	4.83816245810795	2	0.0890033536006276	0.941450755403373
NLK	12.2068242632667	2	0.00223522780120655	0.95141018269642
TMEM97	0.545433457948371	2	0.761308413702494	0.250518192989629
IFT20	0.732243563785854	2	0.693418350268276	

0.149754323443128
TNFAIP1 1.94310053834431 2 0.378495812652105
0.592098222808628
POLDIP2 1.59243489909481 2 0.451031792079853
0.204348392780092
TMEM199 12.2066252115669 2 0.00223545027522387
0.362740560677523
AC005726.1 0.288766110135455 2 0.865556128887485
0.443495656821006
UNC119 9.54219053105703 2 0.00847109697659876
0.535492326624914
PIGS 5.37983476245677 2 0.0678865478432678
0.691343529073978
ALDOC 20.8444897148444 2 2.97629907498598e-05
1.87436014802245
SPAG5 1.87693368742306 2 0.391227188214151
2.33186542854018
AC005726.5 0.623317429025531 2 0.732231385534732
0.258788180880153
SGK494 2.80834337872305 2 0.245570380792349
0.904143172214724
KIAA0100 0.00135684276094502 2 0.99932180869528
0.0142263079077119
SDF2 3.06896147083444 2 0.215567598731627
0.358616852917077
SUPT6H 0.0155753063187631 2 0.992242592047497
0.0362321294506582
PROCA1 0.664141780479121 2 0.717436461809042
0.424541864813094
RAB34 0.346818349920156 2 0.840793512798421
0.26155443942571
RPL23A 77.2940625687167 2 0 0.257335798717091
TLCD1 4.8276082830184 2 0.0894742735393943
2.28591613817149
NEK8 0.35244073532991 2 0.838433199411246
0.118094276438082
TRAF4 19.9913789159555 2 4.55960504570641e-05
0.655105506920052
FAM222B 0.301428123992609 2 0.8600935969478
0.0959712098885543
ERAL1 0.284697499098174 2 0.867318726720562
0.104381165637257
FLOT2 0.00396857907078779 2 0.998017677865573
0.014452087878268
DHRS13 3.43933472813096 2 0.179125721654579
1.05417580362851
PHF12 0.402260954088764 2 0.817805719719101
0.267951238470956
SEZ6 6.41016845565707 2 0.0405554855935243
1.61569135516221

MYO18A 2.66765379604961 2 0.263467067977235
2.02701797375629
NUFIP2 3.09881532450526 2 0.212373733555884
0.413259902483577
TAOK1 0.274593276895706 2 0.871711604598097
0.167726421612171
ABHD15 0.203096542483808 2 0.903437568236246
0.226663433593474
TP53I13 0.496286872036865 2 0.780248019581963
0.215872447047129
GIT1 4.00148807346243 2 0.13523462626574 1.24096415840376
SSH2 0.232836405701424 2 0.890102901878967
0.0984464146843355
EFCAB5 0.140008875461953 2 0.932389682202192
0.443430364448205
NSRP1 29.42934768014 2 4.06909986261361e-07
0.328352162993454
AC104984.3 4.2602843737652 2 0.118820397949277
0.340356076938496
SLC6A4 5.09616067319814 2 0.0782317004794333
1.98927129749976
BLMH 0.122130671642706 2 0.940761772324926
0.0716836489896048
CPD 0.380321443799652 2 0.826826234180514
0.453872881443068
GOSR1 0.596003451909466 2 0.742300058570369
0.155308386124795
CRLF3 0.0629609765668152 2 0.969009863315115
0.0465946588636745
AC127024.5 1.93547325059841 2 0.379942016789955
1.78930333311233
ATAD5 0.159241584107211 2 0.923466465820473
0.184794129623547
TEFM 0.0101900957082286 2 0.994917909886094
0.0253886174813396
ADAP2 4.81895153343831 2 0.0898623910824919
2.00312151876463
RNF135 6.05915509750272 2 0.0483360534396566
1.09607311894495
NF1 3.63347920840397 2 0.1625548811296 0.58929048668268
EVI2B 1.81628600556799 2 0.403272404866899
0.138784509774596
EVI2A 5.75680909161136 2 0.0562243947597474
0.344317573578235
RAB11FIP4 4.98369525751048 2 0.0827569211659199
0.296858728105255
COPRS 3.17303487509292 2 0.204637033479259
0.339339052160151
UTP6 4.10200052679437 2 0.128606199155703
0.27001480304849

SUZ12	2.05566156216327 2	0.357782227379716
0.252870235917333
LRRC37B	2.02922087694103 2	0.362543635379684
0.60703166774704
RHOT1	0.508175228104122	2	0.775623843521652
0.245850766176206
C17orf75 5.23922767628238 2	0.0728309819451876
0.700804192281518
ZNF207	4.49333220281336 2	0.105751201289712
0.221266316077748
PSMD11	9.3570389866947	2	0.00929276170321003
0.226200804626189
CDK5R1	0.837725902859549	2	0.657794338864294
0.668636929824039
MYO1D	1.19179715079885 2	0.551067167924434
0.468178841984954
CCT6B	0.499081077800985	2	0.779158693952259
0.495501981603001
ZNF830	0.513028581329555	2	0.773743937165234
0.157129362341351
LIG3	1.48036837254043 2	0.477026045780916
0.750239213072441
RFFL	0.00719451940008919	2	0.996409202687346
0.0570399990283168
RAD51D	0.46571258271197 2	0.792267421020456
0.34583985891201
NLE1	1.36238279536533 2	0.506013769465423
0.647948166267933
SLFN5	0.00365877102968742	2	0.998172286790918
0.041956613360854
SLFN11	1.87928431664882 2	0.390767643290718
1.21863726860973
SLFN13	0.486005119986847	2	0.784269506044209
0.198629905132285
LINC02001	1.9557033230699	2	0.37611826083955
0.274928636648959
PEX12	1.27162000220097 2	0.529506413787725
1.229151565698
AP2B1	7.10266033673555 2	0.0286864564478723
0.548394011308714
TAF15	0.572301516815563	2	0.751149365328036
0.112178604870806
CCL3	15.7204728284218 2	0.000385782656087907
2.05711224972671
CCL4	1.32573481678017 2	0.515371433393146
0.640417950754519
CCL3L1	4.46556669673397 2	0.107229557182094
8.92728836516489
CCL4L2	0.406214267900756	2	0.816190794997323
0.274250701479526

ZNHIT3	2.70725554365366 2	0.258301499966277
0.208182075162096
MYO19	0.0606240786829228	2	0.970142763603819
0.175609923620009
PIGW	0.223216372514928	2	0.894394624961792
0.299981105617463
GGNBP2	9.39784299151457 2	0.00910509168822393
0.278834815769469
MRM1	0.0224706170009775	2	0.988827571864308
0.0917955734972908
AATF	0.926572417131993	2	0.629212520721362
0.143112385343754
ACACA	0.00172978859211346	2	0.999135479617209
0.0367678213841212
TADA2A	6.08614011550817 2	0.0476882588182821
1.71057341087762
DUSP14	0.00622103412412238	2	0.996894315584161
0.0231985880355529
SYNRG	0.69601096789903 2	0.706095004014412
0.147138461456273
DDX52	3.4124542551836	2	0.181549465069158
0.352364198546613
MRPL45	6.15982709430618 2	0.0459632301293802
0.443035753703036
SOCS7	1.06563944403028 2	0.586947605039032
0.717532817832126
EPOP	1.3471615859692	2	0.5098795319726	1.25535416135699
AC006449.6	0.594138409046173	2	0.742992592134708
0.452043845417429
MLLT6	0.568288726372912	2	0.752657980758434
0.376020586716807
CISD3	3.86276603487668 2	0.144947594745186
0.778047111780354
PSMB3	2.33825382968467 2	0.310638036362665
0.0933893905517048
PIP4K2B	2.81664443708063 2	0.244553246045319
1.78161502861046
CWC25	7.58028181388609 2	0.0225924181982434
0.512705594007693
RPL23	5.47263909399051 2	0.0648084328838048
0.0685962864189313
LASP1	0.594265229745843	2	0.742945480208224
0.110568316548087
LINC00672	0.0368020806738298	2	0.981767225138547
0.127388682120645
CACNB1	0.542212739643591	2	0.762535381338419
0.699306531886238
RPL19	100.161347416425 2	0	0.307105420628832
FBXL20	0.184448309124982	2	0.91190071992712
0.160849675973613

MED1 1.9922411464188 2 0.369309374398501
0.471966805053373
CDK12 1.0736708101467 2 0.584595335630625
0.160078304917438
AC009283.1 0.732123764978308 2 0.693459886858019
0.825358861045334
STARD3 0.270344962860441 2 0.873565224913689
0.127909064350024
MIEN1 0.468771819233482 2 0.791056480680196
0.0762615498940769
IKZF3 2.06094699328046 2 0.356837958986777
0.192471780474275
ZPBP2 0.435320225256524 2 0.804398800202401
0.367792734611732
GSDMB 1.98981485387428 2 0.369757672560004
1.48254573081887
ORMDL3 3.77296260226262 2 0.151604321365401
0.493917969352932
PSMD3 0.266659337922365 2 0.875176526009561
0.0641245629453748
MED24 5.77447673126904 2 0.0557299059148082
0.514801723081855
NR1D1 0.176768370135941 2 0.915409122642316
0.364321016186708
MSL1 0.300550670628057 2 0.860471025746024
0.0932898161253599
CASC3 0.141236337152326 2 0.93181762145811
0.105836654493145
WIPF2 0.615575460311844 2 0.735071334927291
0.255445743444676
RARA 0.000418429762804956 2 0.999790807002505
0.00696113753043849
TOP2A 2.29195428405254 2 0.31791311969825 0.640910304325085
CCR7 1.55092973482967 2 0.460489664595014
0.491790345928033
SMARCE1 0.220075248652794 2 0.895800430774683
0.0936780645567824
AC073508.3 0.324654450646675 2 0.850162964168228
0.56981720664035
KRT10 1.05478489998319 2 0.590141789368307
0.0904560928757038
TMEM99 0.212006698660462 2 0.899421635610805
0.253833864320866
EIF1 0.0746830562161466 2 0.963347069057645
0.00994642355121611
JUP 0.00865044275024818 2 0.995684118923709
0.0330074454997886
NT5C3B 3.11809083703579 2 0.210336758974232
0.208842130594235
ACLY 1.36728790411072 2 0.504774263781323

0.35924527441947
CNP	0.123975266561745	2	0.939894510130634
0.0641635669489767
DNAJC7	1.05822083491526 2	0.589128815351169
0.0466137021095335
NKIRAS2	0.295702846393344	2	0.862559261719778
0.128291951731408
DHX58	2.43377752178752 2	0.296150128933492
1.66111422551172
KAT2A	2.65751824428976 2	0.264805648978916
0.546845538580701
RAB5C	0.78581590351838 2	0.675090885654651
0.0932932948237746
GHDC	0.510374534880015	2	0.77477139491925
0.276716354159199
STAT5B	1.91132616639667 2	0.384557066663687
0.433939009219006
STAT5A	1.46695329821533 2	0.480236470696017
0.208919198057011
STAT3	10.0504040566535 2	0.00657025898240815
0.48941347093116
ATP6V0A1 22.2377664330802 2	1.48296320021668e-05
1.51285848541842
NAGLU	3.43490897767091 2	0.17952254342369 0.992104155835312
HSD17B1	0.0140948648900788	2	0.992977342472977
0.120484984287746
AC067852.2	0.266864138955406	2	0.875086912069586
0.201993272106768
COASY	2.38081479279964 2	0.304097350661626
0.178421495369393
MLX	1.3449589712038 2	0.510441375389815
0.108423007122107
PSMC3IP	0.507890880118617	2	0.775734124899746
0.477637176423665
RETREG3	1.52547965629512 2	0.466386855073565
0.231058581279197
TUBG1	0.0353243432212741	2	0.982492890290317
0.051840513501541
TUBG2	3.7027971517644 2	0.157017411912459
1.29040634419208
PLEKHH3	2.46708353734293 2	0.291259176101258
1.56274644611004
AC100793.2	0.0471550276105702	2	0.976698264135507
0.0574789409999851
EZH1	0.224537997005009	2	0.893803793277625
0.171255788921155
VPS25	0.611620292867109	2	0.736526438354225
0.117655082863563
COA3	0.667014223706705	2	0.716406803641719
0.0404395376980452

BECN1	0.0151900598627232	2	0.992433739427678
0.0201191728690707
PSME3	1.14125587174051	2	0.56517043646202	0.148986038239247
AARSD1	0.0107521067677669	2	0.994638371729473
0.0393051858156573
RUNDC1	1.05618260631548	2	0.589729510988139
0.265009435868177
RPL27	6.86072587367022	2	0.0323751884919209
0.112000396034507
IFI35	4.51940742140892	2	0.104381407267161
0.297898168416564
VAT1	0.113581046077084	2	0.944791960788109
0.155217622691877
BRCA1	1.90315459402008	2	0.386131498821606
0.310879089206608
AC060780.1	1.57539166409795	2	0.454891735614364
0.370526080151499
NBR1	0.745148423708996	2	0.68895852077115
0.297632193883602
TMEM106A	0.488886772857577	2	0.78314032348084
0.35745732634502
LINC00910	0.622090655272363	2	0.732680664434046
0.818735913323257
DHX8	0.921606664697706	2	0.630776718575851
0.249326724076472
TMEM101	0.185891589110143	2	0.911242893283912
0.0327813886814563
LSM12	5.33518004830679	2	0.0694193227692115
0.442984161296363
G6PC3	2.14116988057305	2	0.342807936587871
0.290802358445805
HDAC5	1.74102373144161	2	0.418737157185556
0.38887064492963
ASB16-AS1	1.45029888348136	2	0.484252196056624
0.506694045632089
TMUB2	0.307077538769249	2	0.85766751231259
0.127297107361214
ATXN7L3	0.339160166639593	2	0.844019159917502
0.258530454926303
UBTF	1.13251930180875	2	0.567644662109715
0.170516840349391
AC003102.1	0.000175045026565998	2	0.9999124813167
0.00851099223204572
SLC25A39	2.99017438238989	2	0.224229053079103
0.249973386065728
GRN	6.1724954629777	2	0.045673010679167
0.27021886297239
GPATCH8	0.9288308196919	2	0.628502414139685
0.164669990133045
CCDC43	0.756952704268257	2	0.684904167385456

0.198289643685519
DBF4B	0.22983832991159	2	0.891438200440784	0.604289784004053
EFTUD2	0.428176293601095	2	0.807277222982014	0.0807562528553525
DCAKD	5.14721869887849	2	0.0762597998264165	1.72068279006917
NMT1	0.024664457454606	2	0.98774350157791	0.0185475056535823
ACBD4	7.0283744465194	2	0.0297719911048653	1.2097639143497
AC142472.1	3.8750387917203	2	0.144060864889967	3.00983787320479
HEXIM1	0.135878899486496	2	0.934317045001452	0.153841457495646
AC138150.1	0.0125763278835061	2	0.993731565186135	0.04225718907864
HEXIM2	0.0414344046964463	2	0.979495924553186	0.084252606924567
AC008105.3	0.0465065695757479	2	0.977014989396981	0.161206044722905
FMNL1	0.0468452116764852	2	0.976849574197283	0.0395447240077218
MAP3K14	0.0218671842921895	2	0.989125962326793	0.0795402569370253
ARHGAP27	1.6722015918088	2	0.43339713695843	0.481110803877171
PLEKHM1	4.28538372414806	2	0.117338558300432	0.934098759437646
AC091132.5	4.18694234225775	2	0.123258540896097	0.91409036853486
KANSL1	7.11595523992276	2	0.0284963970225293	0.320647880900644
KANSL1-AS1	1.34283575259485	2	0.510983552442499	0.395015893734267
ARL17B	0.331310712012808	2	0.847338213897947	0.341290221819612
LRRC37A2	0.0256950839490381	2	0.987234635390699	0.0283571015503082
ARL17A	0.417130705603627	2	0.81174798296397	0.0987355931828805
FAM215B	6.37932964443698	2	0.0411856731483975	0.492428759138968
NSF	1.17690394359635	2	0.555186063539725	0.383428072892768
GOSR2	0.00131036114695955	2	0.999345034010446	0.0065388973221802
CDC27	0.368249990771768	2	0.83183182218859	0.12077318711922
MYL4	2.82565183380379	2	0.243454328445954	0.730699188354216

NPEPPS	0.0275462670544893	2	0.98632128211287	0.0347736127684218
KPNB1	5.54183675047196	2	0.0626044839256507	0.210354350479731
OSBPL7	3.41885434870312	2	0.180969426861402	1.89210428281005
MRPL10	9.63270352383517	2	0.00809627044750649	0.472085562880198
LRRC46	0.0414766094266596	2	0.979475255090654	0.21215333326933
SCRN2	0.0662155553768756	2	0.967434286138882	0.0661553793137297
SP2	0.403857792425843	2	0.817153028551578	0.237067384096115
PNPO	2.25480890900874	2	0.32387279299226	0.85083774044773
CDK5RAP3	7.75302455691639	2	0.0207229752561019	0.573902377152756
NFE2L1	1.4629977579927	2	0.481187207894022	0.488452460597584
CBX1	2.81816306704859	2	0.244367623583093	0.192116444650574
SNX11	9.49259162120976	2	0.00868380214913467	0.764490665745127
SKAP1	10.6051706167803	2	0.00497870576439252	1.0329240613667
HOXB2	2.31071302942968	2	0.314945234275446	1.39245067905644
HOXB4	0.0253450494826901	2	0.987407433585969	0.0713207442361976
HOXB7	0.301316903447679	2	0.860141428317017	0.177973210797958
CALCOCO2	0.314460840506789	2	0.854507140379745	0.128711434287162
ATP5MC1	0.444450215297914	2	0.800735092451031	0.0685737931402779
UBE2Z	1.73624876638689	2	0.419738079198356	0.320254706293782
SNF8	6.70664129583339	2	0.0349680445319311	0.292329590505758
GNGT2	1.25877743542074	2	0.532917464468468	0.406800382655194
ABI3	18.4446978872873	2	9.88063243475557e-05	0.47026030753867
ZNF652	0.0658618227016082	2	0.96760540783036	0.084725705039721
PHB	0.14003562187888	2	0.932377213243978	0.0376022827441989
SPOP	0.20493610577452	2	0.902606984979431	0.0641732462199406
SLC35B1	0.405686273510042	2	0.816406295522594	

0.172179848154238
FAM117A 2.39509898852122 2 0.301933195128672
0.306187418149005
KAT7 0.612049593134874 2 0.73636835982202
0.245291863765521
ITGA3 0.640059168577699 2 0.726127554788601
0.899698708098923
PDK2 0.384439056160167 2 0.82512571034474
0.193159962277833
PPP1R9B 2.76517794316396 2 0.250928065745175
0.765978294889798
XYLT2 1.02029417382562e-06 2 0.999999489853043
1.31976599623888e-05
MRPL27 1.19796934019613 2 0.549369143937982
0.126915064029389
LRRC59 18.0866194696681 2 0.000118179047143618
0.396479857994991
ACSF2 0.288870723149888 2 0.865510855853631
0.164248905529443
RSAD1 0.00335491130618276 2 0.998323950489286
0.0289809954646833
SPATA20 0.01231446305498 2 0.993861685377384
0.0468651048712196
ANKRD40 0.0366288134995636 2 0.981852282839364
0.0785337824724688
AC005921.2 2.55128641769778 2 0.279251293540361
1.09678994473114
LUC7L3 0.0676802176114427 2 0.966726063265691
0.0251788699805981
TOB1 3.40510114229843 2 0.182218170437877
0.642865721787317
SPAG9 0.343511403542881 2 0.842184892316377
0.159875906500564
NME1 2.51624558084711 2 0.284187004654046
0.109743519336949
NME2 0.276420773009246 2 0.870915443614003
0.289672981620431
MBTD1 0.462385351312466 2 0.793586546495156
0.271809057629319
UTP18 0.0852407323894622 2 0.958275114649858
0.0483433382507736
COX11 2.20776997480087 2 0.331580392514815
0.188837430027124
MMD 0.203248910658214 2 0.903368743291482
0.115209557789654
PCTP 0.0880510374933991 2 0.956929537518987
0.0953920783916428
C17orf67 8.98610088293416 2 0.0111864680445115
0.83295874620628
DGKE 2.0812308256647 2 0.353237228330588

0.698547004158541
TRIM25	4.42002122114708	2	0.109699484534888
0.931095750272262
COIL	3.14156790784847	2	0.207882148433785
0.215168934603746
SCPEP1	22.772614470925	2	1.13498363180087e-05
0.457572478222425
AC007114.1	0.000667558092578567	2	0.999666276651739
	0.0124542793017824
AKAP1	0.23310159784024	2	0.889984885557232
0.226081436062862
AC007114.2	2.73744939053149	2	0.25443123008925
1.1334018980023
MSI2	7.20438037308876	2	0.0272639438850435
0.270439823967382
MRPS23	0.106687773325475	2	0.948053933315151
0.0480066531272573
VEZF1	0.269866995745897	2	0.873774017586662
0.126018734218521
SRSF1	0.338417386156809	2	0.844332678612332
0.0721777109945227
DYNLL2	0.818532247903445	2	0.664137465915456
0.32077075214619
MKS1	2.53265925702444	2	0.281864272101684
0.560356764062918
TSPOAP1-AS1	5.8524474730197	2	0.0535990606358321
1.37163634782843
AC004687.1	15.1064057723504	2	0.000524427751929624
0.644926523528514
SUPT4H1	6.20887462321687	2	0.0448497478929786
0.218952843346645
MTMR4	6.39327889198281	2	0.0408994179947679
0.672379435918476
RAD51C	0.00807050353072486	2	0.995972878922908
0.018967967114081
TRIM37	1.25607493177375	2	0.533638056905381
0.465264053144098
SKA2	2.354368932115	2	0.308145111398151
0.243867942225434
PRR11	0.00465866280513218	2	0.997673379384643
0.0383718496093487
SMG8	0.715770364555226	2	0.699153345592935
0.578565510304014
YPEL2	1.67242375637765	2	0.433348996888196
0.520646730621259
DHX40	0.339615865979956	2	0.843826872337451
0.221870554412866
AC091271.1	0.0862387565747182	2	0.957797043071253
	0.130573168901582
CLTC	0.436983657039519	2	0.803730047082021

0.145193390701143
PTRH2 6.03922363237324 2 0.0488201658823767
0.739921693859033
VMP1 2.47314400053364 2 0.29037792920881 0.252884968581231
TUBD1 0.0132226661493269 2 0.993410473704091
0.0683021411181964
RPS6KB1 0.0313772898757227 2 0.984433780784747
0.0555916520530767
RNFT1 4.72635933466919 2 0.0941204750990414
0.453380989295023
HEATR6 0.375463372886059 2 0.828837065621688
0.181175467275398
USP32 0.540917976741441 2 0.763029192424908
0.479290220337872
APPBP2 4.1043610741258 2 0.128454498187531
0.602072589893612
PPM1D 2.1761277095496 2 0.336868088252941
0.45002835451413
BCAS3 2.09961915853591 2 0.350004390858296
0.803110183339319
NACA2 0.560180401324334 2 0.755715572336287
0.711873012698634
BRIP1 3.322542496889 2 0.189897419792942
2.16680076875944
INTS2 0.788569690579556 2 0.674161997018762
0.309656991094297
MED13 1.86257760391206 2 0.394045536316115
0.4417127838911
METTL2A 2.0097798662876 2 0.366084926403541
0.374343227053147
TLK2 0.847171194879183 2 0.654695133040814
0.203999785085189
DCAF7 0.635090369202568 2 0.727933788633208
0.0971052296719539
TACO1 4.7487427080763 2 0.0930729807770165
0.604998552300965
MAP3K3 0.328936854935185 2 0.848344540907029
0.290914936623627
STRADA 1.73841221937205 2 0.419284282884141
0.334686388161691
LIMD2 116.510808952197 2 0 0.468358814983721
CCDC47 4.33369288656722 2 0.114538250842413
0.332979265959694
DDX42 0.855991670237252 2 0.651814129521606
0.204927590752989
FTSJ3 0.829334258411014 2 0.660560125286747
0.122194058913986
PSMC5 0.268462487181131 2 0.874387844638459
0.0421011507799743
SMARCD2 0.995787033089293 2 0.607809653128781

0.21070235098347
GH1 2.41858160356309 2 0.298408835417905
2.1154656353289
CD79B 138.375272007949 2 0 0.467082169381001
ICAM2 0.130058116737742 2 0.937040234121016
0.0912181255905516
SNHG25 34.0169918594933 2 4.10491408642244e-08
0.701915261305702
PECAM1 5.59252520523969 2 0.0610377592252572
1.91188973233782
MILR1 3.19842327680783 2 0.202055748213074
0.420870819973681
POLG2 0.395061511026497 2 0.82075489750142
0.214751234495095
DDX5 23.8120921171529 2 6.74947348344723e-06
0.145510987095071
CEP95 0.111558946498262 2 0.945747675557296
0.0519037806765085
SMURF2 2.04470243925668 2 0.359748098217706
0.654979951866769
LRRC37A3 0.472664020737597 2 0.789518502084275
0.483836492832367
GNA13 0.0212782793484048 2 0.989417255795349
0.0372397780155104
RGS9 2.65957259910818 2 0.264533786247984
0.566440708815395
CEP112 3.39956113110294 2 0.182723615509653
1.90722795119689
PRKCA 5.94873204298897 2 0.0510798072409585
1.35738138752052
HELZ 2.56487064199573 2 0.277361014198512
0.328682535128866
PSMD12 1.19505170292336 2 0.550171158738247
0.17172040566063
PITPNC1 0.00925024168058936 2 0.995385558560249
0.0149716657923636
NOL11 12.2927514873125 2 0.0021412280837112
0.704367078252533
BPTF 3.2740219222336 2 0.194560723587016
0.14721730802446
C17orf58 4.54178910096787 2 0.103219803446353
0.483181026285592
KPNA2 6.50357230532738 2 0.0387050129917911
0.341743020867736
AC005332.7 2.02853204505644 2 0.362668522692842
0.890884895909229
AC005332.8 3.43623165354222 2 0.179403857605462
0.984514546237394
AC005332.3 1.61752528852322 2 0.445408854594254
2.16465985130229

AC005332.5 4.90422481064212 2 0.0861114918646262
1.009271216952
AMZ2 23.693149603397 2 7.1630492002317e-06
0.463538008578754
ARSG 0.395538203641912 2 0.820559296913333
0.315433136182081
SLC16A6 0.957057770653852 2 0.619694363040564
0.931819100686224
WIPI1 4.41720071589397 2 0.109854297658235
0.456908112776009
PRKAR1A 0.0327359279409324 2 0.983765263275164
0.0196369269817527
ABCA6 0.613597615498437 2 0.735798622997388
0.357027921219545
ABCA5 0.0536510430321679 2 0.973531086934815
0.143708905530995
MAP2K6 0.432700419141044 2 0.805453175064094
0.252284463840751
LINC01483 0.791082867836744 2 0.673315384752553
0.717466762083708
ROCR 0.0737551549130154 2 0.963794118254382
0.165189762257785
LINC01152 0.0735641201866924 2 0.963886181723948
0.211977178154585
SOX9 1.07547166601312 2 0.584069186576 0.671489676395359
SLC39A11 4.62016006155158 2 0.0992533079225496
0.996943284474119
SSTR2 5.66126995944399 2 0.0589753935984821
1.5653213448216
COG1 1.77576227637061 2 0.411526798032242
0.563836643296311
FAM104A 0.582772628085008 2 0.747226957986573
0.0753034648503337
C17orf80 0.0192946792238247 2 0.990399046680929
0.0557079578151877
RPL38 34.8881255768557 2 2.6554601517681e-08
0.268825776394084
RAB37 7.31507905301702 2 0.025795904847128
2.47887457190669
SLC9A3R1 0.311233741252152 2 0.855887043028962
0.157815843346142
NAT9 0.901959142394629 2 0.63700385527113
0.247690964500481
TMEM104 2.98447060234608 2 0.224869442404124
0.550690660335478
FDXR 5.24592604150315 2 0.0725874657047657
1.04429466510305
MRPL58 2.99028676079614 2 0.224216454181257
0.329800387903806
KCTD2 1.31978382992882 2 0.516907201405718

1.15955108125786
ATP5PD	7.95662332396078	2	0.0187172136596112
0.285825103705173
ARMC7	2.95628775320556	2	0.228060604394043
0.74593494155384
NT5C	28.5158940789484	2	6.42469148326441e-07
0.738696534868148
JPT1	3.37016392502495	2	0.185429233087281
0.188704110515803
SUMO2	8.69543738151475	2	0.0129362906227932
0.151005251961458
NUP85	0.00607859268367619	2	0.996965317643682
0.0230517444208127
GGA3	0.0650564603655863	2	0.967995122766325
0.0979860331099083
MRPS7	0.7113884296953	2	0.700686847114178
0.0982888389020908
MIF4GD	0.151924129081973	2	0.926851366424498
0.0767350560547448
SLC25A19	6.87652620348669	2	0.0321204268185424
0.62984984875951
GRB2	0.297245008943954	2	0.861894414782678
0.0403311698868795
TMEM94	4.44179797935324	2	0.108511514232176
2.38909151324925
TSEN54	0.0616221675375423	2	0.969658740048622
0.0719742198817412
RECQL5	0.412232187397832	2	0.813738600874901
0.207988761543913
SAP30BP	0.058945666956377	2	0.970957254814346
0.0310334675189276
GALK1	0.000197065534275474	2	0.999901472087056
0.00245511974063752
H3F3B	23.0412025701025	2	9.92353563800119e-06
0.125456701037632
UNK	1.44087957318258	2	0.486538235909545
0.344881510231481
UNC13D	1.74797555073145	2	0.417284191314139
0.219148709800536
WBP2	6.95508626025826	2	0.0308831938889048
0.291646121309721
TRIM47	0.307396339742567	2	0.857530810589185
0.515549756048371
MRPL38	0.99576188286194	2	0.607817296452321
0.477223433411553
ACOX1	4.23092147114679	2	0.120577722307212
0.456054764705313
TEN1	0.238221177945365	2	0.887709624438034
0.112949608037108
SRP68	6.03043308656696	2	0.0490352160905804

0.353441246496183
EXOC7 0.0968231749299817 2 0.952741569879662
0.0520844709104986
UBALD2 12.1084305611698 2 0.00234794387509163
0.291687287756131
QRICH2 0.221939308839319 2 0.894965906776795
0.234169982089865
PRPSAP1 3.19060858476658 2 0.202846794375905
0.242006484738507
UBE2O 0.952862630688133 2 0.620995579554284
0.348316613770219
RHBDF2 12.1058994932179 2 0.00235091715883817
0.422003776353842
PRCD 1.29170726860749 2 0.524214863266953
1.34372858890449
JMJD6 0.109197517890471 2 0.946864992852155
0.0785847850897327
METTL23 0.0106749190412198 2 0.994676759407558
0.0131543190101351
SRSF2 0.69391056563256 2 0.706836935308151
0.0668700830561518
MFSD11 0.136161872867027 2 0.93418486092646
0.105543687050161
SEC14L1 7.84441558669822 2 0.0197973380280221
0.311419454538337
SEPT9 16.9610057336983 2 0.00020747434451085
0.429605685374183
TNRC6C 0.0268762412030868 2 0.986651767846678
0.113083257010654
TMC6 18.9502494796637 2 7.67371402077277e-05
0.532351043352567
TMC8 16.7854681303671 2 0.000226507145702493
1.30697906743391
C17orf99 7.63735253312019 2 0.0219568466777651
4.21197431153073
SYNGR2 80.7543482523942 2 0 0.583641105577605
TK1 1.87757842216071 2 0.391101089660994
0.515080638289212
AFMID 2.26594392511299 2 0.322074638872982
0.411787349737107
BIRC5 8.03587626170011 2 0.0179900197920627
1.23637831356849
PGS1 1.61988870483333 2 0.444882822188053
0.381302963775207
LINC02081 0.540659905590778 2 0.763127656688307
0.462858243555475
CYTH1 4.83874585938534 2 0.0889773950517829
0.41112158761097
USP36 0.0743460740758704 2 0.963509398111339
0.132976075026266

LGALS3BP	10.3549606994963	2	0.00564220490228362	1.0075372231359
CANT1	0.586504328767749	2	0.745834044200371	0.177422277296602
ENGASE	0.103344168699227	2	0.949640217681308	0.227826671126217
CBX8	2.839804204837	2	0.241737681273029	0.359222960168461
CBX4	1.06993101632528	2	0.585689491303081	0.651158223585487
EIF4A3	1.57984655548419	2	0.453879616615556	0.0833032696423684
SGSH	1.04979257952661	2	0.591616717895112	0.543834055807064
SLC26A11	2.17758402774761	2	0.336622883974012	2.53165309903736
RNF213	7.68073868916916	2	0.0214856642656789	0.485039768475443
ENDOV	0.520279542506824	2	0.770943822488255	0.206450871526753
RPTOR	1.07362979396014	2	0.584607324689235	0.958909529763872
CHMP6	2.41873871058575	2	0.298385395276736	0.210563677696907
CEP131	0.544679908744028	2	0.761595309421312	0.437232103541166
TEPSIN	0.705023669131871	2	0.702920251005436	0.258690467521501
NDUFAF8	0.607953938460651	2	0.737877860149946	0.0986353289922206
SLC38A10	6.94602265785466	2	0.0310234679911887	0.662273116114942
ACTG1	14.9496162404629	2	0.000567194590364251	0.158798420905539
FAAP100	3.44774868811667e-06	2	0.999998276127142	0.000534621746512771
NPLOC4	1.03095607368285	2	0.597215035580544	0.396108506444634
OXLD1	0.308566570481379	2	0.857029202895667	0.0380260704158289
CCDC137	0.132848418988032	2	0.93573383290954	0.0854400925264558
ARL16	2.312843242116	2	0.314609962690312	0.311636540115456
HGS	0.056575081444125	2	0.972108805742652	0.064844638593505
AC139530.1	0.758826529268643	2	0.684262772621903	0.303192898721113
MRPL12	1.61684304907467	2	0.445560818257351	0.191144063427576

MCRIP1	6.41127266189768 2	0.0405331009632632
0.349916200491117
P4HB	19.9719591258957 2	4.60409397394779e-05
0.328333346709176
ARHGDIA	3.17585918213682 2	0.204348258516989
0.108379185964298
AC145207.5	2.05959638635807 2	0.357079014279142
2.47978645956018
ALYREF	5.48117780657638 2	0.0645323323980926
0.454810954442221
ANAPC11	1.07982634722587 2	0.582798852495993
0.0929559765176788
PCYT2	0.340976279034834	2	0.843253090957901
0.168220508111684
SIRT7	6.60187714805356 2	0.0368485660587925
0.249090262429061
MAFG	1.06088801167943 2	0.588343683645088
0.357139137012434
PYCR1	3.77706527254861 2	0.151293648848821
1.10864535083649
ASPSCR1	1.41453402902523 2	0.492989691688199
0.303289686402803
CENPX	0.355837833606387	2	0.837010288207315
0.105757182563798
LRRC45	0.0315605054928112	2	0.984343603093986
0.11048109760232
DCXR	8.25673437995408 2	0.0161091605590303
0.369553090588661
RFNG	0.0424142587562528	2	0.979016160558338
0.0966194733528921
GPS1	1.00658168060257 2	0.60453794482528 0.155505082250637
DUS1L	0.700895895800153	2	0.704372496858572
0.140275952204301
FASN	0.70150620740412 2	0.704157586296668
0.446274023930907
CCDC57	6.33773593093446 2	0.0420511743507828
0.610971613765748
SLC16A3	0.00484354213659195	2	0.997581159053419
0.0210523926682187
CSNK1D	0.315482737833628	2	0.854070642621954
0.0901298910270461
OGFOD3	0.709933730886266	2	0.701196676664813
0.173959724763193
HEXDC	2.39924690188659 2	0.301307647664306
0.474152434763902
CYBC1	18.4187761272835 2	0.000100095276192369
0.447927288990186
NARF	6.95633841763351 2	0.0308638646308549
0.43467107701885
FOXK2	1.33461843144496 2	0.513087329311902

0.812493781511538
WDR45B 4.52164144946883 2 0.10426487686594 0.314117324587683
FN3KRP 3.54464726450652 2 0.169937657079454
0.56669118444947
TBCD 6.85352779362744 2 0.0324919180221283
1.1733195635238
B3GNTL1 5.05236292080484 2 0.0799637829129255
1.45873916189059
METRNL 0.340045654723442 2 0.843645558174254
0.31042850322137
AP005530.1 0.408327632214092 2 0.815328796257364
0.151961258705353
USP14 5.37332880474565 2 0.0681077409211285
0.227796395231186
THOC1 4.09446273220296 2 0.129091817259457
0.533177844242713
COLEC12 0.456515698945577 2 0.795919006078205
0.438775948442264
TYMS 2.4941957325983 2 0.287337479780766
0.701103037718375
ENOSF1 0.0326549637306357 2 0.983805088970101
0.127834646061096
METTL4 0.114167992137961 2 0.944514730510037
0.153172206668819
NDC80 0.283964446756941 2 0.86763667999771
0.576028862341543
SMCHD1 2.3629036300842 2 0.306832950385633
0.15623488924795
EMILIN2 0.471515981226594 2 0.789971831399349
0.244869585162368
LPIN2 0.232544616702504 2 0.890232772469777
0.251683494920758
AP005329.3 0.141028661152804 2 0.93191438455982
0.206574229768593
MYL12A 3.650820668779 2 0.161151504556795
0.106542939706208
AP005329.1 4.03141730945408 2 0.133225958700175
1.09269697953224
MYL12B 4.55206754673549 2 0.102690694639016
0.0717247846658361
TGIF1 0.705664104809537 2 0.702695199436473
0.144439449143183
DLGAP1 6.87025588454205 2 0.0322212875034882
2.94359530396473
DLGAP1-AS1 3.41818603020829 2 0.181029909573753
0.626024392563475
LINC00526 0.0724537002769252 2 0.964421489517773
0.118384524957121
LINC00667 4.23868615994632 2 0.120110505597212
0.257413009373632

ZBTB14	0.667583321115287	2	0.716202980014247
0.146988811689953
RAB12	1.44907378136903 2	0.484548916119586
0.878614369403541
NDUFV2	7.77717179092256 2	0.0204742783463986
0.213719554720752
NDUFV2-AS1	0.859067787244634	2	0.650812371835315
	0.563222307810594
ANKRD12	0.138883599906527	2	0.932914427467861
0.0454318159765794
TWSG1	0.28589618610375 2	0.866799060621662
0.253697027787035
RALBP1	0.131638831839205	2	0.936299929887697
0.0491841289997662
PPP4R1	2.66747213371981 2	0.263491000084806
0.822498733944452
VAPA	0.000823093837736866	2	0.999588537754949
0.00241356503333709
NAPG	0.716065289009467	2	0.699050254484704
0.185877276058665
LINC01887	13.4665641427034 2	0.00119061884245764
1.3119730622582
CHMP1B	2.88926312679092 2	0.235832950042547
0.295365260490568
MPPE1	0.700788163611972	2	0.704410439675669
0.288721050325837
IMPA2	1.04963637816162 2	0.591662925368949
0.584484846367822
TUBB6	0.00194774518887744	2	0.999026601465573
0.0171697057490758
AFG3L2	2.9661512320003	2	0.226938637823776
0.382032666796537
PSMG2	0.91650064195095 2	0.632389156122331
0.113677256754532
CEP76	0.280829133323489	2	0.868997903153325
0.409842615549962
PTPN2	0.216547718405176	2	0.897381806510108
0.0647010476484009
SEH1L	1.34900140532947 2	0.509410704527952
0.283150000278299
CEP192	0.437930975553646	2	0.803349443050468
0.405484595510561
LDLRAD4	1.10580348578139 2	0.575278077013149
0.406086067258744
FAM210A	0.517275752915032	2	0.772102568943
0.218711178515123
RNMT	1.6711960505266	2	0.43361509110071 0.210270615418435
ZNF519	1.8874891358769	2	0.389167838125766
1.68897923194543
ROCK1	2.04898064668524 2	0.358979382199369

0.18841339444334
ESCO1 0.088457464106896 2 0.956735096460517
0.0823814038391828
SNRPD1 2.55304102499633 2 0.279006412794565
0.123669900690241
ABHD3 2.81921351474643 2 0.244239309579021
0.280767947580393
MIB1 2.76919419022699 2 0.250424676794395
1.13464466814111
RBBP8 0.950634186479514 2 0.621687892179092
0.330000445742132
TMEM241 6.69888791871183 2 0.0351038678524134
1.77512374258274
RIOK3 0.98452247564472 2 0.611242665270595
0.180514018418069
RMC1 3.0650019097009 2 0.215994798015306
0.394813633256955
NPC1 0.13728805058026 2 0.933658979914017
0.253643256183053
TTC39C 16.1136576600789 2 0.000316930253107084
1.02588128303215
IMPACT 6.02415464079603 2 0.0491893904303214
1.30741241581927
LINC01915 1.50930680393966 2 0.470173547718258
1.12010169702395
SS18 0.082415057533591 2 0.959629958459748
0.0423892771359371
PSMA8 8.60229585865845 2 0.013552992201864
1.62777140000756
TAF4B 0.770447172332132 2 0.680298513862501
0.650117518152575
KCTD1 1.18715774602209 2 0.552346963548624
0.427535914347255
TRAPPC8 2.28570950816223 2 0.31890731912122 0.55859603016108
RNF138 1.05079612769497 2 0.591319934423659
0.259210095783452
KLHL14 2.16615493174171 2 0.3385520384765 0.445539777515548
AC012123.1 0.000895076400124945 2 0.99955256193022
0.0192965128561538
MAPRE2 6.50955226584255 2 0.0385894586058302
0.63872664090406
ZNF397 0.789589652379684 2 0.673818274930034
0.134264100843872
ZSCAN30 0.12031874558845 2 0.941614453898657
0.196853726224772
ZNF24 0.286048990324787 2 0.866732837873846
0.0939489091398913
INO80C 0.0241699280442154 2 0.987987765881362
0.0223466433314514
GALNT1 19.0643578746427 2 7.24815191175399e-05

1.09855738098438
C18orf21 2.87970051222777 2 0.236963239821911
0.474728282833752
RPRD1A 1.99639479728758 2 0.368543179198826
0.404708462351598
SLC39A6 2.68569230753168 2 0.261101475091545
0.568949653600404
ELP2 1.31240485660477 2 0.518817846112532
0.135381702758939
FHOD3 1.36084268481556 2 0.5064035781056 0.480011131539462
TPGS2 0.59724228256281 2 0.74184040890882 0.103252839742806
KIAA1328 1.20139465887688 2 0.548429066987873
0.4818061158671
PIK3C3 3.17182991544723 2 0.20476036031017 0.634046660261031
SETBP1 5.68590359539113 2 0.0582534595027014
0.995706228641706
EPG5 3.74636195758198 2 0.153634176649416
1.69748384050744
ATP5F1A 14.1593808150297 2 0.000842033795364627
0.209505842242021
HAUS1 0.0509105376831475 2 0.974865984875276
0.0294463193595761
C18orf25 4.94805013330973 2 0.0842450835741922
0.778600914926405
PIAS2 1.51100665093418 2 0.469774105943783
0.285651781212094
KATNAL2 0.0398566542313687 2 0.980268929490575
0.207872622496631
HDHD2 0.000809911901895062 2 0.999595126032647
0.00615717841536453
IER3IP1 0.7994359647003 2 0.670509114778778
0.140143701093907
SMAD2 1.30812572393858 2 0.519929079664118
0.218845235724406
SMAD7 2.32301702476433 2 0.313013639603901
0.714609999065117
DYM 2.49654689733224 2 0.286999889377707
0.36365519050729
C18orf32 2.34533349496294 2 0.309540373619974
0.274125764460283
RPL17 9.90069366775825 2 0.00708095258885144
0.229339997655471
ACAA2 0.874033547986464 2 0.645960595981083
0.178109081529984
MBD1 0.00381762604562768 2 0.998093007602169
0.0187598317385283
CXXC1 0.0131497261148443 2 0.993446704061851
0.0250733677235896
ME2 3.33472751074832 2 0.188743985667454
0.242231353219713

ELAC1	2.5937157324728	2	0.273389471146582	0.539355003246177
SMAD4	0.147950750423772	2	0.928694562466542	0.175710011232029
MEX3C	0.916778406268036	2	0.632301334649848	0.374939159778082
MBD2	0.422158988423865	2	0.809709697088533	0.154558700588686
AC093462.1	0.176791100152834	2	0.915398719069024	0.276015427479303
POLI	0.52587170881275	2	0.768791210298096	0.478381327083331
TCF4	17.8772956901629	2	0.000131218347814932	0.309217211164895
TXNL1	4.2840216372825	2	0.117418498173148	0.228519929638341
WDR7	1.92653278182727	2	0.381644248510677	1.46732258339852
FECH	4.10187547064091	2	0.128614240905406	0.778695040813668
NARS	2.6746899354443	2	0.262541801000314	0.272189777301086
AC027097.1	8.61508546395401	2	0.0134666000163409	0.938395255713177
NEDD4L	10.4514559129641	2	0.0053764447172383	1.37027914195599
AC104971.3	3.94544421109484	2	0.139077756142417	2.34170606045116
MALT1	6.22936547475421	2	0.0443925890227745	0.281911321754438
ZNF532	5.22773350536992	2	0.0732507528966693	1.06290582097329
SEC11C	45.3419322241604	2	1.42601597197256e-10	0.417758198358156
LMAN1	2.71397470479434	2	0.257435171330965	0.194021173118132
PMAIP1	10.8884264508721	2	0.00432123851107502	0.309596066009307
PIGN	2.34144151961004	2	0.310143321847039	0.436866248956984
KIAA1468	3.63212564364014	2	0.162664932645506	1.30758261357858
ZCCHC2	0.29953962976874	2	0.860906121394271	0.365888327749432
PHLPP1	0.800708118557019	2	0.670082755013792	0.476187513690555
BCL2	5.33124170292307	2	0.0695561560838932	1.05930615051937
KDSR	6.16756845842223	2	0.0457856649500592	1.03918056277131

AC036176.1	4.21790243230502	2	0.121365185555298	1.70523932656658
VPS4B	0.0310758644900662	2	0.984582158631692	0.0270081797733314
SERPINB8	0.656985427701422	2	0.720008174293756	0.218631481697011
TMX3	3.28773950220703	2	0.193230838359489	0.540163957462166
RTTN	0.0411995361468229	2	0.979610957701052	0.145420196645026
CBLN2	15.2846496879534	2	0.000479711899336155	4.22716390714751
TIMM21	3.1202915866818	2	0.210105436994087	0.70558668945838
CYB5A	8.18101403491858	2	0.0167307486260976	0.303836116250888
CNDP2	0.0437363038415472	2	0.97836922265042	0.0244431317706757
LINC00909	1.33571675573284	2	0.512805638528145	0.340519078956274
ZNF407	2.06405445374428	2	0.356283959556288	0.709811065385549
ZADH2	0.236941047022121	2	0.888277998587513	0.287605682046284
ZNF516	3.32177483985783	2	0.18997032182774	4.96331504280105
ZNF236-DT	0.0114932521274289	2	0.994269854208079	0.187548327431591
AC027575.2	0.0196951855028812	2	0.990200736019274	0.18473185985564
ZNF236	3.042122195754	2	0.218479935316753	0.8964434656859
MBP	1.16444653180764	2	0.558654946356465	0.2226075408627
ATP9B	1.25881484023087	2	0.532907497723382	0.624426664524708
NFATC1	5.97105216213863	2	0.0505129226655276	0.314229757995319
CTDP1	1.25273452430969	2	0.534530085907298	0.37079979346277
PQLC1	3.11572547685154	2	0.210585667232121	0.632195275118347
HSBP1L1	0.00504244473501802	2	0.997481953244236	0.0416101566809502
TXNL4A	0.172734700266771	2	0.917257214762229	0.0481694560244422
RBFA	0.029197746487823	2	0.985507173623951	0.050655560715789
ADNP2	1.88419797265348	2	0.389808772774557	8.93336021404822
C20orf96	6.14756671563822	2	0.0462458588311211	

1.42336503700237
ZCCHC3	1.84099600496549 2	0.398320627022082
0.442603027472444
NRSN2-AS1	1.04473838916627 2	0.59311368033804
1.00632152103525
SOX12	2.30157504048309 2	0.316387509675363
2.41284089911925
NRSN2	1.01163613846322 2	0.603012067973832
0.773683173932208
TRIB3	0.000223673038570754	2	0.999888169734185
0.00564871774477396
RBCK1	1.47723220703012 2	0.477774648879476
0.125647172644305
TBC1D20	4.26085909044316 2	0.118786258822401
0.529513185769236
CSNK2A1	3.50690025980109 2	0.173175433833864
0.322593723891908
FAM110A	2.22165768384968 2	0.329285921960306
0.291542646079036
PSMF1	1.08110464067116 2	0.582426477533254
0.131583268783148
SDCBP2	2.22724811189343 2	0.328366782528205
1.39403700436482
SDCBP2-AS1	5.75638563823359 2	0.0562363002249924
1.85862883285061
FKBP1A	21.1753955423476 2	2.52244253949874e-05
0.30372710493429
NSFL1C	1.72274872111762 2	0.422580904507237
0.157475035581727
STK35	16.5555867754379 2	0.000254097276042087
1.26544650584993
SNRPB	0.185591453511942	2	0.911379651760619
0.0328656333361953
ZNF343	0.277869156271941	2	0.870284962260341
0.379035804229907
NOP56	0.452290785288017	2	0.797602127752781
0.0914255555364666
IDH3B	0.346713806490171	2	0.840837463665973
0.067043591085756
CPXM1	3.14227086361488 2	0.207809095295331
1.34561510247767
PCED1A	0.840791135284229	2	0.656786964752299
0.701949607063208
VPS16	3.67867178925963 2	0.158922932642328
0.573338944910429
PTPRA	3.81596700480031 2	0.148379291608952
0.408503903381009
MRPS26	2.88994376059845 2	0.235752705758201
0.26438506602342
UBOX5	1.79184397206661 2	0.408231041758567

0.707061598732487
FASTKD5 2.44951594094544 2 0.293828806965484
0.289113100423296
DDRGK1 0.624452625035335 2 0.731815890389438
0.141838217077232
ITPA 1.39580547972024 2 0.497627865234237
0.166724259639345
C20orf27 3.63498033624901 2 0.162432919076279
0.568247279656619
CENPB 0.000683112645520664 2 0.99965850200096
0.0158101789355045
CDC25B 7.24544412315159 2 0.0267098715328071
0.754616643917022
AP5S1 2.70317740035864 2 0.258828732581229
0.520108050927196
MAVS 1.87807404086217 2 0.39100418316156 0.275681974227041
AL353194.1 4.16162823486241 2 0.124828545724987
0.902035744167111
PANK2 5.0442407226721 2 0.0802891840529661
0.390310112649233
RNF24 3.39718365892554 2 0.182940954819614
1.43057539964017
PRNP 0.0358619465989835 2 0.98222883003363
0.0621001292371271
RASSF2 13.9489811419202 2 0.000935442787776353
1.21769463305452
SLC23A2 1.27352762323934 2 0.529001605784143
0.570323354113512
TMEM230 0.536647422720113 2 0.764660211837379
0.0912530182611947
PCNA 1.54968549312979 2 0.460776233947681
0.273405583634053
CDS2 0.0972793024378186 2 0.952524308836348
0.0751883920233597
GPCPD1 2.63549983160674 2 0.267737055631489
0.377647272620581
SHLD1 0.00593579789568856 2 0.997036500910384
0.0144781550762157
TRMT6 0.0762000120789589 2 0.962616668596811
0.058842052642263
MCM8 2.55971558142447 2 0.278076842751239
1.3782786843486
CRLS1 5.18554732247482 2 0.0748122482134588
0.354144420324058
TMX4 1.38351552036365 2 0.500695193034907
0.15000765208889
MKKS 0.0254170144001376 2 0.987371904877955
0.0622945744354572
SLX4IP 1.18587066621273 2 0.552702535260919
0.311153693782664

TASP1 0.0222701266212976 2 0.988926702040547
0.0559018472961276
ESF1 0.171513420418917 2 0.917817499686835
0.0865204452555986
NDUFAF5 0.736567864296525 2 0.69192069526621
0.359306460621477
MACROD2 0.708043717610656 2 0.701859625374798
0.543787292544471
KIF16B 2.31001335193301 2 0.31505543359687 0.789065687994227
SNRPB2 0.760348813315452 2 0.683742149629762
0.0630468252853406
DSTN 13.4394674011309 2 0.00120685955718702
0.49080073771645
RRBP1 25.7000233901128 2 2.626097392322e-06
0.625045966079475
SNX5 16.6766527273334 2 0.000239172295599777
0.467558021950315
MGME1 5.64261402464808 2 0.0595280878969154
0.443732405102624
PET117 0.178985276187295 2 0.914394996789861
0.14395818723124
KAT14 3.71047634944541 2 0.156415683975933
1.30687230994467
ZNF133 1.36571089974569 2 0.505172436349331
0.89192396092473
DZANK1 3.24084626850502 2 0.197814979079155
5.54585692489851
POLR3F 0.982193575361093 2 0.611954841445203
0.41556485439715
RBBP9 0.111097752511786 2 0.945965787274628
0.118494943484893
SEC23B 1.96211596429896 2 0.374914236382035
0.345376030946027
SMIM26 1.4160143981562 2 0.492624923342074
0.117492195368197
DTD1 1.61522050135713 2 0.445922436767684
0.282957878822635
AL035563.1 0.0262466646293818 2 0.986962403156011
0.11176104260028
NAA20 1.43426621799084 2 0.488149723860963
0.165511595974753
CRNKL1 1.71841166134355 2 0.423498278144931
0.346683313877121
RALGAPA2 0.874740180464174 2 0.645732407926509
0.379010762399961
KIZ 0.144277863294776 2 0.930401624601044
0.143619219154111
AL117332.1 0.0324294114198559 2 0.983916044982167
0.104444073597896
XRN2 1.12377419452677 2 0.570132153224737

0.0887760442861224
NXT1 0.120111985985168 2 0.941711802845994
0.0775761319073212
LINC01431 0.0465401079185886 2 0.976998605802513
0.146272520439584
GZF1 5.14333839951527 2 0.0764078988734725
2.11052866253981
NAPB 8.94935346793654 2 0.0113939047916286
0.656688393694202
APMAP 1.38726898775303 2 0.49975640270064 0.295889719709239
ACSS1 9.16569276349209 2 0.0102257484494076
0.724514614731077
ENTPD6 1.11910409570556 2 0.571464995493763
0.515164797907196
PYGB 0.237937414308398 2 0.887835583229254
0.216467051433807
ABHD12 17.9162619059095 2 0.00012868655031828
1.29726026868894
NANP 1.46726806267341 2 0.480160895957009
1.23636266570626
ZNF337-AS1 2.0387619392625 2 0.360818228495858
1.66579712351027
ZNF337 0.0196549664746599 2 0.990220648675164
0.105595697134332
LINC01597 0.571228060410167 2 0.751552636590695
0.382438934657151
HM13 16.9221498925149 2 0.000211544549387321
0.410511376815594
BCL2L1 0.0175280032546032 2 0.991274290289835
0.0455300894155646
TPX2 3.92562470014755 2 0.140462834255405
0.796178327203071
PDRG1 0.0320491717334733 2 0.984103124729575
0.0359045952508455
HCK 2.15296292606289 2 0.340792509637593
0.387564889567255
TM9SF4 2.3604325604761 2 0.307212287468692
0.64998821698669
PLAGL2 1.51567733265398 2 0.468678303313603
0.683638806439585
POFUT1 0.342105952502018 2 0.842776925127049
0.41648597558842
KIF3B 1.26635188470924 2 0.530903003329911
0.348439541001925
ASXL1 0.0139544924924853 2 0.993047038223947
0.0283686254421267
COMMD7 7.23470787841163 2 0.0268536389269218
0.343394697729826
MAPRE1 0.405254700169322 2 0.816582484127658
0.0352009679117483

AL035071.1	0.0368335625371188	2	0.981751771329395
	0.106780132578147
CDK5RAP1	0.0516086242110335	2	0.974525773847646
0.0490283658009894
SNTA1	11.5382319650385	2	0.00312251665372731
0.452920881648059
CBFA2T2	0.71539770110052	2	0.699283632181389
0.330157901976055
NECAB3	1.65767188203535	2	0.436557168955336
0.417413690242129
ACTL10	4.14665807729818	2	0.125766402812362
1.16331609015412
E2F1	7.95730488055004	2	0.0187108363261502
1.08747257952215
PXMP4	4.07671386059472	2	0.130242532727741
0.444880002936849
ZNF341	1.50303005938708	2	0.471651445228719
1.64789234851
CHMP4B	2.15841969503297	2	0.339863963932542
0.169781816904814
AL034549.1	1.07758430425929	2	0.583452548865455
0.592267430564813
RALY	0.569422450268382	2	0.75223144849336
0.0749957697644325
EIF2S2	4.69426215418552	2	0.0956431618436444
0.184721576976739
AHCY	0.0252562879218451	2	0.987451256470906
0.0292459568368733
ITCH	5.4531366581712	2	0.0654434852702854
0.525854653899792
DYNLRB1	0.737870890056551	2	0.691470046838865
0.0970335479168182
MAP1LC3A	1.42468956842855	2	0.490492748385084
0.49940917704017
PIGU	0.44604855632526	2	0.800095424211924
0.208204178490805
NCOA6	0.169507421241215	2	0.918738532082004
0.191450805256415
TP53INP2	1.5532087766871	2	0.459965225845964
0.697921096056244
GSS	27.46957011105	2	1.08407328025528e-06
0.700545609838587
TRPC4AP	1.3856100994823	2	0.50017109467611	0.12624414839026
EDEM2	11.1704753080585	2	0.00375285778200907
0.8324501452287
MMP24OS	1.19555638871606	2	0.550032344469696
0.217033591352077
EIF6	0.380203269688457	2	0.826875090351552
0.0599884844192384
UQCC1	1.04663021566174	2	0.592552911511537

0.290376485001314
CEP250 3.45587790249641 2 0.177650178585754
0.77542236421026
FO393401.1 0.661839204217215 2 0.718262913542985
0.206453709072285
ERGIC3 0.100867391789343 2 0.95081696965087
0.0374556077487227
CPNE1 1.34364483801609 2 0.510776879577931
0.0830207395104249
RBM12 0.615291202023135 2 0.735175817411991
0.261102273853592
NFS1 0.914502345269669 2 0.633021322459692
0.581245440772985
ROMO1 8.71509423773892 2 0.0128097699883187
0.229198088693775
RBM39 1.3617662369481 2 0.506169787037072
0.0814047880651664
PHF20 7.25574704425403 2 0.0265726304827089
0.504153606683905
SCAND1 0.442620881864169 2 0.801467833244851
0.0609732454341884
CNBD2 3.25250204068413 2 0.196665488717183
0.658916232874218
NORAD 0.473003195527741 2 0.789384621050734
0.215485596122562
AAR2 0.00204934820124522 2 0.998975850698619
0.0116736564006747
DLGAP4 7.65993866374005 2 0.0217102814333718
1.66327690111954
TGIF2 0.0286361805279074 2 0.985783926116807
0.0621377039290445
RAB5IF 2.82535980073204 2 0.243489879399087
0.196991869289642
NDRG3 3.13131612869882 2 0.20895046506864 0.85521454483402
DSN1 0.3559382449853 2 0.836968266583584
0.247912250619461
SOGA1 1.54846017428309 2 0.46105861934367 0.565128504262262
SAMHD1 1.4025567228861 2 0.495950893874841
0.247941505153841
RBL1 4.76063020915801 2 0.0925214189938126
1.94760449288533
MROH8 2.70683892393753 2 0.258355312319701
1.13922860631503
RPN2 5.78839371538221 2 0.0553434559211354
0.157336957585205
MANBAL 1.68638474316176 2 0.430334540515872
0.427257764764818
SRC 16.6142373853706 2 0.000246753994166071
3.23719682028114
BLCAP 14.331177935832 2 0.000772723733929004

0.473810808765848
CTNNBL1 8.09407821851507e-05 2 0.999959530427823
0.00111961491038788
TTI1 0.0465383960057273 2 0.976999442071111
0.0914071812770804
RPRD1B 15.1894901124642 2 0.000503088199543034
0.616876055511083
TGM2 2.08135112608028 2 0.353215981676906
1.18336719581385
RALGAPB 0.677526487512226 2 0.712651153727824
0.376719945197426
ACTR5 0.496458561528528 2 0.780181042263903
0.112931909013318
PPP1R16B 2.30934321936834 2 0.315161015737255
0.811712648316281
FAM83D 0.000299452351968447 2 0.99985028503242
0.0164168711400846
DHX35 0.00276603884351066 2 0.998617936508864
0.0437766177558033
TOP1 14.0146933086104 2 0.000905207232486105
0.388809592396129
PLCG1 3.15701772012539 2 0.206282465006411
2.67261341717982
ZHX3 0.305085222341774 2 0.858522310536084
0.476221580039929
CHD6 1.46143102068722 2 0.48156430255157 0.285361473084037
SRSF6 0.0732420505734405 2 0.964041413447351
0.040776473717834
L3MBTL1 1.34497364871432 2 0.510437629399232
0.885043466286024
IFT52 1.39987247624357 2 0.496616968012548
0.244802844234554
MYBL2 11.2420962700556 2 0.00362084398595264
0.437094027345058
OSER1 3.31941617263619 2 0.190194492373277
0.241316422806132
OSER1-DT 0.337344867429111 2 0.844785581343253
0.335248741886118
TTPAL 1.70285539963099 2 0.42680514712766 0.316642834162589
SERINC3 2.76197814709769 2 0.251329846382341
0.327848745149148
PKIG 1.31664671522603 2 0.517718636221123
0.179540800708283
ADA 0.130906492122838 2 0.936642837477621
0.0399805659339751
YWHAB 4.60753165556599 2 0.0998819962004102
0.0809393101919868
PABPC1L 1.24143539249394 2 0.537558495401933
0.845257919889489
TOMM34 1.67279243111099 2 0.433269121837464

0.396479599641575
STK4 1.78401588185102 2 0.409832007552713
0.136406822658351
SYS1 3.49838762393955 2 0.173914094410042
0.484158773545065
PIGT 9.04527350509791 2 0.0108603498271658
0.596228667198792
DNTTIP1 0.365962027017198 2 0.832783967232621
0.0863626867718451
UBE2C 0.570534982007327 2 0.751813124173177
0.419229573385007
TNNC2 0.730187579547902 2 0.694131545384178
0.44614846056607
ACOT8 0.893452373782923 2 0.639719047757021
0.102749107549975
ZSWIM3 3.4079573167238 2 0.181958132721219
1.18909736362531
ZSWIM1 3.3446178138716 2 0.187812922074662
1.64766355459642
CTSA 5.29539819776562 2 0.0708139616779278
0.591246473542486
PLTP 4.57695090705982 2 0.101420965024548
1.57264734514142
PCIF1 0.065365140975442 2 0.967845733632588
0.0565646607188911
ZNF335 0.0126062299969283 2 0.993716707960214
0.0430383599680564
SLC12A5 1.80600669859617 2 0.405350420854993
1.35799957632279
NCOA5 0.0174908667375448 2 0.991292696698018
0.058881277145398
CD40 7.86802893246926 2 0.019564971769391
0.147741480149365
SLC35C2 4.08064166895416 2 0.129986999876488
0.374090138099464
ELMO2 0.846433239710014 2 0.654936745441682
0.375879807637965
TP53RK 0.0232894388041242 2 0.988422817937599
0.0294129922972218
EYA2 0.960760676844464 2 0.618548089457296
0.925132612628901
ZMYND8 0.555413061776748 2 0.757519097353734
0.163888512774018
LINC01754 3.08329056201707 2 0.214028674272564
0.72125149598723
NCOA3 35.2488074389886 2 2.21727070037758e-08
0.61039598719236
SULF2 26.2234273721191 2 2.0214132022689e-06
0.893568375406893
ARFGEF2 1.06305426248714 2 0.587706778641395

0.357849951273298
CSE1L	0.534812309966494	2	0.765362152676742
0.175759239233754
STAU1	3.37046821350972 2	0.185401023243133
0.32438389848274
DDX27	1.39167407930892 2	0.498656877667481
0.145678115546225
ZNFX1	0.153855875481141	2	0.925956577725445
0.142489809988398
ZFAS1	87.6208343263807 2	0	0.524551435064168
B4GALT5 2.14822383450462 2	0.341600990578382
1.2850289952408
SLC9A8	0.552565615443829	2	0.758598362947081
0.484324709114097
SPATA2	0.00797327411035252	2	0.996021299032725
0.0390888036472145
RNF114	10.7819783341465 2	0.0045574630130214
0.550358594760824
UBE2V1	0.598535138434346	2	0.741361017507311
0.392903860785225
TMEM189 0.75927855114259 2	0.684108139226604
0.361051651031532
CEBPB	0.0390286887567683	2	0.98067482791697
0.0501691834347444
PTPN1	44.138478096459 2	2.60286237008245e-10
0.651584318011899
BCAS4	6.36769484727174 2	0.0414259648837306
0.175624461147841
ADNP	0.366538803961224	2	0.832543836563916
0.197359975418103
ADNP-AS1 2.04952333654169 2	0.358881988178957
0.699283259043587
DPM1	15.7819761438886 2	0.000374099754839596
0.482750308750556
MOCS3	4.34180738934801 2	0.114074481814881
0.732186516367391
KCNG1	0.124016920439264	2	0.939874935209074
0.458034105046571
NFATC2	5.75648427424119 2	0.0562335268313139
0.652038746917194
ZFP64	0.0589299816508196	2	0.970964869724819
0.108186728481622
TSHZ2	19.4207272497327 2	6.06516556286296e-05
1.45435391178516
ZNF217	0.555159628621039	2	0.757615093663444
0.295974888027956
BCAS1	1.85627912101601 2	0.395288436921313
1.34650069585635
PFDN4	20.6029631468753 2	3.35833021803955e-05
0.494869558736174

AURKA	0.382034401238335	2	0.826118378281373	0.258972816424057
CSTF1	0.211793123918123	2	0.899517687611323	0.10771579792840 2
RTF2	1.23418330886496	2	0.53951124321702	0.114913337687817
BMP7	16.8167590973022	2	0.000222990910226484	2.27749923599562
RAE1	0.0998363560732701	2	0.951307259144085	0.0462031741826533
AL109955.1	0.991724817690392	2	0.609045434572813	0.52622540627679
RBM38	34.6087462046066	2	3.05355852825429e-08	0.792783147722788
ZBP1	23.5370605871719	2	7.74447948004298e-06	1.50116884464487
PMEPA1	12.6596819682015	2	0.00178231716333532	2.42034223042124
RAB22A	1.73406587666575	2	0.420196450265554	0.295677923005959
VAPB	0.900723189261634	2	0.637397630385822	0.240779331855804
STX16	0.743794727568434	2	0.689424998866028	0.13397533211272
NPEPL1	0.487079409037581	2	0.783848353092752	0.41388556442306
GNAS	7.0289721370648	2	0.0297630952153781	0.253249245560452
NELFCD	0.275745199797575	2	0.871209676876901	0.0734522630144716
CTSZ	8.57238653293456	2	0.0137571957227265	0.322189109035914
ATP5F1E	88.1130639292089	2	0	0.392014676783722
PRELID3B	9.03983452436494	2	0.0108899246398795	0.498171558087086
ZNF831	14.6371823291661	2	0.000663095746231357	2.19924525205828
FAM217B	0.171051396728856	2	0.918029550893066	0.137236295856878
PPP1R3D	0.210795993270785	2	0.899966267752353	0.295146703318875
TAF4	0.58016647387667	2	0.748201287002191	0.725674147531185
LSM14B	1.51474160712686	2	0.468897631743613	0.305484995152344
PSMA7	4.41799812719045	2	0.109810506859674	0.0899401060672036
SS18L1	0.182524980341836	2	0.912778084175487	0.271024133581472
MTG2	0.334387085866183	2	0.846035851230246	0.212992448332266

OSBPL2	0.992684931206267	2	0.608753128363455	0.25924930444636
ADRM1	0.480023614812831	2	0.786618573086534	0.0240168890180352
LAMA5	0.802850814182996	2	0.669365247738241	0.723347283516658
AL121832.2	1.92429887999631	2	0.382070764557841	1.78193130991736
RPS21	61.8153181072346	2	3.77475828372553e-14	0.370065098083417
SLCO4A1	24.8778982879568	2	3.96125705881545e-06	1.25690711814447
AL357033.4	0.0313614607075912	2	0.984441572199495	0.100068328882442
MRGBP	2.67279127959491	2	0.262791157605269	0.432367958695491
OGFR	8.01508809819717	2	0.0181779846975292	0.24544590622369
COL9A3	44.533105374153	2	2.13677631144549e-10	2.49432555285113
TCFL5	0.751798988322459	2	0.686671344053632	0.483271359179354
DIDO1	0.0872199170082022	2	0.957327282027427	0.0873251757298961
GID8	2.5359505167017	2	0.281400809293775	0.170472062403387
SLC17A9	8.78814742051033	2	0.0123503149988314	1.40968866433172
YTHDF1	0.203846664797367	2	0.903098787432557	0.150456396424182
ARFGAP1	3.19749046628444	2	0.202150010057597	0.590927093178254
PPDPF	7.85505140679329	2	0.0196923370056581	0.237634443290011
HELZ2	1.06076774131684	2	0.588379064862984	0.418566340364892
GMEB2	0.517294539614465	2	0.772095316347616	0.325577915378946
MHENCR	0.018614896784995	2	0.990735731835535	0.0854348654659157
ARFRP1	0.240374528272247	2	0.886754363877994	0.109095383886535
SLC2A4RG	2.39815081417252	2	0.301472822727141	3.11372363587022
AL118506.1	2.20186500489862	2	0.332560825278078	1.89111900017409
TPD52L2	2.48650160131459	2	0.288445014941126	0.331948621717207
DNAJC5	0.0890001293240358	2	0.95647553824598	0.111264643090326

UCKL1	1.47060582822321	2	0.47936023200154	0.275508870812085
ZNF512B	3.67296280054345	2	0.159377225336423	1.69723914070395
SAMD10	0.0959843697058542	2	0.953141235987338	0.191380540749585
PRPF6	0.00985131102096741	2	0.995086455637347	0.0159780599748481
TCEA2	4.73491498375653	2	0.0937187041860303	0.543928697632424
RGS19	14.2785853761746	2	0.000793313017817932	0.466432451428055
PCMTD2	0.0292421318883608	2	0.985485302801321	0.0632588566874149
MIER2	0.212371173557209	2	0.899257742241127	0.240654739387429
TPGS1	1.66470783650383	2	0.43502406905993	0.102046243795097
CDC34	0.00796907568335899	2	0.996023389896274	0.0302504453339111
BSG	13.7907942110656	2	0.00101243485121505	0.32273518446818
POLRMT	0.0545446740661806	2	0.973096195304284	0.0796570711064691
RNF126	1.88133710366096	2	0.390366767682517	0.226803569916645
PTBP1	0.535943924641663	2	0.764929227642478	0.103379340923856
MED16	5.21810606464219	2	0.0736042115806632	0.477518921011443
R3HDM4	7.51370444068914	2	0.0233571479339486	0.401093616057579
ARID3A	6.2102534657025	2	0.0448188381801767	0.657757310558009
WDR18	0.119246211579446	2	0.942119546081276	0.0506082172915348
TMEM259	0.562905517288084	2	0.754686567249921	0.127982641396669
CNN2	27.4220205120716	2	1.11015572901163e-06	0.520746235179553
ABCA7	0.149750294419037	2	0.927859324922908	0.197112769838537
ARHGAP45	19.1093513134652	2	7.08691274829976e-05	0.55036268858285
POLR2E	1.74231266367283	2	0.418467382216153	0.109124563606648
GPX4	14.1568762564086	2	0.000843088917381163	0.331344498298116
SBNO2	0.119632099937235	2	0.941937787134266	0.280837064162132
STK11	0.0279866672056247	2	0.986104118002669	0.0861878934262738

ATP5F1D	0.173975458753453	2	0.916688343901468	0.00940963083209464
MIDN	0.895488305216728	2	0.639068167041289	0.245953413708213
CIRBP	0.156121072795773	2	0.924908434223787	0.0234732825174347
C19orf24	8.24172590756859	2	0.0162305022235562	0.44490777251224
MUM1	4.06233920288178	2	0.13118200073007	0.323794107451815
NDUFS7	1.03511084937461	2	0.595975676085137	0.102855480576902
GAMT	1.05029594725578	2	0.591467836249539	0.311401412005728
DAZAP1	11.6934842508363	2	0.00288929680822447	0.476385172894794
RPS15	88.4602144221623	2	0	0.324497902216285
AC027307.3	3.40231290536725	2	0.182472381312915	0.569744470188263
AC027307.2	2.32932498116546	2	0.312027956636503	2.00563946769147
C19orf25	0.226947024129785	2	0.892727842615178	0.0825677977392343
MBD3	0.00647826225986894	2	0.996766109195743	0.0287673315690331
UQCR11	7.43466571505785	2	0.0242986895632286	0.170536061356367
TCF3	17.2316449871993	2	0.000181215705421511	0.838802132416988
REXO1	1.00213551988545	2	0.605883376203356	0.720506000013016
KLF16	0.241903075048164	2	0.886076900032797	0.363479496024481
AC012615.1	0.659719350615304	2	0.719024623262143	0.589198656252319
ABHD17A	2.52269236699531	2	0.283272433035293	0.484009082488612
SCAMP4	1.39313181051328	2	0.498293556244389	0.215849547052799
CSNK1G2	0.414151610248362	2	0.812958021267958	0.170806889482817
BTBD2	0.501093305351745	2	0.778375165882354	0.478798067081236
MKNK2	13.1404246002235	2	0.00140149984197768	0.539647004906194
MOB3A	7.79123279735425	2	0.0203308386839733	0.335909748699757
IZUMO4	10.6610538216098	2	0.00484151829174329	0.832466825230922
AP3D1	0.977960703199325	2	0.61325137628410 1	0.190067278090469

DOT1L	0.235396471343326	2	0.888964269849192
0.347787383019399
PLEKHJ1	4.33903365238296	2	0.114232797874771
0.238721199729214
SF3A2	4.9278207833438	2	0.0851015191958604
0.338778233909975
JSRP1	2.03540885614683	2	0.361423662626684
1.21809620965957
OAZ1	69.3014688431612	2	8.88178419700125e-16
0.274514066592389
LINGO3	2.81246415955285	2	0.245064930824527
2.40007194259744
LSM7	67.2875546525586	2	2.44249065417534e-15
0.632323767416933
SPPL2B	9.17985795728544	2	0.0101535794687581
0.796733776704006
TIMM13	2.73284266632442	2	0.255017952800619
0.121571958469766
LMNB2	0.185711019047666	2	0.911325168591057
0.210773086117177
GADD45B	4.54762680446913	2	0.102918959415519
0.209433128767313
GNG7	119.958814661677	2	0	1.21236971714393
SLC39A3	2.48227441651041	2	0.289055314868573
0.152377904407639
SGTA	0.785676165578807	2	0.675138055207169
0.230446325929271
THOP1	1.41536013269111	2	0.492786103441572
0.268920488168346
ZNF554	0.0789956853755038	2	0.961272027752281
0.295029181660094
ZNF57	0.62392562447826	2	0.732008749488491
0.728003787627511
AES	39.4375161823993	2	2.73056277588779e-09
0.601972575221149
S1PR4	47.2826887182432	2	5.40374411883704e-11
1.27276146583499
NCLN	9.50248109242547	2	0.00864096902993505
0.577957690091143
NFIC	2.99380833819815	2	0.223822003755993
0.88510660336524
DOHH	0.506685680940554	2	0.776201722837622
0.0983193113964352
FZR1	3.97729659629551	2	0.136880321839539
0.481024539555696
MFSD12	0.73037781289192	2	0.694065525041512
0.344694216887282
HMG20B	2.68836363107747	2	0.26075296463028 0.299763746851282
CACTIN	2.413542479731	2	0.299161642929356
1.06076078523512

PIP5K1C	1.47812275120819	2	0.477561956519979	0.758996298547842
APBA3	2.45128789456448	2	0.293568596743675	0.475757961647858
MRPL54	0.0614105253494002	2	0.969761355826731	0.0276680311980583
DAPK3	1.8896885771445	2	0.388740097464891	0.326388267037458
EEF2	18.0170117566807	2	0.000122364547023857	0.190690621744561
PIAS4	0.00964971821982577	2	0.995186761825498	0.0280088231704846
ZBTB7A	2.87916933717148	2	0.237026182661083	0.403191557081262
MAP2K2	0.0663182387185539	2	0.967384617721245	0.0335171345099884
SIRT6	3.14181485137061	2	0.207856482443368	0.319178060040987
EBI3	11.3017389642982	2	0.00351445968610198	1.60426252702376
YJU2	0.612960437881652	2	0.736033077549221	0.193693335650209
MPND	6.10832797886548	2	0.0471621323347967	0.522815318687456
SH3GL1	0.416641955432579	2	0.811946378187168	0.257573001358291
CHAF1A	1.15902914930114	2	0.560170221402913	0.338290703798827
UBXN6	0.0261154530602742	2	0.987027155722844	0.0344031709778785
HDGFL2	0.747412171777103	2	0.688179147670245	0.163111981747446
SEMA6B	0.4090384010358	2	0.815039092594585	0.492879641395668
TNFAIP8L1	1.61989419161153	2	0.444881601703041	0.462947425289992
MYDGF	16.9769762304053	2	0.00020582420748827	0.283600003005283
DPP9	0.0453740527204947	2	0.977568389037462	0.0774671703530161
FEM1A	3.25682208362522	2	0.196241145499257	0.660822819751538
TICAM1	1.6170334269641	2	0.445518407811788	0.345170461936849
PLIN3	10.955582468824	2	0.00417854894769865	0.28869872616744
ARRDC5	0.101275484847993	2	0.950622978540469	0.217125497504616
UHRF1	0.00534207391463913	2	0.997332527087956	0.0480813019968314

KDM4B	3.41962003436582	2	0.180900157274163	0.373745381050851
PTPRS	2.48277824190991	2	0.288982507334784	0.824849706646668
AC005790.1	0.474023004515703	2	0.788982212888565	0.569473745306652
SAFB2	0.576754799062098	2	0.749478685953439	0.10813776884545
SAFB	5.5270793709562	2	0.0630681314419722	0.298748602146501
RPL36	22.3340191163412	2	1.413283763263e-05	0.161407233251165
C19orf70	1.56455285354297	2	0.457363670445325	0.146462118779273
HSD11B1L	2.78406572424476	2	0.248569483186084	1.88909371355713
LONP1	2.33349111851583	2	0.311378657476408	0.234345281893214
DUS3L	1.23916460524442	2	0.538169182510067	0.248784483853625
NDUFA11	5.00639020065502	2	0.0818231473612021	0.128301983856169
VMAC	1.06587019638877	2	0.586879889173419	0.522990311124681
CAPS	0.856697885810535	2	0.651584009508005	0.637245751758663
RANBP3	0.0774986095377172	2	0.961991845687324	0.0568718561689537
RFX2	0.845627628496274	2	0.655200610774541	0.582804136870886
MLLT1	0.72447219784064	2	0.696117995731288	0.622612710435092
CLPP	1.71519654205921	2	0.424179624391038	0.176308668728903
ALKBH7	3.74193807292455	2	0.153974382707264	0.289658873936334
GTF2F1	0.179855894137538	2	0.913997039064521	0.0706811259820311
KHSRP	0.561959963769558	2	0.755043449875703	0.206160136117777
SLC25A23	2.89030095396722	2	0.235710604866269	2.18165428844237
DENND1C	1.088472154022	2	0.580284907042765	0.212662454388002
CD70	1.21850560840964	2	0.543757010273781	0.508267842550426
GPR108	0.444700052486747	2	0.80063507199612	0.137046366655778
TRIP10	0.67789938202188	2	0.712518294262588	0.346391176522228

SH2D3A	2.17257133881659 2	0.337467635052611
0.622302401006582
VAV1	2.16508026719962 2	0.33873400229534 0.35353584870675
ZNF557	0.00659776476873578	2	0.99670655294964
0.0314999452384422
PEX11G	0.679494240007357	2	0.711950337999379
0.853055185484654
MCOLN1	1.15615593449693 2	0.560975544417418
0.62281460132811
PNPLA6	4.03443435790202 2	0.133025135625534
0.833390616085488
XAB2	0.0020634311035914	2	0.998968816483709
0.0107707734494468
PET100	7.50105832055737 2	0.0235053044910862
0.133497818773735
AC008763.2	1.24696174494695 2	0.536075176826174
1.3357751256863
STXBP2	3.14517732226631 2	0.207507320351106
0.157769815091228
TRAPPC5	0.683212959564983	2	0.710627796098025
0.378821535307699
FCER2	7.58539105469322 2	0.0225347768030382
1.08066250297794
MAP2K7	1.00048647176565 2	0.606383147633002
0.22969615905674
SNAPC2	0.00505510527222953	2	0.997475638935528
0.0203723050880456
TIMM44	1.10259108685303 2	0.576202830822926
0.219471417907482
ELAVL1	11.6142232601763 2	0.00300610027450743
0.297601451635473
AC008946.1	0.999877051925024	2	0.606567946747227
	0.984974780158358
CERS4	3.36749733648232 2	0.185676629711092
0.138597114809159
CD320	2.39660378069974 2	0.301706107214183
0.448560887768449
NDUFA7	2.47434978217731 2	0.290202915782721
0.14151015048968
RPS28	25.779567844982	2	2.52370140518821e-06
0.184358587102211
RAB11B-AS1	2.73926094345792 2	0.254200876609658
0.610082216959898
RAB11B	0.00729950716919981	2	0.996356898670523
0.0129048093508168
MARCH2	3.02813281850273 2	0.22001349155692 0.658654983312164
HNRNPM	25.964104094841	2	2.30126393863106e-06
0.351538358830879
ZNF414	0.313019580989547	2	0.855123145583239
0.148826033661729

MYO1F	0.582379850412126	2	0.747373719430092	1.06934401924493
ZNF558	2.74014232574432	2	0.254088877215126	2.43622467327999
ZNF317	1.36399828076791	2	0.505605205565851	0.721654912210124
ZNF559	0.0216323409508255	2	0.989242113969031	0.103112309756874
ZNF266	7.68662747210864	2	0.0214224951021033	2.18519262925936
ZNF426	0.161112183991972	2	0.922603151480061	0.220600925407733
ZNF121	0.463692730794995	2	0.793067956627811	0.317269563096974
ZNF561	0.0161291902221188	2	0.991967836494943	0.0536121057695753
ZNF562	0.0175455418333681	2	0.991265597556841	0.051670244508947
ZNF846	2.96084489498094	2	0.227541543723084	2.49782206641253
FBXL12	0.115966613121981	2	0.943665700331127	0.0879766818679118
UBL5	20.6541637977467	2	3.27344702603094e-05	0.297118029576794
PIN1	1.31455299068877	2	0.518260900115431	0.160003815784617
C19orf66	2.48544987125728	2	0.288596737976596	0.402126160703961
ANGPTL6	1.73706121789547	2	0.419567605408296	0.557710659963135
PPAN	0.0787101960226247	2	0.961409254010778	0.0594977744046422
P2RY11	11.0639535075259	2	0.00395815704879332	2.00685195477217
EIF3G	11.0344547457933	2	0.00401697007630686	0.182069410982569
DNMT1	2.08586898635642	2	0.352418991961597	0.153851879467027
S1PR2	0.581256213011355	2	0.747793725934551	0.0942636440189597
MRPL4	2.22253739381185	2	0.329141115756531	0.190813534780961
ICAM1	3.81151965655459	2	0.148709605919654	0.755662045269154
ZGLP1	0.040060847010676	2	0.980168852680812	0.160775619104006
AC011511.1	3.1887897957378	2	0.203031346040388	1.18078311105719
FDX2	0.00227726365900795	2	0.998862016165752	0.0108896826225495

RAVER1	0.171780803302989	2	0.917694803543671
0.2370169090824
ICAM3	22.1044990698698 2	1.58514507012653e-05
0.769238866243839
TYK2	0.141434129360505	2	0.931725472882268
0.121184017750196
CDC37	0.140758803017187	2	0.932040135382536
0.0356518603429025
KEAP1	7.48296252502606 2	0.0237189431158412
0.604909249888098
ATG4D	2.58869297941044 2	0.274076917905809
0.38048752726108
KRI1	5.0120398950767 2	0.0815923356290208
0.359753582603794
CDKN2D	0.369837255973946	2	0.831171915232352
0.166661412177195
SLC44A2	0.336284902190678	2	0.845233421681644
0.0867567241706832
ILF3-DT	2.54321102641994 2	0.28038110465221 0.333859960617332
ILF3	0.709183321482419	2	0.701459818317776
0.105902776202233
QTRT1	1.46349724419096 2	0.481067049714425
0.139330789618899
DNM2	0.787212492390385	2	0.674619637999359
0.115245942120299
TMED1	0.181634088144849	2	0.91318476818305
0.0358920698364584
C19orf38 1.08987777273759 2	0.579877220659592
0.491882008535616
CARM1	0.0492666651347854	2	0.97566759198056
0.102832204840677
YIPF2	0.983223636458144	2	0.611639747156098
0.281840876609551
TIMM29	0.61635062854078 2	0.734786488159479
0.170495184198311
SMARCA4	2.15875561851611 2	0.33980688458297 0.146821323347316
LDLR	23.5696319684811 2	7.61937674020885e-06
1.2621848320574
KANK2	27.4801397716196 2	1.07835924911814e-06
1.98895796473372
RAB3D	0.513319886880841	2	0.773631247420135
0.656323996014799
TMEM205	2.4657111105728 2	0.291459110637453
0.368605845864219
CCDC159	6.90436930855517 2	0.0316763588390494
1.39546839125091
SWSAP1	2.20233954909845 2	0.332481927233237
0.391662503037381
PRKCSH	8.63465392783171 2	0.0133354821680767
0.602309285704885

ZNF653	1.38336000040248	2	0.500734128597189	0.788287255527986
ECSIT	0.396337734435894	2	0.820231331259275	0.120035532605925
ELOF1	1.50614943032901	2	0.470916390698179	0.0988178155159494
ZNF627	2.2631722432564	2	0.32252129251346	0.930386870565283
ACP5	3.90306997795433	2	0.142055849981619	0.420989259832892
ZNF823	2.12687125434966	2	0.345267559695092	1.918959596991
ZNF441	2.47705862141193	2	0.289810125322183	1.04909643778736
ZNF440	2.38292950225795	2	0.303775981820153	1.52641137637656
ZNF439	1.70750666326333	2	0.425813708805302	0.415948159680662
ZNF700	0.257424457249799	2	0.879226945490773	0.279303663703589
ZNF433	1.22760810575966	2	0.541287860021789	1.0910087433702
ZNF844	5.23263318583649	2	0.0730715198908611	1.39321437116541
ZNF20	0.442532908080777	2	0.801503088099	0.443452510818107
ZNF136	6.83118029416833	2	0.0328570105098266	0.929628949282716
ZNF44	6.35844789761435	2	0.0416179402442682	0.463734977182998
ZNF563	4.26259532701786	2	0.118683183046288	0.728606306125269
ZNF442	2.36639139964961	2	0.306298335361611	1.7202171409384
ZNF799	5.1443360343747	2	0.0763697947860281	1.39368147052902
ZNF564	0.810442729287705	2	0.666829182114776	0.697955882771751
ZNF791	1.28254067665074	2	0.526623009549576	0.286745323564165
MAN2B1	1.0943616717266	2	0.578578621458701	0.778747795171313
WDR83	4.41696798406637	2	0.109867081697765	0.444157018612833
WDR83OS	0.0475260869034106	2	0.976517074460451	0.0187423811235239
DHPS	3.77895916877664	2	0.151150449425295	0.31226160593815
FBXW9	6.26676404737423	2	0.0435701923691929	1.46816445167721
TNPO2	1.07693094221263	2	0.583643182877746	

0.538261436186992
TRIR 0.345508723435632 2 0.841344255821842
0.0151345775307535
ASNA1 8.26150641366631 2 0.016070769649291
0.186260867690365
HOOK2 0.234282430216072 2 0.889459579163757
0.136973187119757
JUNB 11.2643349636275 2 0.00358080557900864
0.57389219479054
PRDX2 1.04529220349755 2 0.592949465647077
0.108232166001132
RNASEH2A 0.0175835967496821 2 0.991246736471601
0.0682275467263676
DNASE2 10.0142610747489 2 0.00669007270375277
0.773095831904667
GCDH 0.0844052706132665 2 0.958675499385256
0.069737232805755
FARSA 1.15040648787525 2 0.562590514075046
0.173061738658405
CALR 17.4551161329485 2 0.000162057707589591
0.498766399629133
RAD23A 0.00962692372984875 2 0.995198104277468
0.00897316625889875
GADD45GIP1 0.914090191160683 2 0.633151787071676
0.0885849827712118
LYL1 3.39452169384166 2 0.18318460814988 0.273435742265552
TRMT1 7.55985383892312 2 0.0228243593806178
0.502658611353698
NACC1 1.77539187801623 2 0.41160301951449 0.614685381937436
STX10 2.01476872134568 2 0.365172892059843
0.231655612250281
IER2 32.5038134137656 2 8.74755329283161e-08
0.821627005112078
CACNA1A 0.044907325442393 2 0.977796544574717
0.0724503297651714
CCDC130 3.08581512635903 2 0.213758680133194
0.378408644222898
MRI1 1.63190502911669 2 0.442217907693989
0.437502110139432
C19orf53 2.28961498854305 2 0.318285183614354
0.0757191307604154
ZSWIM4 3.79006433702822 2 0.150313499606693
3.16301731157017
AC020916.1 0.015512593003394 2 0.992273705946608
0.0809458529299726
CC2D1A 0.775154594433068 2 0.67869917066704
0.266074336289508
DCAF15 0.0712240443019007 2 0.965014625160799
0.0933096783965993
RFX1 0.0979638521358643 2 0.952198339511083

0.10024262899218
AC022098.4 2.19984733164882 2 0.332896494107661
2.3346677933633
IL27RA 2.64587117712437 2 0.266352252546642
0.828585774095498
SAMD1 0.034272676668027 2 0.983009653599842
0.177985140196637
PRKACA 1.87968787830354 2 0.390688801826964
0.631078167446985
ASF1B 1.04259897795868 2 0.593748476827734
0.604912358435584
AC022098.1 0.369172731337323 2 0.831448128224866
0.479970216685429
ADGRE5 2.58927460729374 2 0.273997224105593
1.06446885271324
DDX39A 21.3249912762464 2 2.34065271110806e-05
0.42238805707235
PKN1 0.622483234927361 2 0.73253686078687
0.140399143537266
GIPC1 2.6805671021081 2 0.26177143249036 1.11045216277496
DNAJB1 2.03193939810465 2 0.362051178871107
0.381085833856666
TECR 1.36580496282769 2 0.505148677869883
0.0984642720676117
NDUFB7 3.90434913634208 2 0.141965023064201
0.15781617948479
CLEC17A 7.64531953412348 2 0.021869555545574
1.82328840077105
ZNF333 4.80926414999405 2 0.0902987126528408
0.714522112200988
ILVBL 1.62958940423369 2 0.442730209606907
0.567047101411305
BRD4 2.37988739137871 2 0.304238393517488
0.230234672089184
AKAP8 3.95282264604628 2 0.138565613337934
0.487485744691121
AKAP8L 0.239664588620096 2 0.887069190794034
0.0858290011290917
WIZ 2.52269328385596 2 0.283272303174649
1.34542295655459
RASAL3 3.33823687540715 2 0.188413090324026
0.347663167175201
TPM4 6.97987370842986 2 0.0305027982335468
0.297060516799077
RAB8A 1.48811927175777 2 0.475180933008581
0.167101851509528
HSH2D 15.9487368514583 2 0.00034417220644134
0.559600345951809
FAM32A 0.153283362893031 2 0.926221676565137
0.0196185944891981

AP1M1	0.800286203375864	2	0.670224128968708	0.190521558331432
KLF2	9.30441365622762	2	0.009540524384405	0.683119455966469
EPS15L1	4.92119188518737	2	0.0853840518090451	0.268862983113242
C19orf44	0.635787661675583	2	0.727680041493937	0.64136675186864
CHERP	0.537957667796551	2	0.764159429753251	0.239435891539129
AC008764.6	0.141002011102705	2	0.931926802425073	0.375523331595475
SLC35E1	2.13547092172551	2	0.343786153792493	0.490341664901667
AC008764.8	1.27746140301928	2	0.52796214046597	0.64296606230595
MED26	0.238810163052113	2	0.887448239053992	0.146607959834839
SMIM7	0.516930149746003	2	0.772236001018626	0.091534125123581
TMEM38A	0.163697932813618	2	0.921411112218741	0.0821146382237472
SIN3B	0.768730096436769	2	0.680882826743903	0.282026058194192
F2RL3	23.3005743737336	2	8.71654872447447e-06	1.12561812290025
HAUS8	3.72398939552705	2	0.155362420011169	0.375629062571494
MYO9B	0.649665070350859	2	0.72264836169061	0.201360249768959
USE1	0.187787094822691	2	0.91037966935513	0.0584137840267752
OCEL1	0.81863226598886	2	0.664104253867018	0.178261312088572
NR2F6	1.85433096956686	2	0.39567366538231	0.610673985506295
BABAM1	3.61482982225398	2	0.164077744586756	0.200558108147446
ANKLE1	3.3404902045151	2	0.188200931512002	0.584069694690626
ABHD8	3.16750784945475	2	0.205203332671675	0.409715136696063
MRPL34	1.63245465544739	2	0.442096397088125	0.0978801897495796
DDA1	0.00061477404720579	2	0.999692660214948	0.00409138327405985
GTPBP3	2.73863316284272	2	0.254280680325161	0.494002138298387
BST2	110.624006315469	2	0	0.751498399886328
BISPR	2.14378488548823	2	0.342360007266458	0.323088337872825

MVB12A	0.903314296610241	2	0.636572382235848
0.192662296370131
AC010618.3	3.61432187308591 2	0.164119421455896
0.95916158164931
PGLS	19.2915468558805 2	6.46984425938246e-05
0.310954090557986
FAM129C	34.070964485042	2	3.99561895791933e-08
1.05655126849793
COLGALT1 0.533013199923193	2	0.766050947802673
0.233694097064531
MAP1S	5.58747827100346 2	0.061191980507955
0.508851746911011
FCHO1	2.66498470413072 2	0.26381891161178 0.384996083315656
JAK3	0.101008788393046	2	0.950749750881899
0.137703054686307
RPL18A	42.860740134339	2	4.93069918228173e-10
0.173122877277357
CCDC124	0.265514358784193	2	0.875677698886244
0.0844792958560979
ARRDC2	1.27991853982318 2	0.527313901147477
0.288910563061135
IL12RB1	3.11167385839513 2	0.211012706023646
0.606449610095631
MAST3	3.33178813974046 2	0.189021583908231
0.520090846141652
PIK3R2	3.24653342547065 2	0.197253275663432
1.64833873781661
IFI30	1.5009711580204	2	0.47213723713781 0.3599593587106
MPV17L2	7.55258351904311 2	0.022907480565362
0.738627330138619
RAB3A	0.899301474876069	2	0.637850890158442
0.594546649740016
JUND	0.65014099054072 2	0.722476420676222
0.152293547988696
LSM4	0.36165395027346 2	0.834579749250854
0.067605508370221
PGPEP1	0.375673250973036	2	0.828750092816314
0.248497714360665
SSBP4	2.55804342716237 2	0.278309433658557
0.512409691096238
ISYNA1	0.502031336558004	2	0.778010181382869
0.532292295585991
ELL	0.137759601525608	2	0.933438871975757
0.136996580788008
FKBP8	0.00122033209621719	2	0.999390020065339
0.00363121527142352
KXD1	0.789915373050117	2	0.673708545595424
0.142558994045324
AC005253.1	0.0700284332412043	2	0.965591688709292
0.194622883912241

UBA52 36.6856045480381 2 1.08099127427153e-08
0.188618572338669
REX1BD 6.83587419247804 2 0.0327799871970337
0.400757812612352
CRTC1 0.231714206958515 2 0.890602478200614
0.153294811175153
UPF1 0.109413019375235 2 0.946762972942699
0.158649786735029
COPE 3.42098243131931 2 0.180776970324729
0.0750727780958052
DDX49 0.343152712078927 2 0.842335948127553
0.115537087909199
SUGP2 1.20786191446119 2 0.546658515709538
0.273129916302188
ARMC6 1.16147290146313 2 0.559486180799626
0.284348771133985
SLC25A42 9.81316439346772 2 0.00739772915558168
0.669646557098153
TMEM161A 5.06152499573012 2 0.0795983036041089
0.56487854725252
BORCS8-MEF2B 32.282619569962 2 9.77053240536208e-08
0.718511267618113
MEF2B 0.0121521674809526 2 0.99394233832611
0.0969927358938233
BORCS8 0.042205077865003 2 0.979118561649839
0.0462356473367941
RFXANK 0.879018426400987 2 0.644352583234769
0.11501107297734
NR2C2AP 1.24867710514519 2 0.535615592931531
0.431684321329235
SUGP1 0.0011954942726855 2 0.999402431478886
0.00871698525241845
MAU2 0.985070030358538 2 0.61107534377473
0.408311211969318
GATAD2A 1.83129970805919 2 0.400256433300257
0.34280582080683
NDUFA13 4.39811467676141 2 0.11090765749294 0.120782853764225
YJEFN3 0.0880863242832062 2 0.956912654182202
0.162689561872822
PBX4 2.46665005947025 2 0.291322310146841
2.5318229966905
LPAR2 0.10788104209142 2 0.947488460448674
0.234028920357655
GMIP 2.46067202789747 2 0.292194379796501
0.251534346222605
ATP13A1 0.0768798206110493 2 0.962289526686204
0.0743043953934399
ZNF101 15.3216398833545 2 0.00047092112459457
0.921371041370196
ZNF14 0.0374670780772598 2 0.981440843074616

0.133339533971033
ZNF506	1.14843222086944 2	0.563146140213376
0.354875353162998
ZNF253	0.37548174350442 2	0.828829452531944
0.244031314828417
ZNF93	0.744620022830308	2	0.689140567962443
0.479821579359187
ZNF682	0.429618966091167	2	0.80669511463445
0.390300994919791
ZNF90	0.734466613359912	2	0.692648026780308
0.608733861392893
AC011447.3	0.587029025710341	2	0.745638401443446
	0.220119076411298
ZNF486	0.178005871461438	2	0.914842887838141
0.246680248249946
ZNF737	0.000495238696530623	2	0.999752411306875
0.0175926348301717
ZNF626	4.22582389386576 2	0.120885441425037
0.51388909372158
ZNF85	0.395718060544783	2	0.820485508604335
0.245687377250022
ZNF430	0.060066103598019	2	0.970413459107846
0.0441555547536811
ZNF714	0.917897355476414	2	0.631947677051364
0.262791189638354
ZNF431	0.005342306548408	2	0.99733241108135
0.0192657973437249
ZNF708	0.266599179722468	2	0.875202850927672
0.200500267279212
ZNF738	0.131940359887358	2	0.936158780182941
0.101590153130831
ZNF493	0.35070737272326 2	0.839160168769133
0.158083627971775
ZNF429	0.424454059168016	2	0.80878105949416
0.253964313884997
ZNF100	0.305512183397633	2	0.85833905230172
0.257474944440235
ZNF43	0.754574467829896	2	0.685719083829258
0.382250548588317
ZNF257	0.718709054237847	2	0.698126802588827
0.625867983317576
ZNF730	0.8132958097666 2	0.665878601635507
0.819554590640857
ZNF724	0.421130887464144	2	0.810126035747245
0.476341080522911
ZNF91	0.285511314478823	2	0.866965879853617
0.233510338445439
ZNF675	0.00825906473712135	2	0.995878982425493
0.0386228347879736
ZNF681	2.39918298373571 2	0.301317277332025

0.717800776509011
ZNF726 3.00648200206315 2 0.222408165693591
1.77488665588193
ZNF254 1.17383741759474 2 0.556037962715361
0.44452777885157
AC092279.1 4.94987931633444 2 0.0841680689596223
1.49450978176452
LINC00662 3.78600990163169 2 0.150618526867253
0.695677726370958
AC006504.5 0.183494918719708 2 0.912335522251448
0.243216356466223
UQCRFS1 1.28583265477296 2 0.525756906829873
0.101203618227178
POP4 0.117201777680309 2 0.94308308904087
0.0563590173011201
PLEKHF1 0.00491489682426451 2 0.997545568642292
0.0721603436448515
C19orf12 0.00228887679532808 2 0.998856216222211
0.0174142396844013
URI1 4.06748030908994 2 0.130845223468257
0.359112687029181
ZNF507 0.00775388541129299 2 0.996130562933947
0.0518384276086996
AC007773.1 2.23369023475792 2 0.327310794563436
1.4891672808963
DPY19L3 4.47107090567513 2 0.106934855948693
3.53266458607085
PDCD5 0.00732355916991995 2 0.996344916554149
0.01237074615595
ANKRD27 2.90142633915617 2 0.234403059338725
0.689145577948745
AC008736.1 1.53542729823249 2 0.464072889749316
1.12993921717044
NUDT19 0.756823086146166 2 0.684948556819854
0.474420590455874
CEP89 0.0528234009428566 2 0.97393403795527
0.0858016647568466
FAAP24 2.74779777089989 2 0.253118154496887
1.06278284010188
GPATCH1 1.84782676901829 2 0.396962530441296
0.561331539873844
CEBPG 1.15033428870363 2 0.562610823726168
0.254430134628463
PEPD 2.5756662820823 2 0.275867902755612
0.256578601134323
LSM14A 0.0199784894312973 2 0.990060482073915
0.012499393795016
KIAA0355 2.32471155886999 2 0.312748545778469
0.201065356756581
GPI 3.49967638032413 2 0.173802064059106

0.408434864972235
PDCD2L 5.72307559269722 2 0.0571807602554307
0.682845586581229
UBA2 3.94825803194946 2 0.138882223777608
0.255006147828754
ZNF302 3.45023720838119 2 0.178151920955434
0.513606321593779
AC020910.4 0.00415946813955448 2 0.997922427078659
0.0540803167281457
ZNF181 0.385428690589388 2 0.824717524935956
0.318026423874656
ZNF599 0.201772461472805 2 0.904035878531583
0.48048106775436
ZNF792 0.847123539395941 2 0.654710733133141
0.412906381912686
GRAMD1A 0.123070794006135 2 0.940319660652424
0.0707427668146218
SCN1B 1.86344704198952 2 0.393874274447445
1.49783100554908
FXYD5 0.0761167969648374 2 0.962656721558025
0.0460111454626948
LSR 0.865308645588582 2 0.648784723137114
0.351490461647298
USF2 6.48691029070939 2 0.0390288116496262
0.357404788466072
CD22 51.9071499641122 2 5.35183009020557e-12
0.631952696110898
FFAR1 1.76413239646548 2 0.413926772743457
0.445534226969248
TMEM147 8.20106593787145 2 0.0165638450339551
0.428270905839962
HAUS5 0.442013276549093 2 0.801711358292428
0.200665910516264
RBM42 2.33997033710773 2 0.310371544490193
0.114969171914429
ETV2 0.000395643089548682 2 0.999802198020617
0.018656228998335
COX6B1 6.04188134631957 2 0.0487553339502359
0.133650864045288
KMT2B 3.59676893462696 2 0.165566149805338
0.922993023438231
IGFLR1 2.405514271259 2 0.300364922376343
0.588566826199264
U2AF1L4 0.676972007585448 2 0.712848756498019
0.215274058421852
PSENEN 0.565002978848883 2 0.753895519088072
0.120468540940547
LIN37 0.748963494090973 2 0.68764556080444
0.777138261506766
PROSER3 0.519723756301371 2 0.771158092229777

0.36759308371691
NFKBID	0.718225394439314	2	0.698295650938639
0.0889082356964305
HCST	13.3541686886782 2	0.00125944473162276
0.683280368146849
SDHAF1	0.344849495857438	2	0.841621620198989
0.13370398615703
ALKBH6	3.14267039465097 2	0.207767586349921
0.551933837289177
THAP8	4.21575446438122 2	0.121495599837006
1.22186456498296
POLR2I	6.99057036859329 2	0.030340094684161
0.188184403513647
TBCB	7.00021458191845 2	0.0301941436898852
0.266558844281058
CAPNS1	1.03851026557783 2	0.594963551801725
0.171031644917162
ZNF146	1.08458032114137 2	0.581415192347163
0.274947492564123
LINC00665	1.5017273008178	2	0.471958769291109
0.382997549932718
ZFP14	0.136001812642776	2	0.934259626837348
0.112063644267024
ZFP82	5.90662862135608 2	0.0521665230516137
0.49322673585048
ZNF566	0.123716278453766	2	0.940016228761822
0.204983053280842
ZNF260	0.000139118873070954	2	0.999930442982666
0.00790690594967465
ZNF529	1.8210088486591	2	0.402321232223108
1.00796779088046
ZNF529-AS1	0.815013762006968	2	0.665306873403364
0.482962984868957
ZNF382	2.83502506689248 2	0.242316020851586
1.22484037603579
ZNF461	1.40124134974836 2	0.496277181402265
1.16628256607088
LINC01534	1.1297046679457	2	0.568444080439326
0.677467229126794
ZNF567	2.33197639658504 2	0.311614572842306
0.643666234219955
AC020928.1	0.0433854089991825	2	0.978540890066361
0.210183814460973
ZNF790	1.69386245148323 2	0.428728586513265
0.644111115388587
ZNF345	0.642274976138908	2	0.725323520803838
0.423822052122289
ZNF568	0.530927171062591	2	0.766850366825893
0.380215574166324
ZNF420	5.17656260265054 2	0.0751490877940963

1.37914877685086
ZNF585A 2.12801043215404 2 0.345070955122011
1.75261640123268
ZNF585B 2.86397502389622 2 0.238833765248023
1.93066445564519
ZNF383 0.000724444307230083 2 0.999637843440909
0.0154893004638771
HKR1 2.73216617586563 2 0.25510422599649 0.870597347407908
ZNF569 3.88527073975222 2 0.143325735303794
1.01155173706952
ZNF570 3.19859338448781 2 0.202038563326627
2.1316113456661
ZNF793 0.0114920007150889 2 0.994270476329056
0.0668617831241235
ZFP30 0.581332567772287 2 0.747765177673918
0.514727505415177
ZNF781 0.622402856987709 2 0.732566301280252
0.529527627150659
ZNF573 0.599687815597348 2 0.740933865653971
0.448889103140142
SIPA1L3 7.4162144889131 2 0.0245238971128429
1.18060021565495
SPINT2 1.00924713988259 2 0.603732795829838
0.0863574824655272
PPP1R14A 8.56723628813919 2 0.0137926678384793
1.96357221610472
YIF1B 0.584498964131288 2 0.746582253854435
0.203296231731287
KCNK6 1.85761489509557 2 0.395024517041461
0.247684014518355
PSMD8 6.12356544038662 2 0.0468041820413347
0.101677240216862
FAM98C 1.86600899862993 2 0.393370053058273
0.381241526429695
MAP4K1 0.610083673497502 2 0.737092536191304
0.096070663055932
EIF3K 8.03014289756753 2 0.0180416654496449
0.1779463452431
ACTN4 0.306629694669237 2 0.857859584483965
0.0788881249967384
CAPN12 0.538449243753318 2 0.763971631632055
0.355713811586191
ECH1 5.67094663145119 2 0.0586907400084643
0.189437751922911
HNRNPL 1.91386931468521 2 0.384068384604304
0.152678117495461
RINL 3.23940379079923 2 0.197957702390363
0.374254136057276
SIRT2 0.0134156883633272 2 0.993314603185927
0.0230071130576744

NFKBIB	0.184017863092181	2	0.912097003072288	0.120023221522271
SARS2	2.92847836721499	2	0.231253866296933	0.442357856436412
MRPS12	1.81453041628533	2	0.403626550633775	0.178514168359425
PAK4	0.0602456649240524	2	0.970326338655011	0.11956806318712
GMFG	32.5214811529892	2	8.67061885623244e-08	0.488166022294821
SAMD4B	0.00396317958727699	2	0.998020372259208	0.0291143227782509
PAF1	2.01259861344414	2	0.365569339393563	0.29092166260336
MED29	1.0748399429123	2	0.584253700713747	0.252229998740787
ZFP36	0.956332589794939	2	0.619919099026887	0.087128535129932
PLEKHG2	0.233279966037175	2	0.889905516596841	0.147220213566824
RPS16	52.0143851854326	2	5.07249797720988e-12	0.2804480515609
SUPT5H	0.0789545367234212	2	0.96129180547985	0.0538217763723991
TIMM50	3.84792947565507	2	0.146026854708631	0.43617766478638
DLL3	0.0578518813083737	2	0.971488409598547	0.139912170398573
EID2B	1.09432682093346	2	0.578588703508469	0.541100160345717
EID2	0.686352092857945	2	0.709513293283232	0.339579810595997
DYRK1B	0.055260315890324	2	0.972748063424294	0.141515796957542
FBL	1.08576709081863	2	0.581070291726594	0.116071944937211
PSMC4	1.39342151109495	2	0.498221383505093	0.133329580854311
ZNF546	0.547103732215561	2	0.760672882191012	0.862909048768727
ZNF780B	1.21498988262528	2	0.544713701163858	0.507364405208712
ZNF780A	0.218781766147213	2	0.896379969253053	0.186414422036392
MAP3K10	2.17720050575981	2	0.336687441302405	2.36730417364295
AKT2	0.104988600513997	2	0.94885972929699	0.0822316647983868
C19orf47	2.37712267079253	2	0.304659251413923	1.05135240667093

PLD3	0.113708339111907	2	0.944731829983705	0.0924950073496341
SERTAD1	0.0565714949056036	2	0.972110548997055	0.0655168492793023
SERTAD3	0.174177258677618	2	0.916595854748481	0.12568321049871
BLVRB	1.22885734945776	2	0.540949865368206	0.561863867597691
SHKBP1	0.0104323656219931	2	0.994797397847236	0.0104372110937873
LTBP4	5.86511137911344	2	0.0532607461266771	2.00386363574421
COQ8B	2.25339446949081	2	0.324101923244027	0.55752005838723
ITPKC	0.0974415943399686	2	0.952447018481327	0.283473488185639
C19orf54	1.62023423015777	2	0.444805969686128	0.455669487275007
SNRPA	1.2617330390795	2	0.532130499696778	0.104820570306103
MIA-RAB4B	1.56681716919745	2	0.456846155595457	1.06767007779919
RAB4B	3.57345773708336	2	0.167507212567151	1.31362584037717
EGLN2	2.84034411123015	2	0.241672432220728	0.671385455483839
HNRNPUL1	3.74765548691857	2	0.153534843618127	0.304318351258791
TGFB1	0.416423538154217	2	0.812035054588244	0.212749732522085
CCDC97	3.01521987166862	2	0.221438598448345	0.28367826091009
TMEM91	0.0278160360507519	2	0.986188251633813	0.106385103021243
B9D2	0.998036523477864	2	0.60712640645362	0.324965940700064
EXOSC5	2.28831808406311	2	0.318491643287001	0.202541126444694
BCKDHA	2.06963104716753	2	0.355291917859481	0.927531576892351
DMAC2	0.0786315153919811	2	0.961447076897962	0.0535663648494117
LINC01480	24.5642470981368	2	4.63384514770571e-06	2.20274178765336
AC243960.1	0.117413760580658	2	0.942983135593826	0.0868346329842186
CEACAM21	0.84240578376332	2	0.656256938695667	0.350844382773764
RPS19	17.2495337378648	2	0.000179602071368135	0.0901716404994729

CD79A	11.8668270221761 2	0.00264942270181512
0.122293618255847
ARHGEF1	0.132782090832006	2	0.935764866173966
0.0410924301799862
RABAC1	1.04593295134325 2	0.59275953052747 0.0857840531710195
ATP1A3	0.46367363972064 2	0.793075526923606
0.673462213165355
ZNF574	0.780776543196279	2	0.676794043571666
0.452704469712493
POU2F2	20.4072526296356 2	3.7035771509375e-05
0.318927597040227
AC010247.2	1.19506018536646 2	0.550168825345421
0.488817941556357
DEDD2	0.498066966401941	2	0.779553870988777
0.152051418417288
ZNF526	1.42912697191764 2	0.489405697635182
0.290568936525763
GSK3A	3.97651754755612 2	0.136933650446349
0.514654229309834
ERF	1.06646586801557 2	0.58670512135161 0.575628274649653
CIC	0.0774598565049686	2	0.962010485918665
0.131182458081732
PAFAH1B3 1.86651717879631 2	0.393270114326025
0.279796574339405
MEGF8	0.682825034642632	2	0.710765644582619
0.743891586251722
LIPE-AS1 3.7313448672438	2	0.154792087477103
0.733624586820374
LIPE	1.23656682727331 2	0.538868658706217
0.472511397116871
CEACAM1	1.10746916035136 2	0.574799163437601
0.919037873921048
PHLDB3	1.03411288486982 2	0.596273131576698
0.818716665442446
ETHE1	20.4288437440625 2	3.66381001233318e-05
1.52542779158568
XRCC1	0.694736187705357	2	0.70654520543912
0.252959577766497
PINLYP	1.24291168573437 2	0.537161844826534
0.884805302324324
IRGQ	0.0469690999746603	2	0.976789065955708
0.143764403746316
ZNF576	4.27751125220294 2	0.117801340767553
0.691438245482463
ZNF428	25.8543834652757 2	2.43103921238053e-06
0.662192558588236
SMG9	0.296789128534406	2	0.862090897564389
0.10607504832876
KCNN4	4.70631437791552 2	0.0950685385622451
0.276604860911812

ZNF283	1.95247873105213	2	0.376725163932618	1.47197812375287
ZNF404	1.57589007315948	2	0.454778388656716	1.20196109835096
ZNF45	2.59248157291511	2	0.273558226323324	1.60466250901921
ZNF155	0.16920836286868	2	0.918875920578637	0.354625816676774
ZNF230	4.61392632855141	2	0.0995631498501587	1.212038065386
ZNF222	1.87360609746825	2	0.391878651845602	0.858766235108525
ZNF223	0.643560314633635	2	0.72485752743847	0.348192076830002
ZNF224	3.8976560747844	2	0.142440909223112	0.803469340468384
ZNF225	0.640266061863049	2	0.726052443215991	0.524430049774399
ZNF234	3.67539903051583	2	0.159183203744078	1.09308256133553
ZNF226	0.00217677673867678	2	0.998912203710459	0.0163580433789225
ZNF227	0.337698067629315	2	0.844636405297663	0.311637683427481
ZNF235	1.04639827510632	2	0.592621634022053	0.596018314977464
ZNF180	0.844311547617093	2	0.655631901160169	0.68323687341004
BCL3	0.429733816498574	2	0.806648791333241	0.217884094863219
TOMM40	1.14573369494347	2	0.56390648528048	0.111439100120107
APOC1	0.0716503175557137	2	0.964808967116087	0.253934625174804
CLPTM1	0.854230993322471	2	0.652388199217635	0.175706209638414
RELB	0.00372046322360214	2	0.998141497546646	0.00141606745809538
CLASRP	0.483888789188803	2	0.785099832132704	0.164096139047748
ZNF296	4.22610460100832	2	0.12086847591223	0.230043441459297
GEMIN7	4.40386534317059	2	0.110589219050413	0.305209349335591
MARK4	0.573503527207727	2	0.750698056289564	0.467011840781663
PPP1R37	0.662873289866808	2	0.717891636848467	0.391258615041271
TRAPPC6A	0.934858838903327	2	0.626610953698099	0.111252740872712
BLOC1S3	1.04329039014632	2	0.593543249837253	0.492506256622892

ERCC2	2.23222858940357	2	0.327550088144525	0.385538853006575
PPP1R13L	0.0902788879975475	2	0.955864183015492	0.247020002104811
CD3EAP	0.903010353141168	2	0.636669130596256	0.457925113273421
ERCC1	2.16878656445071	2	0.338106859118793	0.259057270892604
FOSB	4.04164845090592	2	0.132546172116537	1.48081928083716
RTN2	0.109258161776259	2	0.946836282501193	0.0933218436212953
PPM1N	7.84817253074614	2	0.019760184189536	0.811398473288586
VASP	17.5868529119883	2	0.000151727189445228	0.542765343875031
OPA3	3.21447477169904	2	0.200440589904025	0.615266267178471
EML2	0.199015939573716	2	0.905282734929314	0.145832674525631
SNRPD2	28.4788182966712	2	6.54490250107109e-07	0.313040388697492
FBXO46	0.793096157206845	2	0.672637936431212	0.438461486253291
DMWD	0.662236910446911	2	0.718120098925384	0.181173724303832
SYMPK	0.173162446348914	2	0.917061059149209	0.0941786949096592
IRF2BP1	0.303361609950605	2	0.859262509290475	0.200843481550894
MYPOP	0.285395126424144	2	0.867016246856141	0.182767941534332
CCDC61	0.34189503359035	2	0.842865808609682	0.240447056744064
PPP5C	0.894989804883125	2	0.639227474741432	0.106181549080149
PPP5D1	0.846123358336042	2	0.655038229652766	0.604841330207685
CALM3	0.788958874880397	2	0.674030823149147	0.0914390427669321
PRKD2	15.8768446485192	2	0.000356768900169446	0.611619064287776
STRN4	0.0166358297097174	2	0.991716583281789	0.0540853772230696
FKRP	0.113144737103595	2	0.944998093877007	0.18687739277871
SLC1A5	7.11056937676536	2	0.0285732392889958	0.686248062910165
AP2S1	2.98990133807946e-05	2	0.999985050605053	0.000495146263724593

ARHGAP35	0.636932606916151	2	0.727263583810539	0.369848447285775
NPAS1	0.0907816894117465	2	0.955623908287874	0.0759638268442454
TMEM160	1.72239623227432	2	0.422655388597864	0.113623961145442
ZC3H4	3.32757654566042	2	0.189420044391335	0.472121035598703
SAE1	0.892698911101839	2	0.639960095373698	0.16510191323824
BBC3	5.11183790796906	2	0.07762086927566	0.511870539075108
CCDC9	4.52253342791216e-05	2	0.999977387588525	0.0038604542396292
INAFM1	3.69434480382539	2	0.157682398997244	0.734127077822653
DHX34	0.0466635040065677	2	0.976938328759062	0.11387533586445
KPTN	1.64850956030988	2	0.438561695595841	0.663443838680588
NAPA-AS1	4.00690113469169	2	0.134869104480675	0.946913901545208
NAPA	11.0002827921375	2	0.00408619362590001	0.290397178023687
BICRA	3.29788477325436	2	0.192253130618569	17.0116206126615
NOP53	23.6773349621236	2	7.21991425578494e-06	0.320939772307663
SELENOW	8.01055188247266	2	0.0182192611195354	0.494503230892635
LIG1	0.112271225985766	2	0.945410917192547	0.110069400538526
ZSWIM9	0.335200751250982	2	0.845691726192428	0.193115544033543
CARD8	2.03561034228933	2	0.361387253530903	0.224161796798922
CARD8-AS1	3.03351654781104	2	0.219422041424558	0.754165612907432
EMP3	10.4618856591448	2	0.0053484802193885	0.666314317422503
TMEM143	0.13670776526401	2	0.933929913515028	0.369863462541627
KDELR1	5.57464359887808	2	0.0615859327211044	0.469272660826175
GRWD1	0.256638919813698	2	0.879572346157878	0.100957060450234
CYTH2	1.46749749700112	2	0.480105816420174	0.166282264705741
RPL18	10.146409457789	2	0.00626231891414308	0.114503576307357
SPHK2	0.326116729881203	2	0.849541603522367	

0.155465411483277
DBP 8.41455567215228 2 0.0148868376434282
1.18679203255357
CA11 0.600323407070874 2 0.740698437441265
0.402171333587488
BCAT2 3.74520503289849 2 0.153723073944402
0.57462111307777
PLEKHA4 0.0533680009444399 2 0.973668871819972
0.139111840463447
PPP1R15A 1.57654261501996 2 0.454630031892362
0.099847528516488
NUCB1 4.67659768278387 2 0.0964916462837666
0.202816286603518
BAX 3.37534185459554 2 0.184949784238548
0.196023275380057
FTL 42.6002764851063 2 5.61652280239855e-10
0.293065969592076
GYS1 0.0130427465320141 2 0.993499844740063
0.0621984869808803
RUVBL2 3.81219209856857 2 0.148659615030667
0.219657168250583
SNRNP70 2.74108339927396 2 0.253969347180707
0.174228050293734
LIN7B 2.84268544651768 2 0.241389679660866
1.55245692565524
C19orf73 0.9799762786792 2 0.61263366038112 0.691977353088889
SLC6A16 1.65850214948389 2 0.436375976963986
0.446933067335376
AC011450.1 0.00403201996237033 2 0.997986020802015
0.0501576601334776
CD37 110.998347045103 2 0 0.431294562272292
PIH1D1 9.2811431589158 2 0.00965217906326254
0.421377539633648
ALDH16A1 10.4901023369134 2 0.00527355184820411
0.948239888351922
RPL13A 53.8714054357348 2 2.00439664865826e-12
0.244598281394354
RPS11 30.653238220809 2 2.20665223316097e-07
0.219462586491361
FCGRT 2.84765867514755 2 0.240790182296211
0.421126814670627
NOSIP 0.0344768685340811 2 0.982909297435149
0.0243887854536251
PRRG2 3.14201106877888 2 0.207836090913539
5.93752857079329
PRR12 2.2819408562049 2 0.319508810992831
1.29136073102496
SCAF1 0.768077774398763 2 0.681104940400935
0.301752597946877
IRF3 11.3473386999322 2 0.00343523703465687

0.492530696447624
BCL2L12 23.6231573481512 2 7.41816620086766e-06
1.07366565088723
PRMT1 27.2210953566511 2 1.22747949959923e-06
0.399093845521846
AP2A1 0.11757200651258 2 0.942908526922955
0.105030139470936
FUZ 1.78804397482076 2 0.409007417498603
0.661932914492127
MED25 6.92639325608949 2 0.0313294531701154
0.491103883051022
PTOV1 0.93412213648708 2 0.626841809115243
0.0921685704234585
PNKP 1.07649822085255 2 0.583769473975416
0.185967595687384
AKT1S1 0.000103848630110818 2 0.999948077032988
0.00367797710890057
TBC1D17 6.13311223591593 2 0.0465812994402612
0.836443632573614
IL4I1 10.8756540350931 2 0.00434892314436863
3.60782541240779
NUP62 0.77551200638445 2 0.678577893906309
0.178670643490341
ATF5 6.55051328984124 2 0.0378071649672516
1.14372982437907
VRK3 2.89958352629315 2 0.234619139358637
0.273746528025553
KCNC3 4.50773382139249 2 0.104992442175495
2.48092948621899
NR1H2 0.5914469417112 2 0.743993135361992
0.122087087581729
NAPSA 0.0853248858198689 2 0.958234794429049
0.217584262481093
POLD1 0.123271320401544 2 0.940225385922604
0.132254327903823
SPIB 55.1535837450952 2 1.05571107411606e-12
0.526337442949822
FAM71E1 1.96620544604429 2 0.374148417136938
1.6544464773708
EMC10 5.25453675401524 2 0.072275622584743
0.352051236919726
JOSD2 1.25659471809948 2 0.533499386043534
0.250795794548758
C19orf81 13.2390897551425 2 0.00133403795806586
2.2405547035219
CLEC11A 1.39267473113023 2 0.498407449114047
0.521701625511866
C19orf48 4.9442761717237 2 0.0844042025087749
0.323347521793925
LINC01869 3.50512308393501 2 0.17332938382359

2.4376010429731
KLK1 23.9195732920707 2 6.3963269524292e-06
2.12779665337758
CTU1 10.6546660911589 2 0.00485700616858364
1.25926091588068
ETFB 6.34423473703506 2 0.0419147548975058
0.322575941598762
SIGLEC10 7.02002735667176 2 0.0298965054996856
0.985589270717286
ZNF649 0.204522858733631 2 0.902793504081198
0.44622683668934
ZNF613 1.64166219966233 2 0.440065763899171
0.590414510320526
ZNF350 3.23400752540793 2 0.198492539746152
0.688579106085643
ZNF614 0.0216244166211407 2 0.98924603351712
0.140937043159286
ZNF432 1.18929908147141 2 0.551755899954758
0.346835956370475
ZNF616 7.78450401422587 2 0.0203993547789921
2.5422478220579
ZNF836 3.23269501280345 2 0.198622844478282
1.26225088866706
PPP2R1A 11.2366002476691 2 0.00363080778980718
0.317635541286487
ZNF766 5.37846890733023 2 0.067932925272375
0.79357459051144
ZNF480 11.8654429582573 2 0.00265125682156031
1.02158622442956
ZNF880 3.30973374699068 2 0.191117496816315
0.505083554443174
ZNF528 0.801941446764076 2 0.669669666413928
0.765422168414814
ZNF808 2.79815100154215 2 0.246825048060176
1.32205174448237
ZNF701 1.98046293785253 2 0.371490692517724
0.606136043600494
ZNF83 0.618280362785213 2 0.734077858756787
0.281869097998026
ZNF611 1.49870169912479 2 0.472673289243733
0.940328668919615
ZNF600 5.09816493890364 2 0.0781533411920222
1.05198506561284
ZNF28 0.128310734235872 2 0.93785927571798
0.194565135265502
ZNF468 0.00110567342821695 2 0.999447316071951
0.0190981428438668
ZNF816-ZNF321P 0.365826541940906 2 0.832840384043176
0.531166128227108
ZNF816 1.4952926069644 2 0.473479669707211

1.164483194213
ZNF160	0.0492799126032674	2	0.975661129439126
0.114653579853243
ZNF415	11.5689655415538 2	0.00307490039297453
1.74235031938113
ZNF347	0.00255173677378824	2	0.998724945187135
0.0367302944209113
ZNF677	0.0836197951782849	2	0.95905208135683
0.213407164828871
ZNF845	3.0515147298842	2	0.217456300704246
2.3405176045087
ZNF525	0.0369170069588142	2	0.981710811329468
0.162702439863455
ZNF765	0.219765619442587	2	0.895939124500255
0.346729975290631
ZNF761	0.456136707484449	2	0.796069843622815
0.30296368846477
ZNF331	1.03122525347602 2	0.597134661879533
0.576941940034236
MYADM	1.44846327190528 2	0.484696849546596
0.540891738432068
NDUFA3	4.13407804295536 2	0.12655996880108 0.107596918261101
TFPT	0.837967039053322	2	0.657715034633591
0.167036246100721
PRPF31	0.0120306552689208	2	0.9940027282267
0.0144499942831666
CNOT3	0.176259928404355	2	0.91564186832518
0.0889959939285608
LENG1	0.0820394939527067	2	0.959810176411805
0.0663545261157988
MBOAT7	5.33627102960737 2	0.069381465504022
1.42665028869323
TSEN34	3.22532681000702 2	0.199355940748671
0.455678865903983
RPS9	27.5560609174689 2	1.03819133612237e-06
0.192335254092903
LENG8-AS1	0.0014367940973575	2	0.999281860936699
0.0315618338827402
LENG8	0.130974323625561	2	0.936611071070725
0.119172958410742
LILRB1	7.59516294048524 2	0.0224249417128959
0.513174658192225
NLRP7	0.459292891941295	2	0.794814562724337
0.727942405088247
NLRP2	0.00111521131785508	2	0.999442549774216
0.0170694249708901
RDH13	1.64893936857187 2	0.438467457002257
0.7472786538088
PPP1R12C 0.00172583126276841	2	0.999137456573241
0.0157596123525325

TMEM86B 0.945725996499561 2 0.623215446936204
0.797138607663042
PPP6R1 1.93104950738802 2 0.380783329843737
0.255418147425538
HSPBP1 3.0268336111439 2 0.2201564595618 0.35083713606225
KMT5C 0.00835813072528025 2 0.995829654779435
0.0431871543611882
RPL28 53.596005522504 2 2.30027108472086e-12
0.245173729642077
UBE2S 1.56649441550252 2 0.45691988593685 0.161631210443628
ISOC2 10.435919720647 2 0.00541837209390361
0.704274605548981
ZNF628 2.34722545316392 2 0.309247693352307
0.631769846411522
NAT14 0.344278682160757 2 0.841861859054382
0.283230254148133
ZNF579 2.54953721928381 2 0.279495633334361
1.3529276967217
FIZ1 2.27447806687687 2 0.320703251543945
0.939303095112619
ZNF524 1.23029152254791 2 0.540562096546709
0.288684620314089
ZNF865 4.45099273692529 2 0.108013790687092
2.38256077323032
ZNF784 0.396538484575019 2 0.820149004614183
0.4115705880707
ZNF580 8.22690693793546 2 0.016351208516835
0.32412007477833
ZNF581 19.2023420001989 2 6.76494725517784e-05
0.283488146256778
CCDC106 2.71844878265193 2 0.256859922497968
0.353449784108444
U2AF2 1.58472418142168 2 0.452774037818242
0.13837389783656
EPN1 1.71308364467941 2 0.424627985192897
0.259112456014863
NLRP11 3.83738843942642 2 0.146798523652817
1.69023168743247
NLRP4 8.41610277825813 2 0.0148753263375977
0.770700391458467
ZNF787 1.36646523348352 2 0.504981937970349
0.448588646110534
ZNF444 1.40575995014076 2 0.495157207923506
0.130043016318318
ZSCAN5A 0.474703282146342 2 0.788713895048608
0.355394983552628
ZNF582 0.275565267021784 2 0.8712880599905
0.521691995017001
ZNF583 2.21803576374675 2 0.329882785895736
1.86523057467989

ZNF71 5.66795728631669 2 0.0587785290390906
0.727130318818758
ZNF264 0.096307867332635 2 0.952987078991115
0.157610785832523
AURKC 1.96527188368665 2 0.374323103343088
1.59207940634425
ZNF805 2.1312513303849 2 0.34451223800661 0.590866027744055
ZNF460 2.21611444973298 2 0.330199842372338
1.47659072460608
AC005261.1 2.97660235227672 2 0.225755849381693
0.980607211098143
ZNF543 0.00295773975953938 2 0.998522223109428
0.0429692915464466
ZNF304 1.52993798559735 2 0.465348359900895
1.24234218948638
TRAPPC2B 0.508359222483328 2 0.775552491589999
0.167715635732975
ZNF548 0.137190017162546 2 0.933704745926047
0.236022243286904
ZNF17 0.613198053701062 2 0.735945636192269
0.410126545835646
ZNF419 0.140755126292548 2 0.932041848811576
0.256497546011278
ZNF773 12.2798775127894 2 0.00215505559763052
1.30635544323233
ZNF549 0.741857359191971 2 0.690093157526519
0.65934320468342
ZNF550 3.62902946975503 2 0.162916947123576
4.08388446067636
ZNF416 0.0807530573962068 2 0.960427742454514
0.275668760927098
ZNF134 0.8391216993504 2 0.65733542550539 0.261314956203176
ZNF211 2.77418258215341 2 0.249800846876881
1.11976173085838
ZNF551 0.244777414649956 2 0.884804371709466
0.42979268050753
ZNF154 0.381841233906153 2 0.82619817167628
0.513842233852019
ZNF671 2.1892503410326 2 0.334665025760623
0.617777830688118
ZNF776 1.89737990350271 2 0.387248004867505
1.0323310792035
ZNF586 0.0088255143556283 2 0.995596964729745
0.0443676193571153
ZNF552 0.000257509228256705 2 0.999871253674391
0.00647125320840732
ZNF587B 1.27200798549523 2 0.529403703929098
1.02800536227965
ZNF814 0.0161589033900794 2 0.991953099350947
0.034280722470843

ZNF587	0.115089866262788	2	0.94407946898654	0.122991162374502
ZNF417	0.00941781028341508	2	0.995302164370199	0.0913462070139221
ZNF256	4.06590949084999	2	0.130948030867723	3.00222312988485
ZNF606	1.22889608739449	2	0.540939387828846	0.633681572591448
ZSCAN18	3.59033796619661	2	0.166099381986997	0.795317732224385
ZNF329	1.88610927995263	2	0.389436428542618	1.48994117310125
ZNF274	0.334660183874208	2	0.845920333764479	0.193449122529378
ZNF544	0.565499315275467	2	0.753708449397427	0.192351529328782
AC020915.3	0.281472018352366	2	0.868718615172214	0.360710719933834
ERVK3-1	0.0104380686853083	2	0.994794561155008	0.0387323029846257
AC010642.2	2.4529747478529	2	0.293321097555656	0.306567143990265
A1BG	2.9068148752032	2	0.233772364683078	0.253260750198766
A1BG-AS1	1.72537015813864	2	0.422027382728378	0.607777694600311
ZNF837	7.13426879037717	2	0.028236652944164	1.3566669120964
RPS5	37.1596568744917	2	8.5287038542603e-09	0.204927722954907
ZNF584	0.371699185874595	2	0.830398483386407	0.373405888493042
AC012313.1	0.102854360966112	2	0.949872816723496	0.338065378459457
ZNF324B	1.09840897546503	2	0.577408963639178	0.763953697512049
ZNF324	1.12927140138424	2	0.568567237684882	0.574240389927524
ZNF446	0.820581184181133	2	0.663457426640576	0.784356025285598
SLC27A5	3.34710612188538	2	0.187579399174467	0.780056905598435
ZBTB45	0.492561927516623	2	0.781702563978283	0.305342455608335
TRIM28	2.9637132846985	2	0.227215438715877	0.264761296590389
CHMP2A	15.6425131951984	2	0.000401117322916034	0.395488708012712
UBE2M	0.925269816129733	2	0.629622460633969	0.225269929633283

MZF1	0.30111850747098	2	0.860226756848542	0.187071383722813
RPS4Y1	5.87499812534945	2	0.0529981080797987	0.205137080135694
ZFY	1.32724140098786	2	0.514983354348615	0.483842085847662
TTTY15	0.134344783457692	2	0.935033995314901	0.132847428192114
USP9Y	7.06071791819352	2	0.0292943984886778	1.54891252543053
DDX3Y	1.62912308724947	2	0.442833447949971	0.412696215217016
UTY	0.0498917031460955	2	0.975362724955716	0.072497129731418
TMSB4Y	1.64333262194782	2	0.43969836951692	0.938130047642815
KDM5D	0.556789063804985	2	0.75699810268952	0.259826775469222
EIF1AY	2.11445191373806	2	0.347418227967731	0.180616134216517
IL17RA	0.776851935410312	2	0.678123423054802	0.536109343107813
HDHD5	0.892371680885447	2	0.640064811080246	0.228707054717694
ADA2	1.18427512799183	2	0.553143640197368	0.622778800064681
ATP6V1E1	7.00672948282389	2	0.030095947783832	0.24210762513019
BCL2L13	0.743704124205024	2	0.689456231685331	0.216043477355676
BID	3.03022530406622	2	0.219783424403528	0.184443460693281
LINC00528	0.127737685345255	2	0.938128033827779	0.202760336208869
MICAL3	0.764071494647422	2	0.682470656269857	0.213496840929383
AC016027.1	1.10967830879508	2	0.574164605621541	1.13913039707297
PEX26	1.29312995357219	2	0.52384209956188	0.424013207696272
AC008079.2	0.268355015456946	2	0.874434831885532	0.447185954545012
TMEM191B	0.616927338163441	2	0.734574639485577	0.587029881569288
DGCR2	0.662991888151545	2	0.717849067752256	0.359375505852191
ESS2	4.11577453666804	2	0.127723530587409	0.368820441635693
SLC25A1	0.0269493019455178	2	0.986615725749657	0.0298354006174685
HIRA	1.44727593741942	2	0.4849846836193	0.768090669482026
C22orf39	0.0339573734799559	2	0.9831646388552	

0.0340427955492216
MRPL40	1.75142358642485 2	0.416565405698816
0.124082571634342
UFD1	14.5598631399103 2	0.0006892327263055
0.314030334241703
CDC45	0.360711780469876	2	0.83497299978999
0.655169901486016
GNB1L	0.0414633170125394	2	0.979481764907642
0.0543247420944522
RTL10	1.00015615724974 2	0.606483304481521
0.550577003311906
TXNRD2	0.483060131548035	2	0.785425189017748
0.201924186204118
COMT	0.313172408150343	2	0.855057805058487
0.0968461093337744
TANGO2	2.0233521512516	2	0.363609032319061
0.46865349460172
DGCR8	0.15904573255343 2	0.923556901419486
0.233919118380265
TRMT2A	0.485748332506112	2	0.784370207804145
0.0987936839695888
RANBP1	1.77479561206003 2	0.411725750242622
0.13498090524425
ZDHHC8	1.223441497458	2	0.542416702720426
0.94202856537291
DGCR6L	5.04571681124715 2	0.0802299489410826
0.566126565977794
KLHL22	5.05041104121695 2	0.0800418608442278
0.929741357381634
MED15	1.45732324247255 2	0.482554398652804
0.331351816833446
PI4KA	0.258748580717817	2	0.878645035626037
0.198268145569834
SNAP29	0.924507253806227	2	0.629862569588827
0.16190477334389
CRKL	0.0113641152471881	2	0.994334054734265
0.0434527880318575
AC002470.1	0.506349509515387	2	0.776332202222866
0.651937242810737
LZTR1	2.9705695560645	2	0.226437846969222
1.15349787209452
THAP7	0.313666065861505	2	0.854846778163844
0.055371669730735
THAP7-AS1	6.12334513974569 2	0.0468093378209359
1.78812791236001
TMEM191C 6.27119745963925 2	0.0434737170248223
1.77937470548055
UBE2L3	8.52549641349908 2	0.0140835446848759
0.166260549472414
YDJC	1.80626875629457 2	0.405297311735313

0.125815839276056
SDF2L1	15.2306199665653 2	0.000492847883987624
0.483660346048222
PPIL2	3.27678896897911 2	0.194291730400602
0.402378878873464
YPEL1	0.168718270300241	2	0.919101115299072
0.247846145388239
MAPK1	10.789908884689	2	0.00453942719975053
0.626235893480699
PPM1F	3.26145298769238 2	0.19578728418966 1.03395808620376
AC245452.1	0.670106001851156	2	0.715300173776592
	0.348211197288547
TOP3B	11.0983783646895 2	0.00389061054064488
0.90333425325367
IGLV6-57 6.29833248482227 2	0.0428878700515197
1.04139775252422
VPREB1	11.7210773427643 2	0.00284970821289809
3.48654529954364
AC245060.6	3.20054320050067 2	0.201841690295772
0.315972045080972
AC245060.5	2.08619580739306 2	0.35236140769652
0.402718877953698
IGLV1-51 1.15059510159571 2	0.562537460431769
0.819287030254146
IGLV1-44 10.4404670457869 2	0.00540606653872477
1.88381143233623
IGLV1-40 2.66354723530488 2	0.264008595500339
1.47547152252584
ZNF280B	0.0402531182539674	2	0.980074628068085
0.034085303014639
IGLV3-25 7.81158491710854 2	0.0201249999421053
4.72832476770113
IGLV3-21 0.106821208285707	2	0.947990683655658
0.156560743666416
IGLV2-14 0.871093262370815	2	0.64691094871135
1.02308400957293
IGLV3-10 4.25888134772032 2	0.118903781249585
1.78151514353328
IGLV3-1	0.408609491606037	2	0.815213900314305
0.360985524922134
IGLL5	2.46162732315291 2	0.292054847170524
0.5803126322037
IGLC2	35.1270431414417 2	2.35645688517749e-08
0.589124201289284
IGLC3	17.3058408082224 2	0.00017461615307135
0.693301635326254
IGLC5	3.89642182557551 2	0.14252884014225 0.289961553635716
IGLC6	4.72946215987375 2	0.0939745686176821
22.3474219658354
IGLC7	3.88967513360922 2	0.143010451096108

1.19614372385266
RSPH14	4.08296614422148 2	0.129836011852323
2.8099237296246
GNAZ	6.15624022715452 2	0.0460457360919153
0.975040935647108
BCR	0.761850650547087	2	0.683228907646783
0.491057102369166
IGLL1	41.3669507881449 2	1.04059272310053e-09
1.6486757149946
VPREB3	156.384511690118 2	0	1.28964885140162
CHCHD10	2.32944147098567 2	0.312009783125481
0.160592993364498
MMP11	3.26489541739098 2	0.195450582060286
4.93771823294289
SMARCB1	11.044311241095	2	0.00399722215425913
0.251750226148114
DERL3	4.36058300445326 2	0.11300858358487 0.899482045892287
SLC2A11	4.58171506741338 2	0.101179659672704
0.675109799740605
MIF	1.83659507446918 2	0.399198082770851
0.101793991260036
DDT	5.39711334698401 2	0.067302582272434
0.228514684082162
CABIN1	0.58706729736198 2	0.74562413317339 0.267408750717326
SPECC1L	0.876448037240917	2	0.645181234057088
0.603060800384816
ADORA2A-AS1	0.0418488528929123	2	0.979292970422717
	0.14986199037891
GUCD1	1.57288295779153 2	0.455462688510723
0.325053940976859
SNRPD3	3.05863978524855 2	0.216682984910669
0.172496242871144
GGT1	0.145015849921398	2	0.930058375954995
0.0995866120224409
GRK3	1.69338509158069 2	0.428830927644304
0.383338175838215
ASPHD2	0.0151805046837896	2	0.992438480879984
0.0516925786700986
HPS4	2.35110017348333 2	0.308649149176758
0.237134424860639
SRRD	0.0181868365492167	2	0.990947801814889
0.0394564181068115
TFIP11	2.00430276091297 2	0.367088843274731
0.265346892135984
Z95115.1	4.23076800466729 2	0.120586974981476
1.69715162317897
TPST2	3.77884890316808 2	0.151158783003164
0.331477569750776
MIAT	0.2305571243316	2	0.891117877603669
0.181661400531563

MIATNB 5.2734374655762 2 0.0715958095341152
1.13681814911828
PITPNB 0.0713336082166695 2 0.964961761218737
0.0478910872670929
TTC28 0.000118361588201433 2 0.999940820957048
0.00777543806695081
CHEK2 19.148833388904 2 6.94838160950617e-05
1.00683332440348
HSCB 4.96193830148475 2 0.0836621050982872
0.359888820251958
CCDC117 0.236676262542187 2 0.88839560748654
0.137763655604383
XBP1 354.522646264857 2 0 1.35635088578408
Z93930.2 7.39308485808286 2 0.0248091577702959
0.755075156602518
RHBDD3 6.25869436304181 2 0.0437463463563401
1.52193153246348
EWSR1 10.8523229921409 2 0.0043999526649996
0.277431876376625
AP1B1 0.787604396911754 2 0.674487457707127
0.0856074303230556
THOC5 0.0499375607643834 2 0.975340361306336
0.0774299342926138
NIPSNAP1 0.668226887073904 2 0.715972555160935
0.240152962226399
NF2 5.90258175864426 2 0.0522721852941815
0.659979865634112
ZMAT5 4.83015460025186 2 0.0893604310821925
0.511101605064214
UQCR10 10.9658174226963 2 0.00415721994172824
0.172628124928721
ASCC2 0.0708508411319865 2 0.965194715221384
0.0576255276413427
MTMR3 2.8784315897643 2 0.237113631514728
1.1385307988875
AC003681.1 0.371756595084859 2 0.830374647467941
0.460165824705511
CASTOR1 3.69210233818314 2 0.157859296831609
20.7468785228751
TBC1D10A 0.0318632918027655 2 0.984194591490285
0.0590244804230741
SF3A1 0.978339685524834 2 0.613135181577031
0.17806224608826
MTFP1 1.91377409887957 2 0.384086669729888
0.423714779109077
PES1 1.82979092696697 2 0.400558496892192
0.272702254874706
DUSP18 1.37170074840657 2 0.503661746458781
1.06760645159314
MORC2-AS1 0.290818391863862 2 0.864668401917727

0.200473912559018
MORC2 11.6680480096331 2 0.00292627790000299
1.22208030934461
SMTN 0.678647237885562 2 0.712251913577109
0.526099419818374
SELENOM 9.80630375892924 2 0.00742314928829058
2.59639927319887
RNF185 0.00570545001953718 2 0.997151340143715
0.0319365206453986
LIMK2 0.600855854761471 2 0.740501272101265
0.393161547909606
PIK3IP1 2.06833565636642 2 0.355522113340763
0.777267435246228
PATZ1 2.2731790045881 2 0.320911625959438
0.32518610371967
DRG1 2.7449044441035 2 0.253484596261451
0.184381192213669
EIF4ENIF1 2.10207875598675 2 0.349574220470239
0.444835340570546
SFI1 0.362909409734941 2 0.834056023126002
0.153080582360198
PISD 1.56345836068299 2 0.457614029579008
0.317486725503186
PRR14L 0.360629105735685 2 0.835007516088799
0.207879250456179
DEPDC5 0.101216212958748 2 0.950651151567883
0.106979374625669
YWHAH 2.40471368028064 2 0.300485181167798
0.141884002545666
RTCB 0.807068970321804 2 0.66795499186644
0.130818999453111
FBXO7 0.803158851579875 2 0.669262160912857
0.109660609031803
LARGE1 2.51314313713975 2 0.284628183841128
5.89935353118792
HMGXB4 1.09595701139811 2 0.578117290764004
0.263476778241421
TOM1 0.0356684263775147 2 0.982323875202207
0.0735919240410872
HMOX1 6.25674597985277e-05 2 0.999968716759432
0.0065894896803629
MCM5 14.1550759756976 2 0.000843848157399019
0.570936151594043
APOL6 9.22865247770196 2 0.00990885736151059
2.59936835113062
RBFOX2 10.4144911738009 2 0.00547673813051675
1.02770427029442
APOL3 0.454185178407272 2 0.796846999445347
0.306682057576497
APOL2 0.10589662425674 2 0.948429033493525

0.120327812134466
APOL1	0.897769890637568	2	0.638339538420996
0.816564082814594
MYH9	0.179728329107239	2	0.914055337953717
0.027936094805017
TXN2	0.0206285417833449	2	0.989738738791721
0.0157498411105614
FOXRED2	1.3497719635819	2	0.509214477020433
0.92719934225453
EIF3D	1.20491077303298	2	0.547465744420991
0.0830049428796224
IFT27	0.0286463064314164	2	0.985778935152983
0.0311209315693222
NCF4	1.79217311211155	2	0.408163864694668
0.131865924482451
CSF2RB	10.495616561521	2	0.00525903209900636
1.09946171733593
TST	1.09404887971169	2	0.578669115921399
0.462344893654844
MPST	8.06306346103534	2	0.0177471253104484
0.379724526880457
KCTD17	0.0280190155093775	2	0.986088168733882
0.0537987426751625
RAC2	13.5551319219754	2	0.00113904400443998
0.261983823479976
CYTH4	16.2459804468695	2	0.000296640312110519
1.08855479286831
MFNG	7.6370193727298	2	0.0219605045582272
0.433302752709249
GGA1	2.89847654979173	2	0.234749034239994
0.2837950234625
SH3BP1	2.34748848883675	2	0.309207024439162
0.328662266895592
Z83844.2	1.3216134478134	2	0.516434546303058
1.04785986226847
LGALS1	105.590640879555	2	0	2.35524003955721
NOL12	0.00256958688041686	2	0.99871603155353
0.0103824284953376
TRIOBP	0.747824078799478	2	0.68803742935265
0.303906141600739
H1F0	9.33045630935751	2	0.00941709942309632
1.73741314758425
GCAT	4.70899331239798	2	0.0949412826157821
2.6679493028916
ANKRD54	3.8615177285739	2	0.145038092482519
0.417366364943873
EIF3L	24.8148391464316	2	4.08814362151677e-06
0.261264242942509
MICALL1	5.9545275103269	2	0.0509320058117234
1.2980010171608

POLR2F 14.222111502383 2 0.00081603300934352
0.245184583044488
PICK1 2.94682289579008 2 0.229142442787858
0.552579770795022
PLA2G6 0.356769246831719 2 0.836620577733643
0.302212336158888
AL022322.2 2.34275872867348 2 0.309939127298897
1.71594646524803
MAFF 1.38752751435484 2 0.499691806713412
0.623585918603165
TMEM184B 2.73926905817935 2 0.2541998452271 0.812108868881952
CSNK1E 1.71559510936285 2 0.424095100748832
0.475671207503668
TPTEP2-CSNK1E 1.2372592528301 2 0.538682127782255
0.849188804264156
DDX17 3.05727830486176 2 0.216830539945346
0.178626023442311
DMC1 0.431087379973529 2 0.806103050857133
0.663778951391829
CBY1 1.55622193004044 2 0.459272774710967
0.30137698257702
TOMM22 5.53519121028565 2 0.0628128502174669
0.222823704191168
JOSD1 5.0986030703963 2 0.0781362223471503
0.758789387311285
GTPBP1 2.31935620100909 2 0.313587108169413
0.664019216205206
SUN2 0.14433934672761 2 0.930373022897782
0.0898485170750329
DNAL4 1.89546698751732 2 0.387618568502942
0.784232247743826
CBX6 1.33253353124954 2 0.513622476131942
0.344857583862284
APOBEC3B 0.6352677874983 2 0.7278692171112 0.841489702462725
APOBEC3C 1.07877779623062 2 0.583104479763843
0.165916119231804
APOBEC3D 1.98592457088503 2 0.370477603508643
2.03003486708795
APOBEC3F 1.63021117566474 2 0.442592592501613
0.424632805860432
APOBEC3G 0.186400978470173 2 0.911010834120163
0.0609385988641418
CBX7 0.364944351404926 2 0.833207827028292
0.263063813997074
AL031846.2 4.83662408139882 2 0.0890718402799361
1.61976004193846
RPL3 30.8601043842749 2 1.98981855992209e-07
0.209174379329651
SYNGR1 6.88557419244698 2 0.0319754423865776
0.625010797736896

TAB1	0.498126291524292	2	0.779530747767338
0.230145862996356
MGAT3	1.90663987913296 2	0.385459195601616
0.474378500735595
MIEF1	0.0535476440124634	2	0.973581419315904
0.0768264642164816
ATF4	6.25036993820639 2	0.0439288073997713
0.273297578463825
RPS19BP1 0.00342229305273546	2	0.998290316650161
0.00536130133242646
TNRC6B	1.08037044338182 2	0.582640324752887
0.152286884903465
ADSL	4.04631382375665 2	0.132237343799956
0.203644734014657
SGSM3	4.16255341984438 2	0.124770814331158
0.297884574793387
AL022238.2	5.25929733181739 2	0.0721037903088459
1.06395047222346
MKL1	1.77404528408217 2	0.411880243895844
0.547539163708311
SLC25A17 0.0600813430947308	2	0.970406064829657
0.0614620444786737
ST13	1.40834999020444 2	0.494516384449512
0.0504223853603024
XPNPEP3	1.58898860145807 2	0.451809656975438
0.329289848829277
RBX1	5.89103205982636 2	0.0525749225823281
0.140237096316067
EP300	0.477760218552355	2	0.787509291772177
0.0729707032794531
L3MBTL2	14.9777292003443 2	0.000559277603819464
1.58933534220615
RANGAP1	2.52294706477672 2	0.2832363609021	0.419592388727258
ZC3H7B	0.00910865686047356	2	0.995456026797155
0.0497072470474799
TOB2	1.27258860269071 2	0.529250035788843
0.16956045377407
PHF5A	0.744655957131317	2	0.689128186181373
0.051695581733493
ACO2	10.7283382156961 2	0.00468134832062739
0.346152559933616
POLR3H	7.16605215417218 2	0.0277914717457262
0.807179356098568
PMM1	2.87071417473591 2	0.238030351205485
0.495942025924519
DESI1	0.592472179424741	2	0.743611848187345
0.155141561410016
XRCC6	0.186001114541806	2	0.911192992514657
0.0302708212240177
SNU13	2.05476184375652 2	0.357943215215722

0.0517763963301262
C22orf46 0.519564392554088 2 0.771219541999624
0.374727827589746
MEI1 0.215709665696983 2 0.89775791193026
0.229336384558633
CCDC134 1.00324899076346 2 0.605546153336591
0.749437054873946
AL021453.1 0.015263416089692 2 0.992397339497902
0.0584767443187801
SREBF2 2.10591224271927 2 0.348904818143316
0.255195092629531
SHISA8 5.80086663908277 2 0.0549993825849209
0.906959751466987
TNFRSF13C 14.9625780466846 2 0.000563530543171376
0.383394829947158
CENPM 13.9967381551068 2 0.000913370387741419
0.715126717371172
WBP2NL 0.395378965251266 2 0.820624631785188
0.475327589365273
NAGA 0.448405230513145 2 0.799153197334343
0.310623853613664
SMDT1 5.6445845895898 2 0.0594694648002838
0.157100703716035
NDUFA6 0.0556517332484773 2 0.972557706813555
0.0271598057453537
TCF20 3.61589528390572 2 0.163990358593049
1.70052171074151
RRP7A 1.94369900830103 2 0.378382570409662
0.139597743344726
SERHL2 2.44802478424899 2 0.29404796105016 2.06214555411685
POLDIP3 2.70433748509663 2 0.258678644482954
0.335993463883698
Z93241.1 0.543018880943802 2 0.762228087641229
0.63189052767234
CYB5R3 17.6727822942909 2 0.000145346334873619
0.539325332391452
ATP5MGL 2.76514167929342 2 0.250932615597869
0.89814518978753
A4GALT 24.8704397651828 2 3.97605720148242e-06
0.450100407101967
ARFGAP3 4.06878532336048 2 0.130759873874965
0.398791001122635
PACSIN2 2.58870913329882 2 0.274074704210779
0.297876121603975
TTLL1 0.00481854194416347 2 0.997593628991826
0.0714495918795322
BIK 190.125249274061 2 0 1.16686903164761
MCAT 0.0527920336602417 2 0.973949312907162
0.0655630172318432
TSPO 73.4705168323681 2 1.11022302462516e-16

0.705060854823112
TTLL12	0.000554941018656831	2	0.999722567982053
0.0177638212967029
SAMM50	4.18125353578892	2	0.123609636981195
0.228828170604568
PARVG	3.33294023881719	2	0.188912729467865
0.308864821816193
PRR5	14.7198073519515	2	0.000636259743685863
2.87288036459606
NUP50-DT	2.97769074451967	2	0.225633027346728
0.763887488725891
NUP50	0.0362522528164371	2	0.982037163726714
0.0521775040137593
KIAA0930	1.5538107816883	2	0.459826795997706
0.60514488722444
FAM118A	0.647925006438692	2	0.723277362443993
0.265021413358374
ATXN10	13.6892990077919	2	0.00106513949849518
0.362030004768653
PRR34-AS1	0.204675564081163	2	0.902724576014758
0.150592330729222
MIRLET7BHG	0.00910415009820361	2	0.995458269941514
0.0792868775495913
PPARA	1.10754297878244	2	0.574777948442902
0.571828048062265
CDPF1	0.400173591484728	2	0.818659693818337
0.346993398794707
TTC38	7.34622795080531	2	0.0253972602384691
1.04669252819069
TRMU	0.00312813177931763	2	0.998437156623948
0.0162776972128797
GRAMD4	0.00444674097362536	2	0.99777909937054
0.0384701028761427
CERK	1.26657088360383	2	0.530844872927161
0.427735460104432
AL118516.1	3.18213336594789	2	0.203708203729548
0.600405687026126
TBC1D22A	0.00465415297858767	2	0.997675629054124
0.0103326343611513
C22orf34	1.46156342569023	2	0.481532422845374
1.77684941416728
Z97192.1	3.94993893366902	2	0.138765549129748
0.550326474966008
BRD1	0.299168565224727	2	0.861065862081026
0.328642838483543
ZBED4	4.49810008521555	2	0.105499396906568
2.11262310509965
ALG12	3.32645909275098	2	0.189525907952823
0.666233018686555
CRELD2	4.03585124623265	2	0.132930928118679

0.223655344882231
PIM3 9.05437195618835 2 0.0108110558560117
0.553795429444157
TRABD 1.89475645507449 2 0.387756300751483
0.098558890475044
AL022328.3 3.94949554668598 2 0.138796315959114
1.6298649483603
AL022328.4 1.1922868365581 2 0.550932259568521
0.860226190018224
SELENOO 1.47004011352149 2 0.479495841745074
0.472078928277355
TUBGCP6 2.07579522510115 2 0.354198562334456
0.720163964005206
HDAC10 1.06466997871689 2 0.587232186678399
0.327390364939464
PLXNB2 1.79194285723675 2 0.408210858259524
0.54501496004612
DENND6B 5.41480536898618 2 0.0667098484195502
0.311634374672654
PPP6R2 0.0653902828186044 2 0.96783356699624
0.0593208210775742
SBF1 0.746323650334925 2 0.688553798494009
0.351071882814142
LMF2 2.05952825849361 2 0.357091178001658
0.426055319326437
NCAPH2 0.275295734659142 2 0.871405488067664
0.106056740579467
TYMP 1.27838937542809 2 0.527717230138247
0.253079610336996
ODF3B 0.3430461233037 2 0.842380841102334
0.38086819426216
U62317.5 4.74431440197843 2 0.0932792869135739
1.8540634927489
KLHDC7B 3.70630155792254 2 0.156742526417773
1.57661060574431
CHKB 2.52191675205535 2 0.283382309504943
0.398597511110196
CHKB-DT 1.0477491904965 2 0.592221478338405
0.687512693121567
ARSA 5.27150128462342 2 0.07166515431607 1.19093885593891
RABL2B 2.79468181491709 2 0.247253560678024
0.437074392429675
GATD3B 0.450766042893574 2 0.798210428487839
0.739884684036301
FP565260.1 0.794836429622471 2 0.672052904373674
0.314017419080953
FP565260.6 2.5849527479422 2 0.274589953030352
1.56127093229477
U2AF1L5 0.0485525688482054 2 0.976016014480847
0.132736050094007

HSPA13 4.59540554176703 2 0.100489426006553
1.15672339911636
SAMSN1 19.6713953103687 2 5.35070222758005e-05
0.791681397021927
NRIP1 0.218550149709818 2 0.89648378343172
0.271389121634456
USP25 0.20139296682453 2 0.904207433195963
0.163784238849722
BTG3 0.000107576485235117 2 0.999946213203944
0.00276167708488036
C21orf91 0.648103798806882 2 0.723212707097762
0.282155980850636
MIR155HG 13.193008302819 2 0.00136513200042931
1.09289656869637
MRPL39 4.73743351106933 2 0.0936007619037243
0.41689716593397
ATP5PF 7.3929378108511 2 0.0248109818963395
0.138077818845949
GABPA 5.27126608002018 2 0.0716735827987582
0.458862852103007
N6AMT1 1.52458490790806 2 0.466595551195877
0.661759440672529
LTN1 0.000616568395732049 2 0.999691763316825
0.00698601435923005
RWDD2B 3.48753172312509 2 0.174860658116024
0.644235566471464
USP16 0.219558036822319 2 0.896032120021768
0.0571977164494182
CCT8 0.295983906127498 2 0.862438054898171
0.0351630436959408
MAP3K7CL 8.36642270007252 2 0.0152494575045095
0.613196695577517
BACH1 0.22090078400398 2 0.895430749614542
0.212298076984502
AP000251.1 10.9433517599478 2 0.00420418054883365
2.52022236534383
SOD1 11.54407262167 2 0.00311341118188191
0.180457289867837
SCAF4 13.3268551618491 2 0.00127676265458554
0.61958677103044
MIS18A 0.17804467699388 2 0.914825137527662
0.0695665485958579
URB1 1.87250703930518 2 0.392094059742255
1.18902914859792
URB1-AS1 0.169902750373003 2 0.918556947975853
0.411517659817689
CFAP298 5.73290374997958 2 0.056900458778587
0.174859036380736
PAXBP1 0.0677170946153057 2 0.966708238449639
0.0853170861652962

IFNAR2	0.115994609394224	2	0.943652490862655	0.114504826510662
IL10RB	0.0206022082576391	2	0.989751770532774	0.114843176682527
IFNAR1	0.480179472725079	2	0.78655727511074	0.0720561501445588
IFNGR2	7.63042393822383	2	0.0220330436339358	0.466549219668466
TMEM50B	1.50009264608112	2	0.472344671792832	0.235940192967237
DNAJC28	0.0275009798324348	2	0.986343616241192	0.146945696586645
GART	0.0721282776150561	2	0.964578424589125	0.0574310017463978
SON	0.00320857685032853	2	0.998396997757617	0.00359277841250994
DONSON	1.89366568531037	2	0.387967834854344	1.00549638656569
CRYZL1	0.302717902220787	2	0.8595391107603	0.126524768645229
ATP5PO	5.51840352055551	2	0.0633423105329379	0.14484142645986
LINC00649	2.20905167646584	2	0.331367967018035	1.13762041242005
AP000569.1	0.455380899959419	2	0.796370738262973	0.536354928542251
MRPS6	2.92925072447342	2	0.231164578237526	0.174254358692931
SLC5A3	6.17059324873592	2	0.0457164712694186	0.728218953955584
RUNX1	6.54068505159596	2	0.0379934111226889	0.46476407795242
SETD4	0.416454957927666	2	0.812022297709725	0.238699508035833
CBR1	2.39453857959563	2	0.302017810011593	0.361092680348106
MORC3	0.312676241012545	2	0.855269957165019	0.236979918522277
CHAF1B	0.566995145012383	2	0.753144950392549	0.634748148443367
HLCS	2.46450981135199	2	0.291634228025537	1.50409512921138
PIGP	0.000760354566048064	2	0.999619894975202	0.00669782227873481
TTC3	10.8657279349681	2	0.00437056071741682	0.521079767673164
VPS26C	1.27441524077192	2	0.528766882324132	0.301378180960936
DYRK1A	2.85233069126608	2	0.240228350966523	0.632050689045497

ETS2	2.15263727954773 2	0.340848003101861
1.21975477189125
PSMG1	0.252222243884404	2	0.881516885472294
0.128405781151071
BRWD1	17.9529570500794 2	0.000126346992790904
0.620578097536067
BRWD1-AS2	1.86947945269749 2	0.392688058586674
1.57077807763654
HMGN1	38.0287468924331 2	5.52284096233535e-09
0.255738103527395
WRB	1.30876743948702 2	0.519762283137279
0.148110398771544
SH3BGR	0.895664366115352	2	0.639011912059515
0.748130577053409
BACE2	5.98395464041883 2	0.0501881006007057
2.38036414352457
MX2	3.69927115882455 2	0.15729447721619 0.569176623293919
MX1	28.3734460385964 2	6.89897349470669e-07
0.679615752859644
PRDM15	11.9213045474897 2	0.00257822971189048
0.456956217227271
C2CD2	0.0152266907254043	2	0.992415562742121
0.0971561857178593
ZBTB21	3.42274303199922 2	0.180617902320378
1.29132513461104
ZNF295-AS1	0.0169409144191243	2	0.991565316036601
	0.134656739975125
SLC37A1 2.1841414882199	2	0.335520995730414
0.553150104283993
PDE9A	16.3277513667871 2	0.000284756626848459
1.69881675053409
WDR4	4.77546928504399 2	0.091837493159369
0.691106005247645
NDUFV3	2.31193605514164 2	0.314752700090321
0.303863927545783
PKNOX1	2.17933997431826 2	0.336327467777396
0.382893361349923
U2AF1	0.197594891196724	2	0.9059261887776
0.257349407180623
RRP1B	0.200299761997115	2	0.90470181026298
0.131490472504352
PDXK	94.2184101536687 2	0	0.889500510090762
CSTB	4.93092119035292 2	0.084969696724787
0.18516727203303
RRP1	1.51535999299111 2	0.468752674321048
0.116288211385455
AGPAT3	4.68544388248513 2	0.0960657965796534
1.66219222871155
TRAPPC10 2.1823854047167	2	0.335815726547251
0.521124019071005

PWP2	0.00409495116788944	2	0.997954619064364	0.0328179452623024
GATD3A	1.69591626556926	2	0.428288548086182	0.230039483425388
LINC01678	0.738950797277918	2	0.691096785871267	0.378080794945503
ICOSLG	0.133452120257575	2	0.935451423682945	0.130051741261772
AP001059.3	2.98121546468786	2	0.225235730898211	0.816608226024961
AP001059.2	1.20933970298644	2	0.54625474206005	0.246905856624683
PFKL	8.28994163596935	2	0.015843898301458	0.309975688509884
C21orf2	0.121321018325919	2	0.941142694868158	0.091452010273592
UBE2G2	3.24782129534656	2	0.197126298274582	0.376971163337126
SUMO3	9.25116868967405	2	0.00979792799406431	0.393614431195643
PTTG1IP	3.58927717556658	2	0.166187503688632	0.441163580232027
ITGB2	91.2942865413602	2	0	1.85806886166952
ITGB2-AS1	16.7703507156428	2	0.000228225733902465	2.63859879753588
FAM207A	1.2732357244394	2	0.529078818885556	0.164030363079418
ADARB1	0.552070785618725	2	0.7587860747151	0.327685620563657
POFUT2	0.293638732439486	2	0.863449931555802	0.399179531571068
SPATC1L	0.0850567469702994	2	0.958363273029081	0.0809423750412878
LSS	2.64358660947075	2	0.266656676252997	0.647780103956461
MCM3AP-AS1	0.0187071288301923	2	0.990690044097613	0.107610414257156
MCM3AP	0.903380308492654	2	0.636551371911961	0.160229451499387
YBEY	3.01978171008882	2	0.220934090484968	0.306109567069621
C21orf58	0.0507097829107588	2	0.974963844286082	0.0447961408974205
PCNT	0.166450096472698	2	0.920144047122251	0.158003385013615
DIP2A	0.55152049997315	2	0.758994877981631	0.290682823421434
PRMT2	2.90272226549021	2	0.234251223987206	0.214765892890751
MT-ND1	12.1493600260185	2	0.00230038216073269	

0.207835915155084
MT-ND2 3.06106021666253 2 0.21642091037424 0.0889981133736134
MT-CO1 16.026922448483 2 0.000330977148001343
0.104590363663965
MT-CO2 0.000245428750931775 2 0.999877293153635
0.000605579623528965
MT-ATP8 1.17505537067124 2 0.555699451724779
0.552822303631778
MT-ATP6 2.91860840216186 2 0.232397920708712
0.052374515193507
MT-CO3 35.7318801532442 2 1.74148929721341e-08
0.226532017952941
MT-ND3 11.3924676869824 2 0.00335859064635291
0.101554894061422
MT-ND4L 1.11095172850995 2 0.573799145715661
0.101789030723044
MT-ND4 0.0019153059563055 2 0.999042805425118
0.00198475064034668
MT-ND5 33.0018777180889 2 6.81919810618936e-08
0.436353069367591
MT-ND6 0.0627329703869738 2 0.969120339730903
0.0482591948878375
MT-CYB 1.81799542518242 2 0.402927871246587
0.0669443224195142
AL592183.1 0.370315220493844 2 0.830973303622914
0.271791940708161
FMO1 0.328715661477598 2 0.848438370226656
0.5363751564353
OR2T10 3.43660003568188e-05 2 0.999982817147449
0.00580228614898276
DNASE1L3 0.839768774910453 2 0.65712278706123
0.376506020451841
NMU 1.27234089811546 2 0.529315588675853
1.73779444323259
ENPP3 0.135458345806239 2 0.934513530894845
0.301660858330058
CALD1 0.692461154345457 2 0.707349369683854
0.800134736264545
ATG9B 1.9422159695912 2 0.378663252461909
1.51596327801981
KLF8 1.18594633471164 2 0.552681624570898
0.746220785611074
FGF13 0.0625359363673708 2 0.969215819272013
0.292895769656193
KBTBD11 0.740517840759924 2 0.690555508593348
0.660839926786737
MICU3 2.52452707282648 2 0.283012691398532
1.62356396474762
LINC01506 8.47679506774716 2 0.0144306980295003
2.69517546207388

PALM2	1.90734509701511	2	0.385323303202671	1.45782906016169
AL139099.1	0.864591221540633	2	0.649017491764231	0.915824627506637
GPR135	3.10925174758309	2	0.211268408905941	2.38987302121989
AL022341.1	0.3775392172036	2	0.827977243556645	0.347474866523849
MPV17L	2.51225922922085	2	0.284754004195303	0.564673158047929
CDYL2	1.11339510547898	2	0.573098569936733	1.35881633309821
ZNF286B	2.0910858168763	2	0.351500934742826	1.43236582172004
GNG8	0.805234037344001	2	0.668568099397372	2.08278613004501
CPT1B	0.254391634686615	2	0.880561226553742	0.543208480234578
LINC00158	2.41252452707695	2	0.299313947880081	2.21808833768242
G0S2	0.772147750396368	2	0.67972030935291	24.9283425599971
SPARCL1	1.72719748751405	2	0.421641967308577	228.007653194115
MGARP	0.63150531251159	2	0.729239800765153	0.350904586414906
RPS6KA2	1.62557026500829	2	0.443620801336084	1.53880303312503
SCARF1	7.05178645970203	2	0.0294255118813045	2.95413359085583
TRERNA1	4.83288060336437	2	0.089238715643569	19.3995523359815
CLIP3	0.390320651729888	2	0.822702746952423	0.629050958461669
Z73429.1	1.80689677292592	2	0.405170064988422	9.73503925431461
RNF212	0.0855564886208859	2	0.958123835922537	0.160577375400157
XIST	8.51834881173904	2	0.0141339665143881	0.814265394142491
AC245123.1	2.28808353545531	2	0.318528996363001	2.05674468284287
AL512625.3	5.47933418989068	2	0.0645918462664569	1.58405017517364
AC243829.5	1.20126998533822	2	0.548463255349699	0.568782425799963
PLPP2	6.73173197482041	2	0.0345320988030533	3.23963428018851
CRYBB2	3.63569340503188	2	0.162375016477075	2.60629215476868

TMPRSS15 1.74302622257958 2 0.418318108282791
22.6550689744973
