# Supplementary material for: Distinct stage-specific transcriptional states of B cells derived from human tonsillar tissue
Source: JCI Insight. 2023 Apr 10;8(7):e155199. doi: 10.1172/jci.insight.155199 (PMC10132144; doi:10.1172/jci.insight.155199)
Supplement: Supplemental table 8 [file jciinsight-8-155199-s233.pdf]

TF	Targets
ARID3A	AHNAK
ARID3A	ARID3A
ARID3A	CLDN14
ARID3A	LMNA
ARID3A	TCF4
ATF1	AAAS
ATF1	AAMP
ATF1	AATF
ATF1	ABCC4
ATF1	ABCE1
ATF1	ACIN1
ATF1	ACY3
ATF1	ADSL
ATF1	AMPD3
ATF1	ANAPC10
ATF1	ANAPC2
ATF1	ANKH
ATF1	ANKHD1
ATF1	ANXA5
ATF1	AP5S1
ATF1	APTX
ATF1	ARHGEF2
ATF1	ARL2
ATF1	ATF1
ATF1	ATF3
ATF1	ATL3
ATF1	ATP1B3
ATF1	ATP2A3
ATF1	B3GALNT1
ATF1	BCL11A
ATF1	BDP1
ATF1	BIRC2
ATF1	BLCAP
ATF1	BLOC1S2
ATF1	BLOC1S4
ATF1	BPTF
ATF1	BRAF
ATF1	BUB1B
ATF1	C12orf49
ATF1	C20orf27
ATF1	C2orf49
ATF1	C3orf38
ATF1	CAMK1D
ATF1	CAPNS1
ATF1	CASD1
ATF1	CBFA2T2
ATF1	CC2D1B
ATF1	CCAR1
ATF1	CCAR2

ATF1	CCDC189
ATF1	CCDC59
ATF1	CCT2
ATF1	CCT7
ATF1	CD47
ATF1	CD82
ATF1	CDC16
ATF1	CDC23
ATF1	CDC5L
ATF1	CDCA5
ATF1	CDK4
ATF1	CEP112
ATF1	CEP120
ATF1	CHD1
ATF1	CHD6
ATF1	CHEK1
ATF1	CIR1
ATF1	CMSS1
ATF1	CMTM7
ATF1	CNTROB
ATF1	COA1
ATF1	COMMD2
ATF1	CRLF3
ATF1	CSNK1A1
ATF1	CTPS1
ATF1	CUX1
ATF1	CWC15
ATF1	CXXC1
ATF1	CYHR1
ATF1	DBNL
ATF1	DCAKD
ATF1	DCUN1D5
ATF1	DDX18
ATF1	DDX21
ATF1	DDX5
ATF1	DEPDC5
ATF1	DGKD
ATF1	DHX37
ATF1	DNAJC14
ATF1	DPM1
ATF1	DTL
ATF1	E2F8
ATF1	EDRF1
ATF1	EIF2S1
ATF1	EIF3I
ATF1	EIF5AL1
ATF1	EIF6
ATF1	ELP5
ATF1	EMP3
ATF1	EPB41L2

ATF1	ERF
ATF1	ERI2
ATF1	EYA3
ATF1	FAM122B
ATF1	FAM168B
ATF1	FAM234A
ATF1	FANCD2
ATF1	FBXO22
ATF1	FDXR
ATF1	FIP1L1
ATF1	FOSL1
ATF1	FZD3
ATF1	GABPA
ATF1	GABPB1
ATF1	GAPVD1
ATF1	GAR1
ATF1	GCAT
ATF1	GCC2
ATF1	GEM
ATF1	GLOD4
ATF1	GNL2
ATF1	GOLGA3
ATF1	GPATCH4
ATF1	GPN1
ATF1	GPR160
ATF1	GPS2
ATF1	GTF2H1
ATF1	GTF2H3
ATF1	GTPBP10
ATF1	GTPBP4
ATF1	HAUS5
ATF1	HGH1
ATF1	HGS
ATF1	HIP1R
ATF1	HMGCR
ATF1	HMGCS1
ATF1	HMOX2
ATF1	HNRNPC
ATF1	HNRNPU
ATF1	HOMEZ
ATF1	IDH3G
ATF1	IFT20
ATF1	IKBKB
ATF1	ILF2
ATF1	ILF3
ATF1	IMPDH2
ATF1	INF2
ATF1	INO80D
ATF1	INSIG2
ATF1	INTS10

ATF1	IPO4
ATF1	IRF5
ATF1	IRS1
ATF1	ISG20L2
ATF1	ITPK1
ATF1	JKAMP
ATF1	KBTBD4
ATF1	KCNAB2
ATF1	KCTD6
ATF1	KDSR
ATF1	KIAA0319L
ATF1	KLHL7
ATF1	LACTB
ATF1	LCLAT1
ATF1	LMCD1
ATF1	LRPPRC
ATF1	LRRC47
ATF1	LUZP1
ATF1	LZIC
ATF1	M6PR
ATF1	MAD2L2
ATF1	MAGOH
ATF1	MBD6
ATF1	MBIP
ATF1	MDM4
ATF1	MED7
ATF1	METTL13
ATF1	MFN2
ATF1	MFSD10
ATF1	MITD1
ATF1	MLST8
ATF1	MORF4L1
ATF1	MPHOSPH8
ATF1	MRPL3
ATF1	MRPS10
ATF1	MRPS26
ATF1	MRPS6
ATF1	MRRF
ATF1	MT2A
ATF1	MTA2
ATF1	MVB12A
ATF1	MYL12A
ATF1	MYO9B
ATF1	NAA20
ATF1	NAA40
ATF1	NAA50
ATF1	NAXE
ATF1	NCBP2
ATF1	NCDN
ATF1	NDFIP2

ATF1	NDUFA10
ATF1	NDUFB7
ATF1	NETO2
ATF1	NFKBIZ
ATF1	NGDN
ATF1	NIP7
ATF1	NOL11
ATF1	NOLC1
ATF1	NOP2
ATF1	NOP58
ATF1	NPLOC4
ATF1	NPM1
ATF1	NR1H2
ATF1	NRP2
ATF1	NUDC
ATF1	NUFIP1
ATF1	NUMBL
ATF1	NUP107
ATF1	NUP54
ATF1	NXF1
ATF1	OAT
ATF1	ODC1
ATF1	ORC2
ATF1	ORMDL1
ATF1	PABPC1
ATF1	PABPC4
ATF1	PARM1
ATF1	PARVB
ATF1	PATL1
ATF1	PATZ1
ATF1	PCBP1
ATF1	PCGF1
ATF1	PDCD11
ATF1	PDSS1
ATF1	PFDN2
ATF1	PGM2
ATF1	PHF20
ATF1	PHF5A
ATF1	PICK1
ATF1	PLEKHA1
ATF1	PMAIP1
ATF1	PNO1
ATF1	PNPO
ATF1	PNPT1
ATF1	POC1B
ATF1	POLDIP3
ATF1	POLR2H
ATF1	POLR2K
ATF1	POLR3H
ATF1	PPA1

ATF1	PPIL1
ATF1	PPIL2
ATF1	PPM1A
ATF1	PPP1R11
ATF1	PPP1R37
ATF1	PPP3R1
ATF1	PREPL
ATF1	PRPF18
ATF1	PSMA6
ATF1	PSMG4
ATF1	PTGES3
ATF1	PUF60
ATF1	RAB7A
ATF1	RAD23A
ATF1	RAN
ATF1	RBM22
ATF1	RBM25
ATF1	RBM8A
ATF1	RBPJ
ATF1	REL
ATF1	RER1
ATF1	RFX7
ATF1	RFXAP
ATF1	RIC1
ATF1	RING1
ATF1	RNF139
ATF1	RNF4
ATF1	RNPS1
ATF1	RPL18A
ATF1	RPL21
ATF1	RPL23A
ATF1	RPL38
ATF1	RPRD1A
ATF1	RPS10
ATF1	RPS17
ATF1	RPS21
ATF1	RPS27
ATF1	RPS27A
ATF1	RPS29
ATF1	RPS3A
ATF1	RPS6KA5
ATF1	RPS6KB2
ATF1	RSF1
ATF1	RSL1D1
ATF1	SAFB
ATF1	SCAF11
ATF1	SCNM1
ATF1	SCO1
ATF1	SCYL3
ATF1	SDCBP

ATF1	SEPT7
ATF1	SERPINE2
ATF1	SET
ATF1	SETD4
ATF1	SF3A3
ATF1	SF3B2
ATF1	SFXN1
ATF1	SIKE1
ATF1	SIN3A
ATF1	SLC16A1
ATF1	SLC35F6
ATF1	SLC39A6
ATF1	SLC46A3
ATF1	SLC4A8
ATF1	SLMAP
ATF1	SLU7
ATF1	SMIM14
ATF1	SMYD3
ATF1	SNF8
ATF1	SNHG15
ATF1	SNW1
ATF1	SOAT1
ATF1	SP110
ATF1	SRFBP1
ATF1	SRSF5
ATF1	SRSF6
ATF1	STAU1
ATF1	STK26
ATF1	STYXL1
ATF1	SZRD1
ATF1	TAF2
ATF1	TBC1D7
ATF1	TBC1D9B
ATF1	TCEA2
ATF1	TCOF1
ATF1	TCP1
ATF1	TCP11L1
ATF1	TFE3
ATF1	THRAP3
ATF1	TIAL1
ATF1	TIMM17A
ATF1	TIMM22
ATF1	TLE3
ATF1	TM2D3
ATF1	TMEM147-AS1
ATF1	TMEM237
ATF1	TMEM242
ATF1	TMEM33
ATF1	TMUB1
ATF1	TNFAIP8

ATF1	TOMM40
ATF1	TPR
ATF1	TRIP6
ATF1	TRMT112
ATF1	TSR2
ATF1	TTC17
ATF1	TUFM
ATF1	TWISTNB
ATF1	U2SURP
ATF1	UBAC2-AS1
ATF1	UBC
ATF1	UBE2K
ATF1	UBE2N
ATF1	UBE2V2
ATF1	UBL7
ATF1	UBR5
ATF1	UBXN1
ATF1	USB1
ATF1	USP16
ATF1	USP30
ATF1	UTP18
ATF1	UTP3
ATF1	UTP6
ATF1	VAC14
ATF1	VDAC1
ATF1	VPS4B
ATF1	VRK2
ATF1	WBP11
ATF1	WDR12
ATF1	WDR18
ATF1	WDR74
ATF1	WDR75
ATF1	WDR83
ATF1	WRAP73
ATF1	XPO7
ATF1	XRCC6
ATF1	YBX1
ATF1	YRDC
ATF1	ZC3H18
ATF1	ZFP36
ATF1	ZMYND19
ATF1	ZMYND8
ATF1	ZNF263
ATF1	ZNF408
ATF1	ZNF551
ATF1	ZNRD1
ATF1	ZPR1
ATF1	ZRANB3
ATF1	ZSCAN16
ATF1	ZSWIM7

ATF2	ATF2
ATF2	C14orf28
ATF2	FOXN2
ATF2	SLC46A3
ATF3	ABCE1
ATF3	ACTA2
ATF3	ADGRE5
ATF3	AEN
ATF3	AIMP1
ATF3	AKIRIN1
ATF3	ANKRA2
ATF3	ARL4A
ATF3	ASB16-AS1
ATF3	ATF3
ATF3	ATL2
ATF3	B3GNT7
ATF3	BACH1
ATF3	BBC3
ATF3	BCL2
ATF3	BCL2A1
ATF3	BOLA2B
ATF3	C19orf25
ATF3	CAMTA2
ATF3	CBX6
ATF3	CCDC117
ATF3	CCDC50
ATF3	CCL3
ATF3	CCP110
ATF3	CCSER2
ATF3	CD44
ATF3	CD82
ATF3	CDIP1
ATF3	CDKN1A
ATF3	CENPU
ATF3	CFAP97
ATF3	CHIC2
ATF3	CHMP2A
ATF3	CNOT1
ATF3	COMMD2
ATF3	COQ7
ATF3	COX20
ATF3	CTNNB1
ATF3	CTR9
ATF3	CWC25
ATF3	CYC1
ATF3	DCAF13
ATF3	DCP1B
ATF3	DDIT4
ATF3	DDX20
ATF3	DNPEP

ATF3	DUSP10
ATF3	DUSP22
ATF3	DVL2
ATF3	E2F7
ATF3	EBI3
ATF3	EBNA1BP2
ATF3	ECE1
ATF3	EIF1
ATF3	EIF1B
ATF3	EIF2S2
ATF3	EIF4EBP1
ATF3	EIF5
ATF3	EIF5A2
ATF3	EMP3
ATF3	ESF1
ATF3	FAM120AOS
ATF3	FARSA
ATF3	FBXL5
ATF3	FBXO42
ATF3	FDXR
ATF3	FIGNL1
ATF3	FKRP
ATF3	GADD45A
ATF3	GADD45B
ATF3	GAK
ATF3	GARS
ATF3	GFM1
ATF3	GGNBP2
ATF3	GLTP
ATF3	GNPTG
ATF3	GOLIM4
ATF3	GPATCH3
ATF3	GTF2B
ATF3	HAUS6
ATF3	HDAC9
ATF3	HGSNAT
ATF3	ICAM1
ATF3	IER5
ATF3	IFRD1
ATF3	IFRD2
ATF3	ITFG2
ATF3	KDM3B
ATF3	KDM6B
ATF3	KIAA0232
ATF3	KLF10
ATF3	KPNA1
ATF3	KPNA4
ATF3	LAPTM5
ATF3	LIG3
ATF3	LINC00158

ATF3	LMNA
ATF3	LTA
ATF3	MAD1L1
ATF3	MAGOHB
ATF3	MAP1LC3B
ATF3	MAP4
ATF3	MARCKS
ATF3	MDM2
ATF3	MED28
ATF3	METTL1
ATF3	MFSD2A
ATF3	MIA3
ATF3	MICU2
ATF3	MIOS
ATF3	MIR155HG
ATF3	MIR22HG
ATF3	MRPL49
ATF3	MRPS30
ATF3	MTHFD1L
ATF3	MTHFD2
ATF3	MTRNR2L1
ATF3	MYC
ATF3	MYO9B
ATF3	NADSYN1
ATF3	NAMPT
ATF3	NDUFA10
ATF3	NFIC
ATF3	NFKB1
ATF3	NFKB2
ATF3	NFKBIA
ATF3	NFKBIZ
ATF3	NIFK
ATF3	NINJ2
ATF3	NMT1
ATF3	NOL9
ATF3	NOM1
ATF3	NPW
ATF3	NSA2
ATF3	NUDT9
ATF3	NUP214
ATF3	ODC1
ATF3	PAM
ATF3	PAWR
ATF3	PCK2
ATF3	PDCD10
ATF3	PELI1
ATF3	PHLPP1
ATF3	PICK1
ATF3	PIM3
ATF3	PIP5K1B

ATF3	PLEKHO2
ATF3	PLK2
ATF3	PLK3
ATF3	PMAIP1
ATF3	PNO1
ATF3	POLR1E
ATF3	POLR3C
ATF3	POR
ATF3	PPP1CB
ATF3	PPP1R15A
ATF3	PPP1R8
ATF3	PPP3R1
ATF3	PPP6R3
ATF3	PRAF2
ATF3	PRMT1
ATF3	PRR14L
ATF3	PRRC2A
ATF3	PSAT1
ATF3	PSMC6
ATF3	PTER
ATF3	PTPN11
ATF3	PUS1
ATF3	PUSL1
ATF3	PVT1
ATF3	PYCR1
ATF3	RAB10
ATF3	RAD9A
ATF3	RARA
ATF3	RASSF7
ATF3	RBM18
ATF3	RBM7
ATF3	RCBTB2
ATF3	RCN1
ATF3	RDH14
ATF3	REEP3
ATF3	RIN3
ATF3	RNF11
ATF3	RNF167
ATF3	RNMT
ATF3	RPF2
ATF3	RPIA
ATF3	RPS27L
ATF3	RRAGC
ATF3	RRP7A
ATF3	RSL24D1
ATF3	RUFY1
ATF3	SAFB2
ATF3	SARNP
ATF3	SDCBP
ATF3	SFT2D2

ATF3	SLC16A1
ATF3	SLC1A5
ATF3	SLC20A1
ATF3	SLC25A26
ATF3	SLC25A33
ATF3	SLC29A1
ATF3	SLC35A4
ATF3	SLC38A1
ATF3	SLC3A2
ATF3	SLC4A7
ATF3	SNHG15
ATF3	SNHG8
ATF3	SNN
ATF3	SPPL2A
ATF3	SPTLC1
ATF3	STAMBPL1
ATF3	STAT5A
ATF3	STAU1
ATF3	STRN4
ATF3	TAF9
ATF3	TANK
ATF3	TBC1D23
ATF3	TBC1D9B
ATF3	TCAIM
ATF3	TFEC
ATF3	TGS1
ATF3	THAP9-AS1
ATF3	TIMM44
ATF3	TMEM126A
ATF3	TMEM199
ATF3	TMEM267
ATF3	TMEM38B
ATF3	TNFAIP8
ATF3	TNFRSF18
ATF3	TNIP2
ATF3	TRAF1
ATF3	TRIAP1
ATF3	TRIP6
ATF3	TSPYL2
ATF3	TUBE1
ATF3	UBXN11
ATF3	UPF2
ATF3	USP12
ATF3	VPS41
ATF3	WARS2
ATF3	WDPCP
ATF3	WDR1
ATF3	WDR4
ATF3	WTAP
ATF3	XPC

ATF3	YBX3
ATF3	ZBTB10
ATF3	ZBTB32
ATF3	ZC3H12D
ATF3	ZC3H4
ATF3	ZFAS1
ATF3	ZFR
ATF3	ZHX1
ATF3	ZNF212
ATF3	ZNF830
ATF3	ZRANB2
ATF4	AARS
ATF4	ACSL1
ATF4	ADCY3
ATF4	ADPGK
ATF4	ALG13
ATF4	ANKRD11
ATF4	APLF
ATF4	ARPP19
ATF4	ASNS
ATF4	ATAD1
ATF4	ATF4
ATF4	ATF5
ATF4	ATG101
ATF4	ATG5
ATF4	ATXN2L
ATF4	BBC3
ATF4	BCAS2
ATF4	BET1
ATF4	BUD31
ATF4	C6orf48
ATF4	CARS
ATF4	CASC3
ATF4	CBWD2
ATF4	CCDC59
ATF4	CCL3
ATF4	CCND1
ATF4	CCSER2
ATF4	CD63
ATF4	CDK17
ATF4	CEBPB
ATF4	CEBPG
ATF4	CHAC1
ATF4	CLK1
ATF4	CSNK1D
ATF4	CSTF2T
ATF4	DAP
ATF4	DCTN4
ATF4	DDIT3
ATF4	DDIT4

ATF4	DDX19A
ATF4	DESI1
ATF4	DRAM1
ATF4	DUSP10
ATF4	ECSIT
ATF4	EIF1
ATF4	EIF2S2
ATF4	EIF4A2
ATF4	EIF4EBP1
ATF4	EMD
ATF4	ENC1
ATF4	EPB41
ATF4	EPRS
ATF4	ETF1
ATF4	FAM50A
ATF4	FTH1
ATF4	GARS
ATF4	GHITM
ATF4	GOLGA5
ATF4	GOT1
ATF4	GTF2H1
ATF4	HAX1
ATF4	HERPUD1
ATF4	HSPA9
ATF4	IARS
ATF4	IFRD1
ATF4	IGF2R
ATF4	IL23A
ATF4	IL6
ATF4	IMPDH2
ATF4	INPP5F
ATF4	KLF6
ATF4	KLF7
ATF4	KYNU
ATF4	LIAS
ATF4	LONP1
ATF4	LZTFL1
ATF4	MAFG
ATF4	MAP4
ATF4	MIR155HG
ATF4	MRM3
ATF4	MRPS18B
ATF4	MRRF
ATF4	MSTO1
ATF4	MTHFD2
ATF4	NAMPT
ATF4	NECAP1
ATF4	NFE2L1
ATF4	NFE2L2
ATF4	NFKB2

ATF4	NKRF
ATF4	NOL10
ATF4	NRBF2
ATF4	NRROS
ATF4	OGT
ATF4	PCK2
ATF4	PDAP1
ATF4	PEF1
ATF4	PHGDH
ATF4	PMAIP1
ATF4	PPP1R11
ATF4	PPP1R15A
ATF4	PRNP
ATF4	PRR14
ATF4	PTP4A1
ATF4	PVT1
ATF4	RAD23A
ATF4	RASSF1
ATF4	RBM7
ATF4	RPF1
ATF4	RPL13
ATF4	RPL13A
ATF4	RPL23
ATF4	RPL9
ATF4	RPLP2
ATF4	RPS21
ATF4	RPS29
ATF4	RPS6KB2
ATF4	RSL24D1
ATF4	SARS
ATF4	SBDS
ATF4	SCFD1
ATF4	SECISBP2
ATF4	SESN2
ATF4	SH2B3
ATF4	SHMT2
ATF4	SLC1A5
ATF4	SLC25A39
ATF4	SLC38A2
ATF4	SLC3A2
ATF4	SLC7A1
ATF4	SNHG15
ATF4	SNHG8
ATF4	SPG21
ATF4	STS
ATF4	STX4
ATF4	STX5
ATF4	SYTL1
ATF4	TARS
ATF4	TBL1X

ATF4	TFEC
ATF4	TIMM44
ATF4	TJP1
ATF4	TMEM167B
ATF4	TMEM267
ATF4	TMUB1
ATF4	TNFRSF10B
ATF4	TOX2
ATF4	TRAF1
ATF4	TRIB3
ATF4	TSC22D2
ATF4	TTC19
ATF4	TXNL1
ATF4	UBC
ATF4	UBE2B
ATF4	UBL3
ATF4	USP14
ATF4	USP37
ATF4	WARS
ATF4	WDR82
ATF4	XAB2
ATF4	XPOT
ATF4	YARS
ATF4	YTHDF1
ATF4	ZBED3
ATF4	ZFAND2A
ATF4	ZFAS1
ATF4	ZFPL1
ATF4	ZNF354A
ATF4	ZNF771
ATF4	ZNF839
ATF4	ZSWIM7
ATF5	AARS
ATF5	AFF2
ATF5	ARID5B
ATF5	C11orf24
ATF5	CARS
ATF5	CDK2AP2
ATF5	CEBPB
ATF5	CEBPG
ATF5	CHAC1
ATF5	CRLS1
ATF5	DDIT3
ATF5	DDIT4
ATF5	EEF2
ATF5	EIF1
ATF5	ELMSAN1
ATF5	GARS
ATF5	HERPUD1
ATF5	JOSD2

ATF5	LMO2
ATF5	MIR3681HG
ATF5	NFE2L1
ATF5	PCK2
ATF5	PICALM
ATF5	PLEKHM1
ATF5	PMVK
ATF5	PPDPF
ATF5	PXK
ATF5	RBM3
ATF5	SBDS
ATF5	SH2D3C
ATF5	SHMT2
ATF5	SLC16A3
ATF5	SLC1A5
ATF5	SLC3A2
ATF5	TAF10
ATF5	TMSB4X
ATF5	TRIB3
ATF5	USP36
ATF5	WARS
ATF5	WDR4
ATF5	ZNF354A
ATF6	AAK1
ATF6	AAMP
ATF6	AARS
ATF6	ABCB8
ATF6	ABCB9
ATF6	ABCC4
ATF6	ABCF3
ATF6	ACADVL
ATF6	ACBD3
ATF6	ACO2
ATF6	ACOX1
ATF6	ACSL3
ATF6	ACSL4
ATF6	ACSS1
ATF6	ACTR6
ATF6	ACY3
ATF6	ACYP2
ATF6	ADAM10
ATF6	ADAR
ATF6	ADCK1
ATF6	ADI1
ATF6	ADIPOR1
ATF6	AFF4
ATF6	AGA
ATF6	AGPAT3
ATF6	AK3
ATF6	AKR1A1

ATF6	AKT1S1
ATF6	ALDH18A1
ATF6	ALDH1L2
ATF6	ALG1
ATF6	ALG10B
ATF6	ALG11
ATF6	ALG12
ATF6	ALG13
ATF6	ALG14
ATF6	ALG2
ATF6	ALG3
ATF6	ANAPC13
ATF6	ANAPC5
ATF6	ANKHD1
ATF6	ANKRD28
ATF6	ANKRD37
ATF6	ANKZF1
ATF6	ANO9
ATF6	ANXA5
ATF6	ANXA6
ATF6	ANXA7
ATF6	AP3D1
ATF6	APH1B
ATF6	APMAP
ATF6	AQP3
ATF6	AREG
ATF6	ARF4
ATF6	ARFGAP1
ATF6	ARFGAP3
ATF6	ARFGEF2
ATF6	ARFIP2
ATF6	ARHGAP18
ATF6	ARHGAP27
ATF6	ARID3A
ATF6	ARL1
ATF6	ARL4A
ATF6	ARL5A
ATF6	ARMCX3
ATF6	ARRDC3
ATF6	ARSA
ATF6	ASAH1
ATF6	ASXL1
ATF6	ATF6
ATF6	ATG101
ATF6	ATP13A1
ATF6	ATP2A2
ATF6	ATP2A3
ATF6	ATP6AP1
ATF6	ATP6AP2
ATF6	ATP6V0A1

ATF6	ATP6V0B
ATF6	ATP6V0E1
ATF6	ATP8B2
ATF6	ATRAID
ATF6	AURKAIP1
ATF6	B3GALNT1
ATF6	B3GNTL1
ATF6	B4GALT1
ATF6	B4GALT3
ATF6	B4GALT4
ATF6	BAG3
ATF6	BAZ2A
ATF6	BCAP31
ATF6	BCKDK
ATF6	BET1
ATF6	BET1L
ATF6	BEX5
ATF6	BHLHE41
ATF6	BIRC2
ATF6	BLM
ATF6	BLVRA
ATF6	BORCS5
ATF6	BORCS6
ATF6	BRAP
ATF6	BSDC1
ATF6	BSG
ATF6	BTAF1
ATF6	BTG2
ATF6	C11orf24
ATF6	C11orf80
ATF6	C15orf61
ATF6	C17orf107
ATF6	C18orf32
ATF6	C19orf24
ATF6	C19orf70
ATF6	C19orf81
ATF6	C1GALT1C1
ATF6	C21orf2
ATF6	C2orf88
ATF6	C4orf3
ATF6	C4orf48
ATF6	C5orf15
ATF6	C6orf52
ATF6	CA11
ATF6	CALCOCO1
ATF6	CALR
ATF6	CALU
ATF6	CANX
ATF6	CASP3
ATF6	CASP4

ATF6	CAST
ATF6	CBFA2T2
ATF6	CBFA2T3
ATF6	CBWD1
ATF6	CBWD5
ATF6	CBX4
ATF6	CBX7
ATF6	CCDC144A
ATF6	CCDC167
ATF6	CCDC186
ATF6	CCDC47
ATF6	CCL25
ATF6	CCM2
ATF6	CCNC
ATF6	CCNL1
ATF6	CCPG1
ATF6	CCR10
ATF6	CCS
ATF6	CCT8
ATF6	CD164
ATF6	CD320
ATF6	CD38
ATF6	CD55
ATF6	CD63
ATF6	CDC37
ATF6	CDIP1
ATF6	CDK2AP2
ATF6	CDKN1A
ATF6	CDKN1B
ATF6	CDR2
ATF6	CDV3
ATF6	CEBPB
ATF6	CERS2
ATF6	CFL2
ATF6	CHCHD2
ATF6	CHID1
ATF6	CHPF
ATF6	CHPF2
ATF6	CHRNB1
ATF6	CHST11
ATF6	CHST12
ATF6	CHST2
ATF6	CHUK
ATF6	CINP
ATF6	CISD2
ATF6	CITED2
ATF6	CKAP4
ATF6	CLASP2
ATF6	CLCC1
ATF6	CLCN7

ATF6	CLDN14
ATF6	CLEC2B
ATF6	CLINT1
ATF6	CLPTM1
ATF6	CLPTM1L
ATF6	CNBP
ATF6	CNOT11
ATF6	CNPY2
ATF6	COASY
ATF6	COG3
ATF6	COMMD1
ATF6	COMMD3
ATF6	COPB2
ATF6	COPE
ATF6	COPG1
ATF6	COPZ1
ATF6	COQ10B
ATF6	COQ6
ATF6	COX6A1
ATF6	CPEB4
ATF6	CRADD
ATF6	CREB3
ATF6	CRELD2
ATF6	CRLS1
ATF6	CSAD
ATF6	CSGALNACT2
ATF6	CSNK1E
ATF6	CTBS
ATF6	CTHRC1
ATF6	CTNNA1
ATF6	CTSA
ATF6	CTSB
ATF6	CTSD
ATF6	CUL1
ATF6	CUTA
ATF6	CYB561D2
ATF6	CYB5R1
ATF6	CYCS
ATF6	CYTIP
ATF6	DAD1
ATF6	DAP
ATF6	DAPL1
ATF6	DCTN3
ATF6	DDB1
ATF6	DDIT4
ATF6	DDX31
ATF6	DDX55
ATF6	DEDD2
ATF6	DEF6
ATF6	DENND1B

ATF6	DENND6B
ATF6	DERL1
ATF6	DERL3
ATF6	DESI1
ATF6	DHDDS
ATF6	DHRS7
ATF6	DHRS9
ATF6	DNAJA2
ATF6	DNAJB11
ATF6	DNAJB12
ATF6	DNAJB9
ATF6	DNAJC1
ATF6	DNAJC10
ATF6	DNAJC12
ATF6	DNAJC25
ATF6	DNAJC3
ATF6	DNAJC4
ATF6	DNASE2
ATF6	DNHD1
ATF6	DOHH
ATF6	DPAGT1
ATF6	DPM2
ATF6	DPM3
ATF6	DPP7
ATF6	DUSP12
ATF6	DUSP22
ATF6	DUSP5
ATF6	DYNLL2
ATF6	DYRK4
ATF6	EAF2
ATF6	EBAG9
ATF6	EDEM1
ATF6	EDEM2
ATF6	EDF1
ATF6	EEF1D
ATF6	EIF2A
ATF6	EIF3A
ATF6	EIF3K
ATF6	ELK3
ATF6	ELL
ATF6	ELL2
ATF6	ELMOD2
ATF6	EMC10
ATF6	EMC2
ATF6	EMC3
ATF6	EMC4
ATF6	ENTPD1
ATF6	ENTPD4
ATF6	EPHX1
ATF6	EPRS

ATF6	ERC1
ATF6	ERGIC1
ATF6	ERGIC2
ATF6	ERGIC3
ATF6	ERLEC1
ATF6	ERN1
ATF6	ERO1A
ATF6	ERO1B
ATF6	ERP29
ATF6	ERP44
ATF6	ESR2
ATF6	ESYT2
ATF6	ETNK1
ATF6	EVI2B
ATF6	F2RL3
ATF6	FAM13A
ATF6	FAM160B2
ATF6	FAM174A
ATF6	FAM177A1
ATF6	FAM214A
ATF6	FAM3C
ATF6	FAM49A
ATF6	FAM91A1
ATF6	FAM98A
ATF6	FANCE
ATF6	FASTK
ATF6	FBXL15
ATF6	FBXO11
ATF6	FBXO16
ATF6	FBXW7
ATF6	FDX1
ATF6	FIS1
ATF6	FKBP11
ATF6	FKBP14
ATF6	FKBP2
ATF6	FN3KRP
ATF6	FNDC3A
ATF6	FNDC3B
ATF6	FNTB
ATF6	FOS
ATF6	FOXN3
ATF6	FOXO3
ATF6	FRA10AC1
ATF6	FRZB
ATF6	FTL
ATF6	FTSJ1
ATF6	FUCA2
ATF6	FUNDC2
ATF6	FUT8
ATF6	FUZ

ATF6	G3BP2
ATF6	GABARAP
ATF6	GADD45A
ATF6	GADD45B
ATF6	GALK2
ATF6	GALNT2
ATF6	GAN
ATF6	GAS6
ATF6	GCSH
ATF6	GEMIN7
ATF6	GFPT1
ATF6	GGH
ATF6	GHITM
ATF6	GIGYF2
ATF6	GIT1
ATF6	GLCCI1
ATF6	GLG1
ATF6	GLIPR1
ATF6	GLRX
ATF6	GLS
ATF6	GLT8D1
ATF6	GMPPA
ATF6	GMPPB
ATF6	GNL3
ATF6	GNPTG
ATF6	GOLGA2
ATF6	GOLGA4
ATF6	GOLGA5
ATF6	GOLGB1
ATF6	GOLPH3
ATF6	GOLPH3L
ATF6	GOLT1B
ATF6	GORASP2
ATF6	GOSR2
ATF6	GPCPD1
ATF6	GPLD1
ATF6	GPR108
ATF6	GPR89A
ATF6	GPR89B
ATF6	GPS2
ATF6	GPX7
ATF6	GRINA
ATF6	GRK6
ATF6	GRN
ATF6	GTF2B
ATF6	GUK1
ATF6	GUSB
ATF6	GYS1
ATF6	H1FX
ATF6	HAGH

ATF6	HARBI1
ATF6	HAX1
ATF6	HBP1
ATF6	HCST
ATF6	HDLBP
ATF6	HECW2
ATF6	HERPUD1
ATF6	HEXA
ATF6	HEXB
ATF6	HIBCH
ATF6	HIST1H1C
ATF6	HIST1H2BN
ATF6	HLA-A
ATF6	HLA-B
ATF6	HLA-DQA2
ATF6	HLA-DQB1
ATF6	HLA-DRB5
ATF6	HLA-E
ATF6	HM13
ATF6	HOOK2
ATF6	HOXB2
ATF6	HOXB7
ATF6	HPCAL1
ATF6	HSD17B11
ATF6	HSP90B1
ATF6	HSPA13
ATF6	HSPA5
ATF6	IBTK
ATF6	ICAM1
ATF6	ICAM2
ATF6	ICAM3
ATF6	IDH2
ATF6	IDH3G
ATF6	IER2
ATF6	IER3IP1
ATF6	IFI27L2
ATF6	IFNAR1
ATF6	IFNAR2
ATF6	IFRD1
ATF6	IFT20
ATF6	IGLL5
ATF6	IGSF8
ATF6	IKBIP
ATF6	IL10RA
ATF6	IL15RA
ATF6	IL16
ATF6	IMP3
ATF6	IMPAD1
ATF6	IMPDH1
ATF6	ING2

ATF6	INSIG1
ATF6	INSIG2
ATF6	IPPK
ATF6	IQGAP2
ATF6	IRAK4
ATF6	IRF1
ATF6	IRF4
ATF6	IRF7
ATF6	ISCU
ATF6	ISG20
ATF6	ISL2
ATF6	ISOC2
ATF6	ITM2B
ATF6	ITM2C
ATF6	ITPR2
ATF6	JCHAIN
ATF6	JMJD6
ATF6	JMJD8
ATF6	JMY
ATF6	JUN
ATF6	KCNK6
ATF6	KCNMA1
ATF6	KCNN3
ATF6	KCNN4
ATF6	KDELR1
ATF6	KDELR2
ATF6	KDELR3
ATF6	KDM4C
ATF6	KIAA2013
ATF6	KIF5C
ATF6	KIZ
ATF6	KLC4
ATF6	KLF13
ATF6	KLHDC10
ATF6	KLHDC2
ATF6	KMO
ATF6	KRTCAP2
ATF6	KTI12
ATF6	L3MBTL2
ATF6	LAMTOR2
ATF6	LAPTM4A
ATF6	LARP1B
ATF6	LCORL
ATF6	LEPROTL1
ATF6	LGALS1
ATF6	LINC01485
ATF6	LMAN1
ATF6	LNX1
ATF6	LRIF1
ATF6	LRIG1

ATF6	LRP10
ATF6	LRRC59
ATF6	LRRC8D
ATF6	LSP1
ATF6	LTBP3
ATF6	LTN1
ATF6	LUZP1
ATF6	LYSMD3
ATF6	MAGED1
ATF6	MAGT1
ATF6	MAN1A1
ATF6	MAN1A2
ATF6	MANF
ATF6	MAPK9
ATF6	MARCH6
ATF6	MBNL2
ATF6	MBTPS1
ATF6	MCEE
ATF6	MCFD2
ATF6	MCL1
ATF6	MCUR1
ATF6	ME2
ATF6	MED20
ATF6	MEI1
ATF6	MFF
ATF6	MGAT1
ATF6	MIA2
ATF6	MIA3
ATF6	MIER2
ATF6	MIF
ATF6	MIR193BHG
ATF6	MIR22HG
ATF6	MIR4435-2HG
ATF6	MLEC
ATF6	MLX
ATF6	MMACHC
ATF6	MOGS
ATF6	MON2
ATF6	MORF4L2
ATF6	MOSPD3
ATF6	MPC1
ATF6	MPHOSPH8
ATF6	MPI
ATF6	MPRIP
ATF6	MPZL1
ATF6	MRFAP1L1
ATF6	MRPL36
ATF6	MRPL40
ATF6	MRPL54
ATF6	MRPL55

ATF6	MRPS31
ATF6	MRPS33
ATF6	MSTO1
ATF6	MTDH
ATF6	MTFR1
ATF6	MTRF1L
ATF6	MVP
ATF6	MXD4
ATF6	MYCBP2
ATF6	MYDGF
ATF6	MYL6
ATF6	MYLPF
ATF6	MZB1
ATF6	NAB1
ATF6	NACA
ATF6	NADK
ATF6	NAGK
ATF6	NAGLU
ATF6	NANS
ATF6	NAPA
ATF6	NARF
ATF6	NBR1
ATF6	NCDN
ATF6	NCK1
ATF6	NCLN
ATF6	NDEL1
ATF6	NDFIP1
ATF6	NDFIP2
ATF6	NDUFA1
ATF6	NDUFA11
ATF6	NDUFA13
ATF6	NDUFA4
ATF6	NDUFA7
ATF6	NDUFA9
ATF6	NDUFB4
ATF6	NDUFB6
ATF6	NDUFS5
ATF6	NDUFS6
ATF6	NDUFS8
ATF6	NDUFV3
ATF6	NEAT1
ATF6	NEDD4L
ATF6	NEDD9
ATF6	NENF
ATF6	NET1
ATF6	NETO2
ATF6	NEU1
ATF6	NFE2L3
ATF6	NFKBID
ATF6	NGLY1

ATF6	NIPA2
ATF6	NMD3
ATF6	NOMO3
ATF6	NOP10
ATF6	NOP58
ATF6	NRBF2
ATF6	NRDC
ATF6	NRSN2
ATF6	NSG1
ATF6	NSMCE3
ATF6	NT5C3A
ATF6	NT5DC2
ATF6	NUCB1
ATF6	NUCB2
ATF6	NUPL2
ATF6	NUS1
ATF6	NUTM2B-AS1
ATF6	ODF3B
ATF6	OGFOD2
ATF6	OGFOD3
ATF6	ORMDL1
ATF6	ORMDL2
ATF6	OS9
ATF6	OSBPL7
ATF6	OST4
ATF6	OSTC
ATF6	OTUD1
ATF6	P4HB
ATF6	P4HTM
ATF6	PAGR1
ATF6	PAIP2B
ATF6	PAPSS1
ATF6	PARD6A
ATF6	PARM1
ATF6	PARP4
ATF6	PBXIP1
ATF6	PCMT1
ATF6	PDE4B
ATF6	PDE4DIP
ATF6	PDHA1
ATF6	PDIA3
ATF6	PDIA4
ATF6	PDIA5
ATF6	PDIA6
ATF6	PDK1
ATF6	PDK2
ATF6	PDXK
ATF6	PEBP1
ATF6	PELO
ATF6	PEPD

ATF6	PET100
ATF6	PGM1
ATF6	PGM3
ATF6	PGRMC2
ATF6	PHC3
ATF6	PHF1
ATF6	PHF19
ATF6	PHPT1
ATF6	PIGP
ATF6	PIK3CD-AS2
ATF6	PIK3CG
ATF6	PIM2
ATF6	PIR
ATF6	PJA2
ATF6	PKHD1L1
ATF6	PLD3
ATF6	PLD4
ATF6	PLEKHN1
ATF6	PLOD1
ATF6	PLOD3
ATF6	PLPP5
ATF6	PLTP
ATF6	PMEPA1
ATF6	PNKP
ATF6	PNOC
ATF6	PODXL2
ATF6	POGLUT1
ATF6	POLR3D
ATF6	POMC
ATF6	POP5
ATF6	POR
ATF6	POU2AF1
ATF6	PPCDC
ATF6	PPIB
ATF6	PPIL2
ATF6	PPP2CB
ATF6	PPP2R5B
ATF6	PPP2R5C
ATF6	PPP6R2
ATF6	PRCP
ATF6	PRDM1
ATF6	PRDM15
ATF6	PRDX2
ATF6	PRDX4
ATF6	PREB
ATF6	PRKAG1
ATF6	PRKCSH
ATF6	PRKD2
ATF6	PRKD3
ATF6	PRR14

ATF6	PRR5
ATF6	PRRC1
ATF6	PSMC4
ATF6	PSMC5
ATF6	PSMD12
ATF6	PSMD8
ATF6	PSME1
ATF6	PTP4A2
ATF6	PTP4A3
ATF6	PTPN1
ATF6	PTPN23
ATF6	PTPRN2
ATF6	PTRH2
ATF6	PUS10
ATF6	PUS7L
ATF6	PYGB
ATF6	QPCT
ATF6	QPRT
ATF6	QSOX1
ATF6	QTRT1
ATF6	QTRT2
ATF6	R3HDM1
ATF6	RAB18
ATF6	RAB1A
ATF6	RAB21
ATF6	RAB24
ATF6	RAB2A
ATF6	RAB35
ATF6	RAB3GAP1
ATF6	RAB40B
ATF6	RABAC1
ATF6	RABEPK
ATF6	RAD54B
ATF6	RAD9A
ATF6	RAP1A
ATF6	RARS2
ATF6	RBM19
ATF6	RBM26
ATF6	RBM33
ATF6	RBM47
ATF6	RBPJ
ATF6	RCL1
ATF6	REEP3
ATF6	REEP5
ATF6	RELT
ATF6	RER1
ATF6	REXO2
ATF6	RGCC
ATF6	RGS1
ATF6	RGS16

ATF6	RGS2
ATF6	RHBDD1
ATF6	RING1
ATF6	RNASEH1
ATF6	RNF103
ATF6	RNF11
ATF6	RNF141
ATF6	RNF149
ATF6	RNF168
ATF6	RNF169
ATF6	RNF213
ATF6	RNF32
ATF6	RPL10
ATF6	RPL15
ATF6	RPL3
ATF6	RPL36AL
ATF6	RPLP0
ATF6	RPLP1
ATF6	RPN1
ATF6	RPN2
ATF6	RPS10
ATF6	RPS19
ATF6	RPS28
ATF6	RPS4X
ATF6	RPS6KB2
ATF6	RRAGD
ATF6	RRBP1
ATF6	RREB1
ATF6	RSU1
ATF6	RTN2
ATF6	RTN3
ATF6	RUNX1
ATF6	RWDD2B
ATF6	S100A11
ATF6	SAP18
ATF6	SAR1A
ATF6	SAR1B
ATF6	SARAF
ATF6	SAT1
ATF6	SCAMP2
ATF6	SCAMP3
ATF6	SCFD1
ATF6	SCPEP1
ATF6	SCYL1
ATF6	SDF2L1
ATF6	SDF4
ATF6	SEC11C
ATF6	SEC13
ATF6	SEC14L1
ATF6	SEC22B

ATF6	SEC23B
ATF6	SEC24A
ATF6	SEC24D
ATF6	SEC31A
ATF6	SEC61A1
ATF6	SEC61B
ATF6	SEC61G
ATF6	SEC62
ATF6	SEC63
ATF6	SEL1L
ATF6	SEL1L3
ATF6	SELPLG
ATF6	SERF2
ATF6	SERGEF
ATF6	SERP1
ATF6	SETD3
ATF6	SF3A3
ATF6	SF3B2
ATF6	SF3B5
ATF6	SFMBT2
ATF6	SGK1
ATF6	SGK3
ATF6	SH3GLB1
ATF6	SHC1
ATF6	SHROOM1
ATF6	SIL1
ATF6	SIPA1L3
ATF6	SIRT2
ATF6	SKAP1
ATF6	SLAMF7
ATF6	SLC10A7
ATF6	SLC11A2
ATF6	SLC15A3
ATF6	SLC16A3
ATF6	SLC17A9
ATF6	SLC1A4
ATF6	SLC1A5
ATF6	SLC25A28
ATF6	SLC25A39
ATF6	SLC25A4
ATF6	SLC25A44
ATF6	SLC25A45
ATF6	SLC30A5
ATF6	SLC33A1
ATF6	SLC35A2
ATF6	SLC35A3
ATF6	SLC35B1
ATF6	SLC35C1
ATF6	SLC35C2
ATF6	SLC35F2

ATF6	SLC38A10
ATF6	SLC38A2
ATF6	SLC38A9
ATF6	SLC39A11
ATF6	SLC39A7
ATF6	SLC39A8
ATF6	SLC3A2
ATF6	SLC44A1
ATF6	SLC7A7
ATF6	SLCO3A1
ATF6	SMCHD1
ATF6	SMDT1
ATF6	SMPD1
ATF6	SMUG1
ATF6	SNAI3-AS1
ATF6	SND1
ATF6	SND1-IT1
ATF6	SNHG9
ATF6	SNRPN
ATF6	SNU13
ATF6	SNX13
ATF6	SOCS3
ATF6	SP140L
ATF6	SPAG7
ATF6	SPAG9
ATF6	SPATC1L
ATF6	SPATS2
ATF6	SPCS1
ATF6	SPIN2B
ATF6	SPINT2
ATF6	SPN
ATF6	SPNS1
ATF6	SPOP
ATF6	SPTSSA
ATF6	SQSTM1
ATF6	SRGN
ATF6	SRM
ATF6	SRP19
ATF6	SRP54
ATF6	SRP68
ATF6	SRP72
ATF6	SRPRA
ATF6	SRSF5
ATF6	SSFA2
ATF6	SSR1
ATF6	SSR3
ATF6	SSR4
ATF6	SSSCA1
ATF6	SSU72
ATF6	ST6GAL1

ATF6	ST6GALNAC4
ATF6	STARD10
ATF6	STK17B
ATF6	STK4
ATF6	STT3A
ATF6	STT3B
ATF6	STX11
ATF6	STX5
ATF6	STXBP6
ATF6	SUB1
ATF6	SUCO
ATF6	SURF4
ATF6	SYNE3
ATF6	SYNGR1
ATF6	SYNRG
ATF6	SYTL1
ATF6	SYVN1
ATF6	TAF8
ATF6	TBC1D23
ATF6	TBL2
ATF6	TCF25
ATF6	TCF4
ATF6	TCIRG1
ATF6	TCTA
ATF6	TES
ATF6	TFG
ATF6	THAP11
ATF6	THAP2
ATF6	THAP7
ATF6	THEMIS2
ATF6	THTPA
ATF6	TIMM17A
ATF6	TIMP1
ATF6	TM9SF2
ATF6	TM9SF3
ATF6	TM9SF4
ATF6	TMBIM6
ATF6	TMCC1
ATF6	TMCO1
ATF6	TMED10
ATF6	TMED2
ATF6	TMED3
ATF6	TMED4
ATF6	TMED7
ATF6	TMED9
ATF6	TMEM120B
ATF6	TMEM147
ATF6	TMEM161B
ATF6	TMEM161B-AS1
ATF6	TMEM165

ATF6	TMEM167A
ATF6	TMEM184B
ATF6	TMEM214
ATF6	TMEM219
ATF6	TMEM230
ATF6	TMEM248
ATF6	TMEM258
ATF6	TMEM263
ATF6	TMEM30A
ATF6	TMEM33
ATF6	TMEM39A
ATF6	TMEM59
ATF6	TMEM70
ATF6	TMEM9
ATF6	TMF1
ATF6	TNFAIP3
ATF6	TNFRSF17
ATF6	TNFSF8
ATF6	TNPO2
ATF6	TOLLIP
ATF6	TOR1AIP2
ATF6	TOR3A
ATF6	TPD52
ATF6	TPD52L2
ATF6	TPST2
ATF6	TPT1
ATF6	TRAF3
ATF6	TRAM1
ATF6	TRAM2
ATF6	TRANK1
ATF6	TRAPPC2L
ATF6	TRAPPC6A
ATF6	TRIB1
ATF6	TRIM27
ATF6	TRIM33
ATF6	TRMT44
ATF6	TRPM4
ATF6	TRPT1
ATF6	TSC22D3
ATF6	TSC22D4
ATF6	TSPAN13
ATF6	TTC3
ATF6	TWSG1
ATF6	TXLNA
ATF6	TXNDC11
ATF6	TXNDC15
ATF6	TXNDC5
ATF6	UAP1
ATF6	UBA5
ATF6	UBA6-AS1

ATF6	UBC
ATF6	UBE2D3
ATF6	UBE2H
ATF6	UBE2J1
ATF6	UBE2Q1
ATF6	UBE3A
ATF6	UBL5
ATF6	UBQLN1
ATF6	UBXN4
ATF6	UFC1
ATF6	UFL1
ATF6	UFM1
ATF6	UFSP2
ATF6	UNC50
ATF6	UNKL
ATF6	UPF2
ATF6	UQCC2
ATF6	UQCR11
ATF6	UQCRH
ATF6	UROS
ATF6	USO1
ATF6	USP21
ATF6	USP24
ATF6	USP48
ATF6	UTP11
ATF6	UTP14A
ATF6	VARS2
ATF6	VCP
ATF6	VDAC2
ATF6	VDR
ATF6	VKORC1
ATF6	VKORC1L1
ATF6	VOPP1
ATF6	WDR43
ATF6	WDR89
ATF6	WDR91
ATF6	WIPI1
ATF6	WNT10A
ATF6	WSB2
ATF6	WWOX
ATF6	XRRA1
ATF6	YIF1A
ATF6	YIF1B
ATF6	YIPF1
ATF6	YIPF2
ATF6	YIPF3
ATF6	YIPF5
ATF6	YIPF6
ATF6	YKT6
ATF6	YPEL5

ATF6	ZBP1
ATF6	ZBTB38
ATF6	ZDHHC4
ATF6	ZDHHC7
ATF6	ZFAND2A
ATF6	ZFAT
ATF6	ZFP36L2
ATF6	ZFYVE27
ATF6	ZMYND11
ATF6	ZNF264
ATF6	ZNF275
ATF6	ZNF3
ATF6	ZNF37A
ATF6	ZNF436
ATF6	ZNF593
ATF6	ZNF639
ATF6	ZNF691
ATF6	ZNF706
ATF6	ZNF71
ATF6	ZNF841
ATF6	ZNF844
ATF6	ZNHIT1
ATF6B	C5orf51
ATF6B	CBX8
ATF6B	CEP120
ATF6B	CHSY1
ATF6B	CLCN3
ATF6B	CTHRC1
ATF6B	CTNNA1
ATF6B	EFNA3
ATF6B	FAM78A
ATF6B	GCN1
ATF6B	LEKR1
ATF6B	LPIN1
ATF6B	MTHFR
ATF6B	POPDC2
ATF6B	QKI
ATF6B	RPS29
ATF6B	SLC16A3
ATF6B	TM9SF1
ATF6B	ZFX
BACH1	ABHD5
BACH1	ABHD6
BACH1	ANG
BACH1	ANKHD1
BACH1	ANKRD12
BACH1	ATF3
BACH1	ATP6V1F
BACH1	B9D1
BACH1	BACH1

BACH1	BNIP3L
BACH1	CCDC50
BACH1	CCR7
BACH1	CD63
BACH1	CHD2
BACH1	CHD6
BACH1	CLCN6
BACH1	COCH
BACH1	CSNK2A1
BACH1	DDI2
BACH1	DNAJB12
BACH1	EBI3
BACH1	EIF1AX
BACH1	ERN1
BACH1	FLCN
BACH1	FMR1
BACH1	FOSL1
BACH1	GNL3L
BACH1	GOLIM4
BACH1	GPER1
BACH1	GPR18
BACH1	GRN
BACH1	HEXA
BACH1	IER3
BACH1	IFI30
BACH1	IFRD2
BACH1	KCNG1
BACH1	KLF13
BACH1	KMT2E
BACH1	KPNA4
BACH1	MAPK6
BACH1	MIR155HG
BACH1	NEAT1
BACH1	NUDT19
BACH1	PPP3CA
BACH1	PRKAG2
BACH1	PVT1
BACH1	RAB40C
BACH1	RBBP6
BACH1	RBM15B
BACH1	RBM47
BACH1	RENBP
BACH1	RIN3
BACH1	RNF115
BACH1	SEMA7A
BACH1	SIRT2
BACH1	SLC44A1
BACH1	SPRED2
BACH1	SQSTM1
BACH1	STK4

BACH1	STX6
BACH1	SUN2
BACH1	SYNRG
BACH1	TCAF1
BACH1	TMEM214
BACH1	TNFAIP3
BACH1	TP53INP1
BACH1	TRAF1
BACH1	WDR81
BACH1	XIAP
BACH2	BACH2
BACH2	CENPE
BACH2	MARCH1
BACH2	RIMKLB
BACH2	SYT11
BATF	AHR
BATF	BCL2A1
BATF	CCL4
BATF	DNAJB5
BATF	E4F1
BATF	ETF1
BATF	GPAM
BATF	GTF2H1
BATF	GTF3C2
BATF	NFKBID
BATF	PLK3
BATF	PUM3
BATF	RCL1
BATF	RRP1B
BATF	WDR4
BATF	ZNF593
BATF	ZNF711
BATF3	AARS
BATF3	ABCE1
BATF3	ACSL1
BATF3	ANKRD11
BATF3	ANP32B
BATF3	ASNS
BATF3	ATF5
BATF3	ATP1B3
BATF3	ATXN1
BATF3	B3GLCT
BATF3	BUD31
BATF3	C1orf174
BATF3	CAMK2B
BATF3	CAMKK1
BATF3	CCND2
BATF3	CD44
BATF3	CDIPT
BATF3	CEBPB

BATF3	CSGALNACT2
BATF3	CYCS
BATF3	DDIT3
BATF3	DDIT4
BATF3	DDX49
BATF3	DGAT2
BATF3	DRAM1
BATF3	EIF1
BATF3	EIF2S2
BATF3	EIF4EBP1
BATF3	ETF1
BATF3	FAM110A
BATF3	FIGNL1
BATF3	FOXP1
BATF3	GARS
BATF3	GART
BATF3	GCFC2
BATF3	GHITM
BATF3	GLRX2
BATF3	GNL2
BATF3	GOT1
BATF3	GPRC5C
BATF3	GSPT1
BATF3	HNRNPU
BATF3	HSP90AB1
BATF3	HSPA9
BATF3	HSPBP1
BATF3	HSPD1
BATF3	ID3
BATF3	LARS
BATF3	LDHA
BATF3	LIG3
BATF3	LIN9
BATF3	LSM2
BATF3	MAGOH
BATF3	MAML2
BATF3	MCU
BATF3	MIF
BATF3	MMAB
BATF3	N6AMT1
BATF3	NABP1
BATF3	NEDD1
BATF3	NEDD4L
BATF3	NFE2L1
BATF3	NOLC1
BATF3	NOTCH2
BATF3	NUP133
BATF3	NUP214
BATF3	ODC1
BATF3	OXSR1

BATF3	PAK1IP1
BATF3	PARVB
BATF3	PCK2
BATF3	PDAP1
BATF3	PHGDH
BATF3	PKM
BATF3	PRELID3B
BATF3	PRMT1
BATF3	PRR5
BATF3	PRRC2A
BATF3	PSMD14
BATF3	PUM3
BATF3	RAD23A
BATF3	RAD9A
BATF3	RAN
BATF3	RCN1
BATF3	RNMT
BATF3	RRP1B
BATF3	RUNX3
BATF3	RUVBL1
BATF3	SARS
BATF3	SH3RF1
BATF3	SHMT2
BATF3	SKAP1
BATF3	SLC1A5
BATF3	SLC37A1
BATF3	SLC3A2
BATF3	SLC6A9
BATF3	SMKR1
BATF3	SNHG15
BATF3	SNX12
BATF3	SRSF3
BATF3	SSRP1
BATF3	TBL3
BATF3	TES
BATF3	THOC1
BATF3	TMEM147
BATF3	TMEM147-AS1
BATF3	TRMT61A
BATF3	TWISTNB
BATF3	UBA6
BATF3	UBE2B
BATF3	WARS
BATF3	WDR82
BATF3	ZFP91
BATF3	ZFYVE9
BATF3	ZNF470
BATF3	ZNF593
BATF3	ZNFX1
BCL11A	ABCD4

BCL11A	ABI2
BCL11A	ACSM3
BCL11A	ACTB
BCL11A	ACTR2
BCL11A	ADAM28
BCL11A	ADD3
BCL11A	ADNP
BCL11A	AFF3
BCL11A	AFTPH
BCL11A	AGA
BCL11A	AHNAK
BCL11A	AKAP13
BCL11A	AKAP7
BCL11A	AKNA
BCL11A	ALOX5
BCL11A	ALOX5AP
BCL11A	AMFR
BCL11A	ANGPT2
BCL11A	ANKRD40
BCL11A	ANKRD44
BCL11A	AP1M1
BCL11A	AP4E1
BCL11A	APBB1IP
BCL11A	ARF6
BCL11A	ARHGAP17
BCL11A	ARHGAP22
BCL11A	ARHGAP30
BCL11A	ARHGAP9
BCL11A	ARHGEF7
BCL11A	ARID1A
BCL11A	ARID4B
BCL11A	ARL6IP5
BCL11A	ARPC1B
BCL11A	ARPC3
BCL11A	ARPC4
BCL11A	ASAP1
BCL11A	ASB16-AS1
BCL11A	ASB7
BCL11A	ASPH
BCL11A	ATF7IP
BCL11A	ATP6V1E2
BCL11A	B4GALT1
BCL11A	BACH2
BCL11A	BANK1
BCL11A	BAZ2A
BCL11A	BBX
BCL11A	BCL11A
BCL11A	BEND5
BCL11A	BIRC3
BCL11A	BLCAP

BCL11A	BLK
BCL11A	BLOC1S5
BCL11A	BMF
BCL11A	BNIP3L
BCL11A	BORCS5
BCL11A	BRK1
BCL11A	BROX
BCL11A	BRWD3
BCL11A	BTG1
BCL11A	BTK
BCL11A	C16orf54
BCL11A	C16orf74
BCL11A	C5orf51
BCL11A	C6orf62
BCL11A	CALHM2
BCL11A	CAPZA1
BCL11A	CASP2
BCL11A	CASP4
BCL11A	CBFA2T3
BCL11A	CBL
BCL11A	CBLB
BCL11A	CBWD3
BCL11A	CCDC107
BCL11A	CCDC6
BCL11A	CCDC93
BCL11A	CCNI
BCL11A	CCR6
BCL11A	CCZ1
BCL11A	CD180
BCL11A	CD19
BCL11A	CD22
BCL11A	CD37
BCL11A	CD48
BCL11A	CD52
BCL11A	CD72
BCL11A	CD74
BCL11A	CD79B
BCL11A	CDC42
BCL11A	CDC42SE1
BCL11A	CDK13
BCL11A	CDK14
BCL11A	CFL2
BCL11A	CHI3L2
BCL11A	CHMP1B
BCL11A	CKMT2-AS1
BCL11A	CNEP1R1
BCL11A	CNN2
BCL11A	CNOT8
BCL11A	CNR2
BCL11A	COMMD6

BCL11A	CORO1A
BCL11A	CPD
BCL11A	CPNE5
BCL11A	CPSF2
BCL11A	CREB1
BCL11A	CREBRF
BCL11A	CRIP3
BCL11A	CS
BCL11A	CSK
BCL11A	CSNK1A1
BCL11A	CTDNEP1
BCL11A	CTDSP1
BCL11A	CTDSP2
BCL11A	CTNNB1
BCL11A	CTNND1
BCL11A	CTSS
BCL11A	CTSZ
BCL11A	CXCR4
BCL11A	CXCR5
BCL11A	CXXC5
BCL11A	CYB561A3
BCL11A	CYBB
BCL11A	CYFIP2
BCL11A	CYP20A1
BCL11A	CYTIP
BCL11A	DAAM1
BCL11A	DALRD3
BCL11A	DAPP1
BCL11A	DAZAP2
BCL11A	DBNL
BCL11A	DCLRE1C
BCL11A	DCUN1D1
BCL11A	DDB1
BCL11A	DEDD
BCL11A	DEF8
BCL11A	DENND3
BCL11A	DGKZ
BCL11A	DLEU2
BCL11A	DMXL1
BCL11A	DOCK8
BCL11A	DPEP2
BCL11A	DPYSL2
BCL11A	DSTYK
BCL11A	DYRK1A
BCL11A	E2F3
BCL11A	EEF1A1
BCL11A	EIF2S3
BCL11A	EIF3H
BCL11A	EIF3L
BCL11A	EIF4EBP2

BCL11A	ELF1
BCL11A	ELMSAN1
BCL11A	ELOVL5
BCL11A	EMILIN2
BCL11A	EPC1
BCL11A	EPSTI1
BCL11A	ERP29
BCL11A	EXOC5
BCL11A	EXOC6
BCL11A	EXOC7
BCL11A	FAM102A
BCL11A	FAM107B
BCL11A	FAM184A
BCL11A	FAM20B
BCL11A	FAM76B
BCL11A	FAM81A
BCL11A	FAM96A
BCL11A	FANCI
BCL11A	FAU
BCL11A	FBRS
BCL11A	FBRSL1
BCL11A	FBXL20
BCL11A	FBXW4
BCL11A	FCMR
BCL11A	FCRL1
BCL11A	FCRLA
BCL11A	FGD2
BCL11A	FIS1
BCL11A	FKBP15
BCL11A	FMNL1
BCL11A	FMR1
BCL11A	FNTA
BCL11A	FOS
BCL11A	FOXN2
BCL11A	FOXO1
BCL11A	FOXP1
BCL11A	FRYL
BCL11A	FXYD5
BCL11A	GABPB1-AS1
BCL11A	GALNT3
BCL11A	GBA
BCL11A	GCC2
BCL11A	GCH1
BCL11A	GDI2
BCL11A	GGA2
BCL11A	GIN1
BCL11A	GIT2
BCL11A	GLIPR1
BCL11A	GMFG
BCL11A	GNAI2

BCL11A	GNGT2
BCL11A	GPAM
BCL11A	GPM6B
BCL11A	GPR160
BCL11A	GPR18
BCL11A	GPSM3
BCL11A	GRAP
BCL11A	GRIPAP1
BCL11A	GRN
BCL11A	GSDMD
BCL11A	GSTM4
BCL11A	GTF2I
BCL11A	HAGHL
BCL11A	HCLS1
BCL11A	HDAC7
BCL11A	HERC2
BCL11A	HERC4
BCL11A	HIVEP2
BCL11A	HLA-DMB
BCL11A	HLA-DRA
BCL11A	HMOX1
BCL11A	HNRNPH1
BCL11A	HOXA7
BCL11A	HPCAL1
BCL11A	HVCN1
BCL11A	IFI16
BCL11A	IFIT1
BCL11A	IFITM2
BCL11A	IGF2BP1
BCL11A	IGSF8
BCL11A	IKZF1
BCL11A	IKZF3
BCL11A	IL10RA
BCL11A	IL13RA1
BCL11A	IL16
BCL11A	IL4R
BCL11A	IL6ST
BCL11A	INF2
BCL11A	INPP5B
BCL11A	INPP5D
BCL11A	ISG20
BCL11A	ITGA4
BCL11A	ITGB1
BCL11A	ITSN2
BCL11A	JADE2
BCL11A	JAK2
BCL11A	JAK3
BCL11A	JMJD1C
BCL11A	JUND
BCL11A	KANSL1

BCL11A	KDM2A
BCL11A	KHNYN
BCL11A	KIAA1109
BCL11A	KLF12
BCL11A	KLF2
BCL11A	KLF6
BCL11A	KLF7
BCL11A	KLHL5
BCL11A	L3MBTL2
BCL11A	LAMTOR2
BCL11A	LAPTM5
BCL11A	LBH
BCL11A	LCOR
BCL11A	LDB1
BCL11A	LGALS9
BCL11A	LILRB1
BCL11A	LIMD2
BCL11A	LIMK2
BCL11A	LIPA
BCL11A	LMAN2L
BCL11A	LMO2
BCL11A	LPIN1
BCL11A	LPP
BCL11A	LRBA
BCL11A	LRP10
BCL11A	LRRC75A
BCL11A	LSR
BCL11A	LST1
BCL11A	LTA4H
BCL11A	LTB
BCL11A	LTB4R
BCL11A	LY6E
BCL11A	LY86
BCL11A	LYL1
BCL11A	LYN
BCL11A	LYRM1
BCL11A	LYSMD2
BCL11A	MALT1
BCL11A	MAN2A1
BCL11A	MANBA
BCL11A	MAP4K2
BCL11A	MAPK10
BCL11A	MAPK3
BCL11A	MARCH1
BCL11A	MARCKSL1
BCL11A	MAST3
BCL11A	MBNL1
BCL11A	MBOAT2
BCL11A	MDM4
BCL11A	MED23

BCL11A	MED29
BCL11A	MEF2A
BCL11A	MEF2C
BCL11A	MEIOC
BCL11A	METTL7A
BCL11A	MFN2
BCL11A	MILR1
BCL11A	MLXIP
BCL11A	MORC3
BCL11A	MPZL3
BCL11A	MS4A1
BCL11A	MTMR14
BCL11A	MTPN
BCL11A	MX1
BCL11A	MYH10
BCL11A	MYL12A
BCL11A	MYO1E
BCL11A	N4BP2L2
BCL11A	NACA
BCL11A	NCF1
BCL11A	NCF4
BCL11A	NCKIPSD
BCL11A	NCOA3
BCL11A	NEMF
BCL11A	NIPBL
BCL11A	NLRP1
BCL11A	NPTX2
BCL11A	NR3C1
BCL11A	NT5C3A
BCL11A	NUB1
BCL11A	NUGGC
BCL11A	NUMB
BCL11A	OARD1
BCL11A	ORAI3
BCL11A	OSBPL11
BCL11A	OTUD1
BCL11A	PABPC1
BCL11A	PAFAH1B2
BCL11A	PAN3
BCL11A	PAQR8
BCL11A	PARP3
BCL11A	PBX2
BCL11A	PCBP2
BCL11A	PCF11
BCL11A	PDE7A
BCL11A	PDLIM1
BCL11A	PDLIM2
BCL11A	PFDN5
BCL11A	PHF14
BCL11A	PHF20

BCL11A	PHF3
BCL11A	PHIP
BCL11A	PIK3AP1
BCL11A	PIP4K2A
BCL11A	PKIG
BCL11A	PKN1
BCL11A	PKN2
BCL11A	PLA2G15
BCL11A	PLA2G6
BCL11A	PLAC8
BCL11A	PLEKHA2
BCL11A	PLEKHF2
BCL11A	PLEKHG1
BCL11A	PLSCR1
BCL11A	PLXNC1
BCL11A	PML
BCL11A	PNISR
BCL11A	PNKD
BCL11A	PNP
BCL11A	PNRC1
BCL11A	POGZ
BCL11A	POLD4
BCL11A	POU2F2
BCL11A	PPM1K
BCL11A	PPM1N
BCL11A	PPP1R18
BCL11A	PPP1R3E
BCL11A	PPP2R5E
BCL11A	PPP3CA
BCL11A	PPP6R1
BCL11A	PQLC1
BCL11A	PRADC1
BCL11A	PRDM2
BCL11A	PREX1
BCL11A	PRKCB
BCL11A	PRMT2
BCL11A	PRPF4B
BCL11A	PSMB8
BCL11A	PTAFR
BCL11A	PTBP3
BCL11A	PTK2B
BCL11A	PTPN22
BCL11A	PTPN4
BCL11A	PTPN6
BCL11A	PTPN9
BCL11A	PTPRC
BCL11A	PTTG1IP
BCL11A	PUS10
BCL11A	R3HDM4
BCL11A	RAB11FIP4

BCL11A	RAB1B
BCL11A	RAB28
BCL11A	RAB31
BCL11A	RAB33A
BCL11A	RAB37
BCL11A	RABEP2
BCL11A	RAC2
BCL11A	RACK1
BCL11A	RAD18
BCL11A	RALGPS2
BCL11A	RAP2B
BCL11A	RAP2C
BCL11A	RASA1
BCL11A	RASGRP2
BCL11A	RASGRP3
BCL11A	RBCK1
BCL11A	RBFOX2
BCL11A	RBM38
BCL11A	RCAN3
BCL11A	RCSD1
BCL11A	RERE
BCL11A	RFX1
BCL11A	RFX5
BCL11A	RGL2
BCL11A	RHBDD1
BCL11A	RHBDF2
BCL11A	RHOBTB2
BCL11A	RHOH
BCL11A	RIC1
BCL11A	RINL
BCL11A	RNASE6
BCL11A	RNF13
BCL11A	RNF24
BCL11A	ROCK1
BCL11A	RPL10
BCL11A	RPL10A
BCL11A	RPL11
BCL11A	RPL13
BCL11A	RPL13A
BCL11A	RPL15
BCL11A	RPL17
BCL11A	RPL18A
BCL11A	RPL19
BCL11A	RPL23
BCL11A	RPL26
BCL11A	RPL27
BCL11A	RPL28
BCL11A	RPL32
BCL11A	RPL34
BCL11A	RPL35

BCL11A	RPL35A
BCL11A	RPL37
BCL11A	RPL37A
BCL11A	RPL38
BCL11A	RPL4
BCL11A	RPL41
BCL11A	RPL6
BCL11A	RPL7
BCL11A	RPLP2
BCL11A	RPS10
BCL11A	RPS11
BCL11A	RPS13
BCL11A	RPS15
BCL11A	RPS17
BCL11A	RPS18
BCL11A	RPS19
BCL11A	RPS20
BCL11A	RPS21
BCL11A	RPS25
BCL11A	RPS27
BCL11A	RPS27A
BCL11A	RPS3
BCL11A	RPS3A
BCL11A	RPS5
BCL11A	RPS6
BCL11A	RPS6KA1
BCL11A	RPS8
BCL11A	RXRB
BCL11A	S1PR2
BCL11A	SAMD10
BCL11A	SAMHD1
BCL11A	SCAF11
BCL11A	SCAF4
BCL11A	SCAMP2
BCL11A	SCIMP
BCL11A	SCOC-AS1
BCL11A	SEC22B
BCL11A	SELL
BCL11A	SEPT6
BCL11A	SEPT9
BCL11A	SERTAD2
BCL11A	SESN3
BCL11A	SF1
BCL11A	SGMS1
BCL11A	SH2B1
BCL11A	SH3BGRL
BCL11A	SH3BP5
BCL11A	SH3KBP1
BCL11A	SIAH1
BCL11A	SIPA1

BCL11A	SKAP2
BCL11A	SLC25A22
BCL11A	SLC2A6
BCL11A	SLC35F6
BCL11A	SLC39A13
BCL11A	SLC9A7
BCL11A	SMARCA2
BCL11A	SMARCD2
BCL11A	SMC6
BCL11A	SMIM14
BCL11A	SNAP23
BCL11A	SNRNP200
BCL11A	SNX2
BCL11A	SNX22
BCL11A	SNX3
BCL11A	SOX4
BCL11A	SP1
BCL11A	SP100
BCL11A	SP110
BCL11A	SP140
BCL11A	SPI1
BCL11A	SPTLC2
BCL11A	SRPK2
BCL11A	SRSF6
BCL11A	ST3GAL2
BCL11A	STAG2
BCL11A	STAP1
BCL11A	STIL
BCL11A	STK4
BCL11A	STRN
BCL11A	STX7
BCL11A	SULF2
BCL11A	SUN2
BCL11A	SYK
BCL11A	SYPL1
BCL11A	SYTL1
BCL11A	SYVN1
BCL11A	TAB2
BCL11A	TADA3
BCL11A	TAF13
BCL11A	TAGAP
BCL11A	TBC1D10B
BCL11A	TBC1D10C
BCL11A	TCF3
BCL11A	THOC5
BCL11A	THTPA
BCL11A	TLN1
BCL11A	TLR10
BCL11A	TMC6
BCL11A	TMEM156

BCL11A	TMEM168
BCL11A	TMEM256
BCL11A	TMSB4X
BCL11A	TNFAIP8L2
BCL11A	TNFRSF13C
BCL11A	TNRC6B
BCL11A	TOB1
BCL11A	TPCN1
BCL11A	TRAF3IP3
BCL11A	TRAF5
BCL11A	TRAPPC1
BCL11A	TRIM22
BCL11A	TSC22D3
BCL11A	TSC22D4
BCL11A	TSPAN3
BCL11A	TTC14
BCL11A	TTC17
BCL11A	TTC39C
BCL11A	TTC9C
BCL11A	TTL
BCL11A	TTLL3
BCL11A	TTPAL
BCL11A	UBA6
BCL11A	UBE4B
BCL11A	UBR5
BCL11A	UBR7
BCL11A	UBXN1
BCL11A	UCP2
BCL11A	UNC119
BCL11A	UNC93B1
BCL11A	USF2
BCL11A	USP15
BCL11A	USP6NL
BCL11A	UVRAG
BCL11A	VAV1
BCL11A	VAV2
BCL11A	WAS
BCL11A	WDR66
BCL11A	WIPF1
BCL11A	XAF1
BCL11A	XPR1
BCL11A	YPEL5
BCL11A	YWHAZ
BCL11A	ZBTB20
BCL11A	ZBTB25
BCL11A	ZBTB44
BCL11A	ZC3H12D
BCL11A	ZC3HAV1
BCL11A	ZDHHC2
BCL11A	ZDHHC21

BCL11A	ZFAND6
BCL11A	ZFAS1
BCL11A	ZFX
BCL11A	ZMIZ1
BCL11A	ZNF296
BCL11A	ZNF318
BCL11A	ZNF335
BCL11A	ZNF35
BCL11A	ZNF518A
BCL11A	ZNF524
BCL11A	ZNF652
BCL11A	ZNF780A
BCL11A	ZNF787
BCL11A	ZNF800
BCL11A	ZYX
BHLHE40	ABHD17A
BHLHE40	ABR
BHLHE40	ACP5
BHLHE40	ADGRE5
BHLHE40	AFTPH
BHLHE40	ALDH18A1
BHLHE40	ALG8
BHLHE40	ARF6
BHLHE40	ARHGAP15
BHLHE40	ARL8B
BHLHE40	ASAH1
BHLHE40	ATP6V0E1
BHLHE40	ATP6V1F
BHLHE40	BCAT1
BHLHE40	BCL2
BHLHE40	BHLHE40
BHLHE40	BMI1
BHLHE40	C12orf29
BHLHE40	CD164
BHLHE40	CD200
BHLHE40	CD3EAP
BHLHE40	CD68
BHLHE40	CDK4
BHLHE40	CDK7
BHLHE40	CDK8
BHLHE40	CELF2
BHLHE40	CPOX
BHLHE40	CRY1
BHLHE40	CUL5
BHLHE40	D2HGDH
BHLHE40	DDIT4
BHLHE40	DIAPH2
BHLHE40	DLGAP4
BHLHE40	DNPEP
BHLHE40	DPH2

BHLHE40	EDC3
BHLHE40	EHD3
BHLHE40	EIF4EBP1
BHLHE40	FAS
BHLHE40	FBXO15
BHLHE40	FBXW4
BHLHE40	FLNA
BHLHE40	FTH1
BHLHE40	GNPDA1
BHLHE40	GNPTAB
BHLHE40	GPM6B
BHLHE40	GPR183
BHLHE40	GPX4
BHLHE40	HAUS6
BHLHE40	HBP1
BHLHE40	HIF1A
BHLHE40	HIVEP1
BHLHE40	HSF2
BHLHE40	ID3
BHLHE40	INO80
BHLHE40	KIAA1551
BHLHE40	KLF6
BHLHE40	LITAF
BHLHE40	LY9
BHLHE40	M6PR
BHLHE40	MAP2K7
BHLHE40	MAP4
BHLHE40	MARCH3
BHLHE40	MBP
BHLHE40	MFSD2A
BHLHE40	MOSPD1
BHLHE40	MREG
BHLHE40	MSC
BHLHE40	MTSS1
BHLHE40	MYC
BHLHE40	NABP1
BHLHE40	NAGPA
BHLHE40	NDUFAF5
BHLHE40	NOL8
BHLHE40	NPW
BHLHE40	NUMB
BHLHE40	ODF2L
BHLHE40	ORMDL1
BHLHE40	PARP6
BHLHE40	PEPD
BHLHE40	PET117
BHLHE40	PFKFB3
BHLHE40	PFKP
BHLHE40	PHACTR1
BHLHE40	PHYKPL

BHLHE40	PIM3
BHLHE40	PLEK
BHLHE40	PPAT
BHLHE40	PRDM4
BHLHE40	PSAT1
BHLHE40	PTPN11
BHLHE40	PTPN6
BHLHE40	PUS7
BHLHE40	QPCTL
BHLHE40	RAB27A
BHLHE40	RBM27
BHLHE40	RCAN1
BHLHE40	REL
BHLHE40	RNF146
BHLHE40	S100A6
BHLHE40	SERPINF1
BHLHE40	SLC20A2
BHLHE40	SLC38A5
BHLHE40	SMUG1
BHLHE40	SNX9
BHLHE40	SRFBP1
BHLHE40	STAT5A
BHLHE40	STIP1
BHLHE40	SUN2
BHLHE40	TBC1D9
BHLHE40	TBPL1
BHLHE40	TICAM1
BHLHE40	TIMM23
BHLHE40	TLK2
BHLHE40	TMEM102
BHLHE40	TMEM30A
BHLHE40	TNFRSF14
BHLHE40	TNRC6B
BHLHE40	TOMM40L
BHLHE40	TRAP1
BHLHE40	TRIB2
BHLHE40	TRMT10C
BHLHE40	VAMP1
BHLHE40	VPS13A
BHLHE40	WDR43
BHLHE40	XPOT
BHLHE40	YBX1
BHLHE40	YBX3
BHLHE40	YWHAG
BHLHE40	ZFP82
BHLHE40	ZNF667-AS1
BHLHE41	CTSA
BRF2	APMAP
BRF2	ARF1
BRF2	ATP6V0A1

BRF2	BCOR
BRF2	BIN3
BRF2	BRF2
BRF2	C1orf174
BRF2	CADM1
BRF2	CCND2
BRF2	CHD6
BRF2	CLK2
BRF2	CLTC
BRF2	CNPPD1
BRF2	CYB561
BRF2	DMAP1
BRF2	DUSP4
BRF2	ENO1
BRF2	EXT1
BRF2	FASTKD5
BRF2	FYN
BRF2	GATB
BRF2	HSP90AB1
BRF2	IFT46
BRF2	IL23A
BRF2	KIF9
BRF2	LITAF
BRF2	MAPRE2
BRF2	MCM8
BRF2	MCRS1
BRF2	NEK11
BRF2	NFYC
BRF2	NUP188
BRF2	PIKFYVE
BRF2	POLR3H
BRF2	PPID
BRF2	PRR3
BRF2	PSMD11
BRF2	PYROXD1
BRF2	RARA
BRF2	RASA3
BRF2	RBM47
BRF2	SERTAD2
BRF2	SLC12A4
BRF2	SLC46A3
BRF2	SOX4
BRF2	SQSTM1
BRF2	SSTR2
BRF2	STIL
BRF2	STK10
BRF2	TBCC
BRF2	TEFM
BRF2	TFB1M
BRF2	THAP2

BRF2	TM2D1
BRF2	TMBIM1
BRF2	TMUB2
BRF2	TNIP1
BRF2	TSPAN31
BRF2	VTI1A
BRF2	WDFY1
BRF2	XIAP
BRF2	ZDHHC7
CEBPB	AAGAB
CEBPB	AAMP
CEBPB	AARS
CEBPB	ABCD4
CEBPB	ABCE1
CEBPB	ACBD6
CEBPB	ACIN1
CEBPB	ACOX1
CEBPB	ACOXL
CEBPB	ACTR10
CEBPB	ACTR1B
CEBPB	ADK
CEBPB	ADM2
CEBPB	ADRM1
CEBPB	ADSS
CEBPB	AFF2
CEBPB	AGA
CEBPB	ALDH1L2
CEBPB	ALG5
CEBPB	ANAPC13
CEBPB	ANAPC16
CEBPB	ANGEL2
CEBPB	ANKRD11
CEBPB	ANKRD28
CEBPB	ANKRD37
CEBPB	ANP32B
CEBPB	ANXA7
CEBPB	API5
CEBPB	APP
CEBPB	APPL2
CEBPB	ARCN1
CEBPB	ARHGAP24
CEBPB	ARL2
CEBPB	ARL5B
CEBPB	ARL8B
CEBPB	ARPC5L
CEBPB	ARV1
CEBPB	ASNS
CEBPB	ATF4
CEBPB	ATF5
CEBPB	ATF6

CEBPB	ATL2
CEBPB	ATP1B1
CEBPB	ATP1B3
CEBPB	ATP6V0E1
CEBPB	ATP6V1E2
CEBPB	ATRAID
CEBPB	ATXN1
CEBPB	ATXN2L
CEBPB	AZIN1
CEBPB	B4GALT3
CEBPB	BATF3
CEBPB	BCAS2
CEBPB	BCL2
CEBPB	BET1
CEBPB	BEX2
CEBPB	BEX5
CEBPB	BFAR
CEBPB	BIN2
CEBPB	BLVRB
CEBPB	BMI1
CEBPB	BMT2
CEBPB	BOD1
CEBPB	BSDC1
CEBPB	BTBD7
CEBPB	BTF3L4
CEBPB	BUD31
CEBPB	C11orf1
CEBPB	C12orf29
CEBPB	C15orf61
CEBPB	C19orf70
CEBPB	C1orf52
CEBPB	C1QBP
CEBPB	C20orf27
CEBPB	C6orf48
CEBPB	CALCOCO2
CEBPB	CALU
CEBPB	CARS
CEBPB	CAST
CEBPB	CAV1
CEBPB	CBX6
CEBPB	CCDC144A
CEBPB	CCDC59
CEBPB	CCDC7
CEBPB	CCDC86
CEBPB	CCND2
CEBPB	CCNK
CEBPB	CCPG1
CEBPB	CCR7
CEBPB	CCZ1B
CEBPB	CD63

CEBPB	CDC37L1
CEBPB	CDK4
CEBPB	CDKN1A
CEBPB	CEBPB
CEBPB	CEBPG
CEBPB	CEP131
CEBPB	CEP162
CEBPB	CEP170
CEBPB	CEP70
CEBPB	CFAP97
CEBPB	CFL2
CEBPB	CHAC1
CEBPB	CHERP
CEBPB	CHMP4B
CEBPB	CHP1
CEBPB	CHPF2
CEBPB	CINP
CEBPB	CIRBP
CEBPB	CISD2
CEBPB	CLIP1
CEBPB	CLIP4
CEBPB	CLK2
CEBPB	CLPB
CEBPB	CMSS1
CEBPB	CNN3
CEBPB	CNOT11
CEBPB	COA4
CEBPB	COLGALT1
CEBPB	COMMD3
CEBPB	COPB1
CEBPB	COPG1
CEBPB	COPS2
CEBPB	COX20
CEBPB	CPSF4
CEBPB	CREB5
CEBPB	CRY1
CEBPB	CSNK2A1
CEBPB	CSRP1
CEBPB	CTH
CEBPB	CTNNBIP1
CEBPB	CTR9
CEBPB	CTSB
CEBPB	CYB5R1
CEBPB	CYCS
CEBPB	CYLD
CEBPB	CYTH1
CEBPB	DARS
CEBPB	DDIT3
CEBPB	DDIT4
CEBPB	DDX18

CEBPB	DDX19A
CEBPB	DDX21
CEBPB	DDX39B
CEBPB	DDX55
CEBPB	DEDD2
CEBPB	DERL3
CEBPB	DESI1
CEBPB	DHRS9
CEBPB	DNAJC1
CEBPB	DNAJC10
CEBPB	DNAJC2
CEBPB	DNPEP
CEBPB	DONSON
CEBPB	DPH2
CEBPB	DPM2
CEBPB	DRAM1
CEBPB	DTD2
CEBPB	DTNB
CEBPB	DUSP10
CEBPB	DUSP5
CEBPB	DYNLT3
CEBPB	E2F1
CEBPB	EAPP
CEBPB	EDEM1
CEBPB	EEA1
CEBPB	EEF1A1
CEBPB	EEF1B2
CEBPB	EEF1D
CEBPB	EEF1E1
CEBPB	EFCAB2
CEBPB	EIF1
CEBPB	EIF2A
CEBPB	EIF2S2
CEBPB	EIF3J
CEBPB	EIF4A1
CEBPB	EIF4A2
CEBPB	EIF4EBP1
CEBPB	EIF6
CEBPB	ELAC1
CEBPB	ELL2
CEBPB	EMC2
CEBPB	EMC4
CEBPB	EMC7
CEBPB	EMD
CEBPB	EMG1
CEBPB	EMP3
CEBPB	ENO1
CEBPB	EPB41L2
CEBPB	EPB41L4A-AS1
CEBPB	EPRS

CEBPB	ERGIC2
CEBPB	ERLEC1
CEBPB	ESF1
CEBPB	EVA1B
CEBPB	FAM107B
CEBPB	FAM204A
CEBPB	FAM234A
CEBPB	FBL
CEBPB	FBXO4
CEBPB	FCER2
CEBPB	FDXACB1
CEBPB	FGR
CEBPB	FKBP11
CEBPB	FKBPL
CEBPB	FOXP1
CEBPB	FPGS
CEBPB	FTH1
CEBPB	FTX
CEBPB	FUBP1
CEBPB	FUBP3
CEBPB	FUCA1
CEBPB	FXN
CEBPB	FYN
CEBPB	G6PD
CEBPB	GADD45A
CEBPB	GALNT2
CEBPB	GANAB
CEBPB	GAR1
CEBPB	GARS
CEBPB	GEMIN5
CEBPB	GHITM
CEBPB	GMPPB
CEBPB	GNPNAT1
CEBPB	GNS
CEBPB	GOLGA7
CEBPB	GOT1
CEBPB	GPATCH4
CEBPB	GPN3
CEBPB	GPR65
CEBPB	GPT2
CEBPB	HAX1
CEBPB	HERC2
CEBPB	HERPUD1
CEBPB	HIST1H2BJ
CEBPB	HIST3H2A
CEBPB	HOOK2
CEBPB	HSP90AB1
CEBPB	HSPA5
CEBPB	HSPA8
CEBPB	HSPA9

CEBPB	HUWE1
CEBPB	HYOU1
CEBPB	IARS
CEBPB	ICE1
CEBPB	ID2
CEBPB	IDH3A
CEBPB	IFRD1
CEBPB	IMPDH2
CEBPB	INTS7
CEBPB	IP6K1
CEBPB	IRF1
CEBPB	IRF2
CEBPB	IRF4
CEBPB	ITCH
CEBPB	JMJD1C
CEBPB	JMY
CEBPB	JUN
CEBPB	KDM3A
CEBPB	KDM8
CEBPB	KIAA0232
CEBPB	KIFAP3
CEBPB	KLF7
CEBPB	KLHL28
CEBPB	KMT2E−AS1
CEBPB	KRTCAP2
CEBPB	LAMP1
CEBPB	LAPTM4A
CEBPB	LARP1B
CEBPB	LARS
CEBPB	LEF1
CEBPB	LETMD1
CEBPB	LINC00158
CEBPB	LMAN2
CEBPB	LMBRD1
CEBPB	LMF1
CEBPB	LMF2
CEBPB	LMO4
CEBPB	LONP1
CEBPB	LRRFIP2
CEBPB	LY96
CEBPB	LYSMD3
CEBPB	MACROD2
CEBPB	MAGOH
CEBPB	MAGOHB
CEBPB	MAML2
CEBPB	MAN1B1
CEBPB	MANEA
CEBPB	MANF
CEBPB	MAP4K5
CEBPB	MARCKS

CEBPB	MARS
CEBPB	MCUR1
CEBPB	MDN1
CEBPB	MED15
CEBPB	MED8
CEBPB	MEI1
CEBPB	MFSD14A
CEBPB	MFSD14C
CEBPB	MGAT4B
CEBPB	MIA3
CEBPB	MID1IP1
CEBPB	MIR155HG
CEBPB	MIR22HG
CEBPB	MLH3
CEBPB	MPZL1
CEBPB	MRPL1
CEBPB	MRPL3
CEBPB	MRPL40
CEBPB	MRPL53
CEBPB	MRPS22
CEBPB	MSL3
CEBPB	MSTO1
CEBPB	MTHFD2
CEBPB	MXI1
CEBPB	MYC
CEBPB	N4BP1
CEBPB	N4BP2L2
CEBPB	NAA20
CEBPB	NAGLU
CEBPB	NAMPT
CEBPB	NARS
CEBPB	NCALD
CEBPB	NCAPG2
CEBPB	NCOA2
CEBPB	NCSTN
CEBPB	NDFIP1
CEBPB	NDUFB7
CEBPB	NECAP1
CEBPB	NFE2L1
CEBPB	NFE2L2
CEBPB	NFKB1
CEBPB	NFKBIA
CEBPB	NFKBIB
CEBPB	NGDN
CEBPB	NIP7
CEBPB	NLK
CEBPB	NOC3L
CEBPB	NOLC1
CEBPB	NORAD
CEBPB	NPAT

CEBPB	NPM1
CEBPB	NPM3
CEBPB	NPW
CEBPB	NR1H2
CEBPB	NR2C2AP
CEBPB	NRP2
CEBPB	NSF
CEBPB	NUBP2
CEBPB	NUCB2
CEBPB	NUDT8
CEBPB	NXPH4
CEBPB	NXT1
CEBPB	OCIAD1
CEBPB	ODC1
CEBPB	ODF3B
CEBPB	ORAOV1
CEBPB	OST4
CEBPB	OXR1
CEBPB	PABPC4
CEBPB	PAK1
CEBPB	PAK1IP1
CEBPB	PARG
CEBPB	PARP14
CEBPB	PARP8
CEBPB	PARVB
CEBPB	PARVG
CEBPB	PAXBP1
CEBPB	PBLD
CEBPB	PCK2
CEBPB	PCNP
CEBPB	PDAP1
CEBPB	PDCD11
CEBPB	PDCD4
CEBPB	PDE4A
CEBPB	PDE4B
CEBPB	PDE8A
CEBPB	PEMT
CEBPB	PEX12
CEBPB	PFDN2
CEBPB	PFN2
CEBPB	PHACTR1
CEBPB	PHF1
CEBPB	PHGDH
CEBPB	PHYKPL
CEBPB	PICK1
CEBPB	PIGB
CEBPB	PIGO
CEBPB	PIM3
CEBPB	PIP4K2B
CEBPB	PIP5K1B

CEBPB	PITHD1
CEBPB	PKIG
CEBPB	PLRG1
CEBPB	PMAIP1
CEBPB	PMM2
CEBPB	PNPLA8
CEBPB	POGLUT1
CEBPB	POLQ
CEBPB	POLRMT
CEBPB	PPIP5K2
CEBPB	PPP1R11
CEBPB	PPP1R15A
CEBPB	PPP1R15B
CEBPB	PPP3CC
CEBPB	PQLC1
CEBPB	PRNP
CEBPB	PRPF19
CEBPB	PRR14L
CEBPB	PRRC1
CEBPB	PRRC2C
CEBPB	PSAP
CEBPB	PSAT1
CEBPB	PSENEN
CEBPB	PSMC4
CEBPB	PSMC5
CEBPB	PSMC6
CEBPB	PSMD11
CEBPB	PSMF1
CEBPB	PUM3
CEBPB	PYCR1
CEBPB	PYCR2
CEBPB	QSOX1
CEBPB	RAB10
CEBPB	RAD23A
CEBPB	RAD51D
CEBPB	RALGAPB
CEBPB	RANBP1
CEBPB	RASA2
CEBPB	RBM28
CEBPB	RBM34
CEBPB	RBM39
CEBPB	RCN1
CEBPB	RER1
CEBPB	REV3L
CEBPB	RIC1
CEBPB	RIOK1
CEBPB	RIPK1
CEBPB	RMDN3
CEBPB	RNASEH1
CEBPB	RNF113A

CEBPB	RNF139
CEBPB	RNF145
CEBPB	RNF149
CEBPB	RNF157
CEBPB	RNF170
CEBPB	RNF181
CEBPB	RNF187
CEBPB	RNF216
CEBPB	RNF220
CEBPB	RNF41
CEBPB	RNMT
CEBPB	RPF2
CEBPB	RPL12
CEBPB	RPL13
CEBPB	RPL13A
CEBPB	RPL18
CEBPB	RPL18A
CEBPB	RPL21
CEBPB	RPL32
CEBPB	RPL34
CEBPB	RPL36
CEBPB	RPL5
CEBPB	RPL7
CEBPB	RPL9
CEBPB	RPLP0
CEBPB	RPLP1
CEBPB	RPS10
CEBPB	RPS12
CEBPB	RPS17
CEBPB	RPS19
CEBPB	RPS19BP1
CEBPB	RPS20
CEBPB	RPS21
CEBPB	RPS27
CEBPB	RPS29
CEBPB	RPS3A
CEBPB	RPS6
CEBPB	RPS6KB1
CEBPB	RRP15
CEBPB	RSL1D1
CEBPB	RSL24D1
CEBPB	SACM1L
CEBPB	SARAF
CEBPB	SARS
CEBPB	SBDS
CEBPB	SEC23B
CEBPB	SEC24D
CEBPB	SEC63
CEBPB	SEL1L
CEBPB	SESN2

CEBPB	SFSWAP
CEBPB	SGF29
CEBPB	SGK1
CEBPB	SHARPIN
CEBPB	SHMT2
CEBPB	SIGIRR
CEBPB	SIL1
CEBPB	SLC10A7
CEBPB	SLC16A1
CEBPB	SLC16A3
CEBPB	SLC1A4
CEBPB	SLC1A5
CEBPB	SLC25A39
CEBPB	SLC35B1
CEBPB	SLC38A2
CEBPB	SLC39A1
CEBPB	SLC3A2
CEBPB	SLC44A1
CEBPB	SLC52A2
CEBPB	SLC6A9
CEBPB	SLC7A1
CEBPB	SLC7A5
CEBPB	SLC7A7
CEBPB	SNAP29
CEBPB	SNHG15
CEBPB	SNHG8
CEBPB	SNTB1
CEBPB	SOCS3
CEBPB	SPATC1L
CEBPB	SPATS2
CEBPB	SPEN
CEBPB	SPRY1
CEBPB	SQSTM1
CEBPB	SRFBP1
CEBPB	SRP54
CEBPB	SRSF3
CEBPB	SSBP1
CEBPB	SSR4
CEBPB	SSRP1
CEBPB	STAT3
CEBPB	STAT6
CEBPB	STK4
CEBPB	STOM
CEBPB	STOML2
CEBPB	STT3A
CEBPB	STX11
CEBPB	STX3
CEBPB	STXBP3
CEBPB	SUCO
CEBPB	SYNRG

CEBPB	SYTL1
CEBPB	SYVN1
CEBPB	TAGAP
CEBPB	TARS
CEBPB	TCEA1
CEBPB	TCTN3
CEBPB	TECPR2
CEBPB	TES
CEBPB	TFAM
CEBPB	TFG
CEBPB	THAP1
CEBPB	THAP7
CEBPB	THAP9-AS1
CEBPB	THTPA
CEBPB	TIMM10
CEBPB	TIMM21
CEBPB	TIMM44
CEBPB	TIMM8A
CEBPB	TLCD1
CEBPB	TLE3
CEBPB	TMBIM4
CEBPB	TMBIM6
CEBPB	TMED2
CEBPB	TMED4
CEBPB	TMEM109
CEBPB	TMEM147
CEBPB	TMEM154
CEBPB	TMEM156
CEBPB	TMEM167B
CEBPB	TMEM216
CEBPB	TMEM267
CEBPB	TMEM41B
CEBPB	TMEM50B
CEBPB	TMEM52B
CEBPB	TMEM9B
CEBPB	TNFAIP3
CEBPB	TNFAIP8
CEBPB	TNFRSF9
CEBPB	TNIP2
CEBPB	TOMM40
CEBPB	TP53I13
CEBPB	TPD52L2
CEBPB	TPRKB
CEBPB	TRADD
CEBPB	TRAF3IP2
CEBPB	TRAM1
CEBPB	TREML2
CEBPB	TRIB3
CEBPB	TRIM33
CEBPB	TRIM44

CEBPB	TRMT1
CEBPB	TRNT1
CEBPB	TSC22D3
CEBPB	TSEN15
CEBPB	TSPYL2
CEBPB	TSTA3
CEBPB	TTC19
CEBPB	TTC27
CEBPB	TUFM
CEBPB	TUSC1
CEBPB	TXLNB
CEBPB	TXLNG
CEBPB	TXN
CEBPB	TXNL1
CEBPB	UAP1
CEBPB	UBA5
CEBPB	UBE2B
CEBPB	UBXN1
CEBPB	UCHL5
CEBPB	UGT2B17
CEBPB	UMAD1
CEBPB	URM1
CEBPB	UROS
CEBPB	USP14
CEBPB	USP16
CEBPB	USP36
CEBPB	USPL1
CEBPB	VAMP2
CEBPB	VAV1
CEBPB	VIM
CEBPB	VPS35
CEBPB	VPS37B
CEBPB	WARS
CEBPB	WBP11
CEBPB	WDR45
CEBPB	WDR74
CEBPB	WDR75
CEBPB	WDR83OS
CEBPB	WDR89
CEBPB	WWOX
CEBPB	XBP1
CEBPB	XPO6
CEBPB	XPOT
CEBPB	XRCC5
CEBPB	YARS
CEBPB	YBX1
CEBPB	YBX3
CEBPB	YIF1A
CEBPB	YIPF2
CEBPB	YPEL5

CEBPB	YWHAG
CEBPB	ZC3HC1
CEBPB	ZEB1
CEBPB	ZFAND2A
CEBPB	ZFAND2B
CEBPB	ZFAS1
CEBPB	ZFP36L1
CEBPB	ZFP36L2
CEBPB	ZMAT2
CEBPB	ZNF131
CEBPB	ZNF134
CEBPB	ZNF146
CEBPB	ZNF22
CEBPB	ZNF232
CEBPB	ZNF331
CEBPB	ZNF48
CEBPB	ZNF507
CEBPB	ZNF518B
CEBPB	ZNF667
CEBPB	ZNF75A
CEBPB	ZNF787
CEBPB	ZPR1
CEBPD	NFKBIZ
CEBPG	AASDHPPT
CEBPG	ABCE1
CEBPG	ABHD11
CEBPG	ACSL1
CEBPG	ANKRD11
CEBPG	AP3M2
CEBPG	ARID3A
CEBPG	ARL17A
CEBPG	ASNS
CEBPG	ATAD2
CEBPG	ATF3
CEBPG	ATF4
CEBPG	ATF5
CEBPG	BCAS2
CEBPG	BCAT1
CEBPG	C12orf75
CEBPG	CCND2
CEBPG	CCPG1
CEBPG	CEBPB
CEBPG	CEBPG
CEBPG	CREM
CEBPG	CWC15
CEBPG	CYCS
CEBPG	DDIT4
CEBPG	DPH3
CEBPG	DYRK1A
CEBPG	EIF1

CEBPG	EIF2S2
CEBPG	EIF4EBP1
CEBPG	EPRS
CEBPG	FAM107B
CEBPG	FBXO25
CEBPG	FTH1
CEBPG	GARS
CEBPG	GHITM
CEBPG	GNL1
CEBPG	GOT1
CEBPG	GPT2
CEBPG	HAX1
CEBPG	HNRNPA0
CEBPG	HSPA9
CEBPG	IARS
CEBPG	IFI44L
CEBPG	IKZF3
CEBPG	IRF2BP2
CEBPG	JMY
CEBPG	JUND
CEBPG	KCNA3
CEBPG	KDM6B
CEBPG	KLC2
CEBPG	LOXL2
CEBPG	MAFG
CEBPG	METTL1
CEBPG	MIEF1
CEBPG	MSTO1
CEBPG	MTHFD2
CEBPG	N6AMT1
CEBPG	NAA60
CEBPG	NAMPT
CEBPG	NARS
CEBPG	NFE2L2
CEBPG	NFKB1
CEBPG	NFKBIA
CEBPG	NSUN4
CEBPG	NUP35
CEBPG	NUP98
CEBPG	PDAP1
CEBPG	PDGFA
CEBPG	PHF19
CEBPG	PICALM
CEBPG	PLRG1
CEBPG	PMAIP1
CEBPG	PPID
CEBPG	PPT1
CEBPG	PRNP
CEBPG	PSAT1
CEBPG	PSMD14

CEBPG	PTGIR
CEBPG	PTP4A1
CEBPG	PYCR1
CEBPG	RAB40C
CEBPG	RAD23A
CEBPG	RCN1
CEBPG	RHEB
CEBPG	RHOF
CEBPG	RPF1
CEBPG	RSL24D1
CEBPG	SARS
CEBPG	SEPT11
CEBPG	SESN2
CEBPG	SESTD1
CEBPG	SHMT2
CEBPG	SLC1A5
CEBPG	SLC3A2
CEBPG	SLC6A9
CEBPG	SLC7A11
CEBPG	SNHG15
CEBPG	SNX12
CEBPG	SPIN1
CEBPG	SRSF7
CEBPG	SSX2IP
CEBPG	STARD3NL
CEBPG	STX4
CEBPG	SUMO1
CEBPG	SYTL1
CEBPG	TARS
CEBPG	TBCCD1
CEBPG	TCEA1
CEBPG	TIMM44
CEBPG	TMEM267
CEBPG	TNFAIP3
CEBPG	TRIB3
CEBPG	TSEN15
CEBPG	UBE2B
CEBPG	USP3
CEBPG	USP36
CEBPG	VEGFA
CEBPG	VPS37B
CEBPG	WARS
CEBPG	XPOT
CEBPG	YARS
CEBPG	ZDHHC20
CEBPG	ZNF143
CEBPG	ZNF706
CLOCK	ALDOC
CLOCK	BUB1B
CLOCK	EIF4EBP1

CLOCK	EPB41
CLOCK	ESR2
CLOCK	IQSEC1
CLOCK	KAT5
CLOCK	KAT8
CLOCK	MIR22HG
CLOCK	NABP1
CLOCK	QRICH1
CLOCK	RBM15B
CLOCK	SNAPC1
CLOCK	TMEM214
CLOCK	TRPM7
CLOCK	USP28
CLOCK	ZNF532
CREB1	ABI1
CREB1	ACTR2
CREB1	ADAM17
CREB1	ADO
CREB1	ANKHD1
CREB1	ANKRD11
CREB1	AP1M1
CREB1	ASB8
CREB1	ATM
CREB1	BASP1
CREB1	BAZ2A
CREB1	BBS4
CREB1	BBX
CREB1	C2orf49
CREB1	CBLL1
CREB1	CCNI
CREB1	CDK7
CREB1	CENPF
CREB1	CEP85L
CREB1	CLTC
CREB1	CYB561A3
CREB1	DDX3X
CREB1	DLEU2
CREB1	ELK4
CREB1	EXOC3
CREB1	FADS2
CREB1	FAM110A
CREB1	FAM200A
CREB1	FBRS
CREB1	FBXW4
CREB1	G2E3
CREB1	GABARAPL2
CREB1	GAR1
CREB1	GNAI3
CREB1	GPATCH2L
CREB1	GSKIP

CREB1	HMBS
CREB1	IKZF1
CREB1	KBTBD4
CREB1	KDM2A
CREB1	KLF9
CREB1	KLHL28
CREB1	LATS2
CREB1	LONP1
CREB1	MAGED2
CREB1	MIDN
CREB1	MTPN
CREB1	MYO19
CREB1	NCOA2
CREB1	NEDD4L
CREB1	NFATC1
CREB1	NFKB2
CREB1	NIF3L1
CREB1	NORAD
CREB1	PATL1
CREB1	PDZD11
CREB1	PER2
CREB1	PHIP
CREB1	PHRF1
CREB1	PNISR
CREB1	PRKAG2
CREB1	PRR5
CREB1	PSMA6
CREB1	PTK2B
CREB1	PTPN12
CREB1	QRICH1
CREB1	RAB11A
CREB1	RCSD1
CREB1	ROCK1
CREB1	SHISA8
CREB1	SIN3A
CREB1	SIPA1
CREB1	SKIL
CREB1	SLC23A2
CREB1	SNX16
CREB1	SPDL1
CREB1	SPTLC2
CREB1	SRSF5
CREB1	STAT6
CREB1	SYAP1
CREB1	TAF12
CREB1	TANK
CREB1	TBP
CREB1	TECPR2
CREB1	TLN1
CREB1	TNFAIP8L2

CREB1	TNRC6A
CREB1	TP53BP1
CREB1	TRAF5
CREB1	TUBGCP6
CREB1	VANGL1
CREB1	VEZF1
CREB1	VPS4B
CREB1	VPS51
CREB1	WDR83OS
CREB1	ZBTB20
CREB1	ZCWPW1
CREB1	ZDHHC7
CREB1	ZNF397
CREB1	ZNF655
CREB1	ZNF75A
CREB1	ZSCAN5A
CREB3	AAK1
CREB3	AAMP
CREB3	AARS
CREB3	ABCB8
CREB3	ACADVL
CREB3	ACBD3
CREB3	ACO2
CREB3	ACOT13
CREB3	ACOT9
CREB3	ACOX1
CREB3	ACSL1
CREB3	ACSS1
CREB3	ADAM10
CREB3	ADD1
CREB3	ADI1
CREB3	ADIPOR1
CREB3	ADK
CREB3	ADM
CREB3	ADRM1
CREB3	AEBP1
CREB3	AGPAT1
CREB3	AIFM2
CREB3	AKAP9
CREB3	AKIRIN2
CREB3	AKR1A1
CREB3	AKT1S1
CREB3	ALDH1L2
CREB3	ALG1
CREB3	ALG12
CREB3	ALG14
CREB3	ALG2
CREB3	ALG3
CREB3	ALKBH3
CREB3	ANAPC13

CREB3	ANAPC16
CREB3	ANAPC2
CREB3	ANAPC5
CREB3	ANG
CREB3	ANKHD1
CREB3	ANKMY2
CREB3	ANKRD12
CREB3	ANKRD16
CREB3	ANKRD28
CREB3	ANKRD36
CREB3	ANKRD36C
CREB3	ANKRD37
CREB3	ANKRD42
CREB3	ANXA2
CREB3	ANXA5
CREB3	ANXA6
CREB3	AP2A2
CREB3	AP2B1
CREB3	AP2M1
CREB3	AP2S1
CREB3	AP3B1
CREB3	AP3D1
CREB3	AP3S1
CREB3	AP5B1
CREB3	APMAP
CREB3	APOD
CREB3	APOL3
CREB3	APTX
CREB3	ARCN1
CREB3	ARF1
CREB3	ARF3
CREB3	ARF4
CREB3	ARFGAP1
CREB3	ARFGAP3
CREB3	ARFIP2
CREB3	ARHGDIA
CREB3	ARID3A
CREB3	ARL1
CREB3	ARL2BP
CREB3	ARL4A
CREB3	ARMC10
CREB3	ARMCX3
CREB3	ARMCX5
CREB3	ARRDC3
CREB3	ARSA
CREB3	ARSG
CREB3	ASAH1
CREB3	ASCC3
CREB3	ASNA1
CREB3	ASPHD2

CREB3	ATF4
CREB3	ATF6
CREB3	ATF6B
CREB3	ATG101
CREB3	ATG13
CREB3	ATOX1
CREB3	ATP13A1
CREB3	ATP2C1
CREB3	ATP6AP1
CREB3	ATP6AP2
CREB3	ATP6V0A1
CREB3	ATP6V0B
CREB3	ATP6V0D1
CREB3	ATP6V0E1
CREB3	ATP6V1A
CREB3	ATP6V1E1
CREB3	ATP6V1G1
CREB3	ATRAID
CREB3	ATXN1L
CREB3	ATXN2L
CREB3	AURKAIP1
CREB3	B3GAT3
CREB3	B3GNT9
CREB3	B4GALT2
CREB3	B4GALT3
CREB3	B9D1
CREB3	BABAM1
CREB3	BAZ2B
CREB3	BBX
CREB3	BCAP31
CREB3	BCAS2
CREB3	BCKDK
CREB3	BCLAF1
CREB3	BET1
CREB3	BET1L
CREB3	BEX4
CREB3	BEX5
CREB3	BHLHE41
CREB3	BIRC2
CREB3	BLCAP
CREB3	BLNK
CREB3	BLVRB
CREB3	BMS1
CREB3	BOLA2B
CREB3	BORCS6
CREB3	BRD2
CREB3	BRF2
CREB3	BRK1
CREB3	BSDC1
CREB3	BSG

CREB3	BTG2
CREB3	BTK
CREB3	BUD31
CREB3	C11orf1
CREB3	C11orf68
CREB3	C16orf58
CREB3	C16orf91
CREB3	C17orf107
CREB3	C18orf32
CREB3	C19orf53
CREB3	C19orf70
CREB3	C1GALT1C1
CREB3	C1orf216
CREB3	C1orf43
CREB3	C21orf2
CREB3	C2orf74
CREB3	C4orf48
CREB3	C5orf15
CREB3	C6orf226
CREB3	C6orf62
CREB3	C8orf59
CREB3	C9orf16
CREB3	CACUL1
CREB3	CALCOCO1
CREB3	CALCOCO2
CREB3	CALM1
CREB3	CALR
CREB3	CALU
CREB3	CAMK1
CREB3	CAMK2B
CREB3	CAMTA2
CREB3	CANX
CREB3	CAP1
CREB3	CAPN10
CREB3	CAPRIN1
CREB3	CASP3
CREB3	CASP8
CREB3	CBR1
CREB3	CBWD1
CREB3	CCDC124
CREB3	CCDC144A
CREB3	CCDC159
CREB3	CCDC167
CREB3	CCDC174
CREB3	CCDC186
CREB3	CCDC25
CREB3	CCDC47
CREB3	CCDC57
CREB3	CCDC59
CREB3	CCDC69

CREB3	CCDC85A
CREB3	CCNC
CREB3	CCPG1
CREB3	CCS
CREB3	CCT8
CREB3	CD164
CREB3	CD2AP
CREB3	CD2BP2
CREB3	CD38
CREB3	CD53
CREB3	CD63
CREB3	CD86
CREB3	CDC26
CREB3	CDC34
CREB3	CDC5L
CREB3	CDIPT
CREB3	CDK12
CREB3	CDK16
CREB3	CDK2AP2
CREB3	CDK9
CREB3	CDKN1B
CREB3	CDKN2C
CREB3	CDV3
CREB3	CEP128
CREB3	CEP350
CREB3	CETN2
CREB3	CGRRF1
CREB3	CHD2
CREB3	CHID1
CREB3	CHMP2A
CREB3	CHMP4A
CREB3	CHPF
CREB3	CHPF2
CREB3	CHRNB1
CREB3	CHST12
CREB3	CHSY1
CREB3	CINP
CREB3	CISD2
CREB3	CITED2
CREB3	CKAP4
CREB3	CLCC1
CREB3	CLPB
CREB3	CLPTM1
CREB3	CLPTM1L
CREB3	CLTB
CREB3	CNKSR1
CREB3	CNOT11
CREB3	CNPY2
CREB3	COA1
CREB3	COASY

CREB3	COG1
CREB3	COG3
CREB3	COL4A4
CREB3	COMMD3
CREB3	COMMD8
CREB3	COPA
CREB3	COPB1
CREB3	COPB2
CREB3	COPE
CREB3	COPG1
CREB3	COPS5
CREB3	COPZ1
CREB3	COQ10B
CREB3	COQ9
CREB3	COX14
CREB3	COX5B
CREB3	CPD
CREB3	CPEB4
CREB3	CPSF4
CREB3	CREB3
CREB3	CREB3L2
CREB3	CREBL2
CREB3	CREBZF
CREB3	CRELD2
CREB3	CRLS1
CREB3	CSAD
CREB3	CSDE1
CREB3	CSGALNACT2
CREB3	CSNK1E
CREB3	CSTB
CREB3	CTDNEP1
CREB3	CTDP1
CREB3	CTHRC1
CREB3	CTNNBIP1
CREB3	CTNNBL1
CREB3	CTSA
CREB3	CTSD
CREB3	CTSZ
CREB3	CUTA
CREB3	CYB561D2
CREB3	CYB5R1
CREB3	CYB5R2
CREB3	CYCS
CREB3	CYLD
CREB3	CYP51A1
CREB3	CYTH2
CREB3	CYTIP
CREB3	DAD1
CREB3	DAP
CREB3	DAPL1

CREB3	DCAF11
CREB3	DCTN2
CREB3	DCTN3
CREB3	DCTN4
CREB3	DCXR
CREB3	DDT
CREB3	DDX51
CREB3	DECR1
CREB3	DEDD2
CREB3	DEF6
CREB3	DENND6B
CREB3	DERL1
CREB3	DERL3
CREB3	DESI1
CREB3	DHCR7
CREB3	DHPS
CREB3	DHRS11
CREB3	DHRS7
CREB3	DHX36
CREB3	DLGAP4
CREB3	DNAJB1
CREB3	DNAJB11
CREB3	DNAJB9
CREB3	DNAJC1
CREB3	DNAJC10
CREB3	DNAJC25
CREB3	DNAJC3
CREB3	DNAJC4
CREB3	DNAJC7
CREB3	DNASE2
CREB3	DNPH1
CREB3	DNTTIP1
CREB3	DPAGT1
CREB3	DPM1
CREB3	DPM2
CREB3	DPM3
CREB3	DPP7
CREB3	DSTN
CREB3	DTX3
CREB3	DUSP22
CREB3	DUSP4
CREB3	DUSP5
CREB3	DYNLL1
CREB3	DYRK4
CREB3	EAF2
CREB3	EBAG9
CREB3	EBPL
CREB3	ECI2
CREB3	EDEM1
CREB3	EDEM2

CREB3	EDF1
CREB3	EEF1A1
CREB3	EEF1D
CREB3	EEF2
CREB3	EEF2KMT
CREB3	EGLN2
CREB3	EGR3
CREB3	EHD4
CREB3	EIF1
CREB3	EIF1AD
CREB3	EIF2A
CREB3	EIF2B3
CREB3	EIF3G
CREB3	EIF3K
CREB3	EIF4A3
CREB3	EIF5
CREB3	ELL
CREB3	ELMOD2
CREB3	EMC10
CREB3	EMC2
CREB3	EMC3
CREB3	EMC4
CREB3	EMC9
CREB3	EMD
CREB3	EML2
CREB3	ENO1
CREB3	ENO2
CREB3	ENTPD1
CREB3	EPN2
CREB3	EPRS
CREB3	ERAL1
CREB3	ERCC1
CREB3	ERCC6
CREB3	ERGIC1
CREB3	ERGIC2
CREB3	ERGIC3
CREB3	ERLEC1
CREB3	ERO1A
CREB3	ERO1B
CREB3	ERP29
CREB3	ERP44
CREB3	ESCO1
CREB3	ESRRA
CREB3	EVI2A
CREB3	EVI2B
CREB3	EXOSC1
CREB3	EXT1
CREB3	EXTL2
CREB3	FAAP20
CREB3	FADD

CREB3	FAHD2B
CREB3	FAM118A
CREB3	FAM120AOS
CREB3	FAM174A
CREB3	FAM177A1
CREB3	FAM3C
CREB3	FAM50A
CREB3	FAM69A
CREB3	FAM72C
CREB3	FAM89B
CREB3	FAM8A1
CREB3	FAM98A
CREB3	FAR1
CREB3	FARSA
CREB3	FASTK
CREB3	FAU
CREB3	FBXO16
CREB3	FBXO31
CREB3	FBXW2
CREB3	FBXW7
CREB3	FDXACB1
CREB3	FECH
CREB3	FIBP
CREB3	FIS1
CREB3	FKBP11
CREB3	FKBP1A
CREB3	FKBP2
CREB3	FKBP8
CREB3	FN3K
CREB3	FNDC3A
CREB3	FOXN3
CREB3	FOXO3
CREB3	FPGS
CREB3	FRA10AC1
CREB3	FRMD8
CREB3	FRZB
CREB3	FTH1
CREB3	FTL
CREB3	FTSJ1
CREB3	FUCA2
CREB3	FUNDC2
CREB3	FUT8
CREB3	FUZ
CREB3	FZD3
CREB3	G3BP2
CREB3	G6PD
CREB3	GABARAP
CREB3	GADD45A
CREB3	GADD45B
CREB3	GALK2

CREB3	GALNT14
CREB3	GALNT2
CREB3	GANAB
CREB3	GAS7
CREB3	GBF1
CREB3	GEMIN7
CREB3	GFPT1
CREB3	GGCX
CREB3	GHITM
CREB3	GIPC1
CREB3	GLA
CREB3	GLCCI1
CREB3	GLG1
CREB3	GLIPR1
CREB3	GLMP
CREB3	GLRX
CREB3	GLT8D1
CREB3	GMFG
CREB3	GMPPA
CREB3	GMPPB
CREB3	GNAI2
CREB3	GNAS
CREB3	GNB2
CREB3	GNG7
CREB3	GNL3L
CREB3	GNPNAT1
CREB3	GNPTG
CREB3	GOLGA1
CREB3	GOLGA2
CREB3	GOLGA4
CREB3	GOLGA7
CREB3	GOLGB1
CREB3	GOLIM4
CREB3	GOLT1B
CREB3	GORASP2
CREB3	GOSR2
CREB3	GOT1
CREB3	GPANK1
CREB3	GPR108
CREB3	GPR160
CREB3	GPR89B
CREB3	GRB2
CREB3	GRIPAP1
CREB3	GRK6
CREB3	GTF2A1
CREB3	GTF2B
CREB3	GTF3A
CREB3	GTF3C2
CREB3	GUK1
CREB3	GUSB

CREB3	H1FX
CREB3	HAGH
CREB3	HAX1
CREB3	HBP1
CREB3	HBS1L
CREB3	HCCS
CREB3	HCFC1R1
CREB3	HCST
CREB3	HDLBP
CREB3	HEBP2
CREB3	HELB
CREB3	HERPUD1
CREB3	HEXB
CREB3	HID1
CREB3	HINFP
CREB3	HINT1
CREB3	HIST1H1C
CREB3	HLA-B
CREB3	HLA-DQA1
CREB3	HLA-DQB1
CREB3	HLA-DRB5
CREB3	HLA-E
CREB3	HLA-F
CREB3	HLCS
CREB3	HM13
CREB3	HMGCR
CREB3	HMOX1
CREB3	HOMER2
CREB3	HOOK2
CREB3	HOXA7
CREB3	HOXB7
CREB3	HSD17B10
CREB3	HSD17B7
CREB3	HSP90B1
CREB3	HSPA12A
CREB3	HSPA13
CREB3	HSPA5
CREB3	HTATIP2
CREB3	HYOU1
CREB3	IBTK
CREB3	ICAM2
CREB3	ICAM3
CREB3	IDH2
CREB3	IDH3A
CREB3	IDH3G
CREB3	IER2
CREB3	IER3IP1
CREB3	IFI27L2
CREB3	IFNAR1
CREB3	IFNG-AS1

CREB3	IFT27
CREB3	IFT74
CREB3	IGSF8
CREB3	IKBIP
CREB3	IL16
CREB3	IL4I1
CREB3	ILF2
CREB3	ILVBL
CREB3	IMMT
CREB3	IMPAD1
CREB3	ING3
CREB3	INPP4A
CREB3	INSIG1
CREB3	INSIG2
CREB3	INTS6
CREB3	INTS6L
CREB3	INTS7
CREB3	IQGAP2
CREB3	IRAK1
CREB3	IRF1
CREB3	IRF4
CREB3	ISCU
CREB3	ISG20
CREB3	ISOC2
CREB3	ITCH
CREB3	ITFG2
CREB3	ITGB2
CREB3	ITM2A
CREB3	ITM2B
CREB3	ITM2C
CREB3	JAGN1
CREB3	JCHAIN
CREB3	JMJD6
CREB3	JMJD8
CREB3	JMY
CREB3	JOSD2
CREB3	JUN
CREB3	JUNB
CREB3	KCNAB2
CREB3	KCNK12
CREB3	KCNK6
CREB3	KCNMA1
CREB3	KCNN3
CREB3	KCNN4
CREB3	KCTD20
CREB3	KDELR1
CREB3	KDELR2
CREB3	KDM8
CREB3	KEAP1
CREB3	KIAA0825

CREB3	KIAA0930
CREB3	KLC4
CREB3	KLF13
CREB3	KLHDC2
CREB3	KRTCAP2
CREB3	L3MBTL2
CREB3	LAGE3
CREB3	LAMP2
CREB3	LAMTOR1
CREB3	LAMTOR2
CREB3	LAP3
CREB3	LAPTM4A
CREB3	LARP1B
CREB3	LARP4
CREB3	LARS2
CREB3	LAX1
CREB3	LCORL
CREB3	LDAH
CREB3	LDLR
CREB3	LDLRAP1
CREB3	LEF1
CREB3	LEPROTL1
CREB3	LGALS1
CREB3	LMAN1
CREB3	LMBR1L
CREB3	LMBRD1
CREB3	LMF1
CREB3	LPIN1
CREB3	LRRC59
CREB3	LRRC8D
CREB3	LSP1
CREB3	LTBP3
CREB3	LY75
CREB3	LY96
CREB3	LYSMD3
CREB3	LZTFL1
CREB3	M6PR
CREB3	MAD2L1BP
CREB3	MAGED1
CREB3	MAGEH1
CREB3	MAGOHB
CREB3	MAGT1
CREB3	MAN1A1
CREB3	MAN1A2
CREB3	MANF
CREB3	MAP1LC3A
CREB3	MAP2K2
CREB3	MAP2K3
CREB3	MAP2K7
CREB3	MAPKAP1

CREB3	MAPKAPK2
CREB3	MAPKAPK3
CREB3	MAPKAPK5-AS1
CREB3	MARCH6
CREB3	MARS
CREB3	MAX
CREB3	MBNL2
CREB3	MBTPS1
CREB3	MCCC2
CREB3	MCEE
CREB3	MCFD2
CREB3	MCL1
CREB3	MCUR1
CREB3	MEF2C
CREB3	MEF2D
CREB3	MEI1
CREB3	MESP1
CREB3	METTL1
CREB3	METTL23
CREB3	MFF
CREB3	MFSD14C
CREB3	MGAT1
CREB3	MGLL
CREB3	MGST2
CREB3	MIA3
CREB3	MIEN1
CREB3	MIF
CREB3	MIR4435-2HG
CREB3	MLEC
CREB3	MLF2
CREB3	MLX
CREB3	MLXIP
CREB3	MMAB
CREB3	MOCS2
CREB3	MOGS
CREB3	MON1B
CREB3	MORF4L2
CREB3	MOSPD1
CREB3	MPC1
CREB3	MPG
CREB3	MPHOSPH8
CREB3	MPV17
CREB3	MPV17L2
CREB3	MRPL15
CREB3	MRPL2
CREB3	MRPL23
CREB3	MRPL24
CREB3	MRPL40
CREB3	MRPL52
CREB3	MRPL54

CREB3	MRPL55
CREB3	MRPL57
CREB3	MRPS18A
CREB3	MRPS33
CREB3	MRPS36
CREB3	MSL2
CREB3	MSRB1
CREB3	MT1X
CREB3	MTDH
CREB3	MTFR1
CREB3	MTRF1L
CREB3	MUL1
CREB3	MVP
CREB3	MXD4
CREB3	MYDGF
CREB3	MYL6
CREB3	MYO1G
CREB3	MZB1
CREB3	MZF1
CREB3	N4BP1
CREB3	N4BP2L1
CREB3	N4BP2L2
CREB3	NAA38
CREB3	NAB2
CREB3	NABP1
CREB3	NAGK
CREB3	NANS
CREB3	NAPA
CREB3	NARF
CREB3	NCK2
CREB3	NCLN
CREB3	NCSTN
CREB3	NDEL1
CREB3	NDFIP1
CREB3	NDFIP2
CREB3	NDUFA1
CREB3	NDUFA11
CREB3	NDUFA13
CREB3	NDUFA4
CREB3	NDUFA7
CREB3	NDUFAB1
CREB3	NDUFAF3
CREB3	NDUFB2
CREB3	NDUFB4
CREB3	NDUFB6
CREB3	NDUFS2
CREB3	NDUFS6
CREB3	NDUFS8
CREB3	NDUFV1
CREB3	NDUFV3

CREB3	NEAT1
CREB3	NECAP1
CREB3	NEDD9
CREB3	NEK1
CREB3	NELFE
CREB3	NENF
CREB3	NET1
CREB3	NEU1
CREB3	NFS1
CREB3	NFXL1
CREB3	NGLY1
CREB3	NIPA2
CREB3	NKAP
CREB3	NLRC3
CREB3	NME7
CREB3	NOL7
CREB3	NOMO1
CREB3	NOP10
CREB3	NOSIP
CREB3	NPRL2
CREB3	NRBF2
CREB3	NRBP1
CREB3	NRDC
CREB3	NSD1
CREB3	NSFL1C
CREB3	NT5C2
CREB3	NT5C3A
CREB3	NT5DC2
CREB3	NT5DC3
CREB3	NTMT1
CREB3	NUBPL
CREB3	NUCB2
CREB3	NUDT2
CREB3	NUDT6
CREB3	NUMB
CREB3	NUPL2
CREB3	NUS1
CREB3	NXPE3
CREB3	OAZ1
CREB3	ODF3B
CREB3	OGFOD2
CREB3	ORMDL1
CREB3	ORMDL2
CREB3	OS9
CREB3	OSBP
CREB3	OSBPL2
CREB3	OST4
CREB3	OSTC
CREB3	OSTF1
CREB3	OTUD1

CREB3	OTUD7A
CREB3	P4HB
CREB3	PAIP2B
CREB3	PANK3
CREB3	PAPOLA
CREB3	PAPSS1
CREB3	PARVG
CREB3	PAXBP1
CREB3	PBDC1
CREB3	PCBP1
CREB3	PDAP1
CREB3	PDHB
CREB3	PDIA3
CREB3	PDIA4
CREB3	PDIA6
CREB3	PDK1
CREB3	PDK2
CREB3	PDXK
CREB3	PEBP1
CREB3	PEPD
CREB3	PET100
CREB3	PEX2
CREB3	PGAM1
CREB3	PGK1
CREB3	PGM1
CREB3	PGM3
CREB3	PGP
CREB3	PGPEP1
CREB3	PGRMC2
CREB3	PHACTR1
CREB3	PHF19
CREB3	PHPT1
CREB3	PHYH
CREB3	PICK1
CREB3	PIGH
CREB3	PIGP
CREB3	PIK3C2A
CREB3	PIM2
CREB3	PINK1
CREB3	PITPNA
CREB3	PITPNB
CREB3	PJA1
CREB3	PJA2
CREB3	PKHD1L1
CREB3	PLD3
CREB3	PLD4
CREB3	PLOD3
CREB3	PLPP5
CREB3	PMVK
CREB3	PNKP

CREB3	PNOC
CREB3	PNPLA8
CREB3	PODXL2
CREB3	POGLUT1
CREB3	POLDIP2
CREB3	POLH
CREB3	POLR3D
CREB3	POMP
CREB3	POP5
CREB3	POP7
CREB3	PORCN
CREB3	PPCDC
CREB3	PPIB
CREB3	PPIF
CREB3	PPP1R11
CREB3	PPP1R14B
CREB3	PPP1R15A
CREB3	PPP2R5B
CREB3	PPP3R1
CREB3	PPTC7
CREB3	PRADC1
CREB3	PRAF2
CREB3	PRCP
CREB3	PRDM1
CREB3	PRDX2
CREB3	PRDX4
CREB3	PREB
CREB3	PRKAR2B
CREB3	PRKCSH
CREB3	PRKCZ
CREB3	PRKD2
CREB3	PRMT2
CREB3	PRPF3
CREB3	PRPF4B
CREB3	PRPF6
CREB3	PRPS1
CREB3	PRRC1
CREB3	PSMA1
CREB3	PSMA5
CREB3	PSMB1
CREB3	PSMB10
CREB3	PSMB2
CREB3	PSMB3
CREB3	PSMB4
CREB3	PSMB6
CREB3	PSMC3
CREB3	PSMC4
CREB3	PSMC5
CREB3	PSMC6
CREB3	PSMD12

CREB3	PSMD13
CREB3	PSMD2
CREB3	PSMD3
CREB3	PSMD6
CREB3	PSMD7
CREB3	PSMD8
CREB3	PSME4
CREB3	PSMF1
CREB3	PSMG3
CREB3	PTGIR
CREB3	PTPN23
CREB3	PTPN6
CREB3	PTPRN2
CREB3	PTS
CREB3	PUS7L
CREB3	PYHIN1
CREB3	QPCT
CREB3	QTRT1
CREB3	RAB10
CREB3	RAB11B
CREB3	RAB11B-AS1
CREB3	RAB18
CREB3	RAB1A
CREB3	RAB21
CREB3	RAB27A
CREB3	RAB2A
CREB3	RAB39B
CREB3	RAB6A
CREB3	RAB8A
CREB3	RABAC1
CREB3	RABGGTA
CREB3	RACK1
CREB3	RASAL3
CREB3	RASSF5
CREB3	RBBP5
CREB3	RBBP6
CREB3	RBM18
CREB3	RBM3
CREB3	RBM4
CREB3	RBM47
CREB3	RCN1
CREB3	RDH11
CREB3	REEP3
CREB3	REEP5
CREB3	RER1
CREB3	REXO2
CREB3	RFESD
CREB3	RGS1
CREB3	RGS2
CREB3	RHEB

CREB3	RIDA
CREB3	RIOK2
CREB3	RNASE4
CREB3	RNASEH1
CREB3	RNASEL
CREB3	RNF103
CREB3	RNF11
CREB3	RNF113A
CREB3	RNF114
CREB3	RNF139
CREB3	RNF149
CREB3	RNF181
CREB3	RNH1
CREB3	RPAP1
CREB3	RPL10
CREB3	RPL13A
CREB3	RPL15
CREB3	RPL19
CREB3	RPL23A
CREB3	RPL3
CREB3	RPL32
CREB3	RPL35A
CREB3	RPL36AL
CREB3	RPL41
CREB3	RPL5
CREB3	RPLP0
CREB3	RPLP1
CREB3	RPLP2
CREB3	RPN1
CREB3	RPN2
CREB3	RPS11
CREB3	RPS12
CREB3	RPS13
CREB3	RPS19
CREB3	RPS19BP1
CREB3	RPS26
CREB3	RPS27L
CREB3	RPS28
CREB3	RPS29
CREB3	RPS3
CREB3	RPS4X
CREB3	RPS6KB2
CREB3	RPS9
CREB3	RPSA
CREB3	RRAGD
CREB3	RRBP1
CREB3	RSU1
CREB3	RTCA
CREB3	RTF1
CREB3	RTN3

CREB3	RUNX1
CREB3	RWDD1
CREB3	RWDD2B
CREB3	RWDD4
CREB3	RXRB
CREB3	S100A11
CREB3	SAMD4B
CREB3	SAMM50
CREB3	SAP18
CREB3	SAP30BP
CREB3	SAR1A
CREB3	SAR1B
CREB3	SARAF
CREB3	SARS
CREB3	SART1
CREB3	SASH3
CREB3	SAT1
CREB3	SCAMP1
CREB3	SCAMP2
CREB3	SCAMP3
CREB3	SCAMP4
CREB3	SCAND1
CREB3	SCFD1
CREB3	SCPEP1
CREB3	SCYL1
CREB3	SDAD1
CREB3	SDF2L1
CREB3	SDF4
CREB3	SDHAF1
CREB3	SDHC
CREB3	SEC11C
CREB3	SEC13
CREB3	SEC22A
CREB3	SEC22B
CREB3	SEC22C
CREB3	SEC23B
CREB3	SEC24A
CREB3	SEC24C
CREB3	SEC24D
CREB3	SEC31A
CREB3	SEC61A1
CREB3	SEC61B
CREB3	SEC61G
CREB3	SEC62
CREB3	SEC63
CREB3	SEL1L
CREB3	SEL1L3
CREB3	SELPLG
CREB3	SENP8
CREB3	SERF2

CREB3	SERINC3
CREB3	SERP1
CREB3	SERTAD3
CREB3	SF3B2
CREB3	SF3B5
CREB3	SFMBT2
CREB3	SGK3
CREB3	SGTA
CREB3	SH3GLB1
CREB3	SHANK2
CREB3	SHC1
CREB3	SHMT2
CREB3	SIL1
CREB3	SIRT2
CREB3	SKAP1
CREB3	SLC10A7
CREB3	SLC16A3
CREB3	SLC17A9
CREB3	SLC1A4
CREB3	SLC1A5
CREB3	SLC22A18
CREB3	SLC25A22
CREB3	SLC25A28
CREB3	SLC25A29
CREB3	SLC25A3
CREB3	SLC25A39
CREB3	SLC25A42
CREB3	SLC25A45
CREB3	SLC25A46
CREB3	SLC2A8
CREB3	SLC30A5
CREB3	SLC30A7
CREB3	SLC31A2
CREB3	SLC33A1
CREB3	SLC35B1
CREB3	SLC35B2
CREB3	SLC35C1
CREB3	SLC35C2
CREB3	SLC35E1
CREB3	SLC35F6
CREB3	SLC38A10
CREB3	SLC38A2
CREB3	SLC38A5
CREB3	SLC39A11
CREB3	SLC39A6
CREB3	SLC39A7
CREB3	SLC39A8
CREB3	SLC3A2
CREB3	SLC44A1
CREB3	SLC4A2

CREB3	SLC50A1
CREB3	SLC52A2
CREB3	SLC7A11
CREB3	SLC9A3R1
CREB3	SLCO4A1
CREB3	SMAD5
CREB3	SMDT1
CREB3	SMU1
CREB3	SMUG1
CREB3	SNAP29
CREB3	SNAPC3
CREB3	SND1
CREB3	SND1-IT1
CREB3	SNF8
CREB3	SNHG21
CREB3	SNRPC
CREB3	SNRPD2
CREB3	SNRPN
CREB3	SNU13
CREB3	SNURF
CREB3	SNX16
CREB3	SNX18
CREB3	SNX9
CREB3	SOCS3
CREB3	SPAG7
CREB3	SPAG9
CREB3	SPATS2
CREB3	SPCS1
CREB3	SPINT2
CREB3	SPNS1
CREB3	SPSB1
CREB3	SPSB3
CREB3	SPTSSA
CREB3	SPTY2D1
CREB3	SQLE
CREB3	SQSTM1
CREB3	SRBD1
CREB3	SRD5A3
CREB3	SREBF2
CREB3	SRGN
CREB3	SRM
CREB3	SRP14
CREB3	SRP19
CREB3	SRP54
CREB3	SRP68
CREB3	SRP72
CREB3	SRPRA
CREB3	SRRM2
CREB3	SRSF1
CREB3	SS18L2

CREB3	SSNA1
CREB3	SSR1
CREB3	SSR3
CREB3	SSR4
CREB3	SSSCA1
CREB3	ST6GAL1
CREB3	ST6GALNAC4
CREB3	ST7
CREB3	ST8SIA4
CREB3	STAMBPL1
CREB3	STK10
CREB3	STK17B
CREB3	STK25
CREB3	STT3A
CREB3	STT3B
CREB3	STUB1
CREB3	STX5
CREB3	STXBP6
CREB3	SUB1
CREB3	SUCLG1
CREB3	SUPT5H
CREB3	SURF4
CREB3	SWSAP1
CREB3	SYNGR1
CREB3	SYS1
CREB3	SYTL1
CREB3	SYVN1
CREB3	TAF7
CREB3	TANGO2
CREB3	TARSL2
CREB3	TAX1BP1
CREB3	TBC1D15
CREB3	TBC1D20
CREB3	TBC1D22A
CREB3	TBC1D4
CREB3	TBC1D7
CREB3	TCAF1
CREB3	TCEAL8
CREB3	TCEAL9
CREB3	TCF25
CREB3	TCF4
CREB3	TCTA
CREB3	TDG
CREB3	TDRD3
CREB3	TECR
CREB3	TFG
CREB3	TGDS
CREB3	THAP7
CREB3	THEMIS2
CREB3	TIMM10B

CREB3	TIMM17A
CREB3	TIMM9
CREB3	TIMP1
CREB3	TINF2
CREB3	TIPRL
CREB3	TLR1
CREB3	TM2D2
CREB3	TM7SF2
CREB3	TM9SF1
CREB3	TM9SF2
CREB3	TM9SF3
CREB3	TMBIM1
CREB3	TMBIM4
CREB3	TMBIM6
CREB3	TMC6
CREB3	TMCO1
CREB3	TMED1
CREB3	TMED10
CREB3	TMED2
CREB3	TMED3
CREB3	TMED4
CREB3	TMED7
CREB3	TMED9
CREB3	TMEM101
CREB3	TMEM126A
CREB3	TMEM147
CREB3	TMEM160
CREB3	TMEM161B
CREB3	TMEM165
CREB3	TMEM167A
CREB3	TMEM170A
CREB3	TMEM179B
CREB3	TMEM199
CREB3	TMEM214
CREB3	TMEM219
CREB3	TMEM230
CREB3	TMEM243
CREB3	TMEM248
CREB3	TMEM258
CREB3	TMEM263
CREB3	TMEM30A
CREB3	TMEM39A
CREB3	TMEM50B
CREB3	TMEM59
CREB3	TMEM70
CREB3	TMEM87A
CREB3	TMEM9
CREB3	TMF1
CREB3	TMSB10
CREB3	TMSB4X

CREB3	TMUB1
CREB3	TNFAIP8L2
CREB3	TNFRSF14
CREB3	TNFRSF1B
CREB3	TNFRSF9
CREB3	TOLLIP
CREB3	TOMM20
CREB3	TOP3B
CREB3	TOR3A
CREB3	TP53BP1
CREB3	TP53INP1
CREB3	TPD52
CREB3	TPI1
CREB3	TPP2
CREB3	TPRA1
CREB3	TPST2
CREB3	TPT1
CREB3	TRABD
CREB3	TRAF3
CREB3	TRAF3IP2-AS1
CREB3	TRAF6
CREB3	TRAM1
CREB3	TRAPPC2B
CREB3	TRAPPC2L
CREB3	TRAPPC3
CREB3	TRAPPC6A
CREB3	TRIB1
CREB3	TRIM11
CREB3	TRIM27
CREB3	TRIP11
CREB3	TRIP4
CREB3	TRMT61B
CREB3	TRPV2
CREB3	TSC22D1
CREB3	TSC22D4
CREB3	TSPAN13
CREB3	TSPAN31
CREB3	TSPYL2
CREB3	TSTA3
CREB3	TTC1
CREB3	TTC17
CREB3	TTC3
CREB3	TTC33
CREB3	TUSC2
CREB3	TUT1
CREB3	TVP23B
CREB3	TWSG1
CREB3	TXLNA
CREB3	TXN
CREB3	TXNDC11

CREB3	TXNDC12
CREB3	TXNDC15
CREB3	TXNDC5
CREB3	TXNL1
CREB3	TXNL4A
CREB3	TYMP
CREB3	UAP1
CREB3	UBA5
CREB3	UBA52
CREB3	UBA6-AS1
CREB3	UBALD1
CREB3	UBALD2
CREB3	UBB
CREB3	UBC
CREB3	UBE2D2
CREB3	UBE2D4
CREB3	UBE2J1
CREB3	UBE2K
CREB3	UBE2Q1
CREB3	UBFD1
CREB3	UBL5
CREB3	UBN1
CREB3	UBN2
CREB3	UBXN4
CREB3	UCHL5
CREB3	UFC1
CREB3	UFL1
CREB3	UFM1
CREB3	UFSP2
CREB3	UGDH
CREB3	UNC13D
CREB3	UNC50
CREB3	UNC93B1
CREB3	UPF2
CREB3	UPP1
CREB3	UQCR10
CREB3	UQCR11
CREB3	UQCRB
CREB3	UQCRFS1
CREB3	UROD
CREB3	USO1
CREB3	USP14
CREB3	USP16
CREB3	USP3
CREB3	USP40
CREB3	USP48
CREB3	UTP11
CREB3	UTP23
CREB3	VAMP2
CREB3	VCP

CREB3	VDAC1
CREB3	VDAC3
CREB3	VKORC1
CREB3	VKORC1L1
CREB3	VMA21
CREB3	VMP1
CREB3	VOPP1
CREB3	VPS41
CREB3	VPS53
CREB3	VRK3
CREB3	WDR92
CREB3	WFS1
CREB3	WIPI1
CREB3	WWOX
CREB3	XBP1
CREB3	XIAP
CREB3	XRRA1
CREB3	YBX1
CREB3	YIF1A
CREB3	YIF1B
CREB3	YIPF1
CREB3	YIPF2
CREB3	YIPF3
CREB3	YIPF5
CREB3	YIPF6
CREB3	YKT6
CREB3	YPEL5
CREB3	YWHAB
CREB3	ZAP70
CREB3	ZBP1
CREB3	ZBTB1
CREB3	ZBTB45
CREB3	ZBTB7B
CREB3	ZBTB8OS
CREB3	ZC3H12A
CREB3	ZC3H15
CREB3	ZCWPW1
CREB3	ZDHHC3
CREB3	ZFAND2A
CREB3	ZFAND5
CREB3	ZFAT
CREB3	ZFP36L2
CREB3	ZFPL1
CREB3	ZHX2
CREB3	ZNF215
CREB3	ZNF226
CREB3	ZNF275
CREB3	ZNF276
CREB3	ZNF3
CREB3	ZNF331

CREB3	ZNF335
CREB3	ZNF552
CREB3	ZNF566
CREB3	ZNF574
CREB3	ZNF593
CREB3	ZNF598
CREB3	ZNF670
CREB3	ZNF706
CREB3	ZNF708
CREB3	ZNF720
CREB3	ZNF791
CREB3	ZNHIT1
CREB3	ZNHIT2
CREB3	ZRANB2
CREB3	ZRSR2
CREB3	ZSWIM7
CREB3	ZW10
CREB3L2	AACS
CREB3L2	AARS
CREB3L2	ABCC4
CREB3L2	ABCF3
CREB3L2	ABHD17A
CREB3L2	ABTB1
CREB3L2	ACADVL
CREB3L2	ACBD3
CREB3L2	ACO2
CREB3L2	ACOT13
CREB3L2	ADAM10
CREB3L2	ADAR
CREB3L2	ADD1
CREB3L2	ADPGK
CREB3L2	AGPAT1
CREB3L2	AIFM2
CREB3L2	AKAP5
CREB3L2	AKAP9
CREB3L2	AKR1A1
CREB3L2	ALDH1L2
CREB3L2	ALDOC
CREB3L2	ALG1
CREB3L2	ALG2
CREB3L2	ALG3
CREB3L2	ALKBH5
CREB3L2	AMPD2
CREB3L2	ANAPC13
CREB3L2	ANG
CREB3L2	ANKRD12
CREB3L2	ANKRD28
CREB3L2	ANKRD39
CREB3L2	ANXA11
CREB3L2	ANXA6

CREB3L2	AP2A1
CREB3L2	AP3D1
CREB3L2	APTX
CREB3L2	ARCN1
CREB3L2	AREG
CREB3L2	ARF1
CREB3L2	ARF4
CREB3L2	ARFGAP1
CREB3L2	ARFGAP3
CREB3L2	ARFIP2
CREB3L2	ARHGAP24
CREB3L2	ARID1B
CREB3L2	ARID3A
CREB3L2	ARID3B
CREB3L2	ARL1
CREB3L2	ARMCX3
CREB3L2	ARMCX6
CREB3L2	ATF7
CREB3L2	ATG101
CREB3L2	ATG13
CREB3L2	ATG3
CREB3L2	ATG4C
CREB3L2	ATMIN
CREB3L2	ATP2A2
CREB3L2	ATP6AP1
CREB3L2	ATP6AP2
CREB3L2	ATP6V0A1
CREB3L2	ATP6V0B
CREB3L2	ATP6V0E1
CREB3L2	ATP6V1D
CREB3L2	ATRAID
CREB3L2	ATXN1
CREB3L2	B3GAT3
CREB3L2	B4GALT3
CREB3L2	B9D1
CREB3L2	BAZ2A
CREB3L2	BBIP1
CREB3L2	BCAP31
CREB3L2	BCKDK
CREB3L2	BET1
CREB3L2	BEX5
CREB3L2	BHLHE41
CREB3L2	BLNK
CREB3L2	BLOC1S3
CREB3L2	BRD2
CREB3L2	BSG
CREB3L2	BTG2
CREB3L2	C16orf95
CREB3L2	C19orf24
CREB3L2	C19orf53

CREB3L2	C1GALT1C1
CREB3L2	C21orf2
CREB3L2	C2CD2L
CREB3L2	C2orf74
CREB3L2	CALCOCO1
CREB3L2	CALR
CREB3L2	CALU
CREB3L2	CANX
CREB3L2	CAPN10
CREB3L2	CASP8
CREB3L2	CCDC112
CREB3L2	CCDC144A
CREB3L2	CCDC159
CREB3L2	CCDC167
CREB3L2	CCDC174
CREB3L2	CCDC47
CREB3L2	CCDC57
CREB3L2	CCDC88A
CREB3L2	CCNC
CREB3L2	CCNL1
CREB3L2	CCPG1
CREB3L2	CCR10
CREB3L2	CD2BP2
CREB3L2	CD38
CREB3L2	CD59
CREB3L2	CD63
CREB3L2	CDC42SE1
CREB3L2	CDIPT
CREB3L2	CDK12
CREB3L2	CDK2AP2
CREB3L2	CDK6
CREB3L2	CELF2
CREB3L2	CENPA
CREB3L2	CERCAM
CREB3L2	CFDP1
CREB3L2	CGGBP1
CREB3L2	CHCHD2
CREB3L2	CHID1
CREB3L2	CHP1
CREB3L2	CHPF
CREB3L2	CHRNB1
CREB3L2	CHST11
CREB3L2	CHST12
CREB3L2	CISD2
CREB3L2	CITED2
CREB3L2	CKAP4
CREB3L2	CLCC1
CREB3L2	CLCN3
CREB3L2	CLIC4
CREB3L2	CLINT1

CREB3L2	CLIP4
CREB3L2	CLK1
CREB3L2	CLPTM1
CREB3L2	CLPTM1L
CREB3L2	CNPY2
CREB3L2	COMMD1
CREB3L2	COMMD3
CREB3L2	COPA
CREB3L2	COPB1
CREB3L2	COPB2
CREB3L2	COPE
CREB3L2	COPG1
CREB3L2	COPZ1
CREB3L2	COX18
CREB3L2	COX5B
CREB3L2	CREB3
CREB3L2	CREB3L2
CREB3L2	CREG1
CREB3L2	CRELD2
CREB3L2	CRIPT
CREB3L2	CSNK1E
CREB3L2	CSNK2A2
CREB3L2	CTBP1-AS
CREB3L2	CTDNEP1
CREB3L2	CTSA
CREB3L2	CTSD
CREB3L2	CUTA
CREB3L2	CXorf40B
CREB3L2	CYCS
CREB3L2	CYTIP
CREB3L2	DAD1
CREB3L2	DCAF8
CREB3L2	DCTN5
CREB3L2	DDHD1
CREB3L2	DECR1
CREB3L2	DEDD2
CREB3L2	DENND1B
CREB3L2	DERL1
CREB3L2	DERL3
CREB3L2	DEXI
CREB3L2	DGAT1
CREB3L2	DGKH
CREB3L2	DHRS7
CREB3L2	DHRS9
CREB3L2	DIAPH3
CREB3L2	DIDO1
CREB3L2	DNAJB11
CREB3L2	DNAJB12
CREB3L2	DNAJB9
CREB3L2	DNAJC1

CREB3L2	DNAJC25
CREB3L2	DNAJC3
CREB3L2	DNAJC4
CREB3L2	DNAJC7
CREB3L2	DNASE2
CREB3L2	DPM2
CREB3L2	DPM3
CREB3L2	DSTN
CREB3L2	DYRK4
CREB3L2	EBAG9
CREB3L2	EDEM1
CREB3L2	EDEM2
CREB3L2	EEF2
CREB3L2	EIF3G
CREB3L2	EIF3K
CREB3L2	EIF4G1
CREB3L2	EIF5
CREB3L2	ELL
CREB3L2	ELMOD2
CREB3L2	EMC10
CREB3L2	EMC2
CREB3L2	EMC3
CREB3L2	EMC4
CREB3L2	EMC9
CREB3L2	EMD
CREB3L2	ENAM
CREB3L2	ENTPD1
CREB3L2	EPB41
CREB3L2	EPOR
CREB3L2	EPRS
CREB3L2	ERC1
CREB3L2	ERF
CREB3L2	ERGIC2
CREB3L2	ERGIC3
CREB3L2	ERLEC1
CREB3L2	ERO1A
CREB3L2	ERP29
CREB3L2	ERP44
CREB3L2	ETNK1
CREB3L2	EXD2
CREB3L2	EXOC4
CREB3L2	FADS1
CREB3L2	FAM117A
CREB3L2	FAM174A
CREB3L2	FAM177A1
CREB3L2	FAM193A
CREB3L2	FAM214A
CREB3L2	FAM3C
CREB3L2	FAM50A
CREB3L2	FAM83D

CREB3L2	FAM91A1
CREB3L2	FAM98A
CREB3L2	FASTK
CREB3L2	FAU
CREB3L2	FBXO25
CREB3L2	FBXO33
CREB3L2	FBXW11
CREB3L2	FBXW5
CREB3L2	FCMR
CREB3L2	FECH
CREB3L2	FIS1
CREB3L2	FKBP11
CREB3L2	FKBP14
CREB3L2	FKBP2
CREB3L2	FN3KRP
CREB3L2	FNDC3A
CREB3L2	FOS
CREB3L2	FOXN3
CREB3L2	FOXO3
CREB3L2	FTL
CREB3L2	FTSJ1
CREB3L2	FUCA2
CREB3L2	FUT8
CREB3L2	GABARAP
CREB3L2	GADD45B
CREB3L2	GALNS
CREB3L2	GALNT2
CREB3L2	GANAB
CREB3L2	GAS2L3
CREB3L2	GBF1
CREB3L2	GCLC
CREB3L2	GFPT1
CREB3L2	GHITM
CREB3L2	GLA
CREB3L2	GLMP
CREB3L2	GLRX
CREB3L2	GLT8D1
CREB3L2	GMPPA
CREB3L2	GMPPB
CREB3L2	GNPNAT1
CREB3L2	GOLGA2
CREB3L2	GOLGA4
CREB3L2	GOLGA5
CREB3L2	GOLGB1
CREB3L2	GOLPH3L
CREB3L2	GOPC
CREB3L2	GORASP2
CREB3L2	GOSR2
CREB3L2	GPANK1
CREB3L2	GPLD1

CREB3L2	GPR108
CREB3L2	GPR137B
CREB3L2	GPR146
CREB3L2	GPR160
CREB3L2	GPR65
CREB3L2	GPX7
CREB3L2	GRN
CREB3L2	GUK1
CREB3L2	GUSB
CREB3L2	H1FX
CREB3L2	HAUS5
CREB3L2	HBS1L
CREB3L2	HCST
CREB3L2	HDLBP
CREB3L2	HERPUD1
CREB3L2	HEXA
CREB3L2	HID1
CREB3L2	HIST1H1C
CREB3L2	HIST1H2BJ
CREB3L2	HIST1H2BN
CREB3L2	HIST3H2A
CREB3L2	HLA-B
CREB3L2	HLA-DRB5
CREB3L2	HLA-E
CREB3L2	HM13
CREB3L2	HMG20A
CREB3L2	HMGXB3
CREB3L2	HPCAL1
CREB3L2	HSD17B10
CREB3L2	HSP90B1
CREB3L2	HSPA5
CREB3L2	ICAM2
CREB3L2	ICAM3
CREB3L2	IDH3G
CREB3L2	IER3IP1
CREB3L2	IFI27L2
CREB3L2	IFNAR1
CREB3L2	IFNAR2
CREB3L2	IFNG-AS1
CREB3L2	IFRD1
CREB3L2	IFT27
CREB3L2	IFT81
CREB3L2	IGF2R
CREB3L2	IGSF8
CREB3L2	IKBIP
CREB3L2	IL10RA
CREB3L2	IL16
CREB3L2	IL6R
CREB3L2	INPP4A
CREB3L2	INSIG1

CREB3L2	INSIG2
CREB3L2	IQGAP2
CREB3L2	IRAK1
CREB3L2	IRF1
CREB3L2	IRF2
CREB3L2	IRF4
CREB3L2	IRF7
CREB3L2	IRF9
CREB3L2	ISCU
CREB3L2	ISG20
CREB3L2	ISOC2
CREB3L2	ITGB1
CREB3L2	ITM2B
CREB3L2	ITM2C
CREB3L2	JCHAIN
CREB3L2	JMJD6
CREB3L2	KCNK6
CREB3L2	KCNN3
CREB3L2	KDELR1
CREB3L2	KDELR2
CREB3L2	KDELR3
CREB3L2	KIAA0930
CREB3L2	KIAA2013
CREB3L2	KIF13B
CREB3L2	KLF13
CREB3L2	KLHDC10
CREB3L2	KLHL9
CREB3L2	KMT2E-AS1
CREB3L2	KRTCAP2
CREB3L2	LAMTOR4
CREB3L2	LAP3
CREB3L2	LAPTM4A
CREB3L2	LARP1B
CREB3L2	LAS1L
CREB3L2	LAX1
CREB3L2	LCMT1
CREB3L2	LDLRAD4
CREB3L2	LGMN
CREB3L2	LMAN1
CREB3L2	LMBRD1
CREB3L2	LMF1
CREB3L2	LMF2
CREB3L2	LMTK3
CREB3L2	LPIN1
CREB3L2	LRP10
CREB3L2	LRRC59
CREB3L2	LSP1
CREB3L2	LSR
CREB3L2	LTBP3
CREB3L2	LYSMD3

CREB3L2	LZTFL1
CREB3L2	MACROD2
CREB3L2	MAGED1
CREB3L2	MAGT1
CREB3L2	MAN1A1
CREB3L2	MAN1A2
CREB3L2	MANF
CREB3L2	MAP1LC3A
CREB3L2	MAP1LC3B
CREB3L2	MAP3K14
CREB3L2	MAPK6
CREB3L2	MARCH5
CREB3L2	MARCH6
CREB3L2	MARCKS
CREB3L2	MBTPS1
CREB3L2	MCFD2
CREB3L2	MCL1
CREB3L2	MCUR1
CREB3L2	MEF2C
CREB3L2	MEI1
CREB3L2	METTL15
CREB3L2	MFF
CREB3L2	MGAT1
CREB3L2	MGST2
CREB3L2	MIA2
CREB3L2	MIEF1
CREB3L2	MIR193BHG
CREB3L2	MIR4435−2HG
CREB3L2	MLEC
CREB3L2	MOGS
CREB3L2	MORF4L2
CREB3L2	MPC1
CREB3L2	MPG
CREB3L2	MPHOSPH8
CREB3L2	MPI
CREB3L2	MPV17L2
CREB3L2	MRPL40
CREB3L2	MRPL52
CREB3L2	MRPL54
CREB3L2	MRPL55
CREB3L2	MRPS14
CREB3L2	MRPS18B
CREB3L2	MT1X
CREB3L2	MTDH
CREB3L2	MTFP1
CREB3L2	MTHFD2L
CREB3L2	MXD4
CREB3L2	MYCBP2
CREB3L2	MYDGF
CREB3L2	MYH10

CREB3L2	MYL6
CREB3L2	MZB1
CREB3L2	N4BP2L2
CREB3L2	NAGK
CREB3L2	NANS
CREB3L2	NAPA
CREB3L2	NBAS
CREB3L2	NCK2
CREB3L2	NCSTN
CREB3L2	NDFIP1
CREB3L2	NDUFA1
CREB3L2	NDUFA11
CREB3L2	NDUFA13
CREB3L2	NDUFA4
CREB3L2	NDUFAB1
CREB3L2	NDUFAF3
CREB3L2	NDUFB4
CREB3L2	NDUFB6
CREB3L2	NDUFC2
CREB3L2	NDUFS6
CREB3L2	NDUFV1
CREB3L2	NDUFV3
CREB3L2	NEAT1
CREB3L2	NEDD9
CREB3L2	NEK8
CREB3L2	NELFE
CREB3L2	NET1
CREB3L2	NEU1
CREB3L2	NFATC3
CREB3L2	NGLY1
CREB3L2	NIPA2
CREB3L2	NKIRAS2
CREB3L2	NMRK1
CREB3L2	NNT-AS1
CREB3L2	NPC2
CREB3L2	NRBF2
CREB3L2	NRBP1
CREB3L2	NRDC
CREB3L2	NRIP1
CREB3L2	NSMCE3
CREB3L2	NSUN4
CREB3L2	NT5DC2
CREB3L2	NUCB2
CREB3L2	NUS1
CREB3L2	OAF
CREB3L2	OAS2
CREB3L2	ODF3B
CREB3L2	OGFOD2
CREB3L2	ORMDL1
CREB3L2	ORMDL3

CREB3L2	OS9
CREB3L2	OSBP
CREB3L2	OSTC
CREB3L2	OSTM1
CREB3L2	OTUD5
CREB3L2	OXA1L
CREB3L2	P2RX1
CREB3L2	P4HA1
CREB3L2	P4HB
CREB3L2	PACSIN2
CREB3L2	PANK3
CREB3L2	PAPSS1
CREB3L2	PARP4
CREB3L2	PBXIP1
CREB3L2	PCBP1
CREB3L2	PCGF3
CREB3L2	PDE4B
CREB3L2	PDE4DIP
CREB3L2	PDIA4
CREB3L2	PDIA5
CREB3L2	PDIA6
CREB3L2	PDK1
CREB3L2	PDXDC1
CREB3L2	PDXK
CREB3L2	PECAM1
CREB3L2	PET100
CREB3L2	PEX2
CREB3L2	PEX6
CREB3L2	PGM3
CREB3L2	PGRMC2
CREB3L2	PHACTR1
CREB3L2	PHC3
CREB3L2	PHF1
CREB3L2	PHPT1
CREB3L2	PHTF1
CREB3L2	PIEZO1
CREB3L2	PIM2
CREB3L2	PINK1
CREB3L2	PITRM1
CREB3L2	PJA2
CREB3L2	PLCL2
CREB3L2	PLD3
CREB3L2	PLD4
CREB3L2	PLPP5
CREB3L2	PMEPA1
CREB3L2	PMM2
CREB3L2	PNOC
CREB3L2	POP5
CREB3L2	POP7
CREB3L2	POU2F1

CREB3L2 PPIB
CREB3L2 PPP2R5B
CREB3L2 PPP6R3
CREB3L2 PRCP
CREB3L2 PRDX4
CREB3L2 PREB
CREB3L2 PRKAR2B
CREB3L2 PRKCSH
CREB3L2 PRKD2
CREB3L2 PRKD3
CREB3L2 PRMT5
CREB3L2 PRRC2B
CREB3L2 PSEN2
CREB3L2 PSMA6
CREB3L2 PSMB8-AS1
CREB3L2 PSMC4
CREB3L2 PSMD2
CREB3L2 PSMD7
CREB3L2 PSMF1
CREB3L2 PTPN1
CREB3L2 PUS3
CREB3L2 PUS7L
CREB3L2 PWWP2A
CREB3L2 QPCT
CREB3L2 QPRT
CREB3L2 QSOX1
CREB3L2 RAB11B
CREB3L2 RAB1A
CREB3L2 RAB2A
CREB3L2 RAB2B
CREB3L2 RAB30
CREB3L2 RAB6A
CREB3L2 RABAC1
CREB3L2 RABGGTA
CREB3L2 RAP1A
CREB3L2 RAPGEF6
CREB3L2 RARA
CREB3L2 RARS2
CREB3L2 RASSF5
CREB3L2 RB1
CREB3L2 RBBP6
CREB3L2 RBM33
CREB3L2 REEP5
CREB3L2 REXO2
CREB3L2 RGS1
CREB3L2 RGS16
CREB3L2 RGS2
CREB3L2 RHOT1
CREB3L2 RNASE4
CREB3L2 RNF103

CREB3L2	RNF141
CREB3L2	RNF149
CREB3L2	RNF181
CREB3L2	RNH1
CREB3L2	RPL10
CREB3L2	RPL13
CREB3L2	RPL3
CREB3L2	RPL36AL
CREB3L2	RPLP0
CREB3L2	RPLP1
CREB3L2	RPN1
CREB3L2	RPN2
CREB3L2	RPS11
CREB3L2	RPS19
CREB3L2	RPS28
CREB3L2	RPS29
CREB3L2	RPS4X
CREB3L2	RPS6KB2
CREB3L2	RPS9
CREB3L2	RRBP1
CREB3L2	RREB1
CREB3L2	RSBN1
CREB3L2	RSRP1
CREB3L2	RTN3
CREB3L2	RUNX1
CREB3L2	RXRA
CREB3L2	RYBP
CREB3L2	SAR1A
CREB3L2	SAR1B
CREB3L2	SARAF
CREB3L2	SAT1
CREB3L2	SCAMP2
CREB3L2	SCAMP3
CREB3L2	SCAND1
CREB3L2	SCAPER
CREB3L2	SCFD1
CREB3L2	SCPEP1
CREB3L2	SCYL1
CREB3L2	SDC1
CREB3L2	SDF2L1
CREB3L2	SDF4
CREB3L2	SDHC
CREB3L2	SEC11C
CREB3L2	SEC13
CREB3L2	SEC22B
CREB3L2	SEC23IP
CREB3L2	SEC24A
CREB3L2	SEC24D
CREB3L2	SEC31A
CREB3L2	SEC61A1

CREB3L2	SEC61B
CREB3L2	SEC61G
CREB3L2	SEC62
CREB3L2	SEC63
CREB3L2	SECISBP2L
CREB3L2	SEL1L
CREB3L2	SEL1L3
CREB3L2	SERP1
CREB3L2	SERTAD3
CREB3L2	SET
CREB3L2	SETD7
CREB3L2	SF3B5
CREB3L2	SGK3
CREB3L2	SGPP1
CREB3L2	SIL1
CREB3L2	SIRT2
CREB3L2	SKIL
CREB3L2	SLC17A5
CREB3L2	SLC17A9
CREB3L2	SLC1A4
CREB3L2	SLC25A39
CREB3L2	SLC25A45
CREB3L2	SLC25A46
CREB3L2	SLC30A7
CREB3L2	SLC31A2
CREB3L2	SLC33A1
CREB3L2	SLC35A3
CREB3L2	SLC35B1
CREB3L2	SLC35C1
CREB3L2	SLC38A10
CREB3L2	SLC38A2
CREB3L2	SLC39A7
CREB3L2	SLC39A9
CREB3L2	SLC44A1
CREB3L2	SLCO4A1
CREB3L2	SLFN11
CREB3L2	SMAD4
CREB3L2	SMAD5
CREB3L2	SMAP1
CREB3L2	SMCHD1
CREB3L2	SMIM4
CREB3L2	SMPD1
CREB3L2	SMTN
CREB3L2	SMYD3
CREB3L2	SNAPC2
CREB3L2	SND1
CREB3L2	SNRPN
CREB3L2	SNURF
CREB3L2	SNX16
CREB3L2	SOCS3

CREB3L2	SPAG4
CREB3L2	SPATS2
CREB3L2	SPATS2L
CREB3L2	SPC24
CREB3L2	SPCS1
CREB3L2	SPINT2
CREB3L2	SPON2
CREB3L2	SPRYD3
CREB3L2	SPTBN1
CREB3L2	SPTSSA
CREB3L2	SPTY2D1
CREB3L2	SQSTM1
CREB3L2	SRGN
CREB3L2	SRM
CREB3L2	SRP14
CREB3L2	SRP19
CREB3L2	SRP54
CREB3L2	SRP68
CREB3L2	SRP72
CREB3L2	SRPRA
CREB3L2	SRRM2
CREB3L2	SSR1
CREB3L2	SSR3
CREB3L2	SSR4
CREB3L2	SSSCA1
CREB3L2	ST6GAL1
CREB3L2	ST6GALNAC4
CREB3L2	ST7
CREB3L2	STARD5
CREB3L2	STK17B
CREB3L2	STRN4
CREB3L2	STS
CREB3L2	STT3A
CREB3L2	STT3B
CREB3L2	STUB1
CREB3L2	STX17
CREB3L2	STX5
CREB3L2	SUB1
CREB3L2	SUCO
CREB3L2	SUOX
CREB3L2	SUPT5H
CREB3L2	SUPT7L
CREB3L2	SURF4
CREB3L2	SYNC
CREB3L2	SYNGR1
CREB3L2	SYNRG
CREB3L2	SYTL1
CREB3L2	SYVN1
CREB3L2	TARSL2
CREB3L2	TBC1D7

CREB3L2	TBL2
CREB3L2	TCEAL3
CREB3L2	TCEAL8
CREB3L2	TCEAL9
CREB3L2	TCF25
CREB3L2	TCF4
CREB3L2	TECPR1
CREB3L2	TFG
CREB3L2	THEMIS2
CREB3L2	TIMM17A
CREB3L2	TIMP1
CREB3L2	TJAP1
CREB3L2	TLE1
CREB3L2	TM7SF2
CREB3L2	TM9SF2
CREB3L2	TM9SF3
CREB3L2	TM9SF4
CREB3L2	TMBIM6
CREB3L2	TMED10
CREB3L2	TMED2
CREB3L2	TMED3
CREB3L2	TMED4
CREB3L2	TMED7
CREB3L2	TMED9
CREB3L2	TMEM101
CREB3L2	TMEM134
CREB3L2	TMEM165
CREB3L2	TMEM167A
CREB3L2	TMEM175
CREB3L2	TMEM179B
CREB3L2	TMEM184B
CREB3L2	TMEM214
CREB3L2	TMEM230
CREB3L2	TMEM258
CREB3L2	TMEM263
CREB3L2	TMEM39A
CREB3L2	TMEM45A
CREB3L2	TMEM59
CREB3L2	TMEM9
CREB3L2	TMF1
CREB3L2	TMSB10
CREB3L2	TNFRSF17
CREB3L2	TOR3A
CREB3L2	TOX2
CREB3L2	TPP1
CREB3L2	TPT1
CREB3L2	TRA2A
CREB3L2	TRAM1
CREB3L2	TRAPPC3
CREB3L2	TRIB1

CREB3L2	TRIM27
CREB3L2	TRIP11
CREB3L2	TRNT1
CREB3L2	TRPM4
CREB3L2	TRPV3
CREB3L2	TSC22D3
CREB3L2	TSPAN13
CREB3L2	TSPYL1
CREB3L2	TSPYL2
CREB3L2	TTC3
CREB3L2	TUBG1
CREB3L2	TUSC2
CREB3L2	TVP23B
CREB3L2	TXLNA
CREB3L2	TXNDC11
CREB3L2	TXNDC15
CREB3L2	TXNDC5
CREB3L2	TXNL1
CREB3L2	TYMP
CREB3L2	U2AF1L4
CREB3L2	UAP1
CREB3L2	UBA5
CREB3L2	UBALD1
CREB3L2	UBB
CREB3L2	UBE2D4
CREB3L2	UBE2J1
CREB3L2	UBE2Q1
CREB3L2	UBR5
CREB3L2	UBXN4
CREB3L2	UBXN6
CREB3L2	UFL1
CREB3L2	UFM1
CREB3L2	UFSP2
CREB3L2	UNC50
CREB3L2	UQCR11
CREB3L2	UQCRFS1
CREB3L2	UROS
CREB3L2	USO1
CREB3L2	USP48
CREB3L2	USP7
CREB3L2	USP8
CREB3L2	UTP15
CREB3L2	UTRN
CREB3L2	VAMP2
CREB3L2	VCP
CREB3L2	VDR
CREB3L2	VKORC1
CREB3L2	VMA21
CREB3L2	VOPP1
CREB3L2	VPS11

CREB3L2	VPS25
CREB3L2	VPS51
CREB3L2	WNT6
CREB3L2	WWC3
CREB3L2	XBP1
CREB3L2	XIAP
CREB3L2	XRRA1
CREB3L2	YIF1A
CREB3L2	YIF1B
CREB3L2	YIPF2
CREB3L2	YIPF3
CREB3L2	YIPF5
CREB3L2	YIPF6
CREB3L2	YKT6
CREB3L2	YPEL3
CREB3L2	YPEL5
CREB3L2	YTHDF1
CREB3L2	ZBP1
CREB3L2	ZBTB7B
CREB3L2	ZC3H4
CREB3L2	ZFAND2A
CREB3L2	ZKSCAN1
CREB3L2	ZNF12
CREB3L2	ZNF215
CREB3L2	ZNF275
CREB3L2	ZNF280D
CREB3L2	ZNF3
CREB3L2	ZNF318
CREB3L2	ZNF331
CREB3L2	ZNF358
CREB3L2	ZNF37A
CREB3L2	ZNF529-AS1
CREB3L2	ZNF706
CREB3L2	ZNF791
CREB3L2	ZNF793
CREB3L2	ZSCAN18
CREB3L4	CADPS
CREB3L4	FKBP14
CREB3L4	KLF9
CREB3L4	LSM12
CREB3L4	MAPRE2
CREB3L4	MARCH9
CREB3L4	MBNL2
CREB3L4	NEK1
CREB3L4	RCBTB1
CREB3L4	RNF24
CREB3L4	SEC31A
CREB3L4	TMEM214
CREB5	ABLIM1
CREB5	ACOX3

CREB5	APP
CREB5	ARL8B
CREB5	ARRB2
CREB5	ASNS
CREB5	CCL4
CREB5	CCL4L2
CREB5	CD200
CREB5	CMSS1
CREB5	COA7
CREB5	CREB5
CREB5	CSTF1
CREB5	DDX6
CREB5	DEPDC5
CREB5	EDC3
CREB5	EIF3J
CREB5	FAM184A
CREB5	FGFR1
CREB5	GARS
CREB5	GPR65
CREB5	HRK
CREB5	HSPA9
CREB5	ICE1
CREB5	ID3
CREB5	IL10
CREB5	LARS
CREB5	MAGOHB
CREB5	MAP3K7CL
CREB5	MCM10
CREB5	METTL1
CREB5	MYD88
CREB5	NAMPT
CREB5	NEDD4L
CREB5	OXSR1
CREB5	PAPOLG
CREB5	PCK2
CREB5	PCNX4
CREB5	PFDN2
CREB5	PMAIP1
CREB5	POGZ
CREB5	PRKACB
CREB5	PYCR1
CREB5	RAB3GAP2
CREB5	RBM15B
CREB5	RECQL4
CREB5	REL
CREB5	RNF145
CREB5	RNF19A
CREB5	RRP1B
CREB5	RSL24D1
CREB5	SAMSN1

CREB5	SH3BP2
CREB5	SLC3A2
CREB5	SLC43A3
CREB5	SLC4A7
CREB5	STX4
CREB5	TBC1D5
CREB5	TIPARP
CREB5	TM9SF4
CREB5	TRAF1
CREB5	TRAP1
CREB5	TRIP6
CREB5	TUBE1
CREB5	UBE2B
CREB5	UTP18
CREB5	WDR33
CREB5	WDR74
CREB5	XPO1
CREB5	ZFR
CREB5	ZNF330
CREBZF	KDM6A
CREBZF	PNPLA6
CREBZF	ZNF784
CREM	AARS
CREM	ABRACL
CREM	ABT1
CREM	ACAD9
CREM	ACADVL
CREM	ACAP1
CREM	ACIN1
CREM	ACO2
CREM	ACOX1
CREM	ACTG1
CREM	ADARB1
CREM	ADIPOR1
CREM	ADNP
CREM	AIMP1
CREM	AKAP9
CREM	ALCAM
CREM	ALKBH3
CREM	ANAPC16
CREM	ANKRD11
CREM	ANP32B
CREM	ANXA6
CREM	ANXA7
CREM	AP2B1
CREM	AP2M1
CREM	AP5M1
CREM	APOC1
CREM	APOM
CREM	ARF1

CREM	ARF4
CREM	ARF5
CREM	ARF6
CREM	ARHGAP12
CREM	ARHGEF2
CREM	ARID5B
CREM	ARL1
CREM	ARL17A
CREM	ARMCX3
CREM	ARMCX6
CREM	ARMT1
CREM	ATF4
CREM	ATG3
CREM	ATG5
CREM	ATP6AP2
CREM	ATP6V0D1
CREM	AVPI1
CREM	B3GAT3
CREM	BASP1
CREM	BBIP1
CREM	BCAR3
CREM	BCAS2
CREM	BIRC2
CREM	BRMS1
CREM	BSDC1
CREM	BTF3L4
CREM	BTG1
CREM	BUD31
CREM	C11orf49
CREM	C19orf24
CREM	C1D
CREM	C1orf123
CREM	C1orf174
CREM	C1orf43
CREM	C7orf50
CREM	CALCOCO2
CREM	CAMK1
CREM	CAMK2B
CREM	CAMKK2
CREM	CAP1
CREM	CAPZA2
CREM	CCDC107
CREM	CCDC25
CREM	CCDC92
CREM	CCSER2
CREM	CD164
CREM	CD2BP2
CREM	CD53
CREM	CD83
CREM	CD86

CREM	CDC37
CREM	CDC42
CREM	CDC5L
CREM	CDIPT
CREM	CEBPB
CREM	CEBPG
CREM	CENPM
CREM	CEP295
CREM	CEP57
CREM	CFL1
CREM	CHCHD2
CREM	CHD1
CREM	CHD4
CREM	CHD7
CREM	CHMP2A
CREM	CIRBP
CREM	CLK1
CREM	CLK2
CREM	CMC2
CREM	CMIP
CREM	CNDP2
CREM	CNOT4
CREM	COMMD1
CREM	COMMD3
CREM	COMMD8
CREM	COPB2
CREM	COPE
CREM	COX18
CREM	COX6A1
CREM	COX8A
CREM	CREM
CREM	CSDE1
CREM	CSK
CREM	CSNK1A1
CREM	CTSH
CREM	CYLD
CREM	CYTH1
CREM	DAD1
CREM	DAP
CREM	DAPP1
CREM	DAZAP2
CREM	DCAF11
CREM	DCXR
CREM	DDIT3
CREM	DDIT4
CREM	DDX10
CREM	DDX24
CREM	DDX42
CREM	DDX5
CREM	DENND1B

CREM	DGAT2
CREM	DGUOK
CREM	DHDDS
CREM	DHX36
CREM	DIAPH3
CREM	DNAJA2
CREM	DNAJC10
CREM	DNAJC25
CREM	DOHH
CREM	DPH6
CREM	DPM1
CREM	DSP
CREM	DTNBP1
CREM	DTX3
CREM	DUSP10
CREM	DUSP2
CREM	DUSP3
CREM	DYNLT3
CREM	E2F3
CREM	EBF1
CREM	EEF1A1
CREM	EIF1
CREM	EIF2B1
CREM	EIF3B
CREM	EIF4A2
CREM	EIF4G2
CREM	ELAVL1
CREM	ELL
CREM	EMC4
CREM	EMC8
CREM	ENSA
CREM	EPN1
CREM	ERP44
CREM	EZH1
CREM	FAAP20
CREM	FAF1
CREM	FAM107B
CREM	FAM3C
CREM	FAM49A
CREM	FAM81A
CREM	FAU
CREM	FBXO8
CREM	FDFT1
CREM	FEN1
CREM	FEZ1
CREM	FIS1
CREM	FMR1
CREM	FOXN3
CREM	FPGS
CREM	FTH1

CREM	FXYD5
CREM	FYN
CREM	FYTTD1
CREM	G3BP2
CREM	GABARAPL2
CREM	GADD45B
CREM	GALK2
CREM	GALNT2
CREM	GEM
CREM	GGA1
CREM	GHITM
CREM	GK
CREM	GLA
CREM	GLYR1
CREM	GMDS
CREM	GNAS
CREM	GNG4
CREM	GNL1
CREM	GOT2
CREM	GPATCH8
CREM	GPBP1
CREM	GPER1
CREM	GPR146
CREM	GPR183
CREM	GPR65
CREM	GRB2
CREM	GRPEL1
CREM	GTF2F1
CREM	GUK1
CREM	H3F3A
CREM	H3F3B
CREM	HAT1
CREM	HCCS
CREM	HCFC1
CREM	HECW2
CREM	HERPUD1
CREM	HEXIM1
CREM	HIVEP3
CREM	HLA-DQB1
CREM	HLA-DRA
CREM	HLA-DRB1
CREM	HMCES
CREM	HNRNPA3
CREM	HNRNPK
CREM	HOPX
CREM	HPS4
CREM	HSF2
CREM	HSPA4
CREM	HSPBAP1
CREM	HTATSF1

CREM	HTR3A
CREM	ICE1
CREM	IDH2
CREM	IFI27L2
CREM	IFT74
CREM	IL10RA
CREM	IL21R
CREM	IMPDH2
CREM	INF2
CREM	INIP
CREM	INSIG1
CREM	IQGAP2
CREM	IQSEC1
CREM	ISG15
CREM	ISOC2
CREM	ITGB2
CREM	KAT5
CREM	KAT7
CREM	KCNK12
CREM	KCNN4
CREM	KDM6B
CREM	KMT2E
CREM	KYAT3
CREM	LAP3
CREM	LAPTM4A
CREM	LAPTM5
CREM	LCP1
CREM	LDHA
CREM	LDLRAD4
CREM	LGALS8
CREM	LIMS1
CREM	LINC00909
CREM	LMBR1
CREM	LMF2
CREM	LMO2
CREM	LMTK2
CREM	LPIN1
CREM	LRRC23
CREM	LRRC8A
CREM	LRRC8B
CREM	LYSMD2
CREM	LYST
CREM	MAGOHB
CREM	MAP2K3
CREM	MAP3K8
CREM	MAPK1IP1L
CREM	MARCH1
CREM	MARCH7
CREM	MARCKSL1
CREM	MBTPS1

CREM	MCRIP1
CREM	MED27
CREM	MEF2C
CREM	MET
CREM	MGLL
CREM	MGMT
CREM	MGRN1
CREM	MICU1
CREM	MIF
CREM	MIR193BHG
CREM	MKNK2
CREM	MLF2
CREM	MLX
CREM	MMAB
CREM	MMADHC
CREM	MPC1
CREM	MRPL23
CREM	MRPL4
CREM	MRPS18B
CREM	MS4A1
CREM	MTCH1
CREM	MTSS1
CREM	MYL6
CREM	MYO1G
CREM	NAB1
CREM	NABP1
CREM	NANS
CREM	NAPA
CREM	NARS2
CREM	NBN
CREM	NCOA2
CREM	NDUFA7
CREM	NDUFB2
CREM	NDUFB6
CREM	NDUFS2
CREM	NDUFV1
CREM	NEAT1
CREM	NEK7
CREM	NFE2L2
CREM	NFKB1
CREM	NFKB2
CREM	NIN
CREM	NIPA2
CREM	NOC4L
CREM	NOP58
CREM	NOTCH2
CREM	NPAT
CREM	NPLOC4
CREM	NR3C1
CREM	NSMCE3

CREM	NSRP1
CREM	NTHL1
CREM	NUP133
CREM	NUP62
CREM	NUP93
CREM	NXT2
CREM	OCIAD1
CREM	ORAI1
CREM	ORC4
CREM	ORC5
CREM	OSBP
CREM	OSBPL9
CREM	OXCT2
CREM	PABPC1
CREM	PAK1
CREM	PAN2
CREM	PARK7
CREM	PCIF1
CREM	PDAP1
CREM	PDHA1
CREM	PDK3
CREM	PDXDC1
CREM	PDXK
CREM	PEA15
CREM	PEF1
CREM	PFDN1
CREM	PFKFB3
CREM	PFN1
CREM	PHACTR1
CREM	PHF5A
CREM	PIP4K2A
CREM	PITPNA
CREM	PKM
CREM	PKN2
CREM	PLCL2
CREM	PMVK
CREM	PNRC1
CREM	POLR2M
CREM	POU2F2
CREM	PPFIBP1
CREM	PPM1F
CREM	PPP1R15A
CREM	PPP1R3D
CREM	PPP1R8
CREM	PPP2R1A
CREM	PPP2R2A
CREM	PRDX6
CREM	PRKAR1A
CREM	PRKCD
CREM	PRKCSH

CREM	PRKD2
CREM	PRMT1
CREM	PRPF38A
CREM	PRPSAP2
CREM	PRRC2C
CREM	PRRG4
CREM	PSMA3
CREM	PSMB1
CREM	PSMB2
CREM	PSMB3
CREM	PSMD14
CREM	PTAFR
CREM	PTBP2
CREM	PTMA
CREM	PTP4A1
CREM	PTPN1
CREM	PTPRN2
CREM	PXK
CREM	QRICH1
CREM	RAB11B
CREM	RAB14
CREM	RAB18
CREM	RAB21
CREM	RAB3GAP2
CREM	RAB8B
CREM	RAB9A
CREM	RABEP1
CREM	RABL2B
CREM	RAD23A
CREM	RAD54B
CREM	RALA
CREM	RANBP2
CREM	RANGAP1
CREM	RAP1A
CREM	RASSF5
CREM	RBM3
CREM	RBM39
CREM	RBM42
CREM	RCC1
CREM	REEP3
CREM	REL
CREM	RELB
CREM	RELT
CREM	RFT1
CREM	RFTN1
CREM	RGS13
CREM	RGS20
CREM	RGS3
CREM	RHBDD1
CREM	RHEB

CREM	RHOG
CREM	RNF145
CREM	RNH1
CREM	RPL13A
CREM	RPL18
CREM	RPL3
CREM	RPL36AL
CREM	RPL6
CREM	RPLP1
CREM	RPS19
CREM	RPSA
CREM	RRAS
CREM	RUNX1
CREM	SACM1L
CREM	SAP30BP
CREM	SARAF
CREM	SCAMP1-AS1
CREM	SCAMP2
CREM	SCLT1
CREM	SCRN1
CREM	SDCBP
CREM	SDCCAG8
CREM	SDHAF2
CREM	SDHC
CREM	SEC23B
CREM	SEC61B
CREM	SEC61G
CREM	SEC62
CREM	SENP8
CREM	SERINC1
CREM	SERPINA9
CREM	SERTAD3
CREM	SESN3
CREM	SETD7
CREM	SF3B5
CREM	SFT2D1
CREM	SGF29
CREM	SGSM3
CREM	SH2B2
CREM	SHMT2
CREM	SIRT6
CREM	SKAP1
CREM	SKP1
CREM	SLC1A4
CREM	SLC25A3
CREM	SLC25A39
CREM	SLC37A4
CREM	SLC38A9
CREM	SLCO4A1
CREM	SMAP1

CREM	SMAP2
CREM	SMC5
CREM	SMCHD1
CREM	SMKR1
CREM	SMU1
CREM	SNAPC5
CREM	SND1
CREM	SNF8
CREM	SNHG15
CREM	SNHG8
CREM	SNRNP40
CREM	SNRPG
CREM	SNU13
CREM	SNX11
CREM	SNX8
CREM	SPACA9
CREM	SPG21
CREM	SPICE1
CREM	SPOP
CREM	SPTLC1
CREM	SPTSSA
CREM	SRGN
CREM	SRP54
CREM	SRP68
CREM	SRRD
CREM	SRRM2
CREM	SRSF2
CREM	SRSF5
CREM	SSNA1
CREM	SSSCA1
CREM	SSTR2
CREM	SSU72
CREM	ST6GAL1
CREM	STAM
CREM	STAT3
CREM	STAT4
CREM	STAT5B
CREM	STK4
CREM	STRAP
CREM	STX11
CREM	STX18
CREM	SYBU
CREM	SYNCRIP
CREM	SYNGR1
CREM	SYS1
CREM	TACC1
CREM	TAP1
CREM	TAPT1
CREM	TBC1D9
CREM	TBCC

CREM	TCEAL4
CREM	TCEAL8
CREM	TCF25
CREM	TCP11L2
CREM	TEN1
CREM	THAP9-AS1
CREM	TIMM10B
CREM	TIMM17A
CREM	TJP1
CREM	TMBIM1
CREM	TMC6
CREM	TMEM14A
CREM	TMEM165
CREM	TMEM179B
CREM	TMEM219
CREM	TMEM222
CREM	TMEM259
CREM	TMF1
CREM	TMUB1
CREM	TP53INP2
CREM	TPD52L2
CREM	TPM4
CREM	TPT1
CREM	TRA2B
CREM	TRAF4
CREM	TRMT1
CREM	TRMT10C
CREM	TSC22D3
CREM	TSPO
CREM	TSPYL2
CREM	TTC3
CREM	TUBB3
CREM	TUBGCP2
CREM	TUFM
CREM	TWISTNB
CREM	TXN2
CREM	TYW1
CREM	U2AF1L4
CREM	UBALD1
CREM	UBC
CREM	UBE2B
CREM	UBE2D2
CREM	UBE2D3
CREM	UBE2H
CREM	UBN1
CREM	UBQLN1
CREM	UFL1
CREM	UPF2
CREM	UQCRC2
CREM	UQCRQ

CREM	USP11
CREM	USP8
CREM	USP9X
CREM	UTP23
CREM	VAMP2
CREM	VCP
CREM	WDR1
CREM	WDR33
CREM	WDR91
CREM	WSB1
CREM	XRCC6
CREM	XRN2
CREM	YAE1D1
CREM	YIPF1
CREM	YKT6
CREM	YY1
CREM	ZBTB20
CREM	ZBTB8OS
CREM	ZC3H15
CREM	ZDHHC3
CREM	ZDHHC7
CREM	ZEB1
CREM	ZFAND2B
CREM	ZFHX3
CREM	ZFP36L1
CREM	ZNF143
CREM	ZNF165
CREM	ZNF195
CREM	ZNF331
CREM	ZNF394
CREM	ZYX
CTCF	AAK1
CTCF	AAMP
CTCF	AAR2
CTCF	AATF
CTCF	ABHD13
CTCF	ABHD8
CTCF	ABI3
CTCF	ABL1
CTCF	ABLIM2
CTCF	ABRACL
CTCF	ABT1
CTCF	ACAT2
CTCF	ACBD3
CTCF	ACER3
CTCF	ACIN1
CTCF	ACLY
CTCF	ACOT13
CTCF	ACOT7
CTCF	ACSL4

CTCF	ACTN4
CTCF	ACTR1A
CTCF	ACTR1B
CTCF	ACTR2
CTCF	ACTR6
CTCF	ACYP1
CTCF	ADD1
CTCF	ADD3
CTCF	ADH5
CTCF	ADIPOR2
CTCF	AEBP2
CTCF	AFF3
CTCF	AGBL5
CTCF	AGO2
CTCF	AHCYL1
CTCF	AHDC1
CTCF	AIP
CTCF	AKAP9
CTCF	AKIP1
CTCF	AKIRIN2
CTCF	AKR1B1
CTCF	AKR7A2
CTCF	AKT2
CTCF	ALAS1
CTCF	ALDH16A1
CTCF	ALDH4A1
CTCF	ALG6
CTCF	ALKBH3
CTCF	ALKBH5
CTCF	ALKBH6
CTCF	ALMS1
CTCF	ALOX5
CTCF	ALOX5AP
CTCF	ALYREF
CTCF	AMZ2
CTCF	ANAPC1
CTCF	ANAPC10
CTCF	ANAPC11
CTCF	ANAPC15
CTCF	ANAPC2
CTCF	ANAPC4
CTCF	ANAPC7
CTCF	ANGEL2
CTCF	ANKRD10
CTCF	ANKRD13A
CTCF	ANKRD13D
CTCF	ANKRD26
CTCF	ANKRD34B
CTCF	ANP32A
CTCF	ANP32B

CTCF	ANP32E
CTCF	ANXA2
CTCF	AP1G1
CTCF	AP1M1
CTCF	AP2A1
CTCF	AP2M1
CTCF	AP2S1
CTCF	AP3B1
CTCF	AP3S1
CTCF	AP5M1
CTCF	APEH
CTCF	APLP2
CTCF	ARAF
CTCF	ARAP1
CTCF	ARF1
CTCF	ARF6
CTCF	ARFIP1
CTCF	ARGLU1
CTCF	ARHGAP11A
CTCF	ARHGAP12
CTCF	ARHGAP26
CTCF	ARHGAP27
CTCF	ARHGAP30
CTCF	ARHGAP4
CTCF	ARHGEF39
CTCF	ARID1A
CTCF	ARID1B
CTCF	ARID2
CTCF	ARID4B
CTCF	ARL13B
CTCF	ARL14EP
CTCF	ARL16
CTCF	ARMC1
CTCF	ARPC2
CTCF	ARPC3
CTCF	ARPC5
CTCF	ARPP19
CTCF	ARRDC3-AS1
CTCF	ASAP1
CTCF	ASB1
CTCF	ASB2
CTCF	ASB8
CTCF	ASCC2
CTCF	ASF1A
CTCF	ASF1B
CTCF	ASNSD1
CTCF	ASPSCR1
CTCF	ASXL1
CTCF	ASXL2
CTCF	ATAD2

CTCF	ATAD5
CTCF	ATF1
CTCF	ATF6B
CTCF	ATF7IP
CTCF	ATG2A
CTCF	ATG4B
CTCF	ATG5
CTCF	ATP10D
CTCF	ATP23
CTCF	ATP2A3
CTCF	ATP6V1A
CTCF	ATP6V1B2
CTCF	ATP6V1C1
CTCF	ATP6V1D
CTCF	ATP6V1E1
CTCF	ATP6V1H
CTCF	ATP8A1
CTCF	ATPAF2
CTCF	ATRX
CTCF	ATXN10
CTCF	AURKA
CTCF	AURKB
CTCF	AZIN1
CTCF	B9D2
CTCF	BACH2
CTCF	BAG6
CTCF	BANF1
CTCF	BAP1
CTCF	BARD1
CTCF	BAZ1A
CTCF	BAZ1B
CTCF	BAZ2A
CTCF	BAZ2B
CTCF	BBC3
CTCF	BBIP1
CTCF	BBS2
CTCF	BBS4
CTCF	BBS7
CTCF	BBX
CTCF	BCAS2
CTCF	BCAS3
CTCF	BCL10
CTCF	BCL11A
CTCF	BCL2L11
CTCF	BCL3
CTCF	BCL7A
CTCF	BCL7B
CTCF	BCLAF1
CTCF	BDP1
CTCF	BECN1

CTCF	BICD1
CTCF	BIN3
CTCF	BIRC2
CTCF	BIRC6
CTCF	BLCAP
CTCF	BLMH
CTCF	BLOC1S1
CTCF	BMP2K
CTCF	BNIP2
CTCF	BNIP3L
CTCF	BOD1L1
CTCF	BORA
CTCF	BORCS8
CTCF	BPHL
CTCF	BPTF
CTCF	BRCA1
CTCF	BRCA2
CTCF	BRD3
CTCF	BRD7
CTCF	BRK1
CTCF	BRMS1
CTCF	BROX
CTCF	BRPF1
CTCF	BRWD1
CTCF	BTF3L4
CTCF	BTG2
CTCF	BUB3
CTCF	BUD13
CTCF	BZW1
CTCF	BZW2
CTCF	C11orf49
CTCF	C11orf58
CTCF	C11orf68
CTCF	C12orf10
CTCF	C12orf49
CTCF	C12orf65
CTCF	C12orf75
CTCF	C17orf80
CTCF	C18orf54
CTCF	C19orf25
CTCF	C19orf47
CTCF	C1orf112
CTCF	C1orf131
CTCF	C1orf52
CTCF	C21orf91
CTCF	C2orf68
CTCF	C3orf14
CTCF	C3orf38
CTCF	C4orf33
CTCF	C5orf34

CTCF	C6orf136
CTCF	C6orf48
CTCF	C6orf62
CTCF	C8orf76
CTCF	C9orf78
CTCF	CA5B
CTCF	CAAP1
CTCF	CACTIN
CTCF	CACUL1
CTCF	CACYBP
CTCF	CADPS
CTCF	CALM1
CTCF	CALM2
CTCF	CALM3
CTCF	CAMK2D
CTCF	CAMLG
CTCF	CAMTA1
CTCF	CAPNS1
CTCF	CAPZA1
CTCF	CAPZA2
CTCF	CARD8
CTCF	CARHSP1
CTCF	CASC4
CTCF	CASP2
CTCF	CASP3
CTCF	CASP8AP2
CTCF	CBFB
CTCF	CBR4
CTCF	CBX1
CTCF	CBX2
CTCF	CBX3
CTCF	CBX5
CTCF	CC2D1A
CTCF	CCAR1
CTCF	CCDC102A
CTCF	CCDC12
CTCF	CCDC14
CTCF	CCDC167
CTCF	CCDC174
CTCF	CCDC186
CTCF	CCDC43
CTCF	CCDC82
CTCF	CCDC88A
CTCF	CCHCR1
CTCF	CCM2
CTCF	CCNB1
CTCF	CCNB2
CTCF	CCNDBP1
CTCF	CCNF
CTCF	CCNG2

CTCF	CCNH
CTCF	CCNI
CTCF	CCNK
CTCF	CCNL1
CTCF	CCNL2
CTCF	CCP110
CTCF	CCT7
CTCF	CCZ1
CTCF	CCZ1B
CTCF	CD2AP
CTCF	CD47
CTCF	CD70
CTCF	CDC16
CTCF	CDC27
CTCF	CDC37
CTCF	CDC37L1
CTCF	CDC40
CTCF	CDC42
CTCF	CDC42EP3
CTCF	CDC42SE2
CTCF	CDC73
CTCF	CDCA2
CTCF	CDCA3
CTCF	CDCA5
CTCF	CDCA8
CTCF	CDK1
CTCF	CDK11A
CTCF	CDK11B
CTCF	CDK13
CTCF	CDK16
CTCF	CDK19
CTCF	CDK2AP2
CTCF	CDK5RAP1
CTCF	CDK5RAP2
CTCF	CDK5RAP3
CTCF	CDK9
CTCF	CDKN1B
CTCF	CDKN2A
CTCF	CDKN2C
CTCF	CDKN2D
CTCF	CDKN3
CTCF	CEBPG
CTCF	CENPC
CTCF	CENPF
CTCF	CENPH
CTCF	CENPJ
CTCF	CENPL
CTCF	CENPM
CTCF	CENPP
CTCF	CEP135

CTCF	CEP162
CTCF	CEP350
CTCF	CEP44
CTCF	CEP55
CTCF	CEP57L1
CTCF	CEP63
CTCF	CEP78
CTCF	CEP89
CTCF	CEP95
CTCF	CEP97
CTCF	CETN3
CTCF	CFAP20
CTCF	CFDP1
CTCF	CFL1
CTCF	CHAC2
CTCF	CHAMP1
CTCF	CHCHD5
CTCF	CHD4
CTCF	CHD9
CTCF	CHERP
CTCF	CHMP1A
CTCF	CHMP2B
CTCF	CHMP3
CTCF	CHP1
CTCF	CHST14
CTCF	CHSY1
CTCF	CHTF8
CTCF	CHTOP
CTCF	CHURC1
CTCF	CIAO1
CTCF	CIR1
CTCF	CIT
CTCF	CKAP2
CTCF	CKAP2L
CTCF	CKLF
CTCF	CKS2
CTCF	CLIC1
CTCF	CLIC4
CTCF	CLK1
CTCF	CLK2
CTCF	CLK3
CTCF	CLNS1A
CTCF	CLPTM1
CTCF	CLSTN1
CTCF	CLTA
CTCF	CLTC
CTCF	CMSS1
CTCF	CMTM6
CTCF	CMTM7
CTCF	CMTR1

CTCF	CNIH1
CTCF	CNNM3
CTCF	CNOT6L
CTCF	CNOT8
CTCF	CNPPD1
CTCF	CNTROB
CTCF	COA6
CTCF	COCH
CTCF	COG4
CTCF	COG7
CTCF	COIL
CTCF	COMMD10
CTCF	COMMD2
CTCF	COMMD4
CTCF	COMMD6
CTCF	COMMD8
CTCF	COMTD1
CTCF	COPA
CTCF	COPB1
CTCF	COPE
CTCF	COPS6
CTCF	COPS7A
CTCF	COPS7B
CTCF	COQ10B
CTCF	COQ3
CTCF	COQ4
CTCF	COQ5
CTCF	CORO1A
CTCF	CORO1B
CTCF	CORO6
CTCF	COX17
CTCF	COX5B
CTCF	COX6A1
CTCF	COX7A2
CTCF	COX7A2L
CTCF	COX8A
CTCF	CPSF1
CTCF	CPSF2
CTCF	CPSF3
CTCF	CPSF6
CTCF	CPSF7
CTCF	CPT2
CTCF	CREB1
CTCF	CREBL2
CTCF	CRLF3
CTCF	CRTAP
CTCF	CRYZL1
CTCF	CSE1L
CTCF	CSNK1A1
CTCF	CSNK1D

CTCF	CSNK1G2
CTCF	CSTF1
CTCF	CTCF
CTCF	CTDNEP1
CTCF	CTDP1
CTCF	CTDSP1
CTCF	CTDSP2
CTCF	CTDSPL2
CTCF	CTNNB1
CTCF	CTNNBL1
CTCF	CTSZ
CTCF	CTU1
CTCF	CUEDC2
CTCF	CUL3
CTCF	CWC22
CTCF	CWC25
CTCF	CWF19L2
CTCF	CXCR4
CTCF	CXXC5
CTCF	CYB5B
CTCF	CYB5D2
CTCF	CYB5R3
CTCF	CYSTM1
CTCF	CYTH2
CTCF	DAAM1
CTCF	DARS
CTCF	DAZAP1
CTCF	DAZAP2
CTCF	DBF4
CTCF	DBNL
CTCF	DCAF11
CTCF	DCAF12
CTCF	DCAF15
CTCF	DCAF7
CTCF	DCAKD
CTCF	DCK
CTCF	DCLRE1C
CTCF	DCP1B
CTCF	DCP2
CTCF	DCTN1
CTCF	DCTN2
CTCF	DCTN3
CTCF	DCUN1D1
CTCF	DDA1
CTCF	DDB2
CTCF	DDT
CTCF	DDX19B
CTCF	DDX27
CTCF	DDX39A
CTCF	DDX46

CTCF	DDX5
CTCF	DDX59
CTCF	DEK
CTCF	DENND1B
CTCF	DENND2D
CTCF	DENND3
CTCF	DENND4A
CTCF	DENND4B
CTCF	DESI2
CTCF	DET1
CTCF	DGCR8
CTCF	DGKZ
CTCF	DGUOK
CTCF	DHFR
CTCF	DHPS
CTCF	DHRS7B
CTCF	DHX36
CTCF	DIAPH1
CTCF	DLD
CTCF	DLEU2
CTCF	DLG1
CTCF	DLST
CTCF	DMAP1
CTCF	DMD
CTCF	DNAJA1
CTCF	DNAJB1
CTCF	DNAJC21
CTCF	DNAJC7
CTCF	DNAJC8
CTCF	DNAJC9
CTCF	DNM2
CTCF	DNMT1
CTCF	DNTTIP1
CTCF	DOCK11
CTCF	DOCK8
CTCF	DONSON
CTCF	DOT1L
CTCF	DPAGT1
CTCF	DPH7
CTCF	DPM1
CTCF	DPP9
CTCF	DPY30
CTCF	DR1
CTCF	DRAM2
CTCF	DRAP1
CTCF	DTNBP1
CTCF	DTX1
CTCF	DTX2
CTCF	DTYMK
CTCF	DUSP23

CTCF	DUSP6
CTCF	DUT
CTCF	DVL2
CTCF	DYM
CTCF	DYNC1I2
CTCF	DYNC1LI1
CTCF	DYNLL1
CTCF	DYNLT3
CTCF	DYRK2
CTCF	E2F4
CTCF	E2F8
CTCF	EAPP
CTCF	EBAG9
CTCF	EBF1
CTCF	EBP
CTCF	ECEL1
CTCF	ECSIT
CTCF	ECT2
CTCF	EDC3
CTCF	EDF1
CTCF	EEF1D
CTCF	EFNA4
CTCF	EGLN2
CTCF	EHBP1
CTCF	EID1
CTCF	EID2
CTCF	EIF1B
CTCF	EIF2AK1
CTCF	EIF2B1
CTCF	EIF2S3
CTCF	EIF3A
CTCF	EIF3D
CTCF	EIF3E
CTCF	EIF3I
CTCF	EIF3K
CTCF	EIF3L
CTCF	EIF4E
CTCF	EIF4EBP2
CTCF	EIF4ENIF1
CTCF	EIF4G2
CTCF	ELAVL1
CTCF	ELF1
CTCF	ELMOD2
CTCF	ELOF1
CTCF	ELOVL5
CTCF	ELP5
CTCF	ELP6
CTCF	EMC7
CTCF	EMC9
CTCF	EMD

CTCF	ENOX2
CTCF	ENSA
CTCF	ENTPD4
CTCF	ENY2
CTCF	EPC1
CTCF	EPC2
CTCF	EPN1
CTCF	EPS15
CTCF	EPS15L1
CTCF	ERAP1
CTCF	ERC1
CTCF	ERCC1
CTCF	ERCC3
CTCF	ERGIC1
CTCF	ERGIC3
CTCF	ERP29
CTCF	ESD
CTCF	ETAA1
CTCF	ETS1
CTCF	EVC2
CTCF	EWSR1
CTCF	EXO5
CTCF	EXOC1
CTCF	EXOC3
CTCF	EXOSC10
CTCF	EXOSC3
CTCF	EXOSC9
CTCF	EZH2
CTCF	EZR
CTCF	FAAP20
CTCF	FADD
CTCF	FADS3
CTCF	FAF1
CTCF	FAM104A
CTCF	FAM104B
CTCF	FAM107B
CTCF	FAM110A
CTCF	FAM111A
CTCF	FAM120A
CTCF	FAM120AOS
CTCF	FAM120B
CTCF	FAM122B
CTCF	FAM133B
CTCF	FAM177A1
CTCF	FAM192A
CTCF	FAM204A
CTCF	FAM217B
CTCF	FAM32A
CTCF	FAM49A
CTCF	FAM96A

CTCF	FANCB
CTCF	FANCD2
CTCF	FANCE
CTCF	FANCG
CTCF	FANCI
CTCF	FANCL
CTCF	FBXL12
CTCF	FBXL3
CTCF	FBXO22
CTCF	FBXO3
CTCF	FBXO42
CTCF	FBXO5
CTCF	FBXO7
CTCF	FBXO9
CTCF	FBXW5
CTCF	FCHO1
CTCF	FDPS
CTCF	FDXR
CTCF	FEM1B
CTCF	FEN1
CTCF	FGFR1OP2
CTCF	FIBP
CTCF	FIGNL1
CTCF	FIP1L1
CTCF	FKBP1A
CTCF	FKBP3
CTCF	FLOT1
CTCF	FLOT2
CTCF	FNBP4
CTCF	FNIP1
CTCF	FNTA
CTCF	FOPNL
CTCF	FOXK2
CTCF	FOXM1
CTCF	FOXN2
CTCF	FRA10AC1
CTCF	FSCN1
CTCF	FTH1
CTCF	FTL
CTCF	FTX
CTCF	FUS
CTCF	FZR1
CTCF	G3BP1
CTCF	G6PC3
CTCF	GABARAP
CTCF	GABARAPL2
CTCF	GABPB2
CTCF	GATAD2A
CTCF	GCA
CTCF	GDI1

CTCF	GDI2
CTCF	GEMIN2
CTCF	GEMIN5
CTCF	GEN1
CTCF	GGA1
CTCF	GGA2
CTCF	GGNBP2
CTCF	GHITM
CTCF	GID8
CTCF	GIGYF2
CTCF	GINS3
CTCF	GIT2
CTCF	GLE1
CTCF	GLMN
CTCF	GLRX5
CTCF	GLS
CTCF	GMCL1
CTCF	GMFB
CTCF	GMFG
CTCF	GMNN
CTCF	GNA12
CTCF	GNA13
CTCF	GNAI2
CTCF	GNAI3
CTCF	GNAS
CTCF	GNAZ
CTCF	GNB1L
CTCF	GNB2
CTCF	GNB4
CTCF	GNG5
CTCF	GNL1
CTCF	GNL3L
CTCF	GNPAT
CTCF	GOLGA2
CTCF	GPAA1
CTCF	GPATCH8
CTCF	GPBP1
CTCF	GPCPD1
CTCF	GPD2
CTCF	GPN1
CTCF	GPN3
CTCF	GPS1
CTCF	GPSM2
CTCF	GPSM3
CTCF	GPX4
CTCF	GRAMD1A
CTCF	GRAMD4
CTCF	GRB2
CTCF	GRIPAP1
CTCF	GRK2

CTCF	GRK6
CTCF	GRPEL2
CTCF	GSDMD
CTCF	GSK3A
CTCF	GSS
CTCF	GTDC1
CTCF	GTF2A1
CTCF	GTF2A2
CTCF	GTF2B
CTCF	GTF2E2
CTCF	GTF2F1
CTCF	GTF2F2
CTCF	GTF2I
CTCF	GTSE1
CTCF	H1FX
CTCF	H2AFV
CTCF	H2AFX
CTCF	H2AFY
CTCF	H2AFZ
CTCF	H3F3A
CTCF	H3F3B
CTCF	H6PD
CTCF	HACD2
CTCF	HAGHL
CTCF	HARS
CTCF	HAUS4
CTCF	HAUS5
CTCF	HAUS6
CTCF	HAUS8
CTCF	HBS1L
CTCF	HCFC1
CTCF	HDAC5
CTCF	HDLBP
CTCF	HEBP2
CTCF	HELQ
CTCF	HELZ
CTCF	HENMT1
CTCF	HERC1
CTCF	HERC2
CTCF	HERPUD2
CTCF	HES6
CTCF	HGS
CTCF	HIF1A
CTCF	HIGD2A
CTCF	HINT1
CTCF	HINT2
CTCF	HIRIP3
CTCF	HIST1H1D
CTCF	HIST1H1E
CTCF	HJURP

CTCF	HMBS
CTCF	HMG20B
CTCF	HMGA1
CTCF	HMGB1
CTCF	HMGB2
CTCF	HMGB3
CTCF	HMGN1
CTCF	HMGN2
CTCF	HMGXB3
CTCF	HMGXB4
CTCF	HMMR
CTCF	HNRNPA0
CTCF	HNRNPA1
CTCF	HNRNPA2B1
CTCF	HNRNPA3
CTCF	HNRNPD
CTCF	HNRNPH1
CTCF	HNRNPH2
CTCF	HNRNPH3
CTCF	HNRNPK
CTCF	HNRNPM
CTCF	HNRNPR
CTCF	HNRNPU
CTCF	HNRNPUL1
CTCF	HNRNPUL2
CTCF	HOXB7
CTCF	HP1BP3
CTCF	HPF1
CTCF	HRAS
CTCF	HSBP1
CTCF	HSF1
CTCF	HSF2
CTCF	HSP90AA1
CTCF	HSPBAP1
CTCF	HTATSF1
CTCF	HTRA2
CTCF	HUS1
CTCF	HUWE1
CTCF	HYLS1
CTCF	ID3
CTCF	IDE
CTCF	IDH2
CTCF	IDH3A
CTCF	IDI1
CTCF	IFT122
CTCF	IFT20
CTCF	IFT74
CTCF	IFT88
CTCF	IKBKB
CTCF	IKBKG

CTCF	IKZF1
CTCF	IKZF3
CTCF	ILF3
CTCF	ILKAP
CTCF	IMMT
CTCF	INCENP
CTCF	ING1
CTCF	ING2
CTCF	ING3
CTCF	INIP
CTCF	INO80C
CTCF	INO80E
CTCF	INPPL1
CTCF	INSIG2
CTCF	INTS10
CTCF	IP6K1
CTCF	IPO7
CTCF	IPO9
CTCF	IQGAP1
CTCF	IRF1
CTCF	IRF2BP1
CTCF	IRF9
CTCF	ISCA2
CTCF	ISY1
CTCF	ITFG1
CTCF	ITFG2
CTCF	ITGB3BP
CTCF	ITGB7
CTCF	ITSN2
CTCF	IWS1
CTCF	JADE2
CTCF	JAK1
CTCF	JKAMP
CTCF	JMJD1C
CTCF	KANSL1
CTCF	KANSL2
CTCF	KARS
CTCF	KAT5
CTCF	KATNA1
CTCF	KATNAL1
CTCF	KBTBD2
CTCF	KCNAB2
CTCF	KCNK12
CTCF	KCNMB4
CTCF	KCTD15
CTCF	KCTD17
CTCF	KCTD20
CTCF	KDM4A
CTCF	KDM5A
CTCF	KDM5C

CTCF	KHDRBS1
CTCF	KIAA1586
CTCF	KIAA1841
CTCF	KIAA2026
CTCF	KIF11
CTCF	KIF15
CTCF	KIF18B
CTCF	KIF1BP
CTCF	KIF20B
CTCF	KIF22
CTCF	KIF23
CTCF	KIF2A
CTCF	KIF2C
CTCF	KIF3A
CTCF	KIF5B
CTCF	KIFC1
CTCF	KIN
CTCF	KLC2
CTCF	KLF10
CTCF	KLF2
CTCF	KLHDC2
CTCF	KLHDC3
CTCF	KLHDC4
CTCF	KLHL36
CTCF	KMT2C
CTCF	KMT2E
CTCF	KMT5B
CTCF	KNTC1
CTCF	KPNA3
CTCF	KPNB1
CTCF	KRAS
CTCF	KRR1
CTCF	KTN1
CTCF	KXD1
CTCF	KYAT3
CTCF	LACC1
CTCF	LAMTOR3
CTCF	LAMTOR4
CTCF	LAPTM4B
CTCF	LARP4B
CTCF	LARP7
CTCF	LARS2
CTCF	LAS1L
CTCF	LAT2
CTCF	LATS1
CTCF	LBR
CTCF	LCOR
CTCF	LCORL
CTCF	LEO1
CTCF	LETMD1

CTCF	LIG1
CTCF	LIMK2
CTCF	LINC00487
CTCF	LINC01006
CTCF	LINC01013
CTCF	LINC01134
CTCF	LIPE
CTCF	LIPT2
CTCF	LMF2
CTCF	LMNB1
CTCF	LMNB2
CTCF	LMO7
CTCF	LNPEP
CTCF	LPCAT3
CTCF	LPIN1
CTCF	LPIN2
CTCF	LPP
CTCF	LRCH4
CTCF	LRIF1
CTCF	LRP10
CTCF	LRR1
CTCF	LRRC1
CTCF	LRRC57
CTCF	LRRC58
CTCF	LRRFIP1
CTCF	LRTOMT
CTCF	LSM14A
CTCF	LSM14B
CTCF	LSM2
CTCF	LSM3
CTCF	LSM4
CTCF	LSM5
CTCF	LSM8
CTCF	LTA
CTCF	LTBP4
CTCF	LTN1
CTCF	LUC7L2
CTCF	LY6E
CTCF	LYL1
CTCF	LYPLA2
CTCF	LYRM1
CTCF	LYRM2
CTCF	MAD1L1
CTCF	MAD2L1
CTCF	MAD2L1BP
CTCF	MAD2L2
CTCF	MADD
CTCF	MAEA
CTCF	MAGEF1
CTCF	MAP1LC3B

CTCF	MAP2K1
CTCF	MAP3K7
CTCF	MAP4K1
CTCF	MAP7D1
CTCF	MAPK1
CTCF	MAPK10
CTCF	MAPK8
CTCF	MAPK9
CTCF	MAPRE1
CTCF	MARCH5
CTCF	MARCH7
CTCF	MARCH9
CTCF	MARK3
CTCF	MAT2B
CTCF	MAZ
CTCF	MBD4
CTCF	MBNL1
CTCF	MCM3
CTCF	MCM7
CTCF	MCPH1
CTCF	MCRIP1
CTCF	MDC1
CTCF	MDM1
CTCF	MDM4
CTCF	MEA1
CTCF	MEAF6
CTCF	MED1
CTCF	MED11
CTCF	MED13
CTCF	MED13L
CTCF	MED15
CTCF	MED20
CTCF	MED21
CTCF	MED25
CTCF	MED27
CTCF	MED30
CTCF	MED31
CTCF	MED4
CTCF	MED7
CTCF	MED8
CTCF	MEF2A
CTCF	MEF2C
CTCF	MEF2D
CTCF	MEGF11
CTCF	MELK
CTCF	MEPCE
CTCF	MET
CTCF	METTL14
CTCF	METTL15
CTCF	METTL23

CTCF	METTL4
CTCF	METTL5
CTCF	METTL9
CTCF	MFF
CTCF	MFGE8
CTCF	MFNG
CTCF	MFSD11
CTCF	MFSD14B
CTCF	MFSD14C
CTCF	MFSD5
CTCF	MGME1
CTCF	MIB1
CTCF	MICAL1
CTCF	MICAL3
CTCF	MICU1
CTCF	MID1IP1
CTCF	MIDN
CTCF	MIEN1
CTCF	MIER1
CTCF	MIF4GD
CTCF	MIR181A1HG
CTCF	MIS18A
CTCF	MKI67
CTCF	MKRN1
CTCF	MKRN2
CTCF	MLF2
CTCF	MLLT10
CTCF	MLLT6
CTCF	MLST8
CTCF	MLX
CTCF	MLXIP
CTCF	MMADHC
CTCF	MMGT1
CTCF	MND1
CTCF	MOB1A
CTCF	MOB4
CTCF	MOCS2
CTCF	MORF4L1
CTCF	MPC1
CTCF	MPC2
CTCF	MPDU1
CTCF	MPHOSPH6
CTCF	MPHOSPH8
CTCF	MPLKIP
CTCF	MPND
CTCF	MPP5
CTCF	MRPL17
CTCF	MRPL18
CTCF	MRPL22
CTCF	MRPL27

CTCF	MRPL34
CTCF	MRPL37
CTCF	MRPL40
CTCF	MRPL47
CTCF	MRPL48
CTCF	MRPL51
CTCF	MRPL54
CTCF	MRPS10
CTCF	MRPS14
CTCF	MRPS16
CTCF	MRPS18C
CTCF	MRPS6
CTCF	MRPS9
CTCF	MSANTD2
CTCF	MSI2
CTCF	MSL1
CTCF	MSL3
CTCF	MSMO1
CTCF	MSN
CTCF	MSRA
CTCF	MTA3
CTCF	MTERF3
CTCF	MTF2
CTCF	MTFR2
CTCF	MTG2
CTCF	MTIF2
CTCF	MTIF3
CTCF	MTOR
CTCF	MTPAP
CTCF	MTPN
CTCF	MTX3
CTCF	MVB12A
CTCF	MVK
CTCF	MXD3
CTCF	MYBL1
CTCF	MYBL2
CTCF	MYD88
CTCF	MYEF2
CTCF	MYL12A
CTCF	MYL12B
CTCF	MYO1C
CTCF	MYO9B
CTCF	MZT1
CTCF	N4BP2L2
CTCF	N4BP3
CTCF	NAA38
CTCF	NAA40
CTCF	NAB2
CTCF	NABP2
CTCF	NACC1

CTCF	NAGK
CTCF	NAIF1
CTCF	NAP1L1
CTCF	NAP1L4
CTCF	NBEAL1
CTCF	NCAPD3
CTCF	NCAPG
CTCF	NCAPG2
CTCF	NCAPH2
CTCF	NCOA3
CTCF	NCOR1
CTCF	NDUFA12
CTCF	NDUFA4L2
CTCF	NDUFA6
CTCF	NDUFB4
CTCF	NDUFB6
CTCF	NDUFB7
CTCF	NDUFC1
CTCF	NDUFS1
CTCF	NDUFS3
CTCF	NDUFS7
CTCF	NDUFS8
CTCF	NECAP1
CTCF	NECAP2
CTCF	NEDD1
CTCF	NEIL1
CTCF	NEIL3
CTCF	NEK4
CTCF	NEK9
CTCF	NELFB
CTCF	NELFE
CTCF	NET1
CTCF	NFATC2IP
CTCF	NFKBIL1
CTCF	NFYB
CTCF	NFYC
CTCF	NKAP
CTCF	NKTR
CTCF	NLRC5
CTCF	NME6
CTCF	NMNAT1
CTCF	NNT-AS1
CTCF	NOM1
CTCF	NONO
CTCF	NOP56
CTCF	NPAT
CTCF	NPEPPS
CTCF	NPHP1
CTCF	NR1H2
CTCF	NR3C1

CTCF	NRBP1
CTCF	NRDE2
CTCF	NREP
CTCF	NRGN
CTCF	NSA2
CTCF	NSF
CTCF	NSL1
CTCF	NSMCE4A
CTCF	NSUN5
CTCF	NT5C
CTCF	NTAN1
CTCF	NUCKS1
CTCF	NUDT1
CTCF	NUDT15
CTCF	NUDT16L1
CTCF	NUDT18
CTCF	NUDT2
CTCF	NUDT21
CTCF	NUDT5
CTCF	NUMA1
CTCF	NUP107
CTCF	NUP155
CTCF	NUP37
CTCF	NUP50
CTCF	NUP58
CTCF	NUP62
CTCF	NUP88
CTCF	NUP98
CTCF	NUTF2
CTCF	NXF1
CTCF	NXPH4
CTCF	OARD1
CTCF	OCIAD2
CTCF	ODF2
CTCF	OFD1
CTCF	OGFOD3
CTCF	OGFR
CTCF	ORAI2
CTCF	ORC3
CTCF	ORC6
CTCF	ORMDL2
CTCF	OSBP
CTCF	OSBPL11
CTCF	OSBPL8
CTCF	OSER1
CTCF	OTUB1
CTCF	OTUD6B
CTCF	OXCT1
CTCF	P2RX5
CTCF	PABPC1

CTCF	PABPN1
CTCF	PAFAH1B1
CTCF	PAFAH1B3
CTCF	PAG1
CTCF	PAGR1
CTCF	PAIP2
CTCF	PAIP2B
CTCF	PAN2
CTCF	PAN3
CTCF	PAPOLA
CTCF	PARG
CTCF	PARP6
CTCF	PARVG
CTCF	PATL1
CTCF	PAX5
CTCF	PAXBP1
CTCF	PBK
CTCF	PBRM1
CTCF	PBX2
CTCF	PCBD2
CTCF	PCBP1
CTCF	PCBP2
CTCF	PCDH9
CTCF	PCF11
CTCF	PCGF6
CTCF	PCID2
CTCF	PCIF1
CTCF	PCM1
CTCF	PCMT1
CTCF	PCMTD2
CTCF	PCNT
CTCF	PCSK7
CTCF	PDCD10
CTCF	PDCD2
CTCF	PDCD6
CTCF	PDCD6IP
CTCF	PDCL
CTCF	PDE4DIP
CTCF	PDLIM1
CTCF	PDLIM7
CTCF	PDRG1
CTCF	PDS5A
CTCF	PDS5B
CTCF	PDZD11
CTCF	PECAM1
CTCF	PELP1
CTCF	PER2
CTCF	PET100
CTCF	PEX2
CTCF	PEX26

CTCF	PFDN1
CTCF	PFDN2
CTCF	PFDN5
CTCF	PFKP
CTCF	PGLS
CTCF	PGP
CTCF	PGS1
CTCF	PHACTR4
CTCF	PHAX
CTCF	PHB2
CTCF	PHF13
CTCF	PHF19
CTCF	PHF20L1
CTCF	PHF21A
CTCF	PHF23
CTCF	PHF3
CTCF	PHF5A
CTCF	PHF7
CTCF	PHIP
CTCF	PHKG1
CTCF	PHPT1
CTCF	PHYKPL
CTCF	PIAS1
CTCF	PIAS3
CTCF	PIDD1
CTCF	PIF1
CTCF	PIGBOS1
CTCF	PIGK
CTCF	PIGQ
CTCF	PIH1D1
CTCF	PIK3C2B
CTCF	PILRB
CTCF	PIN1
CTCF	PIN4
CTCF	PIP5K1C
CTCF	PISD
CTCF	PITPNA
CTCF	PJA2
CTCF	PKMYT1
CTCF	PKNOX1
CTCF	PLCG2
CTCF	PLEKHA8
CTCF	PLEKHF2
CTCF	PLK1
CTCF	PLK4
CTCF	PLPP1
CTCF	PMPCB
CTCF	PMVK
CTCF	PNISR
CTCF	PNKD

CTCF	PNKP
CTCF	PNN
CTCF	PNPLA2
CTCF	PNPLA8
CTCF	PNRC2
CTCF	POC1B
CTCF	POC5
CTCF	POGZ
CTCF	POLA2
CTCF	POLD3
CTCF	POLG2
CTCF	POLH
CTCF	POLM
CTCF	POLR2A
CTCF	POLR2B
CTCF	POLR2C
CTCF	POLR2D
CTCF	POLR2E
CTCF	POLR2G
CTCF	POLR2K
CTCF	POMGNT1
CTCF	POP7
CTCF	POU2F2
CTCF	PPDPF
CTCF	PPHLN1
CTCF	PPIA
CTCF	PPIE
CTCF	PPIG
CTCF	PPIH
CTCF	PPIL2
CTCF	PPIP5K2
CTCF	PPM1B
CTCF	PPM1D
CTCF	PPM1G
CTCF	PPP1CA
CTCF	PPP1CB
CTCF	PPP1CC
CTCF	PPP1R12A
CTCF	PPP1R12C
CTCF	PPP1R14A
CTCF	PPP1R15B
CTCF	PPP1R16A
CTCF	PPP1R18
CTCF	PPP1R7
CTCF	PPP2CA
CTCF	PPP2R1A
CTCF	PPP2R2A
CTCF	PPP2R2D
CTCF	PPP2R3C
CTCF	PPP2R5E

CTCF	PPP3CB
CTCF	PPP3CC
CTCF	PPP4C
CTCF	PPP4R2
CTCF	PPP4R3A
CTCF	PPP4R3B
CTCF	PPP6C
CTCF	PPP6R1
CTCF	PPP6R2
CTCF	PRCC
CTCF	PRDM2
CTCF	PRDX2
CTCF	PRIM1
CTCF	PRIM2
CTCF	PRKAR1A
CTCF	PRKCB
CTCF	PRKD3
CTCF	PRKDC
CTCF	PRKRA
CTCF	PRMT2
CTCF	PRPF18
CTCF	PRPF38A
CTCF	PRPF38B
CTCF	PRPF4B
CTCF	PRPF6
CTCF	PRPF8
CTCF	PRPS1
CTCF	PRPSAP1
CTCF	PRR14
CTCF	PRRC2C
CTCF	PSD4
CTCF	PSIP1
CTCF	PSMA4
CTCF	PSMB10
CTCF	PSMB2
CTCF	PSMB3
CTCF	PSMB4
CTCF	PSMC1
CTCF	PSMC2
CTCF	PSMC4
CTCF	PSMD1
CTCF	PSMD13
CTCF	PSMD2
CTCF	PSMD7
CTCF	PSMD8
CTCF	PSMD9
CTCF	PSMF1
CTCF	PSMG2
CTCF	PSPC1
CTCF	PSRC1

CTCF	PTBP1
CTCF	PTEN
CTCF	PTGES3
CTCF	PTK2B
CTCF	PTMA
CTCF	PTP4A1
CTCF	PTP4A2
CTCF	PTPN18
CTCF	PTPRA
CTCF	PTRHD1
CTCF	PTS
CTCF	PTTG1IP
CTCF	PUM2
CTCF	PURA
CTCF	PUS10
CTCF	PYCR2
CTCF	PYGB
CTCF	QRICH1
CTCF	RAB11A
CTCF	RAB11B
CTCF	RAB11B-AS1
CTCF	RAB11FIP4
CTCF	RAB14
CTCF	RAB1B
CTCF	RAB22A
CTCF	RAB27A
CTCF	RAB28
CTCF	RAB2A
CTCF	RAB5C
CTCF	RAB6A
CTCF	RAB7A
CTCF	RABEP1
CTCF	RABIF
CTCF	RABL6
CTCF	RAC1
CTCF	RAC2
CTCF	RACGAP1
CTCF	RACK1
CTCF	RAD1
CTCF	RAD21
CTCF	RAD23B
CTCF	RAD51AP1
CTCF	RAD51C
CTCF	RAE1
CTCF	RAF1
CTCF	RALBP1
CTCF	RALY
CTCF	RANGAP1
CTCF	RAPGEF5
CTCF	RASSF1

CTCF	RBAK-RBAKDN
CTCF	RBBP4
CTCF	RBBP7
CTCF	RBBP9
CTCF	RBL2
CTCF	RBM10
CTCF	RBM12
CTCF	RBM17
CTCF	RBM22
CTCF	RBM23
CTCF	RBM39
CTCF	RBM4
CTCF	RBM45
CTCF	RBM4B
CTCF	RBM5
CTCF	RBM6
CTCF	RBM8A
CTCF	RBMX
CTCF	RBMXL1
CTCF	RBX1
CTCF	RCC2
CTCF	RCCD1
CTCF	RCOR3
CTCF	RCSD1
CTCF	RDM1
CTCF	REEP4
CTCF	RELB
CTCF	REST
CTCF	REV1
CTCF	REV3L
CTCF	RFC3
CTCF	RFC4
CTCF	RFT1
CTCF	RFWD3
CTCF	RFX1
CTCF	RFX2
CTCF	RFX3
CTCF	RFX5
CTCF	RHEB
CTCF	RHNO1
CTCF	RHOH
CTCF	RIC8A
CTCF	RICTOR
CTCF	RIMKLB
CTCF	RING1
CTCF	RIPK2
CTCF	RMDN1
CTCF	RMI1
CTCF	RNASEH2B
CTCF	RNASEH2C

CTCF	RNF13
CTCF	RNF146
CTCF	RNF19A
CTCF	RNF20
CTCF	RNF216
CTCF	RNF219
CTCF	RNF34
CTCF	RNF41
CTCF	RNF8
CTCF	RNFT1
CTCF	ROCK1
CTCF	ROCK2
CTCF	RPA2
CTCF	RPL10
CTCF	RPL11
CTCF	RPL12
CTCF	RPL13A
CTCF	RPL14
CTCF	RPL15
CTCF	RPL18
CTCF	RPL18A
CTCF	RPL22L1
CTCF	RPL23A
CTCF	RPL26
CTCF	RPL27
CTCF	RPL28
CTCF	RPL29
CTCF	RPL3
CTCF	RPL30
CTCF	RPL31
CTCF	RPL32
CTCF	RPL35
CTCF	RPL35A
CTCF	RPL36
CTCF	RPL37
CTCF	RPL37A
CTCF	RPL38
CTCF	RPL4
CTCF	RPL41
CTCF	RPL6
CTCF	RPL8
CTCF	RPLP0
CTCF	RPLP1
CTCF	RPLP2
CTCF	RPP30
CTCF	RPRD1A
CTCF	RPS10
CTCF	RPS11
CTCF	RPS13
CTCF	RPS14

CTCF	RPS15
CTCF	RPS16
CTCF	RPS17
CTCF	RPS18
CTCF	RPS19
CTCF	RPS20
CTCF	RPS21
CTCF	RPS23
CTCF	RPS26
CTCF	RPS27
CTCF	RPS29
CTCF	RPS3
CTCF	RPS3A
CTCF	RPS6
CTCF	RPS6KA1
CTCF	RPS6KB2
CTCF	RPS7
CTCF	RPS8
CTCF	RPS9
CTCF	RPSA
CTCF	RRAGC
CTCF	RRAS2
CTCF	RRM1
CTCF	RRM2
CTCF	RRM2B
CTCF	RSBN1
CTCF	RSF1
CTCF	RSPH14
CTCF	RSRC1
CTCF	RSRC2
CTCF	RSRP1
CTCF	RTCA
CTCF	RTCB
CTCF	RTN3
CTCF	RTN4
CTCF	RUFY3
CTCF	RUNX1
CTCF	RUVBL2
CTCF	RWDD3
CTCF	RXRB
CTCF	RYBP
CTCF	S100A10
CTCF	S1PR2
CTCF	SAC3D1
CTCF	SAE1
CTCF	SAFB
CTCF	SAMHD1
CTCF	SAP18
CTCF	SAP30BP
CTCF	SASS6

CTCF	SAV1
CTCF	SBF1
CTCF	SCAF11
CTCF	SCAF4
CTCF	SCFD2
CTCF	SCMH1
CTCF	SCNM1
CTCF	SCYL3
CTCF	SDCCAG8
CTCF	SDF2
CTCF	SDHA
CTCF	SDHAF2
CTCF	SDR39U1
CTCF	SEC11A
CTCF	SEC14L1
CTCF	SEC22C
CTCF	SECISBP2
CTCF	SENP1
CTCF	SENP6
CTCF	SEPHS1
CTCF	SEPT11
CTCF	SEPT6
CTCF	SEPT7
CTCF	SERINC3
CTCF	SERP2
CTCF	SETD2
CTCF	SETD3
CTCF	SETMAR
CTCF	SF1
CTCF	SF3A2
CTCF	SF3B1
CTCF	SF3B2
CTCF	SF3B4
CTCF	SF3B6
CTCF	SFMBT1
CTCF	SFPQ
CTCF	SFR1
CTCF	SFSWAP
CTCF	SGO1
CTCF	SGO2
CTCF	SGPP1
CTCF	SH2B1
CTCF	SH3GL1
CTCF	SH3GLB2
CTCF	SH3KBP1
CTCF	SHARPIN
CTCF	SHC1
CTCF	SHCBP1
CTCF	SHISA8
CTCF	SHKBP1

CTCF	SHMT2
CTCF	SHOC2
CTCF	SHQ1
CTCF	SIMC1
CTCF	SIPA1
CTCF	SIRT5
CTCF	SIRT6
CTCF	SKA1
CTCF	SKA2
CTCF	SLBP
CTCF	SLC12A2
CTCF	SLC16A4
CTCF	SLC25A1
CTCF	SLC25A14
CTCF	SLC25A19
CTCF	SLC25A42
CTCF	SLC2A1
CTCF	SLC35C2
CTCF	SLC37A4
CTCF	SLC43A2
CTCF	SLC4A2
CTCF	SLC5A3
CTCF	SLC6A16
CTCF	SLC9A3R1
CTCF	SLF1
CTCF	SLU7
CTCF	SMAD2
CTCF	SMARCA2
CTCF	SMARCA4
CTCF	SMARCA5
CTCF	SMARCAL1
CTCF	SMARCB1
CTCF	SMARCC2
CTCF	SMARCD1
CTCF	SMARCE1
CTCF	SMC1A
CTCF	SMC1B
CTCF	SMC2
CTCF	SMC3
CTCF	SMC4
CTCF	SMCHD1
CTCF	SMCR5
CTCF	SMIM19
CTCF	SMIM7
CTCF	SMNDC1
CTCF	SMTN
CTCF	SMU1
CTCF	SMURF2
CTCF	SNF8
CTCF	SNRNP27

CTCF	SNRNP40
CTCF	SNRNP48
CTCF	SNRNP70
CTCF	SNRPA
CTCF	SNRPA1
CTCF	SNRPB
CTCF	SNRPF
CTCF	SNRPG
CTCF	SNX1
CTCF	SNX14
CTCF	SNX16
CTCF	SNX19
CTCF	SNX20
CTCF	SNX3
CTCF	SOCS1
CTCF	SON
CTCF	SOX4
CTCF	SP2
CTCF	SPAG7
CTCF	SPATA2L
CTCF	SPDL1
CTCF	SPG7
CTCF	SPOP
CTCF	SPPL2A
CTCF	SPRTN
CTCF	SPRYD3
CTCF	SPTAN1
CTCF	SQLE
CTCF	SREK1IP1
CTCF	SRGAP2C
CTCF	SRP14
CTCF	SRP19
CTCF	SRP9
CTCF	SRPK1
CTCF	SRRM2
CTCF	SRRT
CTCF	SRSF1
CTCF	SRSF10
CTCF	SRSF3
CTCF	SRSF4
CTCF	SRSF5
CTCF	SRSF7
CTCF	SRSF9
CTCF	SS18
CTCF	SSBP2
CTCF	SSNA1
CTCF	SSRP1
CTCF	STAG1
CTCF	STAMBP
CTCF	STAT2

CTCF	STAU2
CTCF	STIM2
CTCF	STK40
CTCF	STMN1
CTCF	STRBP
CTCF	STRN
CTCF	STRN3
CTCF	STT3B
CTCF	SUGP2
CTCF	SUGT1
CTCF	SULT1A1
CTCF	SUMO2
CTCF	SUPT4H1
CTCF	SUPT5H
CTCF	SUSD1
CTCF	SUSD6
CTCF	SVBP
CTCF	SWAP70
CTCF	SWI5
CTCF	SYNE1
CTCF	SYNE2
CTCF	SYPL1
CTCF	SYT11
CTCF	SYT17
CTCF	SZRD1
CTCF	TAB2
CTCF	TACC1
CTCF	TACC3
CTCF	TADA3
CTCF	TAF11
CTCF	TAF13
CTCF	TAF15
CTCF	TAF1B
CTCF	TAF3
CTCF	TAF5
CTCF	TAF6
CTCF	TAGLN2
CTCF	TANGO2
CTCF	TANK
CTCF	TAP2
CTCF	TARDBP
CTCF	TARS2
CTCF	TARSL2
CTCF	TBC1D1
CTCF	TBCB
CTCF	TBCCD1
CTCF	TBK1
CTCF	TBL1XR1
CTCF	TBP
CTCF	TBPL1

CTCF	TCEA1
CTCF	TCEAL8
CTCF	TCERG1
CTCF	TCF19
CTCF	TCHP
CTCF	TCL1A
CTCF	TCOF1
CTCF	TDG
CTCF	TDP1
CTCF	TDP2
CTCF	TECR
CTCF	TELO2
CTCF	TEN1
CTCF	TERF2IP
CTCF	TES
CTCF	TEX30
CTCF	TFB1M
CTCF	TFCP2
CTCF	TFEB
CTCF	TGIF2
CTCF	TGOLN2
CTCF	THAP1
CTCF	THAP11
CTCF	THOC1
CTCF	THOC3
CTCF	THOC5
CTCF	THOC6
CTCF	THOC7
CTCF	THRAP3
CTCF	TIFA
CTCF	TIGAR
CTCF	TIMM21
CTCF	TIMMDC1
CTCF	TINF2
CTCF	TIPARP
CTCF	TIPRL
CTCF	TJP1
CTCF	TKT
CTCF	TLK1
CTCF	TLN1
CTCF	TM2D3
CTCF	TM9SF3
CTCF	TMA7
CTCF	TMBIM4
CTCF	TMEM107
CTCF	TMEM11
CTCF	TMEM123
CTCF	TMEM126B
CTCF	TMEM14B
CTCF	TMEM14C

CTCF	TMEM150A
CTCF	TMEM19
CTCF	TMEM230
CTCF	TMEM234
CTCF	TMEM242
CTCF	TMEM50A
CTCF	TMEM60
CTCF	TMEM69
CTCF	TMEM87A
CTCF	TMEM8B
CTCF	TMF1
CTCF	TMPO
CTCF	TMPO-AS1
CTCF	TMUB1
CTCF	TMUB2
CTCF	TNF
CTCF	TNFSF8
CTCF	TOB1
CTCF	TOMM7
CTCF	TOP2A
CTCF	TOP2B
CTCF	TOPBP1
CTCF	TOPORS
CTCF	TOX4
CTCF	TP53RK
CTCF	TPCN1
CTCF	TPM1
CTCF	TPM3
CTCF	TPR
CTCF	TPRKB
CTCF	TPX2
CTCF	TRA2A
CTCF	TRA2B
CTCF	TRABD
CTCF	TRAF7
CTCF	TRAIP
CTCF	TRAPPC1
CTCF	TRAPPC10
CTCF	TRAPPC12
CTCF	TRAPPC2B
CTCF	TRAPPC9
CTCF	TRIM11
CTCF	TRIM13
CTCF	TRIM28
CTCF	TRIM37
CTCF	TRIM4
CTCF	TRIOBP
CTCF	TRIP11
CTCF	TRNAU1AP
CTCF	TSC1

CTCF	TSNAX
CTCF	TSR3
CTCF	TSSC4
CTCF	TSTD1
CTCF	TTC1
CTCF	TTC33
CTCF	TTC39C
CTCF	TTF1
CTCF	TTF2
CTCF	TTI2
CTCF	TTK
CTCF	TTLL5
CTCF	TUBA1A
CTCF	TUBA1B
CTCF	TUBA1C
CTCF	TUBA4A
CTCF	TUBB
CTCF	TUBB2A
CTCF	TUBB4B
CTCF	TUBG1
CTCF	TUBGCP2
CTCF	TUBGCP4
CTCF	TUFM
CTCF	TVP23B
CTCF	TXNDC9
CTCF	TYMS
CTCF	U2AF1L5
CTCF	UBA1
CTCF	UBA3
CTCF	UBA52
CTCF	UBAC1
CTCF	UBAC2
CTCF	UBAP1
CTCF	UBAP2
CTCF	UBB
CTCF	UBC
CTCF	UBE2B
CTCF	UBE2C
CTCF	UBE2D2
CTCF	UBE2E1
CTCF	UBE2E3
CTCF	UBE2G1
CTCF	UBE2I
CTCF	UBE2J2
CTCF	UBE2K
CTCF	UBE2L3
CTCF	UBE2L6
CTCF	UBE2O
CTCF	UBE3A
CTCF	UBL4A

CTCF	UBL5
CTCF	UBL7-AS1
CTCF	UBLCP1
CTCF	UBR2
CTCF	UBR5
CTCF	UBXN1
CTCF	UBXN4
CTCF	UBXN7
CTCF	UCHL5
CTCF	UCP2
CTCF	UEVLD
CTCF	UFC1
CTCF	UGCG
CTCF	UGDH
CTCF	UGP2
CTCF	UHRF1BP1
CTCF	UHRF2
CTCF	UMAD1
CTCF	UNG
CTCF	UNKL
CTCF	UPF1
CTCF	UPF3A
CTCF	UQCRB
CTCF	UQCRC1
CTCF	UQCRC2
CTCF	USF2
CTCF	USF3
CTCF	USP1
CTCF	USP10
CTCF	USP13
CTCF	USP14
CTCF	USP16
CTCF	USP30-AS1
CTCF	USP32
CTCF	USP33
CTCF	USP34
CTCF	USP39
CTCF	USP44
CTCF	USP48
CTCF	USP5
CTCF	USP7
CTCF	USP8
CTCF	USPL1
CTCF	UTP18
CTCF	UTP6
CTCF	UVRAG
CTCF	UXT
CTCF	VAMP8
CTCF	VDAC3
CTCF	VEZF1

CTCF	VGLL4
CTCF	VIPAS39
CTCF	VMP1
CTCF	VPS11
CTCF	VPS25
CTCF	VPS26A
CTCF	VPS26B
CTCF	VPS35
CTCF	VPS37C
CTCF	VPS4B
CTCF	VPS51
CTCF	VPS72
CTCF	VRK1
CTCF	WAC
CTCF	WAPL
CTCF	WDFY4
CTCF	WDR33
CTCF	WDR34
CTCF	WDR53
CTCF	WDR61
CTCF	WDR66
CTCF	WDR70
CTCF	WDR82
CTCF	WIPI2
CTCF	WNK1
CTCF	WRAP53
CTCF	WSB1
CTCF	WWOX
CTCF	WWP2
CTCF	XRCC1
CTCF	XRCC4
CTCF	XRCC5
CTCF	XRCC6
CTCF	YEATS4
CTCF	YIF1B
CTCF	YIPF5
CTCF	YLPM1
CTCF	YPEL1
CTCF	YPEL3
CTCF	YWHAB
CTCF	YWHAE
CTCF	YWHAH
CTCF	YWHAQ
CTCF	YWHAZ
CTCF	ZBED5-AS1
CTCF	ZBTB10
CTCF	ZBTB11-AS1
CTCF	ZBTB2
CTCF	ZBTB44
CTCF	ZC3H12A

CTCF	ZC3H12D
CTCF	ZC3H13
CTCF	ZC3H18
CTCF	ZC3H6
CTCF	ZC3HAV1
CTCF	ZCCHC10
CTCF	ZCCHC17
CTCF	ZCCHC7
CTCF	ZCCHC9
CTCF	ZDHHC12
CTCF	ZDHHC4
CTCF	ZEB1
CTCF	ZFAND2B
CTCF	ZFAND6
CTCF	ZFHX3
CTCF	ZFP82
CTCF	ZFP91
CTCF	ZFPL1
CTCF	ZFR
CTCF	ZFX
CTCF	ZHX2
CTCF	ZMYM4
CTCF	ZMYND8
CTCF	ZNF106
CTCF	ZNF143
CTCF	ZNF148
CTCF	ZNF207
CTCF	ZNF213
CTCF	ZNF24
CTCF	ZNF253
CTCF	ZNF281
CTCF	ZNF282
CTCF	ZNF292
CTCF	ZNF347
CTCF	ZNF428
CTCF	ZNF451
CTCF	ZNF48
CTCF	ZNF502
CTCF	ZNF512
CTCF	ZNF561
CTCF	ZNF592
CTCF	ZNF652
CTCF	ZNF655
CTCF	ZNF688
CTCF	ZNF701
CTCF	ZNF711
CTCF	ZNF730
CTCF	ZNF76
CTCF	ZNF775
CTCF	ZNF830

CTCF	ZNF831
CTCF	ZNF880
CTCF	ZSCAN16
CTCF	ZWILCH
CTCF	ZWINT
CTCF	ZZEF1
CUX1	ABHD3
CUX1	ABI1
CUX1	ACAP2
CUX1	ACBD5
CUX1	ACTB
CUX1	ACTR2
CUX1	ACY3
CUX1	ADD3
CUX1	ADIPOR2
CUX1	AFMID
CUX1	ALOX5AP
CUX1	APLP2
CUX1	APOLD1
CUX1	APOPT1
CUX1	ARHGAP17
CUX1	ARID2
CUX1	ARID4B
CUX1	ARL3
CUX1	ARMC1
CUX1	ARPC1A
CUX1	ARPC3
CUX1	ASH1L
CUX1	ASH2L
CUX1	ATF1
CUX1	ATF7IP
CUX1	ATP10D
CUX1	ATP6V1C1
CUX1	BASP1
CUX1	BCL11A
CUX1	BCL3
CUX1	BCL6
CUX1	BCL7A
CUX1	BEX3
CUX1	BICD1
CUX1	BIRC3
CUX1	BPTF
CUX1	BRD4
CUX1	BRK1
CUX1	BRWD1
CUX1	BTG1
CUX1	C12orf65
CUX1	C19orf38
CUX1	C6orf48
CUX1	CALM2

CUX1	CARHSP1
CUX1	CARMIL2
CUX1	CASC4
CUX1	CBX5
CUX1	CCDC171
CUX1	CCDC88C
CUX1	CCM2
CUX1	CCNB2
CUX1	CCND3
CUX1	CCNI
CUX1	CCNL1
CUX1	CD2AP
CUX1	CDC20
CUX1	CDC25B
CUX1	CDC42SE2
CUX1	CDK19
CUX1	CENPM
CUX1	CENPT
CUX1	CHD6
CUX1	CHD9
CUX1	CHTOP
CUX1	CHURC1
CUX1	CKAP5
CUX1	CLASP1
CUX1	CLTA
CUX1	COMMD7
CUX1	CORO1A
CUX1	CORO1B
CUX1	COX8A
CUX1	CPT1A
CUX1	CREB1
CUX1	CREBBP
CUX1	CRLF3
CUX1	CSK
CUX1	CUX1
CUX1	CXCR4
CUX1	CYB5R3
CUX1	DAAM1
CUX1	DAXX
CUX1	DAZAP2
CUX1	DCK
CUX1	DHDDS
CUX1	DNAJB6
CUX1	DNMBP
CUX1	DPF2
CUX1	DPY30
CUX1	DSTN
CUX1	DYNLL1
CUX1	EBF1
CUX1	ECT2

CUX1	EEF2
CUX1	EHBP1
CUX1	EIF2AK1
CUX1	EIF3L
CUX1	EIF4EBP2
CUX1	EIF4H
CUX1	ELAVL1
CUX1	EPS15
CUX1	ERBIN
CUX1	ESYT2
CUX1	EYA3
CUX1	FAM102A
CUX1	FAM107B
CUX1	FAM129A
CUX1	FAM133B
CUX1	FAM200B
CUX1	FAM49B
CUX1	FANCA
CUX1	FBXL20
CUX1	FBXO11
CUX1	FCHO1
CUX1	FDFT1
CUX1	FLAD1
CUX1	FNBP1
CUX1	FXR2
CUX1	GABARAPL2
CUX1	GABPB1
CUX1	GABPB1-AS1
CUX1	GABPB2
CUX1	GATAD2A
CUX1	GBA
CUX1	GCHFR
CUX1	GFOD2
CUX1	GGA1
CUX1	GLG1
CUX1	GLYCTK
CUX1	GMEB1
CUX1	GNA13
CUX1	GNB1
CUX1	GPR160
CUX1	GSK3B
CUX1	H3F3A
CUX1	HAUS4
CUX1	HDAC1
CUX1	HDAC5
CUX1	HERC4
CUX1	HK1
CUX1	HMGB1
CUX1	HNRNPD
CUX1	HOPX

CUX1	HP1BP3
CUX1	HRK
CUX1	IDE
CUX1	IFT81
CUX1	IGF2BP3
CUX1	IKZF3
CUX1	ING4
CUX1	IP6K2
CUX1	IQCD
CUX1	ITGB1
CUX1	JMJD1C
CUX1	KAT2B
CUX1	KDM3B
CUX1	KIAA1191
CUX1	KIF14
CUX1	KIF2C
CUX1	KIFC1
CUX1	KLC1
CUX1	KLF6
CUX1	KLHL20
CUX1	KLHL22
CUX1	KLHL24
CUX1	KLHL42
CUX1	KLHL5
CUX1	KMT2A
CUX1	LCK
CUX1	LCP1
CUX1	LHPP
CUX1	LINC01152
CUX1	LLGL1
CUX1	LMO2
CUX1	LMO4
CUX1	LPP
CUX1	LRBA
CUX1	LRRC37B
CUX1	MAD2L2
CUX1	MAP2K1
CUX1	MAP3K3
CUX1	MAPK14
CUX1	MBD2
CUX1	MBOAT2
CUX1	MED13
CUX1	MED25
CUX1	MEPCE
CUX1	METRNL
CUX1	MFSD13A
CUX1	MICAL3
CUX1	MIEN1
CUX1	MINK1
CUX1	MIR181A1HG

CUX1	MKRN1
CUX1	MPP5
CUX1	MRPS36
CUX1	MRPS6
CUX1	MSH6
CUX1	MTA3
CUX1	MVB12A
CUX1	MYBL2
CUX1	MYO1E
CUX1	MYPOP
CUX1	N4BP3
CUX1	NAP1L4
CUX1	NCOA3
CUX1	NELFB
CUX1	NF1
CUX1	NFYB
CUX1	NFYC
CUX1	NPAS1
CUX1	NR2C1
CUX1	NR3C1
CUX1	NUMB
CUX1	ODF2
CUX1	OPTN
CUX1	ORAI2
CUX1	OSBPL11
CUX1	OSBPL8
CUX1	OTUB1
CUX1	P2RX5
CUX1	PABPN1
CUX1	PAFAH1B3
CUX1	PAIP2
CUX1	PALD1
CUX1	PAN3
CUX1	PARP1
CUX1	PBRM1
CUX1	PCBD2
CUX1	PDPK1
CUX1	PFKFB3
CUX1	PHACTR2
CUX1	PIF1
CUX1	PIGV
CUX1	PIK3C2B
CUX1	PIP5K1A
CUX1	PITPNC1
CUX1	PKNOX1
CUX1	PLCG2
CUX1	PLEKHA2
CUX1	PLEKHM1
CUX1	PLIN3
CUX1	PNISR

CUX1	POM121
CUX1	POP5
CUX1	POU3F1
CUX1	PPIL2
CUX1	PPP1R2
CUX1	PPP2R5C
CUX1	PPP3CC
CUX1	PPP4R2
CUX1	PRDX2
CUX1	PRPSAP2
CUX1	PRRC2B
CUX1	PTK2
CUX1	PTP4A2
CUX1	PTPN11
CUX1	PXK
CUX1	QARS
CUX1	QRICH1
CUX1	R3HDM2
CUX1	RAB11A
CUX1	RAB3GAP2
CUX1	RAB9A
CUX1	RABGAP1
CUX1	RAD21
CUX1	RB1CC1
CUX1	RBFOX2
CUX1	RBM23
CUX1	RBM6
CUX1	RCC1
CUX1	RCC2
CUX1	RCOR1
CUX1	REEP4
CUX1	RFTN1
CUX1	RHBDF2
CUX1	RHOA
CUX1	RHOH
CUX1	RNF169
CUX1	S1PR2
CUX1	SCARB1
CUX1	SCMH1
CUX1	SEC14L1
CUX1	SEL1L3
CUX1	SENP8
CUX1	SEPT6
CUX1	SERF2
CUX1	SETD9
CUX1	SH3KBP1
CUX1	SH3RF1
CUX1	SKA2
CUX1	SLC25A33
CUX1	SLF2

CUX1	SLMAP
CUX1	SMARCA4
CUX1	SMARCC1
CUX1	SMARCD2
CUX1	SMCO4
CUX1	SMG6
CUX1	SMIM14
CUX1	SNAP23
CUX1	SND1-IT1
CUX1	SNTA1
CUX1	SNX10
CUX1	SNX15
CUX1	SNX24
CUX1	SP3
CUX1	SPATS2
CUX1	SPPL3
CUX1	SPTLC2
CUX1	SREK1
CUX1	SSBP3
CUX1	STAG3
CUX1	STARD7
CUX1	STAT5B
CUX1	STAU2
CUX1	STK10
CUX1	STK38
CUX1	STX16
CUX1	SUN2
CUX1	SYNC
CUX1	TADA2A
CUX1	TCF4
CUX1	TERF2
CUX1	TFDP2
CUX1	THAP11
CUX1	TIA1
CUX1	TICAM1
CUX1	TIFA
CUX1	TKT
CUX1	TMEM107
CUX1	TMPO
CUX1	TNFAIP8L1
CUX1	TNFRSF13C
CUX1	TNK2
CUX1	TOMM20
CUX1	TPCN1
CUX1	TPX2
CUX1	TRAF3IP2
CUX1	TRAF5
CUX1	TRAFD1
CUX1	TRAK1
CUX1	TRIOBP

CUX1	TRIP4
CUX1	TRMT10A
CUX1	TROAP
CUX1	TRPC4AP
CUX1	TSC22D1
CUX1	TSC22D3
CUX1	TTC9C
CUX1	TTI2
CUX1	TUBA1C
CUX1	TUBB3
CUX1	UBALD2
CUX1	UBC
CUX1	UBE2E1
CUX1	UBE2E3
CUX1	UBE2J1
CUX1	UBE2R2
CUX1	UBXN6
CUX1	UGP2
CUX1	UIMC1
CUX1	USF2
CUX1	USF3
CUX1	USP12
CUX1	USP34
CUX1	VGLL4
CUX1	VRK1
CUX1	WAPL
CUX1	WDR33
CUX1	WDR76
CUX1	WIPF1
CUX1	YPEL1
CUX1	YY1
CUX1	ZBTB8OS
CUX1	ZC3H14
CUX1	ZC3H6
CUX1	ZDHHC4
CUX1	ZFAND6
CUX1	ZMAT5
CUX1	ZMYND8
CUX1	ZNF106
CUX1	ZNF143
CUX1	ZNF428
CUX1	ZNF608
CUX1	ZNF638
CUX1	ZNF821
DDIT3	AARS
DDIT3	ABHD18
DDIT3	ACP5
DDIT3	AGPAT2
DDIT3	AKTIP
DDIT3	ANK1

DDIT3	ANKRD11
DDIT3	ARID5B
DDIT3	ARIH1
DDIT3	ASAH1
DDIT3	ASNS
DDIT3	ATF3
DDIT3	ATF5
DDIT3	BCL2
DDIT3	BTG1
DDIT3	CARS
DDIT3	CBX7
DDIT3	CCL3
DDIT3	CD63
DDIT3	CDIPT
DDIT3	CEBPB
DDIT3	CHAC1
DDIT3	CHD2
DDIT3	CREB5
DDIT3	DBP
DDIT3	DDIT3
DDIT3	DDIT4
DDIT3	DGAT2
DDIT3	DUSP10
DDIT3	EIF1
DDIT3	EIF2S2
DDIT3	EIF4EBP1
DDIT3	ELMSAN1
DDIT3	ENC1
DDIT3	ENTPD1
DDIT3	EPRS
DDIT3	FAM107B
DDIT3	FAM234A
DDIT3	FAM49A
DDIT3	FCER2
DDIT3	FTH1
DDIT3	GABARAP
DDIT3	GARS
DDIT3	GOT1
DDIT3	GTF3C2
DDIT3	HAX1
DDIT3	HDAC5
DDIT3	HERPUD1
DDIT3	HIPK3
DDIT3	HLA-DRB5
DDIT3	HSPA5
DDIT3	HSPA9
DDIT3	HVCN1
DDIT3	HYOU1
DDIT3	IARS
DDIT3	IRF2

DDIT3	JARID2
DDIT3	KLF7
DDIT3	KLF9
DDIT3	KMT2E
DDIT3	LRMP
DDIT3	MFN2
DDIT3	MGAT5
DDIT3	MID1IP1
DDIT3	MIR3681HG
DDIT3	MOSPD1
DDIT3	MTHFD2
DDIT3	MYC
DDIT3	NFE2L1
DDIT3	NFE2L2
DDIT3	NT5C3A
DDIT3	OSGIN2
DDIT3	PCK2
DDIT3	PDCD4
DDIT3	PHACTR1
DDIT3	PHC1
DDIT3	PHF12
DDIT3	PHF19
DDIT3	PHGDH
DDIT3	PNPLA8
DDIT3	PPP1R12C
DDIT3	PPT1
DDIT3	PSAT1
DDIT3	PTGS1
DDIT3	PYCR1
DDIT3	RHOBTB2
DDIT3	RMND5A
DDIT3	RPL9
DDIT3	RYBP
DDIT3	SARS
DDIT3	SDCBP
DDIT3	SEC62
DDIT3	SESN2
DDIT3	SHMT2
DDIT3	SLC16A7
DDIT3	SLC1A5
DDIT3	SLC38A1
DDIT3	SLC3A2
DDIT3	SLC6A9
DDIT3	SMIM10
DDIT3	SPRYD3
DDIT3	SRF
DDIT3	STARD3NL
DDIT3	STX11
DDIT3	STX3
DDIT3	SUSD6

DDIT3	TAB2
DDIT3	TBPL1
DDIT3	TES
DDIT3	TM6SF1
DDIT3	TMEM168
DDIT3	TRIB3
DDIT3	TSC22D3
DDIT3	TXNL1
DDIT3	UNKL
DDIT3	VEGFA
DDIT3	WARS
DDIT3	XAF1
DDIT3	YARS
DDIT3	ZFP36L1
E2F1	A4GALT
E2F1	AAAS
E2F1	AAK1
E2F1	ABHD10
E2F1	ABHD11
E2F1	ABHD14B
E2F1	ABHD17B
E2F1	ABHD3
E2F1	ABHD5
E2F1	ABI2
E2F1	ABI3
E2F1	ACAA2
E2F1	ACAP2
E2F1	ACAT2
E2F1	ACBD3
E2F1	ACBD4
E2F1	ACD
E2F1	ACER3
E2F1	ACOT8
E2F1	ACP1
E2F1	ACSS1
E2F1	ACTB
E2F1	ACTL6A
E2F1	ACTN4
E2F1	ACTR1B
E2F1	ACTR8
E2F1	ACYP1
E2F1	ADA
E2F1	ADAR
E2F1	ADAT1
E2F1	ADCK5
E2F1	ADH5
E2F1	ADI1
E2F1	ADK
E2F1	ADPRHL2
E2F1	ADRM1

E2F1	ADSS
E2F1	AGBL5
E2F1	AGFG2
E2F1	AGPAT1
E2F1	AGPAT2
E2F1	AGPAT5
E2F1	AHCY
E2F1	AIFM2
E2F1	AIG1
E2F1	AIM2
E2F1	AK2
E2F1	AK3
E2F1	AKAP1
E2F1	AKIP1
E2F1	AKR1B1
E2F1	AKR7A2
E2F1	AKT1S1
E2F1	ALCAM
E2F1	ALDH16A1
E2F1	ALDH5A1
E2F1	ALDH7A1
E2F1	ALG10
E2F1	ALG2
E2F1	ALKBH2
E2F1	ALKBH4
E2F1	ANAPC11
E2F1	ANAPC15
E2F1	ANAPC5
E2F1	ANKH
E2F1	ANKRD10
E2F1	ANKRD26
E2F1	ANKRD27
E2F1	ANO6
E2F1	ANP32A
E2F1	ANP32B
E2F1	ANP32E
E2F1	ANXA11
E2F1	ANXA2
E2F1	ANXA5
E2F1	AP1B1
E2F1	AP1S1
E2F1	AP2A1
E2F1	AP2A2
E2F1	AP2S1
E2F1	APAF1
E2F1	APEX2
E2F1	API5
E2F1	APIP
E2F1	APLP2
E2F1	APOM

E2F1	APOPT1
E2F1	APPL1
E2F1	APTR
E2F1	ARF3
E2F1	ARGLU1
E2F1	ARHGDIA
E2F1	ARHGDIB
E2F1	ARHGEF1
E2F1	ARHGEF9
E2F1	ARID1A
E2F1	ARL13B
E2F1	ARL3
E2F1	ARL6
E2F1	ARL6IP6
E2F1	ARMC6
E2F1	ARNTL2
E2F1	ARPC1A
E2F1	ARPC1B
E2F1	ARPC2
E2F1	ARPC3
E2F1	ARPC4
E2F1	ARPC5
E2F1	ARPC5L
E2F1	ARSK
E2F1	ARV1
E2F1	ASB1
E2F1	ASB16-AS1
E2F1	ASF1A
E2F1	ASF1B
E2F1	ASH2L
E2F1	ASL
E2F1	ASRGL1
E2F1	ATAD2
E2F1	ATAD3A
E2F1	ATAD5
E2F1	ATF2
E2F1	ATF7IP2
E2F1	ATG3
E2F1	ATM
E2F1	ATOX1
E2F1	ATP13A2
E2F1	ATP23
E2F1	ATP2A3
E2F1	ATP6V0D1
E2F1	ATPAF1
E2F1	ATXN10
E2F1	ATXN2
E2F1	AUH
E2F1	AUNIP
E2F1	AURKAIP1

E2F1	AZIN1
E2F1	B3GALNT1
E2F1	B3GAT3
E2F1	B3GLCT
E2F1	B3GNT7
E2F1	BABAM1
E2F1	BAG2
E2F1	BAG5
E2F1	BAG6
E2F1	BANF1
E2F1	BANP
E2F1	BARD1
E2F1	BAX
E2F1	BAZ1A
E2F1	BAZ1B
E2F1	BBIP1
E2F1	BCAS2
E2F1	BCAT2
E2F1	BCKDHB
E2F1	BCL2L11
E2F1	BCL2L12
E2F1	BCL7A
E2F1	BCL7B
E2F1	BCL7C
E2F1	BID
E2F1	BIN3
E2F1	BLM
E2F1	BLMH
E2F1	BLOC1S1
E2F1	BLOC1S2
E2F1	BLOC1S6
E2F1	BLVRB
E2F1	BNIP3
E2F1	BNIP3L
E2F1	BOLA1
E2F1	BPHL
E2F1	BPNT1
E2F1	BPTF
E2F1	BRCA1
E2F1	BRCC3
E2F1	BRD2
E2F1	BRD3
E2F1	BRD4
E2F1	BRD7
E2F1	BRI3
E2F1	BRI3BP
E2F1	BRK1
E2F1	BTF3
E2F1	BTF3L4
E2F1	BTG3

E2F1	BUB3
E2F1	BUD13
E2F1	BZW1
E2F1	C11orf58
E2F1	C12orf49
E2F1	C12orf57
E2F1	C12orf73
E2F1	C12orf75
E2F1	C15orf41
E2F1	C16orf74
E2F1	C16orf87
E2F1	C16orf95
E2F1	C17orf75
E2F1	C19orf25
E2F1	C19orf47
E2F1	C19orf48
E2F1	C19orf53
E2F1	C19orf54
E2F1	C1D
E2F1	C1GALT1
E2F1	C1orf123
E2F1	C1orf35
E2F1	C1orf50
E2F1	C21orf58
E2F1	C21orf91
E2F1	C2orf69
E2F1	C2orf88
E2F1	C3orf38
E2F1	C4orf33
E2F1	C4orf48
E2F1	C5orf24
E2F1	C6orf203
E2F1	C7orf50
E2F1	C8orf76
E2F1	C9orf16
E2F1	C9orf40
E2F1	C9orf78
E2F1	CAB39L
E2F1	CABLES2
E2F1	CACYBP
E2F1	CALM1
E2F1	CALM2
E2F1	CALM3
E2F1	CAMK2D
E2F1	CAMLG
E2F1	CAMTA1
E2F1	CAPN1
E2F1	CAPN10
E2F1	CAPN2
E2F1	CAPN7

E2F1	CARD8
E2F1	CARHSP1
E2F1	CARM1
E2F1	CARNMT1
E2F1	CARS2
E2F1	CASP2
E2F1	CASP8AP2
E2F1	CBFB
E2F1	CBX1
E2F1	CBX2
E2F1	CBX3
E2F1	CBX5
E2F1	CCDC106
E2F1	CCDC136
E2F1	CCDC167
E2F1	CCDC189
E2F1	CCDC191
E2F1	CCDC34
E2F1	CCDC57
E2F1	CCDC58
E2F1	CCDC69
E2F1	CCDC85B
E2F1	CCND3
E2F1	CCNE1
E2F1	CCNE2
E2F1	CCNG2
E2F1	CCNI
E2F1	CCP110
E2F1	CCT2
E2F1	CCT8
E2F1	CD163L1
E2F1	CD2BP2
E2F1	CD70
E2F1	CD79B
E2F1	CD81
E2F1	CDADC1
E2F1	CDC123
E2F1	CDC34
E2F1	CDC37
E2F1	CDC42SE2
E2F1	CDC6
E2F1	CDC7
E2F1	CDCA4
E2F1	CDCA5
E2F1	CDCA7
E2F1	CDCA7L
E2F1	CDK11B
E2F1	CDK2
E2F1	CDK4
E2F1	CDK5

E2F1	CDK6
E2F1	CDK9
E2F1	CDKN2AIP
E2F1	CDKN2AIPNL
E2F1	CDPF1
E2F1	CDT1
E2F1	CEBPB
E2F1	CENPC
E2F1	CENPH
E2F1	CENPI
E2F1	CENPK
E2F1	CENPL
E2F1	CENPM
E2F1	CENPN
E2F1	CENPU
E2F1	CENPW
E2F1	CEP120
E2F1	CEP250
E2F1	CEP41
E2F1	CEP57
E2F1	CEP63
E2F1	CEP78
E2F1	CEP89
E2F1	CETN2
E2F1	CFAP20
E2F1	CFAP73
E2F1	CFL1
E2F1	CHAF1A
E2F1	CHAF1B
E2F1	CHCHD10
E2F1	CHCHD2
E2F1	CHCHD3
E2F1	CHCHD5
E2F1	CHD3
E2F1	CHD4
E2F1	CHD6
E2F1	CHD8
E2F1	CHEK1
E2F1	CHMP1A
E2F1	CHRAC1
E2F1	CHTF8
E2F1	CHTOP
E2F1	CIB1
E2F1	CIC
E2F1	CIRBP
E2F1	CKLF
E2F1	CLASRP
E2F1	CLDN23
E2F1	CLDND1
E2F1	CLEC11A

E2F1	CLIC4
E2F1	CLNS1A
E2F1	CLPP
E2F1	CLSPN
E2F1	CLTA
E2F1	CLTB
E2F1	CLUAP1
E2F1	CMPK2
E2F1	CNBP
E2F1	CNKSR2
E2F1	CNN2
E2F1	CNNM3
E2F1	CNOT10
E2F1	CNOT11
E2F1	CNOT9
E2F1	CNP
E2F1	COA1
E2F1	COA5
E2F1	COCH
E2F1	COG4
E2F1	COMMD4
E2F1	COMMD7
E2F1	COMTD1
E2F1	COPG1
E2F1	COPS3
E2F1	COPS5
E2F1	COPS6
E2F1	COPS7A
E2F1	COQ10A
E2F1	COQ9
E2F1	CORO1A
E2F1	CORO1B
E2F1	CORO7
E2F1	COTL1
E2F1	COX11
E2F1	COX14
E2F1	COX16
E2F1	COX17
E2F1	COX4I1
E2F1	COX5A
E2F1	COX5B
E2F1	COX6A1
E2F1	COX6B1
E2F1	COX6C
E2F1	COX7A2
E2F1	COX7A2L
E2F1	COX7B
E2F1	COX7C
E2F1	COX8A
E2F1	CPSF3

E2F1	CPSF6
E2F1	CRELD1
E2F1	CRY2
E2F1	CSE1L
E2F1	CSK
E2F1	CSNK1G2
E2F1	CSNK2B
E2F1	CSPP1
E2F1	CSRP1
E2F1	CSTF2
E2F1	CSTF2T
E2F1	CSTF3
E2F1	CTCF
E2F1	CTDNEP1
E2F1	CTDSP1
E2F1	CTH
E2F1	CTNNAL1
E2F1	CTNNBIP1
E2F1	CTPS1
E2F1	CTPS2
E2F1	CUEDC2
E2F1	CUL2
E2F1	CUL3
E2F1	CUL4B
E2F1	CUTA
E2F1	CWC27
E2F1	CXorf56
E2F1	CXXC5
E2F1	CYB5B
E2F1	CYB5R3
E2F1	CYBB
E2F1	CYC1
E2F1	CYFIP2
E2F1	CYP20A1
E2F1	CYP2U1
E2F1	CYSTM1
E2F1	D2HGDH
E2F1	DAAM1
E2F1	DAP
E2F1	DARS
E2F1	DAXX
E2F1	DAZAP1
E2F1	DBI
E2F1	DBNDD1
E2F1	DCAKD
E2F1	DCK
E2F1	DCLRE1B
E2F1	DCPS
E2F1	DCTN2
E2F1	DCTN3

E2F1	DCTN5
E2F1	DCTPP1
E2F1	DCUN1D4
E2F1	DDB2
E2F1	DDHD1
E2F1	DDIT3
E2F1	DDT
E2F1	DDX11
E2F1	DDX24
E2F1	DDX39A
E2F1	DDX3X
E2F1	DDX41
E2F1	DDX46
E2F1	DDX55
E2F1	DEF8
E2F1	DEK
E2F1	DENND3
E2F1	DENND4B
E2F1	DESI2
E2F1	DGCR6L
E2F1	DGKA
E2F1	DGUOK
E2F1	DHCR24
E2F1	DHFR
E2F1	DHPS
E2F1	DHRS7B
E2F1	DHX15
E2F1	DHX9
E2F1	DIAPH3
E2F1	DICER1
E2F1	DLAT
E2F1	DLD
E2F1	DLG3
E2F1	DLGAP1-AS1
E2F1	DMC1
E2F1	DMD
E2F1	DMWD
E2F1	DNAJA3
E2F1	DNAJC10
E2F1	DNAJC15
E2F1	DNAJC8
E2F1	DNAJC9
E2F1	DNMT1
E2F1	DOK1
E2F1	DONSON
E2F1	DPCD
E2F1	DPY30
E2F1	DRG1
E2F1	DSN1
E2F1	DSTN

E2F1	DTX1
E2F1	DTYMK
E2F1	DUSP10
E2F1	DUSP23
E2F1	DUT
E2F1	DYM
E2F1	DYNC1H1
E2F1	DYNLT1
E2F1	E2F1
E2F1	E2F7
E2F1	E2F8
E2F1	EBP
E2F1	ECH1
E2F1	EED
E2F1	EEF1AKMT1
E2F1	EEF1B2
E2F1	EEF1D
E2F1	EEF1E1
E2F1	EEF1G
E2F1	EEF2
E2F1	EFHD2
E2F1	EFR3A
E2F1	EFTUD2
E2F1	EHD1
E2F1	EID1
E2F1	EID2B
E2F1	EIF1
E2F1	EIF1AX
E2F1	EIF2AK1
E2F1	EIF2AK2
E2F1	EIF2AK3
E2F1	EIF2B1
E2F1	EIF2D
E2F1	EIF2S3
E2F1	EIF3F
E2F1	EIF3H
E2F1	EIF3I
E2F1	EIF3K
E2F1	EIF3L
E2F1	EIF3M
E2F1	EIF4A3
E2F1	EIF4G2
E2F1	EIF4G3
E2F1	EIF4H
E2F1	EIF5A
E2F1	ELAC1
E2F1	ELAC2
E2F1	ELAVL1
E2F1	ELF1
E2F1	ELF4

E2F1	ELK1
E2F1	ELL
E2F1	ELMO2
E2F1	ELMSAN1
E2F1	ELOVL5
E2F1	ELP5
E2F1	ELP6
E2F1	EMC2
E2F1	EMC6
E2F1	ENO2
E2F1	ENOPH1
E2F1	ENOSF1
E2F1	ENY2
E2F1	EPB41
E2F1	EPN1
E2F1	EPS15
E2F1	EPS8
E2F1	EPS8L2
E2F1	ERGIC1
E2F1	ERH
E2F1	ERI3
E2F1	ERICH1
E2F1	ERN1
E2F1	ERO1A
E2F1	ERP29
E2F1	ESCO1
E2F1	ESCO2
E2F1	ESD
E2F1	ESPL1
E2F1	ETAA1
E2F1	ETFA
E2F1	ETFB
E2F1	EWSR1
E2F1	EXO1
E2F1	EXOC5
E2F1	EXOSC1
E2F1	EXOSC2
E2F1	EXOSC3
E2F1	EXOSC6
E2F1	EXOSC8
E2F1	EXOSC9
E2F1	EYA3
E2F1	EZH2
E2F1	EZR
E2F1	FAAP20
E2F1	FADD
E2F1	FAHD2A
E2F1	FAM111A
E2F1	FAM111B
E2F1	FAM120A

E2F1	FAM120AOS
E2F1	FAM120C
E2F1	FAM167A
E2F1	FAM185A
E2F1	FAM192A
E2F1	FAM193B
E2F1	FAM221A
E2F1	FAM228B
E2F1	FAM49B
E2F1	FAM57A
E2F1	FAM76B
E2F1	FAM96A
E2F1	FANCA
E2F1	FANCB
E2F1	FANCI
E2F1	FANCL
E2F1	FANCM
E2F1	FAU
E2F1	FBL
E2F1	FBXL12
E2F1	FBXL4
E2F1	FBXO11
E2F1	FBXO22
E2F1	FBXO4
E2F1	FBXO43
E2F1	FBXO5
E2F1	FDPS
E2F1	FDX1
E2F1	FEN1
E2F1	FGF14-AS2
E2F1	FGGY
E2F1	FH
E2F1	FIBP
E2F1	FIGNL1
E2F1	FIP1L1
E2F1	FKBP1A
E2F1	FKBP3
E2F1	FKBP8
E2F1	FLAD1
E2F1	FLOT1
E2F1	FLVCR1
E2F1	FLYWCH1
E2F1	FN3KRP
E2F1	FNIP1
E2F1	FNTA
E2F1	FOXM1
E2F1	FOXN3
E2F1	FOXRED2
E2F1	FRAT2
E2F1	FRG1

E2F1	FRY-AS1
E2F1	FSD1L
E2F1	FUBP1
E2F1	FUS
E2F1	G3BP2
E2F1	G6PC3
E2F1	GABPB1-AS1
E2F1	GALE
E2F1	GALK1
E2F1	GALM
E2F1	GAMT
E2F1	GAPDH
E2F1	GBA
E2F1	GCH1
E2F1	GCHFR
E2F1	GDI2
E2F1	GEMIN6
E2F1	GEMIN8
E2F1	GFI1
E2F1	GFOD2
E2F1	GGCT
E2F1	GGH
E2F1	GGNBP2
E2F1	GID8
E2F1	GIN1
E2F1	GINS2
E2F1	GINS3
E2F1	GINS4
E2F1	GIT2
E2F1	GJC1
E2F1	GK
E2F1	GKAP1
E2F1	GLE1
E2F1	GLO1
E2F1	GLRX2
E2F1	GLRX3
E2F1	GLRX5
E2F1	GLUL
E2F1	GMEB1
E2F1	GMNN
E2F1	GMPPB
E2F1	GMPR2
E2F1	GMPS
E2F1	GNA13
E2F1	GNAI2
E2F1	GNAS
E2F1	GNB1
E2F1	GNB2
E2F1	GNB5
E2F1	GNG5

E2F1	GNL1
E2F1	GOT2
E2F1	GPAT4
E2F1	GPD2
E2F1	GPI
E2F1	GPN3
E2F1	GPR160
E2F1	GPS1
E2F1	GPX4
E2F1	GRK2
E2F1	GSDMD
E2F1	GSTO1
E2F1	GSTP1
E2F1	GSTZ1
E2F1	GTF2A2
E2F1	GTF2E2
E2F1	GTF2H1
E2F1	GTF2H5
E2F1	GTF2I
E2F1	GTPBP1
E2F1	GUCA1A
E2F1	GUCD1
E2F1	GUK1
E2F1	GUSB
E2F1	GYG1
E2F1	GYPC
E2F1	H2AFV
E2F1	H2AFX
E2F1	H2AFY
E2F1	H2AFZ
E2F1	H3F3A
E2F1	HACD3
E2F1	HADH
E2F1	HADHA
E2F1	HAGHL
E2F1	HAT1
E2F1	HAUS1
E2F1	HAUS2
E2F1	HAUS4
E2F1	HAUS5
E2F1	HAUS6
E2F1	HAUS8
E2F1	HBS1L
E2F1	HCFC1
E2F1	HCG18
E2F1	HDAC1
E2F1	HDAC2
E2F1	HDDC2
E2F1	HDGF
E2F1	HDLBP

E2F1	HEBP2
E2F1	HELLS
E2F1	HENMT1
E2F1	HERC2
E2F1	HERC3
E2F1	HES6
E2F1	HGS
E2F1	HHEX
E2F1	HIGD1A
E2F1	HIGD2A
E2F1	HILPDA
E2F1	HINT1
E2F1	HINT2
E2F1	HIPK1
E2F1	HIST1H1B
E2F1	HIST1H1E
E2F1	HIST1H4C
E2F1	HK1
E2F1	HLA-DRB1
E2F1	HLCS
E2F1	HMG20A
E2F1	HMG20B
E2F1	HMGA1
E2F1	HMGB1
E2F1	HMGB2
E2F1	HMGN1
E2F1	HMGN2
E2F1	HMGN3
E2F1	HMGXB4
E2F1	HMOX2
E2F1	HNRNPA0
E2F1	HNRNPA1
E2F1	HNRNPA2B1
E2F1	HNRNPA3
E2F1	HNRNPAB
E2F1	HNRNPC
E2F1	HNRNPD
E2F1	HNRNPF
E2F1	HNRNPH3
E2F1	HNRNPK
E2F1	HNRNPL
E2F1	HNRNPM
E2F1	HNRNPR
E2F1	HNRNPU
E2F1	HPF1
E2F1	HPRT1
E2F1	HPS1
E2F1	HRK
E2F1	HS2ST1
E2F1	HSD17B10

E2F1	HSD17B7
E2F1	HSDL1
E2F1	HSDL2
E2F1	HSP90AA1
E2F1	HSPB11
E2F1	HSPD1
E2F1	HTATSF1
E2F1	IAH1
E2F1	ICMT
E2F1	IDH2
E2F1	IDH3A
E2F1	IDH3B
E2F1	IDI1
E2F1	IER2
E2F1	IER3IP1
E2F1	IFI16
E2F1	IFI27L2
E2F1	IFNGR2
E2F1	IFNLR1
E2F1	IFT22
E2F1	IGBP1
E2F1	IGF2BP1
E2F1	IKBKG
E2F1	IL4R
E2F1	ILF3
E2F1	ILK
E2F1	ILVBL
E2F1	IMMT
E2F1	IMP4
E2F1	INAFM1
E2F1	INF2
E2F1	INIP
E2F1	INO80
E2F1	INO80C
E2F1	INPP1
E2F1	INTS10
E2F1	INTS8
E2F1	IPO7
E2F1	IPO9
E2F1	IQCB1
E2F1	IQCD
E2F1	IQGAP1
E2F1	IRAK4
E2F1	IRF8
E2F1	ISG20L2
E2F1	ISOC2
E2F1	ITGB3BP
E2F1	ITPR3
E2F1	ITSN2
E2F1	JADE2

E2F1	JAML
E2F1	KANK1
E2F1	KAT6B
E2F1	KATNAL2
E2F1	KATNB1
E2F1	KCNK12
E2F1	KCNMB4
E2F1	KCTD20
E2F1	KEAP1
E2F1	KHDRBS1
E2F1	KHK
E2F1	KHSRP
E2F1	KIAA0040
E2F1	KIAA0355
E2F1	KIAA1586
E2F1	KIF13A
E2F1	KIF20A
E2F1	KIF22
E2F1	KIF2A
E2F1	KLC2
E2F1	KLHDC2
E2F1	KLHDC3
E2F1	KLHL18
E2F1	KLHL23
E2F1	KLHL25
E2F1	KLHL42
E2F1	KLHL7
E2F1	KPNB1
E2F1	KTN1
E2F1	KXD1
E2F1	KYAT1
E2F1	L2HGDH
E2F1	L3MBTL2
E2F1	LAGE3
E2F1	LAMP1
E2F1	LAMTOR2
E2F1	LAPTM4B
E2F1	LARP4B
E2F1	LAS1L
E2F1	LCMT1
E2F1	LCOR
E2F1	LCP1
E2F1	LDAH
E2F1	LDHA
E2F1	LDHB
E2F1	LETM2
E2F1	LETMD1
E2F1	LGALS8
E2F1	LGALS9
E2F1	LHPP

E2F1	LIG1
E2F1	LIMD2
E2F1	LIMS1
E2F1	LIMS2
E2F1	LIN9
E2F1	LINC00324
E2F1	LINC00426
E2F1	LINC01006
E2F1	LINC01224
E2F1	LINC01431
E2F1	LINC01481
E2F1	LINC01534
E2F1	LIPE-AS1
E2F1	LMBRD2
E2F1	LMCD1
E2F1	LMF2
E2F1	LMNB1
E2F1	LMNB2
E2F1	LNPEP
E2F1	LONP1
E2F1	LOXL2
E2F1	LRP8
E2F1	LRPPRC
E2F1	LRR1
E2F1	LRRC42
E2F1	LRRC45
E2F1	LRRC47
E2F1	LRRC57
E2F1	LRRC58
E2F1	LRRC61
E2F1	LSM14B
E2F1	LSM2
E2F1	LSM3
E2F1	LSM4
E2F1	LSM5
E2F1	LSM6
E2F1	LSM7
E2F1	LSM8
E2F1	LSP1
E2F1	LTBP4
E2F1	LUC7L2
E2F1	LUC7L3
E2F1	LYL1
E2F1	LYPLA1
E2F1	LYPLAL1
E2F1	LYRM1
E2F1	MAD1L1
E2F1	MAD2L1
E2F1	MAD2L2
E2F1	MAGEF1

E2F1	MAGEH1
E2F1	MAGOH
E2F1	MALT1
E2F1	MAML3
E2F1	MAN2A1
E2F1	MAP1LC3A
E2F1	MAP2
E2F1	MAP2K1
E2F1	MAP4K1
E2F1	MAP4K2
E2F1	MAP7D1
E2F1	MAPK1
E2F1	MAPK1IP1L
E2F1	MAPK9
E2F1	MAPRE1
E2F1	MAPRE2
E2F1	MARCH2
E2F1	MARCKSL1
E2F1	MAST2
E2F1	MAT2B
E2F1	MAZ
E2F1	MBD4
E2F1	MCM2
E2F1	MCM3
E2F1	MCM4
E2F1	MCM5
E2F1	MCM6
E2F1	MCM7
E2F1	MCMBP
E2F1	MCOLN2
E2F1	MCPH1
E2F1	MCTS1
E2F1	MCUR1
E2F1	MDH1
E2F1	MDH2
E2F1	MDP1
E2F1	MEA1
E2F1	MED11
E2F1	MED25
E2F1	MED30
E2F1	MED31
E2F1	MED4
E2F1	MEIOC
E2F1	METAP1
E2F1	METAP2
E2F1	METTL15
E2F1	METTL21A
E2F1	METTL2B
E2F1	METTL8
E2F1	METTL9

E2F1	MFAP1
E2F1	MFGE8
E2F1	MFHAS1
E2F1	MFNG
E2F1	MFSD11
E2F1	MFSD13A
E2F1	MFSD14B
E2F1	MGAT3
E2F1	MGAT4A
E2F1	MGME1
E2F1	MGMT
E2F1	MICU2
E2F1	MIDN
E2F1	MIEN1
E2F1	MIF
E2F1	MINCR
E2F1	MINOS1
E2F1	MIS18BP1
E2F1	MKL1
E2F1	MKNK2
E2F1	MKRN2
E2F1	MLLT10
E2F1	MLXIP
E2F1	MMAB
E2F1	MMADHC
E2F1	MME
E2F1	MMS22L
E2F1	MNAT1
E2F1	MND1
E2F1	MOB1A
E2F1	MOB1B
E2F1	MORF4L1
E2F1	MOSPD3
E2F1	MPC2
E2F1	MPDU1
E2F1	MPHOSPH6
E2F1	MRM2
E2F1	MRPL11
E2F1	MRPL13
E2F1	MRPL14
E2F1	MRPL15
E2F1	MRPL16
E2F1	MRPL18
E2F1	MRPL19
E2F1	MRPL20
E2F1	MRPL23
E2F1	MRPL27
E2F1	MRPL28
E2F1	MRPL34
E2F1	MRPL36

E2F1	MRPL37
E2F1	MRPL39
E2F1	MRPL4
E2F1	MRPL48
E2F1	MRPL49
E2F1	MRPL51
E2F1	MRPL57
E2F1	MRPS15
E2F1	MRPS16
E2F1	MRPS18A
E2F1	MRPS21
E2F1	MRPS26
E2F1	MRPS31
E2F1	MRPS33
E2F1	MRPS34
E2F1	MRPS36
E2F1	MRPS5
E2F1	MRPS6
E2F1	MRPS9
E2F1	MRS2
E2F1	MSH2
E2F1	MSH6
E2F1	MSL2
E2F1	MSRB2
E2F1	MTA2
E2F1	MTA3
E2F1	MTERF3
E2F1	MTF2
E2F1	MTFP1
E2F1	MTFR1
E2F1	MTFR1L
E2F1	MTHFD1
E2F1	MTHFD2
E2F1	MTHFD2L
E2F1	MTMR12
E2F1	MTURN
E2F1	MTX1
E2F1	MUT
E2F1	MUTYH
E2F1	MVD
E2F1	MXD3
E2F1	MYBL2
E2F1	MYCBP
E2F1	MYH9
E2F1	MYL12A
E2F1	MYL6
E2F1	MYL6B
E2F1	MYO1C
E2F1	MYO1G
E2F1	MYO9A

E2F1	MZT1
E2F1	MZT2A
E2F1	MZT2B
E2F1	N4BP2
E2F1	N4BP3
E2F1	NAA16
E2F1	NAA38
E2F1	NAA40
E2F1	NAA50
E2F1	NABP2
E2F1	NACA
E2F1	NACC1
E2F1	NADK
E2F1	NAE1
E2F1	NANS
E2F1	NAP1L1
E2F1	NAP1L4
E2F1	NARFL
E2F1	NARS2
E2F1	NASP
E2F1	NBEAL1
E2F1	NCBP1
E2F1	NCBP3
E2F1	NCF4
E2F1	NDUFA1
E2F1	NDUFA11
E2F1	NDUFA12
E2F1	NDUFA13
E2F1	NDUFA3
E2F1	NDUFA4
E2F1	NDUFA6
E2F1	NDUFA8
E2F1	NDUFAB1
E2F1	NDUFAF6
E2F1	NDUFAF7
E2F1	NDUFB10
E2F1	NDUFB11
E2F1	NDUFB2
E2F1	NDUFB3
E2F1	NDUFB5
E2F1	NDUFB6
E2F1	NDUFB8
E2F1	NDUFC1
E2F1	NDUFS3
E2F1	NDUFS4
E2F1	NDUFS5
E2F1	NDUFS6
E2F1	NDUFS7
E2F1	NDUFS8
E2F1	NDUFV1

E2F1	NDUFV2
E2F1	NEDD1
E2F1	NEDD8
E2F1	NEIL1
E2F1	NELFA
E2F1	NELFB
E2F1	NEMP2
E2F1	NENF
E2F1	NET1
E2F1	NETO2
E2F1	NF1
E2F1	NF2
E2F1	NFYB
E2F1	NHLRC3
E2F1	NINJ1
E2F1	NIP7
E2F1	NIPBL
E2F1	NKTR
E2F1	NLK
E2F1	NLRC3
E2F1	NMI
E2F1	NMRAL1
E2F1	NNT-AS1
E2F1	NOA1
E2F1	NOL9
E2F1	NOM1
E2F1	NONO
E2F1	NOP56
E2F1	NORAD
E2F1	NPEPL1
E2F1	NPEPPS
E2F1	NPHP3
E2F1	NPTX2
E2F1	NQO1
E2F1	NQO2
E2F1	NR4A1
E2F1	NRAV
E2F1	NRDE2
E2F1	NSL1
E2F1	NSMCE1
E2F1	NSUN2
E2F1	NSUN5
E2F1	NSUN6
E2F1	NT5DC1
E2F1	NTAN1
E2F1	NTMT1
E2F1	NUBP1
E2F1	NUBP2
E2F1	NUCKS1
E2F1	NUDC

E2F1	NUDT1
E2F1	NUDT15
E2F1	NUDT21
E2F1	NUDT5
E2F1	NUDT8
E2F1	NUP133
E2F1	NUP153
E2F1	NUP155
E2F1	NUP188
E2F1	NUP205
E2F1	NUP210
E2F1	NUP43
E2F1	NUP50
E2F1	NUP62
E2F1	NUP85
E2F1	NVL
E2F1	OAZ1
E2F1	OCIAD2
E2F1	OCLN
E2F1	OFD1
E2F1	OGFRL1
E2F1	OGG1
E2F1	OLA1
E2F1	OPA3
E2F1	ORAI2
E2F1	ORAOV1
E2F1	ORC6
E2F1	OSBPL10
E2F1	OSBPL2
E2F1	OSBPL3
E2F1	OSBPL8
E2F1	OSER1
E2F1	OSTM1
E2F1	OTUD6B
E2F1	OXCT1
E2F1	P2RX5
E2F1	P4HA2
E2F1	P4HTM
E2F1	PA2G4
E2F1	PAAF1
E2F1	PACSIN1
E2F1	PAFAH1B1
E2F1	PAFAH1B3
E2F1	PAG1
E2F1	PAGR1
E2F1	PAICS
E2F1	PAIP2
E2F1	PALB2
E2F1	PANK2
E2F1	PANK4

E2F1	PAQR4
E2F1	PARK7
E2F1	PARN
E2F1	PARP1
E2F1	PARP16
E2F1	PARP2
E2F1	PATL1
E2F1	PAWR
E2F1	PAX5
E2F1	PAXBP1
E2F1	PAXIP1-AS1
E2F1	PBK
E2F1	PBLD
E2F1	PBRM1
E2F1	PBX3
E2F1	PCBD1
E2F1	PCBP1
E2F1	PCCB
E2F1	PCED1B
E2F1	PCIF1
E2F1	PCMT1
E2F1	PCNA
E2F1	PCNP
E2F1	PCYOX1L
E2F1	PCYT2
E2F1	PDAP1
E2F1	PDE9A
E2F1	PDK2
E2F1	PDK3
E2F1	PDLIM1
E2F1	PDPK1
E2F1	PDPR
E2F1	PDS5A
E2F1	PEA15
E2F1	PEBP1
E2F1	PELO
E2F1	PELP1
E2F1	PEX16
E2F1	PEX26
E2F1	PFKFB3
E2F1	PFKL
E2F1	PFKP
E2F1	PFN1
E2F1	PFN2
E2F1	PGAM1
E2F1	PGAP2
E2F1	PGD
E2F1	PGK1
E2F1	PGLS
E2F1	PGM3

E2F1	PGP
E2F1	PGRMC1
E2F1	PHB2
E2F1	PHF10
E2F1	PHF13
E2F1	PHF20
E2F1	PHF5A
E2F1	PHIP
E2F1	PHKG2
E2F1	PHOSPHO2
E2F1	PHPT1
E2F1	PIAS4
E2F1	PIEZO1
E2F1	PIGL
E2F1	PIGO
E2F1	PIH1D1
E2F1	PIN1
E2F1	PITPNA
E2F1	PITPNA-AS1
E2F1	PKD2
E2F1	PKIA
E2F1	PKM
E2F1	PKMYT1
E2F1	PKNOX1
E2F1	PLA2G15
E2F1	PLEKHA3
E2F1	PLEKHF2
E2F1	PLEKHH3
E2F1	PLEKHJ1
E2F1	PLK4
E2F1	PLXNB2
E2F1	PM20D1
E2F1	PMF1
E2F1	PMM1
E2F1	PMPCB
E2F1	PMVK
E2F1	PNKD
E2F1	PNN
E2F1	PNP
E2F1	PNPLA4
E2F1	POFUT1
E2F1	POLA2
E2F1	POLD1
E2F1	POLD3
E2F1	POLDIP3
E2F1	POLE
E2F1	POLE3
E2F1	POLE4
E2F1	POLM
E2F1	POLR1D

E2F1	POLR2C
E2F1	POLR2D
E2F1	POLR2E
E2F1	POLR2F
E2F1	POLR2I
E2F1	POLR2J
E2F1	POLR2K
E2F1	POLR2L
E2F1	POMP
E2F1	POP5
E2F1	POP7
E2F1	POU2AF1
E2F1	PPA1
E2F1	PPA2
E2F1	PPARA
E2F1	PPAT
E2F1	PPDPF
E2F1	PPIA
E2F1	PPIE
E2F1	PPIG
E2F1	PPIH
E2F1	PPIL3
E2F1	PPM1D
E2F1	PPM1G
E2F1	PPM1L
E2F1	PPP1CA
E2F1	PPP1CB
E2F1	PPP1R16B
E2F1	PPP1R2
E2F1	PPP1R35
E2F1	PPP1R7
E2F1	PPP2CA
E2F1	PPP2R1A
E2F1	PPP2R2D
E2F1	PPP2R3C
E2F1	PPP2R5C
E2F1	PPP3R1
E2F1	PPP4C
E2F1	PPP6C
E2F1	PPP6R1
E2F1	PRADC1
E2F1	PRDM15
E2F1	PRDX2
E2F1	PRDX3
E2F1	PRDX5
E2F1	PRDX6
E2F1	PRELID1
E2F1	PRELID3A
E2F1	PRELID3B
E2F1	PRIM1

E2F1	PRIMPOL
E2F1	PRKACB
E2F1	PRKAR2A
E2F1	PRKCE
E2F1	PRKDC
E2F1	PRMT7
E2F1	PRMT9
E2F1	PRPF19
E2F1	PRPF38A
E2F1	PRPF40A
E2F1	PRPF4B
E2F1	PRPS1
E2F1	PRPS2
E2F1	PRPSAP2
E2F1	PRR3
E2F1	PRR7
E2F1	PRRC2B
E2F1	PSIP1
E2F1	PSMA2
E2F1	PSMA3
E2F1	PSMA4
E2F1	PSMA5
E2F1	PSMA8
E2F1	PSMB1
E2F1	PSMB2
E2F1	PSMB3
E2F1	PSMB4
E2F1	PSMB5
E2F1	PSMB8
E2F1	PSMB9
E2F1	PSMC3
E2F1	PSMC3IP
E2F1	PSMC5
E2F1	PSMD12
E2F1	PSMD14
E2F1	PSMD2
E2F1	PSMD6
E2F1	PSMD9
E2F1	PSMG1
E2F1	PSMG2
E2F1	PTAFR
E2F1	PTBP1
E2F1	PTBP3
E2F1	PTDSS1
E2F1	PTEN
E2F1	PTGES3
E2F1	PTMA
E2F1	PTOV1
E2F1	PTP4A1
E2F1	PTPN18

E2F1	PTPN9
E2F1	PTS
E2F1	PUDP
E2F1	PWWP2A
E2F1	PXK
E2F1	PXMP2
E2F1	PYM1
E2F1	QARS
E2F1	QSER1
E2F1	RAB11A
E2F1	RAB14
E2F1	RAB27A
E2F1	RAB28
E2F1	RAB2B
E2F1	RAB4A
E2F1	RAB8A
E2F1	RABGAP1L
E2F1	RABIF
E2F1	RABL6
E2F1	RAC1
E2F1	RACK1
E2F1	RAD1
E2F1	RAD18
E2F1	RAD23A
E2F1	RAD23B
E2F1	RAD51C
E2F1	RAD9A
E2F1	RAE1
E2F1	RAF1
E2F1	RALGAPA2
E2F1	RALY
E2F1	RAN
E2F1	RANBP1
E2F1	RANBP10
E2F1	RANBP3
E2F1	RANGRF
E2F1	RAP1B
E2F1	RAP2B
E2F1	RAPGEF5
E2F1	RASSF1
E2F1	RASSF3
E2F1	RBBP4
E2F1	RBBP7
E2F1	RBM10
E2F1	RBM14
E2F1	RBM17
E2F1	RBM26-AS1
E2F1	RBM28
E2F1	RBM3
E2F1	RBM38

E2F1	RBM4
E2F1	RBM8A
E2F1	RBMX
E2F1	RBMX2
E2F1	RBPJ
E2F1	RBX1
E2F1	RCC1
E2F1	RCC1L
E2F1	RCC2
E2F1	RCCD1
E2F1	RCN2
E2F1	RDH5
E2F1	RDX
E2F1	RECQL
E2F1	RECQL4
E2F1	REV1
E2F1	REV3L
E2F1	RFC1
E2F1	RFC2
E2F1	RFC3
E2F1	RFC4
E2F1	RFTN1
E2F1	RFWD3
E2F1	RFX2
E2F1	RFX3
E2F1	RGCC
E2F1	RGS10
E2F1	RGS12
E2F1	RGS13
E2F1	RGS19
E2F1	RGS20
E2F1	RGS9
E2F1	RHNO1
E2F1	RIC8A
E2F1	RIC8B
E2F1	RIF1
E2F1	RING1
E2F1	RMI1
E2F1	RMI2
E2F1	RMND5A
E2F1	RNASEH1
E2F1	RNASEH2A
E2F1	RNASEH2B
E2F1	RNASEH2C
E2F1	RNF123
E2F1	RNF126
E2F1	RNF139-AS1
E2F1	RNF144B
E2F1	RNF168
E2F1	RNF170

E2F1	RNF2
E2F1	RNF212
E2F1	RNF32
E2F1	RNF34
E2F1	RNF38
E2F1	RNPS1
E2F1	ROCK2
E2F1	ROMO1
E2F1	RPA1
E2F1	RPA2
E2F1	RPA3
E2F1	RPAIN
E2F1	RPE
E2F1	RPIA
E2F1	RPL10A
E2F1	RPL11
E2F1	RPL12
E2F1	RPL13A
E2F1	RPL14
E2F1	RPL15
E2F1	RPL17
E2F1	RPL19
E2F1	RPL21
E2F1	RPL22
E2F1	RPL23
E2F1	RPL23A
E2F1	RPL24
E2F1	RPL26
E2F1	RPL26L1
E2F1	RPL27
E2F1	RPL27A
E2F1	RPL28
E2F1	RPL29
E2F1	RPL3
E2F1	RPL30
E2F1	RPL35
E2F1	RPL36
E2F1	RPL36A
E2F1	RPL37
E2F1	RPL37A
E2F1	RPL38
E2F1	RPL4
E2F1	RPL41
E2F1	RPL5
E2F1	RPL6
E2F1	RPL7
E2F1	RPL8
E2F1	RPLP0
E2F1	RPP25
E2F1	RPP30

E2F1	RPRD1B
E2F1	RPS10
E2F1	RPS11
E2F1	RPS13
E2F1	RPS14
E2F1	RPS15
E2F1	RPS15A
E2F1	RPS16
E2F1	RPS17
E2F1	RPS18
E2F1	RPS19
E2F1	RPS19BP1
E2F1	RPS2
E2F1	RPS20
E2F1	RPS21
E2F1	RPS23
E2F1	RPS24
E2F1	RPS25
E2F1	RPS26
E2F1	RPS27A
E2F1	RPS3
E2F1	RPS3A
E2F1	RPS4X
E2F1	RPS5
E2F1	RPS6
E2F1	RPS6KA4
E2F1	RPS7
E2F1	RPS8
E2F1	RPS9
E2F1	RPSA
E2F1	RPUSD1
E2F1	RRAS2
E2F1	RRM1
E2F1	RRM2
E2F1	RRP7A
E2F1	RSL1D1
E2F1	RSPH3
E2F1	RTCB
E2F1	RTF1
E2F1	RTN3
E2F1	RTN4
E2F1	RUFY2
E2F1	RUSC1
E2F1	RWDD2B
E2F1	S100A10
E2F1	S100PBP
E2F1	SAAL1
E2F1	SAC3D1
E2F1	SAE1
E2F1	SAMD10

E2F1	SAP30
E2F1	SARS2
E2F1	SART3
E2F1	SASH3
E2F1	SBF1
E2F1	SC5D
E2F1	SCAF4
E2F1	SCAND1
E2F1	SCARB1
E2F1	SCCPDH
E2F1	SCP2
E2F1	SCRN1
E2F1	SCYL2
E2F1	SDF2L1
E2F1	SDHA
E2F1	SDHAF1
E2F1	SDHAF2
E2F1	SDHAF3
E2F1	SDHB
E2F1	SDHC
E2F1	SEC11A
E2F1	SEC22C
E2F1	SEC23B
E2F1	SEPHS1
E2F1	SEPHS2
E2F1	SEPT1
E2F1	SEPT11
E2F1	SEPT9
E2F1	SERBP1
E2F1	SERF2
E2F1	SERP2
E2F1	SESTD1
E2F1	SET
E2F1	SETD3
E2F1	SETD4
E2F1	SETD6
E2F1	SETD7
E2F1	SF1
E2F1	SF3A1
E2F1	SF3A2
E2F1	SF3A3
E2F1	SF3B2
E2F1	SF3B3
E2F1	SF3B4
E2F1	SF3B5
E2F1	SF3B6
E2F1	SFMBT1
E2F1	SGCE
E2F1	SGF29
E2F1	SGPP2

E2F1	SH2B2
E2F1	SH3KBP1
E2F1	SH3TC1
E2F1	SHARPIN
E2F1	SHMT1
E2F1	SIMC1
E2F1	SIN3A
E2F1	SIPA1
E2F1	SIRT7
E2F1	SIVA1
E2F1	SKA2
E2F1	SKP1
E2F1	SLBP
E2F1	SLC10A3
E2F1	SLC15A4
E2F1	SLC16A1
E2F1	SLC16A4
E2F1	SLC16A7
E2F1	SLC1A4
E2F1	SLC1A5
E2F1	SLC25A1
E2F1	SLC25A10
E2F1	SLC25A11
E2F1	SLC25A19
E2F1	SLC25A26
E2F1	SLC25A3
E2F1	SLC25A4
E2F1	SLC25A40
E2F1	SLC25A5
E2F1	SLC25A53
E2F1	SLC2A11
E2F1	SLC2A5
E2F1	SLC30A4
E2F1	SLC30A5
E2F1	SLC35B4
E2F1	SLC35F5
E2F1	SLC38A9
E2F1	SLC39A1
E2F1	SLC6A6
E2F1	SLC6A9
E2F1	SLC7A5
E2F1	SLC9A3
E2F1	SLC9A3R1
E2F1	SLC9A7
E2F1	SLF2
E2F1	SLFN13
E2F1	SLIRP
E2F1	SLK
E2F1	SLMAP
E2F1	SLTM

E2F1	SMAD2
E2F1	SMAP1
E2F1	SMARCA2
E2F1	SMARCA4
E2F1	SMARCB1
E2F1	SMARCC1
E2F1	SMARCD2
E2F1	SMC1A
E2F1	SMC2
E2F1	SMC3
E2F1	SMC4
E2F1	SMCHD1
E2F1	SMCR5
E2F1	SMDT1
E2F1	SMIM13
E2F1	SMIM19
E2F1	SMIM20
E2F1	SMIM4
E2F1	SMNDC1
E2F1	SMPD2
E2F1	SMPD4
E2F1	SMS
E2F1	SMURF1
E2F1	SMYD4
E2F1	SNAP23
E2F1	SNAP47
E2F1	SND1
E2F1	SNF8
E2F1	SNHG10
E2F1	SNHG19
E2F1	SNHG21
E2F1	SNHG25
E2F1	SNHG7
E2F1	SNRNP25
E2F1	SNRNP35
E2F1	SNRNP40
E2F1	SNRNP48
E2F1	SNRNP70
E2F1	SNRPA
E2F1	SNRPA1
E2F1	SNRPB
E2F1	SNRPD1
E2F1	SNRPD2
E2F1	SNRPE
E2F1	SNRPF
E2F1	SNRPG
E2F1	SNTA1
E2F1	SNTB2
E2F1	SNU13
E2F1	SNW1

E2F1	SNX1
E2F1	SNX16
E2F1	SNX22
E2F1	SNX3
E2F1	SNX5
E2F1	SOAT1
E2F1	SOD1
E2F1	SOGA1
E2F1	SOX4
E2F1	SP1
E2F1	SP4
E2F1	SPAG16
E2F1	SPAST
E2F1	SPC24
E2F1	SPECC1
E2F1	SPI1
E2F1	SPIN1
E2F1	SPIN2B
E2F1	SPOPL
E2F1	SPPL2A
E2F1	SPTSSA
E2F1	SREK1
E2F1	SREK1IP1
E2F1	SRGAP2
E2F1	SRP14
E2F1	SRP19
E2F1	SRP68
E2F1	SRP9
E2F1	SRPK1
E2F1	SRPRB
E2F1	SRRM1
E2F1	SRRM2
E2F1	SRRT
E2F1	SRSF1
E2F1	SRSF10
E2F1	SRSF2
E2F1	SRSF3
E2F1	SRSF4
E2F1	SRSF6
E2F1	SRSF7
E2F1	SRSF9
E2F1	SS18L2
E2F1	SSB
E2F1	SSNA1
E2F1	SSRP1
E2F1	SSTR2
E2F1	SSU72
E2F1	ST14
E2F1	ST3GAL3
E2F1	STAG1

E2F1	STAG2
E2F1	STAG3
E2F1	STARD7
E2F1	STAT6
E2F1	STIM2
E2F1	STK11
E2F1	STK32C
E2F1	STK38L
E2F1	STK39
E2F1	STK40
E2F1	STMN1
E2F1	STS
E2F1	STUB1
E2F1	STX10
E2F1	STX11
E2F1	STX17
E2F1	STX7
E2F1	STYX
E2F1	SUCLA2
E2F1	SUCLG1
E2F1	SUCLG2
E2F1	SUGCT
E2F1	SUGT1
E2F1	SUMO1
E2F1	SUMO2
E2F1	SUMO3
E2F1	SUPT16H
E2F1	SUPT4H1
E2F1	SUSD3
E2F1	SUZ12
E2F1	SVIP
E2F1	SYAP1
E2F1	SYCE1L
E2F1	SYK
E2F1	SYNC
E2F1	SYNE2
E2F1	SYNGR1
E2F1	SYPL1
E2F1	SZRD1
E2F1	TAB2
E2F1	TADA2A
E2F1	TAF15
E2F1	TAF3
E2F1	TAF6
E2F1	TAF6L
E2F1	TAF9B
E2F1	TAGLN2
E2F1	TALDO1
E2F1	TARBP1
E2F1	TARBP2

E2F1	TARDBP
E2F1	TAX1BP3
E2F1	TBC1D1
E2F1	TBC1D14
E2F1	TBC1D20
E2F1	TBC1D22A
E2F1	TBC1D24
E2F1	TBC1D2B
E2F1	TBCA
E2F1	TBCC
E2F1	TBCK
E2F1	TCEA1
E2F1	TCEAL9
E2F1	TCERG1
E2F1	TCIRG1
E2F1	TCL1A
E2F1	TCL1B
E2F1	TCP1
E2F1	TCTE3
E2F1	TCTEX1D2
E2F1	TDG
E2F1	TDRD3
E2F1	TECR
E2F1	TELO2
E2F1	TERF1
E2F1	TERF2
E2F1	TESC
E2F1	TEX264
E2F1	TEX30
E2F1	TEX9
E2F1	TFAP4
E2F1	TFDP1
E2F1	TFDP2
E2F1	TFEB
E2F1	TGDS
E2F1	TGM2
E2F1	TGS1
E2F1	THAP1
E2F1	THAP11
E2F1	THAP7
E2F1	THAP7-AS1
E2F1	THEM4
E2F1	THEM6
E2F1	THOC3
E2F1	THOC7
E2F1	THOP1
E2F1	THRAP3
E2F1	THUMPD2
E2F1	THUMPD3-AS1
E2F1	TIA1

E2F1	TIAL1
E2F1	TIFA
E2F1	TIGAR
E2F1	TIMELESS
E2F1	TIMM8B
E2F1	TIPIN
E2F1	TIRAP
E2F1	TK1
E2F1	TKFC
E2F1	TKT
E2F1	TLK2
E2F1	TMA7
E2F1	TMC5
E2F1	TMCO3
E2F1	TMCO6
E2F1	TMED1
E2F1	TMED8
E2F1	TMEM106C
E2F1	TMEM107
E2F1	TMEM11
E2F1	TMEM123
E2F1	TMEM126B
E2F1	TMEM141
E2F1	TMEM14A
E2F1	TMEM14B
E2F1	TMEM14C
E2F1	TMEM156
E2F1	TMEM160
E2F1	TMEM186
E2F1	TMEM201
E2F1	TMEM214
E2F1	TMEM223
E2F1	TMEM245
E2F1	TMEM256
E2F1	TMEM258
E2F1	TMEM33
E2F1	TMEM39B
E2F1	TMEM44
E2F1	TMEM60
E2F1	TMEM64
E2F1	TMPO
E2F1	TMPO-AS1
E2F1	TMSB10
E2F1	TMSB4X
E2F1	TMUB1
E2F1	TMX1
E2F1	TNRC6A
E2F1	TNRC6C
E2F1	TOLLIP-AS1
E2F1	TOMM5

E2F1	TOMM7
E2F1	TONSL
E2F1	TOP1
E2F1	TOP3A
E2F1	TOPBP1
E2F1	TOPORS
E2F1	TOX4
E2F1	TP53INP2
E2F1	TPCN1
E2F1	TPD52
E2F1	TPGS2
E2F1	TPI1
E2F1	TPM3
E2F1	TPR
E2F1	TPRA1
E2F1	TRA2B
E2F1	TRABD
E2F1	TRAF5
E2F1	TRAF7
E2F1	TRAPPC1
E2F1	TRAPPC12
E2F1	TRIB3
E2F1	TRIM14
E2F1	TRIM28
E2F1	TRIM41
E2F1	TRIM61
E2F1	TRIM73
E2F1	TRMT10B
E2F1	TRMT11
E2F1	TRMT1L
E2F1	TRNAU1AP
E2F1	TROVE2
E2F1	TRRAP
E2F1	TSC22D1
E2F1	TSEN34
E2F1	TSPAN14
E2F1	TSTD1
E2F1	TTC1
E2F1	TTC13
E2F1	TTC14
E2F1	TTC39C
E2F1	TTC9C
E2F1	TTF1
E2F1	TTF2
E2F1	TTI2
E2F1	TUBA1B
E2F1	TUBA4A
E2F1	TUBB
E2F1	TUBB4B
E2F1	TUBG1

E2F1	TUBGCP4
E2F1	TUBGCP5
E2F1	TWF2
E2F1	TXLNA
E2F1	TXN2
E2F1	TXNDC17
E2F1	TXNDC9
E2F1	TXNRD1
E2F1	TYK2
E2F1	TYMS
E2F1	UBA1
E2F1	UBA2
E2F1	UBA3
E2F1	UBA5
E2F1	UBA52
E2F1	UBAC1
E2F1	UBAP1
E2F1	UBB
E2F1	UBE2A
E2F1	UBE2D1
E2F1	UBE2E1
E2F1	UBE2E3
E2F1	UBE2G1
E2F1	UBE2G2
E2F1	UBE2I
E2F1	UBE2J1
E2F1	UBE2L3
E2F1	UBE2M
E2F1	UBE2N
E2F1	UBE2R2
E2F1	UBE2T
E2F1	UBE2V1
E2F1	UBE2V2
E2F1	UBE3A
E2F1	UBE3D
E2F1	UBL5
E2F1	UBL7
E2F1	UBN1
E2F1	UBR3
E2F1	UBR4
E2F1	UBTF
E2F1	UCHL3
E2F1	UCHL5
E2F1	UCP2
E2F1	UGP2
E2F1	UHRF1
E2F1	UHRF2
E2F1	UIMC1
E2F1	UNC45A
E2F1	UNC93B1

E2F1	UNG
E2F1	UNK
E2F1	UPF3A
E2F1	UPF3B
E2F1	UQCR10
E2F1	UQCRB
E2F1	UQCRC1
E2F1	UQCRC2
E2F1	UQCRFS1
E2F1	UQCRH
E2F1	UQCRQ
E2F1	URM1
E2F1	USB1
E2F1	USP1
E2F1	USP10
E2F1	USP14
E2F1	USP22
E2F1	USP28
E2F1	USP30
E2F1	USP34
E2F1	USP39
E2F1	USP4
E2F1	USP45
E2F1	USP49
E2F1	UTP18
E2F1	UTP4
E2F1	UVRAG
E2F1	VAMP1
E2F1	VAMP8
E2F1	VAPA
E2F1	VARS2
E2F1	VAV2
E2F1	VBP1
E2F1	VDAC1
E2F1	VDAC3
E2F1	VMA21
E2F1	VPS25
E2F1	VPS29
E2F1	VRK1
E2F1	VSIG10
E2F1	VTA1
E2F1	WAC
E2F1	WAS
E2F1	WASL
E2F1	WBP4
E2F1	WDFY2
E2F1	WDR1
E2F1	WDR33
E2F1	WDR34
E2F1	WDR4

E2F1	WDR5
E2F1	WDR53
E2F1	WDR76
E2F1	WDR82
E2F1	WDR83
E2F1	WDR89
E2F1	WDR90
E2F1	WDSUB1
E2F1	WDTC1
E2F1	WDYHV1
E2F1	WEE1
E2F1	WIPI2
E2F1	WRAP53
E2F1	WRN
E2F1	WTAP
E2F1	XPNPEP1
E2F1	XPNPEP3
E2F1	XPO1
E2F1	XRCC2
E2F1	XRCC4
E2F1	XRCC5
E2F1	XRCC6
E2F1	XRN2
E2F1	YARS
E2F1	YBEY
E2F1	YBX1
E2F1	YEATS4
E2F1	YTHDF2
E2F1	YTHDF3-AS1
E2F1	YWHAB
E2F1	YWHAE
E2F1	YWHAH
E2F1	YWHAQ
E2F1	YWHAZ
E2F1	YY1
E2F1	ZBED5-AS1
E2F1	ZBTB7A
E2F1	ZBTB8OS
E2F1	ZC3H13
E2F1	ZC3H14
E2F1	ZC3HAV1
E2F1	ZCCHC17
E2F1	ZCCHC18
E2F1	ZCCHC8
E2F1	ZDHHC12
E2F1	ZDHHC13
E2F1	ZDHHC3
E2F1	ZDHHC4
E2F1	ZDHHC5
E2F1	ZFAND2A

E2F1	ZFAND2B
E2F1	ZFAND3
E2F1	ZFAND6
E2F1	ZFAS1
E2F1	ZFP91
E2F1	ZFYVE19
E2F1	ZKSCAN4
E2F1	ZMYM4
E2F1	ZMYND19
E2F1	ZNF101
E2F1	ZNF106
E2F1	ZNF107
E2F1	ZNF141
E2F1	ZNF16
E2F1	ZNF215
E2F1	ZNF230
E2F1	ZNF326
E2F1	ZNF337-AS1
E2F1	ZNF367
E2F1	ZNF428
E2F1	ZNF444
E2F1	ZNF514
E2F1	ZNF519
E2F1	ZNF529-AS1
E2F1	ZNF544
E2F1	ZNF580
E2F1	ZNF584
E2F1	ZNF595
E2F1	ZNF668
E2F1	ZNF688
E2F1	ZNF689
E2F1	ZNF718
E2F1	ZNF76
E2F1	ZNF776
E2F1	ZNF787
E2F1	ZNF837
E2F1	ZNF93
E2F1	ZNHIT1
E2F1	ZNHIT3
E2F1	ZPBP2
E2F1	ZRANB1
E2F1	ZRANB3
E2F1	ZW10
E2F1	ZWILCH
E2F1	ZWINT
E2F2	AACS
E2F2	ABHD17A
E2F2	ABHD5
E2F2	ACAP1
E2F2	ACAP2

E2F2	ACAP3
E2F2	ADCK5
E2F2	AEBP2
E2F2	AGBL5
E2F2	AGPS
E2F2	AHI1
E2F2	AIG1
E2F2	AKR7A2
E2F2	ALKBH2
E2F2	ALKBH6
E2F2	ANAPC11
E2F2	ANO6
E2F2	ANXA5
E2F2	AP1S1
E2F2	AP4M1
E2F2	APOO
E2F2	ARID1A
E2F2	ARID1B
E2F2	ARL17A
E2F2	ARL6IP6
E2F2	ASB16-AS1
E2F2	ASF1B
E2F2	ASL
E2F2	ASTE1
E2F2	ATAD2
E2F2	ATG16L1
E2F2	ATG4D
E2F2	ATP2A3
E2F2	ATP6V1B2
E2F2	B3GLCT
E2F2	BARD1
E2F2	BAZ1B
E2F2	BCL2L1
E2F2	BCL7B
E2F2	BRI3BP
E2F2	C17orf58
E2F2	C1orf112
E2F2	C20orf27
E2F2	C3orf14
E2F2	C6orf136
E2F2	CADPS
E2F2	CALM1
E2F2	CARHSP1
E2F2	CASP8AP2
E2F2	CBFB
E2F2	CBX2
E2F2	CBX5
E2F2	CCDC150
E2F2	CCDC167
E2F2	CCDC171

E2F2	CCDC61
E2F2	CCDC88C
E2F2	CDC45
E2F2	CDCA5
E2F2	CDK2AP2
E2F2	CDK5RAP3
E2F2	CDKN2AIP
E2F2	CDKN2D
E2F2	CDT1
E2F2	CELF2
E2F2	CENPK
E2F2	CENPM
E2F2	CENPN
E2F2	CENPQ
E2F2	CENPU
E2F2	CEP131
E2F2	CEP295
E2F2	CEP41
E2F2	CEP97
E2F2	CHAF1B
E2F2	CHD4
E2F2	CLDN23
E2F2	CLIC1
E2F2	CLSPN
E2F2	CMPK2
E2F2	CNTLN
E2F2	COCH
E2F2	COQ5
E2F2	COX8A
E2F2	CPSF1
E2F2	CREB3L2
E2F2	CSRP1
E2F2	CTDSPL2
E2F2	CTNNAL1
E2F2	CYTH2
E2F2	DDT
E2F2	DDX11
E2F2	DEK
E2F2	DHCR7
E2F2	DHRS13
E2F2	DIAPH2
E2F2	DIMT1
E2F2	DLG1
E2F2	DMXL1
E2F2	DNA2
E2F2	DNAJC19
E2F2	DNAJC9
E2F2	DONSON
E2F2	DPYSL2
E2F2	DSN1

E2F2	DTL
E2F2	DUT
E2F2	DYRK4
E2F2	E2F2
E2F2	E2F6
E2F2	E2F8
E2F2	EBP
E2F2	EGR3
E2F2	EIF2B5
E2F2	EIF4A3
E2F2	EIF5A2
E2F2	ELF2
E2F2	ELMO1
E2F2	ENOSF1
E2F2	ERBIN
E2F2	ERCC3
E2F2	ERI2
E2F2	EXOG
E2F2	EYA3
E2F2	EZH2
E2F2	FAF1
E2F2	FAM111B
E2F2	FAM50B
E2F2	FAM53C
E2F2	FAM96A
E2F2	FANCL
E2F2	FBXL4
E2F2	FBXW8
E2F2	FEN1
E2F2	FOXM1
E2F2	FSD1L
E2F2	FTH1
E2F2	GFI1
E2F2	GINM1
E2F2	GINS1
E2F2	GINS2
E2F2	GLG1
E2F2	GLMN
E2F2	GMEB1
E2F2	GMNN
E2F2	GOLPH3
E2F2	GPAT2
E2F2	GPR174
E2F2	H2AFV
E2F2	H2AFX
E2F2	H2AFZ
E2F2	H3F3A
E2F2	HAT1
E2F2	HDAC6
E2F2	HDHD2

E2F2	HELLS
E2F2	HERC2
E2F2	HES1
E2F2	HIP1
E2F2	HIRIP3
E2F2	HIST1H2AI
E2F2	HMCES
E2F2	HMGA1
E2F2	HMGB1
E2F2	HMGB2
E2F2	HMGN2
E2F2	HNRNPA0
E2F2	HNRNPF
E2F2	HSP90AB1
E2F2	HSPB11
E2F2	HSPBAP1
E2F2	ICMT
E2F2	IDH2
E2F2	IDI1
E2F2	IFI16
E2F2	IMPA2
E2F2	IMPDH1
E2F2	INO80E
E2F2	IPO9
E2F2	IPPK
E2F2	IQCB1
E2F2	ISCU
E2F2	ITFG1
E2F2	ITGB3BP
E2F2	ITPR3
E2F2	JADE1
E2F2	JAML
E2F2	KAT2B
E2F2	KIF11
E2F2	KIF15
E2F2	KLHDC2
E2F2	KLHDC3
E2F2	KLHL23
E2F2	KRI1
E2F2	LAP3
E2F2	LARP7
E2F2	LCMT1
E2F2	LENG8
E2F2	LILRB1
E2F2	LIPA
E2F2	LRR1
E2F2	LRRC40
E2F2	LSM3
E2F2	LSS
E2F2	LYRM7

E2F2	MAD2L2
E2F2	MAML1
E2F2	MAP2K6
E2F2	MAPK1IP1L
E2F2	MAST3
E2F2	MASTL
E2F2	MBP
E2F2	MCM10
E2F2	MCM2
E2F2	MCM3
E2F2	MCM4
E2F2	MCM5
E2F2	MCM7
E2F2	MFGE8
E2F2	MGME1
E2F2	MIDN
E2F2	MIR3142HG
E2F2	MIS12
E2F2	MIS18A
E2F2	MNAT1
E2F2	MND1
E2F2	MON1B
E2F2	MSRB2
E2F2	MTMR4
E2F2	MYBL2
E2F2	MYL6B
E2F2	MYO1G
E2F2	MYO6
E2F2	NAA38
E2F2	NASP
E2F2	NCAPH2
E2F2	NFATC2IP
E2F2	NFATC4
E2F2	NFYB
E2F2	NINJ1
E2F2	NINJ2
E2F2	NIPSNAP3A
E2F2	NR6A1
E2F2	NUDT1
E2F2	NUDT21
E2F2	NUP210
E2F2	NUP50
E2F2	NUP58
E2F2	NUTM2A-AS1
E2F2	OGFOD1
E2F2	OGG1
E2F2	ORC4
E2F2	OSBPL9
E2F2	PAG1
E2F2	PAK1

E2F2	PAN2
E2F2	PBRM1
E2F2	PCNA
E2F2	PDXDC1
E2F2	PGP
E2F2	PHF10
E2F2	PIGM
E2F2	PKMYT1
E2F2	PKN2
E2F2	PLAC8
E2F2	PLEKHA2
E2F2	PLEKHA8
E2F2	PLK4
E2F2	PMS2
E2F2	POC1A
E2F2	POLE
E2F2	POLQ
E2F2	PPM1N
E2F2	PPP1CA
E2F2	PPP1R37
E2F2	PPP2R3C
E2F2	PPP4R3A
E2F2	PQLC1
E2F2	PRDM2
E2F2	PRIM1
E2F2	PRKAR2B
E2F2	PRPSAP1
E2F2	PSIP1
E2F2	PTMA
E2F2	PTP4A2
E2F2	PTPRA
E2F2	PUSL1
E2F2	RAB11B-AS1
E2F2	RAB9A
E2F2	RALY
E2F2	RBBP5
E2F2	RBBP8
E2F2	RBM7
E2F2	REC8
E2F2	RECQL4
E2F2	RFC4
E2F2	RFC5
E2F2	RGS19
E2F2	RMI1
E2F2	RNASEK
E2F2	RNF141
E2F2	RNF144B
E2F2	RNF2
E2F2	RPA2
E2F2	RPA3

E2F2	RPP30
E2F2	RRM2
E2F2	RUSC1
E2F2	SAE1
E2F2	SAP30
E2F2	SASS6
E2F2	SERGEF
E2F2	SETD9
E2F2	SF3A1
E2F2	SFXN5
E2F2	SGCB
E2F2	SH2B1
E2F2	SH3BP1
E2F2	SH3GLB2
E2F2	SHANK2
E2F2	SIGIRR
E2F2	SIPA1
E2F2	SIPA1L3
E2F2	SIRT1
E2F2	SIVA1
E2F2	SKA3
E2F2	SKP2
E2F2	SLBP
E2F2	SLC20A1
E2F2	SLC25A10
E2F2	SLCO3A1
E2F2	SMAD5
E2F2	SMC1A
E2F2	SMC1B
E2F2	SMC2
E2F2	SMNDC1
E2F2	SMPD4
E2F2	SMYD3
E2F2	ST3GAL2
E2F2	STAC3
E2F2	STMN1
E2F2	STRN
E2F2	SUB1
E2F2	SWSAP1
E2F2	SYCE2
E2F2	TAF4
E2F2	TAF6
E2F2	TAPBPL
E2F2	TBL2
E2F2	TCEA2
E2F2	TCF12
E2F2	TDRD3
E2F2	TEX30
E2F2	TIMELESS
E2F2	TLK1

E2F2	TMEM106C
E2F2	TMEM107
E2F2	TMEM131
E2F2	TMEM14C
E2F2	TMPO
E2F2	TOPBP1
E2F2	TOR1A
E2F2	TOR1AIP2
E2F2	TOR2A
E2F2	TPGS2
E2F2	TRAFD1
E2F2	TRIM8
E2F2	TSEN54
E2F2	TTC37
E2F2	TTC7A
E2F2	TTF1
E2F2	TUBA1B
E2F2	TUBB
E2F2	TYMS
E2F2	UBE2E2
E2F2	UCP2
E2F2	UHRF1
E2F2	UNG
E2F2	USP1
E2F2	USP10
E2F2	USP13
E2F2	USP22
E2F2	VAMP3
E2F2	VNN2
E2F2	VPS4A
E2F2	VPS8
E2F2	WDR34
E2F2	WDYHV1
E2F2	WRAP53
E2F2	WSB2
E2F2	WWOX
E2F2	XRCC1
E2F2	XRCC2
E2F2	XRCC4
E2F2	YEATS2
E2F2	YTHDF3-AS1
E2F2	YWHAH
E2F2	ZC3H10
E2F2	ZCCHC3
E2F2	ZDHHC12
E2F2	ZNF141
E2F2	ZNF282
E2F2	ZNF367
E2F2	ZNF512
E2F2	ZNF529-AS1

E2F2	ZSCAN16
E2F2	ZWILCH
E2F3	AAAS
E2F3	ACOT2
E2F3	ACSL4
E2F3	AHCTF1
E2F3	AHI1
E2F3	ALCAM
E2F3	AP2B1
E2F3	AP5Z1
E2F3	APEX2
E2F3	AQR
E2F3	ARHGAP12
E2F3	ARPC1A
E2F3	ASCL2
E2F3	ASF1B
E2F3	ATAD2
E2F3	ATF2
E2F3	ATG2A
E2F3	BBIP1
E2F3	BMP2K
E2F3	C11orf49
E2F3	C17orf75
E2F3	C18orf54
E2F3	C1orf131
E2F3	C2orf76
E2F3	C4orf46
E2F3	C8orf44
E2F3	CASP2
E2F3	CAV1
E2F3	CCDC125
E2F3	CCDC28B
E2F3	CD151
E2F3	CENPI
E2F3	CEP63
E2F3	CHD9
E2F3	CHERP
E2F3	CIITA
E2F3	CNN2
E2F3	CNOT3
E2F3	CPSF2
E2F3	CREBBP
E2F3	CREM
E2F3	CSNK1G1
E2F3	CSTF2T
E2F3	CUL2
E2F3	CYLD
E2F3	DEXI
E2F3	DHX16
E2F3	DHX36

E2F3	DHX40
E2F3	DLGAP4
E2F3	DNASE1L3
E2F3	DOCK11
E2F3	E2F3
E2F3	EEF2K
E2F3	EID2
E2F3	EIF2S1
E2F3	EIF4A3
E2F3	ELL2
E2F3	ELOF1
E2F3	EXOSC1
E2F3	FAM216A
E2F3	FBXO34
E2F3	FCF1
E2F3	FMR1
E2F3	FOPNL
E2F3	FOXK1
E2F3	FRG1
E2F3	FUCA1
E2F3	FYTTD1
E2F3	GCLM
E2F3	GIGYF2
E2F3	GLYCTK
E2F3	GPALPP1
E2F3	GTSF1
E2F3	HAUS4
E2F3	HAUS6
E2F3	HCFC1R1
E2F3	HELLS
E2F3	HIPK1
E2F3	HIST1H2AJ
E2F3	HPF1
E2F3	IFT74
E2F3	INIP
E2F3	INTS9
E2F3	IQGAP1
E2F3	IRAK1
E2F3	IRS2
E2F3	ISCA1
E2F3	KAT2B
E2F3	KBTBD2
E2F3	KHNYN
E2F3	KLF10
E2F3	KMT2C
E2F3	LATS1
E2F3	LIX1L
E2F3	LOXL2
E2F3	MAN1A1
E2F3	MAP1S

E2F3	MCM4
E2F3	MED26
E2F3	MED27
E2F3	MED6
E2F3	METTL17
E2F3	MGME1
E2F3	MIDN
E2F3	MKLN1
E2F3	MMS19
E2F3	MRPL49
E2F3	MSANTD4
E2F3	MSL1
E2F3	MSRB1
E2F3	MYLK-AS1
E2F3	NBN
E2F3	NCALD
E2F3	NECAP1
E2F3	NENF
E2F3	NGDN
E2F3	NNT
E2F3	NOTCH2
E2F3	NUDC
E2F3	OSBPL3
E2F3	PACS1
E2F3	PAGR1
E2F3	PAN3
E2F3	PAQR4
E2F3	PAX5
E2F3	PCBD1
E2F3	PDPR
E2F3	PFKP
E2F3	PGM2
E2F3	PHACTR4
E2F3	PHF1
E2F3	PHF14
E2F3	PIGW
E2F3	PIKFYVE
E2F3	POLR2I
E2F3	POLR2M
E2F3	PPP1R37
E2F3	PRKCE
E2F3	PRMT1
E2F3	PSMD9
E2F3	PTBP3
E2F3	PUSL1
E2F3	RAD18
E2F3	RAE1
E2F3	RANGAP1
E2F3	RASGEF1B
E2F3	RASSF5

E2F3	RBBP8
E2F3	RCAN1
E2F3	RFC3
E2F3	RFX1
E2F3	RLIM
E2F3	RNF121
E2F3	RNF44
E2F3	RSRC1
E2F3	SENP1
E2F3	SFR1
E2F3	SFXN4
E2F3	SH3BP1
E2F3	SHMT2
E2F3	SLAIN2
E2F3	SLC25A17
E2F3	SLC25A33
E2F3	SMIM13
E2F3	SMKR1
E2F3	SMYD2
E2F3	SNAPC3
E2F3	SNX12
E2F3	SNX13
E2F3	SOCS3
E2F3	SPAG9
E2F3	SPG11
E2F3	STAM2
E2F3	STARD4
E2F3	STK32C
E2F3	SUGP1
E2F3	SYNCRIP
E2F3	TAP1
E2F3	TBL1XR1
E2F3	TGDS
E2F3	TGFB1
E2F3	THAP5
E2F3	THAP9-AS1
E2F3	TIGAR
E2F3	TIPARP-AS1
E2F3	TIPIN
E2F3	TM2D3
E2F3	TM9SF1
E2F3	TMUB1
E2F3	TNFAIP3
E2F3	TOLLIP-AS1
E2F3	TOMM40
E2F3	TRIM13
E2F3	TRPC4AP
E2F3	TTBK2
E2F3	TUBG1
E2F3	TUBGCP5

E2F3	TYW1
E2F3	UFL1
E2F3	UGDH
E2F3	UGT8
E2F3	UNG
E2F3	VAMP2
E2F3	VSIG10
E2F3	WDR24
E2F3	WDR5
E2F3	WWP2
E2F3	YIPF5
E2F3	YIPF6
E2F3	ZBTB48
E2F3	ZFAND3
E2F3	ZFP91
E2F3	ZMAT5
E2F3	ZNF101
E2F3	ZNF107
E2F3	ZNF32
E2F3	ZNF804A
E2F4	AAAS
E2F4	AAMP
E2F4	AAR2
E2F4	AASDHPPT
E2F4	AATF
E2F4	ABCD3
E2F4	ABCF1
E2F4	ABHD10
E2F4	ABRACL
E2F4	ABT1
E2F4	ACACA
E2F4	ACAT2
E2F4	ACBD3
E2F4	ACBD6
E2F4	ACIN1
E2F4	ACTR1B
E2F4	ACTR2
E2F4	ACTR3
E2F4	ACTR6
E2F4	ADAM15
E2F4	ADH5
E2F4	ADIPOR1
E2F4	ADIPOR2
E2F4	ADPGK
E2F4	ADPRHL2
E2F4	ADRM1
E2F4	ADSL
E2F4	ADSS
E2F4	AFTPH
E2F4	AGFG1

E2F4	AHCTF1
E2F4	AHCY
E2F4	AHSA1
E2F4	AIFM1
E2F4	AIMP2
E2F4	AK3
E2F4	AK6
E2F4	AKAP8
E2F4	AKIRIN1
E2F4	AKR1A1
E2F4	AKT2
E2F4	AKTIP
E2F4	ALAS1
E2F4	ALDH18A1
E2F4	ALDOA
E2F4	ALG13
E2F4	ALG8
E2F4	ALKBH2
E2F4	ALKBH3
E2F4	ALKBH5
E2F4	ALYREF
E2F4	AMD1
E2F4	ANAPC11
E2F4	ANAPC13
E2F4	ANK1
E2F4	ANKRD17
E2F4	ANP32A
E2F4	ANP32B
E2F4	AP1S2
E2F4	AP2A1
E2F4	AP2B1
E2F4	AP2S1
E2F4	AP3S1
E2F4	AP5M1
E2F4	APBA3
E2F4	APEH
E2F4	APP
E2F4	APPL2
E2F4	AQR
E2F4	ARCN1
E2F4	ARF1
E2F4	ARF6
E2F4	ARFIP1
E2F4	ARFIP2
E2F4	ARHGDIA
E2F4	ARL14EP
E2F4	ARL2
E2F4	ARL5A
E2F4	ARMC7
E2F4	ARPC4

E2F4	ARPC5L
E2F4	ARRDC3
E2F4	ASNA1
E2F4	ASNSD1
E2F4	ATAD3A
E2F4	ATF4
E2F4	ATF7
E2F4	ATG101
E2F4	ATG12
E2F4	ATG16L2
E2F4	ATG3
E2F4	ATG4B
E2F4	ATOX1
E2F4	ATP1B3
E2F4	ATP6AP2
E2F4	ATP6V0E1
E2F4	ATP6V1B2
E2F4	ATP6V1C1
E2F4	ATP6V1H
E2F4	ATXN10
E2F4	ATXN2L
E2F4	ATXN7L3
E2F4	AUH
E2F4	AURKAIP1
E2F4	BABAM1
E2F4	BAG6
E2F4	BANF1
E2F4	BATF
E2F4	BAZ1A
E2F4	BAZ1B
E2F4	BBS4
E2F4	BCAP31
E2F4	BCAR3
E2F4	BCAT2
E2F4	BCCIP
E2F4	BCL11A
E2F4	BCL2A1
E2F4	BCL2L1
E2F4	BCLAF1
E2F4	BECN1
E2F4	BET1
E2F4	BIRC2
E2F4	BLOC1S2
E2F4	BLOC1S4
E2F4	BMS1
E2F4	BOD1
E2F4	BOLA2B
E2F4	BOP1
E2F4	BORCS8
E2F4	BRCC3

E2F4	BRD2
E2F4	BRF2
E2F4	BRMS1
E2F4	BROX
E2F4	BSG
E2F4	BTAF1
E2F4	BTF3
E2F4	BTF3L4
E2F4	BUD31
E2F4	BZW1
E2F4	BZW2
E2F4	C12orf10
E2F4	C12orf29
E2F4	C14orf119
E2F4	C16orf91
E2F4	C17orf53
E2F4	C18orf21
E2F4	C19orf25
E2F4	C19orf48
E2F4	C19orf53
E2F4	C1orf131
E2F4	C1orf174
E2F4	C1orf52
E2F4	C1QBP
E2F4	C20orf27
E2F4	C21orf2
E2F4	C6orf226
E2F4	C6orf47
E2F4	C8orf33
E2F4	C8orf59
E2F4	C9orf78
E2F4	CACTIN
E2F4	CACYBP
E2F4	CALM2
E2F4	CALR
E2F4	CAMK4
E2F4	CAMTA2
E2F4	CANT1
E2F4	CANX
E2F4	CAP1
E2F4	CAPN10
E2F4	CAPZA2
E2F4	CARM1
E2F4	CARS
E2F4	CASP3
E2F4	CBLL1
E2F4	CBWD1
E2F4	CBX1
E2F4	CBX3
E2F4	CCDC12

E2F4	CCDC134
E2F4	CCDC137
E2F4	CCDC174
E2F4	CCDC25
E2F4	CCDC50
E2F4	CCDC61
E2F4	CCDC85B
E2F4	CCDC97
E2F4	CCNC
E2F4	CCND2
E2F4	CCNL2
E2F4	CCT2
E2F4	CCT5
E2F4	CCT7
E2F4	CCT8
E2F4	CCZ1
E2F4	CD164
E2F4	CD63
E2F4	CD82
E2F4	CDC123
E2F4	CDC37
E2F4	CDC37L1
E2F4	CDC42
E2F4	CDC42SE1
E2F4	CDC5L
E2F4	CDK10
E2F4	CDK11B
E2F4	CDK12
E2F4	CDK4
E2F4	CDK7
E2F4	CDK8
E2F4	CDK9
E2F4	CDKN2A
E2F4	CDR2
E2F4	CEBPG
E2F4	CEBPZ
E2F4	CEBPZOS
E2F4	CELF1
E2F4	CELF2
E2F4	CENPL
E2F4	CEP131
E2F4	CEP63
E2F4	CFAP97
E2F4	CFL1
E2F4	CFLAR
E2F4	CHCHD2
E2F4	CHCHD3
E2F4	CHCHD7
E2F4	CHD1
E2F4	CHD4

E2F4	CHMP1A
E2F4	CHMP2A
E2F4	CHMP2B
E2F4	CHMP4B
E2F4	CHORDC1
E2F4	CHST11
E2F4	CHTOP
E2F4	CIAO1
E2F4	CIB1
E2F4	CINP
E2F4	CIRBP
E2F4	CIZ1
E2F4	CKB
E2F4	CKS2
E2F4	CLASRP
E2F4	CLIC1
E2F4	CLNS1A
E2F4	CLPP
E2F4	CLPTM1
E2F4	CLTA
E2F4	CMC1
E2F4	CMSS1
E2F4	CMTM6
E2F4	CMTM8
E2F4	CMTR2
E2F4	CNBP
E2F4	CNDP2
E2F4	CNEP1R1
E2F4	CNIH1
E2F4	CNN3
E2F4	CNOT4
E2F4	CNPPD1
E2F4	COA4
E2F4	COA6
E2F4	COG2
E2F4	COIL
E2F4	COMMD5
E2F4	COPB1
E2F4	COPB2
E2F4	COPS3
E2F4	COPS5
E2F4	COPZ1
E2F4	COQ3
E2F4	COQ4
E2F4	COQ9
E2F4	COX11
E2F4	COX16
E2F4	COX17
E2F4	COX20
E2F4	COX5B

E2F4	COX6A1
E2F4	COX6B1
E2F4	COX7A2
E2F4	COX7A2L
E2F4	COX7C
E2F4	CPSF4
E2F4	CPSF6
E2F4	CREB1
E2F4	CRIPT
E2F4	CRK
E2F4	CRLS1
E2F4	CRNDE
E2F4	CSDE1
E2F4	CSF1
E2F4	CSK
E2F4	CSNK1A1
E2F4	CSNK1D
E2F4	CSNK2A1
E2F4	CSNK2B
E2F4	CSTF2
E2F4	CSTF3
E2F4	CTDNEP1
E2F4	CTDSP1
E2F4	CTDSPL2
E2F4	CTNNBL1
E2F4	CTPS1
E2F4	CTR9
E2F4	CTSC
E2F4	CTTN
E2F4	CUL2
E2F4	CUL4A
E2F4	CUTC
E2F4	CWC15
E2F4	CWC22
E2F4	CWC25
E2F4	CWC27
E2F4	CYB561
E2F4	CYB5R4
E2F4	CYHR1
E2F4	CYTH2
E2F4	DALRD3
E2F4	DAP3
E2F4	DARS
E2F4	DAXX
E2F4	DAZAP1
E2F4	DAZAP2
E2F4	DCAF10
E2F4	DCAF15
E2F4	DCAF7
E2F4	DCP1A

E2F4	DCUN1D5
E2F4	DDB1
E2F4	DDT
E2F4	DDX10
E2F4	DDX18
E2F4	DDX21
E2F4	DDX23
E2F4	DDX24
E2F4	DDX39A
E2F4	DDX3X
E2F4	DDX42
E2F4	DDX46
E2F4	DDX49
E2F4	DDX5
E2F4	DDX50
E2F4	DDX52
E2F4	DDX56
E2F4	DDX6
E2F4	DENR
E2F4	DESI1
E2F4	DEXI
E2F4	DGCR2
E2F4	DGUOK
E2F4	DHX15
E2F4	DHX30
E2F4	DHX36
E2F4	DHX8
E2F4	DMAP1
E2F4	DNAJA1
E2F4	DNAJA2
E2F4	DNAJA3
E2F4	DNAJA4
E2F4	DNAJB6
E2F4	DNAJC17
E2F4	DNAJC2
E2F4	DNAJC24
E2F4	DNAJC7
E2F4	DNM2
E2F4	DNPEP
E2F4	DNTTIP1
E2F4	DNTTIP2
E2F4	DOCK10
E2F4	DOHH
E2F4	DPH2
E2F4	DPH3
E2F4	DPM1
E2F4	DPP3
E2F4	DPP9
E2F4	DR1
E2F4	DRG2

E2F4	DTD1
E2F4	DUS1L
E2F4	DUS3L
E2F4	DYNC1I2
E2F4	DYNC1LI1
E2F4	E2F4
E2F4	EAPP
E2F4	ECSIT
E2F4	EDF1
E2F4	EEA1
E2F4	EEF1A1
E2F4	EEF1B2
E2F4	EEF1D
E2F4	EEF1E1
E2F4	EEF1G
E2F4	EFHD2
E2F4	EFR3A
E2F4	EFTUD2
E2F4	EIF1AX
E2F4	EIF2A
E2F4	EIF2B1
E2F4	EIF2B2
E2F4	EIF2S1
E2F4	EIF2S2
E2F4	EIF2S3
E2F4	EIF3A
E2F4	EIF3B
E2F4	EIF3D
E2F4	EIF3G
E2F4	EIF3I
E2F4	EIF3K
E2F4	EIF3M
E2F4	EIF4A1
E2F4	EIF4A2
E2F4	EIF4E
E2F4	EIF4EBP1
E2F4	EIF4G1
E2F4	EIF4G2
E2F4	EIF5A
E2F4	EIF6
E2F4	ELAC2
E2F4	ELAVL1
E2F4	ELF2
E2F4	ELMOD2
E2F4	ELOF1
E2F4	ELOVL5
E2F4	ELP2
E2F4	ELP3
E2F4	ELP5
E2F4	EMC3

E2F4	EMC4
E2F4	EMG1
E2F4	ENOPH1
E2F4	ENOX2
E2F4	ENTHD1
E2F4	ENY2
E2F4	EPB41L2
E2F4	EPC1
E2F4	ERH
E2F4	ERI3
E2F4	ERICH1
E2F4	ERLIN2
E2F4	ERP44
E2F4	ESRRA
E2F4	ETF1
E2F4	EXOC7
E2F4	EXOSC3
E2F4	EXOSC4
E2F4	EXOSC5
E2F4	EXOSC6
E2F4	FAAP20
E2F4	FABP5
E2F4	FAM104A
E2F4	FAM160A2
E2F4	FAM168B
E2F4	FAM173A
E2F4	FAM177A1
E2F4	FAM192A
E2F4	FAM204A
E2F4	FAM207A
E2F4	FAM210A
E2F4	FAM217B
E2F4	FAM32A
E2F4	FAM72B
E2F4	FAM96B
E2F4	FAM98A
E2F4	FANCB
E2F4	FANCF
E2F4	FARSA
E2F4	FASTK
E2F4	FASTKD1
E2F4	FASTKD5
E2F4	FBL
E2F4	FBXL12
E2F4	FBXO11
E2F4	FBXO42
E2F4	FBXW2
E2F4	FBXW9
E2F4	FCF1
E2F4	FDFT1

E2F4	FDPS
E2F4	FEM1A
E2F4	FGD2
E2F4	FGFR1
E2F4	FIBP
E2F4	FIP1L1
E2F4	FIS1
E2F4	FKBP1A
E2F4	FLAD1
E2F4	FNBP4
E2F4	FNTA
E2F4	FOSL1
E2F4	FOXP1
E2F4	FPGS
E2F4	FRG1
E2F4	FTH1
E2F4	FTSJ1
E2F4	FUBP1
E2F4	FUBP3
E2F4	FUCA1
E2F4	FUS
E2F4	FYN
E2F4	G3BP1
E2F4	G3BP2
E2F4	G6PC3
E2F4	G6PD
E2F4	GADD45B
E2F4	GADD45GIP1
E2F4	GALNT2
E2F4	GALNT7
E2F4	GAR1
E2F4	GARS
E2F4	GART
E2F4	GAS7
E2F4	GCDH
E2F4	GDAP1
E2F4	GDI2
E2F4	GEMIN4
E2F4	GEMIN5
E2F4	GEMIN6
E2F4	GFM1
E2F4	GFM2
E2F4	GFOD2
E2F4	GGA1
E2F4	GGCT
E2F4	GGPS1
E2F4	GHDC
E2F4	GHITM
E2F4	GHRL
E2F4	GID8

E2F4	GK
E2F4	GLRX3
E2F4	GLRX5
E2F4	GNAI2
E2F4	GNAI3
E2F4	GNB1L
E2F4	GNL1
E2F4	GNL2
E2F4	GNL3
E2F4	GOSR1
E2F4	GPALPP1
E2F4	GPANK1
E2F4	GPATCH4
E2F4	GPLD1
E2F4	GPN1
E2F4	GPR108
E2F4	GPR65
E2F4	GPS2
E2F4	GRB2
E2F4	GRPEL1
E2F4	GRWD1
E2F4	GSDMD
E2F4	GSKIP
E2F4	GSPT1
E2F4	GSTO1
E2F4	GTF2A2
E2F4	GTF2H1
E2F4	GTF2H2
E2F4	GTF2H2C
E2F4	GTF3A
E2F4	GTPBP3
E2F4	GTPBP4
E2F4	H3F3B
E2F4	HARS
E2F4	HAX1
E2F4	HCCS
E2F4	HDAC3
E2F4	HDAC8
E2F4	HDDC2
E2F4	HDHD2
E2F4	HEATR1
E2F4	HECTD4
E2F4	HES6
E2F4	HGH1
E2F4	HGS
E2F4	HIGD1A
E2F4	HIGD2A
E2F4	HINT1
E2F4	HIRA
E2F4	HK1

E2F4	HMBS
E2F4	HMGA1
E2F4	HMOX2
E2F4	HNRNPA0
E2F4	HNRNPA2B1
E2F4	HNRNPA3
E2F4	HNRNPAB
E2F4	HNRNPC
E2F4	HNRNPD
E2F4	HNRNPF
E2F4	HNRNPH1
E2F4	HNRNPH3
E2F4	HNRNPK
E2F4	HNRNPR
E2F4	HNRNPU
E2F4	HSBP1
E2F4	HSD17B10
E2F4	HSP90AA1
E2F4	HSP90AB1
E2F4	HSPA4
E2F4	HSPA8
E2F4	HSPA9
E2F4	HSPBP1
E2F4	HSPD1
E2F4	HSPE1
E2F4	HTATIP2
E2F4	HTATSF1
E2F4	HYAL2
E2F4	ICE2
E2F4	ICMT
E2F4	ID3
E2F4	IDH3A
E2F4	IDH3B
E2F4	IDI1
E2F4	IFIH1
E2F4	IFT20
E2F4	IFT46
E2F4	IL2RA
E2F4	IL4I1
E2F4	ILF2
E2F4	ILF3
E2F4	IMMT
E2F4	IMP3
E2F4	IMP4
E2F4	IMPA1
E2F4	IMPACT
E2F4	IMPDH1
E2F4	ING5
E2F4	INO80
E2F4	INTS10

E2F4	INTS7
E2F4	IPO4
E2F4	IPO7
E2F4	IRF5
E2F4	ISOC2
E2F4	ISY1
E2F4	ITFG2
E2F4	ITGB1BP1
E2F4	ITPA
E2F4	ITPK1
E2F4	JAGN1
E2F4	JARID2
E2F4	JAZF1
E2F4	JMJD8
E2F4	JRK
E2F4	KARS
E2F4	KAT2A
E2F4	KAT5
E2F4	KBTBD4
E2F4	KCMF1
E2F4	KDM3B
E2F4	KEAP1
E2F4	KHNYN
E2F4	KIAA0100
E2F4	KIF5B
E2F4	KLHL18
E2F4	KMT2D
E2F4	KNOP1
E2F4	KPNB1
E2F4	KRR1
E2F4	KRTCAP2
E2F4	KTN1
E2F4	L3MBTL2
E2F4	L3MBTL3
E2F4	LAMTOR1
E2F4	LAMTOR3
E2F4	LARP7
E2F4	LARS
E2F4	LAS1L
E2F4	LDHA
E2F4	LEKR1
E2F4	LEO1
E2F4	LETM1
E2F4	LIMK2
E2F4	LIN7C
E2F4	LINC00426
E2F4	LINC00665
E2F4	LINC00954
E2F4	LINC01353
E2F4	LLPH

E2F4	LMAN2
E2F4	LMO7
E2F4	LONP1
E2F4	LRCH1
E2F4	LRPPRC
E2F4	LRRC47
E2F4	LRRC58
E2F4	LRRC59
E2F4	LRRC61
E2F4	LRRFIP1
E2F4	LRRFIP2
E2F4	LSG1
E2F4	LSM1
E2F4	LSM12
E2F4	LSM14A
E2F4	LSM2
E2F4	LSM3
E2F4	LSM5
E2F4	LTV1
E2F4	LUC7L
E2F4	LUC7L2
E2F4	LUC7L3
E2F4	LYN
E2F4	LYPLA2
E2F4	LYRM2
E2F4	LZTFL1
E2F4	M6PR
E2F4	MAFG
E2F4	MAGOH
E2F4	MALSU1
E2F4	MAP1S
E2F4	MAP2K3
E2F4	MAP3K7
E2F4	MAP3K8
E2F4	MAP9
E2F4	MAPK1IP1L
E2F4	MAPK6
E2F4	MAPK7
E2F4	MAPKAP1
E2F4	MAPKAPK3
E2F4	MAPRE1
E2F4	MARS
E2F4	MATK
E2F4	MAU2
E2F4	MBD1
E2F4	MBD5
E2F4	MCFD2
E2F4	MCRIP1
E2F4	MCRIP2
E2F4	MCRS1

E2F4	MCTS1
E2F4	MDH1
E2F4	MDH2
E2F4	ME2
E2F4	MEA1
E2F4	MEAF6
E2F4	MED10
E2F4	MED12
E2F4	MED19
E2F4	MED27
E2F4	MED28
E2F4	MED6
E2F4	MED8
E2F4	METTL13
E2F4	METTL18
E2F4	METTL23
E2F4	METTL2B
E2F4	METTL5
E2F4	MFAP1
E2F4	MFF
E2F4	MFNG
E2F4	MFSD10
E2F4	MGAT3
E2F4	MIA3
E2F4	MICU2
E2F4	MIER1
E2F4	MIF
E2F4	MINOS1
E2F4	MIR155HG
E2F4	MKRN2
E2F4	MKS1
E2F4	MLEC
E2F4	MLF2
E2F4	MLLT1
E2F4	MLYCD
E2F4	MOB4
E2F4	MOCS3
E2F4	MORF4L2
E2F4	MPDU1
E2F4	MPHOSPH10
E2F4	MRPL1
E2F4	MRPL10
E2F4	MRPL12
E2F4	MRPL13
E2F4	MRPL14
E2F4	MRPL16
E2F4	MRPL17
E2F4	MRPL18
E2F4	MRPL20
E2F4	MRPL21

E2F4	MRPL22
E2F4	MRPL23
E2F4	MRPL24
E2F4	MRPL27
E2F4	MRPL28
E2F4	MRPL3
E2F4	MRPL33
E2F4	MRPL39
E2F4	MRPL40
E2F4	MRPL43
E2F4	MRPL44
E2F4	MRPL47
E2F4	MRPL49
E2F4	MRPL51
E2F4	MRPL52
E2F4	MRPL53
E2F4	MRPL54
E2F4	MRPL57
E2F4	MRPS10
E2F4	MRPS12
E2F4	MRPS16
E2F4	MRPS18A
E2F4	MRPS18B
E2F4	MRPS18C
E2F4	MRPS21
E2F4	MRPS22
E2F4	MRPS23
E2F4	MRPS26
E2F4	MRPS30
E2F4	MRPS34
E2F4	MRRF
E2F4	MSANTD2
E2F4	MSN
E2F4	MSTO1
E2F4	MTA3
E2F4	MTCH2
E2F4	MTF1
E2F4	MTG1
E2F4	MTHFD1L
E2F4	MTHFD2L
E2F4	MTIF2
E2F4	MTMR14
E2F4	MTPN
E2F4	MTX2
E2F4	MYADM
E2F4	MYBBP1A
E2F4	MYCBP
E2F4	MYL12B
E2F4	MYO19
E2F4	MYO9B

E2F4	NAA10
E2F4	NAA15
E2F4	NAA20
E2F4	NAA50
E2F4	NAA60
E2F4	NACA
E2F4	NAF1
E2F4	NAGLU
E2F4	NAP1L1
E2F4	NAP1L4
E2F4	NAPA
E2F4	NARF
E2F4	NARS
E2F4	NARS2
E2F4	NAXE
E2F4	NBN
E2F4	NCBP2
E2F4	NCBP3
E2F4	NCK2
E2F4	NCL
E2F4	NCLN
E2F4	NDUFA1
E2F4	NDUFA10
E2F4	NDUFA13
E2F4	NDUFAF6
E2F4	NDUFAF7
E2F4	NDUFB11
E2F4	NDUFB3
E2F4	NDUFB4
E2F4	NDUFB6
E2F4	NDUFB7
E2F4	NDUFB9
E2F4	NDUFS1
E2F4	NDUFS3
E2F4	NDUFS7
E2F4	NDUFS8
E2F4	NDUFV1
E2F4	NEK9
E2F4	NELFE
E2F4	NFATC1
E2F4	NFE2L2
E2F4	NFKB1
E2F4	NFKBIE
E2F4	NFYB
E2F4	NGDN
E2F4	NGRN
E2F4	NHP2
E2F4	NIFK
E2F4	NIP7
E2F4	NIPA2

E2F4	NKAP
E2F4	NME1
E2F4	NME6
E2F4	NOC2L
E2F4	NOC3L
E2F4	NOL10
E2F4	NOL11
E2F4	NOL12
E2F4	NOL7
E2F4	NOL8
E2F4	NOLC1
E2F4	NOP10
E2F4	NOP56
E2F4	NOP58
E2F4	NOP9
E2F4	NOSIP
E2F4	NPM3
E2F4	NPW
E2F4	NR1H2
E2F4	NR2C2AP
E2F4	NR2F6
E2F4	NR4A1
E2F4	NR4A2
E2F4	NRBF2
E2F4	NSA2
E2F4	NSL1
E2F4	NSMCE4A
E2F4	NSUN2
E2F4	NSUN5
E2F4	NT5C
E2F4	NTMT1
E2F4	NUBP1
E2F4	NUBP2
E2F4	NUDC
E2F4	NUDCD2
E2F4	NUDT4
E2F4	NUDT5
E2F4	NUFIP1
E2F4	NUP107
E2F4	NUP153
E2F4	NUP214
E2F4	NUP50
E2F4	NUP54
E2F4	NUP58
E2F4	NUP88
E2F4	NUP93
E2F4	NUS1
E2F4	NUTF2
E2F4	NVL
E2F4	NXT1

E2F4	OARD1
E2F4	OCIAD1
E2F4	ODC1
E2F4	OGFOD1
E2F4	OGFR
E2F4	OLA1
E2F4	OPA1
E2F4	OPA3
E2F4	ORC4
E2F4	ORC5
E2F4	ORMDL1
E2F4	ORMDL2
E2F4	OSBPL11
E2F4	OSBPL2
E2F4	OSGEPL1
E2F4	OSTC
E2F4	OXNAD1
E2F4	PA2G4
E2F4	PABPC1
E2F4	PABPC4
E2F4	PABPN1
E2F4	PACSIN2
E2F4	PAICS
E2F4	PAIP1
E2F4	PAK1IP1
E2F4	PAK2
E2F4	PAN3
E2F4	PANK3
E2F4	PAPOLA
E2F4	PARK7
E2F4	PARL
E2F4	PARP3
E2F4	PARS2
E2F4	PARVB
E2F4	PAWR
E2F4	PCBP1
E2F4	PCBP2
E2F4	PCGF5
E2F4	PCID2
E2F4	PCNP
E2F4	PDAP1
E2F4	PDCD10
E2F4	PDCD11
E2F4	PDCD2
E2F4	PDCD6
E2F4	PDCD6IP
E2F4	PDCD7
E2F4	PDE6B
E2F4	PDIA3
E2F4	PDLIM2

E2F4	PDLIM5
E2F4	PDRG1
E2F4	PDS5A
E2F4	PDSS2
E2F4	PEF1
E2F4	PES1
E2F4	PET100
E2F4	PET117
E2F4	PEX14
E2F4	PEX16
E2F4	PFDN1
E2F4	PFDN2
E2F4	PFDN5
E2F4	PFDN6
E2F4	PFKP
E2F4	PFN1
E2F4	PGAM1
E2F4	PGGT1B
E2F4	PGK1
E2F4	PGM1
E2F4	PGM2
E2F4	PGP
E2F4	PHB2
E2F4	PHF14
E2F4	PHF23
E2F4	PHF5A
E2F4	PHKG2
E2F4	PI4K2A
E2F4	PIGM
E2F4	PIGT
E2F4	PIGW
E2F4	PIK3R4
E2F4	PILRB
E2F4	PIM3
E2F4	PIP5K1B
E2F4	PISD
E2F4	PITPNB
E2F4	PKIG
E2F4	PKM
E2F4	PLAGL1
E2F4	PLEKHJ1
E2F4	PLRG1
E2F4	PMM2
E2F4	PMPCA
E2F4	PMPCB
E2F4	PMS2
E2F4	PNKP
E2F4	PNN
E2F4	PNO1
E2F4	PNP

E2F4	PNPT1
E2F4	POLDIP2
E2F4	POLE3
E2F4	POLG
E2F4	POLR1B
E2F4	POLR1D
E2F4	POLR2B
E2F4	POLR2C
E2F4	POLR2D
E2F4	POLR2F
E2F4	POLR2G
E2F4	POLR2H
E2F4	POLR2K
E2F4	POLR2L
E2F4	POLR2M
E2F4	POLR3C
E2F4	POLR3D
E2F4	POLR3E
E2F4	POLR3H
E2F4	POLRMT
E2F4	POMP
E2F4	POP4
E2F4	POP7
E2F4	POU2F2
E2F4	PPAN
E2F4	PPID
E2F4	PPIE
E2F4	PPIG
E2F4	PPIH
E2F4	PPIL1
E2F4	PPIL3
E2F4	PPIL4
E2F4	PPM1B
E2F4	PPM1G
E2F4	PPP1CB
E2F4	PPP1R11
E2F4	PPP1R12A
E2F4	PPP2CA
E2F4	PPP2CB
E2F4	PPP2R1A
E2F4	PPP2R2A
E2F4	PPP2R5E
E2F4	PPP4C
E2F4	PPP4R3B
E2F4	PPP6R1
E2F4	PRDX1
E2F4	PRELID1
E2F4	PREP
E2F4	PRKAG1
E2F4	PRKAR1A

E2F4	PRKRA
E2F4	PRMT3
E2F4	PRPF19
E2F4	PRPF31
E2F4	PRPF38A
E2F4	PRPF38B
E2F4	PRPF4
E2F4	PRPF40A
E2F4	PRPF4B
E2F4	PRPF6
E2F4	PRPS2
E2F4	PRR13
E2F4	PRRC2C
E2F4	PSAT1
E2F4	PSMA4
E2F4	PSMA5
E2F4	PSMB1
E2F4	PSMB3
E2F4	PSMB4
E2F4	PSMB7
E2F4	PSMC1
E2F4	PSMC2
E2F4	PSMC3
E2F4	PSMC3IP
E2F4	PSMC4
E2F4	PSMC5
E2F4	PSMC6
E2F4	PSMD11
E2F4	PSMD12
E2F4	PSMD13
E2F4	PSMD14
E2F4	PSMD2
E2F4	PSMD4
E2F4	PSMD6
E2F4	PSMD7
E2F4	PSMD8
E2F4	PSMD9
E2F4	PSME1
E2F4	PSME3
E2F4	PSME4
E2F4	PSMF1
E2F4	PSMG3
E2F4	PSMG3-AS1
E2F4	PTBP1
E2F4	PTDSS2
E2F4	PTGES2
E2F4	PTGES3
E2F4	PTMA
E2F4	PTPN2
E2F4	PTPN6

E2F4	PTRH1
E2F4	PTRH2
E2F4	PUF60
E2F4	PUM1
E2F4	PUM3
E2F4	PUS1
E2F4	PVT1
E2F4	PWP1
E2F4	PYCR2
E2F4	RAB14
E2F4	RAB18
E2F4	RAB21
E2F4	RAB5A
E2F4	RAB5C
E2F4	RAB6A
E2F4	RAB7A
E2F4	RAB8A
E2F4	RAB8B
E2F4	RABEPK
E2F4	RABL2B
E2F4	RACK1
E2F4	RAD18
E2F4	RAD23A
E2F4	RAD23B
E2F4	RAD54L2
E2F4	RAE1
E2F4	RALA
E2F4	RAN
E2F4	RANBP1
E2F4	RANGRF
E2F4	RAP1A
E2F4	RAP2C
E2F4	RARS
E2F4	RBBP6
E2F4	RBCK1
E2F4	RBM14
E2F4	RBM17
E2F4	RBM18
E2F4	RBM22
E2F4	RBM23
E2F4	RBM25
E2F4	RBM28
E2F4	RBM3
E2F4	RBM34
E2F4	RBM39
E2F4	RBM42
E2F4	RBM45
E2F4	RBM7
E2F4	RBM8A
E2F4	RCC1

E2F4	RCC1L
E2F4	RCC2
E2F4	RCHY1
E2F4	RCL1
E2F4	RDH14
E2F4	REL
E2F4	RELA
E2F4	RELB
E2F4	REPS1
E2F4	RER1
E2F4	REXO4
E2F4	RHEB
E2F4	RHOG
E2F4	RHOT2
E2F4	RIC8A
E2F4	RIOK1
E2F4	RIOK2
E2F4	RIOK3
E2F4	RLIM
E2F4	RNASEH1
E2F4	RNASEH2B
E2F4	RNF10
E2F4	RNF126
E2F4	RNF146
E2F4	RNF167
E2F4	RNF181
E2F4	RNF20
E2F4	RNF214
E2F4	RNF34
E2F4	RNF41
E2F4	RNF6
E2F4	RNF7
E2F4	RNMT
E2F4	RNPS1
E2F4	ROCK1
E2F4	RPA2
E2F4	RPAP2
E2F4	RPE
E2F4	RPF1
E2F4	RPF2
E2F4	RPIA
E2F4	RPL10
E2F4	RPL10A
E2F4	RPL11
E2F4	RPL12
E2F4	RPL13A
E2F4	RPL15
E2F4	RPL17
E2F4	RPL18A
E2F4	RPL19

E2F4	RPL21
E2F4	RPL22L1
E2F4	RPL23
E2F4	RPL23A
E2F4	RPL26
E2F4	RPL26L1
E2F4	RPL27
E2F4	RPL27A
E2F4	RPL28
E2F4	RPL29
E2F4	RPL3
E2F4	RPL30
E2F4	RPL31
E2F4	RPL32
E2F4	RPL34
E2F4	RPL35
E2F4	RPL36
E2F4	RPL36AL
E2F4	RPL37
E2F4	RPL37A
E2F4	RPL38
E2F4	RPL39
E2F4	RPL4
E2F4	RPL41
E2F4	RPL6
E2F4	RPL7L1
E2F4	RPL8
E2F4	RPLP1
E2F4	RPLP2
E2F4	RPP38
E2F4	RPS10
E2F4	RPS11
E2F4	RPS12
E2F4	RPS13
E2F4	RPS14
E2F4	RPS15
E2F4	RPS16
E2F4	RPS17
E2F4	RPS18
E2F4	RPS19
E2F4	RPS19BP1
E2F4	RPS2
E2F4	RPS20
E2F4	RPS21
E2F4	RPS25
E2F4	RPS26
E2F4	RPS27
E2F4	RPS27A
E2F4	RPS29
E2F4	RPS3

E2F4	RPS3A
E2F4	RPS4X
E2F4	RPS5
E2F4	RPS6
E2F4	RPS6KA1
E2F4	RPS6KB2
E2F4	RPS7
E2F4	RPS8
E2F4	RPSA
E2F4	RPUSD1
E2F4	RRAGA
E2F4	RRP12
E2F4	RRP15
E2F4	RRP7A
E2F4	RRP8
E2F4	RRP9
E2F4	RRS1
E2F4	RSAD1
E2F4	RSL1D1
E2F4	RSL24D1
E2F4	RSRC1
E2F4	RTCB
E2F4	RUVBL1
E2F4	RWDD1
E2F4	RWDD4
E2F4	RXRB
E2F4	SAAL1
E2F4	SAMM50
E2F4	SAP18
E2F4	SAP30L
E2F4	SAR1A
E2F4	SARS
E2F4	SART1
E2F4	SART3
E2F4	SAYSD1
E2F4	SBDS
E2F4	SCAF1
E2F4	SCAF4
E2F4	SCNM1
E2F4	SCO1
E2F4	SCOC
E2F4	SCRN3
E2F4	SDAD1
E2F4	SDCCAG8
E2F4	SDE2
E2F4	SDF4
E2F4	SDHAF1
E2F4	SDHAF2
E2F4	SDHB
E2F4	SDHD

E2F4	SEC11A
E2F4	SEC24C
E2F4	SEC61G
E2F4	SEPHS1
E2F4	SEPT7
E2F4	SERBP1
E2F4	SERPINE2
E2F4	SERTAD1
E2F4	SET
E2F4	SETBP1
E2F4	SETD3
E2F4	SETD4
E2F4	SETD5
E2F4	SETD6
E2F4	SF1
E2F4	SF3A1
E2F4	SF3A2
E2F4	SF3A3
E2F4	SF3B1
E2F4	SF3B2
E2F4	SF3B4
E2F4	SF3B5
E2F4	SF3B6
E2F4	SFSWAP
E2F4	SFXN1
E2F4	SGPL1
E2F4	SGTA
E2F4	SH3GL1
E2F4	SH3GLB1
E2F4	SHMT2
E2F4	SHQ1
E2F4	SIKE1
E2F4	SIL1
E2F4	SIRT6
E2F4	SLC23A2
E2F4	SLC25A11
E2F4	SLC25A17
E2F4	SLC25A3
E2F4	SLC25A37
E2F4	SLC25A38
E2F4	SLC25A39
E2F4	SLC25A46
E2F4	SLC25A5
E2F4	SLC30A6
E2F4	SLC35A3
E2F4	SLC35A4
E2F4	SLC35B2
E2F4	SLC38A1
E2F4	SLC38A5
E2F4	SLC38A6

E2F4	SLC39A1
E2F4	SLC39A14
E2F4	SLC39A3
E2F4	SLC39A6
E2F4	SLC39A7
E2F4	SLC3A2
E2F4	SLC43A3
E2F4	SLC48A1
E2F4	SLC50A1
E2F4	SLC52A2
E2F4	SLC7A1
E2F4	SLC9A3
E2F4	SLIRP
E2F4	SMAD5
E2F4	SMAP2
E2F4	SMARCB1
E2F4	SMARCC1
E2F4	SMARCD1
E2F4	SMARCE1
E2F4	SMC1A
E2F4	SMC5
E2F4	SMG7
E2F4	SMIM15
E2F4	SMIM7
E2F4	SMN1
E2F4	SMNDC1
E2F4	SMS
E2F4	SMU1
E2F4	SNAPC4
E2F4	SNAPC5
E2F4	SND1
E2F4	SNF8
E2F4	SNHG10
E2F4	SNHG19
E2F4	SNRNP40
E2F4	SNRPA
E2F4	SNRPA1
E2F4	SNRPB
E2F4	SNRPB2
E2F4	SNRPD1
E2F4	SNRPD2
E2F4	SNRPE
E2F4	SNRPF
E2F4	SNRPG
E2F4	SNU13
E2F4	SNX11
E2F4	SNX12
E2F4	SNX17
E2F4	SOD2
E2F4	SON

E2F4	SPAG7
E2F4	SPATA5L1
E2F4	SPEN
E2F4	SPG21
E2F4	SPG7
E2F4	SPHK1
E2F4	SPINT1
E2F4	SPTY2D1
E2F4	SQSTM1
E2F4	SRGN
E2F4	SRP14
E2F4	SRP19
E2F4	SRP54
E2F4	SRP72
E2F4	SRPK1
E2F4	SRRM1
E2F4	SRRM2
E2F4	SRRT
E2F4	SRSF1
E2F4	SRSF10
E2F4	SRSF2
E2F4	SRSF4
E2F4	SRSF6
E2F4	SRSF7
E2F4	SRSF9
E2F4	SSB
E2F4	SSBP1
E2F4	SSRP1
E2F4	SSSCA1
E2F4	ST20-AS1
E2F4	STAT2
E2F4	STAT5A
E2F4	STAT6
E2F4	STIP1
E2F4	STK25
E2F4	STK32C
E2F4	STOML2
E2F4	STRAP
E2F4	STUB1
E2F4	STX10
E2F4	STX11
E2F4	STX4
E2F4	SUB1
E2F4	SUCLG1
E2F4	SUCLG2
E2F4	SUGT1
E2F4	SUMO1
E2F4	SUMO2
E2F4	SUPT16H
E2F4	SUPT3H

E2F4	SUPT4H1
E2F4	SUPT5H
E2F4	SURF2
E2F4	SURF6
E2F4	SYAP1
E2F4	SYMPK
E2F4	SYNCRIP
E2F4	SYNJ2
E2F4	SZRD1
E2F4	TAF11
E2F4	TAF12
E2F4	TAF15
E2F4	TAF1D
E2F4	TAF3
E2F4	TAF7
E2F4	TAF9
E2F4	TAGLN2
E2F4	TALDO1
E2F4	TANK
E2F4	TARBP2
E2F4	TARS
E2F4	TARS2
E2F4	TASP1
E2F4	TATDN1
E2F4	TBC1D7
E2F4	TBCA
E2F4	TBL1X
E2F4	TBL3
E2F4	TBPL1
E2F4	TBRG1
E2F4	TBRG4
E2F4	TBXAS1
E2F4	TCERG1
E2F4	TCF25
E2F4	TCOF1
E2F4	TCP1
E2F4	TDRD3
E2F4	TES
E2F4	TEX10
E2F4	TEX261
E2F4	TFB2M
E2F4	TFE3
E2F4	TFG
E2F4	TFRC
E2F4	TGFB1
E2F4	TGS1
E2F4	THAP4
E2F4	THAP5
E2F4	THAP7
E2F4	THOC3

E2F4	THOP1
E2F4	THUMPD2
E2F4	THUMPD3
E2F4	THUMPD3−AS1
E2F4	TIGAR
E2F4	TIMM10
E2F4	TIMM10B
E2F4	TIMM13
E2F4	TIMM17A
E2F4	TIMM21
E2F4	TIMM22
E2F4	TIMM23
E2F4	TIMM8A
E2F4	TIMM8B
E2F4	TIMMDC1
E2F4	TINF2
E2F4	TIPRL
E2F4	TM2D1
E2F4	TM2D2
E2F4	TM9SF3
E2F4	TMA7
E2F4	TMBIM4
E2F4	TMBIM6
E2F4	TMCO1
E2F4	TMED2
E2F4	TMEM109
E2F4	TMEM115
E2F4	TMEM126A
E2F4	TMEM128
E2F4	TMEM138
E2F4	TMEM14B
E2F4	TMEM167A
E2F4	TMEM189
E2F4	TMEM192
E2F4	TMEM199
E2F4	TMEM208
E2F4	TMEM209
E2F4	TMEM216
E2F4	TMEM222
E2F4	TMEM230
E2F4	TMEM248
E2F4	TMEM251
E2F4	TMEM39A
E2F4	TMEM44
E2F4	TMEM50A
E2F4	TMEM60
E2F4	TMEM69
E2F4	TMEM9B
E2F4	TMX1
E2F4	TMX2

E2F4	TNIP1
E2F4	TNIP2
E2F4	TNPO2
E2F4	TNPO3
E2F4	TOB2
E2F4	TOMM20
E2F4	TOMM22
E2F4	TOMM40
E2F4	TOMM40L
E2F4	TOMM5
E2F4	TOMM7
E2F4	TOP1
E2F4	TOP3B
E2F4	TOR1A
E2F4	TOX4
E2F4	TP53
E2F4	TPGS1
E2F4	TPM3
E2F4	TPRKB
E2F4	TPT1
E2F4	TRA2B
E2F4	TRAF3IP2-AS1
E2F4	TRAF4
E2F4	TRAF7
E2F4	TRAPPC3
E2F4	TRAPPC4
E2F4	TRIM27
E2F4	TRIM4
E2F4	TRIM44
E2F4	TRMT1
E2F4	TRMT10C
E2F4	TRMT112
E2F4	TRMT2A
E2F4	TRMT6
E2F4	TRNT1
E2F4	TROVE2
E2F4	TRUB2
E2F4	TSC2
E2F4	TSC22D2
E2F4	TSC22D4
E2F4	TSEN15
E2F4	TSG101
E2F4	TSR1
E2F4	TSSC4
E2F4	TSTA3
E2F4	TTC1
E2F4	TTC27
E2F4	TTC33
E2F4	TTI2
E2F4	TUBA1C

E2F4	TUBE1
E2F4	TUFM
E2F4	TUSC2
E2F4	TWF1
E2F4	TWF2
E2F4	TXN
E2F4	TXN2
E2F4	TXNDC17
E2F4	TXNDC9
E2F4	TXNL1
E2F4	TXNL4B
E2F4	TXNRD1
E2F4	TYSND1
E2F4	U2AF1L5
E2F4	U2AF2
E2F4	U2SURP
E2F4	UBA1
E2F4	UBA2
E2F4	UBA52
E2F4	UBAC1
E2F4	UBAC2
E2F4	UBALD2
E2F4	UBE2B
E2F4	UBE2D2
E2F4	UBE2D3
E2F4	UBE2E3
E2F4	UBE2F
E2F4	UBE2G2
E2F4	UBE2I
E2F4	UBE2J2
E2F4	UBE2K
E2F4	UBE2L3
E2F4	UBE2L6
E2F4	UBE2M
E2F4	UBE2N
E2F4	UBE2Q1
E2F4	UBE2V2
E2F4	UBE3A
E2F4	UBIAD1
E2F4	UBL5
E2F4	UBLCP1
E2F4	UBOX5
E2F4	UBR7
E2F4	UBTF
E2F4	UBXN1
E2F4	UCHL3
E2F4	UCHL5
E2F4	UFC1
E2F4	UFM1
E2F4	UGDH

E2F4	UMPS
E2F4	UNC50
E2F4	UPF2
E2F4	UPF3A
E2F4	UQCC2
E2F4	UQCR11
E2F4	UQCRC2
E2F4	UQCRFS1
E2F4	UQCRQ
E2F4	URM1
E2F4	UROS
E2F4	USB1
E2F4	USE1
E2F4	USP16
E2F4	USP39
E2F4	USP5
E2F4	USP6NL
E2F4	UTP11
E2F4	UTP14A
E2F4	UTP18
E2F4	UTP23
E2F4	UTP4
E2F4	UTP6
E2F4	UXT
E2F4	VAMP8
E2F4	VASP
E2F4	VCP
E2F4	VDAC1
E2F4	VDAC3
E2F4	VEGFA
E2F4	VMA21
E2F4	VMP1
E2F4	VPS18
E2F4	VPS26A
E2F4	VPS4B
E2F4	VPS72
E2F4	VRK1
E2F4	VTA1
E2F4	VTI1A
E2F4	VTI1B
E2F4	VWCE
E2F4	WAPL
E2F4	WARS
E2F4	WDR1
E2F4	WDR12
E2F4	WDR18
E2F4	WDR24
E2F4	WDR25
E2F4	WDR3
E2F4	WDR33

E2F4	WDR4
E2F4	WDR43
E2F4	WDR46
E2F4	WDR5
E2F4	WDR55
E2F4	WDR61
E2F4	WDR70
E2F4	WDR74
E2F4	WDR77
E2F4	WDR83
E2F4	WDR83OS
E2F4	WRAP53
E2F4	WSB2
E2F4	WTAP
E2F4	XPO1
E2F4	XPOT
E2F4	XPR1
E2F4	XRCC5
E2F4	XRN2
E2F4	XXYLT1
E2F4	YARS2
E2F4	YBX1
E2F4	YIPF4
E2F4	YIPF6
E2F4	YRDC
E2F4	YTHDF2
E2F4	YWHAB
E2F4	YWHAE
E2F4	YWHAQ
E2F4	YWHAZ
E2F4	ZBED5
E2F4	ZBTB2
E2F4	ZBTB32
E2F4	ZBTB6
E2F4	ZC3H13
E2F4	ZC3H15
E2F4	ZDHHC3
E2F4	ZDHHC7
E2F4	ZFAND5
E2F4	ZFAS1
E2F4	ZKSCAN5
E2F4	ZMAT2
E2F4	ZMPSTE24
E2F4	ZNF146
E2F4	ZNF165
E2F4	ZNF22
E2F4	ZNF263
E2F4	ZNF281
E2F4	ZNF330
E2F4	ZNF480

E2F4	ZNF485
E2F4	ZNF518A
E2F4	ZNF562
E2F4	ZNF586
E2F4	ZNF593
E2F4	ZNF622
E2F4	ZNF641
E2F4	ZNF644
E2F4	ZNF672
E2F4	ZNF692
E2F4	ZNF706
E2F4	ZNF740
E2F4	ZNF75A
E2F4	ZNF780A
E2F4	ZNF787
E2F4	ZNF814
E2F4	ZNF830
E2F4	ZNHIT2
E2F4	ZNHIT6
E2F4	ZPR1
E2F4	ZRANB2
E2F4	ZSCAN16-AS1
E2F6	ABCF1
E2F6	ADRM1
E2F6	ADSS
E2F6	ALKBH2
E2F6	APBA3
E2F6	APBB2
E2F6	APRT
E2F6	ARF6
E2F6	ARHGEF2
E2F6	ARL6IP5
E2F6	ARMC7
E2F6	ATAD3A
E2F6	ATP1B3
E2F6	ATP2C1
E2F6	ATXN2L
E2F6	B9D1
E2F6	BCAP31
E2F6	BNIP1
E2F6	BOLA2-SMG1P6
E2F6	BZW1
E2F6	C20orf27
E2F6	C22orf34
E2F6	C8orf33
E2F6	CAB39
E2F6	CAMK1
E2F6	CBWD1
E2F6	CBX6
E2F6	CCDC124

E2F6	CCDC15
E2F6	CCDC6
E2F6	CCDC71L
E2F6	CCNG2
E2F6	CCT7
E2F6	CDC123
E2F6	CFAP97
E2F6	CHRNA1
E2F6	CHST11
E2F6	CMC2
E2F6	CMSS1
E2F6	CNOT4
E2F6	COX20
E2F6	CR2
E2F6	CSNK1D
E2F6	CSNK2A1
E2F6	CTDP1
E2F6	CTNNB1
E2F6	CTPS1
E2F6	CUL1
E2F6	CUL2
E2F6	CYCS
E2F6	DCAF16
E2F6	DCTPP1
E2F6	DDX18
E2F6	DDX19A
E2F6	DDX21
E2F6	DDX52
E2F6	DERL3
E2F6	DGAT2
E2F6	DHCR7
E2F6	DHRS3
E2F6	DKC1
E2F6	DNAJC12
E2F6	DNPEP
E2F6	DNTTIP2
E2F6	DOHH
E2F6	DPYSL2
E2F6	DYNLT3
E2F6	E2F6
E2F6	EBNA1BP2
E2F6	EBPL
E2F6	EEF1E1
E2F6	EGLN2
E2F6	EIF2B2
E2F6	EIF2S1
E2F6	EIF3J
E2F6	EIF4A1
E2F6	EIF4G2
E2F6	EMC1

E2F6	EMC10
E2F6	EMG1
E2F6	ENO1
E2F6	ERCC6L2
E2F6	ERI3
E2F6	EXOSC2
E2F6	EXOSC4
E2F6	FABP5
E2F6	FAM104B
E2F6	FAM114A2
E2F6	FAM136A
E2F6	FAM96B
E2F6	FCER2
E2F6	FH
E2F6	GADD45GIP1
E2F6	GAR1
E2F6	GARS
E2F6	GART
E2F6	GAS6
E2F6	GFM1
E2F6	GNL3
E2F6	GPATCH4
E2F6	GRWD1
E2F6	GSPT2
E2F6	H3F3B
E2F6	HGH1
E2F6	HNRNPDL
E2F6	HRAS
E2F6	HSP90AB1
E2F6	HSPA6
E2F6	HSPA8
E2F6	HSPA9
E2F6	HSPBAP1
E2F6	HSPD1
E2F6	HSPE1
E2F6	HYI
E2F6	ID3
E2F6	IFRD1
E2F6	IFRD2
E2F6	IGFBP4
E2F6	IMMP2L
E2F6	INTS10
E2F6	IPO4
E2F6	IRF2BP2
E2F6	ISCA1
E2F6	JAGN1
E2F6	KAT8
E2F6	KDM5B
E2F6	KLF13
E2F6	LARP1B

E2F6	LDHA
E2F6	LINC00667
E2F6	LINC01184
E2F6	LINS1
E2F6	LRRC42
E2F6	LSM12
E2F6	LTV1
E2F6	LY75
E2F6	LY9
E2F6	LYAR
E2F6	MAEA
E2F6	MAFB
E2F6	MALSU1
E2F6	MAP1B
E2F6	MAP4K5
E2F6	MCCC2
E2F6	MDN1
E2F6	MEST
E2F6	METTL13
E2F6	MFSD13A
E2F6	MICU2
E2F6	MIR155HG
E2F6	MRM1
E2F6	MRPL12
E2F6	MRPL14
E2F6	MRPL20
E2F6	MRPL21
E2F6	MRPL3
E2F6	MRPL47
E2F6	MRPS18A
E2F6	MRPS30
E2F6	MRTO4
E2F6	MSTO1
E2F6	MTHFD1L
E2F6	MYC
E2F6	MYO6
E2F6	MYO9A
E2F6	MZF1-AS1
E2F6	NAA20
E2F6	NADK
E2F6	NAXE
E2F6	NCBP2
E2F6	NCL
E2F6	NCLN
E2F6	NDUFAF4
E2F6	NFAT5
E2F6	NFE2L1
E2F6	NFE2L2
E2F6	NFKB1
E2F6	NHP2

E2F6	NIFK
E2F6	NLE1
E2F6	NME1
E2F6	NOC2L
E2F6	NOL9
E2F6	NOLC1
E2F6	NOP58
E2F6	NPM1
E2F6	NPW
E2F6	NR2F6
E2F6	NSF
E2F6	NUDT14
E2F6	NUDT2
E2F6	NUDT5
E2F6	NUP214
E2F6	NUP35
E2F6	NUP88
E2F6	NUP93
E2F6	ODC1
E2F6	OGFOD2
E2F6	ORMDL1
E2F6	PA2G4
E2F6	PABPC4
E2F6	PAK1IP1
E2F6	PALLD
E2F6	PCCB
E2F6	PDCD5
E2F6	PEMT
E2F6	PHB
E2F6	PIBF1
E2F6	PIKFYVE
E2F6	PIP5K1A
E2F6	PLEKHG2
E2F6	POGK
E2F6	POLD2
E2F6	POLR2I
E2F6	POLR2K
E2F6	POLR3C
E2F6	POLR3H
E2F6	PPAN
E2F6	PPFIA1
E2F6	PPP1R14B
E2F6	PRDX1
E2F6	PRPF4
E2F6	PSMA3
E2F6	PSMA5
E2F6	PSMB1
E2F6	PSMB6
E2F6	PSMC3
E2F6	PSMC6

E2F6	PSMD1
E2F6	PSMD14
E2F6	PSMD7
E2F6	PSME3
E2F6	PTPN6
E2F6	PTPRE
E2F6	PUM1
E2F6	PUM3
E2F6	PYCR1
E2F6	PYCR2
E2F6	PYURF
E2F6	RALBP1
E2F6	RAN
E2F6	RARS
E2F6	RBM18
E2F6	RBM28
E2F6	RCN1
E2F6	RDH14
E2F6	REST
E2F6	RHBDD1
E2F6	RIPK2
E2F6	RNF115
E2F6	RNF145
E2F6	RNH1
E2F6	RPL22L1
E2F6	RPL7L1
E2F6	RPRD1A
E2F6	RRP1B
E2F6	RRP9
E2F6	RSAD1
E2F6	RSL1D1
E2F6	RWDD1
E2F6	RWDD2A
E2F6	RXRB
E2F6	SBDS
E2F6	SCAF1
E2F6	SCFD2
E2F6	SCNM1
E2F6	SCO1
E2F6	SDAD1
E2F6	SDHAF1
E2F6	SEL1L
E2F6	SERPINE2
E2F6	SESN2
E2F6	SFSWAP
E2F6	SHQ1
E2F6	SKAP1
E2F6	SLC26A2
E2F6	SLC39A4
E2F6	SLC39A6

E2F6	SLC7A5
E2F6	SLC9B2
E2F6	SLF1
E2F6	SLIRP
E2F6	SLK
E2F6	SMIM12
E2F6	SNAPC5
E2F6	SORD
E2F6	SRGAP2C
E2F6	SRM
E2F6	SRSF1
E2F6	SRSF2
E2F6	SRSF3
E2F6	SSBP1
E2F6	SSSCA1
E2F6	STAT6
E2F6	STIM1
E2F6	STRADB
E2F6	STX16
E2F6	SUPT3H
E2F6	SYMPK
E2F6	TARS
E2F6	TBC1D10B
E2F6	TBPL1
E2F6	TES
E2F6	TET2
E2F6	TFAP4
E2F6	TFB1M
E2F6	TFEC
E2F6	TFPI2
E2F6	THADA
E2F6	THUMPD3
E2F6	TIMM13
E2F6	TIMM23B
E2F6	TIMM8A
E2F6	TM2D3
E2F6	TMEM126A
E2F6	TMEM161B
E2F6	TMEM167B
E2F6	TMEM230
E2F6	TMEM267
E2F6	TMX2
E2F6	TNPO1
E2F6	TOMM40
E2F6	TPP2
E2F6	TSEN2
E2F6	TSPAN4
E2F6	TSR1
E2F6	TSR2
E2F6	TXN

E2F6	TXNDC5
E2F6	TXNL1
E2F6	U2SURP
E2F6	UACA
E2F6	UBA5
E2F6	UBE2D4
E2F6	UBXN2B
E2F6	URB2
E2F6	URI1
E2F6	UTP14A
E2F6	UTP6
E2F6	UVRAG
E2F6	WDR12
E2F6	WDR46
E2F6	WDR74
E2F6	XRCC4
E2F6	XXYLT1
E2F6	YBX1
E2F6	YBX3
E2F6	YWHAG
E2F6	ZBTB17
E2F6	ZHX1
E2F6	ZNF350
E2F6	ZNF460
E2F6	ZNF507
E2F6	ZNF524
E2F6	ZNF669
E2F6	ZNFX1
E2F7	ACSL3
E2F7	ACSL5
E2F7	ACTA2
E2F7	ADSS
E2F7	AEN
E2F7	AGFG1
E2F7	AGL
E2F7	AGPAT2
E2F7	AP1S2
E2F7	AP5M1
E2F7	ASB16-AS1
E2F7	ASF1B
E2F7	ATAD2
E2F7	ATF3
E2F7	ATP2A3
E2F7	B3GLCT
E2F7	B3GNT2
E2F7	BBC3
E2F7	BCL10
E2F7	BCL6
E2F7	BIN1
E2F7	BTBD7

E2F7	BTG3
E2F7	C16orf74
E2F7	C1GALT1
E2F7	C7orf50
E2F7	CAPNS1
E2F7	CARHSP1
E2F7	CARM1
E2F7	CCDC141
E2F7	CCDC22
E2F7	CCNE2
E2F7	CCNG1
E2F7	CCP110
E2F7	CD2AP
E2F7	CD70
E2F7	CDC45
E2F7	CDC6
E2F7	CDCA5
E2F7	CDIP1
E2F7	CDKN1A
E2F7	CDKN2AIP
E2F7	CDKN2C
E2F7	CENPU
E2F7	CHEK2
E2F7	CHFR
E2F7	CIC
E2F7	CLPX
E2F7	CNOT1
E2F7	COQ7
E2F7	CTDSPL2
E2F7	CTNNAL1
E2F7	CTPS1
E2F7	CYC1
E2F7	DAPP1
E2F7	DCP1B
E2F7	DDX20
E2F7	DDX49
E2F7	DEK
E2F7	DHFR
E2F7	DNA2
E2F7	DNAJC21
E2F7	DNAJC9
E2F7	DSCC1
E2F7	DUSP23
E2F7	DUT
E2F7	E2F1
E2F7	E2F7
E2F7	EIF4H
E2F7	EIF5A2
E2F7	EPB41
E2F7	ESCO2

E2F7	FAM111B
E2F7	FAM81A
E2F7	FBXO42
E2F7	FBXO5
E2F7	FDXR
E2F7	FEN1
E2F7	FIGNL1
E2F7	FKBP5
E2F7	FKRP
E2F7	GADD45A
E2F7	GAK
E2F7	GEMIN8
E2F7	GFOD2
E2F7	GLUD1
E2F7	GMPS
E2F7	GPANK1
E2F7	GTF2IRD2
E2F7	GXYLT1
E2F7	HACD2
E2F7	HAUS6
E2F7	HDAC8
E2F7	HDAC9
E2F7	HIP1R
E2F7	HIST1H1B
E2F7	HPCAL1
E2F7	IDH2
E2F7	IFNLR1
E2F7	ING5
E2F7	KDM2B
E2F7	KDM3B
E2F7	KEAP1
E2F7	KRI1
E2F7	KYNU
E2F7	LAPTM5
E2F7	LARP7
E2F7	LIG1
E2F7	LMCD1
E2F7	LMNA
E2F7	LMNB1
E2F7	LRIF1
E2F7	LTN1
E2F7	LZTS2
E2F7	MACROD2
E2F7	MAD1L1
E2F7	MAZ
E2F7	MCM2
E2F7	MCM7
E2F7	MDM2
E2F7	MED28
E2F7	METTL15

E2F7	MFGE8
E2F7	MFSD11
E2F7	MFSD13A
E2F7	MIA3
E2F7	MIDN
E2F7	MIOS
E2F7	MORC4
E2F7	MRPL49
E2F7	MSL1
E2F7	MTHFR
E2F7	MTRF1
E2F7	MTURN
E2F7	MVB12A
E2F7	MYB
E2F7	MYL12B
E2F7	MYNN
E2F7	MYO9B
E2F7	NAA20
E2F7	NCAPD3
E2F7	NCOA4
E2F7	NEMP2
E2F7	NFYB
E2F7	NINJ2
E2F7	NKAP
E2F7	NLRP4
E2F7	NME7
E2F7	NMT1
E2F7	NMU
E2F7	NOL9
E2F7	NOTCH4
E2F7	NR2C2AP
E2F7	NT5DC1
E2F7	NUFIP2
E2F7	ORC1
E2F7	PARP2
E2F7	PBRM1
E2F7	PCGF6
E2F7	PCNA
E2F7	PCNT
E2F7	PDCL
E2F7	PDS5A
E2F7	PHPT1
E2F7	PICALM
E2F7	PKMYT1
E2F7	PLK3
E2F7	PLSCR1
E2F7	PNPLA8
E2F7	POLA1
E2F7	POLD1
E2F7	POR

E2F7	PPDPF
E2F7	PPM1A
E2F7	PPM1M
E2F7	PPP1R8
E2F7	PPP3R1
E2F7	PRC1
E2F7	PRPS2
E2F7	PRR14L
E2F7	PRRC2A
E2F7	PTPRA
E2F7	PUSL1
E2F7	PYGB
E2F7	RABIF
E2F7	RABL6
E2F7	RAD9A
E2F7	RARA
E2F7	RASSF7
E2F7	RBM4B
E2F7	RFC1
E2F7	RFC5
E2F7	RIF1
E2F7	RNMT
E2F7	RNPEPL1
E2F7	RPP30
E2F7	RRP7A
E2F7	RSBN1L
E2F7	RUFY1
E2F7	RXRB
E2F7	SAE1
E2F7	SAFB2
E2F7	SAMD10
E2F7	SARNP
E2F7	SCCPDH
E2F7	SELL
E2F7	SEPT8
E2F7	SH2B1
E2F7	SH3BP1
E2F7	SLC2A5
E2F7	SLC35A5
E2F7	SLC35C2
E2F7	SLC38A9
E2F7	SLC41A1
E2F7	SLC43A2
E2F7	SMAD2
E2F7	SMAD3
E2F7	SMC2
E2F7	SMCO4
E2F7	SNAP29
E2F7	SNRNP35
E2F7	SP1

E2F7	SPPL2A
E2F7	SRPK1
E2F7	SRRT
E2F7	STAMBPL1
E2F7	SZRD1
E2F7	TAF1
E2F7	TANGO2
E2F7	TCAIM
E2F7	TFDP2
E2F7	THRA
E2F7	THUMPD3
E2F7	TLE4
E2F7	TLR10
E2F7	TMCO3
E2F7	TMEM206
E2F7	TMPO
E2F7	TOE1
E2F7	TOPBP1
E2F7	TPGS1
E2F7	TPRA1
E2F7	TRMT11
E2F7	TSPAN14
E2F7	TTC39C
E2F7	TUBB6
E2F7	TYMS
E2F7	UBXN11
E2F7	UMAD1
E2F7	UPF2
E2F7	USP1
E2F7	USP12
E2F7	UTP20
E2F7	VPS29
E2F7	VPS35
E2F7	WDPCP
E2F7	WDR5
E2F7	WDR76
E2F7	XPC
E2F7	XRCC1
E2F7	ZC3H7A
E2F7	ZDHHC24
E2F7	ZNF292
E2F7	ZNF430
E2F7	ZNF638
E2F7	ZNF804A
E2F8	ACAA2
E2F8	ACTG1
E2F8	ACTL6A
E2F8	ACTN4
E2F8	ADCK2
E2F8	ADD3

E2F8	ADNP2
E2F8	AGTPBP1
E2F8	AHI1
E2F8	AK3
E2F8	AK4
E2F8	ANAPC11
E2F8	ANP32B
E2F8	AP1S1
E2F8	AP2S1
E2F8	APEX2
E2F8	APIP
E2F8	APOO
E2F8	APRT
E2F8	ARF6
E2F8	ARGLU1
E2F8	ARHGAP33
E2F8	ARMC1
E2F8	ARNT
E2F8	ASAP1
E2F8	ASF1B
E2F8	ASPSCR1
E2F8	ASRGL1
E2F8	ASTE1
E2F8	ATAD2
E2F8	ATF7IP2
E2F8	ATG10
E2F8	ATL2
E2F8	ATP10D
E2F8	ATP23
E2F8	ATP6V1A
E2F8	AURKB
E2F8	B3GLCT
E2F8	BAZ1B
E2F8	BAZ2A
E2F8	BCLAF1
E2F8	BDH1
E2F8	BID
E2F8	BLOC1S1
E2F8	BRD3
E2F8	BRMS1L
E2F8	BRWD1
E2F8	C11orf24
E2F8	C14orf93
E2F8	C16orf74
E2F8	C19orf48
E2F8	C21orf58
E2F8	C2orf74
E2F8	C4orf46
E2F8	C6orf106
E2F8	C6orf136

E2F8	C9orf16
E2F8	CAB39
E2F8	CACNA1A
E2F8	CADPS
E2F8	CAMTA1
E2F8	CARHSP1
E2F8	CBL
E2F8	CBLB
E2F8	CBLL1
E2F8	CBX2
E2F8	CBX3
E2F8	CBX5
E2F8	CCDC102A
E2F8	CCDC167
E2F8	CCDC171
E2F8	CCDC22
E2F8	CCDC69
E2F8	CCDC82
E2F8	CCHCR1
E2F8	CCNA2
E2F8	CCND3
E2F8	CCP110
E2F8	CD180
E2F8	CDC23
E2F8	CDC25A
E2F8	CDC42SE2
E2F8	CDC45
E2F8	CDC5L
E2F8	CDC6
E2F8	CDC7
E2F8	CDCA2
E2F8	CDCA5
E2F8	CDCA7L
E2F8	CDK1
E2F8	CDKN2A
E2F8	CDKN2AIP
E2F8	CDKN2D
E2F8	CDKN3
E2F8	CDR2
E2F8	CDT1
E2F8	CENPK
E2F8	CENPM
E2F8	CENPN
E2F8	CENPO
E2F8	CENPQ
E2F8	CEP128
E2F8	CEP152
E2F8	CEP295
E2F8	CEP57
E2F8	CEP68

E2F8	CHAF1A
E2F8	CHAF1B
E2F8	CHERP
E2F8	CIITA
E2F8	CIT
E2F8	CKAP2L
E2F8	CKS1B
E2F8	CLSPN
E2F8	CMPK2
E2F8	CNTLN
E2F8	COPS3
E2F8	COX8A
E2F8	CPSF1
E2F8	CPSF7
E2F8	CREBZF
E2F8	CREM
E2F8	CSRP1
E2F8	CTCF
E2F8	CTH
E2F8	CTNNAL1
E2F8	CWC22
E2F8	DDT
E2F8	DDX46
E2F8	DEK
E2F8	DENND4A
E2F8	DHFR
E2F8	DHRS7B
E2F8	DHX15
E2F8	DIAPH3
E2F8	DICER1
E2F8	DLGAP5
E2F8	DLST
E2F8	DMC1
E2F8	DNAJC21
E2F8	DNAJC9
E2F8	DNMT1
E2F8	DOK1
E2F8	DPH2
E2F8	DRG1
E2F8	DTL
E2F8	DUT
E2F8	DYNLL2
E2F8	E2F1
E2F8	E2F2
E2F8	E2F8
E2F8	EBP
E2F8	ECE1
E2F8	EEPD1
E2F8	EIF1AD
E2F8	EIF4A3

E2F8	ELAVL1
E2F8	ENO2
E2F8	ENOSF1
E2F8	ENOX2
E2F8	ESD
E2F8	EXO1
E2F8	FADS3
E2F8	FAM111A
E2F8	FAM111B
E2F8	FAM161A
E2F8	FAM185A
E2F8	FAM189B
E2F8	FAM192A
E2F8	FAM208B
E2F8	FAM76B
E2F8	FAM81A
E2F8	FAM96A
E2F8	FANCA
E2F8	FANCB
E2F8	FANCE
E2F8	FANCG
E2F8	FANCL
E2F8	FBXO22
E2F8	FBXO30
E2F8	FBXO5
E2F8	FH
E2F8	FHIT
E2F8	FKBP5
E2F8	FLOT1
E2F8	FOXM1
E2F8	FPGS
E2F8	FSD1L
E2F8	FUS
E2F8	FUZ
E2F8	GAMT
E2F8	GAPDH
E2F8	GGH
E2F8	GINS3
E2F8	GLO1
E2F8	GMPS
E2F8	GNB2
E2F8	GNB5
E2F8	GPAT2
E2F8	GRPEL2
E2F8	GSK3A
E2F8	GSTO1
E2F8	GTF2H2C
E2F8	GTPBP1
E2F8	H2AFV
E2F8	H2AFX

E2F8	H2AFY
E2F8	H2AFZ
E2F8	H3F3A
E2F8	HAT1
E2F8	HAUS1
E2F8	HAUS7
E2F8	HELLS
E2F8	HIST1H4C
E2F8	HMGA1
E2F8	HMGB1
E2F8	HMGB2
E2F8	HMGB3
E2F8	HMGN1
E2F8	HMGN2
E2F8	HMGXB4
E2F8	HNRNPA0
E2F8	HNRNPA3
E2F8	HNRNPAB
E2F8	HNRNPF
E2F8	HNRNPH1
E2F8	HNRNPR
E2F8	HNRNPUL1
E2F8	HOXA7
E2F8	HSPA4
E2F8	HSPB11
E2F8	ICMT
E2F8	IFI16
E2F8	ING1
E2F8	INTS4
E2F8	IPO9
E2F8	IRF8
E2F8	ITGB1BP1
E2F8	ITPKA
E2F8	JAK1
E2F8	JUP
E2F8	KATNAL1
E2F8	KCTD18
E2F8	KDM1A
E2F8	KEAP1
E2F8	KIF15
E2F8	KIF2A
E2F8	KIFC1
E2F8	KLF2
E2F8	KLHL14
E2F8	KLHL5
E2F8	KNTC1
E2F8	KPNB1
E2F8	LAGE3
E2F8	LAMP2
E2F8	LDB1

E2F8	LDHB
E2F8	LEMD3
E2F8	LIG1
E2F8	LIMA1
E2F8	LIN52
E2F8	LINC00487
E2F8	LMNB1
E2F8	LMNB2
E2F8	LNX2
E2F8	LPCAT3
E2F8	LRR1
E2F8	LRRC28
E2F8	LRRC59
E2F8	LSM4
E2F8	LUC7L2
E2F8	LYRM7
E2F8	MAD2L1
E2F8	MAP2
E2F8	MAP3K12
E2F8	MAP3K7
E2F8	MAPK8
E2F8	MAPK9
E2F8	MASTL
E2F8	MBD1
E2F8	MBD3
E2F8	MCM3
E2F8	MCM5
E2F8	MCM7
E2F8	MCM8
E2F8	MCUR1
E2F8	MDM1
E2F8	MDP1
E2F8	MED25
E2F8	MED27
E2F8	MELK
E2F8	METTL21A
E2F8	MFGE8
E2F8	MID1IP1
E2F8	MIER2
E2F8	MKI67
E2F8	MLH1
E2F8	MMS22L
E2F8	MROH1
E2F8	MRPL35
E2F8	MRPL49
E2F8	MRPL57
E2F8	MSMO1
E2F8	MTERF3
E2F8	MTFR2
E2F8	MTHFD1

E2F8	MXD3
E2F8	MYBL1
E2F8	MYBL2
E2F8	N4BP2
E2F8	NAA16
E2F8	NAB1
E2F8	NAF1
E2F8	NAIF1
E2F8	NAP1L1
E2F8	NAPEPLD
E2F8	NASP
E2F8	NBN
E2F8	NCAPD3
E2F8	NCAPG
E2F8	NDUFA6
E2F8	NDUFAF6
E2F8	NECTIN1
E2F8	NFKBIL1
E2F8	NONO
E2F8	NT5DC1
E2F8	NUDT1
E2F8	NUDT21
E2F8	NUP133
E2F8	NUP155
E2F8	NUP205
E2F8	NUP43
E2F8	NUP85
E2F8	NUSAP1
E2F8	NUTF2
E2F8	NVL
E2F8	OGFRL1
E2F8	OSBPL3
E2F8	OTUD5
E2F8	OTUD6B
E2F8	PABPN1
E2F8	PAG1
E2F8	PAGR1
E2F8	PANK4
E2F8	PAQR4
E2F8	PARP2
E2F8	PARP6
E2F8	PATL1
E2F8	PAX5
E2F8	PCIF1
E2F8	PCM1
E2F8	PCMTD1
E2F8	PCNA
E2F8	PDS5B
E2F8	PET117
E2F8	PFN1

E2F8	PGM1
E2F8	PHF14
E2F8	PHIP
E2F8	PIN1
E2F8	PJA2
E2F8	PKMYT1
E2F8	PLK4
E2F8	PMF1
E2F8	POLA1
E2F8	POLD3
E2F8	POP7
E2F8	POT1
E2F8	PPIA
E2F8	PPIG
E2F8	PPM1G
E2F8	PPP1R11
E2F8	PPP1R18
E2F8	PPP2R5D
E2F8	PRAF2
E2F8	PRIM2
E2F8	PRKACB
E2F8	PRKCA
E2F8	PRKCD
E2F8	PSMA6
E2F8	PSMC3
E2F8	PSRC1
E2F8	PTBP1
E2F8	PTMA
E2F8	RAB27A
E2F8	RAB8A
E2F8	RAB8B
E2F8	RAD1
E2F8	RAD23A
E2F8	RAD51C
E2F8	RAD54L
E2F8	RAF1
E2F8	RANBP1
E2F8	RANBP3
E2F8	RASSF7
E2F8	RBBP4
E2F8	RBBP7
E2F8	RBM15
E2F8	RC3H2
E2F8	RCC2
E2F8	RCCD1
E2F8	RECQL4
E2F8	RECQL5
E2F8	REV1
E2F8	REV3L
E2F8	RFC1

E2F8	RFC2
E2F8	RFWD3
E2F8	RHBDD1
E2F8	RHOF
E2F8	RNASEH2A
E2F8	RNF138
E2F8	RNF34
E2F8	RPA2
E2F8	RPA3
E2F8	RPE
E2F8	RPP30
E2F8	RPS6KA5
E2F8	RREB1
E2F8	RRM2
E2F8	RSBN1
E2F8	RTKN2
E2F8	RUFY1
E2F8	RYBP
E2F8	SAC3D1
E2F8	SAE1
E2F8	SAP130
E2F8	SCARB1
E2F8	SCCPDH
E2F8	SCYL3
E2F8	SEPHS1
E2F8	SEPT11
E2F8	SERTAD2
E2F8	SET
E2F8	SETD6
E2F8	SF3A2
E2F8	SGO1
E2F8	SH3GLB2
E2F8	SHMT1
E2F8	SIVA1
E2F8	SLBP
E2F8	SLC16A1-AS1
E2F8	SLC1A4
E2F8	SLC25A12
E2F8	SLC25A19
E2F8	SLC29A1
E2F8	SLC35A3
E2F8	SLC35G1
E2F8	SLC39A13
E2F8	SLC43A3
E2F8	SLC44A1
E2F8	SLC6A16
E2F8	SLC7A5
E2F8	SLF2
E2F8	SLFN13
E2F8	SMAD2

E2F8	SMARCA4
E2F8	SMARCC2
E2F8	SMC1A
E2F8	SMC2
E2F8	SMC3
E2F8	SMC4
E2F8	SMIM13
E2F8	SNED1
E2F8	SNRNP70
E2F8	SNRPB
E2F8	SNRPG
E2F8	SNTB2
E2F8	SP3
E2F8	SPATA2L
E2F8	SPC24
E2F8	SPECC1
E2F8	SPEN
E2F8	SPIN1
E2F8	SPSB3
E2F8	SQLE
E2F8	SREBF2
E2F8	SRRM1
E2F8	SRSF10
E2F8	SRSF2
E2F8	SRSF4
E2F8	SRSF7
E2F8	SSBP3
E2F8	SSRP1
E2F8	SSX2IP
E2F8	STAU2
E2F8	STK24
E2F8	STMN1
E2F8	STRBP
E2F8	STRN
E2F8	STT3B
E2F8	SUGP2
E2F8	SUMO1
E2F8	SUMO2
E2F8	SUZ12
E2F8	SVIP
E2F8	SYCE2
E2F8	SYNE2
E2F8	TACC3
E2F8	TAF12
E2F8	TBCA
E2F8	TCERG1
E2F8	TCF3
E2F8	TELO2
E2F8	TEX264
E2F8	TEX30

E2F8	TFDP1
E2F8	TFPT
E2F8	TFRC
E2F8	THAP7
E2F8	TIA1
E2F8	TIMELESS
E2F8	TK1
E2F8	TMEM107
E2F8	TMEM11
E2F8	TMEM161B
E2F8	TMEM91
E2F8	TMUB1
E2F8	TNPO1
E2F8	TOP2A
E2F8	TOPBP1
E2F8	TOPORS
E2F8	TOR2A
E2F8	TP53I13
E2F8	TPGS2
E2F8	TPI1
E2F8	TRA2B
E2F8	TRAF5
E2F8	TRIM41
E2F8	TTC39C
E2F8	TTC7A
E2F8	TTC9C
E2F8	TTN
E2F8	TUBA1B
E2F8	TUBB
E2F8	TUBGCP4
E2F8	TUBGCP5
E2F8	TWF2
E2F8	TYMS
E2F8	UBAC1
E2F8	UBE2E3
E2F8	UBE2I
E2F8	UBE2O
E2F8	UBE2V1
E2F8	UBE3A
E2F8	UBR7
E2F8	UBXN2B
E2F8	UGT8
E2F8	UHRF1
E2F8	UHRF2
E2F8	UPF3B
E2F8	UQCRC1
E2F8	URI1
E2F8	USP1
E2F8	USP28
E2F8	USP4

E2F8	UTY
E2F8	VIPAS39
E2F8	VMA21
E2F8	VPS29
E2F8	WDR34
E2F8	WDR5
E2F8	WDR70
E2F8	WDR90
E2F8	WRAP53
E2F8	XKR6
E2F8	XPO1
E2F8	XRCC5
E2F8	YEATS4
E2F8	YWHAH
E2F8	YWHAQ
E2F8	ZBTB45
E2F8	ZC3H12D
E2F8	ZCCHC17
E2F8	ZFP64
E2F8	ZFP91
E2F8	ZGRF1
E2F8	ZNF133
E2F8	ZNF200
E2F8	ZNF395
E2F8	ZNF502
E2F8	ZNF598
E2F8	ZW10
E4F1	AK4
E4F1	E4F1
EGR1	ABCA7
EGR1	ABCB4
EGR1	ABCB8
EGR1	ABHD17B
EGR1	ABR
EGR1	ABRACL
EGR1	ABT1
EGR1	ACTR6
EGR1	ADA
EGR1	AEN
EGR1	AFTPH
EGR1	AGO1
EGR1	AGPAT5
EGR1	ALCAM
EGR1	ALDOC
EGR1	ANKMY1
EGR1	ANXA6
EGR1	AP3S1
EGR1	ARHGAP33
EGR1	ARHGAP44
EGR1	ARHGEF12

EGR1	ARHGEF7
EGR1	ATG4A
EGR1	ATXN1L
EGR1	B3GALNT1
EGR1	B4GALT3
EGR1	BBIP1
EGR1	BCOR
EGR1	BIK
EGR1	BLNK
EGR1	BLVRA
EGR1	BMPR1A
EGR1	BRPF1
EGR1	BTG2
EGR1	BUB3
EGR1	C18orf21
EGR1	C1orf174
EGR1	C3orf14
EGR1	C6orf47
EGR1	CAMK1
EGR1	CAND1
EGR1	CASC4
EGR1	CCAR1
EGR1	CCDC130
EGR1	CCDC171
EGR1	CCDC82
EGR1	CCDC97
EGR1	CCL3
EGR1	CCNB1IP1
EGR1	CCNL1
EGR1	CCNL2
EGR1	CCNYL1
EGR1	CD69
EGR1	CD70
EGR1	CD72
EGR1	CD9
EGR1	CENPQ
EGR1	CERS2
EGR1	CHCHD2
EGR1	CHD4
EGR1	CHD6
EGR1	CMC2
EGR1	CNIH1
EGR1	CNOT11
EGR1	CNOT2
EGR1	COG7
EGR1	COQ9
EGR1	CPEB4
EGR1	CREBRF
EGR1	CREG1
EGR1	CTNNA1

EGR1	CUL2
EGR1	CUTC
EGR1	CWC22
EGR1	CXCR4
EGR1	CYB5R1
EGR1	CYFIP2
EGR1	DCAF16
EGR1	DCLRE1B
EGR1	DDHD1
EGR1	DDI2
EGR1	DDIT3
EGR1	DDX50
EGR1	DEF6
EGR1	DENND4B
EGR1	DGKA
EGR1	DHX37
EGR1	DMWD
EGR1	DNAJC10
EGR1	DRAM2
EGR1	DUSP1
EGR1	DUSP14
EGR1	DUSP2
EGR1	DYM
EGR1	EGR1
EGR1	EGR2
EGR1	EGR3
EGR1	EHD4
EGR1	EIF2AK2
EGR1	EIF3C
EGR1	EIF4G2
EGR1	EIF5A
EGR1	ELMSAN1
EGR1	ENTPD4
EGR1	ETV5
EGR1	F2RL3
EGR1	FAM3C
EGR1	FAM98B
EGR1	FBXO33
EGR1	FBXW11
EGR1	FCRL5
EGR1	FGFR1
EGR1	FGFR1OP
EGR1	FLCN
EGR1	FOS
EGR1	FOSB
EGR1	FUBP1
EGR1	FXR2
EGR1	GABPB1
EGR1	GCAT
EGR1	GGCX

EGR1	GLRX
EGR1	GLYCTK
EGR1	GMFB
EGR1	GNL1
EGR1	GPCPD1
EGR1	GPER1
EGR1	GPN1
EGR1	GRINA
EGR1	GRN
EGR1	GSS
EGR1	GSTM4
EGR1	GYPC
EGR1	HCFC2
EGR1	HDAC10
EGR1	HDAC7
EGR1	HEATR1
EGR1	HEBP2
EGR1	HERPUD2
EGR1	HES1
EGR1	HEXIM1
EGR1	HIF1AN
EGR1	HIST1H4B
EGR1	HLTF
EGR1	HNRNPH1
EGR1	HYOU1
EGR1	ID2
EGR1	IDH3A
EGR1	IER2
EGR1	IER5
EGR1	IFI30
EGR1	IFI35
EGR1	IFIT2
EGR1	IFT20
EGR1	IGSF8
EGR1	IL16
EGR1	INSIG1
EGR1	INTS5
EGR1	JMJD1C
EGR1	JRKL
EGR1	JUN
EGR1	JUNB
EGR1	JUND
EGR1	KANK1
EGR1	KCNMA1
EGR1	KCTD6
EGR1	KDM4B
EGR1	KDM5B
EGR1	KIAA1841
EGR1	KIAA2026
EGR1	KIF14

EGR1	KIN
EGR1	KLF10
EGR1	KLHL14
EGR1	KLHL18
EGR1	KMO
EGR1	KMT2E-AS1
EGR1	KTI12
EGR1	LASP1
EGR1	LCK
EGR1	LCOR
EGR1	LETM1
EGR1	LGALS1
EGR1	LIMS1
EGR1	LIPE
EGR1	LMBRD1
EGR1	LMCD1
EGR1	LRIF1
EGR1	LRMP
EGR1	LTA
EGR1	MAP1LC3B
EGR1	MAP2K1
EGR1	MAP2K4
EGR1	MAP3K7
EGR1	MAP4
EGR1	MAPKAP1
EGR1	MAPKAPK2
EGR1	MARCH6
EGR1	MARCKSL1
EGR1	MBD3
EGR1	MCL1
EGR1	MDFIC
EGR1	MEF2C
EGR1	MEF2D
EGR1	MFGE8
EGR1	MIATNB
EGR1	MICAL1
EGR1	MICU1
EGR1	MIDN
EGR1	MIER2
EGR1	MIIP
EGR1	MIR22HG
EGR1	MIR3142HG
EGR1	MOCS2
EGR1	MORC4
EGR1	MPG
EGR1	MR1
EGR1	MROH1
EGR1	MS4A1
EGR1	MS4A7
EGR1	MSL1

EGR1	MSRA
EGR1	MTMR14
EGR1	MVD
EGR1	MYB
EGR1	MYO6
EGR1	NAA35
EGR1	NAB2
EGR1	NADK2
EGR1	NCOA5
EGR1	NDEL1
EGR1	NEIL2
EGR1	NEIL3
EGR1	NFATC1
EGR1	NFKBIA
EGR1	NFKBID
EGR1	NFYC
EGR1	NIN
EGR1	NMD3
EGR1	NMU
EGR1	NORAD
EGR1	NR4A1
EGR1	NR4A2
EGR1	NSRP1
EGR1	NTMT1
EGR1	NUBP1
EGR1	NUP93
EGR1	OAT
EGR1	OPTN
EGR1	ORAI2
EGR1	OSBPL10
EGR1	OSBPL9
EGR1	OSTF1
EGR1	OSTM1
EGR1	OTUD1
EGR1	OTUD5
EGR1	P2RY10
EGR1	PAAF1
EGR1	PAFAH1B3
EGR1	PATL1
EGR1	PCSK7
EGR1	PDCL3
EGR1	PDE9A
EGR1	PDIA5
EGR1	PEX2
EGR1	PFKL
EGR1	PGRMC2
EGR1	PHACTR1
EGR1	PHF19
EGR1	PHF6
EGR1	PHLDB3

EGR1	PIAS1
EGR1	PIGA
EGR1	PIGS
EGR1	PIK3CA
EGR1	PITPNB
EGR1	PKIA
EGR1	PMM2
EGR1	PMPCA
EGR1	PNP
EGR1	POLG
EGR1	POLR3E
EGR1	POMGNT1
EGR1	POP1
EGR1	PPID
EGR1	PPP1R14A
EGR1	PPP1R15A
EGR1	PPP1R16B
EGR1	PPP1R2
EGR1	PRKCD
EGR1	PRKRIP1
EGR1	PRPF39
EGR1	PRPS2
EGR1	PSMD2
EGR1	PTP4A1
EGR1	PTPN7
EGR1	PTPRC
EGR1	PTPRJ
EGR1	PYURF
EGR1	QARS
EGR1	QPCT
EGR1	RAB5B
EGR1	RABGAP1
EGR1	RANGAP1
EGR1	RASAL3
EGR1	RASGRF1
EGR1	RASL11A
EGR1	RASSF6
EGR1	RBM42
EGR1	RCAN3
EGR1	RCBTB2
EGR1	RGS13
EGR1	RGS16
EGR1	RGS20
EGR1	RHOQ
EGR1	RMDN3
EGR1	RND1
EGR1	RNF144B
EGR1	RNF20
EGR1	RNPEPL1
EGR1	RPL18A

EGR1	RREB1
EGR1	RSRC2
EGR1	RSU1
EGR1	RUSC1
EGR1	S100A11
EGR1	SACM1L
EGR1	SARS2
EGR1	SBDS
EGR1	SCAMP1
EGR1	SCYL3
EGR1	SDCBP
EGR1	SDR39U1
EGR1	SEC23A
EGR1	SEC24D
EGR1	SERINC3
EGR1	SET
EGR1	SGK1
EGR1	SH3RF1
EGR1	SIN3A
EGR1	SLC16A1
EGR1	SLC25A17
EGR1	SLC25A28
EGR1	SLC25A39
EGR1	SLC25A4
EGR1	SLC35C2
EGR1	SLC35F5
EGR1	SLC37A1
EGR1	SLC9A3R1
EGR1	SLCO4A1
EGR1	SMARCC1
EGR1	SMIM12
EGR1	SNAPIN
EGR1	SNX1
EGR1	SNX9
EGR1	SORL1
EGR1	SP1
EGR1	SP140L
EGR1	SPN
EGR1	SRGAP2
EGR1	STEAP1B
EGR1	STRN4
EGR1	STT3A
EGR1	STX6
EGR1	TACC1
EGR1	TAGAP
EGR1	TBC1D1
EGR1	TBRG1
EGR1	TCF4
EGR1	TEC
EGR1	TERF2IP

EGR1	TFG
EGR1	THAP12
EGR1	THEM4
EGR1	TIGAR
EGR1	TIMM10B
EGR1	TINF2
EGR1	TM2D3
EGR1	TMED8
EGR1	TMEM38A
EGR1	TMEM39A
EGR1	TMEM68
EGR1	TNF
EGR1	TNPO3
EGR1	TOR1AIP1
EGR1	TPGS1
EGR1	TRADD
EGR1	TRAF4
EGR1	TRIM41
EGR1	TRIM61
EGR1	TRIP11
EGR1	TRPC4AP
EGR1	TSG101
EGR1	TSPO
EGR1	TSPYL2
EGR1	TTC14
EGR1	TVP23B
EGR1	UBALD2
EGR1	UBL7-AS1
EGR1	UCHL5
EGR1	UFM1
EGR1	USP42
EGR1	USP44
EGR1	USP5
EGR1	VAV1
EGR1	VEZT
EGR1	VPS72
EGR1	WFS1
EGR1	WSB1
EGR1	WWP1
EGR1	XPC
EGR1	YAE1D1
EGR1	YIPF3
EGR1	ZBTB22
EGR1	ZFAND1
EGR1	ZFP36
EGR1	ZFP36L1
EGR1	ZFPL1
EGR1	ZFYVE16
EGR1	ZMYM2
EGR1	ZNF181

EGR1	ZNF24
EGR1	ZNF296
EGR1	ZNF485
EGR1	ZNF608
EGR1	ZRSR2
EGR1	ZSCAN32
EGR2	ABRACL
EGR2	ALDOC
EGR2	ARRDC2
EGR2	ASNA1
EGR2	ATP13A1
EGR2	BICDL1
EGR2	BIK
EGR2	BZW1
EGR2	CCDC88A
EGR2	CCND2
EGR2	CD83
EGR2	CELF2
EGR2	CERCAM
EGR2	CHD4
EGR2	CREG1
EGR2	CYFIP2
EGR2	DMWD
EGR2	DNAJC5
EGR2	DUSP10
EGR2	DUSP14
EGR2	DUSP2
EGR2	EGR1
EGR2	EGR2
EGR2	EGR3
EGR2	ETV5
EGR2	FAM3C
EGR2	FHOD1
EGR2	FLI1
EGR2	FYN
EGR2	GRINA
EGR2	GSN
EGR2	HES1
EGR2	HOMER2
EGR2	HPCAL1
EGR2	HYOU1
EGR2	IER2
EGR2	IGSF8
EGR2	ING2
EGR2	IP6K2
EGR2	IQSEC1
EGR2	JADE1
EGR2	JUNB
EGR2	KDM6B
EGR2	LGALS1

EGR2	LINC01003
EGR2	LITAF
EGR2	LRMP
EGR2	MAPK8
EGR2	MEA1
EGR2	MEF2C
EGR2	MICAL1
EGR2	MICU1
EGR2	MIR22HG
EGR2	MYC
EGR2	NAB2
EGR2	NAMPT
EGR2	NFATC1
EGR2	NFE2L2
EGR2	NFKBIA
EGR2	NFKBID
EGR2	NIN
EGR2	NR4A1
EGR2	NR4A2
EGR2	NR4A3
EGR2	NTMT1
EGR2	ORC4
EGR2	OTUD1
EGR2	PELI1
EGR2	PEX2
EGR2	PGRMC2
EGR2	PHACTR1
EGR2	PHF19
EGR2	PKIA
EGR2	PLK3
EGR2	PPP1CC
EGR2	PPP1R15A
EGR2	PPP1R2
EGR2	PPP2R2A
EGR2	PTGER4
EGR2	PTPN7
EGR2	RAP1A
EGR2	RASSF2
EGR2	REL
EGR2	SCPEP1
EGR2	SERTAD3
EGR2	SGK1
EGR2	SIPA1
EGR2	SLC16A3
EGR2	SLC25A39
EGR2	SLC37A1
EGR2	SP1
EGR2	SPATA2L
EGR2	STX6
EGR2	TNF

EGR2	TNFAIP3
EGR2	TNFAIP8
EGR2	TNPO3
EGR2	TP53BP1
EGR2	TRAF4
EGR2	TRIP10
EGR2	TSPAN17
EGR2	TVP23B
EGR2	UBIAD1
EGR2	VAT1
EGR2	VAV1
EGR2	WFS1
EGR2	WSB2
EGR2	WWP1
EGR2	ZBTB22
EGR2	ZC3H12A
EGR2	ZFP36
EGR2	ZFP36L1
EGR2	ZFPL1
EGR3	ABRACL
EGR3	ACACA
EGR3	ADAM17
EGR3	ADNP2
EGR3	AGPAT2
EGR3	AGPAT5
EGR3	ARHGEF2
EGR3	ATP6V0A1
EGR3	ATP6V1C1
EGR3	ATXN7L3
EGR3	BAG4
EGR3	BCL2A1
EGR3	BTG2
EGR3	C12orf57
EGR3	CAMK2B
EGR3	CBWD5
EGR3	CCDC107
EGR3	CD274
EGR3	CD83
EGR3	CEP162
EGR3	CEP170
EGR3	CLSTN1
EGR3	CNR2
EGR3	COQ7
EGR3	CREBRF
EGR3	CSF2RB
EGR3	DCP1A
EGR3	DDAH2
EGR3	DLGAP4
EGR3	DUSP10
EGR3	DUSP2

EGR3	EGR1
EGR3	EGR2
EGR3	EGR3
EGR3	ERF
EGR3	ETHE1
EGR3	EVI2A
EGR3	FAM3C
EGR3	FEZ2
EGR3	GRAMD1A
EGR3	HES1
EGR3	HIVEP3
EGR3	HOMER2
EGR3	HOPX
EGR3	HVCN1
EGR3	ICAM1
EGR3	IER2
EGR3	IL21R
EGR3	IL2RG
EGR3	IL6
EGR3	JARID2
EGR3	KLF10
EGR3	LDLR
EGR3	LINC01136
EGR3	LTA
EGR3	MAPK6
EGR3	MCRIP2
EGR3	MEF2C
EGR3	MFN1
EGR3	MIEF1
EGR3	MUT
EGR3	MYC
EGR3	NAB2
EGR3	NCAPG2
EGR3	NFATC1
EGR3	NFE2L2
EGR3	NFKBIA
EGR3	NFKBID
EGR3	NFKBIZ
EGR3	NIN
EGR3	NIPSNAP1
EGR3	NR3C1
EGR3	NR4A1
EGR3	NR4A2
EGR3	NRDE2
EGR3	OLA1
EGR3	OMA1
EGR3	PACSIN2
EGR3	PDCD11
EGR3	PDPK1
EGR3	PHACTR1

EGR3	PIM3
EGR3	PKMYT1
EGR3	PLK3
EGR3	PNMA1
EGR3	PNPLA8
EGR3	POGK
EGR3	PPP1R15A
EGR3	PPP1R2
EGR3	PPP3CA
EGR3	PPTC7
EGR3	PRAF2
EGR3	PRDM1
EGR3	PRDX1
EGR3	PTGER4
EGR3	PTPN12
EGR3	PTPN6
EGR3	PTPRJ
EGR3	QRICH1
EGR3	RASGEF1B
EGR3	RBFA
EGR3	REL
EGR3	RILPL2
EGR3	RIN3
EGR3	RUFY3
EGR3	SBDS
EGR3	SDCBP
EGR3	SEMA7A
EGR3	SIAH2
EGR3	SLC39A10
EGR3	SNX12
EGR3	SNX8
EGR3	SPATA2L
EGR3	SPG21
EGR3	SS18L1
EGR3	STK4
EGR3	STUM
EGR3	TBP
EGR3	TEX10
EGR3	TLK1
EGR3	TMC8
EGR3	TNFAIP3
EGR3	TOMM5
EGR3	TONSL
EGR3	TRAF4
EGR3	TVP23B
EGR3	USP32
EGR3	VAMP4
EGR3	YPEL1
EGR3	ZC3H12A
EGR3	ZC3H7B

EGR3	ZFP36L1
EGR3	ZNF181
EGR3	ZNF273
EGR3	ZNF586
ELF1	A4GALT
ELF1	AAGAB
ELF1	AAMDC
ELF1	AAMP
ELF1	AAR2
ELF1	AASDH
ELF1	AASDHPPT
ELF1	AATF
ELF1	ABCB7
ELF1	ABCD3
ELF1	ABCF1
ELF1	ABHD10
ELF1	ABHD13
ELF1	ABHD14B
ELF1	ABHD2
ELF1	ABHD3
ELF1	ABHD5
ELF1	ABI1
ELF1	ABI3
ELF1	ABL1
ELF1	ABRACL
ELF1	ABT1
ELF1	ABTB1
ELF1	ACAA1
ELF1	ACAA2
ELF1	ACAD9
ELF1	ACADS
ELF1	ACAP1
ELF1	ACAP2
ELF1	ACAT2
ELF1	ACBD3
ELF1	ACBD5
ELF1	ACBD6
ELF1	ACD
ELF1	ACIN1
ELF1	ACLY
ELF1	ACO2
ELF1	ACOT7
ELF1	ACOT8
ELF1	ACP1
ELF1	ACSF2
ELF1	ACSL3
ELF1	ACSL5
ELF1	ACSS1
ELF1	ACTA2
ELF1	ACTB

ELF1	ACTG1
ELF1	ACTL6A
ELF1	ACTN4
ELF1	ACTR1A
ELF1	ACTR1B
ELF1	ACTR2
ELF1	ACTR3
ELF1	ACTR6
ELF1	ACTR8
ELF1	ACY3
ELF1	ACYP1
ELF1	ACYP2
ELF1	ADAM10
ELF1	ADAM17
ELF1	ADAM19
ELF1	ADAM23
ELF1	ADAR
ELF1	ADARB1
ELF1	ADCK5
ELF1	ADD1
ELF1	ADD3
ELF1	ADGRG5
ELF1	ADH5
ELF1	ADI1
ELF1	ADIPOR1
ELF1	ADO
ELF1	ADPRHL2
ELF1	ADPRM
ELF1	ADSL
ELF1	ADSS
ELF1	AEBP2
ELF1	AES
ELF1	AFAP1L2
ELF1	AFF3
ELF1	AFF4
ELF1	AFTPH
ELF1	AGBL5
ELF1	AGFG1
ELF1	AGFG2
ELF1	AGL
ELF1	AGO1
ELF1	AGO2
ELF1	AGPAT2
ELF1	AGPS
ELF1	AGTPBP1
ELF1	AGTRAP
ELF1	AHCTF1
ELF1	AHCYL1
ELF1	AHI1
ELF1	AHSA1

ELF1	AIF1
ELF1	AIFM1
ELF1	AIG1
ELF1	AIM2
ELF1	AIMP1
ELF1	AIP
ELF1	AK3
ELF1	AK6
ELF1	AKAP10
ELF1	AKAP13
ELF1	AKAP8
ELF1	AKAP8L
ELF1	AKAP9
ELF1	AKIRIN1
ELF1	AKIRIN2
ELF1	AKNA
ELF1	AKR1B1
ELF1	AKR7A2
ELF1	AKT2
ELF1	ALAD
ELF1	ALCAM
ELF1	ALDH16A1
ELF1	ALDH18A1
ELF1	ALDH2
ELF1	ALDH5A1
ELF1	ALDH7A1
ELF1	ALDH9A1
ELF1	ALG6
ELF1	ALG8
ELF1	ALKBH4
ELF1	ALKBH5
ELF1	ALKBH7
ELF1	ALOX5
ELF1	ALOX5AP
ELF1	ALPK1
ELF1	AMFR
ELF1	AMZ2
ELF1	ANAPC10
ELF1	ANAPC11
ELF1	ANAPC13
ELF1	ANAPC15
ELF1	ANAPC16
ELF1	ANAPC2
ELF1	ANAPC4
ELF1	ANAPC5
ELF1	ANGEL2
ELF1	ANKFY1
ELF1	ANKH
ELF1	ANKHD1
ELF1	ANKIB1

ELF1	ANKLE1
ELF1	ANKLE2
ELF1	ANKMY2
ELF1	ANKRD10
ELF1	ANKRD11
ELF1	ANKRD12
ELF1	ANKRD13A
ELF1	ANKRD17
ELF1	ANKRD26
ELF1	ANKRD27
ELF1	ANKRD39
ELF1	ANKRD40
ELF1	ANKRD49
ELF1	ANKRD54
ELF1	ANKRD7
ELF1	ANLN
ELF1	ANO6
ELF1	ANP32A
ELF1	ANP32B
ELF1	ANP32E
ELF1	ANXA11
ELF1	ANXA2R
ELF1	ANXA5
ELF1	ANXA6
ELF1	ANXA7
ELF1	AP1B1
ELF1	AP1G2
ELF1	AP1M1
ELF1	AP1S1
ELF1	AP1S2
ELF1	AP2A1
ELF1	AP2A2
ELF1	AP2B1
ELF1	AP2M1
ELF1	AP2S1
ELF1	AP3B1
ELF1	AP3M1
ELF1	AP3S1
ELF1	AP4B1
ELF1	AP4E1
ELF1	AP5B1
ELF1	AP5M1
ELF1	APAF1
ELF1	APBB1
ELF1	APBB1IP
ELF1	APC
ELF1	APEH
ELF1	APEX2
ELF1	APH1A
ELF1	API5

ELF1	APIP
ELF1	APLF
ELF1	APLP2
ELF1	APOLD1
ELF1	APOPT1
ELF1	APPBP2
ELF1	APPL1
ELF1	APTX
ELF1	AQR
ELF1	ARF1
ELF1	ARF3
ELF1	ARF5
ELF1	ARF6
ELF1	ARFGAP2
ELF1	ARFGAP3
ELF1	ARFGEF1
ELF1	ARFGEF2
ELF1	ARFIP1
ELF1	ARG2
ELF1	ARGLU1
ELF1	ARHGAP10
ELF1	ARHGAP11A
ELF1	ARHGAP11B
ELF1	ARHGAP12
ELF1	ARHGAP17
ELF1	ARHGAP18
ELF1	ARHGAP25
ELF1	ARHGAP27
ELF1	ARHGAP30
ELF1	ARHGAP33
ELF1	ARHGAP4
ELF1	ARHGAP9
ELF1	ARHGDIA
ELF1	ARHGDIB
ELF1	ARHGEF1
ELF1	ARHGEF37
ELF1	ARHGEF39
ELF1	ARHGEF6
ELF1	ARHGEF7
ELF1	ARID1A
ELF1	ARID1B
ELF1	ARID2
ELF1	ARID4A
ELF1	ARID4B
ELF1	ARID5A
ELF1	ARID5B
ELF1	ARIH2OS
ELF1	ARL13B
ELF1	ARL14EP
ELF1	ARL15

ELF1	ARL16
ELF1	ARL17A
ELF1	ARL2BP
ELF1	ARL3
ELF1	ARL4A
ELF1	ARL4C
ELF1	ARL5A
ELF1	ARL6IP1
ELF1	ARL6IP5
ELF1	ARL6IP6
ELF1	ARL8A
ELF1	ARMC1
ELF1	ARMC6
ELF1	ARMC8
ELF1	ARMCX3
ELF1	ARMCX6
ELF1	ARMT1
ELF1	ARPC1A
ELF1	ARPC1B
ELF1	ARPC2
ELF1	ARPC3
ELF1	ARPC4
ELF1	ARPC5
ELF1	ARPC5L
ELF1	ARPP19
ELF1	ARRDC1
ELF1	ARRDC1-AS1
ELF1	ARRDC3
ELF1	ARRDC3-AS1
ELF1	ASAP1
ELF1	ASB1
ELF1	ASB13
ELF1	ASB16-AS1
ELF1	ASB2
ELF1	ASB3
ELF1	ASB6
ELF1	ASB7
ELF1	ASB8
ELF1	ASCC2
ELF1	ASF1A
ELF1	ASF1B
ELF1	ASH1L
ELF1	ASH2L
ELF1	ASL
ELF1	ASNA1
ELF1	ASNSD1
ELF1	ASPH
ELF1	ASPM
ELF1	ASPSCR1
ELF1	ASRGL1

ELF1	ASXL1
ELF1	ASXL2
ELF1	ATAD1
ELF1	ATAD2
ELF1	ATAD5
ELF1	ATE1
ELF1	ATF1
ELF1	ATF3
ELF1	ATF4
ELF1	ATF5
ELF1	ATF6B
ELF1	ATF7
ELF1	ATF7IP
ELF1	ATF7IP2
ELF1	ATG101
ELF1	ATG12
ELF1	ATG13
ELF1	ATG16L2
ELF1	ATG3
ELF1	ATG4B
ELF1	ATG4C
ELF1	ATG4D
ELF1	ATG5
ELF1	ATM
ELF1	ATMIN
ELF1	ATP10D
ELF1	ATP11C
ELF1	ATP23
ELF1	ATP2A3
ELF1	ATP2B1
ELF1	ATP6AP1
ELF1	ATP6AP1L
ELF1	ATP6AP2
ELF1	ATP6V0D1
ELF1	ATP6V0E1
ELF1	ATP6V1A
ELF1	ATP6V1B2
ELF1	ATP6V1C1
ELF1	ATP6V1D
ELF1	ATP6V1E1
ELF1	ATP6V1F
ELF1	ATP6V1G1
ELF1	ATP6V1H
ELF1	ATP8A1
ELF1	ATPAF1
ELF1	ATPAF2
ELF1	ATRAID
ELF1	ATRX
ELF1	ATXN10
ELF1	ATXN2

ELF1	ATXN2L
ELF1	ATXN3
ELF1	ATXN7L3
ELF1	ATXN7L3B
ELF1	AURKA
ELF1	AURKAIP1
ELF1	AURKB
ELF1	AVIL
ELF1	AVL9
ELF1	AZI2
ELF1	AZIN1
ELF1	B2M
ELF1	B3GALNT1
ELF1	B3GLCT
ELF1	B3GNT2
ELF1	B9D2
ELF1	BABAM1
ELF1	BACE2
ELF1	BACH2
ELF1	BAG1
ELF1	BAG5
ELF1	BAG6
ELF1	BAIAP2L1
ELF1	BANF1
ELF1	BANP
ELF1	BAP1
ELF1	BARD1
ELF1	BASP1
ELF1	BAX
ELF1	BAZ1A
ELF1	BAZ1B
ELF1	BAZ2A
ELF1	BAZ2B
ELF1	BBIP1
ELF1	BBX
ELF1	BCAR3
ELF1	BCAS2
ELF1	BCAS4
ELF1	BCAT2
ELF1	BCDIN3D
ELF1	BCKDHB
ELF1	BCL10
ELF1	BCL11A
ELF1	BCL2L12
ELF1	BCL2L13
ELF1	BCL6
ELF1	BCL7A
ELF1	BCL7B
ELF1	BCL7C
ELF1	BCLAF1

ELF1	BCOR
ELF1	BDH2
ELF1	BDP1
ELF1	BECN1
ELF1	BET1
ELF1	BEX2
ELF1	BEX4
ELF1	BFAR
ELF1	BHLHE41
ELF1	BICD1
ELF1	BICD2
ELF1	BID
ELF1	BIK
ELF1	BIN1
ELF1	BIN3
ELF1	BIRC2
ELF1	BIRC3
ELF1	BIRC6
ELF1	BISPR
ELF1	BLCAP
ELF1	BLK
ELF1	BLOC1S1
ELF1	BLOC1S2
ELF1	BLOC1S4
ELF1	BLOC1S5
ELF1	BLOC1S6
ELF1	BLZF1
ELF1	BMP2K
ELF1	BNIP2
ELF1	BNIP3L
ELF1	BOD1L1
ELF1	BOLA1
ELF1	BOLA2-SMG1P6
ELF1	BORA
ELF1	BORCS7
ELF1	BORCS8
ELF1	BORCS8-MEF2B
ELF1	BPNT1
ELF1	BPTF
ELF1	BRAT1
ELF1	BRCA1
ELF1	BRCA2
ELF1	BRCC3
ELF1	BRD2
ELF1	BRD4
ELF1	BRD7
ELF1	BRD8
ELF1	BRD9
ELF1	BRI3
ELF1	BRI3BP

ELF1	BRK1
ELF1	BRMS1
ELF1	BRMS1L
ELF1	BROX
ELF1	BRWD1
ELF1	BSDC1
ELF1	BTBD10
ELF1	BTBD6
ELF1	BTBD7
ELF1	BTF3
ELF1	BTF3L4
ELF1	BTG1
ELF1	BTG2
ELF1	BTG3
ELF1	BTK
ELF1	BTNL9
ELF1	BTRC
ELF1	BUB1
ELF1	BUB1B
ELF1	BUB3
ELF1	BUD31
ELF1	BZW1
ELF1	C11orf49
ELF1	C11orf58
ELF1	C12orf10
ELF1	C12orf4
ELF1	C12orf49
ELF1	C12orf57
ELF1	C12orf65
ELF1	C12orf75
ELF1	C14orf119
ELF1	C14orf93
ELF1	C15orf40
ELF1	C16orf54
ELF1	C16orf72
ELF1	C16orf87
ELF1	C16orf91
ELF1	C16orf95
ELF1	C17orf58
ELF1	C17orf75
ELF1	C18orf21
ELF1	C18orf25
ELF1	C19orf12
ELF1	C19orf25
ELF1	C19orf47
ELF1	C19orf48
ELF1	C19orf53
ELF1	C1D
ELF1	C1GALT1
ELF1	C1orf109

ELF1	C1orf123
ELF1	C1orf131
ELF1	C1orf35
ELF1	C1orf43
ELF1	C1orf52
ELF1	C1orf74
ELF1	C21orf91
ELF1	C2CD5
ELF1	C2orf49
ELF1	C2orf68
ELF1	C2orf69
ELF1	C2orf74
ELF1	C2orf76
ELF1	C2orf88
ELF1	C3orf14
ELF1	C3orf38
ELF1	C3orf67
ELF1	C4orf3
ELF1	C4orf33
ELF1	C4orf46
ELF1	C5orf15
ELF1	C5orf24
ELF1	C5orf34
ELF1	C5orf51
ELF1	C5orf63
ELF1	C6orf203
ELF1	C6orf226
ELF1	C6orf47
ELF1	C6orf48
ELF1	C6orf52
ELF1	C6orf62
ELF1	C6orf89
ELF1	C6orf99
ELF1	C7orf26
ELF1	C7orf43
ELF1	C7orf50
ELF1	C8orf44
ELF1	C8orf76
ELF1	C8orf82
ELF1	C9orf16
ELF1	C9orf40
ELF1	C9orf72
ELF1	C9orf78
ELF1	C9orf85
ELF1	CAB39
ELF1	CAB39L
ELF1	CACUL1
ELF1	CACYBP
ELF1	CADPS
ELF1	CALCOCO2

ELF1	CALM1
ELF1	CALM2
ELF1	CALM3
ELF1	CAMK1
ELF1	CAMK2D
ELF1	CAMK2G
ELF1	CAMKK2
ELF1	CAMLG
ELF1	CAMTA1
ELF1	CAND1
ELF1	CAP1
ELF1	CAPN1
ELF1	CAPN7
ELF1	CAPNS1
ELF1	CAPRIN1
ELF1	CAPZA1
ELF1	CAPZA2
ELF1	CAPZB
ELF1	CARD11
ELF1	CARD8
ELF1	CARHSP1
ELF1	CARMIL2
ELF1	CARS
ELF1	CASC3
ELF1	CASC4
ELF1	CASD1
ELF1	CASP2
ELF1	CASP3
ELF1	CASP4
ELF1	CASP8AP2
ELF1	CASP9
ELF1	CAST
ELF1	CAT
ELF1	CBFB
ELF1	CBL
ELF1	CBLL1
ELF1	CBR4
ELF1	CBWD1
ELF1	CBWD2
ELF1	CBWD3
ELF1	CBWD5
ELF1	CBX1
ELF1	CBX3
ELF1	CBX4
ELF1	CBX5
ELF1	CBX8
ELF1	CC2D1A
ELF1	CCAR1
ELF1	CCAR2
ELF1	CCDC102A

ELF1	CCDC106
ELF1	CCDC115
ELF1	CCDC117
ELF1	CCDC12
ELF1	CCDC122
ELF1	CCDC126
ELF1	CCDC127
ELF1	CCDC130
ELF1	CCDC137
ELF1	CCDC14
ELF1	CCDC141
ELF1	CCDC152
ELF1	CCDC171
ELF1	CCDC174
ELF1	CCDC189
ELF1	CCDC191
ELF1	CCDC22
ELF1	CCDC25
ELF1	CCDC34
ELF1	CCDC43
ELF1	CCDC51
ELF1	CCDC6
ELF1	CCDC61
ELF1	CCDC66
ELF1	CCDC69
ELF1	CCDC7
ELF1	CCDC71
ELF1	CCDC77
ELF1	CCDC82
ELF1	CCDC84
ELF1	CCDC88A
ELF1	CCDC88C
ELF1	CCDC90B
ELF1	CCDC91
ELF1	CCDC92
ELF1	CCDC93
ELF1	CCDC97
ELF1	CCHCR1
ELF1	CCL28
ELF1	CCM2
ELF1	CCNA2
ELF1	CCNB1
ELF1	CCNB1IP1
ELF1	CCNB2
ELF1	CCNC
ELF1	CCND3
ELF1	CCNDBP1
ELF1	CCNE2
ELF1	CCNF
ELF1	CCNG1

ELF1	CCNG2
ELF1	CCNH
ELF1	CCNI
ELF1	CCNL1
ELF1	CCNL2
ELF1	CCNT1
ELF1	CCP110
ELF1	CCS
ELF1	CCSAP
ELF1	CCSER2
ELF1	CCT4
ELF1	CCT8
ELF1	CCZ1
ELF1	CCZ1B
ELF1	CD164
ELF1	CD180
ELF1	CD19
ELF1	CD22
ELF1	CD27
ELF1	CD2AP
ELF1	CD2BP2
ELF1	CD37
ELF1	CD40
ELF1	CD47
ELF1	CD53
ELF1	CD59
ELF1	CD72
ELF1	CD79B
ELF1	CD80
ELF1	CD81
ELF1	CD84
ELF1	CDC123
ELF1	CDC16
ELF1	CDC20
ELF1	CDC23
ELF1	CDC25B
ELF1	CDC25C
ELF1	CDC26
ELF1	CDC27
ELF1	CDC34
ELF1	CDC37L1
ELF1	CDC40
ELF1	CDC42
ELF1	CDC42EP3
ELF1	CDC42SE1
ELF1	CDC42SE2
ELF1	CDC45
ELF1	CDC5L
ELF1	CDC6
ELF1	CDC7

ELF1	CDC73
ELF1	CDCA2
ELF1	CDCA3
ELF1	CDCA5
ELF1	CDCA7
ELF1	CDCA7L
ELF1	CDCA8
ELF1	CDIPT
ELF1	CDK1
ELF1	CDK10
ELF1	CDK11A
ELF1	CDK11B
ELF1	CDK13
ELF1	CDK19
ELF1	CDK2
ELF1	CDK2AP2
ELF1	CDK5R1
ELF1	CDK5RAP1
ELF1	CDK5RAP2
ELF1	CDK5RAP3
ELF1	CDK9
ELF1	CDKAL1
ELF1	CDKN2A
ELF1	CDKN2AIP
ELF1	CDKN2C
ELF1	CDKN2D
ELF1	CDKN3
ELF1	CDS2
ELF1	CDT1
ELF1	CDV3
ELF1	CEBPG
ELF1	CEBPZ
ELF1	CEBPZOS
ELF1	CELF1
ELF1	CENPA
ELF1	CENPC
ELF1	CENPE
ELF1	CENPF
ELF1	CENPH
ELF1	CENPI
ELF1	CENPJ
ELF1	CENPK
ELF1	CENPL
ELF1	CENPM
ELF1	CENPN
ELF1	CENPO
ELF1	CENPP
ELF1	CENPQ
ELF1	CENPT
ELF1	CENPU

ELF1	CEP104
ELF1	CEP120
ELF1	CEP128
ELF1	CEP135
ELF1	CEP152
ELF1	CEP295
ELF1	CEP350
ELF1	CEP41
ELF1	CEP44
ELF1	CEP55
ELF1	CEP57
ELF1	CEP63
ELF1	CEP85L
ELF1	CEP89
ELF1	CEP95
ELF1	CEP97
ELF1	CEPT1
ELF1	CERK
ELF1	CERS2
ELF1	CERS5
ELF1	CETN2
ELF1	CETN3
ELF1	CFAP20
ELF1	CFAP73
ELF1	CFAP97
ELF1	CFDP1
ELF1	CFL1
ELF1	CGGBP1
ELF1	CHAC2
ELF1	CHAF1A
ELF1	CHAMP1
ELF1	CHCHD1
ELF1	CHCHD10
ELF1	CHCHD2
ELF1	CHCHD5
ELF1	CHCHD7
ELF1	CHD1
ELF1	CHD1L
ELF1	CHD3
ELF1	CHD4
ELF1	CHD6
ELF1	CHD7
ELF1	CHD8
ELF1	CHD9
ELF1	CHEK1
ELF1	CHEK2
ELF1	CHERP
ELF1	CHFR
ELF1	CHI3L2
ELF1	CHIC2

ELF1	CHKB
ELF1	CHMP1A
ELF1	CHMP1B
ELF1	CHMP2A
ELF1	CHMP2B
ELF1	CHMP3
ELF1	CHMP4B
ELF1	CHMP5
ELF1	CHMP7
ELF1	CHORDC1
ELF1	CHP1
ELF1	CHTF18
ELF1	CHTF8
ELF1	CHTOP
ELF1	CHURC1
ELF1	CIAO1
ELF1	CIAPIN1
ELF1	CIB1
ELF1	CIB2
ELF1	CIC
ELF1	CIITA
ELF1	CIR1
ELF1	CIRBP
ELF1	CIT
ELF1	CITED2
ELF1	CITED4
ELF1	CKAP2
ELF1	CKAP2L
ELF1	CKAP5
ELF1	CKLF
ELF1	CKS1B
ELF1	CKS2
ELF1	CLASRP
ELF1	CLEC11A
ELF1	CLHC1
ELF1	CLIC1
ELF1	CLIC4
ELF1	CLINT1
ELF1	CLIP1
ELF1	CLIP2
ELF1	CLK1
ELF1	CLK2
ELF1	CLK3
ELF1	CLK4
ELF1	CLN5
ELF1	CLNS1A
ELF1	CLP1
ELF1	CLPP
ELF1	CLPX
ELF1	CLSPN

ELF1	CLSTN3
ELF1	CLTA
ELF1	CLTB
ELF1	CLTC
ELF1	CMAS
ELF1	CMC1
ELF1	CMC2
ELF1	CMPK1
ELF1	CMTM6
ELF1	CMTM7
ELF1	CMTR2
ELF1	CNDP2
ELF1	CNIH1
ELF1	CNIH4
ELF1	CNN2
ELF1	CNNM3
ELF1	CNOT1
ELF1	CNOT10
ELF1	CNOT2
ELF1	CNOT4
ELF1	CNOT6L
ELF1	CNOT7
ELF1	CNOT8
ELF1	CNOT9
ELF1	CNP
ELF1	CNPY3
ELF1	CNPY4
ELF1	CNR1
ELF1	CNST
ELF1	COA1
ELF1	COA3
ELF1	COA5
ELF1	COASY
ELF1	COBLL1
ELF1	COCH
ELF1	COG2
ELF1	COG4
ELF1	COG5
ELF1	COG6
ELF1	COG7
ELF1	COIL
ELF1	COL4A3BP
ELF1	COMMD1
ELF1	COMMD10
ELF1	COMMD2
ELF1	COMMD3
ELF1	COMMD4
ELF1	COMMD5
ELF1	COMMD6
ELF1	COMMD7

ELF1	COMMD8
ELF1	COMMD9
ELF1	COMTD1
ELF1	COPB1
ELF1	COPB2
ELF1	COPE
ELF1	COPG2
ELF1	COPS2
ELF1	COPS3
ELF1	COPS4
ELF1	COPS5
ELF1	COPS6
ELF1	COPS7A
ELF1	COPS9
ELF1	COPZ1
ELF1	COQ10A
ELF1	COQ10B
ELF1	COQ2
ELF1	COQ4
ELF1	COQ5
ELF1	COQ7
ELF1	COQ9
ELF1	CORO1A
ELF1	CORO1B
ELF1	CORO7
ELF1	COTL1
ELF1	COX11
ELF1	COX14
ELF1	COX15
ELF1	COX17
ELF1	COX19
ELF1	COX4I1
ELF1	COX5A
ELF1	COX5B
ELF1	COX6A1
ELF1	COX6B1
ELF1	COX6C
ELF1	COX7A2
ELF1	COX7A2L
ELF1	COX7B
ELF1	COX7C
ELF1	COX8A
ELF1	CPM
ELF1	CPNE1
ELF1	CPNE5
ELF1	CPOX
ELF1	CPPED1
ELF1	CPSF1
ELF1	CPSF2
ELF1	CPSF3

ELF1	CPSF4
ELF1	CPSF6
ELF1	CPSF7
ELF1	CPT2
ELF1	CPTP
ELF1	CR1
ELF1	CR2
ELF1	CRADD
ELF1	CRBN
ELF1	CREB1
ELF1	CREB3L4
ELF1	CREB5
ELF1	CREBBP
ELF1	CREBZF
ELF1	CREM
ELF1	CRIPT
ELF1	CRK
ELF1	CRKL
ELF1	CRLF3
ELF1	CRNDE
ELF1	CRNKL1
ELF1	CRYZL1
ELF1	CS
ELF1	CSDE1
ELF1	CSE1L
ELF1	CSGALNACT2
ELF1	CSK
ELF1	CSNK1A1
ELF1	CSNK1D
ELF1	CSNK1G2
ELF1	CSNK1G3
ELF1	CSNK2A1
ELF1	CSNK2B
ELF1	CSPP1
ELF1	CSTF1
ELF1	CSTF2
ELF1	CSTF3
ELF1	CTBP1
ELF1	CTCF
ELF1	CTDNEP1
ELF1	CTDP1
ELF1	CTDSP1
ELF1	CTDSP2
ELF1	CTDSPL2
ELF1	CTNNA1
ELF1	CTNNB1
ELF1	CTNNBIP1
ELF1	CTNNBL1
ELF1	CTPS2
ELF1	CTR9

ELF1	CTSH
ELF1	CTSS
ELF1	CTSZ
ELF1	CTTN
ELF1	CUEDC2
ELF1	CUL1
ELF1	CUL3
ELF1	CUL4A
ELF1	CUL4B
ELF1	CUTA
ELF1	CUTC
ELF1	CUX1
ELF1	CWC15
ELF1	CWC22
ELF1	CWC25
ELF1	CWC27
ELF1	CWF19L1
ELF1	CWF19L2
ELF1	CXCR4
ELF1	CXCR5
ELF1	CXorf21
ELF1	CXorf38
ELF1	CXXC1
ELF1	CYB561A3
ELF1	CYB561D2
ELF1	CYB5B
ELF1	CYB5D2
ELF1	CYB5R3
ELF1	CYB5R4
ELF1	CYBB
ELF1	CYC1
ELF1	CYFIP2
ELF1	CYP2R1
ELF1	CYSTM1
ELF1	CYTH2
ELF1	CYTIP
ELF1	DAAM1
ELF1	DALRD3
ELF1	DAP
ELF1	DAP3
ELF1	DAPP1
ELF1	DARS
ELF1	DAXX
ELF1	DAZAP1
ELF1	DAZAP2
ELF1	DBF4
ELF1	DBI
ELF1	DBNL
ELF1	DBR1
ELF1	DCAF10

ELF1	DCAF11
ELF1	DCAF12
ELF1	DCAF15
ELF1	DCAF5
ELF1	DCAF6
ELF1	DCAF7
ELF1	DCAF8
ELF1	DCK
ELF1	DCLRE1B
ELF1	DCLRE1C
ELF1	DCP1A
ELF1	DCP2
ELF1	DCPS
ELF1	DCTN1
ELF1	DCTN2
ELF1	DCTN3
ELF1	DCTN5
ELF1	DCUN1D1
ELF1	DDA1
ELF1	DDB1
ELF1	DDHD1
ELF1	DDI2
ELF1	DDIT3
ELF1	DDRGK1
ELF1	DDT
ELF1	DDX1
ELF1	DDX17
ELF1	DDX19B
ELF1	DDX23
ELF1	DDX24
ELF1	DDX27
ELF1	DDX39A
ELF1	DDX39B
ELF1	DDX3X
ELF1	DDX3Y
ELF1	DDX42
ELF1	DDX46
ELF1	DDX47
ELF1	DDX5
ELF1	DDX50
ELF1	DDX52
ELF1	DDX58
ELF1	DDX59
ELF1	DDX6
ELF1	DECR1
ELF1	DEDD2
ELF1	DEF6
ELF1	DEF8
ELF1	DEGS1
ELF1	DEK

ELF1	DENND1B
ELF1	DENND1C
ELF1	DENND2D
ELF1	DENND3
ELF1	DENND4A
ELF1	DENND5B
ELF1	DENND6A
ELF1	DENR
ELF1	DEPDC1
ELF1	DEPDC1B
ELF1	DEPDC5
ELF1	DERA
ELF1	DESI2
ELF1	DET1
ELF1	DFFA
ELF1	DGKA
ELF1	DGKD
ELF1	DGKZ
ELF1	DGUOK
ELF1	DHCR24
ELF1	DHFR
ELF1	DHPS
ELF1	DHRS1
ELF1	DHRS11
ELF1	DHRS7B
ELF1	DHTKD1
ELF1	DHX15
ELF1	DHX16
ELF1	DHX30
ELF1	DHX34
ELF1	DHX36
ELF1	DHX38
ELF1	DHX40
ELF1	DHX8
ELF1	DHX9
ELF1	DIAPH1
ELF1	DIAPH2
ELF1	DIAPH3
ELF1	DICER1
ELF1	DIDO1
ELF1	DIP2A
ELF1	DIS3
ELF1	DIS3L
ELF1	DIS3L2
ELF1	DLD
ELF1	DLEU2
ELF1	DLG1
ELF1	DLGAP5
ELF1	DLL3
ELF1	DLST

ELF1	DMAP1
ELF1	DMC1
ELF1	DMD
ELF1	DMTF1
ELF1	DMXL1
ELF1	DNAJA1
ELF1	DNAJA2
ELF1	DNAJB1
ELF1	DNAJB12
ELF1	DNAJB14
ELF1	DNAJB2
ELF1	DNAJB6
ELF1	DNAJC10
ELF1	DNAJC11
ELF1	DNAJC17
ELF1	DNAJC21
ELF1	DNAJC28
ELF1	DNAJC5
ELF1	DNAJC7
ELF1	DNAJC8
ELF1	DNAJC9
ELF1	DNASE1
ELF1	DNASE1L1
ELF1	DNASE2
ELF1	DNER
ELF1	DNM1L
ELF1	DNM2
ELF1	DNMBP
ELF1	DNMT1
ELF1	DNTTIP1
ELF1	DNTTIP2
ELF1	DOCK10
ELF1	DOCK2
ELF1	DOCK8
ELF1	DOCK9
ELF1	DOK1
ELF1	DOK2
ELF1	DONSON
ELF1	DPAGT1
ELF1	DPF2
ELF1	DPH3
ELF1	DPH7
ELF1	DPM1
ELF1	DPP3
ELF1	DPP8
ELF1	DPY30
ELF1	DPYSL2
ELF1	DR1
ELF1	DRAM2
ELF1	DRAP1

ELF1	DRG1
ELF1	DRG2
ELF1	DSCC1
ELF1	DSN1
ELF1	DTD1
ELF1	DTL
ELF1	DTNBP1
ELF1	DTX2
ELF1	DTYMK
ELF1	DUSP1
ELF1	DUSP10
ELF1	DUSP12
ELF1	DUSP14
ELF1	DUSP23
ELF1	DUSP6
ELF1	DUT
ELF1	DVL2
ELF1	DVL3
ELF1	DYM
ELF1	DYNC1H1
ELF1	DYNC1I2
ELF1	DYNC1LI1
ELF1	DYNLL1
ELF1	DYNLL2
ELF1	DYNLRB1
ELF1	DYNLT1
ELF1	DYNLT3
ELF1	DYRK1A
ELF1	DYRK2
ELF1	DZIP3
ELF1	E2F1
ELF1	E2F3
ELF1	E2F4
ELF1	E2F5
ELF1	E2F7
ELF1	EAF2
ELF1	EAPP
ELF1	EBAG9
ELF1	EBF1
ELF1	EBP
ELF1	ECD
ELF1	ECH1
ELF1	ECHDC1
ELF1	ECHDC2
ELF1	ECT2
ELF1	EDF1
ELF1	EDRF1
ELF1	EED
ELF1	EEF1A1
ELF1	EEF1AKMT1

ELF1	EEF1D
ELF1	EEF1G
ELF1	EEF2
ELF1	EEFSEC
ELF1	EEPD1
ELF1	EFCAB11
ELF1	EFCAB14
ELF1	EFHD2
ELF1	EFR3A
ELF1	EFTUD2
ELF1	EGLN1
ELF1	EGR1
ELF1	EGR3
ELF1	EHBP1
ELF1	EHBP1L1
ELF1	EHD1
ELF1	EHD4
ELF1	EHMT1
ELF1	EI24
ELF1	EID1
ELF1	EID2
ELF1	EID2B
ELF1	EID3
ELF1	EIF1
ELF1	EIF1B
ELF1	EIF2A
ELF1	EIF2AK1
ELF1	EIF2AK2
ELF1	EIF2AK3
ELF1	EIF2AK4
ELF1	EIF2B1
ELF1	EIF2B2
ELF1	EIF2B5
ELF1	EIF2D
ELF1	EIF2S2
ELF1	EIF2S3
ELF1	EIF3A
ELF1	EIF3D
ELF1	EIF3E
ELF1	EIF3F
ELF1	EIF3G
ELF1	EIF3H
ELF1	EIF3I
ELF1	EIF3K
ELF1	EIF3L
ELF1	EIF3M
ELF1	EIF4A2
ELF1	EIF4A3
ELF1	EIF4B
ELF1	EIF4E

ELF1	EIF4E2
ELF1	EIF4EBP2
ELF1	EIF4G1
ELF1	EIF4G2
ELF1	EIF4G3
ELF1	EIF4H
ELF1	EIF5
ELF1	EIF5A2
ELF1	ELAC2
ELF1	ELAVL1
ELF1	ELF1
ELF1	ELF2
ELF1	ELF4
ELF1	ELK3
ELF1	ELK4
ELF1	ELMO1
ELF1	ELOVL1
ELF1	ELOVL5
ELF1	ELOVL6
ELF1	ELP3
ELF1	ELP5
ELF1	ELP6
ELF1	EMB
ELF1	EMC2
ELF1	EMC3
ELF1	EMC4
ELF1	EMC6
ELF1	EMC8
ELF1	EMC9
ELF1	EMD
ELF1	EME1
ELF1	EML2
ELF1	EMSY
ELF1	ENDOV
ELF1	ENO2
ELF1	ENOPH1
ELF1	ENOX2
ELF1	ENSA
ELF1	ENTPD4
ELF1	ENY2
ELF1	EP300
ELF1	EP400
ELF1	EPB41L4A-AS1
ELF1	EPC1
ELF1	EPC2
ELF1	EPCAM
ELF1	EPG5
ELF1	EPM2A
ELF1	EPS15
ELF1	EPS15L1

ELF1	EPS8
ELF1	ERBIN
ELF1	ERC1
ELF1	ERCC4
ELF1	ERCC6L
ELF1	ERCC6L2
ELF1	ERGIC1
ELF1	ERH
ELF1	ERI1
ELF1	ERI2
ELF1	ERICH1
ELF1	ERMARD
ELF1	ERP29
ELF1	ERP44
ELF1	ERV3−1
ELF1	ESCO2
ELF1	ESD
ELF1	ESPL1
ELF1	ESR2
ELF1	ETFB
ELF1	ETFDH
ELF1	ETNK1
ELF1	ETS1
ELF1	ETV7
ELF1	EVC2
ELF1	EVI2A
ELF1	EVI2B
ELF1	EVL
ELF1	EWSR1
ELF1	EXO5
ELF1	EXOC1
ELF1	EXOC3
ELF1	EXOC5
ELF1	EXOC6
ELF1	EXOC7
ELF1	EXOG
ELF1	EXOSC1
ELF1	EXOSC10
ELF1	EXOSC3
ELF1	EXOSC6
ELF1	EXOSC9
ELF1	EYA2
ELF1	EYA3
ELF1	EZH2
ELF1	EZR
ELF1	FAAH
ELF1	FAAP20
ELF1	FAAP24
ELF1	FADD
ELF1	FADS1

ELF1	FADS3
ELF1	FAF1
ELF1	FAF2
ELF1	FAHD1
ELF1	FAHD2A
ELF1	FAM102A
ELF1	FAM104A
ELF1	FAM104B
ELF1	FAM107B
ELF1	FAM110A
ELF1	FAM111A
ELF1	FAM111B
ELF1	FAM118B
ELF1	FAM120A
ELF1	FAM120AOS
ELF1	FAM122B
ELF1	FAM126A
ELF1	FAM126B
ELF1	FAM129A
ELF1	FAM133B
ELF1	FAM13B
ELF1	FAM160A2
ELF1	FAM160B1
ELF1	FAM177A1
ELF1	FAM184A
ELF1	FAM185A
ELF1	FAM192A
ELF1	FAM200B
ELF1	FAM204A
ELF1	FAM207A
ELF1	FAM208A
ELF1	FAM208B
ELF1	FAM210A
ELF1	FAM214A
ELF1	FAM219B
ELF1	FAM24B
ELF1	FAM32A
ELF1	FAM3C
ELF1	FAM45A
ELF1	FAM49A
ELF1	FAM49B
ELF1	FAM50A
ELF1	FAM50B
ELF1	FAM53B
ELF1	FAM53C
ELF1	FAM69A
ELF1	FAM72B
ELF1	FAM72C
ELF1	FAM72D
ELF1	FAM76B

ELF1	FAM83D
ELF1	FAM96A
ELF1	FAM96B
ELF1	FAM98A
ELF1	FAM98B
ELF1	FANCA
ELF1	FANCB
ELF1	FANCD2
ELF1	FANCF
ELF1	FANCG
ELF1	FANCI
ELF1	FANCL
ELF1	FANCM
ELF1	FARP2
ELF1	FARS2
ELF1	FAS
ELF1	FAU
ELF1	FBRS
ELF1	FBXL12
ELF1	FBXL15
ELF1	FBXL17
ELF1	FBXL3
ELF1	FBXL4
ELF1	FBXL5
ELF1	FBXO11
ELF1	FBXO21
ELF1	FBXO22
ELF1	FBXO28
ELF1	FBXO3
ELF1	FBXO30
ELF1	FBXO31
ELF1	FBXO34
ELF1	FBXO4
ELF1	FBXO46
ELF1	FBXO48
ELF1	FBXO5
ELF1	FBXO7
ELF1	FBXO8
ELF1	FBXO9
ELF1	FBXW2
ELF1	FBXW5
ELF1	FBXW7
ELF1	FBXW8
ELF1	FCF1
ELF1	FCHO1
ELF1	FCRL3
ELF1	FCRLA
ELF1	FDFT1
ELF1	FDPS
ELF1	FDX1

ELF1	FDXR
ELF1	FECH
ELF1	FEM1A
ELF1	FEM1B
ELF1	FEN1
ELF1	FERMT3
ELF1	FEZ2
ELF1	FGD5-AS1
ELF1	FGFR1OP
ELF1	FGFR1OP2
ELF1	FH
ELF1	FHOD3
ELF1	FIBP
ELF1	FIG4
ELF1	FIP1L1
ELF1	FKBP15
ELF1	FKBP1A
ELF1	FKBP3
ELF1	FKBP5
ELF1	FKBP7
ELF1	FKBP8
ELF1	FKBPL
ELF1	FLI1
ELF1	FLII
ELF1	FLOT1
ELF1	FLOT2
ELF1	FLVCR1
ELF1	FMNL1
ELF1	FMR1
ELF1	FN3KRP
ELF1	FNBP1
ELF1	FNBP4
ELF1	FNIP1
ELF1	FNTA
ELF1	FOPNL
ELF1	FOS
ELF1	FOXM1
ELF1	FOXN2
ELF1	FOXN3
ELF1	FOXP1
ELF1	FRA10AC1
ELF1	FRG1
ELF1	FRMD8
ELF1	FRYL
ELF1	FSD1L
ELF1	FTH1
ELF1	FUBP1
ELF1	FUBP3
ELF1	FUCA1
ELF1	FUNDC1

ELF1	FUNDC2
ELF1	FUS
ELF1	FXR1
ELF1	FYTTD1
ELF1	FZR1
ELF1	G2E3
ELF1	G3BP1
ELF1	G3BP2
ELF1	GABARAP
ELF1	GABARAPL2
ELF1	GABPA
ELF1	GABPB1
ELF1	GABPB1-AS1
ELF1	GABPB2
ELF1	GADD45B
ELF1	GAK
ELF1	GALNT14
ELF1	GALNT6
ELF1	GALNT7
ELF1	GAMT
ELF1	GAPDH
ELF1	GAPVD1
ELF1	GARS
ELF1	GAS2L3
ELF1	GATAD2A
ELF1	GATAD2B
ELF1	GATM
ELF1	GBA
ELF1	GBE1
ELF1	GCA
ELF1	GCC2
ELF1	GCH1
ELF1	GCHFR
ELF1	GCNT2
ELF1	GCSAM
ELF1	GDAP1
ELF1	GDAP2
ELF1	GDE1
ELF1	GDI2
ELF1	GDPD5
ELF1	GEMIN2
ELF1	GEMIN6
ELF1	GEMIN7
ELF1	GFER
ELF1	GFI1
ELF1	GFM1
ELF1	GFM2
ELF1	GFOD1
ELF1	GFOD2
ELF1	GFPT1

ELF1	GGA1
ELF1	GGA2
ELF1	GGCT
ELF1	GGCX
ELF1	GGH
ELF1	GGNBP2
ELF1	GGPS1
ELF1	GHITM
ELF1	GHRL
ELF1	GID8
ELF1	GIGYF2
ELF1	GINM1
ELF1	GINS1
ELF1	GINS2
ELF1	GINS3
ELF1	GINS4
ELF1	GIT2
ELF1	GKAP1
ELF1	GLA
ELF1	GLB1
ELF1	GLCE
ELF1	GLE1
ELF1	GLG1
ELF1	GLO1
ELF1	GLOD4
ELF1	GLRX
ELF1	GLRX3
ELF1	GLRX5
ELF1	GLS
ELF1	GLTP
ELF1	GLUD1
ELF1	GLUL
ELF1	GLYCTK
ELF1	GMCL1
ELF1	GMDS
ELF1	GMEB1
ELF1	GMFB
ELF1	GMFG
ELF1	GMIP
ELF1	GMNN
ELF1	GMPR2
ELF1	GMPS
ELF1	GNA13
ELF1	GNAI2
ELF1	GNAI3
ELF1	GNAS
ELF1	GNAZ
ELF1	GNB1
ELF1	GNB2
ELF1	GNB4

ELF1	GNB5
ELF1	GNG10
ELF1	GNG2
ELF1	GNG5
ELF1	GNGT2
ELF1	GNL1
ELF1	GNPAT
ELF1	GOLGA2
ELF1	GOLGA3
ELF1	GOLGA7
ELF1	GOLGB1
ELF1	GOLPH3
ELF1	GOLT1B
ELF1	GOPC
ELF1	GORASP2
ELF1	GOSR1
ELF1	GOT1
ELF1	GOT2
ELF1	GPAA1
ELF1	GPANK1
ELF1	GPATCH11
ELF1	GPATCH2L
ELF1	GPATCH8
ELF1	GPBP1
ELF1	GPBP1L1
ELF1	GPCPD1
ELF1	GPI
ELF1	GPM6B
ELF1	GPN1
ELF1	GPN2
ELF1	GPN3
ELF1	GPR107
ELF1	GPR143
ELF1	GPR160
ELF1	GPR18
ELF1	GPR180
ELF1	GPR82
ELF1	GPR89A
ELF1	GPS1
ELF1	GPSM2
ELF1	GPSM3
ELF1	GPT2
ELF1	GPX4
ELF1	GPX7
ELF1	GRAMD1A
ELF1	GRAPL
ELF1	GRB2
ELF1	GRIPAP1
ELF1	GRK2
ELF1	GRK6

ELF1	GRSF1
ELF1	GSDMD
ELF1	GSK3B
ELF1	GSKIP
ELF1	GSPT1
ELF1	GSPT2
ELF1	GSS
ELF1	GSTA4
ELF1	GSTCD
ELF1	GSTK1
ELF1	GSTO1
ELF1	GTDC1
ELF1	GTF2A1
ELF1	GTF2A2
ELF1	GTF2B
ELF1	GTF2E2
ELF1	GTF2F1
ELF1	GTF2F2
ELF1	GTF2H5
ELF1	GTF2I
ELF1	GTF2IRD2
ELF1	GTF2IRD2B
ELF1	GTF3C1
ELF1	GTF3C3
ELF1	GTF3C5
ELF1	GTPBP1
ELF1	GTPBP8
ELF1	GTSE1
ELF1	GUCD1
ELF1	GUK1
ELF1	GUSB
ELF1	GXYLT1
ELF1	GYG1
ELF1	GYPC
ELF1	GZF1
ELF1	H1FX
ELF1	H1FX-AS1
ELF1	H2AFJ
ELF1	H2AFV
ELF1	H2AFX
ELF1	H2AFY
ELF1	H2AFZ
ELF1	H3F3A
ELF1	H3F3B
ELF1	HACD1
ELF1	HACD2
ELF1	HACD3
ELF1	HACD4
ELF1	HADHA
ELF1	HADHB

ELF1	HARS
ELF1	HARS2
ELF1	HAT1
ELF1	HAUS1
ELF1	HAUS2
ELF1	HAUS3
ELF1	HAUS4
ELF1	HAUS5
ELF1	HAUS6
ELF1	HAUS8
ELF1	HBP1
ELF1	HBS1L
ELF1	HCCS
ELF1	HCLS1
ELF1	HDAC1
ELF1	HDAC10
ELF1	HDAC2
ELF1	HDAC3
ELF1	HDAC5
ELF1	HDAC6
ELF1	HDAC7
ELF1	HDAC9
ELF1	HDDC2
ELF1	HDGF
ELF1	HDHD2
ELF1	HDHD3
ELF1	HDX
ELF1	HECA
ELF1	HECTD1
ELF1	HECTD4
ELF1	HELLS
ELF1	HELQ
ELF1	HELZ
ELF1	HELZ2
ELF1	HEMK1
ELF1	HERC1
ELF1	HERC2
ELF1	HERC3
ELF1	HERC4
ELF1	HERPUD2
ELF1	HES6
ELF1	HGH1
ELF1	HGSNAT
ELF1	HHEX
ELF1	HHLA3
ELF1	HIBCH
ELF1	HIF1A
ELF1	HIF1AN
ELF1	HIGD1A
ELF1	HIGD2A

ELF1	HINT1
ELF1	HINT2
ELF1	HIP1R
ELF1	HIRIP3
ELF1	HIST1H1A
ELF1	HIST1H1D
ELF1	HIST1H1E
ELF1	HIST2H2AC
ELF1	HIST2H2BE
ELF1	HJURP
ELF1	HK1
ELF1	HKR1
ELF1	HLA-DMA
ELF1	HLA-DMB
ELF1	HLA-DPA1
ELF1	HLA-DPB1
ELF1	HLA-DQA2
ELF1	HLA-DRA
ELF1	HLA-DRB1
ELF1	HLA-DRB5
ELF1	HLTF
ELF1	HMG20A
ELF1	HMG20B
ELF1	HMGA1
ELF1	HMGB1
ELF1	HMGB2
ELF1	HMGB3
ELF1	HMGCS1
ELF1	HMGN1
ELF1	HMGN2
ELF1	HMGN3
ELF1	HMGN4
ELF1	HMGN5
ELF1	HMGXB3
ELF1	HMGXB4
ELF1	HMMR
ELF1	HMOX2
ELF1	HNRNPA0
ELF1	HNRNPA1
ELF1	HNRNPA1L2
ELF1	HNRNPA2B1
ELF1	HNRNPA3
ELF1	HNRNPAB
ELF1	HNRNPC
ELF1	HNRNPD
ELF1	HNRNPF
ELF1	HNRNPH1
ELF1	HNRNPH2
ELF1	HNRNPH3
ELF1	HNRNPK

ELF1	HNRNPL
ELF1	HNRNPM
ELF1	HNRNPR
ELF1	HNRNPU
ELF1	HNRNPUL1
ELF1	HNRNPUL2
ELF1	HOOK2
ELF1	HOPX
ELF1	HOXA7
ELF1	HOXB4
ELF1	HP1BP3
ELF1	HPF1
ELF1	HPRT1
ELF1	HPS1
ELF1	HPS3
ELF1	HPS4
ELF1	HPS5
ELF1	HPS6
ELF1	HRK
ELF1	HS2ST1
ELF1	HSBP1
ELF1	HSCB
ELF1	HSD17B10
ELF1	HSD17B11
ELF1	HSD17B12
ELF1	HSD17B4
ELF1	HSD17B7
ELF1	HSDL2
ELF1	HSF1
ELF1	HSF2
ELF1	HSP90AA1
ELF1	HSPA2
ELF1	HSPA4
ELF1	HSPA8
ELF1	HSPB11
ELF1	HSPBAP1
ELF1	HSPBP1
ELF1	HSPH1
ELF1	HTATSF1
ELF1	HTR3A
ELF1	HTRA2
ELF1	HTT
ELF1	HUS1
ELF1	HUWE1
ELF1	HVCN1
ELF1	HYLS1
ELF1	HYPK
ELF1	IARS2
ELF1	IBTK
ELF1	ICA1

ELF1	ICE1
ELF1	ICE2
ELF1	ICOSLG
ELF1	IDH1
ELF1	IDH2
ELF1	IDH3B
ELF1	IDH3G
ELF1	IDI1
ELF1	IDNK
ELF1	IDS
ELF1	IER2
ELF1	IER3IP1
ELF1	IFI16
ELF1	IFI27L1
ELF1	IFI27L2
ELF1	IFNGR2
ELF1	IFNLR1
ELF1	IFT43
ELF1	IFT74
ELF1	IFT81
ELF1	IFT88
ELF1	IGBP1
ELF1	IGF2BP3
ELF1	IK
ELF1	IKBKE
ELF1	IKBKG
ELF1	IKZF1
ELF1	IKZF3
ELF1	IL10
ELF1	IL12RB1
ELF1	IL16
ELF1	IL17C
ELF1	IL4R
ELF1	ILF2
ELF1	ILF3
ELF1	ILK
ELF1	ILKAP
ELF1	ILVBL
ELF1	IMMP1L
ELF1	IMMT
ELF1	IMP3
ELF1	IMP4
ELF1	IMPA1
ELF1	IMPA2
ELF1	INAFM1
ELF1	INCENP
ELF1	ING1
ELF1	ING2
ELF1	ING3
ELF1	ING4

ELF1	INIP
ELF1	INO80
ELF1	INO80E
ELF1	INPP5B
ELF1	INPP5D
ELF1	INPP5K
ELF1	INTS10
ELF1	INTS12
ELF1	INTS4
ELF1	INTS6
ELF1	INTS6-AS1
ELF1	INTS7
ELF1	INTS8
ELF1	INTS9
ELF1	IP6K1
ELF1	IP6K2
ELF1	IPO5
ELF1	IPO9
ELF1	IPPK
ELF1	IQCB1
ELF1	IQCD
ELF1	IQGAP1
ELF1	IRAK4
ELF1	IREB2
ELF1	IRF2
ELF1	IRF8
ELF1	IRS1
ELF1	IRS2
ELF1	ISCA2
ELF1	ISCU
ELF1	ISG20
ELF1	ISG20L2
ELF1	ISL2
ELF1	IST1
ELF1	ISY1
ELF1	ITFG1
ELF1	ITGAE
ELF1	ITGAL
ELF1	ITGB1
ELF1	ITGB1BP1
ELF1	ITGB2
ELF1	ITGB2-AS1
ELF1	ITGB3BP
ELF1	ITPA
ELF1	ITPKB
ELF1	ITPR1
ELF1	ITPR3
ELF1	ITPRIPL2
ELF1	ITSN2
ELF1	IVD

ELF1	IWS1
ELF1	JADE1
ELF1	JADE2
ELF1	JAK1
ELF1	JMJD1C
ELF1	JMJD6
ELF1	JRK
ELF1	JUND
ELF1	JUP
ELF1	KANSL1
ELF1	KANSL2
ELF1	KANSL3
ELF1	KAT5
ELF1	KAT6A
ELF1	KATNA1
ELF1	KATNAL1
ELF1	KATNBL1
ELF1	KBTBD2
ELF1	KBTBD3
ELF1	KBTBD4
ELF1	KCMF1
ELF1	KCNAB2
ELF1	KCNK12
ELF1	KCNMB3
ELF1	KCNMB4
ELF1	KCNQ1OT1
ELF1	KCTD18
ELF1	KCTD20
ELF1	KCTD5
ELF1	KCTD7
ELF1	KCTD9
ELF1	KDELR1
ELF1	KDM1A
ELF1	KDM1B
ELF1	KDM2B
ELF1	KDM3A
ELF1	KDM4A
ELF1	KDM5A
ELF1	KDM5D
ELF1	KDM6A
ELF1	KDSR
ELF1	KEAP1
ELF1	KHDRBS1
ELF1	KHNYN
ELF1	KHSRP
ELF1	KIAA0232
ELF1	KIAA0355
ELF1	KIAA0586
ELF1	KIAA1143
ELF1	KIAA1217

ELF1	KIAA1328
ELF1	KIAA2026
ELF1	KIDINS220
ELF1	KIF11
ELF1	KIF13A
ELF1	KIF14
ELF1	KIF18B
ELF1	KIF20A
ELF1	KIF20B
ELF1	KIF22
ELF1	KIF23
ELF1	KIF2A
ELF1	KIF2C
ELF1	KIF3A
ELF1	KIF4A
ELF1	KIF5B
ELF1	KIFC1
ELF1	KIN
ELF1	KIZ
ELF1	KLC1
ELF1	KLF12
ELF1	KLF6
ELF1	KLHDC2
ELF1	KLHDC3
ELF1	KLHL18
ELF1	KLHL2
ELF1	KLHL22
ELF1	KLHL23
ELF1	KLHL24
ELF1	KLHL36
ELF1	KLHL42
ELF1	KLHL5
ELF1	KLHL6
ELF1	KLHL7
ELF1	KMT2A
ELF1	KMT2C
ELF1	KMT2E
ELF1	KMT5A
ELF1	KMT5B
ELF1	KMT5C
ELF1	KNSTRN
ELF1	KNTC1
ELF1	KPNA1
ELF1	KPNA2
ELF1	KPNA3
ELF1	KPNA4
ELF1	KPNB1
ELF1	KRAS
ELF1	KRCC1
ELF1	KRI1

ELF1	KRIT1
ELF1	KRR1
ELF1	KRTCAP2
ELF1	KTN1
ELF1	KXD1
ELF1	KYAT3
ELF1	L1TD1
ELF1	L3MBTL2
ELF1	LACC1
ELF1	LAGE3
ELF1	LAMP1
ELF1	LAMTOR1
ELF1	LAMTOR2
ELF1	LAMTOR3
ELF1	LAMTOR4
ELF1	LAMTOR5
ELF1	LAP3
ELF1	LAPTM5
ELF1	LARP4B
ELF1	LARP7
ELF1	LARS
ELF1	LAS1L
ELF1	LASP1
ELF1	LAT2
ELF1	LBR
ELF1	LCK
ELF1	LCMT1
ELF1	LCOR
ELF1	LCORL
ELF1	LCP1
ELF1	LDB1
ELF1	LDHB
ELF1	LDLR
ELF1	LEAP2
ELF1	LEF1
ELF1	LEMD2
ELF1	LENG8
ELF1	LEO1
ELF1	LEPROT
ELF1	LETMD1
ELF1	LGALS8
ELF1	LHPP
ELF1	LIG1
ELF1	LILRB1
ELF1	LIMA1
ELF1	LIMD2
ELF1	LIMK1
ELF1	LIMK2
ELF1	LIMS1
ELF1	LIN52

ELF1	LIN9
ELF1	LINC00426
ELF1	LINC00467
ELF1	LINC00476
ELF1	LINC00487
ELF1	LINC00526
ELF1	LINC00623
ELF1	LINC00667
ELF1	LINC00877
ELF1	LINC00909
ELF1	LINC01013
ELF1	LINC01116
ELF1	LINC01184
ELF1	LINC01215
ELF1	LIPE
ELF1	LITAF
ELF1	LLPH
ELF1	LMBRD1
ELF1	LMNB1
ELF1	LMO2
ELF1	LMO4
ELF1	LMO7
ELF1	LNPEP
ELF1	LONP2
ELF1	LONRF1
ELF1	LOXL2
ELF1	LPCAT3
ELF1	LPGAT1
ELF1	LPIN1
ELF1	LPIN2
ELF1	LPP
ELF1	LPP-AS2
ELF1	LPXN
ELF1	LRBA
ELF1	LRCH1
ELF1	LRCH3
ELF1	LRCH4
ELF1	LRIF1
ELF1	LRMP
ELF1	LRP10
ELF1	LRR1
ELF1	LRRC2
ELF1	LRRC23
ELF1	LRRC28
ELF1	LRRC32
ELF1	LRRC37B
ELF1	LRRC41
ELF1	LRRC47
ELF1	LRRC57
ELF1	LRRC61

ELF1	LRRCC1
ELF1	LRRFIP1
ELF1	LRRFIP2
ELF1	LRRK1
ELF1	LRTOMT
ELF1	LRWD1
ELF1	LSG1
ELF1	LSM1
ELF1	LSM12
ELF1	LSM14A
ELF1	LSM3
ELF1	LSM4
ELF1	LSM5
ELF1	LSM6
ELF1	LSM8
ELF1	LTA4H
ELF1	LTB
ELF1	LTBP1
ELF1	LTN1
ELF1	LUC7L
ELF1	LUC7L2
ELF1	LUC7L3
ELF1	LXN
ELF1	LY86
ELF1	LYL1
ELF1	LYN
ELF1	LYPLA1
ELF1	LYPLAL1
ELF1	LYRM1
ELF1	LYRM2
ELF1	LYSMD2
ELF1	LZIC
ELF1	M6PR
ELF1	MAD1L1
ELF1	MAD2L1
ELF1	MAD2L1BP
ELF1	MAD2L2
ELF1	MADD
ELF1	MAEA
ELF1	MAF1
ELF1	MAFG
ELF1	MAGED2
ELF1	MAGEF1
ELF1	MAGOH
ELF1	MAGOHB
ELF1	MALT1
ELF1	MAP1B
ELF1	MAP1LC3B
ELF1	MAP1S
ELF1	MAP2

ELF1	MAP2K1
ELF1	MAP2K2
ELF1	MAP2K4
ELF1	MAP2K6
ELF1	MAP3K11
ELF1	MAP3K3
ELF1	MAP3K4
ELF1	MAP3K7
ELF1	MAP3K9
ELF1	MAP4
ELF1	MAP4K1
ELF1	MAP4K2
ELF1	MAP4K4
ELF1	MAP4K5
ELF1	MAP7D1
ELF1	MAP7D3
ELF1	MAPK1
ELF1	MAPK10
ELF1	MAPK14
ELF1	MAPK1IP1L
ELF1	MAPK9
ELF1	MAPKAP1
ELF1	MAPRE1
ELF1	MAPRE2
ELF1	MARCH1
ELF1	MARCH5
ELF1	MARCH6
ELF1	MARCH7
ELF1	MARCH8
ELF1	MARCH9
ELF1	MARCKSL1
ELF1	MARK2
ELF1	MARK3
ELF1	MAST2
ELF1	MASTL
ELF1	MAT2A
ELF1	MAT2B
ELF1	MAU2
ELF1	MAX
ELF1	MAZ
ELF1	MBD2
ELF1	MBD4
ELF1	MBD5
ELF1	MBNL1
ELF1	MBP
ELF1	MCCC1
ELF1	MCL1
ELF1	MCM10
ELF1	MCM2
ELF1	MCM3

ELF1	MCM4
ELF1	MCM5
ELF1	MCM6
ELF1	MCM7
ELF1	MCMBP
ELF1	MCPH1
ELF1	MCRS1
ELF1	MCTP2
ELF1	MCTS1
ELF1	MDC1
ELF1	MDFIC
ELF1	MDH1
ELF1	MDH2
ELF1	MDM1
ELF1	MDM2
ELF1	MDM4
ELF1	MDN1
ELF1	MEA1
ELF1	MEAF6
ELF1	MECP2
ELF1	MED10
ELF1	MED11
ELF1	MED16
ELF1	MED17
ELF1	MED18
ELF1	MED20
ELF1	MED21
ELF1	MED24
ELF1	MED27
ELF1	MED28
ELF1	MED30
ELF1	MED31
ELF1	MED4
ELF1	MED6
ELF1	MED7
ELF1	MED8
ELF1	MED9
ELF1	MEF2A
ELF1	MEF2C
ELF1	MEF2D
ELF1	MEIG1
ELF1	MELK
ELF1	MELTF
ELF1	MEMO1
ELF1	MEN1
ELF1	MEPCE
ELF1	METAP1
ELF1	METAP2
ELF1	METRNL
ELF1	METTL17

ELF1	METTL18
ELF1	METTL21A
ELF1	METTL23
ELF1	METTL25
ELF1	METTL2A
ELF1	METTL3
ELF1	METTL4
ELF1	METTL5
ELF1	METTL6
ELF1	METTL7A
ELF1	METTL9
ELF1	MFAP1
ELF1	MFF
ELF1	MFHAS1
ELF1	MFN1
ELF1	MFN2
ELF1	MFSD10
ELF1	MFSD11
ELF1	MFSD13A
ELF1	MFSD14A
ELF1	MFSD14B
ELF1	MFSD14C
ELF1	MFSD5
ELF1	MGA
ELF1	MGME1
ELF1	MGMT
ELF1	MIA-RAB4B
ELF1	MIA3
ELF1	MICAL3
ELF1	MICB
ELF1	MICU1
ELF1	MICU2
ELF1	MICU3
ELF1	MID1IP1
ELF1	MIDN
ELF1	MIEN1
ELF1	MIER1
ELF1	MIF4GD
ELF1	MIIP
ELF1	MILR1
ELF1	MINOS1
ELF1	MIOS
ELF1	MIR181A1HG
ELF1	MIR3681HG
ELF1	MIS12
ELF1	MIS18A
ELF1	MIS18BP1
ELF1	MITD1
ELF1	MKI67
ELF1	MKKS

ELF1	MKLN1
ELF1	MKRN1
ELF1	MKRN2
ELF1	MLF2
ELF1	MLH1
ELF1	MLLT11
ELF1	MLX
ELF1	MLXIP
ELF1	MLYCD
ELF1	MMADHC
ELF1	MMD
ELF1	MME
ELF1	MMS22L
ELF1	MNAT1
ELF1	MND1
ELF1	MOB1A
ELF1	MOB1B
ELF1	MOB2
ELF1	MOB3A
ELF1	MOB4
ELF1	MOCS2
ELF1	MON1A
ELF1	MON2
ELF1	MORC4
ELF1	MORF4L1
ELF1	MORF4L2
ELF1	MOSPD3
ELF1	MPC2
ELF1	MPDU1
ELF1	MPG
ELF1	MPHOSPH8
ELF1	MPHOSPH9
ELF1	MPI
ELF1	MPLKIP
ELF1	MPND
ELF1	MPP5
ELF1	MPP6
ELF1	MPPE1
ELF1	MPV17
ELF1	MPZL1
ELF1	MRGBP
ELF1	MRM2
ELF1	MRM3
ELF1	MROH1
ELF1	MRPL1
ELF1	MRPL10
ELF1	MRPL13
ELF1	MRPL14
ELF1	MRPL15
ELF1	MRPL16

ELF1	MRPL18
ELF1	MRPL21
ELF1	MRPL22
ELF1	MRPL23
ELF1	MRPL27
ELF1	MRPL28
ELF1	MRPL32
ELF1	MRPL33
ELF1	MRPL34
ELF1	MRPL35
ELF1	MRPL37
ELF1	MRPL39
ELF1	MRPL43
ELF1	MRPL44
ELF1	MRPL45
ELF1	MRPL47
ELF1	MRPL48
ELF1	MRPL49
ELF1	MRPL50
ELF1	MRPL51
ELF1	MRPL52
ELF1	MRPL53
ELF1	MRPL54
ELF1	MRPL57
ELF1	MRPS11
ELF1	MRPS14
ELF1	MRPS15
ELF1	MRPS16
ELF1	MRPS18A
ELF1	MRPS18B
ELF1	MRPS18C
ELF1	MRPS2
ELF1	MRPS21
ELF1	MRPS22
ELF1	MRPS23
ELF1	MRPS25
ELF1	MRPS30
ELF1	MRPS31
ELF1	MRPS33
ELF1	MRPS34
ELF1	MRPS35
ELF1	MRPS36
ELF1	MRPS6
ELF1	MRPS7
ELF1	MRPS9
ELF1	MRRF
ELF1	MRS2
ELF1	MS4A1
ELF1	MSANTD2
ELF1	MSANTD4

ELF1	MSH2
ELF1	MSH3
ELF1	MSH6
ELF1	MSI2
ELF1	MSL3
ELF1	MSN
ELF1	MSRA
ELF1	MSRB1
ELF1	MTA1
ELF1	MTA3
ELF1	MTBP
ELF1	MTCH2
ELF1	MTDH
ELF1	MTERF3
ELF1	MTF2
ELF1	MTFMT
ELF1	MTFR2
ELF1	MTG2
ELF1	MTHFD2
ELF1	MTHFR
ELF1	MTHFS
ELF1	MTIF2
ELF1	MTIF3
ELF1	MTMR1
ELF1	MTMR12
ELF1	MTMR14
ELF1	MTMR2
ELF1	MTMR6
ELF1	MTO1
ELF1	MTOR
ELF1	MTPN
ELF1	MTR
ELF1	MTRF1L
ELF1	MTX1
ELF1	MTX2
ELF1	MUS81
ELF1	MUT
ELF1	MUTYH
ELF1	MVB12A
ELF1	MVD
ELF1	MVK
ELF1	MXD3
ELF1	MYBL1
ELF1	MYBL2
ELF1	MYCBP2
ELF1	MYD88
ELF1	MYEF2
ELF1	MYH9
ELF1	MYL12A
ELF1	MYL12B

ELF1	MYL5
ELF1	MYL6
ELF1	MYL6B
ELF1	MYO1E
ELF1	MYO1G
ELF1	MYO6
ELF1	MYO9A
ELF1	MYO9B
ELF1	MYOM2
ELF1	MYPOP
ELF1	MZF1
ELF1	MZT1
ELF1	MZT2A
ELF1	N4BP1
ELF1	N4BP2L2
ELF1	N4BP3
ELF1	NAA15
ELF1	NAA30
ELF1	NAA38
ELF1	NAA40
ELF1	NAA50
ELF1	NAB2
ELF1	NABP2
ELF1	NACA
ELF1	NACC1
ELF1	NADK
ELF1	NAGK
ELF1	NAP1L1
ELF1	NAP1L4
ELF1	NAPA
ELF1	NAPG
ELF1	NARF
ELF1	NARFL
ELF1	NASP
ELF1	NAT14
ELF1	NAXD
ELF1	NBAS
ELF1	NBN
ELF1	NBPF1
ELF1	NBPF20
ELF1	NCALD
ELF1	NCAPD2
ELF1	NCAPG
ELF1	NCAPH
ELF1	NCAPH2
ELF1	NCBP2
ELF1	NCBP2-AS2
ELF1	NCBP3
ELF1	NCF1
ELF1	NCF4

ELF1	NCK1
ELF1	NCKAP1L
ELF1	NCOA1
ELF1	NCOA2
ELF1	NCOA3
ELF1	NCOA4
ELF1	NCOA5
ELF1	NCOA7
ELF1	NCOR1
ELF1	NCOR2
ELF1	NDC1
ELF1	NDC80
ELF1	NDE1
ELF1	NDEL1
ELF1	NDRG3
ELF1	NDUFA1
ELF1	NDUFA10
ELF1	NDUFA12
ELF1	NDUFA13
ELF1	NDUFA2
ELF1	NDUFA4
ELF1	NDUFA5
ELF1	NDUFA6
ELF1	NDUFA7
ELF1	NDUFA8
ELF1	NDUFAB1
ELF1	NDUFAF1
ELF1	NDUFAF3
ELF1	NDUFAF6
ELF1	NDUFB10
ELF1	NDUFB11
ELF1	NDUFB2
ELF1	NDUFB3
ELF1	NDUFB4
ELF1	NDUFB5
ELF1	NDUFB6
ELF1	NDUFB7
ELF1	NDUFB8
ELF1	NDUFB9
ELF1	NDUFC1
ELF1	NDUFS1
ELF1	NDUFS2
ELF1	NDUFS3
ELF1	NDUFS4
ELF1	NDUFS5
ELF1	NDUFS7
ELF1	NDUFS8
ELF1	NDUFV1
ELF1	NDUFV2
ELF1	NEAT1

ELF1	NECAP1
ELF1	NECAP2
ELF1	NEDD8
ELF1	NEDD9
ELF1	NEIL1
ELF1	NEIL3
ELF1	NEK1
ELF1	NEK2
ELF1	NEK4
ELF1	NEK6
ELF1	NEK7
ELF1	NEK8
ELF1	NELFB
ELF1	NELFCD
ELF1	NELFE
ELF1	NEMF
ELF1	NEMP2
ELF1	NET1
ELF1	NF1
ELF1	NFATC2IP
ELF1	NFATC3
ELF1	NFE2L2
ELF1	NFKBIL1
ELF1	NFYA
ELF1	NFYB
ELF1	NFYC
ELF1	NGDN
ELF1	NHLRC2
ELF1	NHLRC3
ELF1	NHP2
ELF1	NIF3L1
ELF1	NIN
ELF1	NINJ2
ELF1	NIPA1
ELF1	NIPAL2
ELF1	NIPBL
ELF1	NIPSNAP3A
ELF1	NISCH
ELF1	NIT1
ELF1	NKAP
ELF1	NKIRAS2
ELF1	NKTR
ELF1	NLK
ELF1	NLRC3
ELF1	NME6
ELF1	NME7
ELF1	NMI
ELF1	NMNAT1
ELF1	NMRAL1
ELF1	NMT1

ELF1	NNT
ELF1	NNT-AS1
ELF1	NOA1
ELF1	NOB1
ELF1	NOL12
ELF1	NOL8
ELF1	NOL9
ELF1	NOMO1
ELF1	NONO
ELF1	NOP10
ELF1	NOP14-AS1
ELF1	NORAD
ELF1	NOSIP
ELF1	NPAS1
ELF1	NPAT
ELF1	NPEPPS
ELF1	NPM1
ELF1	NPRL2
ELF1	NR1H2
ELF1	NR2C1
ELF1	NR2C2
ELF1	NR3C1
ELF1	NR4A3
ELF1	NRAS
ELF1	NRBF2
ELF1	NRBP1
ELF1	NRDC
ELF1	NRDE2
ELF1	NRF1
ELF1	NRM
ELF1	NSD1
ELF1	NSL1
ELF1	NSMCE1
ELF1	NSMCE2
ELF1	NSMCE3
ELF1	NSMCE4A
ELF1	NSRP1
ELF1	NSUN4
ELF1	NSUN5
ELF1	NT5C
ELF1	NT5C2
ELF1	NT5C3B
ELF1	NTAN1
ELF1	NTPCR
ELF1	NUB1
ELF1	NUBP1
ELF1	NUBP2
ELF1	NUBPL
ELF1	NUCKS1
ELF1	NUDCD1

ELF1	NUDCD2
ELF1	NUDT1
ELF1	NUDT15
ELF1	NUDT16
ELF1	NUDT16L1
ELF1	NUDT18
ELF1	NUDT21
ELF1	NUDT22
ELF1	NUDT5
ELF1	NUDT6
ELF1	NUDT7
ELF1	NUF2
ELF1	NUGGC
ELF1	NUMA1
ELF1	NUMB
ELF1	NUP107
ELF1	NUP153
ELF1	NUP205
ELF1	NUP210
ELF1	NUP214
ELF1	NUP37
ELF1	NUP50
ELF1	NUP54
ELF1	NUP58
ELF1	NUP62
ELF1	NUP88
ELF1	NUP98
ELF1	NUSAP1
ELF1	NUTF2
ELF1	NUTM2B-AS1
ELF1	NXF1
ELF1	NXT2
ELF1	OARD1
ELF1	OAT
ELF1	OAZ1
ELF1	OAZ2
ELF1	OCIAD1
ELF1	OCIAD2
ELF1	ODF2
ELF1	ODF2L
ELF1	OFD1
ELF1	OGFOD1
ELF1	OGFR
ELF1	OGFRL1
ELF1	OGG1
ELF1	OLA1
ELF1	OPA3
ELF1	OPTN
ELF1	ORAI2
ELF1	ORAI3

ELF1	ORC1
ELF1	ORC2
ELF1	ORC3
ELF1	ORC4
ELF1	ORC6
ELF1	ORMDL1
ELF1	ORMDL2
ELF1	OSBP
ELF1	OSBPL10
ELF1	OSBPL10-AS1
ELF1	OSBPL11
ELF1	OSBPL2
ELF1	OSBPL8
ELF1	OSBPL9
ELF1	OSER1
ELF1	OST4
ELF1	OSTF1
ELF1	OSTM1
ELF1	OTUB1
ELF1	OTUD1
ELF1	OTULIN
ELF1	OXA1L
ELF1	OXLD1
ELF1	OXNAD1
ELF1	OXR1
ELF1	P2RX5
ELF1	P3H1
ELF1	P4HA1
ELF1	PAAF1
ELF1	PABPC1
ELF1	PABPN1
ELF1	PACS1
ELF1	PACSIN2
ELF1	PAFAH1B1
ELF1	PAFAH1B2
ELF1	PAFAH1B3
ELF1	PAG1
ELF1	PAIP1
ELF1	PAIP2
ELF1	PAK2
ELF1	PAN3
ELF1	PANK2
ELF1	PANK4
ELF1	PAPOLA
ELF1	PAPOLG
ELF1	PAPSS1
ELF1	PAQR4
ELF1	PAQR8
ELF1	PARG
ELF1	PARK7

ELF1	PARN
ELF1	PARP1
ELF1	PARP10
ELF1	PARP11
ELF1	PARP16
ELF1	PARP2
ELF1	PARP8
ELF1	PARVG
ELF1	PATL1
ELF1	PATZ1
ELF1	PAWR
ELF1	PAX5
ELF1	PAXBP1
ELF1	PAXIP1
ELF1	PAXIP1-AS1
ELF1	PBDC1
ELF1	PBK
ELF1	PBLD
ELF1	PBRM1
ELF1	PBX3
ELF1	PCBP1
ELF1	PCBP2
ELF1	PCCB
ELF1	PCED1B
ELF1	PCF11
ELF1	PCGF3
ELF1	PCGF5
ELF1	PCID2
ELF1	PCIF1
ELF1	PCM1
ELF1	PCMT1
ELF1	PCNA
ELF1	PCNP
ELF1	PCNT
ELF1	PCNX4
ELF1	PCSK7
ELF1	PCTP
ELF1	PCYOX1L
ELF1	PCYT2
ELF1	PDAP1
ELF1	PDCD10
ELF1	PDCD2
ELF1	PDCD6
ELF1	PDCD6IP
ELF1	PDCD7
ELF1	PDCL
ELF1	PDCL3
ELF1	PDE12
ELF1	PDE4D
ELF1	PDE6D

ELF1	PDE7A
ELF1	PDGFD
ELF1	PDHA1
ELF1	PDHB
ELF1	PDHX
ELF1	PDK2
ELF1	PDK3
ELF1	PDLIM2
ELF1	PDLIM7
ELF1	PDRG1
ELF1	PDS5A
ELF1	PDS5B
ELF1	PDXDC1
ELF1	PDZD11
ELF1	PDZD8
ELF1	PEA15
ELF1	PEF1
ELF1	PEG10
ELF1	PELI1
ELF1	PEPD
ELF1	PER2
ELF1	PET100
ELF1	PEX11B
ELF1	PEX14
ELF1	PEX16
ELF1	PEX19
ELF1	PEX2
ELF1	PEX3
ELF1	PEX5
ELF1	PEX6
ELF1	PFDN1
ELF1	PFDN2
ELF1	PFDN4
ELF1	PFDN5
ELF1	PFKL
ELF1	PFKP
ELF1	PFN1
ELF1	PFN2
ELF1	PGAM1
ELF1	PGGT1B
ELF1	PGK1
ELF1	PGLS
ELF1	PGM1
ELF1	PGM2
ELF1	PGS1
ELF1	PHACTR4
ELF1	PHAX
ELF1	PHC1
ELF1	PHC3
ELF1	PHF10

ELF1	PHF11
ELF1	PHF14
ELF1	PHF19
ELF1	PHF20
ELF1	PHF20L1
ELF1	PHF21A
ELF1	PHF23
ELF1	PHF3
ELF1	PHF5A
ELF1	PHF6
ELF1	PHGDH
ELF1	PHIP
ELF1	PHKG2
ELF1	PHTF2
ELF1	PHYKPL
ELF1	PI4K2A
ELF1	PIAS1
ELF1	PIAS2
ELF1	PIAS4
ELF1	PICALM
ELF1	PIDD1
ELF1	PIF1
ELF1	PIGB
ELF1	PIGBOS1
ELF1	PIGC
ELF1	PIGG
ELF1	PIGL
ELF1	PIGN
ELF1	PIGO
ELF1	PIGP
ELF1	PIGQ
ELF1	PIGU
ELF1	PIH1D1
ELF1	PIK3AP1
ELF1	PIK3C3
ELF1	PIK3CA
ELF1	PIK3CD
ELF1	PIK3R1
ELF1	PILRB
ELF1	PIN1
ELF1	PIN4
ELF1	PINLYP
ELF1	PIP4K2A
ELF1	PIP4K2B
ELF1	PISD
ELF1	PITPNA-AS1
ELF1	PITPNB
ELF1	PITPNC1
ELF1	PITPNM1
ELF1	PITRM1

ELF1	PJA2
ELF1	PKD2
ELF1	PKHD1L1
ELF1	PKM
ELF1	PKMYT1
ELF1	PKN1
ELF1	PKN2
ELF1	PKNOX1
ELF1	PLA2G6
ELF1	PLAGL2
ELF1	PLCL2
ELF1	PLEK
ELF1	PLEKHA2
ELF1	PLEKHA3
ELF1	PLEKHB2
ELF1	PLEKHF2
ELF1	PLEKHG7
ELF1	PLEKHJ1
ELF1	PLEKHO1
ELF1	PLK1
ELF1	PLK4
ELF1	PLRG1
ELF1	PLSCR1
ELF1	PM20D2
ELF1	PMAIP1
ELF1	PMF1
ELF1	PML
ELF1	PMM1
ELF1	PMPCA
ELF1	PMPCB
ELF1	PMS1
ELF1	PMS2
ELF1	PMVK
ELF1	PNISR
ELF1	PNKD
ELF1	PNKP
ELF1	PNN
ELF1	PNPLA2
ELF1	PNPLA8
ELF1	PNRC1
ELF1	PNRC2
ELF1	POC1A
ELF1	POC1B
ELF1	POC5
ELF1	POGZ
ELF1	POLA1
ELF1	POLA2
ELF1	POLD1
ELF1	POLD3
ELF1	POLD4

ELF1	POLDIP2
ELF1	POLDIP3
ELF1	POLE
ELF1	POLE2
ELF1	POLE3
ELF1	POLE4
ELF1	POLG
ELF1	POLG2
ELF1	POLH
ELF1	POLK
ELF1	POLM
ELF1	POLQ
ELF1	POLR1D
ELF1	POLR1E
ELF1	POLR2A
ELF1	POLR2B
ELF1	POLR2C
ELF1	POLR2D
ELF1	POLR2G
ELF1	POLR2I
ELF1	POLR2J
ELF1	POLR2K
ELF1	POLR2L
ELF1	POLR2M
ELF1	POLR3A
ELF1	POLR3C
ELF1	POM121
ELF1	POMP
ELF1	POP4
ELF1	POP5
ELF1	POT1
ELF1	POU2AF1
ELF1	POU2F1
ELF1	POU2F2
ELF1	POU3F1
ELF1	POU4F1
ELF1	PPA2
ELF1	PPCDC
ELF1	PPCS
ELF1	PPDPF
ELF1	PPFIA1
ELF1	PPHLN1
ELF1	PPIA
ELF1	PPID
ELF1	PPIE
ELF1	PPIG
ELF1	PPIH
ELF1	PPIL2
ELF1	PPIL3
ELF1	PPIL4

ELF1	PPIP5K2
ELF1	PPM1A
ELF1	PPM1B
ELF1	PPM1D
ELF1	PPM1G
ELF1	PPME1
ELF1	PPP1CA
ELF1	PPP1CB
ELF1	PPP1CC
ELF1	PPP1R10
ELF1	PPP1R11
ELF1	PPP1R12A
ELF1	PPP1R16A
ELF1	PPP1R18
ELF1	PPP1R2
ELF1	PPP1R35
ELF1	PPP1R7
ELF1	PPP2CA
ELF1	PPP2R1A
ELF1	PPP2R1B
ELF1	PPP2R2A
ELF1	PPP2R3C
ELF1	PPP2R5A
ELF1	PPP2R5C
ELF1	PPP2R5D
ELF1	PPP2R5E
ELF1	PPP3CB
ELF1	PPP3CC
ELF1	PPP4C
ELF1	PPP4R1
ELF1	PPP4R2
ELF1	PPP4R3A
ELF1	PPP4R3B
ELF1	PPP5C
ELF1	PPP6C
ELF1	PPP6R1
ELF1	PPP6R2
ELF1	PPP6R3
ELF1	PQBP1
ELF1	PRAF2
ELF1	PRDM15
ELF1	PRDM2
ELF1	PRDM4
ELF1	PRDX1
ELF1	PRDX2
ELF1	PRDX3
ELF1	PRDX5
ELF1	PRDX6
ELF1	PRELID3B
ELF1	PRIM1

ELF1	PRIM2
ELF1	PRKAB1
ELF1	PRKACB
ELF1	PRKAG1
ELF1	PRKAR1A
ELF1	PRKAR2A
ELF1	PRKAR2B
ELF1	PRKCA
ELF1	PRKCD
ELF1	PRKCI
ELF1	PRKD2
ELF1	PRKD3
ELF1	PRKDC
ELF1	PRKRA
ELF1	PRKRIP1
ELF1	PRKX
ELF1	PRMT2
ELF1	PRMT7
ELF1	PRMT9
ELF1	PROSER1
ELF1	PRPF18
ELF1	PRPF19
ELF1	PRPF3
ELF1	PRPF31
ELF1	PRPF38A
ELF1	PRPF38B
ELF1	PRPF40A
ELF1	PRPF4B
ELF1	PRPF6
ELF1	PRPF8
ELF1	PRPS1
ELF1	PRPS2
ELF1	PRPSAP1
ELF1	PRPSAP2
ELF1	PRR11
ELF1	PRR13
ELF1	PRR14
ELF1	PRR14L
ELF1	PRRC2A
ELF1	PRRC2B
ELF1	PRRC2C
ELF1	PSEN1
ELF1	PSENEN
ELF1	PSIP1
ELF1	PSMA1
ELF1	PSMA2
ELF1	PSMA3
ELF1	PSMA3-AS1
ELF1	PSMA4
ELF1	PSMA5

ELF1	PSMB1
ELF1	PSMB10
ELF1	PSMB2
ELF1	PSMB3
ELF1	PSMB4
ELF1	PSMB6
ELF1	PSMB7
ELF1	PSMB8
ELF1	PSMB9
ELF1	PSMC1
ELF1	PSMC2
ELF1	PSMC3
ELF1	PSMC3IP
ELF1	PSMC5
ELF1	PSMC6
ELF1	PSMD1
ELF1	PSMD10
ELF1	PSMD11
ELF1	PSMD12
ELF1	PSMD13
ELF1	PSMD14
ELF1	PSMD2
ELF1	PSMD4
ELF1	PSMD6
ELF1	PSMD7
ELF1	PSMD8
ELF1	PSMD9
ELF1	PSME1
ELF1	PSME3
ELF1	PSME4
ELF1	PSMF1
ELF1	PSMG1
ELF1	PSMG2
ELF1	PSMG3
ELF1	PSMG3-AS1
ELF1	PSMG4
ELF1	PSPC1
ELF1	PSRC1
ELF1	PTAFR
ELF1	PTBP1
ELF1	PTBP3
ELF1	PTDSS1
ELF1	PTEN
ELF1	PTER
ELF1	PTGES3
ELF1	PTK2
ELF1	PTK2B
ELF1	PTMA
ELF1	PTP4A1
ELF1	PTP4A2

ELF1	PTPN1
ELF1	PTPN11
ELF1	PTPN18
ELF1	PTPN2
ELF1	PTPN22
ELF1	PTPN6
ELF1	PTPN7
ELF1	PTPRA
ELF1	PTPRC
ELF1	PTPRS
ELF1	PTRHD1
ELF1	PTS
ELF1	PTTG1
ELF1	PTTG1IP
ELF1	PUDP
ELF1	PUF60
ELF1	PUM1
ELF1	PUS10
ELF1	PUS7L
ELF1	PVT1
ELF1	PWP1
ELF1	PWWP2A
ELF1	PXK
ELF1	PXMP2
ELF1	PXN
ELF1	PYCARD
ELF1	PYCR2
ELF1	PYM1
ELF1	PYROXD1
ELF1	PYURF
ELF1	QARS
ELF1	QRICH1
ELF1	QRICH2
ELF1	QSOX2
ELF1	QTRT2
ELF1	R3HDM1
ELF1	R3HDM4
ELF1	RAB10
ELF1	RAB11A
ELF1	RAB11B
ELF1	RAB11B-AS1
ELF1	RAB11FIP1
ELF1	RAB14
ELF1	RAB18
ELF1	RAB1A
ELF1	RAB1B
ELF1	RAB21
ELF1	RAB22A
ELF1	RAB23
ELF1	RAB27A

ELF1	RAB28
ELF1	RAB2A
ELF1	RAB30
ELF1	RAB30-AS1
ELF1	RAB33A
ELF1	RAB35
ELF1	RAB39B
ELF1	RAB3GAP1
ELF1	RAB3GAP2
ELF1	RAB3IP
ELF1	RAB4A
ELF1	RAB5A
ELF1	RAB5B
ELF1	RAB5C
ELF1	RAB6A
ELF1	RAB7A
ELF1	RAB8A
ELF1	RAB8B
ELF1	RAB9A
ELF1	RAB9B
ELF1	RABEP1
ELF1	RABEP2
ELF1	RABGAP1
ELF1	RABGAP1L
ELF1	RABGEF1
ELF1	RABGGTA
ELF1	RABGGTB
ELF1	RABIF
ELF1	RABL3
ELF1	RABL6
ELF1	RAC1
ELF1	RAC2
ELF1	RACGAP1
ELF1	RACK1
ELF1	RAD1
ELF1	RAD17
ELF1	RAD21
ELF1	RAD23A
ELF1	RAD23B
ELF1	RAD51AP1
ELF1	RAD51C
ELF1	RAD51D
ELF1	RAD54L
ELF1	RAE1
ELF1	RAF1
ELF1	RALA
ELF1	RALBP1
ELF1	RALGPS2
ELF1	RALY
ELF1	RAMP1

ELF1	RAN
ELF1	RANBP2
ELF1	RANBP3
ELF1	RANGAP1
ELF1	RANGRF
ELF1	RAP1A
ELF1	RAP1B
ELF1	RAP1GDS1
ELF1	RAP2A
ELF1	RAP2C-AS1
ELF1	RAPGEF1
ELF1	RAPGEF5
ELF1	RARS2
ELF1	RASA2
ELF1	RASAL3
ELF1	RASGEF1B
ELF1	RASGRP1
ELF1	RASGRP2
ELF1	RASGRP3
ELF1	RASL11A
ELF1	RASSF1
ELF1	RASSF1-AS1
ELF1	RASSF2
ELF1	RASSF3
ELF1	RASSF5
ELF1	RASSF6
ELF1	RASSF7
ELF1	RB1
ELF1	RB1CC1
ELF1	RBBP4
ELF1	RBBP5
ELF1	RBBP6
ELF1	RBBP7
ELF1	RBBP8
ELF1	RBCK1
ELF1	RBFOX2
ELF1	RBL1
ELF1	RBL2
ELF1	RBM10
ELF1	RBM14
ELF1	RBM15
ELF1	RBM15B
ELF1	RBM18
ELF1	RBM19
ELF1	RBM22
ELF1	RBM23
ELF1	RBM25
ELF1	RBM26
ELF1	RBM26-AS1
ELF1	RBM3

ELF1	RBM38
ELF1	RBM39
ELF1	RBM4
ELF1	RBM41
ELF1	RBM42
ELF1	RBM45
ELF1	RBM48
ELF1	RBM4B
ELF1	RBM5
ELF1	RBM6
ELF1	RBM7
ELF1	RBM8A
ELF1	RBMX
ELF1	RBMX2
ELF1	RBMXL1
ELF1	RBPJ
ELF1	RBX1
ELF1	RC3H1
ELF1	RCC1
ELF1	RCC1L
ELF1	RCC2
ELF1	RCCD1
ELF1	RCHY1
ELF1	RCN1
ELF1	RCN2
ELF1	RCOR1
ELF1	RCSD1
ELF1	RDH14
ELF1	RECQL
ELF1	REEP3
ELF1	REEP4
ELF1	REEP5
ELF1	REL
ELF1	RELB
ELF1	RELL2
ELF1	RELT
ELF1	REPIN1
ELF1	RER1
ELF1	RERE
ELF1	REST
ELF1	REV1
ELF1	REV3L
ELF1	RFC1
ELF1	RFC2
ELF1	RFC3
ELF1	RFC4
ELF1	RFC5
ELF1	RFK
ELF1	RFTN1
ELF1	RFWD3

ELF1	RFX1
ELF1	RFX5
ELF1	RFX7
ELF1	RFXANK
ELF1	RGP1
ELF1	RGS1
ELF1	RGS10
ELF1	RGS13
ELF1	RGS19
ELF1	RGS2
ELF1	RGS3
ELF1	RHBDD2
ELF1	RHBDF2
ELF1	RHEB
ELF1	RHEBL1
ELF1	RHNO1
ELF1	RHOA
ELF1	RHOG
ELF1	RHOH
ELF1	RHOQ
ELF1	RHOT1
ELF1	RHOT2
ELF1	RIC8A
ELF1	RIF1
ELF1	RIMKLB
ELF1	RING1
ELF1	RINL
ELF1	RINT1
ELF1	RIOK1
ELF1	RIOK3
ELF1	RIPK1
ELF1	RIPK2
ELF1	RIT1
ELF1	RMDN1
ELF1	RMI1
ELF1	RMI2
ELF1	RMND1
ELF1	RMND5A
ELF1	RMND5B
ELF1	RNASEH1
ELF1	RNASEH2A
ELF1	RNASEH2B
ELF1	RNASEH2C
ELF1	RND1
ELF1	RNF10
ELF1	RNF111
ELF1	RNF113A
ELF1	RNF114
ELF1	RNF115
ELF1	RNF126

ELF1	RNF13
ELF1	RNF139
ELF1	RNF14
ELF1	RNF144B
ELF1	RNF166
ELF1	RNF167
ELF1	RNF168
ELF1	RNF170
ELF1	RNF181
ELF1	RNF19A
ELF1	RNF20
ELF1	RNF212
ELF1	RNF214
ELF1	RNF219
ELF1	RNF220
ELF1	RNF25
ELF1	RNF26
ELF1	RNF34
ELF1	RNF38
ELF1	RNF4
ELF1	RNF41
ELF1	RNF44
ELF1	RNF6
ELF1	RNF7
ELF1	RNF8
ELF1	RNFT1
ELF1	RNFT2
ELF1	RNGTT
ELF1	RNMT
ELF1	RNPEPL1
ELF1	RNPS1
ELF1	ROCK1
ELF1	ROCK2
ELF1	ROMO1
ELF1	RP2
ELF1	RPA2
ELF1	RPA3
ELF1	RPAP2
ELF1	RPAP3
ELF1	RPF1
ELF1	RPIA
ELF1	RPL10
ELF1	RPL10A
ELF1	RPL11
ELF1	RPL12
ELF1	RPL13
ELF1	RPL13A
ELF1	RPL14
ELF1	RPL15
ELF1	RPL17

ELF1	RPL18
ELF1	RPL18A
ELF1	RPL19
ELF1	RPL21
ELF1	RPL22
ELF1	RPL23
ELF1	RPL23A
ELF1	RPL24
ELF1	RPL26
ELF1	RPL27
ELF1	RPL27A
ELF1	RPL28
ELF1	RPL29
ELF1	RPL3
ELF1	RPL30
ELF1	RPL31
ELF1	RPL32
ELF1	RPL34
ELF1	RPL35
ELF1	RPL35A
ELF1	RPL36
ELF1	RPL36A
ELF1	RPL36AL
ELF1	RPL37
ELF1	RPL37A
ELF1	RPL38
ELF1	RPL39
ELF1	RPL39L
ELF1	RPL4
ELF1	RPL41
ELF1	RPL5
ELF1	RPL6
ELF1	RPL7
ELF1	RPL7L1
ELF1	RPL8
ELF1	RPL9
ELF1	RPLP0
ELF1	RPLP1
ELF1	RPLP2
ELF1	RPP14
ELF1	RPP25L
ELF1	RPP30
ELF1	RPP38
ELF1	RPRD1A
ELF1	RPRD1B
ELF1	RPRD2
ELF1	RPS10
ELF1	RPS11
ELF1	RPS13
ELF1	RPS14

ELF1	RPS15
ELF1	RPS15A
ELF1	RPS16
ELF1	RPS17
ELF1	RPS18
ELF1	RPS19
ELF1	RPS19BP1
ELF1	RPS2
ELF1	RPS20
ELF1	RPS23
ELF1	RPS24
ELF1	RPS25
ELF1	RPS27
ELF1	RPS27A
ELF1	RPS27L
ELF1	RPS29
ELF1	RPS3
ELF1	RPS3A
ELF1	RPS4X
ELF1	RPS4Y1
ELF1	RPS5
ELF1	RPS6
ELF1	RPS6KA1
ELF1	RPS6KA4
ELF1	RPS6KA5
ELF1	RPS6KB2
ELF1	RPS6KC1
ELF1	RPS7
ELF1	RPS8
ELF1	RPS9
ELF1	RPSA
ELF1	RPUSD2
ELF1	RPUSD3
ELF1	RRAGA
ELF1	RRAGB
ELF1	RRAGC
ELF1	RRAS2
ELF1	RRM1
ELF1	RRM2
ELF1	RRM2B
ELF1	RRNAD1
ELF1	RRP36
ELF1	RRP7A
ELF1	RRP8
ELF1	RSBN1
ELF1	RSBN1L
ELF1	RSF1
ELF1	RSL24D1
ELF1	RSPRY1
ELF1	RSRC1

ELF1	RSRC2
ELF1	RSRP1
ELF1	RSU1
ELF1	RTCA
ELF1	RTCB
ELF1	RTF1
ELF1	RTKN2
ELF1	RTN2
ELF1	RTN3
ELF1	RTN4
ELF1	RTN4IP1
ELF1	RUBCN
ELF1	RUFY1
ELF1	RUFY3
ELF1	RUNDC1
ELF1	RUSC1
ELF1	RUVBL1
ELF1	RUVBL2
ELF1	RWDD4
ELF1	RYK
ELF1	S100A10
ELF1	S100PBP
ELF1	S1PR2
ELF1	S1PR4
ELF1	SAAL1
ELF1	SAC3D1
ELF1	SACM1L
ELF1	SAE1
ELF1	SAFB
ELF1	SAFB2
ELF1	SAMD10
ELF1	SAMM50
ELF1	SAMSN1
ELF1	SAP130
ELF1	SAP18
ELF1	SAP30
ELF1	SAP30BP
ELF1	SAR1A
ELF1	SARS
ELF1	SARS2
ELF1	SART1
ELF1	SART3
ELF1	SASH3
ELF1	SASS6
ELF1	SAV1
ELF1	SBF1
ELF1	SBF2
ELF1	SC5D
ELF1	SCAF1
ELF1	SCAF11

ELF1	SCAF4
ELF1	SCAI
ELF1	SCAMP2
ELF1	SCAND1
ELF1	SCARB1
ELF1	SCFD1
ELF1	SCIMP
ELF1	SCLT1
ELF1	SCLY
ELF1	SCMH1
ELF1	SCNM1
ELF1	SCO1
ELF1	SCOC
ELF1	SCOC-AS1
ELF1	SCPEP1
ELF1	SCRN1
ELF1	SDCBP
ELF1	SDCCAG8
ELF1	SDE2
ELF1	SDF2
ELF1	SDHA
ELF1	SDHAF1
ELF1	SDHAF2
ELF1	SDHAF3
ELF1	SDHB
ELF1	SDHC
ELF1	SDHD
ELF1	SDR16C5
ELF1	SDR39U1
ELF1	SEC11A
ELF1	SEC13
ELF1	SEC14L1
ELF1	SEC22A
ELF1	SEC22B
ELF1	SEC22C
ELF1	SEC23A
ELF1	SEC24C
ELF1	SEC61G
ELF1	SEC62
ELF1	SEC63
ELF1	SECISBP2
ELF1	SECISBP2L
ELF1	SEL1L3
ELF1	SEMA4A
ELF1	SENP1
ELF1	SENP2
ELF1	SENP5
ELF1	SENP6
ELF1	SEPHS1
ELF1	SEPHS2

ELF1	SEPSECS
ELF1	SEPT11
ELF1	SEPT2
ELF1	SEPT6
ELF1	SEPT7
ELF1	SEPT9
ELF1	SERBP1
ELF1	SERF2
ELF1	SERINC3
ELF1	SERP2
ELF1	SERPINB1
ELF1	SERTAD2
ELF1	SESTD1
ELF1	SET
ELF1	SETD2
ELF1	SETD5
ELF1	SETD9
ELF1	SETDB2
ELF1	SETMAR
ELF1	SETX
ELF1	SF1
ELF1	SF3A1
ELF1	SF3A2
ELF1	SF3A3
ELF1	SF3B1
ELF1	SF3B2
ELF1	SF3B4
ELF1	SF3B5
ELF1	SF3B6
ELF1	SFI1
ELF1	SFMBT1
ELF1	SFPQ
ELF1	SFSWAP
ELF1	SFT2D1
ELF1	SFT2D2
ELF1	SFT2D3
ELF1	SFXN1
ELF1	SGK3
ELF1	SGMS1
ELF1	SGO1
ELF1	SGO2
ELF1	SGPP1
ELF1	SGSM3
ELF1	SH2B2
ELF1	SH2D3C
ELF1	SH3BGRL
ELF1	SH3BP1
ELF1	SH3BP5
ELF1	SH3BP5L
ELF1	SH3GLB1

ELF1	SH3GLB2
ELF1	SH3KBP1
ELF1	SHANK2
ELF1	SHARPIN
ELF1	SHC1
ELF1	SHCBP1
ELF1	SHISA5
ELF1	SHISA8
ELF1	SHKBP1
ELF1	SHMT2
ELF1	SHOC2
ELF1	SHQ1
ELF1	SIAH1
ELF1	SIAH2
ELF1	SIGIRR
ELF1	SIK2
ELF1	SIKE1
ELF1	SIPA1
ELF1	SIPA1L1
ELF1	SIPA1L3
ELF1	SIRT1
ELF1	SIRT2
ELF1	SIRT5
ELF1	SIRT6
ELF1	SIRT7
ELF1	SKA2
ELF1	SKA3
ELF1	SKAP2
ELF1	SKIL
ELF1	SKP1
ELF1	SLAIN1
ELF1	SLAIN2
ELF1	SLAMF6
ELF1	SLBP
ELF1	SLC10A3
ELF1	SLC12A2
ELF1	SLC15A4
ELF1	SLC16A1
ELF1	SLC16A4
ELF1	SLC16A7
ELF1	SLC1A4
ELF1	SLC20A1
ELF1	SLC25A1
ELF1	SLC25A11
ELF1	SLC25A12
ELF1	SLC25A13
ELF1	SLC25A17
ELF1	SLC25A19
ELF1	SLC25A20
ELF1	SLC25A25

ELF1	SLC25A26
ELF1	SLC25A3
ELF1	SLC25A36
ELF1	SLC25A37
ELF1	SLC25A38
ELF1	SLC25A40
ELF1	SLC25A45
ELF1	SLC25A5
ELF1	SLC27A3
ELF1	SLC2A6
ELF1	SLC30A4
ELF1	SLC30A5
ELF1	SLC35A1
ELF1	SLC35A4
ELF1	SLC35A5
ELF1	SLC35B2
ELF1	SLC35B3
ELF1	SLC35C1
ELF1	SLC35C2
ELF1	SLC35E3
ELF1	SLC35F5
ELF1	SLC35F6
ELF1	SLC35G1
ELF1	SLC36A4
ELF1	SLC37A1
ELF1	SLC38A1
ELF1	SLC38A2
ELF1	SLC38A5
ELF1	SLC38A9
ELF1	SLC39A1
ELF1	SLC39A3
ELF1	SLC41A1
ELF1	SLC41A3
ELF1	SLC43A2
ELF1	SLC44A2
ELF1	SLC46A3
ELF1	SLC48A1
ELF1	SLC4A1AP
ELF1	SLC4A8
ELF1	SLC5A3
ELF1	SLC9A3R1
ELF1	SLC9A7
ELF1	SLC9A8
ELF1	SLC9B2
ELF1	SLCO3A1
ELF1	SLF1
ELF1	SLF2
ELF1	SLK
ELF1	SLMAP
ELF1	SLTM

ELF1	SLU7
ELF1	SLX4
ELF1	SLX4IP
ELF1	SMAD2
ELF1	SMAD4
ELF1	SMAD5
ELF1	SMAP1
ELF1	SMAP2
ELF1	SMARCA2
ELF1	SMARCA4
ELF1	SMARCA5
ELF1	SMARCAD1
ELF1	SMARCAL1
ELF1	SMARCB1
ELF1	SMARCC1
ELF1	SMARCC2
ELF1	SMARCD1
ELF1	SMARCD2
ELF1	SMARCE1
ELF1	SMC1A
ELF1	SMC2
ELF1	SMC3
ELF1	SMC4
ELF1	SMC5
ELF1	SMCHD1
ELF1	SMCO4
ELF1	SMCR5
ELF1	SMG1
ELF1	SMG6
ELF1	SMG7
ELF1	SMG9
ELF1	SMIM10L1
ELF1	SMIM12
ELF1	SMIM14
ELF1	SMIM15
ELF1	SMIM19
ELF1	SMIM20
ELF1	SMIM7
ELF1	SMN1
ELF1	SMPD1
ELF1	SMPD4
ELF1	SMS
ELF1	SMTN
ELF1	SMU1
ELF1	SMUG1
ELF1	SMURF1
ELF1	SNAP23
ELF1	SNAP29
ELF1	SNAPC1
ELF1	SNAPC3

ELF1	SNAPC5
ELF1	SNAPIN
ELF1	SNF8
ELF1	SNHG21
ELF1	SNHG25
ELF1	SNHG8
ELF1	SNHG9
ELF1	SNRNP200
ELF1	SNRNP25
ELF1	SNRNP27
ELF1	SNRNP35
ELF1	SNRNP40
ELF1	SNRNP48
ELF1	SNRNP70
ELF1	SNRPA1
ELF1	SNRPB
ELF1	SNRPB2
ELF1	SNRPC
ELF1	SNRPD1
ELF1	SNRPD2
ELF1	SNRPF
ELF1	SNRPG
ELF1	SNRPN
ELF1	SNTA1
ELF1	SNTB1
ELF1	SNTB2
ELF1	SNU13
ELF1	SNW1
ELF1	SNX1
ELF1	SNX10
ELF1	SNX13
ELF1	SNX14
ELF1	SNX15
ELF1	SNX17
ELF1	SNX2
ELF1	SNX20
ELF1	SNX22
ELF1	SNX25
ELF1	SNX29
ELF1	SNX3
ELF1	SNX5
ELF1	SNX6
ELF1	SNX8
ELF1	SNX9
ELF1	SOAT1
ELF1	SOBP
ELF1	SOCS1
ELF1	SOD1
ELF1	SON
ELF1	SOS1

ELF1	SOX4
ELF1	SP100
ELF1	SP110
ELF1	SP140L
ELF1	SP2
ELF1	SP3
ELF1	SPA17
ELF1	SPAG5
ELF1	SPAG7
ELF1	SPAG9
ELF1	SPAST
ELF1	SPATA24
ELF1	SPATA33
ELF1	SPATS2
ELF1	SPC24
ELF1	SPC25
ELF1	SPDL1
ELF1	SPEN
ELF1	SPG11
ELF1	SPG21
ELF1	SPG7
ELF1	SPHK2
ELF1	SPI1
ELF1	SPICE1
ELF1	SPIDR
ELF1	SPIN2B
ELF1	SPINK2
ELF1	SPINT1
ELF1	SPINT2
ELF1	SPNS1
ELF1	SPON1
ELF1	SPOP
ELF1	SPOPL
ELF1	SPPL2A
ELF1	SPRED2
ELF1	SPRTN
ELF1	SPSB3
ELF1	SPTAN1
ELF1	SPTBN1
ELF1	SPTLC1
ELF1	SPTLC2
ELF1	SPTSSA
ELF1	SQLE
ELF1	SREBF2
ELF1	SREK1
ELF1	SREK1IP1
ELF1	SRGAP2B
ELF1	SRP14
ELF1	SRP19
ELF1	SRP54

ELF1	SRP72
ELF1	SRP9
ELF1	SRPK1
ELF1	SRPK2
ELF1	SRRM1
ELF1	SRRM2
ELF1	SRRT
ELF1	SRSF1
ELF1	SRSF10
ELF1	SRSF3
ELF1	SRSF4
ELF1	SRSF5
ELF1	SRSF6
ELF1	SRSF7
ELF1	SRSF8
ELF1	SRSF9
ELF1	SS18
ELF1	SS18L2
ELF1	SSB
ELF1	SSBP1
ELF1	SSBP2
ELF1	SSBP3
ELF1	SSBP4
ELF1	SSH2
ELF1	SSNA1
ELF1	SSU72
ELF1	ST13
ELF1	ST14
ELF1	ST20
ELF1	ST3GAL2
ELF1	ST3GAL5
ELF1	ST6GAL1
ELF1	STAG1
ELF1	STAG2
ELF1	STAG3
ELF1	STAM2
ELF1	STAMBP
ELF1	STAP1
ELF1	STARD3
ELF1	STARD3NL
ELF1	STARD4
ELF1	STARD7
ELF1	STAT2
ELF1	STAT3
ELF1	STAT5B
ELF1	STAU1
ELF1	STAU2
ELF1	STIL
ELF1	STIM2
ELF1	STIP1

ELF1	STK10
ELF1	STK11IP
ELF1	STK16
ELF1	STK17B
ELF1	STK24
ELF1	STK25
ELF1	STK26
ELF1	STK38
ELF1	STK38L
ELF1	STK39
ELF1	STK4
ELF1	STK40
ELF1	STMN1
ELF1	STOX1
ELF1	STPG1
ELF1	STRADA
ELF1	STRAP
ELF1	STRBP
ELF1	STRN
ELF1	STRN3
ELF1	STT3B
ELF1	STUB1
ELF1	STX10
ELF1	STX11
ELF1	STX12
ELF1	STX16
ELF1	STX17
ELF1	STX18
ELF1	STX2
ELF1	STX4
ELF1	STX5
ELF1	STX7
ELF1	STX8
ELF1	STXBP2
ELF1	STXBP3
ELF1	STXBP6
ELF1	STYX
ELF1	STYXL1
ELF1	SUCLA2
ELF1	SUCLG1
ELF1	SUCLG2
ELF1	SUDS3
ELF1	SUFU
ELF1	SUGCT
ELF1	SUGP1
ELF1	SUGP2
ELF1	SUGT1
ELF1	SULF2
ELF1	SUMO1
ELF1	SUMO2

ELF1	SUMO3
ELF1	SUN2
ELF1	SUPT16H
ELF1	SUPT20H
ELF1	SUPT4H1
ELF1	SUPT5H
ELF1	SUPV3L1
ELF1	SURF1
ELF1	SUSD3
ELF1	SUSD6
ELF1	SUV39H1
ELF1	SUV39H2
ELF1	SUZ12
ELF1	SVBP
ELF1	SWAP70
ELF1	SWI5
ELF1	SWSAP1
ELF1	SYAP1
ELF1	SYBU
ELF1	SYF2
ELF1	SYK
ELF1	SYMPK
ELF1	SYNC
ELF1	SYNCRIP
ELF1	SYNE1
ELF1	SYNE2
ELF1	SYNJ2BP
ELF1	SYNRG
ELF1	SYPL1
ELF1	SYT17
ELF1	SYTL2
ELF1	SZRD1
ELF1	TAB2
ELF1	TACC1
ELF1	TACC3
ELF1	TADA3
ELF1	TAF1
ELF1	TAF10
ELF1	TAF11
ELF1	TAF12
ELF1	TAF15
ELF1	TAF1B
ELF1	TAF1D
ELF1	TAF2
ELF1	TAF5
ELF1	TAF6
ELF1	TAF7
ELF1	TAF8
ELF1	TAF9
ELF1	TAGAP

ELF1	TALDO1
ELF1	TANGO2
ELF1	TANK
ELF1	TAOK1
ELF1	TAOK3
ELF1	TAP2
ELF1	TAPT1
ELF1	TARDBP
ELF1	TARS2
ELF1	TATDN1
ELF1	TATDN3
ELF1	TAX1BP1
ELF1	TAX1BP3
ELF1	TAZ
ELF1	TBC1D1
ELF1	TBC1D10A
ELF1	TBC1D10C
ELF1	TBC1D15
ELF1	TBC1D17
ELF1	TBC1D20
ELF1	TBC1D22A
ELF1	TBC1D23
ELF1	TBC1D25
ELF1	TBC1D4
ELF1	TBC1D5
ELF1	TBC1D9B
ELF1	TBCA
ELF1	TBCB
ELF1	TBCC
ELF1	TBCCD1
ELF1	TBCK
ELF1	TBK1
ELF1	TBL1X
ELF1	TBL1XR1
ELF1	TBP
ELF1	TBPL1
ELF1	TBRG1
ELF1	TCAIM
ELF1	TCEA1
ELF1	TCEA2
ELF1	TCEAL3
ELF1	TCEAL4
ELF1	TCEAL8
ELF1	TCEAL9
ELF1	TCERG1
ELF1	TCF12
ELF1	TCF19
ELF1	TCF25
ELF1	TCF3
ELF1	TCF4

ELF1	TCHP
ELF1	TCL1A
ELF1	TCL1B
ELF1	TCOF1
ELF1	TCTA
ELF1	TCTE3
ELF1	TCTEX1D2
ELF1	TDG
ELF1	TDP1
ELF1	TDP2
ELF1	TDRD3
ELF1	TDRKH
ELF1	TEC
ELF1	TEFM
ELF1	TELO2
ELF1	TERF1
ELF1	TERF2
ELF1	TERF2IP
ELF1	TESC
ELF1	TESPA1
ELF1	TET2
ELF1	TEX10
ELF1	TEX264
ELF1	TEX30
ELF1	TFB1M
ELF1	TFDP2
ELF1	TFEB
ELF1	TFIP11
ELF1	TFPT
ELF1	TFRC
ELF1	TGFBR1
ELF1	TGIF2
ELF1	TGM2
ELF1	TGOLN2
ELF1	THAP1
ELF1	THAP11
ELF1	THAP12
ELF1	THAP2
ELF1	THAP3
ELF1	THAP4
ELF1	THAP7
ELF1	THAP9
ELF1	THG1L
ELF1	THOC1
ELF1	THOC2
ELF1	THOC3
ELF1	THOC5
ELF1	THOC6
ELF1	THOC7
ELF1	THOP1

ELF1	THRAP3
ELF1	THUMPD1
ELF1	TIA1
ELF1	TIAL1
ELF1	TIAM2
ELF1	TIFA
ELF1	TIGAR
ELF1	TIMELESS
ELF1	TIMM10
ELF1	TIMM10B
ELF1	TIMM17B
ELF1	TIMM21
ELF1	TIMM23
ELF1	TIMM8B
ELF1	TIMMDC1
ELF1	TINF2
ELF1	TIPARP
ELF1	TIPIN
ELF1	TIPRL
ELF1	TKT
ELF1	TLDC1
ELF1	TLE4
ELF1	TLK1
ELF1	TLK2
ELF1	TLN1
ELF1	TLR1
ELF1	TLR10
ELF1	TM2D1
ELF1	TM7SF3
ELF1	TM9SF2
ELF1	TM9SF3
ELF1	TMA16
ELF1	TMA7
ELF1	TMBIM4
ELF1	TMBIM6
ELF1	TMC6
ELF1	TMC8
ELF1	TMCO1
ELF1	TMCO4
ELF1	TMED8
ELF1	TMEM106B
ELF1	TMEM106C
ELF1	TMEM107
ELF1	TMEM11
ELF1	TMEM123
ELF1	TMEM126B
ELF1	TMEM128
ELF1	TMEM131
ELF1	TMEM138
ELF1	TMEM14A

ELF1	TMEM14B
ELF1	TMEM14C
ELF1	TMEM154
ELF1	TMEM156
ELF1	TMEM160
ELF1	TMEM161A
ELF1	TMEM165
ELF1	TMEM167A
ELF1	TMEM170A
ELF1	TMEM18
ELF1	TMEM183A
ELF1	TMEM19
ELF1	TMEM199
ELF1	TMEM206
ELF1	TMEM218
ELF1	TMEM219
ELF1	TMEM222
ELF1	TMEM223
ELF1	TMEM230
ELF1	TMEM234
ELF1	TMEM242
ELF1	TMEM243
ELF1	TMEM245
ELF1	TMEM248
ELF1	TMEM256
ELF1	TMEM258
ELF1	TMEM259
ELF1	TMEM263
ELF1	TMEM33
ELF1	TMEM39B
ELF1	TMEM50A
ELF1	TMEM59
ELF1	TMEM60
ELF1	TMEM69
ELF1	TMEM70
ELF1	TMEM87A
ELF1	TMEM91
ELF1	TMEM97
ELF1	TMEM9B
ELF1	TMF1
ELF1	TMOD3
ELF1	TMPO
ELF1	TMPO-AS1
ELF1	TMSB10
ELF1	TMSB4X
ELF1	TMUB1
ELF1	TMUB2
ELF1	TMX1
ELF1	TMX3
ELF1	TMX4

ELF1	TNFAIP8
ELF1	TNFAIP8L1
ELF1	TNFRSF13C
ELF1	TNKS2
ELF1	TNKS2-AS1
ELF1	TNNC2
ELF1	TNPO1
ELF1	TNPO2
ELF1	TNPO3
ELF1	TNRC6A
ELF1	TNRC6B
ELF1	TNRC6C
ELF1	TOB2
ELF1	TOMM20
ELF1	TOMM34
ELF1	TOMM7
ELF1	TOP1
ELF1	TOP2A
ELF1	TOP2B
ELF1	TOP3B
ELF1	TOPBP1
ELF1	TOPORS
ELF1	TOR2A
ELF1	TOR4A
ELF1	TOX
ELF1	TOX4
ELF1	TP53
ELF1	TP53BP2
ELF1	TP53I13
ELF1	TP53INP2
ELF1	TP53RK
ELF1	TP53TG1
ELF1	TPD52
ELF1	TPD52L2
ELF1	TPGS1
ELF1	TPGS2
ELF1	TPM1
ELF1	TPM3
ELF1	TPM4
ELF1	TPP2
ELF1	TPR
ELF1	TPRA1
ELF1	TPRKB
ELF1	TPT1
ELF1	TPX2
ELF1	TRA2A
ELF1	TRA2B
ELF1	TRABD
ELF1	TRAF2
ELF1	TRAF3IP2

ELF1	TRAF5
ELF1	TRAFD1
ELF1	TRAPPC1
ELF1	TRAPPC11
ELF1	TRAPPC12
ELF1	TRAPPC2
ELF1	TRAPPC2B
ELF1	TRAPPC3
ELF1	TRAPPC4
ELF1	TRAPPC6A
ELF1	TRAPPC6B
ELF1	TRG-AS1
ELF1	TRIB2
ELF1	TRIM13
ELF1	TRIM14
ELF1	TRIM24
ELF1	TRIM26
ELF1	TRIM27
ELF1	TRIM28
ELF1	TRIM33
ELF1	TRIM38
ELF1	TRIM4
ELF1	TRIM44
ELF1	TRIM5
ELF1	TRIM52
ELF1	TRIM56
ELF1	TRIM59
ELF1	TRIM69
ELF1	TRIM8
ELF1	TRIOBP
ELF1	TRIP11
ELF1	TRIP12
ELF1	TRIP13
ELF1	TRIP4
ELF1	TRMT10B
ELF1	TRMT11
ELF1	TRMT112
ELF1	TRMT1L
ELF1	TRMT5
ELF1	TRMU
ELF1	TRNAU1AP
ELF1	TRNT1
ELF1	TROAP
ELF1	TROVE2
ELF1	TRPC4AP
ELF1	TRPT1
ELF1	TRPV2
ELF1	TRRAP
ELF1	TRUB1
ELF1	TSC1

ELF1	TSC2
ELF1	TSC22D2
ELF1	TSC22D3
ELF1	TSC22D4
ELF1	TSEN34
ELF1	TSEN54
ELF1	TSFM
ELF1	TSG101
ELF1	TSN
ELF1	TSNAX
ELF1	TSPAN13
ELF1	TSPAN14
ELF1	TSPO
ELF1	TSPYL4
ELF1	TSR2
ELF1	TSR3
ELF1	TSSC4
ELF1	TSTA3
ELF1	TTC1
ELF1	TTC13
ELF1	TTC14
ELF1	TTC17
ELF1	TTC3
ELF1	TTC30A
ELF1	TTC30B
ELF1	TTC32
ELF1	TTC33
ELF1	TTC37
ELF1	TTC39C
ELF1	TTC5
ELF1	TTC7A
ELF1	TTC9C
ELF1	TTF1
ELF1	TTF2
ELF1	TTI1
ELF1	TTI2
ELF1	TTK
ELF1	TTPAL
ELF1	TTTY15
ELF1	TUBA1A
ELF1	TUBA1B
ELF1	TUBA1C
ELF1	TUBA4A
ELF1	TUBB
ELF1	TUBB2A
ELF1	TUBB2B
ELF1	TUBB4B
ELF1	TUBB6
ELF1	TUBD1
ELF1	TUBG1

ELF1	TUFM
ELF1	TVP23C
ELF1	TWF2
ELF1	TWISTNB
ELF1	TXLNB
ELF1	TXLNG
ELF1	TXN2
ELF1	TXNDC16
ELF1	TXNDC9
ELF1	TXNL1
ELF1	TXNRD1
ELF1	TYMS
ELF1	TYSND1
ELF1	TYW5
ELF1	U2AF2
ELF1	U2SURP
ELF1	UACA
ELF1	UBA1
ELF1	UBA2
ELF1	UBA3
ELF1	UBA52
ELF1	UBA6
ELF1	UBA7
ELF1	UBAC2-AS1
ELF1	UBALD1
ELF1	UBAP1
ELF1	UBAP2
ELF1	UBB
ELF1	UBC
ELF1	UBE2A
ELF1	UBE2B
ELF1	UBE2C
ELF1	UBE2D1
ELF1	UBE2D2
ELF1	UBE2D3
ELF1	UBE2E1
ELF1	UBE2E2
ELF1	UBE2E3
ELF1	UBE2G1
ELF1	UBE2H
ELF1	UBE2I
ELF1	UBE2J1
ELF1	UBE2J2
ELF1	UBE2K
ELF1	UBE2L3
ELF1	UBE2L6
ELF1	UBE2N
ELF1	UBE2O
ELF1	UBE2Q1
ELF1	UBE2Q2

ELF1	UBE2R2
ELF1	UBE2S
ELF1	UBE2T
ELF1	UBE2V1
ELF1	UBE2V2
ELF1	UBE3A
ELF1	UBE3B
ELF1	UBE4A
ELF1	UBE4B
ELF1	UBL5
ELF1	UBL7
ELF1	UBL7-AS1
ELF1	UBLCP1
ELF1	UBN1
ELF1	UBP1
ELF1	UBQLN1
ELF1	UBQLN2
ELF1	UBR2
ELF1	UBR3
ELF1	UBR4
ELF1	UBR5
ELF1	UBR5-AS1
ELF1	UBR7
ELF1	UBTD2
ELF1	UBTF
ELF1	UBXN1
ELF1	UBXN11
ELF1	UBXN2A
ELF1	UBXN2B
ELF1	UBXN4
ELF1	UBXN7
ELF1	UCHL3
ELF1	UCHL5
ELF1	UCK1
ELF1	UCKL1
ELF1	UCP2
ELF1	UFC1
ELF1	UFL1
ELF1	UGDH
ELF1	UGGT1
ELF1	UGP2
ELF1	UHMK1
ELF1	UHRF1
ELF1	UHRF2
ELF1	UIMC1
ELF1	UMPS
ELF1	UNC119
ELF1	UNC45A
ELF1	UNC50
ELF1	UNG

ELF1	UPF2
ELF1	UPF3A
ELF1	UQCC1
ELF1	UQCC2
ELF1	UQCC3
ELF1	UQCR10
ELF1	UQCR11
ELF1	UQCRB
ELF1	UQCRC1
ELF1	UQCRC2
ELF1	UQCRFS1
ELF1	UQCRH
ELF1	UQCRQ
ELF1	URGCP
ELF1	URI1
ELF1	URM1
ELF1	UROD
ELF1	UROS
ELF1	USB1
ELF1	USE1
ELF1	USF2
ELF1	USF3
ELF1	USP1
ELF1	USP10
ELF1	USP11
ELF1	USP12
ELF1	USP13
ELF1	USP14
ELF1	USP15
ELF1	USP16
ELF1	USP22
ELF1	USP24
ELF1	USP3
ELF1	USP30-AS1
ELF1	USP33
ELF1	USP34
ELF1	USP37
ELF1	USP39
ELF1	USP4
ELF1	USP42
ELF1	USP47
ELF1	USP48
ELF1	USP5
ELF1	USP53
ELF1	USP6NL
ELF1	USP7
ELF1	USP8
ELF1	USP9X
ELF1	UTP14A
ELF1	UTP18

ELF1	UTP3
ELF1	UTP6
ELF1	UTY
ELF1	UVRAG
ELF1	UXT
ELF1	VAMP1
ELF1	VAMP2
ELF1	VAMP3
ELF1	VAMP8
ELF1	VANGL1
ELF1	VAPA
ELF1	VASP
ELF1	VAV1
ELF1	VBP1
ELF1	VCP
ELF1	VCPIP1
ELF1	VCPKMT
ELF1	VDAC1
ELF1	VDAC3
ELF1	VEZF1
ELF1	VEZT
ELF1	VGLL4
ELF1	VHL
ELF1	VIPAS39
ELF1	VMA21
ELF1	VMP1
ELF1	VNN2
ELF1	VPS11
ELF1	VPS13A
ELF1	VPS13B
ELF1	VPS16
ELF1	VPS25
ELF1	VPS26A
ELF1	VPS28
ELF1	VPS29
ELF1	VPS33A
ELF1	VPS35
ELF1	VPS36
ELF1	VPS39
ELF1	VPS41
ELF1	VPS45
ELF1	VPS4A
ELF1	VPS4B
ELF1	VPS50
ELF1	VPS51
ELF1	VPS53
ELF1	VPS8
ELF1	VRK1
ELF1	VRK3
ELF1	VTA1

ELF1	VTI1A
ELF1	VTI1B
ELF1	WAC
ELF1	WAPL
ELF1	WAS
ELF1	WASF2
ELF1	WBP1
ELF1	WBP2
ELF1	WBP4
ELF1	WDFY2
ELF1	WDHD1
ELF1	WDPCP
ELF1	WDR1
ELF1	WDR26
ELF1	WDR33
ELF1	WDR34
ELF1	WDR37
ELF1	WDR41
ELF1	WDR45B
ELF1	WDR48
ELF1	WDR53
ELF1	WDR60
ELF1	WDR61
ELF1	WDR66
ELF1	WDR70
ELF1	WDR73
ELF1	WDR76
ELF1	WDR77
ELF1	WDR82
ELF1	WDR83
ELF1	WDR83OS
ELF1	WDR90
ELF1	WDTC1
ELF1	WEE1
ELF1	WIPF1
ELF1	WIPF2
ELF1	WIPI2
ELF1	WNK1
ELF1	WNK2
ELF1	WNT6
ELF1	WRAP53
ELF1	WRAP73
ELF1	WRB
ELF1	WSB1
ELF1	WSB2
ELF1	WTAP
ELF1	WWC3
ELF1	XAB2
ELF1	XIAP
ELF1	XIST

ELF1	XKR6
ELF1	XKRX
ELF1	XPA
ELF1	XPNPEP1
ELF1	XPNPEP3
ELF1	XPO1
ELF1	XPO7
ELF1	XRCC1
ELF1	XRCC2
ELF1	XRCC3
ELF1	XRCC4
ELF1	XRCC5
ELF1	XRCC6
ELF1	XRN1
ELF1	XRN2
ELF1	YAF2
ELF1	YARS
ELF1	YARS2
ELF1	YBEY
ELF1	YBX1
ELF1	YEATS4
ELF1	YIPF1
ELF1	YIPF3
ELF1	YIPF4
ELF1	YIPF5
ELF1	YIPF6
ELF1	YJEFN3
ELF1	YLPM1
ELF1	YME1L1
ELF1	YOD1
ELF1	YPEL3
ELF1	YPEL5
ELF1	YTHDC1
ELF1	YTHDC2
ELF1	YTHDF2
ELF1	YTHDF3
ELF1	YWHAB
ELF1	YWHAE
ELF1	YWHAH
ELF1	YWHAQ
ELF1	YWHAZ
ELF1	YY1
ELF1	YY1AP1
ELF1	ZBED5
ELF1	ZBED5-AS1
ELF1	ZBTB1
ELF1	ZBTB10
ELF1	ZBTB14
ELF1	ZBTB17
ELF1	ZBTB20

ELF1	ZBTB24
ELF1	ZBTB25
ELF1	ZBTB3
ELF1	ZBTB38
ELF1	ZBTB43
ELF1	ZBTB44
ELF1	ZBTB7A
ELF1	ZBTB8OS
ELF1	ZC3H10
ELF1	ZC3H13
ELF1	ZC3H14
ELF1	ZC3H15
ELF1	ZC3H18
ELF1	ZC3H4
ELF1	ZC3H6
ELF1	ZC3H7A
ELF1	ZC3H8
ELF1	ZC3HC1
ELF1	ZCCHC17
ELF1	ZCCHC7
ELF1	ZCCHC9
ELF1	ZDBF2
ELF1	ZDHHC12
ELF1	ZDHHC13
ELF1	ZDHHC17
ELF1	ZDHHC21
ELF1	ZDHHC24
ELF1	ZDHHC3
ELF1	ZDHHC6
ELF1	ZEB1
ELF1	ZFAND1
ELF1	ZFAND2B
ELF1	ZFAND3
ELF1	ZFAND4
ELF1	ZFAND5
ELF1	ZFAND6
ELF1	ZFAS1
ELF1	ZFC3H1
ELF1	ZFP64
ELF1	ZFP91
ELF1	ZFPL1
ELF1	ZFR
ELF1	ZFX
ELF1	ZFYVE16
ELF1	ZGRF1
ELF1	ZHX1-C8orf76
ELF1	ZKSCAN1
ELF1	ZKSCAN4
ELF1	ZKSCAN5
ELF1	ZMAT2

ELF1	ZMAT5
ELF1	ZMYM1
ELF1	ZMYM2
ELF1	ZMYND19
ELF1	ZMYND8
ELF1	ZNF101
ELF1	ZNF106
ELF1	ZNF107
ELF1	ZNF131
ELF1	ZNF141
ELF1	ZNF143
ELF1	ZNF148
ELF1	ZNF165
ELF1	ZNF174
ELF1	ZNF207
ELF1	ZNF212
ELF1	ZNF22
ELF1	ZNF232
ELF1	ZNF24
ELF1	ZNF254
ELF1	ZNF26
ELF1	ZNF267
ELF1	ZNF277
ELF1	ZNF280D
ELF1	ZNF281
ELF1	ZNF282
ELF1	ZNF292
ELF1	ZNF296
ELF1	ZNF318
ELF1	ZNF326
ELF1	ZNF35
ELF1	ZNF362
ELF1	ZNF367
ELF1	ZNF37A
ELF1	ZNF394
ELF1	ZNF395
ELF1	ZNF414
ELF1	ZNF415
ELF1	ZNF428
ELF1	ZNF430
ELF1	ZNF431
ELF1	ZNF451
ELF1	ZNF486
ELF1	ZNF487
ELF1	ZNF506
ELF1	ZNF514
ELF1	ZNF518A
ELF1	ZNF519
ELF1	ZNF557
ELF1	ZNF566

ELF1	ZNF570
ELF1	ZNF580
ELF1	ZNF581
ELF1	ZNF587
ELF1	ZNF592
ELF1	ZNF600
ELF1	ZNF608
ELF1	ZNF609
ELF1	ZNF611
ELF1	ZNF614
ELF1	ZNF622
ELF1	ZNF638
ELF1	ZNF644
ELF1	ZNF652
ELF1	ZNF655
ELF1	ZNF669
ELF1	ZNF672
ELF1	ZNF680
ELF1	ZNF684
ELF1	ZNF688
ELF1	ZNF692
ELF1	ZNF7
ELF1	ZNF706
ELF1	ZNF720
ELF1	ZNF738
ELF1	ZNF740
ELF1	ZNF746
ELF1	ZNF76
ELF1	ZNF761
ELF1	ZNF766
ELF1	ZNF771
ELF1	ZNF775
ELF1	ZNF780A
ELF1	ZNF787
ELF1	ZNF791
ELF1	ZNF800
ELF1	ZNF813
ELF1	ZNF814
ELF1	ZNF821
ELF1	ZNF830
ELF1	ZNF831
ELF1	ZNF85
ELF1	ZNF91
ELF1	ZNF92
ELF1	ZNFX1
ELF1	ZNHIT1
ELF1	ZNHIT3
ELF1	ZPBP2
ELF1	ZPR1
ELF1	ZRANB1

ELF1	ZRANB2
ELF1	ZRSR2
ELF1	ZSCAN16-AS1
ELF1	ZSCAN21
ELF1	ZSCAN25
ELF1	ZSWIM3
ELF1	ZSWIM7
ELF1	ZSWIM8
ELF1	ZW10
ELF1	ZWILCH
ELF1	ZWINT
ELF1	ZXDB
ELF1	ZYX
ELF2	ACBD3
ELF2	ALYREF
ELF2	ARHGAP9
ELF2	ARRB2
ELF2	ATP6V1E1
ELF2	ATP6V1H
ELF2	BANK1
ELF2	BBIP1
ELF2	BMF
ELF2	BNIP3L
ELF2	CADPS
ELF2	CBX5
ELF2	CCDC71
ELF2	CCP110
ELF2	CENPH
ELF2	CEP120
ELF2	CFDP1
ELF2	CGGBP1
ELF2	CHMP1B
ELF2	CLASRP
ELF2	CLK2
ELF2	CRLF3
ELF2	CSRNP1
ELF2	CTDSP1
ELF2	CYSLTR1
ELF2	DIAPH1
ELF2	DPEP2
ELF2	DUSP1
ELF2	E2F2
ELF2	ELF2
ELF2	ERN1
ELF2	FOXO1
ELF2	GOLGA5
ELF2	IFNGR1
ELF2	ING2
ELF2	IQGAP1
ELF2	ISL2

ELF2	JUNB
ELF2	KDM5A
ELF2	LMBRD1
ELF2	LMNA
ELF2	MED18
ELF2	METTL21A
ELF2	MGRN1
ELF2	MTMR2
ELF2	MTMR9
ELF2	MYL6B
ELF2	MYO1F
ELF2	NFKBID
ELF2	NUAK2
ELF2	P2RX1
ELF2	PAFAH1B2
ELF2	PDLIM1
ELF2	PGGT1B
ELF2	PHRF1
ELF2	PLAC8
ELF2	PPP1R35
ELF2	PRDM2
ELF2	PRPF38A
ELF2	R3HCC1L
ELF2	RAB5B
ELF2	RABEP2
ELF2	RDM1
ELF2	RNF214
ELF2	RTKN2
ELF2	S100A11
ELF2	S100A4
ELF2	SETX
ELF2	SLX4IP
ELF2	SPI1
ELF2	SPSB3
ELF2	SUPT20H
ELF2	SZT2
ELF2	TDP1
ELF2	TMEM115
ELF2	TMEM39A
ELF2	TRABD
ELF2	TRAF6
ELF2	TTC17
ELF2	TTI2
ELF2	USP8
ELF2	UVRAG
ELF2	VAMP5
ELF2	VRK3
ELF2	WDFY2
ELF2	ZDHHC20
ELF2	ZFP91

ELF2	ZNF266
ELF2	ZNF408
ELF2	ZNF587B
ELF2	ZNF608
ELF4	ACKR4
ELF4	ACOX1
ELF4	ADAR
ELF4	AP1AR
ELF4	ARF4-AS1
ELF4	ARHGAP5
ELF4	B4GALT1
ELF4	C5orf63
ELF4	CAPG
ELF4	CCDC82
ELF4	CDKN2D
ELF4	CENPN
ELF4	CEP44
ELF4	DCUN1D4
ELF4	DEF8
ELF4	DNAJC14
ELF4	EFL1
ELF4	EIF2S3
ELF4	ELF4
ELF4	ERI2
ELF4	EXOC5
ELF4	FADS2
ELF4	FAM126A
ELF4	FAM72D
ELF4	GHDC
ELF4	GINM1
ELF4	HAUS8
ELF4	HENMT1
ELF4	IGSF10
ELF4	INPP5K
ELF4	INTS4
ELF4	KAT7
ELF4	KDF1
ELF4	KDM6A
ELF4	LAMTOR4
ELF4	LYPD6B
ELF4	MAPK1
ELF4	MAPK8
ELF4	MDM2
ELF4	MED20
ELF4	MEF2A
ELF4	MICAL3
ELF4	MSRA
ELF4	MX1
ELF4	MYH10
ELF4	MYO9B

ELF4	NAF1
ELF4	NARFL
ELF4	NELFB
ELF4	PCSK7
ELF4	PDLIM2
ELF4	PEX26
ELF4	PHC3
ELF4	PIGM
ELF4	PILRB
ELF4	PIP4K2A
ELF4	POM121C
ELF4	RAB11FIP2
ELF4	RAD18
ELF4	RAP2C
ELF4	RBM12
ELF4	RIPK1
ELF4	RNF111
ELF4	RNF13
ELF4	RNFT1
ELF4	S100A11
ELF4	S1PR2
ELF4	SARS2
ELF4	SCMH1
ELF4	SEC24C
ELF4	SEPT11
ELF4	SERINC1
ELF4	SLC25A24
ELF4	SLC39A3
ELF4	SLC41A3
ELF4	SMUG1
ELF4	SPAG5
ELF4	SPRTN
ELF4	SRGAP2B
ELF4	STK10
ELF4	TAF15
ELF4	TCF20
ELF4	TMX4
ELF4	TRAIP
ELF4	TTC7A
ELF4	TTI2
ELF4	TUBB6
ELF4	UGP2
ELF4	UVRAG
ELF4	VPS52
ELF4	VPS8
ELF4	ZBTB24
ELF4	ZNF230
ELF4	ZNF362
ELF4	ZNF761
ELF4	ZSWIM8

ELK1	ABCF1
ELK1	ACTG1
ELK1	APP
ELK1	ATP1B3
ELK1	B3GALNT2
ELK1	BAG3
ELK1	BRCA1
ELK1	C12orf10
ELK1	C18orf54
ELK1	CALHM2
ELK1	CCDC189
ELK1	CCNB1IP1
ELK1	CCND1
ELK1	CD48
ELK1	CDK14
ELK1	CHCHD2
ELK1	COQ3
ELK1	CYB561
ELK1	DCTPP1
ELK1	DENND2D
ELK1	DHRS7B
ELK1	DNAJB1
ELK1	DTL
ELK1	ELK1
ELK1	ELL
ELK1	ENTHD1
ELK1	FAM126A
ELK1	FAM8A1
ELK1	FBXL18
ELK1	FMR1
ELK1	FOXO3
ELK1	FYN
ELK1	GAPVD1
ELK1	HLA-DRA
ELK1	HYOU1
ELK1	KCNN4
ELK1	LTC4S
ELK1	MAPK6
ELK1	MCFD2
ELK1	MLH1
ELK1	MTRF1L
ELK1	MYO1F
ELK1	N4BP3
ELK1	NEDD1
ELK1	PATL1
ELK1	PES1
ELK1	POLR1B
ELK1	PPIL2
ELK1	PPP5C
ELK1	PRR5

ELK1	REXO4
ELK1	RRP9
ELK1	SAV1
ELK1	SERPINI1
ELK1	SLC25A36
ELK1	SLC30A7
ELK1	SLC35E1
ELK1	SMIM12
ELK1	SNAPC5
ELK1	SNX9
ELK1	SPHK1
ELK1	STIM2
ELK1	TBL3
ELK1	TIMM10B
ELK1	TMED7
ELK1	TMEM267
ELK1	TMSB10
ELK1	TOMM22
ELK1	TP53
ELK1	TTC27
ELK1	UBE2D1
ELK1	UBE2M
ELK1	USP48
ELK1	VLDLR
ELK1	VMP1
ELK1	YBX1
ELK1	ZNF22
ELK3	ABHD13
ELK3	ACP5
ELK3	ADAMTS6
ELK3	AHNAK
ELK3	AKT2
ELK3	APOL1
ELK3	ARID4A
ELK3	ATF7IP
ELK3	ATG7
ELK3	ATP6V0E1
ELK3	BLNK
ELK3	BMP2K
ELK3	BORCS7
ELK3	BTNL9
ELK3	C14orf28
ELK3	C17orf49
ELK3	C18orf21
ELK3	C6orf99
ELK3	CAP1
ELK3	CARMIL2
ELK3	CAST
ELK3	CCPG1
ELK3	CD68

ELK3	CENPT
ELK3	CHD2
ELK3	CHPF2
ELK3	CLCN3
ELK3	COBLL1
ELK3	COPS7A
ELK3	CYBB
ELK3	CYTH1
ELK3	CYTIP
ELK3	DHRS9
ELK3	DHX40
ELK3	DNAJB9
ELK3	DOCK8
ELK3	DSTYK
ELK3	DYM
ELK3	EBF1
ELK3	ELK3
ELK3	EPHB6
ELK3	ERGIC2
ELK3	EVI2B
ELK3	FAM49A
ELK3	FCGR2B
ELK3	FRA10AC1
ELK3	FZD6
ELK3	GALNT7
ELK3	GPR137B
ELK3	GRN
ELK3	HELZ
ELK3	HPCAL1
ELK3	IRF2
ELK3	ITM2C
ELK3	JAK1
ELK3	LAPTM5
ELK3	LGALS1
ELK3	LILRB1
ELK3	LRP10
ELK3	LTB
ELK3	LY96
ELK3	MAGED1
ELK3	MAGEH1
ELK3	MED9
ELK3	MORF4L2
ELK3	MPEG1
ELK3	MS4A1
ELK3	MTOR
ELK3	MYL12A
ELK3	NET1
ELK3	NNT-AS1
ELK3	NPAT
ELK3	NXT2

ELK3	ODF2
ELK3	OSBPL2
ELK3	OST4
ELK3	PACSIN2
ELK3	PAG1
ELK3	PARM1
ELK3	PARP4
ELK3	PARP8
ELK3	PGGT1B
ELK3	PGM3
ELK3	PHACTR1
ELK3	PHC1
ELK3	PLD4
ELK3	POU2AF1
ELK3	PRCP
ELK3	PREX1
ELK3	PRPF6
ELK3	PTPRN2
ELK3	PTTG1IP
ELK3	RAB11FIP4
ELK3	RABEP2
ELK3	RALGPS2
ELK3	RGS1
ELK3	RNASE4
ELK3	RRBP1
ELK3	SAMD15
ELK3	SEC24A
ELK3	SERINC1
ELK3	SH3BGRL3
ELK3	SHKBP1
ELK3	SLC35C2
ELK3	SLC39A13
ELK3	SLC43A1
ELK3	SLC43A2
ELK3	SOCS3
ELK3	SRF
ELK3	SSPN
ELK3	STK17B
ELK3	SYMPK
ELK3	TAOK3
ELK3	TFIP11
ELK3	TFRC
ELK3	TGFBR2
ELK3	TMEM175
ELK3	TMEM187
ELK3	TMEM44-AS1
ELK3	TMEM59
ELK3	TMSB4X
ELK3	TOMM34
ELK3	TRAM1

ELK3	TSC22D3
ELK3	TSPAN31
ELK3	TSPYL2
ELK3	UBN2
ELK3	ULK3
ELK3	UNC119
ELK3	USP19
ELK3	USP53
ELK3	WASF2
ELK3	YIPF3
ELK3	YPEL5
ELK3	ZBTB7B
ELK3	ZFC3H1
ELK3	ZNF224
ELK3	ZNF335
ELK4	AFTPH
ELK4	AMIGO2
ELK4	ARHGEF3
ELK4	CAPZA1
ELK4	CDC42
ELK4	CDK11B
ELK4	CHRNA1
ELK4	CNST
ELK4	CUL4B
ELK4	CXXC5
ELK4	ELK4
ELK4	ELMSAN1
ELK4	FOXP1
ELK4	GNPNAT1
ELK4	KCNAB2
ELK4	MAP3K13
ELK4	MAPK10
ELK4	MDM4
ELK4	MIEF1
ELK4	NFKBIE
ELK4	PABPC4
ELK4	RHBDF2
ELK4	RINL
ELK4	SH2D3C
ELK4	SKIL
ELK4	SNX11
ELK4	THTPA
ELK4	TLK1
ELK4	TNRC6B
ELK4	TRPV2
ELK4	XRRA1
ELK4	ZMYND11
ELK4	ZNF768
ERF	C5orf34
ERF	CCDC77

ERF	IL6ST
ERF	NEAT1
ERF	SERP2
ERF	SPTBN1
ERF	UTP6
ESRRA	ABCA5
ESRRA	ABCC4
ESRRA	ACAD9
ESRRA	ACBD3
ESRRA	ACBD6
ESRRA	ACP5
ESRRA	ADO
ESRRA	ADPGK
ESRRA	ADRM1
ESRRA	ADSL
ESRRA	AHCY
ESRRA	AIFM1
ESRRA	AIMP2
ESRRA	AK6
ESRRA	AKAP1
ESRRA	AKAP12
ESRRA	ALDOA
ESRRA	ALDOC
ESRRA	ALG13
ESRRA	ALG3
ESRRA	ALOX5
ESRRA	AMZ2
ESRRA	ANXA7
ESRRA	AP4M1
ESRRA	ARF3
ESRRA	ARHGDIA
ESRRA	ARID3A
ESRRA	ARL17B
ESRRA	ARMCX3
ESRRA	ARPC5L
ESRRA	ASNA1
ESRRA	ASXL1
ESRRA	ATAD3B
ESRRA	ATG3
ESRRA	ATIC
ESRRA	ATN1
ESRRA	ATP1B3
ESRRA	ATP6V0A2
ESRRA	ATP6V0B
ESRRA	ATP6V1F
ESRRA	ATXN2
ESRRA	AXIN1
ESRRA	B3GALT6
ESRRA	BAG5
ESRRA	BAP1

ESRRA	BCAP31
ESRRA	BCAR3
ESRRA	BCAS2
ESRRA	BCAT2
ESRRA	BCKDK
ESRRA	BCL2L13
ESRRA	BET1L
ESRRA	BMF
ESRRA	BMP2K
ESRRA	BSG
ESRRA	C12orf4
ESRRA	C15orf62
ESRRA	C19orf24
ESRRA	C19orf70
ESRRA	C20orf27
ESRRA	C6orf136
ESRRA	C8orf59
ESRRA	CACUL1
ESRRA	CACYBP
ESRRA	CAMK1D
ESRRA	CBWD2
ESRRA	CCAR1
ESRRA	CCDC106
ESRRA	CCDC107
ESRRA	CCDC12
ESRRA	CCDC85B
ESRRA	CCDC86
ESRRA	CCDC88A
ESRRA	CCNE2
ESRRA	CCT2
ESRRA	CCT5
ESRRA	CCT7
ESRRA	CD320
ESRRA	CD46
ESRRA	CD82
ESRRA	CDK11A
ESRRA	CELF1
ESRRA	CELF2
ESRRA	CEP95
ESRRA	CEPT1
ESRRA	CGGBP1
ESRRA	CHAC1
ESRRA	CHD1
ESRRA	CINP
ESRRA	CITED4
ESRRA	CKAP4
ESRRA	CLNS1A
ESRRA	CMSS1
ESRRA	CMTM7
ESRRA	CNDP2

ESRRA	COASY
ESRRA	COG3
ESRRA	COG8
ESRRA	COX5B
ESRRA	CREB3
ESRRA	CRYZL1
ESRRA	CYB5R2
ESRRA	CYC1
ESRRA	CYCS
ESRRA	CYSLTR1
ESRRA	DAPK3
ESRRA	DBP
ESRRA	DCAF13
ESRRA	DCAF4
ESRRA	DCAF6
ESRRA	DCTPP1
ESRRA	DDX21
ESRRA	DDX27
ESRRA	DDX3X
ESRRA	DDX42
ESRRA	DDX51
ESRRA	DENR
ESRRA	DESI1
ESRRA	DGKD
ESRRA	DHCR7
ESRRA	DIMT1
ESRRA	DIS3
ESRRA	DKC1
ESRRA	DMWD
ESRRA	DOLK
ESRRA	DPH2
ESRRA	DPP8
ESRRA	DTD2
ESRRA	DUS1L
ESRRA	DUS3L
ESRRA	DYRK1B
ESRRA	E2F2
ESRRA	EBF1
ESRRA	EBNA1BP2
ESRRA	EEF1G
ESRRA	EFCAB2
ESRRA	EFHD2
ESRRA	EFTUD2
ESRRA	EIF1AX
ESRRA	EIF2S1
ESRRA	EIF3G
ESRRA	EIF3I
ESRRA	EIF3J
ESRRA	EIF4E
ESRRA	EIF4G1

ESRRA	EIF4G2
ESRRA	EIF4H
ESRRA	EIF5B
ESRRA	ELOF1
ESRRA	ELOVL1
ESRRA	ENO1
ESRRA	ENOPH1
ESRRA	EPB41
ESRRA	ERCC3
ESRRA	ERLIN1
ESRRA	ESRRA
ESRRA	ETF1
ESRRA	EXOG
ESRRA	EXOSC5
ESRRA	FABP5
ESRRA	FAM136A
ESRRA	FAM173A
ESRRA	FAM49A
ESRRA	FAM50A
ESRRA	FANCF
ESRRA	FAR1
ESRRA	FARSA
ESRRA	FASTKD1
ESRRA	FBL
ESRRA	FKBP15
ESRRA	FKBP4
ESRRA	FMNL1
ESRRA	FMNL3
ESRRA	GABARAPL1
ESRRA	GADD45G
ESRRA	GAR1
ESRRA	GAS2L3
ESRRA	GIMAP2
ESRRA	GLRX3
ESRRA	GMPPB
ESRRA	GNPTAB
ESRRA	GOLIM4
ESRRA	GOLT1B
ESRRA	GPATCH4
ESRRA	GPN2
ESRRA	GPR146
ESRRA	GPX7
ESRRA	GRPEL1
ESRRA	GRWD1
ESRRA	GTF2F1
ESRRA	GTF2H1
ESRRA	GTF3C6
ESRRA	HACD1
ESRRA	HCG11
ESRRA	HGS

ESRRA	HIP1
ESRRA	HM13
ESRRA	HSBP1
ESRRA	HSF2
ESRRA	HSP90AB1
ESRRA	HSPA8
ESRRA	HSPA9
ESRRA	HSPD1
ESRRA	HSPE1
ESRRA	HUWE1
ESRRA	IBTK
ESRRA	ICE2
ESRRA	ID3
ESRRA	ILF2
ESRRA	IMMP2L
ESRRA	IMPA1
ESRRA	INF2
ESRRA	INTS1
ESRRA	IPO13
ESRRA	IPO7
ESRRA	IRAK1
ESRRA	ISOC2
ESRRA	ITGB2
ESRRA	JAZF1
ESRRA	KCNQ1OT1
ESRRA	KCTD5
ESRRA	KIAA1191
ESRRA	KLF6
ESRRA	KPNB1
ESRRA	KRT18
ESRRA	L3MBTL2
ESRRA	LANCL1
ESRRA	LDHA
ESRRA	LGALS8
ESRRA	LIN37
ESRRA	LINC00467
ESRRA	LINC00926
ESRRA	LPCAT1
ESRRA	LRRC14
ESRRA	LRRC47
ESRRA	LY75
ESRRA	M6PR
ESRRA	MALSU1
ESRRA	MAP3K12
ESRRA	MAP3K13
ESRRA	MAP3K14
ESRRA	MAPKAPK2
ESRRA	MARCKS
ESRRA	MARS2
ESRRA	MAU2

ESRRA	MCTS1
ESRRA	MCUR1
ESRRA	ME2
ESRRA	MED19
ESRRA	METTL18
ESRRA	METTL3
ESRRA	METTL5
ESRRA	MFNG
ESRRA	MFSD2A
ESRRA	MGAT1
ESRRA	MGLL
ESRRA	MGMT
ESRRA	MIF
ESRRA	MIR3142HG
ESRRA	MLF2
ESRRA	MOB4
ESRRA	MOGS
ESRRA	MORC3
ESRRA	MPDU1
ESRRA	MPV17
ESRRA	MRPL12
ESRRA	MRPL20
ESRRA	MRPL21
ESRRA	MRPL3
ESRRA	MRPL52
ESRRA	MRPL55
ESRRA	MRPS16
ESRRA	MRPS18B
ESRRA	MRPS2
ESRRA	MSTO1
ESRRA	MTCH1
ESRRA	MTMR14
ESRRA	MYD88
ESRRA	NAA10
ESRRA	NAMPT
ESRRA	NCL
ESRRA	NCLN
ESRRA	NDOR1
ESRRA	NDUFA1
ESRRA	NDUFA5
ESRRA	NDUFAF1
ESRRA	NDUFB2
ESRRA	NDUFB7
ESRRA	NDUFB9
ESRRA	NDUFS6
ESRRA	NDUFS7
ESRRA	NEDD4L
ESRRA	NEK9
ESRRA	NFE2L1
ESRRA	NFKB1

ESRRA	NHP2
ESRRA	NIFK
ESRRA	NIFK-AS1
ESRRA	NIN
ESRRA	NME1
ESRRA	NOB1
ESRRA	NOC2L
ESRRA	NOL10
ESRRA	NOL11
ESRRA	NOL8
ESRRA	NOLC1
ESRRA	NOP10
ESRRA	NPM1
ESRRA	NPM3
ESRRA	NPW
ESRRA	NR1H2
ESRRA	NR6A1
ESRRA	NSF
ESRRA	NUDC
ESRRA	NUFIP1
ESRRA	NXT1
ESRRA	NXT2
ESRRA	ODC1
ESRRA	OGT
ESRRA	OPA1
ESRRA	OPN3
ESRRA	ORAI1
ESRRA	ORAOV1
ESRRA	ORMDL1
ESRRA	OTUD7B
ESRRA	OXSR1
ESRRA	PA2G4
ESRRA	PAIP1
ESRRA	PAK1IP1
ESRRA	PARVB
ESRRA	PCGF5
ESRRA	PCID2
ESRRA	PCNP
ESRRA	PDCD2L
ESRRA	PDCD5
ESRRA	PDCD6IP
ESRRA	PFDN2
ESRRA	PFDN6
ESRRA	PGAM1
ESRRA	PGAP2
ESRRA	PGK1
ESRRA	PHB
ESRRA	PHB2
ESRRA	PHF5A
ESRRA	PIBF1

ESRRA	PIGF
ESRRA	PIGT
ESRRA	PIGW
ESRRA	PLAA
ESRRA	PLAGL1
ESRRA	PLD6
ESRRA	PLK3
ESRRA	PLP2
ESRRA	PNO1
ESRRA	PNPLA2
ESRRA	PNPLA4
ESRRA	POLB
ESRRA	POLR2A
ESRRA	POLR2I
ESRRA	POLR2M
ESRRA	POLR3H
ESRRA	POMP
ESRRA	PPA1
ESRRA	PPAN
ESRRA	PPP4R2
ESRRA	PRAF2
ESRRA	PRKCZ
ESRRA	PRMT1
ESRRA	PRPF4
ESRRA	PRR13
ESRRA	PRRC2A
ESRRA	PSAT1
ESRRA	PSMA2
ESRRA	PSMA3
ESRRA	PSMB1
ESRRA	PSMC1
ESRRA	PSMC3
ESRRA	PSMD11
ESRRA	PSMD13
ESRRA	PSMD14
ESRRA	PSMD5
ESRRA	PSMD7
ESRRA	PTCD1
ESRRA	PTGES2
ESRRA	PUM3
ESRRA	PXDN
ESRRA	PYCARD
ESRRA	RAB21
ESRRA	RAB33A
ESRRA	RAB3IP
ESRRA	RAB5A
ESRRA	RAB7A
ESRRA	RABEPK
ESRRA	RAI1
ESRRA	RAN

ESRRA	RBM15B
ESRRA	RBM25
ESRRA	RBM3
ESRRA	RBM8A
ESRRA	RBPJ
ESRRA	RCL1
ESRRA	RELB
ESRRA	RELL2
ESRRA	RFWD3
ESRRA	RNASEH2B
ESRRA	RNF121
ESRRA	RNF170
ESRRA	RNF216
ESRRA	RNH1
ESRRA	RPAP1
ESRRA	RPL17
ESRRA	RPL22L1
ESRRA	RPL36AL
ESRRA	RPS19BP1
ESRRA	RRP1
ESRRA	RRP1B
ESRRA	RRP9
ESRRA	RSL24D1
ESRRA	RTCB
ESRRA	RUBCN
ESRRA	RWDD1
ESRRA	S100A4
ESRRA	S100PBP
ESRRA	S1PR4
ESRRA	SAFB2
ESRRA	SAMM50
ESRRA	SAMSN1
ESRRA	SARS
ESRRA	SAYSD1
ESRRA	SBF1
ESRRA	SCAP
ESRRA	SDAD1
ESRRA	SERBP1
ESRRA	SGCE
ESRRA	SIKE1
ESRRA	SLC25A38
ESRRA	SLC26A2
ESRRA	SLC2A11
ESRRA	SLC35C2
ESRRA	SLC35F2
ESRRA	SLC39A10
ESRRA	SLC44A1
ESRRA	SLC4A7
ESRRA	SLC9A3R1
ESRRA	SLIRP

ESRRA	SMIM12
ESRRA	SNRPF
ESRRA	SNRPG
ESRRA	SNU13
ESRRA	SNUPN
ESRRA	SNX14
ESRRA	SORD
ESRRA	SPG21
ESRRA	SPRYD7
ESRRA	SPTBN1
ESRRA	SRFBP1
ESRRA	SRM
ESRRA	SRPRA
ESRRA	SSBP1
ESRRA	SSSCA1
ESRRA	ST13
ESRRA	ST6GALNAC3
ESRRA	STAT1
ESRRA	STAT5A
ESRRA	STIP1
ESRRA	STK4
ESRRA	STOML2
ESRRA	STX18
ESRRA	STX4
ESRRA	STYX
ESRRA	SUB1
ESRRA	SUGCT
ESRRA	TACO1
ESRRA	TBK1
ESRRA	TBL1X
ESRRA	TBL3
ESRRA	TCERG1
ESRRA	TELO2
ESRRA	TEX10
ESRRA	TFAP4
ESRRA	TFCP2
ESRRA	THAP4
ESRRA	THOC5
ESRRA	TIMM13
ESRRA	TIMM44
ESRRA	TIMM9
ESRRA	TKFC
ESRRA	TMEM126A
ESRRA	TMEM126B
ESRRA	TMEM147
ESRRA	TMEM218
ESRRA	TMEM70
ESRRA	TMSB10
ESRRA	TMSB4X
ESRRA	TMX2

ESRRA	TNFAIP3
ESRRA	TOMM22
ESRRA	TOMM40
ESRRA	TPD52L2
ESRRA	TPR
ESRRA	TPST2
ESRRA	TRAFD1
ESRRA	TRIM56
ESRRA	TRMO
ESRRA	TRMT1
ESRRA	TRMT6
ESRRA	TRPM7
ESRRA	TRUB2
ESRRA	TSEN2
ESRRA	TSPAN31
ESRRA	TSR2
ESRRA	TSSC4
ESRRA	TTC1
ESRRA	TTF1
ESRRA	TUBGCP4
ESRRA	TXN
ESRRA	TXNL1
ESRRA	TYMP
ESRRA	UBE2D3
ESRRA	UBE2F
ESRRA	UBE4A
ESRRA	UBQLN4
ESRRA	UBXN8
ESRRA	UPRT
ESRRA	UQCRC1
ESRRA	UQCRQ
ESRRA	UTP18
ESRRA	UTP4
ESRRA	VASP
ESRRA	VCP
ESRRA	VPS13C
ESRRA	VPS26A
ESRRA	VPS4B
ESRRA	WDR4
ESRRA	WDR74
ESRRA	WWP1
ESRRA	XPOT
ESRRA	YARS
ESRRA	YBX1
ESRRA	YIPF2
ESRRA	YWHAG
ESRRA	ZBTB45
ESRRA	ZC3H8
ESRRA	ZDHHC24
ESRRA	ZFP91

ESRRA	ZFR
ESRRA	ZFYVE27
ESRRA	ZNF230
ESRRA	ZNF275
ESRRA	ZNF35
ESRRA	ZNF593
ESRRA	ZNF667-AS1
ESRRA	ZNHIT2
ETS1	A4GALT
ETS1	AAMDC
ETS1	ABCA7
ETS1	ABCB7
ETS1	ABHD14B
ETS1	ABLIM1
ETS1	ACAA1
ETS1	ACAA2
ETS1	ACAD10
ETS1	ACAD8
ETS1	ACAP2
ETS1	ACBD3
ETS1	ACBD4
ETS1	ACLY
ETS1	ACOT13
ETS1	ACOX3
ETS1	ACSF2
ETS1	ACSS1
ETS1	ACTB
ETS1	ACTG1
ETS1	ACTR1A
ETS1	ACTR2
ETS1	ACTR3
ETS1	ACTR8
ETS1	ADA
ETS1	ADD3
ETS1	ADNP2
ETS1	ADPRH
ETS1	AEBP1
ETS1	AES
ETS1	AFF3
ETS1	AFTPH
ETS1	AGBL5
ETS1	AGO3
ETS1	AGO4
ETS1	AGPS
ETS1	AGTRAP
ETS1	AIFM1
ETS1	AIFM2
ETS1	AIM2
ETS1	AIP
ETS1	AKAP13

ETS1	AKIP1
ETS1	AKIRIN1
ETS1	AKIRIN2
ETS1	AKNA
ETS1	ALAS1
ETS1	ALDH16A1
ETS1	ALDH6A1
ETS1	ALG6
ETS1	ALKBH3
ETS1	ALKBH4
ETS1	ALKBH7
ETS1	ALOX5AP
ETS1	ALPK1
ETS1	AMZ2
ETS1	ANAPC10
ETS1	ANAPC15
ETS1	ANAPC7
ETS1	ANKRD13A
ETS1	ANKRD13C
ETS1	ANKRD13D
ETS1	ANKRD40
ETS1	ANKRD44
ETS1	ANKRD49
ETS1	ANXA11
ETS1	ANXA6
ETS1	AP1B1
ETS1	APBB1IP
ETS1	APEH
ETS1	APLP2
ETS1	APPBP2
ETS1	APPL1
ETS1	ARAF
ETS1	ARF3
ETS1	ARF4−AS1
ETS1	ARHGAP11A
ETS1	ARHGAP25
ETS1	ARHGAP30
ETS1	ARHGDIB
ETS1	ARHGEF1
ETS1	ARHGEF6
ETS1	ARHGEF7
ETS1	ARHGEF9
ETS1	ARID1A
ETS1	ARID1B
ETS1	ARID3A
ETS1	ARID4B
ETS1	ARID5A
ETS1	ARL14EP
ETS1	ARL2BP
ETS1	ARL5B

ETS1	ARL8A
ETS1	ARMC1
ETS1	ARMT1
ETS1	ARPC1B
ETS1	ARPC2
ETS1	ARPC3
ETS1	ARPC4
ETS1	ARPC5
ETS1	ARRDC1
ETS1	ASB2
ETS1	ASB6
ETS1	ASH1L-AS1
ETS1	ASPM
ETS1	ATAD1
ETS1	ATF6B
ETS1	ATF7IP
ETS1	ATM
ETS1	ATP10D
ETS1	ATP6V0A1
ETS1	ATP6V0D1
ETS1	ATP6V1D
ETS1	ATP6V1H
ETS1	ATRX
ETS1	ATXN7L3B
ETS1	AUNIP
ETS1	AURKA
ETS1	AURKB
ETS1	AZI2
ETS1	AZIN1
ETS1	B3GALNT1
ETS1	B4GALT1
ETS1	BABAM1
ETS1	BAIAP2L1
ETS1	BASP1
ETS1	BCAS4
ETS1	BCL11A
ETS1	BCL3
ETS1	BCL7A
ETS1	BDH2
ETS1	BECN1
ETS1	BEX4
ETS1	BHLHE41
ETS1	BICD1
ETS1	BIN1
ETS1	BIRC3
ETS1	BIRC5
ETS1	BLCAP
ETS1	BLK
ETS1	BLM
ETS1	BLNK

ETS1	BLOC1S1
ETS1	BLOC1S6
ETS1	BNIP2
ETS1	BOD1L1
ETS1	BORCS8-MEF2B
ETS1	BPTF
ETS1	BRAP
ETS1	BRD4
ETS1	BRD7
ETS1	BRI3
ETS1	BRI3BP
ETS1	BRK1
ETS1	BROX
ETS1	BTG2
ETS1	BTK
ETS1	BUB1
ETS1	BUB1B
ETS1	C12orf49
ETS1	C12orf75
ETS1	C15orf40
ETS1	C15orf61
ETS1	C16orf70
ETS1	C16orf72
ETS1	C16orf74
ETS1	C16orf95
ETS1	C17orf80
ETS1	C19orf53
ETS1	C1D
ETS1	C1GALT1C1
ETS1	C2orf49
ETS1	C3orf67
ETS1	C4orf3
ETS1	C6orf106
ETS1	C6orf62
ETS1	C6orf99
ETS1	C7orf50
ETS1	C9orf16
ETS1	C9orf85
ETS1	CAB39L
ETS1	CACNA1A
ETS1	CALM1
ETS1	CALM3
ETS1	CAND1
ETS1	CAP1
ETS1	CAPN1
ETS1	CAPZB
ETS1	CARD8
ETS1	CARF
ETS1	CARS
ETS1	CARS2

ETS1	CASC4
ETS1	CASP4
ETS1	CASP8
ETS1	CAT
ETS1	CBFA2T3
ETS1	CBFB
ETS1	CBX5
ETS1	CC2D1A
ETS1	CCAR1
ETS1	CCDC106
ETS1	CCDC107
ETS1	CCDC167
ETS1	CCDC34
ETS1	CCDC69
ETS1	CCDC88A
ETS1	CCDC88C
ETS1	CCM2
ETS1	CCNB1
ETS1	CCNB2
ETS1	CCNDBP1
ETS1	CCNF
ETS1	CCNG1
ETS1	CCNI
ETS1	CCNL1
ETS1	CCNY
ETS1	CD151
ETS1	CD19
ETS1	CD2BP2
ETS1	CD37
ETS1	CD52
ETS1	CD53
ETS1	CD79B
ETS1	CD81
ETS1	CD9
ETS1	CDC20
ETS1	CDC27
ETS1	CDC37
ETS1	CDC40
ETS1	CDC42SE2
ETS1	CDK13
ETS1	CDK19
ETS1	CDK5RAP2
ETS1	CDR2
ETS1	CDV3
ETS1	CEBPG
ETS1	CELF1
ETS1	CENPE
ETS1	CENPF
ETS1	CENPN
ETS1	CENPT

ETS1	CEP350
ETS1	CEP55
ETS1	CFAP73
ETS1	CFL1
ETS1	CGGBP1
ETS1	CHCHD1
ETS1	CHCHD7
ETS1	CHD2
ETS1	CHD4
ETS1	CHD8
ETS1	CHD9
ETS1	CHFR
ETS1	CHIC2
ETS1	CHKB
ETS1	CHRNB1
ETS1	CHTF8
ETS1	CHURC1
ETS1	CIR1
ETS1	CIZ1
ETS1	CKAP5
ETS1	CKS1B
ETS1	CKS2
ETS1	CLCN3
ETS1	CLCN6
ETS1	CLIC4
ETS1	CLINT1
ETS1	CLK3
ETS1	CLOCK
ETS1	CLPX
ETS1	CLTC
ETS1	CLTCL1
ETS1	CMAS
ETS1	CMTM8
ETS1	CNDP2
ETS1	CNN2
ETS1	CNOT10
ETS1	CNOT11
ETS1	CNOT6
ETS1	CNOT7
ETS1	COA3
ETS1	COASY
ETS1	COIL
ETS1	COMMD2
ETS1	COMMD4
ETS1	COMMD6
ETS1	COPA
ETS1	COPS6
ETS1	COQ4
ETS1	COQ5
ETS1	CORO1A

ETS1	COTL1
ETS1	COX14
ETS1	COX17
ETS1	COX4I1
ETS1	COX6B1
ETS1	COX6C
ETS1	COX7A2
ETS1	COX7A2L
ETS1	COX8A
ETS1	CPNE5
ETS1	CPSF6
ETS1	CREB1
ETS1	CRIPT
ETS1	CRLF3
ETS1	CRNKL1
ETS1	CSDE1
ETS1	CSK
ETS1	CSNK1G2
ETS1	CSNK2B
ETS1	CSPP1
ETS1	CSRP1
ETS1	CTCF
ETS1	CTDSP2
ETS1	CTHRC1
ETS1	CTSC
ETS1	CTSH
ETS1	CUL3
ETS1	CUTC
ETS1	CUX1
ETS1	CWC27
ETS1	CXCR5
ETS1	CXorf21
ETS1	CXorf38
ETS1	CXorf56
ETS1	CXXC5
ETS1	CYB561A3
ETS1	CYB5B
ETS1	CYB5D2
ETS1	CYB5R3
ETS1	CYBB
ETS1	CYP2R1
ETS1	DAAM1
ETS1	DAP
ETS1	DAPP1
ETS1	DARS
ETS1	DAXX
ETS1	DBI
ETS1	DBNL
ETS1	DCAF11
ETS1	DCAF16

ETS1	DCAF6
ETS1	DCAF7
ETS1	DCP1A
ETS1	DCP2
ETS1	DCTN3
ETS1	DDX17
ETS1	DDX24
ETS1	DDX3X
ETS1	DDX49
ETS1	DDX6
ETS1	DECR1
ETS1	DEF6
ETS1	DEPDC5
ETS1	DGUOK
ETS1	DHRS12
ETS1	DHX16
ETS1	DHX36
ETS1	DICER1
ETS1	DIP2A
ETS1	DIS3L
ETS1	DLAT
ETS1	DMXL1
ETS1	DNAJB2
ETS1	DNAJB6
ETS1	DNAJC27
ETS1	DNAJC7
ETS1	DNASE1L1
ETS1	DNM2
ETS1	DOCK2
ETS1	DOCK8
ETS1	DOK1
ETS1	DOK3
ETS1	DPCD
ETS1	DPY30
ETS1	DTD2
ETS1	DUS1L
ETS1	DUSP2
ETS1	DUSP23
ETS1	DYNC1H1
ETS1	DYNC1I2
ETS1	DYNLL1
ETS1	DYNLRB1
ETS1	DZIP3
ETS1	E2F4
ETS1	E2F5
ETS1	ECH1
ETS1	ECHS1
ETS1	ECT2
ETS1	EDEM2
ETS1	EEF2

ETS1	EFHC1
ETS1	EGR2
ETS1	EHD1
ETS1	EHMT1
ETS1	EID1
ETS1	EID3
ETS1	EIF2A
ETS1	EIF2AK2
ETS1	EIF2B1
ETS1	EIF2D
ETS1	EIF3A
ETS1	EIF3E
ETS1	EIF3F
ETS1	EIF3H
ETS1	EIF3L
ETS1	EIF4B
ETS1	EIF4G2
ETS1	EIF5
ETS1	ELAC1
ETS1	ELF1
ETS1	ELK3
ETS1	ELK4
ETS1	ELP5
ETS1	ENDOG
ETS1	ENOPH1
ETS1	ENSA
ETS1	ENTPD3-AS1
ETS1	ENTPD4
ETS1	ENY2
ETS1	EPC1
ETS1	EPN1
ETS1	ERBIN
ETS1	ERMARD
ETS1	ERP29
ETS1	ESR2
ETS1	ETFA
ETS1	ETFB
ETS1	ETS1
ETS1	EVC2
ETS1	EVI2A
ETS1	EVI2B
ETS1	EVL
ETS1	EWSR1
ETS1	EXOC1
ETS1	EXOC7
ETS1	EZR
ETS1	FADS3
ETS1	FAHD2A
ETS1	FAM107B
ETS1	FAM111B

ETS1	FAM120C
ETS1	FAM172A
ETS1	FAM192A
ETS1	FAM204A
ETS1	FAM49B
ETS1	FAM76B
ETS1	FAM83D
ETS1	FAM96A
ETS1	FAM96B
ETS1	FARS2
ETS1	FARSB
ETS1	FBP1
ETS1	FBXO38
ETS1	FBXO44
ETS1	FBXO9
ETS1	FCRLA
ETS1	FDXR
ETS1	FEZ2
ETS1	FGD3
ETS1	FGFR1OP2
ETS1	FKBP1A
ETS1	FKBP3
ETS1	FKBP5
ETS1	FKBP8
ETS1	FLI1
ETS1	FLVCR1
ETS1	FNBP4
ETS1	FRYL
ETS1	FUT8
ETS1	FXR1
ETS1	G3BP2
ETS1	GABARAPL2
ETS1	GAK
ETS1	GAPDH
ETS1	GATAD2A
ETS1	GCNT2
ETS1	GCSAM
ETS1	GDI1
ETS1	GDI2
ETS1	GEMIN7
ETS1	GFOD1
ETS1	GFPT1
ETS1	GGA2
ETS1	GGA3
ETS1	GGNBP2
ETS1	GIGYF2
ETS1	GIN1
ETS1	GIT2
ETS1	GLRX5
ETS1	GMCL1

ETS1	GMFG
ETS1	GMIP
ETS1	GMPR2
ETS1	GNA13
ETS1	GNAI2
ETS1	GNG5
ETS1	GNPTAB
ETS1	GOLGA3
ETS1	GON4L
ETS1	GOSR1
ETS1	GPANK1
ETS1	GPATCH1
ETS1	GPATCH11
ETS1	GPATCH2
ETS1	GPATCH2L
ETS1	GPN3
ETS1	GPR155
ETS1	GPSM3
ETS1	GRAMD1C
ETS1	GRAP
ETS1	GRAPL
ETS1	GRB2
ETS1	GRIPAP1
ETS1	GRK2
ETS1	GRK3
ETS1	GSDMD
ETS1	GSK3A
ETS1	GSK3B
ETS1	GSKIP
ETS1	GSS
ETS1	GSTZ1
ETS1	GTDC1
ETS1	GTF2A1
ETS1	GTF2B
ETS1	GTF2F1
ETS1	GTF2I
ETS1	GTSE1
ETS1	H1F0
ETS1	H2AFV
ETS1	H3F3A
ETS1	HADHA
ETS1	HADHB
ETS1	HCK
ETS1	HCLS1
ETS1	HDAC1
ETS1	HDAC7
ETS1	HDAC8
ETS1	HDDC2
ETS1	HDGF
ETS1	HEBP2

ETS1	HECTD1
ETS1	HELQ
ETS1	HERC2
ETS1	HERC4
ETS1	HGSNAT
ETS1	HHEX
ETS1	HIBADH
ETS1	HIBCH
ETS1	HIGD2A
ETS1	HINT1
ETS1	HINT3
ETS1	HIRIP3
ETS1	HJURP
ETS1	HLA-DMA
ETS1	HLA-DMB
ETS1	HMG20B
ETS1	HMGB1
ETS1	HMGN1
ETS1	HMGN2
ETS1	HMGN3
ETS1	HMGXB4
ETS1	HMMR
ETS1	HNRNPA1
ETS1	HNRNPA2B1
ETS1	HNRNPA3
ETS1	HNRNPD
ETS1	HNRNPK
ETS1	HNRNPM
ETS1	HNRNPUL2
ETS1	HP1BP3
ETS1	HPS3
ETS1	HRK
ETS1	HS2ST1
ETS1	HSBP1
ETS1	HSCB
ETS1	HSD17B10
ETS1	HSD17B11
ETS1	HSPA4
ETS1	HYPK
ETS1	IBTK
ETS1	ICE2
ETS1	IDH3G
ETS1	IDS
ETS1	IER2
ETS1	IFI16
ETS1	IFI27L2
ETS1	IFNGR2
ETS1	IFNLR1
ETS1	IFT43
ETS1	IFT52

ETS1	IGBP1
ETS1	IKZF1
ETS1	IKZF3
ETS1	IL10RA
ETS1	IL16
ETS1	ILK
ETS1	INIP
ETS1	INO80C
ETS1	INTS10
ETS1	INTS6L
ETS1	IP6K2
ETS1	IQGAP1
ETS1	IQGAP2
ETS1	IRF3
ETS1	IRF8
ETS1	ISCA2
ETS1	ISG20
ETS1	IST1
ETS1	ITGAE
ETS1	ITGAL
ETS1	ITGB1
ETS1	ITGB3BP
ETS1	ITPK1
ETS1	ITPKA
ETS1	ITSN2
ETS1	JADE2
ETS1	JAK1
ETS1	JAK2
ETS1	JRKL
ETS1	JUN
ETS1	KBTBD8
ETS1	KCNA3
ETS1	KCNMB3
ETS1	KCTD20
ETS1	KCTD9
ETS1	KDM2A
ETS1	KDM4C
ETS1	KHDRBS1
ETS1	KIAA0040
ETS1	KIAA0232
ETS1	KIAA0319L
ETS1	KIAA1217
ETS1	KIAA1841
ETS1	KIF18B
ETS1	KIF20B
ETS1	KIF22
ETS1	KIF23
ETS1	KIF5B
ETS1	KIF9
ETS1	KIFAP3

ETS1	KLF2
ETS1	KLHL14
ETS1	KLHL5
ETS1	KMT2A
ETS1	KMT2E
ETS1	KMT2E−AS1
ETS1	KNSTRN
ETS1	KPNA2
ETS1	KPNA5
ETS1	KPTN
ETS1	KRAS
ETS1	KRCC1
ETS1	KTN1
ETS1	LACTB2
ETS1	LAMTOR2
ETS1	LAMTOR4
ETS1	LAMTOR5
ETS1	LAPTM5
ETS1	LBR
ETS1	LCMT1
ETS1	LCORL
ETS1	LCP1
ETS1	LDHB
ETS1	LDLRAD4
ETS1	LENG1
ETS1	LEO1
ETS1	LEPROT
ETS1	LHFPL2
ETS1	LIMD2
ETS1	LIMS1
ETS1	LINC00426
ETS1	LINC00467
ETS1	LINC00667
ETS1	LINC01003
ETS1	LMBRD2
ETS1	LMO7
ETS1	LMTK3
ETS1	LPIN1
ETS1	LRBA
ETS1	LRR1
ETS1	LSG1
ETS1	LSM10
ETS1	LSM12
ETS1	LSM14A
ETS1	LSM3
ETS1	LSM5
ETS1	LSM8
ETS1	LSP1
ETS1	LTB
ETS1	LTN1

ETS1	LY96
ETS1	LYN
ETS1	LYPLA2
ETS1	LYRM2
ETS1	MACROD1
ETS1	MAD1L1
ETS1	MAD2L1BP
ETS1	MADD
ETS1	MAGED2
ETS1	MAGEF1
ETS1	MAN2A2
ETS1	MAP1LC3B
ETS1	MAP3K12
ETS1	MAP3K4
ETS1	MAP7D1
ETS1	MAPRE1
ETS1	MARCH6
ETS1	MARCH7
ETS1	MBD4
ETS1	MBNL1
ETS1	MCL1
ETS1	MDH1
ETS1	MDH2
ETS1	MDM4
ETS1	MED1
ETS1	MED11
ETS1	MED12L
ETS1	MED13
ETS1	MED30
ETS1	MED6
ETS1	MEF2A
ETS1	MEF2C
ETS1	MEN1
ETS1	MET
ETS1	METTL23
ETS1	MFAP1
ETS1	MFSD11
ETS1	MFSD8
ETS1	MGA
ETS1	MGME1
ETS1	MIA3
ETS1	MICAL3
ETS1	MICU2
ETS1	MID1IP1
ETS1	MIEN1
ETS1	MIER2
ETS1	MIF4GD
ETS1	MKRN1
ETS1	MLH1
ETS1	MLH3

ETS1	MLYCD
ETS1	MMADHC
ETS1	MMD
ETS1	MME
ETS1	MOB1A
ETS1	MOB2
ETS1	MOB3A
ETS1	MOV10
ETS1	MPLKIP
ETS1	MRPL23
ETS1	MRPL27
ETS1	MRPL35
ETS1	MRPL40
ETS1	MRPL43
ETS1	MRPL51
ETS1	MRPS10
ETS1	MRPS18B
ETS1	MRPS33
ETS1	MRPS6
ETS1	MRPS9
ETS1	MS4A1
ETS1	MSI2
ETS1	MSL3
ETS1	MSMP
ETS1	MSN
ETS1	MTDH
ETS1	MTERF4
ETS1	MTF2
ETS1	MTIF3
ETS1	MTPN
ETS1	MTRF1
ETS1	MYBL1
ETS1	MYBL2
ETS1	MYEF2
ETS1	MYH9
ETS1	MYL12A
ETS1	MYL12B
ETS1	MYL6B
ETS1	MYO1G
ETS1	MYO7B
ETS1	MZT1
ETS1	MZT2A
ETS1	N4BP2L2
ETS1	NAA15
ETS1	NAA30
ETS1	NACC1
ETS1	NADK
ETS1	NAF1
ETS1	NAPEPLD
ETS1	NARF

ETS1	NCAPH
ETS1	NCBP2
ETS1	NCF1
ETS1	NCKAP1L
ETS1	NCKIPSD
ETS1	NCOA2
ETS1	NCOA3
ETS1	NCOA4
ETS1	NCOR2
ETS1	NDC80
ETS1	NDE1
ETS1	NDUFA1
ETS1	NDUFA10
ETS1	NDUFA12
ETS1	NDUFA2
ETS1	NDUFA4
ETS1	NDUFA8
ETS1	NDUFAF3
ETS1	NDUFAF5
ETS1	NDUFAF7
ETS1	NDUFB3
ETS1	NDUFB4
ETS1	NDUFB5
ETS1	NDUFC1
ETS1	NDUFS2
ETS1	NDUFS5
ETS1	NDUFV2
ETS1	NDUFV3
ETS1	NEDD8
ETS1	NEIL1
ETS1	NEK2
ETS1	NEK4
ETS1	NEK8
ETS1	NEK9
ETS1	NET1
ETS1	NINJ1
ETS1	NIPA1
ETS1	NIPSNAP3A
ETS1	NKTR
ETS1	NMT2
ETS1	NNT
ETS1	NOA1
ETS1	NONO
ETS1	NOSIP
ETS1	NPEPPS
ETS1	NR2C2
ETS1	NRSN2
ETS1	NSD1
ETS1	NSMCE1
ETS1	NSUN3

ETS1	NSUN5
ETS1	NT5C2
ETS1	NUAK2
ETS1	NUDCD2
ETS1	NUDT16L1
ETS1	NUDT2
ETS1	NUDT21
ETS1	NUDT3
ETS1	NUDT5
ETS1	NUF2
ETS1	NUP155
ETS1	NUP37
ETS1	NUPL2
ETS1	OARD1
ETS1	OAS2
ETS1	OAZ1
ETS1	OAZ2
ETS1	OCIAD2
ETS1	ODF2
ETS1	ODF2L
ETS1	OFD1
ETS1	OMA1
ETS1	ORAI2
ETS1	ORC3
ETS1	OSBPL10
ETS1	OSBPL11
ETS1	OSBPL3
ETS1	OSBPL8
ETS1	OSBPL9
ETS1	OST4
ETS1	OSTF1
ETS1	OTUD5
ETS1	OTUD7A
ETS1	OTULIN
ETS1	OXA1L
ETS1	P2RX5
ETS1	PAFAH1B1
ETS1	PAG1
ETS1	PAGE5
ETS1	PAIP2
ETS1	PAK2
ETS1	PAN3
ETS1	PAPSS1
ETS1	PARL
ETS1	PARN
ETS1	PARP1
ETS1	PARP11
ETS1	PARPBP
ETS1	PARVG
ETS1	PAX5

ETS1	PBLD
ETS1	PCED1B
ETS1	PCF11
ETS1	PCIF1
ETS1	PCM1
ETS1	PCMTD2
ETS1	PCNT
ETS1	PCSK7
ETS1	PCYT2
ETS1	PDCD10
ETS1	PDCD2
ETS1	PDCD7
ETS1	PDE4D
ETS1	PDHB
ETS1	PDIK1L
ETS1	PDK2
ETS1	PDLIM1
ETS1	PDS5B
ETS1	PDZD11
ETS1	PECAM1
ETS1	PET117
ETS1	PEX2
ETS1	PEX3
ETS1	PFDN1
ETS1	PFN1
ETS1	PGLS
ETS1	PHF12
ETS1	PHF14
ETS1	PHF19
ETS1	PHF3
ETS1	PHF6
ETS1	PIF1
ETS1	PIGF
ETS1	PIGN
ETS1	PIGT
ETS1	PIGZ
ETS1	PIH1D1
ETS1	PIK3C3
ETS1	PIK3CD
ETS1	PIM1
ETS1	PJA2
ETS1	PKIB
ETS1	PKIG
ETS1	PLA2G15
ETS1	PLEKHA3
ETS1	PLEKHF2
ETS1	PLEKHG1
ETS1	PLK1
ETS1	PLPP5
ETS1	PLXNB2

ETS1	PMF1
ETS1	PMPCB
ETS1	PMVK
ETS1	PNISR
ETS1	PNKD
ETS1	PNKP
ETS1	PNN
ETS1	PNO1
ETS1	PNPLA8
ETS1	PNRC2
ETS1	POC5
ETS1	POLD4
ETS1	POLE
ETS1	POLR1D
ETS1	POLR2B
ETS1	POLR2G
ETS1	POLR3GL
ETS1	POU2F1
ETS1	POU2F2
ETS1	PPARD
ETS1	PPCS
ETS1	PPHLN1
ETS1	PPID
ETS1	PPIG
ETS1	PPIL3
ETS1	PPIP5K2
ETS1	PPM1A
ETS1	PPP1CA
ETS1	PPP1CB
ETS1	PPP1CC
ETS1	PPP1R12A
ETS1	PPP1R14A
ETS1	PPP1R18
ETS1	PPP1R37
ETS1	PPP1R7
ETS1	PPP2CA
ETS1	PPP2R1A
ETS1	PPP2R1B
ETS1	PPP2R5A
ETS1	PPP2R5C
ETS1	PPP2R5E
ETS1	PPP4C
ETS1	PPP4R3B
ETS1	PPP6C
ETS1	PRDM15
ETS1	PRDM2
ETS1	PRDX6
ETS1	PREP
ETS1	PRKACB
ETS1	PRKAR1A

ETS1	PRKCD
ETS1	PRKRIP1
ETS1	PRMT2
ETS1	PRPF38A
ETS1	PRPF40A
ETS1	PRPF8
ETS1	PRR11
ETS1	PRR13
ETS1	PRR5
ETS1	PRRC2B
ETS1	PRRG4
ETS1	PSD4
ETS1	PSMA4
ETS1	PSMB2
ETS1	PSMB3
ETS1	PSMB8
ETS1	PSMB9
ETS1	PSMD13
ETS1	PSMD4
ETS1	PSMF1
ETS1	PSMG1
ETS1	PSPC1
ETS1	PSPH
ETS1	PTEN
ETS1	PTMS
ETS1	PTPN1
ETS1	PTPN18
ETS1	PTPN2
ETS1	PTPRA
ETS1	PTPRC
ETS1	PTPRJ
ETS1	PTRHD1
ETS1	PTTG1
ETS1	PUS10
ETS1	PXK
ETS1	PYGB
ETS1	PYM1
ETS1	PYURF
ETS1	QARS
ETS1	RAB11FIP1
ETS1	RAB11FIP4
ETS1	RAB18
ETS1	RAB1B
ETS1	RAB22A
ETS1	RAB30
ETS1	RAB30-AS1
ETS1	RAB3GAP1
ETS1	RAB3GAP2
ETS1	RAB4A
ETS1	RABEP1

ETS1	RABGAP1L
ETS1	RAC2
ETS1	RAD21
ETS1	RAD51B
ETS1	RALB
ETS1	RALBP1
ETS1	RALGPS2
ETS1	RANGAP1
ETS1	RAP1A
ETS1	RAP1B
ETS1	RAP2C
ETS1	RASAL3
ETS1	RASGEF1B
ETS1	RASGRP2
ETS1	RASGRP3
ETS1	RASSF6
ETS1	RB1
ETS1	RB1CC1
ETS1	RBCK1
ETS1	RBL2
ETS1	RBM17
ETS1	RBM22
ETS1	RBM26-AS1
ETS1	RBM3
ETS1	RBM38
ETS1	RBM39
ETS1	RBM4B
ETS1	RBM5
ETS1	RBX1
ETS1	RCBTB2
ETS1	RCN1
ETS1	RCOR3
ETS1	RCSD1
ETS1	REEP4
ETS1	RELA
ETS1	REPIN1
ETS1	RERE
ETS1	REST
ETS1	RFTN1
ETS1	RFX1
ETS1	RFX7
ETS1	RFXANK
ETS1	RGS13
ETS1	RGS19
ETS1	RHBDF2
ETS1	RHOA
ETS1	RHOH
ETS1	RHOQ
ETS1	RIOK3
ETS1	RLF

ETS1	RMDN1
ETS1	RMND5B
ETS1	RNF10
ETS1	RNF114
ETS1	RNF130
ETS1	RNF14
ETS1	RNF144B
ETS1	RNF170
ETS1	RNF19A
ETS1	RNF26
ETS1	RNF34
ETS1	RNF7
ETS1	RNFT2
ETS1	RNPEPL1
ETS1	ROCK1
ETS1	ROMO1
ETS1	RP2
ETS1	RPL15
ETS1	RPL28
ETS1	RPL39L
ETS1	RPL6
ETS1	RPL8
ETS1	RPS24
ETS1	RPS4X
ETS1	RPUSD3
ETS1	RRM2B
ETS1	RSRC2
ETS1	RTF1
ETS1	RTN4IP1
ETS1	RTP4
ETS1	S100A10
ETS1	S1PR4
ETS1	SAFB2
ETS1	SAMHD1
ETS1	SASH3
ETS1	SAT1
ETS1	SBF2-AS1
ETS1	SCAF11
ETS1	SCAMP1-AS1
ETS1	SCAMP2
ETS1	SCLT1
ETS1	SDHB
ETS1	SDHC
ETS1	SEC14L1
ETS1	SEC23A
ETS1	SECISBP2L
ETS1	SEL1L3
ETS1	SELL
ETS1	SELPLG
ETS1	SEPT2

ETS1	SEPT6
ETS1	SEPT9
ETS1	SERF2
ETS1	SERINC3
ETS1	SERTAD3
ETS1	SETD2
ETS1	SETDB2
ETS1	SETX
ETS1	SF3B1
ETS1	SFPQ
ETS1	SGO2
ETS1	SGSM3
ETS1	SH2B2
ETS1	SH3BGRL
ETS1	SH3KBP1
ETS1	SH3TC1
ETS1	SHCBP1
ETS1	SHF
ETS1	SHKBP1
ETS1	SHOC2
ETS1	SIPA1
ETS1	SIRT1
ETS1	SIRT6
ETS1	SIT1
ETS1	SKAP2
ETS1	SKP1
ETS1	SLC16A6
ETS1	SLC22A5
ETS1	SLC25A13
ETS1	SLC25A5
ETS1	SLC2A5
ETS1	SLC30A6
ETS1	SLC35A1
ETS1	SLC35B4
ETS1	SLC35C2
ETS1	SLC35G2
ETS1	SLC43A2
ETS1	SLC44A2
ETS1	SLC4A1AP
ETS1	SLC8B1
ETS1	SLC9A3R1
ETS1	SLTM
ETS1	SLU7
ETS1	SMAP1
ETS1	SMAP2
ETS1	SMARCA2
ETS1	SMARCA5
ETS1	SMARCC2
ETS1	SMC1A
ETS1	SMCHD1

ETS1	SMDT1
ETS1	SMIM14
ETS1	SMIM19
ETS1	SMU1
ETS1	SMUG1
ETS1	SMYD4
ETS1	SNAP23
ETS1	SNAPIN
ETS1	SNF8
ETS1	SNHG10
ETS1	SNRNP35
ETS1	SNRNP40
ETS1	SNRPB2
ETS1	SNRPN
ETS1	SNTA1
ETS1	SNTB2
ETS1	SNW1
ETS1	SNX1
ETS1	SNX10
ETS1	SNX2
ETS1	SNX25
ETS1	SNX3
ETS1	SNX5
ETS1	SOBP
ETS1	SORL1
ETS1	SOX4
ETS1	SP100
ETS1	SP110
ETS1	SPAG7
ETS1	SPATS2L
ETS1	SPEN
ETS1	SPG7
ETS1	SPHK2
ETS1	SPN
ETS1	SPRYD7
ETS1	SPTBN1
ETS1	SRP14
ETS1	SS18
ETS1	SSH2
ETS1	SSU72
ETS1	ST13
ETS1	ST3GAL2
ETS1	ST3GAL5
ETS1	ST6GAL1
ETS1	STAG2
ETS1	STAM
ETS1	STAP1
ETS1	STARD7
ETS1	STAT5B
ETS1	STIM2

ETS1	STK11IP
ETS1	STK25
ETS1	STK38
ETS1	STRN3
ETS1	STT3B
ETS1	STUB1
ETS1	STX7
ETS1	STX8
ETS1	STXBP2
ETS1	SUGP1
ETS1	SUMO1
ETS1	SUN2
ETS1	SUSD3
ETS1	SUSD6
ETS1	SVBP
ETS1	SWAP70
ETS1	SWI5
ETS1	SYAP1
ETS1	SYK
ETS1	SYNE2
ETS1	SYNE3
ETS1	SYPL1
ETS1	SYT17
ETS1	SYTL2
ETS1	TAB1
ETS1	TACC1
ETS1	TACC3
ETS1	TAF15
ETS1	TAF1B
ETS1	TAF2
ETS1	TAF7
ETS1	TAF8
ETS1	TAGAP
ETS1	TANK
ETS1	TAOK1
ETS1	TAOK3
ETS1	TARDBP
ETS1	TARS2
ETS1	TAX1BP1
ETS1	TAX1BP3
ETS1	TBC1D1
ETS1	TBC1D10C
ETS1	TBC1D23
ETS1	TBC1D7
ETS1	TBCC
ETS1	TBCEL
ETS1	TBCK
ETS1	TCAIM
ETS1	TCEA1
ETS1	TCF3

ETS1	TCF4
ETS1	TCFL5
ETS1	TCHP
ETS1	TCIRG1
ETS1	TCL1A
ETS1	TCP11L2
ETS1	TDP2
ETS1	TECPR1
ETS1	TEFM
ETS1	TEN1
ETS1	TERF2IP
ETS1	TFAP4
ETS1	TFEB
ETS1	TFPT
ETS1	TGDS
ETS1	THAP3
ETS1	THAP7-AS1
ETS1	THG1L
ETS1	THOC2
ETS1	THOC7
ETS1	THUMPD3
ETS1	THUMPD3-AS1
ETS1	THYN1
ETS1	TIFA
ETS1	TIGAR
ETS1	TIMM23
ETS1	TLDC1
ETS1	TLE4
ETS1	TLK1
ETS1	TLK2
ETS1	TLN1
ETS1	TM2D1
ETS1	TM7SF3
ETS1	TMA7
ETS1	TMC6
ETS1	TMEM14C
ETS1	TMEM154
ETS1	TMEM156
ETS1	TMEM205
ETS1	TMEM214
ETS1	TMEM242
ETS1	TMEM243
ETS1	TMEM248
ETS1	TMEM256
ETS1	TMEM263
ETS1	TMEM8A
ETS1	TMF1
ETS1	TMSB10
ETS1	TMSB4X
ETS1	TNFRSF13C

ETS1	TNPO1
ETS1	TNRC6A
ETS1	TOB2
ETS1	TOMM7
ETS1	TOMM70
ETS1	TOP1
ETS1	TOP2A
ETS1	TOP3B
ETS1	TOR1AIP1
ETS1	TOR1AIP2
ETS1	TOX
ETS1	TOX4
ETS1	TP53BP2
ETS1	TPCN1
ETS1	TPGS1
ETS1	TPM3
ETS1	TPM4
ETS1	TPX2
ETS1	TRAF3IP2
ETS1	TRAF3IP2-AS1
ETS1	TRAFD1
ETS1	TRAK1
ETS1	TRANK1
ETS1	TRAPPC1
ETS1	TRIB2
ETS1	TRIM14
ETS1	TRIM26
ETS1	TRIM32
ETS1	TRIM38
ETS1	TRIM52
ETS1	TRIOBP
ETS1	TRMT2B
ETS1	TRPM7
ETS1	TSC22D3
ETS1	TSC22D4
ETS1	TSNAX
ETS1	TSPO
ETS1	TSTD1
ETS1	TTC3
ETS1	TTC37
ETS1	TTLL5
ETS1	TTN
ETS1	TUBA1A
ETS1	TUBA4A
ETS1	TWF1
ETS1	TWF2
ETS1	TXN2
ETS1	TXNDC9
ETS1	TYW5
ETS1	UBA1

ETS1	UBA52
ETS1	UBALD2
ETS1	UBE2D2
ETS1	UBE2E1
ETS1	UBE2G1
ETS1	UBE2J1
ETS1	UBE2J2
ETS1	UBE2Q1
ETS1	UBE2R2
ETS1	UBE2S
ETS1	UBE3A
ETS1	UBE3B
ETS1	UBL5
ETS1	UBL7
ETS1	UBL7-AS1
ETS1	UBN2
ETS1	UBR1
ETS1	UBR5
ETS1	UBXN1
ETS1	UCP2
ETS1	UFC1
ETS1	UFL1
ETS1	UGDH
ETS1	UHRF1BP1L
ETS1	ULK4
ETS1	UNC119
ETS1	UNC13D
ETS1	UNC45A
ETS1	UPF3A
ETS1	UPRT
ETS1	UQCR10
ETS1	UQCRB
ETS1	UQCRC1
ETS1	UQCRFS1
ETS1	UQCRH
ETS1	URI1
ETS1	URM1
ETS1	UROD
ETS1	USP14
ETS1	USP24
ETS1	USP28
ETS1	USP33
ETS1	USP34
ETS1	USP48
ETS1	USP7
ETS1	USP8
ETS1	UTP3
ETS1	UXT
ETS1	VAMP2
ETS1	VAMP8

ETS1	VCP
ETS1	VDAC3
ETS1	VEZF1
ETS1	VNN2
ETS1	VPS13B
ETS1	VPS13C
ETS1	VPS18
ETS1	VPS25
ETS1	VPS28
ETS1	VPS33A
ETS1	VPS39
ETS1	VPS4B
ETS1	VPS51
ETS1	VRK1
ETS1	VRK3
ETS1	WAC
ETS1	WAPL
ETS1	WASF1
ETS1	WASF2
ETS1	WDR1
ETS1	WDR13
ETS1	WDR25
ETS1	WDR33
ETS1	WDR61
ETS1	WDR74
ETS1	WDR83OS
ETS1	WDR92
ETS1	WDTC1
ETS1	WFS1
ETS1	WIPF1
ETS1	WNK1
ETS1	WSB1
ETS1	WTAP
ETS1	XIAP
ETS1	XKR6
ETS1	XKR8
ETS1	XPA
ETS1	XPO1
ETS1	XRCC4
ETS1	YARS2
ETS1	YIPF4
ETS1	YLPM1
ETS1	YPEL5
ETS1	YTHDC1
ETS1	YTHDC2
ETS1	YWHAB
ETS1	YWHAZ
ETS1	YY1
ETS1	ZBED5-AS1
ETS1	ZBP1

ETS1	ZBTB17
ETS1	ZBTB20
ETS1	ZBTB43
ETS1	ZBTB45
ETS1	ZBTB7A
ETS1	ZC3H18
ETS1	ZC3HAV1
ETS1	ZCCHC3
ETS1	ZCCHC7
ETS1	ZDHHC12
ETS1	ZDHHC20
ETS1	ZEB1
ETS1	ZEB2
ETS1	ZFAT
ETS1	ZFC3H1
ETS1	ZFP36L2
ETS1	ZFP82
ETS1	ZFR
ETS1	ZFYVE16
ETS1	ZMYM5
ETS1	ZMYND8
ETS1	ZNF101
ETS1	ZNF106
ETS1	ZNF148
ETS1	ZNF197
ETS1	ZNF227
ETS1	ZNF281
ETS1	ZNF296
ETS1	ZNF330
ETS1	ZNF382
ETS1	ZNF428
ETS1	ZNF581
ETS1	ZNF586
ETS1	ZNF608
ETS1	ZNF611
ETS1	ZNF638
ETS1	ZNF644
ETS1	ZNF655
ETS1	ZNF674-AS1
ETS1	ZNF675
ETS1	ZNF677
ETS1	ZNF687
ETS1	ZNF701
ETS1	ZNF714
ETS1	ZNF720
ETS1	ZNF764
ETS1	ZNF800
ETS1	ZNF804A
ETS1	ZNF880
ETS1	ZNF91

ETS1	ZNHIT1
ETS1	ZNHIT2
ETS1	ZNRD1
ETS1	ZRANB2
ETS1	ZSCAN16-AS1
ETS1	ZSCAN2
ETS1	ZSCAN21
ETS2	DUSP5
ETS2	GTSF1
ETS2	PIK3CD
ETV2	ABHD13
ETV2	AP4M1
ETV2	APOLD1
ETV2	ARHGEF3
ETV2	ASTE1
ETV2	ATAD3A
ETV2	BCOR
ETV2	BTG2
ETV2	C18orf32
ETV2	CASP3
ETV2	CCDC91
ETV2	CENPN
ETV2	CHIC2
ETV2	CHPF
ETV2	CMIP
ETV2	COPB1
ETV2	COPG1
ETV2	CR2
ETV2	CTU1
ETV2	DDX6
ETV2	DNAJC4
ETV2	DOCK9
ETV2	DOK7
ETV2	EHD3
ETV2	EIF2AK2
ETV2	ELAC1
ETV2	ETV2
ETV2	FAM174A
ETV2	FAM200A
ETV2	GIN1
ETV2	GNPAT
ETV2	GPATCH2L
ETV2	GTDC1
ETV2	HEXA
ETV2	HSD17B11
ETV2	ICAM2
ETV2	IFT81
ETV2	INTS5
ETV2	KCNMB3
ETV2	KCNN3

ETV2	KLF10
ETV2	KLHL42
ETV2	LHFPL2
ETV2	MBOAT2
ETV2	MCU
ETV2	MFGE8
ETV2	MGST3
ETV2	MPC2
ETV2	NAXD
ETV2	NCOA6
ETV2	NDN
ETV2	NEK6
ETV2	NFYA
ETV2	NRAS
ETV2	OGFOD1
ETV2	ORMDL2
ETV2	OTULIN
ETV2	P2RX1
ETV2	P2RY10
ETV2	PARG
ETV2	PARP10
ETV2	PBK
ETV2	PHYKPL
ETV2	PISD
ETV2	PLP2
ETV2	POGZ
ETV2	POLE2
ETV2	PRKAR2A
ETV2	PRPS2
ETV2	RAB13
ETV2	RAB40B
ETV2	RAB8B
ETV2	RASGRP1
ETV2	REV1
ETV2	RFT1
ETV2	RNFT1
ETV2	RPL26L1
ETV2	RPLP1
ETV2	RSU1
ETV2	RUNX1
ETV2	RWDD2A
ETV2	SCMH1
ETV2	SERTAD2
ETV2	SHCBP1
ETV2	SIKE1
ETV2	SLC30A7
ETV2	SLC35E2B
ETV2	SNAPC3
ETV2	SNX22
ETV2	SP2

ETV2	SPI1
ETV2	ST7
ETV2	STARD3NL
ETV2	SUPT5H
ETV2	SUV39H1
ETV2	SUZ12
ETV2	SYT17
ETV2	TAX1BP3
ETV2	TBC1D15
ETV2	TBRG1
ETV2	THUMPD3-AS1
ETV2	TIMM17B
ETV2	TMEM106C
ETV2	TMEM161B
ETV2	TMX2
ETV2	TPP2
ETV2	TRPM7
ETV2	TTC33
ETV2	TUBB2A
ETV2	TUBB2B
ETV2	UBL4A
ETV2	USB1
ETV2	YIPF3
ETV2	ZFYVE19
ETV2	ZNF26
ETV2	ZNF322
ETV2	ZNF350
ETV2	ZNF397
ETV2	ZNF652
ETV2	ZNF660
ETV2	ZSCAN16
ETV3	ADTRP
ETV3	AGK
ETV3	AMIGO2
ETV3	ARHGEF7
ETV3	CAMK1D
ETV3	CBWD3
ETV3	CCDC6
ETV3	CD226
ETV3	CEP85L
ETV3	CRK
ETV3	CSPP1
ETV3	CXorf38
ETV3	DCAF16
ETV3	DDB1
ETV3	DOCK11
ETV3	ETV3
ETV3	FAM200B
ETV3	FASTKD1
ETV3	FBXO3

ETV3	FOXO1
ETV3	GABPB1
ETV3	GLYCTK
ETV3	HAAO
ETV3	HELQ
ETV3	HERPUD2
ETV3	IL7
ETV3	JAGN1
ETV3	KIAA0100
ETV3	LRRC61
ETV3	METTL2B
ETV3	MTHFD1L
ETV3	NFYA
ETV3	NPTX2
ETV3	NSUN3
ETV3	P4HA2
ETV3	PEMT
ETV3	PHF8
ETV3	PHPT1
ETV3	PIK3C3
ETV3	PNPT1
ETV3	POP1
ETV3	PTK2B
ETV3	PTMS
ETV3	PYHIN1
ETV3	RFT1
ETV3	RNF111
ETV3	RNF166
ETV3	RPS6KA3
ETV3	SCRN2
ETV3	SLAMF1
ETV3	SLC29A1
ETV3	SPRY1
ETV3	STARD10
ETV3	STAT5A
ETV3	TBC1D31
ETV3	TMEM120A
ETV3	TMEM234
ETV3	TOB2
ETV3	VCPIP1
ETV3	ZNF212
ETV3	ZNF544
ETV5	ABRACL
ETV5	ACSL3
ETV5	ADAM17
ETV5	ADRM1
ETV5	AKIP1
ETV5	ANAPC1
ETV5	ANAPC10
ETV5	ANGEL2

ETV5	APPBP2
ETV5	AQR
ETV5	ARHGAP35
ETV5	ARHGEF7
ETV5	ARID4B
ETV5	ARMCX6
ETV5	ARPP19
ETV5	ARRB2
ETV5	ASCC1
ETV5	ATAD2
ETV5	ATG10
ETV5	ATXN2
ETV5	B9D2
ETV5	BAG1
ETV5	BAP1
ETV5	BBC3
ETV5	BCAR3
ETV5	BCAT1
ETV5	BCLAF1
ETV5	BDP1
ETV5	BIK
ETV5	BRAP
ETV5	C16orf91
ETV5	C19orf48
ETV5	C3orf14
ETV5	C6orf226
ETV5	CAMKK2
ETV5	CAPN10
ETV5	CCDC25
ETV5	CCDC51
ETV5	CCDC61
ETV5	CCNB1IP1
ETV5	CD53
ETV5	CD70
ETV5	CDC34
ETV5	CDC42EP3
ETV5	CEBPD
ETV5	CEP57
ETV5	CEP89
ETV5	CETN3
ETV5	COA1
ETV5	COASY
ETV5	COCH
ETV5	COMMD10
ETV5	CORO1C
ETV5	COX15
ETV5	COX16
ETV5	CPM
ETV5	CTDSP1
ETV5	CTNNBIP1

ETV5	CTPS1
ETV5	CUL4A
ETV5	CYSTM1
ETV5	CYTH2
ETV5	DDHD1
ETV5	DIAPH3
ETV5	DLGAP1-AS1
ETV5	DUS1L
ETV5	DUSP10
ETV5	DYM
ETV5	ECSIT
ETV5	EEFSEC
ETV5	EGR1
ETV5	EGR2
ETV5	EIF5A
ETV5	ETV5
ETV5	EZH2
ETV5	FAAP24
ETV5	FAM120A
ETV5	FBXO25
ETV5	FGD5-AS1
ETV5	FGD6
ETV5	FHOD1
ETV5	FKBP1B
ETV5	FTSJ3
ETV5	G6PD
ETV5	GAMT
ETV5	GANAB
ETV5	GMEB1
ETV5	GMIP
ETV5	GMNN
ETV5	GNPTG
ETV5	GOLGA8A
ETV5	GPSM3
ETV5	GRIPAP1
ETV5	GRK6
ETV5	GSPT2
ETV5	GSTM4
ETV5	H2AFX
ETV5	HADHB
ETV5	HAUS8
ETV5	HDHD2
ETV5	HIBCH
ETV5	HLA-DMB
ETV5	HMGN5
ETV5	HNRNPLL
ETV5	HUWE1
ETV5	HYLS1
ETV5	IDE
ETV5	IDI1

ETV5	IFT122
ETV5	IFT74
ETV5	INSIG2
ETV5	INTS7
ETV5	INTS8
ETV5	IZUMO4
ETV5	KAT8
ETV5	KATNAL1
ETV5	KCNAB2
ETV5	KCTD6
ETV5	KHDC1
ETV5	KIN
ETV5	KLHL20
ETV5	KLHL6
ETV5	KREMEN2
ETV5	LARP4B
ETV5	LEPROT
ETV5	LGALS8
ETV5	LIMK2
ETV5	LMNB1
ETV5	LMO2
ETV5	LPCAT3
ETV5	LPXN
ETV5	LRMP
ETV5	LSAMP
ETV5	LTA4H
ETV5	MAD2L2
ETV5	MAPKAP1
ETV5	MAPRE2
ETV5	MBD6
ETV5	MCM3
ETV5	MDM2
ETV5	MED11
ETV5	MIR3681HG
ETV5	MPC2
ETV5	MPP6
ETV5	MRM2
ETV5	MROH1
ETV5	MRPL39
ETV5	MTBP
ETV5	MTR
ETV5	MTRF1
ETV5	MTX2
ETV5	MYH10
ETV5	NAA60
ETV5	NBN
ETV5	NCK1
ETV5	NCOA7
ETV5	NDFIP2
ETV5	NDUFB5

ETV5	NEDD4L
ETV5	NFAT5
ETV5	NFATC1
ETV5	NFATC3
ETV5	NLK
ETV5	NOMO3
ETV5	NOSIP
ETV5	NSL1
ETV5	NUB1
ETV5	NUP214
ETV5	NUP62
ETV5	ORC6
ETV5	OSBPL10
ETV5	PAICS
ETV5	PANK1
ETV5	PAX5
ETV5	PCGF3
ETV5	PDCD6IP
ETV5	PDGFD
ETV5	PGS1
ETV5	PHACTR2
ETV5	PIGA
ETV5	PITPNA
ETV5	PLCL2
ETV5	POC1A
ETV5	POLDIP3
ETV5	PPM1G
ETV5	PPP1R35
ETV5	PPP4R3A
ETV5	PRCP
ETV5	PRKAA1
ETV5	PRKRA
ETV5	PRPF4B
ETV5	PRPSAP2
ETV5	PRR7
ETV5	PRRC2B
ETV5	PURA
ETV5	PYM1
ETV5	RBBP6
ETV5	RBM22
ETV5	RBM23
ETV5	RGS13
ETV5	RNASEH2A
ETV5	RNF20
ETV5	ROCK1
ETV5	RPA2
ETV5	RUVBL1
ETV5	S100A10
ETV5	S100A13
ETV5	SAR1A

ETV5	SCNM1
ETV5	SEC22A
ETV5	SERP2
ETV5	SETX
ETV5	SFT2D3
ETV5	SGPP1
ETV5	SH3RF1
ETV5	SHC1
ETV5	SHOC2
ETV5	SIRT7
ETV5	SLA
ETV5	SLC16A1
ETV5	SLC25A19
ETV5	SLC35A4
ETV5	SLC4A1AP
ETV5	SLF2
ETV5	SMARCA2
ETV5	SMC5
ETV5	SMS
ETV5	SMURF2
ETV5	SPSB3
ETV5	ST20
ETV5	ST3GAL5
ETV5	STAP1
ETV5	STARD3NL
ETV5	STK24
ETV5	STX16
ETV5	SUCLG1
ETV5	SUPT6H
ETV5	SYNJ2
ETV5	SYTL2
ETV5	TAOK3
ETV5	TCEAL3
ETV5	TIA1
ETV5	TIMM22
ETV5	TIMMDC1
ETV5	TLR10
ETV5	TMBIM1
ETV5	TMEM11
ETV5	TMEM41B
ETV5	TMUB2
ETV5	TP53
ETV5	TP73
ETV5	TPGS1
ETV5	TPM4
ETV5	TRAF7
ETV5	TRAPPC10
ETV5	TRNT1
ETV5	TSEN34
ETV5	TSPO

ETV5	TXLNG
ETV5	TXNRD1
ETV5	TYMS
ETV5	UBA2
ETV5	UBALD1
ETV5	UBAP1
ETV5	UBE2R2
ETV5	UCHL5
ETV5	UHRF2
ETV5	UQCRH
ETV5	USF2
ETV5	VPS35
ETV5	VPS72
ETV5	WASF2
ETV5	WBP4
ETV5	WDR26
ETV5	WDR33
ETV5	WDR55
ETV5	WDTC1
ETV5	WNK2
ETV5	WRNIP1
ETV5	WSB2
ETV5	XKR6
ETV5	XRCC4
ETV5	XRCC5
ETV5	YARS2
ETV5	YTHDF1
ETV5	YWHAG
ETV5	ZBTB2
ETV5	ZC3H14
ETV5	ZDHHC13
ETV5	ZFAND4
ETV5	ZFP36
ETV5	ZNF485
ETV5	ZNF532
ETV5	ZNF544
ETV5	ZNF608
ETV5	ZNF721
ETV6	ADAM28
ETV6	AFF1
ETV6	AGPAT5
ETV6	ANTXR2
ETV6	AP4S1
ETV6	APOBEC3C
ETV6	ARHGAP24
ETV6	ARID3A
ETV6	ARID5B
ETV6	ARL6IP5
ETV6	B2M
ETV6	BMF

ETV6	CBX6
ETV6	CCDC50
ETV6	CCDC71L
ETV6	CCL22
ETV6	CIB2
ETV6	CNN3
ETV6	CNPY3
ETV6	COPA
ETV6	COX7C
ETV6	CRLF3
ETV6	DAPK3
ETV6	DDHD1
ETV6	DDIT4
ETV6	DOK1
ETV6	EEF1A1
ETV6	EEF1B2
ETV6	EMP3
ETV6	EPHB6
ETV6	EPSTI1
ETV6	ETV6
ETV6	FABP5
ETV6	FAM184A
ETV6	FASTKD5
ETV6	FCGR2B
ETV6	FLNA
ETV6	FUCA2
ETV6	FXYD5
ETV6	FZD6
ETV6	GANAB
ETV6	GNPTG
ETV6	GPR183
ETV6	HLA-A
ETV6	IFITM2
ETV6	IFNGR1
ETV6	IL2RG
ETV6	KLF6
ETV6	KREMEN2
ETV6	LDLRAD4
ETV6	LINC00528
ETV6	LMO7
ETV6	LRRC32
ETV6	LY6E
ETV6	MSRA
ETV6	MTBP
ETV6	MX2
ETV6	NCALD
ETV6	NDUFAF1
ETV6	NF2
ETV6	NFE2L2
ETV6	NR4A2

ETV6	P2RX5−TAX1BP3
ETV6	P2RY10
ETV6	PAAF1
ETV6	PABPC1
ETV6	PDCD4
ETV6	PDLIM1
ETV6	PIGK
ETV6	PIP5K1B
ETV6	PLP2
ETV6	PPP1R14B
ETV6	RAB34
ETV6	RACK1
ETV6	RARA
ETV6	RASAL1
ETV6	RASSF5
ETV6	RHOC
ETV6	RNASEH2B
ETV6	RP9
ETV6	RPL10
ETV6	RPL10A
ETV6	RPL11
ETV6	RPL12
ETV6	RPL13
ETV6	RPL13A
ETV6	RPL14
ETV6	RPL15
ETV6	RPL18
ETV6	RPL18A
ETV6	RPL19
ETV6	RPL21
ETV6	RPL23A
ETV6	RPL26
ETV6	RPL27
ETV6	RPL27A
ETV6	RPL28
ETV6	RPL29
ETV6	RPL3
ETV6	RPL30
ETV6	RPL31
ETV6	RPL32
ETV6	RPL34
ETV6	RPL35
ETV6	RPL35A
ETV6	RPL36
ETV6	RPL37
ETV6	RPL37A
ETV6	RPL39
ETV6	RPL4
ETV6	RPL41
ETV6	RPL5

ETV6	RPL6
ETV6	RPL7
ETV6	RPLP1
ETV6	RPLP2
ETV6	RPS10
ETV6	RPS11
ETV6	RPS12
ETV6	RPS13
ETV6	RPS14
ETV6	RPS16
ETV6	RPS17
ETV6	RPS18
ETV6	RPS19
ETV6	RPS2
ETV6	RPS20
ETV6	RPS21
ETV6	RPS24
ETV6	RPS25
ETV6	RPS27
ETV6	RPS27A
ETV6	RPS29
ETV6	RPS3
ETV6	RPS3A
ETV6	RPS4Y1
ETV6	RPS5
ETV6	RPS6
ETV6	RPS7
ETV6	RPS8
ETV6	RPSA
ETV6	RTN4
ETV6	SAT1
ETV6	SERINC1
ETV6	SERINC3
ETV6	SH3BGRL3
ETV6	SIGIRR
ETV6	SLC12A6
ETV6	SNX2
ETV6	SP100
ETV6	STARD10
ETV6	STAT3
ETV6	TARS
ETV6	TCAF1
ETV6	TMSB4X
ETV6	TMTC4
ETV6	TNFRSF12A
ETV6	TNFRSF13B
ETV6	TNRC6B
ETV6	TPT1
ETV6	TRAF3IP3
ETV6	TSHZ2

ETV6	TUBA1A
ETV6	U2AF1L4
ETV6	UBA52
ETV6	VIM
ETV6	VRK2
ETV6	YPEL2
ETV6	ZBTB32
ETV6	ZFP36
ETV6	ZFP36L1
ETV7	ABCB9
ETV7	ABHD2
ETV7	ADAM28
ETV7	ALKBH3
ETV7	ALOX5AP
ETV7	ANG
ETV7	ARID5B
ETV7	ARL6IP5
ETV7	ATP10D
ETV7	B3GALT4
ETV7	BAZ2A
ETV7	BLVRA
ETV7	BSDC1
ETV7	BTNL9
ETV7	C16orf58
ETV7	C16orf74
ETV7	C19orf81
ETV7	C6orf106
ETV7	CADM1
ETV7	CAPG
ETV7	CBWD1
ETV7	CBX4
ETV7	CBX7
ETV7	CCDC88B
ETV7	CCNDBP1
ETV7	CCPG1
ETV7	CD180
ETV7	CD19
ETV7	CD53
ETV7	CD63
ETV7	CD96
ETV7	CDIPT
ETV7	CEP41
ETV7	CHIC2
ETV7	CHID1
ETV7	CHPF
ETV7	CHPF2
ETV7	CHRNA1
ETV7	CHST12
ETV7	CIITA
ETV7	COG8

ETV7	COPA
ETV7	CORO1B
ETV7	CPT2
ETV7	CSGALNACT2
ETV7	CSNK1E
ETV7	CSNK1G3
ETV7	CUTA
ETV7	CYB5A
ETV7	CYTIP
ETV7	DAAM1
ETV7	DCTD
ETV7	DDOST
ETV7	DENND1C
ETV7	DENND3
ETV7	DERL3
ETV7	DHRS7
ETV7	DHRS9
ETV7	DMD
ETV7	DNAJC1
ETV7	DNAJC24
ETV7	EBAG9
ETV7	EDEM2
ETV7	EHD3
ETV7	EMC7
ETV7	ERGIC2
ETV7	ERLEC1
ETV7	ERP44
ETV7	EVI2B
ETV7	EXT1
ETV7	EXTL2
ETV7	FAM102A
ETV7	FAM135A
ETV7	FAM174A
ETV7	FAM3C
ETV7	FBXW7
ETV7	FERMT3
ETV7	FGD2
ETV7	FGFR1
ETV7	FKBP2
ETV7	FLOT1
ETV7	FNDC3A
ETV7	FNDC3B
ETV7	FOXN3
ETV7	FOXO3
ETV7	FUCA2
ETV7	FUT7
ETV7	FUT8-AS1
ETV7	GALE
ETV7	GAS6
ETV7	GLRX

ETV7	GLS
ETV7	GOLGB1
ETV7	GOLT1B
ETV7	GORASP2
ETV7	GOSR2
ETV7	GPAA1
ETV7	GPR137B
ETV7	GPR160
ETV7	GPR34
ETV7	GPR65
ETV7	GUCD1
ETV7	GYPC
ETV7	HEXB
ETV7	HHEX
ETV7	HIST1H4H
ETV7	HLA-DMA
ETV7	HLA-DPA1
ETV7	HLA-DPB1
ETV7	HM13
ETV7	HMOX2
ETV7	HOOK2
ETV7	HOXB2
ETV7	HSP90B1
ETV7	ICAM1
ETV7	ICAM3
ETV7	IFI27L2
ETV7	IFNG-AS1
ETV7	IL12RB1
ETV7	IL16
ETV7	INSIG2
ETV7	IRF1
ETV7	ITFG2
ETV7	ITGB1
ETV7	ITSN2
ETV7	JMJD8
ETV7	JOSD2
ETV7	KCNK6
ETV7	KCNN4
ETV7	KCTD20
ETV7	KDELR3
ETV7	KIAA0232
ETV7	KLHDC2
ETV7	KLHL12
ETV7	KRT18
ETV7	KRTCAP2
ETV7	LAPTM5
ETV7	LMO2
ETV7	LMO4
ETV7	LPP
ETV7	LRP10

ETV7	LSP1
ETV7	LUZP1
ETV7	LY96
ETV7	LYPLAL1
ETV7	MAFG
ETV7	MAGED1
ETV7	MAGEH1
ETV7	MBNL2
ETV7	METTL15
ETV7	MFSD5
ETV7	MGAT1
ETV7	MGAT4A
ETV7	MGMT
ETV7	MGST2
ETV7	MIXL1
ETV7	MKLN1
ETV7	MKS1
ETV7	MPZL1
ETV7	MRPL48
ETV7	MTERF4
ETV7	MTMR14
ETV7	MTMR2
ETV7	MXD4
ETV7	MYO1G
ETV7	MZB1
ETV7	NAA30
ETV7	NAA60
ETV7	NCF4
ETV7	NDUFA1
ETV7	NEK6
ETV7	NINJ2
ETV7	NMRK1
ETV7	NORAD
ETV7	NUP153
ETV7	ORC2
ETV7	ORMDL3
ETV7	OST4
ETV7	P4HB
ETV7	PAG1
ETV7	PARM1
ETV7	PARVG
ETV7	PCED1B
ETV7	PCYT1A
ETV7	PDIA5
ETV7	PGM3
ETV7	PHF1
ETV7	PIGT
ETV7	PIK3CD-AS2
ETV7	PIK3CG
ETV7	PLD3

ETV7	PLD4
ETV7	PLEKHG2
ETV7	PMEPA1
ETV7	PNOC
ETV7	POLR2A
ETV7	POU2AF1
ETV7	PPP2R5D
ETV7	PPP2R5E
ETV7	PRKCSH
ETV7	PRKD2
ETV7	PRPSAP2
ETV7	PRR5
ETV7	PTAFR
ETV7	PTGS1
ETV7	PTP4A2
ETV7	PTPRN2
ETV7	QPCT
ETV7	RAB11B
ETV7	RAB11FIP1
ETV7	RBM18
ETV7	RBM47
ETV7	RBMX2
ETV7	REEP3
ETV7	RGS13
ETV7	RHBDF2
ETV7	RICTOR
ETV7	RNF13
ETV7	RNF4
ETV7	RNFT2
ETV7	RPL10
ETV7	RPN1
ETV7	RPS26
ETV7	RRNAD1
ETV7	S100A10
ETV7	SDC1
ETV7	SEC22B
ETV7	SEL1L3
ETV7	SEMA4D
ETV7	SENP7
ETV7	SERF2
ETV7	SERP1
ETV7	SETBP1
ETV7	SGK1
ETV7	SGMS1
ETV7	SIAE
ETV7	SIL1
ETV7	SIPA1L1
ETV7	SKAP1
ETV7	SLC39A7
ETV7	SLC44A1

ETV7	SMAP2
ETV7	SMIM14
ETV7	SMIM20
ETV7	SNAP23
ETV7	SNX13
ETV7	SNX17
ETV7	SNX22
ETV7	SP3
ETV7	SPATA33
ETV7	SPATS2
ETV7	SPCS3
ETV7	SPIN2B
ETV7	SPINT2
ETV7	SPRED2
ETV7	SPSB3
ETV7	SSR3
ETV7	SSSCA1
ETV7	ST6GAL1
ETV7	ST6GALNAC4
ETV7	STAT1
ETV7	STAT3
ETV7	STK17B
ETV7	STK33
ETV7	STXBP6
ETV7	SYNRG
ETV7	TAF7
ETV7	TBC1D10C
ETV7	TBC1D22A
ETV7	TBC1D9B
ETV7	TCIRG1
ETV7	TERF2IP
ETV7	TEX264
ETV7	THAP2
ETV7	TIMMDC1
ETV7	TM9SF2
ETV7	TMBIM6
ETV7	TMCO3
ETV7	TMED10
ETV7	TMEM156
ETV7	TMEM208
ETV7	TMEM242
ETV7	TMEM59
ETV7	TOP3B
ETV7	TOR4A
ETV7	TOX
ETV7	TP53INP1
ETV7	TPD52
ETV7	TRAPPC2
ETV7	TRAPPC2B
ETV7	TRAPPC6A

ETV7	TRPM7
ETV7	TSC22D3
ETV7	TSPAN13
ETV7	TSPYL2
ETV7	TXNDC5
ETV7	U2AF1L4
ETV7	UAP1
ETV7	UBC
ETV7	UBE2D2
ETV7	UBE2W
ETV7	UBFD1
ETV7	UCP2
ETV7	UGGT2
ETV7	UMAD1
ETV7	UTY
ETV7	UVRAG
ETV7	VGLL4
ETV7	VPREB1
ETV7	VPS37B
ETV7	WIPF1
ETV7	XAB2
ETV7	YIPF1
ETV7	YIPF3
ETV7	YWHAB
ETV7	ZBTB20
ETV7	ZBTB38
ETV7	ZFAND4
ETV7	ZFAND6
ETV7	ZNF200
ETV7	ZNF790
ETV7	ZNF821
FLI1	ABCC4
FLI1	ABTB1
FLI1	ACAP1
FLI1	ACO1
FLI1	ACTG1
FLI1	ADAM23
FLI1	AGPAT1
FLI1	AHNAK
FLI1	ANKS3
FLI1	ANXA4
FLI1	ANXA6
FLI1	AP1S3
FLI1	AP5B1
FLI1	ARHGAP30
FLI1	ARHGDIB
FLI1	ARHGEF1
FLI1	ARID4A
FLI1	ARL6IP5
FLI1	ARMCX5

FLI1	ARPC1B
FLI1	ARPC2
FLI1	ARSA
FLI1	ASCL2
FLI1	ATE1
FLI1	ATF7IP
FLI1	ATG16L2
FLI1	ATM
FLI1	ATP6V0A1
FLI1	ATP6V0E1
FLI1	ATP6V1H
FLI1	ATXN2L
FLI1	AZI2
FLI1	B2M
FLI1	B3GNT9
FLI1	BIN1
FLI1	BLCAP
FLI1	BLK
FLI1	C16orf74
FLI1	C1orf112
FLI1	C2orf49
FLI1	C9orf72
FLI1	CALHM2
FLI1	CAMK2G
FLI1	CAP1
FLI1	CAPN1
FLI1	CAPZB
FLI1	CARS2
FLI1	CASP4
FLI1	CAT
FLI1	CBLB
FLI1	CBR3
FLI1	CCNDBP1
FLI1	CCNI
FLI1	CD180
FLI1	CD37
FLI1	CD52
FLI1	CD53
FLI1	CD69
FLI1	CD74
FLI1	CD79A
FLI1	CD79B
FLI1	CEP85L
FLI1	CHD4
FLI1	CHMP3
FLI1	CHUK
FLI1	CNN2
FLI1	CNPY3
FLI1	CNTN2
FLI1	CORO1A

FLI1	CPT1A
FLI1	CRBN
FLI1	CREBZF
FLI1	CTPS2
FLI1	CTSH
FLI1	CTSS
FLI1	CTSZ
FLI1	CWC22
FLI1	CXXC5
FLI1	CYB561A3
FLI1	CYTH4
FLI1	CYTIP
FLI1	DALRD3
FLI1	DAPK3
FLI1	DBNL
FLI1	DCTN2
FLI1	DDAH2
FLI1	DDX39B
FLI1	DNAJC25
FLI1	DNAJC4
FLI1	DNASE1L3
FLI1	DOCK8
FLI1	DOPEY2
FLI1	DTX3
FLI1	DUSP22
FLI1	ELF2
FLI1	EPSTI1
FLI1	ERCC6L
FLI1	EVI2A
FLI1	EVI2B
FLI1	EVL
FLI1	EXT1
FLI1	FADS3
FLI1	FAM69A
FLI1	FAM72C
FLI1	FANCI
FLI1	FBXO9
FLI1	FBXW4
FLI1	FCGR2B
FLI1	FDFT1
FLI1	FKBP15
FLI1	FLI1
FLI1	FLOT2
FLI1	FRY
FLI1	FYN
FLI1	GABARAPL2
FLI1	GABPA
FLI1	GANC
FLI1	GAPT
FLI1	GAPVD1

FLI1	GLIPR1
FLI1	GLUL
FLI1	GMPR2
FLI1	GNAI3
FLI1	GPSM3
FLI1	GRK6
FLI1	HCK
FLI1	HEBP1
FLI1	HIBCH
FLI1	HLA-DMA
FLI1	HLA-DMB
FLI1	HLA-F
FLI1	HMOX1
FLI1	HP1BP3
FLI1	HPCAL1
FLI1	HVCN1
FLI1	IGBP1
FLI1	IL10RA
FLI1	IL16
FLI1	IL27RA
FLI1	IRF2BP2
FLI1	ISCU
FLI1	ITGB7
FLI1	KIAA2013
FLI1	KLHL12
FLI1	LAPTM5
FLI1	LAT2
FLI1	LBH
FLI1	LINC00877
FLI1	LMBRD1
FLI1	LMO2
FLI1	LRR1
FLI1	LRRC37B
FLI1	LRRFIP1
FLI1	LSP1
FLI1	LY86
FLI1	MARCH1
FLI1	MED23
FLI1	MEF2C
FLI1	MFF
FLI1	MGAT4A
FLI1	MLKL
FLI1	MORC3
FLI1	MPG
FLI1	MS4A1
FLI1	MS4A7
FLI1	MTPN
FLI1	MVB12A
FLI1	MYL12A
FLI1	MYL5

FLI1	MYL6
FLI1	MYO1F
FLI1	N4BP2L2
FLI1	NACC1
FLI1	NBAS
FLI1	NCOA3
FLI1	NCR3
FLI1	NDE1
FLI1	NDEL1
FLI1	NR4A2
FLI1	NUB1
FLI1	OGFR
FLI1	OTUD5
FLI1	P2RX5
FLI1	PCSK7
FLI1	PCYOX1L
FLI1	PDCD6IP
FLI1	PDE4B
FLI1	PDLIM1
FLI1	PDP1
FLI1	PEX16
FLI1	PEX2
FLI1	PIAS3
FLI1	PJA2
FLI1	PLA2G6
FLI1	PLEKHF1
FLI1	PLEKHO1
FLI1	PLEKHO2
FLI1	PNISR
FLI1	PNN
FLI1	POU2AF1
FLI1	PPP1R16A
FLI1	PQLC2
FLI1	PREX1
FLI1	PRKCB
FLI1	PSMB9
FLI1	PTPN18
FLI1	PTPRS
FLI1	PYCARD
FLI1	RAC2
FLI1	RALB
FLI1	RASGRP2
FLI1	RASSF7
FLI1	RB1
FLI1	RCSD1
FLI1	RERE
FLI1	RGS14
FLI1	RHOH
FLI1	RICTOR
FLI1	RNASET2

FLI1	RNF4
FLI1	RPL13A
FLI1	RPL18
FLI1	RPL23A
FLI1	RPL28
FLI1	RPL30
FLI1	RPS15
FLI1	RPS27
FLI1	RPS4X
FLI1	RPSA
FLI1	RUSC1
FLI1	S100A4
FLI1	S1PR4
FLI1	SAPCD2
FLI1	SEC11A
FLI1	SECISBP2L
FLI1	SEPT6
FLI1	SEPT9
FLI1	SGMS1
FLI1	SH3BGRL
FLI1	SH3BGRL3
FLI1	SHKBP1
FLI1	SLC4A2
FLI1	SLC4A7
FLI1	SMIM14
FLI1	SNX2
FLI1	SNX3
FLI1	SP100
FLI1	SPI1
FLI1	SPN
FLI1	SSPN
FLI1	STX12
FLI1	SUSD6
FLI1	SYF2
FLI1	SYMPK
FLI1	TANGO2
FLI1	TATDN1
FLI1	TBC1D10C
FLI1	TBC1D9
FLI1	TBRG1
FLI1	TCEAL1
FLI1	TCF4
FLI1	TM2D1
FLI1	TMEM129
FLI1	TMEM154
FLI1	TMEM156
FLI1	TMSB10
FLI1	TMSB4X
FLI1	TNFRSF13C
FLI1	TPM4

FLI1	TRAF3IP3
FLI1	TRAF4
FLI1	TROAP
FLI1	TSC22D3
FLI1	TSPO
FLI1	TTC13
FLI1	TTC39C
FLI1	TTL
FLI1	TWF2
FLI1	UACA
FLI1	UCP2
FLI1	UNC93B1
FLI1	VAMP2
FLI1	VPS4B
FLI1	WARS2
FLI1	WDR34
FLI1	YPEL3
FLI1	ZAP70
FLI1	ZBTB25
FLI1	ZBTB7A
FLI1	ZCWPW1
FLI1	ZDHHC21
FLI1	ZNF524
FLI1	ZNF596
FLI1	ZNF720
FLI1	ZNF814
FLI1	ZSWIM8
FOS	AASDHPPT
FOS	ABCB8
FOS	ABHD14A
FOS	ACADVL
FOS	ACAP1
FOS	ACAP3
FOS	ACO2
FOS	ACOXL
FOS	ACSS1
FOS	ADAR
FOS	ADGRE5
FOS	ADM
FOS	ADNP−AS1
FOS	AEBP1
FOS	AGFG1
FOS	AIFM2
FOS	AKR1A1
FOS	ALDH6A1
FOS	ALG14
FOS	ALG2
FOS	ALG3
FOS	ALG9
FOS	ALKBH6

FOS	AMOTL1
FOS	ANG
FOS	ANKH
FOS	ANKRD12
FOS	ANKRD28
FOS	ANKRD37
FOS	ANKRD54
FOS	AP3D1
FOS	APH1B
FOS	APOL3
FOS	APOLD1
FOS	APTR
FOS	AREG
FOS	ARFGAP3
FOS	ARHGAP17
FOS	ARHGEF12
FOS	ARID3A
FOS	ARID4B
FOS	ARL1
FOS	ARL6IP1
FOS	ARMC5
FOS	ARMCX3
FOS	ARMCX5
FOS	ARSA
FOS	ARSG
FOS	ASAP1
FOS	ATE1
FOS	ATF6
FOS	ATF6B
FOS	ATL3
FOS	ATP13A1
FOS	ATP6AP1
FOS	ATP6AP2
FOS	ATP6V0A1
FOS	ATP6V1G1
FOS	ATXN2
FOS	AURKB
FOS	B2M
FOS	B3GAT3
FOS	BCKDK
FOS	BCL11A
FOS	BEND5
FOS	BET1
FOS	BEX4
FOS	BICD2
FOS	BLVRA
FOS	BLZF1
FOS	BNIP3L
FOS	BOLA2B
FOS	BORCS6

FOS	BTG2
FOS	BUB3
FOS	C14orf28
FOS	C15orf53
FOS	C17orf107
FOS	C18orf32
FOS	C19orf38
FOS	C1GALT1C1
FOS	C2orf69
FOS	C4orf33
FOS	CADM1
FOS	CALCOCO1
FOS	CALU
FOS	CBX4
FOS	CCDC14
FOS	CCDC144A
FOS	CCDC77
FOS	CCDC88A
FOS	CCDC9
FOS	CCNDBP1
FOS	CCNF
FOS	CCNL1
FOS	CCNYL1
FOS	CCR10
FOS	CD180
FOS	CD22
FOS	CD37
FOS	CD38
FOS	CD46
FOS	CD63
FOS	CD74
FOS	CDADC1
FOS	CDCA3
FOS	CDCA8
FOS	CDK1
FOS	CDK14
FOS	CDKN1B
FOS	CENPA
FOS	CENPL
FOS	CENPT
FOS	CEP89
FOS	CERCAM
FOS	CERS2
FOS	CERS5
FOS	CHAC1
FOS	CHERP
FOS	CHFR
FOS	CHL1
FOS	CHPF
FOS	CHRNB1

FOS	CHST12
FOS	CHST2
FOS	CISD2
FOS	CITED2
FOS	CKAP2
FOS	CKS2
FOS	CLCN3
FOS	CLIP1
FOS	CLK1
FOS	CLK3
FOS	CLK4
FOS	CLNK
FOS	CLOCK
FOS	CNEP1R1
FOS	CNOT6L
FOS	CNPY3
FOS	CNR1
FOS	CORO1C
FOS	CPEB4
FOS	CPM
FOS	CPNE3
FOS	CPNE5
FOS	CREB3L2
FOS	CREBRF
FOS	CSRNP1
FOS	CTHRC1
FOS	CTSB
FOS	CTSZ
FOS	CUL1
FOS	CXCR4
FOS	CXorf56
FOS	CYB561
FOS	CYB5R1
FOS	CYTH2
FOS	CYTH4
FOS	CYTIP
FOS	DCAF11
FOS	DCAF5
FOS	DCP2
FOS	DCXR
FOS	DDAH2
FOS	DDHD1
FOS	DDI2
FOS	DDIT4
FOS	DDOST
FOS	DDX3X
FOS	DDX3Y
FOS	DDX49
FOS	DDX5
FOS	DECR2

FOS	DEDD2
FOS	DERA
FOS	DERL1
FOS	DERL2
FOS	DERL3
FOS	DGKD
FOS	DHCR7
FOS	DHTKD1
FOS	DMXL1
FOS	DNAJB9
FOS	DNAJC1
FOS	DNAJC25
FOS	DNAJC3
FOS	DNAJC4
FOS	DOLK
FOS	DPYD
FOS	DUSP1
FOS	DUSP10
FOS	DUSP6
FOS	DYRK4
FOS	E2F2
FOS	E2F5
FOS	EAF2
FOS	EAPP
FOS	EDEM1
FOS	EDEM3
FOS	EGR1
FOS	EHD4
FOS	EIF1
FOS	EIF2AK4
FOS	ELMO1
FOS	EMC3
FOS	ENTPD1
FOS	EPC1
FOS	EPN2
FOS	ERGIC2
FOS	ERGIC3
FOS	ERO1B
FOS	ERP44
FOS	FADS2
FOS	FAM177A1
FOS	FAM214A
FOS	FAM3C
FOS	FAM50A
FOS	FAM69A
FOS	FAM72D
FOS	FAM89B
FOS	FBXO25
FOS	FBXO33
FOS	FCHO1

FOS	FDFT1
FOS	FGFR1OP2
FOS	FICD
FOS	FKBP11
FOS	FKBP2
FOS	FKBPL
FOS	FNBP4
FOS	FNDC3A
FOS	FOS
FOS	FOSB
FOS	FOXO3
FOS	FTH1
FOS	FUCA2
FOS	FXYD7
FOS	FZD3
FOS	GADD45B
FOS	GANAB
FOS	GCC1
FOS	GCSH
FOS	GDI1
FOS	GFER
FOS	GGA2
FOS	GIGYF2
FOS	GLA
FOS	GLCCI1
FOS	GLIPR1
FOS	GLS
FOS	GLT8D1
FOS	GM2A
FOS	GMPPB
FOS	GMPR
FOS	GNG7
FOS	GOLGA3
FOS	GOLPH3
FOS	GOLT1B
FOS	GON4L
FOS	GPR108
FOS	GPR146
FOS	GPR183
FOS	GPR34
FOS	GPRC5C
FOS	GPSM2
FOS	GRN
FOS	GSS
FOS	GTF2A1
FOS	GYS1
FOS	H1FX
FOS	H3F3A
FOS	H3F3B
FOS	HACL1

FOS	HAGH
FOS	HBP1
FOS	HDAC10
FOS	HERPUD1
FOS	HEXA
FOS	HEXB
FOS	HIST1H1C
FOS	HIST1H2AC
FOS	HIST1H2BG
FOS	HIST1H2BN
FOS	HLA-B
FOS	HLA-DMA
FOS	HLA-DOB
FOS	HLA-DPA1
FOS	HLA-DPB1
FOS	HLA-DQB1
FOS	HLA-DRA
FOS	HLA-DRB1
FOS	HLA-DRB5
FOS	HMCES
FOS	HMGCR
FOS	HMGN4
FOS	HMOX2
FOS	HOOK1
FOS	HPCAL1
FOS	HSD17B7
FOS	HSP90B1
FOS	HSPA13
FOS	HSPA4
FOS	HSPA5
FOS	HTR3A
FOS	HYOU1
FOS	ICOSLG
FOS	ID2
FOS	IDS
FOS	IER2
FOS	IER5
FOS	IFI6
FOS	IFNAR2
FOS	IFT22
FOS	IKBIP
FOS	IKZF1
FOS	IL2RG
FOS	ILVBL
FOS	ING2
FOS	INSIG2
FOS	IQCG
FOS	IQGAP1
FOS	IRF1
FOS	IRS2

FOS	ITFG2
FOS	ITGB2
FOS	ITM2B
FOS	ITM2C
FOS	IVNS1ABP
FOS	JKAMP
FOS	JUN
FOS	JUNB
FOS	JUND
FOS	KANK1
FOS	KAT6A
FOS	KAT6B
FOS	KCNN3
FOS	KDM8
FOS	KIF20A
FOS	KIF3B
FOS	KIF5C
FOS	KLF2
FOS	KLF3
FOS	KLF6
FOS	KLHL24
FOS	KPNA2
FOS	LACC1
FOS	LAIR1
FOS	LAMP2
FOS	LAPTM4A
FOS	LAPTM5
FOS	LARP1B
FOS	LDB1
FOS	LDLRAP1
FOS	LEMD3
FOS	LINC00926
FOS	LINC01485
FOS	LMAN2
FOS	LOXL2
FOS	LPP
FOS	LRMP
FOS	LRP10
FOS	LTBP4
FOS	LTN1
FOS	LY9
FOS	LYSMD3
FOS	M6PR
FOS	MAGED2
FOS	MAGT1
FOS	MAN1A1
FOS	MANF
FOS	MAP2K2
FOS	MAP2K7
FOS	MAP3K11

FOS	MAP3K13
FOS	MAP9
FOS	MAPRE1
FOS	MARCH1
FOS	MARCH8
FOS	MARK2
FOS	MAST3
FOS	MBD1
FOS	MBTD1
FOS	MBTPS1
FOS	MCL1
FOS	MCM8
FOS	MEF2C
FOS	MEPCE
FOS	METRNL
FOS	MGAT1
FOS	MIATNB
FOS	MIR22HG
FOS	MIR4435-2HG
FOS	MMGT1
FOS	MMP23B
FOS	MOB4
FOS	MON2
FOS	MPV17L2
FOS	MRM3
FOS	MRPL40
FOS	MS4A1
FOS	MSANTD2
FOS	MSRA
FOS	MTF1
FOS	MTO1
FOS	MTSS1
FOS	MUM1
FOS	MVD
FOS	MXD3
FOS	MYADM
FOS	MYO18A
FOS	MZB1
FOS	NCAPH
FOS	NCKIPSD
FOS	NCR3
FOS	NEAT1
FOS	NET1
FOS	NEU1
FOS	NKX6-3
FOS	NMNAT1
FOS	NMRK1
FOS	NPC2
FOS	NRGN
FOS	NSMCE3

FOS	NT5C3A
FOS	NTHL1
FOS	NUCB2
FOS	NUDCD2
FOS	NUP153
FOS	NUP54
FOS	NUP98
FOS	NUS1
FOS	OAT
FOS	OCEL1
FOS	OCIAD1
FOS	OIP5
FOS	OS9
FOS	OSTF1
FOS	OTUD1
FOS	OTUD5
FOS	OTUD6B-AS1
FOS	OXR1
FOS	P2RX1
FOS	P4HA1
FOS	PACSIN1
FOS	PAF1
FOS	PAK4
FOS	PARD6A
FOS	PBRM1
FOS	PBXIP1
FOS	PDE4B
FOS	PDE7A
FOS	PDIA4
FOS	PDK1
FOS	PDSS2
FOS	PDXDC1
FOS	PEX2
FOS	PGM3
FOS	PGRMC2
FOS	PHACTR1
FOS	PHF1
FOS	PHGDH
FOS	PHLDB3
FOS	PHYKPL
FOS	PIF1
FOS	PIGN
FOS	PIK3CG
FOS	PIM2
FOS	PINK1
FOS	PIP5K1B
FOS	PJA2
FOS	PKHD1L1
FOS	PLCL2
FOS	PLD3

FOS	PLEKHH3
FOS	PLOD1
FOS	PLPP5
FOS	PLTP
FOS	PMEPA1
FOS	PNOC
FOS	PNRC1
FOS	POLD4
FOS	POP1
FOS	PPAT
FOS	PPP1R10
FOS	PPP1R15A
FOS	PQBP1
FOS	PRCC
FOS	PRDX4
FOS	PRKAR1A
FOS	PRKCB
FOS	PRKCSH
FOS	PRKD3
FOS	PRMT7
FOS	PSAP
FOS	PSAT1
FOS	PTPN18
FOS	PTPRS
FOS	PTTG1IP
FOS	QPCT
FOS	RAB11B
FOS	RAB11FIP1
FOS	RAB1A
FOS	RAB30
FOS	RAB35
FOS	RAB5B
FOS	RAB5C
FOS	RAB6A
FOS	RABAC1
FOS	RAD54L
FOS	RAD9A
FOS	RALGPS2
FOS	RANBP6
FOS	RANGRF
FOS	RDH11
FOS	REC8
FOS	RECK
FOS	REEP3
FOS	RGS1
FOS	RGS14
FOS	RGS16
FOS	RGS2
FOS	RHBDD1
FOS	RHEB

FOS	RILP
FOS	RMDN3
FOS	RNASEH1-AS1
FOS	RNF103
FOS	RNF126
FOS	RNF13
FOS	RNF149
FOS	RNF168
FOS	RNF185
FOS	RNF219
FOS	RNF24
FOS	RPAIN
FOS	RPAP2
FOS	RPN1
FOS	RPN2
FOS	RRBP1
FOS	RRN3
FOS	RSRP1
FOS	RWDD2A
FOS	RYBP
FOS	S100A10
FOS	SAR1A
FOS	SAR1B
FOS	SARAF
FOS	SAT2
FOS	SC5D
FOS	SCAMP4
FOS	SCFD1
FOS	SCPEP1
FOS	SDCBP2
FOS	SDCBP2-AS1
FOS	SDF2L1
FOS	SDF4
FOS	SEC11C
FOS	SEC23B
FOS	SEC62
FOS	SEL1L
FOS	SELPLG
FOS	SEMA4D
FOS	SENP2
FOS	SERPINI1
FOS	SETDB2
FOS	SF3A1
FOS	SGK1
FOS	SGK3
FOS	SGSM3
FOS	SIL1
FOS	SIRT2
FOS	SLC16A3
FOS	SLC17A9

FOS	SLC20A2
FOS	SLC25A42
FOS	SLC26A6
FOS	SLC27A4
FOS	SLC2A6
FOS	SLC30A5
FOS	SLC30A6
FOS	SLC31A2
FOS	SLC33A1
FOS	SLC35B1
FOS	SLC35C1
FOS	SLC37A4
FOS	SLC38A10
FOS	SLC39A11
FOS	SLC39A8
FOS	SLC3A2
FOS	SLC44A1
FOS	SLC4A2
FOS	SLC7A7
FOS	SLC9A3
FOS	SMIM14
FOS	SMYD4
FOS	SNIP1
FOS	SNTB1
FOS	SNX2
FOS	SNX29
FOS	SOCS3
FOS	SOCS5
FOS	SON
FOS	SPAG4
FOS	SPCS2
FOS	SPCS3
FOS	SPDL1
FOS	SPINT2
FOS	SPOCK2
FOS	SPTBN1
FOS	SPTLC2
FOS	SQSTM1
FOS	SRPK2
FOS	SRSF5
FOS	SSH2
FOS	SSR4
FOS	ST3GAL2
FOS	ST6GALNAC4
FOS	STARD5
FOS	STK17B
FOS	STOM
FOS	STRBP
FOS	STT3A
FOS	STT3B

FOS	STX5
FOS	SUDS3
FOS	SUN1
FOS	SUN2
FOS	SWSAP1
FOS	SYNGR1
FOS	SYPL1
FOS	SYVN1
FOS	TAF4B
FOS	TAGAP
FOS	TBC1D10C
FOS	TBC1D15
FOS	TBC1D17
FOS	TBCC
FOS	TECR
FOS	TES
FOS	TEX264
FOS	TFG
FOS	TFRC
FOS	THOP1
FOS	TIMM17B
FOS	TM9SF2
FOS	TMBIM6
FOS	TMED10
FOS	TMED9
FOS	TMEM123
FOS	TMEM129
FOS	TMEM167A
FOS	TMEM167B
FOS	TMEM170A
FOS	TMEM184B
FOS	TMEM184C
FOS	TMEM19
FOS	TMEM205
FOS	TMEM218
FOS	TMEM263
FOS	TMEM39A
FOS	TMEM59
FOS	TMF1
FOS	TNFAIP3
FOS	TOR3A
FOS	TP53BP2
FOS	TP53INP1
FOS	TPD52L2
FOS	TPRA1
FOS	TPST2
FOS	TRABD
FOS	TRAIP
FOS	TRAM1
FOS	TRAM2

FOS	TRAPPC2B
FOS	TRDMT1
FOS	TRIB1
FOS	TRIM39
FOS	TSC22D3
FOS	TSC22D4
FOS	TSPAN13
FOS	TSPAN17
FOS	TTC9C
FOS	TTK
FOS	TUT1
FOS	UAP1
FOS	UBC
FOS	UBE2C
FOS	UBE2F
FOS	UBE2H
FOS	UBE2L6
FOS	UBXN2B
FOS	UMAD1
FOS	UNC93B1
FOS	UNK
FOS	USF3
FOS	USO1
FOS	USPL1
FOS	VEGFB
FOS	VPS35
FOS	VPS4A
FOS	VRK2
FOS	VRK3
FOS	WASL
FOS	WDPCP
FOS	WDR45
FOS	WDR82
FOS	WFS1
FOS	WHAMM
FOS	WIPF1
FOS	XBP1
FOS	XRCC3
FOS	XRN1
FOS	YIF1A
FOS	YIF1B
FOS	YKT6
FOS	YPEL3
FOS	YPEL5
FOS	YTHDF1
FOS	ZAP70
FOS	ZCCHC4
FOS	ZDHHC16
FOS	ZFAND2B
FOS	ZFP36

FOS	ZFP36L2
FOS	ZHX2
FOS	ZMYND11
FOS	ZNF101
FOS	ZNF217
FOS	ZNF24
FOS	ZNF287
FOS	ZNF318
FOS	ZNF44
FOS	ZNF583
FOS	ZNF597
FOS	ZNF622
FOS	ZNF689
FOS	ZNF770
FOS	ZNF791
FOS	ZNF821
FOS	ZNF836
FOSB	ACAP3
FOSB	ACBD5
FOSB	ADAT1
FOSB	ARFGEF2
FOSB	ARMCX3
FOSB	BSDC1
FOSB	C1GALT1
FOSB	CBFA2T2
FOSB	CD63
FOSB	CDK11B
FOSB	CHD2
FOSB	COL19A1
FOSB	CSE1L
FOSB	CYB5D2
FOSB	DHRS7B
FOSB	DUSP1
FOSB	EGR1
FOSB	ELL2
FOSB	FKBPL
FOSB	FOS
FOSB	FOSB
FOSB	FUCA2
FOSB	HAUS5
FOSB	IER2
FOSB	JUN
FOSB	JUNB
FOSB	JUND
FOSB	KANSL1
FOSB	KDELR1
FOSB	KLF6
FOSB	KLF9
FOSB	LMBRD1
FOSB	LMF1

FOSB	MBIP
FOSB	MDN1
FOSB	MED15
FOSB	MIA3
FOSB	MIR22HG
FOSB	NEAT1
FOSB	NFATC2
FOSB	NR4A2
FOSB	P2RX1
FOSB	P2RY10
FOSB	PJA1
FOSB	PKN1
FOSB	PLTP
FOSB	PNRC1
FOSB	PPP1R15A
FOSB	PRR3
FOSB	RRBP1
FOSB	RRNAD1
FOSB	SETD1B
FOSB	SGK1
FOSB	SH3GL1
FOSB	SLC39A13
FOSB	SMARCAD1
FOSB	SPDL1
FOSB	SSR4
FOSB	STAT3
FOSB	TBC1D23
FOSB	TLE3
FOSB	TMUB2
FOSB	TPM4
FOSB	TRIB1
FOSB	TSPAN31
FOSB	UBQLN1
FOSB	YTHDF1
FOSB	ZFP36
FOSB	ZNF335
FOSL1	ADRM1
FOSL1	ANKRD37
FOSL1	ANKRD46
FOSL1	ANXA5
FOSL1	AP1G1
FOSL1	APTX
FOSL1	ARL8B
FOSL1	ARPP19
FOSL1	ATAD3A
FOSL1	ATF1
FOSL1	ATG16L1
FOSL1	ATL3
FOSL1	ATP1A1
FOSL1	ATP1B3

FOSL1	BACH1
FOSL1	BRIX1
FOSL1	BZW2
FOSL1	C20orf27
FOSL1	CACUL1
FOSL1	CCDC130
FOSL1	CCDC59
FOSL1	CCL3
FOSL1	CCL3L1
FOSL1	CCL4
FOSL1	CCL4L2
FOSL1	CCND2
FOSL1	CCSER2
FOSL1	CCT4
FOSL1	CCT6A
FOSL1	CD44
FOSL1	CD55
FOSL1	CD86
FOSL1	CDC23
FOSL1	CDK4
FOSL1	CEBPZ
FOSL1	CHD1
FOSL1	CHST15
FOSL1	CSRNP1
FOSL1	CYCS
FOSL1	DCTD
FOSL1	DCUN1D5
FOSL1	DDX10
FOSL1	DDX21
FOSL1	DDX27
FOSL1	DDX49
FOSL1	DESI1
FOSL1	DNPEP
FOSL1	DNTTIP2
FOSL1	DUSP10
FOSL1	DUSP5
FOSL1	E4F1
FOSL1	EEF1E1
FOSL1	EIF3J
FOSL1	EIF4A1
FOSL1	ERCC2
FOSL1	ESF1
FOSL1	EVI2A
FOSL1	FAM131A
FOSL1	FAM162A
FOSL1	FAM213A
FOSL1	FAM50A
FOSL1	FBL
FOSL1	FOSL1
FOSL1	GFOD1

FOSL1	GNL2
FOSL1	GNL3
FOSL1	GRPEL1
FOSL1	GTDC1
FOSL1	GTPBP4
FOSL1	H3F3B
FOSL1	HDHD2
FOSL1	HGS
FOSL1	HIVEP3
FOSL1	HLA-E
FOSL1	HSP90AB1
FOSL1	HSPD1
FOSL1	IFIH1
FOSL1	IFRD1
FOSL1	IL6
FOSL1	INO80
FOSL1	ISCA1
FOSL1	ITGB2
FOSL1	JUND
FOSL1	KDM5B
FOSL1	KLF6
FOSL1	LDHA
FOSL1	LENG8
FOSL1	LRPPRC
FOSL1	LRRFIP1
FOSL1	LYAR
FOSL1	M6PR
FOSL1	MAGOH
FOSL1	MAGOHB
FOSL1	MAP2K3
FOSL1	MAP4K5
FOSL1	MAPK13
FOSL1	MAT2A
FOSL1	MATK
FOSL1	MED15
FOSL1	METTL1
FOSL1	METTL13
FOSL1	MIDN
FOSL1	MIR155HG
FOSL1	MSANTD2
FOSL1	MTHFD1L
FOSL1	NABP1
FOSL1	NDUFAF4
FOSL1	NEDD4L
FOSL1	NFE2L1
FOSL1	NFKBID
FOSL1	NIFK
FOSL1	NIP7
FOSL1	NOB1
FOSL1	NOC4L

FOSL1	NOLC1
FOSL1	NOP58
FOSL1	NPW
FOSL1	NUP98
FOSL1	ODC1
FOSL1	OSBP
FOSL1	PCGF5
FOSL1	PIGS
FOSL1	PIM3
FOSL1	PITPNB
FOSL1	PLK2
FOSL1	PLRG1
FOSL1	PMAIP1
FOSL1	PNPLA4
FOSL1	POLR3C
FOSL1	PPA2
FOSL1	PPCDC
FOSL1	PPFIA1
FOSL1	PPP1R10
FOSL1	PRKCSH
FOSL1	PRMT5
FOSL1	PRNP
FOSL1	PRR14
FOSL1	PRRG4
FOSL1	PSME3
FOSL1	PTP4A1
FOSL1	PYCR1
FOSL1	RAB27A
FOSL1	RAB7A
FOSL1	RAN
FOSL1	RANBP1
FOSL1	RBM15B
FOSL1	REEP3
FOSL1	RMDN3
FOSL1	RNASEH2B
FOSL1	RNF145
FOSL1	RNF220
FOSL1	RPF2
FOSL1	RSL1D1
FOSL1	RSL24D1
FOSL1	RUNX3
FOSL1	SACS
FOSL1	SBDS
FOSL1	SCAMP3
FOSL1	SERPINE2
FOSL1	SF1
FOSL1	SGK1
FOSL1	SGTA
FOSL1	SLAMF1
FOSL1	SLC1A5

FOSL1	SLC25A39
FOSL1	SLC35A4
FOSL1	SLC3A2
FOSL1	SLC43A1
FOSL1	SMARCAD1
FOSL1	SMG7
FOSL1	SNHG15
FOSL1	SNX8
FOSL1	SNX9
FOSL1	SOD2
FOSL1	SOS1
FOSL1	SPRY1
FOSL1	SPRYD4
FOSL1	SRGN
FOSL1	SRM
FOSL1	SRSF7
FOSL1	ST6GALNAC3
FOSL1	STAT5A
FOSL1	STX4
FOSL1	SUPV3L1
FOSL1	TBRG4
FOSL1	TGIF1
FOSL1	TINF2
FOSL1	TM2D2
FOSL1	TMEM126A
FOSL1	TMEM147
FOSL1	TNFAIP8
FOSL1	TNFSF14
FOSL1	TOMM40
FOSL1	TRAF1
FOSL1	TRAP1
FOSL1	TRAPPC8
FOSL1	TRDMT1
FOSL1	TRIB3
FOSL1	TSPYL2
FOSL1	TUFM
FOSL1	TVP23C
FOSL1	U2SURP
FOSL1	UBA6
FOSL1	UBE2B
FOSL1	USP14
FOSL1	UTP14A
FOSL1	UTP6
FOSL1	VPS33A
FOSL1	VPS37B
FOSL1	WDR12
FOSL1	WDR74
FOSL1	WDR75
FOSL1	WIPI1
FOSL1	XRRA1

FOSL1	ZNF121
FOSL1	ZNF330
FOSL1	ZNF511
FOSL1	ZNF593
FOXJ2	ABTB1
FOXJ2	FAM49B
FOXJ2	FOXJ2
FOXJ2	IFT20
FOXJ2	POU2AF1
FOXJ2	SLC2A1
FOXO1	BCL11A
FOXO1	CDK17
FOXO1	FAM20B
FOXO1	FBXL17
FOXO1	FOXO1
FOXO1	GCNT1
FOXO1	GNB4
FOXO1	HIP1R
FOXO1	ITPKB
FOXO1	KLC1
FOXO1	LIMS1
FOXO1	NFATC2
FOXO1	RCAN3
FOXO1	SDCCAG8
FOXO1	SERPINB8
FOXO1	SLC25A20
FOXO1	STX6
FOXO3	ABR
FOXO3	ACSS1
FOXO3	AEN
FOXO3	CRNDE
FOXO3	FOS
FOXO3	FOXO3
FOXO3	GAS6
FOXO3	LMO4
FOXO3	MBNL2
FOXO3	NEDD9
FOXO3	NUCB2
FOXO3	OTUD1
FOXO3	PLPP5
FOXO3	SGK3
FOXP1	BTG1
FOXP1	FOXP1
FOXP1	PDCD4
FOXP1	PPP3CA
FOXP1	TGIF1
FOXP1	ZEB2
FOXP4	BRD3
FOXP4	CUL4B
FOXP4	FOXP4

FOXP4	MACROD1
FOXP4	SLC7A7
FOXP4	TSHZ1
GABPA	ACER3
GABPA	ADSL
GABPA	AFF2
GABPA	ALAS1
GABPA	ANAPC15
GABPA	ANK1
GABPA	APBB1IP
GABPA	ARL14EP
GABPA	ASNSD1
GABPA	ATF3
GABPA	ATG4D
GABPA	ATP6V1B2
GABPA	ATXN3
GABPA	B4GALT5
GABPA	BBIP1
GABPA	BCKDHB
GABPA	BMP7
GABPA	BNIP2
GABPA	C14orf93
GABPA	C19orf38
GABPA	C2CD2L
GABPA	CAD
GABPA	CALCOCO1
GABPA	CCAR2
GABPA	CCDC122
GABPA	CCDC6
GABPA	CCDC86
GABPA	CCNB1
GABPA	CCNB2
GABPA	CCNJ
GABPA	CD37
GABPA	CDADC1
GABPA	CDC20
GABPA	CDC34
GABPA	CDCA7L
GABPA	CDK9
GABPA	CHEK1
GABPA	COPS2
GABPA	CPSF2
GABPA	CPSF4
GABPA	CRTC2
GABPA	CSNK1A1
GABPA	CTPS2
GABPA	CUL3
GABPA	CUTC
GABPA	CXorf21
GABPA	DCP1B

GABPA	DDX20
GABPA	DDX42
GABPA	DLEU2
GABPA	DNAJB1
GABPA	DOCK8
GABPA	DSCC1
GABPA	DTNBP1
GABPA	DYNC1LI1
GABPA	EEPD1
GABPA	EVI5
GABPA	FAF2
GABPA	FAM217B
GABPA	FBXL17
GABPA	FBXO28
GABPA	FLII
GABPA	FUT11
GABPA	GABPA
GABPA	GABPB1-AS1
GABPA	GBE1
GABPA	GEMIN8
GABPA	GRWD1
GABPA	GSPT2
GABPA	GTDC1
GABPA	GTF2H2
GABPA	GTF3C1
GABPA	HERC4
GABPA	HGH1
GABPA	HIBADH
GABPA	HS2ST1
GABPA	HSDL2
GABPA	HSPA1B
GABPA	IER5
GABPA	IL13RA1
GABPA	IRF5
GABPA	IZUMO4
GABPA	KDM3B
GABPA	KLHL24
GABPA	KYAT3
GABPA	LAMTOR3
GABPA	LRRC28
GABPA	LSM8
GABPA	LSS
GABPA	MCAT
GABPA	MDM1
GABPA	METTL16
GABPA	METTL3
GABPA	METTL4
GABPA	MIEF1
GABPA	MKLN1
GABPA	MKNK2

GABPA	MRPS31
GABPA	MRPS9
GABPA	MSL3
GABPA	MTMR9
GABPA	MYO1F
GABPA	NAA30
GABPA	NAP1L5
GABPA	NARS2
GABPA	NEDD8
GABPA	NUP37
GABPA	NVL
GABPA	NXPH4
GABPA	NXT2
GABPA	OSBPL10
GABPA	OSER1
GABPA	OTUD6B
GABPA	PCGF5
GABPA	PEX14
GABPA	PHF13
GABPA	PLCG2
GABPA	PLEKHG1
GABPA	POLD3
GABPA	PPP1R10
GABPA	PPP5C
GABPA	PRKAG1
GABPA	PRKRA
GABPA	PSMA8
GABPA	PSRC1
GABPA	PWP1
GABPA	RBCK1
GABPA	RBFOX2
GABPA	RC3H1
GABPA	REV1
GABPA	RFC1
GABPA	RHOBTB3
GABPA	RLF
GABPA	RPP21
GABPA	RRN3
GABPA	RRP1
GABPA	SAMD15
GABPA	SEC14L1
GABPA	SESN3
GABPA	SH3TC1
GABPA	SIGLEC10
GABPA	SLC15A4
GABPA	SLC25A26
GABPA	SLC25A36
GABPA	SLC35A5
GABPA	SLC4A1AP
GABPA	SMTN

GABPA	SP2
GABPA	SPI1
GABPA	SPINT1
GABPA	SPN
GABPA	SPON1
GABPA	SPSB3
GABPA	SRP72
GABPA	STARD3NL
GABPA	STAT2
GABPA	STK40
GABPA	TAF2
GABPA	TATDN3
GABPA	TBC1D1
GABPA	TBC1D4
GABPA	TFB2M
GABPA	TFPI2
GABPA	TIMM23B
GABPA	TMED8
GABPA	TMEM120B
GABPA	TMEM170A
GABPA	TOMM70
GABPA	TRAF5
GABPA	TSSC4
GABPA	TUBA4A
GABPA	U2AF2
GABPA	UBE2Q1
GABPA	UEVLD
GABPA	UIMC1
GABPA	USP33
GABPA	UTP4
GABPA	UTP6
GABPA	VMAC
GABPA	VRK3
GABPA	WARS2
GABPA	WDR70
GABPA	WDR74
GABPA	WEE1
GABPA	WSB1
GABPA	XXYLT1-AS2
GABPA	YTHDF1
GABPA	YTHDF3
GABPA	ZBTB7A
GABPA	ZC3H12A
GABPA	ZC3H13
GABPA	ZCCHC3
GABPA	ZNF106
GABPA	ZNF146
GABPA	ZNF331
GABPA	ZNF451
GABPA	ZNF480

GABPA	ZNF567
GABPA	ZNF592
GABPA	ZNF600
GABPA	ZNF714
GABPA	ZNF721
GABPA	ZNF800
GTF2B	ABCA7
GTF2B	ACTL6A
GTF2B	ACTR1A
GTF2B	AGPAT1
GTF2B	ANKRD54
GTF2B	ARL6IP1
GTF2B	BCL2L1
GTF2B	BTBD1
GTF2B	C12orf57
GTF2B	CAMTA1
GTF2B	CATSPER2
GTF2B	CCDC130
GTF2B	CCDC186
GTF2B	CCT7
GTF2B	CDC25A
GTF2B	CDC25C
GTF2B	CDK5RAP3
GTF2B	CDS2
GTF2B	CIRBP
GTF2B	CLINT1
GTF2B	COX7B
GTF2B	CYTH2
GTF2B	DCAF11
GTF2B	DDX5
GTF2B	DNAJC30
GTF2B	DNAJC4
GTF2B	DNAJC7
GTF2B	DPP3
GTF2B	EIF2B2
GTF2B	EMC2
GTF2B	FAM110A
GTF2B	FAM72B
GTF2B	FKBP14
GTF2B	FNIP1
GTF2B	GLS
GTF2B	GMPPB
GTF2B	GOLPH3L
GTF2B	GOLT1B
GTF2B	GORAB
GTF2B	GOT1
GTF2B	H3F3B
GTF2B	HIST1H1A
GTF2B	HSCB
GTF2B	HSPA8

GTF2B	IGF2BP3
GTF2B	ILF2
GTF2B	IMMT
GTF2B	KCNMB3
GTF2B	LANCL1
GTF2B	LAPTM4A
GTF2B	MAGT1
GTF2B	MAP3K13
GTF2B	MORF4L2
GTF2B	MRPS12
GTF2B	MSL3
GTF2B	NARF
GTF2B	NDUFA5
GTF2B	NDUFS8
GTF2B	NFYC
GTF2B	NMT1
GTF2B	NUDCD2
GTF2B	OS9
GTF2B	OSTF1
GTF2B	P4HB
GTF2B	PCF11
GTF2B	PPM1A
GTF2B	PPP2R1A
GTF2B	PRKCZ
GTF2B	PSMA5
GTF2B	RAB6A
GTF2B	RBM14
GTF2B	RBM48
GTF2B	RCCD1
GTF2B	RECQL
GTF2B	RNF181
GTF2B	SBDS
GTF2B	SEC31A
GTF2B	SPAG7
GTF2B	SPCS2
GTF2B	STARD4
GTF2B	STAT6
GTF2B	STX5
GTF2B	TIMM17A
GTF2B	TMEM19
GTF2B	TUBB4B
GTF2B	TUSC2
GTF2B	UBB
GTF2B	UBTD2
GTF2B	UMAD1
GTF2B	VCP
GTF2B	WFS1
GTF2B	YIF1A
HINFP	AKAP8L
HINFP	ANAPC15

HINFP	ASPH
HINFP	BANP
HINFP	BCCIP
HINFP	CD1C
HINFP	CHD8
HINFP	EBPL
HINFP	FAM3B
HINFP	FCGRT
HINFP	GOLT1B
HINFP	HINFP
HINFP	ICAM1
HINFP	LINC00324
HINFP	MTR
HINFP	NPTX2
HINFP	RAMP1
HINFP	SBNO2
HINFP	SCAND1
HINFP	SEMA4B
HINFP	TMEM205
HINFP	TMSB15A
HIVEP1	HIVEP1
HIVEP1	MAP3K11
HIVEP1	PRDM2
HIVEP1	TNFRSF9
HIVEP1	WNT10A
HIVEP3	ABRACL
HIVEP3	ANKHD1
HIVEP3	ANXA6
HIVEP3	ATXN1L
HIVEP3	BEX4
HIVEP3	CAMKK2
HIVEP3	CD274
HIVEP3	CD83
HIVEP3	D2HGDH
HIVEP3	DHRS4L2
HIVEP3	FCRL5
HIVEP3	GATAD1
HIVEP3	HIVEP3
HIVEP3	HLA-DQA1
HIVEP3	HLA-DQB1
HIVEP3	HLA-DRA
HIVEP3	LCK
HIVEP3	MAP2K3
HIVEP3	MELTF
HIVEP3	MIOS
HIVEP3	MYB
HIVEP3	NFATC1
HIVEP3	NFKBIA
HIVEP3	NR4A1
HIVEP3	NR4A2

HIVEP3	OPTN
HIVEP3	PDCD1
HIVEP3	PLEKHG2
HIVEP3	PTPRE
HIVEP3	RELB
HIVEP3	RILPL2
HIVEP3	SIN3A
HIVEP3	SLC17A9
HIVEP3	STAT6
HIVEP3	TANK
HIVEP3	TRPV3
HOXB2	ADAM28
HOXB2	ADAR
HOXB2	AGPAT1
HOXB2	APBA3
HOXB2	C6orf89
HOXB2	CD63
HOXB2	CHPF
HOXB2	CMC1
HOXB2	DPM3
HOXB2	FAM174A
HOXB2	FKBP2
HOXB2	LY9
HOXB2	LY96
HOXB2	PAAF1
HOXB2	PACSIN2
HOXB2	RAB13
HOXB2	RNASE6
HOXB2	SEC24A
HOXB2	TIMM10B
HOXB2	TM9SF3
HOXB2	TMEM208
HOXB2	YIPF5
HOXB7	GRK6
HOXB7	PHF19
IKZF1	AAAS
IKZF1	AAGAB
IKZF1	ABI1
IKZF1	ACBD3
IKZF1	ACSF3
IKZF1	ADA
IKZF1	ADPGK
IKZF1	AFF3
IKZF1	AFTPH
IKZF1	AGGF1
IKZF1	AHDC1
IKZF1	AIP
IKZF1	AK3
IKZF1	AKT2
IKZF1	ALOX5AP

IKZF1	ALPK1
IKZF1	ALYREF
IKZF1	ANAPC11
IKZF1	ANKRD49
IKZF1	AP1B1
IKZF1	AP1G2
IKZF1	AP1M1
IKZF1	AP3B1
IKZF1	AP5M1
IKZF1	APBB1IP
IKZF1	APH1A
IKZF1	APOBEC3C
IKZF1	ARHGAP11B
IKZF1	ARHGAP24
IKZF1	ARHGAP25
IKZF1	ARHGAP30
IKZF1	ARHGEF39
IKZF1	ARHGEF6
IKZF1	ARHGEF7
IKZF1	ARID4A
IKZF1	ARMCX6
IKZF1	ARPC1B
IKZF1	ARPC2
IKZF1	ATF6B
IKZF1	ATF7IP
IKZF1	ATG12
IKZF1	ATG2B
IKZF1	ATG7
IKZF1	ATP6V1D
IKZF1	ATP6V1E1
IKZF1	ATXN2L
IKZF1	AURKAIP1
IKZF1	BAG6
IKZF1	BAZ2A
IKZF1	BBIP1
IKZF1	BCL2A1
IKZF1	BCL2L1
IKZF1	BCLAF1
IKZF1	BDH1
IKZF1	BID
IKZF1	BLNK
IKZF1	BORCS6
IKZF1	BPGM
IKZF1	BRF2
IKZF1	C12orf75
IKZF1	C14orf119
IKZF1	C16orf91
IKZF1	C18orf21
IKZF1	C19orf25
IKZF1	C6orf52

IKZF1	C9orf40
IKZF1	CAAP1
IKZF1	CAB39
IKZF1	CAPNS1
IKZF1	CASP4
IKZF1	CBFA2T3
IKZF1	CCDC91
IKZF1	CCZ1
IKZF1	CD37
IKZF1	CD40
IKZF1	CD72
IKZF1	CD84
IKZF1	CDC123
IKZF1	CDC27
IKZF1	CDC40
IKZF1	CDK6
IKZF1	CDK9
IKZF1	CENPO
IKZF1	CENPQ
IKZF1	CEP135
IKZF1	CEP55
IKZF1	CFDP1
IKZF1	CGGBP1
IKZF1	CHCHD5
IKZF1	CHD2
IKZF1	CHD7
IKZF1	CHMP4B
IKZF1	CHTF8
IKZF1	CLECL1
IKZF1	CLNS1A
IKZF1	CNPPD1
IKZF1	COA6
IKZF1	COG4
IKZF1	COG8
IKZF1	COMMD5
IKZF1	COMMD6
IKZF1	COPS7A
IKZF1	COQ6
IKZF1	COX4I1
IKZF1	COX5B
IKZF1	CPSF7
IKZF1	CRIPT
IKZF1	CSK
IKZF1	CSNK1A1
IKZF1	CSNK1G3
IKZF1	CTDSP1
IKZF1	CTNNA1
IKZF1	CUL4A
IKZF1	CUTC
IKZF1	CWC15

IKZF1	CWC22
IKZF1	CWF19L2
IKZF1	CXXC5
IKZF1	CYB561A3
IKZF1	CYBB
IKZF1	CYFIP2
IKZF1	DALRD3
IKZF1	DAPP1
IKZF1	DBNL
IKZF1	DCAF10
IKZF1	DCAF12
IKZF1	DCAF15
IKZF1	DCLRE1C
IKZF1	DDX19B
IKZF1	DDX27
IKZF1	DDX50
IKZF1	DEF6
IKZF1	DFFA
IKZF1	DGKZ
IKZF1	DHX36
IKZF1	DLG1
IKZF1	DLGAP1-AS1
IKZF1	DLST
IKZF1	DMAP1
IKZF1	DNAJA1
IKZF1	DNAJB14
IKZF1	DNAJC10
IKZF1	DNAJC14
IKZF1	DNAL4
IKZF1	DOCK10
IKZF1	DOCK8
IKZF1	DOK3
IKZF1	DR1
IKZF1	DUSP2
IKZF1	DYNC1I2
IKZF1	E2F4
IKZF1	EAF1
IKZF1	EAPP
IKZF1	EBF1
IKZF1	EFHD2
IKZF1	EGR1
IKZF1	EIF2A
IKZF1	EIF3D
IKZF1	EIF3L
IKZF1	EIF4E2
IKZF1	EIF4EBP2
IKZF1	EIF5A
IKZF1	ELAC1
IKZF1	ELAC2
IKZF1	ELAVL1

IKZF1	ELK4
IKZF1	EML6
IKZF1	ENTPD1-AS1
IKZF1	EP300
IKZF1	ERBIN
IKZF1	EVI2B
IKZF1	EXOC3
IKZF1	EXOC7
IKZF1	FAAP24
IKZF1	FABP5
IKZF1	FAM32A
IKZF1	FAM49B
IKZF1	FAM50B
IKZF1	FAM96A
IKZF1	FBXO11
IKZF1	FBXO22
IKZF1	FBXO34
IKZF1	FBXW11
IKZF1	FBXW9
IKZF1	FCGR2B
IKZF1	FCRL5
IKZF1	FDFT1
IKZF1	FGD2
IKZF1	FIBP
IKZF1	FIG4
IKZF1	FKBP1A
IKZF1	FLII
IKZF1	FMNL1
IKZF1	FRA10AC1
IKZF1	FRMD8
IKZF1	FUT7
IKZF1	G2E3
IKZF1	GABPA
IKZF1	GAK
IKZF1	GAPT
IKZF1	GATB
IKZF1	GCN1
IKZF1	GFI1
IKZF1	GGA1
IKZF1	GIT2
IKZF1	GMFG
IKZF1	GMPR2
IKZF1	GNPAT
IKZF1	GPSM3
IKZF1	GRAPL
IKZF1	GRIPAP1
IKZF1	GTF2E2
IKZF1	GTF2F1
IKZF1	GTF3A
IKZF1	HAT1

IKZF1	HCCS
IKZF1	HECTD1
IKZF1	HERC4
IKZF1	HMBOX1
IKZF1	HOXB4
IKZF1	HPS1
IKZF1	HPS4
IKZF1	HSPA8
IKZF1	ICAM3
IKZF1	ID2
IKZF1	IDH3B
IKZF1	IFT80
IKZF1	IKBKG
IKZF1	IKZF1
IKZF1	IKZF2
IKZF1	IL16
IKZF1	IL17RA
IKZF1	IL2RB
IKZF1	IL4I1
IKZF1	IMP3
IKZF1	INO80C
IKZF1	INO80E
IKZF1	INPP1
IKZF1	IQGAP2
IKZF1	IRF5
IKZF1	ITFG1
IKZF1	ITFG2
IKZF1	ITGAL
IKZF1	ITGB1BP1
IKZF1	ITPA
IKZF1	ITPRIPL1
IKZF1	JAK1
IKZF1	JKAMP
IKZF1	KANK2
IKZF1	KAT7
IKZF1	KBTBD4
IKZF1	KBTBD8
IKZF1	KCMF1
IKZF1	KDM2A
IKZF1	KDM5C
IKZF1	KIAA0040
IKZF1	KIF9
IKZF1	KLF10
IKZF1	KLHDC4
IKZF1	KLHL6
IKZF1	KNOP1
IKZF1	KRR1
IKZF1	LAMTOR4
IKZF1	LAS1L
IKZF1	LCMT1

IKZF1	LCMT2
IKZF1	LEF1
IKZF1	LEPROTL1
IKZF1	LGALS9
IKZF1	LIMK1
IKZF1	LINC00667
IKZF1	LINC01013
IKZF1	LIPT1
IKZF1	LPGAT1
IKZF1	LRP10
IKZF1	LSM2
IKZF1	LSM8
IKZF1	LXN
IKZF1	LYN
IKZF1	LYPLA2
IKZF1	MADD
IKZF1	MAGEF1
IKZF1	MALT1
IKZF1	MANBAL
IKZF1	MAPKAP1
IKZF1	MARCH1
IKZF1	MARK3
IKZF1	MAT2B
IKZF1	MBTPS2
IKZF1	MCRS1
IKZF1	MED11
IKZF1	MED7
IKZF1	MEF2D
IKZF1	METTL17
IKZF1	METTL6
IKZF1	MFAP1
IKZF1	MFN1
IKZF1	MIATNB
IKZF1	MIR3142HG
IKZF1	MKLN1
IKZF1	MKNK1
IKZF1	MLLT10
IKZF1	MPEG1
IKZF1	MPG
IKZF1	MRPL14
IKZF1	MRPL20
IKZF1	MRPL27
IKZF1	MRPL47
IKZF1	MRPL49
IKZF1	MRPL51
IKZF1	MRPL52
IKZF1	MRPS16
IKZF1	MRPS21
IKZF1	MRPS31
IKZF1	MS4A1

IKZF1	MSL3
IKZF1	MSN
IKZF1	MTERF4
IKZF1	MTIF3
IKZF1	MTMR2
IKZF1	MTR
IKZF1	MTRF1L
IKZF1	MUT
IKZF1	MYL12B
IKZF1	MYO1C
IKZF1	N4BP1
IKZF1	NAA35
IKZF1	NAA60
IKZF1	NABP2
IKZF1	NCKAP1L
IKZF1	NDUFA13
IKZF1	NDUFB4
IKZF1	NDUFB7
IKZF1	NDUFS2
IKZF1	NDUFS3
IKZF1	NDUFS4
IKZF1	NDUFV1
IKZF1	NECAP1
IKZF1	NECAP2
IKZF1	NEDD8
IKZF1	NEK6
IKZF1	NFAT5
IKZF1	NFATC1
IKZF1	NFATC3
IKZF1	NFYC
IKZF1	NIF3L1
IKZF1	NIPBL
IKZF1	NME2
IKZF1	NNT
IKZF1	NOL8
IKZF1	NOSIP
IKZF1	NR2C2
IKZF1	NSMCE1
IKZF1	NUDC
IKZF1	NUDT21
IKZF1	NUDT5
IKZF1	NUP107
IKZF1	NUP210
IKZF1	NUP37
IKZF1	NUP93
IKZF1	OCIAD1
IKZF1	ORAI1
IKZF1	ORAI3
IKZF1	ORC2
IKZF1	OST4

IKZF1	OSTF1
IKZF1	OXR1
IKZF1	PAAF1
IKZF1	PABPC1
IKZF1	PARL
IKZF1	PCBP2
IKZF1	PCM1
IKZF1	PDCD6
IKZF1	PDE12
IKZF1	PDLIM2
IKZF1	PDPK1
IKZF1	PFDN5
IKZF1	PHF14
IKZF1	PHF23
IKZF1	PHTF1
IKZF1	PIGN
IKZF1	PIGO
IKZF1	PIK3C3
IKZF1	PIK3CA
IKZF1	PIK3R1
IKZF1	PKM
IKZF1	PLEKHA3
IKZF1	PLEKHG1
IKZF1	PLPP6
IKZF1	PMPCB
IKZF1	PNISR
IKZF1	PNPLA4
IKZF1	POGK
IKZF1	POLQ
IKZF1	POLR2C
IKZF1	POLR2F
IKZF1	POLR2J
IKZF1	POMP
IKZF1	PPHLN1
IKZF1	PPIG
IKZF1	PPM1G
IKZF1	PPP1R12A
IKZF1	PPP4C
IKZF1	PRKACB
IKZF1	PRKCD
IKZF1	PRPF38B
IKZF1	PRPF40A
IKZF1	PRPF4B
IKZF1	PRPF6
IKZF1	PRR14L
IKZF1	PSMA3-AS1
IKZF1	PSMA4
IKZF1	PSMB1
IKZF1	PSMB3
IKZF1	PSMB7

IKZF1	PSMB8
IKZF1	PSMC6
IKZF1	PSMD3
IKZF1	PSMD8
IKZF1	PSMF1
IKZF1	PTBP3
IKZF1	PTPN1
IKZF1	PTPN7
IKZF1	PTPRC
IKZF1	PYCARD
IKZF1	RAB11A
IKZF1	RAB2A
IKZF1	RAB5C
IKZF1	RAB8A
IKZF1	RABEPK
IKZF1	RABGEF1
IKZF1	RAC1
IKZF1	RAC2
IKZF1	RACK1
IKZF1	RAD51D
IKZF1	RALA
IKZF1	RASA1
IKZF1	RASGRP2
IKZF1	RASGRP3
IKZF1	RFTN1
IKZF1	RHOG
IKZF1	RHOT2
IKZF1	RIC8A
IKZF1	RMND5B
IKZF1	RNF121
IKZF1	RNF168
IKZF1	RNF20
IKZF1	RNF214
IKZF1	RNPS1
IKZF1	ROCK1
IKZF1	ROMO1
IKZF1	RPE
IKZF1	RPF2
IKZF1	RPL11
IKZF1	RPL19
IKZF1	RPL23
IKZF1	RPL26
IKZF1	RPL27
IKZF1	RPL28
IKZF1	RPL29
IKZF1	RPL30
IKZF1	RPL31
IKZF1	RPL32
IKZF1	RPL34
IKZF1	RPL38

IKZF1	RPL4
IKZF1	RPL41
IKZF1	RPL6
IKZF1	RPL7
IKZF1	RPL8
IKZF1	RPS13
IKZF1	RPS18
IKZF1	RPS25
IKZF1	RPS8
IKZF1	RRAGA
IKZF1	RRAGB
IKZF1	RRN3
IKZF1	RTN4
IKZF1	S100A4
IKZF1	SAMSN1
IKZF1	SART3
IKZF1	SCOC
IKZF1	SDHAF2
IKZF1	SDHD
IKZF1	SDR42E1
IKZF1	SEC23IP
IKZF1	SEC24C
IKZF1	SEMA4A
IKZF1	SERBP1
IKZF1	SETX
IKZF1	SF1
IKZF1	SF3A1
IKZF1	SF3B1
IKZF1	SF3B2
IKZF1	SH3BGRL
IKZF1	SH3BP5
IKZF1	SHKBP1
IKZF1	SHQ1
IKZF1	SIKE1
IKZF1	SLC35B3
IKZF1	SLC37A1
IKZF1	SLC38A1
IKZF1	SLC39A1
IKZF1	SLC9A3R1
IKZF1	SLF1
IKZF1	SLMAP
IKZF1	SMARCA2
IKZF1	SMC2
IKZF1	SMC4
IKZF1	SMCHD1
IKZF1	SMIM15
IKZF1	SMIM7
IKZF1	SNAP23
IKZF1	SNF8
IKZF1	SNIP1

IKZF1	SNRNP200
IKZF1	SNRPB
IKZF1	SNRPE
IKZF1	SNX10
IKZF1	SNX3
IKZF1	SP100
IKZF1	SPATA2
IKZF1	SPG21
IKZF1	SPI1
IKZF1	SPN
IKZF1	SPTLC2
IKZF1	SRP14
IKZF1	SRP54
IKZF1	SRRM1
IKZF1	SRSF4
IKZF1	SS18
IKZF1	STAP1
IKZF1	STAU1
IKZF1	STK4
IKZF1	STX10
IKZF1	SUB1
IKZF1	SUPT4H1
IKZF1	SYAP1
IKZF1	SYF2
IKZF1	SYK
IKZF1	SYMPK
IKZF1	TACO1
IKZF1	TAF10
IKZF1	TAF11
IKZF1	TAGAP
IKZF1	TAGLN2
IKZF1	TANK
IKZF1	TBC1D1
IKZF1	TBC1D22A
IKZF1	TBK1
IKZF1	TBRG4
IKZF1	TCF25
IKZF1	TCF4
IKZF1	TEFM
IKZF1	TESPA1
IKZF1	TFB1M
IKZF1	TFB2M
IKZF1	TFRC
IKZF1	TGOLN2
IKZF1	THAP11
IKZF1	THAP4
IKZF1	THG1L
IKZF1	THOC5
IKZF1	TIMM23
IKZF1	TIMM8B

IKZF1	TIMMDC1
IKZF1	TINF2
IKZF1	TMA7
IKZF1	TMEM101
IKZF1	TMEM128
IKZF1	TMEM161B
IKZF1	TMEM206
IKZF1	TMEM243
IKZF1	TMX1
IKZF1	TNFAIP8
IKZF1	TNPO3
IKZF1	TNRC6A
IKZF1	TOR4A
IKZF1	TPRKB
IKZF1	TRA2B
IKZF1	TRAF3IP3
IKZF1	TRAM2
IKZF1	TRAPPC4
IKZF1	TRIM22
IKZF1	TRNT1
IKZF1	TRPC4AP
IKZF1	TSC2
IKZF1	TSC22D4
IKZF1	TSNAX
IKZF1	TSSC4
IKZF1	TSTD1
IKZF1	TTC14
IKZF1	TTF2
IKZF1	TUBB
IKZF1	TUBGCP2
IKZF1	TUBGCP4
IKZF1	TYW1
IKZF1	UBA1
IKZF1	UBAP2
IKZF1	UBE2E1
IKZF1	UBE2L3
IKZF1	UBL5
IKZF1	UBXN1
IKZF1	UBXN4
IKZF1	UBXN6
IKZF1	UHMK1
IKZF1	UPF2
IKZF1	UQCR11
IKZF1	URM1
IKZF1	USE1
IKZF1	USP16
IKZF1	USP8
IKZF1	UTP3
IKZF1	UTP4
IKZF1	UVRAG

IKZF1	VAC14
IKZF1	VAMP8
IKZF1	VEZF1
IKZF1	VMA21
IKZF1	VMP1
IKZF1	VNN2
IKZF1	VPS18
IKZF1	VPS51
IKZF1	VPS8
IKZF1	WASF2
IKZF1	WDR41
IKZF1	WDR73
IKZF1	XRN2
IKZF1	YBEY
IKZF1	YKT6
IKZF1	YTHDF3
IKZF1	ZBTB11
IKZF1	ZBTB7A
IKZF1	ZC3H12D
IKZF1	ZDHHC6
IKZF1	ZFC3H1
IKZF1	ZFP90
IKZF1	ZMAT2
IKZF1	ZNF18
IKZF1	ZNF197
IKZF1	ZNF226
IKZF1	ZNF234
IKZF1	ZNF24
IKZF1	ZNF275
IKZF1	ZNF317
IKZF1	ZNF397
IKZF1	ZNF485
IKZF1	ZNF511
IKZF1	ZNF579
IKZF1	ZNF653
IKZF1	ZNF83
IKZF1	ZRANB2
IRF1	ABCB8
IRF1	ABCC4
IRF1	ABCD4
IRF1	ABHD14A
IRF1	ABI2
IRF1	ABTB1
IRF1	ACAA1
IRF1	ACADVL
IRF1	ACAP1
IRF1	ACBD3
IRF1	ACP6
IRF1	ACSS1
IRF1	ADAM28

IRF1	ADAR
IRF1	ADD1
IRF1	ADIPOR1
IRF1	AEBP1
IRF1	AFF1
IRF1	AFF4
IRF1	AGBL5
IRF1	AKAP1
IRF1	AKAP13
IRF1	AKAP8
IRF1	AKAP9
IRF1	AKIRIN2
IRF1	AKT2
IRF1	ALDH18A1
IRF1	ALDH5A1
IRF1	ALG11
IRF1	ALG14
IRF1	ALG2
IRF1	ALG3
IRF1	ALG5
IRF1	ALKBH7
IRF1	AMZ2
IRF1	ANAPC13
IRF1	ANG
IRF1	ANKRD11
IRF1	ANKRD12
IRF1	ANKRD16
IRF1	ANKRD28
IRF1	ANKRD46
IRF1	ANO9
IRF1	ANXA6
IRF1	AP3D1
IRF1	AP4B1
IRF1	APBA3
IRF1	APMAP
IRF1	ARCN1
IRF1	AREG
IRF1	ARF4
IRF1	ARFGAP3
IRF1	ARID4A
IRF1	ARL6IP5
IRF1	ARMCX3
IRF1	ARMCX5
IRF1	ARRDC3
IRF1	ARSA
IRF1	ASL
IRF1	ATF6
IRF1	ATF6B
IRF1	ATG12
IRF1	ATG16L1

IRF1	ATG5
IRF1	ATP13A1
IRF1	ATP23
IRF1	ATP2A2
IRF1	ATP6AP2
IRF1	ATP6V0A1
IRF1	ATP6V1A
IRF1	ATP6V1E1
IRF1	ATP8B2
IRF1	ATRAID
IRF1	ATXN7L3B
IRF1	AUH
IRF1	AZI2
IRF1	AZIN1
IRF1	B2M
IRF1	B3GNT2
IRF1	B4GALT3
IRF1	BAK1
IRF1	BANK1
IRF1	BAZ2A
IRF1	BAZ2B
IRF1	BBX
IRF1	BCKDK
IRF1	BCL2L11
IRF1	BECN1
IRF1	BET1L
IRF1	BEX4
IRF1	BEX5
IRF1	BHLHE41
IRF1	BID
IRF1	BIN2
IRF1	BLVRB
IRF1	BNIP3L
IRF1	BORCS6
IRF1	BRI3
IRF1	BSDC1
IRF1	BTG1
IRF1	BTG2
IRF1	BTK
IRF1	C12orf29
IRF1	C12orf57
IRF1	C12orf65
IRF1	C14orf28
IRF1	C15orf39
IRF1	C15orf61
IRF1	C19orf24
IRF1	C19orf38
IRF1	C19orf66
IRF1	C1GALT1C1
IRF1	C21orf2

IRF1	C6orf47
IRF1	C6orf89
IRF1	C8orf82
IRF1	CADPS
IRF1	CALCOCO1
IRF1	CALR
IRF1	CALU
IRF1	CAMLG
IRF1	CANX
IRF1	CAPN10
IRF1	CAPN12
IRF1	CARNMT1
IRF1	CASP3
IRF1	CASP8
IRF1	CBFA2T2
IRF1	CBX2
IRF1	CBX4
IRF1	CBX7
IRF1	CBX8
IRF1	CCDC107
IRF1	CCDC14
IRF1	CCDC144A
IRF1	CCDC159
IRF1	CCDC186
IRF1	CCDC47
IRF1	CCDC71L
IRF1	CCNL1
IRF1	CCP110
IRF1	CCPG1
IRF1	CCR7
IRF1	CD164
IRF1	CD37
IRF1	CD38
IRF1	CD46
IRF1	CD55
IRF1	CD63
IRF1	CD69
IRF1	CD79B
IRF1	CDC23
IRF1	CDCA5
IRF1	CDK12
IRF1	CDK2AP2
IRF1	CEBPB
IRF1	CELF2
IRF1	CHAC1
IRF1	CHD6
IRF1	CHD9
IRF1	CHID1
IRF1	CHMP3
IRF1	CHPF

IRF1	CHPF2
IRF1	CHRNB1
IRF1	CHST12
IRF1	CHST2
IRF1	CIR1
IRF1	CISD2
IRF1	CITED2
IRF1	CKAP4
IRF1	CLIC1
IRF1	CLINT1
IRF1	CLIP4
IRF1	CLK1
IRF1	CLK4
IRF1	CLN5
IRF1	CLPTM1
IRF1	CLPTM1L
IRF1	CLPX
IRF1	CNNM3
IRF1	CNPY3
IRF1	COASY
IRF1	COBLL1
IRF1	COG1
IRF1	COG3
IRF1	COPA
IRF1	COPB2
IRF1	COPE
IRF1	COPG1
IRF1	COPZ1
IRF1	COQ10A
IRF1	COQ10B
IRF1	CORO1C
IRF1	COX7A2
IRF1	CPEB4
IRF1	CPQ
IRF1	CPT2
IRF1	CREB3
IRF1	CREB3L2
IRF1	CREBRF
IRF1	CRELD2
IRF1	CRIPT
IRF1	CRTC2
IRF1	CSGALNACT2
IRF1	CSNK1E
IRF1	CSRNP1
IRF1	CSRP1
IRF1	CTNNBIP1
IRF1	CUL4B
IRF1	CUTA
IRF1	CXCR4
IRF1	CXorf40B

IRF1	CYB561
IRF1	CYB5A
IRF1	CYB5R4
IRF1	CYBA
IRF1	CYHR1
IRF1	CYTH2
IRF1	CYTH4
IRF1	CYTIP
IRF1	DAD1
IRF1	DAP
IRF1	DCAF10
IRF1	DDIT4
IRF1	DDOST
IRF1	DDRGK1
IRF1	DDT
IRF1	DENND1B
IRF1	DENND1C
IRF1	DERL1
IRF1	DERL2
IRF1	DERL3
IRF1	DESI1
IRF1	DHPS
IRF1	DHRS3
IRF1	DHX36
IRF1	DIS3
IRF1	DNAJB9
IRF1	DNAJC1
IRF1	DNAJC10
IRF1	DNAJC14
IRF1	DNAJC18
IRF1	DNAJC25
IRF1	DNAJC3
IRF1	DNAJC4
IRF1	DNASE2
IRF1	DPM2
IRF1	DPM3
IRF1	DPY19L4
IRF1	DRAP1
IRF1	DUSP1
IRF1	DUSP5
IRF1	DVL3
IRF1	E2F5
IRF1	EAPP
IRF1	EBAG9
IRF1	EDEM2
IRF1	EDEM3
IRF1	EEF1D
IRF1	EIF1AY
IRF1	EIF2A
IRF1	EIF2AK2

IRF1	EIF2AK4
IRF1	EIF4G1
IRF1	ELF2
IRF1	ELL
IRF1	ELL2
IRF1	ELMO1
IRF1	ELMOD2
IRF1	ELOVL1
IRF1	EMC10
IRF1	EMC2
IRF1	EMC3
IRF1	EMC7
IRF1	ENTPD1
IRF1	ENTPD4
IRF1	EPB41
IRF1	ERC1
IRF1	ERCC1
IRF1	ERGIC1
IRF1	ERGIC2
IRF1	ERGIC3
IRF1	ERLEC1
IRF1	ERN1
IRF1	ERO1B
IRF1	ERP29
IRF1	ETV7
IRF1	EVI2B
IRF1	EXD2
IRF1	FADS2
IRF1	FAM117A
IRF1	FAM118A
IRF1	FAM161A
IRF1	FAM173A
IRF1	FAM174A
IRF1	FAM177A1
IRF1	FAM214A
IRF1	FAM3A
IRF1	FAM3C
IRF1	FAM50A
IRF1	FAM50B
IRF1	FAM78A
IRF1	FAM86C1
IRF1	FAM89B
IRF1	FAM96A
IRF1	FAR1
IRF1	FASTK
IRF1	FBXL8
IRF1	FBXO8
IRF1	FBXW5
IRF1	FBXW7
IRF1	FICD

IRF1	FKBP14
IRF1	FKBP2
IRF1	FKBP7
IRF1	FMNL1
IRF1	FNBP4
IRF1	FNDC3A
IRF1	FOS
IRF1	FOXJ3
IRF1	FOXO3
IRF1	FRG1
IRF1	FTH1
IRF1	FTL
IRF1	FUT8
IRF1	FXYD5
IRF1	GAB1
IRF1	GABPB2
IRF1	GALK2
IRF1	GALNS
IRF1	GALT
IRF1	GANAB
IRF1	GAS6
IRF1	GBA2
IRF1	GBF1
IRF1	GBP4
IRF1	GCC2
IRF1	GEMIN7
IRF1	GFER
IRF1	GFPT1
IRF1	GGCX
IRF1	GLCCI1
IRF1	GLG1
IRF1	GLRX
IRF1	GLS
IRF1	GMCL1
IRF1	GMPPA
IRF1	GMPPB
IRF1	GMPR
IRF1	GNG7
IRF1	GNL3
IRF1	GOLGA1
IRF1	GOLGA5
IRF1	GOLGB1
IRF1	GOSR2
IRF1	GPAA1
IRF1	GPD2
IRF1	GPER1
IRF1	GPR137B
IRF1	GPR89A
IRF1	GPS1
IRF1	GPT2

IRF1	GRAP2
IRF1	GRK2
IRF1	GRN
IRF1	GSK3A
IRF1	GTF2A1
IRF1	GTF2B
IRF1	H1F0
IRF1	HAX1
IRF1	HBP1
IRF1	HCFC1
IRF1	HDLBP
IRF1	HEBP2
IRF1	HELB
IRF1	HERPUD1
IRF1	HERPUD2
IRF1	HID1
IRF1	HIP1
IRF1	HIST1H1C
IRF1	HLA-A
IRF1	HLA-B
IRF1	HLA-E
IRF1	HLA-F
IRF1	HM13
IRF1	HNRNPUL2
IRF1	HPF1
IRF1	HS2ST1
IRF1	HSCB
IRF1	HSD17B11
IRF1	HSD17B12
IRF1	HSP90B1
IRF1	HSPA13
IRF1	HSPA5
IRF1	HYOU1
IRF1	ICAM1
IRF1	ICAM3
IRF1	IDH2
IRF1	IDH3G
IRF1	IER2
IRF1	IER3IP1
IRF1	IFI35
IRF1	IFNAR1
IRF1	IFNAR2
IRF1	IFT27
IRF1	IGBP1
IRF1	IGSF8
IRF1	IKBIP
IRF1	IKZF4
IRF1	IL12RB1
IRF1	IL15RA
IRF1	IL16

IRF1	IL2RG
IRF1	INSIG2
IRF1	IRF1
IRF1	IRF2
IRF1	IRF4
IRF1	IRF7
IRF1	ISCA2
IRF1	ISCU
IRF1	ISG20
IRF1	ISOC2
IRF1	ITCH
IRF1	ITFG1
IRF1	ITFG2
IRF1	ITGB2
IRF1	ITGB7
IRF1	ITM2B
IRF1	ITM2C
IRF1	JAGN1
IRF1	JMY
IRF1	JUN
IRF1	JUNB
IRF1	KCNA3
IRF1	KCNAB2
IRF1	KCNK6
IRF1	KDELR1
IRF1	KDELR2
IRF1	KDM5A
IRF1	KDM5D
IRF1	KIAA0930
IRF1	KLF13
IRF1	KLF2
IRF1	KLF3
IRF1	KLF6
IRF1	KLHL24
IRF1	KMT2E
IRF1	KMT2E-AS1
IRF1	KRT18
IRF1	KRTCAP2
IRF1	LACTB
IRF1	LACTB2
IRF1	LAMP2
IRF1	LAPTM4A
IRF1	LARP1B
IRF1	LEPROT
IRF1	LEPROTL1
IRF1	LGALS8
IRF1	LIN54
IRF1	LINC00667
IRF1	LINC00909
IRF1	LINC01485

IRF1	LIPT2
IRF1	LITAF
IRF1	LIX1L
IRF1	LMAN1
IRF1	LMAN2
IRF1	LMBRD1
IRF1	LMF1
IRF1	LMF2
IRF1	LMTK2
IRF1	LONRF1
IRF1	LRBA
IRF1	LRP10
IRF1	LRPAP1
IRF1	LRRC59
IRF1	LSM1
IRF1	LY75
IRF1	LY96
IRF1	LZTFL1
IRF1	MAD2L2
IRF1	MAEA
IRF1	MAFB
IRF1	MAGED1
IRF1	MAGEH1
IRF1	MAGT1
IRF1	MAN1A2
IRF1	MAN2A1
IRF1	MANBA
IRF1	MANBAL
IRF1	MANEA
IRF1	MANF
IRF1	MAP1B
IRF1	MAP4K3
IRF1	MAPKAPK2
IRF1	MAPKAPK3
IRF1	MARCH6
IRF1	MARCH9
IRF1	MARK3
IRF1	MAT2B
IRF1	MBNL2
IRF1	MBTD1
IRF1	MCFD2
IRF1	MCL1
IRF1	MCTP2
IRF1	MDN1
IRF1	MED13L
IRF1	MED31
IRF1	MEF2A
IRF1	MEF2C
IRF1	METTL17
IRF1	METTL23

IRF1	METTL7A
IRF1	MFF
IRF1	MGAT1
IRF1	MIA3
IRF1	MLEC
IRF1	MLX
IRF1	MMP17
IRF1	MOCS2
IRF1	MOGS
IRF1	MORC2-AS1
IRF1	MOSPD1
IRF1	MPC1
IRF1	MPHOSPH8
IRF1	MPRIP
IRF1	MPV17
IRF1	MRPL17
IRF1	MRPL24
IRF1	MRPL52
IRF1	MRPS31
IRF1	MRPS34
IRF1	MSTO1
IRF1	MT1F
IRF1	MTDH
IRF1	MTG1
IRF1	MTG2
IRF1	MTHFSD
IRF1	MVP
IRF1	MXD4
IRF1	MYCBP2
IRF1	MYDGF
IRF1	MZB1
IRF1	MZF1
IRF1	N4BP2L1
IRF1	N4BP2L2
IRF1	NAGK
IRF1	NANS
IRF1	NAPA
IRF1	NBEAL1
IRF1	NBR1
IRF1	NCK1
IRF1	NCKAP1L
IRF1	NCLN
IRF1	NCOA2
IRF1	NCSTN
IRF1	NDST2
IRF1	NDUFA8
IRF1	NDUFAF1
IRF1	NDUFB11
IRF1	NDUFV3
IRF1	NEAT1

IRF1	NEDD9
IRF1	NET1
IRF1	NEU1
IRF1	NFATC3
IRF1	NFYB
IRF1	NGRN
IRF1	NMRK1
IRF1	NPC2
IRF1	NPRL2
IRF1	NRBF2
IRF1	NRDC
IRF1	NSUN6
IRF1	NT5C3A
IRF1	NT5DC1
IRF1	NUBP2
IRF1	NUCB2
IRF1	NUDT22
IRF1	NUDT6
IRF1	NUDT9
IRF1	NUMB
IRF1	NXPE3
IRF1	OAF
IRF1	ODF2L
IRF1	OGFOD2
IRF1	OPTN
IRF1	ORAI2
IRF1	ORAI3
IRF1	ORMDL2
IRF1	ORMDL3
IRF1	OS9
IRF1	OSBPL2
IRF1	OST4
IRF1	OSTC
IRF1	OSTF1
IRF1	OSTM1
IRF1	OXNAD1
IRF1	OXR1
IRF1	P2RX1
IRF1	P4HB
IRF1	PAFAH1B1
IRF1	PAK1
IRF1	PAPSS1
IRF1	PARM1
IRF1	PARP14
IRF1	PARVB
IRF1	PBRM1
IRF1	PBXIP1
IRF1	PCIF1
IRF1	PCMTD1
IRF1	PDE12

IRF1	PDE4B
IRF1	PDE4DIP
IRF1	PDIA3
IRF1	PDIA4
IRF1	PDIA6
IRF1	PDK1
IRF1	PDK2
IRF1	PDPK1
IRF1	PDXDC1
IRF1	PDXK
IRF1	PFDN5
IRF1	PFKL
IRF1	PGM3
IRF1	PGRMC2
IRF1	PHF1
IRF1	PHTF1
IRF1	PIAS3
IRF1	PIGH
IRF1	PIGS
IRF1	PIGT
IRF1	PIK3C3
IRF1	PIK3CG
IRF1	PIK3IP1
IRF1	PIM2
IRF1	PINK1
IRF1	PJA2
IRF1	PLCG2
IRF1	PLD3
IRF1	PLEKHA3
IRF1	PLEKHG1
IRF1	PLEKHH3
IRF1	PLOD1
IRF1	PLOD3
IRF1	PLP2
IRF1	PLTP
IRF1	PML
IRF1	PNISR
IRF1	PNOC
IRF1	PNPLA2
IRF1	PNPLA8
IRF1	PNRC1
IRF1	POLE2
IRF1	POLQ
IRF1	POLR1D
IRF1	POLR3E
IRF1	PPCDC
IRF1	PPIG
IRF1	PPM1K
IRF1	PPP2R5B
IRF1	PQBP1

IRF1	PRDX4
IRF1	PREX1
IRF1	PRKAG1
IRF1	PRKCB
IRF1	PRKCSH
IRF1	PRKD2
IRF1	PRKX
IRF1	PRPF38B
IRF1	PRRC1
IRF1	PSAP
IRF1	PSENEN
IRF1	PSMA5
IRF1	PSMB9
IRF1	PSMD13
IRF1	PSME1
IRF1	PSME2
IRF1	PTAR1
IRF1	PTPN1
IRF1	PTPRN2
IRF1	PUS3
IRF1	PYCARD
IRF1	PYM1
IRF1	PYURF
IRF1	QPCT
IRF1	QPCTL
IRF1	R3HCC1
IRF1	RAB11B
IRF1	RAB1A
IRF1	RAB22A
IRF1	RAB28
IRF1	RAB3GAP1
IRF1	RAB40B
IRF1	RAB6A
IRF1	RAB8A
IRF1	RABAC1
IRF1	RABEP2
IRF1	RABGAP1
IRF1	RABGGTA
IRF1	RAD54L2
IRF1	RAD9A
IRF1	RASGRP2
IRF1	RASSF1
IRF1	RB1CC1
IRF1	RBL2
IRF1	RBM33
IRF1	RBM4
IRF1	RBM45
IRF1	RBM48
IRF1	RCBTB1
IRF1	RCSD1

IRF1	RER1
IRF1	RERE
IRF1	RFXAP
IRF1	RGS2
IRF1	RICTOR
IRF1	RNASE6
IRF1	RNASET2
IRF1	RNF111
IRF1	RNF113A
IRF1	RNF13
IRF1	RNF14
IRF1	RNF149
IRF1	RNF185
IRF1	RNF213
IRF1	RNF32
IRF1	RNF5
IRF1	RNPEPL1
IRF1	RPL10
IRF1	RPL18A
IRF1	RPL36AL
IRF1	RPLP1
IRF1	RPS11
IRF1	RPS19
IRF1	RPS27
IRF1	RPS5
IRF1	RPS6KA3
IRF1	RPS6KA5
IRF1	RPS6KB2
IRF1	RRAGC
IRF1	RRBP1
IRF1	RRP36
IRF1	RSBN1L
IRF1	RSRP1
IRF1	RTN3
IRF1	RWDD2A
IRF1	SAMD4B
IRF1	SAMD9L
IRF1	SAP30
IRF1	SAR1A
IRF1	SAR1B
IRF1	SARAF
IRF1	SAT1
IRF1	SBDS
IRF1	SBF1
IRF1	SCAF1
IRF1	SCAI
IRF1	SCAMP2
IRF1	SCARB2
IRF1	SCFD1
IRF1	SCPEP1

IRF1	SCRN3
IRF1	SDCBP2
IRF1	SDF2L1
IRF1	SDF4
IRF1	SDHAF1
IRF1	SEC11C
IRF1	SEC13
IRF1	SEC14L1
IRF1	SEC23B
IRF1	SEC24A
IRF1	SEC24C
IRF1	SEC24D
IRF1	SEC31A
IRF1	SEC61A1
IRF1	SEC61B
IRF1	SEC61G
IRF1	SEC63
IRF1	SECISBP2L
IRF1	SEL1L
IRF1	SEPT2
IRF1	SEPT6
IRF1	SERINC3
IRF1	SERPINI1
IRF1	SERTAD3
IRF1	SESN3
IRF1	SETD4
IRF1	SF3B2
IRF1	SF3B5
IRF1	SFT2D1
IRF1	SFXN2
IRF1	SGK1
IRF1	SIL1
IRF1	SIRT2
IRF1	SLC17A9
IRF1	SLC1A4
IRF1	SLC25A15
IRF1	SLC25A22
IRF1	SLC25A25
IRF1	SLC25A42
IRF1	SLC25A51
IRF1	SLC30A7
IRF1	SLC33A1
IRF1	SLC35A1
IRF1	SLC35C1
IRF1	SLC35C2
IRF1	SLC38A10
IRF1	SLC38A2
IRF1	SLC39A4
IRF1	SLC50A1
IRF1	SLC52A2

IRF1	SLC9A3R1
IRF1	SMCHD1
IRF1	SMIM7
IRF1	SMPD1
IRF1	SMTN
IRF1	SMUG1
IRF1	SND1
IRF1	SNRNP200
IRF1	SNX14
IRF1	SNX2
IRF1	SOCS3
IRF1	SOCS7
IRF1	SP100
IRF1	SP140L
IRF1	SPATS2
IRF1	SPCS3
IRF1	SPG11
IRF1	SPINT2
IRF1	SPOP
IRF1	SPRED2
IRF1	SPTBN1
IRF1	SPTY2D1
IRF1	SRGN
IRF1	SRM
IRF1	SRP19
IRF1	SRP54
IRF1	SRP68
IRF1	SRP72
IRF1	SRPRA
IRF1	SSPN
IRF1	SSR1
IRF1	SSR2
IRF1	SSR3
IRF1	SSR4
IRF1	ST3GAL1
IRF1	ST6GAL1
IRF1	ST6GALNAC4
IRF1	ST8SIA4
IRF1	STAMBPL1
IRF1	STARD3
IRF1	STARD5
IRF1	STAT1
IRF1	STAT2
IRF1	STK17B
IRF1	STK4
IRF1	STX17
IRF1	STX5
IRF1	STXBP6
IRF1	SUB1
IRF1	SUN2

IRF1	SUPT5H
IRF1	SYVN1
IRF1	TAF3
IRF1	TAGAP
IRF1	TAGLN2
IRF1	TAOK1
IRF1	TAP1
IRF1	TAP2
IRF1	TAPBP
IRF1	TAPBPL
IRF1	TARSL2
IRF1	TAX1BP1
IRF1	TBC1D10A
IRF1	TBC1D20
IRF1	TBL2
IRF1	TBXAS1
IRF1	TCEAL1
IRF1	TCF20
IRF1	TCF25
IRF1	TCIRG1
IRF1	TDRD3
IRF1	TECR
IRF1	TEX264
IRF1	TFG
IRF1	TGIF1
IRF1	THAP11
IRF1	THAP2
IRF1	THEMIS2
IRF1	TIMP1
IRF1	TINF2
IRF1	TJAP1
IRF1	TJP1
IRF1	TM9SF2
IRF1	TM9SF3
IRF1	TMBIM1
IRF1	TMBIM4
IRF1	TMBIM6
IRF1	TMCO1
IRF1	TMED1
IRF1	TMED10
IRF1	TMED2
IRF1	TMED3
IRF1	TMED4
IRF1	TMEM102
IRF1	TMEM106A
IRF1	TMEM109
IRF1	TMEM138
IRF1	TMEM161A
IRF1	TMEM184B
IRF1	TMEM19

IRF1	TMEM199
IRF1	TMEM205
IRF1	TMEM208
IRF1	TMEM230
IRF1	TMEM248
IRF1	TMEM258
IRF1	TMEM263
IRF1	TMEM39A
IRF1	TMEM41B
IRF1	TMEM50A
IRF1	TMEM50B
IRF1	TMEM59
IRF1	TMEM9
IRF1	TMEM91
IRF1	TMF1
IRF1	TMSB10
IRF1	TNFSF10
IRF1	TNRC6B
IRF1	TOLLIP
IRF1	TOMM20
IRF1	TOMM34
IRF1	TOR1B
IRF1	TP53INP1
IRF1	TPRA1
IRF1	TPST2
IRF1	TPT1
IRF1	TRABD
IRF1	TRADD
IRF1	TRAM1
IRF1	TRAM2
IRF1	TRIB1
IRF1	TRIP11
IRF1	TRPC4AP
IRF1	TSC22D1
IRF1	TSC22D3
IRF1	TSC22D4
IRF1	TSPAN13
IRF1	TSPYL2
IRF1	TSSC4
IRF1	TTC17
IRF1	TTC31
IRF1	TTC38
IRF1	TVP23B
IRF1	TXNDC11
IRF1	TXNDC5
IRF1	UAP1
IRF1	UBA5
IRF1	UBALD1
IRF1	UBB
IRF1	UBC

IRF1	UBE2D1
IRF1	UBE2H
IRF1	UBE2J1
IRF1	UBE2L6
IRF1	UBE2Q1
IRF1	UBE2W
IRF1	UBE3B
IRF1	UBE4A
IRF1	UBL7
IRF1	UBXN4
IRF1	UCP2
IRF1	UFC1
IRF1	UFL1
IRF1	UFM1
IRF1	UFSP2
IRF1	UGDH
IRF1	UNC13D
IRF1	UNC50
IRF1	UQCRQ
IRF1	UROS
IRF1	USE1
IRF1	USO1
IRF1	USP3
IRF1	USP36
IRF1	USP4
IRF1	USP46
IRF1	USP8
IRF1	UTP23
IRF1	UTRN
IRF1	UXT
IRF1	VAMP2
IRF1	VAV1
IRF1	VCP
IRF1	VDR
IRF1	VMP1
IRF1	VPS13A
IRF1	WBP4
IRF1	WDFY2
IRF1	WDR25
IRF1	WDR26
IRF1	WDR45
IRF1	WDR92
IRF1	WFS1
IRF1	WIPI1
IRF1	WNT6
IRF1	XRN1
IRF1	XRRA1
IRF1	YIF1A
IRF1	YIF1B
IRF1	YIPF1

IRF1	YIPF2
IRF1	YIPF3
IRF1	YIPF5
IRF1	YKT6
IRF1	YPEL2
IRF1	YPEL3
IRF1	YPEL5
IRF1	YTHDF2
IRF1	ZAP70
IRF1	ZBED5
IRF1	ZBP1
IRF1	ZBTB7A
IRF1	ZBTB8A
IRF1	ZC3HAV1
IRF1	ZDHHC4
IRF1	ZFP36L2
IRF1	ZFPL1
IRF1	ZNF215
IRF1	ZNF224
IRF1	ZNF232
IRF1	ZNF274
IRF1	ZNF292
IRF1	ZNF322
IRF1	ZNF358
IRF1	ZNF562
IRF1	ZNF593
IRF1	ZNF638
IRF1	ZNF671
IRF1	ZNF706
IRF1	ZNF720
IRF1	ZNF771
IRF1	ZNF791
IRF1	ZNF831
IRF1	ZWILCH
IRF2	ADAR
IRF2	AKIRIN2
IRF2	ANKRD28
IRF2	APOL2
IRF2	AREG
IRF2	ARF6
IRF2	ARHGEF37
IRF2	ASAH1
IRF2	B2M
IRF2	CARD16
IRF2	CASP1
IRF2	CCR7
IRF2	CD47
IRF2	CLN5
IRF2	COQ9
IRF2	CYSTM1

IRF2	DEF6
IRF2	DERL1
IRF2	DNAJC1
IRF2	DTX3L
IRF2	ELK3
IRF2	ENTPD1-AS1
IRF2	EPSTI1
IRF2	ERP44
IRF2	ETV7
IRF2	FAR1
IRF2	FBXW7
IRF2	FLOT2
IRF2	FNDC3A
IRF2	FOXO3
IRF2	FXN
IRF2	FXYD5
IRF2	GPRC5C
IRF2	GRK5
IRF2	HEXB
IRF2	HIST1H3G
IRF2	HLA-A
IRF2	IRF2
IRF2	IRF7
IRF2	ITM2C
IRF2	KCNK6
IRF2	KLF9
IRF2	MAP3K14
IRF2	MFSD12
IRF2	MGAT1
IRF2	MICU1
IRF2	MR1
IRF2	MSL2
IRF2	MTG1
IRF2	MTIF3
IRF2	MVP
IRF2	MYO1G
IRF2	NCSTN
IRF2	NPRL3
IRF2	NRAS
IRF2	NT5C3A
IRF2	NUB1
IRF2	PARP14
IRF2	PARP9
IRF2	PCF11
IRF2	PELI1
IRF2	PER3
IRF2	POU2AF1
IRF2	PRDX4
IRF2	PSAP
IRF2	PSMB8-AS1

IRF2	RASSF5
IRF2	RBMS1
IRF2	RNF213
IRF2	RPL17
IRF2	RPS12
IRF2	RPSA
IRF2	RSAD2
IRF2	RTP4
IRF2	SAT1
IRF2	SCARB2
IRF2	SETD1B
IRF2	SP100
IRF2	SPCS3
IRF2	SUB1
IRF2	SUCO
IRF2	TAB1
IRF2	TAF5L
IRF2	TAPBPL
IRF2	TGIF1
IRF2	THTPA
IRF2	TIRAP
IRF2	TMEM52B
IRF2	TPST2
IRF2	TXNDC11
IRF2	UBE2E2
IRF2	UBE2J1
IRF2	UBE2L6
IRF2	UBE4B
IRF2	WBP1L
IRF2	XAF1
IRF2	ZBP1
IRF2	ZC3H4
IRF2	ZFAT
IRF2	ZFP36L1
IRF3	AACS
IRF3	AAMP
IRF3	AARS
IRF3	ABCA5
IRF3	ABCB8
IRF3	ABHD12
IRF3	ABHD17A
IRF3	ACAP1
IRF3	ACAT2
IRF3	ACBD5
IRF3	ACLY
IRF3	ACOT8
IRF3	ACSL4
IRF3	ACTN4
IRF3	ACTR1A
IRF3	ACTR3B

IRF3	ADAMTS6
IRF3	ADAR
IRF3	ADD3
IRF3	ADIPOR1
IRF3	ADIPOR2
IRF3	ADPGK
IRF3	AFF3
IRF3	AFF4
IRF3	AGO3
IRF3	AHCYL1
IRF3	AHDC1
IRF3	AHNAK
IRF3	AKAP13
IRF3	AKAP8
IRF3	AKAP8L
IRF3	AKR1A1
IRF3	AKT1S1
IRF3	ALAS1
IRF3	ALCAM
IRF3	ALDH3A2
IRF3	ALDOA
IRF3	ALKBH7
IRF3	ALKBH8
IRF3	AMD1
IRF3	ANAPC11
IRF3	ANAPC5
IRF3	ANGPTL6
IRF3	ANKRD16
IRF3	ANKRD28
IRF3	ANKS3
IRF3	ANXA6
IRF3	AP1B1
IRF3	AP2M1
IRF3	AP2S1
IRF3	APBB2
IRF3	APPL1
IRF3	AQP3
IRF3	ARF1
IRF3	ARFIP2
IRF3	ARHGAP22
IRF3	ARHGEF12
IRF3	ARID3A
IRF3	ARID4B
IRF3	ARL6IP5
IRF3	ARPC1B
IRF3	ARPC3
IRF3	ARRDC4
IRF3	ASAH1
IRF3	ASH1L
IRF3	ASL

IRF3	ASNA1
IRF3	ATF4
IRF3	ATP6V0E1
IRF3	ATP6V1C1
IRF3	ATP6V1E1
IRF3	ATRX
IRF3	ATXN2L
IRF3	ATXN3
IRF3	AZIN1
IRF3	B3GNT2
IRF3	B4GALT3
IRF3	BAD
IRF3	BAK1
IRF3	BANP
IRF3	BAZ1A
IRF3	BAZ2A
IRF3	BAZ2B
IRF3	BBX
IRF3	BCL7B
IRF3	BICDL1
IRF3	BIK
IRF3	BLM
IRF3	BMPR2
IRF3	BRD4
IRF3	BRK1
IRF3	C11orf24
IRF3	C12orf57
IRF3	C15orf39
IRF3	C16orf74
IRF3	C16orf91
IRF3	C2orf42
IRF3	C9orf16
IRF3	CABIN1
IRF3	CALR
IRF3	CALU
IRF3	CANX
IRF3	CAPG
IRF3	CAPN1
IRF3	CAPNS1
IRF3	CAPZB
IRF3	CARS2
IRF3	CASC4
IRF3	CASP6
IRF3	CAT
IRF3	CBX7
IRF3	CCDC107
IRF3	CCDC167
IRF3	CCDC186
IRF3	CCDC189
IRF3	CCDC88C

IRF3	CCM2
IRF3	CCNL1
IRF3	CCNL2
IRF3	CD19
IRF3	CD27
IRF3	CD37
IRF3	CD48
IRF3	CD52
IRF3	CD63
IRF3	CD74
IRF3	CDIPT
IRF3	CDK2AP2
IRF3	CDK5RAP3
IRF3	CEP152
IRF3	CEP57
IRF3	CERS5
IRF3	CGGBP1
IRF3	CHD8
IRF3	CHKA
IRF3	CHST12
IRF3	CIZ1
IRF3	CLIC1
IRF3	CLIP2
IRF3	CLK2
IRF3	CLK4
IRF3	CLN5
IRF3	CLPP
IRF3	CNNM3
IRF3	CNOT6L
IRF3	CNPY3
IRF3	COPS3
IRF3	COQ5
IRF3	COQ6
IRF3	COTL1
IRF3	COX14
IRF3	COX5A
IRF3	COX7B
IRF3	COX8A
IRF3	CPNE1
IRF3	CPT1A
IRF3	CPTP
IRF3	CREG1
IRF3	CSK
IRF3	CSNK1A1
IRF3	CSNK1G2
IRF3	CSNK2A1
IRF3	CTCF
IRF3	CTSB
IRF3	CUEDC2
IRF3	CUTA

IRF3	CWC25
IRF3	CXXC5
IRF3	CYB5B
IRF3	CYHR1
IRF3	CYP2R1
IRF3	DAPK3
IRF3	DARS
IRF3	DAXX
IRF3	DAZAP2
IRF3	DBI
IRF3	DCAF11
IRF3	DCAF15
IRF3	DCXR
IRF3	DDA1
IRF3	DDOST
IRF3	DDT
IRF3	DDX19B
IRF3	DEF6
IRF3	DENND1C
IRF3	DENND3
IRF3	DENND6A
IRF3	DENND6B
IRF3	DERL2
IRF3	DERL3
IRF3	DHRS4L2
IRF3	DLD
IRF3	DMTF1
IRF3	DMXL1
IRF3	DNAJB11
IRF3	DNAJC3
IRF3	DNAJC4
IRF3	DNASE1L1
IRF3	DNM2
IRF3	DPAGT1
IRF3	DPY30
IRF3	DUSP1
IRF3	EAPP
IRF3	EBP
IRF3	ECH1
IRF3	ECI1
IRF3	EEF1A1
IRF3	EEF2
IRF3	EFNA4
IRF3	EGR1
IRF3	EIF2AK3
IRF3	EIF2B3
IRF3	EIF5B
IRF3	ELL
IRF3	ELL2
IRF3	ELOF1

IRF3	ELP4
IRF3	EMB
IRF3	EMC4
IRF3	ENOX2
IRF3	ENSA
IRF3	ENTPD7
IRF3	ERCC1
IRF3	ERF
IRF3	ERGIC3
IRF3	ESCO1
IRF3	ESR2
IRF3	EXOSC1
IRF3	FAAP20
IRF3	FAF1
IRF3	FAF2
IRF3	FAM117A
IRF3	FAM133B
IRF3	FAM13B
IRF3	FAM162A
IRF3	FAM192A
IRF3	FAM217B
IRF3	FANCG
IRF3	FASN
IRF3	FBXL12
IRF3	FBXO25
IRF3	FBXW4
IRF3	FCGRT
IRF3	FDFT1
IRF3	FDPS
IRF3	FKBP15
IRF3	FKBP8
IRF3	FKBPL
IRF3	FLI1
IRF3	FLNA
IRF3	FLYWCH1
IRF3	FNDC3A
IRF3	FOS
IRF3	FOXN3
IRF3	FRYL
IRF3	FXYD5
IRF3	GABARAPL2
IRF3	GADD45GIP1
IRF3	GALM
IRF3	GATAD2B
IRF3	GBF1
IRF3	GCAT
IRF3	GEMIN7
IRF3	GFOD1
IRF3	GGCX
IRF3	GIGYF2

IRF3	GIPC1
IRF3	GLG1
IRF3	GLO1
IRF3	GLRX5
IRF3	GLS
IRF3	GLT8D1
IRF3	GLTP
IRF3	GMEB1
IRF3	GNAI2
IRF3	GNAS
IRF3	GNB2
IRF3	GOLGA7
IRF3	GOLGB1
IRF3	GOLPH3L
IRF3	GOLT1B
IRF3	GPBP1
IRF3	GPCPD1
IRF3	GPR108
IRF3	GPR89B
IRF3	GPX4
IRF3	GRK6
IRF3	GRN
IRF3	GSDMD
IRF3	GSPT1
IRF3	GTF2B
IRF3	GTF2I
IRF3	GTPBP1
IRF3	GYS1
IRF3	GZF1
IRF3	HADHA
IRF3	HARS
IRF3	HDAC5
IRF3	HERC5
IRF3	HERPUD1
IRF3	HEXB
IRF3	HIP1R
IRF3	HIST2H2AC
IRF3	HLA-DPA1
IRF3	HLA-DPB1
IRF3	HLA-DQB1
IRF3	HLA-DRA
IRF3	HLA-DRB5
IRF3	HLA-E
IRF3	HM13
IRF3	HMG20B
IRF3	HMGCS1
IRF3	HMOX2
IRF3	HNRNPL
IRF3	HNRNPUL2
IRF3	HOMEZ

IRF3	HOOK2
IRF3	HOXB2
IRF3	HPCAL1
IRF3	HSCB
IRF3	HSD17B12
IRF3	HSD17B7
IRF3	HSP90B1
IRF3	HSPA5
IRF3	HYI
IRF3	ICAM3
IRF3	ID3
IRF3	IDH1
IRF3	IDH2
IRF3	IDH3G
IRF3	IFFO1
IRF3	IFNG-AS1
IRF3	IKZF3
IRF3	IL2RG
IRF3	IMMT
IRF3	INSIG1
IRF3	IRF1
IRF3	IRF2
IRF3	ISCA2
IRF3	ISL2
IRF3	ISOC2
IRF3	ITFG2
IRF3	ITGA1
IRF3	ITGAL
IRF3	ITGB7
IRF3	ITM2B
IRF3	ITPR2
IRF3	ITSN1
IRF3	JAGN1
IRF3	JAK1
IRF3	JUND
IRF3	KANSL1
IRF3	KCNAB2
IRF3	KCNK12
IRF3	KDELR1
IRF3	KDM2A
IRF3	KDM4B
IRF3	KIAA0355
IRF3	KIAA1143
IRF3	KIAA1191
IRF3	KIAA2013
IRF3	KIF13B
IRF3	KLF13
IRF3	KMT2E-AS1
IRF3	KXD1
IRF3	KYAT3

IRF3	L1CAM
IRF3	L2HGDH
IRF3	L3MBTL2
IRF3	LAGE3
IRF3	LAMTOR1
IRF3	LANCL1
IRF3	LARP1B
IRF3	LAT2
IRF3	LDLRAD4
IRF3	LEF1
IRF3	LENG1
IRF3	LINC01485
IRF3	LMAN2
IRF3	LMO4
IRF3	LNX2
IRF3	LPCAT4
IRF3	LPIN1
IRF3	LPXN
IRF3	LRCH4
IRF3	LRMP
IRF3	LRP10
IRF3	LRP8
IRF3	LRRC37B
IRF3	LSM1
IRF3	LSM14B
IRF3	LSS
IRF3	LTBP3
IRF3	M6PR
IRF3	MAF1
IRF3	MAGED2
IRF3	MAGT1
IRF3	MANF
IRF3	MAP2K2
IRF3	MAP2K3
IRF3	MAP3K11
IRF3	MAP4K3
IRF3	MAP7D1
IRF3	MAPK8IP3
IRF3	MAPRE1
IRF3	MARK3
IRF3	MAT2B
IRF3	MAZ
IRF3	MBD2
IRF3	MCAT
IRF3	MCCC1
IRF3	MECP2
IRF3	MED10
IRF3	MEN1
IRF3	METTL17
IRF3	METTL2A

IRF3	MFF
IRF3	MGMT
IRF3	MIEF1
IRF3	MIIP
IRF3	MIR22HG
IRF3	MKNK2
IRF3	MLEC
IRF3	MLF2
IRF3	MLYCD
IRF3	MORF4L1
IRF3	MOSPD3
IRF3	MPC1
IRF3	MPHOSPH8
IRF3	MPRIP
IRF3	MPV17L2
IRF3	MRFAP1
IRF3	MRI1
IRF3	MRM3
IRF3	MRPL23
IRF3	MRPL41
IRF3	MRPL52
IRF3	MRPS36
IRF3	MSANTD4
IRF3	MSL3
IRF3	MSMO1
IRF3	MTERF2
IRF3	MTHFD1
IRF3	MTIF3
IRF3	MTMR4
IRF3	MTR
IRF3	MUM1
IRF3	MVD
IRF3	MVP
IRF3	MYB
IRF3	MYL6
IRF3	MYO19
IRF3	MYPOP
IRF3	MZB1
IRF3	NAB2
IRF3	NARF
IRF3	NCAPH
IRF3	NCKAP1L
IRF3	NCOR1
IRF3	NCR3
IRF3	NDEL1
IRF3	NDUFA11
IRF3	NDUFA13
IRF3	NDUFB11
IRF3	NDUFB6
IRF3	NDUFC2

IRF3	NEAT1
IRF3	NEK8
IRF3	NET1
IRF3	NEU1
IRF3	NINJ1
IRF3	NKX6-3
IRF3	NLRP4
IRF3	NME4
IRF3	NME7
IRF3	NONO
IRF3	NPC2
IRF3	NPEPPS
IRF3	NR2C2
IRF3	NR3C1
IRF3	NRDC
IRF3	NSDHL
IRF3	NSFL1C
IRF3	NSUN4
IRF3	NT5DC1
IRF3	NT5DC2
IRF3	NTAN1
IRF3	NUAK2
IRF3	NUBPL
IRF3	NUDT9
IRF3	NUMB
IRF3	NUP107
IRF3	OAZ1
IRF3	OGG1
IRF3	ORC2
IRF3	ORMDL2
IRF3	OS9
IRF3	OSBP
IRF3	OSTF1
IRF3	OTUD5
IRF3	P4HA1
IRF3	P4HB
IRF3	PABPC4
IRF3	PABPN1
IRF3	PAF1
IRF3	PAK2
IRF3	PANK2
IRF3	PCBP1
IRF3	PCF11
IRF3	PDE4B
IRF3	PDE6D
IRF3	PDHA1
IRF3	PDHX
IRF3	PDIA3
IRF3	PDIA6
IRF3	PEBP1

IRF3	PET100
IRF3	PFDN1
IRF3	PGAP2
IRF3	PGK1
IRF3	PGM1
IRF3	PGM3
IRF3	PGP
IRF3	PGRMC2
IRF3	PHC3
IRF3	PHKG2
IRF3	PHPT1
IRF3	PIAS3
IRF3	PIGX
IRF3	PIK3CD-AS2
IRF3	PIK3CG
IRF3	PIK3R4
IRF3	PIN4
IRF3	PITRM1
IRF3	PJA2
IRF3	PNKD
IRF3	PNOC
IRF3	PNP
IRF3	PNPO
IRF3	POLG
IRF3	POLR2A
IRF3	POLR3E
IRF3	POLR3H
IRF3	POLR3K
IRF3	POP5
IRF3	POP7
IRF3	PPIB
IRF3	PPIG
IRF3	PPM1G
IRF3	PPM1M
IRF3	PPP1R16B
IRF3	PPP1R7
IRF3	PPP2R5B
IRF3	PPP3CC
IRF3	PPP6C
IRF3	PPP6R3
IRF3	PQBP1
IRF3	PRCP
IRF3	PRDM2
IRF3	PRKAR2A
IRF3	PRKCSH
IRF3	PRKD2
IRF3	PRPSAP2
IRF3	PRR13
IRF3	PRR5
IRF3	PRRC1

IRF3	PSMD10
IRF3	PSMD9
IRF3	PTPN1
IRF3	PTPN6
IRF3	PURB
IRF3	QPCT
IRF3	R3HDM2
IRF3	RAB11B-AS1
IRF3	RAB5C
IRF3	RAB7A
IRF3	RAB8A
IRF3	RAB9A
IRF3	RABAC1
IRF3	RABL3
IRF3	RAC1
IRF3	RAD9A
IRF3	RAF1
IRF3	RALGPS1
IRF3	RALGPS2
IRF3	RALY
IRF3	RANGRF
IRF3	RAP1A
IRF3	RASA4
IRF3	RASSF5
IRF3	RB1CC1
IRF3	RBM12
IRF3	RBM39
IRF3	RBM42
IRF3	RBM4B
IRF3	RCN2
IRF3	REEP5
IRF3	RFWD3
IRF3	RHNO1
IRF3	RIDA
IRF3	RILPL2
IRF3	RLN2
IRF3	RNF114
IRF3	RNF14
IRF3	RNF181
IRF3	RNF20
IRF3	ROGDI
IRF3	RPL13
IRF3	RPL13A
IRF3	RPL17
IRF3	RPL18A
IRF3	RPL22
IRF3	RPL27
IRF3	RPL29
IRF3	RPL38
IRF3	RPLP2

IRF3	RPN2
IRF3	RPS10
IRF3	RPS11
IRF3	RPS16
IRF3	RPS19
IRF3	RPS21
IRF3	RPS27
IRF3	RPS7
IRF3	RPS8
IRF3	RSBN1L
IRF3	RTF1
IRF3	RTN4
IRF3	RUFY1
IRF3	S1PR4
IRF3	SACM1L
IRF3	SAMD4B
IRF3	SASH3
IRF3	SAT2
IRF3	SC5D
IRF3	SCAMP2
IRF3	SCAMP4
IRF3	SCAP
IRF3	SCARB2
IRF3	SDCCAG8
IRF3	SDF2L1
IRF3	SEC11A
IRF3	SEC23A
IRF3	SEC23B
IRF3	SEC31A
IRF3	SEPT1
IRF3	SEPT6
IRF3	SEPT7
IRF3	SEPT9
IRF3	SERTAD1
IRF3	SETD3
IRF3	SF3B4
IRF3	SGK1
IRF3	SGSM3
IRF3	SH3BGRL3
IRF3	SHKBP1
IRF3	SIGMAR1
IRF3	SIT1
IRF3	SKAP2
IRF3	SLC25A38
IRF3	SLC25A39
IRF3	SLC26A6
IRF3	SLC38A10
IRF3	SLC41A1
IRF3	SLC43A2
IRF3	SLC7A6

IRF3	SLC7A7
IRF3	SLC9A3R1
IRF3	SMARCC2
IRF3	SMCHD1
IRF3	SMDT1
IRF3	SMG1
IRF3	SMIM19
IRF3	SND1
IRF3	SNRNP25
IRF3	SNRNP40
IRF3	SNRPN
IRF3	SNX25
IRF3	SNX3
IRF3	SNX4
IRF3	SNX6
IRF3	SOX4
IRF3	SP100
IRF3	SPATS2
IRF3	SPCS2
IRF3	SPCS3
IRF3	SPG21
IRF3	SPG7
IRF3	SPINT2
IRF3	SPSB3
IRF3	SPTLC2
IRF3	SQLE
IRF3	SQSTM1
IRF3	SRM
IRF3	SRP68
IRF3	SRRM2
IRF3	SRSF4
IRF3	SRSF5
IRF3	SS18L2
IRF3	SSPN
IRF3	SSR1
IRF3	SSR2
IRF3	SSU72
IRF3	STAMBPL1
IRF3	STARD7
IRF3	STEAP1B
IRF3	STIM1
IRF3	STK38
IRF3	STT3A
IRF3	STX5
IRF3	SUB1
IRF3	SUCLG2
IRF3	SUDS3
IRF3	SULF2
IRF3	SUMF2
IRF3	SUMO2

IRF3	SUZ12
IRF3	SYNE1
IRF3	SYNPO
IRF3	SYNRG
IRF3	SZRD1
IRF3	TAF15
IRF3	TAF7
IRF3	TANGO2
IRF3	TBC1D22A
IRF3	TBC1D23
IRF3	TBCCD1
IRF3	TBL2
IRF3	TCF4
IRF3	TDRKH
IRF3	TECR
IRF3	TERF1
IRF3	TESK1
IRF3	THAP7
IRF3	THEMIS2
IRF3	THG1L
IRF3	THTPA
IRF3	TIA1
IRF3	TIMM17B
IRF3	TIMP1
IRF3	TINF2
IRF3	TKT
IRF3	TLE1
IRF3	TM9SF2
IRF3	TMBIM6
IRF3	TMEM160
IRF3	TMEM165
IRF3	TMEM189
IRF3	TMEM19
IRF3	TMEM219
IRF3	TMEM256
IRF3	TMEM30A
IRF3	TMEM33
IRF3	TMEM39B
IRF3	TMEM41B
IRF3	TMEM50B
IRF3	TMEM97
IRF3	TMUB2
IRF3	TNFRSF13C
IRF3	TOMM40L
IRF3	TP53INP1
IRF3	TPM3
IRF3	TPP2
IRF3	TPST2
IRF3	TRAF2
IRF3	TRAF7

IRF3	TRAM2
IRF3	TRAPPC3
IRF3	TRAPPC6A
IRF3	TRIM66
IRF3	TRIOBP
IRF3	TRIT1
IRF3	TRMT10B
IRF3	TRPM4
IRF3	TRUB2
IRF3	TSC22D2
IRF3	TSC22D4
IRF3	TSPAN31
IRF3	TSPAN33
IRF3	TTC17
IRF3	TTC9C
IRF3	TWF2
IRF3	TXLNA
IRF3	TYSND1
IRF3	UBA3
IRF3	UBE2D2
IRF3	UBE2V1
IRF3	UBR5
IRF3	UBTF
IRF3	UBXN1
IRF3	UBXN8
IRF3	UFC1
IRF3	UGDH
IRF3	UNC45A
IRF3	UQCC2
IRF3	UQCRC2
IRF3	URI1
IRF3	USO1
IRF3	USP34
IRF3	VAMP2
IRF3	VCP
IRF3	VPS11
IRF3	VPS33B
IRF3	VRK1
IRF3	WDR45
IRF3	WDR92
IRF3	XAB2
IRF3	YIF1B
IRF3	YKT6
IRF3	YPEL3
IRF3	YWHAB
IRF3	ZBED5
IRF3	ZBTB32
IRF3	ZBTB7A
IRF3	ZC2HC1A
IRF3	ZC3H14

IRF3	ZCCHC17
IRF3	ZDHHC12
IRF3	ZDHHC4
IRF3	ZFAND1
IRF3	ZFAND6
IRF3	ZFP36L2
IRF3	ZMAT3
IRF3	ZMAT5
IRF3	ZMYM3
IRF3	ZMYND8
IRF3	ZNF124
IRF3	ZNF277
IRF3	ZNF292
IRF3	ZNF444
IRF3	ZNF445
IRF3	ZNF473
IRF3	ZNF493
IRF3	ZNF513
IRF3	ZNF518A
IRF3	ZNF580
IRF3	ZNF584
IRF3	ZNF609
IRF3	ZNF638
IRF3	ZNF672
IRF3	ZNF736
IRF3	ZNF791
IRF3	ZNF821
IRF4	ABHD6
IRF4	APOL1
IRF4	APOL6
IRF4	ARL5B
IRF4	ASCL2
IRF4	ATF5
IRF4	ATP1B1
IRF4	B2M
IRF4	BCAS3
IRF4	BTN3A2
IRF4	CHAC1
IRF4	CMIP
IRF4	CSGALNACT2
IRF4	CYP4V2
IRF4	DEXI
IRF4	ERN1
IRF4	FBXO16
IRF4	FBXO38
IRF4	FBXW7
IRF4	FGD2
IRF4	FNDC3A
IRF4	FUCA2
IRF4	GPRIN3

IRF4	HAX1
IRF4	HERC5
IRF4	HERPUD1
IRF4	HLA-B
IRF4	IFIH1
IRF4	INSIG1
IRF4	IRF4
IRF4	ISG15
IRF4	ISOC2
IRF4	ITCH
IRF4	MAN1A2
IRF4	MANF
IRF4	MIA2
IRF4	OAS2
IRF4	P4HB
IRF4	PICALM
IRF4	PLEKHM1
IRF4	PRDM1
IRF4	RARRES3
IRF4	RUNX2
IRF4	SAP30L
IRF4	SCARB2
IRF4	SEC24D
IRF4	SGK3
IRF4	SLC31A2
IRF4	SLC33A1
IRF4	SRGN
IRF4	SRM
IRF4	ST7
IRF4	SUB1
IRF4	TIRAP
IRF4	TUT1
IRF4	TXNDC15
IRF4	UBN2
IRF4	USO1
IRF4	USP18
IRF4	VDR
IRF4	WARS
IRF4	ZBP1
IRF4	ZMYM6
IRF4	ZNF181
IRF4	ZNF3
IRF5	AHDC1
IRF5	ARHGAP5
IRF5	CCR7
IRF5	CD24
IRF5	FGR
IRF5	IRF5
IRF5	MAP2K5
IRF5	MPEG1

IRF5	NFKBIE
IRF5	PARP9
IRF5	PDCD4
IRF5	PLAGL1
IRF5	PPFIBP2
IRF5	RARA
IRF5	RRP12
IRF5	SORBS3
IRF5	TGIF1
IRF5	TNIP1
IRF7	ABHD2
IRF7	ACAA1
IRF7	ACBD4
IRF7	ACKR4
IRF7	ACSS1
IRF7	ADAM15
IRF7	ADAR
IRF7	ADD1
IRF7	ADI1
IRF7	ADIPOR1
IRF7	ADIPOR2
IRF7	ADPGK
IRF7	AKAP8L
IRF7	AKAP9
IRF7	AKR1A1
IRF7	ALCAM
IRF7	ALDH9A1
IRF7	ALDOC
IRF7	ANG
IRF7	ANKIB1
IRF7	ANKRD12
IRF7	ANKRD28
IRF7	ANKRD37
IRF7	ANXA2R
IRF7	ANXA6
IRF7	APBA3
IRF7	APOBEC3G
IRF7	APOD
IRF7	APOL2
IRF7	APOL3
IRF7	APOL6
IRF7	AREG
IRF7	ARF3
IRF7	ARF4
IRF7	ARHGAP15
IRF7	ARHGDIB
IRF7	ARID1B
IRF7	ARL4C
IRF7	ARMC7
IRF7	ARMCX3

IRF7	ARMCX6
IRF7	ARSA
IRF7	ASAH1
IRF7	ASH1L
IRF7	ATF6
IRF7	ATG13
IRF7	ATP10D
IRF7	ATP13A1
IRF7	ATP6AP1
IRF7	ATP6AP2
IRF7	ATP6V0E1
IRF7	ATP6V0E2
IRF7	ATP8B2
IRF7	AZI2
IRF7	B2M
IRF7	B3GNT2
IRF7	B4GALT3
IRF7	BAK1
IRF7	BCAP31
IRF7	BCAR3
IRF7	BCL2L1
IRF7	BET1
IRF7	BHLHE41
IRF7	BISPR
IRF7	BIVM
IRF7	BLNK
IRF7	BORCS5
IRF7	BRD1
IRF7	BROX
IRF7	BTBD1
IRF7	BTG1
IRF7	BTN1A1
IRF7	BTN3A2
IRF7	C11orf68
IRF7	C11orf71
IRF7	C12orf10
IRF7	C14orf93
IRF7	C15orf39
IRF7	C17orf75
IRF7	C18orf25
IRF7	C18orf32
IRF7	C19orf66
IRF7	C1GALT1C1
IRF7	C1orf43
IRF7	C2orf74
IRF7	C3orf52
IRF7	C6orf89
IRF7	CALU
IRF7	CAMK4
IRF7	CAP1

IRF7	CASP4
IRF7	CASP8
IRF7	CBLB
IRF7	CBR1
IRF7	CCDC107
IRF7	CCDC186
IRF7	CCDC69
IRF7	CCDC88A
IRF7	CCDC88B
IRF7	CCL25
IRF7	CCL5
IRF7	CCM2
IRF7	CCNL1
IRF7	CCPG1
IRF7	CCR10
IRF7	CD164
IRF7	CD247
IRF7	CD37
IRF7	CD38
IRF7	CD47
IRF7	CD53
IRF7	CD59
IRF7	CD63
IRF7	CD68
IRF7	CD74
IRF7	CD79A
IRF7	CDIP1
IRF7	CDIPT
IRF7	CDK12
IRF7	CDK14
IRF7	CDR2
IRF7	CDS2
IRF7	CEP97
IRF7	CERCAM
IRF7	CGRRF1
IRF7	CHAC1
IRF7	CHI3L2
IRF7	CHMP5
IRF7	CHST12
IRF7	CHST15
IRF7	CHST2
IRF7	CHSY1
IRF7	CITED2
IRF7	CKAP4
IRF7	CLASP2
IRF7	CLCN4
IRF7	CLEC2B
IRF7	CLIC1
IRF7	CLK1
IRF7	CMPK2

IRF7	CNPY2
IRF7	COG3
IRF7	COG8
IRF7	COMMD3
IRF7	COPA
IRF7	COPB1
IRF7	COPG1
IRF7	COPZ1
IRF7	COX5A
IRF7	CPEB4
IRF7	CPNE5
IRF7	CPXM1
IRF7	CREB3
IRF7	CREB3L2
IRF7	CREBZF
IRF7	CRELD2
IRF7	CRTC3
IRF7	CTHRC1
IRF7	CTNNA1
IRF7	CTSA
IRF7	CTSH
IRF7	CTSS
IRF7	CUTA
IRF7	CYBA
IRF7	CYSTM1
IRF7	CYTH4
IRF7	CYTIP
IRF7	DAPL1
IRF7	DAPP1
IRF7	DCAKD
IRF7	DCLRE1C
IRF7	DCP1A
IRF7	DCTN2
IRF7	DDAH2
IRF7	DDX58
IRF7	DEF6
IRF7	DENND1B
IRF7	DENND6B
IRF7	DERL1
IRF7	DESI1
IRF7	DGKD
IRF7	DHRS7
IRF7	DHRS9
IRF7	DMD
IRF7	DNAJB9
IRF7	DNAJC1
IRF7	DNAJC3
IRF7	DNASE2
IRF7	DOCK8
IRF7	DOK7

IRF7	DPM1
IRF7	DUSP14
IRF7	DYNLRB1
IRF7	DYNLT1
IRF7	EBI3
IRF7	EBP
IRF7	EEF1D
IRF7	EFHC1
IRF7	EHMT1
IRF7	EIF2AK2
IRF7	ELK3
IRF7	EMC3
IRF7	EMC7
IRF7	EMILIN2
IRF7	EML4
IRF7	EML6
IRF7	ENTPD1
IRF7	EPHA1−AS1
IRF7	EPSTI1
IRF7	ERAP1
IRF7	ERGIC2
IRF7	ERGIC3
IRF7	ERLEC1
IRF7	ERO1A
IRF7	ERO1B
IRF7	ERP29
IRF7	ERP44
IRF7	ESCO1
IRF7	ETFBKMT
IRF7	ETV7
IRF7	EVI5
IRF7	FAM118A
IRF7	FAM174A
IRF7	FAM213B
IRF7	FAM78A
IRF7	FAM89B
IRF7	FANCF
IRF7	FBXO11
IRF7	FBXO16
IRF7	FBXO6
IRF7	FBXW7
IRF7	FCRL5
IRF7	FGD2
IRF7	FICD
IRF7	FKBP14
IRF7	FKBP2
IRF7	FNDC3A
IRF7	FOS
IRF7	FOXJ3
IRF7	FOXN3

IRF7	FOXO3
IRF7	FUT8
IRF7	FXR2
IRF7	GABARAP
IRF7	GALK2
IRF7	GBP4
IRF7	GDI2
IRF7	GLCCI1
IRF7	GLRX
IRF7	GLRX2
IRF7	GLS
IRF7	GMPR
IRF7	GOLGA3
IRF7	GOLIM4
IRF7	GORASP2
IRF7	GOSR2
IRF7	GPR146
IRF7	GPR155
IRF7	GPR160
IRF7	GPR65
IRF7	GPSM3
IRF7	GRAP2
IRF7	GRK6
IRF7	GRN
IRF7	GSDMD
IRF7	GSE1
IRF7	GUK1
IRF7	GYPC
IRF7	HELZ2
IRF7	HERC1
IRF7	HERC5
IRF7	HGS
IRF7	HIST1H1C
IRF7	HIST1H2BN
IRF7	HIST1H4H
IRF7	HLA-A
IRF7	HLA-B
IRF7	HLA-DPA1
IRF7	HLA-DQA1
IRF7	HLA-DRA
IRF7	HLA-E
IRF7	HLA-F
IRF7	HM13
IRF7	HMGCS1
IRF7	HOXB2
IRF7	HSCB
IRF7	HSD17B12
IRF7	HSP90B1
IRF7	HSPA13
IRF7	HYI

IRF7	ICAM2
IRF7	IDH2
IRF7	IFI16
IRF7	IFI27L2
IRF7	IFI35
IRF7	IFI44L
IRF7	IFI6
IRF7	IFIH1
IRF7	IFIT1
IRF7	IFIT2
IRF7	IFIT3
IRF7	IFNAR1
IRF7	IFNAR2
IRF7	IFRD1
IRF7	IGFLR1
IRF7	IGSF8
IRF7	IKBIP
IRF7	IKBKG
IRF7	IL12RB1
IRF7	IL16
IRF7	ING1
IRF7	INPP5B
IRF7	INPPL1
IRF7	INSIG2
IRF7	IQGAP2
IRF7	IRAK1
IRF7	IRF1
IRF7	IRF2
IRF7	IRF4
IRF7	IRF7
IRF7	IRF9
IRF7	ISG15
IRF7	ISG20
IRF7	ISOC2
IRF7	ITCH
IRF7	ITGB7
IRF7	ITM2B
IRF7	ITM2C
IRF7	JCHAIN
IRF7	JTB
IRF7	KAT6B
IRF7	KBTBD7
IRF7	KCNA3
IRF7	KCNK6
IRF7	KCNN3
IRF7	KCTD20
IRF7	KDELC2
IRF7	KDELR2
IRF7	KIZ
IRF7	KLF2

IRF7	KLF4
IRF7	KLHDC4
IRF7	KMT2E−AS1
IRF7	KMT5A
IRF7	KRT10
IRF7	LAP3
IRF7	LAPTM4A
IRF7	LAPTM5
IRF7	LARP1B
IRF7	LAX1
IRF7	LDB1
IRF7	LGALS3BP
IRF7	LGALS9
IRF7	LINC00649
IRF7	LINC00847
IRF7	LITAF
IRF7	LMAN2
IRF7	LMO2
IRF7	LMO4
IRF7	LPIN1
IRF7	LRBA
IRF7	LRP10
IRF7	LRRC2
IRF7	LSP1
IRF7	LY6E
IRF7	LZTFL1
IRF7	MAGED1
IRF7	MAGED2
IRF7	MAGT1
IRF7	MAN1A1
IRF7	MAN1A2
IRF7	MANEA
IRF7	MANF
IRF7	MAP3K11
IRF7	MAP3K13
IRF7	MAPKAPK2
IRF7	MARCH6
IRF7	MBD1
IRF7	MBD6
IRF7	MBNL2
IRF7	MBTPS1
IRF7	MCEE
IRF7	MCL1
IRF7	MCTP2
IRF7	MED8
IRF7	MEF2C
IRF7	MEF2D
IRF7	METTL7A
IRF7	MFSD1
IRF7	MFSD12

IRF7	MGAT1
IRF7	MGST2
IRF7	MIA3
IRF7	MIXL1
IRF7	MLEC
IRF7	MLKL
IRF7	MOB3C
IRF7	MORF4L2
IRF7	MPG
IRF7	MPND
IRF7	MPV17L2
IRF7	MRPL44
IRF7	MSL3
IRF7	MTDH
IRF7	MTIF3
IRF7	MTO1
IRF7	MUL1
IRF7	MVB12A
IRF7	MVP
IRF7	MX1
IRF7	MX2
IRF7	MYCBP2
IRF7	MYL4
IRF7	MYO1G
IRF7	MZB1
IRF7	N4BP2L2
IRF7	NAGK
IRF7	NAPA
IRF7	NCF1
IRF7	NCK2
IRF7	NCKAP1L
IRF7	NCLN
IRF7	NDUFAF1
IRF7	NDUFB4
IRF7	NEAT1
IRF7	NECAP2
IRF7	NEDD9
IRF7	NGRN
IRF7	NHLRC2
IRF7	NISCH
IRF7	NKTR
IRF7	NMI
IRF7	NOSIP
IRF7	NPC2
IRF7	NPLOC4
IRF7	NR2C1
IRF7	NR2C2
IRF7	NSMCE3
IRF7	NSUN6
IRF7	NT5C3A

IRF7	NT5E
IRF7	NUCB2
IRF7	OAF
IRF7	OAS1
IRF7	OAS2
IRF7	OGFOD2
IRF7	OPA3
IRF7	ORMDL3
IRF7	OST4
IRF7	OTUD1
IRF7	OXA1L
IRF7	P2RX1
IRF7	P2RX4
IRF7	P4HA1
IRF7	P4HTM
IRF7	PAG1
IRF7	PAOX
IRF7	PARP12
IRF7	PARP4
IRF7	PARP9
IRF7	PARVG
IRF7	PCED1B
IRF7	PDE4DIP
IRF7	PDIA5
IRF7	PDK1
IRF7	PDLIM1
IRF7	PDXK
IRF7	PHF11
IRF7	PIAS1
IRF7	PINK1
IRF7	PITPNM1
IRF7	PLD3
IRF7	PLD4
IRF7	PLSCR1
IRF7	PLTP
IRF7	PMEPA1
IRF7	PNISR
IRF7	PNOC
IRF7	PNPLA8
IRF7	PNPT1
IRF7	PNRC1
IRF7	POLR2A
IRF7	POLR3D
IRF7	POMC
IRF7	POU2F1
IRF7	PPM1K
IRF7	PPP1CA
IRF7	PPP3CC
IRF7	PPP6R1
IRF7	PRDX4

IRF7	PRKAR2B
IRF7	PRKCB
IRF7	PRKCSH
IRF7	PRKD2
IRF7	PRPSAP1
IRF7	PRRC1
IRF7	PSAP
IRF7	PSMB10
IRF7	PSMB8-AS1
IRF7	PSMB9
IRF7	PSME1
IRF7	PSME2
IRF7	PTPN4
IRF7	PTPN6
IRF7	PTPRA
IRF7	PTPRN2
IRF7	PURB
IRF7	PYCARD
IRF7	PYURF
IRF7	QKI
IRF7	QPCT
IRF7	RAB1A
IRF7	RAB5C
IRF7	RAB6A
IRF7	RABAC1
IRF7	RAC2
IRF7	RAD9A
IRF7	RALGPS2
IRF7	RALY
IRF7	RAP2B
IRF7	RASGRP2
IRF7	RBBP5
IRF7	RBCK1
IRF7	RBM18
IRF7	RCN1
IRF7	RCSD1
IRF7	RDM1
IRF7	REEP5
IRF7	RELA
IRF7	RGCC
IRF7	RGS1
IRF7	RNASE6
IRF7	RNASEK
IRF7	RNASET2
IRF7	RNF103
IRF7	RNF114
IRF7	RNF213
IRF7	RNH1
IRF7	RNPEPL1
IRF7	RPL10

IRF7	RPL41
IRF7	RPS11
IRF7	RPS27
IRF7	RPS6KA1
IRF7	RRAGC
IRF7	RRBP1
IRF7	RREB1
IRF7	RSAD2
IRF7	RSPRY1
IRF7	RTP4
IRF7	RUBCN
IRF7	RUNX1
IRF7	S1PR4
IRF7	SAMD9
IRF7	SAMD9L
IRF7	SAR1B
IRF7	SASH3
IRF7	SAT1
IRF7	SCAMP2
IRF7	SCAPER
IRF7	SCPEP1
IRF7	SCRN2
IRF7	SDC1
IRF7	SEC23B
IRF7	SEC24A
IRF7	SEC24D
IRF7	SEC31A
IRF7	SEC61A1
IRF7	SEC62
IRF7	SECISBP2L
IRF7	SELL
IRF7	SELPLG
IRF7	SENP6
IRF7	SERP1
IRF7	SFT2D1
IRF7	SGK3
IRF7	SGSM3
IRF7	SH3BGRL
IRF7	SHISA5
IRF7	SIL1
IRF7	SKAP1
IRF7	SLAMF7
IRF7	SLC15A3
IRF7	SLC22A5
IRF7	SLC25A22
IRF7	SLC30A7
IRF7	SLC33A1
IRF7	SLC38A10
IRF7	SLC38A7
IRF7	SLC38A9

IRF7	SLC39A11
IRF7	SLC39A13
IRF7	SLC39A8
IRF7	SLC44A1
IRF7	SLCO5A1
IRF7	SMCHD1
IRF7	SMIM14
IRF7	SMURF1
IRF7	SMYD2
IRF7	SND1
IRF7	SND1-IT1
IRF7	SNX3
IRF7	SOCS3
IRF7	SOWAHD
IRF7	SP100
IRF7	SP110
IRF7	SP3
IRF7	SPAG9
IRF7	SPATS2
IRF7	SPC24
IRF7	SPCS3
IRF7	SPINT2
IRF7	SPOCK2
IRF7	SPOP
IRF7	SRBD1
IRF7	SRP54
IRF7	SRPK2
IRF7	SRPRB
IRF7	SRSF5
IRF7	SRSF8
IRF7	SSR3
IRF7	SSTR2
IRF7	SSU72
IRF7	ST20-AS1
IRF7	STAP1
IRF7	STARD5
IRF7	STAT1
IRF7	STAT2
IRF7	STAT3
IRF7	STK17B
IRF7	STOM
IRF7	STRN4
IRF7	STT3B
IRF7	STX12
IRF7	SUB1
IRF7	SUCLG2
IRF7	SUCO
IRF7	SUDS3
IRF7	SYTL1
IRF7	TAF5L

IRF7	TAP1
IRF7	TAP2
IRF7	TAPBP
IRF7	TAPBPL
IRF7	TAPT1
IRF7	TAX1BP1
IRF7	TBC1D10C
IRF7	TCEAL1
IRF7	TCF4
IRF7	TFG
IRF7	THEMIS2
IRF7	TICAM1
IRF7	TINF2
IRF7	TJP1
IRF7	TLN1
IRF7	TLR7
IRF7	TM9SF2
IRF7	TM9SF4
IRF7	TMBIM6
IRF7	TMCO1
IRF7	TMED10
IRF7	TMED7
IRF7	TMEM134
IRF7	TMEM167A
IRF7	TMEM214
IRF7	TMEM219
IRF7	TMEM30A
IRF7	TMEM33
IRF7	TMEM50A
IRF7	TMEM50B
IRF7	TMEM59
IRF7	TMEM9
IRF7	TMEM99
IRF7	TMSB10
IRF7	TNFRSF14
IRF7	TNFRSF17
IRF7	TNFSF10
IRF7	TOR3A
IRF7	TP53INP1
IRF7	TPD52
IRF7	TPST2
IRF7	TRABD
IRF7	TRAM1
IRF7	TRAM2
IRF7	TRAM2-AS1
IRF7	TRIB1
IRF7	TRIM22
IRF7	TRIM36
IRF7	TRIM56
IRF7	TRIM73

IRF7	TRMT10B
IRF7	TSC22D1
IRF7	TSC22D3
IRF7	TSPAN13
IRF7	TSPAN31
IRF7	TSPAN5
IRF7	TSPYL2
IRF7	TVP23B
IRF7	TXNDC11
IRF7	TXNDC15
IRF7	UAP1
IRF7	UBASH3B
IRF7	UBB
IRF7	UBC
IRF7	UBE2H
IRF7	UBE2L6
IRF7	UBL7
IRF7	UBTD2
IRF7	UBXN4
IRF7	UCP2
IRF7	UNC45A
IRF7	UNC93B1
IRF7	UROS
IRF7	USO1
IRF7	USP18
IRF7	UTRN
IRF7	VAV1
IRF7	VPS37C
IRF7	VPS41
IRF7	WARS
IRF7	WDR45
IRF7	WDTC1
IRF7	WIPF1
IRF7	XAF1
IRF7	XBP1
IRF7	YIF1B
IRF7	YPEL5
IRF7	ZBP1
IRF7	ZBTB43
IRF7	ZBTB7B
IRF7	ZC3H7B
IRF7	ZC3HAV1
IRF7	ZDHHC5
IRF7	ZFAND1
IRF7	ZFAND2B
IRF7	ZFP36
IRF7	ZFP36L1
IRF7	ZFP36L2
IRF7	ZHX3
IRF7	ZNF133

IRF7	ZNF24
IRF7	ZNF3
IRF7	ZNF654
IRF7	ZNF687
IRF7	ZNF780A
IRF7	ZNF821
IRF7	ZNHIT1
IRF7	ZRANB2
IRF8	ABI1
IRF8	ACAP2
IRF8	ACSL5
IRF8	ACTB
IRF8	ACTR3
IRF8	ADGRG5
IRF8	AGK
IRF8	AIF1
IRF8	AKAP12
IRF8	ALAS1
IRF8	ALG10B
IRF8	ALOX5AP
IRF8	ANKRD13A
IRF8	ANKRD44
IRF8	APLP2
IRF8	ARHGAP17
IRF8	ARHGDIB
IRF8	ARHGEF2
IRF8	ARHGEF7
IRF8	ARID4B
IRF8	ARL14EP
IRF8	ARMCX6
IRF8	ARPC1B
IRF8	ARPC2
IRF8	ARPC3
IRF8	ARPC4
IRF8	ARRDC3-AS1
IRF8	ATF1
IRF8	ATF7
IRF8	ATP6V0A2
IRF8	ATXN10
IRF8	B3GLCT
IRF8	BACH2
IRF8	BAIAP2L1
IRF8	BAZ2B
IRF8	BIN3
IRF8	BIRC3
IRF8	BLCAP
IRF8	BTBD10
IRF8	BZW1
IRF8	C8orf44
IRF8	CADPS

IRF8	CAPZA2
IRF8	CARD11
IRF8	CBWD3
IRF8	CBX3
IRF8	CCND2
IRF8	CCNT2
IRF8	CCNY
IRF8	CD180
IRF8	CD22
IRF8	CD40
IRF8	CD72
IRF8	CD79B
IRF8	CD84
IRF8	CDK13
IRF8	CEP85L
IRF8	CHD7
IRF8	CHI3L2
IRF8	CHIC2
IRF8	CHMP1B
IRF8	CIITA
IRF8	CLASRP
IRF8	CLHC1
IRF8	CNDP2
IRF8	CNFN
IRF8	COMMD6
IRF8	CORO1A
IRF8	CORO1B
IRF8	COX14
IRF8	CPNE5
IRF8	CPSF7
IRF8	CREB1
IRF8	CXCR5
IRF8	CYB561A3
IRF8	DAAM1
IRF8	DBNL
IRF8	DCAF12
IRF8	DDA1
IRF8	DEF8
IRF8	DENND1C
IRF8	DENND3
IRF8	DENND4A
IRF8	DHX36
IRF8	DIAPH2
IRF8	DMTF1
IRF8	DNAJB6
IRF8	DNAJC14
IRF8	DOCK11
IRF8	EIF2B2
IRF8	EIF3H
IRF8	ELF1

IRF8	ELMO1
IRF8	ELMSAN1
IRF8	ELOVL5
IRF8	EPN1
IRF8	EPS15
IRF8	EPS8
IRF8	EXOC1
IRF8	EXOC6
IRF8	EZR
IRF8	FAHD1
IRF8	FAM120AOS
IRF8	FAM129A
IRF8	FAM208B
IRF8	FAM81A
IRF8	FBXO11
IRF8	FBXO28
IRF8	FCRL1
IRF8	FEM1B
IRF8	FERMT3
IRF8	FMNL3
IRF8	FNTA
IRF8	FOXP4
IRF8	GABPB1-AS1
IRF8	GATAD2A
IRF8	GBA
IRF8	GCH1
IRF8	GCNT2
IRF8	GFOD1
IRF8	GHRL
IRF8	GIT2
IRF8	GLYCTK
IRF8	GMIP
IRF8	GNA13
IRF8	GNAI3
IRF8	GNB4
IRF8	GPR107
IRF8	GPR18
IRF8	GPSM3
IRF8	GRAMD1C
IRF8	GRAP
IRF8	GRIPAP1
IRF8	GSKIP
IRF8	H6PD
IRF8	HCLS1
IRF8	HELZ
IRF8	HIF1A
IRF8	HLA-DRA
IRF8	HNRNPA2B1
IRF8	HNRNPD
IRF8	HOPX

IRF8	HRK
IRF8	HSPA4
IRF8	IGSF10
IRF8	INIP
IRF8	INPP5B
IRF8	IQCD
IRF8	ITGB2
IRF8	ITPR1
IRF8	ITSN2
IRF8	JAK1
IRF8	JUP
IRF8	KBTBD8
IRF8	KCTD17
IRF8	KCTD5
IRF8	KLF10
IRF8	KLHL5
IRF8	KLHL6
IRF8	KRAS
IRF8	LAPTM5
IRF8	LARP7
IRF8	LCP1
IRF8	LEAP2
IRF8	LEKR1
IRF8	LIMD2
IRF8	LINC00623
IRF8	LINC00877
IRF8	LINC00886
IRF8	LLPH
IRF8	LPP
IRF8	LRMP
IRF8	LRRC8D
IRF8	LSM5
IRF8	LTB
IRF8	LY86
IRF8	LYL1
IRF8	LYN
IRF8	LYRM1
IRF8	LYSMD2
IRF8	MAP3K11
IRF8	MAPRE2
IRF8	MARCKSL1
IRF8	MCRS1
IRF8	MDM4
IRF8	MED12L
IRF8	MEF2C
IRF8	MGAT4A
IRF8	MICAL3
IRF8	MICU2
IRF8	MID1IP1
IRF8	MIR3681HG

IRF8	MKL1
IRF8	MOB1A
IRF8	MOCS3
IRF8	MPP6
IRF8	MTMR12
IRF8	MTMR14
IRF8	MTMR2
IRF8	MTPN
IRF8	MYO1E
IRF8	NCF1
IRF8	NCF4
IRF8	NCOA3
IRF8	NCOA7
IRF8	NDUFAF5
IRF8	NEK6
IRF8	NFATC2
IRF8	NIN
IRF8	NR6A1
IRF8	NSMCE1
IRF8	NUFIP1
IRF8	NUP153
IRF8	OSBPL10
IRF8	OXLD1
IRF8	PABPC1
IRF8	PAN2
IRF8	PARN
IRF8	PDLIM2
IRF8	PEX5
IRF8	PFKM
IRF8	PHC2
IRF8	PIAS1
IRF8	PIK3AP1
IRF8	PIK3C2B
IRF8	PIP4K2A
IRF8	PISD
IRF8	PITPNC1
IRF8	PLA2G6
IRF8	PLEK
IRF8	PLEKHA2
IRF8	PLEKHF2
IRF8	POLD4
IRF8	POLR2J2
IRF8	POLR3E
IRF8	PPM1N
IRF8	PPP1R18
IRF8	PPP1R21
IRF8	PPP4R3B
IRF8	PQLC1
IRF8	PRKAB1
IRF8	PTAFR

IRF8	PTBP3
IRF8	PTDSS1
IRF8	PTK2
IRF8	PTK2B
IRF8	PTPN6
IRF8	PTPRC
IRF8	PTPRS
IRF8	PVT1
IRF8	QSOX2
IRF8	RAB11FIP1
IRF8	RAB35
IRF8	RABGAP1L
IRF8	RASGEF1B
IRF8	RASGRP3
IRF8	REL
IRF8	RFTN1
IRF8	RFX5
IRF8	RGS19
IRF8	RGS3
IRF8	RHBDF2
IRF8	RHOBTB2
IRF8	RHOH
IRF8	RNF144B
IRF8	RNF19B
IRF8	RNF44
IRF8	RPAP3
IRF8	RUFY3
IRF8	S1PR2
IRF8	SCIMP
IRF8	SEC14L1
IRF8	SERP2
IRF8	SH2B2
IRF8	SH2B3
IRF8	SH2D2A
IRF8	SH3KBP1
IRF8	SH3TC1
IRF8	SHROOM1
IRF8	SIAH2
IRF8	SIK2
IRF8	SKAP2
IRF8	SLAMF6
IRF8	SLC12A2
IRF8	SLC16A6
IRF8	SLC2A5
IRF8	SLC9A7
IRF8	SMIM14
IRF8	SMIM20
IRF8	SMUG1
IRF8	SNAP23
IRF8	SNN

IRF8	SNX3
IRF8	SOCS1
IRF8	SP140
IRF8	SPI1
IRF8	SPPL2A
IRF8	SS18
IRF8	STAP1
IRF8	STAT5B
IRF8	STK40
IRF8	STX7
IRF8	SULF2
IRF8	SUMO1
IRF8	SUSD6
IRF8	SYBU
IRF8	SYPL1
IRF8	SYT17
IRF8	TAB1
IRF8	TADA3
IRF8	TAF7
IRF8	TAGAP
IRF8	TANK
IRF8	TBRG1
IRF8	TESPA1
IRF8	TGIF2
IRF8	TIMM10B
IRF8	TIPARP-AS1
IRF8	TMEM156
IRF8	TMEM161B-AS1
IRF8	TMSB4X
IRF8	TOB2
IRF8	TRAF3IP2
IRF8	TRAF5
IRF8	TRAPPC1
IRF8	TRIB2
IRF8	TRIM39
IRF8	TSG101
IRF8	TUBGCP4
IRF8	UBAC2-AS1
IRF8	UCP2
IRF8	UGGT1
IRF8	UGP2
IRF8	USP6NL
IRF8	VASP
IRF8	VEZF1
IRF8	VGLL4
IRF8	VNN2
IRF8	VPS18
IRF8	VTA1
IRF8	WAS
IRF8	WDR66

IRF8	WIPF1
IRF8	YWHAZ
IRF8	ZBTB1
IRF8	ZFAND4
IRF8	ZFAND6
IRF8	ZFYVE26
IRF8	ZNF718
IRF9	AFF2
IRF9	ANKFY1
IRF9	APOBEC3G
IRF9	ARHGAP12
IRF9	ASB1
IRF9	B2M
IRF9	B4GAT1
IRF9	BBIP1
IRF9	BTAF1
IRF9	BTN3A2
IRF9	C17orf75
IRF9	CARD16
IRF9	CCR10
IRF9	CDC25C
IRF9	CEP85L
IRF9	CHST15
IRF9	CLDN14
IRF9	CLDN23
IRF9	COPS2
IRF9	COX18
IRF9	DCAF6
IRF9	EDEM3
IRF9	EIF1AD
IRF9	EIF5A
IRF9	ELOVL1
IRF9	EP300
IRF9	EPB41
IRF9	EPSTI1
IRF9	ESR2
IRF9	EXOC7
IRF9	FAH
IRF9	FAM129C
IRF9	FAM98B
IRF9	FASTKD2
IRF9	FBXO38
IRF9	FGD3
IRF9	FOXO1
IRF9	GABPB1
IRF9	GABPB1−AS1
IRF9	GALNT1
IRF9	GBA
IRF9	GCSH
IRF9	GGCT

IRF9	HIRA
IRF9	HMG20A
IRF9	HOXB2
IRF9	IFI35
IRF9	IFI44L
IRF9	IFI6
IRF9	IFIT1
IRF9	IL15RA
IRF9	IP6K1
IRF9	IRF7
IRF9	IRF9
IRF9	ISG15
IRF9	ITM2B
IRF9	JCHAIN
IRF9	JOSD1
IRF9	KIAA1551
IRF9	KLHDC4
IRF9	KMO
IRF9	KNOP1
IRF9	LIMK2
IRF9	LTBP3
IRF9	LY6E
IRF9	MAN1A1
IRF9	MARCH8
IRF9	MBNL1-AS1
IRF9	MCTP2
IRF9	MED23
IRF9	MIA3
IRF9	MKLN1
IRF9	MRPL35
IRF9	MRPL43
IRF9	MRPS14
IRF9	MYL4
IRF9	NAA15
IRF9	NAAA
IRF9	NFATC2
IRF9	NPPA-AS1
IRF9	NRROS
IRF9	OAS1
IRF9	OTUD5
IRF9	PARP14
IRF9	PBX2
IRF9	PDGFD
IRF9	PHACTR1
IRF9	PLAC8
IRF9	PLEKHH3
IRF9	POLR3B
IRF9	PPP1R12A
IRF9	PPP1R14A
IRF9	PRIM2

IRF9	PRKX
IRF9	PRR7
IRF9	PSMG3
IRF9	PYCARD
IRF9	RAD51AP1
IRF9	RNF146
IRF9	ROCK2
IRF9	RPL26L1
IRF9	RSAD2
IRF9	RSPH14
IRF9	SAPCD2
IRF9	SELL
IRF9	SGPP1
IRF9	SH3BP5
IRF9	SLC39A11
IRF9	SNX12
IRF9	ST3GAL5
IRF9	STAT1
IRF9	STK26
IRF9	STK4
IRF9	TACO1
IRF9	TGIF1
IRF9	TMEM161B-AS1
IRF9	TMEM9
IRF9	TMX4
IRF9	TP53I11
IRF9	TRIM22
IRF9	TRIM36
IRF9	TRIM4
IRF9	TSEN34
IRF9	TTN-AS1
IRF9	UBA1
IRF9	UBE2L6
IRF9	UBE4A
IRF9	UNC93B1
IRF9	USF2
IRF9	USP18
IRF9	VPS13C
IRF9	WIPF2
IRF9	XAF1
IRF9	YWHAG
IRF9	ZDHHC13
IRF9	ZNF141
IRF9	ZNF804A
IRF9	ZW10
JDP2	BNIP3L
JDP2	JDP2
JDP2	MKL1
JDP2	NSMF
JDP2	POGZ

JUN	ABHD13
JUN	ACAP1
JUN	ADAM28
JUN	AGO2
JUN	AGO3
JUN	AIG1
JUN	AKAP9
JUN	ALG13
JUN	AMIGO2
JUN	AMZ2
JUN	ANKRD11
JUN	ANKRD12
JUN	ANKRD28
JUN	ANKRD37
JUN	AP1S2
JUN	AP4S1
JUN	AREG
JUN	ARHGAP15
JUN	ARHGAP17
JUN	ARHGAP27
JUN	ARHGEF3
JUN	ARHGEF6
JUN	ARL1
JUN	ARL17B
JUN	ARRB2
JUN	ATF2
JUN	ATF6
JUN	ATG13
JUN	ATP6V0A1
JUN	ATXN1L
JUN	ATXN3
JUN	B2M
JUN	B4GALT3
JUN	BAZ2B
JUN	BBX
JUN	BCAR3
JUN	BDP1
JUN	BMP7
JUN	BRAF
JUN	BSDC1
JUN	BTG1
JUN	BTG2
JUN	C12orf42
JUN	C14orf93
JUN	C15orf61
JUN	C2orf74
JUN	CA2
JUN	CALCOCO1
JUN	CAST
JUN	CBX4

JUN	CCDC117
JUN	CCDC144A
JUN	CCDC47
JUN	CCND2
JUN	CCNL1
JUN	CCPG1
JUN	CCR6
JUN	CD47
JUN	CD55
JUN	CD63
JUN	CDCA8
JUN	CDK11B
JUN	CDKN1A
JUN	CEBPB
JUN	CEBPG
JUN	CELF2
JUN	CENPE
JUN	CHD6
JUN	CHMP1B
JUN	CHPF
JUN	CHRNB1
JUN	CITED2
JUN	CLK1
JUN	CLK4
JUN	CMTM6
JUN	COL19A1
JUN	COLGALT1
JUN	COMMD1
JUN	CORO1C
JUN	CREBZF
JUN	CRYBB2
JUN	CSRNP1
JUN	CTBP1
JUN	CTR9
JUN	CXCR4
JUN	CYLD
JUN	CYSLTR1
JUN	CYTIP
JUN	DDIT4
JUN	DDX10
JUN	DDX5
JUN	DEDD2
JUN	DENND4A
JUN	DESI1
JUN	DHCR7
JUN	DHRS7B
JUN	DNAJB1
JUN	DNAJB9
JUN	DNAJC13
JUN	DUSP1

JUN	EGR1
JUN	EIF4A2
JUN	ELL
JUN	EMC10
JUN	EMC2
JUN	EMP3
JUN	EP300
JUN	EPC1
JUN	ESCO1
JUN	ESYT1
JUN	ETS1
JUN	ETV2
JUN	FAM177B
JUN	FANCF
JUN	FBXW7
JUN	FKBP2
JUN	FKBPL
JUN	FLCN
JUN	FMNL1
JUN	FMR1
JUN	FNDC3A
JUN	FOS
JUN	FOSB
JUN	FOXP1
JUN	FUBP1
JUN	G3BP2
JUN	GABPB1
JUN	GAN
JUN	GCC1
JUN	GCC2
JUN	GFOD1
JUN	GIGYF2
JUN	GLIPR1
JUN	GMFB
JUN	GMPPB
JUN	GNB1L
JUN	GNG4
JUN	GPKOW
JUN	GPR146
JUN	GRK6
JUN	GSAP
JUN	GSKIP
JUN	GTSF1
JUN	HBP1
JUN	HECTD4
JUN	HERPUD1
JUN	HERPUD2
JUN	HIP1R
JUN	HIST1H2BG
JUN	HIST2H2BE

JUN	HOOK3
JUN	HOTAIRM1
JUN	HOXB7
JUN	HPS5
JUN	HSPA1A
JUN	HSPA2
JUN	HYPK
JUN	ICAM2
JUN	IER2
JUN	IFNGR1
JUN	IFRD1
JUN	IGSF8
JUN	IL15RA
JUN	ING4
JUN	INSR
JUN	ITM2B
JUN	ITM2C
JUN	JUN
JUN	JUNB
JUN	JUND
JUN	KCNMA1
JUN	KCNN4
JUN	KLF2
JUN	KLF6
JUN	KLF7
JUN	KMT2E
JUN	KMT2E-AS1
JUN	LAPTM4A
JUN	LARP1B
JUN	LDLRAD4
JUN	LETMD1
JUN	LILRB3
JUN	LINC00886
JUN	LINC00926
JUN	LMBRD1
JUN	LPIN1
JUN	LSG1
JUN	LTN1
JUN	LYST
JUN	MAFB
JUN	MAGOHB
JUN	MARCH8
JUN	MBD1
JUN	MBP
JUN	MBTPS1
JUN	MCL1
JUN	MEF2C
JUN	MEST
JUN	MIEF1
JUN	MKRN2

JUN	MLEC
JUN	MOSPD3
JUN	MRPL40
JUN	MRPS18B
JUN	MYADM
JUN	N4BP2L1
JUN	NADK
JUN	NAPA-AS1
JUN	NCBP1
JUN	NCK2
JUN	NDEL1
JUN	NDRG1
JUN	NDUFA5
JUN	NDUFS2
JUN	NDUFV3
JUN	NEAT1
JUN	NEK9
JUN	NEMF
JUN	NF1
JUN	NFKB2
JUN	NMD3
JUN	NME4
JUN	NR4A2
JUN	NR4A3
JUN	NRDC
JUN	NRL
JUN	NSUN4
JUN	NUDCD2
JUN	NUDT3
JUN	NXT2
JUN	ODF2L
JUN	P2RX1
JUN	P2RY10
JUN	PARM1
JUN	PCIF1
JUN	PDE4B
JUN	PDK2
JUN	PFKFB3
JUN	PHF1
JUN	PHF19
JUN	PHF20L1
JUN	PHF21A
JUN	PHRF1
JUN	PKIG
JUN	PLD3
JUN	PLEKHO1
JUN	PLOD1
JUN	PLPP5
JUN	PMAIP1
JUN	PNOC

JUN	PNPLA4
JUN	PNRC1
JUN	PPM1B
JUN	PPM1K
JUN	PPP1R15A
JUN	PPP1R37
JUN	PRCC
JUN	PRDX4
JUN	PRKAB2
JUN	PSAT1
JUN	PSMB8-AS1
JUN	PSME3
JUN	PTPRJ
JUN	PWWP2B
JUN	RALGPS2
JUN	RB1
JUN	RCE1
JUN	RELA
JUN	RFK
JUN	RGS1
JUN	RHBDD1
JUN	RHEB
JUN	RHOB
JUN	RND1
JUN	RNF103
JUN	RNF11
JUN	RPL13
JUN	RPL34
JUN	RPLP2
JUN	RPS27
JUN	RPS27A
JUN	RPS29
JUN	RRBP1
JUN	RSPRY1
JUN	S1PR4
JUN	SARAF
JUN	SCFD1
JUN	SCLT1
JUN	SDCBP
JUN	SEC14L1
JUN	SEC22C
JUN	SELL
JUN	SEPT9
JUN	SETBP1
JUN	SGK1
JUN	SH2D3A
JUN	SIAH1
JUN	SLC25A28
JUN	SLC35A5
JUN	SLC35F5

JUN	SLC38A2
JUN	SLC39A11
JUN	SLC7A7
JUN	SMARCA2
JUN	SMCHD1
JUN	SMIM14
JUN	SNX2
JUN	SOX4
JUN	SPATA2
JUN	SPRY1
JUN	SPTY2D1
JUN	SRD5A3
JUN	SRGAP2
JUN	SSR4
JUN	ST6GAL1
JUN	ST8SIA4
JUN	STX5
JUN	SUB1
JUN	SUN1
JUN	SUN2
JUN	SYNGR1
JUN	TAGLN2
JUN	TAX1BP1
JUN	TCAIM
JUN	TES
JUN	TFRC
JUN	TIGAR
JUN	TIPARP
JUN	TMCO3
JUN	TMED4
JUN	TMED7
JUN	TMSB4X
JUN	TMSB4Y
JUN	TNFAIP3
JUN	TNFAIP8
JUN	TNRC6B
JUN	TOLLIP
JUN	TPM4
JUN	TPT1
JUN	TRA2A
JUN	TRIB1
JUN	TROAP
JUN	TRPM7
JUN	TSC22D3
JUN	TSHZ2
JUN	TSPAN31
JUN	TSPYL2
JUN	TTC21A
JUN	TTC3
JUN	TTI1

JUN	TWF1
JUN	U2AF1L4
JUN	UAP1
JUN	UBB
JUN	UBC
JUN	UBE2S
JUN	UBE2V1
JUN	UBN2
JUN	USP51
JUN	UTP23
JUN	VAMP3
JUN	WDR33
JUN	WDR74
JUN	WIPF2
JUN	WIPI1
JUN	WRB
JUN	XBP1
JUN	XPOT
JUN	XXYLT1
JUN	YIPF6
JUN	YPEL2
JUN	YPEL5
JUN	YTHDC1
JUN	ZBTB1
JUN	ZBTB16
JUN	ZBTB17
JUN	ZBTB48
JUN	ZC3H12D
JUN	ZC3H4
JUN	ZDHHC8
JUN	ZFAND2A
JUN	ZFC3H1
JUN	ZFHX3
JUN	ZFP36
JUN	ZFP36L1
JUN	ZFP36L2
JUN	ZNF184
JUN	ZNF274
JUN	ZNF407
JUN	ZNF426
JUN	ZNF598
JUN	ZNF668
JUN	ZNF746
JUN	ZNF790
JUN	ZNHIT2
JUNB	ACAP1
JUNB	ADAM28
JUNB	AHCYL1
JUNB	AHNAK
JUNB	ANKRD11

JUNB	ANKRD12
JUNB	AP2B1
JUNB	ARHGAP24
JUNB	ARHGAP5
JUNB	ARID5A
JUNB	ARID5B
JUNB	ARL4C
JUNB	ARMCX3
JUNB	ATRX
JUNB	BAZ2B
JUNB	BTAF1
JUNB	BTG1
JUNB	BTG2
JUNB	CCNL1
JUNB	CDC5L
JUNB	CDKN2C
JUNB	CELF2
JUNB	CHD2
JUNB	CIC
JUNB	CLASP2
JUNB	CLEC2B
JUNB	CLIP2
JUNB	CNFN
JUNB	COPB2
JUNB	CREBRF
JUNB	CSNK1D
JUNB	CSNK1G2
JUNB	CSNK1G3
JUNB	CTGF
JUNB	CTNNA1
JUNB	CXCL16
JUNB	CYLD
JUNB	CYSTM1
JUNB	CYTIP
JUNB	DUSP1
JUNB	DUSP14
JUNB	DUSP2
JUNB	EEF1D
JUNB	EGR1
JUNB	EGR2
JUNB	EIF1
JUNB	EIF4A2
JUNB	EPHB6
JUNB	ERF
JUNB	FAM3C
JUNB	FAM91A1
JUNB	FGD2
JUNB	FOS
JUNB	FOSB
JUNB	FOXP1

JUNB	GCC1
JUNB	GOLPH3
JUNB	GPBP1
JUNB	GPR183
JUNB	GPRC5C
JUNB	HERPUD1
JUNB	HES1
JUNB	HOMER2
JUNB	HPCAL1
JUNB	HYOU1
JUNB	ID3
JUNB	IER2
JUNB	IMPDH2
JUNB	INPP1
JUNB	IRF2BP2
JUNB	JOSD1
JUNB	JUN
JUNB	JUNB
JUNB	KCNA3
JUNB	KLF6
JUNB	KMT2E
JUNB	KMT2E−AS1
JUNB	LBH
JUNB	LIG4
JUNB	LMBRD1
JUNB	LTN1
JUNB	LUZP1
JUNB	MAP4
JUNB	MEF2C
JUNB	MEF2D
JUNB	MKL1
JUNB	MXI1
JUNB	NDEL1
JUNB	NEAT1
JUNB	NR4A1
JUNB	NR4A2
JUNB	ODF2L
JUNB	ORMDL3
JUNB	PABPC1
JUNB	PDE4B
JUNB	PDXDC1
JUNB	PHACTR1
JUNB	PLEKHO1
JUNB	PNRC1
JUNB	PPP1R15A
JUNB	PRDM2
JUNB	PRDM8
JUNB	PSD3
JUNB	RALGPS2
JUNB	RARA

JUNB	REL
JUNB	RNF141
JUNB	RNFT1
JUNB	RPL13
JUNB	RPL18
JUNB	RPL21
JUNB	RPL23
JUNB	RPL34
JUNB	RPL38
JUNB	RPL6
JUNB	RPL9
JUNB	RPLP2
JUNB	RPS29
JUNB	RPS3A
JUNB	RRBP1
JUNB	RUNX3
JUNB	SARAF
JUNB	SBNO2
JUNB	SCAF11
JUNB	SCYL2
JUNB	SEC16A
JUNB	SFXN3
JUNB	SGK1
JUNB	SGK3
JUNB	SHISA5
JUNB	SIAH1
JUNB	SIAH2
JUNB	SLC10A3
JUNB	SLC7A6
JUNB	SMAD3
JUNB	SMCHD1
JUNB	SOCS3
JUNB	SP110
JUNB	SPATA2L
JUNB	SPOCK2
JUNB	SSR4
JUNB	STAMBPL1
JUNB	STT3A
JUNB	STXBP3
JUNB	TBL1X
JUNB	TMF1
JUNB	TMSB4X
JUNB	TNFRSF12A
JUNB	TNFRSF1B
JUNB	TP53I11
JUNB	TPT1
JUNB	TRAF1
JUNB	TRIB1
JUNB	TRIM73
JUNB	TSC22D3

JUNB	TSHZ2
JUNB	TSPYL2
JUNB	TTC19
JUNB	TTC3
JUNB	TXNDC5
JUNB	UBC
JUNB	USP8
JUNB	VRK2
JUNB	YPEL2
JUNB	YWHAZ
JUNB	ZC3H12A
JUNB	ZC3H6
JUNB	ZEB2
JUNB	ZFP36
JUNB	ZFP36L1
JUNB	ZFP36L2
JUND	ACBD4
JUND	ACER3
JUND	AGA
JUND	AKT2
JUND	ANAPC1
JUND	ARAP2
JUND	ARID4B
JUND	ARRDC3-AS1
JUND	ASCC1
JUND	ASPSCR1
JUND	ATF7IP
JUND	AURKB
JUND	B3GAT3
JUND	BCL11A
JUND	BOLA3-AS1
JUND	BRAF
JUND	BTG1
JUND	C18orf21
JUND	C5orf30
JUND	CACUL1
JUND	CADPS
JUND	CANT1
JUND	CBLL1
JUND	CCDC59
JUND	CCDC61
JUND	CCNE2
JUND	CCNT1
JUND	CDC37L1
JUND	CDYL
JUND	CEBPG
JUND	CEP83
JUND	CERS6
JUND	CHST15
JUND	CNKSR2

JUND	CXCR4
JUND	CYB5R2
JUND	DDX17
JUND	DDX6
JUND	DEDD
JUND	DESI1
JUND	DUSP1
JUND	DUSP2
JUND	DUSP5
JUND	E4F1
JUND	EEF1D
JUND	EFCAB14
JUND	EGR1
JUND	EIF2S3
JUND	EML3
JUND	ERF
JUND	FAM76A
JUND	FANCC
JUND	FBXL3
JUND	FBXO31
JUND	FBXW2
JUND	FDFT1
JUND	FKBP15
JUND	FOS
JUND	FOSB
JUND	FOSL1
JUND	FOXN3
JUND	FZD3
JUND	GADD45B
JUND	GADD45G
JUND	GALK2
JUND	GAS7
JUND	GATAD2B
JUND	GNG7
JUND	GORASP1
JUND	GOT2
JUND	GRK2
JUND	GTF2A1
JUND	GTPBP1
JUND	HCG18
JUND	HIST1H2AG
JUND	HIST1H2AH
JUND	HIST1H2AJ
JUND	HIST1H2AL
JUND	HIST1H4E
JUND	HMGCS1
JUND	HSPA1A
JUND	HSPA2
JUND	IER2
JUND	IER5

JUND	IFRD1
JUND	IGFBP4
JUND	ING1
JUND	IQCG
JUND	IQGAP2
JUND	JMJD1C
JUND	JUN
JUND	JUND
JUND	KLF10
JUND	KLF3
JUND	KLHL22
JUND	KMT2E
JUND	LANCL2
JUND	LARP1B
JUND	LINS1
JUND	LLGL1
JUND	LMO4
JUND	MAFB
JUND	MAGOHB
JUND	MAP1B
JUND	MAPK10
JUND	MARCH3
JUND	MARCH5
JUND	MBD6
JUND	MED12L
JUND	MEF2C
JUND	MEST
JUND	METRNL
JUND	METTL13
JUND	MLX
JUND	MSANTD2
JUND	MUL1
JUND	NAB2
JUND	NDFIP2
JUND	NEK1
JUND	NELFA
JUND	NFATC3
JUND	NFE2L1
JUND	NFKBID
JUND	NKIRAS1
JUND	NR4A1
JUND	NUDCD3
JUND	NUDT9
JUND	NUP98
JUND	NUTM2A-AS1
JUND	ODF2L
JUND	OSTM1
JUND	PAK1
JUND	PAK1IP1
JUND	PAN3

JUND	PARP2
JUND	PARP6
JUND	PCIF1
JUND	PDE7A
JUND	PDE9A
JUND	PFKFB3
JUND	PGS1
JUND	PIM3
JUND	PITPNB
JUND	PLCG2
JUND	PLEKHO1
JUND	PLK2
JUND	PNMA1
JUND	PNPO
JUND	PPP1R10
JUND	PPP1R2
JUND	PPP4R3A
JUND	PRKCSH
JUND	PTP4A1
JUND	QTRT2
JUND	RAD9A
JUND	RBM15B
JUND	REL
JUND	RFX1
JUND	RICTOR
JUND	RUBCN
JUND	SACM1L
JUND	SACS
JUND	SESN1
JUND	SLC20A1
JUND	SLC27A4
JUND	SLC2A3
JUND	SLC35F5
JUND	SLC38A2
JUND	SMG7
JUND	SMU1
JUND	SNX12
JUND	SOCS3
JUND	SPATA2
JUND	SPHK2
JUND	SPOP
JUND	SPTLC1
JUND	SRGAP2
JUND	SRSF5
JUND	ST3GAL5
JUND	STAMBP
JUND	STIL
JUND	STX4
JUND	SUV39H1
JUND	TBC1D9

JUND	TFDP2
JUND	TIMM10B
JUND	TMEM189
JUND	TMEM33
JUND	TNFAIP8L2
JUND	TPRG1L
JUND	TRAF5
JUND	TRIM33
JUND	TSC22D3
JUND	TSPYL2
JUND	TTC27
JUND	UBAP1
JUND	UGCG
JUND	UGGT2
JUND	USF2
JUND	VPS37B
JUND	WDR82
JUND	WIPI1
JUND	WIZ
JUND	WNK1
JUND	WWP2
JUND	ZFAND3
JUND	ZFP36
JUND	ZNF18
JUND	ZNF2
JUND	ZNF426
JUND	ZNF558
JUND	ZNF747
KLF11	ACP5
KLF11	ARHGAP17
KLF11	CHST11
KLF11	FAM160A2
KLF11	HOMER2
KLF11	IRF5
KLF11	MTSS1
KLF11	PGAP2
KLF11	RNASEH1
KLF11	ZCWPW1
KLF12	AGPAT2
KLF12	AHNAK
KLF12	ALDH2
KLF12	AMZ2
KLF12	ARRDC1
KLF12	BACH2
KLF12	BARD1
KLF12	CARD19
KLF12	CDC42SE2
KLF12	DAAM1
KLF12	DTD2
KLF12	ELL

KLF12	FAAP20
KLF12	FAM3C
KLF12	FLNA
KLF12	FOXO3
KLF12	GDPD5
KLF12	HSDL2
KLF12	KIZ
KLF12	KLF12
KLF12	LAPTM5
KLF12	LEMD2
KLF12	LXN
KLF12	MAPRE2
KLF12	MARCKSL1
KLF12	NEIL3
KLF12	NLK
KLF12	NRDE2
KLF12	PAK1
KLF12	PFKP
KLF12	PIDD1
KLF12	PNKP
KLF12	RABEP2
KLF12	RELB
KLF12	SLAIN2
KLF12	SLC4A7
KLF12	SYT17
KLF12	TARDBP
KLF12	TCF25
KLF12	TRAIP
KLF12	ZFAND6
KLF13	ABCB9
KLF13	ABHD14A
KLF13	ABHD8
KLF13	ACADS
KLF13	ACADVL
KLF13	ACAP1
KLF13	ACO2
KLF13	ACOXL
KLF13	ACSS1
KLF13	ADM
KLF13	AEBP1
KLF13	AGA
KLF13	AGPAT2
KLF13	AHCYL2
KLF13	AIFM2
KLF13	AKAP13
KLF13	AKAP8L
KLF13	AKT1S1
KLF13	ALAD
KLF13	ALDH6A1
KLF13	ALDOC

KLF13	ALG14
KLF13	ALG2
KLF13	ALG3
KLF13	ALKBH7
KLF13	ANAPC16
KLF13	ANG
KLF13	ANKHD1
KLF13	ANKRD10
KLF13	ANKRD28
KLF13	ANKS3
KLF13	ANXA2
KLF13	ANXA6
KLF13	AP3D1
KLF13	ARF1
KLF13	ARF5
KLF13	ARFGAP3
KLF13	ARHGAP18
KLF13	ARID3A
KLF13	ARID4A
KLF13	ARID5A
KLF13	ARMCX3
KLF13	ARMCX5
KLF13	ATG4C
KLF13	ATP13A1
KLF13	ATP6AP2
KLF13	ATP6V0A1
KLF13	ATXN2
KLF13	AUNIP
KLF13	B4GALT3
KLF13	B9D1
KLF13	BACH1
KLF13	BAK1
KLF13	BCAP31
KLF13	BCKDHA
KLF13	BCL2L11
KLF13	BCLAF1
KLF13	BEX4
KLF13	BEX5
KLF13	BHLHE41
KLF13	BNIP1
KLF13	BNIP3L
KLF13	BORCS5
KLF13	BORCS8
KLF13	BSDC1
KLF13	BST2
KLF13	BTAF1
KLF13	BTG2
KLF13	C11orf24
KLF13	C15orf39
KLF13	C19orf81

KLF13	C1GALT1C1
KLF13	C21orf2
KLF13	C4orf33
KLF13	CACTIN
KLF13	CADM1
KLF13	CALCOCO1
KLF13	CALU
KLF13	CAMKK2
KLF13	CAPNS1
KLF13	CBWD5
KLF13	CBX8
KLF13	CCDC167
KLF13	CCDC186
KLF13	CCDC61
KLF13	CCL25
KLF13	CCM2
KLF13	CCNL1
KLF13	CD38
KLF13	CD46
KLF13	CD55
KLF13	CD63
KLF13	CD9
KLF13	CDK12
KLF13	CDK17
KLF13	CDK2AP2
KLF13	CDV3
KLF13	CEBPB
KLF13	CERS2
KLF13	CHAC1
KLF13	CHD2
KLF13	CHD6
KLF13	CHID1
KLF13	CHPF
KLF13	CHRNB1
KLF13	CHST12
KLF13	CHST2
KLF13	CLCC1
KLF13	CLIC1
KLF13	CLINT1
KLF13	CLIP4
KLF13	CNOT6L
KLF13	COPB1
KLF13	COPB2
KLF13	COPZ1
KLF13	COX6B1
KLF13	COX7A2L
KLF13	CPEB4
KLF13	CPQ
KLF13	CREB3L2
KLF13	CRK

KLF13	CSGALNACT2
KLF13	CSNK1E
KLF13	CTR9
KLF13	CTSB
KLF13	CTSD
KLF13	CUTA
KLF13	CYB561D2
KLF13	CYTH2
KLF13	CYTH4
KLF13	DAD1
KLF13	DAP
KLF13	DCAF12
KLF13	DDAH2
KLF13	DDB1
KLF13	DDOST
KLF13	DDRGK1
KLF13	DEDD2
KLF13	DERL3
KLF13	DEXI
KLF13	DGAT1
KLF13	DHDDS
KLF13	DHRS1
KLF13	DHRS9
KLF13	DLAT
KLF13	DNAJC1
KLF13	DNAJC12
KLF13	DNAJC3
KLF13	DNAJC4
KLF13	DNASE2
KLF13	DPAGT1
KLF13	DSTN
KLF13	DYNLL2
KLF13	EDEM1
KLF13	EDEM2
KLF13	EDEM3
KLF13	EFCAB14
KLF13	EFHC1
KLF13	EI24
KLF13	EIF2AK4
KLF13	EIF4G1
KLF13	ELK3
KLF13	ELL
KLF13	ELL2
KLF13	EMC2
KLF13	EMC3
KLF13	EML2
KLF13	ENTPD1
KLF13	EPB41
KLF13	ERC1
KLF13	ERGIC2

KLF13	ERGIC3
KLF13	ERN1
KLF13	ERO1B
KLF13	ETFB
KLF13	EYA3
KLF13	FAF1
KLF13	FAM213A
KLF13	FAM3C
KLF13	FAM91A1
KLF13	FANCE
KLF13	FBXL15
KLF13	FHOD1
KLF13	FICD
KLF13	FKBP2
KLF13	FLYWCH2
KLF13	FN3KRP
KLF13	FOXN3
KLF13	FUCA2
KLF13	FUOM
KLF13	FUT8
KLF13	G6PC3
KLF13	G6PD
KLF13	GALM
KLF13	GANAB
KLF13	GGCX
KLF13	GIGYF2
KLF13	GLCCI1
KLF13	GM2A
KLF13	GMPPA
KLF13	GNAI2
KLF13	GNAS
KLF13	GOLGA2
KLF13	GOLGA3
KLF13	GOLGA5
KLF13	GOLGA8B
KLF13	GOLGB1
KLF13	GPHN
KLF13	GPR108
KLF13	GPR155
KLF13	GPR160
KLF13	GPR89B
KLF13	GPSM3
KLF13	GRAMD4
KLF13	GRK6
KLF13	GRN
KLF13	GSTM3
KLF13	GSTM4
KLF13	GUCD1
KLF13	GYG1
KLF13	H1FX

KLF13	HDAC6
KLF13	HERPUD1
KLF13	HEXDC
KLF13	HID1
KLF13	HINT2
KLF13	HIST1H1C
KLF13	HIST1H2BD
KLF13	HOOK2
KLF13	HPCAL1
KLF13	HSBP1
KLF13	HSD17B8
KLF13	HSPA13
KLF13	HYOU1
KLF13	ICAM3
KLF13	IDH1
KLF13	IER3IP1
KLF13	IFI27L2
KLF13	IFT80
KLF13	IGFBP4
KLF13	IGSF8
KLF13	IKBIP
KLF13	IL10RA
KLF13	IL10RB
KLF13	IMP3
KLF13	IMPDH1
KLF13	INPP4A
KLF13	INSIG2
KLF13	IQSEC1
KLF13	IRF1
KLF13	IRF4
KLF13	IRS1
KLF13	ISCU
KLF13	ISOC2
KLF13	ITCH
KLF13	ITFG2
KLF13	ITGB1
KLF13	ITGB2
KLF13	ITM2B
KLF13	ITM2C
KLF13	ITPRIP
KLF13	JUND
KLF13	KCNK6
KLF13	KCNN3
KLF13	KCNN4
KLF13	KDELR2
KLF13	KIAA2013
KLF13	KIF16B
KLF13	KIFAP3
KLF13	KIZ
KLF13	KLF13

KLF13	KLF2
KLF13	KLHL14
KLF13	KLHL36
KLF13	KRT18
KLF13	LAMP2
KLF13	LAP3
KLF13	LAPTM4A
KLF13	LAPTM5
KLF13	LARP1B
KLF13	LDLRAD4
KLF13	LEPROT
KLF13	LGALS1
KLF13	LGMN
KLF13	LINC01013
KLF13	LMAN1
KLF13	LMAN2
KLF13	LMBRD1
KLF13	LMF1
KLF13	LMF2
KLF13	LMO4
KLF13	LMTK3
KLF13	LRP10
KLF13	LRRC2
KLF13	LSM1
KLF13	LSP1
KLF13	LSR
KLF13	LTBP3
KLF13	LTBP4
KLF13	MACF1
KLF13	MAGED2
KLF13	MAN1A1
KLF13	MAP7D1
KLF13	MAPK7
KLF13	MAPKAPK2
KLF13	MARCH2
KLF13	MARCH3
KLF13	MBNL2
KLF13	MBOAT7
KLF13	MBTPS1
KLF13	MCFD2
KLF13	MECR
KLF13	MEF2A
KLF13	MEF2C
KLF13	METRN
KLF13	MFN2
KLF13	MFSD12
KLF13	MGA
KLF13	MGAT1
KLF13	MIAT
KLF13	MIF4GD

KLF13	MIR22HG
KLF13	MIXL1
KLF13	MLEC
KLF13	MORF4L2
KLF13	MOSPD1
KLF13	MRPL23
KLF13	MRPS14
KLF13	MT1X
KLF13	MTIF3
KLF13	MXD4
KLF13	MYCBP2
KLF13	MYL4
KLF13	MYL6
KLF13	N4BP2L1
KLF13	NAPA
KLF13	NCK2
KLF13	NCOR2
KLF13	NDFIP1
KLF13	NDUFA11
KLF13	NEAT1
KLF13	NEDD9
KLF13	NEK8
KLF13	NMRK1
KLF13	NPC2
KLF13	NSUN2
KLF13	NT5C2
KLF13	NT5C3A
KLF13	NT5DC2
KLF13	NTMT1
KLF13	NUAK2
KLF13	NUCB1
KLF13	NUCB2
KLF13	NUDT19
KLF13	NUDT4
KLF13	OGFOD3
KLF13	ORAI2
KLF13	OSTC
KLF13	OTUD1
KLF13	OTUD5
KLF13	OTUD7B
KLF13	OXR1
KLF13	P4HB
KLF13	PAM
KLF13	PAPSS1
KLF13	PARP10
KLF13	PARP4
KLF13	PBXIP1
KLF13	PCED1B
KLF13	PCYOX1
KLF13	PDE4B

KLF13	PDE4DIP
KLF13	PDIK1L
KLF13	PDK1
KLF13	PDK2
KLF13	PELO
KLF13	PEX10
KLF13	PGM3
KLF13	PGRMC2
KLF13	PHF1
KLF13	PIGT
KLF13	PIH1D1
KLF13	PIM2
KLF13	PINK1
KLF13	PJA2
KLF13	PLD3
KLF13	PLOD3
KLF13	PLTP
KLF13	PNOC
KLF13	PNPLA2
KLF13	PNRC1
KLF13	POLRMT
KLF13	PON2
KLF13	PPP1R10
KLF13	PPP2R5B
KLF13	PRDX2
KLF13	PRKCSH
KLF13	PRKD2
KLF13	PRR5
KLF13	PRSS16
KLF13	PSAP
KLF13	PSENEN
KLF13	PSKH1
KLF13	PTPN1
KLF13	PTPN7
KLF13	PTPRN2
KLF13	PYM1
KLF13	QPCT
KLF13	RAB3GAP1
KLF13	RAB5A
KLF13	RABAC1
KLF13	RAP1A
KLF13	RAP2B
KLF13	RASAL3
KLF13	RBM47
KLF13	RCBTB2
KLF13	RELT
KLF13	RERE
KLF13	RFXANK
KLF13	RGS16
KLF13	RGS2

KLF13	RHBDD1
KLF13	RILP
KLF13	RLIM
KLF13	RNASET2
KLF13	RNF185
KLF13	RNF2
KLF13	RNF34
KLF13	RNF4
KLF13	RPL15
KLF13	RPL41
KLF13	RPS19
KLF13	RPS4X
KLF13	RRAS
KLF13	RRBP1
KLF13	RREB1
KLF13	RSRP1
KLF13	RSU1
KLF13	RTN3
KLF13	RWDD1
KLF13	S100A11
KLF13	SACS
KLF13	SAR1A
KLF13	SARAF
KLF13	SASH3
KLF13	SAT1
KLF13	SBDS
KLF13	SBNO2
KLF13	SCAMP2
KLF13	SCFD1
KLF13	SDCCAG8
KLF13	SDF2
KLF13	SEC11C
KLF13	SEC24D
KLF13	SEC31A
KLF13	SEC61B
KLF13	SEC63
KLF13	SEMA4A
KLF13	SERINC3
KLF13	SH2B3
KLF13	SIL1
KLF13	SKAP1
KLF13	SKIL
KLF13	SLC1A5
KLF13	SLC20A2
KLF13	SLC25A28
KLF13	SLC25A39
KLF13	SLC25A42
KLF13	SLC26A6
KLF13	SLC2A8
KLF13	SLC30A7

KLF13	SLC35A3
KLF13	SLC35C1
KLF13	SLC37A1
KLF13	SLC38A10
KLF13	SLC38A2
KLF13	SLC39A11
KLF13	SLC39A8
KLF13	SLC3A2
KLF13	SMAD5
KLF13	SMCHD1
KLF13	SND1
KLF13	SNX29
KLF13	SOCS3
KLF13	SPATS2
KLF13	SPCS3
KLF13	SPINT2
KLF13	SPRED2
KLF13	SPSB1
KLF13	SPTY2D1
KLF13	SRP54
KLF13	SRP68
KLF13	SSR1
KLF13	SSR4
KLF13	ST6GAL1
KLF13	ST6GALNAC4
KLF13	STARD3
KLF13	STAT2
KLF13	STAT3
KLF13	STK17B
KLF13	STOM
KLF13	STRN4
KLF13	STT3A
KLF13	STT3B
KLF13	STX5
KLF13	SUB1
KLF13	SUCO
KLF13	SUDS3
KLF13	SULT1A1
KLF13	SUPT5H
KLF13	SYNGR1
KLF13	TAP1
KLF13	TARSL2
KLF13	TBC1D10A
KLF13	TBC1D10C
KLF13	TBC1D17
KLF13	TBL2
KLF13	TCEAL9
KLF13	TCF25
KLF13	TCF4
KLF13	TCIRG1

KLF13	TDP1
KLF13	TECR
KLF13	TFG
KLF13	TKFC
KLF13	TLK1
KLF13	TM9SF2
KLF13	TMED1
KLF13	TMED3
KLF13	TMED7
KLF13	TMEM115
KLF13	TMEM161A
KLF13	TMEM205
KLF13	TMEM256
KLF13	TMEM263
KLF13	TMEM50A
KLF13	TMEM50B
KLF13	TMEM9
KLF13	TMX4
KLF13	TNFRSF1B
KLF13	TNRC6B
KLF13	TOB1
KLF13	TOLLIP
KLF13	TP53INP1
KLF13	TP73
KLF13	TPM4
KLF13	TPST2
KLF13	TRABD
KLF13	TRADD
KLF13	TRAK1
KLF13	TRAM1
KLF13	TRAM2
KLF13	TRAPPC3
KLF13	TRAPPC6A
KLF13	TRAPPC8
KLF13	TRIP11
KLF13	TSC22D3
KLF13	TSC22D4
KLF13	TSHZ2
KLF13	TSPO
KLF13	TSPYL2
KLF13	TTC7A
KLF13	TXNDC5
KLF13	U2AF1L4
KLF13	UAP1
KLF13	UBALD1
KLF13	UBALD2
KLF13	UBE2H
KLF13	UBE2Q1
KLF13	UBFD1
KLF13	UBL5

KLF13	UBN2
KLF13	UNC13D
KLF13	UNC93B1
KLF13	UNKL
KLF13	UPF1
KLF13	UQCC2
KLF13	USO1
KLF13	USP3
KLF13	USP36
KLF13	VAMP2
KLF13	VCP
KLF13	VDR
KLF13	VKORC1
KLF13	VOPP1
KLF13	VPS25
KLF13	WBP1L
KLF13	WIPI1
KLF13	XIAP
KLF13	XIST
KLF13	XKR6
KLF13	XPOT
KLF13	YIF1B
KLF13	YPEL5
KLF13	ZAP70
KLF13	ZBTB8A
KLF13	ZDHHC24
KLF13	ZFAND1
KLF13	ZFAND5
KLF13	ZFAT
KLF13	ZFPL1
KLF13	ZHX1−C8orf76
KLF13	ZNF143
KLF13	ZNF174
KLF13	ZNF362
KLF13	ZNF436
KLF13	ZNF526
KLF13	ZNF706
KLF13	ZNF720
KLF13	ZNF791
KLF13	ZNHIT1
KLF2	AAK1
KLF2	ABAT
KLF2	ABCB9
KLF2	ABHD17A
KLF2	ABTB1
KLF2	ACAP1
KLF2	ACBD3
KLF2	ACP5
KLF2	ACSS1
KLF2	ADAP1

KLF2	ADD1
KLF2	ADD3
KLF2	AES
KLF2	AHCYL1
KLF2	AHNAK
KLF2	AIDA
KLF2	AKNA
KLF2	AKT3
KLF2	ALDH16A1
KLF2	ANKRD11
KLF2	ANKRD27
KLF2	ANXA2
KLF2	ANXA4
KLF2	ANXA6
KLF2	AP1B1
KLF2	AP1M1
KLF2	AP1S2
KLF2	AP2A1
KLF2	ARAP2
KLF2	ARGLU1
KLF2	ARHGAP15
KLF2	ARHGAP18
KLF2	ARHGAP27
KLF2	ARHGAP30
KLF2	ARHGAP5
KLF2	ARHGAP9
KLF2	ARHGDIB
KLF2	ARHGEF1
KLF2	ARHGEF2
KLF2	ARID3A
KLF2	ARID5A
KLF2	ARID5B
KLF2	ARL4C
KLF2	ARL4D
KLF2	ARL6IP5
KLF2	ARRB2
KLF2	ASCL2
KLF2	ATP10D
KLF2	ATP11C
KLF2	ATP13A3
KLF2	BAK1
KLF2	BBX
KLF2	BCL11A
KLF2	BCL9L
KLF2	BIN1
KLF2	BLCAP
KLF2	BRD2
KLF2	BRD3
KLF2	BSDC1
KLF2	BTG1

KLF2	BTK
KLF2	C11orf24
KLF2	C12orf66
KLF2	C16orf74
KLF2	C4orf33
KLF2	C5orf34
KLF2	CALCOCO1
KLF2	CALCOCO2
KLF2	CALHM2
KLF2	CALM1
KLF2	CAPG
KLF2	CAPN1
KLF2	CAPN12
KLF2	CAPNS1
KLF2	CARD19
KLF2	CAST
KLF2	CAT
KLF2	CBR3
KLF2	CCNA1
KLF2	CCSER2
KLF2	CD44
KLF2	CD47
KLF2	CD53
KLF2	CD55
KLF2	CDC14B
KLF2	CDC42EP3
KLF2	CDC42SE1
KLF2	CDKN2A
KLF2	CDKN2D
KLF2	CELF2
KLF2	CEP44
KLF2	CHD7
KLF2	CHST14
KLF2	CLIC1
KLF2	CNN2
KLF2	CORO1C
KLF2	COTL1
KLF2	CPNE5
KLF2	CRIP1
KLF2	CRYZL1
KLF2	CSRP1
KLF2	CTNND1
KLF2	CTR9
KLF2	CTSB
KLF2	CTSZ
KLF2	CXCR3
KLF2	CXCR4
KLF2	CXXC5
KLF2	CYTH4
KLF2	CYTIP

KLF2	DBP
KLF2	DDAH2
KLF2	DDX5
KLF2	DENND3
KLF2	DHRS9
KLF2	DIAPH2
KLF2	DLST
KLF2	DMD
KLF2	DOK7
KLF2	DPEP2
KLF2	DPY19L3
KLF2	DRAM2
KLF2	DUSP1
KLF2	DYM
KLF2	ECEL1
KLF2	EEA1
KLF2	EEF1A1
KLF2	EEF1D
KLF2	EEF1E1
KLF2	EFCAB2
KLF2	EIF5A
KLF2	EML4
KLF2	EMP3
KLF2	EP300
KLF2	EPB41
KLF2	EPHB6
KLF2	ERP29
KLF2	ESYT1
KLF2	ETFA
KLF2	ETS1
KLF2	EXT1
KLF2	FAM117B
KLF2	FAM177B
KLF2	FAM43A
KLF2	FAM53B
KLF2	FAM69B
KLF2	FANCB
KLF2	FBRSL1
KLF2	FBXL3
KLF2	FBXO32
KLF2	FCGR2B
KLF2	FCMR
KLF2	FGD3
KLF2	FGR
KLF2	FHOD1
KLF2	FIG4
KLF2	FLI1
KLF2	FLNA
KLF2	FMNL1
KLF2	FOXP1

KLF2	FTH1
KLF2	FXYD5
KLF2	GABARAPL2
KLF2	GALNT2
KLF2	GEMIN6
KLF2	GMFG
KLF2	GMIP
KLF2	GNAI2
KLF2	GOLGA7
KLF2	GPR108
KLF2	GPR146
KLF2	GPSM3
KLF2	GRAP2
KLF2	GRK6
KLF2	GSDMD
KLF2	GSN
KLF2	GUCD1
KLF2	HEBP1
KLF2	HHEX
KLF2	HIP1R
KLF2	HIPK3
KLF2	HOXB4
KLF2	HOXB7
KLF2	HPCAL1
KLF2	HSD17B11
KLF2	HVCN1
KLF2	ID3
KLF2	IDH1
KLF2	IER2
KLF2	IFT57
KLF2	IGFBP4
KLF2	IGSF8
KLF2	IL10RA
KLF2	IL16
KLF2	ING2
KLF2	IQGAP1
KLF2	IQSEC1
KLF2	IRF1
KLF2	IRF2BP1
KLF2	IRF2BP2
KLF2	IRF9
KLF2	ITCH
KLF2	ITGB1
KLF2	ITGB7
KLF2	ITM2B
KLF2	ITPR1
KLF2	ITPR3
KLF2	IVNS1ABP
KLF2	JAK1
KLF2	JUN

KLF2	JUNB
KLF2	KCNMA1
KLF2	KCNN4
KLF2	KDM2A
KLF2	KDM7A
KLF2	KIAA1551
KLF2	KLF13
KLF2	KLF2
KLF2	KLF3
KLF2	KLF6
KLF2	KLHL14
KLF2	KMT2E
KLF2	KRI1
KLF2	KYNU
KLF2	LACTB
KLF2	LAPTM4A
KLF2	LAPTM5
KLF2	LBH
KLF2	LCP1
KLF2	LGALS1
KLF2	LGALS3
KLF2	LGALS9
KLF2	LIMK2
KLF2	LINC00926
KLF2	LINC01013
KLF2	LINC01588
KLF2	LITAF
KLF2	LMBRD1
KLF2	LMNB2
KLF2	LRCH4
KLF2	LRRC28
KLF2	LSP1
KLF2	LST1
KLF2	LTA4H
KLF2	LTB
KLF2	LY6E
KLF2	MAP7D1
KLF2	MAPK7
KLF2	MARCH3
KLF2	MARCH9
KLF2	MAX
KLF2	MBP
KLF2	MEF2C
KLF2	MELK
KLF2	MET
KLF2	MIEF1
KLF2	MORC3
KLF2	MOSPD1
KLF2	MSANTD4
KLF2	MSN

KLF2	MTFR2
KLF2	MTPN
KLF2	MXI1
KLF2	MYH9
KLF2	MYL12A
KLF2	MYO1F
KLF2	MYO1G
KLF2	MYZAP
KLF2	N4BP3
KLF2	NCAPH
KLF2	NCR3
KLF2	NEAT1
KLF2	NPC2
KLF2	NUMB
KLF2	NUTF2
KLF2	NXT2
KLF2	OPN3
KLF2	OPTN
KLF2	ORAI2
KLF2	ORAOV1
KLF2	OSBPL10
KLF2	OSBPL10-AS1
KLF2	OXR1
KLF2	PACSIN2
KLF2	PAICS
KLF2	PARP10
KLF2	PATL2
KLF2	PBXIP1
KLF2	PCBP4
KLF2	PDLIM1
KLF2	PFN1
KLF2	PGM1
KLF2	PGM2L1
KLF2	PHF19
KLF2	PHF21A
KLF2	PITPNA
KLF2	PKM
KLF2	PKMYT1
KLF2	PLAC8
KLF2	PLEC
KLF2	PLEKHA1
KLF2	PLP2
KLF2	PNISR
KLF2	PNKD
KLF2	PNRC1
KLF2	PPA2
KLF2	PPM1K
KLF2	PPM1M
KLF2	PPP1R14A
KLF2	PPP1R18

KLF2	PPP6R3
KLF2	PREX1
KLF2	PRKCB
KLF2	PRMT2
KLF2	PRR13
KLF2	PRR5
KLF2	PSAP
KLF2	PSMB10
KLF2	PSTPIP1
KLF2	PTPN1
KLF2	PTPN6
KLF2	PTPRC
KLF2	PYCARD
KLF2	R3HDM4
KLF2	RAB29
KLF2	RAB2B
KLF2	RAB33A
KLF2	RAB37
KLF2	RAB5B
KLF2	RALB
KLF2	RALGPS2
KLF2	RAP2B
KLF2	RASA3
KLF2	RASGRP1
KLF2	RASGRP2
KLF2	RASSF5
KLF2	RBM12
KLF2	RCSD1
KLF2	RFTN1
KLF2	RGS14
KLF2	RGS3
KLF2	RHBDF2
KLF2	RHOC
KLF2	RHOQ
KLF2	RIT1
KLF2	RNASET2
KLF2	RNF141
KLF2	RNH1
KLF2	RPL29
KLF2	RPL32
KLF2	RPLP2
KLF2	RPS12
KLF2	RPS21
KLF2	RSRP1
KLF2	RTKN
KLF2	RTN4
KLF2	RYBP
KLF2	S100A10
KLF2	S100A11
KLF2	S100A4

KLF2	S1PR4
KLF2	SARAF
KLF2	SATB1
KLF2	SEC22C
KLF2	SEMA4B
KLF2	SESTD1
KLF2	SETD3
KLF2	SH3BGRL3
KLF2	SH3PXD2A
KLF2	SIAH1
KLF2	SIDT2
KLF2	SIGIRR
KLF2	SIGMAR1
KLF2	SIMC1
KLF2	SIPA1
KLF2	SIPA1L1
KLF2	SLC12A9
KLF2	SLC25A12
KLF2	SLC25A28
KLF2	SLC25A29
KLF2	SLC2A6
KLF2	SLC38A2
KLF2	SLC4A7
KLF2	SLC7A7
KLF2	SLC9A3R1
KLF2	SMIM14
KLF2	SMIM20
KLF2	SNAI3
KLF2	SNRNP48
KLF2	SNX10
KLF2	SNX2
KLF2	SNX22
KLF2	SNX3
KLF2	SOCS7
KLF2	SOWAHD
KLF2	SPACA9
KLF2	SPIB
KLF2	SPINT1
KLF2	SPN
KLF2	SPOCK2
KLF2	SPPL2B
KLF2	SRGAP2B
KLF2	SRPK2
KLF2	ST3GAL5
KLF2	STAMBPL1
KLF2	STK26
KLF2	STRN
KLF2	STX7
KLF2	SUB1
KLF2	SULT1A1

KLF2	SUN2
KLF2	SYT11
KLF2	TAGLN
KLF2	TAGLN2
KLF2	TARSL2
KLF2	TBC1D10C
KLF2	TBCC
KLF2	TBXA2R
KLF2	TCF4
KLF2	TCP11L2
KLF2	TGFB1
KLF2	TMBIM1
KLF2	TMEM154
KLF2	TMEM156
KLF2	TMEM218
KLF2	TMEM223
KLF2	TMEM256
KLF2	TMEM41B
KLF2	TMSB10
KLF2	TMSB4X
KLF2	TMX4
KLF2	TNFAIP8L2
KLF2	TNFRSF13C
KLF2	TNFRSF1B
KLF2	TNFSF10
KLF2	TNFSF12
KLF2	TNRC6B
KLF2	TP53I11
KLF2	TRABD
KLF2	TRAF3IP3
KLF2	TRAK2
KLF2	TRIB2
KLF2	TRIB3
KLF2	TRIM39
KLF2	TRIM41
KLF2	TRIM8
KLF2	TRPV2
KLF2	TSC22D3
KLF2	TSC22D4
KLF2	TSPO
KLF2	TST
KLF2	TTC12
KLF2	TUBA1A
KLF2	TXNIP
KLF2	U2AF1L4
KLF2	UCKL1
KLF2	UCP2
KLF2	USP8
KLF2	UTRN
KLF2	VAMP2

KLF2	VAMP5
KLF2	VIM
KLF2	WASF2
KLF2	WDFY2
KLF2	WDFY4
KLF2	XIST
KLF2	YPEL5
KLF2	YWHAZ
KLF2	ZBTB20
KLF2	ZBTB7A
KLF2	ZDHHC19
KLF2	ZDHHC21
KLF2	ZEB2
KLF2	ZFP36
KLF2	ZFP36L2
KLF2	ZNF277
KLF2	ZNF296
KLF2	ZNF7
KLF2	ZNF766
KLF2	ZNF816-ZNF321P
KLF3	ABTB1
KLF3	ARL4A
KLF3	ASCC1
KLF3	C12orf42
KLF3	C17orf67
KLF3	C9orf72
KLF3	CENPC
KLF3	CSPP1
KLF3	CTDSP2
KLF3	CXCR4
KLF3	DDHD2
KLF3	DYRK2
KLF3	EPB41
KLF3	FAAP100
KLF3	ID3
KLF3	IGFBP4
KLF3	ING2
KLF3	IRF2
KLF3	JUNB
KLF3	KLF2
KLF3	KLF3
KLF3	KLF7
KLF3	LBH
KLF3	MBP
KLF3	MLLT10
KLF3	PIAS1
KLF3	PLAC8
KLF3	PLEKHA1
KLF3	PPP1R14A
KLF3	PRKCB

KLF3	RAF1
KLF3	RFX2
KLF3	SARAF
KLF3	SIAH1
KLF3	SIRPA
KLF3	STRN
KLF3	TAF3
KLF3	TARSL2
KLF3	TMEM41A
KLF3	TMSB10
KLF3	TSPAN13
KLF3	TUBGCP2
KLF3	VPS13C
KLF3	ZDHHC13
KLF3	ZEB1
KLF3	ZFP36L2
KLF4	BAIAP2L1
KLF4	BCL10
KLF4	BRI3BP
KLF4	CDK17
KLF4	DNPEP
KLF4	DUSP1
KLF4	ERO1A
KLF4	GPR89A
KLF4	GPR89B
KLF4	HOOK2
KLF4	KLF4
KLF4	MTMR4
KLF4	PRDM1
KLF4	RAB9A
KLF4	SMYD2
KLF4	VDR
KLF6	ABTB2
KLF6	ACAP1
KLF6	ACP5
KLF6	ACTL10
KLF6	ADD1
KLF6	ADGRE5
KLF6	AGAP6
KLF6	AGPAT2
KLF6	AHNAK
KLF6	AIDA
KLF6	AKAP12
KLF6	ANKRD12
KLF6	ANTXR2
KLF6	ANXA2
KLF6	ANXA4
KLF6	AP1G1
KLF6	AP5S1
KLF6	ARF6

KLF6	ARHGAP33
KLF6	ARID2
KLF6	ARID5A
KLF6	ARID5B
KLF6	ARL4A
KLF6	ARL6IP5
KLF6	ARRDC2
KLF6	ASB6
KLF6	ATXN1
KLF6	BCAS1
KLF6	C12orf66
KLF6	CAPG
KLF6	CAPN2
KLF6	CARD19
KLF6	CAST
KLF6	CBX6
KLF6	CBX7
KLF6	CCR7
KLF6	CCSER2
KLF6	CD44
KLF6	CD47
KLF6	CD63
KLF6	CELF2
KLF6	CLIC1
KLF6	CLIP1
KLF6	CLK4
KLF6	CLMN
KLF6	CREBRF
KLF6	CXorf40B
KLF6	CYLD
KLF6	CYSTM1
KLF6	CYTH1
KLF6	CYTH2
KLF6	CYTIP
KLF6	DDAH2
KLF6	DGAT1
KLF6	DPF3
KLF6	DUSP1
KLF6	DYNLT3
KLF6	EEF1A1
KLF6	EMP3
KLF6	EPHB6
KLF6	ESYT1
KLF6	EVI2A
KLF6	FBXL3
KLF6	FGD2
KLF6	FGR
KLF6	FLNA
KLF6	FMNL1
KLF6	FMNL2

KLF6	FOSB
KLF6	FOSL1
KLF6	FSD1L
KLF6	GPRC5C
KLF6	HERC1
KLF6	HIPK3
KLF6	HYAL2
KLF6	IDS
KLF6	IFNGR1
KLF6	IL15
KLF6	IL2RG
KLF6	IL6
KLF6	JUN
KLF6	KDELR3
KLF6	KDM6A
KLF6	KIAA1551
KLF6	KLF6
KLF6	KLF7
KLF6	KLHL26
KLF6	KLK1
KLF6	KYNU
KLF6	LACTB
KLF6	LAMB1
KLF6	LDLRAD4
KLF6	LGALS3
KLF6	LINC00649
KLF6	LMNA
KLF6	LPAR5
KLF6	LRP8
KLF6	LRRC23
KLF6	LRRFIP1
KLF6	MACROD2
KLF6	MAGED2
KLF6	MAP1B
KLF6	MAP4
KLF6	MBP
KLF6	MFSD6
KLF6	MGST3
KLF6	MIATNB
KLF6	MTMR4
KLF6	MTRNR2L1
KLF6	MVP
KLF6	MYADM
KLF6	MYH9
KLF6	MYO1F
KLF6	NPC1
KLF6	NQO2
KLF6	ODF2L
KLF6	OPTN
KLF6	PCK2

KLF6	PHF1
KLF6	PKIG
KLF6	PLEC
KLF6	PLP2
KLF6	PNRC1
KLF6	PPP1R15A
KLF6	PPP3CA
KLF6	PRKAG2
KLF6	PTMS
KLF6	PTPN12
KLF6	PWWP2B
KLF6	RAB13
KLF6	RALB
KLF6	RALGPS2
KLF6	RARA
KLF6	RCAN3
KLF6	RDX
KLF6	REEP4
KLF6	RHOC
KLF6	RHOF
KLF6	RPL34
KLF6	RTN4
KLF6	RUNX3
KLF6	S100A11
KLF6	S100A2
KLF6	S100A4
KLF6	SESN3
KLF6	SESTD1
KLF6	SETX
KLF6	SH3BGRL3
KLF6	SIGIRR
KLF6	SLC25A42
KLF6	SLC2A3
KLF6	SLC2A6
KLF6	SLC39A13
KLF6	SLC4A7
KLF6	SLC9A3R2
KLF6	SMAD3
KLF6	SNX2
KLF6	SPHK1
KLF6	SPTBN1
KLF6	SRPK2
KLF6	ST6GALNAC3
KLF6	STK38
KLF6	SUN2
KLF6	TAGLN2
KLF6	TBCE
KLF6	TCAF1
KLF6	TIMP1
KLF6	TIPARP

KLF6	TMBIM1
KLF6	TNIP2
KLF6	TNRC6B
KLF6	TOX2
KLF6	TRAK1
KLF6	TRIQK
KLF6	TSC22D3
KLF6	TSPAN17
KLF6	TUBA1A
KLF6	UBE4B
KLF6	ULK4
KLF6	UNC119
KLF6	VCL
KLF6	VIM
KLF6	VRK2
KLF6	WASF1
KLF6	WSB1
KLF6	ZBED4
KLF6	ZBTB7A
KLF6	ZEB2
KLF6	ZFP36
KLF6	ZNF267
KLF6	ZNF524
KLF6	ZNF703
KLF6	ZSWIM6
KLF6	ZXDB
KLF7	ACP5
KLF7	AHNAK
KLF7	ATG101
KLF7	BCL3
KLF7	C16orf74
KLF7	CAST
KLF7	CCND2
KLF7	CEP164
KLF7	CERS5
KLF7	CHD2
KLF7	DYRK1A
KLF7	EMP3
KLF7	FBRSL1
KLF7	GNG4
KLF7	GPATCH2L
KLF7	IDH1
KLF7	IL6
KLF7	ITCH
KLF7	JUNB
KLF7	KANSL1
KLF7	KLF3
KLF7	KLF7
KLF7	KLF8
KLF7	LTBP1

KLF7	MEF2C
KLF7	METTL2A
KLF7	MICU1
KLF7	MRRF
KLF7	MTSS1
KLF7	NTAN1
KLF7	PURB
KLF7	RAB5B
KLF7	RALGAPA1
KLF7	RIMKLB
KLF7	SH3BP2
KLF7	SIN3A
KLF7	SLC44A2
KLF7	SPSB1
KLF7	SUN2
KLF7	TBCE
KLF7	TGIF1
KLF7	TMEM256
KLF7	TP53BP2
KLF8	ABTB1
KLF8	BCKDHB
KLF8	C15orf62
KLF8	CENPC
KLF8	CSNK1G1
KLF8	EGLN2
KLF8	GOLGA7
KLF8	GRAP2
KLF8	IDH1
KLF8	IGFBP4
KLF8	ITGB7
KLF8	KLF7
KLF8	KLF8
KLF8	MSC
KLF8	OSBP
KLF8	PNPLA8
KLF8	REXO2
KLF8	RIC1
KLF8	RNASET2
KLF8	RPS6KA2
KLF8	SLC15A3
KLF8	SLC16A3
KLF8	SLC2A3
KLF8	SRGAP2C
KLF8	SRPK2
KLF8	TASP1
KLF8	TXNIP
KLF8	ZEB2
MAFB	AFF4
MAFB	AIG1
MAFB	ANAPC10

MAFB	BCL2L11
MAFB	BRD2
MAFB	C12orf42
MAFB	C1GALT1
MAFB	C2orf49
MAFB	CASP10
MAFB	CDC34
MAFB	CLK4
MAFB	CTNNA1
MAFB	DENND1C
MAFB	ESYT2
MAFB	FAM53C
MAFB	HIST1H4E
MAFB	HSPA2
MAFB	IFRD1
MAFB	IL12RB1
MAFB	JMY
MAFB	JUN
MAFB	JUND
MAFB	LMO4
MAFB	MAFB
MAFB	NFX1
MAFB	NUP153
MAFB	NUP214
MAFB	PABPC1
MAFB	PAK1
MAFB	PHC3
MAFB	PRKACB
MAFB	RAB30
MAFB	RAB40B
MAFB	RGS2
MAFB	RPS29
MAFB	SGK1
MAFB	TPM1
MAFB	TSPYL2
MAFB	UBE4A
MAFB	UBN2
MAFB	ZBTB21
MAFB	ZBTB8A
MAFB	ZDHHC8
MAFB	ZNF507
MAFB	ZNF91
MAFF	GALM
MAFF	HPCAL1
MAFF	LINC00623
MAFF	MAFF
MAFF	MARCH3
MAFF	MICAL3
MAFF	RUFY3
MAFF	SMG7

MAFF	TRIM36
MAFF	VPS11
MAFG	EEPD1
MAFG	FOXN2
MAFG	MAFG
MAFG	MAST2
MAFG	RHBDD3
MAFG	RNF19A
MAFG	RREB1
MAFG	SGK3
MAFG	SKAP1
MAFG	SNX11
MAFG	SQSTM1
MAFG	TRAF1
MAFG	USP44
MAFG	ZBTB20
MAFK	MYC
MAFK	RPS6KB1
MAFK	SLCO4A1
MAFK	SPRY1
MAFK	ZDHHC5
MAX	AACS
MAX	ABCF3
MAX	ABHD5
MAX	ACBD4
MAX	ACO1
MAX	ACTR1B
MAX	ACTR6
MAX	ADCK5
MAX	ADD1
MAX	ADK
MAX	ADSS
MAX	AHI1
MAX	AIDA
MAX	AK9
MAX	AKAP9
MAX	ALG3
MAX	ALG8
MAX	ALKBH1
MAX	ALKBH5
MAX	ANKRD10
MAX	ANXA2
MAX	AP2A1
MAX	AP4E1
MAX	APH1B
MAX	ARAP2
MAX	AREG
MAX	ARFGAP1
MAX	ARFIP1
MAX	ARHGAP25

MAX	ARHGDIB
MAX	ARHGEF1
MAX	ARHGEF12
MAX	ARHGEF2
MAX	ARID4B
MAX	ARMCX6
MAX	ARPC5
MAX	ARSK
MAX	ATAD3A
MAX	ATAD3B
MAX	ATG12
MAX	ATG13
MAX	ATG5
MAX	ATXN1
MAX	ATXN7L3B
MAX	B2M
MAX	BBIP1
MAX	BBX
MAX	BCAP29
MAX	BCAP31
MAX	BCL2L11
MAX	BEX4
MAX	BICD2
MAX	BLOC1S2
MAX	BNIP2
MAX	BORCS7
MAX	BRD2
MAX	BRD9
MAX	BROX
MAX	BTBD7
MAX	BTF3
MAX	BTK
MAX	C12orf10
MAX	C16orf74
MAX	C19orf70
MAX	C1orf174
MAX	C2orf42
MAX	CAMK1D
MAX	CAP1
MAX	CAPZA2
MAX	CBFB
MAX	CCDC86
MAX	CCNA1
MAX	CCNL2
MAX	CCT4
MAX	CCT7
MAX	CD44
MAX	CD47
MAX	CD53
MAX	CD59

MAX	CDC16
MAX	CDC23
MAX	CDC27
MAX	CDC42EP3
MAX	CDC73
MAX	CDIP1
MAX	CDK5RAP3
MAX	CELF1
MAX	CELF2
MAX	CENPL
MAX	CFLAR
MAX	CHCHD5
MAX	CHD1
MAX	CHFR
MAX	CHMP3
MAX	CHMP4A
MAX	CIB1
MAX	CIB2
MAX	CIR1
MAX	CLDN14
MAX	CLDN23
MAX	CLEC16A
MAX	CLK1
MAX	CLNS1A
MAX	CNDP2
MAX	CNKSR1
MAX	CNOT4
MAX	CNOT7
MAX	CNPPD1
MAX	CNPY3
MAX	COA6
MAX	COG4
MAX	COG8
MAX	COMMD6
MAX	COMMD9
MAX	COPA
MAX	CORO1C
MAX	COX20
MAX	COX4I1
MAX	COX7A2L
MAX	COX7C
MAX	CREB1
MAX	CREB3L2
MAX	CTDNEP1
MAX	CTDSP2
MAX	CTU1
MAX	CXXC5
MAX	CYB5A
MAX	DALRD3
MAX	DCAF16

MAX	DDX5
MAX	DDX50
MAX	DHCR7
MAX	DHRS7B
MAX	DMTF1
MAX	DNAJC11
MAX	DNAJC7
MAX	DNMBP
MAX	DPM1
MAX	DPP8
MAX	DUSP1
MAX	DUSP6
MAX	DVL2
MAX	DYNC1I2
MAX	EAPP
MAX	EBAG9
MAX	EEF1A1
MAX	EEF1B2
MAX	EEF1D
MAX	EFCAB7
MAX	EGR2
MAX	EIF1AX
MAX	EIF3E
MAX	EIF3H
MAX	EIF4H
MAX	ELK4
MAX	ELMO1
MAX	ELP3
MAX	EMP3
MAX	ERLIN1
MAX	ETF1
MAX	ETS2
MAX	EVC2
MAX	EXD2
MAX	EXOC2
MAX	FADS3
MAX	FAM122A
MAX	FAM174A
MAX	FAM192A
MAX	FAM193B
MAX	FASTKD5
MAX	FAU
MAX	FBXL17
MAX	FBXO22
MAX	FBXO3
MAX	FBXO7
MAX	FBXW2
MAX	FBXW5
MAX	FCGR2B
MAX	FGGY

MAX	FKBP4
MAX	FNIP1
MAX	FOSL1
MAX	FUCA1
MAX	FXR2
MAX	GANAB
MAX	GATAD2B
MAX	GBA2
MAX	GBF1
MAX	GDAP2
MAX	GGA1
MAX	GGT7
MAX	GLIPR1
MAX	GLOD4
MAX	GMPR
MAX	GNB1L
MAX	GNGT2
MAX	GNL2
MAX	GOLGA7
MAX	GOSR1
MAX	GPATCH11
MAX	GPBP1
MAX	GPN2
MAX	GPR180
MAX	GPSM2
MAX	GPSM3
MAX	GPX7
MAX	GRAMD1C
MAX	GRAP2
MAX	GSPT2
MAX	GSR
MAX	GTF2H5
MAX	GTF2IRD2
MAX	GTF3A
MAX	GXYLT1
MAX	H3F3B
MAX	HCLS1
MAX	HEXA
MAX	HIGD1A
MAX	HIVEP2
MAX	HLA-E
MAX	HMG20B
MAX	HMGCS1
MAX	HNRNPDL
MAX	HOTAIRM1
MAX	HPS3
MAX	HSD17B11
MAX	IARS
MAX	IDE
MAX	IDH3A

MAX	IFI30
MAX	IFNGR1
MAX	IKZF3
MAX	IL12RB1
MAX	IL2RG
MAX	IMP3
MAX	ING3
MAX	INTS1
MAX	INTS10
MAX	INTS9
MAX	IRF2
MAX	IRF8
MAX	ITFG1
MAX	ITPA
MAX	IVNS1ABP
MAX	JADE2
MAX	JOSD1
MAX	JPX
MAX	KBTBD4
MAX	KCTD15
MAX	KCTD17
MAX	KDM3A
MAX	KDM5B
MAX	KIAA0319L
MAX	KLC2
MAX	KLF2
MAX	KLF6
MAX	KLHDC2
MAX	KLHDC4
MAX	KPNA6
MAX	L1CAM
MAX	LACTB2
MAX	LAMP1
MAX	LAMTOR5
MAX	LEMD2
MAX	LENG8
MAX	LGALS3
MAX	LINC00909
MAX	LINC01003
MAX	LINC01116
MAX	LMBR1
MAX	LMF1
MAX	LMO2
MAX	LMTK2
MAX	LSM6
MAX	LTB
MAX	LYN
MAX	LYPLAL1
MAX	LZIC
MAX	M6PR

MAX	MAEA
MAX	MAP2K7
MAX	MAPK6
MAX	MARCH6
MAX	MARS2
MAX	MAST2
MAX	MATK
MAX	MAX
MAX	MAZ
MAX	MBOAT7
MAX	MBP
MAX	MCPH1
MAX	MEAF6
MAX	MED16
MAX	MEN1
MAX	METAP1
MAX	MICALL1
MAX	MIEF1
MAX	MINPP1
MAX	MIS12
MAX	MNT
MAX	MOB1A
MAX	MORF4L2
MAX	MRPL11
MAX	MRPL15
MAX	MRPL20
MAX	MRPL45
MAX	MRPS22
MAX	MTHFR
MAX	MTPN
MAX	MTRF1
MAX	MTRF1L
MAX	MUM1
MAX	MYD88
MAX	MYL12A
MAX	MYL12B
MAX	N4BP2L2
MAX	NACA
MAX	NAP1L1
MAX	NARF
MAX	NBAS
MAX	NBPF12
MAX	NCOA2
MAX	NDUFC1
MAX	NDUFS7
MAX	NEMF
MAX	NF2
MAX	NFE2L2
MAX	NKAP
MAX	NKRF

MAX	NPAS1
MAX	NPHP3
MAX	NPM1
MAX	NR2C1
MAX	NSUN5
MAX	NT5DC3
MAX	NUDT9
MAX	NUP50
MAX	NUTM2A−AS1
MAX	NUTM2B−AS1
MAX	OARD1
MAX	OTUD6B−AS1
MAX	PACSIN3
MAX	PAGR1
MAX	PAIP1
MAX	PAK1IP1
MAX	PCGF1
MAX	PCYOX1
MAX	PCYT1A
MAX	PDLIM1
MAX	PEX11B
MAX	PEX2
MAX	PFDN5
MAX	PFKFB3
MAX	PGBD4
MAX	PHACTR4
MAX	PHB
MAX	PIGQ
MAX	PKD2
MAX	PKN2
MAX	PLA2G6
MAX	PLCL2
MAX	PLEKHA1
MAX	PLEKHO1
MAX	PLP2
MAX	PLRG1
MAX	PMPCB
MAX	PNKP
MAX	PNN
MAX	POC5
MAX	POLB
MAX	POLK
MAX	POLR3D
MAX	POP4
MAX	PPA1
MAX	PPCS
MAX	PPIL1
MAX	PPP1R12A
MAX	PPP1R14A
MAX	PPP2R2D

MAX	PPP4R3A
MAX	PPP6R3
MAX	PRCP
MAX	PRKCB
MAX	PRKCE
MAX	PRKCZ
MAX	PRR13
MAX	PSMB9
MAX	PSTPIP1
MAX	PTOV1
MAX	PUM1
MAX	PUM3
MAX	PUSL1
MAX	PYHIN1
MAX	RAB11B
MAX	RAB2A
MAX	RAB4A
MAX	RABEP1
MAX	RACK1
MAX	RALB
MAX	RANGRF
MAX	RARS
MAX	RASAL3
MAX	RB1CC1
MAX	RBMXL1
MAX	RBPJ
MAX	RCE1
MAX	RCN1
MAX	RDH5
MAX	RELL2
MAX	RFX5
MAX	RICTOR
MAX	RIOK3
MAX	RIT1
MAX	RMDN3
MAX	RNASEK
MAX	RNF103
MAX	RNF13
MAX	RNF167
MAX	RNF168
MAX	RNF216
MAX	RNMT
MAX	RPL10
MAX	RPL10A
MAX	RPL11
MAX	RPL12
MAX	RPL13A
MAX	RPL15
MAX	RPL18A
MAX	RPL19

MAX	RPL23
MAX	RPL23A
MAX	RPL24
MAX	RPL26
MAX	RPL26L1
MAX	RPL27
MAX	RPL3
MAX	RPL30
MAX	RPL31
MAX	RPL32
MAX	RPL34
MAX	RPL35
MAX	RPL35A
MAX	RPL36
MAX	RPL37
MAX	RPL38
MAX	RPL39
MAX	RPL41
MAX	RPL7
MAX	RPL7L1
MAX	RPL8
MAX	RPL9
MAX	RPS11
MAX	RPS13
MAX	RPS14
MAX	RPS15
MAX	RPS18
MAX	RPS19
MAX	RPS21
MAX	RPS25
MAX	RPS27
MAX	RPS27A
MAX	RPS29
MAX	RPS3
MAX	RPS3A
MAX	RPS4X
MAX	RPS5
MAX	RPS6
MAX	RPS7
MAX	RPS8
MAX	RPUSD2
MAX	RRM2B
MAX	RRP1B
MAX	RXRB
MAX	SART1
MAX	SCLT1
MAX	SDCCAG8
MAX	SDR39U1
MAX	SERINC3
MAX	SESTD1

MAX	SFT2D1
MAX	SFXN2
MAX	SGPL1
MAX	SGPP2
MAX	SH2B1
MAX	SH3D19
MAX	SIAH1
MAX	SIK3
MAX	SIMC1
MAX	SIRT7
MAX	SLC25A22
MAX	SLC2A11
MAX	SLC35B3
MAX	SLC38A2
MAX	SLC39A1
MAX	SLC4A1AP
MAX	SLC9A3
MAX	SLCO3A1
MAX	SLCO4A1
MAX	SLF2
MAX	SLU7
MAX	SMC5
MAX	SMIM14
MAX	SMIM19
MAX	SMPD2
MAX	SMPDL3A
MAX	SMYD2
MAX	SNX2
MAX	SP1
MAX	SP100
MAX	SPATA6
MAX	SRGN
MAX	SS18L2
MAX	STAMBPL1
MAX	STAT6
MAX	STK25
MAX	STK33
MAX	STYXL1
MAX	SUCLG1
MAX	SUGP2
MAX	SULF2
MAX	SURF6
MAX	SYNJ2BP
MAX	TAF1C
MAX	TAGLN2
MAX	TAP2
MAX	TASP1
MAX	TATDN3
MAX	TBCA
MAX	TCEAL9

MAX	TCTN3
MAX	TEX261
MAX	TGFB1
MAX	TGFB1I1
MAX	TGOLN2
MAX	THAP9−AS1
MAX	THOC1
MAX	TIPARP
MAX	TJAP1
MAX	TM2D1
MAX	TMBIM1
MAX	TMBIM4
MAX	TMC6
MAX	TMED1
MAX	TMEM11
MAX	TMEM14B
MAX	TMEM186
MAX	TMEM220
MAX	TMF1
MAX	TNFAIP1
MAX	TNRC6A
MAX	TNRC6B
MAX	TOMM7
MAX	TOMM70
MAX	TOP1MT
MAX	TRAF3IP3
MAX	TRAF6
MAX	TRAP1
MAX	TRAPPC4
MAX	TRAPPC9
MAX	TRIM11
MAX	TRIM24
MAX	TRIM8
MAX	TRIP4
MAX	TRMT2B
MAX	TROVE2
MAX	TSC22D1
MAX	TSEN34
MAX	TTBK2
MAX	TTC3
MAX	TTL
MAX	TUBA1A
MAX	TUBB3
MAX	TWISTNB
MAX	TXNL4B
MAX	U2AF1
MAX	UBA5
MAX	UBA52
MAX	UBAP2L
MAX	UBC

MAX	UBE2Q2
MAX	UBIAD1
MAX	UBR5
MAX	UBR7
MAX	UBXN1
MAX	UBXN2A
MAX	UNC50
MAX	UQCRB
MAX	USE1
MAX	USP15
MAX	USP24
MAX	USP25
MAX	USP30-AS1
MAX	USP32
MAX	UTP11
MAX	UTP3
MAX	UTRN
MAX	VCL
MAX	VHL
MAX	VILL
MAX	VMP1
MAX	VPS33B
MAX	VPS36
MAX	VPS72
MAX	VTI1B
MAX	WARS2
MAX	WBP11
MAX	WDR45
MAX	WDR82
MAX	WFS1
MAX	WRAP53
MAX	WRAP73
MAX	XRCC5
MAX	YBX3
MAX	YIF1A
MAX	YIPF4
MAX	YTHDF3-AS1
MAX	YWHAZ
MAX	ZBED5-AS1
MAX	ZDHHC21
MAX	ZDHHC6
MAX	ZDHHC7
MAX	ZFAND5
MAX	ZFC3H1
MAX	ZFP36
MAX	ZFP36L1
MAX	ZFYVE16
MAX	ZHX2
MAX	ZKSCAN1
MAX	ZMYM2

MAX	ZMYND11
MAX	ZNF181
MAX	ZNF202
MAX	ZNF335
MAX	ZNF347
MAX	ZNF414
MAX	ZNF587
MAX	ZNF691
MAX	ZNF768
MAX	ZNF800
MAX	ZNF83
MAX	ZNFX1
MAX	ZRSR2
MAZ	AATF
MAZ	ABCD3
MAZ	ABCD4
MAZ	ABHD11
MAZ	ABHD13
MAZ	ABHD16A
MAZ	ABHD17A
MAZ	ACACA
MAZ	ACAT2
MAZ	ACTL6A
MAZ	ACTR1A
MAZ	ACYP1
MAZ	ADAM10
MAZ	ADH5
MAZ	ADNP
MAZ	AFF4
MAZ	AGL
MAZ	AGPAT5
MAZ	AHCY
MAZ	AK3
MAZ	ALAS1
MAZ	ANAPC11
MAZ	ANK1
MAZ	ANKHD1
MAZ	ANP32B
MAZ	ANP32E
MAZ	ANXA4
MAZ	AP2B1
MAZ	AP2S1
MAZ	AP4S1
MAZ	AP5M1
MAZ	APEH
MAZ	ARF6
MAZ	ARFIP1
MAZ	ARHGAP27
MAZ	ARHGDIA
MAZ	ARHGEF1

MAZ	ARHGEF39
MAZ	ARID1A
MAZ	ARL5B
MAZ	ARL8A
MAZ	ARMCX6
MAZ	ASCC1
MAZ	ASF1B
MAZ	ASPSCR1
MAZ	ATAD2
MAZ	ATAD5
MAZ	ATF6B
MAZ	ATG3
MAZ	ATL3
MAZ	ATP6V0E1
MAZ	ATP6V1A
MAZ	B3GNTL1
MAZ	BAG1
MAZ	BANF1
MAZ	BARD1
MAZ	BAZ1A
MAZ	BBIP1
MAZ	BCL7B
MAZ	BCL7C
MAZ	BCOR
MAZ	BEX4
MAZ	BLOC1S1
MAZ	BLZF1
MAZ	BNIP3L
MAZ	BRAP
MAZ	BTG3
MAZ	C11orf24
MAZ	C11orf58
MAZ	C12orf10
MAZ	C12orf65
MAZ	C15orf61
MAZ	C19orf25
MAZ	C21orf58
MAZ	C3orf38
MAZ	C5orf34
MAZ	C6orf52
MAZ	C9orf16
MAZ	C9orf40
MAZ	CALM1
MAZ	CAMK2D
MAZ	CAMK4
MAZ	CAMLG
MAZ	CAMTA1
MAZ	CAND1
MAZ	CAPN1
MAZ	CAPRIN1

MAZ	CARHSP1
MAZ	CARNMT1
MAZ	CARS2
MAZ	CASP3
MAZ	CASP8AP2
MAZ	CASZ1
MAZ	CBX1
MAZ	CBX2
MAZ	CBX3
MAZ	CCDC15
MAZ	CCDC167
MAZ	CCDC71
MAZ	CCDC77
MAZ	CCDC84
MAZ	CCNE1
MAZ	CCNE2
MAZ	CCZ1
MAZ	CD163L1
MAZ	CD24
MAZ	CD2AP
MAZ	CDC123
MAZ	CDC23
MAZ	CDC34
MAZ	CDC37
MAZ	CDC45
MAZ	CDCA5
MAZ	CDK1
MAZ	CDK14
MAZ	CDK17
MAZ	CDK19
MAZ	CDK2
MAZ	CDKN2A
MAZ	CDKN2AIP
MAZ	CDKN2C
MAZ	CDKN2D
MAZ	CDT1
MAZ	CENPA
MAZ	CENPBD1
MAZ	CENPM
MAZ	CENPP
MAZ	CENPU
MAZ	CEP295
MAZ	CEP72
MAZ	CEP78
MAZ	CEP95
MAZ	CERK
MAZ	CFAP20
MAZ	CFL1
MAZ	CHAF1A
MAZ	CHD4

MAZ	CHEK2
MAZ	CHFR
MAZ	CHIC2
MAZ	CHMP7
MAZ	CIAO1
MAZ	CIB1
MAZ	CIC
MAZ	CIITA
MAZ	CIZ1
MAZ	CKMT2−AS1
MAZ	CLCN6
MAZ	CLIC1
MAZ	CLIC4
MAZ	CLNS1A
MAZ	CLSPN
MAZ	CMC2
MAZ	CNPPD1
MAZ	CNTNAP2
MAZ	COASY
MAZ	COCH
MAZ	COMMD4
MAZ	COMTD1
MAZ	COPS7A
MAZ	COPS7B
MAZ	COX5A
MAZ	COX6B1
MAZ	COX7B
MAZ	COX8A
MAZ	CPNE5
MAZ	CREM
MAZ	CRIPT
MAZ	CRNKL1
MAZ	CSE1L
MAZ	CSTF1
MAZ	CTCF
MAZ	CTDNEP1
MAZ	CTNNBIP1
MAZ	CUEDC2
MAZ	CUTC
MAZ	CXorf38
MAZ	CYB5R1
MAZ	D2HGDH
MAZ	DCAF11
MAZ	DCAF12
MAZ	DCAF15
MAZ	DCAF6
MAZ	DCPS
MAZ	DDT
MAZ	DDX39A
MAZ	DDX46

MAZ	DEK
MAZ	DEPDC5
MAZ	DHPS
MAZ	DHX9
MAZ	DIAPH1
MAZ	DIAPH3
MAZ	DNAJC14
MAZ	DNAJC9
MAZ	DONSON
MAZ	DPF1
MAZ	DPM1
MAZ	DTD1
MAZ	DTX2
MAZ	DUSP23
MAZ	DUT
MAZ	DVL3
MAZ	DYNLL2
MAZ	DYRK1A
MAZ	E2F7
MAZ	EIF2B1
MAZ	EIF3H
MAZ	ELAC2
MAZ	ELMSAN1
MAZ	ELOF1
MAZ	ELP5
MAZ	EMC4
MAZ	EME1
MAZ	EML3
MAZ	EMSY
MAZ	EP400
MAZ	EPN1
MAZ	ERI1
MAZ	ESCO2
MAZ	ESRRA
MAZ	EXO1
MAZ	EXOC3
MAZ	EXOC3-AS1
MAZ	EXOSC2
MAZ	EZH2
MAZ	FAAP20
MAZ	FADD
MAZ	FAM102A
MAZ	FAM111A
MAZ	FAM120A
MAZ	FAM126B
MAZ	FAM53B
MAZ	FAM81A
MAZ	FANCE
MAZ	FANCG
MAZ	FANCI

MAZ	FANCM
MAZ	FBXL18
MAZ	FBXO22
MAZ	FBXO42
MAZ	FBXO5
MAZ	FBXO8
MAZ	FBXW2
MAZ	FDPS
MAZ	FEN1
MAZ	FGF14-AS2
MAZ	FHOD1
MAZ	FIBP
MAZ	FKBP5
MAZ	FLOT1
MAZ	FLYWCH1
MAZ	FOXP4
MAZ	FRA10AC1
MAZ	FRG1
MAZ	FSD1
MAZ	FTH1
MAZ	FUS
MAZ	GABPB1
MAZ	GALE
MAZ	GALK1
MAZ	GAPDH
MAZ	GCNT2
MAZ	GDAP2
MAZ	GEMIN6
MAZ	GFI1
MAZ	GIGYF2
MAZ	GINS2
MAZ	GINS3
MAZ	GLO1
MAZ	GMFG
MAZ	GMNN
MAZ	GMPS
MAZ	GNAI2
MAZ	GNB2
MAZ	GNG5
MAZ	GNPNAT1
MAZ	GPALPP1
MAZ	GPAT4
MAZ	GRK2
MAZ	GTF2IRD2B
MAZ	GUCD1
MAZ	H2AFV
MAZ	H2AFX
MAZ	H2AFY
MAZ	H2AFZ
MAZ	H3F3A

MAZ	HAT1
MAZ	HAUS6
MAZ	HDAC5
MAZ	HDAC8
MAZ	HDDC3
MAZ	HENMT1
MAZ	HES1
MAZ	HES6
MAZ	HINT2
MAZ	HIP1R
MAZ	HIST1H1D
MAZ	HIST1H1E
MAZ	HIST2H2AC
MAZ	HMCES
MAZ	HMGA1
MAZ	HMGB1
MAZ	HMGB2
MAZ	HMGB3
MAZ	HMGCS1
MAZ	HMGN2
MAZ	HMGXB4
MAZ	HMOX2
MAZ	HNRNPA0
MAZ	HNRNPA3
MAZ	HNRNPD
MAZ	HNRNPF
MAZ	HNRNPL
MAZ	HNRNPR
MAZ	HNRNPU
MAZ	HPS3
MAZ	HSPB11
MAZ	HTATSF1
MAZ	HTRA2
MAZ	ID3
MAZ	IFNLR1
MAZ	IKBKB
MAZ	IMPA2
MAZ	INAFM1
MAZ	ING1
MAZ	ING2
MAZ	ING4
MAZ	IPO9
MAZ	ISY1
MAZ	ITGB1
MAZ	ITPKA
MAZ	JMJD8
MAZ	KATNA1
MAZ	KATNB1
MAZ	KCNK12
MAZ	KCTD18

MAZ	KDM8
MAZ	KIF22
MAZ	KIFAP3
MAZ	KLF12
MAZ	KLHDC4
MAZ	KPNB1
MAZ	KRCC1
MAZ	LAMTOR1
MAZ	LAS1L
MAZ	LCMT1
MAZ	LCMT2
MAZ	LCORL
MAZ	LDHA
MAZ	LEMD2
MAZ	LINC01013
MAZ	LIPT2
MAZ	LITAF
MAZ	LMNB2
MAZ	LONP1
MAZ	LPGAT1
MAZ	LRP8
MAZ	LRRC20
MAZ	LRRC47
MAZ	LSM2
MAZ	LSM3
MAZ	LSM4
MAZ	LTV1
MAZ	LYL1
MAZ	MAD2L2
MAZ	MAGOH
MAZ	MAML3
MAZ	MAP1LC3B
MAZ	MAP2K5
MAZ	MAP3K4
MAZ	MAP3K7
MAZ	MAP4
MAZ	MAP4K3
MAZ	MAPK1IP1L
MAZ	MAPK8
MAZ	MAT2A
MAZ	MAZ
MAZ	MCM2
MAZ	MCM3
MAZ	MCM4
MAZ	MCM5
MAZ	MCM7
MAZ	MEA1
MAZ	MED24
MAZ	MELK
MAZ	METRNL

MAZ	METTL3
MAZ	METTL6
MAZ	METTL9
MAZ	MGAT3
MAZ	MGMT
MAZ	MID1IP1
MAZ	MIF
MAZ	MIF4GD
MAZ	MINCR
MAZ	MINPP1
MAZ	MIR193BHG
MAZ	MKLN1
MAZ	MKS1
MAZ	MLST8
MAZ	MLX
MAZ	MORN2
MAZ	MPC1
MAZ	MPC2
MAZ	MPDU1
MAZ	MRPL27
MAZ	MRPL40
MAZ	MRPL43
MAZ	MRPL57
MAZ	MRPS16
MAZ	MRPS18A
MAZ	MRPS26
MAZ	MSANTD2
MAZ	MSANTD4
MAZ	MSH6
MAZ	MTERF3
MAZ	MTHFR
MAZ	MTIF3
MAZ	MUT
MAZ	MYADM
MAZ	MYBL2
MAZ	MYL6B
MAZ	MYLK-AS1
MAZ	MYLPF
MAZ	MYNN
MAZ	N4BP2
MAZ	NAP1L4
MAZ	NAPEPLD
MAZ	NARS2
MAZ	NASP
MAZ	NAT14
MAZ	NAXE
MAZ	NCAPG2
MAZ	NCAPH2
MAZ	NDUFA6
MAZ	NDUFB11

MAZ	NDUFS1
MAZ	NDUFS3
MAZ	NDUFS7
MAZ	NDUFS8
MAZ	NELFE
MAZ	NEMP2
MAZ	NET1
MAZ	NFATC2
MAZ	NFKBIL1
MAZ	NFYB
MAZ	NIT1
MAZ	NKRF
MAZ	NLRC3
MAZ	NME4
MAZ	NONO
MAZ	NOSIP
MAZ	NPTN
MAZ	NR4A3
MAZ	NRDE2
MAZ	NT5DC3
MAZ	NTAN1
MAZ	NUB1
MAZ	NUDC
MAZ	NUDT1
MAZ	NUDT21
MAZ	NUFIP2
MAZ	NUMA1
MAZ	NUP107
MAZ	NUP210
MAZ	NUP214
MAZ	NUP62
MAZ	NXF1
MAZ	ORC1
MAZ	OSBP
MAZ	P3H1
MAZ	PABPC4
MAZ	PABPN1
MAZ	PANK2
MAZ	PAQR4
MAZ	PARD6A
MAZ	PARP3
MAZ	PARP8
MAZ	PBRM1
MAZ	PCBP2
MAZ	PCGF6
MAZ	PDAP1
MAZ	PDCD6IP
MAZ	PDE12
MAZ	PDLIM1
MAZ	PDLIM5

MAZ	PDS5A
MAZ	PDXDC1
MAZ	PEX10
MAZ	PEX19
MAZ	PFN1
MAZ	PGAM1
MAZ	PGM1
MAZ	PGP
MAZ	PGRMC1
MAZ	PHF13
MAZ	PHF19
MAZ	PHF20
MAZ	PICALM
MAZ	PIGG
MAZ	PIH1D1
MAZ	PIK3CD
MAZ	PIN1
MAZ	PIP5K1C
MAZ	PKIG
MAZ	PLEKHM2
MAZ	PLEKHO1
MAZ	PLK4
MAZ	PMM1
MAZ	PNKD
MAZ	POC5
MAZ	POLA1
MAZ	POLDIP2
MAZ	POLG2
MAZ	POLR2G
MAZ	POLRMT
MAZ	POP7
MAZ	PPIA
MAZ	PPIF
MAZ	PPIG
MAZ	PPM1G
MAZ	PPP1CA
MAZ	PPP1R10
MAZ	PPP1R37
MAZ	PPP2R5D
MAZ	PRCC
MAZ	PRIM2
MAZ	PRKCA
MAZ	PRKCD
MAZ	PRKD2
MAZ	PRKDC
MAZ	PRMT7
MAZ	PRPF38A
MAZ	PRPF38B
MAZ	PRPS1
MAZ	PRPS2

MAZ	PRR13
MAZ	PRRC2A
MAZ	PRRC2B
MAZ	PRRG4
MAZ	PSMB3
MAZ	PSMC1
MAZ	PSMC3
MAZ	PSMD14
MAZ	PSMD8
MAZ	PSMG3
MAZ	PTAFR
MAZ	PTBP1
MAZ	PTGES3
MAZ	PTMA
MAZ	PTPN12
MAZ	PTRH1
MAZ	PWP2
MAZ	PXMP2
MAZ	RAB22A
MAZ	RAB27A
MAZ	RAB2A
MAZ	RABEP2
MAZ	RAD1
MAZ	RAD51D
MAZ	RAE1
MAZ	RALB
MAZ	RALY
MAZ	RANBP1
MAZ	RANBP2
MAZ	RANBP3
MAZ	RASSF1
MAZ	RASSF5
MAZ	RBBP7
MAZ	RBL1
MAZ	RBM10
MAZ	RBM12
MAZ	RBMX2
MAZ	RCAN3
MAZ	RCHY1
MAZ	RECQL
MAZ	REEP4
MAZ	REPS1
MAZ	RFC2
MAZ	RFC3
MAZ	RFC4
MAZ	RFC5
MAZ	RFT1
MAZ	RFX2
MAZ	RGCC
MAZ	RHNO1

MAZ	RIC8A
MAZ	RIF1
MAZ	RMI2
MAZ	RNASEH2A
MAZ	RNASEH2B
MAZ	RNASEH2C
MAZ	RNF144B
MAZ	RNF168
MAZ	RNF34
MAZ	RNF4
MAZ	ROGDI
MAZ	RPE
MAZ	RPL11
MAZ	RPL12
MAZ	RPL29
MAZ	RPL31
MAZ	RPL37
MAZ	RPL38
MAZ	RPL41
MAZ	RPLP0
MAZ	RPLP1
MAZ	RPLP2
MAZ	RPS10
MAZ	RPS2
MAZ	RPS21
MAZ	RPS3A
MAZ	RPS5
MAZ	RPS6KB1
MAZ	RPS6KB2
MAZ	RPSA
MAZ	RRM1
MAZ	RRM2
MAZ	RSRP1
MAZ	RSU1
MAZ	RTN4
MAZ	RXRB
MAZ	SAC3D1
MAZ	SAMD15
MAZ	SAMM50
MAZ	SCAI
MAZ	SCNM1
MAZ	SDCCAG8
MAZ	SDHB
MAZ	SDHD
MAZ	SEC11A
MAZ	SEC16A
MAZ	SEC22A
MAZ	SENP7
MAZ	SEPT11
MAZ	SEPT6

MAZ	SEPT7
MAZ	SETD5
MAZ	SETD7
MAZ	SF3A3
MAZ	SF3B4
MAZ	SGO1
MAZ	SHMT2
MAZ	SHROOM1
MAZ	SIAH1
MAZ	SIMC1
MAZ	SIPA1
MAZ	SIVA1
MAZ	SKA3
MAZ	SKP2
MAZ	SLAIN2
MAZ	SLBP
MAZ	SLC10A3
MAZ	SLC18B1
MAZ	SLC1A4
MAZ	SLC25A1
MAZ	SLC25A28
MAZ	SLC25A36
MAZ	SLC25A40
MAZ	SLC2A1
MAZ	SLC2A8
MAZ	SLC35A5
MAZ	SLC46A3
MAZ	SLC52A2
MAZ	SLC6A9
MAZ	SLC7A1
MAZ	SLC9A3R1
MAZ	SLF1
MAZ	SMAD2
MAZ	SMC1B
MAZ	SMC4
MAZ	SMCHD1
MAZ	SMG5
MAZ	SMIM19
MAZ	SMIM7
MAZ	SNAPC5
MAZ	SNRNP40
MAZ	SNRPA
MAZ	SNRPA1
MAZ	SNRPD1
MAZ	SNRPG
MAZ	SNX1
MAZ	SNX17
MAZ	SPAG1
MAZ	SPATA33
MAZ	SPATS2L

MAZ	SPC25
MAZ	SPECC1L
MAZ	SPHK2
MAZ	SPOP
MAZ	SRRM1
MAZ	SRRM2
MAZ	SRRT
MAZ	SRSF10
MAZ	SRSF2
MAZ	SRSF4
MAZ	SSRP1
MAZ	ST14
MAZ	STAG2
MAZ	STAG3
MAZ	STARD7
MAZ	STAU1
MAZ	STK16
MAZ	STK38L
MAZ	STMN1
MAZ	STX11
MAZ	STYXL1
MAZ	SUB1
MAZ	SUMO2
MAZ	SUV39H2
MAZ	SUZ12
MAZ	SVBP
MAZ	SYNJ2BP
MAZ	TACC1
MAZ	TAF12
MAZ	TAF15
MAZ	TAF9B
MAZ	TANGO2
MAZ	TAPT1
MAZ	TARDBP
MAZ	TAX1BP3
MAZ	TBC1D10A
MAZ	TBC1D15
MAZ	TBC1D9B
MAZ	TBCCD1
MAZ	TBPL1
MAZ	TBRG1
MAZ	TCL1A
MAZ	TCOF1
MAZ	TERF1
MAZ	TFDP1
MAZ	TFE3
MAZ	TFEB
MAZ	THAP7
MAZ	THOC3
MAZ	THOP1

MAZ	THUMPD3-AS1
MAZ	TIAL1
MAZ	TIMM10
MAZ	TM7SF3
MAZ	TMCC1
MAZ	TMCO3
MAZ	TMEM107
MAZ	TMEM116
MAZ	TMEM141
MAZ	TMEM216
MAZ	TMEM258
MAZ	TMEM267
MAZ	TMEM41B
MAZ	TMPO
MAZ	TMSB10
MAZ	TMUB1
MAZ	TNPO3
MAZ	TOE1
MAZ	TOMM5
MAZ	TOP1
MAZ	TOPORS
MAZ	TP53I13
MAZ	TPGS1
MAZ	TPI1
MAZ	TPM3
MAZ	TPR
MAZ	TPRA1
MAZ	TRAPPC4
MAZ	TRIM47
MAZ	TRIM52
MAZ	TRIP13
MAZ	TRIT1
MAZ	TRUB2
MAZ	TSPAN14
MAZ	TSTD1
MAZ	TTC21B
MAZ	TTI2
MAZ	TTK
MAZ	TUBA1B
MAZ	TUBA4A
MAZ	TUBB
MAZ	TUBB2A
MAZ	TUBB2B
MAZ	TUBG1
MAZ	TUBGCP6
MAZ	TULP3
MAZ	TYMS
MAZ	U2AF2
MAZ	UBA52
MAZ	UBAC2

MAZ	UBB
MAZ	UBE2B
MAZ	UBE2Q1
MAZ	UBE2Q2
MAZ	UBL4A
MAZ	UBL7
MAZ	UBTF
MAZ	UBXN1
MAZ	UBXN7
MAZ	UHRF1
MAZ	ULK3
MAZ	UNG
MAZ	UPF2
MAZ	UPF3B
MAZ	UQCRFS1
MAZ	UQCRH
MAZ	URB1
MAZ	URM1
MAZ	USP1
MAZ	USP4
MAZ	USP46
MAZ	VDAC1
MAZ	VDAC3
MAZ	VEGFB
MAZ	VPS25
MAZ	VPS45
MAZ	VPS4B
MAZ	WAS
MAZ	WASF1
MAZ	WBP1L
MAZ	WDR1
MAZ	WDR24
MAZ	WDR34
MAZ	WDR70
MAZ	WDR76
MAZ	WDTC1
MAZ	XAB2
MAZ	XPO1
MAZ	XPR1
MAZ	XRCC1
MAZ	XRCC4
MAZ	XRCC5
MAZ	YBX1
MAZ	YIPF5
MAZ	YME1L1
MAZ	YWHAH
MAZ	YY1
MAZ	ZBED5
MAZ	ZBTB48
MAZ	ZBTB8OS

MAZ	ZDHHC12
MAZ	ZDHHC21
MAZ	ZEB1
MAZ	ZFAND2B
MAZ	ZFP91
MAZ	ZFYVE21
MAZ	ZKSCAN4
MAZ	ZMAT5
MAZ	ZNF143
MAZ	ZNF212
MAZ	ZNF292
MAZ	ZNF395
MAZ	ZNF408
MAZ	ZNF507
MAZ	ZNF639
MAZ	ZNF687
MAZ	ZNF706
MAZ	ZSCAN21
MBD4	ARHGDIB
MBD4	HHEX
MBD4	MEF2C
MBD4	PTPRC
MBD4	STX7
MBD4	TMEM156
MEF2A	AAK1
MEF2A	ACIN1
MEF2A	ADD3
MEF2A	AFF3
MEF2A	AGO4
MEF2A	AHNAK
MEF2A	AMFR
MEF2A	AP1G1
MEF2A	AP2S1
MEF2A	AP4S1
MEF2A	APLP2
MEF2A	ARID1A
MEF2A	ATAD5
MEF2A	AZIN1
MEF2A	BBS4
MEF2A	BBX
MEF2A	BCL11A
MEF2A	BCL2L13
MEF2A	BCLAF1
MEF2A	BPTF
MEF2A	C12orf65
MEF2A	C16orf74
MEF2A	CDKN2AIPNL
MEF2A	CEP350
MEF2A	CEP85
MEF2A	CHD3

MEF2A	CLCN4
MEF2A	CPEB4
MEF2A	CREBRF
MEF2A	CRK
MEF2A	CRLF3
MEF2A	CRYZL1
MEF2A	CXXC5
MEF2A	CYTH1
MEF2A	DDX17
MEF2A	EHBP1L1
MEF2A	EIF2S3
MEF2A	EML4
MEF2A	ERBIN
MEF2A	ESR2
MEF2A	FHL3
MEF2A	FNBP1
MEF2A	GABPB1
MEF2A	GCNT2
MEF2A	GDE1
MEF2A	GINS1
MEF2A	GTF2I
MEF2A	HACD1
MEF2A	HDAC9
MEF2A	HELZ
MEF2A	HERC2
MEF2A	HHLA3
MEF2A	IDH1
MEF2A	IKZF3
MEF2A	IPO7
MEF2A	ITPKB
MEF2A	ITSN2
MEF2A	JAK1
MEF2A	JAK3
MEF2A	JMJD1C
MEF2A	KCNMB3
MEF2A	KIAA0040
MEF2A	KIF1BP
MEF2A	KLF7
MEF2A	KLHL24
MEF2A	LAMP2
MEF2A	LINC01003
MEF2A	LRCH1
MEF2A	MDM4
MEF2A	MEF2A
MEF2A	MEF2C
MEF2A	MEPCE
MEF2A	MIA2
MEF2A	MID1IP1
MEF2A	MIEN1
MEF2A	MUL1

MEF2A	MYH9
MEF2A	MYO1C
MEF2A	MYO9A
MEF2A	NPEPPS
MEF2A	OTUB1
MEF2A	PABPN1
MEF2A	PARP1
MEF2A	PHACTR4
MEF2A	PHF3
MEF2A	PHIP
MEF2A	PHKG1
MEF2A	PIAS1
MEF2A	PIP5K1A
MEF2A	PLK3
MEF2A	PNPLA2
MEF2A	POGZ
MEF2A	PPP2R5C
MEF2A	PPP2R5E
MEF2A	PPP3CC
MEF2A	PRDM2
MEF2A	PRKACB
MEF2A	PRPF39
MEF2A	PXMP4
MEF2A	RAB35
MEF2A	RABGAP1
MEF2A	RALGPS2
MEF2A	RCSD1
MEF2A	REV1
MEF2A	RHOT1
MEF2A	RNF166
MEF2A	RPP25
MEF2A	RUBCN
MEF2A	SAMD8
MEF2A	SCMH1
MEF2A	SEPT9
MEF2A	SETD5
MEF2A	SLC23A2
MEF2A	SLC43A2
MEF2A	SLC44A2
MEF2A	SMG1
MEF2A	SMG6
MEF2A	SNX11
MEF2A	SNX8
MEF2A	STAG1
MEF2A	STAG2
MEF2A	STRN3
MEF2A	SWAP70
MEF2A	TAB2
MEF2A	TAF12
MEF2A	TAOK1

MEF2A	TAOK3
MEF2A	TBC1D22A
MEF2A	TBL1XR1
MEF2A	THRAP3
MEF2A	TIA1
MEF2A	TMEM156
MEF2A	TNFRSF13C
MEF2A	TOP2B
MEF2A	TRIM8
MEF2A	TYK2
MEF2A	UGCG
MEF2A	UPF2
MEF2A	USP44
MEF2A	UVRAG
MEF2A	WASF2
MEF2A	XIAP
MEF2A	ZBTB37
MEF2A	ZC3H4
MEF2A	ZC3H7A
MEF2A	ZFAND6
MEF2A	ZFP64
MLX	AFF1
MLX	ALOX12-AS1
MLX	ARFGEF2
MLX	ARL4A
MLX	ATP6V0B
MLX	BACH1
MLX	C16orf72
MLX	CACUL1
MLX	CALR
MLX	CDV3
MLX	CDYL
MLX	CLMN
MLX	CTSB
MLX	CUTA
MLX	DHX30
MLX	DOK7
MLX	DYRK2
MLX	EHMT2
MLX	EP400
MLX	EPB41
MLX	ERP29
MLX	GTF3A
MLX	HOOK2
MLX	KCTD20
MLX	KIAA1217
MLX	KLF13
MLX	MFHAS1
MLX	MFN2
MLX	MIEF1

MLX	MLX
MLX	MORC2
MLX	MOSPD1
MLX	MTDH
MLX	NDUFB6
MLX	NFX1
MLX	NSD1
MLX	PACSIN1
MLX	PARP6
MLX	PDK2
MLX	PEPD
MLX	PLEC
MLX	PPP1R21
MLX	PRDM2
MLX	RNF185
MLX	RPS28
MLX	SCAMP3
MLX	SCPEP1
MLX	SLC25A39
MLX	SLC33A1
MLX	SLC38A10
MLX	SPNS1
MLX	STT3B
MLX	TDG
MLX	TMEM154
MLX	TMEM42
MLX	TRAPPC2L
MLX	TRAPPC6A
MLX	TRMO
MLX	TTC3
MLX	VPS33A
MLX	ZFAND5
MNT	CHD7
MNT	CLCN7
MNT	DUSP1
MNT	FAM117A
MNT	MNT
MNT	NME6
MNT	ST3GAL1
MNT	TXLNG
MXD4	CALR
MXD4	DERL1
MXD4	FKBP11
MXD4	HM13
MXD4	HSP90B1
MXD4	LSP1
MXD4	MXD4
MXD4	MYDGF
MXD4	MZB1
MXD4	PPIB

MXD4	SEC11C
MXD4	SSR3
MXI1	AAED1
MXI1	ABCB7
MXI1	AMMECR1
MXI1	APP
MXI1	ARID5B
MXI1	ARL13B
MXI1	ARRDC2
MXI1	ATXN2L
MXI1	BCL2
MXI1	BTG1
MXI1	CANT1
MXI1	CEBPB
MXI1	CHST15
MXI1	CNN3
MXI1	CRKL
MXI1	CXCR4
MXI1	CYB561
MXI1	DOHH
MXI1	EFCAB14
MXI1	FGR
MXI1	FOXO3
MXI1	GABARAPL1
MXI1	GALNT2
MXI1	HTATIP2
MXI1	IGFBP4
MXI1	INO80
MXI1	IP6K1
MXI1	JUNB
MXI1	KAT6B
MXI1	KDM3A
MXI1	LYST
MXI1	MAML2
MXI1	MBP
MXI1	MXI1
MXI1	NSMCE4A
MXI1	OAF
MXI1	P4HA1
MXI1	PEPD
MXI1	PICK1
MXI1	PNRC1
MXI1	RBFOX2
MXI1	RHOQ
MXI1	RNF2
MXI1	RNPEPL1
MXI1	RPS27
MXI1	SGSM2
MXI1	SIPA1L1
MXI1	SLC35C1

MXI1	SLC7A7
MXI1	SMARCAD1
MXI1	TFAP4
MXI1	TGFBR1
MXI1	TGFBR2
MXI1	TNFAIP8L1
MXI1	UBL3
MXI1	VPS4A
MXI1	YBX3
MYBL1	AGBL5
MYBL1	ASPM
MYBL1	ATF2
MYBL1	BUB1B
MYBL1	CCNG2
MYBL1	CD22
MYBL1	CDC20
MYBL1	CDKN2C
MYBL1	DAAM1
MYBL1	E2F5
MYBL1	ELF1
MYBL1	EZH2
MYBL1	GCSAM
MYBL1	HRK
MYBL1	KATNAL1
MYBL1	KIF4A
MYBL1	LMNB1
MYBL1	LPP
MYBL1	PAG1
MYBL1	PLK1
MYBL1	PSIP1
MYBL1	RAD17
MYBL1	SEC14L1
MYBL1	SGO2
MYBL1	SOX5
MYBL1	SSBP2
MYBL1	SYNE2
MYBL1	TOP2A
MYBL1	WDR62
MYBL1	ZNF608
MYC	AACS
MYC	AAGAB
MYC	AAR2
MYC	AARSD1
MYC	AATF
MYC	ABCE1
MYC	ABCF1
MYC	ABHD5
MYC	ABL2
MYC	ABLIM1
MYC	ABRACL

MYC	ABT1
MYC	ABTB2
MYC	ACADVL
MYC	ACBD6
MYC	ACP1
MYC	ACTR6
MYC	ADAM15
MYC	ADD3
MYC	ADGRE5
MYC	ADK
MYC	ADO
MYC	ADPGK
MYC	ADPRHL2
MYC	ADRM1
MYC	ADSL
MYC	ADSS
MYC	AFG3L2
MYC	AGBL5
MYC	AGO2
MYC	AHCY
MYC	AHSA1
MYC	AIMP1
MYC	AIMP2
MYC	AK6
MYC	AKAP1
MYC	AKIRIN1
MYC	AKR1A1
MYC	AKR1B1
MYC	ALDH18A1
MYC	ALDH1B1
MYC	ALG1
MYC	ALG13
MYC	ALG2
MYC	ALG3
MYC	ALG5
MYC	ALG8
MYC	ALKBH2
MYC	ALKBH8
MYC	AMD1
MYC	AMIGO2
MYC	AMPD2
MYC	ANAPC13
MYC	ANKHD1
MYC	ANKMY2
MYC	ANKRD11
MYC	ANKRD37
MYC	ANKRD46
MYC	ANP32B
MYC	ANXA5
MYC	ANXA7

MYC	AP1B1
MYC	AP1S3
MYC	AP4S1
MYC	APEX1
MYC	APH1A
MYC	API5
MYC	APMAP
MYC	APP
MYC	APRT
MYC	APTX
MYC	ARF1
MYC	ARF4
MYC	ARF6
MYC	ARFGAP2
MYC	ARFGEF1
MYC	ARGLU1
MYC	ARHGAP15
MYC	ARHGAP24
MYC	ARHGDIA
MYC	ARHGEF19
MYC	ARHGEF2
MYC	ARL1
MYC	ARL4A
MYC	ARL8B
MYC	ARMC1
MYC	ARMC10
MYC	ARMC6
MYC	ARMCX1
MYC	ARNTL2
MYC	ARPC5L
MYC	ARPP19
MYC	ASB6
MYC	ASNA1
MYC	ASNS
MYC	ASNSD1
MYC	ATAD1
MYC	ATAD3A
MYC	ATAD3B
MYC	ATF1
MYC	ATF3
MYC	ATF4
MYC	ATG101
MYC	ATG3
MYC	ATG5
MYC	ATIC
MYC	ATL2
MYC	ATL3
MYC	ATN1
MYC	ATOX1
MYC	ATP13A1

MYC	ATP13A2
MYC	ATP13A3
MYC	ATP1A1
MYC	ATP1B3
MYC	ATP6AP2
MYC	ATP6V0A1
MYC	ATP6V0B
MYC	ATP6V1B2
MYC	ATP6V1F
MYC	ATXN1
MYC	ATXN2L
MYC	AUP1
MYC	AURKAIP1
MYC	AVEN
MYC	B3GALT6
MYC	B3GAT3
MYC	B3GNT2
MYC	B3GNTL1
MYC	B4GALT7
MYC	BAG2
MYC	BANF1
MYC	BATF
MYC	BAZ1A
MYC	BBS7
MYC	BCAP31
MYC	BCAR3
MYC	BCAS2
MYC	BCAT1
MYC	BCAT2
MYC	BCCIP
MYC	BCKDK
MYC	BCL2
MYC	BCL2A1
MYC	BCL2L1
MYC	BCL2L13
MYC	BCLAF1
MYC	BDP1
MYC	BEND4
MYC	BET1
MYC	BEX2
MYC	BFAR
MYC	BHLHE40
MYC	BLOC1S2
MYC	BMPR1A
MYC	BMS1
MYC	BNIP2
MYC	BOD1
MYC	BOLA2-SMG1P6
MYC	BOP1
MYC	BRAF

MYC	BRF2
MYC	BRMS1
MYC	BRPF1
MYC	BSG
MYC	BTAF1
MYC	BTF3
MYC	BTF3L4
MYC	BTG3
MYC	BTLA
MYC	BUD31
MYC	BYSL
MYC	BZW1
MYC	BZW2
MYC	C11orf58
MYC	C12orf29
MYC	C12orf45
MYC	C12orf60
MYC	C17orf80
MYC	C18orf25
MYC	C19orf24
MYC	C19orf48
MYC	C19orf70
MYC	C1D
MYC	C1orf131
MYC	C1orf159
MYC	C1orf174
MYC	C1orf43
MYC	C1orf52
MYC	C1QBP
MYC	C20orf27
MYC	C2orf76
MYC	C4orf48
MYC	C8orf33
MYC	C8orf59
MYC	C9orf85
MYC	CA8
MYC	CALM2
MYC	CAMTA2
MYC	CANX
MYC	CAPNS1
MYC	CAPRIN1
MYC	CAPZA2
MYC	CARNMT1
MYC	CASC3
MYC	CAV1
MYC	CBR1
MYC	CBWD1
MYC	CBWD5
MYC	CCDC12
MYC	CCDC124

MYC	CCDC130
MYC	CCDC144A
MYC	CCDC47
MYC	CCDC58
MYC	CCDC59
MYC	CCDC85B
MYC	CCDC86
MYC	CCDC9
MYC	CCL28
MYC	CCL3
MYC	CCL3L1
MYC	CCL4
MYC	CCL4L2
MYC	CCNC
MYC	CCND1
MYC	CCND2
MYC	CCR7
MYC	CCSER2
MYC	CCT2
MYC	CCT4
MYC	CCT5
MYC	CCT6A
MYC	CCT7
MYC	CCT8
MYC	CCZ1
MYC	CD151
MYC	CD164
MYC	CD320
MYC	CD3EAP
MYC	CD40
MYC	CD44
MYC	CD47
MYC	CD63
MYC	CD69
MYC	CD70
MYC	CD82
MYC	CD83
MYC	CDC123
MYC	CDC23
MYC	CDC37
MYC	CDC37L1
MYC	CDC42EP3
MYC	CDC73
MYC	CDIP1
MYC	CDK4
MYC	CDK7
MYC	CDKN1B
MYC	CEBPB
MYC	CEBPG
MYC	CEBPZ

MYC	CELF1
MYC	CENPV
MYC	CEP83
MYC	CERS2
MYC	CERS6
MYC	CFAP97
MYC	CFLAR
MYC	CHCHD2
MYC	CHCHD3
MYC	CHD1
MYC	CHERP
MYC	CHID1
MYC	CHKA
MYC	CHMP1A
MYC	CHMP2B
MYC	CHMP4B
MYC	CHORDC1
MYC	CHPF2
MYC	CHST11
MYC	CHSY1
MYC	CHTOP
MYC	CIAPIN1
MYC	CIB1
MYC	CIRBP
MYC	CISD1
MYC	CKS2
MYC	CLCN7
MYC	CLNS1A
MYC	CLPB
MYC	CLTB
MYC	CLUH
MYC	CMC2
MYC	CMSS1
MYC	CMTM6
MYC	CNBP
MYC	CNDP2
MYC	CNIH1
MYC	CNOT4
MYC	CNPPD1
MYC	CNPY3
MYC	CNST
MYC	COA4
MYC	COA6
MYC	COG7
MYC	COIL
MYC	COPRS
MYC	COPS2
MYC	COPS3
MYC	COPS8
MYC	COQ10B

MYC	COQ3
MYC	COQ5
MYC	COX10
MYC	COX17
MYC	COX19
MYC	COX20
MYC	COX7B
MYC	COX7C
MYC	CPSF4
MYC	CPSF7
MYC	CREB5
MYC	CRLS1
MYC	CRNDE
MYC	CRY1
MYC	CRY2
MYC	CSDE1
MYC	CSF1
MYC	CSNK2A1
MYC	CSNK2B
MYC	CSRNP1
MYC	CTNNBL1
MYC	CTPS1
MYC	CTR9
MYC	CTSC
MYC	CTU2
MYC	CUL1
MYC	CUL4A
MYC	CWC15
MYC	CWC22
MYC	CWC25
MYC	CWF19L1
MYC	CXCR5
MYC	CYB5R2
MYC	CYC1
MYC	CYCS
MYC	CYP51A1
MYC	DAP3
MYC	DAPL1
MYC	DARS
MYC	DBR1
MYC	DCAF13
MYC	DCAF4
MYC	DCP1A
MYC	DCTD
MYC	DCTN6
MYC	DCTPP1
MYC	DCUN1D5
MYC	DDIT3
MYC	DDIT4
MYC	DDX1

MYC	DDX10
MYC	DDX18
MYC	DDX19A
MYC	DDX21
MYC	DDX27
MYC	DDX39A
MYC	DDX3X
MYC	DDX3Y
MYC	DDX46
MYC	DDX47
MYC	DDX49
MYC	DDX5
MYC	DDX50
MYC	DDX51
MYC	DDX55
MYC	DDX56
MYC	DENR
MYC	DESI1
MYC	DGAT2
MYC	DGKZ
MYC	DHCR7
MYC	DHRS12
MYC	DHRS3
MYC	DHX15
MYC	DHX32
MYC	DHX33
MYC	DHX34
MYC	DHX9
MYC	DIMT1
MYC	DKC1
MYC	DLGAP4
MYC	DLST
MYC	DNAAF2
MYC	DNAJA1
MYC	DNAJA2
MYC	DNAJB4
MYC	DNAJB6
MYC	DNAJC17
MYC	DNAJC2
MYC	DNAJC21
MYC	DNAJC24
MYC	DNAJC8
MYC	DNPEP
MYC	DNTTIP2
MYC	DOHH
MYC	DOT1L
MYC	DPH2
MYC	DPH3
MYC	DPH5
MYC	DPM1

MYC	DPM2
MYC	DPP7
MYC	DRG1
MYC	DTD1
MYC	DTD2
MYC	DTWD1
MYC	DUS3L
MYC	DUSP11
MYC	DUSP12
MYC	DUSP2
MYC	DUSP22
MYC	DUSP4
MYC	DUSP5
MYC	DYNC1LI1
MYC	DYNLT3
MYC	E2F4
MYC	E2F6
MYC	EBI3
MYC	EBNA1BP2
MYC	EBPL
MYC	ECE1
MYC	EDEM1
MYC	EEA1
MYC	EEF1A1
MYC	EEF1B2
MYC	EEF1E1
MYC	EEF2KMT
MYC	EFCAB14
MYC	EGR2
MYC	EGR3
MYC	EHBP1L1
MYC	EHD4
MYC	EI24
MYC	EIF1
MYC	EIF1AX
MYC	EIF1AY
MYC	EIF1B
MYC	EIF2A
MYC	EIF2B1
MYC	EIF2B2
MYC	EIF2B3
MYC	EIF2B4
MYC	EIF2B5
MYC	EIF2S1
MYC	EIF2S2
MYC	EIF3A
MYC	EIF3B
MYC	EIF3C
MYC	EIF3E
MYC	EIF3G

MYC	EIF3I
MYC	EIF3J
MYC	EIF3M
MYC	EIF4A1
MYC	EIF4A2
MYC	EIF4A3
MYC	EIF4E
MYC	EIF4EBP1
MYC	EIF4G1
MYC	EIF4G2
MYC	EIF4H
MYC	EIF5
MYC	EIF5A
MYC	EIF5B
MYC	EIF6
MYC	ELAVL1
MYC	ELF2
MYC	ELK1
MYC	ELMO1
MYC	ELP2
MYC	ELP3
MYC	ELP5
MYC	EMC4
MYC	EMC6
MYC	EMC7
MYC	EMC8
MYC	EMG1
MYC	EMILIN2
MYC	EML4
MYC	EML5
MYC	EMP3
MYC	ENO1
MYC	ENO2
MYC	ENOPH1
MYC	ENPP4
MYC	ENSA
MYC	ENY2
MYC	EP400
MYC	EPB41L2
MYC	EPB41L4A-AS1
MYC	EPRS
MYC	ERH
MYC	ERI3
MYC	ERICH1
MYC	ERMARD
MYC	ESCO1
MYC	ESF1
MYC	ESRRA
MYC	ETF1
MYC	ETS2

MYC	EWSR1
MYC	EXOSC10
MYC	EXOSC3
MYC	EXOSC4
MYC	EXOSC6
MYC	EXOSC7
MYC	EXOSC8
MYC	EXT2
MYC	EXTL2
MYC	FAAP100
MYC	FABP5
MYC	FAM114A2
MYC	FAM133B
MYC	FAM136A
MYC	FAM160A2
MYC	FAM207A
MYC	FAM210A
MYC	FAM216A
MYC	FAM234A
MYC	FAM53B
MYC	FAM86B1
MYC	FAM86C1
MYC	FAM96B
MYC	FAM98A
MYC	FAR1
MYC	FARSA
MYC	FARSB
MYC	FASTK
MYC	FASTKD2
MYC	FBL
MYC	FBXL12
MYC	FBXL15
MYC	FBXO11
MYC	FBXO21
MYC	FBXO22
MYC	FBXW11
MYC	FCER2
MYC	FDX1
MYC	FGD5-AS1
MYC	FGFBP3
MYC	FGR
MYC	FH
MYC	FILIP1L
MYC	FIP1L1
MYC	FIS1
MYC	FKBP4
MYC	FLAD1
MYC	FLOT2
MYC	FOXP1
MYC	FPGS

MYC	FTH1
MYC	FTSJ1
MYC	FUBP1
MYC	FUNDC2
MYC	FUS
MYC	FYN
MYC	G3BP1
MYC	G3BP2
MYC	G6PC3
MYC	G6PD
MYC	GABPB1
MYC	GADD45B
MYC	GADD45G
MYC	GADD45GIP1
MYC	GALK2
MYC	GALNT2
MYC	GAR1
MYC	GARS
MYC	GART
MYC	GAS7
MYC	GATAD2A
MYC	GCC1
MYC	GCFC2
MYC	GCN1
MYC	GEM
MYC	GEMIN4
MYC	GEMIN6
MYC	GFM1
MYC	GGCT
MYC	GHDC
MYC	GHITM
MYC	GLO1
MYC	GLRX2
MYC	GLRX3
MYC	GLUD1
MYC	GMEB2
MYC	GMFB
MYC	GNAI3
MYC	GNB5
MYC	GNG2
MYC	GNG4
MYC	GNL1
MYC	GNL2
MYC	GNL3
MYC	GNL3L
MYC	GNPDA1
MYC	GNPNAT1
MYC	GOLGA1
MYC	GOLGA7
MYC	GOT1

MYC	GPATCH4
MYC	GPN1
MYC	GPR183
MYC	GPR65
MYC	GPR89A
MYC	GPT2
MYC	GPX4
MYC	GRAMD1A
MYC	GRIPAP1
MYC	GRK5
MYC	GRPEL1
MYC	GRWD1
MYC	GSPT1
MYC	GTF2A2
MYC	GTF2F2
MYC	GTF2H2
MYC	GTF2H2C
MYC	GTF3A
MYC	GTF3C6
MYC	GTPBP10
MYC	GTPBP3
MYC	GTPBP4
MYC	H3F3B
MYC	HARS
MYC	HAUS6
MYC	HAX1
MYC	HCG11
MYC	HCG18
MYC	HDAC2
MYC	HDDC2
MYC	HEATR1
MYC	HECTD2
MYC	HERPUD1
MYC	HES1
MYC	HEXB
MYC	HGH1
MYC	HIF1A
MYC	HIGD1A
MYC	HINT3
MYC	HIVEP3
MYC	HLA-DQB1
MYC	HMBS
MYC	HMGCR
MYC	HMGCS1
MYC	HNRNPA0
MYC	HNRNPA1
MYC	HNRNPA1L2
MYC	HNRNPAB
MYC	HNRNPC
MYC	HNRNPDL

MYC	HNRNPF
MYC	HNRNPK
MYC	HNRNPM
MYC	HNRNPR
MYC	HNRNPU
MYC	HOMER1
MYC	HPRT1
MYC	HSD17B7
MYC	HSF1
MYC	HSP90AA1
MYC	HSP90AB1
MYC	HSPA14
MYC	HSPA4
MYC	HSPA8
MYC	HSPA9
MYC	HSPBP1
MYC	HSPD1
MYC	HSPE1
MYC	HSPH1
MYC	HYAL2
MYC	HYOU1
MYC	IARS
MYC	ICA1
MYC	ICAM1
MYC	ICMT
MYC	ID3
MYC	IDH3A
MYC	IDI1
MYC	IER2
MYC	IER3
MYC	IER3IP1
MYC	IFRD1
MYC	IFRD2
MYC	IFT46
MYC	IFT80
MYC	IGFBP4
MYC	IL21R
MYC	IL2RA
MYC	IL2RG
MYC	IL6
MYC	ILF2
MYC	ILF3
MYC	ILVBL
MYC	IMMT
MYC	IMP3
MYC	IMP4
MYC	IMPDH1
MYC	IMPDH2
MYC	ING5
MYC	INIP

MYC	INSIG1
MYC	INTS10
MYC	INTS6
MYC	INTS9
MYC	IPO4
MYC	IPO5
MYC	IPO7
MYC	IRF5
MYC	IRF8
MYC	ISCA1
MYC	ISG20L2
MYC	ISOC2
MYC	ITGA1
MYC	JAGN1
MYC	JAM2
MYC	JAML
MYC	JMJD8
MYC	JOSD1
MYC	JTB
MYC	KANSL2
MYC	KARS
MYC	KAT2A
MYC	KCMF1
MYC	KCNG1
MYC	KCTD15
MYC	KDM2B
MYC	KDM5C
MYC	KDM6B
MYC	KIAA1143
MYC	KLF10
MYC	KLF7
MYC	KLHL18
MYC	KMO
MYC	KNOP1
MYC	KPNA1
MYC	KPNA3
MYC	KPNA4
MYC	KPNA6
MYC	KPNB1
MYC	KRI1
MYC	KRR1
MYC	KRTCAP2
MYC	KTI12
MYC	KXD1
MYC	LACTB
MYC	LAMP1
MYC	LAMTOR3
MYC	LAPTM4B
MYC	LARP4
MYC	LARS

MYC	LAS1L
MYC	LASP1
MYC	LAX1
MYC	LCMT2
MYC	LCP1
MYC	LDHA
MYC	LDHB
MYC	LGALS3BP
MYC	LIG3
MYC	LINC00649
MYC	LINC00662
MYC	LINC00665
MYC	LINS1
MYC	LIPA
MYC	LITAF
MYC	LONP1
MYC	LPCAT1
MYC	LRMP
MYC	LRPPRC
MYC	LRRC47
MYC	LRRFIP1
MYC	LSM1
MYC	LSM12
MYC	LSM2
MYC	LSM5
MYC	LTA
MYC	LTV1
MYC	LUC7L
MYC	LUC7L2
MYC	LUC7L3
MYC	LYAR
MYC	LYN
MYC	LYPLA2
MYC	LYRM7
MYC	LYST
MYC	M6PR
MYC	MACROD1
MYC	MAFK
MYC	MAGOH
MYC	MAGOHB
MYC	MALSU1
MYC	MANBAL
MYC	MAP2K3
MYC	MAP3K13
MYC	MAP3K8
MYC	MAP4K5
MYC	MAP7D3
MYC	MAPK13
MYC	MAPK1IP1L
MYC	MAPK6

MYC	MAPKAPK3
MYC	MAPRE1
MYC	MARCH3
MYC	MARCKS
MYC	MARS
MYC	MAT2A
MYC	MATK
MYC	MBIP
MYC	MCAT
MYC	MCRIP1
MYC	MCRIP2
MYC	ME2
MYC	MECR
MYC	MED10
MYC	MED19
MYC	MED23
MYC	MED28
MYC	MED4
MYC	MED8
MYC	MEST
MYC	METRN
MYC	METTL1
MYC	METTL13
MYC	METTL21A
MYC	METTL2A
MYC	METTL2B
MYC	METTL5
MYC	METTL8
MYC	MFN1
MYC	MFNG
MYC	MFSD2A
MYC	MGAT1
MYC	MICAL1
MYC	MIEF1
MYC	MIF
MYC	MINOS1
MYC	MIOS
MYC	MIR155HG
MYC	MIR210HG
MYC	MIR3142HG
MYC	MLF2
MYC	MLST8
MYC	MLYCD
MYC	MMAB
MYC	MMADHC
MYC	MMP7
MYC	MORF4L2
MYC	MPDU1
MYC	MPHOSPH10
MYC	MPHOSPH6

MYC	MPP6
MYC	MREG
MYC	MRM1
MYC	MRM3
MYC	MRPL1
MYC	MRPL11
MYC	MRPL12
MYC	MRPL13
MYC	MRPL15
MYC	MRPL16
MYC	MRPL17
MYC	MRPL19
MYC	MRPL20
MYC	MRPL21
MYC	MRPL22
MYC	MRPL23
MYC	MRPL24
MYC	MRPL3
MYC	MRPL32
MYC	MRPL36
MYC	MRPL39
MYC	MRPL4
MYC	MRPL42
MYC	MRPL44
MYC	MRPL47
MYC	MRPL50
MYC	MRPL52
MYC	MRPL54
MYC	MRPL57
MYC	MRPS10
MYC	MRPS12
MYC	MRPS15
MYC	MRPS16
MYC	MRPS17
MYC	MRPS18C
MYC	MRPS2
MYC	MRPS22
MYC	MRPS23
MYC	MRPS25
MYC	MRPS26
MYC	MRPS30
MYC	MRPS35
MYC	MRPS9
MYC	MRRF
MYC	MRTO4
MYC	MSANTD4
MYC	MSMO1
MYC	MT1X
MYC	MTAP
MYC	MTF1

MYC	MTHFD1L
MYC	MTHFD2
MYC	MTHFD2L
MYC	MTIF2
MYC	MTMR2
MYC	MTPAP
MYC	MTSS1
MYC	MTX2
MYC	MVP
MYC	MYBBP1A
MYC	MYC
MYC	MYO1F
MYC	MYO9B
MYC	NAA10
MYC	NAA15
MYC	NAA20
MYC	NAA25
MYC	NAA35
MYC	NAA50
MYC	NABP1
MYC	NACA
MYC	NAE1
MYC	NAF1
MYC	NAMPT
MYC	NAP1L1
MYC	NARS
MYC	NARS2
MYC	NAT10
MYC	NAT9
MYC	NAXE
MYC	NCBP2
MYC	NCF2
MYC	NCL
MYC	NCLN
MYC	NCSTN
MYC	NDFIP1
MYC	NDUFA13
MYC	NDUFA5
MYC	NDUFAB1
MYC	NDUFAF4
MYC	NDUFAF6
MYC	NDUFB9
MYC	NDUFS6
MYC	NECAP1
MYC	NEDD4L
MYC	NEIL2
MYC	NELFE
MYC	NEPRO
MYC	NETO1
MYC	NETO2

MYC	NEU1
MYC	NFE2L1
MYC	NFE2L2
MYC	NFKB1
MYC	NFKB2
MYC	NFKBIA
MYC	NFKBIB
MYC	NFKBID
MYC	NFKBIE
MYC	NFKBIZ
MYC	NGDN
MYC	NHP2
MYC	NIFK
MYC	NIN
MYC	NINJ1
MYC	NIP7
MYC	NIPA2
MYC	NIPAL4
MYC	NKIRAS2
MYC	NLE1
MYC	NMD3
MYC	NME1
MYC	NOB1
MYC	NOC2L
MYC	NOC3L
MYC	NOC4L
MYC	NOL10
MYC	NOL11
MYC	NOL7
MYC	NOL8
MYC	NOL9
MYC	NOLC1
MYC	NOM1
MYC	NOMO1
MYC	NOP10
MYC	NOP2
MYC	NOP56
MYC	NOP58
MYC	NORAD
MYC	NPAT
MYC	NPM1
MYC	NPM3
MYC	NPW
MYC	NR1D1
MYC	NR2F6
MYC	NR4A1
MYC	NR4A2
MYC	NR4A3
MYC	NRG4
MYC	NRP2

MYC	NRROS
MYC	NSA2
MYC	NSUN2
MYC	NSUN5
MYC	NTMT1
MYC	NUAK2
MYC	NUBP1
MYC	NUBP2
MYC	NUDC
MYC	NUDCD1
MYC	NUDT15
MYC	NUDT19
MYC	NUDT4
MYC	NUDT5
MYC	NUDT9
MYC	NUFIP1
MYC	NUP107
MYC	NUP160
MYC	NUP35
MYC	NUP54
MYC	NUP58
MYC	NUP62
MYC	NUP93
MYC	NUP98
MYC	NUPL2
MYC	NUS1
MYC	NUTF2
MYC	NUTM2B-AS1
MYC	NXT1
MYC	OCIAD1
MYC	ODC1
MYC	OGFOD1
MYC	OLA1
MYC	OPA1
MYC	OPN3
MYC	ORAI1
MYC	ORMDL1
MYC	ORMDL2
MYC	OSTC
MYC	OTUD4
MYC	OTUD6B
MYC	OXNAD1
MYC	OXSR1
MYC	P4HA1
MYC	PA2G4
MYC	PABPC1
MYC	PABPC4
MYC	PACRGL
MYC	PAF1
MYC	PAICS

MYC	PAK1IP1
MYC	PAK2
MYC	PANX1
MYC	PARK7
MYC	PARL
MYC	PARVB
MYC	PAWR
MYC	PBDC1
MYC	PBXIP1
MYC	PCGF5
MYC	PCID2
MYC	PCK2
MYC	PCNP
MYC	PCYT1A
MYC	PDAP1
MYC	PDCD10
MYC	PDCD11
MYC	PDCD2
MYC	PDCD2L
MYC	PDCD4
MYC	PDCD5
MYC	PDCD7
MYC	PDCL3
MYC	PDE12
MYC	PDGFA
MYC	PDHA1
MYC	PDLIM2
MYC	PDRG1
MYC	PEA15
MYC	PEF1
MYC	PELO
MYC	PELP1
MYC	PEMT
MYC	PEPD
MYC	PER2
MYC	PERP
MYC	PES1
MYC	PEX1
MYC	PFDN2
MYC	PFDN6
MYC	PFKP
MYC	PGAM1
MYC	PHACTR1
MYC	PHB
MYC	PHB2
MYC	PHF5A
MYC	PHPT1
MYC	PIGH
MYC	PIGL
MYC	PIGW

MYC	PIKFYVE
MYC	PIM3
MYC	PIP5K1B
MYC	PITHD1
MYC	PITPNB
MYC	PKM
MYC	PLCE1
MYC	PLEKHB2
MYC	PLGRKT
MYC	PLK3
MYC	PLRG1
MYC	PMAIP1
MYC	PMM2
MYC	PMPCB
MYC	PMS1
MYC	PNO1
MYC	PNP
MYC	PNPO
MYC	PNPT1
MYC	POGK
MYC	POLD2
MYC	POLDIP2
MYC	POLE3
MYC	POLR1A
MYC	POLR1C
MYC	POLR1E
MYC	POLR2C
MYC	POLR2D
MYC	POLR2F
MYC	POLR2H
MYC	POLR2I
MYC	POLR2K
MYC	POLR3D
MYC	POLR3G
MYC	POLR3H
MYC	POLRMT
MYC	POMP
MYC	POP1
MYC	POP4
MYC	PPA1
MYC	PPAN
MYC	PPAT
MYC	PPIA
MYC	PPID
MYC	PPIF
MYC	PPIG
MYC	PPIH
MYC	PPIL1
MYC	PPM1G
MYC	PPM1M

MYC	PPP1R11
MYC	PPP1R14B
MYC	PPP1R15A
MYC	PPP1R2
MYC	PPP2CB
MYC	PPP2R2A
MYC	PPP3CA
MYC	PPP4R3B
MYC	PPRC1
MYC	PPT1
MYC	PPWD1
MYC	PRDX1
MYC	PRELID1
MYC	PRELID3B
MYC	PREP
MYC	PREPL
MYC	PRKAB2
MYC	PRKAG2-AS1
MYC	PRKAR1B
MYC	PRMT1
MYC	PRMT3
MYC	PRMT5
MYC	PRNP
MYC	PRPF18
MYC	PRPF19
MYC	PRPF31
MYC	PRPF38A
MYC	PRPF38B
MYC	PRPF39
MYC	PRPF4
MYC	PRPF40A
MYC	PRPF4B
MYC	PRPS1
MYC	PRPS2
MYC	PRR3
MYC	PRR7
MYC	PRRC2C
MYC	PRRG4
MYC	PSAT1
MYC	PSMA1
MYC	PSMA2
MYC	PSMA3
MYC	PSMA4
MYC	PSMA5
MYC	PSMB1
MYC	PSMB2
MYC	PSMB5
MYC	PSMB7
MYC	PSMC1
MYC	PSMC2

MYC	PSMC3
MYC	PSMC4
MYC	PSMC5
MYC	PSMC6
MYC	PSMD1
MYC	PSMD11
MYC	PSMD12
MYC	PSMD13
MYC	PSMD14
MYC	PSMD2
MYC	PSMD3
MYC	PSMD4
MYC	PSMD6
MYC	PSMD7
MYC	PSMD8
MYC	PSME3
MYC	PSMF1
MYC	PSMG1
MYC	PSMG4
MYC	PTBP1
MYC	PTGER4
MYC	PTGES2
MYC	PTGES3
MYC	PTP4A1
MYC	PTPN2
MYC	PTPN6
MYC	PTRH1
MYC	PTRH2
MYC	PUF60
MYC	PUM3
MYC	PUS7
MYC	PWP1
MYC	PYCR1
MYC	PYCR2
MYC	QPCTL
MYC	QTRT1
MYC	QTRT2
MYC	RAB10
MYC	RAB11FIP1
MYC	RAB20
MYC	RAB21
MYC	RAB27A
MYC	RAB28
MYC	RAB7A
MYC	RAB8B
MYC	RABEP1
MYC	RABEPK
MYC	RAD23A
MYC	RAD23B
MYC	RAD51D

MYC	RALA
MYC	RAN
MYC	RANBP1
MYC	RARS
MYC	RASGEF1B
MYC	RB1CC1
MYC	RBM10
MYC	RBM12
MYC	RBM17
MYC	RBM18
MYC	RBM25
MYC	RBM3
MYC	RBM39
MYC	RBM4
MYC	RBM8A
MYC	RBMX
MYC	RCL1
MYC	RCN1
MYC	RCN2
MYC	RDH13
MYC	RDH14
MYC	RDX
MYC	REL
MYC	RELL2
MYC	REV3L
MYC	REXO1
MYC	REXO2
MYC	REXO4
MYC	RFT1
MYC	RFXAP
MYC	RGMB
MYC	RHEB
MYC	RHOB
MYC	RHOBTB3
MYC	RHOC
MYC	RHOF
MYC	RHOG
MYC	RILPL2
MYC	RIN3
MYC	RIOK1
MYC	RIOK2
MYC	RLIM
MYC	RNASEH1
MYC	RNF126
MYC	RNF139
MYC	RNF145
MYC	RNF146
MYC	RNF19A
MYC	RNF2
MYC	RNF220

MYC	RNF41
MYC	RNH1
MYC	RNMT
MYC	RNPS1
MYC	RP9
MYC	RPE
MYC	RPF1
MYC	RPF2
MYC	RPIA
MYC	RPL12
MYC	RPL14
MYC	RPL17
MYC	RPL18A
MYC	RPL22L1
MYC	RPL23
MYC	RPL24
MYC	RPL26L1
MYC	RPL27
MYC	RPL29
MYC	RPL30
MYC	RPL31
MYC	RPL32
MYC	RPL35
MYC	RPL35A
MYC	RPL36
MYC	RPL36AL
MYC	RPL37
MYC	RPL37A
MYC	RPL38
MYC	RPL5
MYC	RPL7
MYC	RPL7L1
MYC	RPL9
MYC	RPLP0
MYC	RPLP2
MYC	RPP25
MYC	RPP38
MYC	RPS10
MYC	RPS12
MYC	RPS13
MYC	RPS17
MYC	RPS19BP1
MYC	RPS21
MYC	RPS24
MYC	RPS25
MYC	RPS29
MYC	RPS5
MYC	RPS6KB1
MYC	RPSA
MYC	RPUSD1

MYC	RRAGD
MYC	RRP1
MYC	RRP15
MYC	RRP1B
MYC	RRP7A
MYC	RRP8
MYC	RRP9
MYC	RRS1
MYC	RSL1D1
MYC	RSL24D1
MYC	RSRC2
MYC	RTCB
MYC	RUSC1
MYC	RUVBL1
MYC	RWDD1
MYC	RWDD4
MYC	RYK
MYC	S100PBP
MYC	SACS
MYC	SAMD1
MYC	SAMSN1
MYC	SAP18
MYC	SAR1A
MYC	SARS
MYC	SART1
MYC	SBDS
MYC	SBF2-AS1
MYC	SCAF4
MYC	SCAMP1-AS1
MYC	SCAMP3
MYC	SCAP
MYC	SCD
MYC	SCML1
MYC	SCO1
MYC	SCRN3
MYC	SDAD1
MYC	SDCBP
MYC	SDF4
MYC	SDHAF3
MYC	SDHB
MYC	SEH1L
MYC	SEMA7A
MYC	SENP3
MYC	SERBP1
MYC	SERPINB8
MYC	SERTAD1
MYC	SESN2
MYC	SET
MYC	SETD6
MYC	SETD7

MYC	SF1
MYC	SF3A3
MYC	SF3B2
MYC	SF3B3
MYC	SF3B4
MYC	SF3B5
MYC	SF3B6
MYC	SFPQ
MYC	SFSWAP
MYC	SGK1
MYC	SGTA
MYC	SH2D4A
MYC	SH3BP2
MYC	SHMT2
MYC	SIAH2
MYC	SIN3A
MYC	SIPA1L1
MYC	SIRPA
MYC	SIRT5
MYC	SLAMF1
MYC	SLC12A6
MYC	SLC16A1
MYC	SLC16A9
MYC	SLC17A9
MYC	SLC19A1
MYC	SLC1A5
MYC	SLC25A13
MYC	SLC25A17
MYC	SLC25A22
MYC	SLC25A25
MYC	SLC25A26
MYC	SLC25A3
MYC	SLC25A33
MYC	SLC25A39
MYC	SLC25A51
MYC	SLC27A4
MYC	SLC29A1
MYC	SLC35A2
MYC	SLC35B2
MYC	SLC35F5
MYC	SLC38A1
MYC	SLC38A2
MYC	SLC38A5
MYC	SLC39A1
MYC	SLC39A6
MYC	SLC39A8
MYC	SLC3A2
MYC	SLC43A3
MYC	SLC4A7
MYC	SLC50A1

MYC	SLC6A9
MYC	SLC7A1
MYC	SLC7A5
MYC	SLC9B2
MYC	SLCO4A1
MYC	SLIRP
MYC	SMARCA5
MYC	SMC5
MYC	SMG5
MYC	SMIM10L1
MYC	SMIM12
MYC	SMIM15
MYC	SMIM19
MYC	SMIM4
MYC	SMKR1
MYC	SMN1
MYC	SMN2
MYC	SMNDC1
MYC	SMS
MYC	SMU1
MYC	SNAPC4
MYC	SNAPC5
MYC	SNHG15
MYC	SNHG8
MYC	SNHG9
MYC	SNIP1
MYC	SNRNP40
MYC	SNRPB
MYC	SNRPB2
MYC	SNRPD1
MYC	SNRPD2
MYC	SNRPE
MYC	SNRPF
MYC	SNRPG
MYC	SNU13
MYC	SNUPN
MYC	SNW1
MYC	SNX18
MYC	SNX8
MYC	SNX9
MYC	SOCS3
MYC	SOD1
MYC	SOD2
MYC	SON
MYC	SORD
MYC	SP2
MYC	SPA17
MYC	SPAG9
MYC	SPATA2
MYC	SPATA5L1

MYC	SPHK1
MYC	SPNS1
MYC	SPRY1
MYC	SPRYD3
MYC	SPRYD4
MYC	SQLE
MYC	SQSTM1
MYC	SREK1IP1
MYC	SRFBP1
MYC	SRGAP2
MYC	SRGN
MYC	SRI
MYC	SRM
MYC	SRP19
MYC	SRP54
MYC	SRP72
MYC	SRPK1
MYC	SRPRB
MYC	SRRM1
MYC	SRRM2
MYC	SRRT
MYC	SRSF1
MYC	SRSF10
MYC	SRSF11
MYC	SRSF2
MYC	SRSF3
MYC	SRSF6
MYC	SRSF7
MYC	SSB
MYC	SSBP1
MYC	SSRP1
MYC	SSSCA1
MYC	ST13
MYC	ST3GAL2
MYC	ST6GALNAC3
MYC	ST8SIA4
MYC	STARD3NL
MYC	STAT5A
MYC	STAU1
MYC	STIP1
MYC	STK4
MYC	STOML1
MYC	STOML2
MYC	STRAP
MYC	STRIP1
MYC	STX11
MYC	STX18
MYC	STX4
MYC	STXBP3
MYC	SUB1

MYC	SUGT1
MYC	SUMO1
MYC	SUMO2
MYC	SUPT16H
MYC	SUPV3L1
MYC	SURF2
MYC	SURF4
MYC	SURF6
MYC	SYNCRIP
MYC	TAF11
MYC	TAF1A
MYC	TAF1D
MYC	TAF5L
MYC	TAF9
MYC	TALDO1
MYC	TAPBP
MYC	TARBP2
MYC	TARS
MYC	TASP1
MYC	TATDN1
MYC	TBC1D24
MYC	TBCA
MYC	TBL3
MYC	TBRG1
MYC	TBRG4
MYC	TCERG1
MYC	TCF4
MYC	TCF7
MYC	TCOF1
MYC	TCP1
MYC	TCTN3
MYC	TDG
MYC	TELO2
MYC	TES
MYC	TEX10
MYC	TFAM
MYC	TFAP4
MYC	TFB2M
MYC	TFEC
MYC	TFPI2
MYC	TFRC
MYC	TGDS
MYC	TGIF1
MYC	TGS1
MYC	THAP1
MYC	THAP12
MYC	THAP7
MYC	THOC1
MYC	THOC6
MYC	THUMPD2

MYC	TIGAR
MYC	TIMM10
MYC	TIMM10B
MYC	TIMM13
MYC	TIMM17A
MYC	TIMM22
MYC	TIMM23
MYC	TIMM23B
MYC	TIMM44
MYC	TIMM50
MYC	TIMM8A
MYC	TIMM9
MYC	TLCD1
MYC	TLK1
MYC	TM2D3
MYC	TM9SF4
MYC	TMA16
MYC	TMBIM1
MYC	TMBIM4
MYC	TMBIM6
MYC	TMCO1
MYC	TMED2
MYC	TMED3
MYC	TMED4
MYC	TMED9
MYC	TMEM109
MYC	TMEM120B
MYC	TMEM123
MYC	TMEM126A
MYC	TMEM141
MYC	TMEM147
MYC	TMEM150A
MYC	TMEM165
MYC	TMEM167A
MYC	TMEM167B
MYC	TMEM168
MYC	TMEM170A
MYC	TMEM183A
MYC	TMEM184C
MYC	TMEM185B
MYC	TMEM192
MYC	TMEM199
MYC	TMEM205
MYC	TMEM208
MYC	TMEM216
MYC	TMEM222
MYC	TMEM248
MYC	TMEM251
MYC	TMEM259
MYC	TMEM260

MYC	TMEM267
MYC	TMEM33
MYC	TMEM38B
MYC	TMEM39B
MYC	TMEM43
MYC	TMEM64
MYC	TMEM70
MYC	TMEM9B
MYC	TMX1
MYC	TMX2
MYC	TMX3
MYC	TNF
MYC	TNFAIP1
MYC	TNFAIP3
MYC	TNFAIP8
MYC	TNFRSF9
MYC	TNIP1
MYC	TNIP2
MYC	TNPO2
MYC	TOE1
MYC	TOLLIP
MYC	TOMM20
MYC	TOMM22
MYC	TOMM34
MYC	TOMM40
MYC	TOMM5
MYC	TOP1MT
MYC	TP53BP2
MYC	TRA2B
MYC	TRAF1
MYC	TRAF2
MYC	TRAF4
MYC	TRAPPC2B
MYC	TRAPPC4
MYC	TRDMT1
MYC	TRIAP1
MYC	TRIB3
MYC	TRIM28
MYC	TRIM44
MYC	TRMO
MYC	TRMT1
MYC	TRMT10C
MYC	TRMT112
MYC	TRMT6
MYC	TRMT61A
MYC	TRNT1
MYC	TRUB1
MYC	TRUB2
MYC	TSC22D2
MYC	TSEN15

MYC	TSEN2
MYC	TSPAN17
MYC	TSPYL5
MYC	TSR1
MYC	TSR2
MYC	TSSC4
MYC	TSTA3
MYC	TSTD2
MYC	TTC1
MYC	TTC19
MYC	TTC27
MYC	TTC33
MYC	TTF1
MYC	TTLL11
MYC	TUBA1C
MYC	TUBB4B
MYC	TUBE1
MYC	TUFM
MYC	TUSC2
MYC	TUT1
MYC	TVP23B
MYC	TWISTNB
MYC	TXLNG
MYC	TXN
MYC	TXNL1
MYC	TXNL4A
MYC	TXNRD1
MYC	TYW3
MYC	U2AF2
MYC	U2SURP
MYC	UAP1
MYC	UBA2
MYC	UBA52
MYC	UBA6
MYC	UBALD2
MYC	UBAP2
MYC	UBB
MYC	UBC
MYC	UBE2B
MYC	UBE2D1
MYC	UBE2D3
MYC	UBE2G2
MYC	UBE2M
MYC	UBE2N
MYC	UBE2Q2
MYC	UBE2V2
MYC	UBE2Z
MYC	UBE3A
MYC	UBE3D
MYC	UBIAD1

MYC	UBLCP1
MYC	UBQLN1
MYC	UBR1
MYC	UBXN6
MYC	UCHL3
MYC	UCHL5
MYC	UCK2
MYC	UEVLD
MYC	UFM1
MYC	UGCG
MYC	UGT8
MYC	UMPS
MYC	UNK
MYC	UPRT
MYC	UQCC2
MYC	UQCRQ
MYC	URI1
MYC	URM1
MYC	USE1
MYC	USP10
MYC	USP12
MYC	USP14
MYC	USP16
MYC	USP25
MYC	USP8
MYC	UTP11
MYC	UTP14A
MYC	UTP15
MYC	UTP18
MYC	UTP23
MYC	UTP3
MYC	UTP4
MYC	UTP6
MYC	UVRAG
MYC	UXS1
MYC	UXT
MYC	VAPA
MYC	VARS2
MYC	VCPKMT
MYC	VDAC1
MYC	VDAC2
MYC	VEGFA
MYC	VKORC1L1
MYC	VLDLR
MYC	VMA21
MYC	VPS26A
MYC	VPS37A
MYC	VPS37B
MYC	VPS4A
MYC	VPS72

MYC	VPS9D1−AS1
MYC	VTI1B
MYC	VWCE
MYC	WARS
MYC	WBP11
MYC	WDFY1
MYC	WDR12
MYC	WDR25
MYC	WDR3
MYC	WDR4
MYC	WDR43
MYC	WDR45B
MYC	WDR46
MYC	WDR5
MYC	WDR55
MYC	WDR59
MYC	WDR70
MYC	WDR74
MYC	WDR75
MYC	WDR77
MYC	WDR82
MYC	WDR83OS
MYC	WDR91
MYC	WIZ
MYC	WRN
MYC	WSB2
MYC	WTAP
MYC	XAB2
MYC	XPO1
MYC	XPO4
MYC	XPO6
MYC	XPOT
MYC	XRCC5
MYC	XRCC6
MYC	XRN2
MYC	XXYLT1
MYC	YARS
MYC	YBX1
MYC	YBX3
MYC	YIF1A
MYC	YIPF4
MYC	YKT6
MYC	YLPM1
MYC	YRDC
MYC	YTHDF2
MYC	YWHAE
MYC	YWHAG
MYC	YWHAQ
MYC	ZAP70
MYC	ZBED3−AS1

MYC	ZBTB11-AS1
MYC	ZBTB2
MYC	ZBTB24
MYC	ZC3H12A
MYC	ZC3H15
MYC	ZC3H8
MYC	ZCCHC4
MYC	ZFAND5
MYC	ZFAS1
MYC	ZFP36L1
MYC	ZFP91
MYC	ZFYVE9
MYC	ZHX1
MYC	ZMAT2
MYC	ZMPSTE24
MYC	ZMYND11
MYC	ZNF131
MYC	ZNF146
MYC	ZNF22
MYC	ZNF263
MYC	ZNF267
MYC	ZNF322
MYC	ZNF330
MYC	ZNF426
MYC	ZNF480
MYC	ZNF485
MYC	ZNF511
MYC	ZNF526
MYC	ZNF567
MYC	ZNF593
MYC	ZNF667
MYC	ZNF706
MYC	ZNF746
MYC	ZNF75A
MYC	ZNF770
MYC	ZNF835
MYC	ZNF85
MYC	ZNFX1
MYC	ZNHIT6
MYC	ZPR1
MYC	ZRANB2
MYC	ZSCAN21
MYC	ZSCAN9
MYC	ZSWIM7
NFATC1	ABRACL
NFATC1	ARHGEF12
NFATC1	BCL2A1
NFATC1	BCL2L1
NFATC1	BLNK
NFATC1	CCDC88A

NFATC1	CD83
NFATC1	EIF5A
NFATC1	FCRL3
NFATC1	HES1
NFATC1	HIVEP3
NFATC1	IGF2BP3
NFATC1	MAP3K8
NFATC1	NFATC1
NFATC1	NFKBID
NFATC1	NIN
NFATC1	PHF6
NFATC1	PIM1
NFATC1	PPAN
NFATC1	PTPRE
NFATC1	RILPL2
NFATC1	SAMSN1
NFATC1	SDCBP
NFATC1	SLC38A1
NFATC1	SSTR2
NFATC1	TESPA1
NFATC1	TGIF1
NFATC1	TLE1
NFATC1	UBALD2
NFE2L1	ANXA7
NFE2L1	ATP1A1
NFE2L1	DNPEP
NFE2L1	EMP3
NFE2L1	ENO1
NFE2L1	FTH1
NFE2L1	GHITM
NFE2L1	HSP90AB1
NFE2L1	HSPA9
NFE2L1	LMNA
NFE2L1	MIR155HG
NFE2L1	NCL
NFE2L1	NFE2L2
NFE2L1	PSMA1
NFE2L1	PSMD11
NFE2L1	PSMD14
NFE2L1	RHOF
NFE2L1	SLC1A5
NFE2L1	SLC3A2
NFE2L1	SQSTM1
NFE2L1	TES
NFE2L1	TFEC
NFE2L1	TRIB3
NFE2L1	TXN
NFE2L1	TXNL1
NFE2L1	UCK2
NFE2L2	AARS

NFE2L2	AASDHPPT
NFE2L2	ABCE1
NFE2L2	ABCF1
NFE2L2	ABT1
NFE2L2	ACOT9
NFE2L2	ADNP2
NFE2L2	ADPGK
NFE2L2	AKIRIN1
NFE2L2	AKTIP
NFE2L2	ALG13
NFE2L2	AMD1
NFE2L2	ANKRD11
NFE2L2	AP2B1
NFE2L2	AP5Z1
NFE2L2	APBB1
NFE2L2	APEX1
NFE2L2	ARHGEF12
NFE2L2	ARPP19
NFE2L2	ASAH1
NFE2L2	ASNS
NFE2L2	ATF4
NFE2L2	ATG5
NFE2L2	ATP11B
NFE2L2	ATP1B3
NFE2L2	BCAS2
NFE2L2	BDP1
NFE2L2	BTG1
NFE2L2	BUD31
NFE2L2	C19orf70
NFE2L2	C1orf174
NFE2L2	C2orf49
NFE2L2	C2orf69
NFE2L2	C6orf99
NFE2L2	CAMK1
NFE2L2	CARS
NFE2L2	CCDC59
NFE2L2	CCL4L2
NFE2L2	CCND1
NFE2L2	CCND2
NFE2L2	CCNL1
NFE2L2	CCT2
NFE2L2	CCT4
NFE2L2	CDC25C
NFE2L2	CDK4
NFE2L2	CEBPB
NFE2L2	CEBPG
NFE2L2	CEBPZ
NFE2L2	CFLAR
NFE2L2	CHAC1
NFE2L2	CHD1

NFE2L2	CIRBP
NFE2L2	CMSS1
NFE2L2	CNOT7
NFE2L2	COMMD1
NFE2L2	CSNK1D
NFE2L2	CTNNA1
NFE2L2	CTU2
NFE2L2	CWC25
NFE2L2	CYCS
NFE2L2	DCTD
NFE2L2	DDIT3
NFE2L2	DDX10
NFE2L2	DDX18
NFE2L2	DDX28
NFE2L2	DDX5
NFE2L2	DNAJA2
NFE2L2	DNPEP
NFE2L2	DNTTIP2
NFE2L2	DPM1
NFE2L2	DUSP2
NFE2L2	DXO
NFE2L2	DYNC1I2
NFE2L2	E4F1
NFE2L2	EBF1
NFE2L2	EGR3
NFE2L2	EIF1
NFE2L2	EIF4A1
NFE2L2	EIF4A2
NFE2L2	ELL2
NFE2L2	ELP5
NFE2L2	ETF1
NFE2L2	ETV6
NFE2L2	FAM234A
NFE2L2	FAM50A
NFE2L2	FBL
NFE2L2	FGFR1
NFE2L2	FOXP1
NFE2L2	GAR1
NFE2L2	GARS
NFE2L2	GHITM
NFE2L2	GNL1
NFE2L2	GNL2
NFE2L2	GOT1
NFE2L2	GPBP1
NFE2L2	GPER1
NFE2L2	GTPBP10
NFE2L2	GTSF1
NFE2L2	HNRNPA3
NFE2L2	HNRNPU
NFE2L2	HSP90AB1

NFE2L2	HSPA9
NFE2L2	IARS
NFE2L2	ID3
NFE2L2	IER2
NFE2L2	ILF3
NFE2L2	IMPDH2
NFE2L2	INSIG1
NFE2L2	IRF4
NFE2L2	ISCA1
NFE2L2	KDM2A
NFE2L2	KDM6B
NFE2L2	KIAA1143
NFE2L2	LAMP1
NFE2L2	LANCL2
NFE2L2	LAPTM4A
NFE2L2	LARP7
NFE2L2	LARS
NFE2L2	LDHA
NFE2L2	LIMK1
NFE2L2	MAFG
NFE2L2	MAFK
NFE2L2	MAGOH
NFE2L2	MAGOHB
NFE2L2	MAPRE1
NFE2L2	MBNL3
NFE2L2	MCRIP1
NFE2L2	MIF
NFE2L2	MIR181A1HG
NFE2L2	MRPL1
NFE2L2	MRPS17
NFE2L2	MRPS18B
NFE2L2	MRRF
NFE2L2	NABP1
NFE2L2	NAMPT
NFE2L2	NCBP2
NFE2L2	NDRG4
NFE2L2	NECAP1
NFE2L2	NELFE
NFE2L2	NFE2L2
NFE2L2	NFKB1
NFE2L2	NFKB2
NFE2L2	NFYB
NFE2L2	NIPA2
NFE2L2	NOC4L
NFE2L2	NOLC1
NFE2L2	NOP58
NFE2L2	NR4A2
NFE2L2	NUP133
NFE2L2	NUP155
NFE2L2	NUP214

NFE2L2	NUP93
NFE2L2	OCIAD1
NFE2L2	OTUD4
NFE2L2	PABPC1
NFE2L2	PAK1IP1
NFE2L2	PARD6A
NFE2L2	PCK2
NFE2L2	PCM1
NFE2L2	PELO
NFE2L2	PEPD
NFE2L2	PFDN2
NFE2L2	PHACTR1
NFE2L2	PIM3
NFE2L2	PLRG1
NFE2L2	PMAIP1
NFE2L2	POLR2H
NFE2L2	POP4
NFE2L2	PPIG
NFE2L2	PPP1R15A
NFE2L2	PRMT1
NFE2L2	PRNP
NFE2L2	PRPF38B
NFE2L2	PRRC2C
NFE2L2	PSMD14
NFE2L2	PTP4A1
NFE2L2	PUM3
NFE2L2	PWP1
NFE2L2	PYCR1
NFE2L2	QTRT1
NFE2L2	RAB21
NFE2L2	RAB27A
NFE2L2	RAD23A
NFE2L2	RAD23B
NFE2L2	RALA
NFE2L2	RALGAPA1
NFE2L2	RBM25
NFE2L2	RCC1
NFE2L2	REL
NFE2L2	RELB
NFE2L2	RHEB
NFE2L2	RING1
NFE2L2	RNH1
NFE2L2	RNPS1
NFE2L2	RPA2
NFE2L2	RPL38
NFE2L2	RPL9
NFE2L2	RPLP2
NFE2L2	RPS29
NFE2L2	RRNAD1
NFE2L2	RRP1

NFE2L2	RRP1B
NFE2L2	RSL24D1
NFE2L2	RTN4
NFE2L2	SBDS
NFE2L2	SDE2
NFE2L2	SEPT7
NFE2L2	SF1
NFE2L2	SH2B3
NFE2L2	SH3RF1
NFE2L2	SHMT2
NFE2L2	SLC25A33
NFE2L2	SLC38A1
NFE2L2	SLC3A2
NFE2L2	SLC7A1
NFE2L2	SMARCA5
NFE2L2	SMNDC1
NFE2L2	SNHG15
NFE2L2	SNX11
NFE2L2	SPG21
NFE2L2	SQSTM1
NFE2L2	SREBF1
NFE2L2	SRRM1
NFE2L2	SRSF3
NFE2L2	SSSCA1
NFE2L2	ST13
NFE2L2	STK19
NFE2L2	STK4
NFE2L2	TBC1D22A
NFE2L2	TBL3
NFE2L2	THOC1
NFE2L2	TMEM120B
NFE2L2	TMEM126B
NFE2L2	TMEM147
NFE2L2	TMEM175
NFE2L2	TMF1
NFE2L2	TMTC4
NFE2L2	TNFAIP3
NFE2L2	TRA2A
NFE2L2	TRA2B
NFE2L2	TRAF4
NFE2L2	TRAP1
NFE2L2	TRIB3
NFE2L2	TRPS1
NFE2L2	TTC1
NFE2L2	TXNL1
NFE2L2	UBAP2
NFE2L2	UBB
NFE2L2	UBC
NFE2L2	UBE2B
NFE2L2	UBE2Z

NFE2L2	UMPS
NFE2L2	USP14
NFE2L2	UTP15
NFE2L2	VPS37B
NFE2L2	WBP11
NFE2L2	XRCC6
NFE2L2	XRN2
NFE2L2	YTHDC1
NFE2L2	ZBTB2
NFE2L2	ZFP36L1
NFE2L2	ZFY
NFE2L2	ZHX2
NFE2L2	ZNF131
NFE2L2	ZNF146
NFE2L2	ZNF281
NFE2L2	ZNF330
NFE2L2	ZNF706
NFE2L2	ZNRD1
NFIC	EEF1B2
NFKB1	ABCE1
NFKB1	ABTB2
NFKB1	BATF3
NFKB1	BCL2
NFKB1	BCL2A1
NFKB1	BCL2L1
NFKB1	BOD1
NFKB1	BTLA
NFKB1	CCND2
NFKB1	CCR7
NFKB1	CD40
NFKB1	CD44
NFKB1	CD82
NFKB1	CD83
NFKB1	CD86
NFKB1	CFLAR
NFKB1	CHMP4B
NFKB1	CMTM6
NFKB1	CREM
NFKB1	CSF1
NFKB1	DLGAP4
NFKB1	DNPEP
NFKB1	DUSP5
NFKB1	EBI3
NFKB1	ECE1
NFKB1	ENO1
NFKB1	EPB41L2
NFKB1	FOXK1
NFKB1	FOXP1
NFKB1	G3BP1
NFKB1	GALNT2

NFKB1	GNG4
NFKB1	HEXIM1
NFKB1	IFRD1
NFKB1	IL21R
NFKB1	IL2RA
NFKB1	IRF2BPL
NFKB1	KDM6B
NFKB1	KRBA1
NFKB1	MAML2
NFKB1	MAP4
NFKB1	MARCKS
NFKB1	MDFIC
NFKB1	MIR155HG
NFKB1	NCF2
NFKB1	NCK2
NFKB1	NDUFAF4
NFKB1	NFATC2
NFKB1	NFE2L1
NFKB1	NFKB1
NFKB1	NFKB2
NFKB1	NFKBIA
NFKB1	NFKBIB
NFKB1	NOC2L
NFKB1	NRP2
NFKB1	NRROS
NFKB1	NUP93
NFKB1	ODC1
NFKB1	PCK2
NFKB1	PDCD4
NFKB1	PMAIP1
NFKB1	PSMA1
NFKB1	PTGER4
NFKB1	REL
NFKB1	RHOF
NFKB1	RHOG
NFKB1	RNF2
NFKB1	SAV1
NFKB1	SIRPA
NFKB1	SLAMF1
NFKB1	SLC39A1
NFKB1	SLC7A1
NFKB1	SLCO5A1
NFKB1	SNX11
NFKB1	STAT5A
NFKB1	TFEC
NFKB1	TNFAIP8
NFKB1	TNFRSF9
NFKB1	TNIP2
NFKB1	TRAF1
NFKB1	TRAF3

NFKB1	ZBTB10
NFKB1	ZHX1
NFKB2	ABCF1
NFKB2	ABTB2
NFKB2	ADCY3
NFKB2	ADGRE5
NFKB2	ADGRG5
NFKB2	AEN
NFKB2	AGFG1
NFKB2	AMMECR1L
NFKB2	AMPD3
NFKB2	ANXA7
NFKB2	APPL1
NFKB2	ARHGAP24
NFKB2	ARHGAP25
NFKB2	ARID5A
NFKB2	ASAP1
NFKB2	ATAD2B
NFKB2	ATF3
NFKB2	ATP2B1
NFKB2	B2M
NFKB2	BASP1
NFKB2	BATF
NFKB2	BCL2
NFKB2	BCL2A1
NFKB2	BCL2L1
NFKB2	BID
NFKB2	BIRC3
NFKB2	BNIP1
NFKB2	C12orf29
NFKB2	C12orf42
NFKB2	C6orf48
NFKB2	CA8
NFKB2	CAMTA2
NFKB2	CAV1
NFKB2	CBX6
NFKB2	CCDC28B
NFKB2	CCDC50
NFKB2	CCDC58
NFKB2	CCND1
NFKB2	CCR7
NFKB2	CCSER2
NFKB2	CD40
NFKB2	CD44
NFKB2	CD58
NFKB2	CD82
NFKB2	CD83
NFKB2	CD86
NFKB2	CDC42SE1
NFKB2	CEBPG

NFKB2	CFLAR
NFKB2	CHMP4B
NFKB2	CIRBP
NFKB2	CMTM6
NFKB2	CREM
NFKB2	CSNK2A2
NFKB2	CYB561
NFKB2	CYB5A
NFKB2	CYLD
NFKB2	CYTH1
NFKB2	DGAT2
NFKB2	DHX40
NFKB2	DNAJB14
NFKB2	DNPEP
NFKB2	DPH2
NFKB2	DUSP10
NFKB2	DYNLT3
NFKB2	EBI3
NFKB2	ECE1
NFKB2	EMP3
NFKB2	EPSTI1
NFKB2	FAM84B
NFKB2	FBRS
NFKB2	FNBP1
NFKB2	FOXP1
NFKB2	GAB1
NFKB2	GADD45B
NFKB2	GALNT2
NFKB2	GNG2
NFKB2	GNG4
NFKB2	GNL2
NFKB2	GPBP1
NFKB2	HCK
NFKB2	HLA-A
NFKB2	HLA-B
NFKB2	HLA-DQA1
NFKB2	HLA-E
NFKB2	HLA-F
NFKB2	ICAM1
NFKB2	IER5
NFKB2	IFIH1
NFKB2	IFRD1
NFKB2	IGF2R
NFKB2	IKBKE
NFKB2	IL15
NFKB2	IL21R
NFKB2	IL2RG
NFKB2	IMMP2L
NFKB2	INO80
NFKB2	KATNA1

NFKB2	KLF10
NFKB2	KLHL18
NFKB2	KLHL36
NFKB2	KMT2E
NFKB2	KYNU
NFKB2	LENG8
NFKB2	LINC00158
NFKB2	LINC00926
NFKB2	LIPE
NFKB2	LITAF
NFKB2	LMNA
NFKB2	LTA
NFKB2	LYST
NFKB2	MAFG
NFKB2	MAP3K8
NFKB2	MAPKAPK5
NFKB2	MARCKS
NFKB2	MDFIC
NFKB2	MED15
NFKB2	MGLL
NFKB2	MIR155HG
NFKB2	MREG
NFKB2	MYO1C
NFKB2	NAMPT
NFKB2	NBPF20
NFKB2	NCF2
NFKB2	NEMF
NFKB2	NFE2L1
NFKB2	NFE2L2
NFKB2	NFKB1
NFKB2	NFKB2
NFKB2	NFKBIA
NFKB2	NFKBIB
NFKB2	NFKBID
NFKB2	NFKBIE
NFKB2	NKRF
NFKB2	NOC2L
NFKB2	NOC3L
NFKB2	NOL11
NFKB2	NR4A2
NFKB2	NRROS
NFKB2	NXT1
NFKB2	OGFOD3
NFKB2	PAK1IP1
NFKB2	PANX1
NFKB2	PARP14
NFKB2	PATL1
NFKB2	PHF1
NFKB2	PIGH
NFKB2	PLAG1

NFKB2	PLCE1
NFKB2	PMAIP1
NFKB2	POMC
NFKB2	PPAN
NFKB2	PPP1R15A
NFKB2	PPTC7
NFKB2	PRNP
NFKB2	PRRC2C
NFKB2	PSMA6
NFKB2	PVT1
NFKB2	RAB27A
NFKB2	RALGPS1
NFKB2	RBM17
NFKB2	RDH14
NFKB2	RDX
NFKB2	REL
NFKB2	RELB
NFKB2	RFTN1
NFKB2	RHOF
NFKB2	RHOG
NFKB2	RHOV
NFKB2	RILPL2
NFKB2	RNASEK
NFKB2	RNF114
NFKB2	RNF145
NFKB2	RPL22L1
NFKB2	RUNX3
NFKB2	SAMSN1
NFKB2	SBF2-AS1
NFKB2	SDCBP
NFKB2	SEMA7A
NFKB2	SH2B3
NFKB2	SIPA1L1
NFKB2	SLC25A37
NFKB2	SLC35F2
NFKB2	SLC38A1
NFKB2	SLC3A2
NFKB2	SLC50A1
NFKB2	SMG9
NFKB2	SNHG7
NFKB2	SNX11
NFKB2	SNX9
NFKB2	SPIB
NFKB2	SQSTM1
NFKB2	SRGN
NFKB2	ST20
NFKB2	STAT5A
NFKB2	TAGLN2
NFKB2	TARS
NFKB2	TBC1D10A

NFKB2	TCF7
NFKB2	TFEB
NFKB2	THUMPD2
NFKB2	TM2D3
NFKB2	TMEM120A
NFKB2	TMEM123
NFKB2	TNFAIP8
NFKB2	TNFRSF18
NFKB2	TNFRSF4
NFKB2	TNIP1
NFKB2	TNIP2
NFKB2	TOMM40L
NFKB2	TRAF1
NFKB2	TRAF2
NFKB2	TRAF3
NFKB2	TRAF4
NFKB2	TRIP10
NFKB2	TRMT61A
NFKB2	TWISTNB
NFKB2	UBAC2
NFKB2	UNC119
NFKB2	UQCC1
NFKB2	USP12
NFKB2	VASP
NFKB2	VOPP1
NFKB2	VWCE
NFKB2	WDR13
NFKB2	XPOT
NFKB2	YAF2
NFKB2	YARS
NFKB2	ZBTB10
NFKB2	ZBTB32
NFKB2	ZC3H12A
NFKB2	ZFAS1
NFKB2	ZFP36L1
NFKB2	ZNF264
NFKB2	ZNF385C
NFKB2	ZNF587B
NFYA	ACADS
NFYA	ACADSB
NFYA	ADGRE5
NFYA	ANXA2
NFYA	ARHGEF3
NFYA	ARL2BP
NFYA	ARMC1
NFYA	ARMC10
NFYA	BATF
NFYA	BLOC1S5
NFYA	BLZF1
NFYA	BZW2

NFYA	C12orf42
NFYA	CA2
NFYA	CD151
NFYA	CD44
NFYA	CHDH
NFYA	CHKB
NFYA	CHST11
NFYA	CLCF1
NFYA	CMIP
NFYA	CNOT9
NFYA	COPRS
NFYA	DOCK9
NFYA	DSCC1
NFYA	EIF2AK3
NFYA	GM2A
NFYA	GON4L
NFYA	GRK3
NFYA	HACD4
NFYA	HBP1
NFYA	HIST1H1D
NFYA	HSPBP1
NFYA	IFNGR1
NFYA	IL6
NFYA	ING5
NFYA	IPO4
NFYA	IQSEC1
NFYA	ISYNA1
NFYA	KCNK6
NFYA	KIAA0040
NFYA	LGALS3
NFYA	LIMS1
NFYA	LIX1L
NFYA	MDM1
NFYA	MFSD3
NFYA	MKNK2
NFYA	MLLT1
NFYA	MORC2
NFYA	NCBP1
NFYA	NEK6
NFYA	NEMP1
NFYA	NF2
NFYA	NFYA
NFYA	NIT1
NFYA	NT5C3A
NFYA	ORMDL3
NFYA	OTUD1
NFYA	PDE4DIP
NFYA	PEA15
NFYA	PIKFYVE
NFYA	PITPNA

NFYA	PLEKHG1
NFYA	PML
NFYA	PPP3CA
NFYA	PSMC3IP
NFYA	RAB13
NFYA	RAD18
NFYA	RCN1
NFYA	RGL2
NFYA	RHOC
NFYA	RPP40
NFYA	RSPH1
NFYA	SEMA7A
NFYA	SH2B3
NFYA	SIN3B
NFYA	SLC16A1
NFYA	SMNDC1
NFYA	SNX22
NFYA	SP3
NFYA	STARD3NL
NFYA	STAT5A
NFYA	SYT17
NFYA	TBC1D15
NFYA	TBCB
NFYA	TCF3
NFYA	TFRC
NFYA	TMBIM1
NFYA	TMEM106C
NFYA	TMEM50B
NFYA	TNFRSF12A
NFYA	TNIP2
NFYA	TNPO2
NFYA	TOR1B
NFYA	TRIP10
NFYA	TUBA1A
NFYA	UBASH3B
NFYA	USB1
NFYA	USP12
NFYA	USP36
NFYA	ZHX1
NFYA	ZNF563
NFYA	ZNF652
NFYA	ZNF740
NFYB	AAMP
NFYB	ABCA6
NFYB	ABI3
NFYB	ABL1
NFYB	ACAT2
NFYB	ACBD5
NFYB	ACIN1
NFYB	ACOT13

NFYB	ACOT4
NFYB	ACOT9
NFYB	ACTA2
NFYB	ACTL6A
NFYB	ACTR1A
NFYB	ACYP1
NFYB	ADAM10
NFYB	ADD3
NFYB	ADPGK
NFYB	AICDA
NFYB	AK2
NFYB	AKAP11
NFYB	AKR1B1
NFYB	ALDH6A1
NFYB	ALDH7A1
NFYB	ALG12
NFYB	ALKBH5
NFYB	ALKBH6
NFYB	AMD1
NFYB	AMMECR1
NFYB	ANAPC11
NFYB	ANAPC15
NFYB	ANKRD54
NFYB	ANP32A
NFYB	ANP32B
NFYB	ANP32E
NFYB	AP2S1
NFYB	APEX2
NFYB	APIP
NFYB	APLP2
NFYB	APOL3
NFYB	APOLD1
NFYB	ARFIP2
NFYB	ARHGAP10
NFYB	ARHGAP11A
NFYB	ARHGAP30
NFYB	ARHGEF6
NFYB	ARID2
NFYB	ARL16
NFYB	ARL6IP1
NFYB	ARL6IP6
NFYB	ARMC1
NFYB	ARMCX6
NFYB	ARPC5
NFYB	ARRB2
NFYB	ASCC1
NFYB	ASF1B
NFYB	ATAD2
NFYB	ATAD5
NFYB	ATF7

NFYB	ATF7IP
NFYB	ATG16L2
NFYB	ATP6V1E1
NFYB	AURKB
NFYB	B3GLCT
NFYB	BABAM1
NFYB	BACH2
NFYB	BAG4
NFYB	BARD1
NFYB	BAZ1B
NFYB	BCKDHA
NFYB	BCL11A
NFYB	BCL2L12
NFYB	BCL7B
NFYB	BNIP3L
NFYB	BRCA1
NFYB	BRD2
NFYB	BRD3
NFYB	BRD7
NFYB	BUB3
NFYB	C12orf65
NFYB	C16orf95
NFYB	C21orf58
NFYB	C3orf67
NFYB	C4orf33
NFYB	C5orf34
NFYB	C6orf99
NFYB	CABIN1
NFYB	CALM1
NFYB	CALM2
NFYB	CALM3
NFYB	CAMK2D
NFYB	CAMTA1
NFYB	CAPZB
NFYB	CARHSP1
NFYB	CASP2
NFYB	CBFB
NFYB	CBWD1
NFYB	CBX3
NFYB	CBX5
NFYB	CCDC117
NFYB	CCDC130
NFYB	CCDC167
NFYB	CCDC34
NFYB	CCND3
NFYB	CCNE2
NFYB	CCNG2
NFYB	CCNL1
NFYB	CCT7
NFYB	CCZ1

NFYB	CD247
NFYB	CDC25A
NFYB	CDC25C
NFYB	CDC6
NFYB	CDCA3
NFYB	CDCA4
NFYB	CDK1
NFYB	CDK17
NFYB	CDK2
NFYB	CDK2AP2
NFYB	CDK5RAP3
NFYB	CDKN1B
NFYB	CDKN2D
NFYB	CDKN3
NFYB	CDT1
NFYB	CENPF
NFYB	CENPH
NFYB	CENPK
NFYB	CENPL
NFYB	CENPM
NFYB	CENPU
NFYB	CEP57
NFYB	CEP97
NFYB	CETN3
NFYB	CGGBP1
NFYB	CHCHD3
NFYB	CHEK1
NFYB	CHEK2
NFYB	CHMP2B
NFYB	CHP1
NFYB	CHURC1
NFYB	CIRBP
NFYB	CIT
NFYB	CKS1B
NFYB	CKS2
NFYB	CLSPN
NFYB	CNIH1
NFYB	CNOT1
NFYB	CNOT2
NFYB	COPS3
NFYB	COPS4
NFYB	COX6A1
NFYB	COX7B
NFYB	COX8A
NFYB	CRNDE
NFYB	CSE1L
NFYB	CSRP1
NFYB	CTDSP2
NFYB	CTNNBIP1
NFYB	CXCR4

NFYB	CYB5A
NFYB	CYCS
NFYB	CYHR1
NFYB	DAAM1
NFYB	DARS
NFYB	DCAF7
NFYB	DCP1A
NFYB	DCP2
NFYB	DCXR
NFYB	DDB2
NFYB	DDIAS
NFYB	DDT
NFYB	DDX39A
NFYB	DDX50
NFYB	DEDD
NFYB	DEK
NFYB	DEPDC5
NFYB	DGAT1
NFYB	DGCR8
NFYB	DHCR24
NFYB	DHX15
NFYB	DLD
NFYB	DLG1
NFYB	DMD
NFYB	DMTF1
NFYB	DNAJB2
NFYB	DNAJC8
NFYB	DOCK9
NFYB	DPP3
NFYB	DPYSL2
NFYB	DRG1
NFYB	DUSP23
NFYB	DVL3
NFYB	DYNLL1
NFYB	DYNLT1
NFYB	E2F1
NFYB	E2F2
NFYB	E2F7
NFYB	EAF2
NFYB	EBAG9
NFYB	EBF1
NFYB	EBP
NFYB	EFCAB11
NFYB	EGLN1
NFYB	EIF2AK3
NFYB	EIF2B2
NFYB	EIF4E
NFYB	ELP4
NFYB	EMC9
NFYB	EML2

NFYB	ERH
NFYB	ERP44
NFYB	ESCO2
NFYB	ESPL1
NFYB	ETFA
NFYB	ETFB
NFYB	EZH2
NFYB	FAF1
NFYB	FAM102A
NFYB	FAM102B
NFYB	FAM111A
NFYB	FAM118A
NFYB	FAM120AOS
NFYB	FAM122B
NFYB	FAM129A
NFYB	FAM133B
NFYB	FAM184A
NFYB	FAM192A
NFYB	FAM208A
NFYB	FAM208B
NFYB	FAM20B
NFYB	FAM221A
NFYB	FAM49A
NFYB	FAM76B
NFYB	FAM81A
NFYB	FANCA
NFYB	FANCI
NFYB	FANCL
NFYB	FBXL12
NFYB	FBXO11
NFYB	FBXO3
NFYB	FBXO5
NFYB	FBXO8
NFYB	FDFT1
NFYB	FDPS
NFYB	FGD6
NFYB	FGGY
NFYB	FH
NFYB	FKBPL
NFYB	FLAD1
NFYB	FN3KRP
NFYB	FNIP1
NFYB	G3BP1
NFYB	GART
NFYB	GCA
NFYB	GCDH
NFYB	GCH1
NFYB	GCHFR
NFYB	GDE1
NFYB	GFOD1

NFYB	GGA2
NFYB	GGCT
NFYB	GGNBP2
NFYB	GID4
NFYB	GLO1
NFYB	GMNN
NFYB	GNB2
NFYB	GNGT2
NFYB	GOLGA8B
NFYB	GORAB
NFYB	GPN3
NFYB	GTF2A1
NFYB	GTF2E2
NFYB	GTF3C5
NFYB	GTSE1
NFYB	H2AFV
NFYB	H2AFX
NFYB	H2AFY
NFYB	H2AFZ
NFYB	H3F3A
NFYB	H3F3B
NFYB	HADH
NFYB	HAGH
NFYB	HAT1
NFYB	HAUS4
NFYB	HAUS7
NFYB	HBS1L
NFYB	HES6
NFYB	HIGD1A
NFYB	HINT2
NFYB	HIST1H1D
NFYB	HIST1H1E
NFYB	HIST1H2AC
NFYB	HIST1H3A
NFYB	HIST1H4C
NFYB	HIST2H2AC
NFYB	HMG20A
NFYB	HMGA1
NFYB	HMGB1
NFYB	HMGB2
NFYB	HMGCS1
NFYB	HMGN1
NFYB	HMGN2
NFYB	HNRNPA0
NFYB	HNRNPA2B1
NFYB	HNRNPA3
NFYB	HNRNPAB
NFYB	HNRNPC
NFYB	HNRNPD
NFYB	HNRNPF

NFYB	HNRNPK
NFYB	HNRNPL
NFYB	HNRNPLL
NFYB	HNRNPR
NFYB	HNRNPU
NFYB	HNRNPUL2
NFYB	HOXB2
NFYB	HS2ST1
NFYB	HSCB
NFYB	HSD17B10
NFYB	HSD17B12
NFYB	HSPA2
NFYB	HSPA4
NFYB	HSPA8
NFYB	HUWE1
NFYB	HYPK
NFYB	IBTK
NFYB	IDH2
NFYB	IDH3A
NFYB	IDH3G
NFYB	IGSF8
NFYB	ILF2
NFYB	IMMP1L
NFYB	IMMT
NFYB	IMP4
NFYB	INCENP
NFYB	ING3
NFYB	INTS8
NFYB	IPO9
NFYB	IQCB1
NFYB	IRF1
NFYB	IRF2
NFYB	ISCA2
NFYB	JADE1
NFYB	JDP2
NFYB	JMJD1C
NFYB	KANK1
NFYB	KATNA1
NFYB	KCNQ5
NFYB	KCTD5
NFYB	KCTD7
NFYB	KIAA1841
NFYB	KIF22
NFYB	KIF4A
NFYB	KLF2
NFYB	KLHL5
NFYB	KMT2C
NFYB	KMT2E
NFYB	KPNB1
NFYB	L2HGDH

NFYB	LBR
NFYB	LDHB
NFYB	LIMS1
NFYB	LMNB1
NFYB	LPP
NFYB	LRIF1
NFYB	LRMP
NFYB	LRRC45
NFYB	LSM1
NFYB	LSM4
NFYB	LSM6
NFYB	LSM8
NFYB	LUC7L2
NFYB	LYPLA1
NFYB	LYRM1
NFYB	MAEA
NFYB	MAP2K1
NFYB	MAPRE1
NFYB	MARK3
NFYB	MAZ
NFYB	MBD6
NFYB	MCM10
NFYB	MCM3
NFYB	MCM4
NFYB	MCM5
NFYB	MCM6
NFYB	MCM7
NFYB	MCTS1
NFYB	MCU
NFYB	MDM2
NFYB	MED19
NFYB	MED30
NFYB	MED8
NFYB	MEF2C-AS1
NFYB	MEGF9
NFYB	MELK
NFYB	MEPCE
NFYB	METAP1
NFYB	METTL17
NFYB	MFF
NFYB	MFGE8
NFYB	MFSD11
NFYB	MGMT
NFYB	MICU2
NFYB	MINK1
NFYB	MIS12
NFYB	MKI67
NFYB	MKL1
NFYB	MKLN1
NFYB	MLXIP

NFYB	MMAB
NFYB	MMD
NFYB	MORF4L1
NFYB	MORF4L2
NFYB	MPC2
NFYB	MPP5
NFYB	MRM3
NFYB	MRPL18
NFYB	MRPL28
NFYB	MRPL37
NFYB	MRPL50
NFYB	MRPL51
NFYB	MRPS22
NFYB	MRPS36
NFYB	MSH2
NFYB	MSL3
NFYB	MTHFD1
NFYB	MTOR
NFYB	MVK
NFYB	MXD3
NFYB	MYBL1
NFYB	MYH10
NFYB	MYO1D
NFYB	MZT1
NFYB	NAA10
NFYB	NAA60
NFYB	NADK2
NFYB	NAP1L4
NFYB	NASP
NFYB	NBR1
NFYB	NCAPD3
NFYB	NCAPG2
NFYB	NCAPH
NFYB	NCK2
NFYB	NCOA7
NFYB	NDUFA11
NFYB	NDUFA2
NFYB	NDUFA5
NFYB	NDUFA6
NFYB	NDUFAF3
NFYB	NDUFB11
NFYB	NDUFB3
NFYB	NFATC2IP
NFYB	NFATC3
NFYB	NFYB
NFYB	NFYC
NFYB	NIPBL
NFYB	NMT1
NFYB	NONO
NFYB	NRF1

NFYB	NT5DC1
NFYB	NT5DC2
NFYB	NUBP2
NFYB	NUCKS1
NFYB	NUDC
NFYB	NUDT21
NFYB	NUDT9
NFYB	NUP50
NFYB	NUP88
NFYB	NUPL2
NFYB	NUSAP1
NFYB	NXT1
NFYB	OAT
NFYB	OCIAD1
NFYB	OIP5
NFYB	ORC4
NFYB	ORC6
NFYB	ORMDL1
NFYB	OSBPL11
NFYB	OSBPL9
NFYB	PABPN1
NFYB	PAFAH1B3
NFYB	PAQR4
NFYB	PARP2
NFYB	PARPBP
NFYB	PBK
NFYB	PBX3
NFYB	PCNA
NFYB	PCYT2
NFYB	PDCL
NFYB	PDRG1
NFYB	PDS5B
NFYB	PDZD8
NFYB	PEG10
NFYB	PELI1
NFYB	PET100
NFYB	PGK1
NFYB	PHAX
NFYB	PHF19
NFYB	PHF3
NFYB	PHF5A
NFYB	PHRF1
NFYB	PIAS4
NFYB	PIF1
NFYB	PIGV
NFYB	PIN1
NFYB	PJA2
NFYB	PKM
NFYB	PKMYT1
NFYB	PMF1

NFYB	PMS1
NFYB	PNN
NFYB	PNP
NFYB	PNRC2
NFYB	POGZ
NFYB	POLA1
NFYB	POLD4
NFYB	POLDIP3
NFYB	POLR3K
NFYB	POP5
NFYB	POU2AF1
NFYB	PPIA
NFYB	PPP2CA
NFYB	PPP2R1A
NFYB	PPP2R5A
NFYB	PPP2R5E
NFYB	PPP5C
NFYB	PPTC7
NFYB	PRADC1
NFYB	PRC1
NFYB	PRDX3
NFYB	PRKCD
NFYB	PRPF40A
NFYB	PRPSAP1
NFYB	PRPSAP2
NFYB	PRRC2A
NFYB	PRRC2C
NFYB	PSMA2
NFYB	PSMA3
NFYB	PSMA3-AS1
NFYB	PSMA5
NFYB	PSMB3
NFYB	PSMC3
NFYB	PSMC3IP
NFYB	PSMD10
NFYB	PSMD14
NFYB	PTBP1
NFYB	PTMA
NFYB	PTMS
NFYB	PTPRA
NFYB	PTTG1
NFYB	PVT1
NFYB	PWWP2A
NFYB	PXMP2
NFYB	PYCR2
NFYB	RAB11B-AS1
NFYB	RAB14
NFYB	RAB1A
NFYB	RAB27A
NFYB	RAB3GAP1

NFYB	RAB5C
NFYB	RABGAP1
NFYB	RABGAP1L
NFYB	RAD17
NFYB	RAD21
NFYB	RAD54L
NFYB	RALY
NFYB	RANBP1
NFYB	RASSF7
NFYB	RBBP4
NFYB	RBBP7
NFYB	RBL1
NFYB	RBM15
NFYB	RBM23
NFYB	RBM4
NFYB	RBM42
NFYB	RBM6
NFYB	RBM8A
NFYB	RBX1
NFYB	RCN1
NFYB	RCN2
NFYB	RECK
NFYB	RECQL4
NFYB	RERE
NFYB	REV3L
NFYB	RFC5
NFYB	RFWD3
NFYB	RGS9
NFYB	RLN2
NFYB	RMI2
NFYB	RNASEH2A
NFYB	RNF13
NFYB	RNF6
NFYB	RNFT2
NFYB	RP2
NFYB	RPA1
NFYB	RPP30
NFYB	RPUSD1
NFYB	RRM2B
NFYB	RSBN1L
NFYB	RTN3
NFYB	RTN4
NFYB	RTN4IP1
NFYB	RUFY1
NFYB	RUVBL1
NFYB	S100A10
NFYB	S100PBP
NFYB	SAP30BP
NFYB	SARNP
NFYB	SDE2

NFYB	SEC22A
NFYB	SEC31A
NFYB	SEMA4B
NFYB	SERP2
NFYB	SETD4
NFYB	SETD9
NFYB	SF1
NFYB	SF3B2
NFYB	SF3B4
NFYB	SFPQ
NFYB	SFR1
NFYB	SFXN1
NFYB	SFXN5
NFYB	SH3GLB2
NFYB	SHCBP1
NFYB	SHOC2
NFYB	SIAH1
NFYB	SIK2
NFYB	SIVA1
NFYB	SKA2
NFYB	SLBP
NFYB	SLC1A4
NFYB	SLC20A1
NFYB	SLC25A12
NFYB	SLC25A19
NFYB	SLC25A5
NFYB	SLC2A1
NFYB	SLC31A1
NFYB	SLC39A8
NFYB	SLC4A8
NFYB	SLC9A3R1
NFYB	SMAD2
NFYB	SMC2
NFYB	SMC3
NFYB	SMG9
NFYB	SND1
NFYB	SNRNP25
NFYB	SNRPF
NFYB	SNRPG
NFYB	SON
NFYB	SPAG5
NFYB	SPC24
NFYB	SPC25
NFYB	SPDL1
NFYB	SPI1
NFYB	SPRED2
NFYB	SPTSSA
NFYB	SQLE
NFYB	SRGAP2C
NFYB	SRP9

NFYB	SRRM1
NFYB	SRSF1
NFYB	SRSF10
NFYB	SRSF2
NFYB	SRSF3
NFYB	SSB
NFYB	SSBP2
NFYB	SSBP3
NFYB	SSX2IP
NFYB	STAG1
NFYB	STAU2
NFYB	STK11
NFYB	STK24
NFYB	STK3
NFYB	STK38L
NFYB	STMN1
NFYB	STOML2
NFYB	STPG1
NFYB	STT3B
NFYB	SUCLG2
NFYB	SUMO2
NFYB	SUN2
NFYB	SUPT20H
NFYB	SUV39H1
NFYB	SUZ12
NFYB	SYVN1
NFYB	TAB2
NFYB	TAF1
NFYB	TAF15
NFYB	TAF1C
NFYB	TALDO1
NFYB	TANK
NFYB	TBC1D10B
NFYB	TBC1D15
NFYB	TBCC
NFYB	TBL1XR1
NFYB	TCF12
NFYB	TCF19
NFYB	TCP1
NFYB	TDP1
NFYB	TDRKH
NFYB	TECR
NFYB	TERF1
NFYB	THOP1
NFYB	TIGAR
NFYB	TIMM17A
NFYB	TIMM17B
NFYB	TK1
NFYB	TLE4
NFYB	TLK2

NFYB	TM7SF3
NFYB	TMA7
NFYB	TMED1
NFYB	TMEM126B
NFYB	TMEM131
NFYB	TMEM14A
NFYB	TMEM14B
NFYB	TMEM161B-AS1
NFYB	TMEM165
NFYB	TMEM167A
NFYB	TMEM218
NFYB	TMEM50B
NFYB	TMEM97
NFYB	TMPO
NFYB	TNK2
NFYB	TNNC2
NFYB	TOP1MT
NFYB	TOP2A
NFYB	TPGS2
NFYB	TPP2
NFYB	TPRA1
NFYB	TRAF2
NFYB	TRIM11
NFYB	TRIM26
NFYB	TRUB1
NFYB	TSC22D1
NFYB	TSPAN13
NFYB	TUBB
NFYB	TUBB2B
NFYB	TUBB4B
NFYB	TUT1
NFYB	TXNDC16
NFYB	TXNDC9
NFYB	UBB
NFYB	UBE2A
NFYB	UBE2C
NFYB	UBE2D3
NFYB	UBE2E2
NFYB	UBE2I
NFYB	UBE2J1
NFYB	UBE2S
NFYB	UBE2T
NFYB	UBR5
NFYB	UCP2
NFYB	UGP2
NFYB	UHRF1
NFYB	UNG
NFYB	UNK
NFYB	UPF3B
NFYB	UQCC2

NFYB	UQCRC2
NFYB	USP1
NFYB	USP16
NFYB	USP5
NFYB	USPL1
NFYB	UTP6
NFYB	VBP1
NFYB	VEZT
NFYB	VGLL4
NFYB	VPS11
NFYB	VPS29
NFYB	VPS72
NFYB	VRK1
NFYB	VRK3
NFYB	WAPL
NFYB	WDR5
NFYB	WIPI1
NFYB	WRNIP1
NFYB	WTAP
NFYB	WWOX
NFYB	WWP2
NFYB	XAB2
NFYB	XIST
NFYB	XPO1
NFYB	XRCC4
NFYB	XRCC6
NFYB	YIPF4
NFYB	YKT6
NFYB	YPEL3
NFYB	YWHAB
NFYB	YWHAQ
NFYB	YWHAZ
NFYB	ZBTB14
NFYB	ZBTB20
NFYB	ZCCHC17
NFYB	ZDHHC12
NFYB	ZFAND4
NFYB	ZFHX3
NFYB	ZFP64
NFYB	ZHX2
NFYB	ZKSCAN1
NFYB	ZMYM2
NFYB	ZNF132
NFYB	ZNF195
NFYB	ZNF326
NFYB	ZNF429
NFYB	ZNF441
NFYB	ZNF638
NFYB	ZNF652
NFYB	ZNF672

NFYB	ZNF7
NFYB	ZNF76
NFYB	ZNF775
NFYB	ZNF787
NFYB	ZNF821
NFYB	ZNF92
NFYB	ZNHIT3
NFYB	ZRSR2
NFYB	ZWINT
NFYC	ABHD15
NFYC	ACAT2
NFYC	ACD
NFYC	ACIN1
NFYC	ACLY
NFYC	ACTL6A
NFYC	ACTR1B
NFYC	ADAR
NFYC	ADD1
NFYC	ADD3
NFYC	AGL
NFYC	AGO1
NFYC	AKR1B1
NFYC	ALAD
NFYC	ALCAM
NFYC	ALDH2
NFYC	ALDH7A1
NFYC	ALG9
NFYC	ALKBH5
NFYC	ALKBH6
NFYC	ANAPC11
NFYC	ANKRD39
NFYC	AP2M1
NFYC	AP2S1
NFYC	AP3D1
NFYC	APIP
NFYC	APLP2
NFYC	APOL3
NFYC	APOLD1
NFYC	ARAP2
NFYC	ARID2
NFYC	ARID4A
NFYC	ARL2
NFYC	ARL2BP
NFYC	ARL4A
NFYC	ARL6IP1
NFYC	ARL6IP5
NFYC	ARMC1
NFYC	ARMCX6
NFYC	ASPSCR1
NFYC	ATF6B

NFYC	ATF7
NFYC	ATG16L2
NFYC	ATXN7
NFYC	ATXN7L3
NFYC	AURKB
NFYC	BCAR1
NFYC	BCAT2
NFYC	BTN2A1
NFYC	BUB3
NFYC	C12orf76
NFYC	C15orf41
NFYC	C18orf32
NFYC	C3orf67
NFYC	CALM1
NFYC	CALM2
NFYC	CALM3
NFYC	CAMTA1
NFYC	CAND1
NFYC	CAPN1
NFYC	CBFB
NFYC	CCDC102A
NFYC	CCDC115
NFYC	CCDC34
NFYC	CCDC77
NFYC	CCNA2
NFYC	CCNB2
NFYC	CCNE2
NFYC	CCNG2
NFYC	CCNL2
NFYC	CDC23
NFYC	CDC27
NFYC	CDCA2
NFYC	CDCA3
NFYC	CDK1
NFYC	CDKN2D
NFYC	CDKN3
NFYC	CELF1
NFYC	CENPK
NFYC	CEP85
NFYC	CETN2
NFYC	CHCHD3
NFYC	CHCHD7
NFYC	CHD6
NFYC	CHD7
NFYC	CHMP2A
NFYC	CHST10
NFYC	CHURC1
NFYC	CIC
NFYC	CKAP2
NFYC	CKS1B

NFYC	CKS2
NFYC	CLK3
NFYC	CLTA
NFYC	CNOT3
NFYC	CNST
NFYC	COMMD10
NFYC	COPS7A
NFYC	COX6B1
NFYC	COX7B
NFYC	COX8A
NFYC	CSNK1G3
NFYC	CTDSP2
NFYC	CUL3
NFYC	CWC22
NFYC	CYCS
NFYC	DBI
NFYC	DCLRE1C
NFYC	DCP2
NFYC	DCTN4
NFYC	DDOST
NFYC	DDX11
NFYC	DDX39A
NFYC	DGKZ
NFYC	DGUOK
NFYC	DLD
NFYC	DMD
NFYC	DNAJB11
NFYC	DNAJB2
NFYC	DNAJC13
NFYC	DNAJC4
NFYC	DNAJC8
NFYC	DOCK9
NFYC	DPYSL2
NFYC	DRG1
NFYC	DUSP10
NFYC	EAF2
NFYC	EBAG9
NFYC	ECT2
NFYC	EGR1
NFYC	EIF3L
NFYC	EIF4G2
NFYC	ELAC2
NFYC	ELAVL1
NFYC	ELK1
NFYC	ELMO2
NFYC	ELP4
NFYC	ELP6
NFYC	EMC2
NFYC	EML4
NFYC	ENTPD3-AS1

NFYC	EPC1
NFYC	ERCC6L
NFYC	ERICH1
NFYC	ETFA
NFYC	FABP5
NFYC	FAM122B
NFYC	FAM192A
NFYC	FAM217B
NFYC	FAM32A
NFYC	FANCL
NFYC	FBXO46
NFYC	FDFT1
NFYC	FDPS
NFYC	FGD5-AS1
NFYC	FLYWCH1
NFYC	FOXN2
NFYC	FPGS
NFYC	G2E3
NFYC	GALK1
NFYC	GALNT14
NFYC	GBF1
NFYC	GCSH
NFYC	GGA2
NFYC	GID8
NFYC	GLRX5
NFYC	GLUD1
NFYC	GNAI2
NFYC	GOLGA8A
NFYC	GOLPH3L
NFYC	GOT1
NFYC	GPR108
NFYC	GSTZ1
NFYC	GTF2B
NFYC	GTF2E2
NFYC	GTF2F2
NFYC	GTF3C5
NFYC	H2AFV
NFYC	H2AFZ
NFYC	H3F3A
NFYC	H3F3B
NFYC	HAGH
NFYC	HCN3
NFYC	HDDC2
NFYC	HENMT1
NFYC	HERC5
NFYC	HES6
NFYC	HIGD1A
NFYC	HIST1H4C
NFYC	HLA-A
NFYC	HLA-DPB1

NFYC	HLA-DRB1
NFYC	HMGB3
NFYC	HMGN1
NFYC	HMGN2
NFYC	HMMR
NFYC	HNRNPA3
NFYC	HNRNPD
NFYC	HNRNPH2
NFYC	HNRNPL
NFYC	HNRNPR
NFYC	HNRNPUL1
NFYC	HOXB2
NFYC	HP1BP3
NFYC	HSD17B12
NFYC	HSD17B7
NFYC	HSPA8
NFYC	HYLS1
NFYC	HYOU1
NFYC	IARS
NFYC	IDH3G
NFYC	IFFO1
NFYC	IKZF1
NFYC	IMMT
NFYC	IQCB1
NFYC	IQSEC1
NFYC	IRF2
NFYC	IRF2BP1
NFYC	IRS2
NFYC	ISCA1
NFYC	IST1
NFYC	IWS1
NFYC	JAK1
NFYC	JMJD1C
NFYC	KDF1
NFYC	KHSRP
NFYC	KIF22
NFYC	KLHL24
NFYC	KMT5B
NFYC	KNTC1
NFYC	KPNA2
NFYC	KXD1
NFYC	LAMTOR1
NFYC	LAMTOR4
NFYC	LANCL1
NFYC	LBR
NFYC	LDB1
NFYC	LIAS
NFYC	LRIF1
NFYC	LSM1
NFYC	LSM6

NFYC	LYPLA1
NFYC	LYRM7
NFYC	LYRM9
NFYC	MADD
NFYC	MAEA
NFYC	MAF1
NFYC	MAML2
NFYC	MAP2K1
NFYC	MAP2K3
NFYC	MAP3K7CL
NFYC	MAT2B
NFYC	MBTD1
NFYC	MCU
NFYC	MDC1
NFYC	MDH1
NFYC	MED24
NFYC	MED8
NFYC	MEF2C
NFYC	METAP1
NFYC	MFF
NFYC	MICAL1
NFYC	MKI67
NFYC	MLF2
NFYC	MMD
NFYC	MMS19
NFYC	MOB1A
NFYC	MPC2
NFYC	MRM3
NFYC	MRPL18
NFYC	MRPL50
NFYC	MRPL51
NFYC	MTFR2
NFYC	MTIF3
NFYC	MXD3
NFYC	MYH9
NFYC	NAA60
NFYC	NAGLU
NFYC	NANS
NFYC	NCALD
NFYC	NCOA2
NFYC	NCOA5
NFYC	NCOA7
NFYC	NDUFA5
NFYC	NDUFB3
NFYC	NDUFS2
NFYC	NDUFS8
NFYC	NEDD9
NFYC	NEK2
NFYC	NFYB
NFYC	NFYC

NFYC	NGDN
NFYC	NMT1
NFYC	NRBP1
NFYC	NSMCE1
NFYC	NSMCE4A
NFYC	NUDT2
NFYC	NUP37
NFYC	NUP88
NFYC	NUSAP1
NFYC	NUTM2A-AS1
NFYC	NUTM2B-AS1
NFYC	OAZ1
NFYC	OGFOD1
NFYC	OSBPL11
NFYC	OSBPL8
NFYC	PAK4
NFYC	PCNT
NFYC	PCYT2
NFYC	PDCD10
NFYC	PDHX
NFYC	PDK2
NFYC	PDLIM7
NFYC	PELO
NFYC	PET100
NFYC	PEX16
NFYC	PGK1
NFYC	PHF19
NFYC	PHF3
NFYC	PHKG2
NFYC	PIN1
NFYC	PKP4
NFYC	PMF1
NFYC	PNP
NFYC	POC1A
NFYC	PPAT
NFYC	PPIL1
NFYC	PPM1A
NFYC	PPP1R12A
NFYC	PPP2CA
NFYC	PPP2R5C
NFYC	PPP2R5D
NFYC	PRKD2
NFYC	PROSER3
NFYC	PRR11
NFYC	PSMA2
NFYC	PSMA5
NFYC	PTK2B
NFYC	PTP4A1
NFYC	PTPRA
NFYC	PTPRC

NFYC	PTRH1
NFYC	PWWP2A
NFYC	PXMP2
NFYC	RAB11B
NFYC	RAB5C
NFYC	RABGAP1L
NFYC	RANGRF
NFYC	RAP1A
NFYC	RASSF2
NFYC	RBM23
NFYC	RBM8A
NFYC	RBX1
NFYC	RERE
NFYC	REV1
NFYC	REV3L
NFYC	RFX1
NFYC	RFX5
NFYC	RGS3
NFYC	RHBDD1
NFYC	RNF219
NFYC	RNF26
NFYC	RNF41
NFYC	ROGDI
NFYC	RRM2B
NFYC	RSRC2
NFYC	RTN4
NFYC	RUNX3
NFYC	RUVBL2
NFYC	SAP130
NFYC	SAP30L
NFYC	SBDS
NFYC	SEC22C
NFYC	SEMA4A
NFYC	SEPT7
NFYC	SFPQ
NFYC	SGO2
NFYC	SHARPIN
NFYC	SHKBP1
NFYC	SIK3
NFYC	SKIL
NFYC	SLC1A4
NFYC	SLC25A3
NFYC	SLC2A1
NFYC	SLC39A3
NFYC	SLCO4A1
NFYC	SLF2
NFYC	SMAD2
NFYC	SMDT1
NFYC	SMIM12
NFYC	SMIM7

NFYC	SMYD4
NFYC	SPATA24
NFYC	SPIN2B
NFYC	SRGAP2C
NFYC	SRP9
NFYC	SSBP3
NFYC	SSR1
NFYC	SSX2IP
NFYC	STAG1
NFYC	STAMBP
NFYC	STIP1
NFYC	STRBP
NFYC	STX5
NFYC	SUMO2
NFYC	SYCE2
NFYC	TAF15
NFYC	TAF1A
NFYC	TBC1D22B
NFYC	TCF19
NFYC	TCF7
NFYC	TCTN3
NFYC	TDP1
NFYC	TDRD3
NFYC	TES
NFYC	TFCP2
NFYC	TM7SF3
NFYC	TMA7
NFYC	TMEM120B
NFYC	TMEM161B-AS1
NFYC	TMEM218
NFYC	TMEM241
NFYC	TMPO-AS1
NFYC	TNFAIP8L2
NFYC	TNRC6B
NFYC	TNS3
NFYC	TOMM70
NFYC	TOX4
NFYC	TP53I11
NFYC	TRAFD1
NFYC	TRAPPC6B
NFYC	TRIP10
NFYC	TSPAN33
NFYC	TTBK2
NFYC	TUBB
NFYC	TUBB4B
NFYC	TXNDC9
NFYC	UBB
NFYC	UBE2F
NFYC	UBE2S
NFYC	UBFD1

NFYC	UBR7
NFYC	USP11
NFYC	USP5
NFYC	VRK3
NFYC	WAPL
NFYC	WDR5
NFYC	WDR6
NFYC	WWOX
NFYC	XPO1
NFYC	XPO6
NFYC	YKT6
NFYC	YPEL1
NFYC	YPEL2
NFYC	YWHAZ
NFYC	ZBED5
NFYC	ZBTB40
NFYC	ZCCHC17
NFYC	ZFAND4
NFYC	ZNF148
NFYC	ZNF253
NFYC	ZNF281
NFYC	ZNF574
NFYC	ZNF680
NFYC	ZNF720
NR1H3	BOLA1
NR1H3	CRB2
NR1H3	EPS15L1
NR1H3	EVI5
NR1H3	GUCY2C
NR1H3	NR1H3
NR1H3	PARP9
NR1H3	PRKAR1B
NR1H3	PTGS1
NR1H3	TET2
NR2C1	DGAT2
NR2C1	DHRS9
NR2C1	NR2C1
NR2C2	ADD3
NR2C2	ANKRD16
NR2C2	AP1B1
NR2C2	ATP2B1
NR2C2	BOD1L1
NR2C2	CDC42EP3
NR2C2	CHKA
NR2C2	CIB2
NR2C2	CLHC1
NR2C2	DIS3L2
NR2C2	ELAC1
NR2C2	FBXO33
NR2C2	GLTP

NR2C2	KANSL1
NR2C2	LDB1
NR2C2	LMNA
NR2C2	MGST3
NR2C2	NLRC3
NR2C2	NR2C2
NR2C2	NUP107
NR2C2	PIK3R1
NR2C2	PIP4K2A
NR2C2	SPATA2L
NR2C2	SSBP3
NR2C2	TOX
NR2C2	USP9X
NR2C2	ZEB2
NR2F6	B4GALT1
NR2F6	MAPKAP1
NR2F6	MOSPD1
NR2F6	NR2F6
NR2F6	USP46
NR3C1	ABCB10
NR3C1	ACBD6
NR3C1	ACOT9
NR3C1	ACOX1
NR3C1	ACSF3
NR3C1	ACTB
NR3C1	ACTG1
NR3C1	ACTR2
NR3C1	ACTR3
NR3C1	ACTR8
NR3C1	ADA
NR3C1	ADD1
NR3C1	ADH5
NR3C1	ADNP2
NR3C1	AEBP2
NR3C1	AFF4
NR3C1	AFTPH
NR3C1	AIM2
NR3C1	AIP
NR3C1	AKAP13
NR3C1	AKAP8L
NR3C1	AKAP9
NR3C1	AKTIP
NR3C1	ALCAM
NR3C1	ALDH2
NR3C1	ALDOA
NR3C1	ALG6
NR3C1	ALKBH5
NR3C1	ALOX5
NR3C1	AMFR
NR3C1	AMMECR1

NR3C1	ANAPC16
NR3C1	ANAPC4
NR3C1	ANKFY1
NR3C1	ANKH
NR3C1	ANKRD10
NR3C1	ANKRD13A
NR3C1	ANKRD33B
NR3C1	ANKRD44
NR3C1	ANP32A
NR3C1	AP1G1
NR3C1	AP1S2
NR3C1	AP4E1
NR3C1	AP5B1
NR3C1	APOPT1
NR3C1	APPL1
NR3C1	AQP3
NR3C1	ARGLU1
NR3C1	ARHGEF1
NR3C1	ARHGEF6
NR3C1	ARHGEF7
NR3C1	ARID1B
NR3C1	ARID4B
NR3C1	ARID5B
NR3C1	ARL17B
NR3C1	ARL2BP
NR3C1	ARL3
NR3C1	ARL5A
NR3C1	ARMC9
NR3C1	ARPC1B
NR3C1	ARRDC2
NR3C1	ARRDC3
NR3C1	ASAP1
NR3C1	ASCC1
NR3C1	ASH1L
NR3C1	ASXL2
NR3C1	ATAD1
NR3C1	ATE1
NR3C1	ATG12
NR3C1	ATG13
NR3C1	ATM
NR3C1	ATR
NR3C1	ATRAID
NR3C1	ATRX
NR3C1	ATXN10
NR3C1	ATXN2L
NR3C1	B4GALT5
NR3C1	BASP1
NR3C1	BAX
NR3C1	BAZ2B
NR3C1	BBX

NR3C1	BCAP29
NR3C1	BCAT1
NR3C1	BCL11A
NR3C1	BEX4
NR3C1	BID
NR3C1	BIRC3
NR3C1	BIRC6
NR3C1	BLCAP
NR3C1	BLOC1S1
NR3C1	BMI1
NR3C1	BNIP2
NR3C1	BNIP3L
NR3C1	BORCS7
NR3C1	BPTF
NR3C1	BRD2
NR3C1	BROX
NR3C1	BTG1
NR3C1	C12orf57
NR3C1	C15orf40
NR3C1	C20orf27
NR3C1	C2orf88
NR3C1	CAB39
NR3C1	CACUL1
NR3C1	CALCOCO1
NR3C1	CALHM2
NR3C1	CALM2
NR3C1	CAMK2B
NR3C1	CAMKK2
NR3C1	CAMLG
NR3C1	CANT1
NR3C1	CAPN1
NR3C1	CAPNS1
NR3C1	CAPZA1
NR3C1	CAPZA2
NR3C1	CAPZB
NR3C1	CARD8
NR3C1	CARS
NR3C1	CASC4
NR3C1	CASP2
NR3C1	CAST
NR3C1	CBFA2T3
NR3C1	CBLB
NR3C1	CCDC12
NR3C1	CCDC144A
NR3C1	CCDC18
NR3C1	CCDC82
NR3C1	CCDC84
NR3C1	CCDC90B
NR3C1	CCDC91
NR3C1	CCDC92

NR3C1	CCNB2
NR3C1	CCNG1
NR3C1	CCNG2
NR3C1	CCNH
NR3C1	CCNI
NR3C1	CCSER2
NR3C1	CCZ1
NR3C1	CD2AP
NR3C1	CD53
NR3C1	CDC16
NR3C1	CDC25B
NR3C1	CDC40
NR3C1	CDC42SE2
NR3C1	CDC6
NR3C1	CDCA2
NR3C1	CDCA7
NR3C1	CDCA7L
NR3C1	CDK17
NR3C1	CDK5R1
NR3C1	CDK5RAP3
NR3C1	CDKN1B
NR3C1	CELF1
NR3C1	CENPC
NR3C1	CENPE
NR3C1	CEP164
NR3C1	CEP44
NR3C1	CEP55
NR3C1	CEP57
NR3C1	CEP57L1
NR3C1	CEP78
NR3C1	CEPT1
NR3C1	CETN3
NR3C1	CFAP36
NR3C1	CHAMP1
NR3C1	CHD4
NR3C1	CHD7
NR3C1	CHFR
NR3C1	CHRNA1
NR3C1	CHST10
NR3C1	CIRBP
NR3C1	CKLF
NR3C1	CKS2
NR3C1	CLIP1
NR3C1	CLK1
NR3C1	CLK3
NR3C1	CLN6
NR3C1	CLSTN1
NR3C1	CMPK2
NR3C1	CNIH1
NR3C1	CNOT2

NR3C1	CNOT4
NR3C1	CNOT6
NR3C1	CNOT8
NR3C1	CNPPD1
NR3C1	COASY
NR3C1	COMMD10
NR3C1	COMMD3
NR3C1	COMMD6
NR3C1	COPS3
NR3C1	COQ10B
NR3C1	COX4I1
NR3C1	CPNE1
NR3C1	CREBBP
NR3C1	CREM
NR3C1	CRIPT
NR3C1	CRYZL1
NR3C1	CSDE1
NR3C1	CSF1
NR3C1	CSK
NR3C1	CSNK1D
NR3C1	CSTF2
NR3C1	CSTF2T
NR3C1	CTPS2
NR3C1	CTSH
NR3C1	CUEDC2
NR3C1	CUL4A
NR3C1	CUX1
NR3C1	CWC25
NR3C1	CXCR4
NR3C1	CXorf56
NR3C1	CYP2R1
NR3C1	CYTH1
NR3C1	CYTH2
NR3C1	DALRD3
NR3C1	DARS
NR3C1	DBR1
NR3C1	DCAF11
NR3C1	DCK
NR3C1	DCP1A
NR3C1	DCP2
NR3C1	DCTN2
NR3C1	DDIAS
NR3C1	DDX17
NR3C1	DDX3Y
NR3C1	DDX5
NR3C1	DDX6
NR3C1	DEGS1
NR3C1	DENND3
NR3C1	DEPDC5
NR3C1	DGCR2

NR3C1	DGCR6L
NR3C1	DGKZ
NR3C1	DGUOK
NR3C1	DHRS1
NR3C1	DHRS11
NR3C1	DHX40
NR3C1	DIMT1
NR3C1	DLGAP5
NR3C1	DMAP1
NR3C1	DMTF1
NR3C1	DMXL1
NR3C1	DNAJB2
NR3C1	DNAJB6
NR3C1	DNAJC7
NR3C1	DNAL4
NR3C1	DNER
NR3C1	DOK1
NR3C1	DPY30
NR3C1	DTD1
NR3C1	DTWD1
NR3C1	DUSP11
NR3C1	DUSP5
NR3C1	DYNC1H1
NR3C1	DYNC1I2
NR3C1	DYNLL2
NR3C1	DZIP3
NR3C1	EBF1
NR3C1	ECE1
NR3C1	ECH1
NR3C1	EEF1A1
NR3C1	EEF1D
NR3C1	EEF2
NR3C1	EEFSEC
NR3C1	EFHC1
NR3C1	EGR3
NR3C1	EHD1
NR3C1	EIF1
NR3C1	EIF1B
NR3C1	EIF3H
NR3C1	EIF3L
NR3C1	EIF4A2
NR3C1	EIF4B
NR3C1	ELAC2
NR3C1	ELF1
NR3C1	ELF2
NR3C1	ELK4
NR3C1	ELMOD3
NR3C1	ELOVL5
NR3C1	ENDOV
NR3C1	ENSA

NR3C1	EPC1
NR3C1	ERBIN
NR3C1	ERCC6L
NR3C1	ESD
NR3C1	EZR
NR3C1	FAF1
NR3C1	FAM107B
NR3C1	FAM111A
NR3C1	FAM172A
NR3C1	FAM184B
NR3C1	FAM20B
NR3C1	FAM213A
NR3C1	FAM49A
NR3C1	FAM49B
NR3C1	FAM72C
NR3C1	FAM76B
NR3C1	FAM98C
NR3C1	FANCD2
NR3C1	FAU
NR3C1	FBXL17
NR3C1	FBXL5
NR3C1	FBXO30
NR3C1	FBXO46
NR3C1	FBXO48
NR3C1	FBXO7
NR3C1	FBXO9
NR3C1	FCHO1
NR3C1	FCHSD2
NR3C1	FCRL3
NR3C1	FEM1A
NR3C1	FEM1B
NR3C1	FERMT3
NR3C1	FGD5-AS1
NR3C1	FHIT
NR3C1	FHOD3
NR3C1	FLYWCH2
NR3C1	FNBP1
NR3C1	FOXO1
NR3C1	FYTTD1
NR3C1	GABARAPL2
NR3C1	GALNT6
NR3C1	GATAD2A
NR3C1	GCA
NR3C1	GCC2
NR3C1	GCDH
NR3C1	GCNT1
NR3C1	GCNT2
NR3C1	GFOD1
NR3C1	GGNBP2
NR3C1	GGPS1

NR3C1	GID8
NR3C1	GIGYF2
NR3C1	GLYR1
NR3C1	GNAI3
NR3C1	GNPAT
NR3C1	GOLGA7
NR3C1	GPALPP1
NR3C1	GPATCH8
NR3C1	GPBP1
NR3C1	GPBP1L1
NR3C1	GPHN
NR3C1	GPN3
NR3C1	GPR143
NR3C1	GPRC5C
NR3C1	GPS2
NR3C1	GRAMD1B
NR3C1	GRB2
NR3C1	GSK3A
NR3C1	GSKIP
NR3C1	GSTA4
NR3C1	GTF2F1
NR3C1	GTF2I
NR3C1	GUSB
NR3C1	HACD2
NR3C1	HAGH
NR3C1	HAUS4
NR3C1	HECTD4
NR3C1	HELZ
NR3C1	HENMT1
NR3C1	HERC4
NR3C1	HERPUD2
NR3C1	HES6
NR3C1	HHLA3
NR3C1	HIF1A
NR3C1	HJURP
NR3C1	HK1
NR3C1	HLA-DMB
NR3C1	HLA-DPA1
NR3C1	HLA-DPB1
NR3C1	HMGN1
NR3C1	HMMR
NR3C1	HNRNPA0
NR3C1	HNRNPH2
NR3C1	HNRNPUL1
NR3C1	HNRNPUL2
NR3C1	HOPX
NR3C1	HOXB7
NR3C1	HSDL1
NR3C1	HSF1
NR3C1	HSPA6

NR3C1	HSPBAP1
NR3C1	HSPH1
NR3C1	HTR3A
NR3C1	IDH3B
NR3C1	IER3
NR3C1	IFNGR2
NR3C1	IFT74
NR3C1	IGF2BP3
NR3C1	IKZF1
NR3C1	IKZF3
NR3C1	IKZF5
NR3C1	IL7
NR3C1	IMPACT
NR3C1	ING3
NR3C1	ING4
NR3C1	INIP
NR3C1	INO80
NR3C1	INPP5K
NR3C1	INTS6
NR3C1	IP6K2
NR3C1	IPO13
NR3C1	IQCB1
NR3C1	IQSEC1
NR3C1	IST1
NR3C1	ISYNA1
NR3C1	ITSN2
NR3C1	IZUMO4
NR3C1	JAK1
NR3C1	JDP2
NR3C1	JMJD1C
NR3C1	JMY
NR3C1	KANSL1
NR3C1	KANSL1-AS1
NR3C1	KAT6A
NR3C1	KATNB1
NR3C1	KATNBL1
NR3C1	KBTBD8
NR3C1	KDM5A
NR3C1	KHDRBS1
NR3C1	KIAA1217
NR3C1	KIAA2026
NR3C1	KIF14
NR3C1	KIF3A
NR3C1	KIF5B
NR3C1	KLF12
NR3C1	KLF6
NR3C1	KLHDC2
NR3C1	KLHDC3
NR3C1	KLHL14
NR3C1	KLHL5

NR3C1	KMT2E
NR3C1	KPNA5
NR3C1	KPNA6
NR3C1	KRAS
NR3C1	KRCC1
NR3C1	KTN1
NR3C1	KYAT1
NR3C1	LAMTOR5
NR3C1	LASP1
NR3C1	LAT2
NR3C1	LHPP
NR3C1	LIMA1
NR3C1	LINC00909
NR3C1	LINC01152
NR3C1	LINC01184
NR3C1	LMO2
NR3C1	LONRF1
NR3C1	LPAR5
NR3C1	LPIN1
NR3C1	LPXN
NR3C1	LRRC20
NR3C1	LRRC41
NR3C1	LRRFIP1
NR3C1	LRSAM1
NR3C1	LSM8
NR3C1	LTA4H
NR3C1	LUC7L3
NR3C1	LYRM2
NR3C1	MAD2L1
NR3C1	MAFG
NR3C1	MAGED2
NR3C1	MAP2K1
NR3C1	MAP2K4
NR3C1	MAP3K7CL
NR3C1	MAP4K3
NR3C1	MAPK10
NR3C1	MAPK3
NR3C1	MARCH7
NR3C1	MARK2
NR3C1	MCOLN2
NR3C1	MDH1
NR3C1	MDM4
NR3C1	MEAF6
NR3C1	METAP2
NR3C1	METTL14
NR3C1	METTL15
NR3C1	METTL17
NR3C1	METTL9
NR3C1	MFAP1
NR3C1	MFSD3

NR3C1	MGRN1
NR3C1	MICAL3
NR3C1	MID1IP1
NR3C1	MKNK2
NR3C1	MKRN2
NR3C1	MME
NR3C1	MOB1A
NR3C1	MOB4
NR3C1	MORF4L1
NR3C1	MPLKIP
NR3C1	MRPS14
NR3C1	MRPS31
NR3C1	MSL3
NR3C1	MTA3
NR3C1	MTCH2
NR3C1	MTF2
NR3C1	MTO1
NR3C1	MYBBP1A
NR3C1	MYEF2
NR3C1	MYH9
NR3C1	MYL12A
NR3C1	MYL12B
NR3C1	MYO1C
NR3C1	MYO1E
NR3C1	MYO9A
NR3C1	N4BP2L2
NR3C1	NAA15
NR3C1	NAP1L4
NR3C1	NARS2
NR3C1	NCAPG2
NR3C1	NCAPH
NR3C1	NCBP2
NR3C1	NCOA3
NR3C1	NCOR1
NR3C1	NDE1
NR3C1	NDUFA10
NR3C1	NDUFS1
NR3C1	NEK6
NR3C1	NEK8
NR3C1	NEK9
NR3C1	NELFA
NR3C1	NFATC2
NR3C1	NFATC3
NR3C1	NFE2L1
NR3C1	NFE2L2
NR3C1	NFKBIA
NR3C1	NFKBID
NR3C1	NIF3L1
NR3C1	NINJ2
NR3C1	NIPAL3

NR3C1	NIPBL
NR3C1	NKTR
NR3C1	NME6
NR3C1	NMI
NR3C1	NMRAL1
NR3C1	NNT
NR3C1	NOL9
NR3C1	NONO
NR3C1	NPAS1
NR3C1	NPRL3
NR3C1	NR3C1
NR3C1	NSMCE2
NR3C1	NT5DC1
NR3C1	NUB1
NR3C1	NUCKS1
NR3C1	NUDCD1
NR3C1	NUDCD2
NR3C1	NUP107
NR3C1	NUP210
NR3C1	NUP58
NR3C1	OARD1
NR3C1	OCIAD1
NR3C1	OFD1
NR3C1	OGG1
NR3C1	OGT
NR3C1	ORC4
NR3C1	OSBPL11
NR3C1	OSBPL9
NR3C1	OSTF1
NR3C1	OTULIN
NR3C1	P4HTM
NR3C1	PABPN1
NR3C1	PAFAH1B2
NR3C1	PAFAH1B3
NR3C1	PAG1
NR3C1	PAK2
NR3C1	PAPOLA
NR3C1	PARP1
NR3C1	PAWR
NR3C1	PAX5
NR3C1	PBLD
NR3C1	PCF11
NR3C1	PCM1
NR3C1	PDCD6IP
NR3C1	PDE4B
NR3C1	PDE7A
NR3C1	PDE9A
NR3C1	PDHB
NR3C1	PDK2
NR3C1	PDK3

NR3C1	PDPK1
NR3C1	PDRG1
NR3C1	PDZD8
NR3C1	PEBP1
NR3C1	PEF1
NR3C1	PFN2
NR3C1	PHF20
NR3C1	PHF20L1
NR3C1	PHF21A
NR3C1	PHF6
NR3C1	PHIP
NR3C1	PHTF2
NR3C1	PIAS1
NR3C1	PIAS2
NR3C1	PIEZO1
NR3C1	PIGU
NR3C1	PIK3R1
NR3C1	PIM3
NR3C1	PINLYP
NR3C1	PIP4K2A
NR3C1	PITPNC1
NR3C1	PKHD1L1
NR3C1	PKMYT1
NR3C1	PKN1
NR3C1	PKN2
NR3C1	PLEKHF2
NR3C1	PLEKHG1
NR3C1	PLEKHO1
NR3C1	PNPLA6
NR3C1	POGK
NR3C1	POLR2M
NR3C1	POR
NR3C1	POU3F1
NR3C1	PPCS
NR3C1	PPDPF
NR3C1	PPIH
NR3C1	PPIL3
NR3C1	PPIL4
NR3C1	PPP1CC
NR3C1	PPP1R12A
NR3C1	PPP1R15A
NR3C1	PPP1R2
NR3C1	PPP1R21
NR3C1	PPP2R1B
NR3C1	PPP2R2A
NR3C1	PPP2R5C
NR3C1	PPP2R5E
NR3C1	PPP4R3A
NR3C1	PRDM2
NR3C1	PRKACB

NR3C1	PRKAR1A
NR3C1	PRKCD
NR3C1	PRMT2
NR3C1	PRMT7
NR3C1	PRPF38B
NR3C1	PRPSAP2
NR3C1	PRRC2B
NR3C1	PRRC2C
NR3C1	PSEN1
NR3C1	PSENEN
NR3C1	PSIP1
NR3C1	PSMC3IP
NR3C1	PTBP3
NR3C1	PTOV1
NR3C1	PTPRC
NR3C1	PTPRS
NR3C1	PTRHD1
NR3C1	PUS10
NR3C1	PYCR2
NR3C1	QRICH1
NR3C1	R3HCC1L
NR3C1	RAB11B
NR3C1	RAB14
NR3C1	RAB21
NR3C1	RAB30
NR3C1	RAB30-AS1
NR3C1	RAB35
NR3C1	RAB5C
NR3C1	RAB8B
NR3C1	RABGAP1
NR3C1	RACK1
NR3C1	RALGAPA1
NR3C1	RALGAPA2
NR3C1	RAP1B
NR3C1	RARA
NR3C1	RASGRP3
NR3C1	RB1CC1
NR3C1	RBBP4
NR3C1	RBBP9
NR3C1	RBFA
NR3C1	RBM15
NR3C1	RBM26-AS1
NR3C1	RBMX
NR3C1	RCC1L
NR3C1	RCSD1
NR3C1	RDH13
NR3C1	RELA
NR3C1	RELB
NR3C1	REV3L
NR3C1	RFC1

NR3C1	RFT1
NR3C1	RFTN1
NR3C1	RFX1
NR3C1	RGS10
NR3C1	RHOA
NR3C1	RHOG
NR3C1	RHOH
NR3C1	RIC3
NR3C1	RIT1
NR3C1	RMDN3
NR3C1	RNF10
NR3C1	RNF167
NR3C1	RNF187
NR3C1	RNF219
NR3C1	RNF4
NR3C1	RNF40
NR3C1	RNF41
NR3C1	ROCK1
NR3C1	RP2
NR3C1	RPA1
NR3C1	RPAP2
NR3C1	RPL10
NR3C1	RPL10A
NR3C1	RPL12
NR3C1	RPL13A
NR3C1	RPL15
NR3C1	RPL17
NR3C1	RPL18
NR3C1	RPL18A
NR3C1	RPL21
NR3C1	RPL23
NR3C1	RPL23A
NR3C1	RPL24
NR3C1	RPL26
NR3C1	RPL27A
NR3C1	RPL28
NR3C1	RPL3
NR3C1	RPL30
NR3C1	RPL32
NR3C1	RPL34
NR3C1	RPL35A
NR3C1	RPL39
NR3C1	RPL4
NR3C1	RPL41
NR3C1	RPL6
NR3C1	RPL7
NR3C1	RPL8
NR3C1	RPL9
NR3C1	RPLP0
NR3C1	RPS11

NR3C1	RPS13
NR3C1	RPS15
NR3C1	RPS15A
NR3C1	RPS16
NR3C1	RPS18
NR3C1	RPS19
NR3C1	RPS2
NR3C1	RPS20
NR3C1	RPS23
NR3C1	RPS27
NR3C1	RPS27A
NR3C1	RPS28
NR3C1	RPS3A
NR3C1	RPS5
NR3C1	RPS6
NR3C1	RPS7
NR3C1	RPS8
NR3C1	RPS9
NR3C1	RRAGA
NR3C1	RRAGC
NR3C1	RSBN1
NR3C1	RSF1
NR3C1	RSL24D1
NR3C1	RSPH3
NR3C1	RTF1
NR3C1	RUBCN
NR3C1	RUFY3
NR3C1	RUSC1
NR3C1	RXRB
NR3C1	SAFB2
NR3C1	SAP30
NR3C1	SBF2
NR3C1	SCAF1
NR3C1	SCAF11
NR3C1	SCAMP1
NR3C1	SCAND1
NR3C1	SCARB1
NR3C1	SCCPDH
NR3C1	SCRN1
NR3C1	SDCBP
NR3C1	SDCCAG8
NR3C1	SEC23A
NR3C1	SEMA4A
NR3C1	SENP2
NR3C1	SEPHS2
NR3C1	SEPT2
NR3C1	SERINC3
NR3C1	SESN3
NR3C1	SESTD1
NR3C1	SETX

NR3C1	SF1
NR3C1	SFI1
NR3C1	SGF29
NR3C1	SGO1
NR3C1	SGSM2
NR3C1	SH2B1
NR3C1	SH3BGRL
NR3C1	SH3BP5
NR3C1	SH3GLB1
NR3C1	SH3KBP1
NR3C1	SHISA8
NR3C1	SHOC2
NR3C1	SLC10A3
NR3C1	SLC11A2
NR3C1	SLC15A4
NR3C1	SLC1A1
NR3C1	SLC25A19
NR3C1	SLC25A3
NR3C1	SLC25A33
NR3C1	SLC46A3
NR3C1	SLC7A1
NR3C1	SLTM
NR3C1	SLU7
NR3C1	SMAP2
NR3C1	SMARCA5
NR3C1	SMARCAD1
NR3C1	SMARCC2
NR3C1	SMARCD2
NR3C1	SMC5
NR3C1	SMCHD1
NR3C1	SMIM14
NR3C1	SMIM7
NR3C1	SNAP23
NR3C1	SNRNP35
NR3C1	SNX1
NR3C1	SNX2
NR3C1	SNX7
NR3C1	SOCS1
NR3C1	SOD1
NR3C1	SON
NR3C1	SORL1
NR3C1	SPAG16
NR3C1	SPECC1
NR3C1	SPG11
NR3C1	SPHK2
NR3C1	SPI1
NR3C1	SPON1
NR3C1	SPSB3
NR3C1	SREK1
NR3C1	SRP14

NR3C1	SRRT
NR3C1	SRSF5
NR3C1	SSU72
NR3C1	STAG2
NR3C1	STAT3
NR3C1	STAT5B
NR3C1	STIM2
NR3C1	STK38L
NR3C1	STK4
NR3C1	STUB1
NR3C1	STX18
NR3C1	STXBP3
NR3C1	SUDS3
NR3C1	SUPT4H1
NR3C1	SUV39H1
NR3C1	SUV39H2
NR3C1	SVBP
NR3C1	SWAP70
NR3C1	SYAP1
NR3C1	SYBU
NR3C1	SYMPK
NR3C1	SYNGR2
NR3C1	TAF12
NR3C1	TAF15
NR3C1	TAF1D
NR3C1	TAF7
NR3C1	TAF8
NR3C1	TANK
NR3C1	TAOK1
NR3C1	TARDBP
NR3C1	TATDN2
NR3C1	TBC1D22A
NR3C1	TBC1D4
NR3C1	TBCA
NR3C1	TBCB
NR3C1	TBL1XR1
NR3C1	TCEAL4
NR3C1	TEX261
NR3C1	TEX9
NR3C1	TFEB
NR3C1	TFRC
NR3C1	TGIF1
NR3C1	TGOLN2
NR3C1	THAP11
NR3C1	THAP9-AS1
NR3C1	THEM6
NR3C1	THOC2
NR3C1	TIAL1
NR3C1	TIMM10B
NR3C1	TIPARP

NR3C1	TLE4
NR3C1	TLR10
NR3C1	TM6SF1
NR3C1	TMC6
NR3C1	TMED8
NR3C1	TMEM101
NR3C1	TMEM102
NR3C1	TMEM123
NR3C1	TMEM14A
NR3C1	TMEM167B
NR3C1	TMEM18
NR3C1	TMEM185A
NR3C1	TMEM216
NR3C1	TMEM218
NR3C1	TMEM219
NR3C1	TMEM38B
NR3C1	TMEM60
NR3C1	TMEM80
NR3C1	TMEM97
NR3C1	TMSB4X
NR3C1	TMUB2
NR3C1	TNIP1
NR3C1	TNPO3
NR3C1	TOLLIP
NR3C1	TOMM5
NR3C1	TOPORS
NR3C1	TOX
NR3C1	TPCN1
NR3C1	TPGS1
NR3C1	TRABD
NR3C1	TRAF5
NR3C1	TRAPPC8
NR3C1	TRIM13
NR3C1	TRIM44
NR3C1	TRIM52
NR3C1	TRIM8
NR3C1	TRIP11
NR3C1	TRIQK
NR3C1	TROVE2
NR3C1	TSC22D2
NR3C1	TSC22D3
NR3C1	TSG101
NR3C1	TSGA10
NR3C1	TSPAN14
NR3C1	TSPAN33
NR3C1	TSPO
NR3C1	TTC14
NR3C1	TTC30A
NR3C1	TTC31
NR3C1	TTI2

NR3C1	TUBGCP5
NR3C1	TWF2
NR3C1	U2AF2
NR3C1	UACA
NR3C1	UBA2
NR3C1	UBAP2
NR3C1	UBAP2L
NR3C1	UBC
NR3C1	UBE2D2
NR3C1	UBE2J2
NR3C1	UBE2K
NR3C1	UBE2R2
NR3C1	UBP1
NR3C1	UBR5
NR3C1	UBXN7
NR3C1	UCHL5
NR3C1	UGP2
NR3C1	UNG
NR3C1	UPF2
NR3C1	USP10
NR3C1	USP13
NR3C1	USP22
NR3C1	USP48
NR3C1	USP6NL
NR3C1	UVRAG
NR3C1	VAC14
NR3C1	VAMP2
NR3C1	VAMP8
NR3C1	VASP
NR3C1	VEZT
NR3C1	VPS28
NR3C1	VPS4B
NR3C1	VRK2
NR3C1	VTI1B
NR3C1	WAC
NR3C1	WAPL
NR3C1	WASF2
NR3C1	WDFY1
NR3C1	WDR1
NR3C1	WDR34
NR3C1	WDR77
NR3C1	WIPF1
NR3C1	WNK1
NR3C1	WRB
NR3C1	WSB1
NR3C1	XIAP
NR3C1	XRN2
NR3C1	YBEY
NR3C1	YKT6
NR3C1	YWHAZ

NR3C1	YY1
NR3C1	ZBTB20
NR3C1	ZC3H14
NR3C1	ZCCHC3
NR3C1	ZCCHC7
NR3C1	ZDHHC2
NR3C1	ZDHHC20
NR3C1	ZDHHC21
NR3C1	ZDHHC24
NR3C1	ZFAND5
NR3C1	ZFAS1
NR3C1	ZFC3H1
NR3C1	ZFR
NR3C1	ZFX
NR3C1	ZGRF1
NR3C1	ZMYM2
NR3C1	ZNF101
NR3C1	ZNF106
NR3C1	ZNF131
NR3C1	ZNF184
NR3C1	ZNF226
NR3C1	ZNF24
NR3C1	ZNF254
NR3C1	ZNF267
NR3C1	ZNF280D
NR3C1	ZNF292
NR3C1	ZNF500
NR3C1	ZNF518A
NR3C1	ZNF580
NR3C1	ZNF638
NR3C1	ZNF644
NR3C1	ZNF681
NR3C1	ZNF75A
NR3C1	ZSCAN16-AS1
NRF1	ACSF3
NRF1	ACTR1B
NRF1	ALDOA
NRF1	ALKBH4
NRF1	ANLN
NRF1	APC
NRF1	ARFGEF1
NRF1	ARHGAP1
NRF1	ARHGAP17
NRF1	ARL17A
NRF1	ARL3
NRF1	ARMC1
NRF1	ARPC1A
NRF1	ATXN2L
NRF1	AURKA
NRF1	BANP

NRF1	BLK
NRF1	C12orf65
NRF1	C5orf15
NRF1	CBX1
NRF1	CCDC71
NRF1	CCNB1
NRF1	CCNF
NRF1	CDCA3
NRF1	CDK14
NRF1	CDK6
NRF1	CDKAL1
NRF1	CENPF
NRF1	CFL2
NRF1	CKAP2
NRF1	CKAP5
NRF1	CKS1B
NRF1	COQ9
NRF1	DPF1
NRF1	DSN1
NRF1	EBF1
NRF1	EFTUD2
NRF1	EHD3
NRF1	EPS15L1
NRF1	ESCO1
NRF1	FKBP4
NRF1	GNL3L
NRF1	GPSM2
NRF1	GSS
NRF1	GTF2A1
NRF1	HERPUD2
NRF1	HIRIP3
NRF1	HSPA8
NRF1	INPP5F
NRF1	ITGA4
NRF1	KDM1B
NRF1	KDM4A
NRF1	KIF14
NRF1	KIF2A
NRF1	KIF2C
NRF1	LPCAT1
NRF1	LRIF1
NRF1	M6PR
NRF1	MAEA
NRF1	MAF1
NRF1	MED13L
NRF1	MRGBP
NRF1	MXD4
NRF1	NARFL
NRF1	NRF1
NRF1	NUP153

NRF1	PACS1
NRF1	PAGR1
NRF1	PDLIM7
NRF1	PHTF2
NRF1	PIGH
NRF1	PLK1
NRF1	PPTC7
NRF1	PRKAR2B
NRF1	PRMT1
NRF1	PSMA3-AS1
NRF1	RACGAP1
NRF1	RAD21
NRF1	RBBP5
NRF1	RBM12
NRF1	RBM27
NRF1	RCSD1
NRF1	RDM1
NRF1	REEP4
NRF1	RGS12
NRF1	RGS3
NRF1	RGS9
NRF1	RHOF
NRF1	RNF14
NRF1	RTCA
NRF1	SCP2
NRF1	SENP2
NRF1	SEPT11
NRF1	SGPP1
NRF1	SLK
NRF1	SOX5
NRF1	SPC24
NRF1	SPN
NRF1	STK10
NRF1	STRADA
NRF1	SUV39H2
NRF1	TBC1D10B
NRF1	TBC1D22A
NRF1	TBL1XR1
NRF1	TELO2
NRF1	TFEB
NRF1	TGIF2
NRF1	TINF2
NRF1	TMEM19
NRF1	TMEM41B
NRF1	TOMM70
NRF1	TTC17
NRF1	TUBB4B
NRF1	UBE2S
NRF1	UBE2T
NRF1	UCK2

NRF1	USP3
NRF1	VPS16
NRF1	WDR5
NRF1	WNT2B
NRF1	ZC3H14
NRF1	ZNF420
PATZ1	ARHGAP33
PATZ1	ARID3A
PATZ1	HOOK2
PATZ1	IGSF8
PATZ1	MTPAP
PATZ1	NAMPT
PAX5	ACAP2
PAX5	ACTB
PAX5	ACTR2
PAX5	AFF3
PAX5	ALOX5AP
PAX5	AP1B1
PAX5	APBB1IP
PAX5	APC
PAX5	APH1A
PAX5	ARHGAP25
PAX5	ARHGAP26
PAX5	ARHGAP30
PAX5	ARHGDIB
PAX5	ARID1B
PAX5	ARPC1B
PAX5	ARPC2
PAX5	ARPC4
PAX5	B9D2
PAX5	BCL11A
PAX5	BCL3
PAX5	BDH1
PAX5	BIN3
PAX5	BLNK
PAX5	BMF
PAX5	BRCA1
PAX5	BRK1
PAX5	BTG1
PAX5	CACNA1A
PAX5	CAT
PAX5	CBL
PAX5	CCDC88B
PAX5	CCNT2
PAX5	CD37
PAX5	CD72
PAX5	CD79B
PAX5	CDK13
PAX5	CELF2
PAX5	CHD3

PAX5	CHERP
PAX5	CNN2
PAX5	CNOT3
PAX5	CNR2
PAX5	CORO1A
PAX5	CREBRF
PAX5	CSK
PAX5	CSNK1G1
PAX5	CTCF
PAX5	CTDSP2
PAX5	CUEDC2
PAX5	CXXC5
PAX5	CYB561A3
PAX5	CYFIP2
PAX5	DCAF12
PAX5	DCAF7
PAX5	DMXL1
PAX5	E2F3
PAX5	EHD1
PAX5	EHD4
PAX5	EIF2B2
PAX5	ELOVL5
PAX5	EPS15
PAX5	ETS1
PAX5	EXOC1
PAX5	FAM126A
PAX5	FAM172A
PAX5	FBXO11
PAX5	FCRL3
PAX5	FCRL5
PAX5	FCRLA
PAX5	FJX1
PAX5	FKBP15
PAX5	FNBP1
PAX5	GAS7
PAX5	GATAD2B
PAX5	GATB
PAX5	GFOD1
PAX5	GIT2
PAX5	GLUL
PAX5	GNAS
PAX5	GPR18
PAX5	GPSM3
PAX5	GRB2
PAX5	GSAP
PAX5	GTF2I
PAX5	GYG1
PAX5	HDAC7
PAX5	HLA-DMB
PAX5	IFI30

PAX5	IKZF1
PAX5	IKZF3
PAX5	IL16
PAX5	IL6R
PAX5	INIP
PAX5	INPP5D
PAX5	IRF5
PAX5	ITGAL
PAX5	ITPR1
PAX5	ITPRIPL1
PAX5	ITSN2
PAX5	JDP2
PAX5	KIAA0040
PAX5	LCP1
PAX5	LILRB1
PAX5	LIMA1
PAX5	LIMD2
PAX5	LMNA
PAX5	LPAR5
PAX5	LYN
PAX5	MAP3K14
PAX5	MBD5
PAX5	MBNL1
PAX5	MEF2A
PAX5	MEF2C
PAX5	MKL1
PAX5	MLEC
PAX5	MORC2
PAX5	MRPL1
PAX5	MTPN
PAX5	MYH9
PAX5	MYO1C
PAX5	N4BP3
PAX5	NCF1
PAX5	NCF4
PAX5	NEIL3
PAX5	NEMF
PAX5	NR3C1
PAX5	NUFIP1
PAX5	NUP214
PAX5	ORC2
PAX5	OSBPL3
PAX5	OSTF1
PAX5	PDLIM1
PAX5	PDLIM2
PAX5	PGS1
PAX5	PHIP
PAX5	PIK3CA
PAX5	PIK3R4
PAX5	PITPNC1

PAX5	PKN1
PAX5	PLA2G15
PAX5	PLCG2
PAX5	PLEKHA2
PAX5	PLEKHB1
PAX5	POLD4
PAX5	PPP1R9B
PAX5	PPP6R1
PAX5	PRRC2C
PAX5	PSD4
PAX5	PTBP3
PAX5	PTK2B
PAX5	PTPRC
PAX5	PVT1
PAX5	RAB11FIP1
PAX5	RAC2
PAX5	RAE1
PAX5	RASSF2
PAX5	RCC2
PAX5	RCSD1
PAX5	RFTN1
PAX5	RGS3
PAX5	RHOG
PAX5	RNF141
PAX5	RNF144B
PAX5	RP2
PAX5	RPA2
PAX5	RPL28
PAX5	RPL4
PAX5	RPS18
PAX5	RPS27A
PAX5	RPS6KA1
PAX5	SAMD4B
PAX5	SCIMP
PAX5	SEC61B
PAX5	SH3BGRL
PAX5	SKIL
PAX5	SLC44A2
PAX5	SLC9A7
PAX5	SNAP23
PAX5	SNX22
PAX5	SP100
PAX5	SPAG7
PAX5	SPI1
PAX5	SSBP4
PAX5	ST3GAL2
PAX5	STAP1
PAX5	STK4
PAX5	STRADA
PAX5	STX7

PAX5	SYK
PAX5	SYT17
PAX5	TADA3
PAX5	TAF6L
PAX5	TBC1D1
PAX5	TBL1X
PAX5	TCF4
PAX5	TEX261
PAX5	TFEB
PAX5	TFRC
PAX5	TIMM8A
PAX5	TLR10
PAX5	TMC8
PAX5	TMEM109
PAX5	TMEM154
PAX5	TMEM156
PAX5	TMSB4X
PAX5	TNFAIP8
PAX5	TNFRSF13C
PAX5	TPRG1L
PAX5	TSPAN33
PAX5	TXNDC16
PAX5	UBE2R2
PAX5	UBP1
PAX5	UNC45A
PAX5	USP15
PAX5	USP9X
PAX5	UVRAG
PAX5	VAV1
PAX5	VEZF1
PAX5	VGLL4
PAX5	WDFY4
PAX5	YARS2
PAX5	YWHAG
PAX5	ZBTB20
PAX5	ZC3H12D
PAX5	ZNF296
PBX3	GSE1
PKNOX1	TTN
PLAGL1	BACE2
PLAGL1	CD44
PLAGL1	LDB1
PLAGL1	NFKBIZ
PLAGL1	RBPJ
PLAGL1	SH3BP5L
POU2F1	ARHGAP27
POU2F1	HIST1H2AE
POU2F1	HIST1H2AG
POU2F1	HIST1H2AH
POU2F1	HIST1H2AJ

POU2F1	HIST1H2BH
POU2F1	HIST1H3B
POU2F1	HIST1H3C
POU2F1	KDM3A
POU2F1	LYST
POU2F1	PLCB2
POU2F1	POU2F1
POU2F1	RBM43
POU2F1	TAB2
POU2F1	TEX261
POU6F1	ALG1
POU6F1	ANKRD17
POU6F1	ARL6IP5
POU6F1	ATF6
POU6F1	BCAR3
POU6F1	ELL
POU6F1	FAM214A
POU6F1	HIST1H2AC
POU6F1	HOOK2
POU6F1	NEDD9
POU6F1	POU6F1
POU6F1	RGS2
POU6F1	SSR1
POU6F1	TSPAN13
PRDM1	FKBP2
PRDM1	PRDM1
REL	AARS2
REL	AATF
REL	ABCF1
REL	ABHD6
REL	ABR
REL	ABT1
REL	ACBD6
REL	ACTN4
REL	ADARB1
REL	ADGRE5
REL	ADIPOR2
REL	ADO
REL	ADPRHL2
REL	ADRM1
REL	ADSL
REL	ADSS
REL	AGFG1
REL	AGO3
REL	AHCY
REL	AHDC1
REL	AHR
REL	AHSA1
REL	AKAP12
REL	ALG3

REL	AMD1
REL	ANKRD11
REL	ANKRD17
REL	ANKRD33B
REL	ANKRD37
REL	ANP32A
REL	ANXA11
REL	AP1AR
REL	AP5Z1
REL	APEX2
REL	APH1A
REL	APLP2
REL	APMAP
REL	ARCN1
REL	ARF6
REL	ARHGAP15
REL	ARHGAP25
REL	ARHGAP9
REL	ARHGEF2
REL	ARID1B
REL	ARID4B
REL	ARID5B
REL	ARL6IP5
REL	ARMC5
REL	ARMCX6
REL	ARPP19
REL	ASCC1
REL	ASH1L
REL	ASNA1
REL	ASXL1
REL	ATAD1
REL	ATAD3B
REL	ATF4
REL	ATF5
REL	ATF7IP
REL	ATG101
REL	ATG5
REL	ATL3
REL	ATM
REL	ATP1B3
REL	ATP6AP2
REL	ATP6V0A1
REL	ATP6V0A2
REL	ATXN1
REL	ATXN10
REL	ATXN2L
REL	AURKAIP1
REL	B3GNT7
REL	BACH1
REL	BACH2

REL	BANF1
REL	BASP1
REL	BATF
REL	BAZ1A
REL	BAZ1B
REL	BAZ2A
REL	BBIP1
REL	BCAR3
REL	BCL2
REL	BCL2A1
REL	BCL2L1
REL	BCL3
REL	BCLAF1
REL	BEX2
REL	BEX4
REL	BHLHE40
REL	BHLHE41
REL	BIRC3
REL	BLCAP
REL	BLOC1S2
REL	BMS1
REL	BOD1
REL	BPTF
REL	BRD2
REL	BRPF1
REL	BTAF1
REL	BTF3
REL	BTF3L4
REL	BTG1
REL	BTLA
REL	BUD31
REL	BZW1
REL	C11orf49
REL	C11orf58
REL	C15orf40
REL	C15orf41
REL	C16orf91
REL	C18orf21
REL	C18orf25
REL	C19orf48
REL	C1orf131
REL	C2orf76
REL	C6orf136
REL	C9orf85
REL	CA8
REL	CAAP1
REL	CABLES1
REL	CALM2
REL	CAMK4
REL	CAMKK1

REL	CAND1
REL	CBLL1
REL	CCDC137
REL	CCDC174
REL	CCDC25
REL	CCDC71
REL	CCL3
REL	CCL4
REL	CCL4L2
REL	CCND1
REL	CCND2
REL	CCNI
REL	CCNK
REL	CCNL1
REL	CCNYL1
REL	CCR7
REL	CCT2
REL	CCT7
REL	CCZ1
REL	CD109
REL	CD164
REL	CD200
REL	CD40
REL	CD48
REL	CD70
REL	CD80
REL	CD83
REL	CD86
REL	CDC123
REL	CDC37
REL	CDC42
REL	CDC42EP3
REL	CDC42SE1
REL	CDK11B
REL	CDK7
REL	CDKN2AIP
REL	CEBPZ
REL	CELF1
REL	CELF2
REL	CFAP97
REL	CFLAR
REL	CGGBP1
REL	CHCHD5
REL	CHD2
REL	CHD4
REL	CHMP2B
REL	CHMP7
REL	CHST11
REL	CIAO1
REL	CIAPIN1

REL	CISD2
REL	CKS2
REL	CLEC2D
REL	CLK4
REL	CLPB
REL	CLTA
REL	CMC2
REL	CMSS1
REL	CMTM6
REL	CNBP
REL	CNDP2
REL	CNIH1
REL	CNOT2
REL	CNOT4
REL	CNOT7
REL	CNST
REL	COIL
REL	COMMD2
REL	COMMD6
REL	COPS3
REL	COPS5
REL	COQ9
REL	COX17
REL	COX7A2L
REL	COX7C
REL	CPSF7
REL	CREB1
REL	CREB5
REL	CREM
REL	CRIM1
REL	CRK
REL	CRLS1
REL	CSNK1D
REL	CSNK2A1
REL	CSNK2B
REL	CTDP1
REL	CTPS1
REL	CUTC
REL	CWC22
REL	CXCR4
REL	CXCR5
REL	CXorf21
REL	CYB561A3
REL	CYC1
REL	CYFIP2
REL	CYLD
REL	CYTH1
REL	DAXX
REL	DCAF10
REL	DCAF4

REL	DCAF6
REL	DCAF7
REL	DCP1A
REL	DCP2
REL	DCTN5
REL	DDB1
REL	DDX18
REL	DDX21
REL	DDX27
REL	DDX3X
REL	DDX50
REL	DDX52
REL	DDX55
REL	DENND1C
REL	DENND4A
REL	DENR
REL	DHRS3
REL	DHX15
REL	DHX16
REL	DHX34
REL	DHX36
REL	DHX37
REL	DIAPH1
REL	DLGAP4
REL	DLST
REL	DMAP1
REL	DNAJA1
REL	DNAJA2
REL	DNAJB1
REL	DNAJB2
REL	DNAJB6
REL	DNAJC25
REL	DNAJC7
REL	DNPEP
REL	DOK1
REL	DPH3
REL	DPM1
REL	DTD2
REL	DTNBP1
REL	DUSP10
REL	DUSP4
REL	DVL3
REL	DYNC1I2
REL	E2F4
REL	E2F7
REL	EBF1
REL	EEA1
REL	EEF1B2
REL	EEF1E1
REL	EFTUD2

REL	EGR2
REL	EGR3
REL	EHD1
REL	EIF1
REL	EIF2B2
REL	EIF2B4
REL	EIF2S3
REL	EIF3A
REL	EIF3D
REL	EIF3F
REL	EIF3G
REL	EIF3H
REL	EIF3K
REL	EIF3M
REL	EIF4A1
REL	EIF4A2
REL	EIF4G1
REL	EIF4G2
REL	EIF5
REL	EIF5B
REL	EIF6
REL	ELP2
REL	EMC7
REL	EMG1
REL	EML3
REL	EML4
REL	ENO1
REL	ENSA
REL	EPB41L4A-AS1
REL	EPC1
REL	EPG5
REL	EPM2A
REL	EPN1
REL	EPS15
REL	ESCO1
REL	ESD
REL	ETF1
REL	ETS1
REL	EXOC7
REL	EXOSC3
REL	EXOSC4
REL	EXOSC5
REL	EXOSC6
REL	EXT2
REL	EZR
REL	FADD
REL	FAM172A
REL	FAM177A1
REL	FAM204A
REL	FAM207A

REL	FAM213B
REL	FAM234A
REL	FAM32A
REL	FAM3C
REL	FAM69A
REL	FAM96B
REL	FASN
REL	FBL
REL	FBXL12
REL	FBXL18
REL	FBXO11
REL	FBXO22
REL	FBXO28
REL	FBXO7
REL	FBXW11
REL	FCER2
REL	FCRL1
REL	FDFT1
REL	FDPS
REL	FDX1
REL	FGF14-AS2
REL	FIS1
REL	FLAD1
REL	FMNL1
REL	FMNL2
REL	FNBP4
REL	FNIP1
REL	FNTA
REL	FOXN3-AS1
REL	FOXP1
REL	FUBP1
REL	FUS
REL	FYN
REL	G3BP2
REL	GABPB1-AS1
REL	GADD45B
REL	GAPVD1
REL	GAR1
REL	GARS
REL	GAS7
REL	GCC2
REL	GDI2
REL	GDPD5
REL	GEMIN6
REL	GFOD1
REL	GGA1
REL	GGNBP2
REL	GLTP
REL	GNAI3
REL	GNL2

REL	GOLGA7
REL	GORAB
REL	GOSR1
REL	GOSR2
REL	GPBP1
REL	GPR137
REL	GPS2
REL	GPSM3
REL	GRAPL
REL	GRIPAP1
REL	GSPT1
REL	GTF2A2
REL	GTF2F1
REL	GTF2H2
REL	GTF2H3
REL	GTPBP10
REL	GTPBP3
REL	GTPBP4
REL	GTPBP8
REL	H3F3A
REL	HDAC5
REL	HEATR1
REL	HGS
REL	HIF1A
REL	HIPK1
REL	HIPK3
REL	HIVEP1
REL	HMGCS1
REL	HMGXB3
REL	HNRNPA0
REL	HNRNPA1L2
REL	HNRNPAB
REL	HNRNPC
REL	HNRNPD
REL	HNRNPF
REL	HNRNPH1
REL	HNRNPH3
REL	HNRNPU
REL	HOPX
REL	HSPA4
REL	HSPA8
REL	HSPBAP1
REL	HSPBP1
REL	ICAM1
REL	ID2
REL	ID3
REL	IDI1
REL	IER5
REL	IFNGR1
REL	IGF2BP3

REL	IGF2R
REL	IGFBP4
REL	IKZF3
REL	IL21R
REL	IL2RA
REL	IL2RG
REL	IL4I1
REL	ILF2
REL	ILF3
REL	ILKAP
REL	IMP3
REL	INF2
REL	ING3
REL	INIP
REL	INO80
REL	INO80B
REL	INO80C
REL	INTS9
REL	IPPK
REL	IRAK1
REL	IREB2
REL	IRF5
REL	ISG20L2
REL	ISY1
REL	ITGB2
REL	ITPA
REL	ITPRIPL2
REL	JAGN1
REL	JAK1
REL	JMJD1C
REL	JUNB
REL	KANSL1
REL	KAT6B
REL	KAT8
REL	KBTBD8
REL	KCNQ5
REL	KCTD10
REL	KCTD12
REL	KCTD5
REL	KDM1B
REL	KDM6B
REL	KHNYN
REL	KIAA0232
REL	KIF20B
REL	KLF10
REL	KLHL18
REL	KMT2E
REL	KPNA4
REL	KPNA6
REL	KPNB1

REL	KRCC1
REL	KRR1
REL	KTI12
REL	KYNU
REL	LAMTOR3
REL	LAPTM5
REL	LARP7
REL	LAS1L
REL	LCOR
REL	LCORL
REL	LCP1
REL	LDHA
REL	LIMD2
REL	LITAF
REL	LLPH
REL	LMNA
REL	LRRC32
REL	LRRC41
REL	LRRC58
REL	LRRC8D
REL	LRRFIP1
REL	LSG1
REL	LSM5
REL	LST1
REL	LTA
REL	LTA4H
REL	LTV1
REL	LUC7L2
REL	LY9
REL	LYPLA2
REL	LYRM2
REL	LYRM7
REL	LYST
REL	M6PR
REL	MAGOH
REL	MANBA
REL	MAP3K1
REL	MAP3K7
REL	MAP3K8
REL	MAP7D3
REL	MAPK1IP1L
REL	MAPK6
REL	MARCKS
REL	MAST4
REL	MAX
REL	MBNL1
REL	MBTPS2
REL	MCRIP1
REL	MDC1
REL	MDN1

REL	MEA1
REL	MED1
REL	MED10
REL	MED19
REL	MED23
REL	MED7
REL	MEF2C
REL	MEMO1
REL	MEPCE
REL	METRN
REL	METTL14
REL	METTL21A
REL	METTL2A
REL	METTL2B
REL	METTL8
REL	METTL9
REL	MFN1
REL	MICU2
REL	MIDN
REL	MIEF1
REL	MINOS1
REL	MIR155HG
REL	MIR3142HG
REL	MKKS
REL	MLF2
REL	MLLT6
REL	MLST8
REL	MME
REL	MOB4
REL	MOCS3
REL	MORC2
REL	MPDU1
REL	MPLKIP
REL	MRPL12
REL	MRPL21
REL	MRPL28
REL	MRPL3
REL	MRPL47
REL	MRPL52
REL	MRPS18B
REL	MRPS22
REL	MRPS23
REL	MS4A1
REL	MTCH2
REL	MTERF2
REL	MTMR14
REL	MTMR9
REL	MTRF1L
REL	MYC
REL	MYNN

REL	NAA10
REL	NAA15
REL	NAA20
REL	NAA35
REL	NAB1
REL	NABP1
REL	NACA
REL	NAP1L1
REL	NARS
REL	NAT9
REL	NBEAL1
REL	NCBP2
REL	NCL
REL	NCOA3
REL	NCOA5
REL	NDUFA12
REL	NDUFAF4
REL	NECAP1
REL	NECAP2
REL	NEDD4L
REL	NEK8
REL	NELFE
REL	NEMF
REL	NFAT5
REL	NFATC1
REL	NFATC3
REL	NFE2L1
REL	NFE2L2
REL	NFKB1
REL	NFKB2
REL	NFKBIA
REL	NFKBIB
REL	NFKBID
REL	NFKBIE
REL	NFKBIZ
REL	NFX1
REL	NGDN
REL	NGRN
REL	NIFK
REL	NIN
REL	NIPBL
REL	NKRF
REL	NME1
REL	NME2
REL	NOC2L
REL	NOC3L
REL	NOL6
REL	NOL8
REL	NOL9
REL	NOP10

REL	NOP2
REL	NOP58
REL	NORAD
REL	NPM3
REL	NPW
REL	NR1H2
REL	NR2F6
REL	NR3C1
REL	NR4A1
REL	NR4A2
REL	NR4A3
REL	NRIP1
REL	NRROS
REL	NRSN2
REL	NSA2
REL	NTMT1
REL	NUBP1
REL	NUCB1
REL	NUCKS1
REL	NUDC
REL	NUFIP1
REL	NUP214
REL	NUP35
REL	NUP62
REL	NUP93
REL	NVL
REL	OCIAD1
REL	OFD1
REL	OGFR
REL	OLA1
REL	ORAI1
REL	ORAOV1
REL	ORC2
REL	OSBPL8
REL	OTUD4
REL	OXSR1
REL	P3H1
REL	PA2G4
REL	PAICS
REL	PAK1IP1
REL	PAK2
REL	PARL
REL	PAWR
REL	PCBP1
REL	PCF11
REL	PCGF5
REL	PCM1
REL	PCNP
REL	PCNX4
REL	PCYT1A

REL	PDCD11
REL	PDCD6
REL	PDCD7
REL	PDE4A
REL	PDE4B
REL	PDLIM5
REL	PDS5B
REL	PDSS1
REL	PEA15
REL	PEF1
REL	PEX5
REL	PFAS
REL	PFDN6
REL	PFKP
REL	PGAM5
REL	PHACTR1
REL	PHB2
REL	PHF11
REL	PHF23
REL	PHF5A
REL	PHTF1
REL	PICALM
REL	PICK1
REL	PIGL
REL	PIK3AP1
REL	PIK3CA
REL	PIKFYVE
REL	PIM3
REL	PITPNM1
REL	PLEKHA1
REL	PLGRKT
REL	PLRG1
REL	PMPCA
REL	PNISR
REL	PNPT1
REL	PNRC1
REL	POGLUT1
REL	POGZ
REL	POLDIP2
REL	POLE3
REL	POLG2
REL	POLR2A
REL	POLR2B
REL	POLR2D
REL	POLR2H
REL	POLR2K
REL	POLR3D
REL	POLR3E
REL	POMGNT1
REL	POMP

REL	POP4
REL	POT1
REL	PPAN
REL	PPIA
REL	PPIG
REL	PPIP5K2
REL	PPP1R10
REL	PPP1R12A
REL	PPP1R18
REL	PPP2CA
REL	PPP2R1A
REL	PPP2R1B
REL	PPP2R2A
REL	PPP2R5A
REL	PPP2R5E
REL	PPP4C
REL	PPP4R3A
REL	PPP6R1
REL	PRDM2
REL	PRKAG1
REL	PRKAR1A
REL	PRKCB
REL	PRNP
REL	PROSER3
REL	PRPF19
REL	PRPF38B
REL	PRPF4
REL	PRPF40A
REL	PRR13
REL	PRR3
REL	PRRC2C
REL	PSEN1
REL	PSMA1
REL	PSMA6
REL	PSMB5
REL	PSMB7
REL	PSMC4
REL	PSMC5
REL	PSMD13
REL	PSMD14
REL	PSMD8
REL	PTBP1
REL	PTBP3
REL	PTDSS2
REL	PTGER4
REL	PTGES3
REL	PTGIR
REL	PTP4A3
REL	PTRH1
REL	PTRH2

REL	PUF60
REL	PUM1
REL	PUM3
REL	PUS1
REL	PWP1
REL	QTRT2
REL	RAB11A
REL	RAB20
REL	RAB21
REL	RAB27A
REL	RAB29
REL	RAB31
REL	RAB34
REL	RAB3GAP2
REL	RAB6A
REL	RAB7A
REL	RAB8B
REL	RACK1
REL	RALB
REL	RALGAPB
REL	RANBP1
REL	RANGAP1
REL	RAP1B
REL	RARA
REL	RARS
REL	RBBP6
REL	RBM14
REL	RBM25
REL	RBM34
REL	RBM39
REL	RBM42
REL	RBM7
REL	RCHY1
REL	RCSD1
REL	RDX
REL	RECQL5
REL	REL
REL	RELB
REL	RELT
REL	REST
REL	REV1
REL	RFK
REL	RHEB
REL	RHOBTB3
REL	RHOC
REL	RHOF
REL	RHOG
REL	RHOT2
REL	RILPL2
REL	RIOK1

REL	RIOK3
REL	RNF138
REL	RNF139
REL	RNF19A
REL	RNF2
REL	RNF20
REL	RNF219
REL	RNF220
REL	RNF41
REL	RNMT
REL	RPA2
REL	RPF1
REL	RPF2
REL	RPL10
REL	RPL10A
REL	RPL11
REL	RPL13A
REL	RPL15
REL	RPL18A
REL	RPL19
REL	RPL21
REL	RPL23
REL	RPL26
REL	RPL27
REL	RPL27A
REL	RPL28
REL	RPL29
REL	RPL3
REL	RPL30
REL	RPL31
REL	RPL32
REL	RPL34
REL	RPL36AL
REL	RPL37
REL	RPL37A
REL	RPL38
REL	RPL4
REL	RPL41
REL	RPL6
REL	RPL7
REL	RPL7L1
REL	RPLP0
REL	RPLP1
REL	RPLP2
REL	RPS11
REL	RPS12
REL	RPS13
REL	RPS14
REL	RPS15
REL	RPS16

REL	RPS17
REL	RPS19
REL	RPS19BP1
REL	RPS20
REL	RPS21
REL	RPS26
REL	RPS27
REL	RPS29
REL	RPS3
REL	RPS3A
REL	RPS6
REL	RPS7
REL	RPSA
REL	RRP12
REL	RRP15
REL	RRP9
REL	RSBN1
REL	RSF1
REL	RUFY3
REL	RUNX3
REL	RUVBL1
REL	RUVBL2
REL	SAMSN1
REL	SARS
REL	SART1
REL	SATB1
REL	SAYSD1
REL	SBDS
REL	SCAF8
REL	SCAMP3
REL	SCD
REL	SCO1
REL	SCYL3
REL	SDAD1
REL	SDCBP
REL	SDE2
REL	SDF4
REL	SDHC
REL	SECISBP2
REL	SEMA7A
REL	SENP1
REL	SEPT9
REL	SERPINB9
REL	SETD4
REL	SETD5
REL	SF1
REL	SF3B1
REL	SF3B2
REL	SF3B3
REL	SF3B5

REL	SFSWAP
REL	SFT2D1
REL	SGTA
REL	SH2B3
REL	SH3BP2
REL	SH3TC1
REL	SHMT2
REL	SIN3A
REL	SIPA1L1
REL	SIRPA
REL	SIRT6
REL	SIRT7
REL	SKIL
REL	SKP2
REL	SLAMF1
REL	SLC12A6
REL	SLC1A5
REL	SLC25A13
REL	SLC25A25
REL	SLC25A3
REL	SLC29A2
REL	SLC35A2
REL	SLC35B3
REL	SLC38A1
REL	SLC39A1
REL	SLC39A14
REL	SLC39A6
REL	SLC3A2
REL	SLC43A3
REL	SLC4A7
REL	SLC6A9
REL	SLC7A1
REL	SLMAP
REL	SLTM
REL	SLU7
REL	SMAP2
REL	SMARCA4
REL	SMARCE1
REL	SMC5
REL	SMCHD1
REL	SMIM12
REL	SMIM19
REL	SMNDC1
REL	SMU1
REL	SNF8
REL	SNHG8
REL	SNRNP200
REL	SNRPB
REL	SNRPB2
REL	SNRPD1

REL	SNRPD2
REL	SNRPE
REL	SNRPF
REL	SNRPG
REL	SNU13
REL	SNW1
REL	SNX11
REL	SNX13
REL	SNX9
REL	SPAG9
REL	SPATA2L
REL	SPIB
REL	SPRTN
REL	SPRY1
REL	SREK1
REL	SRF
REL	SRFBP1
REL	SRP19
REL	SRP72
REL	SRRM1
REL	SRRM2
REL	SRSF1
REL	SRSF5
REL	SRSF7
REL	SRSF9
REL	SS18
REL	SSBP1
REL	SSSCA1
REL	ST14
REL	ST20
REL	ST3GAL4
REL	ST8SIA4
REL	STAG1
REL	STAG2
REL	STAT5A
REL	STAT6
REL	STAU1
REL	STK4
REL	STOML2
REL	STRAP
REL	STT3A
REL	STUM
REL	STX3
REL	STX4
REL	SUGT1
REL	SULF2
REL	SUPT3H
REL	SYF2
REL	SYMPK
REL	SYNCRIP

REL	SYNPO
REL	TACO1
REL	TAF1
REL	TAF7
REL	TANK
REL	TATDN1
REL	TAX1BP1
REL	TBC1D24
REL	TBL1X
REL	TBL3
REL	TBPL1
REL	TBRG1
REL	TBRG4
REL	TCF25
REL	TCF3
REL	TCF7
REL	TCOF1
REL	TCP1
REL	TDG
REL	TDRD3
REL	TES
REL	TESPA1
REL	TFB2M
REL	TFE3
REL	TFPI2
REL	TGFB1
REL	TGFBR2
REL	TGIF1
REL	TGIF2
REL	TGOLN2
REL	THAP1
REL	THAP7
REL	THRAP3
REL	THUMPD3
REL	THUMPD3-AS1
REL	TIGAR
REL	TIMM10B
REL	TIMM17A
REL	TIMM23
REL	TIMM23B
REL	TIMM8A
REL	TINF2
REL	TIPRL
REL	TLK1
REL	TM2D3
REL	TMEM102
REL	TMEM109
REL	TMEM123
REL	TMEM127
REL	TMEM128

REL	TMEM138
REL	TMEM150A
REL	TMEM184C
REL	TMEM199
REL	TMEM208
REL	TMEM216
REL	TMEM251
REL	TMEM33
REL	TMEM65
REL	TMEM69
REL	TMEM97
REL	TMEM9B
REL	TMX1
REL	TMX2
REL	TNF
REL	TNFAIP3
REL	TNFAIP8
REL	TNFRSF14
REL	TNIP1
REL	TNIP2
REL	TNKS2
REL	TNPO2
REL	TNPO3
REL	TNRC6A
REL	TOE1
REL	TOMM20
REL	TOMM22
REL	TOP1
REL	TOX4
REL	TP53BP1
REL	TPM3
REL	TPRKB
REL	TPT1
REL	TRA2B
REL	TRAF1
REL	TRAF4
REL	TRAF7
REL	TRAIP
REL	TRAPPC10
REL	TRIB3
REL	TRIM35
REL	TRIM44
REL	TRMO
REL	TRMT1
REL	TRMT10A
REL	TRMT12
REL	TRMT6
REL	TRUB2
REL	TSC22D2
REL	TSG101

REL	TSHZ2
REL	TSPAN31
REL	TSSC4
REL	TTBK2
REL	TTC1
REL	TTC33
REL	TTF1
REL	TTF2
REL	TTI2
REL	TTPAL
REL	TTTY15
REL	TUFM
REL	TUSC2
REL	TVP23C
REL	TWISTNB
REL	TXNDC16
REL	TXNL1
REL	TXNL4B
REL	U2AF1L4
REL	U2AF2
REL	U2SURP
REL	UBA2
REL	UBA52
REL	UBAC2
REL	UBALD2
REL	UBE2D1
REL	UBE2D3
REL	UBE2E1
REL	UBE2J2
REL	UBE2Z
REL	UBE3A
REL	UBE3C
REL	UBL3
REL	UBLCP1
REL	UBR5
REL	UBXN1
REL	UFM1
REL	UGGT2
REL	UGT8
REL	UNC50
REL	UPP1
REL	UQCRC2
REL	URB1
REL	URM1
REL	USE1
REL	USP12
REL	USP36
REL	USP4
REL	USP8
REL	USP9X

REL	USPL1
REL	UTP14A
REL	UTP18
REL	UTP20
REL	UTP23
REL	UTP3
REL	UTP6
REL	UVRAG
REL	VASP
REL	VIM
REL	VPS26A
REL	VPS51
REL	VTA1
REL	VWA8
REL	WAC
REL	WAPL
REL	WARS
REL	WDR11
REL	WDR12
REL	WDR36
REL	WDR43
REL	WDR46
REL	WDR5B
REL	WDR73
REL	WDR74
REL	WDR75
REL	WDR83
REL	WIPF2
REL	WRAP73
REL	WSB1
REL	WSB2
REL	WTAP
REL	WWP2
REL	XAB2
REL	XIAP
REL	XPO5
REL	XPO6
REL	XRN2
REL	YARS2
REL	YRDC
REL	YTHDF2
REL	YWHAE
REL	YWHAG
REL	YWHAQ
REL	YWHAZ
REL	ZBED2
REL	ZBTB10
REL	ZBTB11-AS1
REL	ZBTB21
REL	ZBTB25

REL	ZBTB7A
REL	ZC3H12A
REL	ZCCHC9
REL	ZFAND6
REL	ZFAS1
REL	ZFC3H1
REL	ZFP36L1
REL	ZFY
REL	ZHX1
REL	ZHX1-C8orf76
REL	ZKSCAN4
REL	ZMAT2
REL	ZMAT3
REL	ZMPSTE24
REL	ZNF131
REL	ZNF184
REL	ZNF207
REL	ZNF232
REL	ZNF235
REL	ZNF276
REL	ZNF324
REL	ZNF33A
REL	ZNF385C
REL	ZNF426
REL	ZNF485
REL	ZNF511
REL	ZNF562
REL	ZNF583
REL	ZNF622
REL	ZNF655
REL	ZNF668
REL	ZNF7
REL	ZNF747
REL	ZNF75A
REL	ZNF800
REL	ZNFX1
REL	ZNHIT2
REL	ZPR1
REL	ZRANB2
RELA	ABR
RELA	ADGRE5
RELA	AGO3
RELA	AGPAT3
RELA	AKAP8
RELA	AKAP9
RELA	ANAPC16
RELA	ANKRD12
RELA	ANTXR2
RELA	ARF1
RELA	ARHGAP11A

RELA	ARID4B
RELA	ARMCX6
RELA	ATP6V1H
RELA	B3GALNT1
RELA	B4GALT3
RELA	BCAP31
RELA	BLOC1S6
RELA	BORCS6
RELA	BRAF
RELA	C11orf71
RELA	CADM1
RELA	CADPS
RELA	CARNMT1
RELA	CASP8
RELA	CCDC102A
RELA	CCSER2
RELA	CDK13
RELA	CDK5RAP3
RELA	CFAP36
RELA	CGRRF1
RELA	CHMP4B
RELA	CHUK
RELA	CINP
RELA	CKAP4
RELA	CNOT3
RELA	COL4A3BP
RELA	COLGALT1
RELA	COMMD2
RELA	CR1
RELA	CRTAP
RELA	CSNK1D
RELA	CSRNP1
RELA	CTDSP2
RELA	CTU1
RELA	CYP2R1
RELA	CYTH1
RELA	DENR
RELA	DHDDS
RELA	DIS3
RELA	DNAAF5
RELA	DTX3L
RELA	EEF1A1
RELA	EEF1B2
RELA	EIF2B1
RELA	ELL2
RELA	EMD
RELA	EMP3
RELA	ENOX2
RELA	FAM122B
RELA	FAM126A

RELA	FAM13B
RELA	FAM204A
RELA	FAM222B
RELA	FBXO16
RELA	FIS1
RELA	FMNL1
RELA	FUZ
RELA	GMDS
RELA	GOPC
RELA	GPATCH2L
RELA	GPR137
RELA	GPX4
RELA	GSKIP
RELA	GSPT2
RELA	HEBP1
RELA	HEG1
RELA	HMGXB3
RELA	HOXB7
RELA	IBTK
RELA	IL15RA
RELA	ING4
RELA	IST1
RELA	JRK
RELA	KAT5
RELA	KAT8
RELA	KIAA2013
RELA	KLF12
RELA	KMT2E
RELA	LMO2
RELA	LPCAT4
RELA	LPGAT1
RELA	MAN1A1
RELA	MAP1S
RELA	MAP4
RELA	MAP9
RELA	MCOLN1
RELA	MCRIP1
RELA	MED11
RELA	MED7
RELA	MIER1
RELA	MKL1
RELA	MLLT10
RELA	MLST8
RELA	MORF4L2-AS1
RELA	MRPL43
RELA	MVB12A
RELA	NCAPH2
RELA	NDUFAF7
RELA	NFATC3
RELA	NPLOC4

RELA	NRM
RELA	NSMCE1
RELA	NUDC
RELA	PASK
RELA	PDE6B
RELA	PEX5
RELA	PIGB
RELA	PLEKHA3
RELA	PLEKHM1
RELA	PNISR
RELA	POLA1
RELA	POLR2B
RELA	PPM1D
RELA	PPP1R18
RELA	PQLC3
RELA	PRKACB
RELA	PRMT5
RELA	PRPF38B
RELA	PTPN4
RELA	RAB40B
RELA	RAB5A
RELA	RBBP6
RELA	RBCK1
RELA	RBMS1
RELA	RELA
RELA	RHOG
RELA	RIC1
RELA	RIC8A
RELA	RNF121
RELA	RPL18A
RELA	RPL21
RELA	RPL32
RELA	RPL37
RELA	RPS27
RELA	RPS27A
RELA	RPS29
RELA	RPS6
RELA	RTCB
RELA	RUFY3
RELA	SACM1L
RELA	SCAMP3
RELA	SDE2
RELA	SEC14L1
RELA	SEC23IP
RELA	SEC24D
RELA	SEC61A1
RELA	SERINC1
RELA	SERTAD3
RELA	SETBP1
RELA	SH3BP2

RELA	SHF
RELA	SLC30A7
RELA	SLC35B3
RELA	SLC39A10
RELA	SLC39A7
RELA	SMCHD1
RELA	SMCO4
RELA	SMG6
RELA	SPG11
RELA	STAT3
RELA	STAT6
RELA	SYNJ2
RELA	TAGLN2
RELA	TBC1D4
RELA	TBC1D7
RELA	TMBIM1
RELA	TMEM101
RELA	TMEM156
RELA	TMEM230
RELA	TMEM68
RELA	TRAF5
RELA	TRAIP
RELA	TRIM28
RELA	TRMT10B
RELA	TRMT61B
RELA	U2AF1
RELA	UBC
RELA	USP36
RELA	VAC14
RELA	VAMP2
RELA	WASF2
RELA	WDR62
RELA	YTHDC1
RELA	ZADH2
RELA	ZBTB11
RELA	ZFAS1
RELA	ZMYM2
RELA	ZNF124
RELA	ZNF234
RELA	ZNF248
RELA	ZNF408
RELA	ZNF780B
RELA	ZNF787
RELB	AATF
RELB	ABCF1
RELB	ABI1
RELB	ACBD5
RELB	ACSL5
RELB	ADO
RELB	ADPRHL2

RELB	ADRM1
RELB	ADSL
RELB	AGL
RELB	AGO1
RELB	AGO3
RELB	AHCY
RELB	AHDC1
RELB	AHSA1
RELB	AKIRIN2
RELB	AKT2
RELB	AMD1
RELB	ANAPC1
RELB	ANKHD1
RELB	ANP32E
RELB	AP1B1
RELB	AP1M1
RELB	APBB1IP
RELB	APMAP
RELB	APOLD1
RELB	APTR
RELB	AQR
RELB	ARHGAP11A
RELB	ARHGAP12
RELB	ARHGDIA
RELB	ARID1A
RELB	ARPC3
RELB	ARPC4
RELB	ARSB
RELB	ARV1
RELB	ASB6
RELB	ASXL2
RELB	ATF2
RELB	ATF7IP
RELB	ATG101
RELB	ATG12
RELB	ATG5
RELB	ATP6V1C2
RELB	ATXN10
RELB	BACE2
RELB	BASP1
RELB	BATF
RELB	BBS10
RELB	BCL2L1
RELB	BCL3
RELB	BECN1
RELB	BIRC3
RELB	BTF3
RELB	BZW1
RELB	C16orf91
RELB	C17orf49

RELB	C3orf67
RELB	C9orf40
RELB	CALM2
RELB	CAMK2B
RELB	CARF
RELB	CASZ1
RELB	CBX4
RELB	CCDC137
RELB	CCDC174
RELB	CCDC28B
RELB	CCDC71
RELB	CCDC9
RELB	CCNB2
RELB	CCNI
RELB	CCNK
RELB	CCR7
RELB	CCT2
RELB	CCT7
RELB	CCZ1
RELB	CD40
RELB	CD70
RELB	CD80
RELB	CD83
RELB	CDC37
RELB	CDK5RAP1
RELB	CDK7
RELB	CENPA
RELB	CENPL
RELB	CEP55
RELB	CETN3
RELB	CFAP20
RELB	CFLAR
RELB	CGGBP1
RELB	CHCHD5
RELB	CHMP2B
RELB	CHMP7
RELB	CKS2
RELB	CLTA
RELB	CNDP2
RELB	CNIH1
RELB	CNOT7
RELB	COG2
RELB	COIL
RELB	COMMD2
RELB	COMMD6
RELB	COPS7A
RELB	COX17
RELB	CREB1
RELB	CREM
RELB	CRK

RELB	CRLS1
RELB	CS
RELB	CSNK2A1
RELB	CSTF2T
RELB	CTDSP1
RELB	CTNNBL1
RELB	CWC22
RELB	CXCR4
RELB	CYLD
RELB	DAAM1
RELB	DBI
RELB	DCAF12
RELB	DCAF7
RELB	DCTN5
RELB	DDX18
RELB	DDX27
RELB	DENND2D
RELB	DHRS3
RELB	DHX15
RELB	DHX36
RELB	DHX38
RELB	DMAP1
RELB	DNAJA1
RELB	DNAJB2
RELB	DNAJB6
RELB	DNAJC7
RELB	DR1
RELB	DTD2
RELB	DTNBP1
RELB	DYNC1I2
RELB	E2F4
RELB	EAPP
RELB	EBF1
RELB	EIF1AD
RELB	EIF2B2
RELB	EIF3D
RELB	EIF3G
RELB	EIF4A1
RELB	EIF5
RELB	EIF6
RELB	ELAVL1
RELB	ELOVL1
RELB	ELP2
RELB	EMC4
RELB	EMG1
RELB	EPS15
RELB	ERCC3
RELB	ETF1
RELB	EXOC2
RELB	EXOC7

RELB	EXOSC3
RELB	EXOSC5
RELB	EXOSC6
RELB	FAM104B
RELB	FAM110A
RELB	FANCL
RELB	FBRS
RELB	FBXO22
RELB	FBXO28
RELB	FBXO34
RELB	FBXO43
RELB	FBXO48
RELB	FBXW2
RELB	FCHO1
RELB	FNIP1
RELB	FNTA
RELB	FOXP1
RELB	FUBP1
RELB	FUBP3
RELB	FUS
RELB	G2E3
RELB	GADD45B
RELB	GALNT6
RELB	GAR1
RELB	GARS
RELB	GAS2L3
RELB	GEMIN7
RELB	GGA1
RELB	GGNBP2
RELB	GLRX5
RELB	GNA13
RELB	GNAI3
RELB	GOSR1
RELB	GPR137
RELB	GRAMD1B
RELB	GRIPAP1
RELB	GSPT1
RELB	GTF2A2
RELB	GTF2F1
RELB	GTPBP4
RELB	HCCS
RELB	HERPUD2
RELB	HIST1H1D
RELB	HIVEP1
RELB	HIVEP3
RELB	HJURP
RELB	HMGB2
RELB	HMHB1
RELB	HNRNPA0
RELB	HNRNPA1L2

RELB	HNRNPC
RELB	HNRNPD
RELB	HNRNPH3
RELB	HOMEZ
RELB	HSBP1
RELB	HSPA4
RELB	HSPA8
RELB	HSPBP1
RELB	ICAM1
RELB	ILF2
RELB	ILF3
RELB	ILKAP
RELB	INO80
RELB	INO80B
RELB	INO80C
RELB	INTS4
RELB	ITGAE
RELB	JADE1
RELB	JAGN1
RELB	KBTBD4
RELB	KBTBD8
RELB	KCP
RELB	KDM2A
RELB	KDM3B
RELB	KIAA0586
RELB	KIF22
RELB	KIF23
RELB	KIF2C
RELB	KIFC1
RELB	LAMTOR3
RELB	LAPTM5
RELB	LARP7
RELB	LAS1L
RELB	LCOR
RELB	LINC00487
RELB	LINC00623
RELB	LLPH
RELB	LMO7
RELB	LONP1
RELB	LRRC32
RELB	LRRC59
RELB	LSM8
RELB	LTB4R
RELB	LYRM1
RELB	LYRM2
RELB	LYRM7
RELB	MAGED2
RELB	MAP3K1
RELB	MAP3K7
RELB	MAP3K8

RELB	MAP4K4
RELB	MAPK1IP1L
RELB	MARK3
RELB	MDC1
RELB	MED10
RELB	MED13L
RELB	MED17
RELB	MELK
RELB	METTL21A
RELB	METTL2A
RELB	METTL3
RELB	METTL9
RELB	MIB1
RELB	MICU2
RELB	MLF2
RELB	MOB4
RELB	MRPL16
RELB	MRPL21
RELB	MRPL27
RELB	MRPL47
RELB	MRPL51
RELB	MRPS18A
RELB	MRPS22
RELB	MS4A1
RELB	MS4A7
RELB	MSRA
RELB	MTFR1L
RELB	MTM1
RELB	MYO1C
RELB	NAA20
RELB	NAB1
RELB	NACA
RELB	NCBP2
RELB	NCL
RELB	NDUFAF3
RELB	NECAP2
RELB	NEK6
RELB	NELFE
RELB	NFAT5
RELB	NFATC1
RELB	NFKB1
RELB	NFKB2
RELB	NFKBIA
RELB	NFKBIB
RELB	NFKBID
RELB	NIFK
RELB	NIPBL
RELB	NKIRAS2
RELB	NMU
RELB	NOC2L

RELB	NOL8
RELB	NOP58
RELB	NORAD
RELB	NR3C1
RELB	NR6A1
RELB	NRBF2
RELB	NSUN6
RELB	NUBP1
RELB	NUCKS1
RELB	NUFIP1
RELB	NUP35
RELB	NUTF2
RELB	OARD1
RELB	OPTN
RELB	OXSR1
RELB	PAK1IP1
RELB	PAK4
RELB	PCBP1
RELB	PCGF3
RELB	PCM1
RELB	PCNP
RELB	PDAP1
RELB	PDCD7
RELB	PDE12
RELB	PDZD11
RELB	PEX5
RELB	PFKP
RELB	PHACTR1
RELB	PHF12
RELB	PHIP
RELB	PIK3CA
RELB	PIM1
RELB	PITPNA-AS1
RELB	PLRG1
RELB	PMPCA
RELB	PNISR
RELB	PNKP
RELB	POLDIP2
RELB	POLE3
RELB	POLR2E
RELB	POLR2L
RELB	POMP
RELB	PPAN
RELB	PPIA
RELB	PPID
RELB	PPIG
RELB	PPM1G
RELB	PPP1R12C
RELB	PPP2CA
RELB	PPP2R1A

RELB	PPP2R2A
RELB	PPP2R5E
RELB	PPP4C
RELB	PPTC7
RELB	PRDM2
RELB	PRELID2
RELB	PRKAB1
RELB	PRKCD
RELB	PRPF19
RELB	PRPF38B
RELB	PRPF4
RELB	PRRC2C
RELB	PSEN1
RELB	PSMB3
RELB	PSMD13
RELB	PSMD6
RELB	PTBP1
RELB	PTBP3
RELB	PTGES3
RELB	PTPRJ
RELB	PTRH1
RELB	PTTG1
RELB	PUF60
RELB	PYROXD1
RELB	RAB11A
RELB	RAB35
RELB	RAB3GAP2
RELB	RAB5C
RELB	RAB7A
RELB	RABGAP1L
RELB	RANGAP1
RELB	RAP1B
RELB	RAP2C
RELB	RAPGEF6
RELB	RARS
RELB	RASGRP3
RELB	RBBP5
RELB	RBM18
RELB	RBM42
RELB	RBM7
RELB	REL
RELB	RELB
RELB	RHEB
RELB	RHOF
RELB	RHOG
RELB	RHOT2
RELB	RIOK1
RELB	RNF20
RELB	RNF41
RELB	RNF6

RELB	RNGTT
RELB	RNMT
RELB	RPF1
RELB	RPGR
RELB	RPL27
RELB	RPL37A
RELB	RPL4
RELB	RPL7L1
RELB	RPP21
RELB	RPS17
RELB	RPS18
RELB	RPS6KA3
RELB	RRAS2
RELB	RSBN1
RELB	RSF1
RELB	RTP4
RELB	RUVBL1
RELB	SAP30L
RELB	SART1
RELB	SCMH1
RELB	SDCBP
RELB	SDHAF2
RELB	SDHC
RELB	SEC14L1
RELB	SENP8
RELB	SERF2
RELB	SETD1A
RELB	SETD5
RELB	SF3B1
RELB	SF3B2
RELB	SGO2
RELB	SH3KBP1
RELB	SHC1
RELB	SHQ1
RELB	SIRT6
RELB	SLC38A1
RELB	SLC39A1
RELB	SLC39A6
RELB	SLMAP
RELB	SLTM
RELB	SMAD3
RELB	SMAP2
RELB	SMARCA4
RELB	SMARCAL1
RELB	SMARCD2
RELB	SMG6
RELB	SMG7
RELB	SMIM19
RELB	SMIM7
RELB	SMU1

RELB	SMURF1
RELB	SNRPB
RELB	SNRPB2
RELB	SNRPD1
RELB	SNRPE
RELB	SNRPF
RELB	SNRPG
RELB	SNX13
RELB	SOGA1
RELB	SOS2
RELB	SP4
RELB	SPN
RELB	SPSB3
RELB	SPTBN1
RELB	SRBD1
RELB	SRRM1
RELB	SRRM2
RELB	SRSF5
RELB	SSBP1
RELB	SSBP2
RELB	ST20
RELB	STAG2
RELB	STK4
RELB	STRADA
RELB	STRAP
RELB	STX16
RELB	STX4
RELB	STXBP3
RELB	SUMO1
RELB	SUPT20H
RELB	SUPT4H1
RELB	SWAP70
RELB	SYAP1
RELB	SYF2
RELB	TAF11
RELB	TAF7
RELB	TANK
RELB	TARS2
RELB	TATDN1
RELB	TAX1BP1
RELB	TBC1D1
RELB	TBRG1
RELB	TCF25
RELB	TCP1
RELB	TEX10
RELB	TFDP2
RELB	THOC5
RELB	THUMPD3
RELB	THUMPD3-AS1
RELB	TIGAR

RELB	TIMM10B
RELB	TINF2
RELB	TM6SF1
RELB	TMED8
RELB	TMEM115
RELB	TMEM199
RELB	TMPO-AS1
RELB	TNFAIP8L1
RELB	TNKS2
RELB	TNRC6A
RELB	TOX
RELB	TPRKB
RELB	TRAF4
RELB	TRAFD1
RELB	TRAPPC10
RELB	TRAPPC8
RELB	TRMO
RELB	TRMT10A
RELB	TRMT6
RELB	TRRAP
RELB	TSG101
RELB	TSSC4
RELB	TTC1
RELB	TTK
RELB	TUFM
RELB	TWF1
RELB	TXNL1
RELB	U2AF2
RELB	U2SURP
RELB	UBA2
RELB	UBALD2
RELB	UBB
RELB	UBE2D3
RELB	UBE2E1
RELB	UBE2J2
RELB	UBL5
RELB	UBR7
RELB	UBXN1
RELB	UCHL3
RELB	UGP2
RELB	UGT8
RELB	URM1
RELB	USE1
RELB	USP4
RELB	UTP14A
RELB	UTP18
RELB	VAC14
RELB	VEZF1
RELB	VPS45
RELB	VPS4B

RELB	VPS72
RELB	VTA1
RELB	WDR73
RELB	XIAP
RELB	XRN1
RELB	YTHDF2
RELB	YTHDF3
RELB	YWHAZ
RELB	ZBTB14
RELB	ZC3H13
RELB	ZCCHC9
RELB	ZEB1
RELB	ZFAS1
RELB	ZNF165
RELB	ZNF408
RELB	ZPR1
RELB	ZSCAN18
RELB	ZWILCH
REST	ABCD4
REST	ABHD14B
REST	ACAA1
REST	ACIN1
REST	ADPGK
REST	AGO3
REST	AGPAT3
REST	AGTPBP1
REST	AIFM1
REST	AIP
REST	AK3
REST	AKAP13
REST	AKAP8
REST	AKAP9
REST	AKNA
REST	ALG11
REST	ALG13
REST	ALKBH7
REST	AMMECR1L
REST	ANAPC4
REST	ANKRD27
REST	ANXA6
REST	AP1B1
REST	AP2S1
REST	AP3D1
REST	AP5M1
REST	APBB2
REST	ARHGAP11B
REST	ARHGAP18
REST	ARHGAP25
REST	ARHGEF9
REST	ARID4B

REST	ARID5B
REST	ARL13B
REST	ARMC10
REST	ARPC3
REST	ASB3
REST	ASB6
REST	ASH1L
REST	ATF2
REST	ATF4
REST	ATF7IP
REST	ATG12
REST	ATG3
REST	ATP6V0D1
REST	ATP6V0E1
REST	ATP6V1H
REST	ATRX
REST	B9D2
REST	BAX
REST	BAZ1B
REST	BAZ2B
REST	BCAT2
REST	BCL10
REST	BCL2L11
REST	BCL3
REST	BCL7A
REST	BECN1
REST	BIRC6
REST	BLNK
REST	BLOC1S2
REST	BLOC1S5
REST	BLZF1
REST	BOD1L1
REST	BPTF
REST	BRD2
REST	BTBD10
REST	BTF3
REST	BTF3L4
REST	C11orf58
REST	C12orf10
REST	C14orf119
REST	C17orf80
REST	C19orf12
REST	C19orf25
REST	C1orf112
REST	C2CD2
REST	C2CD2L
REST	C3orf38
REST	C6orf52
REST	C6orf99
REST	C9orf16

REST	C9orf40
REST	C9orf64
REST	CAAP1
REST	CAB39
REST	CAP1
REST	CAPZA1
REST	CAPZB
REST	CARM1
REST	CASP8AP2
REST	CCDC115
REST	CCDC12
REST	CCDC174
REST	CCDC186
REST	CCM2
REST	CCNDBP1
REST	CCNL1
REST	CCSER2
REST	CDC16
REST	CDC40
REST	CDC42
REST	CDC42EP3
REST	CDCA2
REST	CDK1
REST	CDK12
REST	CDK5RAP2
REST	CDK9
REST	CENPL
REST	CEP135
REST	CEP44
REST	CEP78
REST	CHD6
REST	CHD9
REST	CHMP1B
REST	CHMP5
REST	CHTOP
REST	CHURC1
REST	CIB1
REST	CIR1
REST	CISD2
REST	CKS2
REST	CLK3
REST	CLN5
REST	CLNS1A
REST	CLPTM1
REST	CMTR2
REST	CNOT4
REST	CNOT6
REST	CNP
REST	CNR1
REST	COA5

REST	COBLL1
REST	COMMD6
REST	COPB2
REST	COPS5
REST	COX4I1
REST	CPNE1
REST	CPSF2
REST	CSAD
REST	CSNK1A1
REST	CSNK2A1
REST	CSTF3
REST	CTCF
REST	CTNNB1
REST	CTNNBIP1
REST	CWC27
REST	CXorf38
REST	CYB561A3
REST	DAAM1
REST	DALRD3
REST	DAXX
REST	DBNL
REST	DCAF5
REST	DCP1A
REST	DDB2
REST	DDX17
REST	DDX23
REST	DDX24
REST	DDX5
REST	DDX50
REST	DDX59
REST	DGUOK
REST	DHX36
REST	DHX38
REST	DHX9
REST	DICER1
REST	DNAJB14
REST	DNAJC4
REST	DOCK8
REST	DPAGT1
REST	DYM
REST	E2F5
REST	EAPP
REST	ECH1
REST	ECSIT
REST	EEF1A1
REST	EEF2
REST	EFHD2
REST	EFTUD2
REST	EIF2A
REST	EIF2AK3

REST	EIF3A
REST	EIF3D
REST	EIF3F
REST	EIF3G
REST	EIF3H
REST	EIF3L
REST	EIF4A2
REST	EIF4G3
REST	ELAC2
REST	ELMO1
REST	ELOF1
REST	ELOVL5
REST	EP300
REST	EPB41L4A-AS1
REST	EPC1
REST	EPN2
REST	ERBIN
REST	ESD
REST	ETS1
REST	EVI2B
REST	EXOSC5
REST	FAAP20
REST	FAM107B
REST	FAM160A2
REST	FAM173A
REST	FAM177A1
REST	FAM184A
REST	FAM204A
REST	FAM219B
REST	FAM49B
REST	FAM69A
REST	FAM8A1
REST	FAM96A
REST	FBXL5
REST	FBXW2
REST	FES
REST	FIBP
REST	FMR1
REST	FNBP4
REST	FNTA
REST	FRYL
REST	FUT8
REST	GABARAPL2
REST	GCC2
REST	GFOD2
REST	GGPS1
REST	GIGYF2
REST	GLTP
REST	GNAI3
REST	GNPTG

REST	GOLGA1
REST	GOLGA7
REST	GOLPH3
REST	GOPC
REST	GPATCH11
REST	GPATCH2L
REST	GPATCH8
REST	GPBP1
REST	GPSM3
REST	GRAMD4
REST	GRB2
REST	GRIPAP1
REST	GSTM3
REST	GTF2H3
REST	GTF2IRD2B
REST	GTF3C5
REST	GUCD1
REST	HARS2
REST	HDAC8
REST	HDLBP
REST	HECTD1
REST	HELZ
REST	HIGD2A
REST	HNRNPA3
REST	HNRNPD
REST	HNRNPL
REST	HNRNPLL
REST	HNRNPU
REST	HPF1
REST	HSPA4
REST	IDH3G
REST	IDI1
REST	IER3IP1
REST	IL16
REST	INO80D
REST	INTS5
REST	IQGAP2
REST	IRF2
REST	IRF5
REST	ISCU
REST	ISG20L2
REST	IST1
REST	ISY1
REST	ITGB1
REST	ITSN2
REST	JAK1
REST	JMJD1C
REST	JPX
REST	JTB
REST	JUN

REST	KANSL1
REST	KCMF1
REST	KCNN4
REST	KDELR3
REST	KHNYN
REST	KIAA0040
REST	KIF20B
REST	KIN
REST	KLHL36
REST	KLHL6
REST	KMT2C
REST	KMT2E
REST	LAMTOR1
REST	LAMTOR4
REST	LAP3
REST	LAPTM5
REST	LASP1
REST	LCORL
REST	LEPROTL1
REST	LINC00909
REST	LINC01215
REST	LITAF
REST	LMO7
REST	LPAR5
REST	LRIF1
REST	LRRC58
REST	LSG1
REST	LSP1
REST	LTBP4
REST	LYL1
REST	LYPLA2
REST	MAGED2
REST	MAGEF1
REST	MAP2K1
REST	MAP2K6
REST	MAPK14
REST	MAPK1IP1L
REST	MARCH7
REST	MAT2B
REST	MBTPS2
REST	MCL1
REST	MDH1
REST	MEF2C
REST	MEPCE
REST	METTL18
REST	METTL21A
REST	METTL22
REST	MFSD10
REST	MFSD11
REST	MGA

REST	MIEN1
REST	MINPP1
REST	MITD1
REST	MIXL1
REST	MMADHC
REST	MOB1A
REST	MOCS2
REST	MORC3
REST	MPG
REST	MPV17
REST	MPZL1
REST	MRPL28
REST	MRPL39
REST	MRPS10
REST	MRPS18A
REST	MTIF2
REST	MTIF3
REST	MTMR14
REST	MTR
REST	MUS81
REST	MYO19
REST	N4BP3
REST	NAA60
REST	NABP2
REST	NACA
REST	NAIF1
REST	NCK2
REST	NCKAP1L
REST	NDRG3
REST	NDUFA13
REST	NDUFB3
REST	NDUFB5
REST	NDUFS1
REST	NDUFS2
REST	NEAT1
REST	NEDD9
REST	NF1
REST	NFATC1
REST	NFYB
REST	NKIRAS2
REST	NONO
REST	NOP10
REST	NOSIP
REST	NR1H2
REST	NSD1
REST	NT5C
REST	NT5DC3
REST	NTMT1
REST	NUBP1
REST	NUBP2

REST	NUDCD3
REST	NUP43
REST	OCIAD1
REST	ODF2
REST	ORC4
REST	OSGIN2
REST	OST4
REST	OSTF1
REST	PABPN1
REST	PAK2
REST	PAPOLA
REST	PAPSS1
REST	PARK7
REST	PAWR
REST	PAX5
REST	PCBP1
REST	PCBP2
REST	PCED1B
REST	PCF11
REST	PCNX4
REST	PCSK7
REST	PDCD6
REST	PDCD6IP
REST	PDHX
REST	PDPK1
REST	PDS5B
REST	PEX16
REST	PEX2
REST	PFDN5
REST	PGAP2
REST	PHACTR4
REST	PHF21A
REST	PIGBOS1
REST	PIK3C2B
REST	PIK3CG
REST	PITPNA
REST	PJA2
REST	PMPCB
REST	PNISR
REST	PNPLA8
REST	POLDIP3
REST	POP4
REST	POU2F1
REST	PPID
REST	PPIP5K2
REST	PPP2R1A
REST	PPP2R2A
REST	PPP2R5B
REST	PPP3R1
REST	PPP6R3

REST	PPTC7
REST	PRCP
REST	PRDM2
REST	PRKAG1
REST	PRKAR1A
REST	PRKCD
REST	PRKCI
REST	PRKRA
REST	PRKRIP1
REST	PRPF38A
REST	PRR13
REST	PRRC1
REST	PRRC2C
REST	PSMB1
REST	PSMB7
REST	PTPN12
REST	PTPRA
REST	PTPRC
REST	PTPRE
REST	PTPRJ
REST	PTRHD1
REST	PXN
REST	PYCR2
REST	PYM1
REST	PYROXD1
REST	RAB11B
REST	RAB14
REST	RAB18
REST	RAB1B
REST	RAB3GAP2
REST	RAB5B
REST	RAB5C
REST	RAC1
REST	RACK1
REST	RAD23A
REST	RAD54L2
REST	RAF1
REST	RALY
REST	RAP1A
REST	RASAL3
REST	RASSF5
REST	RB1
REST	RB1CC1
REST	RBBP6
REST	RBM18
REST	RBM25
REST	RBM26
REST	RBM42
REST	RBMX2
REST	RCHY1

REST	RER1
REST	REST
REST	RGP1
REST	RGS3
REST	RHBDD1
REST	RIC8B
REST	RIPK3
REST	RNF20
REST	RNF220
REST	RNF34
REST	RNF6
REST	ROCK1
REST	ROMO1
REST	RPL10
REST	RPL10A
REST	RPL11
REST	RPL12
REST	RPL13A
REST	RPL15
REST	RPL18A
REST	RPL19
REST	RPL21
REST	RPL23
REST	RPL23A
REST	RPL26
REST	RPL26L1
REST	RPL27
REST	RPL27A
REST	RPL28
REST	RPL29
REST	RPL3
REST	RPL30
REST	RPL31
REST	RPL32
REST	RPL34
REST	RPL35A
REST	RPL36
REST	RPL37
REST	RPL37A
REST	RPL38
REST	RPL4
REST	RPL41
REST	RPL6
REST	RPL8
REST	RPP25L
REST	RPRD1A
REST	RPS10
REST	RPS11
REST	RPS13
REST	RPS14

REST	RPS15
REST	RPS15A
REST	RPS16
REST	RPS18
REST	RPS19
REST	RPS2
REST	RPS20
REST	RPS25
REST	RPS27
REST	RPS27A
REST	RPS29
REST	RPS3
REST	RPS3A
REST	RPS4X
REST	RPS6
REST	RPS7
REST	RPS8
REST	RPSA
REST	RRNAD1
REST	RSRC2
REST	RSRP1
REST	RTN4
REST	RWDD4
REST	SASH3
REST	SCAMP4
REST	SCNM1
REST	SCOC
REST	SCRN1
REST	SDCCAG8
REST	SDHB
REST	SDHD
REST	SEC24C
REST	SEPT2
REST	SEPT7
REST	SERINC3
REST	SERTAD1
REST	SERTAD2
REST	SF3B6
REST	SFSWAP
REST	SFT2D1
REST	SFT2D2
REST	SH2B2
REST	SH3BGRL
REST	SH3BP5
REST	SHKBP1
REST	SHOC2
REST	SHPRH
REST	SIKE1
REST	SIMC1
REST	SIRT7

REST	SKAP2
REST	SLC16A3
REST	SLC17A5
REST	SLC20A2
REST	SLC25A4
REST	SLC35A2
REST	SLC35A3
REST	SLC35C2
REST	SLC35E1
REST	SLC44A1
REST	SLC9A3R1
REST	SLF1
REST	SLMAP
REST	SLU7
REST	SLX4IP
REST	SMAD2
REST	SMARCA2
REST	SMARCA4
REST	SMC3
REST	SMCHD1
REST	SMIM14
REST	SMU1
REST	SNN
REST	SNRPN
REST	SNURF
REST	SNX13
REST	SPAG7
REST	SPG21
REST	SPSB1
REST	SPSB2
REST	SQLE
REST	SREBF2
REST	SRP14
REST	SRRM2
REST	SRRT
REST	SRSF4
REST	SRSF5
REST	SRSF6
REST	SS18
REST	SSU72
REST	STARD5
REST	STAT3
REST	STAU2
REST	STIM2
REST	STK10
REST	STK16
REST	STK4
REST	STT3B
REST	STUB1
REST	SUCLA2

REST	SUPT20H
REST	SUPT4H1
REST	SYF2
REST	SYMPK
REST	SYNJ2BP
REST	SZRD1
REST	SZT2
REST	TAF1
REST	TAF12
REST	TAF15
REST	TAF1B
REST	TAF7
REST	TANGO2
REST	TAOK1
REST	TAOK3
REST	TBC1D14
REST	TBK1
REST	TBL1XR1
REST	TCEAL1
REST	TCERG1
REST	TCF25
REST	TCF4
REST	TERF1
REST	TERF2IP
REST	TEX10
REST	TFCP2
REST	TFIP11
REST	TGIF1
REST	THAP4
REST	THOC3
REST	THRAP3
REST	TIA1
REST	TIGAR
REST	TINF2
REST	TLN1
REST	TM2D1
REST	TM9SF3
REST	TMBIM4
REST	TMC6
REST	TMEM161B-AS1
REST	TMEM175
REST	TMEM219
REST	TMEM230
REST	TMEM234
REST	TMEM259
REST	TMEM30A
REST	TMEM59
REST	TMEM60
REST	TMSB4X
REST	TNRC6B

REST	TOP2B
REST	TOX4
REST	TP53I11
REST	TRA2A
REST	TRABD
REST	TRAF6
REST	TRAPPC12
REST	TRAPPC2L
REST	TRAPPC3
REST	TRAPPC6A
REST	TRIM11
REST	TRIM24
REST	TRIM4
REST	TRIP4
REST	TSC1
REST	TSC22D2
REST	TSPO
REST	TTC13
REST	TTC14
REST	TUBGCP2
REST	TXN2
REST	UBA52
REST	UBA6
REST	UBAC1
REST	UBC
REST	UBE2K
REST	UBE3C
REST	UBL7
REST	UBOX5
REST	UBXN11
REST	UBXN6
REST	UCKL1
REST	UFC1
REST	UFSP2
REST	UGDH
REST	UHRF2
REST	UNC13D
REST	UNC45A
REST	UPF2
REST	UQCR11
REST	UQCRC2
REST	UROD
REST	USP10
REST	USP47
REST	USP49
REST	USP8
REST	USPL1
REST	UTP23
REST	UTP3
REST	UTY

REST	UXT
REST	VANGL1
REST	VAPB
REST	VASP
REST	VDR
REST	VMA21
REST	VMP1
REST	VPS4B
REST	VRK3
REST	WAC
REST	WASF2
REST	WBP11
REST	WDR13
REST	WDR70
REST	WSB1
REST	XAB2
REST	XPC
REST	YBEY
REST	YIPF3
REST	YKT6
REST	YME1L1
REST	YPEL3
REST	YPEL5
REST	YTHDC1
REST	YWHAZ
REST	ZBTB20
REST	ZBTB25
REST	ZBTB43
REST	ZBTB44
REST	ZBTB7A
REST	ZC3H12A
REST	ZDHHC5
REST	ZFAND6
REST	ZFAS1
REST	ZFC3H1
REST	ZFP62
REST	ZHX2
REST	ZNF24
REST	ZNF524
REST	ZNF544
REST	ZNF587
REST	ZNF638
REST	ZNF644
REST	ZNF655
REST	ZNF675
REST	ZNF691
REST	ZNF821
REST	ZRANB2
REST	ZRANB3
REST	ZSCAN21

RFX2	RFX2
RFX5	ABCA7
RFX5	ACAP2
RFX5	ACSS1
RFX5	AFF3
RFX5	AGFG1
RFX5	AICDA
RFX5	AIFM1
RFX5	AKTIP
RFX5	ALPK1
RFX5	ANKHD1
RFX5	ANKMY2
RFX5	ANKRD54
RFX5	ANKZF1
RFX5	ANP32E
RFX5	AP1AR
RFX5	APOLD1
RFX5	ARID4B
RFX5	ARL6IP1
RFX5	ASB8
RFX5	ASPH
RFX5	ASPM
RFX5	ATRIP
RFX5	ATXN1
RFX5	AURKA
RFX5	AURKB
RFX5	BASP1
RFX5	BIRC3
RFX5	BTN2A2
RFX5	C12orf49
RFX5	C1orf109
RFX5	C2orf69
RFX5	CALM2
RFX5	CAMK2B
RFX5	CCAR2
RFX5	CCDC102A
RFX5	CCNB1
RFX5	CCNB2
RFX5	CCNG2
RFX5	CCSER2
RFX5	CD180
RFX5	CD40
RFX5	CD74
RFX5	CDC20
RFX5	CDCA2
RFX5	CDCA3
RFX5	CDCA8
RFX5	CDK1
RFX5	CDK14
RFX5	CDKN3

RFX5	CENPA
RFX5	CENPC
RFX5	CENPE
RFX5	CENPF
RFX5	CENPI
RFX5	CEP164
RFX5	CETN3
RFX5	CGGBP1
RFX5	CKAP2
RFX5	CKS1B
RFX5	CKS2
RFX5	COMMD10
RFX5	COMTD1
RFX5	DAZAP2
RFX5	DCAF6
RFX5	DENND4A
RFX5	DEPDC1B
RFX5	DHX15
RFX5	DLGAP5
RFX5	DOCK2
RFX5	DYNC1I2
RFX5	DYNLL1
RFX5	DYNLL2
RFX5	E2F5
RFX5	EFNA4
RFX5	ELF4
RFX5	ELMO2
RFX5	EMC9
RFX5	EML4
RFX5	ERCC6L
RFX5	FAM107B
RFX5	FAM111A
RFX5	FAM120AOS
RFX5	FAM135A
RFX5	FCHO1
RFX5	FDFT1
RFX5	FHIT
RFX5	FRMD8
RFX5	FYTTD1
RFX5	GFOD1
RFX5	GPSM2
RFX5	GRAP2
RFX5	GTF2E2
RFX5	GTSE1
RFX5	GYPC
RFX5	GZF1
RFX5	HDAC9
RFX5	HIST1H1E
RFX5	HIST1H4C
RFX5	HLA-DMA

RFX5	HLA-DMB
RFX5	HLA-DOA
RFX5	HLA-DOB
RFX5	HLA-DPA1
RFX5	HLA-DPB1
RFX5	HLA-DQA1
RFX5	HLA-DQB1
RFX5	HLA-DRA
RFX5	HLA-DRB1
RFX5	HMMR
RFX5	HSD17B11
RFX5	IARS
RFX5	ID2
RFX5	IFT74
RFX5	INCENP
RFX5	ISCA2
RFX5	IVD
RFX5	KIF14
RFX5	KIF20A
RFX5	KIF20B
RFX5	KIF22
RFX5	KIF23
RFX5	KIF4A
RFX5	KIF5B
RFX5	KIFC1
RFX5	KMT5A
RFX5	KPNA2
RFX5	KPNA4
RFX5	LARP7
RFX5	LBR
RFX5	LINC00665
RFX5	LINC00909
RFX5	LMO2
RFX5	LMO7
RFX5	LONRF1
RFX5	LPIN1
RFX5	LPP
RFX5	LRCH4
RFX5	LRMP
RFX5	LRRC37B
RFX5	LSM5
RFX5	LYSMD2
RFX5	MADD
RFX5	MAPK1
RFX5	MARCKSL1
RFX5	MED30
RFX5	MED6
RFX5	MED8
RFX5	MFF
RFX5	MIDN

RFX5	MKI67
RFX5	MME
RFX5	MPRIP
RFX5	MS4A1
RFX5	MTFR1
RFX5	MTFR2
RFX5	MXD3
RFX5	NCAPH
RFX5	NFATC3
RFX5	NFYC
RFX5	NINJ2
RFX5	NR3C1
RFX5	NRF1
RFX5	NUP37
RFX5	NUP88
RFX5	NUTM2B−AS1
RFX5	OIP5
RFX5	OSBPL8
RFX5	P2RX5
RFX5	PARPBP
RFX5	PAX5
RFX5	PBK
RFX5	PDCD10
RFX5	PDPK1
RFX5	PEX16
RFX5	PGAP2
RFX5	PHF6
RFX5	PHTF2
RFX5	PIF1
RFX5	PISD
RFX5	PLK1
RFX5	POLD4
RFX5	POLR2G
RFX5	POU3F1
RFX5	PPM1B
RFX5	PPM1N
RFX5	PPP2R2D
RFX5	PPP2R5E
RFX5	PRC1
RFX5	PRPSAP2
RFX5	PRRC2C
RFX5	PSAT1
RFX5	PSME3
RFX5	PSRC1
RFX5	PTTG1
RFX5	RAD21
RFX5	RALBP1
RFX5	RANGRF
RFX5	RAPGEF1
RFX5	RBMXL1

RFX5	RECQL
RFX5	RELB
RFX5	RFX5
RFX5	RGS16
RFX5	RICTOR
RFX5	RNASEL
RFX5	RNF26
RFX5	RNF44
RFX5	RNF5
RFX5	RSRC2
RFX5	RYBP
RFX5	S100A10
RFX5	SGMS1
RFX5	SGO1
RFX5	SGO2
RFX5	SH2B1
RFX5	SH2B2
RFX5	SLC1A1
RFX5	SMAP2
RFX5	SMARCAL1
RFX5	SMG9
RFX5	SMIM12
RFX5	SNAP23
RFX5	SPAG5
RFX5	SPATA13
RFX5	SPATA24
RFX5	STAMBPL1
RFX5	STARD4
RFX5	STAT6
RFX5	SULF2
RFX5	SUV39H1
RFX5	SYPL1
RFX5	SYT17
RFX5	TACC3
RFX5	TAX1BP3
RFX5	TBC1D22B
RFX5	TBP
RFX5	TGDS
RFX5	THAP8
RFX5	TIPARP
RFX5	TJAP1
RFX5	TLE4
RFX5	TMEM131
RFX5	TMPO
RFX5	TOMM40L
RFX5	TOP2A
RFX5	TP53BP1
RFX5	TPCN1
RFX5	TPX2
RFX5	TRAK1

RFX5	TRAPPC6B
RFX5	TRIM59
RFX5	TRMT1L
RFX5	TROAP
RFX5	TSPAN14
RFX5	TUBA1C
RFX5	TUBB4B
RFX5	UBB
RFX5	UBE2A
RFX5	UBE2C
RFX5	UBE2D3
RFX5	UBE2S
RFX5	UBP1
RFX5	UCP2
RFX5	UNC119B
RFX5	UNG
RFX5	USP32
RFX5	USP53
RFX5	VGLL4
RFX5	VPS16
RFX5	VPS72
RFX5	WDR62
RFX5	XPO6
RFX5	YY1
RFX5	ZMAT3
RFX5	ZMIZ1
RFX5	ZNF444
RFX5	ZNF486
RFX5	ZNF775
RFX5	ZSWIM8
RORA	ACP5
RORA	CAPN2
RORA	LMNA
RORA	LPXN
RORA	PLCD3
RORA	RGS14
RORA	RORA
RORA	SOX12
RORA	TTC39C
RREB1	RREB1
RREB1	SERPINE2
RREB1	SIN3A
RUNX1	RUNX1
RUNX1	TJP1
RUNX2	PRDM2
RUNX2	PSMA6
RUNX2	RUNX1
RUNX2	RUNX2
RUNX2	SULF2
RUNX2	TCF4

RUNX2	ZKSCAN1
RXRA	C18orf25
RXRA	CAMK1D
RXRA	CASP7
RXRA	CCL22
RXRA	CCND2
RXRA	CFLAR
RXRA	CHST15
RXRA	CSF2RB
RXRA	CXorf40B
RXRA	DUS3L
RXRA	DUSP2
RXRA	LGALSL
RXRA	LRBA
RXRA	PDE4B
RXRA	PLAGL1
RXRA	PRKCSH
RXRA	PRR3
RXRA	QSER1
RXRA	RASSF2
RXRA	RNF2
RXRA	RXRA
RXRA	SLA
RXRA	SLC37A1
RXRA	SNX12
RXRA	TESPA1
RXRA	TRIP10
RXRA	TYW3
RXRA	ZBTB32
SETDB1	AHCY
SETDB1	ARL6
SETDB1	BORCS7
SETDB1	CDC25A
SETDB1	CDC40
SETDB1	COX7C
SETDB1	IFRD2
SETDB1	MCTP2
SETDB1	MFSD3
SETDB1	NRDE2
SETDB1	RUNX3
SETDB1	SLF2
SETDB1	SMG1
SETDB1	TP53I11
SETDB1	VPS41
SETDB1	ZNF224
SOX4	TRIB2
SOX5	ADNP
SOX5	AGFG2
SOX5	APBB2
SOX5	CDC42EP3

SOX5	CYP39A1
SOX5	GPATCH2L
SOX5	GPR180
SOX5	NRF1
SOX5	PELI1
SOX5	SOX5
SOX5	TMEM251
SP1	ADIPOR2
SP1	ANKRD44
SP1	ARID4A
SP1	ARIH1
SP1	CDR2
SP1	CHD6
SP1	EFNA4
SP1	EGR2
SP1	FYN
SP1	GFOD1
SP1	GIGYF1
SP1	HES1
SP1	IER5
SP1	IRS2
SP1	KCTD9
SP1	MAP1B
SP1	METTL2A
SP1	RASA3
SP1	RNF24
SP1	RPS6KA5
SP1	SERTAD2
SP1	SHC1
SP1	SLC38A2
SP1	SP1
SP1	ST7
SP1	TFB1M
SP1	ZC2HC1A
SP2	ABHD13
SP2	ACACA
SP2	ACTN4
SP2	ACTR1A
SP2	AGGF1
SP2	ALDH16A1
SP2	ANKRD39
SP2	ARFIP1
SP2	ARHGAP27
SP2	ARHGEF7
SP2	ARRDC3
SP2	ATP6V1B2
SP2	ATR
SP2	AZI2
SP2	BAG4
SP2	BDH1

SP2	BIRC2
SP2	BRAP
SP2	CANT1
SP2	CCDC124
SP2	CCDC14
SP2	CCDC167
SP2	CCM2
SP2	CCNH
SP2	CD2AP
SP2	CDKN1A
SP2	CDS2
SP2	CEP44
SP2	CHEK2
SP2	CHUK
SP2	CMTR2
SP2	COMMD10
SP2	COX17
SP2	COX20
SP2	CTBS
SP2	CTU1
SP2	CYB5R1
SP2	DEDD2
SP2	DEPDC5
SP2	DPP9
SP2	DYNLL1
SP2	ECI2
SP2	EIF4E2
SP2	ELMO2
SP2	EMC3
SP2	EP300
SP2	ERP29
SP2	ETV2
SP2	EXO1
SP2	FAM174A
SP2	FH
SP2	FKBP1B
SP2	G6PC3
SP2	GADD45B
SP2	GAK
SP2	GBA
SP2	GHITM
SP2	GLA
SP2	GM2A
SP2	GPAA1
SP2	GPAT4
SP2	GPR89A
SP2	GTF2H1
SP2	HDAC6
SP2	HID1
SP2	HIST2H2AC

SP2	HMG20B
SP2	HMGA1
SP2	HRAS
SP2	IER3IP1
SP2	IKBIP
SP2	IRF4
SP2	JKAMP
SP2	JOSD1
SP2	KAT6B
SP2	KAT7
SP2	KCNMB3
SP2	KCTD17
SP2	KIAA1551
SP2	KLF6
SP2	KRI1
SP2	L3MBTL3
SP2	LAMP2
SP2	LEPROT
SP2	LIN7B
SP2	LINC01224
SP2	LINC01431
SP2	MAK16
SP2	METTL2B
SP2	MIB2
SP2	MLH1
SP2	MPC2
SP2	MRPL43
SP2	MTIF2
SP2	MTRF1L
SP2	MTX2
SP2	MYC
SP2	NAPA
SP2	NCK2
SP2	NECAP1
SP2	NEK9
SP2	NRDE2
SP2	NT5DC2
SP2	NUP214
SP2	NUTF2
SP2	OGG1
SP2	ORC4
SP2	ORMDL2
SP2	PARP10
SP2	PARVG
SP2	PAXBP1
SP2	PCIF1
SP2	PCYOX1
SP2	PDS5A
SP2	PDXK
SP2	PHRF1

SP2	PI4K2B
SP2	PI4KB
SP2	PIGT
SP2	PILRB
SP2	PLEKHO2
SP2	POLA1
SP2	PRKCD
SP2	PRPS2
SP2	PTPN4
SP2	PTPRS
SP2	PTS
SP2	PWWP2A
SP2	QSOX2
SP2	RAB2A
SP2	RAB3GAP2
SP2	RALGAPB
SP2	RHOQ
SP2	RNF121
SP2	RNF146
SP2	RNF149
SP2	RRM2
SP2	RSU1
SP2	SASS6
SP2	SCMH1
SP2	SDHB
SP2	SEC24D
SP2	SHCBP1
SP2	SHMT1
SP2	SLC35F5
SP2	SLC39A8
SP2	SLF2
SP2	SMARCC2
SP2	SMC2
SP2	SMTN
SP2	SND1
SP2	SNTB2
SP2	SNX1
SP2	SP1
SP2	SP2
SP2	SPAG1
SP2	SPPL2A
SP2	SPSB1
SP2	SRPRA
SP2	STIM1
SP2	STT3A
SP2	SVIP
SP2	TCERG1
SP2	TDG
SP2	TM2D2
SP2	TM9SF1

SP2	TM9SF3
SP2	TMEM106C
SP2	TMEM167B
SP2	TMEM251
SP2	TMEM39B
SP2	TMEM60
SP2	TNFAIP8L1
SP2	TOP2B
SP2	TRIM27
SP2	TRMT112
SP2	TSR2
SP2	TSTD2
SP2	TTK
SP2	TUBB2A
SP2	TUBB2B
SP2	TXLNA
SP2	UBE4A
SP2	UFSP2
SP2	UNC50
SP2	USB1
SP2	VPS45
SP2	VRK2
SP2	XIST
SP2	XRCC4
SP2	ZCCHC8
SP2	ZFAND3
SP2	ZNF397
SP2	ZNF680
SP3	AAAS
SP3	ABCA7
SP3	ABI2
SP3	ACADS
SP3	ACBD3
SP3	ACLY
SP3	ACSF2
SP3	ADAM15
SP3	ADAR
SP3	AMOTL1
SP3	ANAPC11
SP3	ANKRD34B
SP3	ANKRD54
SP3	ANXA4
SP3	AP1AR
SP3	AP2A1
SP3	APEX2
SP3	APOLD1
SP3	ARHGAP11A
SP3	ARHGEF19
SP3	ARMC9
SP3	ARMCX5

SP3	ARRB2
SP3	ARRDC1
SP3	ASCC2
SP3	ATAD5
SP3	ATG4C
SP3	ATG4D
SP3	ATMIN
SP3	ATP11B
SP3	ATP2A3
SP3	ATXN2L
SP3	AVEN
SP3	B4GALT5
SP3	BCKDK
SP3	BRCA2
SP3	BRI3BP
SP3	BUB1B
SP3	BUD31
SP3	C17orf80
SP3	C2CD2L
SP3	C2orf49
SP3	C4orf33
SP3	C8orf76
SP3	CBR4
SP3	CCDC9
SP3	CCNF
SP3	CD24
SP3	CD37
SP3	CDADC1
SP3	CDC25A
SP3	CDC25B
SP3	CDK19
SP3	CDKN2AIPNL
SP3	CENPJ
SP3	CEP55
SP3	CEP57L1
SP3	CGRRF1
SP3	CHDH
SP3	CHKA
SP3	CLSTN3
SP3	CNOT6
SP3	COQ10B
SP3	CRAT
SP3	CRLF3
SP3	CROT
SP3	CYB5B
SP3	CYB5R1
SP3	CYBA
SP3	DDA1
SP3	DEGS2
SP3	DENND1C

SP3	DGCR2
SP3	DIAPH3
SP3	DLAT
SP3	DNAJA2
SP3	DNAJC1
SP3	DNAJC21
SP3	DOCK11
SP3	DOK7
SP3	DUSP1
SP3	DYNLT1
SP3	E2F1
SP3	E2F8
SP3	E4F1
SP3	EFR3A
SP3	EIF2S3
SP3	ERLIN2
SP3	ERP29
SP3	EXOC2
SP3	FAM102A
SP3	FAM200B
SP3	FBXW9
SP3	FCHSD1
SP3	FIGNL1
SP3	FLAD1
SP3	FOXO3
SP3	FZD3
SP3	G6PC3
SP3	GALNT7
SP3	GCLC
SP3	GDE1
SP3	GFI1
SP3	GINS4
SP3	GLCCI1
SP3	GM2A
SP3	GNE
SP3	GOLGA1
SP3	GPCPD1
SP3	GPR65
SP3	GPR89A
SP3	GTF2IRD2
SP3	HCN3
SP3	HERC3
SP3	HIF1AN
SP3	HIGD2A
SP3	HIST1H1D
SP3	HOPX
SP3	HSPB1
SP3	ICMT
SP3	IGF2BP1
SP3	IGSF8

SP3	IMPA2
SP3	INAFM1
SP3	IQCD
SP3	IQGAP1
SP3	IRF2BP1
SP3	ISCU
SP3	ITM2B
SP3	JAK1
SP3	JMJD1C
SP3	KATNAL1
SP3	KBTBD3
SP3	KDM2B
SP3	KIAA0040
SP3	KLF3
SP3	KLHL42
SP3	KMT2E
SP3	KRBOX4
SP3	LIG1
SP3	LIN54
SP3	LIX1L
SP3	LMNB2
SP3	LNX2
SP3	LPCAT3
SP3	LRRC37A2
SP3	LRRC8D
SP3	LTC4S
SP3	MAGEH1
SP3	MARK3
SP3	MDC1
SP3	MEPCE
SP3	MFSD3
SP3	MGA
SP3	MGARP
SP3	MICB
SP3	MIOS
SP3	MIXL1
SP3	MLXIP
SP3	MME
SP3	MOGS
SP3	MORC2
SP3	MORC4
SP3	MRPL28
SP3	MRPS6
SP3	MYO7B
SP3	NAA40
SP3	NCKIPSD
SP3	NCOA6
SP3	NDUFAF6
SP3	NDUFS2
SP3	NECAB3

SP3	NEIL3
SP3	NF1
SP3	NFXL1
SP3	NFYA
SP3	NKIRAS1
SP3	NME7
SP3	NPHP3
SP3	NPRL3
SP3	NRDC
SP3	NUMB
SP3	NUS1
SP3	OPA1
SP3	ORAI2
SP3	ORMDL2
SP3	OSBPL9
SP3	OTUD5
SP3	PAIP2B
SP3	PAQR4
SP3	PARD6A
SP3	PCNT
SP3	PDE4DIP
SP3	PEX11A
SP3	PGM2
SP3	PIF1
SP3	PLD3
SP3	PLEKHA3
SP3	PLEKHH3
SP3	POFUT1
SP3	POGLUT1
SP3	POLR3D
SP3	POLRMT
SP3	PPP1R12B
SP3	PRADC1
SP3	PRKACA
SP3	PRKACB
SP3	PSMC3
SP3	PTCD2
SP3	PTGIR
SP3	PUS7L
SP3	PWWP2A
SP3	QRICH1
SP3	QTRT2
SP3	RAB22A
SP3	RAB3GAP1
SP3	RAB5C
SP3	RALGPS1
SP3	RASA1
SP3	RASGRP2
SP3	RBM6
SP3	RBSN

SP3	RC3H1
SP3	REPS1
SP3	RERE
SP3	REST
SP3	REV3L
SP3	RGS16
SP3	RHBDD2
SP3	RNF168
SP3	RNF2
SP3	RNPEPL1
SP3	ROCK2
SP3	RPS6KB2
SP3	RSPH1
SP3	SAC3D1
SP3	SCAF4
SP3	SCPEP1
SP3	SDCCAG8
SP3	SDHAF1
SP3	SEC13
SP3	SEC22B
SP3	SETD3
SP3	SETD6
SP3	SFT2D2
SP3	SGMS1
SP3	SH3D21
SP3	SHARPIN
SP3	SHF
SP3	SHISA8
SP3	SHOC2
SP3	SIN3B
SP3	SIT1
SP3	SLC19A1
SP3	SLC25A33
SP3	SLC25A40
SP3	SLK
SP3	SLMAP
SP3	SMARCAD1
SP3	SMARCB1
SP3	SMARCD2
SP3	SMC3
SP3	SMCHD1
SP3	SP3
SP3	SPON2
SP3	SPSB2
SP3	STAG2
SP3	STX5
SP3	SUDS3
SP3	SUN1
SP3	SUN2
SP3	SUPT20H

SP3	SVIP
SP3	SYNGR1
SP3	TAF3
SP3	TAOK1
SP3	TARBP2
SP3	TARSL2
SP3	TCF25
SP3	TFDP2
SP3	TGOLN2
SP3	TM7SF2
SP3	TM7SF3
SP3	TMEM143
SP3	TMEM231
SP3	TMPO-AS1
SP3	TMX2
SP3	TNFRSF1B
SP3	TNRC6B
SP3	TP53TG1
SP3	TPRG1L
SP3	TRAPPC6A
SP3	TRIM5
SP3	TRPM7
SP3	TSC22D1
SP3	TSPAN13
SP3	TSPAN5
SP3	TTC14
SP3	TTK
SP3	TTLL3
SP3	TWF1
SP3	TYSND1
SP3	UBASH3B
SP3	UBR2
SP3	UNC45A
SP3	USP11
SP3	USP33
SP3	UTRN
SP3	VAMP1
SP3	VPS33B
SP3	WDR82
SP3	WSB2
SP3	XPO1
SP3	XRCC3
SP3	XXYLT1
SP3	YTHDF2
SP3	ZBTB24
SP3	ZFAND5
SP3	ZFC3H1
SP3	ZFHX3
SP3	ZFP91
SP3	ZFPL1

SP3	ZFX
SP3	ZFYVE21
SP3	ZNF248
SP3	ZNF276
SP3	ZNF281
SP3	ZNF429
SP3	ZNF487
SP3	ZNF506
SP3	ZNF563
SP3	ZNF566
SP3	ZNF598
SP3	ZNF765
SP3	ZNF800
SP3	ZNF804A
SP3	ZNF83
SP3	ZNRF1
SP4	ACLY
SP4	AFF2
SP4	AK4
SP4	BMPR1A
SP4	CACNA1A
SP4	CEP57L1
SP4	FAM110A
SP4	GTF2A1
SP4	KANSL1
SP4	LONRF1
SP4	NR4A2
SP4	SP4
SP4	SPRY1
SP4	WASL
SPI1	ABCB7
SPI1	ABHD13
SPI1	ABI1
SPI1	ABI2
SPI1	ABI3
SPI1	ABTB1
SPI1	ACAA2
SPI1	ACAP1
SPI1	ACAP2
SPI1	ACBD3
SPI1	ACIN1
SPI1	ACLY
SPI1	ACOT8
SPI1	ACP5
SPI1	ACSF3
SPI1	ACSL5
SPI1	ACTB
SPI1	ACTG1
SPI1	ACTR1A
SPI1	ACTR2

SPI1	ACTR3
SPI1	ACYP2
SPI1	ADA
SPI1	ADAM8
SPI1	ADAR
SPI1	ADH5
SPI1	ADPRHL2
SPI1	AFF3
SPI1	AFF4
SPI1	AFTPH
SPI1	AGFG1
SPI1	AGO1
SPI1	AHNAK
SPI1	AIF1
SPI1	AIFM1
SPI1	AIM2
SPI1	AIP
SPI1	AKAP11
SPI1	AKAP13
SPI1	AKIRIN1
SPI1	AKIRIN2
SPI1	AKNA
SPI1	ALAS1
SPI1	ALDH2
SPI1	ALDOA
SPI1	ALKBH5
SPI1	ALOX5
SPI1	ALOX5AP
SPI1	ALPK1
SPI1	AMPD2
SPI1	ANAPC15
SPI1	ANAPC16
SPI1	ANAPC4
SPI1	ANKRA2
SPI1	ANKRD13A
SPI1	ANKRD17
SPI1	ANKRD44
SPI1	ANP32B
SPI1	ANXA11
SPI1	ANXA4
SPI1	ANXA6
SPI1	AP1G2
SPI1	AP1M1
SPI1	AP1S2
SPI1	AP2B1
SPI1	AP2M1
SPI1	AP2S1
SPI1	AP3S1
SPI1	AP3S2
SPI1	AP5M1

SPI1	APAF1
SPI1	APBB1
SPI1	APBB1IP
SPI1	APEX2
SPI1	APOBEC3C
SPI1	APOLD1
SPI1	APOM
SPI1	ARF1
SPI1	ARF3
SPI1	ARF5
SPI1	ARF6
SPI1	ARFGEF1
SPI1	ARHGAP1
SPI1	ARHGAP11A
SPI1	ARHGAP15
SPI1	ARHGAP25
SPI1	ARHGAP30
SPI1	ARHGAP4
SPI1	ARHGAP9
SPI1	ARHGDIA
SPI1	ARHGDIB
SPI1	ARHGEF1
SPI1	ARHGEF2
SPI1	ARHGEF3
SPI1	ARHGEF7
SPI1	ARID1B
SPI1	ARID4A
SPI1	ARID4B
SPI1	ARL5A
SPI1	ARL6IP5
SPI1	ARL6IP6
SPI1	ARMCX6
SPI1	ARMT1
SPI1	ARPC1A
SPI1	ARPC1B
SPI1	ARPC2
SPI1	ARPC3
SPI1	ARPC4
SPI1	ARRB2
SPI1	ARV1
SPI1	ASAP1
SPI1	ASB1
SPI1	ASB2
SPI1	ASB8
SPI1	ASCC1
SPI1	ASNA1
SPI1	ASPH
SPI1	ATAD1
SPI1	ATF4
SPI1	ATF6B

SPI1	ATF7
SPI1	ATF7IP
SPI1	ATF7IP2
SPI1	ATG12
SPI1	ATG2B
SPI1	ATG3
SPI1	ATM
SPI1	ATP23
SPI1	ATP6V0D1
SPI1	ATP6V0E1
SPI1	ATP6V1A
SPI1	ATP6V1D
SPI1	ATP6V1E1
SPI1	ATP6V1H
SPI1	ATXN10
SPI1	ATXN7L3
SPI1	AURKAIP1
SPI1	B9D2
SPI1	BABAM1
SPI1	BACH2
SPI1	BAG6
SPI1	BAK1
SPI1	BANP
SPI1	BASP1
SPI1	BBX
SPI1	BCAR3
SPI1	BCDIN3D
SPI1	BCKDHB
SPI1	BCL11A
SPI1	BCL7C
SPI1	BCOR
SPI1	BDP1
SPI1	BECN1
SPI1	BIK
SPI1	BIN1
SPI1	BIRC3
SPI1	BLCAP
SPI1	BLK
SPI1	BLNK
SPI1	BLOC1S2
SPI1	BLOC1S4
SPI1	BLOC1S6
SPI1	BNIP2
SPI1	BNIP3L
SPI1	BOD1L1
SPI1	BORCS5
SPI1	BORCS6
SPI1	BORCS8
SPI1	BPTF
SPI1	BRD7

SPI1	BRF2
SPI1	BRK1
SPI1	BRMS1
SPI1	BROX
SPI1	BTF3
SPI1	BTG1
SPI1	BTK
SPI1	BTLA
SPI1	BTNL9
SPI1	C11orf24
SPI1	C12orf10
SPI1	C12orf49
SPI1	C12orf75
SPI1	C14orf119
SPI1	C15orf62
SPI1	C16orf58
SPI1	C16orf72
SPI1	C16orf74
SPI1	C18orf54
SPI1	C19orf25
SPI1	C19orf38
SPI1	C1orf131
SPI1	C2orf42
SPI1	C2orf49
SPI1	C2orf68
SPI1	C2orf76
SPI1	C3orf38
SPI1	C4orf46
SPI1	C5orf24
SPI1	C6orf226
SPI1	C7orf50
SPI1	C9orf78
SPI1	CA11
SPI1	CAAP1
SPI1	CABIN1
SPI1	CACNA1A
SPI1	CADPS
SPI1	CALM2
SPI1	CAMK2G
SPI1	CAMK4
SPI1	CAP1
SPI1	CAPG
SPI1	CAPN1
SPI1	CAPZA1
SPI1	CAPZA2
SPI1	CAPZB
SPI1	CARD8
SPI1	CARHSP1
SPI1	CARNMT1
SPI1	CASP4

SPI1	CAST
SPI1	CASZ1
SPI1	CBFA2T2
SPI1	CBFA2T3
SPI1	CBL
SPI1	CBX3
SPI1	CCDC115
SPI1	CCDC12
SPI1	CCDC125
SPI1	CCDC126
SPI1	CCDC174
SPI1	CCDC51
SPI1	CCDC69
SPI1	CCDC71
SPI1	CCDC88C
SPI1	CCDC90B
SPI1	CCM2
SPI1	CCNB1IP1
SPI1	CCND3
SPI1	CCNDBP1
SPI1	CCNG1
SPI1	CCNG2
SPI1	CCNH
SPI1	CCNI
SPI1	CCNK
SPI1	CCP110
SPI1	CCR6
SPI1	CCZ1
SPI1	CD180
SPI1	CD19
SPI1	CD22
SPI1	CD37
SPI1	CD40
SPI1	CD52
SPI1	CD53
SPI1	CD72
SPI1	CD74
SPI1	CD79A
SPI1	CD79B
SPI1	CDC123
SPI1	CDC16
SPI1	CDC25B
SPI1	CDC27
SPI1	CDC34
SPI1	CDC37
SPI1	CDC40
SPI1	CDC42
SPI1	CDC42EP3
SPI1	CDK14
SPI1	CDK19

SPI1	CDK2AP2
SPI1	CDK5RAP1
SPI1	CDK5RAP3
SPI1	CDK7
SPI1	CDKN2A
SPI1	CDV3
SPI1	CEBPD
SPI1	CEBPZOS
SPI1	CELF1
SPI1	CENPL
SPI1	CEP135
SPI1	CEP295
SPI1	CEPT1
SPI1	CETN3
SPI1	CFAP20
SPI1	CFL1
SPI1	CFL2
SPI1	CGGBP1
SPI1	CHCHD5
SPI1	CHIC2
SPI1	CHKB
SPI1	CHM
SPI1	CHMP1A
SPI1	CHMP1B
SPI1	CHMP2A
SPI1	CHMP4B
SPI1	CHMP5
SPI1	CHRNB1
SPI1	CHST10
SPI1	CHTF8
SPI1	CHUK
SPI1	CHURC1
SPI1	CIB1
SPI1	CIITA
SPI1	CIRBP
SPI1	CLASRP
SPI1	CLIC1
SPI1	CLIC4
SPI1	CLIP2
SPI1	CLK2
SPI1	CLK3
SPI1	CLOCK
SPI1	CLSTN3
SPI1	CLTA
SPI1	CLTB
SPI1	CLTC
SPI1	CMAS
SPI1	CMIP
SPI1	CMTM6
SPI1	CMTM7

SPI1	CNDP2
SPI1	CNIH1
SPI1	CNN2
SPI1	CNOT10
SPI1	CNOT2
SPI1	CNOT3
SPI1	CNOT7
SPI1	CNOT8
SPI1	CNPPD1
SPI1	COA4
SPI1	COBLL1
SPI1	COG4
SPI1	COMMD2
SPI1	COMMD4
SPI1	COMMD5
SPI1	COMMD6
SPI1	COPA
SPI1	COPE
SPI1	COPG2
SPI1	COPS5
SPI1	COQ6
SPI1	COQ9
SPI1	CORO1A
SPI1	CORO1B
SPI1	COX14
SPI1	COX16
SPI1	COX17
SPI1	COX4I1
SPI1	COX5B
SPI1	COX6A1
SPI1	COX6B1
SPI1	COX7A2L
SPI1	COX7C
SPI1	COX8A
SPI1	CPNE1
SPI1	CPNE3
SPI1	CPNE5
SPI1	CPSF2
SPI1	CPSF7
SPI1	CPT2
SPI1	CREB1
SPI1	CREBRF
SPI1	CREBZF
SPI1	CRK
SPI1	CRLF3
SPI1	CRYZL1
SPI1	CS
SPI1	CSF2RB
SPI1	CSK
SPI1	CSNK1A1

SPI1	CSNK1D
SPI1	CSNK1G1
SPI1	CSNK1G2
SPI1	CSTF1
SPI1	CSTF2
SPI1	CTDNEP1
SPI1	CTDP1
SPI1	CTDSP1
SPI1	CTDSP2
SPI1	CTDSPL2
SPI1	CTNNA1
SPI1	CTNNBL1
SPI1	CTSB
SPI1	CTSZ
SPI1	CUEDC2
SPI1	CUL3
SPI1	CUL4B
SPI1	CUTC
SPI1	CXCR5
SPI1	CXorf38
SPI1	CXXC1
SPI1	CXXC5
SPI1	CYB561A3
SPI1	CYB561D2
SPI1	CYB5B
SPI1	CYB5R3
SPI1	CYB5R4
SPI1	CYBA
SPI1	CYBB
SPI1	CYFIP2
SPI1	CYSTM1
SPI1	CYTIP
SPI1	DAAM1
SPI1	DAP
SPI1	DAPK3
SPI1	DAPP1
SPI1	DAXX
SPI1	DAZAP1
SPI1	DAZAP2
SPI1	DBNL
SPI1	DCAF11
SPI1	DCAF12
SPI1	DCAF16
SPI1	DCAF5
SPI1	DCAF6
SPI1	DCAF7
SPI1	DCK
SPI1	DCLRE1C
SPI1	DCP1B
SPI1	DCTN3

SPI1	DCTN5
SPI1	DCUN1D1
SPI1	DDA1
SPI1	DDAH2
SPI1	DDIAS
SPI1	DDX19B
SPI1	DDX27
SPI1	DDX3X
SPI1	DDX42
SPI1	DDX5
SPI1	DDX6
SPI1	DEF8
SPI1	DENND1B
SPI1	DENND1C
SPI1	DENND2D
SPI1	DENND4B
SPI1	DENR
SPI1	DEPDC5
SPI1	DFFA
SPI1	DGKZ
SPI1	DGUOK
SPI1	DHPS
SPI1	DHRS4L2
SPI1	DHRS7
SPI1	DHRS7B
SPI1	DIAPH1
SPI1	DICER1
SPI1	DIS3
SPI1	DLAT
SPI1	DLD
SPI1	DLEU2
SPI1	DLGAP1-AS1
SPI1	DMAP1
SPI1	DNAJB6
SPI1	DNAJC4
SPI1	DNAJC5
SPI1	DNAJC7
SPI1	DNAL4
SPI1	DNM2
SPI1	DNMT1
SPI1	DOCK8
SPI1	DOCK9
SPI1	DOK1
SPI1	DPM1
SPI1	DPP3
SPI1	DPP8
SPI1	DPYSL2
SPI1	DR1
SPI1	DSTYK
SPI1	DTNBP1

SPI1	DUSP28
SPI1	DUSP6
SPI1	DYNC1I2
SPI1	DYNC1LI1
SPI1	DYNLL1
SPI1	DYNLRB1
SPI1	DYNLT1
SPI1	DZIP3
SPI1	E2F5
SPI1	EAPP
SPI1	EBAG9
SPI1	EBF1
SPI1	ECH1
SPI1	EEF1B2
SPI1	EEF1G
SPI1	EEPD1
SPI1	EFNA4
SPI1	EGLN1
SPI1	EGR1
SPI1	EHD1
SPI1	EIF1B
SPI1	EIF2A
SPI1	EIF2AK1
SPI1	EIF2B1
SPI1	EIF2B2
SPI1	EIF2S3
SPI1	EIF3D
SPI1	EIF3F
SPI1	EIF3G
SPI1	EIF3H
SPI1	EIF3I
SPI1	EIF3K
SPI1	EIF3L
SPI1	EIF3M
SPI1	EIF4E2
SPI1	EIF4G2
SPI1	EIF4G3
SPI1	EIF4H
SPI1	EIF5A
SPI1	ELAC2
SPI1	ELF1
SPI1	ELF2
SPI1	ELK3
SPI1	ELMO1
SPI1	ELMSAN1
SPI1	ELOF1
SPI1	ELOVL1
SPI1	ELOVL5
SPI1	ELP3
SPI1	EMP3

SPI1	ENTPD1-AS1
SPI1	EP300
SPI1	EPB41
SPI1	EPB41L4A-AS1
SPI1	EPC1
SPI1	EPN1
SPI1	EPS15
SPI1	ERBIN
SPI1	ERCC3
SPI1	ERCC4
SPI1	ERGIC1
SPI1	ERGIC3
SPI1	ERICH1
SPI1	ESD
SPI1	ETFB
SPI1	ETS1
SPI1	ETV2
SPI1	EVI2A
SPI1	EVI2B
SPI1	EXOC1
SPI1	EXOC3
SPI1	EXOC3-AS1
SPI1	EXOC4
SPI1	EXOC7
SPI1	EXT1
SPI1	EYA2
SPI1	FAAP20
SPI1	FADS3
SPI1	FAF2
SPI1	FAM102A
SPI1	FAM107B
SPI1	FAM110A
SPI1	FAM120B
SPI1	FAM122A
SPI1	FAM129A
SPI1	FAM129C
SPI1	FAM13B
SPI1	FAM173A
SPI1	FAM192A
SPI1	FAM193B
SPI1	FAM214A
SPI1	FAM32A
SPI1	FAM49A
SPI1	FAM49B
SPI1	FAM50B
SPI1	FAM53B
SPI1	FAM76B
SPI1	FAM81A
SPI1	FAM91A1
SPI1	FAM96A

SPI1	FAM96B
SPI1	FASTKD5
SPI1	FAU
SPI1	FBRS
SPI1	FBXL20
SPI1	FBXO11
SPI1	FBXO22
SPI1	FBXO25
SPI1	FBXO3
SPI1	FBXO42
SPI1	FBXW2
SPI1	FBXW5
SPI1	FBXW9
SPI1	FCGR2B
SPI1	FCHSD2
SPI1	FCMR
SPI1	FCRL1
SPI1	FCRLA
SPI1	FDFT1
SPI1	FDPS
SPI1	FERMT3
SPI1	FES
SPI1	FEZ2
SPI1	FFAR1
SPI1	FGD2
SPI1	FIBP
SPI1	FIG4
SPI1	FKBP1A
SPI1	FKBP8
SPI1	FLAD1
SPI1	FLI1
SPI1	FLNA
SPI1	FLOT1
SPI1	FMNL1
SPI1	FMNL3
SPI1	FNTA
SPI1	FOXN2
SPI1	FOXO1
SPI1	FPGS
SPI1	FSIP1
SPI1	FUCA1
SPI1	G2E3
SPI1	G6PD
SPI1	GABARAP
SPI1	GABARAPL2
SPI1	GAK
SPI1	GAPDH
SPI1	GATAD2B
SPI1	GBE1
SPI1	GDI2

SPI1	GDPD5
SPI1	GEMIN7
SPI1	GGA2
SPI1	GGNBP2
SPI1	GIN1
SPI1	GIT2
SPI1	GLB1
SPI1	GLE1
SPI1	GLIPR1
SPI1	GLMP
SPI1	GLRX5
SPI1	GLTP
SPI1	GLYCTK
SPI1	GMEB1
SPI1	GMFG
SPI1	GMPR2
SPI1	GNA13
SPI1	GNAI2
SPI1	GNAI3
SPI1	GNAS
SPI1	GNB1L
SPI1	GNB2
SPI1	GNB4
SPI1	GNGT2
SPI1	GNPAT
SPI1	GON4L
SPI1	GOPC
SPI1	GPATCH2
SPI1	GPATCH2L
SPI1	GPATCH8
SPI1	GPBP1
SPI1	GPM6B
SPI1	GPR108
SPI1	GPR160
SPI1	GPR18
SPI1	GPR183
SPI1	GPSM3
SPI1	GRAMD1A
SPI1	GRAP
SPI1	GRB2
SPI1	GRIPAP1
SPI1	GRK2
SPI1	GRK6
SPI1	GRN
SPI1	GSDMD
SPI1	GSK3B
SPI1	GSKIP
SPI1	GSN
SPI1	GSPT2
SPI1	GSTO1

SPI1	GTF2F1
SPI1	GTF2F2
SPI1	GTF2I
SPI1	GTF3C2
SPI1	GTPBP8
SPI1	GTSE1
SPI1	GUCD1
SPI1	GYPC
SPI1	HAGHL
SPI1	HARS
SPI1	HAT1
SPI1	HAUS6
SPI1	HAUS8
SPI1	HBP1
SPI1	HCFC1
SPI1	HCLS1
SPI1	HDAC7
SPI1	HDHD2
SPI1	HECA
SPI1	HECTD1
SPI1	HECTD4
SPI1	HELQ
SPI1	HERC1
SPI1	HERC4
SPI1	HERPUD2
SPI1	HGS
SPI1	HHEX
SPI1	HIF1AN
SPI1	HIGD1A
SPI1	HIGD2A
SPI1	HINT1
SPI1	HIP1R
SPI1	HIPK3
SPI1	HK1
SPI1	HLA-DMB
SPI1	HLA-DOA
SPI1	HLA-DRB5
SPI1	HLA-F
SPI1	HMGA1
SPI1	HMGB1
SPI1	HMGCS1
SPI1	HMGN1
SPI1	HMGN2
SPI1	HMGXB3
SPI1	HNRNPA2B1
SPI1	HNRNPC
SPI1	HNRNPD
SPI1	HNRNPH2
SPI1	HNRNPK
SPI1	HNRNPU

SPI1	HNRNPUL1
SPI1	HOXB4
SPI1	HPS1
SPI1	HPS3
SPI1	HPS6
SPI1	HRK
SPI1	HSBP1
SPI1	HSD17B11
SPI1	HSP90AB1
SPI1	HSPA4
SPI1	HTR3A
SPI1	HVCN1
SPI1	IDH3B
SPI1	IDI1
SPI1	IER2
SPI1	IFI16
SPI1	IFI30
SPI1	IFITM2
SPI1	IFT20
SPI1	IFT22
SPI1	IFT43
SPI1	IFT74
SPI1	IFT81
SPI1	IGBP1
SPI1	IKBKG
SPI1	IKZF1
SPI1	IKZF2
SPI1	IKZF3
SPI1	IL10RA
SPI1	IL13RA1
SPI1	IL16
SPI1	IL2RG
SPI1	IL4R
SPI1	ILK
SPI1	ING4
SPI1	INIP
SPI1	INO80C
SPI1	INO80D
SPI1	INO80E
SPI1	INPP5D
SPI1	INPP5K
SPI1	INTS2
SPI1	INTS4
SPI1	IQGAP1
SPI1	IRF2
SPI1	IRS1
SPI1	ISG20
SPI1	ISG20L2
SPI1	IST1
SPI1	ISY1

SPI1	ITGAE
SPI1	ITGAL
SPI1	ITGB1
SPI1	ITGB1BP1
SPI1	ITGB2
SPI1	ITGB7
SPI1	ITPA
SPI1	ITPR1
SPI1	ITPR2
SPI1	ITPRIP
SPI1	ITSN2
SPI1	JAK1
SPI1	JAK2
SPI1	JMJD1C
SPI1	JOSD2
SPI1	JUP
SPI1	KANK2
SPI1	KANSL1
SPI1	KANSL3
SPI1	KARS
SPI1	KAT5
SPI1	KAT8
SPI1	KBTBD4
SPI1	KCNN4
SPI1	KCTD18
SPI1	KCTD5
SPI1	KDM2A
SPI1	KDM2B
SPI1	KDM3B
SPI1	KDM5A
SPI1	KHDRBS1
SPI1	KHNYN
SPI1	KIAA0100
SPI1	KIAA0355
SPI1	KIAA2013
SPI1	KIDINS220
SPI1	KIF20B
SPI1	KIF23
SPI1	KIF2A
SPI1	KIF5B
SPI1	KIF9
SPI1	KLF12
SPI1	KLF6
SPI1	KLHDC2
SPI1	KLHL18
SPI1	KLHL20
SPI1	KLHL5
SPI1	KLHL6
SPI1	KLRG1
SPI1	KMT2C

SPI1	KMT2E
SPI1	KMT5A
SPI1	KRCC1
SPI1	KRIT1
SPI1	KRR1
SPI1	LACTB2
SPI1	LAMTOR1
SPI1	LAMTOR2
SPI1	LAMTOR4
SPI1	LAPTM5
SPI1	LARP4B
SPI1	LARP7
SPI1	LASP1
SPI1	LCK
SPI1	LCMT1
SPI1	LCP1
SPI1	LEPROT
SPI1	LETMD1
SPI1	LGALS8
SPI1	LGALS9
SPI1	LHPP
SPI1	LILRB1
SPI1	LIMD2
SPI1	LIMK1
SPI1	LIMS1
SPI1	LIMS2
SPI1	LINC00426
SPI1	LINC00467
SPI1	LINC00476
SPI1	LINC00487
SPI1	LINC00623
SPI1	LINC00877
SPI1	LINC01006
SPI1	LINC01215
SPI1	LITAF
SPI1	LIX1L
SPI1	LLPH
SPI1	LMBRD1
SPI1	LMNA
SPI1	LMNB1
SPI1	LMO2
SPI1	LMO4
SPI1	LNPEP
SPI1	LPAR5
SPI1	LPCAT3
SPI1	LPIN1
SPI1	LPP
SPI1	LPXN
SPI1	LRBA
SPI1	LRCH4

SPI1	LRIF1
SPI1	LRMP
SPI1	LRP10
SPI1	LRRC32
SPI1	LRRC45
SPI1	LRRC47
SPI1	LRRC58
SPI1	LRRC61
SPI1	LRRFIP1
SPI1	LRWD1
SPI1	LSM1
SPI1	LSM3
SPI1	LSM6
SPI1	LSP1
SPI1	LST1
SPI1	LTA4H
SPI1	LTB
SPI1	LY6E
SPI1	LY75
SPI1	LY86
SPI1	LYL1
SPI1	LYN
SPI1	LYPLA2
SPI1	LYPLAL1
SPI1	LYRM2
SPI1	LYSMD2
SPI1	LZIC
SPI1	MAD1L1
SPI1	MAD2L1BP
SPI1	MADD
SPI1	MAEA
SPI1	MAFG
SPI1	MAGED2
SPI1	MAGEF1
SPI1	MALT1
SPI1	MAML1
SPI1	MAN2C1
SPI1	MANBA
SPI1	MAP2K1
SPI1	MAP3K1
SPI1	MAP3K7
SPI1	MAP4K1
SPI1	MAP4K2
SPI1	MAP4K4
SPI1	MAP7D1
SPI1	MAP9
SPI1	MAPK9
SPI1	MARCH1
SPI1	MARCKSL1
SPI1	MARK3

SPI1	MAST3
SPI1	MAT2B
SPI1	MAZ
SPI1	MBD5
SPI1	MBD6
SPI1	MBNL1
SPI1	MBNL3
SPI1	MBP
SPI1	MBTD1
SPI1	MCL1
SPI1	MCRS1
SPI1	MCTS1
SPI1	MDH1
SPI1	MDM4
SPI1	MEA1
SPI1	MED11
SPI1	MED23
SPI1	MED25
SPI1	MED6
SPI1	MED9
SPI1	MEF2A
SPI1	MEF2C
SPI1	MEMO1
SPI1	MEN1
SPI1	MEPCE
SPI1	METAP2
SPI1	METTL17
SPI1	METTL23
SPI1	METTL3
SPI1	METTL9
SPI1	MFAP1
SPI1	MFF
SPI1	MFNG
SPI1	MFSD11
SPI1	MGAT3
SPI1	MGAT5
SPI1	MGMT
SPI1	MIB2
SPI1	MICU1
SPI1	MICU2
SPI1	MIDN
SPI1	MIEN1
SPI1	MILR1
SPI1	MINOS1
SPI1	MIR181A1HG
SPI1	MIR3681HG
SPI1	MITD1
SPI1	MKRN1
SPI1	MLF2
SPI1	MLXIP

SPI1	MLYCD
SPI1	MMADHC
SPI1	MOB1A
SPI1	MOB1B
SPI1	MOB3A
SPI1	MOB4
SPI1	MON1B
SPI1	MORC3
SPI1	MORF4L1
SPI1	MPG
SPI1	MPI
SPI1	MPLKIP
SPI1	MPPE1
SPI1	MRM2
SPI1	MRPL10
SPI1	MRPL13
SPI1	MRPL16
SPI1	MRPL27
SPI1	MRPL28
SPI1	MRPL33
SPI1	MRPL38
SPI1	MRPL43
SPI1	MRPL44
SPI1	MRPL48
SPI1	MRPL52
SPI1	MRPL54
SPI1	MRPS18A
SPI1	MRPS18B
SPI1	MRPS21
SPI1	MS4A1
SPI1	MSH3
SPI1	MSL2
SPI1	MSL3
SPI1	MSN
SPI1	MSRA
SPI1	MTA3
SPI1	MTERF4
SPI1	MTF1
SPI1	MTF2
SPI1	MTFMT
SPI1	MTFR2
SPI1	MTIF2
SPI1	MTIF3
SPI1	MTMR12
SPI1	MTMR14
SPI1	MTPN
SPI1	MTRF1L
SPI1	MUTYH
SPI1	MX1
SPI1	MYH9

SPI1	MYL12A
SPI1	MYL12B
SPI1	MYL6
SPI1	MYL6B
SPI1	MYO1C
SPI1	MYO1E
SPI1	MYO1F
SPI1	MYO1G
SPI1	MYO9B
SPI1	N4BP3
SPI1	NAAA
SPI1	NAB2
SPI1	NACA
SPI1	NADK
SPI1	NAGK
SPI1	NAP1L1
SPI1	NARF
SPI1	NARFL
SPI1	NAT1
SPI1	NAT14
SPI1	NBPF12
SPI1	NBR1
SPI1	NCALD
SPI1	NCAPD2
SPI1	NCF1
SPI1	NCF4
SPI1	NCKAP1L
SPI1	NCL
SPI1	NCOA3
SPI1	NCOA7
SPI1	NDUFA1
SPI1	NDUFA13
SPI1	NDUFA4
SPI1	NDUFAF3
SPI1	NDUFAF7
SPI1	NDUFB3
SPI1	NDUFB4
SPI1	NDUFB5
SPI1	NDUFB9
SPI1	NDUFS2
SPI1	NDUFS4
SPI1	NDUFS7
SPI1	NDUFV1
SPI1	NEAT1
SPI1	NECAP2
SPI1	NEDD8
SPI1	NEIL1
SPI1	NEK1
SPI1	NEK6
SPI1	NEK9

SPI1	NEMF
SPI1	NET1
SPI1	NFATC2
SPI1	NFATC3
SPI1	NFATC4
SPI1	NFIC
SPI1	NFYB
SPI1	NFYC
SPI1	NGRN
SPI1	NINJ2
SPI1	NIPBL
SPI1	NIT1
SPI1	NKIRAS2
SPI1	NLK
SPI1	NLRC3
SPI1	NLRP2
SPI1	NMI
SPI1	NNT
SPI1	NOL12
SPI1	NOP10
SPI1	NOSIP
SPI1	NR1H2
SPI1	NR1H3
SPI1	NR3C1
SPI1	NRF1
SPI1	NSMCE1
SPI1	NSUN5
SPI1	NT5C2
SPI1	NT5C3A
SPI1	NTAN1
SPI1	NTPCR
SPI1	NUB1
SPI1	NUBP1
SPI1	NUCB1
SPI1	NUCKS1
SPI1	NUDCD3
SPI1	NUDT1
SPI1	NUGGC
SPI1	NUMB
SPI1	NUP107
SPI1	NUP37
SPI1	NUTM2B-AS1
SPI1	OARD1
SPI1	OAZ1
SPI1	OAZ2
SPI1	OCIAD1
SPI1	OFD1
SPI1	OGFOD1
SPI1	OGG1
SPI1	OMA1

SPI1	OPA3
SPI1	ORAI1
SPI1	ORAI3
SPI1	ORC2
SPI1	ORC4
SPI1	ORMDL2
SPI1	OSBPL2
SPI1	OSBPL8
SPI1	OSER1
SPI1	OST4
SPI1	OSTF1
SPI1	OTUB1
SPI1	OXLD1
SPI1	P2RX5
SPI1	PABPC1
SPI1	PABPN1
SPI1	PACS1
SPI1	PAFAH1B1
SPI1	PAFAH1B2
SPI1	PAFAH1B3
SPI1	PAG1
SPI1	PAIP2
SPI1	PAK2
SPI1	PALD1
SPI1	PALLD
SPI1	PAN2
SPI1	PANK2
SPI1	PAPOLA
SPI1	PARN
SPI1	PARP12
SPI1	PARVG
SPI1	PATZ1
SPI1	PAX5
SPI1	PCBP1
SPI1	PCBP2
SPI1	PCED1B
SPI1	PCF11
SPI1	PCGF1
SPI1	PCNP
SPI1	PDCD10
SPI1	PDCD6
SPI1	PDCD6IP
SPI1	PDCD7
SPI1	PDGFD
SPI1	PDLIM1
SPI1	PDLIM2
SPI1	PDLIM7
SPI1	PDZD11
SPI1	PEA15
SPI1	PEF1

SPI1	PET100
SPI1	PEX16
SPI1	PFDN1
SPI1	PFDN5
SPI1	PFN1
SPI1	PGAP2
SPI1	PGBD4
SPI1	PGGT1B
SPI1	PGK1
SPI1	PHB2
SPI1	PHF11
SPI1	PHF14
SPI1	PHF5A
SPI1	PHF6
SPI1	PHIP
SPI1	PHKG2
SPI1	PHTF2
SPI1	PI4K2B
SPI1	PIBF1
SPI1	PICALM
SPI1	PIGBOS1
SPI1	PIGV
SPI1	PIH1D1
SPI1	PIK3AP1
SPI1	PIK3C3
SPI1	PIK3R1
SPI1	PILRB
SPI1	PIN1
SPI1	PIN4
SPI1	PIP4K2A
SPI1	PITPNA
SPI1	PITPNA-AS1
SPI1	PKIG
SPI1	PKM
SPI1	PKN1
SPI1	PLAC8
SPI1	PLEKHA2
SPI1	PLEKHA3
SPI1	PLEKHF2
SPI1	PLEKHJ1
SPI1	PLEKHO1
SPI1	PLEKHO2
SPI1	PLK1
SPI1	PLRG1
SPI1	PLXNC1
SPI1	PMPCA
SPI1	PMPCB
SPI1	PMVK
SPI1	PNISR
SPI1	PNKD

SPI1	PNN
SPI1	PNOC
SPI1	PNPLA8
SPI1	PNRC1
SPI1	POLD4
SPI1	POLDIP3
SPI1	POLL
SPI1	POLM
SPI1	POLQ
SPI1	POLR2A
SPI1	POLR2G
SPI1	POLR2J
SPI1	POLR2J2
SPI1	POMP
SPI1	POP4
SPI1	POP5
SPI1	POU2AF1
SPI1	POU2F1
SPI1	POU2F2
SPI1	PPHLN1
SPI1	PPID
SPI1	PPIH
SPI1	PPIL3
SPI1	PPM1A
SPI1	PPM1B
SPI1	PPM1M
SPI1	PPP1CA
SPI1	PPP1CC
SPI1	PPP1R12A
SPI1	PPP1R18
SPI1	PPP1R35
SPI1	PPP2R1A
SPI1	PPP2R5A
SPI1	PPP2R5C
SPI1	PPP4C
SPI1	PPP4R3B
SPI1	PPP6R1
SPI1	PPP6R3
SPI1	PRDM2
SPI1	PRKACA
SPI1	PRKAR1A
SPI1	PRKCB
SPI1	PRKD2
SPI1	PRMT2
SPI1	PRMT6
SPI1	PRPF3
SPI1	PRPF38A
SPI1	PRPF38B
SPI1	PRPF6
SPI1	PRPS1

SPI1	PRR13
SPI1	PRR14
SPI1	PRR14L
SPI1	PSD4
SPI1	PSENEN
SPI1	PSIP1
SPI1	PSMA3-AS1
SPI1	PSMA4
SPI1	PSMA8
SPI1	PSMB3
SPI1	PSMB4
SPI1	PSMB8
SPI1	PSMB8-AS1
SPI1	PSMD13
SPI1	PSMD6
SPI1	PSMD8
SPI1	PSRC1
SPI1	PTBP3
SPI1	PTK2
SPI1	PTK2B
SPI1	PTMA
SPI1	PTP4A1
SPI1	PTP4A2
SPI1	PTPDC1
SPI1	PTPN1
SPI1	PTPN23
SPI1	PTPN6
SPI1	PTPN7
SPI1	PTPN9
SPI1	PTPRC
SPI1	PTPRE
SPI1	PTRHD1
SPI1	PUDP
SPI1	PUM2
SPI1	PURA
SPI1	PURB
SPI1	PYCARD
SPI1	PYM1
SPI1	PYROXD1
SPI1	PYURF
SPI1	R3HCC1L
SPI1	R3HDM4
SPI1	RAB11A
SPI1	RAB11B
SPI1	RAB13
SPI1	RAB14
SPI1	RAB18
SPI1	RAB1B
SPI1	RAB2A
SPI1	RAB30

SPI1	RAB33A
SPI1	RAB34
SPI1	RAB35
SPI1	RAB3GAP2
SPI1	RAB5B
SPI1	RAB5C
SPI1	RAB6A
SPI1	RAB7A
SPI1	RAB9A
SPI1	RABEP2
SPI1	RABGAP1L
SPI1	RABGEF1
SPI1	RAC1
SPI1	RAC2
SPI1	RACK1
SPI1	RAD21
SPI1	RALGAPB
SPI1	RALGPS2
SPI1	RALY
SPI1	RANGAP1
SPI1	RAP1A
SPI1	RAP2C
SPI1	RAPGEF1
SPI1	RAPGEF6
SPI1	RASAL1
SPI1	RASAL3
SPI1	RASGRP1
SPI1	RASGRP2
SPI1	RASGRP3
SPI1	RASSF1
SPI1	RASSF2
SPI1	RB1
SPI1	RBBP4
SPI1	RBBP6
SPI1	RBBP7
SPI1	RBCK1
SPI1	RBM14
SPI1	RBM3
SPI1	RBM4
SPI1	RBM42
SPI1	RBM45
SPI1	RBM6
SPI1	RBM8A
SPI1	RCHY1
SPI1	RCN1
SPI1	RCSD1
SPI1	RECQL
SPI1	REL
SPI1	RELA
SPI1	REPIN1

SPI1	RERE
SPI1	REST
SPI1	RFC1
SPI1	RFESD
SPI1	RFTN1
SPI1	RFX5
SPI1	RFXANK
SPI1	RGCC
SPI1	RGL2
SPI1	RGP1
SPI1	RGS13
SPI1	RGS14
SPI1	RGS19
SPI1	RHBDD1
SPI1	RHBDD2
SPI1	RHBDF2
SPI1	RHOG
SPI1	RHOH
SPI1	RIC3
SPI1	RIC8A
SPI1	RING1
SPI1	RINL
SPI1	RIOK3
SPI1	RIPK2
SPI1	RIPK3
SPI1	RMDN1
SPI1	RMI1
SPI1	RMI2
SPI1	RNASEH2C
SPI1	RNF115
SPI1	RNF13
SPI1	RNF141
SPI1	RNF144B
SPI1	RNF167
SPI1	RNF181
SPI1	RNF20
SPI1	RNF220
SPI1	RNF34
SPI1	RNF6
SPI1	RNFT2
SPI1	RNGTT
SPI1	ROCK1
SPI1	ROMO1
SPI1	RP2
SPI1	RPL10
SPI1	RPL10A
SPI1	RPL11
SPI1	RPL12
SPI1	RPL13A
SPI1	RPL15

SPI1	RPL17
SPI1	RPL18
SPI1	RPL18A
SPI1	RPL19
SPI1	RPL21
SPI1	RPL22
SPI1	RPL23
SPI1	RPL23A
SPI1	RPL24
SPI1	RPL26
SPI1	RPL27
SPI1	RPL27A
SPI1	RPL28
SPI1	RPL29
SPI1	RPL3
SPI1	RPL30
SPI1	RPL31
SPI1	RPL32
SPI1	RPL34
SPI1	RPL35
SPI1	RPL36AL
SPI1	RPL37
SPI1	RPL37A
SPI1	RPL38
SPI1	RPL39
SPI1	RPL4
SPI1	RPL41
SPI1	RPL6
SPI1	RPL7
SPI1	RPL8
SPI1	RPLP2
SPI1	RPS10
SPI1	RPS11
SPI1	RPS12
SPI1	RPS13
SPI1	RPS14
SPI1	RPS15
SPI1	RPS16
SPI1	RPS17
SPI1	RPS18
SPI1	RPS19
SPI1	RPS20
SPI1	RPS21
SPI1	RPS25
SPI1	RPS26
SPI1	RPS27
SPI1	RPS27A
SPI1	RPS3
SPI1	RPS3A
SPI1	RPS4X

SPI1	RPS4Y1
SPI1	RPS5
SPI1	RPS6
SPI1	RPS6KA1
SPI1	RPS7
SPI1	RPS8
SPI1	RPSA
SPI1	RRAGA
SPI1	RRM2B
SPI1	RRNAD1
SPI1	RSU1
SPI1	RTCA
SPI1	RTN4
SPI1	RUFY1
SPI1	RUFY3
SPI1	RUNX3
SPI1	RXRB
SPI1	S100A10
SPI1	S100A11
SPI1	S100A4
SPI1	S100A6
SPI1	S1PR2
SPI1	S1PR4
SPI1	SAMD15
SPI1	SASH3
SPI1	SAT1
SPI1	SAV1
SPI1	SCARB1
SPI1	SCIMP
SPI1	SCML4
SPI1	SCNM1
SPI1	SCO1
SPI1	SCOC
SPI1	SCRN2
SPI1	SDCBP
SPI1	SDCCAG8
SPI1	SDF2
SPI1	SDHAF2
SPI1	SDHAF3
SPI1	SDHC
SPI1	SDHD
SPI1	SEC11A
SPI1	SEC14L1
SPI1	SEPT6
SPI1	SEPT7
SPI1	SEPT9
SPI1	SERINC3
SPI1	SERP2
SPI1	SERPINB8
SPI1	SERTAD2

SPI1	SETBP1
SPI1	SETDB1
SPI1	SETX
SPI1	SF1
SPI1	SF3B1
SPI1	SF3B4
SPI1	SF3B6
SPI1	SFI1
SPI1	SFT2D1
SPI1	SFT2D2
SPI1	SGMS1
SPI1	SGO1
SPI1	SGSH
SPI1	SH2B2
SPI1	SH2B3
SPI1	SH3BGRL
SPI1	SH3BGRL3
SPI1	SH3BP1
SPI1	SH3BP5
SPI1	SH3KBP1
SPI1	SH3TC1
SPI1	SHISA5
SPI1	SHKBP1
SPI1	SHOC2
SPI1	SIAE
SPI1	SIN3A
SPI1	SIPA1
SPI1	SIRT6
SPI1	SIRT7
SPI1	SIT1
SPI1	SKA3
SPI1	SKAP2
SPI1	SLAIN2
SPI1	SLC15A2
SPI1	SLC15A4
SPI1	SLC16A3
SPI1	SLC16A4
SPI1	SLC25A1
SPI1	SLC25A11
SPI1	SLC25A40
SPI1	SLC25A44
SPI1	SLC25A53
SPI1	SLC2A1
SPI1	SLC2A6
SPI1	SLC35C2
SPI1	SLC35E4
SPI1	SLC39A1
SPI1	SLC39A13
SPI1	SLC39A3
SPI1	SLC41A1

SPI1	SLC43A2
SPI1	SLC4A2
SPI1	SLC4A8
SPI1	SLC9A3R1
SPI1	SLC9A8
SPI1	SLMAP
SPI1	SLU7
SPI1	SMAD2
SPI1	SMAD4
SPI1	SMAP2
SPI1	SMARCAL1
SPI1	SMARCD2
SPI1	SMC3
SPI1	SMG1
SPI1	SMIM14
SPI1	SMIM15
SPI1	SMIM19
SPI1	SMIM20
SPI1	SMIM7
SPI1	SMUG1
SPI1	SMURF2
SPI1	SNAP23
SPI1	SNF8
SPI1	SNHG19
SPI1	SNHG25
SPI1	SNN
SPI1	SNRNP27
SPI1	SNRNP70
SPI1	SNRPA
SPI1	SNRPA1
SPI1	SNRPB
SPI1	SNRPB2
SPI1	SNRPD2
SPI1	SNRPN
SPI1	SNTB2
SPI1	SNW1
SPI1	SNX1
SPI1	SNX10
SPI1	SNX14
SPI1	SNX20
SPI1	SNX22
SPI1	SNX29
SPI1	SNX3
SPI1	SNX30
SPI1	SOCS1
SPI1	SOS2
SPI1	SP100
SPI1	SP110
SPI1	SP3
SPI1	SPAG7

SPI1	SPATA2L
SPI1	SPC25
SPI1	SPG21
SPI1	SPI1
SPI1	SPON1
SPI1	SPOP
SPI1	SPRED2
SPI1	SPSB3
SPI1	SPTAN1
SPI1	SPTLC2
SPI1	SRD5A1
SPI1	SREK1
SPI1	SRF
SPI1	SRP14
SPI1	SRP9
SPI1	SRPK2
SPI1	SRSF5
SPI1	SRSF9
SPI1	SSBP2
SPI1	SSH2
SPI1	ST14
SPI1	ST20
SPI1	ST6GAL1
SPI1	STAG1
SPI1	STAMBP
SPI1	STAMBPL1
SPI1	STAP1
SPI1	STARD3
SPI1	STAT6
SPI1	STAU2
SPI1	STIL
SPI1	STIM1
SPI1	STIM2
SPI1	STK10
SPI1	STK16
SPI1	STK17B
SPI1	STK24
SPI1	STK26
SPI1	STK38
SPI1	STK4
SPI1	STK40
SPI1	STRADA
SPI1	STRN
SPI1	STUB1
SPI1	STX10
SPI1	STX12
SPI1	STX5
SPI1	STX7
SPI1	STXBP2
SPI1	SUCLG1

SPI1	SUCLG2
SPI1	SUDS3
SPI1	SUGCT
SPI1	SUGT1
SPI1	SULF2
SPI1	SUMO1
SPI1	SUN2
SPI1	SUPT20H
SPI1	SUPT4H1
SPI1	SURF1
SPI1	SUSD3
SPI1	SUSD6
SPI1	SVBP
SPI1	SWI5
SPI1	SYF2
SPI1	SYK
SPI1	SYMPK
SPI1	SYNRG
SPI1	SYT17
SPI1	SYTL1
SPI1	TACC1
SPI1	TADA3
SPI1	TAF1
SPI1	TAF10
SPI1	TAF12
SPI1	TAF15
SPI1	TAF1B
SPI1	TAF1C
SPI1	TAF7
SPI1	TAGAP
SPI1	TAGLN2
SPI1	TALDO1
SPI1	TANGO2
SPI1	TANK
SPI1	TAOK3
SPI1	TARS2
SPI1	TATDN1
SPI1	TATDN3
SPI1	TAX1BP3
SPI1	TBC1D10C
SPI1	TBC1D22A
SPI1	TBCC
SPI1	TBCK
SPI1	TBK1
SPI1	TBPL1
SPI1	TCF3
SPI1	TCIRG1
SPI1	TDP1
SPI1	TEFM
SPI1	TEX261

SPI1	TEX264
SPI1	TFB1M
SPI1	TFEB
SPI1	TFIP11
SPI1	TGIF2
SPI1	TGOLN2
SPI1	THAP1
SPI1	THAP11
SPI1	THAP4
SPI1	THG1L
SPI1	THOC2
SPI1	THRAP3
SPI1	THUMPD3-AS1
SPI1	TIGAR
SPI1	TIMMDC1
SPI1	TINF2
SPI1	TIPARP
SPI1	TIPRL
SPI1	TKT
SPI1	TLN1
SPI1	TLR10
SPI1	TM2D1
SPI1	TMA7
SPI1	TMBIM4
SPI1	TMC6
SPI1	TMC8
SPI1	TMED8
SPI1	TMEM101
SPI1	TMEM128
SPI1	TMEM138
SPI1	TMEM14A
SPI1	TMEM156
SPI1	TMEM161B-AS1
SPI1	TMEM18
SPI1	TMEM206
SPI1	TMEM230
SPI1	TMEM234
SPI1	TMEM242
SPI1	TMEM254
SPI1	TMEM256
SPI1	TMEM30A
SPI1	TMEM60
SPI1	TMF1
SPI1	TMSB4X
SPI1	TMUB1
SPI1	TNFAIP8
SPI1	TNFAIP8L2
SPI1	TNFRSF13B
SPI1	TNFRSF13C
SPI1	TNFRSF1B

SPI1	TNK2
SPI1	TNKS
SPI1	TOB1
SPI1	TOP2A
SPI1	TOR4A
SPI1	TOX4
SPI1	TP53RK
SPI1	TP53TG1
SPI1	TPD52
SPI1	TPGS1
SPI1	TPM3
SPI1	TPM4
SPI1	TPR
SPI1	TPRKB
SPI1	TRA2B
SPI1	TRABD
SPI1	TRADD
SPI1	TRAF3IP3
SPI1	TRAF5
SPI1	TRAF7
SPI1	TRAK1
SPI1	TRAPPC1
SPI1	TRAPPC11
SPI1	TRAPPC6A
SPI1	TRAPPC8
SPI1	TRAPPC9
SPI1	TRIB2
SPI1	TRIM11
SPI1	TRIM22
SPI1	TRIM4
SPI1	TRIM41
SPI1	TRIM8
SPI1	TRIP12
SPI1	TRMO
SPI1	TRMT10A
SPI1	TRMT112
SPI1	TRMT1L
SPI1	TRMT2B
SPI1	TRPT1
SPI1	TRPV2
SPI1	TRRAP
SPI1	TRUB2
SPI1	TSC22D3
SPI1	TSC22D4
SPI1	TSEN34
SPI1	TSG101
SPI1	TSNAX
SPI1	TSPAN33
SPI1	TSPO
SPI1	TTC1

SPI1	TTC14
SPI1	TTC37
SPI1	TTC39C
SPI1	TTC5
SPI1	TTC9C
SPI1	TTF2
SPI1	TUBA1C
SPI1	TUBB2A
SPI1	TUBGCP2
SPI1	TUBGCP4
SPI1	TWF2
SPI1	TXN2
SPI1	TYSND1
SPI1	UBA1
SPI1	UBA3
SPI1	UBA52
SPI1	UBA6-AS1
SPI1	UBAC1
SPI1	UBAP2
SPI1	UBB
SPI1	UBC
SPI1	UBE2C
SPI1	UBE2D2
SPI1	UBE2D3
SPI1	UBE2E1
SPI1	UBE2H
SPI1	UBE2J2
SPI1	UBE2L3
SPI1	UBE2N
SPI1	UBE2Q1
SPI1	UBE2V2
SPI1	UBL5
SPI1	UBL7
SPI1	UBL7-AS1
SPI1	UBLCP1
SPI1	UBTF
SPI1	UBXN1
SPI1	UBXN4
SPI1	UBXN6
SPI1	UCK1
SPI1	UCP2
SPI1	UGGT1
SPI1	UGP2
SPI1	UHMK1
SPI1	UNC119
SPI1	UNC45A
SPI1	UNC50
SPI1	UNC93B1
SPI1	UPF2
SPI1	UQCR11

SPI1	UQCRC2
SPI1	UQCRFS1
SPI1	UQCRH
SPI1	URM1
SPI1	USB1
SPI1	USE1
SPI1	USF2
SPI1	USP53
SPI1	USP7
SPI1	USP9X
SPI1	UTP18
SPI1	UTP3
SPI1	UVRAG
SPI1	UXT
SPI1	VAMP1
SPI1	VAMP2
SPI1	VAMP8
SPI1	VASP
SPI1	VAV1
SPI1	VCP
SPI1	VDAC3
SPI1	VEZF1
SPI1	VGLL4
SPI1	VIM
SPI1	VNN2
SPI1	VPS11
SPI1	VPS13C
SPI1	VPS16
SPI1	VPS25
SPI1	VPS33A
SPI1	VPS4B
SPI1	VPS51
SPI1	VRK3
SPI1	VTA1
SPI1	VTI1A
SPI1	WAC
SPI1	WARS2
SPI1	WAS
SPI1	WASF2
SPI1	WBP1L
SPI1	WBP2
SPI1	WDR1
SPI1	WDR19
SPI1	WDR26
SPI1	WDR34
SPI1	WDR41
SPI1	WDR66
SPI1	WDR70
SPI1	WDR73
SPI1	WDR83OS

SPI1	WDTC1
SPI1	WIPF1
SPI1	WSB1
SPI1	WWP2
SPI1	XKR6
SPI1	YBEY
SPI1	YIPF1
SPI1	YIPF3
SPI1	YPEL5
SPI1	YTHDC1
SPI1	YWHAB
SPI1	YWHAH
SPI1	YWHAZ
SPI1	YY1
SPI1	ZBED5
SPI1	ZBTB20
SPI1	ZBTB43
SPI1	ZBTB44
SPI1	ZBTB7A
SPI1	ZBTB8OS
SPI1	ZC3H10
SPI1	ZC3H12D
SPI1	ZC3H18
SPI1	ZC3HAV1
SPI1	ZC3HC1
SPI1	ZCCHC17
SPI1	ZCCHC7
SPI1	ZCCHC9
SPI1	ZCWPW1
SPI1	ZDHHC12
SPI1	ZDHHC21
SPI1	ZDHHC3
SPI1	ZDHHC4
SPI1	ZDHHC7
SPI1	ZFAND3
SPI1	ZFAND4
SPI1	ZFAND5
SPI1	ZFAND6
SPI1	ZFAS1
SPI1	ZFC3H1
SPI1	ZFP36L1
SPI1	ZFP62
SPI1	ZFYVE27
SPI1	ZKSCAN8
SPI1	ZMAT2
SPI1	ZMIZ1
SPI1	ZMPSTE24
SPI1	ZMYND8
SPI1	ZNF148
SPI1	ZNF180

SPI1	ZNF197
SPI1	ZNF266
SPI1	ZNF267
SPI1	ZNF296
SPI1	ZNF318
SPI1	ZNF330
SPI1	ZNF385A
SPI1	ZNF394
SPI1	ZNF395
SPI1	ZNF48
SPI1	ZNF557
SPI1	ZNF580
SPI1	ZNF596
SPI1	ZNF608
SPI1	ZNF644
SPI1	ZNF652
SPI1	ZNF655
SPI1	ZNF7
SPI1	ZNF701
SPI1	ZNF710
SPI1	ZNF75A
SPI1	ZNF76
SPI1	ZNF775
SPI1	ZNF821
SPI1	ZNF827
SPI1	ZNF83
SPI1	ZNF845
SPI1	ZNHIT1
SPI1	ZPBP2
SPI1	ZRANB2
SPI1	ZRANB3
SPI1	ZRSR2
SPI1	ZYX
SPIB	AAAS
SPIB	AAGAB
SPIB	AAMP
SPIB	AAR2
SPIB	AASDH
SPIB	AASDHPPT
SPIB	AATF
SPIB	ABCB7
SPIB	ABCD3
SPIB	ABCD4
SPIB	ABCF1
SPIB	ABHD10
SPIB	ABHD17B
SPIB	ABHD3
SPIB	ABI1
SPIB	ABI3
SPIB	ABL1

SPIB	ABLIM2
SPIB	ABRACL
SPIB	ABT1
SPIB	ACAA1
SPIB	ACAA2
SPIB	ACAP2
SPIB	ACAT2
SPIB	ACBD3
SPIB	ACBD6
SPIB	ACER3
SPIB	ACIN1
SPIB	ACO2
SPIB	ACOT8
SPIB	ACP6
SPIB	ACSF2
SPIB	ACSF3
SPIB	ACSL5
SPIB	ACTA2
SPIB	ACTB
SPIB	ACTG1
SPIB	ACTN1
SPIB	ACTN4
SPIB	ACTR1A
SPIB	ACTR1B
SPIB	ACTR2
SPIB	ACTR3
SPIB	ACTR6
SPIB	ACTR8
SPIB	ACYP1
SPIB	ACYP2
SPIB	ADA
SPIB	ADAM15
SPIB	ADAM8
SPIB	ADAM9
SPIB	ADAP2
SPIB	ADAR
SPIB	ADAT1
SPIB	ADD3
SPIB	ADGRG5
SPIB	ADH5
SPIB	ADI1
SPIB	ADIPOR1
SPIB	ADIPOR2
SPIB	ADK
SPIB	ADO
SPIB	ADPGK
SPIB	ADPRHL2
SPIB	ADSL
SPIB	ADSS
SPIB	AFF2

SPIB	AFF3
SPIB	AFTPH
SPIB	AGBL5
SPIB	AGFG1
SPIB	AGGF1
SPIB	AGO3
SPIB	AHCY
SPIB	AHCYL1
SPIB	AHDC1
SPIB	AHNAK
SPIB	AHSA1
SPIB	AIF1
SPIB	AIFM1
SPIB	AIM2
SPIB	AIMP2
SPIB	AIP
SPIB	AK2
SPIB	AK3
SPIB	AK6
SPIB	AKAP13
SPIB	AKAP8
SPIB	AKIRIN1
SPIB	AKNA
SPIB	AKR1A1
SPIB	AKT2
SPIB	ALAS1
SPIB	ALDH16A1
SPIB	ALDH18A1
SPIB	ALDH5A1
SPIB	ALDOA
SPIB	ALG13
SPIB	ALG6
SPIB	ALG8
SPIB	ALKBH5
SPIB	ALOX5
SPIB	ALOX5AP
SPIB	ALYREF
SPIB	AMD1
SPIB	AMER1
SPIB	AMFR
SPIB	AMPD2
SPIB	ANAPC11
SPIB	ANAPC13
SPIB	ANAPC15
SPIB	ANAPC16
SPIB	ANAPC4
SPIB	ANKHD1
SPIB	ANKRD13A
SPIB	ANKRD16
SPIB	ANKRD17

SPIB	ANKRD40
SPIB	ANKRD44
SPIB	ANKRD46
SPIB	ANP32B
SPIB	ANP32E
SPIB	ANXA11
SPIB	ANXA4
SPIB	ANXA6
SPIB	AP1AR
SPIB	AP1B1
SPIB	AP1G2
SPIB	AP1M1
SPIB	AP1S1
SPIB	AP1S3
SPIB	AP2A1
SPIB	AP2M1
SPIB	AP2S1
SPIB	AP3B1
SPIB	AP3M1
SPIB	AP3S1
SPIB	AP3S2
SPIB	AP4S1
SPIB	AP5B1
SPIB	AP5M1
SPIB	AP5Z1
SPIB	APAF1
SPIB	APBA3
SPIB	APBB1IP
SPIB	APEX2
SPIB	APH1A
SPIB	APH1B
SPIB	APLP2
SPIB	APOM
SPIB	AQR
SPIB	ARAP2
SPIB	ARCN1
SPIB	ARF1
SPIB	ARF3
SPIB	ARF4
SPIB	ARF5
SPIB	ARF6
SPIB	ARFGEF1
SPIB	ARFGEF2
SPIB	ARGLU1
SPIB	ARHGAP1
SPIB	ARHGAP11A
SPIB	ARHGAP12
SPIB	ARHGAP15
SPIB	ARHGAP17
SPIB	ARHGAP24

SPIB	ARHGAP25
SPIB	ARHGAP27
SPIB	ARHGAP30
SPIB	ARHGAP9
SPIB	ARHGDIA
SPIB	ARHGDIB
SPIB	ARHGEF1
SPIB	ARHGEF2
SPIB	ARHGEF9
SPIB	ARID1B
SPIB	ARID4A
SPIB	ARID4B
SPIB	ARL1
SPIB	ARL14EP
SPIB	ARL5A
SPIB	ARL6IP5
SPIB	ARL6IP6
SPIB	ARMC8
SPIB	ARMC9
SPIB	ARMCX6
SPIB	ARMT1
SPIB	ARPC1A
SPIB	ARPC1B
SPIB	ARPC2
SPIB	ARPC3
SPIB	ARPC4
SPIB	ARPC5L
SPIB	ARPP19
SPIB	ARRB2
SPIB	ARRDC1
SPIB	ARRDC3-AS1
SPIB	ARRDC4
SPIB	ARV1
SPIB	ASB1
SPIB	ASB16-AS1
SPIB	ASB2
SPIB	ASB8
SPIB	ASCC2
SPIB	ASCL2
SPIB	ASH1L
SPIB	ASNA1
SPIB	ASNSD1
SPIB	ASPSCR1
SPIB	ASXL2
SPIB	ATAD1
SPIB	ATAD2
SPIB	ATAD3A
SPIB	ATAT1
SPIB	ATF6B
SPIB	ATF7

SPIB	ATF7IP
SPIB	ATG101
SPIB	ATG12
SPIB	ATG16L2
SPIB	ATG3
SPIB	ATG4B
SPIB	ATG5
SPIB	ATG7
SPIB	ATM
SPIB	ATOX1
SPIB	ATP13A1
SPIB	ATP1A1
SPIB	ATP1B3
SPIB	ATP2A3
SPIB	ATP2C1
SPIB	ATP6V0A2
SPIB	ATP6V0D1
SPIB	ATP6V0E1
SPIB	ATP6V1B2
SPIB	ATP6V1C1
SPIB	ATP6V1D
SPIB	ATP6V1E1
SPIB	ATP6V1H
SPIB	ATRX
SPIB	ATXN10
SPIB	ATXN2
SPIB	ATXN2L
SPIB	ATXN7L3B
SPIB	AURKAIP1
SPIB	B3GALNT1
SPIB	B3GALNT2
SPIB	B3GAT2
SPIB	B3GAT3
SPIB	BABAM1
SPIB	BACE2
SPIB	BACH2
SPIB	BAG5
SPIB	BAG6
SPIB	BAK1
SPIB	BANF1
SPIB	BAP1
SPIB	BASP1
SPIB	BATF
SPIB	BAZ1A
SPIB	BAZ1B
SPIB	BAZ2A
SPIB	BBIP1
SPIB	BCAP31
SPIB	BCAR3
SPIB	BCAT2

SPIB	BCCIP
SPIB	BCDIN3D
SPIB	BCKDHB
SPIB	BCL10
SPIB	BCL11A
SPIB	BCL2A1
SPIB	BCL2L1
SPIB	BCL2L13
SPIB	BCL3
SPIB	BCL6
SPIB	BCL7C
SPIB	BCLAF1
SPIB	BDH1
SPIB	BDH2
SPIB	BDP1
SPIB	BECN1
SPIB	BET1
SPIB	BEX2
SPIB	BFAR
SPIB	BID
SPIB	BIK
SPIB	BIRC2
SPIB	BIRC3
SPIB	BIRC6
SPIB	BIVM
SPIB	BLCAP
SPIB	BLK
SPIB	BLMH
SPIB	BLNK
SPIB	BLOC1S2
SPIB	BLOC1S4
SPIB	BLOC1S6
SPIB	BLZF1
SPIB	BMP2K
SPIB	BMPR1A
SPIB	BNIP2
SPIB	BOD1L1
SPIB	BORCS7
SPIB	BORCS8
SPIB	BORCS8−MEF2B
SPIB	BPTF
SPIB	BRCA1
SPIB	BRCA2
SPIB	BRCC3
SPIB	BRD2
SPIB	BRD7
SPIB	BRF1
SPIB	BRK1
SPIB	BRMS1
SPIB	BROX

SPIB	BRWD3
SPIB	BTAF1
SPIB	BTBD1
SPIB	BTBD19
SPIB	BTF3
SPIB	BTF3L4
SPIB	BTG1
SPIB	BTG2
SPIB	BTK
SPIB	BUB3
SPIB	BUD13
SPIB	BUD31
SPIB	BZW1
SPIB	C11orf24
SPIB	C12orf10
SPIB	C12orf49
SPIB	C12orf73
SPIB	C12orf75
SPIB	C14orf119
SPIB	C15orf40
SPIB	C15orf62
SPIB	C16orf74
SPIB	C16orf91
SPIB	C18orf21
SPIB	C18orf25
SPIB	C18orf54
SPIB	C19orf25
SPIB	C19orf38
SPIB	C19orf48
SPIB	C1GALT1
SPIB	C1orf112
SPIB	C1orf131
SPIB	C1orf50
SPIB	C1orf52
SPIB	C1QBP
SPIB	C22orf34
SPIB	C2orf69
SPIB	C2orf76
SPIB	C3orf14
SPIB	C3orf38
SPIB	C5orf24
SPIB	C5orf34
SPIB	C6orf120
SPIB	C6orf226
SPIB	C6orf48
SPIB	C6orf62
SPIB	C6orf89
SPIB	C6orf99
SPIB	C8orf44
SPIB	C8orf59

SPIB	C8orf76
SPIB	C9orf16
SPIB	C9orf40
SPIB	C9orf78
SPIB	CAAP1
SPIB	CAB39
SPIB	CABIN1
SPIB	CACNA1A
SPIB	CACYBP
SPIB	CALHM2
SPIB	CALM2
SPIB	CALR
SPIB	CAMK2D
SPIB	CAMK2G
SPIB	CAMK4
SPIB	CAMTA1
SPIB	CAND1
SPIB	CAP1
SPIB	CAPG
SPIB	CAPN1
SPIB	CAPNS1
SPIB	CAPZA1
SPIB	CAPZA2
SPIB	CAPZB
SPIB	CARD11
SPIB	CARD8
SPIB	CARF
SPIB	CARHSP1
SPIB	CARM1
SPIB	CARNMT1
SPIB	CARS
SPIB	CARS2
SPIB	CASC4
SPIB	CASP2
SPIB	CASP4
SPIB	CASP8AP2
SPIB	CBFA2T3
SPIB	CBFB
SPIB	CBL
SPIB	CBLL1
SPIB	CBR4
SPIB	CBWD1
SPIB	CBWD5
SPIB	CBX3
SPIB	CBX5
SPIB	CC2D1A
SPIB	CC2D1B
SPIB	CCAR2
SPIB	CCDC102A
SPIB	CCDC106

SPIB	CCDC107
SPIB	CCDC115
SPIB	CCDC12
SPIB	CCDC125
SPIB	CCDC137
SPIB	CCDC146
SPIB	CCDC174
SPIB	CCDC191
SPIB	CCDC22
SPIB	CCDC25
SPIB	CCDC28B
SPIB	CCDC43
SPIB	CCDC51
SPIB	CCDC6
SPIB	CCDC69
SPIB	CCDC7
SPIB	CCDC71
SPIB	CCDC77
SPIB	CCDC85B
SPIB	CCDC86
SPIB	CCDC88A
SPIB	CCDC88C
SPIB	CCDC91
SPIB	CCDC97
SPIB	CCL22
SPIB	CCL4L2
SPIB	CCM2
SPIB	CCNB1IP1
SPIB	CCNB2
SPIB	CCNC
SPIB	CCND2
SPIB	CCND3
SPIB	CCNDBP1
SPIB	CCNG1
SPIB	CCNG2
SPIB	CCNH
SPIB	CCNI
SPIB	CCNK
SPIB	CCNL1
SPIB	CCNL2
SPIB	CCNT2
SPIB	CCSER2
SPIB	CCT2
SPIB	CCT5
SPIB	CCT7
SPIB	CCZ1
SPIB	CD164
SPIB	CD180
SPIB	CD19
SPIB	CD22

SPIB	CD24
SPIB	CD2BP2
SPIB	CD37
SPIB	CD40
SPIB	CD52
SPIB	CD53
SPIB	CD70
SPIB	CD72
SPIB	CD74
SPIB	CD79A
SPIB	CD79B
SPIB	CD82
SPIB	CD83
SPIB	CD9
SPIB	CDC123
SPIB	CDC16
SPIB	CDC25A
SPIB	CDC27
SPIB	CDC34
SPIB	CDC37
SPIB	CDC40
SPIB	CDC42
SPIB	CDC42EP3
SPIB	CDC42SE1
SPIB	CDC42SE2
SPIB	CDC5L
SPIB	CDC7
SPIB	CDK10
SPIB	CDK11A
SPIB	CDK11B
SPIB	CDK13
SPIB	CDK17
SPIB	CDK19
SPIB	CDK2AP2
SPIB	CDK4
SPIB	CDK5RAP1
SPIB	CDK5RAP3
SPIB	CDK6
SPIB	CDK7
SPIB	CDK9
SPIB	CDKL3
SPIB	CDKN2AIP
SPIB	CDV3
SPIB	CDYL
SPIB	CEBPZ
SPIB	CEBPZOS
SPIB	CELF1
SPIB	CELF2
SPIB	CENPH
SPIB	CENPL

SPIB	CENPT
SPIB	CEP112
SPIB	CEP135
SPIB	CEP162
SPIB	CEP350
SPIB	CEP41
SPIB	CEP44
SPIB	CEP57
SPIB	CEP63
SPIB	CEP85L
SPIB	CEP97
SPIB	CEPT1
SPIB	CETN3
SPIB	CFAP20
SPIB	CFAP36
SPIB	CFAP97
SPIB	CFDP1
SPIB	CFL1
SPIB	CFLAR
SPIB	CGGBP1
SPIB	CHAC2
SPIB	CHAF1A
SPIB	CHAMP1
SPIB	CHCHD2
SPIB	CHCHD3
SPIB	CHCHD5
SPIB	CHCHD7
SPIB	CHD4
SPIB	CHD7
SPIB	CHD8
SPIB	CHD9
SPIB	CHEK1
SPIB	CHFR
SPIB	CHMP1A
SPIB	CHMP1B
SPIB	CHMP2A
SPIB	CHMP2B
SPIB	CHMP4B
SPIB	CHMP5
SPIB	CHMP7
SPIB	CHRNB1
SPIB	CHST10
SPIB	CHST11
SPIB	CHTF8
SPIB	CHURC1
SPIB	CIAPIN1
SPIB	CIB1
SPIB	CIB2
SPIB	CIC
SPIB	CIITA

SPIB	CINP
SPIB	CIR1
SPIB	CIRBP
SPIB	CISD2
SPIB	CIZ1
SPIB	CKB
SPIB	CKS2
SPIB	CLASRP
SPIB	CLCN7
SPIB	CLECL1
SPIB	CLHC1
SPIB	CLIC1
SPIB	CLIC4
SPIB	CLINT1
SPIB	CLIP2
SPIB	CLK3
SPIB	CLN5
SPIB	CLNS1A
SPIB	CLPP
SPIB	CLPX
SPIB	CLTA
SPIB	CLTB
SPIB	CLTC
SPIB	CMAS
SPIB	CMC1
SPIB	CMC2
SPIB	CMTM3
SPIB	CMTM6
SPIB	CMTM7
SPIB	CMTR1
SPIB	CMTR2
SPIB	CNDP2
SPIB	CNIH1
SPIB	CNN2
SPIB	CNOT1
SPIB	CNOT10
SPIB	CNOT11
SPIB	CNOT2
SPIB	CNOT7
SPIB	CNOT8
SPIB	CNP
SPIB	CNPPD1
SPIB	CNPY3
SPIB	CNR2
SPIB	CNST
SPIB	CNTLN
SPIB	COA1
SPIB	COA4
SPIB	COA5
SPIB	COA6

SPIB	COASY
SPIB	COCH
SPIB	COG2
SPIB	COG4
SPIB	COMMD1
SPIB	COMMD10
SPIB	COMMD2
SPIB	COMMD4
SPIB	COMMD5
SPIB	COMMD6
SPIB	COPB2
SPIB	COPE
SPIB	COPS3
SPIB	COPS5
SPIB	COPS7A
SPIB	COPZ1
SPIB	COQ3
SPIB	COQ4
SPIB	COQ6
SPIB	COQ9
SPIB	CORO1A
SPIB	CORO1B
SPIB	COX14
SPIB	COX15
SPIB	COX16
SPIB	COX17
SPIB	COX4I1
SPIB	COX5B
SPIB	COX6A1
SPIB	COX6B1
SPIB	COX7A2L
SPIB	COX7B
SPIB	COX7C
SPIB	COX8A
SPIB	CPEB2
SPIB	CPNE1
SPIB	CPNE5
SPIB	CPSF2
SPIB	CPSF3
SPIB	CPSF6
SPIB	CPSF7
SPIB	CRB2
SPIB	CRB3
SPIB	CREB1
SPIB	CRIPT
SPIB	CRLF3
SPIB	CRTC2
SPIB	CRYZL1
SPIB	CS
SPIB	CSDE1

SPIB	CSK
SPIB	CSNK1A1
SPIB	CSNK1D
SPIB	CSNK1G2
SPIB	CSNK1G3
SPIB	CSNK2A1
SPIB	CSNK2A2
SPIB	CSNK2B
SPIB	CSRP1
SPIB	CSTB
SPIB	CSTF1
SPIB	CSTF2
SPIB	CSTF2T
SPIB	CSTF3
SPIB	CTCF
SPIB	CTDNEP1
SPIB	CTDSP1
SPIB	CTDSP2
SPIB	CTDSPL2
SPIB	CTNNB1
SPIB	CTNNBL1
SPIB	CTPS1
SPIB	CTPS2
SPIB	CTR9
SPIB	CTSH
SPIB	CTSS
SPIB	CTSZ
SPIB	CUEDC2
SPIB	CUL1
SPIB	CUL2
SPIB	CUL3
SPIB	CUL4A
SPIB	CUTA
SPIB	CUTC
SPIB	CWC15
SPIB	CWC22
SPIB	CWC25
SPIB	CWC27
SPIB	CXCR5
SPIB	CXorf21
SPIB	CXorf38
SPIB	CXXC1
SPIB	CXXC5
SPIB	CYB561A3
SPIB	CYB5B
SPIB	CYB5R3
SPIB	CYB5R4
SPIB	CYBA
SPIB	CYBB
SPIB	CYFIP2

SPIB	CYLD
SPIB	CYSTM1
SPIB	CYTH1
SPIB	CYTH4
SPIB	DAD1
SPIB	DALRD3
SPIB	DAP
SPIB	DAPK3
SPIB	DAPL1
SPIB	DAPP1
SPIB	DARS
SPIB	DAXX
SPIB	DAZAP1
SPIB	DAZAP2
SPIB	DBNL
SPIB	DCAF10
SPIB	DCAF11
SPIB	DCAF12
SPIB	DCAF15
SPIB	DCAF17
SPIB	DCAF4
SPIB	DCAF6
SPIB	DCAF7
SPIB	DCK
SPIB	DCLRE1C
SPIB	DCP2
SPIB	DCTN3
SPIB	DCTN5
SPIB	DCTPP1
SPIB	DCUN1D1
SPIB	DCUN1D5
SPIB	DDA1
SPIB	DDB1
SPIB	DDIAS
SPIB	DDIT3
SPIB	DDOST
SPIB	DDT
SPIB	DDX10
SPIB	DDX11
SPIB	DDX17
SPIB	DDX18
SPIB	DDX19B
SPIB	DDX20
SPIB	DDX21
SPIB	DDX23
SPIB	DDX24
SPIB	DDX27
SPIB	DDX39A
SPIB	DDX3X
SPIB	DDX46

SPIB	DDX47
SPIB	DDX49
SPIB	DDX5
SPIB	DDX50
SPIB	DDX52
SPIB	DDX56
SPIB	DDX6
SPIB	DECR1
SPIB	DEDD
SPIB	DEF8
SPIB	DENND1B
SPIB	DENND1C
SPIB	DENND2D
SPIB	DENND3
SPIB	DENND4B
SPIB	DENR
SPIB	DEPDC5
SPIB	DERA
SPIB	DFFA
SPIB	DGKA
SPIB	DGKZ
SPIB	DGUOK
SPIB	DHDDS
SPIB	DHFR
SPIB	DHODH
SPIB	DHPS
SPIB	DHRS1
SPIB	DHRS11
SPIB	DHRS7B
SPIB	DHX15
SPIB	DHX16
SPIB	DHX30
SPIB	DHX34
SPIB	DHX36
SPIB	DHX38
SPIB	DHX9
SPIB	DIAPH1
SPIB	DICER1
SPIB	DIDO1
SPIB	DIS3L2
SPIB	DLAT
SPIB	DLD
SPIB	DLEU2
SPIB	DLGAP1-AS1
SPIB	DLST
SPIB	DMAP1
SPIB	DMTF1
SPIB	DNAAF5
SPIB	DNAJA1
SPIB	DNAJA2

SPIB	DNAJA3
SPIB	DNAJB14
SPIB	DNAJB2
SPIB	DNAJB6
SPIB	DNAJC18
SPIB	DNAJC2
SPIB	DNAJC7
SPIB	DNASE1L3
SPIB	DNM1L
SPIB	DNM2
SPIB	DNMT1
SPIB	DNTTIP2
SPIB	DOCK11
SPIB	DOCK2
SPIB	DOCK8
SPIB	DOK2
SPIB	DOK3
SPIB	DOK7
SPIB	DPH2
SPIB	DPH3
SPIB	DPM1
SPIB	DPM2
SPIB	DPM3
SPIB	DPP3
SPIB	DPP8
SPIB	DPP9
SPIB	DPY30
SPIB	DPYSL2
SPIB	DR1
SPIB	DRAM2
SPIB	DSCC1
SPIB	DSTN
SPIB	DSTYK
SPIB	DTD1
SPIB	DTD2
SPIB	DTNBP1
SPIB	DTX1
SPIB	DUS1L
SPIB	DUS3L
SPIB	DUSP12
SPIB	DUSP2
SPIB	DUSP22
SPIB	DUSP28
SPIB	DUSP6
SPIB	DYNC1I2
SPIB	DYNC1LI1
SPIB	DYNLL2
SPIB	DYNLRB1
SPIB	DYNLT1
SPIB	DYRK1A

SPIB	DZIP3
SPIB	E2F4
SPIB	E2F5
SPIB	EAPP
SPIB	EARS2
SPIB	EBAG9
SPIB	EBF1
SPIB	EBI3
SPIB	ECH1
SPIB	ECHDC1
SPIB	ECSIT
SPIB	EDRF1
SPIB	EEF1A1
SPIB	EEF1B2
SPIB	EEF2
SPIB	EEF2K
SPIB	EEFSEC
SPIB	EEPD1
SPIB	EFCAB14
SPIB	EFHC1
SPIB	EFHD2
SPIB	EFR3A
SPIB	EFTUD2
SPIB	EGLN2
SPIB	EGR1
SPIB	EHBP1L1
SPIB	EHD1
SPIB	EHD4
SPIB	EI24
SPIB	EID3
SPIB	EIF1B
SPIB	EIF2A
SPIB	EIF2AK1
SPIB	EIF2B1
SPIB	EIF2B2
SPIB	EIF2B3
SPIB	EIF2B5
SPIB	EIF2D
SPIB	EIF2S2
SPIB	EIF2S3
SPIB	EIF3A
SPIB	EIF3B
SPIB	EIF3D
SPIB	EIF3F
SPIB	EIF3G
SPIB	EIF3H
SPIB	EIF3I
SPIB	EIF3J
SPIB	EIF3K
SPIB	EIF3L

SPIB	EIF3M
SPIB	EIF4A1
SPIB	EIF4A2
SPIB	EIF4E
SPIB	EIF4E2
SPIB	EIF4EBP2
SPIB	EIF4G1
SPIB	EIF4G2
SPIB	EIF4G3
SPIB	EIF4H
SPIB	EIF5A
SPIB	EIF5AL1
SPIB	EIF6
SPIB	ELAC1
SPIB	ELAC2
SPIB	ELAVL1
SPIB	ELF1
SPIB	ELF2
SPIB	ELK4
SPIB	ELMO1
SPIB	ELMSAN1
SPIB	ELOF1
SPIB	ELOVL1
SPIB	ELOVL5
SPIB	ELP2
SPIB	ELP3
SPIB	ELP5
SPIB	EMC4
SPIB	EMC6
SPIB	EMC8
SPIB	EMG1
SPIB	EML3
SPIB	EML6
SPIB	EMP3
SPIB	ENO1
SPIB	ENTPD4
SPIB	ENY2
SPIB	EP300
SPIB	EP400
SPIB	EPB41L4A-AS1
SPIB	EPC1
SPIB	EPN1
SPIB	EPS15
SPIB	EPSTI1
SPIB	ERAP2
SPIB	ERBIN
SPIB	ERCC1
SPIB	ERCC3
SPIB	ERGIC3
SPIB	ERH

SPIB	ERICH1
SPIB	ERP44
SPIB	ESCO1
SPIB	ESD
SPIB	ETF1
SPIB	ETFB
SPIB	ETS1
SPIB	EVI2B
SPIB	EVL
SPIB	EXOC2
SPIB	EXOC3
SPIB	EXOC5
SPIB	EXOC7
SPIB	EXOSC3
SPIB	EXOSC4
SPIB	EXOSC5
SPIB	EXOSC6
SPIB	EZR
SPIB	FAAP20
SPIB	FAAP24
SPIB	FABP5
SPIB	FADS3
SPIB	FAM102A
SPIB	FAM104A
SPIB	FAM104B
SPIB	FAM110A
SPIB	FAM120A
SPIB	FAM120AOS
SPIB	FAM122A
SPIB	FAM122B
SPIB	FAM122C
SPIB	FAM126A
SPIB	FAM129C
SPIB	FAM168B
SPIB	FAM173A
SPIB	FAM177A1
SPIB	FAM192A
SPIB	FAM199X
SPIB	FAM200B
SPIB	FAM204A
SPIB	FAM206A
SPIB	FAM207A
SPIB	FAM208A
SPIB	FAM208B
SPIB	FAM20B
SPIB	FAM228B
SPIB	FAM234A
SPIB	FAM32A
SPIB	FAM43A
SPIB	FAM49B

SPIB	FAM72B
SPIB	FAM81A
SPIB	FAM89A
SPIB	FAM96A
SPIB	FAM96B
SPIB	FAM98A
SPIB	FAM98C
SPIB	FANCB
SPIB	FANCC
SPIB	FANCD2
SPIB	FANCF
SPIB	FANCM
SPIB	FARS2
SPIB	FARSA
SPIB	FAS
SPIB	FASN
SPIB	FASTKD1
SPIB	FAU
SPIB	FBL
SPIB	FBRS
SPIB	FBRSL1
SPIB	FBXL12
SPIB	FBXL20
SPIB	FBXL3
SPIB	FBXO11
SPIB	FBXO22
SPIB	FBXO28
SPIB	FBXO34
SPIB	FBXO42
SPIB	FBXO8
SPIB	FBXW2
SPIB	FBXW5
SPIB	FCF1
SPIB	FCHO1
SPIB	FCHSD2
SPIB	FCRL1
SPIB	FCRL2
SPIB	FCRL3
SPIB	FCRL5
SPIB	FCRLA
SPIB	FDFT1
SPIB	FDPS
SPIB	FDXR
SPIB	FEN1
SPIB	FERMT3
SPIB	FES
SPIB	FEZ2
SPIB	FGD3
SPIB	FGD6
SPIB	FH

SPIB	FHIT
SPIB	FHL3
SPIB	FIBP
SPIB	FIG4
SPIB	FILIP1L
SPIB	FIP1L1
SPIB	FIS1
SPIB	FJX1
SPIB	FKBP1A
SPIB	FKBP1B
SPIB	FKBP5
SPIB	FKBP8
SPIB	FKRP
SPIB	FLAD1
SPIB	FLI1
SPIB	FLNA
SPIB	FLOT1
SPIB	FLOT2
SPIB	FMNL1
SPIB	FMNL3
SPIB	FMO4
SPIB	FNBP1
SPIB	FNBP4
SPIB	FNDC3B
SPIB	FNTA
SPIB	FOPNL
SPIB	FOXO1
SPIB	FOXO3
SPIB	FOXP1
SPIB	FOXP4
SPIB	FPGS
SPIB	FRA10AC1
SPIB	FRG1
SPIB	FRMD8
SPIB	FRS2
SPIB	FTH1
SPIB	FTSJ3
SPIB	FUBP1
SPIB	FUBP3
SPIB	FUCA1
SPIB	FUNDC1
SPIB	FUS
SPIB	FUT7
SPIB	FYN
SPIB	G3BP1
SPIB	G3BP2
SPIB	GAB3
SPIB	GABARAPL2
SPIB	GABPB1
SPIB	GADD45B

SPIB	GALE
SPIB	GALNT14
SPIB	GALNT7
SPIB	GAPDH
SPIB	GAPVD1
SPIB	GAR1
SPIB	GARS
SPIB	GART
SPIB	GAS8
SPIB	GATAD1
SPIB	GATAD2A
SPIB	GCDH
SPIB	GCNT2
SPIB	GDAP1
SPIB	GDI2
SPIB	GEMIN4
SPIB	GEMIN5
SPIB	GEMIN6
SPIB	GEMIN7
SPIB	GEMIN8
SPIB	GEN1
SPIB	GFI1
SPIB	GFM1
SPIB	GFOD1
SPIB	GFOD2
SPIB	GGA1
SPIB	GGNBP2
SPIB	GHITM
SPIB	GID4
SPIB	GIN1
SPIB	GINS2
SPIB	GIT2
SPIB	GLB1
SPIB	GLCE
SPIB	GLRX5
SPIB	GLTP
SPIB	GLUL
SPIB	GMDS
SPIB	GMEB1
SPIB	GMFG
SPIB	GMIP
SPIB	GMNN
SPIB	GMPR2
SPIB	GNA13
SPIB	GNAI2
SPIB	GNAI3
SPIB	GNAS
SPIB	GNB2
SPIB	GNB4
SPIB	GNG11

SPIB	GNG2
SPIB	GNL1
SPIB	GNL2
SPIB	GNPAT
SPIB	GOLGA7
SPIB	GOLGA8A
SPIB	GOLGA8B
SPIB	GOLPH3
SPIB	GON4L
SPIB	GOPC
SPIB	GORASP2
SPIB	GOSR1
SPIB	GOSR2
SPIB	GPALPP1
SPIB	GPATCH2
SPIB	GPATCH2L
SPIB	GPATCH8
SPIB	GPBP1
SPIB	GPM6B
SPIB	GPN1
SPIB	GPN2
SPIB	GPR108
SPIB	GPR137
SPIB	GPR155
SPIB	GPR18
SPIB	GPR34
SPIB	GPR82
SPIB	GPS1
SPIB	GPSM3
SPIB	GRAMD1A
SPIB	GRAMD4
SPIB	GRAP
SPIB	GRB2
SPIB	GRK2
SPIB	GRK6
SPIB	GRN
SPIB	GRPEL1
SPIB	GRPEL2
SPIB	GRWD1
SPIB	GSDMD
SPIB	GSE1
SPIB	GSK3B
SPIB	GSKIP
SPIB	GSPT1
SPIB	GSPT2
SPIB	GTDC1
SPIB	GTF2A2
SPIB	GTF2E2
SPIB	GTF2F1
SPIB	GTF2F2

SPIB	GTF2H1
SPIB	GTF2H2
SPIB	GTF2H3
SPIB	GTF3A
SPIB	GTF3C1
SPIB	GTF3C2
SPIB	GTF3C5
SPIB	GTPBP2
SPIB	GTPBP4
SPIB	GTPBP8
SPIB	GTSF1
SPIB	GUCD1
SPIB	GYG1
SPIB	GYPC
SPIB	H2AFY
SPIB	HAGHL
SPIB	HARS
SPIB	HAT1
SPIB	HCCS
SPIB	HCFC1
SPIB	HCG11
SPIB	HCK
SPIB	HCLS1
SPIB	HCST
SPIB	HDAC7
SPIB	HDDC2
SPIB	HDHD2
SPIB	HEATR1
SPIB	HECA
SPIB	HECTD1
SPIB	HECTD2
SPIB	HERC2
SPIB	HERC4
SPIB	HERPUD1
SPIB	HES1
SPIB	HEXIM2
SPIB	HGH1
SPIB	HHEX
SPIB	HIF1AN
SPIB	HIGD1A
SPIB	HIGD2A
SPIB	HINT1
SPIB	HK1
SPIB	HLA-DMB
SPIB	HLA-DRA
SPIB	HLA-DRB1
SPIB	HMBOX1
SPIB	HMBS
SPIB	HMG20A
SPIB	HMGA1

SPIB	HMGB1
SPIB	HMGB2
SPIB	HMGN1
SPIB	HMGN2
SPIB	HMGXB3
SPIB	HMHB1
SPIB	HNRNPA0
SPIB	HNRNPA2B1
SPIB	HNRNPAB
SPIB	HNRNPC
SPIB	HNRNPD
SPIB	HNRNPF
SPIB	HNRNPH2
SPIB	HNRNPH3
SPIB	HNRNPK
SPIB	HNRNPL
SPIB	HNRNPR
SPIB	HNRNPU
SPIB	HNRNPUL1
SPIB	HOPX
SPIB	HP1BP3
SPIB	HPCAL1
SPIB	HPF1
SPIB	HPS1
SPIB	HPS3
SPIB	HPS4
SPIB	HPS5
SPIB	HSBP1
SPIB	HSCB
SPIB	HSD17B10
SPIB	HSF1
SPIB	HSP90AA1
SPIB	HSP90AB1
SPIB	HSPA4
SPIB	HSPA8
SPIB	HSPBP1
SPIB	HSPD1
SPIB	HSPE1
SPIB	HSPH1
SPIB	HTATSF1
SPIB	HTT
SPIB	HUWE1
SPIB	HVCN1
SPIB	ICAM1
SPIB	ICAM3
SPIB	ICE2
SPIB	ICMT
SPIB	ID2
SPIB	ID3
SPIB	IDE

SPIB	IDH3A
SPIB	IDH3B
SPIB	IDI1
SPIB	IER2
SPIB	IER5
SPIB	IFFO1
SPIB	IFI30
SPIB	IFNGR2
SPIB	IFRD1
SPIB	IFT20
SPIB	IFT22
SPIB	IFT52
SPIB	IGBP1
SPIB	IGF2BP3
SPIB	IGF2R
SPIB	IKBKB
SPIB	IKBKE
SPIB	IKBKG
SPIB	IKZF1
SPIB	IKZF3
SPIB	IL16
SPIB	IL21R
SPIB	IL2RA
SPIB	IL4I1
SPIB	IL4R
SPIB	IL7
SPIB	ILF2
SPIB	ILF3
SPIB	ILK
SPIB	IMP3
SPIB	IMP4
SPIB	IMPA1
SPIB	IMPACT
SPIB	IMPAD1
SPIB	IMPDH1
SPIB	INAFM1
SPIB	INF2
SPIB	ING1
SPIB	ING2
SPIB	ING4
SPIB	INIP
SPIB	INO80C
SPIB	INO80E
SPIB	INPP1
SPIB	INPP5B
SPIB	INPP5D
SPIB	INTS1
SPIB	INTS2
SPIB	INTS3
SPIB	INTS4

SPIB	INTS6
SPIB	INTS9
SPIB	IP6K2
SPIB	IPO4
SPIB	IPO7
SPIB	IPO8
SPIB	IQGAP1
SPIB	IQSEC1
SPIB	IRF2BP2
SPIB	IRF4
SPIB	IRF5
SPIB	IRF8
SPIB	ISCA2
SPIB	ISG20
SPIB	ISG20L2
SPIB	ISL2
SPIB	IST1
SPIB	ISY1
SPIB	ISYNA1
SPIB	ITFG1
SPIB	ITFG2
SPIB	ITGAL
SPIB	ITGB1
SPIB	ITGB1BP1
SPIB	ITGB2
SPIB	ITGB3BP
SPIB	ITGB7
SPIB	ITPA
SPIB	ITPKB
SPIB	ITPR1
SPIB	ITPR3
SPIB	ITSN2
SPIB	IVD
SPIB	IVNS1ABP
SPIB	IZUMO4
SPIB	JADE2
SPIB	JAK1
SPIB	JAK2
SPIB	JAML
SPIB	JDP2
SPIB	JKAMP
SPIB	JMJD1C
SPIB	JMJD8
SPIB	JTB
SPIB	KANSL1
SPIB	KANSL3
SPIB	KARS
SPIB	KAT2A
SPIB	KAT5
SPIB	KAT6A

SPIB	KAT6B
SPIB	KAT8
SPIB	KATNA1
SPIB	KBTBD4
SPIB	KBTBD8
SPIB	KCMF1
SPIB	KCNAB2
SPIB	KCNN4
SPIB	KCTD13
SPIB	KCTD17
SPIB	KCTD18
SPIB	KCTD20
SPIB	KCTD6
SPIB	KDM1B
SPIB	KDM2B
SPIB	KDM3A
SPIB	KDM5A
SPIB	KDM5B
SPIB	KDM6A
SPIB	KDM6B
SPIB	KEAP1
SPIB	KHDRBS1
SPIB	KIAA0040
SPIB	KIAA0232
SPIB	KIAA1217
SPIB	KIAA2013
SPIB	KIDINS220
SPIB	KIF16B
SPIB	KIF1C
SPIB	KIF20B
SPIB	KIF2A
SPIB	KIF3B
SPIB	KIF5B
SPIB	KIN
SPIB	KLC1
SPIB	KLF10
SPIB	KLF8
SPIB	KLHDC4
SPIB	KLHL14
SPIB	KLHL18
SPIB	KLHL42
SPIB	KLHL5
SPIB	KLHL6
SPIB	KLRG1
SPIB	KMO
SPIB	KMT2B
SPIB	KMT2C
SPIB	KMT2E
SPIB	KNOP1
SPIB	KPNB1

SPIB	KRAS
SPIB	KRBOX4
SPIB	KRCC1
SPIB	KRI1
SPIB	KRIT1
SPIB	KRR1
SPIB	KYAT1
SPIB	LAMTOR1
SPIB	LAMTOR3
SPIB	LAMTOR4
SPIB	LAPTM5
SPIB	LARP4
SPIB	LARP4B
SPIB	LARP7
SPIB	LARS
SPIB	LAS1L
SPIB	LASP1
SPIB	LCK
SPIB	LCMT1
SPIB	LCMT2
SPIB	LCOR
SPIB	LCP1
SPIB	LDAH
SPIB	LDHA
SPIB	LEKR1
SPIB	LENG1
SPIB	LENG8
SPIB	LEPROTL1
SPIB	LETMD1
SPIB	LGALS9
SPIB	LHFPL2
SPIB	LHPP
SPIB	LILRB1
SPIB	LILRB3
SPIB	LIMD2
SPIB	LIMK1
SPIB	LIMS1
SPIB	LINC00467
SPIB	LINC00667
SPIB	LINC00954
SPIB	LINC01136
SPIB	LINC01184
SPIB	LINC01215
SPIB	LIPE−AS1
SPIB	LITAF
SPIB	LLPH
SPIB	LMAN2
SPIB	LMCD1
SPIB	LMNB1
SPIB	LMNB2

SPIB	LMO2
SPIB	LNPEP
SPIB	LONP1
SPIB	LONRF1
SPIB	LOXL2
SPIB	LPAR5
SPIB	LPGAT1
SPIB	LPP
SPIB	LPP-AS2
SPIB	LPXN
SPIB	LRCH1
SPIB	LRCH4
SPIB	LRIF1
SPIB	LRMP
SPIB	LRP10
SPIB	LRPPRC
SPIB	LRRC32
SPIB	LRRC45
SPIB	LRRC47
SPIB	LRRC58
SPIB	LRRC59
SPIB	LRRC61
SPIB	LRRC8D
SPIB	LRRFIP1
SPIB	LRRFIP2
SPIB	LRRK2
SPIB	LRWD1
SPIB	LSAMP
SPIB	LSG1
SPIB	LSM1
SPIB	LSM12
SPIB	LSM2
SPIB	LSM3
SPIB	LSM5
SPIB	LSM8
SPIB	LSP1
SPIB	LTA
SPIB	LTA4H
SPIB	LTB
SPIB	LTB4R
SPIB	LTV1
SPIB	LUC7L
SPIB	LUC7L2
SPIB	LXN
SPIB	LY6E
SPIB	LYL1
SPIB	LYN
SPIB	LYPLA2
SPIB	LYPLAL1
SPIB	LYRM1

SPIB	LYRM2
SPIB	LYRM7
SPIB	LYSMD2
SPIB	LZIC
SPIB	M6PR
SPIB	MACROD1
SPIB	MAD1L1
SPIB	MAD2L1
SPIB	MADD
SPIB	MAEA
SPIB	MAFF
SPIB	MAFK
SPIB	MAGED1
SPIB	MAGED2
SPIB	MAGEF1
SPIB	MAGOH
SPIB	MAK16
SPIB	MALSU1
SPIB	MALT1
SPIB	MANBAL
SPIB	MAP1S
SPIB	MAP2K3
SPIB	MAP2K6
SPIB	MAP3K1
SPIB	MAP3K11
SPIB	MAP3K13
SPIB	MAP3K7
SPIB	MAP3K7CL
SPIB	MAP3K8
SPIB	MAP4
SPIB	MAP4K1
SPIB	MAP4K2
SPIB	MAP4K4
SPIB	MAP7D1
SPIB	MAP7D3
SPIB	MAPK1IP1L
SPIB	MAPK3
SPIB	MAPK9
SPIB	MAPKAP1
SPIB	MAPKAPK2
SPIB	MAPKAPK3
SPIB	MAPRE1
SPIB	MAPRE2
SPIB	MARCH1
SPIB	MARCKSL1
SPIB	MARK2
SPIB	MARK3
SPIB	MARS
SPIB	MAST2
SPIB	MAST3

SPIB	MAST4
SPIB	MASTL
SPIB	MAT2A
SPIB	MAT2B
SPIB	MAX
SPIB	MBD2
SPIB	MBD5
SPIB	MBNL1
SPIB	MBOAT2
SPIB	MBP
SPIB	MBTD1
SPIB	MCAT
SPIB	MCCC1
SPIB	MCL1
SPIB	MCM3
SPIB	MCM3AP
SPIB	MCM5
SPIB	MCM7
SPIB	MCM8
SPIB	MCRIP1
SPIB	MCRIP2
SPIB	MCRS1
SPIB	MCTS1
SPIB	MDH1
SPIB	MDM4
SPIB	MDN1
SPIB	ME2
SPIB	MEA1
SPIB	MECR
SPIB	MED10
SPIB	MED11
SPIB	MED12L
SPIB	MED15
SPIB	MED17
SPIB	MED19
SPIB	MED23
SPIB	MED25
SPIB	MED26
SPIB	MED27
SPIB	MED30
SPIB	MED4
SPIB	MED6
SPIB	MED7
SPIB	MEF2A
SPIB	MEF2D
SPIB	MEIG1
SPIB	MELK
SPIB	MEPCE
SPIB	MEST
SPIB	METAP2

SPIB	METRNL
SPIB	METTL14
SPIB	METTL17
SPIB	METTL18
SPIB	METTL21A
SPIB	METTL23
SPIB	METTL2A
SPIB	METTL2B
SPIB	METTL3
SPIB	METTL5
SPIB	METTL6
SPIB	METTL9
SPIB	MEX3C
SPIB	MFAP1
SPIB	MFF
SPIB	MFHAS1
SPIB	MFN1
SPIB	MFNG
SPIB	MFSD1
SPIB	MFSD10
SPIB	MFSD5
SPIB	MFSD9
SPIB	MGA
SPIB	MGAT3
SPIB	MGAT5
SPIB	MGMT
SPIB	MIB2
SPIB	MICAL3
SPIB	MICALL1
SPIB	MICU2
SPIB	MID1IP1
SPIB	MIDN
SPIB	MIEF1
SPIB	MIEN1
SPIB	MIER1
SPIB	MIER2
SPIB	MIER3
SPIB	MILR1
SPIB	MINOS1
SPIB	MIOS
SPIB	MIR3681HG
SPIB	MIS18BP1
SPIB	MITD1
SPIB	MKLN1
SPIB	MKNK1
SPIB	MKRN1
SPIB	MKRN2
SPIB	MLEC
SPIB	MLF2
SPIB	MLLT10

SPIB	MLXIP
SPIB	MLYCD
SPIB	MMADHC
SPIB	MMD
SPIB	MME
SPIB	MMS19
SPIB	MNT
SPIB	MOB1A
SPIB	MOB1B
SPIB	MOB3A
SPIB	MOB4
SPIB	MORC2
SPIB	MORC2-AS1
SPIB	MORF4L1
SPIB	MORN2
SPIB	MPC2
SPIB	MPDU1
SPIB	MPEG1
SPIB	MPG
SPIB	MPI
SPIB	MPLKIP
SPIB	MPV17
SPIB	MPZL1
SPIB	MRM2
SPIB	MROH1
SPIB	MRPL10
SPIB	MRPL12
SPIB	MRPL13
SPIB	MRPL14
SPIB	MRPL16
SPIB	MRPL17
SPIB	MRPL18
SPIB	MRPL20
SPIB	MRPL21
SPIB	MRPL22
SPIB	MRPL23
SPIB	MRPL24
SPIB	MRPL27
SPIB	MRPL28
SPIB	MRPL3
SPIB	MRPL33
SPIB	MRPL40
SPIB	MRPL43
SPIB	MRPL44
SPIB	MRPL47
SPIB	MRPL48
SPIB	MRPL49
SPIB	MRPL51
SPIB	MRPL52
SPIB	MRPL54

SPIB	MRPS10
SPIB	MRPS16
SPIB	MRPS18A
SPIB	MRPS18B
SPIB	MRPS18C
SPIB	MRPS21
SPIB	MRPS22
SPIB	MRPS23
SPIB	MRPS31
SPIB	MRPS33
SPIB	MRPS34
SPIB	MRRF
SPIB	MS4A1
SPIB	MSANTD3
SPIB	MSANTD4
SPIB	MSH2
SPIB	MSH3
SPIB	MSH6
SPIB	MSI2
SPIB	MSL3
SPIB	MSN
SPIB	MSRB1
SPIB	MTA3
SPIB	MTCH2
SPIB	MTERF3
SPIB	MTERF4
SPIB	MTF2
SPIB	MTFMT
SPIB	MTFR1L
SPIB	MTHFD1L
SPIB	MTIF2
SPIB	MTIF3
SPIB	MTMR12
SPIB	MTMR14
SPIB	MTMR2
SPIB	MTMR9
SPIB	MTPN
SPIB	MTR
SPIB	MTRF1
SPIB	MTURN
SPIB	MTX2
SPIB	MUL1
SPIB	MUS81
SPIB	MUTYH
SPIB	MVB12A
SPIB	MYC
SPIB	MYCBP2
SPIB	MYD88
SPIB	MYH10
SPIB	MYH9

SPIB	MYL12A
SPIB	MYL12B
SPIB	MYL5
SPIB	MYL6
SPIB	MYL6B
SPIB	MYNN
SPIB	MYO1C
SPIB	MYO1E
SPIB	MYO1F
SPIB	MYO1G
SPIB	MYO5A
SPIB	MYO9B
SPIB	N4BP3
SPIB	NAA10
SPIB	NAA20
SPIB	NAA30
SPIB	NAA35
SPIB	NAA40
SPIB	NAA50
SPIB	NAA60
SPIB	NAB2
SPIB	NABP2
SPIB	NACA
SPIB	NADK
SPIB	NADSYN1
SPIB	NAGK
SPIB	NAP1L1
SPIB	NAP1L4
SPIB	NAPA
SPIB	NAPEPLD
SPIB	NARF
SPIB	NARFL
SPIB	NASP
SPIB	NAT10
SPIB	NAT9
SPIB	NBEA
SPIB	NBEAL1
SPIB	NBPF20
SPIB	NCBP1
SPIB	NCBP2
SPIB	NCF1
SPIB	NCF2
SPIB	NCF4
SPIB	NCKAP1L
SPIB	NCL
SPIB	NCOA3
SPIB	NCOA5
SPIB	NDE1
SPIB	NDN
SPIB	NDUFA1

SPIB	NDUFA13
SPIB	NDUFA4
SPIB	NDUFA5
SPIB	NDUFAF1
SPIB	NDUFAF3
SPIB	NDUFAF6
SPIB	NDUFB2
SPIB	NDUFB3
SPIB	NDUFB4
SPIB	NDUFB5
SPIB	NDUFB7
SPIB	NDUFB9
SPIB	NDUFS1
SPIB	NDUFS2
SPIB	NDUFS3
SPIB	NDUFS4
SPIB	NDUFS7
SPIB	NDUFV1
SPIB	NEAT1
SPIB	NECAP1
SPIB	NECAP2
SPIB	NEDD8
SPIB	NEIL1
SPIB	NEK6
SPIB	NEK8
SPIB	NELFA
SPIB	NELFB
SPIB	NELFCD
SPIB	NELFE
SPIB	NFAT5
SPIB	NFATC1
SPIB	NFATC2
SPIB	NFATC3
SPIB	NFE2L2
SPIB	NFKB1
SPIB	NFKB2
SPIB	NFKBIB
SPIB	NFKBID
SPIB	NFKBIE
SPIB	NFKBIL1
SPIB	NFKBIZ
SPIB	NFS1
SPIB	NFX1
SPIB	NFYC
SPIB	NGDN
SPIB	NIF3L1
SPIB	NIFK
SPIB	NINJ1
SPIB	NIP7
SPIB	NIPAL3

SPIB	NIPBL
SPIB	NIT1
SPIB	NKAP
SPIB	NKIRAS2
SPIB	NLRC3
SPIB	NLRP2
SPIB	NMD3
SPIB	NME1
SPIB	NME7
SPIB	NMI
SPIB	NMRAL1
SPIB	NMU
SPIB	NNT
SPIB	NNT-AS1
SPIB	NOB1
SPIB	NOC2L
SPIB	NOC3L
SPIB	NOL10
SPIB	NOL11
SPIB	NOL12
SPIB	NOL7
SPIB	NOL8
SPIB	NOL9
SPIB	NOM1
SPIB	NONO
SPIB	NOP10
SPIB	NOP2
SPIB	NOP58
SPIB	NOSIP
SPIB	NPAT
SPIB	NPEPPS
SPIB	NPLOC4
SPIB	NR1H2
SPIB	NR2C2AP
SPIB	NR3C1
SPIB	NR4A1
SPIB	NR4A3
SPIB	NRAS
SPIB	NRBF2
SPIB	NRDC
SPIB	NREP
SPIB	NSA2
SPIB	NSF
SPIB	NSMCE1
SPIB	NSMCE4A
SPIB	NSUN5
SPIB	NSUN6
SPIB	NT5C
SPIB	NT5C2
SPIB	NTHL1

SPIB	NTMT1
SPIB	NTPCR
SPIB	NUB1
SPIB	NUBP1
SPIB	NUBP2
SPIB	NUCKS1
SPIB	NUDC
SPIB	NUDCD2
SPIB	NUDT1
SPIB	NUDT16
SPIB	NUDT17
SPIB	NUDT21
SPIB	NUDT3
SPIB	NUDT5
SPIB	NUFIP1
SPIB	NUFIP2
SPIB	NUMA1
SPIB	NUMB
SPIB	NUP107
SPIB	NUP153
SPIB	NUP155
SPIB	NUP160
SPIB	NUP205
SPIB	NUP214
SPIB	NUP35
SPIB	NUP37
SPIB	NUP54
SPIB	NUP58
SPIB	NUP93
SPIB	NUTF2
SPIB	NXPE3
SPIB	NXT1
SPIB	NXT2
SPIB	OARD1
SPIB	OAS1
SPIB	OAZ2
SPIB	OCIAD1
SPIB	ODC1
SPIB	ODF2
SPIB	OFD1
SPIB	OGFR
SPIB	OGG1
SPIB	OGT
SPIB	OLA1
SPIB	OMA1
SPIB	OPA1
SPIB	OPA3
SPIB	ORAI1
SPIB	ORAI2
SPIB	ORAI3

SPIB	ORC2
SPIB	ORMDL1
SPIB	ORMDL2
SPIB	OSBP
SPIB	OSBPL10
SPIB	OSBPL11
SPIB	OSBPL2
SPIB	OSBPL8
SPIB	OSBPL9
SPIB	OSER1
SPIB	OST4
SPIB	OSTC
SPIB	OSTF1
SPIB	OSTM1
SPIB	OTUB1
SPIB	OTUD4
SPIB	OTUD5
SPIB	OTULIN
SPIB	OXCT1
SPIB	OXLD1
SPIB	OXSR1
SPIB	P2RX5
SPIB	P2RX5-TAX1BP3
SPIB	P4HA1
SPIB	P4HB
SPIB	PAAF1
SPIB	PABPC1
SPIB	PABPN1
SPIB	PACSIN2
SPIB	PACSIN3
SPIB	PAFAH1B1
SPIB	PAFAH1B3
SPIB	PAG1
SPIB	PAICS
SPIB	PAIP2
SPIB	PAK1
SPIB	PAK1IP1
SPIB	PAK2
SPIB	PANK2
SPIB	PANK3
SPIB	PAPOLA
SPIB	PAQR8
SPIB	PARG
SPIB	PARL
SPIB	PARN
SPIB	PARP10
SPIB	PARP11
SPIB	PARP12
SPIB	PARP16
SPIB	PARVG

SPIB	PASK
SPIB	PATL1
SPIB	PATZ1
SPIB	PAWR
SPIB	PAX5
SPIB	PBXIP1
SPIB	PCBP1
SPIB	PCBP2
SPIB	PCED1B
SPIB	PCF11
SPIB	PCGF5
SPIB	PCM1
SPIB	PCNP
SPIB	PCNX4
SPIB	PDAP1
SPIB	PDCD1
SPIB	PDCD10
SPIB	PDCD2
SPIB	PDCD6
SPIB	PDCD6IP
SPIB	PDCD7
SPIB	PDCL3
SPIB	PDE12
SPIB	PDGFD
SPIB	PDHA1
SPIB	PDIA3
SPIB	PDLIM1
SPIB	PDLIM2
SPIB	PDLIM7
SPIB	PDPK1
SPIB	PDRG1
SPIB	PDS5A
SPIB	PDSS1
SPIB	PDXDC1
SPIB	PDZD11
SPIB	PEA15
SPIB	PEF1
SPIB	PER2
SPIB	PES1
SPIB	PET100
SPIB	PEX16
SPIB	PEX2
SPIB	PEX26
SPIB	PEX5
SPIB	PEX6
SPIB	PFDN5
SPIB	PFDN6
SPIB	PFKP
SPIB	PFN1
SPIB	PFN2

SPIB	PGAM1
SPIB	PGAM5
SPIB	PGGT1B
SPIB	PGK1
SPIB	PGLS
SPIB	PGM1
SPIB	PGM2
SPIB	PGP
SPIB	PGS1
SPIB	PHAX
SPIB	PHB2
SPIB	PHF11
SPIB	PHF14
SPIB	PHF20
SPIB	PHF23
SPIB	PHF3
SPIB	PHF5A
SPIB	PHF6
SPIB	PHF8
SPIB	PHLDB3
SPIB	PHOSPHO2
SPIB	PHRF1
SPIB	PHTF1
SPIB	PHTF2
SPIB	PI4KB
SPIB	PIAS4
SPIB	PIBF1
SPIB	PICALM
SPIB	PICK1
SPIB	PIGBOS1
SPIB	PIGC
SPIB	PIGH
SPIB	PIGQ
SPIB	PIGU
SPIB	PIGV
SPIB	PIH1D1
SPIB	PIK3AP1
SPIB	PIK3C2A
SPIB	PIK3CA
SPIB	PIK3CD
SPIB	PIK3R5
SPIB	PIKFYVE
SPIB	PIM1
SPIB	PIM2
SPIB	PIM3
SPIB	PIN1
SPIB	PIP5K1A
SPIB	PIP5K1C
SPIB	PISD
SPIB	PITPNA

SPIB	PITPNA-AS1
SPIB	PITPNC1
SPIB	PITPNM1
SPIB	PKIB
SPIB	PKIG
SPIB	PKM
SPIB	PKN1
SPIB	PLA2G15
SPIB	PLAA
SPIB	PLAGL1
SPIB	PLCL2
SPIB	PLEK
SPIB	PLEKHA2
SPIB	PLEKHA3
SPIB	PLEKHG1
SPIB	PLEKHG2
SPIB	PLEKHJ1
SPIB	PLEKHO2
SPIB	PLRG1
SPIB	PLSCR1
SPIB	PMAIP1
SPIB	PMPCA
SPIB	PMPCB
SPIB	PMS1
SPIB	PMS2
SPIB	PMVK
SPIB	PNISR
SPIB	PNKP
SPIB	PNN
SPIB	PNP
SPIB	PNPLA2
SPIB	PNPLA4
SPIB	PNPLA6
SPIB	PNPO
SPIB	PNRC2
SPIB	POC1A
SPIB	POGK
SPIB	POGZ
SPIB	POLA2
SPIB	POLB
SPIB	POLD3
SPIB	POLD4
SPIB	POLDIP2
SPIB	POLDIP3
SPIB	POLE4
SPIB	POLG2
SPIB	POLM
SPIB	POLR1E
SPIB	POLR2A
SPIB	POLR2C

SPIB	POLR2D
SPIB	POLR2F
SPIB	POLR2G
SPIB	POLR2H
SPIB	POLR2J
SPIB	POLR2K
SPIB	POLR3C
SPIB	POLR3E
SPIB	POLRMT
SPIB	POMC
SPIB	POMP
SPIB	POP4
SPIB	POP5
SPIB	POP7
SPIB	POU2AF1
SPIB	POU2F1
SPIB	POU2F2
SPIB	PPAN
SPIB	PPHLN1
SPIB	PPID
SPIB	PPIF
SPIB	PPIG
SPIB	PPIH
SPIB	PPIL1
SPIB	PPIL2
SPIB	PPM1A
SPIB	PPM1B
SPIB	PPM1G
SPIB	PPM1M
SPIB	PPM1N
SPIB	PPME1
SPIB	PPP1CA
SPIB	PPP1CC
SPIB	PPP1R11
SPIB	PPP1R12A
SPIB	PPP1R14B
SPIB	PPP1R16A
SPIB	PPP1R16B
SPIB	PPP1R18
SPIB	PPP1R35
SPIB	PPP1R9B
SPIB	PPP2R1A
SPIB	PPP2R2A
SPIB	PPP2R5A
SPIB	PPP2R5C
SPIB	PPP2R5E
SPIB	PPP3CC
SPIB	PPP4C
SPIB	PPP4R3A
SPIB	PPP4R3B

SPIB	PPP6R1
SPIB	PPTC7
SPIB	PQLC1
SPIB	PRCC
SPIB	PRDM10
SPIB	PRDM2
SPIB	PRELID1
SPIB	PRELID3B
SPIB	PREP
SPIB	PRKAA1
SPIB	PRKACA
SPIB	PRKACB
SPIB	PRKAG1
SPIB	PRKAR1A
SPIB	PRKAR1B
SPIB	PRKAR2A
SPIB	PRKCB
SPIB	PRKCD
SPIB	PRKCE
SPIB	PRKD2
SPIB	PRKDC
SPIB	PRMT1
SPIB	PRMT2
SPIB	PRMT3
SPIB	PRMT6
SPIB	PRMT9
SPIB	PROSER3
SPIB	PRPF18
SPIB	PRPF19
SPIB	PRPF3
SPIB	PRPF31
SPIB	PRPF38A
SPIB	PRPF38B
SPIB	PRPF4
SPIB	PRPF40A
SPIB	PRPF40B
SPIB	PRPF4B
SPIB	PRPF6
SPIB	PRPF8
SPIB	PRR13
SPIB	PRR14
SPIB	PRR7
SPIB	PRRC2B
SPIB	PRRC2C
SPIB	PSEN1
SPIB	PSIP1
SPIB	PSMA1
SPIB	PSMA3
SPIB	PSMA3-AS1
SPIB	PSMA4

SPIB	PSMA5
SPIB	PSMA6
SPIB	PSMA8
SPIB	PSMB1
SPIB	PSMB3
SPIB	PSMB4
SPIB	PSMB5
SPIB	PSMB7
SPIB	PSMB8
SPIB	PSMC1
SPIB	PSMC2
SPIB	PSMC3
SPIB	PSMC5
SPIB	PSMC6
SPIB	PSMD11
SPIB	PSMD12
SPIB	PSMD13
SPIB	PSMD14
SPIB	PSMD2
SPIB	PSMD3
SPIB	PSMD4
SPIB	PSMD6
SPIB	PSMD7
SPIB	PSMD8
SPIB	PSME1
SPIB	PSME3
SPIB	PSME4
SPIB	PSMF1
SPIB	PSMG3
SPIB	PSMG4
SPIB	PTAR1
SPIB	PTBP1
SPIB	PTBP3
SPIB	PTDSS2
SPIB	PTGES2
SPIB	PTGES3
SPIB	PTGR2
SPIB	PTK2
SPIB	PTK2B
SPIB	PTMA
SPIB	PTP4A2
SPIB	PTP4A3
SPIB	PTPN12
SPIB	PTPN2
SPIB	PTPN6
SPIB	PTPN7
SPIB	PTPN9
SPIB	PTPRC
SPIB	PTPRE
SPIB	PTPRJ

SPIB	PTRH1
SPIB	PTRH2
SPIB	PTRHD1
SPIB	PTTG1
SPIB	PUDP
SPIB	PUF60
SPIB	PUM1
SPIB	PUM2
SPIB	PURB
SPIB	PUS1
SPIB	PUS3
SPIB	PUS7L
SPIB	PVT1
SPIB	PWP1
SPIB	PXN
SPIB	PYCARD
SPIB	PYHIN1
SPIB	PYM1
SPIB	PYROXD1
SPIB	QARS
SPIB	QKI
SPIB	QPCT
SPIB	QRICH2
SPIB	QSER1
SPIB	R3HCC1L
SPIB	R3HDM4
SPIB	RAB11A
SPIB	RAB11B
SPIB	RAB11FIP1
SPIB	RAB11FIP2
SPIB	RAB14
SPIB	RAB18
SPIB	RAB1B
SPIB	RAB21
SPIB	RAB28
SPIB	RAB2A
SPIB	RAB30
SPIB	RAB35
SPIB	RAB3GAP2
SPIB	RAB40C
SPIB	RAB4B
SPIB	RAB5A
SPIB	RAB5B
SPIB	RAB5C
SPIB	RAB6A
SPIB	RAB7A
SPIB	RAB8A
SPIB	RAB8B
SPIB	RABEP1
SPIB	RABEP2

SPIB	RABEPK
SPIB	RABGAP1
SPIB	RABGAP1L
SPIB	RABGGTB
SPIB	RABL2A
SPIB	RABL2B
SPIB	RAC1
SPIB	RAC2
SPIB	RACK1
SPIB	RAD1
SPIB	RAD17
SPIB	RAD18
SPIB	RAD23A
SPIB	RAD51AP1
SPIB	RAD51C
SPIB	RAE1
SPIB	RALA
SPIB	RALGAPB
SPIB	RALY
SPIB	RAN
SPIB	RANBP1
SPIB	RANBP9
SPIB	RAP1A
SPIB	RAP1B
SPIB	RAP2B
SPIB	RAP2C
SPIB	RAPGEF1
SPIB	RARA
SPIB	RARS
SPIB	RARS2
SPIB	RASA1
SPIB	RASA4
SPIB	RASAL1
SPIB	RASAL3
SPIB	RASGRF1
SPIB	RASGRP2
SPIB	RASGRP3
SPIB	RASSF1
SPIB	RASSF2
SPIB	RASSF3
SPIB	RASSF6
SPIB	RASSF7
SPIB	RAVER1
SPIB	RB1
SPIB	RB1CC1
SPIB	RBBP4
SPIB	RBBP6
SPIB	RBBP7
SPIB	RBBP8
SPIB	RBCK1

SPIB	RBL2
SPIB	RBM14
SPIB	RBM17
SPIB	RBM19
SPIB	RBM22
SPIB	RBM23
SPIB	RBM25
SPIB	RBM27
SPIB	RBM28
SPIB	RBM3
SPIB	RBM34
SPIB	RBM38
SPIB	RBM4
SPIB	RBM42
SPIB	RBM45
SPIB	RBM6
SPIB	RBM8A
SPIB	RBMX
SPIB	RBMX2
SPIB	RC3H1
SPIB	RCAN3
SPIB	RCBTB1
SPIB	RCBTB2
SPIB	RCC1
SPIB	RCC2
SPIB	RCCD1
SPIB	RCE1
SPIB	RCHY1
SPIB	RCL1
SPIB	RCN1
SPIB	RCN2
SPIB	RCOR3
SPIB	RCSD1
SPIB	RECQL
SPIB	RECQL5
SPIB	REL
SPIB	RELA
SPIB	RELB
SPIB	RELT
SPIB	REPIN1
SPIB	RER1
SPIB	REST
SPIB	REV1
SPIB	REXO4
SPIB	RFC1
SPIB	RFC2
SPIB	RFC5
SPIB	RFTN1
SPIB	RFX1
SPIB	RFX3

SPIB	RFX5
SPIB	RFXANK
SPIB	RGCC
SPIB	RGP1
SPIB	RGS10
SPIB	RGS13
SPIB	RGS14
SPIB	RGS16
SPIB	RGS19
SPIB	RGS3
SPIB	RHBDD1
SPIB	RHBDF2
SPIB	RHEB
SPIB	RHOBTB2
SPIB	RHOC
SPIB	RHOF
SPIB	RHOG
SPIB	RHOH
SPIB	RHOT2
SPIB	RIC1
SPIB	RIC3
SPIB	RILPL2
SPIB	RIMKLB
SPIB	RIN3
SPIB	RING1
SPIB	RINL
SPIB	RIOK1
SPIB	RIOK3
SPIB	RIPK2
SPIB	RIT1
SPIB	RMDN1
SPIB	RMDN3
SPIB	RMI2
SPIB	RMND5B
SPIB	RNASEH1
SPIB	RNASEH2A
SPIB	RNASEH2B
SPIB	RNASEH2C
SPIB	RNF115
SPIB	RNF121
SPIB	RNF13
SPIB	RNF138
SPIB	RNF14
SPIB	RNF141
SPIB	RNF166
SPIB	RNF167
SPIB	RNF169
SPIB	RNF170
SPIB	RNF181
SPIB	RNF19A

SPIB	RNF214
SPIB	RNF220
SPIB	RNF25
SPIB	RNF34
SPIB	RNF40
SPIB	RNF41
SPIB	RNF7
SPIB	RNGTT
SPIB	RNMT
SPIB	RNPC3
SPIB	RNPS1
SPIB	ROCK1
SPIB	ROCK2
SPIB	ROMO1
SPIB	RPA2
SPIB	RPE
SPIB	RPF1
SPIB	RPF2
SPIB	RPIA
SPIB	RPL10
SPIB	RPL10A
SPIB	RPL11
SPIB	RPL12
SPIB	RPL13A
SPIB	RPL15
SPIB	RPL17
SPIB	RPL18
SPIB	RPL18A
SPIB	RPL19
SPIB	RPL21
SPIB	RPL22
SPIB	RPL23
SPIB	RPL23A
SPIB	RPL26
SPIB	RPL26L1
SPIB	RPL27
SPIB	RPL27A
SPIB	RPL28
SPIB	RPL29
SPIB	RPL3
SPIB	RPL30
SPIB	RPL31
SPIB	RPL32
SPIB	RPL34
SPIB	RPL35
SPIB	RPL36
SPIB	RPL36AL
SPIB	RPL37
SPIB	RPL37A
SPIB	RPL38

SPIB	RPL39
SPIB	RPL4
SPIB	RPL41
SPIB	RPL6
SPIB	RPL7
SPIB	RPL7L1
SPIB	RPL8
SPIB	RPLP2
SPIB	RPS10
SPIB	RPS11
SPIB	RPS12
SPIB	RPS13
SPIB	RPS14
SPIB	RPS15
SPIB	RPS16
SPIB	RPS17
SPIB	RPS18
SPIB	RPS19
SPIB	RPS19BP1
SPIB	RPS20
SPIB	RPS21
SPIB	RPS25
SPIB	RPS26
SPIB	RPS27
SPIB	RPS27A
SPIB	RPS3
SPIB	RPS3A
SPIB	RPS4X
SPIB	RPS4Y1
SPIB	RPS5
SPIB	RPS6
SPIB	RPS6KA1
SPIB	RPS6KA2
SPIB	RPS6KA4
SPIB	RPS6KC1
SPIB	RPS7
SPIB	RPSA
SPIB	RRAGA
SPIB	RRAS2
SPIB	RRM2B
SPIB	RRN3
SPIB	RRP12
SPIB	RRP15
SPIB	RRP36
SPIB	RRP9
SPIB	RRS1
SPIB	RSL1D1
SPIB	RSL24D1
SPIB	RSPRY1
SPIB	RSU1

SPIB	RTCA
SPIB	RTN4
SPIB	RTP4
SPIB	RUBCN
SPIB	RUFY1
SPIB	RUNDC1
SPIB	RUNX3
SPIB	RUVBL1
SPIB	RUVBL2
SPIB	RWDD1
SPIB	RWDD4
SPIB	RXRB
SPIB	S100A10
SPIB	S100A11
SPIB	S100A4
SPIB	S1PR4
SPIB	SAMHD1
SPIB	SAMM50
SPIB	SAMSN1
SPIB	SAP130
SPIB	SAP18
SPIB	SAP30
SPIB	SAP30BP
SPIB	SAR1B
SPIB	SART1
SPIB	SART3
SPIB	SASH3
SPIB	SASS6
SPIB	SAT1
SPIB	SAV1
SPIB	SAYSD1
SPIB	SBDS
SPIB	SBF2-AS1
SPIB	SBNO2
SPIB	SCAF1
SPIB	SCAF11
SPIB	SCAF4
SPIB	SCAND1
SPIB	SCFD1
SPIB	SCFD2
SPIB	SCIMP
SPIB	SCMH1
SPIB	SCNM1
SPIB	SCO1
SPIB	SCRN1
SPIB	SCRN3
SPIB	SDAD1
SPIB	SDCBP
SPIB	SDCBP2-AS1
SPIB	SDE2

SPIB	SDHAF1
SPIB	SDHAF2
SPIB	SDHAF3
SPIB	SDHB
SPIB	SDHC
SPIB	SDHD
SPIB	SDR39U1
SPIB	SDR42E1
SPIB	SEC11A
SPIB	SEC13
SPIB	SEC14L1
SPIB	SEC16A
SPIB	SEC22B
SPIB	SEC22C
SPIB	SEC23B
SPIB	SEC23IP
SPIB	SEC61G
SPIB	SECISBP2
SPIB	SECISBP2L
SPIB	SEMA4A
SPIB	SEMA7A
SPIB	SENP1
SPIB	SEPT2
SPIB	SEPT6
SPIB	SEPT7
SPIB	SEPT9
SPIB	SERBP1
SPIB	SERP1
SPIB	SERP2
SPIB	SERPINA9
SPIB	SERPINB8
SPIB	SERTAD3
SPIB	SESTD1
SPIB	SET
SPIB	SETBP1
SPIB	SETD3
SPIB	SETD4
SPIB	SETD5
SPIB	SETDB1
SPIB	SF1
SPIB	SF3A1
SPIB	SF3A2
SPIB	SF3A3
SPIB	SF3B1
SPIB	SF3B2
SPIB	SF3B3
SPIB	SF3B4
SPIB	SF3B5
SPIB	SF3B6
SPIB	SFI1

SPIB	SFSWAP
SPIB	SFT2D1
SPIB	SFT2D2
SPIB	SFXN1
SPIB	SGMS1
SPIB	SGO1
SPIB	SGPP2
SPIB	SGSH
SPIB	SGSM3
SPIB	SGTA
SPIB	SH2B1
SPIB	SH2B2
SPIB	SH2B3
SPIB	SH2D3C
SPIB	SH3BGRL
SPIB	SH3BP1
SPIB	SH3BP5
SPIB	SH3GLB1
SPIB	SH3KBP1
SPIB	SH3TC1
SPIB	SHC1
SPIB	SHF
SPIB	SHISA5
SPIB	SHISA8
SPIB	SHKBP1
SPIB	SHMT2
SPIB	SHQ1
SPIB	SIAH2
SPIB	SIGLEC10
SPIB	SIN3A
SPIB	SIPA1
SPIB	SIPA1L3
SPIB	SIRPA
SPIB	SIRT3
SPIB	SIRT6
SPIB	SIRT7
SPIB	SIT1
SPIB	SKAP2
SPIB	SKIL
SPIB	SKIV2L
SPIB	SLA
SPIB	SLAMF6
SPIB	SLC15A4
SPIB	SLC16A1
SPIB	SLC16A3
SPIB	SLC1A5
SPIB	SLC23A2
SPIB	SLC25A1
SPIB	SLC25A11
SPIB	SLC25A13

SPIB	SLC25A14
SPIB	SLC25A17
SPIB	SLC25A19
SPIB	SLC25A20
SPIB	SLC25A25
SPIB	SLC25A33
SPIB	SLC25A38
SPIB	SLC25A39
SPIB	SLC25A4
SPIB	SLC25A40
SPIB	SLC25A46
SPIB	SLC25A5
SPIB	SLC25A51
SPIB	SLC29A3
SPIB	SLC2A5
SPIB	SLC2A6
SPIB	SLC30A9
SPIB	SLC35A1
SPIB	SLC35A2
SPIB	SLC35A4
SPIB	SLC35B2
SPIB	SLC35B3
SPIB	SLC35C2
SPIB	SLC35E1
SPIB	SLC37A1
SPIB	SLC38A1
SPIB	SLC38A5
SPIB	SLC39A1
SPIB	SLC39A11
SPIB	SLC39A14
SPIB	SLC43A1
SPIB	SLC43A2
SPIB	SLC43A3
SPIB	SLC44A2
SPIB	SLC50A1
SPIB	SLC6A6
SPIB	SLC9A3R1
SPIB	SLC9A7
SPIB	SLCO4A1
SPIB	SLF1
SPIB	SLIRP
SPIB	SLK
SPIB	SLMAP
SPIB	SLU7
SPIB	SMAD2
SPIB	SMAD3
SPIB	SMAP1
SPIB	SMAP2
SPIB	SMARCA2
SPIB	SMARCAD1

SPIB	SMARCAL1
SPIB	SMARCC1
SPIB	SMARCC2
SPIB	SMARCD1
SPIB	SMARCD2
SPIB	SMARCE1
SPIB	SMC1A
SPIB	SMC3
SPIB	SMCHD1
SPIB	SMCO4
SPIB	SMG1
SPIB	SMG7
SPIB	SMIM12
SPIB	SMIM14
SPIB	SMIM15
SPIB	SMIM19
SPIB	SMIM20
SPIB	SMIM7
SPIB	SMNDC1
SPIB	SMPD2
SPIB	SMS
SPIB	SMU1
SPIB	SMUG1
SPIB	SMYD2
SPIB	SNAP23
SPIB	SNAP29
SPIB	SNAPC4
SPIB	SNAPC5
SPIB	SNF8
SPIB	SNHG19
SPIB	SNHG21
SPIB	SNHG25
SPIB	SNHG8
SPIB	SNN
SPIB	SNRNP200
SPIB	SNRNP27
SPIB	SNRNP35
SPIB	SNRNP40
SPIB	SNRNP48
SPIB	SNRPA
SPIB	SNRPA1
SPIB	SNRPB
SPIB	SNRPB2
SPIB	SNRPD1
SPIB	SNRPD2
SPIB	SNRPE
SPIB	SNRPF
SPIB	SNRPG
SPIB	SNRPN
SPIB	SNTB2

SPIB	SNU13
SPIB	SNUPN
SPIB	SNW1
SPIB	SNX1
SPIB	SNX10
SPIB	SNX11
SPIB	SNX19
SPIB	SNX20
SPIB	SNX3
SPIB	SNX5
SPIB	SNX8
SPIB	SNX9
SPIB	SOBP
SPIB	SOCS1
SPIB	SOD2
SPIB	SORL1
SPIB	SOX4
SPIB	SOX5
SPIB	SP100
SPIB	SP110
SPIB	SP140
SPIB	SP2
SPIB	SPAG7
SPIB	SPAG9
SPIB	SPAST
SPIB	SPATA33
SPIB	SPATA5L1
SPIB	SPECC1
SPIB	SPECC1L
SPIB	SPG21
SPIB	SPHK2
SPIB	SPI1
SPIB	SPIB
SPIB	SPIN2B
SPIB	SPINT1
SPIB	SPINT2
SPIB	SPN
SPIB	SPON1
SPIB	SPOP
SPIB	SPOPL
SPIB	SPPL2A
SPIB	SPTAN1
SPIB	SPTLC1
SPIB	SPTLC2
SPIB	SRD5A1
SPIB	SREK1
SPIB	SREK1IP1
SPIB	SRP14
SPIB	SRP14-AS1
SPIB	SRP19

SPIB	SRP54
SPIB	SRP68
SPIB	SRP9
SPIB	SRPK2
SPIB	SRR
SPIB	SRRM1
SPIB	SRRM2
SPIB	SRRT
SPIB	SRSF1
SPIB	SRSF10
SPIB	SRSF2
SPIB	SRSF3
SPIB	SRSF4
SPIB	SRSF5
SPIB	SRSF6
SPIB	SRSF7
SPIB	SRSF9
SPIB	SS18
SPIB	SSBP1
SPIB	SSBP4
SPIB	SSFA2
SPIB	SSH2
SPIB	SSR2
SPIB	SSSCA1
SPIB	SSTR2
SPIB	ST14
SPIB	ST20
SPIB	ST3GAL2
SPIB	ST3GAL5
SPIB	STAG2
SPIB	STAMBP
SPIB	STAMBPL1
SPIB	STAP1
SPIB	STARD3NL
SPIB	STAT5A
SPIB	STAT5B
SPIB	STAT6
SPIB	STAU1
SPIB	STAU2
SPIB	STIM2
SPIB	STK11
SPIB	STK16
SPIB	STK24
SPIB	STK26
SPIB	STK4
SPIB	STK40
SPIB	STMN1
SPIB	STOML2
SPIB	STRADA
SPIB	STRAP

SPIB	STRIP1
SPIB	STRN4
SPIB	STUB1
SPIB	STX10
SPIB	STX11
SPIB	STX12
SPIB	STX16
SPIB	STX18
SPIB	STX6
SPIB	STX7
SPIB	STXBP2
SPIB	STXBP3
SPIB	STYXL1
SPIB	SUCLG1
SPIB	SUDS3
SPIB	SUGT1
SPIB	SULF2
SPIB	SUMF1
SPIB	SUMO1
SPIB	SUN2
SPIB	SUPT16H
SPIB	SUPT20H
SPIB	SUPT4H1
SPIB	SUPT5H
SPIB	SURF1
SPIB	SURF4
SPIB	SVBP
SPIB	SWI5
SPIB	SYAP1
SPIB	SYF2
SPIB	SYK
SPIB	SYMPK
SPIB	SYNCRIP
SPIB	SYNGR1
SPIB	SYNGR2
SPIB	SYPL1
SPIB	SYS1
SPIB	SYT11
SPIB	SYT17
SPIB	SYTL1
SPIB	SZRD1
SPIB	TAB3
SPIB	TACC1
SPIB	TACO1
SPIB	TADA3
SPIB	TAF1
SPIB	TAF10
SPIB	TAF11
SPIB	TAF12
SPIB	TAF15

SPIB	TAF1B
SPIB	TAF2
SPIB	TAF7
SPIB	TAF9
SPIB	TAGAP
SPIB	TAGLN2
SPIB	TALDO1
SPIB	TANGO2
SPIB	TANK
SPIB	TAOK3
SPIB	TARS2
SPIB	TATDN1
SPIB	TATDN3
SPIB	TAX1BP1
SPIB	TAX1BP3
SPIB	TBC1D1
SPIB	TBC1D10C
SPIB	TBC1D19
SPIB	TBC1D22A
SPIB	TBC1D2B
SPIB	TBC1D5
SPIB	TBCC
SPIB	TBCEL
SPIB	TBK1
SPIB	TBL1XR1
SPIB	TBL3
SPIB	TBPL1
SPIB	TBRG1
SPIB	TBRG4
SPIB	TCEAL8
SPIB	TCF25
SPIB	TCF3
SPIB	TCF4
SPIB	TCIRG1
SPIB	TCOF1
SPIB	TCP1
SPIB	TCP11L1
SPIB	TCTA
SPIB	TCTE3
SPIB	TDG
SPIB	TDP1
SPIB	TDRD3
SPIB	TDRD7
SPIB	TEFM
SPIB	TELO2
SPIB	TESC
SPIB	TESPA1
SPIB	TEX10
SPIB	TFAM
SPIB	TFB1M

SPIB	TFB2M
SPIB	TFDP2
SPIB	TFEB
SPIB	TFIP11
SPIB	TFRC
SPIB	TGFB1
SPIB	TGIF1
SPIB	TGIF2
SPIB	TGOLN2
SPIB	TGS1
SPIB	THAP1
SPIB	THAP11
SPIB	THAP4
SPIB	THAP5
SPIB	THEM6
SPIB	THG1L
SPIB	THOC2
SPIB	THRAP3
SPIB	THTPA
SPIB	THUMPD2
SPIB	THUMPD3
SPIB	THUMPD3-AS1
SPIB	TIAL1
SPIB	TIFA
SPIB	TIGAR
SPIB	TIMM10
SPIB	TIMM10B
SPIB	TIMM17A
SPIB	TIMM23
SPIB	TIMM23B
SPIB	TIMM8A
SPIB	TIMM8B
SPIB	TIMMDC1
SPIB	TINF2
SPIB	TIPARP
SPIB	TIPRL
SPIB	TKT
SPIB	TLCD1
SPIB	TLE1
SPIB	TLE3
SPIB	TLN1
SPIB	TLR10
SPIB	TM2D1
SPIB	TM2D3
SPIB	TMA7
SPIB	TMBIM1
SPIB	TMBIM4
SPIB	TMC6
SPIB	TMC8
SPIB	TMCO3

SPIB	TMCO4
SPIB	TMED2
SPIB	TMED3
SPIB	TMED8
SPIB	TMEM101
SPIB	TMEM115
SPIB	TMEM123
SPIB	TMEM131
SPIB	TMEM138
SPIB	TMEM14A
SPIB	TMEM154
SPIB	TMEM156
SPIB	TMEM161B−AS1
SPIB	TMEM167A
SPIB	TMEM18
SPIB	TMEM186
SPIB	TMEM199
SPIB	TMEM230
SPIB	TMEM231
SPIB	TMEM243
SPIB	TMEM245
SPIB	TMEM248
SPIB	TMEM251
SPIB	TMEM258
SPIB	TMEM259
SPIB	TMEM267
SPIB	TMEM41B
SPIB	TMEM59
SPIB	TMEM60
SPIB	TMEM68
SPIB	TMEM69
SPIB	TMEM8A
SPIB	TMEM9
SPIB	TMEM94
SPIB	TMEM9B
SPIB	TMSB4X
SPIB	TMUB1
SPIB	TMX1
SPIB	TNF
SPIB	TNFAIP1
SPIB	TNFAIP3
SPIB	TNFAIP8
SPIB	TNFAIP8L2
SPIB	TNFRSF13B
SPIB	TNFRSF13C
SPIB	TNFRSF1B
SPIB	TNIP1
SPIB	TNKS2
SPIB	TNPO3
SPIB	TNRC6A

SPIB	TNS3
SPIB	TOE1
SPIB	TOLLIP
SPIB	TOMM20
SPIB	TOMM22
SPIB	TOMM40
SPIB	TOMM5
SPIB	TOMM7
SPIB	TOP1
SPIB	TOP1MT
SPIB	TOR1A
SPIB	TOR4A
SPIB	TOX4
SPIB	TP53
SPIB	TP53I11
SPIB	TP53RK
SPIB	TP53TG1
SPIB	TPCN1
SPIB	TPD52L2
SPIB	TPGS1
SPIB	TPM3
SPIB	TPM4
SPIB	TPMT
SPIB	TPP2
SPIB	TPR
SPIB	TPRKB
SPIB	TPST2
SPIB	TPX2
SPIB	TRA2B
SPIB	TRABD
SPIB	TRADD
SPIB	TRAF3
SPIB	TRAF3IP1
SPIB	TRAF4
SPIB	TRAF5
SPIB	TRAF7
SPIB	TRAFD1
SPIB	TRAK1
SPIB	TRAM1
SPIB	TRAPPC1
SPIB	TRAPPC10
SPIB	TRAPPC11
SPIB	TRAPPC12
SPIB	TRAPPC3
SPIB	TRAPPC4
SPIB	TRAPPC6A
SPIB	TRAPPC8
SPIB	TRIB2
SPIB	TRIM21
SPIB	TRIM22

SPIB	TRIM27
SPIB	TRIM56
SPIB	TRIM61
SPIB	TRIM8
SPIB	TRIP12
SPIB	TRMO
SPIB	TRMT10A
SPIB	TRMT10C
SPIB	TRMT11
SPIB	TRMT112
SPIB	TRMT12
SPIB	TRMT2A
SPIB	TRMT6
SPIB	TRMT61A
SPIB	TRMT61B
SPIB	TRNAU1AP
SPIB	TRNT1
SPIB	TROVE2
SPIB	TRPM4
SPIB	TRPV2
SPIB	TRUB2
SPIB	TSC22D3
SPIB	TSC22D4
SPIB	TSEN34
SPIB	TSG101
SPIB	TSNAX
SPIB	TSPAN14
SPIB	TSPAN3
SPIB	TSPAN31
SPIB	TSPAN33
SPIB	TSPAN4
SPIB	TSPO
SPIB	TSSC4
SPIB	TSTD1
SPIB	TTC1
SPIB	TTC13
SPIB	TTC14
SPIB	TTC32
SPIB	TTC37
SPIB	TTC39C
SPIB	TTC5
SPIB	TTF1
SPIB	TTF2
SPIB	TTI2
SPIB	TUBA1B
SPIB	TUBB
SPIB	TUBB2B
SPIB	TUBB4B
SPIB	TUBB6
SPIB	TUBGCP2

SPIB	TUFM
SPIB	TUSC2
SPIB	TWF1
SPIB	TWF2
SPIB	TXN
SPIB	TXN2
SPIB	TXNDC9
SPIB	TXNL1
SPIB	TXNL4B
SPIB	TXNRD1
SPIB	TYSND1
SPIB	TYW1
SPIB	TYW3
SPIB	U2AF2
SPIB	U2SURP
SPIB	UACA
SPIB	UBA1
SPIB	UBA2
SPIB	UBA52
SPIB	UBA6
SPIB	UBAC1
SPIB	UBAC2
SPIB	UBALD1
SPIB	UBALD2
SPIB	UBAP2
SPIB	UBAP2L
SPIB	UBB
SPIB	UBC
SPIB	UBE2A
SPIB	UBE2C
SPIB	UBE2D1
SPIB	UBE2D2
SPIB	UBE2D3
SPIB	UBE2E1
SPIB	UBE2E3
SPIB	UBE2F
SPIB	UBE2G1
SPIB	UBE2G2
SPIB	UBE2J2
SPIB	UBE2L3
SPIB	UBE2M
SPIB	UBE2N
SPIB	UBE2Q1
SPIB	UBE2V2
SPIB	UBE3A
SPIB	UBFD1
SPIB	UBL5
SPIB	UBL7
SPIB	UBLCP1
SPIB	UBN1

SPIB	UBOX5
SPIB	UBQLN2
SPIB	UBR1
SPIB	UBR2
SPIB	UBR4
SPIB	UBR5
SPIB	UBTF
SPIB	UBXN1
SPIB	UCHL3
SPIB	UCHL5
SPIB	UCK1
SPIB	UCP2
SPIB	UFC1
SPIB	UFM1
SPIB	UFSP2
SPIB	UGCG
SPIB	UGGT2
SPIB	UGP2
SPIB	UHMK1
SPIB	UIMC1
SPIB	UMAD1
SPIB	UMPS
SPIB	UNC13D
SPIB	UNC93B1
SPIB	UPF2
SPIB	UQCC2
SPIB	UQCR11
SPIB	UQCRC2
SPIB	UQCRFS1
SPIB	UQCRH
SPIB	UQCRQ
SPIB	URGCP
SPIB	URI1
SPIB	URM1
SPIB	UROS
SPIB	USB1
SPIB	USE1
SPIB	USP1
SPIB	USP12
SPIB	USP16
SPIB	USP19
SPIB	USP36
SPIB	USP38
SPIB	USP39
SPIB	USP4
SPIB	USP48
SPIB	USP7
SPIB	USP9X
SPIB	USPL1
SPIB	UTP11

SPIB	UTP14A
SPIB	UTP18
SPIB	UTP23
SPIB	UTP3
SPIB	UTP4
SPIB	UTP6
SPIB	UXT
SPIB	VAMP1
SPIB	VAMP2
SPIB	VAMP3
SPIB	VAMP8
SPIB	VARS
SPIB	VASP
SPIB	VAV1
SPIB	VAV2
SPIB	VCP
SPIB	VDAC3
SPIB	VEZF1
SPIB	VGLL4
SPIB	VHL
SPIB	VIM
SPIB	VIPAS39
SPIB	VMA21
SPIB	VOPP1
SPIB	VPS11
SPIB	VPS13A
SPIB	VPS16
SPIB	VPS18
SPIB	VPS25
SPIB	VPS26A
SPIB	VPS33A
SPIB	VPS39
SPIB	VPS4A
SPIB	VPS4B
SPIB	VPS51
SPIB	VPS72
SPIB	VRK1
SPIB	VRK3
SPIB	VTA1
SPIB	WAC
SPIB	WAS
SPIB	WASF2
SPIB	WDFY4
SPIB	WDR1
SPIB	WDR11
SPIB	WDR12
SPIB	WDR3
SPIB	WDR33
SPIB	WDR34
SPIB	WDR37

SPIB	WDR41
SPIB	WDR43
SPIB	WDR44
SPIB	WDR46
SPIB	WDR55
SPIB	WDR61
SPIB	WDR70
SPIB	WDR73
SPIB	WDR81
SPIB	WDR82
SPIB	WDR83
SPIB	WDR83OS
SPIB	WDTC1
SPIB	WDYHV1
SPIB	WEE1
SPIB	WIPF1
SPIB	WIPF2
SPIB	WNK1
SPIB	WNT10A
SPIB	WRAP53
SPIB	WRAP73
SPIB	WSB1
SPIB	WSB2
SPIB	WTAP
SPIB	WWC3
SPIB	WWP2
SPIB	XAB2
SPIB	XIAP
SPIB	XPNPEP1
SPIB	XPO6
SPIB	XRCC4
SPIB	XRCC6
SPIB	XRN2
SPIB	XXYLT1
SPIB	XXYLT1−AS2
SPIB	YARS2
SPIB	YBEY
SPIB	YBX1
SPIB	YEATS4
SPIB	YIPF3
SPIB	YIPF4
SPIB	YME1L1
SPIB	YPEL3
SPIB	YPEL5
SPIB	YRDC
SPIB	YTHDC1
SPIB	YTHDC2
SPIB	YTHDF2
SPIB	YTHDF3
SPIB	YWHAB

SPIB	YWHAE
SPIB	YWHAG
SPIB	YWHAH
SPIB	YWHAZ
SPIB	YY1
SPIB	ZBED5
SPIB	ZBTB1
SPIB	ZBTB17
SPIB	ZBTB24
SPIB	ZBTB25
SPIB	ZBTB32
SPIB	ZBTB45
SPIB	ZBTB5
SPIB	ZBTB7A
SPIB	ZC3H10
SPIB	ZC3H12A
SPIB	ZC3H12D
SPIB	ZC3H13
SPIB	ZC3H14
SPIB	ZC3H15
SPIB	ZC3H18
SPIB	ZC3H7A
SPIB	ZC3HAV1
SPIB	ZC3HC1
SPIB	ZCCHC17
SPIB	ZCCHC7
SPIB	ZCCHC9
SPIB	ZDHHC12
SPIB	ZDHHC13
SPIB	ZDHHC2
SPIB	ZDHHC21
SPIB	ZDHHC24
SPIB	ZDHHC3
SPIB	ZDHHC6
SPIB	ZEB1
SPIB	ZEB2
SPIB	ZFAND3
SPIB	ZFAND6
SPIB	ZFAS1
SPIB	ZFAT
SPIB	ZFC3H1
SPIB	ZFP1
SPIB	ZFP36
SPIB	ZFP36L1
SPIB	ZFP82
SPIB	ZFP91
SPIB	ZFPL1
SPIB	ZFX
SPIB	ZFYVE16
SPIB	ZFYVE27

SPIB	ZGRF1
SPIB	ZKSCAN2
SPIB	ZMAT2
SPIB	ZMYND11
SPIB	ZNF101
SPIB	ZNF106
SPIB	ZNF134
SPIB	ZNF143
SPIB	ZNF16
SPIB	ZNF195
SPIB	ZNF207
SPIB	ZNF213
SPIB	ZNF226
SPIB	ZNF227
SPIB	ZNF24
SPIB	ZNF250
SPIB	ZNF263
SPIB	ZNF280D
SPIB	ZNF281
SPIB	ZNF285
SPIB	ZNF292
SPIB	ZNF296
SPIB	ZNF318
SPIB	ZNF319
SPIB	ZNF324B
SPIB	ZNF330
SPIB	ZNF33A
SPIB	ZNF35
SPIB	ZNF354A
SPIB	ZNF394
SPIB	ZNF408
SPIB	ZNF480
SPIB	ZNF511
SPIB	ZNF512
SPIB	ZNF514
SPIB	ZNF580
SPIB	ZNF584
SPIB	ZNF593
SPIB	ZNF608
SPIB	ZNF644
SPIB	ZNF655
SPIB	ZNF668
SPIB	ZNF672
SPIB	ZNF687
SPIB	ZNF720
SPIB	ZNF721
SPIB	ZNF746
SPIB	ZNF775
SPIB	ZNF791
SPIB	ZNF792

SPIB	ZNF800
SPIB	ZNF804A
SPIB	ZNF827
SPIB	ZNF880
SPIB	ZNFX1
SPIB	ZNHIT1
SPIB	ZP3
SPIB	ZPR1
SPIB	ZRANB2
SPIB	ZRSR2
SPIB	ZSCAN16
SPIB	ZSCAN16-AS1
SPIB	ZSCAN21
SPIB	ZSWIM6
SPIB	ZSWIM8
SPIB	ZWILCH
SPIB	ZYX
SREBF1	AEN
SREBF1	ATAD3B
SREBF1	BBC3
SREBF1	BRF2
SREBF1	CCND1
SREBF1	CLTC
SREBF1	DCP1A
SREBF1	EGR2
SREBF1	IFT22
SREBF1	KDM6B
SREBF1	MSL1
SREBF1	NEK11
SREBF1	NF1
SREBF1	NOL9
SREBF1	NOM1
SREBF1	POLG2
SREBF1	PSEN1
SREBF1	RARA
SREBF1	SARNP
SREBF1	SEMA7A
SREBF1	SREBF1
SREBF1	TCF7
SREBF1	TNFRSF10B
SREBF1	TRAF1
SREBF1	UBC
SREBF1	VIM-AS1
SREBF1	VPS37B
SREBF1	YME1L1
SREBF1	YY1AP1
SREBF1	ZFAS1
SREBF1	ZMPSTE24
SREBF2	ABL2
SREBF2	ACADSB

SREBF2	ACAT2
SREBF2	ACTG1
SREBF2	ADAM19
SREBF2	ADARB1
SREBF2	AHCYL1
SREBF2	AIFM1
SREBF2	AKT1
SREBF2	ALG3
SREBF2	AMDHD2
SREBF2	AMIGO2
SREBF2	ANKRD13A
SREBF2	ANKRD13D
SREBF2	ANKRD46
SREBF2	AQP3
SREBF2	ARF5
SREBF2	ARF6
SREBF2	ARHGAP30
SREBF2	ARMCX3
SREBF2	ARPC1B
SREBF2	ASAH1
SREBF2	ASB1
SREBF2	ATP6V0D1
SREBF2	ATP6V1G1
SREBF2	ATXN2L
SREBF2	ATXN7L3B
SREBF2	AZI2
SREBF2	B3GLCT
SREBF2	BAIAP2L1
SREBF2	BEX4
SREBF2	BIRC6
SREBF2	BLOC1S5
SREBF2	BPHL
SREBF2	BRD3
SREBF2	BTBD7
SREBF2	C12orf43
SREBF2	C19orf38
SREBF2	C1orf43
SREBF2	C21orf58
SREBF2	CAD
SREBF2	CALCOCO2
SREBF2	CAPN1
SREBF2	CASP2
SREBF2	CBFA2T2
SREBF2	CCDC107
SREBF2	CCDC15
SREBF2	CCDC171
SREBF2	CCDC28A
SREBF2	CCDC43
SREBF2	CCNB1IP1
SREBF2	CCNE1

SREBF2	CD164
SREBF2	CD180
SREBF2	CD22
SREBF2	CD52
SREBF2	CD53
SREBF2	CD74
SREBF2	CDK12
SREBF2	CENPBD1
SREBF2	CHCHD10
SREBF2	CHD2
SREBF2	CIRBP
SREBF2	CLCN6
SREBF2	CLK2
SREBF2	CLNS1A
SREBF2	CMTM6
SREBF2	CNOT4
SREBF2	COG2
SREBF2	CORO1A
SREBF2	CPNE5
SREBF2	CREB3
SREBF2	CSNK1G2
SREBF2	CTSH
SREBF2	CUL4A
SREBF2	DDX23
SREBF2	DDX3Y
SREBF2	DDX5
SREBF2	DENND1B
SREBF2	DENND4A
SREBF2	DHPS
SREBF2	DIAPH1
SREBF2	DMD
SREBF2	DNAJC14
SREBF2	DRAM2
SREBF2	DUSP4
SREBF2	EAPP
SREBF2	EEF1A1
SREBF2	EEF2
SREBF2	EIF3H
SREBF2	ERP44
SREBF2	ESCO2
SREBF2	ESYT1
SREBF2	ETHE1
SREBF2	EVC2
SREBF2	EXT1
SREBF2	FAM129A
SREBF2	FAM81A
SREBF2	FAS
SREBF2	FAU
SREBF2	FBXO46
SREBF2	FCER2

SREBF2	FDFT1
SREBF2	FGFR1OP
SREBF2	FLVCR1
SREBF2	FN3KRP
SREBF2	FRAT2
SREBF2	GAB1
SREBF2	GABARAP
SREBF2	GAK
SREBF2	GATAD2A
SREBF2	GATAD2B
SREBF2	GBE1
SREBF2	GIPC1
SREBF2	GLMP
SREBF2	GNA13
SREBF2	GPSM3
SREBF2	GPX4
SREBF2	GRINA
SREBF2	GRK5
SREBF2	HECW2
SREBF2	HEXA
SREBF2	HGSNAT
SREBF2	HK1
SREBF2	HLA-DRA
SREBF2	HMGCS1
SREBF2	HOMER2
SREBF2	HVCN1
SREBF2	IDI1
SREBF2	IL2RG
SREBF2	INTS6
SREBF2	INTS6L
SREBF2	JDP2
SREBF2	JUP
SREBF2	KAT8
SREBF2	KATNB1
SREBF2	KCNN4
SREBF2	KCTD9
SREBF2	KIF15
SREBF2	KLHL5
SREBF2	KRCC1
SREBF2	L3MBTL2
SREBF2	LAGE3
SREBF2	LAPTM5
SREBF2	LAT2
SREBF2	LGALS3
SREBF2	LIMK1
SREBF2	LMAN2L
SREBF2	LMBRD2
SREBF2	LPAR5
SREBF2	LPIN1
SREBF2	LSS

SREBF2	LUC7L2
SREBF2	LY9
SREBF2	LYSMD2
SREBF2	LYST
SREBF2	MADD
SREBF2	MANEA
SREBF2	MAP1B
SREBF2	MAP3K14
SREBF2	MAP3K7CL
SREBF2	MARCKSL1
SREBF2	MAT2A
SREBF2	MBP
SREBF2	MBTD1
SREBF2	MED1
SREBF2	MED13
SREBF2	MED31
SREBF2	METTL9
SREBF2	MKRN1
SREBF2	MS4A1
SREBF2	MSMO1
SREBF2	MSMP
SREBF2	MTIF2
SREBF2	MTSS1
SREBF2	MVB12A
SREBF2	MYCBP2
SREBF2	MYL12B
SREBF2	NBN
SREBF2	NDRG3
SREBF2	NDUFS2
SREBF2	NEK6
SREBF2	NELFB
SREBF2	NFS1
SREBF2	NINJ2
SREBF2	NIT1
SREBF2	NKIRAS2
SREBF2	NLRX1
SREBF2	NMNAT3
SREBF2	NRBF2
SREBF2	NRROS
SREBF2	NSF
SREBF2	NT5DC3
SREBF2	NUDT4
SREBF2	NUGGC
SREBF2	NUMA1
SREBF2	NUP205
SREBF2	NUP43
SREBF2	OCEL1
SREBF2	ORAI3
SREBF2	OSBPL10-AS1
SREBF2	PAFAH1B3

SREBF2	PAN2
SREBF2	PCSK7
SREBF2	PCYOX1L
SREBF2	PDHB
SREBF2	PDS5B
SREBF2	PI4K2B
SREBF2	PIGW
SREBF2	PLCG2
SREBF2	PLEKHF2
SREBF2	PNPLA2
SREBF2	POLL
SREBF2	PPP1R37
SREBF2	PPP4R3B
SREBF2	PRDX1
SREBF2	PREX1
SREBF2	PRKCI
SREBF2	PSMB4
SREBF2	PTK2
SREBF2	PTPN23
SREBF2	PTPN6
SREBF2	PURA
SREBF2	RAB11FIP1
SREBF2	RAB14
SREBF2	RALGAPB
SREBF2	RANBP2
SREBF2	RBM25
SREBF2	RBM47
SREBF2	REST
SREBF2	RFX2
SREBF2	RFXAP
SREBF2	RGS16
SREBF2	RHOT1
SREBF2	RIC3
SREBF2	RIN3
SREBF2	RMDN3
SREBF2	RMND5B
SREBF2	RNF111
SREBF2	RNF138
SREBF2	RNH1
SREBF2	RPL21
SREBF2	RPL28
SREBF2	RPLP2
SREBF2	RPS19
SREBF2	RPS27
SREBF2	RPS28
SREBF2	S1PR1
SREBF2	SCIMP
SREBF2	SEPT11
SREBF2	SFSWAP
SREBF2	SGMS1

SREBF2	SGSM3
SREBF2	SIAH1
SREBF2	SIRT5
SREBF2	SKAP2
SREBF2	SKIV2L
SREBF2	SLC10A3
SREBF2	SLC23A2
SREBF2	SLC35F5
SREBF2	SLC37A4
SREBF2	SLC44A2
SREBF2	SLC6A6
SREBF2	SMAD5
SREBF2	SMAD7
SREBF2	SMARCC2
SREBF2	SMARCD2
SREBF2	SMCHD1
SREBF2	SMG7
SREBF2	SNAPC2
SREBF2	SNX13
SREBF2	SNX25
SREBF2	SOCS4
SREBF2	SPRED2
SREBF2	SPTLC1
SREBF2	SQLE
SREBF2	SREBF2
SREBF2	STAG2
SREBF2	STK11
SREBF2	STK25
SREBF2	STX10
SREBF2	STX16
SREBF2	STX5
SREBF2	STXBP2
SREBF2	SUCLA2
SREBF2	SUGP1
SREBF2	SUPT20H
SREBF2	SUPT4H1
SREBF2	SYS1
SREBF2	SYT11
SREBF2	TAX1BP1
SREBF2	TBC1D10C
SREBF2	TCAF1
SREBF2	TCIRG1
SREBF2	TCOF1
SREBF2	TEFM
SREBF2	TERF2
SREBF2	TFRC
SREBF2	THUMPD2
SREBF2	TICAM1
SREBF2	TMED8
SREBF2	TMEM38B

SREBF2	TMEM63A
SREBF2	TMSB4X
SREBF2	TNFRSF13C
SREBF2	TOMM20
SREBF2	TOPBP1
SREBF2	TPP1
SREBF2	TRAM2
SREBF2	TRAPPC1
SREBF2	TRIM11
SREBF2	TSC22D4
SREBF2	TSTD1
SREBF2	TTC3
SREBF2	TTI1
SREBF2	TUBGCP4
SREBF2	TWF2
SREBF2	UBE2B
SREBF2	UGDH
SREBF2	USF2
SREBF2	USP3
SREBF2	UTY
SREBF2	VAMP2
SREBF2	VASP
SREBF2	VPS26A
SREBF2	VPS33B
SREBF2	VPS35
SREBF2	WAC
SREBF2	WASF2
SREBF2	WDCP
SREBF2	WDFY1
SREBF2	YIPF1
SREBF2	YWHAZ
SREBF2	ZBTB1
SREBF2	ZBTB44
SREBF2	ZC3H12D
SREBF2	ZFAND5
SREBF2	ZFP36L1
SREBF2	ZKSCAN8
SREBF2	ZMAT3
SREBF2	ZMYM5
SREBF2	ZMYND19
SREBF2	ZNF428
SREBF2	ZNF48
SREBF2	ZNF655
SREBF2	ZNF668
SREBF2	ZSCAN30
SREBF2	ZWILCH
SRF	AAAS
SRF	ANTXR2
SRF	APH1A
SRF	ARFGAP1

SRF	ASAP1
SRF	ASPSCR1
SRF	ATF5
SRF	CCDC50
SRF	CDC34
SRF	CLMN
SRF	CNN3
SRF	CNOT8
SRF	COX10
SRF	CUL1
SRF	DDIT3
SRF	DENND4B
SRF	DHX30
SRF	DOCK10
SRF	EFHD2
SRF	EIF2B2
SRF	ENTHD1
SRF	FAM199X
SRF	FAM78A
SRF	GCH1
SRF	GOLGA3
SRF	GPM6B
SRF	GRIPAP1
SRF	GTF3C2
SRF	HCCS
SRF	HOXB4
SRF	IFNGR1
SRF	ITPR3
SRF	JOSD2
SRF	KHSRP
SRF	LHFPL2
SRF	MAD2L1BP
SRF	MSL1
SRF	MSN
SRF	MTF1
SRF	MXI1
SRF	NAB1
SRF	NEDD1
SRF	NEK6
SRF	NPAT
SRF	NSF
SRF	NUDCD3
SRF	PDSS1
SRF	PKN1
SRF	PNOC
SRF	PPME1
SRF	PRPF19
SRF	PTPN9
SRF	RABGGTB
SRF	RGS19

SRF	RPRD1A
SRF	SETBP1
SRF	SLC25A22
SRF	SLC43A1
SRF	SMCR5
SRF	SNHG7
SRF	SQSTM1
SRF	SRF
SRF	SSBP4
SRF	STX16
SRF	TAF15
SRF	TAOK3
SRF	TCF12
SRF	THADA
SRF	TMEM216
SRF	TMEM223
SRF	TSC2
SRF	UBTF
SRF	VEGFA
SRF	VTI1A
SRF	WDR24
SRF	YIPF1
SRF	ZBTB25
SRF	ZBTB7B
SRF	ZNF502
SRF	ZNF644
STAT1	ABCB4
STAT1	ABCF3
STAT1	ACAA1
STAT1	ACSF2
STAT1	ADCY3
STAT1	ADIPOR2
STAT1	ADPGK
STAT1	AFF3
STAT1	AIF1
STAT1	AKAP13
STAT1	ALAD
STAT1	ANKRD10
STAT1	ANKRD27
STAT1	APH1B
STAT1	APOBEC3G
STAT1	AREG
STAT1	ARF3
STAT1	ARHGAP24
STAT1	ARRB2
STAT1	ARRDC1-AS1
STAT1	ARSA
STAT1	ATL1
STAT1	ATP13A1
STAT1	B2M

STAT1	B3GNT2
STAT1	BAG4
STAT1	BBX
STAT1	BCAR3
STAT1	BCKDHB
STAT1	BIRC3
STAT1	BLCAP
STAT1	BOLA2B
STAT1	BORCS6
STAT1	BROX
STAT1	BST2
STAT1	C15orf39
STAT1	C19orf66
STAT1	C1GALT1C1
STAT1	C3orf52
STAT1	CAPN7
STAT1	CASC3
STAT1	CASP7
STAT1	CBR1
STAT1	CCDC125
STAT1	CCDC88A
STAT1	CCR10
STAT1	CD164
STAT1	CD38
STAT1	CD47
STAT1	CD59
STAT1	CD63
STAT1	CD68
STAT1	CEPT1
STAT1	CGRRF1
STAT1	CHAC1
STAT1	CHD2
STAT1	CHMP5
STAT1	CHPF
STAT1	CHST2
STAT1	CKAP4
STAT1	CLASRP
STAT1	CMPK2
STAT1	CNDP2
STAT1	COG8
STAT1	COX5A
STAT1	CPXM1
STAT1	CRTC2
STAT1	CSPP1
STAT1	CTBS
STAT1	CTDSP2
STAT1	CTSA
STAT1	CXXC5
STAT1	CYB5R4
STAT1	CYTH1

STAT1	DAPP1
STAT1	DDIT3
STAT1	DDX58
STAT1	DDX60L
STAT1	DLAT
STAT1	DLST
STAT1	DMD
STAT1	DNAJB14
STAT1	DNAJC3
STAT1	DNASE2
STAT1	DRAP1
STAT1	DYNLT1
STAT1	DYRK1B
STAT1	EDEM2
STAT1	EIF2AK2
STAT1	EPSTI1
STAT1	ERAP1
STAT1	ERO1A
STAT1	ERO1B
STAT1	ERP44
STAT1	ESRRA
STAT1	ETHE1
STAT1	ETV7
STAT1	FADS2
STAT1	FAM3C
STAT1	FBXL5
STAT1	FBXO6
STAT1	FICD
STAT1	FKBP2
STAT1	FLNA
STAT1	FOXO3
STAT1	FPGS
STAT1	GANAB
STAT1	GATM
STAT1	GBP1
STAT1	GBP2
STAT1	GBP4
STAT1	GEN1
STAT1	GIMAP2
STAT1	GMPPA
STAT1	GORASP2
STAT1	GPR146
STAT1	GPX7
STAT1	GRINA
STAT1	GRN
STAT1	GSN
STAT1	HEBP1
STAT1	HERC5
STAT1	HERC6
STAT1	HEXIM2

STAT1	HIP1
STAT1	HIST1H4H
STAT1	HLA-A
STAT1	HLA-B
STAT1	HLA-DRB5
STAT1	HLA-F
STAT1	HM13
STAT1	HMGCS1
STAT1	HSPA13
STAT1	IARS
STAT1	IFI16
STAT1	IFI35
STAT1	IFI6
STAT1	IFIT1
STAT1	IFIT2
STAT1	IFIT3
STAT1	IFITM2
STAT1	IGF2R
STAT1	IGFBP4
STAT1	IGFLR1
STAT1	IKBKG
STAT1	IL15RA
STAT1	IL2RG
STAT1	IMPACT
STAT1	INTS3
STAT1	IRF1
STAT1	IRF7
STAT1	IRF9
STAT1	ISG15
STAT1	ISG20
STAT1	ITM2B
STAT1	JAM2
STAT1	JMY
STAT1	KAT6B
STAT1	KCNN3
STAT1	KLHL14
STAT1	KRT18
STAT1	LANCL1
STAT1	LAP3
STAT1	LGALS9
STAT1	LINC00665
STAT1	LMAN2
STAT1	LTA4H
STAT1	LTC4S
STAT1	LY6E
STAT1	MACROD2
STAT1	MANEA
STAT1	MAP3K11
STAT1	MAP3K13
STAT1	MCL1

STAT1	MCTP2
STAT1	MED24
STAT1	METTL7A
STAT1	MIDN
STAT1	MILR1
STAT1	MORF4L2
STAT1	MPHOSPH10
STAT1	MRPL44
STAT1	MRPS31
STAT1	MX1
STAT1	MX2
STAT1	MYD88
STAT1	MYDGF
STAT1	MZB1
STAT1	N4BP2L1
STAT1	NCKAP1L
STAT1	NCOA7
STAT1	NDST2
STAT1	NEK8
STAT1	NFIC
STAT1	NIT2
STAT1	NMI
STAT1	NOB1
STAT1	NPHP3
STAT1	NT5C3A
STAT1	NUB1
STAT1	OAS1
STAT1	OAS2
STAT1	OAS3
STAT1	OGDH
STAT1	OTUD1
STAT1	P2RX1
STAT1	P4HA1
STAT1	PARP12
STAT1	PDE4DIP
STAT1	PDIA5
STAT1	PDK1
STAT1	PDLIM1
STAT1	PGRMC2
STAT1	PHF11
STAT1	PHPT1
STAT1	PI4KB
STAT1	PIAS3
STAT1	PIGT
STAT1	PIK3AP1
STAT1	PLD3
STAT1	PLD4
STAT1	PLEKHA2
STAT1	PLTP
STAT1	POLE2

STAT1	PPIL2
STAT1	PPM1K
STAT1	PPP2R2A
STAT1	PRDX4
STAT1	PSMB9
STAT1	PSME1
STAT1	PSME2
STAT1	QPCT
STAT1	RAB1A
STAT1	RAB1B
STAT1	RAB29
STAT1	RAB5A
STAT1	RABGAP1L
STAT1	RASA4
STAT1	RASSF5
STAT1	RBM19
STAT1	RCN1
STAT1	RNASE6
STAT1	RND1
STAT1	RNF103
STAT1	RNF14
STAT1	RNF213
STAT1	RNF24
STAT1	RNPEPL1
STAT1	RPL10
STAT1	RPL18A
STAT1	RPL23
STAT1	RPL29
STAT1	RPL32
STAT1	RPL35A
STAT1	RPS11
STAT1	RPS13
STAT1	RPS6KA1
STAT1	RSAD2
STAT1	S100A4
STAT1	SAMD9
STAT1	SAMD9L
STAT1	SARS2
STAT1	SASH3
STAT1	SCAPER
STAT1	SCPEP1
STAT1	SEC22B
STAT1	SEC61A1
STAT1	SECISBP2L
STAT1	SELL
STAT1	SENP6
STAT1	SF3A1
STAT1	SIK3
STAT1	SLAMF7
STAT1	SLC30A7

STAT1	SLC35E2B
STAT1	SLC38A10
STAT1	SLC39A6
STAT1	SLC44A1
STAT1	SLC7A5
STAT1	SLC9A3
STAT1	SLFN5
STAT1	SNX19
STAT1	SP100
STAT1	SP110
STAT1	SP140
STAT1	SPATA20
STAT1	SPINT2
STAT1	SPPL2B
STAT1	SSTR2
STAT1	ST6GAL1
STAT1	ST6GALNAC4
STAT1	STAP1
STAT1	STARD5
STAT1	STAT1
STAT1	STK32C
STAT1	STX12
STAT1	SUB1
STAT1	SUCLG2
STAT1	SYT11
STAT1	TAF1
STAT1	TAP1
STAT1	TAPBPL
STAT1	TARSL2
STAT1	TBC1D31
STAT1	TCTN3
STAT1	TECR
STAT1	TFAP4
STAT1	TGIF1
STAT1	THEMIS2
STAT1	TKFC
STAT1	TM7SF2
STAT1	TM9SF2
STAT1	TMBIM6
STAT1	TMED7
STAT1	TMEM140
STAT1	TMEM185B
STAT1	TMEM219
STAT1	TMSB10
STAT1	TNFRSF13B
STAT1	TNFSF10
STAT1	TP53I13
STAT1	TP53INP1
STAT1	TPST2
STAT1	TRAF3

STAT1	TRAM1
STAT1	TRIB1
STAT1	TRIM44
STAT1	TRIM69
STAT1	TRIM8
STAT1	TRMT10B
STAT1	TRPV2
STAT1	TSPYL1
STAT1	TTN
STAT1	TULP4
STAT1	UBE2L6
STAT1	UNC93B1
STAT1	URI1
STAT1	UROD
STAT1	USP18
STAT1	VARS
STAT1	VKORC1
STAT1	VPS41
STAT1	VRK2
STAT1	WARS
STAT1	WDR26
STAT1	WIPF2
STAT1	XAF1
STAT1	YPEL5
STAT1	ZBTB11-AS1
STAT1	ZBTB20
STAT1	ZBTB43
STAT1	ZBTB7B
STAT1	ZC3H7B
STAT1	ZFP36L2
STAT1	ZNF281
STAT1	ZNF566
STAT1	ZNF821
STAT1	ZNF865
STAT1	ZPBP2
STAT2	ADM
STAT2	AFTPH
STAT2	APOL2
STAT2	ARHGAP30
STAT2	ARL5B
STAT2	ATP1B1
STAT2	CD37
STAT2	CXorf21
STAT2	EVI5
STAT2	FAM172A
STAT2	FBXO11
STAT2	HIST1H1C
STAT2	HLA-B
STAT2	INO80
STAT2	IRF7

STAT2	KCNN3
STAT2	KRT10
STAT2	LEF1
STAT2	LMF1
STAT2	LRRK2
STAT2	ME2
STAT2	MEF2C
STAT2	METTL7A
STAT2	MIA3
STAT2	NCALD
STAT2	NEAT1
STAT2	NEDD9
STAT2	OAF
STAT2	PARP10
STAT2	PATZ1
STAT2	RHOBTB2
STAT2	RTN4
STAT2	SEC61A1
STAT2	SH2B1
STAT2	STAT2
STAT2	TNFRSF17
STAT2	TNRC6B
STAT2	TSC22D1
STAT2	UBE2L6
STAT2	USO1
STAT2	USP3
STAT2	VAMP5
STAT2	VAV1
STAT2	XPO6
STAT2	XRN1
STAT2	ZFX
STAT3	CHD1
STAT3	ETV6
STAT3	MLLT1
STAT3	POU2F2
STAT3	RPL18A
STAT3	SERPINF2
STAT3	SLC25A44
STAT3	SSBP4
STAT3	STAT3
STAT5A	ACTR6
STAT5A	ADIPOR2
STAT5A	ADO
STAT5A	ADSS
STAT5A	AEN
STAT5A	AHCY
STAT5A	AHSA1
STAT5A	AMD1
STAT5A	ARPP19
STAT5A	ASXL1

STAT5A	ATP13A2
STAT5A	AUP1
STAT5A	BOD1
STAT5A	C12orf29
STAT5A	C12orf43
STAT5A	C19orf70
STAT5A	C1orf109
STAT5A	CA11
STAT5A	CAND1
STAT5A	CAPN15
STAT5A	CARS
STAT5A	CCDC25
STAT5A	CCDC77
STAT5A	CCT7
STAT5A	CD163L1
STAT5A	CD3EAP
STAT5A	CDIP1
STAT5A	CFLAR
STAT5A	CHAC2
STAT5A	CHCHD3
STAT5A	CHMP4B
STAT5A	CIRBP
STAT5A	CLPB
STAT5A	CSNK2A1
STAT5A	CSNK2B
STAT5A	CTDP1
STAT5A	DDX49
STAT5A	DFFA
STAT5A	DIMT1
STAT5A	DPH2
STAT5A	DUS3L
STAT5A	EI24
STAT5A	EIF2S2
STAT5A	EIF4E
STAT5A	EIF5A
STAT5A	EIF5B
STAT5A	EML4
STAT5A	EMP3
STAT5A	ENY2
STAT5A	ERI3
STAT5A	FKBP4
STAT5A	FOSL1
STAT5A	FTH1
STAT5A	FTSJ1
STAT5A	FYN
STAT5A	GGPS1
STAT5A	GPD1L
STAT5A	GRWD1
STAT5A	GSPT1
STAT5A	HSPH1

STAT5A	IFRD1
STAT5A	IFRD2
STAT5A	ISG20L2
STAT5A	ISOC2
STAT5A	JARID2
STAT5A	KIF13B
STAT5A	KPNA1
STAT5A	KRR1
STAT5A	LARS
STAT5A	M6PR
STAT5A	MAD2L1BP
STAT5A	MARS
STAT5A	MAU2
STAT5A	MCMBP
STAT5A	MCRIP2
STAT5A	MRPL24
STAT5A	MRPL32
STAT5A	MRPL50
STAT5A	MRPS26
STAT5A	NCLN
STAT5A	NFKBIB
STAT5A	NMT1
STAT5A	NOP10
STAT5A	NOP14
STAT5A	NPL
STAT5A	NRROS
STAT5A	NUDCD1
STAT5A	NUTF2
STAT5A	OGFOD1
STAT5A	OTUD4
STAT5A	PA2G4
STAT5A	PABPC1
STAT5A	PEMT
STAT5A	PHB
STAT5A	PLEKHA1
STAT5A	PMM2
STAT5A	POLR1A
STAT5A	POLR1E
STAT5A	PRCC
STAT5A	PRMT5
STAT5A	PRPF19
STAT5A	PRPF31
STAT5A	PSMA2
STAT5A	PSMC4
STAT5A	PSMD11
STAT5A	PSMD4
STAT5A	RAB29
STAT5A	RAB34
STAT5A	RIC8B
STAT5A	RNF2

STAT5A	RPIA
STAT5A	RPL7
STAT5A	RPP40
STAT5A	RRS1
STAT5A	SCAMP3
STAT5A	SERBP1
STAT5A	SF3B5
STAT5A	SGF29
STAT5A	SHMT2
STAT5A	SLC20A2
STAT5A	SLC25A19
STAT5A	SLC27A4
STAT5A	SLC38A5
STAT5A	SLC7A1
STAT5A	SNHG12
STAT5A	SNW1
STAT5A	SNX11
STAT5A	SRRM2
STAT5A	STX4
STAT5A	TARBP2
STAT5A	TFAP4
STAT5A	THOC6
STAT5A	TIMM13
STAT5A	TMEM109
STAT5A	TMEM147
STAT5A	TMEM150A
STAT5A	TMEM208
STAT5A	TMEM33
STAT5A	TMEM43
STAT5A	TMEM69
STAT5A	TNPO2
STAT5A	TOB2
STAT5A	TOP3A
STAT5A	TP53
STAT5A	TRIP10
STAT5A	TSNAX
STAT5A	TSSC4
STAT5A	TXLNG
STAT5A	UBE2D1
STAT5A	UBE2N
STAT5A	UBQLN4
STAT5A	UBXN8
STAT5A	USP14
STAT5A	UTP11
STAT5A	VAPA
STAT5A	VDAC1
STAT5A	WDR12
STAT5A	WDR46
STAT5A	WRN
STAT5A	XPOT

STAT5A	YBX3
STAT5A	ZFAS1
STAT5A	ZFC3H1
STAT5A	ZFR
STAT5A	ZNF165
STAT5A	ZNF263
STAT5A	ZNF317
STAT5A	ZNF32
STAT5A	ZNF37A
STAT5A	ZNF383
STAT5A	ZNF583
STAT5A	ZNF587B
STAT5A	ZNF667-AS1
STAT5A	ZRANB3
TBP	BMP7
TBP	C16orf54
TBP	CCDC141
TBP	CTNNB1
TBP	FAM185A
TBP	HNRNPH1
TBP	LETMD1
TBP	MPZL1
TBP	NNT
TBP	RASGEF1B
TBP	SLX4IP
TBP	SPRED2
TBP	TBP
TBP	TSPYL1
TBX21	ANGPTL6
TBX21	COPS2
TBX21	CTGF
TBX21	CYSLTR1
TBX21	EMP3
TBX21	HK1
TBX21	RREB1
TBX21	TMBIM1
TBX21	TNFSF10
TCF12	ACP6
TCF12	AHDC1
TCF12	DUSP14
TCF12	GLRX2
TCF12	LDB1
TCF12	MOSPD1
TCF12	PLA2G12A
TCF12	SLC25A45
TCF12	SLC43A2
TCF12	TCF12
TCF12	UBE2V1
TCF4	CD24
TCF4	CXXC5

TCF4	HCK
TCF4	IL16
TCF4	PFDN5
TCF4	RPL10
TCF4	TCF4
TFDP1	A4GALT
TFDP1	AAAS
TFDP1	AASDHPPT
TFDP1	AATF
TFDP1	ABCB10
TFDP1	ABCD3
TFDP1	ABCF1
TFDP1	ABHD10
TFDP1	ABHD11
TFDP1	ABHD12
TFDP1	ABHD17A
TFDP1	ABHD17B
TFDP1	ABHD5
TFDP1	ABI1
TFDP1	ABI2
TFDP1	ABRACL
TFDP1	ABTB1
TFDP1	ACAA1
TFDP1	ACAA2
TFDP1	ACACA
TFDP1	ACAD9
TFDP1	ACAP2
TFDP1	ACAP3
TFDP1	ACAT2
TFDP1	ACBD6
TFDP1	ACLY
TFDP1	ACO2
TFDP1	ACOT13
TFDP1	ACOT7
TFDP1	ACOT8
TFDP1	ACSF2
TFDP1	ACSL5
TFDP1	ACTA2
TFDP1	ACTB
TFDP1	ACTL6A
TFDP1	ACTN4
TFDP1	ACTR1B
TFDP1	ACTR3
TFDP1	ACTR5
TFDP1	ACTR6
TFDP1	ACTR8
TFDP1	ACYP1
TFDP1	ADA
TFDP1	ADAM17
TFDP1	ADAM9

TFDP1	ADAT1
TFDP1	ADD1
TFDP1	ADD3
TFDP1	ADH5
TFDP1	ADIPOR1
TFDP1	ADIPOR2
TFDP1	ADNP
TFDP1	ADPRHL2
TFDP1	ADRM1
TFDP1	ADSL
TFDP1	ADSS
TFDP1	AEBP2
TFDP1	AEN
TFDP1	AGAP2
TFDP1	AGBL5
TFDP1	AGFG1
TFDP1	AGGF1
TFDP1	AGL
TFDP1	AGO1
TFDP1	AGPAT1
TFDP1	AGPAT2
TFDP1	AGTPBP1
TFDP1	AHCY
TFDP1	AHCYL1
TFDP1	AHI1
TFDP1	AHSA1
TFDP1	AIFM1
TFDP1	AIG1
TFDP1	AIP
TFDP1	AK2
TFDP1	AK3
TFDP1	AK4
TFDP1	AKAP1
TFDP1	AKAP13
TFDP1	AKAP8
TFDP1	AKIP1
TFDP1	AKIRIN2
TFDP1	AKR1B1
TFDP1	AKT2
TFDP1	ALAD
TFDP1	ALAS1
TFDP1	ALDH16A1
TFDP1	ALDH6A1
TFDP1	ALDH7A1
TFDP1	ALDH9A1
TFDP1	ALDOA
TFDP1	ALG13
TFDP1	ALG6
TFDP1	ALKBH5
TFDP1	AMD1

TFDP1	AMFR
TFDP1	ANAPC1
TFDP1	ANAPC10
TFDP1	ANAPC11
TFDP1	ANAPC13
TFDP1	ANAPC15
TFDP1	ANAPC4
TFDP1	ANAPC5
TFDP1	ANGEL2
TFDP1	ANK1
TFDP1	ANKH
TFDP1	ANKLE2
TFDP1	ANKRD13A
TFDP1	ANKRD13C
TFDP1	ANKRD17
TFDP1	ANKRD26
TFDP1	ANKRD33B
TFDP1	ANKRD39
TFDP1	ANKRD54
TFDP1	ANLN
TFDP1	ANP32A
TFDP1	ANP32B
TFDP1	ANP32E
TFDP1	ANXA11
TFDP1	ANXA5
TFDP1	ANXA6
TFDP1	AP1AR
TFDP1	AP1G2
TFDP1	AP1M1
TFDP1	AP1S1
TFDP1	AP1S2
TFDP1	AP2B1
TFDP1	AP2M1
TFDP1	AP2S1
TFDP1	AP3D1
TFDP1	AP4E1
TFDP1	AP4M1
TFDP1	AP5M1
TFDP1	APEX2
TFDP1	APIP
TFDP1	APLP2
TFDP1	APOLD1
TFDP1	APOM
TFDP1	APPBP2
TFDP1	APPL1
TFDP1	APPL2
TFDP1	ARF1
TFDP1	ARF3
TFDP1	ARF4-AS1
TFDP1	ARF5

TFDP1	ARF6
TFDP1	ARGLU1
TFDP1	ARHGAP1
TFDP1	ARHGAP10
TFDP1	ARHGAP11A
TFDP1	ARHGAP27
TFDP1	ARHGAP35
TFDP1	ARHGAP4
TFDP1	ARHGAP44
TFDP1	ARHGDIA
TFDP1	ARHGDIB
TFDP1	ARHGEF1
TFDP1	ARHGEF12
TFDP1	ARHGEF2
TFDP1	ARHGEF39
TFDP1	ARHGEF7
TFDP1	ARID1A
TFDP1	ARID1B
TFDP1	ARID4B
TFDP1	ARID5A
TFDP1	ARL13B
TFDP1	ARL16
TFDP1	ARL17A
TFDP1	ARL2
TFDP1	ARL3
TFDP1	ARL6IP1
TFDP1	ARL6IP6
TFDP1	ARMC1
TFDP1	ARPC1A
TFDP1	ARPC1B
TFDP1	ARPC2
TFDP1	ARPC3
TFDP1	ARPC4
TFDP1	ARPP19
TFDP1	ARV1
TFDP1	ASB1
TFDP1	ASB13
TFDP1	ASB16-AS1
TFDP1	ASB3
TFDP1	ASCC1
TFDP1	ASF1A
TFDP1	ASF1B
TFDP1	ASH2L
TFDP1	ASNA1
TFDP1	ASNSD1
TFDP1	ASPH
TFDP1	ASPSCR1
TFDP1	ASRGL1
TFDP1	ASXL1
TFDP1	ATAD2

TFDP1	ATAD3A
TFDP1	ATAD5
TFDP1	ATF1
TFDP1	ATF4
TFDP1	ATF6B
TFDP1	ATF7
TFDP1	ATF7IP2
TFDP1	ATG10
TFDP1	ATG12
TFDP1	ATG16L2
TFDP1	ATG3
TFDP1	ATG4D
TFDP1	ATG5
TFDP1	ATG7
TFDP1	ATM
TFDP1	ATP1A1
TFDP1	ATP23
TFDP1	ATP2A3
TFDP1	ATP6AP2
TFDP1	ATP6V0D1
TFDP1	ATP6V1A
TFDP1	ATP6V1C1
TFDP1	ATP6V1D
TFDP1	ATP6V1E1
TFDP1	ATP6V1F
TFDP1	ATP6V1H
TFDP1	ATPAF1
TFDP1	ATRAID
TFDP1	ATRIP
TFDP1	ATRX
TFDP1	ATXN10
TFDP1	ATXN2L
TFDP1	ATXN7L2
TFDP1	ATXN7L3
TFDP1	AUNIP
TFDP1	AURKB
TFDP1	AZIN1
TFDP1	AZIN1-AS1
TFDP1	B3GALNT1
TFDP1	B3GAT3
TFDP1	B3GNT2
TFDP1	B4GALT4
TFDP1	B9D2
TFDP1	BABAM1
TFDP1	BACH2
TFDP1	BAG5
TFDP1	BAG6
TFDP1	BANF1
TFDP1	BANP
TFDP1	BARD1

TFDP1	BASP1
TFDP1	BATF
TFDP1	BAZ1A
TFDP1	BAZ1B
TFDP1	BAZ2A
TFDP1	BBIP1
TFDP1	BCAP29
TFDP1	BCAP31
TFDP1	BCAR1
TFDP1	BCAR3
TFDP1	BCAS2
TFDP1	BCAT2
TFDP1	BCCIP
TFDP1	BCKDHB
TFDP1	BCL11A
TFDP1	BCL6
TFDP1	BCL7B
TFDP1	BCL7C
TFDP1	BCLAF1
TFDP1	BCOR
TFDP1	BDH1
TFDP1	BECN1
TFDP1	BEX3
TFDP1	BID
TFDP1	BIRC5
TFDP1	BLCAP
TFDP1	BLM
TFDP1	BLMH
TFDP1	BLOC1S1
TFDP1	BLOC1S5
TFDP1	BLVRB
TFDP1	BMP2K
TFDP1	BMP7
TFDP1	BNIP1
TFDP1	BNIP3
TFDP1	BOD1L1
TFDP1	BOLA1
TFDP1	BOLA3
TFDP1	BORA
TFDP1	BPNT1
TFDP1	BPTF
TFDP1	BRAT1
TFDP1	BRCA1
TFDP1	BRCA2
TFDP1	BRD2
TFDP1	BRD7
TFDP1	BRI3BP
TFDP1	BRMS1L
TFDP1	BROX
TFDP1	BRWD1

TFDP1	BTBD1
TFDP1	BTBD10
TFDP1	BTF3
TFDP1	BTF3L4
TFDP1	BTG1
TFDP1	BTG3
TFDP1	BTRC
TFDP1	BUB1B
TFDP1	BUB3
TFDP1	BUD31
TFDP1	BZW1
TFDP1	C11orf24
TFDP1	C11orf58
TFDP1	C12orf42
TFDP1	C12orf49
TFDP1	C12orf65
TFDP1	C12orf75
TFDP1	C15orf39
TFDP1	C16orf72
TFDP1	C16orf87
TFDP1	C16orf91
TFDP1	C17orf58
TFDP1	C17orf75
TFDP1	C18orf25
TFDP1	C19orf38
TFDP1	C19orf48
TFDP1	C19orf54
TFDP1	C1orf112
TFDP1	C1orf35
TFDP1	C1orf43
TFDP1	C1orf52
TFDP1	C1orf74
TFDP1	C1QBP
TFDP1	C21orf58
TFDP1	C21orf91
TFDP1	C2CD2L
TFDP1	C2orf49
TFDP1	C2orf74
TFDP1	C3orf14
TFDP1	C3orf38
TFDP1	C3orf67
TFDP1	C4orf33
TFDP1	C4orf46
TFDP1	C5
TFDP1	C5orf24
TFDP1	C5orf34
TFDP1	C5orf51
TFDP1	C6orf136
TFDP1	C6orf226
TFDP1	C6orf52

TFDP1	C6orf62
TFDP1	C6orf99
TFDP1	C8orf76
TFDP1	C9orf16
TFDP1	C9orf40
TFDP1	C9orf64
TFDP1	CAAP1
TFDP1	CAB39
TFDP1	CABLES2
TFDP1	CACYBP
TFDP1	CALM1
TFDP1	CALM2
TFDP1	CALM3
TFDP1	CAMK2G
TFDP1	CAMKK2
TFDP1	CAMLG
TFDP1	CAMTA1
TFDP1	CAND1
TFDP1	CAP1
TFDP1	CAPN1
TFDP1	CAPN7
TFDP1	CAPNS1
TFDP1	CAPRIN1
TFDP1	CAPZA1
TFDP1	CAPZA2
TFDP1	CAPZB
TFDP1	CARHSP1
TFDP1	CARNMT1
TFDP1	CARS
TFDP1	CASC4
TFDP1	CASP2
TFDP1	CASP3
TFDP1	CASP7
TFDP1	CASP8AP2
TFDP1	CBFA2T3
TFDP1	CBFB
TFDP1	CBL
TFDP1	CBR4
TFDP1	CBWD1
TFDP1	CBX1
TFDP1	CBX2
TFDP1	CBX3
TFDP1	CBX5
TFDP1	CC2D1A
TFDP1	CCAR1
TFDP1	CCAR2
TFDP1	CCDC102A
TFDP1	CCDC107
TFDP1	CCDC124
TFDP1	CCDC126

TFDP1	CCDC150
TFDP1	CCDC152
TFDP1	CCDC167
TFDP1	CCDC174
TFDP1	CCDC189
TFDP1	CCDC25
TFDP1	CCDC28A
TFDP1	CCDC28B
TFDP1	CCDC34
TFDP1	CCDC47
TFDP1	CCDC51
TFDP1	CCDC57
TFDP1	CCDC77
TFDP1	CCDC82
TFDP1	CCDC84
TFDP1	CCDC88A
TFDP1	CCDC90B
TFDP1	CCHCR1
TFDP1	CCM2
TFDP1	CCNC
TFDP1	CCND3
TFDP1	CCNE1
TFDP1	CCNE2
TFDP1	CCNF
TFDP1	CCNG1
TFDP1	CCNG2
TFDP1	CCNI
TFDP1	CCNK
TFDP1	CCNL2
TFDP1	CCNT1
TFDP1	CCP110
TFDP1	CCSAP
TFDP1	CCT2
TFDP1	CCT5
TFDP1	CCT7
TFDP1	CCT8
TFDP1	CCZ1
TFDP1	CCZ1B
TFDP1	CD164
TFDP1	CD2AP
TFDP1	CD2BP2
TFDP1	CD47
TFDP1	CD79B
TFDP1	CDC123
TFDP1	CDC16
TFDP1	CDC23
TFDP1	CDC25B
TFDP1	CDC27
TFDP1	CDC34
TFDP1	CDC37

TFDP1	CDC37L1
TFDP1	CDC40
TFDP1	CDC42
TFDP1	CDC42EP3
TFDP1	CDC42SE2
TFDP1	CDC45
TFDP1	CDC5L
TFDP1	CDC6
TFDP1	CDC7
TFDP1	CDC73
TFDP1	CDCA4
TFDP1	CDCA5
TFDP1	CDCA7
TFDP1	CDCA7L
TFDP1	CDIPT
TFDP1	CDK1
TFDP1	CDK11B
TFDP1	CDK12
TFDP1	CDK16
TFDP1	CDK19
TFDP1	CDK2
TFDP1	CDK2AP2
TFDP1	CDK4
TFDP1	CDK5RAP2
TFDP1	CDK5RAP3
TFDP1	CDK6
TFDP1	CDK9
TFDP1	CDKN2A
TFDP1	CDKN2AIP
TFDP1	CDKN2AIPNL
TFDP1	CDKN2C
TFDP1	CDKN2D
TFDP1	CDKN3
TFDP1	CDS2
TFDP1	CDT1
TFDP1	CDV3
TFDP1	CEBPD
TFDP1	CEBPG
TFDP1	CEBPZOS
TFDP1	CELF1
TFDP1	CENPH
TFDP1	CENPJ
TFDP1	CENPK
TFDP1	CENPL
TFDP1	CENPM
TFDP1	CENPN
TFDP1	CENPO
TFDP1	CENPP
TFDP1	CENPQ
TFDP1	CENPT

TFDP1	CENPU
TFDP1	CENPW
TFDP1	CEP152
TFDP1	CEP192
TFDP1	CEP295
TFDP1	CEP350
TFDP1	CEP44
TFDP1	CEP57
TFDP1	CEP57L1
TFDP1	CEP63
TFDP1	CEP78
TFDP1	CEP89
TFDP1	CEP97
TFDP1	CETN2
TFDP1	CETN3
TFDP1	CFAP20
TFDP1	CFAP73
TFDP1	CFDP1
TFDP1	CFL1
TFDP1	CFL2
TFDP1	CGGBP1
TFDP1	CHAC2
TFDP1	CHAF1A
TFDP1	CHAF1B
TFDP1	CHAMP1
TFDP1	CHCHD1
TFDP1	CHCHD10
TFDP1	CHCHD2
TFDP1	CHCHD3
TFDP1	CHCHD5
TFDP1	CHCHD7
TFDP1	CHD3
TFDP1	CHD4
TFDP1	CHD6
TFDP1	CHD8
TFDP1	CHEK1
TFDP1	CHEK2
TFDP1	CHERP
TFDP1	CHIC2
TFDP1	CHMP1A
TFDP1	CHMP4B
TFDP1	CHMP5
TFDP1	CHP1
TFDP1	CHRAC1
TFDP1	CHST14
TFDP1	CHTF18
TFDP1	CHTF8
TFDP1	CHTOP
TFDP1	CHUK
TFDP1	CHURC1

TFDP1	CIAPIN1
TFDP1	CIB1
TFDP1	CIC
TFDP1	CIRBP
TFDP1	CIT
TFDP1	CIZ1
TFDP1	CKLF
TFDP1	CKS1B
TFDP1	CKS2
TFDP1	CLDN23
TFDP1	CLDND1
TFDP1	CLEC16A
TFDP1	CLIC4
TFDP1	CLINT1
TFDP1	CLK1
TFDP1	CLK3
TFDP1	CLN6
TFDP1	CLNS1A
TFDP1	CLP1
TFDP1	CLPX
TFDP1	CLSPN
TFDP1	CLSTN1
TFDP1	CLTA
TFDP1	CLTB
TFDP1	CLTC
TFDP1	CMC2
TFDP1	CMPK1
TFDP1	CNBP
TFDP1	CNIH1
TFDP1	CNOT1
TFDP1	CNOT10
TFDP1	CNOT11
TFDP1	CNOT2
TFDP1	CNOT3
TFDP1	CNOT4
TFDP1	CNOT6L
TFDP1	CNOT7
TFDP1	CNOT8
TFDP1	CNOT9
TFDP1	CNP
TFDP1	CNPPD1
TFDP1	CNTLN
TFDP1	COA5
TFDP1	COA6
TFDP1	COASY
TFDP1	COCH
TFDP1	COG4
TFDP1	COLGALT1
TFDP1	COMMD1
TFDP1	COMMD4

TFDP1	COMMD5
TFDP1	COMMD6
TFDP1	COMTD1
TFDP1	COPE
TFDP1	COPRS
TFDP1	COPS3
TFDP1	COPS4
TFDP1	COPS5
TFDP1	COPS7A
TFDP1	COPS7B
TFDP1	COQ10A
TFDP1	COQ4
TFDP1	COQ7
TFDP1	COQ9
TFDP1	CORO1A
TFDP1	CORO1B
TFDP1	COTL1
TFDP1	COX11
TFDP1	COX14
TFDP1	COX17
TFDP1	COX4I1
TFDP1	COX5A
TFDP1	COX5B
TFDP1	COX6A1
TFDP1	COX6B1
TFDP1	COX7B
TFDP1	COX7C
TFDP1	COX8A
TFDP1	CPNE1
TFDP1	CPOX
TFDP1	CPPED1
TFDP1	CPSF1
TFDP1	CPSF2
TFDP1	CPSF3
TFDP1	CPSF7
TFDP1	CPT2
TFDP1	CRADD
TFDP1	CRBN
TFDP1	CREB1
TFDP1	CREBBP
TFDP1	CREBZF
TFDP1	CRIP1
TFDP1	CRIPT
TFDP1	CRK
TFDP1	CRLS1
TFDP1	CRNDE
TFDP1	CROT
TFDP1	CRTC2
TFDP1	CRYBB2
TFDP1	CS

TFDP1	CSDE1
TFDP1	CSE1L
TFDP1	CSRP1
TFDP1	CSTF1
TFDP1	CSTF2
TFDP1	CSTF2T
TFDP1	CSTF3
TFDP1	CTCF
TFDP1	CTDNEP1
TFDP1	CTDP1
TFDP1	CTDSP1
TFDP1	CTDSP2
TFDP1	CTDSPL2
TFDP1	CTNNA1
TFDP1	CTNNAL1
TFDP1	CTNNBIP1
TFDP1	CTNNBL1
TFDP1	CTPS1
TFDP1	CTTN
TFDP1	CUEDC2
TFDP1	CUL1
TFDP1	CUL3
TFDP1	CUL4A
TFDP1	CUL4B
TFDP1	CUTA
TFDP1	CUTC
TFDP1	CUX1
TFDP1	CWC22
TFDP1	CWC25
TFDP1	CWC27
TFDP1	CXCR4
TFDP1	CXorf56
TFDP1	CXXC5
TFDP1	CYB5A
TFDP1	CYB5B
TFDP1	CYB5D2
TFDP1	CYB5R3
TFDP1	CYB5R4
TFDP1	CYB5RL
TFDP1	CYC1
TFDP1	CYFIP2
TFDP1	DAD1
TFDP1	DARS
TFDP1	DARS-AS1
TFDP1	DAXX
TFDP1	DAZAP1
TFDP1	DAZAP2
TFDP1	DBF4
TFDP1	DBI
TFDP1	DCAF11

TFDP1	DCAF12
TFDP1	DCAF15
TFDP1	DCAF5
TFDP1	DCAF6
TFDP1	DCAF7
TFDP1	DCAKD
TFDP1	DCK
TFDP1	DCLRE1B
TFDP1	DCLRE1C
TFDP1	DCP2
TFDP1	DCTN2
TFDP1	DCTN3
TFDP1	DCTPP1
TFDP1	DCUN1D4
TFDP1	DCUN1D5
TFDP1	DCXR
TFDP1	DDA1
TFDP1	DDB1
TFDP1	DDB2
TFDP1	DDT
TFDP1	DDX11
TFDP1	DDX17
TFDP1	DDX18
TFDP1	DDX19B
TFDP1	DDX23
TFDP1	DDX27
TFDP1	DDX39A
TFDP1	DDX3X
TFDP1	DDX42
TFDP1	DDX46
TFDP1	DDX49
TFDP1	DDX50
TFDP1	DDX55
TFDP1	DEGS1
TFDP1	DEK
TFDP1	DENND2D
TFDP1	DENND4A
TFDP1	DENR
TFDP1	DEPDC1B
TFDP1	DEPDC5
TFDP1	DESI2
TFDP1	DFFA
TFDP1	DGCR6L
TFDP1	DGCR8
TFDP1	DGKG
TFDP1	DGKH
TFDP1	DGUOK
TFDP1	DHCR24
TFDP1	DHFR
TFDP1	DHPS

TFDP1	DHRS1
TFDP1	DHRS4
TFDP1	DHRS4L2
TFDP1	DHRS7B
TFDP1	DHX15
TFDP1	DHX16
TFDP1	DHX30
TFDP1	DHX36
TFDP1	DHX9
TFDP1	DIAPH3
TFDP1	DICER1
TFDP1	DIDO1
TFDP1	DIS3
TFDP1	DIS3L
TFDP1	DLD
TFDP1	DLEU2
TFDP1	DLG1
TFDP1	DLG3
TFDP1	DLST
TFDP1	DMC1
TFDP1	DMD
TFDP1	DMTF1
TFDP1	DNAJA1
TFDP1	DNAJA2
TFDP1	DNAJA4
TFDP1	DNAJC15
TFDP1	DNAJC19
TFDP1	DNAJC21
TFDP1	DNAJC5
TFDP1	DNAJC9
TFDP1	DNAL1
TFDP1	DNM1L
TFDP1	DNMT1
TFDP1	DNTTIP1
TFDP1	DOCK2
TFDP1	DOHH
TFDP1	DONSON
TFDP1	DOPEY2
TFDP1	DPM1
TFDP1	DPM3
TFDP1	DPP8
TFDP1	DPP9
TFDP1	DPY30
TFDP1	DPYSL2
TFDP1	DR1
TFDP1	DRAM2
TFDP1	DRG1
TFDP1	DSCC1
TFDP1	DSN1
TFDP1	DTNBP1

TFDP1	DTX1
TFDP1	DTX2
TFDP1	DTYMK
TFDP1	DUS1L
TFDP1	DUSP1
TFDP1	DUSP15
TFDP1	DUSP23
TFDP1	DUT
TFDP1	DVL2
TFDP1	DVL3
TFDP1	DYNC1H1
TFDP1	DYNC1I2
TFDP1	DYNC1LI1
TFDP1	DYNLL2
TFDP1	DYNLT1
TFDP1	DYRK1A
TFDP1	E2F1
TFDP1	E2F5
TFDP1	E2F7
TFDP1	E2F8
TFDP1	EBAG9
TFDP1	EBF1
TFDP1	EBNA1BP2
TFDP1	EBP
TFDP1	ECH1
TFDP1	EDF1
TFDP1	EDRF1
TFDP1	EEA1
TFDP1	EED
TFDP1	EEF1A1
TFDP1	EEF1B2
TFDP1	EEF1E1
TFDP1	EEF1G
TFDP1	EEF2
TFDP1	EEFSEC
TFDP1	EEPD1
TFDP1	EFCAB12
TFDP1	EFHD2
TFDP1	EFTUD2
TFDP1	EGLN2
TFDP1	EGR1
TFDP1	EHBP1
TFDP1	EHMT2
TFDP1	EID1
TFDP1	EID2
TFDP1	EIF1
TFDP1	EIF1AD
TFDP1	EIF1AX
TFDP1	EIF1B
TFDP1	EIF2AK1

TFDP1	EIF2AK2
TFDP1	EIF2AK3
TFDP1	EIF2B2
TFDP1	EIF2D
TFDP1	EIF2S1
TFDP1	EIF2S2
TFDP1	EIF2S3
TFDP1	EIF3A
TFDP1	EIF3D
TFDP1	EIF3F
TFDP1	EIF3K
TFDP1	EIF3L
TFDP1	EIF4A2
TFDP1	EIF4A3
TFDP1	EIF4B
TFDP1	EIF4E
TFDP1	EIF4E2
TFDP1	EIF4EBP2
TFDP1	EIF4G1
TFDP1	EIF4G2
TFDP1	EIF4H
TFDP1	EIF5
TFDP1	EIF5A
TFDP1	EIF5AL1
TFDP1	ELAC1
TFDP1	ELAC2
TFDP1	ELAVL1
TFDP1	ELMSAN1
TFDP1	ELOF1
TFDP1	ELOVL1
TFDP1	ELOVL5
TFDP1	ELOVL6
TFDP1	ELP3
TFDP1	ELP4
TFDP1	ELP5
TFDP1	ELP6
TFDP1	EMB
TFDP1	EMC4
TFDP1	EMC8
TFDP1	EME1
TFDP1	EML3
TFDP1	EML6
TFDP1	ENKD1
TFDP1	ENO1
TFDP1	ENO2
TFDP1	ENOPH1
TFDP1	ENSA
TFDP1	ENY2
TFDP1	EP300
TFDP1	EPC1

TFDP1	EPC2
TFDP1	EPN1
TFDP1	EPS15
TFDP1	EPS15L1
TFDP1	ERAL1
TFDP1	ERBIN
TFDP1	ERCC1
TFDP1	ERCC2
TFDP1	ERCC3
TFDP1	ERCC6L
TFDP1	ERGIC1
TFDP1	ERGIC3
TFDP1	ERH
TFDP1	ERI3
TFDP1	ERLIN1
TFDP1	ESCO2
TFDP1	ESD
TFDP1	ESPL1
TFDP1	ESRRA
TFDP1	ETF1
TFDP1	ETFA
TFDP1	ETV5
TFDP1	EWSR1
TFDP1	EXOC1
TFDP1	EXOC5
TFDP1	EXOC6
TFDP1	EXOG
TFDP1	EXOSC1
TFDP1	EXOSC10
TFDP1	EXOSC2
TFDP1	EXOSC8
TFDP1	EXOSC9
TFDP1	EZH2
TFDP1	EZR
TFDP1	FAAP20
TFDP1	FAAP24
TFDP1	FADD
TFDP1	FADS3
TFDP1	FAF1
TFDP1	FAF2
TFDP1	FAHD2A
TFDP1	FAIM
TFDP1	FAM104A
TFDP1	FAM107B
TFDP1	FAM110A
TFDP1	FAM111A
TFDP1	FAM111B
TFDP1	FAM122B
TFDP1	FAM126A
TFDP1	FAM129A

TFDP1	FAM133B
TFDP1	FAM135A
TFDP1	FAM149B1
TFDP1	FAM185A
TFDP1	FAM192A
TFDP1	FAM200A
TFDP1	FAM204A
TFDP1	FAM207A
TFDP1	FAM208A
TFDP1	FAM208B
TFDP1	FAM213A
TFDP1	FAM217B
TFDP1	FAM219B
TFDP1	FAM221A
TFDP1	FAM24B
TFDP1	FAM32A
TFDP1	FAM49B
TFDP1	FAM50A
TFDP1	FAM69A
TFDP1	FAM76A
TFDP1	FAM76B
TFDP1	FAM81A
TFDP1	FAM96A
TFDP1	FAM98C
TFDP1	FANCA
TFDP1	FANCB
TFDP1	FANCD2
TFDP1	FANCE
TFDP1	FANCG
TFDP1	FANCI
TFDP1	FANCL
TFDP1	FARP2
TFDP1	FASTK
TFDP1	FAU
TFDP1	FBL
TFDP1	FBXL19
TFDP1	FBXO22
TFDP1	FBXO33
TFDP1	FBXO34
TFDP1	FBXO38
TFDP1	FBXO4
TFDP1	FBXO45
TFDP1	FBXO46
TFDP1	FBXO48
TFDP1	FBXO5
TFDP1	FBXO7
TFDP1	FBXO9
TFDP1	FBXW8
TFDP1	FCF1
TFDP1	FCHO1

TFDP1	FCHSD2
TFDP1	FDFT1
TFDP1	FDPS
TFDP1	FEM1B
TFDP1	FEN1
TFDP1	FERMT3
TFDP1	FGD6
TFDP1	FGFR1OP
TFDP1	FGFR1OP2
TFDP1	FH
TFDP1	FHIT
TFDP1	FHOD1
TFDP1	FIBP
TFDP1	FIGNL1
TFDP1	FIP1L1
TFDP1	FIS1
TFDP1	FIZ1
TFDP1	FKBP1A
TFDP1	FKBP3
TFDP1	FKBP5
TFDP1	FLAD1
TFDP1	FLI1
TFDP1	FLOT1
TFDP1	FLOT2
TFDP1	FLVCR1
TFDP1	FN3KRP
TFDP1	FNBP1
TFDP1	FNIP1
TFDP1	FNTA
TFDP1	FNTB
TFDP1	FOXK2
TFDP1	FOXM1
TFDP1	FOXO1
TFDP1	FOXRED1
TFDP1	FPGS
TFDP1	FRA10AC1
TFDP1	FRG1
TFDP1	FRYL
TFDP1	FUBP1
TFDP1	FUBP3
TFDP1	FUNDC1
TFDP1	FURIN
TFDP1	FUS
TFDP1	FUZ
TFDP1	FXR2
TFDP1	FYTTD1
TFDP1	FZR1
TFDP1	G3BP1
TFDP1	G3BP2
TFDP1	G6PD

TFDP1	GABARAP
TFDP1	GABARAPL2
TFDP1	GABPB1
TFDP1	GABPB1-AS1
TFDP1	GABPB2
TFDP1	GADD45B
TFDP1	GAK
TFDP1	GALE
TFDP1	GALK1
TFDP1	GALNT7
TFDP1	GANAB
TFDP1	GAPDH
TFDP1	GART
TFDP1	GATAD2A
TFDP1	GATB
TFDP1	GBA
TFDP1	GCAT
TFDP1	GCDH
TFDP1	GCH1
TFDP1	GCHFR
TFDP1	GCLM
TFDP1	GCNT1
TFDP1	GDI1
TFDP1	GDI2
TFDP1	GEMIN2
TFDP1	GEN1
TFDP1	GFI1
TFDP1	GFOD1
TFDP1	GFOD2
TFDP1	GGCT
TFDP1	GGCX
TFDP1	GGH
TFDP1	GGNBP2
TFDP1	GGPS1
TFDP1	GHITM
TFDP1	GID8
TFDP1	GINM1
TFDP1	GINS1
TFDP1	GINS2
TFDP1	GINS3
TFDP1	GINS4
TFDP1	GJC1
TFDP1	GKAP1
TFDP1	GLA
TFDP1	GLI4
TFDP1	GLO1
TFDP1	GLRX2
TFDP1	GLRX3
TFDP1	GLRX5
TFDP1	GLS

TFDP1	GLTP
TFDP1	GLUD1
TFDP1	GLUL
TFDP1	GLYCTK
TFDP1	GLYR1
TFDP1	GMCL1
TFDP1	GMDS
TFDP1	GMIP
TFDP1	GMNN
TFDP1	GMPS
TFDP1	GNA13
TFDP1	GNAI2
TFDP1	GNAI3
TFDP1	GNAS
TFDP1	GNB1
TFDP1	GNB1L
TFDP1	GNB2
TFDP1	GNB4
TFDP1	GNB5
TFDP1	GNL1
TFDP1	GOLGA7
TFDP1	GOLPH3
TFDP1	GOPC
TFDP1	GOSR1
TFDP1	GOSR2
TFDP1	GPALPP1
TFDP1	GPANK1
TFDP1	GPAT4
TFDP1	GPATCH11
TFDP1	GPATCH8
TFDP1	GPBP1
TFDP1	GPD2
TFDP1	GPI
TFDP1	GPKOW
TFDP1	GPN2
TFDP1	GPN3
TFDP1	GPR137
TFDP1	GPR180
TFDP1	GPT2
TFDP1	GRB2
TFDP1	GRIPAP1
TFDP1	GRK2
TFDP1	GRK4
TFDP1	GRPEL1
TFDP1	GRPEL2
TFDP1	GSAP
TFDP1	GSDMD
TFDP1	GSK3A
TFDP1	GSK3B
TFDP1	GSKIP

TFDP1	GSPT1
TFDP1	GSR
TFDP1	GSTO1
TFDP1	GTDC1
TFDP1	GTF2A2
TFDP1	GTF2E2
TFDP1	GTF2F1
TFDP1	GTF2I
TFDP1	GTF2IRD2
TFDP1	GTF3A
TFDP1	GTF3C2
TFDP1	GTF3C3
TFDP1	GTF3C5
TFDP1	GTPBP1
TFDP1	GTPBP3
TFDP1	GTPBP8
TFDP1	GTSF1
TFDP1	GUCD1
TFDP1	GUK1
TFDP1	GYG1
TFDP1	H1FX-AS1
TFDP1	H2AFJ
TFDP1	H2AFV
TFDP1	H2AFX
TFDP1	H2AFY
TFDP1	H2AFZ
TFDP1	H3F3A
TFDP1	H3F3B
TFDP1	HACD2
TFDP1	HACD3
TFDP1	HARS2
TFDP1	HAT1
TFDP1	HAUS1
TFDP1	HAUS2
TFDP1	HAUS3
TFDP1	HAUS4
TFDP1	HAUS5
TFDP1	HAUS6
TFDP1	HAUS7
TFDP1	HAUS8
TFDP1	HBS1L
TFDP1	HCCS
TFDP1	HCFC1
TFDP1	HCLS1
TFDP1	HCN3
TFDP1	HDAC1
TFDP1	HDAC2
TFDP1	HDAC6
TFDP1	HDGF
TFDP1	HDHD2

TFDP1	HDLBP
TFDP1	HECTD4
TFDP1	HELLS
TFDP1	HENMT1
TFDP1	HERC1
TFDP1	HERC2
TFDP1	HERC3
TFDP1	HERC4
TFDP1	HES6
TFDP1	HEXIM2
TFDP1	HIGD1A
TFDP1	HIGD2A
TFDP1	HINT1
TFDP1	HINT2
TFDP1	HIRIP3
TFDP1	HIST1H1D
TFDP1	HIST1H1E
TFDP1	HIST1H2BK
TFDP1	HIST1H2BN
TFDP1	HIST1H3A
TFDP1	HIST1H4B
TFDP1	HIST1H4C
TFDP1	HIST1H4I
TFDP1	HIST2H2AC
TFDP1	HIVEP1
TFDP1	HK1
TFDP1	HLTF
TFDP1	HMBS
TFDP1	HMCES
TFDP1	HMG20B
TFDP1	HMGA1
TFDP1	HMGB1
TFDP1	HMGB2
TFDP1	HMGB3
TFDP1	HMGCR
TFDP1	HMGCS1
TFDP1	HMGN1
TFDP1	HMGN2
TFDP1	HMGN3
TFDP1	HMGXB3
TFDP1	HMGXB4
TFDP1	HNRNPA0
TFDP1	HNRNPA1
TFDP1	HNRNPA2B1
TFDP1	HNRNPA3
TFDP1	HNRNPAB
TFDP1	HNRNPC
TFDP1	HNRNPD
TFDP1	HNRNPF
TFDP1	HNRNPH1

TFDP1	HNRNPH3
TFDP1	HNRNPK
TFDP1	HNRNPL
TFDP1	HNRNPM
TFDP1	HNRNPR
TFDP1	HNRNPU
TFDP1	HNRNPUL1
TFDP1	HNRNPUL2
TFDP1	HOXA7
TFDP1	HP1BP3
TFDP1	HPF1
TFDP1	HPRT1
TFDP1	HPS5
TFDP1	HRAS
TFDP1	HRK
TFDP1	HSD17B12
TFDP1	HSDL2
TFDP1	HSF1
TFDP1	HSF2
TFDP1	HSP90AA1
TFDP1	HSP90AB1
TFDP1	HSPA2
TFDP1	HSPA4
TFDP1	HSPA8
TFDP1	HSPA9
TFDP1	HSPB11
TFDP1	HSPBAP1
TFDP1	HSPBP1
TFDP1	HSPD1
TFDP1	HSPH1
TFDP1	HTATSF1
TFDP1	HUS1
TFDP1	HYI
TFDP1	IARS2
TFDP1	ICE2
TFDP1	ICMT
TFDP1	ID2
TFDP1	IDH2
TFDP1	IDH3A
TFDP1	IDH3B
TFDP1	IDI1
TFDP1	IFI16
TFDP1	IFNGR2
TFDP1	IFT22
TFDP1	IFT52
TFDP1	IFT74
TFDP1	IFT80
TFDP1	IGBP1
TFDP1	IGF2BP1
TFDP1	IGF2BP3

TFDP1	IKBKB
TFDP1	IKBKE
TFDP1	IKBKG
TFDP1	IKZF1
TFDP1	IKZF3
TFDP1	IL4R
TFDP1	ILF2
TFDP1	ILF3
TFDP1	ILKAP
TFDP1	IMMP1L
TFDP1	IMP3
TFDP1	IMPA2
TFDP1	IMPACT
TFDP1	IMPDH2
TFDP1	INAFM1
TFDP1	INF2
TFDP1	ING1
TFDP1	ING2
TFDP1	ING3
TFDP1	ING4
TFDP1	INIP
TFDP1	INO80C
TFDP1	INPP1
TFDP1	INPP5B
TFDP1	INSIG1
TFDP1	INSIG2
TFDP1	INSR
TFDP1	INTS10
TFDP1	INTS4
TFDP1	INTS7
TFDP1	INTS8
TFDP1	IP6K2
TFDP1	IPO11
TFDP1	IPO9
TFDP1	IPPK
TFDP1	IQCB1
TFDP1	IQCD
TFDP1	IQGAP1
TFDP1	IQGAP3
TFDP1	IREB2
TFDP1	IRF1
TFDP1	IRF2
TFDP1	IRF2BP1
TFDP1	IRF8
TFDP1	IST1
TFDP1	ISY1
TFDP1	ISYNA1
TFDP1	ITGB1
TFDP1	ITGB1BP1
TFDP1	ITPA

TFDP1	ITPKB
TFDP1	ITPR3
TFDP1	ITSN2
TFDP1	IWS1
TFDP1	JADE1
TFDP1	JADE2
TFDP1	JAGN1
TFDP1	JAK1
TFDP1	JMJD1C
TFDP1	KANK1
TFDP1	KANK2
TFDP1	KANSL1
TFDP1	KANSL1L
TFDP1	KANSL2
TFDP1	KANSL3
TFDP1	KATNA1
TFDP1	KATNAL1
TFDP1	KATNB1
TFDP1	KATNBL1
TFDP1	KBTBD4
TFDP1	KBTBD7
TFDP1	KCMF1
TFDP1	KCNAB2
TFDP1	KCNK12
TFDP1	KCNMB4
TFDP1	KCTD18
TFDP1	KCTD20
TFDP1	KCTD5
TFDP1	KDELC2
TFDP1	KDM1A
TFDP1	KDM1B
TFDP1	KDM2A
TFDP1	KDM2B
TFDP1	KDM5A
TFDP1	KEAP1
TFDP1	KHDRBS1
TFDP1	KHNYN
TFDP1	KHSRP
TFDP1	KIAA0586
TFDP1	KIAA1468
TFDP1	KIAA1586
TFDP1	KIF11
TFDP1	KIF13A
TFDP1	KIF16B
TFDP1	KIF1BP
TFDP1	KIF20B
TFDP1	KIF22
TFDP1	KIF2A
TFDP1	KIFAP3
TFDP1	KIFC1

TFDP1	KLC1
TFDP1	KLF10
TFDP1	KLHDC2
TFDP1	KLHDC3
TFDP1	KLHL12
TFDP1	KLHL22
TFDP1	KLHL23
TFDP1	KLHL24
TFDP1	KLHL42
TFDP1	KLHL5
TFDP1	KLHL7
TFDP1	KMT2B
TFDP1	KMT5A
TFDP1	KMT5C
TFDP1	KNTC1
TFDP1	KPNA1
TFDP1	KPNA2
TFDP1	KPNA3
TFDP1	KPNA6
TFDP1	KPNB1
TFDP1	KRAS
TFDP1	KREMEN2
TFDP1	KRIT1
TFDP1	KTN1
TFDP1	KXD1
TFDP1	KYAT1
TFDP1	KYAT3
TFDP1	L3MBTL2
TFDP1	LACC1
TFDP1	LACTB2
TFDP1	LAGE3
TFDP1	LAMP1
TFDP1	LAMTOR2
TFDP1	LAMTOR3
TFDP1	LAMTOR4
TFDP1	LAP3
TFDP1	LARP7
TFDP1	LAS1L
TFDP1	LBR
TFDP1	LCMT1
TFDP1	LCMT2
TFDP1	LCP1
TFDP1	LDHA
TFDP1	LDHB
TFDP1	LEF1
TFDP1	LEMD2
TFDP1	LENG1
TFDP1	LETM2
TFDP1	LIG1
TFDP1	LIMS1

TFDP1	LIN52
TFDP1	LIN54
TFDP1	LIN7C
TFDP1	LIN9
TFDP1	LINC00467
TFDP1	LINC00847
TFDP1	LINC01006
TFDP1	LINC01184
TFDP1	LMAN2
TFDP1	LMBR1
TFDP1	LMBRD2
TFDP1	LMCD1
TFDP1	LMNB1
TFDP1	LMNB2
TFDP1	LONP1
TFDP1	LOXL2
TFDP1	LPAR2
TFDP1	LPCAT3
TFDP1	LPGAT1
TFDP1	LPXN
TFDP1	LRBA
TFDP1	LRCH1
TFDP1	LRIF1
TFDP1	LRR1
TFDP1	LRRC1
TFDP1	LRRC14
TFDP1	LRRC28
TFDP1	LRRC40
TFDP1	LRRC45
TFDP1	LRRC58
TFDP1	LRRC59
TFDP1	LRRC8A
TFDP1	LRRCC1
TFDP1	LRRFIP2
TFDP1	LRRK1
TFDP1	LSG1
TFDP1	LSM1
TFDP1	LSM14A
TFDP1	LSM2
TFDP1	LSM3
TFDP1	LSM4
TFDP1	LSM5
TFDP1	LSM6
TFDP1	LSM8
TFDP1	LTA4H
TFDP1	LTN1
TFDP1	LUC7L
TFDP1	LUC7L2
TFDP1	LUC7L3
TFDP1	LYPLA1

TFDP1	LYPLA2
TFDP1	LYRM1
TFDP1	LYRM2
TFDP1	LZIC
TFDP1	M6PR
TFDP1	MAD1L1
TFDP1	MAD2L1
TFDP1	MAD2L1BP
TFDP1	MAD2L2
TFDP1	MAEA
TFDP1	MAGED2
TFDP1	MAGEF1
TFDP1	MAGOH
TFDP1	MAGOHB
TFDP1	MALSU1
TFDP1	MAML1
TFDP1	MAN2A1
TFDP1	MAP2K1
TFDP1	MAP2K2
TFDP1	MAP3K11
TFDP1	MAP3K2
TFDP1	MAP3K3
TFDP1	MAP3K7
TFDP1	MAP3K8
TFDP1	MAP4K1
TFDP1	MAP4K2
TFDP1	MAP4K4
TFDP1	MAP7D1
TFDP1	MAP7D3
TFDP1	MAPK1
TFDP1	MAPK14
TFDP1	MAPK1IP1L
TFDP1	MAPK8
TFDP1	MAPK9
TFDP1	MAPKAP1
TFDP1	MAPRE1
TFDP1	MAPRE2
TFDP1	MARCH7
TFDP1	MARCKSL1
TFDP1	MARK3
TFDP1	MASTL
TFDP1	MAT2A
TFDP1	MAU2
TFDP1	MAX
TFDP1	MAZ
TFDP1	MBD1
TFDP1	MBD2
TFDP1	MBIP
TFDP1	MBNL1
TFDP1	MBNL3

TFDP1	MBOAT2
TFDP1	MCAT
TFDP1	MCM10
TFDP1	MCM2
TFDP1	MCM3
TFDP1	MCM3AP
TFDP1	MCM4
TFDP1	MCM5
TFDP1	MCM6
TFDP1	MCM7
TFDP1	MCM8
TFDP1	MCMBP
TFDP1	MCRS1
TFDP1	MCTS1
TFDP1	MCU
TFDP1	MCUR1
TFDP1	MDH1
TFDP1	MDM1
TFDP1	MDM2
TFDP1	MDM4
TFDP1	MEA1
TFDP1	MEAF6
TFDP1	MECP2
TFDP1	MECR
TFDP1	MED11
TFDP1	MED15
TFDP1	MED17
TFDP1	MED18
TFDP1	MED21
TFDP1	MED25
TFDP1	MED27
TFDP1	MED28
TFDP1	MED30
TFDP1	MED4
TFDP1	MED6
TFDP1	MED7
TFDP1	MEF2A
TFDP1	MEF2C
TFDP1	MEIOC
TFDP1	MELK
TFDP1	MEMO1
TFDP1	MEN1
TFDP1	MEST
TFDP1	METAP1
TFDP1	METAP2
TFDP1	METTL17
TFDP1	METTL21A
TFDP1	METTL2A
TFDP1	METTL2B
TFDP1	METTL3

TFDP1	METTL4
TFDP1	METTL9
TFDP1	MFAP1
TFDP1	MFF
TFDP1	MFGE8
TFDP1	MFHAS1
TFDP1	MFN1
TFDP1	MFSD11
TFDP1	MFSD14B
TFDP1	MFSD14C
TFDP1	MGA
TFDP1	MGME1
TFDP1	MGMT
TFDP1	MICU1
TFDP1	MICU2
TFDP1	MID1IP1
TFDP1	MIER1
TFDP1	MIF
TFDP1	MIF4GD
TFDP1	MINOS1
TFDP1	MINPP1
TFDP1	MIR181A1HG
TFDP1	MIS12
TFDP1	MIS18A
TFDP1	MIS18BP1
TFDP1	MKI67
TFDP1	MKL1
TFDP1	MKNK2
TFDP1	MKS1
TFDP1	MLF2
TFDP1	MLH1
TFDP1	MLLT1
TFDP1	MLLT10
TFDP1	MLLT11
TFDP1	MLST8
TFDP1	MLXIP
TFDP1	MMAB
TFDP1	MMADHC
TFDP1	MMD
TFDP1	MME
TFDP1	MMS19
TFDP1	MMS22L
TFDP1	MND1
TFDP1	MNS1
TFDP1	MOB1A
TFDP1	MOCS2
TFDP1	MORC4
TFDP1	MORF4L1
TFDP1	MORF4L2
TFDP1	MORF4L2-AS1

TFDP1	MOSPD2
TFDP1	MOSPD3
TFDP1	MOV10
TFDP1	MPC1
TFDP1	MPC2
TFDP1	MPDU1
TFDP1	MPHOSPH6
TFDP1	MPHOSPH8
TFDP1	MPI
TFDP1	MPLKIP
TFDP1	MPND
TFDP1	MPP5
TFDP1	MPP6
TFDP1	MPRIP
TFDP1	MROH1
TFDP1	MRPL11
TFDP1	MRPL12
TFDP1	MRPL13
TFDP1	MRPL14
TFDP1	MRPL17
TFDP1	MRPL18
TFDP1	MRPL23
TFDP1	MRPL28
TFDP1	MRPL32
TFDP1	MRPL33
TFDP1	MRPL34
TFDP1	MRPL35
TFDP1	MRPL37
TFDP1	MRPL39
TFDP1	MRPL4
TFDP1	MRPL40
TFDP1	MRPL44
TFDP1	MRPL45
TFDP1	MRPL47
TFDP1	MRPL48
TFDP1	MRPL49
TFDP1	MRPL51
TFDP1	MRPL52
TFDP1	MRPL57
TFDP1	MRPS14
TFDP1	MRPS15
TFDP1	MRPS16
TFDP1	MRPS18B
TFDP1	MRPS2
TFDP1	MRPS21
TFDP1	MRPS22
TFDP1	MRPS23
TFDP1	MRPS25
TFDP1	MRPS31
TFDP1	MRPS33

TFDP1	MRPS5
TFDP1	MRPS6
TFDP1	MRPS7
TFDP1	MRRF
TFDP1	MRS2
TFDP1	MSANTD3
TFDP1	MSANTD4
TFDP1	MSH2
TFDP1	MSH3
TFDP1	MSH6
TFDP1	MSI2
TFDP1	MSL1
TFDP1	MSMO1
TFDP1	MSRA
TFDP1	MSRB2
TFDP1	MTA1
TFDP1	MTA2
TFDP1	MTA3
TFDP1	MTCH2
TFDP1	MTDH
TFDP1	MTERF1
TFDP1	MTERF3
TFDP1	MTF2
TFDP1	MTFMT
TFDP1	MTFP1
TFDP1	MTFR1L
TFDP1	MTFR2
TFDP1	MTHFD1
TFDP1	MTHFD2L
TFDP1	MTMR14
TFDP1	MTMR4
TFDP1	MTMR6
TFDP1	MTO1
TFDP1	MTOR
TFDP1	MTPAP
TFDP1	MTRF1
TFDP1	MTX1
TFDP1	MTX2
TFDP1	MUTYH
TFDP1	MVB12A
TFDP1	MVK
TFDP1	MX1
TFDP1	MXD3
TFDP1	MYBL1
TFDP1	MYBL2
TFDP1	MYCBP
TFDP1	MYH10
TFDP1	MYH9
TFDP1	MYL12B
TFDP1	MYL6

TFDP1	MYL6B
TFDP1	MYNN
TFDP1	MYO1C
TFDP1	MYO6
TFDP1	MYPOP
TFDP1	MZT1
TFDP1	MZT2A
TFDP1	MZT2B
TFDP1	N4BP2
TFDP1	N4BP3
TFDP1	NAA10
TFDP1	NAA20
TFDP1	NAA35
TFDP1	NAA38
TFDP1	NAA40
TFDP1	NAA50
TFDP1	NAB1
TFDP1	NABP2
TFDP1	NACA
TFDP1	NADK
TFDP1	NADK2
TFDP1	NAE1
TFDP1	NAGK
TFDP1	NAIF1
TFDP1	NAP1L1
TFDP1	NAP1L4
TFDP1	NAPA
TFDP1	NAPG
TFDP1	NARF
TFDP1	NARFL
TFDP1	NASP
TFDP1	NAXD
TFDP1	NBEA
TFDP1	NBEAL1
TFDP1	NBN
TFDP1	NBPF1
TFDP1	NCAPD3
TFDP1	NCAPG
TFDP1	NCAPG2
TFDP1	NCAPH
TFDP1	NCAPH2
TFDP1	NCBP1
TFDP1	NCF4
TFDP1	NCKAP1L
TFDP1	NCKIPSD
TFDP1	NCL
TFDP1	NCOA2
TFDP1	NCOA4
TFDP1	NCOA5
TFDP1	NDC1

TFDP1	NDC80
TFDP1	NDRG4
TFDP1	NDUFA12
TFDP1	NDUFA13
TFDP1	NDUFA2
TFDP1	NDUFA4
TFDP1	NDUFA5
TFDP1	NDUFA6
TFDP1	NDUFAF1
TFDP1	NDUFAF3
TFDP1	NDUFAF6
TFDP1	NDUFB10
TFDP1	NDUFB4
TFDP1	NDUFB6
TFDP1	NDUFB9
TFDP1	NDUFC1
TFDP1	NDUFS1
TFDP1	NDUFS2
TFDP1	NDUFS4
TFDP1	NDUFS6
TFDP1	NDUFS7
TFDP1	NDUFS8
TFDP1	NDUFV1
TFDP1	NDUFV3
TFDP1	NECAP2
TFDP1	NEDD1
TFDP1	NEIL1
TFDP1	NEIL3
TFDP1	NEK1
TFDP1	NELFA
TFDP1	NELFB
TFDP1	NEMP1
TFDP1	NEMP2
TFDP1	NENF
TFDP1	NFAT5
TFDP1	NFATC1
TFDP1	NFATC2
TFDP1	NFATC2IP
TFDP1	NFATC3
TFDP1	NFATC4
TFDP1	NFE2L2
TFDP1	NFKBIB
TFDP1	NFKBID
TFDP1	NFKBIL1
TFDP1	NFS1
TFDP1	NFYA
TFDP1	NFYB
TFDP1	NFYC
TFDP1	NGDN
TFDP1	NIF3L1

TFDP1	NIPBL
TFDP1	NIPSNAP3A
TFDP1	NIT1
TFDP1	NKAP
TFDP1	NKIRAS2
TFDP1	NKRF
TFDP1	NKTR
TFDP1	NLK
TFDP1	NLRC3
TFDP1	NMD3
TFDP1	NME6
TFDP1	NMI
TFDP1	NMRAL1
TFDP1	NMT1
TFDP1	NMU
TFDP1	NNT
TFDP1	NOB1
TFDP1	NOC4L
TFDP1	NOL11
TFDP1	NOL8
TFDP1	NONO
TFDP1	NOP10
TFDP1	NOP14
TFDP1	NOP56
TFDP1	NOP58
TFDP1	NOSIP
TFDP1	NPAT
TFDP1	NPM1
TFDP1	NPTX2
TFDP1	NQO2
TFDP1	NR3C1
TFDP1	NRBF2
TFDP1	NRDC
TFDP1	NREP
TFDP1	NRF1
TFDP1	NRM
TFDP1	NRSN2
TFDP1	NSA2
TFDP1	NSD1
TFDP1	NSDHL
TFDP1	NSFL1C
TFDP1	NSL1
TFDP1	NSMCE4A
TFDP1	NSRP1
TFDP1	NSUN4
TFDP1	NSUN6
TFDP1	NT5DC1
TFDP1	NTAN1
TFDP1	NTMT1
TFDP1	NUB1

TFDP1	NUBP1
TFDP1	NUBP2
TFDP1	NUBPL
TFDP1	NUCB1
TFDP1	NUCKS1
TFDP1	NUDC
TFDP1	NUDCD2
TFDP1	NUDT15
TFDP1	NUDT16L1
TFDP1	NUDT19
TFDP1	NUDT21
TFDP1	NUDT8
TFDP1	NUMA1
TFDP1	NUP107
TFDP1	NUP133
TFDP1	NUP155
TFDP1	NUP188
TFDP1	NUP210
TFDP1	NUP214
TFDP1	NUP43
TFDP1	NUP50
TFDP1	NUP58
TFDP1	NUP62
TFDP1	NUP85
TFDP1	NUP88
TFDP1	NUP93
TFDP1	NUP98
TFDP1	NUPL2
TFDP1	NUSAP1
TFDP1	NUTF2
TFDP1	NUTM2A-AS1
TFDP1	NUTM2B-AS1
TFDP1	NVL
TFDP1	NXF1
TFDP1	NXT1
TFDP1	OARD1
TFDP1	OAT
TFDP1	OAZ1
TFDP1	OCIAD1
TFDP1	ODF2
TFDP1	OGFOD1
TFDP1	OGFOD2
TFDP1	OGFRL1
TFDP1	OGG1
TFDP1	OLA1
TFDP1	OPA1
TFDP1	OPA3
TFDP1	ORC1
TFDP1	ORC3
TFDP1	ORC4

TFDP1	ORC5
TFDP1	ORC6
TFDP1	ORMDL1
TFDP1	OSBPL11
TFDP1	OSBPL2
TFDP1	OSBPL8
TFDP1	OSBPL9
TFDP1	OST4
TFDP1	OSTF1
TFDP1	OTUD1
TFDP1	OTULIN
TFDP1	OXCT1
TFDP1	OXR1
TFDP1	OXSR1
TFDP1	PAAF1
TFDP1	PABPC1
TFDP1	PABPC4
TFDP1	PACS1
TFDP1	PACSIN2
TFDP1	PAF1
TFDP1	PAFAH1B1
TFDP1	PAFAH1B2
TFDP1	PAFAH1B3
TFDP1	PAGR1
TFDP1	PAICS
TFDP1	PAIP1
TFDP1	PAIP2
TFDP1	PAK1
TFDP1	PAK2
TFDP1	PAK4
TFDP1	PALLD
TFDP1	PAN2
TFDP1	PAN3
TFDP1	PANK2
TFDP1	PANK3
TFDP1	PANX1
TFDP1	PAPOLA
TFDP1	PAPSS1
TFDP1	PAQR4
TFDP1	PARD6A
TFDP1	PARK7
TFDP1	PARN
TFDP1	PARP1
TFDP1	PARP2
TFDP1	PARP3
TFDP1	PATL1
TFDP1	PATZ1
TFDP1	PAWR
TFDP1	PAX5
TFDP1	PAXBP1

TFDP1	PAXIP1
TFDP1	PAXIP1-AS1
TFDP1	PBK
TFDP1	PBRM1
TFDP1	PBX2
TFDP1	PBX3
TFDP1	PCBD1
TFDP1	PCBP1
TFDP1	PCBP2
TFDP1	PCF11
TFDP1	PCGF5
TFDP1	PCGF6
TFDP1	PCIF1
TFDP1	PCMT1
TFDP1	PCNA
TFDP1	PCNP
TFDP1	PCNT
TFDP1	PCYT1A
TFDP1	PCYT2
TFDP1	PDAP1
TFDP1	PDCD10
TFDP1	PDCD6
TFDP1	PDCL
TFDP1	PDE6D
TFDP1	PDHA1
TFDP1	PDHX
TFDP1	PDIA3
TFDP1	PDIA6
TFDP1	PDK2
TFDP1	PDK3
TFDP1	PDLIM1
TFDP1	PDLIM7
TFDP1	PDPK1
TFDP1	PDS5A
TFDP1	PDS5B
TFDP1	PDSS2
TFDP1	PEA15
TFDP1	PEF1
TFDP1	PEG10
TFDP1	PELP1
TFDP1	PER2
TFDP1	PET100
TFDP1	PET117
TFDP1	PEX11B
TFDP1	PEX16
TFDP1	PEX2
TFDP1	PEX5
TFDP1	PFDN1
TFDP1	PFDN2
TFDP1	PFDN4

TFDP1	PFDN6
TFDP1	PFKP
TFDP1	PFN1
TFDP1	PGAM1
TFDP1	PGAM5
TFDP1	PGAP2
TFDP1	PGD
TFDP1	PGGT1B
TFDP1	PGK1
TFDP1	PGM2
TFDP1	PGP
TFDP1	PGRMC1
TFDP1	PGS1
TFDP1	PHACTR4
TFDP1	PHAX
TFDP1	PHB2
TFDP1	PHC1
TFDP1	PHF11
TFDP1	PHF13
TFDP1	PHF14
TFDP1	PHF19
TFDP1	PHF20
TFDP1	PHF3
TFDP1	PHF5A
TFDP1	PHF6
TFDP1	PHF7
TFDP1	PHGDH
TFDP1	PHIP
TFDP1	PHKG2
TFDP1	PHPT1
TFDP1	PHYHD1
TFDP1	PI4KA
TFDP1	PI4KB
TFDP1	PIAS1
TFDP1	PIAS4
TFDP1	PIDD1
TFDP1	PIGC
TFDP1	PIGF
TFDP1	PIGL
TFDP1	PIGQ
TFDP1	PIGV
TFDP1	PIK3AP1
TFDP1	PIK3C3
TFDP1	PIM1
TFDP1	PIN1
TFDP1	PIN4
TFDP1	PIP4K2A
TFDP1	PIP5K1A
TFDP1	PITPNC1
TFDP1	PKM

TFDP1	PKMYT1
TFDP1	PKN1
TFDP1	PKN2
TFDP1	PKNOX1
TFDP1	PLA2G15
TFDP1	PLAA
TFDP1	PLEKHA3
TFDP1	PLEKHF2
TFDP1	PLEKHJ1
TFDP1	PLEKHO1
TFDP1	PLK4
TFDP1	PLPP1
TFDP1	PLRG1
TFDP1	PLSCR1
TFDP1	PMAIP1
TFDP1	PMF1
TFDP1	PMM1
TFDP1	PMPCB
TFDP1	PMS1
TFDP1	PMVK
TFDP1	PNISR
TFDP1	PNKD
TFDP1	PNN
TFDP1	PNPLA4
TFDP1	PNPLA6
TFDP1	PNPLA8
TFDP1	PNRC2
TFDP1	POC1A
TFDP1	POC1B
TFDP1	POC5
TFDP1	POFUT1
TFDP1	POGZ
TFDP1	POLA1
TFDP1	POLA2
TFDP1	POLD1
TFDP1	POLD3
TFDP1	POLDIP3
TFDP1	POLE
TFDP1	POLE2
TFDP1	POLE4
TFDP1	POLG
TFDP1	POLR2A
TFDP1	POLR2C
TFDP1	POLR2D
TFDP1	POLR2E
TFDP1	POLR2F
TFDP1	POLR2G
TFDP1	POLR2H
TFDP1	POLR2I
TFDP1	POLR2J

TFDP1	POLR2J2
TFDP1	POLR2K
TFDP1	POLR2M
TFDP1	POLR3C
TFDP1	POLRMT
TFDP1	POMC
TFDP1	POMP
TFDP1	POP4
TFDP1	POP5
TFDP1	POP7
TFDP1	POT1
TFDP1	POU2AF1
TFDP1	POU2F1
TFDP1	PPCDC
TFDP1	PPDPF
TFDP1	PPHLN1
TFDP1	PPIA
TFDP1	PPID
TFDP1	PPIE
TFDP1	PPIF
TFDP1	PPIG
TFDP1	PPIH
TFDP1	PPIL2
TFDP1	PPIL3
TFDP1	PPIL4
TFDP1	PPIP5K2
TFDP1	PPM1A
TFDP1	PPM1B
TFDP1	PPM1D
TFDP1	PPM1G
TFDP1	PPP1CA
TFDP1	PPP1CB
TFDP1	PPP1CC
TFDP1	PPP1R10
TFDP1	PPP1R11
TFDP1	PPP1R12A
TFDP1	PPP1R12C
TFDP1	PPP1R13L
TFDP1	PPP1R15A
TFDP1	PPP1R15B
TFDP1	PPP1R18
TFDP1	PPP1R2
TFDP1	PPP1R21
TFDP1	PPP1R35
TFDP1	PPP1R37
TFDP1	PPP1R3E
TFDP1	PPP1R7
TFDP1	PPP1R8
TFDP1	PPP2CA
TFDP1	PPP2R1A

TFDP1	PPP2R2D
TFDP1	PPP2R3C
TFDP1	PPP2R5A
TFDP1	PPP2R5C
TFDP1	PPP2R5E
TFDP1	PPP3CB
TFDP1	PPP3R1
TFDP1	PPP4C
TFDP1	PPP4R2
TFDP1	PPP4R3A
TFDP1	PPP4R3B
TFDP1	PPP5C
TFDP1	PPP6C
TFDP1	PPP6R1
TFDP1	PPP6R2
TFDP1	PPP6R3
TFDP1	PPTC7
TFDP1	PQLC1
TFDP1	PRADC1
TFDP1	PRAF2
TFDP1	PRCC
TFDP1	PRDM15
TFDP1	PRDX1
TFDP1	PRDX2
TFDP1	PRDX6
TFDP1	PRELID1
TFDP1	PRELID3B
TFDP1	PRIM1
TFDP1	PRIM2
TFDP1	PRKAB1
TFDP1	PRKACB
TFDP1	PRKCA
TFDP1	PRKCD
TFDP1	PRKCE
TFDP1	PRKCZ
TFDP1	PRKD3
TFDP1	PRKRA
TFDP1	PRKRIP1
TFDP1	PRMT1
TFDP1	PRMT5
TFDP1	PRMT7
TFDP1	PRMT9
TFDP1	PRPF18
TFDP1	PRPF19
TFDP1	PRPF3
TFDP1	PRPF38B
TFDP1	PRPF40A
TFDP1	PRPF4B
TFDP1	PRPF6
TFDP1	PRPF8

TFDP1	PRPS1
TFDP1	PRPS2
TFDP1	PRPSAP1
TFDP1	PRPSAP2
TFDP1	PRR13
TFDP1	PRR14
TFDP1	PRR14L
TFDP1	PRRC2A
TFDP1	PRRC2C
TFDP1	PSIP1
TFDP1	PSMA1
TFDP1	PSMA2
TFDP1	PSMA3
TFDP1	PSMA3-AS1
TFDP1	PSMA4
TFDP1	PSMA5
TFDP1	PSMA6
TFDP1	PSMB1
TFDP1	PSMB2
TFDP1	PSMB3
TFDP1	PSMB7
TFDP1	PSMB9
TFDP1	PSMC2
TFDP1	PSMC3
TFDP1	PSMC3IP
TFDP1	PSMC4
TFDP1	PSMC5
TFDP1	PSMD1
TFDP1	PSMD11
TFDP1	PSMD12
TFDP1	PSMD13
TFDP1	PSMD14
TFDP1	PSMD2
TFDP1	PSMD3
TFDP1	PSMD4
TFDP1	PSMD5
TFDP1	PSMD6
TFDP1	PSMD7
TFDP1	PSMD9
TFDP1	PSME3
TFDP1	PSMG3
TFDP1	PSPC1
TFDP1	PTAR1
TFDP1	PTBP1
TFDP1	PTBP3
TFDP1	PTDSS1
TFDP1	PTER
TFDP1	PTGES3
TFDP1	PTK2B
TFDP1	PTMA

TFDP1	PTP4A2
TFDP1	PTPMT1
TFDP1	PTPN20
TFDP1	PTPN23
TFDP1	PTPN7
TFDP1	PTPN9
TFDP1	PTRH2
TFDP1	PTTG1
TFDP1	PUF60
TFDP1	PUM1
TFDP1	PURA
TFDP1	PUS3
TFDP1	PUSL1
TFDP1	PWWP2A
TFDP1	PXMP2
TFDP1	PYCR2
TFDP1	PYM1
TFDP1	QARS
TFDP1	QRICH1
TFDP1	QTRT2
TFDP1	RAB10
TFDP1	RAB11B
TFDP1	RAB11B-AS1
TFDP1	RAB14
TFDP1	RAB1A
TFDP1	RAB1B
TFDP1	RAB27A
TFDP1	RAB28
TFDP1	RAB30
TFDP1	RAB33A
TFDP1	RAB34
TFDP1	RAB35
TFDP1	RAB3A
TFDP1	RAB3IP
TFDP1	RAB4A
TFDP1	RAB5C
TFDP1	RAB6A
TFDP1	RAB8A
TFDP1	RAB8B
TFDP1	RABEP1
TFDP1	RABEP2
TFDP1	RABGAP1
TFDP1	RABGEF1
TFDP1	RABIF
TFDP1	RABL6
TFDP1	RAC1
TFDP1	RACK1
TFDP1	RAD18
TFDP1	RAD21
TFDP1	RAD23A

TFDP1	RAD23B
TFDP1	RAD51AP1
TFDP1	RAD51C
TFDP1	RAD52
TFDP1	RAD54L
TFDP1	RAD9A
TFDP1	RAE1
TFDP1	RAI1
TFDP1	RALA
TFDP1	RALBP1
TFDP1	RALGAPA2
TFDP1	RALY
TFDP1	RAN
TFDP1	RANBP1
TFDP1	RANBP3
TFDP1	RANBP9
TFDP1	RANGRF
TFDP1	RAP1B
TFDP1	RAP2B
TFDP1	RAP2C
TFDP1	RAPGEF5
TFDP1	RARS
TFDP1	RARS2
TFDP1	RASA2
TFDP1	RASGRP2
TFDP1	RASSF1
TFDP1	RASSF2
TFDP1	RASSF3
TFDP1	RASSF7
TFDP1	RB1
TFDP1	RBBP4
TFDP1	RBBP7
TFDP1	RBBP8
TFDP1	RBL1
TFDP1	RBM12B
TFDP1	RBM14
TFDP1	RBM15
TFDP1	RBM17
TFDP1	RBM22
TFDP1	RBM23
TFDP1	RBM25
TFDP1	RBM26
TFDP1	RBM27
TFDP1	RBM28
TFDP1	RBM3
TFDP1	RBM38
TFDP1	RBM39
TFDP1	RBM4
TFDP1	RBM41
TFDP1	RBM5

TFDP1	RBM6
TFDP1	RBM8A
TFDP1	RBMX
TFDP1	RBMX2
TFDP1	RBSN
TFDP1	RBX1
TFDP1	RC3H2
TFDP1	RCAN3
TFDP1	RCC1
TFDP1	RCC2
TFDP1	RCCD1
TFDP1	RCHY1
TFDP1	RCOR1
TFDP1	RCSD1
TFDP1	RECQL
TFDP1	RECQL4
TFDP1	REEP4
TFDP1	REEP5
TFDP1	REL
TFDP1	RELA
TFDP1	RELB
TFDP1	RELT
TFDP1	REST
TFDP1	REV1
TFDP1	RFC1
TFDP1	RFC2
TFDP1	RFC3
TFDP1	RFC4
TFDP1	RFC5
TFDP1	RFESD
TFDP1	RFK
TFDP1	RFT1
TFDP1	RFWD3
TFDP1	RFX5
TFDP1	RGCC
TFDP1	RGP1
TFDP1	RGS19
TFDP1	RGS20
TFDP1	RHBDD2
TFDP1	RHBDF2
TFDP1	RHEB
TFDP1	RHNO1
TFDP1	RHOG
TFDP1	RIC1
TFDP1	RIF1
TFDP1	RIOK1
TFDP1	RIPK2
TFDP1	RMDN1
TFDP1	RMI1
TFDP1	RMI2

TFDP1	RMND5A
TFDP1	RMND5B
TFDP1	RNASEH1
TFDP1	RNASEH2A
TFDP1	RNASEH2B
TFDP1	RNF10
TFDP1	RNF115
TFDP1	RNF121
TFDP1	RNF123
TFDP1	RNF126
TFDP1	RNF138
TFDP1	RNF139
TFDP1	RNF144B
TFDP1	RNF146
TFDP1	RNF167
TFDP1	RNF168
TFDP1	RNF181
TFDP1	RNF187
TFDP1	RNF20
TFDP1	RNF212
TFDP1	RNF219
TFDP1	RNF220
TFDP1	RNF26
TFDP1	RNF34
TFDP1	RNF38
TFDP1	RNF4
TFDP1	RNF5
TFDP1	RNF6
TFDP1	RNF8
TFDP1	RNFT1
TFDP1	RNGTT
TFDP1	RNPC3
TFDP1	RNPEP
TFDP1	RNPS1
TFDP1	ROCK2
TFDP1	RP2
TFDP1	RPA1
TFDP1	RPA2
TFDP1	RPA3
TFDP1	RPAIN
TFDP1	RPAP2
TFDP1	RPIA
TFDP1	RPL10
TFDP1	RPL10A
TFDP1	RPL11
TFDP1	RPL12
TFDP1	RPL13A
TFDP1	RPL14
TFDP1	RPL15
TFDP1	RPL17

TFDP1	RPL18A
TFDP1	RPL19
TFDP1	RPL21
TFDP1	RPL22
TFDP1	RPL22L1
TFDP1	RPL23
TFDP1	RPL23A
TFDP1	RPL26
TFDP1	RPL26L1
TFDP1	RPL27
TFDP1	RPL27A
TFDP1	RPL29
TFDP1	RPL3
TFDP1	RPL30
TFDP1	RPL31
TFDP1	RPL32
TFDP1	RPL34
TFDP1	RPL35A
TFDP1	RPL36
TFDP1	RPL36A
TFDP1	RPL37
TFDP1	RPL38
TFDP1	RPL39
TFDP1	RPL4
TFDP1	RPL41
TFDP1	RPL5
TFDP1	RPL6
TFDP1	RPL7
TFDP1	RPL7L1
TFDP1	RPLP1
TFDP1	RPLP2
TFDP1	RPP30
TFDP1	RPRD1B
TFDP1	RPS10
TFDP1	RPS11
TFDP1	RPS12
TFDP1	RPS13
TFDP1	RPS14
TFDP1	RPS15
TFDP1	RPS15A
TFDP1	RPS16
TFDP1	RPS17
TFDP1	RPS19
TFDP1	RPS19BP1
TFDP1	RPS2
TFDP1	RPS20
TFDP1	RPS21
TFDP1	RPS23
TFDP1	RPS24
TFDP1	RPS26

TFDP1	RPS27
TFDP1	RPS27A
TFDP1	RPS27L
TFDP1	RPS29
TFDP1	RPS3
TFDP1	RPS3A
TFDP1	RPS4X
TFDP1	RPS6
TFDP1	RPS6KB2
TFDP1	RPS7
TFDP1	RPS8
TFDP1	RPSA
TFDP1	RPUSD1
TFDP1	RRAGA
TFDP1	RRAS2
TFDP1	RRM1
TFDP1	RRM2
TFDP1	RRN3
TFDP1	RRP7A
TFDP1	RSBN1
TFDP1	RSBN1L
TFDP1	RSF1
TFDP1	RSL24D1
TFDP1	RSRC1
TFDP1	RSRC2
TFDP1	RSU1
TFDP1	RTCA
TFDP1	RTCB
TFDP1	RTF1
TFDP1	RTN3
TFDP1	RTN4
TFDP1	RUBCN
TFDP1	RUFY1
TFDP1	RUFY3
TFDP1	RUNX3
TFDP1	RUSC1
TFDP1	RUVBL1
TFDP1	RYBP
TFDP1	S100A10
TFDP1	S100PBP
TFDP1	S1PR1
TFDP1	S1PR2
TFDP1	SAAL1
TFDP1	SAC3D1
TFDP1	SAE1
TFDP1	SAFB2
TFDP1	SAMD1
TFDP1	SAMM50
TFDP1	SAP130
TFDP1	SAP30

TFDP1	SAR1A
TFDP1	SARNP
TFDP1	SARS
TFDP1	SARS2
TFDP1	SART1
TFDP1	SART3
TFDP1	SASS6
TFDP1	SBDS
TFDP1	SBNO1
TFDP1	SC5D
TFDP1	SCAF1
TFDP1	SCAF11
TFDP1	SCAF4
TFDP1	SCAMP1
TFDP1	SCAND1
TFDP1	SCARB1
TFDP1	SCCPDH
TFDP1	SCLT1
TFDP1	SCLY
TFDP1	SCMH1
TFDP1	SCNM1
TFDP1	SCOC
TFDP1	SCRN1
TFDP1	SDCCAG8
TFDP1	SDHAF1
TFDP1	SDHB
TFDP1	SDHC
TFDP1	SDR39U1
TFDP1	SDR42E1
TFDP1	SEC11A
TFDP1	SEC14L1
TFDP1	SEC22A
TFDP1	SEC22B
TFDP1	SEC22C
TFDP1	SEC23IP
TFDP1	SEC62
TFDP1	SECISBP2
TFDP1	SEL1L3
TFDP1	SENP8
TFDP1	SEPHS1
TFDP1	SEPHS2
TFDP1	SEPT11
TFDP1	SEPT7
TFDP1	SEPT8
TFDP1	SEPT9
TFDP1	SERBP1
TFDP1	SERF2
TFDP1	SERINC2
TFDP1	SERINC3
TFDP1	SERP2

TFDP1	SERTAD2
TFDP1	SESTD1
TFDP1	SET
TFDP1	SETD3
TFDP1	SETD4
TFDP1	SETD5
TFDP1	SETD7
TFDP1	SETDB2
TFDP1	SF1
TFDP1	SF3A1
TFDP1	SF3A2
TFDP1	SF3A3
TFDP1	SF3B1
TFDP1	SF3B2
TFDP1	SF3B3
TFDP1	SF3B5
TFDP1	SFI1
TFDP1	SFMBT1
TFDP1	SFR1
TFDP1	SFSWAP
TFDP1	SFT2D1
TFDP1	SFXN1
TFDP1	SFXN4
TFDP1	SFXN5
TFDP1	SGCE
TFDP1	SGO1
TFDP1	SGTA
TFDP1	SH2B1
TFDP1	SH2B2
TFDP1	SH2B3
TFDP1	SH3BP1
TFDP1	SH3GL1
TFDP1	SH3GLB1
TFDP1	SH3GLB2
TFDP1	SH3KBP1
TFDP1	SHC1
TFDP1	SHCBP1
TFDP1	SHISA5
TFDP1	SHMT1
TFDP1	SHMT2
TFDP1	SHQ1
TFDP1	SIAH1
TFDP1	SIN3A
TFDP1	SIPA1
TFDP1	SIRT1
TFDP1	SIRT6
TFDP1	SIVA1
TFDP1	SKA2
TFDP1	SKAP2
TFDP1	SKP2

TFDP1	SLAIN2
TFDP1	SLBP
TFDP1	SLC11A2
TFDP1	SLC12A5
TFDP1	SLC15A4
TFDP1	SLC16A1
TFDP1	SLC1A4
TFDP1	SLC1A5
TFDP1	SLC20A1
TFDP1	SLC25A1
TFDP1	SLC25A10
TFDP1	SLC25A11
TFDP1	SLC25A12
TFDP1	SLC25A14
TFDP1	SLC25A17
TFDP1	SLC25A19
TFDP1	SLC25A29
TFDP1	SLC25A3
TFDP1	SLC25A33
TFDP1	SLC25A36
TFDP1	SLC25A37
TFDP1	SLC25A39
TFDP1	SLC25A4
TFDP1	SLC25A40
TFDP1	SLC25A5
TFDP1	SLC26A6
TFDP1	SLC27A3
TFDP1	SLC31A1
TFDP1	SLC35A1
TFDP1	SLC35A4
TFDP1	SLC35B2
TFDP1	SLC35B4
TFDP1	SLC35E1
TFDP1	SLC35F5
TFDP1	SLC35G1
TFDP1	SLC37A4
TFDP1	SLC39A3
TFDP1	SLC39A8
TFDP1	SLC41A1
TFDP1	SLC41A3
TFDP1	SLC43A1
TFDP1	SLC43A2
TFDP1	SLC9A3
TFDP1	SLC9A3R1
TFDP1	SLF1
TFDP1	SLF2
TFDP1	SLFN11
TFDP1	SLFN13
TFDP1	SLK
TFDP1	SLMAP

TFDP1	SLTM
TFDP1	SLU7
TFDP1	SLX4
TFDP1	SLX4IP
TFDP1	SMAD2
TFDP1	SMAGP
TFDP1	SMAP1
TFDP1	SMARCA2
TFDP1	SMARCA4
TFDP1	SMARCA5
TFDP1	SMARCAD1
TFDP1	SMARCAL1
TFDP1	SMARCB1
TFDP1	SMARCC1
TFDP1	SMARCD2
TFDP1	SMARCE1
TFDP1	SMC1A
TFDP1	SMC2
TFDP1	SMC3
TFDP1	SMC4
TFDP1	SMC5
TFDP1	SMCHD1
TFDP1	SMCO4
TFDP1	SMCR5
TFDP1	SMG1
TFDP1	SMG5
TFDP1	SMG7
TFDP1	SMG9
TFDP1	SMIM12
TFDP1	SMIM13
TFDP1	SMIM15
TFDP1	SMIM19
TFDP1	SMIM4
TFDP1	SMPD4
TFDP1	SMS
TFDP1	SMUG1
TFDP1	SMYD2
TFDP1	SMYD4
TFDP1	SNAP29
TFDP1	SNAPC1
TFDP1	SNAPC3
TFDP1	SNAPIN
TFDP1	SNF8
TFDP1	SNHG10
TFDP1	SNHG19
TFDP1	SNHG21
TFDP1	SNHG25
TFDP1	SNHG7
TFDP1	SNIP1
TFDP1	SNRNP200

TFDP1	SNRNP27
TFDP1	SNRNP35
TFDP1	SNRNP40
TFDP1	SNRNP48
TFDP1	SNRNP70
TFDP1	SNRPA
TFDP1	SNRPA1
TFDP1	SNRPB
TFDP1	SNRPD1
TFDP1	SNRPD2
TFDP1	SNRPE
TFDP1	SNRPF
TFDP1	SNRPG
TFDP1	SNRPN
TFDP1	SNTA1
TFDP1	SNTB2
TFDP1	SNU13
TFDP1	SNUPN
TFDP1	SNW1
TFDP1	SNX12
TFDP1	SNX14
TFDP1	SNX22
TFDP1	SNX3
TFDP1	SNX5
TFDP1	SOD1
TFDP1	SORL1
TFDP1	SOX4
TFDP1	SP1
TFDP1	SP2
TFDP1	SP4
TFDP1	SPAG1
TFDP1	SPAG16
TFDP1	SPAG7
TFDP1	SPAG9
TFDP1	SPATA33
TFDP1	SPATS2
TFDP1	SPC24
TFDP1	SPC25
TFDP1	SPDL1
TFDP1	SPG21
TFDP1	SPG7
TFDP1	SPHK2
TFDP1	SPIN4
TFDP1	SPOP
TFDP1	SPOPL
TFDP1	SPPL2A
TFDP1	SPSB2
TFDP1	SPSB3
TFDP1	SPTAN1
TFDP1	SPTLC2

TFDP1	SPTSSA
TFDP1	SPTY2D1
TFDP1	SQLE
TFDP1	SRBD1
TFDP1	SREBF2
TFDP1	SREK1IP1
TFDP1	SRGAP2B
TFDP1	SRP14
TFDP1	SRP19
TFDP1	SRP54
TFDP1	SRP9
TFDP1	SRPK1
TFDP1	SRRM1
TFDP1	SRRT
TFDP1	SRSF1
TFDP1	SRSF10
TFDP1	SRSF2
TFDP1	SRSF3
TFDP1	SRSF4
TFDP1	SRSF5
TFDP1	SRSF6
TFDP1	SRSF7
TFDP1	SS18
TFDP1	SSB
TFDP1	SSBP1
TFDP1	SSBP2
TFDP1	SSH2
TFDP1	SSNA1
TFDP1	SSR2
TFDP1	SSRP1
TFDP1	ST20
TFDP1	ST3GAL4
TFDP1	ST7L
TFDP1	STAG1
TFDP1	STAG2
TFDP1	STAG3
TFDP1	STAM
TFDP1	STAM2
TFDP1	STAP1
TFDP1	STARD3NL
TFDP1	STARD7
TFDP1	STAT3
TFDP1	STAT6
TFDP1	STAU1
TFDP1	STAU2
TFDP1	STIM2
TFDP1	STK19
TFDP1	STK24
TFDP1	STK25
TFDP1	STK32C

TFDP1	STK38L
TFDP1	STK39
TFDP1	STK4
TFDP1	STK40
TFDP1	STMN1
TFDP1	STOML2
TFDP1	STPG1
TFDP1	STRADA
TFDP1	STRAP
TFDP1	STRN3
TFDP1	STX11
TFDP1	STX16
TFDP1	STX18
TFDP1	STX5
TFDP1	STXBP3
TFDP1	SUCLA2
TFDP1	SUCLG1
TFDP1	SUCO
TFDP1	SUGP1
TFDP1	SUGP2
TFDP1	SUGT1
TFDP1	SULF2
TFDP1	SUMO1
TFDP1	SUMO2
TFDP1	SUMO3
TFDP1	SUPT16H
TFDP1	SUPT4H1
TFDP1	SUPT5H
TFDP1	SUSD3
TFDP1	SUV39H1
TFDP1	SUV39H2
TFDP1	SUZ12
TFDP1	SVBP
TFDP1	SWI5
TFDP1	SYAP1
TFDP1	SYBU
TFDP1	SYCE1L
TFDP1	SYF2
TFDP1	SYK
TFDP1	SYNC
TFDP1	SYNCRIP
TFDP1	SYNE2
TFDP1	SYVN1
TFDP1	TAB2
TFDP1	TACC1
TFDP1	TACC3
TFDP1	TACO1
TFDP1	TADA1
TFDP1	TADA2A
TFDP1	TADA3

TFDP1	TAF10
TFDP1	TAF11
TFDP1	TAF12
TFDP1	TAF15
TFDP1	TAF1B
TFDP1	TAF3
TFDP1	TAF4B
TFDP1	TAF5
TFDP1	TAF6
TFDP1	TAF7
TFDP1	TAF9B
TFDP1	TALDO1
TFDP1	TANK
TFDP1	TAP1
TFDP1	TARDBP
TFDP1	TATDN2
TFDP1	TAX1BP3
TFDP1	TBC1D1
TFDP1	TBC1D10B
TFDP1	TBC1D13
TFDP1	TBC1D14
TFDP1	TBC1D15
TFDP1	TBC1D22A
TFDP1	TBC1D24
TFDP1	TBC1D31
TFDP1	TBC1D7
TFDP1	TBCA
TFDP1	TBCC
TFDP1	TBK1
TFDP1	TBPL1
TFDP1	TBRG4
TFDP1	TCAIM
TFDP1	TCEA1
TFDP1	TCEAL9
TFDP1	TCEANC2
TFDP1	TCERG1
TFDP1	TCF12
TFDP1	TCF19
TFDP1	TCF25
TFDP1	TCF3
TFDP1	TCF4
TFDP1	TCHP
TFDP1	TCOF1
TFDP1	TCP1
TFDP1	TCTE3
TFDP1	TCTEX1D2
TFDP1	TDG
TFDP1	TDP1
TFDP1	TDP2
TFDP1	TDRD7

TFDP1	TDRKH
TFDP1	TEC
TFDP1	TECR
TFDP1	TELO2
TFDP1	TERF1
TFDP1	TEX10
TFDP1	TEX264
TFDP1	TEX30
TFDP1	TEX9
TFDP1	TFB1M
TFDP1	TFDP1
TFDP1	TFDP2
TFDP1	TFEB
TFDP1	TFIP11
TFDP1	TFPT
TFDP1	TGFB1
TFDP1	TGFBR1
TFDP1	TGOLN2
TFDP1	THAP1
TFDP1	THAP11
TFDP1	THAP2
TFDP1	THAP4
TFDP1	THAP9-AS1
TFDP1	THG1L
TFDP1	THOC1
TFDP1	THOC2
TFDP1	THOC3
TFDP1	THOC5
TFDP1	THOC6
TFDP1	THOC7
TFDP1	THOP1
TFDP1	THRAP3
TFDP1	THUMPD3-AS1
TFDP1	TIA1
TFDP1	TIAL1
TFDP1	TIAM1
TFDP1	TIFA
TFDP1	TIGAR
TFDP1	TIMELESS
TFDP1	TIMM10
TFDP1	TIMM17A
TFDP1	TIMM21
TFDP1	TIMM23
TFDP1	TIMM9
TFDP1	TINF2
TFDP1	TIPARP
TFDP1	TIPIN
TFDP1	TIPRL
TFDP1	TK1
TFDP1	TKFC

TFDP1	TKT
TFDP1	TLCD1
TFDP1	TLE4
TFDP1	TLK1
TFDP1	TLK2
TFDP1	TM2D3
TFDP1	TM7SF3
TFDP1	TM9SF3
TFDP1	TMBIM6
TFDP1	TMCO1
TFDP1	TMED1
TFDP1	TMED8
TFDP1	TMEM102
TFDP1	TMEM106C
TFDP1	TMEM107
TFDP1	TMEM11
TFDP1	TMEM126B
TFDP1	TMEM143
TFDP1	TMEM14A
TFDP1	TMEM14B
TFDP1	TMEM160
TFDP1	TMEM161B
TFDP1	TMEM165
TFDP1	TMEM167A
TFDP1	TMEM18
TFDP1	TMEM183A
TFDP1	TMEM199
TFDP1	TMEM201
TFDP1	TMEM206
TFDP1	TMEM209
TFDP1	TMEM218
TFDP1	TMEM219
TFDP1	TMEM222
TFDP1	TMEM230
TFDP1	TMEM237
TFDP1	TMEM243
TFDP1	TMEM245
TFDP1	TMEM251
TFDP1	TMEM256
TFDP1	TMEM263
TFDP1	TMEM39B
TFDP1	TMEM41B
TFDP1	TMEM60
TFDP1	TMEM64
TFDP1	TMEM68
TFDP1	TMEM80
TFDP1	TMEM91
TFDP1	TMEM97
TFDP1	TMLHE
TFDP1	TMPO

TFDP1	TMSB10
TFDP1	TMSB4X
TFDP1	TMSB4Y
TFDP1	TMTC4
TFDP1	TMUB1
TFDP1	TMUB2
TFDP1	TMX1
TFDP1	TMX4
TFDP1	TNFAIP8
TFDP1	TNIP1
TFDP1	TNK2
TFDP1	TNKS2
TFDP1	TNKS2-AS1
TFDP1	TNPO1
TFDP1	TNPO3
TFDP1	TOB1
TFDP1	TOB2
TFDP1	TOE1
TFDP1	TOMM22
TFDP1	TOMM5
TFDP1	TOMM7
TFDP1	TOP1
TFDP1	TOP2B
TFDP1	TOPBP1
TFDP1	TOPORS
TFDP1	TOR1AIP1
TFDP1	TOR1AIP2
TFDP1	TOR2A
TFDP1	TP53
TFDP1	TP53BP1
TFDP1	TP53I13
TFDP1	TP53INP2
TFDP1	TP53TG1
TFDP1	TPCN1
TFDP1	TPGS2
TFDP1	TPI1
TFDP1	TPK1
TFDP1	TPM1
TFDP1	TPM3
TFDP1	TPM4
TFDP1	TPMT
TFDP1	TPP2
TFDP1	TPR
TFDP1	TPRA1
TFDP1	TRA2A
TFDP1	TRA2B
TFDP1	TRABD
TFDP1	TRAF2
TFDP1	TRAF3IP1
TFDP1	TRAF3IP2-AS1

TFDP1	TRAF4
TFDP1	TRAF7
TFDP1	TRAIP
TFDP1	TRAPPC10
TFDP1	TRAPPC12
TFDP1	TRAPPC2
TFDP1	TRAPPC2B
TFDP1	TRAPPC3
TFDP1	TRAPPC6B
TFDP1	TRAPPC8
TFDP1	TRIM14
TFDP1	TRIM26
TFDP1	TRIM28
TFDP1	TRIM37
TFDP1	TRIM59
TFDP1	TRIOBP
TFDP1	TRIP11
TFDP1	TRIP13
TFDP1	TRIP4
TFDP1	TRMT11
TFDP1	TRMT12
TFDP1	TRMT2A
TFDP1	TRNAU1AP
TFDP1	TRNT1
TFDP1	TROVE2
TFDP1	TRPC4AP
TFDP1	TRUB1
TFDP1	TSC22D4
TFDP1	TSEN34
TFDP1	TSEN54
TFDP1	TSFM
TFDP1	TSG101
TFDP1	TSN
TFDP1	TSNAX
TFDP1	TSPO
TFDP1	TSTD1
TFDP1	TSTD2
TFDP1	TTC13
TFDP1	TTC14
TFDP1	TTC21B
TFDP1	TTC27
TFDP1	TTC30A
TFDP1	TTC5
TFDP1	TTC7A
TFDP1	TTC9C
TFDP1	TTF2
TFDP1	TTI2
TFDP1	TTL
TFDP1	TTLL4
TFDP1	TUBA1B

TFDP1	TUBA4A
TFDP1	TUBB
TFDP1	TUBB2A
TFDP1	TUBB6
TFDP1	TUBG1
TFDP1	TUBGCP3
TFDP1	TUBGCP4
TFDP1	TUBGCP5
TFDP1	TUFM
TFDP1	TUSC2
TFDP1	TWF1
TFDP1	TWF2
TFDP1	TWISTNB
TFDP1	TXLNG
TFDP1	TXN2
TFDP1	TXNDC12
TFDP1	TXNDC16
TFDP1	TXNDC17
TFDP1	TXNDC9
TFDP1	TXNL1
TFDP1	TYMS
TFDP1	U2AF1L5
TFDP1	U2AF2
TFDP1	U2SURP
TFDP1	UBA1
TFDP1	UBA2
TFDP1	UBA3
TFDP1	UBA52
TFDP1	UBA6
TFDP1	UBAC1
TFDP1	UBAC2-AS1
TFDP1	UBAP1
TFDP1	UBAP2
TFDP1	UBAP2L
TFDP1	UBB
TFDP1	UBC
TFDP1	UBE2A
TFDP1	UBE2D2
TFDP1	UBE2D3
TFDP1	UBE2E1
TFDP1	UBE2E3
TFDP1	UBE2G1
TFDP1	UBE2I
TFDP1	UBE2J1
TFDP1	UBE2L3
TFDP1	UBE2L6
TFDP1	UBE2M
TFDP1	UBE2N
TFDP1	UBE2O
TFDP1	UBE2Q1

TFDP1	UBE2R2
TFDP1	UBE2T
TFDP1	UBE3A
TFDP1	UBE3C
TFDP1	UBE4A
TFDP1	UBE4B
TFDP1	UBL4A
TFDP1	UBL5
TFDP1	UBL7
TFDP1	UBL7-AS1
TFDP1	UBLCP1
TFDP1	UBN1
TFDP1	UBP1
TFDP1	UBQLN1
TFDP1	UBQLN2
TFDP1	UBR4
TFDP1	UBR5
TFDP1	UBR7
TFDP1	UBTF
TFDP1	UBXN11
TFDP1	UBXN2A
TFDP1	UBXN2B
TFDP1	UBXN4
TFDP1	UCHL1
TFDP1	UCHL3
TFDP1	UCHL5
TFDP1	UCK1
TFDP1	UCP2
TFDP1	UGGT1
TFDP1	UGP2
TFDP1	UHRF1
TFDP1	UHRF2
TFDP1	ULK4
TFDP1	UMAD1
TFDP1	UMPS
TFDP1	UNG
TFDP1	UNK
TFDP1	UPF1
TFDP1	UPF2
TFDP1	UPF3B
TFDP1	UQCC1
TFDP1	UQCC2
TFDP1	UQCC3
TFDP1	UQCR10
TFDP1	UQCRC1
TFDP1	UQCRH
TFDP1	URGCP
TFDP1	URI1
TFDP1	URM1
TFDP1	UROD

TFDP1	USP1
TFDP1	USP13
TFDP1	USP14
TFDP1	USP22
TFDP1	USP24
TFDP1	USP39
TFDP1	USP4
TFDP1	USP44
TFDP1	USP49
TFDP1	USP6NL
TFDP1	USP7
TFDP1	USP9X
TFDP1	UTP11
TFDP1	UTP18
TFDP1	UTP23
TFDP1	UTP6
TFDP1	UVRAG
TFDP1	UXT
TFDP1	VAMP1
TFDP1	VAMP8
TFDP1	VAPA
TFDP1	VBP1
TFDP1	VCPIP1
TFDP1	VDAC1
TFDP1	VDAC3
TFDP1	VEZF1
TFDP1	VEZT
TFDP1	VGLL4
TFDP1	VMA21
TFDP1	VMP1
TFDP1	VPS13C
TFDP1	VPS16
TFDP1	VPS25
TFDP1	VPS26A
TFDP1	VPS37A
TFDP1	VPS37C
TFDP1	VPS4A
TFDP1	VRK1
TFDP1	VRK3
TFDP1	VTI1B
TFDP1	WAC
TFDP1	WAPL
TFDP1	WASF2
TFDP1	WASL
TFDP1	WBP11
TFDP1	WBP1L
TFDP1	WBP4
TFDP1	WDHD1
TFDP1	WDR1
TFDP1	WDR18

TFDP1	WDR34
TFDP1	WDR37
TFDP1	WDR45
TFDP1	WDR5
TFDP1	WDR61
TFDP1	WDR70
TFDP1	WDR76
TFDP1	WDR77
TFDP1	WDR90
TFDP1	WDR92
TFDP1	WDTC1
TFDP1	WDYHV1
TFDP1	WEE1
TFDP1	WIPF1
TFDP1	WIPI2
TFDP1	WNK1
TFDP1	WRAP53
TFDP1	WRAP73
TFDP1	WRNIP1
TFDP1	WTAP
TFDP1	WWOX
TFDP1	XAB2
TFDP1	XIAP
TFDP1	XKR6
TFDP1	XPNPEP1
TFDP1	XPO1
TFDP1	XRCC1
TFDP1	XRCC3
TFDP1	XRCC4
TFDP1	XRCC5
TFDP1	XRCC6
TFDP1	XRN2
TFDP1	XXYLT1
TFDP1	YAF2
TFDP1	YARS
TFDP1	YBEY
TFDP1	YBX1
TFDP1	YEATS2
TFDP1	YEATS4
TFDP1	YIPF1
TFDP1	YKT6
TFDP1	YLPM1
TFDP1	YME1L1
TFDP1	YPEL3
TFDP1	YTHDC1
TFDP1	YTHDC2
TFDP1	YTHDF2
TFDP1	YTHDF3-AS1
TFDP1	YWHAB
TFDP1	YWHAE

TFDP1	YWHAG
TFDP1	YWHAH
TFDP1	YWHAQ
TFDP1	YWHAZ
TFDP1	YY1
TFDP1	ZBED5
TFDP1	ZBED5-AS1
TFDP1	ZBTB14
TFDP1	ZBTB25
TFDP1	ZBTB45
TFDP1	ZBTB8A
TFDP1	ZBTB8OS
TFDP1	ZC3H13
TFDP1	ZC3H14
TFDP1	ZC3H18
TFDP1	ZC3H6
TFDP1	ZC3H7A
TFDP1	ZCCHC17
TFDP1	ZCCHC3
TFDP1	ZCCHC7
TFDP1	ZCCHC9
TFDP1	ZDHHC13
TFDP1	ZDHHC16
TFDP1	ZDHHC4
TFDP1	ZDHHC5
TFDP1	ZEB1
TFDP1	ZFAND3
TFDP1	ZFAND5
TFDP1	ZFAND6
TFDP1	ZFAS1
TFDP1	ZFP62
TFDP1	ZFP90
TFDP1	ZFP91
TFDP1	ZFR
TFDP1	ZFX
TFDP1	ZFYVE19
TFDP1	ZHX2
TFDP1	ZMAT5
TFDP1	ZMPSTE24
TFDP1	ZMYND8
TFDP1	ZNF100
TFDP1	ZNF101
TFDP1	ZNF106
TFDP1	ZNF107
TFDP1	ZNF131
TFDP1	ZNF143
TFDP1	ZNF180
TFDP1	ZNF207
TFDP1	ZNF212
TFDP1	ZNF232

TFDP1	ZNF264
TFDP1	ZNF273
TFDP1	ZNF280C
TFDP1	ZNF282
TFDP1	ZNF296
TFDP1	ZNF326
TFDP1	ZNF330
TFDP1	ZNF335
TFDP1	ZNF337−AS1
TFDP1	ZNF346
TFDP1	ZNF354A
TFDP1	ZNF414
TFDP1	ZNF428
TFDP1	ZNF444
TFDP1	ZNF511
TFDP1	ZNF518A
TFDP1	ZNF519
TFDP1	ZNF530
TFDP1	ZNF532
TFDP1	ZNF576
TFDP1	ZNF580
TFDP1	ZNF592
TFDP1	ZNF598
TFDP1	ZNF622
TFDP1	ZNF639
TFDP1	ZNF644
TFDP1	ZNF652
TFDP1	ZNF669
TFDP1	ZNF670
TFDP1	ZNF672
TFDP1	ZNF682
TFDP1	ZNF687
TFDP1	ZNF706
TFDP1	ZNF726
TFDP1	ZNF75A
TFDP1	ZNF76
TFDP1	ZNF775
TFDP1	ZNF821
TFDP1	ZNF90
TFDP1	ZNHIT2
TFDP1	ZRANB3
TFDP1	ZSWIM8
TFDP1	ZWILCH
TFDP1	ZWINT
TFDP1	ZXDC
TFDP2	ABTB1
TFDP2	ACSL3
TFDP2	ACTR8
TFDP2	AGFG2
TFDP2	ALG10

TFDP2	ANLN
TFDP2	APPBP2
TFDP2	ARHGAP44
TFDP2	ASB6
TFDP2	ASPM
TFDP2	ATAD2
TFDP2	ATL2
TFDP2	BACE2
TFDP2	BCLAF1
TFDP2	BIRC5
TFDP2	BORA
TFDP2	BORCS6
TFDP2	BRIP1
TFDP2	C6orf47
TFDP2	C6orf99
TFDP2	CALR
TFDP2	CARHSP1
TFDP2	CCAR2
TFDP2	CCNB2
TFDP2	CCNE2
TFDP2	CCNF
TFDP2	CCNG1
TFDP2	CCP110
TFDP2	CD81
TFDP2	CDC25B
TFDP2	CDC27
TFDP2	CDCA3
TFDP2	CDCA7
TFDP2	CDK16
TFDP2	CDK6
TFDP2	CDK9
TFDP2	CDKN2D
TFDP2	CDT1
TFDP2	CENPA
TFDP2	CENPE
TFDP2	CEP290
TFDP2	CGGBP1
TFDP2	CITED2
TFDP2	CKAP2
TFDP2	CKAP2L
TFDP2	CNIH1
TFDP2	CNOT4
TFDP2	CNOT8
TFDP2	COX5A
TFDP2	CREBZF
TFDP2	CTPS2
TFDP2	DCP2
TFDP2	DDX3X
TFDP2	DERL1
TFDP2	DLGAP5

TFDP2	DPYSL2
TFDP2	DUSP6
TFDP2	E2F7
TFDP2	EDEM1
TFDP2	EGR1
TFDP2	EIF3D
TFDP2	EPB41
TFDP2	FAIM
TFDP2	FAM72D
TFDP2	FAM98A
TFDP2	FZR1
TFDP2	GADD45A
TFDP2	GANAB
TFDP2	GDF11
TFDP2	GKAP1
TFDP2	GLG1
TFDP2	GTSE1
TFDP2	HIST1H1B
TFDP2	HMGN3
TFDP2	HMGXB4
TFDP2	HPS3
TFDP2	IBTK
TFDP2	IDE
TFDP2	KDM3B
TFDP2	KIF18B
TFDP2	KIF2C
TFDP2	KIF4A
TFDP2	KLHDC10
TFDP2	KMT2A
TFDP2	KPNA2
TFDP2	LMCD1
TFDP2	MAP3K4
TFDP2	MED25
TFDP2	MED26
TFDP2	MIR4435-2HG
TFDP2	MIS18BP1
TFDP2	MLLT10
TFDP2	MOB1B
TFDP2	MORC4
TFDP2	MPLKIP
TFDP2	MSI2
TFDP2	MT1X
TFDP2	MTA3
TFDP2	MTURN
TFDP2	MTX2
TFDP2	MXD4
TFDP2	N4BP1
TFDP2	NAPEPLD
TFDP2	NDC80
TFDP2	NDUFS2

TFDP2	NHLRC2
TFDP2	NOL12
TFDP2	NUMB
TFDP2	PAN3
TFDP2	PBX3
TFDP2	PCED1B
TFDP2	PCNT
TFDP2	PDS5B
TFDP2	PEG10
TFDP2	PGM2
TFDP2	PHF23
TFDP2	PHIP
TFDP2	PIF1
TFDP2	PKNOX1
TFDP2	PLK1
TFDP2	PMF1
TFDP2	PNN
TFDP2	POLR2D
TFDP2	PPP1R2
TFDP2	PPP2R5D
TFDP2	PPP6R3
TFDP2	PRC1
TFDP2	PRCP
TFDP2	RAB5B
TFDP2	RAD21
TFDP2	RAPGEF5
TFDP2	RBMX
TFDP2	RCCD1
TFDP2	RDM1
TFDP2	REEP4
TFDP2	RPL39L
TFDP2	RPN2
TFDP2	RTN2
TFDP2	RTTN
TFDP2	SCAF11
TFDP2	SCLT1
TFDP2	SLC26A6
TFDP2	SMARCA5
TFDP2	SMNDC1
TFDP2	SNED1
TFDP2	SPSB3
TFDP2	SPTAN1
TFDP2	SSBP2
TFDP2	STIM2
TFDP2	SUGT1
TFDP2	SUZ12
TFDP2	TBC1D1
TFDP2	TCF20
TFDP2	TFDP2
TFDP2	THG1L

TFDP2	TM7SF2
TFDP2	TMEM59
TFDP2	TMEM9
TFDP2	TNRC6B
TFDP2	TOPBP1
TFDP2	TRIM24
TFDP2	TRIM28
TFDP2	TRIM37
TFDP2	TRIM59
TFDP2	TTC9C
TFDP2	UAP1
TFDP2	UBE2C
TFDP2	UBE2E3
TFDP2	UBTF
TFDP2	UCHL1
TFDP2	VEZT
TFDP2	WSB1
TFDP2	XRCC1
TFDP2	XRCC3
TFDP2	YTHDF3-AS1
TFDP2	ZFX
TFDP2	ZNF670
TFDP2	ZNF691
TFDP2	ZNF775
TFDP2	ZNF804A
TFEC	ABLIM1
TFEC	ACP5
TFEC	ADA
TFEC	AHNAK
TFEC	ANXA5
TFEC	AP5Z1
TFEC	ARF5
TFEC	ARF6
TFEC	ARHGAP10
TFEC	ARHGDIA
TFEC	ARID5B
TFEC	ARL8B
TFEC	ASXL2
TFEC	ATF4
TFEC	ATP1A1
TFEC	ATP6V0E1
TFEC	ATP6V1F
TFEC	BBC3
TFEC	BCAT1
TFEC	C19orf48
TFEC	C19orf70
TFEC	CAMK2B
TFEC	CAST
TFEC	CAV1
TFEC	CBX6

TFEC	CD44
TFEC	CD63
TFEC	CDC42
TFEC	CDC42EP3
TFEC	CFAP97
TFEC	CLIP1
TFEC	CREB5
TFEC	CSTB
TFEC	CTSA
TFEC	DCTN4
TFEC	DENR
TFEC	DOCK9
TFEC	DUSP10
TFEC	DYNLT3
TFEC	EEA1
TFEC	EGLN2
TFEC	EIF3A
TFEC	EMILIN2
TFEC	ENO1
TFEC	ENTHD1
TFEC	FAM49A
TFEC	FCMR
TFEC	FLCN
TFEC	FNBP1
TFEC	FOXP1
TFEC	GADD45G
TFEC	GLB1
TFEC	GLRX3
TFEC	GNPDA1
TFEC	GPR183
TFEC	GPRC5C
TFEC	GRN
TFEC	HS1BP3
TFEC	HSPA8
TFEC	IL4I1
TFEC	KAT7
TFEC	KIAA1551
TFEC	KIF13B
TFEC	KIF21A
TFEC	KLF6
TFEC	KPNA1
TFEC	LMNA
TFEC	LRRFIP2
TFEC	LYSMD2
TFEC	MACROD2
TFEC	MAPKAPK3
TFEC	MIR155HG
TFEC	MIR3142HG
TFEC	MVP
TFEC	NFE2L1

TFEC	NME2
TFEC	PDCD4
TFEC	PIAS4
TFEC	PMEPA1
TFEC	PPP1R15A
TFEC	PTPN1
TFEC	RAB13
TFEC	RAB7A
TFEC	RCAN3
TFEC	RDX
TFEC	RHOF
TFEC	RPL17
TFEC	RPS27
TFEC	RPS29
TFEC	RRAGC
TFEC	RRP9
TFEC	RTN4
TFEC	S100A6
TFEC	SCRN1
TFEC	SDHAF1
TFEC	SEPT7
TFEC	SERPINA9
TFEC	SGK1
TFEC	SIRT1
TFEC	SLC16A7
TFEC	SLC25A51
TFEC	SLC35E3
TFEC	SLC38A5
TFEC	SLC39A1
TFEC	SLC4A2
TFEC	SLC4A8
TFEC	SMPDL3A
TFEC	SQSTM1
TFEC	STAT6
TFEC	TAGLN2
TFEC	TCAF1
TFEC	TES
TFEC	TFEC
TFEC	THAP7
TFEC	TMEM120B
TFEC	TMEM192
TFEC	TNIP2
TFEC	TRAF1
TFEC	TRPV2
TFEC	TSC22D2
TFEC	UBC
TFEC	USP28
TFEC	UTP14A
TFEC	VPS72
TFEC	WDFY1

TFEC	YPEL1
TFEC	ZEB2
TFEC	ZFHX3
TFEC	ZNF165
TFEC	ZNF318
TFEC	ZNF322
TFEC	ZNF385C
THAP1	AHCYL1
THAP1	ANAPC2
THAP1	ANKRD13D
THAP1	AP1G2
THAP1	APEH
THAP1	API5
THAP1	ARF1
THAP1	ARHGAP4
THAP1	ARHGEF12
THAP1	ARL5B
THAP1	ARNT
THAP1	ARV1
THAP1	ASB2
THAP1	ATG5
THAP1	ATP23
THAP1	ATP6V0E1
THAP1	ATP6V1H
THAP1	ATXN1
THAP1	B9D2
THAP1	BCL2L13
THAP1	BICD2
THAP1	BRCA1
THAP1	C1orf112
THAP1	C8orf59
THAP1	CAMK2D
THAP1	CBL
THAP1	CCDC107
THAP1	CCDC127
THAP1	CCDC34
THAP1	CCND1
THAP1	CD24
THAP1	CDCA5
THAP1	CDCA7
THAP1	CDKN2D
THAP1	CEBPB
THAP1	CENPI
THAP1	CHD2
THAP1	CHP1
THAP1	CHPF2
THAP1	CHST15
THAP1	CHTOP
THAP1	CIRBP
THAP1	CMTR2

THAP1	COL4A3BP
THAP1	COPB1
THAP1	CORO7
THAP1	CSGALNACT2
THAP1	CTCF
THAP1	CYB561
THAP1	CYP2R1
THAP1	DBF4
THAP1	DCP1A
THAP1	DCTN1
THAP1	DCTN5
THAP1	DDX10
THAP1	DERL3
THAP1	DLG1
THAP1	DMD
THAP1	DNAJC4
THAP1	DOCK9
THAP1	DYM
THAP1	E2F8
THAP1	EBP
THAP1	EFCAB14
THAP1	ELK1
THAP1	ELMO2
THAP1	ELMSAN1
THAP1	ERI2
THAP1	ERP44
THAP1	FAF1
THAP1	FAM173A
THAP1	FAM184B
THAP1	FAM76A
THAP1	FBXO46
THAP1	FBXO7
THAP1	FBXO9
THAP1	FBXW11
THAP1	FGD3
THAP1	FLVCR1
THAP1	FNBP4
THAP1	FTSJ3
THAP1	GADD45B
THAP1	GDAP1
THAP1	GEN1
THAP1	GPI
THAP1	GSN
THAP1	GTF2H5
THAP1	H2AFX
THAP1	HAUS7
THAP1	HGSNAT
THAP1	HOXB4
THAP1	IDI1
THAP1	IFT122

THAP1	IGBP1
THAP1	IKBKE
THAP1	INO80E
THAP1	IRF5
THAP1	KAT8
THAP1	KCNK12
THAP1	KDF1
THAP1	KIFC1
THAP1	KMT5C
THAP1	LEPROT
THAP1	LINC00467
THAP1	LMCD1
THAP1	LPXN
THAP1	LRP10
THAP1	LRRC75A
THAP1	MAK16
THAP1	MAN1B1
THAP1	MAP2K7
THAP1	MAP3K11
THAP1	MAP4K4
THAP1	MAPK10
THAP1	MARCH3
THAP1	MARCH7
THAP1	MEAF6
THAP1	MED16
THAP1	MFSD9
THAP1	MIOS
THAP1	MMS22L
THAP1	MON2
THAP1	MRM1
THAP1	MRPS16
THAP1	MTERF4
THAP1	MTFR1L
THAP1	MTOR
THAP1	MYEF2
THAP1	NAE1
THAP1	NBEAL1
THAP1	NFKB2
THAP1	NOL9
THAP1	NOP10
THAP1	NOSIP
THAP1	ODF2L
THAP1	OSBPL11
THAP1	PAGR1
THAP1	PAK1IP1
THAP1	PCM1
THAP1	PCNT
THAP1	PER1
THAP1	PEX2
THAP1	PI4KA

THAP1	PIBF1
THAP1	PIGO
THAP1	POLR1A
THAP1	PPP1R10
THAP1	PPP1R11
THAP1	PPP1R12A
THAP1	PPP1R35
THAP1	PQLC1
THAP1	PRKAR1A
THAP1	PRKCZ
THAP1	PRPS2
THAP1	PRR13
THAP1	PRR14
THAP1	PSMA3-AS1
THAP1	PSMD1
THAP1	PSMD7
THAP1	PTS
THAP1	PTTG1IP
THAP1	RAB27A
THAP1	RALGPS1
THAP1	RAPGEF1
THAP1	RBL2
THAP1	RBM23
THAP1	RBM7
THAP1	RBM8A
THAP1	RECQL5
THAP1	RFWD3
THAP1	RGMB
THAP1	RIT1
THAP1	RNF219
THAP1	RNF24
THAP1	RNF40
THAP1	RNF41
THAP1	RPGR
THAP1	RRAGC
THAP1	RREB1
THAP1	RSPRY1
THAP1	SACM1L
THAP1	SAP18
THAP1	SAPCD2
THAP1	SC5D
THAP1	SENP5
THAP1	SF3B3
THAP1	SGO2
THAP1	SGPL1
THAP1	SH3BGRL
THAP1	SH3BP2
THAP1	SIGLEC10
THAP1	SLC2A1
THAP1	SLC5A3

THAP1	SLC9B2
THAP1	SMTN
THAP1	SMUG1
THAP1	SPC25
THAP1	SRSF10
THAP1	STAG3
THAP1	TBC1D5
THAP1	TFCP2
THAP1	TFPT
THAP1	THAP1
THAP1	TLE1
THAP1	TMED7
THAP1	TMEM263
THAP1	TMEM33
THAP1	TMEM69
THAP1	TMEM87A
THAP1	TMUB1
THAP1	TNFAIP1
THAP1	TNKS2
THAP1	TOMM34
THAP1	TP53RK
THAP1	TPM3
THAP1	TRAF5
THAP1	TSNAX
THAP1	TTK
THAP1	TUBG1
THAP1	TUT1
THAP1	TVP23C
THAP1	TXNL4B
THAP1	UBE2G2
THAP1	UEVLD
THAP1	UGT8
THAP1	USP53
THAP1	VIPAS39
THAP1	VPS26A
THAP1	VPS26B
THAP1	VPS37A
THAP1	VPS51
THAP1	WDFY1
THAP1	WDR12
THAP1	XAB2
THAP1	YBX1
THAP1	ZBED5-AS1
THAP1	ZC3H12A
THAP1	ZCCHC7
THAP1	ZFP90
THAP1	ZNF526
THAP1	ZNF562
THAP1	ZNF628
THAP1	ZNF678

THAP1	ZNF785
THAP11	ACAT2
THAP11	ACTR1A
THAP11	ADAR
THAP11	ADNP
THAP11	ADPRH
THAP11	ADSS
THAP11	AHCYL1
THAP11	AHDC1
THAP11	AK4
THAP11	ALDOC
THAP11	ARL4C
THAP11	ARMC9
THAP11	ARSK
THAP11	ASH1L-AS1
THAP11	ASPH
THAP11	ASXL2
THAP11	ATP6V1A
THAP11	BANP
THAP11	BCAT2
THAP11	BCKDK
THAP11	BORCS5
THAP11	C12orf43
THAP11	C20orf96
THAP11	CACYBP
THAP11	CANT1
THAP11	CBX1
THAP11	CCNT2
THAP11	CD163L1
THAP11	CELF1
THAP11	CENPT
THAP11	CERS2
THAP11	CHAF1A
THAP11	CHMP1A
THAP11	CLDND1
THAP11	CLOCK
THAP11	CLPB
THAP11	CLSPN
THAP11	CMPK1
THAP11	CNPY3
THAP11	COPE
THAP11	CTHRC1
THAP11	DCTN2
THAP11	DDX24
THAP11	DFFA
THAP11	DHX37
THAP11	DICER1
THAP11	DNAJB5
THAP11	DNAL1
THAP11	DOCK9

THAP11	DOPEY1
THAP11	E2F1
THAP11	EEF1B2
THAP11	EEF2
THAP11	EHD1
THAP11	EMD
THAP11	EXOC5
THAP11	FAM96A
THAP11	FASTKD1
THAP11	FBXO38
THAP11	FHOD1
THAP11	FLVCR1
THAP11	FYN
THAP11	FZD3
THAP11	GABPB1-AS1
THAP11	GATB
THAP11	GLO1
THAP11	GMNN
THAP11	GNAS
THAP11	GPATCH3
THAP11	GPBP1L1
THAP11	HACD3
THAP11	HDAC3
THAP11	ICMT
THAP11	IFT52
THAP11	ING2
THAP11	IRF1
THAP11	JTB
THAP11	KHK
THAP11	KIAA0355
THAP11	KLHL23
THAP11	LCMT2
THAP11	LRBA
THAP11	LSM2
THAP11	LUC7L
THAP11	LYRM1
THAP11	MAGED1
THAP11	MAPK8
THAP11	MAPRE2
THAP11	MAST4
THAP11	MBP
THAP11	MESP1
THAP11	MET
THAP11	METTL2B
THAP11	MFSD3
THAP11	MRPL23
THAP11	MRPS31
THAP11	MTMR2
THAP11	MXD4
THAP11	N4BP2L1

THAP11	NACA
THAP11	NBPF12
THAP11	NDUFB2
THAP11	NOL11
THAP11	NPEPPS
THAP11	NPRL2
THAP11	NR4A1
THAP11	NTPCR
THAP11	NXT2
THAP11	ODF2
THAP11	PALD1
THAP11	PARS2
THAP11	PEX6
THAP11	PIDD1
THAP11	PIK3R1
THAP11	PKN2
THAP11	PLGRKT
THAP11	POGZ
THAP11	POP1
THAP11	PPP1R14B
THAP11	PRH1
THAP11	PRPF19
THAP11	PRR14L
THAP11	PSMC3
THAP11	PSMD14
THAP11	PSMD5
THAP11	RAB29
THAP11	RAB34
THAP11	RAD9A
THAP11	RAPGEF6
THAP11	RBM15B
THAP11	REXO2
THAP11	RFC5
THAP11	RHOT1
THAP11	RPL13
THAP11	RPL15
THAP11	RPL17
THAP11	RPL18A
THAP11	RPL22
THAP11	RPL23A
THAP11	RPL26
THAP11	RPL29
THAP11	RPL30
THAP11	RPL31
THAP11	RPL32
THAP11	RPL34
THAP11	RPL35A
THAP11	RPL38
THAP11	RPL41
THAP11	RPL7

THAP11	RPL8
THAP11	RPLP2
THAP11	RPP40
THAP11	RPS11
THAP11	RPS13
THAP11	RPS15A
THAP11	RPS19
THAP11	RPS23
THAP11	RPS26
THAP11	RPS27
THAP11	RPS27A
THAP11	RPS29
THAP11	RPS3
THAP11	RPS5
THAP11	RSF1
THAP11	RTN3
THAP11	RYBP
THAP11	S100A13
THAP11	SCAF1
THAP11	SCRN2
THAP11	SF1
THAP11	SF3B5
THAP11	SGSM3
THAP11	SKP2
THAP11	SLAIN1
THAP11	SLC10A3
THAP11	SLC16A1
THAP11	SLC25A11
THAP11	SLC39A1
THAP11	SLC5A3
THAP11	SMAP1
THAP11	SMC3
THAP11	SMKR1
THAP11	SMYD2
THAP11	SNHG19
THAP11	SNX3
THAP11	SRP72
THAP11	SRRM2
THAP11	SSTR2
THAP11	STOML2
THAP11	SUCLG1
THAP11	TAGLN2
THAP11	TBC1D24
THAP11	TDRD7
THAP11	TFAP4
THAP11	TFIP11
THAP11	THAP11
THAP11	THAP4
THAP11	THAP7
THAP11	TIAL1

THAP11	TMEM109
THAP11	TMEM14A
THAP11	TOP3A
THAP11	TRIM8
THAP11	TRMT2B
THAP11	TSC22D1
THAP11	TUBGCP5
THAP11	UBA52
THAP11	UBE2E1
THAP11	UBP1
THAP11	UBR5
THAP11	UGDH
THAP11	UNK
THAP11	USP24
THAP11	UVRAG
THAP11	VEZT
THAP11	YTHDC2
THAP11	YWHAG
THAP11	ZMYM3
THAP11	ZNF143
THAP11	ZNF212
THAP11	ZNF318
THAP11	ZNF37A
THAP11	ZNF580
THAP11	ZNF598
THAP11	ZNF680
THAP11	ZNF837
TP53	ADSS
TP53	AHCY
TP53	ALDH1B1
TP53	ARHGAP44
TP53	BCAT2
TP53	C4orf46
TP53	CCDC25
TP53	CCT7
TP53	CDC42
TP53	CDC6
TP53	CHST10
TP53	COBLL1
TP53	COX7B
TP53	CSNK2B
TP53	DHX33
TP53	DPH2
TP53	EIF2B4
TP53	EIF2B5
TP53	EIF2S2
TP53	EIF5A
TP53	ETV5
TP53	FAM189B
TP53	FAM41C

TP53	FAM81A
TP53	FKBP4
TP53	FYN
TP53	GIT1
TP53	GLO1
TP53	GPT2
TP53	H1F0
TP53	HCFC1R1
TP53	HSP90AB1
TP53	IFRD2
TP53	INO80D
TP53	IQGAP3
TP53	KAT2A
TP53	KCTD17
TP53	KPNA3
TP53	KRBA1
TP53	LARS
TP53	LTA4H
TP53	MCM3
TP53	MCMBP
TP53	MCRIP2
TP53	MDM4
TP53	MRPL11
TP53	MRPS26
TP53	MTA2
TP53	MVB12A
TP53	NCLN
TP53	NDUFB2
TP53	NFKBIB
TP53	NOP14
TP53	NR6A1
TP53	NUTF2
TP53	ORC4
TP53	OTUD4
TP53	PA2G4
TP53	PDCD2L
TP53	PDE4D
TP53	PDE4DIP
TP53	PHB
TP53	POP1
TP53	PPP1CB
TP53	PPP1R14B
TP53	PPTC7
TP53	PRPF19
TP53	PTDSS2
TP53	RBL1
TP53	RDH14
TP53	RPIA
TP53	RPL6
TP53	RPL7

TP53	RPP40
TP53	RPS13
TP53	RPS5
TP53	RRP7A
TP53	RRS1
TP53	SERBP1
TP53	SH2B1
TP53	SHMT2
TP53	SIPA1L3
TP53	SLC20A2
TP53	SLC25A19
TP53	SNX11
TP53	SYMPK
TP53	TCOF1
TP53	TIMM13
TP53	TIMM22
TP53	TMEM69
TP53	TP53
TP53	TSC2
TP53	TXLNG
TP53	UBE2D1
TP53	VIPAS39
TP53	WRAP73
TP53	WRNIP1
TP53	YBX1
TP53	ZFC3H1
TP53	ZNF566
TP53	ZNF567
TP53	ZNF587
TP53	ZRANB3
USF2	ABHD17A
USF2	ACAP3
USF2	ACBD3
USF2	ACO2
USF2	ACP5
USF2	ACTG1
USF2	ADORA2A
USF2	ADPGK
USF2	AGO2
USF2	AGPAT1
USF2	AGPAT2
USF2	AHI1
USF2	AKAP10
USF2	ALKBH3
USF2	ANAPC10
USF2	ANKH
USF2	ANKRD17
USF2	ANKRD46
USF2	ANXA4
USF2	ANXA6

USF2	AP5M1
USF2	APPL2
USF2	ARFGEF1
USF2	ARHGAP15
USF2	ARHGAP17
USF2	ARHGAP24
USF2	ARHGAP27
USF2	ARHGAP4
USF2	ARHGDIA
USF2	ARHGDIB
USF2	ARID5B
USF2	ARMCX6
USF2	ARPC1B
USF2	ARRDC1
USF2	ASAH1
USF2	ASB6
USF2	ASF1A
USF2	ASPSCR1
USF2	ATP11B
USF2	ATP6V0D1
USF2	ATP6V0E1
USF2	ATP6V1C1
USF2	ATP6V1D
USF2	ATP6V1F
USF2	ATP6V1G1
USF2	B3GAT3
USF2	B9D2
USF2	BABAM1
USF2	BATF
USF2	BAX
USF2	BBS7
USF2	BBX
USF2	BCAS3
USF2	BCL11A
USF2	BDP1
USF2	BET1
USF2	BEX4
USF2	BFSP2
USF2	BICDL1
USF2	BIK
USF2	BLCAP
USF2	BLOC1S1
USF2	BPTF
USF2	BRI3
USF2	BRK1
USF2	BTAF1
USF2	BTBD7
USF2	BTG1
USF2	C12orf65
USF2	C1orf43

USF2	C4orf3
USF2	C6orf52
USF2	C7orf50
USF2	CAMK2B
USF2	CC2D1B
USF2	CCR6
USF2	CCR7
USF2	CD180
USF2	CD2AP
USF2	CEP164
USF2	CEP85L
USF2	CERK
USF2	CFDP1
USF2	CHFR
USF2	CHIC2
USF2	CHMP4A
USF2	CIB1
USF2	CLK3
USF2	CLK4
USF2	CLN3
USF2	CLTB
USF2	CLTC
USF2	CLUAP1
USF2	CMC1
USF2	CNOT4
USF2	CNR1
USF2	COMMD3
USF2	COMMD4
USF2	COMMD7
USF2	COMMD9
USF2	CORO1B
USF2	CORO7
USF2	CPNE1
USF2	CREM
USF2	CSNK1G2
USF2	CSTB
USF2	CTNNBIP1
USF2	CTNS
USF2	CTSA
USF2	CYSTM1
USF2	CYTIP
USF2	DAP
USF2	DAZAP2
USF2	DBI
USF2	DBP
USF2	DCAF5
USF2	DDT
USF2	DENND1C
USF2	DENND4A
USF2	DENND4C

USF2	DFFA
USF2	DGCR2
USF2	DGKD
USF2	DHDDS
USF2	DIAPH1
USF2	DICER1
USF2	DNAJA4
USF2	DNAJB5
USF2	DNAJC8
USF2	DOK1
USF2	DOK3
USF2	DPP7
USF2	DRAM2
USF2	DTX2
USF2	DTX3
USF2	DUSP23
USF2	DYM
USF2	EEF1D
USF2	EFHD2
USF2	EGLN2
USF2	EIF2AK4
USF2	EIF2B1
USF2	EIF3D
USF2	ELF1
USF2	ELF4
USF2	ELMO1
USF2	ELP5
USF2	ENO2
USF2	ENTPD1-AS1
USF2	EPC1
USF2	ERMARD
USF2	ERP29
USF2	ETV5
USF2	EYA3
USF2	FAM102A
USF2	FAM172A
USF2	FAM24B
USF2	FAM3C
USF2	FAM50A
USF2	FAM53B
USF2	FAM53C
USF2	FAU
USF2	FBXO25
USF2	FBXO34
USF2	FBXO42
USF2	FCER2
USF2	FCMR
USF2	FDFT1
USF2	FKBP8
USF2	FLI1

USF2	FNIP1
USF2	FOXN3
USF2	FOXRED2
USF2	FRYL
USF2	FTH1
USF2	FURIN
USF2	GABARAP
USF2	GABARAPL2
USF2	GAS6-AS1
USF2	GATAD2B
USF2	GBA
USF2	GCNT1
USF2	GHDC
USF2	GMDS
USF2	GNB1
USF2	GNB2
USF2	GNG5
USF2	GNG7
USF2	GPAT4
USF2	GPM6B
USF2	GPR160
USF2	GPR18
USF2	GPR89A
USF2	GRB2
USF2	GRN
USF2	GTF2F1
USF2	GTF2IRD1
USF2	GZF1
USF2	H3F3A
USF2	HEXA
USF2	HEXB
USF2	HIF1A
USF2	HLA-DMB
USF2	HMGN1
USF2	HOOK2
USF2	HPCAL1
USF2	HPS4
USF2	HSD17B7
USF2	HSDL1
USF2	HTR3A
USF2	ICA1L
USF2	ICE1
USF2	IDS
USF2	IGF2R
USF2	INPP5D
USF2	INPP5F
USF2	INTS3
USF2	IRF4
USF2	IRF9
USF2	ITGB2

USF2	JADE1
USF2	JAK1
USF2	JMJD1C
USF2	JUND
USF2	KANSL1
USF2	KAT6A
USF2	KCNN4
USF2	KDM4C
USF2	KHSRP
USF2	KIAA0355
USF2	KIAA2013
USF2	KLF6
USF2	KLHL12
USF2	KLHL5
USF2	KMT5A
USF2	LAMTOR1
USF2	LARP7
USF2	LBH
USF2	LCK
USF2	LCMT1
USF2	LGALS8
USF2	LINC00426
USF2	LMBRD1
USF2	LMO2
USF2	LMO4
USF2	LPP
USF2	LRIF1
USF2	LRRFIP1
USF2	LTA4H
USF2	LUZP1
USF2	M6PR
USF2	MAD2L2
USF2	MANBA
USF2	MAP1LC3B
USF2	MAP3K12
USF2	MAPK8
USF2	MAST3
USF2	MBD2
USF2	MCM10
USF2	MCRIP1
USF2	MED12L
USF2	MEF2C
USF2	MEF2D
USF2	MFF
USF2	MFSD14C
USF2	MIB2
USF2	MIR3681HG
USF2	MLXIP
USF2	MMS19
USF2	MRPL23

USF2	MS4A1
USF2	MSL2
USF2	MSRA
USF2	MTCH1
USF2	MTHFR
USF2	MYH9
USF2	MYL12A
USF2	MYL5
USF2	MYO1G
USF2	MYO6
USF2	NAA60
USF2	NAPA
USF2	NCF4
USF2	NDE1
USF2	NDUFB2
USF2	NEAT1
USF2	NELFA
USF2	NFAT5
USF2	NINJ2
USF2	NNT-AS1
USF2	NOM1
USF2	NPC2
USF2	NRAS
USF2	NRGN
USF2	NTHL1
USF2	NUDT18
USF2	NUP50
USF2	NUTM2A-AS1
USF2	OPA3
USF2	ORAI2
USF2	OSBP
USF2	OSBPL9
USF2	OTUD1
USF2	OTULIN
USF2	PAFAH1B3
USF2	PAG1
USF2	PCM1
USF2	PCYT2
USF2	PDK2
USF2	PDPK1
USF2	PEPD
USF2	PER2
USF2	PGBD2
USF2	PHF21A
USF2	PHF8
USF2	PHYKPL
USF2	PI4KB
USF2	PIAS4
USF2	PIK3R1
USF2	PKIG

USF2	PLEKHF2
USF2	PLIN2
USF2	POLD4
USF2	POM121
USF2	POP4
USF2	POU2F1
USF2	PPM1B
USF2	PPP6R1
USF2	PPRC1
USF2	PPT1
USF2	PQLC1
USF2	PRKCD
USF2	PRMT9
USF2	PRPSAP2
USF2	PSMB8-AS1
USF2	PTAFR
USF2	PTCH1
USF2	PTPN6
USF2	PTPRN2
USF2	PTPRS
USF2	QRICH1
USF2	QTRT1
USF2	RAB11B
USF2	RAB2A
USF2	RAB7A
USF2	RAB9A
USF2	RABGAP1L
USF2	RABL6
USF2	RALGPS2
USF2	RASAL3
USF2	RASSF5
USF2	RB1
USF2	RBM19
USF2	RBM23
USF2	RBM42
USF2	RC3H2
USF2	RFX1
USF2	RGS14
USF2	RGS20
USF2	RHBDF2
USF2	RHOBTB2
USF2	RMDN3
USF2	RNF139
USF2	RNF14
USF2	RNF141
USF2	RNF19B
USF2	ROR1
USF2	RPL13
USF2	RPL13A
USF2	RPL15

USF2	RPL18
USF2	RPL19
USF2	RPL21
USF2	RPL24
USF2	RPL37A
USF2	RPL38
USF2	RPL41
USF2	RPLP2
USF2	RPS11
USF2	RPS27
USF2	RPS28
USF2	RPS29
USF2	RPS3A
USF2	RPS6KB2
USF2	RRAGC
USF2	RRBP1
USF2	S100PBP
USF2	S1PR2
USF2	SAP130
USF2	SCAF4
USF2	SCFD2
USF2	SCIMP
USF2	SCPEP1
USF2	SDHC
USF2	SEC11A
USF2	SEL1L3
USF2	SEPHS2
USF2	SEPT6
USF2	SERF2
USF2	SGF29
USF2	SH3BP5
USF2	SH3KBP1
USF2	SIDT2
USF2	SIK3
USF2	SIRT1
USF2	SKAP2
USF2	SKIV2L
USF2	SLC10A3
USF2	SLC12A8
USF2	SLC19A1
USF2	SLC23A2
USF2	SLC25A25
USF2	SLC25A36
USF2	SLC25A37
USF2	SLC25A46
USF2	SLC2A3
USF2	SLC2A6
USF2	SLC46A3
USF2	SLC4A7
USF2	SLC9A3

USF2	SLF2
USF2	SMAD7
USF2	SMCHD1
USF2	SMDT1
USF2	SMIM14
USF2	SNRK
USF2	SNX2
USF2	SP100
USF2	SPACA9
USF2	SPG21
USF2	SPI1
USF2	SREBF2
USF2	SRPK2
USF2	SRSF5
USF2	SSBP3
USF2	STAG1
USF2	STAT3
USF2	STK16
USF2	STK17B
USF2	STK26
USF2	STRBP
USF2	STX12
USF2	STX7
USF2	STXBP3
USF2	SULF2
USF2	SUMO3
USF2	SUN2
USF2	SUPT6H
USF2	SYMPK
USF2	SYNE1
USF2	SYT11
USF2	TAF10
USF2	TAX1BP1
USF2	TBL1XR1
USF2	TCEAL3
USF2	TCEAL4
USF2	TCEAL8
USF2	TCF25
USF2	TEN1
USF2	TEX264
USF2	TFPT
USF2	TGFBRAP1
USF2	TGOLN2
USF2	THOC3
USF2	TJP1
USF2	TM9SF2
USF2	TMBIM1
USF2	TMC6
USF2	TMC8
USF2	TMEM131

USF2	TMEM219
USF2	TMEM256
USF2	TMEM60
USF2	TMEM9B
USF2	TMSB4X
USF2	TMX4
USF2	TNFRSF13C
USF2	TNFRSF14
USF2	TOLLIP
USF2	TOMM20
USF2	TOPORS
USF2	TPP1
USF2	TPT1
USF2	TRIB2
USF2	TRIM11
USF2	TRIP12
USF2	TSC22D3
USF2	TSEN2
USF2	TTC9
USF2	TUBA1A
USF2	TUBB2A
USF2	TUBB3
USF2	TWF2
USF2	TXN2
USF2	U2AF1
USF2	UBA52
USF2	UBE4A
USF2	UBN1
USF2	UBTF
USF2	UROD
USF2	USF2
USF2	USP11
USF2	USP44
USF2	USP8
USF2	VAPA
USF2	VASP
USF2	VAV1
USF2	VPS11
USF2	VPS35
USF2	WASF2
USF2	WDR13
USF2	WDR26
USF2	WIPF1
USF2	WIPI2
USF2	WWC3
USF2	XXYLT1
USF2	YPEL2
USF2	YWHAZ
USF2	ZBTB20
USF2	ZDHHC13

USF2	ZDHHC17
USF2	ZDHHC3
USF2	ZFAND6
USF2	ZFP62
USF2	ZFX
USF2	ZHX2
USF2	ZMAT3
USF2	ZMYM3
USF2	ZNF318
USF2	ZNF580
USF2	ZNF608
USF2	ZNF621
USF2	ZNF680
USF2	ZNF720
USF2	ZNF76
USF2	ZSWIM8
VEZF1	ARAP2
VEZF1	L3MBTL1
VEZF1	MARK2
VEZF1	PATZ1
VEZF1	PLEKHA1
VEZF1	RNF44
VEZF1	VEZF1
XBP1	AAAS
XBP1	AACS
XBP1	AAK1
XBP1	AAMP
XBP1	AARS
XBP1	AASDHPPT
XBP1	ABCA5
XBP1	ABCB8
XBP1	ABCC4
XBP1	ABCF3
XBP1	ABL2
XBP1	ABTB1
XBP1	ACACB
XBP1	ACAD10
XBP1	ACADVL
XBP1	ACBD3
XBP1	ACBD4
XBP1	ACO2
XBP1	ACOX1
XBP1	ACOX3
XBP1	ACSL3
XBP1	ACSL4
XBP1	ACSS1
XBP1	ACYP2
XBP1	ADA
XBP1	ADAM10
XBP1	ADAR

XBP1	ADGRG5
XBP1	ADI1
XBP1	ADIPOR1
XBP1	ADM
XBP1	ADORA2A
XBP1	ADRM1
XBP1	AEBP1
XBP1	AFF1
XBP1	AGA
XBP1	AGAP5
XBP1	AGO1
XBP1	AGPAT1
XBP1	AGPAT3
XBP1	AGTRAP
XBP1	AIFM2
XBP1	AIG1
XBP1	AK3
XBP1	AK9
XBP1	AKAP1
XBP1	AKAP13
XBP1	AKAP8L
XBP1	AKAP9
XBP1	AKIRIN1
XBP1	AKIRIN2
XBP1	AKR1A1
XBP1	AKT1S1
XBP1	ALDH18A1
XBP1	ALDH3A2
XBP1	ALDOA
XBP1	ALDOC
XBP1	ALG1
XBP1	ALG11
XBP1	ALG12
XBP1	ALG13
XBP1	ALG14
XBP1	ALG2
XBP1	ALG3
XBP1	ALG9
XBP1	ALKBH5
XBP1	ALKBH7
XBP1	ALOX12-AS1
XBP1	AMN
XBP1	AMOTL1
XBP1	AMPD2
XBP1	AMPD3
XBP1	ANAPC10
XBP1	ANAPC11
XBP1	ANAPC13
XBP1	ANAPC16
XBP1	ANAPC2

XBP1	ANAPC5
XBP1	ANG
XBP1	ANKHD1
XBP1	ANKRD12
XBP1	ANKRD28
XBP1	ANKRD36
XBP1	ANKRD36B
XBP1	ANKRD37
XBP1	ANKRD39
XBP1	ANKS3
XBP1	ANKZF1
XBP1	ANO9
XBP1	ANXA11
XBP1	ANXA5
XBP1	ANXA6
XBP1	AP2A1
XBP1	AP3B1
XBP1	AP3D1
XBP1	APBB3
XBP1	APH1B
XBP1	APMAP
XBP1	APOL3
XBP1	APP
XBP1	APPBP2
XBP1	APTX
XBP1	AQP3
XBP1	ARCN1
XBP1	AREG
XBP1	ARF1
XBP1	ARF4
XBP1	ARFGAP1
XBP1	ARFGAP3
XBP1	ARFGEF2
XBP1	ARFIP1
XBP1	ARFIP2
XBP1	ARHGEF6
XBP1	ARID3A
XBP1	ARID3B
XBP1	ARID4A
XBP1	ARL1
XBP1	ARL5A
XBP1	ARMCX3
XBP1	ARMCX5
XBP1	ARRDC3
XBP1	ARSA
XBP1	ARV1
XBP1	ASB6
XBP1	ASB8
XBP1	ASCC2
XBP1	ASCC3

XBP1	ASNA1
XBP1	ASPHD2
XBP1	ATF4
XBP1	ATF5
XBP1	ATF6
XBP1	ATF6B
XBP1	ATG101
XBP1	ATG13
XBP1	ATG16L1
XBP1	ATG4C
XBP1	ATG5
XBP1	ATP13A1
XBP1	ATP1B1
XBP1	ATP2B4
XBP1	ATP6AP1
XBP1	ATP6AP2
XBP1	ATP6V0A1
XBP1	ATP6V0B
XBP1	ATP6V0D1
XBP1	ATP6V0E1
XBP1	ATP6V1D
XBP1	ATP6V1G1
XBP1	ATP8B2
XBP1	ATRAID
XBP1	ATRX
XBP1	AURKAIP1
XBP1	AZIN1
XBP1	B3GAT2
XBP1	B3GAT3
XBP1	B4GALT3
XBP1	B4GALT4
XBP1	B4GAT1
XBP1	B9D1
XBP1	BACH1
XBP1	BAIAP2L1
XBP1	BAZ2B
XBP1	BBX
XBP1	BCAP31
XBP1	BCAR1
XBP1	BCAR3
XBP1	BCAS3
XBP1	BCKDK
XBP1	BDH2
XBP1	BECN1
XBP1	BET1
XBP1	BET1L
XBP1	BEX4
XBP1	BEX5
XBP1	BFSP2-AS1
XBP1	BHLHE41

XBP1	BICD1
XBP1	BIRC2
XBP1	BLNK
XBP1	BLOC1S3
XBP1	BLVRB
XBP1	BLZF1
XBP1	BMI1
XBP1	BMT2
XBP1	BNIP3
XBP1	BOLA1
XBP1	BORCS5
XBP1	BORCS6
XBP1	BPGM
XBP1	BRD1
XBP1	BRD2
XBP1	BRI3
XBP1	BSDC1
XBP1	BSG
XBP1	BTBD7
XBP1	BTG2
XBP1	BTLA
XBP1	BUB3
XBP1	C11orf24
XBP1	C11orf68
XBP1	C11orf71
XBP1	C14orf28
XBP1	C15orf61
XBP1	C16orf58
XBP1	C16orf72
XBP1	C16orf91
XBP1	C16orf95
XBP1	C17orf53
XBP1	C18orf32
XBP1	C19orf12
XBP1	C19orf24
XBP1	C19orf53
XBP1	C19orf81
XBP1	C1GALT1
XBP1	C1GALT1C1
XBP1	C1orf43
XBP1	C21orf2
XBP1	C2orf74
XBP1	C2orf88
XBP1	C4orf3
XBP1	C4orf48
XBP1	C5orf15
XBP1	C6orf106
XBP1	C6orf52
XBP1	C6orf89
XBP1	C7orf50

XBP1	CAAP1
XBP1	CACUL1
XBP1	CADM1
XBP1	CALCOCO1
XBP1	CALCOCO2
XBP1	CALR
XBP1	CALU
XBP1	CAMP
XBP1	CAMTA1
XBP1	CANX
XBP1	CAP1
XBP1	CAPN10
XBP1	CAPN7
XBP1	CASP3
XBP1	CASP4
XBP1	CASP7
XBP1	CASP8
XBP1	CASP9
XBP1	CBR1
XBP1	CBWD1
XBP1	CBWD5
XBP1	CBWD6
XBP1	CBX4
XBP1	CBX7
XBP1	CCDC107
XBP1	CCDC112
XBP1	CCDC130
XBP1	CCDC144A
XBP1	CCDC167
XBP1	CCDC186
XBP1	CCDC47
XBP1	CCDC57
XBP1	CCDC66
XBP1	CCDC69
XBP1	CCDC88A
XBP1	CCDC88C
XBP1	CCL25
XBP1	CCM2
XBP1	CCNC
XBP1	CCNL1
XBP1	CCNT2
XBP1	CCPG1
XBP1	CCR10
XBP1	CCRL2
XBP1	CD164
XBP1	CD200
XBP1	CD2BP2
XBP1	CD38
XBP1	CD55
XBP1	CD59

XBP1	CD63
XBP1	CD74
XBP1	CDADC1
XBP1	CDC16
XBP1	CDC26
XBP1	CDIP1
XBP1	CDIPT
XBP1	CDK12
XBP1	CDK14
XBP1	CDK2AP2
XBP1	CDK5
XBP1	CDK5RAP3
XBP1	CDK6
XBP1	CDKN1A
XBP1	CDKN1B
XBP1	CDR2
XBP1	CDV3
XBP1	CEBPB
XBP1	CEP120
XBP1	CEP128
XBP1	CEP63
XBP1	CEP70
XBP1	CERCAM
XBP1	CERS6
XBP1	CETN2
XBP1	CFL2
XBP1	CHAC1
XBP1	CHCHD2
XBP1	CHCHD7
XBP1	CHD6
XBP1	CHD9
XBP1	CHID1
XBP1	CHMP2A
XBP1	CHMP2B
XBP1	CHP1
XBP1	CHPF
XBP1	CHPF2
XBP1	CHRNB1
XBP1	CHST11
XBP1	CHST12
XBP1	CHST7
XBP1	CISD2
XBP1	CITED2
XBP1	CKAP4
XBP1	CLCC1
XBP1	CLCN3
XBP1	CLCN4
XBP1	CLCN7
XBP1	CLIC4
XBP1	CLINT1

XBP1	CLIP4
XBP1	CLK1
XBP1	CLN5
XBP1	CLOCK
XBP1	CLP1
XBP1	CLPB
XBP1	CLPTM1
XBP1	CLPTM1L
XBP1	CLSTN3
XBP1	CMIP
XBP1	CNOT6L
XBP1	CNPY2
XBP1	CNR1
XBP1	COG3
XBP1	COG5
XBP1	COG6
XBP1	COMMD1
XBP1	COMMD3
XBP1	COMT
XBP1	COPA
XBP1	COPB1
XBP1	COPB2
XBP1	COPE
XBP1	COPG1
XBP1	COPS6
XBP1	COPS8
XBP1	COPS9
XBP1	COPZ1
XBP1	COPZ2
XBP1	COQ10B
XBP1	COQ4
XBP1	COQ6
XBP1	CORO1C
XBP1	COX10
XBP1	COX18
XBP1	COX6C
XBP1	COX7A2L
XBP1	CPEB4
XBP1	CR1
XBP1	CRB3
XBP1	CREB3
XBP1	CREB3L2
XBP1	CREBZF
XBP1	CRELD2
XBP1	CRIPT
XBP1	CRK
XBP1	CRLS1
XBP1	CRNKL1
XBP1	CSDE1
XBP1	CSF2RB

XBP1	CSGALNACT2
XBP1	CSNK1A1
XBP1	CSNK1E
XBP1	CSRP1
XBP1	CSTB
XBP1	CTBP1
XBP1	CTBP1-AS
XBP1	CTC1
XBP1	CTDNEP1
XBP1	CTGF
XBP1	CTHRC1
XBP1	CTNNBIP1
XBP1	CTSA
XBP1	CTSB
XBP1	CTSD
XBP1	CUEDC2
XBP1	CUTA
XBP1	CWC25
XBP1	CYB561
XBP1	CYB561D2
XBP1	CYB5R1
XBP1	CYCS
XBP1	CYLD
XBP1	CYTH2
XBP1	CYTIP
XBP1	DAD1
XBP1	DAP
XBP1	DCAF11
XBP1	DCAF5
XBP1	DCAF8
XBP1	DCP1A
XBP1	DCPS
XBP1	DCTN4
XBP1	DCXR
XBP1	DDB1
XBP1	DDHD1
XBP1	DDIT4
XBP1	DDX1
XBP1	DDX17
XBP1	DDX20
XBP1	DDX23
XBP1	DDX3X
XBP1	DDX3Y
XBP1	DDX49
XBP1	DDX5
XBP1	DDX51
XBP1	DDX59
XBP1	DECR1
XBP1	DEDD2
XBP1	DEF6

XBP1	DENND1B
XBP1	DENND1C
XBP1	DENND6B
XBP1	DERL1
XBP1	DERL3
XBP1	DEXI
XBP1	DHCR7
XBP1	DHDDS
XBP1	DHPS
XBP1	DHRS4L2
XBP1	DHRS7
XBP1	DHX37
XBP1	DLAT
XBP1	DNAJB1
XBP1	DNAJB11
XBP1	DNAJB12
XBP1	DNAJB9
XBP1	DNAJC1
XBP1	DNAJC10
XBP1	DNAJC12
XBP1	DNAJC16
XBP1	DNAJC25
XBP1	DNAJC3
XBP1	DNAJC30
XBP1	DNAJC4
XBP1	DNASE2
XBP1	DPAGT1
XBP1	DPM1
XBP1	DPM2
XBP1	DPM3
XBP1	DPP7
XBP1	DRAP1
XBP1	DSTN
XBP1	DUSP1
XBP1	DUSP12
XBP1	DUSP22
XBP1	DUSP5
XBP1	DVL3
XBP1	DYNLL1
XBP1	DYRK4
XBP1	E2F5
XBP1	E4F1
XBP1	EAF2
XBP1	EBAG9
XBP1	EBPL
XBP1	EDEM1
XBP1	EDEM2
XBP1	EDEM3
XBP1	EDRF1
XBP1	EEF2

XBP1	EFHC1
XBP1	EGR1
XBP1	EHD3
XBP1	EHMT1
XBP1	EIF1
XBP1	EIF1AD
XBP1	EIF2A
XBP1	EIF2AK2
XBP1	EIF2AK3
XBP1	EIF2AK4
XBP1	EIF3G
XBP1	EIF4E3
XBP1	EIF4ENIF1
XBP1	EIF4G1
XBP1	EIF5
XBP1	ELAC2
XBP1	ELK1
XBP1	ELK3
XBP1	ELL
XBP1	ELL2
XBP1	ELMOD2
XBP1	ELP3
XBP1	EMC10
XBP1	EMC2
XBP1	EMC3
XBP1	EMC4
XBP1	EMC7
XBP1	EML6
XBP1	ENAM
XBP1	ENDOG
XBP1	ENTPD7
XBP1	EPHX1
XBP1	EPOR
XBP1	EPRS
XBP1	ERC1
XBP1	ERCC1
XBP1	ERCC4
XBP1	ERCC8
XBP1	ERGIC1
XBP1	ERGIC2
XBP1	ERGIC3
XBP1	ERLEC1
XBP1	ERN1
XBP1	ERO1A
XBP1	ERO1B
XBP1	ERP29
XBP1	ERP44
XBP1	ESCO1
XBP1	ESR2
XBP1	ETNK1

XBP1	ETS1
XBP1	ETV2
XBP1	EVI2A
XBP1	EVI2B
XBP1	EXD2
XBP1	EXOC5
XBP1	EXOSC1
XBP1	EXTL2
XBP1	FADD
XBP1	FAF1
XBP1	FAM114A1
XBP1	FAM117A
XBP1	FAM120AOS
XBP1	FAM174A
XBP1	FAM177A1
XBP1	FAM199X
XBP1	FAM214A
XBP1	FAM3A
XBP1	FAM3C
XBP1	FAM50A
XBP1	FAM53C
XBP1	FAM78A
XBP1	FAM91A1
XBP1	FAM98A
XBP1	FAR1
XBP1	FASTK
XBP1	FAU
XBP1	FBXL15
XBP1	FBXO11
XBP1	FBXO16
XBP1	FBXO25
XBP1	FBXO3
XBP1	FBXO34
XBP1	FBXO4
XBP1	FBXO42
XBP1	FBXO46
XBP1	FBXO8
XBP1	FBXW5
XBP1	FBXW7
XBP1	FDX1
XBP1	FECH
XBP1	FEM1C
XBP1	FGFR1OP2
XBP1	FIS1
XBP1	FKBP11
XBP1	FKBP14
XBP1	FKBP2
XBP1	FKBP8
XBP1	FKBPL
XBP1	FLYWCH2

XBP1	FMNL2
XBP1	FN3KRP
XBP1	FNDC3A
XBP1	FNDC3B
XBP1	FOS
XBP1	FOSB
XBP1	FOSL1
XBP1	FOXN3
XBP1	FOXO3
XBP1	FPGS
XBP1	FRZB
XBP1	FTH1
XBP1	FTL
XBP1	FUBP3
XBP1	FUCA2
XBP1	FUT8
XBP1	FZD3
XBP1	FZD6
XBP1	G6PC3
XBP1	GABARAP
XBP1	GABARAPL1
XBP1	GABARAPL2
XBP1	GADD45A
XBP1	GADD45B
XBP1	GADD45G
XBP1	GAK
XBP1	GALC
XBP1	GALK2
XBP1	GALNT2
XBP1	GANAB
XBP1	GAS6
XBP1	GCC1
XBP1	GDI1
XBP1	GEMIN7
XBP1	GFER
XBP1	GFPT1
XBP1	GGCX
XBP1	GGH
XBP1	GHITM
XBP1	GIGYF2
XBP1	GLCCI1
XBP1	GLG1
XBP1	GLIPR1
XBP1	GLMP
XBP1	GLRX
XBP1	GLS
XBP1	GLT8D1
XBP1	GMCL1
XBP1	GMFG
XBP1	GMPPA

XBP1	GMPPB
XBP1	GNAS
XBP1	GNB3
XBP1	GNG7
XBP1	GNPTG
XBP1	GOLGA2
XBP1	GOLGA3
XBP1	GOLGA4
XBP1	GOLGA5
XBP1	GOLGB1
XBP1	GOLIM4
XBP1	GOLPH3
XBP1	GOLPH3L
XBP1	GOLT1B
XBP1	GON4L
XBP1	GORASP2
XBP1	GOSR1
XBP1	GOSR2
XBP1	GPANK1
XBP1	GPER1
XBP1	GPKOW
XBP1	GPLD1
XBP1	GPM6B
XBP1	GPR108
XBP1	GPR137B
XBP1	GPR155
XBP1	GPR160
XBP1	GPR180
XBP1	GPR183
XBP1	GPR65
XBP1	GPR89B
XBP1	GPRIN3
XBP1	GPX7
XBP1	GRB2
XBP1	GRINA
XBP1	GRK5
XBP1	GRK6
XBP1	GRN
XBP1	GSK3B
XBP1	GSTM3
XBP1	GTF2A1
XBP1	GTF2B
XBP1	GTF3A
XBP1	GUK1
XBP1	GUSB
XBP1	GYPC
XBP1	H1FX
XBP1	H3F3B
XBP1	HABP4
XBP1	HAGH

XBP1	HARBI1
XBP1	HAX1
XBP1	HBP1
XBP1	HBS1L
XBP1	HCCS
XBP1	HCFC2
XBP1	HCST
XBP1	HDLBP
XBP1	HECW2
XBP1	HELZ2
XBP1	HERPUD1
XBP1	HES1
XBP1	HEXA
XBP1	HEXB
XBP1	HIBCH
XBP1	HIST1H1C
XBP1	HIST1H2AE
XBP1	HIST1H2BD
XBP1	HIST1H2BG
XBP1	HIST1H2BJ
XBP1	HIST1H2BN
XBP1	HLA-A
XBP1	HLA-B
XBP1	HLA-DMA
XBP1	HLA-DPA1
XBP1	HLA-DQA1
XBP1	HLA-DQA2
XBP1	HLA-DQB1
XBP1	HLA-DRB5
XBP1	HLA-E
XBP1	HLA-F
XBP1	HM13
XBP1	HMBOX1
XBP1	HMGN4
XBP1	HMGXB3
XBP1	HMOX2
XBP1	HNRNPH2
XBP1	HOOK2
XBP1	HOTAIRM1
XBP1	HOXB2
XBP1	HOXB7
XBP1	HSCB
XBP1	HSD17B10
XBP1	HSP90B1
XBP1	HSPA13
XBP1	HSPA1A
XBP1	HSPA5
XBP1	HTR3A
XBP1	HYOU1
XBP1	IBTK

XBP1	ICAM1
XBP1	ICAM2
XBP1	ICAM3
XBP1	IDH1
XBP1	IDH2
XBP1	IDH3G
XBP1	IDUA
XBP1	IER2
XBP1	IER3IP1
XBP1	IFI27L2
XBP1	IFNAR1
XBP1	IFNAR2
XBP1	IFNG-AS1
XBP1	IFNLR1
XBP1	IFRD1
XBP1	IFT20
XBP1	IFT27
XBP1	IGLL5
XBP1	IGSF8
XBP1	IKBIP
XBP1	IL10RA
XBP1	IL12RB1
XBP1	IL16
XBP1	IL6
XBP1	IL6R
XBP1	IL6ST
XBP1	IMP3
XBP1	IMPAD1
XBP1	ING2
XBP1	INPP4A
XBP1	INSIG1
XBP1	INSIG2
XBP1	INTS6
XBP1	INTS7
XBP1	IQGAP2
XBP1	IQSEC1
XBP1	IRAK1
XBP1	IRF1
XBP1	IRF2
XBP1	IRF2BP1
XBP1	IRF4
XBP1	IRF7
XBP1	IRF9
XBP1	IRS1
XBP1	ISCU
XBP1	ISG15
XBP1	ISG20
XBP1	ISOC2
XBP1	ITCH
XBP1	ITFG2

XBP1	ITGB2
XBP1	ITM2B
XBP1	ITM2C
XBP1	ITPR2
XBP1	ITPR3
XBP1	ITPRIPL2
XBP1	JAGN1
XBP1	JCHAIN
XBP1	JDP2
XBP1	JMJD6
XBP1	JMJD8
XBP1	JMY
XBP1	JOSD2
XBP1	JUN
XBP1	JUNB
XBP1	KBTBD2
XBP1	KCNA3
XBP1	KCNK12
XBP1	KCNK6
XBP1	KCNMA1
XBP1	KCNN3
XBP1	KCNN4
XBP1	KDELR1
XBP1	KDELR2
XBP1	KDM5A
XBP1	KDM5C
XBP1	KHK
XBP1	KIAA0232
XBP1	KIAA2013
XBP1	KIAA2026
XBP1	KIF5C
XBP1	KIFAP3
XBP1	KIZ
XBP1	KLF13
XBP1	KLF6
XBP1	KLHDC10
XBP1	KLHDC2
XBP1	KLHDC7B
XBP1	KLHL14
XBP1	KLHL28
XBP1	KMT2E
XBP1	KMT2E-AS1
XBP1	KMT5B
XBP1	KRT18
XBP1	KRTCAP2
XBP1	KTI12
XBP1	L3MBTL2
XBP1	LAMTOR4
XBP1	LAP3
XBP1	LAPTM4A

XBP1	LARP1B
XBP1	LAX1
XBP1	LBH
XBP1	LDLR
XBP1	LDLRAD4
XBP1	LDLRAP1
XBP1	LEPROTL1
XBP1	LETM1
XBP1	LGALS1
XBP1	LIG3
XBP1	LIMD1
XBP1	LIN52
XBP1	LIN54
XBP1	LINC00501
XBP1	LINC01013
XBP1	LMAN1
XBP1	LMBR1
XBP1	LMBRD1
XBP1	LMF1
XBP1	LMF2
XBP1	LMO4
XBP1	LMTK2
XBP1	LNX1
XBP1	LPIN1
XBP1	LRIG1
XBP1	LRIG2
XBP1	LRP10
XBP1	LRRC14
XBP1	LRRC2
XBP1	LRRC59
XBP1	LSP1
XBP1	LSR
XBP1	LST1
XBP1	LTB4R
XBP1	LTBP1
XBP1	LTBP3
XBP1	LTC4S
XBP1	LTN1
XBP1	LUZP1
XBP1	LY6E
XBP1	LY6G5C
XBP1	LYSMD3
XBP1	LZTFL1
XBP1	MAEA
XBP1	MAGED1
XBP1	MAGED2
XBP1	MAGOHB
XBP1	MAGT1
XBP1	MAN1A1
XBP1	MAN1A2

XBP1	MAN2A1
XBP1	MAN2C1
XBP1	MANBAL
XBP1	MANF
XBP1	MAP1LC3B
XBP1	MAP2K2
XBP1	MAP3K13
XBP1	MAP4K5
XBP1	MAP7D1
XBP1	MAP9
XBP1	MAPK6
XBP1	MAPK8
XBP1	MAPKAPK2
XBP1	MAPKAPK5−AS1
XBP1	MAPRE1
XBP1	MARCH3
XBP1	MARCH5
XBP1	MARCH6
XBP1	MARCH8
XBP1	MAX
XBP1	MBNL2
XBP1	MBTPS1
XBP1	MCEE
XBP1	MCFD2
XBP1	MCL1
XBP1	MCOLN1
XBP1	MDM2
XBP1	MDP1
XBP1	MECR
XBP1	MED18
XBP1	MEF2C
XBP1	MELK
XBP1	MESP1
XBP1	METTL15
XBP1	MFF
XBP1	MFN2
XBP1	MFSD1
XBP1	MFSD5
XBP1	MGAT1
XBP1	MGAT4A
XBP1	MIA2
XBP1	MIA3
XBP1	MIAT
XBP1	MIB2
XBP1	MIEF1
XBP1	MIF
XBP1	MIR193BHG
XBP1	MIR22HG
XBP1	MIR3142HG
XBP1	MIR4435−2HG

XBP1	MIS12
XBP1	MKKS
XBP1	MKRN2
XBP1	MLEC
XBP1	MLF2
XBP1	MLX
XBP1	MMP25-AS1
XBP1	MOAP1
XBP1	MOB3C
XBP1	MOGS
XBP1	MON2
XBP1	MORC2
XBP1	MORF4L2
XBP1	MOSPD1
XBP1	MOSPD2
XBP1	MOSPD3
XBP1	MPC1
XBP1	MPEG1
XBP1	MPG
XBP1	MPHOSPH8
XBP1	MPI
XBP1	MPP5
XBP1	MPV17
XBP1	MPV17L2
XBP1	MRPL2
XBP1	MRPL23
XBP1	MRPL36
XBP1	MRPL40
XBP1	MRPL48
XBP1	MRPL52
XBP1	MRPL54
XBP1	MRPL55
XBP1	MRPL57
XBP1	MRPS18A
XBP1	MRPS31
XBP1	MRPS33
XBP1	MSI2
XBP1	MSL3
XBP1	MSMO1
XBP1	MSN
XBP1	MSRA
XBP1	MSTO1
XBP1	MTDH
XBP1	MTG1
XBP1	MTIF3
XBP1	MTRF1L
XBP1	MUL1
XBP1	MUT
XBP1	MVB12A
XBP1	MVD

XBP1	MVP
XBP1	MXD4
XBP1	MYCBP2
XBP1	MYDGF
XBP1	MYL4
XBP1	MYL6
XBP1	MYO1D
XBP1	MYO1G
XBP1	MZB1
XBP1	MZF1
XBP1	N4BP1
XBP1	N4BP2L2
XBP1	NAA38
XBP1	NADK
XBP1	NAGK
XBP1	NANS
XBP1	NAP1L4
XBP1	NAPA
XBP1	NAPA−AS1
XBP1	NARF
XBP1	NAT1
XBP1	NBAS
XBP1	NCALD
XBP1	NCK1
XBP1	NCK2
XBP1	NCLN
XBP1	NCOR2
XBP1	NDEL1
XBP1	NDFIP1
XBP1	NDFIP2
XBP1	NDUFA1
XBP1	NDUFA11
XBP1	NDUFA13
XBP1	NDUFA4
XBP1	NDUFA8
XBP1	NDUFAB1
XBP1	NDUFAF3
XBP1	NDUFAF6
XBP1	NDUFB1
XBP1	NDUFB2
XBP1	NDUFB4
XBP1	NDUFB6
XBP1	NDUFC1
XBP1	NDUFS1
XBP1	NDUFS2
XBP1	NDUFS5
XBP1	NDUFS6
XBP1	NDUFS8
XBP1	NDUFV1
XBP1	NDUFV3

XBP1	NEAT1
XBP1	NECAP1
XBP1	NEDD9
XBP1	NET1
XBP1	NETO2
XBP1	NEU1
XBP1	NFU1
XBP1	NGLY1
XBP1	NGRN
XBP1	NHLRC3
XBP1	NKIRAS1
XBP1	NKIRAS2
XBP1	NKTR
XBP1	NKX6-3
XBP1	NMD3
XBP1	NMRK1
XBP1	NOL6
XBP1	NOL7
XBP1	NOP10
XBP1	NORAD
XBP1	NOSIP
XBP1	NPIPB11
XBP1	NPRL2
XBP1	NR1H2
XBP1	NR2F6
XBP1	NRBF2
XBP1	NRBP1
XBP1	NRDC
XBP1	NSD1
XBP1	NSG1
XBP1	NSMCE3
XBP1	NSRP1
XBP1	NSUN4
XBP1	NT5C3A
XBP1	NT5DC2
XBP1	NUCB2
XBP1	NUDCD3
XBP1	NUDT18
XBP1	NUDT6
XBP1	NUDT9
XBP1	NUMB
XBP1	NUS1
XBP1	NUTM2B-AS1
XBP1	OAF
XBP1	OAZ1
XBP1	OCIAD1
XBP1	ODF3B
XBP1	OGFOD2
XBP1	ORAI3
XBP1	ORMDL1

XBP1	ORMDL2
XBP1	ORMDL3
XBP1	OS9
XBP1	OSBP
XBP1	OSBPL2
XBP1	OSBPL3
XBP1	OST4
XBP1	OSTC
XBP1	OSTM1
XBP1	OTUD1
XBP1	OTUD4
XBP1	OTUD5
XBP1	OTUD6B-AS1
XBP1	OXA1L
XBP1	OXR1
XBP1	P2RX1
XBP1	P4HA1
XBP1	P4HB
XBP1	P4HTM
XBP1	PAAF1
XBP1	PACSIN2
XBP1	PAFAH2
XBP1	PAIP2B
XBP1	PAN2
XBP1	PANK3
XBP1	PAPOLA
XBP1	PAPSS1
XBP1	PARD6A
XBP1	PARM1
XBP1	PARP1
XBP1	PARP2
XBP1	PARVG
XBP1	PBXIP1
XBP1	PCBP1
XBP1	PCED1B
XBP1	PCF11
XBP1	PCYOX1
XBP1	PDE12
XBP1	PDE4B
XBP1	PDE4DIP
XBP1	PDHB
XBP1	PDIA3
XBP1	PDIA4
XBP1	PDIA5
XBP1	PDIA6
XBP1	PDK1
XBP1	PDK2
XBP1	PDXDC1
XBP1	PDXK
XBP1	PEBP1

XBP1	PECAM1
XBP1	PELO
XBP1	PEMT
XBP1	PET100
XBP1	PET117
XBP1	PEX2
XBP1	PEX26
XBP1	PGBD2
XBP1	PGK1
XBP1	PGM1
XBP1	PGM3
XBP1	PHACTR1
XBP1	PHF1
XBP1	PHF19
XBP1	PHF20L1
XBP1	PHPT1
XBP1	PI4K2A
XBP1	PIAS3
XBP1	PIGP
XBP1	PIK3CD-AS2
XBP1	PIK3CG
XBP1	PIM2
XBP1	PINK1
XBP1	PITPNA
XBP1	PITRM1
XBP1	PJA1
XBP1	PJA2
XBP1	PKIB
XBP1	PLBD2
XBP1	PLCL2
XBP1	PLD3
XBP1	PLD4
XBP1	PLEKHB1
XBP1	PLIN2
XBP1	PLOD3
XBP1	PLPP5
XBP1	PLTP
XBP1	PMEPA1
XBP1	PNISR
XBP1	PNKP
XBP1	PNOC
XBP1	PNPLA2
XBP1	PNPLA4
XBP1	PNPLA8
XBP1	PNRC1
XBP1	PODXL2
XBP1	POLK
XBP1	POLR2K
XBP1	POLR3D
XBP1	POMC

XBP1	PON2
XBP1	POP5
XBP1	POU2AF1
XBP1	POU2F1
XBP1	PPARA
XBP1	PPHLN1
XBP1	PPIB
XBP1	PPIP5K2
XBP1	PPM1F
XBP1	PPM1K
XBP1	PPP1R10
XBP1	PPP1R13B
XBP1	PPP1R15B
XBP1	PPP1R2
XBP1	PPP2R5B
XBP1	PPP2R5C
XBP1	PQBP1
XBP1	PQLC1
XBP1	PQLC3
XBP1	PRADC1
XBP1	PRCC
XBP1	PRCP
XBP1	PRDM1
XBP1	PRDX1
XBP1	PRDX2
XBP1	PRDX4
XBP1	PREB
XBP1	PRKAG1
XBP1	PRKCSH
XBP1	PRKD2
XBP1	PRKD3
XBP1	PRKRIP1
XBP1	PRPS1
XBP1	PRR5
XBP1	PRRC1
XBP1	PSEN2
XBP1	PSMA3
XBP1	PSMA5
XBP1	PSMB1
XBP1	PSMB10
XBP1	PSMB2
XBP1	PSMB4
XBP1	PSMB5
XBP1	PSMB8-AS1
XBP1	PSMD7
XBP1	PSMF1
XBP1	PTK2B
XBP1	PTP4A2
XBP1	PTP4A3
XBP1	PTPN1

XBP1	PTPN2
XBP1	PTPN22
XBP1	PTPN23
XBP1	PTPN7
XBP1	PTPRN2
XBP1	PTS
XBP1	PUM1
XBP1	PURB
XBP1	PUS7L
XBP1	PUSL1
XBP1	PXMP4
XBP1	PYGB
XBP1	QKI
XBP1	QPCT
XBP1	QPCTL
XBP1	QPRT
XBP1	QRICH1
XBP1	QSOX1
XBP1	QTRT1
XBP1	R3HDM1
XBP1	RAB11B
XBP1	RAB11FIP2
XBP1	RAB18
XBP1	RAB1A
XBP1	RAB24
XBP1	RAB2A
XBP1	RAB30
XBP1	RAB39B
XBP1	RAB5A
XBP1	RAB6A
XBP1	RAB8A
XBP1	RABAC1
XBP1	RALGPS1
XBP1	RALGPS2
XBP1	RAP1A
XBP1	RARS2
XBP1	RASAL3
XBP1	RASSF5
XBP1	RBBP6
XBP1	RBFA
XBP1	RBM15B
XBP1	RBM18
XBP1	RBM19
XBP1	RBM26
XBP1	RBM33
XBP1	RBM39
XBP1	RBM41
XBP1	RCE1
XBP1	RCN2
XBP1	REEP3

XBP1	REEP5
XBP1	RELT
XBP1	REPIN1
XBP1	RER1
XBP1	REXO2
XBP1	RGS1
XBP1	RGS13
XBP1	RGS14
XBP1	RGS16
XBP1	RGS2
XBP1	RHBDD1
XBP1	RIDA
XBP1	RIOK2
XBP1	RIPK1
XBP1	RNASEH1
XBP1	RNF10
XBP1	RNF103
XBP1	RNF113A
XBP1	RNF13
XBP1	RNF14
XBP1	RNF149
XBP1	RNF166
XBP1	RNF168
XBP1	RNF181
XBP1	RNF185
XBP1	RNF219
XBP1	RNF7
XBP1	RNGTT
XBP1	RNH1
XBP1	RPL10
XBP1	RPL36AL
XBP1	RPN1
XBP1	RPN2
XBP1	RPP25
XBP1	RPRD2
XBP1	RPS11
XBP1	RPS19BP1
XBP1	RPS6KB2
XBP1	RPUSD2
XBP1	RRAGD
XBP1	RRAS
XBP1	RRBP1
XBP1	RREB1
XBP1	RRNAD1
XBP1	RSAD1
XBP1	RSRP1
XBP1	RTKN
XBP1	RTN3
XBP1	RUNDC1
XBP1	RUNX1

XBP1	RWDD1
XBP1	RYBP
XBP1	S100A11
XBP1	S100A13
XBP1	SAMD8
XBP1	SAP18
XBP1	SAP30
XBP1	SAP30BP
XBP1	SAR1A
XBP1	SAR1B
XBP1	SARAF
XBP1	SASH3
XBP1	SAT1
XBP1	SBDS
XBP1	SCAF1
XBP1	SCAMP1
XBP1	SCAMP2
XBP1	SCAMP3
XBP1	SCAND1
XBP1	SCAP
XBP1	SCARB2
XBP1	SCFD1
XBP1	SCPEP1
XBP1	SCRN2
XBP1	SCYL1
XBP1	SDC1
XBP1	SDCBP2
XBP1	SDCCAG8
XBP1	SDF2L1
XBP1	SDF4
XBP1	SDHB
XBP1	SDHC
XBP1	SEC11C
XBP1	SEC13
XBP1	SEC14L1
XBP1	SEC16A
XBP1	SEC22B
XBP1	SEC23A
XBP1	SEC23B
XBP1	SEC23IP
XBP1	SEC24A
XBP1	SEC24D
XBP1	SEC31A
XBP1	SEC61A1
XBP1	SEC61B
XBP1	SEC61G
XBP1	SEC62
XBP1	SEC63
XBP1	SECISBP2L
XBP1	SEL1L

XBP1	SEL1L3
XBP1	SELPLG
XBP1	SENP2
XBP1	SENP7
XBP1	SEPHS1
XBP1	SEPT2
XBP1	SEPT6
XBP1	SERF2
XBP1	SERINC3
XBP1	SERP1
XBP1	SETD3
XBP1	SETD5
XBP1	SETD7
XBP1	SF3B2
XBP1	SF3B5
XBP1	SFMBT2
XBP1	SFXN2
XBP1	SGK1
XBP1	SGK3
XBP1	SGSM3
XBP1	SH3BGR
XBP1	SH3BP5L
XBP1	SH3GLB1
XBP1	SIAH1
XBP1	SIKE1
XBP1	SIL1
XBP1	SIPA1L3
XBP1	SIRT2
XBP1	SKIV2L
XBP1	SLC10A7
XBP1	SLC12A4
XBP1	SLC12A8
XBP1	SLC15A3
XBP1	SLC17A5
XBP1	SLC17A9
XBP1	SLC1A4
XBP1	SLC1A5
XBP1	SLC25A22
XBP1	SLC25A25
XBP1	SLC25A28
XBP1	SLC25A36
XBP1	SLC25A39
XBP1	SLC25A4
XBP1	SLC25A42
XBP1	SLC25A46
XBP1	SLC26A11
XBP1	SLC26A6
XBP1	SLC27A4
XBP1	SLC30A5
XBP1	SLC30A7

XBP1	SLC31A2
XBP1	SLC33A1
XBP1	SLC35B1
XBP1	SLC35B3
XBP1	SLC35C1
XBP1	SLC35C2
XBP1	SLC38A10
XBP1	SLC38A2
XBP1	SLC38A9
XBP1	SLC39A11
XBP1	SLC39A13
XBP1	SLC39A7
XBP1	SLC39A8
XBP1	SLC39A9
XBP1	SLC3A2
XBP1	SLC43A2
XBP1	SLC44A1
XBP1	SLC4A1AP
XBP1	SLC50A1
XBP1	SLC6A9
XBP1	SLC7A6
XBP1	SLC7A7
XBP1	SLCO3A1
XBP1	SLCO4A1
XBP1	SLCO5A1
XBP1	SLU7
XBP1	SMAD5
XBP1	SMAP1
XBP1	SMARCA2
XBP1	SMCHD1
XBP1	SMDT1
XBP1	SMG7
XBP1	SMIM7
XBP1	SMPD1
XBP1	SMUG1
XBP1	SMYD2
XBP1	SMYD3
XBP1	SNAP29
XBP1	SNAPC3
XBP1	SND1
XBP1	SND1-IT1
XBP1	SNF8
XBP1	SNHG9
XBP1	SNRPN
XBP1	SNU13
XBP1	SNURF
XBP1	SNX13
XBP1	SOCS3
XBP1	SOX4
XBP1	SPAG7

XBP1	SPATC1L
XBP1	SPATS2
XBP1	SPCS1
XBP1	SPIN2B
XBP1	SPINT2
XBP1	SPNS1
XBP1	SPOCK2
XBP1	SPOP
XBP1	SPPL2B
XBP1	SPRYD3
XBP1	SPSB3
XBP1	SPTBN1
XBP1	SPTSSA
XBP1	SPTY2D1
XBP1	SQLE
XBP1	SQSTM1
XBP1	SRBD1
XBP1	SRD5A3
XBP1	SRGN
XBP1	SRM
XBP1	SRP14
XBP1	SRP19
XBP1	SRP54
XBP1	SRP68
XBP1	SRP72
XBP1	SRPRA
XBP1	SRRM2
XBP1	SRSF5
XBP1	SS18
XBP1	SS18L2
XBP1	SSPN
XBP1	SSR1
XBP1	SSR3
XBP1	SSR4
XBP1	SSSCA1
XBP1	SSTR2
XBP1	SSU72
XBP1	ST3GAL1
XBP1	ST3GAL2
XBP1	ST6GAL1
XBP1	ST6GALNAC4
XBP1	ST8SIA4
XBP1	STARD3
XBP1	STARD3NL
XBP1	STARD5
XBP1	STAT1
XBP1	STAT3
XBP1	STK17B
XBP1	STRN4
XBP1	STT3A

XBP1	STT3B
XBP1	STUB1
XBP1	STX11
XBP1	STX17
XBP1	STX5
XBP1	STXBP2
XBP1	STXBP6
XBP1	SUB1
XBP1	SUCLG1
XBP1	SUCLG2
XBP1	SUCO
XBP1	SUOX
XBP1	SUPT5H
XBP1	SURF4
XBP1	SYNGR1
XBP1	SYNRG
XBP1	SYS1
XBP1	SYT15
XBP1	SYTL1
XBP1	SYVN1
XBP1	TAF10
XBP1	TAF13
XBP1	TAF4B
XBP1	TAF6
XBP1	TAF7
XBP1	TAGAP
XBP1	TARSL2
XBP1	TAX1BP1
XBP1	TBC1D14
XBP1	TBC1D15
XBP1	TBC1D20
XBP1	TBC1D23
XBP1	TBC1D25
XBP1	TBC1D2B
XBP1	TBC1D7
XBP1	TBCC
XBP1	TBL2
XBP1	TCAIM
XBP1	TCEAL8
XBP1	TCF25
XBP1	TCF4
XBP1	TCIRG1
XBP1	TCP11L2
XBP1	TCTA
XBP1	TDG
XBP1	TDRD3
XBP1	TECPR2
XBP1	TEX264
XBP1	TFG
XBP1	TGDS

XBP1	TGOLN2
XBP1	THADA
XBP1	THAP11
XBP1	THAP2
XBP1	THAP4
XBP1	THAP5
XBP1	THAP7
XBP1	THAP8
XBP1	THNSL2
XBP1	THUMPD3
XBP1	TIMM10B
XBP1	TIMM17A
XBP1	TIMP1
XBP1	TJAP1
XBP1	TLE1
XBP1	TLN1
XBP1	TM7SF2
XBP1	TM7SF3
XBP1	TM9SF1
XBP1	TM9SF2
XBP1	TM9SF3
XBP1	TM9SF4
XBP1	TMBIM6
XBP1	TMC6
XBP1	TMCC1
XBP1	TMCO3
XBP1	TMED1
XBP1	TMED10
XBP1	TMED2
XBP1	TMED3
XBP1	TMED4
XBP1	TMED7
XBP1	TMED9
XBP1	TMEM101
XBP1	TMEM106A
XBP1	TMEM134
XBP1	TMEM147
XBP1	TMEM154
XBP1	TMEM164
XBP1	TMEM165
XBP1	TMEM167A
XBP1	TMEM179B
XBP1	TMEM184B
XBP1	TMEM184C
XBP1	TMEM19
XBP1	TMEM199
XBP1	TMEM206
XBP1	TMEM214
XBP1	TMEM219
XBP1	TMEM230

XBP1	TMEM248
XBP1	TMEM256
XBP1	TMEM258
XBP1	TMEM263
XBP1	TMEM30A
XBP1	TMEM33
XBP1	TMEM39A
XBP1	TMEM41A
XBP1	TMEM41B
XBP1	TMEM45A
XBP1	TMEM50B
XBP1	TMEM59
XBP1	TMEM70
XBP1	TMEM8B
XBP1	TMEM9
XBP1	TMF1
XBP1	TMSB10
XBP1	TMUB1
XBP1	TMUB2
XBP1	TMX4
XBP1	TNFAIP8L2
XBP1	TNFRSF1B
XBP1	TNFSF8
XBP1	TNK2
XBP1	TNKS
XBP1	TNS3
XBP1	TOLLIP
XBP1	TOM1L2
XBP1	TOMM20
XBP1	TOP1
XBP1	TOP3B
XBP1	TOPORS
XBP1	TOR1AIP1
XBP1	TOR2A
XBP1	TOR3A
XBP1	TP53INP1
XBP1	TP73
XBP1	TPD52
XBP1	TPP2
XBP1	TPST2
XBP1	TPT1
XBP1	TRABD
XBP1	TRAF3
XBP1	TRAF3IP2
XBP1	TRAM1
XBP1	TRAM2
XBP1	TRAPPC2L
XBP1	TRAPPC6A
XBP1	TRIB1
XBP1	TRIM27

XBP1	TRIM52
XBP1	TRIM73
XBP1	TRIP11
XBP1	TRIP12
XBP1	TRMT2A
XBP1	TRMT61B
XBP1	TRPC4AP
XBP1	TRPM4
XBP1	TRPM7
XBP1	TRPT1
XBP1	TSC22D3
XBP1	TSC22D4
XBP1	TSPAN13
XBP1	TSPAN31
XBP1	TSPAN4
XBP1	TSPYL2
XBP1	TTC1
XBP1	TTC13
XBP1	TTC14
XBP1	TTC17
XBP1	TTC3
XBP1	TTC7A
XBP1	TTC9
XBP1	TUSC2
XBP1	TVP23B
XBP1	TVP23C
XBP1	TWF1
XBP1	TXLNA
XBP1	TXLNB
XBP1	TXNDC11
XBP1	TXNDC15
XBP1	TXNDC5
XBP1	TXNL1
XBP1	TXNL4A
XBP1	U2AF1L4
XBP1	UAP1
XBP1	UBA5
XBP1	UBA6−AS1
XBP1	UBALD1
XBP1	UBB
XBP1	UBC
XBP1	UBE2D2
XBP1	UBE2D4
XBP1	UBE2H
XBP1	UBE2J1
XBP1	UBE2Q1
XBP1	UBE3A
XBP1	UBE4A
XBP1	UBFD1
XBP1	UBL3

XBP1	UBL5
XBP1	UBN1
XBP1	UBN2
XBP1	UBQLN1
XBP1	UBR1
XBP1	UBXN4
XBP1	UBXN6
XBP1	UFC1
XBP1	UFL1
XBP1	UFM1
XBP1	UFSP2
XBP1	UGDH
XBP1	UNC13D
XBP1	UNC50
XBP1	UNC93B1
XBP1	UPF2
XBP1	UQCC1
XBP1	UQCC2
XBP1	UQCR11
XBP1	UQCRB
XBP1	UQCRFS1
XBP1	USO1
XBP1	USP11
XBP1	USP16
XBP1	USP3
XBP1	USP36
XBP1	USP40
XBP1	USP48
XBP1	USP8
XBP1	UTRN
XBP1	UVSSA
XBP1	VAMP1
XBP1	VAMP2
XBP1	VAMP3
XBP1	VAMP4
XBP1	VANGL1
XBP1	VAPA
XBP1	VAV1
XBP1	VCP
XBP1	VDAC2
XBP1	VEGFA
XBP1	VKORC1
XBP1	VOPP1
XBP1	VPS33A
XBP1	VPS37A
XBP1	VPS37C
XBP1	VPS51
XBP1	VWA5A
XBP1	WBP2
XBP1	WDR33

XBP1	WDR92
XBP1	WHAMM
XBP1	WIPI1
XBP1	WNT10A
XBP1	WNT6
XBP1	WRAP73
XBP1	WRB
XBP1	WWOX
XBP1	XAF1
XBP1	XIAP
XBP1	XIST
XBP1	XPO4
XBP1	XRRA1
XBP1	YIF1A
XBP1	YIF1B
XBP1	YIPF1
XBP1	YIPF2
XBP1	YIPF3
XBP1	YIPF5
XBP1	YIPF6
XBP1	YKT6
XBP1	YPEL3
XBP1	YPEL5
XBP1	YTHDC1
XBP1	YTHDF1
XBP1	YWHAB
XBP1	ZBP1
XBP1	ZBTB11
XBP1	ZBTB11-AS1
XBP1	ZBTB7B
XBP1	ZBTB8A
XBP1	ZBTB8OS
XBP1	ZC3H12D
XBP1	ZC3H15
XBP1	ZC3H7A
XBP1	ZCCHC17
XBP1	ZDBF2
XBP1	ZDHHC3
XBP1	ZDHHC4
XBP1	ZDHHC5
XBP1	ZEB1
XBP1	ZEB1-AS1
XBP1	ZFAND1
XBP1	ZFAND2A
XBP1	ZFAT
XBP1	ZFC3H1
XBP1	ZFP36L2
XBP1	ZFPL1
XBP1	ZFX
XBP1	ZFYVE27

XBP1	ZKSCAN1
XBP1	ZNF133
XBP1	ZNF215
XBP1	ZNF24
XBP1	ZNF274
XBP1	ZNF275
XBP1	ZNF276
XBP1	ZNF281
XBP1	ZNF296
XBP1	ZNF3
XBP1	ZNF322
XBP1	ZNF354A
XBP1	ZNF37A
XBP1	ZNF407
XBP1	ZNF500
XBP1	ZNF544
XBP1	ZNF566
XBP1	ZNF568
XBP1	ZNF576
XBP1	ZNF587
XBP1	ZNF593
XBP1	ZNF628
XBP1	ZNF652
XBP1	ZNF655
XBP1	ZNF706
XBP1	ZNF780A
XBP1	ZNF790
XBP1	ZNF791
XBP1	ZNF804A
XBP1	ZNF816
XBP1	ZNF841
XBP1	ZNHIT1
XBP1	ZRANB2
XBP1	ZRSR2
XBP1	ZSCAN5A
XBP1	ZW10
YBX1	ABCA7
YBX1	ABCB8
YBX1	ABHD17B
YBX1	ABR
YBX1	ACACA
YBX1	ACAP1
YBX1	ACAT2
YBX1	ACBD4
YBX1	ACIN1
YBX1	ACOT8
YBX1	ACSL5
YBX1	ACTL6A
YBX1	ACTR3B
YBX1	ACYP1

YBX1	ADD1
YBX1	ADD3
YBX1	AEN
YBX1	AFF3
YBX1	AGBL5
YBX1	AGFG1
YBX1	AGFG2
YBX1	AGO1
YBX1	AGPAT1
YBX1	AHCY
YBX1	AHDC1
YBX1	AIFM1
YBX1	AIMP2
YBX1	AK2
YBX1	AKAP11
YBX1	AKR1B1
YBX1	AKT1S1
YBX1	ALCAM
YBX1	ALDH4A1
YBX1	ALDH6A1
YBX1	ALG12
YBX1	ALKBH6
YBX1	AMD1
YBX1	ANKRD49
YBX1	ANKRD54
YBX1	ANP32A
YBX1	AP1AR
YBX1	AP2A1
YBX1	AP2S1
YBX1	AP3M2
YBX1	AP4M1
YBX1	ARAF
YBX1	ARFGAP2
YBX1	ARFIP2
YBX1	ARHGAP4
YBX1	ARHGEF12
YBX1	ARHGEF9
YBX1	ARID1B
YBX1	ARID3A
YBX1	ARID4A
YBX1	ARID4B
YBX1	ARL17B
YBX1	ARL4A
YBX1	ARL6IP1
YBX1	ARL6IP6
YBX1	ARMC1
YBX1	ARRB2
YBX1	ASB7
YBX1	ASCL2
YBX1	ASNA1

YBX1	ASPM
YBX1	ATAD2
YBX1	ATF4
YBX1	ATF7
YBX1	ATG2A
YBX1	ATP2A1-AS1
YBX1	ATP2A2
YBX1	ATXN2L
YBX1	ATXN7L3
YBX1	AURKA
YBX1	AVIL
YBX1	AXIN1
YBX1	B4GALT3
YBX1	BAZ1A
YBX1	BBX
YBX1	BCKDHB
YBX1	BCL11A
YBX1	BCL2L1
YBX1	BCL2L11
YBX1	BCLAF1
YBX1	BDH1
YBX1	BICDL1
YBX1	BMF
YBX1	BPGM
YBX1	BRCA1
YBX1	BRD2
YBX1	BTBD1
YBX1	BUB3
YBX1	C12orf49
YBX1	C12orf65
YBX1	C19orf54
YBX1	C19orf73
YBX1	C1orf159
YBX1	C20orf27
YBX1	C21orf58
YBX1	C2orf69
YBX1	C5orf51
YBX1	C9orf85
YBX1	CABIN1
YBX1	CABLES2
YBX1	CALM2
YBX1	CALR
YBX1	CAMTA1
YBX1	CAND1
YBX1	CAT
YBX1	CBX1
YBX1	CBX5
YBX1	CBX8
YBX1	CCDC102A
YBX1	CCDC167

YBX1	CCDC189
YBX1	CCDC51
YBX1	CCDC61
YBX1	CCDC71L
YBX1	CCNA2
YBX1	CCNB2
YBX1	CCND1
YBX1	CCNE1
YBX1	CCNE2
YBX1	CCNF
YBX1	CCNG1
YBX1	CCNG2
YBX1	CCNK
YBX1	CCSER2
YBX1	CCT7
YBX1	CD48
YBX1	CDC23
YBX1	CDC45
YBX1	CDC6
YBX1	CDCA2
YBX1	CDCA3
YBX1	CDK1
YBX1	CDK19
YBX1	CDK2
YBX1	CDK2AP2
YBX1	CDK5RAP3
YBX1	CDKN2D
YBX1	CDKN3
YBX1	CDV3
YBX1	CEBPG
YBX1	CENPF
YBX1	CENPM
YBX1	CEP41
YBX1	CEP57
YBX1	CEP85
YBX1	CEP89
YBX1	CEP95
YBX1	CETN2
YBX1	CFAP36
YBX1	CGGBP1
YBX1	CHAF1A
YBX1	CHD2
YBX1	CHD6
YBX1	CHEK2
YBX1	CHKA
YBX1	CHST10
YBX1	CHTF18
YBX1	CIC
YBX1	CIRBP
YBX1	CKAP2

YBX1	CKAP2L
YBX1	CKAP5
YBX1	CKS1B
YBX1	CKS2
YBX1	CLEC16A
YBX1	CLK3
YBX1	CLPX
YBX1	CLTA
YBX1	CNBP
YBX1	CNN3
YBX1	CNNM3
YBX1	CNOT1
YBX1	CNOT3
YBX1	COQ10B
YBX1	COTL1
YBX1	COX7B
YBX1	CREB3L2
YBX1	CREB5
YBX1	CRIM1
YBX1	CRY2
YBX1	CSE1L
YBX1	CSNK1G3
YBX1	CTPS1
YBX1	CUL4B
YBX1	CUTA
YBX1	CYB5B
YBX1	CYHR1
YBX1	CYLD
YBX1	CYSTM1
YBX1	DAXX
YBX1	DBI
YBX1	DBP
YBX1	DCAF11
YBX1	DCAF7
YBX1	DCAKD
YBX1	DCLRE1C
YBX1	DCP2
YBX1	DCUN1D4
YBX1	DDA1
YBX1	DDIT4
YBX1	DDT
YBX1	DDX11
YBX1	DDX19B
YBX1	DDX5
YBX1	DDX54
YBX1	DDX6
YBX1	DENR
YBX1	DERL2
YBX1	DEXI
YBX1	DHCR24

YBX1	DHCR7
YBX1	DHODH
YBX1	DHX34
YBX1	DHX57
YBX1	DICER1
YBX1	DLD
YBX1	DLG1
YBX1	DLGAP5
YBX1	DMPK
YBX1	DNAJB1
YBX1	DNAJB11
YBX1	DNAJB2
YBX1	DNAJC30
YBX1	DNAJC4
YBX1	DNAJC7
YBX1	DNAL1
YBX1	DNM2
YBX1	DOLPP1
YBX1	DPF1
YBX1	DPP3
YBX1	DRG1
YBX1	DUSP10
YBX1	DYM
YBX1	DYNLL1
YBX1	DYNLT3
YBX1	DYRK1B
YBX1	E2F3
YBX1	E2F8
YBX1	E4F1
YBX1	EAF2
YBX1	EBP
YBX1	ECSIT
YBX1	EDEM1
YBX1	EEA1
YBX1	EFCAB11
YBX1	EFNA4
YBX1	EFNB1
YBX1	EHD4
YBX1	EIF2B2
YBX1	EIF3L
YBX1	EIF5
YBX1	ELAC2
YBX1	ELAVL1
YBX1	ELK1
YBX1	ELMO1
YBX1	ELOVL5
YBX1	ELP4
YBX1	EMC2
YBX1	EMD
YBX1	EML2

YBX1	ENSA
YBX1	EPB41
YBX1	EPC1
YBX1	EPM2AIP1
YBX1	ERCC8
YBX1	ETAA1
YBX1	ETF1
YBX1	ETFB
YBX1	ETV5
YBX1	EXOSC1
YBX1	EXOSC2
YBX1	EXT1
YBX1	EZH2
YBX1	F11R
YBX1	FAAP24
YBX1	FADS1
YBX1	FADS2
YBX1	FAM107B
YBX1	FAM110A
YBX1	FAM126A
YBX1	FAM161A
YBX1	FAM185A
YBX1	FAM204A
YBX1	FAM72A
YBX1	FAM72B
YBX1	FAM76B
YBX1	FANCA
YBX1	FAR1
YBX1	FARP2
YBX1	FARSA
YBX1	FBXL20
YBX1	FBXL3
YBX1	FBXO11
YBX1	FBXO5
YBX1	FDFT1
YBX1	FDPS
YBX1	FDXR
YBX1	FGD6
YBX1	FH
YBX1	FLNA
YBX1	FLYWCH1
YBX1	FNBP1
YBX1	FNBP4
YBX1	FNIP1
YBX1	FOXN3
YBX1	FOXO1
YBX1	FOXP1
YBX1	FPGS
YBX1	FUT8
YBX1	FXYD5

YBX1	FYN
YBX1	FZD3
YBX1	G3BP1
YBX1	GAN
YBX1	GANAB
YBX1	GART
YBX1	GATAD2A
YBX1	GATAD2B
YBX1	GBF1
YBX1	GCA
YBX1	GCDH
YBX1	GCHFR
YBX1	GCNT1
YBX1	GFOD1
YBX1	GGCT
YBX1	GGPS1
YBX1	GID4
YBX1	GIPC1
YBX1	GLA
YBX1	GLRX5
YBX1	GLS
YBX1	GLTP
YBX1	GOLGA2
YBX1	GOLGB1
YBX1	GOLPH3L
YBX1	GOLT1B
YBX1	GOT1
YBX1	GPR108
YBX1	GPR180
YBX1	GPR89A
YBX1	GPR89B
YBX1	GPX4
YBX1	GRPEL1
YBX1	GSK3B
YBX1	GTDC1
YBX1	GTF2E1
YBX1	GTF2IRD1
YBX1	GTF2IRD2
YBX1	GTF3C5
YBX1	GTPBP1
YBX1	H2AFJ
YBX1	H2AFX
YBX1	H2AFY
YBX1	H2AFZ
YBX1	H3F3B
YBX1	HAGH
YBX1	HAUS7
YBX1	HDAC1
YBX1	HERC2
YBX1	HERPUD1

YBX1	HES1
YBX1	HEXIM2
YBX1	HIBADH
YBX1	HIF1A
YBX1	HIGD1A
YBX1	HIST2H2AC
YBX1	HIVEP3
YBX1	HMBS
YBX1	HMGB1
YBX1	HMGB2
YBX1	HMGB3
YBX1	HMGCR
YBX1	HMGCS1
YBX1	HMGN2
YBX1	HMGN3
YBX1	HMMR
YBX1	HNRNPA0
YBX1	HNRNPC
YBX1	HNRNPL
YBX1	HNRNPLL
YBX1	HNRNPR
YBX1	HNRNPUL1
YBX1	HNRNPUL2
YBX1	HOOK1
YBX1	HSCB
YBX1	HSD17B10
YBX1	HSD17B12
YBX1	HSPA8
YBX1	HSPH1
YBX1	HTATIP2
YBX1	HYLS1
YBX1	HYOU1
YBX1	IARS
YBX1	IBTK
YBX1	ID2
YBX1	IER5
YBX1	IFNLR1
YBX1	IFT27
YBX1	IGF2BP3
YBX1	IL12RB1
YBX1	ILF2
YBX1	IMMP1L
YBX1	IMP3
YBX1	IPO13
YBX1	IQCB1
YBX1	ISOC2
YBX1	ITSN1
YBX1	IVNS1ABP
YBX1	JARID2
YBX1	JOSD1

YBX1	JUN
YBX1	JUP
YBX1	KANSL2
YBX1	KAT6B
YBX1	KAT7
YBX1	KATNAL2
YBX1	KATNB1
YBX1	KBTBD8
YBX1	KCNAB2
YBX1	KCNMB3
YBX1	KCNQ1OT1
YBX1	KCTD20
YBX1	KCTD9
YBX1	KDM3B
YBX1	KDM4B
YBX1	KDM6B
YBX1	KHSRP
YBX1	KIF1BP
YBX1	KLF10
YBX1	KLF6
YBX1	KLF9
YBX1	KLHL25
YBX1	KLHL5
YBX1	KMT2E
YBX1	KMT5B
YBX1	KMT5C
YBX1	KPNA2
YBX1	KPNA4
YBX1	KPNB1
YBX1	LAMTOR1
YBX1	LANCL1
YBX1	LAPTM4A
YBX1	LBR
YBX1	LDB1
YBX1	LEMD3
YBX1	LENG1
YBX1	LGALS1
YBX1	LGMN
YBX1	LIMK2
YBX1	LIN52
YBX1	LINC01003
YBX1	LMCD1
YBX1	LONRF3
YBX1	LPCAT3
YBX1	LPXN
YBX1	LRCH4
YBX1	LRP8
YBX1	LRRC20
YBX1	LRRC45
YBX1	LSS

YBX1	LTBP4
YBX1	LY75
YBX1	M6PR
YBX1	MACROD2
YBX1	MAEA
YBX1	MAF1
YBX1	MAP3K14
YBX1	MAP3K3
YBX1	MAP4
YBX1	MAPK7
YBX1	MAPKAPK3
YBX1	MAPRE2
YBX1	MAST3
YBX1	MAT2B
YBX1	MBTD1
YBX1	MCM3
YBX1	MCM3AP-AS1
YBX1	MCM7
YBX1	MDC1
YBX1	MDM1
YBX1	MED24
YBX1	MED29
YBX1	MEF2C
YBX1	MEF2D
YBX1	MEPCE
YBX1	METRN
YBX1	METTL13
YBX1	MFSD2A
YBX1	MICU2
YBX1	MID1IP1
YBX1	MIER3
YBX1	MIIP
YBX1	MIR22HG
YBX1	MIS12
YBX1	MKL1
YBX1	MKNK2
YBX1	MLEC
YBX1	MMAA
YBX1	MMAB
YBX1	MNS1
YBX1	MOCS2
YBX1	MON1A
YBX1	MORC2
YBX1	MORF4L2
YBX1	MORN2
YBX1	MOSPD3
YBX1	MPND
YBX1	MPRIP
YBX1	MPV17L2
YBX1	MRM3

YBX1	MROH1
YBX1	MRPL51
YBX1	MRPS12
YBX1	MRPS18B
YBX1	MRPS30
YBX1	MRPS36
YBX1	MSL3
YBX1	MSMO1
YBX1	MTA2
YBX1	MTFR2
YBX1	MTMR4
YBX1	MTR
YBX1	MVD
YBX1	MVK
YBX1	MXD4
YBX1	MYB
YBX1	MYL6
YBX1	N4BP2
YBX1	NAA60
YBX1	NADK2
YBX1	NAP1L4
YBX1	NARF
YBX1	NCAPD2
YBX1	NCAPD3
YBX1	NCAPH
YBX1	NCDN
YBX1	NCK2
YBX1	NCOA2
YBX1	NCOA5
YBX1	NDE1
YBX1	NDUFA5
YBX1	NDUFAF2
YBX1	NDUFB11
YBX1	NDUFB3
YBX1	NDUFS8
YBX1	NEDD4L
YBX1	NEMP1
YBX1	NET1
YBX1	NEU1
YBX1	NF2
YBX1	NFATC3
YBX1	NFE2L1
YBX1	NFKB2
YBX1	NFKBIB
YBX1	NFYA
YBX1	NFYC
YBX1	NFYC-AS1
YBX1	NLK
YBX1	NLRP11
YBX1	NMI

YBX1	NMT1
YBX1	NNT
YBX1	NNT-AS1
YBX1	NOP56
YBX1	NR2C2
YBX1	NR4A2
YBX1	NRAV
YBX1	NRBP1
YBX1	NRDC
YBX1	NRGN
YBX1	NRL
YBX1	NRP2
YBX1	NSDHL
YBX1	NT5DC1
YBX1	NT5DC2
YBX1	NUBP2
YBX1	NUDC
YBX1	NUDCD2
YBX1	NUDT2
YBX1	NUMA1
YBX1	NUMB
YBX1	NUP214
YBX1	NUP93
YBX1	NUS1
YBX1	NUSAP1
YBX1	OAT
YBX1	OCIAD1
YBX1	OGFOD1
YBX1	OGT
YBX1	OIP5
YBX1	OLA1
YBX1	OPA3
YBX1	ORC6
YBX1	ORMDL2
YBX1	OSBP
YBX1	OSBPL3
YBX1	OSBPL9
YBX1	OTUD5
YBX1	P4HB
YBX1	PAK4
YBX1	PALLD
YBX1	PASK
YBX1	PAX5
YBX1	PBXIP1
YBX1	PCDH9
YBX1	PCF11
YBX1	PCK2
YBX1	PCNA
YBX1	PCYOX1L
YBX1	PDCD10

YBX1	PDCD4
YBX1	PDE6D
YBX1	PDIA6
YBX1	PDK4
YBX1	PEBP1
YBX1	PELP1
YBX1	PES1
YBX1	PET100
YBX1	PEX2
YBX1	PGP
YBX1	PGRMC2
YBX1	PHF19
YBX1	PHF20
YBX1	PHKG2
YBX1	PHPT1
YBX1	PI4K2A
YBX1	PIGA
YBX1	PIGS
YBX1	PIM3
YBX1	PIN1
YBX1	PIP4K2B
YBX1	PITPNC1
YBX1	PITRM1
YBX1	PKP4
YBX1	PMF1
YBX1	PMM1
YBX1	PMS2
YBX1	PNN
YBX1	PNP
YBX1	PNPO
YBX1	PNRC2
YBX1	POLA1
YBX1	POLD4
YBX1	POLR3H
YBX1	POLR3K
YBX1	PPM1A
YBX1	PPM1B
YBX1	PPP1R10
YBX1	PPP1R12B
YBX1	PPP1R7
YBX1	PPP2R5A
YBX1	PPP2R5C
YBX1	PPP2R5D
YBX1	PPP3CA
YBX1	PPP6R3
YBX1	PRADC1
YBX1	PRDM10
YBX1	PRKCSH
YBX1	PRKCZ
YBX1	PRMT1

YBX1	PROSER3
YBX1	PRPF40A
YBX1	PRPSAP2
YBX1	PSAT1
YBX1	PSMA3-AS1
YBX1	PSMA5
YBX1	PSMB3
YBX1	PTBP1
YBX1	PTGER4
YBX1	PTMA
YBX1	PTMS
YBX1	PWWP2A
YBX1	PXMP4
YBX1	QPCTL
YBX1	RAB33A
YBX1	RAB6A
YBX1	RAB8A
YBX1	RAB8B
YBX1	RABGAP1
YBX1	RABL3
YBX1	RACGAP1
YBX1	RAD18
YBX1	RALB
YBX1	RALY
YBX1	RANBP9
YBX1	RAP2A
YBX1	RASSF2
YBX1	RASSF7
YBX1	RAVER1
YBX1	RBBP4
YBX1	RBM14
YBX1	RBM15
YBX1	RBM23
YBX1	RBM3
YBX1	RBM4
YBX1	RBM45
YBX1	RBM6
YBX1	RCL1
YBX1	RCN1
YBX1	RCOR1
YBX1	REC8
YBX1	RECQL
YBX1	RECQL5
YBX1	RERE
YBX1	RFWD3
YBX1	RFX2
YBX1	RFX5
YBX1	RGS16
YBX1	RHEB
YBX1	RHEBL1

YBX1	RHOF
YBX1	RHOQ
YBX1	RIN3
YBX1	RIPK2
YBX1	RLN1
YBX1	RLN2
YBX1	RMDN3
YBX1	RMI2
YBX1	RNF121
YBX1	RNF181
YBX1	RNF19A
YBX1	RNF220
YBX1	RNF26
YBX1	RNF34
YBX1	RNF40
YBX1	RNF6
YBX1	ROGDI
YBX1	RPA2
YBX1	RPE
YBX1	RPL13
YBX1	RPL13A
YBX1	RPL17
YBX1	RPLP0
YBX1	RPRD2
YBX1	RPS6KA2
YBX1	RPUSD1
YBX1	RUFY1
YBX1	RUNX1
YBX1	S1PR2
YBX1	SAP130
YBX1	SAP30BP
YBX1	SARS2
YBX1	SCD
YBX1	SEC23B
YBX1	SEC31A
YBX1	SERPINE2
YBX1	SERPINI1
YBX1	SERTAD1
YBX1	SERTAD3
YBX1	SESN2
YBX1	SETD3
YBX1	SETD5
YBX1	SF1
YBX1	SF3B4
YBX1	SFXN5
YBX1	SH2D3C
YBX1	SH3GL1
YBX1	SH3GLB2
YBX1	SHARPIN
YBX1	SHC1

YBX1	SHKBP1
YBX1	SIGMAR1
YBX1	SIN3A
YBX1	SIRT2
YBX1	SKA2
YBX1	SLC11A2
YBX1	SLC16A4
YBX1	SLC16A9
YBX1	SLC1A4
YBX1	SLC25A23
YBX1	SLC25A25
YBX1	SLC25A28
YBX1	SLC2A8
YBX1	SLC30A1
YBX1	SLC37A1
YBX1	SLC43A2
YBX1	SLC46A3
YBX1	SLC9A3R1
YBX1	SMAD2
YBX1	SMAP2
YBX1	SMC2
YBX1	SMIM4
YBX1	SMYD2
YBX1	SND1
YBX1	SNHG10
YBX1	SNRNP25
YBX1	SNRPA
YBX1	SNX11
YBX1	SOD2
YBX1	SON
YBX1	SP2
YBX1	SPAG7
YBX1	SPC25
YBX1	SPSB3
YBX1	SPTLC2
YBX1	SRD5A3
YBX1	SRF
YBX1	SRGAP2C
YBX1	SRM
YBX1	SRP9
YBX1	SRSF1
YBX1	SRSF3
YBX1	SRSF6
YBX1	ST20
YBX1	ST8SIA4
YBX1	STAG1
YBX1	STARD3NL
YBX1	STARD7
YBX1	STAT3
YBX1	STAT6

YBX1	STIL
YBX1	STMN1
YBX1	STRAP
YBX1	STX5
YBX1	STX7
YBX1	SUCLG2
YBX1	SUFU
YBX1	SUN2
YBX1	SWSAP1
YBX1	SYVN1
YBX1	TACC3
YBX1	TADA2B
YBX1	TAF1C
YBX1	TAF8
YBX1	TANGO2
YBX1	TARS2
YBX1	TBC1D10B
YBX1	TBCC
YBX1	TBL1XR1
YBX1	TCEAL4
YBX1	TCERG1
YBX1	TCF19
YBX1	TCF4
YBX1	TCTN3
YBX1	TDP1
YBX1	TDRD3
YBX1	TECR
YBX1	TERF1
YBX1	TES
YBX1	TGDS
YBX1	TGM2
YBX1	THAP7
YBX1	THAP7−AS1
YBX1	THAP8
YBX1	THAP9
YBX1	THAP9−AS1
YBX1	THUMPD3−AS1
YBX1	TIGAR
YBX1	TLE1
YBX1	TLE3
YBX1	TLE4
YBX1	TLK1
YBX1	TLK2
YBX1	TM2D2
YBX1	TMEM129
YBX1	TMEM14A
YBX1	TMEM161B−AS1
YBX1	TMEM189
YBX1	TMEM218
YBX1	TMEM256

YBX1	TMEM50B
YBX1	TMEM97
YBX1	TMTC4
YBX1	TNFAIP3
YBX1	TNFAIP8
YBX1	TNRC6B
YBX1	TOB1
YBX1	TOB2
YBX1	TOMM40
YBX1	TOMM40L
YBX1	TOP1MT
YBX1	TOP2A
YBX1	TP53BP1
YBX1	TPCN1
YBX1	TPD52L2
YBX1	TPX2
YBX1	TRAFD1
YBX1	TRAPPC6A
YBX1	TRAPPC6B
YBX1	TRIM59
YBX1	TRIM68
YBX1	TRIM73
YBX1	TRIOBP
YBX1	TRIP4
YBX1	TRMT10B
YBX1	TRUB1
YBX1	TSC22D1
YBX1	TSPAN33
YBX1	TTBK2
YBX1	TTC39C
YBX1	TTC9C
YBX1	TTF2
YBX1	TUBA1C
YBX1	TUBB2B
YBX1	TUBB4B
YBX1	TULP3
YBX1	TUSC2
YBX1	TXNDC16
YBX1	TYK2
YBX1	TYSND1
YBX1	TYW1
YBX1	TYW1B
YBX1	UBALD2
YBX1	UBB
YBX1	UBE2C
YBX1	UBE2F
YBX1	UBE2S
YBX1	UBE2T
YBX1	UBP1
YBX1	UBR5

YBX1	UBTD2
YBX1	UBXN11
YBX1	UCP2
YBX1	UHRF1
YBX1	UNG
YBX1	UNK
YBX1	UQCRC2
YBX1	USF3
YBX1	USP13
YBX1	USP16
YBX1	VAMP1
YBX1	VCP
YBX1	VEZT
YBX1	VLDLR
YBX1	VPS35
YBX1	VRK1
YBX1	WAS
YBX1	WASF2
YBX1	WBP1L
YBX1	WDR6
YBX1	WDR62
YBX1	WDR81
YBX1	WDR89
YBX1	WFS1
YBX1	WHAMM
YBX1	WNT16
YBX1	XAB2
YBX1	XPO1
YBX1	XRCC3
YBX1	YBX1
YBX1	YIF1A
YBX1	YJEFN3
YBX1	YWHAQ
YBX1	YWHAZ
YBX1	ZBTB10
YBX1	ZBTB20
YBX1	ZBTB26
YBX1	ZBTB32
YBX1	ZC3H14
YBX1	ZCWPW1
YBX1	ZDHHC16
YBX1	ZDHHC24
YBX1	ZDHHC5
YBX1	ZFAND6
YBX1	ZFP82
YBX1	ZFP91
YBX1	ZFYVE21
YBX1	ZMYM2
YBX1	ZMYND8
YBX1	ZNF124

YBX1	ZNF213
YBX1	ZNF253
YBX1	ZNF263
YBX1	ZNF267
YBX1	ZNF326
YBX1	ZNF330
YBX1	ZNF367
YBX1	ZNF397
YBX1	ZNF43
YBX1	ZNF430
YBX1	ZNF431
YBX1	ZNF444
YBX1	ZNF519
YBX1	ZNF526
YBX1	ZNF574
YBX1	ZNF584
YBX1	ZNF608
YBX1	ZNF672
YBX1	ZNF680
YBX1	ZNF687
YBX1	ZNF689
YBX1	ZNF711
YBX1	ZNF787
YBX1	ZNF8
YBX1	ZSCAN2
YBX1	ZSWIM1
YBX1	ZSWIM3
YY1	AAAS
YY1	AAMP
YY1	AARS2
YY1	AASDH
YY1	AASDHPPT
YY1	AATF
YY1	ABCB7
YY1	ABCF1
YY1	ABHD11
YY1	ABHD17B
YY1	ABHD3
YY1	ABI1
YY1	ABI2
YY1	ABT1
YY1	ACAA1
YY1	ACAA2
YY1	ACADVL
YY1	ACAP2
YY1	ACAT2
YY1	ACBD3
YY1	ACBD4
YY1	ACD
YY1	ACIN1

YY1	ACLY
YY1	ACO2
YY1	ACOT13
YY1	ACOT7
YY1	ACOT8
YY1	ACOT9
YY1	ACP5
YY1	ACSL1
YY1	ACSL3
YY1	ACSL4
YY1	ACSS1
YY1	ACTB
YY1	ACTG1
YY1	ACTL6A
YY1	ACTN4
YY1	ACTR1A
YY1	ACTR1B
YY1	ACTR2
YY1	ACTR3
YY1	ACTR3C
YY1	ACTR6
YY1	ACTR8
YY1	ADAM10
YY1	ADAM15
YY1	ADAM17
YY1	ADAM9
YY1	ADAR
YY1	ADAT1
YY1	ADD1
YY1	ADD3
YY1	ADH5
YY1	ADI1
YY1	ADIPOR1
YY1	ADK
YY1	ADO
YY1	ADPGK
YY1	ADPRHL2
YY1	ADRM1
YY1	ADSS
YY1	AEBP2
YY1	AES
YY1	AFF3
YY1	AFF4
YY1	AFTPH
YY1	AGO3
YY1	AGPAT1
YY1	AGPAT2
YY1	AGPAT3
YY1	AGPS
YY1	AHCY

YY1	AHI1
YY1	AHNAK
YY1	AHR
YY1	AHSA1
YY1	AIDA
YY1	AIF1
YY1	AIFM1
YY1	AIFM2
YY1	AIM2
YY1	AIMP1
YY1	AIP
YY1	AK3
YY1	AKAP1
YY1	AKAP10
YY1	AKAP11
YY1	AKAP13
YY1	AKAP8
YY1	AKAP8L
YY1	AKAP9
YY1	AKIP1
YY1	AKIRIN1
YY1	AKIRIN2
YY1	AKR1B1
YY1	AKT2
YY1	AKTIP
YY1	ALDH4A1
YY1	ALDH6A1
YY1	ALDH9A1
YY1	ALDOA
YY1	ALG11
YY1	ALG13
YY1	ALG3
YY1	ALKBH5
YY1	ALPK1
YY1	ALYREF
YY1	AMD1
YY1	AMMECR1
YY1	ANAPC11
YY1	ANAPC15
YY1	ANAPC16
YY1	ANAPC5
YY1	ANAPC7
YY1	ANG
YY1	ANGPT2
YY1	ANKHD1
YY1	ANKLE2
YY1	ANKRD10
YY1	ANKRD11
YY1	ANKRD13A
YY1	ANKRD26

YY1	ANKRD34B
YY1	ANKRD46
YY1	ANKZF1
YY1	ANP32A
YY1	ANP32B
YY1	ANP32E
YY1	ANXA11
YY1	ANXA2
YY1	AP1AR
YY1	AP1B1
YY1	AP1S1
YY1	AP2B1
YY1	AP2M1
YY1	AP2S1
YY1	AP3B1
YY1	AP3D1
YY1	AP3S1
YY1	AP4B1
YY1	AP4E1
YY1	AP5B1
YY1	AP5M1
YY1	APEH
YY1	APEX2
YY1	APH1A
YY1	API5
YY1	APLP2
YY1	APOPT1
YY1	APPL1
YY1	APRT
YY1	APTX
YY1	ARAF
YY1	ARAP1
YY1	ARCN1
YY1	ARF1
YY1	ARF3
YY1	ARF5
YY1	ARF6
YY1	ARFGAP2
YY1	ARFGEF1
YY1	ARFIP2
YY1	ARG2
YY1	ARGLU1
YY1	ARHGAP12
YY1	ARHGAP19
YY1	ARHGAP22
YY1	ARHGAP24
YY1	ARHGAP25
YY1	ARHGAP30
YY1	ARHGAP4
YY1	ARHGAP5

YY1	ARHGDIA
YY1	ARHGDIB
YY1	ARHGEF1
YY1	ARID1A
YY1	ARID1B
YY1	ARID4A
YY1	ARID4B
YY1	ARID5A
YY1	ARID5B
YY1	ARL14EP
YY1	ARL2
YY1	ARL2BP
YY1	ARL3
YY1	ARL5A
YY1	ARL6IP1
YY1	ARL8A
YY1	ARMCX3
YY1	ARMCX5
YY1	ARMCX6
YY1	ARNT
YY1	ARPC1A
YY1	ARPC1B
YY1	ARPC2
YY1	ARPC3
YY1	ARPC4
YY1	ARPC5L
YY1	ARPP19
YY1	ARRDC3
YY1	ARRDC3-AS1
YY1	ARV1
YY1	ASB16-AS1
YY1	ASB2
YY1	ASB3
YY1	ASB7
YY1	ASB8
YY1	ASCC1
YY1	ASF1B
YY1	ASH1L
YY1	ASH1L-AS1
YY1	ASL
YY1	ASNA1
YY1	ASNSD1
YY1	ASPM
YY1	ASTE1
YY1	ASXL1
YY1	ATAD2
YY1	ATAD5
YY1	ATF1
YY1	ATF4
YY1	ATF7

YY1	ATF7IP
YY1	ATF7IP2
YY1	ATG10
YY1	ATG101
YY1	ATG12
YY1	ATG3
YY1	ATG4B
YY1	ATG4D
YY1	ATG5
YY1	ATIC
YY1	ATL3
YY1	ATOX1
YY1	ATP11B
YY1	ATP13A3
YY1	ATP2A3
YY1	ATP2C1
YY1	ATP5S
YY1	ATP6V0D1
YY1	ATP6V0E1
YY1	ATP6V1A
YY1	ATP6V1C1
YY1	ATP6V1E1
YY1	ATP6V1F
YY1	ATP6V1G1
YY1	ATP6V1H
YY1	ATPAF2
YY1	ATRAID
YY1	ATRX
YY1	ATXN10
YY1	ATXN2
YY1	ATXN2L
YY1	ATXN3
YY1	ATXN7L3B
YY1	AUH
YY1	AURKAIP1
YY1	AZI2
YY1	AZIN1
YY1	AZIN1-AS1
YY1	B2M
YY1	B3GALNT1
YY1	B3GLCT
YY1	B9D2
YY1	BABAM1
YY1	BAG5
YY1	BAG6
YY1	BAK1
YY1	BANF1
YY1	BANP
YY1	BASP1
YY1	BAX

YY1	BAZ1A
YY1	BAZ1B
YY1	BAZ2A
YY1	BAZ2B
YY1	BBIP1
YY1	BBX
YY1	BCAP29
YY1	BCAP31
YY1	BCAS2
YY1	BCAS3
YY1	BCCIP
YY1	BCKDHB
YY1	BCL10
YY1	BCL11A
YY1	BCL2L11
YY1	BCL2L13
YY1	BCL6
YY1	BCL7B
YY1	BCLAF1
YY1	BDH1
YY1	BDH2
YY1	BDP1
YY1	BEX1
YY1	BEX4
YY1	BFAR
YY1	BID
YY1	BIRC2
YY1	BIRC3
YY1	BIRC5
YY1	BIRC6
YY1	BLK
YY1	BLMH
YY1	BLOC1S1
YY1	BLOC1S2
YY1	BLOC1S5
YY1	BMF
YY1	BMP2K
YY1	BMP7
YY1	BMS1
YY1	BNIP2
YY1	BNIP3L
YY1	BOLA2-SMG1P6
YY1	BORCS6
YY1	BORCS7
YY1	BORCS8
YY1	BPHL
YY1	BPNT1
YY1	BPTF
YY1	BRAF
YY1	BRAP

YY1	BRCA2
YY1	BRD1
YY1	BRD2
YY1	BRD4
YY1	BRD7
YY1	BRD9
YY1	BRI3BP
YY1	BRK1
YY1	BRMS1
YY1	BROX
YY1	BRWD1
YY1	BSDC1
YY1	BTF3
YY1	BTF3L4
YY1	BTG2
YY1	BTK
YY1	BUB3
YY1	BUD13
YY1	BUD31
YY1	BZW1
YY1	C11orf24
YY1	C11orf49
YY1	C11orf54
YY1	C11orf58
YY1	C11orf71
YY1	C12orf10
YY1	C12orf43
YY1	C12orf49
YY1	C12orf65
YY1	C12orf66
YY1	C12orf75
YY1	C14orf119
YY1	C15orf40
YY1	C16orf58
YY1	C16orf72
YY1	C16orf87
YY1	C17orf53
YY1	C17orf75
YY1	C18orf21
YY1	C18orf32
YY1	C19orf25
YY1	C19orf38
YY1	C19orf48
YY1	C19orf53
YY1	C19orf54
YY1	C19orf66
YY1	C19orf70
YY1	C1D
YY1	C1GALT1
YY1	C1orf123

YY1	C1orf131
YY1	C1orf174
YY1	C1orf43
YY1	C1orf52
YY1	C1QBP
YY1	C20orf27
YY1	C2orf42
YY1	C2orf49
YY1	C2orf69
YY1	C2orf74
YY1	C3orf38
YY1	C4orf46
YY1	C4orf48
YY1	C5orf24
YY1	C5orf34
YY1	C5orf51
YY1	C5orf63
YY1	C6orf106
YY1	C6orf48
YY1	C6orf62
YY1	C8orf59
YY1	C9orf16
YY1	C9orf78
YY1	CAB39
YY1	CABLES2
YY1	CACUL1
YY1	CACYBP
YY1	CALCOCO2
YY1	CALM1
YY1	CALM2
YY1	CAMK1
YY1	CAMK2D
YY1	CAMKK2
YY1	CAMLG
YY1	CAMTA1
YY1	CAND1
YY1	CAP1
YY1	CAPN15
YY1	CAPNS1
YY1	CAPRIN1
YY1	CAPZA1
YY1	CAPZA2
YY1	CAPZB
YY1	CARHSP1
YY1	CARMIL2
YY1	CARS
YY1	CARS2
YY1	CASC3
YY1	CASC4
YY1	CASD1

YY1	CASP2
YY1	CASP3
YY1	CASP8AP2
YY1	CAST
YY1	CATSPER2
YY1	CBFB
YY1	CBR4
YY1	CBWD2
YY1	CBX1
YY1	CBX3
YY1	CBX5
YY1	CBX7
YY1	CC2D1A
YY1	CCAR1
YY1	CCAR2
YY1	CCDC115
YY1	CCDC117
YY1	CCDC12
YY1	CCDC124
YY1	CCDC137
YY1	CCDC142
YY1	CCDC150
YY1	CCDC174
YY1	CCDC186
YY1	CCDC189
YY1	CCDC22
YY1	CCDC25
YY1	CCDC28B
YY1	CCDC50
YY1	CCDC6
YY1	CCDC61
YY1	CCDC66
YY1	CCDC69
YY1	CCDC71
YY1	CCDC71L
YY1	CCDC84
YY1	CCDC88C
YY1	CCDC90B
YY1	CCNA2
YY1	CCNB1
YY1	CCNB1IP1
YY1	CCNB2
YY1	CCND3
YY1	CCNDBP1
YY1	CCNH
YY1	CCNI
YY1	CCNK
YY1	CCNL1
YY1	CCNL2
YY1	CCSAP

YY1	CCT2
YY1	CCT4
YY1	CCT5
YY1	CCT6A
YY1	CCT7
YY1	CCT8
YY1	CCZ1
YY1	CD164
YY1	CD2BP2
YY1	CD40
YY1	CD47
YY1	CD59
YY1	CD68
YY1	CD79B
YY1	CDC123
YY1	CDC16
YY1	CDC20
YY1	CDC25A
YY1	CDC26
YY1	CDC27
YY1	CDC37
YY1	CDC40
YY1	CDC42
YY1	CDC42SE2
YY1	CDC45
YY1	CDC5L
YY1	CDC7
YY1	CDC73
YY1	CDCA2
YY1	CDCA4
YY1	CDCA7
YY1	CDCA7L
YY1	CDK10
YY1	CDK11B
YY1	CDK12
YY1	CDK13
YY1	CDK14
YY1	CDK16
YY1	CDK17
YY1	CDK2
YY1	CDK2AP2
YY1	CDK5RAP2
YY1	CDK5RAP3
YY1	CDK9
YY1	CDKAL1
YY1	CDKN2D
YY1	CDS2
YY1	CDT1
YY1	CDV3
YY1	CEBPZOS

YY1	CELF1
YY1	CENPE
YY1	CENPF
YY1	CENPH
YY1	CENPM
YY1	CENPN
YY1	CENPQ
YY1	CENPT
YY1	CENPU
YY1	CEP104
YY1	CEP164
YY1	CEP19
YY1	CEP295
YY1	CEP55
YY1	CEP57
YY1	CEP63
YY1	CEP72
YY1	CEP76
YY1	CEP78
YY1	CEPT1
YY1	CERS5
YY1	CETN2
YY1	CFAP20
YY1	CFDP1
YY1	CFL1
YY1	CGGBP1
YY1	CGRRF1
YY1	CHAC2
YY1	CHAF1A
YY1	CHAF1B
YY1	CHAMP1
YY1	CHCHD1
YY1	CHCHD2
YY1	CHCHD3
YY1	CHCHD5
YY1	CHD3
YY1	CHD4
YY1	CHD8
YY1	CHD9
YY1	CHFR
YY1	CHKA
YY1	CHM
YY1	CHMP1A
YY1	CHMP1B
YY1	CHMP2A
YY1	CHMP2B
YY1	CHMP3
YY1	CHMP4B
YY1	CHMP5
YY1	CHMP7

YY1	CHRAC1
YY1	CHTF18
YY1	CHTF8
YY1	CHTOP
YY1	CHURC1
YY1	CIAO1
YY1	CIB1
YY1	CIB2
YY1	CINP
YY1	CIR1
YY1	CIRBP
YY1	CISD2
YY1	CKAP2L
YY1	CKS1B
YY1	CKS2
YY1	CLASRP
YY1	CLCN7
YY1	CLIC4
YY1	CLINT1
YY1	CLK1
YY1	CLK3
YY1	CLK4
YY1	CLNS1A
YY1	CLPP
YY1	CLPTM1
YY1	CLPX
YY1	CLSPN
YY1	CLTA
YY1	CLTB
YY1	CLTC
YY1	CMC1
YY1	CMC2
YY1	CMPK1
YY1	CMTM6
YY1	CNBP
YY1	CNDP2
YY1	CNIH1
YY1	CNIH3
YY1	CNN2
YY1	CNOT1
YY1	CNOT10
YY1	CNOT11
YY1	CNOT2
YY1	CNOT3
YY1	CNOT6
YY1	CNOT7
YY1	CNOT8
YY1	CNP
YY1	CNPPD1
YY1	CNPY3

YY1	CNR1
YY1	COA4
YY1	COA6
YY1	COASY
YY1	COG2
YY1	COG3
YY1	COIL
YY1	COL4A3BP
YY1	COMMD1
YY1	COMMD2
YY1	COMMD4
YY1	COMMD5
YY1	COMMD6
YY1	COMMD7
YY1	COMMD8
YY1	COPA
YY1	COPB1
YY1	COPB2
YY1	COPE
YY1	COPS2
YY1	COPS3
YY1	COPS4
YY1	COPS5
YY1	COPS6
YY1	COPS7B
YY1	COPS9
YY1	COPZ1
YY1	COQ10A
YY1	COQ2
YY1	COQ5
YY1	COQ6
YY1	COQ9
YY1	CORO1A
YY1	CORO1B
YY1	COTL1
YY1	COX11
YY1	COX14
YY1	COX15
YY1	COX17
YY1	COX18
YY1	COX20
YY1	COX4I1
YY1	COX5A
YY1	COX5B
YY1	COX6A1
YY1	COX6B1
YY1	COX7A2L
YY1	COX7B
YY1	COX7C
YY1	COX8A

YY1	CPNE1
YY1	CPNE2
YY1	CPNE3
YY1	CPSF2
YY1	CPSF3
YY1	CPSF4
YY1	CPSF6
YY1	CPSF7
YY1	CRBN
YY1	CREB1
YY1	CREM
YY1	CRK
YY1	CRKL
YY1	CRYZL1
YY1	CS
YY1	CSDE1
YY1	CSE1L
YY1	CSK
YY1	CSNK1A1
YY1	CSNK1D
YY1	CSNK1G2
YY1	CSNK2A1
YY1	CSNK2B
YY1	CSTF1
YY1	CSTF2
YY1	CSTF3
YY1	CTCF
YY1	CTDNEP1
YY1	CTDSP1
YY1	CTDSPL2
YY1	CTNNAL1
YY1	CTNNBL1
YY1	CTPS2
YY1	CTR9
YY1	CTSH
YY1	CTSZ
YY1	CUEDC2
YY1	CUL1
YY1	CUL4B
YY1	CUL5
YY1	CUTC
YY1	CUX1
YY1	CWC15
YY1	CWC22
YY1	CWC25
YY1	CWC27
YY1	CWF19L2
YY1	CXCR4
YY1	CXCR5
YY1	CXorf56

YY1	CXXC1
YY1	CXXC5
YY1	CYB561A3
YY1	CYB561D2
YY1	CYB5B
YY1	CYB5R4
YY1	CYC1
YY1	CYSLTR1
YY1	CYSTM1
YY1	CYTH1
YY1	DALRD3
YY1	DAP
YY1	DAP3
YY1	DAPK3
YY1	DAPP1
YY1	DARS
YY1	DAXX
YY1	DAZAP1
YY1	DAZAP2
YY1	DBF4
YY1	DBI
YY1	DBNL
YY1	DCAF11
YY1	DCAF12
YY1	DCAF13
YY1	DCAF7
YY1	DCAF8
YY1	DCK
YY1	DCLRE1C
YY1	DCP2
YY1	DCTN5
YY1	DCTN6
YY1	DCTPP1
YY1	DCUN1D1
YY1	DCUN1D5
YY1	DDA1
YY1	DDB1
YY1	DDB2
YY1	DDHD1
YY1	DDIAS
YY1	DDT
YY1	DDX1
YY1	DDX17
YY1	DDX19A
YY1	DDX19B
YY1	DDX23
YY1	DDX24
YY1	DDX27
YY1	DDX39A
YY1	DDX39B

YY1	DDX3X
YY1	DDX3Y
YY1	DDX42
YY1	DDX46
YY1	DDX5
YY1	DDX52
YY1	DDX56
YY1	DDX6
YY1	DECR1
YY1	DEDD
YY1	DEGS1
YY1	DEK
YY1	DENND2D
YY1	DENND4A
YY1	DENND4B
YY1	DENR
YY1	DEPDC1
YY1	DEPDC5
YY1	DEPTOR
YY1	DESI2
YY1	DET1
YY1	DFFA
YY1	DGCR8
YY1	DGUOK
YY1	DHFR
YY1	DHODH
YY1	DHPS
YY1	DHRS7B
YY1	DHX15
YY1	DHX30
YY1	DHX36
YY1	DHX38
YY1	DHX40
YY1	DHX8
YY1	DHX9
YY1	DICER1
YY1	DIS3
YY1	DLD
YY1	DLG1
YY1	DLGAP5
YY1	DLST
YY1	DMAP1
YY1	DMD
YY1	DNAJA1
YY1	DNAJA3
YY1	DNAJB1
YY1	DNAJB12
YY1	DNAJB14
YY1	DNAJB2
YY1	DNAJB6

YY1	DNAJC21
YY1	DNAJC7
YY1	DNAJC8
YY1	DNAJC9
YY1	DNAL4
YY1	DNASE1
YY1	DNM1L
YY1	DNMBP
YY1	DNMT1
YY1	DNTTIP2
YY1	DOCK2
YY1	DOCK7
YY1	DOCK8
YY1	DOK1
YY1	DOK2
YY1	DONSON
YY1	DPCD
YY1	DPF2
YY1	DPH3
YY1	DPH5
YY1	DPP3
YY1	DPP8
YY1	DPY30
YY1	DPYSL2
YY1	DR1
YY1	DRG1
YY1	DRG2
YY1	DSCC1
YY1	DTD1
YY1	DTL
YY1	DTNBP1
YY1	DTX1
YY1	DTYMK
YY1	DUS1L
YY1	DUS3L
YY1	DUSP1
YY1	DUSP11
YY1	DUSP14
YY1	DUT
YY1	DYM
YY1	DYNC1H1
YY1	DYNC1I2
YY1	DYNC1LI2
YY1	DYNLT1
YY1	DZIP3
YY1	E2F1
YY1	E2F3
YY1	E2F4
YY1	EAPP
YY1	EARS2

YY1	EBAG9
YY1	EBP
YY1	ECE1
YY1	ECSIT
YY1	EDF1
YY1	EDRF1
YY1	EEA1
YY1	EED
YY1	EEF1A1
YY1	EEF1B2
YY1	EEF1D
YY1	EEF1G
YY1	EEF2
YY1	EFCAB14
YY1	EFTUD2
YY1	EGLN2
YY1	EHD1
YY1	EHD4
YY1	EID1
YY1	EID2
YY1	EIF1
YY1	EIF1AD
YY1	EIF1AX
YY1	EIF1AY
YY1	EIF1B
YY1	EIF2A
YY1	EIF2AK1
YY1	EIF2AK2
YY1	EIF2B1
YY1	EIF2B2
YY1	EIF2B5
YY1	EIF2S1
YY1	EIF2S2
YY1	EIF2S3
YY1	EIF3A
YY1	EIF3B
YY1	EIF3D
YY1	EIF3E
YY1	EIF3F
YY1	EIF3G
YY1	EIF3H
YY1	EIF3I
YY1	EIF3K
YY1	EIF3L
YY1	EIF3M
YY1	EIF4A2
YY1	EIF4A3
YY1	EIF4B
YY1	EIF4E
YY1	EIF4E2

YY1	EIF4EBP2
YY1	EIF4G1
YY1	EIF4G2
YY1	EIF4H
YY1	EIF5
YY1	EIF5A2
YY1	EIF5B
YY1	EIF6
YY1	ELAC1
YY1	ELAC2
YY1	ELF1
YY1	ELK4
YY1	ELOF1
YY1	ELOVL5
YY1	ELP2
YY1	ELP5
YY1	ELP6
YY1	EMB
YY1	EMC10
YY1	EMC4
YY1	EMC6
YY1	EMC9
YY1	EMD
YY1	EME1
YY1	ENDOV
YY1	ENO1
YY1	ENOPH1
YY1	ENOX2
YY1	ENSA
YY1	ENY2
YY1	EPB41
YY1	EPB41L4A-AS1
YY1	EPB41L5
YY1	EPC1
YY1	EPRS
YY1	EPS15
YY1	ERBIN
YY1	ERCC1
YY1	ERCC3
YY1	ERH
YY1	ERI1
YY1	ERI2
YY1	ERLIN1
YY1	ERP44
YY1	ESCO1
YY1	ESCO2
YY1	ESD
YY1	ETFB
YY1	ETS1
YY1	EVI5

YY1	EWSR1
YY1	EXO5
YY1	EXOC1
YY1	EXOC5
YY1	EXOSC10
YY1	EXOSC2
YY1	EXOSC3
YY1	EXOSC4
YY1	EXOSC6
YY1	EXOSC9
YY1	EYA3
YY1	EZH2
YY1	EZR
YY1	FAAP20
YY1	FABP5
YY1	FAF1
YY1	FAF2
YY1	FAIM
YY1	FAM104A
YY1	FAM107B
YY1	FAM111A
YY1	FAM120A
YY1	FAM122B
YY1	FAM133B
YY1	FAM136A
YY1	FAM13B
YY1	FAM173A
YY1	FAM177A1
YY1	FAM185A
YY1	FAM192A
YY1	FAM199X
YY1	FAM204A
YY1	FAM208A
YY1	FAM20B
YY1	FAM219B
YY1	FAM222B
YY1	FAM24B
YY1	FAM32A
YY1	FAM3A
YY1	FAM49B
YY1	FAM50A
YY1	FAM76B
YY1	FAM78A
YY1	FAM91A1
YY1	FAM96A
YY1	FAM96B
YY1	FANCE
YY1	FASTK
YY1	FAU
YY1	FBL

YY1	FBRS
YY1	FBRSL1
YY1	FBXL3
YY1	FBXL5
YY1	FBXO11
YY1	FBXO21
YY1	FBXO25
YY1	FBXO30
YY1	FBXO34
YY1	FBXO7
YY1	FBXO9
YY1	FBXW2
YY1	FBXW4
YY1	FBXW5
YY1	FBXW9
YY1	FCF1
YY1	FDFT1
YY1	FDPS
YY1	FEM1A
YY1	FEN1
YY1	FERMT3
YY1	FGD5-AS1
YY1	FGFR1OP
YY1	FGFR1OP2
YY1	FHIT
YY1	FIBP
YY1	FICD
YY1	FIP1L1
YY1	FIS1
YY1	FKBP1A
YY1	FKBP3
YY1	FKBP5
YY1	FKBP8
YY1	FLAD1
YY1	FLI1
YY1	FLII
YY1	FLNA
YY1	FLNB
YY1	FLOT1
YY1	FMR1
YY1	FN3KRP
YY1	FNBP1
YY1	FNBP4
YY1	FNIP1
YY1	FNTA
YY1	FOPNL
YY1	FOXK2
YY1	FOXM1
YY1	FOXO1
YY1	FOXP1

YY1	FRA10AC1
YY1	FRAT2
YY1	FRG1
YY1	FTH1
YY1	FUBP1
YY1	FUCA1
YY1	FUS
YY1	FUT11
YY1	FUZ
YY1	FXR1
YY1	FXR2
YY1	FYTTD1
YY1	FZD3
YY1	G2E3
YY1	G3BP1
YY1	G3BP2
YY1	G6PC3
YY1	G6PD
YY1	GABARAP
YY1	GABARAPL2
YY1	GABPA
YY1	GADD45GIP1
YY1	GANAB
YY1	GAPDH
YY1	GAPVD1
YY1	GART
YY1	GAS2L3
YY1	GATAD2A
YY1	GATAD2B
YY1	GATB
YY1	GBA
YY1	GBF1
YY1	GCA
YY1	GCC2
YY1	GCDH
YY1	GCHFR
YY1	GCNT2
YY1	GDI1
YY1	GDI2
YY1	GEMIN4
YY1	GEMIN7
YY1	GEMIN8
YY1	GFER
YY1	GFOD2
YY1	GGA1
YY1	GGCT
YY1	GGNBP2
YY1	GHITM
YY1	GID8
YY1	GIGYF2

YY1	GIMAP2
YY1	GIN1
YY1	GINM1
YY1	GINS2
YY1	GINS3
YY1	GIPC1
YY1	GJC1
YY1	GLA
YY1	GLE1
YY1	GLO1
YY1	GLOD4
YY1	GLRX3
YY1	GLRX5
YY1	GLS
YY1	GLTP
YY1	GLUL
YY1	GLYCTK
YY1	GLYR1
YY1	GMCL1
YY1	GMEB2
YY1	GMFB
YY1	GMNN
YY1	GMPR2
YY1	GNAI2
YY1	GNAI3
YY1	GNAS
YY1	GNB1
YY1	GNB2
YY1	GNB4
YY1	GNB5
YY1	GNG5
YY1	GNGT2
YY1	GNL1
YY1	GNPAT
YY1	GNPTG
YY1	GOLGA4
YY1	GOLGA7
YY1	GON4L
YY1	GOPC
YY1	GORASP2
YY1	GOT1
YY1	GOT2
YY1	GPALPP1
YY1	GPANK1
YY1	GPAT4
YY1	GPATCH1
YY1	GPATCH2
YY1	GPATCH2L
YY1	GPATCH8
YY1	GPBP1

YY1	GPBP1L1
YY1	GPCPD1
YY1	GPI
YY1	GPM6B
YY1	GPN2
YY1	GPR137
YY1	GPS1
YY1	GPX4
YY1	GRAMD1C
YY1	GRAP2
YY1	GRB2
YY1	GRINA
YY1	GSDMD
YY1	GSKIP
YY1	GSPT1
YY1	GSTO1
YY1	GSTP1
YY1	GSTZ1
YY1	GTDC1
YY1	GTF2A2
YY1	GTF2B
YY1	GTF2E2
YY1	GTF2F1
YY1	GTF2F2
YY1	GTF2H5
YY1	GTF2I
YY1	GTF3A
YY1	GTF3C1
YY1	GTF3C3
YY1	GTF3C5
YY1	GTF3C6
YY1	GTPBP2
YY1	GTPBP3
YY1	GTSE1
YY1	GUCD1
YY1	GUK1
YY1	GYG1
YY1	GZF1
YY1	H2AFV
YY1	H2AFY
YY1	H2AFZ
YY1	H3F3A
YY1	H3F3B
YY1	H6PD
YY1	HACD2
YY1	HACD3
YY1	HADHA
YY1	HADHB
YY1	HARS2
YY1	HAT1

YY1	HAUS5
YY1	HAUS7
YY1	HAX1
YY1	HBS1L
YY1	HCFC2
YY1	HCLS1
YY1	HDAC1
YY1	HDAC2
YY1	HDAC3
YY1	HDAC5
YY1	HDAC7
YY1	HDAC9
YY1	HDDC2
YY1	HDGF
YY1	HDLBP
YY1	HEBP2
YY1	HECA
YY1	HECTD1
YY1	HELLS
YY1	HELZ
YY1	HENMT1
YY1	HERC1
YY1	HERC2
YY1	HERC4
YY1	HES1
YY1	HGS
YY1	HIBADH
YY1	HIF1A
YY1	HIGD1A
YY1	HIGD2A
YY1	HINT1
YY1	HINT2
YY1	HIST1H4D
YY1	HK1
YY1	HKR1
YY1	HLA-DMB
YY1	HLA-DPA1
YY1	HLA-DRA
YY1	HLA-E
YY1	HMG20B
YY1	HMGA1
YY1	HMGB1
YY1	HMGB2
YY1	HMGCL
YY1	HMGN2
YY1	HMGN3
YY1	HMGXB3
YY1	HMGXB4
YY1	HMMR
YY1	HMOX2

YY1	HNRNPA0
YY1	HNRNPA1
YY1	HNRNPA2B1
YY1	HNRNPA3
YY1	HNRNPAB
YY1	HNRNPC
YY1	HNRNPD
YY1	HNRNPF
YY1	HNRNPH1
YY1	HNRNPH2
YY1	HNRNPH3
YY1	HNRNPK
YY1	HNRNPL
YY1	HNRNPM
YY1	HNRNPR
YY1	HNRNPU
YY1	HOMEZ
YY1	HOOK2
YY1	HOOK3
YY1	HOTAIRM1
YY1	HP1BP3
YY1	HPCAL1
YY1	HPF1
YY1	HPRT1
YY1	HPS1
YY1	HPS6
YY1	HSBP1
YY1	HSD17B10
YY1	HSF1
YY1	HSF2
YY1	HSP90AA1
YY1	HSPA14
YY1	HSPA1A
YY1	HSPA1B
YY1	HSPA4
YY1	HSPA8
YY1	HSPB11
YY1	HSPBP1
YY1	HSPD1
YY1	HSPE1
YY1	HSPH1
YY1	HTATIP2
YY1	HTRA2
YY1	HTT
YY1	HUWE1
YY1	IARS
YY1	ICMT
YY1	ICOSLG
YY1	ID3
YY1	IDH3A

YY1	IDH3B
YY1	IER3IP1
YY1	IFI16
YY1	IFNGR2
YY1	IFT20
YY1	IFT22
YY1	IFT57
YY1	IFT74
YY1	IGBP1
YY1	IGF2BP1
YY1	IGF2R
YY1	IKBKE
YY1	IKBKG
YY1	IKZF1
YY1	IKZF3
YY1	IL15RA
YY1	IL16
YY1	IL2RG
YY1	ILF2
YY1	ILF3
YY1	ILKAP
YY1	ILVBL
YY1	IMMT
YY1	IMP3
YY1	IMP4
YY1	IMPDH2
YY1	INCENP
YY1	ING5
YY1	INIP
YY1	INO80
YY1	INO80C
YY1	INO80E
YY1	INPP5K
YY1	INTS1
YY1	INTS10
YY1	INTS4
YY1	INTS7
YY1	IP6K1
YY1	IP6K2
YY1	IPO11
YY1	IPO5
YY1	IPO9
YY1	IPPK
YY1	IQCG
YY1	IQGAP1
YY1	IRAK1
YY1	IRAK1BP1
YY1	IREB2
YY1	IRF1
YY1	IRF2

YY1	IRF8
YY1	ISCU
YY1	ISG20L2
YY1	ISL2
YY1	IST1
YY1	ISY1
YY1	ITGB1
YY1	ITGB1BP1
YY1	ITGB3BP
YY1	ITPA
YY1	ITPK1
YY1	ITPKB
YY1	ITPRIPL1
YY1	ITSN2
YY1	JADE1
YY1	JAK1
YY1	JAML
YY1	JAZF1
YY1	JKAMP
YY1	JMJD1C
YY1	JOSD1
YY1	JPX
YY1	JTB
YY1	JUND
YY1	KANSL1-AS1
YY1	KANSL2
YY1	KARS
YY1	KAT14
YY1	KAT6A
YY1	KAT6B
YY1	KATNA1
YY1	KATNAL1
YY1	KATNB1
YY1	KCMF1
YY1	KCNG1
YY1	KCNQ1OT1
YY1	KCTD10
YY1	KCTD18
YY1	KCTD5
YY1	KDM2A
YY1	KDM2B
YY1	KDM3B
YY1	KHDRBS1
YY1	KIAA0355
YY1	KIAA1191
YY1	KIAA1551
YY1	KIF14
YY1	KIF20A
YY1	KIF20B
YY1	KIF23

YY1	KIF2A
YY1	KIF4A
YY1	KIF5B
YY1	KLC1
YY1	KLF6
YY1	KLHDC3
YY1	KLHDC4
YY1	KLHL2
YY1	KLHL24
YY1	KLHL42
YY1	KLHL5
YY1	KLHL7
YY1	KLHL9
YY1	KMT2A
YY1	KMT2B
YY1	KMT2C
YY1	KMT2E
YY1	KMT5A
YY1	KMT5B
YY1	KMT5C
YY1	KNTC1
YY1	KPNA3
YY1	KPNB1
YY1	KRAS
YY1	KRCC1
YY1	KRIT1
YY1	KRR1
YY1	KTN1
YY1	KXD1
YY1	KYAT3
YY1	L3MBTL2
YY1	LACTB2
YY1	LAGE3
YY1	LAMTOR1
YY1	LAMTOR3
YY1	LAMTOR4
YY1	LAMTOR5
YY1	LARP4
YY1	LARP7
YY1	LARS
YY1	LAS1L
YY1	LBR
YY1	LCOR
YY1	LCORL
YY1	LCP1
YY1	LDHA
YY1	LDHB
YY1	LGMN
YY1	LIAS
YY1	LIMA1

YY1	LINC00467
YY1	LINC00476
YY1	LINC00623
YY1	LINC00663
YY1	LIPE
YY1	LITAF
YY1	LLPH
YY1	LMF1
YY1	LNPEP
YY1	LONP1
YY1	LONP2
YY1	LOXL2
YY1	LPAR2
YY1	LPIN2
YY1	LPP-AS2
YY1	LPXN
YY1	LRIF1
YY1	LRPPRC
YY1	LRR1
YY1	LRRC37B
YY1	LRRC41
YY1	LRRC47
YY1	LRRC58
YY1	LRRFIP1
YY1	LRRFIP2
YY1	LRSAM1
YY1	LSM1
YY1	LSM12
YY1	LSM14A
YY1	LSM2
YY1	LSM3
YY1	LSM4
YY1	LSM5
YY1	LSM6
YY1	LSP1
YY1	LTA4H
YY1	LTBP1
YY1	LUC7L
YY1	LUC7L2
YY1	LUC7L3
YY1	LUZP1
YY1	LYN
YY1	LYPLA1
YY1	LYPLA2
YY1	LYPLAL1
YY1	LYRM1
YY1	LZIC
YY1	M6PR
YY1	MAD1L1
YY1	MAD2L1

YY1	MAD2L1BP
YY1	MAD2L2
YY1	MAEA
YY1	MAFG
YY1	MAGED2
YY1	MAGEF1
YY1	MAGEH1
YY1	MAGOH
YY1	MALT1
YY1	MANBAL
YY1	MAP1LC3B
YY1	MAP1S
YY1	MAP2
YY1	MAP2K1
YY1	MAP2K2
YY1	MAP2K3
YY1	MAP2K5
YY1	MAP2K7
YY1	MAP3K1
YY1	MAP3K6
YY1	MAP3K7
YY1	MAP4
YY1	MAP4K4
YY1	MAP7D1
YY1	MAP9
YY1	MAPK1IP1L
YY1	MAPKAP1
YY1	MAPKAPK5
YY1	MAPRE1
YY1	MARCH1
YY1	MARCH7
YY1	MARCH9
YY1	MARK3
YY1	MASTL
YY1	MAT2A
YY1	MAT2B
YY1	MAVS
YY1	MAX
YY1	MAZ
YY1	MBD2
YY1	MBD3
YY1	MBD4
YY1	MBD5
YY1	MBIP
YY1	MBNL1
YY1	MBNL3
YY1	MCAT
YY1	MCCC1
YY1	MCL1
YY1	MCM2

YY1	MCM3
YY1	MCM3AP
YY1	MCM6
YY1	MCM7
YY1	MCM8
YY1	MCMBP
YY1	MCOLN2
YY1	MCRIP1
YY1	MCRS1
YY1	MCTS1
YY1	MCUR1
YY1	MDC1
YY1	MDH1
YY1	MDH2
YY1	MDM1
YY1	MDN1
YY1	MDP1
YY1	MEA1
YY1	MEAF6
YY1	MECP2
YY1	MED10
YY1	MED13
YY1	MED19
YY1	MED25
YY1	MED26
YY1	MED27
YY1	MED28
YY1	MED30
YY1	MED31
YY1	MED4
YY1	MED6
YY1	MED7
YY1	MED8
YY1	MED9
YY1	MEF2A
YY1	MELK
YY1	MEN1
YY1	MESP1
YY1	METAP1
YY1	METAP2
YY1	METTL14
YY1	METTL16
YY1	METTL17
YY1	METTL21A
YY1	METTL23
YY1	METTL5
YY1	METTL9
YY1	MFAP1
YY1	MFF
YY1	MFN2

YY1	MFNG
YY1	MFSD14B
YY1	MFSD14C
YY1	MGAT4B
YY1	MGME1
YY1	MGMT
YY1	MIA-RAB4B
YY1	MIB2
YY1	MICU1
YY1	MID1IP1
YY1	MIDN
YY1	MIEF2
YY1	MIER1
YY1	MIF
YY1	MIF4GD
YY1	MINOS1
YY1	MINPP1
YY1	MIR210HG
YY1	MIS18A
YY1	MIS18BP1
YY1	MITD1
YY1	MKL1
YY1	MKNK2
YY1	MKRN1
YY1	MKRN2
YY1	MLF2
YY1	MLLT1
YY1	MLLT10
YY1	MLYCD
YY1	MMADHC
YY1	MMD
YY1	MMGT1
YY1	MND1
YY1	MOB1A
YY1	MOB2
YY1	MOB4
YY1	MON1B
YY1	MORC2-AS1
YY1	MORF4L1
YY1	MORF4L2
YY1	MPC2
YY1	MPDU1
YY1	MPG
YY1	MPHOSPH10
YY1	MPHOSPH6
YY1	MPHOSPH8
YY1	MPLKIP
YY1	MPND
YY1	MRFAP1L1
YY1	MRGBP

YY1	MRM2
YY1	MRM3
YY1	MROH1
YY1	MRPL10
YY1	MRPL11
YY1	MRPL13
YY1	MRPL14
YY1	MRPL15
YY1	MRPL16
YY1	MRPL17
YY1	MRPL18
YY1	MRPL19
YY1	MRPL20
YY1	MRPL21
YY1	MRPL22
YY1	MRPL23
YY1	MRPL27
YY1	MRPL28
YY1	MRPL30
YY1	MRPL32
YY1	MRPL34
YY1	MRPL35
YY1	MRPL37
YY1	MRPL39
YY1	MRPL40
YY1	MRPL43
YY1	MRPL44
YY1	MRPL45
YY1	MRPL47
YY1	MRPL48
YY1	MRPL49
YY1	MRPL51
YY1	MRPL52
YY1	MRPL54
YY1	MRPL57
YY1	MRPS10
YY1	MRPS15
YY1	MRPS16
YY1	MRPS18A
YY1	MRPS18B
YY1	MRPS18C
YY1	MRPS21
YY1	MRPS26
YY1	MRPS30
YY1	MRPS31
YY1	MRPS33
YY1	MRPS34
YY1	MRPS35
YY1	MRPS36
YY1	MRPS6

YY1	MRPS9
YY1	MRRF
YY1	MSH2
YY1	MSH3
YY1	MSH6
YY1	MSL3
YY1	MSN
YY1	MT1F
YY1	MTA2
YY1	MTA3
YY1	MTCH2
YY1	MTDH
YY1	MTERF1
YY1	MTERF4
YY1	MTF2
YY1	MTFMT
YY1	MTFR1
YY1	MTG1
YY1	MTG2
YY1	MTHFD1
YY1	MTIF2
YY1	MTIF3
YY1	MTMR14
YY1	MTMR4
YY1	MTMR6
YY1	MTO1
YY1	MTPN
YY1	MTX1
YY1	MTX2
YY1	MUS81
YY1	MUTYH
YY1	MVB12A
YY1	MVD
YY1	MYBL1
YY1	MYBL2
YY1	MYCBP2
YY1	MYEF2
YY1	MYH9
YY1	MYL12A
YY1	MYL12B
YY1	MYL6B
YY1	MYLPF
YY1	MYO19
YY1	MYO1G
YY1	MZT1
YY1	MZT2A
YY1	N4BP2
YY1	N4BP2L1
YY1	N4BP2L2
YY1	NAA10

YY1	NAA15
YY1	NAA25
YY1	NAA30
YY1	NAA35
YY1	NAA60
YY1	NAB2
YY1	NABP2
YY1	NACA
YY1	NADK
YY1	NADK2
YY1	NAE1
YY1	NAGK
YY1	NAP1L1
YY1	NAP1L4
YY1	NARFL
YY1	NASP
YY1	NAXE
YY1	NBEAL1
YY1	NCAPD3
YY1	NCAPH
YY1	NCBP2
YY1	NCBP2-AS2
YY1	NCBP3
YY1	NCF4
YY1	NCKAP1L
YY1	NCL
YY1	NCOA3
YY1	NCOA4
YY1	NCOR1
YY1	NCOR2
YY1	NDE1
YY1	NDN
YY1	NDUFA1
YY1	NDUFA10
YY1	NDUFA12
YY1	NDUFA13
YY1	NDUFA4
YY1	NDUFA4L2
YY1	NDUFA5
YY1	NDUFA6
YY1	NDUFA7
YY1	NDUFA8
YY1	NDUFAB1
YY1	NDUFAF6
YY1	NDUFB10
YY1	NDUFB2
YY1	NDUFB3
YY1	NDUFB4
YY1	NDUFB5
YY1	NDUFB6

YY1	NDUFB7
YY1	NDUFB8
YY1	NDUFB9
YY1	NDUFC1
YY1	NDUFS3
YY1	NDUFS4
YY1	NDUFS5
YY1	NDUFS6
YY1	NDUFS7
YY1	NDUFS8
YY1	NDUFV1
YY1	NDUFV2
YY1	NEAT1
YY1	NEDD1
YY1	NEDD4L
YY1	NEDD8
YY1	NELFB
YY1	NELFE
YY1	NEMF
YY1	NEMP1
YY1	NEU3
YY1	NF1
YY1	NFAT5
YY1	NFATC1
YY1	NFATC2IP
YY1	NFATC3
YY1	NFE2L2
YY1	NFKBIB
YY1	NGDN
YY1	NIN
YY1	NIP7
YY1	NIPA2
YY1	NIPBL
YY1	NIPSNAP3A
YY1	NKAP
YY1	NKIRAS2
YY1	NKTR
YY1	NLK
YY1	NLRP2
YY1	NME6
YY1	NMI
YY1	NMU
YY1	NOL10
YY1	NOL12
YY1	NOL7
YY1	NOL8
YY1	NOL9
YY1	NOM1
YY1	NONO
YY1	NOP10

YY1	NOP14-AS1
YY1	NOP58
YY1	NOSIP
YY1	NPAT
YY1	NPEPPS
YY1	NPHP3
YY1	NPIPB15
YY1	NPLOC4
YY1	NPM1
YY1	NPM3
YY1	NPRL2
YY1	NR1H2
YY1	NR2C1
YY1	NR2C2AP
YY1	NR3C1
YY1	NRAS
YY1	NRAV
YY1	NRBF2
YY1	NRBP1
YY1	NRDC
YY1	NRDE2
YY1	NSA2
YY1	NSD1
YY1	NSDHL
YY1	NSMCE3
YY1	NSMCE4A
YY1	NT5C
YY1	NTAN1
YY1	NTPCR
YY1	NUBP2
YY1	NUBPL
YY1	NUCB1
YY1	NUCKS1
YY1	NUDC
YY1	NUDCD2
YY1	NUDCD3
YY1	NUDT1
YY1	NUDT16L1
YY1	NUDT21
YY1	NUDT5
YY1	NUDT8
YY1	NUFIP2
YY1	NUP107
YY1	NUP133
YY1	NUP153
YY1	NUP160
YY1	NUP210
YY1	NUP214
YY1	NUP37
YY1	NUP50

YY1	NUP58
YY1	NUP93
YY1	NUSAP1
YY1	NUTF2
YY1	NUTM2B-AS1
YY1	NXF1
YY1	NXT1
YY1	NXT2
YY1	OARD1
YY1	OAZ1
YY1	OCIAD1
YY1	OCIAD2
YY1	ODF2
YY1	OGG1
YY1	OGT
YY1	OLA1
YY1	OPA1
YY1	ORAI2
YY1	ORC5
YY1	ORC6
YY1	ORMDL1
YY1	ORMDL2
YY1	OSBPL11
YY1	OSBPL8
YY1	OSBPL9
YY1	OST4
YY1	OSTC
YY1	OTUB1
YY1	OTUD1
YY1	OTUD3
YY1	OTUD4
YY1	OTUD6B
YY1	OTULIN
YY1	OXA1L
YY1	OXCT1
YY1	OXNAD1
YY1	P4HB
YY1	PA2G4
YY1	PABPC1
YY1	PABPC4
YY1	PABPN1
YY1	PACS1
YY1	PACSIN2
YY1	PADI4
YY1	PAFAH1B3
YY1	PAIP1
YY1	PAIP2
YY1	PAK1
YY1	PAK2
YY1	PAK4

YY1	PALLD
YY1	PANK2
YY1	PANK3
YY1	PAPOLA
YY1	PAPSS1
YY1	PARG
YY1	PARK7
YY1	PARL
YY1	PAXBP1
YY1	PAXIP1
YY1	PBK
YY1	PBRM1
YY1	PCBD2
YY1	PCBP1
YY1	PCBP2
YY1	PCF11
YY1	PCGF1
YY1	PCGF3
YY1	PCGF6
YY1	PCIF1
YY1	PCM1
YY1	PCMT1
YY1	PCNA
YY1	PCNP
YY1	PCNT
YY1	PCNX1
YY1	PCNX4
YY1	PCSK7
YY1	PDAP1
YY1	PDCD10
YY1	PDCD2
YY1	PDCD6
YY1	PDCD7
YY1	PDE4D
YY1	PDE4DIP
YY1	PDE6D
YY1	PDHB
YY1	PDK2
YY1	PDLIM1
YY1	PDRG1
YY1	PDS5A
YY1	PDS5B
YY1	PDSS2
YY1	PDXDC1
YY1	PDZD11
YY1	PEF1
YY1	PET100
YY1	PEX1
YY1	PEX10
YY1	PEX11B

YY1	PEX16
YY1	PEX19
YY1	PEX3
YY1	PFDN1
YY1	PFDN5
YY1	PFDN6
YY1	PFN1
YY1	PGAM1
YY1	PGAM5
YY1	PGK1
YY1	PGLS
YY1	PGM1
YY1	PGM2
YY1	PGP
YY1	PGRMC1
YY1	PGS1
YY1	PHACTR4
YY1	PHB
YY1	PHB2
YY1	PHF10
YY1	PHF12
YY1	PHF13
YY1	PHF14
YY1	PHF20
YY1	PHF20L1
YY1	PHF21A
YY1	PHF23
YY1	PHF5A
YY1	PHF7
YY1	PHIP
YY1	PHLPP1
YY1	PHRF1
YY1	PHTF2
YY1	PI4K2A
YY1	PIAS2
YY1	PIGA
YY1	PIGBOS1
YY1	PIGC
YY1	PIGF
YY1	PIGG
YY1	PIGP
YY1	PIGV
YY1	PIH1D1
YY1	PIK3CD
YY1	PILRB
YY1	PIM1
YY1	PIN1
YY1	PIN4
YY1	PIP4K2A
YY1	PISD

YY1	PITHD1
YY1	PITPNA
YY1	PITPNM1
YY1	PJA2
YY1	PKIG
YY1	PKM
YY1	PKN1
YY1	PKN2
YY1	PLAA
YY1	PLAGL2
YY1	PLCD1
YY1	PLCL2
YY1	PLEKHA3
YY1	PLEKHB1
YY1	PLEKHG2
YY1	PLEKHJ1
YY1	PLK4
YY1	PLPP1
YY1	PMAIP1
YY1	PMF1
YY1	PMM1
YY1	PMPCB
YY1	PMS1
YY1	PMVK
YY1	PNISR
YY1	PNKD
YY1	PNKP
YY1	PNMA1
YY1	PNN
YY1	PNP
YY1	PNPLA4
YY1	PNPLA6
YY1	PNPLA8
YY1	PNRC1
YY1	PNRC2
YY1	POC1A
YY1	POC1B
YY1	POLA2
YY1	POLD3
YY1	POLD4
YY1	POLDIP2
YY1	POLDIP3
YY1	POLE3
YY1	POLG
YY1	POLR1D
YY1	POLR1E
YY1	POLR2B
YY1	POLR2C
YY1	POLR2D
YY1	POLR2E

YY1	POLR2F
YY1	POLR2G
YY1	POLR2H
YY1	POLR2I
YY1	POLR2J
YY1	POLR2K
YY1	POLR2L
YY1	POLR2M
YY1	POLR3E
YY1	POLR3GL
YY1	POMP
YY1	POP4
YY1	POP5
YY1	POP7
YY1	POU2F2
YY1	PPA1
YY1	PPA2
YY1	PPCS
YY1	PPHLN1
YY1	PPIA
YY1	PPID
YY1	PPIE
YY1	PPIG
YY1	PPIH
YY1	PPIL2
YY1	PPIL4
YY1	PPIP5K2
YY1	PPM1A
YY1	PPM1D
YY1	PPM1G
YY1	PPME1
YY1	PPP1CA
YY1	PPP1CB
YY1	PPP1CC
YY1	PPP1R10
YY1	PPP1R11
YY1	PPP1R12A
YY1	PPP1R12C
YY1	PPP1R14B
YY1	PPP1R15A
YY1	PPP1R2
YY1	PPP1R21
YY1	PPP1R35
YY1	PPP2CA
YY1	PPP2R1A
YY1	PPP2R1B
YY1	PPP2R2A
YY1	PPP2R5A
YY1	PPP2R5B
YY1	PPP2R5C

YY1	PPP2R5E
YY1	PPP3CB
YY1	PPP3R1
YY1	PPP4C
YY1	PPP4R2
YY1	PPP4R3A
YY1	PPP4R3B
YY1	PPP5C
YY1	PPP6C
YY1	PPP6R1
YY1	PPP6R2
YY1	PPP6R3
YY1	PQLC1
YY1	PRCC
YY1	PRDM2
YY1	PRDX6
YY1	PRELID1
YY1	PRELID3B
YY1	PRH1
YY1	PRIM1
YY1	PRKACA
YY1	PRKACB
YY1	PRKAG1
YY1	PRKAR1A
YY1	PRKAR2A
YY1	PRKCD
YY1	PRKCE
YY1	PRKCZ
YY1	PRKD2
YY1	PRKD3
YY1	PRKDC
YY1	PRKRA
YY1	PRKRIP1
YY1	PRMT2
YY1	PRPF19
YY1	PRPF3
YY1	PRPF31
YY1	PRPF38A
YY1	PRPF38B
YY1	PRPF39
YY1	PRPF4
YY1	PRPF40A
YY1	PRPF4B
YY1	PRPF8
YY1	PRPS1
YY1	PRPS2
YY1	PRPSAP1
YY1	PRR13
YY1	PRR7-AS1
YY1	PRRC2A

YY1	PRRC2B
YY1	PRRC2C
YY1	PSD4
YY1	PSEN1
YY1	PSIP1
YY1	PSKH1
YY1	PSMA1
YY1	PSMA2
YY1	PSMA3
YY1	PSMA3-AS1
YY1	PSMA4
YY1	PSMA5
YY1	PSMA7
YY1	PSMB1
YY1	PSMB2
YY1	PSMB3
YY1	PSMB4
YY1	PSMB5
YY1	PSMB7
YY1	PSMB8
YY1	PSMB9
YY1	PSMC1
YY1	PSMC2
YY1	PSMC3
YY1	PSMC4
YY1	PSMC5
YY1	PSMC6
YY1	PSMD1
YY1	PSMD11
YY1	PSMD12
YY1	PSMD13
YY1	PSMD14
YY1	PSMD2
YY1	PSMD3
YY1	PSMD4
YY1	PSMD6
YY1	PSMD7
YY1	PSMD8
YY1	PSMD9
YY1	PSME1
YY1	PSME3
YY1	PSME4
YY1	PSMF1
YY1	PSMG2
YY1	PSPC1
YY1	PSRC1
YY1	PSTK
YY1	PTBP1
YY1	PTBP3
YY1	PTDSS1

YY1	PTEN
YY1	PTGES3
YY1	PTMA
YY1	PTOV1
YY1	PTP4A1
YY1	PTP4A2
YY1	PTPN12
YY1	PTPN2
YY1	PTPRA
YY1	PTRH2
YY1	PTRHD1
YY1	PTTG1
YY1	PTTG1IP
YY1	PUDP
YY1	PUF60
YY1	PUM1
YY1	PUM2
YY1	PURA
YY1	PUS10
YY1	PWP1
YY1	PXK
YY1	PYGB
YY1	PYURF
YY1	QARS
YY1	QKI
YY1	QSER1
YY1	QSOX2
YY1	R3HDM4
YY1	RAB10
YY1	RAB11B
YY1	RAB11FIP1
YY1	RAB14
YY1	RAB1A
YY1	RAB21
YY1	RAB22A
YY1	RAB2A
YY1	RAB30
YY1	RAB35
YY1	RAB37
YY1	RAB3GAP1
YY1	RAB5A
YY1	RAB5C
YY1	RAB6A
YY1	RAB7A
YY1	RAB8A
YY1	RAB8B
YY1	RAB9A
YY1	RABEP1
YY1	RABIF
YY1	RABL2B

YY1	RABL6
YY1	RAC1
YY1	RACGAP1
YY1	RACK1
YY1	RAD17
YY1	RAD18
YY1	RAD21
YY1	RAD23A
YY1	RAD23B
YY1	RAD51D
YY1	RAD54B
YY1	RAD9A
YY1	RAE1
YY1	RAF1
YY1	RALA
YY1	RALB
YY1	RALBP1
YY1	RALGAPB
YY1	RALGPS2
YY1	RALY
YY1	RAN
YY1	RANBP1
YY1	RANBP3
YY1	RANGRF
YY1	RAP1A
YY1	RAP1B
YY1	RAPGEF1
YY1	RAPGEF5
YY1	RAPGEF6
YY1	RARS
YY1	RARS2
YY1	RASAL3
YY1	RASGRF1
YY1	RASSF1
YY1	RASSF5
YY1	RB1
YY1	RB1CC1
YY1	RBBP6
YY1	RBBP7
YY1	RBBP8
YY1	RBCK1
YY1	RBM10
YY1	RBM12
YY1	RBM12B
YY1	RBM14
YY1	RBM15
YY1	RBM15B
YY1	RBM17
YY1	RBM18
YY1	RBM22

YY1	RBM23
YY1	RBM25
YY1	RBM26
YY1	RBM26-AS1
YY1	RBM27
YY1	RBM28
YY1	RBM3
YY1	RBM39
YY1	RBM4
YY1	RBM41
YY1	RBM42
YY1	RBM45
YY1	RBM7
YY1	RBM8A
YY1	RBMX
YY1	RBMX2
YY1	RBMXL1
YY1	RBPJ
YY1	RBX1
YY1	RCC1
YY1	RCC1L
YY1	RCC2
YY1	RCHY1
YY1	RCSD1
YY1	RDH14
YY1	RECQL
YY1	RELA
YY1	RELB
YY1	RELL2
YY1	REPS1
YY1	RER1
YY1	RERE
YY1	REST
YY1	REV1
YY1	REXO2
YY1	REXO4
YY1	RFC1
YY1	RFC2
YY1	RFC3
YY1	RFC4
YY1	RFC5
YY1	RFK
YY1	RFT1
YY1	RFTN1
YY1	RFX5
YY1	RFX7
YY1	RFXANK
YY1	RGS10
YY1	RGS14
YY1	RGS3

YY1	RHBDD2
YY1	RHEB
YY1	RHNO1
YY1	RHOBTB2
YY1	RHOG
YY1	RHOH
YY1	RHOT2
YY1	RIC8A
YY1	RICTOR
YY1	RIF1
YY1	RING1
YY1	RIOK3
YY1	RIPK2
YY1	RMI1
YY1	RMI2
YY1	RMND1
YY1	RMND5B
YY1	RNASEH1
YY1	RNASEH2B
YY1	RNASEH2C
YY1	RNASEL
YY1	RNF10
YY1	RNF114
YY1	RNF115
YY1	RNF126
YY1	RNF138
YY1	RNF139
YY1	RNF14
YY1	RNF146
YY1	RNF166
YY1	RNF167
YY1	RNF168
YY1	RNF181
YY1	RNF187
YY1	RNF2
YY1	RNF20
YY1	RNF216
YY1	RNF219
YY1	RNF25
YY1	RNF26
YY1	RNF4
YY1	RNF41
YY1	RNF6
YY1	RNF7
YY1	RNF8
YY1	RNMT
YY1	RNPEPL1
YY1	RNPS1
YY1	ROCK1
YY1	ROCK2

YY1	RPA2
YY1	RPE
YY1	RPIA
YY1	RPL10
YY1	RPL10A
YY1	RPL11
YY1	RPL12
YY1	RPL13
YY1	RPL13A
YY1	RPL14
YY1	RPL15
YY1	RPL17
YY1	RPL18A
YY1	RPL19
YY1	RPL21
YY1	RPL22
YY1	RPL22L1
YY1	RPL23
YY1	RPL23A
YY1	RPL24
YY1	RPL26
YY1	RPL26L1
YY1	RPL27
YY1	RPL27A
YY1	RPL28
YY1	RPL29
YY1	RPL3
YY1	RPL30
YY1	RPL31
YY1	RPL32
YY1	RPL34
YY1	RPL35
YY1	RPL35A
YY1	RPL36
YY1	RPL36AL
YY1	RPL37
YY1	RPL37A
YY1	RPL38
YY1	RPL39
YY1	RPL4
YY1	RPL41
YY1	RPL5
YY1	RPL6
YY1	RPL7
YY1	RPL7L1
YY1	RPL8
YY1	RPL9
YY1	RPLP0
YY1	RPLP1
YY1	RPLP2

YY1	RPP25L
YY1	RPP30
YY1	RPP38
YY1	RPRD2
YY1	RPS10
YY1	RPS11
YY1	RPS12
YY1	RPS13
YY1	RPS14
YY1	RPS15
YY1	RPS15A
YY1	RPS16
YY1	RPS17
YY1	RPS18
YY1	RPS19
YY1	RPS19BP1
YY1	RPS2
YY1	RPS20
YY1	RPS21
YY1	RPS23
YY1	RPS24
YY1	RPS25
YY1	RPS26
YY1	RPS27
YY1	RPS27A
YY1	RPS27L
YY1	RPS28
YY1	RPS29
YY1	RPS3
YY1	RPS3A
YY1	RPS4X
YY1	RPS5
YY1	RPS6
YY1	RPS6KA3
YY1	RPS6KB2
YY1	RPS7
YY1	RPS8
YY1	RPS9
YY1	RPSA
YY1	RPUSD1
YY1	RPUSD2
YY1	RRAGC
YY1	RRM1
YY1	RRM2B
YY1	RRP36
YY1	RSBN1
YY1	RSBN1L
YY1	RSF1
YY1	RSL1D1
YY1	RSL24D1

YY1	RSRC1
YY1	RSRC2
YY1	RSRP1
YY1	RSU1
YY1	RTCA
YY1	RTCB
YY1	RTF1
YY1	RTN3
YY1	RTN4
YY1	RUFY1
YY1	RUFY3
YY1	RUNX3
YY1	RUVBL1
YY1	RXRB
YY1	RYBP
YY1	S100PBP
YY1	SAAL1
YY1	SACM1L
YY1	SAE1
YY1	SAFB
YY1	SAFB2
YY1	SAMD4A
YY1	SAMD4B
YY1	SAMM50
YY1	SAP18
YY1	SAP30BP
YY1	SAP30L
YY1	SARNP
YY1	SART1
YY1	SART3
YY1	SASH3
YY1	SAT1
YY1	SC5D
YY1	SCAF1
YY1	SCAF11
YY1	SCAF4
YY1	SCAND1
YY1	SCCPDH
YY1	SCD
YY1	SCFD1
YY1	SCLT1
YY1	SCML4
YY1	SCNM1
YY1	SCO1
YY1	SCOC
YY1	SCRN2
YY1	SDAD1
YY1	SDCBP
YY1	SDCCAG8
YY1	SDF2

YY1	SDHA
YY1	SDHB
YY1	SDHC
YY1	SDHD
YY1	SEC11A
YY1	SEC16A
YY1	SEC22C
YY1	SEC23A
YY1	SEC61A2
YY1	SEC62
YY1	SECISBP2L
YY1	SEMA4A
YY1	SENP2
YY1	SENP5
YY1	SENP6
YY1	SEPHS2
YY1	SEPT11
YY1	SEPT2
YY1	SEPT6
YY1	SEPT7
YY1	SEPT9
YY1	SERBP1
YY1	SERF2
YY1	SERPINB8
YY1	SET
YY1	SETD2
YY1	SETD5
YY1	SETD9
YY1	SF1
YY1	SF3A1
YY1	SF3A2
YY1	SF3A3
YY1	SF3B1
YY1	SF3B2
YY1	SF3B4
YY1	SF3B5
YY1	SF3B6
YY1	SFPQ
YY1	SFR1
YY1	SFSWAP
YY1	SFT2D1
YY1	SFT2D2
YY1	SFXN1
YY1	SFXN2
YY1	SGCB
YY1	SGO1
YY1	SGO2
YY1	SGPL1
YY1	SGTA
YY1	SH2B3

YY1	SH2D3C
YY1	SH3BGRL
YY1	SH3BP1
YY1	SH3BP5
YY1	SH3GL1
YY1	SH3GLB1
YY1	SH3KBP1
YY1	SHANK2
YY1	SHARPIN
YY1	SHISA5
YY1	SHISA8
YY1	SHKBP1
YY1	SHMT2
YY1	SHOC2
YY1	SHPRH
YY1	SHROOM1
YY1	SIKE1
YY1	SIMC1
YY1	SIN3A
YY1	SIRT6
YY1	SIRT7
YY1	SIT1
YY1	SIVA1
YY1	SKA1
YY1	SKA3
YY1	SKAP2
YY1	SKP1
YY1	SKP2
YY1	SLC15A4
YY1	SLC1A1
YY1	SLC25A1
YY1	SLC25A11
YY1	SLC25A16
YY1	SLC25A17
YY1	SLC25A19
YY1	SLC25A20
YY1	SLC25A24
YY1	SLC25A3
YY1	SLC25A33
YY1	SLC25A36
YY1	SLC25A37
YY1	SLC25A39
YY1	SLC25A46
YY1	SLC25A5
YY1	SLC29A1
YY1	SLC35A4
YY1	SLC35A5
YY1	SLC35E3
YY1	SLC35F5
YY1	SLC36A4

YY1	SLC38A1
YY1	SLC39A3
YY1	SLC39A6
YY1	SLC41A1
YY1	SLC44A2
YY1	SLC4A2
YY1	SLC4A7
YY1	SLC50A1
YY1	SLC6A6
YY1	SLC7A7
YY1	SLC9A3R1
YY1	SLC9A7
YY1	SLIRP
YY1	SLK
YY1	SLTM
YY1	SLU7
YY1	SMAD4
YY1	SMAP2
YY1	SMARCA2
YY1	SMARCA4
YY1	SMARCA5
YY1	SMARCAL1
YY1	SMARCB1
YY1	SMARCC1
YY1	SMARCC2
YY1	SMARCD1
YY1	SMARCD2
YY1	SMC1A
YY1	SMC2
YY1	SMC3
YY1	SMC4
YY1	SMC5
YY1	SMCHD1
YY1	SMCO4
YY1	SMDT1
YY1	SMG1
YY1	SMG7
YY1	SMIM10L1
YY1	SMIM12
YY1	SMIM15
YY1	SMIM20
YY1	SMIM4
YY1	SMIM7
YY1	SMIM8
YY1	SMNDC1
YY1	SMPD2
YY1	SMPD4
YY1	SMU1
YY1	SMYD4
YY1	SNAP23

YY1	SNAPC1
YY1	SNAPC2
YY1	SNAPC3
YY1	SNAPC5
YY1	SNAPIN
YY1	SND1
YY1	SNF8
YY1	SNHG7
YY1	SNHG9
YY1	SNIP1
YY1	SNRNP200
YY1	SNRNP27
YY1	SNRNP35
YY1	SNRNP40
YY1	SNRNP70
YY1	SNRPA
YY1	SNRPA1
YY1	SNRPB
YY1	SNRPB2
YY1	SNRPD1
YY1	SNRPD2
YY1	SNRPE
YY1	SNRPF
YY1	SNRPG
YY1	SNU13
YY1	SNW1
YY1	SNX1
YY1	SNX10
YY1	SNX14
YY1	SNX15
YY1	SNX17
YY1	SNX2
YY1	SNX5
YY1	SOD1
YY1	SOGA1
YY1	SON
YY1	SP100
YY1	SP3
YY1	SPA17
YY1	SPAG7
YY1	SPAG9
YY1	SPATA24
YY1	SPATA33
YY1	SPATA5L1
YY1	SPECC1L
YY1	SPEN
YY1	SPG21
YY1	SPG7
YY1	SPI1
YY1	SPIN2B

YY1	SPINT1
YY1	SPINT2
YY1	SPOP
YY1	SPOPL
YY1	SPPL2A
YY1	SPSB2
YY1	SPSB3
YY1	SPTAN1
YY1	SPTBN1
YY1	SPTLC1
YY1	SPTLC2
YY1	SPTSSA
YY1	SQLE
YY1	SREBF2
YY1	SREK1
YY1	SREK1IP1
YY1	SRFBP1
YY1	SRGAP2C
YY1	SRP14
YY1	SRP19
YY1	SRP9
YY1	SRPK1
YY1	SRPK2
YY1	SRRM1
YY1	SRRM2
YY1	SRRM2-AS1
YY1	SRRT
YY1	SRSF1
YY1	SRSF10
YY1	SRSF11
YY1	SRSF2
YY1	SRSF3
YY1	SRSF4
YY1	SRSF5
YY1	SRSF6
YY1	SRSF9
YY1	SS18L2
YY1	SSB
YY1	SSBP1
YY1	SSBP4
YY1	SSH2
YY1	SSNA1
YY1	SSRP1
YY1	SSU72
YY1	ST13
YY1	ST14
YY1	ST20-AS1
YY1	ST3GAL3
YY1	ST3GAL4
YY1	STAG1

YY1	STAG2
YY1	STAMBPL1
YY1	STARD7
YY1	STAT2
YY1	STAT3
YY1	STAT6
YY1	STAU1
YY1	STAU2
YY1	STIM1
YY1	STIP1
YY1	STK11
YY1	STK11IP
YY1	STK16
YY1	STK24
YY1	STK25
YY1	STK3
YY1	STK38L
YY1	STK4
YY1	STMN1
YY1	STOML1
YY1	STOML2
YY1	STRADA
YY1	STRAP
YY1	STRBP
YY1	STUB1
YY1	STX10
YY1	STX11
YY1	STX18
YY1	STX5
YY1	STX6
YY1	STX8
YY1	STXBP2
YY1	STXBP3
YY1	SUCLA2
YY1	SUCLG1
YY1	SUCLG2
YY1	SUCO
YY1	SUDS3
YY1	SUGP1
YY1	SUGP2
YY1	SUGT1
YY1	SULF2
YY1	SUMO1
YY1	SUMO2
YY1	SUMO3
YY1	SUMO4
YY1	SUPT16H
YY1	SUPT4H1
YY1	SUPT5H
YY1	SUPT6H

YY1	SURF1
YY1	SUSD1
YY1	SUV39H1
YY1	SUZ12
YY1	SVBP
YY1	SWAP70
YY1	SYCE1L
YY1	SYCE2
YY1	SYCP2
YY1	SYF2
YY1	SYMPK
YY1	SYNC
YY1	SYNCRIP
YY1	SYNE3
YY1	SYNJ2BP
YY1	SYPL1
YY1	SYS1
YY1	SZRD1
YY1	TAB2
YY1	TACC1
YY1	TACC3
YY1	TADA1
YY1	TADA2A
YY1	TAF10
YY1	TAF11
YY1	TAF12
YY1	TAF15
YY1	TAF1A
YY1	TAF1C
YY1	TAF2
YY1	TAF3
YY1	TAF4
YY1	TAF7
YY1	TAF9
YY1	TAF9B
YY1	TAGLN2
YY1	TANGO2
YY1	TAOK2
YY1	TAOK3
YY1	TAPBP
YY1	TARBP2
YY1	TARDBP
YY1	TAX1BP1
YY1	TBC1D1
YY1	TBC1D15
YY1	TBC1D2
YY1	TBC1D23
YY1	TBC1D25
YY1	TBC1D4
YY1	TBC1D5

YY1	TBCA
YY1	TBCB
YY1	TBCC
YY1	TBL1X
YY1	TBL1XR1
YY1	TBPL1
YY1	TBRG1
YY1	TBRG4
YY1	TCAIM
YY1	TCEA2
YY1	TCERG1
YY1	TCF12
YY1	TCF25
YY1	TCF3
YY1	TCF4
YY1	TCOF1
YY1	TCP1
YY1	TCTA
YY1	TCTN3
YY1	TDG
YY1	TDP2
YY1	TDRKH
YY1	TELO2
YY1	TERF1
YY1	TERF2IP
YY1	TES
YY1	TEX10
YY1	TEX264
YY1	TFB1M
YY1	TFDP2
YY1	TFEB
YY1	TFG
YY1	TFIP11
YY1	TGDS
YY1	TGFB1
YY1	TGOLN2
YY1	TGS1
YY1	THAP1
YY1	THAP11
YY1	THAP12
YY1	THAP2
YY1	THAP7
YY1	THG1L
YY1	THOC1
YY1	THOC2
YY1	THOC3
YY1	THOC7
YY1	THOP1
YY1	THRAP3
YY1	THUMPD3-AS1

YY1	TIA1
YY1	TIAL1
YY1	TIGD1
YY1	TIMM10
YY1	TIMM21
YY1	TIMM8A
YY1	TIMM8B
YY1	TIMMDC1
YY1	TINF2
YY1	TIPARP
YY1	TIPARP-AS1
YY1	TIPIN
YY1	TIPRL
YY1	TJAP1
YY1	TLE4
YY1	TLK2
YY1	TLN1
YY1	TM2D2
YY1	TM7SF2
YY1	TM9SF3
YY1	TM9SF4
YY1	TMA16
YY1	TMA7
YY1	TMBIM4
YY1	TMBIM6
YY1	TMCO1
YY1	TMED7
YY1	TMED8
YY1	TMEM101
YY1	TMEM106C
YY1	TMEM109
YY1	TMEM11
YY1	TMEM116
YY1	TMEM123
YY1	TMEM126A
YY1	TMEM126B
YY1	TMEM131
YY1	TMEM134
YY1	TMEM14A
YY1	TMEM14B
YY1	TMEM160
YY1	TMEM165
YY1	TMEM167A
YY1	TMEM170A
YY1	TMEM183A
YY1	TMEM184B
YY1	TMEM186
YY1	TMEM203
YY1	TMEM219
YY1	TMEM222

YY1	TMEM223
YY1	TMEM230
YY1	TMEM242
YY1	TMEM243
YY1	TMEM256
YY1	TMEM258
YY1	TMEM259
YY1	TMEM39B
YY1	TMEM44
YY1	TMEM50A
YY1	TMEM59
YY1	TMEM60
YY1	TMEM79
YY1	TMEM8A
YY1	TMF1
YY1	TMPO
YY1	TMSB10
YY1	TMSB4X
YY1	TMUB2
YY1	TMX1
YY1	TNFAIP8
YY1	TNFRSF14
YY1	TNIP1
YY1	TNKS2-AS1
YY1	TNPO1
YY1	TNPO2
YY1	TNPO3
YY1	TNRC6B
YY1	TNS3
YY1	TOB2
YY1	TOMM20
YY1	TOMM22
YY1	TOMM34
YY1	TOMM40L
YY1	TOMM7
YY1	TOP1
YY1	TOP1MT
YY1	TOP2B
YY1	TOP3A
YY1	TOPBP1
YY1	TOPORS
YY1	TOR1A
YY1	TOR4A
YY1	TP53
YY1	TP53BP1
YY1	TP53RK
YY1	TPD52
YY1	TPD52L2
YY1	TPGS1
YY1	TPM3

YY1	TPM4
YY1	TPR
YY1	TPRKB
YY1	TPT1
YY1	TPX2
YY1	TRA2A
YY1	TRA2B
YY1	TRABD
YY1	TRAF3IP1
YY1	TRAF3IP2-AS1
YY1	TRAF4
YY1	TRAF5
YY1	TRAF7
YY1	TRAK1
YY1	TRAPPC1
YY1	TRAPPC10
YY1	TRAPPC13
YY1	TRAPPC4
YY1	TRAPPC6A
YY1	TREML2
YY1	TRIM13
YY1	TRIM28
YY1	TRIM32
YY1	TRIM41
YY1	TRIM59
YY1	TRIOBP
YY1	TRIP4
YY1	TRMT10C
YY1	TRMT11
YY1	TRMT112
YY1	TRMT12
YY1	TRMT1L
YY1	TRMT2A
YY1	TRMT6
YY1	TRMT61B
YY1	TRMU
YY1	TROVE2
YY1	TRPM7
YY1	TRPV2
YY1	TRUB2
YY1	TSC1
YY1	TSC2
YY1	TSC22D4
YY1	TSEN34
YY1	TSG101
YY1	TSPAN14
YY1	TSPO
YY1	TSPYL5
YY1	TSR2
YY1	TSTD1

YY1	TTC1
YY1	TTC13
YY1	TTC14
YY1	TTC30B
YY1	TTC32
YY1	TTF1
YY1	TTF2
YY1	TUBA1A
YY1	TUBA1B
YY1	TUBA1C
YY1	TUBA4A
YY1	TUBB
YY1	TUBGCP6
YY1	TUFM
YY1	TUSC2
YY1	TUT1
YY1	TWF1
YY1	TWF2
YY1	TXLNA
YY1	TXLNG
YY1	TXNDC12
YY1	TXNDC17
YY1	TXNDC9
YY1	TXNL1
YY1	TXNL4B
YY1	TXNRD1
YY1	TYMS
YY1	TYSND1
YY1	TYW3
YY1	U2AF1
YY1	U2AF2
YY1	U2SURP
YY1	UAP1L1
YY1	UBA1
YY1	UBA2
YY1	UBA3
YY1	UBA52
YY1	UBAC1
YY1	UBAC2
YY1	UBAC2−AS1
YY1	UBALD2
YY1	UBAP2
YY1	UBAP2L
YY1	UBB
YY1	UBC
YY1	UBE2A
YY1	UBE2B
YY1	UBE2D1
YY1	UBE2D2
YY1	UBE2D3

YY1	UBE2E1
YY1	UBE2E2
YY1	UBE2E3
YY1	UBE2F
YY1	UBE2G1
YY1	UBE2G2
YY1	UBE2H
YY1	UBE2I
YY1	UBE2J2
YY1	UBE2K
YY1	UBE2L3
YY1	UBE2L6
YY1	UBE2M
YY1	UBE2N
YY1	UBE2Q1
YY1	UBE2R2
YY1	UBE2S
YY1	UBE2T
YY1	UBE2V2
YY1	UBE2Z
YY1	UBE3A
YY1	UBE3B
YY1	UBIAD1
YY1	UBL3
YY1	UBL4A
YY1	UBL5
YY1	UBL7
YY1	UBL7-AS1
YY1	UBLCP1
YY1	UBN1
YY1	UBP1
YY1	UBR4
YY1	UBR5-AS1
YY1	UBR7
YY1	UBTD2
YY1	UBTF
YY1	UBXN1
YY1	UBXN2A
YY1	UBXN4
YY1	UCHL3
YY1	UCHL5
YY1	UCK1
YY1	UCKL1
YY1	UFC1
YY1	UFL1
YY1	UGCG
YY1	UGP2
YY1	UHRF1
YY1	UHRF2
YY1	UIMC1

YY1	UMPS
YY1	UNC45A
YY1	UNC50
YY1	UNG
YY1	UPF2
YY1	UPF3A
YY1	UPF3B
YY1	UQCC1
YY1	UQCC2
YY1	UQCC3
YY1	UQCR10
YY1	UQCR11
YY1	UQCRB
YY1	UQCRC1
YY1	UQCRC2
YY1	UQCRFS1
YY1	UQCRH
YY1	UQCRQ
YY1	URM1
YY1	UROD
YY1	USE1
YY1	USF2
YY1	USP1
YY1	USP10
YY1	USP12
YY1	USP13
YY1	USP14
YY1	USP15
YY1	USP16
YY1	USP22
YY1	USP24
YY1	USP28
YY1	USP3
YY1	USP34
YY1	USP39
YY1	USP47
YY1	USP48
YY1	USP5
YY1	USP53
YY1	USP7
YY1	USP8
YY1	UTP14A
YY1	UTP18
YY1	UTP20
YY1	UTP23
YY1	UTP6
YY1	UXT
YY1	VAMP1
YY1	VAMP2
YY1	VAMP5

YY1	VAMP8
YY1	VAPA
YY1	VASP
YY1	VAV1
YY1	VCP
YY1	VCPIP1
YY1	VCPKMT
YY1	VDAC1
YY1	VDAC2
YY1	VDAC3
YY1	VEZF1
YY1	VHL
YY1	VIM
YY1	VKORC1
YY1	VMA21
YY1	VMP1
YY1	VOPP1
YY1	VPS13B
YY1	VPS16
YY1	VPS26A
YY1	VPS28
YY1	VPS29
YY1	VPS35
YY1	VPS37A
YY1	VPS39
YY1	VPS41
YY1	VPS4A
YY1	VPS4B
YY1	VPS50
YY1	VPS51
YY1	VPS53
YY1	VRK1
YY1	VRK3
YY1	VTA1
YY1	VTI1A
YY1	VTI1B
YY1	WAC
YY1	WAPL
YY1	WARS2
YY1	WAS
YY1	WASF1
YY1	WASF2
YY1	WBP11
YY1	WBP1L
YY1	WBP2
YY1	WBP4
YY1	WDHD1
YY1	WDR1
YY1	WDR11
YY1	WDR13

YY1	WDR18
YY1	WDR33
YY1	WDR45B
YY1	WDR48
YY1	WDR59
YY1	WDR6
YY1	WDR61
YY1	WDR70
YY1	WDR82
YY1	WDR83OS
YY1	WDR89
YY1	WDR90
YY1	WEE1
YY1	WHAMM
YY1	WIPF1
YY1	WIPI2
YY1	WNK1
YY1	WRAP53
YY1	WRAP73
YY1	WRB
YY1	WRNIP1
YY1	WSB1
YY1	WTAP
YY1	XIAP
YY1	XPNPEP1
YY1	XPNPEP3
YY1	XPO1
YY1	XPR1
YY1	XRCC1
YY1	XRCC2
YY1	XRCC3
YY1	XRCC5
YY1	XRCC6
YY1	XRN2
YY1	YAF2
YY1	YARS
YY1	YARS2
YY1	YBEY
YY1	YBX1
YY1	YDJC
YY1	YEATS2
YY1	YEATS4
YY1	YIPF4
YY1	YIPF6
YY1	YKT6
YY1	YLPM1
YY1	YME1L1
YY1	YPEL1
YY1	YPEL3
YY1	YPEL5

YY1	YTHDC1
YY1	YTHDC2
YY1	YTHDF2
YY1	YTHDF3-AS1
YY1	YWHAB
YY1	YWHAE
YY1	YWHAH
YY1	YWHAQ
YY1	YWHAZ
YY1	YY1
YY1	ZBED5
YY1	ZBTB1
YY1	ZBTB11
YY1	ZBTB2
YY1	ZBTB21
YY1	ZBTB25
YY1	ZBTB37
YY1	ZBTB7A
YY1	ZBTB8OS
YY1	ZC3H12A
YY1	ZC3H13
YY1	ZC3H14
YY1	ZC3H8
YY1	ZC3HAV1
YY1	ZC3HC1
YY1	ZCCHC17
YY1	ZCCHC3
YY1	ZCCHC9
YY1	ZDHHC12
YY1	ZDHHC3
YY1	ZDHHC4
YY1	ZEB2
YY1	ZFAND1
YY1	ZFAND5
YY1	ZFAS1
YY1	ZFC3H1
YY1	ZFP36L1
YY1	ZFP64
YY1	ZFP82
YY1	ZFP91
YY1	ZFPL1
YY1	ZFR
YY1	ZFX
YY1	ZFYVE19
YY1	ZKSCAN1
YY1	ZMAT2
YY1	ZMAT5
YY1	ZMYND19
YY1	ZMYND8
YY1	ZNF106

YY1	ZNF121
YY1	ZNF143
YY1	ZNF148
YY1	ZNF207
YY1	ZNF22
YY1	ZNF24
YY1	ZNF248
YY1	ZNF264
YY1	ZNF28
YY1	ZNF281
YY1	ZNF292
YY1	ZNF318
YY1	ZNF32
YY1	ZNF322
YY1	ZNF326
YY1	ZNF335
YY1	ZNF35
YY1	ZNF367
YY1	ZNF395
YY1	ZNF408
YY1	ZNF428
YY1	ZNF430
YY1	ZNF444
YY1	ZNF48
YY1	ZNF480
YY1	ZNF500
YY1	ZNF506
YY1	ZNF511
YY1	ZNF512B
YY1	ZNF544
YY1	ZNF566
YY1	ZNF574
YY1	ZNF579
YY1	ZNF580
YY1	ZNF581
YY1	ZNF593
YY1	ZNF622
YY1	ZNF638
YY1	ZNF639
YY1	ZNF664
YY1	ZNF668
YY1	ZNF669
YY1	ZNF680
YY1	ZNF687
YY1	ZNF706
YY1	ZNF714
YY1	ZNF726
YY1	ZNF75A
YY1	ZNF776
YY1	ZNF827

YY1	ZNF837
YY1	ZNF85
YY1	ZNF879
YY1	ZNFX1
YY1	ZNHIT1
YY1	ZRANB1
YY1	ZRANB2
YY1	ZRANB3
YY1	ZSCAN16-AS1
YY1	ZSWIM7
YY1	ZW10
YY1	ZWILCH
YY1	ZWINT
YY1	ZXDC
YY1	ZYX
YY1	ZZEF1
YY2	AHCYL1
YY2	ARID5B
YY2	CNOT6
YY2	CXCR4
YY2	EGR2
YY2	ETS2
YY2	FAM53C
YY2	GLUL
YY2	IGFBP4
YY2	LCOR
YY2	NIPBL
YY2	RERE
YY2	SF3A1
YY2	SLC38A1
YY2	SP3
YY2	STAG2
YY2	TIA1
YY2	TRAK1
YY2	USP3
YY2	WDR33
YY2	ZSCAN18
ZBTB18	LINC00623
ZBTB18	LINC01588
ZBTB18	LTBP1
ZBTB18	RINL
ZBTB18	ZBTB18
ZBTB33	ACTN1
ZBTB33	AFTPH
ZBTB33	ARHGAP18
ZBTB33	ASB2
ZBTB33	BACE2
ZBTB33	C12orf29
ZBTB33	CAMK2G
ZBTB33	CAMLG

ZBTB33	CCNG1
ZBTB33	DNPH1
ZBTB33	DPYSL2
ZBTB33	EEF1A1
ZBTB33	FOXJ3
ZBTB33	GART
ZBTB33	GDI1
ZBTB33	GPR137
ZBTB33	HES1
ZBTB33	HTT
ZBTB33	KIAA0040
ZBTB33	MED1
ZBTB33	MTURN
ZBTB33	NACA
ZBTB33	NTPCR
ZBTB33	PDPR
ZBTB33	PFDN5
ZBTB33	PPP1R18
ZBTB33	PRNP
ZBTB33	RCSD1
ZBTB33	RNF2
ZBTB33	RPL10A
ZBTB33	RPL12
ZBTB33	RPL15
ZBTB33	RPL23
ZBTB33	RPL26
ZBTB33	RPL27A
ZBTB33	RPL28
ZBTB33	RPL29
ZBTB33	RPL32
ZBTB33	RPL34
ZBTB33	RPL36
ZBTB33	RPL37
ZBTB33	RPL4
ZBTB33	RPLP2
ZBTB33	RPS21
ZBTB33	RPS28
ZBTB33	SEC22C
ZBTB33	SKIL
ZBTB33	STX12
ZBTB33	TEX261
ZBTB33	ZBTB33
ZBTB7A	SEMA4B
ZNF134	ARRDC2
ZNF134	DDX19A
ZNF134	LIMK2
ZNF134	TIMM8A
ZNF134	ZNF800
ZNF136	EPC2
ZNF136	FKBP5

ZNF136	PKN2
ZNF136	PLCL2
ZNF136	SIPA1L1
ZNF136	SLC25A53
ZNF136	UPRT
ZNF136	ZNF831
ZNF143	ABHD14A
ZNF143	ACAD9
ZNF143	ACY3
ZNF143	AFF4
ZNF143	AKAP9
ZNF143	ALG13
ZNF143	AP4M1
ZNF143	APBB3
ZNF143	APOLD1
ZNF143	APTX
ZNF143	AQP3
ZNF143	ARHGAP18
ZNF143	ARL17A
ZNF143	ARMCX3
ZNF143	ARRDC3
ZNF143	ARSG
ZNF143	ASB3
ZNF143	ATAD1
ZNF143	ATF6B
ZNF143	ATG4C
ZNF143	AZIN1
ZNF143	B9D1
ZNF143	BAG5
ZNF143	BANP
ZNF143	BAX
ZNF143	BBC3
ZNF143	C19orf25
ZNF143	C19orf38
ZNF143	C2CD2L
ZNF143	C3orf14
ZNF143	CALCOCO1
ZNF143	CBR4
ZNF143	CBX6
ZNF143	CBX8
ZNF143	CCDC174
ZNF143	CCDC93
ZNF143	CCNL1
ZNF143	CD2AP
ZNF143	CDC42SE2
ZNF143	CDCA5
ZNF143	CEBPG
ZNF143	CENPQ
ZNF143	CFAP20
ZNF143	CHST12

ZNF143	CLCN6
ZNF143	CLDN14
ZNF143	CLDND1
ZNF143	CLTC
ZNF143	CMAS
ZNF143	CPEB4
ZNF143	CPSF7
ZNF143	CRTAP
ZNF143	CSDE1
ZNF143	CTDSPL2
ZNF143	CTHRC1
ZNF143	CTU1
ZNF143	CWF19L1
ZNF143	CXorf56
ZNF143	DCUN1D4
ZNF143	DEDD2
ZNF143	DEPDC5
ZNF143	DERA
ZNF143	DHX15
ZNF143	DIAPH1
ZNF143	DLGAP4
ZNF143	DMTF1
ZNF143	DMXL1
ZNF143	DNAJB4
ZNF143	DNAJC4
ZNF143	DPY19L4
ZNF143	DVL2
ZNF143	EIF2AK3
ZNF143	ELP5
ZNF143	EPM2AIP1
ZNF143	ERCC1
ZNF143	ESCO1
ZNF143	EXOC7
ZNF143	FAM122C
ZNF143	FAM131C
ZNF143	FAM208B
ZNF143	FAM72D
ZNF143	FAM78A
ZNF143	FBXO22
ZNF143	FBXO7
ZNF143	FCF1
ZNF143	FNDC3B
ZNF143	GANAB
ZNF143	GART
ZNF143	GATAD2A
ZNF143	GATB
ZNF143	GEMIN5
ZNF143	GLYR1
ZNF143	GOLGA2
ZNF143	GOLGA8B

ZNF143	GPN2
ZNF143	GPR107
ZNF143	HAUS1
ZNF143	HDAC10
ZNF143	HECTD1
ZNF143	HP1BP3
ZNF143	IFT20
ZNF143	IL13RA1
ZNF143	INO80
ZNF143	INO80E
ZNF143	ISOC2
ZNF143	KANSL1
ZNF143	KCTD13
ZNF143	KDM4B
ZNF143	KIF13A
ZNF143	KLHL18
ZNF143	KMT2B
ZNF143	KMT2E-AS1
ZNF143	KNSTRN
ZNF143	LAX1
ZNF143	LIMK2
ZNF143	LIN54
ZNF143	LINC00891
ZNF143	LINC01588
ZNF143	LNPEP
ZNF143	LPIN1
ZNF143	LRRC8D
ZNF143	LTA4H
ZNF143	LYL1
ZNF143	MAP2K5
ZNF143	MAP3K14
ZNF143	MAPK9
ZNF143	MAPRE2
ZNF143	MAST2
ZNF143	MAST3
ZNF143	MAST4
ZNF143	MECP2
ZNF143	MED17
ZNF143	MED25
ZNF143	MED6
ZNF143	METTL18
ZNF143	METTL23
ZNF143	MLLT10
ZNF143	MTA2
ZNF143	MTHFD2L
ZNF143	MYO19
ZNF143	N4BP2L1
ZNF143	NDE1
ZNF143	NETO1
ZNF143	NOTCH2

ZNF143	NSMAF
ZNF143	NUDT4
ZNF143	NUMA1
ZNF143	OXSR1
ZNF143	P2RX5
ZNF143	PANK3
ZNF143	PARP14
ZNF143	PAWR
ZNF143	PCM1
ZNF143	PDCD11
ZNF143	PGP
ZNF143	PHF20
ZNF143	PHTF2
ZNF143	PITPNA-AS1
ZNF143	PITPNC1
ZNF143	PLD3
ZNF143	POLB
ZNF143	POLR3A
ZNF143	POLR3C
ZNF143	POU3F1
ZNF143	PPP4R3B
ZNF143	PRIM2
ZNF143	PRKAR2B
ZNF143	PTBP3
ZNF143	RANGRF
ZNF143	RASGRF1
ZNF143	RASSF7
ZNF143	RBM41
ZNF143	RBM7
ZNF143	RDX
ZNF143	RFX3
ZNF143	RGS19
ZNF143	RNF169
ZNF143	RPAP1
ZNF143	RPS27L
ZNF143	RTCA
ZNF143	SAE1
ZNF143	SAMSN1
ZNF143	SAPCD2
ZNF143	SEC24C
ZNF143	SEMA4D
ZNF143	SETD3
ZNF143	SETX
ZNF143	SFT2D1
ZNF143	SGK3
ZNF143	SIN3B
ZNF143	SKIL
ZNF143	SKIV2L
ZNF143	SLC10A3
ZNF143	SLC25A13

ZNF143	SLC25A17
ZNF143	SLC26A2
ZNF143	SLC29A1
ZNF143	SLC2A13
ZNF143	SLC31A2
ZNF143	SLC37A4
ZNF143	SLC39A3
ZNF143	SLC39A9
ZNF143	SLC43A2
ZNF143	SLMAP
ZNF143	SMCR8
ZNF143	SMG5
ZNF143	SMIM20
ZNF143	SMNDC1
ZNF143	SNAPC5
ZNF143	SQSTM1
ZNF143	ST3GAL2
ZNF143	STIM1
ZNF143	STX3
ZNF143	SUCLG1
ZNF143	SYNJ2BP
ZNF143	TBXAS1
ZNF143	TCHP
ZNF143	TEX264
ZNF143	TFEB
ZNF143	THAP2
ZNF143	TMCO1
ZNF143	TMEM167A
ZNF143	TMEM206
ZNF143	TMEM216
ZNF143	TMEM242
ZNF143	TNRC6B
ZNF143	TRAIP
ZNF143	TRAK1
ZNF143	TRIM56
ZNF143	TRMT12
ZNF143	TRMT1L
ZNF143	TSG101
ZNF143	TSPAN31
ZNF143	TTC21B
ZNF143	TTC9C
ZNF143	TTN
ZNF143	UACA
ZNF143	UBE4B
ZNF143	UHMK1
ZNF143	USP28
ZNF143	WDFY1
ZNF143	WDR25
ZNF143	WDR59
ZNF143	YIPF5

ZNF143	YPEL1
ZNF143	ZDHHC17
ZNF143	ZFAND6
ZNF143	ZFPL1
ZNF143	ZMAT5
ZNF143	ZNF143
ZNF143	ZNF181
ZNF143	ZNF230
ZNF143	ZNF680
ZNF143	ZYX
ZNF235	CLEC2B
ZNF235	PREPL
ZNF235	ZNF226
ZNF274	AAAS
ZNF274	ACLY
ZNF274	ADD1
ZNF274	ASTE1
ZNF274	BBS4
ZNF274	C12orf66
ZNF274	CCPG1
ZNF274	CELF2
ZNF274	CHMP1A
ZNF274	CYTH1
ZNF274	DNAJB9
ZNF274	EPB41
ZNF274	EPC1
ZNF274	EPHB6
ZNF274	FBXL12
ZNF274	FEM1A
ZNF274	FKBP15
ZNF274	G6PD
ZNF274	GXYLT1
ZNF274	IGFBP4
ZNF274	JAK2
ZNF274	JARID2
ZNF274	JUNB
ZNF274	KRAS
ZNF274	MAN1A1
ZNF274	MANF
ZNF274	MAP1S
ZNF274	MEX3C
ZNF274	NMNAT1
ZNF274	PACSIN2
ZNF274	PDE4D
ZNF274	POLR2A
ZNF274	PSENEN
ZNF274	PTDSS2
ZNF274	RABEPK
ZNF274	RASA4
ZNF274	RIC8B

ZNF274	RNF38
ZNF274	RPAP2
ZNF274	RPL38
ZNF274	RPS27
ZNF274	RPS29
ZNF274	RRNAD1
ZNF274	RUNX1
ZNF274	SARAF
ZNF274	SEMA4B
ZNF274	SH2B1
ZNF274	SLC1A5
ZNF274	SLC25A38
ZNF274	SLC35A3
ZNF274	SLC35A5
ZNF274	SLC38A10
ZNF274	SNX25
ZNF274	STAT6
ZNF274	TM9SF4
ZNF274	TMEM62
ZNF274	TSC2
ZNF274	TSC22D2
ZNF274	VTA1
ZNF274	WDYHV1
ZNF274	ZFYVE16
ZNF274	ZNF213
ZNF274	ZNF274
ZNF274	ZNF395
ZNF274	ZNF518A
ZNF274	ZNF644
ZNF274	ZNHIT2
ZNF274	ZSCAN32
ZNF425	EDEM1
ZNF425	ELL2
ZNF425	FAM174A
ZNF425	FAM214A
ZNF425	MTRF1L
ZNF425	SERINC5
ZNF425	STXBP6
ZNF76	ADORA2A
ZNF76	APEX2
ZNF76	ARHGAP17
ZNF76	ARHGAP44
ZNF76	ARHGEF3
ZNF76	ARV1
ZNF76	ASF1B
ZNF76	ATP13A2
ZNF76	ATP2A1-AS1
ZNF76	B3GALT4
ZNF76	BIK
ZNF76	BLOC1S5

ZNF76	C2orf49
ZNF76	C6orf99
ZNF76	C8orf76
ZNF76	CAND1
ZNF76	CAPN15
ZNF76	CARF
ZNF76	CCNF
ZNF76	CENPJ
ZNF76	CEP85L
ZNF76	CHAF1A
ZNF76	CHCHD1
ZNF76	CHEK2
ZNF76	CKAP4
ZNF76	CNKSR1
ZNF76	CNOT4
ZNF76	COA1
ZNF76	DAPK2
ZNF76	DDX3X
ZNF76	DOCK2
ZNF76	E2F1
ZNF76	EDRF1
ZNF76	EPC2
ZNF76	EPS15L1
ZNF76	ERCC6L2
ZNF76	EXOSC10
ZNF76	EXOSC6
ZNF76	EYA3
ZNF76	FAM131C
ZNF76	FAM210A
ZNF76	FBXO46
ZNF76	FBXO7
ZNF76	FES
ZNF76	FICD
ZNF76	FLI1
ZNF76	FLNA
ZNF76	GABPA
ZNF76	GALNT6
ZNF76	GEMIN6
ZNF76	GID4
ZNF76	GM2A
ZNF76	GNAZ
ZNF76	GSK3A
ZNF76	GSTA4
ZNF76	GTF2H2
ZNF76	GTPBP8
ZNF76	HDAC8
ZNF76	HIPK1
ZNF76	IL6R
ZNF76	IRF2BP2
ZNF76	KCNK12

ZNF76	KLHL24
ZNF76	KPNA3
ZNF76	KRR1
ZNF76	LCOR
ZNF76	LEMD2
ZNF76	LRPPRC
ZNF76	MAP2K6
ZNF76	MBTPS1
ZNF76	MCTP2
ZNF76	MEN1
ZNF76	MICAL3
ZNF76	MOCS3
ZNF76	MYH9
ZNF76	NAXD
ZNF76	NDUFA7
ZNF76	NFKBIB
ZNF76	NLK
ZNF76	NME7
ZNF76	NOL11
ZNF76	NUDT3
ZNF76	NUDT9
ZNF76	OGFOD1
ZNF76	OSBPL2
ZNF76	PAQR8
ZNF76	PEX14
ZNF76	PHTF1
ZNF76	PLEKHO1
ZNF76	POMGNT1
ZNF76	PPP1R12B
ZNF76	PRDM1
ZNF76	PRDM15
ZNF76	PTBP3
ZNF76	QPRT
ZNF76	QRICH1
ZNF76	RABL6
ZNF76	RASSF2
ZNF76	RBM14
ZNF76	RELT
ZNF76	RNF144B
ZNF76	ROCK2
ZNF76	SAP130
ZNF76	SDHAF1
ZNF76	SERP2
ZNF76	SH3TC1
ZNF76	SLAMF6
ZNF76	SLC20A1
ZNF76	SLC29A3
ZNF76	SLC38A9
ZNF76	SMIM19
ZNF76	SNHG21

ZNF76	SNRPA
ZNF76	SNTB2
ZNF76	STAU1
ZNF76	STK26
ZNF76	TAF8
ZNF76	TCAF1
ZNF76	TEX30
ZNF76	TGDS
ZNF76	THOP1
ZNF76	TIGAR
ZNF76	TIMM23
ZNF76	TOX
ZNF76	TP53TG1
ZNF76	TPCN2
ZNF76	TRAF3IP1
ZNF76	TRAIP
ZNF76	TRAPPC13
ZNF76	UBAC1
ZNF76	UNK
ZNF76	VPS52
ZNF76	WDFY4
ZNF76	WDR34
ZNF76	WRAP53
ZNF76	WTAP
ZNF76	XPO6
ZNF76	ZCCHC7
ZNF76	ZNF148
ZNF76	ZNF317
ZNF76	ZNF444
ZNF76	ZNF76
ZNF76	ZNF780B
ZXDB	GPRC5C
ZXDB	PIK3CD
ZXDB	RNF2
ZXDB	TNFRSF1B
